# Inferring drug-disease associations based on known protein complexes

**DOI:** 10.1186/1755-8794-8-S2-S2

**Published:** 2015-05-29

**Authors:** Liang Yu, Jianbin Huang, Zhixin Ma, Jing Zhang, Yapeng Zou, Lin Gao

**Affiliations:** 1 School of Computer Science and Technology, Xidian University, Xi'an 710071, PR China

**Keywords:** Drug-disease associations, protein complexes, drug repositioning, weighted network

## Abstract

Inferring drug-disease associations is critical in unveiling disease mechanisms, as well as discovering novel functions of available drugs, or drug repositioning. Previous work is primarily based on drug-gene-disease relationship, which throws away many important information since genes execute their functions through interacting others. To overcome this issue, we propose a novel methodology that discover the drug-disease association based on protein complexes. Firstly, the integrated heterogeneous network consisting of drugs, protein complexes, and disease are constructed, where we assign weights to the drug-disease association by using probability. Then, from the tripartite network, we get the indirect weighted relationships between drugs and diseases. The larger the weight, the higher the reliability of the correlation. We apply our method to mental disorders and hypertension, and validate the result by using comparative toxicogenomics database. Our ranked results can be directly reinforced by existing biomedical literature, suggesting that our proposed method obtains higher specificity and sensitivity. The proposed method offers new insight into drug-disease discovery. Our method is publicly available at http://1.complexdrug.sinaapp.com/Drug_Complex_Disease/Data_Download.html.

## Background

Diseases are often caused by congenital disorder or expression of abnormal genes, which induces multi-factor-driven alterations and disrupts functional modules [1]. Drugs accomplish their therapeutic effect by changing downstream processes of their targets, which contend with the alterations of the abnormal genes. Drug development is expensive, time consuming and has a high risk of failures. By conservative estimates, it now takes ~15 years [2] and $800 ~ $1000 million to bring a single drug to market [3]. This situation hampers the pharmaceutical industry to find innovative strategies against currently incurable diseases. Drug repositioning (or drug repurposing) attempts to find previously unknown targets for drugs already established on the market or drugs currently in advanced development stages. Several examples throughout history have shown that such repositioning can be very successful (one example is Sildenafil, also known as Viagra) [4]. Therefore, more and more research is focusing on inferring drug-disease associations by computational methods.

Several network-based methods have been studied to infer the relationships between drugs and disease (for a review, see [5]). Matteo indicated that the combination of bipartite network projections, weighted integration of different pharmacological spaces and kernelized score functions with random walk kernels play a key role in significantly improving the drug ranking results with respect to DrugBank therapeutic categories [6]. Cheng [7] integrated three networks, chemical, gene and disease, to infer chemical hazard profiles, identify exposure data gaps, and incorporate genes and disease networks into chemical safety evaluations. Lee established a database PharmDB, an integrated tripartite database, coupled with Shared Neighborhood Scoring (SNS) algorithm, to find new indication of known drugs [8]. With increasing evidence in genetic and molecular biology, we know that protein complexes and pathways are not affected by a single gene, instead a group of interacting genes underlying similar diseases, which point out the therapeutic importance of those modules [9]. Therefore, it is of great importance to investigate how drugs and disease phenotypes are associated on the basis of gene modules [10]. In 2004, different tumor types were tentatively characterized by predefined gene modules using gene expression data [11]. Wong et al. defined a module map to connect gene modules with human cancers, which was shown to guide new disease therapies [12]. PREDICT is based on the observation that similar drugs are indicated for similar diseases, and utilizes multiple drug-drug and disease-disease similarity measures for the prediction task [13]. It allows easy integration of additional similarity measures among diseases and drugs. In 2012, Daminelli constructed a drug-target-disease network and extracted the bi-cliques where every drug is linked to every target and disease [14]. This method can reposition drugs and predict a drug's off targets simultaneously. Ye integrated known drug target information and proposed a disease-oriented strategy for evaluating the relationships between drugs and specific diseases based on their pathway profile [15]. Zhao et al. developed a Bayesian partition method to discover drug-gene-disease co-modules. Such a co-module approach offered a systematic and holistic view to study drug-disease relationships and their molecular basis [16]. A huge amount of chemical, genomic and disease phenotype data is rapidly accumulated, but the drug-diseases associations are still not clear.

Protein complexes are key molecular entities that integrate multiple gene products to perform cellular functions. CORUM provides a comprehensive dataset of protein complexes for discoveries in systems biology, analyses of protein networks and protein complex-associated diseases [17]. Therefore, based on the known complexes in CORUM database, we design a method to infer drug-complex-disease phenotype relationships using a network model, where protein complexes are related to not only drugs but also to the disease phenotype.

In our study, based on a symmetrical conditional probability model, we construct a weighted tripartite hetero-network of drugs, protein complexes, and diseases. From this drug-complex-disease tripartite network, we are able to obtain indirect weighted relationships between drugs and diseases, which is a bipartite hetero-network. A drug which has high correlation with a complex set receives a higher closeness score with disease, which also highly related to the same complex set. We rank the associations between drugs and diseases in descending order, by edge weights, in drug-disease network. The larger the weight of the association, the greater the degree of reliability, thus the greater the possibility of relation of drug to disease. We select mental disorders and hypertension as our test data. We use the both curated and inferred drug-disease associations from Comparative Toxicogenomics Database (CTD; http://ctd.mdibl.org)[18]as our benchmark. Our ranked results show that our proposed method obtain higher specificity and sensitivity. Our approach renders a promising perspective to investigate drug-disease associations and provides computational evidence in revealing their mechanism basis.

## Materials and methods

**Figure 1:**
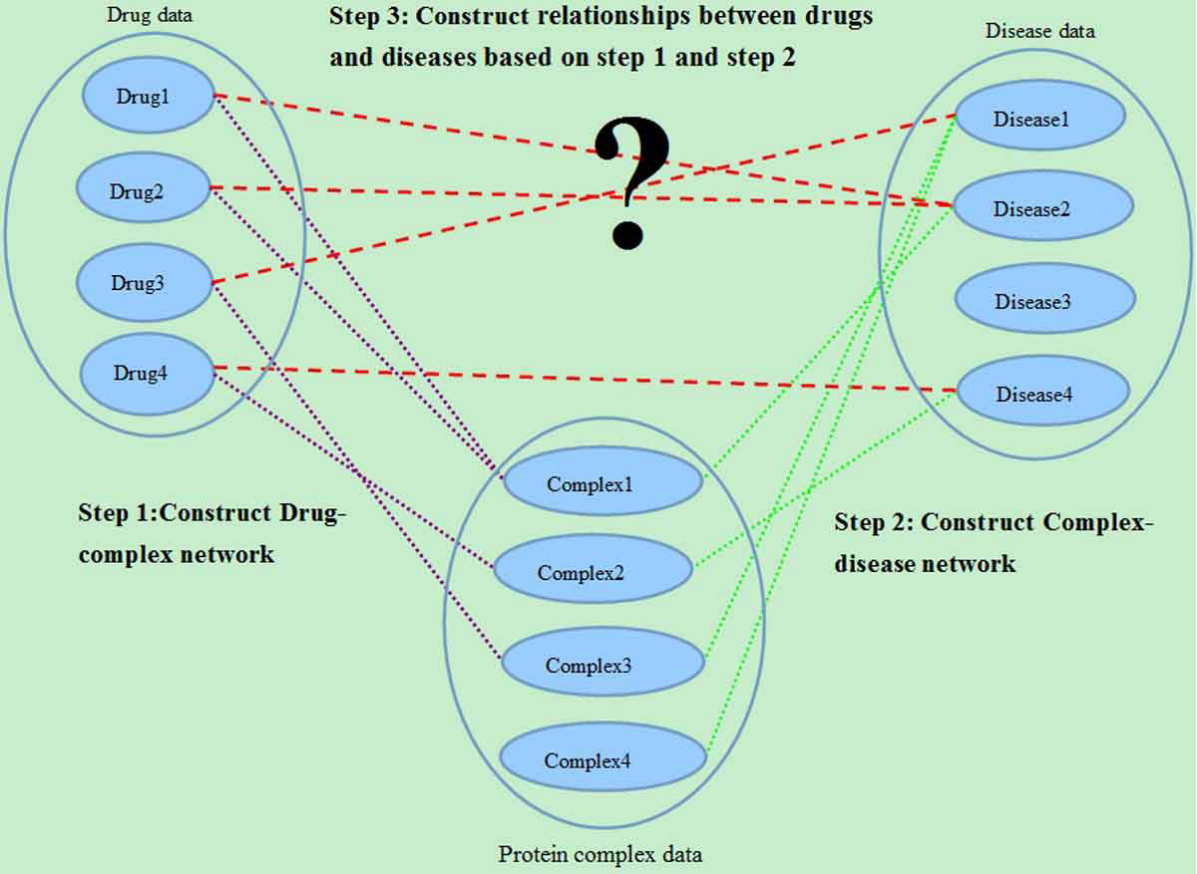
**The overview of our proposed method**. Firstly, we construct a drug-complex network. If the target set of a drug has at least one common protein with a complex, there will be an edge between the drug and the complex. Then, we construct a complex-disease network. If there is an edge between a complex and a disease, at least one protein of the complex is also a protein related to the disease. In this way, we get a drug-complex-disease tripartite network. Based on the tripartite, we can extract the associations between drugs and diseases. If a drug and a disease have at least one common protein complex neighbor, there will be a connection between them.

The integrated network, including three heterogeneous data of drug, disease, protein complex are illustrated in Figure 1.

### Materials

#### Data sources

##### Drug data

The DrugBank database combines detailed drug (i.e. chemical, pharmacological and pharmaceutical) data with comprehensive drug target (i.e. sequence, structure, and pathway) information [19,20]. We collect FDA-approved drugs in the latest release of DrugBank database (version 4.0) [21].

##### Protein complexes data

The CORUM database is a comprehensive resource of manually annotated protein complexes from mammalian organisms. All the information is obtained from individual experiments published in scientific articles, and data from high-throughput experiments are excluded. We download the all Complexes from CORUM [17](the release February 2012).

##### Disease data

The disease data is downloaded from FunDO (http://django.nubic.northwestern.edu/fundo/) [22]. FunDO takes a list of genes and finds relevant diseases based on statistical analysis of the Disease Ontology annotation database [23].

##### Protein-protein interaction network

We obtain relationships between genes (or equivalently, proteins) as demonstrated by Liu et al. [24]. The final binary protein-protein interaction network contains 7,533 nodes and 22, 345 edges. Genes are identified by their NCBI gene IDs. We use the PPI network to filter the predicted drug-disease associations. If a drug and a disease are associated with two different genes in a same complex, and there is a direct connection between the two genes in the PPI network, we will track the association, or else we discard it.

#### Benchmark of drug-disease associations

We extract all the known associations between chemicals (or equivalently, drugs) and disorders or its descendants from Comparative Toxicogenomics Database (CTD) in May 2014 as our benchmark [25]. CTD contains curated and inferred chemical-disease associations. Curated chemical-disease associations are extracted from the published literature by CTD biocurators. Inferred associations are established via CTD-curated chemical-gene interactions. In our research the curated and inferred associations have been identified, and they can help researchers develop hypotheses about environmental diseases and their underlying mechanisms.

#### Functional enrichment analysis

In order to evaluate our method further, we perform functional enrichment analysis using DAVID [26,27] on the target sets of predicted drugs. With the target genes as inputs, we observe gene-disease associations and the enriched KEGG pathways on the related biological process. With Benjamin multiple testing correction method [28], the enrichment p-value was corrected to control family-wide false discovery rate under certain rate (e.g. ≥ 0.05).

### Methods

#### Weighted network construction

To construct a weighted tripartite network of drugs, protein complexes, and diseases, we map the UniProt ID of each drug target to the Entrez gene ID. We obtain a list of gene targets for each drug. There are 6,039 relations between 1,481 drugs and 1,583 targets (additional file 1). We collect the list of protein subunits for each complex in the all Complexes set, which are referenced by their Entrez IDs (additional file 2). The same operation is conducted for all genes related to diseases, resulting in a list of Entrez gene identifiers for each disease (additional file 3). The relations between drugs, protein complexes, and diseases can be represented as a tripartite network, which can be expressed as:

(1) $${\text{G}}_{\text{TPD}}=\left(\text{T},\text{P},\text{D},{\text{E}}_{\text{T}},{\text{E}}_{\text{D}}\right)$$

*T, P*, and *D *are finite sets of drug, protein complex, and disease; ***E_T _***and ***E_D _***denote the two types of undirected links in the network: drug-complex and complex-disease. The relevance between drug *t_i _*(***t_i _***∈ ***T***, ***i ***= 1,...,|***T***|) and complex *p_j _*(***p_j _***∈ ***P***, ***j ***= 1,...,|***P***| ), ***w_T_***(***t_i_***, ***p_j_***) is calculated by symmetrical conditional probability, as in equation (2).

(2) $${\text{w}}_{\text{T}}\left({\text{t}}_{\text{i}},{\text{p}}_{\text{j}}\right)=\sqrt{\text{pro}\left({\text{t}}_{\text{i}}|{\text{p}}_{\text{j }}\right)\cdot \text{pro}\left({\text{p}}_{\text{j }}|{\text{t}}_{\text{i}}\right)}$$

Equation (2) indicates that the relevance between *t_i _*and *p_j _*is determined jointly by their conditional probabilities on each other.

Suppose that ***g***(***t_i_***, ***p_j_***) denotes the number of elements shared by the target set of the drug *t_i _*and the complex set *p_j_*, ***g***(***t_i_***) and ***g***(***p_j_***) stand for the number of targets of the drug *t_i _*and the number of proteins in complex *p_j _*respectively. Accordingly, equation (2) can be expressed as:

(3) $${\text{w}}_{\text{T}}\left({\text{t}}_{\text{i}},{\text{p}}_{\text{j}}\right)=\sqrt{\frac{\text{g}\left({\text{t}}_{\text{i}},{\text{p}}_{\text{j }}\right)}{\text{g}\left({\text{t}}_{\text{i}}\right)}\cdot \frac{\text{g}\left({\text{t}}_{\text{i}},{\text{p}}_{\text{j}}\right)}{\text{g}\left({\text{p}}_{\text{j}}\right)}}$$

Similarly, we can obtain the weight ***w_D_***(***p_i_***, ***d_j_***) (***p_i _***∈ ***P***, ***d_j _***∈ ***D***, ***i ***= 1,...,|***P***|, ***j ***= 1,...,|***D***|) to the links between complexes and diseases. (***p_i_***, ***d_j_***) ∈ ***E_D _***if at least one protein of the complex *p_i _*is also a protein related to the disease *d_j_*, where ***p_i _***∈ ***P***, ***d_j _***∈ ***D***, ***i ***= 1,...,|***P***|, ***j ***= 1,...,|***D***|.

#### Derivative Network

To identify the drug-disease association, a derived drug-disease network can be extract with an immediate purpose to facilitate the association identification. A bipartite network ***G_TD _***= (***T***, ***D***, ***E_TD_***) is used to illustrate their associations, where *T, D *are finite sets of drug and disease respectively. ***E_TD _***denotes the undirected links between drugs and diseases. The drug-disease interaction exists if and only if the following two constraints are met simultaneously: i) the drug and the disease have at least one common protein complex neighbor in *G_TPD _*network; ii) at least one protein target of the drug was also a subunit of the protein complex. Specifically, it is defined as

(4) $${\text{E}}_{\text{TD}}=\left\{\left(\text{t},\text{d}\right)|\left(\exists \text{p}\in \text{P}\right)\left(\left(\text{t},\text{p}\right)\in {\text{E}}_{\text{T}}\wedge \left(\text{p},\text{d}\right)\in {\text{E}}_{\text{D}}\right)\wedge \text{t}\in \text{T}\wedge \text{d}\in \text{D}\right\}$$

where *P *is the set of protein complexes. For each edge (***t***, ***d***) ∈ ***E_TD_***, its weight ***w_TD_***(***t***, ***d***) can be calculated by equation (5):

(5) $${\text{w}}_{\text{TD}}\left(\text{t},\text{d}\right)=\sqrt{\frac{{\text{g}}_{\text{T}}\left(\text{t},\text{C}\right)}{\text{g}\left(\text{t}\right)}\cdot \frac{{\text{g}}_{\text{D}}\left(\text{d},\text{C}\right)}{\text{g}\left(\text{d}\right)}}$$

Suppose *C *represents the set of protein complexes that both drug *t *and disease *d *connect to in *G_TPD _*network, then:

(6) $$\text{C}=\left\{\text{p}|\text{p}\in \text{P}\wedge \left(\text{t},\text{p}\right)\in {\text{E}}_{\text{T}}\wedge \left(\text{p},\text{d}\right)\in {\text{E}}_{\text{D}}\wedge \text{t}\in \text{T}\wedge \text{d}\in \text{D}\right\}$$

***g_T_***(***t***, ***C***) represents the sum of edge weights between drug *t *and protein complexes in set *C*. The formulas of ***g_T_***(***t***, ***C***) and ***g_D_***(***d***, ***C***) are given as follows:

(7) $${\text{g}}_{\text{T}}\left(\text{t},\text{C}\right)=\underset{{\text{p}}^{\prime }\in \text{C}}{\sum }{\text{w}}_{\text{T}}\left(\text{t},{\text{p}}^{\prime }\right)$$

(8) $${\text{g}}_{\text{D}}\left(\text{d},\text{C}\right)=\underset{{\text{p}}^{\prime }\in \text{C}}{\sum }{\text{w}}_{\text{D}}\left({\text{p}}^{\prime },\text{d}\right)$$

***g***(***t***) and ***g***(***d***) in equation (5) respectively indicate the sum of edge weights between drug *t*, disease *d *and protein complexes in set *P*. Therefore:

(9) $$\text{g}\left(\text{t}\right)=\underset{{\text{p}}^{\prime }\in \text{P}}{\sum }{\text{w}}_{\text{T}}\left(\text{t},{\text{p}}^{\prime }\right)$$

(10) $$\text{g}\left(\text{d}\right)=\underset{{\text{p}}^{\prime }\in \text{P}}{\sum }{\text{w}}_{\text{D}}\left({\text{p}}^{\prime },\text{d}\right)$$

If drug ***t^' ^***and disease ***d^' ^***cannot be connected by common complex neighbors, but at least one protein target of drug ***t^' ^***is also a protein related to disease ***d^'^***, a connection will be created between ***t^' ^***and ***d^'^***. Similarly, the weight of edge (***t^'^***, ***d^'^***) can be calculated by equation (3).

#### Network conversions

In order to verify the predicted drug-disease correlations by modularity, we first need to convert *G_TD _*into two networks. Each converted network is composed of a single type of node. The bipartite network for drugs and diseases *G_TD _*is converted into two independent networks, which are denoted by ***G***_1 _= (***V***_1_,***E***_1_) and ***G***_2 _= (***V***_2_,***E***_2_). ***G***_1 _and ***G***_2 _are the drugs and the diseases networks respectively. In ***G***_1_, nodes of *V*_1 _are connected together if they have at least one common neighbor (*D*) in *G_TD_*. The set of edges ***E***_1 _can be defined as:

(11) $${\text{E}}_{1}=\left\{\left(\text{t},{\text{t}}^{\prime }\right)|\left(\exists \text{d}\in \text{D}\right)\left(\left(\text{t},\text{d}\right)\in {\text{E}}_{\text{TD}}\wedge \left({\text{t}}^{\prime },\text{d}\right)\in {\text{E}}_{\text{TD}}\wedge \text{t}\neq {\text{t}}^{\prime }\right)\right\}$$

The set of edges ***E***_2 _is defined similarly. The weight of edge (***t***, ***t^'^***) ∈ ***E***_1_, ***w***(***t***, ***t***^'^) is defined as:

(12) $$\text{w}\left(\text{t},{\text{t}}^{\prime }\right)=\underset{\text{d}\in \text{D}}{\sum }\text{min}\left({\text{w}}_{\text{TD}}\left(\text{t},\text{d}\right),{\text{w}}_{\text{TD}}\left({\text{t}}^{\prime },\text{d}\right)\right)$$

Edge weights in ***G***2 have a similar definition. Therefore, we get two weighted networks: a drug-drug network and a disease-disease network.

#### Module structure in converted network

We use ClusterONE (Clustering with Overlapping Neighborhood Expansion) [29] to obtain modules in converted networks. ClusterONE is a graph clustering algorithm that is able to handle weighted graphs. Owing to these properties, ClusterONE is especially useful for detecting modules in networks with associated confidence values.

## Results

### Bipartite network of drugs and diseases

**Figure 2:**
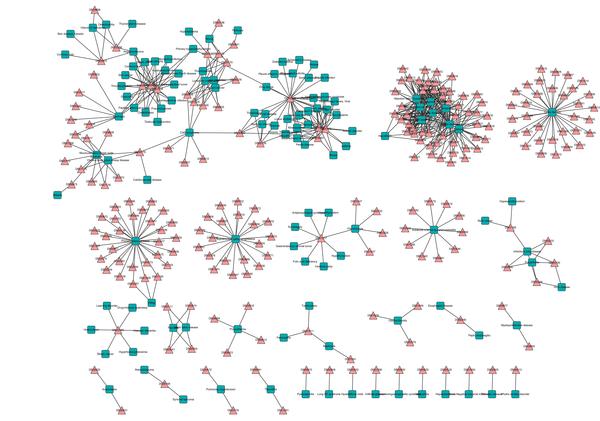
**Bipartite network of drugs and diseases**. A drug is connected to a disease if they share at least one complex and the value of relationship is not lower than 0.5. Drugs are represented by triangles and diseases by squares. Different types of nodes also distinguish from each other by color. Every connected subgraph is a module. Drugs and diseases are labeled by their DrugBank identifier and name in FunDO, respectively.

The weighted tripartite network of drug-complex-disease consists of two bipartite networks: drug-complex and complex-disease. The drug-complex network contains 1,229 nodes (628 drugs and 601 complexes) and 3,405 weighted edges (additional file 4). The complex-disease network contains 1932 nodes (1,472 complexes and 460 diseases) and 14,848 weighted edges (additional file 5). The bipartite network of drug-disease obtained from the tripartite network includes 1,634 nodes (1,127 drugs and 507 diseases) and 30,722 weighted edges (additional file 6). In order to improve the reliability of the predicted correlations between drugs and diseases, we first use PPI network to filter the results, then we discard the edges whose weights are lower than 0.50. The final network consists of 353 nodes (231 drugs and 122 diseases) and 594 weighted edges (***weight ***≥ 0.50) (additional file 7). This is a scale-free network, with a small number of nodes connected to many edges and the majority of nodes connected to few edges (Figure 2).

All network visualizations were produced using the Cytoscape software [30]. Every connected subgraph represents a module, resulting in 29 modules with bipartite structure as shown in Figure 2. Nodes with a large degree can be seen among both drugs and diseases (See Table 1).

**Table 1:** Top diseases and drugs with a large degree in the bipartite drug-disease network

Disease Name	Number of direct neighbors	Sum of weight on edges	Drug ID	Drug name	Number of directed edges	Sum of weight on edges
Mental disorders	51	39.58	DB00098	Antithymocyte globulin	27	19.90

Cystic fibrosis	33	18.56	DB01259	Lapatinib	18	15.07

Primary biliary cirrhosis	30	20.28	DB08916	Afatinib	17	13.79

Attention deficit hyperactivity disorder	26	16.40	DB00054	Abciximab	16	13.49

Anorexia nervosa	25	17.14	DB00775	Tirofiban	15	15.31

Panic disorder	25	16.77	DB00072	Trastuzumab	12	9.13

Sudden infant death syndrome	25	18.27				

Epilepsy	24	13.37				

Hypertension	24	13.26				

Migraine	21	11.10				

Supranuclear palsy, progressive	21	11.51				

Mucocutaneous lymph node syndrome	11	8.45				

Subacute sclerosing panencephalitis	10	5				

Table 1 shows the number of edges directly related to the hubs (column: Number of directed edges) and the sum of weight on these edges (column: Sum of weight on edges). We find that the sum of weights of edges may more accurately reflect the role of nodes in the network. For example, cystic fibrosis has more direct neighbors than primary biliary cirrhosis in bipartite network. But, the correlation between the drugs and primary biliary cirrhosis is greater than that between the drugs and cystic fibrosis. In Table 1 the most connected disease is mental disorders (Synonym: behavior disease), which is a mental or behavioral pattern, or an anomaly that causes either suffering or an impaired ability to function in ordinary life (disability). The most connected drug is anti-thymocyte globulin (ATG). It is an infusion of horse or rabbit-derived antibodies against human T cells, which is used in the prevention and treatment of acute rejection in organ transplantation and therapy of aplastic anemia.

### Case study: Mental Disorders

#### Potential drugs and Mental Disorders relations

Mental disorders are one aspect of mental health [31], which are generally defined by a combination of how a person feels, acts, thinks and perceives. This may be associated with particular regions or functions of the brain, or any part of the nervous system, often in a social context. 226 drug-mental disorders relations are found in our candidate sets (additional file 8). In order to improve the accuracy of the prediction, an association will not be considered if its weight is below 0.5. The reason is that based on the experiments, 0.5 as threshold can conserve more real correlations, as well as avoid including too many false-positive ones. Finally, 51 drug-mental disorders correlations are obtained (see Table 2).

**Table 2:** Drug-mental disorders associations (*weight *≥ 0.5)

ID	Drug ID	Drug Name	Weight	ID	Drug ID	Drug Name	Weight
1	DB00904	Ondansetron	0.89	27	DB00334	Olanzapine	0.82

2	DB00669	Sumatriptan	0.85	28	DB01186	Pergolide	0.82

3	DB00734	Risperidone	0.73	29	DB01618	Molindone	0.82

4	DB00490	Buspirone	0.77	***30***	***DB06684***	***Vilazodone***	***0.82 ***

5	DB01149	Nefazodone	0.93	***31***	***DB01621***	***Pipotiazine***	***0.82 ***

6	DB01142	Doxepin	0.83	32	DB01616	Alverine	0.82

7	DB01392	Yohimbine	0.87	33	DB01200	Bromocriptine	0.82

8	DB00540	Nortriptyline	0.89	34	DB00216	Eletriptan	0.81

9	DB01224	Quetiapine	0.82	***35***	***DB01622***	***Thioproperazine***	***0.79 ***

10	DB00363	Clozapine	0.82	***36***	***DB01614***	***Acepromazine***	***0.79 ***

11	DB00477	Chlorpromazine	0.78	37	DB00960	Pindolol	0.77

12	DB00571	Propranolol	0.75	***38***	***DB04946***	***Iloperidone***	***0.76 ***

13	DB00321	Amitriptyline	0.69	***39***	***DB08807***	***Bopindolol***	***0.75 ***

14	DB00726	Trimipramine	0.90	40	DB08815	Lurasidone	0.75

15	DB00247	Methysergide	0.87	41	DB06216	Asenapine	0.74

16	DB00656	Trazodone	0.86	***42***	***DB01049***	***Ergoloid mesylate***	***0.74 ***

17	DB00315	Zolmitriptan	0.85	***43***	***DB05271***	***Rotigotine***	***0.72 ***

18	DB00952	Naratriptan	0.85	***44***	***DB01267***	***Paliperidone***	***0.72 ***

**19**	**DB08810**	**Cinitapride**	**0.84 **	**45**	**DB01359**	**Penbutolol**	**0.60 **

20	DB00589	Lisuride	0.84	46	DB00866	Alprenolol	0.60

21	DB00268	Ropinirole	0.84	47	DB00696	Ergotamine	0.56

22	DB00413	Pramipexole	0.84	48	DB00998	Frovatriptan	0.55

23	DB00714	Apomorphine	0.83	49	DB00918	Almotriptan	0.55

24	DB00248	Cabergoline	0.83	50	DB00953	Rizatriptan	0.53

25	DB01238	aripiprazole	0.82	51	DB00320	Dihydroergotamine	0.50

26	DB00246	ziprasidone	0.82				

Drug ID represents the unique DrugBank accession number of a drug. Drug Name represents the corresponding name of a Drug ID. Weight represents the correlation between a drug and the mental disorder. 40 drugs are approved by our benchmark, 9 predicted drugs are supported by literature (in bold italic), and 2 are not directly supported by the literatures (in underlined bold).

Since the predictions are merely assumptions, we need to further examine these predictions using external literature support: 40 known associations agree with the benchmark (CTD), 9 predicted associations are supported by the literature (in bold italic). We find the 9 predicted drugs for the treatment of mental disorders may have a good effect. For example, vilazodone [32] (ID = 30) is approved for treatment of acute episodes of major depression. Major depressive disorder (MDD) is a mental disorders characterized by a pervasive and persistent low mood that is accompanied by low self-esteem and by a loss of interest or pleasure in normally enjoyable activities.

Pipotiazine (ID = 31) is a typical antipsychotic of the phenothiazine class [33] used in the United Kingdom and other countries for the treatment of schizophrenia. Thioproperazine (ID = 35) is an antipsychotic. Antipsychotics [34] are a class of psychiatric medication primarily used to manage psychosis, in and concentration [35,36]. Certain mental health problems, such as depression and disturbances, including hallucinations, delusions and paranoia, are possible complications of Parkinson's disease and/or its treatment. Rotigotine (ID = 43) is for treatment in neurologic disorders and Parkinson's disease, as well as moderate-to-severe primary Restless Legs Syndrome [37]. Paliperidone (ID = 44) is the major active metabolite of risperidone. It is used for schizophrenia and schizoaffective cinitapride (ID = 19) and penbutolol (ID = 45), there is no direct support in literature. However, we are confident that they maybe effective in the treatment of mental disorders. Cinitapride is a substituted benzamide with 5-HT receptor antagonist and agonist activity [38]. The 5-HT receptors are the target of a variety of pharmaceutical drugs, including many antidepressants, antipsychotics, etc [39], so cinitapride may be effective in the treatment of mental disorders. Similarly, penbutolol is able to bind both β-1 adrenergic receptors (ARs) and β-2 adrenergic receptors [40], and the interaction between β-1 ARs and testosterone has been shown in anxiolytic behaviors in the basolateral amygdale [41]. β-2 receptor is also involved in brain-immune-communication [42]. Therefore, we can conclude that penbutolol has a high correlation with mental disorders.

#### The significant modules related to mental disorders in drug-drug network

**Figure 3:**
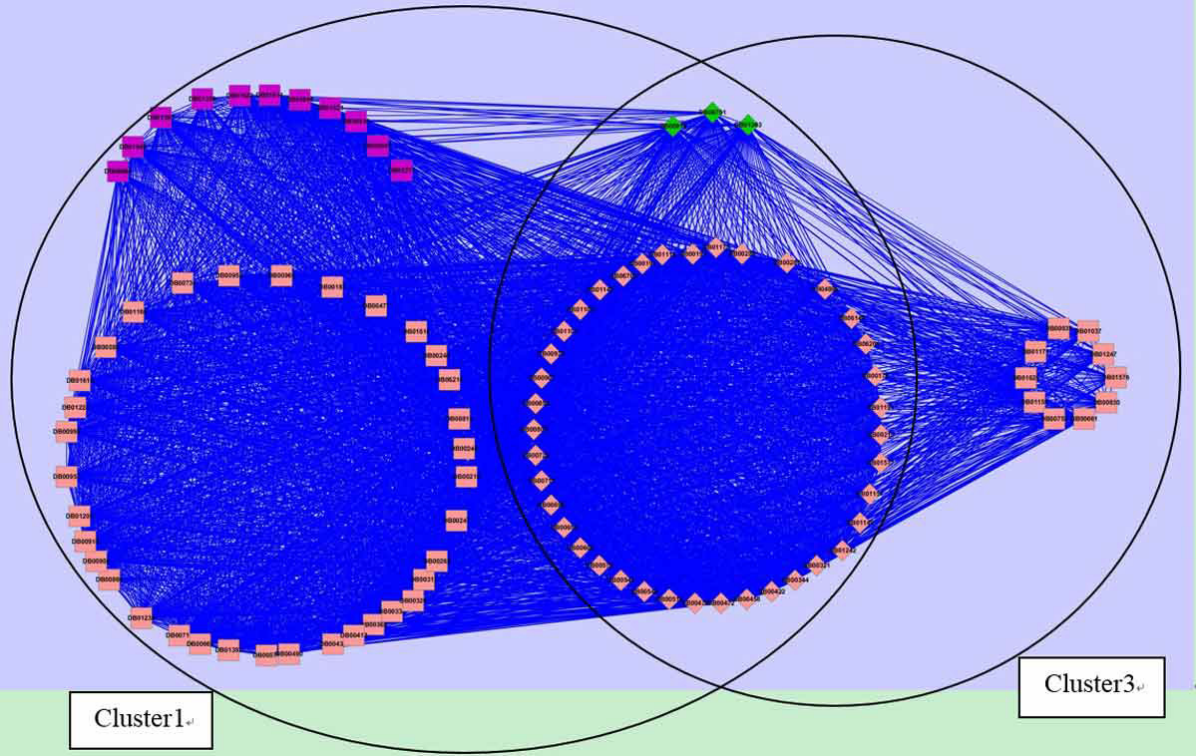
**Drugs associated with mental disorder within the module after merging cluster 1 and cluster 3**. Nodes represent drugs. Diamond nodes represent the overlap of cluster 1 and cluster 3. Nodes colored pink represent drugs that have been shown to be associated with mental disorder by the benchmark (CTD). Purple nodes represent drugs predicted by our method. Green nodes are newly predicted drugs related to mental disorder. Drugs are labeled by their DrugBank identifiers.

Modular structure is one of the emerging properties of complex networks. A module is associated to sets of nodes with specific function. In order to further validate the effectiveness of our algorithm, we run ClusterONE with parameter Minimum density set to 0.35 and other parameters using default values in drug-drug network. We get 23 clusters from drug-drug network (additional file 9); nodes representing drugs. All drugs associated with mental disorders are scattered into two overlapping modules (cluster 1 and cluster 3, i.e. Cluster Label = 1 and Cluster Label = 3 in additional file 9). To analyze drugs associated with mental disorders, we merge these two modules (shown in Figure 3). Diamonds represent overlapping drugs of cluster 1 and cluster 3. In Figure 3, drugs colored pink have been shown to be associated with mental disorders by the benchmark (CTD). Purple nodes are drugs predicted by our method. They are listed in Table 2, and their correlations with mental disorders are not lower than 0.5 in drug-disease network. They are closely linked with known drugs (pink nodes), which further confirms that they have a high functional similarity with known drugs. That is, the 11 predicted drugs also have a strong association with mental disorders. The 3 green nodes are new predicted drugs by clustering the drug-drug network. They are also closely connected with known drugs, and are supported by literature. For example, dexmethylphenidate (DB06701) is used as a treatment for Attention Deficit Hyperactivity Disorder (ADHD), ideally in conjunction with psychological, educational, behavioral or other forms of treatment [43] Levomilnacipran (DB08918) is an antidepressant developed by Forest Laboratories and Pierre Fabre Group for the treatment of depression [44-46]. For ephedra (DB01363), studies have shown that it may cause serious mental illness [47]. Maglione et al. reviewed all 1,820 adverse event reports related to dietary supplements containing herbal ephedra from FDA MedWatch files as of Sept. 30, 2001. Fifty-seven serious psychiatric events were reported. Therefore, clinicians should be aware that serious psychiatric symptoms could be associated with ephedra use.

#### Functional enrichment analysis on target genes of potential drugs of mental disorder

Functional analysis are performed on the target sets of eleven drugs, which are not approved by CTD (see Table 2, drugs in bold italic and underlined bold). Gene-disease associations and KEGG pathway enrichment analysis are made on them with the functional annotation tool of DAVID. We find ten target sets of them are directly associated with mental disorder or the same type of diseases, such as depressive disorder, and personality disorders. In addition, the same ten target sets of drugs are significantly enriched in the mental disorder related pathways: neuroactive ligand-receptor interaction. Adkins et al. systematically screened associations between 58 neuroactive ligand-receptor interaction pathways and antipsychotic treatment efficacy by bioinformatics tools [48]. The target set of vilazodone (Drug ID=DB06684) is not obtained annotations from DAVID. We infer the reason is that the set only includes one gene (HTR1A). In fact, vilazodone is already approved for treatment of acute episodes of major depression [32].

### Case study: Hypertension

#### Potential drugs and Hypertension relations

Hypertension, also referred to as high blood pressure, is a condition in which the arteries have persistently elevated blood pressure. A blood pressure of 140/90 or above is considered hypertension. Hypertension can lead to damaged organs, as well as several illnesses, such as renal failure (kidney failure), aneurysm, heart failure, stroke, or heart attack [49].

We find 339 drug-hypertension relations in our candidate sets in all (additional file 10). 69.3% of the weight is less than 0.1, and there are 31 associations with high confidence (***weight ***≥ 0.5, see Table 3). Among them, 26 known associations agree with the benchmark (CTD). Through in-depth analysis of the other 5 associations (in bold italic), there are two types of correlation between diseases and drugs: positive and negative correlations. Positive correlations refer to the positive effect of drugs on diseases. For example, drugs can treat diseases. Negative correlations, for example, are that drugs can cause diseases, namely, side effects of drugs, or drugs that worsen diseases, etc. Both are very important in discovering the causes of a disease or in using drugs safely, so that we can treat diseases more effectively. Using SIDER (Side Effect Resource, http://sideeffects.embl.de) [50], we find asenapine (ID = 1) has the side effect of hypertension [50]. For trimipramine (ID = 29) and paliperidone (ID = 31), although there is no clear evidence showing they have side effect of hypertension, there have been some indications that they are likely to lead to high blood pressure [50,51]. Mehtysergide (ID = 20) is metabolised into methylergometrine in humans [52]. Adverse effects of methylergometrine include cholinergic effects, pulmonary hypertension, and severe systemic hypertension, etc [53]. The last drug, iloperidone (ID = 30), plays an active role in the treatment of hypertension. Considering the alpha1 antagonism characteristics of iloperidone, the effect of anti-hypertensive agents would be potentiated when administered concomitantly [54]. This shows that iloperidone has certain effects on lower blood pressure.

**Table 3:** Drug-hypertension associations (*weight *≥ 0.5)

ID	Drug ID	Drug Name	Weight	ID	Drug ID	Drug Name	Weight
***1***	***DB06216***	***Asenapine***	***0.71 ***	17	DB00413	Pramipexole	0.52

2	DB00571	Propranolol	0.66	18	DB00589	Lisuride	0.52

3	DB08807	Bopindolol	0.66	19	DB01149	Nefazodone	0.52

4	DB00960	Pindolol	0.64	***20***	***DB00247***	***Methysergide***	***0.52 ***

5	DB00866	Alprenolol	0.59	21	DB01049	Ergoloid mesylate	0.52

6	DB01359	Penbutolol	0.59	22	DB00714	Apomorphine	0.52

7	DB01200	Bromocriptine	0.54	23	DB00656	Trazodone	0.51

8	DB00248	Cabergoline	0.54	24	DB01142	Doxepin	0.50

9	DB00246	Ziprasidone	0.53	25	DB00904	Ondansetron	0.50

10	DB00334	Olanzapine	0.53	26	DB08815	Lurasidone	0.50

11	DB01238	Aripiprazole	0.53	27	DB00216	Eletriptan	0.50

12	DB00363	Clozapine	0.53	28	DB00734	Risperidone	0.50

13	DB01224	Quetiapine	0.53	***29***	***DB00726***	***Trimipramine***	***0.50 ***

14	DB01186	Pergolide	0.53	**30**	**DB04946**	**Iloperidone**	**0.50 **

15	DB01392	Yohimbine	0.52	***31***	***DB01267***	***Paliperidone***	***0.50 ***

16	DB00268	Ropinirole	0.52				

Drug ID represents the unique DrugBank accession number of a drug. Drug Name represents the corresponding name of a Drug ID. Weight represents the value of correlation between a drug and the mental disorders. 26 drugs are approved by our benchmark. Among the remaining 5 drugs, 4 (in bold italic) have negative relationships with the mental disorders and 1 (in underlined bold) has a positive relationship with the mental disorders.

#### The significant modules related to hypertension in drug-drug network

**Figure 4:**
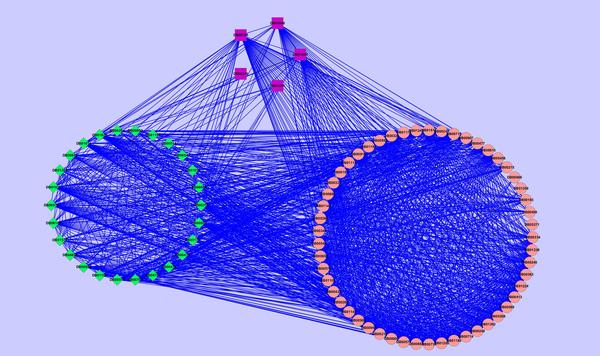
**Drugs associated with hypertension**. Nodes represent drugs. Circular nodes represent drugs that have been shown to be associated with hypertension by the benchmark (CTD). Rectangle and diamond nodes respectively represent our predicted drugs whose relationship with hypertension are higher than 0.5 and lower than 0.5. Drugs are labeled by their DrugBank identifiers.

Of the 23 drug modules, 11 are found to be related to hypertension. Five predicted drugs (purple rectangle nodes: DB06216, DB00247, DB00726, DB04946, DB01267) are in the same cluster (Figure 4). They are listed in Table 2 and their associations with hypertension is not lower than 0.5. The pink circular nodes have been confirmed to be associated with hypertension by CTD. It can be seen that the interactions between the five predicted drugs and the known drugs are very frequent. These results further indicate that they are highly correlated with hypertension. In addition, twenty-six nodes in Figure 4 are shown in Table 4. They includes two types of drugs: (1) predicted by our method, but their association with hypertension is lower than 0.5; (2) new drugs predicted by clustering drug-drug network. The first sixteen drugs (ID = 1 to ID = 16) were predicted by our method previously. The remaining ten drugs (ID = 17 to ID = 26) are newly predicted by clustering drug-drug network. They have high accuracy: nine of them are approved by CTD database (Correlation=CTD, see Table 4); one is supported by literature [55] (ephedra (ID = 17)). Ephedra containing products (ECPs), which are most often found in sources of caffeine alkaloids, may be an under-recognized cause of hypertension. For the previously predicted drugs with lower weights (ID = 1 to ID = 16), seven of them may cause high blood pressure, and are negatively correlated with hypertension (Correlation = N, see Table 4). Milnacipran (ID = 3) for example, researchers presented the case of a patient with major depressive disorder (MDD) who developed hypertension during treatment with regular therapeutic doses of milnacipran [56]. Desvenlafaxine (ID = 6) is similar to venlafaxine, its use may worsen preexisting hypertension [57]. For the remaining eight drugs, there are no evidence suggesting drug-hypertension relations. From the results, we derived two indications: 1) as a metric, our definition of weight is reasonable in assessing the credibility of drug-disease correlation - the greater the degree of reliability, the larger the weight, while the smaller the weight, the lower the reliability; 2) combined with modularity in projected network, our method is very effective in predicting drug-disease associations.

**Table 4:** Correlations of twenty-six Drugs with hypertension

ID	Drug ID	Drug Name	Correlation (CTD, N, or Unknown)	ID	Drug ID	Drug Name	Correlation (CTD, N, or Unknown)
1	DB00315	Zolmitriptan[60]	N	14	DB01622	Thioproperazine	Unknown

2	DB00476	Duloxetine[61]	N	15	DB06701	Dexmethylphenidate	Unknown

3	DB04896	Milnacipran[56]	N	16	DB08810	Cinitapride	Unknown

4	DB06204	Tapentadol[62]	N	17	DB01363	Ephedra [55]	N

5	DB06684	Vilazodone[63]	N	18	DB00472	Fluoxetine	CTD

6	DB06700	Desvenlafaxine [57]	N	19	DB00176	Fluvoxamine	CTD

7	DB08918	Levomilnacipran [64]	N	20	DB00543	Amoxapine	CTD

8	DB00805	Minaprine	Unknown	21	DB01104	Sertraline	CTD

9	DB00952	Naratriptan	Unknown	22	DB06148	Mianserin	CTD

10	DB00998	Frovatriptan	Unknown	23	DB01577	Methamphetamine	CTD

11	DB01614	Acepromazine	Unknown	24	DB01151	Desipramine	CTD

12	DB01616	Alverine	Unknown	25	DB00852	Pseudoephedrine	CTD

13	DB01621	Pipotiazine	Unknown	26	DB00514	Dextromethorphan	CTD

Drug ID represents the unique DrugBank accession number of a drug. Drug Name represents the corresponding name of a Drug ID. There are three types of correlation: (a) CTD represents the drug-hypertension correlation that can be found in the CTD database; (b) N represents the drug-hypertension correlation that may be negative, supported by literature; (c) Unknown means that there is no evidence suggesting the drug-hypertension relation as yet.

#### Functional enrichment analysis on target genes of potential drugs of hypertension

There are five drugs predicted by our method, but not approved by the benchmark (see Table 3, drugs in bold italic and underlined bold). We perform the gene-disease associations and KEGG pathway enrichment analysis on their target sets with DAVID. The enrichment result thus obtained show that three target sets of them are directly associated with hypertension. But all of them are significantly enriched in the hypertension related pathway, such as gap junction. It is instructive to note that the gap junction has been proved to be relevant to hypertension [58].

### Comparison with other method

To evaluate the performance of our method, we compare it with a popular web tool, PROMISCUOUS [59]. PROMISCUOUS contains three different types of entities: drugs, proteins and side-effects as well as relations between them. It is kind of knowledge-based drug repositioning method, which offers exploits known interactions between a drug and a target and combine this information with new knowledge about the target's role in a new indication.

We compare our method and PROMISCUOUS on eleven potential drugs of mental disorder one by one. They are shown in Table 2 (drugs in bold italic and underlined bold). By experimentation, five of them, pipotiazine, thioproperazine, acepromazine, ergoloid mesylate and paliperidone, are found to be antipsychotic medications by PROMISCUOUS, which are consistent with our prediction. Penbutolol (ID = 45) and bopindolol (ID = 39) are not shown associated with the treatments of mental disorders directly by PROMISCUOUS. However, for penbutolol, based on the fact that similar drugs often act on the same targets, PROMISCUOUS finds eight drugs similar to it. One of them is pemoline, which is a kind of antipsychotic drugs. Moreover, PROMISCUOUS also find penbutolol and bopindolol are related to KEGG pathways: neuroactive ligand-receptor interaction, which is proved associated with antipsychotic treatment [48]. Therefore, one can assume that penbutolol and bopindolol may also be effective for treatment of mental disorders. Because PROMISCUOUS integrated multiple public database, such as Drugbank, Protein Data Bank, KEGG, UniProt, SIDER, etc., the comparative results show the validity of our algorithm from another side. The last four drugs, cinitapride, vilazodone, iloperidone and rotigotine, are not found closely related to mental disorders by PROMISCUOUS. But with the exception of cinitapride (ID = 19), the other three drugs are all directly supported by the literatures.

A comparison also is made between PROMISCUOUS and our method on five potential drugs of hypertension (see Table 3, drugs in bold italic and underlined bold). Among the five drugs, PROMISCUOUS finds methysergide and paliperidone related to gap junction pathway, which is supported to be associated with hypertension [58]. The other three drugs, asenapine, trimipramine and iloperidone, are not found by PROMISCUOUS. More likely the reason is that they may have the side effect of hypertension. This is also consistent with our inference.

## Conclusions

We integrate the information of drugs, protein complexes and diseases from available experimental data and knowledge as weighted drug-complex-disease tripartite networks and obtain a derived connected relationships network, i.e. drug-disease bipartite network. One of the advantages of our model is its relative simplicity. It is not like other existing algorithms that first need to construct drug and disease similarity networks. With protein complexes as the bridge, we apply drug-complex-disease approach for inferring and evaluating the likelihood of the probability between drugs and diseases. In our simulation experiment, we take mental disorders and hypertension as our case study. The results of the experiment are encouraging. Both the positive and negative associations can be predicted and are found to be reinforced by existing biomedical literature. The success of our methods can be attributed to the following factors: first, we integrate heterogeneous data and knowledge about drugs, protein complexes, and diseases into our model; next, we use symmetric probability modelling dependencies between drugs, protein complexes, and diseases; last, our method combines the information derived from other connected hetero-networks to infer the drug-disease associations. We believe that the integration of networks and heterogeneous data sources will help us bring about new hypotheses to infer the drug-disease associations and even speed up drug development processes. Our study provides opportunities for future toxicogenomics and drug discovery applications. However, we find that it is difficult to automatically distinguish the positive and negative associations between drug and disease. For the next step, we suggest: 1) for commonly used data, such as drugs, targets, protein complexes, and diseases, we need to integrate data sources with higher confidence to improve the accuracy of the prediction; 2) in order to predict the positive and negative associations automatically as much as possible, we need to integrate data sources that can offer information about the side effects of drugs, such as drug side effect resources, response profiles, pharmacological data and therapeutic/toxicological expression profiles.

## Competing interests

The authors declare that they have no competing interests.

## Authors' contributions

LY carried out the study. LY, JZ, and YZ designed the study. LY wrote the first draft of the manuscript. JH, ZM, and LG revised the manuscript. All the authors read and approved the final manuscript.

## Supplementary Material

Additional file 1 **Table illustrating the relations between drugs and targets**. Click here for file

Additional file 2 **Table illustrating the list of protein complexes**. Click here for file

Additional file 3 **Table illustrating disease-gene dataset**. Click here for file

Additional file 4 **Table illustrating the information of drug-complex network**. Click here for file

Additional file 5 **Table illustrating the information of complex-disease network**. Click here for file

Additional file 6 **Table illustrating the information of drug-disease network before being filtered by PPI network and weight**. Click here for file

Additional file 7 **Table illustrating the information of drug-disease network after being filtered by PPI network and weight**. Click here for file

Additional file 8 **Table illustrating the drug-mental disorders relations predicted by our method**. Click here for file

Additional file 9 **Table illustrating 23 clusters got from drug-drug network**. Click here for file

Additional file 10 **Table illustrating the drug-hypertension relations predicted by our method**. Click here for file

## Declarations

Publication of this article has been funded by the National Natural Science Foundation of China (No. 61202174).

This article has been published as part of *BMC Medical Genomics *Volume 8 Supplement 2, 2015: Selected articles from the 4th Translational Bioinformatics Conference and the 8th International Conference on Systems Biology (TBC/ISB 2014). The full contents of the supplement are available online at http://www.biomedcentral.com/bmcmedgenomics/supplements/8/S2.
