# Supplementary material for: Inferring drug-disease associations based on known protein complexes
Source: BMC Med Genomics. 2015 May 29;8(Suppl 2):S2. doi: 10.1186/1755-8794-8-S2-S2 (PMC4460611; doi:10.1186/1755-8794-8-S2-S2)
Supplement: Additional file 1 — Table illustrating the relations between drugs and targets. [file 1755-8794-8-S2-S2-S1.pdf]

**Drug ID**	**List of related genes (names)**	**List of related genes (Entrez IDs)**	**Species**	**Number of related genes**
DB00303	ftsI mrdA mrcB mrcA dacB ftsI mrdA dacC	950626 950929 944843 947907 947693 12932643 12930918 12933831	Haemophilus influenzae (strain ATCC 51907 / DSM 11121 / KW20 / Rd)	8
DB00117	HDC HAL SLC38A3 HARS	3067 3034 10991 3035	Human	4
DB00125	NOS2 SLC7A4 SLC7A3 SLC7A1 NOS3 ADC ASL ASS1 ARG2	4843 6545 84889 6541 4846 113451 435 445 384	Human	9
DB00155	NOS2 NOS1 NOS3 OTC DDAH2 ASS1 DDAH1 ASS1 PADI4 PADI6 PADI1 PADI3 PADI2	4843 4842 4846 5009 23564 445 23576 445 23569 353238 29943 51702 11240	Human	13
DB01110	NOS2 KCNH2 ERG11 NOS3 KCNN4 KCNH6 KCNMA1 KCNH7 KCNMB2 KCNMB1 KCNMB3 KCNMB4 KCNN1	4843 3757 3641571 4846 3783 81033 3778 90134 10242 3779 27094 27345 3780 3781 3782	Human	15
DB01234	NOS2 NR0B1 ANXA1 NR3C1	4843 190 301 2908	Human	4
DB08814	NOS2 PTGS1 PDE10A NFKB1	4843 5742 10846 4790	Human	4
DB00131	PYGL PRKAB1 ADCY1 ACSS2 PDE4B ACSL1 PRKAA1 ACSS1 PRKAB2 CREB1 PIM1 PDE4D HINT1 FBP1 ADK	5836 5564 107 55902 5142 2180 5562 84532 5565 1385 5292 5144 3094 2203 132	Human	15
DB00116	AMT MTHFD2 SHMT2 SHMT1 MTHFD1 MTR FTCD ALDH1L1 ATIC MTHFR MTFMT	275 10797 6472 6470 4522 4548 10841 10840 471 4524 123263	Human	11
DB00568	CACNA1I CACNA1C HRH1 CACNA1G DRD2 CACNA1H CACNA1S CACNA1F CACNA1D	8911 775 3269 8913 1813 8912 779 778 776	Human	9
DB00617	CACNA1I	8911	Human	1
DB00661	CACNA1I KCNH2 SCN5A CACNB1 CACNA1A CACNA1C CACNA1G KCNJ11 SLC6A4 CACNA1B CACNB2 CACNA1S CACNB4 CACNA1F CACNB3	8911 3757 6331 782 773 775 8913 3767 6532 774 783 779 785 778 784 776	Human	16
DB00909	CACNA1I SCN1A SCN11A SCN5A CA1 CA2 CACNA1G CA4 CACNA1H SCN9A CA12 SCN2A SCN3A SCN4A MAOB MAOA CA9 CA3 CA5A CA5B CA6 CA7 CA8 CA10 CA11 CA13 CA14 SCN1B SCN2B SCN3B SCN4B	8911 6323 11280 6331 759 760 8913 762 8912 6335 771 6326 6328 6329 4129 4128 768 761 763 11238 765 766 767 56934 770 377677 23632 6324 6327 55800 6330	Human	31
DB04841	CACNA1I CALM1 HRH1 CACNA1G CACNA1H	8911 805 3269 8913 8912	Human	5
DB00201	ADORA1 PDE4B RYR1	134 5142 6261 135	Human	4
DB00277	ADORA1 PDE5A ADORA2B PDE3A PDE4B ADORA2A PDE4A HDAC2	134 8654 136 5139 5142 135 5141 3066	Human	8
DB00640	ADORA1 ADORA2B ADORA3 ADORA2A	134 136 140 135	Human	4

DB00651	ADORA1 PDE4B PDE7B PDE7A ADORA2A PDE4A PDE4D	134 5142 27115 5150 135 5141 5144 5143	Human	8
DB00806	ADORA1 PDE5A PDE4B ADORA2A NT5E PDE4A	134 8654 5142 135 4907 5141	Human	6
DB00996	ADORA1 CACNA2D1 CACNA1B CACNA2D2	134 781 774 9254	Human	4
DB01223	ADORA1 ADORA3 PDE3A	134 140 5139 3066	Human	4
DB01303	ADORA1 PDE3A ADORA2A PDE4A HDAC2	134 5139 135 5141 3066	Human	5
DB01412	ADORA1 PDE4B ADORA2A	134 5142 135	Human	3
DB04932	ADORA1 ADORA2B ADORA2A	134 136 135	Human	3
DB00171	ABL1 ADRBK2 ABCA1 AFG3L2 NAE1 ABCC8 ASNS NT5C2 ACVR1 ADCY1 APAF1 ADRBK1 ACSS2 AKT1 ARAF ABL2 CDK15 ACVRL1 ABCC9 SLC25A4 ACSL1 ACVR1B ASS1 AMHR2 PRKAA1 ABCB11 ACSS1 ASNA1 TNK2 ABCB1	25 157 19 10939 8883 6833 440 22978 90 107 317 156 55902 238 207 369 27 65061 94 10060 291 2180 91 445 269 5562 8647 84532 439 10188 5243 1244 9619	Human	33
DB00619	ABL1 PDGFRB KIT PDGFRA CSF1R NTRK1 DDR1	25 5159 3815 5156 1436 4914 780	Human	7
DB01254	ABL1 PDGFRB ABL2 KIT SRC FYN YES1 EPHA2 LCK STAT5B	25 5159 27 3815 6714 2534 7525 1969 3932 6777	Human	10
DB04868	ABL1 KIT	25 3815	Human	2
DB06616	ABL1 SRC MAP2K1 CDK2deltaT HCK LYN CAMK2G	25 6714 5604 1017 3055 4067 818 613 5605 10746	Human	10
DB08896	ABL1 FLT4 FLT1 RAF1 PDGFRB KDR KIT BRAF FGFR2 EPHA2 PDGFRA NTRK1 FGFR1 RET TEK MAPK11	25 2324 2321 5894 5159 3791 3815 673 2263 1969 5156 4914 2260 5979 7010 5600 4921 2444	Human	18
DB08901	ABL1 FLT3 KDR KIT FGFR2 SRC LCK PDGFRA FGFR4 FGFR1 FGFR3 RET TEK LYN	25 2322 3791 3815 2263 6714 3932 5156 2264 2260 2261 5979 7010 4067 613	Human	15
DB00043	FCER1A MS4A2	2205 2206	Human	2
DB00895	FCER1A FCER1G	2205 2207	Human	2
DB00055	F8 F5 F2 SERPINE1 GGCX PROS1 PF4 CP THBD PROCR SERPINA5 SERPINB6	2157 2153 2147 5054 2677 5627 5196 1356 7056 10544 5104 5269	Human	12
DB00100	F8 F2 F10 F7 GGCX F11 LRP1	2157 2147 2159 2155 2677 2160 4035	Human	7
DB00154	PTGS1 PTGS2	5742 5743	Human	2
DB00159	PTGS1 PPARG PTGS2 FFAR1 FADS1 SLC8A1 ACSL4 PPARD TRPV1 ACSL3	5742 5468 5743 2864 3992 6546 2182 5467 7442 2181	Human	10
DB00244	PTGS1 PPARG ALOX5 PTGS2 MPO IKBKB nat CHUK	5742 5468 240 5743 4353 3551 888005 1147	Human	8
DB00316	PTGS1 PTGS2	5742 5743	Human	2
DB00328	PTGS1 PPARG PTGS2 GLO1 PLA2G2A PTGR2 GPR44	5742 5468 5743 2739 5320 145482 11251	Human	7
DB00350	PTGS1 KCNJ1	5742 3758	Human	2
DB00461	PTGS1 PTGS2	5742 5743	Human	2
DB00465	PTGS1 PTGS2	5742 5743	Human	2

DB00469	PTGS1 PTGS2	5742 5743	Human	2
DB00500	PTGS1 PTGS2	5742 5743	Human	2
DB00554	PTGS1 PTGS2	5742 5743	Human	2
DB00573	PTGS1 PTGS2	5742 5743	Human	2
DB00586	PTGS1 ALOX5 PTGS2 ACCN2 KCNQ2 PLA2G2A SCN4A	5742 240 5743 41 3785 5320 6329 3786	Human	8
DB00605	PTGS1 PTGS2 AKR1B1 PPARD MAPK3 GPR44	5742 5743 231 5467 5595 11251	Human	6
DB00711	PTGS1 ALOX5	5742 240	Human	2
DB00712	PTGS1 PTGS2	5742 5743	Human	2
DB00749	PTGS1 PTGS2 RXRA	5742 5743 6256	Human	3
DB00784	PTGS1 PTGS2	5742 5743	Human	2
DB00788	PTGS1 PTGS2	5742 5743	Human	2
DB00795	PTGS1 SLC7A11 ACAT1 PPARG ALOX5 PTGS2 IKBKB CHUK	5742 23657 38 5468 240 5743 3551 1147	Human	8
DB00812	PTGS1 PTGIS PTGS2	5742 5740 5743	Human	3
DB00814	PTGS1 PTGS2	5742 5743	Human	2
DB00821	PTGS1 PTGS2	5742 5743	Human	2
DB00861	PTGS1 PTGS2	5742 5743	Human	2
DB00870	PTGS1 PTGS2	5742 5743	Human	2
DB00936	PTGS1 AKR1C1 PTGS2	5742 1645 5743	Human	3
DB00939	PTGS1 ALOX5 PTGS2 KCNQ2 KCNQ3	5742 240 5743 3785 3786	Human	5
DB00945	PTGS1 AKR1C1 PTGS2	5742 1645 5743	Human	3
DB00963	PTGS1 PTGS2	5742 5743	Human	2
DB00991	PTGS1 PTGS2	5742 5743	Human	2
DB01009	PTGS1 PTGS2 CXCR1	5742 5743 3577	Human	3
DB01014	PTGS1 PPARG ALOX5 PTGS2	5742 5468 240 5743	Human	4
DB01050	PTGS1 PPARG BCL2 PTGS2 PLAT THBD CFTR FABP2	5742 5468 596 5743 5327 7056 1080 2169	Human	8
DB01283	PTGS1 PTGS2	5742 5743	Human	2
DB01397	PTGS1 PTGS2	5742 5743	Human	2
DB01398	PTGS1 PTGS2	5742 5743	Human	2
DB01399	PTGS1 PTGS2	5742 5743	Human	2
DB01401	PTGS1 PTGS2	5742 5743	Human	2
DB01419	PTGS1 PTGS2	5742 5743	Human	2
DB01435	PTGS1 PTGS2	5742 5743	Human	2
DB01600	PTGS1 PTGS2	5742 5743	Human	2
DB04552	PTGS1 PTGS2 PLA2G4A CLCNKA UGT1A9 PLA2G1B	5742 5743 5321 1187 54600 5319	Human	6
DB06725	PTGS1 PTGS2	5742 5743	Human	2
DB06802	PTGS1 PTGS2	5742 5743	Human	2
DB00254	rpsD rpsI	947793 949000	Escherichia coli (strain K12)	2
DB00256	rpsD rpsI	947793 949000	Escherichia coli (strain K12)	2

DB00453	rpsD rpsI	947793 949000	Escherichia coli (strain K12)	2
DB00595	rpsD rpsI	947793 949000	Escherichia coli (strain K12)	2
DB00618	rpsD rpsI	947793 949000	Escherichia coli (strain K12)	2
DB01017	rpsD rpsI VEGFA ALOX5 CASP1 CYCS IL1B CASP3	947793 4318 949000 7422 240 834 54205 3553 836	Escherichia coli (strain K12)	9
DB00246	DRD1 CHRM3 CHRM1 DRD5 CHRM5 ADRA2A HTR1A HTR3A ADRA2C DRD4 CHRM4 HRH1 HTR2A HTR1E ADRA1A HTR2C CHRM2 ADRA2B ADRA1B DRD3 HTR7 HTR1D DRD2 HTR1B	1812 1131 1128 1816 1133 150 3350 3359 152 1815 1132 3269 3356 3354 148 3358 1129 151 147 1814 3363 3352 1813 3351 3362	Human	25
DB00248	DRD1 DRD5 ADRA2A HTR1A ADRA2C DRD4 HTR2B HTR2A HTR2C ADRA2B DRD3 HTR7 HTR1D DRD2 HTR1B	1812 1816 150 3350 152 1815 3357 3356 3358 151 1814 3363 3352 1813 3351	Human	15
DB00268	DRD1 DRD5 ADRA2A HTR1A ADRA2C DRD4 HTR2B HTR2A HTR2C ADRA2B DRD3 HTR1D DRD2 HTR1B	1812 1816 150 3350 152 1815 3357 3356 3358 151 1814 3352 1813 3351	Human	14
DB00334	DRD1 CHRM3 CHRM1 DRD5 CHRM5 ADRA2A HTR1A HTR3A ADRA2C DRD4 CHRM4 HRH1 HTR2A HTR1E ADRA1A HTR2C CHRM2 ADRA2B ADRA1B DRD3 HTR7 HTR1D DRD2 HTR1B	1812 1131 1128 1816 1133 150 3350 3359 152 1815 1132 3269 3356 3354 148 3358 1129 151 147 1814 3363 3352 1813 3351 3362	Human	25
DB00353	DRD1	1812	Human	1
DB00363	DRD1 CHRM3 CHRM1 HRH4 CHRM5 ADRA2A HTR1A HTR3A ADRA2C DRD4 CHRM4 HRH1 HTR2A HTR1E ADRA1A HTR2C CHRM2 ADRA2B ADRA1B DRD3 HTR7 HTR1D CALY DRD2 HTR1B HTR6	1812 1131 1128 59340 1133 150 3350 3359 152 1815 1132 3269 3356 3354 148 3358 1129 151 147 1814 3363 3352 50632 1813 3351 3362	Human	26
DB00372	DRD1 DRD4 DRD2	1812 1815 1813	Human	3
DB00397	DRD1 ADRB1 ADRA2A ADRA1A	1812 153 150 148	Human	4
DB00408	DRD1 HTR2A HTR2C DRD2	1812 3356 3358 1813	Human	4
DB00413	DRD1 DRD5 ADRA2A HTR1A ADRA2C DRD4 HTR2B HTR2A HTR2C ADRA2B DRD3 HTR1D DRD2 HTR1B	1812 1816 150 3350 152 1815 3357 3356 3358 151 1814 3352 1813 3351	Human	14
DB00420	DRD1 CHRM3 CHRM1 CHRM5 DRD4 CHRM4 HRH1 HTR2A ADRA1A HTR2C CHRM2 ADRA1B ADRA1D DRD2	1812 1131 1128 1133 1815 1132 3269 3356 148 3358 1129 147 146 1813	Human	14

DB00477	DRD1 HTR1A HRH1 HTR2A ADRA1A ADRA1B DRD2	1812 3350 3269 3356 148 147 1813	Human	7
DB00502	DRD1 GRIN2B HTR2A DRD3	1812 2904 3356 1814 1813	Human	5
DB00508	DRD1 CHRM1 HTR2B CHRM2 DRD2	1812 1128 3357 1129 1813	Human	5
DB00543	DRD1 CHRM1 ADRA2A SLC6A2 ADRA1A SLC6A4 DRD2	1812 1128 150 6530 148 6532 1813 2554	Human	8
DB00589	DRD1 DRD5 ADRA2A HTR1A ADRA2C DRD4 HTR2B HTR2A HTR2C ADRA2B DRD3 HTR1D DRD2 HTR1B	1812 1816 150 3350 152 1815 3357 3356 3358 151 1814 3352 1813 3351	Human	14
DB00623	DRD1 CALM1 DRD2	1812 805 1813	Human	3
DB00679	DRD1 KCNH2 HTR2A ADRA1A ADRA1B DRD2	1812 3757 3356 148 147 1813	Human	6
DB00714	DRD1 DRD5 ADRA2A HTR1A ADRA2C DRD4 HTR2B HTR2A HTR2C ADRA2B DRD3 HTR1D CALY DRD2 HTR1B	1812 1816 150 3350 152 1815 3357 3356 3358 151 1814 3352 50632 1813 3351	Human	15
DB00726	DRD1 HTR1A HRH1 HTR2A SLC6A2 ADRA1A ADRA2B ADRA1B SLC6A3 SLC6A4 DRD2	1812 3350 3269 3356 6530 148 151 147 6531 6532 1813	Human	11
DB00734	DRD1 ADRA2A HTR1A ADRA2C DRD4 HRH1 HTR2A ADRA1A HTR2C ADRA2B ADRA1B DRD3 HTR1D DRD2	1812 150 3350 152 1815 3269 3356 148 3358 151 147 1814 3352 1813	Human	14
DB00777	DRD1 CHRM3 CHRM1 CHRM5 DRD4 CHRM4 HRH1 HTR2A ADRA1A HTR2C CHRM2 ADRA1B ADRA1D DRD2	1812 1131 1128 1133 1815 1132 3269 3356 148 3358 1129 147 146 1813	Human	14
DB00800	DRD1 DRD5 ADRA2A ADRA2C ADRA2B	1812 1816 150 152 151	Human	5
DB00805	DRD1 CHRM1 HTR2B ACHE HTR2A HTR2C SLC6A4 DRD2 MAOA	1812 1128 3357 43 3356 3358 6532 1813 4128	Human	9
DB00850	DRD1 CALM1 DRD2	1812 805 1813	Human	3
DB00875	DRD1 CHRM1 HTR2A ADRA1A DRD2	1812 1128 3356 148 1813	Human	5
DB00988	DRD1 DBH DRD5 DRD4 DRD3 SLC6A3 DRD2	1812 1621 1816 1815 1814 6531 1813	Human	7
DB01038	DRD1 DRD5 DRD2	1812 1816 1813	Human	3
DB01049	DRD1 DRD2 SLCO2B1 GABRA1 GABRA2 GABRA3 GABRA4 GABRA5 GABRA6 GABRB1 GABRB2 GABRB3 GABRD GABRE GABRG1 GABRG2 GABRG3 GABRP GABRQ HTR1A HTR1B HTR1D HTR1E HTR1F HTR2A HTR2B HTR2C HTR3A HTR4 HTR6 HTR7 ADRA1A ADRA1B ADRA1D	1812 1813 11309 2554 2555 2556 2557 2558 2559 2560 2561 2562 2563 2564 2565 2567 2568 2568 55879 3350 3351 3352 3354 3355 3356 3357 3358 3359 9177 170572 200909 285242 3360 3362 3363 148 147 146 150 151 152	Human	41
DB01063	DRD1 DRD2	1812 1813	Human	2

DB01186	DRD1 DRD5 ADRA2A HTR1A ADRA2C DRD4 HTR2B HTR2A ADRA1A HTR2C ADRA2B ADRA1B DRD3 HTR1D ADRA1D DRD2 HTR1B	1812 1816 150 3350 152 1815 3357 3356 148 3358 151 147 1814 3352 146 1813 3351	Human	17
DB01200	DRD1 DRD5 ADRA2A HTR1A ADRA2C DRD4 HTR2B HTR2A ADRA1A HTR2C ADRA2B ADRA1B DRD3 HTR7 HTR1D ADRA1D DRD2 HTR1B	1812 1816 150 3350 152 1815 3357 3356 148 3358 151 147 1814 3363 3352 146 1813 3351	Human	18
DB01224	DRD1 CHRM3 CHRM1 DRD5 CHRM5 ADRA2A HTR1A HTR3A ADRA2C DRD4 CHRM4 HRH1 HTR2A HTR1E ADRA1A HTR2C CHRM2 ADRA2B ADRA1B DRD3 HTR7 HTR1D ADRA1D DRD2 HTR1B HTR6	1812 1131 1128 1816 1133 150 3350 3359 152 1815 1132 3269 3356 3354 148 3358 1129 151 147 1814 3363 3352 146 1813 3351 3362	Human	26
DB01235	DRD1 DRD5 DRD4 DRD3 DRD2	1812 1816 1815 1814 1813	Human	5
DB01238	DRD1 CHRM3 CHRM1 DRD5 CHRM5 ADRA2A HTR1A HTR3A ADRA2C DRD4 CHRM4 HRH1 HTR2A HTR1E ADRA1A HTR2C CHRM2 ADRA2B ADRA1B DRD3 HTR7 HTR1D DRD2 HTR1B	1812 1131 1128 1816 1133 150 3350 3359 152 1815 1132 3269 3356 3354 148 3358 1129 151 147 1814 3363 3352 1813 3351 3362	Human	25
DB01239	DRD1 CHRM3 CHRM1 CHRM5 HTR2B CHRM4 HRH1 HTR2A HTR2C CHRM2 DRD3 DRD2	1812 1131 1128 1133 3357 1132 3269 3356 3358 1129 1814 1813	Human	12
DB01267	DRD1 ADRA2A HTR1A ADRA2C DRD4 HRH1 HTR2A ADRA1A HTR2C ADRA2B ADRA1B DRD3 HTR1D DRD2 CYP3A5 CYP2D6 CYP3A4	1812 150 3350 152 1815 3269 3356 148 3358 151 147 1814 3352 1813 1577 1565 1576	Human	17
DB01403	DRD1 CHRM3 CHRM1 DRD5 CHRM5 ADRA2A ADRA2C DRD4 CHRM4 HRH1 HTR2A ADRA1A HTR2C CHRM2 ADRA2B ADRA1B DRD3	1812 1131 1128 1816 1133 150 152 1815 1132 3269 3356 148 3358 1129 151 147 1814 146 1813	Human	19
DB01608	DRD1 ADRA2A ADRA1B	1812 150 147	Human	3
DB01614	DRD1 HTR1A HTR2A ADRA1A ADRA1B DRD2	1812 3350 3356 148 147 1813	Human	6
DB01621	DRD1 HTR1A HTR2A DRD2	1812 3350 3356 1813	Human	4
DB01622	DRD1 HTR1A HTR2A ADRA1A ADRA1B DRD2	1812 3350 3356 148 147 1813	Human	6
DB01623	DRD1 HTR2A DRD2	1812 3356 1813	Human	3
DB04946	DRD1 HTR1A ADRA2C DRD4 HRH1 HTR2A ADRA1A DRD3 HTR7 DRD2 HTR6	1812 3350 152 1815 3269 3356 148 1814 3363 1813 3362	Human	11
DB05271	DRD1 DRD5 HTR1A DRD4 ADRA2B DRD3 DRD2	1812 1816 3350 1815 151 1814 1813	Human	7

DB06216	DRD1 HRH2 ADRB1 ADRA2A HTR1A ADRA2C DRD4 HTR2B HRH1 HTR2A ADRA1A HTR2C ADRA2B DRD3 HTR7 ADRB2 DRD2 HTR1B HTR6	1812 3274 153 150 3350 152 1815 3357 3269 3356 148 3358 151 1814 3363 154 1813 3351 3362 3361	Human	20
DB01099	TMP1 DNMT1	3643367 1786	Yeast	2
DB00139	SLC13A1 P4HA1 PLOD1 ALDH5A1 SDHA SUCLG2 SLC13A2 ASPH SLC13A3 HSD17B6 PLOD3 BBOX1 LEPRE1 LEPREL1 LEPREL2 TMLHE SDHD P4HA2 SDHB SUCLG1 OXCT1 SDHC SUCNR1	6561 5033 5351 7915 6389 8801 9058 444 64849 8630 8985 8424 64175 55214 10536 55217 6392 8974 6390 8802 5019 6391 56670 64064 8803 1468	Human	26
DB00398	FLT4 FLT1 RAF1 FLT3 PDGFRB KDR KIT BRAF FGFR1 RET	2324 2321 5894 2322 5159 3791 3815 673 2260 5979	Human	10
DB01268	FLT4 FLT1 FLT3 PDGFRB KDR KIT PDGFRA CSF1R	2324 2321 2322 5159 3791 3815 5156 1436	Human	8
DB06589	FLT4 FLT1 PDGFRB KDR KIT FGF1 PDGFRA ITK FGFR3	2324 2321 5159 3791 3815 2246 5156 3702 2261 10019	Human	10
DB06626	FLT4 FLT1 KDR	2324 2321 3791	Human	3
DB00384	SCNN1B SCNN1A SCNN1D SCNN1G	6338 6337 6339 6340	Human	4
DB00594	SCNN1B SCNN1A ABP1 ACCN2 SCNN1D SCNN1G ACCN1 SLC9A1 PLAU	6338 6337 26 41 6339 6340 40 6548 5328	Human	9
DB00048	COL1A1 COL3A1 COL2A1	1277 1281 1280 1278	Human	4
DB00309	TUBB1	81027	Human	1
DB01229	TUBB1 BCL2 NR1I2 MAP2 MAPT MAP4	81027 596 8856 4133 4137 4134	Human	6
DB01248	TUBB1 BCL2 MAP2 MAPT	81027 596 4133 4137 4134	Human	5
DB01394	TUBB1 TUBB	81027 203068	Human	2
DB06772	TUBB1 TUBA4A	81027 7277	Human	2
DB00120	FARS2 FARSA FARSB TH TAT PAH SLC7A8	10667 2193 10056 7054 6898 5053 23428	Human	7
DB00138	SLC7A11 CTNS SLC3A1 SLC7A9	23657 1497 6519 11136	Human	4
DB00740	SLC7A11 SCN5A	23657 6331	Human	2
DB00162	DHRS4 RBP3 RBP1 ALDH1A2 ALDH1A1 RDH5 ALDH1A3 RDH13 RDH12 RLBP1 LRAT DHRS3 RETSAT RDH11 RDH14	10901 5949 5947 8854 216 5959 220 112724 145226 6017 9227 9249 54884 51109 57665 50700	Human	16
DB00030	INSR IGF1R CTSD LRP2 CPE NOV IDE RB1 PCSK2 PCSK1 IGFBP7 SYTL4	3634 3480 1509 4036 1363 4856 3416 5925 5126 5122 3490 94121	Human	12
DB00046	INSR IGF1R	3634 3480	Human	2
DB00047	INSR IGF1R	3634 3480	Human	2
DB00071	INSR IGF1R CTSD LRP2 CPE NOV IDE HLA-DQA2 RB1 PCSK2 PCSK1 IGFBP7 SYTL4 HLA-	3634 3480 1509 4036 1363 4856 3416 3118 5925 5126 5122 3490 94121 3119	Human	14
DB01277	INSR IGF1R IGFBP3 IGF2R	3634 3480 3486 3482	Human	4
DB01306	INSR	3634	Human	1
DB01307	INSR	3634	Human	1

DB01309	INSR	3634	Human	1
DB08914	INSR	3634	Human	1
DB00330	embA embB embC	886123 886126 886112	Mycobacterium tuberculosis	3
DB08912	RAF1 BRAF	5894 673 150094 79858 3984	Human	5
DB00166	LIPT1 SLC5A6 LIAS	51601 8884 11019	Human	3
DB05777	F5 F2	2153 2147	Human	2
DB00147	PDXK	8566	Human	1
DB00165	PDXK	8566	Human	1
DB00559	EDNRB EDNRA	1910 1909	Human	2
DB06268	EDNRB EDNRA	1910 1909	Human	2
DB08932	EDNRB EDNRA	1910 1909	Human	2
DB00185	CHRM3 CHRM1	1131 1128	Human	2
DB00193	CHRM3 OPRD1 SLC6A2 HTR2C OPRK1 GRIN3A SLC6A4 OPRM1	1131 4985 6530 3358 4986 116443 6532 4988	Human	8
DB00202	CHRM3 CHRM1 CHRNA10	1131 1128 57053 1129	Human	4
DB00280	CHRM3 CHRM1 SCN5A CHRM2 KCND2 KCND3	1131 1128 6331 1129 3751 3752	Human	6
DB00321	CHRM3 CHRM1 CHRM5 ADRA2A HTR1A CHRM4 OPRD1 HRH1 HTR2A SLC6A2 ADRA1A CHRM2 OPRK1 ADRA1D SLC6A4 KCNA1 KCNQ2 NTRK1 KCND2 KCND3 NTRK2	1131 1128 1133 150 3350 1132 4985 3269 3356 6530 148 1129 4986 146 6532 3736 3785 4914 3751 3752 4915	Human	21
DB00332	CHRM3 CHRM1 CHRM2	1131 1128 1129	Human	3
DB00340	CHRM3 CHRM1 CHRM5 CHRM4 CHRM2	1131 1128 1133 1132 1129	Human	5
DB00342	CHRM3 KCNH2 HRH1	1131 3757 3269	Human	3
DB00376	CHRM3 CHRM1 CHRM5 CHRM4 CHRM2	1131 1128 1133 1132 1129	Human	5
DB00383	CHRM3 CHRM1 CHRM2	1131 1128 1129	Human	3
DB00387	CHRM3 CHRM1 CHRM4 CHRM2	1131 1128 1132 1129	Human	4
DB00424	CHRM3 CHRM1 CHRM4 CHRM2	1131 1128 1132 1129	Human	4
DB00434	CHRM3 CHRM1 HRH1 HTR2A HTR2C CHRM2	1131 1128 3269 3356 3358 1129	Human	6
DB00458	CHRM3 CHRM1 CHRM5 CHRM4 HRH1 HTR2A SLC6A2 ADRA1A CHRM2 ADRA1D SLC6A4 KCND2 KCND3 snf	1131 1128 1133 1132 3269 3356 6530 148 1129 146 6532 3751 3752 1193843	Human	14
DB00462	CHRM3 CHRM1 CHRM2	1131 1128 1129	Human	3
DB00496	CHRM3 CHRM1 CHRM5 CHRM4 CHRM2	1131 1128 1133 1132 1129	Human	5
DB00517	CHRM3 CHRM1 CHRM2	1131 1128 1129	Human	3
DB00540	CHRM3 CHRM1 CHRM5 HTR1A CHRM4 HRH1 HTR2A SLC6A2 ADRA1A CHRM2 ADRA1D SLC6A4	1131 1128 1133 3350 1132 3269 3356 6530 148 1129 146 6532	Human	12
DB00572	CHRM3 CHRM1 CHRM5 CHRM4 CHRM2	1131 1128 1133 1132 1129	Human	5

DB00622	CHRM3 CHRM1 CHRM5 CHRM4 CALM1 CACNA1C ADRA1A CHRM2 ADRA1B CACNA2D1 ADRA1D CACNB2 CACNA1D PDE1B PDE1A	1131 1128 1133 1132 805 775 148 1129 147 781 146 783 776 5153 5136	Human	15
DB00715	CHRM3 CHRM1 CHRM5 CHRM4 HTR2A SLC6A2 CHRM2 SLC6A4	1131 1128 1133 1132 3356 6530 1129 6532	Human	8
DB00725	CHRM3 CHRM1 CHRM5 CHRM4 CHRM2	1131 1128 1133 1132 1129	Human	5
DB00729	CHRM3	1131	Human	1
DB00747	CHRM3 CHRM1 CHRM5 CHRM4 CHRM2 SI	1131 1128 1133 1132 1129 6476	Human	6
DB00785	CHRM3 CHRM1 CHRM5 CHRM4 CHRM2	1131 1128 1133 1132 1129	Human	5
DB00809	CHRM3 CHRM1 CHRM4 CHRM2	1131 1128 1132 1129	Human	4
DB00835	CHRM3 CHRM1 CHRM5 CHRM4 HRH1 CHRM2	1131 1128 1133 1132 3269 1129	Human	6
DB00934	CHRM3 CHRM1 CHRM5 CHRM4 HRH1 SLC6A2 ADRA1A CHRM2	1131 1128 1133 1132 3269 6530 148 1129	Human	8
DB01036	CHRM3 CHRM1 CHRM5 CHRM4 CHRM2	1131 1128 1133 1132 1129	Human	5
DB01062	CHRM3 CHRM1 CHRM2	1131 1128 1129	Human	3
DB01069	CHRM3 CHRM1 CHRM5 CHRM4 CALM1 HRH1 HTR2A ADRA1A CHRM2 DRD2	1131 1128 1133 1132 805 3269 3356 148 1129 1813	Human	10
DB01085	CHRM3 CHRM1 CHRM2	1131 1128 1129	Human	3
DB01142	CHRM3 CHRM1 HRH2 CHRM5 ADRA2A HTR1A ADRA2C HTR2B CHRM4 HRH1 HTR2A SLC6A2 ADRA1A HTR2C CHRM2 ADRA2B ADRA1B	1131 1128 3274 1133 150 3350 152 3357 1132 3269 3356 6530 148 3358 1129 151 147 146 6532 1813	Human	20
DB01151	CHRM3 CHRM1 ADRB1 CHRM5 CHRM4 HRH1 HTR2A SLC6A2 ADRA1A CHRM2 ADRB2 SLC6A4 SMPD1 snf	1131 1128 153 1133 1132 3269 3356 6530 148 1129 154 6532 6609 1193843	Human	14
DB01226	CHRM3 CHRM2 CHRNA2 BCHE	1131 1129 1135 590	Human	4
DB01231	CHRM3 CHRM1 CHRM2	1131 1128 1129	Human	3
DB01337	CHRM3 CHRM2 CHRNA2	1131 1129 1135	Human	3
DB01338	CHRM3 CHRM2 CHRNA2	1131 1129 1135	Human	3
DB01409	CHRM3 CHRM1 CHRM2	1131 1128 1129	Human	3
DB01591	CHRM3 CHRM1 CHRM5 CHRM4 CHRM2	1131 1128 1133 1132 1129	Human	5
DB01625	CHRM3 CHRM4	1131 1132	Human	2
DB04843	CHRM3 CHRM1	1131 1128	Human	2
DB06702	CHRM3 CHRM1 CHRM5 CHRM4 CHRM2	1131 1128 1133 1132 1129	Human	5
DB06709	CHRM3	1131	Human	1
DB08897	CHRM3 CHRM1 CHRM5 CHRM4 CHRM2	1131 1128 1133 1132 1129	Human	5

DB00119	PKLR PKM2 PDHB AGXT2 SLC16A6 SLC16A2 SLC16A4 ABAT SLC16A8 SLC16A5 SLC16A7 SLC16A3 SLC16A1 PC	5313 5315 5162 64902 9120 6567 9122 18 23539 9121 9194 9123 6566 5091	Human	14
DB00761	SLC12A5 SLC12A6 SLC12A7 SLC12A2 SLC12A1 SLC12A4	57468 9990 10723 6558 6557 6560	Human	6
DB00887	SLC12A5 SLC12A2 SLC12A1 SLC12A4 CFTR	57468 6558 6557 6560 1080	Human	5
DB00001	F2	2147	Human	1
DB00006	F2	2147	Human	1
DB00170	F2 F10 NQO2 F7 F9 PROC GGCX PROZ VKORC1 BGLAP PROS1 NQO1 VKORC1L1	2147 2159 4835 2155 2158 5624 2677 8858 79001 632 5627 1728 154807	Human	13
DB00278	F2	2147	Human	1
DB01123	F2 qacR lfrR	2147 9487076 4534500	Human	3
DB04786	F2 FSHR RYR1 C3L PLA2G2A P2RY2 SIRT5	2147 2492 6261 3707640 5320 5029 23408	Human	7
DB04898	F2	2147	Human	1
DB06695	F2	2147	Human	1
DB00855	ALAD	210	Human	1
DB01108	HSD3B1 HSD3B2 ESR1 ESR2	3283 3284 2099 2100	Human	4
DB00123	SLC7A4 SLC7A3 KARS SLC7A1 SLC7A2	6545 84889 3735 6541 6542	Human	5
DB00129	SLC7A4 SLC7A3 SLC25A15 SLC7A1 SLC25A2 ARG1 OTC GATM OAZ1 OAT SLC7A2 ARG2 OAZ2 OAZ3	6545 84889 10166 6541 83884 383 5009 2628 4946 4942 6542 384 4947 51686	Human	14
DB00233	folK ALOX5 PTGS2 PLA2G2E CHUK	885848 240 5743 30814 1147	Mycobacterium tuberculosis	5
DB00145	GLRB AGXT2 GSS SHMT2 AGXT SHMT1 GATM GNMT ALAS1 GLRA3 GLRA1 GRIN2C GCAT GRIN3B SLC36A1 GLDC GLRA2 GRIN2A ALAS2 SLC6A9 GARS GCSH BAAT GPR18 GLYAT GLYATL2 GLYATL1 SLC32A1 PIPOX SLC6A5	2743 64902 2937 6472 189 6470 2628 27232 211 8001 2741 2905 23464 116444 206358 2731 2742 2903 212 6536 2617 2653 570 2841 10249 219970 92292 140679 51268 9152	Human	30
DB00431	GLRB GLRA3 GLRA1 GLRA2 GABRB1	2743 8001 2741 2742 2560	Human	5
DB00336	mdh gor poxB citC	12931785 948014 946132 945231	Escherichia coli (strain K12)	4
DB00558	NA NEU2	947854 4759	Influenza A virus (strain A/Bangkok/1/1979 H3N2)	2

DB00786	MMP2 MMP1 MMP13 MMP3 MMP8 MMP12 MMP16 MMP10 MMP7 MMP11 MMP14 MMP15 MMP17 MMP19 MMP20 MMP21 MMP23A MMP24 MMP25 MMP26 MMP27 MMP28	4318 4313 4312 4322 4314 4317 4321 4325 4319 4316 4320 4323 4324 4326 4327 9313 118856 8510 10893 64386 56547 64066 79148	Human	23
DB01197	ACE MMP2	4318 1636 4313	Human	3
DB01296	TNF IFNG NFKB2	4318 7124 3458 4791	Human	4
DB00126	P4HA1 PLOD1 DBH SLC23A1 SP_0314 PHYH PLOD2 PLOD3 BBOX1 PAM LEPRE1 OGFOD2 ALKBH2 LEPREL1 LEPREL2 OGFOD1 EGLN2 ALKBH3 KDM5D EGLN1 EGLN3 TMLHE	5033 5351 1621 9963 930127 5264 5352 8985 8424 5066 64175 79676 121642 55214 10536 55239 112398 221120 8284 54583 112399 55217 54681 3938	Human	24
DB00172	P4HA1 EPRS PYCR1 SLC6A14 SLC6A7 PYCR2 PPIA PPIF PROSC LEPRE1 LEPREL1 LEPREL2 P4HA2 PRODH PPIH PPIB PPIC PPIG PARS2	5033 2058 5831 11254 6534 29920 5478 10105 11212 64175 55214 10536 8974 5625 10465 5479 5480 9360 25973 112849 65263	Human	21
DB01275	P4HA1 AOC3	5033 8639	Human	2
DB00470	CNR1 CNR2	1268 1269	Human	2
DB00486	CNR1 CNR2	1268 1269	Human	2
DB06155	CNR1	1268	Human	1
DB00177	AGTR1	185	Human	1
DB00275	AGTR1	185	Human	1
DB00678	AGTR1	185	Human	1
DB00796	AGTR1	185	Human	1
DB00876	AGTR1	185	Human	1
DB00966	AGTR1 PPARG	185 5468	Human	2
DB01029	AGTR1 JUN	185 3725	Human	2
DB01342	AGTR1	185	Human	1
DB01347	AGTR1	185	Human	1
DB01349	AGTR1 AGTR2	185 186	Human	2
DB08822	AGTR1	185	Human	1
DB00133	SARS SDS AGXT CBS SRR SPTLC1 SPTLC2	6301 10993 189 875 63826 10558 9517	Human	7
DB00297	PTGER1 SCN10A	5731 6336	Human	2
DB00429	PTGER1	5731	Human	1
DB00770	PTGER1 PTGER2	5731 5732	Human	2
DB00905	PTGER1 AKR1C3 PTGFR	5731 8644 5737 5733	Human	4
DB00917	PTGER1 PTGER2 PTGER3	5731 5732 5733 5734	Human	4
DB01088	PTGER1 PTGIR PDE4B PLAT PDE4A PDE4D PDE4C	5731 5739 5142 5327 5141 5144 5143	Human	7
DB00073	MS4A1 FCGR1A FCGR3B C1S C1R C1QA C1QB C1QC FCGR3A FCGR2A FCGR2B	931 2209 2215 716 715 712 713 714 2214 2212 2213	Human	11
DB00078	MS4A1 FCGR1A FCGR3B C1S C1R C1QA C1QB C1QC FCGR3A FCGR2A FCGR2B	931 2209 2215 716 715 712 713 714 2214 2212 2213	Human	11

DB00081	MS4A1 FCGR1A FCGR3B C1R C1QA C1QB C1QC FCGR3A FCGR2A FCGR2B	931 2209 2215 715 712 713 714 2214 2212 2213	Human	10
DB08935	MS4A1	931	Human	1
DB03147	NOS1 dld CYB5R3 NQO2 gor IVD ACAD8 GSR DLD ACADS DPYD AIFM1 NQO1 pox5 fprA thyX SO_0970 pobA thiO hmp glf ACADM xecC putA GCDH fadH phr TXNRD1 metF trxB MAOB MAOA XDH POR npr ifcA hmp cysJ PSPTO1126 phrB phr nadB cry murB murB lpdA glf ahpF pamO benC TT_C0779 ahpF petH fpr ACOX1 DAO FDXR CYB5R1 IL4I1 ERO1LB GFER	4842 946653 1727 4835 948014 3712 27034 2936 1738 35 1806 9131 1728 1062065 888839 897468 1168814 882128 939377 2656642 945235 34 5420640 945600 2639 947594 3169528 7296 948432 949054 4129 4128 7498 5447 1200111 4279493 947018 947239 1182762 947005 3199486 947049 954400 948470 1002811 887659 886142 1252129 3581683 2880892 2775096 947540 1107723 948414 51 1610 2232 51706 [illegible]	Human	61
DB00636	PPARA	5465	Human	1
DB01039	PPARA	5465	Human	1
DB01241	PPARA	5465	Human	1
DB01393	PPARA PPARG PPARD	5465 5468 5467	Human	3
DB00121	MCCC2 SLC5A6 HLCS MCCC1 PCCA PCCB ACACA PC ACACB	64087 8884 3141 56922 5095 5096 31 5091 32	Human	9
DB00755	NR0B1 RXRB RARG ALDH1A2 ALDH1A1 RARRES1 GPRC5A RXRG	190 6257 5916 8854 216 5918 9052 6258	Human	8
DB00052	GHR PRLR	2690 5618	Human	2
DB00082	GHR	2690	Human	1
DB00163	SEC14L4 ALOX5 SEC14L2 SEC14L3 PRKCB NR1I2 PRKCA DGKA PPP2CB PPP2CA	284904 240 23541 266629 5579 8856 5578 1606 5516 5515	Human	10
DB00993	HPRT1	3251	Human	1
DB01033	HPRT1	3251	Human	1
DB00210	RXRB RARG RARB RXRA RARA RXRG	6257 5916 5915 6256 5914 6258	Human	6
DB00307	RXRB RXRA RXRG	6257 6256 6258	Human	3
DB00459	RXRB RARG RARB RBP1 RXRA RARA RXRG	6257 5916 5915 5947 6256 5914 6258	Human	7
DB00523	RXRB RARG RARB RXRA RARA RXRG	6257 5916 5915 6256 5914 6258	Human	6
DB00799	RXRB RARG RARB RARA	6257 5916 5915 5914	Human	4
DB00151	CSAD NFS1 GCLC GSS CBS GOT1 CDO1 GCLM CARS CTH SLC19A3 MGMT CARS2	51380 9054 2729 2937 875 2805 1036 2730 833 1491 80704 4255 79587	Human	13
DB00148	CKB CKMT1A SLC6A8 CKMT2 GAMT CKM	1152 1159 6535 1160 2593 1158	Human	6
DB00087	CD52 FCGR1A FCGR3B C1R C1QA C1QB C1QC FCGR3A FCGR2A FCGR2B	1043 2209 2215 715 712 713 714 2214 2212 2213	Human	10
DB00604	HTR4 KCNH2 HTR3A HTR2A	1043 3757 3359 3356	Human	4
DB00904	HTR4 HTR1A HTR3A OPRM1 HTR1B	1043 3350 3359 4988 3351	Human	5

DB01233	HTR4 CHRM1 DRD2	1043 1128 1813	Human	3
DB08810	HTR4 HTR1A HTR2A	1043 3350 3356	Human	3
DB00893	FTL HBA1 FTH1 HBB	2512 3039 2495 3043	Human	4
DB00204	KCNH2 KCNK2 KCNJ12	3757 3776 3768	Human	3
DB00276	KCNH2 TOP2A	3757 7153	Human	2
DB00308	KCNH2 KCNK1 CACNB1 CACNA1C KCNH6 CACNG1 KCNK6 CACNA2D1 KCNH7 KCNJ11	3757 3775 782 775 81033 786 9424 781 90134 3767	Human	10
DB00457	KCNH2 KCNH6 ADRA1A ADRA1B KCNH7 ADRA1D	3757 81033 148 147 90134 146	Human	6
DB00489	KCNH2 ADRB1 ADRB2	3757 153 154	Human	3
DB00590	KCNH2 KCNH6 ADRA1A ADRA1B KCNH7 ADRA1D	3757 81033 148 147 90134 146	Human	6
DB00637	KCNH2 HRH1	3757 3269	Human	2
DB00908	KCNH2 KCNK1 SCN5A KCNK6	3757 3775 6331 9424	Human	4
DB01100	KCNH2 CALM1 DRD3 DRD2	3757 805 1814 1813	Human	4
DB01118	KCNH2 ADRB1 CACNA1H CACNA2D2	3757 153 8912 9254	Human	4
DB01136	KCNH2 VEGFA ADRB1 NDUFC2 ADRA1A ADRB2 NPPB GJA1 VCAM1	3757 7422 153 4718 148 154 4879 2697 7412	Human	9
DB01162	KCNH2 KCNH6 ADRA1A ADRA1B KCNH7 ADRA1D	3757 81033 148 147 90134 146	Human	6
DB01182	KCNH2 SCN5A	3757 6331	Human	2
DB01218	KCNH2	3757	Human	1
DB04855	KCNH2 SCN1A ADRB1 KCNK2 ADRA2A CACNB1 ADRA2C CACNA1C ADRA1A ADRA2B ADRA1B ADRA1D CACNB2 CACNA1S CACNB4 CACNA1F CACNB3 CACNA1D	3757 6323 153 3776 150 782 152 775 148 151 147 146 783 779 785 778 784 776	Human	18
DB06144	KCNH2 HTR2A ADRA1A HTR2C ADRA1B ADRA1D DRD2 HTR6	3757 3356 148 3358 147 146 1813 3362	Human	8
DB00762	TOP1MT TOP1	116447 7150	Human	2
DB01030	TOP1MT TOP1	116447 7150	Human	2
DB00209	CHRM1	1128	Human	1
DB00215	CHRM1 HRH1 ADRA1A SLC6A4	1128 3269 148 6532	Human	4
DB00219	CHRM1	1128	Human	1
DB00245	CHRM1 HRH1 SLC6A3	1128 3269 6531	Human	3
DB00354	CHRM1 HRH1	1128 3269	Human	2
DB00366	CHRM1 HRH1	1128 3269	Human	2
DB00392	CHRM1 CHRM2 GRIN3A	1128 1129 116443	Human	3
DB00411	CHRM1 CHRM2 CHRNA2	1128 1129 1135	Human	3
DB00670	CHRM1	1128	Human	1
DB00771	CHRM1	1128	Human	1
DB00782	CHRM1	1128	Human	1
DB00804	CHRM1 CHRM2	1128 1129	Human	2
DB00810	CHRM1 CHRNA2	1128 1135	Human	2

DB00907	CHRM1 SCN10A SCN11A SCN5A SLC6A2 CHRM2 SLC6A3	1128 6336 11280 6331 6530 1129 6531 6532	Human	8
DB00940	CHRM1 HRH2	1128 3274	Human	2
DB00942	CHRM1	1128	Human	1
DB00979	CHRM1	1128	Human	1
DB00986	CHRM1	1128	Human	1
DB01148	CHRM1 CHRM2	1128 1129	Human	2
DB01175	CHRM1 HRH1 SLC6A2 ADRA1A SLC6A3 SLC6A4	1128 3269 6530 148 6531 6532	Human	6
DB01618	CHRM1 HTR1A HTR2A DRD2	1128 3350 3356 1813	Human	4
DB00583	SLC25A29 SLC22A5 CPT2 CROT CPT1A CRAT SLC25A20 SLC22A4 MPO XDH CES1	123096 6584 1376 54677 1374 1384 788 6583 4353 7498 1066	Human	11
DB00716	CYSLTR2 PTGDR CYSLTR1 FPR1 HSP90AA1	57105 5729 10800 2357 3320	Human	5
DB00118	AMD1 MAT2A CBS GNMT MAT1A COMT	262 4144 875 27232 4143 1312	Human	6
DB00160	AGXT2 NFS1 ABAT AARS AGXT KYNU SLC1A4 SLC36A1 GPT SLC7A8 AGXT2L2 GPT2 AARS2	64902 9054 18 16 189 8942 6509 206358 2875 23428 85007 84706 57505	Human	13
DB00671	mrdA	950929	Haemophilus influenzae (strain ATCC 51907 / DSM 11121 / KW20 / Rd)	1
DB00973	SOAT1 NPC1L1 ANPEP	6646 29881 290	Human	3
DB01094	SOAT1 MTTP SOAT2	6646 4547 8435	Human	3
DB00113	CEACAM1	634	Human	1
DB00141	RENBP B4GALT1 NAGLU B4GALT3 B4GALT4 B4GALT2 NAGK NAGPA	5973 2683 4669 8703 8702 8704 55577 51172	Human	8
DB00208	P2RY12	64805	Human	1
DB00374	P2RY12 PTGIR PPARD	64805 5739 5467	Human	3
DB00758	P2RY12	64805	Human	1
DB01240	P2RY12 PTGIS PTGIR	64805 5740 5739	Human	3
DB06209	P2RY12	64805	Human	1
DB08816	P2RY12	64805	Human	1
DB00272	HRH2	3274	Human	1
DB00501	HRH2	3274	Human	1
DB00585	HRH2	3274	Human	1
DB00667	HRH2 HRH4 HRH1 HRH3	3274 59340 3269 11255	Human	4
DB00751	HRH2 ADRA2A HRH1 HTR2A ADRA1A HTR7	3274 150 3269 3356 148 3363	Human	6
DB00863	HRH2	3274	Human	1
DB00927	HRH2	3274	Human	1
DB08806	HRH2	3274	Human	1
DB00987	POLB	5423	Human	1

DB00756	dld GLUD1 SDHD	946653 2746 6392	Escherichia coli (strain K12)	3
DB00173	APRT ACACB MTAP cobT mtnN ptd mutY PECR ruvB ACP1 SRPK2	353 32 4507 1253537 948542 16795090 947447 55825 3168960 52	Human	11
DB00581	ebgA	947583	Escherichia coli (strain K12)	1
DB00182	SLC18A2 SLC6A3 CARTPT	6571 6531 9607 134864	Human	4
DB00206	SLC18A2	6571	Human	1
DB00386	SLC18A2	6571	Human	1
DB00865	SLC18A2 ADRA2A ADRA1A SLC6A3	6571 150 148 6531	Human	4
DB01089	SLC18A2	6571	Human	1
DB01363	SLC18A2 ADRB1 ADRA2A ADRA2C SLC6A2 ADRA2B SLC6A3 ADRB2 SLC6A4 ADRB3 MAOB MAOA SLC18A1	6571 153 150 152 6530 151 6531 154 6532 155 4129 4128 6570	Human	13
DB01364	SLC18A2 ACHE SLC6A2	6571 43 6530 148	Human	4
DB01576	SLC18A2 SLC6A2 ADRA1A ADRA1B SLC6A3 TAAR1	6571 6530 148 147 6531 134864	Human	6
DB01577	SLC18A2 ADRA2A ADRA2C SLC6A2 ADRA2B SLC6A3 SLC6A4 TAAR1 MAOB MAOA	6571 150 152 6530 151 6531 6532 134864 4129 4128 6570	Human	11
DB04844	SLC18A2 DRD2	6571 1813	Human	2
DB06706	SLC18A2 ADRA1A	6571 148	Human	2
DB06714	SLC18A2 TAAR1	6571 134864	Human	2
DB01016	ABCA1 ABCC8 ABCC9 KCNJ1 ABCB11 KCNJ11 CFTR KCNJ5	19 6833 10060 3758 8647 3767 1080 3762	Human	8
DB01599	ABCA1 CES1	19 1066	Human	2
DB00360	TPH1 NOS3 TH PAH	7166 4846 7054 5053	Human	4
DB00255	ESR1 ESR2 ESRRG	2099 2100 2104	Human	3
DB00269	ESR1	2099	Human	1
DB00286	ESR1	2099	Human	1
DB00294	ESR1 PGR	2099 5241	Human	2
DB00304	ESR1 PGR	2099 5241	Human	2
DB00367	ESR1 AR SRD5A1 PGR	2099 367 6715 5241	Human	4
DB00396	ESR1 CYP17A1 PGR NR3C2	2099 1586 5241 4306	Human	4
DB00481	ESR1 ESR2	2099 2100	Human	2
DB00539	ESR1	2099	Human	1
DB00603	ESR1 PGR	2099 5241	Human	2
DB00655	ESR1	2099	Human	1
DB00675	ESR1 ESR2	2099 2100	Human	2
DB00783	ESR1 ESR2 NR1I2	2099 2100 8856	Human	3
DB00823	ESR1 PGR	2099 5241	Human	2
DB00882	ESR1	2099	Human	1
DB00890	ESR1	2099	Human	1
DB00947	ESR1	2099	Human	1
DB00957	ESR1 PGR	2099 5241	Human	2
DB00977	ESR1 NR1I2	2099 8856	Human	2

DB01065	ESR1 NQO2 MTNR1B CALM1 MTNR1A MPO CALR ASMT EPX	2099 4835 4544 805 4543 4353 811 438 8288	Human	9
DB01183	ESR1 OPRD1 OPRK1 OPRM1 CREB1 TLR4	2099 4985 4986 4988 1385 7099	Human	6
DB01185	ESR1 AR PRLR NR3C1	2099 367 5618 2908	Human	4
DB01196	ESR1 ESR2 MAP2 MAP1A	2099 2100 4133 4130	Human	4
DB01357	ESR1	2099	Human	1
DB01406	ESR1 AR GNRHR PGR CCL2	2099 367 2798 5241 6347	Human	5
DB01431	ESR1 PGR	2099 5241	Human	2
DB04573	ESR1 ESR2	2099 2100	Human	2
DB04574	ESR1 ESR2	2099 2100	Human	2
DB04575	ESR1	2099	Human	1
DB04938	ESR1	2099	Human	1
DB01015	folC folP	945451 947691	Escherichia coli (strain K12)	2
DB01025	IL3 FGF1 S100A13 S100A12	3562 2246 6284 6283	Human	4
DB00560	rpsI rpsL rpsN rpsM rpsS	949000 947845 947801 947791 947811	Escherichia coli (strain K12)	5
DB01301	rpsI	949000	Escherichia coli (strain K12)	1
DB00231	GABRR1 GABRA2 GABRA5 GABRA4 GABRA3 TSPO GABRA6 GABRA1 GABRB1 GABRB3 GABRB2 GABRG2 GABRG1 GABRG3 GABRE GABRP GABRQ GABRR2 GABRD GABRR3 GABRA1 GABRA2 GABRA3 GABRA4 GABRA5 GABRA6 GABRB1 GABRB2 GABRB3 GABRD GABRE GABRG1 GABRG2 [illegible]	2569 2555 2558 2557 2556 706 2559 2554 2560 2562 2561 2566 2565 2567 2564 2568 55879 2570 2563 200959 2554 2555 2556 2557 2558 2559 2560 2561 2562 2563 2564 2565 2567 2568 2568 55879	Human	36
DB00466	GABRR1 GLRA3 GLRA2	2569 8001 2742 2554	Human	4
DB00683	GABRR1 GABRA2 GABRA5 GABRA4 GABRA3 GABRA6 GABRA1 GABRB1 GABRB3 GABRB2 GABRG2 GABRG1 GABRG3 GABRE GABRP GABRQ GABRR2 GABRD	2569 2555 2558 2557 2556 2559 2554 2560 2562 2561 2566 2565 2567 2564 2568 55879 2570 2563 200959	Human	19
DB00690	GABRR1 GABRA2 GABRA5 GABRA4 GABRA3 GABRA6 GABRA1 GABRB1 GABRB3 GABRB2 GABRG2 GABRG1 GABRG3 GABRE GABRP GABRQ GABRR2 GABRD	2569 2555 2558 2557 2556 2559 2554 2560 2562 2561 2566 2565 2567 2564 2568 55879 2570 2563 200959	Human	19

DB00801	GABRR1 GABRA2 GABRA5 GABRA3 GABRA1 GABRB1 GABRB3 GABRB2 GABRG2 GABRG1 GABRG3 GABRE GABRP GABRR2 GABRD GABRR3 GABRA1 GABRA2 GABRA3 GABRA4 GABRA5 GABRA6 GABRB1 GABRB2 GABRB3 GABRD GABRE GABRG1 GABRG2 GABRG3	2569 2555 2558 2556 2554 2560 2562 2561 2566 2565 2567 2564 2568 2570 2563 200959 2554 2555 2556 2557 2558 2559 2560 2561 2562 2563 2564 2565 2567 2568 2568 55879	Human	32
DB00829	GABRR1 GABRA2 GABRA5 GABRA3 TSPO GABRA1 GABRB1 GABRB3 GABRB2 GABRG2 GABRG1 GABRG3 GABRE GABRP GABRQ GABRR2 GABRD GABRR3 GABRA1 GABRA2 GABRA3 GABRA4 GABRA5 GABRA6 GABRB1 GABRB2 GABRB3 GABRD GABRE GABRG1	2569 2555 2558 2556 706 2554 2560 2562 2561 2566 2565 2567 2564 2568 55879 2570 2563 200959 2554 2555 2556 2557 2558 2559 2560 2561 2562 2563 2564 2565 2567 2568 2568 55879	Human	34
DB00842	GABRR1 GABRA2 GABRA5 GABRA4 GABRA3 GABRA6 GABRA1 GABRB1 GABRB3 GABRB2 GABRG2 GABRG1 GABRG3 GABRE GABRP GABRQ GABRR2 GABRD GABRR3 GABRA1 GABRA2 GABRA3 GABRA4 GABRA5 GABRA6 GABRB1 GABRB2 GABRB3 GABRD GABRE GABRG1 GABRG2 GABRG3 [illegible]	2569 2555 2558 2557 2556 2559 2554 2560 2562 2561 2566 2565 2567 2564 2568 55879 2570 2563 200959 2554 2555 2556 2557 2558 2559 2560 2561 2562 2563 2564 2565 2567 2568 2568 55879	Human	35
DB00897	GABRR1 GABRA2 GABRA5 GABRA4 GABRA3 TSPO GABRA6 GABRA1 GABRB1 GABRB3 GABRB2 GABRG2 GABRG1 GABRG3 GABRE GABRP GABRQ GABRR2 GABRD GABRR3 GABRA1 GABRA2 GABRA3 GABRA4 GABRA5 GABRA6 GABRB1 GABRB2 GABRB3 GABRD GABRE GABRG1 GABRG2 [illegible]	2569 2555 2558 2557 2556 706 2559 2554 2560 2562 2561 2566 2565 2567 2564 2568 55879 2570 2563 200959 2554 2555 2556 2557 2558 2559 2560 2561 2562 2563 2564 2565 2567 2568 2568 55879	Human	36
DB01215	GABRR1 GABRA2 GABRA5 GABRA3 GABRA1 GABRB1 GABRB3 GABRB2 GABRG2 GABRG1 GABRG3 GABRE GABRP GABRR2 GABRD GABRR3 GABRA1 GABRA2 GABRA3 GABRA4 GABRA5 GABRA6 GABRB1 GABRB2 GABRB3 GABRD GABRE GABRG1 GABRG2 GABRG3	2569 2555 2558 2556 2554 2560 2562 2561 2566 2565 2567 2564 2568 2570 2563 200959 2554 2555 2556 2557 2558 2559 2560 2561 2562 2563 2564 2565 2567 2568 2568 55879	Human	32

DB01558	GABRR1 GABRA2 GABRA5 GABRA4 GABRA3 GABRA6 GABRA1 GABRB1 GABRB3 GABRB2 GABRG2 GABRG1 GABRG3 GABRE GABRP GABRQ GABRR2 GABRD	2569 2555 2558 2557 2556 2559 2554 2560 2562 2561 2566 2565 2567 2564 2568 55879 2570 2563 200959	Human	19
DB01559	GABRR1 GABRA2 GABRA5 GABRA3 GABRA1 GABRB1 GABRB3 GABRB2 GABRG2 GABRG1 GABRG3 GABRE GABRP GABRR2 GABRD	2569 2555 2558 2556 2554 2560 2562 2561 2566 2565 2567 2564 2568 2570 2563 200959	Human	16
DB01567	GABRR1 GABRA2 GABRA5 GABRA3 GABRA1 GABRB1 GABRB3 GABRB2 GABRG2 GABRG1 GABRG3 GABRE GABRP GABRR2 GABRD	2569 2555 2558 2556 2554 2560 2562 2561 2566 2565 2567 2564 2568 2570 2563 200959	Human	16
DB01588	GABRR1 GABRA2 GABRA5 GABRA3 GABRA1 GABRB1 GABRB3 GABRB2 GABRG2 GABRG1 GABRG3 GABRE GABRP GABRR2 GABRD GABRR3 GABRA1 GABRA2 GABRA3 GABRA4 GABRA5 GABRA6 GABRB1 GABRB2 GABRB3 GABRD GABRE GABRG1 GABRG2 GABRG3	2569 2555 2558 2556 2554 2560 2562 2561 2566 2565 2567 2564 2568 2570 2563 200959 2554 2555 2556 2557 2558 2559 2560 2561 2562 2563 2564 2565 2567 2568 2568 55879	Human	32
DB01589	GABRR1 GABRA2 GABRA5 GABRA3 GABRA1 GABRB1 GABRB3 GABRG2 GABRG1 GABRG3 GABRE GABRP GABRR2 GABRD GABRR3 GABRA1 GABRA2 GABRA3 GABRA4 GABRA5 GABRA6 GABRB1 GABRB2 GABRB3 GABRD GABRE GABRG1 GABRG2 GABRG3 GABRP	2569 2555 2558 2556 2554 2560 2562 2566 2565 2567 2564 2568 2570 2563 200959 2554 2555 2556 2557 2558 2559 2560 2561 2562 2563 2564 2565 2567 2568 2568 55879	Human	31
DB01594	GABRR1 GABRA2 GABRA5 GABRA3 GABRA1 GABRB1 GABRB3 GABRB2 GABRG2 GABRG1 GABRG3 GABRE GABRP GABRR2 GABRD	2569 2555 2558 2556 2554 2560 2562 2561 2566 2565 2567 2564 2568 2570 2563 200959	Human	16
DB01595	GABRR1 SCN1A GABRA2 GABRA5 GABRA4 GABRA3 GABRA6 GABRA1 GABRB1 GABRB3 GABRB2 GABRG2 GABRG1 GABRG3 GABRE GABRP GABRQ GABRR2	2569 6323 2555 2558 2557 2556 2559 2554 2560 2562 2561 2566 2565 2567 2564 2568 55879 2570 2563 200959	Human	20
DB00358	HBA1 ADORA2A	3039 135	Human	2
DB00421	AR NR3C2	367 4306	Human	2
DB00499	AR AHR	367 196	Human	2
DB00621	AR	367	Human	1
DB00624	AR	367	Human	1

DB00665	AR	367	Human	1
DB00687	AR NR3C2 NR3C1	367 4306 2908	Human	3
DB00858	AR	367	Human	1
DB00984	AR	367	Human	1
DB01128	AR	367	Human	1
DB01395	AR PGR NR3C2	367 5241 4306	Human	3
DB01420	AR	367	Human	1
DB04839	AR	367	Human	1
DB06710	AR	367	Human	1
DB08804	AR	367	Human	1
DB08899	AR	367	Human	1
DB00536	ALDH2 GAMT E argG RNASE1 DLG4	217 2593 1258585 947590 6035 1742	Human	6
DB00822	ALDH2 DBH	217 1621	Human	2
DB00014	LHCGR GNRHR	3973 2798	Human	2
DB00032	LHCGR FSHR	3973 2492	Human	2
DB00044	LHCGR	3973	Human	1
DB00050	LHCGR GNRHR	3973 2798	Human	2
DB00097	LHCGR FSHR	3973 2492	Human	2
DB06719	LHCGR GNRHR	3973 2798	Human	2
DB00252	SCN1A SCN5A	6323 6331	Human	2
DB00273	SCN1A CA2 GRIK1 CA4 GABRA1 GABRA1 GABRA2 GABRA3 GABRA4 GABRA5 GABRA6 GABRB1 GABRB2 GABRB3 GABRD GABRE GABRG1 GABRG2 GABRG3	6323 760 2897 762 2554 2554 2555 2556 2557 2558 2559 2560 2561 2562 2563 2564 2565 2567 2568 2568 55879	Human	21
DB01121	SCN1A	6323	Human	1
DB01438	SCN1A	6323	Human	1
DB04930	SCN1A	6323	Human	1
DB01147	penA pbpA ampC dacA pbp2a pbpB	931164 990276 948669 1036908 933569 881247	Streptococcus pneumoniae serotype 4 (strain ATCC BAA-334 / TIGR4)	6
DB01150	penA pbpX ponA	931164 930153 930269	Streptococcus pneumoniae serotype 4 (strain ATCC BAA-334 / TIGR4)	3
DB00361	TUBB	203068	Human	1
DB00541	TUBB TUBA4A	203068 7277	Human	2
DB00570	TUBB JUN TUBA1A TUBD1 TUBE1 TUBG1	203068 3725 7846 51174 51175 7283	Human	6
DB01179	TUBB TOP2A TUBA4A	203068 7153 7277	Human	3

DB00313	ALDH5A1 OGDH ABAT ACADSB HDAC9	7915 4967 18 36 9734	Human	5
DB00534	ALDH5A1 SLC12A1	7915 6557	Human	2
DB01346	KCNK1 SCN5A GABRA2 GABRA1 GRIA2	3775 6331 2555 2554 2891	Human	5
DB00724	TLR7 TLR8	51284 51311	Human	2
DB01611	TLR7 TLR9	51284 54106	Human	2
DB00409	SIGMAR1 DRD4 HTR2A DRD3 DRD2	10280 1815 3356 1814 1813	Human	5
DB00514	SIGMAR1 GRIN3A CHRNA2 SLC6A4	10280 116443 1135 6532	Human	4
DB00652	SIGMAR1 OPRK1 OPRM1	10280 4986 4988	Human	3
DB00152	TPK1 SLC19A2	27010 10560	Human	2
DB00698	rpsJ nfsA ydbK	947816 945483 946587	Escherichia coli (strain K12)	3
DB00112	VEGFA FCGR1A FCGR3B C1R C1QA C1QB C1QC FCGR3A FCGR2A FCGR2B	7422 2209 2215 715 712 713 714 2214 2212 2213	Human	10
DB01120	VEGFA ABCC8	7422 6833	Human	2
DB01270	VEGFA	7422	Human	1
DB05294	VEGFA EGFR TEK	7422 1956 7010 5753	Human	4
DB06779	VEGFA SERPINC1 TFPI SELP	7422 462 7035 6403	Human	4
DB08885	VEGFA PGF	7422 5228 7423	Human	3
DB00035	AVPR1A AVPR2 AVPR1B	552 554 553	Human	3
DB00067	AVPR1A AVPR2 AVPR1B	552 554 553	Human	3
DB00093	AVPR1A	552	Human	1
DB00872	AVPR1A AVPR2	552 554	Human	2
DB02638	AVPR1A AVPR2 AVPR1B	552 554 553	Human	3
DB06212	AVPR1A AVPR2	552 554	Human	2
DB00290	LIG3 LIG1	3980 3978	Human	2
DB00187	ADRB1	153	Human	1
DB00195	ADRB1 ADRB2	153 154	Human	2
DB00221	ADRB1	153	Human	1
DB00264	ADRB1 ADRB2	153 154	Human	2
DB00335	ADRB1	153	Human	1
DB00368	ADRB1 ADRA2A ADRA2C ADRA1A ADRA2B ADRA1B ADRB2 ADRA1D PAH ADRB3	153 150 152 148 151 147 154 146 5053 155	Human	10
DB00373	ADRB1 ADRB2 E	153 154 1258585	Human	3
DB00521	ADRB1 ADRB2	153 154	Human	2
DB00571	ADRB1 HTR1A ADRB2 HTR1B ADRB3	153 3350 154 3351 155	Human	5
DB00598	ADRB1 ADRA1A ADRA1B	153 148 147 154	Human	4
DB00612	ADRB1 ADRB2	153 154	Human	2
DB00668	ADRB1 ADRA2A ADRA1A ADRA2B ADRA1B ADRB2 ADRA1D PAH	153 150 148 151 147 154 146 5053	Human	8
DB00841	ADRB1 ADRB2	153 154	Human	2

DB00852	ADRB1 ADRA2A SLC6A2 ADRA1A SLC6A3 ADRB2	153 150 6530 148 6531 154 6532	Human	7
DB00866	ADRB1 HTR1A ADRB2	153 3350 154	Human	3
DB00960	ADRB1 HTR1A ADRB2 HTR1B	153 3350 154 3351	Human	4
DB01001	ADRB1 ADRB2	153 154	Human	2
DB01064	ADRB1 ADRB2 MAPK1 PIK3R1 ADRB3 PIK3R2 PIK3R3	153 154 5594 5295 155 5296 8503	Human	7
DB01102	ADRB1 ADRB2 ADRB3	153 154 155	Human	3
DB01193	ADRB1 ADRB2	153 154	Human	2
DB01203	ADRB1 ADRB2	153 154	Human	2
DB01210	ADRB1 ADRB2	153 154	Human	2
DB01214	ADRB1 ADRB2	153 154	Human	2
DB01288	ADRB1 ADRB2 ADRB3	153 154 155	Human	3
DB01291	ADRB1 ADRB2	153 154	Human	2
DB01295	ADRB1 ADRA1A ADRB2	153 148 154	Human	3
DB01297	ADRB1	153	Human	1
DB01359	ADRB1 HTR1A ADRB2	153 3350 154	Human	3
DB01407	ADRB1 ADRB2 TNF ADRB3 NGF	153 154 7124 155 4803	Human	5
DB01580	ADRB1 ADRB2	153 154	Human	2
DB04861	ADRB1 ADRB2	153 154	Human	2
DB06262	ADRB1 ADRA2A ADRA2C ADRA1A ADRA2B ADRA1B ADRB2 ADRA1D PAH ADRB3	153 150 152 148 151 147 154 146 5053 155	Human	10
DB08807	ADRB1 HTR1A ADRB2 HTR1B ADRB3	153 3350 154 3351 155	Human	5
DB08808	ADRB1 ADRB2 ADRB3	153 154 155	Human	3
DB00281	SCN10A SCN5A EGFR SCN9A	6336 6331 1956 6335	Human	4
DB00296	SCN10A	6336	Human	1
DB00473	SCN10A SCN5A	6336 6331	Human	2
DB00527	SCN10A SCN5A CALM1	6336 6331 805	Human	3
DB00645	SCN10A	6336	Human	1
DB00721	SCN10A HTR3A GRIN3A SLC6A3 CHRNA2	6336 3359 116443 6531 1135	Human	5
DB00807	SCN10A	6336	Human	1
DB00892	SCN10A	6336	Human	1
DB00961	SCN10A	6336	Human	1
DB01002	SCN10A	6336	Human	1
DB01086	SCN10A	6336	Human	1
DB01161	SCN10A HTR3A CHRNA10 GRIN3A SLC6A3	6336 3359 57053 116443 6531	Human	5
DB01173	SCN10A GRIN1 GRIN2D HRH1 SLC6A2 GRIN3B GRIN3A	6336 2902 2906 3269 6530 116444 116443	Human	7
DB06218	SCN10A SCN9A SCN3A	6336 6335 6328	Human	3
DB01074	CPT2 CPT1A	1376 1374	Human	2
DB00203	PDE5A PDE6G PDE6H	8654 5148 5149	Human	3
DB00820	PDE5A PDE11A	8654 50940	Human	2
DB00862	PDE5A PDE6G PDE6H	8654 5148 5149	Human	3
DB00975	PDE5A PDE10A PDE4A ADA	8654 10846 5141 100	Human	4
DB06237	PDE5A	8654	Human	1

DB06267	PDE5A	8654	Human	1
DB00143	GSS GSR GSTM1 GSTK1 GSTA3 GSTM3 GSTA4 GSTM4 GSTA5 GSTP1 GSTO1 GPX1 GPX2 GSTT1 GSTZ1 GPX5 GPX3 GGT1 GLO1 LTC4S GPX4 HPGDS GSTM2 HAGH GSTA2 MGST3 TXNDC12 GSTA1 MGST1 ESD GLRX GSTM5 GPX6 GPX8 GPX7	2937 2936 2944 373156 2940 2947 2941 2948 221357 2950 9446 2876 2877 2952 2954 2880 2878 2678 2739 4056 2879 27306 2946 3029 2939 4259 51060 2938 4257 2098 2745 2949 257202 493869 2882 4258 119391 51022	Human	38
DB01201	rpoC	888177	Mycobacterium tuberculosis	1
DB00128	ASPA ASNS SLC25A12 SLC25A13 GOT2 ACY1 ASPH GOT1 ASS1 DARS ASS1 SLC1A1 PAICS CAD ADSS DARS2 ASRGL1 ADSSL1 ACY3 RNASE1	443 440 8604 10165 2806 95 444 2805 445 1615 445 6505 10606 790 159 55157 80150 122622 91703 6035 4069	Human	21
DB00144	SCARB1 PISD ATP8A1 PTDSS1 PRKCA DGKG DGKD PTDSS2 SMPD4 SMPD3	949 23761 10396 9791 5578 1608 8527 81490 55627 55512	Human	10
DB02187	HSD17B1	3292	Human	1
DB00192	SCN5A	6331	Human	1
DB00243	SCN5A SCN9A	6331 6335	Human	2
DB00379	SCN5A AHR	6331 196	Human	2
DB00564	SCN5A	6331	Human	1
DB00680	SCN5A	6331	Human	1
DB00750	SCN5A	6331	Human	1
DB00754	SCN5A	6331	Human	1
DB00776	SCN5A	6331	Human	1
DB00868	SCN5A	6331	Human	1
DB01035	SCN5A DNMT1	6331 1786	Human	2
DB01056	SCN5A	6331	Human	1
DB01195	SCN5A SCN4A	6331 6329	Human	2
DB01228	SCN5A	6331	Human	1
DB01320	SCN5A	6331	Human	1
DB01426	SCN5A	6331	Human	1
DB01429	SCN5A CALM1	6331 805	Human	2
DB00007	GNRHR	2798	Human	1
DB00106	GNRHR	2798	Human	1
DB00644	GNRHR	2798	Human	1
DB00666	GNRHR	2798	Human	1
DB06699	GNRHR	2798	Human	1
DB00149	LARS2 LCMT1 BCAT1 LCMT2 BCAT2 LARS	23395 51451 586 9836 587 51520	Human	6
DB00102	PDGFRB PDGFRA A2M	5159 5156 2	Human	3
DB04942	RARB RARA	5915 5914	Human	2
DB00222	ABCC8 KCNJ1 KCNJ11	6833 3758 3767	Human	3
DB00672	ABCC8	6833	Human	1
DB00731	ABCC8 PPARG	6833 5468	Human	2
DB00912	ABCC8 PPARG	6833 5468	Human	2

DB01067	ABCC8 PPARG	6833 5468	Human	2
DB01124	ABCC8 KCNJ1	6833 3758	Human	2
DB01251	ABCC8 KCNJ8	6833 3764	Human	2
DB01252	ABCC8 PPARG	6833 5468	Human	2
DB01382	ABCC8 KCNJ1	6833 3758	Human	2
DB00009	PLG SERPINE1 FGA PLAUR	5340 5054 2243 5329	Human	4
DB00013	PLG SERPINE1 PLAUR PLAU LRP2 SERPINB2 PLAT ST14 SERPINA5 NID1	5340 5054 5329 5328 4036 5055 5327 6768 5104 4811	Human	10
DB00015	PLG SERPINE1 FGA PLAUR	5340 5054 2243 5329	Human	4
DB00029	PLG SERPINE1 FGA PLAUR	5340 5054 2243 5329	Human	4
DB00031	PLG SERPINE1 FGA PLAUR SERPINB2 CANX ANXA2 CLEC3B KRT8 CALR LRP1	5340 5054 2243 5329 5055 821 302 7123 3856 811 4035	Human	11
DB00086	PLG F2R	5340 2149	Human	2
DB00302	PLG	5340	Human	1
DB00513	PLG PLAT	5340 5327	Human	2
DB06692	PLG KLK1 PRSS1	5340 3816 5644	Human	3
DB00412	PPARG ACSL4	5468 2182	Human	2
DB01132	PPARG	5468	Human	1
DB00025	F10 F9 CANX VWF HSPA5 CALR LRP1 PHYH ASGR2 LMAN1 MCFD2	2159 2158 821 7450 3309 811 4035 5264 433 3998 90411	Human	11
DB00036	F10 F7 GGCX TFPI F3 HPN	2159 2155 2677 7035 2152 3249	Human	6
DB00569	F10 SERPINC1	2159 462	Human	2
DB01109	F10 SERPINC1 SELP	2159 462 6403	Human	3
DB01225	F10 SERPINC1	2159 462	Human	2
DB06228	F10	2159	Human	1
DB06605	F10	2159	Human	1
DB00181	GABBR1 GABBR2	2550 9568	Human	2
DB00837	GABBR1 GABRA1	2550 2554	Human	2
DB01080	GABBR1 ABAT	2550 18	Human	2
DB00228	ATP2C1 KCNN4 ATP5D GRIA1 KCNA1 GABRA1 GABRA2 GABRA3 GABRA4 GABRA5 GABRA6 GABRB1 GABRB2 GABRB3 GABRD GABRE GABRG1 GABRG2 GABRG3 GABRP GABRQ GLRA1 GLRB	27032 3783 513 2890 3736 2554 2555 2556 2557 2558 2559 2560 2561 2562 2563 2564 2565 2567 2568 2568 55879 2741 2743	Human	23
DB00753	ATP2C1 CALM1 GLRA1 ATP5D GRIA1 KCNA1 GABRA1 GABRA1 GABRA2 GABRA3 GABRA4 GABRA5 GABRA6 GABRB1 GABRB2 GABRB3 GABRD GABRE GABRG1 GABRG2 GABRG3 GABRP	27032 805 2741 513 2890 3736 2554 2554 2555 2556 2557 2558 2559 2560 2561 2562 2563 2564 2565 2567 2568 2568 55879	Human	23

DB01028	ATP2C1 GLRA1 ATP5D MT-ND1 GRIA1 KCNA1 GABRA1 GABRA1 GABRA2 GABRA3 GABRA4 GABRA5 GABRA6 GABRB1 GABRB2 GABRB3 GABRD GABRE GABRG1 GABRG2 GABRG3 GABRP	27032 2741 513 4535 2890 3736 2554 2554 2555 2556 2557 2558 2559 2560 2561 2562 2563 2564 2565 2567 2568 2568 55879	Human	23
DB01159	ATP2C1 KCNN4 GLRA1 ATP5D MT-ND1 GRIN3B KCNMA1 GRIN3A GRIN2A GABRA1 NPSR1 KCNJ3 KCNJ6 GNG2 RHO KCNK3 KCNK9 GABRA1 GABRA2 GABRA3 GABRA4 GABRA5 GABRA6 GABRB1 GABRB2 GABRB3 GABRD GABRE GABRG1 GABRG2	27032 3783 2741 513 4535 116444 3778 116443 2903 2554 387129 3760 3763 54331 6010 3777 51305 2554 2555 2556 2557 2558 2559 2560 2561 2562 2563 2564 2565 2567 2568 2568 55879	Human	33
DB01189	ATP2C1 GLRA1 ATP5D MT-ND1 GRIA1 KCNA1 GABRA1 GABRA2 GABRA3 GABRA4 GABRA5 GABRA6 GABRB1 GABRB2 GABRB3 GABRD GABRE GABRG1 GABRG2	27032 2741 513 4535 2890 3736 2554 2555 2556 2557 2558 2559 2560 2561 2562 2563 2564 2565 2567 2568 2568 55879	Human	22
DB01236	ATP2C1 GLRA1 ATP5D MT-ND1 GRIA1 KCNA1 GABRA1 GABRA1 GABRA2 GABRA3 GABRA4 GABRA5 GABRA6 GABRB1 GABRB2 GABRB3 GABRD GABRE GABRG1 GABRG2 GABRG3 GABRP	27032 2741 513 4535 2890 3736 2554 2554 2555 2556 2557 2558 2559 2560 2561 2562 2563 2564 2565 2567 2568 2568 55879	Human	23
DB00174	ASNS SLC1A5 SLC38A3 NARS ASRGL1 NARS2	440 6510 10991 4677 80150 79731	Human	6
DB01087	NQO2 KRT7	4835 3855	Human	2
DB00178	ACE	1636	Human	1
DB00492	ACE	1636	Human	1
DB00519	ACE	1636	Human	1
DB00542	ACE	1636	Human	1
DB00584	ACE	1636	Human	1
DB00616	ACE MME	1636 4311	Human	2
DB00691	ACE ACE2	1636 59272	Human	2
DB00722	ACE ACE2	1636 59272	Human	2
DB00790	ACE	1636	Human	1
DB00881	ACE	1636	Human	1
DB01180	ACE	1636	Human	1
DB01340	ACE	1636	Human	1
DB01348	ACE	1636	Human	1
DB00161	VARS BCAT1 PCCB	7407 586 5096	Human	3
DB00898	ADH1A ADH1B GLRA1 ADH1C GRIN3A GLRA2 GABRA1	124 125 2741 126 116443 2742 2554	Human	7
DB01213	ADH1A ADH1B ADH1C CAT	124 125 126 847	Human	4
DB00150	WARS WARS2	7453 10352	Human	2
DB00606	FXYD2 CA1 CA2 CA4	486 759 760 762	Human	4

DB00135	YARS YARS2 TH TAT	8565 51067 7054 6898	Human	4
DB08827	MTTP	4547	Human	1
DB00196	ERG11	3641571	Yeast	1
DB00239	ERG11 ERG7	3641571 3635761	Yeast	2
DB00251	ERG11	3641571	Yeast	1
DB00257	ERG11 KCNN4	3641571 3783	Yeast	2
DB00582	ERG11	3641571	Yeast	1
DB01007	ERG11 CYP51A1	3641571 1595	Yeast	2
DB01026	ERG11	3641571	Yeast	1
DB01127	ERG11	3641571	Yeast	1
DB01153	ERG11	3641571	Yeast	1
DB01263	ERG11	3641571	Yeast	1
DB04794	ERG11 CYP2B6	3641571 1555	Yeast	2
DB08933	ERG11	3641571	Yeast	1
DB00156	TARS THNSL1 TARS2	6897 79896 80222	Human	3
DB00200	TCN1 MMAA AMN MMAB MTR MTRR MUT CUBN MMACHC	6947 166785 81693 326625 4548 4552 4594 8029 25974	Human	9
DB05269	LPL	4023	Human	1
DB06439	LPL	4023	Human	1
DB00824	ADORA2B PDE4B PDE4A	136 5142 5141	Human	3
DB00648	FDX1 CYP11B1	2230 1584	Human	2
DB01367	BCL2 MAOB	596 4129	Human	2
DB00179	ALOX5	240	Human	1
DB00471	ALOX5 CYSLTR1	240 10800	Human	2
DB00744	ALOX5	240	Human	1
DB00906	SLC6A1	6529	Human	1
DB00242	PNP RRM1 POLA1 RRM2 RRM2B POLE POLE2 POLE4	4860 6240 5422 6241 50484 5426 5427 56655 54107	Human	9
DB00900	PNP	4860	Human	1
DB00548	TYR trxB polA SRD5A2 AKR1D1	7299 1120737 948356 6716 6718	Human	5
DB00600	TYR	7299	Human	1
DB01055	TYR SHMT1 CCL2	7299 6470 6347	Human	3
DB00780	ABAT GPT AOC3 GPT2 MAOB MAOA	18 2875 8639 84706 4129 4128	Human	6
DB00331	PRKAB1	5564	Human	1
DB00615	rpoB rpoA HSP90AA1 HSP90B1 rpoC	948488 947794 3320 7184 948487	Escherichia coli (strain K12)	5
DB01045	rpoB NR1I2 rpoC	948488 8856 948487	Escherichia coli (strain K12)	3
DB01220	rpoB NR1I2	948488 8856	Escherichia coli (strain K12)	2
DB00811	NT5C2 ENPP1 L IMPDH1 ADK	22978 5167 935189 3614 132	Human	5
DB00480	PTGS2 CDH5 TNFSF11	5743 1003 8600 51185	Human	4
DB00482	PTGS2 PDPK1	5743 5170	Human	2
DB01041	PTGS2 TNF FGFR2 NFKB1	5743 7124 2263 4790 51185	Human	5
DB01404	PTGS2 IL6 AHR	5743 3569 196	Human	3

DB01628	PTGS2	5743	Human	1
DB08910	PTGS2 TNF	5743 7124 51185	Human	3
DB00232	CA1 CA2 SLC12A1 CA4	759 760 6557 762	Human	4
DB00311	CA1 CA2 CA4 CA7	759 760 762 766	Human	4
DB00381	CA1 CACNA1C CACNA2D1 CACNA1B CACNB2 CACNA1S CACNA1D CACNA2D3	759 775 781 774 783 779 776 55799	Human	8
DB00423	CA1	759	Human	1
DB00436	CA1 CA2 SLC12A3 CA4	759 760 6559 762 3778	Human	5
DB00562	CA1 CA2 SLC12A3 CA4 CA12	759 760 6559 762 771 768	Human	6
DB00703	CA1 CA2 CA4 CA7	759 760 762 766	Human	4
DB00774	CA1 CA2 SLC12A1 CA4 KCNMA1 ATP1A1 CA12 CA9	759 760 6557 762 3778 476 771 768	Human	8
DB00819	CA1 CA2 CA4 AQP1 CA3 CA7 CA14	759 760 762 358 761 766 23632	Human	7
DB00869	CA1 CA2 CA4	759 760 762	Human	3
DB00880	CA1 CA2 SLC12A3 CA4	759 760 6559 762	Human	4
DB00999	CA1 CA2 SLC12A3 CA4 KCNMA1 CA12 CA9	759 760 6559 762 3778 771 768	Human	7
DB01021	CA1 CA2 SLC12A1 CA4 ATP1A1	759 760 6557 762 476	Human	5
DB01031	CA1 CA2	759 760	Human	2
DB01119	CA1 CA2 SLC12A3 KCNMA1 KCNJ11 ATP1A1	759 760 6559 3778 3767 476	Human	6
DB01144	CA1 CA2 CA4 CA7	759 760 762 766	Human	4
DB01194	CA1 CA2 CA4 CA5A	759 760 762 763	Human	4
DB01325	CA1 SLC12A2 CA2 SLC12A3 SLC12A1	759 6558 760 6559 6557	Human	5
DB00212	REN	5972	Human	1
DB01258	REN	5972	Human	1
DB00158	FOLR2 FOLR3	2350 2352	Human	2
DB00075	CD3E FCGR1A FCGR3B C1S C1R C1QA C1QB C1QC FCGR3A FCGR2A FCGR2B CD3D CD3G CD247	916 2209 2215 716 715 712 713 714 2214 2212 2213 915 917 919	Human	14
DB00452	rpsL CXCR4	947845 7852	Escherichia coli (strain K12)	2
DB00479	rpsL	947845	Escherichia coli (strain K12)	1
DB00684	rpsL	947845	Escherichia coli (strain K12)	1
DB00798	rpsL LRP2	947845 4036	Escherichia coli (strain K12)	2
DB00919	rpsL	947845	Escherichia coli (strain K12)	1

DB00955	rpsL	947845	Escherichia coli (strain K12)	1
DB00994	rpsL	947845	Escherichia coli (strain K12)	1
DB01082	rpsL	947845	Escherichia coli (strain K12)	1
DB01172	rpsL	947845	Escherichia coli (strain K12)	1
DB06696	rpsL	947845	Escherichia coli (strain K12)	1
DB00407	SERPINC1 SERPIND1	462 3053	Human	2
DB06271	SERPINC1 SERPIND1	462 3053	Human	2
DB06822	SERPINC1 ITGA4 CXCL12	462 3676 6387	Human	3
DB08813	SERPINC1 FOS SELP	462 2353 6403 4609	Human	4
DB00217	ADRA2A ADRA2C ADRA2B KCNJ1	150 152 151 3758	Human	4
DB00320	ADRA2A HTR2B HTR1D HTR1B	150 3357 3352 3351	Human	4
DB00370	ADRA2A HTR3A HRH1 HTR2A HTR2C OPRK1	150 3359 3269 3356 3358 4986	Human	6
DB00449	ADRA2A ADRA1A ADRB2 BCHE	150 148 154 590	Human	4
DB00484	ADRA2A ADRA2C ADRA2B	150 152 151	Human	3
DB00575	ADRA2A ADRA2C ADRA2B	150 152 151	Human	3
DB00629	ADRA2A	150	Human	1
DB00633	ADRA2A	150	Human	1
DB00656	ADRA2A HTR1A HRH1 HTR2A ADRA1A HTR2C SLC6A4	150 3350 3269 3356 148 3358 6532	Human	7
DB00692	ADRA2A ADRA1A	150 148	Human	2
DB00696	ADRA2A HTR2A SLC6A2 ADRA1A ADRA2B ADRA1B HTR1D ADRA1D DRD2 HTR1B	150 3356 6530 148 151 147 3352 146 1813 3351	Human	10
DB00697	ADRA2A ADRA2C ADRA2B NISCH	150 152 151 11188	Human	4
DB00797	ADRA2A HRH1 ADRA1A	150 3269 148	Human	3
DB00925	ADRA2A ADRA2C CALM1 ADRA1A ADRA2B ADRB2	150 152 805 148 151 154	Human	6
DB00935	ADRA2A ADRA1A	150 148	Human	2
DB00964	ADRA2A ADRA1A	150 148	Human	2
DB00968	ADRA2A	150	Human	1
DB01018	ADRA2A	150	Human	1
DB01149	ADRA2A HTR1A HTR2A SLC6A2 ADRA1A HTR2C ADRA1B SLC6A3 SLC6A4	150 3350 3356 6530 148 3358 147 6531 6532	Human	9
DB01392	ADRA2A HTR1A ADRA2C HTR2A HTR2C ADRA2B DRD3 HTR1D DRD2 HTR1B	150 3350 152 3356 3358 151 1814 3352 1813 3351	Human	10
DB04948	ADRA2A	150	Human	1

DB06148	ADRA2A HRH1 HTR2A SLC6A2 HTR2C SLC6A4	150 3269 3356 6530 3358 6532	Human	6
DB06623	ADRA2A	150	Human	1
DB06694	ADRA2A ADRA2C ADRA1A ADRA2B ADRA1B ADRA1D	150 152 148 151 147 146	Human	6
DB06711	ADRA2A ADRA1A	150 148	Human	2
DB08815	ADRA2A HTR1A ADRA2C HTR2A HTR7 DRD2	150 3350 152 3356 3363 1813	Human	6
DB00216	HTR1A HTR2B HTR1F HTR1E HTR7 HTR1D HTR1B	3350 3357 3355 3354 3363 3352 3351	Human	7
DB00247	HTR1A HTR2B HTR2A HTR2C HTR7	3350 3357 3356 3358 3363	Human	5
DB00315	HTR1A HTR1F HTR1D HTR1B	3350 3355 3352 3351	Human	4
DB00490	HTR1A DRD2	3350 1813	Human	2
DB00669	HTR1A HTR1F HTR1D HTR1B	3350 3355 3352 3351	Human	4
DB00952	HTR1A HTR1F HTR1D HTR1B	3350 3355 3352 3351	Human	4
DB01616	HTR1A	3350	Human	1
DB06684	HTR1A	3350	Human	1
DB00766	blaZ	8655740	Staphylococcus aureus	1
DB01598	blaZ mrcB mrcA pbpC mrdA	8655740 944843 947907 940139 12930918	Staphylococcus aureus	5
DB00393	CACNB1 CACNA1C NR3C2 CACNB2 CACNA1S CACNB4 CACNA1F CACNB3 CACNA1D AHR	782 775 4306 783 779 785 778 784 776 196	Human	10
DB00653	CACNB1 CACNA1C CACNG1 CACNA2D1 CACNB2 CACNA1S	782 775 786 781 783 779	Human	6
DB00299	UL30	2703462	HHV-1	1
DB00426	UL30	2703462	HHV-1	1
DB00529	UL30	2703462	HHV-1	1
DB00577	UL30	2703462	HHV-1	1
DB00787	UL30 TK	2703462 2703374	HHV-1	2
DB01004	UL30 TK	2703462 2703374	HHV-1	2
DB00377	HTR3A	3359	Human	1
DB00728	HTR3A CHRM2 CHRNA2	3359 1129 1135	Human	3
DB00757	HTR3A	3359	Human	1
DB00889	HTR3A	3359	Human	1
DB00969	HTR3A	3359	Human	1
DB01043	HTR3A GRIN2B GRIN3A	3359 2904 116443 2903	Human	4
DB01199	HTR3A ACHE CHRNA2	3359 43 1135	Human	3
DB06204	HTR3A OPRD1 SLC6A2 OPRK1 SLC6A4 OPRM1	3359 4985 6530 4986 6532 4988	Human	6
DB00279	THRB THRA	7068 7067	Human	2
DB00451	THRB THRA	7068 7067	Human	2
DB00509	THRB TPO THRA	7068 7173 7067	Human	3
DB01583	THRB THRA	7068 7067	Human	2
DB00021	SCTR	6344	Human	1
DB00115	MMAA MTR MTRR MUT MMACHC MTHFR	166785 4548 4552 4594 25974 4524	Human	6

DB00695	CA2 SLC12A1	760 6557	Human	2
DB00293	TYMS FPGS	7298 2356	Human	2
DB00322	TYMS	7298	Human	1
DB00432	TYMS	7298	Human	1
DB00440	TYMS DHFR	7298 1719	Human	2
DB00441	TYMS RRM1 CMPK1	7298 6240 51727	Human	3
DB00544	TYMS	7298	Human	1
DB00642	TYMS DHFR GART ATIC	7298 1719 2618 471	Human	4
DB00650	TYMS	7298	Human	1
DB01101	TYMS	7298	Human	1
DB06813	TYMS DHFR	7298 1719	Human	2
DB00631	RRM1 POLA1	6240 5422	Human	2
DB01005	RRM1	6240	Human	1
DB01073	RRM1 POLA1 DCK	6240 5422 1633	Human	3
DB00686	FGF4 FGF1 FGF2	2249 2246 2247	Human	3
DB00980	MTNR1B MTNR1A	4544 4543	Human	2
DB06594	MTNR1B MTNR1A HTR2C	4544 4543 3358	Human	3
DB00635	HSD11B1 NR3C1	3290 2908	Human	2
DB00205	DHFR	1719	Human	1
DB00563	DHFR	1719	Human	1
DB01131	DHFR	1719	Human	1
DB01157	DHFR	1719	Human	1
DB00609	inhA katG	886523 885638	Mycobacterium tuberculosis	2
DB00951	inhA katG	886523 885638	Mycobacterium tuberculosis	2
DB08604	inhA fabI fabI fabI	886523 899156 945870 2859621	Mycobacterium tuberculosis	4
DB05812	CYP17A1	1586	Human	1
DB00213	ATP4A	495	Human	1
DB00338	ATP4A	495	Human	1
DB00448	ATP4A	495	Human	1
DB00736	ATP4A	495	Human	1
DB01129	ATP4A	495	Human	1
DB00230	CACNA1A	773	Human	1
DB00836	CACNA1A CALM1 OPRD1 OPRK1 OPRM1 POMC	773 805 4985 4986 4988 5443	Human	6
DB01244	CACNA1A CALM1 KCNQ1 ATP1A1 CACNA1H TNNC1 CACNA2D2 PDE1B PDE1A	773 805 3784 476 8912 7134 9254 5153 5136	Human	9
DB00454	GRIN1 GRIN2D GRIN2B GRIN2C OPRK1 GRIN2A	2902 2906 2904 2905 4986 2903	Human	6
DB04896	GRIN1 SLC6A2 SLC6A4	2902 6530 6532	Human	3

DB00218	gyrA parC TOP2A	950199 950391 7153	Haemophilus influenzae (strain ATCC 51907 / DSM 11121 / KW20 / Rd)	3
DB00467	gyrA parC TOP2A	950199 950391 7153	Haemophilus influenzae (strain ATCC 51907 / DSM 11121 / KW20 / Rd)	3
DB00487	gyrA parC TOP2A	950199 950391 7153	Haemophilus influenzae (strain ATCC 51907 / DSM 11121 / KW20 / Rd)	3
DB00537	gyrA parC TOP2A	950199 950391 7153	Haemophilus influenzae (strain ATCC 51907 / DSM 11121 / KW20 / Rd)	3
DB00685	gyrA parC TOP2A	950199 950391 7153	Haemophilus influenzae (strain ATCC 51907 / DSM 11121 / KW20 / Rd)	3
DB00827	gyrA	950199	Haemophilus influenzae (strain ATCC 51907 / DSM 11121 / KW20 / Rd)	1
DB00978	gyrA parC TOP2A	950199 950391 7153	Haemophilus influenzae (strain ATCC 51907 / DSM 11121 / KW20 / Rd)	3
DB01059	gyrA parC TOP2A	950199 950391 7153	Haemophilus influenzae (strain ATCC 51907 / DSM 11121 / KW20 / Rd)	3

DB01137	gyrA parC TOP2A	950199 950391 7153	Haemophilus influenzae (strain ATCC 51907 / DSM 11121 / KW20 / Rd)	3
DB01155	gyrA parC	950199 950391	Haemophilus influenzae (strain ATCC 51907 / DSM 11121 / KW20 / Rd)	2
DB01165	gyrA parC TOP2A	950199 950391 7153	Haemophilus influenzae (strain ATCC 51907 / DSM 11121 / KW20 / Rd)	3
DB01208	gyrA parC TOP2A	950199 950391 7153	Haemophilus influenzae (strain ATCC 51907 / DSM 11121 / KW20 / Rd)	3
DB04576	gyrA parC TOP2A	950199 950391 7153	Haemophilus influenzae (strain ATCC 51907 / DSM 11121 / KW20 / Rd)	3
DB06771	gyrA parC gyrA parC	950199 950391 931733 930805	Haemophilus influenzae (strain ATCC 51907 / DSM 11121 / KW20 / Rd)	4
DB00929	PTGER2 PTGER3 PTGER4	5732 5733 5734	Human	3
DB08875	KDR MET RET	3791 4233 5979	Human	3
DB00140	RFK BLVRB ribE	55312 645 945848	Human	3
DB00130	PPAT CTPS GLUL	5471 1503 2752	Human	3
DB00091	CAMLG PPIA PPP3R2	819 5478 5535	Human	3
DB00464	PROC PROS1	5624 5627	Human	2
DB00241	GABRA2 GABRA5 GABRA4 GABRA3 GABRA6 GABRA1 GRIK2 GRIA2 CHRNA4 CHRNA7 GABRA1 GABRA2 GABRA3 GABRA4 GABRA5 GABRA6 GABRB1 GABRB2 GABRB3 GABRD GABRE GABRG1 GABRG2 GABRG3 GABRP	2555 2558 2557 2556 2559 2554 2898 2891 1137 1139 2554 2555 2556 2557 2558 2559 2560 2561 2562 2563 2564 2565 2567 2568 2568 55879	Human	26

DB00306	GABRA2 GABRA5 GABRA4 GABRA3 GABRA6 GABRA1 GRIK2 GRIA2 CHRNA4 CHRNA7 GABRA1 GABRA2 GABRA3 GABRA4 GABRA5 GABRA6 GABRB1 GABRB2 GABRB3 GABRD GABRE GABRG1 GABRG2 GABRG3 GABRP	2555 2558 2557 2556 2559 2554 2898 2891 1137 1139 2554 2555 2556 2557 2558 2559 2560 2561 2562 2563 2564 2565 2567 2568 2568 55879	Human	26
DB00312	GABRA2 GABRA5 GABRA4 GABRA3 GABRA6 GABRA1 GRIK2 GRIA2 CHRNA4 CHRNA7 GABRA1 GABRA2 GABRA3 GABRA4 GABRA5 GABRA6 GABRB1 GABRB2 GABRB3 GABRD GABRE GABRG1 GABRG2 GABRG3 GABRP	2555 2558 2557 2556 2559 2554 2898 2891 1137 1139 2554 2555 2556 2557 2558 2559 2560 2561 2562 2563 2564 2565 2567 2568 2568 55879	Human	26
DB00371	GABRA2 GABRA5 GABRA4 GABRA3 GABRA6 GABRA1 GABRA1 GABRA2 GABRA3 GABRA4 GABRA5 GABRA6 GABRB1 GABRB2 GABRB3 GABRD GABRE GABRG1 GABRG2 GABRG3 GABRP	2555 2558 2557 2556 2559 2554 2554 2555 2556 2557 2558 2559 2560 2561 2562 2563 2564 2565 2567 2568 2568 55879	Human	22
DB00402	GABRA2 GABRA5 GABRA3 TSPO GABRA1 GABRA1 GABRA2 GABRA3 GABRA4 GABRA5 GABRA6 GABRB1 GABRB2 GABRB3 GABRD GABRE GABRG1 GABRG2	2555 2558 2556 706 2554 2554 2555 2556 2557 2558 2559 2560 2561 2562 2563 2564 2565 2567 2568 2568 55879	Human	21
DB00418	GABRA2 GABRA5 GABRA4 GABRA3 GABRA6 GABRA1 GRIK2 GRIA2 CHRNA4 CHRNA7	2555 2558 2557 2556 2559 2554 2898 2891 1137 1139	Human	10
DB00425	GABRA2 GABRA3 GABRA1	2555 2556 2554	Human	3
DB00463	GABRA2 GABRA5 GABRA4 GABRA3 GABRA6 GABRA1 GRIK2 GRIA2 CHRNA4 CHRNA7 GABRA1 GABRA2 GABRA3 GABRA4 GABRA5 GABRA6 GABRB1 GABRB2 GABRB3 GABRD GABRE GABRG1 GABRG2 GABRG3 GABRP	2555 2558 2557 2556 2559 2554 2898 2891 1137 1139 2554 2555 2556 2557 2558 2559 2560 2561 2562 2563 2564 2565 2567 2568 2568 55879	Human	26
DB00599	GABRA2 GABRA5 GABRA4 GABRA3 GABRA6 GABRA1 GRIK2 GRIA2 CHRNA4 FAAH	2555 2558 2557 2556 2559 2554 2898 2891 1137 2166 1139	Human	11
DB00794	GABRA2 GABRA5 GABRA4 GABRA3 GABRA6 GABRA1 GRIK2 GRIA2 CHRNA4 CHRNA7 GABRA1 GABRA2 GABRA3 GABRA4 GABRA5 GABRA6 GABRB1 GABRB2 GABRB3 GABRD GABRE GABRG1 GABRG2 GABRG3 GABRP	2555 2558 2557 2556 2559 2554 2898 2891 1137 1139 2554 2555 2556 2557 2558 2559 2560 2561 2562 2563 2564 2565 2567 2568 2568 55879	Human	26

DB00849	GABRA2 GABRA5 GABRA4 GABRA3 GABRA6 GABRA1 GRIK2 GRIA2 CHRNA4 CHRNA7	2555 2558 2557 2556 2559 2554 2898 2891 1137 1139	Human	10
DB01174	GABRA2 GABRA5 GABRA4 GABRA3 GABRA6 GABRA1 GRIK2 GRIA2 CHRNA4 CHRNA7	2555 2558 2557 2556 2559 2554 2898 2891 1137 1139	Human	10
DB01198	GABRA2 GABRA5 GABRA3 TSPO GABRA1	2555 2558 2556 706 2554	Human	5
DB01351	GABRA2 GABRA5 GABRA4 GABRA3 GABRA6 GABRA1 GRIK2 GRIA2 CHRNA4 CHRNA7	2555 2558 2557 2556 2559 2554 2898 2891 1137 1139	Human	10
DB01352	GABRA2 GABRA5 GABRA4 GABRA3 GABRA6 GABRA1 GRIK2 GRIA2 CHRNA4 CHRNA7	2555 2558 2557 2556 2559 2554 2898 2891 1137 1139	Human	10
DB01353	GABRA2 GABRA5 GABRA4 GABRA3 GABRA6 GABRA1 GRIK2 GRIA2 CHRNA4 CHRNA7	2555 2558 2557 2556 2559 2554 2898 2891 1137 1139	Human	10
DB01354	GABRA2 GABRA5 GABRA4 GABRA3 GABRA6 GABRA1 GRIK2 GRIA2 CHRNA4 CHRNA7	2555 2558 2557 2556 2559 2554 2898 2891 1137 1139	Human	10
DB01355	GABRA2 GABRA5 GABRA4 GABRA3 GABRA6 GABRA1 GRIK2 GRIA2 CHRNA4 CHRNA7	2555 2558 2557 2556 2559 2554 2898 2891 1137 1139	Human	10
DB01544	GABRA2 GABRA5 GABRA4 GABRA3 TSPO GABRA6	2555 2558 2557 2556 706 2559	Human	6
DB01097	DHODH PTK2B AHR	1723 2185 196	Human	3
DB01117	DHODH PFF0160c MT-CYB	1723 3885966 2655541	Human	3
DB08880	DHODH	1723	Human	1
DB00673	TACR1	6869	Human	1
DB01221	TACR1 GRIN3A DRD2	6869 116443 1813	Human	3
DB04894	TACR1 SSTR5 SSTR2	6869 6755 6752	Human	3
DB00008	IFNAR2 IFNAR1	3455 3454	Human	2
DB00011	IFNAR2 IFNAR1	3455 3454	Human	2
DB00018	IFNAR2 IFNAR1	3455 3454	Human	2
DB00022	IFNAR2 IFNAR1	3455 3454	Human	2
DB00034	IFNAR2 IFNAR1	3455 3454	Human	2
DB00060	IFNAR2 IFNAR1	3455 3454	Human	2
DB00068	IFNAR2 IFNAR1	3455 3454	Human	2
DB00069	IFNAR2 IFNAR1	3455 3454	Human	2
DB00105	IFNAR2 IFNAR1	3455 3454	Human	2
DB00066	FSHR	2492	Human	1
DB00094	FSHR	2492	Human	1
DB00083	SNAP25 RHOB	6616 388	Human	2
DB01242	HTR2B HTR2A SLC6A2 HTR2C SLC6A4 GSTP1 snf	3357 3356 6530 3358 6532 2950 1193843	Human	7
DB00019	ELANE CSF3R	1991 1441	Human	2
DB00058	ELANE	1991	Human	1
DB00099	ELANE CSF3R	1991 1441	Human	2
DB00168	TAS1R2 TRPV1	80834 7442	Human	2
DB00403	CCKAR	886	Human	1

DB01022	GGCX BGLAP	2677 632	Human	2
DB01125	GGCX	2677	Human	1
DB00127	ODC1 SMOX SMS	4953 54498 6611	Human	3
DB00184	CHRNA10 CHRNA2 CHRNA4 CHRNB2 CHRNA7 CHRNA9 CHRNB4 CHRNA3 CHRNA5 CHRNA6 CHRNB3	57053 1135 1137 1141 1139 55584 1143 1136 1138 8973 1142	Human	11
DB00333	CHRNA10 OPRD1 GRIN3A	57053 4985 116443 4988	Human	4
DB00674	CHRNA10 ACHE CHRNA2 CHRNA4 CHRNB2 BCHE CHRNA7 CHRNA9 CHRNB4 CHRNA3 CHRNA5 CHRNA6 CHRNB3 CHRNA1 CHRNB1 CHRND CHRNG CHRNE	57053 43 1135 1137 1141 590 1139 55584 1143 1136 1138 8973 1142 1134 1140 1144 1146 1145	Human	18
DB01090	CHRNA10 CHRNB4 CHRNA3	57053 1143 1136	Human	3
DB01116	CHRNA10	57053	Human	1
DB00602	GLRA3 GABRB3	8001 2562	Human	2
DB00468	KCNN4 GP9	3783 2815	Human	2
DB00038	IL11RA	3590	Human	1
DB00949	GRIN2B GRIN3A GRIN2A	2904 116443 2903	Human	3
DB00831	CALM1 ADRA1A CALY DRD2 TNNC1 S100A4	805 148 50632 1813 7134 6275	Human	6
DB01023	CALM1 CACNA1C NR3C2 CACNA2D1 CACNA1H TNNC1 CACNB2 CACNA1S CACNA1D CACNA2D2 PDE1B PDE1A	805 775 4306 781 8912 7134 783 779 776 9254 5153 5136 7125	Human	13
DB01115	CALM1 CACNA1C CACNA2D1 KCNA1 CACNA1H CACNB2 CACNA1S CACNA1D	805 775 781 3736 8912 783 779 776	Human	8
DB00524	SLC12A3	6559	Human	1
DB01324	SLC12A3	6559	Human	1
DB00295	OPRD1 OPRK1 OPRM1	4985 4986 4988	Human	3
DB00318	OPRD1 OPRK1 OPRM1	4985 4986 4988	Human	3
DB00327	OPRD1 OPRK1 OPRM1	4985 4986 4988	Human	3
DB00497	OPRD1 OPRK1 OPRM1	4985 4986 4988	Human	3
DB00611	OPRD1 OPRK1 OPRM1	4985 4986 4988	Human	3
DB00647	OPRD1 OPRK1 OPRM1	4985 4986 4988	Human	3
DB00704	OPRD1 OPRK1 OPRM1	4985 4986 4988	Human	3
DB00708	OPRD1 OPRK1 OPRM1	4985 4986 4988	Human	3
DB00813	OPRD1 OPRK1 OPRM1	4985 4986 4988	Human	3
DB00844	OPRD1 OPRK1 OPRM1	4985 4986 4988	Human	3
DB00854	OPRD1 OPRK1 OPRM1	4985 4986 4988	Human	3
DB00899	OPRD1 OPRK1 OPRM1	4985 4986 4988	Human	3
DB00921	OPRD1 OPRK1 OPRM1	4985 4986 4988	Human	3
DB00956	OPRD1 OPRM1	4985 4988	Human	2
DB01081	OPRD1 OPRM1	4985 4988	Human	2
DB01192	OPRD1 OPRM1	4985 4988	Human	2
DB01452	OPRD1 OPRK1 OPRM1	4985 4986 4988	Human	3
DB06274	OPRD1 OPRK1 OPRM1	4985 4986 4988	Human	3

DB06738	OPRD1 OPRK1 OPRM1	4985 4986 4988	Human	3
DB00288	ANXA1 NR3C1	301 2908	Human	2
DB00741	ANXA1 NR3C1	301 2908	Human	2
DB00122	ACHE PCYT1B PCYT1A PLD2 BCHE PLD1 PHOSPHO1	43 9468 5130 5338 590 5337 162466	Human	7
DB00382	ACHE BCHE	43 590	Human	2
DB00483	ACHE CHRM2 CHRNA2	43 1129 1135	Human	3
DB00545	ACHE BCHE	43 590	Human	2
DB00677	ACHE BCHE	43 590	Human	2
DB00733	ACHE BCHE	43 590	Human	2
DB00843	ACHE HTR2A	43 3356	Human	2
DB00944	ACHE BCHE	43 590	Human	2
DB00981	ACHE	43	Human	1
DB00989	ACHE BCHE	43 590	Human	2
DB01010	ACHE BCHE	43 590	Human	2
DB01122	ACHE	43	Human	1
DB01245	ACHE CHRNA2	43 1135	Human	2
DB01400	ACHE	43	Human	1
DB01169	AKT1 MAPK1 CCND1 JUN MAPK3 IKBKB TXNRD1	207 5594 595 3725 5595 3551 7296	Human	7
DB00270	CACNA1C CACNA2D1 CACNA1H CACNB2 CACNA1S CACNA1D CACNA2D2	775 781 8912 783 779 776 9254	Human	7
DB00401	CACNA1C CACNA2D1 CACNB2 CACNA1S CACNA1D	775 781 783 779 776	Human	5
DB01054	CACNA1C CACNG1 CACNA2D1 CACNA1H CACNB2 CACNA1S CACNA1D CACNA2D2	775 786 781 8912 783 779 776 9254	Human	8
DB04920	CACNA1C CACNA1S CACNA1F CACNA1D	775 779 778 776	Human	4
DB06712	CACNA1C CACNA2D1 CACNB2 CACNA1S CACNA1D	775 781 783 779 776 55799	Human	6
DB06751	CACNA1C PDE4A	775 5141	Human	2
DB00017	CALCR	799	Human	1
DB01278	CALCR RAMP1 RAMP2 RAMP3	799 10267 10266 10268	Human	4
DB01381	GLRA1 SLC6A2 GABRA1 PLA2G2A GABRB2 GABRG2	2741 6530 2554 5320 2561 2566	Human	6
DB00235	PDE3A	5139	Human	1
DB00261	PDE3A	5139	Human	1
DB00922	PDE3A KCNJ11 TNNC1 KCNJ8	5139 3767 7134 3764	Human	4
DB01166	PDE3A	5139	Human	1
DB01427	PDE3A PDE4B TNF	5139 5142 7124	Human	3
DB04880	PDE3A	5139	Human	1
DB05266	PDE3A PDE4B PDE4A PDE4D PDE4C	5139 5142 5141 5144 5143	Human	5
DB08811	PDE3A PDE10A PDE4A PDE2A	5139 10846 5141 5138	Human	4
DB00054	ITGA2B FCGR1A FCGR3B VTN ITGB3 C1S C1R C1QA C1QB C1QC FCGR3A FCGR2A FCGR2B	3674 2209 2215 7448 3690 716 715 712 713 714 2214 2212 2213	Human	13
DB00775	ITGA2B ITGB3	3674 3690	Human	2

DB00283	HRH1	3269	Human	1
DB00341	HRH1	3269	Human	1
DB00405	HRH1	3269	Human	1
DB00427	HRH1	3269	Human	1
DB00455	HRH1	3269	Human	1
DB00557	HRH1	3269	Human	1
DB00719	HRH1	3269	Human	1
DB00737	HRH1	3269	Human	1
DB00748	HRH1	3269	Human	1
DB00768	HRH1 S100A13 S100A1 S100B S100A12 S100A2	3269 6284 6271 6285 6283 6273	Human	6
DB00792	HRH1	3269	Human	1
DB00902	HRH1	3269	Human	1
DB00920	HRH1 PDE4B PDE7B PDE7A PDE4A PDE4D PDE4C PGD	3269 5142 27115 5150 5141 5144 5143 5226 5151 8622	Human	10
DB00950	HRH1	3269	Human	1
DB00967	HRH1	3269	Human	1
DB00972	HRH1	3269	Human	1
DB00985	HRH1	3269	Human	1
DB01071	HRH1	3269	Human	1
DB01075	HRH1	3269	Human	1
DB01084	HRH1	3269	Human	1
DB01106	HRH1 NTSR2	3269 23620	Human	2
DB01114	HRH1 SLC6A2 SLC6A3 SLC6A4	3269 6530 6531 6532	Human	4
DB01146	HRH1 SLC6A3	3269 6531	Human	2
DB01176	HRH1 SULT1E1	3269 6783	Human	2
DB01237	HRH1	3269	Human	1
DB01246	HRH1	3269	Human	1
DB01615	HRH1	3269	Human	1
DB01619	HRH1	3269	Human	1
DB01620	HRH1	3269	Human	1
DB04837	HRH1	3269	Human	1
DB04890	HRH1	3269	Human	1
DB06691	HRH1	3269	Human	1
DB06698	HRH1 HRH3	3269 11255	Human	2
DB06766	HRH1	3269	Human	1
DB08799	HRH1	3269	Human	1
DB08800	HRH1	3269	Human	1
DB08801	HRH1 CHRM2	3269 1129	Human	2
DB08802	HRH1	3269	Human	1
DB08936	HRH1	3269	Human	1
DB01436	CYP27B1 VDR	1594 7421	Human	2
DB00924	HTR2A	3356	Human	1
DB00933	HTR2A DRD2	3356 1813	Human	2
DB04842	HTR2A CACNG1 DRD2	3356 786 1813	Human	3
DB06288	HTR2A DRD3 HTR7 DRD2	3356 1814 3363 1813	Human	4
DB00104	SSTR1 SSTR5 SSTR2	6751 6755 6752	Human	3
DB06663	SSTR1 SSTR5 SSTR2 SSTR3	6751 6755 6752 6753	Human	4

DB00348	HPD	3242	Human	1
DB01143	ENPP1 ALPPL2	5167 251	Human	2
DB00953	HTR1F HTR1D HTR1B	3355 3352 3351	Human	3
DB00188	PSMD1 PSMD2 PSMB1 PSMB5 PSMB2	5707 5708 5689 5693 5690	Human	5
DB00033	IFNGR2 IFNGR1	3460 3459	Human	2
DB00828	murA	947703	Escherichia coli (strain K12)	1
DB01205	GABRA5 GABRA1 GABRG2 GABRA1 GABRA2 GABRA3 GABRA4 GABRA5 GABRA6 GABRB1 GABRB2 GABRB3 GABRD GABRE GABRG1 GABRG2 GABRG3 GABRP	2558 2554 2566 2554 2555 2556 2557 2558 2559 2560 2561 2562 2563 2564 2565 2567 2568 2568 55879	Human	19
DB01160	PTGIR PTGFR	5739 5737	Human	2
DB00419	UGCG	7357	Human	1
DB00347	CACNA1G	8913	Human	1
DB00593	CACNA1G	8913	Human	1
DB05246	CACNA1G	8913	Human	1
DB00191	SLC6A2 SLC6A3 SLC6A4 MAOB MAOA	6530 6531 6532 4129 4128	Human	5
DB00226	SLC6A2	6530	Human	1
DB00234	SLC6A2	6530	Human	1
DB00285	SLC6A2 SLC6A3 SLC6A4	6530 6531 6532	Human	3
DB00289	SLC6A2 SLC6A4	6530 6532	Human	2
DB00344	SLC6A2 SLC6A4	6530 6532	Human	2
DB00422	SLC6A2 SLC6A3 SLC6A4	6530 6531 6532	Human	3
DB00476	SLC6A2 SLC6A3 SLC6A4	6530 6531 6532	Human	3
DB00579	SLC6A2 SLC6A3 SLC6A4	6530 6531 6532	Human	3
DB00830	SLC6A2 SLC6A3	6530 6531	Human	2
DB00937	SLC6A2 SLC6A3	6530 6531	Human	2
DB01105	SLC6A2 SLC6A3 SLC6A4	6530 6531 6532	Human	3
DB01156	SLC6A2 SLC6A3 CHRNA3	6530 6531 1136	Human	3
DB01170	SLC6A2	6530	Human	1
DB01579	SLC6A2 ADRA1A ADRA1B	6530 148 147	Human	3
DB04840	SLC6A2	6530	Human	1
DB06700	SLC6A2 SLC6A4	6530 6532	Human	2
DB06701	SLC6A2 SLC6A3 SLC6A4	6530 6531 6532	Human	3
DB06707	SLC6A2 ADRA1A	6530 148	Human	2
DB08918	SLC6A2 SLC6A4	6530 6532	Human	2
DB01113	PDE4B PDE10A	5142 10846	Human	2
DB01656	PDE4B PDE4A PDE4D PDE4C	5142 5141 5144 5143	Human	4
DB00274	mrcB mrcA dacA ftsI dacC	944843 947907 945222 12932643 12933831	Escherichia coli (strain K12)	5
DB00430	mrcB mrcA ftsI dacC mrcA ponB	944843 947907 12932643 12933831 881163 881511	Escherichia coli (strain K12)	6

DB00438	mrcB mrcA ftsI mrdA ponB pbpA pbp3 pbpA pbpG	944843 947907 12932643 12930918 881511 934791 7329159 878962 877694	Escherichia coli (strain K12)	9
DB01327	mrcB mrcA PON1 ftsI mrdA pbpC	944843 947907 5444 12932643 12930918 947152	Escherichia coli (strain K12)	6
DB01328	mrcB mrcA dacB ftsI mrdA	944843 947907 947693 12932643 12930918	Escherichia coli (strain K12)	5
DB01329	mrcB mrcA dacB dacA ftsI mrdA dacC mrcA ponB	944843 947907 947693 945222 12932643 12930918 12933831 881163 881511	Escherichia coli (strain K12)	9
DB01331	mrcB mrcA dacB dacA ftsI penA dacC pbpA pbp1b pbpG pbp3 pbp2a	944843 947907 947693 945222 12932643 933948 12933831 934791 934893 946662 7329159 933569	Escherichia coli (strain K12)	12
DB01332	mrcB mrcA ftsI	944843 947907 12932643	Escherichia coli (strain K12)	3
DB01414	mrcB mrcA	944843 947907	Escherichia coli (strain K12)	2
DB01415	mrcB mrcA ftsI mrdA	944843 947907 12932643 12930918	Escherichia coli (strain K12)	4
DB04570	mrcB mrcA dacB pbpC	944843 947907 947693 940139	Escherichia coli (strain K12)	4
DB00808	KCNE1 KCNQ1	3753 3784	Human	2
DB00024	TSHR	7253	Human	1
DB00167	IARS BCAT1 ACADSB BCAT2 IARS2	3376 586 36 587 55699	Human	5
DB00707	LDLR FCGR1A	3949 2209	Human	2
DB00211	ADRA1A ADRA1B	148 147	Human	2
DB00298	ADRA1A ADRA1B ADRA1D	148 147 146	Human	3
DB00346	ADRA1A ADRA1B ADRA1D	148 147 146	Human	3
DB00388	ADRA1A ADRA1B ADRA1D	148 147 146	Human	3
DB00450	ADRA1A DRD2	148 1813	Human	2
DB00610	ADRA1A	148	Human	1
DB00699	ADRA1A	148	Human	1
DB00706	ADRA1A ADRA1B ADRA1D	148 147 146	Human	3
DB00723	ADRA1A ADRA1B	148 147	Human	2
DB01253	ADRA1A	148	Human	1
DB01365	ADRA1A	148	Human	1
DB06207	ADRA1A ADRA1B ADRA1D	148 147 146	Human	3
DB00214	SLC12A1	6557	Human	1
DB00310	SLC12A1	6557	Human	1
DB00903	SLC12A1 ATP1A1	6557 476	Human	2
DB00389	TPO	7173	Human	1
DB00550	TPO	7173	Human	1
DB00763	TPO	7173	Human	1

DB01012	CASR	846	Human	1
DB01370	TF ATP1A1	7018 476	Human	2
DB00072	ERBB2 FCGR1A EGFR FCGR3B C1S C1R C1QA C1QB C1QC FCGR3A FCGR2A FCGR2B	2064 2209 1956 2215 716 715 712 713 714 2214 2212 2213	Human	12
DB01259	ERBB2 EGFR	2064 1956	Human	2
DB05773	ERBB2	2064	Human	1
DB06366	ERBB2	2064	Human	1
DB08916	ERBB2 EGFR	2064 1956 2066	Human	3
DB00095	ITGAL FCGR1A FCGR3B C1R C1QA C1QB C1QC FCGR3A FCGR2A FCGR2B	3683 2209 2215 715 712 713 714 2214 2212 2213	Human	10
DB00098	ITGAL CD1A CD86 ITGB3 FCGR2B CD4 MR1 ITGB1 ITGAV	3683 909 942 3690 2213 920 3140 3688 3685	Human	9
DB00227	ITGAL HMGCR HDAC2	3683 3156 3066	Human	3
DB00627	QPRT GPR109A NNMT GPR109B	23475 338442 4837 8843	Human	4
DB00040	GCGR GLP1R GLP2R	2642 2740 9340	Human	3
DB00445	CHD1 TOP2A	1105 7153	Human	2
DB01191	HTR2C SLC6A4	3358 6532	Human	2
DB04871	HTR2C	3358	Human	1
DB00364	FGA EGF FGF2 FGB FGG	2243 1950 2247 2244 2266	Human	5
DB01126	SRD5A1 SRD5A2	6715 6716	Human	2
DB01216	SRD5A1 SRD5A2 AKR1D1	6715 6716 6718	Human	3
DB01280	POLA1	5422	Human	1
DB00730	frdA	948667	Escherichia coli (strain K12)	1
DB00356	KCNMA1	3778	Human	1
DB01003	KCNMA1 S100P	3778 6286	Human	2
DB00325	NPR1	4881	Human	1
DB00727	NPR1	4881	Human	1
DB00883	NPR1	4881	Human	1
DB01612	NPR1	4881	Human	1
DB01613	NPR1 NPR2	4881 4882	Human	2
DB04899	NPR1 NPR2 NPR3	4881 4882 4883	Human	3
DB00351	PGR NR3C1	5241 2908	Human	2
DB00378	PGR	5241	Human	1
DB00588	PGR PLA2G4A NR3C2 NR3C1	5241 5321 4306 2908	Human	4
DB00717	PGR	5241	Human	1
DB00834	PGR NR3C1	5241 2908	Human	2
DB06713	PGR	5241	Human	1
DB01034	fabH fabF fabB FASN	946003 946665 946799 2194	Escherichia coli (strain K12)	4
DB01019	CHRM2	1129	Human	1
DB01135	CHRM2 CHRNA2	1129 1135	Human	2
DB01336	CHRM2 CHRNA2	1129 1135	Human	2
DB00026	IL1R1	3554	Human	1
DB00089	FOLH1	2346	Human	1

DB00292	ADRA2B GABRA1 GABRA1 GABRA2 GABRA3 GABRA4 GABRA5 GABRA6 GABRB1 GABRB2 GABRB3 GABRD GABRE GABRG1 GABRG2 GABRG3 GABRP GABRQ	151 2554 2554 2555 2556 2557 2558 2559 2560 2561 2562 2563 2564 2565 2567 2568 2568 55879	Human	18
DB00175	HMGCR	3156	Human	1
DB00641	HMGCR ITGB2	3156 3689	Human	2
DB01076	HMGCR AHR	3156 196 397492	Human	3
DB01095	HMGCR	3156	Human	1
DB01098	HMGCR	3156	Human	1
DB08860	HMGCR	3156	Human	1
DB00745	ADRA1B SLC6A3	147 6531	Human	2
DB01255	ADRA1B SLC6A3	147 6531	Human	2
DB00229	pbpA	990276	Clostridium perfringens (strain 13 / Type A)	1
DB00267	pbpA ftsI	990276 12932643	Clostridium perfringens (strain 13 / Type A)	2
DB00301	pbpA	990276	Clostridium perfringens (strain 13 / Type A)	1
DB00417	pbpA dacB	990276 947693	Clostridium perfringens (strain 13 / Type A)	2
DB00447	pbpA pbp3	990276 7329159	Clostridium perfringens (strain 13 / Type A)	2
DB00456	pbpA ampC penA pbpA pbp1b pbp3 pbp2a	990276 948669 933948 934791 934893 7329159 933569	Clostridium perfringens (strain 13 / Type A)	7
DB00713	pbpA penA pbpA pbp1b pbp3 pbp2a	990276 933948 934791 934893 7329159 933569	Clostridium perfringens (strain 13 / Type A)	6
DB00833	pbpA pbp3	990276 7329159	Clostridium perfringens (strain 13 / Type A)	2
DB00948	pbpA mrdA pbp3	990276 12930918 7329159	Clostridium perfringens (strain 13 / Type A)	3

DB01000	pbpA pbpA pbp3	990276 934791 7329159	Clostridium perfringens (strain 13 / Type A)	3
DB01060	pbpA	990276	Clostridium perfringens (strain 13 / Type A)	1
DB01061	pbpA	990276	Clostridium perfringens (strain 13 / Type A)	1
DB01066	pbpA penA	990276 933948	Clostridium perfringens (strain 13 / Type A)	2
DB01112	pbpA	990276	Clostridium perfringens (strain 13 / Type A)	1
DB01139	pbpA	990276	Clostridium perfringens (strain 13 / Type A)	1
DB01602	pbpA	990276	Clostridium perfringens (strain 13 / Type A)	1
DB01604	pbpA	990276	Clostridium perfringens (strain 13 / Type A)	1
DB01605	pbpA	990276	Clostridium perfringens (strain 13 / Type A)	1
DB00735	SQLE	6713	Human	1
DB00857	SQLE	6713	Human	1
DB01091	SQLE	6713	Human	1
DB00391	DRD3 DRD2	1814 1813	Human	2
DB01184	DRD3 DRD2	1814 1813	Human	2
DB01333	mrcA	947907	Escherichia coli (strain K12)	1
DB00108	ITGA4 FCGR1A FCGR3B ICAM1 C1R C1QA C1QB C1QC FCGR3A FCGR2A FCGR2B	3676 2209 2215 3383 715 712 713 714 2214 2212 2213	Human	11
DB00632	BLLF1	3783713	HHV-4	1
DB00720	SLC25A4 SLC25A6 SLC25A5	291 293 292	Human	3
DB00287	PTGFR	5737	Human	1
DB00654	PTGFR	5737	Human	1
DB08819	PTGFR	5737	Human	1

DB00765	TH	7054	Human	1
DB00845	GK0582	3184233	Geobacillus kaustophilus (strain HTA426)	1
DB01586	AKR1C2	1646	Human	1
DB01133	PTPN1 ATP6V1A	5770 523	Human	2
DB00010	GHRHR	2692	Human	1
DB08869	GHRHR	2692	Human	1
DB00020	CSF2RA IL3RA PRG2 CSF2RB	1438 3563 5553 1439 6383	Human	5
DB00825	OPRK1 TRPA1 TRPM8 TRPV3	4986 8989 79054 162514	Human	4
DB01209	OPRK1 OPRM1	4986 4988	Human	2
DB06800	OPRK1 OPRM1	4986 4988	Human	2
DB08881	BRAF	673	Human	1
DB08883	GRIA1	2890	Human	1
DB00915	GRIN3A DRD2	116443 1813	Human	2
DB00414	KCNJ1	3758	Human	1
DB00839	KCNJ1	3758	Human	1
DB01104	SLC6A3 SLC6A4	6531 6532	Human	2
DB01463	SLC6A3	6531	Human	1
DB00262	GSR	2936	Human	1
DB00004	IL2RB IL2RA IL2RG	3560 3559 3561	Human	3
DB00041	IL2RB IL2RA IL2RG	3560 3559 3561	Human	3
DB00074	IL2RB IL2RA FCGR1A FCGR3B C1S C1R C1QA C1QB C1QC FCGR3A FCGR2A FCGR2B	3560 3559 2209 2215 716 715 712 713 714 2214 2212 2213	Human	12
DB00111	IL2RB IL2RA FCGR1A FCGR3B C1R C1QA C1QB C1QC FCGR3A FCGR2A FCGR2B	3560 3559 2209 2215 715 712 713 714 2214 2212 2213	Human	11
DB00012	EPOR	2057	Human	1
DB00016	EPOR	2057	Human	1
DB08894	EPOR	2057	Human	1
DB08923	EPOR	2057	Human	1
DB01103	PLA2G4A PLA2G6 PLCL1	5321 8398 5334	Human	3
DB00918	HTR1D HTR1B	3352 3351	Human	2
DB00998	HTR1D HTR1B	3352 3351	Human	2
DB05258	IFNAR1	3454	Human	1
DB00982	RARA	5914	Human	1
DB00700	NR3C2	4306	Human	1
DB00056	CD33 FCGR1A FCGR3B C1S C1R C1QA C1QB C1QC FCGR3A FCGR2A FCGR2B	945 2209 2215 716 715 712 713 714 2214 2212 2213	Human	11
DB00914	PRKAA1 KCNJ8	5562 3764	Human	2
DB00343	CACNG1	786	Human	1
DB00528	CACNG1	786	Human	1
DB01083	PNLIP FASN LIPF	5406 2194 8513	Human	3
DB01167	ERG11 CYP51A1	2887532 1595	Yeast	2
DB04838	CACNA2D1 CES1	781 1066	Human	2
DB00816	ADRB2	154	Human	1

DB00867	ADRB2	154	Human	1
DB00871	ADRB2	154	Human	1
DB00938	ADRB2	154	Human	1
DB00983	ADRB2	154	Human	1
DB01274	ADRB2	154	Human	1
DB01366	ADRB2	154	Human	1
DB01408	ADRB2	154	Human	1
DB05039	ADRB2	154	Human	1
DB00337	FKBP1A MTOR	2280 2475	Human	2
DB00864	FKBP1A	2280	Human	1
DB00877	FKBP1A FGF2 MTOR	2280 2247 2475	Human	3
DB00005	TNF FCGR1A FCGR3B TNFRSF1B LTA C1S C1R C1QA C1QB C1QC FCGR3A FCGR2A	7124 2209 2215 7133 4049 716 715 712 713 714 2214 2212 2213	Human	13
DB00051	TNF FCGR1A FCGR3B C1S C1R C1QA C1QB C1QC FCGR3A FCGR2A FCGR2B	7124 2209 2215 716 715 712 713 714 2214 2212 2213	Human	11
DB00065	TNF	7124	Human	1
DB00608	TNF TLR9 GSTA2	7124 54106 2939	Human	3
DB01411	TNF CYSLTR1 RNASE3 MUC2 NFKB1 IL5	7124 10800 6037 4583 4790 3567	Human	6
DB06674	TNF	7124	Human	1
DB08904	TNF	7124	Human	1
DB00549	CYSLTR1	10800	Human	1
DB00587	CYSLTR1	10800	Human	1
DB01154	KCNJ11 GABRA1 KCNJ8	3767 2554 3764	Human	3
DB00002	FCGR1A EGFR FCGR3B C1S C1R C1QA C1QB C1QC FCGR3A FCGR2A FCGR2B	2209 1956 2215 716 715 712 713 714 2214 2212 2213	Human	11
DB00028	FCGR1A C5 FCGR3B FCGR3A FCGR2A FCGR2B C3 FCGR1B C4A C4B	2209 727 2215 2214 2212 2213 718 2210 720 721	Human	10
DB00092	FCGR1A CD2 FCGR3B C1R C1QA C1QB C1QC FCGR3A FCGR2A FCGR2B	2209 914 2215 715 712 713 714 2214 2212 2213	Human	10
DB00110	FCGR1A FCGR3B C1R C1QA C1QB C1QC FCGR3A FCGR2A	2209 2215 715 712 713 714 2214 2212 2213	Human	9
DB00992	FCGR1A	2209	Human	1
DB00266	VKORC1 NQO1 CRYZ	79001 1728 1429	Human	3
DB00498	VKORC1	79001	Human	1
DB00682	VKORC1	79001	Human	1
DB00946	VKORC1	79001	Human	1
DB01418	VKORC1	79001	Human	1
DB00851	POLA2 PGD	23649 5226	Human	2
DB00688	IMPDH2 IMPDH1	3615 3614	Human	2
DB01024	IMPDH2 IMPDH1	3615 3614	Human	2
DB05260	BGLAP IL1B RRM2 ATP6V1B2	632 3553 6241 526	Human	4
DB01011	CYP11B1	1584	Human	1
DB00390	ATP1A1	476	Human	1

DB00511	ATP1A1	476	Human	1
DB01078	ATP1A1	476	Human	1
DB01092	ATP1A1	476	Human	1
DB01158	ATP1A1	476	Human	1
DB01188	ATP1A1	476	Human	1
DB01396	ATP1A1	476	Human	1
DB01430	ATP1A1	476	Human	1
DB00260	ddlA	945313	Escherichia coli (strain K12)	1
DB00134	MTR MTRR BHMT METAP2 BHMT2	4548 4552 635 10988 23743	Human	5
DB00186	TSPO GABRA1 GABRA2 GABRA3 GABRA4 GABRA5 GABRA6 GABRB1 GABRB2 GABRB3 GABRD GABRE GABRG1 GABRG2 GABRG3	706 2554 2555 2556 2557 2558 2559 2560 2561 2562 2563 2564 2565 2567 2568 2568 55879	Human	17
DB00628	TSPO GABRA1 GABRA2 GABRA3 GABRA4 GABRA5 GABRA6 GABRB1 GABRB2 GABRB3 GABRD GABRE GABRG1 GABRG2 GABRG3	706 2554 2555 2556 2557 2558 2559 2560 2561 2562 2563 2564 2565 2567 2568 2568 55879	Human	17
DB00962	TSPO GABRA1	706 2554	Human	2
DB01068	TSPO GABRA1 GABRA2 GABRA3 GABRA4 GABRA5 GABRA6 GABRB1 GABRB2 GABRB3 GABRD GABRE GABRG1 GABRG2 GABRG3	706 2554 2555 2556 2557 2558 2559 2560 2561 2562 2563 2564 2565 2567 2568 2568 55879	Human	17
DB01178	TSPO	706	Human	1
DB01587	TSPO GABRA1 GABRB1 GABRG1 GABRE GABRD	706 2554 2560 2565 2564 2563	Human	6
DB00416	CHRNA2	1135	Human	1
DB00565	CHRNA2	1135	Human	1
DB00657	CHRNA2	1135	Human	1
DB00732	CHRNA2	1135	Human	1
DB01339	CHRNA2	1135	Human	1
DB01219	RYR1	6261	Human	1
DB00380	TOP2A TOP2B	7153 7155	Human	2
DB00385	TOP2A	7153	Human	1
DB00444	TOP2A	7153	Human	1
DB00694	TOP2A TOP2B	7153 7155	Human	2
DB00773	TOP2A TOP2B	7153 7155	Human	2
DB00997	TOP2A	7153	Human	1
DB01177	TOP2A	7153	Human	1
DB01204	TOP2A	7153	Human	1
DB04967	TOP2A TOP1 APEX1	7153 7150 328	Human	3
DB00778	rplJ ABCB1	1025180 5243	Shigella flexneri	2
DB01190	rplJ	1025180	Shigella flexneri	1

DB01211	rplJ	1025180	Shigella flexneri	1
DB01369	rplJ rplV	1025180 915968	Shigella flexneri	2
DB01627	rplJ	1025180	Shigella flexneri	1
DB00760	dacB	947693	Escherichia coli (strain K12)	1
DB00039	FGFR2 HSPG2 FGFR4 NRP1 FGFR1 FGFR3	2263 3339 2264 8829 2260 2261	Human	6
DB00176	SLC6A4	6532	Human	1
DB00472	SLC6A4	6532	Human	1
DB00107	OXT OXTR	5020 5021	Human	2
DB00433	DRD2	1813	Human	1
DB01425	DRD2	1813	Human	1
DB01207	TBXA2R TBXAS1	6915 6916	Human	2
DB01046	CLCN2	1181	Human	1
DB00317	EGFR	1956	Human	1
DB00530	EGFR NR1I2	1956 8856	Human	2
DB01269	EGFR	1956	Human	1
DB00504	OPRM1	4988	Human	1
DB00802	OPRM1	4988	Human	1
DB00913	OPRM1	4988	Human	1
DB01227	OPRM1 CHRNB4 CHRNA3	4988 1143 1136	Human	3
DB01433	OPRM1	4988	Human	1
DB01466	OPRM1	4988	Human	1
DB00282	FDPS	2224	Human	1
DB00399	FDPS GGPS1	2224 9453	Human	2
DB00630	FDPS PTPN4 PTPRS PTPRE ATP6V1A	2224 5775 5802 5791 523	Human	5
DB00710	FDPS	2224	Human	1
DB00884	FDPS	2224	Human	1
DB00136	VDR	7421	Human	1
DB00146	VDR	7421	Human	1
DB00153	VDR	7421	Human	1
DB00169	VDR	7421	Human	1
DB00910	VDR	7421	Human	1
DB01070	VDR	7421	Human	1
DB02300	VDR	7421	Human	1
DB06637	KCNA1 KCND2 KCND3 KCNA4	3736 3751 3752 3739 3737 3738 3741 3742 3743 3744 3745 9312 3746 3747 3748 3750	Human	16
DB01138	ABCC1 ABCC2	4363 1244	Human	2
DB00355	pbpC	940139	Bacillus subtilis (strain 168)	1
DB00493	pbpC penA pbpA pbp1b pbp2a	940139 933948 934791 934893 933569	Bacillus subtilis (strain 168)	5

DB00180	NR3C1	2908	Human	1
DB00223	NR3C1	2908	Human	1
DB00240	NR3C1	2908	Human	1
DB00253	NR3C1	2908	Human	1
DB00324	NR3C1	2908	Human	1
DB00394	NR3C1	2908	Human	1
DB00443	NR3C1	2908	Human	1
DB00547	NR3C1	2908	Human	1
DB00591	NR3C1	2908	Human	1
DB00596	NR3C1	2908	Human	1
DB00620	NR3C1	2908	Human	1
DB00663	NR3C1	2908	Human	1
DB00764	NR3C1	2908	Human	1
DB00769	NR3C1	2908	Human	1
DB00838	NR3C1	2908	Human	1
DB00846	NR3C1	2908	Human	1
DB00860	NR3C1	2908	Human	1
DB00873	NR3C1	2908	Human	1
DB00896	NR3C1	2908	Human	1
DB00959	NR3C1	2908	Human	1
DB01013	NR3C1	2908	Human	1
DB01047	NR3C1 SMO	2908 6608	Human	2
DB01130	NR3C1	2908	Human	1
DB01222	NR3C1	2908	Human	1
DB01260	NR3C1	2908	Human	1
DB01380	NR3C1	2908	Human	1
DB01384	NR3C1	2908	Human	1
DB01410	NR3C1	2908	Human	1
DB06781	NR3C1	2908	Human	1
DB08906	NR3C1	2908	Human	1
DB00474	GABRA1	2554	Human	1
DB01107	GABRA1 GABRA1 GABRA2 GABRA3 GABRA4 GABRA5 GABRA6 GABRB1 GABRB2 GABRB3 GABRD GABRE GABRG1 GABRG2 GABRG3	2554 2554 2555 2556 2557 2558 2559 2560 2561 2562 2563 2564 2565 2567 2568 2568 55879	Human	17
DB01437	GABRA1 GABRA1 GABRA2 GABRA3 GABRA4 GABRA5 GABRA6 GABRB1 GABRB2 GABRB3 GABRD GABRE GABRG1 GABRG2 GABRG3	2554 2554 2555 2556 2557 2558 2559 2560 2561 2562 2563 2564 2565 2567 2568 2568 55879	Human	17
DB00817	parC gyrB	947499 948211	Escherichia coli (strain K12)	2
DB00259	folP	947691	Escherichia coli (strain K12)	1

DB00263	folP	947691	Escherichia coli (strain K12)	1
DB00576	folP	947691	Escherichia coli (strain K12)	1
DB00634	folP sulI FOL1	947691 10752055 855465	Escherichia coli (strain K12)	3
DB01298	folP	947691	Escherichia coli (strain K12)	1
DB01581	folP	947691	Escherichia coli (strain K12)	1
DB01582	folP	947691	Escherichia coli (strain K12)	1
DB06729	folP	947691	Escherichia coli (strain K12)	1
DB00237	GRIK2 GRIA2 CHRNA4 CHRNA7 GABRA1 GABRA2 GABRA3 GABRA4 GABRA5 GABRA6 GABRB1 GABRB2 GABRB3 GABRD GABRE GABRG1 GABRG2 GABRG3 GABRP	2898 2891 1137 1139 2554 2555 2556 2557 2558 2559 2560 2561 2562 2563 2564 2565 2567 2568 2568 55879	Human	20
DB01356	GRIA3 IMPA2 GSK3B IMPA1	2892 3613 2932 3612	Human	4
DB00284	MGAM GAA SI AMY2A	8972 2548 6476 279	Human	4
DB00491	MGAM GAA	8972 2548 23193 2595	Human	4
DB04878	MGAM	8972	Human	1
DB01256	rplC	900403	Streptococcus pyogenes serotype M1	1
DB01282	OXTR	5021	Human	1
DB01281	CD86 CD80	942 941	Human	2
DB06681	CD86 CD80	942 941	Human	2
DB01284	MC2R	4158	Human	1
DB01285	MC2R CRH	4158 1392	Human	2
DB01273	CHRNA4 CHRNA7 CHRNA3 CHRNA6	1137 1139 1136 8973	Human	4
DB01261	DPP4	1803	Human	1
DB04876	DPP4	1803	Human	1
DB06203	DPP4	1803	Human	1
DB06335	DPP4	1803	Human	1
DB08882	DPP4	1803	Human	1
DB01257	C5	727	Human	1
DB01271	PLIN3	10226	Human	1
DB01279	PLIN3	10226	Human	1
DB01276	GLP1R	2740	Human	1
DB06655	GLP1R	2740	Human	1

DB08911	MAP2K1	5604 5605	Human	2
DB05278	CRP	1401	Human	1
DB01032	SLC22A11 SLC22A6 SLC22A8 PANX1	55867 9356 9376 24145	Human	4
DB06186	CTLA4	1493	Human	1
DB00132	FADS1 SLC8A1 TRPV1 FADS2 ELOVL4	3992 6546 7442 9415 6785	Human	5
DB00928	DNMT1	1786	Human	1
DB01262	DNMT1	1786	Human	1
DB04953	KCNQ2 KCNQ3 KCNQ4 KCNQ5	3785 3786 9132 56479	Human	4
DB00995	PRDX5 IKBKB	25824 3551	Human	2
DB00626	A2M IDE	2 3416	Human	2
DB08888	A2M SERPINF2 FN1	2 5345 2335	Human	3
DB04835	CCR5	1234	Human	1
DB00198	NEU1 CES1 NEU2	4758 1066 4759	Human	3
DB00061	GRB2	2885	Human	1
DB06809	CXCR4	7852	Human	1
DB06213	ADORA2A	135	Human	1
DB00446	CD55 rplP	1604 947806	Human	2
DB00062	AMBP APOE SAA1	259 348 6288	Human	3
DB00064	AMBP APOE SAA1	259 348 6288	Human	3
DB01250	IFNG TPMT	3458 7172	Human	2
DB08864	NR1I2	8856	Human	1
DB05630	TOP1	7150	Human	1
DB06720	GBA	2629	Human	1
DB00070	TGFB1	7040	Human	1
DB01202	SV2A CACNA1B	9900 774	Human	2
DB08879	TNFSF13B	10673	Human	1
DB08893	ADRB3	155	Human	1
DB01375	TNNC1 VDAC1 VDAC2 VDAC3	7134 7416 7417 7419	Human	4
DB04877	ABCB1	5243	Human	1
DB00428	SLC2A2 BT_4395	6514 1074035	Human	2
DB06168	IL1B	3553	Human	1
DB06372	IL1B	3553 3552 3557	Human	3
DB00847	SST NPY2R	6750 4887	Human	2
DB00535	MPO	4353	Human	1
DB06196	ANPEP BDKRB2	290 624	Human	2
DB08818	ICAM1 CD44	3383 960 3161	Human	3
DB00759	PRNP rpsN rpsS rpsC rpsH rpsG	5621 947801 947811 947814 947802 947846	Human	6
DB05013	PRKCA PRKCD	5578 5580	Human	2
DB06201	SCN9A GRM5	6335 2915	Human	2
DB01597	DPEP1	1800	Human	1
DB00063	ITGB3	3690	Human	1
DB00183	CCKBR	887	Human	1
DB00045	TLR2	7097	Human	1

DB00659	GRM5 GABRA1 GABRA2 GABRA3 GABRA4 GABRA5 GABRA6 GABRB1 GABRB2 GABRB3 GABRD GABRE GABRG1 GABRG2 GABRG3 GABRP GABRQ GRIN1 GRIN2A GRIN2B GRIN2C GRIN2D	2915 2554 2555 2556 2557 2558 2559 2560 2561 2562 2563 2564 2565 2567 2568 2568 55879 2902 2903 2904 2905 2906 116443 116444	Human	24
DB04941	CFTR	1080 55107	Human	2
DB08820	CFTR	1080	Human	1
DB00551	MMP12	4321	Human	1
DB03255	cobT E	1253537 1258585	Salmonella typhimurium (strain LT2 / SGSC1412 / ATCC 700720)	2
DB00518	TUBA1A TUBB2C SO_0970	7846 10383 1168814	Human	3
DB00643	TUBA1A TUBB2C	7846 10383	Human	2
DB04845	TUBB3	10381	Human	1
DB01606	bla	1251335	Salmonella typhi	1
DB02546	HDAC8 HDAC1 HDAC2 HDAC3 HDAC6 acuC1	55869 3065 3066 8841 10013 1193769	Human	6
DB06777	NR1H4	9971	Human	1
DB02703	cat	1251342	Salmonella typhi	1
DB02959	trpS2	1797478	Deinococcus radiodurans (strain ATCC 13939 / DSM 20539 / JCM 16871 / LMG 4051 / NBRC 15346 / NCIMB 9279 / R1 / VKM B- [illegible]	1
DB00555	SCN2A	6326	Human	1
DB00818	SCN2A SCN4A GABRB3 GABRB2 GABRA1 GABRA2 GABRA3 GABRA4 GABRA5 GABRA6 GABRB1 GABRB2 GABRB3 GABRD GABRE GABRG1 GABRG2 GABRG3	6326 6329 2562 2561 2554 2555 2556 2557 2558 2559 2560 2561 2562 2563 2564 2565 2567 2568 2568 55879	Human	20
DB00250	folP1 folP2	908646 910150	Mycobacterium leprae (strain TN)	2
DB00323	COMT	1312	Human	1
DB00494	COMT	1312	Human	1
DB00357	CYP19A1 CYP11A1	1588 1583	Human	2
DB00990	CYP19A1	1588	Human	1

DB01006	CYP19A1	1588	Human	1
DB01217	CYP19A1	1588	Human	1
DB04895	NRP1	8829	Human	1
DB08900	GLP2R	9340	Human	1
DB00042	VAMP1 VAMP2 SYT2	6843 6844 127833	Human	3
DB00190	DDC	1644	Human	1
DB00772	BCHE	590	Human	1
DB00941	BCHE	590	Human	1
DB01057	BCHE	590	Human	1
DB00752	MAOB MAOA	4129 4128	Human	2
DB01037	MAOB MAOA	4129 4128	Human	2
DB01247	MAOB MAOA	4129 4128	Human	2
DB01626	MAOB MAOA	4129 4128	Human	2
DB01171	MAOA	4128	Human	1
DB00437	XDH	7498	Human	1
DB00552	ADA	100	Human	1
DB01440	GABRB1	2560 79581	Human	2
DB00592	GABRB3	2562	Human	1
DB06716	GABRB3 GABRB2	2562 2561	Human	2
DB08889	PSMB1 PSMB5 PSMB2	5689 5693 5690 5698 5699 5696	Human	6
DB00194	BALF5	3783681	HHV-4	1
DB01590	MTOR	2475	Human	1
DB06287	MTOR	2475	Human	1
DB01413	ftsI mrdA pbpB pbpA	12932643 12930918 881247 878962	Escherichia coli (strain K12)	4
DB01416	ftsI	12932643	Escherichia coli (strain K12)	1
DB08877	JAK2 JAK1	3717 3716	Human	2
DB08895	JAK2 JAK3 JAK1	3717 3718 3716	Human	3
DB05265	NOXO1	124056	Human	1
DB08868	S1PR5	53637	Human	1
DB00613	HNMT	3176	Human	1
DB01289	KCNJ8	3764	Human	1
DB00495	TERT	7015	Human	1
DB00199	rplV rplD	915968 915964	Escherichia coli O157:H7	2
DB00207	rplV rplD	915968 915964	Escherichia coli O157:H7	2
DB00738	TRDMT1	1787	Human	1
DB06273	IL6R	3570	Human	1
DB05389	MAEA CD163	10296 9332	Human	2
DB05332	MPL	4352	Human	1
DB06210	MPL	4352	Human	1
DB08870	TNFRSF8	943	Human	1
DB08890	GUCY2C	2984	Human	1
DB05829	PTH1R	5745 5746	Human	2

DB06285	PTH1R	5745	Human	1
DB06643	TNFSF11	8600	Human	1
DB00789	PGD	5226	Human	1
DB01077	PTPRS ATP6V1A	5802 523	Human	2
DB00435	GUCY1A2	2977	Human	1
DB01020	GUCY1A2	2977	Human	1
DB01163	mrdA penA pbpA pbp1b pbp3 pbp2a	12930918 933948 934791 934893 7329159 933569	Escherichia coli (strain K12)	6
DB00561	KCNK3 KCNK9	3777 51305	Human	2
DB00319	penA pbp1b pbp3 pbp2a	933948 934893 7329159 933569	Streptococcus pneumoniae (strain ATCC BAA-255 / R6)	4
DB00415	penA pbpA pbp1b pbp3 pbp2a	933948 934791 934893 7329159 933569	Streptococcus pneumoniae (strain ATCC BAA-255 / R6)	5
DB00485	penA pbpA pbp1b pbp3 pbp2a LMHCC_2184 LMHCC_2773	933948 934791 934893 7329159 933569 7079532 7079540	Streptococcus pneumoniae (strain ATCC BAA-255 / R6)	7
DB00567	penA pbpA pbp1b pbp3 pbp2a	933948 934791 934893 7329159 933569	Streptococcus pneumoniae (strain ATCC BAA-255 / R6)	5
DB00607	penA pbpA pbp1b pbp3 pbp2a	933948 934791 934893 7329159 933569	Streptococcus pneumoniae (strain ATCC BAA-255 / R6)	5
DB00739	penA pbpA pbp1b pbp3 pbp2a	933948 934791 934893 7329159 933569	Streptococcus pneumoniae (strain ATCC BAA-255 / R6)	5
DB01140	penA pbpA pbp1b pbp3	933948 934791 934893 7329159	Streptococcus pneumoniae (strain ATCC BAA-255 / R6)	4

DB01212	penA	933948	Streptococcus pneumoniae (strain ATCC BAA-255 / R6)	1
DB01603	penA pbpA pbp1b pbp3 pbp2a	933948 934791 934893 7329159 933569	Streptococcus pneumoniae (strain ATCC BAA-255 / R6)	5
DB08795	penA pbpA pbp1b pbp3 pbp2a	933948 934791 934893 7329159 933569	Streptococcus pneumoniae (strain ATCC BAA-255 / R6)	5
DB01361	rplD rpmF	12932297 1800201	Escherichia coli (strain K12)	2
DB01421	rpsJ	3167932	Thermus thermophilus (strain HB8 / ATCC 27634 / DSM 579)	1
DB01321	rplD	950806	Haemophilus influenzae (strain ATCC 51907 / DSM 11121 / KW20 / Rd)	1
DB01044	gyrA gyrB parC parE	931733 930756 930805 930802	Streptococcus pneumoniae serotype 4 (strain ATCC BAA-334 / TIGR4)	4
DB04865	RPL3	6122 3128427	Human	2
DB01206	STMN4	81551	Human	1
DB01422	METAP1	23173	Human	1
DB00916	rdxA	899487	Helicobacter pylori (strain ATCC 700392 /	1
DB01272	M6PR	4074	Human	1
DB01330	pbp3	7329159	Streptococcus pneumoniae	1

DB01053	pbp3	3915175	Staphylococcus aureus (strain USA300)	1
DB00689	pbpA	3097544	Bacillus licheniformis (strain DSM 13 / ATCC 14580)	1
DB08828	SMO	6608	Human	1
DB00400	KRT12	3859	Human	1
DB00362	fksA	4981154	Aspergillus niger (strain CBS 513.88 / FGSC	1
DB00520	fksA	4981154	Aspergillus niger (strain CBS 513.88 / FGSC	1
DB01141	fksA	4981154	Aspergillus niger (strain CBS 513.88 / FGSC	1
DB00525	ERG1	3646458	Yeast	1
DB06775	CPS1	1373	Human	1
DB06689		2161	Human	1
DB08874		4915727	Clostridium difficile (strain 630)	1
DB08902		1158723	Bacillus anthracis	1
DB08903		886937	Mycobacterium tuberculosis	1
DB06292		6524	Human	1
DB08907		6524 6523	Human	2
DB08908		9817	Human	1
DB00189	GABRA1 GABRA2 GABRA3 GABRA4 GABRA5 GABRA6 GABRB1 GABRB2 GABRB3 GABRD GABRE GABRG1 GABRG2 GABRG3 GABRP	2554 2555 2556 2557 2558 2559 2560 2561 2562 2563 2564 2565 2567 2568 2568 55879	Human	16
DB00349	GABRA1 GABRA2 GABRA3 GABRA4 GABRA5 GABRA6 GABRB1 GABRB2 GABRB3 GABRD GABRE GABRG1 GABRG2 GABRG3 GABRP	2554 2555 2556 2557 2558 2559 2560 2561 2562 2563 2564 2565 2567 2568 2568 55879	Human	16
DB00404	GABRA1 GABRA2 GABRA3 GABRA4 GABRA5 GABRA6 GABRB1 GABRB2 GABRB3 GABRD GABRE GABRG1 GABRG2 GABRG3 GABRP	2554 2555 2556 2557 2558 2559 2560 2561 2562 2563 2564 2565 2567 2568 2568 55879	Human	16

DB00475	GABRA1 GABRA2 GABRA3 GABRA4 GABRA5 GABRA6 GABRB1 GABRB2 GABRB3 GABRD GABRE GABRG1 GABRG2 GABRG3 GABRP	2554 2555 2556 2557 2558 2559 2560 2561 2562 2563 2564 2565 2567 2568 2568 55879	Human	16
