# Supplementary material for: Inferring drug-disease associations based on known protein complexes
Source: BMC Med Genomics. 2015 May 29;8(Suppl 2):S2. doi: 10.1186/1755-8794-8-S2-S2 (PMC4460611; doi:10.1186/1755-8794-8-S2-S2)
Supplement: Additional file 2 — Table illustrating the list of protein complexes. [file 1755-8794-8-S2-S2-S2.pdf]

Complex id	Complex name	Subunits (Entrez IDs)	Organism	Number of subunit
1	BCL6-HDAC4 complex	604 9759	Human	2
2	BCL6-HDAC5 complex	604 10014	Human	2
3	BCL6-HDAC7 complex	604 51564	Human	2
4	Multisubunit ACTR coactivator complex	1387 2033 8202 8850	Human	4
9	6S-nuclear aryl hydrocarbon (Ah) receptor ligand-activated complex	11622 11863	Mouse	2
10	13S condensin complex	9918 64151 23397 10592 10051	Human	5
11	BLOC-3 (biogenesis of lysosome-related organelles complex 3)	3257 89781	Human	2
12	BLOC-2 (biogenesis of lysosome-related organelles complex 2)	84343 11234 79803	Human	3
13	MUS81-CDS1 complex	1040 80198	Human	2
14	BLOC-2 (biogenesis of lysosome-related organelles complex 2)	12807 246694 20170	Mouse	3
15	NCOR complex	7464 2874 8841 9611 6907 79718	Human	6
23	BLOC-1 (biogenesis of lysosome-related organelles complex 1)	2647 282991 388552 55330 84062 63915 26258 23557	Human	8
24	BLOC-1 (biogenesis of lysosome-related organelles complex 1)	14533 73689 232946 117197 94245 17828 18457 20615	Mouse	8
25	9S-cytosolic aryl hydrocarbon (Ah) receptor non-ligand activated complex	11622 11632 15519 15516	Mouse	4
26	Bestrophin-protein phosphatase 2A complex	397169 397656 397136 397088 397470	Pig	5
27	Arp2/3 protein complex	10097 10096 10095 10109 10094 10093 10092	Human	7
28	Modulator	508448 518637 513315	Bovine	3
29	PA28gamma complex	10197	Human	1
30	PA28 complex	5720 5721	Human	2
31	PA28 complex	510041 509857	Bovine	2
32	PA700 complex	5700 5701 5702 5704 5705 5706 5707 5716 5717 5718 5719 10213 5708 5709 5710 5711 9861 5713 5714 5715	Human	20
33	Prefoldin	5201 5202 5203 5204 10471 7411	Human	6
34	Aqp4-Dag1-Dmd-Snta1 complex	25293 114489 362242 24907 24907	Rat	6
36	AP1 adaptor complex	162 164 8906 8907 10053 1174 8905 130340	Human	8
37	CDH23-harmonin transmembrane complex	22295 72088	Mouse	2
38	20S proteasome	26440 19166 19167 26441 26442 26443 26444 19170 26445 26446 19172 19173 19175 19177	Mouse	14
39	immunoproteasome	26440 19166 19167 26441 26442 26443 26444 19170 19171 26445 26446 19172 16913 16912	Mouse	14
40	COP9 signalosome complex (Gps1 Cops1 Cops2 Cops3 Cops4 Cops5 Cops6 Cops7a Cops7b Cops8)	12848 26572 26891 26754 26893 26894 26895 108679 209318	Mouse	9
41	Mi-2/NuRD-MTA2 complex	604 3065 53615 9219 57504	Human	5

42	CASK-MINT-VELI tripartite complex	83589 29647 85327 60377 60442	Rat	5
43	Gamma-secretase complex (APH1A PSEN1 PSENEN NCSTN variant)	51107 23385 5663 55851	Human	4
44	CASK-MINT complex	319924 12361	Mouse	2
46	AP2 adaptor complex	11772 163 116563 232910	Mammalia	4
47	DNA polymerase alpha-primase complex	18968 18969 19075 19076	Mouse	4
48	RGS7-Gbeta5-Galphaq complex	14682 14697 281452	Mammalia	3
49	DNMT3B complex	1789 3065 24137 25942 8467 10592 10051	Human	7
50	Rab5 GDP/GTP exchange factor complex	54189 56715	Mouse	2
51	CCT micro-complex	21454 12461 12462 12464 12465 12466 12468 12469	Mouse	8
52	CCT complex (chaperonin containing TCP1 complex)	21454 12461 12462 12464 12465 12466 12468 12469	Mouse	8
53	Gbeta5-rgs7 complex	83579 54296	Rat	2
54	SIN3 complex	3065 3066 5928 5931 10284 8819	Human	7
55	HDAC4-ERK1 complex	9759 5595	Human	2
56	Elongin (SIII) complex	64525 81807 25562	Rat	3
57	HDAC4-ERK2 complex	9759 5594	Human	2
58	SMRT complex	2874 8841 9612 6907 79718	Human	5
59	AP3 adapter complex	8546 8120 8943 26985 10947 1176	Human	7
60	Interferon-stimulated gene factor 3 transcription complex ISGF3	10379 6772 6773	Human	3
61	Mi2/NuRD complex	1108 3065 3066 53615 9219 5928	Human	7
62	MeCP1 complex	1108 3065 3066 8932 53615 9219 5928 5931	Human	8
63	Mitotic 14S cohesin 1 complex	5885 8243 9126 10274	Human	4
64	Mitotic 14S cohesin 2 complex	5885 8243 9126 10735	Human	4
65	Multiprotein trafficking complex	83589 29647 25252 117052 60442	Rat	5
66	TRAP complex	5469 9968 9282 10025 9440 9862 7067 9967	Human	8
67	AP4 adaptor complex	10717 23431 9179 11154	Human	4
68	BCDX2 complex	5889 5890 5892 7516	Human	4
69	ApoB RNA editing enzyme complex	170912 25383 171137	Rat	3
70	CLIC4 complex	81822 24264 83718 25751 64158 29753 25578	Rat	7
71	MRN complex (MRE11-RAD50-NBS1 complex)	4361 4683 10111	Human	3
72	R/M complex (RAD50-MRE11	4361 10111	Human	2
73	MRN complex (MRE11-RAD50-NBN complex)	4361 4683 10111	Human	3
74	TRPC1-Homer3-IP3R complex	29548 3708 7220	Mammalia	3
75	TSC1-TSC2 complex	7248 7249	Human	2
76	Cytoplasmic dynein complex	65028 29489 29564 116659 252902 65209 58945 170714 83462	Rat	9
77	nNos-Capon-Dexras1 complex	18125 70729 19416	Mouse	3
78	nNos-Capon-Dexras1 complex	24598 192363 64455	Rat	3
79	nNos-Capon-Syn1 complex	24598 192363 24949	Rat	3

80	Ubiquitin E3 ligase (Skp1A Skp2 Cul1 Rbx1)	8454 9978 6500 6502	Human	4
81	Ubiquitin E3 ligase (SKP1A FBXW8 CUL7 RBX1)	9820 26259 9978 6500	Human	4
82	Ubiquitin E3 ligase (Vhl Tceb1 Tceb2 Cul2 Rbx1)	71745 300084 64525 81807 24874	Rat	5
83	Tubulin:stathmin complex	616317 777775 281555	Bovine	3
84	Nucleic acid channel complex	24551 245922	Rat	2
86	NUMAC complex (nucleosomal methylation activator complex)	60 8289 10498 57410 6597 6598 6599 6601 6602 6605	Human	10
87	Nup 107-160 subcomplex	57122 55746 23279 79023 348995 79902 4928 6396 81929	Human	9
88	SNARE complex (Scfd1 Sec22b Stx5 Bet1 Gosr2 Gosr1)	29631 94189 64154 54350 310710 65134	Rat	6
89	Sec6/8 exocyst complex	55763 55770 11336 60412 10640 54536 23265 149371	Human	8
91	FA complex (Fanconi anemia complex) cytoplasmic	2175 2176 2188 2189	Human	4
92	CD28-transactivation complex	940 4691	Human	2
93	Anaphase-promoting complex	64682 29882 29945 51433 51434 8881 8697 996	Human	8
94	ATP-utilizing chromatin assembly and remodeling factor (hACF) complex	11177 8467	Human	2
95	Ku antigen-NARG1 complex	80155 7520 2547	Human	3
96	Anaphase-promoting complex	64682 10393 29882 29945 51433 51434 8881 8697 996	Human	9
97	Anaphase-promoting complex	17222 68999 99152 52206 59008 56317 381580 69957 52563	Mouse	9
98	p300-MDM2-p53 protein complex	2033 4193 7157	Human	3
100	hNURF complex	2186 5928 5931 6594	Human	4
101	Nucleolar remodeling complex (NoRC complex)	116848 93762	Mouse	2
102	Meiotic cohesin complex	56739 140557 13006 20962	Mouse	4
103	RNA polymerase II holoenzyme complex	2068 2071 2959 2960 2961 2962 2963 2965 2966 2967 2968 5430 5431 5432 5433 5434 5435 5436 5437 5438 5439 5440 5441 6908	Human	24
104	RNA polymerase II core complex	5430 5431 5432 5433 5434 5435 5436 5437 5438 5439 5440 5441	Human	12
105	Polycomb repressive complex 2 (PRC	8726 2146 5928 5931 23512	Human	5
106	Srf-myogenin-E12 complex	17928 20807 21423	Mouse	3
107	TFIIH transcription factor complex	902 1022 2068 2071 2965 2966 2967 2968 4331	Human	9
108	ApoB mRNA editing enzyme complex	170912 25383 171137	Rat	3
109	Fertilin complex (Adam1b Adam2)	280667 11495	Mouse	2
112	Prefoldin complex	5201 5202 5203 5204 10471 7411	Human	6
114	SNAPc (small nuclear RNA-activating protein) complex	6617 6618 6619 6621 10302	Human	5
115	Polycomb repressive complex 1 (PRC1 hPRC-H)	648 84733 8535 57332 3303 1911 1912 80012 6015 6045 22955 8467	Human	12

116	Polycomb repressive complex 1 (PRC1 hPRC-H)	648 84733 8535 57332 3303 1911 1912 80012 6015 6045 22955 8467	Human	13
117	GPR56-CD81-Galphaq/11-Gbeta complex	975 9289 2767 2776 2782 2783 2784 59345	Human	8
118	GPI-GnT activity complex	5277 5279 5283 9091	Human	4
120	Lymphotoxin beta receptor complex	4049 4050 4055	Human	3
122	MCM complex	17216 17215 17217 17218 17219	Mouse	6
123	Rnase/Mrp complex	10940 10775 51367 10248 11102 79897 54913 10556 10557 10799	Human	10
124	RGS9-GNB5-GNAT1-R9AP complex	281794 281453 282338	Bovine	3
126	CCT micro-complex	6950 10576 7203 10575 22948 908 10574 10694	Human	8
127	NDC80 kinetochore complex	10403 83540 147841 57405	Human	4
128	ORC complex (origin recognition complex)	18392 18393 50793 26428 26429 56452	Mouse	6
129	PIDDsome complex	835 8738 55367	Human	3
130	Gamma-secretase complex (Aph1a Psen1 Psenen Ncstn)	226548 59287 19164 66340	Mouse	4
131	Skeletal muscle sarcoglycan complex SGC alpha-beta-gamma-delta	20391 24051 24052 24053	Mouse	4
136	Troponin complex	100009341 100009581	Rabbit	2
138	Telosome complex	65057 25913 7013 7014 54386 26277	Human	6
139	SDH-mABC1-PIC-ANT-ATPase complex	362302 65262 157074 298596 245959 85333 25176	Rat	7
140	E-box sequence-binding complex	84707 8861 4005 4808	Human	4
141	Sos1-Abi1-Eps8 complex	11308 13860 20662	Mouse	3
142	CD147-gamma-secretase complex (APH-1a PS-1 PEN-2 NCT variant)	51107 682 23385 5663 55851	Human	5
143	APP-FE65-LRP complex	322 351 4035	Human	3
144	Gamma-BAR-AP1 complex	162 164 8906 8907 10053 1174 8905 130340 55435	Human	9
145	CCT:PFD complex	512043 505313 504735 613336 533784 521540 514355 281047 5201 5202 5203 5204 10471 7411	Mammalia	14
146	Src-dynamin-synapsin complex	20779 20964 13429 13430	Mouse	4
147	Src-dynamin-synapsin complex	140694 83805 24949	Rat	3
148	Src-dynamin-synapsin-alpha-adaptin complex	81637 140694 83805 24949	Rat	4
149	PBAF complex (Polybromo- and BAF containing complex)	196528 55193 6597 6598 6599 6601 6602 6605 60 71 86 51412	Human	12
150	CCT complex (chaperonin containing TCP1 complex) testis specific	512043 505313 504735 613336 533784 538090 514355 281047	Bovine	8
152	TFIIE complex	2960 2961	Human	2
153	TFIIF complex (transcription factor	2962 2963	Human	2
154	Src1-Ep300-Crebbp complex	12914 328572 17977	Mouse	3
155	TFIID complex	6872 6881 6882 6873 6874 6877 6878 6880 6908	Human	9
156	Retrotranslocation complex	79139 50628 84447	Human	3
157	Condensin I complex	9918 64151 23397 10592 10051	Human	5

158	ATP synthasome	65262 171374 116550 245965 245958 171375 29754 171082 114630 641434 140608 94271 192241 26197 26196 245959 25176	Rat	17
159	Condensin I-PARP-1-XRCC1 complex	9918 64151 23397 142 10592 10051	Human	7
160	Condensin II	23310 54892 29781 10592 10051	Human	5
161	SWAP complex	17975 18148 11545 20947	Mouse	4
162	Conserved oligomeric Golgi (COG) complex	9382 22796 83548 25839 10466 57511 91949 84342	Human	8
163	Cohesin-SA2 complex	5885 8243 9126 10735	Human	4
164	Cohesin-SA1 complex	5885 8243 9126 10274	Human	4
168	Protein-sorting complex (Stam2 Hgs Eps15)	13858 15239 56324	Mouse	3
169	Protein-sorting complex (Stam1 Hgs Eps15)	13858 15239 8027	Mammalia	3
170	Ric-8A G(i) alpha-1 subunit complex	25686 293614	Rat	2
171	Ric-8A G(i) alpha-2 subunit complex	81664 293614	Rat	2
172	Ric-8A G(o) alpha-1 subunit complex	50664 293614	Rat	2
174	Ric-8A G(o) alpha-2 subunit complex	50664 293614	Rat	2
175	Ric-8A G(q) alpha subunit complex	81666 293614	Rat	2
176	Ric-8A G alpha 13 complex	303634 293614	Rat	2
177	OGT-TRAK1-TRAK2 complex	8473 22906 66008	Human	3
178	Respiratory chain complex I (holoenzyme) mitochondrial	4535 4536 4537 4539 4540 4541 4694 4705 126328 55967 51079 4695 4696 4697 4698 4700 4701 4702 4704 4706 4707 4716 54539 4708 4709 4710 4711 4712 4713 4714 4715 4717 4718 4719 4720 4722 4724 4725 4726 374291 4728 4723 4729 4731	Human	44
179	Nephrin multiprotein complex	29647 361598 113970 64563 305614 64159 81634 63836	Rat	8
180	PNUTS-PP1 complex	5499 5514	Human	2
181	26S proteasome	5682 5683 5684 5685 5686 5687 5688 5689 5690 5691 5692 5693 5694 5695 5700 5701 5702 5704 5705 5706 5719 5710	Human	22
182	Synaptotagmin-sodium channel complex (Syt1-Scn1a-Scn1b)	81574 29686 25716	Rat	3
183	Synaptotagmin-sodium channel complex (Syt2-Scn1a-Scn1b)	81574 29686 24805	Rat	3
184	Synaptotagmin-sodium channel complex (Syt1-Scn2a-Scn1b)	29686 24766 25716	Rat	3
185	Synaptotagmin-sodium channel complex (Syt2-Scn2a-Scn1b)	29686 24766 24805	Rat	3
186	Wave-2 complex	10006 55845 23191 10787 10163	Human	5
187	Wave-2 complex (Rac-activated)	11308 20430 50884 19353 242687	Mouse	5
188	Wave-2 complex	11308 20430 50884 242687	Mouse	4
189	BAF complex	6595 6597 6598 6599 6601 6602 6605 60 71 86 51412 8289 57492	Human	13
190	Mitotic checkpoint complex (MCC)	701 9184 991 4085	Human	4

191	20S proteasome	5682 5683 5684 5685 5686 5687 5688 5689 5690 5691 5692 5693 5694 5695	Human	14
192	PA28-20S proteasome	5682 5683 5684 5685 5686 5687 5688 5689 5690 5691 5692 5693 5694 5695 5720 5721	Human	16
193	PA700-20S-PA28 complex	5682 5683 5684 5685 5686 5687 5688 5689 5690 5691 5692 5693 5694 5695 5700 5701 5702 5704 5705 5706 5707 5716 5717 5718 5719 10213 5708 5709 5710 5711 9861 5713 5714 5715 5720 5721	Human	36
194	PA28gamma-20S proteasome	5682 5683 5684 5685 5686 5687 5688 5689 5690 5691 5692 5693 5694 5695 10197	Human	15
195	Ubiquitin E3 ligase (Lrrc41 Tceb1 Tceb2 Cul5 Rbx1)	64624 362566 300084 64525 81807	Rat	5
196	Ubiquitin E3 ligase (Vhl Tceb1 Tceb2 Cul5 Rbx1)	64624 300084 64525 81807 24874	Rat	5
198	Ubiquitin E3 ligase (Tceb1 Tceb2 Tceb3 Cul5 Rbx1)	64624 300084 64525 81807 25562	Rat	5
199	Ubiquitin E3 ligase (Socs1 Tceb1 Tceb2 Cul5 Rbx1)	64624 300084 252971 64525 81807	Rat	5
200	Ubiquitin E3 ligase (Wsb1 Tceb1 Tceb2 Cul5 Rbx1)	64624 300084 64525 81807 303336	Rat	5
201	HUIC complex	580 672	Human	2
202	BRCA1-RAD50-MRE11-NBS1	672 4361 4683 10111	Human	4
203	Ubiquitin E3 ligase (Med8 Tceb1 Tceb2 Cul2 Rbx1)	71745 80509 300084 64525 81807	Rat	5
204	Ubiquitin E3 ligase (Asb2 Tceb1 Tceb2 Cul5 Rbx1)	299266 64624 300084 64525 81807	Rat	5
205	Ubiquitin E3 ligase (VHL TCEB1 TCEB2 CUL2 RBX1)	8453 9978 6921 6923 7428	Human	5
206	DNA ligase IV-XRCC4 complex	3981 7518	Human	2
207	Ubiquitin E3 ligase (ASB2 TCEB1 TCEB2 CUL5 RNF7)	51676 8065 9616 6921 6923	Human	5
208	Ubiquitin E3 ligase (ASB1 TCEB1 TCEB2 CUL5 RNF7)	51665 8065 9616 6921 6923	Human	5
209	Ubiquitin E3 ligase (ASB6 TCEB1 TCEB2 CUL5 RNF7)	140459 8065 9616 6921 6923	Human	5
210	Ubiquitin E3 ligase (ASB7 TCEB1 TCEB2 CUL5 RNF7)	140460 8065 9616 6921 6923	Human	5
211	Ubiquitin E3 ligase (ASB12 TCEB1 TCEB2 CUL5 RNF7)	142689 8065 9616 6921 6923	Human	5
212	DNA ligase III-XRCC1 complex	3980 7515	Human	2
213	DNA ligase IV-XRCC1 complex	3981 7515	Human	2
214	Ubiquitin E3 ligase (WSB1 TCEB1 TCEB2 CUL5 RBX1)	8065 9978 6921 6923 26118	Human	5
216	PDZK1-NaPiIIa-MAP17 complex	59020 67182 20505	Mouse	3
217	CRSP complex	5469 9282 9440 9439 9862 9441 9442 9443	Human	8
219	CAND1-CUL1-RBX1 complex	55832 8454 9978	Human	3
220	ARF-Mule complex	1029 10075 4869	Human	3

221	CAND1-CUL2-RBX1 complex	55832 8453 9978	Human	3
222	CAND1-CUL3-RBX1 complex	55832 8452 9978	Human	3
223	CAND1-CUL4A-RBX1 complex	55832 8451 9978	Human	3
224	CAND1-CUL4B-RBX1 complex	55832 8450 9978	Human	3
226	Ubiquitin E3 ligase (SKP1A SKP2 CUL1)	8454 6500 6502	Human	3
227	Ubiquitin E3 ligase (SKP1A BTRC CUL1)	8945 8454 6500	Human	3
228	SMCC complex	892 1024 5469 9282 9412 9862 51003 10001	Human	8
229	NAT complex	892 1024 84246 9282 9412 9439	Human	7
230	Mediator complex	892 23097 1024 5469 84246 400569 9968 9969 23389 9282 51586 10025 9440 54797 9477 9412 6837 9439 9862 81857 9441 9442 80306 55588 90390 51003 29079 10001 9443	Human	32
232	ARC complex	27034 5469 9968 9969 9282 51586 9440 9439 9862 81857 9441 29079 10001 9443 112950	Human	15
234	HuCHRAC complex	11177 54108 54107 8467	Human	4
235	WICH complex	22385 93762	Mouse	2
236	WICH complex	9031 8467	Human	2
237	Ags3-Lkb1-Gnai3 complex	25643 246254	Rat	2
238	SWI-SNF chromatin remodeling-related-BRCA1 complex	86 672 6595 6597 6598 6599 6601 6603 6605 8289 57492	Human	11
239	Sin3-Hdac1-Sds3 complex	433759 20467 71954	Mouse	3
240	BRCA1-CTIP-ZBRK1 repressor	672 5932 59348	Human	3
242	BRCA1-BACH1 complex	672 83990	Human	2
243	RalBP1-CDC2-CCNB1 complex	891 983 10928	Human	3
244	BRAFT complex	641 2175 2176 2178 2188 2189 55120 4292 80010 6117 6118 6119 7156	Human	13
245	FA core complex (Fanconi anemia core complex)	80233 2175 2187 2176 2178 2188 2189 55120 80010	Human	9
246	BLM complex III	641 4292 80010 7156	Human	4
247	RalBP1-CCNB1-AP2A-NUMB-EPN1 complex	891 29924 8650 10928 160 161	Human	6
249	Caspase-2-TRAF2-RIP1 complex	12366 19765 22030	Mouse	3
252	RAD51C-XRCC3 complex	5889 7517	Human	2
254	TBPIP/HOP2-MND1 complex	84057 29893	Human	2
255	TBPIP/HOP2-Mnd1 complex	76915 19183	Mouse	2
256	RAD51B-RAD51C complex	5889 5890	Human	2
257	Hop2-Mnd1 complex	76915 19183	Mouse	2
261	RAD51B-RAD51C-RAD51D-XRCC2-XRCC3 complex	5889 5890 5892 7516 7517	Human	5
262	RAD51L3-XRCC2 complex	5892 7516	Human	2
265	ATR-ATRIP complex	545 84126	Human	2
266	RAD17-RFC complex	5884 5982 5983 5984 5985	Human	5
267	Checkpoint 9-1-1 complex	3364 5810 5883	Human	3
268	Checkpoint Rad complex	3364 5810 5884 5883 5982 5983 5984 5985	Human	8

272	Dysbindin-pallidin-muted-beta-dystrobrevin complex	13528 94245 17828 18457	Mouse	4
274	RAD17-RFC-9-1-1 checkpoint supercomplex	3364 5810 5884 5883 5982 5983 5984 5985	Human	8
277	RFC complex	5981 5982 5983 5984 5985	Human	5
278	RFC core complex	5982 5984 5985	Human	3
279	RFC complex (activator A 1 complex)	5981 5982 5983 5984 5985	Human	5
280	HMGB1-HMGB2-HSC70-ERP60-GAPDH complex	2597 3146 3148 3312 2923	Human	5
281	NELF complex (Negative elongation factor complex)	25920 7936 51497 7469	Human	4
282	SNF2h-cohesin-NuRD complex	11177 1107 3065 3066 8932 53615 9112 9219 5885 5928 5931 8467 8243 9126 10274 10735	Human	16
283	Sin3 complex	3065 3066 5928 5931 10284 8819	Human	7
284	CRSP complex	5469 9282 51586 9440 9412 9439 9862 81857 9441 10001 9443	Human	11
285	PCNA-MLH1-PMS1 complex	4292 5111 5378	Human	3
286	PCNA-MSH2-MSH6 complex	4436 2956 5111	Human	3
287	ARC-L complex	892 1024 5469 9968 9969 9282 51586 9440 9412 9439 9862 81857 10001	Human	14
288	ARC complex	892 1024 5469 9968 9969 9282 51586 9440 9412 9439 9862 81857 9441 10001 9443	Human	15
290	MSH2-MLH1-PMS2-PCNA DNA-repair initiation complex	4292 4436 5111 5395	Human	4
291	MSH2-MLH1-PMS2 DNA-repair initiation complex	4292 4436 5395	Human	3
292	MutL-alpha complex	4292 5395	Human	2
293	PCNA-DNA polymerase delta complex	18538 18971 18972 67967 69745	Mouse	5
294	DNA polymerase delta complex	18971 18972 67967 69745	Mouse	4
295	PCNA-DNA ligase complex	16881 18538 18972 67967	Mouse	4
296	Pcna-Msh2-Msh6 complex	17685 17688 18538	Mouse	3
297	PCNA-DNA polymerase delta complex	5111 5424 5425 10714 57804	Human	5
298	VEGF transcriptional complex	328 3091 6774 4435 10370 163732	Human	6
299	IRF3-CBP complex	3661 4435 10370 163732	Human	4
300	PC2 complex	84246 9282 10025 9440 9477 9412 9862 9442 51003 10001 9443 9967	Human	12
301	SMCC complex	892 1024 5469 84246 9968 9969 9282 10025 9440 9477 9412 9862 9442 51003 10001 9443 9967	Human	17
302	INO80 chromatin remodeling complex	86 79913 93973 125476 283899 54891 54617 10445 4798 8607 10856 6929 83444	Human	13
304	SRCAP-associated chromatin remodeling complex	86 64431 55840 3015 8607 10856 10847 6944 8089 10467	Human	10
305	40S ribosomal subunit cytoplasmic	2197 6204 6205 6206 6207 6208 6209 6210 6217 6218 6222 6223 6187 6224 6227 6228 6229 6230 6231 6232 6233 6234 6235 6188 6189 6191 6193 6194 6201 6202 6203 3921 6192 140032	Human	34

306	Ribosome cytoplasmic	2197 6134 4736 6135 6136 6137 23521 9045 6138 6139 6141 6142 6143 6144 6146 9349 6147 6152 6154 6155 6157 6158 6159 6122 6156 6160 6161 6164 11224 6165 25873 6173 6167 6168 6169 6170 6124 6171 6125 6128 6129 6130 6132 6133 6175 6176 6181 6204 6205 6206 6207 6208 6209 6210 6217 6218 6222 6223 6187 6224 6227 6228 6229 6230 6231 6232 6233 6234 6235 6188 6189 6191 6193 6194 6201 6202 6203 3921 7311 6192 [illegible]	Human	81
307	Epithelial utrophin-associated protein complex	13528 20650 22288	Mammalia	3
308	60S ribosomal subunit cytoplasmic	6134 4736 6135 6136 6137 23521 9045 6138 6139 6141 6142 6143 6144 6146 9349 6147 6152 6154 6155 6157 6158 6159 6122 6156 6160 6161 6164 11224 6165 25873 6173 6167 6168 6169 6170 6124 6171 6125 6128 6129 6130 6132 6133 6175 6176 6181 7311	Human	47
309	RC complex	18968 514793 281990 281991 19075 19076 19687 19718 515602 106344	Bovine	11
310	Cell cycle kinase complex CDC2	891 9133 595 983 1026 5111	Human	6
311	Cell cycle kinase complex CDK2	595 1017 1026 5111	Human	4
312	Cell cycle kinase complex CDK4	595 1019 1026 5111	Human	4
313	Cell cycle kinase complex CDK5	595 896 1020 1026 5111	Human	5
314	PCNA-p21 complex	1026 5111	Human	2
315	28S ribosomal subunit mitochondrial	7818 55173 64963 6183 63931 64960 51021 51373 55168 28973 51023 51116 54460 56945 51649 64951 64432 64949 23107 28957 10884 10240 51650 65993 60488 92259	Human	30
316	Dystrophin associated complex DPC (Dmd Dtnb) brain-derived	362715 24907 24907 24907	Rat	4
317	Brain-derived dystrobrevin-syntrophin complex	13405 13527 13528 20648 20649 20650 71096 268534	Mouse	8
318	Muscle-derived dystrobrevin-syntrophin complex	13527 20648 20649 20650 71096 268534	Mouse	6

320	55S ribosome mitochondrial	7818 114294 65008 124995 65003 6182 28998 64928 29088 54948 63875 29074 9801 51069 55052 219927 29093 6150 79590 51264 10573 11222 51263 64983 9553 64981 51318 64979 51253 64978 54148 51073 64976 64975 28977 84545 65080 84311 26589 57129 51642 740 54534 51258 122704 116540 116541 128308 65005 55173 64963 6183 63931 64960 51021 51373 55168 28973 51023 51116 54460 56945 51649 64951 64432 64949 23107 28957 10884 10240 [illegible]	Human	78
321	p27-cyclinD2-Cdk4 complex	12444 12567	Mammalia	2
322	DNA-PK-Ku complex	5591 7520 2547	Human	3
323	28S ribosomal subunit mitochondrial	504320 515885 509816 445421 510899 508143 614102 510824 613561 505681 614343 532044 515228 617466 533011 516004 514740 535290 516084 534185 614148 523435 618357 513438	Bovine	29
324	39S ribosomal subunit mitochondrial	114294 65008 124995 65003 6182 28998 64928 29088 54948 63875 29074 9801 51069 55052 219927 29093 6150 79590 51264 10573 11222 51263 64983 9553 64981 51318 64979 51253 64978 54148 51073 64976 64975 28977 84545 65080 84311 26589 57129 51642 740 54534 51258 122704 116540 116541	Human	48
325	Skeletal muscle sarcoglycan complex SGC beta-gamma-delta-zeta	24051 24052 24053 244431	Mouse	4
326	Smooth muscle sarcoglycan complex SGC beta-delta-zeta	24051 24052 244431	Mouse	3
328	Ku antigen complex	7520 2547	Human	2
330	PSF-p54(nrb) complex	4841 6421	Human	2
332	Skeletal muscle sarcoglycan complex SGC alpha-beta-epsilon-gamma	20391 24051 20392 24053	Mouse	4
5856	AK2-FADD-caspase-10 (AFAC10) complex	204 843 8772	Human	3
334	Skeletal muscle sarcoglycan complex SGC epsilon-beta-gamma-delta	24051 24052 20392 24053	Mouse	4
335	p54(nrb)-PSF-matrin3 complex	9782 4841 6421	Human	3
336	DNA ligase IV-XRCC4-AHNK	79026 3981 7518	Human	3
337	Dystrophin-sarcoglycan-syntrophin complex skeletal muscle	13405 20391 24051 24052 24053 20648 20649 20650 71096 268534	Mouse	10
339	Smooth muscle dystroglycan complex	100009214 100009278 100009208 24052 20392 100008714 22288	Rabbit	7
340	Dystrophin-glycoprotein complex DGC skeletal muscle	100009214 100009278 13405 100009178 100009208 24052	Rabbit	8

342	Skeletal muscle sarcoglycan-sarcospan complex SG-SPN	100009214 100009178 100009208 24052 100008714	Rabbit	5
343	Sarcoglycan-sarcospan complex SG-SPN	6442 6443 6444 6445 8082	Human	5
344	DNA ligase IV-XRCC4 complex (LX complex)	3981 7518	Human	2
346	Sarcoglycan-sarcospan-dystroglycan complex	13138 20391 24051 24052 24053 16651	Mouse	6
347	Sarcoglycan-sarcospan-complex SG-SPN	20391 24051 24052 24053 16651	Mouse	5
349	Sarcoglycan-sarcospan-syntrophin-dystrobrevin complex	20391 24051 24052 24053 20648 20649 16651 13527 13528	Mouse	9
350	DNA ligase IV-XRCC4-PNK complex	3981 11284 7518	Human	3
351	Spliceosome	22985 9716 7919 10286 84811 8896 29105 58509 79002 10421 51362 988 1153 11052 51755 51340 51747 56259 51503 57703 10521 9416 1654 51428 9879 1655 8220 1665 8449 9785 1659 1660 22826 9343 9775 55660 94104 27336 10643 3550 57461 25962 57819 27258 25804 11157 51690 4116 4236 4343 4686 22916 4809 11051 26986 5356 10450 10465 51645 23759 53938 5496 23398 8559 27339 9129 26121 9128 55660 8899 24148 10594 22827 64783 84991 55696 58517 9584 9939 10921 9092 7536 10291 8175 10946 23451 51639 10992 23450 10262 6426 9295 6427 6428 6429 6430 6432 23517 10569 10285 55234 23020 11017 9410 6625 6626 6627 6628 6629 6631 6632 6633 6634 6635 6636 6637 22938 23013 23350 10250 23524 51593 6431 8683 25949 10915 24144 9984 57187 84321 10189 8563 79228 80145 6434 10907 7307 11338	Human	143
353	DNA ligase IV-condensin complex	3981 10592 10051	Human	3
355	DNA-PK-Ku antigen complex	5591 7520 2547	Human	3
358	DNA ligase IV-Xrcc4-DNA-protein kinase complex	319583 19090 108138	Mouse	3
359	DNA ligase IV-XRCC4-XLF complex	3981 79840 7518	Human	3
360	Artemis-DNA-PK complex	64421 5591	Human	2
362	DNA ligase III-XRCC1-PNK-DNA-pol III multiprotein complex	3980 11284 5423 7515	Human	4
364	RC-1 complex (recombination complex 1)	16882 18973 18974 282370 14211	Bovine	5
366	Rag1-Rag2 protein-DNA complex	19373 19374	Mouse	2
367	Rag1-Rag2-Ku70-Ku80 protein-DNA complex	15289 19373 19374 22596 14375	Mouse	5
368	ERCC1-ERCC4-MSH2 complex	2067 2072 4436	Human	3
369	MSH2-MSH6-PMS2-MLH1 complex	4292 4436 2956 5395	Human	4
370	MSH2-MSH6-PMS1-MLH1 complex	4292 4436 2956 5378	Human	4

371	Structure-specific endonulease complex	2067	Mammalia	1
372	F0F1 ATP synthase mitochondrial	65262 171374 116550 245965 245958 171375 641434 140608 94271 57423 25392 300677 26197 26196 29754 171082 114630	Rat	17
373	Vacuolar ATPase	327687 513520 286768 550622 282148 338075 282147 338089 404152 287017 282405 281641	Bovine	14
374	MSH2-MSH6 complex	4436 2956	Human	2
375	MSH2-MSH3 complex	4436 4437	Human	2
376	PCNA-MutS-alpha-MutL-alpha-DNA complex	4292 4436 2956 5111 5395	Human	5
377	PCNA-MutS-alpha-DNA initial	4436 2956 5111	Human	3
378	MutS-beta complex	4436 4437	Human	2
380	MutL-beta complex	4292 5378	Human	2
381	Respiratory chain complex I mitochondrial	78330 17717 17721 17716 17719 67273 66414 67184 17991 66091 17992 68202 67130 66416 68375 66108 70316 68342 104130 66495 68194 66046 230075 66916 67264 66218 68197 227197 226646 68349 17993 595136 407785 75406 225887	Mouse	37
382	Respiratory chain complex I mitochondrial	78330 17721 17716 17718 17719 54405 67273 69875 66414 67184 17991 66091 17992 68202 67130 66416 68375 66108 70316 68342 104130 68198 66495 68194 66046 230075 66916 67264 66218 68197 227197 226646 68349 17993 595136	Mouse	40
384	Caveolar macromolecular signaling complex	11514 11555 12288 12391 14683 51792 19088	Mouse	7
385	Caveolar macromolecular signaling complex using anti-Cav-3	11514 11555 12288 12391 14683 51792 19088 14677 14678 14679	Mouse	10
386	Ubiquitin E3 ligase (UBADC1	63891 10422	Human	2
387	MCM complex	4171 4172 4173 4174 4175 4176	Human	6
388	Respiratory chain complex I mitochondrial	3283884 3283886 3283885 3283888 327673 338060 326346 281742 338084 327698 338064 327704 327714 327670 338063 327710 404188 327702 327690 327701 404161 327713 338073 327706 338061 327665 338065 282517 327660 282289 338046 288380 327697 287327 327680 338057	Bovine	42
389	ORC complex (origin recognition complex)	4998 4999 23595 5000 5001 23594	Human	6
390	Ubiquitin E3 ligase (SKP1A FBXW2 CUL1)	8454 26190 6500	Human	3
391	Ubiquitin E3 ligase (DDB1 CUL4A RBX1)	8451 1642 9978	Human	3
392	Succinyl-CoA synthetase GDP-	399539 397026	Pig	2
393	Succinyl-CoA synthetase GDP-	8802 8801	Human	2

394	Succinyl-CoA synthetase ADP-forming	8803 8802	Human	2
395	Succinyl-CoA synthetase ADP-forming	20916 56451	Mouse	2
396	Succinyl-CoA synthetase GDP-	56451 20917	Mouse	2
397	Succinyl-CoA synthetase ADP-forming	114597 362404	Rat	2
398	Egasyn-glucuronidase complex	13897 110006	Mouse	2
399	Respiratory chain complex I mitochondrial	3283884 3283886 3283885 3283888 327673 338060 326346 281742 338084 327698 338064 327704 327714 327670 338063 327710 404188 327702 327690 327701 404161 327713 338073 327706 338061 327665 338065 282517 327660 282289 338046 288380 327697 287327 327680 338057	Bovine	42
400	Respiratory chain complex I (subcomplex I alpha) mitochondrial	3283888 327673 338060 326346 281742 338084 327698 338064 327714 327670 338063 327710 404188 327702 327706 338065 288380 327697 287327 327691	Bovine	25
401	Respiratory chain complex I (subcomplex I lambda) mitochondrial	281742 338084 327698 327714 338063 338065 288380 327697 287327 327691 338079 287027	Bovine	15
402	Respiratory chain complex I (subcomplex I beta) mitochondrial	3283886 327702 327690 327701 404161 327713 338073 327706 338061 327665 338065 282517	Bovine	13
403	Cytochrome bc1-complex (EC 1.10.2.2) mitochondrial	512500 3283889 281570 616109 616871 282393 282394 287020	Bovine	10
404	Isocitrate dehydrogenase cytoplasmic	3417	Human	1
405	Glucosidase 2	23193 5589	Rat	2
406	N-acetylglucosamine-1-phosphotransferase	79158 508713	Bovine	2
407	Guanylyl cyclase soluble (GUCY1A2 GUCY1B3)	2977 2983	Human	2
408	Guanylyl cyclase soluble (GUCY1A3 GUCY1B3)	2982 2983	Human	2
409	Gamma-glutamylcysteine synthetase	25283 29739	Rat	2
413	(ER)-localized multiprotein complex Ig heavy chains associated	29867 67838 22027 14828 12282 18453 12304 19035 64136 320011	Mouse	10
414	(ER)-localized multiprotein complex in absence of Ig heavy chains	29867 22027 14828 12282 18453 12304 19035 64136 320011	Mouse	9
415	EXO1-MLH1-PMS2 complex	9156 4292 5395	Human	3
416	Nitric oxide synthase-dystrophin complex skeletal muscle	13405 18125	Mouse	2
417	COX-2-Cav-3 complex	29161 29527	Rat	2
418	H/ACA ribonucleoprotein complex	170944 499709 52530 66181	Rat	4
419	Farnesyltransferase	2339 2342	Human	2
420	SAP97-Cav-3-Kv1.5 complex	12391 13383 25470	Mammalia	3
5850	p65-p65 Nf(kappa)B complex Il-1-beta induced	309165	Rat	1

421	SAP97-Cav-3-Kv1.5 complex	29161 25252 25470	Rat	3
422	Beta-dystroglycan-caveolin-3 complex	859 1605	Human	2
424	EXO1-MLH1-PCNA complex	9156 4292 5111	Human	3
425	MSH4-MSH5-GPS2 complex	2874 4438 4439	Human	3
426	Meprin A	4224 4225	Human	2
427	Tripeptidyl peptidase	22019	Mouse	1
428	Molybdopterin synthase	4338 4338	Human	2
429	Phosphatidylinositol 3-kinase complex (PIK3C3 PIK3R4)	5289 30849	Human	2
430	18S U11/U12 snRNP	79622 154007 1665 10081 10594 55599 23451 51639 10992 23450 10262 83443 6628 6632 6633 6634 6635 6636 6637 11066 4904 85437	Human	24
432	N-NOS-CHIP-HSP70-1 complex	3303 4842 10273	Human	3
433	BASC complex (BRCA1-associated genome surveillance complex)	472 641 672 4292 4361 4436 2956 4683 10111 5981 5982 5984	Human	12
434	BASC (Ab 80) complex (BRCA1-associated genome surveillance complex)	641 672 4292 4436 2956 5981 5982 5984	Human	8
435	BASC (Ab 81) complex (BRCA1-associated genome surveillance complex)	472 672 4292 5981 5982 5984	Human	6
436	BASC (Ab C-20) complex (BRCA1-associated genome surveillance complex)	672 2956 10111	Human	3
437	AQP2-binding multiprotein force generator complex	63836 56611 79125 25386 296654 108897 25745 50685 501203 298975 361710 64159 59069 81822 287876	Rat	15
438	GCN5-TRRAP histone acetyltransferase complex	672 2648 4436 2956 8464 10474 6881 27097 6880 8295	Human	10
439	Phosphatidylinositol 3-kinase complex (PIK3CA PIK3R1)	282306 282307	Bovine	2
440	Succinate dehydrogenase complex II (EC 1.3.5.1) mitochondrial	66945 67680 66052 66925	Mouse	4
441	TFTC-type histone acetyl transferase complex	2099 2648 5469 9968 9969 9282 10025 9440 9439 9862 8295	Human	11
442	Vacuolar ATPase	550622 282148 282147 338089 404152 287017 282405 281641 282657 286768 338038 338059	Bovine	13
443	BP-SMAD complex	571 2071 10804 7071 4601 5702 4093 7045	Human	8
444	Signal peptidase complex	404004 404003 404016 404005	Dog	4
445	TFTC complex (TATA-binding protein-free TAF-II-containing complex)	6314 2648 23450 8464 10474 6881 6883 6873 6874 6877 27097 6878 10629 6879 6880 8295	Human	16
446	Succinate dehydrogenase complex II (EC 1.3.5.1) mitochondrial	281480 286840 327696 281481	Bovine	4
447	Kir4.1-dystrophin complex retinal Mueller cells	29718 24907 24907 24907	Rat	4
448	Kir4.1-alpha-syntrophin complex retinal Mueller cells	29718 362242	Rat	2

449	Kir4.1-beta-dystroglycan complex retinal Mueller cells	114489 29718	Rat	2
450	Kir4.1-Aqp4 complex retinal Mueller cells	25293 29718	Rat	2
451	Kir4.1-dystrobrevin complex retinal Mueller cells	362715 29718	Rat	2
452	Retinal Mueller cells Aqp4-alpha-syntrophin complex	25293 362242	Rat	2
453	Retinal Mueller cells Aqp4-beta-dystroglycan complex	25293 114489	Rat	2
454	MKP3-CK2alpha complex	12995 67603	Mouse	2
457	Dystrophin-associated glycoprotein complex DGC (Dag1 Dp71f Sgcd Snta1) retinal glia cells	114489 13405 24052 362242	Rat	4
458	Dystrophin-associated glycoprotein complex DGC (Dag1 Dp71f Utrn) retinal glia cells	114489 13405 25600	Rat	3
460	CRB1-MPP5-INADL complex	23418 10207 64398	Human	3
461	MPP4-MPP5-CRB1 complex	23418 58538 64398	Human	3
463	TFIID-beta complex	6872 6881 6882 6883 6884 6874 6877 6878 6879 6880 6908	Human	11
464	InsP3R1-HAP1A-Htt complex	29430 29424 25262	Rat	3
465	InsP3R1-HAP1A-Htt complex	15114 15194 16438	Mouse	3
466	Gamma-secretase complex (APH1A PSEN1 PSENEN NCSTN)	51107 23385 5663 55851	Human	4
467	Gamma-secretase complex (APH1B PSEN2 PSENEN NCSTN)	83464 23385 5664 55851	Human	4
468	Gamma-secretase complex (APH1A PSEN2 PSENEN NCSTN)	51107 23385 5664 55851	Human	4
469	TFIID-beta complex	6872 6881 6882 6883 6884 6873 6874 6877 6878 6879 6880 6908	Human	12
470	TFTC complex (TATA-binding protein-free TAF-II-containing complex)	6314 2648 23450 8464 10474 6881 6883 6884 6873 6874 6877 27097 6878 10629 6879 6880 8295	Human	17
471	PCAF complex	8850 8464 6871 10474 6881 6883 27097 10629 6880 8295	Human	10
472	Prolyl 4-hydroxylase (alpha(I)-type)	5033 5034	Human	2
473	Prolyl 4-hydroxylase (alpha(II)-type)	18452 5034	Mammalia	2
474	Prolyl 4-hydroxylase (alpha(III)-type)	283208 5034	Human	2
475	STAGA complex (SPT3-TAF9-GCN5 acetyltransferase complex)	2648 23450 8464 9913 117143 10474 6881 6883 27097 10629 6880 8295	Human	12
476	STAGA complex (SPT3-TAF9-GCN5 acetyltransferase complex)	6314 2648 23450 8464 9913 117143 10474 6881 6883 27097 10629 6880	Human	13
478	STAGA core complex (SPT3-TAF9-GCN5 acetyltransferase complex)	2648 8464 6880	Human	3
479	Mupp1-Dlg4-Syngap1-CamkII complex	25400 24245 29495 29365 192117	Rat	5
480	TIF-IB complex	21339 21340 21341 21374	Mouse	4
482	cRET-Shc-Grb2-Gab2-P85PI3K-Shp2 complex	14389 14784 18708 19247 19713 20416	Mouse	6
484	TFIID complex	6872 6881 6882 6883 6884 6874 6877 6878 6879 6880 6908	Human	11

485	TFIID complex B-cell specific	6872 6881 6882 6883 6884 6874 6875 6877 6878 6879 6880 6908	Human	12
486	WIP-WASp-actin-myosin-IIa complex	59 10627 4627 4637 7454 7456	Human	6
488	Vezf1-p68RacGap complex	239027 22344	Mouse	2
489	TFIIA complex	2957 2958	Human	2
492	DA complex	2957 2958 6872 6881 6882 6883 6884 6873 83860 6874 6877 6878 6879 6880 6908	Human	15
493	DAB complex	2957 2958 2959 6872 6881 6882 6883 6884 6873 83860 6874 6877 6878 6879 6880 6908	Human	16
495	Cytochrome bc1-complex (EC 1.10.2.2) mitochondrial	66445 17711 66594 66152 67530 22273 67003 66694 66576 22272	Mouse	10
496	TFIID complex	270627 24075 68776 66464 319944 228980 226182 21343 24074 63856 108143 21374	Mouse	12
497	TAF4-TAF12 subcomplex of TFIID complex	6883 6874	Human	2
498	Ryk-Wny1-Fzd8 complex	14370 20187 22408	Mouse	3
499	Ryk-Wny1 complex	20187 22408	Mouse	2
500	Ryk-Wnt3a complex	20187 22416	Mouse	2
501	Ryk-Dvl1 complex	13542 20187	Mouse	2
502	Kir4.1-dystrophin complex whole brain lysate	13405 16513	Mouse	2
503	Kir4.1-alpha-1-syntrophin complex whole brain lysate	16513 20648	Mouse	2
504	Kir4.1-beta-dystroglycan complex whole brain lysate	13138 16513	Mouse	2
505	Kir4.1-dystrophin complex cortical astrocytes	13405 16513	Mouse	2
506	Kir4.1-alpha-1-syntrophin complex cortical astrocytes	16513 20648	Mouse	2
507	Kir4.1-beta-dystroglycan complex cortical astrocytes	13138 16513	Mouse	2
508	TFIID complex	6872 6881 6882 6883 6874 6877 6878 6879 6880 6908	Human	10
509	TFIID complex	6872 6881 6882 6883 6884 8148 6874 6877 6878 6879 6880 6908	Human	12
510	TFIID subcomplex testis-specific	74469 21374	Mouse	2
511	TFIID subcomplex testis-specific	270627 74469	Mouse	2
512	AQP-1 regulatory complex	25240 29232 25643 25472 29692	Rat	5
513	TFTC complex (TATA-binding protein-free TAF-II-containing complex)	6314 2648 23450 8464 10474 6881 6883 6873 6874 6877 27097 6878 10629 6879 6880 51616 8295	Human	17
514	TFIID-beta complex	6872 6881 6882 6883 6884 6874 6877 6878 6879 6880 51616 6908	Human	12
515	Inner medullary AKAP-signaling complex	25522 24674 24675 29699 24679	Rat	5
516	Cytochrome bc1-complex (EC 1.10.2.2) mitochondrial	66445 690848 66152 362897 301011 293448 291103 366448 497902	Rat	9
517	Kinase-scaffold-phosphatase complex PKA-AKAP79-CaN	9495 5530 5576	Human	3

518	AKAP250-PKA-PDE4D complex	9590 5144 5566 5567 5568	Human	5
519	AMY-1-S-AKAP84-RII-beta complex	8165 26292 5577	Human	3
520	KCNQ1 macromolecular complex	10142 3784 5499 5500 5501 5566 5567 5568 5576 5577	Human	10
521	Polycystin-1-E-cadherin-beta-catenin complex	999 1499 5310	Human	3
522	Polycystin-1-E-cadherin-beta-catenin-Flotillin-2 complex	999 1499 2319 5310	Human	4
523	Yotiao-RII-NR1 complex	246150 24408 29699 24679	Rat	4
525	TIP60 histone acetylase complex	86 10524 8607 10856 8295	Human	5
526	TRPC3-TRPC4 channel complex redox-sensitive	22065 22066	Pig	2
527	TRPC3-TRPC4 channel complex redox-sensitive	7222 7223	Human	2
528	NuA4/Tip60 HAT complex	86 10902 64769 55929 57634 80314 10524 54556 10933 9643 55257 8607 10856 8295 8089	Human	15
529	NuA4/Tip60 HAT complex	86 10902 64769 55929 57634 80314 10524 54556 10933 9643 55257 8607 10856 8295 6944 8089	Human	16
530	Cytochrome c oxidase (EC 1.9.3.1) mitochondrial	281090 444878 287012 282200 100270792 782486 338086 327674 327718 615757 3283880	Bovine	11
531	XPA-ERCC1-ERCC4 complex	2067 2072 7507	Human	3
532	M1 mAChR-TRPC6-multiprotein complex	24244 25229 25639 24680 89823 24674 24675	Rat	7
533	IMP3-IMP4-MPP10 complex	55272 92856 10199	Human	3
534	M1 mAChR-TRPC6-PKC complex	25229 24680 89823	Rat	3
535	TRAP complex	892 5469 84246 9968 9969 9282 10025 9440 9412 9862 9442 51003 29079 10001 7067 9967	Human	16
536	TRPC1-TRPC3-TRPC7 complex	7220 7222 57113	Human	3
537	Mediator complex	57261 26896 216154 234959 56771 108098 67381 69792 66213	Mouse	9
538	Cytochrome c oxidase (EC 1.9.3.1) mitochondrial	12857 12858 12859 12861 110323 12864 12866 66142 12867 12868	Mouse	12
540	HMG1-TBP-TATA complex	282691 6908	Mammalia	2
541	IGF1-IGFBP3-ALS complex	3479 3483 3486	Human	3
543	Cytochrome c oxidase (EC 1.9.3.1) mitochondrial	281090 444878 287012 282199 100270792 782486 327688 327674 327718 281091 3283880	Bovine	11
544	TRPC3-TRPC6-Ca2+-signaling complex	81662 25262 25679 24654 85240 29322 81802 60395 89823 89823	Dog	11
546	Cytochrome c oxidase (EC 1.9.3.1) mitochondrial	29445 252934 94194 25282 286962 29507 303393 100188937 171335 26195 26198 26204	Rat	12
547	SMCC complex	892 1024 5469 9968 9969 9282 10025 9440 9412 9862 9442 29079 10001	Human	14
548	DRIP complex	5469 9968 9969 9282 10025 9440 9439 9862 9441 29079 10001 9443	Human	14
550	NOS3-CAV1-NOSTRIN complex	857 4846 115677	Human	3

551	TDT-TDIF2-core-histone complex	1791 30836 9555 55506	Human	4
552	IFNB1-IFNAR1-IFNAR2- complex	3454 3455 3456	Human	3
553	RHOA-IP3R-TRPC1 complex	3708 387 7220	Human	3
554	PBAF complex (Polybromo- and BAF containing complex)	86 196528 55193 6597 6598 6599 6601 6602 6603 6605	Human	10
555	BAF complex	86 8289 6597 6598 6599 6601 6602 6603 6605	Human	9
557	TRP1-G alpha-11-IP3R3-CAV1 signaling complex	857 2767 3710 7220	Human	4
558	Channel complex TRPM6-TRPM7	140803 54822	Human	2
559	Decapping complex	55802 167227 1656 80153 23644	Human	5
560	Kir6.2-Sur1 complex	25559 16514	Mouse	2
561	LSm1-7 complex	27257 57819 27258 25804 23658 11157 51690	Human	7
562	LSm2-8 complex	57819 27258 25804 23658 11157 51690 51691	Human	7
563	F1F0-ATP synthase (EC 3.6.3.14) mitochondrial	498 506 509 513 514 515 516 10476 521 522 9551 10632 539 93974 4508	Human	16
564	BAF complex	86 8289 6598 6599 6601 6605 6595 6597 6602 6603 6604	Human	11
565	PBAF complex (Polybromo- and BAF containing complex)	86 196528 55193 6597 6598 6599 6601 6605 6602 6603 6604	Human	11
566	BAF complex	60 86 8289 6597 6598 6599 6601 6605 6602 6603 6604	Human	11
567	Clathrin-CAP-1A-Na(v)1.8 channel complex	266809 29571 83800 116561 54241	Rat	5
568	Nuclear pore complex	8086 57122 55746 25281 117021 23279 366016 23165 8021 295692 79023 348995 25497 53372 65274 79902 113929 291874 4928 245922 11097 113975 58958 362281 5903	Rat	28
569	PSD95-Nos1-Grin2b complex	29495 24410 24598	Rat	3
570	p300-CBP-p270-SWI/SNF complex	8289 1387 2033 6597 6598 6599 6601	Human	7
571	p300-CBP-p270 complex	8289 1387 2033	Human	3
572	PYR complex	107932 15182 15289 22778 245688 20587 20588 68094 83797 57376 11461 11465 56456 83766	Mouse	14
574	F1-ATP synthase (EC 3.6.3.14) - IF1 (inhibitor protein) complex mitochondrial	282578 327675 327668 338081 617230 327699	Bovine	6
575	ABIN2-NFKB1-MAP3K8 complex	1326 4790 79155	Human	3
576	F1F0-ATP synthase (EC 3.6.3.14) mitochondrial	282578 327675 327668 338081 617230 282701 338053 282710 338040 282690 281640 327699	Bovine	14
577	FHL2-p53-HIPK2 complex	2274 28996 7157	Human	3
579	Epo-R-PLC-gamma-TRPC2 complex	13857 18803 22064	Mouse	3
580	Epo-R-IP(3)R type II-TRPC2 complex	13857 16439 22064	Mouse	3
581	Epo-R-PLC-gamma 1-IP(3)R type II-TRPC2 signaling complex	13857 16439 18803 22064	Mouse	4
582	Ikaros complex	107932 433759 15182 22778 22779 22780 19646 20586 20588 83797	Mouse	12

583	Ikaros-NuRD complex	107932 433759 15182 22778 22779	Mouse	6
584	Ppp3cb-RyR2-mAkap complex	64553 24675 689560	Rat	3
585	Mi2/NuRD-BCL6-MTA3 complex	604 1108 3065 53615 57504	Human	5
586	Ccnb1-Itpr1-Cdc2 signaling complex	25203 54237 25262	Rat	3
587	NuRD.1 complex	1108 3065 3066 53615 9112 57504 5928 5931	Human	8
588	Respiratory chain supercomplex (complex I III IV) mitochondrial heart	281090 444878 287012 282199 100270792 782486 327688 327674 327718 615757 512500 3283880 3283889 3283884 3283886 3283885 3283888 327673 338060 326346 281742 338084 327698 338064 327704 327714 327670 338063 327710 404188 327702 327690 327701 404161 327713 338073 327706 338061 327665 338065 282517 327660 282289 338046 288380 327697 287327 327680 338057 327691 338079 287027 287014 282290 327717 281570 [illegible]	Bovine	63
590	Kv4.2-KChIP3-Dpp10 channel	363972 65180 65199	Rat	3
591	SAP complex (Sin3-associated protein complex)	51742 3065 3066 5928 5931 79595 8819 25942	Human	8
592	SAP complex (Sin3-associated protein complex)	51742 3065 3066 5928 5931 79595 8819 25942 64426	Human	9
593	Kv4.2-Dpp10 channel complex	363972 65180	Rat	2
594	Kv4.2-DPPX channel complex	29272 65180	Rat	2
595	Kv4.2-DPP10 channel complex	57628 3751	Human	2
596	SIN3-HDAC-SAP30-ARID4 complex	51742 3065 3066 5928 5931 8819	Human	7
597	Cx43/Gja1-Tom20-Hsp90ab complex	24392 301252 266601	Rat	3
598	Kv4.3-Dpp10 channel complex	363972 65195	Rat	2
599	Kv4.3-Dpp6 channel complex	29272 65195	Rat	2
600	Kv4.1-Dpp6 channel complex	29272 16506	Rat	2
601	Kv4.1-Dpp10 channel complex	363972 16506	Rat	2
603	Kv4.2-Dpp6 channel complex	29272 65180	Rat	2
604	Kv4.2-DPPX-KChIP channel complex	29272 65180 65023 56817 65199	Rat	6
605	TIM (TIMM17ab TIMM23 TIMM44) complex mitochondrial	10440 10245 10431 10469	Human	4
606	Kv4.2-Kchip1 channel complex	65180 65023	Rat	2
607	Kv4.2-Kchip2 channel complex	65180 56817	Rat	2
608	Kv4.2-Kchip3 channel complex	65180 65199	Rat	2
609	Kv4.2-Kv4.3 channel complex	65180 65195	Rat	2
610	MSL complex	284058 55167 10943 84148	Human	4
611	Exocyst complex	69940 171455 252881 116654 60627 50556 64632 245709	Rat	8
612	CTF18-CTF8-DCC1 complex	63922 54921 79075	Human	3
613	CTF18-CTF8-DCC1-RFC3 complex	63922 54921 79075 5983	Human	4
614	NRD complex (Nucleosome remodeling and deacetylation complex)	23028 1107 1108 3065 3066 9112 5928	Human	7

616	TIM (Timm17a Timm23 Timm44 Hsp70) complex mitochondrial	24963 54311 54312 29635	Rat	4
617	CASK-LIN7C-APBA1 complex	320 8573 55327	Human	3
619	MRE11A-RAD50-NBN-TRF2	4361 4683 10111 7014	Human	4
620	CoREST-HDAC complex	23028 3065 3066 10362 51317 23186 7764	Human	7
622	Ubiquitin E3 ligase (VHL TCEB1 TCEB2 CUL2)	8453 6921 6923 7428	Human	4
623	TIM (TIMM9 TIMM10) complex mitochondrial	26519 26520	Human	2
624	LSD1-CoREST complex	23028 23186	Human	2
626	LSD1 complex	23028 1487 3065 10363 10362 3303 51317 112885 23186 55758 6239 7750 7764	Human	13
627	MRN-TRRAP complex (MRE11A-RAD50-NBN-TRRAP complex)	4361 4683 10111 8295	Human	4
628	SNARE complex (Stx Stx8 Vti1b VAMP8)	60466 59074 83730 366673	Rat	4
629	BLM-TRF2 complex	641 9519	Human	2
630	RyR2 macromolecular complex	58950 689560 24668 25594 24669 24672 24673 25636 293508 29699	Rat	11
631	Tacc1-chTOG-AuroraA complex	6790 9793 6867	Human	3
632	Anti-HDAC2 complex	23028 1107 1108 23199 2969 3065 3066 10362 9112 9219 51317 5928 5931 23186 7750 9203 25942 23309	Human	18
633	anti-BHC110 complex	23028 23199 2969 3065 3066 10362 51317 23186 7750 9203 7764	Human	11
634	XFIM complex	23028 2969 3065 3066 9203	Human	5
635	AGPS-GNPAT complex	8540 8443	Human	2
636	BHC complex	23028 3065 3066 10362 51317 23186	Human	6
638	cGMP kinase signaling complex (IP3RI IRAG cGKI-beta)	317697 281918 282004	Bovine	3
639	RANBPM-Muskelin-TWA1-HSMpp8 complex	54994 4289 54737 10048	Human	4
640	SNARE complex (Snap47 Stx1a	303183 116470 24803	Rat	3
641	RANBPM-SMP1 complex	10048 23585	Human	2
642	CtBP complex	23028 8535 9425 1487 1488 79813 10919 3065 3066 84458 23186 55758 6239 6935 9839 7764 9658	Human	17
643	CtBP core complex	23028 1487 1488 79813 10919 3065 3066 23186 6935	Human	9
644	ASPP1-SAM68 complex	10657 23368	Human	2
646	HDAC1-associated protein complex	23028 1107 3065 8932 53615 9219 5928 5931 23186	Human	9
647	HPFH(-198) complex	13433 98053 14885 3047	Mouse	4
648	HDAC1-associated core complex cI	23028 3065 23186	Human	3
649	HDAC1-associated core complex cII	1107 3065 8932 53615 9219 5928 5931 23186 54815 57459	Human	10
650	HDAC2-asscociated core complex	1107 3066 53615 9219 5928 5931 54815 57459	Human	8
651	SNARE complex (Snap25 Stx1a	25012 116470 24803	Rat	3

652	AP3-BLOC1 complex	8546 8120 8943 26985 10947 1176 10239 2647 282991 388552 55330 84062 63915 26258 23557	Human	15
653	SNARE complex (Ykt6 Stx5 Gosr2 Bet1)	29631 64154 65134 64351	Rat	4
654	BLOC1-BLOC2 complex	2647 282991 388552 55330 84062 84343 11234 79803 63915 26258	Human	11
655	HSF1-HSF2 complex	3297 3298	Human	2
656	VPS35-VPS29-VPS26A complex	9559 51699 55737	Human	3
657	Retromer complex (SNX1 SNX2 VPS35 VPS29 VPS26A)	6642 6643 9559 51699 55737	Human	5
658	SNARE complex (Sec22b Stx5 Gosr2 Bet1)	29631 64154 310710 65134	Rat	4
659	MeCP1 complex	1108 57459 3065 3066 8932 53615 9219 5928 5931	Human	9
660	TIM (TIMM23 TIMM50) complex mitochondrial	10431 92609	Human	2
661	Karyopherin complex	288064 24917 56813	Rat	3
662	Hoxa9-PBX2-Meis1 complex	15405 17268 5089	Mammalia	3
663	SNARE complex (Stx5 Ykt6 Gosr1 Bet1)	54400 94189 65134 64351	Rat	4
664	SNARE complex (Stx5 Gosr2 Sec22b Bet1)	29631 64154 310710 65134	Rat	4
665	BKCA alpha-Cav1.2 channel complex	24239 83731	Rat	2
666	BKCA alpha-Cav2.1 channel complex	25398 83731	Rat	2
667	BKCA alpha-Cav2.2 channel complex	257648 83731	Rat	2
668	BKCA-beta2AR-AKAP79 signaling complex	154 9495 3778	Human	3
669	beta2AR-AKAP79/150 complex	24176 171026	Rat	2
670	BKCa-AKAP79/150 complex	171026 83731	Rat	2
671	BKCa-beta2AR complex	24176 83731	Rat	2
672	BKCA-beta2AR complex	154 3778	Human	2
673	SNARE complex (Stx5 p97 Nsfl1c)	83809 65134 116643	Rat	3
674	Gamma-secretase complex (Aph1a Ncstn Psen1 Psenen Tmp21)	226548 59287 19164 66340 68581	Mouse	5
675	TMP21-P24 complex	22360 68581	Mouse	2
676	Metallothionein-3 complex	11461 12709 12934 15516 193740	Mouse	6
677	Mt3-Hsp84-Ck complex	12709 15516 17751	Mouse	3
678	GINS complex	9837 51659 64785 84296	Human	4
679	Tis7-Sin3-Hdac1-Ncor1-Sap30	433759 15982 20185 60406 20467	Mouse	5
680	RANBMP-CD39 complex	953 5902	Human	2
681	(C-CFTR)2-NHERF-ezrin complex	1080 7430 9368	Human	3
682	C-CFTR-NHERF(PDZ1 domain)-ezrin complex	1080 7430 9368	Human	3
683	C-CFTR-NHERF(PDZ2 domain)-ezrin complex	1080 7430 9368	Human	3
684	PAX9-MSX1 complex	4487 5083	Human	2
685	MeCP1 complex	1108 57459 3065 3066 8932 53615 9219 5928 5931	Human	9
686	Apg16L-Apg12-Apg5 complex	67526 77040 11793	Mouse	3

687	CFTR-NHERF-beta(2)AR signaling complex	154 1080 9368	Human	3
688	Cd2ap-Fyn complex	12488 14360	Mouse	2
690	Cd2ap-Synpo complex	12488 104027	Mouse	2
691	SIN3-SAP25 complex	51742 3065 3066 5928 5931 79595 10284 751865 8819 25942 64426	Human	11
692	LysRS-Hint-Mitf complex	690660 292028 25094	Rat	3
693	TOM (Tomm70 Tomm20 Tomm22) complex mitochondrial	266601 300075 304017	Rat	3
694	Bad-Gk-Wave1-Pkaca-Ppp1cc complex	12015 103988 19047 18747 83767	Mouse	5
696	BRMS1-SIN3-HDAC complex	25855 3065 3066 5928 5931 8819 25942 23309	Human	8
697	BRMS1-RBP1 complex	51742 25855	Human	2
700	Snap25-caveolin complex	25404 25012	Rat	2
701	HuR-Tia1-Tiar-Hrnpu complex	363854 117280 312510 361655	Rat	4
702	ARD1-NATH complex	80155 373	Human	2
703	SNARE complex (Vamp7 Stx5 Bet1 Vti1a)	29631 65134 85491 65277	Rat	4
704	SNARE complex (RINT1 ZW10 p31 Stx18)	5714 60561 360953 9183	Mammalia	4
705	SNARE complex (RINT1 ZW10 p31)	5714 60561 9183	Human	3
706	SNARE complex (HGS SNAP25 STX13)	9146 6616 23673	Human	3
707	SNARE complex (VAMP2 SNAP25 STX13)	6616 23673 6844	Human	3
710	Brg1-associated complex I	86 8289 10419 6597 6598 6599 6601 6605 6602 6603 6604	Human	11
711	Brm-associated complex	86 8289 10419 6595 6598 6599 6601 6605 6602 6603 6604	Human	11
712	cGMP kinase signaling complex (IP3RI IRAG cGKI-beta alpha-actin calponin H1 PLN RhoA)	515610 534583 317697 281918 18821 282004 338049	Bovine	7
713	BRG1-SIN3A complex	86 8289 3066 10419 5928 25942 6597 6598 6599 6601 6602 6603 6604 6605	Human	14
714	BRM-SIN3A complex	86 8289 3065 3066 10419 5928 25942 6595 6598 6599 6601 6602 6603 6604 6605	Human	15
716	PU.1-associated protein complex	26365 319177 17257 17975 20375	Mouse	5
717	PU.1-Sin3A-Hdac-MeCP2 complex	433759 17257 20466 20375	Mouse	4
718	TRPV5-S100A10-annexin 2 complex	12306 20194 194352	Mouse	3
719	S100A10-annexin 2 complex	12306 20194	Mouse	2
720	PU.1-SIN3A-HDAC complex	3065 25942 6688	Human	3
721	PU.1-TBP complex	6688 6908	Human	2
722	MRG15-PAM14-RB complex	10933 93621 5925	Human	3
723	MAF1 complex	10933 93621 5925	Human	3
724	MAF2 complex	10933 84148	Human	2
725	P2X7 receptor signalling complex	60 81 3326 3303 3312 3689 3909 4356 29665 5297 5787 6840	Human	12
726	DDB2 complex	9318 8533 51138 10987 10980 10920 8451 1642 1643 2873 9978 50813	Human	13

727	CSA complex	9318 8533 51138 10987 10980 10920 8451 1642 1161 2873 9978 50813	Human	13
728	CSA-POLIIa complex	9318 8533 51138 10987 10980 10920 8451 1642 1161 2873 5430 9978 50813 64708	Human	14
729	Ubiquitin E3 ligase (FBXO31 SKP1A CUL1 RBX1)	8454 79791 9978 6500	Human	4
730	SNARE complex (STX4 SNAP23 VAMP1)	8773 6810 6843	Human	3
731	SNARE complex (STX4 SNAP23 VAMP2)	8773 6810 6844	Human	3
733	SNARE complex (STX4 VAMP1 VAMP7)	6810 6843 6845	Human	3
734	SNARE complex (STX4 SNAP23 VAMP2)	8773 6810 24803	Mammalia	3
735	SNARE complex (Vti1b Stx6 Stx7)	58244 53331 53612	Mouse	3
736	SNARE complex (Vti1b Stx7 Stx8 Vamp8)	53331 55943 22320 53612	Mouse	4
737	Ppp2c-PKA-Ryr2-mAkap-Pde4d3 complex	64553 689560 24672 24673 25636 293508	Rat	6
738	SIN3-ING1b complex I	51742 3065 3066 3621 5928 5931 10284 8819 25942	Human	9
739	SIN3-ING1b complex II	86 8289 51742 3065 3066 3621 5928 5931 10284 8819 25942 6597 6598 6599 6601 6602	Human	16
740	Exon junction complex EIF4A3-MLN51-MAGOH-Y14 (RNA-protein	22794 9775 4116 9939	Human	4
741	NCOR-HDAC3 complex	2874 8841 9611 6907 79718	Human	5
742	eIF3 complex (EIF3S6 EIF3S5 EIF3S4 EIF3S3 EIF3S6IP EIF3S2 EIF3S9 EIF3S12 EIF3S10 EIF3S8 EIF3S1 EIF3S7)	8661 8662 8663 8664 3646 51386 8665 8666 8667 8668 8669 27335	Human	12
744	Exon junction complex (EIF4A3-MLN51-MAGOH-Y14)	22794 9775 4116 9939	Human	4
745	NCOR-SIN3-RPD3 complex	3066 9612 25942 23309	Human	4
746	C/EBPalpha-HNF6 complex	1050 3175	Human	2
747	NCOR-SIN3-HDAC1 complex	3065 9612 25942	Human	3
748	TRAF6-TAK1 complex	6885 7189	Human	2
749	MeCP2-SIN3A-HDAC complex	3065 3066 4204 25942	Human	4
750	MeCP2-Sin3a-Hdac complex	84576 84577 29386 20466	Rat	4
751	Trpc1-dystrophin-alpha1-syntrophin complex	13405 20648 22063	Mouse	3
752	SMRT core complex	8841 9612 6907	Human	3
753	UTM-SGCE-DAG1-CAV1-NOS3 complex	857 1605 4846 8910 7402	Human	5
754	Exon junction complex (EIF4A3-MLN51-UPF3B-MAGOH-Y14)	22794 9775 4116 9939 65109	Human	5
755	Exon junction complex (F4A3-MLN51-UPF3B-MAGOH-Y14-PYM)	22794 9775 4116 9939 65109 84305	Human	6
756	Prune-Gelsolin complex	2934 58497	Human	2
757	Prune-GSK3beta complex	2932 58497	Human	2

758	Prune/Nm23-H1 complex	4830 58497	Human	2
759	Fgfr1-Kal1 complex	79114 3730	Rat	2
760	Apoptosis- and splicing-associated protein complex (ASAP-L) SAP18-RNPS1-Acinus-L	22985 10921 10284	Human	3
761	Apoptosis- and splicing-associated protein complex (ASAP-S) SAP18-RNPS1-Acinus-S	22985 10921 10284	Human	3
762	Ahnak1-dysferlin complex	191572 26903	Rat	2
763	Y14-Magoh complex	4116 9939	Human	2
764	Dysferlin-affixin complex	8291 29780	Human	2
765	SNARE complex (Stx6 Snap29)	116500 60562	Rat	2
766	Cask-Grip1-Prkce-Rgs4-Gria2 complex	29647 29627 84016 29340 29480	Rat	5
767	Cask-Grip1-Gria2-Mint1 complex	83589 29647 29627 84016	Rat	4
768	NdpkA-Ampkalpha1 complex	191575 65248	Rat	2
769	Exon junction complex (EIF4A3-MLN51-MAGOH-Y14) RNA-protein complex	22794 9775 4116 9939	Human	4
770	TREX complex	7919 9984 57187 84321 10189 8563 79228 80145	Human	8
771	NDPKA-AMPKalpha1 complex	4830 5562	Human	2
772	eIF4AIII-Btz complex	22794 9775	Human	2
774	THO complex	9984 57187 8563 79228 80145	Human	5
775	CBC complex (cap binding complex)	4686 22916	Human	2
776	Plp1-Calr-Itgav complex	64202 16410 24943	Rat	3
777	Exon junction complex	10482 9939 10921 10250 10189 26019 65109	Mammalia	7
778	LARC complex (LCR-associated remodeling complex)	60 86 8289 1108 5977 57459 3065 3066 3183 8932 53615 9219 5928 6597 6598 6599 6601 6603 6605	Human	19
779	K(ATP) macromolecular complex (Kir6.2 Pkm2 Gapdh Tpi1)	24383 83535 25630 24849	Rat	4
781	URI complex (Unconventional prefoldin RPB5 Interactor)	8454 5202 5434 8725 8607 10856 6500 6502 26228	Human	9
782	Itch-Fam/Usp9x complex	311567 22284	Rat	2
783	ITCH-FAM/USP9x complex	83737 8239	Human	2
784	SMG-1-Upf1-eRF1-eRF3 complex (SURF)	2107 23049 5976 2935 23708	Human	5
785	Exon junction complex	7919 9775 4116 9939 10921 10250 10189 65109	Human	8
786	MR-UBC9-SRC1 complex	8648 4306 5530	Human	3
787	NuA4/Tip60-HAT complex B	86 55929 80314 10524 10933 8607 10856 8295	Human	8
788	Exosome	22894 51013 23404 51010 54512 56915 118460 23016 11340 5393	Human	10
789	Exosome	22894 51013 5394 23404 51010 54512 56915 118460 23016 11340	Human	11
790	DDB complex	1642 1643	Human	2
791	SNARE complex (Vamp2 Snap25 Stx1a Cplx1)	64832 25012 116470 24803	Rat	4

792	SNARE complex (Vamp2 Snap25 Cplx2 Stx1a)	116657 25012 116470 24803	Rat	4
793	SNARE complex (VAMP2 SNAP25 STX1a CPLX1)	10815 6616 6804 6844	Human	4
794	SNARE complex (VAMP2 SNAP25 STX1a CPLX2)	10814 6616 6804 6844	Human	4
795	SNARE complex (Vamp2 Snap25 Stx1a Cplx1)	12889 20614 20907 22318	Mouse	4
796	SNARE complex (Vamp2 Snap25 Stx1a Cplx2)	12890 20614 20907 22318	Mouse	4
797	SNARE complex (STX11 VAMP2 SNAP23)	8773 8676 6844	Human	3
798	NuA4/Tip60-HAT complex A	86 10902 64769 55929 57634 80314 26122 10524 54556 10933 55257 8607 10856 8295 8089	Human	15
799	DMAP1-associated complex	86 10902 55929 57634 80314 8607 10856 10847 8295 6944	Human	10
800	DNMT1-HDAC1-DMAP1 complex	66233 13433 15182	Mammalia	3
801	SNARE complex (STX2 SNAP23)	8773 2054	Human	2
802	Cdc42-Par6b-Par3-Prkci complex	12540 93742 58220 18759	Mammalia	4
803	BRG1-SIN3A-HDAC containing SWI/SNF remodeling complex I	86 8289 3066 10419 25942 6597 6598 6599 6601 6602 6605	Human	11
804	CDC42-Par6c-Par3-Prkcz complex	403934 93742 56513 18762	Dog	4
805	SNARE complex (STX4 SNAP23)	8773 6810	Human	2
806	BRM-SIN3A-HDAC complex	86 8289 3066 10419 25942 6595 6598 6599 6601 6602 6603 6605	Human	12
807	BRG1-associated complex	86 8289 10419 6597 6598 6599 6601 6602 6605	Human	9
808	BRM-associated complex	86 8289 10419 6595 6598 6599 6601 6602 6603 6605	Human	10
809	eRF1-eRF3-GTP-Mg(2+) complex	2107 2935 23708	Human	3
810	FCP1-associated protein complex	9150 2079 2962 5431 10419 11329	Human	7
812	Upf complex (UPF1 UPF2 UPF3a)	5976 26019 65110	Human	3
813	Upf complex (UPF1 UPF2 UPF3b)	5976 26019 65109	Human	3
814	Postsplicing complex	10482 9939 10921 5976 26019 65110 65109	Human	7
815	MRIP-MBS-RHOA complex	23164 4659 387	Human	3
816	MRIP-RHOA complex	23164 387	Human	2
817	MRIP-MBS complex	23164 4659	Human	2
819	20S methylosome-SmD complex	1207 10419 6632 6634	Human	4
820	SNARE complex (STX6 SNAP23)	8773 10228	Human	2
821	Par-3-VE-cadherin-alpha-beta-catenin complex	12562 12385 12387 93742	Hamster	4
822	mRNA decay complex (UPF1 UPF2 UPF3B DCP2 XRN1 XRN2 EXOSC2 EXOSC4 EXOSC10	167227 5394 23404 54512 5073 5976 26019 65109 54464 22803	Human	10
823	PAR-3-VE-cadherin complex endothelial	12562 93742	Mouse	2
824	Anti-SMN protein complex	11218 50628 8487 6606 6628	Human	5
825	JBP1-pICln complex	1207 10419 6628	Human	3

826	PAR-3-VE-cadherin-beta-catenin complex	1003 1499 56288	Human	3
827	NgR-TROY-LINGO1 complex	84894 65078 55504	Human	3
828	TRPC1-STIM1-ORAI1 complex	1501 84876 7220	Human	3
829	PAR-6-VE-cadherin complex endothelial	1003 50855 84552	Human	3
830	Trpc1-Stim1-Orai1 complex	304496 361618 89821	Rat	3
831	PAR-6-PAR-3-VE-cadherin complex endothelial	1003 56288 50855 84552	Human	4
832	Anti-Sm protein complex	1207 11218 50628 10419 8487 6606	Human	7
833	6S methyltransferase complex	1207 6632 6634	Human	3
834	20S methylosome and RG-containing Sm protein complex	1207 10419 6628 6632 6633 6634	Human	6
835	6S methyltransferase and RG-containing Sm proteins complex	1207 6628 6632 6633 6634 6635 6636 6637	Human	8
836	20S methyltransferase core complex	1207 10419	Human	2
837	20S methyltransferase complex	1207 10419 79084	Human	3
838	p27-Cdk2-Fgf2 complex	100009068	Rabbit	1
839	LIN9-BMYB complex	286826 4605	Human	2
840	Disc1-Grb2-Kif5a complex	307940 81504 314906	Rat	3
841	SNARE complex (Stx1a Snap25	25012 116470 24803	Rat	3
842	SNARE complex (Stx3 Snap25 Vamp2 Cplx1)	64832 25012 81802 24803	Rat	4
843	SNARE complex (STX1A SNAP25 VAMP2)	540853 788566 282116	Bovine	3
844	SNARE complex (Stx1a Snap25	25012 116470 24803	Rat	3
845	PCI-PSA-SCG2 complex	354 7857 5104	Human	3
846	RICH1/AMOT polarity complex Flag-Rich1 precipitated	154796 55114 832 23607 10207 64398 30011 829 830	Human	9
847	RICH1-PAR3-aPKC polarity complex	55114 56288 5584	Human	3
848	RICH1/AMOT polarity complex Flag-Amot precipitated	154796 154810 51421 55114 10207 8777 64398 143098	Human	8
851	SNARE complex (Stx2 Snap25	25012 25130 24803	Rat	3
852	SNARE complex (Stx3 Snap25	25012 81802 24803	Rat	3
853	SNARE complex (Stx4 Snap25	25012 81803 24803	Rat	3
854	SNARE complex (Stx1a Snap25	25012 116470 83730	Rat	3
855	SNARE complex (Stx2 Snap25	25012 25130 83730	Rat	3
856	SNARE complex (Stx3 Snap25	25012 81802 83730	Rat	3
857	SNARE complex (Stx4 Snap25	25012 81803 83730	Rat	3
858	CRB3-PALS1-PATJ cell polarity complex	224912 12695 56217	Dog	3
859	SNARE complex (Stx4 Stx6 Stx7 Vamp3 Vamp7 Vamp8 Vti1b)	20909 58244 53331 22319 20955 22320 53612	Mouse	7
860	DNMT1-G9a-PCNA complex	1786 10919 5111	Human	3
861	Par-3-p75NTR complex	18053 93742	Mouse	2
862	DNMT1-G9a complex	1786 10919	Human	2
863	Srf-Myocd-Msx1 complex	81710 246297 20807	Rat	3
864	Srf-Myocd-Msx2 complex	25483 246297 20807	Rat	3
867	TRAP-SMCC mediator complex	5469 400569 9440 54797 6837 81857 55588 29079 112950 219541	Human	10

868	aPKC-PAR-6-PAR-3 cell polarity complex	93742 58220 18759	Dog	3
871	BRAF53-BRCA2 complex	23028 675 3065 3066 10362 51317	Human	7
872	SNARE complex (Stx1a Snap29)	116500 116470	Rat	2
873	SNARE complex (STX1A SNAP29)	9342 6804	Human	2
874	SNARE complex (VAMP3 VAMP4 VAMP8 STX6)	10228 9341 8674 8673	Human	4
875	SNARE complex (VAMP3 VAMP4 STX16)	8675 9341 8674	Human	3
876	SNARE complex (VAMP3 STX6 VTI1A)	10228 9341 143187	Human	3
877	SNARE complex (VAMP4 STX6 STX16 VTI1a VTI1b)	8675 10228 8674 143187 10490	Human	5
878	Prkac-Akap5-Adrb1 complex	24925 171026 25636 293508	Rat	4
879	PRKAC-AKAP5-ADRB1 complex	153 9495 5566 5567 5568	Human	5
880	SNARE complex (Vamp4 Stx6 Stx16 Vti1b)	362283 60562 53330 366673	Rat	4
881	SNARE complex (Vamp4 Stx6 Stx16 Vti1a)	362283 60562 53330 65277	Rat	4
882	PAR-6-aPKC-PAR-3 complex	93742 18759 56513 58220	Dog	4
883	PAR-6-aPKC-PAR-3-Cdc42-Rac1 complex	12540 93742 18759 19353 56513 58220	Dog	6
884	GluR1-cadherin-catenin complex	83501 84353 50592	Rat	3
885	Glur4-cadherin-catenin complex	83501 84353 29629	Rat	3
886	MTA1 complex	3065 3066 53615 9112 5928 5931	Human	6
887	Glur2-Glur4-Plp-Itgav complex	29627 29629 16410 24943	Rat	4
888	MTA2 complex	1108 3065 3066 53615 9219 5928 5931 25942 23309	Human	9
889	MTA1-HDAC core complex	3065 3066 9112 5928 5931	Human	5
890	PAR-3-PKCz-Tiam2 complex	81918 25522 24001	Rat	3
891	PAR-3-PKC-zeta-Tiam1 complex	81918 25522 21844	Rat	3
892	PAR-3-aPKC-Tiam2 complex	81918 18759 24001	Rat	3
893	PAR-3-aPKC-Tiam1 complex	81918 18759 21844	Rat	3
894	Tiam2-PAR-3-aPKC-PAR-6-Cdc42-GTP complex	64465 81918 307799 84006 24001	Rat	5
895	hMediator complex (MED23 CDK8 CCNC MED7)	892 1024 9439 9443	Human	4
896	hMediator complex (MED23 CDK8 CCNC)	892 1024 9439	Human	3
897	CDK8-CyclinC-Mediator complex	892 1024	Human	2
898	Mediator complex 1	892 1024 9282 9439 10001	Human	5
899	Par-3-KIF3A-PKC-zeta complex	84392 81918 25522	Rat	3
900	Mediator complex 2	9282 9439 10001	Human	3
901	Par-3-KIF3A-aPKC complex	84392 81918 18759	Rat	3
902	SNARE complex (Stx5 Gosr1 GS15)	54400 94189 65134	Rat	3
903	RET-Rai complex	5979 53358	Human	2
904	SHC3-GAB1 complex	2549 53358	Human	2
905	KIF3A/B-PAR-3-aPKC-PAR-6	11127 9371 56288 5584 50855 84612	Mammalia	6
906	ADAR1-CDK2 complex	103 1017	Human	2

907	SNARE complex (Stx5 Gosr1 Bet1 Gef2)	29631 64670 94189 65134	Rat	4
909	ARC92-Mediator complex	1024 5469 9968 9969 9282 51586 10025 9440 9439 81857 9441 10001	Human	13
910	CRSP-Mediator 2 complex	9282 51586 10025 9440 54797 9439 9862 29079 10001 9443	Human	10
911	Par3-Apc-Kif3a complex	24205 84392 81918	Rat	3
912	Kal1-Fgfr1 complex	79114 3730	Rat	2
913	SNARE complex (Stx1a Snap25 Cplx2 Vamp2)	116657 25012 116470 24803	Rat	4
914	PALS1-PATJ-CRB3-PAR3-PAR6-aPKC-14-3-3 zeta complex	224912 12695 56217 93742 56513 18759 22631	Dog	7
915	SNARE complex (SNAP23 STX1A)	8773 6804	Human	2
916	PALS1-Par3-aPKC-14-3-3 zeta	56217 93742 18759 22631	Dog	4
917	CERF complex (CECR2-containing remodeling factor complex)	27443 6594	Human	2
918	PAR3-PAR6-PALS1 complex	64398 56288 50855	Human	3
920	PAR3-PATJ-PALS1 complex	12695 56217 93742	Dog	3
921	Remodeling and spacing factor (RSF) complex	51773 8467	Human	2
923	SNF2L-RSF1 complex	51773 6594	Human	2
924	Toposome	9188 1660 3183 10594 6732 6749	Human	7
925	WCRF complex	11177 8467	Human	2
926	SCRIB-LGL2 complex	3993 23513	Human	2
927	CENP-A nucleosome associated complex	1060 64946 79019 55839 80152 79682	Human	6
928	SCRIB-VANGL2 complex	23513 57216	Human	2
929	CEN complex	648 55717 11335 57332 1058 1059 1060 64946 2491 64105 91687 79019 79172 22837 8451 1642 9785 2091 3309 3312 25962 9493 79948 79682 9688 29127 6015 6045 51773 79801 8467 51593 6749 11198 9589 23091	Human	37
930	Scrib-beta-PIX-GIT1 complex	8874 28964 23513	Human	3
931	Scrib-beta-PIX-GIT1 complex	54126 216963 105782	Mouse	3
932	Ppar(alpha)-Pric320 complex	109151 25747	Rat	2
933	SCRIB-APC complex	324 23513	Human	2
934	FACT-NEK9 complex	91754 6749 11198	Human	3
935	Scrib-APC-beta-catenin complex	11789 12387 105782	Mouse	3
936	FACT complex	6749 11198	Human	2
938	FACT complex UV-activated	1457 1459 1460 6749 11198	Human	5
939	Dysferlin-Dhpr complex	682930 26903	Rat	2
940	AnnexinVI-Fyn-Pyk2-RasGAP	79125 25150 50646 25676	Rat	4
941	Arf1-Ap1g1-Ap1b1 complex	29663 171494 64310	Rat	3
942	Cd44-Ots8 complex	25406 54320	Rat	2
943	Class C Vps complex (VPS11 VPS18 STX7)	8417 55823 57617	Human	3
944	Class C Vps complex (VPS11 VPS18 VPS16)	55823 64601 57617	Human	3

945	Class C Vps complex (hVPS11 hVPS18 rVPS33a)	55823 57617 65081	Mammalia	3
946	Class C Vps complex (hVPS11 hVPS18 hVPS16 rVPS33a)	55823 64601 57617 65081	Mammalia	4
947	CDC7-DBF7 complex	8317 10926	Human	2
948	Kif17-Lin10 complex	234678 16559	Mouse	2
950	SNARE complex (Stx1a Snap25	25012 116470 81022	Rat	3
951	Fmrp-Lgl complex	14265 16897	Mouse	2
952	Kif13a-AP1 complex	11764 11767 16553 11765 11766	Mouse	5
953	p97-Ufd1-Npl4-IP3 receptor complex	16438 217365 22230 269523	Mouse	4
954	p97-Ufd1-Npl4-IP3 receptor complex	25262 140639 84478 116643	Rat	4
955	LLGL2-PAR-6B-PRKCI complex	3993 84612 5584	Human	3
956	PAR-3-PAR-6B-PRKCI complex	56288 84612 5584	Human	3
957	p97-Ufd1-Npl4 complex	140639 84478 116643	Rat	3
958	Ufd1-Npl4 complex	140639 84478	Rat	2
959	LLGL1-PAR-6B-PRKCI complex	3996 84612 5584	Human	3
960	p97-p47 oligomer complex	83809 116643	Rat	2
961	Tamalin-mGluR1a-cytohesin complex	192254 24414 116692	Rat	3
962	Tamalin-mGluR1a complex	192254 24414	Rat	2
963	Tamalin-mGluR5 complex	192254 29559	Rat	2
965	mPar6c-Mlgl-aPKC complex	16897 56513 18759	Mouse	3
966	Rab27A-Melanophilin-MyosinVa complex	171531 17918 11891	Mouse	3
967	Pallidin-Mu complex	17828 18457	Mouse	2
968	Crb1-Mupp1-Pals1-Mpp4 complex	170788 17475 227157 56217	Mouse	4
970	Rab11Fip2-Ap2a complex	74998 11771 11772	Mouse	3
971	Rab11-Fip2-Reps1 complex	74998 19707	Mammalia	2
973	Sec61alpha-CollagenIV-Hsp47	12826 53421 12406	Mouse	3
974	EED-EZH2 complex	121536 8726 2146 5928 23512	Human	5
975	Ocrl-Cdc42 complex	12540 320634	Mouse	2
980	SNARE complex (Stx4 Snap25 Vamp2 Syt1)	25012 81803 25716 24803	Rat	4
981	SNARE complex (Stx3 Snap25 Vamp2 Syt1)	25012 81802 25716 24803	Rat	4
982	SNARE complex (Stx2 Snap25 Vamp2 Syt1)	25012 25130 25716 24803	Rat	4
983	SNARE complex (Stx1a Snap25 Vamp2 Syt1)	25012 116470 25716 24803	Rat	4
984	SNARE complex (Stx1a Snap25 Vamp2 Cplx1)	64832 25012 116470 24803	Rat	4
985	SNARE complex (Stx1a Snap25 Vamp2 Cplx2)	116657 25012 116470 24803	Rat	4
986	SNARE complex (Stx2 Snap25 Vamp2 Cplx1)	64832 25012 25130 24803	Rat	4
987	SNARE complex (Stx2 Snap25 Vamp2 Cplx2)	116657 25012 25130 24803	Rat	4
988	SNARE complex (Stx3 Snap25 Vamp2 Cplx2)	116657 25012 81802 24803	Rat	4
990	Agap11-AP3 complex	347722 11774 55946 64933 11778	Mouse	5
991	Tubulin polyglutamylase complex	66648 110012 102747 66257 319953	Mouse	5

992	Kif5a-Dtnb-Dtna complex	13527 362715 314906	Rat	3
993	Kif5b-Dtnb complex	13528 16573	Mammalia	2
994	GAA1-GPI8-PIGT-PIG-PIGS complex	8733 10026 94005 51604 128869	Human	5
995	Polycomb repressive complex 3	8726 2146 5928 5931 23512	Human	5
996	Polycomb repressive complex 2	8726 2146 5928 5931 23512	Human	5
997	KIN17-PCNA-RPA70 complex	22944 5111 6117	Human	3
999	p23 protein complex	3181 10016 10728	Human	3
1000	TorsinA-TorsinB complex	1861 27348	Human	2
1001	DNA synthesome complex	16881 18538 18968 18969 18971 18972 67967 69745 19075 19076 21969 21973 21974	Mouse	13
1002	Cask-Dlg1 complex	12361 13383	Mouse	2
1003	RC complex (Replication competent complex)	890 1017 5422 23649 5981 5982 5983 5984 5985	Human	9
1004	RC complex during S-phase of cell cycle	890 1017 3978 142 5422 5424 5426 5981 5982 6117 6118 6119 7150	Human	13
1005	RC complex during G2/M-phase of cell cycle	890 891 983 142 5422 5424 5426 5981 5982 6117 6118 6119 7150	Human	13
1006	Caveolin complex	81822 287876 25404 25035 83764	Rat	6
1007	CAK core complex (Cdk-activating kinase core complex)	902 1022	Human	2
1008	CAK complex (Cdk-activating kinase complex)	902 1022 4331	Human	3
1009	TFIIH transcription factor complex	902 1022 2068 2071 2965 2966 2967 2968 404672 4331	Human	10
1011	SNARE complex (Stx1a 1b 7 13 Rtn1 Vamp2)	116644 65033 116470 24923 60466 24803	Rat	6
1014	Pcdhga1-Pcdha4 complex	12936 93709	Mouse	2
1015	Pcdhga1-Pcdhgb2 complex	12936 93700	Mouse	2
1016	Pcdhga1-Pcdhga3 complex	12936 93711	Mouse	2
1017	Pcdhga1-Pcdhgb4 complex	12936 93701	Mouse	2
1018	Pcdha7-Pcdhgb4 complex	12939 93701	Mouse	2
1019	Pcdha7-Pcdhga3 complex	12939 93711	Mouse	2
1020	Pcdha7-Pcdhgb2 complex	12939 93700	Mouse	2
1021	Pcdha7-Pcdhga1 complex	12939 93709	Mouse	2
1022	Fgr-Pyk2-p190RhoGap complex	14191 232906 19229	Mouse	3
1023	Tiam1-Par-3-aPKC-zeta complex	93742 18762 21844	Mouse	3
1024	Tiam1-IRSp53 complex	117542 21844	Rat	2
1025	Calreticulin-Tnfr1-Tradd complex	100009050 21937 71609	Rabbit	3
1026	Tiam1-spinophilin complex	84686 21844	Rat	2
1027	SNARE complex (Stx4 Vamp7 Snap23 Syt7)	64630 81803 59267 85491	Rat	4
1028	HNF4A-SUB1 complex	3172 10923	Human	2
1029	TFIIH transcription factor complex	902 1022 2068 2071 2965 2966 2967 2968 404672 4331	Human	10
1030	CAK-ERCC2 complex	902 1022 2068 4331	Human	4
1031	ORC complex (origin recognition complex)	4998 4999 23595 5000 5001 23594	Human	6
1032	ORC 2-4 complex (origin recognition 2-4 complex)	4999 23595 5000	Human	3

1033	ORC complex (origin recognition complex)	4998 4999 23595 5000 5001 23594	Human	6
1034	ORC 2-5 complex (origin recognition 2-5 complex)	4999 23595 5000 5001	Human	4
1035	OAP-1-OSP/claudin-11-ITGB1	18417 16412 56434	Mouse	3
1037	TFIIH transcription factor core	2071 2965 2966 2967 2968	Human	5
1038	ORC 1-5 complex (origin recognition 1-5 complex)	4998 4999 23595 5000 5001	Human	5
1039	PCNA-PAF complex	9768 5111	Human	2
1040	p33ING1b-PCNA complex	3621 9768	Human	2
1041	Alpha-dystrobrevin-ZO-1-actin	1837 7082 60 71	Mammalia	4
1042	SRA-SRC-1 ribonucleoprotein complex	8648 10011	Human	2
1043	Car-Lnx2 complex	13052 140887	Mouse	2
1044	Car-Jam3 complex	13052 83964	Mouse	2
1045	Snurportin-CRM1-RanGTP complex	5901 10073 7514	Human	3
1046	Lnx1-Jam4 complex	72058 16924	Mouse	2
1047	Lnx1-Jam4-Numb complex	72058 16924 18222	Mammalia	3
1048	Hnf4-Pc4 complex	15378 20024	Mouse	2
1050	Vdr-Med4 complex	67381 22337	Mouse	2
1051	Ubiquitin E3 ligase (SKP1A SKP2 CUL1 RBX1)	8454 9978 6500 6502	Human	4
1052	Ubiquitin E3 ligase (FBXW11 SKP1A CUL1 RBX1)	8454 23291 9978 6500	Human	4
1054	ESR1-RELA-BCL3-NCOA3 complex	602 2099 8202 5970	Human	4
1055	ZNF198-PML complex	5371 7750	Human	2
1056	ZNF198-SUMO1 complex	7341 7750	Human	2
1057	DIPA-MCRS1 complex	11007 10445	Human	2
1058	SNX complex (SNX1 SNX6)	6642 58533	Human	2
1060	Retromer complex (SNX1 SNX2 VPS35 VPS29 VPS26B)	6642 6643 112936 51699 55737	Human	5
1062	BAR-BCL2-CASP8 complex	596 51283 841	Human	3
1063	GRASP-GRP1 complex	160622 9265	Human	2
1064	IFP35-NMI complex	3430 9111	Human	2
1066	mPar6c-Mlgl-aPKCz complex	16897 56513 18762	Mouse	3
1067	CD8A-LCK complex	925 3932	Human	2
1068	12S U11 snRNP	79622 154007 10081 79706 6432 6628 6632 6633 6634 6635 6636 6637 114034 11066 4904	Human	15
1069	FIF-FGR2 complex	8539 2247	Human	2
1070	SNX complex (SNX1a SNX2 SNX4)	6642 6643 8723	Human	3
1071	PKD2-FPC complex	5311 5314	Human	2
1072	SNX complex (SNX2) oligomeric	6643	Human	1
1073	Pkd2-Fpc complex	18764 241035	Mouse	2
1074	Pkd2-Fpc-Kif3b complex	16569 18764 241035	Mouse	3
1076	Pkhd1-Pkd2 complex	18764 241035	Mouse	2
1077	Pkd2-Fpc-Kif3a complex	16568 18764 241035	Dog	3
1079	P-TEFb.1 complex	904 1025	Human	2
1080	P-TEFb.2 complex	905 1025	Human	2
1083	P-TEFb.4 complex	8812 1025	Human	2

1084	Tiam2-PAR-3-aPKC-PAR-6-Rac1-GTP complex	81918 307799 84006 363875 24001	Rat	5
1085	DNA repair complex NEIL2-PNK-Pol(beta)-LigIII(alpha)-XRCC1	3980 252969 8566 5423 7515	Human	5
1086	DNA repair complex NEIL1-PNK-Pol(beta)-LigIII(alpha)-XRCC1	3980 79661 8566 5423 7515	Human	5
1087	BIRC5-AURKB-INCENP-EVI5	9212 332 7813 3619	Human	4
1088	PRNP-ApolopoproteinE3 complex	348 5621	Human	2
1089	VAM1-VELI1 complex	8825 51678	Human	2
1090	APLG1-Rababtin5 complex	164 9135 79874	Human	3
1091	SNX complex (SNX1a SNX2 SNX4 LEPR)	3953 6642 6643 8723	Human	4
1092	PCNA-KU antigen complex	5111 7520 2547	Human	3
1093	SNX complex (SNX1a SNX2 SNX4 INSR)	3643 6642 6643 8723	Human	4
1094	Frataxin complex	10939 10632 2395 3313 3329 57128	Mammalia	7
1095	SNX complex (SNX1a SNX2 SNX4 EGFR)	1956 6642 6643 8723	Human	4
1096	SNX complex (SNX1 1a 2 4 PDGF receptor)	5156 6642 6643 8723	Human	4
1097	eIF3 complex (EIF3S6 EIF3S5 EIF3S4 EIF3S3 EIF3S6IP EIF3S2 EIF3S9 EIF3S12 EIF3S10 EIF3S8 EIF3S1 EIF3S7 PCID1)	8661 8662 8663 8664 3646 51386 8665 8666 8667 8668 8669 27335 10480	Human	13
1098	DNA synthesome complex (13 subunits)	5111 5422 23649 5424 5425 10714 57804 5557 5558 5981 6117 7150	Human	14
1099	DNA synthesome complex (17 subunits)	5111 5422 23649 5424 5425 10714 57804 5426 5427 54107 56655 5557 5558 5981 6117 7150 7153 7155	Human	18
1100	DNA polymerase alpha-primase complex	5422 23649 5557 5558	Human	4
1101	TFIIIC containing complex TFIIIC2	2975 2976 9330 9329 9328	Human	5
1103	Wave1-Bcl-xl-Pancortin-2 complex focal ischemic stroke induced	12015 56177 83767	Mouse	3
1104	SNX complex (SNX1a SNX2 SNX4 TFRC)	6642 6643 8723 7037	Human	4
1105	TFIIIC containing complex	2975 2976 9330 9329 9328	Human	5
1106	TFIIIC containing-TOP1-SUB1	2975 2976 9330 9329 9328 10923	Human	7
1107	DNA synthesome core complex	5111 5422 23649 5424 5425 10714 57804 5557 5558 5981	Human	10
1108	DNA synthesome complex (15 subunits)	3978 5111 5422 23649 5424 5425 10714 57804 5426 5427 54107 56655 5557 5558 5981	Human	15
1109	DNA polymerase alpha	18968 18969	Mouse	2
1112	Psd3-Actn1 complex	109711 234353	Mouse	2
1113	5S-DNA-TFIIIA-TFIIIC2 subcomplex	2971 2975 2976 9330 9329 9328	Human	6
1114	5S-DNA-TFIIIA-TFIIIC2-TFIIIB subcomplex	55814 2972 2971 2975 2976 9330 9329 9328 6908	Human	9
1115	Cdk5/p35 complex (Cdk5-Cdk5r1 complex)	12568 12569	Mouse	2

1116	CRM1-Survivin-AuroraB mitotic complex	9212 332 7514	Human	3
1117	CRM1-Survivin mitotic complex	332 7514	Human	2
1118	Chromosomal passenger complex CPC (INCENP CDCA8 BIRC5 AURKB)	9212 332 55143 3619	Human	4
1120	Chromosomal passenger complex CPC (INCENP CDCA8 BIRC5)	332 55143 3619	Human	3
1121	WNK1-OSR1 complex	9943 65125	Human	2
1122	Smcb-Smcd-PW29 complex	19357 24061 13006	Mouse	3
1123	WNK1-SPAk complex	27347 65125	Human	2
1124	SNARE complex (Snap25 Stx1a Vamp2 Cplx1 Cplx3)	64832 235415 25012 116470 24803	Mammalia	5
1125	SNARE complex (Snap25 Stx1a Vamp2 Cplx3 Cplx4)	235415 225644 25012 116470 24803	Mammalia	5
1126	SNARE complex (Snap25 Stx1a 3 Vamp2 Cplx1 Cplx3 Cplx4)	64832 235415 225644 25012 116470 81802 24803	Mammalia	7
1127	Shps1-Fyb-Skap55r complex	23880 19261 54353	Mammalia	3
1128	Shps1-Pyk2 complex	19229 19261	Mammalia	2
1129	Complexin complex (Stx3 Cplx1	64832 235415 81802	Mammalia	3
1130	Hip1R-cortactin complex	2017 9026	Human	2
1131	RFC2-RIalpha complex	5518 5982	Human	2
1132	RFC2-RIalpha complex	5518 5984	Human	2
1133	ATR-HDAC2 complex	545 3066	Human	2
1134	ATR-HDAC2-CHD4 complex	545 1108 3066	Human	3
1135	Mss4-Itga3 complex	16400 98710	Mouse	2
1136	Mss4-Itga7 complex	16404 98710	Mouse	2
1137	SNARE complex (VAMP2 SNAP25 STX1a CPLX1 CPLX3)	10815 594855 6616 6804 6844	Human	5
1138	SNARE complex (VAMP2 SNAP25 STX1a CPLX3 CPLX4)	594855 339302 6616 6804 6844	Human	5
1139	SNARE complex (VAMP2 SNAP25 STX1a STX3 CPLX1 CPLX3	10815 594855 339302 6616 6804 6809 6844	Human	7
1140	Complexin complex (STX3 CPLX1 CPLX3)	10815 594855 6809	Human	3
1141	CF IIAm complex (Cleavage factor IIAm complex)	4076 10978 51692 11052 79869 8449 2071 2968 4361 11051 51585 5725 6421 7082 7307 11338	Human	16
1142	SMN complex	11218 8487 6606 6628 6632 6633 6634 6635 6636 6637	Human	10
1143	SMN complex	11218 50628 25929 79833 79760 54960 8487 6606 6628 6632 6633 6634 6635 6636 6637 11171	Human	16
1144	Cleavage and polyadenylation factor (CPSF)	29894 53981 51692 10898 81608	Human	5
1145	Mammalian cleavage factor I (CF Im)	11052 11051	Mammalia	2
1146	Cleavage stimulation factor	1477 1478 1479	Human	3
1147	Polyadenylation complex (CSTF1 CSTF2 CSTF3 SYMPK CPSF1 CPSF2 CPSF3)	29894 53981 51692 1477 1478 1479 8189	Human	7
1148	snRNP-free U1A (SF-A) complex	1655 4841 6421 6626	Human	4

1149	Histone H3.1 complex	25842 55723 10036 8208 8520 8350 554313 79711 4678 5928	Human	10
1150	Histone H3.3 complex	25842 55723 3020 8520 7290 554313 79711 4678 5928	Human	9
1151	Sycp1-Syce1-Syce2 complex	74075 71846 20957	Mouse	3
1152	FA complex (Fanconi anemia complex)	2175 2187 2176 2178 2188 2189 55120 57697	Human	8
1153	Integrator complex	54973 26173 55174 57117 57508 65123 92105 80789 26512 25896	Human	12
1154	DSS1 complex	675 54973 55174 57508 92105 80789 26512 25896 55656 55756 114823 66406 7979	Human	13
1155	Integrator-RNAPII complex	54973 26173 55174 57117 57508 65123 92105 80789 26512 25896 55656 55756 5430 5431	Human	14
1156	PS1-E-cadherin-catenin complex epithelial	12550 12385 12387 19164	Dog	4
1157	PS1-E-cadherin-catenin complex brain	12550 12558 12387 19164	Mouse	4
1158	p33ING1b-p300 complex	2033 3621	Human	2
1159	p33ING1b-HDAC1 complex	3065 3621	Human	2
1160	ING1-p300-PCNA complex	2033 3621 5111	Human	3
1161	Smrt-Sin3A-Hdac7 complex	56233 20602 20466	Mouse	3
1162	Ubiquitin E3 ligase (DDB1 DDB2 CUL4A CUL4B RBX1)	8451 8450 1642 1643 9978	Human	5
1163	ING1-PCNA complex	3621 5111	Human	2
1164	ActRIIA-ActRIB-Smad3-Arip1	11479 11480 50791 17127	Mouse	4
1165	RNF20-RNF40-UbE2E1 complex	56254 9810 7324	Human	3
1166	p400-associated complex	60 57634 8607 10856 8295 86 51412	Human	7
1167	Paf complex	79577 9646 123169 54623 80349	Human	5
1168	SKI complex	6499 9652 80349	Human	3
1169	SNARE complex (STX4 VAMP8 VAMP3 SNAP23)	8773 6810 9341 8673	Human	4
1170	cMYC-ATPase-helicase complex	86 4609 8607 10856 8295	Human	5
1171	c-MYC-ATPase-helicase complex	86 4609 8607 10856 8295	Human	5
1173	TIP49-TIP48-BAF53 complex	86 8607 10856	Human	3
1174	PHAX-CBC complex (cap binding complex)	4686 22916 51808	Human	3
1175	TRRAP-BAF53-HAT complex	86 8295	Human	2
1176	CRM1-RAN-PHAX-CBC complex (cap binding complex)	4686 22916 5901 51808 7514	Human	5
1177	Polycomb repressive complex 4	8726 2146 23411	Human	3
1178	BCOR complex	54880 84678 3312 84759 6015 6045 23429 6500	Human	8
1179	CENP-A NAC-CAD complex	378708 1060 64946 64105 91687 79019 55839 79172 401541 55166 80152 23421 79682	Human	13
1180	Sur2 subcomplex of mediator	216154 70208 23989	Mouse	3

1181	C complex spliceosome	9716 58509 51362 988 51340 51503 57703 9416 51428 1655 8220 60625 9785 1659 9343 9775 2483 55094 3178 3181 220988 3183 3185 3187 3190 4670 10236 3192 57461 57819 27258 4116 84292 26986 5356 5411 10450 51645 53938 23398 27339 8899 24148 10594 22913 55696 9939 27316 9092 10283 10291 8175 10946 23451 10992 23450 6426 23517 10569 23020 9410 6627 6628 6629 6632 6633 6634 6635 6636 6637 22938 10250 23524 25949 10492 [illegible]	Human	80
1182	CDC5L core complex	10286 988 10985 3312 5356 27339	Human	6
1183	CDC5L complex	10286 9689 988 51503 1778 10985 3312 3608 5356 8493 5499 5591 27339 22828 27316 23451 10992 10262 9814 6421 6426 6427 6626 6627 6632 6633 6634 10250 6925	Human	30
1184	Mediator complex	51813 264064 19014 216154 70208 23989 69792 230753 59024	Mouse	9
1185	EGFR-containing signaling complex	1956 2064 5286 5287	Human	4
1186	ESCRT-III complex	5119 57132 27243 25978 29082 128866 92421 51510 79643 51652	Human	10
1187	ESCRT-II complex	11267 84313 51028	Human	3
1189	DNA double-strand break end-joining complex	3981 4361 4683 10111 7518 7520 2547	Human	7
1190	NCBP-NIP1 complex	4686 63941	Human	2
1191	RNA pol II containing coactivator complex Tat-SF	1025 27336 4691 5430 6829	Human	5
1192	ESCRT-I complex	7251 51160 55048	Human	3
1193	Rap1 complex	4361 142 10111 7014 54386 7520	Human	7
1194	E2F-6 complex	1876 79813 10919 2707 83746 4149 23269 84108 6015 6045 7027 10138	Human	12
1195	Exon junction complex (mRNA splicing-dependent)	10594 10250	Human	2
1197	TRF1-TIN2 complex	65057 25913 7013 7014 54386 26277	Human	6
1198	TIN2 complex	65057 25913 7014 26277	Human	4
1199	Oligosaccharyltransferase complex (Stt3A variant)	1603 1650 84061 6184 6185 3703	Human	6
1200	Oligosaccharyltransferase complex (Stt3B variant)	1603 1650 84061 6184 6185 201595 7991	Human	7
1202	TRF1 telomere length regulation complex	7013 26277 8658	Human	3
1203	Oligosaccharyltransferase OSTC-I	1603 58505 404012 200185 6184 6185 3703	Dog	7
1204	Rap1 complex	65057 25913 10111 7013 7014 54386 26277	Human	7
1205	TRF2-Rap1 complex III	7014 54386	Human	2
1206	TRF-Rap1 complex I 2MD	25913 7013 7014 54386 26277 8658	Human	6
1207	TRF2-Rap1 complex II	25913 7014 54386 26277	Human	4

1208	Oligosaccharyltransferase OSTC-II	1603 58505 404012 200185 6184 6185 404006 404018 404017 3703	Dog	10
1209	Oligosaccharyltransferase OSTC-III	1603 58505 404012 200185 6184 6185 404006 404018 403951 6747	Dog	11
1210	Sec61-Sec62-Sec63 complex	10952 615778 7095 11231 505064	Bovine	6
1211	Ubiquitin E3 ligase (AHR ARNT DDB1 TBL3 CUL4B RBX1)	196 405 8450 1642 9978 10607	Human	6
1212	SNARE complex (Stx4 Vamp8	20619 20909 22320	Mouse	3
1213	SNARE complex (Stx4 Vamp2	20619 20909 22318	Mouse	3
1214	Ubiquitin E3 ligase (DET1 DDB1 CUL4A RBX1 COP1)	8451 1642 55070 9978 64326	Human	5
1215	Ubiquitin E3 ligase (FBXW7 CUL1 SKP1A RBX1)	8454 55294 9978 6500	Human	4
1216	Hspb2-Hspb3 complex	161476 78951	Rat	2
1217	WRN-TRF2 complex	7014 7486	Human	2
1218	BLM-TRF2 complex	641 7014	Human	2
1219	Tankyrin 1-tankyrin 2-TRF1 complex	7013 8658 80351	Human	3
1220	TRF2-Ku complex	7014 7520 2547	Human	3
1221	TFIIA-TRF2 complex	2957 2958 7014	Human	3
1222	Irs1-Grb2-Ptpn1 complex	81504 25467 24697	Rat	3
1223	H2AX complex isolated from cells without IR exposure	805 811 51386 3014 554313 128312 10236 3309 4869 26986 6741 6749	Human	13
1224	Ubiquitin E3 ligase (BMI1 SPOP	648 8452 8405	Human	3
1225	Ubiquitin E3 ligase (H2AFY SPOP CUL3)	8452 9555 8405	Human	3
1226	H2AX complex I	811 22907 3014 128312 3309 4869	Human	7
1227	H2AX complex II	811 8531 3014 3018 8342 554313 128312 3309 4869 55037	Human	10
1228	Epsin-clathrin complex	60668 81637 116563 117277 65178	Rat	5
1229	RACK1-containing mRNP complex	83427 171350 305240 266780	Rat	4
1230	WINAC complex	86 8289 9031 10036 6595 6597 6598 6599 6601 6602 6605 11198 7155	Human	14
1231	FIB-associated protein complex	708 2091 3276 10419 7846 81027	Human	6
1232	REST-CoREST-mSIN3A complex	23186 5978 25942	Human	3
1233	CoREST-HDAC2 complex	3066 23186	Human	2
1235	SNARE complex (Vamp2 Snap25 Stx1a Syt1 Cplx1)	64832 25012 116470 25716 24803	Rat	5
1236	SNARE complex (Vamp2 Snap25 Stx1a Syt1 Cplx2)	116657 25012 116470 25716 24803	Rat	5
1239	EBAFb complex	86 57492 4298 6597 6598 6599 6601 6602 6603 6605	Human	10
1240	SC5bp-7 complex	15139 12274 730 100009197 100009128 100009076 100009385	Rabbit	7
1241	Esr1-Pit1 complex	24890 25517	Rat	2
1243	Ubiquitin E3 ligase (SPOP DAXX CUL3)	8452 1616 8405	Human	3
1244	SNARE complex (STX1A STX1B SNAP25 RAB3A SYT1 VAMP2)	540853 282029 788566 282377 281511 282116	Bovine	6
1245	Protein phosphatase 4 complex	5531 151987	Human	2
1246	ERdj3-BiP complex	67838 14828	Mouse	2

1247	SNARE complex (STX1a STX1b SNAP25 RAB3a SYT1 VAMP2 CPLX2)	540853 281711 282029 788566 282377 281511 282116	Bovine	7
1248	Apoptosome	317 54205	Human	2
1250	pRB-E2F-1 complex	1869 5925	Human	2
1252	EBAFa complex	86 8289 4298 6597 6598 6599 6601 6602 6603 6605	Human	10
1254	Menin-associated histone methyltransferase complex	9070 84661 4221 5431 5929 9757 11091	Human	7
1255	Ubiquitin E3 ligase (SIAH1 SIP SKP1A TBL1X)	27101 6477 6500 6907	Human	4
1256	MLL-HCF complex	9070 3054 4221 4297 5929 3053	Human	7
1257	ALL-1 supercomplex	23028 1107 53981 9343 3065 3066 53615 4297 9219 5901 5928 5929 5931 10284 8819 25942 6595 8467 6598 6599 6601 8189 6872 6883 6878	Human	28
1258	Ubiquitin E3 ligase (GLMN FBXW8 SKP1A RBX1)	26259 11146 9978 6500	Human	4
1259	Chromatin assembly complex (CAF-1 complex)	10036 8208 5928	Human	3
1260	Neddylin ligase (FBXO11 SKP1 CUL1 RBX1)	8454 80204 9978 6500	Human	4
1261	SRm160/300 complex	6625 6627 10250 23524 6434	Human	5
1287	HNRPF-HNRPH1 complex	3185 3187	Human	2
1288	DCS complex (PTBP1 PTBP2 HNRPH1 HNRPF)	3185 3187 5725 58155	Human	4
1293	IPO13-RBM8A-MAGOH complex	9670 4116 9939	Human	3
1296	DCP1A-DCP2 Decapping complex	55802 167227	Human	2
1297	MKK4-ARRB2-ASK1 complex	409 6416 4217	Human	3
1298	MKK4-ARRB2-JNK3 complex	409 6416 5602	Human	3
1299	POSH-MLK3 complex	4296 57630	Human	2
1300	CRLR-RAMP1 complex	10203 10267	Human	2
1306	PIN1-AUF1 complex	3184 5300	Human	2
1307	Multiprotein complex (mRNA	7812 3184 26986 10605 10492	Human	5
1308	PABPC1-HSPA8-HNRPD-EIF4G1 complex	3303 1981 3184 3312 26986	Human	5
1310	Rab27a-Mlph-Myo5a complex	171531 17918 11891	Mouse	3
1318	Transcription initiation factor complex (TAF1 TAF5 TAF11 TAF12 TBP)	6872 6882 6883 6877 6908	Human	5
5703	Bash-Bnas2/Cmtm3-Btk-Erk2 complex	17060 12229 68119 26413	Mouse	4
1321	Transcription initiation factor complex (TAF5 TAF6 TAF9 TAF11 TBP)	6882 6877 6878 6880 6908	Human	5
1332	Large Drosha complex	1653 10521 1654 1655 54487 1665 2130 2521 3187 4670 3192 11100 9987 3608 3609 22913 29102 6732	Human	20
1335	SNW1 complex	10061 988 1642 9416 1915 1937 9343 3192 3309 9782 4436 5211 10594 6421 23020 22938 203068 51366	Human	18
1338	FOXO3-PCAF complex oxidative stress stimulated	2309 8850	Human	2
1345	Septin complex	55752 4735 989 23176 10801	Human	5

1347	NPC subcomplex (NUP98 NUP107 NUP133 NUP160)	57122 55746 23279 4928	Human	4
1348	GLE1-NUPL2-NUP155 complex	2733 9631 11097	Human	3
1352	ING4 complex (ING4 MYST2 C1orf149 PHF17)	64769 51147 11143 79960	Human	4
1371	Gata1-Tal1-Tcf3-Lmo2 complex	14460 16909 21349 21423	Mouse	4
1372	Rb-tal-1-E2A-Lmo2-Ldb1 complex	8861 4005 5925 6886 6929	Human	5
1373	Ldb1-Lmo2-Gata-1-Tal1-E47 complex	14460 16825 16909 21349 21423	Mouse	5
1375	Pyruvate dehydrogenase complex	1738 5160 613610 517402	Bovine	4
1377	hASC-1 complex	51008 84164 10973 9325	Human	4
1378	Translocon-associated protein (TRAP) complex	403951 403950 6747 6748	Dog	4
1379	GALNS-lysosomal hydrolase 1.27 MDa complex	5476 2588 2720 4758	Human	4
1380	Elongator holo complex	55250 55140 26610 8518	Human	4
1385	DICER1-NCOA6-AGO2 complex	23405 27161 23054	Human	3
1386	TTP-AGO4 complex	192670 7538	Human	2
1387	TTP-AGO2 complex	27161 7538	Human	2
1388	RB1-TFAP2A complex	7020	Dog	1
1390	Rb1-Runx2 complex	19645 12393	Mouse	2
1399	WDR5-ASH2L-RBBP5-MLL2	9070 8085 5929 11091	Human	4
1400	ASCOM complex	9070 8085 58508 23054 5929 7277 203068	Human	7
1401	MOF complex	4297 8085 84148 5929 6045 9739 6872 6878 6880 11091	Human	10
1408	Ets2-Smarca4-Smarcb1-Smarce1 complex	23872 20586 20587 57376	Mouse	4
1413	NCOR1 complex	8841 9611 10291 23450 6597 6598 6599 6601 10847 10155	Human	10
1423	L-periaxin-Drp2 complex	13138 13405 13497 19153 22288	Mouse	5
1439	PTGS2 homodimer complex	5743	Human	1
1442	Transporter (Ncx1) - receptor complex	362036 29693 25262 29715 64159	Rat	5
1448	LIN2-LIN7-SAP97 complex	8573 1739 8825	Mammalia	3
1449	Dlg3-Lin7-SAP97 complex	13383 53310 108030	Mammalia	3
1452	MCM2-MCM6-MCM7 complex	4171 4175 4176	Human	3
1457	AFF1-MLLT1-CBX8 complex	4299 57332 4298	Human	3
1458	SNF2h-HDAC12 complex	3066 8467	Human	2
1462	hPRC1L complex	648 1912 6015 6045	Human	4
1464	Mis12 centromere complex	23468 79980 79003 25936 11243	Human	6
1469	E2f5-Rbl2-Hdac1 complex	13559 433759 19651	Mouse	3
1470	pRb2/p130-multimolecular complex (DNMT1 E2F5 SuV39H1 HDAC1 RBL2)	1786 1875 3065 5934 6839	Human	5
1471	pRb2/p130-multimolecular complex (RB2 E2F5 HDAC1 SUV39H1	1875 2033 3065 5934 6839	Human	5
1473	E2F5-RB2-DP1 complex	1875 5934 7027	Human	3
1474	SMAD3/4-E2F4/5-p107-DP1 complex	1874 1875 5933 4088 4089 7027	Human	6
1475	S-phase-specific E2F-p107 complex	12566 104394 19650 21781 12427	Mouse	6
5658	Nrp1-PlexinD1 complex	8829 23129	Human	2

5708	Dok1-Map4k4-Nck1-Rasa1 complex	13448 26921 17973 218397	Mouse	4
1488	DNMT1-RB1-HDAC1-E2F1 complex	1786 1869 3065 5925	Human	4
1490	DAXX-DNMT1-DMAP1 complex	1616 55929 1786	Human	3
1491	RGS6-DNMT1-DMAP1 complex	55929 1786 9628	Human	3
1492	BHC110 complex	23028 23199 3065 3066 10362 51317 23186 7750 7764 9658	Human	10
1495	PID complex	1108 3065 53615 9219 5928	Human	5
1503	Hdac1-Mecp2-Rcor1-Sin3a complex	433759 17257 217864 20466	Mouse	4
1505	NCOR2 complex	3065 3066 8841 9611 9612 8819	Human	7
1508	BCL6-ZBTB17 complex	604 7709	Human	2
1514	IL4-IL4R complex	3565 3566	Human	2
1515	IL4-IL4R-IL2RG complex	3561 3565 3566	Human	3
1519	IL6ST-PRKCD-STAT3 complex	3572 5580 6774	Human	3
1520	STAT3-NLK-MAP3K7 complex	6885 51701 6774	Human	3
1521	p300-SMAD1-STAT3 complex	2033 4086 6774	Mammalia	3
1522	STAT3 homodimer copmplex	6774	Human	1
1523	Stat3-Crebbp complex	12914 20848	Mouse	2
1530	Girdin homo-oligomer complex	55704	Human	1
1537	Heterotrimer complex (Rnd1 Rras Plxnb1)	235611 223881 20130	Mammalia	3
1539	G protein complex (GNG2 GNB2L1 RAF1)	10399 54331 5894	Human	3
1543	PPP2CA-PPP2R1A complex	5515 5518	Human	2
1544	PPP2CA-PPP2R1A-PPP2R3A complex	5515 5518 5523	Human	3
1549	Kpna2-Kpnb1 complex	16647 16211	Mouse	2
1551	IPO13-RAN-EIF1AX complex	1964 9670 5901	Human	3
1552	TNPO2-RAN-NXF1 complex	10482 5901 30000	Human	3
1554	RANBP1-RAN-KPNB1 complex	3837 5901 5902	Human	3
1555	Nmi-Rpa2-Rrn3 complex	64685 19891 106298	Mouse	3
1556	Rpa2-Rrn3 complex	19891 106298	Mouse	2
1557	NMI-POLR1B-RRN3 complex	9111 84172 54700	Human	3
1558	POLR1B-RRN3 complex	84172 54700	Human	2
1560	Nmi-Rrn3 complex	64685 106298	Mouse	2
1561	NMI homodimer complex	9111	Human	1
1571	Vigilin-DNA-PK-Ku antigen complex	3069 5591 7520 2547	Human	4
5757	PLXNA1-RANBPM complex	5361 10048	Human	2
1589	Junb-Sumo1 complex	16477 22218	Mouse	2
1595	Cdk5-c-Abl-Cables complex	11350 63955 12568	Mouse	3
1602	Cdk5-p35-CaMKII(alpha)-(alpha)actinin1 complex	81634 25400 140908 116671	Rat	4
1608	Kir3.2 homotetramer complex	16522	Mouse	1
1609	G protein complex (Gngt2 Kcnj3	14688 14710 16519	Mouse	3
1610	G protein complex (Gngt2 Kcnj6	14688 14710 16522	Mouse	3
1612	Heterotrimeric G protein complex (GNG2 GNB1 GNAS)	2778 2782 54331	Human	3
1614	G protein complex (MCF2 GNB1	2782 54331 4168	Human	3
1615	G protein complex (BTK GNG1	695 2782 54331	Human	3

1617	G protein complex (CACNA1A GNB1 GNG2)	773 2782 54331	Human	3
1618	G protein complex (PTHR1 GNB1 GNG2)	2782 54331 5745	Human	3
1619	G protein complex (HDAC5 GNB1 GNG2)	2782 54331 10014	Human	3
1620	G protein complex (HDAC4 GNB1 GNG2)	2782 54331 9759	Human	3
1623	FA core complex 1 (Fanconi anemia core complex 1)	2175 2176 2178 2188 2189	Human	5
1624	FA core complex (Fanconi anemia core complex)	80233 2175 2187 2176 2178 2188 2189 55120 57697	Human	9
1625	FA core complex (Fanconi anemia core complex)	2175 2187 2176 2178 2188 2189 57697	Human	7
1633	CyclinD1-CDK4-CDK6 complex	595 1019 1021	Human	3
1634	CyclinD1-CDK4-p21 complex	595 1019 1026	Mammalia	3
1637	Ccnd1-Cdk4 complex	12443 12567	Mouse	2
1642	p16-cyclin D2-CDK4 complex	894 1019 1029	Human	3
1652	Ccna2-Cdk2 complex	12428 12566	Mouse	2
1656	p27-cyclinE-CDK2 complex	898 1017 1027	Human	3
1661	E2F4-p107-cyclinA complex	890 1874 5933	Human	3
5712	FAK-beta5 integrin complex VEGF induced	3693 5747	Human	2
1695	SCAMP1-SCAMP2 complex	9522 10066	Human	2
1700	ABL2-HRAS-RIN1 complex	27 3265 9610	Human	3
1703	IGHM-VPREB1-IGLL1 complex	3507 3543 7441	Human	3
1707	IL2-IL2RA-IL2RB complex	3558 3559 3560	Human	3
1714	TICAM1-TICAM2-TLR4 complex	148022 353376 7099	Human	3
1723	Pax3-Lef1-Tle4 complex	16842 18505 21888	Mouse	3
1724	Spen-Hivep1 complex	110521 56381	Mouse	2
1728	CTCF-nucleophosmin-PARP-HIS-KPNA-LMNA-TOP complex	10664 3015 723790 3836 3839 4000 4869 142 7153	Human	9
1729	TLE1 corepressor complex (MASH1 promoter-corepressor complex)	60 3308 4628 4691 4841 4869 142 10111 7088 7155	Human	10
1730	Nfatc2ip-Prmt1	18019 18020 15469	Mouse	3
1731	PRMT1 complex	3276	Human	1
1734	SF3A1-SF3A2-SF3A3 complex	10291 8175 10946	Human	3
1737	SF3b complex	11325 84844 23451 51639 10992 23450 10262 83443	Human	8
1743	(E.F.G) complex	6635 6636 6637	Human	3
1745	SMN complex	11218 50628 25929 79833 79760 8487 6626 6628 6632 6633 6634 6635 6636 6637 6638	Human	15
1746	SMN containing complex	50628 3956 3958 8487 6606 6632 6633 6634	Human	8
1748	PRMT5 complex	10419	Human	1
1749	SMN-PolII-RHA complex	11218 1660 50628 2959 5430 8487 6628 6629 6638 6908	Human	10
1750	PPP4C-PPP4R2-Gemin3-Gemin4	11218 50628 5531 151987	Human	4

1751	SMN complex	11218 50628 25929 79833 79760 8487 6606 6626 6628 11171	Human	10
1752	SMN complex	11218 50628 25929 79833 79760 8487 6606	Human	7
1760	TOP1-PSF-P54 complex	4841 6421 7150	Human	3
1767	CPSF6-ITCH-NUDT21-POLR2A-UBAP2L complex	11052 83737 11051 5430 9898	Human	5
1768	CPSF6-EWSR1-ITCH-NUDT21-POLR2A-UBAP2L complex	11052 2130 83737 11051 5430 9898	Human	6
1769	CPSF6-ITCH-NUDT21-POLR2A complex	11052 83737 11051 5430	Human	4
1772	MICB-KLRK1-HCST complex	10870 22914 4277	Human	3
1773	ULBP3-KLRK1-HCST complex	10870 22914 79465	Human	3
1774	MICA-KLRK1-HCST complex	10870 22914 4276	Human	3
1775	ULBP2-KLRK1-HCST complex	10870 22914 80328	Human	3
1777	TGF-beta-receptor-SMAD7-SMURF2 complex	4092 64750 7046 7048	Mammalia	4
1778	TGF-beta-receptor-Strap complex	20901 21812 21813	Mammalia	3
5852	p65-p50 Nf(kappa)B complex Il-1-beta induced	81736 309165	Rat	2
1779	TGF-beta-receptor-PAR6 complex	50855 7046 7048	Human	3
1782	TGF-beta receptor	21812 21813	Mouse	2
1783	TGF-beta receptor I-SMAD7-SMURF1 complex	4092 57154 7046	Mammalia	3
1784	RNF11-SMURF2-STAMBP complex	26994 64750 10617	Human	3
1787	Nogo-potassium channel complex	8506 3736 3737 57142	Human	4
1791	Nbr1-Sqstm1-Trim55 complex	303554 113894 365751	Rat	3
1792	Lingo1-Rtn4r-Tnfrsf19 complex	235402 65079 29820	Mouse	3
1793	LINGO-1-Nogo-66-p75 signaling complex	84894 4804 65078	Human	3
1794	SORT1-NGFR complex	4804 6272	Human	2
1795	SORT1-NGFR-NGFB complex	4803 4804 6272	Human	3
5718	eNOS-HSP90-AKT complex VEGF induced	207 3320 4846	Human	3
1804	Smurf1-Par6 complex	56513 75788	Mouse	2
1810	ITGA4-PXN-GIT1 complex	28964 3676 5829	Human	3
1812	AXIN-MEKK4-CCD1 complex	8312 85458 4216	Human	3
1813	MAD1L1 homodimer complex	8379	Human	1
1814	MAD1L1-MAD2L1 complex	8379 4085	Human	2
1816	JUN-TCF4-CTNNB1 complex	1499 3725 6925	Human	3
1826	SMAD3-HEF1-APC10-CDH1 complex	10393 999 4739 4088	Human	4
1827	PML-SMAD2/3-SARA complex	5371 4087 4088 9372	Human	4
1828	TGF-beta receptor I-Axin-SMAD3 complex	8312 4088 7046	Human	3
1829	SMAD3-VDR complex	17127 22337	Mouse	2
1830	Smad3-Hdac-Runx2 complex	363287 84580 367218 25631	Rat	4
1831	PIAS3-SMAD3-P300 complex	2033 10401 4088	Human	3
1834	ITGAE-ITGB7-CDH1 complex	999 3682 3695	Human	3
1839	SDCBP-CTNNB1-CTNNA1-CDH1 complex	999 1495 1499 6386	Dog	4

1843	EB1-APC-mDia2 complex	11789 56419 13589	Mouse	3
1844	APC-IQGAP1 complex	324 8826	Mammalia	2
1845	APC-IQGAP1-CLIP-170 complex	324 6249 8826	Mammalia	3
1847	Axin-GSK-3-beta-beta-catenin complex	79257 84353 84027	Mammalia	3
1851	BUB1-BUB3 complex	699 9184	Human	2
1856	CDCA5-PDS5A-RAD21-SMC1A-PDS5B-SMC3 complex	113130 23244 23047 5885 8243 9126	Human	6
1863	TSG101-VPS37B-VPS28 complex	7251 51160 79720	Human	3
1867	ERC2-RIMS1-UNC13B complex	259269 84556 64830	Rat	3
1873	SNARE complex (Snap25 Syt1 Unc13b Vamp2 Stx1b2 Stx1a)	25012 116470 24923 25716 64830 24803	Rat	6
1874	SNARE complex (SNAP25 VAMP3 VAMP2 NAPB STX13)	63908 6616 23673 6844 9341	Human	5
1875	STX12-SNAP25-HGS-VAMP2	56084 25012 65033 24803	Rat	4
1876	SNARE complex (Stx4 Napa Vamp3 Nsf Vamp2)	140673 60355 81803 24803 29528	Rat	5
1879	Nsf-Stx1a-NAPB complex	499903 60355 116470	Rat	3
1880	SNARE complex (Stx1a Napa Snap25 Vamp2 Nsf)	140673 60355 25012 116470 24803	Rat	5
1883	Stx7-Vti1b-Vamp8-Stx8 complex	60466 59074 83730 366673	Rat	4
1890	ELK1-SRF-ELK4 complex	2002 2005 6722	Human	3
1893	mTOR-RICTOR complex	2475 64223 253260	Human	3
1895	RICTOR-mTOR complex	2475 64223 253260	Human	3
1897	RAPTOR-mTOR complex	2475 64223 57521	Human	3
1899	NTF4 homodimer complex	4909	Human	1
1900	PSD-95-TrkB complex	29495 24408 25054 25738 85385	Rat	5
1901	Drd1-Nmdar1 complex	24316 24408	Rat	2
5719	eNos3-Calmodulin complex	24244 24600	Rat	2
1907	NMDA receptor complex (NR2B NR1 PSD-95 SynGAP CaMK-II)	25400 29495 24408 24410 192117	Rat	5
1908	NMDA receptor complex (NR2A NR2B NR1 PSD-95 SynGAP	25400 29495 24408 24409 24410 192117	Rat	6
1909	APC-DLG4 complex	324 1742	Human	2
1913	GluR delta-2 complex postsynaptic (GluR delta-2 Shank1 Shank2 mGluR1 alpha Homer PSD-95)	13385 14804 14816 26556 78957 210274	Mouse	6
1914	KIF1B-alpha-PSD-95-SAP-97-S-SCAM complex	25252 29495 117548 113970	Rat	4
5707	Ternary complex (Abl1 Dok1 Nck1)	11350 13448 17973	Mouse	3
1923	CaMKII-densin180-NR2B-(alpha)actinin-2 complex	11472 25400 24410 117284	Rat	4
1926	Foxh1-Nkx2-5 complex	14106 18091	Mouse	2
1927	PKD2-PACS1 complex	55690 5311	Human	2
5721	CIN85-CBL-SH3GL2 complex	867 6456 30011	Human	3
1932	NEK2-NEK11 complex	79858 4751	Human	2
5713	SH3P2/OSTF1-CBL-SRC complex	867 26578 6714	Human	3
1937	Olfr992-Rtp1 complex	258865 239766	Mouse	2
1938	Olfr992-Reep1 complex	258865 52250	Mouse	2
1941	Sirpa-Pik3cg complex	30955 19261	Mouse	2

1944	IRAK1-IRAK2 complex	3654 3656	Human	2
1945	IRAK1-IRAK3 complex	3654 11213	Human	2
1949	MAML2-RBP-Jkappa-Notch1 complex	270118 4851 19664	Mammalia	3
1957	DAP12 signaling homodimer complex	7305	Human	1
5716	eNOS-HSP90 complex VEGF induced	3320 4846	Human	2
1970	BMP4-TWSG1 complex	652 57045	Human	2
1972	BMP4-BGN complex	633 652	Human	2
1975	GT198 homodimer complex	140938	Rat	1
1976	MTNR1A homodimer complex	4543	Human	1
1977	MTNR1B homodimer complex	4544	Human	1
1978	MTNR1A-MTNR1B complex	4543 4544	Human	2
1982	CACY homodimer complex	6277	Human	1
1983	Retn homohexamer complex	57264	Mouse	1
1984	PEX14 homodimer complex	5195	Human	1
1985	AIRE homodimer complex	326	Human	1
1986	Endoglin homodimer complex	2022	Human	1
1987	MYOM1 homodimer complex	8736	Human	1
1988	Bcl2 homodimer complex	12043	Mouse	1
1989	BANF1 homodimer protein	8815	Human	1
1990	PGRMC1-SCAP complex	10857 22937	Human	2
1991	PRMT2 homodimer complex	3275	Human	1
1992	LEPR homodimer complex	3953	Human	1
1993	SLC1A2 homotrimer complex	6506	Human	1
1995	Sgk3 homodimer complex	170755	Mouse	1
1997	Adiponectin homotrimer complex	11450	Mouse	1
1998	Adiponectin homohexamer complex	11450	Mouse	1
2000	BAX homo-oligomer complex	581	Human	1
2001	NOD1 homodimer complex	10392	Human	1
2003	COX1 homodimer complex	5742	Human	1
2004	C1D homodimer protein	10438	Human	1
2005	PTPRA homodimer complex	5786	Human	1
2006	Ptpra homodimer complex	19262	Mouse	1
2007	PRNP homo-oligomer complex	5621	Human	1
2008	Prnp homodimer complex	19122	Mouse	1
2009	Birc6 homodimer complex	12211	Mouse	1
2010	AXL homodimer complex	558	Human	1
2013	STAT5B homodimer complex	6777	Human	1
2014	Whrn homodimer complex	73750	Mouse	1
2016	IL12A homodimer complex	3592	Human	1
2018	IL12A-IL12B complex	3592 3593	Human	2
2019	IL12A-IL12B-IL12RB1 complex	3592 3593 3594	Human	3
2020	IL12B-IL12RB1-IL12RB2 complex	3593 3594 3595	Human	3
2021	IL12A-IL12B-IL12RB2 complex	3592 3593 3595	Human	3
2022	Stat5a-Jak2 complex	16452 20850	Mouse	2
2023	STAT5A homodimer complex	6776	Human	1
2024	Agtr1a-Jak2 complex	11607 16452	Mouse	2
2026	IL12RB1-IL12RB2 complex	3594 3595	Human	2

2028	JAK2-IL12RB2 complex	3595 3717	Human	2
2029	BNIP2-ARHGAP8 complex	23779 663	Human	2
2034	Apaf1 homo-oligomer complex	11783	Mouse	1
2051	MALT1 oligomer complex	10892	Human	1
2052	BCL10 oligomer complex	8915	Human	1
2053	BCL10-MALT1 complex	8915 10892	Human	2
2054	CASP8-FADD-MALT1-BCL10	8915 841 8772 10892	Human	4
2055	CASP8-CHUK-IKBKB-MALT1-BCL10 complex	8915 841 1147 3551 10892	Human	5
2056	BCL10-CHUK-BCL10-IKBKB	8915 841 1147 3551	Human	4
2073	TNFRSF11A-TRAF6-SRC complex	6714 8792 7189	Human	3
2074	TRAF6 oligomer complex	7189	Human	1
2077	B-raf-MAP3K11 complex	6635 4296	Human	2
2084	NFKB1-NFKB2-REL-RELA-RELB complex	4790 4791 5966 5970 5971	Human	5
2086	NFKB1-NFKB2-RELA-RELB	4790 4791 5970 5971	Human	4
5723	Cbl-Cd2ap-Flt1 complex	12402 12488 14254	Mouse	3
2100	CHUK-IKBKB-MAP3K14 complex	1147 3551 9020	Human	3
2101	IKKA-IKKB complex	1147 3551	Human	2
2104	IKKB-NIK complex	3551 9020	Human	2
2105	IkappaB kinase complex (IKBKB CHUK IKBKAP NFKBIA RELA	1147 8518 3551 9020 4792 5970	Human	6
2112	CDC37-HSP90AA1-HSP90AB1-MAP3K11 complex	11140 3320 3326 4296	Human	4
2118	CHUK-ERC1-IKBKB-IKBKG	1147 23085 3551 8517	Human	4
2120	Chuk-Ikbkb-Ikbkg complex	12675 16150 16151	Mouse	3
2121	CHUK-IKBKB-IKBKG complex	1147 3551 8517	Human	3
2123	IKBKG homodimer complex	8517	Human	1
2124	IKK-alpha--ER-alpha-AIB1 complex	1147 2099 8202	Human	3
2129	DNAJB2-HSPA8-PSMA3 complex	3300 3312 5684	Human	3
2142	Map2k5-Map3k3 complex	23938 26406	Mouse	2
2143	MAP2K5-PRKCI-SQSTM1 complex	5607 5584 8878	Human	3
2145	HSF1-YWHAE complex	3297 7531	Human	2
2147	Aip-Hsp90-Ahr complex	11622 11632 15516	Mouse	3
2149	Arnt-Sim2 complex	11863 20465	Mouse	2
2151	Arnt-Sim1 complex	11863 20464	Mouse	2
2152	ARNT-HLF complex	405 3131	Human	2
2153	ITGAM-ITGB2-CD11 complex	967 3684 3689	Human	3
2156	YBX1-AKT1 complex	207 4904	Human	2
2159	AR-AKT-APPL complex	207 26060 367	Human	3
2160	AOF2-AR complex	23028 367	Human	2
2162	APPBP1-UBA3 complex	8883 9039	Human	2
2169	Ubiquitin E3 ligase (Neurl2 Tceb1 Tceb2 Cul5 Rbx1)	75717 415115 56438 67923 67673	Mouse	5
2170	Ubiquitin E3 ligase (CDC34 CUL1 RBX1)	997 8454 9978	Human	3
2171	Ubiquitin E3 ligase (CHEK1 CUL4A)	1111 8451	Human	2
2172	Ubiquitin E3 ligase (CDT1 DDB1 CUL4A RBX1)	81620 8451 1642 9978	Human	4

2173	COP9 signalosome complex (CSN)	12848 26754 108679 11692 209318	Mammalia	5
2174	COP9 signalosome complex	9318 8533 51138 10987 10980 10920 2873 50813 64708	Human	9
2179	CNS-P53 complex	9318 8533 51138 10987 10980 50813 10920 2873 7157	Human	9
2183	Kaiso-NCOR complex	7464 2874 8841 9682 3832 9611 6907 79718 51592 10009	Human	10
2184	Gata1-Gfi1b complex	14460 14582	Mouse	2
2185	Gata1-Ldb1-Tal1 complex	14460 16825 21349	Mouse	3
2186	Gata1-Snf2h complex	14460 93762	Mouse	2
2187	Ubiquitin E3 ligase (NFKBIA FBXW11 BTRC CUL1 SKP1A)	8945 8454 23291 4792 6500	Human	5
2188	Ubiquitin E3 ligase (CDC34 NEDD8 BTRC CUL1 SKP1A RBX1)	8945 8454 4738 9978 6500	Human	5
2189	Ubiquitin E3 ligase (SMAD3 BTRC CUL1 SKP1A RBX1)	8945 8454 9978 6500 4088	Human	5
2190	Ubiquitin E3 ligase (Fbxo15 Cul1 Skp1a)	26965 50764 21402	Mouse	3
2191	Ubiquitin E3 ligase (FBXO18 SKP1A CUL1 RBX1)	8454 84893 9978 6500	Human	4
2192	Ubiquitin E3 ligase (NIPA SKP1A CUL1 RBX1)	8454 9978 6500 51530	Human	4
2193	Ubiquitin E3 ligase (Fbxo32 Skp1a Cul1 Rbx1)	26965 67731 56438 21402	Mouse	4
2194	Ubiquitin E3 ligase (Fbxo2 Skp1a Cul1 Rbx1)	26965 230904 56438 21402	Mouse	4
2196	LIG1-9-1-1 complex	3364 3978 5810 5883	Human	4
2197	FEN1-9-1-1 complex	2237 3364 5810 5883	Human	4
2198	RAD9-RAD1-HUS1-POLB complex	3364 5423 5810 5883	Human	4
2199	CTF18-RFC subcomplex	63922 5982 5983 5984 5985	Human	5
2200	RFC2-5 subcomplex	5982 5983 5984 5985	Human	4
2201	PCNA-RFC2-5 complex	5111 5982 5983 5984 5985	Human	5
2202	CHL12-RFC2-5 complex	63922 5982 5983 5984 5985	Human	5
2203	BRD4-RFC complex	23476 5981 5982 5983 5984 5985	Human	6
2204	MCM8-ORC2-CDC6 complex	990 84515 4999	Human	3
2205	ORC5-ORC1 complex	4998 5001	Human	2
2210	BRCA1-IRIS-pre-replication complex	672 990 51053 4998	Human	4
2211	BARD1-BRCA1-CSTF complex	580 672 1477 1478 1479	Human	5
2213	BRCA1-BARD1-POLR2A complex	580 672 5430	Human	3
2214	LMO4-BRCA1-CTIP-LDB1 complex	672 8861 8543 5932	Human	4
2215	BRCA1-LMO4-CTIP complex	672 8543 5932	Human	3
2217	MDC1-MRN-ATM-FANCD2 complex	472 2177 9656 4361 4683 10111	Human	6
2218	MDC1-MRE11-RAD50-NBS1	9656 4361 4683 10111	Human	4
2219	TERF1 homodimer complex	7013	Human	1
2220	RAD52-ERCC4-ERCC1 complex	2067 2072 5893	Human	3
2221	RPA-MSH4-BLM complex	12144 55993 68275 19891 68240	Mouse	5
2222	BLM complex II	641 80010 6117 6118 6119 7156	Human	6
2223	BLM-TOP3A complex	641 7156	Human	2
2224	MSH2/6-BLM-p53-RAD51 complex	641 4436 2956 5888 7157	Human	5

2225	MutS-alpha-histone H4 complex	97122 17685 17688	Mouse	3
2226	MutS-alpha-PK-zeta complex	4436 2956 5590	Human	3
2228	BLM-RAD51L3-XRCC2 complex	641 5892 7516	Human	3
2230	PCNA complex	1019 1020 1026 1660 5111 6117 7150	Rabbit	7
2231	PCNA homotrimer complex	5111	Human	1
2233	Replication-coupled CAF-1-MBD1-ETDB1 complex	10036 4152 9869	Human	3
2235	ASF1-interacting protein complex	25842 55723 10036 8208 7290 8350	Human	6
2236	ASF1-histone containing complex	25842 55723 8208 11200 8350 4678	Human	7
2237	SP1-MCAF2 complex	80063 6667	Human	2
2238	MBD1-MCAF1-SETDB1 complex	55729 4152 9869	Human	3
2240	Hd-Hap1-Dctn1 complex	29167 29430 29424	Rat	3
2241	HD-RAB8A-OPTN complex	3064 10133 4218	Human	3
2242	TGM2-HD-CALM1 complex	805 3064 7052	Human	3
2243	TGM2-HD complex	3064 7052	Human	2
2247	Dynactin complex (DCTN1 DCTN2 DCTN3 DCTN4 DCTN6 CAPZA1 CAPZB ACTR1A)	10121 829 832 1639 10540 11258 51164 10671	Bovine	8
2254	CTGF/Hcs24-actin complex	1490 60 71	Human	3
2255	Cofilin-actin-CAP1 complex	60 10487 1072	Human	3
2256	RIAM-Rap1-GTP complex	54518 5906	Human	2
2258	VILIP-1-AChR-alpha-4-AChR-beta-2 complex	1137 1141 7447	Human	3
2261	GluR6a-GluR6b heterodimer complex	14806	Mammalia	1
2266	PPP3CA-PPP3CA-RCAN1 complex	5530 5532 1827	Human	3
2267	Pick1 homodimer complex	84591	Rat	1
2268	Pick1-Glur2-Pkca complex TPA (tissue plasminogen activator) treated	29627 84591 24680	Rat	3
2270	Hippocalcin-beta2-adaptin-Glur2 complex Ca(2+) dependent	140670 29627 29177	Rat	3
2271	Grip-Glur2/3-liprin-alpha-Lar complex	29627 29628 140592 360406 84016 171571	Rat	6
2272	PICK1-GRIP1-GLUR2 complex	2891 23426 9463	Human	3
2273	Nsg1-Glur2-Grip1 complex	29627 84016 25247	Rat	3
2274	Beta-catenin-Cadherin-Grip-liprin-alpha-GluR2/3 complex	83501 84353 29627 29628 84016 171571 140591 140592	Rat	8
2275	GluR receptor complex	50592 29627 29628 29629	Rat	4
2276	GluR1 homomer complex	50592	Rat	1
2277	D2 receptor-GluR2-GluR1 complex	24318 50592 29627	Rat	3
2281	Protein kinase A II alpha homodimer complex	19087	Mouse	1
2294	ABI1-WASF2 complex	10006 10163	Human	2
2296	Abi1-Wasl complex	11308 73178	Mouse	2
2297	ABI1-WASL complex	10006 8976	Human	2
2300	Profilin 2 complex	60 23191 1759 3312 10787 5217 9475 6853 6854	Human	9
2307	AP3D1-AP3S2 complex	8943 10239	Human	2
2308	AP3D1-AP3S1 complex	8943 1176	Human	2
2318	ITGA6-ITGB4-Laminin10/12 complex	3655 3691 3911 3912 3915	Human	5

2319	ITGA6-ITGB4-Laminin10/12 complex	3655 3691 3911 3913 3915	Human	5
2320	ITGA6-ITGB4-CD151 complex	977 3655 3691	Human	3
2321	ITGA6-ITGB4-FYN complex	2534 3655 3691	Human	3
2322	ITGA6-ITGB4-LAMA5 complex	3655 3691 3911	Human	3
2323	ITGA6-ITGB4 complex	3655 3691	Human	2
2342	ITGAV-ITGB8-MMP14-TGFB1	3685 3696 4323 7040	Human	4
2343	ITGAV-ITGB5-PLAUR complex	3685 3693 5329	Human	3
2345	ITGAV-ITGB5-ICAM4 complex	3386 3685 3693	Human	3
2346	ITGAV-ITGB5-ADAM9 complex	8754 3685 3693	Human	3
2347	ITGAV-ITGB5-SPP1 complex	3685 3693 6696	Human	3
2348	ITGAV-ITGB5-CYR61 complex	3491 3685 3693	Human	3
2350	ITGAV-ITGB5 complex	3685 3693	Human	2
2351	ITGB6-FYN-FN1 complex	2335 2534 3694	Human	3
2352	ITGAV-ITGB6-SPP1 complex	3685 3694 6696	Human	3
2353	ITGAV-ITGB6-TGFB3 complex	3685 3694 7043	Human	3
2354	ITGAV-ITGB6 complex	3685 3694	Human	2
2355	ITGAV-ITGB3-CD47-FCER2 complex	961 2208 3685 3690	Human	4
2356	ITGB3-ITGAV-CD47 complex	961 3685 3690	Human	3
2358	ITGAV-ITGB3-SPP1 complex	3685 3690 6696	Human	3
2359	ITGAV-ITGB3-ADAM15 complex	8751 3685 3690	Human	3
2360	Itgav-Itgb3-Gsn complex	227753 16410 16416	Mouse	3
2362	ITAGV-ITGB3-F11R complex	50848 3685 3690	Human	3
2363	ITGAV-ITGB3-PXN-PTK2b complex	3685 3690 2185 5829	Human	4
2364	ITGAV-ITGB3-ADAM23 complex	8745 3685 3690	Human	3
2365	ITGAV-ITGB3-COL4A3 complex	1285 3685 3690	Human	3
2366	ITGAV-ITGB3-PPAP2b complex	3685 3690 8613	Human	3
2369	ITGAV-ITGB3-EGFR complex	1956 3685 3690	Human	3
2370	ITGA2b-ITGB3-CD9 complex	928 3674 3690	Human	3
2374	ITGAV-ITGB3-LAMA4 complex	3685 3690 3910	Human	3
2375	FN1-TGM2 complex	2335 7052	Human	2
2376	ITGA2B-ITGB3-FN1-TGM2 complex	2335 3674 3690 7052	Human	4
2377	ITGA2b-ITGB3-CD47-SRC complex	961 3674 3690 6714	Human	4
2378	ITGA2b-ITGB3-TLN1 complex	3674 3690 7094	Human	3
2379	ITGA2B-ITGB3-CIB1 complex	10519 3674 3690	Human	3
2381	ITGA2B-ITGB3 complex	3674 3690	Human	2
2382	ITGA2B-ITGB3-F11R complex	50848 3674 3690	Human	3
2383	ITGA5-ITGB1-FN1-TGM2 complex	2335 3678 3688 7052	Human	4
2384	ITGA5-ITGB1-ADAM15 complex	8751 3678 3688	Human	3
2385	ITGA5-ITGB4 complex	3678 3688	Human	2
2386	Itga-Itgb1-Ppap2b complex	16402 16412 67916	Mouse	3
2388	Itga5-Itgb1-Fn1-Sfrp2 complex	403845 3678 3688 475471	Dog	4
2390	CD98-LAT2-ITGB1 complex	3688 23428 6520 8140	Human	4
2395	ITGA7-ITGB1-CD151 complex	977 3679 3688	Human	3
2396	ITGA7-ITGB1-CD9 complex	928 3679 3688	Human	3
2397	ITGA7-ITGB1-ITGB1BP3 complex	3679 3688 27231	Human	3
2398	ITGA3-ITGB1-BSG complex	682 3675 3688	Human	3
2399	ITGA3-ITGB1-CD63 complex	967 3675 3688	Human	3

2400	ITGA3-ITGB1-CD151 complex	977 3675 3688	Human	3
2401	ITGA3-ITGB1-THBS1 complex	3675 3688 7057	Human	3
2402	Itga3-Itgb1-Tgm2 complex	16400 24511 56083	Rat	3
2406	ITGA3-ITGB1 complex	3675 3688	Human	2
2411	ITGA6-ITGB1-CD151 complex	977 3655 3688	Human	3
2413	ITGA6-ITGB1 complex	3655 3688	Human	2
2415	Itgb1-Rap1a-Prkd1 complex	16412 18760 109905	Mouse	3
2416	ITGB1-RAP1A-PKD1 complex	3688 5310 5906	Human	3
2417	ITGA4-ITGB1-EMILIN1 complex	11117 3676 3688	Human	3
2418	ITGA4-ITGB1 complex	3676 3688	Human	2
2419	ITGA4-ITGB1-CD81 complex	975 3676 3688	Human	3
2420	ITGA4-ITGB1-CD53 complex	963 3676 3688	Human	3
2421	ITGA4-ITGB1-VCAM1 complex	3676 3688 7412	Human	3
2422	ITGA4-ITGB1-JAM2 complex	3676 3688 58494	Human	3
2423	ITGA4-ITGB1-CD47 complex	961 3676 3688	Human	3
2424	ITGA4-ITGB1-CD63 complex	967 3676 3688	Human	3
2425	ITGA4-ITGB1-PXN complex	3676 3688 5829	Hamster	3
2426	ITGA4-ITGB1-THBS1 complex	3676 3688 7057	Human	3
2427	Itga4-Itgb1-Adam2 complex	11495 16401 16412	Mouse	3
2428	ITGA4-ITGB1-THBS2 complex	3676 3688 7058	Human	3
2429	ITGA2-ITGB1-CD47 complex	961 3673 3688	Human	3
2430	ITGA2-ITGB1-CHAD complex	1101 3673 3688	Human	3
2431	ITGA2-ITGB1-COL6A3 complex	1293 3673 3688	Human	3
2432	ITGA2-ITGB1 complex	3673 3688	Human	2
2433	Itga5-Itgb1-Tgm2 complex	315346 24511 56083	Rat	3
2434	ITGA1-ITGB1-COL6A3 complex	1293 3672 3688	Human	3
2435	ITGA1-ITGB1-PTPN2 complex	3672 3688 5771	Human	3
2436	ITGAV-ITGB1 complex	3685 3688	Human	2
2437	ITGA6-ITGB1-CYR61 complex	3491 3655 3688	Human	3
2439	ITGA8-ITGB1 complex	8516 3688	Human	2
2440	ITGA9-ITGB1-ADAM9 complex	8754 3680 3688	Human	3
2441	Itga9-Itgb1-Adam2 complex	11495 3680 3688	Mammalia	3
2442	ITGA9-ITGB1-VCAM1 complex	3680 3688 7412	Human	3
2443	ITGA9-ITGB1-TNC complex	3680 3688 3371	Human	3
2444	ITGB1-ITGA9 complex	3680 3688	Human	2
2445	ITGA9-ITGB1-ADAM15 complex	8751 3680 3688	Human	3
2446	ITGA9-ITGB1-FIGF complex	2277 3680 3688	Human	3
2447	ITGA9-ITGB1-ADAM12 complex	8038 3680 3688	Human	3
2453	Multiprotein complex (monoubiquitination)	868 1956 30011 7314	Human	4
2454	CIN85-CBL-SH3GL2-EGFR complex EGF stimulated	867 1956 6456 30011	Human	4
2455	CIN85-SH3GL2 complex	6456 30011	Human	2
2456	MET-CIN85-SH3GL3-CBL complex HGF stimulated	867 4233 6457 30011	Human	4
2457	CIN85-SH3GL3 complex	6457 30011	Human	2
5722	Cbl-Cd2ap complex	12402 12488	Mouse	2
5714	eNOS-CAV1 complex	857 4846	Human	2

2462	Caveolin-1 homodimer complex	857	Human	1
2470	p130Cas-ER-alpha-cSrc-kinase- PI3-kinase p85-subunit complex	9564 2099 5295 6714	Human	4
2471	SRC-PRKCD-CDCP1 complex	64866 5580 6714	Human	3
2475	Cbl-Crkl-Rapgef1 complex	12402 12929 107746	Mouse	3
2476	CRKL-PDGFRA-CRK-RAPGEF1 complex	1398 1399 5156 2889	Human	4
2480	CIN85 complex (CIN85 CRK BCAR1 CBL PIK3R1 GRB2 SOS1)	9564 867 1398 2885 5295 30011 6654	Human	7
2486	GIPC1-LHCGR complex	10755 3973	Human	2
2487	GIPC1-NTRK1-RGS19 complex	10755 4914 10287	Human	3
2489	NCR3-CD247 complex	919 259197	Human	2
5709	ArgBP2a-CBL-PTK2B complex	8470 867 2185	Human	3
2510	ZAP70-CRKL-WIPF1-WAS complex	1399 7454 7456 7535	Human	4
2511	CRKL-WIPF1-WAS complex	1399 7454 7456	Human	3
2513	N-WASp homomer	8976	Human	1
5720	CIN85-CBL complex	867 30011	Human	2
2528	ERBB2-MEMO-SHC complex	2064 51072 6464	Human	3
2529	LAT-PLC-gamma-1-p85-GRB2-CBL-VAV-SLP-76 signaling complex C305 activated	867 2885 27040 3937 5295 5335 7409	Human	7
2534	Cbl-SLP-76-Grb2 complex Fc receptor gamma-R1 stimulated	867 2885 3937	Human	3
2535	SLP-76-Cbl-Grb2-Shc complex Fc receptor gamma-R1 stimulated	867 2885 3937 6464	Human	4
2536	PLC-gamma-2-SLP-76-Lyn-Grb2 complex	2885 3937 4067 5336	Human	4
2537	PKC-alpha-PLD1-PLC-gamma-2 signaling complex lacritin stimulated	5336 5337 5578	Human	3
2539	Lab-Grb2 complex BCR stimulated	14784 56743	Mouse	2
2540	BCR-ABL (p210 fusion protein)-GRB2 complex	2885	Human	1
2541	HGF-Met complex	3082 4233	Human	2
2542	EGFR-CBL-GRB2 complex	867 1956 2885	Human	3
2545	Grb2-mSos1 complex	14784 20662	Mouse	2
2547	PLC-gamma-1-SLP-76-SOS1-LAT complex	27040 3937 5335 6654	Human	4
2548	Egfr-Grb2-mSos1 complex EGF stimulated	13649 14784 20662	Mouse	3
2550	Frs2-Grb2-Shp2 complex FGF stimulated	327826 14784 19247	Mouse	3
2551	PDGFRA-PLC-gamma-1-PI3K-SHP-2 complex PDGF stimulated	5156 5295 5335 5781	Human	4
2552	Il3rb1-Shc complex IL-3 stimulated	12983 20416	Mouse	2
2553	Shc-Grb2-mSos1 complex EGF stimulated	14784 20416 20662	Mouse	3
2558	p56(Lck)-CAML complex	12328 16818	Mouse	2
2559	p56(LCK)-CAML complex	819 3932	Human	2
2560	Nephrin-cadherin complex (Nphs1 Ctnnd1 ZO-1 Cd2ap)	12488 12388 54631 403752	Dog	4

2561	Nephrin-cadherin complex (Nphs1 Cask Cd2ap)	29647 316258 64563	Rat	3
2562	Nephrin-cadherin complex (Nphs1 Ctnnd1 Cdh3 Cd2ap)	12488 12560 12388 54631	Dog	4
2563	FGFR2-c-Cbl-Lyn-Fyn complex	867 2263 2534 4067	Human	4
2564	p21(ras)GAP-Fyn-Lyn-Yes complex thrombin stimulated	2534 4067 5921 7525	Human	4
2565	CD20-LCK-LYN-FYN-p75/80 complex (Raji human B cell line)	2534 3932 4067 931	Human	4
2567	NCAM140-p59(fyn)-p125(fak) signaling complex	25150 24586 25614	Mammalia	3
2568	Slam-SAP-FynT complex	14360 20400 27218	Mouse	3
2569	Slam-SAP-SHIP complex	16331 20400 27218	Mouse	3
2570	Slam-SAP complex	20400 27218	Mouse	2
2572	RAB5-EEA1 complex	8411 5868	Human	2
2573	Class C VPS/HOPS complex	8411 5868 55823 64601 57617 23339	Human	6
2574	CD19-Vav-PI 3-kinase (p85 subunit) complex	930 5295 7409	Human	3
2575	PI3-kinase p85-subunit alpha- PI3-kinase p110 complex	282306 282307	Bovine	2
2577	Sam68-p85 P13K-IRS-1-IR signaling complex	3643 3667 10657 5295	Human	4
2578	Sam68-p120GAP complex	10657 5921	Human	2
2579	Chromosomal passenger complex CPC (INCENP BIRC5 AURKB)	9212 332 3619	Human	3
2580	Survivin homodimer complex	332	Human	1
2581	RasGAP-AURKA/AURKB-survivin complex	332 5921 6790 9212	Human	4
2582	Chromosomal passenger complex CPC (CDCA8 AURKB BIRC5)	9212 332 55143	Human	3
2583	CSK-GAP-A.p62 complex	12988 20218	Mouse	2
2584	c-Src-Muc1 complex	12988 17829	Mouse	2
2585	KLC1-nKHC-uKHC kinesin complex	16572 16573 16593	Mouse	3
2586	KLC2-nKHC-uKHC kinesin complex	16572 16573 16594	Mouse	3
2587	nKHC-KLC1-KLC2 kinesin complex	16572 16593 16594	Mouse	3
2589	PGC-1-SRp40-SRp55-SRp75 complex	10891 6429 6430 6431	Human	4
2590	FOXO1-FHL2-SIRT1 complex	2274 2308 23411	Human	3
2591	SIRT1-HNF4-alpha-PGC-1-alpha complex in response to fasting	15378 19017 93759	Mouse	3
2592	SIRT1 homotrimer complex	23411	Human	1
2593	FOXO3-SIRT1 complex oxidative stress stimulated	2309 23411	Human	2
2599	POLR2A-CCNT1-CDK9-NCL-LEM6-CPSF2 complex	904 1025 53981 4691 5430 10891	Human	6
2600	BRD4 complex	23476 904 1025 5469 9968 9282 9440 9862	Human	8
2601	P-TEFb-BRD4-TRAP220 complex	23476 904 1025 5469	Human	4
2602	P-TEFb-7SKRNA-HEXIM1 complex	904 1025 10614	Human	3
2603	Transcription elongation factor complex (SUPT5H CDK9 CCNT1)	904 1025 6829	Human	3
2604	P-TEFb-SKP2 complex	904 1025 6502	Human	3

2605	Heterotrimeric complex (CCNT1 CDK9 GRN)	904 1025 2896	Human	3
2608	Brd4-P-TEFb complex	57261 12455 107951	Mouse	3
2618	TUBA1A-TUBB2A complex	281555	Bovine	1
2625	CDK8-MED6-PARP1 complex	1024 10001 142	Human	3
2626	CCNC-CDK8-MED1-MED6-MED7 xcomplex	892 1024 5469 10001 9443	Human	5
2628	CCNC-CDK3 complex	892 1018	Human	2
2635	BETA2-Cyclin D1 complex	595 4760	Human	2
2638	HES1 promoter corepressor complex	1022 1387 2033 5430 3516 6830	Human	6
2639	HES1 promoter-Notch enhancer complex	904 1022 1024 1025 2033 9794 5469 4851 5430 3516 51763 11198 6830	Human	13
2641	p300/CBP-PCAF-MyoD complex	4654 8850 1387 2033	Human	4
2642	SMAD1-P300 complex	2033 4086	Human	2
2649	MYC-DNMT3A-ZBTB17 complex	1788 4609 7709	Human	3
2650	DNMT3B-DNMT3L complex	1789 29947	Human	2
2651	DNMT3L-DNMT3A complex	1788 29947	Human	2
2653	MYC-MAX-BLOC1S1 complex	2647 4149 4609	Human	3
2655	MYC-MAX complex	4149 4609	Human	2
2657	ESR1-CDK7-CCNH-MNAT1-MTA1-HDAC2 complex	902 1022 2099 3066 4331 9112	Human	6
2660	ERCC2/CAK complex	902 1022 2068 4331	Human	4
2670	Er-alpha-p53-hdm2 complex	2099 4193 7157	Human	3
2678	FOXO3-TP53 complex oxidative stress stimulated	2309 7157	Human	2
2679	p53-SP1 complex	6667 7157	Human	2
2681	p53 homotetramer complex	7157	Human	1
2682	Bcl-xL-p53-PUMA complex DNA damage induced	170770 12048 22059	Mouse	3
2683	PUMA-Bcl-xL complex DNA-damage induced	170770 12048	Mouse	2
2684	p53-Bcl-xL complex DNA-damage induced	12048 22059	Mouse	2
2685	RNA polymerase II (RNAPII)	9150 2959 2962 2963 5430 5431 5432 5433 5434 5435 5436 5437 5438 5439 5440 5441 26015	Human	17
2686	BRCA1-core RNA polymerase II complex	672 5430 5431 5432 5433 5434 5435 5436 5437 5438 5439 5440 5441	Human	13
2688	MT1-MMP-claudin-1 complex	9076 4323	Human	2
2689	Fra1-JunB DNA-protein complex	25445 24517	Rat	2
2690	Atf2-c-Jun-c-Myc complex	81647 24516 24577	Rat	3
2692	SMAD3-SMAD4-cJun-cFos complex	2353 3725 4088 4089	Human	4
2693	NFAT-JUN-FOS DNA-protein	2353 3725 4773	Human	3
2694	ERG-JUN-FOS DNA-protein complex	2078 2353 3725	Human	3
2695	ETS2-FOS-JUN complex	2114 2353 3725	Human	3
2699	ER-alpha-GRIP1-c-Jun complex	2099 23426 3725	Human	3
2700	ER-alpha-c-Jun complex	2099 3725	Human	2
2704	Ectodermin-SMAD4 complex	4089 51592	Human	2
2705	SMAD3-SMAD4-CTCF protein-DNA complex	10664 4088 4089	Human	3

2706	SMAD3-SMAD4-SP1 complex	4088 4089 6667	Human	3
2707	SMAD3-SMAD4-FOXO3-FOXG1 complex	2290 2309 4088 4089	Human	4
2708	SMAD3-SMAD4-cJUN complex	3725 4088 4089	Human	3
2709	MMP-9-TIMP-1-LRP complex	4035 4318 7076	Human	3
2710	LRP-1-Alpha-2-M-annexin VI complex	2 309 4035	Human	3
2711	Amyloid beta protein oligomer	351	Human	1
2712	Aph1a-Psen1-Ncstn complex	226548 59287 19164	Mouse	3
2714	Ubiquitin E3 ligase (CHEK1 CUL1)	1111 8454	Human	2
2715	Ubiquitin E3 ligase (CSN1 CSN8 HRT1 SKP1 SKP2 CUL1 CUL2	10920 8454 8453 8452 2873 9978 6500 6502	Human	8
2716	Elongator core complex	55250 55140 8518	Human	3
2717	Ubiquitin E3 ligase (TRIM25 DDX58)	23586 7706	Human	2
2718	MAD2-CDC20 complex	991 4085	Human	2
2719	Casein kinase II-HMG1 complex	1457 1459 1460 3146	Human	4
2720	Casein kinase II complex	1457 1459 1460	Human	3
2721	HCF-1 complex	9070 3054 3065 3066 3308 3309 3312 8473 5928 5931 8819 9739 25942 23309 6667 64426 11091 3320 3326	Human	19
2723	ATM-NBS1 complex	472 4683	Human	2
2724	Ubiquitin E3 ligase (NFKBIA BTRC CUL1 SKP1A)	8945 8454 4792 6500	Human	4
2725	Ubiquitin E3 ligase (NFKBIA FBXW11 CUL1 SKP1A)	8454 23291 4792 6500	Human	4
2726	PXN-ITGB5-PTK2 complex	3693 5747 5829	Human	3
2727	SRC-3 complex	1147 1387 3551 8517 10499 8202	Human	7
2728	SRC-1 complex	1387 8648 10499 6738	Human	4
2729	PIP complex	16202 110829 57342	Mouse	3
2730	Set1B complex	9070 219771 5929 23067 11091	Human	6
2731	Set1A complex	9070 219771 5929 9739 11091 80335	Human	6
2732	Translocon-associated protein complex (TRAP complex)	403951 403950 6747 6748	Dog	4
2733	Aph1a-pPsen1-nNcstn complex	365872 289231 29192	Rat	3
2734	APH1A-PSEN1-NCSTN complex	51107 23385 5663	Human	3
2735	APH1A-PSEN2-NCSTN complex	51107 23385 5664	Human	3
2736	TAJ-NgR1-LINGO-1 signaling	84894 65078 55504	Human	3
2739	FA complex (Fanconi anemia complex)	641 2175 2187 2176 2178 2188 2189 55120 80010 6117 7156	Human	11
2740	MutS-alpha complex	4436 2956	Human	2
2742	Gata2-Tal1-Tcf3-Lmo2 complex	14461 16909 21349 21423	Mouse	4
2743	TRAF6-MALT1 complex	10892 7189	Human	2
2744	TRAF2-MALT1 complex	10892 7186	Human	2
2745	Ubiquitin ligase complex (TRAF6 TAB2 MALT1 UEV1A BCL10)	8915 10892 23118 7189 387522	Human	5
2746	NMDA receptor complex	24408 24409 191573	Rat	3
2747	Gata1-Fog1 complex	14460 22761	Mouse	2
2748	Ubiquitin-protein ligase (UBE2N UBE2V2/MMS2)	7334 387522	Human	2
2749	SETDB1-containing HMTase complex	55729 9869	Human	2

2750	Gata1-Fog1-MeCP1 complex	107932 14460 229542 433759 15182 17191 17192 116870 23942 116871 19646 245688 22761	Mouse	13
2752	CARMA1-BCL10-MALT1 complex	8915 84433 10892	Human	3
2753	FYB-CARMA1-BCL-10-MALT1 complex	8915 84433 2533 10892	Human	4
2754	JUND-FOSB-SMAD3-SMAD4	2354 3727 4088 4089	Human	4
2755	17S U2 snRNP	10523 9879 1665 22826 10362 3329 84844 22827 84991 10291 8175 10946 23451 51639 10992 23450 10262 83443 6426 10285 6627 6628 6629 6632 6633 6634 6635 6636 6637 23350 10131 7307 11338	Human	33
2756	Ubiquitin-protein-ligase (UBE2N UBE2V2/MMS2)	7334 7336	Human	2
2757	SMN complex U7 snRNA specific	84967 134353 6628 6634 6635 6636	Human	7
2758	TRIKA2 protein kinase complex (TAK1 TAB1 TAB2)	10454 23118 6885	Human	3
2759	MBD1-MCAF complex	55729 4152	Human	2
2760	SMAD3-SMAD4-FOXO3 complex	2309 4088 4089	Human	3
2761	SMAD3-SMAD4-FOXO1 complex	2308 4088 4089	Human	3
2762	SMAD3-SMAD4-FOXO4 complex	4303 4088 4089	Human	3
2763	MBD1-Suv39h1-HP1 complex	23468 4152 6839	Human	3
2766	TERF2-RAP1 complex	4361 10111 7014 54386 26277 7520	Human	7
2767	RAD50-MRE11-NBN-p200-p350 complex	4361 4683 10111	Human	3
2768	IKK complex (NEMO IKKB)	84351 309295	Rat	2
2769	IKK complex (NEMO)	309295	Rat	1
2770	ITGA6-ITGB4-CD9 complex	928 3655 3691	Human	3
2771	RAD51-DMC1 complex	13404 19361	Mouse	2
2772	Ubiquitin E3 ligase (CRY1 SKP1A CUL1 FBXL3)	1407 8454 26224 6500	Human	4
2773	Ubiquitin E3 ligase (CRY2 SKP1A CUL1 FBXL3)	1408 8454 26224 6500	Human	4
2774	MDC1-H2AFX-TP53BP1 complex	3014 9656 7158	Human	3
2775	MDC1-p53BP1-SMC1 complex	9656 8243 7158	Human	3
2776	RAD50-BRCA1 complex	672 10111	Human	2
2777	Ecsit complex (Ecsit2-Smad4)	26940 17128	Mouse	2
2778	Ecsit complex (Ecsit2-Smad1)	26940 17125	Mouse	2
2779	Ecsit complex (Smad1-Smad4-Ecsit2)	26940 17125 17128	Mouse	3
2780	Rab1-GTP-GM130 complex	64528 81754	Rat	2
2781	Rab1-GTP-p115 complex	81754 56042	Rat	2
2782	Rab1-GTP-GM130-GRASP65 complex	64528 56082 81754	Rat	3
2783	BARD1-BRCA1-CSTF64 complex	580 672 1478	Human	3
2784	Alpha-2-M enhanceosome DNA-protein complex	314322 24516 24413 171068 25125	Rat	5
2786	BRCA1 A complex	580 672 84142 51720	Human	4
2787	BRCA1 C complex	580 672 5932 51720	Human	4
2788	BRCA1 B complex	571 580 672	Human	3
2789	ETS2-ERG complex	2078 2114	Human	2

2790	ETS2-ETS1 complex	2113 2114	Human	2
2791	MCM4-MCM6-MCM7 complex	4173 4175 4176	Human	3
2792	MCM2-MCM4-MCM6-MCM7	4171 4173 4175 4176	Human	4
2793	Brd4-Rfc complex	57261 19687 19718 69263 106344	Mouse	6
2794	ATF2-c-Jun complex	81647 24516	Rat	2
2796	FosB-JunB DNA-protein complex	14282 24517	Rat	2
2797	PCNA-CHL12-RFC2-5 complex	63922 5111 5982 5983 5984 5985	Human	6
2798	MMP-2-claudin-1 complex	9076 4313	Human	2
2800	SNARE complex (STX1A CPLX2 SNAP-25 VAMP2)	540853 281711 788566 282116	Bovine	4
2801	OCT4-SOX2 DNA-protein complex	5460 6657	Human	2
2802	OCT1-SOX2 DNA-protein complex	5451 6657	Human	2
2803	PAX6-SOX2 DNA-protein complex	5080 6657	Human	2
2804	CTF18-cohesion-RFC complex	63922 54921 79075 5982 5983 5984	Human	7
2805	CTF8-DCC1 subcomplex	54921 79075	Human	2
2806	CTF8-CTF18-DCC1 subcomplex	63922 54921 79075	Human	3
2808	RAD9-RAD1-HUS1-APE1 complex	328 3364 5810 5883	Human	4
2809	9-1-1 complex	3364 5810 5883	Human	3
2810	Rad17-RFC complex	5884 5982 5983 5984 5985	Human	5
2811	BRCA1-cABL complex	25 672	Human	2
2812	RPAP1-RPB2-RPB3 complex	5431 5432 26015	Human	3
2813	BRCA1-SMAD3 complex	672 4088	Human	2
2814	BRCA1-HDAC1-HDAC2 complex	672 3065 3066	Human	3
2815	BRCA1-BARD1-BACH1-DNA damage complex II	571 580 672 4361 4683 10111 5932 11073	Human	8
2816	ITGAV-ITGB3 complex	3685 3690	Human	2
2817	BRCA1-BARD1-BACH1-DNA damage complex I	571 580 672 4292 2956 11073	Human	6
2818	BRCA1-BARD1-BRCA2-DNA damage complex III	580 672 675	Human	3
2819	BRCA1-CtIP-CtBP complex	672 1487 5932	Human	3
2820	BRCA1-VCP complex	672 7415	Human	2
2821	hSIR2-p53 complex	23411 7157	Human	2
2822	BRCA1-BARD1-UbcH5c complex	580 672 7323	Human	3
2823	BRCA1-BARD1-UbcH7c complex	580 672 7332	Human	3
2824	BRCA1-RAD51 complex	672 5888	Human	2
2825	BRCA1-RNA polymerase II complex	672 2068 2071 2959 2960 2961 2962 2963 2965 2966 2967 2968 9412 5430 5431 5432 5433 5434 5435 5436 5437 5438 5439 5440 5441 6908	Human	26
2826	ITGB3-ITGAV-VTN complex	3685 3690 7448	Human	3
2829	RSmad complex	57492 1387 8202 4087 4088 4089 6597 6599 6601 51592	Human	10
2830	TIF1gamma-SMAD2-SMAD3 complex	4087 4088 51592	Human	3
2831	TIF1gamma-Smad2-Smad3 complex	17126 17127 94093	Mouse	3
2832	Smad4-Smad2-Smad3 complex	17126 17127 17128	Mouse	3
2833	SRm160-SRm300 complex	10250 23524	Human	2
2834	SMAD4-SMAD2-SMAD3 complex	4087 4088 4089	Human	3

2835	Profilin 2 complex	11461 20430 13429 15481 50884 18645 19878 20964 20965	Mouse	9
2836	Profilin 1 complex	11465 67300 15481 18643 73710	Mouse	6
2837	Profilin 1 complex	71 1213 3312 5216 347733 7415	Human	6
2838	AR coactivator complex	1207 10419 79084	Human	3
2839	ATRX-DAXX complex	546 1616	Human	2
2840	uKHC-KLC1-KLC2 kinesin complex	16573 16593 16594	Mouse	3
2842	DAXX-Axin-p53-HIPK2 complex	8312 1616 28996 7157	Human	4
2844	Axin-p53-HIPK2 complex	8312 28996 7157	Human	3
2845	BAX-BAK-IRE1alpha complex	12018 12028 78943	Mouse	3
2846	ITGAV-ITGB3-THBS1 complex	3685 3690 7057	Human	3
2847	ING4 complex (ING4 MYST2 C1orf149 PHF15)	64769 51147 11143 23338	Human	4
2848	ING4 complex (ING4 MYST2 C1orf149 PHF16)	64769 51147 11143 9767	Human	4
2849	ITGAV-ITGB3-NOV complex	3685 3690 4856	Human	3
2850	ITGA5-ITGB1-FN-1-NOV complex	2335 3678 3688 4856	Human	4
2851	ING2 complex	5926 25855 84312 3065 3066 3622 5928 5931 79595 8819 25942 64426	Human	12
2852	Brg1-based SWI/SNF chromatin remodeling complex	86 6598 6599 6601	Human	4
2853	ITGA5-ITGB1-CAL4A3 complex	80781 3678 3688	Human	3
2854	PI3-kinase p85-subunit beta- PI3-kinase p110 complex	282306 282308	Bovine	2
2857	NuA4/Tip60 HAT complex	86 10902 64769 55929 57634 80314 26122 10524 54556 10933 8607 10856 8295 8089	Human	14
2858	HBO1 complex	64769 51147 84289 11143 23338 9767 79960	Human	7
2859	ING5 complex	23774 7862 27154 64769 84289 11143 7994 23522 23338 9767 79960	Human	11
2860	DSIF complex (DRB sensitivity-inducing factor complex)	6827 6829	Human	2
2863	Serine-palmitoyltransferase (SPT) complex	10558 9517 55304	Human	3
2866	TEAD2-YAP DNA-protein complex	21677 22601	Mouse	2
2867	TEAD1-YAP DNA-protein complex	21676 22601	Mouse	2
2868	TEAD3-YAP DNA-protein complex	21678 22601	Mouse	2
2869	TEAD4-YAP DNA-protein complex	21679 22601	Mouse	2
2870	TEAD2-multiprotein complex	17475 21677 22601 22630	Mouse	4
2872	ITGA2b-ITGB3-CD9-GP1b-CD47 complex	961 928 3674 3690 2811 2812	Human	6
2875	BRD4-P-TEFb complex	23476 904 1025	Human	3
2876	SNARE complex (Snap25 Vamp3 Vamp2 Napa Stx12/13)	140673 25012 65033 24803 29528	Rat	5
2879	CD20-LCK-FYN-p75/80 complex	2534 3932 931	Human	3
2880	SCF subcomplex (WEE1 SKP2	8945 6502 7465	Human	3
2881	Ubiquitin E3 ligase (CUL1 RBX1 SKP1A)	8454 9978 6500	Human	3
2882	ITGA5-ITGB3-COL6A3 complex	1293 3678 3690	Human	3

2883	Nephrin-cadherin complex (Nphs1 Ctnnd1 Cdh3 Cd2ap)	316258 116777 12388 64563	Rat	4
2884	Respiratory chain complex I (early intermediate NDUFAF1 assembly) mitochondrial	4535 4700 4704 51103 4712 4722 374291	Human	7
2885	ITGAV-ITGB1-SPP1 complex	3685 3688 6696	Human	3
2886	Respiratory chain complex I (incomplete intermediate ND1 ND2 ND3 CIA30 assembly) mitochondrial	4535 4536 4537 51103	Human	4
2887	Shc-Egfr complex EGF stimulated	24329 85385	Rat	2
2888	Grb2-Egfr complex EGF stimulated	24329 81504	Rat	2
2889	Grb2-Shc complex EGF stimulated	81504 85385	Rat	2
2890	Notch1-fraction 30 complex	9794 4851 3516	Human	3
2892	BCR-ABL (p185 fusion protein)-GRB2 complex	2885	Human	1
2893	BCR-ABL (p210 fusion protein)-GRB2-SOS1 complex	2885 6654	Human	2
2894	Itga1-Itgb1-Tgm2 complex	25118 24511 56083	Rat	3
2895	SHC-GRB2 complex	2885 6464	Human	2
2896	ITGA2b-ITGB3-CD47-FAK complex	961 3674 3690 5747	Human	4
2897	RBPJ-NotchIC-Mastermind complex	9794 4851 3516	Human	3
2898	Respiratory chain complex I (intermediate I/200kD and III/250kD) mitochondrial	4704 4720 4722	Human	3
2899	PLC-gamma-1-Lab-Blnk complex BCR stimulated	17060 56743 18803	Mouse	3
2900	PLC-gamma-2-Lab-Blnk complex BCR stimulated	17060 56743 234779	Mouse	3
2901	Respiratory chain complex I (intermediate IV/310kD)	4535 4704 4720 4722	Human	4
2902	Grb2-Lab-Blnk complex BCR	17060 14784 56743	Mouse	3
2903	Respiratory chain complex I (intermediate V/380kD and VI/480kD) mitochondrial	4535 4695 4704 4720 4722	Human	5
2904	Respiratory chain complex I (intermediate VII/650kD)	4535 4695 4700 4704 4720 4722 4724 4725 374291 4729	Human	10
2906	Respiratory chain complex I (intermediate II/230kD) mitochondrial	4724 374291 4729	Human	3
2907	RBP-Jkappa-Notch1 complex	4851 3516	Human	2
2908	RBP-Jkappa-SHARP complex	3516 23013	Human	2
2909	PLC-gamma-2-Syk-LAT-FcR-gamma complex	2209 27040 5336 6850	Human	4
2910	PLC-gamma-2-Lyn-FcR-gamma	2209 4067 5336	Human	3
2911	SMRT-SKIP-CBF1 complex	9612 3516 22938	Human	3
2912	PLC-gamma-2-SLP-76 complex	3937 5336	Human	2
2913	PLC-gamma-2-LAT complex	27040 5336	Human	2
2914	Respiratory chain complex I (beta subunit) mitochondrial	4538 4540 4697 4706 4707 4716 54539 4708 4709 4710 4711 4712 4713 4714 4715 4718	Human	16
2915	CASK-Caskin1 complex	29647 140722	Rat	2
2916	CASK-Caskin1-Velis complex	29647 140722 85327 60377 60442	Rat	5

2917	Grb2-Sos complex Fc receptor gamma-R1 stimulated	2885 6654	Human	2
2918	Ku antigen-YY1-alphaMyHC promoter complex	7520 2547 7528	Human	3
2919	Respiratory chain complex I (gamma subunit) mitochondrial	4535 4536 4537 4539 4541 4694 4705 4696 4700 4702 4704 4717 4725	Human	13
2920	Respiratory chain complex I (lambda subunit) mitochondrial	126328 55967 51079 4695 4698 4701 4719 4720 4722 4724 4726 374291 4728 4723 4729 4731	Human	16
2921	SHARP-CtBP complex	1487 1488 23013	Human	3
2922	LAT-PLC-gamma-1-p85-GRB2-SOS signaling complex C305 activated	2885 27040 5295 5335 6654	Human	5
2923	SHARP-CtBP1-CtIP complex	1487 5932 23013	Human	3
2924	Respiratory chain complex I (beta subunit) mitochondrial	3283886 327704 327702 327690 327701 404161 327713 338073 327706 338061 327665 338065	Bovine	15
2925	Respiratory chain complex I (lambda subunit) mitochondrial	326346 281742 338084 327698 327714 338063 288380 327697 287327 327680 327691 338079 287027 287014 282290 327717	Bovine	16
2926	Respiratory chain complex I (gamma subunit) mitochondrial	3283884 3283885 3283888 327673 338060 338064 327670 327710 404188 282289 338057	Bovine	11
2927	Respiratory chain complex I (incomplete gamma subunit)	3283884 3283885 282289	Bovine	3
2928	Respiratory chain complex I (lambda subunit) mitochondrial	327698 327714 327670 338063 327706 288380 327697 287327 327680 327691 338079 287027	Bovine	15
2929	Respiratory chain complex I (beta subunit) mitochondrial	3283886 327702 327690 327701 327713 327706 338061 327665 338065 282517 327660	Bovine	11
2930	SHARP-CtIP-RBP-Jkappa complex	5932 3516 23013	Human	3
2931	SHARP-CtBP1-CtIP-RBP-Jkappa corepressor complex	1487 5932 3516 23013	Human	4
2932	PTF1-L complex (Ptf1a Tcf12 Rbpjl)	117034 19668 25720	Rat	3
2933	PTF1-L complex (Ptf1a Tcf4 Rbpjl)	117034 19668 84382	Rat	3
2934	PTF1-L complex (Ptf1a Tcf3 Rbpjl)	117034 19668 171046	Rat	3
2935	PTF1 complex (Ptf1a Tcf12 Rbpj)	117034 19664 25720	Rat	3
2936	Ecsit complex (ECSIT MT-CO2 GAPDH TRAF6 NDUFAF1)	51295 2597 4513 51103 7189	Human	5
2937	PTF1-bHLH complex	256297 6938	Human	2
2938	Ecsit complex (ECSIT NDUFS3 TOM20)	51295 4722 9804	Human	3
2939	Ecsit complex (ECSIT MT-CO2 NDUFA1 MT-ND1 TRAF6 NDUFAF1)	51295 4513 4535 4694 51103 7189	Human	6
2940	Acinar cell-specific C complex	17536 18514 18609	Mouse	3
2942	Ecsit complex (ECSIT NDUFS3 NDUFAF1)	51295 51103 4722	Human	3
2943	Respiratory chain complex I (incomplete NDUFAF1 assembly)	4535 51103	Human	2
2944	Notch1-p56lck-PI3K complex	3932 4851 5295	Human	3

2945	RBP-Jkappa-RING1-KyoT2 complex	2273 3516 6015	Human	3
2946	YY1-Notch1-RBP-Jkappa complex	4851 3516 7528	Human	3
2947	YY1-Notch1 complex	4851 7528	Human	2
2948	Respiratory chain complex I (incomplete intermediate)	4705 4704 4714 4719 4720 4724 4726 374291 4723 4729 4731	Human	11
2949	MAML1-RBP-Jkappa-Notch1 complex	103806 18128 19664	Mammalia	3
2950	MAML1-RBP-Jkappa-Notch2 complex	103806 18129 19664	Mammalia	3
2951	MAML1-RBP-Jkappa-Notch3 complex	103806 18131 19664	Mammalia	3
2952	MAML1-RBP-Jkappa-Notch4 complex	103806 18132 19664	Mammalia	3
2953	Respiratory chain complex I (nuclear encoded subunits) mitochondrial	327673 338060 326346 281742 338084 327698 338064 327704 327714 327670 338063 327710 404188 327702 327690 327701 404161 327713 338073 327706 338061 327665 338065 282517 327660 282289 338046 288380 327697 287327 327680 338057	Bovine	38
2954	Smad1-Notch1-p300-Pcaf complex	2033 18128 8850 17125	Mouse	4
2955	LCK-SLP76-PLC-gamma-1-LAT complex pervanadate-activated	27040 3932 3937 5335	Human	4
2956	PLC-gamma-1-LAT-c-CBL complex OKT3 stimulated	867 27040 5335	Human	3
2957	LAT-GRB2 complex Fyn-mLck(KA) or Syk kinase activated	2885 27040	Human	2
2958	SMAD1-CBP complex	1387 4086	Human	2
2959	SMAD1-OAZ-HsN3 complex	4946 5692 4086	Mammalia	3
2960	SLP-76-PLC-gamma-1-ITK complex alpha-TCR stimulated	3702 3937 5335	Human	3
2961	SLP-76-PLC-gamma-1-VAV complex alpha-TCR stimulated	3937 5335 7409	Human	3
2962	CRK-BCAR1-DOCK1 complex	9564 1398 1793	Human	3
2963	ITK-SLP-76 complex anti-TCR stimulated	3702 3937	Human	2
2964	ITGA9-ITGB1-ADAM1 complex	280668 3680 3688	Mammalia	3
2965	ITGA9-ITGB1-ADAM3 complex	11497 3680 3688	Mammalia	3
2966	NuMA-LGN-G-alpha-i-1 complex	2770 29899 4926	Human	3
2967	Itga9-Itgb1-Thbs1 complex	104099 16412 21825	Mouse	3
2968	Axin-SMAD3 complex	8312 4088	Human	2
2969	mTORC2 complex (mTOR/FRAP1 LST8 mAVO3/RICTOR)	2475 64223 253260	Human	3
2970	mTORC1 complex (mTOR/FRAP1 LST8 RAPTOR)	2475 64223 57521	Human	3
2971	ITGA9-ITGB1-VEGFC complex	3680 3688 7424	Human	3
2972	ITGA9-ITGB1-VEGFA complex	3680 3688 7422	Human	3
2973	Ccd1-Dvl2-Rac complex	330938 13543 19353	Mouse	3
2974	Dvl2-Rac complex	13543 19353	Mouse	2
2975	SMAD3-E2F4/5-p107-DP1 complex	1874 1875 5933 4088 7027	Human	5
2976	Ccd1-Dvl2 complex	330938 13543	Mouse	2
2977	Ccd1-Axin complex	12005 330938	Mouse	2
2978	Mekk1-Axin complex	12005 26401	Mouse	2

2979	Axin-Mekk4 complex	12005 26407	Mouse	2
2980	Ccd1-Mekk4 complex	330938 26407	Mouse	2
2981	Nkx3.2-SMAD1 complex	12020 17125	Mammalia	2
2982	Nkx3.2-SMAD1-SMAD4 complex	12020 17125 17128	Mammalia	3
2983	Nkx3.2-SMAD1-SMAD4-HDAC1 complex	433759 12020 17125 17128	Mammalia	4
2984	Nkx3.2-SMAD1-SMAD4-HDAC-Sin3A complex	433759 12020 19646 245688 20466 17125 17128	Mammalia	7
2985	mTOR-signaling complex	2475 57521	Human	2
2986	FKBP12-FK506 complex	25639 56718	Rat	2
2987	FKBP12-FK506 complex	14225 56717	Mouse	2
2989	ITGA9-ITGB1-ADAM8 complex	101 3680 3688	Human	3
2990	mTOR-signaling complex (FRAP1/mTOR GBL RAPTOR)	2475 64223 57521	Human	3
2991	mTOR-signaling complex (mTOR/FRAP1 RAPTOR)	2475 57521	Human	2
2992	SMAD7-SMURF2 complex	4092 64750	Human	2
2993	Axin-GSK-3-beta complex	79257 84027	Mammalia	2
2994	Axin-GSK-3-alpha complex	79257 50686	Mammalia	2
2995	Axin-beta-catenin complex	79257 84353	Mammalia	2
2996	SMAD7-SMURF1 complex	4092 57154	Mammalia	2
2997	SMAD7-SMURF1-TGF-beta receptor complex	4092 57154 7046 7048	Mammalia	4
2998	Axin-PP2A A-PP2A C-GSK3-beta-beta-catenin complex	8312 1499 2932 5525	Human	4
2999	Smad2 homotrimer complex	17126	Mammalia	1
3000	Smad2-Smad3 complex	17126 17127	Mammalia	2
3001	Smad2-Smad4 heteromer complex	17126 17128	Mammalia	2
3002	Smad3-Smad4 complex	17127 17128	Mammalia	2
3003	Smad3 homotrimer complex	17127	Mammalia	1
3004	APC-Axin-1-beta-catenin complex	324 8312 1499	Human	3
3005	TGF-beta-receptor type I homodimer complex	29591	Rat	1
3006	TGF-beta-receptor type II homodimer complex	21813	Mammalia	1
3007	TGF-beta-receptor type I homodimer complex	21812	Mammalia	1
3008	60S APC containing complex	324 8826 7846 10376 84790 7283	Human	7
3009	TFIID complex	6872 6882 6883 6873 6874 6877 6878 6880 6908	Human	9
3010	TFIID subcomplex	6883 6874 6877 6878 6880	Human	5
3011	APC-IQGAP1-Rac1 complex	324 8826 5879	Mammalia	3
3012	APC-IQGAP1-Cdc42 complex	324 998 8826	Mammalia	3
3013	EB1-APC-mDia1 complex	11789 13367 13589	Mouse	3
3014	Cohesin complex incomplete (Stag3 Scp1 Ss181)	269397 50878 20957	Mouse	3
3015	p27-cyclinE-Cdk2 - Ubiquitin E3 ligase (SKP1A SKP2 CUL1 CKS1B RBX1) complex	898 1017 1027 1163 8454 9978 6500 6502	Human	8

3016	Cohesin complex incomplete (Stag3 Sycp3)	50878 20962	Mouse	2
3017	Cohesin complex incomplete (Stag3 Crest/Ss18I1)	269397 50878	Mouse	2
3018	Cohesin complex incomplete (Sycp2 Crest/Ss18I1)	269397 320558	Mouse	2
3019	Cohesin complex incomplete (Smc4 Crest/Ss18I1)	70099 269397	Mouse	2
3020	Cohesin complex incomplete (Rec8 Crest/Ss18I1)	56739 269397	Mouse	2
3021	Cohesin complex incomplete (Smc1b Crest/Ss18I1)	140557 269397	Mouse	2
3022	Cohesin complex incomplete (Sycp1 Crest/Ss18I1 Rec8)	56739 269397 20957	Mouse	3
3023	Cohesin complex incomplete (Smc1b Sycp1 Crest/Ss18I1)	140557 269397 20957	Mouse	3
3024	Cohesin complex incomplete (Smc3 Sycp1 Crest/Ss18I1)	13006 269397 20957	Mouse	3
3025	TGF-beta receptor II-TGF-beta3 complex	7043 7048	Human	2
3026	TGF-beta receptor II-TGF-beta1 complex	7040 7048	Human	2
3027	TGF-beta receptor II-TGF-beta receptor I-TGF-beta1 complex	7040 7046 7048	Human	3
3028	TGF-beta receptor II-TGF-beta receptor I-TGF-beta3 complex	7043 7046 7048	Human	3
3029	Drosha complex	67040 13207 94223 13204 59013 76936 14000 230908	Mouse	8
3032	RNA-induced silencing complex RISC	23405 27161 3326 6895	Human	4
3033	PAC3-PAC4 complex	84262 389362	Human	2
3034	PAC1-PAC2 complex	8624 56984	Human	2
3035	LAT2-ITGB1 complex	3688 23428	Human	2
3036	Ubiquitin E3 ligase (SKP1A SKP2 CUL1 CKS1B RBX1)	1163 8454 9978 6500 6502	Human	5
3037	Ubiquitin E3 ligase (Fbxl20 Skp1	26965 72194 21402	Mouse	3
3038	SMAD2-SMAD4-FAST1 complex	8928 4087 4089	Human	3
3039	SMAD2-FAST1 complex	8928 4087	Human	2
3040	Multisynthetase complex	1615 9521 2058 3376 7965 3735 51520 4141 5859 5917 9255	Human	11
3041	TGF-beta-receptor type II homodimer complex	7048	Human	1
3042	TGF-beta-receptor II-TGF-beta1 complex	7040 7048	Human	2
3043	BMP2-BRIA complex	650 652 657	Human	3
3044	SKI-NCOR1-SIN3A-HDAC1 complex	3065 9611 25942 6497	Human	4
3045	hs4 enhancer complex (faster migrating complex)	4790 5451 5452 5970 7528	Human	5
3046	hs4 enhancer complex (slow migrating complex)	5971 7528	Human	2

3047	Parvulin-associated pre-rRNP complex	12181 67239 984 12607 66942 56200 27225 228889 13207 52513 1665 69072 67160 14113 14268 56095 2622 230737 69237 67949 84057 69902 18432 17975 110109 55989 67134 64934 5303 55131 6134 4736 270106 22121 67115 66480 19899 76808 19921 19933 19941 27367 67891 19983 19988 19989 27176 26961 11837 27050 20091 20102 20104 20116 6203 18114 59014 [illegible]	Mouse	62
3048	mSin3A complex	3065 3066 5928 5931 25942	Human	5
3049	Mad-Max-mSin3a complex	17187 17119 20466	Mouse	3
3050	Mad-Max-mSin3B complex	17187 17119 20467	Mouse	3
3051	MAD-MAX complex	4149 4084	Human	2
3053	mSin3A-HDAC1-HDAC2 complex	3065 3066 25942	Human	3
3054	MAD1-mSin3A-HDAC2 complex	3066 4084 25942	Human	3
3055	Nop56p-associated pre-rRNA complex	59 72 55299 9188 1660 10969 1915 1938 2091 26354 8971 3007 8342 3178 4670 3192 10642 3608 3609 51631 55646 10514 4673 4691 4809 51602 10528 9221 4869 11137 55131 6134 4736 6135 6136 6137 23521 9045 6138 6139 6141 6142 6143 6144 6146 9349 6147 6152 6154 6155 6157 6158 6159 6122 6156 6160 6161 11224 6165 25873 6173 6168 6170 6124 6125 6128 6129 6130 6132 6133 6175 6176 6181 6205 6206 6207 6208 6209 6210 6217 6218 6222 6187 6228 6229 6235 6189 6194 6201 6202 6203 26156 55681 292 6727 6732 6949 [illegible]	Human	104
3056	Microprocessor complex	54487 29102	Human	2
3057	ITGA10-ITGB1 complex	8515 3688	Human	2
3058	ITGA11-ITGB1 complex	22801 3688	Human	2
3059	ITGA11-ITGB1-COL1A1 complex	1277 22801 3688	Human	3
3060	RNA polymerase II complex (RPB1 RAP74 CDK8 CYCC SRB7 BAF190 BAF47) chromatin structure	892 1024 2962 9412 5430 6598 6595 6597	Human	8
3061	RNA polymerase II complex (CBP PCAF RPB1 BAF47 CYCC CDK8) chromatin structure modifying	892 1024 1387 8850 5430 6598	Human	6
3062	RNA polymerase II complex incomplete (CBP RPBI PCAF BAF47) chromatin structure modifying	1387 8850 5430 6598	Human	4
3063	Brg1-associated complex II	86 8289 10419 6597 6598 6599 6601	Human	7
3064	RNA polymerase II complex chromatin structure modifying	892 1024 10589 2959 2960 2962 2965 9412 54760 5430 6598 6599 6601 6908 6595 6597 6602 6603 6604	Human	19
3065	RNA polymerase II complex chromatin structure modifying	86 892 1024 9412 6598 6599 6601 6605 6602 6603 6604	Human	11

3066	RNA polymerase II complex chromatin structure modifying	892 1024 1387 2071 2959 2962 2967 9412 8850 5430 6595 6597 6598	Human	13
3067	RNA polymerase II complex incomplete (CDK8 complex) chromatin structure modifying	892 902 1024 2962 9412 6598 6599 6601	Human	8
3068	Fertilin complex (Adam1a Adam2)	280668 11495	Mouse	2
3069	Rich1-Amot-Par-3 polarity complex	27494 63994 81918	Rat	3
3070	CTF18-cohesion-RFC-POLH complex	63922 54921 79075 5429 5982 5983 5984 5985	Human	8
3071	CTLH complex	25852 10296 4289 10048 64795	Human	5
3072	CCT complex (chaperonin containing TCP1 complex) testis specific	21454 12461 12462 12464 12465 12467 12468 12469	Mouse	8
3073	CCT complex (chaperonin containing TCP1 complex)	512043 505313 504735 613336 533784 521540 514355 281047	Bovine	8
3074	CCT:PFD complex testis specific	512043 505313 504735 613336 533784 538090 514355 281047 5201 5202 5203 5204 10471 7411	Mammalia	14
3075	UTX-MLL2/3 complex	9070 80209 8085 58508 55728 23054 22976 5929 55291 7403 11091 23528	Human	12
3076	Srf-Elk1 complex	314436 20807	Rat	2
3077	Srf-Myocd complex	246297 20807	Rat	2
3078	DGCR8-NCL complex	54487 4691	Human	2
3079	DGCR8-ILF3 complex	54487 3609	Human	2
3080	ILF3-XPO5 complex	3609 57510	Human	2
3082	DGCR8 multiprotein complex	10521 1655 54487 1660 2521 3187 10236 3192 3309 3609 4691	Human	11
3083	Nucleic and chromatin Fanconi	2175 2176 2178 2188 2189	Human	5
3084	CCND1-CDK4 complex	595 1019	Human	2
3085	CCND2-CDK4 complex	894 1019	Human	2
3086	CCND3-CDK4 complex	896 1019	Human	2
3087	CCND1-CDK6 complex	595 1021	Human	2
3088	CCND2-CDK6 complex	894 1021	Human	2
3089	CCND3-CDK6 complex	896 1021	Human	2
3090	Kv4.2-Kchip4 channel complex	65180 259243	Rat	2
3091	Kv4.3-Kchip1 channel complex	65195 65023	Rat	2
3092	APP-TOMM40 complex	351 10452	Human	2
3093	APP-TIMM23 complex	351 10431	Human	2
3094	Metaxin complex	17827 53375	Mouse	2
3095	Itgav-Itgb3-Tgm2 complex	16410 29302 56083	Rat	3
3096	ITGA6-ITGB4-SHC1-GRB2 complex	2885 3655 3691 6464	Human	4
3097	TIMM17-TIMM23 complex	10440 10431	Human	2
3098	TIM50a-SMN1 complex	6606 92609	Human	2
3099	TIM50a-coilin complex	8161 92609	Human	2
3100	Yy1-Ppargc1a complex	19017 22632	Mouse	2
3101	Yy1-Ppargc1a-Frap1 complex	56717 19017 22632	Mouse	3
3102	DHX9-ADAR-vigilin-DNA-PK-Ku antigen complex	103 1660 3069 5591 7520 2547	Human	6
3103	ITGAV-ITGB3-SLC3A2 complex	3685 3690 6520	Human	3
3104	ITGB1-NRP1 complex	3688 8829	Human	2

3105	Itga7-Itgb11-Lama2 complex	81008 24511 16773	Rat	3
3106	Itga7-Itgb11-Ptk2 complex	81008 24511 25614	Rat	3
3110	ITGAV-P2RY2-GNA12 complex	2768 3685 5029	Human	3
3111	ITGA9-ITGB1-SPP1 complex	3680 3688 6696	Human	3
3112	ITGA5-ITGB1-SPP1 complex	3678 3688 6696	Human	3
3113	MAML1-RBP-Jkappa-Notch1 complex	9794 4851 3516	Human	3
3114	Itgax-Itgb2-Icam4 complex	78369 16411 16414	Mouse	3
3115	ITGA2B-ITGB3-ICAM4 complex	3386 3674 3690	Human	3
3116	Rab27a-melanophilin-myosin-Va complex	171531 17918 11891	Mouse	3
3117	ITGB5-ITGAV-VTN complex	3685 3693 7448	Human	3
3118	SMN1-SIP1-SNRP complex	8487 6606 6628 6632 6633 6634 6635	Human	7
3119	Kif17-Lin10-Lin2-Lin7-NR2B complex	12361 234678 14812 16559 108030	Mouse	6
3120	OCT1-OBF1-DNA-TLE1 complex	5450 5451 7088	Human	3
3121	OCT2-TLE4 complex	5452 7091	Human	2
3122	OCT1-OBF1-DNA complex	5450 5451	Human	2
3123	TLE2 homodimer complex	7089	Human	1
3124	TLE1-TLE2 complex	7088 7089	Human	2
3125	TLE1 homodimer complex	7088	Human	1
3126	Tle2-Hes1 complex	29577 299636	Rat	2
3127	TLE-Histone H3 complex	3020 8350 126961 7088 7089 7090	Human	7
3128	Gamma-secretase complex (APH1B PSEN1 PSENEN NCSTN)	83464 23385 5663 55851	Human	4
3129	STAT6-p100-RHA complex	1660 27044 6778	Human	3
3130	Tle3-Aes complex	14797 21887	Mouse	2
3131	Hes1-TLE1 complex	29577 7088	Mammalia	2
3133	Phosphatidylinositol 3-kinase (PIK3CA PIK3R1)	5290 5295	Human	2
3134	Na(+)/K(+) ATPase	397481 396898 11936	Pig	3
3136	Hes1-Tle1 complex	29577 21885	Rat	2
3137	MASH1 promoter-coactivator complex	1387 3280 554313 4841 142 8850 5430 6714 3020 8350 126961	Human	11
3138	POSH-AKT2 complex	208 57630	Human	2
3139	CRLR-RAMP1-ARRB2 complex	25388 10203 10267	Mammalia	3
3140	CRLR-RAMP2 complex	10203 10266	Human	2
3141	CRLR-RAMP3 complex	10203 10268	Human	2
3142	CAMK2-delta-MASH1 promoter-coactivator complex	817 1387 3280 4841 142 8850 5430 6714	Human	8
3144	Sos1-Grb2 complex	2885 6654	Mammalia	2
3145	Coatomer complex	213827 12847 70349 50797 59042 54161 56447	Rabbit	7
3146	Coatomer-Arf1 complex	213827 11840 12847 70349 50797 59042 54161 56447	Rabbit	8
3147	Beta/delta-coat protein subcomplex	213827 70349	Rabbit	2
3148	Arf1-beta/delta-coat protein subcomplex	213827 11840 70349	Rabbit	3
3149	NK-3-Groucho-HIPK2-SIN3A-RbpA48-HDAC1 complex	3065 28996 5928 25942 4824 579 7088 7089 7090 7091 79816	Human	11
3150	NK-3-Groucho complex	4824 579 7088 7089 7090 7091 79816	Human	7

3151	Sulphiredoxin-peroxiredoxin complex	5052 140809	Human	2
3152	Notch1(N-TM)-Notch1(N-EC) heterodimer complex	18128	Mouse	1
3153	GNAQ-GEFT-RHOA complex	115557 2776 387	Human	3
3154	Notch2(N-TM)-Notch2(N-EC)-Delta complex	4853 28514 10683 54567	Human	4
3155	Bipartite complex (TFC4 CTNNB1)	1499 6934	Human	2
3156	CBF1-HDAC1-SMRT complex	3065 9612 3516	Human	3
3157	Prolactin (PRL) - PRL receptor (PRLR) complex	5617 5618	Human	2
3158	RIAM-Rap1-GTP-profilin complex	54518 5216 5906	Mammalia	3
3159	RIAM-profilin complex	54518 5216	Human	2
3160	RIAM-VASP complex	54518 7408	Human	2
3161	PRL receptor (PRLR) dimer complex	5618	Human	1
3162	TF-FVIIa-FXa-TFPI complex	2159 2152 2155 7035	Human	4
3163	VILIP-1-AChR-alpha-4-AChR-beta-2 complex	25590 54239 24877	Rat	3
3164	HESX1-TLE1 complex	8820 7088	Human	2
3166	AXIN-APC-betaCatenin-GSK3B complex	324 8312 1499 2932	Human	4
3167	NCOR-SIN3-HDAC-HESX1 complex	3065 3066 8820 9612 25942 23309	Human	6
3168	DAXX-AXIN complex	8312 1616	Human	2
3169	Daxx-Axin complex	12005 13163	Mouse	2
3170	Daxx-Axin-p53 complex	8312 1616 7157	Human	3
3171	GluR6a-GluR6b-KA2 complex	14806 14809	Mouse	2
3172	NUMB-TP53-MDM2 complex	4193 8650 7157	Human	3
3173	CIN85 homotetramer complex	30011	Human	1
3174	CIN85-BLNK complex	29760 30011	Human	2
3175	CIN85-c-CBL complex	867 30011	Human	2
3176	USP1-UAF1 complex	7398 57599	Human	2
3177	GNA14-p115RhoGEF complex	9138 9630	Human	2
3178	Frs2-Shp2 complex FGF stimulated	327826 19247	Mouse	2
3179	Grb2-Shp2 complex FGF stimulated	14784 19247	Mouse	2
3180	Shp2-Sos complex FGF stimulated	19247 20662	Mouse	2
3181	LMO4-CREB complex	1385 8543	Human	2
3182	FHL2 homodimer complex	2274	Human	1
3183	PDGFRA-SHP-2 complex PDGF stimulated	5156 5781	Human	2
3184	FHL3 homodimer complex	2275	Human	1
3185	ACT homodimer complex	9457	Human	1
3186	GRB2-SHP-2 complex PDGF	2885 5781	Human	2
3187	FHL2-FHL3 complex	2274 2275	Human	2
3188	FHL2-ACT complex	2274 9457	Human	2
3189	FHL2-CREB complex	1385 2274	Human	2
3190	FHL3-CREB complex	1385 2275	Human	2
3191	ACT-CREB complex	1385 9457	Human	2
3192	G protein complex (Hdac4 Gnb1	14688 14702 208727	Mouse	3
3193	G protein complex (Hdac5 Gnb1	14688 14702 15184	Mouse	3

3194	G protein complex (HDAC5 GNB1 GNG2)	281201 281203 504242	Bovine	3
3195	G protein complex (Btk Gng2 Gnb1)	12229 14688 14702	Mouse	3
3196	FHL4/STX11-ACT complex	9457 8676	Human	2
3197	SMAD4-SNO-SKI complex	6497 6498 4089	Human	3
3198	SMAD2-SKI complex	6497 4087	Human	2
3199	SMAD3-SKI complex	6497 4088	Human	2
3200	SMAD4-SKI complex	6497 4089	Human	2
3201	Ship-Shc complex IL-3 stimulated	16331 20416	Mouse	2
3202	Il3rb1-Shc-Ship complex IL-3	12983 16331 20416	Mouse	3
3203	Shc-Ship-Grb2 complex IL-3	14784 16331 20416	Mouse	3
3204	SMAD2-SKI-NCOR complex	9611 6497 4087	Human	3
3205	SMAD3-SKI-NCOR complex	9611 6497 4088	Human	3
3206	SMAD4-SKI-NCOR complex	9611 6497 4089	Human	3
3207	LIN2-LIN7-SAP97-MINT1 complex	320 8573 1739 8825	Human	4
3208	LIN2-LIN7 complex	8573 8825	Human	2
3209	LIN2-SAP97 complex	8573 1739	Mammalia	2
3210	Dlg3-SAP97 complex	25252 58948	Rat	2
3211	Lin2/CASK-SAP97 complex	12361 13383	Mammalia	2
3212	Pax7-HMT complex	23808 18509 75410 140858	Mouse	4
3213	ULBP1-KLRK1-HCST complex	10870 22914 80329	Human	3
3214	G protein complex (Mcf2l Gnb1	24400 80850 117020	Rat	3
3215	G protein complex (Kalrn Gnb1	24400 80850 84009	Rat	3
3216	G protein complex (CACNA1A GNB1 GNG2)	282648 281201 281203	Bovine	3
3217	MAML2-RBP-Jkappa-Notch2 complex	270118 4853 19664	Mammalia	3
3218	MAML2-RBP-Jkappa-Notch3 complex	270118 18131 19664	Mouse	3
3219	MAML2-RBP-Jkappa-Notch4 complex	270118 4855 19664	Mammalia	3
3220	MAML3-RBP-Jkappa-Notch1 complex	55534 4851 19664	Mammalia	3
3221	MAML3-RBP-Jkappa-Notch2 complex	55534 4853 19664	Mammalia	3
3222	MAML3-RBP-Jkappa-Notch3 complex	433586 18131 19664	Mouse	3
3223	MAML3-RBP-Jkappa-Notch4 complex	55534 4855 19664	Mammalia	3
3224	G protein complex (NME2 GNB1 GNGT1)	281201 281796 615447	Bovine	3
3225	NMDA receptor complex (NR2A NR2B NR1 PSD-95)	29495 24408 24409 24410	Rat	4
3226	Cd74-Cd44 receptor complex	12505 16149	Mouse	2
3227	Pick1-Pkca complex TPA (tissue plasminogen activator) treated	84591 24680	Rat	2
3228	Pick1-Glur2 complex	29627 84591	Rat	2
3229	Heterodimer complex (CDK9 IL6ST)	1025 3572	Human	2
3230	Heterodimer complex (Cdk9 Il6st)	107951 16195	Mouse	2
3231	Eps15-stonin2 complex	2060 85439	Human	2
3232	Hippocalcin-beta2-adaptin complex	140670 29177	Rat	2
3233	SMAD2-SMAD4-FAST1-TGIF complex TGF(beta) induced	8928 4087 4089 7050	Mammalia	4
3234	SMAD2-SMAD4-FAST1-TGIF-HDAC1 complex TGF(beta) induced	8928 3065 4087 4089 7050	Mammalia	5
3235	PTF1 complex (Ptf1a Tcf12 Rbpj)	19213 19664 21406	Mouse	3

3240	Nsg1-Glur2-Grip1-Stx13 complex	29627 84016 25247 100226	Rat	4
3262	SCAMP1-SCAMP2-SCAMP3 complex	9522 10066 10067	Human	3
3263	HERP1/HEY2-NCOR-SIN3A complex	3065 23493 9612 25942	Human	4
3269	RB1-HDAC1-BRG1 complex	3065 5925 6597	Human	3
3270	Delta1 homodimer complex	28514	Human	1
3271	Gamma-secretase-Delta1 complex	51107 28514 23385 5663 55851	Human	5
3276	GluR1-GluR2 complex	50592 29627	Rat	2
3277	D2 receptor-Nsf-GluR2 complex	24318 29627 60355	Rat	3
3284	SMN complex (GEMIN5 2 3 4 SMN)	11218 50628 25929 8487 6606	Human	5
3289	Ng2-Grip1-Glur2 complex	121021 14800 74053	Mouse	3
3296	SMN complex (GEMIN5 4 3) SMN-independent intermediate	11218 50628 25929	Human	3
3297	SMN complex (GEMIN6 7 UNRIP) SMN-independent intermediate	79833 79760 11171	Human	3
3298	SMN complex (GEMIN2 5 SMN)	25929 8487 6606	Human	3
3335	Homotetrameric complex NIAP	4671	Human	1
3439	CCT:PhLP complex	504735 533784 287007	Bovine	3
3457	B23-NPM3 complex	18148 18150	Mouse	2
3489	Klf5-Pias1 complex	12224 56469	Mouse	2
3492	Bax homooligomeric complex after apoptotic stimulation	581	Human	1
3522	COG complex	9382 22796 83548 25839 10466 57511 91949 84342	Human	8
3525	Tetrameric COG subcomplex	10466 57511 91949 84342	Human	4
3529	Ternary COG subcomplex	10466 57511 91949	Human	3
3530	Binary COG subcomplex	10466 91949	Human	2
3532	COG1-COG8 subcomplex	9382 84342	Human	2
3539	Limd1-p62-Traf6-Prkcz complex	29806 18762 18412 22034	Mouse	4
3544	COG2-COG3-COG4 subcomplex	22796 83548 25839	Human	3
3545	COG5-COG6-COG7 subcomplex	10466 57511 91949	Human	3
3551	COG1-COG8-COG2-COG3-COG4 subcomplex	9382 22796 83548 25839 84342	Human	5
3552	COG1-COG8-COG5-COG6-COG7 subcomplex	9382 10466 57511 91949 84342	Human	5
3553	Traf6-p62-aPKC complex RANK-L stimulated	18759 18412 22034	Mouse	3
3558	Fgf2-Ck2 complex	13001 14173	Mouse	2
3563	Fgf2-Rsk2 complex	14173 110651	Mouse	2
3569	Angiomotin isoform p80-Angiostatin complex	27494 18815	Mouse	2
3618	GammaH2AFX-NDHII-Ku70-DNA complex	1660 3014 2547	Human	3
3634	NR3C2-UBC9-SRC-1 complex	8648 4306 7329	Human	3
3677	RIN1-STAM2-HRS complex	9146 9610 10254	Human	3
3678	RIN1-STAM2-EGFR complex EGF stimulated	1956 9610 10254	Human	3
3710	CHL2-BMP2 complex	650 25884	Human	2
3711	CHL2-BMP2-TSG complex	650 25884 57045	Human	3
3712	Smad2-Smad4 complex	17126 17128	Mouse	2

3714	Pericentrin-GCP complex	5116 10844 10426	Human	3
3715	Pericentrin-GCP complex	18541 74237 259279	Mouse	3
3729	SKI-SMAD2 hexameric complex	6497 4087	Human	2
3733	SKI-SMAD3 hexameric complex	6497 4088	Human	2
3739	SKI-SMAD2-SMAD4 pentameric complex	6497 4087 4089	Human	3
3740	SKI-SMAD3-SMAD4 pentameric complex	6497 4088 4089	Human	3
3741	Slp5-Rab27A complex	11891 236643	Mouse	2
3749	CREBBP-SMAD2 hexameric complex	1387 4087	Human	2
3750	CREBBP-SMAD3 hexameric complex	1387 4088	Human	2
3753	CREBBP-SMAD2-SMAD4 pentameric complex	1387 4087 4089	Human	3
3754	CREBBP-SMAD3-SMAD4 pentameric complex	1387 4088 4089	Human	3
3828	DRIP78-PHLP complex	85406 5082	Human	2
3830	ADRB2 homodimer complex	154	Human	1
3837	SP1-E2F1 complex	13555 20683	Mouse	2
3838	SP1-E2F2 complex	242705 6667	Mammalia	2
3839	SP1-E2F3 complex	13557 6667	Mammalia	2
3847	TCL1(trimer)-AKT1 complex	207 8115	Human	2
3848	TCL1(trimer)-AKT2 complex	208 8115	Human	2
3849	TCL1(homotrimer) complex	8115	Human	1
3852	Rb-HDAC1 complex	3065 5925	Mammalia	2
3867	Xin-Cdh2-Ctnnb1-Ctnnd1 complex	12558 12387 12388 22437	Mouse	4
3882	Abcg5-Abcg8 complex	27409 67470	Mouse	2
3883	Abcg5-Abcg1 complex	11307 27409	Mouse	2
3884	Abcg5-Abcg2 complex	26357 27409	Mouse	2
3886	Abcg5-Abcg4 complex	192663 27409	Mouse	2
3888	Abcg8-Abcg4 complex	192663 67470	Mouse	2
3900	GABP(gamma)1-E2F1-DP1 complex	1869 126626 7027	Human	3
3903	Grip-Glur2/3-liprin-alpha complex	29627 29628 140592 84016 171571	Rat	5
3917	Ternary complex (GATA4 SRF MYOCD)	2626 93649 6722	Human	3
3929	Ternary complex (CCD1 Dvl Rac)	11651 85458 13542	Mammalia	3
3930	CCD1-Axin complex	12005 85458	Mammalia	2
3941	RCP-Rab11 complex	8766 80223	Human	2
3942	Beta-catenin-Cadherin-LAR complex	83501 84353 360406	Rat	3
3943	MondoA-Mlx complex	21428 208104	Mouse	2
3952	Mlx-Mad1 complex	21428 17119	Mouse	2
3959	SMAD3-SMAD4-cSKI TGF(beta)-dependent	6497 4088 4089	Human	3
3961	SMAD3-cSKI-SIN3A-HDAC1	3065 25942 6497 4088	Human	4
3967	SMURF2-SMAD2 complex TGF(beta)-dependent	4087 64750	MINK	2
3971	SMURF2-SMAD3 complex TGF(beta)-dependent	4088 64750	Mammalia	2
3972	SMURF2-SMAD3-SnoN complex TGF(beta)-dependent	6498 4088 64750	Human	3

3979	mTORC2 complex (mTOR/FRAP1 LST8 mAVO3/RICTOR SIN1)	2475 64223 79109 253260	Human	4
3980	mTOR-RAPTOR complex	2475 64223 57521	Human	3
3988	ClpP complex heptameric	8192	Human	1
3989	ClpP complex heptameric	53895	Rat	1
3990	ClpXP complex	53895 270166	Rat	2
3991	ClpX complex hexameric	10845	Human	1
3992	ClpXP complex	8192 10845	Human	2
4025	Affixin-actinin(alpha) complex	87 29780	Human	2
4039	PAR4-BACE1 complex	23621 5074	Human	2
4043	NEMO-HIF2(alpha)-ARNT complex	405 2034 8517	Human	3
4055	Axin-Dvl-Gsk complex	12005 13542 56637	Mouse	3
4056	Axin-Dvl-Gsk-Frat1 complex	12005 13542 14296 56637	Mouse	4
4062	NRP1-VEGFR2-VEGF(165) complex	3791 8829 7422	Human	3
4072	Heterotrimeric SKP1-CUL1-ROC1 complex	8454 9978 6500	Human	3
4081	Ku70/Ku86 complex	7520 2547	Human	2
4082	Ku70/Ku86/Werner complex	7486 7520 2547	Human	3
4089	SMAD6-HOXC8 complex	3224 4091	Human	2
4090	SMAD6-HOXA9 complex	3205 4091	Human	2
4095	Catulin (alpha) - catenin (beta) complex	8727 1499	Human	2
4096	Catenin (alpha) - catenin (beta)	1495 1499	Human	2
4149	5'-AMP-activated protein kinase complex (AMPK)	78975 108097 19082	Mammalia	3
4151	TSC complex	79257 50686 60445 24855	Rat	4
4158	HSP90-FKBP38-CAM-Ca(2+)	805 23770 3320 3326	Human	4
4200	DLP1-hFIS1 complex	10059 51024	Human	2
4216	GR-hnRNP U complex	3192 2908	Human	2
4389	EIF3 core complex (EIF3A EIF3B EIF3G EIF3I)	8661 8662 8666 8668	Human	4
4392	EIF3 complex (EIF3A EIF3B EIF3G EIF3I EIF3C)	8661 8662 8663 8666 8668	Human	5
4395	EIF3 complex (EIF3B EIF3G EIF3I)	8662 8666 8668	Human	3
4399	EIF3 complex (EIF3B EIF3J EIF3I)	8662 8668 8669	Human	3
4403	EIF3 complex (EIF3A EIF3B EIF3G EIF3I EIF3J)	8661 8662 8666 8668 8669	Human	5
4478	CBF-DNA complex	4800 4801 4802	Human	3
4498	p32-CBF-DNA complex	708 4800 4801 4802	Human	4
4869	beta(1)-AR receptosome (ADRB1-SAP97-AKAP79-PRKAR2A)	153 9495 1739 5576	Human	4
4976	SVIP-p97/VCP-DERL1 complex	79139 258010 7415	Human	3
4977	gp78-p97/VCP-DERL1 complex	267 79139 7415	Human	3
4997	p97/VCP-VIMP-DERL1 complex	79139 55829 7415	Human	3
4998	p97/VCP-VIMP-DERL2 complex	51009 55829 7415	Human	3
4999	p97/VCP-VIMP-DERL1-DERL2-HRD1-SEL1L complex	79139 51009 6400 55829 84447 7415	Human	6
5000	p47-p97 complex	83809 116643	Rat	2
5003	p97/VCP homohexamer complex	269523	Mouse	1

5772	ZO1-(beta)cadherin-(VE)cadherin-VEGFR2 complex	1003 1499 3791 7082	Human	4
5092	Cdk5-p39-CaMKII(alpha)-(alpha)actinin1 complex	81634 25400 140908 12570	Rat	4
5099	RB1(hypophosphorylated)-E2F4 complex	1874 5925	Human	2
5100	CyclinD3-CDK4-CDK6 complex	896 1019 1021	Human	3
5101	CyclinD3-CDK4-CDK6-p21 complex	896 1019 1021 1026	Human	4
5107	p34(SEI-1)-CDK4-CyclinD2 complex	894 1019 29950	Human	3
5115	Trip(Br1)-Dp1-E2F1 complex	13555 55942 21781	Mouse	3
5116	Trip(Br2)-Dp1-E2F1 complex	13555 58172 21781	Mouse	3
5117	pRb2/p130-multimolecular complex (DNMT1 E2F4 SuV39H1 HDAC1 RBL2)	1786 1874 3065 5934 6839	Human	5
5118	pRb2/p130-multimolecular complex (RB2 E2F4 HDAC1 SUV39H1	1874 2033 3065 5934 6839	Human	5
5119	p19-Cdk4 complex	12567 12581	Mouse	2
5120	p19-Cdk6 complex	12571 12581	Mouse	2
5127	p18-Cdk4 complex	12567 12580	Mouse	2
5128	p18-Cdk6 complex	12571 12580	Mouse	2
5142	DCS complex (Ptbp1 Ptbp2 Hnrph1 Hnrpf)	98758 59013 19205 56195	Mouse	4
5143	E2F1-Rb complex	1869 5925	Human	2
5144	E2F1-p107-cyclinA complex	890 1869 5933	Human	3
5145	p19-Cdk4-cyclinD2 complex	12444 12567 12581	Mouse	3
5146	RB1-TFAP2A complex	5925 7020	Human	2
5151	Nsf-Stx1a-Napa complex	140673 60355 116470	Rat	3
5152	Nsf-Stx1a-NAPG complex	8774 60355 116470	Rat	3
5153	CTFC-TAF1 complex	10664 6418	Human	2
5154	CTCF-nucleophosmin complex	10664 4869	Human	2
5158	SMARCA2/BRM-BAF57-MECP2 complex	4204 6595 6605	Human	3
5159	E2F4-p107-cyclinE complex	898 1874 5933	Human	3
5160	E2F4-p130 complex	1874 5934	Human	2
5162	ELK1-SRF-ELK3 complex	2002 2004 6722	Human	3
5165	AP1G1-PACS1-FURIN complex	164 5045 55690	Human	3
5166	C3G-Crkl-Shp2 complex	287942 25622 63881	Rat	3
5167	C3G-Crkl-Shp2-Cbl-Egfr complex	12402 287942 24329 25622 63881	Rat	5
5168	C3G-Crkl-Shp2-Gab2-TrkA complex	287942 84477 59109 25622 63881	Rat	5
5171	SH3KBP1-CBLB-EGFR complex	868 1956 30011	Human	3
5172	Sh3kbp1-Cblb-Egfr complex	208650 13649 58194	Mouse	3
5173	Sh3kbp1-Cblb-Pdgfrb complex	208650 18596 58194	Mouse	3
5174	Smarcb1/Ini1-Smarca2-Mecp2 complex	17257 67155 20587	Mouse	3
5175	SWI/SNF-related complex	15182 17257 20466 67155 20587 20588 57376	Mouse	7
5176	MGC1-DNA-PKcs-Ku complex	9656 5591 7520 2547	Human	4

5177	Polycystin-1 multiprotein complex (ACTN1 CDH1 SRC JUP VCL CTNNB1 PXN BCAR1 PKD1 PTK2 TLN1)	87 9564 999 1499 3728 5310 5747 5829 6714 7094 7414	Human	11
5178	JAK2-PAFR-TYK2 complex	3717 5724 7297	Mammalia	3
5179	NCOA6-DNA-PK-Ku-PARP1 complex	23054 142 5591 7520 2547	Human	5
5183	DNA-PK-Ku-eIF2-NF90-NF45 complex	1965 8894 1968 3608 3609 5591 7520 2547	Human	8
5184	SWI/SNF chromatin-remodeling complex	3065 4204 25942 6595 6605	Human	5
5185	SWI/SNF chromatin-remodeling complex (Mecp2 Smarc)	15182 17257 20466 67155 20587 57376	Mouse	6
5189	YWHAQ-CALM1-CABIN1 complex	23523 805 10971	Human	3
5190	TIAM1-EFNB1-EPHA2 complex	1947 1969 7074	Human	3
5191	Ezh2 methyltransferase complex cytosolic	8726 14056 23512	Mammalia	3
5192	Tiam1-Efnb1-Epha2 complex	13641 13836 21844	Mouse	3
5193	TNF-alpha/NF-kappa B signaling complex (CHUK KPNA3 NFKB2 NFKBIB REL IKBKG NFKB1 NFKBIE RELB NFKBIA RELA TNIP2)	1147 8517 3839 4790 4791 4792 4793 4794 5966 5970 5971 79155	Human	12
5194	TNF-alpha/NF-kappa B signaling complex (SEC16A CHUK IKBKB NFKB2 REL IKBKG MAP3K14 RELA FBXW7 USP2)	1147 55294 3551 8517 9020 4791 5966 5970 9919 9099	Human	10
5195	PTIP-HMT complex	9070 84661 58508 23054 79447 22976 5929 7403 9757 11091	Human	10
5196	TNF-alpha/NF-kappa B signaling complex (CHUK BTRC NFKB2 PPP6C REL CUL1 IKBKE SAPS2 SAPS1 ANKRD28 RELA SKP1)	23243 8945 1147 8454 9641 4791 5537 5966 5970 22870 9701 6500	Human	12
5197	PTIP-DNA damage response complex	641 4361 4683 22976 10111 7158	Human	6
5198	CBP-RARA-RXRA-DNA complex ligand stimulated	1387 5914 6256	Human	3
5199	Kinase maturation complex 1	11140 3320 3326 3308 5607 4215 2011 81572 5202 84231 7529 7531 7532 7533 10971 7534	Human	16
5727	TBP-TAF complex	9015 9014 9013 6908	Human	4
5206	Pentraxin complex	266777 288475 81005 29218	Rat	4
5209	Ubiquilin-proteasome complex	5682 5683 5687 7337 29979 29978	Human	6
5210	TANK-TRAF2-TRAF3 complex	10010 7186 7187	Human	3
5211	RAF1-PPP2-PIN1 complex	5300 5516 5518 5520 5894	Human	5
5212	Kinase maturation complex 2	64343 11140 3320 3326 10010 29110 9755 7186	Human	8
5213	Pex26-Pex6-Pex1 complex	5189 55670 5190	Human	3
5215	CS-MAP3K7IP1-MAP3K7IP2	1431 10454 23118	Human	3
5216	Casein kinase II (beta-dimer alpha-dimer)	282419 539235	Bovine	2
5217	Calreticulin oligomer complex	811	Human	1

5218	Casein kinase II (beta-dimer alpha alpha')	282419 282420 539235	Bovine	3
5219	Casein kinase II (beta-dimer alpha'-dimer)	282420 539235	Bovine	2
5220	CHUK-IQGAP2-AKAP8L-RELA-TNIP2 complex	26993 1147 10788 5970 79155	Human	5
5222	p14-Mp1-MEK1 complex	5604 28956 8649	Human	3
5223	Casein kinase II (beta-dimer alpha'-dimer)	1459 1460	Human	2
5224	Casein kinase II (beta-dimer alpha alpha')	1457 1459 1460	Human	3
5225	Casein kinase II (beta-dimer alpha-dimer)	1457 1460	Human	2
5226	p14-Mp1-ERK2 complex	28956 5594 8649	Human	3
5227	p14-Mp1-Erk1/2 complex	56692 83409 26413 26417	Mouse	4
5228	REL-MAP3K8-RELA-TNIP2-PAPOLA complex	1326 10914 5966 5970 79155	Human	5
5229	RPA complex	6117 6118 6119	Human	3
5230	CHUK-NFKB2-REL-IKBKG-SPAG9-NFKB1-NFKBIE-COPB2-TNIP1-NFKBIA-RELA-TNIP2 complex	1147 9276 8517 4790 4791 4792 4794 5966 5970 9043 10318 79155	Human	12
5231	53BP1-containing complex	6117 6118 7158 7520 2547	Human	5
5232	TNF-alpha/Nf-kappa B signaling complex (RPL6 RPL30 RPS13 CHUK DDX3X NFKB2 NFKBIB REL IKBKG NFKB1 MAP3K8 RELB GLG1 NFKBIA RELA	1147 1654 2734 2969 8517 1326 4790 4791 4792 4793 5966 5970 5971 6156 6128 6207 79155	Human	17
5233	TNF-alpha/NF-kappa B signaling complex 5	10849 1147 8454 23291 3551 8517 10788 3838 10128 4163 4528 4790 4791 4793 5134 25885 84172 51082 64425 5437 5441 9462 5966 5970	Human	25
5234	IKBKB-CDC37-KIAA1967-HSP90AB1-HSP90AA1 complex	11140 3320 3326 3551 57805	Human	5
5235	WRN-Ku70-Ku80-PARP1 complex	142 7486 7520 2547	Human	4
5237	c-Myc-Max-Arf complex	12578 17187 17869	Mouse	3
5239	CAP(C)-CAP(E) complex	10592 10051	Human	2
5241	SMC1-SMC3 complex	8243 9126	Human	2
5243	XRCC1-LIG3-PNK-TDP1 complex	3980 11284 55775 7515	Human	4
5244	Dolichol-phosphate mannose (DPM) synthase	8813 8818 54344	Human	3
5248	Iqgap1-Cdc42-Ctnnb1-Cdh2 complex	64465 83501 84353 29875	Rat	4
5249	Iqgap1-Cdh1-Ctnnb1-Cdh2 complex	83502 83501 84353 29875	Rat	4
5250	Iqgap1-Actb-Vim complex	81822 29875 81818	Rat	3
5251	Ku-ORC complex	4999 23595 5000 23594 7520 2547	Human	6
5252	Htt-Dctn1-Hap1 complex	13191 15114 15194	Mouse	3
5253	MNK1-eIF4F complex	1973 1977 8569 1981 1982	Human	5
5260	TCF4-CTNNB1-SUMO1-EP300-HADAC6 complex	1499 2033 10013 7341 6925	Human	5
5261	TCF4-CTNNB1-EP300 complex	1499 2033 6925	Human	3
5262	TCF4-CTNNB1 complex	1499 6925	Human	2
5264	TCF4-CTNNB1-CREBBP complex	1387 1499 6925	Human	3

5266	TNF-alpha/NF-kappa B signaling complex 6	11140 1147 2091 3320 3326 3551 8517 9020 6156 6124 6128 6132 6205	Human	14
5267	VHL-VDU1-TCEB1-TCEB2 complex	6921 6923 23032 7428	Human	4
5268	TNF-alpha/NF-kappa B signaling complex 7	11140 2289 3320 3326 6885 10454 23118 257397	Human	8
5269	TNF-alpha/NF-kappa B signaling complex 8	11140 2289 3320 3326 9641 3895	Human	6
5270	VHL-TCEB1-TCEB2 complex	6921 6923 7428	Human	3
5271	Kif3-cadherin-catenin complex	12558 12387 12388 16569 16579	Mouse	5
5273	VHL-TBP1-HIF1A complex	3091 5702 7428	Human	3
5274	Cell-cell junction complex (ARHGAP10-CTNNA1)	79658 1495	Human	2
5276	HIF1A-OS9-EGLN1 complex	54583 3091 10956	Human	3
5277	HIF1A-OS9-EGLN3 complex	112399 3091 10956	Human	3
5280	RAB9-TIP47-MPRI complex	3482 10226 9367	Human	3
5281	Cell-cell junction complex (CDH1-CTNNB1)	999 1499	Human	2
5282	CAS-SRC-FAK complex	9564 5747 6714	Human	3
5283	tRNA splicing endonuclease	10978 116461 80746 79042 283989	Human	5
5284	RNA endonuclease (SEN2deltaEx8 SEN54 CLP1)	10978 80746 283989	Human	3
5285	TNF-alpha/NF-kappa B signaling complex 9	11140 1147 2289 3326 4793	Human	5
5286	TNF-alpha/NF-kappa B signaling complex 10	55054 55749 11140 1147 3320 3326 8517 4793 29110 200081	Human	10
5287	CDK4-CCND1 complex	595 1019	Human	2
5288	P53-BARD1-Ku70 complex	580 7157 2547	Human	3
5291	Cd3e-Nck1-Mink1 complex	12501 50932 17973	Mouse	3
5292	Ets2-Ini1-Smarce1-Smarca4 complex	23872 20586 20587 57376	Mouse	4
5293	ETS2-SMARCA4-INI1 complex	2114 6597 6598	Human	3
5295	Cd247 homodimer complex	12503	Mouse	1
5296	Cd3e homodimer complex	12501	Mouse	1
5301	Striatin-SG2NA-zinedin-caveolin1 complex	25404 29149 114520 97387	Rat	4
5302	Rab3a-Rims2-Rapgef4 complex	19339 56508 116838	Mouse	3
5308	Rb-NeuroD1-Ngfi-B complex	18012 15370 19650	Mouse	3
5309	Cd3g-Cd3e complex	12501 12502	Mouse	2
5310	Cd3d-Cd3g-Cd3e-Cd247 complex	12503 12500 12501 12502	Mouse	4
5311	Cd3g-Cd3e-Cd247-Canx complex	12330 12503 12501 12502	Mouse	4
5315	Munc13-1-Rim2-Rab3a complex	25531 116839 64829	Rat	3
5317	LATS1-HTRA2-BIRC4 complex	27429 9113 331	Human	3
5318	DDEF1-CTTN-PXN complex	2017 50807 5829	Human	3
5319	CDH1-CKS1B complex	999 1163	Human	2
5320	CDH1-SKP2 complex	999 6502	Human	2
5321	Cebpa-Smarca2 complex	12606 67155	Mouse	2
5330	Lef1-Tle1 complex	16842 21885	Mouse	2
5331	YY1-MDM2-p53 complex	4193 7157 7528	Human	3
5333	Lef1-Tle1-Ctnnb1 complex	12387 16842 21885	Mouse	3
5337	ELMO1-DOCK1 complex	1793 9844	Human	2

5341	ELMO1-DOCK2 complex	1794 9844	Human	2
5342	ELMO1-DOCK1-RAC1 complex	1793 9844 5879	Human	3
5343	ELMO1-DOCK1-CRKII complex	1398 1793 9844	Human	3
5344	Endocytic coat complex (11 subunits)	60668 11771 140670 117028 54241 140694 313474 81922 116743 65178 85238	Rat	11
5345	Endocytic coat complex (7 subunits)	60668 11771 140670 117028 54241 140694 85238	Rat	7
5348	Pik3r5-Pik3cg complex	30955 320207	Mouse	2
5358	GTP-Rho-Rhpn1-Ropn1 complex	11848 14787 76378	Mouse	3
5360	AGO2-FXR1-TNF(alpha)ARE-RNP complex	27161 8087	Human	2
5361	Cell division cycle complex (CDC27 CDC16 ANAPC7)	51434 8881 996	Human	3
5363	Actin-ribonucleoprotein complex (POLR2A GTF2F1 HNRNPU)	2962 3192 5430	Human	3
5365	Ripk2-Nod2 complex	257632 192656	Mouse	2
5366	Calm1-Ryr1 complex	24244 114207	Rat	2
5367	THRB-RXRB complex	6257 7068	Human	2
5368	Nfkb1-Rela complex	18033 19697	Mouse	2
5369	ATM homodimer complex	472	Human	1
5370	ERAP1-ERAP2 complex	51752 64167	Human	2
5371	Grancalcin-sorcin complex	25801 6717	Human	2
5372	SRI homodimer complex	6717	Human	1
5373	Chromatin remodeling complex (TACC2 TACC3 PCAF)	8850 10579 10460	Human	3
5375	EGR-EP300 complex	1958 2033	Human	2
5376	Rock1-Pfn2 complex	81531 81762	Rat	2
5378	TRBP containing complex (DICER TRBP AGO2 RPL7A EIF6 MOV10)	23405 27161 3692 4343 6130 6895	Human	6
5379	Kcna2-Kcnab2 complex	25468 29738	Rat	2
5380	TRBP containing complex (DICER RPL7A EIF6 MOV10 and subunits of the 60S ribosomal particle)	51187 23405 3692 4343 4736 6135 6136 6137 9045 6138 6142 6143 6144 6155 6158 6156 6161 6164 6165 25873 6168 6129 6130 6176 6181	Human	25
5382	ARNT-HIF1A complex	405 3091	Human	2
5383	TRIB3-DDIT3 complex	1649 57761	Human	2
5384	RAG1-RAG2 tetramer complex	5896 5897	Human	2
5385	GAIT complex	2058 2597 23521 10492	Human	4
5386	MLL1-WDR5 complex	9070 124944 125476 57680 1876 3054 3308 284058 81887 10445 8972 4297 84148 27043 51230 26121 5929 6045 8607 26168 6872 6874 6878 6879 6880 54881 11091	Human	27
5388	SERPINA1-ELA2 complex	1991 5265	Human	2
5389	SERPINA3-CTSG complex	1511 12	Human	2
5391	SERPINA1-CTSG complex	1511 5265	Human	2
5400	BRCC complex	672 675 79184 9577 5888	Human	5
5401	PLEKHM2-KIF5B complex	3799 23207	Human	2
5402	Cdk5-Cdk5r1-Pctk1 complex	12568 12569 18555	Mouse	3

5404	Myod1-Tcf3 complex	17927 21423	Mouse	2
5405	Myod1 homodimer complex	17927	Mouse	1
5407	NGF-TrkA complex	4803 4914	Human	2
5408	Neurotrophin-3-p75 complex	4804 4908	Human	2
5409	TIAM1-GRIN1 complex	2902 7074	Human	2
5411	EDG1-HTR1D complex	1901 3352	Human	2
5412	HTR1D homodimer complex	3352	Human	1
5414	HTR1A-HTR1D complex	3350 3352	Human	2
5415	HTR1B homodimer complex	3351	Human	1
5416	HTR1A-HTR1B complex	3350 3351	Human	2
5417	HTR1D-HTR1B complex	3351 3352	Human	2
5418	GABBR2-HTR1A complex	9568 3350	Human	2
5419	HTR1A-GPR26 complex	2849 3350	Human	2
5420	HTR1A-EDG3 complex	1903 3350	Human	2
5421	HTR1A homodimer complex	3350	Human	1
5422	HTR1A-EDG1 complex	1901 3350	Human	2
5423	HSP70-BAG5-PARK2 complex	9529 5071 3303 3308	Human	4
5424	NGF-p75 complex	4803 4804	Human	2
5426	ANCO1-HDAC3 complex	29123 8841	Human	2
5432	Sororin-cohesin complex	113130 23244 23047 5885 8243 9126	Human	6
5435	CycD1-Cdk4 complex	12443 12567	Mouse	2
5436	Cyclin D1-associated protein complex	12443 12567 12575 15511	Mouse	4
5439	Eya1/3-Dach1/2-Six1 complex	20471 13134 93837 14048 14050	Mouse	5
5441	NRG1-IKZF4 complex	64375 3084	Human	2
5442	EPOR receptor complex	2057	Human	1
5444	CRKII-C3G complex	1398 2889	Human	2
5446	EPO-EPOR complex	2056 2057	Human	2
5447	Grin2a-Lrp8 complex	14811 16975	Mouse	2
5448	Grin2b-Lrp8 complex	14812 16975	Mouse	2
5449	Splicing-associated factors complex	7913 9939 10921 10250 10189	Human	5
5450	Mediator complex	5469 84246 400569 9969 23389 9282 51586 10025 9440 54797 9477 9412 6837 9439 9862 81857 9441 9442 55588 90390 51003 29079 10001 9443 112950 219541 4976	Human	27
5451	MED18-MED20-MED29 mediator subcomplex	54797 9477 55588	Human	3
5452	Foxo4-Srf-Myocd complex	54601 214384 20807	Mouse	3
5459	Axin2-Ctnnb1-Apc complex	11789 12006 12387	Mouse	3
5460	p50-p65 NF(kappa)B complex	4790 5970	Human	2
5461	p50-p65 NF(kappa)B-SRC1 complex	8648 4790 5970	Human	3
5464	I(kappa)B(alpha)-NF(kappa)Bp50-NF(kappa)Bp65 complex	4790 4792 5970	Human	3
5465	IKB(epsilon)-RELA-cREL complex	4794 5966 5970	Human	3
5466	IKB(beta)-RELA-cREL complex	4793 5966 5970	Human	3
5467	IKB(alpha)-RELA-cREL complex	4792 5966 5970	Human	3
5473	FAS-FADD-CASP8 complex	841 8772 355	Human	3
5475	MURR1-NF(kappa)Bp65-IKBA	150684 4792 5970	Human	3

5492	IKBA-NF(kappa)Bp65-NF(kappa)Bp50 complex	4790 4792 5970	Human	3
5493	Tmsb4x-Lims1-Ilk complex	16202 110829 19241	Mouse	3
5495	TFIIH transcription factor complex (ERCC2 ERCC3 GTF2H1 CDK7 CCNH GTF2H2)	902 1022 2068 2071 2965 2966	Human	6
5497	Thyroglobulin folding complex (Tg Hspa5 Pdi Erp29 Grp94)	117030 25617 29511 24826 362862	Rat	5
5498	ILK-PARVB-ARHGEF6 complex	9459 3611 29780	Human	3
5499	PARVB-ARHGEF6 complex	9459 29780	Human	2
5500	Thyroglobulin folding complex (Tg Pdi Erp29)	117030 29511 24826	Rat	3
5501	Thyroglobulin folding complex (Tg Hspa5 Erp29 Grp94)	117030 25617 24826 362862	Rat	4
5502	EED-EZH-YY1 polycomb complex	8726 2146 7528	Human	3
5511	Tctex1-channel complex	257648 29564 83462	Rat	3
5513	Polycomb repressive complex	648 8535 1911 6015	Human	4
5518	BMI1-HPH1-HPH2 complex	648 1911 1912	Human	3
5521	EED-EZH polycomb complex	8726 2146	Human	2
5524	Bmi1-Mel18-Mph1-M33 polycomb repressor complex	12151 12416 22658 13619	Mouse	4
5526	CALM1-FKBP38-BCL2 complex	596 805 23770	Human	3
5528	Homodimeric complex Adrb2	24176	Rat	1
5529	TRAF2-cIAP1/BIRC2 complex	329 7186	Human	2
5530	Homodimeric complex LTBR	4055	Human	1
5531	Tumor necrosis factor receptor 1 signaling complex	329 7132 8717 7186	Human	4
5532	p35-Cdk5 complex	140908 116671	Rat	2
5533	p35-Cdk5 complex	281066 282173	Bovine	2
5534	PLCB1-PARD3-PARD6A complex	56288 50855 23236	Human	3
5535	PLCB3-PARD3-PARD6A complex	56288 50855 5331	Human	3
5540	TRAF2-TRADD complex	8717 7186	Human	2
5541	Ternary complex (TRAF2 FADD TRADD)	8772 8717 7186	Human	3
5542	CCNB2-CDC2 complex	9133 983	Human	2
5543	CCNB1-CDC2 complex	891 983	Human	2
5544	CDC2-PCNA-CCNB1-GADD45A complex	891 983 1647 5111	Human	4
5545	CDC2-PCNA-CCNB1-GADD45B complex	891 983 4616 5111	Human	4
5546	CDC2-PCNA-CCNB1-GADD45G complex	891 983 10912 5111	Human	4
5547	Cdc2-Ccnb1 complex	268697 12534	Mouse	2
5548	IL-12 heterodimer complex	3592 3593	Human	2
5549	IL-12 subunit p40 homodimer complex	3593	Human	1
5550	CDC2-CCNB1-CCNF complex	891 899 983	Human	3
5551	CDC2-CCNB1-PTCH1 complex	891 983 5727	Human	3
5556	CDK2-CCNA2 complex	890 1017	Human	2
5557	CDC2-CCNA2 complex	890 983	Human	2
5558	PAR6-CDC42 complex	998 84612	Human	2

5559	CDC2-CCNA2-CDK2 complex	890 983 1017	Human	3
5560	CDK2-CCNE1 complex	898 1017	Human	2
5564	LMO4-gp130 complex	3572 3716 8543 5781 9021	Human	5
5566	Isl1-Jak1-Stat3 complex	16392 16451 20848	Mouse	3
5573	Stat1-alpha-dimer-CBP DNA-protein complex	1387 6772	Human	2
5574	Vps29-Vps35-Vps26a complex	30930 56433 65114	Mouse	3
5575	SHP-NR5A2 complex	8431 2494	Human	2
5576	Pigr-Vps35-Vps26a-Vps29 complex	25046 361846 56433 65114	Rat	4
5577	Ephb1-Sdc2 complex	24338 25615	Rat	2
5578	Sdc2-Ephb2 complex	13844 15529	Mouse	2
5579	CNTF-CNTFR-gp130-LIFR complex	1270 1271 3572 3977	Human	4
5582	LIFR-LIF-gp130 complex	3572 3976 3977	Human	3
5583	Per1-Cry1-Cry2-Nono-Wdr5 complex	299691 170917 317259 287422	Rat	5
5584	Per1-Nono-Wdr5 complex	317259 287422 362093	Rat	3
5585	Per2-Nono-Wdr5 complex	317259 63840 362093	Rat	3
5589	LINC complex S-phase	55957 91750 132660 286826 4605 5928 5933	Human	7
5590	Zip-Prkcz-Kcnab2 complex	29738 25522 113894	Rat	3
5593	LINC core complex	55957 91750 132660 286826 5928	Human	5
5596	LINC complex quiescent cells	1874 55957 91750 132660 286826 5928 5934	Human	7
5597	Zip3-Prkcz-Gabrr3 complex	192258 25522 113894	Rat	3
5604	Emerin complex 1	60 1266 2010 4008 4627 4643 80143	Human	8
5606	Emerin-actin-NMI-(alphaII)spectrin complex	60 2010 9111 6709	Human	4
5607	Emerin-actin-NMI complex	60 2010 9111	Human	3
5608	Emerin architectural complex	60 2010 4000 4001 9111 6709	Human	6
5609	Emerin regulatory complex	60 8815 2010 3065 8841 3024 8350 4008 5933	Human	9
5610	Alpha-GDI-Hsp90 chaperone complex ATP dependent	79130 25183 299331 24468	Rat	4
5611	Emerin complex 24	140836 708 2010 3024 8350 3192 4000 4001 4171 4173 4175 9111 5925 5934 79595	Human	15
5612	Alpha-GDI-Rab3a complex cytosolic	25183 25531	Rat	2
5613	Emerin complex 25	60 8815 56647 11140 2010 10146 3190 4001 57819 51691 79595 6632 6741 7529 7531 9406	Human	16
5614	Emerin complex 32	60 140836 1108 84661 2010 3065 8841 8350 4001 29079 9111 5933 5934 23186 10284 6598 6599 6601 6709 79718 90665 10155	Human	22
5615	Emerin complex 52	60 8539 708 1152 10212 2010 3068 3024 3190 3191 3192 3609 8826 23592 4001 51691 9111 27250 5934 6632 7067 7531 10971	Human	23
5616	Hsp110-Hsc70-Hsp25 complex	15481 15507 15505	Mouse	3
5622	HSP90-CIP1-FKBPL complex	1026 63943 3320	Human	3
5623	Ask1-HSP90-AKT1 complex	280991 26408 281832 767874	Bovine	4

5624	Sema3E-PlexinD1 complex	67784 20349	Mammalia	2
5626	Sema3E-PlexinD1-Nrp1 complex	18186 67784 20349	Mammalia	3
5627	Cdh23-Myo1c complex	22295 17913	Mouse	2
5628	Ask1-Traf6 complex LPS induced	26408 22034	Mouse	2
5641	PSD95-FYN-NR2A complex	1742 2534 2903	Human	3
5642	Securin-separase complex	9700 9232	Human	2
5643	ESPL1-CDC2 complex	983 9700	Human	2
5644	Sharpin homo-oligomer complex	81859	Rat	1
5645	Sharpin-Shank1 complex	78957 81859	Rat	2
5646	FARP2-NRP1-PlexinA1 complex	9855 8829 5361	Human	3
5647	FARP2-NRP1-PlexinA2 complex	9855 8829 5362	Human	3
5648	FARP2-NRP1-PlexinA3 complex	9855 8829 55558	Human	3
5649	FARP2-NRP1-PlexinA4 complex	9855 8829 91584	Human	3
5654	SEMA4A-PlexinD1 complex	23129 64218	Human	2
5655	Ternary complex (LRRC7 CAMK2a ACTN4)	81 815 57554	Human	3
5656	CEBPE-E2F1-RB1 complex	1053 1869 5925	Human	3
5659	SEMA3C-PlexinD1-Nrp1 complex	8829 23129 10512	Mammalia	3
5660	PlexinC1-SEMA7A complex	10154 8482	Mammalia	2
5661	PlexinC1-SEMA7A complex	5364 10507	Mammalia	2
5663	TRIM27-RB1 complex	5925 5987	Human	2
5668	PlexinA1-Nrp1 complex	8829 5361	Mammalia	2
5669	PlexinA3-Nrp1 complex	8829 55558	Mammalia	2
5670	PlexinB1-Nrp1 complex	8829 5364	Mammalia	2
5673	PlexinA1-Nrp2 complex	8828 5361	Mammalia	2
5728	VEGFR2-STAT3 complex VEGF induced	407170 508541	Bovine	2
5682	Retn homotrimer complex	57264	Mouse	1
5683	hRAD51C-hXRCC3 complex	5888 7516	Human	2
5684	Membrane protein complex (DERL1 SELS VCP)	79139 55829 7415	Human	3
5685	Membrane protein complex (VCP UFD1L SEC61B)	10952 7353 7415	Human	3
5689	SEMA6D-PlexinA1-NRP1 complex	8829 5361 80031	Human	3
5691	TALL1 homo-oligomer complex	10673	Human	1
5693	Tip5-Dnmt-Hdac1 complex	116848 13433 13436 433759	Mouse	4
5694	Nucleolar remodeling complex (NoRC complex)	11176 8467	Human	2
5695	TIP5-DNMT-HDAC1 complex	11176 1786 1789 3065	Human	4
5696	VEGFA(165)-KDR-NRP1 complex	3791 8829 7422	Human	3
5697	VEGFA(165)-KDR-NRP1 complex	8829 397157	Pig	2
5698	VEGFA(165)-VEGFR2-NRP1	3791 8829 7422	Human	3
5701	NRP1-VEGF(165/121) complex	8829 7422	Human	2
5702	VEGF-KDR-NRP2 complex	397157	Pig	1
5705	CyclinA2-Cdk2 complex	12428 12566	Mouse	2
5706	CyclinB1-Cdc2 complex	268697 12534	Mouse	2
5710	PRMT2 homo-oligomer complex	3275	Human	1
5724	CIN85-SH3GL3-CBL complex	867 6457 30011	Human	3

5725	Mdm2-P53 complex	12578 17246 67025 22059	Mouse	4
5729	VEGF-VEGFR2 complex	407170 281572	Bovine	2
5730	IKBKG homotrimer complex	8517	Human	1
5731	NRP1-VEGFC complex heparin dependent	8829 7424	Human	2
5732	NRP2-VEGFC complex	8828 7424	Human	2
5733	NRP2-VEGFD complex heparin dependent	2277 8828	Human	2
5734	NRP1-VEGFD complex heparin dependent	2277 8829	Human	2
5735	TGF-beta receptor-SMAD3 complex	4088 7046 7048	Human	3
5736	Pre-initiation complex (PIC)	60 5430 6908	Human	3
5737	Multicomponent signaling complex anti-CD40 stimulated (Birc2 Birc3 Cd40 Ikbkg Map2k4 Map2k7 Map3k1 Traf2 Ube2n)	11797 11796 21939 16151 26398 26400 26401 22030 93765	Mouse	9
5740	NRP2-VEGFR3 complex	2324 8828	Human	2
5742	Multicomponent signaling complex anti-CD40 stimulated (Birc2 Birc3 Cd40 Ikbkg Map3k1 Traf2 Ube2n)	11797 11796 21939 16151 26401 22030 93765	Mouse	7
5743	Membrane-associated multicomponent signaling complex anti-CD40 stimulated (Cd40 Ikbkg Map3k1	21939 16151 26401 22030 93765	Mouse	5
5744	Cytosolic multicomponent signaling complex anti-CD40 stimulated (Ikbkg Map3k1 Traf2 Ube2n)	16151 26401 22030 93765	Mouse	4
5745	PlexinA1-NRP1 complex	8829 5361	Human	2
5746	PlexinA1-NRP1-SEMA3A complex	8829 5361 10371	Human	3
5747	2AR-mGluR2 complex	150 2891	Human	2
5749	MRIT complex	598 841 8837	Human	3
5755	SUMO1-SUA1-UBA2 complex	10055 7341 10054	Human	3
5756	SUA1-UBA2 complex	10055 10054	Human	2
5758	PLXNA2-RANBPM complex	5362 10048	Human	2
5759	PLXNA3-RANBPM complex	55558 10048	Human	2
5760	PLXNA4-RANBPM complex	91584 10048	Human	2
5762	CRMP-MICAL-PlexinA1 complex induced by SEMA3A	1400 64780 5361	Human	3
5770	RUNX1-CBF-beta-DNA complex	865 861	Human	2
5791	PlexinA1-PlexinB1 complex	18844 235611	Mouse	2
5798	Death induced signaling complex II (FADD CASP8 CFLAR) cytosolic CD95L induced	841 8837 8772	Human	3
5799	Death induced signaling complex DISC (FAS FADD CASP8 CFLAR) membrane-associated CD95L induced	841 8837 8772 355	Human	4
5800	Death-inducing signaling complex DISC (type I cells associated)	841 8772 355	Human	3
5805	PGAM5-KEAP1-NRF2 complex	9817 4780 192111	Human	3
5806	Keap1-Nrf2-Cul3 complex	26554 50868 18024	Mouse	3
5807	Keap1-Nrf2 complex	50868 18024	Mammalia	2

5808	DISC complex	841 355 8772	Mammalia	3
5809	GABAA receptor	2554 2561 2566	Human	3
5811	p53-BCL2 complex	596 7157	Mammalia	2
5812	p53-BCL2 complex	598 7157	Mammalia	2
5813	tBID-BAK1 complex	12018 12122	Mouse	2
5814	Tp53-BAK1 complex	12018 22059	Mouse	2
5815	Quaternary complex (Dvl Gsk3b Frat1 Axin1)	12005 13542 14296 56637	Mouse	4
5816	Apoptosome-procaspase 9 complex	317 842 54205	Human	3
5817	tBID-BCL2 complex	596 637	Mammalia	2
5818	BIM-BCL2 complex	596 10018	Mammalia	2
5819	BIM-BCL2xL complex	598 10018	Mammalia	2
5820	tBID-BCL2xL complex	598 637	Mammalia	2
5821	BAD-BCL2xL complex	12015 12048	Mouse	2
5822	MCL1-NOXA complex	4170 5366	Mammalia	2
5823	MCL1-BAK1 complex	578 4170	Human	2
5827	IKBKG tetramer complex	8517	Human	1
5828	IKBKG-IKBKB complex	3551 8517	Human	2
5829	IKBKG-CHUK complex	1147 8517	Human	2
5830	DJ-1-SNCA complex high molecular weight complex	11315 6622	Human	2
5877	MAP2K1-BRAF-RAF1-YWHAE-KSR1 complex	673 8844 5604 5894 7531	Human	5
5832	PINK1-MIRO2-Milton complex	65018 89941 22906	Human	3
5836	MIRO2-Milton complex	89941 22906	Human	2
5837	PPD complex	5071 11315 65018	Human	3
5838	Heterotrimeric complex (Rnd1 Rras Plxnb1)	235611 223881 20130	Mammalia	3
5843	AIF-CYPA-DNA complex	9131 5478	Human	2
5844	I-kappa-B kinase (IKK) complex	1147 3551 8517	Human	3
5849	HSP90-CDC37-LRRK2 complex	11140 3326 120892	Human	3
5859	FAS-FADD-CASP8-CASP10 complex	843 841 8772 355	Human	4
5861	FAS-FADD-CASP10 complex	843 8772 355	Human	3
5862	CAV1-VDAC1-ESR1 complex	857 2099 7416	Human	3
5870	FE65-TSHZ3-HDAC1 complex	322 3065 57616	Human	3
5872	BRAF-MAP2K1-MAP2K2-YWHAE complex	673 5604 5605 7531	Human	4
5873	RAF1-MAP2K1-YWHAE complex	5604 5894 7531	Human	3
5876	PPP2R1A-PPP2R1B-PPP2CA-PPME1-EIF4A1 complex	1973 51400 5515 5518 5519	Human	5
5879	Ksr1-PP2A holoenzyme complex (Ppp2r1a Ppp2r2b Ppp2ca) PDGF stimulated	16706 19052 51792 72930	Mouse	4
5880	Ksr1-PP2A core enzyme complex (Ppp2r1a Ppp2ca) untreated	16706 19052 51792	Mouse	3
5882	Raf1-PP2A holoenzyme complex (Ppp2r1a Ppp2r2b Ppp2ca) PDGF stimulated	19052 51792 72930 110157	Mouse	4

5883	Raf1-PP2A core enzyme complex (Ppp2r1a Ppp2ca) untreated	19052 51792 110157	Mouse	3
5886	Ksr1 complex (Ksr1 Mek 14-3-3) unstimulated	16706 26395 26396 55948 54401 22627 22628 22629 22630 22631	Mouse	10
5909	Ksr1 complex (Ksr1 Mek 14-3-3 Mapk) EGF stimulated	16706 26413 26395 26396 55948 54401 22627 22628 22629 22630	Mouse	11
5899	Lebercilin complex (Lca5 Ncl Npm1 Ywhae HSPA1A/B Dctn1 Dctn2)	13191 69654 75782 17975 18148 22627 396648	Pig	7
5910	Ksr1-CK2 complex	12995 13000 13001 16706	Mouse	4
5915	Ksr1-Brap (IMP) complex	72399 16706	Rat	2
5918	KSR1 homooligomer complex	8844	Human	1
5919	BRAF-RAF1-14-3-3 complex	673 5894 2810 7529 7531 7532 7533 10971 7534	Human	9
5920	KSR1-RAF1-MEK complex	8844 5894 5604 5605	Human	4
5921	KSR1-BRAF-MEK complex	673 8844 5604 5605	Human	4
5922	RAF1-RAS complex EGF induced	5894 3265 3845 4893	Human	4
5923	RAF1-BRAF complex RAS stimulated	673 5894	Human	2
5924	RAF1-CNK1 complex RAS stimulated	10256 5894	Human	2
5925	BRAF-CNK1 complex not RAS stimulated	673 10256	Human	2
5926	CNK1 homodimer complex	10256	Human	1
5928	CNK1-SRC-RAF1 complex	10256 5894 6714	Human	3
5936	Ksr1-CK2-MEK-14-3-3 complex PDGF treated	12995 13000 13001 16706 26395 26396 54401 22627 22628 22629	Mouse	12
5937	B-Ksr1-MEK-MAPK-14-3-3 complex	16706 26413 26395 26396 54401 22627 22628 22629 22630 22631	Mouse	10
5946	Ksr1-Mek-Braf complex EGF induced	109880 16706 26395 26396	Mouse	4
5947	Ksr1-Mek-Braf-Erk complex EGF induced	109880 16706 26395 26396 26413 26417	Mouse	6
