# Supplementary material for: Inferring drug-disease associations based on known protein complexes
Source: BMC Med Genomics. 2015 May 29;8(Suppl 2):S2. doi: 10.1186/1755-8794-8-S2-S2 (PMC4460611; doi:10.1186/1755-8794-8-S2-S2)
Supplement: Additional file 3 — Table illustrating disease-gene dataset. [file 1755-8794-8-S2-S2-S3.pdf]

| **Disease Name** | **Lists of related gene names** | **Lists of related gene Entrez IDs** | **Number of genes** |
|---|---|---|---|
| Adenocarcinoma, Mucinous | SEPT9 CHST4 KRT13 | 10801 10164 3860 | 3 |
| Hemorrhagic fevers, Viral | ICAM1 IL6 CCL2 ITGB3 ICAM2 | 3383 3569 6347 3690 3384 3661 | 6 |
| Hepatitis, Autoimmune | GSTT1 DBT ARG1 | 2952 1629 383 | 3 |
| Hypertension, Pulmonary | SEPN1 TNC ENG SLC6A4 HIF1A JUN CX3CL1 F3 TRPC6 BMP7 GREM1 BMP2 KCNA5 BMPR2 TRPC3 BMPR1B ARHGEF6 ARG2 | 57190 3371 2022 6532 3091 3725 6376 2152 7225 655 26585 650 3741 659 7222 658 9459 384 7223 | 19 |
| Infertility, Male | DAZ1 MTRR MTHFR CFTR AHRR CREM APOB TSSK4 AR BRCA2 POLG HSPA2 REC8 DAZL CYP21A2 ESR1 INSL3 DDX25 KIF2C SOX11 GSTM1 BRDT | 1617 4552 4524 1080 57491 1390 338 283629 367 675 5428 3306 9985 1618 1589 2099 3640 29118 11004 6664 2944 676 79582 4282 | 24 |
| Kidney tubular necrosis, acute | PARP1 TYRO3 AXL L1CAM IL6R MERTK | 142 7301 558 3897 3570 10461 | 6 |
| Labor, Premature | DEFA3 PTGES IL1B CRH S100A8 BPI DEFA1 ITIH2 RHOA IL1RN REN S100A9 SELE ICAM1 CCL2 | 1668 9536 3553 1392 6279 671 1667 3698 387 3557 5972 6280 6401 3383 6347 | 15 |
| Mycobacterium infection, Atypical | ICOS IRF1 SLC11A1 CCL2 IFNG SP110 IL8 NOD2 SLC22A4 | 29851 3659 6556 6347 3458 3431 3576 64127 6583 | 9 |
| Myoclonic epilepsies, Progressive | BRD2 NHLRC1 CSTB | 6046 378884 1476 | 3 |
| Myoclonic epilepsy, Juvenile | SCN1A KCNQ3 CHRNA7 GJD2 BRD2 EFHC2 | 6323 3786 1139 57369 6046 80258 | 6 |
| Myopathies, Nemaline | TPM2 ACTA1 TPM3 RYR1 ACTC1 NEB | 7169 58 7170 6261 70 4703 | 6 |
| Pemphigoid, Bullous | SELL SELE MIF DST ITGA6 ITGB1 ITGB3 ICAM1 ITGB4 FCGR3A | 6402 6401 4282 667 3655 3688 3690 3383 3691 2214 | 10 |
| Pleural effusion, Malignant | ITGAV CCL2 CCL11 CXCL11 CCL22 EGFR WT1 ITGB1 CD247 MMP9 CALB2 | 3685 6347 6356 6373 6367 1956 7490 3688 919 4318 794 | 11 |
| Prostatic hypertrophy, Benign | IL4 IL1RN EDN1 CST3 FGF7 A2M MT1A CHIT1 VCAN MAGEA1 ADAMTS9 MT3 RARG ADAMTS5 MAGEA4 CSN1S1 UBC FGF17 | 3565 3557 1906 1471 2252 2 4489 1118 1462 4100 56999 4504 5916 11096 4103 1446 7316 8822 | 18 |
| Purpura, Thrombocytopenic, Idiopathic | LEP LTA CX3CR1 ITGA2B TNFSF13B ITGA4 ITGB3 IL4 FCGR2C NMNAT3 ITGB1 IL1RN | 3952 4049 1524 3674 10673 3676 3690 3565 9103 349565 3688 3557 931 3458 | 14 |
| Rhabdomyosarcoma, Embryonal | TSPAN31 | 6302 | 1 |
| Skin disease, Genetic | PTPN11 KRT10 SNAP29 RAF1 JUP KRT14 ALOX12B KRTAP5-1 KRT1 TMC6 IGFBP3 ATP2A2 CCL11 FECH IGFBP1 DCN ABCG2 CYP1A2 VIM LAMC2 TINF2 ITGB4 ELN SERPINH1 B4GALT7 PLOD2 LAMA3 KRT5 FBLN5 ALDH3A2 | 5781 3858 9342 5894 3728 3861 242 387264 3848 11322 3486 488 6356 2235 3484 1634 9429 1544 7431 3918 26277 3691 2006 871 11285 5352 3909 3852 10516 224 3914 | 31 |
| Spastic paraplegia, Hereditary | SPAST SPG11 KIF5A HSPD1 SPG7 HSPE1 | 6683 80208 3798 3329 6687 3336 | 6 |
| Stress disorder, post-traumatic | IL8 FKBP5 SLC6A3 APOE MAOB CRH DBH IL2 | 3576 2289 6531 348 4129 1392 1621 3558 | 8 |

| | | | |
|---|---|---|---|
| Supranuclear palsy, progressive | CRHR1 CHRM1 CHRM4 | 1394 1128 1132 | 3 |
| Thrombocythemia, Hemorrhagic | LDHA TYK2 LDHC | 3939 7297 3948 | 3 |
| AIDS | CASP8 ARPC1B NOL3 ARPC5 CAT VPS33B INSIG1 | 841 10095 8996 10092 847 26276 3638 | 7 |
| Abortion | MTHFR IL10 F2 IL1RN ABO AGTR1 IFNG CTLA4 SERPINE1 ADFP IL6 HLA-A NOD2 CALR IL4 FASLG TLR2 ITGAL NOD1 F5 FCGR3A APOH JAK2 CD28 MUC1 MC4R BAX GAD2 POMC ACP1 MME LNPEP ABCG2 ASPH BRCA2 LDLR TYMS ASCL2 CGA INSR BRCA1 TRO F2RL1 CXCR5 CGB MUSK TSHR SELPLG KIR2DL1 CRYAB PAPPA GNLY TUBA1B S1PR1 TRIM28 WDR1 SNX2 RPS6 MSMB IL10RB DSG3 HPBP HOXA11 NR5A1 | 4524 3586 2147 3557 28 185 3458 1493 5054 123 3569 3105 64127 811 3565 356 7097 3683 10392 2153 2214 350 3717 940 4582 4160 581 2572 5443 52 4311 4012 9429 444 675 3949 7298 430 1081 3643 672 7216 2150 643 1082 4593 7253 6404 3802 1410 5069 10578 10376 1901 10155 9948 6643 6194 4477 3588 1830 100144632 3207 2649 | 64 |
| Abruption placentae | MTHFR BHMT F3 AGT F5 MTHFD1 SLC19A1 MTRR | 4524 635 2152 183 2153 4522 6573 4552 | 8 |
| Achalasia and cardiospasm | PTPN22 PLN ATP2A2 CALR CASQ1 | 26191 5350 488 811 844 | 5 |
| Acne | LCN2 ANPEP MMP2 S100A7 MME NOD1 | 3934 290 4313 6278 4311 10392 | 6 |
| Acromegaly | INS IGF1 AIP GHRHR GH1 GHR IGFBP3 IGFALS ADIPOQ | 3630 3479 9049 2692 2688 2690 3486 3483 9370 | 9 |
| Actinic keratosis | HRAS SDHD TRPC1 CDKN2A CDKN2B GSTM1 MMP12 | 3265 6392 7220 1029 1030 2944 4321 | 7 |
| Adenocarcinoma | AGTR1 TNFRSF10B AGT REG1A CYP2A6 TLR4 KRT18 VIP FGF1 AQP3 CAT ADORA2B CHGA ADCY3 PROZ TSN WNT8B MYEOV ADAM8 SKAP2 S100PBP EPHA4 BTF3 DYRK1B HOXA1 ABCC6 HTR7 F12 ADCY6 CLDN5 CLCN1 KNG1 LAMP3 ATP2A3 PTMA ASAH1 FKBP5 PYGB CRTC3 IGAD1 GKN2 NET1 GKN1 | 185 8795 183 5967 1548 7099 3875 7432 2246 360 847 136 1113 109 8858 7247 7479 26579 101 8935 64766 2043 689 9149 3198 368 3363 2161 112 7122 1180 3827 27074 489 5757 427 2289 5834 64784 10986 200504 10276 56287 7123 10003 10981 113 | 47 |
| Adenoid cystic cancer | PIN1 CYLD ACCN2 SOX4 | 5300 1540 41 6659 | 4 |
| Adenoma | INS AGTR1 IL6 BDKRB1 IL12B IL10 MIF MMP3 EPHX1 ALDH2 ADM HSD11B1 GHR NOX1 SMAD3 SEPP1 CGA EXTL3 CD3EAP MRE11A FABP6 MAL SLC9A3R2 LPAR1 XRCC2 CGB KCNE1 MC2R GPR182 HGS | 3630 185 3569 623 3593 3586 4282 4314 2052 217 133 3290 2690 27035 4088 6414 1081 2137 10849 4361 2172 4118 9351 1902 7516 1082 3753 4158 11318 9146 63892 7296 7201 11284 | 34 |
| Adenoma of thyroid | FHL5 RBL1 | 9457 5933 | 2 |

| | | | |
|---|---|---|---|
| Adenovirus infection | IL8 CDKN1B RAC1 RLN2 MDM2 NR1I2 CASP9 F10 EIF4G1 CCR5 EP400 RAD50 HTR4 ATF1 IL1F7 SOCS3 AKT1 BCL2L1 CSK C1QBP APOB CD86 IL13 TNFRSF10A FAS CAT ATM APP HNRNPUL1 MRE11A U2AF1 RB1 CD46 MYC CAV3 CXADR CCND1 PAX6 LOC728931 CFLAR CD80 SIN3A EGFR LTF WWP2 SGMS1 MED23 IL24 BIRC5 SOD1 NBN CDKN2A APOA1 EIF4E PTK2 CIAPIN1 BAX [illegible] | 3576 1027 5879 6019 4193 8856 842 2159 1981 1234 57634 10111 3360 466 27178 9021 207 598 1445 708 338 942 3596 8797 355 847 472 351 11100 4361 7307 5925 4179 4609 859 1525 595 5080 728931 8837 941 25942 1956 4057 11060 259230 9439 11009 332 6647 4683 1029 335 1977 5747 57019 581 5430 3716 | 59 |
| Adrenal gland hyperfunction | CDKN2A SELP POMC KLK3 SMARCA4 MC2R DUSP2 | 1029 6403 5443 354 6597 4158 1844 | 7 |
| Adrenal gland hypofunction | ACD PTPN22 NR0B1 | 65057 26191 190 | 3 |
| Adrenal gland tumor | ACADVL AKR1B1 CYP19A1 SMARCA2 AGTR1 AGTR2 IL6 HSD11B2 HSD11B1 UTS2 LHCGR | 37 231 1588 6595 185 186 3569 3291 3290 10911 3973 | 11 |
| Adrenoleukodystrophy | ACD ABCD1 TRIM37 BCAP31 ANXA1 TJP1 ADM TTPA ANXA2 PLG ABCD4 ABCD2 ABCD3 | 65057 215 4591 10134 301 7082 133 7274 302 5340 5826 225 5825 406994 | 14 |
| Advanced cancer | ADIPOQ IGF1 IL12B LEP FGF1 IRAK1 BCAT1 BCL2A1 MSMB | 9370 3479 3593 3952 2246 3654 586 597 4477 9185 | 10 |
| Albers-Schonberg disease | PLEKHM1 TCIRG1 CLCN7 LRP5 ACP5 OSTM1 CSF1 CLCN1 | 9842 10312 1186 4041 54 28962 1435 1180 | 8 |
| Alcoholic liver disease | TNF IL1RN CCL5 CTLA4 ALDH2 CD14 | 7124 3557 6352 1493 217 929 | 6 |
| Alimentary system disease | IL1B GCG IL6 IL8 MUC2 CDX2 REG1A IL2 IL10 IL18 CD14 TLR2 SPINK1 CFTR PDX1 MUC1 KLF4 MUC4 VIM CEACAM5 CTSW | 3553 2641 3569 3576 4583 1045 5967 3558 3586 3606 929 7097 6690 1080 3651 4582 9314 4585 7431 1048 1521 | 21 |
| Alopecia | GJA1 LPAR6 LIPH CDSN ICAM1 IFNG PTPN22 CCL2 MICA FCGR1A IL1RN IL16 TNFSF13B IL1F5 AIRE PSIP1 FCGR1B HLA-B IGF1 IL6 HLA-A IL6ST HLA-DQA1 NF1 OSM NR3C2 POMC ITGB1 LIF | 2697 10161 200879 1041 3383 3458 26191 6347 4276 2209 3557 3603 10673 26525 326 11168 2210 3106 3479 3569 3105 3572 3117 4763 5008 4306 5443 3688 3976 1489 | 30 |
| Alveolar bone loss | MMP13 P2RY1 SPARC IL1B CCL3L3 CCL3 CCL3L1 | 4322 5028 6678 3553 414062 6348 6349 | 7 |

| | | | |
|---|---|---|---|
| Alzheimer's disease | PIN1; SP1; HSPB2; HSP90AA1; CDK5R1; MARK2; ADRBK1; STUB1; RBM4; IL8; NAT2; MMP3; BCL2; APP; BAX; NES; F2RL1; ABCD1; JPH3; GNE; SQSTM1; SLC1A3; CHAT; CHMP2B; ITM2B; PCSK1N; STH; IL1A; IL1B; ICAM1; INS; MTHFR; SOD2; IGF1; IFNG; TNF; IL6; TSC2; CTSD; IL10; CCR5; FAS; IL18; CCL2; FASLG; TLR4; S100A12; CFTR; MTRR; TIMP1; CRP; LPL; ACE; CDKN1B; MTR; CTNNB1; CST3; APOC1; ESR1; FGF1; C3; PLA2G2A; APOD; ESR2; PTGS2; SORL1; PAK1; PLAU; CCL3; MPO; HSPG2; TIMP2; CSF1; A2M; GNB2L1; RPS6KB1; BCHE; GH1; PCK1; ABCG1; ABCA1; APOA1; ITGAM; PRDX6; TTR; S100A9; CETP; CHGA; ITGB1; MME; PTGES2; IGF2R; APOM; CYBB; ENO1; BDKRB2; MSI1; NDRG2; WT1; LDLR; BCL2L2; GLUL; ITGAV; PRDX1; UCHL1; ALOX12; LRP1; BRCA1; ERBB4; MRE11A; ALOX15; CASP6; E2F1; CSNK1D; ADAM10; FABP3; POLB; CLU; CKB; ABL1; HNRNPA2B1; CAST; CBS; BACH1; HSPB8; SLC1A2; PCSK9; C5AR1; RTN4; ADRB1; KALRN; EPOR; LAMC1; LAMA1; EIF2S1; RAF1; LHCGR; DBN1; BLMH; STMN1; | 5300 6667 3316 3320 8851 2011 156 10273 5936 3576 10 4314 596 351 581 10763 2150 215 57338 10020 8878 6507 1103 25978 9445 27344 246744 3552 3553 3383 3630 4524 6648 3479 3458 7124 3569 7249 1509 3586 1234 355 3606 6347 356 7099 6283 1080 4552 7076 1401 4023 1636 1027 4548 1499 1471 341 2099 2246 718 5320 347 2100 5743 6653 5058 5328 6348 4353 3339 7077 1435 2 10399 6198 590 2688 5105 9619 19 335 3684 9588 7276 6280 1071 1113 3688 4311 80142 3482 55937 1536 2023 624 4440 57447 7490 3949 599 2752 3685 5052 7345 239 4035 672 2066 4361 246 839 1869 1453 102 2170 5423 1191 1152 25 3181 831 875 571 26353 6506 255738 728 57142 153 8997 2057 3915 284217 1965 5894 3973 1627 642 3925 3831 4741 9482 10858 2078 6400 1978 29119 2618 9420 65078 4666 9821 20 375790 347731 11033 29904 255022 4543 837 26088 4223 815 27163 1136 755 7447 10524 6453 3658 3749 10211 6646 10776 10280 892 1938 323 334 715 64137 9479 618 9546 320 5870 410 498 6655 9261 4241 285672 | 193 |
| Amaurosis congenita of leber I | RPGRIP1; CRX; LRAT; TULP1; CEP290; RPE65; IMPDH1; PDC | 57096 1406 9227 7287 80184 6121 3614 5132 | 8 |
| Amnionitis | EDN1; IL1B; IL6; LTA; GSTT1; MMP9; CYP1A1; ADM; ADRB3; IL8RB; DEFB103A; BMP2; ANXA2 | 1906 3553 3569 4049 2952 4318 1543 133 155 3579 55894 650 302 | 13 |
| Amyloidosis | MEFV; MAZ; IAPP; TTR; APP; MME; APCS; SAA1; MAG; CALR; SNCA; SAA2; MBL2; ICAM3; APOA1; APOC2; TNF; MMP3; SEMG1; B2M; PARK7; HSPB8; CST3; SOD1; FGA; LYZ; CD14; | 4210 4150 3375 7276 351 4311 325 6288 4099 811 6622 6289 4153 3385 335 344 7124 4314 6406 567 11315 26353 1471 6647 2243 4069 929 643387 | 28 |

| | | | |
|---|---|---|---|
| Amyotrophic lateral sclerosis | SPAST; SOD1; CSF2; CHMP2B; PARP1; IFT74; TIMP1; IGFALS; ALS2; SLC1A2; DCTN1; MET; ANG; ENG; RNF19A; LOC643387; RTN4; LOX; KDR; HTR1A; VAPB; ANKRD2; NEFL; MAPT; VGF; APP; APEX1; KIF3A; SMN1; GSTP1; ITPR2; C3; PARK7; CACNA1S; IGHMBP2; RAB5A; ANKRD1; SMAD2; AR; SMAD3; MMP9; NEFH; APOE; SMN2; IL13; LAMC1; CST3; CASP3; MAPK14; ALS2CL; CCL2; BDNF; CTSB; P4HB; CCS | 6683 6647 1437 25978 142 80173 7076 3483 57679 6506 1639 4233 283 2022 25897 643387 57142 4015 3791 3350 9217 26287 4747 4137 7425 351 328 11127 6606 2950 3709 718 11315 779 3508 5868 27063 4087 367 4088 4318 4744 348 6607 3596 3915 1471 836 1432 259173 6347 627 1508 5034 9973 | 55 |
| Anemia | FABP1; TLR4; EPO; CA2; HAMP; FASLG; IL12B; FGF2; TNF; IL10; IFNG; ACE; AQP3; LEP; ADAMTS13; ALB; CA1; IL6; PTPN6; CCL5; SLC11A1; ACO1 | 2168 7099 2056 760 57817 356 3593 2247 7124 3586 3458 1636 360 3952 11093 213 759 3569 5777 6352 6556 48 | 22 |
| Angiomyolipoma | TSC2; IFNG; MFI2; EGFR; CSPG4; CHL1; BRAF | 7249 3458 4241 1956 1464 10752 673 | 7 |
| Ankylosing spondylitis | SOD2; HLA-A; CRP; ACE; LEP; KIR3DL1; MSX2 | 6648 3105 1401 1636 3952 3811 4488 | 7 |
| Ankylosis | BMP2; NOG; BMP7 | 650 9241 655 | 3 |
| Anorexia nervosa | LEPR; BDNF; HTR2C; CLOCK; SLC6A4; MAOA; LEP; SLC6A2; MC3R; RETN; ARVCF; AGRP; IL6; HTR2A; TNF; SHBG; ADIPOQ; UCP3; INHBB; IFNG; UCP2; GCG; S100B; HTR1D | 3953 627 3358 9575 6532 4128 3952 6530 4159 56729 421 181 3569 3356 7124 6462 9370 7352 3625 3458 7351 2641 6285 3352 | 24 |
| Antiphospholipid syndrome | APOH; SERPINB2; IGF1; PLAT; MTHFR; PROZ; FCGR2A; ANXA2; IGFBP1 | 350 5055 3479 5327 4524 8858 2212 302 3484 | 9 |
| Anxiety disorder | GAD1; GAD2; ACCN2 | 2571 2572 41 | 3 |
| Aortic aneurysm | HSPB1; CMA1; ITGB1; MMP8; ITGA5; RTN4; ACTA2; TIMP3; IL10; CCR5; CX3CR1; MIF; TIMP1; LEP; HLA-DQA1; MMP10; CX3CL1; HPSE; XYLT1; IGKC; | 3315 1215 3688 4317 3678 57142 59 7078 3586 1234 1524 4282 7076 3952 3117 4319 6376 10855 64131 3514 5104 368 | 22 |
| Aortic valve disease | MBL2; MASP2; HSPB1; APOB; C3; APOA1; PCNA; ELN; C5 | 4153 10747 3315 338 718 335 5111 2006 727 | 9 |
| Aplastic anemia | FASLG; IL10; RMRP; HLA-DQA1; GATA2; GSTT1; ITGA2B; CYP1A1; ITGB3; HLA-DQB1; TNF; GSTP1; CD55; GSTM1; WT1; GATA3; GATA1; NFATC2; RPS24; RPS19; FLVCR1; RPL35A; RPS17 | 356 3586 6023 3117 2624 2952 3674 1543 3690 3119 7124 2950 1604 2944 7490 2625 2623 4773 6229 6223 28982 6165 6218 | 23 |
| Arteriopathy | MTHFR; INS; CST3; IL6; MYH11; RETN; MMP9; ELN; SCN9A | 4524 3630 1471 3569 4629 56729 4318 2006 6335 | 9 |

| | | | |
|---|---|---|---|
| Arthritis | CCR5; IL18; S100A12; UNC13D; SH2D2A; IL1A; IL1B; PITX1; ACE; LPAR1; PRG4; MMP2; CCL2; CD14; MMP9; IL11; ADRB2; IFNA1; IFNB1; BMP7; CCL4; ELF3; ANKH; TNAP; NAMPT; RETN; SOD2; IFNG; TNF; NOD2; IL15; PTPN22; PTGES; FGF2; TLR2; IL8RA; MIF; PDCD1; TNFRSF9; VIP; SLC11A1; NOD1; CRP; IL17A; CXCL13; CYP1A1; OSM; MMP10; CXCL12; KL; HRH4; CTSB; HPRT1; IL8RB; VIPR2; MUC5AC; BIRC5; WISP1; BSG; SAA1; CCL23; MUC3B; [illegible] | 1234 3606 6283 201294 9047 3552 3553 5307 1636 1902 10216 4313 6347 929 4318 3589 154 3439 3456 655 6351 1999 56172 445341 10135 56729 6648 3458 7124 64127 3600 26191 9536 2247 7097 3577 4282 5133 3604 7432 6556 10392 1401 3605 10563 1543 5008 4319 6387 9365 59340 1508 3251 3579 7434 4586 332 8840 682 6288 6368 57876 4584 353 11240 | 65 |
| Aseptic necrosis of bone | ANXA2; CTNNB1; F2; F5; SERPINE1; MTHFR; BMP6; | 302 1499 2147 2153 5054 4524 654 1387 9365 3091 | 10 |
| Asthma | CTLA4; CALCA; IL10; TJP1; IL16; PAFAH1B1; TNFRSF10A; CMA1; CCL18; LAMA5; CTNNA1; SELPLG; CHIA; CHML; ITK; IL1B; ICAM1; MTHFR; SOD2; MMP2; TNC; IFNG; SERPINE1; TNF; BDNF; IL8; BDKRB1; LTA; GSTM1; HSPB1; GSTT1; GSTP1; EGFR; CCL28; IL4; CCR5; CXCL10; IDO1; IL18; CX3CR1; FGF2; TLR4; IRF1; TLR3; TLR2; CCR1; MBL2; HSPA1B; IRAK3; SLC11A1; TIMP1; IL1RN; HLA-DQB1; CD86; CRP; EDN1; IL17A; CCL24; ACE; IL5; IL18RAP; AREG; IL4R; LEP; HLA-DQA1; FCGR2A; CCR9; BCL2; HNMT; ALOX5; EGR1; CX3CL1; PTGS2; BCL2L1; ELANE; PLAU; SOD1; CLCA1; ADORA2A; CAT; HSPA1A; XDH; BAX; ITGB3; IFNA1; CYBA; CHRM3; ANGPT1; PIK3CG; LGALS3; ADA; HAVCR1; BDKRB2; MUC5AC; SERPINB3; ETS1; SERPINB4; SMAD2; KDR; MAF; LTC4S; IL12A; CCL22; GATA3; EFNA1; ITGA4; ANXA2; CYSLTR1; F2RL1; ARG1; CHI3L1; ADAM8; ADH5; SERBP1; INHA; TLR1; TNFSF4; C5AR1; BMPR2; IL9; ADAM33; C4A; CHRM1; NPSR1; BMPR1B; MYLK; FCER1A; ACVR1; GPR44; CYSLTR2; C3AR1; IGHE; IGES; IL9R; IL13RA1; | 1493 796 3586 7082 3603 5048 8797 1215 6362 3911 1495 6404 27159 1122 3702 3553 3383 4524 6648 4313 3371 3458 5054 7124 627 3576 623 4049 2944 3315 2952 2950 1956 56477 3565 1234 3627 3620 3606 1524 2247 7099 3659 7098 7097 1230 4153 3304 11213 6556 7076 3557 3119 942 1401 1906 3605 6369 1636 3567 8807 374 3566 3952 3117 2212 10803 596 3176 240 1958 6376 5743 598 1991 5328 6647 1179 135 847 3303 7498 581 3690 3439 1535 1131 284 5294 3958 100 26762 624 4586 6317 2113 6318 4087 3791 4094 4056 3592 6367 2625 1942 3676 302 10800 2150 383 1116 101 128 26135 3623 7096 7292 728 659 3578 80332 720 1128 387129 658 4638 2205 90 11251 57105 719 3497 3478 3581 3597 3568 6036 3631 57628 10333 29949 6346 1395 246778 594857 7177 5367 8809 64806 9173 | 150 |
| Ataxia telangiectasia | HPRT1; MRE11A; UPF1; PMS1; RPS6KA3 | 3251 4361 5976 5378 6197 | 5 |

| | | | |
|---|---|---|---|
| Atherosclerosis | CCR5; F8; ICAM1; TLR2; ITGAL; ITGA2; HIF1A; MMP9; MMP14; BSG; IFNG; MMP3; CX3CL1; CSF1; LCAT; MCAM; RAMP1; ALOX5AP; AIF1; SPEG; RASL11B; TNFSF4; AGTR1; LPL; IGFBP1; APOB; EDNRA; ABCA1; SOD3; MEF2A; EDNRB; ATXN1; ATP5J; TTN; ZC3H12A; IL1B; IGFBP3; FCAR; CXCL10; CX3CR1; NOD1; IL1RN; IL18BP; APOA4; MTR; EPHX1; APOC1; ADRB2; MGP; KCNJ11; VWF; APOC3; CYBA; PLTP; PON1; LDLR; CLU; PCSK9; VKORC1; SELPLG; KIF6; APOC2; SELL; BDNF; IL8; LTA; GSTM1; HSPB1; GSTT1; CCL11; IL4; IL10; CXCL11; HSPA1B; F13A1; HBEGF; CD80; CST3; LEP; CYP1A1; ESR1; PLA2G2A; ANGPT2; ALOX5; CASR; ALDH2; ADH1B; KLF2; ADRB3; BCHE; AR; WRN; SHC1; ECE1; ITGA2B; APOA2; ADA; APOM; LIPG; KIT; LGALS2; LRP6; CBS; IL7; SLC2A9; GCH1; TLR1; ABCC6; NCF1C; KALRN; GATA2; PAFAH2; LSAMP; GJA4; IFNE; NCF2; GCLM; F12; VTN; F11; IL1A; INS; CCL5; VEGFA; ENG; AKR1B1; MMP1; AKR1B10; TNF; SLC6A4; IL18; SPP1; CCL2; FASLG; TLR4; CD14; ALB; CXCL16; TIMP1; CD86; TNFSF15; FCGR3A; EDN1; | 1234 2157 3383 7097 3683 3673 3091 4318 4323 682 3458 4314 6376 1435 3931 4162 10267 241 199 10290 65997 7292 185 4023 3484 338 1909 19 6649 4205 1910 6310 522 7273 80149 3553 3486 2204 3627 1524 10392 3557 10068 337 4548 2052 341 154 4256 3767 7450 345 1535 5360 5444 3949 1191 255738 79001 6404 221458 344 6402 627 3576 4049 2944 3315 2952 6356 3565 3586 6373 3304 2162 1839 941 1471 3952 1543 2099 5320 285 240 846 217 125 10365 155 590 367 7486 6464 1889 3674 336 100 55937 9388 3815 3957 4040 875 3574 56606 2643 7096 368 654817 8997 2624 5051 4045 2701 338376 4688 2730 2161 7448 2160 3552 3630 6352 7422 2022 231 4312 57016 7124 6532 3606 6696 6347 356 7099 929 213 58191 7076 942 9966 2214 1906 350 3572 5327 3717 257313 2212 29851 3329 2100 5743 6489 2152 1991 5328 4353 3339 4321 6279 6647 820 7295 6387 11132 197 7498 4547 9365 7351 3690 207 6280 4317 27035 1118 10062 3480 5329 3685 1028 4035 246 1994 1116 80310 215 5144 2053 9446 4332 9572 6095 55911 3693 5155 775 339479 5029 64805 325 1593 5136 | 204 |
| Atopic rhinitis | GATA3; ICAM1; ADIPOQ; BDKRB1; EGFR; CX3CR1; EDN1; IL17A; CX3CL1; UCN; HRH2; HRH1; F2RL1; S100A7; FOXJ1 | 2625 3383 9370 623 1956 1524 1906 3605 6376 7349 3274 3269 2150 6278 2302 | 15 |
| Attention deficit hyperactivity disorder | FADS2; MTHFR; IL1RN; NSD1; STS; ADRA2A; HTR4; SLC1A3; LMO3; SNAP25; GNAL; HTR1D | 9415 4524 3557 64324 412 150 3360 6507 55885 6616 2774 3352 | 12 |

| | | | |
|---|---|---|---|
| Autistic disorder | EXT1; WNT2; COPG2; MLPH; MEST; MOG; NSD1; EN2; NLGN4Y; SLC6A4; GFAP; CHRNA7; HOXA1; NRP2; GAD1; NBEA; MED12; HLA-A; DISC1; EGF; UBE3A; PITX1; MBD1; NLGN4X; AVPR1A; EFHC2; TPH1; MECP2; APOE; GJA1; OXTR; ABAT; HOXB1; ITGB3; TSC1; CNTNAP2; CYP21A2; C4B; BDNF; ASMT; DDC; REEP3; SEMA5A; RYR3; TSC2; RAB3A; HTR2A; GLO1; MET; AQP4; RAPGEF4; NRXN1; ADSL; CHRNB2; BCL2; SCN2A; GSTM1; ADRB2; STS; ADA; SCT; LEP; SCN1A; MAOA | 2131 7472 26958 79083 4232 4340 64324 2020 22829 6532 2670 1139 3198 8828 2571 26960 9968 3105 27185 1950 7337 5307 4152 57502 552 80258 7166 4204 348 2697 5021 18 3211 3690 7248 26047 1589 721 627 438 1644 221035 9037 6263 7249 5864 3356 2739 4233 361 11069 9378 158 1141 596 6326 2944 154 412 100 6343 3952 6323 4128 6328 1137 7328 221037 | 68 |
| Autoimmune disease | KCNA3; PTPN22; HLA-DOB; IRF5; MAN2A1; IL21; CYP27B1; MMP9; IL6R; BCL2L1; GSTT1; FCGR3B; SERPINH1; C4B; CTLA4; CCL22; IL2RA; MBL2; S100A8; PDCD1; FASLG; C4A; IER3; SUMO4; BCL2; JAK1; CASP8; WFS1; TNFSF13B; IL4; CEACAM1; BAX; KCNK3; CD274; IL10; TLR4; SPRR1B; FAS; S100A9; TNF; CBLB; SLC11A1; BAK1; MSH6; DBH; CCL17; IL32; KRT14; IL8; IL6; MYO9B; PRSS16; CIITA; FCGR2B; CXCL10; CXCL9; IFI16; TYK2; HLA-DQA1; HNRNPD; IFIH1; HLA-DOA; CCL21; CCL3L1; ITGA2B; AIRE; MICA; AGER; HLA-B; CCL2; MIF; IL1RN; HLA-DQB1; IL4R; FCGR2A; CASR; KIR2DS2; PTPRC; IFNB1; GATA3; CUGBP2; EXOSC9; EXOSC10; CCL7; CCM2 | 3738 26191 3112 3663 4124 59067 1594 4318 3570 598 2952 2215 871 721 1493 6367 3559 4153 6279 5133 356 720 8870 387082 596 3716 841 7466 10673 3565 634 581 3777 29126 3586 7099 6699 355 6280 7124 868 6556 578 2956 1621 6361 9235 3861 3576 3569 4650 10279 4261 2213 3627 4283 3428 7297 3117 3184 64135 3111 6366 6349 3674 326 4276 177 3106 6347 4282 3557 3119 3566 2212 846 100132285 5788 3456 2625 10659 5393 5394 6354 9247 | 85 |
| Autoimmune thyroiditis | INS; HLA-A | 3630 3105 | 2 |
| Azoospermia | DAZ1; AR; ERCC1; DDX25; RAP1A; NR0B1; CFTR; ART3; DAZL; BOLL; GGT1; INHBA; TIMP2; XRCC1; TIMP1; BRCA2; HLA-DQB1; ERCC2; GSTT1; KITLG; AMH; CLU; RBMY1A1; | 1617 367 2067 29118 5906 190 1080 419 1618 66037 2678 3624 7077 7515 7076 675 3119 2068 2952 4254 268 1191 5940 2768 | 24 |
| Bacterial infection | PLA2G4A; CXCL16; OSM; MUC1; ABL1; SNCA; F3; VWF; HSP90B1; ADAMTS13; IL12RB1; PRDM1; ARAP3; EMILIN1 | 5321 58191 5008 4582 25 6622 2152 7450 7184 11093 3594 639 64411 11117 | 14 |
| Bacterial vaginosis | IL1B; MBL2; IL8; MMP8; TLR2; IL1RN | 3553 4153 3576 4317 7097 3557 | 6 |
| Bardet-Biedl syndrome | BBS2; BBS10; CCDC28B; BBS9; BBS1; BBS4; CEP290 | 583 79738 79140 27241 582 585 80184 | 7 |

| | | | |
|---|---|---|---|
| Barrett's esophagus | RUNX3; XRCC1; IGF2BP3; MAD2L1; GSTM1; BUB1; SKIL; ERCC2; APC; SFRP1; MUC2; HSPB1; CDX2; AKR1B10; NR1H4; HIF1A; WIF1; GSTT1; GSTP1; IL6; REG1A; CDKN2A; EGFR; CES2; AKR1C2; CCNA2; CTSD; SKI; | 864 7515 10643 4085 2944 699 6498 2068 324 6422 4583 3315 1045 57016 9971 3091 11197 2952 2950 3569 5967 1029 1956 8824 1646 890 1509 6497 5307 6648 | 30 |
| Basal cell carcinoma | CCL5; TNF; IL6; IL8; EDN1; ASIP; S100A9; GREM1; MSH3; XPA; NDUFA1; GLI2; ERP29 | 6352 7124 3569 3576 1906 434 6280 26585 4437 7507 4694 2736 10961 | 13 |
| Beckwith-Wiedemann syndrome | KCNQ1OT1; H19; NSD1 | 10984 283120 64324 | 3 |
| Behavior disease | WFS1; BDNF; SLC6A4; HTR2A; HTR1A; SNCA; S100B; MAPT; SLC6A2; CLOCK; SLC6A3; CHRNA7; MECP2; DBH; MAOA; CHRM2; HTR1B; HTR2C; ANKK1; FAAH; CHRNA4; TPH1; MAOB; CNTNAP2; CHRNB2; SLC6A8; GFAP; SLC18A2; STH; MEIS1; PDE4B; YKT6; PDLIM5; DAOA; DISC1; NOS1AP; CPLX2; CPLX1; DISC2; EN2; HMGXB4; TOM1 | 7466 627 6532 3356 3350 6622 6285 4137 6530 9575 6531 1139 4204 1621 4128 1129 3351 3358 255239 2166 1137 7166 4129 26047 1141 6535 2670 6571 246744 4211 5142 10652 10611 267012 27185 9722 10814 10815 27184 2020 10042 10043 | 42 |
| Behcet syndrome | IL17A; HLA-B; IL4; CXCL13; ADIPOQ; APOH; MEFV; IL8; IRF1; GSTM1; IL2; CCR5; GSTT1; MICA; F5; LEP; TXK; IL18; SAG; CYP1A1; VWF; PTPN22; ACE; ENO1; F2; IFNG; MMP2; TLR4; NOD2; MBL2; TNF; SOD2; ADARB1; SELL; IL1A; ICAM1; IL12B; CXCL12; KLRC1; LGALS3BP; MMP9; SLC11A1; ITGA2; KLRD1; CCL21; GSTP1; IL1B; PROZ; ARR3 | 3605 3106 3565 10563 9370 350 4210 3576 3659 2944 3558 1234 2952 4276 2153 3952 7294 3606 6295 1543 7450 26191 1636 2023 2147 3458 4313 7099 64127 4153 7124 6648 104 6402 3552 3383 3593 6387 3821 3959 4318 6556 3673 3824 6366 2950 3553 8858 407 | 49 |
| Biliary Atresia | ICAM1; CTGF; SPP1; CFC1; MX1; TIMP1; CD14; MMP7 | 3383 1490 6696 55997 4599 7076 929 4316 | 8 |
| Biliary cancer | CCK; HOXB7; APOE; APOB; LDLR | 885 3217 348 338 3949 | 5 |

| | | | |
|---|---|---|---|
| Bipolar disorder | BDNF; ESR1; CHRNA7; SRR; ADARB1; SLC6A5; DAOA; CIT; SLC6A3; RFX4; NCAM1; ALOX12; ADRBK2; HTR1B; HTR2C; GNAL; DISC1; NDUFV2; SLC6A4; IMPA1; PCNT; HTR2A; DAO; ATF5; HSPA5; DNAJB1; MTRR; VAMP3; SLC6A2; MAOA; TPH1; OTX2; ADCYAP1; AMD1; SLC18A1; DUSP6; IGFBP2; TRPM2; G30; TAAR6; HSP90B1; ST3GAL1; SYNJ1; CTLA4; INPP1; S100B; ATF4; GPR50; XBP1; TSNAX; PAWR; CAMK2A; RGS4; GPR78; IMPA2; FAT1; PDLIM5; SLC25A4; RTN4; SYNGR1; CRHBP; IL1B; GFAP; MTHFR; NTNG1; CCL2; CHRM2; CLOCK; GCH1; SLC18A2; PER3; MC5R; NOS1AP; PCDHA1; DDC; GAD1; ADCY9; PER2 | 627 2099 1139 63826 104 9152 267012 11113 6531 5992 4684 239 157 3351 3358 2774 27185 4729 6532 3612 5116 3356 1610 22809 3309 3337 4552 9341 6530 4128 7166 5015 116 262 6570 1848 3485 7226 282706 319100 7184 6482 8867 1493 3628 6285 468 9248 7494 7257 5074 815 5999 27201 3613 2195 10611 291 57142 9145 1393 3553 2670 4524 22854 6347 1129 9575 2643 6571 8863 4161 9722 56147 1644 2571 115 8864 | 78 |
| Bladder cancer | MAGEA4; UPK1A; MDM2; CAV1; FGF10; CHRM2; CAGE1; FABP4; EFNA1; PREPL; RTKN; CASP5; UPK1B; ICAM1; ITGAL; MSH3; SOX4; SP4; SP3; TRIO; ABO; CD274; FASLG; PDCD1; NF1; DUSP1; CD276; CD3E; BCL2L14; TMOD1; KRT34; TMOD3; TMOD2; IL1A; TNF; HLA-A; IL8; LTA; CALR; AKR1C2; IRF1; EDN1; LEPR; MTR; KLF5; CSF3; MTSS1; TSGA10; ETS2; CLU; MGAT5; H19; CGB; PAX6; IFN1@; TPPP; ZDHHC11; CSF3R; CSMD1; UCA1 | 4103 11045 4193 857 2255 1129 285782 2167 1942 9581 6242 838 7348 3383 3683 4437 6659 6671 6670 7204 28 29126 356 5133 4763 1843 80381 916 79370 7111 3885 29766 29767 3552 7124 3105 3576 4049 811 1646 3659 1906 3953 4548 688 1440 9788 80705 2114 1191 4249 283120 1082 5080 3438 11076 79844 1441 64478 652995 | 60 |
| Bone disease | IL11; BMP6; FAM20C; MPL; SH3BP2; FGFR3; MMP2; IGF1; WISP3; PTH1R; RUNX2; EXT1; TSC2; NF1; PKD1; SMO; SUMO3; FBN1; FBN2; DLL3; LFNG; USP6; IL17A; ALPL; EZR; LRP5; BGLAP; MAF; S100A6; CHI3L1; CLCN7; ACP5; IL1F6; WNT10B | 3589 654 56975 4352 6452 2261 4313 3479 8838 5745 860 2131 7249 4763 5310 6608 6612 2200 2201 10683 3955 9098 3605 249 7430 4041 632 4094 6277 1116 1186 54 27179 7480 | 34 |
| Bone marrow disease | CX3CR1; ETV6; RUNX1; MYB; JAK3; ABL1; BCR; DPYSL5; PDGFB; GATA1; MPL; HLA-C; IRF4; CD177; IKZF1 | 1524 2120 861 4602 3718 25 613 56896 5155 2623 4352 3107 3662 57126 10320 | 15 |
| Bone metastases | IL6; IL4; EDN1; VIM; ITGA5; NOG; MAPKAP1; FGF8 | 3569 3565 1906 7431 3678 9241 79109 2253 | 8 |

| | | | |
|---|---|---|---|
| Brain disease | APP; PIN1; IDE; PRDX3; SQSTM1; CHMP2B; SLC18A2; PCSK1N; STH; SNCA; S100B; MAPT; SLC6A3; SNCG; AQP1; SCN1A; GBA; GLS2; SLC19A2; MYT1; ASCL1; CDK5R1; ACVRL1; LGI1; OLIG2; MYOG; SMARCB1; DNAJC15; MIB1; DAB1; WASF3 | 351 5300 3416 10935 8878 25978 6571 27344 246744 6622 6285 4137 6531 6623 358 6323 2629 27165 10560 4661 429 8851 94 9211 10215 4656 6598 29103 57534 1600 10810 | 31 |
| Brain ischemia | BCL2; MDK; KCNK2; EPO; AGTR1 | 596 4192 3776 2056 185 | 5 |
| Brain tumor | NAMPT; HNRNPA2B1; PANX2; MBP; HIPK2; PTN; DNAJC15; MIB1; TSC2; ADAMTS13; EMP3; SCML2; ARHGDIA; PRDX6; PTPRZ1; EDNRB; PARVG; AJAP1; INS; LRRC4; BAI1; B4GALT5; BCAN; SCG5; EEF1D; NUDT6; FOXG1; SNCB; PDGFC; RPL30; LDHB; OTX2; RPS20; FGF6; ICAM1; IL6; PTGES; MMP3; NF1; IFNB1; ADARB1; CASP9; ADORA3; PLD2; P2RY2; CTSS; ADAR; LATS2; LATS1; PLD1; PCDHGA11; LPAR6; PTTG1IP; DBC1; SLC6A12; HOXC9; APP; CAT; APLN; ACHE; MATK; CXCR5; APLNR; STS; PIAS3; SEPT2; ADAMTS8; SLC38A3; DCX; RAC2; FGF5; GLG1; AQP9; ARL4D; DAB1; CTSL1; IL8; CCL2; TLR3; MIF; PTPN11; HBEGF; EDN1; MTR; CST3; AHSG; HOXB7; VIM; ATF5; ASPM; EFNB3; CHI3L1; LAMC1; CRYAB; GAPDH; ADAMTS4; LAMA3; ELK1; MIR222; MIR221; MIR7-1; ECHS1; TNFRSF10B; TRPV1; CTGF; IL16; HYAL1; CALD1; ELMO1; CLCN2; VCAN; CCK; LAMA5; EFNA1; DLL1; HSPA8; TRAF1; IER3; PAX6; PTPRJ; GAS6; LAMA4; CLCN3; KCNMB1; C5ORF13; LIMS1; S100A13; XRCC4; KCNA3; | 10135 3181 56666 4155 28996 5764 29103 57534 7249 11093 2014 10389 396 9588 5803 1910 64098 55966 3630 64101 575 9334 63827 6447 1936 11162 2290 6620 56034 6156 3945 5015 6224 2251 3383 3569 9536 4314 4763 3456 104 842 140 5338 5029 1520 103 26524 9113 5337 56105 10161 754 1620 6539 3225 351 847 8862 43 4145 643 187 412 10401 4735 11095 10991 1641 5880 2250 2734 366 379 1600 1514 3576 6347 7098 4282 5781 1839 1906 4548 1471 197 3217 7431 22809 259266 1949 1116 3915 1410 2597 9507 3909 2002 407007 407006 407043 1892 8795 7442 1490 3603 3373 800 9844 1181 1462 885 3911 1942 28514 3312 7185 8870 5080 5795 2621 3910 1182 3779 9315 3987 6284 7518 3738 11096 7447 40 9093 10512 482 3981 2173 11010 55859 253260 80309 60672 1032 2289 57787 816 9806 5989 6660 6663 10124 10963 65065 9294 10362 | 155 |

| | | | |
|---|---|---|---|
| Breast cancer | SERPINE1; ICAM1; IL6; IL8; TIMP1; FCGR3A; EDN1; NFATC1; TXNIP; BGLAP; CHI3L1; C1QA; MIR10B; CSNK1A1; KRT8; F3; S100A8; CSK; BTC; KLF4; S100A9; ACTN4; DUSP6; HIST2H3A; NOL3; TRPS1; KIAA0100; ANXA8L2; WFS1; PINK1; SLC6A3; PARK2; TMPRSS3; FOXL2; MAOA; ND3; HTR4; CYP21A2; ATXN2; MUS81; HOXA2; ZFHX4; RSPO1; IGF1; MICA; CSF1R; EPHX1; EDNRA; GH1; APLN; INHBA; HTATIP2; GATA3; PLXNA1; CLU; E2F4; GH2; EDNRB; HOXA1; CYP19A1; CRYAB; HSPA9; KCNJ3; ADAM28; RBBP7; CYP4Z1; PIP; USP28; EIF4G1; ADAMTS8; NCL; RHEB; CCL16; MIER1; TOPBP1; SDC4; SELE; NEUROD1; INS; CTSL1; PPARGC1A; ADIPOQ; AGER; HLA-B; FADS2; AGTR1; TNFRSF10B; ARNT; HP; TNC; IFNG; CTLA4; ITGA2; AKR1B10; CD55; TNF; NOD2; RNF5; SLC16A1; SCN5A; TSC2; LTA; AKR1C2; IL4; IL10; CCR5; IDO1; CD274; FASLG; IRF1; ADIPOR2; KRT18; CCL21; MIF; MBL2; VIP; MTRR; MMP3; XBP1; HBEGF; LEPR; ACE; IL11; SOCS3; AREG; IGFBP1; PAK2; CST3; APOE; LEP; SHBG; HLA-DQA1; PTPN13; CTGF; ARNT2; FCGR2A; | 5054 3383 3569 3576 7076 2214 1906 4772 10628 632 1116 712 406903 1452 3856 2152 6279 1445 685 9314 6280 81 1848 333932 8996 7227 9703 244 7466 65018 6531 5071 64699 668 4128 4537 3360 1589 6311 80198 3199 79776 284654 3479 4276 1436 2052 1909 2688 8862 3624 10553 2625 5361 1191 1874 2689 1910 3198 1588 1410 3313 3760 10863 5931 199974 5304 57646 1981 11095 4691 6009 6360 57708 11073 6385 6401 4760 3630 1514 10891 9370 177 3106 9415 185 8795 405 3240 3371 3458 1493 3673 57016 1604 7124 64127 6048 6566 6331 7249 4049 1646 3565 3586 1234 3620 29126 356 3659 79602 3875 6366 4282 4153 7432 4552 4314 7494 1839 3953 1636 3589 9021 374 3484 5062 1471 348 3952 6462 3117 5783 1490 9915 2212 142 578 9622 5300 5058 4322 6348 4353 4057 3559 920 3297 6647 9368 5045 6446 133 4306 6515 847 3291 1594 7498 186 2088 7450 688 5770 6464 3373 9588 6319 8835 3932 136 7071 654 2033 2690 4317 824 8976 1462 11214 7431 5887 1843 3625 5371 6093 463 8932 203068 842 8838 6495 3551 182 140 29948 28234 255313 2274 1936 4179 10861 2583 1119 1081 3643 7079 63923 4361 27250 1084 835 3294 5803 25920 196 6351 5423 6548 3590 10111 5914 2114 5333 2192 3934 | 432 |
| Bronchial disease | CCL5; CD14; EDNRB; RNASE3; KCNMB1; TIMP3; AKR1B10; CCL11; DAPK1; ADRB2; S100A9; GCLC; CXADR; DNAH7; RP11-529I10.4; CCR3; RPS3A | 6352 929 1910 6037 3779 7078 57016 6356 1612 154 6280 2729 1525 56171 25911 1232 6189 | 17 |
| Bronchial hyperreactivity | CHI3L1; ADAM33; ESR1; IL13; IL10; NPSR1; CRP; IL5 | 1116 80332 2099 3596 3586 387129 1401 3567 | 8 |
| Bronchiectasis | IFNG; SCNN1B; TLR2; IL8RA; SCNN1G; MMP8 | 3458 6338 7097 3577 6340 4317 | 6 |
| Bronchiolitis | IFI27; CCL3; ICAM1; CLC; CCL2; CTLA4; BDNF; CCL5 | 3429 6348 3383 1178 6347 1493 627 6352 | 8 |
| Bronchiolitis obliterans | NOD2; MMP8; TIMP1; IL6; SFTPA1; IL17A; MMP9; IL8; IFNG | 64127 4317 7076 3569 653509 3605 4318 3576 3458 | 9 |
| Bronchopulmonary dysplasia | TIMP1; IL1RN; F2RL1; MMP2; ACE; FGF7; TIMP2; MMP9; IL10; TNC; IL1B | 7076 3557 2150 4313 1636 2252 7077 4318 3586 3371 3553 | 11 |
| Brucellosis | SELL; IL10; IL4; MEFV; TLR4; IFNG; IL1RN; IL6; SLC11A1; CCR5; | 6402 3586 3565 4210 7099 3458 3557 3569 6556 1234 2212 | 11 |
| Bulimia | ADIPOQ; MC4R | 9370 4160 | 2 |
| CNS lymphoma | SERPINC1 | 462 | 1 |

| | | | |
|---|---|---|---|
| CNS metastases | PARK2; GCH1; CCL2; TIMP2; IDS; MMP2; DGKG; NUPR1 | 5071 2643 6347 7077 3423 4313 1608 26471 | 8 |
| Cancer | MTHFR; CD9; XRCC1; IGF2BP3; GSTM1; BUB1; ERCC2; CTSD; ANXA1; NAT2; FECH; CYP1A1; ADH1B; TNFRSF10A; RPS6KB1; ABCA1; SMAD7; ANG; CD24; HSPA5; CYP24A1; IL7R; FAP; CDKN2B; TSC22D1; MDK; PROM1; RBP1; SFRP5; MAT2A; DLGAP5; BNIP3; ADH1C; LCMT2; SOX2; HSP90B1; PODXL; TACSTD2; GPC3; SPRED2; ENDOG; XRCC3; MIR21; IL24; DDR1; COPS5; MAGEA3; SMYD3; PEBP1; ANPEP; ZEB2; PIK3R1; CCNG1; WNT2B; CEACAM5; ERCC1; ALOX15B; CTTN; LRP1; NRP2; DCC; EXO1; PPP1R13L; XPC; CLDN2; CYP1B1; TK1; BAD; HNRNPF; LRIG1; EPHB2; RIN1; NEIL1; GATA6; ST3GAL6; BIRC2; ATOH1; KCNA5; TNK2; PTPRA; HPSE2; SHMT1; RARRES1; MMP26; FXYD3; HNRNPK; ADCYAP1R1; BID; BCAR1; MIRLET7A1; MYB; BMPR1A; PTHLH; BCL2L11; ETV4; CEBPA; ONECUT1; S100P; MSLN; PTH1R; TRAF2; RRM2; KIF14; S100A2; CYP27A1; CCR3; IL9R; RYR1; PAPPA; OSTM1; CCNT1; IGFBP5; C1D; GPNMB; PTPN6; NCR2; SCGB2A2; XAGE1D; CASC5; NMU; INTS6; ITIH1; PLCB2; | 4524 928 7515 10643 2944 699 2068 1509 301 10 2235 1543 125 8797 6198 19 4092 283 100133941 3309 1591 3575 2191 1030 8848 4192 8842 5947 6425 4144 9787 664 126 9836 6657 7184 5420 4070 2719 200734 2021 7517 406991 11009 780 10987 4102 64754 5037 290 9839 5295 900 7482 1048 2067 247 2017 4035 8828 1630 9156 10848 7508 9075 1545 7083 572 3185 26018 2048 9610 79661 2627 10402 329 474 3741 10188 5786 60495 6470 5918 56547 5349 3190 117 637 9564 406881 4602 657 5744 10018 2118 1050 3175 6286 10232 5745 7186 6241 9928 6273 1593 1232 3581 6261 5069 28962 904 3488 10438 10457 5777 9436 4250 9503 57082 10874 26512 3697 5330 58480 26959 8915 25875 4752 822 7163 80760 9922 5036 382 56892 10451 931 9510 4751 10158 10507 397 6484 3487 56988 26799 27173 9833 2250 10381 4133 1031 79682 1620 407040 3963 26118 5996 6386 9168 2208 339883 10452 10180 64693 1894 387103 3921 5100 5316 5005 10072 10109 100133944 56475 55975 200576 22801 10486 56915 10927 4216 29941 64326 6125 26147 3964 56954 7174 10403 10919 10887 9181 22795 6135 5965 6520 64782 10572 10549 57650 6045 273 10413 3714 2241 6674 316 10274 1108 55837 | 736 |
| Capillaries disease | VEGFA; MMP2; CRP; EDN1; CD14; EDNRA; ENG; SMAD4; F8; BMPR2 | 7422 4313 1401 1906 929 1909 2022 4089 2157 659 | 10 |
| Carcinoma | IGF2BP3; ITGAE; SGK1; HSP90B1; MSLN; C19ORF33; RAD9A; SET; PGRMC1; BCL2L10; INPPL1; ATP7B; ECM1; MT1A; IRF8; POLR2F; PDCD4; ID3; TSHR; XPO1; LAMB1; TPM3; TOPORS; ERP29; THADA; MDC1; MAP4K4; MCM6; TRIP6; ATP12A; VASH1; RPL22; SAV1; CHD1L; C15ORF55 | 10643 3682 6446 7184 10232 64073 5883 6418 10857 10017 3636 540 1893 4489 3394 5435 27250 3399 7253 7514 3912 7170 10210 10961 63892 9656 9448 4175 7205 479 22846 6146 60485 9557 256646 | 35 |

| | | | |
|---|---|---|---|
| Cardiovascular disease | SELE; ADIPOQ; AGER; AGTR1; MTHFR; MMP2; AGT; IL6; F5; F2; CRP; ACE; APOE; SELP; LIPC; LPA; AGTR2; UTS2; CYBA; ANGPT1; ADD1; F7; ATP5J; GSTM2; AMPD1; PDE4D; ACVRL1; IGF1; SERPINE1; FGB; MGP; FURIN; ABCA1; APOA1; FOXC2; PLTP; PTX3; CETP; KDR; ANXA2; | 6401 9370 177 185 4524 4313 183 3569 2153 2147 1401 1636 348 6403 3990 4018 186 10911 1535 284 118 2155 522 2946 270 5144 94 3479 5054 2244 4256 5045 19 335 2303 5360 5806 1071 3791 302 9342 10928 2266 | 43 |
| Celiac disease | ABP1; MBL2; TLR4; HLA-DQA1; MYO9B; S100B; SELE; HP; MICA; FCGR2A; IL10; IL12B; CTLA4; CELIAC2; IL1B; CCR9; FAS; MIF; IL2; PARP1; HLA-DQB1; IFNGR2; IRF1; DLG5; CD28; PTPN22; IL21; ICAM1; BAK1; KIR2DL5B; MLN; FASLG; IFNG; IL18RAP; TNF; BCL2; HNMT | 26 4153 7099 3117 4650 6285 6401 3240 4276 2212 3586 3593 1493 317782 3553 10803 355 4282 3558 142 3119 3460 3659 9231 940 26191 59067 3383 578 553128 4295 356 3458 8807 7124 596 3176 | 37 |
| Central nervous system disease | SNCA; APP; MAPT; CASP3; TYMS; NEFL; SCN1A; SPAST; CHRM2; LGI1; CLN9; IDS; GJA3; STH; OLIG2; MIB1 | 6622 351 4137 836 7298 4747 6323 6683 1129 9211 497231 3423 2700 246744 10215 57534 | 16 |
| Cerebellar disease | MET; MAZ; CACNA1A; REST; ATXN3 | 4233 4150 773 5978 4287 | 5 |
| Cerebral palsy | MBL2; IL8; IL6; IGF1; PLP1; IL18; F5; IGFBP3 | 4153 3576 3569 3479 5354 3606 2153 3486 | 8 |
| Cerebrovascular disorder | GSTM1; MTHFR; CYBA; F5; APP; LTC4S; APOB; IL1RN; APOE; ADIPOQ; FGA; BCR; ESR1; APOA4; GSTT1; F13A1; IL2; CLOCK; TRNS1; MYT1; SLC1A2 | 2944 4524 1535 2153 351 4056 338 3557 348 9370 2243 613 2099 337 2952 2162 3558 9575 4574 4661 6506 | 21 |
| Cervical cancer | SLPI; INS; TNF; SLC2A3; GATA3; HES1; DHRS4; SERPINH1; BLCAP; HES5; SGMS1; ERPL2; CTLA4; IL6; ATP2A2; TLR3; TIMP1; SOCS3; IL6ST; PKP1; CSF3; CSF2; ST6GAL1; IFI27; CAMK2A; EBF3; RPS12; MRC2; ATP2A3; SPINK5; RHOBTB1; CCNL1; IRX1; IFI16; IL1A; IL1B; HLA-B; IGF1; IFNG; AKR1B10; HLA-A; IL10; CCR5; FASLG; IRF1; MICA; IL1RN; CD28; HSPD1; MPO; MMP12; SIX1; RNASEN; TIMP4; MYOD1; CA12; SLC4A2; SLC9A1; C1GALT1C1; ITGA5; COX2; ICAM3; IL18R1; IFNA17; ENOX2; LAMP3; KLK8; SLC46A2; HOXC10; EPHA1; [illegible] | 6590 3630 7124 6515 2625 3280 10901 871 10904 388585 259230 2083 1493 3569 488 7098 7076 9021 3572 5317 1440 1437 6480 3429 815 253738 6206 9902 489 11005 9886 57018 79192 3428 3552 3553 3106 3479 3458 57016 3105 3586 1234 356 3659 4276 3557 940 3329 4353 4321 6495 29102 7079 4654 771 6522 6548 29071 3678 4513 3385 8809 3451 10495 27074 11202 57864 3226 2041 4714 2059 2188 29947 | 74 |
| Charcot-Marie-Tooth disease | NDRG1; GJB1; GARS; EMP2; NEFL; LMNA; EGR2; ERBB2; MFN2; LITAF; MPZ; ERBB3; PRX; PMP22; MTM1; KIF20A; HSPB1; | 10397 2705 2617 2013 4747 4000 1959 2064 9927 9516 4359 2065 57716 5376 4534 10112 3315 3236 8898 | 19 |

| | | | |
|---|---|---|---|
| Cholangiocarcinoma | FASLG; MMP3; MICA; SOCS3; TTR; RAD51AP1; SAFB | 356 4314 4276 9021 7276 10635 6294 | 7 |
| Cholangitis | FAS; TLR3; KRT18; CXCL16 | 355 7098 3875 58191 | 4 |
| Cholelithiasis | ESR1; LRPAP1; ABCG8; ABCG5; LEPR; CCK; APOC1; APOE; NR1H3; VIPR2; LEP; APOB; APOA1; AR; ESR2; MUC5AC; APOC3; EGFR; NR1H4; MICA; MLN; PLA2G2A; VIP; ADRB3; | 2099 4043 64241 64240 3953 885 341 348 10062 7434 3952 338 335 367 2100 4586 345 1956 9971 4276 4295 5320 7432 155 339 | 25 |
| Cholestasis | MX1; FASLG; CASP3; MUC5AC; CLDN1; SOX2; NR1H4; ABCC4; GH1; MME; ACTG2; NR1H3; | 4599 356 836 4586 9076 6657 9971 10257 2688 4311 72 10062 200931 | 13 |
| Chondrosarcoma | AGER; TNC; MMP3; INHBA; SMO; TFG; SIX3 | 177 3371 4314 3624 6608 10342 6496 | 7 |
| Chordoma | LOC652799 | 652799 | 1 |
| Choriocarcinoma | JAK2; LNPEP; ASCL2; INSR; TIMP3; IGF2; IFNG; ETS2; LEP; ANG; RORA; HTR2A | 3717 4012 430 3643 7078 3481 3458 2114 3952 283 6095 3356 | 12 |
| Chorioretinitis | IL8; CFLAR | 3576 8837 | 2 |
| Chronic fatigue syndrome | HTR2A; PTGS2; ACE; SMAD7; WEE1; RNASEL; SLC6A4; | 3356 5743 1636 4092 7465 6041 6532 866 | 8 |
| Chronic obstructive airway disease | ADM; GC; IL1B; GSTP1; IL13; MMP9; GSTO1; IL6; CXCR6; CD8A; ADRB2; CYP1A2; SLPI; ATP2A2; SPINT1; NAT2; ITGAM; EPHX1; CAT; IL8; MMP1; IL10; CAMP; IL27; PARP1; CXCL10; MUC5AC; C3AR1; ATP2A1; ADORA2A; GSTT1; COPD; SLC16A4; PLAU; TLR4; PLA2G2D; LEP; SLC2A4; SOD2; KDR; IL10RA; CXCL11; GSTM1; ICAM1; CCR5; IL18; IL1RN; MAPK14; EPO; ITGAL; ACE; ADIPOQ; SFTPD; C5AR1; MBL2; FGFR1; PDE4A; TLR2; TIMP1; C4A; PDE4D; SLC6A4; SFTPB; GCLC; CKMT1B; ITGB2; SOD3; VEGFA; CLCA1; CCL11; MMP12; CCL5; CYP1A1; SFTPA1; ELN; GPR182; PARK7; | 133 2638 3553 2950 3596 4318 9446 3569 10663 925 154 1544 6590 488 6692 10 3684 2052 847 3576 4312 3586 820 246778 142 3627 4586 719 487 135 2952 260431 9122 5328 7099 26279 3952 6517 6648 3791 3587 6373 2944 3383 1234 3606 3557 1432 2056 3683 1636 9370 6441 728 4153 2260 5141 7097 7076 720 5144 6532 6439 2729 1159 3689 6649 7422 1179 6356 4321 6352 1543 653509 2006 11318 11315 6556 1401 4323 | 80 |
| Chronic progressive external ophthalmoplegia | SLC25A4; POLG; C10ORF2; POLRMT; RNR2; TRNI | 291 5428 56652 5442 4550 4565 | 6 |
| Chronic rejection of renal transplant | PDGFD; CCL5; CTGF; GAS6; TNFRSF8; AGT; CCR5; CDKN1A; BMP7; MARK2; CXCL9; ACE; MMP2; MTHFR; CXCL10; ICOS | 80310 6352 1490 2621 943 183 1234 1026 655 2011 4283 1636 4313 4524 3627 29851 | 16 |
| Chronic simple glaucoma | FAS; CYP1B1; OPTN; MYOC; TBK1; ACE; GSTM1; LOXL1; GLC1H; FASLG; OPTC; APOE; IL1RN; MTHFR; CDKN1A; GSTT1; EPO; CPNE1; SERPINE1; ELN; NRG2; GLC1C | 355 1545 10133 4653 29110 1636 2944 4016 399564 356 26254 348 3557 4524 1026 2952 2056 8904 5054 2006 9542 2723 | 22 |

| | | | |
|---|---|---|---|
| Cirrhosis | IGF1; RBP4; FIBP; APOE; CRP; WT1; INS; FGF2; SOD2; BMP7; ADAMTS13; ADIPOQ; KRT8; APOH; GFER; KRT18; MTHFR; LEP; IGFBP1; SELP; MMP3; TYMP; SLC2A4; RETN; AQP2; MDM2; RBP1; ADIPOR2; IGFBP3; AGT; F3; F8; TNFRSF8; EGF; CDKN2A | 3479 5950 9158 348 1401 7490 3630 2247 6648 655 11093 9370 3856 350 2671 3875 4524 3952 3484 6403 4314 1890 6517 56729 359 4193 5947 79602 3486 183 2152 2157 943 1950 1029 | 35 |
| Clear cell carcinoma | ADFP; KLF10; INHBA; CD3E; MITF; ATF1; ABCF2 | 123 7071 3624 916 4286 466 10061 | 7 |
| Cleft palate | IRF6; FGFR1; MSX1; FOXE1; FGF1; BMP4; MTR; ARNT2; RYK; PVRL1; GAD1; MTHFD1; NAT1; MTHFR | 3664 2260 4487 2304 2246 652 4548 9915 6259 5818 2571 4522 9 4524 | 14 |
| Cockayne syndrome | ERCC6; APEX1; ERCC5; LMNA; ERCC8; ERCC2 | 2074 328 2073 4000 1161 2068 | 6 |
| Colon cancer | SLC26A1; B4GALNT1; ASCL2; GLP2R; LTA; SLC6A4; HTR2A; HTR3A; HTR2B; TLR4; CEACAM5; CALR; BCHE; APOA1; SLC4A1; ACSL4; ANXA5; PMP22; PPM1H; LGALS4; MMP3; SLC16A4; DEFA3; DEFA1; SEPT4; UCHL1; SLC6A14; SLC16A7; FES; IL4; CRP; NOX1; FGF19; TNKS2; DLL1; CALB2; CKB; FBLN1; SAA1; GGT1; SELL; TNF; FASLG; NFATC1; SELP; HSPD1; CASR; CLCA1; LMNA; EP300; SDC2; CASP6; OLFM4; RNF14; HACE1; LGALS2; CXCR5; TMSB4X; JAK3; MAP4K1; AATF; EFNB3; SNAI1L1; CTBP1; NFATC2; RPS6KA6; SELE; ICAM1; IGF1; AKR1B1; CTLA4; ITGA2; HLA-A; IL15; PLA2G4A; ATP2A2; NOD1; TIMP1; EDN1; EPHX1; LEP; CAV2; MMP13; HSF1; ADCYAP1; DDIT3; HSPA1A; PTPN1; UCP2; SHC1; VWA2; ACP1; HRH4; HPRT1; LGALS3BP; ROCK1; CDC16; TSGA10; PRDX1; GCNT3; HPGD; HLTF; CLU; HSPA8; C1GALT1C1; OGFR; DTYMK; ENPP7; ADAMTS13; PMEPA1; RPL29; FDPS; SRPRB; METAP2; LCP1; ACHE; TRAF1; NKRF; E2F4; DKK4; DDX5; SLC9A3R2; IFITM3; HRH2; DSTN; AIFM1; CDCP1; MGAT5; LPAR1; MSR1; CITED2; | 10861 2583 430 9340 4049 6532 3356 3359 3357 7099 1048 811 590 335 6521 2182 308 5376 57460 3960 4314 9122 1668 1667 5414 7345 11254 9194 2242 3565 1401 27035 9965 80351 28514 794 1152 2192 6288 2678 6402 7124 356 4772 6403 3329 846 1179 4000 2033 6383 839 10562 9604 57531 3957 643 7114 3718 11184 26574 1949 10415 1487 4773 27330 6401 3383 3479 231 1493 3673 3105 3600 5321 488 10392 7076 1906 2052 3952 858 4322 3297 116 1649 3303 5770 7351 6464 340706 52 59340 3251 3959 6093 8881 80705 5052 9245 3248 6596 1191 3312 29071 11054 1841 339221 11093 56937 6159 2224 58477 10988 3936 43 7185 55922 1874 27121 1655 9351 10410 3274 11034 9131 64866 4249 1902 4481 10370 6059 3269 10521 64682 79816 1072 10825 28517 3816 3934 27429 3678 6642 10800 4145 80279 2150 64386 374907 27044 8817 767 1139 8996 10659 2729 3092 10953 657 5424 9170 3694 9370 177 3371 3458 5054 1604 64127 10097 4629 8824 1548 1234 3620 1524 3577 4282 3604 3557 3689 3953 1636 1044 4319 7082 2252 1435 217 1445 7295 567 11132 3291 3290 5788 688 7293 3456 57817 3579 140 27030 255313 6303 1119 586 27076 29117 9069 8836 10666 57188 338377 10984 55872 1081 | 284 |
| Combined immunodeficiency | JAK3; DCLRE1C; JAK2; ADA; CD3E; CD3D | 3718 64421 3717 100 916 915 | 6 |
| Common cold | DHRS4; SCN4A; IL8RB; BRAF; SELP; CLCN1; PARP1; AKR1B1; ROCK1; TRPM8; KCNJ6; ADRA2C; HSPA1A; LPL | 10901 6329 3579 673 6403 1180 142 231 6093 79054 3763 152 3303 4023 | 14 |

| | | | |
|---|---|---|---|
| Common variable immunodeficiency | SH2D1A; TNFSF13B; BTK; TNFRSF8; ICOS; CD19; CD86; IL7 | 4068 10673 695 943 29851 930 942 3574 | 8 |
| Common wart | HLA-DQB1; HTRA2; EGFR; LATS1; HLA-DQA1 | 3119 27429 1956 9113 3117 | 5 |
| Communicable disease | IFNG; IL12B; IL13; CCR5; TLR4; CD14; TLR3; TLR2; MBL2; SLAMF1; FCGR2A; IFNGR1; NR1H3; IL12RB1; MRC1; RAG2; RAG1; LBP; SLC22A18AS; LUZP6; | 3458 3593 3596 1234 7099 929 7098 7097 4153 6504 2212 3459 10062 3594 4360 5897 5896 3929 5003 767558 79132 | 21 |
| Conduct disorder | CFTR; ALDH2; CLCNKA; GJB6; KCNQ4; TMPRSS3; MYO1A; AVPR1A; DYX8 | 1080 217 1187 10804 9132 64699 4640 552 406874 | 9 |
| Congenital abnormality | GCG; ATP7B; TIMP3; NOD2; SCN5A; MLH1; SHH; MGMT; APC; HIF1A; REG1A; CDKN2A; IL10; TLR4; IL8RA; RB1; HSPA1B; EFNB2; MMP3; CTNNB1; EPO; IL6ST; ALPL; SNCA; CTGF; MSX1; FOXE1; BCL2; CCND1; NF1; EGR1; TNFSF13B; PAFAH1B1; SFTPB; IAPP; HSD11B2; REG1B; AKT2; HSD11B1; PTPRC; VWF; EZR; SERPINB2; FOXC2; RDX; PINK1; CD24; TTR; MSN; EP300; IL7R; GHR; CDKN2B; HPSE; GJB2; ABCA4; BIRC5; FHIT; SOX2; SPINT2; PARK2; LAMA5; JAG1; ZEB2; RARB; CDKN1C; CD226; KCNQ1OT1; ATF3; CYP1B1; EPHB2; BTRC; CD247; KCNA5; VHL; KLK1; ABL1; LILRB4; PLG; CFC1; H19; CGB; INSL3; TPM2; FOXO1; MUSK; FANCD2; LAMB1; BCR; BACH1; SFTPC; PLP1; NDP; FOXC1; RS1; PAX6; SALL4; HOXA1; SPAST; GHRHR; CACNA1A; GALE; TOR1A; ACTB; NFIA; MYH7; ABCA3; ATP2A1; LMX1B; PKD2; CLCN5; LHB; WNT7A; LAMC1; NLRP7; KAL1; PROK2; GNA12; RYR1; AP3M2; CLN8; GLI3; LAMA2; ACTC1; MLL; EIF2B5; ROBO3; CHAT; ATP7A; TYR; IL10RB; KCNJ8; TUSC2; AIRE; LIG4; PRDM1; | 2641 540 7078 64127 6331 4292 6469 4255 324 3091 5967 1029 3586 7099 3577 5925 3304 1948 4314 1499 2056 3572 249 6622 1490 4487 2304 596 595 4763 1958 10673 5048 6439 3375 3291 5968 208 3290 5788 7450 7430 5055 2303 5962 65018 100133941 7276 4478 2033 3575 2690 1030 10855 2706 24 332 2272 6657 10653 5071 3911 182 9839 5915 1028 10666 10984 467 1545 2048 8945 919 3741 7428 3816 25 11006 5340 55997 283120 1082 3640 7169 2308 4593 2177 3912 613 571 6440 5354 4693 2296 6247 5080 57167 3198 6683 2692 773 2582 1861 60 4774 4625 21 487 4010 5311 1184 3972 7476 3915 199713 3730 60675 2768 6261 10947 2055 2737 3908 70 4297 8893 64221 1103 538 7299 3588 3764 11334 326 3981 639 6385 7480 9401 2131 1441 3284 378708 242 387264 3848 4014 79840 10059 5359 3195 3239 10652 57761 9444 64220 4291 2138 5318 326625 10736 348938 845 3295 5309 10585 11146 4990 889 6468 7305 4594 2132 6500 10008 100049707 | 176 |
| Congenital heart disease | GATA4; ACTC1; SLC6A4; PTPN11; BMPR2; OXTR; CACNA1H; NKX2-5; ASMT | 2626 70 6532 5781 659 5021 8912 1482 438 | 9 |
| Connective tissue disease | FBN1; MMP1; ACE; SNRNP70; ACVRL1; CRTAP; CEBPB; MUC2; ELANE; VIM; BSG; TIAM1; PLAG1; NAGLU; SGSH; IDUA | 2200 4312 1636 6625 94 10491 1051 4583 1991 7431 682 7074 5324 4669 6448 3425 | 16 |
| Copper metabolism disorder | XIAP; ATP7B | 331 540 | 2 |

| | | | |
|---|---|---|---|
| Corneal disease | SP1; SOD3; BIRC5; TACSTD2; KRT12; CHST6; PITX2 | 6667 6649 332 4070 3859 4166 5308 | 7 |
| Coronavirus infection | ACE2; ANPEP; NUFIP2; | 59272 290 57532 9727 | 4 |
| Craniosynostosis | FGFR3; TWIST1; FGFR1; FGFR2; JUN; NELL1 | 2261 7291 2260 2263 3725 4745 | 6 |
| Creutzfeldt-Jakob syndrome | CTSD; CHGA; ADAM10; AIF1 | 1509 1113 102 199 | 4 |
| Cushing syndrome | GIPR; HSD11B2; IL18; IL1RN; IL6; POMC; IL8; HSD11B1; LHCGR; GH1; GIP | 2696 3291 3606 3557 3569 5443 3576 3290 3973 2688 2695 | 11 |
| Cutaneous fibrous histiocytoma | SFTPB; SFTPA1B; SERPINH1 | 6439 6435 871 | 3 |
| Cystic fibrosis | ADRB2; CSK; CFTR; BPI; IL10; CLCA1; ADORA2A; TLR5; IL1B; IL1A; GC; TLR4; IL8; GSTP1; ND4; GOPC; MUC1; SCNN1G; TLR2; MMP9; CCL3; GNB2L1; DDIT3; FCGR2A; ARSB; BEST1; KLF2; SFTPB; ICAM1; VAMP3; SLC9A3R1; CCL17; SFTPD; CAMP; PLAU; CLCA4; TRPV4; SLC15A2; SP1; SCNN1B; SFTPA1B; FURIN; IL1RN; SLPI; IL17A; IL18; PLA2G2A; TXN; MASP2 | 154 1445 1080 671 3586 1179 135 7100 3553 3552 2638 7099 3576 2950 4538 57120 4582 6340 7097 4318 6348 10399 1649 2212 411 7439 10365 6439 3383 9341 9368 6361 6441 820 5328 22802 59341 6565 6667 6338 6435 5045 3557 6590 3605 3606 5320 7295 10747 | 49 |
| Cystitis | ITIH4 | 3700 | 1 |
| Cytomegalovirus infection | B2M; TLR2; RAB8A; SLPI; EGF; CX3CL1; EGFR; PML; CD83; CX3CR1; TYMS; ALOX5; EIF4G1; | 567 7097 4218 6590 1950 6376 1956 5371 9308 1524 7298 240 1981 9118 7099 | 15 |
| Deafness | F5; DFNB40; PTPRJ; TMIE; CRYM; TRIOBP; USH1C; MYO7A; SLC17A8; DFNB72; DPH3; DFNA53; ACTG1; GSTM1; GSTT1; GSTP1; PTPN11; EPO; ND4; CAT; RDX; GJB2; JAG1; RNR1; GATA3; PMP22; TRNS1; GJD2; DFNB59; OTOF; CDH23; EYA4; CLCNKA; GJB6; KCNQ4; TMPRSS3; KCNQ1; ATP6; SLC12A7; MYO6; MYH9; MYO1A; SALL4; CLCNKB; [illegible] | 2153 379003 5795 259236 1428 11078 10083 4647 246213 791116 285381 353347 71 2944 2952 2950 5781 2056 4538 847 5962 2706 182 4549 2625 5376 4574 57369 494513 9381 64072 2070 1187 10804 9132 64699 3784 4508 10723 4646 4627 4640 57167 1188 448962 65217 3198 3753 | 48 |
| Degenerative disc disease | SLC2A3; SLC2A9; SLC2A1; ACAN; IL1R1; MMP2 | 6515 56606 6513 176 3554 4313 | 6 |
| Demyelinating disease | GJC2; S100B; ITGA4; PTPRZ1; IGFBP2 | 57165 6285 3676 5803 3485 | 5 |
| Dental enamel hypoplasia | NF1; KLK4; ENAM; MMP20; AMELX; CKAP4 | 4763 9622 10117 9313 265 10970 | 6 |

| | | | |
|---|---|---|---|
| Dental plaque | APP; ADIPOQ; CX3CR1; FASLG; CRP; ICOS; GJA1; OSM; SELP; IL6; C3; MAPT; SERPINA3; IL1B; SERPINE1; PLA2G2A; INS; MMP10; APOD; BCL2; LEP; PROCR; IL18; BAK1; MMP1; BDNF; TIMP1; ANGPT2; HSPD1; ESR2; EPS15; SNCA; ALOX5; PKP1; EGR1; SCN2A; CX3CL1; ICAM1; PTGS2; PTGES; ST8SIA1; SLC30A5; SORL1; HSPB1; PIN1; ESR1; IL10; FAS; LTA4H; BCL2L1; SPP1; F3; PAK1; TJP1; SELE; MMP9; ELANE; IL8; F2; APOE; GPR132; CD163 | 351 9370 1524 356 1401 29851 2697 5008 6403 3569 718 4137 12 3553 5054 5320 3630 4319 347 596 3952 10544 3606 578 4312 627 7076 285 3329 2100 2060 6622 240 5317 1958 6326 6376 3383 5743 9536 6489 64924 6653 3315 5300 2099 3586 355 4048 598 6696 2152 5058 7082 6401 4318 1991 3576 2147 348 29933 9332 | 62 |
| Depression | C5; SNCA; PTX3; AGT; BDNF; HTR1A; SLC6A3; S100A10; SLC6A4; CTNNB1; PDLIM5; SGCE; IGF1R; HTR1B; FKBP5; GFAP; ARRB1; DISC1; APOE; AKR1C4; CAMK2B; P2RX7; ITGB1; C3; IL8; TPH1; MTNR1A; MTHFR; CRHR1; PDE1A; CAMK2A; TTR; RTN4; CCL2; DUSP1; ADIPOQ; BAG4; CHRM2; MAOA; CLOCK; HTR2A; KCNK2; OXT; ADORA2A; ITGA2; SELP; ESR1; PDE4B; GDF15; TPPP; AGTR1; AR; MAPT; APAF1; POMC; WFS1; APOA4; ACE | 727 6622 5806 183 627 3350 6531 6281 6532 1499 10611 8910 3480 3351 2289 2670 408 27185 348 1109 816 5027 3688 718 3576 7166 4543 4524 1394 5136 815 7276 57142 6347 1843 9370 9530 1129 4128 9575 3356 3776 5020 135 3673 6403 2099 5142 9518 11076 185 367 4137 317 5443 7466 337 1636 | 58 |

| | | | |
|---|---|---|---|
| Dermatitis | IL1B; TNF; HTR1A; NAT2; ACE; IL16; HIF1A; IL1RN; HLA-DQB1; CTNNB1; BAX; KDR; RXRA; ESRRA; ESRRG; RXRG; SELE; HLA-B; IL6; NOD2; CCL26; HSPB1; CD274; CCL21; MAPK14; CD83; MYO9B; IL6ST; APOE; OSM; HSPD1; LTF; CCL18; IL24; LIF; SELPLG; CTF1; IL19; CCL1; CCL19; IL1F6; IL31; NANOS2; CTSL1; CCL5; IFNG; CTLA4; BDNF; IL15; IL12B; CALCA; SLC6A4; HTR2A; GSTM1; GSTT1; GSTP1; IL13; IL4; IL18; CX3CR1; CD14; TLR2; MBL2; VIP; CXCL9; TIMP1; IL5; IL4R; APOC1; ADRB2; SELP; CX3CL1; PLAU; CASP3; AQP3; CCL17; CMA1; CSF2; SMAD3; HAVCR1; DEFB103A; ALDH1A1; CDKN1A; KITLG; SMAD2; PLAUR; MIR21; S100A11; CCL22; GATA3; CYSLTR1; F2RL1; IFNA2; RNASE3; TNFSF8; IL12RB1; BCL2A1; NPSR1; FCER1A; GPR44; CCR3; IL13RA1; RNASE2; IL1RL1; CCL13; TBC1D4; CCL23; CSTA; SMPD1; REL; TNFSF18; SPINK5; MIR146A; COL29A1; IVL; SMPD2; CTSE; | 3553 7124 3350 10 1636 3603 3091 3557 3119 1499 581 3791 6256 2101 2104 6258 6401 3106 3569 64127 10344 3315 29126 6366 1432 9308 4650 3572 348 5008 3329 4057 6362 11009 3976 6404 1489 29949 6346 6363 27179 386653 339345 1514 6352 3458 1493 627 3600 3593 796 6532 3356 2944 2952 2950 3596 3565 3606 1524 929 7097 4153 7432 4283 7076 3567 3566 341 154 6403 6376 5328 836 360 6361 1215 1437 4088 26762 55894 216 1026 4254 4087 5329 406991 6282 6367 2625 10800 2150 3440 6037 944 3594 597 387129 2205 11251 1232 3597 6036 9173 6357 9882 6368 1475 6609 5966 8995 11005 406938 256076 3713 6610 1510 79924 4014 406911 1233 | 121 |
| Diabetes insipidus | AVP | 551 | 1 |

| | | | |
|---|---|---|---|
| Diabetes mellitus | FGF2; EPO; CTGF; ALDRL2; PLXDC1; VEGFA; AKR1B1; SUMO4; RELA; PLAT; PARP1; TXN; KCNJ11; UCP2; GFPT1; AQP2; CCR5; CD86; IGFBP1; CST3; EGF; HLA-DPB1; GAD2; AGTR2; ERVK3; IDDM2; VWF; EIF2AK3; G6PC2; ERVK2; NEUROD1; INS; NAMPT; AGER; SLC2A4; HP; LPL; HNF4A; GIP; GCK; IAPP; CAPN10; UCP1; JUN; MAPK14; APOE; GJA1; MPO; TIMP2; SLC2A1; TRPC1; TSC22D1; BMP7; MMP8; SLC12A3; DEFA3; ELMO1; FABP1; DEFA1; GREM1; ANKRD1; PON1; XYLT2; HPSE; XYLT1; NOX1; MCAM; ACTG2; MDK; SDC2; DCN; SELE; ICAM1; GGT1; WFS1; MTHFR; SELL; CTLA4; SERPINE1; TNF; PLA2G4A; PTPN22; ATP2A2; GSTP1; IL13; CCL2; ALB; NAT2; MICA; HLA-DQB1; CRP; ITGB2; TXNIP; IL4R; HLA-DQA1; F3; CD163; GC; LIPE; ADM; CAT; CAPN1; AHSG; IMPDH2; IP6K1; BCHE; KIR2DS2; GH1; ADRA2B; LMX1A; LIPC; REG1B; KIR2DL2; AKT2; FGF21; PCK1; LPA; ND1; HSPA1A; ABCG1; NUDT1; PCBD1; ABCA1; CYP27B1; TRNL1; MT1A; PBX1; BTC; HSD11B1; WRN; IL1A; SCG5; TNMD; FTO; RBP4; RETN; CASQ1; INPPL1; PLIN; AGTR1; | 2247 2056 1490 233 57125 7422 231 387082 5970 5327 142 7295 3767 7351 2673 359 1234 942 3484 1471 1950 3115 2572 186 2088 3401 7450 9451 57818 2087 4760 3630 10135 177 6517 3240 4023 3172 2695 2645 3375 11132 7350 3725 1432 348 2697 4353 7077 6513 7220 8848 655 4317 6559 1668 9844 2168 1667 26585 27063 5444 64132 10855 64131 27035 4162 72 4192 6383 1634 6401 3383 2678 7466 4524 6402 1493 5054 7124 5321 26191 488 2950 3596 6347 213 10 4276 3119 1401 3689 10628 3566 3117 2152 9332 2638 3991 133 847 823 197 3615 9807 590 100132285 2688 151 4009 3990 5968 3803 208 26291 5105 4018 4535 3303 9619 4521 5092 19 1594 4567 4489 5087 685 3290 7486 3552 6447 64102 79068 5950 56729 844 3636 5346 185 2169 6352 405 4313 57016 3600 5325 3593 6566 4049 6469 4255 2944 3315 3091 2952 1029 1956 3565 6696 3350 7099 7098 79602 4055 56244 6583 4153 3304 6584 2153 2147 7076 7494 59067 337 1906 3953 4277 341 1622 1543 2099 154 6403 5320 285 240 7082 920 1909 6647 10365 3667 6387 3291 367 1990 335 688 4036 3426 7430 5055 4547 9365 2650 345 3690 364 2303 6464 60412 5078 4092 283 10911 5443 3670 777 3416 3373 5798 2646 3684 9588 340706 7274 6319 434 | 361 |
| Disseminated cancer | IQGAP1; REG1A; HSPG2; MMP8; IL11RA; CHI3L1; B4GALT1; PAK6; WAS; WIPF1 | 8826 5967 3339 4317 3590 1116 2683 56924 7454 7456 | 10 |
| Disseminated intravascular coagulation | APOH; ELANE | 350 1991 | 2 |

| | | | |
|---|---|---|---|
| Down syndrome | GATA1; ARPP19; MTHFR; APP; PRDX6; DYRK1A; PRDX3; DOPEY2; TPTE; CASP3; MATR3; CCL2; SLC19A1; MTRR; AQP4; ENSA; RBM4; FABP3; JAK3; APOE; CHRNA7; IL2RA; MAPT; CAMP; REST; MTR; RUNX1; S100B; KCNJ15; BACH1; GFAP; CA2; KRT8; CBS; STMN1; INHA; NACA; ITSN1; SOD1; GDI2; NCAM1; ABCG1; SIM2; MAOA; PHYHIP; ESR1; KCNJ6; NPAS4; CHRNA4; LEP; CHRNB2; FAAH; NDUFS3; PRDX1; HINT1; HSPD1; IL6; ERF; DPYSL4; C21ORF2; ARSA; GNB2L1; HNRNPA2B1; ADAMTS1; PAK1; MTHFD1L; EDNRB; JAK2; CHRNA3; ERG; RFC1; GNLY; TNF; SNAP25; IMMT; PKNOX1; FABP7 | 2623 10776 4524 351 9588 1859 10935 9980 7179 836 9782 6347 6573 4552 361 2029 5936 2170 3718 348 1139 3559 4137 820 5978 4548 861 6285 3772 571 2670 760 3856 875 3925 3623 4666 6453 6647 2665 4684 9619 6493 4128 9796 2099 3763 266743 1137 3952 1141 2166 4722 5052 3094 3329 3569 2077 10570 755 410 10399 3181 9510 5058 25902 1910 3717 1136 2078 5981 10578 7124 6616 10989 5316 2173 | 77 |
| Drug abuse | ALDH2; GSTM1; GSTP1; BDNF; HTR2B; CHRNA7; ITGA2; ITGB1; DEFA1; ITGAV; CCKAR; HSPA4; MC2R; HS3ST2; PER3; HS3ST4; GDNF; BCL2; PIK3CG; SLC6A3; CARTPT; ANKK1; MITF; SLC4A7; IL1B; CALCA; HSPA1B; IL1RN; APOE; DBI; ADH1B; GAD2; POMC; ALDH1A1; ADH1C; CRH; NCAM1; DUSP8; SGCE; ADH4; RXRB; MGLL; GALR2; GABRG3; NRXN3; ZNF699; GALR1; ACN9; PNOC; REN; SCN5A; SLC6A4; MLH1; SHH; HIF1A; EGFR; CCR5; CD14; TLR2; IGF2; EPO; ABCC1; SH2D1A; CTGF; EGF; CD28; CCND1; ADRB2; C3; PLA2G2A; F3; HRAS; CD4; HSF1; B2M; APOA1; APOC3; UCP2; SLC6A2; TRPC1; SLC5A5; ERBB2; EPHA2; KCNH2; BRCA1; TNKS2; SLC22A3; INHA; SCN1A; DCLRE1C; FBN1; TP73; CACNA1F; SPAST; MAOA; HTR1B; FAAH; SLC1A2; KCND3; LMAN2; SCN4A; EPOR; KCNJ2; KAT2B; VTNR; HGS; GJA8; SLC6A9; DUSP3; CABP1; SLC6A1; | 217 2944 2950 627 3357 1139 3673 3688 1667 3685 886 3308 4158 9956 8863 9951 2668 596 5294 6531 9607 255239 4286 9497 3553 796 3304 3557 348 1622 125 2572 5443 216 126 1392 4684 1850 8910 127 6257 11343 8811 2567 9369 374879 2587 57001 5368 5972 6331 6532 4292 6469 3091 1956 1234 929 7097 3481 2056 4363 4068 1490 1950 940 595 154 718 5320 2152 3265 920 3297 567 335 345 7351 6530 7220 6528 2064 1969 3757 672 80351 6581 3623 6323 64421 2200 7161 778 6683 4128 3351 2166 6506 3752 10960 6329 2057 3759 8850 7449 9146 2703 6536 1845 9478 6529 4188 64781 2107 | 114 |
| Drug-Induced dyskinesia | GSTM1; GSTP1; ANKK1; CYP1A2; HTR2A; SOD2; APOE | 2944 2950 255239 1544 3356 6648 348 | 7 |
| Duane retraction syndrome | CHN1; HOXD3; SALL4; BHLHE22; FOXL2 | 1123 3232 57167 27319 668 | 5 |

| | | | |
|---|---|---|---|
| Eating disorder | LEPR; LEP; GHSR; INS; IL8; IL15; HTR3A; GSTP1; CDKN2A; SPP1; FGF2; MDM2; ITGAL; BCL2; APP; S100A8; HRAS; IAPP; GH1; ARHGEF1; SMAD3; VCAN; HSP90AA1; ATP2C1; ITIH2; NANOG; ADORA1; AKAP13; RAP1A; HTR3B; PCSK1; LMO3; | 3953 3952 2693 3630 3576 3600 3359 2950 1029 6696 2247 4193 3683 596 351 6279 3265 3375 2688 9138 4088 1462 3320 27032 3698 79923 134 11214 5906 9177 5122 55885 6492 | 33 |
| Ectodermal dysplasia | WNT10A; KRT14; PKP1; TRAF6; TRAF3; EDA2R; KRT85; GJB2; | 80326 3861 5317 7189 7187 60401 3891 2706 3248 | 9 |
| Embryoma | IL1A; NEUROD1; CTSL1; ADIPOQ; GGT1; INPPL1; AGTR1; TNC; IGF1; IFNG; AGT; CASP10; SERPINE1; TNF; IL6; HLA-A; IL8; IL15; ACTR3; SLC16A1; HTR3A; CES2; PITX1; IL4; IL2; IL10; IDO1; IL18; CD274; FASLG; TLR4; IRF1; KRT8; KRT18; SPINK1; SLC11A1; F5; MICA; F2; TIMP1; MASP2; IL21; CRP; EDN1; ACE; MICB; ECM1; MTR; IL6ST; IGFBP1; EPHX1; CST3; APOE; LEP; CD27; CTGF; FCGR2A; MUC5B; PLA2G2A; PIN1; F3; PAK1; CCL20; TNFSF13B; IL2RA; CD4; HSF1; ADCYAP1; GNB2L1; SLPI; SGK1; MC4R; B2M; CAT; GH1; BTC; AGTR2; ERVK3; KLF5; SHC1; IFNA1; TNFRSF4; TRPC6; CSF3; ACP1; CALD1; KEAP1; CHGA; PCSK2; P2RX7; GHR; GREM1; MCAM; DCN; HAMP; HPRT1; SMAD3; MT1F; ACSL4; TP53AIP1; GSTM3; ROCK1; BCAM; GLUL; FOXN3; FHL2; UCHL1; ASCL2; KCNQ1OT1; ATF3; BLM; GDF11; TRO; JAK1; HSPE1; CEACAM7; TMEM97; ARPC1B; CD247; ADH7; CSNK1E; MALT1; TNKS2; TRAF1; CFL1; LCN2; F2RL1; RNF14; JAK3; PCSK1; CHI3L1; B3GNT2; RNASEL; GIPC1; RAP1GAP; PDGFD; LILRB4; PLG; H19; | 3552 4760 1514 9370 2678 3636 185 3371 3479 3458 183 843 5054 7124 3569 3105 3576 3600 10096 6566 3359 8824 5307 3565 3558 3586 3620 3606 29126 356 7099 3659 3856 3875 6690 6556 2153 4276 2147 7076 10747 59067 1401 1906 1636 4277 1893 4548 3572 3484 2052 1471 348 3952 939 1490 2212 727897 5320 5300 2152 5058 6364 10673 3559 920 3297 116 10399 6590 6446 4160 567 847 2688 685 186 2088 688 6464 3439 7293 7225 1440 52 800 9817 1113 5126 5027 2690 26585 4162 1634 57817 3251 4088 4494 2182 63970 2947 6093 4059 2752 1112 2274 7345 430 10984 467 641 10220 7216 3716 3336 1087 27346 10095 919 131 1454 10892 80351 7185 1072 3934 2150 9604 3718 5122 1116 10678 6041 10755 5909 80310 11006 5340 283120 10761 4534 7173 4513 875 7514 79728 4204 361 11315 3305 5957 10461 8929 445 3438 255738 65997 57142 874 9173 10663 7301 3560 3206 6654 60675 4512 6368 3738 8878 4671 9821 3313 912 218 1553 317716 3838 10286 7320 3276 347252 6130 11279 11059 4691 2131 57099 10121 29947 1441 5364 4286 5116 26298 606293 25898 2734 366 10362 3225 407043 288 26038 6656 7272 79840 3561 752 3195 3601 282991 29128 79885 26468 7473 2619 468 | 263 |
| Emphysema | EMP1; C5ORF13; MMP1; IFNG; TNFRSF10A; CDKN2A; MMP9; MAEA; EPHX1; MMP12; MMP2; BAX; CTSS; EMP2; FGFR2; PCNA; FGF7; EGR1; GSTP1; ITGB6; TNFRSF10B; MET; MMP14; RAP1A | 2012 9315 4312 3458 8797 1029 4318 10296 2052 4321 4313 581 1520 2013 2263 5111 2252 1958 2950 3694 8795 4233 4323 5906 | 24 |
| Encephalitis | F2R; TLR3; APP; IFNB1; CCL3; ENO2; CLDN11; IL1RN; CCR5; MOG; MBP; APOE; BRSK2 | 2149 7098 351 3456 6348 2026 5010 3557 1234 4340 4155 348 9024 | 13 |

| | | | |
|---|---|---|---|
| Encephalopathies | SLC2A4; ATP7B; LOX; LOXL2; ATOX1; PNOC; MTRR; XBP1; APOE; APOB; DBT; PEX16; PEX5; GLUD1; ND1; TRNL1; MTTP; HPRT1; CHIT1; ENO1; OTC; CBS; FBN1; MECP2; C10ORF2; GLDC; EIF2B1; NDUFV2; NTNG1; GLS; FAH; ASS1; SLC25A13; GALE; GALT; PAH; MAOB; BCKDHA; BCKDHB; GLB1; ALS2; TRNA; APRT; GNPTAB; MCOLN1; OS9; ATP7A; SMPD1; NPC1L1; NPC2; [illegible] | 6517 540 4015 4017 475 5368 4552 7494 348 338 1629 9409 5830 2746 4535 4567 4547 3251 1118 2023 5009 875 2200 4204 56652 2731 1967 4729 22854 2744 2184 445 10165 2582 2592 5053 4129 593 594 2720 57679 4553 353 79158 57192 10956 538 6609 29881 10577 4580 | 51 |
| Endemic goiter | EDN1; CCND1; KCNJ6 | 1906 595 3763 | 3 |
| Endocrine gland cancer | PRPH2 | 5961 | 1 |
| Endocrine system disease | MEN1; IGF1; SHBG; BLM; RET; RYR1; CACNA1S | 4221 3479 6462 641 5979 6261 779 | 7 |
| Endometrial cancer | IRF1; CLU; ACTN1; MUC5B; MMP12; P2RX7; CRHR1; INHA; FOXP1; MUC8; ESRRA; ESRRG; ADIPOQ; IL6; TSC2; CRP; ACE; S100A8; GH1; SERPINB2; EP300; MSI1; FST; HSPE1; F2RL1; NR0B1; HOXA10; CASC2; KIAA1324; ARMC3; FSTL1; SLC29A2; PELP1 | 3659 1191 87 727897 4321 5027 1394 3623 27086 4590 2101 2104 9370 3569 7249 1401 1636 6279 2688 5055 2033 4440 10468 3336 2150 190 3206 255082 57535 219681 11167 3177 27043 | 33 |

| | | | |
|---|---|---|---|
| Endometriosis | IL6; IL8; CDKN2A; MDM2; CCND1; CSF2; CDKN1A; KIR3DL1; KIR2DL3; IL1A; IL1B; ICAM1; ADIPOQ; HLA-B; CCL5; ARNT; HP; TNC; ENG; IGF1; IFNG; IGFBP3; MMP1; TNF; HLA-A; IL15; LTA; PTPN22; XRCC1; GSTM1; GSTT1; GSTP1; EGFR; IL4; PTGES; IL10; IL18; CCL2; FGF2; FASLG; KLRC1; KLRD1; CCR1; IL8RA; MIF; RB1; MMP9; MST1R; NAT2; MMP3; F2; TIMP1; MAPK14; IL1RN; IL17A; LEPR; ACE; RELA; IL11; MMP7; CTNNB1; EPO; IGFBP1; CYP1A1; ESR1; FGF1; EGF; C3; ANGPT2; ESR2; PTGS2; MMP13; PLAU; IL16; TNFSF13B; TIMP2; MMP12; LTF; KRAS; GC; CAT; AHSG; GH1; AR; CFI; EZR; ANG; IL6R; ANGPT1; CLOCK; MMP14; HPSE; MDK; AKAP13; IL8RB; MSI1; FSTL3; BIRC5; BRCA2; FST; L1CAM; WT1; EBAG9; CYR61; F2R; KDR; CXCL5; BRCA1; TRO; FGFR2; CYP1B1; HSD17B2; AHR; F2RL1; IL1R1; PDGFA; LIF; STS; LAMB1; HSD17B1; C5; GAS6; NR0B1; CYP19A1; FOXP1; IL12RB1; CCL1; AXL; AHRR; C9; LHB; S100A13; WNT7A; GAP43; IL2RB; HOXA10; MST1; EMX2; OXTR; EPOR; | 3569 3576 1029 4193 595 1437 1026 3811 3804 3552 3553 3383 9370 3106 6352 405 3240 3371 2022 3479 3458 3486 4312 7124 3105 3600 4049 26191 7515 2944 2952 2950 1956 3565 9536 3586 3606 6347 2247 356 3821 3824 1230 3577 4282 5925 4318 4486 10 4314 2147 7076 1432 3557 3605 3953 1636 5970 3589 4316 1499 2056 3484 1543 2099 2246 1950 718 285 2100 5743 4322 5328 3603 10673 7077 4321 4057 3845 2638 847 197 2688 367 3426 7430 283 3570 284 9575 4323 10855 4192 11214 3579 4440 10272 332 675 10468 3897 7490 9166 3491 2149 3791 6374 672 7216 2263 1545 3294 196 2150 3554 5154 3976 412 3912 3292 727 2621 190 1588 27086 3594 6346 558 57491 735 3972 6284 7476 2596 3560 3206 4485 2018 5021 2057 87 3915 284217 7518 2254 | 145 |
| Endometrium cancer | ADRB3; INHBA; JAZF1; IL1A; AKR1B10; NR1I2; LEPR; CYP27B1; VCAN; LLGL1; HSD17B1; KCNN4; COX1; LHCGR; ETV5; CASC2; KIAA1324 | 155 3624 221895 3552 57016 8856 3953 1594 1462 3996 3292 3783 4512 3973 2119 255082 57535 | 17 |

| | | | |
|---|---|---|---|
| Enteritis | ALPI; BTNL2; NOD2; TNFRSF9; TLR4; ITGAL; SLC22A4; S100A12; CCL5; MEFV; LTA; MBL2; CFTR; HSPA1B; TLR5; F13A1; VIP; IDO1; IRGM; IL13; IRAK3; SLC11A1; NOD1; SLC22A5; PDX1; ICAM1; EFNB2; MST1R; IL8; CXCL16; IL4; SPP1; CX3CR1; ARHGDIA; NR1I2; IL18; NAT2; MTRR; CD14; CDX2; CXCL6; CXCL9; TLR2; MMP3; IFNG; PTPN22; CCNY; IL6; DLG5; F5; KCNN1; MICA; KRT8; F2; MMP1; IL10; TIMP1; MAPK14; TNF; TWIST1; IL3RA; FCGR1A; IL1RN; HLA-DQB1; XBP1; CD86; CSF1R; CD83; MASP2; TNFSF15; IL21; VIPR1; MYO9B; CEACAM6; IL18BP; C13ORF23; FCGR3A; APOA4; CRP; CTLA4; ITGAE; MLH1; ITGB2 | 248 56244 64127 3604 7099 3683 6583 6283 6352 4210 4049 4153 1080 3304 7100 2162 7432 3620 345611 3596 11213 6556 10392 6584 3651 3383 1948 4486 3576 58191 3565 6696 1524 396 8856 3606 10 4552 929 1045 6372 4283 7097 4314 3458 26191 219771 3569 9231 2153 3780 4276 3856 2147 4312 3586 7076 1432 7124 7291 3563 2209 3557 3119 7494 942 1436 9308 10747 9966 59067 7433 4650 4680 10068 80209 2214 337 1401 1493 3682 4292 3689 | 83 |
| Eosinophilia | CCL17; IL25; ETV6; IL2RA; IL5; ABL1; CHIC2; BIRC3; MUC1; BIRC5; CCNT1 | 6361 64806 2120 3559 3567 25 26511 330 4582 332 904 | 11 |
| Ependymoma | EPB41 | 2035 | 1 |
| Epidermolysis bullosa | KRT18; PLEC1 | 3875 5339 | 2 |
| Epilepsy | BRD2; NDUFA1; BDNF; CHRNA4; CHRNB2; CHRNA2; EGR1; CACNA1G; RCN2; COX1; IL1B; SLC6A4; HTR1A; MDM2; DAPK1; RTN4; EIF2S1; ACOT7; GABBR2; MTHFR; IL4; NR1I2; CST3; ABCC1; CASR; UCP2; ATP1A2; CLCN2; GLUL; VHL; HSPA8; MUSK; MECP2; PHOX2A; DBH; NTNG1; KCNMA1; RALBP1; EMX2; PVALB; SCN1B; LGI1; AP3M2; ANKH; CACNA1H; NHLRC1; CLN5; CNTNAP2; CLN8; PCDH19 | 6046 4694 627 1137 1141 1135 1958 8913 5955 4512 3553 6532 3350 4193 1612 57142 1965 11332 9568 4524 3565 8856 1471 4363 846 7351 477 1181 2752 7428 3312 4593 4204 401 1621 22854 3778 10928 2018 5816 6324 9211 10947 56172 8912 378884 1203 26047 2055 57526 | 50 |
| Epstein-Barr virus infection | IL1B; SH2D1A; IL10; BAX; CCL17; C15ORF55; GBP1; MYC; EIF2S1; STMN1; BIRC5; CCL2; DHRS9; GPR183; TLR2; TRAF3; BCL2; TNFSF13B; CCL22; EBI3; FHIT | 3553 4068 3586 581 6361 256646 2633 4609 1965 3925 332 6347 10170 1880 7097 7187 596 10673 6367 10148 2272 | 21 |
| Esophageal disease | CCL26; MGMT; RUNX3; XRCC1; AKR1C2; PRDX1 | 10344 4255 864 7515 1646 5052 | 6 |
| Esophageal tumor | CD274; SLC11A1; FGF7; ALDH2; S100A9; MLH3; TSPAN8; TMPRSS11A; CT47A11; ADAMTS9; RNF34; PABPC1; MT2A; ERAS; PRDX1; EVPL; CSAG2; FRAT1; PDCD1LG2; | 29126 6556 2252 217 6280 27030 7103 339967 255313 56999 80196 26986 4502 3266 5052 2125 728461 10023 80380 2071 65117 | 21 |
| Esophagitis | CCL11; IL6; IL1B; NR1H4; IL13 | 6356 3569 3553 9971 3596 | 5 |

| | | | |
|---|---|---|---|
| Esophagus cancer | ADIPOQ; AKR1B10; NR1H4; NOX5; NUDT6; CD55; CCL2; MMP3; G3BP1; EEF1D; CD46; IGF1; ACE; TMPRSS11A; RNF34; PABPC1; EVPL; RSRC2; RNASEN; SELE; SERPINE1; SKI; CRP; CAV2; S100A8; PBX1; CHGA; INHBA; LYPD3; HLTF; MT3; RARA; ETS2; CLU; SPRR3; PLCD1; MYEOV; RARRES1; GAEC1; MAPRE2; HOXD9; CREBBP; ITGA4; MAL; [illegible] | 9370 57016 9971 79400 11162 1604 6347 4314 10146 1936 4179 3479 1636 339967 80196 26986 2125 65117 29102 6401 5054 6497 1401 858 6279 5087 1113 3624 27076 6596 4504 5914 2114 1191 6707 5333 26579 5918 100126794 10982 3235 1387 3676 4118 9659 | 45 |
| Esotropia | EGFR; HSD17B1; BCL2; MMP11; STS; MMP9; AGT; IGF1; LDLR; HPSE; AGTR1; CBL | 1956 3292 596 4320 412 4318 183 3479 3949 10855 185 867 | 12 |
| Ewings sarcoma | HSPB1; MMP3; PARP1; CCK; PLD2; KCMF1; NR0B1; ERG; | 3315 4314 142 885 5338 56888 190 2078 56034 | 9 |
| Extramammary Paget's disease | MMP19 | 4327 | 1 |
| Eye cancer | RB1; BCL2 | 5925 596 | 2 |
| Eye disease | ABCA4; SAG; ARMS2; OPTN; RLBP1; LOXL1; MYOC; WFS1; EVC; CISD2; CRMP1; LPIN2; PRPH2; NDUFA1; DPYSL5; PTPN12; CHM; CLK1; SFRS1; CRYBB2; CRYAA; OPA3; MYP12; NYX; MYP11; PHOX2A; PHOX2B; | 24 6295 387715 10133 6017 4016 4653 7466 2121 493856 1400 9663 5961 4694 56896 5782 1121 1195 6426 1415 1409 80207 664780 60506 594832 401 8929 57165 692222 | 29 |
| Familial Mediterranean fever | MEFV; CCL3; SAA1; TNF; IL18; CD14; LMX1B; IL12A; LEP; SAA2; MICA; ACE; ABCC6; IFNG; VEGFA | 4210 6348 6288 7124 3606 929 4010 3592 3952 6289 4276 1636 368 3458 7422 | 15 |
| Fanconi's anemia | PARP1; ATR; FANCC; SPTAN1; FANCD2; BLM; UBE2T; TP73; ATM; BRCA2; XPO1; HES1; CHEK1; FANCA; FANCG; NBN; PALB2; FANCE; FANCB; TNF; HSP90AA1; EVI1; ERCC4; BAX; | 142 545 2176 6709 2177 641 29089 7161 472 675 7514 3280 1111 2175 2189 4683 79728 2178 2187 7124 3320 2122 2072 581 3458 | 25 |
| Fatty liver | BHMT; ALB; MET; ADIPOR2; ADIPOQ; IL10; FAS; APOC1; DBI; GGT1; LEP; APOB | 635 213 4233 79602 9370 3586 355 341 1622 2678 3952 338 | 12 |
| Female reproductive cancer | MAN1B1; AMHR2; MSH2; BARD1; SLC12A4; SLC12A6; SLC12A7; HOXB13 | 11253 269 4436 580 6560 9990 10723 10481 | 8 |
| Female reproductive system disorder | LEP; SHBG; LIPE; AMH; PAEP | 3952 6462 3991 268 5047 | 5 |
| Fetal disease | DEFA3; DEFA1; S100A8; GLE1; BPI; S100A9; CCR5; HSD11B2; PTPRC; TRNE | 1668 1667 6279 2733 671 6280 1234 3291 5788 4556 | 10 |
| Fibroadenoma | INHBA; RHEB | 3624 6009 | 2 |
| Fibroid tumor | SHBG; OXTR; IGF1; ACE; CTGF; BAK1; TIE1; FBLN1; ABI2; PDGFC; FERMT2 | 6462 5021 3479 1636 1490 578 7075 2192 10152 56034 10979 | 11 |
| Fibroma | H19 | 283120 | 1 |

| | | | |
|---|---|---|---|
| Fibromyalgia | MAOB; BDNF; IL10; MAOA; IGF1; IGFBP3; IL8; SLC6A4 | 4129 627 3586 4128 3479 3486 3576 6532 | 8 |
| Fibrosarcoma | CMA1; TPSAB1; SLC26A2; ARFIP1 | 1215 7177 1836 27236 | 4 |
| Filariasis | EDN1; IL2 | 1906 3558 | 2 |
| Folic acid deficiency | TYMS; MTHFR; IGF1; SLC19A1; F2RL2; MTHFD1L | 7298 4524 3479 6573 2151 25902 | 6 |
| Gallbaldder cancer | CTGF; GSTM3; FASLG; CD83; PML | 1490 2947 356 9308 5371 | 5 |
| Gallbladder disease | APOA1; APOB; BAX; ABCG2; | 335 338 581 9429 7434 | 5 |
| Gastritis | SHH; FAS; FASLG; IL1RN; AREG; IL4R; CDX1; DAPK1; IL1A; SELE; SELL; IL12B; MLH1; TLR4; MIF; RB1; MMP9; EDN1; LEPR; FGF7; CCL20; SLPI; B2M; UCN; DEFB103A; CRK; ATP4A; MSH2 | 6469 355 356 3557 374 3566 1044 1612 3552 6401 6402 3593 4292 7099 4282 5925 4318 1906 3953 2252 6364 6590 567 7349 55894 1398 495 4436 | 28 |
| Gastrointestinal cancer | CIITA; KLF4; SPINT1; FHL2; | 4261 9314 6692 2274 11107 | 5 |
| Gastrointestinal infection | CCL28 | 56477 | 1 |
| Gastrointestinal stromal tumor | IGF1; ADIPOR2; NF1; TLX2; TIE1; LCMT2 | 3479 79602 4763 3196 7075 9836 | 6 |
| Gastrointestinal tumor | EGFR; KIT; ACTA1; ITGA1; SDHC; ITGA6; EFNA2; MICB; FHIT; NAT2; KLF4; HSP90B1; CTNNA1 | 1956 3815 58 3672 6391 3655 1943 4277 2272 10 9314 7184 1495 | 13 |
| Generalized anxiety disorder | GRAP2; GAD2; ADIPOQ; CTLA4; SLC6A4; GAD1; MAOA; LCP2; IL6; PTPN22; LAT; CD28 | 9402 2572 9370 1493 6532 2571 4128 3937 3569 26191 27040 940 | 12 |
| Germ cell tumor | NANOG; XIST; TSPY1; TSP50; MIR373; MDC1; MIR372; DND1 | 79923 7503 7258 29122 442918 9656 442917 373863 | 8 |
| Gestational diabetes | IGFBP3; FGF2; MBL2; IGFBP1; UTS2D; SLC29A1; LIPG; PAX3 | 3486 2247 4153 3484 257313 2030 9388 5077 | 8 |
| Gestational trophoblastic tumor | IGFBP1; HSD3B1 | 3484 3283 | 2 |
| Gigantism | GAPDH | 2597 | 1 |
| Gilles de la Tourette syndrome | ROBO3; SGCE; SLC6A3; HTR3B; HTR3A; CNTNAP2; PKM2 | 64221 8910 6531 9177 3359 26047 5315 | 7 |
| Gingival overgrowth | SPARC; HSPG2; EDN1; CTLA4; IL15; FGF7; MMP1 | 6678 3339 1906 1493 3600 2252 4312 | 7 |
| Glaucoma | APOE; ESR1; ESR2; EDNRA; ADRB1; TYR; TYRP1; IL1A; IL1B; MTHFR; SOD2; SFRP1; EGFR; AKR1C2; MMP9; PEX5; BAK1; BCL2; ADRB2; GJA1; BCL2L1; CAPN1; BAX; TTR; AKR1C1; CYP1B1; CA12; FBN1; PITX2; LOXL1; MYOC; FOXC1; OCLM; | 348 2099 2100 1909 153 7299 7306 3552 3553 4524 6648 6422 1956 1646 4318 5830 578 596 154 2697 598 823 581 7276 1645 1545 771 2200 5308 4016 4653 2296 10896 2723 10218 338917 | 36 |
| Glomerulonephritis | SERPINE1; FCAR; KLK1; ELANE; SGK1; MPO; MMP2; JUND; TIMP1; CTLA4; FCGR3B; P2RX7; CD46; IL6; APOE; MMP9 | 5054 2204 3816 1991 6446 4353 4313 3727 7076 1493 2215 5027 4179 3569 348 4318 | 16 |
| Glucose intolerance | SCG5; SELE; NEUROD1; ICAM1; INS; CTSL1; GCG; NAMPT; PPARGC1A; TNMD; ADIPOQ; FTO; AGER; GGT1; RBP4; RETN; CASQ1; SLC2A4; INPPL1 | 6447 6401 4760 3383 3630 1514 2641 10135 10891 64102 9370 79068 177 2678 5950 56729 844 6517 3636 | 19 |

| | | | |
|---|---|---|---|
| Glycogen storage disease | AGL; VWF; IL8; G6PC2; KCNJ12; GBE1 | 178 7450 3576 57818 3768 2632 | 6 |
| Gonadal dysgenesis | FGF9 | 2254 | 1 |
| Gouts | SLC2A9; CTGF; IL4; BMP2; TLR2; MAZ; MMP3; IL1B; BMP6; FAP; HPRT1; PLA2G4A; PTGS2 | 56606 1490 3565 650 7097 4150 4314 3553 654 2191 3251 5321 5743 | 13 |
| Gram-Negative bacterial infection | CFTR; MICA; LTF; SLC4A1; DEFB103A; BAD; CEACAM1; BCL2L11; MYD88; YWHAQ; MVK; C6 | 1080 4276 4057 6521 55894 572 634 10018 4615 10971 4598 729 | 12 |
| Granulomatous disease | MLL; CYBB; TNF; CYBA; NOX5; NCF2; DECR1; SETBP1; NOX1; MDS1; PRDM16; NCF1C | 4297 1536 7124 1535 79400 4688 1666 26040 27035 4197 63976 654817 | 12 |
| Graves' disease | CTLA4; FGFR1; CCND3; HLA-C; IL1B; SELE; ICAM1; ADIPOQ; CCL5; HLA-A; IL13; CCR5; TNFRSF8; CD274; IRF1; TNFRSF9; EDN1; CD80; BCL2; ADRB2; IL16; GC; BAX; TNFRSF4; IGF1R; ST6GAL1; TSHR; CD1A; TNFSF8; CXCR6; CD1C; ST3GAL1 | 1493 2260 896 3107 3553 6401 3383 9370 6352 3105 3596 1234 943 29126 3659 3604 1906 941 596 154 3603 2638 581 7293 3480 6480 7253 909 944 10663 911 6482 | 32 |
| Growth retardation | IGF2; CST3; APOE; LEP; FSTL3; MBL2; HSD11B2; ADIPOQ; IGF1; FAS; ATP1A1; EDN1; EGF; PTGS2; IGFBP1; CCL2; AGT; MMP9; CGB; S100B; ENG; ANXA5; PTX3; SLC6A6; LGALS13; IGFBP3; MMP2 | 3481 1471 348 3952 10272 4153 3291 9370 3479 355 476 1906 1950 5743 3484 6347 183 4318 1082 6285 2022 308 5806 6533 29124 3486 4313 | 27 |
| HELLP syndrome | FASLG; CD46 | 356 4179 | 2 |
| HIV infection | BCL2L1; MSN; CTBP1; IL8; CD163; PCNA; GRK1; MLH1; FAS; TNFRSF8; FASLG; CXCL11; BCL2; BAX; CYBA; MEF2C; MME; CCL18; LRP1; BLM; CXCR6; FEN1; CDK9; TSG101; LAMB3; DDB1; ICAM1; ADIPOQ; RETN; SOD2; SELL; TNF; HLA-A; IL15; HSPB1; SPP1; CD274; CCL2; CCL21; PDCD1; MAPK14; VIPR1; ITGB2; LEPR; CD80; CXCL13; LEP; CD28; CX3CL1; IL16; CCL3; CCL3L1; IL2RA; CD4; B2M; CXCL12; GH1; PTPRC; IFNA1; TNFRSF4; CEBPB; HSPA5; IL7R; SLC29A1; APOBEC3G; BIRC5; CD34; PLAUR; F2R; CD226; E2F1; CCL4; CTNNA1; CXCR5; PTN; SLC28A1; EMD; NEFL; GLB1; IL18R1; ITK; IL2RB; CCL19; SIGLEC1; MPL; CCNT1; HLA-C; GNLY; CYP2B6; SLC28A3; HDAC6; NCL; SLC29A2; SMARCB1; BAG3; BACH2; SLC28A2; PLEC1; KIR3DS1; AP3D1; MAN1A1; CD8B; TRIM22; | 598 4478 1487 3576 9332 5111 6011 4292 355 943 356 6373 596 581 1535 4208 4311 6362 4035 641 10663 2237 1025 7251 3914 1642 3383 9370 56729 6648 6402 7124 3105 3600 3315 6696 29126 6347 6366 5133 1432 7433 3689 3953 941 10563 3952 940 6376 3603 6348 6349 3559 920 567 6387 2688 5788 3439 7293 1051 3309 3575 2030 60489 332 947 5329 2149 10666 1869 6351 1495 643 5764 9154 2010 4747 2720 8809 3702 3560 6363 6614 4352 904 3107 10578 1555 64078 10013 4691 3177 6598 9531 60468 9153 5339 3813 8943 4121 926 10346 5867 200316 11262 9560 11018 10657 | 109 |

| | | | |
|---|---|---|---|
| HTLV-I infection | FAS; CD3G; IL6; MMP9; CFLAR; TXN; CCL5; IL6R; IL10 | 355 917 3569 4318 8837 7295 6352 3570 3586 | 9 |
| Hamman-Rich syndrome | SHH; IL4R; FOXF1; IL1A; AGER; AGTR1; MMP2; IGFBP3; AGT; TIMP3; MMP1; AKR1B10; IL6; CDKN2A; CCL21; ANXA1; IL11; CCND1; PLAU; MUC1; CMA1; SOD3; RHOA; METAP2; FSD1; IGFBP5; ELMOD2 | 6469 3566 2294 3552 177 185 4313 3486 183 7078 4312 57016 3569 1029 6366 301 3589 595 5328 4582 1215 6649 387 10988 79187 3488 255520 | 27 |
| Heart disease | IL1RN; ALDH2; SCN5A; ICAM1; IL6; AGTR1; ACE; APOD; LMNA; MEF2A; AMPD1 | 3557 217 6331 3383 3569 185 1636 347 4000 4205 270 | 11 |
| Heart failure | TIMP3; TNF; CCR5; CCL2; MMP9; IGFBP1; EDNRA; ADORA2A; NR3C2; FABP3; POLB; KCNA1; EDNRB; KCNQ1; KCNE1; NUP85; CTF1; CBR3; GIT2; RETN; SLC2A4; LOX; HLA-B; IGF1; MMP1; BDKRB1; SLC6A4; PLN; IL18; SPP1; FGF2; TLR4; CFTR; TIMP1; MAPK14; EDN1; IL6ST; PARP1; ADRB2; GJA1; HSPD1; F3; TJP1; MMP13; CTSG; CASP1; ADM; SLC2A1; FOXC2; CHGA; SOD3; ANKRD1; AQP2; ABCG2; DUSP1; NOX5; KCNH2; NOL3; PTHLH; RAPGEF3; FOXC1; AMPD1; RTN4; NISCH; ADRB1; ADCY6; CAMK2D; KCND3; MYH7; NUPR1; FOXP1; RYR2; MYBPC3; FKBP1B; LAMA4; MIR1-1; HDAC4; MYH6; CLDN5; S100A1; PURA; HDAC5; PURB; MAP4; GATM; MYL9; TRPC3; ACY1 | 7078 7124 1234 6347 4318 3484 1909 135 4306 2170 5423 3736 1910 3784 3753 79902 1489 874 9815 56729 6517 4015 3106 3479 4312 623 6532 5350 3606 6696 2247 7099 1080 7076 1432 1906 3572 142 154 2697 3329 2152 7082 4322 1511 834 133 6513 2303 1113 6649 27063 359 9429 1843 79400 3757 8996 5744 10411 2296 270 57142 11188 153 112 817 3752 4625 26471 27086 6262 4607 2281 3910 406904 9759 4624 7122 6271 5813 10014 5814 4134 2628 10398 7222 95 | 88 |
| Heart valve disease | LECT1; CXCL16 | 11061 58191 | 2 |
| Helicobacter infection | IFNG; MMP2; CD274; MSI1; IL8; MPO; TLR4; IL18; IL1RL1; LEP; SMAD5; IFNGR1; SMAD7; CCL20; MMP9; CASP3; IL10; IL1B; AICDA; PCNA | 3458 4313 29126 4440 3576 4353 7099 3606 9173 3952 4090 3459 4092 6364 4318 836 3586 3553 57379 5111 | 20 |
| Helminthiasis | IL4; IL5; CCL17; CHIT1 | 3565 3567 6361 1118 | 4 |
| Hemangioma | AIF1 | 199 | 1 |
| Hematopoietic system disease | JAK2; CLDN1; RUNX1; ADAMTS13; GATA1; MPL; CHIC2; | 3717 9076 861 11093 2623 4352 26511 80324 10320 | 9 |
| Hemoglobinopathies | SLC4A1; HBB@; CD1B; CD1C | 6521 64162 910 911 | 4 |
| Hemolytic anemia | IFNG; CTLA4; SLC16A1; ERCC2; IL10; H6PD; SLC2A1; PKLR; SLC4A1; ERCC1; TRNE; ANK1; EPB42; GANC; HBB@; ATRX | 3458 1493 6566 2068 3586 9563 6513 5313 6521 2067 4556 286 2038 2595 64162 546 | 16 |
| Hemolytic-Uremic syndrome | CD46; CDKN1A; IL7; MCM4; CX3CL1; UTS2; IL2RA; ELAVL4; ADAMTS13; NF1; ELAVL2; FGFBP1; IL4R; IL15; CFB; F3; | 4179 1026 3574 4173 6376 10911 3559 1996 11093 4763 1993 9982 3566 3600 629 2152 3190 | 17 |

| | | | |
|---|---|---|---|
| Hemophilia | CCR5; MYC; F8 | 1234 4609 2157 | 3 |
| Hemorrhagic disorder | PTPN11; F5; PLAT; VWF; ADAMTS13; PLG; F7; FGG; FGA; KLKB1; SERPINF2; F9; F13A1; F2; FGB; ABCC4; CFI; ITGB3; ITGA2B; LRPAP1; ANXA2; FBLN1; F8; F12; F11; MPL; F10; HPS1; BLOC1S3; VPS33A; LYST; ANO2 | 5781 2153 5327 7450 11093 5340 2155 2266 2243 3818 5345 2158 2162 2147 2244 10257 3426 3690 3674 4043 302 2192 2157 2161 2160 4352 2159 3257 388552 65082 1130 57101 | 32 |
| Henoch-Schoenlein purpura | IL6; ADM; AGT; IL1B; HLA-A; CAT; MMP9; PTPN22; C4B; HLA-B | 3569 133 183 3553 3105 847 4318 26191 721 3106 | 10 |
| Hepatitis | IL1B; LMAN1; APOBEC3G; IL1R1; GGT1; GSTM1; GSTT1; LTBR; IL8RA; ANXA1; FGL2; CXCL16; PDCD1; JUN; APP | 3553 3998 60489 3554 2678 2944 2952 4055 3577 301 10875 58191 5133 3725 351 | 15 |
| Hepatitis B | HLA-A; CCR1; MIF; IFNA2; CIITA; MMP2; AGT; TNF; CD274; CCL2; FASLG; MMP9; TIMP1; ANGPT2; TIMP2; APOA1; CHI3L1 | 3105 1230 4282 3440 4261 4313 183 7124 29126 6347 356 4318 7076 285 7077 335 1116 | 17 |
| Hepatitis C | CCR5; FCAR; MTHFR; IL10; MX1; IL2; CXCL10; IDO1; FAS; CTLA4; IL18; SPP1; TNFRSF8; CD274; LAMB2; IGF1; HTR1A; CCL2; CX3CR1; RBP4; FGF2; FASLG; KLRC1; IL1A; TLR4; MKI67; IRF1; ENG; CD14; TLR3; KLRD1; REXO1L1; IFNG; TLR2; ADIPOQ; CCR1; ADIPOR2; CCL5; IL1B; KRT8; KRT18; IL6; MDM2; | 1234 2204 4524 3586 4599 3558 3627 3620 355 1493 3606 6696 943 29126 3913 3479 3350 6347 1524 5950 2247 356 3821 3552 7099 4288 3659 2022 929 7098 3824 254958 3458 7097 9370 1230 79602 6352 3553 3856 3875 3569 4193 6373 6366 3576 | 46 |
| Hepatoblastoma | MPO; EP300; DLL1 | 4353 2033 28514 | 3 |
| Hereditary disease | MYH11; SNCA; RUNX2; PAFAH1B1; NBN; APP; GJB3; TNAP; NOX1; FEN1; SMARCA4; VLDLR; CHM; TTN; GATA1; GAS1; LMNA; DSPP; ICOS; | 4629 6622 860 5048 4683 351 2707 445341 27035 2237 6597 7436 1121 7273 2623 2619 4000 1834 29851 57379 2071 10111 | 22 |
| Hereditary nonpolyposis colorectal tumor | CASP1 | 834 | 1 |
| Herpes | EIF5B; IL1A; PTPN11; APP; RAF1; ICAM1; CD55; GAD2; ITGAV; ITGB6; CD86; CD80; SP1; EIF2AK3; IFNB1; HSPA8; DNAJB1; HLA-A; IL10; APOE; ESR1; PAEP; ARVCF; IZUMO1; IL15; SLC6A4; HTR3A; GSTP1; CDKN2A; SPP1; FGF2; MDM2; ITGAL; BCL2; S100A8; HRAS; IAPP; ARHGEF1; SMAD3; VCAN; HSP90AA1; ATP2C1; ITIH2; NANOG; ADORA1; AKAP13; RAP1A; SLC6A3; HTR3B | 9669 3552 5781 351 5894 3383 1604 2572 3685 3694 942 941 6667 9451 3456 3312 3337 3105 3586 348 2099 5047 421 284359 3600 6532 3359 2950 1029 6696 2247 4193 3683 596 6279 3265 3375 9138 4088 1462 3320 27032 3698 79923 134 11214 5906 6531 9177 | 49 |
| Histiocytosis | TNF; IL1RN; IL17A; CSF1; APOE; SMPD1; NPC1L1; NPC2; SEMA6A | 7124 3557 3605 1435 348 6609 29881 10577 57556 | 9 |

| | | | |
|---|---|---|---|
| Hodgkin's disease | IL1B; SELE; ICAM1; SELL; HLA-A; IL8; IL13; IL4; IL10; TNFRSF8; CD274; PDCD1; TIMP1; IL1RN; HLA-DQB1; APOH; SLC29A1; ADARB1; CYLD; PDCD1LG2; MRE11A; IL11RA; RAD50; EIF2S1; REL; LY75; IGL@; BCL3; JUNB; | 3553 6401 3383 6402 3105 3576 3596 3565 3586 943 29126 5133 7076 3557 3119 350 2030 104 1540 80380 4361 3590 10111 1965 5966 4065 3535 602 3726 3219 9936 3902 | 32 |
| Huntington disease | JPH3; BDNF; LEP; TRIP10; SETDB1; ADORA2A; GIT1; GDNF; SGK1; MSX1; MMP2; FAAH; MAOB; GAPDH; TCERG1; STH; MTHFR; HAP1; DNAJB2; TIMP2; | 57338 627 3952 9322 9869 135 28964 2668 6446 4487 4313 2166 4129 2597 10915 246744 4524 9001 3300 7077 207 | 21 |
| Hydatidiform mole | ADA; NLRP7 | 100 199713 | 2 |
| Hydrocephalus | FAS; NFIA; CASP3; ADCYAP1R1; L1CAM; FASLG; B4GALT1; MAPT; INVS | 355 4774 836 117 3897 356 2683 4137 27130 | 9 |
| Hyperaldosteronism | GNA12; MEN1; AGTR1; RBAK; PMS2; CLCNKA; CLCNKB; RGS2; KCNJ1; SLC12A1 | 2768 4221 185 57786 5395 1187 1188 5997 3758 6557 | 10 |
| Hypercholesterolemia | AGT; EGFR; SOD1; MTTP; SELE; PLIN; AGTR1; IL6; IL8; CCL2; FASLG; TLR4; CRP; LEPR; SELP; ABCA1; VWF; CYBA; ABCG8; ABCG5; CLU; PCSK9; APOB48R; TRNI; NPC1L1; C7ORF16; | 183 1956 6647 4547 6401 5346 185 3569 3576 6347 356 7099 1401 3953 6403 19 7450 1535 64241 64240 1191 255738 55911 4565 29881 10842 26119 | 27 |
| Hyperglycemia | CYBA; AKT1; TRPC6; CCL5; LPL; GIP; GCK; IL6; IL8; NCF1; IGF1; NAMPT; GLDC; SERPINE1; AKT2; SGK1; AGT; SOD3; KCNJ11; PRDX6; HIF1A; GCG; SMO; IL1A; INSR; SPHKAP; IL4; CAPN10; GLO1; SHC1; ROCK1; PIK3R2; REN; INS; GCNT1; MTHFR; | 1535 207 7225 6352 4023 2695 2645 3569 3576 653361 3479 10135 2731 5054 208 6446 183 6649 3767 9588 3091 2641 6608 3552 3643 80309 3565 11132 2739 6464 6093 5296 5972 3630 2650 4524 345 3339 6647 | 39 |
| Hyperhomocysteinemia | MTHFR; F5; CBS; ATF3; MTR; BHMT; MTRR; F2; TPMT; CCL2; ACE; APOE | 4524 2153 875 467 4548 635 4552 2147 7172 6347 1636 348 | 12 |
| Hyperinsulinism | KCNJ11; INS; IRS1; SLC16A1; SGK1; SELE; PNPLA2; LEP; ADIPOQ; LIPE; CRP; GLUD1; NAMPT; ADM; HNF4A; CCL2; MC4R; APOB; ICAM1; GIP; REN; SERPINE1; EDN1; GCK; LPL; | 3767 3630 3667 6566 6446 6401 57104 3952 9370 3991 1401 2746 10135 133 3172 6347 4160 338 3383 2695 5972 5054 1906 2645 4023 6517 6198 | 27 |
| Hyperlipidemia | FABP2; TXNIP; IRS1; SCD; APOA2; RXRG; CCL5; REN; CCR5; APOA4; APOE; APOC1; ESR1; OSM; C3; F3; MPO; LIPC; APOA1; APOC3; LCAT; MMP14; ACSL3; SLC10A2; F7; PAPPA | 2169 10628 3667 6319 336 6258 6352 5972 1234 337 348 341 2099 5008 718 2152 4353 3990 335 345 3931 4323 2181 6555 2155 5069 | 26 |
| Hyperopia | SOX2; PAX6; FGFR3; TWIST1; MBL2; APOBEC3G; CD247; NOG; GLRA1 | 6657 5080 2261 7291 4153 60489 919 9241 2741 | 9 |
| Hyperparathyroidism | CCND1; CDC73; CASR; GCM2; IL6R; WT1; MEN1; CDKN1B; | 595 79577 846 9247 3570 7490 4221 1027 1026 | 9 |

| | | | |
|---|---|---|---|
| Hypertension | IL1B; INS; BMP4; HTR2A; CDKN2A; CCR5; TIMP1; LEPR; ESR1; ESR2; TIMP2; AKT1; WNK1; WNK4; CORIN; SLC6A19; BMP10; ATP5B; SUCNR1; KNG1; IL15; IL12B; IL10; MMP9; VIP; CST3; APOB; PLAT; ADORA2A; CAT; ADRB3; ADA; ADORA1; INSR; ALOX12; KCNA5; CLU; SLC8A1; SLC22A2; BMPR2; CALCRL; SLC7A1; CTH; PNMT; ADRA1A; AVPR1A; CYP19A1; CTSL1; PPARGC1A; RETN; INPPL1; AGTR1; HP; REN; CALCA; LTA; HTR3A; HTR2B; ATP2A2; GSTT1; TNFRSF8; HTR1A; MBL2; CFTR; IL1RN; EDN1; LPL; ACE; SCNN1G; SCNN1B; CAV1; CTGF; UTS2D; ADRB2; SELP; ANGPT2; ALDH2; SGK1; ADM; NR3C2; IAPP; CAPN10; HSD11B2; GH1; ADRA2B; ND1; HSD11B1; UCP1; XDH; VWF; PTPN1; APOC3; UCP2; UTS2; IL6R; SLC6A2; TTR; ADORA2B; LRP5; ECE1; GCGR; CHGA; RLN2; ADD1; SOD3; GHR; ACE2; SLC12A3; NOX1; AQP2; VNN1; HTR3B; BDKRB2; ANPEP; TYMS; TSPO; ANXA5; ADAMTS13; KLK1; LCN2; GCLC; ARG1; ROS1; EDNRB; ND2; ADRA2A; CLCNKA; CLCNKB; PHOX2A; DBH; HTR1B; HTR2C; | 3553 3630 652 3356 1029 1234 7076 3953 2099 2100 7077 207 65125 65266 10699 340024 27302 506 56670 3827 3600 3593 3586 4318 7432 1471 338 5327 135 847 155 100 134 3643 239 3741 1191 6546 6582 659 10203 6541 1491 5409 148 552 1588 1514 10891 56729 3636 185 3240 5972 796 4049 3359 3357 488 2952 943 3350 4153 1080 3557 1906 4023 1636 6340 6338 857 1490 257313 154 6403 285 217 6446 133 4306 3375 11132 3291 2688 151 4535 3290 7350 7498 7450 5770 345 7351 10911 3570 6530 7276 136 4041 1889 2642 1113 6019 118 6649 2690 59272 6559 27035 359 8876 9177 624 290 7298 706 308 11093 3816 3934 2729 383 6098 1910 4536 150 1187 1188 401 1621 3351 3358 7166 2053 7503 3360 3818 476 1114 409 9475 2028 944 3362 5997 3363 3283 9530 79608 6296 654817 6013 4565 11188 4069 2185 153 1589 190 408 | 160 |
| Hyperthyroidism | TSHR; SLC2A3; SLC2A4; CTLA4; RETN; IGFBP1; IFNG; IGF1; SERPINE1 | 7253 6515 6517 1493 56729 3484 3458 3479 5054 | 9 |
| Hyperuricemia | MTHFR; APOA1; SLC22A11 | 4524 335 55867 | 3 |
| Hypogammaglobulinemia | IL10; CD19 | 3586 930 | 2 |
| Hypoglycemia | SLC16A1; ACE; APOE; SLC2A1; NR3C2; SLC2A3 | 6566 1636 348 6513 4306 6515 | 6 |
| Hypogonadism | TSPY1; NMT2; GABRG3; AMH; AR | 7258 9397 2567 268 367 | 5 |
| Hypopituitarism | SOX3; HESX1; GLI2; TBX19 | 6658 8820 2736 9095 | 4 |
| Hypothyroidism | IGFBP1; PTPRJ; INS; LEP; CXCL10; TPO; IYD; IGFBP3; ALAD; LRPAP1; CCL2; IGF1; SLC5A5; RETN; IL4; CRP; TNF; | 3484 5795 3630 3952 3627 7173 389434 3486 210 4043 6347 3479 6528 56729 3565 1401 7124 2304 | 18 |

| | | | |
|---|---|---|---|
| IGA glomerulonephritis | IFNA1; EDN1; IFNG; CD80; HLA-A; RHOA; AGTR1; MIF; ADM; TNFSF13B; CCR5; IL10; PTPRO; AGTR2; SELL; AGT; SELE; PTX3; MEFV; CTGF; XCL1; EGF; ACE; ACSM3; IL4; MMP9; CCL2; LCN2; IGHMBP2; CYBA; IL6; CD86; FCGR3B; IL5RA | 3439 1906 3458 941 3105 387 185 4282 133 10673 1234 3586 5800 186 6402 183 6401 5806 4210 1490 6375 1950 1636 6296 3565 4318 6347 3934 3508 1535 3569 942 2215 3568 | 34 |
| Ichthyoses | PNPLA2; TGM5; VPS33B; KRT2; ALDH3A2 | 57104 9333 26276 3849 224 | 5 |
| Immune complex disease | FCGR1A; TNFSF13B | 2209 10673 | 2 |
| Immunologic deficiency syndrome | THY1; NBN; PDCD1; RAG2; CASP8; ATM; CXCL12; RAG1; TNFSF13B; SP110; CIITA; IL9; LIG4; CTSC; AIRE; MLL; NHEJ1; XIAP; NOX1; MLLT3; ITGAL; IL17A; APOA1; CUGBP2; RAP1GAP; IL7; IGHG2; BTK; | 7070 4683 5133 5897 841 472 6387 5896 10673 3431 4261 3578 3981 1075 326 4297 79840 331 27035 4300 3683 3605 335 10659 5909 3574 3501 695 3561 5994 647275 | 31 |
| Infantile spasms | TSC1; TSC2; SCN1A; KCNJ11; NDP; MC4R; PDCD6IP | 7248 7249 6323 3767 4693 4160 10015 | 7 |
| Infection | NAMPT; ABO; HLA-B; PLIN; CCL5; IFNG; RUNX3; HIF1A; IL4; IL2; IL10; MX1; CXCL10; IL18; IRF1; REXO1L1; IL8RA; LMAN1; PTPN11; MBL2; NOD1; CD83; IL21; CCL24; ABCC1; CAV2; PVRL1; EGF; S100B; PDLIM7; EPS15; SFTPD; IL12RB2; SFTPA1B; EZR; POMC; IDE; RDX; IRF8; MSN; APOM; FAP; DEFA1; IFNGR1; VIPR2; AFP; CDKN1A; CLDN1; FHIT; ITGA6; LDLR; HSP90B1; RASSF1; IFNAR2; ANPEP; SDC1; CASP9; LAMA5; PDCD1LG2; PRDM5; CD46; CTTN; CXCL5; ATF3; ATM; JAK1; HDAC3; HSPA8; CTBP1; ABL1; RNASEL; EMP2; TPO; C4B; FLVCR1; SFTPC; VLDLR; SELPLG; SP110; IL12RB1; CLEC4M; IRF3; MAVS; C4A; IL27; IL10RA; KIR2DL3; TIA1; EIF2S1; SMN1; TRAF6; IKBKE; LPO; TBK1; CSNK2A1; EIF4G1; IFI16; RICTOR; RPTOR; CD33; C1QBP; LBP; APOBEC3C; HPR; C7ORF49; KLRG1; IFNA4; CALCOCO2; CD6; SGPL1; M6PR; THOC1; SIGLEC7; | 10135 28 3106 5346 6352 3458 864 3091 3565 3558 3586 4599 3627 3606 3659 254958 3577 3998 5781 4153 10392 9308 59067 6369 4363 858 5818 1950 6285 9260 2060 6441 3595 6435 7430 5443 3416 5962 3394 4478 55937 2191 1667 3459 7434 174 1026 9076 2272 3655 3949 7184 11186 3455 290 6382 842 3911 80380 11107 4179 2017 6374 467 472 3716 8841 3312 1487 25 6041 2013 7173 721 28982 6440 7436 6404 3431 3594 10332 3661 57506 720 246778 3587 3804 7072 1965 6606 7189 9641 4025 29110 1457 1981 3428 253260 57521 945 708 3929 27350 3250 78996 10219 3441 10241 923 8879 4074 9984 27036 9956 10538 | 115 |
| Infection by cryptococcus neoformans | SELL; FCGR3A; FCGR3B | 6402 2214 2215 | 3 |
| Infectious lung disease | CYLD; C2; IL1B; SERPINE1; IL8; CALCA; MASP2; ACE; IL6ST; APOE; EGR1; ELANE; FGF7; SLPI; TXN; HSD11B2; MUC5AC; IKBKB; FGG; CHUK | 1540 717 3553 5054 3576 796 10747 1636 3572 348 1958 1991 2252 6590 7295 3291 4586 3551 2266 1147 | 20 |

| | | | |
|---|---|---|---|
| Infertility | ARNT; TRO; CYP21A2; AHRR; C9; ND4; C3; BOLL; CGA; MTHFR; HRH1; INHBA; LIF; LEP; F5; TNF; HRH2; IL1A; MICA; IGF1R; GALT; AR; CCL2; IL1B; DAZ2; LIPE; IL11; AMH; GTF2A1L; PAEP; IL6; GSTM1; IL6R; MIF; EIF5A2; BIRC5; LMNA; CFTR; FAS; SGK1; DAZL; HSPA2; CRISP2; ADM; DAZ1; AHR; CD9; CFI; CX3CL1; | 405 7216 1589 57491 735 4538 718 66037 1081 4524 3269 3624 3976 3952 2153 7124 3274 3552 4276 3480 2592 367 6347 3553 57055 3991 3589 268 11036 5047 3569 2944 3570 4282 56648 332 4000 1080 355 6446 1618 3306 7180 133 1617 196 928 3426 6376 2099 6352 727 3972 | 53 |
| Infiltrating cancer | AGER; SERPINE1; TIMP1; CSK; HYAL1; TIMP4; GALT; CYP19A1; MAPKAPK2; RNF11; KPNA2; INSL4; MEST; REL | 177 5054 7076 1445 3373 7079 2592 1588 9261 26994 3838 3641 4232 5966 | 14 |
| Inflammation of the central nervous system | ICAM1; IL8; SPP1; TLR2; MBL2; CXCL5; ENO2; GBP1 | 3383 3576 6696 7097 4153 6374 2026 2633 | 8 |
| Influenza | CCL5; MBL2; CCL3; SERPINE1; MX1; TNFSF4; KIR2DL1; NR1I3; IFNG; GCC2; TLR3; LILRB1; IGHD; GCC1; CRKL; CCL11; TLR4; ICAM1; CPSF4; IL10; IFNA1; APCS; CRK; TLR2; CCL2 | 6352 4153 6348 5054 4599 7292 3802 9970 3458 9648 7098 10859 3495 79571 1399 6356 7099 3383 10898 3586 3439 325 1398 7097 6347 | 25 |
| Intermediate coronary syndrome | AGT; TLR4; TNF; CD14; CRP; IL18; ITGB3; PTX3; LEP | 183 7099 7124 929 1401 3606 3690 5806 3952 | 9 |
| Intestinal disease | ND5; ABO; TLR5; TRPV1; PLAGL1; SLC16A1; CALCA; SCN5A; TSC2; CCL11; HNMT; ACO1; WASL; ABCA4; CEACAM5; | 4540 28 7100 7442 5325 6566 796 6331 7249 6356 3176 48 8976 24 1048 317 | 16 |
| Intracranial aneurysm | FGF1; ELN; LOX; ENG; FBN2; IL6; CASP3; TIMP1; TIMP2; VCAN; TIMP3 | 2246 2006 4015 2022 2201 3569 836 7076 7077 1462 7078 | 11 |
| Intracranial hypertension | CASP8; FGFR3 | 841 2261 | 2 |
| Intractable epilepsy | PHLDA1; AQP1; SCN1A; CCL2; GLI3; SCN2A; GSTM1; HSPBAP1 | 22822 358 6323 6347 2737 6326 2944 79663 | 8 |
| Intraocular melanoma | CIITA; ICAM1; IFNG; S100B; NF1; PAK1; EDNRB; PARK7; LZTS1; APITD1 | 4261 3383 3458 6285 4763 5058 1910 11315 11178 378708 | 10 |
| Iron overload | UBE2D1 | 7321 | 1 |
| Ischemia | MFGE8; MAPT; ADORA1; NOL3; HIF1A; ALB; S100B; MAPK14; NTNG1; BCL2L1; HSPE1; FAS; HSPA4; XRCC1; PARP1; SOD1; KCNK2; AGTR2; EDN1; ADIPOQ; TRPC5; XIAP; BAI2; BMX; MPO; PLAT; EPO; MMP14; ANXA5; ADM; SNCA; SLC8A1; MAG; KCNMA1; SLC9A1; SCN5A; SELP; TRAP1; FASLG; CRP; ANGPT1; ERN1; BCL2; ADORA3; IL1RN; SLC29A4; MMP9; APOE; CSF3; AMPD1; IL8; ACCN2; AKR1B1; PTGS2; AIFM1; ROCK1; VHL; LIF; [illegible] | 4240 4137 134 8996 3091 213 6285 1432 22854 598 3336 355 3308 7515 142 6647 3776 186 1906 9370 7224 331 576 660 4353 5327 2056 4323 308 133 6622 6546 4099 3778 6548 6331 6403 10131 356 1401 284 2081 596 140 3557 222962 4318 348 1440 270 3576 41 231 5743 9131 6093 7428 3976 185 4205 522 | 61 |

| | | | |
|---|---|---|---|
| Kaposi sarcoma | ICAM1; IL8; CD86; PARP1; OSM; EDNRA; RBPJ; MBD2; PAX2; BACH1; LYN; PCBP1; EDNRB | 3383 3576 942 142 5008 1909 3516 8932 5076 571 4067 5093 1910 | 13 |
| Keratoconjunctivitis Sicca | GJB2; IL6; FASLG; MMP9; SOD1; CCL17; AQP5; ADAR; HNRNPA1 | 2706 3569 356 4318 6647 6361 362 103 3178 | 9 |
| Keratoconus | S100A2; EMP3; SOD3; SLPI; CLC; SP1; AQP5 | 6273 2014 6649 6590 1178 6667 362 | 7 |
| Keratosis | CTSL1; FGFR3; CTSD; EGF; BCL2; CCND1; TRPC1; CDKN1A; SNAP29 | 1514 2261 1509 1950 596 595 7220 1026 9342 | 9 |
| Kidney cancer | MUC3A; CD3E; SFPQ | 4584 916 6421 | 3 |
| Kidney disease | AGTR1; AGT; LIPE; UTS2; PAX2; PKD2; TNF; CDKN2A; MBL2; TIMP1; MME; GREM1; CLU; COL4A3BP; ICAM2; MYH9; AIF1; CTNS; ADIPOQ; RBP4; WFS1; EVC; CISD2; CRMP1; FABP2; IL8; MUC2; CD274; CX3CR1; PDCD1; HBEGF; EDN1; ACE; SCNN1B; ALPL; LEP; CTGF; PEX16; PEX5; FCGR2A; C3; SGK1; LRP2; CYBA; ACE2; AQP2; BDKRB2; MUC5AC; PTK2; SIX1; FGF10; INHA; SLC1A5; TPO; SLC7A9; SLC3A1; PREPL; SLC5A2; AGXT; GRHPR; FGFBP1; SCNN1A; LMX1B; SLC22A6; SLC22A8; LIMS1; [illegible] | 185 183 3991 10911 5076 5311 7124 1029 4153 7076 4311 26585 1191 10087 3384 4627 199 1497 9370 5950 7466 2121 493856 1400 2169 3576 4583 29126 1524 5133 1839 1906 1636 6338 249 3952 1490 9409 5830 2212 718 6446 4036 1535 59272 359 624 4586 5747 6495 2255 3623 6510 7173 11136 6519 9581 6524 189 9380 9982 6337 4010 9356 9376 3987 6092 462 3758 54 | 70 |
| Kidney failure | IL10; CRP; HSPA1A; HPRT1; HYOU1; REN; CST3; APOE; S100A8; ALDH2; ADORA2A; S100A9; MCAM; MYC; ADAMTS13; ATXN7; AMBP; AVPR2; ARHGAP4; SELE; ICAM1; RETN; VEGFA; TLR4; F3; CD163; MPO; KL; PTX3; SLC4A1; HAMP; ICAM3; CCL15; IL6; ADIPOR2; ALB; ITGB2; CCND1; CASR; SLC2A1; ABCG1; ABCA1; ITGAM; PON1; PTH1R; IL19; DAO; RUNX2; IL1A; IL1B; MTHFR; CCL5; GSTM1; GSTT1; GSTP1; IL4; CCR5; SPP1; CCL2; TLR2; IL1RN; EPO; APOB; ESR1; EGF; FCGR3B; SHC1; PVT1; CHGA; ADD1; ADA; KLK1; INVS; ATP5J; ADRA1A; [illegible] | 3586 1401 3303 3251 10525 5972 1471 348 6279 217 135 6280 4162 4609 11093 6314 259 554 393 6401 3383 56729 7422 7099 2152 9332 4353 9365 5806 6521 57817 3385 6359 3569 79602 213 3689 595 846 6513 9619 19 3684 5444 5745 29949 1610 860 3552 3553 4524 6352 2944 2952 2950 3565 1234 6696 6347 7097 3557 2056 338 2099 1950 2215 6464 5820 1113 118 100 3816 27130 522 148 146 6573 147 | 78 |
| Kuhnt-Junius degeneration | GSTT1; GSTM1; ARMS2; CST3; SOD2; GSTP1 | 2952 2944 387715 1471 6648 2950 | 6 |
| Larynx cancer | HLA-B; IL6; IL8; FURIN; CLU; MIR184; GGNBP2; EPHX1; CAPN10; MGST1 | 3106 3569 3576 5045 1191 406960 79893 2052 11132 4257 | 10 |
| Late pregnancy | KCNJ11; ESR2; ABCC9; PLAT; INS; KCNJ8; PLAU; ADIPOQ; RBP4; IGF1; ESR1; CRP | 3767 2100 10060 5327 3630 3764 5328 9370 5950 3479 2099 1401 | 12 |
| Learning disorder | APOE; MRPL19; DYX3 | 348 9801 11192 | 3 |

| | | | |
|---|---|---|---|
| Leigh disease | NDUFS7; SFRS2; NDUFS1; ND3; ATP6; NDUFS4; ATP5J; ND2 | 374291 6427 4719 4537 4508 4724 522 4536 | 8 |
| Leiomyosarcoma | CD163; CALD1; TSPAN31 | 9332 800 6302 | 3 |
| Leprosy | TNF; HLA-A; LTA; TLR2; CD1B; SLC11A1; SLAMF1; MICA; IL10; PARK2; CCL11; IL12B; MBL2; | 7124 3105 4049 7097 910 6556 6504 4276 3586 5071 6356 3593 4153 56244 | 14 |
| Leukemia | IL8; ABI1; IKZF2; NKX2-5; IL2RA; PVRL2; HOXA11; BACH2; PVR; IL1A; FASLG; IL8RA; SYNJ2; ITGAX; RHOH; TNFRSF8; AHR; ENO2; PIAS3; RTN4; JUND; CCR4; CISH; MICB; IGF2BP2; RUNX1; MYB; MLL; PTPN6; IRF4; ZNF521; IKZF1; IGH@; IKZF3; ETV6; PAX4; PBK; CEBPA; SPANXB1; TLE1; RUNX1T1; GCET2; CD52; TLX1; SOCS3; ELANE; SOCS2; IFNA1; PDCD5; EVI1; LYN; CRKL; HOXA10; PER3; ORM1; P2RX5; PRDM12; ARNT; SERPINE1; CALCA; IL10; CCL2; HLA-DPB1; TSGA10; JAK1; RAPGEF3; IL7; AFF3; LIG4; AFF1; AFF4; ASNS; SELL; IL6; NOD2; IL4; IL2; MTRR; MPO; HPRT1; PML; TNFSF8; NCOA2; CD96; MLLT1; HOXD13; FRYL; IL18; TIMP1; ITGAM; CAMK2G; SDC2; JAG1; PRKD2; DLL1; H19; CBL; GATA1; TPT1; PRDM16; CD33; PLSCR1; MN1; L3MBTL; MNX1; PLEKHG2; MLL5; IL1B; IFNG; CTLA4; TNF; CCL11; CCL21; ALB; CD83; IL21; LPL; PLAT; IFNGR1; SLC4A1; LAMA5; IKBKB; PDCD1LG2; PMAIP1; ITGA4; TRAF1; BGN; LILRB4; TPO; PDE4D; BCL2A1; IGHD; CHUK; PDE4A; CCL19; RHD; PTPRO; RAD52; IGHM; | 3576 10006 22807 1482 3559 5819 3207 60468 5817 3552 356 3577 8871 3687 399 943 196 2026 10401 57142 3727 1233 1154 4277 10644 861 4602 4297 5777 3662 25925 10320 3492 22806 2120 5078 55872 1050 728695 7088 862 257144 1043 3195 9021 1991 8835 3439 9141 2122 4067 1399 3206 8863 5004 5026 59335 405 5054 796 3586 6347 3115 80705 3716 10411 3574 3899 3981 4299 27125 440 6402 3569 64127 3565 3558 4552 4353 3251 5371 944 10499 10225 4298 3239 285527 3606 7076 3684 818 6383 182 25865 28514 283120 867 2623 7178 63976 945 5359 4330 26013 3110 64857 55904 3553 3458 1493 7124 6356 6366 213 9308 59067 4023 5327 3459 6521 3911 3551 80380 5366 3676 7185 633 11006 7173 5144 597 3495 1147 5141 6363 6007 5800 5893 3507 11040 5910 5142 752 574028 3509 11086 28444 10725 79368 914 5140 3506 3903 3201 9214 6401 9370 5972 3479 5321 4629 811 6497 3620 3683 5781 3563 942 1436 3953 1636 3952 4763 671 6348 1435 1511 820 567 3394 1440 868 2030 1118 865 5914 1387 3934 2175 64324 2308 2177 1178 57167 4069 21 930 2078 8864 863 1052 9846 2249 6670 1441 6418 1053 80010 11168 5896 924 4211 4005 79870 27036 9402 64919 1849 4066 3805 | 315 |
| Leukodystrophy NOS | EIF2B4; MPV17; HSPD1; EIF2B2; EIF2B5; ARSB; CHIT1; ARSA | 8890 4358 3329 8892 8893 411 1118 410 | 8 |
| Leukoencephalopathy | VEGFA; CXCL12; EIF2B5; GCDH; EIF4E; IL6; CSF1R; F3; ELANE; TNFRSF10A; BIRC5; MYC; PML; CHEK2; BLM; MRE11A; RARA; CREBBP; NFATC2; PAWR; TP73; LYZ; MNAT1; CCNH; SPI1; TOPBP1; CSNK1A1; HIPK2; UBE2D3; DHX9; SP100; DAPK3; ELF4; CEBPE; UBA7; SATB1 | 7422 6387 8893 2639 1977 3569 1436 2152 1991 8797 332 4609 5371 11200 641 4361 5914 1387 4773 5074 7161 4069 4331 902 6688 11073 1452 28996 7323 1660 6672 1613 2000 1053 7318 6304 | 36 |
| Leukopenia | SLCO1B3; CEBPA | 28234 1050 | 2 |
| Lewy body disease | TUBA1B | 10376 | 1 |

| | | | |
|---|---|---|---|
| Lichen planus | DEFB103A; TNF; IFNG; MMP9; BCL2; MMP3; IDO1; MMP2 | 55894 7124 3458 4318 596 4314 3620 4313 | 8 |
| Lipodystrophy | SLC29A2; ADIPOQ; LMNA; GH1; SLC28A1; LEP; SLC29A1; SLC28A3; FAS; RETN; SLC28A2; | 3177 9370 4000 2688 9154 3952 2030 64078 355 56729 9153 7124 6347 | 13 |
| Lipoidosis | SNCB; RAB4A; PNPLA2; ECM1 | 6620 5867 57104 1893 | 4 |
| Liver cancer | TNF; PLA2G4A; SLC9A3R1; GNL3; RBMY1A1; MT1F; MSI1; ALDH1A1; GSDMB; ACSL4; FSTL3; ITGA7; TP53AIP1; PSMG2; SELE; ICAM1; GGT1; HLA-B; IGF1; IFNG; SERPINE1; ADFP; HLA-A; HSPB1; IL4; PTGES; CCR5; CCL2; CX3CR1; FASLG; CCR1; ALB; TNFRSF9; MBL2; MMP3; MICA; F2; TIMP1; HLA-DQB1; CRP; ACE; CD80; GNMT; IGFBP1; EPHX1; APOE; LEP; SHBG; ELF1; HLA-DQA1; CTGF; PARP1; HSPD1; PIN1; CCL20; CCL3; IL2RA; ALDH2; SOD1; GH1; LPA; HSPA1A; APOA1; MTTP; APOC3; SHC1; IL6R; ASIP; MEF2C; ALAD; BMP6; ACO1; SLC12A2; GJB1; RAD23B; POLL; RBPJ; DUSP1; ZNF443; ACSL3; GSTM3; PML; MIR122; CYLD; DLEC1; IFNAR2; BCL2L2; SPRED2; RPL36A; NR1I3; CCT3; WEE1; DUSP6; TSPAN1; MAD2L1BP; EEF1A1; ATF5; CIAPIN1; MEF2A; CSE1L; PARK2; OTC; AMFR; MMP16; SPRY2; MAGEA1; EFNB1; SMAD5; ROCK1; KIAA0101; VEGFB; ASPM; BRE; INHBA; MTSS1; F2RL3; ING2; ACTR1B; C14ORF68; RELB; FKBP4; ZFHX3; SIAH1; HIST2H3A; SEPP1; MBD2; CLDN10; MIA3; DDX6; TUBB; | 7124 5321 9368 26354 5940 4494 4440 216 55876 2182 10272 3679 63970 56984 6401 3383 2678 3106 3479 3458 5054 123 3105 3315 3565 9536 1234 6347 1524 356 1230 213 3604 4153 4314 4276 2147 7076 3119 1401 1636 941 27232 3484 2052 348 3952 6462 1997 3117 1490 142 3329 5300 6364 6348 3559 217 6647 2688 4018 3303 335 4547 345 6464 3570 434 4208 210 654 48 6558 2705 5887 27343 3516 1843 10224 2181 2947 5371 406906 1540 9940 3455 599 200734 6173 9970 7203 7465 1848 10103 9587 1915 22809 57019 4205 1434 5071 5009 267 4325 10253 4100 1947 4090 6093 9768 7423 259266 9577 3624 9788 9002 3622 10120 283600 5971 2288 463 6477 333932 6414 8932 9071 375056 1656 203068 10553 728655 407008 10459 5794 9032 4059 2752 3811 10912 842 283518 3308 8881 1465 8838 1112 3911 5414 6495 3551 182 140 10935 6016 9863 10587 4616 1917 10899 80705 3692 29948 3858 10783 28234 | 166 |
| Liver disease | IFNG; KRT18; EPO; FECH; GNMT; IGFBP6; PLA2G5; IL6ST; CXCL10; IGFBP1; PKHD1; GSTP1; IL18; MPV17; HSPB1; CCR5; ALPL; TLR2; KRT8; IGFBP3; ADIPOQ; IGF1; EPHX1; PAK2; FGB; GGT1; IGFBP2; IGF2; H6PD; CST3; IL6; ATP7B; APOE; MTHFR; SERPINE1; CCL11; CCL2; F5; F2; PLAT; CTGF; | 3458 3875 2056 2235 27232 3489 5322 3572 3627 3484 5314 2950 3606 4358 3315 1234 249 7097 3856 3486 9370 3479 2052 5062 2244 2678 3485 3481 9563 1471 3569 540 348 4524 5054 6356 6347 2153 2147 5327 1490 257313 | 42 |
| Liver failure | FAS; ALAS1; ACADM; GC; IL18; RGN; POLG | 355 211 34 2638 3606 9104 5428 | 7 |
| Liver metastases | IL1A; ACTR2; TIMP1; F3; TJP1; CCL20; TXN; CHGA; IFNB1; PCSK2; WASF2; PCSK1; ARG1 | 3552 10097 7076 2152 7082 6364 7295 1113 3456 5126 10163 5122 383 | 13 |

| | | | |
|---|---|---|---|
| Liver tumor | TNFRSF10B; ADFP; HSPB1; SOCS3; IGFBP1; HSPD1; GH1; LPA; APOA1; MTTP; APOC3; IL6R; ASIP; ALAD; BMP6; ACO1; SLC12A2; SYT13; LOC652799; GJB1; RAD51AP1; RASGRF1; ALDOB; RAD23B; POLL; RBPJ; FGF19; DUSP1; INHBB; ZNF443; | 8795 123 3315 9021 3484 3329 2688 4018 335 4547 345 3570 434 210 654 48 6558 57586 652799 2705 10635 5923 229 5887 27343 3516 9965 1843 3625 10224 2181 10468 | 32 |
| Long QT syndrome | KCNQ1; KCNH2; SCN5A; KCNE1; ANK2; KCND2; SNTA1; RYR2; KCND3; SCN4B; CAV3; AKAP9 | 3784 3757 6331 3753 287 3751 6640 6262 3752 6330 859 10142 | 12 |
| Lung cancer | TNC; ALB; CEBPG; LGALS3BP; EIF3E; CTAG1A; MAGEA4; MAGEC1; TIMP1; AQP3; MRE11A; PAWR; BMPR2; AMBP; RASA1; TK2; DMTF1; ITIH3; HUS1; ADAM28; ICAM1; GDNF; IL8; CALCA; NUDT1; HYAL1; CACNA2D2; DLEC1; NUDT6; SAT1; RCVRN; ENO2; BRSK2; IL10RA; MARCKSL1; RAP1GDS1; RBM5; PYGB; SOX1; PLA2G16; DSP; AGER; SERPINE1; ITGA2; IL2; MBL2; PLAT; OSM; MMP10; MPO; MMP12; IL2RA; SOD1; CSTB; PDCD6; TUSC2; ESM1; CSTA; SEMA3F; ACACA; CXCR7; ACACB; EML4; CHRNB1; CRMP1; CCL5; IL6; IL4; CCR5; LPL; IL6ST; CTGF; ADRB2; AMFR; EEF1A2; AHR; FABP3; CDCP1; CDK5RAP3; EIF5A; NAPSA; STMN1; LAMA3; CYP2A13; CD3E; CCNB2; AZGP1; IL1B; SELE; ADIPOQ; HLA-B; HP; IGF1; IFNG; AKR1B10; NOD2; LTA; IL10; CYP2A6; FASLG; TLR4; KRT8; MIF; MMP3; IL1RN; CD83; SOCS3; EPHX1; LEP; ABCC4; SELP; ST8SIA1; LTF; ALDH2; ADCYAP1; SLPI; TXN; CYP27B1; APOA1; ARHGEF11; TTR; CSF3; KEAP1; GHR; CSF2; GJB1; POLL; SMAD5; ING2; CASP9; CT47A11; PRDX1; ERCC3; UCHL1; BLM; | 3371 213 1054 3959 3646 246100 4103 9947 7076 360 4361 5074 659 259 5921 7084 9988 3699 3364 10863 3383 2668 3576 796 4521 3373 9254 9940 11162 6303 5957 2026 9024 3587 65108 5910 10181 5834 6656 11145 1832 177 5054 3673 3558 4153 5327 5008 4319 4353 4321 3559 6647 1476 10016 11334 11082 1475 6405 31 57007 32 27436 1140 1400 6352 3569 3565 1234 4023 3572 1490 154 267 1917 196 2170 64866 80279 1984 9476 3925 3909 1553 916 9133 563 3553 6401 9370 3106 3240 3479 3458 57016 64127 4049 3586 1548 356 7099 3856 4282 4314 3557 9308 9021 2052 3952 10257 6403 6489 4057 217 116 6590 7295 1594 335 9826 7276 1440 9817 2690 1437 2705 27343 4090 3622 842 255313 5052 2071 7345 641 7507 9770 3248 8945 6367 7280 1387 43 9131 3678 3554 101 128 4086 5340 10761 391533 875 29089 1910 56896 2202 2074 551 10401 10928 5029 2730 3763 384 6573 57491 2073 5800 10971 1136 64840 25942 2042 3820 79723 9403 10140 4149 257019 3490 406913 10664 9700 10266 2948 218 11116 79365 11035 3762 11228 1138 5031 838 2237 4331 3765 25890 3760 902 5426 57060 26524 6235 | 205 |
| Lung disease | DEFA3; ITGA4; BDNF; NOD2; ITGAE; INS; IL15; IL12B; CCL11; PDCD1; MUC5B; SFTPD; SFTPB; CCL17; SFTPA1B; LCK; DDR1; GCLC; FANCB; SFTPC; MYCN; KCNQ1; SFTPA2B; SFTPA2; CDR2; ABCA3; CCNT1; STX2; AMY1A; LPP; RAD51L1; RPS3A | 1668 3676 627 64127 3682 3630 3600 3593 6356 5133 727897 6441 6439 6361 6435 3932 780 2729 2187 6440 4613 3784 6436 729238 1039 21 904 2054 276 4026 5890 6189 | 32 |

| | | | |
|---|---|---|---|
| Lupus erythematosus | AGTR1; CCL5; AGT; SPP1; TIMP1; CRP; ACE; ELF1; ESR1; CD27; ICOS; IL16; CHGA; P2RX7; CD247; IER3; TPI1; C4A; CREM; IGHG1; SNRNP70; CDR1; IGHG2; IL1B; SELE; ICAM1; RETN; TLR5; HLA-B; CIITA; SOD2; SELL; IFNG; CTLA4; TNF; HLA-A; CALCA; PTPN22; EGFR; IL4; IL2; IL10; CCR5; FAS; IL18; TNFRSF8; CD274; FASLG; MIF; PDCD1; JUN; BTNL2; MBL2; NAT2; F2; FCGR1A; IL1RN; HLA-DQB1; CD83; MYO9B; FCGR3A; IL17A; NAT1; MTR; IL4R; APOE; CYP1A1; S100B; FCGR2A; IFNGR2; CD28; BCL2; SELP; C3; PROCR; ELANE; FCGR3B; TNFSF13B; CASP3; IL2RA; CD4; SOD1; CAT; CXCL12; KIR2DS2; SERPINB2; APOC3; ITGB3; ITGAM; IRAK1; CD24; ITPR3; IRF5; HLA-DMA; ITGB1; CEBPB; ADA; IFNGR1; HAMP; HAVCR1; IL8RB; EFNA2; PTK2; KDR; PDCD1LG2; CD226; GATA3; CLU; TRAF1; ELAVL1; LCN2; CGB; C4B; LYN; MECP2; P2RY12; LILRB1; CD8A; IL10RA; FCGR2B; ADAR; LTK; PAPPA; KIR2DS1; LAT; TREX1; PHRF1; SEPT2; TYK2; MAN2A1; KCNA3; SLEB3; SIGLEC1; HRES1; CAMK4; LY9; | 185 6352 183 6696 7076 1401 1636 1997 2099 939 29851 3603 1113 5027 919 8870 7167 720 1390 3500 6625 1038 3501 3553 6401 3383 56729 7100 3106 4261 6648 6402 3458 1493 7124 3105 796 26191 1956 3565 3558 3586 1234 355 3606 943 29126 356 4282 5133 3725 56244 4153 10 2147 2209 3557 3119 9308 4650 2214 3605 9 4548 3566 348 1543 6285 2212 3460 940 596 6403 718 10544 1991 2215 10673 836 3559 920 6647 847 6387 100132285 5055 345 3690 3684 3654 100133941 3710 3663 3108 3688 1051 100 3459 57817 26762 3579 1943 5747 3791 80380 10666 2625 1191 7185 1994 3934 1082 721 4067 4204 64805 10859 925 3587 2213 103 4058 5069 3806 27040 11277 57661 4735 7297 4124 3738 64695 6614 3272 814 4063 11027 | 137 |
| Lupus vulgaris | ESR1; FCGR2A; CD27; TIMP1; FAS; IER3; CCL5; IGHG1; CREM; IL6; AGT; SNRNP70; FASLG; TPI1; P2RX7; PDCD1; CDR1; MMP9; CRP; ACE; AGTR1; CCL2; ELF1; SPP1; CD247; IL16; IGHG2; MIF | 2099 2212 939 7076 355 8870 6352 3500 1390 3569 183 6625 356 7167 5027 5133 1038 4318 1401 1636 185 6347 1997 6696 919 3603 3501 4282 | 28 |
| Lyme disease | ENO2; HSPD1; CXCL11; IL6; CCL2; MMP1; TLR2; IL1B; MMP13 | 2026 3329 6373 3569 6347 4312 7097 3553 4322 | 9 |
| Lymphatic metastasis | MMP3; F3 | 4314 2152 | 2 |

| | | | |
|---|---|---|---|
| Lymphoma | CTLA4; CASP7; IL10; IL21; B2M; SOX5; GCET2; SERPINA9; IFNAR2; PBK; NFATC2; POLI; POLM; ETV6; TNFSF13B; NR3C2; CSF2; RUNX1; BCL2L11; GATA1; MLL; HLA-C; PRDM1; SPI1; RUNX1T1; PRDM16; IKZF1; P2RY8; IGH@; IKZF3; MN1; MNX1; SLC16A1; IL4; IL18; TNFRSF8; CX3CR1; CXCL11; MMP3; IL2RA; GCNT1; LCK; IRF8; PCSK2; SPTA1; TSN; IL12A; GATA3; AHR; TRAF1; PCSK1; BRD2; DCLRE1C; RFC1; ENO2; RTN4; FOXP1; IRF3; SMC4; SMC2; SLC19A1; KCNA3; PTPRO; PER2; PTK6; LAMP1; CCR4; CD5; IKZF2; IL15RA; SLC22A2; IRF7; SLC22A1; | 1493 840 3586 59067 567 6660 257144 327657 3455 55872 4773 11201 27434 2120 10673 4306 1437 861 10018 2623 4297 3107 639 6688 862 63976 10320 286530 3492 22806 4330 3110 6566 3565 3606 943 1524 6373 4314 3559 2650 3932 3394 5126 6708 7247 3592 2625 196 7185 5122 6046 64421 5981 2026 57142 27086 3661 10051 10592 6573 3738 5800 8864 5753 3916 1233 921 22807 3601 9962 3665 9963 602 5134 2262 3726 | 77 |
| Lymphopenia | BCL2L1; FAS; XCL1; CD28; IL7; IL15; IL2 | 598 355 6375 940 3574 3600 3558 | 7 |
| Lymphoproliferative disorder | CCR9; FOXC2; CUGBP2; TLR1; IKZF1; PTPN11; CXCL9; CXCL13; SH2D1A; CCL17; BCL6; FSCN1; XCL1; LY75; BTK | 10803 2303 10659 7096 10320 5781 4283 10563 4068 6361 604 6624 6375 4065 695 | 15 |
| Lysosomal storage disease | UCHL1; MCOLN1; TPP1; SNCA | 7345 57192 1200 6622 | 4 |
| Macular degeneration | BEST1; EFEMP1; SELE; ICAM1; HP; VEGFA; TIMP3; CCL2; CX3CR1; TLR4; CRP; ACE; MMP7; CST3; APOE; C3; KLF5; LRP6; PLEKHA1; CFHR3; VLDLR; C2; | 7439 2202 6401 3383 3240 7422 7078 6347 1524 7099 1401 1636 4316 1471 348 718 688 4040 59338 10878 7436 717 2074 | 23 |
| Malaria | IL1RN; CX3CL1; VEGFA; MMP1; CTGF; IFNAR1; HAVCR1; ICAM1; HP; TNF; MIF; APOE; MET; ADRB2; P2RY1; AQP3; CAPN1; AHSG; PKLR; CLCN2; SLC4A1; HAMP; CAPN2; VNN1; NCR3; CCL18; CYBB; HPRT1; SPTA1 | 3557 6376 7422 4312 1490 3454 26762 3383 3240 7124 4282 348 4233 154 5028 360 823 197 5313 1181 6521 57817 824 8876 259197 6362 1536 3251 6708 | 29 |
| Malignant childhood tumor | F13A1; F8 | 2162 2157 | 2 |
| Malignant fibroxanthoma | HBEGF; BTC | 1839 685 | 2 |
| Malignant glioma | SERPINE1; TNF; CALR; IL4; CD274; FASLG; MTRR; F2; IL4R; IL6ST; OSM; CCL20; MMP12; FURIN; DCN; JAG1; ITGA5; SUMO1; PARK7; IL13RA1; STMN1; SP3; BEX1; CDKN2D; SPOCK2; ARL4A; STIP1; NBEAL1; S1PR2 | 5054 7124 811 3565 29126 356 4552 2147 3566 3572 5008 6364 4321 5045 1634 182 3678 7341 11315 3597 3925 6670 55859 1032 9806 10124 10963 65065 9294 | 29 |
| Malnutrition | TTPA; SCT; SLC41A1; IL6; IL1RN; CST3; MC4R; CAT | 7274 6343 254428 3569 3557 1471 4160 847 | 8 |
| Mastocytosis | TPSAB1; TPSB2 | 7177 64499 | 2 |

| | | | |
|---|---|---|---|
| Melanoma | TNF; LTA; MMP13; AMFR; ICAM1; CCR5; TIMP1; EDN1; CD80; IL6ST; CASP7; ING2; TMSB4X; MAGEC1; EDN3; IL1B; AGER; CCL5; SERPINE1; ITGA2; HLA-A; IL8; CALR; SKIL; SKI; IL2; IL10; IL18; CCL2; FASLG; TLR4; CCL21; LTBR; IL8RA; MMP3; MICA; CD86; ITGB2; SOCS3; CCR9; PARP1; BAK1; ICOS; F3; TJP1; CASP1; GNB2L1; DDIT3; B2M; POMC; IFIH1; ITGAM; ASIP; TNFRSF4; CDC2L2; IFNB1; P2RX7; MCAM; PAX3; VCAN; IL8RB; FST; SPRY2; MAGEA1; TSGA10; SLC26A1; B4GALNT1; PMAIP1; LLGL1; JAK1; BTRC; IL12A; SLC9A1; CLU; ITGA4; C1GALT1C1; LCP1; HRH2; JAK3; MAGED1; CRH; HSPB8; NOG; HOXD3; GLS; TPI1; ELN; RYR2; IL27; CD8A; MARK2; HNRNPD; PHLDA1; FCGR2B; F10; HLA-C; CTSK; OS9; MFI2; NEDD9; PDCD6; DCT; HOXB13; LGALS9; PLD1; CUX1; LAMP3; MITF; MAGEA6; SOX10; RNH1; MAGEB2; SILV; EPHB6; CSPG4; RGS1; KIR2DS4; ABCB5; TRH; IFI30; DGKA; IFIT2; CCL25; ABCA12; DBF4; MAGEB1; ITGAX; LTBP2; TTK; AIM1; PLXNC1; SCNN1D; IRF4; SCFV; UBE2I; RMI1; CANX; RALGDS; | 7124 4049 4322 267 3383 1234 7076 1906 941 3572 840 3622 7114 9947 1908 3553 177 6352 5054 3673 3105 3576 811 6498 6497 3558 3586 3606 6347 356 7099 6366 4055 3577 4314 4276 942 3689 9021 10803 142 578 29851 2152 7082 834 10399 1649 567 5443 64135 3684 434 7293 728642 3456 5027 4162 5077 1462 3579 10468 10253 4100 80705 10861 2583 5366 3996 3716 8945 3592 6548 1191 3676 29071 3936 3274 3718 9500 1392 26353 9241 3232 2744 7167 2006 6262 246778 925 2011 3184 22822 2213 2159 3107 1513 10956 4241 4739 10016 1638 10481 3965 5337 1523 27074 4286 4105 6663 6050 4113 6490 2051 1464 5996 3809 340273 7200 10437 1606 3433 6370 26154 10926 4112 3687 4053 7272 202 10154 6339 3662 652070 7329 80010 821 5900 9913 7268 3916 9636 57498 27123 | 144 |
| Meningioma | CTSL1; HLA-A; MTRR; TIMP1; MTR; ALPL; S100B; F3; CBS; RFC1; STS; EPB41; LAMB1; ROS1; | 1514 3105 4552 7076 4548 249 6285 2152 875 5981 412 2035 3912 6098 200895 | 15 |
| Mental retardation | GH1; AGTR2; ACSL4; PHOX2A; MED12; PQBP1; PJA1; MRX82; SMARCA1; ZNF674; PAK3; MRX81; REST; WNK3; NXF5; HUWE1; PLP2; FGD1; KIAA2022; SMS; RAC1; LAMB2; NF1; ADA; ATP1A2; AQP2; EEF1A1; SMC3; SIM2; NSD1; NKX2-1; RPS6KA3; IGF2BP1; HOXA1; SPAST; SOX3; CACNA1A; GLDC; CDK5R1; ATRX; FKRP; LAMA2; POMT2; SPG11; SEMA3F; KLF8; DCX; NLGN4Y; RAB3A; DOPEY2; IL1RAPL1; STRA6; MCRS1; CMD1B; CC2D2A; TSPAN7; [illegible] | 2688 186 2182 401 9968 10084 64219 378484 6594 641339 5063 266678 5978 65267 55998 10075 5355 2245 340533 6611 5879 3913 4763 100 477 359 1915 9126 6493 64324 7080 6197 10642 3198 6683 6658 773 2731 8851 546 79147 3908 29954 80208 6405 11279 1641 22829 5864 9980 11141 64220 10445 1218 57545 7102 266743 406874 9856 9901 | 60 |
| Mesothelioma | KRT5; AVEN; ACTR1A; ITGA2; POMC; CLNS1A | 3852 57099 10121 3673 5443 1207 | 6 |

| | | | |
|---|---|---|---|
| Metabolic syndrome X | ADIPOQ; CRP; IGFBP3; IL10; CTF1; APOC3; ACE; AHSG | 9370 1401 3486 3586 1489 345 1636 197 | 8 |
| Metabolism disease | ADIPOQ; FGB; ACE; ADRB3; CRP; PPARGC1A; AKT2; BCS1L; ADSL; GCG; SERPINE1; MMP9; PTPN1; IL6; MMP3; ADRB2; HIF1A; SLC25A13; PYGM; INS; NAMPT; SLC2A4; AKR1B1; SUMO4; LPL; APOB; KCNJ11; HNF4A; GIP; GCK; B2M; IAPP; AHSG; LMNA; ACO1; XPA; SLCO1B1; TNAP; ANK3 | 9370 2244 1636 155 1401 10891 208 617 158 2641 5054 4318 5770 3569 4314 154 3091 10165 5837 3630 10135 6517 231 387082 4023 338 3767 3172 2695 2645 567 3375 197 4000 48 7507 10599 445341 288 | 39 |
| Metaplastic polyp | CEACAM1; PTGS2; BRAF; SFRP1; CDKN2B; MLH1; INS; CDKN1A; MUC6 | 634 5743 673 6422 1030 4292 3630 1026 4588 | 9 |
| Metastasis to lymph nodes | IL8; TIMP1; SOCS3; CTGF; SERPINA3; HSPD1; MMP13; CSF1; CD4; ADM; SLC2A3; B2M; MSN; MMP8; VCAN; ZFHX3; ADAMTS9; G3BP1; EEF1D; LLGL1; HSPE1; RASSF2; FABP6; KIF2C; ANXA7; DSG3 | 3576 7076 9021 1490 12 3329 4322 1435 920 133 6515 567 4478 4317 1462 463 56999 10146 1936 3996 3336 9770 2172 11004 310 1830 | 26 |
| Metastatic cancer to brain | HP; APOA1; TTR | 3240 335 7276 | 3 |
| Migraine | MEP1A; ESR1; RHAG; HTR1A; ACE; SLC6A4; IL6; MTHFR; HTR2C; TNF; AR; ICAM1; SLC6A3; ITGB2; IL1A; ITGAL; CALCA; CTLA4; LTA; CACNA1A; BDNF; ATP1A2; KCNN3; LDLR; SCN1A; RAMP1; TPH1; INSR; AQP1 | 4224 2099 6005 3350 1636 6532 3569 4524 3358 7124 367 3383 6531 3689 3552 3683 796 1493 4049 773 627 477 3782 3949 6323 10267 7166 3643 358 | 29 |
| Mitochondrial disease | TRNF; CYTB; POLG; SCO2; BCS1L; MB | 4558 4519 5428 9997 617 4151 | 6 |
| Mitochondrial encephalomyopathies | COX2; NDUFAF4; TYMP; BRD2 | 4513 29078 1890 6046 | 4 |
| Mitral valve disease | PLAU; IL2RA; CD24; IL1R1; FBN1 | 5328 3559 100133941 3554 2200 | 5 |
| Mixed salivary gland tumor | FSD1 | 79187 | 1 |
| Movement disorder | ATP7B; LOX; LOXL2; ATOX1; PNOC; SNCA; GAD1; ADH1B; GAD2; PARK2; MECP2; SGCE; KCNMA1; JPH3; TOR1A; HS1BP3; ETM2; PANK2; UBE3A; SLC9A6; CST6; NPFFR2; ATXN2 | 540 4015 4017 475 5368 6622 2571 125 2572 5071 4204 8910 3778 57338 1861 64342 2112 80025 7337 10479 1474 10886 6311 | 23 |
| Moyamoya disease | MTHFR; TIMP2; TTR; HIF1A; ENG; CASP3 | 4524 7077 7276 3091 2022 836 | 6 |
| Mucocutaneous lymph node syndrome | MMP3; CRP; ACE; HLA-C; HLA-A; SLC11A1; CCL3L1; MMP9; MBL2; AGTR1; IL10; KDR; TIMP1; CCR5; S100A12; MMP13; ITPKC; TNF; VEGFA; IL4; HLA-B | 4314 1401 1636 3107 3105 6556 6349 4318 4153 185 3586 3791 7076 1234 6283 4322 80271 7124 7422 3565 3106 | 21 |
| Mucopolysaccharidosis | GALNS; CREBBP; RPS27 | 2588 1387 6232 | 3 |

| | | | |
|---|---|---|---|
| Multiple endocrine neoplasia | MEN1; SLC6A2; RET; AMHR2; CHGA; SLC18A2; SLC18A1; RPA2; CDKN1B | 4221 6530 5979 269 1113 6571 6570 6118 1027 | 9 |
| Multiple malignancy | CTLA4; ZFHX3; RASSF2; HACE1; ABCA12 | 1493 463 9770 57531 26154 | 5 |
| Multiple myeloma | IL6ST; IL1B; CIITA; IFNG; TNF; BDNF; HLA-A; LTA; HSPB1; CCR5; FASLG; MBL2; MTRR; MICA; TIMP1; IL1RN; XBP1; AREG; CD28; OSM; CCL20; IL16; CCL3; IL12RB2; CASR; PTPRC; IL6R; BGLAP; NCR3; IFNAR1; CYLD; IFNAR2; CD226; PMAIP1; MAF; CCL4; IL11RA; CHI3L1; LYN; NCR1; AMBP; XRCC4; ACP5; CALCR; CTSK; MAGEC1; WWTR1; GAB2; SPANXB1; WHSC1; SULF2; LIG4; SLAMF7; TNFRSF17; DAZAP2; TRAF3; IGL@; IGHM | 3572 3553 4261 3458 7124 627 3105 4049 3315 1234 356 4153 4552 4276 7076 3557 7494 374 940 5008 6364 3603 6348 3595 846 5788 3570 632 259197 3454 1540 3455 10666 5366 4094 6351 3590 1116 4067 9437 259 7518 54 799 1513 9947 25937 9846 728695 7468 55959 3981 57823 608 9802 7187 3535 3507 | 58 |
| Multiple sclerosis | IL1B; ICAM1; ND5; CCL5; IFNG; SERPINE1; TNF; BDNF; HLA-A; IL8; NOD2; BDKRB1; IL12B; SHH; GSTM1; IL13; IL2; CCR5; MX1; IDO1; IL18; SPP1; CX3CR1; TLR4; IRF1; CD14; BTNL2; MST1R; MMP3; TIMP1; FCGR3A; IL17A; LEPR; RELA; MICB; BIRC3; CD80; LEP; PLAT; CD28; ICOS; CX3CL1; BCL2L1; MPO; TIMP2; MMP12; CASP1; CD4; A2M; CXCL12; TRNL1; UCP2; CD24; TNFRSF4; IL7R; IL8RB; VIPR2; ITGA1; XIAP; CD46; BIRC2; CALB2; F2RL1; ATP6; MOG; MBP; SLC1A2; TPI1; RTN4; PNMT; SELPLG; GLO1; IL5RA; TPSAB1; CD8A; PVALB; KCNA3; PADI2; HLA-C; IL17C; PVRL2; STMN1; SLC1A1; SLC1A3; KLC1; TRBV5-2; PTPRCAP; EIF2B5; EBF1; PTPN6; TPMT; C10ORF27; IFNA17; ATP8 | 3553 3383 4540 6352 3458 5054 7124 627 3105 3576 64127 623 3593 6469 2944 3596 3558 1234 4599 3620 3606 6696 1524 7099 3659 929 56244 4486 4314 7076 2214 3605 3953 5970 4277 330 941 3952 5327 940 29851 6376 598 4353 7077 4321 834 920 2 6387 4567 7351 100133941 7293 3575 3579 7434 3672 331 4179 329 794 2150 4508 4340 4155 6506 7167 57142 5409 6404 2739 3568 7177 925 5816 3738 11240 3107 27189 5819 3925 6505 6507 3831 28613 5790 8893 1879 5777 7172 219793 3451 4509 | 94 |
| Multiple system atrophy | ICAM1; IL8; DBH | 3383 3576 1621 | 3 |
| Muscular atrophy | BCLAF1; TRNS1; TPM2; FHL1; CAST; IL15; FOXO1; MUSK; SOD1; EMD; AR; LMNA; CBLB | 9774 4574 7169 2273 831 3600 2308 4593 6647 2010 367 4000 868 | 13 |
| Muscular dystrophies | LMNA; FKRP; TTN; LAMA2; SEPN1; SMN1; SHOX; CTGF; FST; ANXA2; EMD; ITGA7; ANXA1; CAPN3; CAV3; TNF; INSR; SMC3; HNRNPA2B1; BCLAF1; GNE; AMPD3; HNRNPA1; DNAJA1; CDC42BPB; CUGBP1 | 4000 79147 7273 3908 57190 6606 6473 1490 10468 302 2010 3679 301 825 859 7124 3643 9126 3181 9774 10020 272 3178 3301 9578 10658 | 26 |

| | | | |
|---|---|---|---|
| Muscular dystrophy | CFLAR; SGCB; POMT2; SGCG; MMP2; TIMP1; HSPG2; TIMP2; CAPN2; PROM1; MSTN; PAMR1; SDC3 | 8837 6443 29954 6445 4313 7076 3339 7077 824 8842 2660 25891 9672 | 13 |
| Myasthenia Gravis | IL12B; CXCL13; ESR1; ESR2; CTSL2; MUSK; CD276; MYAS1 | 3593 10563 2099 2100 1515 4593 80381 246750 | 8 |
| Mycoplasma pneumonia | TLR1; TLR6 | 7096 10333 | 2 |
| Mycoses | IFNA1; APOE; TNF; CALCA; TLR4 | 3439 348 7124 796 7099 | 5 |
| Mycosis fungoides | CTLA4; TLR4; TLR2; HLA-DPB1; DEFB103A; ICAM3; CCL27; CDO1; DNM3; CCL21; IL21; CXCL13; CD4; EPHA4; KIR2DL1; LILRB1; KIR2DL3; CCL19; PLS3; KIR3DL2 | 1493 7099 7097 3115 55894 3385 10850 1036 26052 6366 59067 10563 920 2043 3802 10859 3804 6363 5358 3812 | 20 |
| Myelofibrosis | IL8; IL8RA; MMP13; BMP6; MMP8; IL8RB; AMFR; FBN1; HDAC4; FKBP5; BMP1 | 3576 3577 4322 654 4317 3579 267 2200 9759 2289 649 | 11 |
| Myeloid metaplasia | IL8; IL8RA; IL8RB | 3576 3577 3579 | 3 |
| Myeloproliferative disease | IL3RA; JAK2; PTPN11; GATA1; FGFR1; JAK3; BCR; EP300; EPOR; NOG; DIDO1; MYB; CD177; MPL | 3563 3717 5781 2623 2260 3718 613 2033 2057 9241 11083 4602 57126 4352 | 14 |
| Myopathy | SELE; ATOX1; XBP1; BRAF; SNCA; GAD1; MAPT; SCN2A; ACSL4; RET; NEFL; SCN1A; SCN8A; SPTLC1; HSN2; SPAST; ANKK1; GLB1; ALS2; PANK2; DOK7; RAPSN; CHRNA1; KCNQ2; SMN1; SQSTM1; NAIP; STH; ATXN3; BSCL2; NEFM; SMN2; SCO2; UBA1; OS9; ATP7A; ATP2A2; IL11; ADRB2; SOD1; PNPLA2; CAPN1; ITGA7; FHL1; EMD; SPTAN1; LAMP2; NCAM1; CACNA1A; MYH7; SCN4A; TRNE; CAV3; TK2; MYH2; COX3; RNR2; CHRNE; SLCO1B1; TRNK; ETFDH; MYH3; RYR1; CRYAB; FKRP; KCNJ2; UBB; PAX7 | 6401 475 7494 673 6622 2571 4137 6326 2182 5979 4747 6323 6334 10558 378465 6683 255239 2720 57679 80025 285489 5913 1134 3785 6606 8878 4671 246744 4287 26580 4741 6607 9997 7317 10956 538 488 3589 154 6647 57104 823 3679 2273 2010 6709 3920 4684 773 4625 6329 4556 859 7084 4620 4514 4550 1145 10599 4566 2110 4621 6261 1410 79147 3759 7314 5081 | 68 |
| Myotonic disorder | HSPG2; CLCN1; ND1; CACNA1A; ND3; GCH1; ATP1A3; TPI1; ACTB; GCHFR; SPR | 3339 1180 4535 773 4537 2643 478 7167 60 2644 6697 | 11 |
| Narcolepsy | LEP; CLOCK; NCRNA00163; HLA-DQB1; MX2; IFN1@; TNF | 3952 9575 727699 3119 4600 3438 7124 | 7 |
| Nasopharyngeal cancer | HLA-B; IFNG; TNF; IL6; HLA-A; IL8; IL10; CCL2; TLR4; TLR3; HSPA1B; MICA; CD86; CD80; LTF; DLEC1; ADAMTS9; BCAT1; RASSF2; CENPH; PAWR; H19; EDNRB; LGALS9; TRAF3; TUBB2C; LOC344967 | 3106 3458 7124 3569 3105 3576 3586 6347 7099 7098 3304 4276 942 941 4057 9940 56999 586 9770 64946 5074 283120 1910 3965 7187 10383 344967 | 27 |
| Neck cancer | SELL; CDKN1A; BBC3; RET; CYP1A1; CCND1; BCL2L1; HYAL1; XRCC3; S100A2 | 6402 1026 27113 5979 1543 595 598 3373 7517 6273 | 10 |
| Necrotizing enterocolitis | EGF; TLR4; MMP3; TNF; IL4R; MIF | 1950 7099 4314 7124 3566 4282 | 6 |

| | | | |
|---|---|---|---|
| Neoplasm metastasis | IL1B; SELE; ICAM1; CTSL1; PPARGC1A; FTO; AGER; INPPL1; CRMP1; IFNG; CASP10; SERPINE1; TSC2; PTGES; IL10; IL18; FASLG; CCR1; PDX1; CXCL6; F2; CSF1R; MASP2; CCL24; LEPR; ACE; ECM1; CST3; PTPN13; S100B; CCR9; OSM; SELP; PLA2G2A; ST8SIA1; CCL3; FURIN; SLPI; VWF; FOXC2; IL6R; LRP5; BMP6; PLXDC1; SDC2; PAX3; LGALS3BP; GJB1; FST; AMFR; MMP16; ROCK1; MTSS1; HTATIP2; BCAM; HSPA4; JAG1; EIF6; TSPAN8; LYPD3; MSH3; CTSH; ATF3; PLD2; MAF; NTN1; PKD1; S100A6; IL12A; RNASE1; EPHB1; ADAMTS13; METAP2; CDCP1; LPAR1; LCN2; F2RL1; ITGB6; PRPH2; MAGED1; WISP2; SERBP1; F7; TPO; STS; LAMB1; EDNRB; SERPINA5; ARRB1; CBR3; CHUK; LIMS1; MARK2; AMBP; MST1; GNA12; CRYAB; LTBP1; FCGR2B; MLL; IBSP; BMP5; CTSK; STMN1; TPP1; VSNL1; TYR; KNG1; SEMA3F; ITIH3; LGALS9; ARID4A; DRG1; DNAJB6; DNAJA3; PAX7; ABI2; PELP1; KLK8; SLC38A3; RAN; DACH1; TBC1D3B; FABP5; GNA13; TMSB15A; SMARCC1; LZTS1; RAD21; SATB1; SILV; EPHB6; RALGDS; MSRA; PTPRD; | 3553 6401 3383 1514 10891 79068 177 3636 1400 3458 843 5054 7249 9536 3586 3606 356 1230 3651 6372 2147 1436 10747 6369 3953 1636 1893 1471 5783 6285 10803 5008 6403 5320 6489 6348 5045 6590 7450 2303 3570 4041 654 57125 6383 5077 3959 2705 10468 267 4325 6093 9788 10553 4059 3308 182 3692 7103 27076 4437 1512 467 5338 4094 9423 5310 6277 3592 6035 2047 11093 10988 64866 1902 3934 2150 3694 5961 9500 8839 26135 2155 7173 412 3912 1910 5104 408 874 1147 3987 2011 259 4485 2768 1410 4052 2213 4297 3381 653 1513 3925 1200 7447 7299 3827 6405 3699 3965 5926 4733 10049 9093 5081 10152 27043 11202 10991 5901 1602 414059 2171 10672 11013 6599 11178 5885 6304 6490 2051 5900 4482 5789 28444 3417 6281 6520 4071 57504 680 55898 2770 64090 27069 9097 9439 3993 3696 5110 | 151 |
| Nephritis | CCL2; KIT; ANKRD1; KITLG; WT1 | 6347 3815 27063 4254 7490 | 5 |
| Nephroblastoma | ATF3; BBS9; TRIM37; RBM4; CTCF; POU6F2; WTAP | 467 27241 4591 5936 10664 11281 9589 | 7 |
| Nephrosis | IL18; RELA; MAF; LAMB2; LEPR; HSPC159; NDST1; CCL13; HPX; IL1B; TNF; F5; APOA4; BRAF; APOE; F3; CETP; ADA; CLU; IL1R1 | 3606 5970 4094 3913 3953 29094 3340 6357 3263 3553 7124 2153 337 673 348 2152 1071 100 1191 3554 | 20 |
| Nervous system disease | FASLG; ICAM1; FAS; MECP2; HSPB8; B4GALT1; AQP4; SNCA; APP; MAPT; SCN1A; POLG; OTSC5; OTSC4; ADRA2A | 356 3383 355 4204 26353 2683 361 6622 351 4137 6323 5428 317682 286751 150 | 15 |
| Nervous system tumor | PTN; DNAJC15; MIB1; INS; LRRC4; BAI1; B4GALT5; BCAN; TNC; PTK2B; GFAP; MXI1 | 5764 29103 57534 3630 64101 575 9334 63827 3371 2185 2670 4601 | 12 |
| Neurilemmoma | CXCL16; TIE1; CXCR6; ABCA2; | 58191 7075 10663 20 9146 | 5 |
| Neuritis | CCR5; FAS; FASLG; TIMP2; SEPT4; SPHK1; SEPT9; RTKN | 1234 355 356 7077 5414 8877 10801 6242 | 8 |

| | | | |
|---|---|---|---|
| Neuroblastoma | WFS1; GDNF; CCL2; TLR4; LEP; ABCC4; PIN1; ADCYAP1; SOD1; DDC; CHRM3; TTR; ACP1; TRPC1; DUSP1; PML; CASP9; PMAIP1; CACYBP; CREBBP; CFL1; CHRNA7; PAWR; ELAVL4; EPB41; ENO2; PHOX2B; ALOX5AP; CHRM1; CD276; CDK9; TMSB15A; UBE2D3; PCTK1; APITD1; INSRR; GMEB1; ATP1B1; DDX1; BNIP2; INA; FLJ22536; ELAVL3; MYBL2; PIK3CD; MIR34A; DHX9; SP100; CHD5; LMO3; PRAF2; ALX3; [illegible] | 7466 2668 6347 7099 3952 10257 5300 116 6647 1644 1131 7276 52 7220 1843 5371 842 5366 27101 1387 1072 1139 5074 1996 2035 2026 8929 241 1128 80381 1025 11013 7323 5127 378708 3645 10691 481 1653 663 9118 401237 1995 4605 5293 407040 1660 6672 26038 55885 11230 257 1613 | 53 |
| Neurodegenerative disorder | SNCA; APP; MAPT; PIN1; NEFL; LOC643387; PARK7; SQSTM1; ADRBK1; ATXN2; CHMP2B; STH; STUB1; GDNF; APOE; AHSG; PINK1; CASP8; CAPN2; SCT; KITLG; WNT1; SEPT4; ACHE; SUMO1; KCNMA1; GAPDH; DYRK1A; KCNC3; SFRS7; REEP1; PQBP1; SNCAIP; CLN3; DBN1; | 6622 351 4137 5300 4747 643387 11315 8878 156 6311 25978 246744 10273 2668 348 197 65018 841 824 6343 4254 7471 5414 43 7341 3778 2597 1859 3748 6432 65055 10084 9627 1201 1627 642 11076 | 37 |
| Neuroendocrine tumor | IL6; SLC6A2; CHGA; GGH; B3GAT1; CGB; TRPM8; APLP1 | 3569 6530 1113 8836 27087 1082 79054 333 | 8 |
| Neurofibromatosis | NF1; KITLG; KIT; NF2; ENO2; GDNF; APOBEC1; KIF5B; MDK; | 4763 4254 3815 4771 2026 2668 339 3799 4192 338 | 10 |
| Neuropathy | LRP2; CKB; SLC12A6; FIG4; PRX; HSN2; SPTLC2; MAPT; SCN2A; LMNA; MFN2; MPZ; SCN1A; SH3TC2; NAB2; MYOT; SPTLC1; NAB1; POLG; C10ORF2; SMN1; STH; SMN2; AGER; TRPV1; ALB; SNCA; GAD1; ND1; LITAF; MTMR2; DFNB59; OTOF; CD1A; SNAP29; SERPING1; DPYSL5; | 4036 1152 9990 9896 57716 378465 9517 4137 6326 4000 9927 4359 6323 79628 4665 9499 10558 4664 5428 56652 6606 246744 6607 177 7442 213 6622 2571 4535 9516 8898 494513 9381 909 9342 710 56896 5354 4541 | 39 |
| Neurotic disorder | SLC6A4; ANKK1; XBP1; DAOA; MAOA; MBL2; APOBEC3G; CD247; GLRA1; MC2R | 6532 255239 7494 267012 4128 4153 60489 919 2741 4158 | 10 |
| Neutropenia | ELANE; IFNG; MASP2; CSF3; RECQL4; CSF3R | 1991 3458 10747 1440 9401 1441 | 6 |
| Nevus | ASIP; F2; IGFBP1; HLTF; GLB1; RHCE; RHD; F10 | 434 2147 3484 6596 2720 6006 6007 2159 | 8 |

| | | | |
|---|---|---|---|
| Obesity | SELE; ICAM1; TNF; ADRA2B; SCG5; TNMD; FTO; GGT1; RBP4; RETN; FADS2; PLIN; AGTR1; MTHFR; FABP2; MMP2; VEGFA; REN; IGF1; IGFBP3; AGT; SERPINE1; BDNF; IL6; IL8; TRPV1; SLC16A1; CALCA; SLC6A4; HTR2A; HIF1A; IL10; FAS; IL18; SPP1; CCL2; ADIPOR2; MIF; MMP9; HSPA1B; MTRR; MMP3; IL1RN; APOA4; CRP; ITGB2; EDN1; LEPR; IGF2; ACE; SOCS3; MTR; IGFBP6; IL6ST; LEP; SHBG; ESR1; CCND1; ADRB2; APP; A2M; GC; IRS1; PNPLA2; LIPE; ADM; MC4R; SLC2A1; CAPN10; HSD11B2; BCHE; GH1; LIPC; AKT2; LPA; ABCA1; PBX1; HSD11B1; UCP1; GAD2; MTTP; UCP2; AQP7; FOXC2; POMC; ISL1; GCKR; SCD; AKT1; APLN; PINK1; MC3R; SLC2A2; FABP4; GFPT1; CLOCK; APOA2; SLC16A4; ADCY3; AGRP; HAMP; ADORA1; SLC6A3; BDKRB2; NR1H3; FGF19; INHBB; RASSF1; AKR1C1; INSR; RARA; SLC6A14; SAA1; LCN2; CEBPA; PCSK1; CCKAR; HRH3; INHA; MFN2; BBS4; ADRA2A; MAOA; CARTPT; HTR2C; ANKK1; FAAH; GALR1; NPFFR2; FGA; RORA; ACSM3; ADRB1; PNMT; CYP19A1; PMCH; CHUK; CTSS; | 6401 3383 7124 151 6447 64102 79068 2678 5950 56729 9415 5346 185 4524 2169 4313 7422 5972 3479 3486 183 5054 627 3569 3576 7442 6566 796 6532 3356 3091 3586 355 3606 6696 6347 79602 4282 4318 3304 4552 4314 3557 337 1401 3689 1906 3953 3481 1636 9021 4548 3489 3572 3952 6462 2099 595 154 351 2 2638 3667 57104 3991 133 4160 6513 11132 3291 590 2688 3990 208 4018 19 5087 3290 7350 2572 4547 7351 364 2303 5443 3670 2646 6319 207 8862 65018 4159 6514 2167 2673 9575 336 9122 109 181 57817 134 6531 624 10062 9965 3625 11186 1645 3643 5914 11254 6288 3934 1050 5122 886 11255 3623 9927 585 150 4128 9607 3358 255239 2166 2587 10886 2243 6095 6296 153 5409 1588 5367 1147 1520 6357 726 825 2660 9672 54 2194 1513 4671 2693 10874 563 10400 366 55885 6287 6289 100125288 797 29106 3098 56259 7275 1149 866 6492 286753 79827 79689 66036 10999 | 169 |
| Obsessive-compulsive disorder | PKM2; IL6; SLC6A4; CNTNAP2; HTR2A; SLC22A3; HTR3A; BDNF; SGCE; SLC1A1; TOR1A | 5315 3569 6532 26047 3356 6581 3359 627 8910 6505 1861 | 11 |
| Oligospermia | BRCA2; AR; DAZ1; DDX25; BPY2B; H19; ADM; DAZL; GSTT1; BPY2C; CFTR; POLG; GGT1; | 675 367 1617 29118 442867 283120 133 1618 2952 442868 1080 5428 2678 3623 9083 | 15 |
| Optic atrophy | CACNA1F; OPA3; EGFR; EPHX1; ND6 | 778 80207 1956 2052 4541 | 5 |
| Oral cancer | EGFR; KLRC1; PDCD1; PTGS2; ERBB2; VHL; IL1B; TNC; ITGA2; IL8; MGMT; XRCC1; GSTM1; GSTT1; GSTP1; CDKN2A; IL4; IL10; CYP2A6; IL18; TLR4; MMP9; MMP3; IGF2; CDKN1B; MMP7; CTNNB1; BMI1; CAV1; CCND1; MMP13; PLAU; CXCL12; CDKN2B; DCN; NANOG; IGF1R; MYC; CDKN1A; FHIT; GSTM3; XRCC3; BSG; SNAI1; TYMP; POSTN; XRCC2; PTH1R; XRCC4; LGALS9; PCBP2; MAP2; FAT1; ORAOV1 | 1956 3821 5133 5743 2064 7428 3553 3371 3673 3576 4255 7515 2944 2952 2950 1029 3565 3586 1548 3606 7099 4318 4314 3481 1027 4316 1499 648 857 595 4322 5328 6387 1030 1634 79923 3480 4609 1026 2272 2947 7517 682 6615 1890 10631 7516 5745 7518 3965 5094 4133 2195 220064 | 54 |

| | | | |
|---|---|---|---|
| Osteitis deformans | SQSTM1; CCND1; EDN1; FASN; ERBB2; GNE | 8878 595 1906 2194 2064 10020 | 6 |
| Osteomyelitis | IFNGR1; F10; MLL; RAF1; TLR5; RUNX1; CLCN7; BAX; DUSP1 | 3459 2159 4297 5894 7100 861 1186 581 1843 | 9 |
| Osteoporosis | MTHFR; CALCA; ALPL; LTF; BMP7; CST3; ALOX15; KIT; LEP; IBSP; LRP5; ITGA6; ESR1; IGF1; ICAM1; BMP2; MMP13; SPP1; IL1A; ESR2; GH1; TRAF6; AHSG; RUNX2; PTK2B; SHBG; GC; CYP1A1; POMC; IGF1R; ACAN; INSL3; HSD11B1; SLC22A11; CYP27B1; BGLAP; CALCR; | 4524 796 249 4057 655 1471 246 3815 3952 3381 4041 3655 2099 3479 3383 650 4322 6696 3552 2100 2688 7189 197 860 2185 6462 2638 1543 5443 3480 176 3640 3290 55867 1594 632 799 1588 3672 367 | 40 |
| Osteosarcoma | IL8; MICA; CTGF; RAPH1; BUB3; IGF1; NR1I2; IMPDH2; DCN; RASGRF1; EFNB1; ITGA4; ANXA5; FDPS; SUMO1; ACTA2; SLC19A1; MTNR1A; CAMK2A; FAU; TOPORS; RECQL4 | 3576 4276 1490 65059 9184 3479 8856 3615 1634 5923 1947 3676 308 2224 7341 59 6573 4543 815 2197 10210 9401 | 22 |
| Otitis media | FBXO11; SFTPA1B; IL1B; TNF; EYA4; BCL6; IL8; MUC5B; MUC4 | 80204 6435 3553 7124 2070 604 3576 727897 4585 | 9 |
| Ovarian cancer | IQGAP1; FRAT1; CLU; MCM5; PPARGC1A; GGT1; HLA-DQA1; MCAM; ERCC3; XRCC2; CBR3; MYD88; CAMK4; RAD52; IL1A; IL1B; ATP7B; AGTR1; IFNG; SERPINE1; IL6; CALCA; IL4; IL18; CD274; FASLG; MIF; MMP3; IL1RN; CRP; EDN1; EPHX1; APOE; PLAT; CTGF; FGF1; PAK1; EDNRA; SLPI; ADM; HSD11B2; ITIH2; DLEC1; PARK2; ROCK1; JAG1; EEF1A2; SAT1; MSH3; INSR; RUNX1; CTNNA1; ACHE; ITGA5; LPAR2; RNASEL; FANCA; LIF; CRHR1; SERBP1; CRH; INSL3; COX2; IL7; GALT; GAS6; CYP19A1; VTN; RNASE2; EIF5A2; LTBP1; ATP7A; ESRRA; C11ORF30; GAB2; KLF8; KLK8; EPHA1; FANCF; TSG101; FGF8; DNAJC15; ZNF350; BACH2; SCAF1; CLIP1; AGPAT2; RAB25; EPHA5; SELENBP1; EYA2; C1ORF38; USP36; CARD10; LOC390998; CHP2; PCSK6; | 8826 10023 1191 4174 10891 2678 3117 4162 2071 7516 874 4615 814 5893 3552 3553 540 185 3458 5054 3569 796 3565 3606 29126 356 4282 4314 3557 1401 1906 2052 348 5327 1490 2246 5058 1909 6590 133 3291 3698 9940 5071 6093 182 1917 6303 4437 3643 861 1495 43 3678 9170 6041 2175 3976 1394 26135 1392 3640 4513 3574 2592 2621 1588 7448 6036 56648 4052 538 2101 56946 9846 11279 11202 2041 2188 7251 2253 29103 59348 60468 58506 6249 10555 57111 2044 8991 2139 9473 57602 29775 390998 63928 5046 406883 4756 | 99 |
| Ovarian disease | GPC3; GATA4; CDKN1C; ELN; IL10; MMP9; KRT7; ESR1; ESR2; GH1; ACTN4; MIR223; ERCC1; XPA; AMH; SOX9; AMHR2; FH | 2719 2626 1028 2006 3586 4318 3855 2099 2100 2688 81 407008 2067 7507 268 6662 269 2271 | 18 |

| | | | |
|---|---|---|---|
| Ovarian failure | GDF9; LHX8; MSH4; GALT; POF1B; INHA; BMP15; BCKDHB; FOXL2; AMH; INHBB; FOXE1; MSH5; KITLG; FIGLA; H19; ESR1; NANOS3; DMC1 | 2661 431707 4438 2592 79983 3623 9210 594 668 268 3625 2304 4439 4254 344018 283120 2099 342977 11144 | 19 |
| Ovary cancer | TNF; IL8; IL15; IL13; TIMP1; FGF7; CASP1; CAMP; FURIN; DUSP1; LCN2; CASP4; HUS1; C19ORF33; WFDC2; RAD9A; SET; PGRMC1; FBXO5; LTB4R2 | 7124 3576 3600 3596 7076 2252 834 820 5045 1843 3934 837 3364 64073 10406 5883 6418 10857 26271 56413 | 20 |
| Overnutrition | IGFBP1; APOB; CETP; ATP1A2; ALDOB; LDLR; PIK3R1; ITGA4; KCNJ6; KCNJ3; CD52; AGL; KCNJ12; GBE1; BTD | 3484 338 1071 477 229 3949 5295 3676 3763 3760 1043 178 3768 2632 686 | 15 |
| PEComa | TSC2; CD1A | 7249 909 | 2 |
| Palmoplantar keratosis | GJA1; SNAP29; RSPO1; DSP; KRT9 | 2697 9342 284654 1832 3857 | 5 |
| Pancreas cancer | PLAT; WISP2; SLC28A1; SERPINE1; KRT8; S100A6; BGN; CCKAR; HNRNPA2B1; PAWR; S100P; IFNG; HLA-A; HRH4; SMO; ABCC5; LIF; RAPGEF3; U2AF1; GDI2; CELA3A; CCNI; LIFR; HRH3; IL1A; AGER; GDNF; ITGA2; TNF; TSC2; IL4; FASLG; CXCL16; MTRR; DLG5; CA2; NFATC1; TXNIP; IL4R; APOC1; CTGF; F3; FGF7; MPO; CSF1; IL2RA; ADM; IL6R; GLP1R; BGLAP; KLF10; IFNAR1; IFNAR2; DUSP6; ROCK1; F2RL3; SIAH1; EIF4A3; GATA3; OGFR; LCN2; F2RL1; TMSB4X; B3GNT2; MGAT4B; XRCC2; MAGED1; IHH; MAP3K5; ADAM8; PMS1; RNASEL; CS; MAG; KIF20A; SPOCK1; GIPC1; ADH5; RAP1GAP; MSX2; SMURF1; SMAD1; RARG; EIF5A; MGAT4A; FGF10; PDGFD; FANCA; IER3; RPL19; LILRB4; PTN; PLG | 5327 8839 9154 5054 3856 6277 633 886 3181 5074 6286 3458 3105 59340 6608 10057 3976 10411 7307 2665 10136 10983 3977 11255 3552 177 2668 3673 7124 7249 3565 356 58191 4552 9231 760 4772 10628 3566 341 1490 2152 2252 4353 1435 3559 133 3570 2740 632 7071 3454 3455 1848 6093 9002 6477 9775 2625 11054 3934 2150 7114 10678 11282 7516 9500 3549 4217 101 5378 6041 1431 4099 10112 6695 10755 128 5909 4488 57154 4086 5916 1984 11320 2255 80310 2175 8870 6143 11006 5764 5340 | 93 |
| Pancreas disease | INS; ADIPOQ; SOD2; AKR1B1; SUMO4; PDX1; PTPRS; LEP; CASP1; HRAS; RPLP2; ALDH2; DDIT3; KCNJ11; GLUD1; HNF4A; GCK; DDC; IAPP; CELA1; ACTN4; | 3630 9370 6648 231 387082 3651 5802 3952 834 3265 6181 217 1649 3767 2746 3172 2645 1644 3375 1990 81 64399 79679 | 23 |
| Pancreatitis | CCL2; HSPA1B; CEL; ADH1B; CDKN2A; CXCL10; SPP1; RELA; MET; TNFRSF10A; CRISP3; KRAS; ADCYAP1; ENG; IFNG; CTLA4; GSTM1; GSTP1; KRT8; CA2; CYP1A1; IL2RA; CASR; CTRC; ABCF1; CD4; ALDH2; HSF1; SELE; TNF; LTA; GSTT1; TLR4; MIF; MBL2; EDN1; LPL; SELP; EDNRA; A2M; SOD1 | 6347 3304 1056 125 1029 3627 6696 5970 4233 8797 10321 3845 116 2022 3458 1493 2944 2950 3856 760 1543 3559 846 11330 23 920 217 3297 6401 7124 4049 2952 7099 4282 4153 1906 4023 6403 1909 2 6647 | 41 |

| | | | |
|---|---|---|---|
| Panic disorder | CRHR2; HTR1A; SLC6A4; CRH; HTR3A; DAOA; CCK; ESR1; ELN; HTR2A; MAOA; CCKAR; ADORA2A; CCKBR; GLO1; CREM; SLC6A2; LDHA; TSPO; G30; | 1395 3350 6532 1392 3359 267012 885 2099 2006 3356 4128 886 135 887 2739 1390 6530 3939 706 282706 387129 | 21 |
| Papillary adenocarcinoma | F2RL1; EGFR; CHST2 | 2150 1956 9435 | 3 |
| Papillary cancer | FAM38A; PAX2; EIF2S1; MAGEA4; AKAP9; TRPS1; RBL1; S100A10 | 9780 5076 1965 4103 10142 7227 5933 6281 | 8 |
| Papillomavirus infection | ERBB2; CDKN1A; CDKN2A | 2064 1026 1029 | 3 |
| Parasitic disease | ALAD; IFNGR1; BIRC5; SERPINB4 | 210 3459 332 6318 | 4 |
| Parkinson disease | ND5; TIMP1; APOH; PINK1; UCHL1; CHRNA7; GBA; PARK7; CHRNA4; ATP1A3; CHAT; ATXN3; IREB2; FLOT1; DYT3; IL1B; INS; MTHFR; HP; IFNG; IL8; TSC2; GSTM1; CDKN2A; IL10; FAS; SPP1; RB1; NAT2; IGF2; ACE; NAT1; EPHX1; CYP1A1; ESR1; PARP1; APOD; ESR2; PTGS2; BCL2L1; CCK; ADH1C; SIAH1; CASP9; CCKBR; FGF20; E2F1; HRH1; HTRA2; CD200; CCKAR; MAP3K5; NDUFA1; ELAVL4; ND2; HS1BP3; GCH1; SPR; EPHX2; PVALB; SNCAIP; ATXN8OS; RNF19A; PARK3; NEDD9; RNF11; HSPA9; LRRK1; HSP90AB2P; AMIGO2; GFRA1; CALB1; SNCB; CDNF; TYR; MIR133B; FUBP1; LPO; MTIF3; ACCN1; [illegible]; | 4540 7076 350 65018 7345 1139 2629 11315 1137 478 1103 4287 3658 10211 1863 3553 3630 4524 3240 3458 3576 7249 2944 1029 3586 355 6696 5925 10 3481 1636 9 2052 1543 2099 142 347 2100 5743 598 885 126 6477 842 887 26281 1869 3269 27429 4345 886 4217 4694 1996 4536 64342 2643 6697 2053 5816 9627 6315 25897 5072 4739 26994 3313 79705 391634 347902 2674 793 6620 441549 7299 442890 8880 4025 219402 40 26058 59269 | 82 |
| Pelvic inflammatory disease | LOC643387; MTA2; SNCA; MAN1B1; IFNG | 643387 9219 6622 11253 3458 | 5 |
| Penile disease | CCL2; CRP; HRAS; KRAS; ITGB3; FHIT; ITGA1; CD82 | 6347 1401 3265 3845 3690 2272 3672 3732 | 8 |
| Peptic esophagitis | XRCC1; ERCC2; GSTM1; IL8RA; IL8RB; GSTP1; GSTT1; IL8; IL1F5; CCL2 | 7515 2068 2944 3577 3579 2950 2952 3576 26525 6347 | 10 |
| Peptic ulcer | BDKRB1; IL12B; NOD1; HNMT; ADA; LDHA; SCT; CSTF2; BDKRB2; SELE; VEGFA; SELL; LTA; IL1RN; PLAU; CACNA2D2; APOBEC3G; KLK3 | 623 3593 10392 3176 100 3939 6343 1478 624 6401 7422 6402 4049 3557 5328 9254 60489 354 | 18 |
| Periodontal disease | IL1RN; IL11; CST3; MPO; SERPINE1; NAT2; CRP; IL17A; ALPL; MMP13; PLAU; FGF7; CCL20; FCGR3B; IL16; BPI; CTSC; | 3557 3589 1471 4353 5054 10 1401 3605 249 4322 5328 2252 6364 2215 3603 671 1075 4256 | 18 |

| | | | |
|---|---|---|---|
| Periodontitis | TNFSF13B; IL1A; SFTPD; CASP3; FCGR2A; IL11; TIMP2; IL10; IL4; CD14; HLA-B; MMP12; IL17A; GSTM1; TLR4; TNF; IL1B; NOD2; IFNG; IL12RB2; MMP2; CYP1A1; AQP3; CTSC; MMP9; HLA-A; MMP3; LTF; CSF1; GSTT1; PLAT; TLR2; FCGR3A; IL13; CASP7; MMP1; PTGS2; MPO; IL16; CTSG; IL2RA; S100A8; PAFAH1B1 | 10673 3552 6441 836 2212 3589 7077 3586 3565 929 3106 4321 3605 2944 7099 7124 3553 64127 3458 3595 4313 1543 360 1075 4318 3105 4314 4057 1435 2952 5327 7097 2214 3596 840 4312 5743 4353 3603 1511 3559 6279 5048 | 43 |
| Peripheral nerve sheath cancer | MMP13; CXCR6; BMP5; BMP3 | 4322 10663 653 651 | 4 |
| Peripheral nerve sheath tumor | NF1 | 4763 | 1 |
| Pertussis | ITGB2; PIK3CG; ICAM1; ITGAM; SERPING1; ITGA5 | 3689 5294 3383 3684 710 3678 | 6 |
| Pervasive development disorder | NLGN4X; PCDH19; AVPR1A; DAOA; HOXA1; CHDS3; SLC6A4; ASMT; MET; MIF; MARK1; OXT; DAO; EN2; APC | 57502 57526 552 267012 3198 387573 6532 438 4233 4282 4139 5020 1610 2020 324 | 15 |
| Phobic anxiety disorder | ADORA2A; AR; P2RX7; CAMKK2 | 135 367 5027 10645 | 4 |
| Pick disease of the brain | STH; PRDX6; ADORA1; MSI1; PRDX1 | 246744 9588 134 4440 5052 | 5 |
| Pilomatrixoma | KRT35; KRT31 | 3886 3881 | 2 |
| Pituitary tumor | NEUROD1; LEPR; HSD11B2; SCGB1D2; SCGN; IL6; CALCA; TLR4; PARP1; GH1; GHR; | 4760 3953 3291 10647 10590 3569 796 7099 142 2688 2690 10912 9049 | 13 |
| Placenta disease | ANGPT2; CDKN1C | 285 1028 | 2 |
| Pneumoconiosis | ERBB2; MMP3 | 2064 4314 | 2 |
| Polyarthritis | MBL2; HLA-DPB1; C4B; ADIPOQ; BMP4; TNC; SERPINE1; MMP1; IL8; TRPV1; PLA2G4A; HIF1A; CCL11; PTGES; MX1; TLR4; TLR2; IL8RA; VIP; MMP3; EDN1; IL17A; RELA; SOCS3; CXCL13; LEP; ESR1; PTGS2; BCL2L1; MMP13; PLAU; B2M; CXCL12; CYBA; CHGA; FAP; HPSE; SMAD3; VIPR2; CDKN1A; EPAS1; WISP3; IKBKB; GADD45B; FHL2; S100A11; CD247; ELAVL1; LCN2; ITGA5; IL7; ACAN; MAZ; TPI1; NR1D1; CYP19A1; TPSAB1; CHUK; TIA1; IL29; ADAMTS4; MATN3; P4HA2; BMP5; ADAMTS5; GPNMB; RHOB; TM2D3; CYP2B6; IL28A; GALNS; CTSK; SOX9; IKBKE; SLC20A1; | 4153 3115 721 9370 652 3371 5054 4312 3576 7442 5321 3091 6356 9536 4599 7099 7097 3577 7432 4314 1906 3605 5970 9021 10563 3952 2099 5743 598 4322 5328 567 6387 1535 1113 2191 10855 4088 7434 1026 2034 8838 3551 4616 2274 6282 919 1994 3934 3678 3574 176 4150 7167 9572 1588 7177 1147 7072 282618 9507 4148 8974 653 11096 10457 388 80213 1555 282616 2588 1513 6662 9641 6574 5033 801 | 77 |
| Polycystic kidney | PKD1; NEK1; TSC2; PKHD1; INVS; IGF1; CFTR; APOE; ADD1; IL8RB; SLC12A2; LCN2; EDNRB; UBD | 5310 4750 7249 5314 27130 3479 1080 348 118 3579 6558 3934 1910 10537 | 14 |

| | | | |
|---|---|---|---|
| Polycystic ovary syndrome | BRCA1; FOXL2; MMP1; LTF; INS; NAMPT; SERPINE1; IL6; MC4R; GH1; INSR; HSD3B1; CYP21A2; SORBS1; IL1A; PPARGC1A; FTO; RETN; SLC2A4; MTHFR; MMP2; TNF; GSTM1; GSTT1; ADIPOR2; F5; TIMP1; IL1RN; CRP; SOCS3; IGFBP1; PLAT; CYP1A1; ADRB2; KRAS; KCNJ11; IRS1; CAPN10; ADRA2B; AKT1; KLK3; CCK; INHBB; FST; PLAUR; CYR61; GATA6; HSD17B2; STS; HSD17B1; HOXA10; CAPN5; TBC1D4; CYP3A7; BMP15; RASA1; SOS1 | 672 668 4312 4057 3630 10135 5054 3569 4160 2688 3643 3283 1589 10580 3552 10891 79068 56729 6517 4524 4313 7124 2944 2952 79602 2153 7076 3557 1401 9021 3484 5327 1543 154 3845 3767 3667 11132 151 207 354 885 3625 10468 5329 3491 2627 3294 412 3292 3206 726 9882 1551 9210 5921 6654 | 57 |
| Polycythemia | VHL; ACE; JAK2; EPO; EPOR; HIF1A; MPL; EDN1; IGF1 | 7428 1636 3717 2056 2057 3091 4352 1906 3479 | 9 |
| Polymyositis | IL15; GNLY; C1D; IL1B; CCR5; TLR3; CCL21; CD83; IL17A; S100A8; CTSS; CKM; IL1RAPL2; | 3600 10578 10438 3553 1234 7098 6366 9308 3605 6279 1520 1158 26280 6363 322 | 15 |
| Polyneuropathy | TTR; MFN2; MPV17; NF2; PARK7; POLG; MAG; GALK1; VEGFA; S100A12; ABCA1; APOA1; EGR2; ABCD1 | 7276 9927 4358 4771 11315 5428 4099 2584 7422 6283 19 335 1959 215 | 14 |
| Porcine reproductive and respiratory syndrome | TLR2; NCF1C; TLR4; NOD1; NCF2; CYBA | 7097 654817 7099 10392 4688 1535 | 6 |
| Pre-Eclampsia | ICAM1; SOD2; VEGFA; ENG; SELL; IFNG; NOD2; IL15; CALR; GSTM1; HIF1A; GSTP1; IL4; IL10; CXCL10; FAS; FGF2; TLR4; TLR2; MIF; MMP9; NAT2; MAPK14; IGF2; IGFBP1; EPHX1; IFNGR2; IL16; CSF1; EDNRA; SOD1; ADRB3; POMC; APLN; CD24; FABP4; ADA; ACTG2; DCN; CHIT1; VCAN; PROZ; INHBB; FST; CD34; RASSF1; EBAG9; CYR61; INHBA; EFNA1; CLU; CHI3L1; BGN; LIF; PLAC1; GH2; LOC391533; CRHR1; APLNR; SLC22A3; LGALS13; INHBC; TPBG; SYNPO; SERBP1; SLC1A5; CRH; CGB; INSL3; BOK; [illegible] | 3383 6648 7422 2022 6402 3458 64127 3600 811 2944 3091 2950 3565 3586 3627 355 2247 7099 7097 4282 4318 10 1432 3481 3484 2052 3460 3603 1435 1909 6647 155 5443 8862 100133941 2167 100 72 1634 1118 1462 8858 3625 10468 947 11186 9166 3491 3624 1942 1191 1116 633 3976 10761 2689 391533 1394 187 6581 29124 3626 7162 11346 26135 6510 1392 1082 3640 666 5837 219736 | 72 |
| Premature birth | IL1B; LTA; F2R; CRH; CYP1A1; EPO; IL1RN; ADRB2; ACE; AGTR1; MBL2; GSTT1; LGALS13; TLR4; REN; GSTM1; CRP | 3553 4049 2149 1392 1543 2056 3557 154 1636 185 4153 2952 29124 7099 5972 2944 1401 | 17 |
| Primary biliary cirrhosis | ICAM1; ITGAL; CTLA4; HLA-A; PTPN22; GSTP1; CXCL10; IDO1; FAS; TLR4; F13A1; CXCL9; LEP; JAK2; CYP1A1; ABCC1; ELF1; HLA-DQA1; CD180; ETV6; CAV2; SLAMF1; SH2D1A; SNCA; ESR1; BMI1; ALPP; DBT; CD27; CAV1; | 3383 3683 1493 3105 26191 2950 3627 3620 355 7099 2162 4283 3952 3717 1543 4363 1997 3117 4064 2120 858 6504 4068 6622 2099 648 250 1629 939 857 5783 | 31 |

| | | | |
|---|---|---|---|
| Primary hyperparathyroidism | ADM; CASR; KCNQ1; RET; EGFR; ITGB1; PTK2 | 133 846 3784 5979 1956 3688 5747 | 7 |
| Primary tumor | IGF1; KRT8; SPINK1; TIMP1; EDN1; F3; EDNRA; LCK; FAP; PARK2; AMFR; ATF3; PTPRG; PMEPA1; GIPC1; PALB2; STS; PAX2; CD1A; CCNA1; NOG; IL10RA; TSPY1; CALCR; VSNL1; MIR184; SEMA3F; CDK9; CCNE2; | 3479 3856 6690 7076 1906 2152 1909 3932 2191 5071 267 467 5793 56937 10755 79728 412 5076 909 8900 9241 3587 7258 799 7447 406960 6405 1025 9134 7168 7112 4175 | 32 |
| Prion disease | APP; MAPT; HSPD1; PTGS2; YWHAQ; A2M | 351 4137 3329 5743 10971 2 | 6 |
| Prostate cancer | ITPR1; NCOA2; CHAF1A; TNF; AR; KLK3; BRCA2; ERCC1; ATP2A1; DAZL; DAZ1; ATP2B1; DDX25; CCR5; XBP1; ADRB2; CRISP3; SCD; SQSTM1; ERG; CSMD1; UBC; RPL10; GSTP1; IGFBP2; EGR1; PLAU; KCNRG; ERBB3; SCN9A; SERPINA5; CARM1; ETV1; TBC1D3B; IL15; FGF1; SERPINA3; S100A8; HSPA1A; S100A9; MMP16; LCT; CREBBP; INHA; CGB; MIR222; MMP24; KIAA0196; MIR221; MMP17; C15ORF21; MMP15; RAD21; IGF1; TIMP1; CSF1R; RLN2; MT2A; ATF3; PLCG1; RNR1; ITGA5; F2RL1; COX2; ATP6; ADRA1A; FOXP1; IL10RA; TSPY1; CALCR; BMP5; DRG1; PBOV1; CDC37; TSG101; TES; RCBTB2; WDR19; STEAP2; NUDC; PCNT; RASL11A; WFDC2; FABP5; ICAM1; INS; ADIPOQ; AGER; CCL5; IFNG; CTLA4; SERPINE1; ITGA2; CD55; IL6; HLA-A; IL8; CALCA; HSPB1; NR1H4; IL4; IL2; IL10; CXCL10; IL18; CCL2; CX3CR1; TLR4; TLR3; CCR1; ADIPOR2; MIF; CXCL16; SPINK1; VIP; PDX1; MTRR; IL1RN; VIPR1; CRP; EDN1; ACE; SOCS3; AREG; IGFBP1; ALPL; PAK2; CST3; APOE; LEP; ABCC4; CCR9; KLK4; OSM; HSPD1; PIN1; F3; | 3708 10499 10036 7124 367 354 675 2067 487 1618 1617 490 29118 1234 7494 154 10321 6319 8878 2078 64478 7316 6134 2950 3485 1958 5328 283518 2065 6335 5104 10498 2115 414059 3600 2246 12 6279 3303 6280 4325 3938 1387 3623 1082 407007 10893 9897 407006 4326 283651 4324 5885 3479 7076 1436 6019 4502 467 5335 4549 3678 2150 4513 4508 148 27086 3587 7258 799 653 4733 59351 11140 7251 26136 1102 57728 261729 10726 5116 387496 10406 2171 3383 3630 9370 177 6352 3458 1493 5054 3673 1604 3569 3105 3576 796 3315 9971 3565 3558 3586 3627 3606 6347 1524 7099 7098 1230 79602 4282 58191 6690 7432 3651 4552 3557 7433 1401 1906 1636 9021 374 3484 249 5062 1471 348 3952 10257 10803 9622 5008 3329 5300 2152 3603 4353 3339 4321 360 1435 116 1909 2 6647 1445 2638 7295 133 567 823 4521 1594 5087 688 4036 2650 5770 6464 3373 1113 3456 6649 654 2033 2690 27035 4162 1118 4088 1462 2023 10062 3679 7431 5371 10253 4100 9002 5971 463 10553 3911 3551 182 80705 28234 56999 6692 2274 4437 7079 3716 92 8945 9506 3590 5914 1191 4118 11093 56937 9131 64866 4249 1902 4481 6059 10800 9604 1116 6041 80310 6143 5764 5340 2308 7341 6197 79728 | 367 |
| Protein deficiency | EPB42; PROZ; ABCD1 | 2038 8858 215 | 3 |
| Protein-energy malnutrition | MCM2; CDKN2A; CD9; VCAN; PCM1; IGF1; LEPR; LEP; SHBG | 4171 1029 928 1462 5108 3479 3953 3952 6462 | 9 |
| Proteinuria | HPSE; PTX3; HP; F3; WT1; SYNPO; AGT; HPX; ACTN4; CTGF; CCL2; CLCN5; ACE; NR3C2; LPA; ADM; ADIPOQ; ACE2; SLC9A3; CDKN1B | 10855 5806 3240 2152 7490 11346 183 3263 81 1490 6347 1184 1636 4306 4018 133 9370 59272 6550 1027 | 20 |
| Protozoan infection | IL5; CTSB | 3567 1508 | 2 |

| | | | |
|---|---|---|---|
| Pseudoxanthoma elasticum | ICAM1; SELL; ABCC1; XYLT1; ABCC6 | 3383 6402 4363 64131 368 | 5 |
| Psoriasis | S100A9; HLA-C; MICA; CYP1A1; NOD2; KIR2DS1; HLA-B; PTPN22; HLA-A; S100A12; ACE; TNF; ENG; LTA; IL1B; AREG; S100A8; IL4R | 6280 3107 4276 1543 64127 3806 3106 26191 3105 6283 1636 7124 2022 4049 3553 374 6279 3566 | 18 |
| Psychotic disorder | HTR2A; ANKK1; ATXN2; APOE; B3GAT1; SLC6A4; SLC6A9; DAOA; DISC1; S100B; BDNF; SERPINA3; AKT1; PICK1; MAOB; SOD2; GSTP1; KCNN3; GAD1; APP; SLC6A3; PRDX3; CHRNA7; PAWR; DBH; CHRNA4; TOR1A; CHGB; HTR6; DAO; CHRNB2; SQSTM1; CHMP2B; SLC18A2; PCSK1N; STH; RGS4; PDLIM5; AVPR1B | 3356 255239 6311 348 27087 6532 6536 267012 27185 6285 627 12 207 9463 4129 6648 2950 3782 2571 351 6531 10935 1139 5074 1621 1137 1861 1114 3362 1610 1141 8878 25978 6571 27344 246744 5999 10611 553 | 39 |
| Ptosis | ESR2; FOXL2; TWIST1; FGFR3; MMP2; ELN; MMP1; DCN; LAMC1 | 2100 668 7291 2261 4313 2006 4312 1634 3915 | 9 |
| Pulmonary alveolar proteinosis | CTSH; SFTPA1B; IL10; CST3; SFTPD; NAPSA; CSF2; ABCG1 | 1512 6435 3586 1471 6441 9476 1437 9619 | 8 |
| Pulmonary embolism | F13A1; CRP; P2RY12; SERPINF2; ALB; F5 | 2162 1401 64805 5345 213 2153 | 6 |
| Pulmonary fibrosis | CCL2; FASLG; CCL18; F2R; SFTPA1B; MMP2; MMP7; IL5; IGF1; CAV1; EDN1; CCL5; ERBB2; CSF1; TNF; ERBB3; CCL11; AREG; SLIT2; IL7; SGK1; FAS; SERPINB3; IGF2; F2; CXCL12; BDNF; STX2 | 6347 356 6362 2149 6435 4313 4316 3567 3479 857 1906 6352 2064 1435 7124 2065 6356 374 9353 3574 6446 355 6317 3481 2147 6387 627 2054 | 28 |
| Pulmonary hypertension | SLC6A4; VIP; KCNA5; BMPR2 | 6532 7432 3741 659 | 4 |
| Pyelonephritis | TNFRSF8 | 943 | 1 |
| Rabies | F8; PPARGC1A; LRP6; BMPR2; ANXA11; MOXD1; CRYAA; SOAT1; APOH; TPP1; IGFBP6; LAMA1; ATP5A1; KCNQ1; SLC9A1; SFTPB; RHCE; MVK; ADFP; LOC729991-MEF2B; VKORC1; AVPR2; FGG; CTSH; PITX2; COPG; RHD; CACNA1E; BRCA1; TMPRSS11E; ELN; ESR1; SFTPC; TSHR; APP; M6PRBP1; IGFBP1; GDF9; CRYGD; CD1D; CAMP; FURIN; F9; F7; CLCN1; UNC119; DEFA1; RNASE1; GCNT2; PTBP2; ITGA7; VHL; VIPR1; LYZ; CYP2A6; CAST; RTN1; AR; EDN1; STOM; MMP7; SNCA; PDCD6; TRPC1; SMAD3; CHM; SERPINC1; BCAT2; BCL2L1; CASP3; CAV1; CALR; PEX19; ACO2; TIAL1; IAPP; SCN1B; HDAC9; MORF4L1; CD55; SLC28A3 | 2157 10891 4040 659 311 26002 1409 6646 350 1200 3489 284217 498 3784 6548 6439 6006 4598 123 4207 79001 554 2266 1512 5308 22820 6007 777 672 28983 2006 2099 6440 7253 351 10226 3484 2661 1421 912 820 5045 2158 2155 1180 9094 1667 6035 2651 58155 3679 7428 7433 4069 1548 831 6252 367 1906 2040 4316 6622 10016 7220 4088 1121 462 587 598 836 857 811 5824 50 7073 3375 6324 9734 10933 1604 64078 | 81 |
| Rectal tumor | PRPH2 | 5961 | 1 |
| Rectum cancer | APOE; F3 | 348 2152 | 2 |

| | | | |
|---|---|---|---|
| Renal Cell cancer | PAK1; LCK; LNPEP; CD247; CACYBP; ESM1; EDNRA; HLTF; FLCN; GLO1; PHF17; LRAT; IGF1; CTLA4; TNF; IL6; HSPB1; IL2; TLR3; CXCL16; MMP3; CRP; IGFBP6; FCGR3B; LTF; NR3C2; B2M; HSD11B2; HAVCR1; GJB1; DUSP1; IFNAR2; MAD2L2; HSPA4; UCHL1; CLU; NEU3; HTRA2; NOL3; CGB; EDNRB; ASS1; SERPINA5; BMPR2; LAMA4; S100A1; TRPC4; IL10RA; CD276; ANKRD2; SOAT1; CUX1; FABP7; SLC38A3; KHK; RAN; WNT11; FAM107A; AP1M2; AGMAT; KCNIP4; C3ORF10; ACAT2; PLXNB1; MT1G; ANXA4; LOC442459; ST3GAL4; MITF; MAPKAP1; MAGEA6; HPN; DACH1; MAGEA9 | 5058 3932 4012 919 27101 11082 1909 6596 201163 2739 79960 9227 3479 1493 7124 3569 3315 3558 7098 58191 4314 1401 3489 2215 4057 4306 567 3291 26762 2705 1843 3455 10459 3308 7345 1191 10825 27429 8996 1082 1910 445 5104 659 3910 6271 7223 3587 80381 26287 6646 1523 2173 10991 3795 5901 7481 11170 10053 79814 80333 55845 39 5364 4495 307 442459 6484 4286 79109 4105 3249 1602 4108 | 74 |
| Renal tubular acidosis | RBPJ; IL6; SMARCA4; SLC4A1; SUMO1; ESR1; MED12; PARP1; PIAS2 | 3516 3569 6597 6521 7341 2099 9968 142 9063 | 9 |
| Respiratory distress syndrome | MIF; SFTPB; HSD11B2; ACE; IL1B; F3; SERPINE1; IL6; FASLG; IL8; SFTPD; EDN1; FAS | 4282 6439 3291 1636 3553 2152 5054 3569 356 3576 6441 1906 355 | 13 |
| Respiratory failure | MYH10; SGCA; IL10; HSPA1A; SCNN1A; TNC; F2RL1; NPSR1 | 4628 6442 3586 3303 6337 3371 2150 387129 | 8 |
| Respiratory tract disease | IL13; S100B; SFTPD; SFTPB; CAMP; SFTPA1B; SFTPC; SFTPA2B; SLC34A2; MMP9; CFTR; APOD; IFNB1; ADORA1; FLCN; DNAH7; RP11-529I10.4; RXRB; IRF3; MAVS; SMC4; SMC2 | 3596 6285 6441 6439 820 6435 6440 6436 10568 4318 1080 347 3456 134 201163 56171 25911 6257 3661 57506 10051 10592 | 22 |
| Reticulosarcoma | IL9; ALK; CASP9; KIAA1618; CARS; JAK3; CASP3; LYVE1 | 3578 238 842 57714 833 3718 836 10894 | 8 |
| Retinal disease | SFRP1; SAG; FBLN1; PRPH2; GRK1; RLBP1; RDH5; RPE65; MERTK; ATXN7; GDNF; DFNB59; NR2E3; RPGR; RRH; PITPNM1; TULP1; IMPDH1; NPHP3; BBS1; PRPF3; RGR; LOXL1; MYOC; RS1; HIF1A; ALB; F5; HBEGF; HLA-DQA1; CYBA; PON1; ABCA4; IGF1R; PROM1; KDR; EDNRB; RD3; ZNF385A; SEMA4A; NDP; ACAD9; LAMP2; RPGRIP1; RHO; RCVRN; CACNA1F; NARG1; [illegible] | 6422 6295 2192 5961 6011 6017 5959 6121 10461 6314 2668 494513 10002 6103 10692 9600 7287 3614 27031 582 9129 5995 4016 4653 6247 3091 213 2153 1839 3117 1535 5444 24 3480 8842 3791 1910 343035 25946 64218 4693 28976 3920 57096 6010 5957 778 80155 6310 4356 1527 | 51 |
| Retinitis pigmentosa | RP1; RRH; RHO; PRPF8; PRPF3; SAG; RPGR; IMPDH1; CERKL; INVS; PRPF31; CA4; RPGRIP1; RLBP1; PRPH2; RPE65; BBS4; RP9; SMC3; MPP4; RP33; CDH23; RP2; PDC; ABCA4 | 6101 10692 6010 10594 9129 6295 6103 3614 375298 27130 26121 762 57096 6017 5961 6121 585 6100 9126 58538 692221 64072 6102 5132 24 | 25 |

| | | | |
|---|---|---|---|
| Retinoblastoma | IL1B; KRT8; CCNA1 | 3553 3856 8900 | 3 |
| Rett syndrome | MECP2; ID2; ID1; BDNF; NTNG1; ID3; ID4; FXYD1; MBD1; LEP; | 4204 3398 3397 627 22854 3399 3400 5348 4152 3952 2290 | 11 |
| Rhabdomyosarcoma | AGER; FABP2; TNFRSF10B; IGFBP6; TNFSF13B; PAX3; MYOD1; NOG; ANKRD2; KAT2B; | 177 2169 8795 3489 10673 5077 4654 9241 26287 8850 4656 | 11 |
| Rheumatic fever | ICAM1; MUC5AC; PLAUR; HLA-DQB1; MASP2; HLA-DQA1; FCGR3B; HLA-B; IL10; MAPK14; DUSP1; RHAG; RHCE; RHD; | 3383 4586 5329 3119 10747 3117 2215 3106 3586 1432 1843 6005 6006 6007 4052 | 15 |
| Rheumatism | SUMO4; MEFV; TWIST1; MAPT; SCN2A; F3; CREBBP; PTH1R; SCN1A; BMX; KLRC2; MYD88; SERPINH1; FCN1; HNRNPD; SMN1; STH; SMN2 | 387082 4210 7291 4137 6326 2152 1387 5745 6323 660 3822 4615 871 2219 3184 6606 246744 6607 | 18 |
| Rheumatoid arthritis | IL1A; IL1B; SELE; ICAM1; ADIPOQ; RETN; BMP4; AGTR1; MTHFR; CD9; CCL5; MMP2; CASP10; TIMP3; SERPINE1; MMP1; TNF; IL8; NOD2; TRPV1; IL15; PLA2G4A; RAC1; IL12B; LTA; HTR2A; XRCC1; GSTM1; GSTT1; CCL11; IL4; PTGES; IL2; SPP1; TNFRSF8; CD274; CX3CR1; CD14; TLR2; MDM2; ANXA1; RB1; CXCL16; JUN; BTNL2; TNFRSF9; SLC22A4; MBL2; VIP; SLC22A5; NAT2; MTRR; MMP3; MAPK14; CD86; CD83; TNFSF15; IL21; VIPR1; FCGR3A; ITGB2; EDN1; IL17A; LEPR; IGF2; NFATC1; CD80; CXCL13; CST3; APOE; PLAT; JAK2; PTPN13; EGF; PARP1; CD28; BCL2; ADRB2; ICOS; OSM; SELP; ANGPT2; HSPD1; EGR1; CX3CL1; PTGS2; BCL2L1; MMP13; PLAU; CCL20; IL16; BPI; CCL3; CCL3L1; SFTPD; CSF1; CTSG; TNFRSF10A; SOD1; CSK; ADM; CAT; ADRB3; CXCL12; HSD11B2; AR; APOA1; LRP2; GCNT1; HLA-DMB; HLA-DMA; ANGPT1; CHGA; ITGB1; PIK3CG; HRH4; P2RX7; SELS; TRPC1; DEFA3; DEFA1; HPSE; MDK; IFNGR1; HAMP; NCR3; CCL18; ENO1; VIPR2; MUC5AC; SLC5A5; RBP1; ALDH1A1; HOXB7; BIRC5; | 3552 3553 6401 3383 9370 56729 652 185 4524 928 6352 4313 843 7078 5054 4312 7124 3576 64127 7442 3600 5321 5879 3593 4049 3356 7515 2944 2952 6356 3565 9536 3558 6696 943 29126 1524 929 7097 4193 301 5925 58191 3725 56244 3604 6583 4153 7432 6584 10 4552 4314 1432 942 9308 9966 59067 7433 2214 3689 1906 3605 3953 3481 4772 941 10563 1471 348 5327 3717 5783 1950 142 940 596 154 29851 5008 6403 285 3329 1958 6376 5743 598 4322 5328 6364 3603 671 6348 6349 6441 1435 1511 8797 6647 1445 133 847 155 6387 3291 367 335 4036 2650 3109 3108 284 1113 3688 5294 59340 5027 55829 7220 1668 1667 10855 4192 3459 57817 259197 6362 2023 7434 4586 6528 5947 216 3217 332 4609 1026 2064 3672 4170 6275 7424 5371 3491 11009 1947 682 2034 7298 387 8838 182 140 10587 3685 80380 5915 79400 2956 8837 472 9518 5347 9141 861 5914 1191 3235 3676 4118 28514 6288 7185 4249 1902 1994 6416 2150 3554 1116 3181 5979 5916 8839 283120 1392 7341 3574 26353 127 7224 660 270 727 728 476 10203 2624 896 3385 3802 10859 3661 7177 6357 6573 871 7072 9672 6368 54 11240 6375 3107 80381 9507 653 11096 1513 9641 3709 9420 4739 4331 902 3965 10512 9846 608 1981 11167 | 267 |
| Salmonella infection | RAC1; RNF5; ACTR3; ACTG1; ACTR2; TLR5; IL12B; IQGAP1 | 5879 6048 10096 71 10097 7100 3593 8826 | 8 |

| | | | |
|---|---|---|---|
| Sarcoidosis | ACE2; CCL18; CXCL5; IL1B; SELE; TNC; TNF; NOD2; LTA; IL18; BTNL2; NOD1; CD86; ACE; CD80; CCL3; CMA1; CHIT1; CDKN1A; IFNA2; CHI3L1; HSPA1L; IGKC; IL18R1; TYK2; IGHG1 | 59272 6362 6374 3553 6401 3371 7124 64127 4049 3606 56244 10392 942 1636 941 6348 1215 1118 1026 3440 1116 3305 3514 8809 7297 3500 | 26 |
| Sarcoma | VIM; MC1R; F13A1; DDIT3; WRN; IGF2BP2; S100A6; FES; IHH; F8; ERCC5; SALL2; WNT10B; ATF1; TFG; SIX3; ASPSCR1 | 7431 4157 2162 1649 7486 10644 6277 2242 3549 2157 2073 6297 7480 466 10342 6496 79058 | 17 |
| Schistosoma mansonii infection | ICAM1; MAPK14; CCL3 | 3383 1432 6348 | 3 |
| Schistosomiasis | CSF2; BCL2; IL10; MDM2 | 1437 596 3586 4193 | 4 |
| Schizophrenia | IL1A; IL1B; ICAM1; ND5; HLA-B; MTHFR; SOD2; IGF1; GDNF; TNF; HLA-A; PLA2G4A; IL12B; LTA; HTR3A; GSTM1; APC; GSTP1; EGFR; IL4; IL2; IL10; CCR5; IL18; CCL2; JUN; MTRR; IL3RA; IL1RN; HLA-DQB1; XBP1; MYO9B; LEPR; IGF2; ACE; MICB; DBI; LEP; ESR1; MTHFD1; EGF; PLA2G2A; APOD; ESR2; ADCYAP1; SOD1; ADORA2A; SP1; ADM; DDC; ND1; AR; APOA1; GAD2; KLF5; UCP2; POMC; AKT1; ARHGEF11; TTR; CHGA; ITGA2B; PON1; CYP1A2; DUSP6; DDR1; SMAD5; SEPT4; ERBB3; ERBB4; BIRC2; DKK4; HRH1; IL1R1; CHI3L1; CCKAR; MAG; SEMA3A; GJD2; PLP1; VLDLR; ADRA2A; NCAM1; MOG; RXRB; NDUFV2; NTNG1; RTN4; HTR4; RGS2; HTR7; GCLM; CLDN5; CHRM1; PMCH; IL10RA; MED12; GAP43; PVALB; RHD; MLL; SLC1A1; SLC1A3; CHRM4; RTN4R; CAMK2A; DKK3; KCNJ5; PEMT; OLIG2; SMARCA2; AMD1; MAP2; CAMK2B; SOX10; PANX2; CHL1; FEZ1; SLC18A1; TAAR6; SYNGR1; CRHBP; EN1; SRR; CRY1; G30; PICK1; SLIT3; CELSR1; KPNA3; ZDHHC8; ME2; LRRTM1; PIP4K2A; DNAJB1; PLXNA2; TRMT2A; PCM1; | 3552 3553 3383 4540 3106 4524 6648 3479 2668 7124 3105 5321 3593 4049 3359 2944 324 2950 1956 3565 3558 3586 1234 3606 6347 3725 4552 3563 3557 3119 7494 4650 3953 3481 1636 4277 1622 3952 2099 4522 1950 5320 347 2100 116 6647 135 6667 133 1644 4535 367 335 2572 688 7351 5443 207 9826 7276 1113 3674 5444 1544 1848 780 4090 5414 2065 2066 329 27121 3269 3554 1116 886 4099 10371 57369 5354 7436 150 4684 4340 6257 4729 22854 57142 3360 5997 3363 2730 7122 1128 5367 3587 9968 2596 5816 6007 4297 6505 6507 1132 65078 815 27122 3762 10400 10215 6595 262 4133 816 6663 56666 10752 9638 6570 319100 9145 1393 2019 63826 1407 282706 9463 6586 9620 3839 29801 4200 347730 5305 3337 5362 27037 5108 9764 259217 57030 9444 284890 9456 3843 9685 3720 1133 6616 1960 29114 4762 9378 9152 9016 2703 56147 5100 3796 9993 406920 6511 64067 56990 9481 9248 338442 321 2774 1438 10814 9019 27020 | 173 |
| Scleroderma | IL1A; EXOSC10; SKIL; C1D; EXOSC9; UBE4A; ENG; SMAD7; SERPINB3; SERPINB2; KIR2DS2; KIR2DS1; ROCK2; SMURF1; KIR2DL2; ROCK1; CAV1; MIF; SKI; SMAD3; EP300; CTGF | 3552 5394 6498 10438 5393 9354 2022 4092 6317 5055 100132285 3806 9475 57154 3803 6093 857 4282 6497 4088 2033 1490 | 22 |

| | | | |
|---|---|---|---|
| Sella turcica tumor | POMC; PCSK1; LIF; MC2R; AIP; HGS; SCGN | 5443 5122 3976 4158 9049 9146 10590 | 7 |
| Seminoma | CYP27B1; FASLG; CGB; NLRP7; SOX5; DAD1L; LDHAL1 | 1594 356 1082 199713 6660 56286 3940 | 7 |
| Septicemia | IL6; CRP; IGF1R; FST; CD6 | 3569 1401 3480 10468 923 | 5 |
| Serous cancer | IFNG; FRAT1; CLU; SRA1 | 3458 10023 1191 10011 | 4 |
| Severe acute respiratory syndrome | IL6; HLA-A; RB1; MAPK14; ACE; BCL2L1; CD34; CCND3; ICAM3; | 3569 3105 5925 1432 1636 598 947 896 3385 1999 | 10 |
| Shigella infection | ITGB1; IL1B; ELANE; ELMO1; GJB2; IL8 | 3688 3553 1991 9844 2706 3576 | 6 |
| Sicca syndrome | PDCD1; IL1B; TNFSF13B; IL10; SPTAN1; TXN; CRISP3; IFNB1; FAS; LCN1; IFNA1; CXCL9; MALT1; MAP2K4; IL17A; CXCL13; GRAP; CD274; XDH; FASLG; APOH; ITPR1; CCL21; JUN; | 5133 3553 10673 3586 6709 7295 10321 3456 355 3933 3439 4283 10892 6416 3605 10563 10750 29126 7498 356 350 3708 6366 3725 57379 3383 | 26 |
| Sickle cell disease | RHCE; MPO; IL18; ARG2; SCD; KCNN4; PTGS2; ARG1; ITGB1; BCAM; MTHFR; CSF2; CD1A; ICAM4; IGFBP3; LEP; SELP; ITGA4; SCD5; ADRB2; IGF1R; LTF; CD1C; CD1B; F5; IFT81; IGF1 | 6006 4353 3606 384 6319 3783 5743 383 3688 4059 4524 1437 909 3386 3486 3952 6403 3676 79966 154 3480 4057 911 910 2153 28981 3479 | 27 |
| Silicosis | FAS; GSTP1; TNF; SFTPD; HLA-DQB1; IL12B; FASLG | 355 2950 7124 6441 3119 3593 356 | 7 |
| Sinusitis | CAMP; MUC5B; MUC8; IL12B; CLCN3; TNFSF13B; CLCN2; IL22RA1; SFTPB; CFTR; IL12A; MMP8; MMP2; LTC4S; IL5; IL8; MMP9; IL13; MUC5AC; IL4 | 820 727897 4590 3593 1182 10673 1181 58985 6439 1080 3592 4317 4313 4056 3567 3576 4318 3596 4586 3565 | 20 |
| Skin cancer | MMP2; MC1R; SOD2; TIMP1; SMAD2; XRCC1; MAPK14; SERPINB4; TIMP2; S100A7; EGFR; MMP9; LGALS3; IL10; PTPN11; S100B; CXCR5; SMO; WASF3 | 4313 4157 6648 7076 4087 7515 1432 6318 7077 6278 1956 4318 3958 3586 5781 6285 643 6608 10810 | 19 |
| Skin disease | CCL27; IL18; GJB3; MIF; SPINK5; MMP9; GJB2; GJB6; AIF1; CCL28; IL1A; MCM5; ABCC6; IL1F6; IDO1; NR1H4; F13A1; LPL; CAV2; PKLR; RHOC; SKP2; SNAI1; TSC1; BRCA1; SUMO3; CYP27A1; IL2RB; TYR; TYRP1; MC1R; ANGPTL1; APOBEC3A; APOBEC3B | 10850 3606 2707 4282 11005 4318 2706 10804 199 56477 3552 4174 368 27179 3620 9971 2162 4023 858 5313 389 6502 6615 7248 672 6612 1593 3560 7299 7306 4157 9068 200315 9582 | 34 |
| Skin tumor | BCAM; S100A7; LAT; MAGEA4; MC1R; MMP19; POLH | 4059 6278 27040 4103 4157 4327 5429 | 7 |
| Solid tumor | IGF1; IFNG; IL4; PTPN11; IL1RN; EPHA7; F10; ERCC5; FANCF; IGHV3-21; AIMP1; EDIL3 | 3479 3458 3565 5781 3557 2045 2159 2073 2188 28444 9255 10085 | 12 |
| Spinal cord disease | LOC647859; PLS3; TRA2B; DDX20; ICAM1; CX3CR1; S100B; GAD2; SAT1; NF2; HLA-C; UBA1; INS; IGF1; HSPB1; GH1; SPG7; NEFH; DCTN1; PNPLA6 | 647859 5358 6434 11218 3383 1524 6285 2572 6303 4771 3107 7317 3630 3479 3315 2688 6687 4744 1639 10908 | 20 |

| | | | |
|---|---|---|---|
| Spinal dysraphism | MTHFR; MTRR; HK1; CYP26A1; PCYT1A; PAX3; NAT1; SLC19A1; UCP2; BMP4; SLC2A1; CHKA; MTHFD1; LEPR; PCMT1; JARID2; CFL1; BRCA1; MTR | 4524 4552 3098 1592 5130 5077 9 6573 7351 652 6513 1119 4522 3953 5110 3720 1072 672 4548 | 19 |
| Spinocerebellar ataxias | ATXN3; ITPR1; ATXN1; STUB1; ATXN8OS; CACNA1A; C10ORF2; ATXN7; PLEKHG4; TTPA; FGF14; ATXN2 | 4287 3708 6310 10273 6315 773 56652 6314 25894 7274 2259 6311 | 12 |
| Splenic disease | CFC1 | 55997 | 1 |
| Spondylarthropathies | HLA-B; NOD2; SLC11A1; CYP1A1; CD163; KL; VCAN; WISP1; PADI2 | 3106 64127 6556 1543 9332 9365 1462 8840 11240 | 9 |
| Squamous cell cancer | PTPRD; BCL2L14; LGALS7B; S100A7; REL; DVL1; TCEAL1; IL1B; CTSL1; AGER; GGT1; LOXL2; ABO; IFNG; AKR1B10; TSC2; HSPB1; IL4; PTGES; CCL2; CCR1; KRT18; MMP3; MICA; EDN1; CD80; AREG; EPHX1; CTGF; BAK1; CKAP4; PIN1; MMP13; CCL3; CSF1; IL2RA; CD4; ALDH2; SOD1; ADM; CAT; HYAL1; MSN; S100A9; NANOG; RAP1A; DEFB103A; SAFB; TSPAN1; MAGEA1; KRT10; G3BP1; XPA; RASSF2; NEIL2; ITGA5; ITGB6; CHI3L1; SUMO1; HES1; EDNRB; CLDN5; ELF3; DHRS4; SERPINF2; TK2; IKBKE; STMN1; CHAT; KLRB1; CHRNA5; RSPO1; MAGEA4; LGALS9; KRT5; LYNX1; PTK6; NEDD8; KRT17; EIF4G1; HES5; MAGEA6; FABP5; LZTS1; LAMB3; FAT1; SERTAD1; AP2M1; ART1; MAGEA12; MAGEA2; CCT6A; MAGEA5; | 5789 79370 653499 6278 5966 1855 9338 3553 1514 177 2678 4017 28 3458 57016 7249 3315 3565 9536 6347 1230 3875 4314 4276 1906 941 374 2052 1490 578 10970 5300 4322 6348 1435 3559 920 217 6647 133 847 3373 4478 6280 79923 5906 55894 6294 10103 4100 3858 10146 7507 9770 252969 3678 3694 1116 7341 3280 1910 7122 1999 10901 5345 7084 9641 3925 1103 3820 1138 284654 4103 3965 3852 66004 5753 4738 3872 1981 388585 4105 2171 11178 3914 2195 29950 1173 417 4111 4101 908 4104 5908 9252 220064 | 96 |

| | | | |
|---|---|---|---|
| Stomach cancer | IL1RN; DDIT3; PDCD5; CA12; RPL15; ICAM1; TLR5; ITGA2; CD274; IL11; MUC5B; CASP1; BLM; MYCT1; PLXNA1; FABP3; CCL4; POLB; FAF1; CCDC136; OLFM4; SLC9A1; IL11RA; KRT81; MALT1; MT3; EPHB1; BMP8B; CASP8AP2; EPHA7; RAD50; IL1A; SELE; ADIPOQ; AGER; AGTR1; CCL5; SELL; AGT; CTLA4; SERPINE1; TNF; BDKRB1; IL12B; LTA; PITX1; CCL2; FASLG; TLR4; ADIPOR2; MIF; MBL2; TIMP1; HLA-DQB1; HBEGF; LEPR; ACE; CD80; LEP; HLA-DQA1; CTGF; IFNGR2; BAK1; PLA2G2A; MMP10; PIN1; F3; FGF7; CASP7; CCL17; SLPI; CFI; IRAK1; ITPR3; SMAD3; LGALS3BP; BDKRB2; GNL3; DUSP1; CIAPIN1; MAGEA1; EFNB1; ZFHX3; SIAH1; CXCL5; MRE11A; RASSF2; KIF2C; PIWIL1; PKD1; PLCG2; MIR25; RPL23; ARPC1B; EIF4A3; ST3GAL6; UPF1; HPGD; S100A6; KCMF1; CASP6; BTRC; TP53I11; PRMT5; IL12A; CD247; RPS27; CASP2; RPL6; PAGE4; CBFB; HLTF; CCL22; IQGAP2; RUNX1; RNASE1; CSNK1D; SLC4A2; RNR1; HSD17B2; WNT8B; PTPRZ1; B3GAT1; GATA3; INSIG1; PRKD2; ID3; RPS13; TINF2; CACYBP; | 3557 1649 9141 771 6138 3383 7100 3673 29126 3589 727897 834 641 80177 5361 2170 6351 5423 11124 64753 10562 6548 3590 3887 10892 4504 2047 656 9994 2045 10111 3552 6401 9370 177 185 6352 6402 183 1493 5054 7124 623 3593 4049 5307 6347 356 7099 79602 4282 4153 7076 3119 1839 3953 1636 941 3952 3117 1490 3460 578 5320 4319 5300 2152 2252 840 6361 6590 3426 3654 3710 4088 3959 624 26354 1843 57019 4100 1947 463 6477 6374 4361 9770 11004 9271 5310 5336 407014 9349 10095 9775 10402 5976 3248 6277 56888 839 8945 9537 10419 3592 919 6232 835 6128 9506 865 6596 6367 10788 861 6035 1453 6522 4549 3294 7479 5803 27087 2625 3638 25865 3399 6207 26277 27101 406948 406950 131 25920 196 1454 7280 5793 201163 310 1942 | 141 |
| Stomach disease | IL8; REG1A; MICB; MPO; SOD1; XDH; PGA3; BCL6 | 3576 5967 4277 4353 6647 7498 643834 604 | 8 |
| Stroke | MPO; APLNR; ICAM1; FABP2; IGFBP3; TNF; GSTM1; FGF2; RELA; CYP1A1; ADH1B; FABP1; MDK; LGALS2; ROS1; IL1B; PLIN; REN; ITGA2; BDNF; LTA; SLC6A4; HTR2A; XRCC1; ERCC2; HIF1A; EGFR; IL4; FAS; IL18; HTR1A; CX3CR1; TLR4; CD14; MMP9; IL1RN; ITGB2; EDN1; APOB; PLAT; JAK2; ESR1; BCL2; C3; SERPINA3; PLA2G2A; PROCR; PTGS2; MMP13; KL; ITGB3; IL6R; TTPA; ITGA2B; GHR; SELS; HPSE; PROZ; LDLR; APEX1; ROCK1; F2R; MMP11; MFN2; CBS; STS; HSD17B1; CBL; ACCN2; F13B; ALOX5AP; EPHX2; GSTO1; C5; GAS6; PCSK9; F8; SLC25A15 | 4353 187 3383 2169 3486 7124 2944 2247 5970 1543 125 2168 4192 3957 6098 3553 5346 5972 3673 627 4049 6532 3356 7515 2068 3091 1956 3565 355 3606 3350 1524 7099 929 4318 3557 3689 1906 338 5327 3717 2099 596 718 12 5320 10544 5743 4322 9365 3690 3570 7274 3674 2690 55829 10855 8858 3949 328 6093 2149 4320 9927 875 412 3292 867 41 2165 241 2053 9446 727 2621 255738 2157 10166 | 78 |

| | | | |
|---|---|---|---|
| Subacute sclerosing panencephalitis | IL6; MMP9; TIMP1; ACE | 3569 4318 7076 1636 | 4 |
| Subarachnoid hemorrhage | AQP1; ITGB3; ADRB2; APOE; ADRB1; ROCK1; EDN1; BDNF; GFAP; ELN; AQP4 | 358 3690 154 348 153 6093 1906 627 2670 2006 361 | 11 |
| Sudden infant death syndrome | SLC9A3; RMRP; IL6; SLC6A4; KCNE2; SCN5A; KCNJ2; KCNE1; KCNQ4; RYR2; KCNQ2; CAV3; KCNQ1; PHOX2B; IL10; CASP3; KCNH2; SRA1; HTR1A; KCNQ3; MAOA | 6550 6023 3569 6532 9992 6331 3759 3753 9132 6262 3785 859 3784 8929 3586 836 3757 10011 3350 3786 4128 | 21 |
| Synovial sarcoma | IL1B; TLE1; SS18L1; SALL2 | 3553 7088 26039 6297 | 4 |
| Synovitis | F2RL1; TYMP; CCL5; MYC; ESR1; CD163; MMP1; CSF1 | 2150 1890 6352 4609 2099 9332 4312 1435 | 8 |
| Systemic infection | IL6; EDN1; MEFV; ADM; F5; IL10; MPO; KNG1; GPR182; WNT5A; ANGPT1; ITGAM; SFTPD; SMPD1; TIMP1; TNF; CALCA; CAMP; TLR1; NOD2; IL11; GPR77; F2R; PLAT; IL2RA; TLR2; IRF1; LTF; ITGB1; SERPINE1; TLR4; TIMP2; IL18; APOC1; ITGA4; CD163; ZBTB7C; MYLK; CRP; FGB; FCGR1A; ANGPT2; MMP9; CXCL10; F3; VEGFA; MMP2; HSPD1; ICAM1; ADAMTS13; NTN1; IL1RN; CD14; CSF3; BPI; VWF; F7; ACE; ADRB3; CX3CR1; MBL2; F2; RELB; SOD2; IL1RL1; PTPRC; IFNG; F2RL1; MIF; ELANE; CD4; TNIP3; PROCR; AKT1; PDE3A; PYGM | 3569 1906 4210 133 2153 3586 4353 3827 11318 7474 284 3684 6441 6609 7076 7124 796 820 7096 64127 3589 27202 2149 5327 3559 7097 3659 4057 3688 5054 7099 7077 3606 341 3676 9332 201501 4638 1401 2244 2209 285 4318 3627 2152 7422 4313 3329 3383 11093 9423 3557 929 1440 671 7450 2155 1636 155 1524 4153 2147 5971 6648 9173 5788 3458 2150 4282 1991 920 79931 10544 207 5138 5837 | 76 |
| Systemic scleroderma | CASP3; KIR2DS2; SCN2B; MAPK14; CCL7; CTGF; IL1RN; CTLA4; SKIL; FAS; UBE4A; IL1A; TIMP1; PMAIP1; CASP8; SKI; ACE; CCL2; SELP; FBN1; AIF1; PAK2; CTNNB1; ENG; MMP2; PDGFB; NDN; CD19; PTPN22; SMAD7; ROCK2; SERPINA5; IL1B; SFTPB; IL13RA2; F10; ADAM10; SMAD3; HAX1; F5; KIR2DS1; KIR2DL2; IL4; F2; EP300; AKT1; EXOSC9; DLL1; SOD1; MMP1; IL2; IL10; SERPINB3; AGTR1; EXOSC10; CRB2; WNT1; SMURF1; MIF; CAV1; C1D; SIGLEC1; SPAG9; SERPINB2; TIMP2; EDN1; HAVCR1; PLAU; CCL18; APCS; PTPRF; IL17A; CRP; PTPRC; MMP9; FGF2; ANXA5; BPI; SLC11A1; ROCK1; JAG1; INSR; IL13; MET; SNCA; CTSC; HRAS; | 836 100132285 6327 1432 6354 1490 3557 1493 6498 355 9354 3552 7076 5366 841 6497 1636 6347 6403 2200 199 5062 1499 2022 4313 5155 4692 930 26191 4092 9475 5104 3553 6439 3598 2159 102 4088 10456 2153 3806 3803 3565 2147 2033 207 5393 28514 6647 4312 3558 3586 6317 185 5394 286204 7471 57154 4282 857 10438 6614 9043 5055 7077 1906 26762 5328 6362 325 5792 3605 1401 5788 4318 2247 308 671 6556 6093 182 3643 3596 4233 6622 1511 3265 10673 79658 7486 6375 | 91 |

| | | | |
|---|---|---|---|
| Takayasu's arteritis | HLA-B; ITGA2B; ITGB3; MMP3; HLA-A; MMP2; SELP; MMP9; MICA; MTHFR | 3106 3674 3690 4314 3105 4313 6403 4318 4276 4524 | 10 |
| Temporal arteritis | IL4; ESR2; ITGB3; CD24; CXCL5 | 3565 2100 3690 100133941 6374 | 5 |
| Testicular dysfunction | POU5F1P3; KRAS; CFTR; CDKN1B; MSH5; TSPY1; GGNBP1; RNF14; CD4; PRSS21; GSTM1; MCL1; LRRC7; EGFR; MSH2; CCR5; KIT; BIRC7; ESR2; ACE; CCNE1; FGF4; BPY2B; XRCC1; BPY2C; ERBB2; PDILT; SUMO1; SPAG9; GSTP1; BIRC5; BRAF; MC2R; RBMY1A1; CTAG1B; IGF1; MLH1; MIR373; TSP50; GAPDH; FAS; SHBG; FHIT; NANOG; MDC1; [illegible] | 642559 3845 1080 1027 4439 7258 449520 9604 920 10942 2944 4170 57554 1956 4436 1234 3815 79444 2100 1636 898 2249 442867 7515 442868 2064 204474 7341 9043 2950 332 673 4158 5940 1485 3479 4292 442918 29122 2597 355 6462 2272 79923 9656 442917 9083 7503 894 | 49 |
| Testicular tumor | ERCC4; WDR77; PRMT5; XPA; FH; MAPK8IP1; GHSR; ERCC1 | 2072 79084 10419 7507 2271 9479 2693 2067 | 8 |
| Thalassemia | INS; MTHFR; MUC1; GDF15; ARID1B; TSPYL1; IGF1; IL6; IL8; HLA-DQB1; ITGB1; SPTA1; CHIT1; KIR3DL1; ITGA4; LCN2; TPH1; | 3630 4524 4582 9518 57492 7259 3479 3569 3576 3119 3688 6708 1118 3811 3676 3934 7166 287 | 18 |
| Thromboangiitis obliterans | CD14; CSF1; ICAM1; MMP3; SELE; ITGA2 | 929 1435 3383 4314 6401 3673 | 6 |
| Thrombocytopenia | RUNX1; ADAMTS13; FAS; ITGA2B; MPL; FCGR3B; WAS; CCL5; MBL2; MYH9; GATA1; ITGB3; WIPF1; IL11; IL8; IL6 | 861 11093 355 3674 4352 2215 7454 6352 4153 4627 2623 3690 7456 3589 3576 3569 | 16 |
| Thrombocytosis | IL6; ADORA2A; IFNA1; TPO; MPL | 3569 135 3439 7173 4352 | 5 |
| Thrombophilia | F2; F5; PROCR; FGG; CRP; ACE; F9; F3; MTHFR; F11; JAK2; TNF; SERPINE1; F8; ITGAM; ITGB2; SERPINC1; PAFAH1B2 | 2147 2153 10544 2266 1401 1636 2158 2152 4524 2160 3717 7124 5054 2157 3684 3689 462 5049 | 18 |
| Thrombophlebitis | TK1; TYMP; PCTP; EGFR; LRP6; MYH8; BNIP3; SP1; TYMS; ACOT13; PAX3 | 7083 1890 58488 1956 4040 4626 664 6667 7298 55856 5077 | 11 |
| Thymoma | LY75; ESR1; PTPN22; GAPDH; AIRE; IL12B; VNN2; ABL2; CTLA4; | 4065 2099 26191 2597 326 3593 8875 27 1493 4276 | 10 |
| Thyroid cancer | RBL1; KIAA0101; DUSP26; TCEAL4; INSL3; RLN1; NUPR1; GFRA1; P2RX7; EIF2S1; GAPDH; PTTG1IP; TIMP1; NARG1; KRT17; MT1G; MIR146A; DHRS3; IL4; IL10; TJP1; P2RY1; P2RY2; THADA; TNF; CD86; RLN2; DCN; FBN1; PARK7; PTPRJ; HLA-C; | 5933 9768 78986 79921 3640 6013 26471 2674 5027 1965 2597 754 7076 80155 3872 4495 406938 9249 3565 3586 7082 5028 5029 63892 7124 942 6019 1634 2200 11315 5795 3107 6258 | 33 |
| Thyroid gland disease | INSL3; LRP2; LGALS3; CCL5; BCL2; BAX; BCL2L1; LGALS1; CXCL10; PTGS2; KIT; TPO; BAK1; BRAF; SLC5A5; RET; TSHR; | 3640 4036 3958 6352 596 581 598 3956 3627 5743 3815 7173 578 673 6528 5979 7253 7080 389434 | 19 |
| Thyroiditis | CD80; ICAM1 | 941 3383 | 2 |
| Tic disorder | HSPD1; BRCA1 | 3329 672 | 2 |
| Tooth disease | MUC5B; MUC7; PDLIM7 | 727897 4589 9260 | 3 |

| | | | |
|---|---|---|---|
| Transient hypertension of pregnancy | MMP9; LIPE; LIPC; ACE; TIMP1; LEP; TIMP2; MMP2; APOC3; AGT | 4318 3991 3990 1636 7076 3952 7077 4313 345 183 | 10 |
| Tropical spastic paraparesis | MICA; MMP9; IL6; AKT1; ACAN; SETDB1; MICB | 4276 4318 3569 207 176 9869 4277 | 7 |
| Tuberculosis | SLC11A1; SLPI; CCL2; RELA; CCL3; PTX3; CD247; CCL4; IFN1@; IL1A; IL1B; ICAM1; HP; TNC; MMP1; TNF; IL8; NOD2; PTPN22; GSTM1; ITGAL; MIF; BTNL2; MAPK14; SOCS3; CD80; HLA-DQA1; SH2D1A; BCL2; C3; ALOX5; P2RY1; CTSG; IL2RA; GC; AGTR2; MC3R; ACP1; P2RX7; CHIT1; VIM; MCL1; PARK2; FBN1; ESD; UBE3A; NCR1; RAB33A; LMAN2; SFTPA1; CTSZ; BCL2A1; [illegible] | 6556 6590 6347 5970 6348 5806 919 6351 3438 3552 3553 3383 3240 3371 4312 7124 3576 64127 26191 2944 3683 4282 56244 1432 9021 941 3117 4068 596 718 240 5028 1511 3559 2638 186 4159 52 5027 1118 7431 4170 5071 2200 2098 7337 9437 9363 10960 653509 1522 597 2739 3578 58190 | 55 |
| Tuberous sclerosis | ABCC1; CCL2; TSC2; PCNA; TSC1; IKBKB; PKD1; RPTOR; RPS6KB1; RPS6; TNF; RBBP4; ICAM1; RHEB; IRS1 | 4363 6347 7249 5111 7248 3551 5310 57521 6198 6194 7124 5928 3383 6009 3667 | 15 |
| Tumor virus infection | FAS | 355 | 1 |
| Turner's syndrome | FGFR3; PLCG1; SHOX | 2261 5335 6473 | 3 |
| Ulcerative colitis | FABP2; CD14; NOD2; IL17A; BRAF; CCL24; TNF; LEPR; MIF; CTLA4; ICAM1; NR1I2; CXCL10; LPL; TNFSF15; TLR4; PTPRS; HSPA1B; MYO9B; IGF2; XBP1; ACE; KRT8; CARD9; MICA; HLA-A; C13ORF23; TIMP1; RELA; ITGAL; IL11; KCNN1; IL1B; MICB; SPP1; CA2; IL1RN; MLH1; MTHFR; MMP1; IL6; TLR2; SLC22A5; NFATC1; MMP3; CCNA2; SCNN1G; APOA4; ECM1; IFNG; MEFV; NAMPT; SLC22A4; IRAK3; ALPI; TWIST1; SOCS3; NOD1; CCL26; HERC2; AQP8; SCNN1B; CD55; NAT1; IL8; IL5; BTNL2; ARHGDIA; RNF128; CCNY; CDKN1B; BIRC3; CD86; APC; SLC6A4; MST1R; IL18RAP; IDO1; MTR; PTPN2; CCL21; IL4; IL12B; VIPR1; IL13; CDKN2A; CD80; KRT7; TXNIP; UBE4A; CXCL13; MMP7; CTNNB1; APOH | 2169 929 64127 3605 673 6369 7124 3953 4282 1493 3383 8856 3627 4023 9966 7099 5802 3304 4650 3481 7494 1636 3856 64170 4276 3105 80209 7076 5970 3683 3589 3780 3553 4277 6696 760 3557 4292 4524 4312 3569 7097 6584 4772 4314 890 6340 337 1893 3458 4210 10135 6583 11213 248 7291 9021 10392 10344 8924 343 6338 1604 9 3576 3567 56244 396 79589 219771 1027 330 942 324 6532 4486 8807 3620 4548 5771 6366 3565 3593 7433 3596 1029 941 3855 10628 9354 10563 4316 1499 350 | 94 |
| Urinary tract infection | ICAM1; SLPI | 3383 6590 | 2 |
| Urogenital abnormalities | BDNF; PKHD1; SHBG; FGFR1; AR; WT1; INHBA; NR0B1; CYP19A1; ROBO2; KAL1; NELF; PALM2-AKAP2; LHCGR | 627 5314 6462 2260 367 7490 3624 190 1588 6092 3730 26012 445815 3973 | 14 |
| Uterine cancer | CXCL10; MALAT1 | 3627 378938 | 2 |
| Uterine disease | MALAT1; MMP2; CTNNB1; ESR1; STS; CYP19A1; OVGP1 | 378938 4313 1499 2099 412 1588 5016 | 7 |

| | | | |
|---|---|---|---|
| Uterine fibroids | ARNT; IL6; PARP1; VWF; ALDH1A1; AHR; LAMB1; CUX1 | 405 3569 142 7450 216 196 3912 1523 | 8 |
| Uveitis | ADM; NOD2; TRIB2; ESD; CKB; FAS; IFNG; IL17A; HSPA1L; RLBP1; CX3CR1; SOCS3; ARR3; MUC5AC; SOCS5; NCR2 | 133 64127 28951 2098 1152 355 3458 3605 3305 6017 1524 9021 407 4586 9655 9436 | 16 |
| Uveomeningoencephalitic syndrome | TYR; TYRP1; DCT | 7299 7306 1638 | 3 |
| Vaccinia | RHOA; KLRC1; VRK1; DUSP3; BAX; IFNG; BCL2L11; BAK1; JUN; GAPDH; VRK2 | 387 3821 7443 1845 581 3458 10018 578 3725 2597 7444 | 11 |
| Varicosity | P2RX1; MGP; MMP9; MMP3; GSTM1; INHBB; HSPA2 | 5023 4256 4318 4314 2944 3625 3306 | 7 |
| Vascular dementia | APOE; PLA2G2A; AGT; AGER; AVP; ACE; C5AR1; GSTO1; CST3 | 348 5320 183 177 551 1636 728 9446 1471 | 9 |
| Vascular disease | RETN; GSTM1; MAPK14; GJA1; ADM; INHBB; AVP; HSPA2; GGT1; PTPN22; CX3CR1; IGFBP1; FCGR3B; TXN; DCN; AQP1; SMAD4; CLU; VHL; F2RL1; F8; P2RY12; VKORC1; BMPR2; TPSAB1; B4GALT7; PLOD2; NAIP; | 56729 2944 1432 2697 133 3625 551 3306 2678 26191 1524 3484 2215 7295 1634 358 4089 1191 7428 2150 2157 64805 79001 659 7177 11285 5352 4671 64499 2717 | 30 |
| Vasculitis | MMP2; MPO; ICAM1; HIF1A; CXCL13; MEFV; SERPING1; AGER; S100A9; DDR1; RNASE3 | 4313 4353 3383 3091 10563 4210 710 177 6280 780 6037 | 11 |
| Viral hepatitis | PTGES; CYP2A6 | 9536 1548 | 2 |
| Viremia | PDCD1; CXCR6; CCR5; APOBEC3G; HLA-B; BACH2 | 5133 10663 1234 60489 3106 60468 | 6 |
| Virus disease | HLA-B; IFNG; CALCA; CCL26; MX1; KLRC1; IL21; IL5; IL4R; PLA2G5; CD27; PAK1; LMNA; IFIH1; CYR61; PMAIP1; ITGA4; RNASEL; IL12RB1; MAVS; CHUK; ADAR; IL29; IL28A; RAB5A; TBK1; KPNA2; RBBP8; DAXX; IRF7; KLRG1; KPNB1; DDX24; TANK; | 3106 3458 796 10344 4599 3821 59067 3567 3566 5322 939 5058 4000 64135 3491 5366 3676 6041 3594 57506 1147 103 282618 282616 5868 29110 3838 5932 1616 3665 10219 3837 57062 10010 4001 3609 | 36 |
| Vitamin B deficiency | MTHFD1; MIR122; SLC19A2; GCH1; ETFDH; CDKN3; ETFA | 4522 406906 10560 2643 2110 1033 2108 | 7 |
| Vitamin D deficiency | CASR; DUSP1; RUNX1; F10; MLL; RAF1 | 846 1843 861 2159 4297 5894 | 6 |
| Vitiligo | MYO5A; MLPH; GSTM1; GSTT1; EDN1; ESR1; FURIN; CAT; BCHE; POMC; AKT1; ACHE; HLA-C; AIRE; SILV; AIS; MSRA; | 4644 79083 2944 2952 1906 2099 5045 847 590 5443 207 43 3107 326 6490 260402 4482 60314 | 18 |
| Von Hippel-Lindau syndrome | CLU; PHF17 | 1191 79960 | 2 |
| Vulvar disease | TNC; CRP; PTGS2; CASP3; CA9 | 3371 1401 5743 836 768 | 5 |
| Werner syndrome | PRPF19; WRN; MYC; PARP1; ADIPOQ; POLB; NBN | 27339 7486 4609 142 9370 5423 4683 | 7 |
| Wiskott-Aldrich syndrome | WIPF1; WAS; ACTR2; BCAR1; IL2; CXCL12; NCK1; PTK2; ACTR3; WASF1 | 7456 7454 10097 9564 3558 6387 4690 5747 10096 8936 | 10 |

| | | | |
|---|---|---|---|
| Yersinia infection | APOBEC3G; UCHL1; ICAM2; SPANXB2; VCX; IGF2; EPHX1; CHEK2; MICA; ATP1A3; SP3; AMELY; HLA-DQA1; CSAG2; CAMK2A; SP1; CEBPA; EP300; APOBEC3F; CIITA; ACAT1; LAT; MSH6; VWF; IKBKB; KCNQ1; EGFR; SFTPA1; AHRR; HOXB4; KCNQ3; STMN1; APOA1; AHR; FTO; VIPR1; AR; RFX1; CCNB2; POLI; TSPY1; MSH2; DYRK1A; IL6; SCNN1B; LCP2; GABRG3; SOX11; WT1; CTSK; RS1; CEBPB; IVL; ZNF267; GADD45A; HSPD1; SOX3; POLH; CD247; ABO; KLF2; ITGAM; SLC7A7; APOE; PLD2; IL10; ICAM1; ABCA1; ERBB2; HIPK2; NPDC1 | 60489 7345 3384 100133171 26609 3481 2052 11200 4276 478 6670 266 3117 728461 815 6667 1050 2033 200316 4261 38 27040 2956 7450 3551 3784 1956 653509 57491 3214 3786 3925 335 196 79068 7433 367 5989 9133 11201 7258 4436 1859 3569 6338 3937 2567 6664 7490 1513 6247 1051 3713 10308 1647 3329 6658 5429 919 28 10365 3684 9056 348 5338 3586 3383 19 2064 28996 56654 | 71 |
