# Supplementary material for: Inferring drug-disease associations based on known protein complexes
Source: BMC Med Genomics. 2015 May 29;8(Suppl 2):S2. doi: 10.1186/1755-8794-8-S2-S2 (PMC4460611; doi:10.1186/1755-8794-8-S2-S2)
Supplement: Additional file 4 — Table illustrating the information of drug-complex network. [file 1755-8794-8-S2-S2-S4.PDF]

**Drug ID**	**Related Gene Entrez ID**	**Complex Name**	**Weight**
DB00125	550	NOS3-CAV1-NOSTRIN complex	0.19245
DB00125	753	UTM-SGCE-DAG1-CAV1-NOS3 complex	0.14907
DB00125	5718	eNOS-HSP90-AKT complex VEGF induced	0.19245
DB00125	5716	eNOS-HSP90 complex VEGF induced	0.2357
DB00125	5714	eNOS-CAV1 complex	0.2357
DB00155	432	N-NOS-CHIP-HSP70-1 complex	0.16013
DB00155	550	NOS3-CAV1-NOSTRIN complex	0.16013
DB00155	753	UTM-SGCE-DAG1-CAV1-NOS3 complex	0.12403
DB00155	5718	eNOS-HSP90-AKT complex VEGF induced	0.16013
DB00155	5716	eNOS-HSP90 complex VEGF induced	0.19612
DB00155	5714	eNOS-CAV1 complex	0.19612
DB01110	550	NOS3-CAV1-NOSTRIN complex	0.14907
DB01110	668	BKCA-beta2AR-AKAP79 signaling complex	0.14907
DB01110	672	BKCA-beta2AR complex	0.18257
DB01110	753	UTM-SGCE-DAG1-CAV1-NOS3 complex	0.11547
DB01110	5718	eNOS-HSP90-AKT complex VEGF induced	0.14907
DB01110	5716	eNOS-HSP90 complex VEGF induced	0.18257
DB01110	5714	eNOS-CAV1 complex	0.18257
DB01234	4216	GR-hnRNP U complex	0.35355
DB08814	575	ABIN2-NFKB1-MAP3K8 complex	0.28868
DB08814	2003	COX1 homodimer complex	0.5
DB08814	2084	NFKB1-NFKB2-REL-RELA-RELB complex	0.22361
DB08814	2086	NFKB1-NFKB2-RELA-RELB complex	0.25
DB08814	3045	hs4 enhancer complex (faster migrating complex)	0.22361
DB08814	5193	TNF-alpha/NF-kappa B signaling complex (CHUK KPNA3 NFKB2 NFKBIB REL IKBKG NFKB1 NFKBIE RELB NFKBIA RELA TNIP2)	0.14434
DB08814	5230	CHUK-NFKB2-REL-IKBKG-SPAG9-NFKB1-NFKBIE-COPB2-TNIP1-NFKBIA-RELA-TNIP2 complex	0.14434
DB08814	5232	TNF-alpha/Nf-kappa B signaling complex (RPL6 RPL30 RPS13 CHUK DDX3X NFKB2 NFKBIB REL IKBKG NFKB1 MAP3K8 RELB GLG1 NFKBIA RELA TNIP2 GTF2I)	0.12127
DB08814	5233	TNF-alpha/NF-kappa B signaling complex 5	0.1
DB08814	5460	p50-p65 NF(kappa)B complex	0.35355
DB08814	5461	p50-p65 NF(kappa)B-SRC1 complex	0.28868
DB08814	5464	I(kappa)B(alpha)-NF(kappa)Bp50-NF(kappa)Bp65 complex	0.28868
DB08814	5492	IKBA-NF(kappa)Bp65-NF(kappa)Bp50 complex	0.28868
DB00131	518	AKAP250-PKA-PDE4D complex	0.11547
DB00131	771	NDPKA-AMPKalpha1 complex	0.18257
DB00131	3181	LMO4-CREB complex	0.18257
DB00131	3189	FHL2-CREB complex	0.18257
DB00131	3190	FHL3-CREB complex	0.18257
DB00131	3191	ACT-CREB complex	0.18257
DB00661	1617	G protein complex (CACNA1A GNB1 GNG2)	0.14434
DB04841	1223	H2AX complex isolated from cells without IR exposure	0.12403
DB04841	2242	TGM2-HD-CALM1 complex	0.2582
DB04841	4158	HSP90-FKBP38-CAM-Ca(2+) complex	0.22361
DB04841	5189	YWHAQ-CALM1-CABIN1 complex	0.2582
DB04841	5526	CALM1-FKBP38-BCL2 complex	0.2582
DB00277	54	SIN3 complex	0.13363
DB00277	61	Mi2/NuRD complex	0.13363
DB00277	62	MeCP1 complex	0.125
DB00277	282	SNF2h-cohesin-NuRD complex	0.08839
DB00277	283	Sin3 complex	0.13363

DB00277	587	NuRD.1 complex	0.125
DB00277	591	SAP complex (Sin3-associated protein complex)	0.125
DB00277	592	SAP complex (Sin3-associated protein complex)	0.11785
DB00277	596	SIN3-HDAC-SAP30-ARID4 complex	0.13363
DB00277	614	NRD complex (Nucleosome remodeling and deacetylation complex)	0.13363
DB00277	620	CoREST-HDAC complex	0.13363
DB00277	632	Anti-HDAC2 complex	0.08333
DB00277	633	anti-BHC110 complex	0.1066
DB00277	634	XFIM complex	0.15811
DB00277	636	BHC complex	0.14434
DB00277	642	CtBP complex	0.08575
DB00277	643	CtBP core complex	0.11785
DB00277	650	HDAC2-asscociated core complex	0.125
DB00277	659	MeCP1 complex	0.11785
DB00277	685	MeCP1 complex	0.11785
DB00277	691	SIN3-SAP25 complex	0.1066
DB00277	696	BRMS1-SIN3-HDAC complex	0.125
DB00277	713	BRG1-SIN3A complex	0.09449
DB00277	714	BRM-SIN3A complex	0.09129
DB00277	738	SIN3-ING1b complex I	0.11785
DB00277	739	SIN3-ING1b complex II	0.08839
DB00277	745	NCOR-SIN3-RPD3 complex	0.17678
DB00277	749	MeCP2-SIN3A-HDAC complex	0.17678
DB00277	778	LARC complex (LCR-associated remodeling complex)	0.08111
DB00277	803	BRG1-SIN3A-HDAC containing SWI/SNF remodeling complex I	0.1066
DB00277	806	BRM-SIN3A-HDAC complex	0.10206
DB00277	871	BRAF53-BRCA2 complex	0.13363
DB00277	886	MTA1 complex	0.14434
DB00277	888	MTA2 complex	0.11785
DB00277	889	MTA1-HDAC core complex	0.15811
DB00277	1133	ATR-HDAC2 complex	0.25
DB00277	1134	ATR-HDAC2-CHD4 complex	0.20412
DB00277	1233	CoREST-HDAC2 complex	0.25
DB00277	1257	ALL-1 supercomplex	0.06682
DB00277	1458	SNF2h-HDAC12 complex	0.25
DB00277	1492	BHC110 complex	0.1118
DB00277	1505	NCOR2 complex	0.13363
DB00277	2657	ESR1-CDK7-CCNH-MNAT1-MTA1-HDAC2 complex	0.14434
DB00277	2721	HCF-1 complex	0.08111
DB00277	2814	BRCA1-HDAC1-HDAC2 complex	0.20412
DB00277	2851	ING2 complex	0.10206
DB00277	3048	mSin3A complex	0.15811
DB00277	3053	mSin3A-HDAC1-HDAC2 complex	0.20412
DB00277	3054	MAD1-mSin3A-HDAC2 complex	0.20412
DB00277	3167	NCOR-SIN3-HDAC-HESX1 complex	0.14434
DB00651	518	AKAP250-PKA-PDE4D complex	0.15811
DB01223	54	SIN3 complex	0.18898
DB01223	61	Mi2/NuRD complex	0.18898
DB01223	62	MeCP1 complex	0.17678
DB01223	282	SNF2h-cohesin-NuRD complex	0.125
DB01223	283	Sin3 complex	0.18898
DB01223	587	NuRD.1 complex	0.17678
DB01223	591	SAP complex (Sin3-associated protein complex)	0.17678
DB01223	592	SAP complex (Sin3-associated protein complex)	0.16667

DB01223	596	SIN3-HDAC-SAP30-ARID4 complex	0.18898
DB01223	614	NRD complex (Nucleosome remodeling and deacetylation complex)	0.18898
DB01223	620	CoREST-HDAC complex	0.18898
DB01223	632	Anti-HDAC2 complex	0.11785
DB01223	633	anti-BHC110 complex	0.15076
DB01223	634	XFIM complex	0.22361
DB01223	636	BHC complex	0.20412
DB01223	642	CtBP complex	0.12127
DB01223	643	CtBP core complex	0.16667
DB01223	650	HDAC2-asscociated core complex	0.17678
DB01223	659	MeCP1 complex	0.16667
DB01223	685	MeCP1 complex	0.16667
DB01223	691	SIN3-SAP25 complex	0.15076
DB01223	696	BRMS1-SIN3-HDAC complex	0.17678
DB01223	713	BRG1-SIN3A complex	0.13363
DB01223	714	BRM-SIN3A complex	0.1291
DB01223	738	SIN3-ING1b complex I	0.16667
DB01223	739	SIN3-ING1b complex II	0.125
DB01223	745	NCOR-SIN3-RPD3 complex	0.25
DB01223	749	MeCP2-SIN3A-HDAC complex	0.25
DB01223	778	LARC complex (LCR-associated remodeling complex)	0.11471
DB01223	803	BRG1-SIN3A-HDAC containing SWI/SNF remodeling complex I	0.15076
DB01223	806	BRM-SIN3A-HDAC complex	0.14434
DB01223	871	BRAF53-BRCA2 complex	0.18898
DB01223	886	MTA1 complex	0.20412
DB01223	888	MTA2 complex	0.16667
DB01223	889	MTA1-HDAC core complex	0.22361
DB01223	1133	ATR-HDAC2 complex	0.35355
DB01223	1134	ATR-HDAC2-CHD4 complex	0.28868
DB01223	1233	CoREST-HDAC2 complex	0.35355
DB01223	1257	ALL-1 supercomplex	0.09449
DB01223	1458	SNF2h-HDAC12 complex	0.35355
DB01223	1492	BHC110 complex	0.15811
DB01223	1505	NCOR2 complex	0.18898
DB01223	2657	ESR1-CDK7-CCNH-MNAT1-MTA1-HDAC2 complex	0.20412
DB01223	2721	HCF-1 complex	0.11471
DB01223	2814	BRCA1-HDAC1-HDAC2 complex	0.28868
DB01223	2851	ING2 complex	0.14434
DB01223	3048	mSin3A complex	0.22361
DB01223	3053	mSin3A-HDAC1-HDAC2 complex	0.28868
DB01223	3054	MAD1-mSin3A-HDAC2 complex	0.28868
DB01223	3167	NCOR-SIN3-HDAC-HESX1 complex	0.20412
DB01303	54	SIN3 complex	0.16903
DB01303	61	Mi2/NuRD complex	0.16903
DB01303	62	MeCP1 complex	0.15811
DB01303	282	SNF2h-cohesin-NuRD complex	0.1118
DB01303	283	Sin3 complex	0.16903
DB01303	587	NuRD.1 complex	0.15811
DB01303	591	SAP complex (Sin3-associated protein complex)	0.15811
DB01303	592	SAP complex (Sin3-associated protein complex)	0.14907
DB01303	596	SIN3-HDAC-SAP30-ARID4 complex	0.16903
DB01303	614	NRD complex (Nucleosome remodeling and deacetylation complex)	0.16903
DB01303	620	CoREST-HDAC complex	0.16903
DB01303	632	Anti-HDAC2 complex	0.10541

DB01303	633	anti-BHC110 complex	0.13484
DB01303	634	XFIM complex	0.2
DB01303	636	BHC complex	0.18257
DB01303	642	CtBP complex	0.10847
DB01303	643	CtBP core complex	0.14907
DB01303	650	HDAC2-asscociated core complex	0.15811
DB01303	659	MeCP1 complex	0.14907
DB01303	685	MeCP1 complex	0.14907
DB01303	691	SIN3-SAP25 complex	0.13484
DB01303	696	BRMS1-SIN3-HDAC complex	0.15811
DB01303	713	BRG1-SIN3A complex	0.11952
DB01303	714	BRM-SIN3A complex	0.11547
DB01303	738	SIN3-ING1b complex I	0.14907
DB01303	739	SIN3-ING1b complex II	0.1118
DB01303	745	NCOR-SIN3-RPD3 complex	0.22361
DB01303	749	MeCP2-SIN3A-HDAC complex	0.22361
DB01303	778	LARC complex (LCR-associated remodeling complex)	0.1026
DB01303	803	BRG1-SIN3A-HDAC containing SWI/SNF remodeling complex I	0.13484
DB01303	806	BRM-SIN3A-HDAC complex	0.1291
DB01303	871	BRAF53-BRCA2 complex	0.16903
DB01303	886	MTA1 complex	0.18257
DB01303	888	MTA2 complex	0.14907
DB01303	889	MTA1-HDAC core complex	0.2
DB01303	1133	ATR-HDAC2 complex	0.31623
DB01303	1134	ATR-HDAC2-CHD4 complex	0.2582
DB01303	1233	CoREST-HDAC2 complex	0.31623
DB01303	1257	ALL-1 supercomplex	0.08452
DB01303	1458	SNF2h-HDAC12 complex	0.31623
DB01303	1492	BHC110 complex	0.14142
DB01303	1505	NCOR2 complex	0.16903
DB01303	2657	ESR1-CDK7-CCNH-MNAT1-MTA1-HDAC2 complex	0.18257
DB01303	2721	HCF-1 complex	0.1026
DB01303	2814	BRCA1-HDAC1-HDAC2 complex	0.2582
DB01303	2851	ING2 complex	0.1291
DB01303	3048	mSin3A complex	0.2
DB01303	3053	mSin3A-HDAC1-HDAC2 complex	0.2582
DB01303	3054	MAD1-mSin3A-HDAC2 complex	0.2582
DB01303	3167	NCOR-SIN3-HDAC-HESX1 complex	0.18257
DB00171	771	NDPKA-AMPKalpha1 complex	0.12309
DB00171	1094	Frataxin complex	0.0658
DB00171	1248	Apoptosome	0.12309
DB00171	1700	ABL2-HRAS-RIN1 complex	0.1005
DB00171	5718	eNOS-HSP90-AKT complex VEGF induced	0.1005
DB00171	2156	YBX1-AKT1 complex	0.12309
DB00171	2159	AR-AKT-APPL complex	0.1005
DB00171	2162	APPBP1-UBA3 complex	0.12309
DB00171	2811	BRCA1-cABL complex	0.12309
DB00171	3847	TCL1(trimer)-AKT1 complex	0.12309
DB00171	5816	Apoptosome-procaspase 9 complex	0.1005
DB00619	1096	SNX complex (SNX1 1a 2 4 PDGF receptor)	0.18898
DB00619	2476	CRKL-PDGFRA-CRK-RAPGEF1 complex	0.18898
DB00619	2487	GIPC1-NTRK1-RGS19 complex	0.21822
DB00619	2551	PDGFRA-PLC-gamma-1-PI3K-SHP-2 complex PDGF stimulated	0.18898
DB00619	2811	BRCA1-cABL complex	0.26726

DB00619	3183	PDGFRA-SHP-2 complex PDGF stimulated	0.26726
DB00619	5407	NGF-TrkA complex	0.26726
DB01254	1067	CD8A-LCK complex	0.22361
DB01254	1700	ABL2-HRAS-RIN1 complex	0.18257
DB01254	5713	SH3P2/OSTF1-CBL-SRC complex	0.18257
DB01254	2013	STAT5B homodimer complex	0.31623
DB01254	2073	TNFRSF11A-TRAF6-SRC complex	0.18257
DB01254	2321	ITGA6-ITGB4-FYN complex	0.18257
DB01254	2351	ITGB6-FYN-FN1 complex	0.18257
DB01254	2377	ITGA2b-ITGB3-CD47-SRC complex	0.15811
DB01254	2470	p130Cas-ER-alpha-cSrc-kinase- PI3-kinase p85-subunit complex	0.15811
DB01254	2471	SRC-PRKCD-CDCP1 complex	0.18257
DB01254	2559	p56(LCK)-CAML complex	0.22361
DB01254	2563	FGFR2-c-Cbl-Lyn-Fyn complex	0.15811
DB01254	2564	p21(ras)GAP-Fyn-Lyn-Yes complex thrombin stimulated	0.31623
DB01254	2565	CD20-LCK-LYN-FYN-p75/80 complex (Raji human B cell line)	0.31623
DB01254	2811	BRCA1-cABL complex	0.22361
DB01254	2879	CD20-LCK-FYN-p75/80 complex	0.36515
DB01254	2944	Notch1-p56lck-PI3K complex	0.18257
DB01254	2955	LCK-SLP76-PLC-gamma-1-LAT complex pervanadate-activated	0.15811
DB01254	3137	MASH1 promoter-coactivator complex	0.09535
DB01254	3142	CAMK2-delta-MASH1 promoter-coactivator complex	0.1118
DB01254	5177	Polycystin-1 multiprotein complex (ACTN1 CDH1 SRC JUP VCL CTNNB1 PXN BCAR1 PKD1 PTK2 TLN1)	0.09535
DB01254	5190	TIAM1-EFNB1-EPHA2 complex	0.18257
DB01254	5282	CAS-SRC-FAK complex	0.18257
DB01254	5641	PSD95-FYN-NR2A complex	0.18257
DB01254	5928	CNK1-SRC-RAF1 complex	0.18257
DB04868	2811	BRCA1-cABL complex	0.5
DB06616	311	Cell cycle kinase complex CDK2	0.15811
DB06616	906	ADAR1-CDK2 complex	0.22361
DB06616	1003	RC complex (Replication competent complex)	0.10541
DB06616	1004	RC complex during S-phase of cell cycle	0.08771
DB06616	1656	p27-cyclinE-CDK2 complex	0.18257
DB06616	5713	SH3P2/OSTF1-CBL-SRC complex	0.18257
DB06616	2073	TNFRSF11A-TRAF6-SRC complex	0.18257
DB06616	2377	ITGA2b-ITGB3-CD47-SRC complex	0.15811
DB06616	2470	p130Cas-ER-alpha-cSrc-kinase- PI3-kinase p85-subunit complex	0.15811
DB06616	2471	SRC-PRKCD-CDCP1 complex	0.18257
DB06616	2536	PLC-gamma-2-SLP-76-Lyn-Grb2 complex	0.15811
DB06616	2563	FGFR2-c-Cbl-Lyn-Fyn complex	0.15811
DB06616	2564	p21(ras)GAP-Fyn-Lyn-Yes complex thrombin stimulated	0.15811
DB06616	2565	CD20-LCK-LYN-FYN-p75/80 complex (Raji human B cell line)	0.15811
DB06616	2811	BRCA1-cABL complex	0.22361
DB06616	2910	PLC-gamma-2-Lyn-FcR-gamma complex	0.18257
DB06616	3015	p27-cyclinE-Cdk2 - Ubiquitin E3 ligase (SKP1A SKP2 CUL1 CKS1B RBX1) complex	0.1118
DB06616	3137	MASH1 promoter-coactivator complex	0.09535
DB06616	3142	CAMK2-delta-MASH1 promoter-coactivator complex	0.1118
DB06616	5177	Polycystin-1 multiprotein complex (ACTN1 CDH1 SRC JUP VCL CTNNB1 PXN BCAR1 PKD1 PTK2 TLN1)	0.09535
DB06616	5222	p14-Mp1-MEK1 complex	0.18257
DB06616	5282	CAS-SRC-FAK complex	0.18257
DB06616	5556	CDK2-CCNA2 complex	0.22361
DB06616	5559	CDC2-CCNA2-CDK2 complex	0.18257

DB06616	5560	CDK2-CCNE1 complex	0.22361
DB06616	5877	MAP2K1-BRAF-RAF1-YWHAE-KSR1 complex	0.14142
DB06616	5872	BRAF-MAP2K1-MAP2K2-YWHAE complex	0.31623
DB06616	5873	RAF1-MAP2K1-YWHAE complex	0.18257
DB06616	5920	KSR1-RAF1-MEK complex	0.31623
DB06616	5921	KSR1-BRAF-MEK complex	0.31623
DB06616	5928	CNK1-SRC-RAF1 complex	0.18257
DB08896	903	RET-Rai complex	0.16667
DB08896	1096	SNX complex (SNX1 1a 2 4 PDGF receptor)	0.11785
DB08896	1539	G protein complex (GNG2 GNB2L1 RAF1)	0.13608
DB08896	2476	CRKL-PDGFRA-CRK-RAPGEF1 complex	0.11785
DB08896	2487	GIPC1-NTRK1-RGS19 complex	0.13608
DB08896	2551	PDGFRA-PLC-gamma-1-PI3K-SHP-2 complex PDGF stimulated	0.11785
DB08896	2563	FGFR2-c-Cbl-Lyn-Fyn complex	0.11785
DB08896	2811	BRCA1-cABL complex	0.16667
DB08896	3183	PDGFRA-SHP-2 complex PDGF stimulated	0.16667
DB08896	4062	NRP1-VEGFR2-VEGF(165) complex	0.13608
DB08896	5772	ZO1-(beta)cadherin-(VE)cadherin-VEGFR2 complex	0.11785
DB08896	5190	TIAM1-EFNB1-EPHA2 complex	0.13608
DB08896	5211	RAF1-PPP2-PIN1 complex	0.10541
DB08896	5407	NGF-TrkA complex	0.16667
DB08896	5696	VEGFA(165)-KDR-NRP1 complex	0.13608
DB08896	5698	VEGFA(165)-VEGFR2-NRP1 complex	0.13608
DB08896	5740	NRP2-VEGFR3 complex	0.16667
DB08896	5877	MAP2K1-BRAF-RAF1-YWHAE-KSR1 complex	0.21082
DB08896	5872	BRAF-MAP2K1-MAP2K2-YWHAE complex	0.11785
DB08896	5873	RAF1-MAP2K1-YWHAE complex	0.13608
DB08896	5919	BRAF-RAF1-14-3-3 complex	0.15713
DB08896	5920	KSR1-RAF1-MEK complex	0.11785
DB08896	5921	KSR1-BRAF-MEK complex	0.11785
DB08896	5922	RAF1-RAS complex EGF induced	0.11785
DB08896	5923	RAF1-BRAF complex RAS stimulated	0.33333
DB08896	5924	RAF1-CNK1 complex RAS stimulated	0.16667
DB08896	5925	BRAF-CNK1 complex not RAS stimulated	0.16667
DB08896	5928	CNK1-SRC-RAF1 complex	0.13608
DB08901	903	RET-Rai complex	0.18257
DB08901	1067	CD8A-LCK complex	0.18257
DB08901	1096	SNX complex (SNX1 1a 2 4 PDGF receptor)	0.1291
DB08901	5713	SH3P2/OSTF1-CBL-SRC complex	0.14907
DB08901	2073	TNFRSF11A-TRAF6-SRC complex	0.14907
DB08901	2377	ITGA2b-ITGB3-CD47-SRC complex	0.1291
DB08901	2470	p130Cas-ER-alpha-cSrc-kinase- PI3-kinase p85-subunit complex	0.1291
DB08901	2471	SRC-PRKCD-CDCP1 complex	0.14907
DB08901	2476	CRKL-PDGFRA-CRK-RAPGEF1 complex	0.1291
DB08901	2536	PLC-gamma-2-SLP-76-Lyn-Grb2 complex	0.1291
DB08901	2551	PDGFRA-PLC-gamma-1-PI3K-SHP-2 complex PDGF stimulated	0.1291
DB08901	2559	p56(LCK)-CAML complex	0.18257
DB08901	2563	FGFR2-c-Cbl-Lyn-Fyn complex	0.2582
DB08901	2564	p21(ras)GAP-Fyn-Lyn-Yes complex thrombin stimulated	0.1291
DB08901	2565	CD20-LCK-LYN-FYN-p75/80 complex (Raji human B cell line)	0.2582
DB08901	2811	BRCA1-cABL complex	0.18257
DB08901	2879	CD20-LCK-FYN-p75/80 complex	0.14907
DB08901	2910	PLC-gamma-2-Lyn-FcR-gamma complex	0.14907
DB08901	2944	Notch1-p56lck-PI3K complex	0.14907

DB08901	2955	LCK-SLP76-PLC-gamma-1-LAT complex pervanadate-activated	0.1291
DB08901	3137	MASH1 promoter-coactivator complex	0.07785
DB08901	3142	CAMK2-delta-MASH1 promoter-coactivator complex	0.09129
DB08901	3183	PDGFRA-SHP-2 complex PDGF stimulated	0.18257
DB08901	4062	NRP1-VEGFR2-VEGF(165) complex	0.14907
DB08901	5772	ZO1-(beta)cadherin-(VE)cadherin-VEGFR2 complex	0.1291
DB08901	5177	Polycystin-1 multiprotein complex (ACTN1 CDH1 SRC JUP VCL CTNNB1 PXN BCAR1 PKD1 PTK2 TLN1)	0.07785
DB08901	5282	CAS-SRC-FAK complex	0.14907
DB08901	5696	VEGFA(165)-KDR-NRP1 complex	0.14907
DB08901	5698	VEGFA(165)-VEGFR2-NRP1 complex	0.14907
DB08901	5928	CNK1-SRC-RAF1 complex	0.14907
DB00055	845	PCI-PSA-SCG2 complex	0.16667
DB00100	143	APP-FE65-LRP complex	0.21822
DB00100	2709	MMP-9-TIMP-1-LRP complex	0.21822
DB00100	2710	LRP-1-Alpha-2-M-annexin VI complex	0.21822
DB00100	3162	TF-FVIIa-FXa-TFPI complex	0.37796
DB00154	1439	PTGS2 homodimer complex	0.70711
DB00154	2003	COX1 homodimer complex	0.70711
DB00159	1439	PTGS2 homodimer complex	0.31623
DB00159	2003	COX1 homodimer complex	0.31623
DB00244	1439	PTGS2 homodimer complex	0.35355
DB00244	2003	COX1 homodimer complex	0.35355
DB00244	2055	CASP8-CHUK-IKBKB-MALT1-BCL10 complex	0.31623
DB00244	2056	BCL10-CHUK-BCL10-IKBKB complex	0.35355
DB00244	2100	CHUK-IKBKB-MAP3K14 complex	0.40825
DB00244	2101	IKKA-IKKB complex	0.5
DB00244	2104	IKKB-NIK complex	0.25
DB00244	2105	IkappaB kinase complex (IKBKB CHUK IKBKAP NFKBIA RELA MAP3K14)	0.28868
DB00244	2118	CHUK-ERC1-IKBKB-IKBKG	0.35355
DB00244	2121	CHUK-IKBKB-IKBKG complex	0.40825
DB00244	2124	IKK-alpha--ER-alpha-AIB1 complex	0.20412
DB00244	2727	SRC-3 complex	0.26726
DB00244	5193	TNF-alpha/NF-kappa B signaling complex (CHUK KPNA3 NFKB2 NFKBIB REL IKBKG NFKB1 NFKBIE RELB NFKBIA RELA TNIP2)	0.10206
DB00244	5194	TNF-alpha/NF-kappa B signaling complex (SEC16A CHUK IKBKB NFKB2 REL IKBKG MAP3K14 RELA FBXW7 USP2)	0.22361
DB00244	5196	TNF-alpha/NF-kappa B signaling complex (CHUK BTRC NFKB2 PPP6C REL CUL1 IKBKE SAPS2 SAPS1 ANKRD28 RELA SKP1)	0.10206
DB00244	5220	CHUK-IQGAP2-AKAP8L-RELA-TNIP2 complex	0.15811
DB00244	5230	CHUK-NFKB2-REL-IKBKG-SPAG9-NFKB1-NFKBIE-COPB2-TNIP1-NFKBIA-RELA-TNIP2 complex	0.10206
DB00244	5232	TNF-alpha/Nf-kappa B signaling complex (RPL6 RPL30 RPS13 CHUK DDX3X NFKB2 NFKBIB REL IKBKG NFKB1 MAP3K8 RELB GLG1 NFKBIA RELA TNIP2 GTF2I)	0.08575
DB00244	5233	TNF-alpha/NF-kappa B signaling complex 5	0.14142
DB00244	5234	IKBKB-CDC37-KIAA1967-HSP90AB1-HSP90AA1 complex	0.15811
DB00244	5266	TNF-alpha/NF-kappa B signaling complex 6	0.18898
DB00244	5285	TNF-alpha/NF-kappa B signaling complex 9	0.15811
DB00244	5286	TNF-alpha/NF-kappa B signaling complex 10	0.1118
DB00244	5828	IKBKG-IKBKB complex	0.25
DB00244	5829	IKBKG-CHUK complex	0.25
DB00244	5844	I-kappa-B kinase (IKK) complex	0.40825
DB00316	1439	PTGS2 homodimer complex	0.70711

DB00316	2003	COX1 homodimer complex	0.70711
DB00328	1439	PTGS2 homodimer complex	0.37796
DB00328	2003	COX1 homodimer complex	0.37796
DB00350	2003	COX1 homodimer complex	0.70711
DB00461	1439	PTGS2 homodimer complex	0.70711
DB00461	2003	COX1 homodimer complex	0.70711
DB00465	1439	PTGS2 homodimer complex	0.70711
DB00465	2003	COX1 homodimer complex	0.70711
DB00469	1439	PTGS2 homodimer complex	0.70711
DB00469	2003	COX1 homodimer complex	0.70711
DB00500	1439	PTGS2 homodimer complex	0.70711
DB00500	2003	COX1 homodimer complex	0.70711
DB00554	1439	PTGS2 homodimer complex	0.70711
DB00554	2003	COX1 homodimer complex	0.70711
DB00573	1439	PTGS2 homodimer complex	0.70711
DB00573	2003	COX1 homodimer complex	0.70711
DB00586	1439	PTGS2 homodimer complex	0.35355
DB00586	2003	COX1 homodimer complex	0.35355
DB00605	55	HDAC4-ERK1 complex	0.28868
DB00605	1439	PTGS2 homodimer complex	0.40825
DB00605	2003	COX1 homodimer complex	0.40825
DB00711	2003	COX1 homodimer complex	0.70711
DB00712	1439	PTGS2 homodimer complex	0.70711
DB00712	2003	COX1 homodimer complex	0.70711
DB00749	1439	PTGS2 homodimer complex	0.57735
DB00749	2003	COX1 homodimer complex	0.57735
DB00749	5198	CBP-RARA-RXRA-DNA complex ligand stimulated	0.33333
DB00784	1439	PTGS2 homodimer complex	0.70711
DB00784	2003	COX1 homodimer complex	0.70711
DB00788	1439	PTGS2 homodimer complex	0.70711
DB00788	2003	COX1 homodimer complex	0.70711
DB00795	1439	PTGS2 homodimer complex	0.35355
DB00795	2003	COX1 homodimer complex	0.35355
DB00795	2055	CASP8-CHUK-IKBKB-MALT1-BCL10 complex	0.31623
DB00795	2056	BCL10-CHUK-BCL10-IKBKB complex	0.35355
DB00795	2100	CHUK-IKBKB-MAP3K14 complex	0.40825
DB00795	2101	IKKA-IKKB complex	0.5
DB00795	2104	IKKB-NIK complex	0.25
DB00795	2105	IkappaB kinase complex (IKBKB CHUK IKBKAP NFKBIA RELA MAP3K14)	0.28868
DB00795	2118	CHUK-ERC1-IKBKB-IKBKG	0.35355
DB00795	2121	CHUK-IKBKB-IKBKG complex	0.40825
DB00795	2124	IKK-alpha--ER-alpha-AIB1 complex	0.20412
DB00795	2727	SRC-3 complex	0.26726
DB00795	5193	TNF-alpha/NF-kappa B signaling complex (CHUK KPNA3 NFKB2 NFKBIB REL IKBKG NFKB1 NFKBIE RELB NFKBIA RELA TNIP2)	0.10206
DB00795	5194	TNF-alpha/NF-kappa B signaling complex (SEC16A CHUK IKBKB NFKB2 REL IKBKG MAP3K14 RELA FBXW7 USP2)	0.22361
DB00795	5196	TNF-alpha/NF-kappa B signaling complex (CHUK BTRC NFKB2 PPP6C REL CUL1 IKBKE SAPS2 SAPS1 ANKRD28 RELA SKP1)	0.10206
DB00795	5220	CHUK-IQGAP2-AKAP8L-RELA-TNIP2 complex	0.15811
DB00795	5230	CHUK-NFKB2-REL-IKBKG-SPAG9-NFKB1-NFKBIE-COPB2-TNIP1-NFKBIA-RELA-TNIP2 complex	0.10206

DB00795	5232	TNF-alpha/Nf-kappa B signaling complex (RPL6 RPL30 RPS13 CHUK DDX3X NFKB2 NFKBIB REL IKBKG NFKB1 MAP3K8 RELB GLG1 NFKBIA RELA TNIP2 GTF2I)	0.08575
DB00795	5233	TNF-alpha/NF-kappa B signaling complex 5	0.14142
DB00795	5234	IKBKB-CDC37-KIAA1967-HSP90AB1-HSP90AA1 complex	0.15811
DB00795	5266	TNF-alpha/NF-kappa B signaling complex 6	0.18898
DB00795	5285	TNF-alpha/NF-kappa B signaling complex 9	0.15811
DB00795	5286	TNF-alpha/NF-kappa B signaling complex 10	0.1118
DB00795	5828	IKBKG-IKBKB complex	0.25
DB00795	5829	IKBKG-CHUK complex	0.25
DB00795	5844	I-kappa-B kinase (IKK) complex	0.40825
DB00812	1439	PTGS2 homodimer complex	0.57735
DB00812	2003	COX1 homodimer complex	0.57735
DB00814	1439	PTGS2 homodimer complex	0.70711
DB00814	2003	COX1 homodimer complex	0.70711
DB00821	1439	PTGS2 homodimer complex	0.70711
DB00821	2003	COX1 homodimer complex	0.70711
DB00861	1439	PTGS2 homodimer complex	0.70711
DB00861	2003	COX1 homodimer complex	0.70711
DB00870	1439	PTGS2 homodimer complex	0.70711
DB00870	2003	COX1 homodimer complex	0.70711
DB00936	1439	PTGS2 homodimer complex	0.57735
DB00936	2003	COX1 homodimer complex	0.57735
DB00939	1439	PTGS2 homodimer complex	0.44721
DB00939	2003	COX1 homodimer complex	0.44721
DB00945	1439	PTGS2 homodimer complex	0.57735
DB00945	2003	COX1 homodimer complex	0.57735
DB00963	1439	PTGS2 homodimer complex	0.70711
DB00963	2003	COX1 homodimer complex	0.70711
DB00991	1439	PTGS2 homodimer complex	0.70711
DB00991	2003	COX1 homodimer complex	0.70711
DB01009	1439	PTGS2 homodimer complex	0.57735
DB01009	2003	COX1 homodimer complex	0.57735
DB01014	1439	PTGS2 homodimer complex	0.5
DB01014	2003	COX1 homodimer complex	0.5
DB01050	681	(C-CFTR)2-NHERF-ezrin complex	0.20412
DB01050	682	C-CFTR-NHERF(PDZ1 domain)-ezrin complex	0.20412
DB01050	683	C-CFTR-NHERF(PDZ2 domain)-ezrin complex	0.20412
DB01050	687	CFTR-NHERF-beta(2)AR signaling complex	0.20412
DB01050	1062	BAR-BCL2-CASP8 complex	0.20412
DB01050	1439	PTGS2 homodimer complex	0.35355
DB01050	2003	COX1 homodimer complex	0.35355
DB01050	5526	CALM1-FKBP38-BCL2 complex	0.20412
DB01050	5811	p53-BCL2 complex	0.25
DB01050	5817	tBID-BCL2 complex	0.25
DB01050	5818	BIM-BCL2 complex	0.25
DB01283	1439	PTGS2 homodimer complex	0.70711
DB01283	2003	COX1 homodimer complex	0.70711
DB01397	1439	PTGS2 homodimer complex	0.70711
DB01397	2003	COX1 homodimer complex	0.70711
DB01398	1439	PTGS2 homodimer complex	0.70711
DB01398	2003	COX1 homodimer complex	0.70711
DB01399	1439	PTGS2 homodimer complex	0.70711
DB01399	2003	COX1 homodimer complex	0.70711

DB01401	1439	PTGS2 homodimer complex	0.70711
DB01401	2003	COX1 homodimer complex	0.70711
DB01419	1439	PTGS2 homodimer complex	0.70711
DB01419	2003	COX1 homodimer complex	0.70711
DB01435	1439	PTGS2 homodimer complex	0.70711
DB01435	2003	COX1 homodimer complex	0.70711
DB01600	1439	PTGS2 homodimer complex	0.70711
DB01600	2003	COX1 homodimer complex	0.70711
DB04552	1439	PTGS2 homodimer complex	0.40825
DB04552	2003	COX1 homodimer complex	0.40825
DB06725	1439	PTGS2 homodimer complex	0.70711
DB06725	2003	COX1 homodimer complex	0.70711
DB06802	1439	PTGS2 homodimer complex	0.70711
DB06802	2003	COX1 homodimer complex	0.70711
DB01017	1248	Apoptosome	0.2357
DB01017	2709	MMP-9-TIMP-1-LRP complex	0.19245
DB01017	2972	ITGA9-ITGB1-VEGFA complex	0.19245
DB01017	4062	NRP1-VEGFR2-VEGF(165) complex	0.19245
DB01017	5696	VEGFA(165)-KDR-NRP1 complex	0.19245
DB01017	5698	VEGFA(165)-VEGFR2-NRP1 complex	0.19245
DB01017	5701	NRP1-VEGF(165/121) complex	0.2357
DB01017	5816	Apoptosome-procaspase 9 complex	0.19245
DB00246	5411	EDG1-HTR1D complex	0.14142
DB00246	5412	HTR1D homodimer complex	0.2
DB00246	5414	HTR1A-HTR1D complex	0.28284
DB00246	5415	HTR1B homodimer complex	0.2
DB00246	5416	HTR1A-HTR1B complex	0.28284
DB00246	5417	HTR1D-HTR1B complex	0.28284
DB00246	5418	GABBR2-HTR1A complex	0.14142
DB00246	5419	HTR1A-GPR26 complex	0.14142
DB00246	5420	HTR1A-EDG3 complex	0.14142
DB00246	5421	HTR1A homodimer complex	0.2
DB00246	5422	HTR1A-EDG1 complex	0.14142
DB00246	5747	2AR-mGluR2 complex	0.14142
DB00248	5411	EDG1-HTR1D complex	0.18257
DB00248	5412	HTR1D homodimer complex	0.2582
DB00248	5414	HTR1A-HTR1D complex	0.36515
DB00248	5415	HTR1B homodimer complex	0.2582
DB00248	5416	HTR1A-HTR1B complex	0.36515
DB00248	5417	HTR1D-HTR1B complex	0.36515
DB00248	5418	GABBR2-HTR1A complex	0.18257
DB00248	5419	HTR1A-GPR26 complex	0.18257
DB00248	5420	HTR1A-EDG3 complex	0.18257
DB00248	5421	HTR1A homodimer complex	0.2582
DB00248	5422	HTR1A-EDG1 complex	0.18257
DB00248	5747	2AR-mGluR2 complex	0.18257
DB00268	5411	EDG1-HTR1D complex	0.18898
DB00268	5412	HTR1D homodimer complex	0.26726
DB00268	5414	HTR1A-HTR1D complex	0.37796
DB00268	5415	HTR1B homodimer complex	0.26726
DB00268	5416	HTR1A-HTR1B complex	0.37796
DB00268	5417	HTR1D-HTR1B complex	0.37796
DB00268	5418	GABBR2-HTR1A complex	0.18898
DB00268	5419	HTR1A-GPR26 complex	0.18898

DB00268	5420	HTR1A-EDG3 complex	0.18898
DB00268	5421	HTR1A homodimer complex	0.26726
DB00268	5422	HTR1A-EDG1 complex	0.18898
DB00268	5747	2AR-mGluR2 complex	0.18898
DB00334	5411	EDG1-HTR1D complex	0.14142
DB00334	5412	HTR1D homodimer complex	0.2
DB00334	5414	HTR1A-HTR1D complex	0.28284
DB00334	5415	HTR1B homodimer complex	0.2
DB00334	5416	HTR1A-HTR1B complex	0.28284
DB00334	5417	HTR1D-HTR1B complex	0.28284
DB00334	5418	GABBR2-HTR1A complex	0.14142
DB00334	5419	HTR1A-GPR26 complex	0.14142
DB00334	5420	HTR1A-EDG3 complex	0.14142
DB00334	5421	HTR1A homodimer complex	0.2
DB00334	5422	HTR1A-EDG1 complex	0.14142
DB00334	5747	2AR-mGluR2 complex	0.14142
DB00363	5411	EDG1-HTR1D complex	0.13868
DB00363	5412	HTR1D homodimer complex	0.19612
DB00363	5414	HTR1A-HTR1D complex	0.27735
DB00363	5415	HTR1B homodimer complex	0.19612
DB00363	5416	HTR1A-HTR1B complex	0.27735
DB00363	5417	HTR1D-HTR1B complex	0.27735
DB00363	5418	GABBR2-HTR1A complex	0.13868
DB00363	5419	HTR1A-GPR26 complex	0.13868
DB00363	5420	HTR1A-EDG3 complex	0.13868
DB00363	5421	HTR1A homodimer complex	0.19612
DB00363	5422	HTR1A-EDG1 complex	0.13868
DB00363	5747	2AR-mGluR2 complex	0.13868
DB00397	879	PRKAC-AKAP5-ADRB1 complex	0.22361
DB00397	4869	beta(1)-AR receptosome (ADRB1-SAP97-AKAP79-PRKAR2A)	0.25
DB00397	5747	2AR-mGluR2 complex	0.35355
DB00413	5411	EDG1-HTR1D complex	0.18898
DB00413	5412	HTR1D homodimer complex	0.26726
DB00413	5414	HTR1A-HTR1D complex	0.37796
DB00413	5415	HTR1B homodimer complex	0.26726
DB00413	5416	HTR1A-HTR1B complex	0.37796
DB00413	5417	HTR1D-HTR1B complex	0.37796
DB00413	5418	GABBR2-HTR1A complex	0.18898
DB00413	5419	HTR1A-GPR26 complex	0.18898
DB00413	5420	HTR1A-EDG3 complex	0.18898
DB00413	5421	HTR1A homodimer complex	0.26726
DB00413	5422	HTR1A-EDG1 complex	0.18898
DB00413	5747	2AR-mGluR2 complex	0.18898
DB00477	5414	HTR1A-HTR1D complex	0.26726
DB00477	5416	HTR1A-HTR1B complex	0.26726
DB00477	5418	GABBR2-HTR1A complex	0.26726
DB00477	5419	HTR1A-GPR26 complex	0.26726
DB00477	5420	HTR1A-EDG3 complex	0.26726
DB00477	5421	HTR1A homodimer complex	0.37796
DB00477	5422	HTR1A-EDG1 complex	0.26726
DB00543	5747	2AR-mGluR2 complex	0.25
DB00543	5809	GABAA receptor	0.20412
DB00589	5411	EDG1-HTR1D complex	0.18898
DB00589	5412	HTR1D homodimer complex	0.26726

DB00589	5414	HTR1A-HTR1D complex	0.37796
DB00589	5415	HTR1B homodimer complex	0.26726
DB00589	5416	HTR1A-HTR1B complex	0.37796
DB00589	5417	HTR1D-HTR1B complex	0.37796
DB00589	5418	GABBR2-HTR1A complex	0.18898
DB00589	5419	HTR1A-GPR26 complex	0.18898
DB00589	5420	HTR1A-EDG3 complex	0.18898
DB00589	5421	HTR1A homodimer complex	0.26726
DB00589	5422	HTR1A-EDG1 complex	0.18898
DB00589	5747	2AR-mGluR2 complex	0.18898
DB00623	1223	H2AX complex isolated from cells without IR exposure	0.16013
DB00623	2242	TGM2-HD-CALM1 complex	0.33333
DB00623	4158	HSP90-FKBP38-CAM-Ca(2+) complex	0.28868
DB00623	5189	YWHAQ-CALM1-CABIN1 complex	0.33333
DB00623	5526	CALM1-FKBP38-BCL2 complex	0.33333
DB00714	5411	EDG1-HTR1D complex	0.18257
DB00714	5412	HTR1D homodimer complex	0.2582
DB00714	5414	HTR1A-HTR1D complex	0.36515
DB00714	5415	HTR1B homodimer complex	0.2582
DB00714	5416	HTR1A-HTR1B complex	0.36515
DB00714	5417	HTR1D-HTR1B complex	0.36515
DB00714	5418	GABBR2-HTR1A complex	0.18257
DB00714	5419	HTR1A-GPR26 complex	0.18257
DB00714	5420	HTR1A-EDG3 complex	0.18257
DB00714	5421	HTR1A homodimer complex	0.2582
DB00714	5422	HTR1A-EDG1 complex	0.18257
DB00714	5747	2AR-mGluR2 complex	0.18257
DB00726	5414	HTR1A-HTR1D complex	0.2132
DB00726	5416	HTR1A-HTR1B complex	0.2132
DB00726	5418	GABBR2-HTR1A complex	0.2132
DB00726	5419	HTR1A-GPR26 complex	0.2132
DB00726	5420	HTR1A-EDG3 complex	0.2132
DB00726	5421	HTR1A homodimer complex	0.30151
DB00726	5422	HTR1A-EDG1 complex	0.2132
DB00734	5411	EDG1-HTR1D complex	0.18898
DB00734	5412	HTR1D homodimer complex	0.26726
DB00734	5414	HTR1A-HTR1D complex	0.37796
DB00734	5416	HTR1A-HTR1B complex	0.18898
DB00734	5417	HTR1D-HTR1B complex	0.18898
DB00734	5418	GABBR2-HTR1A complex	0.18898
DB00734	5419	HTR1A-GPR26 complex	0.18898
DB00734	5420	HTR1A-EDG3 complex	0.18898
DB00734	5421	HTR1A homodimer complex	0.26726
DB00734	5422	HTR1A-EDG1 complex	0.18898
DB00734	5747	2AR-mGluR2 complex	0.18898
DB00800	5747	2AR-mGluR2 complex	0.31623
DB00850	1223	H2AX complex isolated from cells without IR exposure	0.16013
DB00850	2242	TGM2-HD-CALM1 complex	0.33333
DB00850	4158	HSP90-FKBP38-CAM-Ca(2+) complex	0.28868
DB00850	5189	YWHAQ-CALM1-CABIN1 complex	0.33333
DB00850	5526	CALM1-FKBP38-BCL2 complex	0.33333
DB01049	5411	EDG1-HTR1D complex	0.11043
DB01049	5412	HTR1D homodimer complex	0.15617
DB01049	5414	HTR1A-HTR1D complex	0.22086

DB01049	5415	HTR1B homodimer complex	0.15617
DB01049	5416	HTR1A-HTR1B complex	0.22086
DB01049	5417	HTR1D-HTR1B complex	0.22086
DB01049	5418	GABBR2-HTR1A complex	0.11043
DB01049	5419	HTR1A-GPR26 complex	0.11043
DB01049	5420	HTR1A-EDG3 complex	0.11043
DB01049	5421	HTR1A homodimer complex	0.15617
DB01049	5422	HTR1A-EDG1 complex	0.11043
DB01049	5747	2AR-mGluR2 complex	0.11043
DB01049	5809	GABAA receptor	0.18033
DB01186	5411	EDG1-HTR1D complex	0.1715
DB01186	5412	HTR1D homodimer complex	0.24254
DB01186	5414	HTR1A-HTR1D complex	0.343
DB01186	5415	HTR1B homodimer complex	0.24254
DB01186	5416	HTR1A-HTR1B complex	0.343
DB01186	5417	HTR1D-HTR1B complex	0.343
DB01186	5418	GABBR2-HTR1A complex	0.1715
DB01186	5419	HTR1A-GPR26 complex	0.1715
DB01186	5420	HTR1A-EDG3 complex	0.1715
DB01186	5421	HTR1A homodimer complex	0.24254
DB01186	5422	HTR1A-EDG1 complex	0.1715
DB01186	5747	2AR-mGluR2 complex	0.1715
DB01200	5411	EDG1-HTR1D complex	0.16667
DB01200	5412	HTR1D homodimer complex	0.2357
DB01200	5414	HTR1A-HTR1D complex	0.33333
DB01200	5415	HTR1B homodimer complex	0.2357
DB01200	5416	HTR1A-HTR1B complex	0.33333
DB01200	5417	HTR1D-HTR1B complex	0.33333
DB01200	5418	GABBR2-HTR1A complex	0.16667
DB01200	5419	HTR1A-GPR26 complex	0.16667
DB01200	5420	HTR1A-EDG3 complex	0.16667
DB01200	5421	HTR1A homodimer complex	0.2357
DB01200	5422	HTR1A-EDG1 complex	0.16667
DB01200	5747	2AR-mGluR2 complex	0.16667
DB01224	5411	EDG1-HTR1D complex	0.13868
DB01224	5412	HTR1D homodimer complex	0.19612
DB01224	5414	HTR1A-HTR1D complex	0.27735
DB01224	5415	HTR1B homodimer complex	0.19612
DB01224	5416	HTR1A-HTR1B complex	0.27735
DB01224	5417	HTR1D-HTR1B complex	0.27735
DB01224	5418	GABBR2-HTR1A complex	0.13868
DB01224	5419	HTR1A-GPR26 complex	0.13868
DB01224	5420	HTR1A-EDG3 complex	0.13868
DB01224	5421	HTR1A homodimer complex	0.19612
DB01224	5422	HTR1A-EDG1 complex	0.13868
DB01224	5747	2AR-mGluR2 complex	0.13868
DB01238	5411	EDG1-HTR1D complex	0.14142
DB01238	5412	HTR1D homodimer complex	0.2
DB01238	5414	HTR1A-HTR1D complex	0.28284
DB01238	5415	HTR1B homodimer complex	0.2
DB01238	5416	HTR1A-HTR1B complex	0.28284
DB01238	5417	HTR1D-HTR1B complex	0.28284
DB01238	5418	GABBR2-HTR1A complex	0.14142
DB01238	5419	HTR1A-GPR26 complex	0.14142

DB01238	5420	HTR1A-EDG3 complex	0.14142
DB01238	5421	HTR1A homodimer complex	0.2
DB01238	5422	HTR1A-EDG1 complex	0.14142
DB01238	5747	2AR-mGluR2 complex	0.14142
DB01267	5411	EDG1-HTR1D complex	0.1715
DB01267	5412	HTR1D homodimer complex	0.24254
DB01267	5414	HTR1A-HTR1D complex	0.343
DB01267	5416	HTR1A-HTR1B complex	0.1715
DB01267	5417	HTR1D-HTR1B complex	0.1715
DB01267	5418	GABBR2-HTR1A complex	0.1715
DB01267	5419	HTR1A-GPR26 complex	0.1715
DB01267	5420	HTR1A-EDG3 complex	0.1715
DB01267	5421	HTR1A homodimer complex	0.24254
DB01267	5422	HTR1A-EDG1 complex	0.1715
DB01267	5747	2AR-mGluR2 complex	0.1715
DB01403	5747	2AR-mGluR2 complex	0.16222
DB01608	5747	2AR-mGluR2 complex	0.40825
DB01614	5414	HTR1A-HTR1D complex	0.28868
DB01614	5416	HTR1A-HTR1B complex	0.28868
DB01614	5418	GABBR2-HTR1A complex	0.28868
DB01614	5419	HTR1A-GPR26 complex	0.28868
DB01614	5420	HTR1A-EDG3 complex	0.28868
DB01614	5421	HTR1A homodimer complex	0.40825
DB01614	5422	HTR1A-EDG1 complex	0.28868
DB01621	5414	HTR1A-HTR1D complex	0.35355
DB01621	5416	HTR1A-HTR1B complex	0.35355
DB01621	5418	GABBR2-HTR1A complex	0.35355
DB01621	5419	HTR1A-GPR26 complex	0.35355
DB01621	5420	HTR1A-EDG3 complex	0.35355
DB01621	5421	HTR1A homodimer complex	0.5
DB01621	5422	HTR1A-EDG1 complex	0.35355
DB01622	5414	HTR1A-HTR1D complex	0.28868
DB01622	5416	HTR1A-HTR1B complex	0.28868
DB01622	5418	GABBR2-HTR1A complex	0.28868
DB01622	5419	HTR1A-GPR26 complex	0.28868
DB01622	5420	HTR1A-EDG3 complex	0.28868
DB01622	5421	HTR1A homodimer complex	0.40825
DB01622	5422	HTR1A-EDG1 complex	0.28868
DB04946	5414	HTR1A-HTR1D complex	0.2132
DB04946	5416	HTR1A-HTR1B complex	0.2132
DB04946	5418	GABBR2-HTR1A complex	0.2132
DB04946	5419	HTR1A-GPR26 complex	0.2132
DB04946	5420	HTR1A-EDG3 complex	0.2132
DB04946	5421	HTR1A homodimer complex	0.30151
DB04946	5422	HTR1A-EDG1 complex	0.2132
DB05271	5414	HTR1A-HTR1D complex	0.26726
DB05271	5416	HTR1A-HTR1B complex	0.26726
DB05271	5418	GABBR2-HTR1A complex	0.26726
DB05271	5419	HTR1A-GPR26 complex	0.26726
DB05271	5420	HTR1A-EDG3 complex	0.26726
DB05271	5421	HTR1A homodimer complex	0.37796
DB05271	5422	HTR1A-EDG1 complex	0.26726
DB06216	668	BKCA-beta2AR-AKAP79 signaling complex	0.1291
DB06216	672	BKCA-beta2AR complex	0.15811

DB06216	687	CFTR-NHERF-beta(2)AR signaling complex	0.1291
DB06216	879	PRKAC-AKAP5-ADRB1 complex	0.1
DB06216	3830	ADRB2 homodimer complex	0.22361
DB06216	4869	beta(1)-AR receptosome (ADRB1-SAP97-AKAP79-PRKAR2A)	0.1118
DB06216	5414	HTR1A-HTR1D complex	0.15811
DB06216	5415	HTR1B homodimer complex	0.22361
DB06216	5416	HTR1A-HTR1B complex	0.31623
DB06216	5417	HTR1D-HTR1B complex	0.15811
DB06216	5418	GABBR2-HTR1A complex	0.15811
DB06216	5419	HTR1A-GPR26 complex	0.15811
DB06216	5420	HTR1A-EDG3 complex	0.15811
DB06216	5421	HTR1A homodimer complex	0.22361
DB06216	5422	HTR1A-EDG1 complex	0.15811
DB06216	5747	2AR-mGluR2 complex	0.15811
DB01099	860	DNMT1-G9a-PCNA complex	0.40825
DB01099	862	DNMT1-G9a complex	0.5
DB01099	1470	pRb2/p130-multimolecular complex (DNMT1 E2F5 SuV39H1 HDAC1 RBL2)	0.31623
DB01099	1488	DNMT1-RB1-HDAC1-E2F1 complex	0.35355
DB01099	1490	DAXX-DNMT1-DMAP1 complex	0.40825
DB01099	1491	RGS6-DNMT1-DMAP1 complex	0.40825
DB01099	5117	pRb2/p130-multimolecular complex (DNMT1 E2F4 SuV39H1 HDAC1 RBL2)	0.31623
DB01099	5695	TIP5-DNMT-HDAC1 complex	0.35355
DB00139	393	Succinyl-CoA synthetase GDP-forming	0.27735
DB00139	394	Succinyl-CoA synthetase ADP-forming	0.27735
DB00139	472	Prolyl 4-hydroxylase (alpha(I)-type)	0.13868
DB00139	1094	Frataxin complex	0.07412
DB00398	903	RET-Rai complex	0.22361
DB00398	1539	G protein complex (GNG2 GNB2L1 RAF1)	0.18257
DB00398	4062	NRP1-VEGFR2-VEGF(165) complex	0.18257
DB00398	5772	ZO1-(beta)cadherin-(VE)cadherin-VEGFR2 complex	0.15811
DB00398	5211	RAF1-PPP2-PIN1 complex	0.14142
DB00398	5696	VEGFA(165)-KDR-NRP1 complex	0.18257
DB00398	5698	VEGFA(165)-VEGFR2-NRP1 complex	0.18257
DB00398	5740	NRP2-VEGFR3 complex	0.22361
DB00398	5877	MAP2K1-BRAF-RAF1-YWHAE-KSR1 complex	0.28284
DB00398	5872	BRAF-MAP2K1-MAP2K2-YWHAE complex	0.15811
DB00398	5873	RAF1-MAP2K1-YWHAE complex	0.18257
DB00398	5919	BRAF-RAF1-14-3-3 complex	0.21082
DB00398	5920	KSR1-RAF1-MEK complex	0.15811
DB00398	5921	KSR1-BRAF-MEK complex	0.15811
DB00398	5922	RAF1-RAS complex EGF induced	0.15811
DB00398	5923	RAF1-BRAF complex RAS stimulated	0.44721
DB00398	5924	RAF1-CNK1 complex RAS stimulated	0.22361
DB00398	5925	BRAF-CNK1 complex not RAS stimulated	0.22361
DB00398	5928	CNK1-SRC-RAF1 complex	0.18257
DB01268	1096	SNX complex (SNX1 1a 2 4 PDGF receptor)	0.17678
DB01268	2476	CRKL-PDGFRA-CRK-RAPGEF1 complex	0.17678
DB01268	2551	PDGFRA-PLC-gamma-1-PI3K-SHP-2 complex PDGF stimulated	0.17678
DB01268	3183	PDGFRA-SHP-2 complex PDGF stimulated	0.25
DB01268	4062	NRP1-VEGFR2-VEGF(165) complex	0.20412
DB01268	5772	ZO1-(beta)cadherin-(VE)cadherin-VEGFR2 complex	0.17678
DB01268	5696	VEGFA(165)-KDR-NRP1 complex	0.20412

DB01268	5698	VEGFA(165)-VEGFR2-NRP1 complex	0.20412
DB01268	5740	NRP2-VEGFR3 complex	0.25
DB06589	1096	SNX complex (SNX1 1a 2 4 PDGF receptor)	0.15811
DB06589	2476	CRKL-PDGFRA-CRK-RAPGEF1 complex	0.15811
DB06589	2551	PDGFRA-PLC-gamma-1-PI3K-SHP-2 complex PDGF stimulated	0.15811
DB06589	2960	SLP-76-PLC-gamma-1-ITK complex alpha-TCR stimulated	0.18257
DB06589	2963	ITK-SLP-76 complex anti-TCR stimulated	0.22361
DB06589	3183	PDGFRA-SHP-2 complex PDGF stimulated	0.22361
DB06589	4062	NRP1-VEGFR2-VEGF(165) complex	0.18257
DB06589	5772	ZO1-(beta)cadherin-(VE)cadherin-VEGFR2 complex	0.15811
DB06589	5696	VEGFA(165)-KDR-NRP1 complex	0.18257
DB06589	5698	VEGFA(165)-VEGFR2-NRP1 complex	0.18257
DB06589	5740	NRP2-VEGFR3 complex	0.22361
DB06626	4062	NRP1-VEGFR2-VEGF(165) complex	0.33333
DB06626	5772	ZO1-(beta)cadherin-(VE)cadherin-VEGFR2 complex	0.28868
DB06626	5696	VEGFA(165)-KDR-NRP1 complex	0.33333
DB06626	5698	VEGFA(165)-VEGFR2-NRP1 complex	0.33333
DB06626	5740	NRP2-VEGFR3 complex	0.40825
DB00048	3059	ITGA11-ITGB1-COL1A1 complex	0.28868
DB00309	1231	FIB-associated protein complex	0.40825
DB00309	3055	Nop56p-associated pre-rRNA complex	0.09806
DB01229	1062	BAR-BCL2-CASP8 complex	0.2357
DB01229	1231	FIB-associated protein complex	0.16667
DB01229	3055	Nop56p-associated pre-rRNA complex	0.04003
DB01229	5526	CALM1-FKBP38-BCL2 complex	0.2357
DB01229	5811	p53-BCL2 complex	0.28868
DB01229	5817	tBID-BCL2 complex	0.28868
DB01229	5818	BIM-BCL2 complex	0.28868
DB01248	1062	BAR-BCL2-CASP8 complex	0.2582
DB01248	1231	FIB-associated protein complex	0.18257
DB01248	3055	Nop56p-associated pre-rRNA complex	0.04385
DB01248	5526	CALM1-FKBP38-BCL2 complex	0.2582
DB01248	5811	p53-BCL2 complex	0.31623
DB01248	5817	tBID-BCL2 complex	0.31623
DB01248	5818	BIM-BCL2 complex	0.31623
DB01394	1231	FIB-associated protein complex	0.28868
DB01394	1335	SNW1 complex	0.16667
DB01394	1400	ASCOM complex	0.26726
DB01394	3055	Nop56p-associated pre-rRNA complex	0.06934
DB06772	1231	FIB-associated protein complex	0.28868
DB06772	1400	ASCOM complex	0.26726
DB06772	3055	Nop56p-associated pre-rRNA complex	0.06934
DB00120	2390	CD98-LAT2-ITGB1 complex	0.18898
DB00120	3035	LAT2-ITGB1 complex	0.26726
DB00030	722	MRG15-PAM14-RB complex	0.16667
DB00030	723	MAF1 complex	0.16667
DB00030	1250	pRB-E2F-1 complex	0.20412
DB00030	1372	Rb-tal-1-E2A-Lmo2-Ldb1 complex	0.1291
DB00030	1488	DNMT1-RB1-HDAC1-E2F1 complex	0.14434
DB00030	2849	ITGAV-ITGB3-NOV complex	0.16667
DB00030	2850	ITGA5-ITGB1-FN-1-NOV complex	0.14434
DB00030	3269	RB1-HDAC1-BRG1 complex	0.16667
DB00030	3852	Rb-HDAC1 complex	0.20412
DB00030	5099	RB1(hypophosphorylated)-E2F4 complex	0.20412

DB00030	5143	E2F1-Rb complex	0.20412
DB00030	5146	RB1-TFAP2A complex	0.20412
DB00030	5611	Emerin complex 24	0.07454
DB00030	5656	CEBPE-E2F1-RB1 complex	0.16667
DB00030	5663	TRIM27-RB1 complex	0.20412
DB00071	722	MRG15-PAM14-RB complex	0.1543
DB00071	723	MAF1 complex	0.1543
DB00071	1250	pRB-E2F-1 complex	0.18898
DB00071	1372	Rb-tal-1-E2A-Lmo2-Ldb1 complex	0.11952
DB00071	1488	DNMT1-RB1-HDAC1-E2F1 complex	0.13363
DB00071	2849	ITGAV-ITGB3-NOV complex	0.1543
DB00071	2850	ITGA5-ITGB1-FN-1-NOV complex	0.13363
DB00071	3269	RB1-HDAC1-BRG1 complex	0.1543
DB00071	3852	Rb-HDAC1 complex	0.18898
DB00071	5099	RB1(hypophosphorylated)-E2F4 complex	0.18898
DB00071	5143	E2F1-Rb complex	0.18898
DB00071	5146	RB1-TFAP2A complex	0.18898
DB00071	5611	Emerin complex 24	0.06901
DB00071	5656	CEBPE-E2F1-RB1 complex	0.1543
DB00071	5663	TRIM27-RB1 complex	0.18898
DB01277	541	IGF1-IGFBP3-ALS complex	0.28868
DB01277	5280	RAB9-TIP47-MPRI complex	0.28868
DB08912	1539	G protein complex (GNG2 GNB2L1 RAF1)	0.2582
DB08912	1932	NEK2-NEK11 complex	0.31623
DB08912	5211	RAF1-PPP2-PIN1 complex	0.2
DB08912	5877	MAP2K1-BRAF-RAF1-YWHAE-KSR1 complex	0.4
DB08912	5872	BRAF-MAP2K1-MAP2K2-YWHAE complex	0.22361
DB08912	5873	RAF1-MAP2K1-YWHAE complex	0.2582
DB08912	5919	BRAF-RAF1-14-3-3 complex	0.29814
DB08912	5920	KSR1-RAF1-MEK complex	0.22361
DB08912	5921	KSR1-BRAF-MEK complex	0.22361
DB08912	5922	RAF1-RAS complex EGF induced	0.22361
DB08912	5923	RAF1-BRAF complex RAS stimulated	0.63246
DB08912	5924	RAF1-CNK1 complex RAS stimulated	0.31623
DB08912	5925	BRAF-CNK1 complex not RAS stimulated	0.31623
DB08912	5928	CNK1-SRC-RAF1 complex	0.2582
DB00147	1085	DNA repair complex NEIL2-PNK-Pol(beta)-LigIII(alpha)-XRCC1	0.44721
DB00147	1086	DNA repair complex NEIL1-PNK-Pol(beta)-LigIII(alpha)-XRCC1	0.44721
DB00165	1085	DNA repair complex NEIL2-PNK-Pol(beta)-LigIII(alpha)-XRCC1	0.44721
DB00165	1086	DNA repair complex NEIL1-PNK-Pol(beta)-LigIII(alpha)-XRCC1	0.44721
DB00280	595	Kv4.2-DPP10 channel complex	0.28868
DB00321	595	Kv4.2-DPP10 channel complex	0.1543
DB00321	1787	Nogo-potassium channel complex	0.10911
DB00321	2487	GIPC1-NTRK1-RGS19 complex	0.12599
DB00321	5407	NGF-TrkA complex	0.1543
DB00321	5414	HTR1A-HTR1D complex	0.1543
DB00321	5416	HTR1A-HTR1B complex	0.1543
DB00321	5418	GABBR2-HTR1A complex	0.1543
DB00321	5419	HTR1A-GPR26 complex	0.1543
DB00321	5420	HTR1A-EDG3 complex	0.1543
DB00321	5421	HTR1A homodimer complex	0.21822
DB00321	5422	HTR1A-EDG1 complex	0.1543
DB00321	5747	2AR-mGluR2 complex	0.1543
DB00458	595	Kv4.2-DPP10 channel complex	0.18898

DB00540	5414	HTR1A-HTR1D complex	0.20412
DB00540	5416	HTR1A-HTR1B complex	0.20412
DB00540	5418	GABBR2-HTR1A complex	0.20412
DB00540	5419	HTR1A-GPR26 complex	0.20412
DB00540	5420	HTR1A-EDG3 complex	0.20412
DB00540	5421	HTR1A homodimer complex	0.28868
DB00540	5422	HTR1A-EDG1 complex	0.20412
DB00622	1223	H2AX complex isolated from cells without IR exposure	0.07161
DB00622	2242	TGM2-HD-CALM1 complex	0.14907
DB00622	4158	HSP90-FKBP38-CAM-Ca(2+) complex	0.1291
DB00622	5189	YWHAQ-CALM1-CABIN1 complex	0.14907
DB00622	5526	CALM1-FKBP38-BCL2 complex	0.14907
DB01069	1223	H2AX complex isolated from cells without IR exposure	0.08771
DB01069	2242	TGM2-HD-CALM1 complex	0.18257
DB01069	4158	HSP90-FKBP38-CAM-Ca(2+) complex	0.15811
DB01069	5189	YWHAQ-CALM1-CABIN1 complex	0.18257
DB01069	5526	CALM1-FKBP38-BCL2 complex	0.18257
DB01142	5414	HTR1A-HTR1D complex	0.15811
DB01142	5416	HTR1A-HTR1B complex	0.15811
DB01142	5418	GABBR2-HTR1A complex	0.15811
DB01142	5419	HTR1A-GPR26 complex	0.15811
DB01142	5420	HTR1A-EDG3 complex	0.15811
DB01142	5421	HTR1A homodimer complex	0.22361
DB01142	5422	HTR1A-EDG1 complex	0.15811
DB01142	5747	2AR-mGluR2 complex	0.15811
DB01151	668	BKCA-beta2AR-AKAP79 signaling complex	0.1543
DB01151	672	BKCA-beta2AR complex	0.18898
DB01151	687	CFTR-NHERF-beta(2)AR signaling complex	0.1543
DB01151	879	PRKAC-AKAP5-ADRB1 complex	0.11952
DB01151	3830	ADRB2 homodimer complex	0.26726
DB01151	4869	beta(1)-AR receptosome (ADRB1-SAP97-AKAP79-PRKAR2A)	0.13363
DB00887	681	(C-CFTR)2-NHERF-ezrin complex	0.2582
DB00887	682	C-CFTR-NHERF(PDZ1 domain)-ezrin complex	0.2582
DB00887	683	C-CFTR-NHERF(PDZ2 domain)-ezrin complex	0.2582
DB00887	687	CFTR-NHERF-beta(2)AR signaling complex	0.2582
DB00170	3162	TF-FVIIa-FXa-TFPI complex	0.27735
DB04786	3110	ITGAV-P2RY2-GNA12 complex	0.21822
DB01108	441	TFTC-type histone acetyl transferase complex	0.15076
DB01108	1054	ESR1-RELA-BCL3-NCOA3 complex	0.25
DB01108	2124	IKK-alpha--ER-alpha-AIB1 complex	0.28868
DB01108	2470	p130Cas-ER-alpha-cSrc-kinase- PI3-kinase p85-subunit complex	0.25
DB01108	2657	ESR1-CDK7-CCNH-MNAT1-MTA1-HDAC2 complex	0.20412
DB01108	2670	Er-alpha-p53-hdm2 complex	0.28868
DB01108	2699	ER-alpha-GRIP1-c-Jun complex	0.28868
DB01108	2700	ER-alpha-c-Jun complex	0.35355
DB01108	5862	CAV1-VDAC1-ESR1 complex	0.28868
DB00123	3040	Multisynthetase complex	0.13484
DB00129	2959	SMAD1-OAZ-HsN3 complex	0.1543
DB00233	1439	PTGS2 homodimer complex	0.44721
DB00233	2055	CASP8-CHUK-IKBKB-MALT1-BCL10 complex	0.2
DB00233	2056	BCL10-CHUK-BCL10-IKBKB complex	0.22361
DB00233	2100	CHUK-IKBKB-MAP3K14 complex	0.2582
DB00233	2101	IKKA-IKKB complex	0.31623

DB00233	2105	IkappaB kinase complex (IKBKB CHUK IKBKAP NFKBIA RELA MAP3K14)	0.18257
DB00233	2118	CHUK-ERC1-IKBKB-IKBKG	0.22361
DB00233	2121	CHUK-IKBKB-IKBKG complex	0.2582
DB00233	2124	IKK-alpha--ER-alpha-AIB1 complex	0.2582
DB00233	2727	SRC-3 complex	0.16903
DB00233	5193	TNF-alpha/NF-kappa B signaling complex (CHUK KPNA3 NFKB2 NFKBIB REL IKBKG NFKB1 NFKBIE RELB NFKBIA RELA TNIP2)	0.1291
DB00233	5194	TNF-alpha/NF-kappa B signaling complex (SEC16A CHUK IKBKB NFKB2 REL IKBKG MAP3K14 RELA FBXW7 USP2)	0.14142
DB00233	5196	TNF-alpha/NF-kappa B signaling complex (CHUK BTRC NFKB2 PPP6C REL CUL1 IKBKE SAPS2 SAPS1 ANKRD28 RELA SKP1)	0.1291
DB00233	5220	CHUK-IQGAP2-AKAP8L-RELA-TNIP2 complex	0.2
DB00233	5230	CHUK-NFKB2-REL-IKBKG-SPAG9-NFKB1-NFKBIE-COPB2-TNIP1-NFKBIA-RELA-TNIP2 complex	0.1291
DB00233	5232	TNF-alpha/Nf-kappa B signaling complex (RPL6 RPL30 RPS13 CHUK DDX3X NFKB2 NFKBIB REL IKBKG NFKB1 MAP3K8 RELB GLG1 NFKBIA RELA TNIP2 GTF2I)	0.10847
DB00233	5233	TNF-alpha/NF-kappa B signaling complex 5	0.08944
DB00233	5266	TNF-alpha/NF-kappa B signaling complex 6	0.11952
DB00233	5285	TNF-alpha/NF-kappa B signaling complex 9	0.2
DB00233	5286	TNF-alpha/NF-kappa B signaling complex 10	0.14142
DB00233	5829	IKBKG-CHUK complex	0.31623
DB00233	5844	I-kappa-B kinase (IKK) complex	0.2582
DB00145	5641	PSD95-FYN-NR2A complex	0.10541
DB00786	2342	ITGAV-ITGB8-MMP14-TGFB1 complex	0.10426
DB00786	2688	MT1-MMP-claudin-1 complex	0.14744
DB00786	2709	MMP-9-TIMP-1-LRP complex	0.12039
DB00786	2798	MMP-2-claudin-1 complex	0.14744
DB01197	2709	MMP-9-TIMP-1-LRP complex	0.33333
DB01197	2798	MMP-2-claudin-1 complex	0.40825
DB01296	2084	NFKB1-NFKB2-REL-RELA-RELB complex	0.22361
DB01296	2086	NFKB1-NFKB2-RELA-RELB complex	0.25
DB01296	2709	MMP-9-TIMP-1-LRP complex	0.28868
DB01296	5193	TNF-alpha/NF-kappa B signaling complex (CHUK KPNA3 NFKB2 NFKBIB REL IKBKG NFKB1 NFKBIE RELB NFKBIA RELA TNIP2)	0.14434
DB01296	5194	TNF-alpha/NF-kappa B signaling complex (SEC16A CHUK IKBKB NFKB2 REL IKBKG MAP3K14 RELA FBXW7 USP2)	0.15811
DB01296	5196	TNF-alpha/NF-kappa B signaling complex (CHUK BTRC NFKB2 PPP6C REL CUL1 IKBKE SAPS2 SAPS1 ANKRD28 RELA SKP1)	0.14434
DB01296	5230	CHUK-NFKB2-REL-IKBKG-SPAG9-NFKB1-NFKBIE-COPB2-TNIP1-NFKBIA-RELA-TNIP2 complex	0.14434
DB01296	5232	TNF-alpha/Nf-kappa B signaling complex (RPL6 RPL30 RPS13 CHUK DDX3X NFKB2 NFKBIB REL IKBKG NFKB1 MAP3K8 RELB GLG1 NFKBIA RELA TNIP2 GTF2I)	0.12127
DB01296	5233	TNF-alpha/NF-kappa B signaling complex 5	0.1
DB00126	472	Prolyl 4-hydroxylase (alpha(I)-type)	0.14434
DB00126	5276	HIF1A-OS9-EGLN1 complex	0.11785
DB00126	5277	HIF1A-OS9-EGLN3 complex	0.11785
DB00172	351	Spliceosome	0.01825
DB00172	472	Prolyl 4-hydroxylase (alpha(I)-type)	0.1543
DB00172	3040	Multisynthetase complex	0.0658
DB00172	5385	GAIT complex	0.10911
DB00172	5843	AIF-CYPA-DNA complex	0.1543
DB01275	472	Prolyl 4-hydroxylase (alpha(I)-type)	0.5

DB01029	1816	JUN-TCF4-CTNNB1 complex	0.40825
DB01029	2692	SMAD3-SMAD4-cJun-cFos complex	0.35355
DB01029	2693	NFAT-JUN-FOS DNA-protein complex	0.40825
DB01029	2694	ERG-JUN-FOS DNA-protein complex	0.40825
DB01029	2695	ETS2-FOS-JUN complex	0.40825
DB01029	2699	ER-alpha-GRIP1-c-Jun complex	0.40825
DB01029	2700	ER-alpha-c-Jun complex	0.5
DB01029	2708	SMAD3-SMAD4-cJUN complex	0.40825
DB00133	2863	Serine-palmitoyltransferase (SPT) complex	0.43644
DB01088	518	AKAP250-PKA-PDE4D complex	0.16903
DB00073	2565	CD20-LCK-LYN-FYN-p75/80 complex (Raji human B cell line)	0.15076
DB00073	2879	CD20-LCK-FYN-p75/80 complex	0.17408
DB00073	2909	PLC-gamma-2-Syk-LAT-FcR-gamma complex	0.15076
DB00073	2910	PLC-gamma-2-Lyn-FcR-gamma complex	0.17408
DB00078	2565	CD20-LCK-LYN-FYN-p75/80 complex (Raji human B cell line)	0.15076
DB00078	2879	CD20-LCK-FYN-p75/80 complex	0.17408
DB00078	2909	PLC-gamma-2-Syk-LAT-FcR-gamma complex	0.15076
DB00078	2910	PLC-gamma-2-Lyn-FcR-gamma complex	0.17408
DB00081	2565	CD20-LCK-LYN-FYN-p75/80 complex (Raji human B cell line)	0.15811
DB00081	2879	CD20-LCK-FYN-p75/80 complex	0.18257
DB00081	2909	PLC-gamma-2-Syk-LAT-FcR-gamma complex	0.15811
DB00081	2910	PLC-gamma-2-Lyn-FcR-gamma complex	0.18257
DB08935	2565	CD20-LCK-LYN-FYN-p75/80 complex (Raji human B cell line)	0.5
DB08935	2879	CD20-LCK-FYN-p75/80 complex	0.57735
DB03147	232	ARC complex	0.03306
DB03147	432	N-NOS-CHIP-HSP70-1 complex	0.07392
DB03147	1375	Pyruvate dehydrogenase complex	0.06402
DB03147	5843	AIF-CYPA-DNA complex	0.09054
DB00755	548	DRIP complex	0.09449
DB00755	5367	THRB-RXRB complex	0.25
DB00052	3157	Prolactin (PRL) - PRL receptor (PRLR) complex	0.5
DB00052	3161	PRL receptor (PRLR) dimer complex	0.70711
DB00163	1543	PPP2CA-PPP2R1A complex	0.22361
DB00163	1544	PPP2CA-PPP2R1A-PPP2R3A complex	0.18257
DB00163	2537	PKC-alpha-PLD1-PLC-gamma-2 signaling complex lactiti[illegible] stimulated	0.18257
DB00163	5211	RAF1-PPP2-PIN1 complex	0.14142
DB00163	5876	PPP2R1A-PPP2R1B-PPP2CA-PPME1-EIF4A1 complex	0.14142
DB00210	548	DRIP complex	0.10911
DB00210	5198	CBP-RARA-RXRA-DNA complex ligand stimulated	0.4714
DB00210	5367	THRB-RXRB complex	0.28868
DB00307	548	DRIP complex	0.1543
DB00307	5198	CBP-RARA-RXRA-DNA complex ligand stimulated	0.33333
DB00307	5367	THRB-RXRB complex	0.40825
DB00459	548	DRIP complex	0.10102
DB00459	5198	CBP-RARA-RXRA-DNA complex ligand stimulated	0.43644
DB00459	5367	THRB-RXRB complex	0.26726
DB00523	548	DRIP complex	0.10911
DB00523	5198	CBP-RARA-RXRA-DNA complex ligand stimulated	0.4714
DB00523	5367	THRB-RXRB complex	0.28868
DB00799	548	DRIP complex	0.13363
DB00799	5198	CBP-RARA-RXRA-DNA complex ligand stimulated	0.28868
DB00799	5367	THRB-RXRB complex	0.35355
DB00148	5615	Emerin complex 52	0.08513
DB00087	2909	PLC-gamma-2-Syk-LAT-FcR-gamma complex	0.15811

DB00087	2910	PLC-gamma-2-Lyn-FcR-gamma complex	0.18257
DB00904	5414	HTR1A-HTR1D complex	0.31623
DB00904	5415	HTR1B homodimer complex	0.44721
DB00904	5416	HTR1A-HTR1B complex	0.63246
DB00904	5417	HTR1D-HTR1B complex	0.31623
DB00904	5418	GABBR2-HTR1A complex	0.31623
DB00904	5419	HTR1A-GPR26 complex	0.31623
DB00904	5420	HTR1A-EDG3 complex	0.31623
DB00904	5421	HTR1A homodimer complex	0.44721
DB00904	5422	HTR1A-EDG1 complex	0.31623
DB08810	5414	HTR1A-HTR1D complex	0.40825
DB08810	5416	HTR1A-HTR1B complex	0.40825
DB08810	5418	GABBR2-HTR1A complex	0.40825
DB08810	5419	HTR1A-GPR26 complex	0.40825
DB08810	5420	HTR1A-EDG3 complex	0.40825
DB08810	5421	HTR1A homodimer complex	0.57735
DB08810	5422	HTR1A-EDG1 complex	0.40825
DB00276	924	Toposome	0.26726
DB00276	1098	DNA synthesome complex (13 subunits)	0.18898
DB00276	1099	DNA synthesome complex (17 subunits)	0.16667
DB00276	1183	CDC5L complex	0.1291
DB00276	1728	CTCF-nucleophosmin-PARP-HIS-KPNA-LMNA-TOP complex	0.2357
DB00489	668	BKCA-beta2AR-AKAP79 signaling complex	0.33333
DB00489	672	BKCA-beta2AR complex	0.40825
DB00489	687	CFTR-NHERF-beta(2)AR signaling complex	0.33333
DB00489	879	PRKAC-AKAP5-ADRB1 complex	0.2582
DB00489	3830	ADRB2 homodimer complex	0.57735
DB00489	4869	beta(1)-AR receptosome (ADRB1-SAP97-AKAP79-PRKAR2A)	0.28868
DB01100	1223	H2AX complex isolated from cells without IR exposure	0.13868
DB01100	2242	TGM2-HD-CALM1 complex	0.28868
DB01100	4158	HSP90-FKBP38-CAM-Ca(2+) complex	0.25
DB01100	5189	YWHAQ-CALM1-CABIN1 complex	0.28868
DB01100	5526	CALM1-FKBP38-BCL2 complex	0.28868
DB01118	879	PRKAC-AKAP5-ADRB1 complex	0.22361
DB01118	4869	beta(1)-AR receptosome (ADRB1-SAP97-AKAP79-PRKAR2A)	0.25
DB01136	178	Respiratory chain complex I (holoenzyme) mitochondrial	0.05025
DB01136	668	BKCA-beta2AR-AKAP79 signaling complex	0.19245
DB01136	672	BKCA-beta2AR complex	0.2357
DB01136	687	CFTR-NHERF-beta(2)AR signaling complex	0.19245
DB01136	879	PRKAC-AKAP5-ADRB1 complex	0.14907
DB01136	2421	ITGA4-ITGB1-VCAM1 complex	0.19245
DB01136	2442	ITGA9-ITGB1-VCAM1 complex	0.19245
DB01136	2914	Respiratory chain complex I (beta subunit) mitochondrial	0.08333
DB01136	2972	ITGA9-ITGB1-VEGFA complex	0.19245
DB01136	3830	ADRB2 homodimer complex	0.33333
DB01136	4062	NRP1-VEGFR2-VEGF(165) complex	0.19245
DB01136	4869	beta(1)-AR receptosome (ADRB1-SAP97-AKAP79-PRKAR2A)	0.16667
DB01136	5696	VEGFA(165)-KDR-NRP1 complex	0.19245
DB01136	5698	VEGFA(165)-VEGFR2-NRP1 complex	0.19245
DB01136	5701	NRP1-VEGF(165/121) complex	0.2357
DB04855	879	PRKAC-AKAP5-ADRB1 complex	0.10541
DB04855	4869	beta(1)-AR receptosome (ADRB1-SAP97-AKAP79-PRKAR2A)	0.11785
DB04855	5747	2AR-mGluR2 complex	0.16667
DB00762	1004	RC complex during S-phase of cell cycle	0.19612

DB00762	1005	RC complex during G2/M-phase of cell cycle	0.19612
DB00762	1098	DNA synthesome complex (13 subunits)	0.18898
DB00762	1099	DNA synthesome complex (17 subunits)	0.16667
DB00762	1106	TFIIIC containing-TOP1-SUB1 complex	0.26726
DB00762	1760	TOP1-PSF-P54 complex	0.40825
DB00762	2230	PCNA complex	0.26726
DB00762	3055	Nop56p-associated pre-rRNA complex	0.06934
DB01030	1004	RC complex during S-phase of cell cycle	0.19612
DB01030	1005	RC complex during G2/M-phase of cell cycle	0.19612
DB01030	1098	DNA synthesome complex (13 subunits)	0.18898
DB01030	1099	DNA synthesome complex (17 subunits)	0.16667
DB01030	1106	TFIIIC containing-TOP1-SUB1 complex	0.26726
DB01030	1760	TOP1-PSF-P54 complex	0.40825
DB01030	2230	PCNA complex	0.26726
DB01030	3055	Nop56p-associated pre-rRNA complex	0.06934
DB01618	5414	HTR1A-HTR1D complex	0.35355
DB01618	5416	HTR1A-HTR1B complex	0.35355
DB01618	5418	GABBR2-HTR1A complex	0.35355
DB01618	5419	HTR1A-GPR26 complex	0.35355
DB01618	5420	HTR1A-EDG3 complex	0.35355
DB01618	5421	HTR1A homodimer complex	0.5
DB01618	5422	HTR1A-EDG1 complex	0.35355
DB00716	5718	eNOS-HSP90-AKT complex VEGF induced	0.2582
DB00716	5716	eNOS-HSP90 complex VEGF induced	0.31623
DB00716	2112	CDC37-HSP90AA1-HSP90AB1-MAP3K11 complex	0.22361
DB00716	2721	HCF-1 complex	0.1026
DB00716	4158	HSP90-FKBP38-CAM-Ca(2+) complex	0.22361
DB00716	5199	Kinase maturation complex 1	0.1118
DB00716	5212	Kinase maturation complex 2	0.15811
DB00716	5234	IKBKB-CDC37-KIAA1967-HSP90AB1-HSP90AA1 complex	0.2
DB00716	5266	TNF-alpha/NF-kappa B signaling complex 6	0.11952
DB00716	5268	TNF-alpha/NF-kappa B signaling complex 7	0.15811
DB00716	5269	TNF-alpha/NF-kappa B signaling complex 8	0.18257
DB00716	5286	TNF-alpha/NF-kappa B signaling complex 10	0.14142
DB00716	5622	HSP90-CIP1-FKBPL complex	0.2582
DB00160	2390	CD98-LAT2-ITGB1 complex	0.13868
DB00160	3035	LAT2-ITGB1 complex	0.19612
DB00751	5747	2AR-mGluR2 complex	0.28868
DB00987	362	DNA ligase III-XRCC1-PNK-DNA-pol III multiprotein complex	0.5
DB00987	1085	DNA repair complex NEIL2-PNK-Pol(beta)-LigIII(alpha)-XRCC1	0.44721
DB00987	1086	DNA repair complex NEIL1-PNK-Pol(beta)-LigIII(alpha)-XRCC1	0.44721
DB00987	2198	RAD9-RAD1-HUS1-POLB complex	0.5
DB00865	5747	2AR-mGluR2 complex	0.35355
DB01363	668	BKCA-beta2AR-AKAP79 signaling complex	0.16013
DB01363	672	BKCA-beta2AR complex	0.19612
DB01363	687	CFTR-NHERF-beta(2)AR signaling complex	0.16013
DB01363	879	PRKAC-AKAP5-ADRB1 complex	0.12403
DB01363	3830	ADRB2 homodimer complex	0.27735
DB01363	4869	beta(1)-AR receptosome (ADRB1-SAP97-AKAP79-PRKAR2A)	0.13868
DB01363	5747	2AR-mGluR2 complex	0.19612
DB01577	5747	2AR-mGluR2 complex	0.2132
DB01016	681	(C-CFTR)2-NHERF-ezrin complex	0.20412
DB01016	682	C-CFTR-NHERF(PDZ1 domain)-ezrin complex	0.20412
DB01016	683	C-CFTR-NHERF(PDZ2 domain)-ezrin complex	0.20412

DB01016	687	CFTR-NHERF-beta(2)AR signaling complex	0.20412
DB00360	550	NOS3-CAV1-NOSTRIN complex	0.28868
DB00360	753	UTM-SGCE-DAG1-CAV1-NOS3 complex	0.22361
DB00360	5718	eNOS-HSP90-AKT complex VEGF induced	0.28868
DB00360	5716	eNOS-HSP90 complex VEGF induced	0.35355
DB00360	5714	eNOS-CAV1 complex	0.35355
DB00255	441	TFTC-type histone acetyl transferase complex	0.17408
DB00255	1054	ESR1-RELA-BCL3-NCOA3 complex	0.28868
DB00255	2124	IKK-alpha--ER-alpha-AIB1 complex	0.33333
DB00255	2470	p130Cas-ER-alpha-cSrc-kinase- PI3-kinase p85-subunit complex	0.28868
DB00255	2657	ESR1-CDK7-CCNH-MNAT1-MTA1-HDAC2 complex	0.2357
DB00255	2670	Er-alpha-p53-hdm2 complex	0.33333
DB00255	2699	ER-alpha-GRIP1-c-Jun complex	0.33333
DB00255	2700	ER-alpha-c-Jun complex	0.40825
DB00255	5862	CAV1-VDAC1-ESR1 complex	0.33333
DB00269	441	TFTC-type histone acetyl transferase complex	0.30151
DB00269	1054	ESR1-RELA-BCL3-NCOA3 complex	0.5
DB00269	2124	IKK-alpha--ER-alpha-AIB1 complex	0.57735
DB00269	2470	p130Cas-ER-alpha-cSrc-kinase- PI3-kinase p85-subunit complex	0.5
DB00269	2657	ESR1-CDK7-CCNH-MNAT1-MTA1-HDAC2 complex	0.40825
DB00269	2670	Er-alpha-p53-hdm2 complex	0.57735
DB00269	2699	ER-alpha-GRIP1-c-Jun complex	0.57735
DB00269	2700	ER-alpha-c-Jun complex	0.70711
DB00269	5862	CAV1-VDAC1-ESR1 complex	0.57735
DB00286	441	TFTC-type histone acetyl transferase complex	0.30151
DB00286	1054	ESR1-RELA-BCL3-NCOA3 complex	0.5
DB00286	2124	IKK-alpha--ER-alpha-AIB1 complex	0.57735
DB00286	2470	p130Cas-ER-alpha-cSrc-kinase- PI3-kinase p85-subunit complex	0.5
DB00286	2657	ESR1-CDK7-CCNH-MNAT1-MTA1-HDAC2 complex	0.40825
DB00286	2670	Er-alpha-p53-hdm2 complex	0.57735
DB00286	2699	ER-alpha-GRIP1-c-Jun complex	0.57735
DB00286	2700	ER-alpha-c-Jun complex	0.70711
DB00286	5862	CAV1-VDAC1-ESR1 complex	0.57735
DB00294	441	TFTC-type histone acetyl transferase complex	0.2132
DB00294	1054	ESR1-RELA-BCL3-NCOA3 complex	0.35355
DB00294	2124	IKK-alpha--ER-alpha-AIB1 complex	0.40825
DB00294	2470	p130Cas-ER-alpha-cSrc-kinase- PI3-kinase p85-subunit complex	0.35355
DB00294	2657	ESR1-CDK7-CCNH-MNAT1-MTA1-HDAC2 complex	0.28868
DB00294	2670	Er-alpha-p53-hdm2 complex	0.40825
DB00294	2699	ER-alpha-GRIP1-c-Jun complex	0.40825
DB00294	2700	ER-alpha-c-Jun complex	0.5
DB00294	5862	CAV1-VDAC1-ESR1 complex	0.40825
DB00304	441	TFTC-type histone acetyl transferase complex	0.2132
DB00304	1054	ESR1-RELA-BCL3-NCOA3 complex	0.35355
DB00304	2124	IKK-alpha--ER-alpha-AIB1 complex	0.40825
DB00304	2470	p130Cas-ER-alpha-cSrc-kinase- PI3-kinase p85-subunit complex	0.35355
DB00304	2657	ESR1-CDK7-CCNH-MNAT1-MTA1-HDAC2 complex	0.28868
DB00304	2670	Er-alpha-p53-hdm2 complex	0.40825
DB00304	2699	ER-alpha-GRIP1-c-Jun complex	0.40825
DB00304	2700	ER-alpha-c-Jun complex	0.5
DB00304	5862	CAV1-VDAC1-ESR1 complex	0.40825
DB00367	441	TFTC-type histone acetyl transferase complex	0.15076
DB00367	1054	ESR1-RELA-BCL3-NCOA3 complex	0.25
DB00367	2124	IKK-alpha--ER-alpha-AIB1 complex	0.28868

DB00367	2159	AR-AKT-APPL complex	0.28868
DB00367	2160	AOF2-AR complex	0.35355
DB00367	2470	p130Cas-ER-alpha-cSrc-kinase- PI3-kinase p85-subunit complex	0.25
DB00367	2657	ESR1-CDK7-CCNH-MNAT1-MTA1-HDAC2 complex	0.20412
DB00367	2670	Er-alpha-p53-hdm2 complex	0.28868
DB00367	2699	ER-alpha-GRIP1-c-Jun complex	0.28868
DB00367	2700	ER-alpha-c-Jun complex	0.35355
DB00367	5862	CAV1-VDAC1-ESR1 complex	0.28868
DB00396	441	TFTC-type histone acetyl transferase complex	0.15076
DB00396	786	MR-UBC9-SRC1 complex	0.28868
DB00396	1054	ESR1-RELA-BCL3-NCOA3 complex	0.25
DB00396	2124	IKK-alpha--ER-alpha-AIB1 complex	0.28868
DB00396	2470	p130Cas-ER-alpha-cSrc-kinase- PI3-kinase p85-subunit complex	0.25
DB00396	2657	ESR1-CDK7-CCNH-MNAT1-MTA1-HDAC2 complex	0.20412
DB00396	2670	Er-alpha-p53-hdm2 complex	0.28868
DB00396	2699	ER-alpha-GRIP1-c-Jun complex	0.28868
DB00396	2700	ER-alpha-c-Jun complex	0.35355
DB00396	3634	NR3C2-UBC9-SRC-1 complex	0.28868
DB00396	5862	CAV1-VDAC1-ESR1 complex	0.28868
DB00481	441	TFTC-type histone acetyl transferase complex	0.2132
DB00481	1054	ESR1-RELA-BCL3-NCOA3 complex	0.35355
DB00481	2124	IKK-alpha--ER-alpha-AIB1 complex	0.40825
DB00481	2470	p130Cas-ER-alpha-cSrc-kinase- PI3-kinase p85-subunit complex	0.35355
DB00481	2657	ESR1-CDK7-CCNH-MNAT1-MTA1-HDAC2 complex	0.28868
DB00481	2670	Er-alpha-p53-hdm2 complex	0.40825
DB00481	2699	ER-alpha-GRIP1-c-Jun complex	0.40825
DB00481	2700	ER-alpha-c-Jun complex	0.5
DB00481	5862	CAV1-VDAC1-ESR1 complex	0.40825
DB00539	441	TFTC-type histone acetyl transferase complex	0.30151
DB00539	1054	ESR1-RELA-BCL3-NCOA3 complex	0.5
DB00539	2124	IKK-alpha--ER-alpha-AIB1 complex	0.57735
DB00539	2470	p130Cas-ER-alpha-cSrc-kinase- PI3-kinase p85-subunit complex	0.5
DB00539	2657	ESR1-CDK7-CCNH-MNAT1-MTA1-HDAC2 complex	0.40825
DB00539	2670	Er-alpha-p53-hdm2 complex	0.57735
DB00539	2699	ER-alpha-GRIP1-c-Jun complex	0.57735
DB00539	2700	ER-alpha-c-Jun complex	0.70711
DB00539	5862	CAV1-VDAC1-ESR1 complex	0.57735
DB00603	441	TFTC-type histone acetyl transferase complex	0.2132
DB00603	1054	ESR1-RELA-BCL3-NCOA3 complex	0.35355
DB00603	2124	IKK-alpha--ER-alpha-AIB1 complex	0.40825
DB00603	2470	p130Cas-ER-alpha-cSrc-kinase- PI3-kinase p85-subunit complex	0.35355
DB00603	2657	ESR1-CDK7-CCNH-MNAT1-MTA1-HDAC2 complex	0.28868
DB00603	2670	Er-alpha-p53-hdm2 complex	0.40825
DB00603	2699	ER-alpha-GRIP1-c-Jun complex	0.40825
DB00603	2700	ER-alpha-c-Jun complex	0.5
DB00603	5862	CAV1-VDAC1-ESR1 complex	0.40825
DB00655	441	TFTC-type histone acetyl transferase complex	0.30151
DB00655	1054	ESR1-RELA-BCL3-NCOA3 complex	0.5
DB00655	2124	IKK-alpha--ER-alpha-AIB1 complex	0.57735
DB00655	2470	p130Cas-ER-alpha-cSrc-kinase- PI3-kinase p85-subunit complex	0.5
DB00655	2657	ESR1-CDK7-CCNH-MNAT1-MTA1-HDAC2 complex	0.40825
DB00655	2670	Er-alpha-p53-hdm2 complex	0.57735
DB00655	2699	ER-alpha-GRIP1-c-Jun complex	0.57735
DB00655	2700	ER-alpha-c-Jun complex	0.70711

DB00655	5862	CAV1-VDAC1-ESR1 complex	0.57735
DB00675	441	TFTC-type histone acetyl transferase complex	0.2132
DB00675	1054	ESR1-RELA-BCL3-NCOA3 complex	0.35355
DB00675	2124	IKK-alpha--ER-alpha-AIB1 complex	0.40825
DB00675	2470	p130Cas-ER-alpha-cSrc-kinase- PI3-kinase p85-subunit complex	0.35355
DB00675	2657	ESR1-CDK7-CCNH-MNAT1-MTA1-HDAC2 complex	0.28868
DB00675	2670	Er-alpha-p53-hdm2 complex	0.40825
DB00675	2699	ER-alpha-GRIP1-c-Jun complex	0.40825
DB00675	2700	ER-alpha-c-Jun complex	0.5
DB00675	5862	CAV1-VDAC1-ESR1 complex	0.40825
DB00783	441	TFTC-type histone acetyl transferase complex	0.17408
DB00783	1054	ESR1-RELA-BCL3-NCOA3 complex	0.28868
DB00783	2124	IKK-alpha--ER-alpha-AIB1 complex	0.33333
DB00783	2470	p130Cas-ER-alpha-cSrc-kinase- PI3-kinase p85-subunit complex	0.28868
DB00783	2657	ESR1-CDK7-CCNH-MNAT1-MTA1-HDAC2 complex	0.2357
DB00783	2670	Er-alpha-p53-hdm2 complex	0.33333
DB00783	2699	ER-alpha-GRIP1-c-Jun complex	0.33333
DB00783	2700	ER-alpha-c-Jun complex	0.40825
DB00783	5862	CAV1-VDAC1-ESR1 complex	0.33333
DB00823	441	TFTC-type histone acetyl transferase complex	0.2132
DB00823	1054	ESR1-RELA-BCL3-NCOA3 complex	0.35355
DB00823	2124	IKK-alpha--ER-alpha-AIB1 complex	0.40825
DB00823	2470	p130Cas-ER-alpha-cSrc-kinase- PI3-kinase p85-subunit complex	0.35355
DB00823	2657	ESR1-CDK7-CCNH-MNAT1-MTA1-HDAC2 complex	0.28868
DB00823	2670	Er-alpha-p53-hdm2 complex	0.40825
DB00823	2699	ER-alpha-GRIP1-c-Jun complex	0.40825
DB00823	2700	ER-alpha-c-Jun complex	0.5
DB00823	5862	CAV1-VDAC1-ESR1 complex	0.40825
DB00882	441	TFTC-type histone acetyl transferase complex	0.30151
DB00882	1054	ESR1-RELA-BCL3-NCOA3 complex	0.5
DB00882	2124	IKK-alpha--ER-alpha-AIB1 complex	0.57735
DB00882	2470	p130Cas-ER-alpha-cSrc-kinase- PI3-kinase p85-subunit complex	0.5
DB00882	2657	ESR1-CDK7-CCNH-MNAT1-MTA1-HDAC2 complex	0.40825
DB00882	2670	Er-alpha-p53-hdm2 complex	0.57735
DB00882	2699	ER-alpha-GRIP1-c-Jun complex	0.57735
DB00882	2700	ER-alpha-c-Jun complex	0.70711
DB00882	5862	CAV1-VDAC1-ESR1 complex	0.57735
DB00890	441	TFTC-type histone acetyl transferase complex	0.30151
DB00890	1054	ESR1-RELA-BCL3-NCOA3 complex	0.5
DB00890	2124	IKK-alpha--ER-alpha-AIB1 complex	0.57735
DB00890	2470	p130Cas-ER-alpha-cSrc-kinase- PI3-kinase p85-subunit complex	0.5
DB00890	2657	ESR1-CDK7-CCNH-MNAT1-MTA1-HDAC2 complex	0.40825
DB00890	2670	Er-alpha-p53-hdm2 complex	0.57735
DB00890	2699	ER-alpha-GRIP1-c-Jun complex	0.57735
DB00890	2700	ER-alpha-c-Jun complex	0.70711
DB00890	5862	CAV1-VDAC1-ESR1 complex	0.57735
DB00947	441	TFTC-type histone acetyl transferase complex	0.30151
DB00947	1054	ESR1-RELA-BCL3-NCOA3 complex	0.5
DB00947	2124	IKK-alpha--ER-alpha-AIB1 complex	0.57735
DB00947	2470	p130Cas-ER-alpha-cSrc-kinase- PI3-kinase p85-subunit complex	0.5
DB00947	2657	ESR1-CDK7-CCNH-MNAT1-MTA1-HDAC2 complex	0.40825
DB00947	2670	Er-alpha-p53-hdm2 complex	0.57735
DB00947	2699	ER-alpha-GRIP1-c-Jun complex	0.57735
DB00947	2700	ER-alpha-c-Jun complex	0.70711

DB00947	5862	CAV1-VDAC1-ESR1 complex	0.57735
DB00957	441	TFTC-type histone acetyl transferase complex	0.2132
DB00957	1054	ESR1-RELA-BCL3-NCOA3 complex	0.35355
DB00957	2124	IKK-alpha--ER-alpha-AIB1 complex	0.40825
DB00957	2470	p130Cas-ER-alpha-cSrc-kinase- PI3-kinase p85-subunit complex	0.35355
DB00957	2657	ESR1-CDK7-CCNH-MNAT1-MTA1-HDAC2 complex	0.28868
DB00957	2670	Er-alpha-p53-hdm2 complex	0.40825
DB00957	2699	ER-alpha-GRIP1-c-Jun complex	0.40825
DB00957	2700	ER-alpha-c-Jun complex	0.5
DB00957	5862	CAV1-VDAC1-ESR1 complex	0.40825
DB00977	441	TFTC-type histone acetyl transferase complex	0.2132
DB00977	1054	ESR1-RELA-BCL3-NCOA3 complex	0.35355
DB00977	2124	IKK-alpha--ER-alpha-AIB1 complex	0.40825
DB00977	2470	p130Cas-ER-alpha-cSrc-kinase- PI3-kinase p85-subunit complex	0.35355
DB00977	2657	ESR1-CDK7-CCNH-MNAT1-MTA1-HDAC2 complex	0.28868
DB00977	2670	Er-alpha-p53-hdm2 complex	0.40825
DB00977	2699	ER-alpha-GRIP1-c-Jun complex	0.40825
DB00977	2700	ER-alpha-c-Jun complex	0.5
DB00977	5862	CAV1-VDAC1-ESR1 complex	0.40825
DB01065	441	TFTC-type histone acetyl transferase complex	0.1005
DB01065	1054	ESR1-RELA-BCL3-NCOA3 complex	0.16667
DB01065	1223	H2AX complex isolated from cells without IR exposure	0.1849
DB01065	1226	H2AX complex I	0.12599
DB01065	1227	H2AX complex II	0.10541
DB01065	1976	MTNR1A homodimer complex	0.33333
DB01065	1977	MTNR1B homodimer complex	0.33333
DB01065	1978	MTNR1A-MTNR1B complex	0.4714
DB01065	2124	IKK-alpha--ER-alpha-AIB1 complex	0.19245
DB01065	2242	TGM2-HD-CALM1 complex	0.19245
DB01065	2470	p130Cas-ER-alpha-cSrc-kinase- PI3-kinase p85-subunit complex	0.16667
DB01065	2657	ESR1-CDK7-CCNH-MNAT1-MTA1-HDAC2 complex	0.13608
DB01065	2670	Er-alpha-p53-hdm2 complex	0.19245
DB01065	2699	ER-alpha-GRIP1-c-Jun complex	0.19245
DB01065	2700	ER-alpha-c-Jun complex	0.2357
DB01065	4158	HSP90-FKBP38-CAM-Ca(2+) complex	0.16667
DB01065	5189	YWHAQ-CALM1-CABIN1 complex	0.19245
DB01065	5217	Calreticulin oligomer complex	0.33333
DB01065	5526	CALM1-FKBP38-BCL2 complex	0.19245
DB01065	5862	CAV1-VDAC1-ESR1 complex	0.19245
DB01183	441	TFTC-type histone acetyl transferase complex	0.12309
DB01183	1054	ESR1-RELA-BCL3-NCOA3 complex	0.20412
DB01183	1714	TICAM1-TICAM2-TLR4 complex	0.2357
DB01183	2124	IKK-alpha--ER-alpha-AIB1 complex	0.2357
DB01183	2470	p130Cas-ER-alpha-cSrc-kinase- PI3-kinase p85-subunit complex	0.20412
DB01183	2657	ESR1-CDK7-CCNH-MNAT1-MTA1-HDAC2 complex	0.16667
DB01183	2670	Er-alpha-p53-hdm2 complex	0.2357
DB01183	2699	ER-alpha-GRIP1-c-Jun complex	0.2357
DB01183	2700	ER-alpha-c-Jun complex	0.28868
DB01183	3181	LMO4-CREB complex	0.28868
DB01183	3189	FHL2-CREB complex	0.28868
DB01183	3190	FHL3-CREB complex	0.28868
DB01183	3191	ACT-CREB complex	0.28868
DB01183	5862	CAV1-VDAC1-ESR1 complex	0.2357
DB01185	441	TFTC-type histone acetyl transferase complex	0.15076

DB01185	1054	ESR1-RELA-BCL3-NCOA3 complex	0.25
DB01185	2124	IKK-alpha--ER-alpha-AIB1 complex	0.28868
DB01185	2159	AR-AKT-APPL complex	0.28868
DB01185	2160	AOF2-AR complex	0.35355
DB01185	2470	p130Cas-ER-alpha-cSrc-kinase- PI3-kinase p85-subunit complex	0.25
DB01185	2657	ESR1-CDK7-CCNH-MNAT1-MTA1-HDAC2 complex	0.20412
DB01185	2670	Er-alpha-p53-hdm2 complex	0.28868
DB01185	2699	ER-alpha-GRIP1-c-Jun complex	0.28868
DB01185	2700	ER-alpha-c-Jun complex	0.35355
DB01185	3157	Prolactin (PRL) - PRL receptor (PRLR) complex	0.35355
DB01185	3161	PRL receptor (PRLR) dimer complex	0.5
DB01185	4216	GR-hnRNP U complex	0.35355
DB01185	5862	CAV1-VDAC1-ESR1 complex	0.28868
DB01196	441	TFTC-type histone acetyl transferase complex	0.15076
DB01196	1054	ESR1-RELA-BCL3-NCOA3 complex	0.25
DB01196	2124	IKK-alpha--ER-alpha-AIB1 complex	0.28868
DB01196	2470	p130Cas-ER-alpha-cSrc-kinase- PI3-kinase p85-subunit complex	0.25
DB01196	2657	ESR1-CDK7-CCNH-MNAT1-MTA1-HDAC2 complex	0.20412
DB01196	2670	Er-alpha-p53-hdm2 complex	0.28868
DB01196	2699	ER-alpha-GRIP1-c-Jun complex	0.28868
DB01196	2700	ER-alpha-c-Jun complex	0.35355
DB01196	5862	CAV1-VDAC1-ESR1 complex	0.28868
DB01357	441	TFTC-type histone acetyl transferase complex	0.30151
DB01357	1054	ESR1-RELA-BCL3-NCOA3 complex	0.5
DB01357	2124	IKK-alpha--ER-alpha-AIB1 complex	0.57735
DB01357	2470	p130Cas-ER-alpha-cSrc-kinase- PI3-kinase p85-subunit complex	0.5
DB01357	2657	ESR1-CDK7-CCNH-MNAT1-MTA1-HDAC2 complex	0.40825
DB01357	2670	Er-alpha-p53-hdm2 complex	0.57735
DB01357	2699	ER-alpha-GRIP1-c-Jun complex	0.57735
DB01357	2700	ER-alpha-c-Jun complex	0.70711
DB01357	5862	CAV1-VDAC1-ESR1 complex	0.57735
DB01406	441	TFTC-type histone acetyl transferase complex	0.13484
DB01406	1054	ESR1-RELA-BCL3-NCOA3 complex	0.22361
DB01406	2124	IKK-alpha--ER-alpha-AIB1 complex	0.2582
DB01406	2159	AR-AKT-APPL complex	0.2582
DB01406	2160	AOF2-AR complex	0.31623
DB01406	2470	p130Cas-ER-alpha-cSrc-kinase- PI3-kinase p85-subunit complex	0.22361
DB01406	2657	ESR1-CDK7-CCNH-MNAT1-MTA1-HDAC2 complex	0.18257
DB01406	2670	Er-alpha-p53-hdm2 complex	0.2582
DB01406	2699	ER-alpha-GRIP1-c-Jun complex	0.2582
DB01406	2700	ER-alpha-c-Jun complex	0.31623
DB01406	5862	CAV1-VDAC1-ESR1 complex	0.2582
DB01431	441	TFTC-type histone acetyl transferase complex	0.2132
DB01431	1054	ESR1-RELA-BCL3-NCOA3 complex	0.35355
DB01431	2124	IKK-alpha--ER-alpha-AIB1 complex	0.40825
DB01431	2470	p130Cas-ER-alpha-cSrc-kinase- PI3-kinase p85-subunit complex	0.35355
DB01431	2657	ESR1-CDK7-CCNH-MNAT1-MTA1-HDAC2 complex	0.28868
DB01431	2670	Er-alpha-p53-hdm2 complex	0.40825
DB01431	2699	ER-alpha-GRIP1-c-Jun complex	0.40825
DB01431	2700	ER-alpha-c-Jun complex	0.5
DB01431	5862	CAV1-VDAC1-ESR1 complex	0.40825
DB04573	441	TFTC-type histone acetyl transferase complex	0.2132
DB04573	1054	ESR1-RELA-BCL3-NCOA3 complex	0.35355
DB04573	2124	IKK-alpha--ER-alpha-AIB1 complex	0.40825

DB04573	2470	p130Cas-ER-alpha-cSrc-kinase- PI3-kinase p85-subunit complex	0.35355
DB04573	2657	ESR1-CDK7-CCNH-MNAT1-MTA1-HDAC2 complex	0.28868
DB04573	2670	Er-alpha-p53-hdm2 complex	0.40825
DB04573	2699	ER-alpha-GRIP1-c-Jun complex	0.40825
DB04573	2700	ER-alpha-c-Jun complex	0.5
DB04573	5862	CAV1-VDAC1-ESR1 complex	0.40825
DB04574	441	TFTC-type histone acetyl transferase complex	0.2132
DB04574	1054	ESR1-RELA-BCL3-NCOA3 complex	0.35355
DB04574	2124	IKK-alpha--ER-alpha-AIB1 complex	0.40825
DB04574	2470	p130Cas-ER-alpha-cSrc-kinase- PI3-kinase p85-subunit complex	0.35355
DB04574	2657	ESR1-CDK7-CCNH-MNAT1-MTA1-HDAC2 complex	0.28868
DB04574	2670	Er-alpha-p53-hdm2 complex	0.40825
DB04574	2699	ER-alpha-GRIP1-c-Jun complex	0.40825
DB04574	2700	ER-alpha-c-Jun complex	0.5
DB04574	5862	CAV1-VDAC1-ESR1 complex	0.40825
DB04575	441	TFTC-type histone acetyl transferase complex	0.30151
DB04575	1054	ESR1-RELA-BCL3-NCOA3 complex	0.5
DB04575	2124	IKK-alpha--ER-alpha-AIB1 complex	0.57735
DB04575	2470	p130Cas-ER-alpha-cSrc-kinase- PI3-kinase p85-subunit complex	0.5
DB04575	2657	ESR1-CDK7-CCNH-MNAT1-MTA1-HDAC2 complex	0.40825
DB04575	2670	Er-alpha-p53-hdm2 complex	0.57735
DB04575	2699	ER-alpha-GRIP1-c-Jun complex	0.57735
DB04575	2700	ER-alpha-c-Jun complex	0.70711
DB04575	5862	CAV1-VDAC1-ESR1 complex	0.57735
DB04938	441	TFTC-type histone acetyl transferase complex	0.30151
DB04938	1054	ESR1-RELA-BCL3-NCOA3 complex	0.5
DB04938	2124	IKK-alpha--ER-alpha-AIB1 complex	0.57735
DB04938	2470	p130Cas-ER-alpha-cSrc-kinase- PI3-kinase p85-subunit complex	0.5
DB04938	2657	ESR1-CDK7-CCNH-MNAT1-MTA1-HDAC2 complex	0.40825
DB04938	2670	Er-alpha-p53-hdm2 complex	0.57735
DB04938	2699	ER-alpha-GRIP1-c-Jun complex	0.57735
DB04938	2700	ER-alpha-c-Jun complex	0.70711
DB04938	5862	CAV1-VDAC1-ESR1 complex	0.57735
DB00231	5809	GABAA receptor	0.28868
DB00466	5809	GABAA receptor	0.28868
DB00683	5809	GABAA receptor	0.39736
DB00690	5809	GABAA receptor	0.39736
DB00801	5809	GABAA receptor	0.30619
DB00829	5809	GABAA receptor	0.29704
DB00842	5809	GABAA receptor	0.29277
DB00897	5809	GABAA receptor	0.28868
DB01215	5809	GABAA receptor	0.30619
DB01558	5809	GABAA receptor	0.39736
DB01559	5809	GABAA receptor	0.43301
DB01567	5809	GABAA receptor	0.43301
DB01588	5809	GABAA receptor	0.30619
DB01589	5809	GABAA receptor	0.31109
DB01594	5809	GABAA receptor	0.43301
DB01595	5809	GABAA receptor	0.3873
DB00421	786	MR-UBC9-SRC1 complex	0.40825
DB00421	2159	AR-AKT-APPL complex	0.40825
DB00421	2160	AOF2-AR complex	0.5
DB00421	3634	NR3C2-UBC9-SRC-1 complex	0.40825
DB00499	1211	Ubiquitin E3 ligase (AHR ARNT DDB1 TBL3 CUL4B RBX1)	0.28868

DB00499	2159	AR-AKT-APPL complex	0.40825
DB00499	2160	AOF2-AR complex	0.5
DB00621	2159	AR-AKT-APPL complex	0.57735
DB00621	2160	AOF2-AR complex	0.70711
DB00624	2159	AR-AKT-APPL complex	0.57735
DB00624	2160	AOF2-AR complex	0.70711
DB00665	2159	AR-AKT-APPL complex	0.57735
DB00665	2160	AOF2-AR complex	0.70711
DB00687	786	MR-UBC9-SRC1 complex	0.33333
DB00687	2159	AR-AKT-APPL complex	0.33333
DB00687	2160	AOF2-AR complex	0.40825
DB00687	3634	NR3C2-UBC9-SRC-1 complex	0.33333
DB00687	4216	GR-hnRNP U complex	0.40825
DB00858	2159	AR-AKT-APPL complex	0.57735
DB00858	2160	AOF2-AR complex	0.70711
DB00984	2159	AR-AKT-APPL complex	0.57735
DB00984	2160	AOF2-AR complex	0.70711
DB01128	2159	AR-AKT-APPL complex	0.57735
DB01128	2160	AOF2-AR complex	0.70711
DB01395	786	MR-UBC9-SRC1 complex	0.33333
DB01395	2159	AR-AKT-APPL complex	0.33333
DB01395	2160	AOF2-AR complex	0.40825
DB01395	3634	NR3C2-UBC9-SRC-1 complex	0.33333
DB01420	2159	AR-AKT-APPL complex	0.57735
DB01420	2160	AOF2-AR complex	0.70711
DB04839	2159	AR-AKT-APPL complex	0.57735
DB04839	2160	AOF2-AR complex	0.70711
DB06710	2159	AR-AKT-APPL complex	0.57735
DB06710	2160	AOF2-AR complex	0.70711
DB08804	2159	AR-AKT-APPL complex	0.57735
DB08804	2160	AOF2-AR complex	0.70711
DB08899	2159	AR-AKT-APPL complex	0.57735
DB08899	2160	AOF2-AR complex	0.70711
DB00536	1909	APC-DLG4 complex	0.28868
DB00536	5641	PSD95-FYN-NR2A complex	0.2357
DB00014	2486	GIPC1-LHCGR complex	0.5
DB00032	2486	GIPC1-LHCGR complex	0.5
DB00044	2486	GIPC1-LHCGR complex	0.70711
DB00050	2486	GIPC1-LHCGR complex	0.5
DB00097	2486	GIPC1-LHCGR complex	0.5
DB06719	2486	GIPC1-LHCGR complex	0.5
DB00273	5809	GABAA receptor	0.25198
DB00361	1335	SNW1 complex	0.2357
DB00361	1400	ASCOM complex	0.37796
DB00541	1335	SNW1 complex	0.16667
DB00541	1400	ASCOM complex	0.53452
DB00570	1231	FIB-associated protein complex	0.16667
DB00570	1335	SNW1 complex	0.09623
DB00570	1400	ASCOM complex	0.1543
DB00570	1816	JUN-TCF4-CTNNB1 complex	0.2357
DB00570	2692	SMAD3-SMAD4-cJun-cFos complex	0.20412
DB00570	2693	NFAT-JUN-FOS DNA-protein complex	0.2357
DB00570	2694	ERG-JUN-FOS DNA-protein complex	0.2357
DB00570	2695	ETS2-FOS-JUN complex	0.2357

DB00570	2699	ER-alpha-GRIP1-c-Jun complex	0.2357
DB00570	2700	ER-alpha-c-Jun complex	0.28868
DB00570	2708	SMAD3-SMAD4-cJUN complex	0.2357
DB00570	3008	60S APC containing complex	0.30861
DB00570	3055	Nop56p-associated pre-rRNA complex	0.04003
DB01179	924	Toposome	0.21822
DB01179	1098	DNA synthesome complex (13 subunits)	0.1543
DB01179	1099	DNA synthesome complex (17 subunits)	0.13608
DB01179	1183	CDC5L complex	0.10541
DB01179	1335	SNW1 complex	0.13608
DB01179	1400	ASCOM complex	0.43644
DB01179	1728	CTCF-nucleophosmin-PARP-HIS-KPNA-LMNA-TOP complex	0.19245
DB01346	2272	PICK1-GRIP1-GLUR2 complex	0.2582
DB01346	5747	2AR-mGluR2 complex	0.31623
DB01346	5809	GABAA receptor	0.2582
DB00112	2909	PLC-gamma-2-Syk-LAT-FcR-gamma complex	0.15811
DB00112	2910	PLC-gamma-2-Lyn-FcR-gamma complex	0.18257
DB00112	2972	ITGA9-ITGB1-VEGFA complex	0.18257
DB00112	4062	NRP1-VEGFR2-VEGF(165) complex	0.18257
DB00112	5696	VEGFA(165)-KDR-NRP1 complex	0.18257
DB00112	5698	VEGFA(165)-VEGFR2-NRP1 complex	0.18257
DB00112	5701	NRP1-VEGF(165/121) complex	0.22361
DB01120	2972	ITGA9-ITGB1-VEGFA complex	0.40825
DB01120	4062	NRP1-VEGFR2-VEGF(165) complex	0.40825
DB01120	5696	VEGFA(165)-KDR-NRP1 complex	0.40825
DB01120	5698	VEGFA(165)-VEGFR2-NRP1 complex	0.40825
DB01120	5701	NRP1-VEGF(165/121) complex	0.5
DB01270	2972	ITGA9-ITGB1-VEGFA complex	0.57735
DB01270	4062	NRP1-VEGFR2-VEGF(165) complex	0.57735
DB01270	5696	VEGFA(165)-KDR-NRP1 complex	0.57735
DB01270	5698	VEGFA(165)-VEGFR2-NRP1 complex	0.57735
DB01270	5701	NRP1-VEGF(165/121) complex	0.70711
DB05294	1095	SNX complex (SNX1a SNX2 SNX4 EGFR)	0.25
DB05294	1185	EGFR-containing signaling complex	0.25
DB05294	2369	ITGAV-ITGB3-EGFR complex	0.28868
DB05294	2453	Multiprotein complex (monoubiquitination)	0.25
DB05294	2454	CIN85-CBL-SH3GL2-EGFR complex EGF stimulated	0.25
DB05294	2542	EGFR-CBL-GRB2 complex	0.28868
DB05294	2972	ITGA9-ITGB1-VEGFA complex	0.28868
DB05294	3678	RIN1-STAM2-EGFR complex EGF stimulated	0.28868
DB05294	4062	NRP1-VEGFR2-VEGF(165) complex	0.28868
DB05294	5171	SH3KBP1-CBLB-EGFR complex	0.28868
DB05294	5696	VEGFA(165)-KDR-NRP1 complex	0.28868
DB05294	5698	VEGFA(165)-VEGFR2-NRP1 complex	0.28868
DB05294	5701	NRP1-VEGF(165/121) complex	0.35355
DB06779	2972	ITGA9-ITGB1-VEGFA complex	0.28868
DB06779	3162	TF-FVIIa-FXa-TFPI complex	0.25
DB06779	4062	NRP1-VEGFR2-VEGF(165) complex	0.28868
DB06779	5696	VEGFA(165)-KDR-NRP1 complex	0.28868
DB06779	5698	VEGFA(165)-VEGFR2-NRP1 complex	0.28868
DB06779	5701	NRP1-VEGF(165/121) complex	0.35355
DB08885	2972	ITGA9-ITGB1-VEGFA complex	0.33333
DB08885	4062	NRP1-VEGFR2-VEGF(165) complex	0.33333
DB08885	5696	VEGFA(165)-KDR-NRP1 complex	0.33333

DB08885	5698	VEGFA(165)-VEGFR2-NRP1 complex	0.33333
DB08885	5701	NRP1-VEGF(165/121) complex	0.40825
DB00290	212	DNA ligase III-XRCC1 complex	0.5
DB00290	362	DNA ligase III-XRCC1-PNK-DNA-pol III multiprotein complex	0.35355
DB00290	1004	RC complex during S-phase of cell cycle	0.19612
DB00290	1085	DNA repair complex NEIL2-PNK-Pol(beta)-LigIII(alpha)-XRCC1	0.31623
DB00290	1086	DNA repair complex NEIL1-PNK-Pol(beta)-LigIII(alpha)-XRCC1	0.31623
DB00290	1108	DNA synthesome complex (15 subunits)	0.18257
DB00290	2196	LIG1-9-1-1 complex	0.35355
DB00290	5243	XRCC1-LIG3-PNK-TDP1 complex	0.35355
DB00187	879	PRKAC-AKAP5-ADRB1 complex	0.44721
DB00187	4869	beta(1)-AR receptosome (ADRB1-SAP97-AKAP79-PRKAR2A)	0.5
DB00195	668	BKCA-beta2AR-AKAP79 signaling complex	0.40825
DB00195	672	BKCA-beta2AR complex	0.5
DB00195	687	CFTR-NHERF-beta(2)AR signaling complex	0.40825
DB00195	879	PRKAC-AKAP5-ADRB1 complex	0.31623
DB00195	3830	ADRB2 homodimer complex	0.70711
DB00195	4869	beta(1)-AR receptosome (ADRB1-SAP97-AKAP79-PRKAR2A)	0.35355
DB00221	879	PRKAC-AKAP5-ADRB1 complex	0.44721
DB00221	4869	beta(1)-AR receptosome (ADRB1-SAP97-AKAP79-PRKAR2A)	0.5
DB00264	668	BKCA-beta2AR-AKAP79 signaling complex	0.40825
DB00264	672	BKCA-beta2AR complex	0.5
DB00264	687	CFTR-NHERF-beta(2)AR signaling complex	0.40825
DB00264	879	PRKAC-AKAP5-ADRB1 complex	0.31623
DB00264	3830	ADRB2 homodimer complex	0.70711
DB00264	4869	beta(1)-AR receptosome (ADRB1-SAP97-AKAP79-PRKAR2A)	0.35355
DB00335	879	PRKAC-AKAP5-ADRB1 complex	0.44721
DB00335	4869	beta(1)-AR receptosome (ADRB1-SAP97-AKAP79-PRKAR2A)	0.5
DB00368	668	BKCA-beta2AR-AKAP79 signaling complex	0.18257
DB00368	672	BKCA-beta2AR complex	0.22361
DB00368	687	CFTR-NHERF-beta(2)AR signaling complex	0.18257
DB00368	879	PRKAC-AKAP5-ADRB1 complex	0.14142
DB00368	3830	ADRB2 homodimer complex	0.31623
DB00368	4869	beta(1)-AR receptosome (ADRB1-SAP97-AKAP79-PRKAR2A)	0.15811
DB00368	5747	2AR-mGluR2 complex	0.22361
DB00373	668	BKCA-beta2AR-AKAP79 signaling complex	0.33333
DB00373	672	BKCA-beta2AR complex	0.40825
DB00373	687	CFTR-NHERF-beta(2)AR signaling complex	0.33333
DB00373	879	PRKAC-AKAP5-ADRB1 complex	0.2582
DB00373	3830	ADRB2 homodimer complex	0.57735
DB00373	4869	beta(1)-AR receptosome (ADRB1-SAP97-AKAP79-PRKAR2A)	0.28868
DB00521	668	BKCA-beta2AR-AKAP79 signaling complex	0.40825
DB00521	672	BKCA-beta2AR complex	0.5
DB00521	687	CFTR-NHERF-beta(2)AR signaling complex	0.40825
DB00521	879	PRKAC-AKAP5-ADRB1 complex	0.31623
DB00521	3830	ADRB2 homodimer complex	0.70711
DB00521	4869	beta(1)-AR receptosome (ADRB1-SAP97-AKAP79-PRKAR2A)	0.35355
DB00571	668	BKCA-beta2AR-AKAP79 signaling complex	0.2582
DB00571	672	BKCA-beta2AR complex	0.31623
DB00571	687	CFTR-NHERF-beta(2)AR signaling complex	0.2582
DB00571	879	PRKAC-AKAP5-ADRB1 complex	0.2
DB00571	3830	ADRB2 homodimer complex	0.44721
DB00571	4869	beta(1)-AR receptosome (ADRB1-SAP97-AKAP79-PRKAR2A)	0.22361
DB00571	5414	HTR1A-HTR1D complex	0.31623

DB00571	5415	HTR1B homodimer complex	0.44721
DB00571	5416	HTR1A-HTR1B complex	0.63246
DB00571	5417	HTR1D-HTR1B complex	0.31623
DB00571	5418	GABBR2-HTR1A complex	0.31623
DB00571	5419	HTR1A-GPR26 complex	0.31623
DB00571	5420	HTR1A-EDG3 complex	0.31623
DB00571	5421	HTR1A homodimer complex	0.44721
DB00571	5422	HTR1A-EDG1 complex	0.31623
DB00598	668	BKCA-beta2AR-AKAP79 signaling complex	0.28868
DB00598	672	BKCA-beta2AR complex	0.35355
DB00598	687	CFTR-NHERF-beta(2)AR signaling complex	0.28868
DB00598	879	PRKAC-AKAP5-ADRB1 complex	0.22361
DB00598	3830	ADRB2 homodimer complex	0.5
DB00598	4869	beta(1)-AR receptosome (ADRB1-SAP97-AKAP79-PRKAR2A)	0.25
DB00612	668	BKCA-beta2AR-AKAP79 signaling complex	0.40825
DB00612	672	BKCA-beta2AR complex	0.5
DB00612	687	CFTR-NHERF-beta(2)AR signaling complex	0.40825
DB00612	879	PRKAC-AKAP5-ADRB1 complex	0.31623
DB00612	3830	ADRB2 homodimer complex	0.70711
DB00612	4869	beta(1)-AR receptosome (ADRB1-SAP97-AKAP79-PRKAR2A)	0.35355
DB00668	668	BKCA-beta2AR-AKAP79 signaling complex	0.20412
DB00668	672	BKCA-beta2AR complex	0.25
DB00668	687	CFTR-NHERF-beta(2)AR signaling complex	0.20412
DB00668	879	PRKAC-AKAP5-ADRB1 complex	0.15811
DB00668	3830	ADRB2 homodimer complex	0.35355
DB00668	4869	beta(1)-AR receptosome (ADRB1-SAP97-AKAP79-PRKAR2A)	0.17678
DB00668	5747	2AR-mGluR2 complex	0.25
DB00841	668	BKCA-beta2AR-AKAP79 signaling complex	0.40825
DB00841	672	BKCA-beta2AR complex	0.5
DB00841	687	CFTR-NHERF-beta(2)AR signaling complex	0.40825
DB00841	879	PRKAC-AKAP5-ADRB1 complex	0.31623
DB00841	3830	ADRB2 homodimer complex	0.70711
DB00841	4869	beta(1)-AR receptosome (ADRB1-SAP97-AKAP79-PRKAR2A)	0.35355
DB00852	668	BKCA-beta2AR-AKAP79 signaling complex	0.21822
DB00852	672	BKCA-beta2AR complex	0.26726
DB00852	687	CFTR-NHERF-beta(2)AR signaling complex	0.21822
DB00852	879	PRKAC-AKAP5-ADRB1 complex	0.16903
DB00852	3830	ADRB2 homodimer complex	0.37796
DB00852	4869	beta(1)-AR receptosome (ADRB1-SAP97-AKAP79-PRKAR2A)	0.18898
DB00852	5747	2AR-mGluR2 complex	0.26726
DB00866	668	BKCA-beta2AR-AKAP79 signaling complex	0.33333
DB00866	672	BKCA-beta2AR complex	0.40825
DB00866	687	CFTR-NHERF-beta(2)AR signaling complex	0.33333
DB00866	879	PRKAC-AKAP5-ADRB1 complex	0.2582
DB00866	3830	ADRB2 homodimer complex	0.57735
DB00866	4869	beta(1)-AR receptosome (ADRB1-SAP97-AKAP79-PRKAR2A)	0.28868
DB00866	5414	HTR1A-HTR1D complex	0.40825
DB00866	5416	HTR1A-HTR1B complex	0.40825
DB00866	5418	GABBR2-HTR1A complex	0.40825
DB00866	5419	HTR1A-GPR26 complex	0.40825
DB00866	5420	HTR1A-EDG3 complex	0.40825
DB00866	5421	HTR1A homodimer complex	0.57735
DB00866	5422	HTR1A-EDG1 complex	0.40825
DB00960	668	BKCA-beta2AR-AKAP79 signaling complex	0.28868

DB00960	672	BKCA-beta2AR complex	0.35355
DB00960	687	CFTR-NHERF-beta(2)AR signaling complex	0.28868
DB00960	879	PRKAC-AKAP5-ADRB1 complex	0.22361
DB00960	3830	ADRB2 homodimer complex	0.5
DB00960	4869	beta(1)-AR receptosome (ADRB1-SAP97-AKAP79-PRKAR2A)	0.25
DB00960	5414	HTR1A-HTR1D complex	0.35355
DB00960	5415	HTR1B homodimer complex	0.5
DB00960	5416	HTR1A-HTR1B complex	0.70711
DB00960	5417	HTR1D-HTR1B complex	0.35355
DB00960	5418	GABBR2-HTR1A complex	0.35355
DB00960	5419	HTR1A-GPR26 complex	0.35355
DB00960	5420	HTR1A-EDG3 complex	0.35355
DB00960	5421	HTR1A homodimer complex	0.5
DB00960	5422	HTR1A-EDG1 complex	0.35355
DB01001	668	BKCA-beta2AR-AKAP79 signaling complex	0.40825
DB01001	672	BKCA-beta2AR complex	0.5
DB01001	687	CFTR-NHERF-beta(2)AR signaling complex	0.40825
DB01001	879	PRKAC-AKAP5-ADRB1 complex	0.31623
DB01001	3830	ADRB2 homodimer complex	0.70711
DB01001	4869	beta(1)-AR receptosome (ADRB1-SAP97-AKAP79-PRKAR2A)	0.35355
DB01064	57	HDAC4-ERK2 complex	0.26726
DB01064	668	BKCA-beta2AR-AKAP79 signaling complex	0.21822
DB01064	672	BKCA-beta2AR complex	0.26726
DB01064	687	CFTR-NHERF-beta(2)AR signaling complex	0.21822
DB01064	879	PRKAC-AKAP5-ADRB1 complex	0.16903
DB01064	2470	p130Cas-ER-alpha-cSrc-kinase- PI3-kinase p85-subunit complex	0.18898
DB01064	2480	CIN85 complex (CIN85 CRK BCAR1 CBL PIK3R1 GRB2 SOS1)	0.14286
DB01064	2529	LAT-PLC-gamma-1-p85-GRB2-CBL-VAV-SLP-76 signaling complex C305 activated	0.14286
DB01064	2551	PDGFRA-PLC-gamma-1-PI3K-SHP-2 complex PDGF stimulated	0.18898
DB01064	2574	CD19-Vav-PI 3-kinase (p85 subunit) complex	0.21822
DB01064	2577	Sam68-p85 P13K-IRS-1-IR signaling complex	0.18898
DB01064	2922	LAT-PLC-gamma-1-p85-GRB2-SOS signaling complex C305 activated	0.16903
DB01064	2944	Notch1-p56lck-PI3K complex	0.21822
DB01064	3133	Phosphatidylinositol 3-kinase (PIK3CA PIK3R1)	0.26726
DB01064	3830	ADRB2 homodimer complex	0.37796
DB01064	4869	beta(1)-AR receptosome (ADRB1-SAP97-AKAP79-PRKAR2A)	0.18898
DB01064	5226	p14-Mp1-ERK2 complex	0.21822
DB01102	668	BKCA-beta2AR-AKAP79 signaling complex	0.33333
DB01102	672	BKCA-beta2AR complex	0.40825
DB01102	687	CFTR-NHERF-beta(2)AR signaling complex	0.33333
DB01102	879	PRKAC-AKAP5-ADRB1 complex	0.2582
DB01102	3830	ADRB2 homodimer complex	0.57735
DB01102	4869	beta(1)-AR receptosome (ADRB1-SAP97-AKAP79-PRKAR2A)	0.28868
DB01193	668	BKCA-beta2AR-AKAP79 signaling complex	0.40825
DB01193	672	BKCA-beta2AR complex	0.5
DB01193	687	CFTR-NHERF-beta(2)AR signaling complex	0.40825
DB01193	879	PRKAC-AKAP5-ADRB1 complex	0.31623
DB01193	3830	ADRB2 homodimer complex	0.70711
DB01193	4869	beta(1)-AR receptosome (ADRB1-SAP97-AKAP79-PRKAR2A)	0.35355
DB01203	668	BKCA-beta2AR-AKAP79 signaling complex	0.40825
DB01203	672	BKCA-beta2AR complex	0.5
DB01203	687	CFTR-NHERF-beta(2)AR signaling complex	0.40825
DB01203	879	PRKAC-AKAP5-ADRB1 complex	0.31623

DB01203	3830	ADRB2 homodimer complex	0.70711
DB01203	4869	beta(1)-AR receptosome (ADRB1-SAP97-AKAP79-PRKAR2A)	0.35355
DB01210	668	BKCA-beta2AR-AKAP79 signaling complex	0.40825
DB01210	672	BKCA-beta2AR complex	0.5
DB01210	687	CFTR-NHERF-beta(2)AR signaling complex	0.40825
DB01210	879	PRKAC-AKAP5-ADRB1 complex	0.31623
DB01210	3830	ADRB2 homodimer complex	0.70711
DB01210	4869	beta(1)-AR receptosome (ADRB1-SAP97-AKAP79-PRKAR2A)	0.35355
DB01214	668	BKCA-beta2AR-AKAP79 signaling complex	0.40825
DB01214	672	BKCA-beta2AR complex	0.5
DB01214	687	CFTR-NHERF-beta(2)AR signaling complex	0.40825
DB01214	879	PRKAC-AKAP5-ADRB1 complex	0.31623
DB01214	3830	ADRB2 homodimer complex	0.70711
DB01214	4869	beta(1)-AR receptosome (ADRB1-SAP97-AKAP79-PRKAR2A)	0.35355
DB01288	668	BKCA-beta2AR-AKAP79 signaling complex	0.33333
DB01288	672	BKCA-beta2AR complex	0.40825
DB01288	687	CFTR-NHERF-beta(2)AR signaling complex	0.33333
DB01288	879	PRKAC-AKAP5-ADRB1 complex	0.2582
DB01288	3830	ADRB2 homodimer complex	0.57735
DB01288	4869	beta(1)-AR receptosome (ADRB1-SAP97-AKAP79-PRKAR2A)	0.28868
DB01291	668	BKCA-beta2AR-AKAP79 signaling complex	0.40825
DB01291	672	BKCA-beta2AR complex	0.5
DB01291	687	CFTR-NHERF-beta(2)AR signaling complex	0.40825
DB01291	879	PRKAC-AKAP5-ADRB1 complex	0.31623
DB01291	3830	ADRB2 homodimer complex	0.70711
DB01291	4869	beta(1)-AR receptosome (ADRB1-SAP97-AKAP79-PRKAR2A)	0.35355
DB01295	668	BKCA-beta2AR-AKAP79 signaling complex	0.33333
DB01295	672	BKCA-beta2AR complex	0.40825
DB01295	687	CFTR-NHERF-beta(2)AR signaling complex	0.33333
DB01295	879	PRKAC-AKAP5-ADRB1 complex	0.2582
DB01295	3830	ADRB2 homodimer complex	0.57735
DB01295	4869	beta(1)-AR receptosome (ADRB1-SAP97-AKAP79-PRKAR2A)	0.28868
DB01297	879	PRKAC-AKAP5-ADRB1 complex	0.44721
DB01297	4869	beta(1)-AR receptosome (ADRB1-SAP97-AKAP79-PRKAR2A)	0.5
DB01359	668	BKCA-beta2AR-AKAP79 signaling complex	0.33333
DB01359	672	BKCA-beta2AR complex	0.40825
DB01359	687	CFTR-NHERF-beta(2)AR signaling complex	0.33333
DB01359	879	PRKAC-AKAP5-ADRB1 complex	0.2582
DB01359	3830	ADRB2 homodimer complex	0.57735
DB01359	4869	beta(1)-AR receptosome (ADRB1-SAP97-AKAP79-PRKAR2A)	0.28868
DB01359	5414	HTR1A-HTR1D complex	0.40825
DB01359	5416	HTR1A-HTR1B complex	0.40825
DB01359	5418	GABBR2-HTR1A complex	0.40825
DB01359	5419	HTR1A-GPR26 complex	0.40825
DB01359	5420	HTR1A-EDG3 complex	0.40825
DB01359	5421	HTR1A homodimer complex	0.57735
DB01359	5422	HTR1A-EDG1 complex	0.40825
DB01407	668	BKCA-beta2AR-AKAP79 signaling complex	0.2582
DB01407	672	BKCA-beta2AR complex	0.31623
DB01407	687	CFTR-NHERF-beta(2)AR signaling complex	0.2582
DB01407	879	PRKAC-AKAP5-ADRB1 complex	0.2
DB01407	1795	SORT1-NGFR-NGFB complex	0.2582
DB01407	3830	ADRB2 homodimer complex	0.44721
DB01407	4869	beta(1)-AR receptosome (ADRB1-SAP97-AKAP79-PRKAR2A)	0.22361

DB01407	5407	NGF-TrkA complex	0.31623
DB01407	5424	NGF-p75 complex	0.31623
DB01580	668	BKCA-beta2AR-AKAP79 signaling complex	0.40825
DB01580	672	BKCA-beta2AR complex	0.5
DB01580	687	CFTR-NHERF-beta(2)AR signaling complex	0.40825
DB01580	879	PRKAC-AKAP5-ADRB1 complex	0.31623
DB01580	3830	ADRB2 homodimer complex	0.70711
DB01580	4869	beta(1)-AR receptosome (ADRB1-SAP97-AKAP79-PRKAR2A)	0.35355
DB04861	668	BKCA-beta2AR-AKAP79 signaling complex	0.40825
DB04861	672	BKCA-beta2AR complex	0.5
DB04861	687	CFTR-NHERF-beta(2)AR signaling complex	0.40825
DB04861	879	PRKAC-AKAP5-ADRB1 complex	0.31623
DB04861	3830	ADRB2 homodimer complex	0.70711
DB04861	4869	beta(1)-AR receptosome (ADRB1-SAP97-AKAP79-PRKAR2A)	0.35355
DB06262	668	BKCA-beta2AR-AKAP79 signaling complex	0.18257
DB06262	672	BKCA-beta2AR complex	0.22361
DB06262	687	CFTR-NHERF-beta(2)AR signaling complex	0.18257
DB06262	879	PRKAC-AKAP5-ADRB1 complex	0.14142
DB06262	3830	ADRB2 homodimer complex	0.31623
DB06262	4869	beta(1)-AR receptosome (ADRB1-SAP97-AKAP79-PRKAR2A)	0.15811
DB06262	5747	2AR-mGluR2 complex	0.22361
DB08807	668	BKCA-beta2AR-AKAP79 signaling complex	0.2582
DB08807	672	BKCA-beta2AR complex	0.31623
DB08807	687	CFTR-NHERF-beta(2)AR signaling complex	0.2582
DB08807	879	PRKAC-AKAP5-ADRB1 complex	0.2
DB08807	3830	ADRB2 homodimer complex	0.44721
DB08807	4869	beta(1)-AR receptosome (ADRB1-SAP97-AKAP79-PRKAR2A)	0.22361
DB08807	5414	HTR1A-HTR1D complex	0.31623
DB08807	5415	HTR1B homodimer complex	0.44721
DB08807	5416	HTR1A-HTR1B complex	0.63246
DB08807	5417	HTR1D-HTR1B complex	0.31623
DB08807	5418	GABBR2-HTR1A complex	0.31623
DB08807	5419	HTR1A-GPR26 complex	0.31623
DB08807	5420	HTR1A-EDG3 complex	0.31623
DB08807	5421	HTR1A homodimer complex	0.44721
DB08807	5422	HTR1A-EDG1 complex	0.31623
DB08808	668	BKCA-beta2AR-AKAP79 signaling complex	0.33333
DB08808	672	BKCA-beta2AR complex	0.40825
DB08808	687	CFTR-NHERF-beta(2)AR signaling complex	0.33333
DB08808	879	PRKAC-AKAP5-ADRB1 complex	0.2582
DB08808	3830	ADRB2 homodimer complex	0.57735
DB08808	4869	beta(1)-AR receptosome (ADRB1-SAP97-AKAP79-PRKAR2A)	0.28868
DB00281	1095	SNX complex (SNX1a SNX2 SNX4 EGFR)	0.25
DB00281	1185	EGFR-containing signaling complex	0.25
DB00281	2369	ITGAV-ITGB3-EGFR complex	0.28868
DB00281	2453	Multiprotein complex (monoubiquitination)	0.25
DB00281	2454	CIN85-CBL-SH3GL2-EGFR complex EGF stimulated	0.25
DB00281	2542	EGFR-CBL-GRB2 complex	0.28868
DB00281	3678	RIN1-STAM2-EGFR complex EGF stimulated	0.28868
DB00281	5171	SH3KBP1-CBLB-EGFR complex	0.28868
DB00527	1223	H2AX complex isolated from cells without IR exposure	0.16013
DB00527	2242	TGM2-HD-CALM1 complex	0.33333
DB00527	4158	HSP90-FKBP38-CAM-Ca(2+) complex	0.28868
DB00527	5189	YWHAQ-CALM1-CABIN1 complex	0.33333

DB00527	5526	CALM1-FKBP38-BCL2 complex	0.33333
DB01173	5409	TIAM1-GRIN1 complex	0.26726
DB00128	3040	Multisynthetase complex	0.0658
DB00144	2537	PKC-alpha-PLD1-PLC-gamma-2 signaling complex lacritin stimulated	0.18257
DB00379	1211	Ubiquitin E3 ligase (AHR ARNT DDB1 TBL3 CUL4B RBX1)	0.28868
DB01035	860	DNMT1-G9a-PCNA complex	0.40825
DB01035	862	DNMT1-G9a complex	0.5
DB01035	1470	pRb2/p130-multimolecular complex (DNMT1 E2F5 SuV39H1 HDAC1 RBL2)	0.31623
DB01035	1488	DNMT1-RB1-HDAC1-E2F1 complex	0.35355
DB01035	1490	DAXX-DNMT1-DMAP1 complex	0.40825
DB01035	1491	RGS6-DNMT1-DMAP1 complex	0.40825
DB01035	5117	pRb2/p130-multimolecular complex (DNMT1 E2F4 SuV39H1 HDAC1 RBL2)	0.31623
DB01035	5695	TIP5-DNMT-HDAC1 complex	0.35355
DB01429	1223	H2AX complex isolated from cells without IR exposure	0.19612
DB01429	2242	TGM2-HD-CALM1 complex	0.40825
DB01429	4158	HSP90-FKBP38-CAM-Ca(2+) complex	0.35355
DB01429	5189	YWHAQ-CALM1-CABIN1 complex	0.40825
DB01429	5526	CALM1-FKBP38-BCL2 complex	0.40825
DB00149	3040	Multisynthetase complex	0.12309
DB00102	1096	SNX complex (SNX1 1a 2 4 PDGF receptor)	0.28868
DB00102	2476	CRKL-PDGFRA-CRK-RAPGEF1 complex	0.28868
DB00102	2551	PDGFRA-PLC-gamma-1-PI3K-SHP-2 complex PDGF stimulated	0.28868
DB00102	2710	LRP-1-Alpha-2-M-annexin VI complex	0.33333
DB00102	3183	PDGFRA-SHP-2 complex PDGF stimulated	0.40825
DB04942	5198	CBP-RARA-RXRA-DNA complex ligand stimulated	0.40825
DB00009	2343	ITGAV-ITGB5-PLAUR complex	0.28868
DB00013	845	PCI-PSA-SCG2 complex	0.18257
DB00013	2343	ITGAV-ITGB5-PLAUR complex	0.18257
DB00015	2343	ITGAV-ITGB5-PLAUR complex	0.28868
DB00029	2343	ITGAV-ITGB5-PLAUR complex	0.28868
DB00031	143	APP-FE65-LRP complex	0.17408
DB00031	1223	H2AX complex isolated from cells without IR exposure	0.08362
DB00031	1226	H2AX complex I	0.11396
DB00031	1227	H2AX complex II	0.09535
DB00031	2343	ITGAV-ITGB5-PLAUR complex	0.17408
DB00031	2709	MMP-9-TIMP-1-LRP complex	0.17408
DB00031	2710	LRP-1-Alpha-2-M-annexin VI complex	0.17408
DB00031	5217	Calreticulin oligomer complex	0.30151
DB00025	143	APP-FE65-LRP complex	0.17408
DB00025	929	CEN complex	0.04957
DB00025	1223	H2AX complex isolated from cells without IR exposure	0.16725
DB00025	1226	H2AX complex I	0.22792
DB00025	1227	H2AX complex II	0.19069
DB00025	1335	SNW1 complex	0.07107
DB00025	2709	MMP-9-TIMP-1-LRP complex	0.17408
DB00025	2710	LRP-1-Alpha-2-M-annexin VI complex	0.17408
DB00025	2721	HCF-1 complex	0.06917
DB00025	3082	DGCR8 multiprotein complex	0.09091
DB00025	3162	TF-FVIIa-FXa-TFPI complex	0.15076
DB00025	5217	Calreticulin oligomer complex	0.30151
DB00036	3162	TF-FVIIa-FXa-TFPI complex	0.8165
DB00569	3162	TF-FVIIa-FXa-TFPI complex	0.35355

DB01109	3162	TF-FVIIa-FXa-TFPI complex	0.28868
DB01225	3162	TF-FVIIa-FXa-TFPI complex	0.35355
DB06228	3162	TF-FVIIa-FXa-TFPI complex	0.5
DB06605	3162	TF-FVIIa-FXa-TFPI complex	0.5
DB00181	5418	GABBR2-HTR1A complex	0.5
DB00837	5809	GABAA receptor	0.40825
DB00228	563	F1F0-ATP synthase (EC 3.6.3.14) mitochondrial	0.05213
DB00228	1787	Nogo-potassium channel complex	0.10426
DB00228	5809	GABAA receptor	0.24077
DB00753	563	F1F0-ATP synthase (EC 3.6.3.14) mitochondrial	0.05213
DB00753	1223	H2AX complex isolated from cells without IR exposure	0.05783
DB00753	1787	Nogo-potassium channel complex	0.10426
DB00753	2242	TGM2-HD-CALM1 complex	0.12039
DB00753	4158	HSP90-FKBP38-CAM-Ca(2+) complex	0.10426
DB00753	5189	YWHAQ-CALM1-CABIN1 complex	0.12039
DB00753	5526	CALM1-FKBP38-BCL2 complex	0.12039
DB00753	5809	GABAA receptor	0.24077
DB01028	178	Respiratory chain complex I (holoenzyme) mitochondrial	0.03143
DB01028	563	F1F0-ATP synthase (EC 3.6.3.14) mitochondrial	0.05213
DB01028	1787	Nogo-potassium channel complex	0.10426
DB01028	2884	Respiratory chain complex I (early intermediate NDUFAF1 assembly) mitochondrial	0.07881
DB01028	2886	Respiratory chain complex I (incomplete intermediate ND1 ND2 ND3 CIA30 assembly) mitochondrial	0.10426
DB01028	2901	Respiratory chain complex I (intermediate IV/310kD) mitochondrial	0.10426
DB01028	2903	Respiratory chain complex I (intermediate V/380kD and VI/480kD) mitochondrial	0.09325
DB01028	2904	Respiratory chain complex I (intermediate VII/650kD) mitochondrial	0.06594
DB01028	2919	Respiratory chain complex I (gamma subunit) mitochondrial	0.05783
DB01028	2939	Ecsit complex (ECSIT MT-CO2 NDUFA1 MT-ND1 TRAF6 NDUFAF1)	0.08513
DB01028	2943	Respiratory chain complex I (incomplete NDUFAF1 assembly) mitochondrial	0.14744
DB01028	5809	GABAA receptor	0.24077
DB01159	178	Respiratory chain complex I (holoenzyme) mitochondrial	0.02624
DB01159	563	F1F0-ATP synthase (EC 3.6.3.14) mitochondrial	0.04352
DB01159	668	BKCA-beta2AR-AKAP79 signaling complex	0.1005
DB01159	672	BKCA-beta2AR complex	0.12309
DB01159	1539	G protein complex (GNG2 GNB2L1 RAF1)	0.1005
DB01159	1612	Heterotrimeric G protein complex (GNG2 GNB1 GNAS)	0.1005
DB01159	1614	G protein complex (MCF2 GNB1 GNG2)	0.1005
DB01159	1615	G protein complex (BTK GNG1 GNG2)	0.1005
DB01159	1617	G protein complex (CACNA1A GNB1 GNG2)	0.1005
DB01159	1618	G protein complex (PTHR1 GNB1 GNG2)	0.1005
DB01159	1619	G protein complex (HDAC5 GNB1 GNG2)	0.1005
DB01159	1620	G protein complex (HDAC4 GNB1 GNG2)	0.1005
DB01159	2884	Respiratory chain complex I (early intermediate NDUFAF1 assembly) mitochondrial	0.0658
DB01159	2886	Respiratory chain complex I (incomplete intermediate ND1 ND2 ND3 CIA30 assembly) mitochondrial	0.08704
DB01159	2901	Respiratory chain complex I (intermediate IV/310kD) mitochondrial	0.08704
DB01159	2903	Respiratory chain complex I (intermediate V/380kD and VI/480kD) mitochondrial	0.07785
DB01159	2904	Respiratory chain complex I (intermediate VII/650kD) mitochondrial	0.05505
DB01159	2919	Respiratory chain complex I (gamma subunit) mitochondrial	0.04828

DB01159	2939	Ecsit complex (ECSIT MT-CO2 NDUFA1 MT-ND1 TRAF6 NDUFAF1)	0.07107
DB01159	2943	Respiratory chain complex I (incomplete NDUFAF1 assembly) mitochondrial	0.12309
DB01159	5641	PSD95-FYN-NR2A complex	0.1005
DB01159	5809	GABAA receptor	0.20101
DB01189	178	Respiratory chain complex I (holoenzyme) mitochondrial	0.03214
DB01189	563	F1F0-ATP synthase (EC 3.6.3.14) mitochondrial	0.0533
DB01189	1787	Nogo-potassium channel complex	0.1066
DB01189	2884	Respiratory chain complex I (early intermediate NDUFAF1 assembly) mitochondrial	0.08058
DB01189	2886	Respiratory chain complex I (incomplete intermediate ND1 ND2 ND3 CIA30 assembly) mitochondrial	0.1066
DB01189	2901	Respiratory chain complex I (intermediate IV/310kD) mitochondrial	0.1066
DB01189	2903	Respiratory chain complex I (intermediate V/380kD and VI/480kD) mitochondrial	0.09535
DB01189	2904	Respiratory chain complex I (intermediate VII/650kD) mitochondrial	0.06742
DB01189	2919	Respiratory chain complex I (gamma subunit) mitochondrial	0.05913
DB01189	2939	Ecsit complex (ECSIT MT-CO2 NDUFA1 MT-ND1 TRAF6 NDUFAF1)	0.08704
DB01189	2943	Respiratory chain complex I (incomplete NDUFAF1 assembly) mitochondrial	0.15076
DB01189	5809	GABAA receptor	0.24618
DB01236	178	Respiratory chain complex I (holoenzyme) mitochondrial	0.03143
DB01236	563	F1F0-ATP synthase (EC 3.6.3.14) mitochondrial	0.05213
DB01236	1787	Nogo-potassium channel complex	0.10426
DB01236	2884	Respiratory chain complex I (early intermediate NDUFAF1 assembly) mitochondrial	0.07881
DB01236	2886	Respiratory chain complex I (incomplete intermediate ND1 ND2 ND3 CIA30 assembly) mitochondrial	0.10426
DB01236	2901	Respiratory chain complex I (intermediate IV/310kD) mitochondrial	0.10426
DB01236	2903	Respiratory chain complex I (intermediate V/380kD and VI/480kD) mitochondrial	0.09325
DB01236	2904	Respiratory chain complex I (intermediate VII/650kD) mitochondrial	0.06594
DB01236	2919	Respiratory chain complex I (gamma subunit) mitochondrial	0.05783
DB01236	2939	Ecsit complex (ECSIT MT-CO2 NDUFA1 MT-ND1 TRAF6 NDUFAF1)	0.08513
DB01236	2943	Respiratory chain complex I (incomplete NDUFAF1 assembly) mitochondrial	0.14744
DB01236	5809	GABAA receptor	0.24077
DB00898	5809	GABAA receptor	0.21822
DB01367	1062	BAR-BCL2-CASP8 complex	0.40825
DB01367	5526	CALM1-FKBP38-BCL2 complex	0.40825
DB01367	5811	p53-BCL2 complex	0.5
DB01367	5817	tBID-BCL2 complex	0.5
DB01367	5818	BIM-BCL2 complex	0.5
DB00242	234	HuCHRAC complex	0.16667
DB00242	1003	RC complex (Replication competent complex)	0.11111
DB00242	1004	RC complex during S-phase of cell cycle	0.1849
DB00242	1005	RC complex during G2/M-phase of cell cycle	0.1849
DB00242	1098	DNA synthesome complex (13 subunits)	0.08909
DB00242	1099	DNA synthesome complex (17 subunits)	0.39284
DB00242	1100	DNA polymerase alpha-primase complex	0.16667
DB00242	1107	DNA synthesome core complex	0.10541
DB00242	1108	DNA synthesome complex (15 subunits)	0.43033
DB00615	5718	eNOS-HSP90-AKT complex VEGF induced	0.2582
DB00615	5716	eNOS-HSP90 complex VEGF induced	0.31623

DB00615	2112	CDC37-HSP90AA1-HSP90AB1-MAP3K11 complex	0.22361
DB00615	2721	HCF-1 complex	0.1026
DB00615	4158	HSP90-FKBP38-CAM-Ca(2+) complex	0.22361
DB00615	5199	Kinase maturation complex 1	0.1118
DB00615	5212	Kinase maturation complex 2	0.15811
DB00615	5234	IKBKB-CDC37-KIAA1967-HSP90AB1-HSP90AA1 complex	0.2
DB00615	5266	TNF-alpha/NF-kappa B signaling complex 6	0.11952
DB00615	5268	TNF-alpha/NF-kappa B signaling complex 7	0.15811
DB00615	5269	TNF-alpha/NF-kappa B signaling complex 8	0.18257
DB00615	5286	TNF-alpha/NF-kappa B signaling complex 10	0.14142
DB00615	5622	HSP90-CIP1-FKBPL complex	0.2582
DB00480	826	PAR-3-VE-cadherin-beta-catenin complex	0.28868
DB00480	829	PAR-6-VE-cadherin complex endothelial	0.28868
DB00480	831	PAR-6-PAR-3-VE-cadherin complex endothelial	0.25
DB00480	1439	PTGS2 homodimer complex	0.5
DB00480	5772	ZO1-(beta)cadherin-(VE)cadherin-VEGFR2 complex	0.25
DB00482	1439	PTGS2 homodimer complex	0.70711
DB01041	575	ABIN2-NFKB1-MAP3K8 complex	0.2582
DB01041	1439	PTGS2 homodimer complex	0.44721
DB01041	2084	NFKB1-NFKB2-REL-RELA-RELB complex	0.2
DB01041	2086	NFKB1-NFKB2-RELA-RELB complex	0.22361
DB01041	2563	FGFR2-c-Cbl-Lyn-Fyn complex	0.22361
DB01041	3045	hs4 enhancer complex (faster migrating complex)	0.2
DB01041	5193	TNF-alpha/NF-kappa B signaling complex (CHUK KPNA3 NFKB2 NFKBIB REL IKBKG NFKB1 NFKBIE RELB NFKBIA RELA TNIP2)	0.1291
DB01041	5230	CHUK-NFKB2-REL-IKBKG-SPAG9-NFKB1-NFKBIE-COPB2-TNIP1-NFKBIA-RELA-TNIP2 complex	0.1291
DB01041	5232	TNF-alpha/Nf-kappa B signaling complex (RPL6 RPL30 RPS13 CHUK DDX3X NFKB2 NFKBIB REL IKBKG NFKB1 MAP3K8 RELB GLG1 NFKBIA RELA TNIP2 GTF2I)	0.10847
DB01041	5233	TNF-alpha/NF-kappa B signaling complex 5	0.08944
DB01041	5460	p50-p65 NF(kappa)B complex	0.31623
DB01041	5461	p50-p65 NF(kappa)B-SRC1 complex	0.2582
DB01041	5464	I(kappa)B(alpha)-NF(kappa)Bp50-NF(kappa)Bp65 complex	0.2582
DB01041	5492	IKBA-NF(kappa)Bp65-NF(kappa)Bp50 complex	0.2582
DB01404	1211	Ubiquitin E3 ligase (AHR ARNT DDB1 TBL3 CUL4B RBX1)	0.2357
DB01404	1439	PTGS2 homodimer complex	0.57735
DB01628	1439	PTGS2 homodimer complex	1
DB08910	1439	PTGS2 homodimer complex	0.57735
DB00436	668	BKCA-beta2AR-AKAP79 signaling complex	0.2582
DB00436	672	BKCA-beta2AR complex	0.31623
DB00774	668	BKCA-beta2AR-AKAP79 signaling complex	0.20412
DB00774	672	BKCA-beta2AR complex	0.25
DB00999	668	BKCA-beta2AR-AKAP79 signaling complex	0.21822
DB00999	672	BKCA-beta2AR complex	0.26726
DB01119	668	BKCA-beta2AR-AKAP79 signaling complex	0.2357
DB01119	672	BKCA-beta2AR complex	0.28868
DB00075	2489	NCR3-CD247 complex	0.18898
DB00075	2909	PLC-gamma-2-Syk-LAT-FcR-gamma complex	0.13363
DB00075	2910	PLC-gamma-2-Lyn-FcR-gamma complex	0.1543
DB00407	1256	MLL-HCF complex	0.26726
DB06271	1256	MLL-HCF complex	0.26726
DB06822	1810	ITGA4-PXN-GIT1 complex	0.33333
DB06822	2417	ITGA4-ITGB1-EMILIN1 complex	0.33333

DB06822	2418	ITGA4-ITGB1 complex	0.40825
DB06822	2419	ITGA4-ITGB1-CD81 complex	0.33333
DB06822	2420	ITGA4-ITGB1-CD53 complex	0.33333
DB06822	2421	ITGA4-ITGB1-VCAM1 complex	0.33333
DB06822	2422	ITGA4-ITGB1-JAM2 complex	0.33333
DB06822	2423	ITGA4-ITGB1-CD47 complex	0.33333
DB06822	2424	ITGA4-ITGB1-CD63 complex	0.33333
DB06822	2425	ITGA4-ITGB1-PXN complex	0.33333
DB06822	2426	ITGA4-ITGB1-THBS1 complex	0.33333
DB06822	2428	ITGA4-ITGB1-THBS2 complex	0.33333
DB08813	1170	cMYC-ATPase-helicase complex	0.22361
DB08813	1171	c-MYC-ATPase-helicase complex	0.22361
DB08813	2649	MYC-DNMT3A-ZBTB17 complex	0.28868
DB08813	2653	MYC-MAX-BLOC1S1 complex	0.28868
DB08813	2655	MYC-MAX complex	0.35355
DB08813	2692	SMAD3-SMAD4-cJun-cFos complex	0.25
DB08813	2693	NFAT-JUN-FOS DNA-protein complex	0.28868
DB08813	2694	ERG-JUN-FOS DNA-protein complex	0.28868
DB08813	2695	ETS2-FOS-JUN complex	0.28868
DB00217	5747	2AR-mGluR2 complex	0.35355
DB00320	5411	EDG1-HTR1D complex	0.35355
DB00320	5412	HTR1D homodimer complex	0.5
DB00320	5414	HTR1A-HTR1D complex	0.35355
DB00320	5415	HTR1B homodimer complex	0.5
DB00320	5416	HTR1A-HTR1B complex	0.35355
DB00320	5417	HTR1D-HTR1B complex	0.70711
DB00320	5747	2AR-mGluR2 complex	0.35355
DB00370	5747	2AR-mGluR2 complex	0.28868
DB00449	668	BKCA-beta2AR-AKAP79 signaling complex	0.28868
DB00449	672	BKCA-beta2AR complex	0.35355
DB00449	687	CFTR-NHERF-beta(2)AR signaling complex	0.28868
DB00449	3830	ADRB2 homodimer complex	0.5
DB00449	5747	2AR-mGluR2 complex	0.35355
DB00484	5747	2AR-mGluR2 complex	0.40825
DB00575	5747	2AR-mGluR2 complex	0.40825
DB00629	5747	2AR-mGluR2 complex	0.70711
DB00633	5747	2AR-mGluR2 complex	0.70711
DB00656	5414	HTR1A-HTR1D complex	0.26726
DB00656	5416	HTR1A-HTR1B complex	0.26726
DB00656	5418	GABBR2-HTR1A complex	0.26726
DB00656	5419	HTR1A-GPR26 complex	0.26726
DB00656	5420	HTR1A-EDG3 complex	0.26726
DB00656	5421	HTR1A homodimer complex	0.37796
DB00656	5422	HTR1A-EDG1 complex	0.26726
DB00656	5747	2AR-mGluR2 complex	0.26726
DB00692	5747	2AR-mGluR2 complex	0.5
DB00696	5411	EDG1-HTR1D complex	0.22361
DB00696	5412	HTR1D homodimer complex	0.31623
DB00696	5414	HTR1A-HTR1D complex	0.22361
DB00696	5415	HTR1B homodimer complex	0.31623
DB00696	5416	HTR1A-HTR1B complex	0.22361
DB00696	5417	HTR1D-HTR1B complex	0.44721
DB00696	5747	2AR-mGluR2 complex	0.22361
DB00697	5747	2AR-mGluR2 complex	0.35355

DB00797	5747	2AR-mGluR2 complex	0.40825
DB00925	668	BKCA-beta2AR-AKAP79 signaling complex	0.2357
DB00925	672	BKCA-beta2AR complex	0.28868
DB00925	687	CFTR-NHERF-beta(2)AR signaling complex	0.2357
DB00925	1223	H2AX complex isolated from cells without IR exposure	0.11323
DB00925	2242	TGM2-HD-CALM1 complex	0.2357
DB00925	3830	ADRB2 homodimer complex	0.40825
DB00925	4158	HSP90-FKBP38-CAM-Ca(2+) complex	0.20412
DB00925	5189	YWHAQ-CALM1-CABIN1 complex	0.2357
DB00925	5526	CALM1-FKBP38-BCL2 complex	0.2357
DB00925	5747	2AR-mGluR2 complex	0.28868
DB00935	5747	2AR-mGluR2 complex	0.5
DB00964	5747	2AR-mGluR2 complex	0.5
DB00968	5747	2AR-mGluR2 complex	0.70711
DB01018	5747	2AR-mGluR2 complex	0.70711
DB01149	5414	HTR1A-HTR1D complex	0.2357
DB01149	5416	HTR1A-HTR1B complex	0.2357
DB01149	5418	GABBR2-HTR1A complex	0.2357
DB01149	5419	HTR1A-GPR26 complex	0.2357
DB01149	5420	HTR1A-EDG3 complex	0.2357
DB01149	5421	HTR1A homodimer complex	0.33333
DB01149	5422	HTR1A-EDG1 complex	0.2357
DB01149	5747	2AR-mGluR2 complex	0.2357
DB01392	5411	EDG1-HTR1D complex	0.22361
DB01392	5412	HTR1D homodimer complex	0.31623
DB01392	5414	HTR1A-HTR1D complex	0.44721
DB01392	5415	HTR1B homodimer complex	0.31623
DB01392	5416	HTR1A-HTR1B complex	0.44721
DB01392	5417	HTR1D-HTR1B complex	0.44721
DB01392	5418	GABBR2-HTR1A complex	0.22361
DB01392	5419	HTR1A-GPR26 complex	0.22361
DB01392	5420	HTR1A-EDG3 complex	0.22361
DB01392	5421	HTR1A homodimer complex	0.31623
DB01392	5422	HTR1A-EDG1 complex	0.22361
DB01392	5747	2AR-mGluR2 complex	0.22361
DB04948	5747	2AR-mGluR2 complex	0.70711
DB06148	5747	2AR-mGluR2 complex	0.28868
DB06623	5747	2AR-mGluR2 complex	0.70711
DB06694	5747	2AR-mGluR2 complex	0.28868
DB06711	5747	2AR-mGluR2 complex	0.5
DB08815	5414	HTR1A-HTR1D complex	0.28868
DB08815	5416	HTR1A-HTR1B complex	0.28868
DB08815	5418	GABBR2-HTR1A complex	0.28868
DB08815	5419	HTR1A-GPR26 complex	0.28868
DB08815	5420	HTR1A-EDG3 complex	0.28868
DB08815	5421	HTR1A homodimer complex	0.40825
DB08815	5422	HTR1A-EDG1 complex	0.28868
DB08815	5747	2AR-mGluR2 complex	0.28868
DB00216	5411	EDG1-HTR1D complex	0.26726
DB00216	5412	HTR1D homodimer complex	0.37796
DB00216	5414	HTR1A-HTR1D complex	0.53452
DB00216	5415	HTR1B homodimer complex	0.37796
DB00216	5416	HTR1A-HTR1B complex	0.53452
DB00216	5417	HTR1D-HTR1B complex	0.53452

DB00216	5418	GABBR2-HTR1A complex	0.26726
DB00216	5419	HTR1A-GPR26 complex	0.26726
DB00216	5420	HTR1A-EDG3 complex	0.26726
DB00216	5421	HTR1A homodimer complex	0.37796
DB00216	5422	HTR1A-EDG1 complex	0.26726
DB00247	5414	HTR1A-HTR1D complex	0.31623
DB00247	5416	HTR1A-HTR1B complex	0.31623
DB00247	5418	GABBR2-HTR1A complex	0.31623
DB00247	5419	HTR1A-GPR26 complex	0.31623
DB00247	5420	HTR1A-EDG3 complex	0.31623
DB00247	5421	HTR1A homodimer complex	0.44721
DB00247	5422	HTR1A-EDG1 complex	0.31623
DB00315	5411	EDG1-HTR1D complex	0.35355
DB00315	5412	HTR1D homodimer complex	0.5
DB00315	5414	HTR1A-HTR1D complex	0.70711
DB00315	5415	HTR1B homodimer complex	0.5
DB00315	5416	HTR1A-HTR1B complex	0.70711
DB00315	5417	HTR1D-HTR1B complex	0.70711
DB00315	5418	GABBR2-HTR1A complex	0.35355
DB00315	5419	HTR1A-GPR26 complex	0.35355
DB00315	5420	HTR1A-EDG3 complex	0.35355
DB00315	5421	HTR1A homodimer complex	0.5
DB00315	5422	HTR1A-EDG1 complex	0.35355
DB00490	5414	HTR1A-HTR1D complex	0.5
DB00490	5416	HTR1A-HTR1B complex	0.5
DB00490	5418	GABBR2-HTR1A complex	0.5
DB00490	5419	HTR1A-GPR26 complex	0.5
DB00490	5420	HTR1A-EDG3 complex	0.5
DB00490	5421	HTR1A homodimer complex	0.70711
DB00490	5422	HTR1A-EDG1 complex	0.5
DB00669	5411	EDG1-HTR1D complex	0.35355
DB00669	5412	HTR1D homodimer complex	0.5
DB00669	5414	HTR1A-HTR1D complex	0.70711
DB00669	5415	HTR1B homodimer complex	0.5
DB00669	5416	HTR1A-HTR1B complex	0.70711
DB00669	5417	HTR1D-HTR1B complex	0.70711
DB00669	5418	GABBR2-HTR1A complex	0.35355
DB00669	5419	HTR1A-GPR26 complex	0.35355
DB00669	5420	HTR1A-EDG3 complex	0.35355
DB00669	5421	HTR1A homodimer complex	0.5
DB00669	5422	HTR1A-EDG1 complex	0.35355
DB00952	5411	EDG1-HTR1D complex	0.35355
DB00952	5412	HTR1D homodimer complex	0.5
DB00952	5414	HTR1A-HTR1D complex	0.70711
DB00952	5415	HTR1B homodimer complex	0.5
DB00952	5416	HTR1A-HTR1B complex	0.70711
DB00952	5417	HTR1D-HTR1B complex	0.70711
DB00952	5418	GABBR2-HTR1A complex	0.35355
DB00952	5419	HTR1A-GPR26 complex	0.35355
DB00952	5420	HTR1A-EDG3 complex	0.35355
DB00952	5421	HTR1A homodimer complex	0.5
DB00952	5422	HTR1A-EDG1 complex	0.35355
DB01616	5414	HTR1A-HTR1D complex	0.70711
DB01616	5416	HTR1A-HTR1B complex	0.70711

DB01616	5418	GABBR2-HTR1A complex	0.70711
DB01616	5419	HTR1A-GPR26 complex	0.70711
DB01616	5420	HTR1A-EDG3 complex	0.70711
DB01616	5421	HTR1A homodimer complex	1
DB01616	5422	HTR1A-EDG1 complex	0.70711
DB06684	5414	HTR1A-HTR1D complex	0.70711
DB06684	5416	HTR1A-HTR1B complex	0.70711
DB06684	5418	GABBR2-HTR1A complex	0.70711
DB06684	5419	HTR1A-GPR26 complex	0.70711
DB06684	5420	HTR1A-EDG3 complex	0.70711
DB06684	5421	HTR1A homodimer complex	1
DB06684	5422	HTR1A-EDG1 complex	0.70711
DB00393	786	MR-UBC9-SRC1 complex	0.18257
DB00393	1211	Ubiquitin E3 ligase (AHR ARNT DDB1 TBL3 CUL4B RBX1)	0.1291
DB00393	3634	NR3C2-UBC9-SRC-1 complex	0.18257
DB01043	5641	PSD95-FYN-NR2A complex	0.28868
DB00279	66	TRAP complex	0.25
DB00279	535	TRAP complex	0.17678
DB00279	5367	THRB-RXRB complex	0.5
DB00279	5615	Emerin complex 52	0.14744
DB00451	66	TRAP complex	0.25
DB00451	535	TRAP complex	0.17678
DB00451	5367	THRB-RXRB complex	0.5
DB00451	5615	Emerin complex 52	0.14744
DB00509	66	TRAP complex	0.20412
DB00509	535	TRAP complex	0.14434
DB00509	5367	THRB-RXRB complex	0.40825
DB00509	5615	Emerin complex 52	0.12039
DB01583	66	TRAP complex	0.25
DB01583	535	TRAP complex	0.17678
DB01583	5367	THRB-RXRB complex	0.5
DB01583	5615	Emerin complex 52	0.14744
DB00631	1003	RC complex (Replication competent complex)	0.2357
DB00631	1004	RC complex during S-phase of cell cycle	0.19612
DB00631	1005	RC complex during G2/M-phase of cell cycle	0.19612
DB00631	1098	DNA synthesome complex (13 subunits)	0.18898
DB00631	1099	DNA synthesome complex (17 subunits)	0.16667
DB00631	1100	DNA polymerase alpha-primase complex	0.35355
DB00631	1107	DNA synthesome core complex	0.22361
DB00631	1108	DNA synthesome complex (15 subunits)	0.18257
DB01073	1003	RC complex (Replication competent complex)	0.19245
DB01073	1004	RC complex during S-phase of cell cycle	0.16013
DB01073	1005	RC complex during G2/M-phase of cell cycle	0.16013
DB01073	1098	DNA synthesome complex (13 subunits)	0.1543
DB01073	1099	DNA synthesome complex (17 subunits)	0.13608
DB01073	1100	DNA polymerase alpha-primase complex	0.28868
DB01073	1107	DNA synthesome core complex	0.18257
DB01073	1108	DNA synthesome complex (15 subunits)	0.14907
DB00686	1069	FIF-FGR2 complex	0.40825
DB00980	1976	MTNR1A homodimer complex	0.70711
DB00980	1977	MTNR1B homodimer complex	0.70711
DB00980	1978	MTNR1A-MTNR1B complex	1
DB06594	1976	MTNR1A homodimer complex	0.57735
DB06594	1977	MTNR1B homodimer complex	0.57735

DB06594	1978	MTNR1A-MTNR1B complex	0.8165
DB00635	4216	GR-hnRNP U complex	0.5
DB00230	1617	G protein complex (CACNA1A GNB1 GNG2)	0.57735
DB00836	1223	H2AX complex isolated from cells without IR exposure	0.11323
DB00836	1617	G protein complex (CACNA1A GNB1 GNG2)	0.2357
DB00836	2242	TGM2-HD-CALM1 complex	0.2357
DB00836	4158	HSP90-FKBP38-CAM-Ca(2+) complex	0.20412
DB00836	5189	YWHAQ-CALM1-CABIN1 complex	0.2357
DB00836	5526	CALM1-FKBP38-BCL2 complex	0.2357
DB01244	520	KCNQ1 macromolecular complex	0.10541
DB01244	1223	H2AX complex isolated from cells without IR exposure	0.09245
DB01244	1617	G protein complex (CACNA1A GNB1 GNG2)	0.19245
DB01244	2242	TGM2-HD-CALM1 complex	0.19245
DB01244	4158	HSP90-FKBP38-CAM-Ca(2+) complex	0.16667
DB01244	5189	YWHAQ-CALM1-CABIN1 complex	0.19245
DB01244	5526	CALM1-FKBP38-BCL2 complex	0.19245
DB00454	5409	TIAM1-GRIN1 complex	0.28868
DB00454	5641	PSD95-FYN-NR2A complex	0.2357
DB04896	5409	TIAM1-GRIN1 complex	0.40825
DB00218	924	Toposome	0.21822
DB00218	1098	DNA synthesome complex (13 subunits)	0.1543
DB00218	1099	DNA synthesome complex (17 subunits)	0.13608
DB00218	1183	CDC5L complex	0.10541
DB00218	1728	CTCF-nucleophosmin-PARP-HIS-KPNA-LMNA-TOP complex	0.19245
DB00467	924	Toposome	0.21822
DB00467	1098	DNA synthesome complex (13 subunits)	0.1543
DB00467	1099	DNA synthesome complex (17 subunits)	0.13608
DB00467	1183	CDC5L complex	0.10541
DB00467	1728	CTCF-nucleophosmin-PARP-HIS-KPNA-LMNA-TOP complex	0.19245
DB00487	924	Toposome	0.21822
DB00487	1098	DNA synthesome complex (13 subunits)	0.1543
DB00487	1099	DNA synthesome complex (17 subunits)	0.13608
DB00487	1183	CDC5L complex	0.10541
DB00487	1728	CTCF-nucleophosmin-PARP-HIS-KPNA-LMNA-TOP complex	0.19245
DB00537	924	Toposome	0.21822
DB00537	1098	DNA synthesome complex (13 subunits)	0.1543
DB00537	1099	DNA synthesome complex (17 subunits)	0.13608
DB00537	1183	CDC5L complex	0.10541
DB00537	1728	CTCF-nucleophosmin-PARP-HIS-KPNA-LMNA-TOP complex	0.19245
DB00685	924	Toposome	0.21822
DB00685	1098	DNA synthesome complex (13 subunits)	0.1543
DB00685	1099	DNA synthesome complex (17 subunits)	0.13608
DB00685	1183	CDC5L complex	0.10541
DB00685	1728	CTCF-nucleophosmin-PARP-HIS-KPNA-LMNA-TOP complex	0.19245
DB00978	924	Toposome	0.21822
DB00978	1098	DNA synthesome complex (13 subunits)	0.1543
DB00978	1099	DNA synthesome complex (17 subunits)	0.13608
DB00978	1183	CDC5L complex	0.10541
DB00978	1728	CTCF-nucleophosmin-PARP-HIS-KPNA-LMNA-TOP complex	0.19245
DB01059	924	Toposome	0.21822
DB01059	1098	DNA synthesome complex (13 subunits)	0.1543
DB01059	1099	DNA synthesome complex (17 subunits)	0.13608
DB01059	1183	CDC5L complex	0.10541
DB01059	1728	CTCF-nucleophosmin-PARP-HIS-KPNA-LMNA-TOP complex	0.19245

DB01137	924	Toposome	0.21822
DB01137	1098	DNA synthesome complex (13 subunits)	0.1543
DB01137	1099	DNA synthesome complex (17 subunits)	0.13608
DB01137	1183	CDC5L complex	0.10541
DB01137	1728	CTCF-nucleophosmin-PARP-HIS-KPNA-LMNA-TOP complex	0.19245
DB01165	924	Toposome	0.21822
DB01165	1098	DNA synthesome complex (13 subunits)	0.1543
DB01165	1099	DNA synthesome complex (17 subunits)	0.13608
DB01165	1183	CDC5L complex	0.10541
DB01165	1728	CTCF-nucleophosmin-PARP-HIS-KPNA-LMNA-TOP complex	0.19245
DB01208	924	Toposome	0.21822
DB01208	1098	DNA synthesome complex (13 subunits)	0.1543
DB01208	1099	DNA synthesome complex (17 subunits)	0.13608
DB01208	1183	CDC5L complex	0.10541
DB01208	1728	CTCF-nucleophosmin-PARP-HIS-KPNA-LMNA-TOP complex	0.19245
DB04576	924	Toposome	0.21822
DB04576	1098	DNA synthesome complex (13 subunits)	0.1543
DB04576	1099	DNA synthesome complex (17 subunits)	0.13608
DB04576	1183	CDC5L complex	0.10541
DB04576	1728	CTCF-nucleophosmin-PARP-HIS-KPNA-LMNA-TOP complex	0.19245
DB08875	903	RET-Rai complex	0.40825
DB08875	2456	MET-CIN85-SH3GL3-CBL complex HGF stimulated	0.28868
DB08875	2541	HGF-Met complex	0.40825
DB08875	4062	NRP1-VEGFR2-VEGF(165) complex	0.33333
DB08875	5772	ZO1-(beta)cadherin-(VE)cadherin-VEGFR2 complex	0.28868
DB08875	5696	VEGFA(165)-KDR-NRP1 complex	0.33333
DB08875	5698	VEGFA(165)-VEGFR2-NRP1 complex	0.33333
DB00091	2559	p56(LCK)-CAML complex	0.40825
DB00091	5843	AIF-CYPA-DNA complex	0.40825
DB00241	2258	VILIP-1-AChR-alpha-4-AChR-beta-2 complex	0.11323
DB00241	2272	PICK1-GRIP1-GLUR2 complex	0.11323
DB00241	5747	2AR-mGluR2 complex	0.13868
DB00241	5809	GABAA receptor	0.22646
DB00306	2258	VILIP-1-AChR-alpha-4-AChR-beta-2 complex	0.11323
DB00306	2272	PICK1-GRIP1-GLUR2 complex	0.11323
DB00306	5747	2AR-mGluR2 complex	0.13868
DB00306	5809	GABAA receptor	0.22646
DB00312	2258	VILIP-1-AChR-alpha-4-AChR-beta-2 complex	0.11323
DB00312	2272	PICK1-GRIP1-GLUR2 complex	0.11323
DB00312	5747	2AR-mGluR2 complex	0.13868
DB00312	5809	GABAA receptor	0.22646
DB00371	5809	GABAA receptor	0.24618
DB00402	5809	GABAA receptor	0.25198
DB00418	2258	VILIP-1-AChR-alpha-4-AChR-beta-2 complex	0.18257
DB00418	2272	PICK1-GRIP1-GLUR2 complex	0.18257
DB00418	5747	2AR-mGluR2 complex	0.22361
DB00418	5809	GABAA receptor	0.18257
DB00425	5809	GABAA receptor	0.33333
DB00463	2258	VILIP-1-AChR-alpha-4-AChR-beta-2 complex	0.11323
DB00463	2272	PICK1-GRIP1-GLUR2 complex	0.11323
DB00463	5747	2AR-mGluR2 complex	0.13868
DB00463	5809	GABAA receptor	0.22646
DB00599	2258	VILIP-1-AChR-alpha-4-AChR-beta-2 complex	0.17408
DB00599	2272	PICK1-GRIP1-GLUR2 complex	0.17408

DB00599	5747	2AR-mGluR2 complex	0.2132
DB00599	5809	GABAA receptor	0.17408
DB00794	2258	VILIP-1-AChR-alpha-4-AChR-beta-2 complex	0.11323
DB00794	2272	PICK1-GRIP1-GLUR2 complex	0.11323
DB00794	5747	2AR-mGluR2 complex	0.13868
DB00794	5809	GABAA receptor	0.22646
DB00849	2258	VILIP-1-AChR-alpha-4-AChR-beta-2 complex	0.18257
DB00849	2272	PICK1-GRIP1-GLUR2 complex	0.18257
DB00849	5747	2AR-mGluR2 complex	0.22361
DB00849	5809	GABAA receptor	0.18257
DB01174	2258	VILIP-1-AChR-alpha-4-AChR-beta-2 complex	0.18257
DB01174	2272	PICK1-GRIP1-GLUR2 complex	0.18257
DB01174	5747	2AR-mGluR2 complex	0.22361
DB01174	5809	GABAA receptor	0.18257
DB01198	5809	GABAA receptor	0.2582
DB01351	2258	VILIP-1-AChR-alpha-4-AChR-beta-2 complex	0.18257
DB01351	2272	PICK1-GRIP1-GLUR2 complex	0.18257
DB01351	5747	2AR-mGluR2 complex	0.22361
DB01351	5809	GABAA receptor	0.18257
DB01352	2258	VILIP-1-AChR-alpha-4-AChR-beta-2 complex	0.18257
DB01352	2272	PICK1-GRIP1-GLUR2 complex	0.18257
DB01352	5747	2AR-mGluR2 complex	0.22361
DB01352	5809	GABAA receptor	0.18257
DB01353	2258	VILIP-1-AChR-alpha-4-AChR-beta-2 complex	0.18257
DB01353	2272	PICK1-GRIP1-GLUR2 complex	0.18257
DB01353	5747	2AR-mGluR2 complex	0.22361
DB01353	5809	GABAA receptor	0.18257
DB01354	2258	VILIP-1-AChR-alpha-4-AChR-beta-2 complex	0.18257
DB01354	2272	PICK1-GRIP1-GLUR2 complex	0.18257
DB01354	5747	2AR-mGluR2 complex	0.22361
DB01354	5809	GABAA receptor	0.18257
DB01355	2258	VILIP-1-AChR-alpha-4-AChR-beta-2 complex	0.18257
DB01355	2272	PICK1-GRIP1-GLUR2 complex	0.18257
DB01355	5747	2AR-mGluR2 complex	0.22361
DB01355	5809	GABAA receptor	0.18257
DB01097	1211	Ubiquitin E3 ligase (AHR ARNT DDB1 TBL3 CUL4B RBX1)	0.2357
DB01097	2363	ITGAV-ITGB3-PXN-PTK2b complex	0.28868
DB01097	5709	ArgBP2a-CBL-PTK2B complex	0.33333
DB00008	552	IFNB1-IFNAR1-IFNAR2- complex	0.8165
DB00011	552	IFNB1-IFNAR1-IFNAR2- complex	0.8165
DB00018	552	IFNB1-IFNAR1-IFNAR2- complex	0.8165
DB00022	552	IFNB1-IFNAR1-IFNAR2- complex	0.8165
DB00034	552	IFNB1-IFNAR1-IFNAR2- complex	0.8165
DB00060	552	IFNB1-IFNAR1-IFNAR2- complex	0.8165
DB00068	552	IFNB1-IFNAR1-IFNAR2- complex	0.8165
DB00069	552	IFNB1-IFNAR1-IFNAR2- complex	0.8165
DB00105	552	IFNB1-IFNAR1-IFNAR2- complex	0.8165
DB00083	706	SNARE complex (HGS SNAP25 STX13)	0.40825
DB00083	707	SNARE complex (VAMP2 SNAP25 STX13)	0.40825
DB00083	793	SNARE complex (VAMP2 SNAP25 STX1a CPLX1)	0.35355
DB00083	794	SNARE complex (VAMP2 SNAP25 STX1a CPLX2)	0.35355
DB00083	1137	SNARE complex (VAMP2 SNAP25 STX1a CPLX1 CPLX3)	0.31623
DB00083	1138	SNARE complex (VAMP2 SNAP25 STX1a CPLX3 CPLX4)	0.31623

DB00083	1139	SNARE complex (VAMP2 SNAP25 STX1a STX3 CPLX1 CPLX3 CPLX4)	0.26726
DB00083	1874	SNARE complex (SNAP25 VAMP3 VAMP2 NAPB STX13)	0.31623
DB00019	5388	SERPINA1-ELA2 complex	0.5
DB00058	5388	SERPINA1-ELA2 complex	0.70711
DB00099	5388	SERPINA1-ELA2 complex	0.5
DB00184	2258	VILIP-1-AChR-alpha-4-AChR-beta-2 complex	0.34816
DB00674	2258	VILIP-1-AChR-alpha-4-AChR-beta-2 complex	0.27217
DB00949	5641	PSD95-FYN-NR2A complex	0.33333
DB00831	1223	H2AX complex isolated from cells without IR exposure	0.11323
DB00831	2242	TGM2-HD-CALM1 complex	0.2357
DB00831	4158	HSP90-FKBP38-CAM-Ca(2+) complex	0.20412
DB00831	5189	YWHAQ-CALM1-CABIN1 complex	0.2357
DB00831	5526	CALM1-FKBP38-BCL2 complex	0.2357
DB01023	786	MR-UBC9-SRC1 complex	0.16013
DB01023	1223	H2AX complex isolated from cells without IR exposure	0.07692
DB01023	2242	TGM2-HD-CALM1 complex	0.16013
DB01023	3634	NR3C2-UBC9-SRC-1 complex	0.16013
DB01023	4158	HSP90-FKBP38-CAM-Ca(2+) complex	0.13868
DB01023	5189	YWHAQ-CALM1-CABIN1 complex	0.16013
DB01023	5526	CALM1-FKBP38-BCL2 complex	0.16013
DB01115	1223	H2AX complex isolated from cells without IR exposure	0.09806
DB01115	1787	Nogo-potassium channel complex	0.17678
DB01115	2242	TGM2-HD-CALM1 complex	0.20412
DB01115	4158	HSP90-FKBP38-CAM-Ca(2+) complex	0.17678
DB01115	5189	YWHAQ-CALM1-CABIN1 complex	0.20412
DB01115	5526	CALM1-FKBP38-BCL2 complex	0.20412
DB00288	4216	GR-hnRNP U complex	0.5
DB00741	4216	GR-hnRNP U complex	0.5
DB00122	2537	PKC-alpha-PLD1-PLC-gamma-2 signaling complex lacri[illegible] stimulated	0.21822
DB01169	55	HDAC4-ERK1 complex	0.26726
DB01169	57	HDAC4-ERK2 complex	0.26726
DB01169	310	Cell cycle kinase complex CDC2	0.1543
DB01169	311	Cell cycle kinase complex CDK2	0.18898
DB01169	312	Cell cycle kinase complex CDK4	0.18898
DB01169	313	Cell cycle kinase complex CDK5	0.16903
DB01169	1633	CyclinD1-CDK4-CDK6 complex	0.21822
DB01169	1634	CyclinD1-CDK4-p21 complex	0.21822
DB01169	5718	eNOS-HSP90-AKT complex VEGF induced	0.21822
DB01169	1816	JUN-TCF4-CTNNB1 complex	0.21822
DB01169	2055	CASP8-CHUK-IKBKB-MALT1-BCL10 complex	0.16903
DB01169	2056	BCL10-CHUK-BCL10-IKBKB complex	0.18898
DB01169	2100	CHUK-IKBKB-MAP3K14 complex	0.21822
DB01169	2101	IKKA-IKKB complex	0.26726
DB01169	2104	IKKB-NIK complex	0.26726
DB01169	2105	IkappaB kinase complex (IKBKB CHUK IKBKAP NFKBIA RELA MAP3K14)	0.1543
DB01169	2118	CHUK-ERC1-IKBKB-IKBKG	0.18898
DB01169	2121	CHUK-IKBKB-IKBKG complex	0.21822
DB01169	2156	YBX1-AKT1 complex	0.26726
DB01169	2159	AR-AKT-APPL complex	0.21822
DB01169	2635	BETA2-Cyclin D1 complex	0.26726
DB01169	2692	SMAD3-SMAD4-cJun-cFos complex	0.18898
DB01169	2693	NFAT-JUN-FOS DNA-protein complex	0.21822

DB01169	2694	ERG-JUN-FOS DNA-protein complex	0.21822
DB01169	2695	ETS2-FOS-JUN complex	0.21822
DB01169	2699	ER-alpha-GRIP1-c-Jun complex	0.21822
DB01169	2700	ER-alpha-c-Jun complex	0.26726
DB01169	2708	SMAD3-SMAD4-cJUN complex	0.21822
DB01169	2727	SRC-3 complex	0.14286
DB01169	3084	CCND1-CDK4 complex	0.26726
DB01169	3087	CCND1-CDK6 complex	0.26726
DB01169	3847	TCL1(trimer)-AKT1 complex	0.26726
DB01169	5194	TNF-alpha/NF-kappa B signaling complex (SEC16A CHUK IKBKB NFKB2 REL IKBKG MAP3K14 RELA FBXW7 USP2)	0.11952
DB01169	5226	p14-Mp1-ERK2 complex	0.21822
DB01169	5233	TNF-alpha/NF-kappa B signaling complex 5	0.07559
DB01169	5234	IKBKB-CDC37-KIAA1967-HSP90AB1-HSP90AA1 complex	0.16903
DB01169	5266	TNF-alpha/NF-kappa B signaling complex 6	0.10102
DB01169	5287	CDK4-CCND1 complex	0.26726
DB01169	5828	IKBKG-IKBKB complex	0.26726
DB01169	5844	I-kappa-B kinase (IKK) complex	0.21822
DB01278	1300	CRLR-RAMP1 complex	0.35355
DB01278	3139	CRLR-RAMP1-ARRB2 complex	0.28868
DB01278	3140	CRLR-RAMP2 complex	0.35355
DB01278	3141	CRLR-RAMP3 complex	0.35355
DB01381	5809	GABAA receptor	0.70711
DB05266	518	AKAP250-PKA-PDE4D complex	0.2
DB00054	2355	ITGAV-ITGB3-CD47-FCER2 complex	0.13868
DB00054	2356	ITGB3-ITGAV-CD47 complex	0.16013
DB00054	2358	ITGAV-ITGB3-SPP1 complex	0.16013
DB00054	2359	ITGAV-ITGB3-ADAM15 complex	0.16013
DB00054	2362	ITAGV-ITGB3-F11R complex	0.16013
DB00054	2363	ITGAV-ITGB3-PXN-PTK2b complex	0.13868
DB00054	2364	ITGAV-ITGB3-ADAM23 complex	0.16013
DB00054	2365	ITGAV-ITGB3-COL4A3 complex	0.16013
DB00054	2366	ITGAV-ITGB3-PPAP2b complex	0.16013
DB00054	2369	ITGAV-ITGB3-EGFR complex	0.16013
DB00054	2370	ITGA2b-ITGB3-CD9 complex	0.32026
DB00054	2374	ITGAV-ITGB3-LAMA4 complex	0.16013
DB00054	2376	ITGA2B-ITGB3-FN1-TGM2 complex	0.27735
DB00054	2377	ITGA2b-ITGB3-CD47-SRC complex	0.27735
DB00054	2378	ITGA2b-ITGB3-TLN1 complex	0.32026
DB00054	2379	ITGA2B-ITGB3-CIB1 complex	0.32026
DB00054	2381	ITGA2B-ITGB3 complex	0.39223
DB00054	2382	ITGA2B-ITGB3-F11R complex	0.32026
DB00054	2816	ITGAV-ITGB3 complex	0.19612
DB00054	2826	ITGB3-ITGAV-VTN complex	0.32026
DB00054	2846	ITGAV-ITGB3-THBS1 complex	0.16013
DB00054	2849	ITGAV-ITGB3-NOV complex	0.16013
DB00054	2872	ITGA2b-ITGB3-CD9-GP1b-CD47 complex	0.22646
DB00054	2882	ITGA5-ITGB3-COL6A3 complex	0.16013
DB00054	2896	ITGA2b-ITGB3-CD47-FAK complex	0.27735
DB00054	2909	PLC-gamma-2-Syk-LAT-FcR-gamma complex	0.13868
DB00054	2910	PLC-gamma-2-Lyn-FcR-gamma complex	0.16013
DB00054	3103	ITGAV-ITGB3-SLC3A2 complex	0.16013
DB00054	3115	ITGA2B-ITGB3-ICAM4 complex	0.32026
DB00054	3117	ITGB5-ITGAV-VTN complex	0.16013

DB00775	2355	ITGAV-ITGB3-CD47-FCER2 complex	0.35355
DB00775	2356	ITGB3-ITGAV-CD47 complex	0.40825
DB00775	2358	ITGAV-ITGB3-SPP1 complex	0.40825
DB00775	2359	ITGAV-ITGB3-ADAM15 complex	0.40825
DB00775	2362	ITAGV-ITGB3-F11R complex	0.40825
DB00775	2363	ITGAV-ITGB3-PXN-PTK2b complex	0.35355
DB00775	2364	ITGAV-ITGB3-ADAM23 complex	0.40825
DB00775	2365	ITGAV-ITGB3-COL4A3 complex	0.40825
DB00775	2366	ITGAV-ITGB3-PPAP2b complex	0.40825
DB00775	2369	ITGAV-ITGB3-EGFR complex	0.40825
DB00775	2370	ITGA2b-ITGB3-CD9 complex	0.8165
DB00775	2374	ITGAV-ITGB3-LAMA4 complex	0.40825
DB00775	2376	ITGA2B-ITGB3-FN1-TGM2 complex	0.70711
DB00775	2377	ITGA2b-ITGB3-CD47-SRC complex	0.70711
DB00775	2378	ITGA2b-ITGB3-TLN1 complex	0.8165
DB00775	2379	ITGA2B-ITGB3-CIB1 complex	0.8165
DB00775	2381	ITGA2B-ITGB3 complex	1
DB00775	2382	ITGA2B-ITGB3-F11R complex	0.8165
DB00775	2816	ITGAV-ITGB3 complex	0.5
DB00775	2826	ITGB3-ITGAV-VTN complex	0.40825
DB00775	2846	ITGAV-ITGB3-THBS1 complex	0.40825
DB00775	2849	ITGAV-ITGB3-NOV complex	0.40825
DB00775	2872	ITGA2b-ITGB3-CD9-GP1b-CD47 complex	0.57735
DB00775	2882	ITGA5-ITGB3-COL6A3 complex	0.40825
DB00775	2896	ITGA2b-ITGB3-CD47-FAK complex	0.70711
DB00775	3103	ITGAV-ITGB3-SLC3A2 complex	0.40825
DB00775	3115	ITGA2B-ITGB3-ICAM4 complex	0.8165
DB00920	518	AKAP250-PKA-PDE4D complex	0.14142
DB01436	548	DRIP complex	0.18898
DB01436	1230	WINAC complex	0.18898
DB00953	5411	EDG1-HTR1D complex	0.40825
DB00953	5412	HTR1D homodimer complex	0.57735
DB00953	5414	HTR1A-HTR1D complex	0.40825
DB00953	5415	HTR1B homodimer complex	0.57735
DB00953	5416	HTR1A-HTR1B complex	0.40825
DB00953	5417	HTR1D-HTR1B complex	0.8165
DB00188	32	PA700 complex	0.2
DB00188	181	26S proteasome	0.28604
DB00188	191	20S proteasome	0.35857
DB00188	192	PA28-20S proteasome	0.33541
DB00188	193	PA700-20S-PA28 complex	0.37268
DB00188	194	PA28gamma-20S proteasome	0.34641
DB01205	5809	GABAA receptor	0.39736
DB01656	518	AKAP250-PKA-PDE4D complex	0.22361
DB00808	520	KCNQ1 macromolecular complex	0.22361
DB00167	3040	Multisynthetase complex	0.13484
DB00707	2909	PLC-gamma-2-Syk-LAT-FcR-gamma complex	0.35355
DB00707	2910	PLC-gamma-2-Lyn-FcR-gamma complex	0.40825
DB00072	1095	SNX complex (SNX1a SNX2 SNX4 EGFR)	0.14434
DB00072	1185	EGFR-containing signaling complex	0.28868
DB00072	2369	ITGAV-ITGB3-EGFR complex	0.16667
DB00072	2453	Multiprotein complex (monoubiquitination)	0.14434
DB00072	2454	CIN85-CBL-SH3GL2-EGFR complex EGF stimulated	0.14434
DB00072	2528	ERBB2-MEMO-SHC complex	0.16667

DB00072	2542	EGFR-CBL-GRB2 complex	0.16667
DB00072	2909	PLC-gamma-2-Syk-LAT-FcR-gamma complex	0.14434
DB00072	2910	PLC-gamma-2-Lyn-FcR-gamma complex	0.16667
DB00072	3678	RIN1-STAM2-EGFR complex EGF stimulated	0.16667
DB00072	5171	SH3KBP1-CBLB-EGFR complex	0.16667
DB01259	1095	SNX complex (SNX1a SNX2 SNX4 EGFR)	0.35355
DB01259	1185	EGFR-containing signaling complex	0.70711
DB01259	2369	ITGAV-ITGB3-EGFR complex	0.40825
DB01259	2453	Multiprotein complex (monoubiquitination)	0.35355
DB01259	2454	CIN85-CBL-SH3GL2-EGFR complex EGF stimulated	0.35355
DB01259	2528	ERBB2-MEMO-SHC complex	0.40825
DB01259	2542	EGFR-CBL-GRB2 complex	0.40825
DB01259	3678	RIN1-STAM2-EGFR complex EGF stimulated	0.40825
DB01259	5171	SH3KBP1-CBLB-EGFR complex	0.40825
DB05773	1185	EGFR-containing signaling complex	0.5
DB05773	2528	ERBB2-MEMO-SHC complex	0.57735
DB06366	1185	EGFR-containing signaling complex	0.5
DB06366	2528	ERBB2-MEMO-SHC complex	0.57735
DB08916	1095	SNX complex (SNX1a SNX2 SNX4 EGFR)	0.28868
DB08916	1185	EGFR-containing signaling complex	0.57735
DB08916	2369	ITGAV-ITGB3-EGFR complex	0.33333
DB08916	2453	Multiprotein complex (monoubiquitination)	0.28868
DB08916	2454	CIN85-CBL-SH3GL2-EGFR complex EGF stimulated	0.28868
DB08916	2528	ERBB2-MEMO-SHC complex	0.33333
DB08916	2542	EGFR-CBL-GRB2 complex	0.33333
DB08916	3678	RIN1-STAM2-EGFR complex EGF stimulated	0.33333
DB08916	5171	SH3KBP1-CBLB-EGFR complex	0.33333
DB00095	2909	PLC-gamma-2-Syk-LAT-FcR-gamma complex	0.15811
DB00095	2910	PLC-gamma-2-Lyn-FcR-gamma complex	0.18257
DB00098	2342	ITGAV-ITGB8-MMP14-TGFB1 complex	0.16667
DB00098	2343	ITGAV-ITGB5-PLAUR complex	0.19245
DB00098	2345	ITGAV-ITGB5-ICAM4 complex	0.19245
DB00098	2346	ITGAV-ITGB5-ADAM9 complex	0.19245
DB00098	2347	ITGAV-ITGB5-SPP1 complex	0.19245
DB00098	2348	ITGAV-ITGB5-CYR61 complex	0.19245
DB00098	2350	ITGAV-ITGB5 complex	0.2357
DB00098	2352	ITGAV-ITGB6-SPP1 complex	0.19245
DB00098	2353	ITGAV-ITGB6-TGFB3 complex	0.19245
DB00098	2354	ITGAV-ITGB6 complex	0.2357
DB00098	2355	ITGAV-ITGB3-CD47-FCER2 complex	0.33333
DB00098	2356	ITGB3-ITGAV-CD47 complex	0.3849
DB00098	2358	ITGAV-ITGB3-SPP1 complex	0.3849
DB00098	2359	ITGAV-ITGB3-ADAM15 complex	0.3849
DB00098	2362	ITAGV-ITGB3-F11R complex	0.3849
DB00098	2363	ITGAV-ITGB3-PXN-PTK2b complex	0.33333
DB00098	2364	ITGAV-ITGB3-ADAM23 complex	0.3849
DB00098	2365	ITGAV-ITGB3-COL4A3 complex	0.3849
DB00098	2366	ITGAV-ITGB3-PPAP2b complex	0.3849
DB00098	2369	ITGAV-ITGB3-EGFR complex	0.3849
DB00098	2370	ITGA2b-ITGB3-CD9 complex	0.19245
DB00098	2374	ITGAV-ITGB3-LAMA4 complex	0.3849
DB00098	2376	ITGA2B-ITGB3-FN1-TGM2 complex	0.16667
DB00098	2377	ITGA2b-ITGB3-CD47-SRC complex	0.16667
DB00098	2378	ITGA2b-ITGB3-TLN1 complex	0.19245

DB00098	2379	ITGA2B-ITGB3-CIB1 complex	0.19245
DB00098	2381	ITGA2B-ITGB3 complex	0.2357
DB00098	2382	ITGA2B-ITGB3-F11R complex	0.19245
DB00098	2383	ITGA5-ITGB1-FN1-TGM2 complex	0.16667
DB00098	2384	ITGA5-ITGB1-ADAM15 complex	0.19245
DB00098	2385	ITGA5-ITGB4 complex	0.2357
DB00098	2388	Itga5-Itgb1-Fn1-Sfrp2 complex	0.16667
DB00098	2390	CD98-LAT2-ITGB1 complex	0.16667
DB00098	2395	ITGA7-ITGB1-CD151 complex	0.19245
DB00098	2396	ITGA7-ITGB1-CD9 complex	0.19245
DB00098	2397	ITGA7-ITGB1-ITGB1BP3 complex	0.19245
DB00098	2398	ITGA3-ITGB1-BSG complex	0.19245
DB00098	2399	ITGA3-ITGB1-CD63 complex	0.19245
DB00098	2400	ITGA3-ITGB1-CD151 complex	0.19245
DB00098	2401	ITGA3-ITGB1-THBS1 complex	0.19245
DB00098	2406	ITGA3-ITGB1 complex	0.2357
DB00098	2411	ITGA6-ITGB1-CD151 complex	0.19245
DB00098	2413	ITGA6-ITGB1 complex	0.2357
DB00098	2416	ITGB1-RAP1A-PKD1 complex	0.19245
DB00098	2417	ITGA4-ITGB1-EMILIN1 complex	0.19245
DB00098	2418	ITGA4-ITGB1 complex	0.2357
DB00098	2419	ITGA4-ITGB1-CD81 complex	0.19245
DB00098	2420	ITGA4-ITGB1-CD53 complex	0.19245
DB00098	2421	ITGA4-ITGB1-VCAM1 complex	0.19245
DB00098	2422	ITGA4-ITGB1-JAM2 complex	0.19245
DB00098	2423	ITGA4-ITGB1-CD47 complex	0.19245
DB00098	2424	ITGA4-ITGB1-CD63 complex	0.19245
DB00098	2425	ITGA4-ITGB1-PXN complex	0.19245
DB00098	2426	ITGA4-ITGB1-THBS1 complex	0.19245
DB00098	2428	ITGA4-ITGB1-THBS2 complex	0.19245
DB00098	2429	ITGA2-ITGB1-CD47 complex	0.19245
DB00098	2430	ITGA2-ITGB1-CHAD complex	0.19245
DB00098	2431	ITGA2-ITGB1-COL6A3 complex	0.19245
DB00098	2432	ITGA2-ITGB1 complex	0.2357
DB00098	2434	ITGA1-ITGB1-COL6A3 complex	0.19245
DB00098	2435	ITGA1-ITGB1-PTPN2 complex	0.19245
DB00098	2436	ITGAV-ITGB1 complex	0.4714
DB00098	2437	ITGA6-ITGB1-CYR61 complex	0.19245
DB00098	2439	ITGA8-ITGB1 complex	0.2357
DB00098	2440	ITGA9-ITGB1-ADAM9 complex	0.19245
DB00098	2441	Itga9-Itgb1-Adam2 complex	0.19245
DB00098	2442	ITGA9-ITGB1-VCAM1 complex	0.19245
DB00098	2443	ITGA9-ITGB1-TNC complex	0.19245
DB00098	2444	ITGB1-ITGA9 complex	0.2357
DB00098	2445	ITGA9-ITGB1-ADAM15 complex	0.19245
DB00098	2446	ITGA9-ITGB1-FIGF complex	0.19245
DB00098	2447	ITGA9-ITGB1-ADAM12 complex	0.19245
DB00098	2816	ITGAV-ITGB3 complex	0.4714
DB00098	2826	ITGB3-ITGAV-VTN complex	0.3849
DB00098	2846	ITGAV-ITGB3-THBS1 complex	0.3849
DB00098	2849	ITGAV-ITGB3-NOV complex	0.3849
DB00098	2850	ITGA5-ITGB1-FN-1-NOV complex	0.16667
DB00098	2853	ITGA5-ITGB1-CAL4A3 complex	0.19245
DB00098	2872	ITGA2b-ITGB3-CD9-GP1b-CD47 complex	0.13608

DB00098	2882	ITGA5-ITGB3-COL6A3 complex	0.19245
DB00098	2885	ITGAV-ITGB1-SPP1 complex	0.3849
DB00098	2896	ITGA2b-ITGB3-CD47-FAK complex	0.16667
DB00098	2964	ITGA9-ITGB1-ADAM1 complex	0.19245
DB00098	2965	ITGA9-ITGB1-ADAM3 complex	0.19245
DB00098	2971	ITGA9-ITGB1-VEGFC complex	0.19245
DB00098	2972	ITGA9-ITGB1-VEGFA complex	0.19245
DB00098	2989	ITGA9-ITGB1-ADAM8 complex	0.19245
DB00098	3035	LAT2-ITGB1 complex	0.2357
DB00098	3057	ITGA10-ITGB1 complex	0.2357
DB00098	3058	ITGA11-ITGB1 complex	0.2357
DB00098	3059	ITGA11-ITGB1-COL1A1 complex	0.19245
DB00098	3103	ITGAV-ITGB3-SLC3A2 complex	0.3849
DB00098	3104	ITGB1-NRP1 complex	0.2357
DB00098	3110	ITGAV-P2RY2-GNA12 complex	0.19245
DB00098	3111	ITGA9-ITGB1-SPP1 complex	0.19245
DB00098	3112	ITGA5-ITGB1-SPP1 complex	0.19245
DB00098	3115	ITGA2B-ITGB3-ICAM4 complex	0.19245
DB00098	3117	ITGB5-ITGAV-VTN complex	0.19245
DB00227	54	SIN3 complex	0.21822
DB00227	61	Mi2/NuRD complex	0.21822
DB00227	62	MeCP1 complex	0.20412
DB00227	282	SNF2h-cohesin-NuRD complex	0.14434
DB00227	283	Sin3 complex	0.21822
DB00227	587	NuRD.1 complex	0.20412
DB00227	591	SAP complex (Sin3-associated protein complex)	0.20412
DB00227	592	SAP complex (Sin3-associated protein complex)	0.19245
DB00227	596	SIN3-HDAC-SAP30-ARID4 complex	0.21822
DB00227	614	NRD complex (Nucleosome remodeling and deacetylation complex)	0.21822
DB00227	620	CoREST-HDAC complex	0.21822
DB00227	632	Anti-HDAC2 complex	0.13608
DB00227	633	anti-BHC110 complex	0.17408
DB00227	634	XFIM complex	0.2582
DB00227	636	BHC complex	0.2357
DB00227	642	CtBP complex	0.14003
DB00227	643	CtBP core complex	0.19245
DB00227	650	HDAC2-asscociated core complex	0.20412
DB00227	659	MeCP1 complex	0.19245
DB00227	685	MeCP1 complex	0.19245
DB00227	691	SIN3-SAP25 complex	0.17408
DB00227	696	BRMS1-SIN3-HDAC complex	0.20412
DB00227	713	BRG1-SIN3A complex	0.1543
DB00227	714	BRM-SIN3A complex	0.14907
DB00227	738	SIN3-ING1b complex I	0.19245
DB00227	739	SIN3-ING1b complex II	0.14434
DB00227	745	NCOR-SIN3-RPD3 complex	0.28868
DB00227	749	MeCP2-SIN3A-HDAC complex	0.28868
DB00227	778	LARC complex (LCR-associated remodeling complex)	0.13245
DB00227	803	BRG1-SIN3A-HDAC containing SWI/SNF remodeling complex I	0.17408
DB00227	806	BRM-SIN3A-HDAC complex	0.16667
DB00227	871	BRAF53-BRCA2 complex	0.21822
DB00227	886	MTA1 complex	0.2357
DB00227	888	MTA2 complex	0.19245
DB00227	889	MTA1-HDAC core complex	0.2582

DB00227	1133	ATR-HDAC2 complex	0.40825
DB00227	1134	ATR-HDAC2-CHD4 complex	0.33333
DB00227	1233	CoREST-HDAC2 complex	0.40825
DB00227	1257	ALL-1 supercomplex	0.10911
DB00227	1458	SNF2h-HDAC12 complex	0.40825
DB00227	1492	BHC110 complex	0.18257
DB00227	1505	NCOR2 complex	0.21822
DB00227	2657	ESR1-CDK7-CCNH-MNAT1-MTA1-HDAC2 complex	0.2357
DB00227	2721	HCF-1 complex	0.13245
DB00227	2814	BRCA1-HDAC1-HDAC2 complex	0.33333
DB00227	2851	ING2 complex	0.16667
DB00227	3048	mSin3A complex	0.2582
DB00227	3053	mSin3A-HDAC1-HDAC2 complex	0.33333
DB00227	3054	MAD1-mSin3A-HDAC2 complex	0.33333
DB00227	3167	NCOR-SIN3-HDAC-HESX1 complex	0.2357
DB00445	924	Toposome	0.26726
DB00445	1098	DNA synthesome complex (13 subunits)	0.18898
DB00445	1099	DNA synthesome complex (17 subunits)	0.16667
DB00445	1183	CDC5L complex	0.1291
DB00445	1728	CTCF-nucleophosmin-PARP-HIS-KPNA-LMNA-TOP complex	0.2357
DB00364	1069	FIF-FGR2 complex	0.31623
DB01280	1003	RC complex (Replication competent complex)	0.33333
DB01280	1004	RC complex during S-phase of cell cycle	0.27735
DB01280	1005	RC complex during G2/M-phase of cell cycle	0.27735
DB01280	1098	DNA synthesome complex (13 subunits)	0.26726
DB01280	1099	DNA synthesome complex (17 subunits)	0.2357
DB01280	1100	DNA polymerase alpha-primase complex	0.5
DB01280	1107	DNA synthesome core complex	0.31623
DB01280	1108	DNA synthesome complex (15 subunits)	0.2582
DB00356	668	BKCA-beta2AR-AKAP79 signaling complex	0.57735
DB00356	672	BKCA-beta2AR complex	0.70711
DB01003	668	BKCA-beta2AR-AKAP79 signaling complex	0.40825
DB01003	672	BKCA-beta2AR complex	0.5
DB00351	4216	GR-hnRNP U complex	0.5
DB00588	786	MR-UBC9-SRC1 complex	0.28868
DB00588	3634	NR3C2-UBC9-SRC-1 complex	0.28868
DB00588	4216	GR-hnRNP U complex	0.35355
DB00834	4216	GR-hnRNP U complex	0.5
DB00292	5809	GABAA receptor	0.27217
DB00641	725	P2X7 receptor signalling complex	0.20412
DB00641	2153	ITGAM-ITGB2-CD11 complex	0.40825
DB01076	1211	Ubiquitin E3 ligase (AHR ARNT DDB1 TBL3 CUL4B RBX1)	0.2357
DB00108	1810	ITGA4-PXN-GIT1 complex	0.17408
DB00108	2417	ITGA4-ITGB1-EMILIN1 complex	0.17408
DB00108	2418	ITGA4-ITGB1 complex	0.2132
DB00108	2419	ITGA4-ITGB1-CD81 complex	0.17408
DB00108	2420	ITGA4-ITGB1-CD53 complex	0.17408
DB00108	2421	ITGA4-ITGB1-VCAM1 complex	0.17408
DB00108	2422	ITGA4-ITGB1-JAM2 complex	0.17408
DB00108	2423	ITGA4-ITGB1-CD47 complex	0.17408
DB00108	2424	ITGA4-ITGB1-CD63 complex	0.17408
DB00108	2425	ITGA4-ITGB1-PXN complex	0.17408
DB00108	2426	ITGA4-ITGB1-THBS1 complex	0.17408
DB00108	2428	ITGA4-ITGB1-THBS2 complex	0.17408

DB00108	2909	PLC-gamma-2-Syk-LAT-FcR-gamma complex	0.15076
DB00108	2910	PLC-gamma-2-Lyn-FcR-gamma complex	0.17408
DB00720	3055	Nop56p-associated pre-rRNA complex	0.05661
DB08881	5877	MAP2K1-BRAF-RAF1-YWHAE-KSR1 complex	0.44721
DB08881	5872	BRAF-MAP2K1-MAP2K2-YWHAE complex	0.5
DB08881	5919	BRAF-RAF1-14-3-3 complex	0.33333
DB08881	5921	KSR1-BRAF-MEK complex	0.5
DB08881	5923	RAF1-BRAF complex RAS stimulated	0.70711
DB08881	5925	BRAF-CNK1 complex not RAS stimulated	0.70711
DB00004	1515	IL4-IL4R-IL2RG complex	0.33333
DB00004	1707	IL2-IL2RA-IL2RB complex	0.66667
DB00041	1515	IL4-IL4R-IL2RG complex	0.33333
DB00041	1707	IL2-IL2RA-IL2RB complex	0.66667
DB00074	1707	IL2-IL2RA-IL2RB complex	0.33333
DB00074	2909	PLC-gamma-2-Syk-LAT-FcR-gamma complex	0.14434
DB00074	2910	PLC-gamma-2-Lyn-FcR-gamma complex	0.16667
DB00111	1707	IL2-IL2RA-IL2RB complex	0.34816
DB00111	2909	PLC-gamma-2-Syk-LAT-FcR-gamma complex	0.15076
DB00111	2910	PLC-gamma-2-Lyn-FcR-gamma complex	0.17408
DB00012	5442	EPOR receptor complex	1
DB00012	5446	EPO-EPOR complex	0.70711
DB00016	5442	EPOR receptor complex	1
DB00016	5446	EPO-EPOR complex	0.70711
DB08894	5442	EPOR receptor complex	1
DB08894	5446	EPO-EPOR complex	0.70711
DB08923	5442	EPOR receptor complex	1
DB08923	5446	EPO-EPOR complex	0.70711
DB00918	5411	EDG1-HTR1D complex	0.5
DB00918	5412	HTR1D homodimer complex	0.70711
DB00918	5414	HTR1A-HTR1D complex	0.5
DB00918	5415	HTR1B homodimer complex	0.70711
DB00918	5416	HTR1A-HTR1B complex	0.5
DB00918	5417	HTR1D-HTR1B complex	1
DB00998	5411	EDG1-HTR1D complex	0.5
DB00998	5412	HTR1D homodimer complex	0.70711
DB00998	5414	HTR1A-HTR1D complex	0.5
DB00998	5415	HTR1B homodimer complex	0.70711
DB00998	5416	HTR1A-HTR1B complex	0.5
DB00998	5417	HTR1D-HTR1B complex	1
DB05258	552	IFNB1-IFNAR1-IFNAR2- complex	0.57735
DB00982	5198	CBP-RARA-RXRA-DNA complex ligand stimulated	0.57735
DB00700	786	MR-UBC9-SRC1 complex	0.57735
DB00700	3634	NR3C2-UBC9-SRC-1 complex	0.57735
DB00056	2909	PLC-gamma-2-Syk-LAT-FcR-gamma complex	0.15076
DB00056	2910	PLC-gamma-2-Lyn-FcR-gamma complex	0.17408
DB00914	771	NDPKA-AMPKalpha1 complex	0.5
DB00816	668	BKCA-beta2AR-AKAP79 signaling complex	0.57735
DB00816	672	BKCA-beta2AR complex	0.70711
DB00816	687	CFTR-NHERF-beta(2)AR signaling complex	0.57735
DB00816	3830	ADRB2 homodimer complex	1
DB00867	668	BKCA-beta2AR-AKAP79 signaling complex	0.57735
DB00867	672	BKCA-beta2AR complex	0.70711
DB00867	687	CFTR-NHERF-beta(2)AR signaling complex	0.57735
DB00867	3830	ADRB2 homodimer complex	1

DB00871	668	BKCA-beta2AR-AKAP79 signaling complex	0.57735
DB00871	672	BKCA-beta2AR complex	0.70711
DB00871	687	CFTR-NHERF-beta(2)AR signaling complex	0.57735
DB00871	3830	ADRB2 homodimer complex	1
DB00938	668	BKCA-beta2AR-AKAP79 signaling complex	0.57735
DB00938	672	BKCA-beta2AR complex	0.70711
DB00938	687	CFTR-NHERF-beta(2)AR signaling complex	0.57735
DB00938	3830	ADRB2 homodimer complex	1
DB00983	668	BKCA-beta2AR-AKAP79 signaling complex	0.57735
DB00983	672	BKCA-beta2AR complex	0.70711
DB00983	687	CFTR-NHERF-beta(2)AR signaling complex	0.57735
DB00983	3830	ADRB2 homodimer complex	1
DB01274	668	BKCA-beta2AR-AKAP79 signaling complex	0.57735
DB01274	672	BKCA-beta2AR complex	0.70711
DB01274	687	CFTR-NHERF-beta(2)AR signaling complex	0.57735
DB01274	3830	ADRB2 homodimer complex	1
DB01366	668	BKCA-beta2AR-AKAP79 signaling complex	0.57735
DB01366	672	BKCA-beta2AR complex	0.70711
DB01366	687	CFTR-NHERF-beta(2)AR signaling complex	0.57735
DB01366	3830	ADRB2 homodimer complex	1
DB01408	668	BKCA-beta2AR-AKAP79 signaling complex	0.57735
DB01408	672	BKCA-beta2AR complex	0.70711
DB01408	687	CFTR-NHERF-beta(2)AR signaling complex	0.57735
DB01408	3830	ADRB2 homodimer complex	1
DB05039	668	BKCA-beta2AR-AKAP79 signaling complex	0.57735
DB05039	672	BKCA-beta2AR complex	0.70711
DB05039	687	CFTR-NHERF-beta(2)AR signaling complex	0.57735
DB05039	3830	ADRB2 homodimer complex	1
DB00337	1893	mTOR-RICTOR complex	0.40825
DB00337	1895	RICTOR-mTOR complex	0.40825
DB00337	1897	RAPTOR-mTOR complex	0.40825
DB00337	2969	mTORC2 complex (mTOR/FRAP1 LST8 mAVO3/RICTOR)	0.40825
DB00337	2970	mTORC1 complex (mTOR/FRAP1 LST8 RAPTOR)	0.40825
DB00337	2985	mTOR-signaling complex	0.5
DB00337	2990	mTOR-signaling complex (FRAP1/mTOR GBL RAPTOR)	0.40825
DB00337	2991	mTOR-signaling complex (mTOR/FRAP1 RAPTOR)	0.5
DB00337	3979	mTORC2 complex (mTOR/FRAP1 LST8 mAVO3/RICTOR SIN1)	0.35355
DB00337	3980	mTOR-RAPTOR complex	0.40825
DB00877	1069	FIF-FGR2 complex	0.40825
DB00877	1893	mTOR-RICTOR complex	0.33333
DB00877	1895	RICTOR-mTOR complex	0.33333
DB00877	1897	RAPTOR-mTOR complex	0.33333
DB00877	2969	mTORC2 complex (mTOR/FRAP1 LST8 mAVO3/RICTOR)	0.33333
DB00877	2970	mTORC1 complex (mTOR/FRAP1 LST8 RAPTOR)	0.33333
DB00877	2985	mTOR-signaling complex	0.40825
DB00877	2990	mTOR-signaling complex (FRAP1/mTOR GBL RAPTOR)	0.33333
DB00877	2991	mTOR-signaling complex (mTOR/FRAP1 RAPTOR)	0.40825
DB00877	3979	mTORC2 complex (mTOR/FRAP1 LST8 mAVO3/RICTOR SIN1)	0.28868
DB00877	3980	mTOR-RAPTOR complex	0.33333
DB00005	120	Lymphotoxin beta receptor complex	0.16013
DB00005	2909	PLC-gamma-2-Syk-LAT-FcR-gamma complex	0.13868
DB00005	2910	PLC-gamma-2-Lyn-FcR-gamma complex	0.16013
DB00051	2909	PLC-gamma-2-Syk-LAT-FcR-gamma complex	0.15076
DB00051	2910	PLC-gamma-2-Lyn-FcR-gamma complex	0.17408

DB01411	575	ABIN2-NFKB1-MAP3K8 complex	0.2357
DB01411	2084	NFKB1-NFKB2-REL-RELA-RELB complex	0.18257
DB01411	2086	NFKB1-NFKB2-RELA-RELB complex	0.20412
DB01411	3045	hs4 enhancer complex (faster migrating complex)	0.18257
DB01411	5193	TNF-alpha/NF-kappa B signaling complex (CHUK KPNA3 NFKB2 NFKBIB REL IKBKG NFKB1 NFKBIE RELB NFKBIA RELA TNIP2)	0.11785
DB01411	5230	CHUK-NFKB2-REL-IKBKG-SPAG9-NFKB1-NFKBIE-COPB2-TNIP1-NFKBIA-RELA-TNIP2 complex	0.11785
DB01411	5232	TNF-alpha/Nf-kappa B signaling complex (RPL6 RPL30 RPS13 CHUK DDX3X NFKB2 NFKBIB REL IKBKG NFKB1 MAP3K8 RELB GLG1 NFKBIA RELA TNIP2 GTF2I)	0.09901
DB01411	5233	TNF-alpha/NF-kappa B signaling complex 5	0.08165
DB01411	5460	p50-p65 NF(kappa)B complex	0.28868
DB01411	5461	p50-p65 NF(kappa)B-SRC1 complex	0.2357
DB01411	5464	I(kappa)B(alpha)-NF(kappa)Bp50-NF(kappa)Bp65 complex	0.2357
DB01411	5492	IKBA-NF(kappa)Bp65-NF(kappa)Bp50 complex	0.2357
DB01154	5809	GABAA receptor	0.33333
DB00002	1095	SNX complex (SNX1a SNX2 SNX4 EGFR)	0.15076
DB00002	1185	EGFR-containing signaling complex	0.15076
DB00002	2369	ITGAV-ITGB3-EGFR complex	0.17408
DB00002	2453	Multiprotein complex (monoubiquitination)	0.15076
DB00002	2454	CIN85-CBL-SH3GL2-EGFR complex EGF stimulated	0.15076
DB00002	2542	EGFR-CBL-GRB2 complex	0.17408
DB00002	2909	PLC-gamma-2-Syk-LAT-FcR-gamma complex	0.15076
DB00002	2910	PLC-gamma-2-Lyn-FcR-gamma complex	0.17408
DB00002	3678	RIN1-STAM2-EGFR complex EGF stimulated	0.17408
DB00002	5171	SH3KBP1-CBLB-EGFR complex	0.17408
DB00028	2909	PLC-gamma-2-Syk-LAT-FcR-gamma complex	0.15811
DB00028	2910	PLC-gamma-2-Lyn-FcR-gamma complex	0.18257
DB00092	2909	PLC-gamma-2-Syk-LAT-FcR-gamma complex	0.15811
DB00092	2910	PLC-gamma-2-Lyn-FcR-gamma complex	0.18257
DB00110	2909	PLC-gamma-2-Syk-LAT-FcR-gamma complex	0.16667
DB00110	2910	PLC-gamma-2-Lyn-FcR-gamma complex	0.19245
DB00992	2909	PLC-gamma-2-Syk-LAT-FcR-gamma complex	0.5
DB00992	2910	PLC-gamma-2-Lyn-FcR-gamma complex	0.57735
DB00851	1003	RC complex (Replication competent complex)	0.2357
DB00851	1098	DNA synthesome complex (13 subunits)	0.18898
DB00851	1099	DNA synthesome complex (17 subunits)	0.16667
DB00851	1100	DNA polymerase alpha-primase complex	0.35355
DB00851	1107	DNA synthesome core complex	0.22361
DB00851	1108	DNA synthesome complex (15 subunits)	0.18257
DB00186	5809	GABAA receptor	0.28006
DB00628	5809	GABAA receptor	0.28006
DB00962	5809	GABAA receptor	0.40825
DB01068	5809	GABAA receptor	0.28006
DB01587	5809	GABAA receptor	0.2357
DB00380	924	Toposome	0.26726
DB00380	1098	DNA synthesome complex (13 subunits)	0.37796
DB00380	1099	DNA synthesome complex (17 subunits)	0.33333
DB00380	1183	CDC5L complex	0.1291
DB00380	1230	WINAC complex	0.18898
DB00380	1728	CTCF-nucleophosmin-PARP-HIS-KPNA-LMNA-TOP complex	0.2357
DB00380	1729	TLE1 corepressor complex (MASH1 promoter-corepressor complex)	0.22361
DB00385	924	Toposome	0.37796

DB00385	1098	DNA synthesome complex (13 subunits)	0.26726
DB00385	1099	DNA synthesome complex (17 subunits)	0.2357
DB00385	1183	CDC5L complex	0.18257
DB00385	1728	CTCF-nucleophosmin-PARP-HIS-KPNA-LMNA-TOP complex	0.33333
DB00444	924	Toposome	0.37796
DB00444	1098	DNA synthesome complex (13 subunits)	0.26726
DB00444	1099	DNA synthesome complex (17 subunits)	0.2357
DB00444	1183	CDC5L complex	0.18257
DB00444	1728	CTCF-nucleophosmin-PARP-HIS-KPNA-LMNA-TOP complex	0.33333
DB00694	924	Toposome	0.26726
DB00694	1098	DNA synthesome complex (13 subunits)	0.37796
DB00694	1099	DNA synthesome complex (17 subunits)	0.33333
DB00694	1183	CDC5L complex	0.1291
DB00694	1230	WINAC complex	0.18898
DB00694	1728	CTCF-nucleophosmin-PARP-HIS-KPNA-LMNA-TOP complex	0.2357
DB00694	1729	TLE1 corepressor complex (MASH1 promoter-corepressor complex)	0.22361
DB00773	924	Toposome	0.26726
DB00773	1098	DNA synthesome complex (13 subunits)	0.37796
DB00773	1099	DNA synthesome complex (17 subunits)	0.33333
DB00773	1183	CDC5L complex	0.1291
DB00773	1230	WINAC complex	0.18898
DB00773	1728	CTCF-nucleophosmin-PARP-HIS-KPNA-LMNA-TOP complex	0.2357
DB00773	1729	TLE1 corepressor complex (MASH1 promoter-corepressor complex)	0.22361
DB00997	924	Toposome	0.37796
DB00997	1098	DNA synthesome complex (13 subunits)	0.26726
DB00997	1099	DNA synthesome complex (17 subunits)	0.2357
DB00997	1183	CDC5L complex	0.18257
DB00997	1728	CTCF-nucleophosmin-PARP-HIS-KPNA-LMNA-TOP complex	0.33333
DB01177	924	Toposome	0.37796
DB01177	1098	DNA synthesome complex (13 subunits)	0.26726
DB01177	1099	DNA synthesome complex (17 subunits)	0.2357
DB01177	1183	CDC5L complex	0.18257
DB01177	1728	CTCF-nucleophosmin-PARP-HIS-KPNA-LMNA-TOP complex	0.33333
DB01204	924	Toposome	0.37796
DB01204	1098	DNA synthesome complex (13 subunits)	0.26726
DB01204	1099	DNA synthesome complex (17 subunits)	0.2357
DB01204	1183	CDC5L complex	0.18257
DB01204	1728	CTCF-nucleophosmin-PARP-HIS-KPNA-LMNA-TOP complex	0.33333
DB04967	298	VEGF transcriptional complex	0.2357
DB04967	924	Toposome	0.21822
DB04967	1004	RC complex during S-phase of cell cycle	0.16013
DB04967	1005	RC complex during G2/M-phase of cell cycle	0.16013
DB04967	1098	DNA synthesome complex (13 subunits)	0.30861
DB04967	1099	DNA synthesome complex (17 subunits)	0.27217
DB04967	1106	TFIIIC containing-TOP1-SUB1 complex	0.21822
DB04967	1183	CDC5L complex	0.10541
DB04967	1728	CTCF-nucleophosmin-PARP-HIS-KPNA-LMNA-TOP complex	0.19245
DB04967	1760	TOP1-PSF-P54 complex	0.33333
DB04967	2230	PCNA complex	0.21822
DB04967	2808	RAD9-RAD1-HUS1-APE1 complex	0.28868
DB04967	3055	Nop56p-associated pre-rRNA complex	0.05661
DB00039	5658	Nrp1-PlexinD1 complex	0.28868
DB00039	2563	FGFR2-c-Cbl-Lyn-Fyn complex	0.20412
DB00039	3104	ITGB1-NRP1 complex	0.28868

DB00039	4062	NRP1-VEGFR2-VEGF(165) complex	0.2357
DB00039	5646	FARP2-NRP1-PlexinA1 complex	0.2357
DB00039	5647	FARP2-NRP1-PlexinA2 complex	0.2357
DB00039	5648	FARP2-NRP1-PlexinA3 complex	0.2357
DB00039	5649	FARP2-NRP1-PlexinA4 complex	0.2357
DB00039	5659	SEMA3C-PlexinD1-Nrp1 complex	0.2357
DB00039	5668	PlexinA1-Nrp1 complex	0.28868
DB00039	5669	PlexinA3-Nrp1 complex	0.28868
DB00039	5670	PlexinB1-Nrp1 complex	0.28868
DB00039	5689	SEMA6D-PlexinA1-NRP1 complex	0.2357
DB00039	5696	VEGFA(165)-KDR-NRP1 complex	0.2357
DB00039	5697	VEGFA(165)-KDR-NRP1 complex	0.28868
DB00039	5698	VEGFA(165)-VEGFR2-NRP1 complex	0.2357
DB00039	5701	NRP1-VEGF(165/121) complex	0.28868
DB00039	5731	NRP1-VEGFC complex heparin dependent	0.28868
DB00039	5734	NRP1-VEGFD complex heparin dependent	0.28868
DB00039	5745	PlexinA1-NRP1 complex	0.28868
DB00039	5746	PlexinA1-NRP1-SEMA3A complex	0.2357
DB00317	1095	SNX complex (SNX1a SNX2 SNX4 EGFR)	0.5
DB00317	1185	EGFR-containing signaling complex	0.5
DB00317	2369	ITGAV-ITGB3-EGFR complex	0.57735
DB00317	2453	Multiprotein complex (monoubiquitination)	0.5
DB00317	2454	CIN85-CBL-SH3GL2-EGFR complex EGF stimulated	0.5
DB00317	2542	EGFR-CBL-GRB2 complex	0.57735
DB00317	3678	RIN1-STAM2-EGFR complex EGF stimulated	0.57735
DB00317	5171	SH3KBP1-CBLB-EGFR complex	0.57735
DB00530	1095	SNX complex (SNX1a SNX2 SNX4 EGFR)	0.35355
DB00530	1185	EGFR-containing signaling complex	0.35355
DB00530	2369	ITGAV-ITGB3-EGFR complex	0.40825
DB00530	2453	Multiprotein complex (monoubiquitination)	0.35355
DB00530	2454	CIN85-CBL-SH3GL2-EGFR complex EGF stimulated	0.35355
DB00530	2542	EGFR-CBL-GRB2 complex	0.40825
DB00530	3678	RIN1-STAM2-EGFR complex EGF stimulated	0.40825
DB00530	5171	SH3KBP1-CBLB-EGFR complex	0.40825
DB01269	1095	SNX complex (SNX1a SNX2 SNX4 EGFR)	0.5
DB01269	1185	EGFR-containing signaling complex	0.5
DB01269	2369	ITGAV-ITGB3-EGFR complex	0.57735
DB01269	2453	Multiprotein complex (monoubiquitination)	0.5
DB01269	2454	CIN85-CBL-SH3GL2-EGFR complex EGF stimulated	0.5
DB01269	2542	EGFR-CBL-GRB2 complex	0.57735
DB01269	3678	RIN1-STAM2-EGFR complex EGF stimulated	0.57735
DB01269	5171	SH3KBP1-CBLB-EGFR complex	0.57735
DB00136	548	DRIP complex	0.26726
DB00136	1230	WINAC complex	0.26726
DB00146	548	DRIP complex	0.26726
DB00146	1230	WINAC complex	0.26726
DB00153	548	DRIP complex	0.26726
DB00153	1230	WINAC complex	0.26726
DB00169	548	DRIP complex	0.26726
DB00169	1230	WINAC complex	0.26726
DB00910	548	DRIP complex	0.26726
DB00910	1230	WINAC complex	0.26726
DB01070	548	DRIP complex	0.26726
DB01070	1230	WINAC complex	0.26726

DB02300	548	DRIP complex	0.26726
DB02300	1230	WINAC complex	0.26726
DB06637	595	Kv4.2-DPP10 channel complex	0.17678
DB06637	1787	Nogo-potassium channel complex	0.25
DB00180	4216	GR-hnRNP U complex	0.70711
DB00223	4216	GR-hnRNP U complex	0.70711
DB00240	4216	GR-hnRNP U complex	0.70711
DB00253	4216	GR-hnRNP U complex	0.70711
DB00324	4216	GR-hnRNP U complex	0.70711
DB00394	4216	GR-hnRNP U complex	0.70711
DB00443	4216	GR-hnRNP U complex	0.70711
DB00547	4216	GR-hnRNP U complex	0.70711
DB00591	4216	GR-hnRNP U complex	0.70711
DB00596	4216	GR-hnRNP U complex	0.70711
DB00620	4216	GR-hnRNP U complex	0.70711
DB00663	4216	GR-hnRNP U complex	0.70711
DB00764	4216	GR-hnRNP U complex	0.70711
DB00769	4216	GR-hnRNP U complex	0.70711
DB00838	4216	GR-hnRNP U complex	0.70711
DB00846	4216	GR-hnRNP U complex	0.70711
DB00860	4216	GR-hnRNP U complex	0.70711
DB00873	4216	GR-hnRNP U complex	0.70711
DB00896	4216	GR-hnRNP U complex	0.70711
DB00959	4216	GR-hnRNP U complex	0.70711
DB01013	4216	GR-hnRNP U complex	0.70711
DB01047	4216	GR-hnRNP U complex	0.5
DB01130	4216	GR-hnRNP U complex	0.70711
DB01222	4216	GR-hnRNP U complex	0.70711
DB01260	4216	GR-hnRNP U complex	0.70711
DB01380	4216	GR-hnRNP U complex	0.70711
DB01384	4216	GR-hnRNP U complex	0.70711
DB01410	4216	GR-hnRNP U complex	0.70711
DB06781	4216	GR-hnRNP U complex	0.70711
DB08906	4216	GR-hnRNP U complex	0.70711
DB00474	5809	GABAA receptor	0.57735
DB01107	5809	GABAA receptor	0.28006
DB01437	5809	GABAA receptor	0.28006
DB00237	2258	VILIP-1-AChR-alpha-4-AChR-beta-2 complex	0.1291
DB00237	2272	PICK1-GRIP1-GLUR2 complex	0.1291
DB00237	5747	2AR-mGluR2 complex	0.15811
DB00237	5809	GABAA receptor	0.2582
DB01356	757	Prune-GSK3beta complex	0.35355
DB01356	2998	Axin-PP2A A-PP2A C-GSK3-beta-beta-catenin complex	0.25
DB01356	3166	AXIN-APC-betaCatenin-GSK3B complex	0.25
DB00284	5386	MLL1-WDR5 complex	0.09623
DB00491	405	Glucosidase 2	0.35355
DB00491	5386	MLL1-WDR5 complex	0.09623
DB04878	5386	MLL1-WDR5 complex	0.19245
DB01273	2258	VILIP-1-AChR-alpha-4-AChR-beta-2 complex	0.28868
DB01271	5280	RAB9-TIP47-MPRI complex	0.57735
DB01279	5280	RAB9-TIP47-MPRI complex	0.57735
DB08911	5222	p14-Mp1-MEK1 complex	0.40825
DB08911	5877	MAP2K1-BRAF-RAF1-YWHAE-KSR1 complex	0.31623
DB08911	5872	BRAF-MAP2K1-MAP2K2-YWHAE complex	0.70711

DB08911	5873	RAF1-MAP2K1-YWHAE complex	0.40825
DB08911	5920	KSR1-RAF1-MEK complex	0.70711
DB08911	5921	KSR1-BRAF-MEK complex	0.70711
DB00928	860	DNMT1-G9a-PCNA complex	0.57735
DB00928	862	DNMT1-G9a complex	0.70711
DB00928	1470	pRb2/p130-multimolecular complex (DNMT1 E2F5 SuV39H1 HDAC1 RBL2)	0.44721
DB00928	1488	DNMT1-RB1-HDAC1-E2F1 complex	0.5
DB00928	1490	DAXX-DNMT1-DMAP1 complex	0.57735
DB00928	1491	RGS6-DNMT1-DMAP1 complex	0.57735
DB00928	5117	pRb2/p130-multimolecular complex (DNMT1 E2F4 SuV39H1 HDAC1 RBL2)	0.44721
DB00928	5695	TIP5-DNMT-HDAC1 complex	0.5
DB01262	860	DNMT1-G9a-PCNA complex	0.57735
DB01262	862	DNMT1-G9a complex	0.70711
DB01262	1470	pRb2/p130-multimolecular complex (DNMT1 E2F5 SuV39H1 HDAC1 RBL2)	0.44721
DB01262	1488	DNMT1-RB1-HDAC1-E2F1 complex	0.5
DB01262	1490	DAXX-DNMT1-DMAP1 complex	0.57735
DB01262	1491	RGS6-DNMT1-DMAP1 complex	0.57735
DB01262	5117	pRb2/p130-multimolecular complex (DNMT1 E2F4 SuV39H1 HDAC1 RBL2)	0.44721
DB01262	5695	TIP5-DNMT-HDAC1 complex	0.5
DB00995	2055	CASP8-CHUK-IKBKB-MALT1-BCL10 complex	0.31623
DB00995	2056	BCL10-CHUK-BCL10-IKBKB complex	0.35355
DB00995	2100	CHUK-IKBKB-MAP3K14 complex	0.40825
DB00995	2101	IKKA-IKKB complex	0.5
DB00995	2104	IKKB-NIK complex	0.5
DB00995	2105	IkappaB kinase complex (IKBKB CHUK IKBKAP NFKBIA RELA MAP3K14)	0.28868
DB00995	2118	CHUK-ERC1-IKBKB-IKBKG	0.35355
DB00995	2121	CHUK-IKBKB-IKBKG complex	0.40825
DB00995	2727	SRC-3 complex	0.26726
DB00995	5194	TNF-alpha/NF-kappa B signaling complex (SEC16A CHUK IKBKB NFKB2 REL IKBKG MAP3K14 RELA FBXW7 USP2)	0.22361
DB00995	5233	TNF-alpha/NF-kappa B signaling complex 5	0.14142
DB00995	5234	IKBKB-CDC37-KIAA1967-HSP90AB1-HSP90AA1 complex	0.31623
DB00995	5266	TNF-alpha/NF-kappa B signaling complex 6	0.18898
DB00995	5828	IKBKG-IKBKB complex	0.5
DB00995	5844	I-kappa-B kinase (IKK) complex	0.40825
DB00626	2710	LRP-1-Alpha-2-M-annexin VI complex	0.40825
DB08888	2351	ITGB6-FYN-FN1 complex	0.33333
DB08888	2375	FN1-TGM2 complex	0.40825
DB08888	2376	ITGA2B-ITGB3-FN1-TGM2 complex	0.28868
DB08888	2383	ITGA5-ITGB1-FN1-TGM2 complex	0.28868
DB08888	2710	LRP-1-Alpha-2-M-annexin VI complex	0.33333
DB08888	2850	ITGA5-ITGB1-FN-1-NOV complex	0.28868
DB00198	1379	GALNS-lysosomal hydrolase 1.27 MDa complex	0.28868
DB00061	2480	CIN85 complex (CIN85 CRK BCAR1 CBL PIK3R1 GRB2 SOS1)	0.37796
DB00061	2529	LAT-PLC-gamma-1-p85-GRB2-CBL-VAV-SLP-76 signaling complex C305 activated	0.37796
DB00061	2534	Cbl-SLP-76-Grb2 complex Fc receptor gamma-R1 stimulated	0.57735
DB00061	2535	SLP-76-Cbl-Grb2-Shc complex Fc receptor gamma-R1 stimulated	0.5
DB00061	2536	PLC-gamma-2-SLP-76-Lyn-Grb2 complex	0.5
DB00061	2540	BCR-ABL (p210 fusion protein)-GRB2 complex	1

DB00061	2542	EGFR-CBL-GRB2 complex	0.57735
DB00061	2892	BCR-ABL (p185 fusion protein)-GRB2 complex	1
DB00061	2893	BCR-ABL (p210 fusion protein)-GRB2-SOS1 complex	0.70711
DB00061	2895	SHC-GRB2 complex	0.70711
DB00061	2917	Grb2-Sos complex Fc receptor gamma-R1 stimulated	0.70711
DB00061	2922	LAT-PLC-gamma-1-p85-GRB2-SOS signaling complex C305 activated	0.44721
DB00061	2957	LAT-GRB2 complex Fyn-mLck(KA) or Syk kinase activated	0.70711
DB00061	3096	ITGA6-ITGB4-SHC1-GRB2 complex	0.5
DB00061	3144	Sos1-Grb2 complex	0.70711
DB00061	3186	GRB2-SHP-2 complex PDGF stimulated	0.70711
DB00062	1088	PRNP-ApolopoproteinE3 complex	0.40825
DB00064	1088	PRNP-ApolopoproteinE3 complex	0.40825
DB05630	1004	RC complex during S-phase of cell cycle	0.27735
DB05630	1005	RC complex during G2/M-phase of cell cycle	0.27735
DB05630	1098	DNA synthesome complex (13 subunits)	0.26726
DB05630	1099	DNA synthesome complex (17 subunits)	0.2357
DB05630	1106	TFIIIC containing-TOP1-SUB1 complex	0.37796
DB05630	1760	TOP1-PSF-P54 complex	0.57735
DB05630	2230	PCNA complex	0.37796
DB05630	3055	Nop56p-associated pre-rRNA complex	0.09806
DB00070	2342	ITGAV-ITGB8-MMP14-TGFB1 complex	0.5
DB00070	3026	TGF-beta receptor II-TGF-beta1 complex	0.70711
DB00070	3027	TGF-beta receptor II-TGF-beta receptor I-TGF-beta1 complex	0.57735
DB00070	3042	TGF-beta-receptor II-TGF-beta1 complex	0.70711
DB08879	5691	TALL1 homo-oligomer complex	1
DB01375	5862	CAV1-VDAC1-ESR1 complex	0.28868
DB00759	1088	PRNP-ApolopoproteinE3 complex	0.28868
DB00759	2007	PRNP homo-oligomer complex	0.40825
DB05013	1519	IL6ST-PRKCD-STAT3 complex	0.40825
DB05013	2471	SRC-PRKCD-CDCP1 complex	0.40825
DB05013	2537	PKC-alpha-PLD1-PLC-gamma-2 signaling complex lacriti[illegible] stimulated	0.40825
DB00063	2355	ITGAV-ITGB3-CD47-FCER2 complex	0.5
DB00063	2356	ITGB3-ITGAV-CD47 complex	0.57735
DB00063	2358	ITGAV-ITGB3-SPP1 complex	0.57735
DB00063	2359	ITGAV-ITGB3-ADAM15 complex	0.57735
DB00063	2362	ITAGV-ITGB3-F11R complex	0.57735
DB00063	2363	ITGAV-ITGB3-PXN-PTK2b complex	0.5
DB00063	2364	ITGAV-ITGB3-ADAM23 complex	0.57735
DB00063	2365	ITGAV-ITGB3-COL4A3 complex	0.57735
DB00063	2366	ITGAV-ITGB3-PPAP2b complex	0.57735
DB00063	2369	ITGAV-ITGB3-EGFR complex	0.57735
DB00063	2370	ITGA2b-ITGB3-CD9 complex	0.57735
DB00063	2374	ITGAV-ITGB3-LAMA4 complex	0.57735
DB00063	2376	ITGA2B-ITGB3-FN1-TGM2 complex	0.5
DB00063	2377	ITGA2b-ITGB3-CD47-SRC complex	0.5
DB00063	2378	ITGA2b-ITGB3-TLN1 complex	0.57735
DB00063	2379	ITGA2B-ITGB3-CIB1 complex	0.57735
DB00063	2381	ITGA2B-ITGB3 complex	0.70711
DB00063	2382	ITGA2B-ITGB3-F11R complex	0.57735
DB00063	2816	ITGAV-ITGB3 complex	0.70711
DB00063	2826	ITGB3-ITGAV-VTN complex	0.57735
DB00063	2846	ITGAV-ITGB3-THBS1 complex	0.57735
DB00063	2849	ITGAV-ITGB3-NOV complex	0.57735
DB00063	2872	ITGA2b-ITGB3-CD9-GP1b-CD47 complex	0.40825

DB00063	2882	ITGA5-ITGB3-COL6A3 complex	0.57735
DB00063	2896	ITGA2b-ITGB3-CD47-FAK complex	0.5
DB00063	3103	ITGAV-ITGB3-SLC3A2 complex	0.57735
DB00063	3115	ITGA2B-ITGB3-ICAM4 complex	0.57735
DB00659	5409	TIAM1-GRIN1 complex	0.14434
DB00659	5641	PSD95-FYN-NR2A complex	0.11785
DB00659	5809	GABAA receptor	0.2357
DB04941	681	(C-CFTR)2-NHERF-ezrin complex	0.40825
DB04941	682	C-CFTR-NHERF(PDZ1 domain)-ezrin complex	0.40825
DB04941	683	C-CFTR-NHERF(PDZ2 domain)-ezrin complex	0.40825
DB04941	687	CFTR-NHERF-beta(2)AR signaling complex	0.40825
DB08820	681	(C-CFTR)2-NHERF-ezrin complex	0.57735
DB08820	682	C-CFTR-NHERF(PDZ1 domain)-ezrin complex	0.57735
DB08820	683	C-CFTR-NHERF(PDZ2 domain)-ezrin complex	0.57735
DB08820	687	CFTR-NHERF-beta(2)AR signaling complex	0.57735
DB00518	1231	FIB-associated protein complex	0.2357
DB00518	3008	60S APC containing complex	0.21822
DB00518	3055	Nop56p-associated pre-rRNA complex	0.05661
DB00643	1231	FIB-associated protein complex	0.28868
DB00643	3008	60S APC containing complex	0.26726
DB00643	3055	Nop56p-associated pre-rRNA complex	0.06934
DB02546	15	NCOR complex	0.16667
DB02546	41	Mi-2/NuRD-MTA2 complex	0.18257
DB02546	49	DNMT3B complex	0.1543
DB02546	54	SIN3 complex	0.30861
DB02546	58	SMRT complex	0.18257
DB02546	61	Mi2/NuRD complex	0.30861
DB02546	62	MeCP1 complex	0.28868
DB02546	282	SNF2h-cohesin-NuRD complex	0.20412
DB02546	283	Sin3 complex	0.30861
DB02546	585	Mi2/NuRD-BCL6-MTA3 complex	0.18257
DB02546	587	NuRD.1 complex	0.28868
DB02546	591	SAP complex (Sin3-associated protein complex)	0.28868
DB02546	592	SAP complex (Sin3-associated protein complex)	0.27217
DB02546	596	SIN3-HDAC-SAP30-ARID4 complex	0.30861
DB02546	614	NRD complex (Nucleosome remodeling and deacetylation complex)	0.30861
DB02546	620	CoREST-HDAC complex	0.30861
DB02546	626	LSD1 complex	0.11323
DB02546	632	Anti-HDAC2 complex	0.19245
DB02546	633	anti-BHC110 complex	0.24618
DB02546	634	XFIM complex	0.36515
DB02546	636	BHC complex	0.33333
DB02546	642	CtBP complex	0.19803
DB02546	643	CtBP core complex	0.27217
DB02546	646	HDAC1-associated protein complex	0.13608
DB02546	648	HDAC1-associated core complex cI	0.2357
DB02546	649	HDAC1-associated core complex cII	0.1291
DB02546	650	HDAC2-asscociated core complex	0.14434
DB02546	659	MeCP1 complex	0.27217
DB02546	685	MeCP1 complex	0.27217
DB02546	691	SIN3-SAP25 complex	0.24618
DB02546	696	BRMS1-SIN3-HDAC complex	0.28868
DB02546	713	BRG1-SIN3A complex	0.10911
DB02546	714	BRM-SIN3A complex	0.21082

DB02546	720	PU.1-SIN3A-HDAC complex	0.2357
DB02546	738	SIN3-ING1b complex I	0.27217
DB02546	739	SIN3-ING1b complex II	0.20412
DB02546	741	NCOR-HDAC3 complex	0.18257
DB02546	745	NCOR-SIN3-RPD3 complex	0.20412
DB02546	747	NCOR-SIN3-HDAC1 complex	0.2357
DB02546	749	MeCP2-SIN3A-HDAC complex	0.40825
DB02546	752	SMRT core complex	0.2357
DB02546	778	LARC complex (LCR-associated remodeling complex)	0.18732
DB02546	803	BRG1-SIN3A-HDAC containing SWI/SNF remodeling complex I	0.12309
DB02546	806	BRM-SIN3A-HDAC complex	0.11785
DB02546	871	BRAF53-BRCA2 complex	0.30861
DB02546	886	MTA1 complex	0.33333
DB02546	888	MTA2 complex	0.27217
DB02546	889	MTA1-HDAC core complex	0.36515
DB02546	1133	ATR-HDAC2 complex	0.28868
DB02546	1134	ATR-HDAC2-CHD4 complex	0.2357
DB02546	1159	p33ING1b-HDAC1 complex	0.28868
DB02546	1233	CoREST-HDAC2 complex	0.28868
DB02546	1257	ALL-1 supercomplex	0.1543
DB02546	1413	NCOR1 complex	0.1291
DB02546	1458	SNF2h-HDAC12 complex	0.28868
DB02546	1470	pRb2/p130-multimolecular complex (DNMT1 E2F5 SuV39H1 HDAC1 RBL2)	0.18257
DB02546	1471	pRb2/p130-multimolecular complex (RB2 E2F5 HDAC1 SUV39H1 P300)	0.18257
DB02546	1488	DNMT1-RB1-HDAC1-E2F1 complex	0.20412
DB02546	1492	BHC110 complex	0.2582
DB02546	1495	PID complex	0.18257
DB02546	1505	NCOR2 complex	0.46291
DB02546	2183	Kaiso-NCOR complex	0.1291
DB02546	2657	ESR1-CDK7-CCNH-MNAT1-MTA1-HDAC2 complex	0.16667
DB02546	2721	HCF-1 complex	0.18732
DB02546	2814	BRCA1-HDAC1-HDAC2 complex	0.4714
DB02546	2851	ING2 complex	0.2357
DB02546	3044	SKI-NCOR1-SIN3A-HDAC1 complex	0.20412
DB02546	3048	mSin3A complex	0.36515
DB02546	3053	mSin3A-HDAC1-HDAC2 complex	0.4714
DB02546	3054	MAD1-mSin3A-HDAC2 complex	0.2357
DB02546	3149	NK-3-Groucho-HIPK2-SIN3A-RbpA48-HDAC1 complex	0.12309
DB02546	3156	CBF1-HDAC1-SMRT complex	0.2357
DB02546	3167	NCOR-SIN3-HDAC-HESX1 complex	0.33333
DB02546	3234	SMAD2-SMAD4-FAST1-TGIF-HDAC1 complex TGF(beta) induced	0.18257
DB02546	3263	HERP1/HEY2-NCOR-SIN3A complex	0.20412
DB02546	3269	RB1-HDAC1-BRG1 complex	0.2357
DB02546	3852	Rb-HDAC1 complex	0.28868
DB02546	3961	SMAD3-cSKI-SIN3A-HDAC1 complex	0.20412
DB02546	5117	pRb2/p130-multimolecular complex (DNMT1 E2F4 SuV39H1 HDAC1 RBL2)	0.18257
DB02546	5118	pRb2/p130-multimolecular complex (RB2 E2F4 HDAC1 SUV39H1 P300)	0.18257
DB02546	5184	SWI/SNF chromatin-remodeling complex	0.18257
DB02546	5260	TCF4-CTNNB1-SUMO1-EP300-HADAC6 complex	0.18257
DB02546	5426	ANCO1-HDAC3 complex	0.28868
DB02546	5609	Emerin regulatory complex	0.27217

DB02546	5614	Emerin complex 32	0.17408
DB02546	5695	TIP5-DNMT-HDAC1 complex	0.20412
DB02546	5870	FE65-TSHZ3-HDAC1 complex	0.2357
DB00818	5809	GABAA receptor	0.2582
DB04895	5658	Nrp1-PlexinD1 complex	0.70711
DB04895	3104	ITGB1-NRP1 complex	0.70711
DB04895	4062	NRP1-VEGFR2-VEGF(165) complex	0.57735
DB04895	5646	FARP2-NRP1-PlexinA1 complex	0.57735
DB04895	5647	FARP2-NRP1-PlexinA2 complex	0.57735
DB04895	5648	FARP2-NRP1-PlexinA3 complex	0.57735
DB04895	5649	FARP2-NRP1-PlexinA4 complex	0.57735
DB04895	5659	SEMA3C-PlexinD1-Nrp1 complex	0.57735
DB04895	5668	PlexinA1-Nrp1 complex	0.70711
DB04895	5669	PlexinA3-Nrp1 complex	0.70711
DB04895	5670	PlexinB1-Nrp1 complex	0.70711
DB04895	5689	SEMA6D-PlexinA1-NRP1 complex	0.57735
DB04895	5696	VEGFA(165)-KDR-NRP1 complex	0.57735
DB04895	5697	VEGFA(165)-KDR-NRP1 complex	0.70711
DB04895	5698	VEGFA(165)-VEGFR2-NRP1 complex	0.57735
DB04895	5701	NRP1-VEGF(165/121) complex	0.70711
DB04895	5731	NRP1-VEGFC complex heparin dependent	0.70711
DB04895	5734	NRP1-VEGFD complex heparin dependent	0.70711
DB04895	5745	PlexinA1-NRP1 complex	0.70711
DB04895	5746	PlexinA1-NRP1-SEMA3A complex	0.57735
DB00042	707	SNARE complex (VAMP2 SNAP25 STX13)	0.33333
DB00042	730	SNARE complex (STX4 SNAP23 VAMP1)	0.33333
DB00042	731	SNARE complex (STX4 SNAP23 VAMP2)	0.33333
DB00042	733	SNARE complex (STX4 VAMP1 VAMP7)	0.33333
DB00042	793	SNARE complex (VAMP2 SNAP25 STX1a CPLX1)	0.28868
DB00042	794	SNARE complex (VAMP2 SNAP25 STX1a CPLX2)	0.28868
DB00042	797	SNARE complex (STX11 VAMP2 SNAP23)	0.33333
DB00042	1137	SNARE complex (VAMP2 SNAP25 STX1a CPLX1 CPLX3)	0.2582
DB00042	1138	SNARE complex (VAMP2 SNAP25 STX1a CPLX3 CPLX4)	0.2582
DB00042	1139	SNARE complex (VAMP2 SNAP25 STX1a STX3 CPLX1 CPLX3 CPLX4)	0.21822
DB00042	1874	SNARE complex (SNAP25 VAMP3 VAMP2 NAPB STX13)	0.2582
DB06716	5809	GABAA receptor	0.40825
DB08889	181	26S proteasome	0.26112
DB08889	191	20S proteasome	0.32733
DB08889	192	PA28-20S proteasome	0.30619
DB08889	193	PA700-20S-PA28 complex	0.20412
DB08889	194	PA28gamma-20S proteasome	0.31623
DB01590	1893	mTOR-RICTOR complex	0.57735
DB01590	1895	RICTOR-mTOR complex	0.57735
DB01590	1897	RAPTOR-mTOR complex	0.57735
DB01590	2969	mTORC2 complex (mTOR/FRAP1 LST8 mAVO3/RICTOR)	0.57735
DB01590	2970	mTORC1 complex (mTOR/FRAP1 LST8 RAPTOR)	0.57735
DB01590	2985	mTOR-signaling complex	0.70711
DB01590	2990	mTOR-signaling complex (FRAP1/mTOR GBL RAPTOR)	0.57735
DB01590	2991	mTOR-signaling complex (mTOR/FRAP1 RAPTOR)	0.70711
DB01590	3979	mTORC2 complex (mTOR/FRAP1 LST8 mAVO3/RICTOR SIN1)	0.5
DB01590	3980	mTOR-RAPTOR complex	0.57735
DB06287	1893	mTOR-RICTOR complex	0.57735
DB06287	1895	RICTOR-mTOR complex	0.57735

DB06287	1897	RAPTOR-mTOR complex	0.57735
DB06287	2969	mTORC2 complex (mTOR/FRAP1 LST8 mAVO3/RICTOR)	0.57735
DB06287	2970	mTORC1 complex (mTOR/FRAP1 LST8 RAPTOR)	0.57735
DB06287	2985	mTOR-signaling complex	0.70711
DB06287	2990	mTOR-signaling complex (FRAP1/mTOR GBL RAPTOR)	0.57735
DB06287	2991	mTOR-signaling complex (mTOR/FRAP1 RAPTOR)	0.70711
DB06287	3979	mTORC2 complex (mTOR/FRAP1 LST8 mAVO3/RICTOR SIN1)	0.5
DB06287	3980	mTOR-RAPTOR complex	0.57735
DB08877	2028	JAK2-IL12RB2 complex	0.5
DB08877	5178	JAK2-PAFR-TYK2 complex	0.40825
DB08877	5564	LMO4-gp130 complex	0.31623
DB08895	2028	JAK2-IL12RB2 complex	0.40825
DB08895	5178	JAK2-PAFR-TYK2 complex	0.33333
DB08895	5564	LMO4-gp130 complex	0.2582
DB05389	3071	CTLH complex	0.31623
DB05829	1618	G protein complex (PTHR1 GNB1 GNG2)	0.40825
DB06285	1618	G protein complex (PTHR1 GNB1 GNG2)	0.57735
DB00435	407	Guanylyl cyclase soluble (GUCY1A2 GUCY1B3)	0.70711
DB01020	407	Guanylyl cyclase soluble (GUCY1A2 GUCY1B3)	0.70711
DB04865	306	Ribosome cytoplasmic	0.07857
DB04865	308	60S ribosomal subunit cytoplasmic	0.10314
DB04865	3055	Nop56p-associated pre-rRNA complex	0.06934
DB08908	5805	PGAM5-KEAP1-NRF2 complex	0.57735
DB00189	5809	GABAA receptor	0.28868
DB00349	5809	GABAA receptor	0.28868
DB00404	5809	GABAA receptor	0.28868
DB00475	5809	GABAA receptor	0.28868
