# Supplementary material for: Inferring drug-disease associations based on known protein complexes
Source: BMC Med Genomics. 2015 May 29;8(Suppl 2):S2. doi: 10.1186/1755-8794-8-S2-S2 (PMC4460611; doi:10.1186/1755-8794-8-S2-S2)
Supplement: Additional file 5 — Table illustrating the information of complex-disease network. [file 1755-8794-8-S2-S2-S5.PDF]

Disease Name	Related Gene Entrez ID	Complex Name	Weight
Adenocarcinoma, Mucinous	1345	Septin complex	0.2582
Hemorrhagic fevers, Viral	299	IRF3-CBP complex	0.20412
Hemorrhagic fevers, Viral	2355	ITGAV-ITGB3-CD47-FCER2 complex	0.20412
Hemorrhagic fevers, Viral	2356	ITGB3-ITGAV-CD47 complex	0.2357
Hemorrhagic fevers, Viral	2358	ITGAV-ITGB3-SPP1 complex	0.2357
Hemorrhagic fevers, Viral	2359	ITGAV-ITGB3-ADAM15 complex	0.2357
Hemorrhagic fevers, Viral	2362	ITAGV-ITGB3-F11R complex	0.2357
Hemorrhagic fevers, Viral	2363	ITGAV-ITGB3-PXN-PTK2b complex	0.20412
Hemorrhagic fevers, Viral	2364	ITGAV-ITGB3-ADAM23 complex	0.2357
Hemorrhagic fevers, Viral	2365	ITGAV-ITGB3-COL4A3 complex	0.2357
Hemorrhagic fevers, Viral	2366	ITGAV-ITGB3-PPAP2b complex	0.2357
Hemorrhagic fevers, Viral	2369	ITGAV-ITGB3-EGFR complex	0.2357
Hemorrhagic fevers, Viral	2370	ITGA2b-ITGB3-CD9 complex	0.2357
Hemorrhagic fevers, Viral	2374	ITGAV-ITGB3-LAMA4 complex	0.2357
Hemorrhagic fevers, Viral	2376	ITGA2B-ITGB3-FN1-TGM2 complex	0.20412
Hemorrhagic fevers, Viral	2377	ITGA2b-ITGB3-CD47-SRC complex	0.20412
Hemorrhagic fevers, Viral	2378	ITGA2b-ITGB3-TLN1 complex	0.2357
Hemorrhagic fevers, Viral	2379	ITGA2B-ITGB3-CIB1 complex	0.2357
Hemorrhagic fevers, Viral	2381	ITGA2B-ITGB3 complex	0.28868
Hemorrhagic fevers, Viral	2382	ITGA2B-ITGB3-F11R complex	0.2357
Hemorrhagic fevers, Viral	2816	ITGAV-ITGB3 complex	0.28868
Hemorrhagic fevers, Viral	2826	ITGB3-ITGAV-VTN complex	0.2357
Hemorrhagic fevers, Viral	2846	ITGAV-ITGB3-THBS1 complex	0.2357
Hemorrhagic fevers, Viral	2849	ITGAV-ITGB3-NOV complex	0.2357
Hemorrhagic fevers, Viral	2872	ITGA2b-ITGB3-CD9-GP1b-CD47 complex	0.16667
Hemorrhagic fevers, Viral	2882	ITGA5-ITGB3-COL6A3 complex	0.2357
Hemorrhagic fevers, Viral	2896	ITGA2b-ITGB3-CD47-FAK complex	0.20412
Hemorrhagic fevers, Viral	3103	ITGAV-ITGB3-SLC3A2 complex	0.2357
Hemorrhagic fevers, Viral	3115	ITGA2B-ITGB3-ICAM4 complex	0.2357
Hypertension, Pulmonary	298	VEGF transcriptional complex	0.09366
Hypertension, Pulmonary	527	TRPC3-TRPC4 channel complex redox-sensitive	0.32444
Hypertension, Pulmonary	536	TRPC1-TRPC3-TRPC7 complex	0.13245
Hypertension, Pulmonary	1816	JUN-TCF4-CTNNB1 complex	0.13245
Hypertension, Pulmonary	1986	Endoglin homodimer complex	0.22942
Hypertension, Pulmonary	2443	ITGA9-ITGB1-TNC complex	0.13245
Hypertension, Pulmonary	2692	SMAD3-SMAD4-cJun-cFos complex	0.11471
Hypertension, Pulmonary	2693	NFAT-JUN-FOS DNA-protein complex	0.13245
Hypertension, Pulmonary	2694	ERG-JUN-FOS DNA-protein complex	0.13245
Hypertension, Pulmonary	2695	ETS2-FOS-JUN complex	0.13245
Hypertension, Pulmonary	2699	ER-alpha-GRIP1-c-Jun complex	0.13245
Hypertension, Pulmonary	2700	ER-alpha-c-Jun complex	0.16222
Hypertension, Pulmonary	2708	SMAD3-SMAD4-cJUN complex	0.13245
Hypertension, Pulmonary	3043	BMP2-BRIA complex	0.13245
Hypertension, Pulmonary	3162	TF-FVIIa-FXa-TFPI complex	0.11471

Hypertension, Pulmonary	3710	CHL2-BMP2 complex	0.16222
Hypertension, Pulmonary	3711	CHL2-BMP2-TSG complex	0.13245
Hypertension, Pulmonary	5273	VHL-TBP1-HIF1A complex	0.13245
Hypertension, Pulmonary	5276	HIF1A-OS9-EGLN1 complex	0.13245
Hypertension, Pulmonary	5277	HIF1A-OS9-EGLN3 complex	0.13245
Hypertension, Pulmonary	5382	ARNT-HIF1A complex	0.16222
Hypertension, Pulmonary	5498	ILK-PARVB-ARHGEF6 complex	0.13245
Hypertension, Pulmonary	5499	PARVB-ARHGEF6 complex	0.16222
Infertility, Male	441	TFTC-type histone acetyl transferase complex	0.06155
Infertility, Male	681	(C-CFTR)2-NHERF-ezrin complex	0.11785
Infertility, Male	682	C-CFTR-NHERF(PDZ1 domain)-ezrin complex	0.11785
Infertility, Male	683	C-CFTR-NHERF(PDZ2 domain)-ezrin complex	0.11785
Infertility, Male	687	CFTR-NHERF-beta(2)AR signaling complex	0.11785
Infertility, Male	871	BRAF53-BRCA2 complex	0.07715
Infertility, Male	1054	ESR1-RELA-BCL3-NCOA3 complex	0.10206
Infertility, Male	1154	DSS1 complex	0.05661
Infertility, Male	2124	IKK-alpha--ER-alpha-AIB1 complex	0.11785
Infertility, Male	2159	AR-AKT-APPL complex	0.11785
Infertility, Male	2160	AOF2-AR complex	0.14434
Infertility, Male	2470	p130Cas-ER-alpha-cSrc-kinase- PI3-kinase p85-subunit complex	0.10206
Infertility, Male	2657	ESR1-CDK7-CCNH-MNAT1-MTA1-HDAC2 complex	0.08333
Infertility, Male	2670	Er-alpha-p53-hdm2 complex	0.11785
Infertility, Male	2699	ER-alpha-GRIP1-c-Jun complex	0.11785
Infertility, Male	2700	ER-alpha-c-Jun complex	0.14434
Infertility, Male	2818	BRCA1-BARD1-BRCA2-DNA damage complex III	0.11785
Infertility, Male	5400	BRCC complex	0.09129
Infertility, Male	5862	CAV1-VDAC1-ESR1 complex	0.11785
Kidney tubular necrosis, acute	159	Condensin I-PARP-1-XRCC1 complex	0.1543
Kidney tubular necrosis, acute	1004	RC complex during S-phase of cell cycle	0.11323
Kidney tubular necrosis, acute	1005	RC complex during G2/M-phase of cell cycle	0.11323
Kidney tubular necrosis, acute	1193	Rap1 complex	0.1543
Kidney tubular necrosis, acute	1226	H2AX complex I	0.1543
Kidney tubular necrosis, acute	1728	CTCF-nucleophosmin-PARP-HIS-KPNA-LMNA-TOP complex	0.13608
Kidney tubular necrosis, acute	1729	TLE1 corepressor complex (MASH1 promoter-corepressor complex)	0.1291
Kidney tubular necrosis, acute	2010	AXL homodimer complex	0.40825
Kidney tubular necrosis, acute	2625	CDK8-MED6-PARP1 complex	0.2357
Kidney tubular necrosis, acute	3137	MASH1 promoter-coactivator complex	0.12309
Kidney tubular necrosis, acute	3142	CAMK2-delta-MASH1 promoter-coactivator complex	0.14434
Kidney tubular necrosis, acute	5179	NCOA6-DNA-PK-Ku-PARP1 complex	0.18257
Kidney tubular necrosis, acute	5235	WRN-Ku70-Ku80-PARP1 complex	0.20412
Labor, Premature	553	RHOA-IP3R-TRPC1 complex	0.14907
Labor, Premature	815	MRIP-MBS-RHOA complex	0.14907
Labor, Premature	816	MRIP-RHOA complex	0.18257
Labor, Premature	3153	GNAQ-GEFT-RHOA complex	0.14907
Pemphigoid, Bullous	2318	ITGA6-ITGB4-Laminin10/12 complex	0.28284

Pemphigoid, Bullous	2319	ITGA6-ITGB4-Laminin10/12 complex	0.28284
Pemphigoid, Bullous	2320	ITGA6-ITGB4-CD151 complex	0.36515
Pemphigoid, Bullous	2321	ITGA6-ITGB4-FYN complex	0.36515
Pemphigoid, Bullous	2322	ITGA6-ITGB4-LAMA5 complex	0.36515
Pemphigoid, Bullous	2323	ITGA6-ITGB4 complex	0.44721
Pemphigoid, Bullous	2355	ITGAV-ITGB3-CD47-FCER2 complex	0.15811
Pemphigoid, Bullous	2356	ITGB3-ITGAV-CD47 complex	0.18257
Pemphigoid, Bullous	2358	ITGAV-ITGB3-SPP1 complex	0.18257
Pemphigoid, Bullous	2359	ITGAV-ITGB3-ADAM15 complex	0.18257
Pemphigoid, Bullous	2362	ITAGV-ITGB3-F11R complex	0.18257
Pemphigoid, Bullous	2363	ITGAV-ITGB3-PXN-PTK2b complex	0.15811
Pemphigoid, Bullous	2364	ITGAV-ITGB3-ADAM23 complex	0.18257
Pemphigoid, Bullous	2365	ITGAV-ITGB3-COL4A3 complex	0.18257
Pemphigoid, Bullous	2366	ITGAV-ITGB3-PPAP2b complex	0.18257
Pemphigoid, Bullous	2369	ITGAV-ITGB3-EGFR complex	0.18257
Pemphigoid, Bullous	2370	ITGA2b-ITGB3-CD9 complex	0.18257
Pemphigoid, Bullous	2374	ITGAV-ITGB3-LAMA4 complex	0.18257
Pemphigoid, Bullous	2376	ITGA2B-ITGB3-FN1-TGM2 complex	0.15811
Pemphigoid, Bullous	2377	ITGA2b-ITGB3-CD47-SRC complex	0.15811
Pemphigoid, Bullous	2378	ITGA2b-ITGB3-TLN1 complex	0.18257
Pemphigoid, Bullous	2379	ITGA2B-ITGB3-CIB1 complex	0.18257
Pemphigoid, Bullous	2381	ITGA2B-ITGB3 complex	0.22361
Pemphigoid, Bullous	2382	ITGA2B-ITGB3-F11R complex	0.18257
Pemphigoid, Bullous	2383	ITGA5-ITGB1-FN1-TGM2 complex	0.15811
Pemphigoid, Bullous	2384	ITGA5-ITGB1-ADAM15 complex	0.18257
Pemphigoid, Bullous	2385	ITGA5-ITGB4 complex	0.22361
Pemphigoid, Bullous	2388	Itga5-Itgb1-Fn1-Sfrp2 complex	0.15811
Pemphigoid, Bullous	2390	CD98-LAT2-ITGB1 complex	0.15811
Pemphigoid, Bullous	2395	ITGA7-ITGB1-CD151 complex	0.18257
Pemphigoid, Bullous	2396	ITGA7-ITGB1-CD9 complex	0.18257
Pemphigoid, Bullous	2397	ITGA7-ITGB1-ITGB1BP3 complex	0.18257
Pemphigoid, Bullous	2398	ITGA3-ITGB1-BSG complex	0.18257
Pemphigoid, Bullous	2399	ITGA3-ITGB1-CD63 complex	0.18257
Pemphigoid, Bullous	2400	ITGA3-ITGB1-CD151 complex	0.18257
Pemphigoid, Bullous	2401	ITGA3-ITGB1-THBS1 complex	0.18257
Pemphigoid, Bullous	2406	ITGA3-ITGB1 complex	0.22361
Pemphigoid, Bullous	2411	ITGA6-ITGB1-CD151 complex	0.36515
Pemphigoid, Bullous	2413	ITGA6-ITGB1 complex	0.44721
Pemphigoid, Bullous	2416	ITGB1-RAP1A-PKD1 complex	0.18257
Pemphigoid, Bullous	2417	ITGA4-ITGB1-EMILIN1 complex	0.18257
Pemphigoid, Bullous	2418	ITGA4-ITGB1 complex	0.22361
Pemphigoid, Bullous	2419	ITGA4-ITGB1-CD81 complex	0.18257
Pemphigoid, Bullous	2420	ITGA4-ITGB1-CD53 complex	0.18257
Pemphigoid, Bullous	2421	ITGA4-ITGB1-VCAM1 complex	0.18257
Pemphigoid, Bullous	2422	ITGA4-ITGB1-JAM2 complex	0.18257
Pemphigoid, Bullous	2423	ITGA4-ITGB1-CD47 complex	0.18257
Pemphigoid, Bullous	2424	ITGA4-ITGB1-CD63 complex	0.18257

Pemphigoid, Bullous	2425	ITGA4-ITGB1-PXN complex	0.18257
Pemphigoid, Bullous	2426	ITGA4-ITGB1-THBS1 complex	0.18257
Pemphigoid, Bullous	2428	ITGA4-ITGB1-THBS2 complex	0.18257
Pemphigoid, Bullous	2429	ITGA2-ITGB1-CD47 complex	0.18257
Pemphigoid, Bullous	2430	ITGA2-ITGB1-CHAD complex	0.18257
Pemphigoid, Bullous	2431	ITGA2-ITGB1-COL6A3 complex	0.18257
Pemphigoid, Bullous	2432	ITGA2-ITGB1 complex	0.22361
Pemphigoid, Bullous	2434	ITGA1-ITGB1-COL6A3 complex	0.18257
Pemphigoid, Bullous	2435	ITGA1-ITGB1-PTPN2 complex	0.18257
Pemphigoid, Bullous	2436	ITGAV-ITGB1 complex	0.22361
Pemphigoid, Bullous	2437	ITGA6-ITGB1-CYR61 complex	0.36515
Pemphigoid, Bullous	2439	ITGA8-ITGB1 complex	0.22361
Pemphigoid, Bullous	2440	ITGA9-ITGB1-ADAM9 complex	0.18257
Pemphigoid, Bullous	2441	Itga9-Itgb1-Adam2 complex	0.18257
Pemphigoid, Bullous	2442	ITGA9-ITGB1-VCAM1 complex	0.18257
Pemphigoid, Bullous	2443	ITGA9-ITGB1-TNC complex	0.18257
Pemphigoid, Bullous	2444	ITGB1-ITGA9 complex	0.22361
Pemphigoid, Bullous	2445	ITGA9-ITGB1-ADAM15 complex	0.18257
Pemphigoid, Bullous	2446	ITGA9-ITGB1-FIGF complex	0.18257
Pemphigoid, Bullous	2447	ITGA9-ITGB1-ADAM12 complex	0.18257
Pemphigoid, Bullous	2770	ITGA6-ITGB4-CD9 complex	0.36515
Pemphigoid, Bullous	2816	ITGAV-ITGB3 complex	0.22361
Pemphigoid, Bullous	2826	ITGB3-ITGAV-VTN complex	0.18257
Pemphigoid, Bullous	2846	ITGAV-ITGB3-THBS1 complex	0.18257
Pemphigoid, Bullous	2849	ITGAV-ITGB3-NOV complex	0.18257
Pemphigoid, Bullous	2850	ITGA5-ITGB1-FN-1-NOV complex	0.15811
Pemphigoid, Bullous	2853	ITGA5-ITGB1-CAL4A3 complex	0.18257
Pemphigoid, Bullous	2872	ITGA2b-ITGB3-CD9-GP1b-CD47 complex	0.1291
Pemphigoid, Bullous	2882	ITGA5-ITGB3-COL6A3 complex	0.18257
Pemphigoid, Bullous	2885	ITGAV-ITGB1-SPP1 complex	0.18257
Pemphigoid, Bullous	2896	ITGA2b-ITGB3-CD47-FAK complex	0.15811
Pemphigoid, Bullous	2964	ITGA9-ITGB1-ADAM1 complex	0.18257
Pemphigoid, Bullous	2965	ITGA9-ITGB1-ADAM3 complex	0.18257
Pemphigoid, Bullous	2971	ITGA9-ITGB1-VEGFC complex	0.18257
Pemphigoid, Bullous	2972	ITGA9-ITGB1-VEGFA complex	0.18257
Pemphigoid, Bullous	2989	ITGA9-ITGB1-ADAM8 complex	0.18257
Pemphigoid, Bullous	3035	LAT2-ITGB1 complex	0.22361
Pemphigoid, Bullous	3057	ITGA10-ITGB1 complex	0.22361
Pemphigoid, Bullous	3058	ITGA11-ITGB1 complex	0.22361
Pemphigoid, Bullous	3059	ITGA11-ITGB1-COL1A1 complex	0.18257
Pemphigoid, Bullous	3096	ITGA6-ITGB4-SHC1-GRB2 complex	0.31623
Pemphigoid, Bullous	3103	ITGAV-ITGB3-SLC3A2 complex	0.18257
Pemphigoid, Bullous	3104	ITGB1-NRP1 complex	0.22361
Pemphigoid, Bullous	3111	ITGA9-ITGB1-SPP1 complex	0.18257
Pemphigoid, Bullous	3112	ITGA5-ITGB1-SPP1 complex	0.18257
Pemphigoid, Bullous	3115	ITGA2B-ITGB3-ICAM4 complex	0.18257
Pleural effusion, Malignant	1095	SNX complex (SNX1a SNX2 SNX4 EGFR)	0.15076

Pleural effusion, Malignant	1185	EGFR-containing signaling complex	0.15076
Pleural effusion, Malignant	2342	ITGAV-ITGB8-MMP14-TGFB1 complex	0.15076
Pleural effusion, Malignant	2343	ITGAV-ITGB5-PLAUR complex	0.17408
Pleural effusion, Malignant	2345	ITGAV-ITGB5-ICAM4 complex	0.17408
Pleural effusion, Malignant	2346	ITGAV-ITGB5-ADAM9 complex	0.17408
Pleural effusion, Malignant	2347	ITGAV-ITGB5-SPP1 complex	0.17408
Pleural effusion, Malignant	2348	ITGAV-ITGB5-CYR61 complex	0.17408
Pleural effusion, Malignant	2350	ITGAV-ITGB5 complex	0.2132
Pleural effusion, Malignant	2352	ITGAV-ITGB6-SPP1 complex	0.17408
Pleural effusion, Malignant	2353	ITGAV-ITGB6-TGFB3 complex	0.17408
Pleural effusion, Malignant	2354	ITGAV-ITGB6 complex	0.2132
Pleural effusion, Malignant	2355	ITGAV-ITGB3-CD47-FCER2 complex	0.15076
Pleural effusion, Malignant	2356	ITGB3-ITGAV-CD47 complex	0.17408
Pleural effusion, Malignant	2358	ITGAV-ITGB3-SPP1 complex	0.17408
Pleural effusion, Malignant	2359	ITGAV-ITGB3-ADAM15 complex	0.17408
Pleural effusion, Malignant	2362	ITAGV-ITGB3-F11R complex	0.17408
Pleural effusion, Malignant	2363	ITGAV-ITGB3-PXN-PTK2b complex	0.15076
Pleural effusion, Malignant	2364	ITGAV-ITGB3-ADAM23 complex	0.17408
Pleural effusion, Malignant	2365	ITGAV-ITGB3-COL4A3 complex	0.17408
Pleural effusion, Malignant	2366	ITGAV-ITGB3-PPAP2b complex	0.17408
Pleural effusion, Malignant	2369	ITGAV-ITGB3-EGFR complex	0.34816
Pleural effusion, Malignant	2374	ITGAV-ITGB3-LAMA4 complex	0.17408
Pleural effusion, Malignant	2383	ITGA5-ITGB1-FN1-TGM2 complex	0.15076
Pleural effusion, Malignant	2384	ITGA5-ITGB1-ADAM15 complex	0.17408
Pleural effusion, Malignant	2385	ITGA5-ITGB4 complex	0.2132
Pleural effusion, Malignant	2388	Itga5-Itgb1-Fn1-Sfrp2 complex	0.15076
Pleural effusion, Malignant	2390	CD98-LAT2-ITGB1 complex	0.15076
Pleural effusion, Malignant	2395	ITGA7-ITGB1-CD151 complex	0.17408
Pleural effusion, Malignant	2396	ITGA7-ITGB1-CD9 complex	0.17408
Pleural effusion, Malignant	2397	ITGA7-ITGB1-ITGB1BP3 complex	0.17408
Pleural effusion, Malignant	2398	ITGA3-ITGB1-BSG complex	0.17408
Pleural effusion, Malignant	2399	ITGA3-ITGB1-CD63 complex	0.17408
Pleural effusion, Malignant	2400	ITGA3-ITGB1-CD151 complex	0.17408
Pleural effusion, Malignant	2401	ITGA3-ITGB1-THBS1 complex	0.17408
Pleural effusion, Malignant	2406	ITGA3-ITGB1 complex	0.2132
Pleural effusion, Malignant	2411	ITGA6-ITGB1-CD151 complex	0.17408
Pleural effusion, Malignant	2413	ITGA6-ITGB1 complex	0.2132
Pleural effusion, Malignant	2416	ITGB1-RAP1A-PKD1 complex	0.17408
Pleural effusion, Malignant	2417	ITGA4-ITGB1-EMILIN1 complex	0.17408
Pleural effusion, Malignant	2418	ITGA4-ITGB1 complex	0.2132
Pleural effusion, Malignant	2419	ITGA4-ITGB1-CD81 complex	0.17408
Pleural effusion, Malignant	2420	ITGA4-ITGB1-CD53 complex	0.17408
Pleural effusion, Malignant	2421	ITGA4-ITGB1-VCAM1 complex	0.17408
Pleural effusion, Malignant	2422	ITGA4-ITGB1-JAM2 complex	0.17408
Pleural effusion, Malignant	2423	ITGA4-ITGB1-CD47 complex	0.17408
Pleural effusion, Malignant	2424	ITGA4-ITGB1-CD63 complex	0.17408
Pleural effusion, Malignant	2425	ITGA4-ITGB1-PXN complex	0.17408

Pleural effusion, Malignant	2426	ITGA4-ITGB1-THBS1 complex	0.17408
Pleural effusion, Malignant	2428	ITGA4-ITGB1-THBS2 complex	0.17408
Pleural effusion, Malignant	2429	ITGA2-ITGB1-CD47 complex	0.17408
Pleural effusion, Malignant	2430	ITGA2-ITGB1-CHAD complex	0.17408
Pleural effusion, Malignant	2431	ITGA2-ITGB1-COL6A3 complex	0.17408
Pleural effusion, Malignant	2432	ITGA2-ITGB1 complex	0.2132
Pleural effusion, Malignant	2434	ITGA1-ITGB1-COL6A3 complex	0.17408
Pleural effusion, Malignant	2435	ITGA1-ITGB1-PTPN2 complex	0.17408
Pleural effusion, Malignant	2436	ITGAV-ITGB1 complex	0.4264
Pleural effusion, Malignant	2437	ITGA6-ITGB1-CYR61 complex	0.17408
Pleural effusion, Malignant	2439	ITGA8-ITGB1 complex	0.2132
Pleural effusion, Malignant	2440	ITGA9-ITGB1-ADAM9 complex	0.17408
Pleural effusion, Malignant	2441	Itga9-Itgb1-Adam2 complex	0.17408
Pleural effusion, Malignant	2442	ITGA9-ITGB1-VCAM1 complex	0.17408
Pleural effusion, Malignant	2443	ITGA9-ITGB1-TNC complex	0.17408
Pleural effusion, Malignant	2444	ITGB1-ITGA9 complex	0.2132
Pleural effusion, Malignant	2445	ITGA9-ITGB1-ADAM15 complex	0.17408
Pleural effusion, Malignant	2446	ITGA9-ITGB1-FIGF complex	0.17408
Pleural effusion, Malignant	2447	ITGA9-ITGB1-ADAM12 complex	0.17408
Pleural effusion, Malignant	2453	Multiprotein complex (monoubiquitination)	0.15076
Pleural effusion, Malignant	2454	CIN85-CBL-SH3GL2-EGFR complex EGF stimulated	0.15076
Pleural effusion, Malignant	2489	NCR3-CD247 complex	0.2132
Pleural effusion, Malignant	2542	EGFR-CBL-GRB2 complex	0.17408
Pleural effusion, Malignant	2709	MMP-9-TIMP-1-LRP complex	0.17408
Pleural effusion, Malignant	2816	ITGAV-ITGB3 complex	0.2132
Pleural effusion, Malignant	2826	ITGB3-ITGAV-VTN complex	0.17408
Pleural effusion, Malignant	2846	ITGAV-ITGB3-THBS1 complex	0.17408
Pleural effusion, Malignant	2849	ITGAV-ITGB3-NOV complex	0.17408
Pleural effusion, Malignant	2850	ITGA5-ITGB1-FN-1-NOV complex	0.15076
Pleural effusion, Malignant	2853	ITGA5-ITGB1-CAL4A3 complex	0.17408
Pleural effusion, Malignant	2885	ITGAV-ITGB1-SPP1 complex	0.34816
Pleural effusion, Malignant	2964	ITGA9-ITGB1-ADAM1 complex	0.17408
Pleural effusion, Malignant	2965	ITGA9-ITGB1-ADAM3 complex	0.17408
Pleural effusion, Malignant	2971	ITGA9-ITGB1-VEGFC complex	0.17408
Pleural effusion, Malignant	2972	ITGA9-ITGB1-VEGFA complex	0.17408
Pleural effusion, Malignant	2989	ITGA9-ITGB1-ADAM8 complex	0.17408
Pleural effusion, Malignant	3035	LAT2-ITGB1 complex	0.2132
Pleural effusion, Malignant	3057	ITGA10-ITGB1 complex	0.2132
Pleural effusion, Malignant	3058	ITGA11-ITGB1 complex	0.2132
Pleural effusion, Malignant	3059	ITGA11-ITGB1-COL1A1 complex	0.17408
Pleural effusion, Malignant	3103	ITGAV-ITGB3-SLC3A2 complex	0.17408
Pleural effusion, Malignant	3104	ITGB1-NRP1 complex	0.2132
Pleural effusion, Malignant	3110	ITGAV-P2RY2-GNA12 complex	0.17408
Pleural effusion, Malignant	3111	ITGA9-ITGB1-SPP1 complex	0.17408
Pleural effusion, Malignant	3112	ITGA5-ITGB1-SPP1 complex	0.17408
Pleural effusion, Malignant	3117	ITGB5-ITGAV-VTN complex	0.17408
Pleural effusion, Malignant	3678	RIN1-STAM2-EGFR complex EGF stimulated	0.17408

Pleural effusion, Malignant	5171	SH3KBP1-CBLB-EGFR complex	0.17408
Prostatic hypertrophy, Benign	1514	IL4-IL4R complex	0.16667
Prostatic hypertrophy, Benign	1515	IL4-IL4R-IL2RG complex	0.13608
Prostatic hypertrophy, Benign	2710	LRP-1-Alpha-2-M-annexin VI complex	0.13608
Purpura, Thrombocytopenic, Idiopathic	120	Lymphotoxin beta receptor complex	0.1543
Purpura, Thrombocytopenic, Idiopathic	1514	IL4-IL4R complex	0.18898
Purpura, Thrombocytopenic, Idiopathic	1515	IL4-IL4R-IL2RG complex	0.1543
Purpura, Thrombocytopenic, Idiopathic	1810	ITGA4-PXN-GIT1 complex	0.1543
Purpura, Thrombocytopenic, Idiopathic	2355	ITGAV-ITGB3-CD47-FCER2 complex	0.13363
Purpura, Thrombocytopenic, Idiopathic	2356	ITGB3-ITGAV-CD47 complex	0.1543
Purpura, Thrombocytopenic, Idiopathic	2358	ITGAV-ITGB3-SPP1 complex	0.1543
Purpura, Thrombocytopenic, Idiopathic	2359	ITGAV-ITGB3-ADAM15 complex	0.1543
Purpura, Thrombocytopenic, Idiopathic	2362	ITAGV-ITGB3-F11R complex	0.1543
Purpura, Thrombocytopenic, Idiopathic	2363	ITGAV-ITGB3-PXN-PTK2b complex	0.13363
Purpura, Thrombocytopenic, Idiopathic	2364	ITGAV-ITGB3-ADAM23 complex	0.1543
Purpura, Thrombocytopenic, Idiopathic	2365	ITGAV-ITGB3-COL4A3 complex	0.1543
Purpura, Thrombocytopenic, Idiopathic	2366	ITGAV-ITGB3-PPAP2b complex	0.1543
Purpura, Thrombocytopenic, Idiopathic	2369	ITGAV-ITGB3-EGFR complex	0.1543
Purpura, Thrombocytopenic, Idiopathic	2370	ITGA2b-ITGB3-CD9 complex	0.30861
Purpura, Thrombocytopenic, Idiopathic	2374	ITGAV-ITGB3-LAMA4 complex	0.1543
Purpura, Thrombocytopenic, Idiopathic	2376	ITGA2B-ITGB3-FN1-TGM2 complex	0.26726
Purpura, Thrombocytopenic, Idiopathic	2377	ITGA2b-ITGB3-CD47-SRC complex	0.26726
Purpura, Thrombocytopenic, Idiopathic	2378	ITGA2b-ITGB3-TLN1 complex	0.30861
Purpura, Thrombocytopenic, Idiopathic	2379	ITGA2B-ITGB3-CIB1 complex	0.30861
Purpura, Thrombocytopenic, Idiopathic	2381	ITGA2B-ITGB3 complex	0.37796
Purpura, Thrombocytopenic, Idiopathic	2382	ITGA2B-ITGB3-F11R complex	0.30861
Purpura, Thrombocytopenic, Idiopathic	2383	ITGA5-ITGB1-FN1-TGM2 complex	0.13363
Purpura, Thrombocytopenic, Idiopathic	2384	ITGA5-ITGB1-ADAM15 complex	0.1543

Purpura, Thrombocytopenic, Idiopathic	2385	ITGA5-ITGB4 complex	0.18898
Purpura, Thrombocytopenic, Idiopathic	2388	Itga5-Itgb1-Fn1-Sfrp2 complex	0.13363
Purpura, Thrombocytopenic, Idiopathic	2390	CD98-LAT2-ITGB1 complex	0.13363
Purpura, Thrombocytopenic, Idiopathic	2395	ITGA7-ITGB1-CD151 complex	0.1543
Purpura, Thrombocytopenic, Idiopathic	2396	ITGA7-ITGB1-CD9 complex	0.1543
Purpura, Thrombocytopenic, Idiopathic	2397	ITGA7-ITGB1-ITGB1BP3 complex	0.1543
Purpura, Thrombocytopenic, Idiopathic	2398	ITGA3-ITGB1-BSG complex	0.1543
Purpura, Thrombocytopenic, Idiopathic	2399	ITGA3-ITGB1-CD63 complex	0.1543
Purpura, Thrombocytopenic, Idiopathic	2400	ITGA3-ITGB1-CD151 complex	0.1543
Purpura, Thrombocytopenic, Idiopathic	2401	ITGA3-ITGB1-THBS1 complex	0.1543
Purpura, Thrombocytopenic, Idiopathic	2406	ITGA3-ITGB1 complex	0.18898
Purpura, Thrombocytopenic, Idiopathic	2411	ITGA6-ITGB1-CD151 complex	0.1543
Purpura, Thrombocytopenic, Idiopathic	2413	ITGA6-ITGB1 complex	0.18898
Purpura, Thrombocytopenic, Idiopathic	2416	ITGB1-RAP1A-PKD1 complex	0.1543
Purpura, Thrombocytopenic, Idiopathic	2417	ITGA4-ITGB1-EMILIN1 complex	0.30861
Purpura, Thrombocytopenic, Idiopathic	2418	ITGA4-ITGB1 complex	0.37796
Purpura, Thrombocytopenic, Idiopathic	2419	ITGA4-ITGB1-CD81 complex	0.30861
Purpura, Thrombocytopenic, Idiopathic	2420	ITGA4-ITGB1-CD53 complex	0.30861
Purpura, Thrombocytopenic, Idiopathic	2421	ITGA4-ITGB1-VCAM1 complex	0.30861
Purpura, Thrombocytopenic, Idiopathic	2422	ITGA4-ITGB1-JAM2 complex	0.30861
Purpura, Thrombocytopenic, Idiopathic	2423	ITGA4-ITGB1-CD47 complex	0.30861
Purpura, Thrombocytopenic, Idiopathic	2424	ITGA4-ITGB1-CD63 complex	0.30861
Purpura, Thrombocytopenic, Idiopathic	2425	ITGA4-ITGB1-PXN complex	0.30861
Purpura, Thrombocytopenic, Idiopathic	2426	ITGA4-ITGB1-THBS1 complex	0.30861
Purpura, Thrombocytopenic, Idiopathic	2428	ITGA4-ITGB1-THBS2 complex	0.30861
Purpura, Thrombocytopenic, Idiopathic	2429	ITGA2-ITGB1-CD47 complex	0.1543

Purpura, Thrombocytopenic, Idiopathic	2430	ITGA2-ITGB1-CHAD complex	0.1543
Purpura, Thrombocytopenic, Idiopathic	2431	ITGA2-ITGB1-COL6A3 complex	0.1543
Purpura, Thrombocytopenic, Idiopathic	2432	ITGA2-ITGB1 complex	0.18898
Purpura, Thrombocytopenic, Idiopathic	2434	ITGA1-ITGB1-COL6A3 complex	0.1543
Purpura, Thrombocytopenic, Idiopathic	2435	ITGA1-ITGB1-PTPN2 complex	0.1543
Purpura, Thrombocytopenic, Idiopathic	2436	ITGAV-ITGB1 complex	0.18898
Purpura, Thrombocytopenic, Idiopathic	2437	ITGA6-ITGB1-CYR61 complex	0.1543
Purpura, Thrombocytopenic, Idiopathic	2439	ITGA8-ITGB1 complex	0.18898
Purpura, Thrombocytopenic, Idiopathic	2440	ITGA9-ITGB1-ADAM9 complex	0.1543
Purpura, Thrombocytopenic, Idiopathic	2441	Itga9-Itgb1-Adam2 complex	0.1543
Purpura, Thrombocytopenic, Idiopathic	2442	ITGA9-ITGB1-VCAM1 complex	0.1543
Purpura, Thrombocytopenic, Idiopathic	2443	ITGA9-ITGB1-TNC complex	0.1543
Purpura, Thrombocytopenic, Idiopathic	2444	ITGB1-ITGA9 complex	0.18898
Purpura, Thrombocytopenic, Idiopathic	2445	ITGA9-ITGB1-ADAM15 complex	0.1543
Purpura, Thrombocytopenic, Idiopathic	2446	ITGA9-ITGB1-FIGF complex	0.1543
Purpura, Thrombocytopenic, Idiopathic	2447	ITGA9-ITGB1-ADAM12 complex	0.1543
Purpura, Thrombocytopenic, Idiopathic	2565	CD20-LCK-LYN-FYN-p75/80 complex (Raji human B cell line)	0.13363
Purpura, Thrombocytopenic, Idiopathic	2816	ITGAV-ITGB3 complex	0.18898
Purpura, Thrombocytopenic, Idiopathic	2826	ITGB3-ITGAV-VTN complex	0.1543
Purpura, Thrombocytopenic, Idiopathic	2846	ITGAV-ITGB3-THBS1 complex	0.1543
Purpura, Thrombocytopenic, Idiopathic	2849	ITGAV-ITGB3-NOV complex	0.1543
Purpura, Thrombocytopenic, Idiopathic	2850	ITGA5-ITGB1-FN-1-NOV complex	0.13363
Purpura, Thrombocytopenic, Idiopathic	2853	ITGA5-ITGB1-CAL4A3 complex	0.1543
Purpura, Thrombocytopenic, Idiopathic	2872	ITGA2b-ITGB3-CD9-GP1b-CD47 complex	0.21822
Purpura, Thrombocytopenic, Idiopathic	2879	CD20-LCK-FYN-p75/80 complex	0.1543
Purpura, Thrombocytopenic, Idiopathic	2882	ITGA5-ITGB3-COL6A3 complex	0.1543

Purpura, Thrombocytopenic, Idiopathic	2885	ITGAV-ITGB1-SPP1 complex	0.1543
Purpura, Thrombocytopenic, Idiopathic	2896	ITGA2b-ITGB3-CD47-FAK complex	0.26726
Purpura, Thrombocytopenic, Idiopathic	2964	ITGA9-ITGB1-ADAM1 complex	0.1543
Purpura, Thrombocytopenic, Idiopathic	2965	ITGA9-ITGB1-ADAM3 complex	0.1543
Purpura, Thrombocytopenic, Idiopathic	2971	ITGA9-ITGB1-VEGFC complex	0.1543
Purpura, Thrombocytopenic, Idiopathic	2972	ITGA9-ITGB1-VEGFA complex	0.1543
Purpura, Thrombocytopenic, Idiopathic	2989	ITGA9-ITGB1-ADAM8 complex	0.1543
Purpura, Thrombocytopenic, Idiopathic	3035	LAT2-ITGB1 complex	0.18898
Purpura, Thrombocytopenic, Idiopathic	3057	ITGA10-ITGB1 complex	0.18898
Purpura, Thrombocytopenic, Idiopathic	3058	ITGA11-ITGB1 complex	0.18898
Purpura, Thrombocytopenic, Idiopathic	3059	ITGA11-ITGB1-COL1A1 complex	0.1543
Purpura, Thrombocytopenic, Idiopathic	3103	ITGAV-ITGB3-SLC3A2 complex	0.1543
Purpura, Thrombocytopenic, Idiopathic	3104	ITGB1-NRP1 complex	0.18898
Purpura, Thrombocytopenic, Idiopathic	3111	ITGA9-ITGB1-SPP1 complex	0.1543
Purpura, Thrombocytopenic, Idiopathic	3112	ITGA5-ITGB1-SPP1 complex	0.1543
Purpura, Thrombocytopenic, Idiopathic	3115	ITGA2B-ITGB3-ICAM4 complex	0.30861
Purpura, Thrombocytopenic, Idiopathic	5691	TALL1 homo-oligomer complex	0.26726
Skin disease, Genetic	138	Telosome complex	0.07332
Skin disease, Genetic	541	IGF1-IGFBP3-ALS complex	0.1037
Skin disease, Genetic	725	P2X7 receptor signalling complex	0.05185
Skin disease, Genetic	873	SNARE complex (STX1A SNAP29)	0.127
Skin disease, Genetic	1197	TRF1-TIN2 complex	0.07332
Skin disease, Genetic	1198	TIN2 complex	0.0898
Skin disease, Genetic	1202	TRF1 telomere length regulation complex	0.1037
Skin disease, Genetic	1204	Rap1 complex	0.06788
Skin disease, Genetic	1206	TRF-Rap1 complex I 2MD	0.07332
Skin disease, Genetic	1207	TRF2-Rap1 complex II	0.0898
Skin disease, Genetic	1539	G protein complex (GNG2 GNB2L1 RAF1)	0.1037
Skin disease, Genetic	2318	ITGA6-ITGB4-Laminin10/12 complex	0.08032
Skin disease, Genetic	2319	ITGA6-ITGB4-Laminin10/12 complex	0.08032
Skin disease, Genetic	2320	ITGA6-ITGB4-CD151 complex	0.1037
Skin disease, Genetic	2321	ITGA6-ITGB4-FYN complex	0.1037
Skin disease, Genetic	2322	ITGA6-ITGB4-LAMA5 complex	0.1037
Skin disease, Genetic	2323	ITGA6-ITGB4 complex	0.127

Skin disease, Genetic	2551	PDGFRA-PLC-gamma-1-PI3K-SHP-2 complex PDGF stimulated	0.0898
Skin disease, Genetic	2766	TERF2-RAP1 complex	0.06788
Skin disease, Genetic	2770	ITGA6-ITGB4-CD9 complex	0.1037
Skin disease, Genetic	3096	ITGA6-ITGB4-SHC1-GRB2 complex	0.0898
Skin disease, Genetic	3183	PDGFRA-SHP-2 complex PDGF stimulated	0.127
Skin disease, Genetic	3186	GRB2-SHP-2 complex PDGF stimulated	0.127
Skin disease, Genetic	5177	Polycystin-1 multiprotein complex (ACTN1 CDH1 SRC JUP VCL CTNNB1 PXN BCAR1 PKD1	0.05415
Skin disease, Genetic	5211	RAF1-PPP2-PIN1 complex	0.08032
Skin disease, Genetic	5564	LMO4-gp130 complex	0.08032
Skin disease, Genetic	5877	MAP2K1-BRAF-RAF1-YWHAE-KSR1 complex	0.08032
Skin disease, Genetic	5873	RAF1-MAP2K1-YWHAE complex	0.1037
Skin disease, Genetic	5919	BRAF-RAF1-14-3-3 complex	0.05987
Skin disease, Genetic	5920	KSR1-RAF1-MEK complex	0.0898
Skin disease, Genetic	5922	RAF1-RAS complex EGF induced	0.0898
Skin disease, Genetic	5923	RAF1-BRAF complex RAS stimulated	0.127
Skin disease, Genetic	5924	RAF1-CNK1 complex RAS stimulated	0.127
Skin disease, Genetic	5928	CNK1-SRC-RAF1 complex	0.1037
Spastic paraplegia, Hereditary	1094	Frataxin complex	0.1543
Spastic paraplegia, Hereditary	2755	17S U2 snRNP	0.07107
Stress disorder, post-traumatic	1088	PRNP-ApolopoproteinE3 complex	0.25
Stress disorder, post-traumatic	1707	IL2-IL2RA-IL2RB complex	0.20412
Stress disorder, post-traumatic	5268	TNF-alpha/NF-kappa B signaling complex 7	0.125
Stress disorder, post-traumatic	5269	TNF-alpha/NF-kappa B signaling complex 8	0.14434
Stress disorder, post-traumatic	5285	TNF-alpha/NF-kappa B signaling complex 9	0.15811
Thrombocythemia, Hemorrhagic	5178	JAK2-PAFR-TYK2 complex	0.33333
AIDS	27	Arp2/3 protein complex	0.28571
AIDS	1062	BAR-BCL2-CASP8 complex	0.21822
AIDS	2054	CASP8-FADD-MALT1-BCL10 complex	0.18898
AIDS	2055	CASP8-CHUK-IKBKB-MALT1-BCL10 complex	0.16903
AIDS	2056	BCL10-CHUK-BCL10-IKBKB complex	0.18898
AIDS	5473	FAS-FADD-CASP8 complex	0.21822
AIDS	5749	MRIT complex	0.21822
AIDS	5798	Death induced signaling complex II (FADD CASP8 CFLAR) cytosolic CD95L induced	0.21822
AIDS	5799	Death induced signaling complex DISC (FAS FADD CASP8 CFLAR) membrane-associated CD95L	0.18898
AIDS	5800	Death-inducing signaling complex DISC (type I cells associated) stimulated	0.21822
AIDS	5808	DISC complex	0.21822
AIDS	5859	FAS-FADD-CASP8-CASP10 complex	0.18898
Abortion	92	CD28-transactivation complex	0.08839
Abortion	201	HUIC complex	0.08839
Abortion	202	BRCA1-RAD50-MRE11-NBS1 complex	0.0625
Abortion	238	SWI-SNF chromatin remodeling-related-BRCA1	0.03769
Abortion	240	BRCA1-CTIP-ZBRK1 repressor complex	0.07217
Abortion	242	BRCA1-BACH1 complex	0.08839

Abortion	305	40S ribosomal subunit cytoplasmic	0.02144
Abortion	306	Ribosome cytoplasmic	0.01389
Abortion	433	BASC complex (BRCA1-associated genome surveillance complex)	0.03608
Abortion	434	BASC (Ab 80) complex (BRCA1-associated genome surveillance complex)	0.04419
Abortion	435	BASC (Ab 81) complex (BRCA1-associated genome surveillance complex)	0.05103
Abortion	436	BASC (Ab C-20) complex (BRCA1-associated genome surveillance complex)	0.07217
Abortion	438	GCN5-TRRAP histone acetyltransferase complex	0.03953
Abortion	657	Retromer complex (SNX1 SNX2 VPS35 VPS29 VPS26A)	0.0559
Abortion	871	BRAF53-BRCA2 complex	0.04725
Abortion	1060	Retromer complex (SNX1 SNX2 VPS35 VPS29	0.0559
Abortion	1070	SNX complex (SNX1a SNX2 SNX4)	0.07217
Abortion	1072	SNX complex (SNX2) oligomeric	0.125
Abortion	1091	SNX complex (SNX1a SNX2 SNX4 LEPR)	0.0625
Abortion	1093	SNX complex (SNX1a SNX2 SNX4 INSR)	0.125
Abortion	1095	SNX complex (SNX1a SNX2 SNX4 EGFR)	0.0625
Abortion	1096	SNX complex (SNX1 1a 2 4 PDGF receptor)	0.0625
Abortion	1104	SNX complex (SNX1a SNX2 SNX4 TFRC)	0.0625
Abortion	1154	DSS1 complex	0.03467
Abortion	1223	H2AX complex isolated from cells without IR exposure	0.03467
Abortion	1226	H2AX complex I	0.04725
Abortion	1227	H2AX complex II	0.03953
Abortion	1413	NCOR1 complex	0.03953
Abortion	1514	IL4-IL4R complex	0.08839
Abortion	1515	IL4-IL4R-IL2RG complex	0.07217
Abortion	2000	BAX homo-oligomer complex	0.125
Abortion	2001	NOD1 homodimer complex	0.125
Abortion	2028	JAK2-IL12RB2 complex	0.08839
Abortion	2210	BRCA1-IRIS-pre-replication complex	0.0625
Abortion	2211	BARD1-BRCA1-CSTF complex	0.0559
Abortion	2213	BRCA1-BARD1-POLR2A complex	0.07217
Abortion	2214	LMO4-BRCA1-CTIP-LDB1 complex	0.0625
Abortion	2215	BRCA1-LMO4-CTIP complex	0.07217
Abortion	2577	Sam68-p85 P13K-IRS-1-IR signaling complex	0.0625
Abortion	2686	BRCA1-core RNA polymerase II complex	0.03467
Abortion	2776	RAD50-BRCA1 complex	0.08839
Abortion	2783	BARD1-BRCA1-CSTF64 complex	0.07217
Abortion	2786	BRCA1 A complex	0.0625
Abortion	2787	BRCA1 C complex	0.0625
Abortion	2788	BRCA1 B complex	0.07217
Abortion	2811	BRCA1-cABL complex	0.08839
Abortion	2813	BRCA1-SMAD3 complex	0.08839
Abortion	2814	BRCA1-HDAC1-HDAC2 complex	0.07217
Abortion	2815	BRCA1-BARD1-BACH1-DNA damage complex II	0.04419

Abortion	2817	BRCA1-BARD1-BACH1-DNA damage complex I	0.05103
Abortion	2818	BRCA1-BARD1-BRCA2-DNA damage complex III	0.14434
Abortion	2819	BRCA1-CtIP-CtBP complex	0.07217
Abortion	2820	BRCA1-VCP complex	0.08839
Abortion	2822	BRCA1-BARD1-UbcH5c complex	0.07217
Abortion	2823	BRCA1-BARD1-UbcH7c complex	0.07217
Abortion	2824	BRCA1-RAD51 complex	0.08839
Abortion	2825	BRCA1-RNA polymerase II complex	0.02451
Abortion	3008	60S APC containing complex	0.04725
Abortion	3047	Parvulin-associated pre-rRNP complex	0.01588
Abortion	3055	Nop56p-associated pre-rRNA complex	0.01226
Abortion	3492	Bax homooligomeric complex after apoptotic	0.125
Abortion	5178	JAK2-PAFR-TYK2 complex	0.07217
Abortion	5217	Calreticulin oligomer complex	0.125
Abortion	5400	BRCC complex	0.1118
Abortion	5411	EDG1-HTR1D complex	0.08839
Abortion	5422	HTR1A-EDG1 complex	0.08839
Abortion	5614	Emerin complex 32	0.02665
Abruption placentae	3162	TF-FVIIa-FXa-TFPI complex	0.17678
Achalasia and cardiospasm	1223	H2AX complex isolated from cells without IR exposure	0.12403
Achalasia and cardiospasm	1226	H2AX complex I	0.16903
Achalasia and cardiospasm	1227	H2AX complex II	0.14142
Achalasia and cardiospasm	5217	Calreticulin oligomer complex	0.44721
Acne	2001	NOD1 homodimer complex	0.40825
Acne	2798	MMP-2-claudin-1 complex	0.28868
Acromegaly	541	IGF1-IGFBP3-ALS complex	0.57735
Actinic keratosis	74	TRPC1-Homer3-IP3R complex	0.21822
Actinic keratosis	220	ARF-Mule complex	0.21822
Actinic keratosis	536	TRPC1-TRPC3-TRPC7 complex	0.21822
Actinic keratosis	553	RHOA-IP3R-TRPC1 complex	0.21822
Actinic keratosis	557	TRP1-G alpha-11-IP3R3-CAV1 signaling complex	0.18898
Actinic keratosis	828	TRPC1-STIM1-ORAI1 complex	0.21822
Actinic keratosis	1642	p16-cyclin D2-CDK4 complex	0.21822
Actinic keratosis	1700	ABL2-HRAS-RIN1 complex	0.21822
Actinic keratosis	5922	RAF1-RAS complex EGF induced	0.18898
Adenocarcinoma	1714	TICAM1-TICAM2-TLR4 complex	0.08422
Adenocarcinoma	2989	ITGA9-ITGB1-ADAM8 complex	0.08422
Adenocarcinoma	5268	TNF-alpha/NF-kappa B signaling complex 7	0.05157
Adenocarcinoma	5269	TNF-alpha/NF-kappa B signaling complex 8	0.05955
Adenocarcinoma	5285	TNF-alpha/NF-kappa B signaling complex 9	0.06523
Adenoid cystic cancer	1306	PIN1-AUF1 complex	0.35355
Adenoid cystic cancer	5211	RAF1-PPP2-PIN1 complex	0.22361
Adenoma	68	BCDX2 complex	0.08575
Adenoma	71	MRN complex (MRE11-RAD50-NBS1 complex)	0.09901
Adenoma	72	R/M complex (RAD50-MRE11 complex)	0.12127
Adenoma	73	MRN complex (MRE11-RAD50-NBN complex)	0.09901
Adenoma	202	BRCA1-RAD50-MRE11-NBS1 complex	0.08575

Adenoma	261	RAD51B-RAD51C-RAD51D-XRCC2-XRCC3	0.0767
Adenoma	262	RAD51L3-XRCC2 complex	0.12127
Adenoma	350	DNA ligase IV-XRCC4-PNK complex	0.09901
Adenoma	362	DNA ligase III-XRCC1-PNK-DNA-pol III multiprotein complex	0.08575
Adenoma	433	BASC complex (BRCA1-associated genome surveillance complex)	0.04951
Adenoma	619	MRE11A-RAD50-NBN-TRF2 complex	0.08575
Adenoma	627	MRN-TRRAP complex (MRE11A-RAD50-NBN-TRRAP complex)	0.08575
Adenoma	706	SNARE complex (HGS SNAP25 STX13)	0.09901
Adenoma	1141	CF IIAm complex (Cleavage factor IIAm complex)	0.04287
Adenoma	1189	DNA double-strand break end-joining complex	0.06482
Adenoma	1193	Rap1 complex	0.06482
Adenoma	1474	SMAD3/4-E2F4/5-p107-DP1 complex	0.07001
Adenoma	1826	SMAD3-HEF1-APC10-CDH1 complex	0.08575
Adenoma	1827	PML-SMAD2/3-SARA complex	0.08575
Adenoma	1828	TGF-beta receptor I-Axin-SMAD3 complex	0.09901
Adenoma	1831	PIAS3-SMAD3-P300 complex	0.09901
Adenoma	2018	IL12A-IL12B complex	0.12127
Adenoma	2019	IL12A-IL12B-IL12RB1 complex	0.09901
Adenoma	2020	IL12B-IL12RB1-IL12RB2 complex	0.09901
Adenoma	2021	IL12A-IL12B-IL12RB2 complex	0.09901
Adenoma	2189	Ubiquitin E3 ligase (SMAD3 BTRC CUL1 SKP1A RBX1)	0.0767
Adenoma	2217	MDC1-MRN-ATM-FANCD2 complex	0.07001
Adenoma	2218	MDC1-MRE11-RAD50-NBS1 complex	0.08575
Adenoma	2228	BLM-RAD51L3-XRCC2 complex	0.09901
Adenoma	2692	SMAD3-SMAD4-cJun-cFos complex	0.08575
Adenoma	2705	SMAD3-SMAD4-CTCF protein-DNA complex	0.09901
Adenoma	2706	SMAD3-SMAD4-SP1 complex	0.09901
Adenoma	2707	SMAD3-SMAD4-FOXO3-FOXG1 complex	0.08575
Adenoma	2708	SMAD3-SMAD4-cJUN complex	0.09901
Adenoma	2754	JUND-FOSB-SMAD3-SMAD4 complex	0.08575
Adenoma	2760	SMAD3-SMAD4-FOXO3 complex	0.09901
Adenoma	2761	SMAD3-SMAD4-FOXO1 complex	0.09901
Adenoma	2762	SMAD3-SMAD4-FOXO4 complex	0.09901
Adenoma	2766	TERF2-RAP1 complex	0.06482
Adenoma	2767	RAD50-MRE11-NBN-p200-p350 complex	0.09901
Adenoma	2813	BRCA1-SMAD3 complex	0.12127
Adenoma	2815	BRCA1-BARD1-BACH1-DNA damage complex II	0.06063
Adenoma	2829	RSmad complex	0.05423
Adenoma	2830	TIF1gamma-SMAD2-SMAD3 complex	0.09901
Adenoma	2834	SMAD4-SMAD2-SMAD3 complex	0.09901
Adenoma	2968	Axin-SMAD3 complex	0.12127
Adenoma	2975	SMAD3-E2F4/5-p107-DP1 complex	0.0767
Adenoma	3199	SMAD3-SKI complex	0.12127
Adenoma	3205	SMAD3-SKI-NCOR complex	0.09901

Adenoma	3677	RIN1-STAM2-HRS complex	0.09901
Adenoma	3733	SKI-SMAD3 hexameric complex	0.12127
Adenoma	3740	SKI-SMAD3-SMAD4 pentameric complex	0.09901
Adenoma	3750	CREBBP-SMAD3 hexameric complex	0.12127
Adenoma	3754	CREBBP-SMAD3-SMAD4 pentameric complex	0.09901
Adenoma	3959	SMAD3-SMAD4-cSKI TGF(beta)-dependent	0.09901
Adenoma	3961	SMAD3-cSKI-SIN3A-HDAC1 complex	0.08575
Adenoma	3971	SMURF2-SMAD3 complex TGF(beta)-dependent	0.12127
Adenoma	3972	SMURF2-SMAD3-SnoN complex TGF(beta)-	0.09901
Adenoma	5197	PTIP-DNA damage response complex	0.07001
Adenoma	5233	TNF-alpha/NF-kappa B signaling complex 5	0.0343
Adenoma	5243	XRCC1-LIG3-PNK-TDP1 complex	0.08575
Adenoma	5548	IL-12 heterodimer complex	0.12127
Adenoma	5549	IL-12 subunit p40 homodimer complex	0.1715
Adenoma	5683	hRAD51C-hXRCC3 complex	0.12127
Adenoma	5735	TGF-beta receptor-SMAD3 complex	0.09901
Adenoma of thyroid	1474	SMAD3/4-E2F4/5-p107-DP1 complex	0.28868
Adenoma of thyroid	1661	E2F4-p107-cyclinA complex	0.40825
Adenoma of thyroid	2975	SMAD3-E2F4/5-p107-DP1 complex	0.31623
Adenoma of thyroid	3185	ACT homodimer complex	0.70711
Adenoma of thyroid	3188	FHL2-ACT complex	0.5
Adenoma of thyroid	3191	ACT-CREB complex	0.5
Adenoma of thyroid	3196	FHL4/STX11-ACT complex	0.5
Adenoma of thyroid	5144	E2F1-p107-cyclinA complex	0.40825
Adenoma of thyroid	5159	E2F4-p107-cyclinE complex	0.40825
Adenoma of thyroid	5589	LINC complex S-phase	0.26726
Adenoma of thyroid	5609	Emerin regulatory complex	0.2357
Adenoma of thyroid	5614	Emerin complex 32	0.15076
Adenovirus infection	49	DNMT3B complex	0.04921
Adenovirus infection	54	SIN3 complex	0.04921
Adenovirus infection	71	MRN complex (MRE11-RAD50-NBS1 complex)	0.22549
Adenovirus infection	72	R/M complex (RAD50-MRE11 complex)	0.18411
Adenovirus infection	73	MRN complex (MRE11-RAD50-NBN complex)	0.22549
Adenovirus infection	98	p300-MDM2-p53 protein complex	0.07516
Adenovirus infection	103	RNA polymerase II holoenzyme complex	0.02657
Adenovirus infection	104	RNA polymerase II core complex	0.03758
Adenovirus infection	143	APP-FE65-LRP complex	0.07516
Adenovirus infection	202	BRCA1-RAD50-MRE11-NBS1 complex	0.19528
Adenovirus infection	217	CRSP complex	0.04603
Adenovirus infection	220	ARF-Mule complex	0.07516
Adenovirus infection	229	NAT complex	0.04921
Adenovirus infection	230	Mediator complex	0.02301
Adenovirus infection	232	ARC complex	0.03361
Adenovirus infection	283	Sin3 complex	0.04921
Adenovirus infection	284	CRSP complex	0.03925
Adenovirus infection	287	ARC-L complex	0.03479
Adenovirus infection	288	ARC complex	0.03361

Adenovirus infection	310	Cell cycle kinase complex CDC2	0.05315
Adenovirus infection	311	Cell cycle kinase complex CDK2	0.06509
Adenovirus infection	312	Cell cycle kinase complex CDK4	0.06509
Adenovirus infection	313	Cell cycle kinase complex CDK5	0.05822
Adenovirus infection	351	Spliceosome	0.01089
Adenovirus infection	422	Beta-dystroglycan-caveolin-3 complex	0.09206
Adenovirus infection	433	BASC complex (BRCA1-associated genome surveillance complex)	0.15033
Adenovirus infection	435	BASC (Ab 81) complex (BRCA1-associated genome surveillance complex)	0.05315
Adenovirus infection	436	BASC (Ab C-20) complex (BRCA1-associated genome surveillance complex)	0.07516
Adenovirus infection	441	TFTC-type histone acetyl transferase complex	0.03925
Adenovirus infection	528	NuA4/Tip60 HAT complex	0.03361
Adenovirus infection	529	NuA4/Tip60 HAT complex	0.03255
Adenovirus infection	548	DRIP complex	0.03479
Adenovirus infection	591	SAP complex (Sin3-associated protein complex)	0.04603
Adenovirus infection	592	SAP complex (Sin3-associated protein complex)	0.0434
Adenovirus infection	596	SIN3-HDAC-SAP30-ARID4 complex	0.04921
Adenovirus infection	619	MRE11A-RAD50-NBN-TRF2 complex	0.19528
Adenovirus infection	627	MRN-TRRAP complex (MRE11A-RAD50-NBN-TRRAP complex)	0.19528
Adenovirus infection	632	Anti-HDAC2 complex	0.03069
Adenovirus infection	691	SIN3-SAP25 complex	0.03925
Adenovirus infection	696	BRMS1-SIN3-HDAC complex	0.04603
Adenovirus infection	713	BRG1-SIN3A complex	0.03479
Adenovirus infection	714	BRM-SIN3A complex	0.03361
Adenovirus infection	720	PU.1-SIN3A-HDAC complex	0.07516
Adenovirus infection	722	MRG15-PAM14-RB complex	0.07516
Adenovirus infection	723	MAF1 complex	0.07516
Adenovirus infection	728	CSA-POLIIa complex	0.03479
Adenovirus infection	738	SIN3-ING1b complex I	0.0434
Adenovirus infection	739	SIN3-ING1b complex II	0.03255
Adenovirus infection	745	NCOR-SIN3-RPD3 complex	0.06509
Adenovirus infection	747	NCOR-SIN3-HDAC1 complex	0.07516
Adenovirus infection	749	MeCP2-SIN3A-HDAC complex	0.06509
Adenovirus infection	798	NuA4/Tip60-HAT complex A	0.03361
Adenovirus infection	799	DMAP1-associated complex	0.04117
Adenovirus infection	803	BRG1-SIN3A-HDAC containing SWI/SNF remodeling complex I	0.03925
Adenovirus infection	806	BRM-SIN3A-HDAC complex	0.03758
Adenovirus infection	888	MTA2 complex	0.0434
Adenovirus infection	895	hMediator complex (MED23 CDK8 CCNC MED7)	0.06509
Adenovirus infection	896	hMediator complex (MED23 CDK8 CCNC)	0.07516
Adenovirus infection	898	Mediator complex 1	0.05822
Adenovirus infection	900	Mediator complex 2	0.07516
Adenovirus infection	909	ARC92-Mediator complex	0.03611
Adenovirus infection	910	CRSP-Mediator 2 complex	0.04117

Adenovirus infection	1087	BIRC5-AURKB-INCENP-EVI5 complex	0.06509
Adenovirus infection	1095	SNX complex (SNX1a SNX2 SNX4 EGFR)	0.06509
Adenovirus infection	1116	CRM1-Survivin-AuroraB mitotic complex	0.07516
Adenovirus infection	1117	CRM1-Survivin mitotic complex	0.09206
Adenovirus infection	1118	Chromosomal passenger complex CPC (INCENP CDCA8 BIRC5 AURKB)	0.06509
Adenovirus infection	1120	Chromosomal passenger complex CPC (INCENP CDCA8 BIRC5)	0.07516
Adenovirus infection	1141	CF IIAm complex (Cleavage factor IIAm complex)	0.06509
Adenovirus infection	1155	Integrator-RNAPII complex	0.03479
Adenovirus infection	1166	p400-associated complex	0.04921
Adenovirus infection	1170	cMYC-ATPase-helicase complex	0.05822
Adenovirus infection	1171	c-MYC-ATPase-helicase complex	0.05822
Adenovirus infection	1181	C complex spliceosome	0.01456
Adenovirus infection	1185	EGFR-containing signaling complex	0.06509
Adenovirus infection	1189	DNA double-strand break end-joining complex	0.14762
Adenovirus infection	1191	RNA pol II containing coactivator complex Tat-SF	0.05822
Adenovirus infection	1193	Rap1 complex	0.09841
Adenovirus infection	1204	Rap1 complex	0.04921
Adenovirus infection	1231	FIB-associated protein complex	0.05315
Adenovirus infection	1232	REST-CoREST-mSIN3A complex	0.07516
Adenovirus infection	1250	pRB-E2F-1 complex	0.09206
Adenovirus infection	1257	ALL-1 supercomplex	0.0246
Adenovirus infection	1308	PABPC1-HSPA8-HNRPD-EIF4G1 complex	0.05822
Adenovirus infection	1332	Large Drosha complex	0.02911
Adenovirus infection	1372	Rb-tal-1-E2A-Lmo2-Ldb1 complex	0.05822
Adenovirus infection	1488	DNMT1-RB1-HDAC1-E2F1 complex	0.06509
Adenovirus infection	1505	NCOR2 complex	0.04921
Adenovirus infection	1633	CyclinD1-CDK4-CDK6 complex	0.07516
Adenovirus infection	1634	CyclinD1-CDK4-p21 complex	0.07516
Adenovirus infection	1642	p16-cyclin D2-CDK4 complex	0.07516
Adenovirus infection	1656	p27-cyclinE-CDK2 complex	0.07516
Adenovirus infection	5712	FAK-beta5 integrin complex VEGF induced	0.09206
Adenovirus infection	1729	TLE1 corepressor complex (MASH1 promoter-corepressor complex)	0.04117
Adenovirus infection	1749	SMN-PolII-RHA complex	0.04117
Adenovirus infection	1767	CPSF6-ITCH-NUDT21-POLR2A-UBAP2L complex	0.05822
Adenovirus infection	1768	CPSF6-EWSR1-ITCH-NUDT21-POLR2A-UBAP2L complex	0.05315
Adenovirus infection	1769	CPSF6-ITCH-NUDT21-POLR2A complex	0.06509
Adenovirus infection	5718	eNOS-HSP90-AKT complex VEGF induced	0.07516
Adenovirus infection	2000	BAX homo-oligomer complex	0.13019
Adenovirus infection	2156	YBX1-AKT1 complex	0.09206
Adenovirus infection	2159	AR-AKT-APPL complex	0.07516
Adenovirus infection	2213	BRCA1-BARD1-POLR2A complex	0.07516
Adenovirus infection	2217	MDC1-MRN-ATM-FANCD2 complex	0.2126
Adenovirus infection	2218	MDC1-MRE11-RAD50-NBS1 complex	0.19528
Adenovirus infection	2369	ITGAV-ITGB3-EGFR complex	0.07516

Adenovirus infection	2453	Multiprotein complex (monoubiquitination)	0.06509
Adenovirus infection	2454	CIN85-CBL-SH3GL2-EGFR complex EGF stimulated	0.06509
Adenovirus infection	2542	EGFR-CBL-GRB2 complex	0.07516
Adenovirus infection	2579	Chromosomal passenger complex CPC (INCENP BIRC5 AURKB)	0.07516
Adenovirus infection	2580	Survivin homodimer complex	0.13019
Adenovirus infection	2581	RasGAP-AURKA/AURKB-survivin complex	0.06509
Adenovirus infection	2582	Chromosomal passenger complex CPC (CDCA8 AURKB BIRC5)	0.07516
Adenovirus infection	2599	POLR2A-CCNT1-CDK9-NCL-LEM6-CPSF2 complex	0.05315
Adenovirus infection	2635	BETA2-Cyclin D1 complex	0.09206
Adenovirus infection	2638	HES1 promoter corepressor complex	0.05315
Adenovirus infection	2639	HES1 promoter-Notch enhancer complex	0.03611
Adenovirus infection	2649	MYC-DNMT3A-ZBTB17 complex	0.07516
Adenovirus infection	2653	MYC-MAX-BLOC1S1 complex	0.07516
Adenovirus infection	2655	MYC-MAX complex	0.09206
Adenovirus infection	2670	Er-alpha-p53-hdm2 complex	0.07516
Adenovirus infection	2685	RNA polymerase II (RNAPII)	0.03158
Adenovirus infection	2686	BRCA1-core RNA polymerase II complex	0.03611
Adenovirus infection	2711	Amyloid beta protein oligomer	0.13019
Adenovirus infection	2721	HCF-1 complex	0.02987
Adenovirus infection	2723	ATM-NBS1 complex	0.18411
Adenovirus infection	2726	PXN-ITGB5-PTK2 complex	0.07516
Adenovirus infection	2755	17S U2 snRNP	0.02266
Adenovirus infection	2766	TERF2-RAP1 complex	0.09841
Adenovirus infection	2767	RAD50-MRE11-NBN-p200-p350 complex	0.22549
Adenovirus infection	2776	RAD50-BRCA1 complex	0.09206
Adenovirus infection	2803	PAX6-SOX2 DNA-protein complex	0.09206
Adenovirus infection	2815	BRCA1-BARD1-BACH1-DNA damage complex II	0.13809
Adenovirus infection	2825	BRCA1-RNA polymerase II complex	0.02553
Adenovirus infection	2851	ING2 complex	0.03758
Adenovirus infection	2857	NuA4/Tip60 HAT complex	0.03479
Adenovirus infection	2896	ITGA2b-ITGB3-CD47-FAK complex	0.06509
Adenovirus infection	3011	APC-IQGAP1-Rac1 complex	0.07516
Adenovirus infection	3015	p27-cyclinE-Cdk2 - Ubiquitin E3 ligase (SKP1A SKP2 CUL1 CKS1B RBX1) complex	0.04603
Adenovirus infection	3044	SKI-NCOR1-SIN3A-HDAC1 complex	0.06509
Adenovirus infection	3048	mSin3A complex	0.05822
Adenovirus infection	3053	mSin3A-HDAC1-HDAC2 complex	0.07516
Adenovirus infection	3054	MAD1-mSin3A-HDAC2 complex	0.07516
Adenovirus infection	3055	Nop56p-associated pre-rRNA complex	0.01277
Adenovirus infection	3060	RNA polymerase II complex (RPB1 RAP74 CDK8 CYCC SRB7 BAF190 BAF47) chromatin structure modifying	0.04603
Adenovirus infection	3061	RNA polymerase II complex (CBP PCAF RPB1 BAF47 CYCC CDK8) chromatin structure modifying	0.05315
Adenovirus infection	3062	RNA polymerase II complex incomplete (CBP RPBI PCAF BAF47) chromatin structure modifying	0.06509

Adenovirus infection	3064	RNA polymerase II complex chromatin structure modifying	0.02987
Adenovirus infection	3066	RNA polymerase II complex chromatin structure modifying	0.03611
Adenovirus infection	3084	CCND1-CDK4 complex	0.09206
Adenovirus infection	3087	CCND1-CDK6 complex	0.09206
Adenovirus infection	3092	APP-TOMM40 complex	0.09206
Adenovirus infection	3093	APP-TIMM23 complex	0.09206
Adenovirus infection	3137	MASH1 promoter-coactivator complex	0.03925
Adenovirus infection	3142	CAMK2-delta-MASH1 promoter-coactivator complex	0.04603
Adenovirus infection	3149	NK-3-Groucho-HIPK2-SIN3A-RbpA48-HDAC1	0.03925
Adenovirus infection	3162	TF-FVIIa-FXa-TFPI complex	0.06509
Adenovirus infection	3167	NCOR-SIN3-HDAC-HESX1 complex	0.05315
Adenovirus infection	3172	NUMB-TP53-MDM2 complex	0.07516
Adenovirus infection	3263	HERP1/HEY2-NCOR-SIN3A complex	0.06509
Adenovirus infection	3269	RB1-HDAC1-BRG1 complex	0.07516
Adenovirus infection	3492	Bax homooligomeric complex after apoptotic	0.13019
Adenovirus infection	3678	RIN1-STAM2-EGFR complex EGF stimulated	0.07516
Adenovirus infection	3847	TCL1(trimer)-AKT1 complex	0.09206
Adenovirus infection	3852	Rb-HDAC1 complex	0.09206
Adenovirus infection	3961	SMAD3-cSKI-SIN3A-HDAC1 complex	0.06509
Adenovirus infection	4498	p32-CBF-DNA complex	0.06509
Adenovirus infection	5099	RB1(hypophosphorylated)-E2F4 complex	0.09206
Adenovirus infection	5143	E2F1-Rb complex	0.09206
Adenovirus infection	5146	RB1-TFAP2A complex	0.09206
Adenovirus infection	5171	SH3KBP1-CBLB-EGFR complex	0.07516
Adenovirus infection	5177	Polycystin-1 multiprotein complex (ACTN1 CDH1 SRC JUP VCL CTNNB1 PXN BCAR1 PKD1	0.03925
Adenovirus infection	5184	SWI/SNF chromatin-remodeling complex	0.05822
Adenovirus infection	5197	PTIP-DNA damage response complex	0.15945
Adenovirus infection	5253	MNK1-eIF4F complex	0.11644
Adenovirus infection	5282	CAS-SRC-FAK complex	0.07516
Adenovirus infection	5287	CDK4-CCND1 complex	0.09206
Adenovirus infection	5331	YY1-MDM2-p53 complex	0.07516
Adenovirus infection	5342	ELMO1-DOCK1-RAC1 complex	0.07516
Adenovirus infection	5363	Actin-ribonucleoprotein complex (POLR2A GTF2F1 HNRNPU)	0.07516
Adenovirus infection	5369	ATM homodimer complex	0.13019
Adenovirus infection	5450	Mediator complex	0.02505
Adenovirus infection	5473	FAS-FADD-CASP8 complex	0.07516
Adenovirus infection	5564	LMO4-gp130 complex	0.11644
Adenovirus infection	5611	Emerin complex 24	0.06723
Adenovirus infection	5615	Emerin complex 52	0.02715
Adenovirus infection	5656	CEBPE-E2F1-RB1 complex	0.07516
Adenovirus infection	5663	TRIM27-RB1 complex	0.09206
Adenovirus infection	5736	Pre-initiation complex (PIC)	0.07516
Adenovirus infection	5749	MRIT complex	0.15033

Adenovirus infection	5798	Death induced signaling complex II (FADD CASP8 CFLAR) cytosolic CD95L induced	0.07516
Adenovirus infection	5799	Death induced signaling complex DISC (FAS FADD CASP8 CFLAR) membrane-associated CD95L	0.13019
Adenovirus infection	5800	Death-inducing signaling complex DISC (type I cells associated) stimulated	0.07516
Adenovirus infection	5808	DISC complex	0.07516
Adenovirus infection	5812	p53-BCL2 complex	0.09206
Adenovirus infection	5816	Apoptosome-procaspase 9 complex	0.07516
Adenovirus infection	5819	BIM-BCL2xL complex	0.09206
Adenovirus infection	5820	tBID-BCL2xL complex	0.09206
Adenovirus infection	5859	FAS-FADD-CASP8-CASP10 complex	0.06509
Adenovirus infection	5861	FAS-FADD-CASP10 complex	0.07516
Adrenal gland hyperfunction	86	NUMAC complex (nucleosomal methylation activator complex)	0.11952
Adrenal gland hyperfunction	149	PBAF complex (Polybromo- and BAF containing complex)	0.10911
Adrenal gland hyperfunction	189	BAF complex	0.10483
Adrenal gland hyperfunction	220	ARF-Mule complex	0.21822
Adrenal gland hyperfunction	238	SWI-SNF chromatin remodeling-related-BRCA1	0.11396
Adrenal gland hyperfunction	554	PBAF complex (Polybromo- and BAF containing complex)	0.11952
Adrenal gland hyperfunction	555	BAF complex	0.12599
Adrenal gland hyperfunction	564	BAF complex	0.11396
Adrenal gland hyperfunction	565	PBAF complex (Polybromo- and BAF containing complex)	0.11396
Adrenal gland hyperfunction	566	BAF complex	0.11396
Adrenal gland hyperfunction	570	p300-CBP-p270-SWI/SNF complex	0.14286
Adrenal gland hyperfunction	710	Brg1-associated complex I	0.11396
Adrenal gland hyperfunction	713	BRG1-SIN3A complex	0.10102
Adrenal gland hyperfunction	739	SIN3-ING1b complex II	0.09449
Adrenal gland hyperfunction	778	LARC complex (LCR-associated remodeling complex)	0.08671
Adrenal gland hyperfunction	803	BRG1-SIN3A-HDAC containing SWI/SNF remodeling complex I	0.11396
Adrenal gland hyperfunction	807	BRG1-associated complex	0.12599
Adrenal gland hyperfunction	845	PCI-PSA-SCG2 complex	0.21822
Adrenal gland hyperfunction	1230	WINAC complex	0.10102
Adrenal gland hyperfunction	1239	EBAFb complex	0.11952
Adrenal gland hyperfunction	1252	EBAFa complex	0.11952
Adrenal gland hyperfunction	1413	NCOR1 complex	0.11952
Adrenal gland hyperfunction	1642	p16-cyclin D2-CDK4 complex	0.21822
Adrenal gland hyperfunction	2829	RSmad complex	0.11952
Adrenal gland hyperfunction	3060	RNA polymerase II complex (RPB1 RAP74 CDK8 CYCC SRB7 BAF190 BAF47) chromatin structure modifying	0.13363
Adrenal gland hyperfunction	3063	Brg1-associated complex II	0.14286
Adrenal gland hyperfunction	3064	RNA polymerase II complex chromatin structure modifying	0.08671
Adrenal gland hyperfunction	3066	RNA polymerase II complex chromatin structure modifying	0.10483

Adrenal gland hyperfunction	3269	RB1-HDAC1-BRG1 complex	0.21822
Adrenal gland hyperfunction	5293	ETS2-SMARCA4-INI1 complex	0.21822
Adrenal gland hypofunction	138	Telosome complex	0.2357
Adrenal gland hypofunction	1197	TRF1-TIN2 complex	0.2357
Adrenal gland hypofunction	1198	TIN2 complex	0.28868
Adrenal gland hypofunction	1204	Rap1 complex	0.21822
Adrenal gland tumor	189	BAF complex	0.08362
Adrenal gland tumor	238	SWI-SNF chromatin remodeling-related-BRCA1	0.09091
Adrenal gland tumor	564	BAF complex	0.09091
Adrenal gland tumor	711	Brm-associated complex	0.09091
Adrenal gland tumor	714	BRM-SIN3A complex	0.07785
Adrenal gland tumor	806	BRM-SIN3A-HDAC complex	0.08704
Adrenal gland tumor	808	BRM-associated complex	0.09535
Adrenal gland tumor	1230	WINAC complex	0.08058
Adrenal gland tumor	1257	ALL-1 supercomplex	0.05698
Adrenal gland tumor	2486	GIPC1-LHCGR complex	0.2132
Adrenal gland tumor	3060	RNA polymerase II complex (RPB1 RAP74 CDK8 CYCC SRB7 BAF190 BAF47) chromatin structure modifying	0.1066
Adrenal gland tumor	3064	RNA polymerase II complex chromatin structure modifying	0.06917
Adrenal gland tumor	3066	RNA polymerase II complex chromatin structure modifying	0.08362
Adrenal gland tumor	5158	SMARCA2/BRM-BAF57-MECP2 complex	0.17408
Adrenal gland tumor	5184	SWI/SNF chromatin-remodeling complex	0.13484
Adrenoleukodystrophy	138	Telosome complex	0.10911
Adrenoleukodystrophy	1041	Alpha-dystrobrevin-ZO-1-actin complex	0.13363
Adrenoleukodystrophy	1141	CF IIAm complex (Cleavage factor IIAm complex)	0.06682
Adrenoleukodystrophy	1197	TRF1-TIN2 complex	0.10911
Adrenoleukodystrophy	1198	TIN2 complex	0.13363
Adrenoleukodystrophy	1204	Rap1 complex	0.10102
Adrenoleukodystrophy	5772	ZO1-(beta)cadherin-(VE)cadherin-VEGFR2 complex	0.13363
Advanced cancer	541	IGF1-IGFBP3-ALS complex	0.18257
Advanced cancer	1944	IRAK1-IRAK2 complex	0.22361
Advanced cancer	1945	IRAK1-IRAK3 complex	0.22361
Advanced cancer	2018	IL12A-IL12B complex	0.22361
Advanced cancer	2019	IL12A-IL12B-IL12RB1 complex	0.18257
Advanced cancer	2020	IL12B-IL12RB1-IL12RB2 complex	0.18257
Advanced cancer	2021	IL12A-IL12B-IL12RB2 complex	0.18257
Advanced cancer	5548	IL-12 heterodimer complex	0.22361
Advanced cancer	5549	IL-12 subunit p40 homodimer complex	0.31623
Alimentary system disease	681	(C-CFTR)2-NHERF-ezrin complex	0.12599
Alimentary system disease	682	C-CFTR-NHERF(PDZ1 domain)-ezrin complex	0.12599
Alimentary system disease	683	C-CFTR-NHERF(PDZ2 domain)-ezrin complex	0.12599
Alimentary system disease	687	CFTR-NHERF-beta(2)AR signaling complex	0.12599
Alimentary system disease	1707	IL2-IL2RA-IL2RB complex	0.12599
Alopecia	541	IGF1-IGFBP3-ALS complex	0.10541
Alopecia	786	MR-UBC9-SRC1 complex	0.10541

Alopecia	1519	IL6ST-PRKCD-STAT3 complex	0.10541
Alopecia	1774	MICA-KLRK1-HCST complex	0.10541
Alopecia	1985	AIRE homodimer complex	0.18257
Alopecia	2383	ITGA5-ITGB1-FN1-TGM2 complex	0.09129
Alopecia	2384	ITGA5-ITGB1-ADAM15 complex	0.10541
Alopecia	2385	ITGA5-ITGB4 complex	0.1291
Alopecia	2388	Itga5-Itgb1-Fn1-Sfrp2 complex	0.09129
Alopecia	2390	CD98-LAT2-ITGB1 complex	0.09129
Alopecia	2395	ITGA7-ITGB1-CD151 complex	0.10541
Alopecia	2396	ITGA7-ITGB1-CD9 complex	0.10541
Alopecia	2397	ITGA7-ITGB1-ITGB1BP3 complex	0.10541
Alopecia	2398	ITGA3-ITGB1-BSG complex	0.10541
Alopecia	2399	ITGA3-ITGB1-CD63 complex	0.10541
Alopecia	2400	ITGA3-ITGB1-CD151 complex	0.10541
Alopecia	2401	ITGA3-ITGB1-THBS1 complex	0.10541
Alopecia	2406	ITGA3-ITGB1 complex	0.1291
Alopecia	2411	ITGA6-ITGB1-CD151 complex	0.10541
Alopecia	2413	ITGA6-ITGB1 complex	0.1291
Alopecia	2416	ITGB1-RAP1A-PKD1 complex	0.10541
Alopecia	2417	ITGA4-ITGB1-EMILIN1 complex	0.10541
Alopecia	2418	ITGA4-ITGB1 complex	0.1291
Alopecia	2419	ITGA4-ITGB1-CD81 complex	0.10541
Alopecia	2420	ITGA4-ITGB1-CD53 complex	0.10541
Alopecia	2421	ITGA4-ITGB1-VCAM1 complex	0.10541
Alopecia	2422	ITGA4-ITGB1-JAM2 complex	0.10541
Alopecia	2423	ITGA4-ITGB1-CD47 complex	0.10541
Alopecia	2424	ITGA4-ITGB1-CD63 complex	0.10541
Alopecia	2425	ITGA4-ITGB1-PXN complex	0.10541
Alopecia	2426	ITGA4-ITGB1-THBS1 complex	0.10541
Alopecia	2428	ITGA4-ITGB1-THBS2 complex	0.10541
Alopecia	2429	ITGA2-ITGB1-CD47 complex	0.10541
Alopecia	2430	ITGA2-ITGB1-CHAD complex	0.10541
Alopecia	2431	ITGA2-ITGB1-COL6A3 complex	0.10541
Alopecia	2432	ITGA2-ITGB1 complex	0.1291
Alopecia	2434	ITGA1-ITGB1-COL6A3 complex	0.10541
Alopecia	2435	ITGA1-ITGB1-PTPN2 complex	0.10541
Alopecia	2436	ITGAV-ITGB1 complex	0.1291
Alopecia	2437	ITGA6-ITGB1-CYR61 complex	0.10541
Alopecia	2439	ITGA8-ITGB1 complex	0.1291
Alopecia	2440	ITGA9-ITGB1-ADAM9 complex	0.10541
Alopecia	2441	Itga9-Itgb1-Adam2 complex	0.10541
Alopecia	2442	ITGA9-ITGB1-VCAM1 complex	0.10541
Alopecia	2443	ITGA9-ITGB1-TNC complex	0.10541
Alopecia	2444	ITGB1-ITGA9 complex	0.1291
Alopecia	2445	ITGA9-ITGB1-ADAM15 complex	0.10541
Alopecia	2446	ITGA9-ITGB1-FIGF complex	0.10541
Alopecia	2447	ITGA9-ITGB1-ADAM12 complex	0.10541

Alopecia	2850	ITGA5-ITGB1-FN-1-NOV complex	0.09129
Alopecia	2853	ITGA5-ITGB1-CAL4A3 complex	0.10541
Alopecia	2885	ITGAV-ITGB1-SPP1 complex	0.10541
Alopecia	2909	PLC-gamma-2-Syk-LAT-FcR-gamma complex	0.09129
Alopecia	2910	PLC-gamma-2-Lyn-FcR-gamma complex	0.10541
Alopecia	2964	ITGA9-ITGB1-ADAM1 complex	0.10541
Alopecia	2965	ITGA9-ITGB1-ADAM3 complex	0.10541
Alopecia	2971	ITGA9-ITGB1-VEGFC complex	0.10541
Alopecia	2972	ITGA9-ITGB1-VEGFA complex	0.10541
Alopecia	2989	ITGA9-ITGB1-ADAM8 complex	0.10541
Alopecia	3035	LAT2-ITGB1 complex	0.1291
Alopecia	3057	ITGA10-ITGB1 complex	0.1291
Alopecia	3058	ITGA11-ITGB1 complex	0.1291
Alopecia	3059	ITGA11-ITGB1-COL1A1 complex	0.10541
Alopecia	3104	ITGB1-NRP1 complex	0.1291
Alopecia	3111	ITGA9-ITGB1-SPP1 complex	0.10541
Alopecia	3112	ITGA5-ITGB1-SPP1 complex	0.10541
Alopecia	3229	Heterodimer complex (CDK9 IL6ST)	0.1291
Alopecia	3634	NR3C2-UBC9-SRC-1 complex	0.10541
Alopecia	5564	LMO4-gp130 complex	0.08165
Alopecia	5579	CNTF-CNTFR-gp130-LIFR complex	0.09129
Alopecia	5582	LIFR-LIF-gp130 complex	0.21082
Alopecia	5691	TALL1 homo-oligomer complex	0.18257
Alzheimer's disease	71	MRN complex (MRE11-RAD50-NBS1 complex)	0.04156
Alzheimer's disease	72	R/M complex (RAD50-MRE11 complex)	0.0509
Alzheimer's disease	73	MRN complex (MRE11-RAD50-NBN complex)	0.04156
Alzheimer's disease	75	TSC1-TSC2 complex	0.0509
Alzheimer's disease	143	APP-FE65-LRP complex	0.08312
Alzheimer's disease	201	HUIC complex	0.0509
Alzheimer's disease	202	BRCA1-RAD50-MRE11-NBS1 complex	0.07198
Alzheimer's disease	228	SMCC complex	0.02545
Alzheimer's disease	229	NAT complex	0.02721
Alzheimer's disease	230	Mediator complex	0.01272
Alzheimer's disease	238	SWI-SNF chromatin remodeling-related-BRCA1	0.0217
Alzheimer's disease	240	BRCA1-CTIP-ZBRK1 repressor complex	0.04156
Alzheimer's disease	242	BRCA1-BACH1 complex	0.0509
Alzheimer's disease	287	ARC-L complex	0.01924
Alzheimer's disease	288	ARC complex	0.01859
Alzheimer's disease	301	SMCC complex	0.01746
Alzheimer's disease	362	DNA ligase III-XRCC1-PNK-DNA-pol III multiprotein complex	0.03599
Alzheimer's disease	432	N-NOS-CHIP-HSP70-1 complex	0.04156
Alzheimer's disease	433	BASC complex (BRCA1-associated genome surveillance complex)	0.04156
Alzheimer's disease	434	BASC (Ab 80) complex (BRCA1-associated genome surveillance complex)	0.02545
Alzheimer's disease	435	BASC (Ab 81) complex (BRCA1-associated genome surveillance complex)	0.02939

Alzheimer's disease	436	BASC (Ab C-20) complex (BRCA1-associated genome surveillance complex)	0.04156
Alzheimer's disease	438	GCN5-TRRAP histone acetyltransferase complex	0.02276
Alzheimer's disease	441	TFTC-type histone acetyl transferase complex	0.0217
Alzheimer's disease	443	BP-SMAD complex	0.02545
Alzheimer's disease	521	Polycystin-1-E-cadherin-beta-catenin complex	0.04156
Alzheimer's disease	522	Polycystin-1-E-cadherin-beta-catenin-Flotillin-2	0.03599
Alzheimer's disease	525	TIP60 histone acetylase complex	0.03219
Alzheimer's disease	528	NuA4/Tip60 HAT complex	0.01859
Alzheimer's disease	529	NuA4/Tip60 HAT complex	0.018
Alzheimer's disease	535	TRAP complex	0.018
Alzheimer's disease	541	IGF1-IGFBP3-ALS complex	0.04156
Alzheimer's disease	547	SMCC complex	0.01924
Alzheimer's disease	563	F1F0-ATP synthase (EC 3.6.3.14) mitochondrial	0.018
Alzheimer's disease	617	CASK-LIN7C-APBA1 complex	0.04156
Alzheimer's disease	619	MRE11A-RAD50-NBN-TRF2 complex	0.03599
Alzheimer's disease	627	MRN-TRRAP complex (MRE11A-RAD50-NBN-TRRAP complex)	0.03599
Alzheimer's disease	681	(C-CFTR)2-NHERF-ezrin complex	0.04156
Alzheimer's disease	682	C-CFTR-NHERF(PDZ1 domain)-ezrin complex	0.04156
Alzheimer's disease	683	C-CFTR-NHERF(PDZ2 domain)-ezrin complex	0.04156
Alzheimer's disease	687	CFTR-NHERF-beta(2)AR signaling complex	0.04156
Alzheimer's disease	787	NuA4/Tip60-HAT complex B	0.02545
Alzheimer's disease	798	NuA4/Tip60-HAT complex A	0.01859
Alzheimer's disease	826	PAR-3-VE-cadherin-beta-catenin complex	0.04156
Alzheimer's disease	827	NgR-TROY-LINGO1 complex	0.04156
Alzheimer's disease	879	PRKAC-AKAP5-ADRB1 complex	0.03219
Alzheimer's disease	895	hMediator complex (MED23 CDK8 CCNC MED7)	0.03599
Alzheimer's disease	896	hMediator complex (MED23 CDK8 CCNC)	0.04156
Alzheimer's disease	897	CDK8-CyclinC-Mediator complex	0.0509
Alzheimer's disease	898	Mediator complex 1	0.03219
Alzheimer's disease	999	p23 protein complex	0.04156
Alzheimer's disease	1054	ESR1-RELA-BCL3-NCOA3 complex	0.03599
Alzheimer's disease	1062	BAR-BCL2-CASP8 complex	0.04156
Alzheimer's disease	1085	DNA repair complex NEIL2-PNK-Pol(beta)-LigIII(alpha)-XRCC1	0.03219
Alzheimer's disease	1086	DNA repair complex NEIL1-PNK-Pol(beta)-LigIII(alpha)-XRCC1	0.03219
Alzheimer's disease	1141	CF IIAm complex (Cleavage factor IIAm complex)	0.018
Alzheimer's disease	1181	C complex spliceosome	0.00805
Alzheimer's disease	1186	ESCRT-III complex	0.02276
Alzheimer's disease	1189	DNA double-strand break end-joining complex	0.02721
Alzheimer's disease	1193	Rap1 complex	0.02721
Alzheimer's disease	1250	pRB-E2F-1 complex	0.0509
Alzheimer's disease	1306	PIN1-AUF1 complex	0.0509
Alzheimer's disease	1439	PTGS2 homodimer complex	0.07198
Alzheimer's disease	1488	DNMT1-RB1-HDAC1-E2F1 complex	0.03599
Alzheimer's disease	1539	G protein complex (GNG2 GNB2L1 RAF1)	0.08312

Alzheimer's disease	1656	p27-cyclinE-CDK2 complex	0.04156
Alzheimer's disease	1714	TICAM1-TICAM2-TLR4 complex	0.04156
Alzheimer's disease	1787	Nogo-potassium channel complex	0.03599
Alzheimer's disease	1793	LINGO-1-Nogo-66-p75 signaling complex	0.04156
Alzheimer's disease	5718	eNOS-HSP90-AKT complex VEGF induced	0.04156
Alzheimer's disease	1816	JUN-TCF4-CTNNB1 complex	0.04156
Alzheimer's disease	1839	SDCBP-CTNNB1-CTNNA1-CDH1 complex	0.03599
Alzheimer's disease	5716	eNOS-HSP90 complex VEGF induced	0.0509
Alzheimer's disease	1976	MTNR1A homodimer complex	0.07198
Alzheimer's disease	1978	MTNR1A-MTNR1B complex	0.0509
Alzheimer's disease	1993	SLC1A2 homotrimer complex	0.07198
Alzheimer's disease	2000	BAX homo-oligomer complex	0.07198
Alzheimer's disease	2112	CDC37-HSP90AA1-HSP90AB1-MAP3K11 complex	0.03599
Alzheimer's disease	2124	IKK-alpha--ER-alpha-AIB1 complex	0.04156
Alzheimer's disease	2143	MAP2K5-PRKCI-SQSTM1 complex	0.04156
Alzheimer's disease	2153	ITGAM-ITGB2-CD11 complex	0.04156
Alzheimer's disease	2198	RAD9-RAD1-HUS1-POLB complex	0.03599
Alzheimer's disease	2210	BRCA1-IRIS-pre-replication complex	0.03599
Alzheimer's disease	2211	BARD1-BRCA1-CSTF complex	0.03219
Alzheimer's disease	2213	BRCA1-BARD1-POLR2A complex	0.04156
Alzheimer's disease	2214	LMO4-BRCA1-CTIP-LDB1 complex	0.03599
Alzheimer's disease	2215	BRCA1-LMO4-CTIP complex	0.04156
Alzheimer's disease	2217	MDC1-MRN-ATM-FANCD2 complex	0.02939
Alzheimer's disease	2218	MDC1-MRE11-RAD50-NBS1 complex	0.03599
Alzheimer's disease	2237	SP1-MCAF2 complex	0.0509
Alzheimer's disease	2258	VILIP-1-AChR-alpha-4-AChR-beta-2 complex	0.04156
Alzheimer's disease	2318	ITGA6-ITGB4-Laminin10/12 complex	0.03219
Alzheimer's disease	2319	ITGA6-ITGB4-Laminin10/12 complex	0.03219
Alzheimer's disease	2342	ITGAV-ITGB8-MMP14-TGFB1 complex	0.03599
Alzheimer's disease	2343	ITGAV-ITGB5-PLAUR complex	0.04156
Alzheimer's disease	2345	ITGAV-ITGB5-ICAM4 complex	0.04156
Alzheimer's disease	2346	ITGAV-ITGB5-ADAM9 complex	0.04156
Alzheimer's disease	2347	ITGAV-ITGB5-SPP1 complex	0.04156
Alzheimer's disease	2348	ITGAV-ITGB5-CYR61 complex	0.04156
Alzheimer's disease	2350	ITGAV-ITGB5 complex	0.0509
Alzheimer's disease	2352	ITGAV-ITGB6-SPP1 complex	0.04156
Alzheimer's disease	2353	ITGAV-ITGB6-TGFB3 complex	0.04156
Alzheimer's disease	2354	ITGAV-ITGB6 complex	0.0509
Alzheimer's disease	2355	ITGAV-ITGB3-CD47-FCER2 complex	0.03599
Alzheimer's disease	2356	ITGB3-ITGAV-CD47 complex	0.04156
Alzheimer's disease	2358	ITGAV-ITGB3-SPP1 complex	0.04156
Alzheimer's disease	2359	ITGAV-ITGB3-ADAM15 complex	0.04156
Alzheimer's disease	2362	ITAGV-ITGB3-F11R complex	0.04156
Alzheimer's disease	2363	ITGAV-ITGB3-PXN-PTK2b complex	0.03599
Alzheimer's disease	2364	ITGAV-ITGB3-ADAM23 complex	0.04156
Alzheimer's disease	2365	ITGAV-ITGB3-COL4A3 complex	0.04156
Alzheimer's disease	2366	ITGAV-ITGB3-PPAP2b complex	0.04156

Alzheimer's disease	2369	ITGAV-ITGB3-EGFR complex	0.04156
Alzheimer's disease	2374	ITGAV-ITGB3-LAMA4 complex	0.04156
Alzheimer's disease	2383	ITGA5-ITGB1-FN1-TGM2 complex	0.03599
Alzheimer's disease	2384	ITGA5-ITGB1-ADAM15 complex	0.04156
Alzheimer's disease	2385	ITGA5-ITGB4 complex	0.0509
Alzheimer's disease	2388	Itga5-Itgb1-Fn1-Sfrp2 complex	0.03599
Alzheimer's disease	2390	CD98-LAT2-ITGB1 complex	0.03599
Alzheimer's disease	2395	ITGA7-ITGB1-CD151 complex	0.04156
Alzheimer's disease	2396	ITGA7-ITGB1-CD9 complex	0.04156
Alzheimer's disease	2397	ITGA7-ITGB1-ITGB1BP3 complex	0.04156
Alzheimer's disease	2398	ITGA3-ITGB1-BSG complex	0.04156
Alzheimer's disease	2399	ITGA3-ITGB1-CD63 complex	0.04156
Alzheimer's disease	2400	ITGA3-ITGB1-CD151 complex	0.04156
Alzheimer's disease	2401	ITGA3-ITGB1-THBS1 complex	0.04156
Alzheimer's disease	2406	ITGA3-ITGB1 complex	0.0509
Alzheimer's disease	2411	ITGA6-ITGB1-CD151 complex	0.04156
Alzheimer's disease	2413	ITGA6-ITGB1 complex	0.0509
Alzheimer's disease	2416	ITGB1-RAP1A-PKD1 complex	0.04156
Alzheimer's disease	2417	ITGA4-ITGB1-EMILIN1 complex	0.04156
Alzheimer's disease	2418	ITGA4-ITGB1 complex	0.0509
Alzheimer's disease	2419	ITGA4-ITGB1-CD81 complex	0.04156
Alzheimer's disease	2420	ITGA4-ITGB1-CD53 complex	0.04156
Alzheimer's disease	2421	ITGA4-ITGB1-VCAM1 complex	0.04156
Alzheimer's disease	2422	ITGA4-ITGB1-JAM2 complex	0.04156
Alzheimer's disease	2423	ITGA4-ITGB1-CD47 complex	0.04156
Alzheimer's disease	2424	ITGA4-ITGB1-CD63 complex	0.04156
Alzheimer's disease	2425	ITGA4-ITGB1-PXN complex	0.04156
Alzheimer's disease	2426	ITGA4-ITGB1-THBS1 complex	0.04156
Alzheimer's disease	2428	ITGA4-ITGB1-THBS2 complex	0.04156
Alzheimer's disease	2429	ITGA2-ITGB1-CD47 complex	0.04156
Alzheimer's disease	2430	ITGA2-ITGB1-CHAD complex	0.04156
Alzheimer's disease	2431	ITGA2-ITGB1-COL6A3 complex	0.04156
Alzheimer's disease	2432	ITGA2-ITGB1 complex	0.0509
Alzheimer's disease	2434	ITGA1-ITGB1-COL6A3 complex	0.04156
Alzheimer's disease	2435	ITGA1-ITGB1-PTPN2 complex	0.04156
Alzheimer's disease	2436	ITGAV-ITGB1 complex	0.1018
Alzheimer's disease	2437	ITGA6-ITGB1-CYR61 complex	0.04156
Alzheimer's disease	2439	ITGA8-ITGB1 complex	0.0509
Alzheimer's disease	2440	ITGA9-ITGB1-ADAM9 complex	0.04156
Alzheimer's disease	2441	Itga9-Itgb1-Adam2 complex	0.04156
Alzheimer's disease	2442	ITGA9-ITGB1-VCAM1 complex	0.04156
Alzheimer's disease	2443	ITGA9-ITGB1-TNC complex	0.04156
Alzheimer's disease	2444	ITGB1-ITGA9 complex	0.0509
Alzheimer's disease	2445	ITGA9-ITGB1-ADAM15 complex	0.04156
Alzheimer's disease	2446	ITGA9-ITGB1-FIGF complex	0.04156
Alzheimer's disease	2447	ITGA9-ITGB1-ADAM12 complex	0.04156

Alzheimer's disease	2470	p130Cas-ER-alpha-cSrc-kinase- PI3-kinase p85-subunit complex	0.03599
Alzheimer's disease	2486	GIPC1-LHCGR complex	0.0509
Alzheimer's disease	2626	CCNC-CDK8-MED1-MED6-MED7 xcomplex	0.03219
Alzheimer's disease	2628	CCNC-CDK3 complex	0.0509
Alzheimer's disease	2657	ESR1-CDK7-CCNH-MNAT1-MTA1-HDAC2 complex	0.02939
Alzheimer's disease	2670	Er-alpha-p53-hdm2 complex	0.04156
Alzheimer's disease	2679	p53-SP1 complex	0.0509
Alzheimer's disease	2686	BRCA1-core RNA polymerase II complex	0.01996
Alzheimer's disease	2694	ERG-JUN-FOS DNA-protein complex	0.04156
Alzheimer's disease	2699	ER-alpha-GRIP1-c-Jun complex	0.04156
Alzheimer's disease	2700	ER-alpha-c-Jun complex	0.0509
Alzheimer's disease	2706	SMAD3-SMAD4-SP1 complex	0.04156
Alzheimer's disease	2709	MMP-9-TIMP-1-LRP complex	0.08312
Alzheimer's disease	2710	LRP-1-Alpha-2-M-annexin VI complex	0.08312
Alzheimer's disease	2711	Amyloid beta protein oligomer	0.07198
Alzheimer's disease	2721	HCF-1 complex	0.03303
Alzheimer's disease	2736	TAJ-NgR1-LINGO-1 signaling complex	0.04156
Alzheimer's disease	2766	TERF2-RAP1 complex	0.02721
Alzheimer's disease	2767	RAD50-MRE11-NBN-p200-p350 complex	0.04156
Alzheimer's disease	2776	RAD50-BRCA1 complex	0.0509
Alzheimer's disease	2783	BARD1-BRCA1-CSTF64 complex	0.04156
Alzheimer's disease	2786	BRCA1 A complex	0.03599
Alzheimer's disease	2787	BRCA1 C complex	0.03599
Alzheimer's disease	2788	BRCA1 B complex	0.08312
Alzheimer's disease	2789	ETS2-ERG complex	0.0509
Alzheimer's disease	2811	BRCA1-cABL complex	0.1018
Alzheimer's disease	2813	BRCA1-SMAD3 complex	0.0509
Alzheimer's disease	2814	BRCA1-HDAC1-HDAC2 complex	0.04156
Alzheimer's disease	2815	BRCA1-BARD1-BACH1-DNA damage complex II	0.07635
Alzheimer's disease	2816	ITGAV-ITGB3 complex	0.0509
Alzheimer's disease	2817	BRCA1-BARD1-BACH1-DNA damage complex I	0.05877
Alzheimer's disease	2818	BRCA1-BARD1-BRCA2-DNA damage complex III	0.04156
Alzheimer's disease	2819	BRCA1-CtIP-CtBP complex	0.04156
Alzheimer's disease	2820	BRCA1-VCP complex	0.0509
Alzheimer's disease	2822	BRCA1-BARD1-UbcH5c complex	0.04156
Alzheimer's disease	2823	BRCA1-BARD1-UbcH7c complex	0.04156
Alzheimer's disease	2824	BRCA1-RAD51 complex	0.0509
Alzheimer's disease	2825	BRCA1-RNA polymerase II complex	0.01412
Alzheimer's disease	2826	ITGB3-ITGAV-VTN complex	0.04156
Alzheimer's disease	2846	ITGAV-ITGB3-THBS1 complex	0.04156
Alzheimer's disease	2849	ITGAV-ITGB3-NOV complex	0.04156
Alzheimer's disease	2850	ITGA5-ITGB1-FN-1-NOV complex	0.03599
Alzheimer's disease	2853	ITGA5-ITGB1-CAL4A3 complex	0.04156
Alzheimer's disease	2857	NuA4/Tip60 HAT complex	0.01924
Alzheimer's disease	2885	ITGAV-ITGB1-SPP1 complex	0.08312
Alzheimer's disease	2964	ITGA9-ITGB1-ADAM1 complex	0.04156

Alzheimer's disease	2965	ITGA9-ITGB1-ADAM3 complex	0.04156
Alzheimer's disease	2971	ITGA9-ITGB1-VEGFC complex	0.04156
Alzheimer's disease	2972	ITGA9-ITGB1-VEGFA complex	0.04156
Alzheimer's disease	2989	ITGA9-ITGB1-ADAM8 complex	0.04156
Alzheimer's disease	2998	Axin-PP2A A-PP2A C-GSK3-beta-beta-catenin	0.03599
Alzheimer's disease	3004	APC-Axin-1-beta-catenin complex	0.04156
Alzheimer's disease	3015	p27-cyclinE-Cdk2 - Ubiquitin E3 ligase (SKP1A SKP2 CUL1 CKS1B RBX1) complex	0.02545
Alzheimer's disease	3035	LAT2-ITGB1 complex	0.0509
Alzheimer's disease	3055	Nop56p-associated pre-rRNA complex	0.00706
Alzheimer's disease	3057	ITGA10-ITGB1 complex	0.0509
Alzheimer's disease	3058	ITGA11-ITGB1 complex	0.0509
Alzheimer's disease	3059	ITGA11-ITGB1-COL1A1 complex	0.04156
Alzheimer's disease	3060	RNA polymerase II complex (RPB1 RAP74 CDK8 CYCC SRB7 BAF190 BAF47) chromatin structure modifying	0.02545
Alzheimer's disease	3061	RNA polymerase II complex (CBP PCAF RPB1 BAF47 CYCC CDK8) chromatin structure modifying	0.02939
Alzheimer's disease	3064	RNA polymerase II complex chromatin structure modifying	0.01651
Alzheimer's disease	3065	RNA polymerase II complex chromatin structure modifying	0.0217
Alzheimer's disease	3066	RNA polymerase II complex chromatin structure modifying	0.01996
Alzheimer's disease	3067	RNA polymerase II complex incomplete (CDK8 complex) chromatin structure modifying	0.02545
Alzheimer's disease	3092	APP-TOMM40 complex	0.0509
Alzheimer's disease	3093	APP-TIMM23 complex	0.0509
Alzheimer's disease	3103	ITGAV-ITGB3-SLC3A2 complex	0.04156
Alzheimer's disease	3104	ITGB1-NRP1 complex	0.0509
Alzheimer's disease	3110	ITGAV-P2RY2-GNA12 complex	0.04156
Alzheimer's disease	3111	ITGA9-ITGB1-SPP1 complex	0.04156
Alzheimer's disease	3112	ITGA5-ITGB1-SPP1 complex	0.04156
Alzheimer's disease	3117	ITGB5-ITGAV-VTN complex	0.04156
Alzheimer's disease	3151	Sulphiredoxin-peroxiredoxin complex	0.0509
Alzheimer's disease	3155	Bipartite complex (TFC4 CTNNB1)	0.0509
Alzheimer's disease	3166	AXIN-APC-betaCatenin-GSK3B complex	0.03599
Alzheimer's disease	3207	LIN2-LIN7-SAP97-MINT1 complex	0.03599
Alzheimer's disease	3492	Bax homooligomeric complex after apoptotic	0.07198
Alzheimer's disease	3838	SP1-E2F2 complex	0.0509
Alzheimer's disease	3839	SP1-E2F3 complex	0.0509
Alzheimer's disease	3900	GABP(gamma)1-E2F1-DP1 complex	0.04156
Alzheimer's disease	4095	Catulin (alpha) - catenin (beta) complex	0.0509
Alzheimer's disease	4096	Catenin (alpha) - catenin (beta) complex	0.0509
Alzheimer's disease	4158	HSP90-FKBP38-CAM-Ca(2+) complex	0.03599
Alzheimer's disease	4869	beta(1)-AR receptosome (ADRB1-SAP97-AKAP79-PRKAR2A)	0.03599
Alzheimer's disease	4999	p97/VCP-VIMP-DERL1-DERL2-HRD1-SEL1L	0.02939
Alzheimer's disease	5772	ZO1-(beta)cadherin-(VE)cadherin-VEGFR2 complex	0.03599

Alzheimer's disease	5143	E2F1-Rb complex	0.0509
Alzheimer's disease	5144	E2F1-p107-cyclinA complex	0.04156
Alzheimer's disease	5177	Polycystin-1 multiprotein complex (ACTN1 CDH1 SRC JUP VCL CTNNB1 PXN BCAR1 PKD1	0.0217
Alzheimer's disease	5183	DNA-PK-Ku-eIF2-NF90-NF45 complex	0.02545
Alzheimer's disease	5197	PTIP-DNA damage response complex	0.02939
Alzheimer's disease	5199	Kinase maturation complex 1	0.03599
Alzheimer's disease	5211	RAF1-PPP2-PIN1 complex	0.06438
Alzheimer's disease	5212	Kinase maturation complex 2	0.02545
Alzheimer's disease	5234	IKBKB-CDC37-KIAA1967-HSP90AB1-HSP90AA1 complex	0.03219
Alzheimer's disease	5260	TCF4-CTNNB1-SUMO1-EP300-HADAC6 complex	0.03219
Alzheimer's disease	5261	TCF4-CTNNB1-EP300 complex	0.04156
Alzheimer's disease	5262	TCF4-CTNNB1 complex	0.0509
Alzheimer's disease	5264	TCF4-CTNNB1-CREBBP complex	0.04156
Alzheimer's disease	5266	TNF-alpha/NF-kappa B signaling complex 6	0.01924
Alzheimer's disease	5268	TNF-alpha/NF-kappa B signaling complex 7	0.02545
Alzheimer's disease	5269	TNF-alpha/NF-kappa B signaling complex 8	0.02939
Alzheimer's disease	5280	RAB9-TIP47-MPRI complex	0.04156
Alzheimer's disease	5281	Cell-cell junction complex (CDH1-CTNNB1)	0.0509
Alzheimer's disease	5286	TNF-alpha/NF-kappa B signaling complex 10	0.02276
Alzheimer's disease	5400	BRCC complex	0.03219
Alzheimer's disease	5442	EPOR receptor complex	0.07198
Alzheimer's disease	5446	EPO-EPOR complex	0.0509
Alzheimer's disease	5473	FAS-FADD-CASP8 complex	0.04156
Alzheimer's disease	5526	CALM1-FKBP38-BCL2 complex	0.04156
Alzheimer's disease	5615	Emerin complex 52	0.01501
Alzheimer's disease	5622	HSP90-CIP1-FKBPL complex	0.04156
Alzheimer's disease	5655	Ternary complex (LRRC7 CAMK2a ACTN4)	0.04156
Alzheimer's disease	5656	CEBPE-E2F1-RB1 complex	0.04156
Alzheimer's disease	5799	Death induced signaling complex DISC (FAS FADD CASP8 CFLAR) membrane-associated CD95L	0.03599
Alzheimer's disease	5800	Death-inducing signaling complex DISC (type I cells associated) stimulated	0.04156
Alzheimer's disease	5808	DISC complex	0.04156
Alzheimer's disease	5811	p53-BCL2 complex	0.0509
Alzheimer's disease	5817	tBID-BCL2 complex	0.0509
Alzheimer's disease	5818	BIM-BCL2 complex	0.0509
Alzheimer's disease	5877	MAP2K1-BRAF-RAF1-YWHAE-KSR1 complex	0.03219
Alzheimer's disease	5859	FAS-FADD-CASP8-CASP10 complex	0.03599
Alzheimer's disease	5861	FAS-FADD-CASP10 complex	0.04156
Alzheimer's disease	5862	CAV1-VDAC1-ESR1 complex	0.04156
Alzheimer's disease	5873	RAF1-MAP2K1-YWHAE complex	0.04156
Alzheimer's disease	5919	BRAF-RAF1-14-3-3 complex	0.02399
Alzheimer's disease	5920	KSR1-RAF1-MEK complex	0.03599
Alzheimer's disease	5922	RAF1-RAS complex EGF induced	0.03599
Alzheimer's disease	5923	RAF1-BRAF complex RAS stimulated	0.0509
Alzheimer's disease	5924	RAF1-CNK1 complex RAS stimulated	0.0509

Alzheimer's disease	5928	CNK1-SRC-RAF1 complex	0.04156
Amnionitis	120	Lymphotoxin beta receptor complex	0.16013
Amnionitis	2709	MMP-9-TIMP-1-LRP complex	0.16013
Amnionitis	3043	BMP2-BRIA complex	0.16013
Amnionitis	3710	CHL2-BMP2 complex	0.19612
Amnionitis	3711	CHL2-BMP2-TSG complex	0.16013
Amyloidosis	143	APP-FE65-LRP complex	0.10911
Amyloidosis	1223	H2AX complex isolated from cells without IR exposure	0.05241
Amyloidosis	1226	H2AX complex I	0.07143
Amyloidosis	1227	H2AX complex II	0.05976
Amyloidosis	2711	Amyloid beta protein oligomer	0.18898
Amyloidosis	3092	APP-TOMM40 complex	0.13363
Amyloidosis	3093	APP-TIMM23 complex	0.13363
Amyloidosis	5217	Calreticulin oligomer complex	0.18898
Amyloidosis	5830	DJ-1-SNCA complex high molecular weight complex	0.26726
Amyloidosis	5837	PPD complex	0.10911
Amyotrophic lateral sclerosis	143	APP-FE65-LRP complex	0.07785
Amyotrophic lateral sclerosis	159	Condensin I-PARP-1-XRCC1 complex	0.05096
Amyotrophic lateral sclerosis	298	VEGF transcriptional complex	0.05505
Amyotrophic lateral sclerosis	472	Prolyl 4-hydroxylase (alpha(I)-type)	0.09535
Amyotrophic lateral sclerosis	473	Prolyl 4-hydroxylase (alpha(II)-type)	0.09535
Amyotrophic lateral sclerosis	474	Prolyl 4-hydroxylase (alpha(III)-type)	0.09535
Amyotrophic lateral sclerosis	541	IGF1-IGFBP3-ALS complex	0.07785
Amyotrophic lateral sclerosis	824	Anti-SMN protein complex	0.0603
Amyotrophic lateral sclerosis	832	Anti-Sm protein complex	0.05096
Amyotrophic lateral sclerosis	905	KIF3A/B-PAR-3-aPKC-PAR-6 complex	0.05505
Amyotrophic lateral sclerosis	1004	RC complex during S-phase of cell cycle	0.0374
Amyotrophic lateral sclerosis	1005	RC complex during G2/M-phase of cell cycle	0.0374
Amyotrophic lateral sclerosis	1088	PRNP-ApolopoproteinE3 complex	0.09535
Amyotrophic lateral sclerosis	1142	SMN complex	0.04264
Amyotrophic lateral sclerosis	1143	SMN complex	0.03371
Amyotrophic lateral sclerosis	1186	ESCRT-III complex	0.04264
Amyotrophic lateral sclerosis	1193	Rap1 complex	0.05096
Amyotrophic lateral sclerosis	1226	H2AX complex I	0.05096
Amyotrophic lateral sclerosis	1474	SMAD3/4-E2F4/5-p107-DP1 complex	0.05505
Amyotrophic lateral sclerosis	1728	CTCF-nucleophosmin-PARP-HIS-KPNA-LMNA-TOP complex	0.04495
Amyotrophic lateral sclerosis	1729	TLE1 corepressor complex (MASH1 promoter-corepressor complex)	0.04264
Amyotrophic lateral sclerosis	1746	SMN containing complex	0.04767
Amyotrophic lateral sclerosis	1751	SMN complex	0.04264
Amyotrophic lateral sclerosis	1752	SMN complex	0.05096
Amyotrophic lateral sclerosis	1787	Nogo-potassium channel complex	0.06742
Amyotrophic lateral sclerosis	1826	SMAD3-HEF1-APC10-CDH1 complex	0.06742
Amyotrophic lateral sclerosis	1827	PML-SMAD2/3-SARA complex	0.13484
Amyotrophic lateral sclerosis	1828	TGF-beta receptor I-Axin-SMAD3 complex	0.07785
Amyotrophic lateral sclerosis	1831	PIAS3-SMAD3-P300 complex	0.07785
Amyotrophic lateral sclerosis	1986	Endoglin homodimer complex	0.13484

Amyotrophic lateral sclerosis	1993	SLC1A2 homotrimer complex	0.13484
Amyotrophic lateral sclerosis	2159	AR-AKT-APPL complex	0.07785
Amyotrophic lateral sclerosis	2160	AOF2-AR complex	0.09535
Amyotrophic lateral sclerosis	2189	Ubiquitin E3 ligase (SMAD3 BTRC CUL1 SKP1A RBX1)	0.0603
Amyotrophic lateral sclerosis	2247	Dynactin complex (DCTN1 DCTN2 DCTN3 DCTN4 DCTN6 CAPZA1 CAPZB ACTR1A)	0.04767
Amyotrophic lateral sclerosis	2318	ITGA6-ITGB4-Laminin10/12 complex	0.0603
Amyotrophic lateral sclerosis	2319	ITGA6-ITGB4-Laminin10/12 complex	0.0603
Amyotrophic lateral sclerosis	2456	MET-CIN85-SH3GL3-CBL complex HGF stimulated	0.06742
Amyotrophic lateral sclerosis	2541	HGF-Met complex	0.09535
Amyotrophic lateral sclerosis	2572	RAB5-EEA1 complex	0.09535
Amyotrophic lateral sclerosis	2573	Class C VPS/HOPS complex	0.05505
Amyotrophic lateral sclerosis	2625	CDK8-MED6-PARP1 complex	0.07785
Amyotrophic lateral sclerosis	2692	SMAD3-SMAD4-cJun-cFos complex	0.06742
Amyotrophic lateral sclerosis	2705	SMAD3-SMAD4-CTCF protein-DNA complex	0.07785
Amyotrophic lateral sclerosis	2706	SMAD3-SMAD4-SP1 complex	0.07785
Amyotrophic lateral sclerosis	2707	SMAD3-SMAD4-FOXO3-FOXG1 complex	0.06742
Amyotrophic lateral sclerosis	2708	SMAD3-SMAD4-cJUN complex	0.07785
Amyotrophic lateral sclerosis	2709	MMP-9-TIMP-1-LRP complex	0.1557
Amyotrophic lateral sclerosis	2711	Amyloid beta protein oligomer	0.13484
Amyotrophic lateral sclerosis	2754	JUND-FOSB-SMAD3-SMAD4 complex	0.06742
Amyotrophic lateral sclerosis	2760	SMAD3-SMAD4-FOXO3 complex	0.07785
Amyotrophic lateral sclerosis	2761	SMAD3-SMAD4-FOXO1 complex	0.07785
Amyotrophic lateral sclerosis	2762	SMAD3-SMAD4-FOXO4 complex	0.07785
Amyotrophic lateral sclerosis	2808	RAD9-RAD1-HUS1-APE1 complex	0.06742
Amyotrophic lateral sclerosis	2813	BRCA1-SMAD3 complex	0.09535
Amyotrophic lateral sclerosis	2829	RSmad complex	0.08528
Amyotrophic lateral sclerosis	2830	TIF1gamma-SMAD2-SMAD3 complex	0.1557
Amyotrophic lateral sclerosis	2834	SMAD4-SMAD2-SMAD3 complex	0.1557
Amyotrophic lateral sclerosis	2968	Axin-SMAD3 complex	0.09535
Amyotrophic lateral sclerosis	2975	SMAD3-E2F4/5-p107-DP1 complex	0.0603
Amyotrophic lateral sclerosis	3038	SMAD2-SMAD4-FAST1 complex	0.07785
Amyotrophic lateral sclerosis	3039	SMAD2-FAST1 complex	0.09535
Amyotrophic lateral sclerosis	3092	APP-TOMM40 complex	0.09535
Amyotrophic lateral sclerosis	3093	APP-TIMM23 complex	0.09535
Amyotrophic lateral sclerosis	3098	TIM50a-SMN1 complex	0.09535
Amyotrophic lateral sclerosis	3118	SMN1-SIP1-SNRP complex	0.05096
Amyotrophic lateral sclerosis	3137	MASH1 promoter-coactivator complex	0.04066
Amyotrophic lateral sclerosis	3142	CAMK2-delta-MASH1 promoter-coactivator complex	0.04767
Amyotrophic lateral sclerosis	3198	SMAD2-SKI complex	0.09535
Amyotrophic lateral sclerosis	3199	SMAD3-SKI complex	0.09535
Amyotrophic lateral sclerosis	3204	SMAD2-SKI-NCOR complex	0.07785
Amyotrophic lateral sclerosis	3205	SMAD3-SKI-NCOR complex	0.07785
Amyotrophic lateral sclerosis	3233	SMAD2-SMAD4-FAST1-TGIF complex TGF(beta) induced	0.06742
Amyotrophic lateral sclerosis	3234	SMAD2-SMAD4-FAST1-TGIF-HDAC1 complex TGF(beta) induced	0.0603

Amyotrophic lateral sclerosis	3284	SMN complex (GEMIN5 2 3 4 SMN)	0.0603
Amyotrophic lateral sclerosis	3298	SMN complex (GEMIN2 5 SMN)	0.07785
Amyotrophic lateral sclerosis	3729	SKI-SMAD2 hexameric complex	0.09535
Amyotrophic lateral sclerosis	3733	SKI-SMAD3 hexameric complex	0.09535
Amyotrophic lateral sclerosis	3739	SKI-SMAD2-SMAD4 pentameric complex	0.07785
Amyotrophic lateral sclerosis	3740	SKI-SMAD3-SMAD4 pentameric complex	0.07785
Amyotrophic lateral sclerosis	3749	CREBBP-SMAD2 hexameric complex	0.09535
Amyotrophic lateral sclerosis	3750	CREBBP-SMAD3 hexameric complex	0.09535
Amyotrophic lateral sclerosis	3753	CREBBP-SMAD2-SMAD4 pentameric complex	0.07785
Amyotrophic lateral sclerosis	3754	CREBBP-SMAD3-SMAD4 pentameric complex	0.07785
Amyotrophic lateral sclerosis	3959	SMAD3-SMAD4-cSKI TGF(beta)-dependent	0.07785
Amyotrophic lateral sclerosis	3961	SMAD3-cSKI-SIN3A-HDAC1 complex	0.06742
Amyotrophic lateral sclerosis	3967	SMURF2-SMAD2 complex TGF(beta)-dependent	0.09535
Amyotrophic lateral sclerosis	3971	SMURF2-SMAD3 complex TGF(beta)-dependent	0.09535
Amyotrophic lateral sclerosis	3972	SMURF2-SMAD3-SnoN complex TGF(beta)-	0.07785
Amyotrophic lateral sclerosis	4062	NRP1-VEGFR2-VEGF(165) complex	0.07785
Amyotrophic lateral sclerosis	5772	ZO1-(beta)cadherin-(VE)cadherin-VEGFR2 complex	0.06742
Amyotrophic lateral sclerosis	5179	NCOA6-DNA-PK-Ku-PARP1 complex	0.0603
Amyotrophic lateral sclerosis	5235	WRN-Ku70-Ku80-PARP1 complex	0.06742
Amyotrophic lateral sclerosis	5414	HTR1A-HTR1D complex	0.09535
Amyotrophic lateral sclerosis	5416	HTR1A-HTR1B complex	0.09535
Amyotrophic lateral sclerosis	5418	GABBR2-HTR1A complex	0.09535
Amyotrophic lateral sclerosis	5419	HTR1A-GPR26 complex	0.09535
Amyotrophic lateral sclerosis	5420	HTR1A-EDG3 complex	0.09535
Amyotrophic lateral sclerosis	5421	HTR1A homodimer complex	0.13484
Amyotrophic lateral sclerosis	5422	HTR1A-EDG1 complex	0.09535
Amyotrophic lateral sclerosis	5696	VEGFA(165)-KDR-NRP1 complex	0.07785
Amyotrophic lateral sclerosis	5698	VEGFA(165)-VEGFR2-NRP1 complex	0.07785
Amyotrophic lateral sclerosis	5735	TGF-beta receptor-SMAD3 complex	0.07785
Amyotrophic lateral sclerosis	5830	DJ-1-SNCA complex high molecular weight complex	0.09535
Amyotrophic lateral sclerosis	5837	PPD complex	0.07785
Anemia	1069	FIF-FGR2 complex	0.15076
Anemia	1714	TICAM1-TICAM2-TLR4 complex	0.12309
Anemia	2018	IL12A-IL12B complex	0.15076
Anemia	2019	IL12A-IL12B-IL12RB1 complex	0.12309
Anemia	2020	IL12B-IL12RB1-IL12RB2 complex	0.12309
Anemia	2021	IL12A-IL12B-IL12RB2 complex	0.12309
Anemia	5446	EPO-EPOR complex	0.15076
Anemia	5548	IL-12 heterodimer complex	0.15076
Anemia	5549	IL-12 subunit p40 homodimer complex	0.2132
Angiomyolipoma	75	TSC1-TSC2 complex	0.26726
Angiomyolipoma	1095	SNX complex (SNX1a SNX2 SNX4 EGFR)	0.18898
Angiomyolipoma	1185	EGFR-containing signaling complex	0.18898
Angiomyolipoma	2369	ITGAV-ITGB3-EGFR complex	0.21822
Angiomyolipoma	2453	Multiprotein complex (monoubiquitination)	0.18898
Angiomyolipoma	2454	CIN85-CBL-SH3GL2-EGFR complex EGF stimulated	0.18898
Angiomyolipoma	2542	EGFR-CBL-GRB2 complex	0.21822

Angiomyolipoma	3678	RIN1-STAM2-EGFR complex EGF stimulated	0.21822
Angiomyolipoma	5171	SH3KBP1-CBLB-EGFR complex	0.21822
Angiomyolipoma	5877	MAP2K1-BRAF-RAF1-YWHAE-KSR1 complex	0.16903
Angiomyolipoma	5872	BRAF-MAP2K1-MAP2K2-YWHAE complex	0.18898
Angiomyolipoma	5919	BRAF-RAF1-14-3-3 complex	0.12599
Angiomyolipoma	5921	KSR1-BRAF-MEK complex	0.18898
Angiomyolipoma	5923	RAF1-BRAF complex RAS stimulated	0.26726
Angiomyolipoma	5925	BRAF-CNK1 complex not RAS stimulated	0.26726
Ankylosis	3043	BMP2-BRIA complex	0.33333
Ankylosis	3710	CHL2-BMP2 complex	0.40825
Ankylosis	3711	CHL2-BMP2-TSG complex	0.33333
Anorexia nervosa	1091	SNX complex (SNX1a SNX2 SNX4 LEPR)	0.10206
Anorexia nervosa	1992	LEPR homodimer complex	0.20412
Anorexia nervosa	5411	EDG1-HTR1D complex	0.14434
Anorexia nervosa	5412	HTR1D homodimer complex	0.20412
Anorexia nervosa	5414	HTR1A-HTR1D complex	0.14434
Anorexia nervosa	5417	HTR1D-HTR1B complex	0.14434
Antiphospholipid syndrome	541	IGF1-IGFBP3-ALS complex	0.19245
Aortic aneurysm	486	WIP-WASp-actin-myosin-IIa complex	0.08704
Aortic aneurysm	845	PCI-PSA-SCG2 complex	0.12309
Aortic aneurysm	1787	Nogo-potassium channel complex	0.1066
Aortic aneurysm	2383	ITGA5-ITGB1-FN1-TGM2 complex	0.2132
Aortic aneurysm	2384	ITGA5-ITGB1-ADAM15 complex	0.24618
Aortic aneurysm	2385	ITGA5-ITGB4 complex	0.30151
Aortic aneurysm	2388	Itga5-Itgb1-Fn1-Sfrp2 complex	0.2132
Aortic aneurysm	2390	CD98-LAT2-ITGB1 complex	0.1066
Aortic aneurysm	2395	ITGA7-ITGB1-CD151 complex	0.12309
Aortic aneurysm	2396	ITGA7-ITGB1-CD9 complex	0.12309
Aortic aneurysm	2397	ITGA7-ITGB1-ITGB1BP3 complex	0.12309
Aortic aneurysm	2398	ITGA3-ITGB1-BSG complex	0.12309
Aortic aneurysm	2399	ITGA3-ITGB1-CD63 complex	0.12309
Aortic aneurysm	2400	ITGA3-ITGB1-CD151 complex	0.12309
Aortic aneurysm	2401	ITGA3-ITGB1-THBS1 complex	0.12309
Aortic aneurysm	2406	ITGA3-ITGB1 complex	0.15076
Aortic aneurysm	2411	ITGA6-ITGB1-CD151 complex	0.12309
Aortic aneurysm	2413	ITGA6-ITGB1 complex	0.15076
Aortic aneurysm	2416	ITGB1-RAP1A-PKD1 complex	0.12309
Aortic aneurysm	2417	ITGA4-ITGB1-EMILIN1 complex	0.12309
Aortic aneurysm	2418	ITGA4-ITGB1 complex	0.15076
Aortic aneurysm	2419	ITGA4-ITGB1-CD81 complex	0.12309
Aortic aneurysm	2420	ITGA4-ITGB1-CD53 complex	0.12309
Aortic aneurysm	2421	ITGA4-ITGB1-VCAM1 complex	0.12309
Aortic aneurysm	2422	ITGA4-ITGB1-JAM2 complex	0.12309
Aortic aneurysm	2423	ITGA4-ITGB1-CD47 complex	0.12309
Aortic aneurysm	2424	ITGA4-ITGB1-CD63 complex	0.12309
Aortic aneurysm	2425	ITGA4-ITGB1-PXN complex	0.12309
Aortic aneurysm	2426	ITGA4-ITGB1-THBS1 complex	0.12309

Aortic aneurysm	2428	ITGA4-ITGB1-THBS2 complex	0.12309
Aortic aneurysm	2429	ITGA2-ITGB1-CD47 complex	0.12309
Aortic aneurysm	2430	ITGA2-ITGB1-CHAD complex	0.12309
Aortic aneurysm	2431	ITGA2-ITGB1-COL6A3 complex	0.12309
Aortic aneurysm	2432	ITGA2-ITGB1 complex	0.15076
Aortic aneurysm	2434	ITGA1-ITGB1-COL6A3 complex	0.12309
Aortic aneurysm	2435	ITGA1-ITGB1-PTPN2 complex	0.12309
Aortic aneurysm	2436	ITGAV-ITGB1 complex	0.15076
Aortic aneurysm	2437	ITGA6-ITGB1-CYR61 complex	0.12309
Aortic aneurysm	2439	ITGA8-ITGB1 complex	0.15076
Aortic aneurysm	2440	ITGA9-ITGB1-ADAM9 complex	0.12309
Aortic aneurysm	2441	Itga9-Itgb1-Adam2 complex	0.12309
Aortic aneurysm	2442	ITGA9-ITGB1-VCAM1 complex	0.12309
Aortic aneurysm	2443	ITGA9-ITGB1-TNC complex	0.12309
Aortic aneurysm	2444	ITGB1-ITGA9 complex	0.15076
Aortic aneurysm	2445	ITGA9-ITGB1-ADAM15 complex	0.12309
Aortic aneurysm	2446	ITGA9-ITGB1-FIGF complex	0.12309
Aortic aneurysm	2447	ITGA9-ITGB1-ADAM12 complex	0.12309
Aortic aneurysm	2709	MMP-9-TIMP-1-LRP complex	0.12309
Aortic aneurysm	2850	ITGA5-ITGB1-FN-1-NOV complex	0.2132
Aortic aneurysm	2853	ITGA5-ITGB1-CAL4A3 complex	0.24618
Aortic aneurysm	2882	ITGA5-ITGB3-COL6A3 complex	0.12309
Aortic aneurysm	2885	ITGAV-ITGB1-SPP1 complex	0.12309
Aortic aneurysm	2964	ITGA9-ITGB1-ADAM1 complex	0.12309
Aortic aneurysm	2965	ITGA9-ITGB1-ADAM3 complex	0.12309
Aortic aneurysm	2971	ITGA9-ITGB1-VEGFC complex	0.12309
Aortic aneurysm	2972	ITGA9-ITGB1-VEGFA complex	0.12309
Aortic aneurysm	2989	ITGA9-ITGB1-ADAM8 complex	0.12309
Aortic aneurysm	3035	LAT2-ITGB1 complex	0.15076
Aortic aneurysm	3055	Nop56p-associated pre-rRNA complex	0.02091
Aortic aneurysm	3057	ITGA10-ITGB1 complex	0.15076
Aortic aneurysm	3058	ITGA11-ITGB1 complex	0.15076
Aortic aneurysm	3059	ITGA11-ITGB1-COL1A1 complex	0.12309
Aortic aneurysm	3104	ITGB1-NRP1 complex	0.15076
Aortic aneurysm	3111	ITGA9-ITGB1-SPP1 complex	0.12309
Aortic aneurysm	3112	ITGA5-ITGB1-SPP1 complex	0.24618
Aortic valve disease	285	PCNA-MLH1-PMS1 complex	0.19245
Aortic valve disease	286	PCNA-MSH2-MSH6 complex	0.19245
Aortic valve disease	290	MSH2-MLH1-PMS2-PCNA DNA-repair initiation complex	0.16667
Aortic valve disease	297	PCNA-DNA polymerase delta complex	0.14907
Aortic valve disease	310	Cell cycle kinase complex CDC2	0.13608
Aortic valve disease	311	Cell cycle kinase complex CDK2	0.16667
Aortic valve disease	312	Cell cycle kinase complex CDK4	0.16667
Aortic valve disease	313	Cell cycle kinase complex CDK5	0.14907
Aortic valve disease	314	PCNA-p21 complex	0.2357
Aortic valve disease	376	PCNA-MutS-alpha-MutL-alpha-DNA complex	0.14907

Aortic valve disease	377	PCNA-MutS-alpha-DNA initial complex	0.19245
Aortic valve disease	424	EXO1-MLH1-PCNA complex	0.19245
Aortic valve disease	860	DNMT1-G9a-PCNA complex	0.19245
Aortic valve disease	997	KIN17-PCNA-RPA70 complex	0.19245
Aortic valve disease	1039	PCNA-PAF complex	0.2357
Aortic valve disease	1092	PCNA-KU antigen complex	0.19245
Aortic valve disease	1098	DNA synthesome complex (13 subunits)	0.08909
Aortic valve disease	1099	DNA synthesome complex (17 subunits)	0.07857
Aortic valve disease	1107	DNA synthesome core complex	0.10541
Aortic valve disease	1108	DNA synthesome complex (15 subunits)	0.08607
Aortic valve disease	1160	ING1-p300-PCNA complex	0.19245
Aortic valve disease	1163	ING1-PCNA complex	0.2357
Aortic valve disease	2201	PCNA-RFC2-5 complex	0.14907
Aortic valve disease	2230	PCNA complex	0.12599
Aortic valve disease	2231	PCNA homotrimer complex	0.33333
Aortic valve disease	2797	PCNA-CHL12-RFC2-5 complex	0.13608
Aortic valve disease	5544	CDC2-PCNA-CCNB1-GADD45A complex	0.16667
Aortic valve disease	5545	CDC2-PCNA-CCNB1-GADD45B complex	0.16667
Aortic valve disease	5546	CDC2-PCNA-CCNB1-GADD45G complex	0.16667
Aplastic anemia	305	40S ribosomal subunit cytoplasmic	0.10728
Aplastic anemia	306	Ribosome cytoplasmic	0.09267
Aplastic anemia	308	60S ribosomal subunit cytoplasmic	0.03041
Aplastic anemia	2355	ITGAV-ITGB3-CD47-FCER2 complex	0.10426
Aplastic anemia	2356	ITGB3-ITGAV-CD47 complex	0.12039
Aplastic anemia	2358	ITGAV-ITGB3-SPP1 complex	0.12039
Aplastic anemia	2359	ITGAV-ITGB3-ADAM15 complex	0.12039
Aplastic anemia	2362	ITAGV-ITGB3-F11R complex	0.12039
Aplastic anemia	2363	ITGAV-ITGB3-PXN-PTK2b complex	0.10426
Aplastic anemia	2364	ITGAV-ITGB3-ADAM23 complex	0.12039
Aplastic anemia	2365	ITGAV-ITGB3-COL4A3 complex	0.12039
Aplastic anemia	2366	ITGAV-ITGB3-PPAP2b complex	0.12039
Aplastic anemia	2369	ITGAV-ITGB3-EGFR complex	0.12039
Aplastic anemia	2370	ITGA2b-ITGB3-CD9 complex	0.24077
Aplastic anemia	2374	ITGAV-ITGB3-LAMA4 complex	0.12039
Aplastic anemia	2376	ITGA2B-ITGB3-FN1-TGM2 complex	0.20851
Aplastic anemia	2377	ITGA2b-ITGB3-CD47-SRC complex	0.20851
Aplastic anemia	2378	ITGA2b-ITGB3-TLN1 complex	0.24077
Aplastic anemia	2379	ITGA2B-ITGB3-CIB1 complex	0.24077
Aplastic anemia	2381	ITGA2B-ITGB3 complex	0.29488
Aplastic anemia	2382	ITGA2B-ITGB3-F11R complex	0.24077
Aplastic anemia	2693	NFAT-JUN-FOS DNA-protein complex	0.12039
Aplastic anemia	2816	ITGAV-ITGB3 complex	0.14744
Aplastic anemia	2826	ITGB3-ITGAV-VTN complex	0.12039
Aplastic anemia	2846	ITGAV-ITGB3-THBS1 complex	0.12039
Aplastic anemia	2849	ITGAV-ITGB3-NOV complex	0.12039
Aplastic anemia	2872	ITGA2b-ITGB3-CD9-GP1b-CD47 complex	0.17025
Aplastic anemia	2882	ITGA5-ITGB3-COL6A3 complex	0.12039

Aplastic anemia	2896	ITGA2b-ITGB3-CD47-FAK complex	0.20851
Aplastic anemia	3055	Nop56p-associated pre-rRNA complex	0.06134
Aplastic anemia	3103	ITGAV-ITGB3-SLC3A2 complex	0.12039
Aplastic anemia	3115	ITGA2B-ITGB3-ICAM4 complex	0.24077
Aplastic anemia	5380	TRBP containing complex (DICER RPL7A EIF6 MOV10 and subunits of the 60S ribosomal particle)	0.0417
Arteriopathy	2709	MMP-9-TIMP-1-LRP complex	0.19245
Arthritis	142	CD147-gamma-secretase complex (APH-1a PS-1 PEN-2 NCT variant)	0.05547
Arthritis	552	IFNB1-IFNAR1-IFNAR2- complex	0.07161
Arthritis	668	BKCA-beta2AR-AKAP79 signaling complex	0.07161
Arthritis	672	BKCA-beta2AR complex	0.08771
Arthritis	687	CFTR-NHERF-beta(2)AR signaling complex	0.07161
Arthritis	1069	FIF-FGR2 complex	0.08771
Arthritis	1087	BIRC5-AURKB-INCENP-EVI5 complex	0.06202
Arthritis	1116	CRM1-Survivin-AuroraB mitotic complex	0.07161
Arthritis	1117	CRM1-Survivin mitotic complex	0.08771
Arthritis	1118	Chromosomal passenger complex CPC (INCENP CDCA8 BIRC5 AURKB)	0.06202
Arthritis	1120	Chromosomal passenger complex CPC (INCENP CDCA8 BIRC5)	0.07161
Arthritis	2001	NOD1 homodimer complex	0.12403
Arthritis	2398	ITGA3-ITGB1-BSG complex	0.07161
Arthritis	2579	Chromosomal passenger complex CPC (INCENP BIRC5 AURKB)	0.07161
Arthritis	2580	Survivin homodimer complex	0.12403
Arthritis	2581	RasGAP-AURKA/AURKB-survivin complex	0.06202
Arthritis	2582	Chromosomal passenger complex CPC (CDCA8 AURKB BIRC5)	0.07161
Arthritis	2709	MMP-9-TIMP-1-LRP complex	0.07161
Arthritis	2798	MMP-2-claudin-1 complex	0.08771
Arthritis	3830	ADRB2 homodimer complex	0.12403
Aseptic necrosis of bone	4	Multisubunit ACTR coactivator complex	0.15811
Aseptic necrosis of bone	298	VEGF transcriptional complex	0.1291
Aseptic necrosis of bone	521	Polycystin-1-E-cadherin-beta-catenin complex	0.18257
Aseptic necrosis of bone	522	Polycystin-1-E-cadherin-beta-catenin-Flotillin-2	0.15811
Aseptic necrosis of bone	570	p300-CBP-p270-SWI/SNF complex	0.11952
Aseptic necrosis of bone	571	p300-CBP-p270 complex	0.18257
Aseptic necrosis of bone	826	PAR-3-VE-cadherin-beta-catenin complex	0.18257
Aseptic necrosis of bone	1816	JUN-TCF4-CTNNB1 complex	0.18257
Aseptic necrosis of bone	1839	SDCBP-CTNNB1-CTNNA1-CDH1 complex	0.15811
Aseptic necrosis of bone	2638	HES1 promoter corepressor complex	0.1291
Aseptic necrosis of bone	2641	p300/CBP-PCAF-MyoD complex	0.15811
Aseptic necrosis of bone	2727	SRC-3 complex	0.11952
Aseptic necrosis of bone	2728	SRC-1 complex	0.15811
Aseptic necrosis of bone	2829	RSmad complex	0.1
Aseptic necrosis of bone	2958	SMAD1-CBP complex	0.22361
Aseptic necrosis of bone	2998	Axin-PP2A A-PP2A C-GSK3-beta-beta-catenin	0.15811

Aseptic necrosis of bone	3004	APC-Axin-1-beta-catenin complex	0.18257
Aseptic necrosis of bone	3061	RNA polymerase II complex (CBP PCAF RPB1 BAF47 CYCC CDK8) chromatin structure modifying	0.1291
Aseptic necrosis of bone	3062	RNA polymerase II complex incomplete (CBP RPBI PCAF BAF47) chromatin structure modifying	0.15811
Aseptic necrosis of bone	3066	RNA polymerase II complex chromatin structure modifying	0.08771
Aseptic necrosis of bone	3137	MASH1 promoter-coactivator complex	0.09535
Aseptic necrosis of bone	3142	CAMK2-delta-MASH1 promoter-coactivator complex	0.1118
Aseptic necrosis of bone	3155	Bipartite complex (TFC4 CTNNB1)	0.22361
Aseptic necrosis of bone	3166	AXIN-APC-betaCatenin-GSK3B complex	0.15811
Aseptic necrosis of bone	3749	CREBBP-SMAD2 hexameric complex	0.22361
Aseptic necrosis of bone	3750	CREBBP-SMAD3 hexameric complex	0.22361
Aseptic necrosis of bone	3753	CREBBP-SMAD2-SMAD4 pentameric complex	0.18257
Aseptic necrosis of bone	3754	CREBBP-SMAD3-SMAD4 pentameric complex	0.18257
Aseptic necrosis of bone	4095	Catulin (alpha) - catenin (beta) complex	0.22361
Aseptic necrosis of bone	4096	Catenin (alpha) - catenin (beta) complex	0.22361
Aseptic necrosis of bone	5772	ZO1-(beta)cadherin-(VE)cadherin-VEGFR2 complex	0.15811
Aseptic necrosis of bone	5177	Polycystin-1 multiprotein complex (ACTN1 CDH1 SRC JUP VCL CTNNB1 PXN BCAR1 PKD1	0.09535
Aseptic necrosis of bone	5198	CBP-RARA-RXRA-DNA complex ligand stimulated	0.18257
Aseptic necrosis of bone	5260	TCF4-CTNNB1-SUMO1-EP300-HADAC6 complex	0.14142
Aseptic necrosis of bone	5261	TCF4-CTNNB1-EP300 complex	0.18257
Aseptic necrosis of bone	5262	TCF4-CTNNB1 complex	0.22361
Aseptic necrosis of bone	5264	TCF4-CTNNB1-CREBBP complex	0.36515
Aseptic necrosis of bone	5273	VHL-TBP1-HIF1A complex	0.18257
Aseptic necrosis of bone	5276	HIF1A-OS9-EGLN1 complex	0.18257
Aseptic necrosis of bone	5277	HIF1A-OS9-EGLN3 complex	0.18257
Aseptic necrosis of bone	5281	Cell-cell junction complex (CDH1-CTNNB1)	0.22361
Aseptic necrosis of bone	5382	ARNT-HIF1A complex	0.22361
Aseptic necrosis of bone	5573	Stat1-alpha-dimer-CBP DNA-protein complex	0.22361
Asthma	115	Polycomb repressive complex 1 (PRC1 hPRC-H)	0.02357
Asthma	116	Polycomb repressive complex 1 (PRC1 hPRC-H)	0.02265
Asthma	120	Lymphotoxin beta receptor complex	0.04714
Asthma	432	N-NOS-CHIP-HSP70-1 complex	0.04714
Asthma	595	Kv4.2-DPP10 channel complex	0.05774
Asthma	626	LSD1 complex	0.02265
Asthma	725	P2X7 receptor signalling complex	0.02357
Asthma	1041	Alpha-dystrobrevin-ZO-1-actin complex	0.04082
Asthma	1062	BAR-BCL2-CASP8 complex	0.04714
Asthma	1069	FIF-FGR2 complex	0.05774
Asthma	1095	SNX complex (SNX1a SNX2 SNX4 EGFR)	0.04082
Asthma	1141	CF IIAm complex (Cleavage factor IIAm complex)	0.02041
Asthma	1185	EGFR-containing signaling complex	0.04082
Asthma	1308	PABPC1-HSPA8-HNRPD-EIF4G1 complex	0.03651
Asthma	1439	PTGS2 homodimer complex	0.08165
Asthma	1514	IL4-IL4R complex	0.11547
Asthma	1515	IL4-IL4R-IL2RG complex	0.09428

Asthma	1714	TICAM1-TICAM2-TLR4 complex	0.04714
Asthma	1746	SMN containing complex	0.02887
Asthma	1810	ITGA4-PXN-GIT1 complex	0.04714
Asthma	1827	PML-SMAD2/3-SARA complex	0.04082
Asthma	1839	SDCBP-CTNNB1-CTNNA1-CDH1 complex	0.04082
Asthma	1945	IRAK1-IRAK3 complex	0.05774
Asthma	2000	BAX homo-oligomer complex	0.08165
Asthma	2016	IL12A homodimer complex	0.08165
Asthma	2018	IL12A-IL12B complex	0.05774
Asthma	2019	IL12A-IL12B-IL12RB1 complex	0.04714
Asthma	2021	IL12A-IL12B-IL12RB2 complex	0.04714
Asthma	2318	ITGA6-ITGB4-Laminin10/12 complex	0.03651
Asthma	2319	ITGA6-ITGB4-Laminin10/12 complex	0.03651
Asthma	2322	ITGA6-ITGB4-LAMA5 complex	0.04714
Asthma	2355	ITGAV-ITGB3-CD47-FCER2 complex	0.04082
Asthma	2356	ITGB3-ITGAV-CD47 complex	0.04714
Asthma	2358	ITGAV-ITGB3-SPP1 complex	0.04714
Asthma	2359	ITGAV-ITGB3-ADAM15 complex	0.04714
Asthma	2362	ITAGV-ITGB3-F11R complex	0.04714
Asthma	2363	ITGAV-ITGB3-PXN-PTK2b complex	0.04082
Asthma	2364	ITGAV-ITGB3-ADAM23 complex	0.04714
Asthma	2365	ITGAV-ITGB3-COL4A3 complex	0.04714
Asthma	2366	ITGAV-ITGB3-PPAP2b complex	0.04714
Asthma	2369	ITGAV-ITGB3-EGFR complex	0.09428
Asthma	2370	ITGA2b-ITGB3-CD9 complex	0.04714
Asthma	2374	ITGAV-ITGB3-LAMA4 complex	0.04714
Asthma	2376	ITGA2B-ITGB3-FN1-TGM2 complex	0.04082
Asthma	2377	ITGA2b-ITGB3-CD47-SRC complex	0.04082
Asthma	2378	ITGA2b-ITGB3-TLN1 complex	0.04714
Asthma	2379	ITGA2B-ITGB3-CIB1 complex	0.04714
Asthma	2381	ITGA2B-ITGB3 complex	0.05774
Asthma	2382	ITGA2B-ITGB3-F11R complex	0.04714
Asthma	2417	ITGA4-ITGB1-EMILIN1 complex	0.04714
Asthma	2418	ITGA4-ITGB1 complex	0.05774
Asthma	2419	ITGA4-ITGB1-CD81 complex	0.04714
Asthma	2420	ITGA4-ITGB1-CD53 complex	0.04714
Asthma	2421	ITGA4-ITGB1-VCAM1 complex	0.04714
Asthma	2422	ITGA4-ITGB1-JAM2 complex	0.04714
Asthma	2423	ITGA4-ITGB1-CD47 complex	0.04714
Asthma	2424	ITGA4-ITGB1-CD63 complex	0.04714
Asthma	2425	ITGA4-ITGB1-PXN complex	0.04714
Asthma	2426	ITGA4-ITGB1-THBS1 complex	0.04714
Asthma	2428	ITGA4-ITGB1-THBS2 complex	0.04714
Asthma	2443	ITGA9-ITGB1-TNC complex	0.04714
Asthma	2453	Multiprotein complex (monoubiquitination)	0.04082
Asthma	2454	CIN85-CBL-SH3GL2-EGFR complex EGF stimulated	0.04082
Asthma	2542	EGFR-CBL-GRB2 complex	0.04714

Asthma	2709	MMP-9-TIMP-1-LRP complex	0.04714
Asthma	2790	ETS2-ETS1 complex	0.05774
Asthma	2798	MMP-2-claudin-1 complex	0.05774
Asthma	2816	ITGAV-ITGB3 complex	0.05774
Asthma	2826	ITGB3-ITGAV-VTN complex	0.04714
Asthma	2829	RSmad complex	0.02582
Asthma	2830	TIF1gamma-SMAD2-SMAD3 complex	0.04714
Asthma	2834	SMAD4-SMAD2-SMAD3 complex	0.04714
Asthma	2846	ITGAV-ITGB3-THBS1 complex	0.04714
Asthma	2849	ITGAV-ITGB3-NOV complex	0.04714
Asthma	2872	ITGA2b-ITGB3-CD9-GP1b-CD47 complex	0.03333
Asthma	2882	ITGA5-ITGB3-COL6A3 complex	0.04714
Asthma	2896	ITGA2b-ITGB3-CD47-FAK complex	0.04082
Asthma	2960	SLP-76-PLC-gamma-1-ITK complex alpha-TCR stimulated	0.04714
Asthma	2963	ITK-SLP-76 complex anti-TCR stimulated	0.05774
Asthma	2989	ITGA9-ITGB1-ADAM8 complex	0.04714
Asthma	3038	SMAD2-SMAD4-FAST1 complex	0.04714
Asthma	3039	SMAD2-FAST1 complex	0.05774
Asthma	3103	ITGAV-ITGB3-SLC3A2 complex	0.04714
Asthma	3115	ITGA2B-ITGB3-ICAM4 complex	0.04714
Asthma	3198	SMAD2-SKI complex	0.05774
Asthma	3204	SMAD2-SKI-NCOR complex	0.04714
Asthma	3233	SMAD2-SMAD4-FAST1-TGIF complex TGF(beta) induced	0.04082
Asthma	3234	SMAD2-SMAD4-FAST1-TGIF-HDAC1 complex TGF(beta) induced	0.03651
Asthma	3492	Bax homooligomeric complex after apoptotic	0.08165
Asthma	3678	RIN1-STAM2-EGFR complex EGF stimulated	0.04714
Asthma	3729	SKI-SMAD2 hexameric complex	0.05774
Asthma	3739	SKI-SMAD2-SMAD4 pentameric complex	0.04714
Asthma	3749	CREBBP-SMAD2 hexameric complex	0.05774
Asthma	3753	CREBBP-SMAD2-SMAD4 pentameric complex	0.04714
Asthma	3967	SMURF2-SMAD2 complex TGF(beta)-dependent	0.05774
Asthma	4062	NRP1-VEGFR2-VEGF(165) complex	0.04714
Asthma	4096	Catenin (alpha) - catenin (beta) complex	0.05774
Asthma	5772	ZO1-(beta)cadherin-(VE)cadherin-VEGFR2 complex	0.08165
Asthma	5171	SH3KBP1-CBLB-EGFR complex	0.04714
Asthma	5274	Cell-cell junction complex (ARHGAP10-CTNNA1)	0.05774
Asthma	5375	EGR-EP300 complex	0.05774
Asthma	5388	SERPINA1-ELA2 complex	0.05774
Asthma	5423	HSP70-BAG5-PARK2 complex	0.04082
Asthma	5526	CALM1-FKBP38-BCL2 complex	0.04714
Asthma	5548	IL-12 heterodimer complex	0.05774
Asthma	5696	VEGFA(165)-KDR-NRP1 complex	0.04714
Asthma	5698	VEGFA(165)-VEGFR2-NRP1 complex	0.04714
Asthma	5749	MRIT complex	0.04714
Asthma	5811	p53-BCL2 complex	0.05774

Asthma	5812	p53-BCL2 complex	0.05774
Asthma	5817	tBID-BCL2 complex	0.05774
Asthma	5818	BIM-BCL2 complex	0.05774
Asthma	5819	BIM-BCL2xL complex	0.05774
Asthma	5820	tBID-BCL2xL complex	0.05774
Ataxia telangiectasia	71	MRN complex (MRE11-RAD50-NBS1 complex)	0.2582
Ataxia telangiectasia	72	R/M complex (RAD50-MRE11 complex)	0.31623
Ataxia telangiectasia	73	MRN complex (MRE11-RAD50-NBN complex)	0.2582
Ataxia telangiectasia	202	BRCA1-RAD50-MRE11-NBS1 complex	0.22361
Ataxia telangiectasia	285	PCNA-MLH1-PMS1 complex	0.2582
Ataxia telangiectasia	370	MSH2-MSH6-PMS1-MLH1 complex	0.22361
Ataxia telangiectasia	380	MutL-beta complex	0.31623
Ataxia telangiectasia	433	BASC complex (BRCA1-associated genome surveillance complex)	0.1291
Ataxia telangiectasia	619	MRE11A-RAD50-NBN-TRF2 complex	0.22361
Ataxia telangiectasia	627	MRN-TRRAP complex (MRE11A-RAD50-NBN-TRRAP complex)	0.22361
Ataxia telangiectasia	784	SMG-1-Upf1-eRF1-eRF3 complex (SURF)	0.2
Ataxia telangiectasia	812	Upf complex (UPF1 UPF2 UPF3a)	0.2582
Ataxia telangiectasia	813	Upf complex (UPF1 UPF2 UPF3b)	0.2582
Ataxia telangiectasia	814	Postsplicing complex	0.16903
Ataxia telangiectasia	822	mRNA decay complex (UPF1 UPF2 UPF3B DCP2 XRN1 XRN2 EXOSC2 EXOSC4 EXOSC10 PARN)	0.14142
Ataxia telangiectasia	1141	CF IIAm complex (Cleavage factor IIAm complex)	0.1118
Ataxia telangiectasia	1189	DNA double-strand break end-joining complex	0.16903
Ataxia telangiectasia	1193	Rap1 complex	0.16903
Ataxia telangiectasia	2217	MDC1-MRN-ATM-FANCD2 complex	0.18257
Ataxia telangiectasia	2218	MDC1-MRE11-RAD50-NBS1 complex	0.22361
Ataxia telangiectasia	2766	TERF2-RAP1 complex	0.16903
Ataxia telangiectasia	2767	RAD50-MRE11-NBN-p200-p350 complex	0.2582
Ataxia telangiectasia	2815	BRCA1-BARD1-BACH1-DNA damage complex II	0.15811
Ataxia telangiectasia	5197	PTIP-DNA damage response complex	0.18257
Atherosclerosis	120	Lymphotoxin beta receptor complex	0.04042
Atherosclerosis	142	CD147-gamma-secretase complex (APH-1a PS-1 PEN-2 NCT variant)	0.03131
Atherosclerosis	143	APP-FE65-LRP complex	0.04042
Atherosclerosis	298	VEGF transcriptional complex	0.02858
Atherosclerosis	441	TFTC-type histone acetyl transferase complex	0.02111
Atherosclerosis	518	AKAP250-PKA-PDE4D complex	0.03131
Atherosclerosis	541	IGF1-IGFBP3-ALS complex	0.04042
Atherosclerosis	563	F1F0-ATP synthase (EC 3.6.3.14) mitochondrial	0.0175
Atherosclerosis	668	BKCA-beta2AR-AKAP79 signaling complex	0.04042
Atherosclerosis	672	BKCA-beta2AR complex	0.04951
Atherosclerosis	687	CFTR-NHERF-beta(2)AR signaling complex	0.04042
Atherosclerosis	1054	ESR1-RELA-BCL3-NCOA3 complex	0.03501
Atherosclerosis	1094	Frataxin complex	0.02646
Atherosclerosis	1217	WRN-TRF2 complex	0.04951
Atherosclerosis	1300	CRLR-RAMP1 complex	0.04951

Atherosclerosis	1439	PTGS2 homodimer complex	0.07001
Atherosclerosis	1514	IL4-IL4R complex	0.04951
Atherosclerosis	1515	IL4-IL4R-IL2RG complex	0.04042
Atherosclerosis	1519	IL6ST-PRKCD-STAT3 complex	0.04042
Atherosclerosis	5712	FAK-beta5 integrin complex VEGF induced	0.04951
Atherosclerosis	1714	TICAM1-TICAM2-TLR4 complex	0.04042
Atherosclerosis	5718	eNOS-HSP90-AKT complex VEGF induced	0.04042
Atherosclerosis	1986	Endoglin homodimer complex	0.07001
Atherosclerosis	2001	NOD1 homodimer complex	0.07001
Atherosclerosis	2028	JAK2-IL12RB2 complex	0.04951
Atherosclerosis	2124	IKK-alpha--ER-alpha-AIB1 complex	0.04042
Atherosclerosis	2156	YBX1-AKT1 complex	0.04951
Atherosclerosis	2159	AR-AKT-APPL complex	0.08085
Atherosclerosis	2160	AOF2-AR complex	0.04951
Atherosclerosis	2342	ITGAV-ITGB8-MMP14-TGFB1 complex	0.07001
Atherosclerosis	2343	ITGAV-ITGB5-PLAUR complex	0.12127
Atherosclerosis	2345	ITGAV-ITGB5-ICAM4 complex	0.08085
Atherosclerosis	2346	ITGAV-ITGB5-ADAM9 complex	0.08085
Atherosclerosis	2347	ITGAV-ITGB5-SPP1 complex	0.12127
Atherosclerosis	2348	ITGAV-ITGB5-CYR61 complex	0.08085
Atherosclerosis	2350	ITGAV-ITGB5 complex	0.09901
Atherosclerosis	2352	ITGAV-ITGB6-SPP1 complex	0.08085
Atherosclerosis	2353	ITGAV-ITGB6-TGFB3 complex	0.04042
Atherosclerosis	2354	ITGAV-ITGB6 complex	0.04951
Atherosclerosis	2355	ITGAV-ITGB3-CD47-FCER2 complex	0.07001
Atherosclerosis	2356	ITGB3-ITGAV-CD47 complex	0.08085
Atherosclerosis	2358	ITGAV-ITGB3-SPP1 complex	0.12127
Atherosclerosis	2359	ITGAV-ITGB3-ADAM15 complex	0.08085
Atherosclerosis	2362	ITAGV-ITGB3-F11R complex	0.08085
Atherosclerosis	2363	ITGAV-ITGB3-PXN-PTK2b complex	0.07001
Atherosclerosis	2364	ITGAV-ITGB3-ADAM23 complex	0.08085
Atherosclerosis	2365	ITGAV-ITGB3-COL4A3 complex	0.08085
Atherosclerosis	2366	ITGAV-ITGB3-PPAP2b complex	0.08085
Atherosclerosis	2369	ITGAV-ITGB3-EGFR complex	0.08085
Atherosclerosis	2370	ITGA2b-ITGB3-CD9 complex	0.08085
Atherosclerosis	2374	ITGAV-ITGB3-LAMA4 complex	0.08085
Atherosclerosis	2376	ITGA2B-ITGB3-FN1-TGM2 complex	0.07001
Atherosclerosis	2377	ITGA2b-ITGB3-CD47-SRC complex	0.07001
Atherosclerosis	2378	ITGA2b-ITGB3-TLN1 complex	0.08085
Atherosclerosis	2379	ITGA2B-ITGB3-CIB1 complex	0.08085
Atherosclerosis	2381	ITGA2B-ITGB3 complex	0.09901
Atherosclerosis	2382	ITGA2B-ITGB3-F11R complex	0.08085
Atherosclerosis	2398	ITGA3-ITGB1-BSG complex	0.04042
Atherosclerosis	2429	ITGA2-ITGB1-CD47 complex	0.04042
Atherosclerosis	2430	ITGA2-ITGB1-CHAD complex	0.04042
Atherosclerosis	2431	ITGA2-ITGB1-COL6A3 complex	0.04042
Atherosclerosis	2432	ITGA2-ITGB1 complex	0.04951

Atherosclerosis	2436	ITGAV-ITGB1 complex	0.04951
Atherosclerosis	2470	p130Cas-ER-alpha-cSrc-kinase- PI3-kinase p85-subunit complex	0.03501
Atherosclerosis	2528	ERBB2-MEMO-SHC complex	0.04042
Atherosclerosis	2535	SLP-76-Cbl-Grb2-Shc complex Fc receptor gamma-R1 stimulated	0.03501
Atherosclerosis	2657	ESR1-CDK7-CCNH-MNAT1-MTA1-HDAC2 complex	0.02858
Atherosclerosis	2670	Er-alpha-p53-hdm2 complex	0.04042
Atherosclerosis	2688	MT1-MMP-claudin-1 complex	0.04951
Atherosclerosis	2699	ER-alpha-GRIP1-c-Jun complex	0.04042
Atherosclerosis	2700	ER-alpha-c-Jun complex	0.04951
Atherosclerosis	2709	MMP-9-TIMP-1-LRP complex	0.12127
Atherosclerosis	2710	LRP-1-Alpha-2-M-annexin VI complex	0.04042
Atherosclerosis	2726	PXN-ITGB5-PTK2 complex	0.04042
Atherosclerosis	2755	17S U2 snRNP	0.01219
Atherosclerosis	2816	ITGAV-ITGB3 complex	0.09901
Atherosclerosis	2826	ITGB3-ITGAV-VTN complex	0.12127
Atherosclerosis	2846	ITGAV-ITGB3-THBS1 complex	0.08085
Atherosclerosis	2849	ITGAV-ITGB3-NOV complex	0.08085
Atherosclerosis	2872	ITGA2b-ITGB3-CD9-GP1b-CD47 complex	0.05717
Atherosclerosis	2882	ITGA5-ITGB3-COL6A3 complex	0.04042
Atherosclerosis	2885	ITGAV-ITGB1-SPP1 complex	0.08085
Atherosclerosis	2895	SHC-GRB2 complex	0.04951
Atherosclerosis	2896	ITGA2b-ITGB3-CD47-FAK complex	0.07001
Atherosclerosis	2972	ITGA9-ITGB1-VEGFA complex	0.04042
Atherosclerosis	3096	ITGA6-ITGB4-SHC1-GRB2 complex	0.03501
Atherosclerosis	3103	ITGAV-ITGB3-SLC3A2 complex	0.08085
Atherosclerosis	3110	ITGAV-P2RY2-GNA12 complex	0.08085
Atherosclerosis	3111	ITGA9-ITGB1-SPP1 complex	0.04042
Atherosclerosis	3112	ITGA5-ITGB1-SPP1 complex	0.04042
Atherosclerosis	3115	ITGA2B-ITGB3-ICAM4 complex	0.08085
Atherosclerosis	3117	ITGB5-ITGAV-VTN complex	0.12127
Atherosclerosis	3139	CRLR-RAMP1-ARRB2 complex	0.04042
Atherosclerosis	3162	TF-FVIIa-FXa-TFPI complex	0.03501
Atherosclerosis	3229	Heterodimer complex (CDK9 IL6ST)	0.04951
Atherosclerosis	3830	ADRB2 homodimer complex	0.07001
Atherosclerosis	3847	TCL1(trimer)-AKT1 complex	0.04951
Atherosclerosis	4062	NRP1-VEGFR2-VEGF(165) complex	0.04042
Atherosclerosis	4082	Ku70/Ku86/Werner complex	0.04042
Atherosclerosis	5178	JAK2-PAFR-TYK2 complex	0.04042
Atherosclerosis	5235	WRN-Ku70-Ku80-PARP1 complex	0.03501
Atherosclerosis	5273	VHL-TBP1-HIF1A complex	0.04042
Atherosclerosis	5276	HIF1A-OS9-EGLN1 complex	0.04042
Atherosclerosis	5277	HIF1A-OS9-EGLN3 complex	0.04042
Atherosclerosis	5382	ARNT-HIF1A complex	0.04951
Atherosclerosis	5388	SERPINA1-ELA2 complex	0.04951
Atherosclerosis	5564	LMO4-gp130 complex	0.03131
Atherosclerosis	5579	CNTF-CNTFR-gp130-LIFR complex	0.03501

Atherosclerosis	5582	LIFR-LIF-gp130 complex	0.04042
Atherosclerosis	5696	VEGFA(165)-KDR-NRP1 complex	0.04042
Atherosclerosis	5698	VEGFA(165)-VEGFR2-NRP1 complex	0.04042
Atherosclerosis	5701	NRP1-VEGF(165/121) complex	0.04951
Atherosclerosis	5862	CAV1-VDAC1-ESR1 complex	0.04042
Atopic rhinitis	1095	SNX complex (SNX1a SNX2 SNX4 EGFR)	0.1291
Atopic rhinitis	1185	EGFR-containing signaling complex	0.1291
Atopic rhinitis	2369	ITGAV-ITGB3-EGFR complex	0.14907
Atopic rhinitis	2453	Multiprotein complex (monoubiquitination)	0.1291
Atopic rhinitis	2454	CIN85-CBL-SH3GL2-EGFR complex EGF stimulated	0.1291
Atopic rhinitis	2542	EGFR-CBL-GRB2 complex	0.14907
Atopic rhinitis	3678	RIN1-STAM2-EGFR complex EGF stimulated	0.14907
Atopic rhinitis	5171	SH3KBP1-CBLB-EGFR complex	0.14907
Attention deficit hyperactivity disorder	706	SNARE complex (HGS SNAP25 STX13)	0.16667
Attention deficit hyperactivity disorder	707	SNARE complex (VAMP2 SNAP25 STX13)	0.16667
Attention deficit hyperactivity disorder	793	SNARE complex (VAMP2 SNAP25 STX1a CPLX1)	0.14434
Attention deficit hyperactivity disorder	794	SNARE complex (VAMP2 SNAP25 STX1a CPLX2)	0.14434
Attention deficit hyperactivity disorder	1137	SNARE complex (VAMP2 SNAP25 STX1a CPLX1 CPLX3)	0.1291
Attention deficit hyperactivity disorder	1138	SNARE complex (VAMP2 SNAP25 STX1a CPLX3 CPLX4)	0.1291
Attention deficit hyperactivity disorder	1139	SNARE complex (VAMP2 SNAP25 STX1a STX3 CPLX1 CPLX3 CPLX4)	0.10911
Attention deficit hyperactivity disorder	1874	SNARE complex (SNAP25 VAMP3 VAMP2 NAPB STX13)	0.1291
Attention deficit hyperactivity disorder	5411	EDG1-HTR1D complex	0.20412
Attention deficit hyperactivity disorder	5412	HTR1D homodimer complex	0.28868
Attention deficit hyperactivity disorder	5414	HTR1A-HTR1D complex	0.20412
Attention deficit hyperactivity disorder	5417	HTR1D-HTR1B complex	0.20412
Attention deficit hyperactivity disorder	5747	2AR-mGluR2 complex	0.20412
Autistic disorder	66	TRAP complex	0.04287
Autistic disorder	75	TSC1-TSC2 complex	0.1715
Autistic disorder	230	Mediator complex	0.02144
Autistic disorder	232	ARC complex	0.03131
Autistic disorder	287	ARC-L complex	0.03241
Autistic disorder	288	ARC complex	0.03131
Autistic disorder	301	SMCC complex	0.02941
Autistic disorder	441	TFTC-type histone acetyl transferase complex	0.03656
Autistic disorder	535	TRAP complex	0.03032
Autistic disorder	547	SMCC complex	0.03241
Autistic disorder	548	DRIP complex	0.03241

Autistic disorder	668	BKCA-beta2AR-AKAP79 signaling complex	0.07001
Autistic disorder	672	BKCA-beta2AR complex	0.08575
Autistic disorder	687	CFTR-NHERF-beta(2)AR signaling complex	0.07001
Autistic disorder	749	MeCP2-SIN3A-HDAC complex	0.06063
Autistic disorder	909	ARC92-Mediator complex	0.03363
Autistic disorder	1062	BAR-BCL2-CASP8 complex	0.07001
Autistic disorder	1088	PRNP-ApolopoproteinE3 complex	0.08575
Autistic disorder	2233	Replication-coupled CAF-1-MBD1-ETDB1 complex	0.07001
Autistic disorder	2238	MBD1-MCAF1-SETDB1 complex	0.07001
Autistic disorder	2258	VILIP-1-AChR-alpha-4-AChR-beta-2 complex	0.14003
Autistic disorder	2355	ITGAV-ITGB3-CD47-FCER2 complex	0.06063
Autistic disorder	2356	ITGB3-ITGAV-CD47 complex	0.07001
Autistic disorder	2358	ITGAV-ITGB3-SPP1 complex	0.07001
Autistic disorder	2359	ITGAV-ITGB3-ADAM15 complex	0.07001
Autistic disorder	2362	ITAGV-ITGB3-F11R complex	0.07001
Autistic disorder	2363	ITGAV-ITGB3-PXN-PTK2b complex	0.06063
Autistic disorder	2364	ITGAV-ITGB3-ADAM23 complex	0.07001
Autistic disorder	2365	ITGAV-ITGB3-COL4A3 complex	0.07001
Autistic disorder	2366	ITGAV-ITGB3-PPAP2b complex	0.07001
Autistic disorder	2369	ITGAV-ITGB3-EGFR complex	0.07001
Autistic disorder	2370	ITGA2b-ITGB3-CD9 complex	0.07001
Autistic disorder	2374	ITGAV-ITGB3-LAMA4 complex	0.07001
Autistic disorder	2376	ITGA2B-ITGB3-FN1-TGM2 complex	0.06063
Autistic disorder	2377	ITGA2b-ITGB3-CD47-SRC complex	0.06063
Autistic disorder	2378	ITGA2b-ITGB3-TLN1 complex	0.07001
Autistic disorder	2379	ITGA2B-ITGB3-CIB1 complex	0.07001
Autistic disorder	2381	ITGA2B-ITGB3 complex	0.08575
Autistic disorder	2382	ITGA2B-ITGB3-F11R complex	0.07001
Autistic disorder	2456	MET-CIN85-SH3GL3-CBL complex HGF stimulated	0.06063
Autistic disorder	2541	HGF-Met complex	0.08575
Autistic disorder	2600	BRD4 complex	0.04287
Autistic disorder	2759	MBD1-MCAF complex	0.08575
Autistic disorder	2763	MBD1-Suv39h1-HP1 complex	0.07001
Autistic disorder	2816	ITGAV-ITGB3 complex	0.08575
Autistic disorder	2826	ITGB3-ITGAV-VTN complex	0.07001
Autistic disorder	2846	ITGAV-ITGB3-THBS1 complex	0.07001
Autistic disorder	2849	ITGAV-ITGB3-NOV complex	0.07001
Autistic disorder	2872	ITGA2b-ITGB3-CD9-GP1b-CD47 complex	0.04951
Autistic disorder	2882	ITGA5-ITGB3-COL6A3 complex	0.07001
Autistic disorder	2896	ITGA2b-ITGB3-CD47-FAK complex	0.06063
Autistic disorder	3103	ITGAV-ITGB3-SLC3A2 complex	0.07001
Autistic disorder	3115	ITGA2B-ITGB3-ICAM4 complex	0.07001
Autistic disorder	3830	ADRB2 homodimer complex	0.12127
Autistic disorder	5158	SMARCA2/BRM-BAF57-MECP2 complex	0.07001
Autistic disorder	5184	SWI/SNF chromatin-remodeling complex	0.05423
Autistic disorder	5209	Ubiquilin-proteasome complex	0.04951
Autistic disorder	5526	CALM1-FKBP38-BCL2 complex	0.07001

Autistic disorder	5673	PlexinA1-Nrp2 complex	0.08575
Autistic disorder	5732	NRP2-VEGFC complex	0.08575
Autistic disorder	5733	NRP2-VEGFD complex heparin dependent	0.08575
Autistic disorder	5740	NRP2-VEGFR3 complex	0.08575
Autistic disorder	5811	p53-BCL2 complex	0.08575
Autistic disorder	5817	tBID-BCL2 complex	0.08575
Autistic disorder	5818	BIM-BCL2 complex	0.08575
Autoimmune disease	286	PCNA-MSH2-MSH6 complex	0.06262
Autoimmune disease	369	MSH2-MSH6-PMS2-MLH1 complex	0.05423
Autoimmune disease	370	MSH2-MSH6-PMS1-MLH1 complex	0.05423
Autoimmune disease	374	MSH2-MSH6 complex	0.0767
Autoimmune disease	376	PCNA-MutS-alpha-MutL-alpha-DNA complex	0.04851
Autoimmune disease	377	PCNA-MutS-alpha-DNA initial complex	0.06262
Autoimmune disease	433	BASC complex (BRCA1-associated genome surveillance complex)	0.03131
Autoimmune disease	434	BASC (Ab 80) complex (BRCA1-associated genome surveillance complex)	0.03835
Autoimmune disease	436	BASC (Ab C-20) complex (BRCA1-associated genome surveillance complex)	0.06262
Autoimmune disease	438	GCN5-TRRAP histone acetyltransferase complex	0.0343
Autoimmune disease	552	IFNB1-IFNAR1-IFNAR2- complex	0.06262
Autoimmune disease	788	Exosome	0.0343
Autoimmune disease	789	Exosome	0.06541
Autoimmune disease	822	mRNA decay complex (UPF1 UPF2 UPF3B DCP2 XRN1 XRN2 EXOSC2 EXOSC4 EXOSC10 PARN)	0.0343
Autoimmune disease	1062	BAR-BCL2-CASP8 complex	0.12524
Autoimmune disease	1306	PIN1-AUF1 complex	0.0767
Autoimmune disease	1307	Multiprotein complex (mRNA turnover)	0.04851
Autoimmune disease	1308	PABPC1-HSPA8-HNRPD-EIF4G1 complex	0.04851
Autoimmune disease	1514	IL4-IL4R complex	0.15339
Autoimmune disease	1515	IL4-IL4R-IL2RG complex	0.12524
Autoimmune disease	1707	IL2-IL2RA-IL2RB complex	0.06262
Autoimmune disease	1714	TICAM1-TICAM2-TLR4 complex	0.06262
Autoimmune disease	1774	MICA-KLRK1-HCST complex	0.06262
Autoimmune disease	1985	AIRE homodimer complex	0.10847
Autoimmune disease	2000	BAX homo-oligomer complex	0.10847
Autoimmune disease	2054	CASP8-FADD-MALT1-BCL10 complex	0.05423
Autoimmune disease	2055	CASP8-CHUK-IKBKB-MALT1-BCL10 complex	0.04851
Autoimmune disease	2056	BCL10-CHUK-BCL10-IKBKB complex	0.05423
Autoimmune disease	2224	MSH2/6-BLM-p53-RAD51 complex	0.04851
Autoimmune disease	2226	MutS-alpha-PK-zeta complex	0.06262
Autoimmune disease	2370	ITGA2b-ITGB3-CD9 complex	0.06262
Autoimmune disease	2376	ITGA2B-ITGB3-FN1-TGM2 complex	0.05423
Autoimmune disease	2377	ITGA2b-ITGB3-CD47-SRC complex	0.05423
Autoimmune disease	2378	ITGA2b-ITGB3-TLN1 complex	0.06262
Autoimmune disease	2379	ITGA2B-ITGB3-CIB1 complex	0.06262
Autoimmune disease	2381	ITGA2B-ITGB3 complex	0.0767
Autoimmune disease	2382	ITGA2B-ITGB3-F11R complex	0.06262

Autoimmune disease	2453	Multiprotein complex (monoubiquitination)	0.05423
Autoimmune disease	2709	MMP-9-TIMP-1-LRP complex	0.06262
Autoimmune disease	2740	MutS-alpha complex	0.0767
Autoimmune disease	2817	BRCA1-BARD1-BACH1-DNA damage complex I	0.04428
Autoimmune disease	2872	ITGA2b-ITGB3-CD9-GP1b-CD47 complex	0.04428
Autoimmune disease	2896	ITGA2b-ITGB3-CD47-FAK complex	0.05423
Autoimmune disease	3115	ITGA2B-ITGB3-ICAM4 complex	0.06262
Autoimmune disease	3492	Bax homooligomeric complex after apoptotic	0.10847
Autoimmune disease	5171	SH3KBP1-CBLB-EGFR complex	0.06262
Autoimmune disease	5178	JAK2-PAFR-TYK2 complex	0.06262
Autoimmune disease	5473	FAS-FADD-CASP8 complex	0.12524
Autoimmune disease	5526	CALM1-FKBP38-BCL2 complex	0.06262
Autoimmune disease	5564	LMO4-gp130 complex	0.04851
Autoimmune disease	5691	TALL1 homo-oligomer complex	0.10847
Autoimmune disease	5749	MRIT complex	0.12524
Autoimmune disease	5798	Death induced signaling complex II (FADD CASP8 CFLAR) cytosolic CD95L induced	0.06262
Autoimmune disease	5799	Death induced signaling complex DISC (FAS FADD CASP8 CFLAR) membrane-associated CD95L	0.10847
Autoimmune disease	5800	Death-inducing signaling complex DISC (type I cells associated) stimulated	0.12524
Autoimmune disease	5808	DISC complex	0.12524
Autoimmune disease	5811	p53-BCL2 complex	0.0767
Autoimmune disease	5812	p53-BCL2 complex	0.0767
Autoimmune disease	5817	tBID-BCL2 complex	0.0767
Autoimmune disease	5818	BIM-BCL2 complex	0.0767
Autoimmune disease	5819	BIM-BCL2xL complex	0.0767
Autoimmune disease	5820	tBID-BCL2xL complex	0.0767
Autoimmune disease	5823	MCL1-BAK1 complex	0.0767
Autoimmune disease	5859	FAS-FADD-CASP8-CASP10 complex	0.10847
Autoimmune disease	5861	FAS-FADD-CASP10 complex	0.06262
Azoospermia	103	RNA polymerase II holoenzyme complex	0.04167
Azoospermia	107	TFIIH transcription factor complex	0.06804
Azoospermia	159	Condensin I-PARP-1-XRCC1 complex	0.07715
Azoospermia	212	DNA ligase III-XRCC1 complex	0.14434
Azoospermia	213	DNA ligase IV-XRCC1 complex	0.14434
Azoospermia	362	DNA ligase III-XRCC1-PNK-DNA-pol III multiprotein complex	0.10206
Azoospermia	368	ERCC1-ERCC4-MSH2 complex	0.11785
Azoospermia	371	Structure-specific endonulease complex	0.20412
Azoospermia	531	XPA-ERCC1-ERCC4 complex	0.11785
Azoospermia	681	(C-CFTR)2-NHERF-ezrin complex	0.11785
Azoospermia	682	C-CFTR-NHERF(PDZ1 domain)-ezrin complex	0.11785
Azoospermia	683	C-CFTR-NHERF(PDZ2 domain)-ezrin complex	0.11785
Azoospermia	687	CFTR-NHERF-beta(2)AR signaling complex	0.11785
Azoospermia	871	BRAF53-BRCA2 complex	0.07715
Azoospermia	1009	TFIIH transcription factor complex	0.06455
Azoospermia	1029	TFIIH transcription factor complex	0.06455

Azoospermia	1030	CAK-ERCC2 complex	0.10206
Azoospermia	1085	DNA repair complex NEIL2-PNK-Pol(beta)-LigIII(alpha)-XRCC1	0.09129
Azoospermia	1086	DNA repair complex NEIL1-PNK-Pol(beta)-LigIII(alpha)-XRCC1	0.09129
Azoospermia	1154	DSS1 complex	0.05661
Azoospermia	2159	AR-AKT-APPL complex	0.11785
Azoospermia	2160	AOF2-AR complex	0.14434
Azoospermia	2220	RAD52-ERCC4-ERCC1 complex	0.11785
Azoospermia	2256	RIAM-Rap1-GTP complex	0.14434
Azoospermia	2416	ITGB1-RAP1A-PKD1 complex	0.11785
Azoospermia	2660	ERCC2/CAK complex	0.10206
Azoospermia	2709	MMP-9-TIMP-1-LRP complex	0.11785
Azoospermia	2818	BRCA1-BARD1-BRCA2-DNA damage complex III	0.11785
Azoospermia	2825	BRCA1-RNA polymerase II complex	0.04003
Azoospermia	3110	ITGAV-P2RY2-GNA12 complex	0.11785
Azoospermia	3158	RIAM-Rap1-GTP-profilin complex	0.11785
Azoospermia	5243	XRCC1-LIG3-PNK-TDP1 complex	0.10206
Azoospermia	5400	BRCC complex	0.09129
Azoospermia	5495	TFIIH transcription factor complex (ERCC2 ERCC3 GTF2H1 CDK7 CCNH GTF2H2)	0.08333
Bacterial infection	2019	IL12A-IL12B-IL12RB1 complex	0.1543
Bacterial infection	2020	IL12B-IL12RB1-IL12RB2 complex	0.1543
Bacterial infection	2026	IL12RB1-IL12RB2 complex	0.18898
Bacterial infection	2417	ITGA4-ITGB1-EMILIN1 complex	0.1543
Bacterial infection	2811	BRCA1-cABL complex	0.18898
Bacterial infection	3162	TF-FVIIa-FXa-TFPI complex	0.13363
Bacterial infection	5830	DJ-1-SNCA complex high molecular weight complex	0.18898
Barrett's esophagus	103	RNA polymerase II holoenzyme complex	0.03727
Barrett's esophagus	107	TFIIH transcription factor complex	0.06086
Barrett's esophagus	159	Condensin I-PARP-1-XRCC1 complex	0.06901
Barrett's esophagus	190	Mitotic checkpoint complex (MCC)	0.09129
Barrett's esophagus	212	DNA ligase III-XRCC1 complex	0.1291
Barrett's esophagus	213	DNA ligase IV-XRCC1 complex	0.1291
Barrett's esophagus	220	ARF-Mule complex	0.10541
Barrett's esophagus	298	VEGF transcriptional complex	0.07454
Barrett's esophagus	351	Spliceosome	0.01527
Barrett's esophagus	362	DNA ligase III-XRCC1-PNK-DNA-pol III multiprotein complex	0.09129
Barrett's esophagus	933	SCRIB-APC complex	0.1291
Barrett's esophagus	1003	RC complex (Replication competent complex)	0.06086
Barrett's esophagus	1004	RC complex during S-phase of cell cycle	0.05064
Barrett's esophagus	1005	RC complex during G2/M-phase of cell cycle	0.05064
Barrett's esophagus	1009	TFIIH transcription factor complex	0.05774
Barrett's esophagus	1029	TFIIH transcription factor complex	0.05774
Barrett's esophagus	1030	CAK-ERCC2 complex	0.09129
Barrett's esophagus	1085	DNA repair complex NEIL2-PNK-Pol(beta)-LigIII(alpha)-XRCC1	0.08165

Barrett's esophagus	1086	DNA repair complex NEIL1-PNK-Pol(beta)-LigIII(alpha)-XRCC1	0.08165
Barrett's esophagus	1095	SNX complex (SNX1a SNX2 SNX4 EGFR)	0.09129
Barrett's esophagus	1185	EGFR-containing signaling complex	0.09129
Barrett's esophagus	1642	p16-cyclin D2-CDK4 complex	0.10541
Barrett's esophagus	1661	E2F4-p107-cyclinA complex	0.10541
Barrett's esophagus	1814	MAD1L1-MAD2L1 complex	0.1291
Barrett's esophagus	1844	APC-IQGAP1 complex	0.1291
Barrett's esophagus	1845	APC-IQGAP1-CLIP-170 complex	0.10541
Barrett's esophagus	1851	BUB1-BUB3 complex	0.1291
Barrett's esophagus	1909	APC-DLG4 complex	0.1291
Barrett's esophagus	2369	ITGAV-ITGB3-EGFR complex	0.10541
Barrett's esophagus	2453	Multiprotein complex (monoubiquitination)	0.09129
Barrett's esophagus	2454	CIN85-CBL-SH3GL2-EGFR complex EGF stimulated	0.09129
Barrett's esophagus	2542	EGFR-CBL-GRB2 complex	0.10541
Barrett's esophagus	2660	ERCC2/CAK complex	0.09129
Barrett's esophagus	2718	MAD2-CDC20 complex	0.1291
Barrett's esophagus	2825	BRCA1-RNA polymerase II complex	0.03581
Barrett's esophagus	3004	APC-Axin-1-beta-catenin complex	0.10541
Barrett's esophagus	3008	60S APC containing complex	0.06901
Barrett's esophagus	3011	APC-IQGAP1-Rac1 complex	0.10541
Barrett's esophagus	3012	APC-IQGAP1-Cdc42 complex	0.10541
Barrett's esophagus	3044	SKI-NCOR1-SIN3A-HDAC1 complex	0.09129
Barrett's esophagus	3166	AXIN-APC-betaCatenin-GSK3B complex	0.09129
Barrett's esophagus	3197	SMAD4-SNO-SKI complex	0.21082
Barrett's esophagus	3198	SMAD2-SKI complex	0.1291
Barrett's esophagus	3199	SMAD3-SKI complex	0.1291
Barrett's esophagus	3200	SMAD4-SKI complex	0.1291
Barrett's esophagus	3204	SMAD2-SKI-NCOR complex	0.10541
Barrett's esophagus	3205	SMAD3-SKI-NCOR complex	0.10541
Barrett's esophagus	3206	SMAD4-SKI-NCOR complex	0.10541
Barrett's esophagus	3678	RIN1-STAM2-EGFR complex EGF stimulated	0.10541
Barrett's esophagus	3729	SKI-SMAD2 hexameric complex	0.1291
Barrett's esophagus	3733	SKI-SMAD3 hexameric complex	0.1291
Barrett's esophagus	3739	SKI-SMAD2-SMAD4 pentameric complex	0.10541
Barrett's esophagus	3740	SKI-SMAD3-SMAD4 pentameric complex	0.10541
Barrett's esophagus	3959	SMAD3-SMAD4-cSKI TGF(beta)-dependent	0.10541
Barrett's esophagus	3961	SMAD3-cSKI-SIN3A-HDAC1 complex	0.09129
Barrett's esophagus	3972	SMURF2-SMAD3-SnoN complex TGF(beta)-	0.10541
Barrett's esophagus	5144	E2F1-p107-cyclinA complex	0.10541
Barrett's esophagus	5171	SH3KBP1-CBLB-EGFR complex	0.10541
Barrett's esophagus	5243	XRCC1-LIG3-PNK-TDP1 complex	0.09129
Barrett's esophagus	5273	VHL-TBP1-HIF1A complex	0.10541
Barrett's esophagus	5276	HIF1A-OS9-EGLN1 complex	0.10541
Barrett's esophagus	5277	HIF1A-OS9-EGLN3 complex	0.10541
Barrett's esophagus	5382	ARNT-HIF1A complex	0.1291
Barrett's esophagus	5495	TFIIH transcription factor complex (ERCC2 ERCC3 GTF2H1 CDK7 CCNH GTF2H2)	0.07454

Barrett's esophagus	5556	CDK2-CCNA2 complex	0.1291
Barrett's esophagus	5557	CDC2-CCNA2 complex	0.1291
Barrett's esophagus	5559	CDC2-CCNA2-CDK2 complex	0.10541
Basal cell carcinoma	178	Respiratory chain complex I (holoenzyme)	0.04181
Basal cell carcinoma	375	MSH2-MSH3 complex	0.19612
Basal cell carcinoma	378	MutS-beta complex	0.19612
Basal cell carcinoma	531	XPA-ERCC1-ERCC4 complex	0.16013
Basal cell carcinoma	2919	Respiratory chain complex I (gamma subunit) mitochondrial	0.07692
Basal cell carcinoma	2939	Ecsit complex (ECSIT MT-CO2 NDUFA1 MT-ND1 TRAF6 NDUFAF1)	0.11323
Behavior disease	749	MeCP2-SIN3A-HDAC complex	0.07715
Behavior disease	793	SNARE complex (VAMP2 SNAP25 STX1a CPLX1)	0.07715
Behavior disease	794	SNARE complex (VAMP2 SNAP25 STX1a CPLX2)	0.07715
Behavior disease	1137	SNARE complex (VAMP2 SNAP25 STX1a CPLX1 CPLX3)	0.06901
Behavior disease	1139	SNARE complex (VAMP2 SNAP25 STX1a STX3 CPLX1 CPLX3 CPLX4)	0.05832
Behavior disease	1140	Complexin complex (STX3 CPLX1 CPLX3)	0.08909
Behavior disease	2258	VILIP-1-AChR-alpha-4-AChR-beta-2 complex	0.17817
Behavior disease	5158	SMARCA2/BRM-BAF57-MECP2 complex	0.08909
Behavior disease	5184	SWI/SNF chromatin-remodeling complex	0.06901
Behavior disease	5414	HTR1A-HTR1D complex	0.10911
Behavior disease	5415	HTR1B homodimer complex	0.1543
Behavior disease	5416	HTR1A-HTR1B complex	0.21822
Behavior disease	5417	HTR1D-HTR1B complex	0.10911
Behavior disease	5418	GABBR2-HTR1A complex	0.10911
Behavior disease	5419	HTR1A-GPR26 complex	0.10911
Behavior disease	5420	HTR1A-EDG3 complex	0.10911
Behavior disease	5421	HTR1A homodimer complex	0.1543
Behavior disease	5422	HTR1A-EDG1 complex	0.10911
Behavior disease	5830	DJ-1-SNCA complex high molecular weight complex	0.10911
Behcet syndrome	1514	IL4-IL4R complex	0.10102
Behcet syndrome	1515	IL4-IL4R-IL2RG complex	0.08248
Behcet syndrome	1707	IL2-IL2RA-IL2RB complex	0.08248
Behcet syndrome	1714	TICAM1-TICAM2-TLR4 complex	0.08248
Behcet syndrome	1774	MICA-KLRK1-HCST complex	0.08248
Behcet syndrome	2018	IL12A-IL12B complex	0.10102
Behcet syndrome	2019	IL12A-IL12B-IL12RB1 complex	0.08248
Behcet syndrome	2020	IL12B-IL12RB1-IL12RB2 complex	0.08248
Behcet syndrome	2021	IL12A-IL12B-IL12RB2 complex	0.08248
Behcet syndrome	2429	ITGA2-ITGB1-CD47 complex	0.08248
Behcet syndrome	2430	ITGA2-ITGB1-CHAD complex	0.08248
Behcet syndrome	2431	ITGA2-ITGB1-COL6A3 complex	0.08248
Behcet syndrome	2432	ITGA2-ITGB1 complex	0.10102
Behcet syndrome	2709	MMP-9-TIMP-1-LRP complex	0.08248
Behcet syndrome	2798	MMP-2-claudin-1 complex	0.10102
Behcet syndrome	5548	IL-12 heterodimer complex	0.10102

Behcet syndrome	5549	IL-12 subunit p40 homodimer complex	0.14286
Biliary Atresia	2254	CTGF/Hcs24-actin complex	0.20412
Biliary Atresia	2347	ITGAV-ITGB5-SPP1 complex	0.20412
Biliary Atresia	2352	ITGAV-ITGB6-SPP1 complex	0.20412
Biliary Atresia	2358	ITGAV-ITGB3-SPP1 complex	0.20412
Biliary Atresia	2709	MMP-9-TIMP-1-LRP complex	0.20412
Biliary Atresia	2885	ITGAV-ITGB1-SPP1 complex	0.20412
Biliary Atresia	3111	ITGA9-ITGB1-SPP1 complex	0.20412
Biliary Atresia	3112	ITGA5-ITGB1-SPP1 complex	0.20412
Biliary cancer	1088	PRNP-ApolopoproteinE3 complex	0.31623
Bipolar disorder	178	Respiratory chain complex I (holoenzyme)	0.01707
Bipolar disorder	441	TFTC-type histone acetyl transferase complex	0.03414
Bipolar disorder	874	SNARE complex (VAMP3 VAMP4 VAMP8 STX6)	0.05661
Bipolar disorder	875	SNARE complex (VAMP3 VAMP4 STX16)	0.06537
Bipolar disorder	876	SNARE complex (VAMP3 STX6 VTI1A)	0.06537
Bipolar disorder	929	CEN complex	0.01861
Bipolar disorder	1054	ESR1-RELA-BCL3-NCOA3 complex	0.05661
Bipolar disorder	1169	SNARE complex (STX4 VAMP8 VAMP3 SNAP23)	0.05661
Bipolar disorder	1223	H2AX complex isolated from cells without IR exposure	0.0314
Bipolar disorder	1226	H2AX complex I	0.0428
Bipolar disorder	1227	H2AX complex II	0.03581
Bipolar disorder	1335	SNW1 complex	0.02669
Bipolar disorder	1787	Nogo-potassium channel complex	0.05661
Bipolar disorder	1874	SNARE complex (SNAP25 VAMP3 VAMP2 NAPB STX13)	0.05064
Bipolar disorder	2124	IKK-alpha--ER-alpha-AIB1 complex	0.06537
Bipolar disorder	2470	p130Cas-ER-alpha-cSrc-kinase- PI3-kinase p85-subunit complex	0.05661
Bipolar disorder	2657	ESR1-CDK7-CCNH-MNAT1-MTA1-HDAC2 complex	0.04623
Bipolar disorder	2670	Er-alpha-p53-hdm2 complex	0.06537
Bipolar disorder	2699	ER-alpha-GRIP1-c-Jun complex	0.06537
Bipolar disorder	2700	ER-alpha-c-Jun complex	0.08006
Bipolar disorder	2721	HCF-1 complex	0.02598
Bipolar disorder	2904	Respiratory chain complex I (intermediate VII/650kD) mitochondrial	0.03581
Bipolar disorder	2906	Respiratory chain complex I (intermediate II/230kD) mitochondrial	0.06537
Bipolar disorder	2920	Respiratory chain complex I (lambda subunit) mitochondrial	0.02831
Bipolar disorder	2948	Respiratory chain complex I (incomplete intermediate) mitochondrial	0.03414
Bipolar disorder	3082	DGCR8 multiprotein complex	0.03414
Bipolar disorder	3714	Pericentrin-GCP complex	0.06537
Bipolar disorder	4039	PAR4-BACE1 complex	0.08006
Bipolar disorder	5415	HTR1B homodimer complex	0.11323
Bipolar disorder	5416	HTR1A-HTR1B complex	0.08006
Bipolar disorder	5417	HTR1D-HTR1B complex	0.08006
Bipolar disorder	5655	Ternary complex (LRRC7 CAMK2a ACTN4)	0.06537

Bipolar disorder	5862	CAV1-VDAC1-ESR1 complex	0.06537
Bladder cancer	98	p300-MDM2-p53 protein complex	0.07454
Bladder cancer	120	Lymphotoxin beta receptor complex	0.07454
Bladder cancer	375	MSH2-MSH3 complex	0.09129
Bladder cancer	378	MutS-beta complex	0.09129
Bladder cancer	550	NOS3-CAV1-NOSTRIN complex	0.07454
Bladder cancer	557	TRP1-G alpha-11-IP3R3-CAV1 signaling complex	0.06455
Bladder cancer	753	UTM-SGCE-DAG1-CAV1-NOS3 complex	0.05774
Bladder cancer	1091	SNX complex (SNX1a SNX2 SNX4 LEPR)	0.06455
Bladder cancer	1223	H2AX complex isolated from cells without IR exposure	0.03581
Bladder cancer	1226	H2AX complex I	0.0488
Bladder cancer	1227	H2AX complex II	0.04082
Bladder cancer	1992	LEPR homodimer complex	0.1291
Bladder cancer	5714	eNOS-CAV1 complex	0.09129
Bladder cancer	2462	Caveolin-1 homodimer complex	0.1291
Bladder cancer	2670	Er-alpha-p53-hdm2 complex	0.07454
Bladder cancer	2695	ETS2-FOS-JUN complex	0.07454
Bladder cancer	2789	ETS2-ERG complex	0.09129
Bladder cancer	2790	ETS2-ETS1 complex	0.09129
Bladder cancer	2803	PAX6-SOX2 DNA-protein complex	0.09129
Bladder cancer	3172	NUMB-TP53-MDM2 complex	0.07454
Bladder cancer	5217	Calreticulin oligomer complex	0.1291
Bladder cancer	5293	ETS2-SMARCA4-INI1 complex	0.07454
Bladder cancer	5331	YY1-MDM2-p53 complex	0.07454
Bladder cancer	5862	CAV1-VDAC1-ESR1 complex	0.07454
Bone disease	75	TSC1-TSC2 complex	0.12127
Bone disease	521	Polycystin-1-E-cadherin-beta-catenin complex	0.09901
Bone disease	522	Polycystin-1-E-cadherin-beta-catenin-Flotillin-2	0.08575
Bone disease	541	IGF1-IGFBP3-ALS complex	0.09901
Bone disease	681	(C-CFTR)2-NHERF-ezrin complex	0.09901
Bone disease	682	C-CFTR-NHERF(PDZ1 domain)-ezrin complex	0.09901
Bone disease	683	C-CFTR-NHERF(PDZ2 domain)-ezrin complex	0.09901
Bone disease	1618	G protein complex (PTHR1 GNB1 GNG2)	0.09901
Bone disease	1982	CACY homodimer complex	0.1715
Bone disease	2416	ITGB1-RAP1A-PKD1 complex	0.09901
Bone disease	2798	MMP-2-claudin-1 complex	0.12127
Bone disease	3154	Notch2(N-TM)-Notch2(N-EC)-Delta complex	0.08575
Bone disease	5177	Polycystin-1 multiprotein complex (ACTN1 CDH1 SRC JUP VCL CTNNB1 PXN BCAR1 PKD1	0.05171
Bone marrow disease	2811	BRCA1-cABL complex	0.18257
Bone marrow disease	5770	RUNX1-CBF-beta-DNA complex	0.18257
Bone metastases	1514	IL4-IL4R complex	0.25
Bone metastases	1515	IL4-IL4R-IL2RG complex	0.20412
Bone metastases	2383	ITGA5-ITGB1-FN1-TGM2 complex	0.17678
Bone metastases	2384	ITGA5-ITGB1-ADAM15 complex	0.20412
Bone metastases	2385	ITGA5-ITGB4 complex	0.25
Bone metastases	2388	Itga5-Itgb1-Fn1-Sfrp2 complex	0.17678

Bone metastases	2850	ITGA5-ITGB1-FN-1-NOV complex	0.17678
Bone metastases	2853	ITGA5-ITGB1-CAL4A3 complex	0.20412
Bone metastases	2882	ITGA5-ITGB3-COL6A3 complex	0.20412
Bone metastases	3112	ITGA5-ITGB1-SPP1 complex	0.20412
Bone metastases	3979	mTORC2 complex (mTOR/FRAP1 LST8 mAVO3/RICTOR SIN1)	0.17678
Brain disease	86	NUMAC complex (nucleosomal methylation activator complex)	0.0568
Brain disease	143	APP-FE65-LRP complex	0.1037
Brain disease	149	PBAF complex (Polybromo- and BAF containing complex)	0.05185
Brain disease	189	BAF complex	0.04981
Brain disease	238	SWI-SNF chromatin remodeling-related-BRCA1	0.05415
Brain disease	554	PBAF complex (Polybromo- and BAF containing complex)	0.0568
Brain disease	555	BAF complex	0.05987
Brain disease	564	BAF complex	0.05415
Brain disease	565	PBAF complex (Polybromo- and BAF containing complex)	0.05415
Brain disease	566	BAF complex	0.05415
Brain disease	570	p300-CBP-p270-SWI/SNF complex	0.06788
Brain disease	710	Brg1-associated complex I	0.05415
Brain disease	711	Brm-associated complex	0.05415
Brain disease	713	BRG1-SIN3A complex	0.048
Brain disease	714	BRM-SIN3A complex	0.04637
Brain disease	739	SIN3-ING1b complex II	0.0449
Brain disease	778	LARC complex (LCR-associated remodeling complex)	0.0412
Brain disease	803	BRG1-SIN3A-HDAC containing SWI/SNF remodeling complex I	0.05415
Brain disease	806	BRM-SIN3A-HDAC complex	0.05185
Brain disease	807	BRG1-associated complex	0.05987
Brain disease	808	BRM-associated complex	0.0568
Brain disease	1186	ESCRT-III complex	0.0568
Brain disease	1230	WINAC complex	0.048
Brain disease	1239	EBAFb complex	0.0568
Brain disease	1252	EBAFa complex	0.0568
Brain disease	1257	ALL-1 supercomplex	0.03394
Brain disease	1306	PIN1-AUF1 complex	0.127
Brain disease	1413	NCOR1 complex	0.0568
Brain disease	2143	MAP2K5-PRKCI-SQSTM1 complex	0.1037
Brain disease	2711	Amyloid beta protein oligomer	0.17961
Brain disease	2852	Brg1-based SWI/SNF chromatin remodeling complex	0.0898
Brain disease	3060	RNA polymerase II complex (RPB1 RAP74 CDK8 CYCC SRB7 BAF190 BAF47) chromatin structure modifying	0.0635
Brain disease	3061	RNA polymerase II complex (CBP PCAF RPB1 BAF47 CYCC CDK8) chromatin structure modifying	0.07332
Brain disease	3062	RNA polymerase II complex incomplete (CBP RPBI PCAF BAF47) chromatin structure modifying	0.0898

Brain disease	3063	Brg1-associated complex II	0.06788
Brain disease	3064	RNA polymerase II complex chromatin structure modifying	0.0412
Brain disease	3065	RNA polymerase II complex chromatin structure modifying	0.05415
Brain disease	3066	RNA polymerase II complex chromatin structure modifying	0.04981
Brain disease	3067	RNA polymerase II complex incomplete (CDK8 complex) chromatin structure modifying	0.0635
Brain disease	3092	APP-TOMM40 complex	0.127
Brain disease	3093	APP-TIMM23 complex	0.127
Brain disease	5211	RAF1-PPP2-PIN1 complex	0.08032
Brain disease	5293	ETS2-SMARCA4-INI1 complex	0.1037
Brain disease	5614	Emerin complex 32	0.03829
Brain disease	5830	DJ-1-SNCA complex high molecular weight complex	0.127
Brain ischemia	1062	BAR-BCL2-CASP8 complex	0.2582
Brain ischemia	5446	EPO-EPOR complex	0.31623
Brain ischemia	5526	CALM1-FKBP38-BCL2 complex	0.2582
Brain ischemia	5811	p53-BCL2 complex	0.31623
Brain ischemia	5817	tBID-BCL2 complex	0.31623
Brain ischemia	5818	BIM-BCL2 complex	0.31623
Brain tumor	75	TSC1-TSC2 complex	0.0568
Brain tumor	143	APP-FE65-LRP complex	0.04637
Brain tumor	206	DNA ligase IV-XRCC4 complex	0.11359
Brain tumor	213	DNA ligase IV-XRCC1 complex	0.0568
Brain tumor	280	HMGB1-HMGB2-HSC70-ERP60-GAPDH complex	0.07184
Brain tumor	305	40S ribosomal subunit cytoplasmic	0.01378
Brain tumor	306	Ribosome cytoplasmic	0.01785
Brain tumor	308	60S ribosomal subunit cytoplasmic	0.01172
Brain tumor	336	DNA ligase IV-XRCC4-AHNK complex	0.09275
Brain tumor	344	DNA ligase IV-XRCC4 complex (LX complex)	0.11359
Brain tumor	350	DNA ligase IV-XRCC4-PNK complex	0.09275
Brain tumor	353	DNA ligase IV-condensin complex	0.04637
Brain tumor	359	DNA ligase IV-XRCC4-XLF complex	0.09275
Brain tumor	552	IFNB1-IFNAR1-IFNAR2- complex	0.04637
Brain tumor	577	FHL2-p53-HIPK2 complex	0.04637
Brain tumor	620	CoREST-HDAC complex	0.03036
Brain tumor	626	LSD1 complex	0.02228
Brain tumor	632	Anti-HDAC2 complex	0.01893
Brain tumor	633	anti-BHC110 complex	0.02422
Brain tumor	636	BHC complex	0.03279
Brain tumor	725	P2X7 receptor signalling complex	0.04637
Brain tumor	871	BRAF53-BRCA2 complex	0.03036
Brain tumor	906	ADAR1-CDK2 complex	0.0568
Brain tumor	929	CEN complex	0.0132
Brain tumor	999	p23 protein complex	0.04637
Brain tumor	1178	BCOR complex	0.0284
Brain tumor	1181	C complex spliceosome	0.00898

Brain tumor	1182	CDC5L core complex	0.03279
Brain tumor	1183	CDC5L complex	0.01466
Brain tumor	1189	DNA double-strand break end-joining complex	0.06072
Brain tumor	1308	PABPC1-HSPA8-HNRPD-EIF4G1 complex	0.03592
Brain tumor	1345	Septin complex	0.03592
Brain tumor	1492	BHC110 complex	0.0254
Brain tumor	1831	PIAS3-SMAD3-P300 complex	0.04637
Brain tumor	1890	ELK1-SRF-ELK4 complex	0.04637
Brain tumor	1893	mTOR-RICTOR complex	0.04637
Brain tumor	1895	RICTOR-mTOR complex	0.04637
Brain tumor	2129	DNAJB2-HSPA8-PSMA3 complex	0.04637
Brain tumor	2254	CTGF/Hcs24-actin complex	0.04637
Brain tumor	2258	VILIP-1-AChR-alpha-4-AChR-beta-2 complex	0.04637
Brain tumor	2300	Profilin 2 complex	0.02677
Brain tumor	2318	ITGA6-ITGB4-Laminin10/12 complex	0.07184
Brain tumor	2319	ITGA6-ITGB4-Laminin10/12 complex	0.07184
Brain tumor	2322	ITGA6-ITGB4-LAMA5 complex	0.04637
Brain tumor	2374	ITGAV-ITGB3-LAMA4 complex	0.04637
Brain tumor	2537	PKC-alpha-PLD1-PLC-gamma-2 signaling complex lacritin stimulated	0.04637
Brain tumor	2551	PDGFRA-PLC-gamma-1-PI3K-SHP-2 complex PDGF stimulated	0.04016
Brain tumor	2707	SMAD3-SMAD4-FOXO3-FOXG1 complex	0.04016
Brain tumor	2711	Amyloid beta protein oligomer	0.08032
Brain tumor	2721	HCF-1 complex	0.01843
Brain tumor	2755	17S U2 snRNP	0.01398
Brain tumor	2803	PAX6-SOX2 DNA-protein complex	0.0568
Brain tumor	2837	Profilin 1 complex	0.03279
Brain tumor	2842	DAXX-Axin-p53-HIPK2 complex	0.04016
Brain tumor	2844	Axin-p53-HIPK2 complex	0.04637
Brain tumor	2936	Ecsit complex (ECSIT MT-CO2 GAPDH TRAF6 NDUFAF1)	0.03592
Brain tumor	2969	mTORC2 complex (mTOR/FRAP1 LST8 mAVO3/RICTOR)	0.04637
Brain tumor	3055	Nop56p-associated pre-rRNA complex	0.00788
Brain tumor	3092	APP-TOMM40 complex	0.0568
Brain tumor	3093	APP-TIMM23 complex	0.0568
Brain tumor	3102	DHX9-ADAR-vigilin-DNA-PK-Ku antigen complex	0.03279
Brain tumor	3110	ITGAV-P2RY2-GNA12 complex	0.04637
Brain tumor	3149	NK-3-Groucho-HIPK2-SIN3A-RbpA48-HDAC1	0.02422
Brain tumor	3154	Notch2(N-TM)-Notch2(N-EC)-Delta complex	0.04016
Brain tumor	3183	PDGFRA-SHP-2 complex PDGF stimulated	0.0568
Brain tumor	3186	GRB2-SHP-2 complex PDGF stimulated	0.0568
Brain tumor	3270	Delta1 homodimer complex	0.08032
Brain tumor	3271	Gamma-secretase-Delta1 complex	0.03592
Brain tumor	3979	mTORC2 complex (mTOR/FRAP1 LST8 mAVO3/RICTOR SIN1)	0.04016
Brain tumor	5162	ELK1-SRF-ELK3 complex	0.04637

Brain tumor	5232	TNF-alpha/Nf-kappa B signaling complex (RPL6 RPL30 RPS13 CHUK DDX3X NFKB2 NFKBIB REL IKBKG NFKB1 MAP3K8 RELB GLG1 NFKBIA RELA TNIP2 GTF2I)	0.03896
Brain tumor	5266	TNF-alpha/NF-kappa B signaling complex 6	0.02147
Brain tumor	5268	TNF-alpha/NF-kappa B signaling complex 7	0.0284
Brain tumor	5269	TNF-alpha/NF-kappa B signaling complex 8	0.03279
Brain tumor	5285	TNF-alpha/NF-kappa B signaling complex 9	0.03592
Brain tumor	5317	LATS1-HTRA2-BIRC4 complex	0.04637
Brain tumor	5337	ELMO1-DOCK1 complex	0.0568
Brain tumor	5341	ELMO1-DOCK2 complex	0.0568
Brain tumor	5342	ELMO1-DOCK1-RAC1 complex	0.04637
Brain tumor	5343	ELMO1-DOCK1-CRKII complex	0.04637
Brain tumor	5380	TRBP containing complex (DICER RPL7A EIF6 MOV10 and subunits of the 60S ribosomal particle)	0.01606
Brain tumor	5385	GAIT complex	0.04016
Brain tumor	5564	LMO4-gp130 complex	0.03592
Brain tumor	5659	SEMA3C-PlexinD1-Nrp1 complex	0.04637
Brain tumor	5816	Apoptosome-procaspase 9 complex	0.04637
Breast cancer	1	BCL6-HDAC4 complex	0.03402
Breast cancer	4	Multisubunit ACTR coactivator complex	0.02406
Breast cancer	13	MUS81-CDS1 complex	0.03402
Breast cancer	15	NCOR complex	0.01964
Breast cancer	54	SIN3 complex	0.01818
Breast cancer	55	HDAC4-ERK1 complex	0.03402
Breast cancer	57	HDAC4-ERK2 complex	0.03402
Breast cancer	58	SMRT complex	0.02152
Breast cancer	61	Mi2/NuRD complex	0.01818
Breast cancer	62	MeCP1 complex	0.03402
Breast cancer	66	TRAP complex	0.01701
Breast cancer	68	BCDX2 complex	0.02406
Breast cancer	71	MRN complex (MRE11-RAD50-NBS1 complex)	0.05556
Breast cancer	72	R/M complex (RAD50-MRE11 complex)	0.06804
Breast cancer	73	MRN complex (MRE11-RAD50-NBN complex)	0.05556
Breast cancer	75	TSC1-TSC2 complex	0.03402
Breast cancer	86	NUMAC complex (nucleosomal methylation activator complex)	0.03043
Breast cancer	91	FA complex (Fanconi anemia complex) cytoplasmic	0.02406
Breast cancer	92	CD28-transactivation complex	0.03402
Breast cancer	98	p300-MDM2-p53 protein complex	0.02778
Breast cancer	100	hNURF complex	0.02406
Breast cancer	105	Polycomb repressive complex 2 (PRC 2)	0.02152
Breast cancer	117	GPR56-CD81-Galphaq/11-Gbeta complex	0.01701
Breast cancer	120	Lymphotoxin beta receptor complex	0.02778
Breast cancer	129	PIDDsome complex	0.02778
Breast cancer	149	PBAF complex (Polybromo- and BAF containing complex)	0.01389
Breast cancer	156	Retrotranslocation complex	0.02778

Breast cancer	159	Condensin I-PARP-1-XRCC1 complex	0.01818
Breast cancer	178	Respiratory chain complex I (holoenzyme)	0.00725
Breast cancer	186	Wave-2 complex	0.04303
Breast cancer	189	BAF complex	0.01334
Breast cancer	202	BRCA1-RAD50-MRE11-NBS1 complex	0.04811
Breast cancer	206	DNA ligase IV-XRCC4 complex	0.03402
Breast cancer	217	CRSP complex	0.01701
Breast cancer	228	SMCC complex	0.01701
Breast cancer	229	NAT complex	0.01818
Breast cancer	230	Mediator complex	0.00851
Breast cancer	232	ARC complex	0.01242
Breast cancer	238	SWI-SNF chromatin remodeling-related-BRCA1	0.01451
Breast cancer	240	BRCA1-CTIP-ZBRK1 repressor complex	0.02778
Breast cancer	244	BRAFT complex	0.01334
Breast cancer	245	FA core complex (Fanconi anemia core complex)	0.01604
Breast cancer	261	RAD51B-RAD51C-RAD51D-XRCC2-XRCC3	0.02152
Breast cancer	262	RAD51L3-XRCC2 complex	0.03402
Breast cancer	266	RAD17-RFC complex	0.02152
Breast cancer	268	Checkpoint Rad complex	0.01701
Breast cancer	274	RAD17-RFC-9-1-1 checkpoint supercomplex	0.01701
Breast cancer	281	NELF complex (Negative elongation factor complex)	0.02406
Breast cancer	282	SNF2h-cohesin-NuRD complex	0.02406
Breast cancer	283	Sin3 complex	0.01818
Breast cancer	284	CRSP complex	0.01451
Breast cancer	287	ARC-L complex	0.01286
Breast cancer	288	ARC complex	0.01242
Breast cancer	300	PC2 complex	0.01389
Breast cancer	301	SMCC complex	0.01167
Breast cancer	306	Ribosome cytoplasmic	0.00535
Breast cancer	308	60S ribosomal subunit cytoplasmic	0.00702
Breast cancer	336	DNA ligase IV-XRCC4-AHNK complex	0.02778
Breast cancer	344	DNA ligase IV-XRCC4 complex (LX complex)	0.03402
Breast cancer	350	DNA ligase IV-XRCC4-PNK complex	0.02778
Breast cancer	351	Spliceosome	0.00402
Breast cancer	359	DNA ligase IV-XRCC4-XLF complex	0.02778
Breast cancer	362	DNA ligase III-XRCC1-PNK-DNA-pol III multiprotein complex	0.02406
Breast cancer	368	ERCC1-ERCC4-MSH2 complex	0.02778
Breast cancer	433	BASC complex (BRCA1-associated genome surveillance complex)	0.02778
Breast cancer	436	BASC (Ab C-20) complex (BRCA1-associated genome surveillance complex)	0.02778
Breast cancer	438	GCN5-TRRAP histone acetyltransferase complex	0.01521
Breast cancer	441	TFTC-type histone acetyl transferase complex	0.01451
Breast cancer	443	BP-SMAD complex	0.01701
Breast cancer	445	TFTC complex (TATA-binding protein-free TAF-II-containing complex)	0.01203

Breast cancer	470	TFTC complex (TATA-binding protein-free TAF-II-containing complex)	0.01167
Breast cancer	471	PCAF complex	0.01521
Breast cancer	475	STAGA complex (SPT3-TAF9-GCN5 acetyltransferase complex)	0.01389
Breast cancer	476	STAGA complex (SPT3-TAF9-GCN5 acetyltransferase complex)	0.01334
Breast cancer	513	TFTC complex (TATA-binding protein-free TAF-II-containing complex)	0.01167
Breast cancer	520	KCNQ1 macromolecular complex	0.01521
Breast cancer	531	XPA-ERCC1-ERCC4 complex	0.02778
Breast cancer	535	TRAP complex	0.01203
Breast cancer	541	IGF1-IGFBP3-ALS complex	0.05556
Breast cancer	547	SMCC complex	0.01286
Breast cancer	548	DRIP complex	0.01286
Breast cancer	554	PBAF complex (Polybromo- and BAF containing complex)	0.01521
Breast cancer	555	BAF complex	0.01604
Breast cancer	557	TRP1-G alpha-11-IP3R3-CAV1 signaling complex	0.02406
Breast cancer	561	LSm1-7 complex	0.01818
Breast cancer	564	BAF complex	0.01451
Breast cancer	565	PBAF complex (Polybromo- and BAF containing complex)	0.01451
Breast cancer	566	BAF complex	0.01451
Breast cancer	570	p300-CBP-p270-SWI/SNF complex	0.01818
Breast cancer	571	p300-CBP-p270 complex	0.02778
Breast cancer	577	FHL2-p53-HIPK2 complex	0.02778
Breast cancer	587	NuRD.1 complex	0.01701
Breast cancer	591	SAP complex (Sin3-associated protein complex)	0.01701
Breast cancer	592	SAP complex (Sin3-associated protein complex)	0.01604
Breast cancer	596	SIN3-HDAC-SAP30-ARID4 complex	0.01818
Breast cancer	619	MRE11A-RAD50-NBN-TRF2 complex	0.04811
Breast cancer	627	MRN-TRRAP complex (MRE11A-RAD50-NBN-TRRAP complex)	0.04811
Breast cancer	632	Anti-HDAC2 complex	0.01134
Breast cancer	646	HDAC1-associated protein complex	0.03208
Breast cancer	649	HDAC1-associated core complex cII	0.03043
Breast cancer	650	HDAC2-asscociated core complex	0.01701
Breast cancer	655	HSF1-HSF2 complex	0.03402
Breast cancer	659	MeCP1 complex	0.03208
Breast cancer	681	(C-CFTR)2-NHERF-ezrin complex	0.02778
Breast cancer	682	C-CFTR-NHERF(PDZ1 domain)-ezrin complex	0.02778
Breast cancer	683	C-CFTR-NHERF(PDZ2 domain)-ezrin complex	0.02778
Breast cancer	685	MeCP1 complex	0.03208
Breast cancer	687	CFTR-NHERF-beta(2)AR signaling complex	0.02778
Breast cancer	691	SIN3-SAP25 complex	0.01451
Breast cancer	696	BRMS1-SIN3-HDAC complex	0.01701
Breast cancer	710	Brg1-associated complex I	0.01451
Breast cancer	711	Brm-associated complex	0.01451

Breast cancer	713	BRG1-SIN3A complex	0.01286
Breast cancer	714	BRM-SIN3A complex	0.01242
Breast cancer	725	P2X7 receptor signalling complex	0.04167
Breast cancer	726	DDB2 complex	0.01334
Breast cancer	729	Ubiquitin E3 ligase (FBXO31 SKP1A CUL1 RBX1)	0.02406
Breast cancer	738	SIN3-ING1b complex I	0.01604
Breast cancer	739	SIN3-ING1b complex II	0.01203
Breast cancer	741	NCOR-HDAC3 complex	0.02152
Breast cancer	745	NCOR-SIN3-RPD3 complex	0.02406
Breast cancer	747	NCOR-SIN3-HDAC1 complex	0.02778
Breast cancer	752	SMRT core complex	0.02778
Breast cancer	764	Dysferlin-affixin complex	0.03402
Breast cancer	778	LARC complex (LCR-associated remodeling complex)	0.02208
Breast cancer	786	MR-UBC9-SRC1 complex	0.02778
Breast cancer	790	DDB complex	0.03402
Breast cancer	803	BRG1-SIN3A-HDAC containing SWI/SNF remodeling complex I	0.01451
Breast cancer	806	BRM-SIN3A-HDAC complex	0.01389
Breast cancer	807	BRG1-associated complex	0.01604
Breast cancer	808	BRM-associated complex	0.01521
Breast cancer	845	PCI-PSA-SCG2 complex	0.02778
Breast cancer	886	MTA1 complex	0.01964
Breast cancer	888	MTA2 complex	0.01604
Breast cancer	889	MTA1-HDAC core complex	0.02152
Breast cancer	898	Mediator complex 1	0.02152
Breast cancer	900	Mediator complex 2	0.02778
Breast cancer	909	ARC92-Mediator complex	0.01334
Breast cancer	910	CRSP-Mediator 2 complex	0.01521
Breast cancer	929	CEN complex	0.00791
Breast cancer	938	FACT complex UV-activated	0.02152
Breast cancer	995	Polycomb repressive complex 3 (PRC3)	0.02152
Breast cancer	996	Polycomb repressive complex 2 (PRC2)	0.02152
Breast cancer	1004	RC complex during S-phase of cell cycle	0.01334
Breast cancer	1005	RC complex during G2/M-phase of cell cycle	0.01334
Breast cancer	1042	SRA-SRC-1 ribonucleoprotein complex	0.03402
Breast cancer	1055	ZNF198-PML complex	0.03402
Breast cancer	1064	IFP35-NMI complex	0.03402
Breast cancer	1067	CD8A-LCK complex	0.03402
Breast cancer	1079	P-TEFb.1 complex	0.03402
Breast cancer	1080	P-TEFb.2 complex	0.03402
Breast cancer	1083	P-TEFb.4 complex	0.03402
Breast cancer	1085	DNA repair complex NEIL2-PNK-Pol(beta)-LigIII(alpha)-XRCC1	0.02152
Breast cancer	1086	DNA repair complex NEIL1-PNK-Pol(beta)-LigIII(alpha)-XRCC1	0.02152
Breast cancer	1088	PRNP-ApolopoproteinE3 complex	0.03402
Breast cancer	1091	SNX complex (SNX1a SNX2 SNX4 LEPR)	0.02406
Breast cancer	1093	SNX complex (SNX1a SNX2 SNX4 INSR)	0.02406

Breast cancer	1094	Frataxin complex	0.01818
Breast cancer	1141	CF IIAm complex (Cleavage factor IIAm complex)	0.01203
Breast cancer	1152	FA complex (Fanconi anemia complex)	0.01701
Breast cancer	1158	p33ING1b-p300 complex	0.03402
Breast cancer	1160	ING1-p300-PCNA complex	0.02778
Breast cancer	1162	Ubiquitin E3 ligase (DDB1 DDB2 CUL4A CUL4B RBX1)	0.02152
Breast cancer	1181	C complex spliceosome	0.00538
Breast cancer	1182	CDC5L core complex	0.01964
Breast cancer	1183	CDC5L complex	0.01757
Breast cancer	1189	DNA double-strand break end-joining complex	0.05455
Breast cancer	1191	RNA pol II containing coactivator complex Tat-SF	0.04303
Breast cancer	1193	Rap1 complex	0.05455
Breast cancer	1204	Rap1 complex	0.01818
Breast cancer	1211	Ubiquitin E3 ligase (AHR ARNT DDB1 TBL3 CUL4B RBX1)	0.03928
Breast cancer	1226	H2AX complex I	0.01818
Breast cancer	1230	WINAC complex	0.01286
Breast cancer	1231	FIB-associated protein complex	0.01964
Breast cancer	1239	EBAFb complex	0.01521
Breast cancer	1252	EBAFa complex	0.01521
Breast cancer	1257	ALL-1 supercomplex	0.00909
Breast cancer	1306	PIN1-AUF1 complex	0.03402
Breast cancer	1308	PABPC1-HSPA8-HNRPD-EIF4G1 complex	0.02152
Breast cancer	1335	SNW1 complex	0.01134
Breast cancer	1400	ASCOM complex	0.03637
Breast cancer	1413	NCOR1 complex	0.01521
Breast cancer	1471	pRb2/p130-multimolecular complex (RB2 E2F5 HDAC1 SUV39H1 P300)	0.02152
Breast cancer	1474	SMAD3/4-E2F4/5-p107-DP1 complex	0.03928
Breast cancer	1505	NCOR2 complex	0.03637
Breast cancer	1514	IL4-IL4R complex	0.03402
Breast cancer	1515	IL4-IL4R-IL2RG complex	0.02778
Breast cancer	1521	p300-SMAD1-STAT3 complex	0.02778
Breast cancer	1557	NMI-POLR1B-RRN3 complex	0.02778
Breast cancer	1561	NMI homodimer complex	0.04811
Breast cancer	5757	PLXNA1-RANBPM complex	0.03402
Breast cancer	1620	G protein complex (HDAC4 GNB1 GNG2)	0.02778
Breast cancer	1623	FA core complex 1 (Fanconi anemia core complex 1)	0.02152
Breast cancer	1624	FA core complex (Fanconi anemia core complex)	0.01604
Breast cancer	1625	FA core complex (Fanconi anemia core complex)	0.01818
Breast cancer	1661	E2F4-p107-cyclinA complex	0.05556
Breast cancer	1707	IL2-IL2RA-IL2RB complex	0.02778
Breast cancer	1728	CTCF-nucleophosmin-PARP-HIS-KPNA-LMNA-TOP complex	0.03208
Breast cancer	1729	TLE1 corepressor complex (MASH1 promoter-corepressor complex)	0.04564
Breast cancer	1731	PRMT1 complex	0.04811

Breast cancer	1774	MICA-KLRK1-HCST complex	0.02778
Breast cancer	1784	RNF11-SMURF2-STAMBP complex	0.02778
Breast cancer	1827	PML-SMAD2/3-SARA complex	0.02406
Breast cancer	1831	PIAS3-SMAD3-P300 complex	0.02778
Breast cancer	5721	CIN85-CBL-SH3GL2 complex	0.02778
Breast cancer	1992	LEPR homodimer complex	0.04811
Breast cancer	2055	CASP8-CHUK-IKBKB-MALT1-BCL10 complex	0.02152
Breast cancer	2056	BCL10-CHUK-BCL10-IKBKB complex	0.02406
Breast cancer	2084	NFKB1-NFKB2-REL-RELA-RELB complex	0.02152
Breast cancer	2100	CHUK-IKBKB-MAP3K14 complex	0.02778
Breast cancer	2101	IKKA-IKKB complex	0.03402
Breast cancer	2104	IKKB-NIK complex	0.03402
Breast cancer	2105	IkappaB kinase complex (IKBKB CHUK IKBKAP NFKBIA RELA MAP3K14)	0.01964
Breast cancer	2118	CHUK-ERC1-IKBKB-IKBKG	0.02406
Breast cancer	2121	CHUK-IKBKB-IKBKG complex	0.02778
Breast cancer	2143	MAP2K5-PRKCI-SQSTM1 complex	0.02778
Breast cancer	2145	HSF1-YWHAE complex	0.03402
Breast cancer	2152	ARNT-HLF complex	0.03402
Breast cancer	2183	Kaiso-NCOR complex	0.01521
Breast cancer	2188	Ubiquitin E3 ligase (CDC34 NEDD8 BTRC CUL1 SKP1A RBX1)	0.02152
Breast cancer	2198	RAD9-RAD1-HUS1-POLB complex	0.02406
Breast cancer	2214	LMO4-BRCA1-CTIP-LDB1 complex	0.02406
Breast cancer	2215	BRCA1-LMO4-CTIP complex	0.02778
Breast cancer	2217	MDC1-MRN-ATM-FANCD2 complex	0.05893
Breast cancer	2218	MDC1-MRE11-RAD50-NBS1 complex	0.04811
Breast cancer	2220	RAD52-ERCC4-ERCC1 complex	0.05556
Breast cancer	2228	BLM-RAD51L3-XRCC2 complex	0.02778
Breast cancer	2254	CTGF/Hcs24-actin complex	0.02778
Breast cancer	2294	ABI1-WASF2 complex	0.06804
Breast cancer	2297	ABI1-WASL complex	0.06804
Breast cancer	2429	ITGA2-ITGB1-CD47 complex	0.02778
Breast cancer	2430	ITGA2-ITGB1-CHAD complex	0.02778
Breast cancer	2431	ITGA2-ITGB1-COL6A3 complex	0.02778
Breast cancer	2432	ITGA2-ITGB1 complex	0.03402
Breast cancer	2443	ITGA9-ITGB1-TNC complex	0.02778
Breast cancer	2454	CIN85-CBL-SH3GL2-EGFR complex EGF stimulated	0.02406
Breast cancer	2455	CIN85-SH3GL2 complex	0.03402
Breast cancer	2486	GIPC1-LHCGR complex	0.03402
Breast cancer	2513	N-WASp homomer	0.04811
Breast cancer	2528	ERBB2-MEMO-SHC complex	0.02778
Breast cancer	2535	SLP-76-Cbl-Grb2-Shc complex Fc receptor gamma-R1 stimulated	0.02406
Breast cancer	2537	PKC-alpha-PLD1-PLC-gamma-2 signaling complex lacritin stimulated	0.02778
Breast cancer	2559	p56(LCK)-CAML complex	0.03402

Breast cancer	2565	CD20-LCK-LYN-FYN-p75/80 complex (Raji human B cell line)	0.02406
Breast cancer	2577	Sam68-p85 P13K-IRS-1-IR signaling complex	0.02406
Breast cancer	2589	PGC-1-SRp40-SRp55-SRp75 complex	0.02406
Breast cancer	2590	FOXO1-FHL2-SIRT1 complex	0.02778
Breast cancer	2599	POLR2A-CCNT1-CDK9-NCL-LEM6-CPSF2 complex	0.05893
Breast cancer	2600	BRD4 complex	0.03402
Breast cancer	2601	P-TEFb-BRD4-TRAP220 complex	0.02406
Breast cancer	2602	P-TEFb-7SKRNA-HEXIM1 complex	0.02778
Breast cancer	2603	Transcription elongation factor complex (SUPT5H CDK9 CCNT1)	0.02778
Breast cancer	2604	P-TEFb-SKP2 complex	0.02778
Breast cancer	2605	Heterotrimeric complex (CCNT1 CDK9 GRN)	0.02778
Breast cancer	2625	CDK8-MED6-PARP1 complex	0.02778
Breast cancer	2635	BETA2-Cyclin D1 complex	0.03402
Breast cancer	2638	HES1 promoter corepressor complex	0.01964
Breast cancer	2639	HES1 promoter-Notch enhancer complex	0.02669
Breast cancer	2641	p300/CBP-PCAF-MyoD complex	0.02406
Breast cancer	2642	SMAD1-P300 complex	0.03402
Breast cancer	2693	NFAT-JUN-FOS DNA-protein complex	0.02778
Breast cancer	2695	ETS2-FOS-JUN complex	0.02778
Breast cancer	2705	SMAD3-SMAD4-CTCF protein-DNA complex	0.02778
Breast cancer	2709	MMP-9-TIMP-1-LRP complex	0.02778
Breast cancer	2719	Casein kinase II-HMG1 complex	0.02406
Breast cancer	2720	Casein kinase II complex	0.02778
Breast cancer	2721	HCF-1 complex	0.01104
Breast cancer	2727	SRC-3 complex	0.03637
Breast cancer	2728	SRC-1 complex	0.02406
Breast cancer	2739	FA complex (Fanconi anemia complex)	0.01451
Breast cancer	2758	TRIKA2 protein kinase complex (TAK1 TAB1 TAB2)	0.02778
Breast cancer	2766	TERF2-RAP1 complex	0.03637
Breast cancer	2767	RAD50-MRE11-NBN-p200-p350 complex	0.05556
Breast cancer	2776	RAD50-BRCA1 complex	0.03402
Breast cancer	2787	BRCA1 C complex	0.02406
Breast cancer	2789	ETS2-ERG complex	0.03402
Breast cancer	2790	ETS2-ETS1 complex	0.03402
Breast cancer	2810	Rad17-RFC complex	0.02152
Breast cancer	2815	BRCA1-BARD1-BACH1-DNA damage complex II	0.06804
Breast cancer	2817	BRCA1-BARD1-BACH1-DNA damage complex I	0.01964
Breast cancer	2819	BRCA1-CtIP-CtBP complex	0.02778
Breast cancer	2826	ITGB3-ITGAV-VTN complex	0.02778
Breast cancer	2851	ING2 complex	0.02778
Breast cancer	2875	BRD4-P-TEFb complex	0.02778
Breast cancer	2879	CD20-LCK-FYN-p75/80 complex	0.02778
Breast cancer	2886	Respiratory chain complex I (incomplete intermediate ND1 ND2 ND3 CIA30 assembly) mitochondrial	0.02406
Breast cancer	2895	SHC-GRB2 complex	0.03402
Breast cancer	2911	SMRT-SKIP-CBF1 complex	0.02778

Breast cancer	2919	Respiratory chain complex I (gamma subunit) mitochondrial	0.01334
Breast cancer	2923	SHARP-CtBP1-CtIP complex	0.02778
Breast cancer	2930	SHARP-CtIP-RBP-Jkappa complex	0.02778
Breast cancer	2931	SHARP-CtBP1-CtIP-RBP-Jkappa corepressor complex	0.02406
Breast cancer	2944	Notch1-p56lck-PI3K complex	0.02778
Breast cancer	2954	Smad1-Notch1-p300-Pcaf complex	0.02406
Breast cancer	2955	LCK-SLP76-PLC-gamma-1-LAT complex pervanadate-activated	0.02406
Breast cancer	2975	SMAD3-E2F4/5-p107-DP1 complex	0.04303
Breast cancer	3008	60S APC containing complex	0.01818
Breast cancer	3044	SKI-NCOR1-SIN3A-HDAC1 complex	0.02406
Breast cancer	3047	Parvulin-associated pre-rRNP complex	0.00611
Breast cancer	3048	mSin3A complex	0.02152
Breast cancer	3055	Nop56p-associated pre-rRNA complex	0.02359
Breast cancer	3065	RNA polymerase II complex chromatin structure modifying	0.01451
Breast cancer	3078	DGCR8-NCL complex	0.03402
Breast cancer	3082	DGCR8 multiprotein complex	0.01451
Breast cancer	3083	Nucleic and chromatin Fanconi complex	0.02152
Breast cancer	3096	ITGA6-ITGB4-SHC1-GRB2 complex	0.02406
Breast cancer	3110	ITGAV-P2RY2-GNA12 complex	0.02778
Breast cancer	3117	ITGB5-ITGAV-VTN complex	0.02778
Breast cancer	3137	MASH1 promoter-coactivator complex	0.02901
Breast cancer	3142	CAMK2-delta-MASH1 promoter-coactivator complex	0.03402
Breast cancer	3156	CBF1-HDAC1-SMRT complex	0.02778
Breast cancer	3162	TF-FVIIa-FXa-TFPI complex	0.07217
Breast cancer	3167	NCOR-SIN3-HDAC-HESX1 complex	0.01964
Breast cancer	3182	FHL2 homodimer complex	0.04811
Breast cancer	3187	FHL2-FHL3 complex	0.03402
Breast cancer	3188	FHL2-ACT complex	0.03402
Breast cancer	3189	FHL2-CREB complex	0.03402
Breast cancer	3204	SMAD2-SKI-NCOR complex	0.02778
Breast cancer	3205	SMAD3-SKI-NCOR complex	0.02778
Breast cancer	3206	SMAD4-SKI-NCOR complex	0.02778
Breast cancer	3229	Heterodimer complex (CDK9 IL6ST)	0.03402
Breast cancer	3263	HERP1/HEY2-NCOR-SIN3A complex	0.02406
Breast cancer	3634	NR3C2-UBC9-SRC-1 complex	0.02778
Breast cancer	4025	Affixin-actinin(alpha) complex	0.03402
Breast cancer	4043	NEMO-HIF2(alpha)-ARNT complex	0.02778
Breast cancer	4976	SVIP-p97/VCP-DERL1 complex	0.02778
Breast cancer	4977	gp78-p97/VCP-DERL1 complex	0.02778
Breast cancer	4997	p97/VCP-VIMP-DERL1 complex	0.02778
Breast cancer	4999	p97/VCP-VIMP-DERL1-DERL2-HRD1-SEL1L	0.01964
Breast cancer	5099	RB1(hypophosphorylated)-E2F4 complex	0.03402
Breast cancer	5117	pRb2/p130-multimolecular complex (DNMT1 E2F4 SuV39H1 HDAC1 RBL2)	0.02152

Breast cancer	5118	pRb2/p130-multimolecular complex (RB2 E2F4 HDAC1 SUV39H1 P300)	0.04303
Breast cancer	5144	E2F1-p107-cyclinA complex	0.02778
Breast cancer	5153	CTFC-TAF1 complex	0.03402
Breast cancer	5154	CTCF-nucleophosmin complex	0.03402
Breast cancer	5158	SMARCA2/BRM-BAF57-MECP2 complex	0.02778
Breast cancer	5159	E2F4-p107-cyclinE complex	0.05556
Breast cancer	5160	E2F4-p130 complex	0.03402
Breast cancer	5165	AP1G1-PACS1-FURIN complex	0.02778
Breast cancer	5179	NCOA6-DNA-PK-Ku-PARP1 complex	0.02152
Breast cancer	5184	SWI/SNF chromatin-remodeling complex	0.02152
Breast cancer	5193	TNF-alpha/NF-kappa B signaling complex (CHUK KPNA3 NFKB2 NFKBIB REL IKBKG NFKB1 NFKBIE RELB NFKBIA RELA TNIP2)	0.01389
Breast cancer	5194	TNF-alpha/NF-kappa B signaling complex (SEC16A CHUK IKBKB NFKB2 REL IKBKG MAP3K14 RELA FBXW7 USP2)	0.03043
Breast cancer	5196	TNF-alpha/NF-kappa B signaling complex (CHUK BTRC NFKB2 PPP6C REL CUL1 IKBKE SAPS2 SAPS1 ANKRD28 RELA SKP1)	0.02778
Breast cancer	5197	PTIP-DNA damage response complex	0.03928
Breast cancer	5198	CBP-RARA-RXRA-DNA complex ligand stimulated	0.02778
Breast cancer	5209	Ubiquilin-proteasome complex	0.01964
Breast cancer	5211	RAF1-PPP2-PIN1 complex	0.02152
Breast cancer	5215	CS-MAP3K7IP1-MAP3K7IP2 complex	0.02778
Breast cancer	5224	Casein kinase II (beta-dimer alpha alpha')	0.02778
Breast cancer	5225	Casein kinase II (beta-dimer alpha-dimer)	0.03402
Breast cancer	5228	REL-MAP3K8-RELA-TNIP2-PAPOLA complex	0.02152
Breast cancer	5230	CHUK-NFKB2-REL-IKBKG-SPAG9-NFKB1-NFKBIE-COPB2-TNIP1-NFKBIA-RELA-TNIP2	0.01389
Breast cancer	5232	TNF-alpha/Nf-kappa B signaling complex (RPL6 RPL30 RPS13 CHUK DDX3X NFKB2 NFKBIB REL IKBKG NFKB1 MAP3K8 RELB GLG1 NFKBIA RELA TNIP2 GTF2I)	0.01167
Breast cancer	5233	TNF-alpha/NF-kappa B signaling complex 5	0.02887
Breast cancer	5234	IKBKB-CDC37-KIAA1967-HSP90AB1-HSP90AA1 complex	0.04303
Breast cancer	5235	WRN-Ku70-Ku80-PARP1 complex	0.02406
Breast cancer	5253	MNK1-eIF4F complex	0.02152
Breast cancer	5260	TCF4-CTNNB1-SUMO1-EP300-HADAC6 complex	0.04303
Breast cancer	5261	TCF4-CTNNB1-EP300 complex	0.02778
Breast cancer	5266	TNF-alpha/NF-kappa B signaling complex 6	0.01286
Breast cancer	5268	TNF-alpha/NF-kappa B signaling complex 7	0.01701
Breast cancer	5269	TNF-alpha/NF-kappa B signaling complex 8	0.01964
Breast cancer	5293	ETS2-SMARCA4-INI1 complex	0.02778
Breast cancer	5317	LATS1-HTRA2-BIRC4 complex	0.02778
Breast cancer	5375	EGR-EP300 complex	0.03402
Breast cancer	5378	TRBP containing complex (DICER TRBP AGO2 RPL7A EIF6 MOV10)	0.01964

Breast cancer	5380	TRBP containing complex (DICER RPL7A EIF6 MOV10 and subunits of the 60S ribosomal particle)	0.00962
Breast cancer	5382	ARNT-HIF1A complex	0.03402
Breast cancer	5423	HSP70-BAG5-PARK2 complex	0.02406
Breast cancer	5450	Mediator complex	0.00926
Breast cancer	5465	IKB(epsilon)-RELA-cREL complex	0.02778
Breast cancer	5466	IKB(beta)-RELA-cREL complex	0.02778
Breast cancer	5467	IKB(alpha)-RELA-cREL complex	0.02778
Breast cancer	5498	ILK-PARVB-ARHGEF6 complex	0.02778
Breast cancer	5499	PARVB-ARHGEF6 complex	0.03402
Breast cancer	5564	LMO4-gp130 complex	0.02152
Breast cancer	5589	LINC complex S-phase	0.01818
Breast cancer	5596	LINC complex quiescent cells	0.01818
Breast cancer	5606	Emerin-actin-NMI-(alphaII)spectrin complex	0.02406
Breast cancer	5607	Emerin-actin-NMI complex	0.02778
Breast cancer	5608	Emerin architectural complex	0.01964
Breast cancer	5609	Emerin regulatory complex	0.01604
Breast cancer	5611	Emerin complex 24	0.01242
Breast cancer	5613	Emerin complex 25	0.01203
Breast cancer	5614	Emerin complex 32	0.02052
Breast cancer	5615	Emerin complex 52	0.02006
Breast cancer	5646	FARP2-NRP1-PlexinA1 complex	0.02778
Breast cancer	5655	Ternary complex (LRRC7 CAMK2a ACTN4)	0.02778
Breast cancer	5659	SEMA3C-PlexinD1-Nrp1 complex	0.02778
Breast cancer	5668	PlexinA1-Nrp1 complex	0.03402
Breast cancer	5673	PlexinA1-Nrp2 complex	0.03402
Breast cancer	5683	hRAD51C-hXRCC3 complex	0.03402
Breast cancer	5684	Membrane protein complex (DERL1 SELS VCP)	0.02778
Breast cancer	5689	SEMA6D-PlexinA1-NRP1 complex	0.02778
Breast cancer	5745	PlexinA1-NRP1 complex	0.03402
Breast cancer	5746	PlexinA1-NRP1-SEMA3A complex	0.02778
Breast cancer	5762	CRMP-MICAL-PlexinA1 complex induced by	0.02778
Breast cancer	5816	Apoptosome-procaspase 9 complex	0.02778
Breast cancer	5823	MCL1-BAK1 complex	0.03402
Breast cancer	5828	IKBKG-IKBKB complex	0.03402
Breast cancer	5832	PINK1-MIRO2-Milton complex	0.02778
Breast cancer	5837	PPD complex	0.05556
Breast cancer	5844	I-kappa-B kinase (IKK) complex	0.02778
Bronchial disease	305	40S ribosomal subunit cytoplasmic	0.04159
Bronchial disease	306	Ribosome cytoplasmic	0.02695
Bronchial disease	668	BKCA-beta2AR-AKAP79 signaling complex	0.14003
Bronchial disease	672	BKCA-beta2AR complex	0.1715
Bronchial disease	687	CFTR-NHERF-beta(2)AR signaling complex	0.14003
Bronchial disease	3055	Nop56p-associated pre-rRNA complex	0.02378
Bronchial disease	3830	ADRB2 homodimer complex	0.24254
Bronchial hyperreactivity	441	TFTC-type histone acetyl transferase complex	0.1066
Bronchial hyperreactivity	1054	ESR1-RELA-BCL3-NCOA3 complex	0.17678

Bronchial hyperreactivity	2124	IKK-alpha--ER-alpha-AIB1 complex	0.20412
Bronchial hyperreactivity	2470	p130Cas-ER-alpha-cSrc-kinase- PI3-kinase p85-subunit complex	0.17678
Bronchial hyperreactivity	2657	ESR1-CDK7-CCNH-MNAT1-MTA1-HDAC2 complex	0.14434
Bronchial hyperreactivity	2670	Er-alpha-p53-hdm2 complex	0.20412
Bronchial hyperreactivity	2699	ER-alpha-GRIP1-c-Jun complex	0.20412
Bronchial hyperreactivity	2700	ER-alpha-c-Jun complex	0.25
Bronchial hyperreactivity	5862	CAV1-VDAC1-ESR1 complex	0.20412
Bronchiolitis obliterans	2709	MMP-9-TIMP-1-LRP complex	0.3849
Bronchopulmonary dysplasia	2443	ITGA9-ITGB1-TNC complex	0.17408
Bronchopulmonary dysplasia	2709	MMP-9-TIMP-1-LRP complex	0.34816
Bronchopulmonary dysplasia	2798	MMP-2-claudin-1 complex	0.2132
Brucellosis	1514	IL4-IL4R complex	0.2132
Brucellosis	1515	IL4-IL4R-IL2RG complex	0.17408
Brucellosis	1714	TICAM1-TICAM2-TLR4 complex	0.17408
CNS metastases	2798	MMP-2-claudin-1 complex	0.25
CNS metastases	5423	HSP70-BAG5-PARK2 complex	0.17678
CNS metastases	5837	PPD complex	0.20412
Cancer	1	BCL6-HDAC4 complex	0.02606
Cancer	2	BCL6-HDAC5 complex	0.05213
Cancer	3	BCL6-HDAC7 complex	0.02606
Cancer	15	NCOR complex	0.01505
Cancer	27	Arp2/3 protein complex	0.01393
Cancer	33	Prefoldin	0.01505
Cancer	41	Mi-2/NuRD-MTA2 complex	0.01648
Cancer	54	SIN3 complex	0.01393
Cancer	58	SMRT complex	0.01648
Cancer	61	Mi2/NuRD complex	0.02786
Cancer	62	MeCP1 complex	0.02606
Cancer	63	Mitotic 14S cohesin 1 complex	0.01843
Cancer	71	MRN complex (MRE11-RAD50-NBS1 complex)	0.02128
Cancer	73	MRN complex (MRE11-RAD50-NBN complex)	0.02128
Cancer	75	TSC1-TSC2 complex	0.02606
Cancer	80	Ubiquitin E3 ligase (Skp1A Skp2 Cul1 Rbx1)	0.01843
Cancer	86	NUMAC complex (nucleosomal methylation activator complex)	0.03497
Cancer	98	p300-MDM2-p53 protein complex	0.02128
Cancer	100	hNURF complex	0.01843
Cancer	103	RNA polymerase II holoenzyme complex	0.00752
Cancer	105	Polycomb repressive complex 2 (PRC 2)	0.03297
Cancer	107	TFIIH transcription factor complex	0.01229
Cancer	112	Prefoldin complex	0.01505
Cancer	115	Polycomb repressive complex 1 (PRC1 hPRC-H)	0.02128
Cancer	116	Polycomb repressive complex 1 (PRC1 hPRC-H)	0.02045
Cancer	117	GPR56-CD81-Galphaq/11-Gbeta complex	0.01303
Cancer	126	CCT micro-complex	0.01303
Cancer	127	NDC80 kinetochore complex	0.01843
Cancer	138	Telosome complex	0.01505

Cancer	142	CD147-gamma-secretase complex (APH-1a PS-1 PEN-2 NCT variant)	0.01648
Cancer	143	APP-FE65-LRP complex	0.02128
Cancer	145	CCT:PFD complex	0.00985
Cancer	149	PBAF complex (Polybromo- and BAF containing complex)	0.03192
Cancer	159	Condensin I-PARP-1-XRCC1 complex	0.01393
Cancer	164	Cohesin-SA1 complex	0.01843
Cancer	189	BAF complex	0.03067
Cancer	190	Mitotic checkpoint complex (MCC)	0.03686
Cancer	201	HUIC complex	0.05213
Cancer	202	BRCA1-RAD50-MRE11-NBS1 complex	0.03686
Cancer	205	Ubiquitin E3 ligase (VHL TCEB1 TCEB2 CUL2	0.01648
Cancer	212	DNA ligase III-XRCC1 complex	0.02606
Cancer	213	DNA ligase IV-XRCC1 complex	0.02606
Cancer	214	Ubiquitin E3 ligase (WSB1 TCEB1 TCEB2 CUL5 RBX1)	0.01648
Cancer	220	ARF-Mule complex	0.02128
Cancer	226	Ubiquitin E3 ligase (SKP1A SKP2 CUL1)	0.02128
Cancer	238	SWI-SNF chromatin remodeling-related-BRCA1	0.03334
Cancer	240	BRCA1-CTIP-ZBRK1 repressor complex	0.02128
Cancer	242	BRCA1-BACH1 complex	0.02606
Cancer	243	RalBP1-CDC2-CCNB1 complex	0.02128
Cancer	244	BRAFT complex	0.01022
Cancer	246	BLM complex III	0.01843
Cancer	247	RalBP1-CCNB1-AP2A-NUMB-EPN1 complex	0.01505
Cancer	252	RAD51C-XRCC3 complex	0.02606
Cancer	261	RAD51B-RAD51C-RAD51D-XRCC2-XRCC3	0.01648
Cancer	265	ATR-ATRIP complex	0.02606
Cancer	282	SNF2h-cohesin-NuRD complex	0.02765
Cancer	283	Sin3 complex	0.01393
Cancer	285	PCNA-MLH1-PMS1 complex	0.04256
Cancer	286	PCNA-MSH2-MSH6 complex	0.06384
Cancer	290	MSH2-MLH1-PMS2-PCNA DNA-repair initiation complex	0.07372
Cancer	291	MSH2-MLH1-PMS2 DNA-repair initiation complex	0.06384
Cancer	292	MutL-alpha complex	0.05213
Cancer	297	PCNA-DNA polymerase delta complex	0.01648
Cancer	298	VEGF transcriptional complex	0.0301
Cancer	304	SRCAP-associated chromatin remodeling complex	0.01166
Cancer	305	40S ribosomal subunit cytoplasmic	0.01264
Cancer	306	Ribosome cytoplasmic	0.02867
Cancer	308	60S ribosomal subunit cytoplasmic	0.02688
Cancer	310	Cell cycle kinase complex CDC2	0.06019
Cancer	311	Cell cycle kinase complex CDK2	0.05529
Cancer	312	Cell cycle kinase complex CDK4	0.05529
Cancer	313	Cell cycle kinase complex CDK5	0.06594
Cancer	314	PCNA-p21 complex	0.05213

Cancer	351	Spliceosome	0.00925
Cancer	362	DNA ligase III-XRCC1-PNK-DNA-pol III multiprotein complex	0.01843
Cancer	368	ERCC1-ERCC4-MSH2 complex	0.04256
Cancer	369	MSH2-MSH6-PMS2-MLH1 complex	0.07372
Cancer	370	MSH2-MSH6-PMS1-MLH1 complex	0.05529
Cancer	371	Structure-specific endonulease complex	0.03686
Cancer	374	MSH2-MSH6 complex	0.05213
Cancer	375	MSH2-MSH3 complex	0.02606
Cancer	376	PCNA-MutS-alpha-MutL-alpha-DNA complex	0.08242
Cancer	377	PCNA-MutS-alpha-DNA initial complex	0.06384
Cancer	378	MutS-beta complex	0.02606
Cancer	380	MutL-beta complex	0.02606
Cancer	387	MCM complex	0.04514
Cancer	404	Isocitrate dehydrogenase cytoplasmic	0.03686
Cancer	415	EXO1-MLH1-PMS2 complex	0.06384
Cancer	422	Beta-dystroglycan-caveolin-3 complex	0.05213
Cancer	424	EXO1-MLH1-PCNA complex	0.06384
Cancer	433	BASC complex (BRCA1-associated genome surveillance complex)	0.06384
Cancer	434	BASC (Ab 80) complex (BRCA1-associated genome surveillance complex)	0.05213
Cancer	435	BASC (Ab 81) complex (BRCA1-associated genome surveillance complex)	0.04514
Cancer	436	BASC (Ab C-20) complex (BRCA1-associated genome surveillance complex)	0.04256
Cancer	438	GCN5-TRRAP histone acetyltransferase complex	0.03497
Cancer	441	TFTC-type histone acetyl transferase complex	0.01111
Cancer	475	STAGA complex (SPT3-TAF9-GCN5 acetyltransferase complex)	0.01064
Cancer	476	STAGA complex (SPT3-TAF9-GCN5 acetyltransferase complex)	0.01022
Cancer	518	AKAP250-PKA-PDE4D complex	0.01648
Cancer	521	Polycystin-1-E-cadherin-beta-catenin complex	0.02128
Cancer	522	Polycystin-1-E-cadherin-beta-catenin-Flotillin-2	0.01843
Cancer	531	XPA-ERCC1-ERCC4 complex	0.02128
Cancer	541	IGF1-IGFBP3-ALS complex	0.02128
Cancer	550	NOS3-CAV1-NOSTRIN complex	0.02128
Cancer	553	RHOA-IP3R-TRPC1 complex	0.02128
Cancer	554	PBAF complex (Polybromo- and BAF containing complex)	0.02331
Cancer	555	BAF complex	0.02457
Cancer	557	TRP1-G alpha-11-IP3R3-CAV1 signaling complex	0.01843
Cancer	564	BAF complex	0.02223
Cancer	565	PBAF complex (Polybromo- and BAF containing complex)	0.02223
Cancer	566	BAF complex	0.03334
Cancer	570	p300-CBP-p270-SWI/SNF complex	0.02786
Cancer	585	Mi2/NuRD-BCL6-MTA3 complex	0.03297

Cancer	587	NuRD.1 complex	0.0391
Cancer	591	SAP complex (Sin3-associated protein complex)	0.01303
Cancer	592	SAP complex (Sin3-associated protein complex)	0.01229
Cancer	596	SIN3-HDAC-SAP30-ARID4 complex	0.01393
Cancer	614	NRD complex (Nucleosome remodeling and deacetylation complex)	0.0418
Cancer	619	MRE11A-RAD50-NBN-TRF2 complex	0.01843
Cancer	622	Ubiquitin E3 ligase (VHL TCEB1 TCEB2 CUL2)	0.01843
Cancer	627	MRN-TRRAP complex (MRE11A-RAD50-NBN-TRRAP complex)	0.01843
Cancer	632	Anti-HDAC2 complex	0.02606
Cancer	642	CtBP complex	0.01788
Cancer	643	CtBP core complex	0.01229
Cancer	644	ASPP1-SAM68 complex	0.02606
Cancer	646	HDAC1-associated protein complex	0.01229
Cancer	649	HDAC1-associated core complex cII	0.01166
Cancer	650	HDAC2-asscociated core complex	0.01303
Cancer	659	MeCP1 complex	0.02457
Cancer	662	Hoxa9-PBX2-Meis1 complex	0.02128
Cancer	681	(C-CFTR)2-NHERF-ezrin complex	0.02128
Cancer	682	C-CFTR-NHERF(PDZ1 domain)-ezrin complex	0.02128
Cancer	683	C-CFTR-NHERF(PDZ2 domain)-ezrin complex	0.02128
Cancer	685	MeCP1 complex	0.02457
Cancer	691	SIN3-SAP25 complex	0.01111
Cancer	696	BRMS1-SIN3-HDAC complex	0.02606
Cancer	697	BRMS1-RBP1 complex	0.02606
Cancer	710	Brg1-associated complex I	0.02223
Cancer	711	Brm-associated complex	0.01111
Cancer	713	BRG1-SIN3A complex	0.02955
Cancer	714	BRM-SIN3A complex	0.01903
Cancer	722	MRG15-PAM14-RB complex	0.02128
Cancer	723	MAF1 complex	0.02128
Cancer	725	P2X7 receptor signalling complex	0.01064
Cancer	726	DDB2 complex	0.02045
Cancer	727	CSA complex	0.02045
Cancer	728	CSA-POLIIa complex	0.0197
Cancer	738	SIN3-ING1b complex I	0.02457
Cancer	739	SIN3-ING1b complex II	0.03686
Cancer	741	NCOR-HDAC3 complex	0.01648
Cancer	746	C/EBPalpha-HNF6 complex	0.05213
Cancer	752	SMRT core complex	0.02128
Cancer	753	UTM-SGCE-DAG1-CAV1-NOS3 complex	0.03297
Cancer	756	Prune-Gelsolin complex	0.02606
Cancer	757	Prune-GSK3beta complex	0.02606
Cancer	758	Prune/Nm23-H1 complex	0.02606
Cancer	764	Dysferlin-affixin complex	0.02606
Cancer	778	LARC complex (LCR-associated remodeling complex)	0.04228

Cancer	781	URI complex (Unconventional prefoldin RPB5 Interactor)	0.01229
Cancer	788	Exosome	0.01166
Cancer	789	Exosome	0.01111
Cancer	803	BRG1-SIN3A-HDAC containing SWI/SNF remodeling complex I	0.02223
Cancer	806	BRM-SIN3A-HDAC complex	0.01064
Cancer	807	BRG1-associated complex	0.02457
Cancer	808	BRM-associated complex	0.01166
Cancer	810	FCP1-associated protein complex	0.01393
Cancer	815	MRIP-MBS-RHOA complex	0.02128
Cancer	816	MRIP-RHOA complex	0.02606
Cancer	822	mRNA decay complex (UPF1 UPF2 UPF3B DCP2 XRN1 XRN2 EXOSC2 EXOSC4 EXOSC10 PARN)	0.01166
Cancer	826	PAR-3-VE-cadherin-beta-catenin complex	0.02128
Cancer	839	LIN9-BMYB complex	0.02606
Cancer	845	PCI-PSA-SCG2 complex	0.02128
Cancer	860	DNMT1-G9a-PCNA complex	0.04256
Cancer	862	DNMT1-G9a complex	0.02606
Cancer	871	BRAF53-BRCA2 complex	0.01393
Cancer	886	MTA1 complex	0.0301
Cancer	888	MTA2 complex	0.02457
Cancer	889	MTA1-HDAC core complex	0.03297
Cancer	903	RET-Rai complex	0.02606
Cancer	927	CENP-A nucleosome associated complex	0.01505
Cancer	929	CEN complex	0.02424
Cancer	933	SCRIB-APC complex	0.02606
Cancer	974	EED-EZH2 complex	0.03297
Cancer	995	Polycomb repressive complex 3 (PRC3)	0.03297
Cancer	996	Polycomb repressive complex 2 (PRC2)	0.03297
Cancer	997	KIN17-PCNA-RPA70 complex	0.02128
Cancer	999	p23 protein complex	0.02128
Cancer	1003	RC complex (Replication competent complex)	0.01229
Cancer	1004	RC complex during S-phase of cell cycle	0.01022
Cancer	1005	RC complex during G2/M-phase of cell cycle	0.02045
Cancer	1009	TFIIH transcription factor complex	0.01166
Cancer	1029	TFIIH transcription factor complex	0.01166
Cancer	1030	CAK-ERCC2 complex	0.01843
Cancer	1039	PCNA-PAF complex	0.02606
Cancer	1040	p33ING1b-PCNA complex	0.02606
Cancer	1041	Alpha-dystrobrevin-ZO-1-actin complex	0.01843
Cancer	1051	Ubiquitin E3 ligase (SKP1A SKP2 CUL1 RBX1)	0.01843
Cancer	1054	ESR1-RELA-BCL3-NCOA3 complex	0.03686
Cancer	1062	BAR-BCL2-CASP8 complex	0.04256
Cancer	1069	FIF-FGR2 complex	0.02606
Cancer	1079	P-TEFb.1 complex	0.02606
Cancer	1085	DNA repair complex NEIL2-PNK-Pol(beta)-LigIII(alpha)-XRCC1	0.01648

Cancer	1086	DNA repair complex NEIL1-PNK-Pol(beta)-LigIII(alpha)-XRCC1	0.03297
Cancer	1087	BIRC5-AURKB-INCENP-EVI5 complex	0.03686
Cancer	1090	APLG1-Rababtin5 complex	0.02128
Cancer	1092	PCNA-KU antigen complex	0.02128
Cancer	1094	Frataxin complex	0.01393
Cancer	1095	SNX complex (SNX1a SNX2 SNX4 EGFR)	0.01843
Cancer	1098	DNA synthesome complex (13 subunits)	0.00985
Cancer	1099	DNA synthesome complex (17 subunits)	0.00869
Cancer	1107	DNA synthesome core complex	0.01166
Cancer	1108	DNA synthesome complex (15 subunits)	0.00952
Cancer	1116	CRM1-Survivin-AuroraB mitotic complex	0.04256
Cancer	1117	CRM1-Survivin mitotic complex	0.02606
Cancer	1118	Chromosomal passenger complex CPC (INCENP CDCA8 BIRC5 AURKB)	0.03686
Cancer	1120	Chromosomal passenger complex CPC (INCENP CDCA8 BIRC5)	0.02128
Cancer	1130	Hip1R-cortactin complex	0.02606
Cancer	1133	ATR-HDAC2 complex	0.02606
Cancer	1134	ATR-HDAC2-CHD4 complex	0.04256
Cancer	1143	SMN complex	0.01843
Cancer	1148	snRNP-free U1A (SF-A) complex	0.01843
Cancer	1149	Histone H3.1 complex	0.01166
Cancer	1150	Histone H3.3 complex	0.01229
Cancer	1153	Integrator complex	0.01064
Cancer	1154	DSS1 complex	0.02045
Cancer	1155	Integrator-RNAPII complex	0.00985
Cancer	1158	p33ING1b-p300 complex	0.02606
Cancer	1159	p33ING1b-HDAC1 complex	0.02606
Cancer	1160	ING1-p300-PCNA complex	0.04256
Cancer	1163	ING1-PCNA complex	0.05213
Cancer	1166	p400-associated complex	0.01393
Cancer	1167	Paf complex	0.01648
Cancer	1170	cMYC-ATPase-helicase complex	0.01648
Cancer	1171	c-MYC-ATPase-helicase complex	0.01648
Cancer	1177	Polycomb repressive complex 4 (PRC4)	0.02128
Cancer	1178	BCOR complex	0.01303
Cancer	1179	CENP-A NAC-CAD complex	0.01022
Cancer	1181	C complex spliceosome	0.02061
Cancer	1183	CDC5L complex	0.01346
Cancer	1185	EGFR-containing signaling complex	0.03686
Cancer	1189	DNA double-strand break end-joining complex	0.01393
Cancer	1194	E2F-6 complex	0.02128
Cancer	1197	TRF1-TIN2 complex	0.01505
Cancer	1198	TIN2 complex	0.01843
Cancer	1204	Rap1 complex	0.01393
Cancer	1206	TRF-Rap1 complex I 2MD	0.01505
Cancer	1207	TRF2-Rap1 complex II	0.01843

Cancer	1214	Ubiquitin E3 ligase (DET1 DDB1 CUL4A RBX1	0.01648
Cancer	1223	H2AX complex isolated from cells without IR exposure	0.01022
Cancer	1224	Ubiquitin E3 ligase (BMI1 SPOP CUL3)	0.02128
Cancer	1226	H2AX complex I	0.01393
Cancer	1227	H2AX complex II	0.01166
Cancer	1230	WINAC complex	0.0197
Cancer	1231	FIB-associated protein complex	0.01505
Cancer	1239	EBAFb complex	0.02331
Cancer	1248	Apoptosome	0.02606
Cancer	1250	pRB-E2F-1 complex	0.05213
Cancer	1252	EBAFa complex	0.02331
Cancer	1254	Menin-associated histone methyltransferase complex	0.02786
Cancer	1256	MLL-HCF complex	0.02786
Cancer	1257	ALL-1 supercomplex	0.01393
Cancer	1259	Chromatin assembly complex (CAF-1 complex)	0.02128
Cancer	1287	HNRPF-HNRPH1 complex	0.02606
Cancer	1288	DCS complex (PTBP1 PTBP2 HNRPH1 HNRPF)	0.01843
Cancer	1297	MKK4-ARRB2-ASK1 complex	0.02128
Cancer	1298	MKK4-ARRB2-JNK3 complex	0.02128
Cancer	1332	Large Drosha complex	0.01648
Cancer	1335	SNW1 complex	0.01738
Cancer	1372	Rb-tal-1-E2A-Lmo2-Ldb1 complex	0.01648
Cancer	1399	WDR5-ASH2L-RBBP5-MLL2 complex	0.01843
Cancer	1400	ASCOM complex	0.02786
Cancer	1401	MOF complex	0.01166
Cancer	1413	NCOR1 complex	0.03497
Cancer	1439	PTGS2 homodimer complex	0.03686
Cancer	1452	MCM2-MCM6-MCM7 complex	0.04256
Cancer	1462	hPRC1L complex	0.03686
Cancer	1470	pRb2/p130-multimolecular complex (DNMT1 E2F5 SuV39H1 HDAC1 RBL2)	0.01648
Cancer	1471	pRb2/p130-multimolecular complex (RB2 E2F5 HDAC1 SUV39H1 P300)	0.01648
Cancer	1473	E2F5-RB2-DP1 complex	0.02128
Cancer	1474	SMAD3/4-E2F4/5-p107-DP1 complex	0.01505
Cancer	5658	Nrp1-PlexinD1 complex	0.02606
Cancer	1488	DNMT1-RB1-HDAC1-E2F1 complex	0.03686
Cancer	1495	PID complex	0.03297
Cancer	1505	NCOR2 complex	0.01393
Cancer	1508	BCL6-ZBTB17 complex	0.02606
Cancer	1539	G protein complex (GNG2 GNB2L1 RAF1)	0.02128
Cancer	1618	G protein complex (PTHR1 GNB1 GNG2)	0.02128
Cancer	1619	G protein complex (HDAC5 GNB1 GNG2)	0.02128
Cancer	1633	CyclinD1-CDK4-CDK6 complex	0.02128
Cancer	1634	CyclinD1-CDK4-p21 complex	0.04256
Cancer	1642	p16-cyclin D2-CDK4 complex	0.04256
Cancer	1656	p27-cyclinE-CDK2 complex	0.04256
Cancer	1661	E2F4-p107-cyclinA complex	0.02128

Cancer	5712	FAK-beta5 integrin complex VEGF induced	0.02606
Cancer	1700	ABL2-HRAS-RIN1 complex	0.04256
Cancer	1729	TLE1 corepressor complex (MASH1 promoter-corepressor complex)	0.02331
Cancer	1745	SMN complex	0.00952
Cancer	1746	SMN containing complex	0.02606
Cancer	1751	SMN complex	0.02331
Cancer	1752	SMN complex	0.01393
Cancer	1768	CPSF6-EWSR1-ITCH-NUDT21-POLR2A-UBAP2L complex	0.01505
Cancer	1777	TGF-beta-receptor-SMAD7-SMURF2 complex	0.03686
Cancer	1783	TGF-beta receptor I-SMAD7-SMURF1 complex	0.02128
Cancer	1784	RNF11-SMURF2-STAMBP complex	0.02128
Cancer	1787	Nogo-potassium channel complex	0.03686
Cancer	5718	eNOS-HSP90-AKT complex VEGF induced	0.04256
Cancer	1810	ITGA4-PXN-GIT1 complex	0.02128
Cancer	1812	AXIN-MEKK4-CCD1 complex	0.02128
Cancer	1814	MAD1L1-MAD2L1 complex	0.02606
Cancer	1816	JUN-TCF4-CTNNB1 complex	0.04256
Cancer	1827	PML-SMAD2/3-SARA complex	0.01843
Cancer	1834	ITGAE-ITGB7-CDH1 complex	0.02128
Cancer	1839	SDCBP-CTNNB1-CTNNA1-CDH1 complex	0.03686
Cancer	1844	APC-IQGAP1 complex	0.02606
Cancer	1845	APC-IQGAP1-CLIP-170 complex	0.02128
Cancer	1851	BUB1-BUB3 complex	0.02606
Cancer	1909	APC-DLG4 complex	0.02606
Cancer	1932	NEK2-NEK11 complex	0.02606
Cancer	1945	IRAK1-IRAK3 complex	0.02606
Cancer	5716	eNOS-HSP90 complex VEGF induced	0.02606
Cancer	1970	BMP4-TWSG1 complex	0.02606
Cancer	1972	BMP4-BGN complex	0.02606
Cancer	1986	Endoglin homodimer complex	0.03686
Cancer	2000	BAX homo-oligomer complex	0.03686
Cancer	2004	C1D homodimer protein	0.03686
Cancer	2005	PTPRA homodimer complex	0.03686
Cancer	2010	AXL homodimer complex	0.03686
Cancer	2028	JAK2-IL12RB2 complex	0.02606
Cancer	2052	BCL10 oligomer complex	0.03686
Cancer	2053	BCL10-MALT1 complex	0.02606
Cancer	2054	CASP8-FADD-MALT1-BCL10 complex	0.03686
Cancer	2055	CASP8-CHUK-IKBKB-MALT1-BCL10 complex	0.03297
Cancer	2056	BCL10-CHUK-BCL10-IKBKB complex	0.03686
Cancer	2084	NFKB1-NFKB2-REL-RELA-RELB complex	0.01648
Cancer	2086	NFKB1-NFKB2-RELA-RELB complex	0.01843
Cancer	2105	IkappaB kinase complex (IKBKB CHUK IKBKAP NFKBIA RELA MAP3K14)	0.01505
Cancer	2112	CDC37-HSP90AA1-HSP90AB1-MAP3K11 complex	0.03686
Cancer	2124	IKK-alpha--ER-alpha-AIB1 complex	0.02128

Cancer	2156	YBX1-AKT1 complex	0.02606
Cancer	2159	AR-AKT-APPL complex	0.04256
Cancer	2160	AOF2-AR complex	0.02606
Cancer	2171	Ubiquitin E3 ligase (CHEK1 CUL4A)	0.02606
Cancer	2174	COP9 signalosome complex	0.02457
Cancer	2179	CNS-P53 complex	0.02457
Cancer	2183	Kaiso-NCOR complex	0.02331
Cancer	2201	PCNA-RFC2-5 complex	0.01648
Cancer	2210	BRCA1-IRIS-pre-replication complex	0.01843
Cancer	2211	BARD1-BRCA1-CSTF complex	0.03297
Cancer	2213	BRCA1-BARD1-POLR2A complex	0.04256
Cancer	2214	LMO4-BRCA1-CTIP-LDB1 complex	0.01843
Cancer	2215	BRCA1-LMO4-CTIP complex	0.02128
Cancer	2217	MDC1-MRN-ATM-FANCD2 complex	0.0301
Cancer	2218	MDC1-MRE11-RAD50-NBS1 complex	0.01843
Cancer	2220	RAD52-ERCC4-ERCC1 complex	0.02128
Cancer	2224	MSH2/6-BLM-p53-RAD51 complex	0.04945
Cancer	2226	MutS-alpha-PK-zeta complex	0.04256
Cancer	2230	PCNA complex	0.02786
Cancer	2231	PCNA homotrimer complex	0.03686
Cancer	2236	ASF1-histone containing complex	0.01393
Cancer	2237	SP1-MCAF2 complex	0.02606
Cancer	2254	CTGF/Hcs24-actin complex	0.02128
Cancer	2255	Cofilin-actin-CAP1 complex	0.02128
Cancer	2300	Profilin 2 complex	0.01229
Cancer	2318	ITGA6-ITGB4-Laminin10/12 complex	0.03297
Cancer	2319	ITGA6-ITGB4-Laminin10/12 complex	0.03297
Cancer	2320	ITGA6-ITGB4-CD151 complex	0.04256
Cancer	2321	ITGA6-ITGB4-FYN complex	0.04256
Cancer	2322	ITGA6-ITGB4-LAMA5 complex	0.04256
Cancer	2323	ITGA6-ITGB4 complex	0.05213
Cancer	2342	ITGAV-ITGB8-MMP14-TGFB1 complex	0.03686
Cancer	2343	ITGAV-ITGB5-PLAUR complex	0.04256
Cancer	2345	ITGAV-ITGB5-ICAM4 complex	0.02128
Cancer	2346	ITGAV-ITGB5-ADAM9 complex	0.02128
Cancer	2347	ITGAV-ITGB5-SPP1 complex	0.04256
Cancer	2348	ITGAV-ITGB5-CYR61 complex	0.04256
Cancer	2350	ITGAV-ITGB5 complex	0.02606
Cancer	2352	ITGAV-ITGB6-SPP1 complex	0.04256
Cancer	2353	ITGAV-ITGB6-TGFB3 complex	0.02128
Cancer	2354	ITGAV-ITGB6 complex	0.02606
Cancer	2355	ITGAV-ITGB3-CD47-FCER2 complex	0.05529
Cancer	2356	ITGB3-ITGAV-CD47 complex	0.04256
Cancer	2358	ITGAV-ITGB3-SPP1 complex	0.06384
Cancer	2359	ITGAV-ITGB3-ADAM15 complex	0.04256
Cancer	2362	ITAGV-ITGB3-F11R complex	0.04256
Cancer	2363	ITGAV-ITGB3-PXN-PTK2b complex	0.05529

Cancer	2364	ITGAV-ITGB3-ADAM23 complex	0.04256
Cancer	2365	ITGAV-ITGB3-COL4A3 complex	0.04256
Cancer	2366	ITGAV-ITGB3-PPAP2b complex	0.04256
Cancer	2369	ITGAV-ITGB3-EGFR complex	0.06384
Cancer	2370	ITGA2b-ITGB3-CD9 complex	0.04256
Cancer	2374	ITGAV-ITGB3-LAMA4 complex	0.04256
Cancer	2376	ITGA2B-ITGB3-FN1-TGM2 complex	0.01843
Cancer	2377	ITGA2b-ITGB3-CD47-SRC complex	0.01843
Cancer	2378	ITGA2b-ITGB3-TLN1 complex	0.02128
Cancer	2379	ITGA2B-ITGB3-CIB1 complex	0.02128
Cancer	2381	ITGA2B-ITGB3 complex	0.02606
Cancer	2382	ITGA2B-ITGB3-F11R complex	0.02128
Cancer	2383	ITGA5-ITGB1-FN1-TGM2 complex	0.01843
Cancer	2384	ITGA5-ITGB1-ADAM15 complex	0.02128
Cancer	2385	ITGA5-ITGB4 complex	0.02606
Cancer	2388	Itga5-Itgb1-Fn1-Sfrp2 complex	0.01843
Cancer	2390	CD98-LAT2-ITGB1 complex	0.03686
Cancer	2395	ITGA7-ITGB1-CD151 complex	0.02128
Cancer	2396	ITGA7-ITGB1-CD9 complex	0.04256
Cancer	2397	ITGA7-ITGB1-ITGB1BP3 complex	0.02128
Cancer	2398	ITGA3-ITGB1-BSG complex	0.06384
Cancer	2399	ITGA3-ITGB1-CD63 complex	0.04256
Cancer	2400	ITGA3-ITGB1-CD151 complex	0.04256
Cancer	2401	ITGA3-ITGB1-THBS1 complex	0.04256
Cancer	2406	ITGA3-ITGB1 complex	0.05213
Cancer	2411	ITGA6-ITGB1-CD151 complex	0.04256
Cancer	2413	ITGA6-ITGB1 complex	0.05213
Cancer	2416	ITGB1-RAP1A-PKD1 complex	0.02128
Cancer	2417	ITGA4-ITGB1-EMILIN1 complex	0.02128
Cancer	2418	ITGA4-ITGB1 complex	0.02606
Cancer	2419	ITGA4-ITGB1-CD81 complex	0.02128
Cancer	2420	ITGA4-ITGB1-CD53 complex	0.02128
Cancer	2421	ITGA4-ITGB1-VCAM1 complex	0.02128
Cancer	2422	ITGA4-ITGB1-JAM2 complex	0.02128
Cancer	2423	ITGA4-ITGB1-CD47 complex	0.02128
Cancer	2424	ITGA4-ITGB1-CD63 complex	0.02128
Cancer	2425	ITGA4-ITGB1-PXN complex	0.04256
Cancer	2426	ITGA4-ITGB1-THBS1 complex	0.02128
Cancer	2428	ITGA4-ITGB1-THBS2 complex	0.02128
Cancer	2429	ITGA2-ITGB1-CD47 complex	0.02128
Cancer	2430	ITGA2-ITGB1-CHAD complex	0.02128
Cancer	2431	ITGA2-ITGB1-COL6A3 complex	0.02128
Cancer	2432	ITGA2-ITGB1 complex	0.02606
Cancer	2434	ITGA1-ITGB1-COL6A3 complex	0.02128
Cancer	2435	ITGA1-ITGB1-PTPN2 complex	0.02128
Cancer	2436	ITGAV-ITGB1 complex	0.05213
Cancer	2437	ITGA6-ITGB1-CYR61 complex	0.06384

Cancer	2439	ITGA8-ITGB1 complex	0.02606
Cancer	2440	ITGA9-ITGB1-ADAM9 complex	0.02128
Cancer	2441	Itga9-Itgb1-Adam2 complex	0.02128
Cancer	2442	ITGA9-ITGB1-VCAM1 complex	0.02128
Cancer	2443	ITGA9-ITGB1-TNC complex	0.02128
Cancer	2444	ITGB1-ITGA9 complex	0.02606
Cancer	2445	ITGA9-ITGB1-ADAM15 complex	0.02128
Cancer	2446	ITGA9-ITGB1-FIGF complex	0.04256
Cancer	2447	ITGA9-ITGB1-ADAM12 complex	0.02128
Cancer	2453	Multiprotein complex (monoubiquitination)	0.01843
Cancer	2454	CIN85-CBL-SH3GL2-EGFR complex EGF stimulated	0.01843
Cancer	2456	MET-CIN85-SH3GL3-CBL complex HGF stimulated	0.01843
Cancer	5714	eNOS-CAV1 complex	0.02606
Cancer	2462	Caveolin-1 homodimer complex	0.03686
Cancer	2470	p130Cas-ER-alpha-cSrc-kinase- PI3-kinase p85-subunit complex	0.05529
Cancer	2476	CRKL-PDGFRA-CRK-RAPGEF1 complex	0.01843
Cancer	2480	CIN85 complex (CIN85 CRK BCAR1 CBL PIK3R1 GRB2 SOS1)	0.0418
Cancer	2528	ERBB2-MEMO-SHC complex	0.02128
Cancer	2529	LAT-PLC-gamma-1-p85-GRB2-CBL-VAV-SLP-76 signaling complex C305 activated	0.01393
Cancer	2541	HGF-Met complex	0.02606
Cancer	2542	EGFR-CBL-GRB2 complex	0.02128
Cancer	2551	PDGFRA-PLC-gamma-1-PI3K-SHP-2 complex PDGF stimulated	0.01843
Cancer	2563	FGFR2-c-Cbl-Lyn-Fyn complex	0.01843
Cancer	2565	CD20-LCK-LYN-FYN-p75/80 complex (Raji human B cell line)	0.01843
Cancer	2574	CD19-Vav-PI 3-kinase (p85 subunit) complex	0.02128
Cancer	2577	Sam68-p85 P13K-IRS-1-IR signaling complex	0.05529
Cancer	2578	Sam68-p120GAP complex	0.02606
Cancer	2579	Chromosomal passenger complex CPC (INCENP BIRC5 AURKB)	0.04256
Cancer	2580	Survivin homodimer complex	0.03686
Cancer	2581	RasGAP-AURKA/AURKB-survivin complex	0.03686
Cancer	2582	Chromosomal passenger complex CPC (CDCA8 AURKB BIRC5)	0.04256
Cancer	2599	POLR2A-CCNT1-CDK9-NCL-LEM6-CPSF2 complex	0.01505
Cancer	2600	BRD4 complex	0.01303
Cancer	2601	P-TEFb-BRD4-TRAP220 complex	0.01843
Cancer	2602	P-TEFb-7SKRNA-HEXIM1 complex	0.02128
Cancer	2603	Transcription elongation factor complex (SUPT5H CDK9 CCNT1)	0.02128
Cancer	2604	P-TEFb-SKP2 complex	0.04256
Cancer	2605	Heterotrimeric complex (CCNT1 CDK9 GRN)	0.02128
Cancer	2635	BETA2-Cyclin D1 complex	0.02606
Cancer	2639	HES1 promoter-Notch enhancer complex	0.01022
Cancer	2649	MYC-DNMT3A-ZBTB17 complex	0.02128

Cancer	2653	MYC-MAX-BLOC1S1 complex	0.02128
Cancer	2655	MYC-MAX complex	0.02606
Cancer	2657	ESR1-CDK7-CCNH-MNAT1-MTA1-HDAC2 complex	0.0301
Cancer	2660	ERCC2/CAK complex	0.01843
Cancer	2670	Er-alpha-p53-hdm2 complex	0.04256
Cancer	2679	p53-SP1 complex	0.02606
Cancer	2686	BRCA1-core RNA polymerase II complex	0.01022
Cancer	2688	MT1-MMP-claudin-1 complex	0.05213
Cancer	2692	SMAD3-SMAD4-cJun-cFos complex	0.03686
Cancer	2693	NFAT-JUN-FOS DNA-protein complex	0.02128
Cancer	2694	ERG-JUN-FOS DNA-protein complex	0.02128
Cancer	2695	ETS2-FOS-JUN complex	0.02128
Cancer	2699	ER-alpha-GRIP1-c-Jun complex	0.04256
Cancer	2700	ER-alpha-c-Jun complex	0.05213
Cancer	2704	Ectodermin-SMAD4 complex	0.02606
Cancer	2705	SMAD3-SMAD4-CTCF protein-DNA complex	0.02128
Cancer	2706	SMAD3-SMAD4-SP1 complex	0.04256
Cancer	2707	SMAD3-SMAD4-FOXO3-FOXG1 complex	0.01843
Cancer	2708	SMAD3-SMAD4-cJUN complex	0.04256
Cancer	2709	MMP-9-TIMP-1-LRP complex	0.04256
Cancer	2710	LRP-1-Alpha-2-M-annexin VI complex	0.02128
Cancer	2714	Ubiquitin E3 ligase (CHEK1 CUL1)	0.02606
Cancer	2715	Ubiquitin E3 ligase (CSN1 CSN8 HRT1 SKP1 SKP2 CUL1 CUL2 CUL3)	0.01303
Cancer	2718	MAD2-CDC20 complex	0.02606
Cancer	2721	HCF-1 complex	0.04228
Cancer	2723	ATM-NBS1 complex	0.05213
Cancer	2726	PXN-ITGB5-PTK2 complex	0.04256
Cancer	2730	Set1B complex	0.01505
Cancer	2731	Set1A complex	0.01505
Cancer	2740	MutS-alpha complex	0.05213
Cancer	2744	TRAF2-MALT1 complex	0.02606
Cancer	2745	Ubiquitin ligase complex (TRAF6 TAB2 MALT1 UEV1A BCL10)	0.01648
Cancer	2752	CARMA1-BCL10-MALT1 complex	0.02128
Cancer	2753	FYB-CARMA1-BCL-10-MALT1 complex	0.01843
Cancer	2754	JUND-FOSB-SMAD3-SMAD4 complex	0.01843
Cancer	2755	17S U2 snRNP	0.00642
Cancer	2760	SMAD3-SMAD4-FOXO3 complex	0.02128
Cancer	2761	SMAD3-SMAD4-FOXO1 complex	0.02128
Cancer	2762	SMAD3-SMAD4-FOXO4 complex	0.02128
Cancer	2767	RAD50-MRE11-NBN-p200-p350 complex	0.02128
Cancer	2770	ITGA6-ITGB4-CD9 complex	0.06384
Cancer	2776	RAD50-BRCA1 complex	0.02606
Cancer	2783	BARD1-BRCA1-CSTF64 complex	0.04256
Cancer	2786	BRCA1 A complex	0.03686
Cancer	2787	BRCA1 C complex	0.03686
Cancer	2788	BRCA1 B complex	0.04256

Cancer	2790	ETS2-ETS1 complex	0.02606
Cancer	2791	MCM4-MCM6-MCM7 complex	0.02128
Cancer	2792	MCM2-MCM4-MCM6-MCM7 complex	0.03686
Cancer	2797	PCNA-CHL12-RFC2-5 complex	0.01505
Cancer	2798	MMP-2-claudin-1 complex	0.05213
Cancer	2801	OCT4-SOX2 DNA-protein complex	0.02606
Cancer	2802	OCT1-SOX2 DNA-protein complex	0.02606
Cancer	2803	PAX6-SOX2 DNA-protein complex	0.02606
Cancer	2808	RAD9-RAD1-HUS1-APE1 complex	0.01843
Cancer	2811	BRCA1-cABL complex	0.05213
Cancer	2813	BRCA1-SMAD3 complex	0.02606
Cancer	2814	BRCA1-HDAC1-HDAC2 complex	0.02128
Cancer	2815	BRCA1-BARD1-BACH1-DNA damage complex II	0.0391
Cancer	2816	ITGAV-ITGB3 complex	0.05213
Cancer	2817	BRCA1-BARD1-BACH1-DNA damage complex I	0.06019
Cancer	2818	BRCA1-BARD1-BRCA2-DNA damage complex III	0.06384
Cancer	2819	BRCA1-CtIP-CtBP complex	0.02128
Cancer	2820	BRCA1-VCP complex	0.02606
Cancer	2822	BRCA1-BARD1-UbcH5c complex	0.04256
Cancer	2823	BRCA1-BARD1-UbcH7c complex	0.04256
Cancer	2824	BRCA1-RAD51 complex	0.05213
Cancer	2825	BRCA1-RNA polymerase II complex	0.01446
Cancer	2826	ITGB3-ITGAV-VTN complex	0.04256
Cancer	2829	RSmad complex	0.03497
Cancer	2830	TIF1gamma-SMAD2-SMAD3 complex	0.02128
Cancer	2834	SMAD4-SMAD2-SMAD3 complex	0.04256
Cancer	2846	ITGAV-ITGB3-THBS1 complex	0.04256
Cancer	2849	ITGAV-ITGB3-NOV complex	0.04256
Cancer	2850	ITGA5-ITGB1-FN-1-NOV complex	0.01843
Cancer	2851	ING2 complex	0.02128
Cancer	2852	Brg1-based SWI/SNF chromatin remodeling complex	0.01843
Cancer	2853	ITGA5-ITGB1-CAL4A3 complex	0.02128
Cancer	2872	ITGA2b-ITGB3-CD9-GP1b-CD47 complex	0.0301
Cancer	2875	BRD4-P-TEFb complex	0.02128
Cancer	2879	CD20-LCK-FYN-p75/80 complex	0.02128
Cancer	2880	SCF subcomplex (WEE1 SKP2 BTRC)	0.02128
Cancer	2882	ITGA5-ITGB3-COL6A3 complex	0.02128
Cancer	2885	ITGAV-ITGB1-SPP1 complex	0.06384
Cancer	2896	ITGA2b-ITGB3-CD47-FAK complex	0.03686
Cancer	2922	LAT-PLC-gamma-1-p85-GRB2-SOS signaling complex C305 activated	0.01648
Cancer	2944	Notch1-p56lck-PI3K complex	0.02128
Cancer	2962	CRK-BCAR1-DOCK1 complex	0.04256
Cancer	2964	ITGA9-ITGB1-ADAM1 complex	0.02128
Cancer	2965	ITGA9-ITGB1-ADAM3 complex	0.02128
Cancer	2971	ITGA9-ITGB1-VEGFC complex	0.04256
Cancer	2972	ITGA9-ITGB1-VEGFA complex	0.04256

Cancer	2989	ITGA9-ITGB1-ADAM8 complex	0.02128
Cancer	2992	SMAD7-SMURF2 complex	0.05213
Cancer	2996	SMAD7-SMURF1 complex	0.02606
Cancer	2997	SMAD7-SMURF1-TGF-beta receptor complex	0.01843
Cancer	2998	Axin-PP2A A-PP2A C-GSK3-beta-beta-catenin	0.01843
Cancer	3004	APC-Axin-1-beta-catenin complex	0.04256
Cancer	3008	60S APC containing complex	0.02786
Cancer	3011	APC-IQGAP1-Rac1 complex	0.04256
Cancer	3012	APC-IQGAP1-Cdc42 complex	0.02128
Cancer	3015	p27-cyclinE-Cdk2 - Ubiquitin E3 ligase (SKP1A SKP2 CUL1 CKS1B RBX1) complex	0.05213
Cancer	3035	LAT2-ITGB1 complex	0.02606
Cancer	3036	Ubiquitin E3 ligase (SKP1A SKP2 CUL1 CKS1B RBX1)	0.03297
Cancer	3038	SMAD2-SMAD4-FAST1 complex	0.04256
Cancer	3039	SMAD2-FAST1 complex	0.02606
Cancer	3040	Multisynthetase complex	0.01111
Cancer	3043	BMP2-BRIA complex	0.06384
Cancer	3045	hs4 enhancer complex (faster migrating complex)	0.01648
Cancer	3047	Parvulin-associated pre-rRNP complex	0.00468
Cancer	3048	mSin3A complex	0.01648
Cancer	3055	Nop56p-associated pre-rRNA complex	0.02169
Cancer	3057	ITGA10-ITGB1 complex	0.02606
Cancer	3058	ITGA11-ITGB1 complex	0.05213
Cancer	3059	ITGA11-ITGB1-COL1A1 complex	0.04256
Cancer	3060	RNA polymerase II complex (RPB1 RAP74 CDK8 CYCC SRB7 BAF190 BAF47) chromatin structure modifying	0.02606
Cancer	3061	RNA polymerase II complex (CBP PCAF RPB1 BAF47 CYCC CDK8) chromatin structure modifying	0.01505
Cancer	3062	RNA polymerase II complex incomplete (CBP RPBI PCAF BAF47) chromatin structure modifying	0.01843
Cancer	3063	Brg1-associated complex II	0.02786
Cancer	3064	RNA polymerase II complex chromatin structure modifying	0.01691
Cancer	3065	RNA polymerase II complex chromatin structure modifying	0.01111
Cancer	3066	RNA polymerase II complex chromatin structure modifying	0.02045
Cancer	3067	RNA polymerase II complex incomplete (CDK8 complex) chromatin structure modifying	0.01303
Cancer	3074	CCT:PFD complex testis specific	0.00985
Cancer	3075	UTX-MLL2/3 complex	0.01064
Cancer	3082	DGCR8 multiprotein complex	0.02223
Cancer	3084	CCND1-CDK4 complex	0.02606
Cancer	3085	CCND2-CDK4 complex	0.02606
Cancer	3086	CCND3-CDK4 complex	0.02606
Cancer	3087	CCND1-CDK6 complex	0.02606
Cancer	3088	CCND2-CDK6 complex	0.02606

Cancer	3089	CCND3-CDK6 complex	0.02606
Cancer	3092	APP-TOMM40 complex	0.02606
Cancer	3096	ITGA6-ITGB4-SHC1-GRB2 complex	0.03686
Cancer	3103	ITGAV-ITGB3-SLC3A2 complex	0.06384
Cancer	3104	ITGB1-NRP1 complex	0.05213
Cancer	3110	ITGAV-P2RY2-GNA12 complex	0.02128
Cancer	3111	ITGA9-ITGB1-SPP1 complex	0.04256
Cancer	3112	ITGA5-ITGB1-SPP1 complex	0.04256
Cancer	3115	ITGA2B-ITGB3-ICAM4 complex	0.02128
Cancer	3117	ITGB5-ITGAV-VTN complex	0.02128
Cancer	3133	Phosphatidylinositol 3-kinase (PIK3CA PIK3R1)	0.02606
Cancer	3138	POSH-AKT2 complex	0.02606
Cancer	3149	NK-3-Groucho-HIPK2-SIN3A-RbpA48-HDAC1	0.01111
Cancer	3153	GNAQ-GEFT-RHOA complex	0.02128
Cancer	3155	Bipartite complex (TFC4 CTNNB1)	0.02606
Cancer	3166	AXIN-APC-betaCatenin-GSK3B complex	0.03686
Cancer	3172	NUMB-TP53-MDM2 complex	0.02128
Cancer	3197	SMAD4-SNO-SKI complex	0.02128
Cancer	3198	SMAD2-SKI complex	0.02606
Cancer	3200	SMAD4-SKI complex	0.02606
Cancer	3204	SMAD2-SKI-NCOR complex	0.02128
Cancer	3206	SMAD4-SKI-NCOR complex	0.02128
Cancer	3233	SMAD2-SMAD4-FAST1-TGIF complex TGF(beta) induced	0.03686
Cancer	3234	SMAD2-SMAD4-FAST1-TGIF-HDAC1 complex TGF(beta) induced	0.03297
Cancer	3269	RB1-HDAC1-BRG1 complex	0.04256
Cancer	3284	SMN complex (GEMIN5 2 3 4 SMN)	0.01648
Cancer	3296	SMN complex (GEMIN5 4 3) SMN-independent intermediate	0.02128
Cancer	3297	SMN complex (GEMIN6 7 UNRIP) SMN-independent intermediate	0.02128
Cancer	3298	SMN complex (GEMIN2 5 SMN)	0.02128
Cancer	3492	Bax homooligomeric complex after apoptotic	0.03686
Cancer	3677	RIN1-STAM2-HRS complex	0.02128
Cancer	3678	RIN1-STAM2-EGFR complex EGF stimulated	0.04256
Cancer	3710	CHL2-BMP2 complex	0.02606
Cancer	3711	CHL2-BMP2-TSG complex	0.02128
Cancer	3729	SKI-SMAD2 hexameric complex	0.02606
Cancer	3739	SKI-SMAD2-SMAD4 pentameric complex	0.04256
Cancer	3740	SKI-SMAD3-SMAD4 pentameric complex	0.02128
Cancer	3749	CREBBP-SMAD2 hexameric complex	0.02606
Cancer	3753	CREBBP-SMAD2-SMAD4 pentameric complex	0.04256
Cancer	3754	CREBBP-SMAD3-SMAD4 pentameric complex	0.02128
Cancer	3838	SP1-E2F2 complex	0.02606
Cancer	3839	SP1-E2F3 complex	0.02606
Cancer	3847	TCL1(trimer)-AKT1 complex	0.02606
Cancer	3848	TCL1(trimer)-AKT2 complex	0.02606

Cancer	3852	Rb-HDAC1 complex	0.02606
Cancer	3900	GABP(gamma)1-E2F1-DP1 complex	0.02128
Cancer	3917	Ternary complex (GATA4 SRF MYOCD)	0.02128
Cancer	3959	SMAD3-SMAD4-cSKI TGF(beta)-dependent	0.02128
Cancer	3967	SMURF2-SMAD2 complex TGF(beta)-dependent	0.05213
Cancer	3971	SMURF2-SMAD3 complex TGF(beta)-dependent	0.02606
Cancer	3972	SMURF2-SMAD3-SnoN complex TGF(beta)-	0.02128
Cancer	4025	Affixin-actinin(alpha) complex	0.02606
Cancer	4043	NEMO-HIF2(alpha)-ARNT complex	0.02128
Cancer	4062	NRP1-VEGFR2-VEGF(165) complex	0.06384
Cancer	4089	SMAD6-HOXC8 complex	0.02606
Cancer	4090	SMAD6-HOXA9 complex	0.02606
Cancer	4095	Catulin (alpha) - catenin (beta) complex	0.02606
Cancer	4096	Catenin (alpha) - catenin (beta) complex	0.02606
Cancer	4158	HSP90-FKBP38-CAM-Ca(2+) complex	0.01843
Cancer	4498	p32-CBF-DNA complex	0.01843
Cancer	5772	ZO1-(beta)cadherin-(VE)cadherin-VEGFR2 complex	0.03686
Cancer	5099	RB1(hypophosphorylated)-E2F4 complex	0.02606
Cancer	5100	CyclinD3-CDK4-CDK6 complex	0.02128
Cancer	5101	CyclinD3-CDK4-CDK6-p21 complex	0.03686
Cancer	5107	p34(SEI-1)-CDK4-CyclinD2 complex	0.02128
Cancer	5117	pRb2/p130-multimolecular complex (DNMT1 E2F4 SuV39H1 HDAC1 RBL2)	0.01648
Cancer	5118	pRb2/p130-multimolecular complex (RB2 E2F4 HDAC1 SUV39H1 P300)	0.01648
Cancer	5143	E2F1-Rb complex	0.05213
Cancer	5144	E2F1-p107-cyclinA complex	0.04256
Cancer	5146	RB1-TFAP2A complex	0.02606
Cancer	5159	E2F4-p107-cyclinE complex	0.02128
Cancer	5160	E2F4-p130 complex	0.02606
Cancer	5171	SH3KBP1-CBLB-EGFR complex	0.02128
Cancer	5177	Polycystin-1 multiprotein complex (ACTN1 CDH1 SRC JUP VCL CTNNB1 PXN BCAR1 PKD1	0.05557
Cancer	5178	JAK2-PAFR-TYK2 complex	0.02128
Cancer	5190	TIAM1-EFNB1-EPHA2 complex	0.04256
Cancer	5193	TNF-alpha/NF-kappa B signaling complex (CHUK KPNA3 NFKB2 NFKBIB REL IKBKG NFKB1 NFKBIE RELB NFKBIA RELA TNIP2)	0.01064
Cancer	5194	TNF-alpha/NF-kappa B signaling complex (SEC16A CHUK IKBKB NFKB2 REL IKBKG MAP3K14 RELA FBXW7 USP2)	0.01166
Cancer	5195	PTIP-HMT complex	0.01166
Cancer	5196	TNF-alpha/NF-kappa B signaling complex (CHUK BTRC NFKB2 PPP6C REL CUL1 IKBKE SAPS2 SAPS1 ANKRD28 RELA SKP1)	0.01064
Cancer	5197	PTIP-DNA damage response complex	0.01505
Cancer	5198	CBP-RARA-RXRA-DNA complex ligand stimulated	0.02128
Cancer	5199	Kinase maturation complex 1	0.01843
Cancer	5210	TANK-TRAF2-TRAF3 complex	0.02128

Cancer	5211	RAF1-PPP2-PIN1 complex	0.01648
Cancer	5212	Kinase maturation complex 2	0.0391
Cancer	5220	CHUK-IQGAP2-AKAP8L-RELA-TNIP2 complex	0.01648
Cancer	5228	REL-MAP3K8-RELA-TNIP2-PAPOLA complex	0.01648
Cancer	5230	CHUK-NFKB2-REL-IKBKG-SPAG9-NFKB1-NFKBIE-COPB2-TNIP1-NFKBIA-RELA-TNIP2	0.02128
Cancer	5232	TNF-alpha/Nf-kappa B signaling complex (RPL6 RPL30 RPS13 CHUK DDX3X NFKB2 NFKBIB REL IKBKG NFKB1 MAP3K8 RELB GLG1 NFKBIA RELA TNIP2 GTF2I)	0.00894
Cancer	5233	TNF-alpha/NF-kappa B signaling complex 5	0.01474
Cancer	5234	IKBKB-CDC37-KIAA1967-HSP90AB1-HSP90AA1 complex	0.03297
Cancer	5243	XRCC1-LIG3-PNK-TDP1 complex	0.01843
Cancer	5253	MNK1-eIF4F complex	0.03297
Cancer	5260	TCF4-CTNNB1-SUMO1-EP300-HADAC6 complex	0.01648
Cancer	5261	TCF4-CTNNB1-EP300 complex	0.02128
Cancer	5262	TCF4-CTNNB1 complex	0.02606
Cancer	5264	TCF4-CTNNB1-CREBBP complex	0.02128
Cancer	5266	TNF-alpha/NF-kappa B signaling complex 6	0.0197
Cancer	5267	VHL-VDU1-TCEB1-TCEB2 complex	0.01843
Cancer	5268	TNF-alpha/NF-kappa B signaling complex 7	0.02606
Cancer	5269	TNF-alpha/NF-kappa B signaling complex 8	0.0301
Cancer	5270	VHL-TCEB1-TCEB2 complex	0.02128
Cancer	5273	VHL-TBP1-HIF1A complex	0.04256
Cancer	5276	HIF1A-OS9-EGLN1 complex	0.02128
Cancer	5277	HIF1A-OS9-EGLN3 complex	0.02128
Cancer	5280	RAB9-TIP47-MPRI complex	0.02128
Cancer	5281	Cell-cell junction complex (CDH1-CTNNB1)	0.02606
Cancer	5282	CAS-SRC-FAK complex	0.04256
Cancer	5285	TNF-alpha/NF-kappa B signaling complex 9	0.01648
Cancer	5286	TNF-alpha/NF-kappa B signaling complex 10	0.02331
Cancer	5287	CDK4-CCND1 complex	0.02606
Cancer	5288	P53-BARD1-Ku70 complex	0.02128
Cancer	5293	ETS2-SMARCA4-INI1 complex	0.04256
Cancer	5317	LATS1-HTRA2-BIRC4 complex	0.02128
Cancer	5318	DDEF1-CTTN-PXN complex	0.04256
Cancer	5319	CDH1-CKS1B complex	0.02606
Cancer	5320	CDH1-SKP2 complex	0.02606
Cancer	5331	YY1-MDM2-p53 complex	0.02128
Cancer	5342	ELMO1-DOCK1-RAC1 complex	0.02128
Cancer	5343	ELMO1-DOCK1-CRKII complex	0.02128
Cancer	5369	ATM homodimer complex	0.03686
Cancer	5373	Chromatin remodeling complex (TACC2 TACC3	0.02128
Cancer	5375	EGR-EP300 complex	0.02606
Cancer	5380	TRBP containing complex (DICER RPL7A EIF6 MOV10 and subunits of the 60S ribosomal particle)	0.01474
Cancer	5382	ARNT-HIF1A complex	0.02606

Cancer	5383	TRIB3-DDIT3 complex	0.02606
Cancer	5386	MLL1-WDR5 complex	0.01419
Cancer	5400	BRCC complex	0.04945
Cancer	5409	TIAM1-GRIN1 complex	0.02606
Cancer	5426	ANCO1-HDAC3 complex	0.02606
Cancer	5442	EPOR receptor complex	0.03686
Cancer	5444	CRKII-C3G complex	0.02606
Cancer	5446	EPO-EPOR complex	0.05213
Cancer	5460	p50-p65 NF(kappa)B complex	0.02606
Cancer	5461	p50-p65 NF(kappa)B-SRC1 complex	0.02128
Cancer	5464	I(kappa)B(alpha)-NF(kappa)Bp50-NF(kappa)Bp65 complex	0.02128
Cancer	5465	IKB(epsilon)-RELA-cREL complex	0.02128
Cancer	5466	IKB(beta)-RELA-cREL complex	0.02128
Cancer	5467	IKB(alpha)-RELA-cREL complex	0.02128
Cancer	5473	FAS-FADD-CASP8 complex	0.04256
Cancer	5475	MURR1-NF(kappa)Bp65-IKBA complex	0.02128
Cancer	5492	IKBA-NF(kappa)Bp65-NF(kappa)Bp50 complex	0.02128
Cancer	5495	TFIIH transcription factor complex (ERCC2 ERCC3 GTF2H1 CDK7 CCNH GTF2H2)	0.01505
Cancer	5498	ILK-PARVB-ARHGEF6 complex	0.06384
Cancer	5499	PARVB-ARHGEF6 complex	0.05213
Cancer	5502	EED-EZH-YY1 polycomb complex	0.02128
Cancer	5513	Polycomb repressive complex	0.01843
Cancer	5518	BMI1-HPH1-HPH2 complex	0.02128
Cancer	5521	EED-EZH polycomb complex	0.02606
Cancer	5526	CALM1-FKBP38-BCL2 complex	0.02128
Cancer	5529	TRAF2-cIAP1/BIRC2 complex	0.05213
Cancer	5531	Tumor necrosis factor receptor 1 signaling complex	0.03686
Cancer	5540	TRAF2-TRADD complex	0.02606
Cancer	5541	Ternary complex (TRAF2 FADD TRADD)	0.02128
Cancer	5543	CCNB1-CDC2 complex	0.02606
Cancer	5544	CDC2-PCNA-CCNB1-GADD45A complex	0.05529
Cancer	5545	CDC2-PCNA-CCNB1-GADD45B complex	0.03686
Cancer	5546	CDC2-PCNA-CCNB1-GADD45G complex	0.03686
Cancer	5550	CDC2-CCNB1-CCNF complex	0.02128
Cancer	5551	CDC2-CCNB1-PTCH1 complex	0.02128
Cancer	5556	CDK2-CCNA2 complex	0.02606
Cancer	5557	CDC2-CCNA2 complex	0.02606
Cancer	5559	CDC2-CCNA2-CDK2 complex	0.02128
Cancer	5560	CDK2-CCNE1 complex	0.02606
Cancer	5589	LINC complex S-phase	0.02786
Cancer	5593	LINC core complex	0.01648
Cancer	5596	LINC complex quiescent cells	0.02786
Cancer	5604	Emerin complex 1	0.01303
Cancer	5606	Emerin-actin-NMI-(alphaII)spectrin complex	0.01843
Cancer	5607	Emerin-actin-NMI complex	0.02128
Cancer	5608	Emerin architectural complex	0.01505

Cancer	5609	Emerin regulatory complex	0.02457
Cancer	5611	Emerin complex 24	0.03807
Cancer	5613	Emerin complex 25	0.02765
Cancer	5614	Emerin complex 32	0.03929
Cancer	5615	Emerin complex 52	0.03843
Cancer	5622	HSP90-CIP1-FKBPL complex	0.04256
Cancer	5642	Securin-separase complex	0.02606
Cancer	5646	FARP2-NRP1-PlexinA1 complex	0.02128
Cancer	5647	FARP2-NRP1-PlexinA2 complex	0.02128
Cancer	5648	FARP2-NRP1-PlexinA3 complex	0.02128
Cancer	5649	FARP2-NRP1-PlexinA4 complex	0.02128
Cancer	5656	CEBPE-E2F1-RB1 complex	0.04256
Cancer	5659	SEMA3C-PlexinD1-Nrp1 complex	0.02128
Cancer	5661	PlexinC1-SEMA7A complex	0.02606
Cancer	5663	TRIM27-RB1 complex	0.02606
Cancer	5668	PlexinA1-Nrp1 complex	0.02606
Cancer	5669	PlexinA3-Nrp1 complex	0.02606
Cancer	5670	PlexinB1-Nrp1 complex	0.02606
Cancer	5673	PlexinA1-Nrp2 complex	0.02606
Cancer	5683	hRAD51C-hXRCC3 complex	0.02606
Cancer	5689	SEMA6D-PlexinA1-NRP1 complex	0.02128
Cancer	5696	VEGFA(165)-KDR-NRP1 complex	0.06384
Cancer	5697	VEGFA(165)-KDR-NRP1 complex	0.02606
Cancer	5698	VEGFA(165)-VEGFR2-NRP1 complex	0.06384
Cancer	5701	NRP1-VEGF(165/121) complex	0.05213
Cancer	5731	NRP1-VEGFC complex heparin dependent	0.05213
Cancer	5732	NRP2-VEGFC complex	0.05213
Cancer	5733	NRP2-VEGFD complex heparin dependent	0.05213
Cancer	5734	NRP1-VEGFD complex heparin dependent	0.05213
Cancer	5736	Pre-initiation complex (PIC)	0.02128
Cancer	5740	NRP2-VEGFR3 complex	0.02606
Cancer	5745	PlexinA1-NRP1 complex	0.02606
Cancer	5746	PlexinA1-NRP1-SEMA3A complex	0.04256
Cancer	5749	MRIT complex	0.06384
Cancer	5798	Death induced signaling complex II (FADD CASP8 CFLAR) cytosolic CD95L induced	0.04256
Cancer	5799	Death induced signaling complex DISC (FAS FADD CASP8 CFLAR) membrane-associated CD95L	0.05529
Cancer	5800	Death-inducing signaling complex DISC (type I cells associated) stimulated	0.04256
Cancer	5808	DISC complex	0.04256
Cancer	5811	p53-BCL2 complex	0.02606
Cancer	5812	p53-BCL2 complex	0.02606
Cancer	5816	Apoptosome-procaspase 9 complex	0.02128
Cancer	5817	tBID-BCL2 complex	0.05213
Cancer	5818	BIM-BCL2 complex	0.05213
Cancer	5819	BIM-BCL2xL complex	0.05213
Cancer	5820	tBID-BCL2xL complex	0.05213

Cancer	5822	MCL1-NOXA complex	0.02606
Cancer	5823	MCL1-BAK1 complex	0.02606
Cancer	5877	MAP2K1-BRAF-RAF1-YWHAE-KSR1 complex	0.03297
Cancer	5849	HSP90-CDC37-LRRK2 complex	0.02128
Cancer	5859	FAS-FADD-CASP8-CASP10 complex	0.03686
Cancer	5861	FAS-FADD-CASP10 complex	0.02128
Cancer	5862	CAV1-VDAC1-ESR1 complex	0.04256
Cancer	5872	BRAF-MAP2K1-MAP2K2-YWHAE complex	0.01843
Cancer	5873	RAF1-MAP2K1-YWHAE complex	0.02128
Cancer	5919	BRAF-RAF1-14-3-3 complex	0.02457
Cancer	5920	KSR1-RAF1-MEK complex	0.01843
Cancer	5921	KSR1-BRAF-MEK complex	0.01843
Cancer	5922	RAF1-RAS complex EGF induced	0.05529
Cancer	5923	RAF1-BRAF complex RAS stimulated	0.05213
Cancer	5924	RAF1-CNK1 complex RAS stimulated	0.02606
Cancer	5925	BRAF-CNK1 complex not RAS stimulated	0.02606
Cancer	5928	CNK1-SRC-RAF1 complex	0.02128
Capillaries disease	1474	SMAD3/4-E2F4/5-p107-DP1 complex	0.1291
Capillaries disease	1986	Endoglin homodimer complex	0.31623
Capillaries disease	2692	SMAD3-SMAD4-cJun-cFos complex	0.15811
Capillaries disease	2704	Ectodermin-SMAD4 complex	0.22361
Capillaries disease	2705	SMAD3-SMAD4-CTCF protein-DNA complex	0.18257
Capillaries disease	2706	SMAD3-SMAD4-SP1 complex	0.18257
Capillaries disease	2707	SMAD3-SMAD4-FOXO3-FOXG1 complex	0.15811
Capillaries disease	2708	SMAD3-SMAD4-cJUN complex	0.18257
Capillaries disease	2754	JUND-FOSB-SMAD3-SMAD4 complex	0.15811
Capillaries disease	2760	SMAD3-SMAD4-FOXO3 complex	0.18257
Capillaries disease	2761	SMAD3-SMAD4-FOXO1 complex	0.18257
Capillaries disease	2762	SMAD3-SMAD4-FOXO4 complex	0.18257
Capillaries disease	2798	MMP-2-claudin-1 complex	0.22361
Capillaries disease	2829	RSmad complex	0.1
Capillaries disease	2834	SMAD4-SMAD2-SMAD3 complex	0.18257
Capillaries disease	2972	ITGA9-ITGB1-VEGFA complex	0.18257
Capillaries disease	3038	SMAD2-SMAD4-FAST1 complex	0.18257
Capillaries disease	3197	SMAD4-SNO-SKI complex	0.18257
Capillaries disease	3200	SMAD4-SKI complex	0.22361
Capillaries disease	3206	SMAD4-SKI-NCOR complex	0.18257
Capillaries disease	3233	SMAD2-SMAD4-FAST1-TGIF complex TGF(beta) induced	0.15811
Capillaries disease	3234	SMAD2-SMAD4-FAST1-TGIF-HDAC1 complex TGF(beta) induced	0.14142
Capillaries disease	3739	SKI-SMAD2-SMAD4 pentameric complex	0.18257
Capillaries disease	3740	SKI-SMAD3-SMAD4 pentameric complex	0.18257
Capillaries disease	3753	CREBBP-SMAD2-SMAD4 pentameric complex	0.18257
Capillaries disease	3754	CREBBP-SMAD3-SMAD4 pentameric complex	0.18257
Capillaries disease	3959	SMAD3-SMAD4-cSKI TGF(beta)-dependent	0.18257
Capillaries disease	4062	NRP1-VEGFR2-VEGF(165) complex	0.18257
Capillaries disease	5696	VEGFA(165)-KDR-NRP1 complex	0.18257

Capillaries disease	5698	VEGFA(165)-VEGFR2-NRP1 complex	0.18257
Capillaries disease	5701	NRP1-VEGF(165/121) complex	0.22361
Carcinoma	103	RNA polymerase II holoenzyme complex	0.0345
Carcinoma	104	RNA polymerase II core complex	0.0488
Carcinoma	267	Checkpoint 9-1-1 complex	0.09759
Carcinoma	268	Checkpoint Rad complex	0.05976
Carcinoma	274	RAD17-RFC-9-1-1 checkpoint supercomplex	0.05976
Carcinoma	306	Ribosome cytoplasmic	0.01878
Carcinoma	308	60S ribosomal subunit cytoplasmic	0.02466
Carcinoma	351	Spliceosome	0.01414
Carcinoma	387	MCM complex	0.06901
Carcinoma	1045	Snurportin-CRM1-RanGTP complex	0.09759
Carcinoma	1116	CRM1-Survivin-AuroraB mitotic complex	0.09759
Carcinoma	1117	CRM1-Survivin mitotic complex	0.11952
Carcinoma	1176	CRM1-RAN-PHAX-CBC complex (cap binding	0.07559
Carcinoma	1452	MCM2-MCM6-MCM7 complex	0.09759
Carcinoma	1834	ITGAE-ITGB7-CDH1 complex	0.09759
Carcinoma	1990	PGRMC1-SCAP complex	0.11952
Carcinoma	2196	LIG1-9-1-1 complex	0.08452
Carcinoma	2197	FEN1-9-1-1 complex	0.08452
Carcinoma	2198	RAD9-RAD1-HUS1-POLB complex	0.08452
Carcinoma	2217	MDC1-MRN-ATM-FANCD2 complex	0.06901
Carcinoma	2218	MDC1-MRE11-RAD50-NBS1 complex	0.08452
Carcinoma	2318	ITGA6-ITGB4-Laminin10/12 complex	0.07559
Carcinoma	2685	RNA polymerase II (RNAPII)	0.041
Carcinoma	2686	BRCA1-core RNA polymerase II complex	0.04688
Carcinoma	2774	MDC1-H2AFX-TP53BP1 complex	0.09759
Carcinoma	2775	MDC1-p53BP1-SMC1 complex	0.09759
Carcinoma	2791	MCM4-MCM6-MCM7 complex	0.09759
Carcinoma	2792	MCM2-MCM4-MCM6-MCM7 complex	0.08452
Carcinoma	2808	RAD9-RAD1-HUS1-APE1 complex	0.08452
Carcinoma	2809	9-1-1 complex	0.09759
Carcinoma	2825	BRCA1-RNA polymerase II complex	0.03315
Carcinoma	3055	Nop56p-associated pre-rRNA complex	0.01657
Carcinoma	5153	CTFC-TAF1 complex	0.11952
Carcinoma	5176	MGC1-DNA-PKcs-Ku complex	0.08452
Carcinoma	5611	Emerin complex 24	0.04364
Carcinoma	5615	Emerin complex 52	0.03525
Cardiovascular disease	243	RalBP1-CDC2-CCNB1 complex	0.08805
Cardiovascular disease	247	RalBP1-CCNB1-AP2A-NUMB-EPN1 complex	0.06226
Cardiovascular disease	518	AKAP250-PKA-PDE4D complex	0.0682
Cardiovascular disease	541	IGF1-IGFBP3-ALS complex	0.08805
Cardiovascular disease	563	F1F0-ATP synthase (EC 3.6.3.14) mitochondrial	0.03812
Cardiovascular disease	873	SNARE complex (STX1A SNAP29)	0.10783
Cardiovascular disease	1088	PRNP-ApolopoproteinE3 complex	0.10783
Cardiovascular disease	2798	MMP-2-claudin-1 complex	0.10783
Cardiovascular disease	3162	TF-FVIIa-FXa-TFPI complex	0.07625

Cardiovascular disease	4062	NRP1-VEGFR2-VEGF(165) complex	0.08805
Cardiovascular disease	5772	ZO1-(beta)cadherin-(VE)cadherin-VEGFR2 complex	0.07625
Cardiovascular disease	5165	AP1G1-PACS1-FURIN complex	0.08805
Cardiovascular disease	5696	VEGFA(165)-KDR-NRP1 complex	0.08805
Cardiovascular disease	5698	VEGFA(165)-VEGFR2-NRP1 complex	0.08805
Celiac disease	92	CD28-transactivation complex	0.11625
Celiac disease	159	Condensin I-PARP-1-XRCC1 complex	0.06214
Celiac disease	1004	RC complex during S-phase of cell cycle	0.0456
Celiac disease	1005	RC complex during G2/M-phase of cell cycle	0.0456
Celiac disease	1062	BAR-BCL2-CASP8 complex	0.09492
Celiac disease	1193	Rap1 complex	0.06214
Celiac disease	1226	H2AX complex I	0.06214
Celiac disease	1707	IL2-IL2RA-IL2RB complex	0.09492
Celiac disease	1714	TICAM1-TICAM2-TLR4 complex	0.09492
Celiac disease	1728	CTCF-nucleophosmin-PARP-HIS-KPNA-LMNA-TOP complex	0.0548
Celiac disease	1729	TLE1 corepressor complex (MASH1 promoter-corepressor complex)	0.05199
Celiac disease	1774	MICA-KLRK1-HCST complex	0.09492
Celiac disease	2018	IL12A-IL12B complex	0.11625
Celiac disease	2019	IL12A-IL12B-IL12RB1 complex	0.09492
Celiac disease	2020	IL12B-IL12RB1-IL12RB2 complex	0.09492
Celiac disease	2021	IL12A-IL12B-IL12RB2 complex	0.09492
Celiac disease	2625	CDK8-MED6-PARP1 complex	0.09492
Celiac disease	3137	MASH1 promoter-coactivator complex	0.04957
Celiac disease	3142	CAMK2-delta-MASH1 promoter-coactivator complex	0.05812
Celiac disease	5179	NCOA6-DNA-PK-Ku-PARP1 complex	0.07352
Celiac disease	5235	WRN-Ku70-Ku80-PARP1 complex	0.0822
Celiac disease	5473	FAS-FADD-CASP8 complex	0.09492
Celiac disease	5526	CALM1-FKBP38-BCL2 complex	0.09492
Celiac disease	5548	IL-12 heterodimer complex	0.11625
Celiac disease	5549	IL-12 subunit p40 homodimer complex	0.1644
Celiac disease	5799	Death induced signaling complex DISC (FAS FADD CASP8 CFLAR) membrane-associated CD95L	0.0822
Celiac disease	5800	Death-inducing signaling complex DISC (type I cells associated) stimulated	0.09492
Celiac disease	5808	DISC complex	0.09492
Celiac disease	5811	p53-BCL2 complex	0.11625
Celiac disease	5817	tBID-BCL2 complex	0.11625
Celiac disease	5818	BIM-BCL2 complex	0.11625
Celiac disease	5823	MCL1-BAK1 complex	0.11625
Celiac disease	5859	FAS-FADD-CASP8-CASP10 complex	0.0822
Celiac disease	5861	FAS-FADD-CASP10 complex	0.09492
Central nervous system disease	143	APP-FE65-LRP complex	0.14434
Central nervous system disease	2711	Amyloid beta protein oligomer	0.25
Central nervous system disease	3092	APP-TOMM40 complex	0.17678
Central nervous system disease	3093	APP-TIMM23 complex	0.17678
Central nervous system disease	5830	DJ-1-SNCA complex high molecular weight complex	0.17678

Cerebellar disease	1232	REST-CoREST-mSIN3A complex	0.2582
Cerebellar disease	1617	G protein complex (CACNA1A GNB1 GNG2)	0.2582
Cerebellar disease	2456	MET-CIN85-SH3GL3-CBL complex HGF stimulated	0.22361
Cerebellar disease	2541	HGF-Met complex	0.31623
Cerebral palsy	541	IGF1-IGFBP3-ALS complex	0.40825
Cerebrovascular disorder	143	APP-FE65-LRP complex	0.12599
Cerebrovascular disorder	441	TFTC-type histone acetyl transferase complex	0.0658
Cerebrovascular disorder	1054	ESR1-RELA-BCL3-NCOA3 complex	0.10911
Cerebrovascular disorder	1088	PRNP-ApolopoproteinE3 complex	0.1543
Cerebrovascular disorder	1707	IL2-IL2RA-IL2RB complex	0.12599
Cerebrovascular disorder	1993	SLC1A2 homotrimer complex	0.21822
Cerebrovascular disorder	2124	IKK-alpha--ER-alpha-AIB1 complex	0.12599
Cerebrovascular disorder	2470	p130Cas-ER-alpha-cSrc-kinase- PI3-kinase p85-subunit complex	0.10911
Cerebrovascular disorder	2657	ESR1-CDK7-CCNH-MNAT1-MTA1-HDAC2 complex	0.08909
Cerebrovascular disorder	2670	Er-alpha-p53-hdm2 complex	0.12599
Cerebrovascular disorder	2699	ER-alpha-GRIP1-c-Jun complex	0.12599
Cerebrovascular disorder	2700	ER-alpha-c-Jun complex	0.1543
Cerebrovascular disorder	2711	Amyloid beta protein oligomer	0.21822
Cerebrovascular disorder	3092	APP-TOMM40 complex	0.1543
Cerebrovascular disorder	3093	APP-TIMM23 complex	0.1543
Cerebrovascular disorder	5862	CAV1-VDAC1-ESR1 complex	0.12599
Cervical cancer	91	FA complex (Fanconi anemia complex) cytoplasmic	0.05812
Cervical cancer	92	CD28-transactivation complex	0.0822
Cervical cancer	178	Respiratory chain complex I (holoenzyme)	0.01752
Cervical cancer	244	BRAFT complex	0.03224
Cervical cancer	245	FA core complex (Fanconi anemia core complex)	0.03875
Cervical cancer	305	40S ribosomal subunit cytoplasmic	0.01994
Cervical cancer	306	Ribosome cytoplasmic	0.01292
Cervical cancer	541	IGF1-IGFBP3-ALS complex	0.06712
Cervical cancer	1094	Frataxin complex	0.04394
Cervical cancer	1152	FA complex (Fanconi anemia complex)	0.0411
Cervical cancer	1332	Large Drosha complex	0.02599
Cervical cancer	1519	IL6ST-PRKCD-STAT3 complex	0.06712
Cervical cancer	1623	FA core complex 1 (Fanconi anemia core complex 1)	0.05199
Cervical cancer	1624	FA core complex (Fanconi anemia core complex)	0.03875
Cervical cancer	1625	FA core complex (Fanconi anemia core complex)	0.04394
Cervical cancer	1774	MICA-KLRK1-HCST complex	0.06712
Cervical cancer	2383	ITGA5-ITGB1-FN1-TGM2 complex	0.05812
Cervical cancer	2384	ITGA5-ITGB1-ADAM15 complex	0.06712
Cervical cancer	2385	ITGA5-ITGB4 complex	0.0822
Cervical cancer	2388	Itga5-Itgb1-Fn1-Sfrp2 complex	0.05812
Cervical cancer	2641	p300/CBP-PCAF-MyoD complex	0.05812
Cervical cancer	2650	DNMT3B-DNMT3L complex	0.0822
Cervical cancer	2651	DNMT3L-DNMT3A complex	0.0822
Cervical cancer	2709	MMP-9-TIMP-1-LRP complex	0.06712
Cervical cancer	2739	FA complex (Fanconi anemia complex)	0.03505

Cervical cancer	2755	17S U2 snRNP	0.02024
Cervical cancer	2850	ITGA5-ITGB1-FN-1-NOV complex	0.05812
Cervical cancer	2853	ITGA5-ITGB1-CAL4A3 complex	0.06712
Cervical cancer	2882	ITGA5-ITGB3-COL6A3 complex	0.06712
Cervical cancer	2914	Respiratory chain complex I (beta subunit)	0.02906
Cervical cancer	2936	Ecsit complex (ECSIT MT-CO2 GAPDH TRAF6 NDUFAF1)	0.05199
Cervical cancer	2939	Ecsit complex (ECSIT MT-CO2 NDUFA1 MT-ND1 TRAF6 NDUFAF1)	0.04746
Cervical cancer	2948	Respiratory chain complex I (incomplete intermediate) mitochondrial	0.03505
Cervical cancer	3055	Nop56p-associated pre-rRNA complex	0.0114
Cervical cancer	3056	Microprocessor complex	0.0822
Cervical cancer	3083	Nucleic and chromatin Fanconi complex	0.05199
Cervical cancer	3112	ITGA5-ITGB1-SPP1 complex	0.06712
Cervical cancer	3137	MASH1 promoter-coactivator complex	0.03505
Cervical cancer	3142	CAMK2-delta-MASH1 promoter-coactivator complex	0.0411
Cervical cancer	3229	Heterodimer complex (CDK9 IL6ST)	0.0822
Cervical cancer	5564	LMO4-gp130 complex	0.10398
Cervical cancer	5579	CNTF-CNTFR-gp130-LIFR complex	0.05812
Cervical cancer	5582	LIFR-LIF-gp130 complex	0.06712
Cervical cancer	5655	Ternary complex (LRRC7 CAMK2a ACTN4)	0.06712
Charcot-Marie-Tooth disease	1185	EGFR-containing signaling complex	0.11471
Charcot-Marie-Tooth disease	1728	CTCF-nucleophosmin-PARP-HIS-KPNA-LMNA-TOP complex	0.07647
Charcot-Marie-Tooth disease	2528	ERBB2-MEMO-SHC complex	0.13245
Charcot-Marie-Tooth disease	5608	Emerin architectural complex	0.09366
Charcot-Marie-Tooth disease	5611	Emerin complex 24	0.05923
Cholangiocarcinoma	1774	MICA-KLRK1-HCST complex	0.21822
Cholangiocarcinoma	5564	LMO4-gp130 complex	0.16903
Cholangitis	5473	FAS-FADD-CASP8 complex	0.28868
Cholangitis	5799	Death induced signaling complex DISC (FAS FADD CASP8 CFLAR) membrane-associated CD95L	0.25
Cholangitis	5800	Death-inducing signaling complex DISC (type I cells associated) stimulated	0.28868
Cholangitis	5808	DISC complex	0.28868
Cholangitis	5859	FAS-FADD-CASP8-CASP10 complex	0.25
Cholangitis	5861	FAS-FADD-CASP10 complex	0.28868
Cholelithiasis	441	TFTC-type histone acetyl transferase complex	0.0603
Cholelithiasis	1054	ESR1-RELA-BCL3-NCOA3 complex	0.1
Cholelithiasis	1088	PRNP-ApolopoproteinE3 complex	0.14142
Cholelithiasis	1091	SNX complex (SNX1a SNX2 SNX4 LEPR)	0.1
Cholelithiasis	1095	SNX complex (SNX1a SNX2 SNX4 EGFR)	0.1
Cholelithiasis	1185	EGFR-containing signaling complex	0.1
Cholelithiasis	1774	MICA-KLRK1-HCST complex	0.11547
Cholelithiasis	1992	LEPR homodimer complex	0.2
Cholelithiasis	2124	IKK-alpha--ER-alpha-AIB1 complex	0.11547
Cholelithiasis	2159	AR-AKT-APPL complex	0.11547

Cholelithiasis	2160	AOF2-AR complex	0.14142
Cholelithiasis	2369	ITGAV-ITGB3-EGFR complex	0.11547
Cholelithiasis	2453	Multiprotein complex (monoubiquitination)	0.1
Cholelithiasis	2454	CIN85-CBL-SH3GL2-EGFR complex EGF stimulated	0.1
Cholelithiasis	2470	p130Cas-ER-alpha-cSrc-kinase- PI3-kinase p85-subunit complex	0.1
Cholelithiasis	2542	EGFR-CBL-GRB2 complex	0.11547
Cholelithiasis	2657	ESR1-CDK7-CCNH-MNAT1-MTA1-HDAC2 complex	0.08165
Cholelithiasis	2670	Er-alpha-p53-hdm2 complex	0.11547
Cholelithiasis	2699	ER-alpha-GRIP1-c-Jun complex	0.11547
Cholelithiasis	2700	ER-alpha-c-Jun complex	0.14142
Cholelithiasis	3678	RIN1-STAM2-EGFR complex EGF stimulated	0.11547
Cholelithiasis	5171	SH3KBP1-CBLB-EGFR complex	0.11547
Cholelithiasis	5862	CAV1-VDAC1-ESR1 complex	0.11547
Cholestasis	2688	MT1-MMP-claudin-1 complex	0.19612
Cholestasis	2798	MMP-2-claudin-1 complex	0.19612
Cholestasis	2801	OCT4-SOX2 DNA-protein complex	0.19612
Cholestasis	2802	OCT1-SOX2 DNA-protein complex	0.19612
Cholestasis	2803	PAX6-SOX2 DNA-protein complex	0.19612
Cholestasis	3055	Nop56p-associated pre-rRNA complex	0.0272
Chondrosarcoma	2443	ITGA9-ITGB1-TNC complex	0.21822
Choriocarcinoma	1093	SNX complex (SNX1a SNX2 SNX4 INSR)	0.14434
Choriocarcinoma	2028	JAK2-IL12RB2 complex	0.20412
Choriocarcinoma	2577	Sam68-p85 P13K-IRS-1-IR signaling complex	0.14434
Choriocarcinoma	2695	ETS2-FOS-JUN complex	0.16667
Choriocarcinoma	2789	ETS2-ERG complex	0.20412
Choriocarcinoma	2790	ETS2-ETS1 complex	0.20412
Choriocarcinoma	5178	JAK2-PAFR-TYK2 complex	0.16667
Choriocarcinoma	5293	ETS2-SMARCA4-INI1 complex	0.16667
Chorioretinitis	5749	MRIT complex	0.40825
Chorioretinitis	5798	Death induced signaling complex II (FADD CASP8 CFLAR) cytosolic CD95L induced	0.40825
Chorioretinitis	5799	Death induced signaling complex DISC (FAS FADD CASP8 CFLAR) membrane-associated CD95L	0.35355
Chronic fatigue syndrome	1439	PTGS2 homodimer complex	0.35355
Chronic fatigue syndrome	1777	TGF-beta-receptor-SMAD7-SMURF2 complex	0.17678
Chronic fatigue syndrome	1783	TGF-beta receptor I-SMAD7-SMURF1 complex	0.20412
Chronic fatigue syndrome	2880	SCF subcomplex (WEE1 SKP2 BTRC)	0.20412
Chronic fatigue syndrome	2992	SMAD7-SMURF2 complex	0.25
Chronic fatigue syndrome	2996	SMAD7-SMURF1 complex	0.25
Chronic fatigue syndrome	2997	SMAD7-SMURF1-TGF-beta receptor complex	0.17678
Chronic obstructive airway disease	159	Condensin I-PARP-1-XRCC1 complex	0.04226
Chronic obstructive airway disease	518	AKAP250-PKA-PDE4D complex	0.05
Chronic obstructive airway disease	668	BKCA-beta2AR-AKAP79 signaling complex	0.06455
Chronic obstructive airway disease	672	BKCA-beta2AR complex	0.07906

Chronic obstructive airway disease	687	CFTR-NHERF-beta(2)AR signaling complex	0.06455
Chronic obstructive airway disease	725	P2X7 receptor signalling complex	0.03227
Chronic obstructive airway disease	1004	RC complex during S-phase of cell cycle	0.03101
Chronic obstructive airway disease	1005	RC complex during G2/M-phase of cell cycle	0.03101
Chronic obstructive airway disease	1067	CD8A-LCK complex	0.07906
Chronic obstructive airway disease	1193	Rap1 complex	0.04226
Chronic obstructive airway disease	1226	H2AX complex I	0.04226
Chronic obstructive airway disease	1714	TICAM1-TICAM2-TLR4 complex	0.06455
Chronic obstructive airway disease	1728	CTCF-nucleophosmin-PARP-HIS-KPNA-LMNA-TOP complex	0.03727
Chronic obstructive airway disease	1729	TLE1 corepressor complex (MASH1 promoter-corepressor complex)	0.03536
Chronic obstructive airway disease	2153	ITGAM-ITGB2-CD11 complex	0.1291
Chronic obstructive airway disease	2342	ITGAV-ITGB8-MMP14-TGFB1 complex	0.0559
Chronic obstructive airway disease	2625	CDK8-MED6-PARP1 complex	0.06455
Chronic obstructive airway disease	2688	MT1-MMP-claudin-1 complex	0.07906
Chronic obstructive airway disease	2709	MMP-9-TIMP-1-LRP complex	0.1291
Chronic obstructive airway disease	2972	ITGA9-ITGB1-VEGFA complex	0.06455
Chronic obstructive airway disease	3137	MASH1 promoter-coactivator complex	0.03371
Chronic obstructive airway disease	3142	CAMK2-delta-MASH1 promoter-coactivator complex	0.03953
Chronic obstructive airway disease	3830	ADRB2 homodimer complex	0.1118
Chronic obstructive airway disease	4062	NRP1-VEGFR2-VEGF(165) complex	0.1291
Chronic obstructive airway disease	5772	ZO1-(beta)cadherin-(VE)cadherin-VEGFR2 complex	0.0559
Chronic obstructive airway disease	5179	NCOA6-DNA-PK-Ku-PARP1 complex	0.05
Chronic obstructive airway disease	5235	WRN-Ku70-Ku80-PARP1 complex	0.0559
Chronic obstructive airway disease	5446	EPO-EPOR complex	0.07906
Chronic obstructive airway disease	5696	VEGFA(165)-KDR-NRP1 complex	0.1291
Chronic obstructive airway disease	5698	VEGFA(165)-VEGFR2-NRP1 complex	0.1291

Chronic obstructive airway disease	5701	NRP1-VEGF(165/121) complex	0.07906
Chronic obstructive airway disease	5830	DJ-1-SNCA complex high molecular weight complex	0.07906
Chronic obstructive airway disease	5837	PPD complex	0.06455
Chronic rejection of renal transplant	310	Cell cycle kinase complex CDC2	0.10206
Chronic rejection of renal transplant	311	Cell cycle kinase complex CDK2	0.125
Chronic rejection of renal transplant	312	Cell cycle kinase complex CDK4	0.125
Chronic rejection of renal transplant	313	Cell cycle kinase complex CDK5	0.1118
Chronic rejection of renal transplant	314	PCNA-p21 complex	0.17678
Chronic rejection of renal transplant	1634	CyclinD1-CDK4-p21 complex	0.14434
Chronic rejection of renal transplant	2230	PCNA complex	0.09449
Chronic rejection of renal transplant	2254	CTGF/Hcs24-actin complex	0.14434
Chronic rejection of renal transplant	2798	MMP-2-claudin-1 complex	0.17678
Chronic rejection of renal transplant	5101	CyclinD3-CDK4-CDK6-p21 complex	0.125
Chronic rejection of renal transplant	5199	Kinase maturation complex 1	0.0625
Chronic rejection of renal transplant	5622	HSP90-CIP1-FKBPL complex	0.14434
Chronic simple glaucoma	310	Cell cycle kinase complex CDC2	0.08704
Chronic simple glaucoma	311	Cell cycle kinase complex CDK2	0.1066
Chronic simple glaucoma	312	Cell cycle kinase complex CDK4	0.1066
Chronic simple glaucoma	313	Cell cycle kinase complex CDK5	0.09535
Chronic simple glaucoma	314	PCNA-p21 complex	0.15076
Chronic simple glaucoma	1088	PRNP-ApolopoproteinE3 complex	0.15076
Chronic simple glaucoma	1634	CyclinD1-CDK4-p21 complex	0.12309
Chronic simple glaucoma	2230	PCNA complex	0.08058
Chronic simple glaucoma	2241	HD-RAB8A-OPTN complex	0.12309
Chronic simple glaucoma	5101	CyclinD3-CDK4-CDK6-p21 complex	0.1066
Chronic simple glaucoma	5212	Kinase maturation complex 2	0.07538
Chronic simple glaucoma	5286	TNF-alpha/NF-kappa B signaling complex 10	0.06742
Chronic simple glaucoma	5446	EPO-EPOR complex	0.15076
Chronic simple glaucoma	5473	FAS-FADD-CASP8 complex	0.12309
Chronic simple glaucoma	5622	HSP90-CIP1-FKBPL complex	0.12309
Chronic simple glaucoma	5799	Death induced signaling complex DISC (FAS FADD CASP8 CFLAR) membrane-associated CD95L	0.1066
Chronic simple glaucoma	5800	Death-inducing signaling complex DISC (type I cells associated) stimulated	0.12309
Chronic simple glaucoma	5808	DISC complex	0.12309
Chronic simple glaucoma	5859	FAS-FADD-CASP8-CASP10 complex	0.1066

Chronic simple glaucoma	5861	FAS-FADD-CASP10 complex	0.12309
Cirrhosis	98	p300-MDM2-p53 protein complex	0.09759
Cirrhosis	220	ARF-Mule complex	0.09759
Cirrhosis	541	IGF1-IGFBP3-ALS complex	0.19518
Cirrhosis	1069	FIF-FGR2 complex	0.11952
Cirrhosis	1088	PRNP-ApolopoproteinE3 complex	0.11952
Cirrhosis	1642	p16-cyclin D2-CDK4 complex	0.09759
Cirrhosis	2670	Er-alpha-p53-hdm2 complex	0.09759
Cirrhosis	3162	TF-FVIIa-FXa-TFPI complex	0.08452
Cirrhosis	3172	NUMB-TP53-MDM2 complex	0.09759
Cirrhosis	5331	YY1-MDM2-p53 complex	0.09759
Clear cell carcinoma	443	BP-SMAD complex	0.13363
Clear cell carcinoma	1335	SNW1 complex	0.08909
Cleft palate	684	PAX9-MSX1 complex	0.18898
Cleft palate	1970	BMP4-TWSG1 complex	0.18898
Cleft palate	1972	BMP4-BGN complex	0.18898
Cleft palate	3043	BMP2-BRIA complex	0.1543
Cockayne syndrome	103	RNA polymerase II holoenzyme complex	0.08333
Cockayne syndrome	107	TFIIH transcription factor complex	0.13608
Cockayne syndrome	298	VEGF transcriptional complex	0.16667
Cockayne syndrome	727	CSA complex	0.11323
Cockayne syndrome	728	CSA-POLIIa complex	0.10911
Cockayne syndrome	1009	TFIIH transcription factor complex	0.1291
Cockayne syndrome	1029	TFIIH transcription factor complex	0.1291
Cockayne syndrome	1030	CAK-ERCC2 complex	0.20412
Cockayne syndrome	1728	CTCF-nucleophosmin-PARP-HIS-KPNA-LMNA-TOP complex	0.13608
Cockayne syndrome	2660	ERCC2/CAK complex	0.20412
Cockayne syndrome	2808	RAD9-RAD1-HUS1-APE1 complex	0.20412
Cockayne syndrome	2825	BRCA1-RNA polymerase II complex	0.08006
Cockayne syndrome	5495	TFIIH transcription factor complex (ERCC2 ERCC3 GTF2H1 CDK7 CCNH GTF2H2)	0.16667
Cockayne syndrome	5608	Emerin architectural complex	0.16667
Cockayne syndrome	5611	Emerin complex 24	0.10541
Colon cancer	4	Multisubunit ACTR coactivator complex	0.02967
Colon cancer	27	Arp2/3 protein complex	0.02243
Colon cancer	63	Mitotic 14S cohesin 1 complex	0.02967
Colon cancer	64	Mitotic 14S cohesin 2 complex	0.02967
Colon cancer	71	MRN complex (MRE11-RAD50-NBS1 complex)	0.03426
Colon cancer	72	R/M complex (RAD50-MRE11 complex)	0.04196
Colon cancer	73	MRN complex (MRE11-RAD50-NBN complex)	0.03426
Colon cancer	93	Anaphase-promoting complex	0.04196
Colon cancer	96	Anaphase-promoting complex	0.03956
Colon cancer	98	p300-MDM2-p53 protein complex	0.03426
Colon cancer	103	RNA polymerase II holoenzyme complex	0.01211
Colon cancer	104	RNA polymerase II core complex	0.01713
Colon cancer	115	Polycomb repressive complex 1 (PRC1 hPRC-H)	0.01713
Colon cancer	116	Polycomb repressive complex 1 (PRC1 hPRC-H)	0.01646

Colon cancer	120	Lymphotoxin beta receptor complex	0.03426
Colon cancer	163	Cohesin-SA2 complex	0.02967
Colon cancer	164	Cohesin-SA1 complex	0.02967
Colon cancer	202	BRCA1-RAD50-MRE11-NBS1 complex	0.02967
Colon cancer	244	BRAFT complex	0.01646
Colon cancer	246	BLM complex III	0.02967
Colon cancer	280	HMGB1-HMGB2-HSC70-ERP60-GAPDH complex	0.02654
Colon cancer	282	SNF2h-cohesin-NuRD complex	0.01483
Colon cancer	297	PCNA-DNA polymerase delta complex	0.02654
Colon cancer	298	VEGF transcriptional complex	0.02423
Colon cancer	299	IRF3-CBP complex	0.02967
Colon cancer	306	Ribosome cytoplasmic	0.00659
Colon cancer	308	60S ribosomal subunit cytoplasmic	0.00866
Colon cancer	351	Spliceosome	0.00992
Colon cancer	375	MSH2-MSH3 complex	0.04196
Colon cancer	378	MutS-beta complex	0.04196
Colon cancer	426	Meprin A	0.04196
Colon cancer	432	N-NOS-CHIP-HSP70-1 complex	0.03426
Colon cancer	433	BASC complex (BRCA1-associated genome surveillance complex)	0.03426
Colon cancer	434	BASC (Ab 80) complex (BRCA1-associated genome surveillance complex)	0.02098
Colon cancer	531	XPA-ERCC1-ERCC4 complex	0.03426
Colon cancer	541	IGF1-IGFBP3-ALS complex	0.03426
Colon cancer	552	IFNB1-IFNAR1-IFNAR2- complex	0.03426
Colon cancer	570	p300-CBP-p270-SWI/SNF complex	0.02243
Colon cancer	571	p300-CBP-p270 complex	0.03426
Colon cancer	619	MRE11A-RAD50-NBN-TRF2 complex	0.02967
Colon cancer	626	LSD1 complex	0.03292
Colon cancer	627	MRN-TRRAP complex (MRE11A-RAD50-NBN-TRRAP complex)	0.02967
Colon cancer	629	BLM-TRF2 complex	0.04196
Colon cancer	642	CtBP complex	0.01439
Colon cancer	643	CtBP core complex	0.01978
Colon cancer	655	HSF1-HSF2 complex	0.04196
Colon cancer	657	Retromer complex (SNX1 SNX2 VPS35 VPS29 VPS26A)	0.02654
Colon cancer	725	P2X7 receptor signalling complex	0.05139
Colon cancer	927	CENP-A nucleosome associated complex	0.02423
Colon cancer	929	CEN complex	0.02927
Colon cancer	959	LLGL1-PAR-6B-PRKCI complex	0.03426
Colon cancer	1004	RC complex during S-phase of cell cycle	0.01646
Colon cancer	1005	RC complex during G2/M-phase of cell cycle	0.01646
Colon cancer	1041	Alpha-dystrobrevin-ZO-1-actin complex	0.02967
Colon cancer	1058	SNX complex (SNX1 SNX6)	0.04196
Colon cancer	1060	Retromer complex (SNX1 SNX2 VPS35 VPS29	0.02654
Colon cancer	1070	SNX complex (SNX1a SNX2 SNX4)	0.03426

Colon cancer	1085	DNA repair complex NEIL2-PNK-Pol(beta)-LigIII(alpha)-XRCC1	0.02654
Colon cancer	1091	SNX complex (SNX1a SNX2 SNX4 LEPR)	0.05934
Colon cancer	1093	SNX complex (SNX1a SNX2 SNX4 INSR)	0.05934
Colon cancer	1094	Frataxin complex	0.02243
Colon cancer	1095	SNX complex (SNX1a SNX2 SNX4 EGFR)	0.02967
Colon cancer	1096	SNX complex (SNX1 1a 2 4 PDGF receptor)	0.02967
Colon cancer	1098	DNA synthesome complex (13 subunits)	0.01586
Colon cancer	1099	DNA synthesome complex (17 subunits)	0.01399
Colon cancer	1104	SNX complex (SNX1a SNX2 SNX4 TFRC)	0.02967
Colon cancer	1107	DNA synthesome core complex	0.01876
Colon cancer	1108	DNA synthesome complex (15 subunits)	0.01532
Colon cancer	1141	CF IIAm complex (Cleavage factor IIAm complex)	0.02967
Colon cancer	1143	SMN complex	0.01483
Colon cancer	1148	snRNP-free U1A (SF-A) complex	0.02967
Colon cancer	1158	p33ING1b-p300 complex	0.04196
Colon cancer	1160	ING1-p300-PCNA complex	0.03426
Colon cancer	1165	RNF20-RNF40-UbE2E1 complex	0.03426
Colon cancer	1178	BCOR complex	0.02098
Colon cancer	1179	CENP-A NAC-CAD complex	0.01646
Colon cancer	1181	C complex spliceosome	0.01327
Colon cancer	1182	CDC5L core complex	0.02423
Colon cancer	1183	CDC5L complex	0.01083
Colon cancer	1189	DNA double-strand break end-joining complex	0.02243
Colon cancer	1193	Rap1 complex	0.02243
Colon cancer	1218	BLM-TRF2 complex	0.04196
Colon cancer	1219	Tankyrin 1-tankyrin 2-TRF1 complex	0.03426
Colon cancer	1223	H2AX complex isolated from cells without IR exposure	0.01646
Colon cancer	1226	H2AX complex I	0.02243
Colon cancer	1227	H2AX complex II	0.01876
Colon cancer	1287	HNRPF-HNRPH1 complex	0.04196
Colon cancer	1288	DCS complex (PTBP1 PTBP2 HNRPH1 HNRPF)	0.02967
Colon cancer	1308	PABPC1-HSPA8-HNRPD-EIF4G1 complex	0.05307
Colon cancer	1332	Large Drosha complex	0.02654
Colon cancer	1471	pRb2/p130-multimolecular complex (RB2 E2F5 HDAC1 SUV39H1 P300)	0.02654
Colon cancer	1474	SMAD3/4-E2F4/5-p107-DP1 complex	0.02423
Colon cancer	1514	IL4-IL4R complex	0.04196
Colon cancer	1515	IL4-IL4R-IL2RG complex	0.03426
Colon cancer	1521	p300-SMAD1-STAT3 complex	0.03426
Colon cancer	1661	E2F4-p107-cyclinA complex	0.03426
Colon cancer	1714	TICAM1-TICAM2-TLR4 complex	0.03426
Colon cancer	1728	CTCF-nucleophosmin-PARP-HIS-KPNA-LMNA-TOP complex	0.01978
Colon cancer	1751	SMN complex	0.01876
Colon cancer	1831	PIAS3-SMAD3-P300 complex	0.03426
Colon cancer	1856	CDCA5-PDS5A-RAD21-SMC1A-PDS5B-SMC3	0.02423
Colon cancer	1992	LEPR homodimer complex	0.05934

Colon cancer	2001	NOD1 homodimer complex	0.05934
Colon cancer	2129	DNAJB2-HSPA8-PSMA3 complex	0.03426
Colon cancer	2145	HSF1-YWHAE complex	0.04196
Colon cancer	2153	ITGAM-ITGB2-CD11 complex	0.03426
Colon cancer	2217	MDC1-MRN-ATM-FANCD2 complex	0.02423
Colon cancer	2218	MDC1-MRE11-RAD50-NBS1 complex	0.02967
Colon cancer	2222	BLM complex II	0.02423
Colon cancer	2223	BLM-TOP3A complex	0.04196
Colon cancer	2224	MSH2/6-BLM-p53-RAD51 complex	0.02654
Colon cancer	2228	BLM-RAD51L3-XRCC2 complex	0.03426
Colon cancer	2255	Cofilin-actin-CAP1 complex	0.03426
Colon cancer	2300	Profilin 2 complex	0.01978
Colon cancer	2351	ITGB6-FYN-FN1 complex	0.03426
Colon cancer	2352	ITGAV-ITGB6-SPP1 complex	0.03426
Colon cancer	2353	ITGAV-ITGB6-TGFB3 complex	0.03426
Colon cancer	2354	ITGAV-ITGB6 complex	0.04196
Colon cancer	2383	ITGA5-ITGB1-FN1-TGM2 complex	0.02967
Colon cancer	2384	ITGA5-ITGB1-ADAM15 complex	0.03426
Colon cancer	2385	ITGA5-ITGB4 complex	0.04196
Colon cancer	2388	Itga5-Itgb1-Fn1-Sfrp2 complex	0.02967
Colon cancer	2429	ITGA2-ITGB1-CD47 complex	0.03426
Colon cancer	2430	ITGA2-ITGB1-CHAD complex	0.03426
Colon cancer	2431	ITGA2-ITGB1-COL6A3 complex	0.03426
Colon cancer	2432	ITGA2-ITGB1 complex	0.04196
Colon cancer	2443	ITGA9-ITGB1-TNC complex	0.03426
Colon cancer	2471	SRC-PRKCD-CDCP1 complex	0.03426
Colon cancer	2528	ERBB2-MEMO-SHC complex	0.03426
Colon cancer	2529	LAT-PLC-gamma-1-p85-GRB2-CBL-VAV-SLP-76 signaling complex C305 activated	0.02243
Colon cancer	2535	SLP-76-Cbl-Grb2-Shc complex Fc receptor gamma-R1 stimulated	0.02967
Colon cancer	2547	PLC-gamma-1-SLP-76-SOS1-LAT complex	0.02967
Colon cancer	2551	PDGFRA-PLC-gamma-1-PI3K-SHP-2 complex PDGF stimulated	0.02967
Colon cancer	2577	Sam68-p85 P13K-IRS-1-IR signaling complex	0.02967
Colon cancer	2638	HES1 promoter corepressor complex	0.02423
Colon cancer	2639	HES1 promoter-Notch enhancer complex	0.01646
Colon cancer	2641	p300/CBP-PCAF-MyoD complex	0.05934
Colon cancer	2642	SMAD1-P300 complex	0.04196
Colon cancer	2685	RNA polymerase II (RNAPII)	0.01439
Colon cancer	2686	BRCA1-core RNA polymerase II complex	0.01646
Colon cancer	2693	NFAT-JUN-FOS DNA-protein complex	0.03426
Colon cancer	2709	MMP-9-TIMP-1-LRP complex	0.03426
Colon cancer	2721	HCF-1 complex	0.01361
Colon cancer	2739	FA complex (Fanconi anemia complex)	0.01789
Colon cancer	2755	17S U2 snRNP	0.01033
Colon cancer	2766	TERF2-RAP1 complex	0.02243
Colon cancer	2767	RAD50-MRE11-NBN-p200-p350 complex	0.03426

Colon cancer	2815	BRCA1-BARD1-BACH1-DNA damage complex II	0.02098
Colon cancer	2819	BRCA1-CtIP-CtBP complex	0.03426
Colon cancer	2825	BRCA1-RNA polymerase II complex	0.01164
Colon cancer	2837	Profilin 1 complex	0.02423
Colon cancer	2850	ITGA5-ITGB1-FN-1-NOV complex	0.02967
Colon cancer	2853	ITGA5-ITGB1-CAL4A3 complex	0.03426
Colon cancer	2882	ITGA5-ITGB3-COL6A3 complex	0.03426
Colon cancer	2895	SHC-GRB2 complex	0.04196
Colon cancer	2921	SHARP-CtBP complex	0.03426
Colon cancer	2922	LAT-PLC-gamma-1-p85-GRB2-SOS signaling complex C305 activated	0.02654
Colon cancer	2923	SHARP-CtBP1-CtIP complex	0.03426
Colon cancer	2931	SHARP-CtBP1-CtIP-RBP-Jkappa corepressor complex	0.02967
Colon cancer	2954	Smad1-Notch1-p300-Pcaf complex	0.02967
Colon cancer	2955	LCK-SLP76-PLC-gamma-1-LAT complex pervanadate-activated	0.02967
Colon cancer	2956	PLC-gamma-1-LAT-c-CBL complex OKT3 stimulated	0.03426
Colon cancer	2960	SLP-76-PLC-gamma-1-ITK complex alpha-TCR stimulated	0.03426
Colon cancer	2961	SLP-76-PLC-gamma-1-VAV complex alpha-TCR stimulated	0.03426
Colon cancer	2975	SMAD3-E2F4/5-p107-DP1 complex	0.02654
Colon cancer	3043	BMP2-BRIA complex	0.03426
Colon cancer	3055	Nop56p-associated pre-rRNA complex	0.00582
Colon cancer	3082	DGCR8 multiprotein complex	0.03578
Colon cancer	3096	ITGA6-ITGB4-SHC1-GRB2 complex	0.02967
Colon cancer	3112	ITGA5-ITGB1-SPP1 complex	0.03426
Colon cancer	3129	STAT6-p100-RHA complex	0.03426
Colon cancer	3149	NK-3-Groucho-HIPK2-SIN3A-RbpA48-HDAC1	0.01789
Colon cancer	3150	NK-3-Groucho complex	0.02243
Colon cancer	3151	Sulphiredoxin-peroxiredoxin complex	0.04196
Colon cancer	3154	Notch2(N-TM)-Notch2(N-EC)-Delta complex	0.02967
Colon cancer	3270	Delta1 homodimer complex	0.05934
Colon cancer	3271	Gamma-secretase-Delta1 complex	0.02654
Colon cancer	3297	SMN complex (GEMIN6 7 UNRIP) SMN-independent intermediate	0.03426
Colon cancer	5772	ZO1-(beta)cadherin-(VE)cadherin-VEGFR2 complex	0.02967
Colon cancer	5099	RB1(hypophosphorylated)-E2F4 complex	0.04196
Colon cancer	5117	pRb2/p130-multimolecular complex (DNMT1 E2F4 SuV39H1 HDAC1 RBL2)	0.02654
Colon cancer	5118	pRb2/p130-multimolecular complex (RB2 E2F4 HDAC1 SUV39H1 P300)	0.05307
Colon cancer	5159	E2F4-p107-cyclinE complex	0.03426
Colon cancer	5160	E2F4-p130 complex	0.04196
Colon cancer	5197	PTIP-DNA damage response complex	0.04845
Colon cancer	5217	Calreticulin oligomer complex	0.05934
Colon cancer	5233	TNF-alpha/NF-kappa B signaling complex 5	0.01187
Colon cancer	5241	SMC1-SMC3 complex	0.04196
Colon cancer	5260	TCF4-CTNNB1-SUMO1-EP300-HADAC6 complex	0.02654

Colon cancer	5261	TCF4-CTNNB1-EP300 complex	0.03426
Colon cancer	5317	LATS1-HTRA2-BIRC4 complex	0.03426
Colon cancer	5361	Cell division cycle complex (CDC27 CDC16	0.03426
Colon cancer	5375	EGR-EP300 complex	0.04196
Colon cancer	5383	TRIB3-DDIT3 complex	0.04196
Colon cancer	5423	HSP70-BAG5-PARK2 complex	0.02967
Colon cancer	5432	Sororin-cohesin complex	0.02423
Colon cancer	5564	LMO4-gp130 complex	0.02654
Colon cancer	5596	LINC complex quiescent cells	0.02243
Colon cancer	5608	Emerin architectural complex	0.02423
Colon cancer	5611	Emerin complex 24	0.01532
Colon cancer	5615	Emerin complex 52	0.01237
Colon cancer	5822	MCL1-NOXA complex	0.04196
Colon cancer	5843	AIF-CYPA-DNA complex	0.04196
Combined immunodeficiency	360	Artemis-DNA-PK complex	0.28868
Combined immunodeficiency	2028	JAK2-IL12RB2 complex	0.28868
Combined immunodeficiency	5178	JAK2-PAFR-TYK2 complex	0.2357
Common cold	115	Polycomb repressive complex 1 (PRC1 hPRC-H)	0.07715
Common cold	116	Polycomb repressive complex 1 (PRC1 hPRC-H)	0.07412
Common cold	159	Condensin I-PARP-1-XRCC1 complex	0.10102
Common cold	432	N-NOS-CHIP-HSP70-1 complex	0.1543
Common cold	626	LSD1 complex	0.07412
Common cold	725	P2X7 receptor signalling complex	0.07715
Common cold	1004	RC complex during S-phase of cell cycle	0.07412
Common cold	1005	RC complex during G2/M-phase of cell cycle	0.07412
Common cold	1193	Rap1 complex	0.10102
Common cold	1226	H2AX complex I	0.10102
Common cold	1308	PABPC1-HSPA8-HNRPD-EIF4G1 complex	0.11952
Common cold	1728	CTCF-nucleophosmin-PARP-HIS-KPNA-LMNA-TOP complex	0.08909
Common cold	1729	TLE1 corepressor complex (MASH1 promoter-corepressor complex)	0.08452
Common cold	2625	CDK8-MED6-PARP1 complex	0.1543
Common cold	3137	MASH1 promoter-coactivator complex	0.08058
Common cold	3142	CAMK2-delta-MASH1 promoter-coactivator complex	0.09449
Common cold	5179	NCOA6-DNA-PK-Ku-PARP1 complex	0.11952
Common cold	5235	WRN-Ku70-Ku80-PARP1 complex	0.13363
Common cold	5423	HSP70-BAG5-PARK2 complex	0.13363
Common cold	5877	MAP2K1-BRAF-RAF1-YWHAE-KSR1 complex	0.11952
Common cold	5872	BRAF-MAP2K1-MAP2K2-YWHAE complex	0.13363
Common cold	5919	BRAF-RAF1-14-3-3 complex	0.08909
Common cold	5921	KSR1-BRAF-MEK complex	0.13363
Common cold	5923	RAF1-BRAF complex RAS stimulated	0.18898
Common cold	5925	BRAF-CNK1 complex not RAS stimulated	0.18898
Common variable immunodeficiency	1615	G protein complex (BTK GNG1 GNG2)	0.20412
Common variable immunodeficiency	2574	CD19-Vav-PI 3-kinase (p85 subunit) complex	0.20412

Common variable immunodeficiency	5691	TALL1 homo-oligomer complex	0.35355
Common wart	1095	SNX complex (SNX1a SNX2 SNX4 EGFR)	0.22361
Common wart	1185	EGFR-containing signaling complex	0.22361
Common wart	2369	ITGAV-ITGB3-EGFR complex	0.2582
Common wart	2453	Multiprotein complex (monoubiquitination)	0.22361
Common wart	2454	CIN85-CBL-SH3GL2-EGFR complex EGF stimulated	0.22361
Common wart	2542	EGFR-CBL-GRB2 complex	0.2582
Common wart	3678	RIN1-STAM2-EGFR complex EGF stimulated	0.2582
Common wart	5171	SH3KBP1-CBLB-EGFR complex	0.2582
Common wart	5317	LATS1-HTRA2-BIRC4 complex	0.5164
Communicable disease	1714	TICAM1-TICAM2-TLR4 complex	0.12599
Communicable disease	2018	IL12A-IL12B complex	0.1543
Communicable disease	2019	IL12A-IL12B-IL12RB1 complex	0.25198
Communicable disease	2020	IL12B-IL12RB1-IL12RB2 complex	0.25198
Communicable disease	2021	IL12A-IL12B-IL12RB2 complex	0.12599
Communicable disease	2026	IL12RB1-IL12RB2 complex	0.1543
Communicable disease	5384	RAG1-RAG2 tetramer complex	0.30861
Communicable disease	5548	IL-12 heterodimer complex	0.1543
Communicable disease	5549	IL-12 subunit p40 homodimer complex	0.21822
Conduct disorder	443	BP-SMAD complex	0.11785
Conduct disorder	681	(C-CFTR)2-NHERF-ezrin complex	0.19245
Conduct disorder	682	C-CFTR-NHERF(PDZ1 domain)-ezrin complex	0.19245
Conduct disorder	683	C-CFTR-NHERF(PDZ2 domain)-ezrin complex	0.19245
Conduct disorder	687	CFTR-NHERF-beta(2)AR signaling complex	0.19245
Congenital abnormality	4	Multisubunit ACTR coactivator complex	0.03769
Congenital abnormality	59	AP3 adapter complex	0.02849
Congenital abnormality	80	Ubiquitin E3 ligase (Skp1A Skp2 Cul1 Rbx1)	0.03769
Congenital abnormality	81	Ubiquitin E3 ligase (SKP1A FBXW8 CUL7 RBX1)	0.03769
Congenital abnormality	86	NUMAC complex (nucleosomal methylation activator complex)	0.02384
Congenital abnormality	98	p300-MDM2-p53 protein complex	0.04352
Congenital abnormality	149	PBAF complex (Polybromo- and BAF containing complex)	0.02176
Congenital abnormality	189	BAF complex	0.02091
Congenital abnormality	205	Ubiquitin E3 ligase (VHL TCEB1 TCEB2 CUL2	0.03371
Congenital abnormality	206	DNA ligase IV-XRCC4 complex	0.0533
Congenital abnormality	213	DNA ligase IV-XRCC1 complex	0.0533
Congenital abnormality	220	ARF-Mule complex	0.04352
Congenital abnormality	226	Ubiquitin E3 ligase (SKP1A SKP2 CUL1)	0.04352
Congenital abnormality	227	Ubiquitin E3 ligase (SKP1A BTRC CUL1)	0.08704
Congenital abnormality	244	BRAFT complex	0.02091
Congenital abnormality	246	BLM complex III	0.03769
Congenital abnormality	285	PCNA-MLH1-PMS1 complex	0.04352
Congenital abnormality	290	MSH2-MLH1-PMS2-PCNA DNA-repair initiation complex	0.03769
Congenital abnormality	291	MSH2-MLH1-PMS2 DNA-repair initiation complex	0.04352
Congenital abnormality	292	MutL-alpha complex	0.0533

Congenital abnormality	298	VEGF transcriptional complex	0.03077
Congenital abnormality	310	Cell cycle kinase complex CDC2	0.03077
Congenital abnormality	311	Cell cycle kinase complex CDK2	0.03769
Congenital abnormality	312	Cell cycle kinase complex CDK4	0.03769
Congenital abnormality	313	Cell cycle kinase complex CDK5	0.03371
Congenital abnormality	336	DNA ligase IV-XRCC4-AHNK complex	0.04352
Congenital abnormality	344	DNA ligase IV-XRCC4 complex (LX complex)	0.0533
Congenital abnormality	350	DNA ligase IV-XRCC4-PNK complex	0.04352
Congenital abnormality	353	DNA ligase IV-condensin complex	0.04352
Congenital abnormality	359	DNA ligase IV-XRCC4-XLF complex	0.08704
Congenital abnormality	369	MSH2-MSH6-PMS2-MLH1 complex	0.03769
Congenital abnormality	370	MSH2-MSH6-PMS1-MLH1 complex	0.03769
Congenital abnormality	376	PCNA-MutS-alpha-MutL-alpha-DNA complex	0.03371
Congenital abnormality	380	MutL-beta complex	0.0533
Congenital abnormality	390	Ubiquitin E3 ligase (SKP1A FBXW2 CUL1)	0.04352
Congenital abnormality	415	EXO1-MLH1-PMS2 complex	0.04352
Congenital abnormality	424	EXO1-MLH1-PCNA complex	0.04352
Congenital abnormality	433	BASC complex (BRCA1-associated genome surveillance complex)	0.02176
Congenital abnormality	434	BASC (Ab 80) complex (BRCA1-associated genome surveillance complex)	0.02665
Congenital abnormality	435	BASC (Ab 81) complex (BRCA1-associated genome surveillance complex)	0.03077
Congenital abnormality	443	BP-SMAD complex	0.02665
Congenital abnormality	521	Polycystin-1-E-cadherin-beta-catenin complex	0.04352
Congenital abnormality	522	Polycystin-1-E-cadherin-beta-catenin-Flotillin-2	0.03769
Congenital abnormality	566	BAF complex	0.02273
Congenital abnormality	570	p300-CBP-p270-SWI/SNF complex	0.02849
Congenital abnormality	571	p300-CBP-p270 complex	0.04352
Congenital abnormality	622	Ubiquitin E3 ligase (VHL TCEB1 TCEB2 CUL2)	0.03769
Congenital abnormality	642	CtBP complex	0.01828
Congenital abnormality	652	AP3-BLOC1 complex	0.01946
Congenital abnormality	681	(C-CFTR)2-NHERF-ezrin complex	0.04352
Congenital abnormality	682	C-CFTR-NHERF(PDZ1 domain)-ezrin complex	0.04352
Congenital abnormality	683	C-CFTR-NHERF(PDZ2 domain)-ezrin complex	0.04352
Congenital abnormality	684	PAX9-MSX1 complex	0.0533
Congenital abnormality	722	MRG15-PAM14-RB complex	0.04352
Congenital abnormality	723	MAF1 complex	0.04352
Congenital abnormality	725	P2X7 receptor signalling complex	0.02176
Congenital abnormality	729	Ubiquitin E3 ligase (FBXO31 SKP1A CUL1 RBX1)	0.03769
Congenital abnormality	759	Fgfr1-Kal1 complex	0.0533
Congenital abnormality	778	LARC complex (LCR-associated remodeling complex)	0.01729
Congenital abnormality	781	URI complex (Unconventional prefoldin RPB5 Interactor)	0.02513
Congenital abnormality	826	PAR-3-VE-cadherin-beta-catenin complex	0.04352
Congenital abnormality	912	Kal1-Fgfr1 complex	0.0533
Congenital abnormality	933	SCRIB-APC complex	0.0533
Congenital abnormality	1000	TorsinA-TorsinB complex	0.0533

Congenital abnormality	1041	Alpha-dystrobrevin-ZO-1-actin complex	0.03769
Congenital abnormality	1051	Ubiquitin E3 ligase (SKP1A SKP2 CUL1 RBX1)	0.03769
Congenital abnormality	1052	Ubiquitin E3 ligase (FBXW11 SKP1A CUL1 RBX1)	0.03769
Congenital abnormality	1062	BAR-BCL2-CASP8 complex	0.04352
Congenital abnormality	1071	PKD2-FPC complex	0.0533
Congenital abnormality	1087	BIRC5-AURKB-INCENP-EVI5 complex	0.03769
Congenital abnormality	1116	CRM1-Survivin-AuroraB mitotic complex	0.04352
Congenital abnormality	1117	CRM1-Survivin mitotic complex	0.0533
Congenital abnormality	1118	Chromosomal passenger complex CPC (INCENP CDCA8 BIRC5 AURKB)	0.03769
Congenital abnormality	1120	Chromosomal passenger complex CPC (INCENP CDCA8 BIRC5)	0.04352
Congenital abnormality	1158	p33ING1b-p300 complex	0.0533
Congenital abnormality	1160	ING1-p300-PCNA complex	0.04352
Congenital abnormality	1166	p400-associated complex	0.02849
Congenital abnormality	1178	BCOR complex	0.02665
Congenital abnormality	1179	CENP-A NAC-CAD complex	0.02091
Congenital abnormality	1189	DNA double-strand break end-joining complex	0.02849
Congenital abnormality	1215	Ubiquitin E3 ligase (FBXW7 CUL1 SKP1A RBX1)	0.03769
Congenital abnormality	1250	pRB-E2F-1 complex	0.0533
Congenital abnormality	1255	Ubiquitin E3 ligase (SIAH1 SIP SKP1A TBL1X)	0.03769
Congenital abnormality	1256	MLL-HCF complex	0.02849
Congenital abnormality	1257	ALL-1 supercomplex	0.01425
Congenital abnormality	1258	Ubiquitin E3 ligase (GLMN FBXW8 SKP1A RBX1)	0.07538
Congenital abnormality	1260	Neddylin ligase (FBXO11 SKP1 CUL1 RBX1)	0.03769
Congenital abnormality	1372	Rb-tal-1-E2A-Lmo2-Ldb1 complex	0.03371
Congenital abnormality	1401	MOF complex	0.02384
Congenital abnormality	1471	pRb2/p130-multimolecular complex (RB2 E2F5 HDAC1 SUV39H1 P300)	0.03371
Congenital abnormality	1488	DNMT1-RB1-HDAC1-E2F1 complex	0.03769
Congenital abnormality	1519	IL6ST-PRKCD-STAT3 complex	0.04352
Congenital abnormality	1521	p300-SMAD1-STAT3 complex	0.04352
Congenital abnormality	1617	G protein complex (CACNA1A GNB1 GNG2)	0.04352
Congenital abnormality	1633	CyclinD1-CDK4-CDK6 complex	0.04352
Congenital abnormality	1634	CyclinD1-CDK4-p21 complex	0.04352
Congenital abnormality	1642	p16-cyclin D2-CDK4 complex	0.04352
Congenital abnormality	1714	TICAM1-TICAM2-TLR4 complex	0.04352
Congenital abnormality	1729	TLE1 corepressor complex (MASH1 promoter-corepressor complex)	0.02384
Congenital abnormality	1816	JUN-TCF4-CTNNB1 complex	0.04352
Congenital abnormality	1831	PIAS3-SMAD3-P300 complex	0.04352
Congenital abnormality	1839	SDCBP-CTNNB1-CTNNA1-CDH1 complex	0.03769
Congenital abnormality	1844	APC-IQGAP1 complex	0.0533
Congenital abnormality	1845	APC-IQGAP1-CLIP-170 complex	0.04352
Congenital abnormality	1909	APC-DLG4 complex	0.0533
Congenital abnormality	1927	PKD2-PACS1 complex	0.0533
Congenital abnormality	1957	DAP12 signaling homodimer complex	0.07538
Congenital abnormality	1985	AIRE homodimer complex	0.07538

Congenital abnormality	2187	Ubiquitin E3 ligase (NFKBIA FBXW11 BTRC CUL1 SKP1A)	0.06742
Congenital abnormality	2188	Ubiquitin E3 ligase (CDC34 NEDD8 BTRC CUL1 SKP1A RBX1)	0.06742
Congenital abnormality	2189	Ubiquitin E3 ligase (SMAD3 BTRC CUL1 SKP1A RBX1)	0.06742
Congenital abnormality	2191	Ubiquitin E3 ligase (FBXO18 SKP1A CUL1 RBX1)	0.03769
Congenital abnormality	2192	Ubiquitin E3 ligase (NIPA SKP1A CUL1 RBX1)	0.03769
Congenital abnormality	2217	MDC1-MRN-ATM-FANCD2 complex	0.03077
Congenital abnormality	2254	CTGF/Hcs24-actin complex	0.08704
Congenital abnormality	2255	Cofilin-actin-CAP1 complex	0.04352
Congenital abnormality	2300	Profilin 2 complex	0.02513
Congenital abnormality	2318	ITGA6-ITGB4-Laminin10/12 complex	0.10113
Congenital abnormality	2319	ITGA6-ITGB4-Laminin10/12 complex	0.06742
Congenital abnormality	2322	ITGA6-ITGB4-LAMA5 complex	0.04352
Congenital abnormality	2489	NCR3-CD247 complex	0.0533
Congenital abnormality	2579	Chromosomal passenger complex CPC (INCENP BIRC5 AURKB)	0.04352
Congenital abnormality	2580	Survivin homodimer complex	0.07538
Congenital abnormality	2581	RasGAP-AURKA/AURKB-survivin complex	0.03769
Congenital abnormality	2582	Chromosomal passenger complex CPC (CDCA8 AURKB BIRC5)	0.04352
Congenital abnormality	2590	FOXO1-FHL2-SIRT1 complex	0.04352
Congenital abnormality	2635	BETA2-Cyclin D1 complex	0.0533
Congenital abnormality	2638	HES1 promoter corepressor complex	0.03077
Congenital abnormality	2639	HES1 promoter-Notch enhancer complex	0.02091
Congenital abnormality	2641	p300/CBP-PCAF-MyoD complex	0.03769
Congenital abnormality	2642	SMAD1-P300 complex	0.0533
Congenital abnormality	2715	Ubiquitin E3 ligase (CSN1 CSN8 HRT1 SKP1 SKP2 CUL1 CUL2 CUL3)	0.02665
Congenital abnormality	2724	Ubiquitin E3 ligase (NFKBIA BTRC CUL1 SKP1A)	0.07538
Congenital abnormality	2725	Ubiquitin E3 ligase (NFKBIA FBXW11 CUL1	0.03769
Congenital abnormality	2761	SMAD3-SMAD4-FOXO1 complex	0.04352
Congenital abnormality	2772	Ubiquitin E3 ligase (CRY1 SKP1A CUL1 FBXL3)	0.03769
Congenital abnormality	2773	Ubiquitin E3 ligase (CRY2 SKP1A CUL1 FBXL3)	0.03769
Congenital abnormality	2788	BRCA1 B complex	0.04352
Congenital abnormality	2801	OCT4-SOX2 DNA-protein complex	0.0533
Congenital abnormality	2802	OCT1-SOX2 DNA-protein complex	0.0533
Congenital abnormality	2803	PAX6-SOX2 DNA-protein complex	0.1066
Congenital abnormality	2811	BRCA1-cABL complex	0.0533
Congenital abnormality	2815	BRCA1-BARD1-BACH1-DNA damage complex II	0.02665
Congenital abnormality	2817	BRCA1-BARD1-BACH1-DNA damage complex I	0.06155
Congenital abnormality	2880	SCF subcomplex (WEE1 SKP2 BTRC)	0.04352
Congenital abnormality	2881	Ubiquitin E3 ligase (CUL1 RBX1 SKP1A)	0.04352
Congenital abnormality	2954	Smad1-Notch1-p300-Pcaf complex	0.03769
Congenital abnormality	2998	Axin-PP2A A-PP2A C-GSK3-beta-beta-catenin	0.03769
Congenital abnormality	3004	APC-Axin-1-beta-catenin complex	0.08704
Congenital abnormality	3008	60S APC containing complex	0.02849

Congenital abnormality	3011	APC-IQGAP1-Rac1 complex	0.04352
Congenital abnormality	3012	APC-IQGAP1-Cdc42 complex	0.04352
Congenital abnormality	3015	p27-cyclinE-Cdk2 - Ubiquitin E3 ligase (SKP1A SKP2 CUL1 CKS1B RBX1) complex	0.02665
Congenital abnormality	3036	Ubiquitin E3 ligase (SKP1A SKP2 CUL1 CKS1B RBX1)	0.03371
Congenital abnormality	3084	CCND1-CDK4 complex	0.0533
Congenital abnormality	3087	CCND1-CDK6 complex	0.0533
Congenital abnormality	3110	ITGAV-P2RY2-GNA12 complex	0.04352
Congenital abnormality	3138	POSH-AKT2 complex	0.0533
Congenital abnormality	3155	Bipartite complex (TFC4 CTNNB1)	0.0533
Congenital abnormality	3166	AXIN-APC-betaCatenin-GSK3B complex	0.07538
Congenital abnormality	3229	Heterodimer complex (CDK9 IL6ST)	0.0533
Congenital abnormality	3269	RB1-HDAC1-BRG1 complex	0.04352
Congenital abnormality	3848	TCL1(trimer)-AKT2 complex	0.0533
Congenital abnormality	3852	Rb-HDAC1 complex	0.0533
Congenital abnormality	4072	Heterotrimeric SKP1-CUL1-ROC1 complex	0.04352
Congenital abnormality	4095	Catulin (alpha) - catenin (beta) complex	0.0533
Congenital abnormality	4096	Catenin (alpha) - catenin (beta) complex	0.0533
Congenital abnormality	4200	DLP1-hFIS1 complex	0.0533
Congenital abnormality	5772	ZO1-(beta)cadherin-(VE)cadherin-VEGFR2 complex	0.03769
Congenital abnormality	5099	RB1(hypophosphorylated)-E2F4 complex	0.0533
Congenital abnormality	5118	pRb2/p130-multimolecular complex (RB2 E2F4 HDAC1 SUV39H1 P300)	0.03371
Congenital abnormality	5143	E2F1-Rb complex	0.0533
Congenital abnormality	5146	RB1-TFAP2A complex	0.0533
Congenital abnormality	5177	Polycystin-1 multiprotein complex (ACTN1 CDH1 SRC JUP VCL CTNNB1 PXN BCAR1 PKD1	0.02273
Congenital abnormality	5196	TNF-alpha/NF-kappa B signaling complex (CHUK BTRC NFKB2 PPP6C REL CUL1 IKBKE SAPS2 SAPS1 ANKRD28 RELA SKP1)	0.04352
Congenital abnormality	5233	TNF-alpha/NF-kappa B signaling complex 5	0.01508
Congenital abnormality	5260	TCF4-CTNNB1-SUMO1-EP300-HADAC6 complex	0.06742
Congenital abnormality	5261	TCF4-CTNNB1-EP300 complex	0.08704
Congenital abnormality	5262	TCF4-CTNNB1 complex	0.0533
Congenital abnormality	5264	TCF4-CTNNB1-CREBBP complex	0.04352
Congenital abnormality	5267	VHL-VDU1-TCEB1-TCEB2 complex	0.03769
Congenital abnormality	5270	VHL-TCEB1-TCEB2 complex	0.04352
Congenital abnormality	5273	VHL-TBP1-HIF1A complex	0.08704
Congenital abnormality	5276	HIF1A-OS9-EGLN1 complex	0.04352
Congenital abnormality	5277	HIF1A-OS9-EGLN3 complex	0.04352
Congenital abnormality	5281	Cell-cell junction complex (CDH1-CTNNB1)	0.0533
Congenital abnormality	5287	CDK4-CCND1 complex	0.0533
Congenital abnormality	5375	EGR-EP300 complex	0.1066
Congenital abnormality	5382	ARNT-HIF1A complex	0.0533
Congenital abnormality	5383	TRIB3-DDIT3 complex	0.0533
Congenital abnormality	5386	MLL1-WDR5 complex	0.01451
Congenital abnormality	5423	HSP70-BAG5-PARK2 complex	0.03769

Congenital abnormality	5446	EPO-EPOR complex	0.0533
Congenital abnormality	5526	CALM1-FKBP38-BCL2 complex	0.04352
Congenital abnormality	5564	LMO4-gp130 complex	0.03371
Congenital abnormality	5579	CNTF-CNTFR-gp130-LIFR complex	0.03769
Congenital abnormality	5582	LIFR-LIF-gp130 complex	0.04352
Congenital abnormality	5604	Emerin complex 1	0.02665
Congenital abnormality	5606	Emerin-actin-NMI-(alphaII)spectrin complex	0.03769
Congenital abnormality	5607	Emerin-actin-NMI complex	0.04352
Congenital abnormality	5608	Emerin architectural complex	0.03077
Congenital abnormality	5609	Emerin regulatory complex	0.02513
Congenital abnormality	5611	Emerin complex 24	0.01946
Congenital abnormality	5613	Emerin complex 25	0.01884
Congenital abnormality	5614	Emerin complex 32	0.01607
Congenital abnormality	5615	Emerin complex 52	0.01572
Congenital abnormality	5656	CEBPE-E2F1-RB1 complex	0.04352
Congenital abnormality	5663	TRIM27-RB1 complex	0.0533
Congenital abnormality	5691	TALL1 homo-oligomer complex	0.07538
Congenital abnormality	5736	Pre-initiation complex (PIC)	0.04352
Congenital abnormality	5811	p53-BCL2 complex	0.0533
Congenital abnormality	5817	tBID-BCL2 complex	0.0533
Congenital abnormality	5818	BIM-BCL2 complex	0.0533
Congenital abnormality	5830	DJ-1-SNCA complex high molecular weight complex	0.0533
Congenital abnormality	5832	PINK1-MIRO2-Milton complex	0.04352
Congenital abnormality	5837	PPD complex	0.08704
Congenital heart disease	2551	PDGFRA-PLC-gamma-1-PI3K-SHP-2 complex PDGF stimulated	0.16667
Congenital heart disease	3183	PDGFRA-SHP-2 complex PDGF stimulated	0.2357
Congenital heart disease	3186	GRB2-SHP-2 complex PDGF stimulated	0.2357
Congenital heart disease	3917	Ternary complex (GATA4 SRF MYOCD)	0.19245
Congenital heart disease	5564	LMO4-gp130 complex	0.14907
Connective tissue disease	142	CD147-gamma-secretase complex (APH-1a PS-1 PEN-2 NCT variant)	0.1118
Connective tissue disease	351	Spliceosome	0.02091
Connective tissue disease	1261	SRm160/300 complex	0.1118
Connective tissue disease	2398	ITGA3-ITGB1-BSG complex	0.14434
Connective tissue disease	5190	TIAM1-EFNB1-EPHA2 complex	0.14434
Connective tissue disease	5388	SERPINA1-ELA2 complex	0.17678
Connective tissue disease	5409	TIAM1-GRIN1 complex	0.17678
Copper metabolism disorder	5317	LATS1-HTRA2-BIRC4 complex	0.40825
Corneal disease	1087	BIRC5-AURKB-INCENP-EVI5 complex	0.18898
Corneal disease	1116	CRM1-Survivin-AuroraB mitotic complex	0.21822
Corneal disease	1117	CRM1-Survivin mitotic complex	0.26726
Corneal disease	1118	Chromosomal passenger complex CPC (INCENP CDCA8 BIRC5 AURKB)	0.18898
Corneal disease	1120	Chromosomal passenger complex CPC (INCENP CDCA8 BIRC5)	0.21822
Corneal disease	2237	SP1-MCAF2 complex	0.26726

Corneal disease	2579	Chromosomal passenger complex CPC (INCENP BIRC5 AURKB)	0.21822
Corneal disease	2580	Survivin homodimer complex	0.37796
Corneal disease	2581	RasGAP-AURKA/AURKB-survivin complex	0.18898
Corneal disease	2582	Chromosomal passenger complex CPC (CDCA8 AURKB BIRC5)	0.21822
Corneal disease	2679	p53-SP1 complex	0.26726
Corneal disease	2706	SMAD3-SMAD4-SP1 complex	0.21822
Corneal disease	2721	HCF-1 complex	0.08671
Corneal disease	3838	SP1-E2F2 complex	0.26726
Corneal disease	3839	SP1-E2F3 complex	0.26726
Craniosynostosis	1816	JUN-TCF4-CTNNB1 complex	0.2357
Craniosynostosis	2563	FGFR2-c-Cbl-Lyn-Fyn complex	0.20412
Craniosynostosis	2692	SMAD3-SMAD4-cJun-cFos complex	0.20412
Craniosynostosis	2693	NFAT-JUN-FOS DNA-protein complex	0.2357
Craniosynostosis	2694	ERG-JUN-FOS DNA-protein complex	0.2357
Craniosynostosis	2695	ETS2-FOS-JUN complex	0.2357
Craniosynostosis	2699	ER-alpha-GRIP1-c-Jun complex	0.2357
Craniosynostosis	2700	ER-alpha-c-Jun complex	0.28868
Craniosynostosis	2708	SMAD3-SMAD4-cJUN complex	0.2357
Cushing syndrome	2486	GIPC1-LHCGR complex	0.2132
Cystic fibrosis	668	BKCA-beta2AR-AKAP79 signaling complex	0.08248
Cystic fibrosis	672	BKCA-beta2AR complex	0.10102
Cystic fibrosis	681	(C-CFTR)2-NHERF-ezrin complex	0.16496
Cystic fibrosis	682	C-CFTR-NHERF(PDZ1 domain)-ezrin complex	0.16496
Cystic fibrosis	683	C-CFTR-NHERF(PDZ2 domain)-ezrin complex	0.16496
Cystic fibrosis	687	CFTR-NHERF-beta(2)AR signaling complex	0.24744
Cystic fibrosis	874	SNARE complex (VAMP3 VAMP4 VAMP8 STX6)	0.07143
Cystic fibrosis	875	SNARE complex (VAMP3 VAMP4 STX16)	0.08248
Cystic fibrosis	876	SNARE complex (VAMP3 STX6 VTI1A)	0.08248
Cystic fibrosis	1169	SNARE complex (STX4 VAMP8 VAMP3 SNAP23)	0.07143
Cystic fibrosis	1539	G protein complex (GNG2 GNB2L1 RAF1)	0.08248
Cystic fibrosis	1714	TICAM1-TICAM2-TLR4 complex	0.08248
Cystic fibrosis	1874	SNARE complex (SNAP25 VAMP3 VAMP2 NAPB STX13)	0.06389
Cystic fibrosis	2237	SP1-MCAF2 complex	0.10102
Cystic fibrosis	2679	p53-SP1 complex	0.10102
Cystic fibrosis	2706	SMAD3-SMAD4-SP1 complex	0.08248
Cystic fibrosis	2709	MMP-9-TIMP-1-LRP complex	0.08248
Cystic fibrosis	2721	HCF-1 complex	0.03277
Cystic fibrosis	2914	Respiratory chain complex I (beta subunit)	0.03571
Cystic fibrosis	3830	ADRB2 homodimer complex	0.14286
Cystic fibrosis	3838	SP1-E2F2 complex	0.10102
Cystic fibrosis	3839	SP1-E2F3 complex	0.10102
Cystic fibrosis	5165	AP1G1-PACS1-FURIN complex	0.08248
Cystic fibrosis	5383	TRIB3-DDIT3 complex	0.10102
Cytomegalovirus infection	1055	ZNF198-PML complex	0.18257
Cytomegalovirus infection	1095	SNX complex (SNX1a SNX2 SNX4 EGFR)	0.1291

Cytomegalovirus infection	1185	EGFR-containing signaling complex	0.1291
Cytomegalovirus infection	1308	PABPC1-HSPA8-HNRPD-EIF4G1 complex	0.11547
Cytomegalovirus infection	1714	TICAM1-TICAM2-TLR4 complex	0.14907
Cytomegalovirus infection	1827	PML-SMAD2/3-SARA complex	0.1291
Cytomegalovirus infection	2241	HD-RAB8A-OPTN complex	0.14907
Cytomegalovirus infection	2369	ITGAV-ITGB3-EGFR complex	0.14907
Cytomegalovirus infection	2453	Multiprotein complex (monoubiquitination)	0.1291
Cytomegalovirus infection	2454	CIN85-CBL-SH3GL2-EGFR complex EGF stimulated	0.1291
Cytomegalovirus infection	2542	EGFR-CBL-GRB2 complex	0.14907
Cytomegalovirus infection	3678	RIN1-STAM2-EGFR complex EGF stimulated	0.14907
Cytomegalovirus infection	5171	SH3KBP1-CBLB-EGFR complex	0.14907
Cytomegalovirus infection	5253	MNK1-eIF4F complex	0.11547
Deafness	149	PBAF complex (Polybromo- and BAF containing complex)	0.04167
Deafness	189	BAF complex	0.04003
Deafness	443	BP-SMAD complex	0.05103
Deafness	486	WIP-WASp-actin-myosin-IIa complex	0.05893
Deafness	520	KCNQ1 macromolecular complex	0.04564
Deafness	563	F1F0-ATP synthase (EC 3.6.3.14) mitochondrial	0.03608
Deafness	1041	Alpha-dystrobrevin-ZO-1-actin complex	0.07217
Deafness	2254	CTGF/Hcs24-actin complex	0.08333
Deafness	2551	PDGFRA-PLC-gamma-1-PI3K-SHP-2 complex PDGF stimulated	0.07217
Deafness	2837	Profilin 1 complex	0.05893
Deafness	2914	Respiratory chain complex I (beta subunit)	0.03608
Deafness	3183	PDGFRA-SHP-2 complex PDGF stimulated	0.10206
Deafness	3186	GRB2-SHP-2 complex PDGF stimulated	0.10206
Deafness	5446	EPO-EPOR complex	0.10206
Deafness	5564	LMO4-gp130 complex	0.06455
Deafness	5604	Emerin complex 1	0.05103
Degenerative disc disease	2798	MMP-2-claudin-1 complex	0.28868
Demyelinating disease	1810	ITGA4-PXN-GIT1 complex	0.2582
Demyelinating disease	2417	ITGA4-ITGB1-EMILIN1 complex	0.2582
Demyelinating disease	2418	ITGA4-ITGB1 complex	0.31623
Demyelinating disease	2419	ITGA4-ITGB1-CD81 complex	0.2582
Demyelinating disease	2420	ITGA4-ITGB1-CD53 complex	0.2582
Demyelinating disease	2421	ITGA4-ITGB1-VCAM1 complex	0.2582
Demyelinating disease	2422	ITGA4-ITGB1-JAM2 complex	0.2582
Demyelinating disease	2423	ITGA4-ITGB1-CD47 complex	0.2582
Demyelinating disease	2424	ITGA4-ITGB1-CD63 complex	0.2582
Demyelinating disease	2425	ITGA4-ITGB1-PXN complex	0.2582
Demyelinating disease	2426	ITGA4-ITGB1-THBS1 complex	0.2582
Demyelinating disease	2428	ITGA4-ITGB1-THBS2 complex	0.2582
Dental plaque	143	APP-FE65-LRP complex	0.07332
Dental plaque	441	TFTC-type histone acetyl transferase complex	0.03829
Dental plaque	1041	Alpha-dystrobrevin-ZO-1-actin complex	0.0635
Dental plaque	1054	ESR1-RELA-BCL3-NCOA3 complex	0.0635
Dental plaque	1062	BAR-BCL2-CASP8 complex	0.07332

Dental plaque	1088	PRNP-ApolopoproteinE3 complex	0.0898
Dental plaque	1094	Frataxin complex	0.048
Dental plaque	1141	CF IIAm complex (Cleavage factor IIAm complex)	0.03175
Dental plaque	1306	PIN1-AUF1 complex	0.0898
Dental plaque	1439	PTGS2 homodimer complex	0.127
Dental plaque	2124	IKK-alpha--ER-alpha-AIB1 complex	0.07332
Dental plaque	2347	ITGAV-ITGB5-SPP1 complex	0.07332
Dental plaque	2352	ITGAV-ITGB6-SPP1 complex	0.07332
Dental plaque	2358	ITGAV-ITGB3-SPP1 complex	0.07332
Dental plaque	2470	p130Cas-ER-alpha-cSrc-kinase- PI3-kinase p85-subunit complex	0.0635
Dental plaque	2657	ESR1-CDK7-CCNH-MNAT1-MTA1-HDAC2 complex	0.05185
Dental plaque	2670	Er-alpha-p53-hdm2 complex	0.07332
Dental plaque	2699	ER-alpha-GRIP1-c-Jun complex	0.07332
Dental plaque	2700	ER-alpha-c-Jun complex	0.0898
Dental plaque	2709	MMP-9-TIMP-1-LRP complex	0.14665
Dental plaque	2711	Amyloid beta protein oligomer	0.127
Dental plaque	2755	17S U2 snRNP	0.02211
Dental plaque	2885	ITGAV-ITGB1-SPP1 complex	0.07332
Dental plaque	3092	APP-TOMM40 complex	0.0898
Dental plaque	3093	APP-TIMM23 complex	0.0898
Dental plaque	3111	ITGA9-ITGB1-SPP1 complex	0.07332
Dental plaque	3112	ITGA5-ITGB1-SPP1 complex	0.07332
Dental plaque	3162	TF-FVIIa-FXa-TFPI complex	0.0635
Dental plaque	3231	Eps15-stonin2 complex	0.0898
Dental plaque	5772	ZO1-(beta)cadherin-(VE)cadherin-VEGFR2 complex	0.0635
Dental plaque	5211	RAF1-PPP2-PIN1 complex	0.0568
Dental plaque	5375	EGR-EP300 complex	0.0898
Dental plaque	5388	SERPINA1-ELA2 complex	0.0898
Dental plaque	5389	SERPINA3-CTSG complex	0.0898
Dental plaque	5473	FAS-FADD-CASP8 complex	0.07332
Dental plaque	5526	CALM1-FKBP38-BCL2 complex	0.07332
Dental plaque	5749	MRIT complex	0.07332
Dental plaque	5799	Death induced signaling complex DISC (FAS FADD CASP8 CFLAR) membrane-associated CD95L	0.0635
Dental plaque	5800	Death-inducing signaling complex DISC (type I cells associated) stimulated	0.07332
Dental plaque	5808	DISC complex	0.07332
Dental plaque	5811	p53-BCL2 complex	0.0898
Dental plaque	5812	p53-BCL2 complex	0.0898
Dental plaque	5817	tBID-BCL2 complex	0.0898
Dental plaque	5818	BIM-BCL2 complex	0.0898
Dental plaque	5819	BIM-BCL2xL complex	0.0898
Dental plaque	5820	tBID-BCL2xL complex	0.0898
Dental plaque	5823	MCL1-BAK1 complex	0.0898
Dental plaque	5830	DJ-1-SNCA complex high molecular weight complex	0.0898
Dental plaque	5859	FAS-FADD-CASP8-CASP10 complex	0.0635
Dental plaque	5861	FAS-FADD-CASP10 complex	0.07332

Dental plaque	5862	CAV1-VDAC1-ESR1 complex	0.07332
Depression	441	TFTC-type histone acetyl transferase complex	0.03959
Depression	521	Polycystin-1-E-cadherin-beta-catenin complex	0.07581
Depression	522	Polycystin-1-E-cadherin-beta-catenin-Flotillin-2	0.06565
Depression	753	UTM-SGCE-DAG1-CAV1-NOS3 complex	0.05872
Depression	826	PAR-3-VE-cadherin-beta-catenin complex	0.07581
Depression	1054	ESR1-RELA-BCL3-NCOA3 complex	0.06565
Depression	1088	PRNP-ApolopoproteinE3 complex	0.09285
Depression	1248	Apoptosome	0.09285
Depression	1787	Nogo-potassium channel complex	0.06565
Depression	1816	JUN-TCF4-CTNNB1 complex	0.07581
Depression	1839	SDCBP-CTNNB1-CTNNA1-CDH1 complex	0.06565
Depression	1976	MTNR1A homodimer complex	0.13131
Depression	1978	MTNR1A-MTNR1B complex	0.09285
Depression	2124	IKK-alpha--ER-alpha-AIB1 complex	0.07581
Depression	2159	AR-AKT-APPL complex	0.07581
Depression	2160	AOF2-AR complex	0.09285
Depression	2383	ITGA5-ITGB1-FN1-TGM2 complex	0.06565
Depression	2384	ITGA5-ITGB1-ADAM15 complex	0.07581
Depression	2385	ITGA5-ITGB4 complex	0.09285
Depression	2388	Itga5-Itgb1-Fn1-Sfrp2 complex	0.06565
Depression	2390	CD98-LAT2-ITGB1 complex	0.06565
Depression	2395	ITGA7-ITGB1-CD151 complex	0.07581
Depression	2396	ITGA7-ITGB1-CD9 complex	0.07581
Depression	2397	ITGA7-ITGB1-ITGB1BP3 complex	0.07581
Depression	2398	ITGA3-ITGB1-BSG complex	0.07581
Depression	2399	ITGA3-ITGB1-CD63 complex	0.07581
Depression	2400	ITGA3-ITGB1-CD151 complex	0.07581
Depression	2401	ITGA3-ITGB1-THBS1 complex	0.07581
Depression	2406	ITGA3-ITGB1 complex	0.09285
Depression	2411	ITGA6-ITGB1-CD151 complex	0.07581
Depression	2413	ITGA6-ITGB1 complex	0.09285
Depression	2416	ITGB1-RAP1A-PKD1 complex	0.07581
Depression	2417	ITGA4-ITGB1-EMILIN1 complex	0.07581
Depression	2418	ITGA4-ITGB1 complex	0.09285
Depression	2419	ITGA4-ITGB1-CD81 complex	0.07581
Depression	2420	ITGA4-ITGB1-CD53 complex	0.07581
Depression	2421	ITGA4-ITGB1-VCAM1 complex	0.07581
Depression	2422	ITGA4-ITGB1-JAM2 complex	0.07581
Depression	2423	ITGA4-ITGB1-CD47 complex	0.07581
Depression	2424	ITGA4-ITGB1-CD63 complex	0.07581
Depression	2425	ITGA4-ITGB1-PXN complex	0.07581
Depression	2426	ITGA4-ITGB1-THBS1 complex	0.07581
Depression	2428	ITGA4-ITGB1-THBS2 complex	0.07581
Depression	2429	ITGA2-ITGB1-CD47 complex	0.15162
Depression	2430	ITGA2-ITGB1-CHAD complex	0.15162
Depression	2431	ITGA2-ITGB1-COL6A3 complex	0.15162

Depression	2432	ITGA2-ITGB1 complex	0.1857
Depression	2434	ITGA1-ITGB1-COL6A3 complex	0.07581
Depression	2435	ITGA1-ITGB1-PTPN2 complex	0.07581
Depression	2436	ITGAV-ITGB1 complex	0.09285
Depression	2437	ITGA6-ITGB1-CYR61 complex	0.07581
Depression	2439	ITGA8-ITGB1 complex	0.09285
Depression	2440	ITGA9-ITGB1-ADAM9 complex	0.07581
Depression	2441	Itga9-Itgb1-Adam2 complex	0.07581
Depression	2442	ITGA9-ITGB1-VCAM1 complex	0.07581
Depression	2443	ITGA9-ITGB1-TNC complex	0.07581
Depression	2444	ITGB1-ITGA9 complex	0.09285
Depression	2445	ITGA9-ITGB1-ADAM15 complex	0.07581
Depression	2446	ITGA9-ITGB1-FIGF complex	0.07581
Depression	2447	ITGA9-ITGB1-ADAM12 complex	0.07581
Depression	2470	p130Cas-ER-alpha-cSrc-kinase- PI3-kinase p85-subunit complex	0.06565
Depression	2657	ESR1-CDK7-CCNH-MNAT1-MTA1-HDAC2 complex	0.05361
Depression	2670	Er-alpha-p53-hdm2 complex	0.07581
Depression	2699	ER-alpha-GRIP1-c-Jun complex	0.07581
Depression	2700	ER-alpha-c-Jun complex	0.09285
Depression	2850	ITGA5-ITGB1-FN-1-NOV complex	0.06565
Depression	2853	ITGA5-ITGB1-CAL4A3 complex	0.07581
Depression	2885	ITGAV-ITGB1-SPP1 complex	0.07581
Depression	2964	ITGA9-ITGB1-ADAM1 complex	0.07581
Depression	2965	ITGA9-ITGB1-ADAM3 complex	0.07581
Depression	2971	ITGA9-ITGB1-VEGFC complex	0.07581
Depression	2972	ITGA9-ITGB1-VEGFA complex	0.07581
Depression	2989	ITGA9-ITGB1-ADAM8 complex	0.07581
Depression	2998	Axin-PP2A A-PP2A C-GSK3-beta-beta-catenin	0.06565
Depression	3004	APC-Axin-1-beta-catenin complex	0.07581
Depression	3035	LAT2-ITGB1 complex	0.09285
Depression	3057	ITGA10-ITGB1 complex	0.09285
Depression	3058	ITGA11-ITGB1 complex	0.09285
Depression	3059	ITGA11-ITGB1-COL1A1 complex	0.07581
Depression	3104	ITGB1-NRP1 complex	0.09285
Depression	3111	ITGA9-ITGB1-SPP1 complex	0.07581
Depression	3112	ITGA5-ITGB1-SPP1 complex	0.07581
Depression	3155	Bipartite complex (TFC4 CTNNB1)	0.09285
Depression	3166	AXIN-APC-betaCatenin-GSK3B complex	0.06565
Depression	4095	Catulin (alpha) - catenin (beta) complex	0.09285
Depression	4096	Catenin (alpha) - catenin (beta) complex	0.09285
Depression	5772	ZO1-(beta)cadherin-(VE)cadherin-VEGFR2 complex	0.06565
Depression	5177	Polycystin-1 multiprotein complex (ACTN1 CDH1 SRC JUP VCL CTNNB1 PXN BCAR1 PKD1	0.03959
Depression	5260	TCF4-CTNNB1-SUMO1-EP300-HADAC6 complex	0.05872
Depression	5261	TCF4-CTNNB1-EP300 complex	0.07581
Depression	5262	TCF4-CTNNB1 complex	0.09285
Depression	5264	TCF4-CTNNB1-CREBBP complex	0.07581

Depression	5268	TNF-alpha/NF-kappa B signaling complex 7	0.04642
Depression	5269	TNF-alpha/NF-kappa B signaling complex 8	0.05361
Depression	5281	Cell-cell junction complex (CDH1-CTNNB1)	0.09285
Depression	5285	TNF-alpha/NF-kappa B signaling complex 9	0.05872
Depression	5414	HTR1A-HTR1D complex	0.09285
Depression	5415	HTR1B homodimer complex	0.13131
Depression	5416	HTR1A-HTR1B complex	0.1857
Depression	5417	HTR1D-HTR1B complex	0.09285
Depression	5418	GABBR2-HTR1A complex	0.09285
Depression	5419	HTR1A-GPR26 complex	0.09285
Depression	5420	HTR1A-EDG3 complex	0.09285
Depression	5421	HTR1A homodimer complex	0.13131
Depression	5422	HTR1A-EDG1 complex	0.09285
Depression	5655	Ternary complex (LRRC7 CAMK2a ACTN4)	0.07581
Depression	5816	Apoptosome-procaspase 9 complex	0.07581
Depression	5830	DJ-1-SNCA complex high molecular weight complex	0.09285
Depression	5862	CAV1-VDAC1-ESR1 complex	0.07581
Dermatitis	298	VEGF transcriptional complex	0.03711
Dermatitis	310	Cell cycle kinase complex CDC2	0.03711
Dermatitis	311	Cell cycle kinase complex CDK2	0.04545
Dermatitis	312	Cell cycle kinase complex CDK4	0.04545
Dermatitis	313	Cell cycle kinase complex CDK5	0.04066
Dermatitis	314	PCNA-p21 complex	0.06428
Dermatitis	521	Polycystin-1-E-cadherin-beta-catenin complex	0.05249
Dermatitis	522	Polycystin-1-E-cadherin-beta-catenin-Flotillin-2	0.04545
Dermatitis	668	BKCA-beta2AR-AKAP79 signaling complex	0.05249
Dermatitis	672	BKCA-beta2AR complex	0.06428
Dermatitis	687	CFTR-NHERF-beta(2)AR signaling complex	0.05249
Dermatitis	826	PAR-3-VE-cadherin-beta-catenin complex	0.05249
Dermatitis	1088	PRNP-ApolopoproteinE3 complex	0.06428
Dermatitis	1094	Frataxin complex	0.03436
Dermatitis	1474	SMAD3/4-E2F4/5-p107-DP1 complex	0.03711
Dermatitis	1514	IL4-IL4R complex	0.12856
Dermatitis	1515	IL4-IL4R-IL2RG complex	0.10497
Dermatitis	1519	IL6ST-PRKCD-STAT3 complex	0.05249
Dermatitis	1634	CyclinD1-CDK4-p21 complex	0.05249
Dermatitis	1816	JUN-TCF4-CTNNB1 complex	0.05249
Dermatitis	1826	SMAD3-HEF1-APC10-CDH1 complex	0.04545
Dermatitis	1827	PML-SMAD2/3-SARA complex	0.09091
Dermatitis	1828	TGF-beta receptor I-Axin-SMAD3 complex	0.05249
Dermatitis	1831	PIAS3-SMAD3-P300 complex	0.05249
Dermatitis	1839	SDCBP-CTNNB1-CTNNA1-CDH1 complex	0.04545
Dermatitis	2000	BAX homo-oligomer complex	0.09091
Dermatitis	2018	IL12A-IL12B complex	0.06428
Dermatitis	2019	IL12A-IL12B-IL12RB1 complex	0.10497
Dermatitis	2020	IL12B-IL12RB1-IL12RB2 complex	0.10497
Dermatitis	2021	IL12A-IL12B-IL12RB2 complex	0.05249

Dermatitis	2026	IL12RB1-IL12RB2 complex	0.06428
Dermatitis	2084	NFKB1-NFKB2-REL-RELA-RELB complex	0.04066
Dermatitis	2189	Ubiquitin E3 ligase (SMAD3 BTRC CUL1 SKP1A RBX1)	0.04066
Dermatitis	2230	PCNA complex	0.03436
Dermatitis	2343	ITGAV-ITGB5-PLAUR complex	0.05249
Dermatitis	2692	SMAD3-SMAD4-cJun-cFos complex	0.04545
Dermatitis	2705	SMAD3-SMAD4-CTCF protein-DNA complex	0.05249
Dermatitis	2706	SMAD3-SMAD4-SP1 complex	0.05249
Dermatitis	2707	SMAD3-SMAD4-FOXO3-FOXG1 complex	0.04545
Dermatitis	2708	SMAD3-SMAD4-cJUN complex	0.05249
Dermatitis	2709	MMP-9-TIMP-1-LRP complex	0.05249
Dermatitis	2754	JUND-FOSB-SMAD3-SMAD4 complex	0.04545
Dermatitis	2755	17S U2 snRNP	0.01583
Dermatitis	2760	SMAD3-SMAD4-FOXO3 complex	0.05249
Dermatitis	2761	SMAD3-SMAD4-FOXO1 complex	0.05249
Dermatitis	2762	SMAD3-SMAD4-FOXO4 complex	0.05249
Dermatitis	2813	BRCA1-SMAD3 complex	0.06428
Dermatitis	2829	RSmad complex	0.0575
Dermatitis	2830	TIF1gamma-SMAD2-SMAD3 complex	0.10497
Dermatitis	2834	SMAD4-SMAD2-SMAD3 complex	0.10497
Dermatitis	2968	Axin-SMAD3 complex	0.06428
Dermatitis	2975	SMAD3-E2F4/5-p107-DP1 complex	0.04066
Dermatitis	2998	Axin-PP2A A-PP2A C-GSK3-beta-beta-catenin	0.04545
Dermatitis	3004	APC-Axin-1-beta-catenin complex	0.05249
Dermatitis	3038	SMAD2-SMAD4-FAST1 complex	0.05249
Dermatitis	3039	SMAD2-FAST1 complex	0.06428
Dermatitis	3155	Bipartite complex (TFC4 CTNNB1)	0.06428
Dermatitis	3166	AXIN-APC-betaCatenin-GSK3B complex	0.04545
Dermatitis	3198	SMAD2-SKI complex	0.06428
Dermatitis	3199	SMAD3-SKI complex	0.06428
Dermatitis	3204	SMAD2-SKI-NCOR complex	0.05249
Dermatitis	3205	SMAD3-SKI-NCOR complex	0.05249
Dermatitis	3229	Heterodimer complex (CDK9 IL6ST)	0.06428
Dermatitis	3233	SMAD2-SMAD4-FAST1-TGIF complex TGF(beta) induced	0.04545
Dermatitis	3234	SMAD2-SMAD4-FAST1-TGIF-HDAC1 complex TGF(beta) induced	0.04066
Dermatitis	3492	Bax homooligomeric complex after apoptotic	0.09091
Dermatitis	3729	SKI-SMAD2 hexameric complex	0.06428
Dermatitis	3733	SKI-SMAD3 hexameric complex	0.06428
Dermatitis	3739	SKI-SMAD2-SMAD4 pentameric complex	0.05249
Dermatitis	3740	SKI-SMAD3-SMAD4 pentameric complex	0.05249
Dermatitis	3749	CREBBP-SMAD2 hexameric complex	0.06428
Dermatitis	3750	CREBBP-SMAD3 hexameric complex	0.06428
Dermatitis	3753	CREBBP-SMAD2-SMAD4 pentameric complex	0.05249
Dermatitis	3754	CREBBP-SMAD3-SMAD4 pentameric complex	0.05249
Dermatitis	3830	ADRB2 homodimer complex	0.09091

Dermatitis	3959	SMAD3-SMAD4-cSKI TGF(beta)-dependent	0.05249
Dermatitis	3961	SMAD3-cSKI-SIN3A-HDAC1 complex	0.04545
Dermatitis	3967	SMURF2-SMAD2 complex TGF(beta)-dependent	0.06428
Dermatitis	3971	SMURF2-SMAD3 complex TGF(beta)-dependent	0.06428
Dermatitis	3972	SMURF2-SMAD3-SnoN complex TGF(beta)-	0.05249
Dermatitis	4062	NRP1-VEGFR2-VEGF(165) complex	0.05249
Dermatitis	4095	Catulin (alpha) - catenin (beta) complex	0.06428
Dermatitis	4096	Catenin (alpha) - catenin (beta) complex	0.06428
Dermatitis	5772	ZO1-(beta)cadherin-(VE)cadherin-VEGFR2 complex	0.09091
Dermatitis	5101	CyclinD3-CDK4-CDK6-p21 complex	0.04545
Dermatitis	5177	Polycystin-1 multiprotein complex (ACTN1 CDH1 SRC JUP VCL CTNNB1 PXN BCAR1 PKD1	0.02741
Dermatitis	5193	TNF-alpha/NF-kappa B signaling complex (CHUK KPNA3 NFKB2 NFKBIB REL IKBKG NFKB1 NFKBIE RELB NFKBIA RELA TNIP2)	0.02624
Dermatitis	5194	TNF-alpha/NF-kappa B signaling complex (SEC16A CHUK IKBKB NFKB2 REL IKBKG MAP3K14 RELA FBXW7 USP2)	0.02875
Dermatitis	5196	TNF-alpha/NF-kappa B signaling complex (CHUK BTRC NFKB2 PPP6C REL CUL1 IKBKE SAPS2 SAPS1 ANKRD28 RELA SKP1)	0.02624
Dermatitis	5198	CBP-RARA-RXRA-DNA complex ligand stimulated	0.05249
Dermatitis	5228	REL-MAP3K8-RELA-TNIP2-PAPOLA complex	0.04066
Dermatitis	5230	CHUK-NFKB2-REL-IKBKG-SPAG9-NFKB1-NFKBIE-COPB2-TNIP1-NFKBIA-RELA-TNIP2	0.02624
Dermatitis	5232	TNF-alpha/Nf-kappa B signaling complex (RPL6 RPL30 RPS13 CHUK DDX3X NFKB2 NFKBIB REL IKBKG NFKB1 MAP3K8 RELB GLG1 NFKBIA RELA TNIP2 GTF2I)	0.02205
Dermatitis	5233	TNF-alpha/NF-kappa B signaling complex 5	0.01818
Dermatitis	5260	TCF4-CTNNB1-SUMO1-EP300-HADAC6 complex	0.04066
Dermatitis	5261	TCF4-CTNNB1-EP300 complex	0.05249
Dermatitis	5262	TCF4-CTNNB1 complex	0.06428
Dermatitis	5264	TCF4-CTNNB1-CREBBP complex	0.05249
Dermatitis	5273	VHL-TBP1-HIF1A complex	0.05249
Dermatitis	5276	HIF1A-OS9-EGLN1 complex	0.05249
Dermatitis	5277	HIF1A-OS9-EGLN3 complex	0.05249
Dermatitis	5281	Cell-cell junction complex (CDH1-CTNNB1)	0.06428
Dermatitis	5382	ARNT-HIF1A complex	0.06428
Dermatitis	5414	HTR1A-HTR1D complex	0.06428
Dermatitis	5416	HTR1A-HTR1B complex	0.06428
Dermatitis	5418	GABBR2-HTR1A complex	0.06428
Dermatitis	5419	HTR1A-GPR26 complex	0.06428
Dermatitis	5420	HTR1A-EDG3 complex	0.06428
Dermatitis	5421	HTR1A homodimer complex	0.09091
Dermatitis	5422	HTR1A-EDG1 complex	0.06428
Dermatitis	5465	IKB(epsilon)-RELA-cREL complex	0.05249
Dermatitis	5466	IKB(beta)-RELA-cREL complex	0.05249
Dermatitis	5467	IKB(alpha)-RELA-cREL complex	0.05249

Dermatitis	5548	IL-12 heterodimer complex	0.06428
Dermatitis	5549	IL-12 subunit p40 homodimer complex	0.09091
Dermatitis	5564	LMO4-gp130 complex	0.04066
Dermatitis	5579	CNTF-CNTFR-gp130-LIFR complex	0.04545
Dermatitis	5582	LIFR-LIF-gp130 complex	0.10497
Dermatitis	5622	HSP90-CIP1-FKBPL complex	0.05249
Dermatitis	5696	VEGFA(165)-KDR-NRP1 complex	0.05249
Dermatitis	5698	VEGFA(165)-VEGFR2-NRP1 complex	0.05249
Dermatitis	5735	TGF-beta receptor-SMAD3 complex	0.05249
Diabetes mellitus	4	Multisubunit ACTR coactivator complex	0.02632
Diabetes mellitus	74	TRPC1-Homer3-IP3R complex	0.03039
Diabetes mellitus	89	Sec6/8 exocyst complex	0.01861
Diabetes mellitus	98	p300-MDM2-p53 protein complex	0.03039
Diabetes mellitus	115	Polycomb repressive complex 1 (PRC1 hPRC-H)	0.01519
Diabetes mellitus	116	Polycomb repressive complex 1 (PRC1 hPRC-H)	0.0146
Diabetes mellitus	120	Lymphotoxin beta receptor complex	0.06077
Diabetes mellitus	159	Condensin I-PARP-1-XRCC1 complex	0.01989
Diabetes mellitus	178	Respiratory chain complex I (holoenzyme)	0.00793
Diabetes mellitus	220	ARF-Mule complex	0.03039
Diabetes mellitus	298	VEGF transcriptional complex	0.02149
Diabetes mellitus	5856	AK2-FADD-caspase-10 (AFAC10) complex	0.03039
Diabetes mellitus	432	N-NOS-CHIP-HSP70-1 complex	0.03039
Diabetes mellitus	438	GCN5-TRRAP histone acetyltransferase complex	0.01664
Diabetes mellitus	441	TFTC-type histone acetyl transferase complex	0.01587
Diabetes mellitus	443	BP-SMAD complex	0.01861
Diabetes mellitus	445	TFTC complex (TATA-binding protein-free TAF-II-containing complex)	0.01316
Diabetes mellitus	470	TFTC complex (TATA-binding protein-free TAF-II-containing complex)	0.01277
Diabetes mellitus	471	PCAF complex	0.01664
Diabetes mellitus	475	STAGA complex (SPT3-TAF9-GCN5 acetyltransferase complex)	0.01519
Diabetes mellitus	476	STAGA complex (SPT3-TAF9-GCN5 acetyltransferase complex)	0.0146
Diabetes mellitus	513	TFTC complex (TATA-binding protein-free TAF-II-containing complex)	0.01277
Diabetes mellitus	536	TRPC1-TRPC3-TRPC7 complex	0.03039
Diabetes mellitus	541	IGF1-IGFBP3-ALS complex	0.03039
Diabetes mellitus	553	RHOA-IP3R-TRPC1 complex	0.03039
Diabetes mellitus	557	TRP1-G alpha-11-IP3R3-CAV1 signaling complex	0.05263
Diabetes mellitus	570	p300-CBP-p270-SWI/SNF complex	0.01989
Diabetes mellitus	571	p300-CBP-p270 complex	0.03039
Diabetes mellitus	626	LSD1 complex	0.0146
Diabetes mellitus	668	BKCA-beta2AR-AKAP79 signaling complex	0.03039
Diabetes mellitus	672	BKCA-beta2AR complex	0.03722
Diabetes mellitus	681	(C-CFTR)2-NHERF-ezrin complex	0.03039
Diabetes mellitus	682	C-CFTR-NHERF(PDZ1 domain)-ezrin complex	0.03039
Diabetes mellitus	683	C-CFTR-NHERF(PDZ2 domain)-ezrin complex	0.03039

Diabetes mellitus	687	CFTR-NHERF-beta(2)AR signaling complex	0.03039
Diabetes mellitus	725	P2X7 receptor signalling complex	0.03039
Diabetes mellitus	828	TRPC1-STIM1-ORAI1 complex	0.03039
Diabetes mellitus	929	CEN complex	0.00865
Diabetes mellitus	1004	RC complex during S-phase of cell cycle	0.0146
Diabetes mellitus	1005	RC complex during G2/M-phase of cell cycle	0.0146
Diabetes mellitus	1028	HNF4A-SUB1 complex	0.03722
Diabetes mellitus	1041	Alpha-dystrobrevin-ZO-1-actin complex	0.02632
Diabetes mellitus	1054	ESR1-RELA-BCL3-NCOA3 complex	0.05263
Diabetes mellitus	1062	BAR-BCL2-CASP8 complex	0.03039
Diabetes mellitus	1067	CD8A-LCK complex	0.03722
Diabetes mellitus	1069	FIF-FGR2 complex	0.03722
Diabetes mellitus	1088	PRNP-ApolopoproteinE3 complex	0.03722
Diabetes mellitus	1091	SNX complex (SNX1a SNX2 SNX4 LEPR)	0.02632
Diabetes mellitus	1095	SNX complex (SNX1a SNX2 SNX4 EGFR)	0.02632
Diabetes mellitus	1141	CF IIAm complex (Cleavage factor IIAm complex)	0.01316
Diabetes mellitus	1158	p33ING1b-p300 complex	0.03722
Diabetes mellitus	1160	ING1-p300-PCNA complex	0.03039
Diabetes mellitus	1185	EGFR-containing signaling complex	0.02632
Diabetes mellitus	1193	Rap1 complex	0.01989
Diabetes mellitus	1211	Ubiquitin E3 ligase (AHR ARNT DDB1 TBL3 CUL4B RBX1)	0.02149
Diabetes mellitus	1217	WRN-TRF2 complex	0.03722
Diabetes mellitus	1223	H2AX complex isolated from cells without IR exposure	0.0146
Diabetes mellitus	1226	H2AX complex I	0.03979
Diabetes mellitus	1227	H2AX complex II	0.01664
Diabetes mellitus	1308	PABPC1-HSPA8-HNRPD-EIF4G1 complex	0.02354
Diabetes mellitus	1335	SNW1 complex	0.01241
Diabetes mellitus	1471	pRb2/p130-multimolecular complex (RB2 E2F5 HDAC1 SUV39H1 P300)	0.02354
Diabetes mellitus	1514	IL4-IL4R complex	0.07443
Diabetes mellitus	1515	IL4-IL4R-IL2RG complex	0.06077
Diabetes mellitus	1521	p300-SMAD1-STAT3 complex	0.03039
Diabetes mellitus	1642	p16-cyclin D2-CDK4 complex	0.03039
Diabetes mellitus	1714	TICAM1-TICAM2-TLR4 complex	0.03039
Diabetes mellitus	1728	CTCF-nucleophosmin-PARP-HIS-KPNA-LMNA-TOP complex	0.01754
Diabetes mellitus	1729	TLE1 corepressor complex (MASH1 promoter-corepressor complex)	0.01664
Diabetes mellitus	1746	SMN containing complex	0.01861
Diabetes mellitus	1772	MICB-KLRK1-HCST complex	0.03039
Diabetes mellitus	1774	MICA-KLRK1-HCST complex	0.03039
Diabetes mellitus	1777	TGF-beta-receptor-SMAD7-SMURF2 complex	0.02632
Diabetes mellitus	1783	TGF-beta receptor I-SMAD7-SMURF1 complex	0.03039
Diabetes mellitus	5718	eNOS-HSP90-AKT complex VEGF induced	0.03039
Diabetes mellitus	1816	JUN-TCF4-CTNNB1 complex	0.03039
Diabetes mellitus	1831	PIAS3-SMAD3-P300 complex	0.03039
Diabetes mellitus	1944	IRAK1-IRAK2 complex	0.03722

Diabetes mellitus	1945	IRAK1-IRAK3 complex	0.03722
Diabetes mellitus	1970	BMP4-TWSG1 complex	0.03722
Diabetes mellitus	1972	BMP4-BGN complex	0.03722
Diabetes mellitus	1986	Endoglin homodimer complex	0.05263
Diabetes mellitus	1992	LEPR homodimer complex	0.05263
Diabetes mellitus	2018	IL12A-IL12B complex	0.03722
Diabetes mellitus	2019	IL12A-IL12B-IL12RB1 complex	0.03039
Diabetes mellitus	2020	IL12B-IL12RB1-IL12RB2 complex	0.03039
Diabetes mellitus	2021	IL12A-IL12B-IL12RB2 complex	0.03039
Diabetes mellitus	2054	CASP8-FADD-MALT1-BCL10 complex	0.02632
Diabetes mellitus	2055	CASP8-CHUK-IKBKB-MALT1-BCL10 complex	0.02354
Diabetes mellitus	2056	BCL10-CHUK-BCL10-IKBKB complex	0.02632
Diabetes mellitus	2084	NFKB1-NFKB2-REL-RELA-RELB complex	0.02354
Diabetes mellitus	2086	NFKB1-NFKB2-RELA-RELB complex	0.02632
Diabetes mellitus	2105	IkappaB kinase complex (IKBKB CHUK IKBKAP NFKBIA RELA MAP3K14)	0.02149
Diabetes mellitus	2124	IKK-alpha--ER-alpha-AIB1 complex	0.03039
Diabetes mellitus	2152	ARNT-HLF complex	0.03722
Diabetes mellitus	2153	ITGAM-ITGB2-CD11 complex	0.06077
Diabetes mellitus	2156	YBX1-AKT1 complex	0.03722
Diabetes mellitus	2159	AR-AKT-APPL complex	0.06077
Diabetes mellitus	2160	AOF2-AR complex	0.03722
Diabetes mellitus	2183	Kaiso-NCOR complex	0.01664
Diabetes mellitus	2254	CTGF/Hcs24-actin complex	0.03039
Diabetes mellitus	2342	ITGAV-ITGB8-MMP14-TGFB1 complex	0.02632
Diabetes mellitus	2347	ITGAV-ITGB5-SPP1 complex	0.03039
Diabetes mellitus	2352	ITGAV-ITGB6-SPP1 complex	0.03039
Diabetes mellitus	2355	ITGAV-ITGB3-CD47-FCER2 complex	0.02632
Diabetes mellitus	2356	ITGB3-ITGAV-CD47 complex	0.03039
Diabetes mellitus	2358	ITGAV-ITGB3-SPP1 complex	0.06077
Diabetes mellitus	2359	ITGAV-ITGB3-ADAM15 complex	0.03039
Diabetes mellitus	2362	ITAGV-ITGB3-F11R complex	0.03039
Diabetes mellitus	2363	ITGAV-ITGB3-PXN-PTK2b complex	0.02632
Diabetes mellitus	2364	ITGAV-ITGB3-ADAM23 complex	0.03039
Diabetes mellitus	2365	ITGAV-ITGB3-COL4A3 complex	0.03039
Diabetes mellitus	2366	ITGAV-ITGB3-PPAP2b complex	0.03039
Diabetes mellitus	2369	ITGAV-ITGB3-EGFR complex	0.06077
Diabetes mellitus	2370	ITGA2b-ITGB3-CD9 complex	0.09116
Diabetes mellitus	2374	ITGAV-ITGB3-LAMA4 complex	0.03039
Diabetes mellitus	2376	ITGA2B-ITGB3-FN1-TGM2 complex	0.05263
Diabetes mellitus	2377	ITGA2b-ITGB3-CD47-SRC complex	0.05263
Diabetes mellitus	2378	ITGA2b-ITGB3-TLN1 complex	0.06077
Diabetes mellitus	2379	ITGA2B-ITGB3-CIB1 complex	0.06077
Diabetes mellitus	2381	ITGA2B-ITGB3 complex	0.07443
Diabetes mellitus	2382	ITGA2B-ITGB3-F11R complex	0.06077
Diabetes mellitus	2383	ITGA5-ITGB1-FN1-TGM2 complex	0.02632
Diabetes mellitus	2384	ITGA5-ITGB1-ADAM15 complex	0.03039

Diabetes mellitus	2385	ITGA5-ITGB4 complex	0.03722
Diabetes mellitus	2388	Itga5-Itgb1-Fn1-Sfrp2 complex	0.02632
Diabetes mellitus	2390	CD98-LAT2-ITGB1 complex	0.02632
Diabetes mellitus	2395	ITGA7-ITGB1-CD151 complex	0.03039
Diabetes mellitus	2396	ITGA7-ITGB1-CD9 complex	0.06077
Diabetes mellitus	2397	ITGA7-ITGB1-ITGB1BP3 complex	0.03039
Diabetes mellitus	2398	ITGA3-ITGB1-BSG complex	0.03039
Diabetes mellitus	2399	ITGA3-ITGB1-CD63 complex	0.03039
Diabetes mellitus	2400	ITGA3-ITGB1-CD151 complex	0.03039
Diabetes mellitus	2401	ITGA3-ITGB1-THBS1 complex	0.03039
Diabetes mellitus	2406	ITGA3-ITGB1 complex	0.03722
Diabetes mellitus	2411	ITGA6-ITGB1-CD151 complex	0.03039
Diabetes mellitus	2413	ITGA6-ITGB1 complex	0.03722
Diabetes mellitus	2416	ITGB1-RAP1A-PKD1 complex	0.03039
Diabetes mellitus	2417	ITGA4-ITGB1-EMILIN1 complex	0.03039
Diabetes mellitus	2418	ITGA4-ITGB1 complex	0.03722
Diabetes mellitus	2419	ITGA4-ITGB1-CD81 complex	0.03039
Diabetes mellitus	2420	ITGA4-ITGB1-CD53 complex	0.03039
Diabetes mellitus	2421	ITGA4-ITGB1-VCAM1 complex	0.03039
Diabetes mellitus	2422	ITGA4-ITGB1-JAM2 complex	0.03039
Diabetes mellitus	2423	ITGA4-ITGB1-CD47 complex	0.03039
Diabetes mellitus	2424	ITGA4-ITGB1-CD63 complex	0.03039
Diabetes mellitus	2425	ITGA4-ITGB1-PXN complex	0.03039
Diabetes mellitus	2426	ITGA4-ITGB1-THBS1 complex	0.03039
Diabetes mellitus	2428	ITGA4-ITGB1-THBS2 complex	0.03039
Diabetes mellitus	2429	ITGA2-ITGB1-CD47 complex	0.06077
Diabetes mellitus	2430	ITGA2-ITGB1-CHAD complex	0.06077
Diabetes mellitus	2431	ITGA2-ITGB1-COL6A3 complex	0.06077
Diabetes mellitus	2432	ITGA2-ITGB1 complex	0.07443
Diabetes mellitus	2434	ITGA1-ITGB1-COL6A3 complex	0.03039
Diabetes mellitus	2435	ITGA1-ITGB1-PTPN2 complex	0.03039
Diabetes mellitus	2436	ITGAV-ITGB1 complex	0.03722
Diabetes mellitus	2437	ITGA6-ITGB1-CYR61 complex	0.03039
Diabetes mellitus	2439	ITGA8-ITGB1 complex	0.03722
Diabetes mellitus	2440	ITGA9-ITGB1-ADAM9 complex	0.03039
Diabetes mellitus	2441	Itga9-Itgb1-Adam2 complex	0.03039
Diabetes mellitus	2442	ITGA9-ITGB1-VCAM1 complex	0.03039
Diabetes mellitus	2443	ITGA9-ITGB1-TNC complex	0.06077
Diabetes mellitus	2444	ITGB1-ITGA9 complex	0.03722
Diabetes mellitus	2445	ITGA9-ITGB1-ADAM15 complex	0.03039
Diabetes mellitus	2446	ITGA9-ITGB1-FIGF complex	0.03039
Diabetes mellitus	2447	ITGA9-ITGB1-ADAM12 complex	0.03039
Diabetes mellitus	2453	Multiprotein complex (monoubiquitination)	0.05263
Diabetes mellitus	2454	CIN85-CBL-SH3GL2-EGFR complex EGF stimulated	0.02632
Diabetes mellitus	2470	p130Cas-ER-alpha-cSrc-kinase- PI3-kinase p85-subunit complex	0.02632
Diabetes mellitus	2528	ERBB2-MEMO-SHC complex	0.03039

Diabetes mellitus	2535	SLP-76-Cbl-Grb2-Shc complex Fc receptor gamma-R1 stimulated	0.02632
Diabetes mellitus	2542	EGFR-CBL-GRB2 complex	0.03039
Diabetes mellitus	2559	p56(LCK)-CAML complex	0.03722
Diabetes mellitus	2565	CD20-LCK-LYN-FYN-p75/80 complex (Raji human B cell line)	0.02632
Diabetes mellitus	2577	Sam68-p85 P13K-IRS-1-IR signaling complex	0.02632
Diabetes mellitus	2625	CDK8-MED6-PARP1 complex	0.03039
Diabetes mellitus	2635	BETA2-Cyclin D1 complex	0.03722
Diabetes mellitus	2638	HES1 promoter corepressor complex	0.02149
Diabetes mellitus	2639	HES1 promoter-Notch enhancer complex	0.0146
Diabetes mellitus	2641	p300/CBP-PCAF-MyoD complex	0.02632
Diabetes mellitus	2642	SMAD1-P300 complex	0.03722
Diabetes mellitus	2657	ESR1-CDK7-CCNH-MNAT1-MTA1-HDAC2 complex	0.02149
Diabetes mellitus	2670	Er-alpha-p53-hdm2 complex	0.03039
Diabetes mellitus	2688	MT1-MMP-claudin-1 complex	0.03722
Diabetes mellitus	2692	SMAD3-SMAD4-cJun-cFos complex	0.02632
Diabetes mellitus	2693	NFAT-JUN-FOS DNA-protein complex	0.03039
Diabetes mellitus	2694	ERG-JUN-FOS DNA-protein complex	0.03039
Diabetes mellitus	2695	ETS2-FOS-JUN complex	0.03039
Diabetes mellitus	2699	ER-alpha-GRIP1-c-Jun complex	0.06077
Diabetes mellitus	2700	ER-alpha-c-Jun complex	0.07443
Diabetes mellitus	2708	SMAD3-SMAD4-cJUN complex	0.03039
Diabetes mellitus	2709	MMP-9-TIMP-1-LRP complex	0.03039
Diabetes mellitus	2721	HCF-1 complex	0.01207
Diabetes mellitus	2770	ITGA6-ITGB4-CD9 complex	0.03039
Diabetes mellitus	2798	MMP-2-claudin-1 complex	0.03722
Diabetes mellitus	2816	ITGAV-ITGB3 complex	0.03722
Diabetes mellitus	2826	ITGB3-ITGAV-VTN complex	0.03039
Diabetes mellitus	2846	ITGAV-ITGB3-THBS1 complex	0.03039
Diabetes mellitus	2849	ITGAV-ITGB3-NOV complex	0.03039
Diabetes mellitus	2850	ITGA5-ITGB1-FN-1-NOV complex	0.02632
Diabetes mellitus	2853	ITGA5-ITGB1-CAL4A3 complex	0.03039
Diabetes mellitus	2872	ITGA2b-ITGB3-CD9-GP1b-CD47 complex	0.06446
Diabetes mellitus	2879	CD20-LCK-FYN-p75/80 complex	0.03039
Diabetes mellitus	2882	ITGA5-ITGB3-COL6A3 complex	0.03039
Diabetes mellitus	2884	Respiratory chain complex I (early intermediate NDUFAF1 assembly) mitochondrial	0.01989
Diabetes mellitus	2885	ITGAV-ITGB1-SPP1 complex	0.06077
Diabetes mellitus	2886	Respiratory chain complex I (incomplete intermediate ND1 ND2 ND3 CIA30 assembly) mitochondrial	0.02632
Diabetes mellitus	2895	SHC-GRB2 complex	0.03722
Diabetes mellitus	2896	ITGA2b-ITGB3-CD47-FAK complex	0.05263
Diabetes mellitus	2901	Respiratory chain complex I (intermediate IV/310kD) mitochondrial	0.02632
Diabetes mellitus	2903	Respiratory chain complex I (intermediate V/380kD and VI/480kD) mitochondrial	0.02354
Diabetes mellitus	2904	Respiratory chain complex I (intermediate VII/650kD) mitochondrial	0.01664

Diabetes mellitus	2919	Respiratory chain complex I (gamma subunit) mitochondrial	0.0146
Diabetes mellitus	2939	Ecsit complex (ECSIT MT-CO2 NDUFA1 MT-ND1 TRAF6 NDUFAF1)	0.02149
Diabetes mellitus	2943	Respiratory chain complex I (incomplete NDUFAF1 assembly) mitochondrial	0.03722
Diabetes mellitus	2944	Notch1-p56lck-PI3K complex	0.03039
Diabetes mellitus	2954	Smad1-Notch1-p300-Pcaf complex	0.02632
Diabetes mellitus	2955	LCK-SLP76-PLC-gamma-1-LAT complex pervanadate-activated	0.02632
Diabetes mellitus	2964	ITGA9-ITGB1-ADAM1 complex	0.03039
Diabetes mellitus	2965	ITGA9-ITGB1-ADAM3 complex	0.03039
Diabetes mellitus	2971	ITGA9-ITGB1-VEGFC complex	0.03039
Diabetes mellitus	2972	ITGA9-ITGB1-VEGFA complex	0.06077
Diabetes mellitus	2989	ITGA9-ITGB1-ADAM8 complex	0.03039
Diabetes mellitus	2992	SMAD7-SMURF2 complex	0.03722
Diabetes mellitus	2996	SMAD7-SMURF1 complex	0.03722
Diabetes mellitus	2997	SMAD7-SMURF1-TGF-beta receptor complex	0.02632
Diabetes mellitus	3035	LAT2-ITGB1 complex	0.03722
Diabetes mellitus	3043	BMP2-BRIA complex	0.03039
Diabetes mellitus	3045	hs4 enhancer complex (faster migrating complex)	0.02354
Diabetes mellitus	3055	Nop56p-associated pre-rRNA complex	0.00516
Diabetes mellitus	3057	ITGA10-ITGB1 complex	0.03722
Diabetes mellitus	3058	ITGA11-ITGB1 complex	0.03722
Diabetes mellitus	3059	ITGA11-ITGB1-COL1A1 complex	0.03039
Diabetes mellitus	3082	DGCR8 multiprotein complex	0.01587
Diabetes mellitus	3096	ITGA6-ITGB4-SHC1-GRB2 complex	0.02632
Diabetes mellitus	3103	ITGAV-ITGB3-SLC3A2 complex	0.03039
Diabetes mellitus	3104	ITGB1-NRP1 complex	0.03722
Diabetes mellitus	3111	ITGA9-ITGB1-SPP1 complex	0.06077
Diabetes mellitus	3112	ITGA5-ITGB1-SPP1 complex	0.06077
Diabetes mellitus	3115	ITGA2B-ITGB3-ICAM4 complex	0.06077
Diabetes mellitus	3137	MASH1 promoter-coactivator complex	0.01587
Diabetes mellitus	3138	POSH-AKT2 complex	0.03722
Diabetes mellitus	3142	CAMK2-delta-MASH1 promoter-coactivator complex	0.01861
Diabetes mellitus	3162	TF-FVIIa-FXa-TFPI complex	0.02632
Diabetes mellitus	3678	RIN1-STAM2-EGFR complex EGF stimulated	0.03039
Diabetes mellitus	3830	ADRB2 homodimer complex	0.05263
Diabetes mellitus	3847	TCL1(trimer)-AKT1 complex	0.03722
Diabetes mellitus	3848	TCL1(trimer)-AKT2 complex	0.03722
Diabetes mellitus	4043	NEMO-HIF2(alpha)-ARNT complex	0.03039
Diabetes mellitus	4062	NRP1-VEGFR2-VEGF(165) complex	0.03039
Diabetes mellitus	4082	Ku70/Ku86/Werner complex	0.03039
Diabetes mellitus	4997	p97/VCP-VIMP-DERL1 complex	0.03039
Diabetes mellitus	4998	p97/VCP-VIMP-DERL2 complex	0.03039
Diabetes mellitus	4999	p97/VCP-VIMP-DERL1-DERL2-HRD1-SEL1L	0.02149
Diabetes mellitus	5772	ZO1-(beta)cadherin-(VE)cadherin-VEGFR2 complex	0.02632

Diabetes mellitus	5118	pRb2/p130-multimolecular complex (RB2 E2F4 HDAC1 SUV39H1 P300)	0.02354
Diabetes mellitus	5171	SH3KBP1-CBLB-EGFR complex	0.06077
Diabetes mellitus	5179	NCOA6-DNA-PK-Ku-PARP1 complex	0.02354
Diabetes mellitus	5193	TNF-alpha/NF-kappa B signaling complex (CHUK KPNA3 NFKB2 NFKBIB REL IKBKG NFKB1 NFKBIE RELB NFKBIA RELA TNIP2)	0.01519
Diabetes mellitus	5194	TNF-alpha/NF-kappa B signaling complex (SEC16A CHUK IKBKB NFKB2 REL IKBKG MAP3K14 RELA FBXW7 USP2)	0.01664
Diabetes mellitus	5196	TNF-alpha/NF-kappa B signaling complex (CHUK BTRC NFKB2 PPP6C REL CUL1 IKBKE SAPS2 SAPS1 ANKRD28 RELA SKP1)	0.01519
Diabetes mellitus	5220	CHUK-IQGAP2-AKAP8L-RELA-TNIP2 complex	0.02354
Diabetes mellitus	5228	REL-MAP3K8-RELA-TNIP2-PAPOLA complex	0.02354
Diabetes mellitus	5230	CHUK-NFKB2-REL-IKBKG-SPAG9-NFKB1-NFKBIE-COPB2-TNIP1-NFKBIA-RELA-TNIP2	0.01519
Diabetes mellitus	5232	TNF-alpha/Nf-kappa B signaling complex (RPL6 RPL30 RPS13 CHUK DDX3X NFKB2 NFKBIB REL IKBKG NFKB1 MAP3K8 RELB GLG1 NFKBIA RELA TNIP2 GTF2I)	0.01277
Diabetes mellitus	5233	TNF-alpha/NF-kappa B signaling complex 5	0.01053
Diabetes mellitus	5235	WRN-Ku70-Ku80-PARP1 complex	0.05263
Diabetes mellitus	5253	MNK1-eIF4F complex	0.02354
Diabetes mellitus	5260	TCF4-CTNNB1-SUMO1-EP300-HADAC6 complex	0.02354
Diabetes mellitus	5261	TCF4-CTNNB1-EP300 complex	0.03039
Diabetes mellitus	5273	VHL-TBP1-HIF1A complex	0.03039
Diabetes mellitus	5276	HIF1A-OS9-EGLN1 complex	0.03039
Diabetes mellitus	5277	HIF1A-OS9-EGLN3 complex	0.03039
Diabetes mellitus	5280	RAB9-TIP47-MPRI complex	0.03039
Diabetes mellitus	5337	ELMO1-DOCK1 complex	0.03722
Diabetes mellitus	5341	ELMO1-DOCK2 complex	0.03722
Diabetes mellitus	5342	ELMO1-DOCK1-RAC1 complex	0.03039
Diabetes mellitus	5343	ELMO1-DOCK1-CRKII complex	0.03039
Diabetes mellitus	5375	EGR-EP300 complex	0.03722
Diabetes mellitus	5382	ARNT-HIF1A complex	0.07443
Diabetes mellitus	5414	HTR1A-HTR1D complex	0.03722
Diabetes mellitus	5416	HTR1A-HTR1B complex	0.03722
Diabetes mellitus	5418	GABBR2-HTR1A complex	0.03722
Diabetes mellitus	5419	HTR1A-GPR26 complex	0.03722
Diabetes mellitus	5420	HTR1A-EDG3 complex	0.03722
Diabetes mellitus	5421	HTR1A homodimer complex	0.05263
Diabetes mellitus	5422	HTR1A-EDG1 complex	0.03722
Diabetes mellitus	5423	HSP70-BAG5-PARK2 complex	0.02632
Diabetes mellitus	5446	EPO-EPOR complex	0.03722
Diabetes mellitus	5460	p50-p65 NF(kappa)B complex	0.03722
Diabetes mellitus	5461	p50-p65 NF(kappa)B-SRC1 complex	0.03039
Diabetes mellitus	5464	I(kappa)B(alpha)-NF(kappa)Bp50-NF(kappa)Bp65 complex	0.03039

Diabetes mellitus	5465	IKB(epsilon)-RELA-cREL complex	0.03039
Diabetes mellitus	5466	IKB(beta)-RELA-cREL complex	0.03039
Diabetes mellitus	5467	IKB(alpha)-RELA-cREL complex	0.03039
Diabetes mellitus	5473	FAS-FADD-CASP8 complex	0.03039
Diabetes mellitus	5475	MURR1-NF(kappa)Bp65-IKBA complex	0.03039
Diabetes mellitus	5492	IKBA-NF(kappa)Bp65-NF(kappa)Bp50 complex	0.03039
Diabetes mellitus	5530	Homodimeric complex LTBR	0.05263
Diabetes mellitus	5548	IL-12 heterodimer complex	0.03722
Diabetes mellitus	5549	IL-12 subunit p40 homodimer complex	0.05263
Diabetes mellitus	5655	Ternary complex (LRRC7 CAMK2a ACTN4)	0.03039
Diabetes mellitus	5684	Membrane protein complex (DERL1 SELS VCP)	0.03039
Diabetes mellitus	5696	VEGFA(165)-KDR-NRP1 complex	0.03039
Diabetes mellitus	5698	VEGFA(165)-VEGFR2-NRP1 complex	0.03039
Diabetes mellitus	5701	NRP1-VEGF(165/121) complex	0.03722
Diabetes mellitus	5749	MRIT complex	0.03039
Diabetes mellitus	5798	Death induced signaling complex II (FADD CASP8 CFLAR) cytosolic CD95L induced	0.03039
Diabetes mellitus	5799	Death induced signaling complex DISC (FAS FADD CASP8 CFLAR) membrane-associated CD95L	0.02632
Diabetes mellitus	5800	Death-inducing signaling complex DISC (type I cells associated) stimulated	0.03039
Diabetes mellitus	5805	PGAM5-KEAP1-NRF2 complex	0.03039
Diabetes mellitus	5808	DISC complex	0.03039
Diabetes mellitus	5832	PINK1-MIRO2-Milton complex	0.03039
Diabetes mellitus	5837	PPD complex	0.03039
Diabetes mellitus	5859	FAS-FADD-CASP8-CASP10 complex	0.05263
Diabetes mellitus	5861	FAS-FADD-CASP10 complex	0.03039
Diabetes mellitus	5862	CAV1-VDAC1-ESR1 complex	0.03039
Disseminated cancer	486	WIP-WASp-actin-myosin-IIa complex	0.2582
Disseminated cancer	1844	APC-IQGAP1 complex	0.22361
Disseminated cancer	1845	APC-IQGAP1-CLIP-170 complex	0.18257
Disseminated cancer	2510	ZAP70-CRKL-WIPF1-WAS complex	0.31623
Disseminated cancer	2511	CRKL-WIPF1-WAS complex	0.36515
Disseminated cancer	3008	60S APC containing complex	0.11952
Disseminated cancer	3011	APC-IQGAP1-Rac1 complex	0.18257
Disseminated cancer	3012	APC-IQGAP1-Cdc42 complex	0.18257
Disseminated cancer	5615	Emerin complex 52	0.06594
Disseminated intravascular coagulation	5388	SERPINA1-ELA2 complex	0.5
Down syndrome	143	APP-FE65-LRP complex	0.0658
Down syndrome	178	Respiratory chain complex I (holoenzyme)	0.01718
Down syndrome	277	RFC complex	0.05096
Down syndrome	279	RFC complex (activator A 1 complex)	0.05096
Down syndrome	335	p54(nrb)-PSF-matrin3 complex	0.0658
Down syndrome	433	BASC complex (BRCA1-associated genome surveillance complex)	0.0329
Down syndrome	434	BASC (Ab 80) complex (BRCA1-associated genome surveillance complex)	0.04029

Down syndrome	435	BASC (Ab 81) complex (BRCA1-associated genome surveillance complex)	0.04652
Down syndrome	441	TFTC-type histone acetyl transferase complex	0.03436
Down syndrome	443	BP-SMAD complex	0.04029
Down syndrome	706	SNARE complex (HGS SNAP25 STX13)	0.0658
Down syndrome	707	SNARE complex (VAMP2 SNAP25 STX13)	0.0658
Down syndrome	793	SNARE complex (VAMP2 SNAP25 STX1a CPLX1)	0.05698
Down syndrome	794	SNARE complex (VAMP2 SNAP25 STX1a CPLX2)	0.05698
Down syndrome	999	p23 protein complex	0.0658
Down syndrome	1003	RC complex (Replication competent complex)	0.03799
Down syndrome	1004	RC complex during S-phase of cell cycle	0.03161
Down syndrome	1005	RC complex during G2/M-phase of cell cycle	0.03161
Down syndrome	1054	ESR1-RELA-BCL3-NCOA3 complex	0.05698
Down syndrome	1088	PRNP-ApolopoproteinE3 complex	0.08058
Down syndrome	1094	Frataxin complex	0.04307
Down syndrome	1098	DNA synthesome complex (13 subunits)	0.03046
Down syndrome	1099	DNA synthesome complex (17 subunits)	0.02686
Down syndrome	1107	DNA synthesome core complex	0.03604
Down syndrome	1108	DNA synthesome complex (15 subunits)	0.02942
Down syndrome	1137	SNARE complex (VAMP2 SNAP25 STX1a CPLX1 CPLX3)	0.05096
Down syndrome	1138	SNARE complex (VAMP2 SNAP25 STX1a CPLX3 CPLX4)	0.05096
Down syndrome	1139	SNARE complex (VAMP2 SNAP25 STX1a STX3 CPLX1 CPLX3 CPLX4)	0.04307
Down syndrome	1181	C complex spliceosome	0.01274
Down syndrome	1232	REST-CoREST-mSIN3A complex	0.0658
Down syndrome	1335	SNW1 complex	0.02686
Down syndrome	1539	G protein complex (GNG2 GNB2L1 RAF1)	0.0658
Down syndrome	1707	IL2-IL2RA-IL2RB complex	0.0658
Down syndrome	1874	SNARE complex (SNAP25 VAMP3 VAMP2 NAPB STX13)	0.05096
Down syndrome	2028	JAK2-IL12RB2 complex	0.08058
Down syndrome	2124	IKK-alpha--ER-alpha-AIB1 complex	0.0658
Down syndrome	2203	BRD4-RFC complex	0.04652
Down syndrome	2258	VILIP-1-AChR-alpha-4-AChR-beta-2 complex	0.13159
Down syndrome	2470	p130Cas-ER-alpha-cSrc-kinase- PI3-kinase p85-subunit complex	0.05698
Down syndrome	2657	ESR1-CDK7-CCNH-MNAT1-MTA1-HDAC2 complex	0.04652
Down syndrome	2670	Er-alpha-p53-hdm2 complex	0.0658
Down syndrome	2694	ERG-JUN-FOS DNA-protein complex	0.0658
Down syndrome	2699	ER-alpha-GRIP1-c-Jun complex	0.0658
Down syndrome	2700	ER-alpha-c-Jun complex	0.08058
Down syndrome	2711	Amyloid beta protein oligomer	0.11396
Down syndrome	2755	17S U2 snRNP	0.01984
Down syndrome	2788	BRCA1 B complex	0.0658
Down syndrome	2789	ETS2-ERG complex	0.08058
Down syndrome	2815	BRCA1-BARD1-BACH1-DNA damage complex II	0.04029

Down syndrome	2817	BRCA1-BARD1-BACH1-DNA damage complex I	0.04652
Down syndrome	2884	Respiratory chain complex I (early intermediate NDUFAF1 assembly) mitochondrial	0.04307
Down syndrome	2898	Respiratory chain complex I (intermediate I/200kD and III/250kD) mitochondrial	0.0658
Down syndrome	2901	Respiratory chain complex I (intermediate IV/310kD) mitochondrial	0.05698
Down syndrome	2903	Respiratory chain complex I (intermediate V/380kD and VI/480kD) mitochondrial	0.05096
Down syndrome	2904	Respiratory chain complex I (intermediate VII/650kD) mitochondrial	0.03604
Down syndrome	2920	Respiratory chain complex I (lambda subunit) mitochondrial	0.02849
Down syndrome	2938	Ecsit complex (ECSIT NDUFS3 TOM20)	0.0658
Down syndrome	2942	Ecsit complex (ECSIT NDUFS3 NDUFAF1)	0.0658
Down syndrome	3092	APP-TOMM40 complex	0.08058
Down syndrome	3093	APP-TIMM23 complex	0.08058
Down syndrome	3151	Sulphiredoxin-peroxiredoxin complex	0.08058
Down syndrome	5178	JAK2-PAFR-TYK2 complex	0.0658
Down syndrome	5770	RUNX1-CBF-beta-DNA complex	0.08058
Down syndrome	5862	CAV1-VDAC1-ESR1 complex	0.0658
Drug abuse	4	Multisubunit ACTR coactivator complex	0.04683
Drug abuse	74	TRPC1-Homer3-IP3R complex	0.05407
Drug abuse	92	CD28-transactivation complex	0.06623
Drug abuse	201	HUIC complex	0.06623
Drug abuse	202	BRCA1-RAD50-MRE11-NBS1 complex	0.04683
Drug abuse	238	SWI-SNF chromatin remodeling-related-BRCA1	0.02824
Drug abuse	240	BRCA1-CTIP-ZBRK1 repressor complex	0.05407
Drug abuse	242	BRCA1-BACH1 complex	0.06623
Drug abuse	244	BRAFT complex	0.02598
Drug abuse	246	BLM complex III	0.04683
Drug abuse	285	PCNA-MLH1-PMS1 complex	0.05407
Drug abuse	290	MSH2-MLH1-PMS2-PCNA DNA-repair initiation complex	0.04683
Drug abuse	291	MSH2-MLH1-PMS2 DNA-repair initiation complex	0.05407
Drug abuse	292	MutL-alpha complex	0.06623
Drug abuse	298	VEGF transcriptional complex	0.03824
Drug abuse	310	Cell cycle kinase complex CDC2	0.03824
Drug abuse	311	Cell cycle kinase complex CDK2	0.04683
Drug abuse	312	Cell cycle kinase complex CDK4	0.04683
Drug abuse	313	Cell cycle kinase complex CDK5	0.04189
Drug abuse	360	Artemis-DNA-PK complex	0.06623
Drug abuse	369	MSH2-MSH6-PMS2-MLH1 complex	0.04683
Drug abuse	370	MSH2-MSH6-PMS1-MLH1 complex	0.04683
Drug abuse	376	PCNA-MutS-alpha-MutL-alpha-DNA complex	0.04189
Drug abuse	380	MutL-beta complex	0.06623
Drug abuse	415	EXO1-MLH1-PMS2 complex	0.05407
Drug abuse	424	EXO1-MLH1-PCNA complex	0.05407

Drug abuse	433	BASC complex (BRCA1-associated genome surveillance complex)	0.05407
Drug abuse	434	BASC (Ab 80) complex (BRCA1-associated genome surveillance complex)	0.06623
Drug abuse	435	BASC (Ab 81) complex (BRCA1-associated genome surveillance complex)	0.07647
Drug abuse	436	BASC (Ab C-20) complex (BRCA1-associated genome surveillance complex)	0.05407
Drug abuse	438	GCN5-TRRAP histone acetyltransferase complex	0.02962
Drug abuse	471	PCAF complex	0.02962
Drug abuse	536	TRPC1-TRPC3-TRPC7 complex	0.05407
Drug abuse	548	DRIP complex	0.02503
Drug abuse	553	RHOA-IP3R-TRPC1 complex	0.05407
Drug abuse	557	TRP1-G alpha-11-IP3R3-CAV1 signaling complex	0.04683
Drug abuse	655	HSF1-HSF2 complex	0.06623
Drug abuse	668	BKCA-beta2AR-AKAP79 signaling complex	0.05407
Drug abuse	672	BKCA-beta2AR complex	0.06623
Drug abuse	687	CFTR-NHERF-beta(2)AR signaling complex	0.05407
Drug abuse	706	SNARE complex (HGS SNAP25 STX13)	0.05407
Drug abuse	753	UTM-SGCE-DAG1-CAV1-NOS3 complex	0.04189
Drug abuse	784	SMG-1-Upf1-eRF1-eRF3 complex (SURF)	0.04189
Drug abuse	809	eRF1-eRF3-GTP-Mg(2+) complex	0.05407
Drug abuse	828	TRPC1-STIM1-ORAI1 complex	0.05407
Drug abuse	1062	BAR-BCL2-CASP8 complex	0.05407
Drug abuse	1088	PRNP-ApolopoproteinE3 complex	0.06623
Drug abuse	1095	SNX complex (SNX1a SNX2 SNX4 EGFR)	0.04683
Drug abuse	1185	EGFR-containing signaling complex	0.09366
Drug abuse	1219	Tankyrin 1-tankyrin 2-TRF1 complex	0.05407
Drug abuse	1338	FOXO3-PCAF complex oxidative stress stimulated	0.06623
Drug abuse	1633	CyclinD1-CDK4-CDK6 complex	0.05407
Drug abuse	1634	CyclinD1-CDK4-p21 complex	0.05407
Drug abuse	1700	ABL2-HRAS-RIN1 complex	0.05407
Drug abuse	1729	TLE1 corepressor complex (MASH1 promoter-corepressor complex)	0.02962
Drug abuse	1993	SLC1A2 homotrimer complex	0.09366
Drug abuse	2145	HSF1-YWHAE complex	0.06623
Drug abuse	2210	BRCA1-IRIS-pre-replication complex	0.04683
Drug abuse	2211	BARD1-BRCA1-CSTF complex	0.04189
Drug abuse	2213	BRCA1-BARD1-POLR2A complex	0.05407
Drug abuse	2214	LMO4-BRCA1-CTIP-LDB1 complex	0.04683
Drug abuse	2215	BRCA1-LMO4-CTIP complex	0.05407
Drug abuse	2254	CTGF/Hcs24-actin complex	0.05407
Drug abuse	2342	ITGAV-ITGB8-MMP14-TGFB1 complex	0.04683
Drug abuse	2343	ITGAV-ITGB5-PLAUR complex	0.05407
Drug abuse	2345	ITGAV-ITGB5-ICAM4 complex	0.05407
Drug abuse	2346	ITGAV-ITGB5-ADAM9 complex	0.05407
Drug abuse	2347	ITGAV-ITGB5-SPP1 complex	0.05407
Drug abuse	2348	ITGAV-ITGB5-CYR61 complex	0.05407

Drug abuse	2350	ITGAV-ITGB5 complex	0.06623
Drug abuse	2352	ITGAV-ITGB6-SPP1 complex	0.05407
Drug abuse	2353	ITGAV-ITGB6-TGFB3 complex	0.05407
Drug abuse	2354	ITGAV-ITGB6 complex	0.06623
Drug abuse	2355	ITGAV-ITGB3-CD47-FCER2 complex	0.04683
Drug abuse	2356	ITGB3-ITGAV-CD47 complex	0.05407
Drug abuse	2358	ITGAV-ITGB3-SPP1 complex	0.05407
Drug abuse	2359	ITGAV-ITGB3-ADAM15 complex	0.05407
Drug abuse	2362	ITAGV-ITGB3-F11R complex	0.05407
Drug abuse	2363	ITGAV-ITGB3-PXN-PTK2b complex	0.04683
Drug abuse	2364	ITGAV-ITGB3-ADAM23 complex	0.05407
Drug abuse	2365	ITGAV-ITGB3-COL4A3 complex	0.05407
Drug abuse	2366	ITGAV-ITGB3-PPAP2b complex	0.05407
Drug abuse	2369	ITGAV-ITGB3-EGFR complex	0.10815
Drug abuse	2374	ITGAV-ITGB3-LAMA4 complex	0.05407
Drug abuse	2383	ITGA5-ITGB1-FN1-TGM2 complex	0.04683
Drug abuse	2384	ITGA5-ITGB1-ADAM15 complex	0.05407
Drug abuse	2385	ITGA5-ITGB4 complex	0.06623
Drug abuse	2388	Itga5-Itgb1-Fn1-Sfrp2 complex	0.04683
Drug abuse	2390	CD98-LAT2-ITGB1 complex	0.04683
Drug abuse	2395	ITGA7-ITGB1-CD151 complex	0.05407
Drug abuse	2396	ITGA7-ITGB1-CD9 complex	0.05407
Drug abuse	2397	ITGA7-ITGB1-ITGB1BP3 complex	0.05407
Drug abuse	2398	ITGA3-ITGB1-BSG complex	0.05407
Drug abuse	2399	ITGA3-ITGB1-CD63 complex	0.05407
Drug abuse	2400	ITGA3-ITGB1-CD151 complex	0.05407
Drug abuse	2401	ITGA3-ITGB1-THBS1 complex	0.05407
Drug abuse	2406	ITGA3-ITGB1 complex	0.06623
Drug abuse	2411	ITGA6-ITGB1-CD151 complex	0.05407
Drug abuse	2413	ITGA6-ITGB1 complex	0.06623
Drug abuse	2416	ITGB1-RAP1A-PKD1 complex	0.05407
Drug abuse	2417	ITGA4-ITGB1-EMILIN1 complex	0.05407
Drug abuse	2418	ITGA4-ITGB1 complex	0.06623
Drug abuse	2419	ITGA4-ITGB1-CD81 complex	0.05407
Drug abuse	2420	ITGA4-ITGB1-CD53 complex	0.05407
Drug abuse	2421	ITGA4-ITGB1-VCAM1 complex	0.05407
Drug abuse	2422	ITGA4-ITGB1-JAM2 complex	0.05407
Drug abuse	2423	ITGA4-ITGB1-CD47 complex	0.05407
Drug abuse	2424	ITGA4-ITGB1-CD63 complex	0.05407
Drug abuse	2425	ITGA4-ITGB1-PXN complex	0.05407
Drug abuse	2426	ITGA4-ITGB1-THBS1 complex	0.05407
Drug abuse	2428	ITGA4-ITGB1-THBS2 complex	0.05407
Drug abuse	2429	ITGA2-ITGB1-CD47 complex	0.10815
Drug abuse	2430	ITGA2-ITGB1-CHAD complex	0.10815
Drug abuse	2431	ITGA2-ITGB1-COL6A3 complex	0.10815
Drug abuse	2432	ITGA2-ITGB1 complex	0.13245
Drug abuse	2434	ITGA1-ITGB1-COL6A3 complex	0.05407

Drug abuse	2435	ITGA1-ITGB1-PTPN2 complex	0.05407
Drug abuse	2436	ITGAV-ITGB1 complex	0.13245
Drug abuse	2437	ITGA6-ITGB1-CYR61 complex	0.05407
Drug abuse	2439	ITGA8-ITGB1 complex	0.06623
Drug abuse	2440	ITGA9-ITGB1-ADAM9 complex	0.05407
Drug abuse	2441	Itga9-Itgb1-Adam2 complex	0.05407
Drug abuse	2442	ITGA9-ITGB1-VCAM1 complex	0.05407
Drug abuse	2443	ITGA9-ITGB1-TNC complex	0.05407
Drug abuse	2444	ITGB1-ITGA9 complex	0.06623
Drug abuse	2445	ITGA9-ITGB1-ADAM15 complex	0.05407
Drug abuse	2446	ITGA9-ITGB1-FIGF complex	0.05407
Drug abuse	2447	ITGA9-ITGB1-ADAM12 complex	0.05407
Drug abuse	2453	Multiprotein complex (monoubiquitination)	0.04683
Drug abuse	2454	CIN85-CBL-SH3GL2-EGFR complex EGF stimulated	0.04683
Drug abuse	2528	ERBB2-MEMO-SHC complex	0.05407
Drug abuse	2542	EGFR-CBL-GRB2 complex	0.05407
Drug abuse	2635	BETA2-Cyclin D1 complex	0.06623
Drug abuse	2641	p300/CBP-PCAF-MyoD complex	0.04683
Drug abuse	2686	BRCA1-core RNA polymerase II complex	0.02598
Drug abuse	2721	HCF-1 complex	0.02149
Drug abuse	2776	RAD50-BRCA1 complex	0.06623
Drug abuse	2783	BARD1-BRCA1-CSTF64 complex	0.05407
Drug abuse	2786	BRCA1 A complex	0.04683
Drug abuse	2787	BRCA1 C complex	0.04683
Drug abuse	2788	BRCA1 B complex	0.05407
Drug abuse	2811	BRCA1-cABL complex	0.06623
Drug abuse	2813	BRCA1-SMAD3 complex	0.06623
Drug abuse	2814	BRCA1-HDAC1-HDAC2 complex	0.05407
Drug abuse	2815	BRCA1-BARD1-BACH1-DNA damage complex II	0.03311
Drug abuse	2816	ITGAV-ITGB3 complex	0.06623
Drug abuse	2817	BRCA1-BARD1-BACH1-DNA damage complex I	0.07647
Drug abuse	2818	BRCA1-BARD1-BRCA2-DNA damage complex III	0.05407
Drug abuse	2819	BRCA1-CtIP-CtBP complex	0.05407
Drug abuse	2820	BRCA1-VCP complex	0.06623
Drug abuse	2822	BRCA1-BARD1-UbcH5c complex	0.05407
Drug abuse	2823	BRCA1-BARD1-UbcH7c complex	0.05407
Drug abuse	2824	BRCA1-RAD51 complex	0.06623
Drug abuse	2825	BRCA1-RNA polymerase II complex	0.01837
Drug abuse	2826	ITGB3-ITGAV-VTN complex	0.05407
Drug abuse	2846	ITGAV-ITGB3-THBS1 complex	0.05407
Drug abuse	2849	ITGAV-ITGB3-NOV complex	0.05407
Drug abuse	2850	ITGA5-ITGB1-FN-1-NOV complex	0.04683
Drug abuse	2853	ITGA5-ITGB1-CAL4A3 complex	0.05407
Drug abuse	2885	ITGAV-ITGB1-SPP1 complex	0.10815
Drug abuse	2954	Smad1-Notch1-p300-Pcaf complex	0.04683
Drug abuse	2964	ITGA9-ITGB1-ADAM1 complex	0.05407
Drug abuse	2965	ITGA9-ITGB1-ADAM3 complex	0.05407

Drug abuse	2971	ITGA9-ITGB1-VEGFC complex	0.05407
Drug abuse	2972	ITGA9-ITGB1-VEGFA complex	0.05407
Drug abuse	2989	ITGA9-ITGB1-ADAM8 complex	0.05407
Drug abuse	3035	LAT2-ITGB1 complex	0.06623
Drug abuse	3057	ITGA10-ITGB1 complex	0.06623
Drug abuse	3058	ITGA11-ITGB1 complex	0.06623
Drug abuse	3059	ITGA11-ITGB1-COL1A1 complex	0.05407
Drug abuse	3061	RNA polymerase II complex (CBP PCAF RPB1 BAF47 CYCC CDK8) chromatin structure modifying	0.03824
Drug abuse	3062	RNA polymerase II complex incomplete (CBP RPBI PCAF BAF47) chromatin structure modifying	0.04683
Drug abuse	3066	RNA polymerase II complex chromatin structure modifying	0.02598
Drug abuse	3084	CCND1-CDK4 complex	0.06623
Drug abuse	3087	CCND1-CDK6 complex	0.06623
Drug abuse	3103	ITGAV-ITGB3-SLC3A2 complex	0.05407
Drug abuse	3104	ITGB1-NRP1 complex	0.06623
Drug abuse	3110	ITGAV-P2RY2-GNA12 complex	0.05407
Drug abuse	3111	ITGA9-ITGB1-SPP1 complex	0.05407
Drug abuse	3112	ITGA5-ITGB1-SPP1 complex	0.05407
Drug abuse	3117	ITGB5-ITGAV-VTN complex	0.05407
Drug abuse	3137	MASH1 promoter-coactivator complex	0.02824
Drug abuse	3142	CAMK2-delta-MASH1 promoter-coactivator complex	0.03311
Drug abuse	3162	TF-FVIIa-FXa-TFPI complex	0.04683
Drug abuse	3677	RIN1-STAM2-HRS complex	0.05407
Drug abuse	3678	RIN1-STAM2-EGFR complex EGF stimulated	0.05407
Drug abuse	3830	ADRB2 homodimer complex	0.09366
Drug abuse	5171	SH3KBP1-CBLB-EGFR complex	0.05407
Drug abuse	5190	TIAM1-EFNB1-EPHA2 complex	0.05407
Drug abuse	5199	Kinase maturation complex 1	0.02341
Drug abuse	5273	VHL-TBP1-HIF1A complex	0.05407
Drug abuse	5276	HIF1A-OS9-EGLN1 complex	0.05407
Drug abuse	5277	HIF1A-OS9-EGLN3 complex	0.05407
Drug abuse	5287	CDK4-CCND1 complex	0.06623
Drug abuse	5367	THRB-RXRB complex	0.06623
Drug abuse	5373	Chromatin remodeling complex (TACC2 TACC3	0.05407
Drug abuse	5382	ARNT-HIF1A complex	0.06623
Drug abuse	5386	MLL1-WDR5 complex	0.01802
Drug abuse	5400	BRCC complex	0.04189
Drug abuse	5415	HTR1B homodimer complex	0.09366
Drug abuse	5416	HTR1A-HTR1B complex	0.06623
Drug abuse	5417	HTR1D-HTR1B complex	0.06623
Drug abuse	5423	HSP70-BAG5-PARK2 complex	0.04683
Drug abuse	5442	EPOR receptor complex	0.09366
Drug abuse	5446	EPO-EPOR complex	0.13245
Drug abuse	5526	CALM1-FKBP38-BCL2 complex	0.05407
Drug abuse	5811	p53-BCL2 complex	0.06623
Drug abuse	5817	tBID-BCL2 complex	0.06623

Drug abuse	5818	BIM-BCL2 complex	0.06623
Drug abuse	5922	RAF1-RAS complex EGF induced	0.04683
Drug-Induced dyskinesia	1088	PRNP-ApolopoproteinE3 complex	0.26726
Eating disorder	98	p300-MDM2-p53 protein complex	0.1005
Eating disorder	143	APP-FE65-LRP complex	0.1005
Eating disorder	220	ARF-Mule complex	0.1005
Eating disorder	1062	BAR-BCL2-CASP8 complex	0.1005
Eating disorder	1069	FIF-FGR2 complex	0.12309
Eating disorder	1091	SNX complex (SNX1a SNX2 SNX4 LEPR)	0.08704
Eating disorder	1474	SMAD3/4-E2F4/5-p107-DP1 complex	0.07107
Eating disorder	1642	p16-cyclin D2-CDK4 complex	0.1005
Eating disorder	1700	ABL2-HRAS-RIN1 complex	0.1005
Eating disorder	5718	eNOS-HSP90-AKT complex VEGF induced	0.1005
Eating disorder	1826	SMAD3-HEF1-APC10-CDH1 complex	0.08704
Eating disorder	1827	PML-SMAD2/3-SARA complex	0.08704
Eating disorder	1828	TGF-beta receptor I-Axin-SMAD3 complex	0.1005
Eating disorder	1831	PIAS3-SMAD3-P300 complex	0.1005
Eating disorder	5716	eNOS-HSP90 complex VEGF induced	0.12309
Eating disorder	1992	LEPR homodimer complex	0.17408
Eating disorder	2112	CDC37-HSP90AA1-HSP90AB1-MAP3K11 complex	0.08704
Eating disorder	2189	Ubiquitin E3 ligase (SMAD3 BTRC CUL1 SKP1A RBX1)	0.07785
Eating disorder	2256	RIAM-Rap1-GTP complex	0.12309
Eating disorder	2347	ITGAV-ITGB5-SPP1 complex	0.1005
Eating disorder	2352	ITGAV-ITGB6-SPP1 complex	0.1005
Eating disorder	2358	ITGAV-ITGB3-SPP1 complex	0.1005
Eating disorder	2416	ITGB1-RAP1A-PKD1 complex	0.1005
Eating disorder	2670	Er-alpha-p53-hdm2 complex	0.1005
Eating disorder	2692	SMAD3-SMAD4-cJun-cFos complex	0.08704
Eating disorder	2705	SMAD3-SMAD4-CTCF protein-DNA complex	0.1005
Eating disorder	2706	SMAD3-SMAD4-SP1 complex	0.1005
Eating disorder	2707	SMAD3-SMAD4-FOXO3-FOXG1 complex	0.08704
Eating disorder	2708	SMAD3-SMAD4-cJUN complex	0.1005
Eating disorder	2711	Amyloid beta protein oligomer	0.17408
Eating disorder	2721	HCF-1 complex	0.03994
Eating disorder	2754	JUND-FOSB-SMAD3-SMAD4 complex	0.08704
Eating disorder	2760	SMAD3-SMAD4-FOXO3 complex	0.1005
Eating disorder	2761	SMAD3-SMAD4-FOXO1 complex	0.1005
Eating disorder	2762	SMAD3-SMAD4-FOXO4 complex	0.1005
Eating disorder	2813	BRCA1-SMAD3 complex	0.12309
Eating disorder	2829	RSmad complex	0.05505
Eating disorder	2830	TIF1gamma-SMAD2-SMAD3 complex	0.1005
Eating disorder	2834	SMAD4-SMAD2-SMAD3 complex	0.1005
Eating disorder	2885	ITGAV-ITGB1-SPP1 complex	0.1005
Eating disorder	2968	Axin-SMAD3 complex	0.12309
Eating disorder	2975	SMAD3-E2F4/5-p107-DP1 complex	0.07785
Eating disorder	3092	APP-TOMM40 complex	0.12309

Eating disorder	3093	APP-TIMM23 complex	0.12309
Eating disorder	3111	ITGA9-ITGB1-SPP1 complex	0.1005
Eating disorder	3112	ITGA5-ITGB1-SPP1 complex	0.1005
Eating disorder	3158	RIAM-Rap1-GTP-profilin complex	0.1005
Eating disorder	3172	NUMB-TP53-MDM2 complex	0.1005
Eating disorder	3177	GNA14-p115RhoGEF complex	0.12309
Eating disorder	3199	SMAD3-SKI complex	0.12309
Eating disorder	3205	SMAD3-SKI-NCOR complex	0.1005
Eating disorder	3733	SKI-SMAD3 hexameric complex	0.12309
Eating disorder	3740	SKI-SMAD3-SMAD4 pentameric complex	0.1005
Eating disorder	3750	CREBBP-SMAD3 hexameric complex	0.12309
Eating disorder	3754	CREBBP-SMAD3-SMAD4 pentameric complex	0.1005
Eating disorder	3959	SMAD3-SMAD4-cSKI TGF(beta)-dependent	0.1005
Eating disorder	3961	SMAD3-cSKI-SIN3A-HDAC1 complex	0.08704
Eating disorder	3971	SMURF2-SMAD3 complex TGF(beta)-dependent	0.12309
Eating disorder	3972	SMURF2-SMAD3-SnoN complex TGF(beta)-	0.1005
Eating disorder	4158	HSP90-FKBP38-CAM-Ca(2+) complex	0.08704
Eating disorder	5199	Kinase maturation complex 1	0.04352
Eating disorder	5212	Kinase maturation complex 2	0.06155
Eating disorder	5234	IKBKB-CDC37-KIAA1967-HSP90AB1-HSP90AA1 complex	0.07785
Eating disorder	5266	TNF-alpha/NF-kappa B signaling complex 6	0.04652
Eating disorder	5268	TNF-alpha/NF-kappa B signaling complex 7	0.06155
Eating disorder	5269	TNF-alpha/NF-kappa B signaling complex 8	0.07107
Eating disorder	5286	TNF-alpha/NF-kappa B signaling complex 10	0.05505
Eating disorder	5331	YY1-MDM2-p53 complex	0.1005
Eating disorder	5526	CALM1-FKBP38-BCL2 complex	0.1005
Eating disorder	5622	HSP90-CIP1-FKBPL complex	0.1005
Eating disorder	5735	TGF-beta receptor-SMAD3 complex	0.1005
Eating disorder	5811	p53-BCL2 complex	0.12309
Eating disorder	5817	tBID-BCL2 complex	0.12309
Eating disorder	5818	BIM-BCL2 complex	0.12309
Eating disorder	5922	RAF1-RAS complex EGF induced	0.08704
Ectodermal dysplasia	748	TRAF6-TAK1 complex	0.2357
Ectodermal dysplasia	2073	TNFRSF11A-TRAF6-SRC complex	0.19245
Ectodermal dysplasia	2074	TRAF6 oligomer complex	0.33333
Ectodermal dysplasia	2743	TRAF6-MALT1 complex	0.2357
Ectodermal dysplasia	2745	Ubiquitin ligase complex (TRAF6 TAB2 MALT1 UEV1A BCL10)	0.14907
Ectodermal dysplasia	2936	Ecsit complex (ECSIT MT-CO2 GAPDH TRAF6 NDUFAF1)	0.14907
Ectodermal dysplasia	2939	Ecsit complex (ECSIT MT-CO2 NDUFA1 MT-ND1 TRAF6 NDUFAF1)	0.13608
Ectodermal dysplasia	5210	TANK-TRAF2-TRAF3 complex	0.19245
Embryoma	23	BLOC-1 (biogenesis of lysosome-related organelles complex 1)	0.0218
Embryoma	27	Arp2/3 protein complex	0.06992
Embryoma	33	Prefoldin	0.02517

Embryoma	61	Mi2/NuRD complex	0.02331
Embryoma	62	MeCP1 complex	0.0218
Embryoma	63	Mitotic 14S cohesin 1 complex	0.03083
Embryoma	92	CD28-transactivation complex	0.0436
Embryoma	112	Prefoldin complex	0.02517
Embryoma	115	Polycomb repressive complex 1 (PRC1 hPRC-H)	0.0178
Embryoma	116	Polycomb repressive complex 1 (PRC1 hPRC-H)	0.0171
Embryoma	127	NDC80 kinetochore complex	0.03083
Embryoma	145	CCT:PFD complex	0.01648
Embryoma	164	Cohesin-SA1 complex	0.03083
Embryoma	244	BRAFT complex	0.0171
Embryoma	246	BLM complex III	0.03083
Embryoma	282	SNF2h-cohesin-NuRD complex	0.01542
Embryoma	305	40S ribosomal subunit cytoplasmic	0.01058
Embryoma	306	Ribosome cytoplasmic	0.02741
Embryoma	308	60S ribosomal subunit cytoplasmic	0.02698
Embryoma	5856	AK2-FADD-caspase-10 (AFAC10) complex	0.0356
Embryoma	351	Spliceosome	0.00516
Embryoma	359	DNA ligase IV-XRCC4-XLF complex	0.0356
Embryoma	433	BASC complex (BRCA1-associated genome surveillance complex)	0.0178
Embryoma	434	BASC (Ab 80) complex (BRCA1-associated genome surveillance complex)	0.0218
Embryoma	541	IGF1-IGFBP3-ALS complex	0.0356
Embryoma	577	FHL2-p53-HIPK2 complex	0.0356
Embryoma	585	Mi2/NuRD-BCL6-MTA3 complex	0.02758
Embryoma	587	NuRD.1 complex	0.0218
Embryoma	614	NRD complex (Nucleosome remodeling and deacetylation complex)	0.02331
Embryoma	620	CoREST-HDAC complex	0.02331
Embryoma	626	LSD1 complex	0.0171
Embryoma	629	BLM-TRF2 complex	0.0436
Embryoma	632	Anti-HDAC2 complex	0.02907
Embryoma	633	anti-BHC110 complex	0.01859
Embryoma	636	BHC complex	0.02517
Embryoma	652	AP3-BLOC1 complex	0.01592
Embryoma	654	BLOC1-BLOC2 complex	0.01859
Embryoma	655	HSF1-HSF2 complex	0.0436
Embryoma	659	MeCP1 complex	0.02055
Embryoma	685	MeCP1 complex	0.02055
Embryoma	749	MeCP2-SIN3A-HDAC complex	0.03083
Embryoma	778	LARC complex (LCR-associated remodeling complex)	0.01415
Embryoma	871	BRAF53-BRCA2 complex	0.02331
Embryoma	888	MTA2 complex	0.02055
Embryoma	929	CEN complex	0.01014
Embryoma	1045	Snurportin-CRM1-RanGTP complex	0.0356
Embryoma	1088	PRNP-ApolopoproteinE3 complex	0.0436
Embryoma	1094	Frataxin complex	0.02331

Embryoma	1116	CRM1-Survivin-AuroraB mitotic complex	0.0356
Embryoma	1117	CRM1-Survivin mitotic complex	0.0436
Embryoma	1134	ATR-HDAC2-CHD4 complex	0.0356
Embryoma	1176	CRM1-RAN-PHAX-CBC complex (cap binding	0.02758
Embryoma	1178	BCOR complex	0.0218
Embryoma	1182	CDC5L core complex	0.02517
Embryoma	1183	CDC5L complex	0.01126
Embryoma	1191	RNA pol II containing coactivator complex Tat-SF	0.02758
Embryoma	1194	E2F-6 complex	0.0178
Embryoma	1218	BLM-TRF2 complex	0.0436
Embryoma	1219	Tankyrin 1-tankyrin 2-TRF1 complex	0.0356
Embryoma	1231	FIB-associated protein complex	0.02517
Embryoma	1306	PIN1-AUF1 complex	0.0436
Embryoma	1401	MOF complex	0.0195
Embryoma	1462	hPRC1L complex	0.03083
Embryoma	1474	SMAD3/4-E2F4/5-p107-DP1 complex	0.02517
Embryoma	1492	BHC110 complex	0.0195
Embryoma	1495	PID complex	0.02758
Embryoma	1514	IL4-IL4R complex	0.0436
Embryoma	1515	IL4-IL4R-IL2RG complex	0.0712
Embryoma	1519	IL6ST-PRKCD-STAT3 complex	0.0356
Embryoma	1539	G protein complex (GNG2 GNB2L1 RAF1)	0.0356
Embryoma	1707	IL2-IL2RA-IL2RB complex	0.1068
Embryoma	1714	TICAM1-TICAM2-TLR4 complex	0.0356
Embryoma	1729	TLE1 corepressor complex (MASH1 promoter-corepressor complex)	0.0195
Embryoma	1731	PRMT1 complex	0.06166
Embryoma	1772	MICB-KLRK1-HCST complex	0.0356
Embryoma	1774	MICA-KLRK1-HCST complex	0.0356
Embryoma	1777	TGF-beta-receptor-SMAD7-SMURF2 complex	0.03083
Embryoma	1784	RNF11-SMURF2-STAMBP complex	0.0356
Embryoma	1787	Nogo-potassium channel complex	0.03083
Embryoma	1812	AXIN-MEKK4-CCD1 complex	0.0356
Embryoma	1826	SMAD3-HEF1-APC10-CDH1 complex	0.03083
Embryoma	1827	PML-SMAD2/3-SARA complex	0.03083
Embryoma	1828	TGF-beta receptor I-Axin-SMAD3 complex	0.0356
Embryoma	1831	PIAS3-SMAD3-P300 complex	0.0356
Embryoma	2051	MALT1 oligomer complex	0.06166
Embryoma	2053	BCL10-MALT1 complex	0.0436
Embryoma	2054	CASP8-FADD-MALT1-BCL10 complex	0.03083
Embryoma	2055	CASP8-CHUK-IKBKB-MALT1-BCL10 complex	0.02758
Embryoma	2143	MAP2K5-PRKCI-SQSTM1 complex	0.0356
Embryoma	2145	HSF1-YWHAE complex	0.0436
Embryoma	2189	Ubiquitin E3 ligase (SMAD3 BTRC CUL1 SKP1A RBX1)	0.02758
Embryoma	2222	BLM complex II	0.02517
Embryoma	2223	BLM-TOP3A complex	0.0436
Embryoma	2224	MSH2/6-BLM-p53-RAD51 complex	0.02758

Embryoma	2228	BLM-RAD51L3-XRCC2 complex	0.0356
Embryoma	2247	Dynactin complex (DCTN1 DCTN2 DCTN3 DCTN4 DCTN6 CAPZA1 CAPZB ACTR1A)	0.0218
Embryoma	2254	CTGF/Hcs24-actin complex	0.0356
Embryoma	2255	Cofilin-actin-CAP1 complex	0.0356
Embryoma	2443	ITGA9-ITGB1-TNC complex	0.0356
Embryoma	2480	CIN85 complex (CIN85 CRK BCAR1 CBL PIK3R1 GRB2 SOS1)	0.02331
Embryoma	2486	GIPC1-LHCGR complex	0.0436
Embryoma	2487	GIPC1-NTRK1-RGS19 complex	0.0356
Embryoma	2489	NCR3-CD247 complex	0.0436
Embryoma	2528	ERBB2-MEMO-SHC complex	0.0356
Embryoma	2535	SLP-76-Cbl-Grb2-Shc complex Fc receptor gamma-R1 stimulated	0.03083
Embryoma	2547	PLC-gamma-1-SLP-76-SOS1-LAT complex	0.03083
Embryoma	2590	FOXO1-FHL2-SIRT1 complex	0.0356
Embryoma	2599	POLR2A-CCNT1-CDK9-NCL-LEM6-CPSF2 complex	0.02517
Embryoma	2635	BETA2-Cyclin D1 complex	0.0436
Embryoma	2650	DNMT3B-DNMT3L complex	0.0436
Embryoma	2651	DNMT3L-DNMT3A complex	0.0436
Embryoma	2692	SMAD3-SMAD4-cJun-cFos complex	0.03083
Embryoma	2705	SMAD3-SMAD4-CTCF protein-DNA complex	0.0356
Embryoma	2706	SMAD3-SMAD4-SP1 complex	0.0356
Embryoma	2707	SMAD3-SMAD4-FOXO3-FOXG1 complex	0.03083
Embryoma	2708	SMAD3-SMAD4-cJUN complex	0.0356
Embryoma	2709	MMP-9-TIMP-1-LRP complex	0.0356
Embryoma	2739	FA complex (Fanconi anemia complex)	0.01859
Embryoma	2743	TRAF6-MALT1 complex	0.0436
Embryoma	2744	TRAF2-MALT1 complex	0.0436
Embryoma	2745	Ubiquitin ligase complex (TRAF6 TAB2 MALT1 UEV1A BCL10)	0.02758
Embryoma	2752	CARMA1-BCL10-MALT1 complex	0.0356
Embryoma	2753	FYB-CARMA1-BCL-10-MALT1 complex	0.03083
Embryoma	2754	JUND-FOSB-SMAD3-SMAD4 complex	0.03083
Embryoma	2755	17S U2 snRNP	0.01073
Embryoma	2760	SMAD3-SMAD4-FOXO3 complex	0.0356
Embryoma	2761	SMAD3-SMAD4-FOXO1 complex	0.0356
Embryoma	2762	SMAD3-SMAD4-FOXO4 complex	0.0356
Embryoma	2813	BRCA1-SMAD3 complex	0.0436
Embryoma	2829	RSmad complex	0.0195
Embryoma	2830	TIF1gamma-SMAD2-SMAD3 complex	0.0356
Embryoma	2834	SMAD4-SMAD2-SMAD3 complex	0.0356
Embryoma	2893	BCR-ABL (p210 fusion protein)-GRB2-SOS1 complex	0.0436
Embryoma	2895	SHC-GRB2 complex	0.0436
Embryoma	2917	Grb2-Sos complex Fc receptor gamma-R1 stimulated	0.0436
Embryoma	2922	LAT-PLC-gamma-1-p85-GRB2-SOS signaling complex C305 activated	0.02758

Embryoma	2936	Ecsit complex (ECSIT MT-CO2 GAPDH TRAF6 NDUFAF1)	0.02758
Embryoma	2939	Ecsit complex (ECSIT MT-CO2 NDUFA1 MT-ND1 TRAF6 NDUFAF1)	0.02517
Embryoma	2968	Axin-SMAD3 complex	0.0436
Embryoma	2975	SMAD3-E2F4/5-p107-DP1 complex	0.02758
Embryoma	2992	SMAD7-SMURF2 complex	0.0436
Embryoma	3055	Nop56p-associated pre-rRNA complex	0.02419
Embryoma	3058	ITGA11-ITGB1 complex	0.0436
Embryoma	3059	ITGA11-ITGB1-COL1A1 complex	0.0356
Embryoma	3074	CCT:PFD complex testis specific	0.01648
Embryoma	3078	DGCR8-NCL complex	0.0436
Embryoma	3082	DGCR8 multiprotein complex	0.01859
Embryoma	3096	ITGA6-ITGB4-SHC1-GRB2 complex	0.03083
Embryoma	3144	Sos1-Grb2 complex	0.0436
Embryoma	3162	TF-FVIIa-FXa-TFPI complex	0.03083
Embryoma	3182	FHL2 homodimer complex	0.06166
Embryoma	3187	FHL2-FHL3 complex	0.0436
Embryoma	3188	FHL2-ACT complex	0.0436
Embryoma	3189	FHL2-CREB complex	0.0436
Embryoma	3199	SMAD3-SKI complex	0.0436
Embryoma	3205	SMAD3-SKI-NCOR complex	0.0356
Embryoma	3229	Heterodimer complex (CDK9 IL6ST)	0.0436
Embryoma	3335	Homotetrameric complex NIAP	0.06166
Embryoma	3714	Pericentrin-GCP complex	0.0356
Embryoma	3733	SKI-SMAD3 hexameric complex	0.0436
Embryoma	3740	SKI-SMAD3-SMAD4 pentameric complex	0.0356
Embryoma	3750	CREBBP-SMAD3 hexameric complex	0.0436
Embryoma	3754	CREBBP-SMAD3-SMAD4 pentameric complex	0.0356
Embryoma	3959	SMAD3-SMAD4-cSKI TGF(beta)-dependent	0.0356
Embryoma	3961	SMAD3-cSKI-SIN3A-HDAC1 complex	0.03083
Embryoma	3967	SMURF2-SMAD2 complex TGF(beta)-dependent	0.0436
Embryoma	3971	SMURF2-SMAD3 complex TGF(beta)-dependent	0.0872
Embryoma	3972	SMURF2-SMAD3-SnoN complex TGF(beta)-	0.0712
Embryoma	5158	SMARCA2/BRM-BAF57-MECP2 complex	0.0356
Embryoma	5184	SWI/SNF chromatin-remodeling complex	0.02758
Embryoma	5197	PTIP-DNA damage response complex	0.02517
Embryoma	5211	RAF1-PPP2-PIN1 complex	0.02758
Embryoma	5232	TNF-alpha/Nf-kappa B signaling complex (RPL6 RPL30 RPS13 CHUK DDX3X NFKB2 NFKBIB REL IKBKG NFKB1 MAP3K8 RELB GLG1 NFKBIA RELA TNIP2 GTF2I)	0.01496
Embryoma	5233	TNF-alpha/NF-kappa B signaling complex 5	0.01233
Embryoma	5378	TRBP containing complex (DICER TRBP AGO2 RPL7A EIF6 MOV10)	0.02517
Embryoma	5380	TRBP containing complex (DICER RPL7A EIF6 MOV10 and subunits of the 60S ribosomal particle)	0.02467
Embryoma	5386	MLL1-WDR5 complex	0.01187

Embryoma	5564	LMO4-gp130 complex	0.05515
Embryoma	5579	CNTF-CNTFR-gp130-LIFR complex	0.03083
Embryoma	5582	LIFR-LIF-gp130 complex	0.0356
Embryoma	5614	Emerin complex 32	0.01315
Embryoma	5615	Emerin complex 52	0.01286
Embryoma	5661	PlexinC1-SEMA7A complex	0.0436
Embryoma	5670	PlexinB1-Nrp1 complex	0.0436
Embryoma	5691	TALL1 homo-oligomer complex	0.06166
Embryoma	5735	TGF-beta receptor-SMAD3 complex	0.0356
Embryoma	5805	PGAM5-KEAP1-NRF2 complex	0.0356
Embryoma	5830	DJ-1-SNCA complex high molecular weight complex	0.0436
Embryoma	5837	PPD complex	0.0356
Embryoma	5859	FAS-FADD-CASP8-CASP10 complex	0.03083
Embryoma	5861	FAS-FADD-CASP10 complex	0.0356
Emphysema	220	ARF-Mule complex	0.11785
Emphysema	285	PCNA-MLH1-PMS1 complex	0.11785
Emphysema	286	PCNA-MSH2-MSH6 complex	0.11785
Emphysema	290	MSH2-MLH1-PMS2-PCNA DNA-repair initiation complex	0.10206
Emphysema	297	PCNA-DNA polymerase delta complex	0.09129
Emphysema	310	Cell cycle kinase complex CDC2	0.08333
Emphysema	311	Cell cycle kinase complex CDK2	0.10206
Emphysema	312	Cell cycle kinase complex CDK4	0.10206
Emphysema	313	Cell cycle kinase complex CDK5	0.09129
Emphysema	314	PCNA-p21 complex	0.14434
Emphysema	376	PCNA-MutS-alpha-MutL-alpha-DNA complex	0.09129
Emphysema	377	PCNA-MutS-alpha-DNA initial complex	0.11785
Emphysema	424	EXO1-MLH1-PCNA complex	0.11785
Emphysema	860	DNMT1-G9a-PCNA complex	0.11785
Emphysema	997	KIN17-PCNA-RPA70 complex	0.11785
Emphysema	1039	PCNA-PAF complex	0.14434
Emphysema	1092	PCNA-KU antigen complex	0.11785
Emphysema	1098	DNA synthesome complex (13 subunits)	0.05455
Emphysema	1099	DNA synthesome complex (17 subunits)	0.04811
Emphysema	1107	DNA synthesome core complex	0.06455
Emphysema	1108	DNA synthesome complex (15 subunits)	0.0527
Emphysema	1160	ING1-p300-PCNA complex	0.11785
Emphysema	1163	ING1-PCNA complex	0.14434
Emphysema	1642	p16-cyclin D2-CDK4 complex	0.11785
Emphysema	2000	BAX homo-oligomer complex	0.20412
Emphysema	2201	PCNA-RFC2-5 complex	0.09129
Emphysema	2230	PCNA complex	0.07715
Emphysema	2231	PCNA homotrimer complex	0.20412
Emphysema	2256	RIAM-Rap1-GTP complex	0.14434
Emphysema	2342	ITGAV-ITGB8-MMP14-TGFB1 complex	0.10206
Emphysema	2351	ITGB6-FYN-FN1 complex	0.11785
Emphysema	2352	ITGAV-ITGB6-SPP1 complex	0.11785

Emphysema	2353	ITGAV-ITGB6-TGFB3 complex	0.11785
Emphysema	2354	ITGAV-ITGB6 complex	0.14434
Emphysema	2416	ITGB1-RAP1A-PKD1 complex	0.11785
Emphysema	2456	MET-CIN85-SH3GL3-CBL complex HGF stimulated	0.10206
Emphysema	2541	HGF-Met complex	0.14434
Emphysema	2563	FGFR2-c-Cbl-Lyn-Fyn complex	0.10206
Emphysema	2688	MT1-MMP-claudin-1 complex	0.14434
Emphysema	2709	MMP-9-TIMP-1-LRP complex	0.11785
Emphysema	2797	PCNA-CHL12-RFC2-5 complex	0.08333
Emphysema	2798	MMP-2-claudin-1 complex	0.14434
Emphysema	3071	CTLH complex	0.09129
Emphysema	3158	RIAM-Rap1-GTP-profilin complex	0.11785
Emphysema	3492	Bax homooligomeric complex after apoptotic	0.20412
Emphysema	5375	EGR-EP300 complex	0.14434
Emphysema	5544	CDC2-PCNA-CCNB1-GADD45A complex	0.10206
Emphysema	5545	CDC2-PCNA-CCNB1-GADD45B complex	0.10206
Emphysema	5546	CDC2-PCNA-CCNB1-GADD45G complex	0.10206
Encephalitis	143	APP-FE65-LRP complex	0.16013
Encephalitis	552	IFNB1-IFNAR1-IFNAR2- complex	0.16013
Encephalitis	1088	PRNP-ApolopoproteinE3 complex	0.19612
Encephalitis	2711	Amyloid beta protein oligomer	0.27735
Encephalitis	3092	APP-TOMM40 complex	0.19612
Encephalitis	3093	APP-TIMM23 complex	0.19612
Encephalopathies	178	Respiratory chain complex I (holoenzyme)	0.04222
Encephalopathies	406	N-acetylglucosamine-1-phosphotransferase	0.09901
Encephalopathies	749	MeCP2-SIN3A-HDAC complex	0.07001
Encephalopathies	1088	PRNP-ApolopoproteinE3 complex	0.09901
Encephalopathies	1379	GALNS-lysosomal hydrolase 1.27 MDa complex	0.07001
Encephalopathies	2884	Respiratory chain complex I (early intermediate NDUFAF1 assembly) mitochondrial	0.05293
Encephalopathies	2886	Respiratory chain complex I (incomplete intermediate ND1 ND2 ND3 CIA30 assembly) mitochondrial	0.07001
Encephalopathies	2901	Respiratory chain complex I (intermediate IV/310kD) mitochondrial	0.07001
Encephalopathies	2903	Respiratory chain complex I (intermediate V/380kD and VI/480kD) mitochondrial	0.06262
Encephalopathies	2904	Respiratory chain complex I (intermediate VII/650kD) mitochondrial	0.08856
Encephalopathies	2906	Respiratory chain complex I (intermediate II/230kD) mitochondrial	0.08085
Encephalopathies	2919	Respiratory chain complex I (gamma subunit) mitochondrial	0.03884
Encephalopathies	2920	Respiratory chain complex I (lambda subunit) mitochondrial	0.03501
Encephalopathies	2939	Ecsit complex (ECSIT MT-CO2 NDUFA1 MT-ND1 TRAF6 NDUFAF1)	0.05717
Encephalopathies	2943	Respiratory chain complex I (incomplete NDUFAF1 assembly) mitochondrial	0.09901

Encephalopathies	2948	Respiratory chain complex I (incomplete intermediate) mitochondrial	0.04222
Encephalopathies	5158	SMARCA2/BRM-BAF57-MECP2 complex	0.08085
Encephalopathies	5184	SWI/SNF chromatin-remodeling complex	0.06262
Encephalopathies	5276	HIF1A-OS9-EGLN1 complex	0.08085
Encephalopathies	5277	HIF1A-OS9-EGLN3 complex	0.08085
Endemic goiter	310	Cell cycle kinase complex CDC2	0.2357
Endemic goiter	311	Cell cycle kinase complex CDK2	0.28868
Endemic goiter	312	Cell cycle kinase complex CDK4	0.28868
Endemic goiter	313	Cell cycle kinase complex CDK5	0.2582
Endemic goiter	1633	CyclinD1-CDK4-CDK6 complex	0.33333
Endemic goiter	1634	CyclinD1-CDK4-p21 complex	0.33333
Endemic goiter	2635	BETA2-Cyclin D1 complex	0.40825
Endemic goiter	3084	CCND1-CDK4 complex	0.40825
Endemic goiter	3087	CCND1-CDK6 complex	0.40825
Endemic goiter	5287	CDK4-CCND1 complex	0.40825
Endocrine system disease	244	BRAFT complex	0.10483
Endocrine system disease	246	BLM complex III	0.18898
Endocrine system disease	433	BASC complex (BRCA1-associated genome surveillance complex)	0.10911
Endocrine system disease	434	BASC (Ab 80) complex (BRCA1-associated genome surveillance complex)	0.13363
Endocrine system disease	541	IGF1-IGFBP3-ALS complex	0.21822
Endocrine system disease	629	BLM-TRF2 complex	0.26726
Endocrine system disease	903	RET-Rai complex	0.26726
Endocrine system disease	1218	BLM-TRF2 complex	0.26726
Endocrine system disease	1254	Menin-associated histone methyltransferase complex	0.14286
Endocrine system disease	1256	MLL-HCF complex	0.14286
Endocrine system disease	2222	BLM complex II	0.1543
Endocrine system disease	2223	BLM-TOP3A complex	0.26726
Endocrine system disease	2224	MSH2/6-BLM-p53-RAD51 complex	0.16903
Endocrine system disease	2228	BLM-RAD51L3-XRCC2 complex	0.21822
Endocrine system disease	2739	FA complex (Fanconi anemia complex)	0.11396
Endocrine system disease	5197	PTIP-DNA damage response complex	0.1543
Endometrial cancer	4	Multisubunit ACTR coactivator complex	0.08704
Endometrial cancer	75	TSC1-TSC2 complex	0.12309
Endometrial cancer	98	p300-MDM2-p53 protein complex	0.1005
Endometrial cancer	570	p300-CBP-p270-SWI/SNF complex	0.0658
Endometrial cancer	571	p300-CBP-p270 complex	0.1005
Endometrial cancer	1158	p33ING1b-p300 complex	0.12309
Endometrial cancer	1160	ING1-p300-PCNA complex	0.1005
Endometrial cancer	1471	pRb2/p130-multimolecular complex (RB2 E2F5 HDAC1 SUV39H1 P300)	0.07785
Endometrial cancer	1521	p300-SMAD1-STAT3 complex	0.1005
Endometrial cancer	1831	PIAS3-SMAD3-P300 complex	0.1005
Endometrial cancer	2638	HES1 promoter corepressor complex	0.07107
Endometrial cancer	2639	HES1 promoter-Notch enhancer complex	0.04828
Endometrial cancer	2641	p300/CBP-PCAF-MyoD complex	0.08704

Endometrial cancer	2642	SMAD1-P300 complex	0.12309
Endometrial cancer	2954	Smad1-Notch1-p300-Pcaf complex	0.08704
Endometrial cancer	4025	Affixin-actinin(alpha) complex	0.12309
Endometrial cancer	5118	pRb2/p130-multimolecular complex (RB2 E2F4 HDAC1 SUV39H1 P300)	0.07785
Endometrial cancer	5177	Polycystin-1 multiprotein complex (ACTN1 CDH1 SRC JUP VCL CTNNB1 PXN BCAR1 PKD1	0.05249
Endometrial cancer	5260	TCF4-CTNNB1-SUMO1-EP300-HADAC6 complex	0.07785
Endometrial cancer	5261	TCF4-CTNNB1-EP300 complex	0.1005
Endometrial cancer	5375	EGR-EP300 complex	0.12309
Endometrial cancer	5386	MLL1-WDR5 complex	0.0335
Endometriosis	98	p300-MDM2-p53 protein complex	0.04795
Endometriosis	120	Lymphotoxin beta receptor complex	0.04795
Endometriosis	159	Condensin I-PARP-1-XRCC1 complex	0.03139
Endometriosis	201	HUIC complex	0.05872
Endometriosis	202	BRCA1-RAD50-MRE11-NBS1 complex	0.04152
Endometriosis	206	DNA ligase IV-XRCC4 complex	0.05872
Endometriosis	212	DNA ligase III-XRCC1 complex	0.05872
Endometriosis	213	DNA ligase IV-XRCC1 complex	0.05872
Endometriosis	220	ARF-Mule complex	0.04795
Endometriosis	238	SWI-SNF chromatin remodeling-related-BRCA1	0.02504
Endometriosis	240	BRCA1-CTIP-ZBRK1 repressor complex	0.04795
Endometriosis	242	BRCA1-BACH1 complex	0.05872
Endometriosis	310	Cell cycle kinase complex CDC2	0.06781
Endometriosis	311	Cell cycle kinase complex CDK2	0.08305
Endometriosis	312	Cell cycle kinase complex CDK4	0.08305
Endometriosis	313	Cell cycle kinase complex CDK5	0.07428
Endometriosis	314	PCNA-p21 complex	0.05872
Endometriosis	336	DNA ligase IV-XRCC4-AHNK complex	0.04795
Endometriosis	344	DNA ligase IV-XRCC4 complex (LX complex)	0.05872
Endometriosis	350	DNA ligase IV-XRCC4-PNK complex	0.04795
Endometriosis	359	DNA ligase IV-XRCC4-XLF complex	0.04795
Endometriosis	362	DNA ligase III-XRCC1-PNK-DNA-pol III multiprotein complex	0.04152
Endometriosis	433	BASC complex (BRCA1-associated genome surveillance complex)	0.02397
Endometriosis	434	BASC (Ab 80) complex (BRCA1-associated genome surveillance complex)	0.02936
Endometriosis	435	BASC (Ab 81) complex (BRCA1-associated genome surveillance complex)	0.0339
Endometriosis	436	BASC (Ab C-20) complex (BRCA1-associated genome surveillance complex)	0.04795
Endometriosis	438	GCN5-TRRAP histone acetyltransferase complex	0.02626
Endometriosis	441	TFTC-type histone acetyl transferase complex	0.02504
Endometriosis	521	Polycystin-1-E-cadherin-beta-catenin complex	0.04795
Endometriosis	522	Polycystin-1-E-cadherin-beta-catenin-Flotillin-2	0.04152
Endometriosis	541	IGF1-IGFBP3-ALS complex	0.09589
Endometriosis	681	(C-CFTR)2-NHERF-ezrin complex	0.04795
Endometriosis	682	C-CFTR-NHERF(PDZ1 domain)-ezrin complex	0.04795

Endometriosis	683	C-CFTR-NHERF(PDZ2 domain)-ezrin complex	0.04795
Endometriosis	722	MRG15-PAM14-RB complex	0.04795
Endometriosis	723	MAF1 complex	0.04795
Endometriosis	826	PAR-3-VE-cadherin-beta-catenin complex	0.04795
Endometriosis	871	BRAF53-BRCA2 complex	0.03139
Endometriosis	1054	ESR1-RELA-BCL3-NCOA3 complex	0.08305
Endometriosis	1069	FIF-FGR2 complex	0.05872
Endometriosis	1085	DNA repair complex NEIL2-PNK-Pol(beta)-LigIII(alpha)-XRCC1	0.03714
Endometriosis	1086	DNA repair complex NEIL1-PNK-Pol(beta)-LigIII(alpha)-XRCC1	0.03714
Endometriosis	1087	BIRC5-AURKB-INCENP-EVI5 complex	0.04152
Endometriosis	1091	SNX complex (SNX1a SNX2 SNX4 LEPR)	0.04152
Endometriosis	1095	SNX complex (SNX1a SNX2 SNX4 EGFR)	0.04152
Endometriosis	1116	CRM1-Survivin-AuroraB mitotic complex	0.04795
Endometriosis	1117	CRM1-Survivin mitotic complex	0.05872
Endometriosis	1118	Chromosomal passenger complex CPC (INCENP CDCA8 BIRC5 AURKB)	0.04152
Endometriosis	1120	Chromosomal passenger complex CPC (INCENP CDCA8 BIRC5)	0.04795
Endometriosis	1154	DSS1 complex	0.02303
Endometriosis	1185	EGFR-containing signaling complex	0.04152
Endometriosis	1189	DNA double-strand break end-joining complex	0.03139
Endometriosis	1211	Ubiquitin E3 ligase (AHR ARNT DDB1 TBL3 CUL4B RBX1)	0.06781
Endometriosis	1250	pRB-E2F-1 complex	0.05872
Endometriosis	1372	Rb-tal-1-E2A-Lmo2-Ldb1 complex	0.03714
Endometriosis	1439	PTGS2 homodimer complex	0.08305
Endometriosis	1488	DNMT1-RB1-HDAC1-E2F1 complex	0.04152
Endometriosis	1514	IL4-IL4R complex	0.05872
Endometriosis	1515	IL4-IL4R-IL2RG complex	0.04795
Endometriosis	1633	CyclinD1-CDK4-CDK6 complex	0.04795
Endometriosis	1634	CyclinD1-CDK4-p21 complex	0.09589
Endometriosis	1642	p16-cyclin D2-CDK4 complex	0.04795
Endometriosis	1707	IL2-IL2RA-IL2RB complex	0.04795
Endometriosis	1816	JUN-TCF4-CTNNB1 complex	0.04795
Endometriosis	1839	SDCBP-CTNNB1-CTNNA1-CDH1 complex	0.04152
Endometriosis	1986	Endoglin homodimer complex	0.08305
Endometriosis	1992	LEPR homodimer complex	0.08305
Endometriosis	2010	AXL homodimer complex	0.08305
Endometriosis	2019	IL12A-IL12B-IL12RB1 complex	0.04795
Endometriosis	2020	IL12B-IL12RB1-IL12RB2 complex	0.04795
Endometriosis	2026	IL12RB1-IL12RB2 complex	0.05872
Endometriosis	2084	NFKB1-NFKB2-REL-RELA-RELB complex	0.03714
Endometriosis	2086	NFKB1-NFKB2-RELA-RELB complex	0.04152
Endometriosis	2105	IkappaB kinase complex (IKBKB CHUK IKBKAP NFKBIA RELA MAP3K14)	0.0339
Endometriosis	2124	IKK-alpha--ER-alpha-AIB1 complex	0.04795

Endometriosis	2152	ARNT-HLF complex	0.05872
Endometriosis	2159	AR-AKT-APPL complex	0.04795
Endometriosis	2160	AOF2-AR complex	0.05872
Endometriosis	2210	BRCA1-IRIS-pre-replication complex	0.04152
Endometriosis	2211	BARD1-BRCA1-CSTF complex	0.03714
Endometriosis	2213	BRCA1-BARD1-POLR2A complex	0.04795
Endometriosis	2214	LMO4-BRCA1-CTIP-LDB1 complex	0.04152
Endometriosis	2215	BRCA1-LMO4-CTIP complex	0.04795
Endometriosis	2230	PCNA complex	0.03139
Endometriosis	2318	ITGA6-ITGB4-Laminin10/12 complex	0.07428
Endometriosis	2319	ITGA6-ITGB4-Laminin10/12 complex	0.03714
Endometriosis	2342	ITGAV-ITGB8-MMP14-TGFB1 complex	0.04152
Endometriosis	2348	ITGAV-ITGB5-CYR61 complex	0.04795
Endometriosis	2369	ITGAV-ITGB3-EGFR complex	0.04795
Endometriosis	2437	ITGA6-ITGB1-CYR61 complex	0.04795
Endometriosis	2443	ITGA9-ITGB1-TNC complex	0.04795
Endometriosis	2453	Multiprotein complex (monoubiquitination)	0.04152
Endometriosis	2454	CIN85-CBL-SH3GL2-EGFR complex EGF stimulated	0.04152
Endometriosis	2470	p130Cas-ER-alpha-cSrc-kinase- PI3-kinase p85-subunit complex	0.04152
Endometriosis	2542	EGFR-CBL-GRB2 complex	0.04795
Endometriosis	2563	FGFR2-c-Cbl-Lyn-Fyn complex	0.04152
Endometriosis	2579	Chromosomal passenger complex CPC (INCENP BIRC5 AURKB)	0.04795
Endometriosis	2580	Survivin homodimer complex	0.08305
Endometriosis	2581	RasGAP-AURKA/AURKB-survivin complex	0.04152
Endometriosis	2582	Chromosomal passenger complex CPC (CDCA8 AURKB BIRC5)	0.04795
Endometriosis	2635	BETA2-Cyclin D1 complex	0.05872
Endometriosis	2657	ESR1-CDK7-CCNH-MNAT1-MTA1-HDAC2 complex	0.0339
Endometriosis	2670	Er-alpha-p53-hdm2 complex	0.09589
Endometriosis	2686	BRCA1-core RNA polymerase II complex	0.02303
Endometriosis	2688	MT1-MMP-claudin-1 complex	0.05872
Endometriosis	2699	ER-alpha-GRIP1-c-Jun complex	0.04795
Endometriosis	2700	ER-alpha-c-Jun complex	0.05872
Endometriosis	2709	MMP-9-TIMP-1-LRP complex	0.09589
Endometriosis	2776	RAD50-BRCA1 complex	0.05872
Endometriosis	2783	BARD1-BRCA1-CSTF64 complex	0.04795
Endometriosis	2786	BRCA1 A complex	0.04152
Endometriosis	2787	BRCA1 C complex	0.04152
Endometriosis	2788	BRCA1 B complex	0.04795
Endometriosis	2811	BRCA1-cABL complex	0.05872
Endometriosis	2813	BRCA1-SMAD3 complex	0.05872
Endometriosis	2814	BRCA1-HDAC1-HDAC2 complex	0.04795
Endometriosis	2815	BRCA1-BARD1-BACH1-DNA damage complex II	0.02936
Endometriosis	2817	BRCA1-BARD1-BACH1-DNA damage complex I	0.0339
Endometriosis	2818	BRCA1-BARD1-BRCA2-DNA damage complex III	0.09589
Endometriosis	2819	BRCA1-CtIP-CtBP complex	0.04795

Endometriosis	2820	BRCA1-VCP complex	0.05872
Endometriosis	2822	BRCA1-BARD1-UbcH5c complex	0.04795
Endometriosis	2823	BRCA1-BARD1-UbcH7c complex	0.04795
Endometriosis	2824	BRCA1-RAD51 complex	0.05872
Endometriosis	2825	BRCA1-RNA polymerase II complex	0.01629
Endometriosis	2998	Axin-PP2A A-PP2A C-GSK3-beta-beta-catenin	0.04152
Endometriosis	3004	APC-Axin-1-beta-catenin complex	0.04795
Endometriosis	3045	hs4 enhancer complex (faster migrating complex)	0.03714
Endometriosis	3084	CCND1-CDK4 complex	0.05872
Endometriosis	3087	CCND1-CDK6 complex	0.05872
Endometriosis	3155	Bipartite complex (TFC4 CTNNB1)	0.05872
Endometriosis	3166	AXIN-APC-betaCatenin-GSK3B complex	0.04152
Endometriosis	3172	NUMB-TP53-MDM2 complex	0.04795
Endometriosis	3269	RB1-HDAC1-BRG1 complex	0.04795
Endometriosis	3678	RIN1-STAM2-EGFR complex EGF stimulated	0.04795
Endometriosis	3852	Rb-HDAC1 complex	0.05872
Endometriosis	4025	Affixin-actinin(alpha) complex	0.05872
Endometriosis	4043	NEMO-HIF2(alpha)-ARNT complex	0.04795
Endometriosis	4062	NRP1-VEGFR2-VEGF(165) complex	0.04795
Endometriosis	4095	Catulin (alpha) - catenin (beta) complex	0.05872
Endometriosis	4096	Catenin (alpha) - catenin (beta) complex	0.05872
Endometriosis	5772	ZO1-(beta)cadherin-(VE)cadherin-VEGFR2 complex	0.08305
Endometriosis	5099	RB1(hypophosphorylated)-E2F4 complex	0.05872
Endometriosis	5101	CyclinD3-CDK4-CDK6-p21 complex	0.04152
Endometriosis	5143	E2F1-Rb complex	0.05872
Endometriosis	5146	RB1-TFAP2A complex	0.05872
Endometriosis	5171	SH3KBP1-CBLB-EGFR complex	0.04795
Endometriosis	5177	Polycystin-1 multiprotein complex (ACTN1 CDH1 SRC JUP VCL CTNNB1 PXN BCAR1 PKD1	0.05008
Endometriosis	5193	TNF-alpha/NF-kappa B signaling complex (CHUK KPNA3 NFKB2 NFKBIB REL IKBKG NFKB1 NFKBIE RELB NFKBIA RELA TNIP2)	0.02397
Endometriosis	5194	TNF-alpha/NF-kappa B signaling complex (SEC16A CHUK IKBKB NFKB2 REL IKBKG MAP3K14 RELA FBXW7 USP2)	0.02626
Endometriosis	5196	TNF-alpha/NF-kappa B signaling complex (CHUK BTRC NFKB2 PPP6C REL CUL1 IKBKE SAPS2 SAPS1 ANKRD28 RELA SKP1)	0.02397
Endometriosis	5220	CHUK-IQGAP2-AKAP8L-RELA-TNIP2 complex	0.03714
Endometriosis	5228	REL-MAP3K8-RELA-TNIP2-PAPOLA complex	0.03714
Endometriosis	5230	CHUK-NFKB2-REL-IKBKG-SPAG9-NFKB1-NFKBIE-COPB2-TNIP1-NFKBIA-RELA-TNIP2	0.02397
Endometriosis	5232	TNF-alpha/Nf-kappa B signaling complex (RPL6 RPL30 RPS13 CHUK DDX3X NFKB2 NFKBIB REL IKBKG NFKB1 MAP3K8 RELB GLG1 NFKBIA RELA TNIP2 GTF2I)	0.02014
Endometriosis	5233	TNF-alpha/NF-kappa B signaling complex 5	0.01661
Endometriosis	5243	XRCC1-LIG3-PNK-TDP1 complex	0.04152
Endometriosis	5260	TCF4-CTNNB1-SUMO1-EP300-HADAC6 complex	0.03714

Endometriosis	5261	TCF4-CTNNB1-EP300 complex	0.04795
Endometriosis	5262	TCF4-CTNNB1 complex	0.05872
Endometriosis	5264	TCF4-CTNNB1-CREBBP complex	0.04795
Endometriosis	5281	Cell-cell junction complex (CDH1-CTNNB1)	0.05872
Endometriosis	5287	CDK4-CCND1 complex	0.05872
Endometriosis	5331	YY1-MDM2-p53 complex	0.04795
Endometriosis	5382	ARNT-HIF1A complex	0.05872
Endometriosis	5400	BRCC complex	0.07428
Endometriosis	5442	EPOR receptor complex	0.08305
Endometriosis	5446	EPO-EPOR complex	0.11744
Endometriosis	5460	p50-p65 NF(kappa)B complex	0.05872
Endometriosis	5461	p50-p65 NF(kappa)B-SRC1 complex	0.04795
Endometriosis	5464	I(kappa)B(alpha)-NF(kappa)Bp50-NF(kappa)Bp65 complex	0.04795
Endometriosis	5465	IKB(epsilon)-RELA-cREL complex	0.04795
Endometriosis	5466	IKB(beta)-RELA-cREL complex	0.04795
Endometriosis	5467	IKB(alpha)-RELA-cREL complex	0.04795
Endometriosis	5475	MURR1-NF(kappa)Bp65-IKBA complex	0.04795
Endometriosis	5492	IKBA-NF(kappa)Bp65-NF(kappa)Bp50 complex	0.04795
Endometriosis	5582	LIFR-LIF-gp130 complex	0.04795
Endometriosis	5611	Emerin complex 24	0.02144
Endometriosis	5622	HSP90-CIP1-FKBPL complex	0.04795
Endometriosis	5656	CEBPE-E2F1-RB1 complex	0.04795
Endometriosis	5663	TRIM27-RB1 complex	0.05872
Endometriosis	5691	TALL1 homo-oligomer complex	0.08305
Endometriosis	5696	VEGFA(165)-KDR-NRP1 complex	0.04795
Endometriosis	5698	VEGFA(165)-VEGFR2-NRP1 complex	0.04795
Endometriosis	5862	CAV1-VDAC1-ESR1 complex	0.04795
Endometriosis	5922	RAF1-RAS complex EGF induced	0.04152
Endometrium cancer	959	LLGL1-PAR-6B-PRKCI complex	0.14003
Endometrium cancer	1091	SNX complex (SNX1a SNX2 SNX4 LEPR)	0.12127
Endometrium cancer	1992	LEPR homodimer complex	0.24254
Endometrium cancer	2486	GIPC1-LHCGR complex	0.1715
Enteritis	120	Lymphotoxin beta receptor complex	0.06337
Enteritis	244	BRAFT complex	0.03044
Enteritis	246	BLM complex III	0.05488
Enteritis	285	PCNA-MLH1-PMS1 complex	0.06337
Enteritis	290	MSH2-MLH1-PMS2-PCNA DNA-repair initiation complex	0.05488
Enteritis	291	MSH2-MLH1-PMS2 DNA-repair initiation complex	0.06337
Enteritis	292	MutL-alpha complex	0.07762
Enteritis	369	MSH2-MSH6-PMS2-MLH1 complex	0.05488
Enteritis	370	MSH2-MSH6-PMS1-MLH1 complex	0.05488
Enteritis	376	PCNA-MutS-alpha-MutL-alpha-DNA complex	0.04909
Enteritis	380	MutL-beta complex	0.07762
Enteritis	415	EXO1-MLH1-PMS2 complex	0.06337
Enteritis	424	EXO1-MLH1-PCNA complex	0.06337

Enteritis	433	BASC complex (BRCA1-associated genome surveillance complex)	0.03169
Enteritis	434	BASC (Ab 80) complex (BRCA1-associated genome surveillance complex)	0.03881
Enteritis	435	BASC (Ab 81) complex (BRCA1-associated genome surveillance complex)	0.04481
Enteritis	681	(C-CFTR)2-NHERF-ezrin complex	0.06337
Enteritis	682	C-CFTR-NHERF(PDZ1 domain)-ezrin complex	0.06337
Enteritis	683	C-CFTR-NHERF(PDZ2 domain)-ezrin complex	0.06337
Enteritis	687	CFTR-NHERF-beta(2)AR signaling complex	0.06337
Enteritis	725	P2X7 receptor signalling complex	0.03169
Enteritis	1514	IL4-IL4R complex	0.07762
Enteritis	1515	IL4-IL4R-IL2RG complex	0.06337
Enteritis	1714	TICAM1-TICAM2-TLR4 complex	0.06337
Enteritis	1774	MICA-KLRK1-HCST complex	0.06337
Enteritis	1834	ITGAE-ITGB7-CDH1 complex	0.06337
Enteritis	1945	IRAK1-IRAK3 complex	0.07762
Enteritis	2001	NOD1 homodimer complex	0.10976
Enteritis	2153	ITGAM-ITGB2-CD11 complex	0.06337
Enteritis	2347	ITGAV-ITGB5-SPP1 complex	0.06337
Enteritis	2352	ITGAV-ITGB6-SPP1 complex	0.06337
Enteritis	2358	ITGAV-ITGB3-SPP1 complex	0.06337
Enteritis	2709	MMP-9-TIMP-1-LRP complex	0.06337
Enteritis	2730	Set1B complex	0.04481
Enteritis	2731	Set1A complex	0.04481
Enteritis	2817	BRCA1-BARD1-BACH1-DNA damage complex I	0.04481
Enteritis	2885	ITGAV-ITGB1-SPP1 complex	0.06337
Enteritis	2909	PLC-gamma-2-Syk-LAT-FcR-gamma complex	0.05488
Enteritis	2910	PLC-gamma-2-Lyn-FcR-gamma complex	0.06337
Enteritis	3075	UTX-MLL2/3 complex	0.03169
Enteritis	3111	ITGA9-ITGB1-SPP1 complex	0.06337
Enteritis	3112	ITGA5-ITGB1-SPP1 complex	0.06337
Eosinophilia	1079	P-TEFb.1 complex	0.2132
Eosinophilia	1087	BIRC5-AURKB-INCENP-EVI5 complex	0.15076
Eosinophilia	1116	CRM1-Survivin-AuroraB mitotic complex	0.17408
Eosinophilia	1117	CRM1-Survivin mitotic complex	0.2132
Eosinophilia	1118	Chromosomal passenger complex CPC (INCENP CDCA8 BIRC5 AURKB)	0.15076
Eosinophilia	1120	Chromosomal passenger complex CPC (INCENP CDCA8 BIRC5)	0.17408
Eosinophilia	1707	IL2-IL2RA-IL2RB complex	0.17408
Eosinophilia	2579	Chromosomal passenger complex CPC (INCENP BIRC5 AURKB)	0.17408
Eosinophilia	2580	Survivin homodimer complex	0.30151
Eosinophilia	2581	RasGAP-AURKA/AURKB-survivin complex	0.15076
Eosinophilia	2582	Chromosomal passenger complex CPC (CDCA8 AURKB BIRC5)	0.17408
Eosinophilia	2599	POLR2A-CCNT1-CDK9-NCL-LEM6-CPSF2 complex	0.12309
Eosinophilia	2600	BRD4 complex	0.1066

Eosinophilia	2601	P-TEFb-BRD4-TRAP220 complex	0.15076
Eosinophilia	2602	P-TEFb-7SKRNA-HEXIM1 complex	0.17408
Eosinophilia	2603	Transcription elongation factor complex (SUPT5H CDK9 CCNT1)	0.17408
Eosinophilia	2604	P-TEFb-SKP2 complex	0.17408
Eosinophilia	2605	Heterotrimeric complex (CCNT1 CDK9 GRN)	0.17408
Eosinophilia	2639	HES1 promoter-Notch enhancer complex	0.08362
Eosinophilia	2811	BRCA1-cABL complex	0.2132
Eosinophilia	2875	BRD4-P-TEFb complex	0.17408
Epilepsy	59	AP3 adapter complex	0.05345
Epilepsy	98	p300-MDM2-p53 protein complex	0.08165
Epilepsy	178	Respiratory chain complex I (holoenzyme)	0.02132
Epilepsy	205	Ubiquitin E3 ligase (VHL TCEB1 TCEB2 CUL2	0.06325
Epilepsy	243	RalBP1-CDC2-CCNB1 complex	0.08165
Epilepsy	247	RalBP1-CCNB1-AP2A-NUMB-EPN1 complex	0.05774
Epilepsy	280	HMGB1-HMGB2-HSC70-ERP60-GAPDH complex	0.06325
Epilepsy	622	Ubiquitin E3 ligase (VHL TCEB1 TCEB2 CUL2)	0.07071
Epilepsy	652	AP3-BLOC1 complex	0.03651
Epilepsy	668	BKCA-beta2AR-AKAP79 signaling complex	0.08165
Epilepsy	672	BKCA-beta2AR complex	0.1
Epilepsy	725	P2X7 receptor signalling complex	0.04082
Epilepsy	749	MeCP2-SIN3A-HDAC complex	0.07071
Epilepsy	929	CEN complex	0.02325
Epilepsy	1178	BCOR complex	0.05
Epilepsy	1182	CDC5L core complex	0.05774
Epilepsy	1183	CDC5L complex	0.02582
Epilepsy	1308	PABPC1-HSPA8-HNRPD-EIF4G1 complex	0.06325
Epilepsy	1514	IL4-IL4R complex	0.1
Epilepsy	1515	IL4-IL4R-IL2RG complex	0.08165
Epilepsy	1787	Nogo-potassium channel complex	0.07071
Epilepsy	2129	DNAJB2-HSPA8-PSMA3 complex	0.08165
Epilepsy	2258	VILIP-1-AChR-alpha-4-AChR-beta-2 complex	0.1633
Epilepsy	2300	Profilin 2 complex	0.04714
Epilepsy	2670	Er-alpha-p53-hdm2 complex	0.08165
Epilepsy	2721	HCF-1 complex	0.03244
Epilepsy	2837	Profilin 1 complex	0.05774
Epilepsy	2919	Respiratory chain complex I (gamma subunit) mitochondrial	0.03922
Epilepsy	2939	Ecsit complex (ECSIT MT-CO2 NDUFA1 MT-ND1 TRAF6 NDUFAF1)	0.05774
Epilepsy	3172	NUMB-TP53-MDM2 complex	0.08165
Epilepsy	5158	SMARCA2/BRM-BAF57-MECP2 complex	0.08165
Epilepsy	5183	DNA-PK-Ku-eIF2-NF90-NF45 complex	0.05
Epilepsy	5184	SWI/SNF chromatin-remodeling complex	0.06325
Epilepsy	5267	VHL-VDU1-TCEB1-TCEB2 complex	0.07071
Epilepsy	5270	VHL-TCEB1-TCEB2 complex	0.08165
Epilepsy	5273	VHL-TBP1-HIF1A complex	0.08165
Epilepsy	5331	YY1-MDM2-p53 complex	0.08165

Epilepsy	5375	EGR-EP300 complex	0.1
Epilepsy	5414	HTR1A-HTR1D complex	0.1
Epilepsy	5416	HTR1A-HTR1B complex	0.1
Epilepsy	5418	GABBR2-HTR1A complex	0.2
Epilepsy	5419	HTR1A-GPR26 complex	0.1
Epilepsy	5420	HTR1A-EDG3 complex	0.1
Epilepsy	5421	HTR1A homodimer complex	0.14142
Epilepsy	5422	HTR1A-EDG1 complex	0.1
Epstein-Barr virus infection	1062	BAR-BCL2-CASP8 complex	0.12599
Epstein-Barr virus infection	1087	BIRC5-AURKB-INCENP-EVI5 complex	0.10911
Epstein-Barr virus infection	1116	CRM1-Survivin-AuroraB mitotic complex	0.12599
Epstein-Barr virus infection	1117	CRM1-Survivin mitotic complex	0.1543
Epstein-Barr virus infection	1118	Chromosomal passenger complex CPC (INCENP CDCA8 BIRC5 AURKB)	0.10911
Epstein-Barr virus infection	1120	Chromosomal passenger complex CPC (INCENP CDCA8 BIRC5)	0.12599
Epstein-Barr virus infection	1170	cMYC-ATPase-helicase complex	0.09759
Epstein-Barr virus infection	1171	c-MYC-ATPase-helicase complex	0.09759
Epstein-Barr virus infection	2000	BAX homo-oligomer complex	0.21822
Epstein-Barr virus infection	2579	Chromosomal passenger complex CPC (INCENP BIRC5 AURKB)	0.12599
Epstein-Barr virus infection	2580	Survivin homodimer complex	0.21822
Epstein-Barr virus infection	2581	RasGAP-AURKA/AURKB-survivin complex	0.10911
Epstein-Barr virus infection	2582	Chromosomal passenger complex CPC (CDCA8 AURKB BIRC5)	0.12599
Epstein-Barr virus infection	2649	MYC-DNMT3A-ZBTB17 complex	0.12599
Epstein-Barr virus infection	2653	MYC-MAX-BLOC1S1 complex	0.12599
Epstein-Barr virus infection	2655	MYC-MAX complex	0.1543
Epstein-Barr virus infection	3492	Bax homooligomeric complex after apoptotic	0.21822
Epstein-Barr virus infection	5183	DNA-PK-Ku-eIF2-NF90-NF45 complex	0.07715
Epstein-Barr virus infection	5210	TANK-TRAF2-TRAF3 complex	0.12599
Epstein-Barr virus infection	5526	CALM1-FKBP38-BCL2 complex	0.12599
Epstein-Barr virus infection	5691	TALL1 homo-oligomer complex	0.21822
Epstein-Barr virus infection	5811	p53-BCL2 complex	0.1543
Epstein-Barr virus infection	5817	tBID-BCL2 complex	0.1543
Epstein-Barr virus infection	5818	BIM-BCL2 complex	0.1543
Esophageal disease	159	Condensin I-PARP-1-XRCC1 complex	0.1543
Esophageal disease	212	DNA ligase III-XRCC1 complex	0.28868
Esophageal disease	213	DNA ligase IV-XRCC1 complex	0.28868
Esophageal disease	362	DNA ligase III-XRCC1-PNK-DNA-pol III multiprotein complex	0.20412
Esophageal disease	1085	DNA repair complex NEIL2-PNK-Pol(beta)-LigIII(alpha)-XRCC1	0.18257
Esophageal disease	1086	DNA repair complex NEIL1-PNK-Pol(beta)-LigIII(alpha)-XRCC1	0.18257
Esophageal disease	3151	Sulphiredoxin-peroxiredoxin complex	0.28868
Esophageal disease	5243	XRCC1-LIG3-PNK-TDP1 complex	0.20412
Esophageal tumor	103	RNA polymerase II holoenzyme complex	0.04454
Esophageal tumor	107	TFIIH transcription factor complex	0.07274

Esophageal tumor	351	Spliceosome	0.01825
Esophageal tumor	443	BP-SMAD complex	0.07715
Esophageal tumor	1009	TFIIH transcription factor complex	0.06901
Esophageal tumor	1029	TFIIH transcription factor complex	0.06901
Esophageal tumor	1037	TFIIH transcription factor core complex	0.09759
Esophageal tumor	1141	CF IIAm complex (Cleavage factor IIAm complex)	0.05455
Esophageal tumor	1181	C complex spliceosome	0.0244
Esophageal tumor	1223	H2AX complex isolated from cells without IR exposure	0.06052
Esophageal tumor	1307	Multiprotein complex (mRNA turnover)	0.09759
Esophageal tumor	1308	PABPC1-HSPA8-HNRPD-EIF4G1 complex	0.09759
Esophageal tumor	2825	BRCA1-RNA polymerase II complex	0.0428
Esophageal tumor	3066	RNA polymerase II complex chromatin structure modifying	0.06052
Esophageal tumor	3151	Sulphiredoxin-peroxiredoxin complex	0.1543
Esophageal tumor	5495	TFIIH transcription factor complex (ERCC2 ERCC3 GTF2H1 CDK7 CCNH GTF2H2)	0.08909
Esophagus cancer	4	Multisubunit ACTR coactivator complex	0.07454
Esophagus cancer	351	Spliceosome	0.01247
Esophagus cancer	541	IGF1-IGFBP3-ALS complex	0.08607
Esophagus cancer	570	p300-CBP-p270-SWI/SNF complex	0.05634
Esophagus cancer	571	p300-CBP-p270 complex	0.08607
Esophagus cancer	1181	C complex spliceosome	0.01667
Esophagus cancer	1223	H2AX complex isolated from cells without IR exposure	0.04134
Esophagus cancer	1307	Multiprotein complex (mRNA turnover)	0.06667
Esophagus cancer	1308	PABPC1-HSPA8-HNRPD-EIF4G1 complex	0.06667
Esophagus cancer	1332	Large Drosha complex	0.03333
Esophagus cancer	1810	ITGA4-PXN-GIT1 complex	0.08607
Esophagus cancer	2417	ITGA4-ITGB1-EMILIN1 complex	0.08607
Esophagus cancer	2418	ITGA4-ITGB1 complex	0.10541
Esophagus cancer	2419	ITGA4-ITGB1-CD81 complex	0.08607
Esophagus cancer	2420	ITGA4-ITGB1-CD53 complex	0.08607
Esophagus cancer	2421	ITGA4-ITGB1-VCAM1 complex	0.08607
Esophagus cancer	2422	ITGA4-ITGB1-JAM2 complex	0.08607
Esophagus cancer	2423	ITGA4-ITGB1-CD47 complex	0.08607
Esophagus cancer	2424	ITGA4-ITGB1-CD63 complex	0.08607
Esophagus cancer	2425	ITGA4-ITGB1-PXN complex	0.08607
Esophagus cancer	2426	ITGA4-ITGB1-THBS1 complex	0.08607
Esophagus cancer	2428	ITGA4-ITGB1-THBS2 complex	0.08607
Esophagus cancer	2638	HES1 promoter corepressor complex	0.06086
Esophagus cancer	2641	p300/CBP-PCAF-MyoD complex	0.07454
Esophagus cancer	2695	ETS2-FOS-JUN complex	0.08607
Esophagus cancer	2727	SRC-3 complex	0.05634
Esophagus cancer	2728	SRC-1 complex	0.07454
Esophagus cancer	2789	ETS2-ERG complex	0.10541
Esophagus cancer	2790	ETS2-ETS1 complex	0.10541
Esophagus cancer	2829	RSmad complex	0.04714
Esophagus cancer	2958	SMAD1-CBP complex	0.10541
Esophagus cancer	3044	SKI-NCOR1-SIN3A-HDAC1 complex	0.07454

Esophagus cancer	3056	Microprocessor complex	0.10541
Esophagus cancer	3061	RNA polymerase II complex (CBP PCAF RPB1 BAF47 CYCC CDK8) chromatin structure modifying	0.06086
Esophagus cancer	3062	RNA polymerase II complex incomplete (CBP RPBI PCAF BAF47) chromatin structure modifying	0.07454
Esophagus cancer	3066	RNA polymerase II complex chromatin structure modifying	0.04134
Esophagus cancer	3137	MASH1 promoter-coactivator complex	0.04495
Esophagus cancer	3142	CAMK2-delta-MASH1 promoter-coactivator complex	0.0527
Esophagus cancer	3197	SMAD4-SNO-SKI complex	0.08607
Esophagus cancer	3198	SMAD2-SKI complex	0.10541
Esophagus cancer	3199	SMAD3-SKI complex	0.10541
Esophagus cancer	3200	SMAD4-SKI complex	0.10541
Esophagus cancer	3204	SMAD2-SKI-NCOR complex	0.08607
Esophagus cancer	3205	SMAD3-SKI-NCOR complex	0.08607
Esophagus cancer	3206	SMAD4-SKI-NCOR complex	0.08607
Esophagus cancer	3729	SKI-SMAD2 hexameric complex	0.10541
Esophagus cancer	3733	SKI-SMAD3 hexameric complex	0.10541
Esophagus cancer	3739	SKI-SMAD2-SMAD4 pentameric complex	0.08607
Esophagus cancer	3740	SKI-SMAD3-SMAD4 pentameric complex	0.08607
Esophagus cancer	3749	CREBBP-SMAD2 hexameric complex	0.10541
Esophagus cancer	3750	CREBBP-SMAD3 hexameric complex	0.10541
Esophagus cancer	3753	CREBBP-SMAD2-SMAD4 pentameric complex	0.08607
Esophagus cancer	3754	CREBBP-SMAD3-SMAD4 pentameric complex	0.08607
Esophagus cancer	3959	SMAD3-SMAD4-cSKI TGF(beta)-dependent	0.08607
Esophagus cancer	3961	SMAD3-cSKI-SIN3A-HDAC1 complex	0.07454
Esophagus cancer	5198	CBP-RARA-RXRA-DNA complex ligand stimulated	0.17213
Esophagus cancer	5264	TCF4-CTNNB1-CREBBP complex	0.08607
Esophagus cancer	5293	ETS2-SMARCA4-INI1 complex	0.08607
Esophagus cancer	5573	Stat1-alpha-dimer-CBP DNA-protein complex	0.10541
Esophagus cancer	5613	Emerin complex 25	0.03727
Esotropia	541	IGF1-IGFBP3-ALS complex	0.16667
Esotropia	1062	BAR-BCL2-CASP8 complex	0.16667
Esotropia	1095	SNX complex (SNX1a SNX2 SNX4 EGFR)	0.14434
Esotropia	1185	EGFR-containing signaling complex	0.14434
Esotropia	5721	CIN85-CBL-SH3GL2 complex	0.16667
Esotropia	5713	SH3P2/OSTF1-CBL-SRC complex	0.16667
Esotropia	2369	ITGAV-ITGB3-EGFR complex	0.16667
Esotropia	2453	Multiprotein complex (monoubiquitination)	0.14434
Esotropia	2454	CIN85-CBL-SH3GL2-EGFR complex EGF stimulated	0.28868
Esotropia	2456	MET-CIN85-SH3GL3-CBL complex HGF stimulated	0.14434
Esotropia	2480	CIN85 complex (CIN85 CRK BCAR1 CBL PIK3R1 GRB2 SOS1)	0.10911
Esotropia	5709	ArgBP2a-CBL-PTK2B complex	0.16667
Esotropia	5720	CIN85-CBL complex	0.20412
Esotropia	2529	LAT-PLC-gamma-1-p85-GRB2-CBL-VAV-SLP-76 signaling complex C305 activated	0.10911

Esotropia	2534	Cbl-SLP-76-Grb2 complex Fc receptor gamma-R1 stimulated	0.16667
Esotropia	2535	SLP-76-Cbl-Grb2-Shc complex Fc receptor gamma-R1 stimulated	0.14434
Esotropia	2542	EGFR-CBL-GRB2 complex	0.33333
Esotropia	2563	FGFR2-c-Cbl-Lyn-Fyn complex	0.14434
Esotropia	2709	MMP-9-TIMP-1-LRP complex	0.16667
Esotropia	2956	PLC-gamma-1-LAT-c-CBL complex OKT3 stimulated	0.16667
Esotropia	3175	CIN85-c-CBL complex	0.20412
Esotropia	3678	RIN1-STAM2-EGFR complex EGF stimulated	0.16667
Esotropia	5171	SH3KBP1-CBLB-EGFR complex	0.16667
Esotropia	5526	CALM1-FKBP38-BCL2 complex	0.16667
Esotropia	5724	CIN85-SH3GL3-CBL complex	0.16667
Esotropia	5811	p53-BCL2 complex	0.20412
Esotropia	5817	tBID-BCL2 complex	0.20412
Esotropia	5818	BIM-BCL2 complex	0.20412
Ewings sarcoma	159	Condensin I-PARP-1-XRCC1 complex	0.12599
Ewings sarcoma	1004	RC complex during S-phase of cell cycle	0.09245
Ewings sarcoma	1005	RC complex during G2/M-phase of cell cycle	0.09245
Ewings sarcoma	1193	Rap1 complex	0.12599
Ewings sarcoma	1226	H2AX complex I	0.12599
Ewings sarcoma	1728	CTCF-nucleophosmin-PARP-HIS-KPNA-LMNA-TOP complex	0.11111
Ewings sarcoma	1729	TLE1 corepressor complex (MASH1 promoter-corepressor complex)	0.10541
Ewings sarcoma	2625	CDK8-MED6-PARP1 complex	0.19245
Ewings sarcoma	2694	ERG-JUN-FOS DNA-protein complex	0.19245
Ewings sarcoma	2789	ETS2-ERG complex	0.2357
Ewings sarcoma	3137	MASH1 promoter-coactivator complex	0.1005
Ewings sarcoma	3142	CAMK2-delta-MASH1 promoter-coactivator complex	0.11785
Ewings sarcoma	5179	NCOA6-DNA-PK-Ku-PARP1 complex	0.14907
Ewings sarcoma	5235	WRN-Ku70-Ku80-PARP1 complex	0.16667
Eye cancer	722	MRG15-PAM14-RB complex	0.40825
Eye cancer	723	MAF1 complex	0.40825
Eye cancer	1062	BAR-BCL2-CASP8 complex	0.40825
Eye cancer	1250	pRB-E2F-1 complex	0.5
Eye cancer	1372	Rb-tal-1-E2A-Lmo2-Ldb1 complex	0.31623
Eye cancer	1488	DNMT1-RB1-HDAC1-E2F1 complex	0.35355
Eye cancer	3269	RB1-HDAC1-BRG1 complex	0.40825
Eye cancer	3852	Rb-HDAC1 complex	0.5
Eye cancer	5099	RB1(hypophosphorylated)-E2F4 complex	0.5
Eye cancer	5143	E2F1-Rb complex	0.5
Eye cancer	5146	RB1-TFAP2A complex	0.5
Eye cancer	5526	CALM1-FKBP38-BCL2 complex	0.40825
Eye cancer	5611	Emerin complex 24	0.18257
Eye cancer	5656	CEBPE-E2F1-RB1 complex	0.40825
Eye cancer	5663	TRIM27-RB1 complex	0.5
Eye cancer	5811	p53-BCL2 complex	0.5

Eye cancer	5817	tBID-BCL2 complex	0.5
Eye cancer	5818	BIM-BCL2 complex	0.5
Eye disease	178	Respiratory chain complex I (holoenzyme)	0.02799
Eye disease	351	Spliceosome	0.01553
Eye disease	1181	C complex spliceosome	0.02076
Eye disease	1183	CDC5L complex	0.0339
Eye disease	2241	HD-RAB8A-OPTN complex	0.10721
Eye disease	2755	17S U2 snRNP	0.03233
Eye disease	2919	Respiratory chain complex I (gamma subunit) mitochondrial	0.0515
Eye disease	2939	Ecsit complex (ECSIT MT-CO2 NDUFA1 MT-ND1 TRAF6 NDUFAF1)	0.07581
Eye disease	5762	CRMP-MICAL-PlexinA1 complex induced by	0.10721
Familial Mediterranean fever	1774	MICA-KLRK1-HCST complex	0.14907
Familial Mediterranean fever	2016	IL12A homodimer complex	0.2582
Familial Mediterranean fever	2018	IL12A-IL12B complex	0.18257
Familial Mediterranean fever	2019	IL12A-IL12B-IL12RB1 complex	0.14907
Familial Mediterranean fever	2021	IL12A-IL12B-IL12RB2 complex	0.14907
Familial Mediterranean fever	2972	ITGA9-ITGB1-VEGFA complex	0.14907
Familial Mediterranean fever	4062	NRP1-VEGFR2-VEGF(165) complex	0.14907
Familial Mediterranean fever	5548	IL-12 heterodimer complex	0.18257
Familial Mediterranean fever	5696	VEGFA(165)-KDR-NRP1 complex	0.14907
Familial Mediterranean fever	5698	VEGFA(165)-VEGFR2-NRP1 complex	0.14907
Familial Mediterranean fever	5701	NRP1-VEGF(165/121) complex	0.18257
Fanconi's anemia	71	MRN complex (MRE11-RAD50-NBS1 complex)	0.11547
Fanconi's anemia	73	MRN complex (MRE11-RAD50-NBN complex)	0.11547
Fanconi's anemia	91	FA complex (Fanconi anemia complex) cytoplasmic	0.3
Fanconi's anemia	159	Condensin I-PARP-1-XRCC1 complex	0.07559
Fanconi's anemia	202	BRCA1-RAD50-MRE11-NBS1 complex	0.1
Fanconi's anemia	244	BRAFT complex	0.27735
Fanconi's anemia	245	FA core complex (Fanconi anemia core complex)	0.33333
Fanconi's anemia	246	BLM complex III	0.1
Fanconi's anemia	265	ATR-ATRIP complex	0.14142
Fanconi's anemia	368	ERCC1-ERCC4-MSH2 complex	0.11547
Fanconi's anemia	433	BASC complex (BRCA1-associated genome surveillance complex)	0.17321
Fanconi's anemia	434	BASC (Ab 80) complex (BRCA1-associated genome surveillance complex)	0.07071
Fanconi's anemia	435	BASC (Ab 81) complex (BRCA1-associated genome surveillance complex)	0.08165
Fanconi's anemia	531	XPA-ERCC1-ERCC4 complex	0.11547
Fanconi's anemia	619	MRE11A-RAD50-NBN-TRF2 complex	0.1
Fanconi's anemia	627	MRN-TRRAP complex (MRE11A-RAD50-NBN-TRRAP complex)	0.1
Fanconi's anemia	629	BLM-TRF2 complex	0.14142
Fanconi's anemia	871	BRAF53-BRCA2 complex	0.07559
Fanconi's anemia	1004	RC complex during S-phase of cell cycle	0.05547
Fanconi's anemia	1005	RC complex during G2/M-phase of cell cycle	0.05547

Fanconi's anemia	1045	Snurportin-CRM1-RanGTP complex	0.11547
Fanconi's anemia	1116	CRM1-Survivin-AuroraB mitotic complex	0.11547
Fanconi's anemia	1117	CRM1-Survivin mitotic complex	0.14142
Fanconi's anemia	1133	ATR-HDAC2 complex	0.14142
Fanconi's anemia	1134	ATR-HDAC2-CHD4 complex	0.11547
Fanconi's anemia	1152	FA complex (Fanconi anemia complex)	0.35355
Fanconi's anemia	1154	DSS1 complex	0.05547
Fanconi's anemia	1176	CRM1-RAN-PHAX-CBC complex (cap binding	0.08944
Fanconi's anemia	1189	DNA double-strand break end-joining complex	0.07559
Fanconi's anemia	1193	Rap1 complex	0.07559
Fanconi's anemia	1218	BLM-TRF2 complex	0.14142
Fanconi's anemia	1226	H2AX complex I	0.07559
Fanconi's anemia	1623	FA core complex 1 (Fanconi anemia core complex 1)	0.35777
Fanconi's anemia	1624	FA core complex (Fanconi anemia core complex)	0.33333
Fanconi's anemia	1625	FA core complex (Fanconi anemia core complex)	0.37796
Fanconi's anemia	1728	CTCF-nucleophosmin-PARP-HIS-KPNA-LMNA-TOP complex	0.06667
Fanconi's anemia	1729	TLE1 corepressor complex (MASH1 promoter-corepressor complex)	0.06325
Fanconi's anemia	5718	eNOS-HSP90-AKT complex VEGF induced	0.11547
Fanconi's anemia	5716	eNOS-HSP90 complex VEGF induced	0.14142
Fanconi's anemia	2000	BAX homo-oligomer complex	0.2
Fanconi's anemia	2112	CDC37-HSP90AA1-HSP90AB1-MAP3K11 complex	0.1
Fanconi's anemia	2171	Ubiquitin E3 ligase (CHEK1 CUL4A)	0.14142
Fanconi's anemia	2217	MDC1-MRN-ATM-FANCD2 complex	0.24495
Fanconi's anemia	2218	MDC1-MRE11-RAD50-NBS1 complex	0.1
Fanconi's anemia	2220	RAD52-ERCC4-ERCC1 complex	0.11547
Fanconi's anemia	2222	BLM complex II	0.08165
Fanconi's anemia	2223	BLM-TOP3A complex	0.14142
Fanconi's anemia	2224	MSH2/6-BLM-p53-RAD51 complex	0.08944
Fanconi's anemia	2228	BLM-RAD51L3-XRCC2 complex	0.11547
Fanconi's anemia	2625	CDK8-MED6-PARP1 complex	0.11547
Fanconi's anemia	2714	Ubiquitin E3 ligase (CHEK1 CUL1)	0.14142
Fanconi's anemia	2721	HCF-1 complex	0.04588
Fanconi's anemia	2723	ATM-NBS1 complex	0.28284
Fanconi's anemia	2739	FA complex (Fanconi anemia complex)	0.36181
Fanconi's anemia	2767	RAD50-MRE11-NBN-p200-p350 complex	0.11547
Fanconi's anemia	2815	BRCA1-BARD1-BACH1-DNA damage complex II	0.07071
Fanconi's anemia	2818	BRCA1-BARD1-BRCA2-DNA damage complex III	0.11547
Fanconi's anemia	3083	Nucleic and chromatin Fanconi complex	0.35777
Fanconi's anemia	3137	MASH1 promoter-coactivator complex	0.1206
Fanconi's anemia	3142	CAMK2-delta-MASH1 promoter-coactivator complex	0.14142
Fanconi's anemia	3492	Bax homooligomeric complex after apoptotic	0.2
Fanconi's anemia	4158	HSP90-FKBP38-CAM-Ca(2+) complex	0.1
Fanconi's anemia	5179	NCOA6-DNA-PK-Ku-PARP1 complex	0.08944
Fanconi's anemia	5197	PTIP-DNA damage response complex	0.1633
Fanconi's anemia	5199	Kinase maturation complex 1	0.05
Fanconi's anemia	5212	Kinase maturation complex 2	0.07071

Fanconi's anemia	5234	IKBKB-CDC37-KIAA1967-HSP90AB1-HSP90AA1 complex	0.08944
Fanconi's anemia	5235	WRN-Ku70-Ku80-PARP1 complex	0.1
Fanconi's anemia	5266	TNF-alpha/NF-kappa B signaling complex 6	0.05345
Fanconi's anemia	5268	TNF-alpha/NF-kappa B signaling complex 7	0.07071
Fanconi's anemia	5269	TNF-alpha/NF-kappa B signaling complex 8	0.08165
Fanconi's anemia	5286	TNF-alpha/NF-kappa B signaling complex 10	0.06325
Fanconi's anemia	5369	ATM homodimer complex	0.2
Fanconi's anemia	5400	BRCC complex	0.08944
Fanconi's anemia	5604	Emerin complex 1	0.07071
Fanconi's anemia	5606	Emerin-actin-NMI-(alphaII)spectrin complex	0.1
Fanconi's anemia	5608	Emerin architectural complex	0.08165
Fanconi's anemia	5614	Emerin complex 32	0.04264
Fanconi's anemia	5622	HSP90-CIP1-FKBPL complex	0.11547
Fatty liver	2456	MET-CIN85-SH3GL3-CBL complex HGF stimulated	0.14434
Fatty liver	2541	HGF-Met complex	0.20412
Fatty liver	5473	FAS-FADD-CASP8 complex	0.16667
Fatty liver	5799	Death induced signaling complex DISC (FAS FADD CASP8 CFLAR) membrane-associated CD95L	0.14434
Fatty liver	5800	Death-inducing signaling complex DISC (type I cells associated) stimulated	0.16667
Fatty liver	5808	DISC complex	0.16667
Fatty liver	5859	FAS-FADD-CASP8-CASP10 complex	0.14434
Fatty liver	5861	FAS-FADD-CASP10 complex	0.16667
Female reproductive cancer	201	HUIC complex	0.25
Female reproductive cancer	286	PCNA-MSH2-MSH6 complex	0.20412
Female reproductive cancer	290	MSH2-MLH1-PMS2-PCNA DNA-repair initiation complex	0.17678
Female reproductive cancer	291	MSH2-MLH1-PMS2 DNA-repair initiation complex	0.20412
Female reproductive cancer	368	ERCC1-ERCC4-MSH2 complex	0.20412
Female reproductive cancer	369	MSH2-MSH6-PMS2-MLH1 complex	0.17678
Female reproductive cancer	370	MSH2-MSH6-PMS1-MLH1 complex	0.17678
Female reproductive cancer	374	MSH2-MSH6 complex	0.25
Female reproductive cancer	375	MSH2-MSH3 complex	0.25
Female reproductive cancer	376	PCNA-MutS-alpha-MutL-alpha-DNA complex	0.15811
Female reproductive cancer	377	PCNA-MutS-alpha-DNA initial complex	0.20412
Female reproductive cancer	378	MutS-beta complex	0.25
Female reproductive cancer	433	BASC complex (BRCA1-associated genome surveillance complex)	0.10206
Female reproductive cancer	434	BASC (Ab 80) complex (BRCA1-associated genome surveillance complex)	0.125
Female reproductive cancer	438	GCN5-TRRAP histone acetyltransferase complex	0.1118
Female reproductive cancer	1335	SNW1 complex	0.08333
Female reproductive cancer	2211	BARD1-BRCA1-CSTF complex	0.15811
Female reproductive cancer	2213	BRCA1-BARD1-POLR2A complex	0.20412
Female reproductive cancer	2224	MSH2/6-BLM-p53-RAD51 complex	0.15811
Female reproductive cancer	2226	MutS-alpha-PK-zeta complex	0.20412
Female reproductive cancer	2740	MutS-alpha complex	0.25

Female reproductive cancer	2783	BARD1-BRCA1-CSTF64 complex	0.20412
Female reproductive cancer	2786	BRCA1 A complex	0.17678
Female reproductive cancer	2787	BRCA1 C complex	0.17678
Female reproductive cancer	2788	BRCA1 B complex	0.20412
Female reproductive cancer	2815	BRCA1-BARD1-BACH1-DNA damage complex II	0.125
Female reproductive cancer	2817	BRCA1-BARD1-BACH1-DNA damage complex I	0.14434
Female reproductive cancer	2818	BRCA1-BARD1-BRCA2-DNA damage complex III	0.20412
Female reproductive cancer	2822	BRCA1-BARD1-UbcH5c complex	0.20412
Female reproductive cancer	2823	BRCA1-BARD1-UbcH7c complex	0.20412
Female reproductive cancer	5288	P53-BARD1-Ku70 complex	0.20412
Fetal disease	1348	GLE1-NUPL2-NUP155 complex	0.18257
Fibroid tumor	541	IGF1-IGFBP3-ALS complex	0.17408
Fibroid tumor	2254	CTGF/Hcs24-actin complex	0.17408
Fibroid tumor	5823	MCL1-BAK1 complex	0.2132
Fibromyalgia	541	IGF1-IGFBP3-ALS complex	0.40825
Filariasis	1707	IL2-IL2RA-IL2RB complex	0.40825
Folic acid deficiency	541	IGF1-IGFBP3-ALS complex	0.2357
Gallbaldder cancer	1055	ZNF198-PML complex	0.31623
Gallbaldder cancer	1827	PML-SMAD2/3-SARA complex	0.22361
Gallbaldder cancer	2254	CTGF/Hcs24-actin complex	0.2582
Gallbladder disease	2000	BAX homo-oligomer complex	0.44721
Gallbladder disease	3492	Bax homooligomeric complex after apoptotic	0.44721
Gastritis	244	BRAFT complex	0.05241
Gastritis	246	BLM complex III	0.09449
Gastritis	285	PCNA-MLH1-PMS1 complex	0.10911
Gastritis	286	PCNA-MSH2-MSH6 complex	0.10911
Gastritis	290	MSH2-MLH1-PMS2-PCNA DNA-repair initiation complex	0.18898
Gastritis	291	MSH2-MLH1-PMS2 DNA-repair initiation complex	0.21822
Gastritis	292	MutL-alpha complex	0.13363
Gastritis	368	ERCC1-ERCC4-MSH2 complex	0.10911
Gastritis	369	MSH2-MSH6-PMS2-MLH1 complex	0.18898
Gastritis	370	MSH2-MSH6-PMS1-MLH1 complex	0.18898
Gastritis	374	MSH2-MSH6 complex	0.13363
Gastritis	375	MSH2-MSH3 complex	0.13363
Gastritis	376	PCNA-MutS-alpha-MutL-alpha-DNA complex	0.16903
Gastritis	377	PCNA-MutS-alpha-DNA initial complex	0.10911
Gastritis	378	MutS-beta complex	0.13363
Gastritis	380	MutL-beta complex	0.13363
Gastritis	415	EXO1-MLH1-PMS2 complex	0.10911
Gastritis	424	EXO1-MLH1-PCNA complex	0.10911
Gastritis	433	BASC complex (BRCA1-associated genome surveillance complex)	0.10911
Gastritis	434	BASC (Ab 80) complex (BRCA1-associated genome surveillance complex)	0.13363
Gastritis	435	BASC (Ab 81) complex (BRCA1-associated genome surveillance complex)	0.07715
Gastritis	438	GCN5-TRRAP histone acetyltransferase complex	0.05976

Gastritis	722	MRG15-PAM14-RB complex	0.10911
Gastritis	723	MAF1 complex	0.10911
Gastritis	1091	SNX complex (SNX1a SNX2 SNX4 LEPR)	0.09449
Gastritis	1250	pRB-E2F-1 complex	0.13363
Gastritis	1335	SNW1 complex	0.04454
Gastritis	1372	Rb-tal-1-E2A-Lmo2-Ldb1 complex	0.08452
Gastritis	1488	DNMT1-RB1-HDAC1-E2F1 complex	0.09449
Gastritis	1514	IL4-IL4R complex	0.13363
Gastritis	1515	IL4-IL4R-IL2RG complex	0.10911
Gastritis	1714	TICAM1-TICAM2-TLR4 complex	0.10911
Gastritis	1992	LEPR homodimer complex	0.18898
Gastritis	2018	IL12A-IL12B complex	0.13363
Gastritis	2019	IL12A-IL12B-IL12RB1 complex	0.10911
Gastritis	2020	IL12B-IL12RB1-IL12RB2 complex	0.10911
Gastritis	2021	IL12A-IL12B-IL12RB2 complex	0.10911
Gastritis	2224	MSH2/6-BLM-p53-RAD51 complex	0.08452
Gastritis	2226	MutS-alpha-PK-zeta complex	0.10911
Gastritis	2476	CRKL-PDGFRA-CRK-RAPGEF1 complex	0.09449
Gastritis	2480	CIN85 complex (CIN85 CRK BCAR1 CBL PIK3R1 GRB2 SOS1)	0.07143
Gastritis	2709	MMP-9-TIMP-1-LRP complex	0.10911
Gastritis	2740	MutS-alpha complex	0.13363
Gastritis	2817	BRCA1-BARD1-BACH1-DNA damage complex I	0.07715
Gastritis	2962	CRK-BCAR1-DOCK1 complex	0.10911
Gastritis	3269	RB1-HDAC1-BRG1 complex	0.10911
Gastritis	3852	Rb-HDAC1 complex	0.13363
Gastritis	5099	RB1(hypophosphorylated)-E2F4 complex	0.13363
Gastritis	5143	E2F1-Rb complex	0.13363
Gastritis	5146	RB1-TFAP2A complex	0.13363
Gastritis	5343	ELMO1-DOCK1-CRKII complex	0.10911
Gastritis	5444	CRKII-C3G complex	0.13363
Gastritis	5473	FAS-FADD-CASP8 complex	0.10911
Gastritis	5548	IL-12 heterodimer complex	0.13363
Gastritis	5549	IL-12 subunit p40 homodimer complex	0.18898
Gastritis	5611	Emerin complex 24	0.0488
Gastritis	5656	CEBPE-E2F1-RB1 complex	0.10911
Gastritis	5663	TRIM27-RB1 complex	0.13363
Gastritis	5799	Death induced signaling complex DISC (FAS FADD CASP8 CFLAR) membrane-associated CD95L	0.09449
Gastritis	5800	Death-inducing signaling complex DISC (type I cells associated) stimulated	0.10911
Gastritis	5808	DISC complex	0.10911
Gastritis	5859	FAS-FADD-CASP8-CASP10 complex	0.09449
Gastritis	5861	FAS-FADD-CASP10 complex	0.10911
Gastrointestinal cancer	577	FHL2-p53-HIPK2 complex	0.2582
Gastrointestinal cancer	2590	FOXO1-FHL2-SIRT1 complex	0.2582
Gastrointestinal cancer	3182	FHL2 homodimer complex	0.44721
Gastrointestinal cancer	3187	FHL2-FHL3 complex	0.31623

Gastrointestinal cancer	3188	FHL2-ACT complex	0.31623
Gastrointestinal cancer	3189	FHL2-CREB complex	0.31623
Gastrointestinal stromal tumor	541	IGF1-IGFBP3-ALS complex	0.2357
Gastrointestinal tumor	1095	SNX complex (SNX1a SNX2 SNX4 EGFR)	0.13868
Gastrointestinal tumor	1185	EGFR-containing signaling complex	0.13868
Gastrointestinal tumor	1772	MICB-KLRK1-HCST complex	0.16013
Gastrointestinal tumor	1839	SDCBP-CTNNB1-CTNNA1-CDH1 complex	0.13868
Gastrointestinal tumor	2318	ITGA6-ITGB4-Laminin10/12 complex	0.12403
Gastrointestinal tumor	2319	ITGA6-ITGB4-Laminin10/12 complex	0.12403
Gastrointestinal tumor	2320	ITGA6-ITGB4-CD151 complex	0.16013
Gastrointestinal tumor	2321	ITGA6-ITGB4-FYN complex	0.16013
Gastrointestinal tumor	2322	ITGA6-ITGB4-LAMA5 complex	0.16013
Gastrointestinal tumor	2323	ITGA6-ITGB4 complex	0.19612
Gastrointestinal tumor	2369	ITGAV-ITGB3-EGFR complex	0.16013
Gastrointestinal tumor	2411	ITGA6-ITGB1-CD151 complex	0.16013
Gastrointestinal tumor	2413	ITGA6-ITGB1 complex	0.19612
Gastrointestinal tumor	2434	ITGA1-ITGB1-COL6A3 complex	0.16013
Gastrointestinal tumor	2435	ITGA1-ITGB1-PTPN2 complex	0.16013
Gastrointestinal tumor	2437	ITGA6-ITGB1-CYR61 complex	0.16013
Gastrointestinal tumor	2453	Multiprotein complex (monoubiquitination)	0.13868
Gastrointestinal tumor	2454	CIN85-CBL-SH3GL2-EGFR complex EGF stimulated	0.13868
Gastrointestinal tumor	2542	EGFR-CBL-GRB2 complex	0.16013
Gastrointestinal tumor	2770	ITGA6-ITGB4-CD9 complex	0.16013
Gastrointestinal tumor	3096	ITGA6-ITGB4-SHC1-GRB2 complex	0.13868
Gastrointestinal tumor	3678	RIN1-STAM2-EGFR complex EGF stimulated	0.16013
Gastrointestinal tumor	4096	Catenin (alpha) - catenin (beta) complex	0.19612
Gastrointestinal tumor	5171	SH3KBP1-CBLB-EGFR complex	0.16013
Gastrointestinal tumor	5274	Cell-cell junction complex (ARHGAP10-CTNNA1)	0.19612
Generalized anxiety disorder	92	CD28-transactivation complex	0.20412
Generalized anxiety disorder	2529	LAT-PLC-gamma-1-p85-GRB2-CBL-VAV-SLP-76 signaling complex C305 activated	0.21822
Generalized anxiety disorder	2534	Cbl-SLP-76-Grb2 complex Fc receptor gamma-R1 stimulated	0.16667
Generalized anxiety disorder	2535	SLP-76-Cbl-Grb2-Shc complex Fc receptor gamma-R1 stimulated	0.14434
Generalized anxiety disorder	2536	PLC-gamma-2-SLP-76-Lyn-Grb2 complex	0.14434
Generalized anxiety disorder	2547	PLC-gamma-1-SLP-76-SOS1-LAT complex	0.28868
Generalized anxiety disorder	2909	PLC-gamma-2-Syk-LAT-FcR-gamma complex	0.14434
Generalized anxiety disorder	2912	PLC-gamma-2-SLP-76 complex	0.20412
Generalized anxiety disorder	2913	PLC-gamma-2-LAT complex	0.20412
Generalized anxiety disorder	2922	LAT-PLC-gamma-1-p85-GRB2-SOS signaling complex C305 activated	0.1291
Generalized anxiety disorder	2955	LCK-SLP76-PLC-gamma-1-LAT complex pervanadate-activated	0.28868
Generalized anxiety disorder	2956	PLC-gamma-1-LAT-c-CBL complex OKT3 stimulated	0.16667
Generalized anxiety disorder	2957	LAT-GRB2 complex Fyn-mLck(KA) or Syk kinase activated	0.20412
Generalized anxiety disorder	2960	SLP-76-PLC-gamma-1-ITK complex alpha-TCR stimulated	0.16667

Generalized anxiety disorder	2961	SLP-76-PLC-gamma-1-VAV complex alpha-TCR stimulated	0.16667
Generalized anxiety disorder	2963	ITK-SLP-76 complex anti-TCR stimulated	0.20412
Germ cell tumor	2217	MDC1-MRN-ATM-FANCD2 complex	0.14434
Germ cell tumor	2218	MDC1-MRE11-RAD50-NBS1 complex	0.17678
Germ cell tumor	2774	MDC1-H2AFX-TP53BP1 complex	0.20412
Germ cell tumor	2775	MDC1-p53BP1-SMC1 complex	0.20412
Germ cell tumor	5176	MGC1-DNA-PKcs-Ku complex	0.17678
Gestational diabetes	541	IGF1-IGFBP3-ALS complex	0.20412
Gestational diabetes	1069	FIF-FGR2 complex	0.25
Gigantism	280	HMGB1-HMGB2-HSC70-ERP60-GAPDH complex	0.44721
Gigantism	2936	Ecsit complex (ECSIT MT-CO2 GAPDH TRAF6 NDUFAF1)	0.44721
Gigantism	5385	GAIT complex	0.5
Gilles de la Tourette syndrome	753	UTM-SGCE-DAG1-CAV1-NOS3 complex	0.16903
Glaucoma	441	TFTC-type histone acetyl transferase complex	0.05025
Glaucoma	668	BKCA-beta2AR-AKAP79 signaling complex	0.09623
Glaucoma	672	BKCA-beta2AR complex	0.11785
Glaucoma	687	CFTR-NHERF-beta(2)AR signaling complex	0.09623
Glaucoma	879	PRKAC-AKAP5-ADRB1 complex	0.07454
Glaucoma	1054	ESR1-RELA-BCL3-NCOA3 complex	0.08333
Glaucoma	1062	BAR-BCL2-CASP8 complex	0.09623
Glaucoma	1088	PRNP-ApolopoproteinE3 complex	0.11785
Glaucoma	1095	SNX complex (SNX1a SNX2 SNX4 EGFR)	0.08333
Glaucoma	1185	EGFR-containing signaling complex	0.08333
Glaucoma	2000	BAX homo-oligomer complex	0.16667
Glaucoma	2124	IKK-alpha--ER-alpha-AIB1 complex	0.09623
Glaucoma	2369	ITGAV-ITGB3-EGFR complex	0.09623
Glaucoma	2453	Multiprotein complex (monoubiquitination)	0.08333
Glaucoma	2454	CIN85-CBL-SH3GL2-EGFR complex EGF stimulated	0.08333
Glaucoma	2470	p130Cas-ER-alpha-cSrc-kinase- PI3-kinase p85-subunit complex	0.08333
Glaucoma	2542	EGFR-CBL-GRB2 complex	0.09623
Glaucoma	2657	ESR1-CDK7-CCNH-MNAT1-MTA1-HDAC2 complex	0.06804
Glaucoma	2670	Er-alpha-p53-hdm2 complex	0.09623
Glaucoma	2699	ER-alpha-GRIP1-c-Jun complex	0.09623
Glaucoma	2700	ER-alpha-c-Jun complex	0.11785
Glaucoma	2709	MMP-9-TIMP-1-LRP complex	0.09623
Glaucoma	3492	Bax homooligomeric complex after apoptotic	0.16667
Glaucoma	3678	RIN1-STAM2-EGFR complex EGF stimulated	0.09623
Glaucoma	3830	ADRB2 homodimer complex	0.16667
Glaucoma	4869	beta(1)-AR receptosome (ADRB1-SAP97-AKAP79-PRKAR2A)	0.08333
Glaucoma	5171	SH3KBP1-CBLB-EGFR complex	0.09623
Glaucoma	5526	CALM1-FKBP38-BCL2 complex	0.09623
Glaucoma	5749	MRIT complex	0.09623
Glaucoma	5811	p53-BCL2 complex	0.11785
Glaucoma	5812	p53-BCL2 complex	0.11785

Glaucoma	5817	tBID-BCL2 complex	0.11785
Glaucoma	5818	BIM-BCL2 complex	0.11785
Glaucoma	5819	BIM-BCL2xL complex	0.11785
Glaucoma	5820	tBID-BCL2xL complex	0.11785
Glaucoma	5823	MCL1-BAK1 complex	0.11785
Glaucoma	5862	CAV1-VDAC1-ESR1 complex	0.09623
Glomerulonephritis	1088	PRNP-ApolopoproteinE3 complex	0.17678
Glomerulonephritis	2709	MMP-9-TIMP-1-LRP complex	0.28868
Glomerulonephritis	2754	JUND-FOSB-SMAD3-SMAD4 complex	0.125
Glomerulonephritis	2798	MMP-2-claudin-1 complex	0.17678
Glomerulonephritis	5388	SERPINA1-ELA2 complex	0.17678
Glucose intolerance	2589	PGC-1-SRp40-SRp55-SRp75 complex	0.11471
Glucose intolerance	2599	POLR2A-CCNT1-CDK9-NCL-LEM6-CPSF2 complex	0.09366
Glucose intolerance	2635	BETA2-Cyclin D1 complex	0.16222
Gouts	1439	PTGS2 homodimer complex	0.27735
Gouts	1514	IL4-IL4R complex	0.19612
Gouts	1515	IL4-IL4R-IL2RG complex	0.16013
Gouts	2254	CTGF/Hcs24-actin complex	0.16013
Gouts	3043	BMP2-BRIA complex	0.16013
Gouts	3710	CHL2-BMP2 complex	0.19612
Gouts	3711	CHL2-BMP2-TSG complex	0.16013
Gram-Negative bacterial	681	(C-CFTR)2-NHERF-ezrin complex	0.16667
Gram-Negative bacterial	682	C-CFTR-NHERF(PDZ1 domain)-ezrin complex	0.16667
Gram-Negative bacterial	683	C-CFTR-NHERF(PDZ2 domain)-ezrin complex	0.16667
Gram-Negative bacterial	687	CFTR-NHERF-beta(2)AR signaling complex	0.16667
Gram-Negative bacterial	1774	MICA-KLRK1-HCST complex	0.16667
Gram-Negative bacterial	5189	YWHAQ-CALM1-CABIN1 complex	0.16667
Gram-Negative bacterial	5199	Kinase maturation complex 1	0.07217
Gram-Negative bacterial	5615	Emerin complex 52	0.06019
Gram-Negative bacterial	5818	BIM-BCL2 complex	0.20412
Gram-Negative bacterial	5819	BIM-BCL2xL complex	0.20412
Gram-Negative bacterial	5919	BRAF-RAF1-14-3-3 complex	0.09623
Granulomatous disease	1256	MLL-HCF complex	0.10911
Granulomatous disease	1257	ALL-1 supercomplex	0.05455
Granulomatous disease	1401	MOF complex	0.09129
Granulomatous disease	5386	MLL1-WDR5 complex	0.05556
Graves' disease	313	Cell cycle kinase complex CDK5	0.07906
Graves' disease	668	BKCA-beta2AR-AKAP79 signaling complex	0.10206
Graves' disease	672	BKCA-beta2AR complex	0.125
Graves' disease	687	CFTR-NHERF-beta(2)AR signaling complex	0.10206
Graves' disease	1062	BAR-BCL2-CASP8 complex	0.10206
Graves' disease	2000	BAX homo-oligomer complex	0.17678
Graves' disease	3086	CCND3-CDK4 complex	0.125
Graves' disease	3089	CCND3-CDK6 complex	0.125
Graves' disease	3492	Bax homooligomeric complex after apoptotic	0.17678
Graves' disease	3830	ADRB2 homodimer complex	0.17678
Graves' disease	5100	CyclinD3-CDK4-CDK6 complex	0.10206

Graves' disease	5101	CyclinD3-CDK4-CDK6-p21 complex	0.08839
Graves' disease	5526	CALM1-FKBP38-BCL2 complex	0.10206
Graves' disease	5811	p53-BCL2 complex	0.125
Graves' disease	5817	tBID-BCL2 complex	0.125
Graves' disease	5818	BIM-BCL2 complex	0.125
Growth retardation	541	IGF1-IGFBP3-ALS complex	0.22222
Growth retardation	1088	PRNP-ApolopoproteinE3 complex	0.13608
Growth retardation	1439	PTGS2 homodimer complex	0.19245
Growth retardation	1986	Endoglin homodimer complex	0.19245
Growth retardation	2709	MMP-9-TIMP-1-LRP complex	0.11111
Growth retardation	2798	MMP-2-claudin-1 complex	0.13608
Growth retardation	5473	FAS-FADD-CASP8 complex	0.11111
Growth retardation	5799	Death induced signaling complex DISC (FAS FADD CASP8 CFLAR) membrane-associated CD95L	0.09623
Growth retardation	5800	Death-inducing signaling complex DISC (type I cells associated) stimulated	0.11111
Growth retardation	5808	DISC complex	0.11111
Growth retardation	5859	FAS-FADD-CASP8-CASP10 complex	0.09623
Growth retardation	5861	FAS-FADD-CASP10 complex	0.11111
HIV infection	59	AP3 adapter complex	0.0362
HIV infection	86	NUMAC complex (nucleosomal methylation activator complex)	0.03029
HIV infection	92	CD28-transactivation complex	0.13546
HIV infection	143	APP-FE65-LRP complex	0.0553
HIV infection	149	PBAF complex (Polybromo- and BAF containing complex)	0.02765
HIV infection	189	BAF complex	0.02657
HIV infection	238	SWI-SNF chromatin remodeling-related-BRCA1	0.02888
HIV infection	244	BRAFT complex	0.05313
HIV infection	246	BLM complex III	0.09578
HIV infection	285	PCNA-MLH1-PMS1 complex	0.1106
HIV infection	286	PCNA-MSH2-MSH6 complex	0.0553
HIV infection	290	MSH2-MLH1-PMS2-PCNA DNA-repair initiation complex	0.09578
HIV infection	291	MSH2-MLH1-PMS2 DNA-repair initiation complex	0.0553
HIV infection	292	MutL-alpha complex	0.06773
HIV infection	297	PCNA-DNA polymerase delta complex	0.04284
HIV infection	310	Cell cycle kinase complex CDC2	0.0391
HIV infection	311	Cell cycle kinase complex CDK2	0.04789
HIV infection	312	Cell cycle kinase complex CDK4	0.04789
HIV infection	313	Cell cycle kinase complex CDK5	0.04284
HIV infection	314	PCNA-p21 complex	0.06773
HIV infection	369	MSH2-MSH6-PMS2-MLH1 complex	0.04789
HIV infection	370	MSH2-MSH6-PMS1-MLH1 complex	0.04789
HIV infection	376	PCNA-MutS-alpha-MutL-alpha-DNA complex	0.08567
HIV infection	377	PCNA-MutS-alpha-DNA initial complex	0.0553
HIV infection	380	MutL-beta complex	0.06773
HIV infection	391	Ubiquitin E3 ligase (DDB1 CUL4A RBX1)	0.0553

HIV infection	415	EXO1-MLH1-PMS2 complex	0.0553
HIV infection	424	EXO1-MLH1-PCNA complex	0.1106
HIV infection	433	BASC complex (BRCA1-associated genome surveillance complex)	0.0553
HIV infection	434	BASC (Ab 80) complex (BRCA1-associated genome surveillance complex)	0.06773
HIV infection	435	BASC (Ab 81) complex (BRCA1-associated genome surveillance complex)	0.0391
HIV infection	554	PBAF complex (Polybromo- and BAF containing complex)	0.03029
HIV infection	555	BAF complex	0.03193
HIV infection	564	BAF complex	0.02888
HIV infection	565	PBAF complex (Polybromo- and BAF containing complex)	0.02888
HIV infection	566	BAF complex	0.02888
HIV infection	570	p300-CBP-p270-SWI/SNF complex	0.0362
HIV infection	626	LSD1 complex	0.02657
HIV infection	629	BLM-TRF2 complex	0.06773
HIV infection	642	CtBP complex	0.02323
HIV infection	643	CtBP core complex	0.03193
HIV infection	644	ASPP1-SAM68 complex	0.06773
HIV infection	652	AP3-BLOC1 complex	0.02473
HIV infection	710	Brg1-associated complex I	0.02888
HIV infection	711	Brm-associated complex	0.02888
HIV infection	713	BRG1-SIN3A complex	0.0256
HIV infection	714	BRM-SIN3A complex	0.02473
HIV infection	725	P2X7 receptor signalling complex	0.02765
HIV infection	726	DDB2 complex	0.02657
HIV infection	727	CSA complex	0.02657
HIV infection	728	CSA-POLIIa complex	0.0256
HIV infection	739	SIN3-ING1b complex II	0.02395
HIV infection	778	LARC complex (LCR-associated remodeling complex)	0.02197
HIV infection	790	DDB complex	0.06773
HIV infection	803	BRG1-SIN3A-HDAC containing SWI/SNF remodeling complex I	0.02888
HIV infection	806	BRM-SIN3A-HDAC complex	0.02765
HIV infection	807	BRG1-associated complex	0.03193
HIV infection	808	BRM-associated complex	0.03029
HIV infection	860	DNMT1-G9a-PCNA complex	0.0553
HIV infection	929	CEN complex	0.03149
HIV infection	997	KIN17-PCNA-RPA70 complex	0.0553
HIV infection	1039	PCNA-PAF complex	0.06773
HIV infection	1062	BAR-BCL2-CASP8 complex	0.0553
HIV infection	1079	P-TEFb.1 complex	0.13546
HIV infection	1080	P-TEFb.2 complex	0.06773
HIV infection	1083	P-TEFb.4 complex	0.06773
HIV infection	1087	BIRC5-AURKB-INCENP-EVI5 complex	0.04789
HIV infection	1091	SNX complex (SNX1a SNX2 SNX4 LEPR)	0.04789

HIV infection	1092	PCNA-KU antigen complex	0.0553
HIV infection	1098	DNA synthesome complex (13 subunits)	0.0256
HIV infection	1099	DNA synthesome complex (17 subunits)	0.02258
HIV infection	1107	DNA synthesome core complex	0.03029
HIV infection	1108	DNA synthesome complex (15 subunits)	0.02473
HIV infection	1116	CRM1-Survivin-AuroraB mitotic complex	0.0553
HIV infection	1117	CRM1-Survivin mitotic complex	0.06773
HIV infection	1118	Chromosomal passenger complex CPC (INCENP CDCA8 BIRC5 AURKB)	0.04789
HIV infection	1120	Chromosomal passenger complex CPC (INCENP CDCA8 BIRC5)	0.0553
HIV infection	1160	ING1-p300-PCNA complex	0.0553
HIV infection	1162	Ubiquitin E3 ligase (DDB1 DDB2 CUL4A CUL4B RBX1)	0.04284
HIV infection	1163	ING1-PCNA complex	0.06773
HIV infection	1191	RNA pol II containing coactivator complex Tat-SF	0.08567
HIV infection	1192	ESCRT-I complex	0.0553
HIV infection	1211	Ubiquitin E3 ligase (AHR ARNT DDB1 TBL3 CUL4B RBX1)	0.0391
HIV infection	1214	Ubiquitin E3 ligase (DET1 DDB1 CUL4A RBX1	0.04284
HIV infection	1218	BLM-TRF2 complex	0.06773
HIV infection	1223	H2AX complex isolated from cells without IR exposure	0.02657
HIV infection	1226	H2AX complex I	0.0362
HIV infection	1227	H2AX complex II	0.03029
HIV infection	1230	WINAC complex	0.0256
HIV infection	1239	EBAFb complex	0.03029
HIV infection	1250	pRB-E2F-1 complex	0.06773
HIV infection	1252	EBAFa complex	0.03029
HIV infection	1257	ALL-1 supercomplex	0.0181
HIV infection	1335	SNW1 complex	0.04515
HIV infection	1379	GALNS-lysosomal hydrolase 1.27 MDa complex	0.04789
HIV infection	1413	NCOR1 complex	0.03029
HIV infection	1488	DNMT1-RB1-HDAC1-E2F1 complex	0.04789
HIV infection	1707	IL2-IL2RA-IL2RB complex	0.1106
HIV infection	1729	TLE1 corepressor complex (MASH1 promoter-corepressor complex)	0.03029
HIV infection	1839	SDCBP-CTNNB1-CTNNA1-CDH1 complex	0.04789
HIV infection	1863	TSG101-VPS37B-VPS28 complex	0.0553
HIV infection	1992	LEPR homodimer complex	0.09578
HIV infection	2000	BAX homo-oligomer complex	0.09578
HIV infection	2153	ITGAM-ITGB2-CD11 complex	0.0553
HIV infection	2172	Ubiquitin E3 ligase (CDT1 DDB1 CUL4A RBX1)	0.04789
HIV infection	2197	FEN1-9-1-1 complex	0.04789
HIV infection	2201	PCNA-RFC2-5 complex	0.04284
HIV infection	2222	BLM complex II	0.0391
HIV infection	2223	BLM-TOP3A complex	0.06773
HIV infection	2224	MSH2/6-BLM-p53-RAD51 complex	0.04284
HIV infection	2228	BLM-RAD51L3-XRCC2 complex	0.0553

HIV infection	2230	PCNA complex	0.0362
HIV infection	2231	PCNA homotrimer complex	0.09578
HIV infection	2307	AP3D1-AP3S2 complex	0.06773
HIV infection	2308	AP3D1-AP3S1 complex	0.06773
HIV infection	2343	ITGAV-ITGB5-PLAUR complex	0.0553
HIV infection	2347	ITGAV-ITGB5-SPP1 complex	0.0553
HIV infection	2352	ITGAV-ITGB6-SPP1 complex	0.0553
HIV infection	2358	ITGAV-ITGB3-SPP1 complex	0.0553
HIV infection	2577	Sam68-p85 P13K-IRS-1-IR signaling complex	0.04789
HIV infection	2578	Sam68-p120GAP complex	0.06773
HIV infection	2579	Chromosomal passenger complex CPC (INCENP BIRC5 AURKB)	0.0553
HIV infection	2580	Survivin homodimer complex	0.09578
HIV infection	2581	RasGAP-AURKA/AURKB-survivin complex	0.04789
HIV infection	2582	Chromosomal passenger complex CPC (CDCA8 AURKB BIRC5)	0.0553
HIV infection	2599	POLR2A-CCNT1-CDK9-NCL-LEM6-CPSF2 complex	0.11731
HIV infection	2600	BRD4 complex	0.06773
HIV infection	2601	P-TEFb-BRD4-TRAP220 complex	0.09578
HIV infection	2602	P-TEFb-7SKRNA-HEXIM1 complex	0.1106
HIV infection	2603	Transcription elongation factor complex (SUPT5H CDK9 CCNT1)	0.1106
HIV infection	2604	P-TEFb-SKP2 complex	0.1106
HIV infection	2605	Heterotrimeric complex (CCNT1 CDK9 GRN)	0.1106
HIV infection	2639	HES1 promoter-Notch enhancer complex	0.05313
HIV infection	2709	MMP-9-TIMP-1-LRP complex	0.0553
HIV infection	2710	LRP-1-Alpha-2-M-annexin VI complex	0.0553
HIV infection	2721	HCF-1 complex	0.02197
HIV infection	2739	FA complex (Fanconi anemia complex)	0.02888
HIV infection	2797	PCNA-CHL12-RFC2-5 complex	0.0391
HIV infection	2817	BRCA1-BARD1-BACH1-DNA damage complex I	0.0391
HIV infection	2819	BRCA1-CtIP-CtBP complex	0.0553
HIV infection	2852	Brg1-based SWI/SNF chromatin remodeling complex	0.04789
HIV infection	2875	BRD4-P-TEFb complex	0.1106
HIV infection	2885	ITGAV-ITGB1-SPP1 complex	0.0553
HIV infection	2921	SHARP-CtBP complex	0.0553
HIV infection	2923	SHARP-CtBP1-CtIP complex	0.0553
HIV infection	2931	SHARP-CtBP1-CtIP-RBP-Jkappa corepressor complex	0.04789
HIV infection	2960	SLP-76-PLC-gamma-1-ITK complex alpha-TCR stimulated	0.0553
HIV infection	2963	ITK-SLP-76 complex anti-TCR stimulated	0.06773
HIV infection	3055	Nop56p-associated pre-rRNA complex	0.00939
HIV infection	3060	RNA polymerase II complex (RPB1 RAP74 CDK8 CYCC SRB7 BAF190 BAF47) chromatin structure modifying	0.03386
HIV infection	3061	RNA polymerase II complex (CBP PCAF RPB1 BAF47 CYCC CDK8) chromatin structure modifying	0.0391
HIV infection	3062	RNA polymerase II complex incomplete (CBP RPBI PCAF BAF47) chromatin structure modifying	0.04789

HIV infection	3063	Brg1-associated complex II	0.0362
HIV infection	3064	RNA polymerase II complex chromatin structure modifying	0.02197
HIV infection	3065	RNA polymerase II complex chromatin structure modifying	0.02888
HIV infection	3066	RNA polymerase II complex chromatin structure modifying	0.02657
HIV infection	3067	RNA polymerase II complex incomplete (CDK8 complex) chromatin structure modifying	0.03386
HIV infection	3078	DGCR8-NCL complex	0.06773
HIV infection	3082	DGCR8 multiprotein complex	0.05776
HIV infection	3111	ITGA9-ITGB1-SPP1 complex	0.0553
HIV infection	3112	ITGA5-ITGB1-SPP1 complex	0.0553
HIV infection	3229	Heterodimer complex (CDK9 IL6ST)	0.06773
HIV infection	3492	Bax homooligomeric complex after apoptotic	0.09578
HIV infection	3900	GABP(gamma)1-E2F1-DP1 complex	0.0553
HIV infection	4096	Catenin (alpha) - catenin (beta) complex	0.06773
HIV infection	5143	E2F1-Rb complex	0.06773
HIV infection	5144	E2F1-p107-cyclinA complex	0.0553
HIV infection	5197	PTIP-DNA damage response complex	0.0391
HIV infection	5260	TCF4-CTNNB1-SUMO1-EP300-HADAC6 complex	0.04284
HIV infection	5274	Cell-cell junction complex (ARHGAP10-CTNNA1)	0.06773
HIV infection	5293	ETS2-SMARCA4-INI1 complex	0.0553
HIV infection	5473	FAS-FADD-CASP8 complex	0.0553
HIV infection	5526	CALM1-FKBP38-BCL2 complex	0.0553
HIV infection	5544	CDC2-PCNA-CCNB1-GADD45A complex	0.04789
HIV infection	5545	CDC2-PCNA-CCNB1-GADD45B complex	0.04789
HIV infection	5546	CDC2-PCNA-CCNB1-GADD45G complex	0.04789
HIV infection	5604	Emerin complex 1	0.03386
HIV infection	5606	Emerin-actin-NMI-(alphaII)spectrin complex	0.04789
HIV infection	5607	Emerin-actin-NMI complex	0.0553
HIV infection	5608	Emerin architectural complex	0.0391
HIV infection	5609	Emerin regulatory complex	0.03193
HIV infection	5611	Emerin complex 24	0.02473
HIV infection	5613	Emerin complex 25	0.02395
HIV infection	5614	Emerin complex 32	0.04084
HIV infection	5615	Emerin complex 52	0.01997
HIV infection	5656	CEBPE-E2F1-RB1 complex	0.0553
HIV infection	5749	MRIT complex	0.0553
HIV infection	5799	Death induced signaling complex DISC (FAS FADD CASP8 CFLAR) membrane-associated CD95L	0.04789
HIV infection	5800	Death-inducing signaling complex DISC (type I cells associated) stimulated	0.0553
HIV infection	5808	DISC complex	0.0553
HIV infection	5811	p53-BCL2 complex	0.06773
HIV infection	5812	p53-BCL2 complex	0.06773
HIV infection	5817	tBID-BCL2 complex	0.06773
HIV infection	5818	BIM-BCL2 complex	0.06773

HIV infection	5819	BIM-BCL2xL complex	0.06773
HIV infection	5820	tBID-BCL2xL complex	0.06773
HIV infection	5859	FAS-FADD-CASP8-CASP10 complex	0.04789
HIV infection	5861	FAS-FADD-CASP10 complex	0.0553
HTLV-I infection	2709	MMP-9-TIMP-1-LRP complex	0.19245
HTLV-I infection	5473	FAS-FADD-CASP8 complex	0.19245
HTLV-I infection	5749	MRIT complex	0.19245
HTLV-I infection	5798	Death induced signaling complex II (FADD CASP8 CFLAR) cytosolic CD95L induced	0.19245
HTLV-I infection	5799	Death induced signaling complex DISC (FAS FADD CASP8 CFLAR) membrane-associated CD95L	0.33333
HTLV-I infection	5800	Death-inducing signaling complex DISC (type I cells associated) stimulated	0.19245
HTLV-I infection	5808	DISC complex	0.19245
HTLV-I infection	5859	FAS-FADD-CASP8-CASP10 complex	0.16667
HTLV-I infection	5861	FAS-FADD-CASP10 complex	0.19245
Hamman-Rich syndrome	220	ARF-Mule complex	0.11111
Hamman-Rich syndrome	310	Cell cycle kinase complex CDC2	0.07857
Hamman-Rich syndrome	311	Cell cycle kinase complex CDK2	0.09623
Hamman-Rich syndrome	312	Cell cycle kinase complex CDK4	0.09623
Hamman-Rich syndrome	313	Cell cycle kinase complex CDK5	0.08607
Hamman-Rich syndrome	541	IGF1-IGFBP3-ALS complex	0.11111
Hamman-Rich syndrome	553	RHOA-IP3R-TRPC1 complex	0.11111
Hamman-Rich syndrome	815	MRIP-MBS-RHOA complex	0.11111
Hamman-Rich syndrome	816	MRIP-RHOA complex	0.13608
Hamman-Rich syndrome	1514	IL4-IL4R complex	0.13608
Hamman-Rich syndrome	1515	IL4-IL4R-IL2RG complex	0.11111
Hamman-Rich syndrome	1633	CyclinD1-CDK4-CDK6 complex	0.11111
Hamman-Rich syndrome	1634	CyclinD1-CDK4-p21 complex	0.11111
Hamman-Rich syndrome	1642	p16-cyclin D2-CDK4 complex	0.11111
Hamman-Rich syndrome	2635	BETA2-Cyclin D1 complex	0.13608
Hamman-Rich syndrome	2798	MMP-2-claudin-1 complex	0.13608
Hamman-Rich syndrome	3084	CCND1-CDK4 complex	0.13608
Hamman-Rich syndrome	3087	CCND1-CDK6 complex	0.13608
Hamman-Rich syndrome	3153	GNAQ-GEFT-RHOA complex	0.11111
Hamman-Rich syndrome	5287	CDK4-CCND1 complex	0.13608
Heart disease	1728	CTCF-nucleophosmin-PARP-HIS-KPNA-LMNA-TOP complex	0.1005
Heart disease	5608	Emerin architectural complex	0.12309
Heart disease	5611	Emerin complex 24	0.07785
Heart failure	1	BCL6-HDAC4 complex	0.07538
Heart failure	2	BCL6-HDAC5 complex	0.07538
Heart failure	55	HDAC4-ERK1 complex	0.07538
Heart failure	57	HDAC4-ERK2 complex	0.07538
Heart failure	87	Nup 107-160 subcomplex	0.03553
Heart failure	159	Condensin I-PARP-1-XRCC1 complex	0.04029
Heart failure	362	DNA ligase III-XRCC1-PNK-DNA-pol III multiprotein complex	0.0533

Heart failure	520	KCNQ1 macromolecular complex	0.03371
Heart failure	527	TRPC3-TRPC4 channel complex redox-sensitive	0.07538
Heart failure	536	TRPC1-TRPC3-TRPC7 complex	0.06155
Heart failure	541	IGF1-IGFBP3-ALS complex	0.06155
Heart failure	568	Nuclear pore complex	0.02015
Heart failure	668	BKCA-beta2AR-AKAP79 signaling complex	0.06155
Heart failure	672	BKCA-beta2AR complex	0.07538
Heart failure	681	(C-CFTR)2-NHERF-ezrin complex	0.06155
Heart failure	682	C-CFTR-NHERF(PDZ1 domain)-ezrin complex	0.06155
Heart failure	683	C-CFTR-NHERF(PDZ2 domain)-ezrin complex	0.06155
Heart failure	687	CFTR-NHERF-beta(2)AR signaling complex	0.12309
Heart failure	786	MR-UBC9-SRC1 complex	0.06155
Heart failure	879	PRKAC-AKAP5-ADRB1 complex	0.04767
Heart failure	1004	RC complex during S-phase of cell cycle	0.02957
Heart failure	1005	RC complex during G2/M-phase of cell cycle	0.02957
Heart failure	1041	Alpha-dystrobrevin-ZO-1-actin complex	0.0533
Heart failure	1069	FIF-FGR2 complex	0.07538
Heart failure	1085	DNA repair complex NEIL2-PNK-Pol(beta)-LigIII(alpha)-XRCC1	0.04767
Heart failure	1086	DNA repair complex NEIL1-PNK-Pol(beta)-LigIII(alpha)-XRCC1	0.04767
Heart failure	1094	Frataxin complex	0.04029
Heart failure	1141	CF IIAm complex (Cleavage factor IIAm complex)	0.02665
Heart failure	1193	Rap1 complex	0.04029
Heart failure	1226	H2AX complex I	0.04029
Heart failure	1519	IL6ST-PRKCD-STAT3 complex	0.06155
Heart failure	1619	G protein complex (HDAC5 GNB1 GNG2)	0.06155
Heart failure	1620	G protein complex (HDAC4 GNB1 GNG2)	0.06155
Heart failure	1714	TICAM1-TICAM2-TLR4 complex	0.06155
Heart failure	1728	CTCF-nucleophosmin-PARP-HIS-KPNA-LMNA-TOP complex	0.03553
Heart failure	1729	TLE1 corepressor complex (MASH1 promoter-corepressor complex)	0.03371
Heart failure	1787	Nogo-potassium channel complex	0.1066
Heart failure	2198	RAD9-RAD1-HUS1-POLB complex	0.0533
Heart failure	2347	ITGAV-ITGB5-SPP1 complex	0.06155
Heart failure	2352	ITGAV-ITGB6-SPP1 complex	0.06155
Heart failure	2358	ITGAV-ITGB3-SPP1 complex	0.06155
Heart failure	2374	ITGAV-ITGB3-LAMA4 complex	0.06155
Heart failure	2625	CDK8-MED6-PARP1 complex	0.06155
Heart failure	2709	MMP-9-TIMP-1-LRP complex	0.12309
Heart failure	2755	17S U2 snRNP	0.01856
Heart failure	2885	ITGAV-ITGB1-SPP1 complex	0.06155
Heart failure	3111	ITGA9-ITGB1-SPP1 complex	0.06155
Heart failure	3112	ITGA5-ITGB1-SPP1 complex	0.06155
Heart failure	3137	MASH1 promoter-coactivator complex	0.03214
Heart failure	3142	CAMK2-delta-MASH1 promoter-coactivator complex	0.07538
Heart failure	3162	TF-FVIIa-FXa-TFPI complex	0.0533

Heart failure	3229	Heterodimer complex (CDK9 IL6ST)	0.07538
Heart failure	3634	NR3C2-UBC9-SRC-1 complex	0.06155
Heart failure	3830	ADRB2 homodimer complex	0.1066
Heart failure	4869	beta(1)-AR receptosome (ADRB1-SAP97-AKAP79-PRKAR2A)	0.0533
Heart failure	5772	ZO1-(beta)cadherin-(VE)cadherin-VEGFR2 complex	0.0533
Heart failure	5179	NCOA6-DNA-PK-Ku-PARP1 complex	0.04767
Heart failure	5235	WRN-Ku70-Ku80-PARP1 complex	0.0533
Heart failure	5389	SERPINA3-CTSG complex	0.07538
Heart failure	5391	SERPINA1-CTSG complex	0.07538
Heart failure	5564	LMO4-gp130 complex	0.04767
Heart failure	5579	CNTF-CNTFR-gp130-LIFR complex	0.0533
Heart failure	5582	LIFR-LIF-gp130 complex	0.06155
Helicobacter infection	285	PCNA-MLH1-PMS1 complex	0.1291
Helicobacter infection	286	PCNA-MSH2-MSH6 complex	0.1291
Helicobacter infection	290	MSH2-MLH1-PMS2-PCNA DNA-repair initiation complex	0.1118
Helicobacter infection	297	PCNA-DNA polymerase delta complex	0.1
Helicobacter infection	310	Cell cycle kinase complex CDC2	0.09129
Helicobacter infection	311	Cell cycle kinase complex CDK2	0.1118
Helicobacter infection	312	Cell cycle kinase complex CDK4	0.1118
Helicobacter infection	313	Cell cycle kinase complex CDK5	0.1
Helicobacter infection	314	PCNA-p21 complex	0.15811
Helicobacter infection	376	PCNA-MutS-alpha-MutL-alpha-DNA complex	0.1
Helicobacter infection	377	PCNA-MutS-alpha-DNA initial complex	0.1291
Helicobacter infection	424	EXO1-MLH1-PCNA complex	0.1291
Helicobacter infection	860	DNMT1-G9a-PCNA complex	0.1291
Helicobacter infection	997	KIN17-PCNA-RPA70 complex	0.1291
Helicobacter infection	1039	PCNA-PAF complex	0.15811
Helicobacter infection	1092	PCNA-KU antigen complex	0.1291
Helicobacter infection	1098	DNA synthesome complex (13 subunits)	0.05976
Helicobacter infection	1099	DNA synthesome complex (17 subunits)	0.0527
Helicobacter infection	1107	DNA synthesome core complex	0.07071
Helicobacter infection	1108	DNA synthesome complex (15 subunits)	0.05774
Helicobacter infection	1160	ING1-p300-PCNA complex	0.1291
Helicobacter infection	1163	ING1-PCNA complex	0.15811
Helicobacter infection	1714	TICAM1-TICAM2-TLR4 complex	0.1291
Helicobacter infection	1777	TGF-beta-receptor-SMAD7-SMURF2 complex	0.1118
Helicobacter infection	1783	TGF-beta receptor I-SMAD7-SMURF1 complex	0.1291
Helicobacter infection	2201	PCNA-RFC2-5 complex	0.1
Helicobacter infection	2230	PCNA complex	0.08452
Helicobacter infection	2231	PCNA homotrimer complex	0.22361
Helicobacter infection	2709	MMP-9-TIMP-1-LRP complex	0.1291
Helicobacter infection	2797	PCNA-CHL12-RFC2-5 complex	0.09129
Helicobacter infection	2798	MMP-2-claudin-1 complex	0.15811
Helicobacter infection	2992	SMAD7-SMURF2 complex	0.15811
Helicobacter infection	2996	SMAD7-SMURF1 complex	0.15811
Helicobacter infection	2997	SMAD7-SMURF1-TGF-beta receptor complex	0.1118

Helicobacter infection	5544	CDC2-PCNA-CCNB1-GADD45A complex	0.1118
Helicobacter infection	5545	CDC2-PCNA-CCNB1-GADD45B complex	0.1118
Helicobacter infection	5546	CDC2-PCNA-CCNB1-GADD45G complex	0.1118
Helminthiasis	1514	IL4-IL4R complex	0.35355
Helminthiasis	1515	IL4-IL4R-IL2RG complex	0.28868
Hematopoietic system disease	2028	JAK2-IL12RB2 complex	0.2357
Hematopoietic system disease	2688	MT1-MMP-claudin-1 complex	0.2357
Hematopoietic system disease	2798	MMP-2-claudin-1 complex	0.2357
Hematopoietic system disease	5178	JAK2-PAFR-TYK2 complex	0.19245
Hematopoietic system disease	5770	RUNX1-CBF-beta-DNA complex	0.2357
Hemolytic anemia	103	RNA polymerase II holoenzyme complex	0.05103
Hemolytic anemia	107	TFIIH transcription factor complex	0.08333
Hemolytic anemia	368	ERCC1-ERCC4-MSH2 complex	0.14434
Hemolytic anemia	371	Structure-specific endonulease complex	0.25
Hemolytic anemia	531	XPA-ERCC1-ERCC4 complex	0.14434
Hemolytic anemia	1009	TFIIH transcription factor complex	0.07906
Hemolytic anemia	1029	TFIIH transcription factor complex	0.07906
Hemolytic anemia	1030	CAK-ERCC2 complex	0.125
Hemolytic anemia	2220	RAD52-ERCC4-ERCC1 complex	0.14434
Hemolytic anemia	2660	ERCC2/CAK complex	0.125
Hemolytic anemia	2825	BRCA1-RNA polymerase II complex	0.04903
Hemolytic anemia	2839	ATRX-DAXX complex	0.17678
Hemolytic anemia	5495	TFIIH transcription factor complex (ERCC2 ERCC3 GTF2H1 CDK7 CCNH GTF2H2)	0.10206
Hemolytic-Uremic syndrome	310	Cell cycle kinase complex CDC2	0.09901
Hemolytic-Uremic syndrome	311	Cell cycle kinase complex CDK2	0.12127
Hemolytic-Uremic syndrome	312	Cell cycle kinase complex CDK4	0.12127
Hemolytic-Uremic syndrome	313	Cell cycle kinase complex CDK5	0.10847
Hemolytic-Uremic syndrome	314	PCNA-p21 complex	0.1715
Hemolytic-Uremic syndrome	387	MCM complex	0.09901
Hemolytic-Uremic syndrome	1181	C complex spliceosome	0.02712
Hemolytic-Uremic syndrome	1514	IL4-IL4R complex	0.1715
Hemolytic-Uremic syndrome	1515	IL4-IL4R-IL2RG complex	0.14003
Hemolytic-Uremic syndrome	1634	CyclinD1-CDK4-p21 complex	0.14003
Hemolytic-Uremic syndrome	1707	IL2-IL2RA-IL2RB complex	0.14003
Hemolytic-Uremic syndrome	2230	PCNA complex	0.09167
Hemolytic-Uremic syndrome	2791	MCM4-MCM6-MCM7 complex	0.14003
Hemolytic-Uremic syndrome	2792	MCM2-MCM4-MCM6-MCM7 complex	0.12127
Hemolytic-Uremic syndrome	3162	TF-FVIIa-FXa-TFPI complex	0.12127
Hemolytic-Uremic syndrome	5101	CyclinD3-CDK4-CDK6-p21 complex	0.12127
Hemolytic-Uremic syndrome	5611	Emerin complex 24	0.06262
Hemolytic-Uremic syndrome	5613	Emerin complex 25	0.06063
Hemolytic-Uremic syndrome	5615	Emerin complex 52	0.05057
Hemolytic-Uremic syndrome	5622	HSP90-CIP1-FKBPL complex	0.14003
Hemophilia	1170	cMYC-ATPase-helicase complex	0.2582
Hemophilia	1171	c-MYC-ATPase-helicase complex	0.2582
Hemophilia	2649	MYC-DNMT3A-ZBTB17 complex	0.33333

Hemophilia	2653	MYC-MAX-BLOC1S1 complex	0.33333
Hemophilia	2655	MYC-MAX complex	0.40825
Hemorrhagic disorder	11	BLOC-3 (biogenesis of lysosome-related organelles complex 3)	0.125
Hemorrhagic disorder	23	BLOC-1 (biogenesis of lysosome-related organelles complex 1)	0.0625
Hemorrhagic disorder	652	AP3-BLOC1 complex	0.04564
Hemorrhagic disorder	654	BLOC1-BLOC2 complex	0.0533
Hemorrhagic disorder	2355	ITGAV-ITGB3-CD47-FCER2 complex	0.08839
Hemorrhagic disorder	2356	ITGB3-ITGAV-CD47 complex	0.10206
Hemorrhagic disorder	2358	ITGAV-ITGB3-SPP1 complex	0.10206
Hemorrhagic disorder	2359	ITGAV-ITGB3-ADAM15 complex	0.10206
Hemorrhagic disorder	2362	ITAGV-ITGB3-F11R complex	0.10206
Hemorrhagic disorder	2363	ITGAV-ITGB3-PXN-PTK2b complex	0.08839
Hemorrhagic disorder	2364	ITGAV-ITGB3-ADAM23 complex	0.10206
Hemorrhagic disorder	2365	ITGAV-ITGB3-COL4A3 complex	0.10206
Hemorrhagic disorder	2366	ITGAV-ITGB3-PPAP2b complex	0.10206
Hemorrhagic disorder	2369	ITGAV-ITGB3-EGFR complex	0.10206
Hemorrhagic disorder	2370	ITGA2b-ITGB3-CD9 complex	0.20412
Hemorrhagic disorder	2374	ITGAV-ITGB3-LAMA4 complex	0.10206
Hemorrhagic disorder	2376	ITGA2B-ITGB3-FN1-TGM2 complex	0.17678
Hemorrhagic disorder	2377	ITGA2b-ITGB3-CD47-SRC complex	0.17678
Hemorrhagic disorder	2378	ITGA2b-ITGB3-TLN1 complex	0.20412
Hemorrhagic disorder	2379	ITGA2B-ITGB3-CIB1 complex	0.20412
Hemorrhagic disorder	2381	ITGA2B-ITGB3 complex	0.25
Hemorrhagic disorder	2382	ITGA2B-ITGB3-F11R complex	0.20412
Hemorrhagic disorder	2551	PDGFRA-PLC-gamma-1-PI3K-SHP-2 complex PDGF stimulated	0.08839
Hemorrhagic disorder	2816	ITGAV-ITGB3 complex	0.125
Hemorrhagic disorder	2826	ITGB3-ITGAV-VTN complex	0.10206
Hemorrhagic disorder	2846	ITGAV-ITGB3-THBS1 complex	0.10206
Hemorrhagic disorder	2849	ITGAV-ITGB3-NOV complex	0.10206
Hemorrhagic disorder	2872	ITGA2b-ITGB3-CD9-GP1b-CD47 complex	0.14434
Hemorrhagic disorder	2882	ITGA5-ITGB3-COL6A3 complex	0.10206
Hemorrhagic disorder	2896	ITGA2b-ITGB3-CD47-FAK complex	0.17678
Hemorrhagic disorder	3103	ITGAV-ITGB3-SLC3A2 complex	0.10206
Hemorrhagic disorder	3115	ITGA2B-ITGB3-ICAM4 complex	0.20412
Hemorrhagic disorder	3162	TF-FVIIa-FXa-TFPI complex	0.17678
Hemorrhagic disorder	3183	PDGFRA-SHP-2 complex PDGF stimulated	0.125
Hemorrhagic disorder	3186	GRB2-SHP-2 complex PDGF stimulated	0.125
Hemorrhagic disorder	5564	LMO4-gp130 complex	0.07906
Henoch-Schoenlein purpura	2709	MMP-9-TIMP-1-LRP complex	0.18257
Hepatitis	120	Lymphotoxin beta receptor complex	0.14907
Hepatitis	143	APP-FE65-LRP complex	0.14907
Hepatitis	1816	JUN-TCF4-CTNNB1 complex	0.14907
Hepatitis	2692	SMAD3-SMAD4-cJun-cFos complex	0.1291
Hepatitis	2693	NFAT-JUN-FOS DNA-protein complex	0.14907
Hepatitis	2694	ERG-JUN-FOS DNA-protein complex	0.14907

Hepatitis	2695	ETS2-FOS-JUN complex	0.14907
Hepatitis	2699	ER-alpha-GRIP1-c-Jun complex	0.14907
Hepatitis	2700	ER-alpha-c-Jun complex	0.18257
Hepatitis	2708	SMAD3-SMAD4-cJUN complex	0.14907
Hepatitis	2711	Amyloid beta protein oligomer	0.2582
Hepatitis	3092	APP-TOMM40 complex	0.18257
Hepatitis	3093	APP-TIMM23 complex	0.18257
Hepatitis	5530	Homodimeric complex LTBR	0.2582
Hepatitis B	2709	MMP-9-TIMP-1-LRP complex	0.28006
Hepatitis B	2798	MMP-2-claudin-1 complex	0.1715
Hepatitis C	98	p300-MDM2-p53 protein complex	0.08513
Hepatitis C	541	IGF1-IGFBP3-ALS complex	0.08513
Hepatitis C	1069	FIF-FGR2 complex	0.10426
Hepatitis C	1707	IL2-IL2RA-IL2RB complex	0.08513
Hepatitis C	1714	TICAM1-TICAM2-TLR4 complex	0.08513
Hepatitis C	1986	Endoglin homodimer complex	0.14744
Hepatitis C	2319	ITGA6-ITGB4-Laminin10/12 complex	0.06594
Hepatitis C	2347	ITGAV-ITGB5-SPP1 complex	0.08513
Hepatitis C	2352	ITGAV-ITGB6-SPP1 complex	0.08513
Hepatitis C	2358	ITGAV-ITGB3-SPP1 complex	0.08513
Hepatitis C	2670	Er-alpha-p53-hdm2 complex	0.08513
Hepatitis C	2885	ITGAV-ITGB1-SPP1 complex	0.08513
Hepatitis C	3111	ITGA9-ITGB1-SPP1 complex	0.08513
Hepatitis C	3112	ITGA5-ITGB1-SPP1 complex	0.08513
Hepatitis C	3172	NUMB-TP53-MDM2 complex	0.08513
Hepatitis C	5331	YY1-MDM2-p53 complex	0.08513
Hepatitis C	5414	HTR1A-HTR1D complex	0.10426
Hepatitis C	5416	HTR1A-HTR1B complex	0.10426
Hepatitis C	5418	GABBR2-HTR1A complex	0.10426
Hepatitis C	5419	HTR1A-GPR26 complex	0.10426
Hepatitis C	5420	HTR1A-EDG3 complex	0.10426
Hepatitis C	5421	HTR1A homodimer complex	0.14744
Hepatitis C	5422	HTR1A-EDG1 complex	0.10426
Hepatitis C	5473	FAS-FADD-CASP8 complex	0.08513
Hepatitis C	5799	Death induced signaling complex DISC (FAS FADD CASP8 CFLAR) membrane-associated CD95L	0.07372
Hepatitis C	5800	Death-inducing signaling complex DISC (type I cells associated) stimulated	0.08513
Hepatitis C	5808	DISC complex	0.08513
Hepatitis C	5859	FAS-FADD-CASP8-CASP10 complex	0.07372
Hepatitis C	5861	FAS-FADD-CASP10 complex	0.08513
Hepatoblastoma	4	Multisubunit ACTR coactivator complex	0.28868
Hepatoblastoma	98	p300-MDM2-p53 protein complex	0.33333
Hepatoblastoma	570	p300-CBP-p270-SWI/SNF complex	0.21822
Hepatoblastoma	571	p300-CBP-p270 complex	0.33333
Hepatoblastoma	1158	p33ING1b-p300 complex	0.40825
Hepatoblastoma	1160	ING1-p300-PCNA complex	0.33333

Hepatoblastoma	1471	pRb2/p130-multimolecular complex (RB2 E2F5 HDAC1 SUV39H1 P300)	0.2582
Hepatoblastoma	1521	p300-SMAD1-STAT3 complex	0.33333
Hepatoblastoma	1831	PIAS3-SMAD3-P300 complex	0.33333
Hepatoblastoma	2638	HES1 promoter corepressor complex	0.2357
Hepatoblastoma	2639	HES1 promoter-Notch enhancer complex	0.16013
Hepatoblastoma	2641	p300/CBP-PCAF-MyoD complex	0.28868
Hepatoblastoma	2642	SMAD1-P300 complex	0.40825
Hepatoblastoma	2954	Smad1-Notch1-p300-Pcaf complex	0.28868
Hepatoblastoma	3154	Notch2(N-TM)-Notch2(N-EC)-Delta complex	0.28868
Hepatoblastoma	3270	Delta1 homodimer complex	0.57735
Hepatoblastoma	3271	Gamma-secretase-Delta1 complex	0.2582
Hepatoblastoma	5118	pRb2/p130-multimolecular complex (RB2 E2F4 HDAC1 SUV39H1 P300)	0.2582
Hepatoblastoma	5260	TCF4-CTNNB1-SUMO1-EP300-HADAC6 complex	0.2582
Hepatoblastoma	5261	TCF4-CTNNB1-EP300 complex	0.33333
Hepatoblastoma	5375	EGR-EP300 complex	0.40825
Hereditary disease	71	MRN complex (MRE11-RAD50-NBS1 complex)	0.24618
Hereditary disease	72	R/M complex (RAD50-MRE11 complex)	0.15076
Hereditary disease	73	MRN complex (MRE11-RAD50-NBN complex)	0.24618
Hereditary disease	86	NUMAC complex (nucleosomal methylation activator complex)	0.06742
Hereditary disease	103	RNA polymerase II holoenzyme complex	0.04352
Hereditary disease	107	TFIIH transcription factor complex	0.07107
Hereditary disease	143	APP-FE65-LRP complex	0.12309
Hereditary disease	149	PBAF complex (Polybromo- and BAF containing complex)	0.06155
Hereditary disease	189	BAF complex	0.05913
Hereditary disease	202	BRCA1-RAD50-MRE11-NBS1 complex	0.2132
Hereditary disease	238	SWI-SNF chromatin remodeling-related-BRCA1	0.06428
Hereditary disease	433	BASC complex (BRCA1-associated genome surveillance complex)	0.12309
Hereditary disease	436	BASC (Ab C-20) complex (BRCA1-associated genome surveillance complex)	0.12309
Hereditary disease	443	BP-SMAD complex	0.07538
Hereditary disease	554	PBAF complex (Polybromo- and BAF containing complex)	0.06742
Hereditary disease	555	BAF complex	0.07107
Hereditary disease	564	BAF complex	0.06428
Hereditary disease	565	PBAF complex (Polybromo- and BAF containing complex)	0.06428
Hereditary disease	566	BAF complex	0.06428
Hereditary disease	570	p300-CBP-p270-SWI/SNF complex	0.08058
Hereditary disease	619	MRE11A-RAD50-NBN-TRF2 complex	0.2132
Hereditary disease	627	MRN-TRRAP complex (MRE11A-RAD50-NBN-TRRAP complex)	0.2132
Hereditary disease	710	Brg1-associated complex I	0.06428
Hereditary disease	713	BRG1-SIN3A complex	0.05698
Hereditary disease	739	SIN3-ING1b complex II	0.0533

Hereditary disease	778	LARC complex (LCR-associated remodeling complex)	0.04891
Hereditary disease	803	BRG1-SIN3A-HDAC containing SWI/SNF remodeling complex I	0.06428
Hereditary disease	807	BRG1-associated complex	0.07107
Hereditary disease	1009	TFIIH transcription factor complex	0.06742
Hereditary disease	1029	TFIIH transcription factor complex	0.06742
Hereditary disease	1037	TFIIH transcription factor core complex	0.09535
Hereditary disease	1141	CF IIAm complex (Cleavage factor IIAm complex)	0.0533
Hereditary disease	1189	DNA double-strand break end-joining complex	0.16116
Hereditary disease	1193	Rap1 complex	0.08058
Hereditary disease	1194	E2F-6 complex	0.06155
Hereditary disease	1204	Rap1 complex	0.08058
Hereditary disease	1230	WINAC complex	0.05698
Hereditary disease	1239	EBAFb complex	0.06742
Hereditary disease	1252	EBAFa complex	0.06742
Hereditary disease	1413	NCOR1 complex	0.06742
Hereditary disease	1728	CTCF-nucleophosmin-PARP-HIS-KPNA-LMNA-TOP complex	0.07107
Hereditary disease	1729	TLE1 corepressor complex (MASH1 promoter-corepressor complex)	0.06742
Hereditary disease	2197	FEN1-9-1-1 complex	0.1066
Hereditary disease	2217	MDC1-MRN-ATM-FANCD2 complex	0.17408
Hereditary disease	2218	MDC1-MRE11-RAD50-NBS1 complex	0.2132
Hereditary disease	2711	Amyloid beta protein oligomer	0.2132
Hereditary disease	2723	ATM-NBS1 complex	0.15076
Hereditary disease	2766	TERF2-RAP1 complex	0.08058
Hereditary disease	2767	RAD50-MRE11-NBN-p200-p350 complex	0.24618
Hereditary disease	2776	RAD50-BRCA1 complex	0.15076
Hereditary disease	2815	BRCA1-BARD1-BACH1-DNA damage complex II	0.15076
Hereditary disease	2825	BRCA1-RNA polymerase II complex	0.04181
Hereditary disease	2829	RSmad complex	0.06742
Hereditary disease	3060	RNA polymerase II complex (RPB1 RAP74 CDK8 CYCC SRB7 BAF190 BAF47) chromatin structure modifying	0.07538
Hereditary disease	3063	Brg1-associated complex II	0.08058
Hereditary disease	3064	RNA polymerase II complex chromatin structure modifying	0.04891
Hereditary disease	3066	RNA polymerase II complex chromatin structure modifying	0.11826
Hereditary disease	3092	APP-TOMM40 complex	0.15076
Hereditary disease	3093	APP-TIMM23 complex	0.15076
Hereditary disease	3269	RB1-HDAC1-BRG1 complex	0.12309
Hereditary disease	5197	PTIP-DNA damage response complex	0.17408
Hereditary disease	5293	ETS2-SMARCA4-INI1 complex	0.12309
Hereditary disease	5495	TFIIH transcription factor complex (ERCC2 ERCC3 GTF2H1 CDK7 CCNH GTF2H2)	0.08704
Hereditary disease	5608	Emerin architectural complex	0.08704
Hereditary disease	5611	Emerin complex 24	0.05505
Hereditary disease	5830	DJ-1-SNCA complex high molecular weight complex	0.15076

Herpes	98	p300-MDM2-p53 protein complex	0.08248
Herpes	143	APP-FE65-LRP complex	0.08248
Herpes	220	ARF-Mule complex	0.08248
Herpes	280	HMGB1-HMGB2-HSC70-ERP60-GAPDH complex	0.06389
Herpes	441	TFTC-type histone acetyl transferase complex	0.04307
Herpes	552	IFNB1-IFNAR1-IFNAR2- complex	0.08248
Herpes	725	P2X7 receptor signalling complex	0.04124
Herpes	929	CEN complex	0.02349
Herpes	1054	ESR1-RELA-BCL3-NCOA3 complex	0.07143
Herpes	1062	BAR-BCL2-CASP8 complex	0.08248
Herpes	1069	FIF-FGR2 complex	0.10102
Herpes	1088	PRNP-ApolopoproteinE3 complex	0.10102
Herpes	1178	BCOR complex	0.05051
Herpes	1182	CDC5L core complex	0.05832
Herpes	1183	CDC5L complex	0.02608
Herpes	1308	PABPC1-HSPA8-HNRPD-EIF4G1 complex	0.06389
Herpes	1474	SMAD3/4-E2F4/5-p107-DP1 complex	0.05832
Herpes	1539	G protein complex (GNG2 GNB2L1 RAF1)	0.08248
Herpes	1642	p16-cyclin D2-CDK4 complex	0.08248
Herpes	1700	ABL2-HRAS-RIN1 complex	0.08248
Herpes	5718	eNOS-HSP90-AKT complex VEGF induced	0.08248
Herpes	1826	SMAD3-HEF1-APC10-CDH1 complex	0.07143
Herpes	1827	PML-SMAD2/3-SARA complex	0.07143
Herpes	1828	TGF-beta receptor I-Axin-SMAD3 complex	0.08248
Herpes	1831	PIAS3-SMAD3-P300 complex	0.08248
Herpes	5716	eNOS-HSP90 complex VEGF induced	0.10102
Herpes	2112	CDC37-HSP90AA1-HSP90AB1-MAP3K11 complex	0.07143
Herpes	2124	IKK-alpha--ER-alpha-AIB1 complex	0.08248
Herpes	2129	DNAJB2-HSPA8-PSMA3 complex	0.08248
Herpes	2189	Ubiquitin E3 ligase (SMAD3 BTRC CUL1 SKP1A RBX1)	0.06389
Herpes	2237	SP1-MCAF2 complex	0.10102
Herpes	2256	RIAM-Rap1-GTP complex	0.10102
Herpes	2300	Profilin 2 complex	0.04762
Herpes	2342	ITGAV-ITGB8-MMP14-TGFB1 complex	0.07143
Herpes	2343	ITGAV-ITGB5-PLAUR complex	0.08248
Herpes	2345	ITGAV-ITGB5-ICAM4 complex	0.08248
Herpes	2346	ITGAV-ITGB5-ADAM9 complex	0.08248
Herpes	2347	ITGAV-ITGB5-SPP1 complex	0.16496
Herpes	2348	ITGAV-ITGB5-CYR61 complex	0.08248
Herpes	2350	ITGAV-ITGB5 complex	0.10102
Herpes	2351	ITGB6-FYN-FN1 complex	0.08248
Herpes	2352	ITGAV-ITGB6-SPP1 complex	0.24744
Herpes	2353	ITGAV-ITGB6-TGFB3 complex	0.16496
Herpes	2354	ITGAV-ITGB6 complex	0.20203
Herpes	2355	ITGAV-ITGB3-CD47-FCER2 complex	0.07143
Herpes	2356	ITGB3-ITGAV-CD47 complex	0.08248

Herpes	2358	ITGAV-ITGB3-SPP1 complex	0.16496
Herpes	2359	ITGAV-ITGB3-ADAM15 complex	0.08248
Herpes	2362	ITAGV-ITGB3-F11R complex	0.08248
Herpes	2363	ITGAV-ITGB3-PXN-PTK2b complex	0.07143
Herpes	2364	ITGAV-ITGB3-ADAM23 complex	0.08248
Herpes	2365	ITGAV-ITGB3-COL4A3 complex	0.08248
Herpes	2366	ITGAV-ITGB3-PPAP2b complex	0.08248
Herpes	2369	ITGAV-ITGB3-EGFR complex	0.08248
Herpes	2374	ITGAV-ITGB3-LAMA4 complex	0.08248
Herpes	2416	ITGB1-RAP1A-PKD1 complex	0.08248
Herpes	2436	ITGAV-ITGB1 complex	0.10102
Herpes	2470	p130Cas-ER-alpha-cSrc-kinase- PI3-kinase p85-subunit complex	0.07143
Herpes	2551	PDGFRA-PLC-gamma-1-PI3K-SHP-2 complex PDGF stimulated	0.07143
Herpes	2657	ESR1-CDK7-CCNH-MNAT1-MTA1-HDAC2 complex	0.05832
Herpes	2670	Er-alpha-p53-hdm2 complex	0.16496
Herpes	2679	p53-SP1 complex	0.10102
Herpes	2692	SMAD3-SMAD4-cJun-cFos complex	0.07143
Herpes	2699	ER-alpha-GRIP1-c-Jun complex	0.08248
Herpes	2700	ER-alpha-c-Jun complex	0.10102
Herpes	2705	SMAD3-SMAD4-CTCF protein-DNA complex	0.08248
Herpes	2706	SMAD3-SMAD4-SP1 complex	0.16496
Herpes	2707	SMAD3-SMAD4-FOXO3-FOXG1 complex	0.07143
Herpes	2708	SMAD3-SMAD4-cJUN complex	0.08248
Herpes	2711	Amyloid beta protein oligomer	0.14286
Herpes	2721	HCF-1 complex	0.09832
Herpes	2754	JUND-FOSB-SMAD3-SMAD4 complex	0.07143
Herpes	2760	SMAD3-SMAD4-FOXO3 complex	0.08248
Herpes	2761	SMAD3-SMAD4-FOXO1 complex	0.08248
Herpes	2762	SMAD3-SMAD4-FOXO4 complex	0.08248
Herpes	2813	BRCA1-SMAD3 complex	0.10102
Herpes	2816	ITGAV-ITGB3 complex	0.10102
Herpes	2826	ITGB3-ITGAV-VTN complex	0.08248
Herpes	2829	RSmad complex	0.04518
Herpes	2830	TIF1gamma-SMAD2-SMAD3 complex	0.08248
Herpes	2834	SMAD4-SMAD2-SMAD3 complex	0.08248
Herpes	2837	Profilin 1 complex	0.05832
Herpes	2846	ITGAV-ITGB3-THBS1 complex	0.08248
Herpes	2849	ITGAV-ITGB3-NOV complex	0.08248
Herpes	2885	ITGAV-ITGB1-SPP1 complex	0.16496
Herpes	2968	Axin-SMAD3 complex	0.10102
Herpes	2975	SMAD3-E2F4/5-p107-DP1 complex	0.06389
Herpes	3092	APP-TOMM40 complex	0.10102
Herpes	3093	APP-TIMM23 complex	0.10102
Herpes	3103	ITGAV-ITGB3-SLC3A2 complex	0.08248
Herpes	3110	ITGAV-P2RY2-GNA12 complex	0.08248
Herpes	3111	ITGA9-ITGB1-SPP1 complex	0.08248

Herpes	3112	ITGA5-ITGB1-SPP1 complex	0.08248
Herpes	3117	ITGB5-ITGAV-VTN complex	0.08248
Herpes	3158	RIAM-Rap1-GTP-profilin complex	0.08248
Herpes	3172	NUMB-TP53-MDM2 complex	0.08248
Herpes	3177	GNA14-p115RhoGEF complex	0.10102
Herpes	3183	PDGFRA-SHP-2 complex PDGF stimulated	0.10102
Herpes	3186	GRB2-SHP-2 complex PDGF stimulated	0.10102
Herpes	3199	SMAD3-SKI complex	0.10102
Herpes	3205	SMAD3-SKI-NCOR complex	0.08248
Herpes	3733	SKI-SMAD3 hexameric complex	0.10102
Herpes	3740	SKI-SMAD3-SMAD4 pentameric complex	0.08248
Herpes	3750	CREBBP-SMAD3 hexameric complex	0.10102
Herpes	3754	CREBBP-SMAD3-SMAD4 pentameric complex	0.08248
Herpes	3838	SP1-E2F2 complex	0.10102
Herpes	3839	SP1-E2F3 complex	0.10102
Herpes	3959	SMAD3-SMAD4-cSKI TGF(beta)-dependent	0.08248
Herpes	3961	SMAD3-cSKI-SIN3A-HDAC1 complex	0.07143
Herpes	3971	SMURF2-SMAD3 complex TGF(beta)-dependent	0.10102
Herpes	3972	SMURF2-SMAD3-SnoN complex TGF(beta)-	0.08248
Herpes	4158	HSP90-FKBP38-CAM-Ca(2+) complex	0.07143
Herpes	5199	Kinase maturation complex 1	0.03571
Herpes	5211	RAF1-PPP2-PIN1 complex	0.06389
Herpes	5212	Kinase maturation complex 2	0.05051
Herpes	5234	IKBKB-CDC37-KIAA1967-HSP90AB1-HSP90AA1 complex	0.06389
Herpes	5266	TNF-alpha/NF-kappa B signaling complex 6	0.03818
Herpes	5268	TNF-alpha/NF-kappa B signaling complex 7	0.05051
Herpes	5269	TNF-alpha/NF-kappa B signaling complex 8	0.05832
Herpes	5286	TNF-alpha/NF-kappa B signaling complex 10	0.04518
Herpes	5331	YY1-MDM2-p53 complex	0.08248
Herpes	5526	CALM1-FKBP38-BCL2 complex	0.08248
Herpes	5564	LMO4-gp130 complex	0.06389
Herpes	5622	HSP90-CIP1-FKBPL complex	0.08248
Herpes	5735	TGF-beta receptor-SMAD3 complex	0.08248
Herpes	5811	p53-BCL2 complex	0.10102
Herpes	5817	tBID-BCL2 complex	0.10102
Herpes	5818	BIM-BCL2 complex	0.10102
Herpes	5877	MAP2K1-BRAF-RAF1-YWHAE-KSR1 complex	0.06389
Herpes	5862	CAV1-VDAC1-ESR1 complex	0.08248
Herpes	5873	RAF1-MAP2K1-YWHAE complex	0.08248
Herpes	5919	BRAF-RAF1-14-3-3 complex	0.04762
Herpes	5920	KSR1-RAF1-MEK complex	0.07143
Herpes	5922	RAF1-RAS complex EGF induced	0.14286
Herpes	5923	RAF1-BRAF complex RAS stimulated	0.10102
Herpes	5924	RAF1-CNK1 complex RAS stimulated	0.10102
Herpes	5928	CNK1-SRC-RAF1 complex	0.08248
Histiocytosis	1088	PRNP-ApolopoproteinE3 complex	0.2357

Hodgkin's disease	71	MRN complex (MRE11-RAD50-NBS1 complex)	0.20412
Hodgkin's disease	72	R/M complex (RAD50-MRE11 complex)	0.25
Hodgkin's disease	73	MRN complex (MRE11-RAD50-NBN complex)	0.20412
Hodgkin's disease	202	BRCA1-RAD50-MRE11-NBS1 complex	0.17678
Hodgkin's disease	433	BASC complex (BRCA1-associated genome surveillance complex)	0.10206
Hodgkin's disease	436	BASC (Ab C-20) complex (BRCA1-associated genome surveillance complex)	0.10206
Hodgkin's disease	619	MRE11A-RAD50-NBN-TRF2 complex	0.17678
Hodgkin's disease	627	MRN-TRRAP complex (MRE11A-RAD50-NBN-TRRAP complex)	0.17678
Hodgkin's disease	1054	ESR1-RELA-BCL3-NCOA3 complex	0.08839
Hodgkin's disease	1141	CF IIAm complex (Cleavage factor IIAm complex)	0.04419
Hodgkin's disease	1189	DNA double-strand break end-joining complex	0.13363
Hodgkin's disease	1193	Rap1 complex	0.13363
Hodgkin's disease	1204	Rap1 complex	0.06682
Hodgkin's disease	1514	IL4-IL4R complex	0.125
Hodgkin's disease	1515	IL4-IL4R-IL2RG complex	0.10206
Hodgkin's disease	1729	TLE1 corepressor complex (MASH1 promoter-corepressor complex)	0.0559
Hodgkin's disease	2084	NFKB1-NFKB2-REL-RELA-RELB complex	0.07906
Hodgkin's disease	2217	MDC1-MRN-ATM-FANCD2 complex	0.14434
Hodgkin's disease	2218	MDC1-MRE11-RAD50-NBS1 complex	0.17678
Hodgkin's disease	2709	MMP-9-TIMP-1-LRP complex	0.10206
Hodgkin's disease	2766	TERF2-RAP1 complex	0.13363
Hodgkin's disease	2767	RAD50-MRE11-NBN-p200-p350 complex	0.20412
Hodgkin's disease	2776	RAD50-BRCA1 complex	0.125
Hodgkin's disease	2815	BRCA1-BARD1-BACH1-DNA damage complex II	0.125
Hodgkin's disease	5183	DNA-PK-Ku-eIF2-NF90-NF45 complex	0.0625
Hodgkin's disease	5193	TNF-alpha/NF-kappa B signaling complex (CHUK KPNA3 NFKB2 NFKBIB REL IKBKG NFKB1 NFKBIE RELB NFKBIA RELA TNIP2)	0.05103
Hodgkin's disease	5194	TNF-alpha/NF-kappa B signaling complex (SEC16A CHUK IKBKB NFKB2 REL IKBKG MAP3K14 RELA FBXW7 USP2)	0.0559
Hodgkin's disease	5196	TNF-alpha/NF-kappa B signaling complex (CHUK BTRC NFKB2 PPP6C REL CUL1 IKBKE SAPS2 SAPS1 ANKRD28 RELA SKP1)	0.05103
Hodgkin's disease	5197	PTIP-DNA damage response complex	0.14434
Hodgkin's disease	5228	REL-MAP3K8-RELA-TNIP2-PAPOLA complex	0.07906
Hodgkin's disease	5230	CHUK-NFKB2-REL-IKBKG-SPAG9-NFKB1-NFKBIE-COPB2-TNIP1-NFKBIA-RELA-TNIP2	0.05103
Hodgkin's disease	5232	TNF-alpha/Nf-kappa B signaling complex (RPL6 RPL30 RPS13 CHUK DDX3X NFKB2 NFKBIB REL IKBKG NFKB1 MAP3K8 RELB GLG1 NFKBIA RELA TNIP2 GTF2I)	0.04287
Hodgkin's disease	5233	TNF-alpha/NF-kappa B signaling complex 5	0.03536
Hodgkin's disease	5465	IKB(epsilon)-RELA-cREL complex	0.10206
Hodgkin's disease	5466	IKB(beta)-RELA-cREL complex	0.10206
Hodgkin's disease	5467	IKB(alpha)-RELA-cREL complex	0.10206

Huntington disease	280	HMGB1-HMGB2-HSC70-ERP60-GAPDH complex	0.09759
Huntington disease	351	Spliceosome	0.01825
Huntington disease	684	PAX9-MSX1 complex	0.1543
Huntington disease	930	Scrib-beta-PIX-GIT1 complex	0.12599
Huntington disease	5718	eNOS-HSP90-AKT complex VEGF induced	0.12599
Huntington disease	1810	ITGA4-PXN-GIT1 complex	0.12599
Huntington disease	2129	DNAJB2-HSPA8-PSMA3 complex	0.12599
Huntington disease	2156	YBX1-AKT1 complex	0.1543
Huntington disease	2159	AR-AKT-APPL complex	0.12599
Huntington disease	2233	Replication-coupled CAF-1-MBD1-ETDB1 complex	0.12599
Huntington disease	2238	MBD1-MCAF1-SETDB1 complex	0.12599
Huntington disease	2749	SETDB1-containing HMTase complex	0.1543
Huntington disease	2798	MMP-2-claudin-1 complex	0.1543
Huntington disease	2936	Ecsit complex (ECSIT MT-CO2 GAPDH TRAF6 NDUFAF1)	0.09759
Huntington disease	3847	TCL1(trimer)-AKT1 complex	0.1543
Huntington disease	5385	GAIT complex	0.10911
Hydrocephalus	5473	FAS-FADD-CASP8 complex	0.19245
Hydrocephalus	5799	Death induced signaling complex DISC (FAS FADD CASP8 CFLAR) membrane-associated CD95L	0.16667
Hydrocephalus	5800	Death-inducing signaling complex DISC (type I cells associated) stimulated	0.19245
Hydrocephalus	5808	DISC complex	0.19245
Hydrocephalus	5859	FAS-FADD-CASP8-CASP10 complex	0.16667
Hydrocephalus	5861	FAS-FADD-CASP10 complex	0.19245
Hyperaldosteronism	290	MSH2-MLH1-PMS2-PCNA DNA-repair initiation complex	0.15811
Hyperaldosteronism	291	MSH2-MLH1-PMS2 DNA-repair initiation complex	0.18257
Hyperaldosteronism	292	MutL-alpha complex	0.22361
Hyperaldosteronism	369	MSH2-MSH6-PMS2-MLH1 complex	0.15811
Hyperaldosteronism	376	PCNA-MutS-alpha-MutL-alpha-DNA complex	0.14142
Hyperaldosteronism	415	EXO1-MLH1-PMS2 complex	0.18257
Hyperaldosteronism	1254	Menin-associated histone methyltransferase complex	0.11952
Hyperaldosteronism	1256	MLL-HCF complex	0.11952
Hyperaldosteronism	3110	ITGAV-P2RY2-GNA12 complex	0.18257
Hypercholesterolemia	1091	SNX complex (SNX1a SNX2 SNX4 LEPR)	0.09623
Hypercholesterolemia	1095	SNX complex (SNX1a SNX2 SNX4 EGFR)	0.09623
Hypercholesterolemia	1185	EGFR-containing signaling complex	0.09623
Hypercholesterolemia	1714	TICAM1-TICAM2-TLR4 complex	0.11111
Hypercholesterolemia	1992	LEPR homodimer complex	0.19245
Hypercholesterolemia	2369	ITGAV-ITGB3-EGFR complex	0.11111
Hypercholesterolemia	2453	Multiprotein complex (monoubiquitination)	0.09623
Hypercholesterolemia	2454	CIN85-CBL-SH3GL2-EGFR complex EGF stimulated	0.09623
Hypercholesterolemia	2542	EGFR-CBL-GRB2 complex	0.11111
Hypercholesterolemia	3678	RIN1-STAM2-EGFR complex EGF stimulated	0.11111
Hypercholesterolemia	5171	SH3KBP1-CBLB-EGFR complex	0.11111
Hyperglycemia	298	VEGF transcriptional complex	0.06537
Hyperglycemia	541	IGF1-IGFBP3-ALS complex	0.09245

Hyperglycemia	1093	SNX complex (SNX1a SNX2 SNX4 INSR)	0.08006
Hyperglycemia	1514	IL4-IL4R complex	0.11323
Hyperglycemia	1515	IL4-IL4R-IL2RG complex	0.09245
Hyperglycemia	5718	eNOS-HSP90-AKT complex VEGF induced	0.09245
Hyperglycemia	2156	YBX1-AKT1 complex	0.11323
Hyperglycemia	2159	AR-AKT-APPL complex	0.09245
Hyperglycemia	2528	ERBB2-MEMO-SHC complex	0.09245
Hyperglycemia	2535	SLP-76-Cbl-Grb2-Shc complex Fc receptor gamma-R1 stimulated	0.08006
Hyperglycemia	2577	Sam68-p85 P13K-IRS-1-IR signaling complex	0.08006
Hyperglycemia	2895	SHC-GRB2 complex	0.11323
Hyperglycemia	3096	ITGA6-ITGB4-SHC1-GRB2 complex	0.08006
Hyperglycemia	3138	POSH-AKT2 complex	0.11323
Hyperglycemia	3847	TCL1(trimer)-AKT1 complex	0.11323
Hyperglycemia	3848	TCL1(trimer)-AKT2 complex	0.11323
Hyperglycemia	5273	VHL-TBP1-HIF1A complex	0.09245
Hyperglycemia	5276	HIF1A-OS9-EGLN1 complex	0.09245
Hyperglycemia	5277	HIF1A-OS9-EGLN3 complex	0.09245
Hyperglycemia	5382	ARNT-HIF1A complex	0.11323
Hyperhomocysteinemia	1088	PRNP-ApolopoproteinE3 complex	0.20412
Hyperinsulinism	1028	HNF4A-SUB1 complex	0.13608
Hyperinsulinism	2577	Sam68-p85 P13K-IRS-1-IR signaling complex	0.09623
Hyperlipidemia	441	TFTC-type histone acetyl transferase complex	0.05913
Hyperlipidemia	1054	ESR1-RELA-BCL3-NCOA3 complex	0.09806
Hyperlipidemia	1088	PRNP-ApolopoproteinE3 complex	0.13868
Hyperlipidemia	2124	IKK-alpha--ER-alpha-AIB1 complex	0.11323
Hyperlipidemia	2342	ITGAV-ITGB8-MMP14-TGFB1 complex	0.09806
Hyperlipidemia	2470	p130Cas-ER-alpha-cSrc-kinase- PI3-kinase p85-subunit complex	0.09806
Hyperlipidemia	2577	Sam68-p85 P13K-IRS-1-IR signaling complex	0.09806
Hyperlipidemia	2657	ESR1-CDK7-CCNH-MNAT1-MTA1-HDAC2 complex	0.08006
Hyperlipidemia	2670	Er-alpha-p53-hdm2 complex	0.11323
Hyperlipidemia	2688	MT1-MMP-claudin-1 complex	0.13868
Hyperlipidemia	2699	ER-alpha-GRIP1-c-Jun complex	0.11323
Hyperlipidemia	2700	ER-alpha-c-Jun complex	0.13868
Hyperlipidemia	3162	TF-FVIIa-FXa-TFPI complex	0.19612
Hyperlipidemia	5862	CAV1-VDAC1-ESR1 complex	0.11323
Hyperopia	2489	NCR3-CD247 complex	0.2357
Hyperopia	2801	OCT4-SOX2 DNA-protein complex	0.2357
Hyperopia	2802	OCT1-SOX2 DNA-protein complex	0.2357
Hyperopia	2803	PAX6-SOX2 DNA-protein complex	0.4714
Hyperparathyroidism	310	Cell cycle kinase complex CDC2	0.27217
Hyperparathyroidism	311	Cell cycle kinase complex CDK2	0.33333
Hyperparathyroidism	312	Cell cycle kinase complex CDK4	0.33333
Hyperparathyroidism	313	Cell cycle kinase complex CDK5	0.29814
Hyperparathyroidism	314	PCNA-p21 complex	0.2357
Hyperparathyroidism	1167	Paf complex	0.14907
Hyperparathyroidism	1254	Menin-associated histone methyltransferase complex	0.12599

Hyperparathyroidism	1256	MLL-HCF complex	0.12599
Hyperparathyroidism	1633	CyclinD1-CDK4-CDK6 complex	0.19245
Hyperparathyroidism	1634	CyclinD1-CDK4-p21 complex	0.3849
Hyperparathyroidism	1656	p27-cyclinE-CDK2 complex	0.19245
Hyperparathyroidism	2230	PCNA complex	0.12599
Hyperparathyroidism	2635	BETA2-Cyclin D1 complex	0.2357
Hyperparathyroidism	3015	p27-cyclinE-Cdk2 - Ubiquitin E3 ligase (SKP1A SKP2 CUL1 CKS1B RBX1) complex	0.11785
Hyperparathyroidism	3084	CCND1-CDK4 complex	0.2357
Hyperparathyroidism	3087	CCND1-CDK6 complex	0.2357
Hyperparathyroidism	5101	CyclinD3-CDK4-CDK6-p21 complex	0.16667
Hyperparathyroidism	5287	CDK4-CCND1 complex	0.2357
Hyperparathyroidism	5622	HSP90-CIP1-FKBPL complex	0.19245
Hypertension	120	Lymphotoxin beta receptor complex	0.04564
Hypertension	178	Respiratory chain complex I (holoenzyme)	0.02384
Hypertension	220	ARF-Mule complex	0.04564
Hypertension	441	TFTC-type histone acetyl transferase complex	0.02384
Hypertension	550	NOS3-CAV1-NOSTRIN complex	0.04564
Hypertension	557	TRP1-G alpha-11-IP3R3-CAV1 signaling complex	0.03953
Hypertension	563	F1F0-ATP synthase (EC 3.6.3.14) mitochondrial	0.01976
Hypertension	668	BKCA-beta2AR-AKAP79 signaling complex	0.04564
Hypertension	672	BKCA-beta2AR complex	0.0559
Hypertension	681	(C-CFTR)2-NHERF-ezrin complex	0.04564
Hypertension	682	C-CFTR-NHERF(PDZ1 domain)-ezrin complex	0.04564
Hypertension	683	C-CFTR-NHERF(PDZ2 domain)-ezrin complex	0.04564
Hypertension	687	CFTR-NHERF-beta(2)AR signaling complex	0.09129
Hypertension	753	UTM-SGCE-DAG1-CAV1-NOS3 complex	0.03536
Hypertension	786	MR-UBC9-SRC1 complex	0.04564
Hypertension	879	PRKAC-AKAP5-ADRB1 complex	0.03536
Hypertension	1054	ESR1-RELA-BCL3-NCOA3 complex	0.03953
Hypertension	1091	SNX complex (SNX1a SNX2 SNX4 LEPR)	0.03953
Hypertension	1093	SNX complex (SNX1a SNX2 SNX4 INSR)	0.03953
Hypertension	1121	WNK1-OSR1 complex	0.0559
Hypertension	1123	WNK1-SPAk complex	0.0559
Hypertension	1297	MKK4-ARRB2-ASK1 complex	0.04564
Hypertension	1298	MKK4-ARRB2-JNK3 complex	0.04564
Hypertension	1300	CRLR-RAMP1 complex	0.0559
Hypertension	1642	p16-cyclin D2-CDK4 complex	0.04564
Hypertension	5718	eNOS-HSP90-AKT complex VEGF induced	0.04564
Hypertension	1970	BMP4-TWSG1 complex	0.0559
Hypertension	1972	BMP4-BGN complex	0.0559
Hypertension	1992	LEPR homodimer complex	0.07906
Hypertension	2018	IL12A-IL12B complex	0.0559
Hypertension	2019	IL12A-IL12B-IL12RB1 complex	0.04564
Hypertension	2020	IL12B-IL12RB1-IL12RB2 complex	0.04564
Hypertension	2021	IL12A-IL12B-IL12RB2 complex	0.04564
Hypertension	2124	IKK-alpha--ER-alpha-AIB1 complex	0.04564

Hypertension	2156	YBX1-AKT1 complex	0.0559
Hypertension	2159	AR-AKT-APPL complex	0.04564
Hypertension	2254	CTGF/Hcs24-actin complex	0.04564
Hypertension	2300	Profilin 2 complex	0.02635
Hypertension	2363	ITGAV-ITGB3-PXN-PTK2b complex	0.03953
Hypertension	5714	eNOS-CAV1 complex	0.0559
Hypertension	2462	Caveolin-1 homodimer complex	0.07906
Hypertension	2470	p130Cas-ER-alpha-cSrc-kinase- PI3-kinase p85-subunit complex	0.03953
Hypertension	5709	ArgBP2a-CBL-PTK2B complex	0.04564
Hypertension	2577	Sam68-p85 P13K-IRS-1-IR signaling complex	0.03953
Hypertension	2589	PGC-1-SRp40-SRp55-SRp75 complex	0.03953
Hypertension	2599	POLR2A-CCNT1-CDK9-NCL-LEM6-CPSF2 complex	0.03227
Hypertension	2657	ESR1-CDK7-CCNH-MNAT1-MTA1-HDAC2 complex	0.03227
Hypertension	2670	Er-alpha-p53-hdm2 complex	0.04564
Hypertension	2699	ER-alpha-GRIP1-c-Jun complex	0.04564
Hypertension	2700	ER-alpha-c-Jun complex	0.0559
Hypertension	2709	MMP-9-TIMP-1-LRP complex	0.09129
Hypertension	2884	Respiratory chain complex I (early intermediate NDUFAF1 assembly) mitochondrial	0.02988
Hypertension	2886	Respiratory chain complex I (incomplete intermediate ND1 ND2 ND3 CIA30 assembly) mitochondrial	0.07906
Hypertension	2901	Respiratory chain complex I (intermediate IV/310kD) mitochondrial	0.03953
Hypertension	2903	Respiratory chain complex I (intermediate V/380kD and VI/480kD) mitochondrial	0.03536
Hypertension	2904	Respiratory chain complex I (intermediate VII/650kD) mitochondrial	0.025
Hypertension	2919	Respiratory chain complex I (gamma subunit) mitochondrial	0.04385
Hypertension	2939	Ecsit complex (ECSIT MT-CO2 NDUFA1 MT-ND1 TRAF6 NDUFAF1)	0.03227
Hypertension	2943	Respiratory chain complex I (incomplete NDUFAF1 assembly) mitochondrial	0.0559
Hypertension	3043	BMP2-BRIA complex	0.04564
Hypertension	3139	CRLR-RAMP1-ARRB2 complex	0.04564
Hypertension	3140	CRLR-RAMP2 complex	0.0559
Hypertension	3141	CRLR-RAMP3 complex	0.0559
Hypertension	3634	NR3C2-UBC9-SRC-1 complex	0.04564
Hypertension	3830	ADRB2 homodimer complex	0.07906
Hypertension	3847	TCL1(trimer)-AKT1 complex	0.0559
Hypertension	4869	beta(1)-AR receptosome (ADRB1-SAP97-AKAP79-PRKAR2A)	0.03953
Hypertension	5414	HTR1A-HTR1D complex	0.0559
Hypertension	5415	HTR1B homodimer complex	0.07906
Hypertension	5416	HTR1A-HTR1B complex	0.1118
Hypertension	5417	HTR1D-HTR1B complex	0.0559
Hypertension	5418	GABBR2-HTR1A complex	0.0559
Hypertension	5419	HTR1A-GPR26 complex	0.0559

Hypertension	5420	HTR1A-EDG3 complex	0.0559
Hypertension	5421	HTR1A homodimer complex	0.07906
Hypertension	5422	HTR1A-EDG1 complex	0.0559
Hypertension	5548	IL-12 heterodimer complex	0.0559
Hypertension	5549	IL-12 subunit p40 homodimer complex	0.07906
Hypertension	5747	2AR-mGluR2 complex	0.0559
Hypertension	5862	CAV1-VDAC1-ESR1 complex	0.09129
Hyperthyroidism	541	IGF1-IGFBP3-ALS complex	0.19245
Hypogammaglobulinemia	2574	CD19-Vav-PI 3-kinase (p85 subunit) complex	0.40825
Hypoglycemia	786	MR-UBC9-SRC1 complex	0.2357
Hypoglycemia	1088	PRNP-ApolopoproteinE3 complex	0.28868
Hypoglycemia	3634	NR3C2-UBC9-SRC-1 complex	0.2357
Hypogonadism	2159	AR-AKT-APPL complex	0.2582
Hypogonadism	2160	AOF2-AR complex	0.31623
Hypopituitarism	3164	HESX1-TLE1 complex	0.35355
Hypopituitarism	3167	NCOR-SIN3-HDAC-HESX1 complex	0.20412
Hypothyroidism	541	IGF1-IGFBP3-ALS complex	0.27217
Hypothyroidism	1514	IL4-IL4R complex	0.16667
Hypothyroidism	1515	IL4-IL4R-IL2RG complex	0.13608
IGA glomerulonephritis	553	RHOA-IP3R-TRPC1 complex	0.09901
IGA glomerulonephritis	815	MRIP-MBS-RHOA complex	0.09901
IGA glomerulonephritis	816	MRIP-RHOA complex	0.12127
IGA glomerulonephritis	1514	IL4-IL4R complex	0.12127
IGA glomerulonephritis	1515	IL4-IL4R-IL2RG complex	0.09901
IGA glomerulonephritis	2254	CTGF/Hcs24-actin complex	0.09901
IGA glomerulonephritis	2709	MMP-9-TIMP-1-LRP complex	0.09901
IGA glomerulonephritis	3153	GNAQ-GEFT-RHOA complex	0.09901
IGA glomerulonephritis	5691	TALL1 homo-oligomer complex	0.1715
Immune complex disease	2909	PLC-gamma-2-Syk-LAT-FcR-gamma complex	0.35355
Immune complex disease	2910	PLC-gamma-2-Lyn-FcR-gamma complex	0.40825
Immune complex disease	5691	TALL1 homo-oligomer complex	0.70711
Immunologic deficiency syndrome	71	MRN complex (MRE11-RAD50-NBS1 complex)	0.1037
Immunologic deficiency syndrome	73	MRN complex (MRE11-RAD50-NBN complex)	0.1037
Immunologic deficiency syndrome	202	BRCA1-RAD50-MRE11-NBS1 complex	0.0898
Immunologic deficiency syndrome	206	DNA ligase IV-XRCC4 complex	0.127
Immunologic deficiency syndrome	213	DNA ligase IV-XRCC1 complex	0.127
Immunologic deficiency syndrome	336	DNA ligase IV-XRCC4-AHNK complex	0.1037
Immunologic deficiency syndrome	344	DNA ligase IV-XRCC4 complex (LX complex)	0.127
Immunologic deficiency syndrome	350	DNA ligase IV-XRCC4-PNK complex	0.1037
Immunologic deficiency syndrome	353	DNA ligase IV-condensin complex	0.1037

Immunologic deficiency syndrome	359	DNA ligase IV-XRCC4-XLF complex	0.20739
Immunologic deficiency syndrome	433	BASC complex (BRCA1-associated genome surveillance complex)	0.1037
Immunologic deficiency syndrome	435	BASC (Ab 81) complex (BRCA1-associated genome surveillance complex)	0.07332
Immunologic deficiency syndrome	619	MRE11A-RAD50-NBN-TRF2 complex	0.0898
Immunologic deficiency syndrome	627	MRN-TRRAP complex (MRE11A-RAD50-NBN-TRRAP complex)	0.0898
Immunologic deficiency syndrome	1062	BAR-BCL2-CASP8 complex	0.1037
Immunologic deficiency syndrome	1189	DNA double-strand break end-joining complex	0.13577
Immunologic deficiency syndrome	1256	MLL-HCF complex	0.06788
Immunologic deficiency syndrome	1257	ALL-1 supercomplex	0.03394
Immunologic deficiency syndrome	1401	MOF complex	0.0568
Immunologic deficiency syndrome	1515	IL4-IL4R-IL2RG complex	0.1037
Immunologic deficiency syndrome	1615	G protein complex (BTK GNG1 GNG2)	0.1037
Immunologic deficiency syndrome	1985	AIRE homodimer complex	0.17961
Immunologic deficiency syndrome	2054	CASP8-FADD-MALT1-BCL10 complex	0.0898
Immunologic deficiency syndrome	2055	CASP8-CHUK-IKBKB-MALT1-BCL10 complex	0.08032
Immunologic deficiency syndrome	2056	BCL10-CHUK-BCL10-IKBKB complex	0.0898
Immunologic deficiency syndrome	2217	MDC1-MRN-ATM-FANCD2 complex	0.14665
Immunologic deficiency syndrome	2218	MDC1-MRE11-RAD50-NBS1 complex	0.0898
Immunologic deficiency syndrome	2723	ATM-NBS1 complex	0.254
Immunologic deficiency syndrome	2767	RAD50-MRE11-NBN-p200-p350 complex	0.1037
Immunologic deficiency syndrome	2815	BRCA1-BARD1-BACH1-DNA damage complex II	0.0635
Immunologic deficiency syndrome	5197	PTIP-DNA damage response complex	0.07332
Immunologic deficiency syndrome	5317	LATS1-HTRA2-BIRC4 complex	0.1037
Immunologic deficiency syndrome	5369	ATM homodimer complex	0.17961
Immunologic deficiency syndrome	5384	RAG1-RAG2 tetramer complex	0.254
Immunologic deficiency syndrome	5386	MLL1-WDR5 complex	0.03457

Immunologic deficiency syndrome	5473	FAS-FADD-CASP8 complex	0.1037
Immunologic deficiency syndrome	5691	TALL1 homo-oligomer complex	0.17961
Immunologic deficiency syndrome	5749	MRIT complex	0.1037
Immunologic deficiency syndrome	5798	Death induced signaling complex II (FADD CASP8 CFLAR) cytosolic CD95L induced	0.1037
Immunologic deficiency syndrome	5799	Death induced signaling complex DISC (FAS FADD CASP8 CFLAR) membrane-associated CD95L	0.0898
Immunologic deficiency syndrome	5800	Death-inducing signaling complex DISC (type I cells associated) stimulated	0.1037
Immunologic deficiency syndrome	5808	DISC complex	0.1037
Immunologic deficiency syndrome	5859	FAS-FADD-CASP8-CASP10 complex	0.0898
Infantile spasms	75	TSC1-TSC2 complex	0.53452
Infection	15	NCOR complex	0.03807
Infection	58	SMRT complex	0.0417
Infection	280	HMGB1-HMGB2-HSC70-ERP60-GAPDH complex	0.0417
Infection	298	VEGF transcriptional complex	0.03807
Infection	299	IRF3-CBP complex	0.04663
Infection	310	Cell cycle kinase complex CDC2	0.03807
Infection	311	Cell cycle kinase complex CDK2	0.04663
Infection	312	Cell cycle kinase complex CDK4	0.04663
Infection	313	Cell cycle kinase complex CDK5	0.0417
Infection	314	PCNA-p21 complex	0.06594
Infection	351	Spliceosome	0.0078
Infection	433	BASC complex (BRCA1-associated genome surveillance complex)	0.02692
Infection	435	BASC (Ab 81) complex (BRCA1-associated genome surveillance complex)	0.03807
Infection	552	IFNB1-IFNAR1-IFNAR2- complex	0.05384
Infection	626	LSD1 complex	0.02586
Infection	642	CtBP complex	0.02262
Infection	643	CtBP core complex	0.03108
Infection	681	(C-CFTR)2-NHERF-ezrin complex	0.05384
Infection	682	C-CFTR-NHERF(PDZ1 domain)-ezrin complex	0.05384
Infection	683	C-CFTR-NHERF(PDZ2 domain)-ezrin complex	0.05384
Infection	725	P2X7 receptor signalling complex	0.02692
Infection	741	NCOR-HDAC3 complex	0.0417
Infection	748	TRAF6-TAK1 complex	0.06594
Infection	752	SMRT core complex	0.05384
Infection	770	TREX complex	0.03297
Infection	774	THO complex	0.0417
Infection	824	Anti-SMN protein complex	0.0417
Infection	832	Anti-Sm protein complex	0.03525
Infection	929	CEN complex	0.01533
Infection	938	FACT complex UV-activated	0.0417

Infection	1130	Hip1R-cortactin complex	0.06594
Infection	1142	SMN complex	0.02949
Infection	1143	SMN complex	0.02331
Infection	1178	BCOR complex	0.03297
Infection	1182	CDC5L core complex	0.03807
Infection	1183	CDC5L complex	0.01703
Infection	1231	FIB-associated protein complex	0.03807
Infection	1308	PABPC1-HSPA8-HNRPD-EIF4G1 complex	0.08341
Infection	1413	NCOR1 complex	0.02949
Infection	1505	NCOR2 complex	0.03525
Infection	1514	IL4-IL4R complex	0.06594
Infection	1515	IL4-IL4R-IL2RG complex	0.05384
Infection	1634	CyclinD1-CDK4-p21 complex	0.05384
Infection	1707	IL2-IL2RA-IL2RB complex	0.05384
Infection	1746	SMN containing complex	0.03297
Infection	1751	SMN complex	0.02949
Infection	1752	SMN complex	0.03525
Infection	1893	mTOR-RICTOR complex	0.05384
Infection	1895	RICTOR-mTOR complex	0.05384
Infection	1897	RAPTOR-mTOR complex	0.05384
Infection	2001	NOD1 homodimer complex	0.09325
Infection	2019	IL12A-IL12B-IL12RB1 complex	0.05384
Infection	2020	IL12B-IL12RB1-IL12RB2 complex	0.10768
Infection	2021	IL12A-IL12B-IL12RB2 complex	0.05384
Infection	2026	IL12RB1-IL12RB2 complex	0.13188
Infection	2028	JAK2-IL12RB2 complex	0.06594
Infection	2073	TNFRSF11A-TRAF6-SRC complex	0.05384
Infection	2074	TRAF6 oligomer complex	0.09325
Infection	2129	DNAJB2-HSPA8-PSMA3 complex	0.05384
Infection	2183	Kaiso-NCOR complex	0.02949
Infection	2217	MDC1-MRN-ATM-FANCD2 complex	0.03807
Infection	2230	PCNA complex	0.03525
Infection	2300	Profilin 2 complex	0.03108
Infection	2318	ITGA6-ITGB4-Laminin10/12 complex	0.08341
Infection	2319	ITGA6-ITGB4-Laminin10/12 complex	0.08341
Infection	2320	ITGA6-ITGB4-CD151 complex	0.05384
Infection	2321	ITGA6-ITGB4-FYN complex	0.05384
Infection	2322	ITGA6-ITGB4-LAMA5 complex	0.10768
Infection	2323	ITGA6-ITGB4 complex	0.06594
Infection	2411	ITGA6-ITGB1-CD151 complex	0.05384
Infection	2413	ITGA6-ITGB1 complex	0.06594
Infection	2437	ITGA6-ITGB1-CYR61 complex	0.05384
Infection	2551	PDGFRA-PLC-gamma-1-PI3K-SHP-2 complex PDGF stimulated	0.04663
Infection	2688	MT1-MMP-claudin-1 complex	0.06594
Infection	2719	Casein kinase II-HMG1 complex	0.04663
Infection	2720	Casein kinase II complex	0.05384

Infection	2721	HCF-1 complex	0.02139
Infection	2723	ATM-NBS1 complex	0.06594
Infection	2743	TRAF6-MALT1 complex	0.06594
Infection	2745	Ubiquitin ligase complex (TRAF6 TAB2 MALT1 UEV1A BCL10)	0.0417
Infection	2770	ITGA6-ITGB4-CD9 complex	0.05384
Infection	2798	MMP-2-claudin-1 complex	0.06594
Infection	2811	BRCA1-cABL complex	0.06594
Infection	2819	BRCA1-CtIP-CtBP complex	0.05384
Infection	2837	Profilin 1 complex	0.03807
Infection	2921	SHARP-CtBP complex	0.05384
Infection	2923	SHARP-CtBP1-CtIP complex	0.05384
Infection	2931	SHARP-CtBP1-CtIP-RBP-Jkappa corepressor complex	0.04663
Infection	2936	Ecsit complex (ECSIT MT-CO2 GAPDH TRAF6 NDUFAF1)	0.0417
Infection	2939	Ecsit complex (ECSIT MT-CO2 NDUFA1 MT-ND1 TRAF6 NDUFAF1)	0.03807
Infection	2969	mTORC2 complex (mTOR/FRAP1 LST8 mAVO3/RICTOR)	0.05384
Infection	2970	mTORC1 complex (mTOR/FRAP1 LST8 RAPTOR)	0.05384
Infection	2985	mTOR-signaling complex	0.06594
Infection	2990	mTOR-signaling complex (FRAP1/mTOR GBL RAPTOR)	0.05384
Infection	2991	mTOR-signaling complex (mTOR/FRAP1 RAPTOR)	0.06594
Infection	3096	ITGA6-ITGB4-SHC1-GRB2 complex	0.04663
Infection	3098	TIM50a-SMN1 complex	0.06594
Infection	3118	SMN1-SIP1-SNRP complex	0.03525
Infection	3183	PDGFRA-SHP-2 complex PDGF stimulated	0.06594
Infection	3186	GRB2-SHP-2 complex PDGF stimulated	0.06594
Infection	3231	Eps15-stonin2 complex	0.06594
Infection	3284	SMN complex (GEMIN5 2 3 4 SMN)	0.0417
Infection	3298	SMN complex (GEMIN2 5 SMN)	0.05384
Infection	3979	mTORC2 complex (mTOR/FRAP1 LST8 mAVO3/RICTOR SIN1)	0.04663
Infection	3980	mTOR-RAPTOR complex	0.05384
Infection	4498	p32-CBF-DNA complex	0.04663
Infection	5101	CyclinD3-CDK4-CDK6-p21 complex	0.04663
Infection	5183	DNA-PK-Ku-eIF2-NF90-NF45 complex	0.03297
Infection	5196	TNF-alpha/NF-kappa B signaling complex (CHUK BTRC NFKB2 PPP6C REL CUL1 IKBKE SAPS2 SAPS1 ANKRD28 RELA SKP1)	0.02692
Infection	5212	Kinase maturation complex 2	0.03297
Infection	5224	Casein kinase II (beta-dimer alpha alpha')	0.05384
Infection	5225	Casein kinase II (beta-dimer alpha-dimer)	0.06594
Infection	5253	MNK1-eIF4F complex	0.0417
Infection	5269	TNF-alpha/NF-kappa B signaling complex 8	0.03807
Infection	5273	VHL-TBP1-HIF1A complex	0.05384
Infection	5276	HIF1A-OS9-EGLN1 complex	0.05384
Infection	5277	HIF1A-OS9-EGLN3 complex	0.05384

Infection	5286	TNF-alpha/NF-kappa B signaling complex 10	0.02949
Infection	5318	DDEF1-CTTN-PXN complex	0.05384
Infection	5369	ATM homodimer complex	0.09325
Infection	5382	ARNT-HIF1A complex	0.06594
Infection	5426	ANCO1-HDAC3 complex	0.06594
Infection	5564	LMO4-gp130 complex	0.08341
Infection	5609	Emerin regulatory complex	0.03108
Infection	5611	Emerin complex 24	0.02408
Infection	5614	Emerin complex 32	0.01988
Infection	5615	Emerin complex 52	0.01944
Infection	5622	HSP90-CIP1-FKBPL complex	0.05384
Infection	5816	Apoptosome-procaspase 9 complex	0.05384
Infectious lung disease	1088	PRNP-ApolopoproteinE3 complex	0.15811
Infectious lung disease	1519	IL6ST-PRKCD-STAT3 complex	0.1291
Infectious lung disease	2055	CASP8-CHUK-IKBKB-MALT1-BCL10 complex	0.2
Infectious lung disease	2056	BCL10-CHUK-BCL10-IKBKB complex	0.22361
Infectious lung disease	2100	CHUK-IKBKB-MAP3K14 complex	0.2582
Infectious lung disease	2101	IKKA-IKKB complex	0.31623
Infectious lung disease	2104	IKKB-NIK complex	0.15811
Infectious lung disease	2105	IkappaB kinase complex (IKBKB CHUK IKBKAP NFKBIA RELA MAP3K14)	0.18257
Infectious lung disease	2118	CHUK-ERC1-IKBKB-IKBKG	0.22361
Infectious lung disease	2121	CHUK-IKBKB-IKBKG complex	0.2582
Infectious lung disease	2124	IKK-alpha--ER-alpha-AIB1 complex	0.1291
Infectious lung disease	2727	SRC-3 complex	0.16903
Infectious lung disease	3229	Heterodimer complex (CDK9 IL6ST)	0.15811
Infectious lung disease	5193	TNF-alpha/NF-kappa B signaling complex (CHUK KPNA3 NFKB2 NFKBIB REL IKBKG NFKB1 NFKBIE RELB NFKBIA RELA TNIP2)	0.06455
Infectious lung disease	5194	TNF-alpha/NF-kappa B signaling complex (SEC16A CHUK IKBKB NFKB2 REL IKBKG MAP3K14 RELA FBXW7 USP2)	0.14142
Infectious lung disease	5196	TNF-alpha/NF-kappa B signaling complex (CHUK BTRC NFKB2 PPP6C REL CUL1 IKBKE SAPS2 SAPS1 ANKRD28 RELA SKP1)	0.06455
Infectious lung disease	5220	CHUK-IQGAP2-AKAP8L-RELA-TNIP2 complex	0.1
Infectious lung disease	5230	CHUK-NFKB2-REL-IKBKG-SPAG9-NFKB1-NFKBIE-COPB2-TNIP1-NFKBIA-RELA-TNIP2	0.06455
Infectious lung disease	5232	TNF-alpha/Nf-kappa B signaling complex (RPL6 RPL30 RPS13 CHUK DDX3X NFKB2 NFKBIB REL IKBKG NFKB1 MAP3K8 RELB GLG1 NFKBIA RELA TNIP2 GTF2I)	0.05423
Infectious lung disease	5233	TNF-alpha/NF-kappa B signaling complex 5	0.08944
Infectious lung disease	5234	IKBKB-CDC37-KIAA1967-HSP90AB1-HSP90AA1 complex	0.1
Infectious lung disease	5266	TNF-alpha/NF-kappa B signaling complex 6	0.11952
Infectious lung disease	5285	TNF-alpha/NF-kappa B signaling complex 9	0.1
Infectious lung disease	5286	TNF-alpha/NF-kappa B signaling complex 10	0.07071
Infectious lung disease	5375	EGR-EP300 complex	0.15811

Infectious lung disease	5388	SERPINA1-ELA2 complex	0.15811
Infectious lung disease	5564	LMO4-gp130 complex	0.1
Infectious lung disease	5579	CNTF-CNTFR-gp130-LIFR complex	0.1118
Infectious lung disease	5582	LIFR-LIF-gp130 complex	0.1291
Infectious lung disease	5828	IKBKG-IKBKB complex	0.15811
Infectious lung disease	5829	IKBKG-CHUK complex	0.15811
Infectious lung disease	5844	I-kappa-B kinase (IKK) complex	0.2582
Infertility	441	TFTC-type histone acetyl transferase complex	0.04142
Infertility	681	(C-CFTR)2-NHERF-ezrin complex	0.07931
Infertility	682	C-CFTR-NHERF(PDZ1 domain)-ezrin complex	0.07931
Infertility	683	C-CFTR-NHERF(PDZ2 domain)-ezrin complex	0.07931
Infertility	687	CFTR-NHERF-beta(2)AR signaling complex	0.07931
Infertility	1054	ESR1-RELA-BCL3-NCOA3 complex	0.06868
Infertility	1087	BIRC5-AURKB-INCENP-EVI5 complex	0.06868
Infertility	1116	CRM1-Survivin-AuroraB mitotic complex	0.07931
Infertility	1117	CRM1-Survivin mitotic complex	0.09713
Infertility	1118	Chromosomal passenger complex CPC (INCENP CDCA8 BIRC5 AURKB)	0.06868
Infertility	1120	Chromosomal passenger complex CPC (INCENP CDCA8 BIRC5)	0.07931
Infertility	1211	Ubiquitin E3 ligase (AHR ARNT DDB1 TBL3 CUL4B RBX1)	0.11215
Infertility	1728	CTCF-nucleophosmin-PARP-HIS-KPNA-LMNA-TOP complex	0.04579
Infertility	1774	MICA-KLRK1-HCST complex	0.07931
Infertility	2124	IKK-alpha--ER-alpha-AIB1 complex	0.07931
Infertility	2152	ARNT-HLF complex	0.09713
Infertility	2159	AR-AKT-APPL complex	0.07931
Infertility	2160	AOF2-AR complex	0.09713
Infertility	2370	ITGA2b-ITGB3-CD9 complex	0.07931
Infertility	2396	ITGA7-ITGB1-CD9 complex	0.07931
Infertility	2470	p130Cas-ER-alpha-cSrc-kinase- PI3-kinase p85-subunit complex	0.06868
Infertility	2579	Chromosomal passenger complex CPC (INCENP BIRC5 AURKB)	0.07931
Infertility	2580	Survivin homodimer complex	0.13736
Infertility	2581	RasGAP-AURKA/AURKB-survivin complex	0.06868
Infertility	2582	Chromosomal passenger complex CPC (CDCA8 AURKB BIRC5)	0.07931
Infertility	2657	ESR1-CDK7-CCNH-MNAT1-MTA1-HDAC2 complex	0.05608
Infertility	2670	Er-alpha-p53-hdm2 complex	0.07931
Infertility	2699	ER-alpha-GRIP1-c-Jun complex	0.07931
Infertility	2700	ER-alpha-c-Jun complex	0.09713
Infertility	2770	ITGA6-ITGB4-CD9 complex	0.07931
Infertility	2872	ITGA2b-ITGB3-CD9-GP1b-CD47 complex	0.05608
Infertility	2914	Respiratory chain complex I (beta subunit)	0.03434
Infertility	4043	NEMO-HIF2(alpha)-ARNT complex	0.07931
Infertility	5382	ARNT-HIF1A complex	0.09713
Infertility	5473	FAS-FADD-CASP8 complex	0.07931

Infertility	5582	LIFR-LIF-gp130 complex	0.07931
Infertility	5608	Emerin architectural complex	0.05608
Infertility	5611	Emerin complex 24	0.03547
Infertility	5799	Death induced signaling complex DISC (FAS FADD CASP8 CFLAR) membrane-associated CD95L	0.06868
Infertility	5800	Death-inducing signaling complex DISC (type I cells associated) stimulated	0.07931
Infertility	5808	DISC complex	0.07931
Infertility	5859	FAS-FADD-CASP8-CASP10 complex	0.06868
Infertility	5861	FAS-FADD-CASP10 complex	0.07931
Infertility	5862	CAV1-VDAC1-ESR1 complex	0.07931
Infiltrating cancer	1784	RNF11-SMURF2-STAMBP complex	0.1543
Infiltrating cancer	2084	NFKB1-NFKB2-REL-RELA-RELB complex	0.11952
Infiltrating cancer	2709	MMP-9-TIMP-1-LRP complex	0.1543
Infiltrating cancer	5193	TNF-alpha/NF-kappa B signaling complex (CHUK KPNA3 NFKB2 NFKBIB REL IKBKG NFKB1 NFKBIE RELB NFKBIA RELA TNIP2)	0.07715
Infiltrating cancer	5194	TNF-alpha/NF-kappa B signaling complex (SEC16A CHUK IKBKB NFKB2 REL IKBKG MAP3K14 RELA FBXW7 USP2)	0.08452
Infiltrating cancer	5196	TNF-alpha/NF-kappa B signaling complex (CHUK BTRC NFKB2 PPP6C REL CUL1 IKBKE SAPS2 SAPS1 ANKRD28 RELA SKP1)	0.07715
Infiltrating cancer	5228	REL-MAP3K8-RELA-TNIP2-PAPOLA complex	0.11952
Infiltrating cancer	5230	CHUK-NFKB2-REL-IKBKG-SPAG9-NFKB1-NFKBIE-COPB2-TNIP1-NFKBIA-RELA-TNIP2	0.07715
Infiltrating cancer	5232	TNF-alpha/Nf-kappa B signaling complex (RPL6 RPL30 RPS13 CHUK DDX3X NFKB2 NFKBIB REL IKBKG NFKB1 MAP3K8 RELB GLG1 NFKBIA RELA TNIP2 GTF2I)	0.06482
Infiltrating cancer	5233	TNF-alpha/NF-kappa B signaling complex 5	0.1069
Infiltrating cancer	5465	IKB(epsilon)-RELA-cREL complex	0.1543
Infiltrating cancer	5466	IKB(beta)-RELA-cREL complex	0.1543
Infiltrating cancer	5467	IKB(alpha)-RELA-cREL complex	0.1543
Inflammation of the central nervous system	2347	ITGAV-ITGB5-SPP1 complex	0.20412
Inflammation of the central nervous system	2352	ITGAV-ITGB6-SPP1 complex	0.20412
Inflammation of the central nervous system	2358	ITGAV-ITGB3-SPP1 complex	0.20412
Inflammation of the central nervous system	2885	ITGAV-ITGB1-SPP1 complex	0.20412
Inflammation of the central nervous system	3111	ITGA9-ITGB1-SPP1 complex	0.20412
Inflammation of the central nervous system	3112	ITGA5-ITGB1-SPP1 complex	0.20412
Influenza	1144	Cleavage and polyadenylation factor (CPSF)	0.08944
Influenza	1714	TICAM1-TICAM2-TLR4 complex	0.11547
Influenza	2476	CRKL-PDGFRA-CRK-RAPGEF1 complex	0.2
Influenza	2480	CIN85 complex (CIN85 CRK BCAR1 CBL PIK3R1 GRB2 SOS1)	0.07559

Influenza	2510	ZAP70-CRKL-WIPF1-WAS complex	0.1
Influenza	2511	CRKL-WIPF1-WAS complex	0.11547
Influenza	2962	CRK-BCAR1-DOCK1 complex	0.11547
Influenza	5343	ELMO1-DOCK1-CRKII complex	0.11547
Influenza	5444	CRKII-C3G complex	0.14142
Intermediate coronary syndrome	1714	TICAM1-TICAM2-TLR4 complex	0.19245
Intermediate coronary syndrome	2355	ITGAV-ITGB3-CD47-FCER2 complex	0.16667
Intermediate coronary syndrome	2356	ITGB3-ITGAV-CD47 complex	0.19245
Intermediate coronary syndrome	2358	ITGAV-ITGB3-SPP1 complex	0.19245
Intermediate coronary syndrome	2359	ITGAV-ITGB3-ADAM15 complex	0.19245
Intermediate coronary syndrome	2362	ITAGV-ITGB3-F11R complex	0.19245
Intermediate coronary syndrome	2363	ITGAV-ITGB3-PXN-PTK2b complex	0.16667
Intermediate coronary syndrome	2364	ITGAV-ITGB3-ADAM23 complex	0.19245
Intermediate coronary syndrome	2365	ITGAV-ITGB3-COL4A3 complex	0.19245
Intermediate coronary syndrome	2366	ITGAV-ITGB3-PPAP2b complex	0.19245
Intermediate coronary syndrome	2369	ITGAV-ITGB3-EGFR complex	0.19245
Intermediate coronary syndrome	2370	ITGA2b-ITGB3-CD9 complex	0.19245
Intermediate coronary syndrome	2374	ITGAV-ITGB3-LAMA4 complex	0.19245
Intermediate coronary syndrome	2376	ITGA2B-ITGB3-FN1-TGM2 complex	0.16667
Intermediate coronary syndrome	2377	ITGA2b-ITGB3-CD47-SRC complex	0.16667
Intermediate coronary syndrome	2378	ITGA2b-ITGB3-TLN1 complex	0.19245
Intermediate coronary syndrome	2379	ITGA2B-ITGB3-CIB1 complex	0.19245
Intermediate coronary syndrome	2381	ITGA2B-ITGB3 complex	0.2357
Intermediate coronary syndrome	2382	ITGA2B-ITGB3-F11R complex	0.19245
Intermediate coronary syndrome	2816	ITGAV-ITGB3 complex	0.2357
Intermediate coronary syndrome	2826	ITGB3-ITGAV-VTN complex	0.19245
Intermediate coronary syndrome	2846	ITGAV-ITGB3-THBS1 complex	0.19245
Intermediate coronary syndrome	2849	ITGAV-ITGB3-NOV complex	0.19245
Intermediate coronary syndrome	2872	ITGA2b-ITGB3-CD9-GP1b-CD47 complex	0.13608
Intermediate coronary syndrome	2882	ITGA5-ITGB3-COL6A3 complex	0.19245
Intermediate coronary syndrome	2896	ITGA2b-ITGB3-CD47-FAK complex	0.16667
Intermediate coronary syndrome	3103	ITGAV-ITGB3-SLC3A2 complex	0.19245
Intermediate coronary syndrome	3115	ITGA2B-ITGB3-ICAM4 complex	0.19245
Intestinal disease	75	TSC1-TSC2 complex	0.17678
Intestinal disease	178	Respiratory chain complex I (holoenzyme)	0.03769
Intestinal disease	1248	Apoptosome	0.17678
Intestinal disease	2297	ABI1-WASL complex	0.17678
Intestinal disease	2513	N-WASp homomer	0.25
Intestinal disease	2914	Respiratory chain complex I (beta subunit)	0.0625
Intestinal disease	5816	Apoptosome-procaspase 9 complex	0.14434
Intracranial aneurysm	1986	Endoglin homodimer complex	0.30151
Intracranial aneurysm	2709	MMP-9-TIMP-1-LRP complex	0.17408
Intracranial hypertension	1062	BAR-BCL2-CASP8 complex	0.40825
Intracranial hypertension	2054	CASP8-FADD-MALT1-BCL10 complex	0.35355
Intracranial hypertension	2055	CASP8-CHUK-IKBKB-MALT1-BCL10 complex	0.31623
Intracranial hypertension	2056	BCL10-CHUK-BCL10-IKBKB complex	0.35355
Intracranial hypertension	5473	FAS-FADD-CASP8 complex	0.40825

Intracranial hypertension	5749	MRIT complex	0.40825
Intracranial hypertension	5798	Death induced signaling complex II (FADD CASP8 CFLAR) cytosolic CD95L induced	0.40825
Intracranial hypertension	5799	Death induced signaling complex DISC (FAS FADD CASP8 CFLAR) membrane-associated CD95L	0.35355
Intracranial hypertension	5800	Death-inducing signaling complex DISC (type I cells associated) stimulated	0.40825
Intracranial hypertension	5808	DISC complex	0.40825
Intracranial hypertension	5859	FAS-FADD-CASP8-CASP10 complex	0.35355
Intraocular melanoma	1179	CENP-A NAC-CAD complex	0.08771
Intraocular melanoma	5830	DJ-1-SNCA complex high molecular weight complex	0.22361
Intraocular melanoma	5837	PPD complex	0.18257
Ischemia	159	Condensin I-PARP-1-XRCC1 complex	0.09679
Ischemia	205	Ubiquitin E3 ligase (VHL TCEB1 TCEB2 CUL2	0.05726
Ischemia	212	DNA ligase III-XRCC1 complex	0.09054
Ischemia	213	DNA ligase IV-XRCC1 complex	0.09054
Ischemia	298	VEGF transcriptional complex	0.05227
Ischemia	362	DNA ligase III-XRCC1-PNK-DNA-pol III multiprotein complex	0.06402
Ischemia	563	F1F0-ATP synthase (EC 3.6.3.14) mitochondrial	0.03201
Ischemia	622	Ubiquitin E3 ligase (VHL TCEB1 TCEB2 CUL2)	0.06402
Ischemia	668	BKCA-beta2AR-AKAP79 signaling complex	0.07392
Ischemia	672	BKCA-beta2AR complex	0.09054
Ischemia	1004	RC complex during S-phase of cell cycle	0.03551
Ischemia	1005	RC complex during G2/M-phase of cell cycle	0.03551
Ischemia	1062	BAR-BCL2-CASP8 complex	0.07392
Ischemia	1085	DNA repair complex NEIL2-PNK-Pol(beta)-LigIII(alpha)-XRCC1	0.05726
Ischemia	1086	DNA repair complex NEIL1-PNK-Pol(beta)-LigIII(alpha)-XRCC1	0.05726
Ischemia	1088	PRNP-ApolopoproteinE3 complex	0.09054
Ischemia	1193	Rap1 complex	0.04839
Ischemia	1226	H2AX complex I	0.04839
Ischemia	1439	PTGS2 homodimer complex	0.12804
Ischemia	1728	CTCF-nucleophosmin-PARP-HIS-KPNA-LMNA-TOP complex	0.04268
Ischemia	1729	TLE1 corepressor complex (MASH1 promoter-corepressor complex)	0.08098
Ischemia	2342	ITGAV-ITGB8-MMP14-TGFB1 complex	0.06402
Ischemia	2625	CDK8-MED6-PARP1 complex	0.07392
Ischemia	2688	MT1-MMP-claudin-1 complex	0.09054
Ischemia	2709	MMP-9-TIMP-1-LRP complex	0.07392
Ischemia	2721	HCF-1 complex	0.02937
Ischemia	2755	17S U2 snRNP	0.02229
Ischemia	3137	MASH1 promoter-coactivator complex	0.0386
Ischemia	3142	CAMK2-delta-MASH1 promoter-coactivator complex	0.04527
Ischemia	5179	NCOA6-DNA-PK-Ku-PARP1 complex	0.05726
Ischemia	5199	Kinase maturation complex 1	0.03201
Ischemia	5235	WRN-Ku70-Ku80-PARP1 complex	0.06402

Ischemia	5243	XRCC1-LIG3-PNK-TDP1 complex	0.06402
Ischemia	5267	VHL-VDU1-TCEB1-TCEB2 complex	0.06402
Ischemia	5270	VHL-TCEB1-TCEB2 complex	0.07392
Ischemia	5273	VHL-TBP1-HIF1A complex	0.14784
Ischemia	5276	HIF1A-OS9-EGLN1 complex	0.07392
Ischemia	5277	HIF1A-OS9-EGLN3 complex	0.07392
Ischemia	5317	LATS1-HTRA2-BIRC4 complex	0.07392
Ischemia	5382	ARNT-HIF1A complex	0.09054
Ischemia	5386	MLL1-WDR5 complex	0.02464
Ischemia	5423	HSP70-BAG5-PARK2 complex	0.06402
Ischemia	5446	EPO-EPOR complex	0.09054
Ischemia	5473	FAS-FADD-CASP8 complex	0.07392
Ischemia	5526	CALM1-FKBP38-BCL2 complex	0.07392
Ischemia	5582	LIFR-LIF-gp130 complex	0.07392
Ischemia	5749	MRIT complex	0.07392
Ischemia	5799	Death induced signaling complex DISC (FAS FADD CASP8 CFLAR) membrane-associated CD95L	0.06402
Ischemia	5800	Death-inducing signaling complex DISC (type I cells associated) stimulated	0.07392
Ischemia	5808	DISC complex	0.07392
Ischemia	5811	p53-BCL2 complex	0.09054
Ischemia	5812	p53-BCL2 complex	0.09054
Ischemia	5817	tBID-BCL2 complex	0.09054
Ischemia	5818	BIM-BCL2 complex	0.09054
Ischemia	5819	BIM-BCL2xL complex	0.09054
Ischemia	5820	tBID-BCL2xL complex	0.09054
Ischemia	5830	DJ-1-SNCA complex high molecular weight complex	0.09054
Ischemia	5843	AIF-CYPA-DNA complex	0.09054
Ischemia	5859	FAS-FADD-CASP8-CASP10 complex	0.06402
Ischemia	5861	FAS-FADD-CASP10 complex	0.07392
Kaposi sarcoma	62	MeCP1 complex	0.09806
Kaposi sarcoma	159	Condensin I-PARP-1-XRCC1 complex	0.10483
Kaposi sarcoma	282	SNF2h-cohesin-NuRD complex	0.06934
Kaposi sarcoma	443	BP-SMAD complex	0.09806
Kaposi sarcoma	646	HDAC1-associated protein complex	0.09245
Kaposi sarcoma	649	HDAC1-associated core complex cII	0.08771
Kaposi sarcoma	659	MeCP1 complex	0.09245
Kaposi sarcoma	685	MeCP1 complex	0.09245
Kaposi sarcoma	778	LARC complex (LCR-associated remodeling complex)	0.06363
Kaposi sarcoma	1004	RC complex during S-phase of cell cycle	0.07692
Kaposi sarcoma	1005	RC complex during G2/M-phase of cell cycle	0.07692
Kaposi sarcoma	1193	Rap1 complex	0.10483
Kaposi sarcoma	1226	H2AX complex I	0.10483
Kaposi sarcoma	1728	CTCF-nucleophosmin-PARP-HIS-KPNA-LMNA-TOP complex	0.09245
Kaposi sarcoma	1729	TLE1 corepressor complex (MASH1 promoter-corepressor complex)	0.08771
Kaposi sarcoma	2536	PLC-gamma-2-SLP-76-Lyn-Grb2 complex	0.13868

Kaposi sarcoma	2563	FGFR2-c-Cbl-Lyn-Fyn complex	0.13868
Kaposi sarcoma	2564	p21(ras)GAP-Fyn-Lyn-Yes complex thrombin	0.13868
Kaposi sarcoma	2565	CD20-LCK-LYN-FYN-p75/80 complex (Raji human B cell line)	0.13868
Kaposi sarcoma	2625	CDK8-MED6-PARP1 complex	0.16013
Kaposi sarcoma	2638	HES1 promoter corepressor complex	0.11323
Kaposi sarcoma	2639	HES1 promoter-Notch enhancer complex	0.07692
Kaposi sarcoma	2788	BRCA1 B complex	0.16013
Kaposi sarcoma	2815	BRCA1-BARD1-BACH1-DNA damage complex II	0.09806
Kaposi sarcoma	2817	BRCA1-BARD1-BACH1-DNA damage complex I	0.11323
Kaposi sarcoma	2890	Notch1-fraction 30 complex	0.16013
Kaposi sarcoma	2897	RBPJ-NotchIC-Mastermind complex	0.16013
Kaposi sarcoma	2907	RBP-Jkappa-Notch1 complex	0.19612
Kaposi sarcoma	2908	RBP-Jkappa-SHARP complex	0.19612
Kaposi sarcoma	2910	PLC-gamma-2-Lyn-FcR-gamma complex	0.16013
Kaposi sarcoma	2911	SMRT-SKIP-CBF1 complex	0.16013
Kaposi sarcoma	2930	SHARP-CtIP-RBP-Jkappa complex	0.16013
Kaposi sarcoma	2931	SHARP-CtBP1-CtIP-RBP-Jkappa corepressor complex	0.13868
Kaposi sarcoma	2945	RBP-Jkappa-RING1-KyoT2 complex	0.16013
Kaposi sarcoma	2946	YY1-Notch1-RBP-Jkappa complex	0.16013
Kaposi sarcoma	3113	MAML1-RBP-Jkappa-Notch1 complex	0.16013
Kaposi sarcoma	3137	MASH1 promoter-coactivator complex	0.08362
Kaposi sarcoma	3142	CAMK2-delta-MASH1 promoter-coactivator complex	0.09806
Kaposi sarcoma	3156	CBF1-HDAC1-SMRT complex	0.16013
Kaposi sarcoma	5179	NCOA6-DNA-PK-Ku-PARP1 complex	0.12403
Kaposi sarcoma	5235	WRN-Ku70-Ku80-PARP1 complex	0.13868
Keratoconjunctivitis Sicca	906	ADAR1-CDK2 complex	0.2357
Keratoconjunctivitis Sicca	1181	C complex spliceosome	0.03727
Keratoconjunctivitis Sicca	2709	MMP-9-TIMP-1-LRP complex	0.19245
Keratoconjunctivitis Sicca	3055	Nop56p-associated pre-rRNA complex	0.03269
Keratoconjunctivitis Sicca	3102	DHX9-ADAR-vigilin-DNA-PK-Ku antigen complex	0.13608
Keratoconus	2237	SP1-MCAF2 complex	0.26726
Keratoconus	2679	p53-SP1 complex	0.26726
Keratoconus	2706	SMAD3-SMAD4-SP1 complex	0.21822
Keratoconus	2721	HCF-1 complex	0.08671
Keratoconus	3838	SP1-E2F2 complex	0.26726
Keratoconus	3839	SP1-E2F3 complex	0.26726
Keratosis	74	TRPC1-Homer3-IP3R complex	0.19245
Keratosis	310	Cell cycle kinase complex CDC2	0.27217
Keratosis	311	Cell cycle kinase complex CDK2	0.33333
Keratosis	312	Cell cycle kinase complex CDK4	0.33333
Keratosis	313	Cell cycle kinase complex CDK5	0.29814
Keratosis	314	PCNA-p21 complex	0.2357
Keratosis	536	TRPC1-TRPC3-TRPC7 complex	0.19245
Keratosis	553	RHOA-IP3R-TRPC1 complex	0.19245
Keratosis	557	TRP1-G alpha-11-IP3R3-CAV1 signaling complex	0.16667
Keratosis	828	TRPC1-STIM1-ORAI1 complex	0.19245

Keratosis	873	SNARE complex (STX1A SNAP29)	0.2357
Keratosis	1062	BAR-BCL2-CASP8 complex	0.19245
Keratosis	1633	CyclinD1-CDK4-CDK6 complex	0.19245
Keratosis	1634	CyclinD1-CDK4-p21 complex	0.3849
Keratosis	2230	PCNA complex	0.12599
Keratosis	2635	BETA2-Cyclin D1 complex	0.2357
Keratosis	3084	CCND1-CDK4 complex	0.2357
Keratosis	3087	CCND1-CDK6 complex	0.2357
Keratosis	5101	CyclinD3-CDK4-CDK6-p21 complex	0.16667
Keratosis	5287	CDK4-CCND1 complex	0.2357
Keratosis	5526	CALM1-FKBP38-BCL2 complex	0.19245
Keratosis	5622	HSP90-CIP1-FKBPL complex	0.19245
Keratosis	5811	p53-BCL2 complex	0.2357
Keratosis	5817	tBID-BCL2 complex	0.2357
Keratosis	5818	BIM-BCL2 complex	0.2357
Kidney cancer	330	PSF-p54(nrb) complex	0.40825
Kidney cancer	335	p54(nrb)-PSF-matrin3 complex	0.33333
Kidney cancer	1141	CF IIAm complex (Cleavage factor IIAm complex)	0.14434
Kidney cancer	1148	snRNP-free U1A (SF-A) complex	0.28868
Kidney cancer	1183	CDC5L complex	0.10541
Kidney cancer	1335	SNW1 complex	0.13608
Kidney cancer	1760	TOP1-PSF-P54 complex	0.33333
Kidney disease	220	ARF-Mule complex	0.06901
Kidney disease	486	WIP-WASp-actin-myosin-IIa complex	0.0488
Kidney disease	1071	PKD2-FPC complex	0.08452
Kidney disease	1642	p16-cyclin D2-CDK4 complex	0.06901
Kidney disease	5712	FAK-beta5 integrin complex VEGF induced	0.08452
Kidney disease	1927	PKD2-PACS1 complex	0.08452
Kidney disease	2254	CTGF/Hcs24-actin complex	0.06901
Kidney disease	2709	MMP-9-TIMP-1-LRP complex	0.06901
Kidney disease	2726	PXN-ITGB5-PTK2 complex	0.06901
Kidney disease	2896	ITGA2b-ITGB3-CD47-FAK complex	0.05976
Kidney disease	5177	Polycystin-1 multiprotein complex (ACTN1 CDH1 SRC JUP VCL CTNNB1 PXN BCAR1 PKD1	0.03604
Kidney disease	5282	CAS-SRC-FAK complex	0.06901
Kidney disease	5604	Emerin complex 1	0.04226
Kidney disease	5762	CRMP-MICAL-PlexinA1 complex induced by	0.06901
Kidney failure	115	Polycomb repressive complex 1 (PRC1 hPRC-H)	0.03269
Kidney failure	116	Polycomb repressive complex 1 (PRC1 hPRC-H)	0.0314
Kidney failure	310	Cell cycle kinase complex CDC2	0.04623
Kidney failure	311	Cell cycle kinase complex CDK2	0.05661
Kidney failure	312	Cell cycle kinase complex CDK4	0.05661
Kidney failure	313	Cell cycle kinase complex CDK5	0.05064
Kidney failure	432	N-NOS-CHIP-HSP70-1 complex	0.06537
Kidney failure	441	TFTC-type histone acetyl transferase complex	0.03414
Kidney failure	445	TFTC complex (TATA-binding protein-free TAF-II-containing complex)	0.02831

Kidney failure	470	TFTC complex (TATA-binding protein-free TAF-II-containing complex)	0.02746
Kidney failure	476	STAGA complex (SPT3-TAF9-GCN5 acetyltransferase complex)	0.0314
Kidney failure	513	TFTC complex (TATA-binding protein-free TAF-II-containing complex)	0.02746
Kidney failure	563	F1F0-ATP synthase (EC 3.6.3.14) mitochondrial	0.02831
Kidney failure	626	LSD1 complex	0.0314
Kidney failure	725	P2X7 receptor signalling complex	0.06537
Kidney failure	1054	ESR1-RELA-BCL3-NCOA3 complex	0.05661
Kidney failure	1088	PRNP-ApolopoproteinE3 complex	0.08006
Kidney failure	1170	cMYC-ATPase-helicase complex	0.05064
Kidney failure	1171	c-MYC-ATPase-helicase complex	0.05064
Kidney failure	1308	PABPC1-HSPA8-HNRPD-EIF4G1 complex	0.05064
Kidney failure	1514	IL4-IL4R complex	0.08006
Kidney failure	1515	IL4-IL4R-IL2RG complex	0.06537
Kidney failure	1618	G protein complex (PTHR1 GNB1 GNG2)	0.06537
Kidney failure	1633	CyclinD1-CDK4-CDK6 complex	0.06537
Kidney failure	1634	CyclinD1-CDK4-p21 complex	0.06537
Kidney failure	1714	TICAM1-TICAM2-TLR4 complex	0.06537
Kidney failure	2124	IKK-alpha--ER-alpha-AIB1 complex	0.06537
Kidney failure	2153	ITGAM-ITGB2-CD11 complex	0.13074
Kidney failure	2347	ITGAV-ITGB5-SPP1 complex	0.06537
Kidney failure	2352	ITGAV-ITGB6-SPP1 complex	0.06537
Kidney failure	2358	ITGAV-ITGB3-SPP1 complex	0.06537
Kidney failure	2470	p130Cas-ER-alpha-cSrc-kinase- PI3-kinase p85-subunit complex	0.05661
Kidney failure	2528	ERBB2-MEMO-SHC complex	0.06537
Kidney failure	2535	SLP-76-Cbl-Grb2-Shc complex Fc receptor gamma-R1 stimulated	0.05661
Kidney failure	2635	BETA2-Cyclin D1 complex	0.08006
Kidney failure	2649	MYC-DNMT3A-ZBTB17 complex	0.06537
Kidney failure	2653	MYC-MAX-BLOC1S1 complex	0.06537
Kidney failure	2655	MYC-MAX complex	0.08006
Kidney failure	2657	ESR1-CDK7-CCNH-MNAT1-MTA1-HDAC2 complex	0.04623
Kidney failure	2670	Er-alpha-p53-hdm2 complex	0.06537
Kidney failure	2699	ER-alpha-GRIP1-c-Jun complex	0.06537
Kidney failure	2700	ER-alpha-c-Jun complex	0.08006
Kidney failure	2885	ITGAV-ITGB1-SPP1 complex	0.06537
Kidney failure	2895	SHC-GRB2 complex	0.08006
Kidney failure	2972	ITGA9-ITGB1-VEGFA complex	0.06537
Kidney failure	3084	CCND1-CDK4 complex	0.08006
Kidney failure	3087	CCND1-CDK6 complex	0.08006
Kidney failure	3096	ITGA6-ITGB4-SHC1-GRB2 complex	0.05661
Kidney failure	3111	ITGA9-ITGB1-SPP1 complex	0.06537
Kidney failure	3112	ITGA5-ITGB1-SPP1 complex	0.06537
Kidney failure	3162	TF-FVIIa-FXa-TFPI complex	0.05661
Kidney failure	4062	NRP1-VEGFR2-VEGF(165) complex	0.06537

Kidney failure	5287	CDK4-CCND1 complex	0.08006
Kidney failure	5423	HSP70-BAG5-PARK2 complex	0.05661
Kidney failure	5446	EPO-EPOR complex	0.08006
Kidney failure	5696	VEGFA(165)-KDR-NRP1 complex	0.06537
Kidney failure	5698	VEGFA(165)-VEGFR2-NRP1 complex	0.06537
Kidney failure	5701	NRP1-VEGF(165/121) complex	0.08006
Kidney failure	5862	CAV1-VDAC1-ESR1 complex	0.06537
Larynx cancer	5165	AP1G1-PACS1-FURIN complex	0.18257
Late pregnancy	441	TFTC-type histone acetyl transferase complex	0.08704
Late pregnancy	541	IGF1-IGFBP3-ALS complex	0.16667
Late pregnancy	1054	ESR1-RELA-BCL3-NCOA3 complex	0.14434
Late pregnancy	2124	IKK-alpha--ER-alpha-AIB1 complex	0.16667
Late pregnancy	2470	p130Cas-ER-alpha-cSrc-kinase- PI3-kinase p85-subunit complex	0.14434
Late pregnancy	2657	ESR1-CDK7-CCNH-MNAT1-MTA1-HDAC2 complex	0.11785
Late pregnancy	2670	Er-alpha-p53-hdm2 complex	0.16667
Late pregnancy	2699	ER-alpha-GRIP1-c-Jun complex	0.16667
Late pregnancy	2700	ER-alpha-c-Jun complex	0.20412
Late pregnancy	5862	CAV1-VDAC1-ESR1 complex	0.16667
Learning disorder	320	55S ribosome mitochondrial	0.06537
Learning disorder	324	39S ribosomal subunit mitochondrial	0.08333
Learning disorder	1088	PRNP-ApolopoproteinE3 complex	0.40825
Leigh disease	178	Respiratory chain complex I (holoenzyme)	0.2665
Leigh disease	351	Spliceosome	0.02957
Leigh disease	563	F1F0-ATP synthase (EC 3.6.3.14) mitochondrial	0.17678
Leigh disease	1183	CDC5L complex	0.06455
Leigh disease	2884	Respiratory chain complex I (early intermediate NDUFAF1 assembly) mitochondrial	0.13363
Leigh disease	2886	Respiratory chain complex I (incomplete intermediate ND1 ND2 ND3 CIA30 assembly) mitochondrial	0.35355
Leigh disease	2904	Respiratory chain complex I (intermediate VII/650kD) mitochondrial	0.22361
Leigh disease	2906	Respiratory chain complex I (intermediate II/230kD) mitochondrial	0.40825
Leigh disease	2919	Respiratory chain complex I (gamma subunit) mitochondrial	0.19612
Leigh disease	2920	Respiratory chain complex I (lambda subunit) mitochondrial	0.26517
Leigh disease	2948	Respiratory chain complex I (incomplete intermediate) mitochondrial	0.3198
Leprosy	120	Lymphotoxin beta receptor complex	0.1543
Leprosy	1774	MICA-KLRK1-HCST complex	0.1543
Leprosy	2018	IL12A-IL12B complex	0.18898
Leprosy	2019	IL12A-IL12B-IL12RB1 complex	0.1543
Leprosy	2020	IL12B-IL12RB1-IL12RB2 complex	0.1543
Leprosy	2021	IL12A-IL12B-IL12RB2 complex	0.1543
Leprosy	5423	HSP70-BAG5-PARK2 complex	0.13363
Leprosy	5548	IL-12 heterodimer complex	0.18898
Leprosy	5549	IL-12 subunit p40 homodimer complex	0.26726

Leprosy	5837	PPD complex	0.1543
Leukemia	1	BCL6-HDAC4 complex	0.03984
Leukemia	4	Multisubunit ACTR coactivator complex	0.05634
Leukemia	55	HDAC4-ERK1 complex	0.03984
Leukemia	57	HDAC4-ERK2 complex	0.03984
Leukemia	91	FA complex (Fanconi anemia complex) cytoplasmic	0.02817
Leukemia	92	CD28-transactivation complex	0.03984
Leukemia	140	E-box sequence-binding complex	0.02817
Leukemia	143	APP-FE65-LRP complex	0.03253
Leukemia	159	Condensin I-PARP-1-XRCC1 complex	0.0213
Leukemia	186	Wave-2 complex	0.0252
Leukemia	206	DNA ligase IV-XRCC4 complex	0.03984
Leukemia	213	DNA ligase IV-XRCC1 complex	0.03984
Leukemia	244	BRAFT complex	0.04688
Leukemia	245	FA core complex (Fanconi anemia core complex)	0.03756
Leukemia	246	BLM complex III	0.05634
Leukemia	336	DNA ligase IV-XRCC4-AHNK complex	0.03253
Leukemia	344	DNA ligase IV-XRCC4 complex (LX complex)	0.03984
Leukemia	350	DNA ligase IV-XRCC4-PNK complex	0.03253
Leukemia	353	DNA ligase IV-condensin complex	0.03253
Leukemia	359	DNA ligase IV-XRCC4-XLF complex	0.03253
Leukemia	433	BASC complex (BRCA1-associated genome surveillance complex)	0.01627
Leukemia	434	BASC (Ab 80) complex (BRCA1-associated genome surveillance complex)	0.01992
Leukemia	471	PCAF complex	0.01782
Leukemia	518	AKAP250-PKA-PDE4D complex	0.0252
Leukemia	525	TIP60 histone acetylase complex	0.0252
Leukemia	528	NuA4/Tip60 HAT complex	0.01455
Leukemia	529	NuA4/Tip60 HAT complex	0.01409
Leukemia	541	IGF1-IGFBP3-ALS complex	0.03253
Leukemia	570	p300-CBP-p270-SWI/SNF complex	0.0213
Leukemia	571	p300-CBP-p270 complex	0.03253
Leukemia	577	FHL2-p53-HIPK2 complex	0.03253
Leukemia	629	BLM-TRF2 complex	0.03984
Leukemia	725	P2X7 receptor signalling complex	0.01627
Leukemia	746	C/EBPalpha-HNF6 complex	0.03984
Leukemia	787	NuA4/Tip60-HAT complex B	0.01992
Leukemia	798	NuA4/Tip60-HAT complex A	0.01455
Leukemia	1004	RC complex during S-phase of cell cycle	0.01563
Leukemia	1005	RC complex during G2/M-phase of cell cycle	0.01563
Leukemia	1055	ZNF198-PML complex	0.03984
Leukemia	1067	CD8A-LCK complex	0.03984
Leukemia	1091	SNX complex (SNX1a SNX2 SNX4 LEPR)	0.02817
Leukemia	1152	FA complex (Fanconi anemia complex)	0.01992
Leukemia	1189	DNA double-strand break end-joining complex	0.0213
Leukemia	1191	RNA pol II containing coactivator complex Tat-SF	0.0252
Leukemia	1193	Rap1 complex	0.0213

Leukemia	1211	Ubiquitin E3 ligase (AHR ARNT DDB1 TBL3 CUL4B RBX1)	0.046
Leukemia	1218	BLM-TRF2 complex	0.03984
Leukemia	1223	H2AX complex isolated from cells without IR exposure	0.01563
Leukemia	1226	H2AX complex I	0.04259
Leukemia	1227	H2AX complex II	0.01782
Leukemia	1239	EBAFb complex	0.01782
Leukemia	1252	EBAFa complex	0.01782
Leukemia	1256	MLL-HCF complex	0.0213
Leukemia	1257	ALL-1 supercomplex	0.01065
Leukemia	1297	MKK4-ARRB2-ASK1 complex	0.03253
Leukemia	1332	Large Drosha complex	0.0126
Leukemia	1338	FOXO3-PCAF complex oxidative stress stimulated	0.03984
Leukemia	1372	Rb-tal-1-E2A-Lmo2-Ldb1 complex	0.0252
Leukemia	1401	MOF complex	0.01782
Leukemia	1457	AFF1-MLLT1-CBX8 complex	0.06506
Leukemia	1514	IL4-IL4R complex	0.03984
Leukemia	1515	IL4-IL4R-IL2RG complex	0.06506
Leukemia	1620	G protein complex (HDAC4 GNB1 GNG2)	0.03253
Leukemia	1623	FA core complex 1 (Fanconi anemia core complex 1)	0.0252
Leukemia	1624	FA core complex (Fanconi anemia core complex)	0.01878
Leukemia	1625	FA core complex (Fanconi anemia core complex)	0.0213
Leukemia	1703	IGHM-VPREB1-IGLL1 complex	0.03253
Leukemia	1707	IL2-IL2RA-IL2RB complex	0.09759
Leukemia	1728	CTCF-nucleophosmin-PARP-HIS-KPNA-LMNA-TOP complex	0.01878
Leukemia	1729	TLE1 corepressor complex (MASH1 promoter-corepressor complex)	0.07127
Leukemia	1772	MICB-KLRK1-HCST complex	0.03253
Leukemia	1774	MICA-KLRK1-HCST complex	0.03253
Leukemia	1775	ULBP2-KLRK1-HCST complex	0.03253
Leukemia	1787	Nogo-potassium channel complex	0.02817
Leukemia	1810	ITGA4-PXN-GIT1 complex	0.03253
Leukemia	1827	PML-SMAD2/3-SARA complex	0.02817
Leukemia	1831	PIAS3-SMAD3-P300 complex	0.03253
Leukemia	5721	CIN85-CBL-SH3GL2 complex	0.03253
Leukemia	5713	SH3P2/OSTF1-CBL-SRC complex	0.03253
Leukemia	1972	BMP4-BGN complex	0.03984
Leukemia	1992	LEPR homodimer complex	0.05634
Leukemia	2055	CASP8-CHUK-IKBKB-MALT1-BCL10 complex	0.0504
Leukemia	2056	BCL10-CHUK-BCL10-IKBKB complex	0.05634
Leukemia	2100	CHUK-IKBKB-MAP3K14 complex	0.06506
Leukemia	2101	IKKA-IKKB complex	0.07968
Leukemia	2104	IKKB-NIK complex	0.03984
Leukemia	2105	IkappaB kinase complex (IKBKB CHUK IKBKAP NFKBIA RELA MAP3K14)	0.046
Leukemia	2112	CDC37-HSP90AA1-HSP90AB1-MAP3K11 complex	0.02817
Leukemia	2118	CHUK-ERC1-IKBKB-IKBKG	0.05634

Leukemia	2121	CHUK-IKBKB-IKBKG complex	0.06506
Leukemia	2124	IKK-alpha--ER-alpha-AIB1 complex	0.03253
Leukemia	2152	ARNT-HLF complex	0.03984
Leukemia	2153	ITGAM-ITGB2-CD11 complex	0.03253
Leukemia	2217	MDC1-MRN-ATM-FANCD2 complex	0.023
Leukemia	2220	RAD52-ERCC4-ERCC1 complex	0.03253
Leukemia	2222	BLM complex II	0.046
Leukemia	2223	BLM-TOP3A complex	0.03984
Leukemia	2224	MSH2/6-BLM-p53-RAD51 complex	0.0252
Leukemia	2228	BLM-RAD51L3-XRCC2 complex	0.03253
Leukemia	2294	ABI1-WASF2 complex	0.03984
Leukemia	2297	ABI1-WASL complex	0.03984
Leukemia	2318	ITGA6-ITGB4-Laminin10/12 complex	0.0252
Leukemia	2319	ITGA6-ITGB4-Laminin10/12 complex	0.0252
Leukemia	2322	ITGA6-ITGB4-LAMA5 complex	0.03253
Leukemia	2417	ITGA4-ITGB1-EMILIN1 complex	0.03253
Leukemia	2418	ITGA4-ITGB1 complex	0.03984
Leukemia	2419	ITGA4-ITGB1-CD81 complex	0.03253
Leukemia	2420	ITGA4-ITGB1-CD53 complex	0.03253
Leukemia	2421	ITGA4-ITGB1-VCAM1 complex	0.03253
Leukemia	2422	ITGA4-ITGB1-JAM2 complex	0.03253
Leukemia	2423	ITGA4-ITGB1-CD47 complex	0.03253
Leukemia	2424	ITGA4-ITGB1-CD63 complex	0.03253
Leukemia	2425	ITGA4-ITGB1-PXN complex	0.03253
Leukemia	2426	ITGA4-ITGB1-THBS1 complex	0.03253
Leukemia	2428	ITGA4-ITGB1-THBS2 complex	0.03253
Leukemia	2453	Multiprotein complex (monoubiquitination)	0.02817
Leukemia	2454	CIN85-CBL-SH3GL2-EGFR complex EGF stimulated	0.02817
Leukemia	2456	MET-CIN85-SH3GL3-CBL complex HGF stimulated	0.02817
Leukemia	2476	CRKL-PDGFRA-CRK-RAPGEF1 complex	0.02817
Leukemia	2480	CIN85 complex (CIN85 CRK BCAR1 CBL PIK3R1 GRB2 SOS1)	0.0213
Leukemia	5709	ArgBP2a-CBL-PTK2B complex	0.03253
Leukemia	2510	ZAP70-CRKL-WIPF1-WAS complex	0.02817
Leukemia	2511	CRKL-WIPF1-WAS complex	0.03253
Leukemia	5720	CIN85-CBL complex	0.03984
Leukemia	2529	LAT-PLC-gamma-1-p85-GRB2-CBL-VAV-SLP-76 signaling complex C305 activated	0.0213
Leukemia	2534	Cbl-SLP-76-Grb2 complex Fc receptor gamma-R1 stimulated	0.03253
Leukemia	2535	SLP-76-Cbl-Grb2-Shc complex Fc receptor gamma-R1 stimulated	0.02817
Leukemia	2536	PLC-gamma-2-SLP-76-Lyn-Grb2 complex	0.02817
Leukemia	2542	EGFR-CBL-GRB2 complex	0.03253
Leukemia	2551	PDGFRA-PLC-gamma-1-PI3K-SHP-2 complex PDGF stimulated	0.02817
Leukemia	2559	p56(LCK)-CAML complex	0.03984
Leukemia	2563	FGFR2-c-Cbl-Lyn-Fyn complex	0.05634

Leukemia	2564	p21(ras)GAP-Fyn-Lyn-Yes complex thrombin	0.02817
Leukemia	2565	CD20-LCK-LYN-FYN-p75/80 complex (Raji human B cell line)	0.02817
Leukemia	2574	CD19-Vav-PI 3-kinase (p85 subunit) complex	0.03253
Leukemia	2590	FOXO1-FHL2-SIRT1 complex	0.03253
Leukemia	2599	POLR2A-CCNT1-CDK9-NCL-LEM6-CPSF2 complex	0.023
Leukemia	2625	CDK8-MED6-PARP1 complex	0.03253
Leukemia	2638	HES1 promoter corepressor complex	0.023
Leukemia	2641	p300/CBP-PCAF-MyoD complex	0.05634
Leukemia	2694	ERG-JUN-FOS DNA-protein complex	0.03253
Leukemia	2709	MMP-9-TIMP-1-LRP complex	0.03253
Leukemia	2711	Amyloid beta protein oligomer	0.05634
Leukemia	2721	HCF-1 complex	0.02585
Leukemia	2727	SRC-3 complex	0.08518
Leukemia	2728	SRC-1 complex	0.05634
Leukemia	2739	FA complex (Fanconi anemia complex)	0.05096
Leukemia	2754	JUND-FOSB-SMAD3-SMAD4 complex	0.02817
Leukemia	2761	SMAD3-SMAD4-FOXO1 complex	0.03253
Leukemia	2789	ETS2-ERG complex	0.03984
Leukemia	2829	RSmad complex	0.01782
Leukemia	2842	DAXX-Axin-p53-HIPK2 complex	0.02817
Leukemia	2844	Axin-p53-HIPK2 complex	0.03253
Leukemia	2857	NuA4/Tip60 HAT complex	0.01506
Leukemia	2910	PLC-gamma-2-Lyn-FcR-gamma complex	0.03253
Leukemia	2954	Smad1-Notch1-p300-Pcaf complex	0.02817
Leukemia	2956	PLC-gamma-1-LAT-c-CBL complex OKT3 stimulated	0.03253
Leukemia	2958	SMAD1-CBP complex	0.03984
Leukemia	3032	RNA-induced silencing complex RISC	0.02817
Leukemia	3044	SKI-NCOR1-SIN3A-HDAC1 complex	0.02817
Leukemia	3055	Nop56p-associated pre-rRNA complex	0.00552
Leukemia	3056	Microprocessor complex	0.03984
Leukemia	3061	RNA polymerase II complex (CBP PCAF RPB1 BAF47 CYCC CDK8) chromatin structure modifying	0.046
Leukemia	3062	RNA polymerase II complex incomplete (CBP RPBI PCAF BAF47) chromatin structure modifying	0.05634
Leukemia	3066	RNA polymerase II complex chromatin structure modifying	0.03125
Leukemia	3078	DGCR8-NCL complex	0.03984
Leukemia	3082	DGCR8 multiprotein complex	0.01699
Leukemia	3083	Nucleic and chromatin Fanconi complex	0.0252
Leukemia	3092	APP-TOMM40 complex	0.03984
Leukemia	3093	APP-TIMM23 complex	0.03984
Leukemia	3110	ITGAV-P2RY2-GNA12 complex	0.03253
Leukemia	3120	OCT1-OBF1-DNA-TLE1 complex	0.03253
Leukemia	3124	TLE1-TLE2 complex	0.03984
Leukemia	3125	TLE1 homodimer complex	0.05634
Leukemia	3127	TLE-Histone H3 complex	0.0213
Leukemia	3131	Hes1-TLE1 complex	0.03984

Leukemia	3137	MASH1 promoter-coactivator complex	0.05096
Leukemia	3142	CAMK2-delta-MASH1 promoter-coactivator complex	0.05976
Leukemia	3149	NK-3-Groucho-HIPK2-SIN3A-RbpA48-HDAC1	0.03398
Leukemia	3150	NK-3-Groucho complex	0.0213
Leukemia	3154	Notch2(N-TM)-Notch2(N-EC)-Delta complex	0.02817
Leukemia	3162	TF-FVIIa-FXa-TFPI complex	0.02817
Leukemia	3164	HESX1-TLE1 complex	0.03984
Leukemia	3175	CIN85-c-CBL complex	0.03984
Leukemia	3183	PDGFRA-SHP-2 complex PDGF stimulated	0.03984
Leukemia	3186	GRB2-SHP-2 complex PDGF stimulated	0.03984
Leukemia	3197	SMAD4-SNO-SKI complex	0.03253
Leukemia	3198	SMAD2-SKI complex	0.03984
Leukemia	3199	SMAD3-SKI complex	0.03984
Leukemia	3200	SMAD4-SKI complex	0.03984
Leukemia	3204	SMAD2-SKI-NCOR complex	0.03253
Leukemia	3205	SMAD3-SKI-NCOR complex	0.03253
Leukemia	3206	SMAD4-SKI-NCOR complex	0.03253
Leukemia	3270	Delta1 homodimer complex	0.05634
Leukemia	3271	Gamma-secretase-Delta1 complex	0.0252
Leukemia	3335	Homotetrameric complex NIAP	0.05634
Leukemia	3729	SKI-SMAD2 hexameric complex	0.03984
Leukemia	3733	SKI-SMAD3 hexameric complex	0.03984
Leukemia	3739	SKI-SMAD2-SMAD4 pentameric complex	0.03253
Leukemia	3740	SKI-SMAD3-SMAD4 pentameric complex	0.03253
Leukemia	3749	CREBBP-SMAD2 hexameric complex	0.03984
Leukemia	3750	CREBBP-SMAD3 hexameric complex	0.03984
Leukemia	3753	CREBBP-SMAD2-SMAD4 pentameric complex	0.03253
Leukemia	3754	CREBBP-SMAD3-SMAD4 pentameric complex	0.03253
Leukemia	3959	SMAD3-SMAD4-cSKI TGF(beta)-dependent	0.03253
Leukemia	3961	SMAD3-cSKI-SIN3A-HDAC1 complex	0.02817
Leukemia	4043	NEMO-HIF2(alpha)-ARNT complex	0.03253
Leukemia	4158	HSP90-FKBP38-CAM-Ca(2+) complex	0.02817
Leukemia	4200	DLP1-hFIS1 complex	0.03984
Leukemia	4977	gp78-p97/VCP-DERL1 complex	0.03253
Leukemia	4999	p97/VCP-VIMP-DERL1-DERL2-HRD1-SEL1L	0.023
Leukemia	5153	CTFC-TAF1 complex	0.03984
Leukemia	5171	SH3KBP1-CBLB-EGFR complex	0.03253
Leukemia	5179	NCOA6-DNA-PK-Ku-PARP1 complex	0.0252
Leukemia	5193	TNF-alpha/NF-kappa B signaling complex (CHUK KPNA3 NFKB2 NFKBIB REL IKBKG NFKB1 NFKBIE RELB NFKBIA RELA TNIP2)	0.01627
Leukemia	5194	TNF-alpha/NF-kappa B signaling complex (SEC16A CHUK IKBKB NFKB2 REL IKBKG MAP3K14 RELA FBXW7 USP2)	0.03563
Leukemia	5196	TNF-alpha/NF-kappa B signaling complex (CHUK BTRC NFKB2 PPP6C REL CUL1 IKBKE SAPS2 SAPS1 ANKRD28 RELA SKP1)	0.01627
Leukemia	5197	PTIP-DNA damage response complex	0.023

Leukemia	5198	CBP-RARA-RXRA-DNA complex ligand stimulated	0.06506
Leukemia	5199	Kinase maturation complex 1	0.02817
Leukemia	5212	Kinase maturation complex 2	0.01992
Leukemia	5217	Calreticulin oligomer complex	0.05634
Leukemia	5220	CHUK-IQGAP2-AKAP8L-RELA-TNIP2 complex	0.0252
Leukemia	5230	CHUK-NFKB2-REL-IKBKG-SPAG9-NFKB1-NFKBIE-COPB2-TNIP1-NFKBIA-RELA-TNIP2	0.01627
Leukemia	5232	TNF-alpha/Nf-kappa B signaling complex (RPL6 RPL30 RPS13 CHUK DDX3X NFKB2 NFKBIB REL IKBKG NFKB1 MAP3K8 RELB GLG1 NFKBIA RELA TNIP2 GTF2I)	0.01367
Leukemia	5233	TNF-alpha/NF-kappa B signaling complex 5	0.02254
Leukemia	5234	IKBKB-CDC37-KIAA1967-HSP90AB1-HSP90AA1 complex	0.0504
Leukemia	5235	WRN-Ku70-Ku80-PARP1 complex	0.02817
Leukemia	5264	TCF4-CTNNB1-CREBBP complex	0.03253
Leukemia	5266	TNF-alpha/NF-kappa B signaling complex 6	0.04518
Leukemia	5268	TNF-alpha/NF-kappa B signaling complex 7	0.01992
Leukemia	5269	TNF-alpha/NF-kappa B signaling complex 8	0.023
Leukemia	5285	TNF-alpha/NF-kappa B signaling complex 9	0.0504
Leukemia	5286	TNF-alpha/NF-kappa B signaling complex 10	0.03563
Leukemia	5373	Chromatin remodeling complex (TACC2 TACC3	0.03253
Leukemia	5382	ARNT-HIF1A complex	0.03984
Leukemia	5383	TRIB3-DDIT3 complex	0.03984
Leukemia	5384	RAG1-RAG2 tetramer complex	0.07968
Leukemia	5386	MLL1-WDR5 complex	0.02169
Leukemia	5388	SERPINA1-ELA2 complex	0.03984
Leukemia	5389	SERPINA3-CTSG complex	0.03984
Leukemia	5391	SERPINA1-CTSG complex	0.03984
Leukemia	5423	HSP70-BAG5-PARK2 complex	0.05634
Leukemia	5564	LMO4-gp130 complex	0.07559
Leukemia	5573	Stat1-alpha-dimer-CBP DNA-protein complex	0.03984
Leukemia	5656	CEBPE-E2F1-RB1 complex	0.03253
Leukemia	5724	CIN85-SH3GL3-CBL complex	0.03253
Leukemia	5770	RUNX1-CBF-beta-DNA complex	0.07968
Leukemia	5822	MCL1-NOXA complex	0.03984
Leukemia	5828	IKBKG-IKBKB complex	0.03984
Leukemia	5829	IKBKG-CHUK complex	0.03984
Leukemia	5837	PPD complex	0.03253
Leukemia	5844	I-kappa-B kinase (IKK) complex	0.06506
Leukemia	5849	HSP90-CDC37-LRRK2 complex	0.03253
Leukodystrophy NOS	1094	Frataxin complex	0.13363
Leukodystrophy NOS	2755	17S U2 snRNP	0.06155
Leukoencephalopathy	4	Multisubunit ACTR coactivator complex	0.08333
Leukoencephalopathy	71	MRN complex (MRE11-RAD50-NBS1 complex)	0.09623
Leukoencephalopathy	72	R/M complex (RAD50-MRE11 complex)	0.11785
Leukoencephalopathy	73	MRN complex (MRE11-RAD50-NBN complex)	0.09623
Leukoencephalopathy	107	TFIIH transcription factor complex	0.11111

Leukoencephalopathy	202	BRCA1-RAD50-MRE11-NBS1 complex	0.08333
Leukoencephalopathy	244	BRAFT complex	0.04623
Leukoencephalopathy	246	BLM complex III	0.08333
Leukoencephalopathy	351	Spliceosome	0.01394
Leukoencephalopathy	433	BASC complex (BRCA1-associated genome surveillance complex)	0.09623
Leukoencephalopathy	434	BASC (Ab 80) complex (BRCA1-associated genome surveillance complex)	0.05893
Leukoencephalopathy	570	p300-CBP-p270-SWI/SNF complex	0.06299
Leukoencephalopathy	571	p300-CBP-p270 complex	0.09623
Leukoencephalopathy	577	FHL2-p53-HIPK2 complex	0.09623
Leukoencephalopathy	619	MRE11A-RAD50-NBN-TRF2 complex	0.08333
Leukoencephalopathy	627	MRN-TRRAP complex (MRE11A-RAD50-NBN-TRRAP complex)	0.08333
Leukoencephalopathy	629	BLM-TRF2 complex	0.11785
Leukoencephalopathy	720	PU.1-SIN3A-HDAC complex	0.09623
Leukoencephalopathy	721	PU.1-TBP complex	0.11785
Leukoencephalopathy	924	Toposome	0.06299
Leukoencephalopathy	1007	CAK core complex (Cdk-activating kinase core	0.11785
Leukoencephalopathy	1008	CAK complex (Cdk-activating kinase complex)	0.19245
Leukoencephalopathy	1009	TFIIH transcription factor complex	0.10541
Leukoencephalopathy	1029	TFIIH transcription factor complex	0.10541
Leukoencephalopathy	1030	CAK-ERCC2 complex	0.16667
Leukoencephalopathy	1055	ZNF198-PML complex	0.11785
Leukoencephalopathy	1087	BIRC5-AURKB-INCENP-EVI5 complex	0.08333
Leukoencephalopathy	1116	CRM1-Survivin-AuroraB mitotic complex	0.09623
Leukoencephalopathy	1117	CRM1-Survivin mitotic complex	0.11785
Leukoencephalopathy	1118	Chromosomal passenger complex CPC (INCENP CDCA8 BIRC5 AURKB)	0.08333
Leukoencephalopathy	1120	Chromosomal passenger complex CPC (INCENP CDCA8 BIRC5)	0.09623
Leukoencephalopathy	1141	CF IIAm complex (Cleavage factor IIAm complex)	0.04167
Leukoencephalopathy	1170	cMYC-ATPase-helicase complex	0.07454
Leukoencephalopathy	1171	c-MYC-ATPase-helicase complex	0.07454
Leukoencephalopathy	1189	DNA double-strand break end-joining complex	0.06299
Leukoencephalopathy	1193	Rap1 complex	0.06299
Leukoencephalopathy	1218	BLM-TRF2 complex	0.11785
Leukoencephalopathy	1749	SMN-PolII-RHA complex	0.0527
Leukoencephalopathy	1827	PML-SMAD2/3-SARA complex	0.08333
Leukoencephalopathy	2217	MDC1-MRN-ATM-FANCD2 complex	0.06804
Leukoencephalopathy	2218	MDC1-MRE11-RAD50-NBS1 complex	0.08333
Leukoencephalopathy	2222	BLM complex II	0.06804
Leukoencephalopathy	2223	BLM-TOP3A complex	0.11785
Leukoencephalopathy	2224	MSH2/6-BLM-p53-RAD51 complex	0.07454
Leukoencephalopathy	2228	BLM-RAD51L3-XRCC2 complex	0.09623
Leukoencephalopathy	2230	PCNA complex	0.06299
Leukoencephalopathy	2236	ASF1-histone containing complex	0.06299

Leukoencephalopathy	2579	Chromosomal passenger complex CPC (INCENP BIRC5 AURKB)	0.09623
Leukoencephalopathy	2580	Survivin homodimer complex	0.16667
Leukoencephalopathy	2581	RasGAP-AURKA/AURKB-survivin complex	0.08333
Leukoencephalopathy	2582	Chromosomal passenger complex CPC (CDCA8 AURKB BIRC5)	0.09623
Leukoencephalopathy	2638	HES1 promoter corepressor complex	0.06804
Leukoencephalopathy	2641	p300/CBP-PCAF-MyoD complex	0.08333
Leukoencephalopathy	2649	MYC-DNMT3A-ZBTB17 complex	0.09623
Leukoencephalopathy	2653	MYC-MAX-BLOC1S1 complex	0.09623
Leukoencephalopathy	2655	MYC-MAX complex	0.11785
Leukoencephalopathy	2657	ESR1-CDK7-CCNH-MNAT1-MTA1-HDAC2 complex	0.13608
Leukoencephalopathy	2660	ERCC2/CAK complex	0.16667
Leukoencephalopathy	2693	NFAT-JUN-FOS DNA-protein complex	0.09623
Leukoencephalopathy	2727	SRC-3 complex	0.06299
Leukoencephalopathy	2728	SRC-1 complex	0.08333
Leukoencephalopathy	2739	FA complex (Fanconi anemia complex)	0.05025
Leukoencephalopathy	2766	TERF2-RAP1 complex	0.06299
Leukoencephalopathy	2767	RAD50-MRE11-NBN-p200-p350 complex	0.09623
Leukoencephalopathy	2815	BRCA1-BARD1-BACH1-DNA damage complex II	0.11785
Leukoencephalopathy	2817	BRCA1-BARD1-BACH1-DNA damage complex I	0.06804
Leukoencephalopathy	2822	BRCA1-BARD1-UbcH5c complex	0.09623
Leukoencephalopathy	2829	RSmad complex	0.0527
Leukoencephalopathy	2842	DAXX-Axin-p53-HIPK2 complex	0.08333
Leukoencephalopathy	2844	Axin-p53-HIPK2 complex	0.09623
Leukoencephalopathy	2958	SMAD1-CBP complex	0.11785
Leukoencephalopathy	2972	ITGA9-ITGB1-VEGFA complex	0.09623
Leukoencephalopathy	3055	Nop56p-associated pre-rRNA complex	0.01634
Leukoencephalopathy	3061	RNA polymerase II complex (CBP PCAF RPB1 BAF47 CYCC CDK8) chromatin structure modifying	0.06804
Leukoencephalopathy	3062	RNA polymerase II complex incomplete (CBP RPBI PCAF BAF47) chromatin structure modifying	0.08333
Leukoencephalopathy	3066	RNA polymerase II complex chromatin structure modifying	0.04623
Leukoencephalopathy	3067	RNA polymerase II complex incomplete (CDK8 complex) chromatin structure modifying	0.05893
Leukoencephalopathy	3082	DGCR8 multiprotein complex	0.05025
Leukoencephalopathy	3102	DHX9-ADAR-vigilin-DNA-PK-Ku antigen complex	0.06804
Leukoencephalopathy	3129	STAT6-p100-RHA complex	0.09623
Leukoencephalopathy	3137	MASH1 promoter-coactivator complex	0.05025
Leukoencephalopathy	3142	CAMK2-delta-MASH1 promoter-coactivator complex	0.05893
Leukoencephalopathy	3149	NK-3-Groucho-HIPK2-SIN3A-RbpA48-HDAC1	0.05025
Leukoencephalopathy	3162	TF-FVIIa-FXa-TFPI complex	0.08333
Leukoencephalopathy	3618	GammaH2AFX-NDHII-Ku70-DNA complex	0.09623
Leukoencephalopathy	3749	CREBBP-SMAD2 hexameric complex	0.11785
Leukoencephalopathy	3750	CREBBP-SMAD3 hexameric complex	0.11785
Leukoencephalopathy	3753	CREBBP-SMAD2-SMAD4 pentameric complex	0.09623
Leukoencephalopathy	3754	CREBBP-SMAD3-SMAD4 pentameric complex	0.09623

Leukoencephalopathy	4039	PAR4-BACE1 complex	0.11785
Leukoencephalopathy	4062	NRP1-VEGFR2-VEGF(165) complex	0.09623
Leukoencephalopathy	5197	PTIP-DNA damage response complex	0.13608
Leukoencephalopathy	5198	CBP-RARA-RXRA-DNA complex ligand stimulated	0.19245
Leukoencephalopathy	5253	MNK1-eIF4F complex	0.07454
Leukoencephalopathy	5264	TCF4-CTNNB1-CREBBP complex	0.09623
Leukoencephalopathy	5388	SERPINA1-ELA2 complex	0.11785
Leukoencephalopathy	5495	TFIIH transcription factor complex (ERCC2 ERCC3 GTF2H1 CDK7 CCNH GTF2H2)	0.06804
Leukoencephalopathy	5573	Stat1-alpha-dimer-CBP DNA-protein complex	0.11785
Leukoencephalopathy	5656	CEBPE-E2F1-RB1 complex	0.09623
Leukoencephalopathy	5696	VEGFA(165)-KDR-NRP1 complex	0.09623
Leukoencephalopathy	5698	VEGFA(165)-VEGFR2-NRP1 complex	0.09623
Leukoencephalopathy	5701	NRP1-VEGF(165/121) complex	0.11785
Leukopenia	746	C/EBPalpha-HNF6 complex	0.5
Lewy body disease	3008	60S APC containing complex	0.37796
Lewy body disease	3047	Parvulin-associated pre-rRNP complex	0.127
Lichen planus	1062	BAR-BCL2-CASP8 complex	0.20412
Lichen planus	2709	MMP-9-TIMP-1-LRP complex	0.20412
Lichen planus	2798	MMP-2-claudin-1 complex	0.25
Lichen planus	5526	CALM1-FKBP38-BCL2 complex	0.20412
Lichen planus	5811	p53-BCL2 complex	0.25
Lichen planus	5817	tBID-BCL2 complex	0.25
Lichen planus	5818	BIM-BCL2 complex	0.25
Lipodystrophy	1728	CTCF-nucleophosmin-PARP-HIS-KPNA-LMNA-TOP complex	0.09245
Lipodystrophy	5473	FAS-FADD-CASP8 complex	0.16013
Lipodystrophy	5608	Emerin architectural complex	0.11323
Lipodystrophy	5611	Emerin complex 24	0.07161
Lipodystrophy	5799	Death induced signaling complex DISC (FAS FADD CASP8 CFLAR) membrane-associated CD95L	0.13868
Lipodystrophy	5800	Death-inducing signaling complex DISC (type I cells associated) stimulated	0.16013
Lipodystrophy	5808	DISC complex	0.16013
Lipodystrophy	5859	FAS-FADD-CASP8-CASP10 complex	0.13868
Lipodystrophy	5861	FAS-FADD-CASP10 complex	0.16013
Liver cancer	62	MeCP1 complex	0.02744
Liver cancer	93	Anaphase-promoting complex	0.02744
Liver cancer	96	Anaphase-promoting complex	0.02587
Liver cancer	115	Polycomb repressive complex 1 (PRC1 hPRC-H)	0.02241
Liver cancer	116	Polycomb repressive complex 1 (PRC1 hPRC-H)	0.02153
Liver cancer	126	CCT micro-complex	0.02744
Liver cancer	159	Condensin I-PARP-1-XRCC1 complex	0.02934
Liver cancer	282	SNF2h-cohesin-NuRD complex	0.0194
Liver cancer	306	Ribosome cytoplasmic	0.00862
Liver cancer	308	60S ribosomal subunit cytoplasmic	0.01132
Liver cancer	432	N-NOS-CHIP-HSP70-1 complex	0.04481
Liver cancer	541	IGF1-IGFBP3-ALS complex	0.04481

Liver cancer	552	IFNB1-IFNAR1-IFNAR2- complex	0.04481
Liver cancer	559	Decapping complex	0.03471
Liver cancer	626	LSD1 complex	0.02153
Liver cancer	646	HDAC1-associated protein complex	0.02587
Liver cancer	649	HDAC1-associated core complex cII	0.02454
Liver cancer	659	MeCP1 complex	0.02587
Liver cancer	681	(C-CFTR)2-NHERF-ezrin complex	0.04481
Liver cancer	682	C-CFTR-NHERF(PDZ1 domain)-ezrin complex	0.04481
Liver cancer	683	C-CFTR-NHERF(PDZ2 domain)-ezrin complex	0.04481
Liver cancer	685	MeCP1 complex	0.02587
Liver cancer	687	CFTR-NHERF-beta(2)AR signaling complex	0.04481
Liver cancer	725	P2X7 receptor signalling complex	0.02241
Liver cancer	778	LARC complex (LCR-associated remodeling complex)	0.01781
Liver cancer	1004	RC complex during S-phase of cell cycle	0.02153
Liver cancer	1005	RC complex during G2/M-phase of cell cycle	0.02153
Liver cancer	1039	PCNA-PAF complex	0.05488
Liver cancer	1040	p33ING1b-PCNA complex	0.05488
Liver cancer	1055	ZNF198-PML complex	0.05488
Liver cancer	1088	PRNP-ApolopoproteinE3 complex	0.05488
Liver cancer	1094	Frataxin complex	0.02934
Liver cancer	1193	Rap1 complex	0.02934
Liver cancer	1226	H2AX complex I	0.02934
Liver cancer	1255	Ubiquitin E3 ligase (SIAH1 SIP SKP1A TBL1X)	0.03881
Liver cancer	1306	PIN1-AUF1 complex	0.05488
Liver cancer	1308	PABPC1-HSPA8-HNRPD-EIF4G1 complex	0.03471
Liver cancer	1335	SNW1 complex	0.03659
Liver cancer	1400	ASCOM complex	0.02934
Liver cancer	1514	IL4-IL4R complex	0.05488
Liver cancer	1515	IL4-IL4R-IL2RG complex	0.04481
Liver cancer	1707	IL2-IL2RA-IL2RB complex	0.04481
Liver cancer	1728	CTCF-nucleophosmin-PARP-HIS-KPNA-LMNA-TOP complex	0.02587
Liver cancer	1729	TLE1 corepressor complex (MASH1 promoter-corepressor complex)	0.04909
Liver cancer	1774	MICA-KLRK1-HCST complex	0.04481
Liver cancer	1827	PML-SMAD2/3-SARA complex	0.03881
Liver cancer	2055	CASP8-CHUK-IKBKB-MALT1-BCL10 complex	0.03471
Liver cancer	2056	BCL10-CHUK-BCL10-IKBKB complex	0.03881
Liver cancer	2084	NFKB1-NFKB2-REL-RELA-RELB complex	0.03471
Liver cancer	2086	NFKB1-NFKB2-RELA-RELB complex	0.03881
Liver cancer	2100	CHUK-IKBKB-MAP3K14 complex	0.04481
Liver cancer	2101	IKKA-IKKB complex	0.05488
Liver cancer	2104	IKKB-NIK complex	0.05488
Liver cancer	2105	IkappaB kinase complex (IKBKB CHUK IKBKAP NFKBIA RELA MAP3K14)	0.03169
Liver cancer	2118	CHUK-ERC1-IKBKB-IKBKG	0.03881
Liver cancer	2121	CHUK-IKBKB-IKBKG complex	0.04481
Liver cancer	2254	CTGF/Hcs24-actin complex	0.04481

Liver cancer	2318	ITGA6-ITGB4-Laminin10/12 complex	0.03471
Liver cancer	2319	ITGA6-ITGB4-Laminin10/12 complex	0.03471
Liver cancer	2322	ITGA6-ITGB4-LAMA5 complex	0.04481
Liver cancer	2395	ITGA7-ITGB1-CD151 complex	0.04481
Liver cancer	2396	ITGA7-ITGB1-CD9 complex	0.04481
Liver cancer	2397	ITGA7-ITGB1-ITGB1BP3 complex	0.04481
Liver cancer	2528	ERBB2-MEMO-SHC complex	0.04481
Liver cancer	2535	SLP-76-Cbl-Grb2-Shc complex Fc receptor gamma-R1 stimulated	0.03881
Liver cancer	2625	CDK8-MED6-PARP1 complex	0.04481
Liver cancer	2638	HES1 promoter corepressor complex	0.03169
Liver cancer	2639	HES1 promoter-Notch enhancer complex	0.02153
Liver cancer	2709	MMP-9-TIMP-1-LRP complex	0.04481
Liver cancer	2721	HCF-1 complex	0.01781
Liver cancer	2727	SRC-3 complex	0.02934
Liver cancer	2755	17S U2 snRNP	0.01351
Liver cancer	2851	ING2 complex	0.02241
Liver cancer	2880	SCF subcomplex (WEE1 SKP2 BTRC)	0.04481
Liver cancer	2890	Notch1-fraction 30 complex	0.04481
Liver cancer	2895	SHC-GRB2 complex	0.05488
Liver cancer	2897	RBPJ-NotchIC-Mastermind complex	0.04481
Liver cancer	2907	RBP-Jkappa-Notch1 complex	0.05488
Liver cancer	2908	RBP-Jkappa-SHARP complex	0.05488
Liver cancer	2911	SMRT-SKIP-CBF1 complex	0.04481
Liver cancer	2930	SHARP-CtIP-RBP-Jkappa complex	0.04481
Liver cancer	2931	SHARP-CtBP1-CtIP-RBP-Jkappa corepressor complex	0.03881
Liver cancer	2945	RBP-Jkappa-RING1-KyoT2 complex	0.04481
Liver cancer	2946	YY1-Notch1-RBP-Jkappa complex	0.04481
Liver cancer	3034	PAC1-PAC2 complex	0.05488
Liver cancer	3046	hs4 enhancer complex (slow migrating complex)	0.05488
Liver cancer	3055	Nop56p-associated pre-rRNA complex	0.02283
Liver cancer	3096	ITGA6-ITGB4-SHC1-GRB2 complex	0.03881
Liver cancer	3113	MAML1-RBP-Jkappa-Notch1 complex	0.04481
Liver cancer	3137	MASH1 promoter-coactivator complex	0.0234
Liver cancer	3142	CAMK2-delta-MASH1 promoter-coactivator complex	0.02744
Liver cancer	3156	CBF1-HDAC1-SMRT complex	0.04481
Liver cancer	4977	gp78-p97/VCP-DERL1 complex	0.04481
Liver cancer	5179	NCOA6-DNA-PK-Ku-PARP1 complex	0.03471
Liver cancer	5190	TIAM1-EFNB1-EPHA2 complex	0.04481
Liver cancer	5193	TNF-alpha/NF-kappa B signaling complex (CHUK KPNA3 NFKB2 NFKBIB REL IKBKG NFKB1 NFKBIE RELB NFKBIA RELA TNIP2)	0.02241
Liver cancer	5194	TNF-alpha/NF-kappa B signaling complex (SEC16A CHUK IKBKB NFKB2 REL IKBKG MAP3K14 RELA FBXW7 USP2)	0.02454
Liver cancer	5199	Kinase maturation complex 1	0.0194
Liver cancer	5211	RAF1-PPP2-PIN1 complex	0.03471

Liver cancer	5232	TNF-alpha/Nf-kappa B signaling complex (RPL6 RPL30 RPS13 CHUK DDX3X NFKB2 NFKBIB REL IKBKG NFKB1 MAP3K8 RELB GLG1 NFKBIA RELA TNIP2 GTF2I)	0.01882
Liver cancer	5233	TNF-alpha/NF-kappa B signaling complex 5	0.01552
Liver cancer	5234	IKBKB-CDC37-KIAA1967-HSP90AB1-HSP90AA1 complex	0.03471
Liver cancer	5235	WRN-Ku70-Ku80-PARP1 complex	0.03881
Liver cancer	5266	TNF-alpha/NF-kappa B signaling complex 6	0.02074
Liver cancer	5361	Cell division cycle complex (CDC27 CDC16	0.04481
Liver cancer	5378	TRBP containing complex (DICER TRBP AGO2 RPL7A EIF6 MOV10)	0.03169
Liver cancer	5380	TRBP containing complex (DICER RPL7A EIF6 MOV10 and subunits of the 60S ribosomal particle)	0.01552
Liver cancer	5386	MLL1-WDR5 complex	0.01494
Liver cancer	5400	BRCC complex	0.03471
Liver cancer	5423	HSP70-BAG5-PARK2 complex	0.11642
Liver cancer	5545	CDC2-PCNA-CCNB1-GADD45B complex	0.03881
Liver cancer	5546	CDC2-PCNA-CCNB1-GADD45G complex	0.03881
Liver cancer	5816	Apoptosome-procaspase 9 complex	0.04481
Liver cancer	5828	IKBKG-IKBKB complex	0.05488
Liver cancer	5837	PPD complex	0.04481
Liver cancer	5844	I-kappa-B kinase (IKK) complex	0.04481
Liver disease	541	IGF1-IGFBP3-ALS complex	0.17817
Liver disease	1071	PKD2-FPC complex	0.10911
Liver disease	1088	PRNP-ApolopoproteinE3 complex	0.10911
Liver disease	1519	IL6ST-PRKCD-STAT3 complex	0.08909
Liver disease	2254	CTGF/Hcs24-actin complex	0.08909
Liver disease	3229	Heterodimer complex (CDK9 IL6ST)	0.10911
Liver disease	5446	EPO-EPOR complex	0.10911
Liver disease	5564	LMO4-gp130 complex	0.06901
Liver disease	5579	CNTF-CNTFR-gp130-LIFR complex	0.07715
Liver disease	5582	LIFR-LIF-gp130 complex	0.08909
Liver failure	5473	FAS-FADD-CASP8 complex	0.21822
Liver failure	5799	Death induced signaling complex DISC (FAS FADD CASP8 CFLAR) membrane-associated CD95L	0.18898
Liver failure	5800	Death-inducing signaling complex DISC (type I cells associated) stimulated	0.21822
Liver failure	5808	DISC complex	0.21822
Liver failure	5859	FAS-FADD-CASP8-CASP10 complex	0.18898
Liver failure	5861	FAS-FADD-CASP10 complex	0.21822
Liver metastases	27	Arp2/3 protein complex	0.10483
Liver metastases	186	Wave-2 complex	0.12403
Liver metastases	552	IFNB1-IFNAR1-IFNAR2- complex	0.16013
Liver metastases	1041	Alpha-dystrobrevin-ZO-1-actin complex	0.13868
Liver metastases	1141	CF IIAm complex (Cleavage factor IIAm complex)	0.06934
Liver metastases	2294	ABI1-WASF2 complex	0.19612
Liver metastases	2709	MMP-9-TIMP-1-LRP complex	0.16013
Liver metastases	3162	TF-FVIIa-FXa-TFPI complex	0.13868

Liver metastases	5772	ZO1-(beta)cadherin-(VE)cadherin-VEGFR2 complex	0.13868
Liver tumor	1094	Frataxin complex	0.06682
Liver tumor	2638	HES1 promoter corepressor complex	0.07217
Liver tumor	2639	HES1 promoter-Notch enhancer complex	0.04903
Liver tumor	2755	17S U2 snRNP	0.03077
Liver tumor	2890	Notch1-fraction 30 complex	0.10206
Liver tumor	2897	RBPJ-NotchIC-Mastermind complex	0.10206
Liver tumor	2907	RBP-Jkappa-Notch1 complex	0.125
Liver tumor	2908	RBP-Jkappa-SHARP complex	0.125
Liver tumor	2911	SMRT-SKIP-CBF1 complex	0.10206
Liver tumor	2930	SHARP-CtIP-RBP-Jkappa complex	0.10206
Liver tumor	2931	SHARP-CtBP1-CtIP-RBP-Jkappa corepressor complex	0.08839
Liver tumor	2945	RBP-Jkappa-RING1-KyoT2 complex	0.10206
Liver tumor	2946	YY1-Notch1-RBP-Jkappa complex	0.10206
Liver tumor	3113	MAML1-RBP-Jkappa-Notch1 complex	0.10206
Liver tumor	3156	CBF1-HDAC1-SMRT complex	0.10206
Liver tumor	5564	LMO4-gp130 complex	0.07906
Long QT syndrome	422	Beta-dystroglycan-caveolin-3 complex	0.20412
Long QT syndrome	520	KCNQ1 macromolecular complex	0.18257
Long QT syndrome	595	Kv4.2-DPP10 channel complex	0.20412
Lung cancer	4	Multisubunit ACTR coactivator complex	0.03492
Lung cancer	49	DNMT3B complex	0.0264
Lung cancer	54	SIN3 complex	0.0264
Lung cancer	71	MRN complex (MRE11-RAD50-NBS1 complex)	0.04032
Lung cancer	72	R/M complex (RAD50-MRE11 complex)	0.04939
Lung cancer	73	MRN complex (MRE11-RAD50-NBN complex)	0.04032
Lung cancer	103	RNA polymerase II holoenzyme complex	0.01426
Lung cancer	107	TFIIH transcription factor complex	0.06984
Lung cancer	120	Lymphotoxin beta receptor complex	0.04032
Lung cancer	202	BRCA1-RAD50-MRE11-NBS1 complex	0.03492
Lung cancer	227	Ubiquitin E3 ligase (SKP1A BTRC CUL1)	0.04032
Lung cancer	243	RalBP1-CDC2-CCNB1 complex	0.04032
Lung cancer	244	BRAFT complex	0.01937
Lung cancer	246	BLM complex III	0.03492
Lung cancer	247	RalBP1-CCNB1-AP2A-NUMB-EPN1 complex	0.02851
Lung cancer	267	Checkpoint 9-1-1 complex	0.04032
Lung cancer	268	Checkpoint Rad complex	0.02469
Lung cancer	274	RAD17-RFC-9-1-1 checkpoint supercomplex	0.02469
Lung cancer	283	Sin3 complex	0.0264
Lung cancer	305	40S ribosomal subunit cytoplasmic	0.01198
Lung cancer	306	Ribosome cytoplasmic	0.00776
Lung cancer	310	Cell cycle kinase complex CDC2	0.02851
Lung cancer	433	BASC complex (BRCA1-associated genome surveillance complex)	0.04032
Lung cancer	434	BASC (Ab 80) complex (BRCA1-associated genome surveillance complex)	0.02469
Lung cancer	443	BP-SMAD complex	0.02469
Lung cancer	531	XPA-ERCC1-ERCC4 complex	0.04032

Lung cancer	541	IGF1-IGFBP3-ALS complex	0.04032
Lung cancer	570	p300-CBP-p270-SWI/SNF complex	0.0264
Lung cancer	571	p300-CBP-p270 complex	0.04032
Lung cancer	591	SAP complex (Sin3-associated protein complex)	0.02469
Lung cancer	592	SAP complex (Sin3-associated protein complex)	0.02328
Lung cancer	596	SIN3-HDAC-SAP30-ARID4 complex	0.0264
Lung cancer	619	MRE11A-RAD50-NBN-TRF2 complex	0.03492
Lung cancer	627	MRN-TRRAP complex (MRE11A-RAD50-NBN-TRRAP complex)	0.03492
Lung cancer	629	BLM-TRF2 complex	0.04939
Lung cancer	632	Anti-HDAC2 complex	0.01646
Lung cancer	668	BKCA-beta2AR-AKAP79 signaling complex	0.04032
Lung cancer	672	BKCA-beta2AR complex	0.04939
Lung cancer	687	CFTR-NHERF-beta(2)AR signaling complex	0.04032
Lung cancer	691	SIN3-SAP25 complex	0.02106
Lung cancer	696	BRMS1-SIN3-HDAC complex	0.02469
Lung cancer	713	BRG1-SIN3A complex	0.01867
Lung cancer	714	BRM-SIN3A complex	0.01803
Lung cancer	720	PU.1-SIN3A-HDAC complex	0.04032
Lung cancer	725	P2X7 receptor signalling complex	0.02016
Lung cancer	738	SIN3-ING1b complex I	0.02328
Lung cancer	739	SIN3-ING1b complex II	0.01746
Lung cancer	742	eIF3 complex (EIF3S6 EIF3S5 EIF3S4 EIF3S3 EIF3S6IP EIF3S2 EIF3S9 EIF3S12 EIF3S10 EIF3S8 EIF3S1 EIF3S7)	0.02016
Lung cancer	745	NCOR-SIN3-RPD3 complex	0.03492
Lung cancer	747	NCOR-SIN3-HDAC1 complex	0.04032
Lung cancer	749	MeCP2-SIN3A-HDAC complex	0.03492
Lung cancer	803	BRG1-SIN3A-HDAC containing SWI/SNF remodeling complex I	0.02106
Lung cancer	806	BRM-SIN3A-HDAC complex	0.02016
Lung cancer	888	MTA2 complex	0.02328
Lung cancer	999	p23 protein complex	0.04032
Lung cancer	1004	RC complex during S-phase of cell cycle	0.01937
Lung cancer	1005	RC complex during G2/M-phase of cell cycle	0.01937
Lung cancer	1007	CAK core complex (Cdk-activating kinase core	0.04939
Lung cancer	1008	CAK complex (Cdk-activating kinase complex)	0.08065
Lung cancer	1009	TFIIH transcription factor complex	0.06626
Lung cancer	1029	TFIIH transcription factor complex	0.06626
Lung cancer	1030	CAK-ERCC2 complex	0.06984
Lung cancer	1037	TFIIH transcription factor core complex	0.03123
Lung cancer	1097	eIF3 complex (EIF3S6 EIF3S5 EIF3S4 EIF3S3 EIF3S6IP EIF3S2 EIF3S9 EIF3S12 EIF3S10 EIF3S8 EIF3S1 EIF3S7 PCID1)	0.01937
Lung cancer	1099	DNA synthesome complex (17 subunits)	0.01646
Lung cancer	1108	DNA synthesome complex (15 subunits)	0.01803
Lung cancer	1141	CF IIAm complex (Cleavage factor IIAm complex)	0.03492
Lung cancer	1189	DNA double-strand break end-joining complex	0.0264

Lung cancer	1193	Rap1 complex	0.0264
Lung cancer	1194	E2F-6 complex	0.02016
Lung cancer	1211	Ubiquitin E3 ligase (AHR ARNT DDB1 TBL3 CUL4B RBX1)	0.02851
Lung cancer	1218	BLM-TRF2 complex	0.04939
Lung cancer	1232	REST-CoREST-mSIN3A complex	0.04032
Lung cancer	1257	ALL-1 supercomplex	0.0132
Lung cancer	1505	NCOR2 complex	0.0264
Lung cancer	1514	IL4-IL4R complex	0.04939
Lung cancer	1515	IL4-IL4R-IL2RG complex	0.04032
Lung cancer	1519	IL6ST-PRKCD-STAT3 complex	0.04032
Lung cancer	1521	p300-SMAD1-STAT3 complex	0.04032
Lung cancer	1707	IL2-IL2RA-IL2RB complex	0.08065
Lung cancer	1714	TICAM1-TICAM2-TLR4 complex	0.04032
Lung cancer	1728	CTCF-nucleophosmin-PARP-HIS-KPNA-LMNA-TOP complex	0.02328
Lung cancer	1831	PIAS3-SMAD3-P300 complex	0.04032
Lung cancer	2187	Ubiquitin E3 ligase (NFKBIA FBXW11 BTRC CUL1 SKP1A)	0.03123
Lung cancer	2188	Ubiquitin E3 ligase (CDC34 NEDD8 BTRC CUL1 SKP1A RBX1)	0.03123
Lung cancer	2189	Ubiquitin E3 ligase (SMAD3 BTRC CUL1 SKP1A RBX1)	0.03123
Lung cancer	2196	LIG1-9-1-1 complex	0.03492
Lung cancer	2197	FEN1-9-1-1 complex	0.06984
Lung cancer	2198	RAD9-RAD1-HUS1-POLB complex	0.03492
Lung cancer	2217	MDC1-MRN-ATM-FANCD2 complex	0.02851
Lung cancer	2218	MDC1-MRE11-RAD50-NBS1 complex	0.03492
Lung cancer	2222	BLM complex II	0.02851
Lung cancer	2223	BLM-TOP3A complex	0.04939
Lung cancer	2224	MSH2/6-BLM-p53-RAD51 complex	0.03123
Lung cancer	2228	BLM-RAD51L3-XRCC2 complex	0.04032
Lung cancer	2254	CTGF/Hcs24-actin complex	0.04032
Lung cancer	2383	ITGA5-ITGB1-FN1-TGM2 complex	0.03492
Lung cancer	2384	ITGA5-ITGB1-ADAM15 complex	0.04032
Lung cancer	2385	ITGA5-ITGB4 complex	0.04939
Lung cancer	2388	Itga5-Itgb1-Fn1-Sfrp2 complex	0.03492
Lung cancer	2429	ITGA2-ITGB1-CD47 complex	0.04032
Lung cancer	2430	ITGA2-ITGB1-CHAD complex	0.04032
Lung cancer	2431	ITGA2-ITGB1-COL6A3 complex	0.04032
Lung cancer	2432	ITGA2-ITGB1 complex	0.04939
Lung cancer	2443	ITGA9-ITGB1-TNC complex	0.04032
Lung cancer	2471	SRC-PRKCD-CDCP1 complex	0.04032
Lung cancer	2564	p21(ras)GAP-Fyn-Lyn-Yes complex thrombin	0.03492
Lung cancer	2578	Sam68-p120GAP complex	0.04939
Lung cancer	2581	RasGAP-AURKA/AURKB-survivin complex	0.03492
Lung cancer	2638	HES1 promoter corepressor complex	0.02851
Lung cancer	2641	p300/CBP-PCAF-MyoD complex	0.03492

Lung cancer	2642	SMAD1-P300 complex	0.04939
Lung cancer	2653	MYC-MAX-BLOC1S1 complex	0.04032
Lung cancer	2655	MYC-MAX complex	0.04939
Lung cancer	2657	ESR1-CDK7-CCNH-MNAT1-MTA1-HDAC2 complex	0.05703
Lung cancer	2660	ERCC2/CAK complex	0.06984
Lung cancer	2705	SMAD3-SMAD4-CTCF protein-DNA complex	0.04032
Lung cancer	2709	MMP-9-TIMP-1-LRP complex	0.04032
Lung cancer	2721	HCF-1 complex	0.01602
Lung cancer	2724	Ubiquitin E3 ligase (NFKBIA BTRC CUL1 SKP1A)	0.03492
Lung cancer	2727	SRC-3 complex	0.0264
Lung cancer	2728	SRC-1 complex	0.03492
Lung cancer	2739	FA complex (Fanconi anemia complex)	0.02106
Lung cancer	2766	TERF2-RAP1 complex	0.0264
Lung cancer	2767	RAD50-MRE11-NBN-p200-p350 complex	0.04032
Lung cancer	2808	RAD9-RAD1-HUS1-APE1 complex	0.03492
Lung cancer	2809	9-1-1 complex	0.04032
Lung cancer	2815	BRCA1-BARD1-BACH1-DNA damage complex II	0.02469
Lung cancer	2825	BRCA1-RNA polymerase II complex	0.0137
Lung cancer	2829	RSmad complex	0.02209
Lung cancer	2850	ITGA5-ITGB1-FN-1-NOV complex	0.03492
Lung cancer	2851	ING2 complex	0.04032
Lung cancer	2853	ITGA5-ITGB1-CAL4A3 complex	0.04032
Lung cancer	2880	SCF subcomplex (WEE1 SKP2 BTRC)	0.04032
Lung cancer	2882	ITGA5-ITGB3-COL6A3 complex	0.04032
Lung cancer	2958	SMAD1-CBP complex	0.09877
Lung cancer	2959	SMAD1-OAZ-HsN3 complex	0.04032
Lung cancer	2989	ITGA9-ITGB1-ADAM8 complex	0.04032
Lung cancer	3044	SKI-NCOR1-SIN3A-HDAC1 complex	0.03492
Lung cancer	3048	mSin3A complex	0.03123
Lung cancer	3051	MAD-MAX complex	0.04939
Lung cancer	3053	mSin3A-HDAC1-HDAC2 complex	0.04032
Lung cancer	3054	MAD1-mSin3A-HDAC2 complex	0.04032
Lung cancer	3055	Nop56p-associated pre-rRNA complex	0.00685
Lung cancer	3061	RNA polymerase II complex (CBP PCAF RPB1 BAF47 CYCC CDK8) chromatin structure modifying	0.02851
Lung cancer	3062	RNA polymerase II complex incomplete (CBP RPBI PCAF BAF47) chromatin structure modifying	0.03492
Lung cancer	3066	RNA polymerase II complex chromatin structure modifying	0.03874
Lung cancer	3067	RNA polymerase II complex incomplete (CDK8 complex) chromatin structure modifying	0.02469
Lung cancer	3110	ITGAV-P2RY2-GNA12 complex	0.04032
Lung cancer	3112	ITGA5-ITGB1-SPP1 complex	0.04032
Lung cancer	3137	MASH1 promoter-coactivator complex	0.02106
Lung cancer	3140	CRLR-RAMP2 complex	0.04939
Lung cancer	3142	CAMK2-delta-MASH1 promoter-coactivator complex	0.02469
Lung cancer	3149	NK-3-Groucho-HIPK2-SIN3A-RbpA48-HDAC1	0.02106
Lung cancer	3151	Sulphiredoxin-peroxiredoxin complex	0.04939

Lung cancer	3167	NCOR-SIN3-HDAC-HESX1 complex	0.02851
Lung cancer	3229	Heterodimer complex (CDK9 IL6ST)	0.04939
Lung cancer	3263	HERP1/HEY2-NCOR-SIN3A complex	0.03492
Lung cancer	3749	CREBBP-SMAD2 hexameric complex	0.04939
Lung cancer	3750	CREBBP-SMAD3 hexameric complex	0.04939
Lung cancer	3753	CREBBP-SMAD2-SMAD4 pentameric complex	0.04032
Lung cancer	3754	CREBBP-SMAD3-SMAD4 pentameric complex	0.04032
Lung cancer	3830	ADRB2 homodimer complex	0.06984
Lung cancer	3961	SMAD3-cSKI-SIN3A-HDAC1 complex	0.03492
Lung cancer	4039	PAR4-BACE1 complex	0.04939
Lung cancer	4977	gp78-p97/VCP-DERL1 complex	0.04032
Lung cancer	5153	CTFC-TAF1 complex	0.04939
Lung cancer	5154	CTCF-nucleophosmin complex	0.04939
Lung cancer	5184	SWI/SNF chromatin-remodeling complex	0.03123
Lung cancer	5189	YWHAQ-CALM1-CABIN1 complex	0.04032
Lung cancer	5196	TNF-alpha/NF-kappa B signaling complex (CHUK BTRC NFKB2 PPP6C REL CUL1 IKBKE SAPS2 SAPS1 ANKRD28 RELA SKP1)	0.02016
Lung cancer	5197	PTIP-DNA damage response complex	0.05703
Lung cancer	5198	CBP-RARA-RXRA-DNA complex ligand stimulated	0.04032
Lung cancer	5199	Kinase maturation complex 1	0.01746
Lung cancer	5264	TCF4-CTNNB1-CREBBP complex	0.04032
Lung cancer	5495	TFIIH transcription factor complex (ERCC2 ERCC3 GTF2H1 CDK7 CCNH GTF2H2)	0.05703
Lung cancer	5542	CCNB2-CDC2 complex	0.04939
Lung cancer	5564	LMO4-gp130 complex	0.06247
Lung cancer	5573	Stat1-alpha-dimer-CBP DNA-protein complex	0.04939
Lung cancer	5579	CNTF-CNTFR-gp130-LIFR complex	0.03492
Lung cancer	5582	LIFR-LIF-gp130 complex	0.04032
Lung cancer	5615	Emerin complex 52	0.01456
Lung cancer	5642	Securin-separase complex	0.04939
Lung cancer	5643	ESPL1-CDC2 complex	0.04939
Lung cancer	5762	CRMP-MICAL-PlexinA1 complex induced by	0.04032
Lung cancer	5805	PGAM5-KEAP1-NRF2 complex	0.04032
Lung cancer	5816	Apoptosome-procaspase 9 complex	0.04032
Lung cancer	5843	AIF-CYPA-DNA complex	0.04939
Lung cancer	5919	BRAF-RAF1-14-3-3 complex	0.02328
Lung disease	68	BCDX2 complex	0.08839
Lung disease	245	FA core complex (Fanconi anemia core complex)	0.05893
Lung disease	256	RAD51B-RAD51C complex	0.125
Lung disease	261	RAD51B-RAD51C-RAD51D-XRCC2-XRCC3	0.07906
Lung disease	305	40S ribosomal subunit cytoplasmic	0.03032
Lung disease	306	Ribosome cytoplasmic	0.01964
Lung disease	520	KCNQ1 macromolecular complex	0.0559
Lung disease	801	SNARE complex (STX2 SNAP23)	0.125
Lung disease	1067	CD8A-LCK complex	0.125
Lung disease	1079	P-TEFb.1 complex	0.125
Lung disease	1152	FA complex (Fanconi anemia complex)	0.0625

Lung disease	1624	FA core complex (Fanconi anemia core complex)	0.05893
Lung disease	1625	FA core complex (Fanconi anemia core complex)	0.06682
Lung disease	1810	ITGA4-PXN-GIT1 complex	0.10206
Lung disease	1834	ITGAE-ITGB7-CDH1 complex	0.10206
Lung disease	2018	IL12A-IL12B complex	0.125
Lung disease	2019	IL12A-IL12B-IL12RB1 complex	0.10206
Lung disease	2020	IL12B-IL12RB1-IL12RB2 complex	0.10206
Lung disease	2021	IL12A-IL12B-IL12RB2 complex	0.10206
Lung disease	2417	ITGA4-ITGB1-EMILIN1 complex	0.10206
Lung disease	2418	ITGA4-ITGB1 complex	0.125
Lung disease	2419	ITGA4-ITGB1-CD81 complex	0.10206
Lung disease	2420	ITGA4-ITGB1-CD53 complex	0.10206
Lung disease	2421	ITGA4-ITGB1-VCAM1 complex	0.10206
Lung disease	2422	ITGA4-ITGB1-JAM2 complex	0.10206
Lung disease	2423	ITGA4-ITGB1-CD47 complex	0.10206
Lung disease	2424	ITGA4-ITGB1-CD63 complex	0.10206
Lung disease	2425	ITGA4-ITGB1-PXN complex	0.10206
Lung disease	2426	ITGA4-ITGB1-THBS1 complex	0.10206
Lung disease	2428	ITGA4-ITGB1-THBS2 complex	0.10206
Lung disease	2559	p56(LCK)-CAML complex	0.125
Lung disease	2565	CD20-LCK-LYN-FYN-p75/80 complex (Raji human B cell line)	0.08839
Lung disease	2599	POLR2A-CCNT1-CDK9-NCL-LEM6-CPSF2 complex	0.07217
Lung disease	2600	BRD4 complex	0.0625
Lung disease	2601	P-TEFb-BRD4-TRAP220 complex	0.08839
Lung disease	2602	P-TEFb-7SKRNA-HEXIM1 complex	0.10206
Lung disease	2603	Transcription elongation factor complex (SUPT5H CDK9 CCNT1)	0.10206
Lung disease	2604	P-TEFb-SKP2 complex	0.10206
Lung disease	2605	Heterotrimeric complex (CCNT1 CDK9 GRN)	0.10206
Lung disease	2639	HES1 promoter-Notch enhancer complex	0.04903
Lung disease	2739	FA complex (Fanconi anemia complex)	0.0533
Lung disease	2875	BRD4-P-TEFb complex	0.10206
Lung disease	2879	CD20-LCK-FYN-p75/80 complex	0.10206
Lung disease	2944	Notch1-p56lck-PI3K complex	0.10206
Lung disease	2955	LCK-SLP76-PLC-gamma-1-LAT complex pervanadate-activated	0.08839
Lung disease	3055	Nop56p-associated pre-rRNA complex	0.01733
Lung disease	5548	IL-12 heterodimer complex	0.125
Lung disease	5549	IL-12 subunit p40 homodimer complex	0.17678
Lupus erythematosus	92	CD28-transactivation complex	0.06041
Lupus erythematosus	351	Spliceosome	0.00714
Lupus erythematosus	441	TFTC-type histone acetyl transferase complex	0.02576
Lupus erythematosus	557	TRP1-G alpha-11-IP3R3-CAV1 signaling complex	0.04272
Lupus erythematosus	749	MeCP2-SIN3A-HDAC complex	0.04272
Lupus erythematosus	906	ADAR1-CDK2 complex	0.06041
Lupus erythematosus	1054	ESR1-RELA-BCL3-NCOA3 complex	0.04272
Lupus erythematosus	1062	BAR-BCL2-CASP8 complex	0.04933

Lupus erythematosus	1067	CD8A-LCK complex	0.06041
Lupus erythematosus	1088	PRNP-ApolopoproteinE3 complex	0.06041
Lupus erythematosus	1095	SNX complex (SNX1a SNX2 SNX4 EGFR)	0.04272
Lupus erythematosus	1185	EGFR-containing signaling complex	0.04272
Lupus erythematosus	1261	SRm160/300 complex	0.03821
Lupus erythematosus	1345	Septin complex	0.03821
Lupus erythematosus	1514	IL4-IL4R complex	0.12082
Lupus erythematosus	1515	IL4-IL4R-IL2RG complex	0.09865
Lupus erythematosus	5712	FAK-beta5 integrin complex VEGF induced	0.06041
Lupus erythematosus	1707	IL2-IL2RA-IL2RB complex	0.09865
Lupus erythematosus	1816	JUN-TCF4-CTNNB1 complex	0.04933
Lupus erythematosus	1944	IRAK1-IRAK2 complex	0.06041
Lupus erythematosus	1945	IRAK1-IRAK3 complex	0.06041
Lupus erythematosus	2124	IKK-alpha--ER-alpha-AIB1 complex	0.04933
Lupus erythematosus	2153	ITGAM-ITGB2-CD11 complex	0.04933
Lupus erythematosus	2347	ITGAV-ITGB5-SPP1 complex	0.04933
Lupus erythematosus	2352	ITGAV-ITGB6-SPP1 complex	0.04933
Lupus erythematosus	2355	ITGAV-ITGB3-CD47-FCER2 complex	0.04272
Lupus erythematosus	2356	ITGB3-ITGAV-CD47 complex	0.04933
Lupus erythematosus	2358	ITGAV-ITGB3-SPP1 complex	0.09865
Lupus erythematosus	2359	ITGAV-ITGB3-ADAM15 complex	0.04933
Lupus erythematosus	2362	ITAGV-ITGB3-F11R complex	0.04933
Lupus erythematosus	2363	ITGAV-ITGB3-PXN-PTK2b complex	0.04272
Lupus erythematosus	2364	ITGAV-ITGB3-ADAM23 complex	0.04933
Lupus erythematosus	2365	ITGAV-ITGB3-COL4A3 complex	0.04933
Lupus erythematosus	2366	ITGAV-ITGB3-PPAP2b complex	0.04933
Lupus erythematosus	2369	ITGAV-ITGB3-EGFR complex	0.09865
Lupus erythematosus	2370	ITGA2b-ITGB3-CD9 complex	0.04933
Lupus erythematosus	2374	ITGAV-ITGB3-LAMA4 complex	0.04933
Lupus erythematosus	2376	ITGA2B-ITGB3-FN1-TGM2 complex	0.04272
Lupus erythematosus	2377	ITGA2b-ITGB3-CD47-SRC complex	0.04272
Lupus erythematosus	2378	ITGA2b-ITGB3-TLN1 complex	0.04933
Lupus erythematosus	2379	ITGA2B-ITGB3-CIB1 complex	0.04933
Lupus erythematosus	2381	ITGA2B-ITGB3 complex	0.06041
Lupus erythematosus	2382	ITGA2B-ITGB3-F11R complex	0.04933
Lupus erythematosus	2383	ITGA5-ITGB1-FN1-TGM2 complex	0.04272
Lupus erythematosus	2384	ITGA5-ITGB1-ADAM15 complex	0.04933
Lupus erythematosus	2385	ITGA5-ITGB4 complex	0.06041
Lupus erythematosus	2388	Itga5-Itgb1-Fn1-Sfrp2 complex	0.04272
Lupus erythematosus	2390	CD98-LAT2-ITGB1 complex	0.04272
Lupus erythematosus	2395	ITGA7-ITGB1-CD151 complex	0.04933
Lupus erythematosus	2396	ITGA7-ITGB1-CD9 complex	0.04933
Lupus erythematosus	2397	ITGA7-ITGB1-ITGB1BP3 complex	0.04933
Lupus erythematosus	2398	ITGA3-ITGB1-BSG complex	0.04933
Lupus erythematosus	2399	ITGA3-ITGB1-CD63 complex	0.04933
Lupus erythematosus	2400	ITGA3-ITGB1-CD151 complex	0.04933
Lupus erythematosus	2401	ITGA3-ITGB1-THBS1 complex	0.04933

Lupus erythematosus	2406	ITGA3-ITGB1 complex	0.06041
Lupus erythematosus	2411	ITGA6-ITGB1-CD151 complex	0.04933
Lupus erythematosus	2413	ITGA6-ITGB1 complex	0.06041
Lupus erythematosus	2416	ITGB1-RAP1A-PKD1 complex	0.04933
Lupus erythematosus	2417	ITGA4-ITGB1-EMILIN1 complex	0.04933
Lupus erythematosus	2418	ITGA4-ITGB1 complex	0.06041
Lupus erythematosus	2419	ITGA4-ITGB1-CD81 complex	0.04933
Lupus erythematosus	2420	ITGA4-ITGB1-CD53 complex	0.04933
Lupus erythematosus	2421	ITGA4-ITGB1-VCAM1 complex	0.04933
Lupus erythematosus	2422	ITGA4-ITGB1-JAM2 complex	0.04933
Lupus erythematosus	2423	ITGA4-ITGB1-CD47 complex	0.04933
Lupus erythematosus	2424	ITGA4-ITGB1-CD63 complex	0.04933
Lupus erythematosus	2425	ITGA4-ITGB1-PXN complex	0.04933
Lupus erythematosus	2426	ITGA4-ITGB1-THBS1 complex	0.04933
Lupus erythematosus	2428	ITGA4-ITGB1-THBS2 complex	0.04933
Lupus erythematosus	2429	ITGA2-ITGB1-CD47 complex	0.04933
Lupus erythematosus	2430	ITGA2-ITGB1-CHAD complex	0.04933
Lupus erythematosus	2431	ITGA2-ITGB1-COL6A3 complex	0.04933
Lupus erythematosus	2432	ITGA2-ITGB1 complex	0.06041
Lupus erythematosus	2434	ITGA1-ITGB1-COL6A3 complex	0.04933
Lupus erythematosus	2435	ITGA1-ITGB1-PTPN2 complex	0.04933
Lupus erythematosus	2436	ITGAV-ITGB1 complex	0.06041
Lupus erythematosus	2437	ITGA6-ITGB1-CYR61 complex	0.04933
Lupus erythematosus	2439	ITGA8-ITGB1 complex	0.06041
Lupus erythematosus	2440	ITGA9-ITGB1-ADAM9 complex	0.04933
Lupus erythematosus	2441	Itga9-Itgb1-Adam2 complex	0.04933
Lupus erythematosus	2442	ITGA9-ITGB1-VCAM1 complex	0.04933
Lupus erythematosus	2443	ITGA9-ITGB1-TNC complex	0.04933
Lupus erythematosus	2444	ITGB1-ITGA9 complex	0.06041
Lupus erythematosus	2445	ITGA9-ITGB1-ADAM15 complex	0.04933
Lupus erythematosus	2446	ITGA9-ITGB1-FIGF complex	0.04933
Lupus erythematosus	2447	ITGA9-ITGB1-ADAM12 complex	0.04933
Lupus erythematosus	2453	Multiprotein complex (monoubiquitination)	0.04272
Lupus erythematosus	2454	CIN85-CBL-SH3GL2-EGFR complex EGF stimulated	0.04272
Lupus erythematosus	2470	p130Cas-ER-alpha-cSrc-kinase- PI3-kinase p85-subunit complex	0.04272
Lupus erythematosus	2489	NCR3-CD247 complex	0.06041
Lupus erythematosus	2529	LAT-PLC-gamma-1-p85-GRB2-CBL-VAV-SLP-76 signaling complex C305 activated	0.03229
Lupus erythematosus	2536	PLC-gamma-2-SLP-76-Lyn-Grb2 complex	0.04272
Lupus erythematosus	2542	EGFR-CBL-GRB2 complex	0.04933
Lupus erythematosus	2547	PLC-gamma-1-SLP-76-SOS1-LAT complex	0.04272
Lupus erythematosus	2563	FGFR2-c-Cbl-Lyn-Fyn complex	0.04272
Lupus erythematosus	2564	p21(ras)GAP-Fyn-Lyn-Yes complex thrombin	0.04272
Lupus erythematosus	2565	CD20-LCK-LYN-FYN-p75/80 complex (Raji human B cell line)	0.04272
Lupus erythematosus	2657	ESR1-CDK7-CCNH-MNAT1-MTA1-HDAC2 complex	0.03488
Lupus erythematosus	2670	Er-alpha-p53-hdm2 complex	0.04933

Lupus erythematosus	2692	SMAD3-SMAD4-cJun-cFos complex	0.04272
Lupus erythematosus	2693	NFAT-JUN-FOS DNA-protein complex	0.04933
Lupus erythematosus	2694	ERG-JUN-FOS DNA-protein complex	0.04933
Lupus erythematosus	2695	ETS2-FOS-JUN complex	0.04933
Lupus erythematosus	2699	ER-alpha-GRIP1-c-Jun complex	0.09865
Lupus erythematosus	2700	ER-alpha-c-Jun complex	0.12082
Lupus erythematosus	2708	SMAD3-SMAD4-cJUN complex	0.04933
Lupus erythematosus	2709	MMP-9-TIMP-1-LRP complex	0.04933
Lupus erythematosus	2726	PXN-ITGB5-PTK2 complex	0.04933
Lupus erythematosus	2816	ITGAV-ITGB3 complex	0.06041
Lupus erythematosus	2826	ITGB3-ITGAV-VTN complex	0.04933
Lupus erythematosus	2846	ITGAV-ITGB3-THBS1 complex	0.04933
Lupus erythematosus	2849	ITGAV-ITGB3-NOV complex	0.04933
Lupus erythematosus	2850	ITGA5-ITGB1-FN-1-NOV complex	0.04272
Lupus erythematosus	2853	ITGA5-ITGB1-CAL4A3 complex	0.04933
Lupus erythematosus	2872	ITGA2b-ITGB3-CD9-GP1b-CD47 complex	0.03488
Lupus erythematosus	2882	ITGA5-ITGB3-COL6A3 complex	0.04933
Lupus erythematosus	2885	ITGAV-ITGB1-SPP1 complex	0.09865
Lupus erythematosus	2896	ITGA2b-ITGB3-CD47-FAK complex	0.08544
Lupus erythematosus	2909	PLC-gamma-2-Syk-LAT-FcR-gamma complex	0.08544
Lupus erythematosus	2910	PLC-gamma-2-Lyn-FcR-gamma complex	0.09865
Lupus erythematosus	2913	PLC-gamma-2-LAT complex	0.06041
Lupus erythematosus	2922	LAT-PLC-gamma-1-p85-GRB2-SOS signaling complex C305 activated	0.03821
Lupus erythematosus	2955	LCK-SLP76-PLC-gamma-1-LAT complex pervanadate-activated	0.04272
Lupus erythematosus	2956	PLC-gamma-1-LAT-c-CBL complex OKT3 stimulated	0.04933
Lupus erythematosus	2957	LAT-GRB2 complex Fyn-mLck(KA) or Syk kinase activated	0.06041
Lupus erythematosus	2964	ITGA9-ITGB1-ADAM1 complex	0.04933
Lupus erythematosus	2965	ITGA9-ITGB1-ADAM3 complex	0.04933
Lupus erythematosus	2971	ITGA9-ITGB1-VEGFC complex	0.04933
Lupus erythematosus	2972	ITGA9-ITGB1-VEGFA complex	0.04933
Lupus erythematosus	2989	ITGA9-ITGB1-ADAM8 complex	0.04933
Lupus erythematosus	3035	LAT2-ITGB1 complex	0.06041
Lupus erythematosus	3057	ITGA10-ITGB1 complex	0.06041
Lupus erythematosus	3058	ITGA11-ITGB1 complex	0.06041
Lupus erythematosus	3059	ITGA11-ITGB1-COL1A1 complex	0.04933
Lupus erythematosus	3102	DHX9-ADAR-vigilin-DNA-PK-Ku antigen complex	0.03488
Lupus erythematosus	3103	ITGAV-ITGB3-SLC3A2 complex	0.04933
Lupus erythematosus	3104	ITGB1-NRP1 complex	0.06041
Lupus erythematosus	3111	ITGA9-ITGB1-SPP1 complex	0.09865
Lupus erythematosus	3112	ITGA5-ITGB1-SPP1 complex	0.09865
Lupus erythematosus	3115	ITGA2B-ITGB3-ICAM4 complex	0.04933
Lupus erythematosus	3678	RIN1-STAM2-EGFR complex EGF stimulated	0.04933
Lupus erythematosus	4062	NRP1-VEGFR2-VEGF(165) complex	0.04933
Lupus erythematosus	5772	ZO1-(beta)cadherin-(VE)cadherin-VEGFR2 complex	0.04272
Lupus erythematosus	5158	SMARCA2/BRM-BAF57-MECP2 complex	0.04933

Lupus erythematosus	5171	SH3KBP1-CBLB-EGFR complex	0.04933
Lupus erythematosus	5177	Polycystin-1 multiprotein complex (ACTN1 CDH1 SRC JUP VCL CTNNB1 PXN BCAR1 PKD1	0.02576
Lupus erythematosus	5178	JAK2-PAFR-TYK2 complex	0.04933
Lupus erythematosus	5184	SWI/SNF chromatin-remodeling complex	0.03821
Lupus erythematosus	5282	CAS-SRC-FAK complex	0.04933
Lupus erythematosus	5388	SERPINA1-ELA2 complex	0.06041
Lupus erythematosus	5473	FAS-FADD-CASP8 complex	0.04933
Lupus erythematosus	5526	CALM1-FKBP38-BCL2 complex	0.04933
Lupus erythematosus	5691	TALL1 homo-oligomer complex	0.08544
Lupus erythematosus	5696	VEGFA(165)-KDR-NRP1 complex	0.04933
Lupus erythematosus	5698	VEGFA(165)-VEGFR2-NRP1 complex	0.04933
Lupus erythematosus	5799	Death induced signaling complex DISC (FAS FADD CASP8 CFLAR) membrane-associated CD95L	0.04272
Lupus erythematosus	5800	Death-inducing signaling complex DISC (type I cells associated) stimulated	0.04933
Lupus erythematosus	5808	DISC complex	0.04933
Lupus erythematosus	5811	p53-BCL2 complex	0.06041
Lupus erythematosus	5817	tBID-BCL2 complex	0.06041
Lupus erythematosus	5818	BIM-BCL2 complex	0.06041
Lupus erythematosus	5859	FAS-FADD-CASP8-CASP10 complex	0.04272
Lupus erythematosus	5861	FAS-FADD-CASP10 complex	0.04933
Lupus erythematosus	5862	CAV1-VDAC1-ESR1 complex	0.04933
Lupus vulgaris	351	Spliceosome	0.0158
Lupus vulgaris	441	TFTC-type histone acetyl transferase complex	0.05698
Lupus vulgaris	1054	ESR1-RELA-BCL3-NCOA3 complex	0.09449
Lupus vulgaris	1261	SRm160/300 complex	0.08452
Lupus vulgaris	2124	IKK-alpha--ER-alpha-AIB1 complex	0.10911
Lupus vulgaris	2347	ITGAV-ITGB5-SPP1 complex	0.10911
Lupus vulgaris	2352	ITGAV-ITGB6-SPP1 complex	0.10911
Lupus vulgaris	2358	ITGAV-ITGB3-SPP1 complex	0.10911
Lupus vulgaris	2470	p130Cas-ER-alpha-cSrc-kinase- PI3-kinase p85-subunit complex	0.09449
Lupus vulgaris	2489	NCR3-CD247 complex	0.13363
Lupus vulgaris	2657	ESR1-CDK7-CCNH-MNAT1-MTA1-HDAC2 complex	0.07715
Lupus vulgaris	2670	Er-alpha-p53-hdm2 complex	0.10911
Lupus vulgaris	2699	ER-alpha-GRIP1-c-Jun complex	0.10911
Lupus vulgaris	2700	ER-alpha-c-Jun complex	0.13363
Lupus vulgaris	2709	MMP-9-TIMP-1-LRP complex	0.21822
Lupus vulgaris	2885	ITGAV-ITGB1-SPP1 complex	0.10911
Lupus vulgaris	3111	ITGA9-ITGB1-SPP1 complex	0.10911
Lupus vulgaris	3112	ITGA5-ITGB1-SPP1 complex	0.10911
Lupus vulgaris	5473	FAS-FADD-CASP8 complex	0.10911
Lupus vulgaris	5799	Death induced signaling complex DISC (FAS FADD CASP8 CFLAR) membrane-associated CD95L	0.09449
Lupus vulgaris	5800	Death-inducing signaling complex DISC (type I cells associated) stimulated	0.10911
Lupus vulgaris	5808	DISC complex	0.10911

Lupus vulgaris	5859	FAS-FADD-CASP8-CASP10 complex	0.09449
Lupus vulgaris	5861	FAS-FADD-CASP10 complex	0.10911
Lupus vulgaris	5862	CAV1-VDAC1-ESR1 complex	0.10911
Lyme disease	1094	Frataxin complex	0.12599
Lyme disease	2755	17S U2 snRNP	0.05803
Lymphatic metastasis	3162	TF-FVIIa-FXa-TFPI complex	0.35355
Lymphoma	10	13S condensin complex	0.10193
Lymphoma	49	DNMT3B complex	0.08615
Lymphoma	157	Condensin I complex	0.10193
Lymphoma	159	Condensin I-PARP-1-XRCC1 complex	0.08615
Lymphoma	160	Condensin II	0.10193
Lymphoma	277	RFC complex	0.05096
Lymphoma	279	RFC complex (activator A 1 complex)	0.05096
Lymphoma	299	IRF3-CBP complex	0.05698
Lymphoma	353	DNA ligase IV-condensin complex	0.13159
Lymphoma	360	Artemis-DNA-PK complex	0.08058
Lymphoma	433	BASC complex (BRCA1-associated genome surveillance complex)	0.0329
Lymphoma	434	BASC (Ab 80) complex (BRCA1-associated genome surveillance complex)	0.04029
Lymphoma	435	BASC (Ab 81) complex (BRCA1-associated genome surveillance complex)	0.04652
Lymphoma	552	IFNB1-IFNAR1-IFNAR2- complex	0.0658
Lymphoma	720	PU.1-SIN3A-HDAC complex	0.0658
Lymphoma	721	PU.1-TBP complex	0.08058
Lymphoma	786	MR-UBC9-SRC1 complex	0.0658
Lymphoma	1003	RC complex (Replication competent complex)	0.03799
Lymphoma	1004	RC complex during S-phase of cell cycle	0.03161
Lymphoma	1005	RC complex during G2/M-phase of cell cycle	0.03161
Lymphoma	1054	ESR1-RELA-BCL3-NCOA3 complex	0.05698
Lymphoma	1067	CD8A-LCK complex	0.08058
Lymphoma	1098	DNA synthesome complex (13 subunits)	0.03046
Lymphoma	1099	DNA synthesome complex (17 subunits)	0.02686
Lymphoma	1107	DNA synthesome core complex	0.03604
Lymphoma	1108	DNA synthesome complex (15 subunits)	0.02942
Lymphoma	1211	Ubiquitin E3 ligase (AHR ARNT DDB1 TBL3 CUL4B RBX1)	0.04652
Lymphoma	1256	MLL-HCF complex	0.04307
Lymphoma	1257	ALL-1 supercomplex	0.02154
Lymphoma	1401	MOF complex	0.03604
Lymphoma	1514	IL4-IL4R complex	0.08058
Lymphoma	1515	IL4-IL4R-IL2RG complex	0.0658
Lymphoma	1707	IL2-IL2RA-IL2RB complex	0.0658
Lymphoma	1787	Nogo-potassium channel complex	0.05698
Lymphoma	2016	IL12A homodimer complex	0.11396
Lymphoma	2018	IL12A-IL12B complex	0.08058
Lymphoma	2019	IL12A-IL12B-IL12RB1 complex	0.0658
Lymphoma	2021	IL12A-IL12B-IL12RB2 complex	0.0658

Lymphoma	2203	BRD4-RFC complex	0.04652
Lymphoma	2559	p56(LCK)-CAML complex	0.08058
Lymphoma	2565	CD20-LCK-LYN-FYN-p75/80 complex (Raji human B cell line)	0.05698
Lymphoma	2693	NFAT-JUN-FOS DNA-protein complex	0.0658
Lymphoma	2879	CD20-LCK-FYN-p75/80 complex	0.0658
Lymphoma	2944	Notch1-p56lck-PI3K complex	0.0658
Lymphoma	2955	LCK-SLP76-PLC-gamma-1-LAT complex pervanadate-activated	0.05698
Lymphoma	3634	NR3C2-UBC9-SRC-1 complex	0.0658
Lymphoma	5233	TNF-alpha/NF-kappa B signaling complex 5	0.02279
Lymphoma	5239	CAP(C)-CAP(E) complex	0.16116
Lymphoma	5386	MLL1-WDR5 complex	0.02193
Lymphoma	5548	IL-12 heterodimer complex	0.08058
Lymphoma	5691	TALL1 homo-oligomer complex	0.11396
Lymphoma	5770	RUNX1-CBF-beta-DNA complex	0.08058
Lymphoma	5818	BIM-BCL2 complex	0.08058
Lymphoma	5819	BIM-BCL2xL complex	0.08058
Lymphopenia	92	CD28-transactivation complex	0.26726
Lymphopenia	1707	IL2-IL2RA-IL2RB complex	0.21822
Lymphopenia	5473	FAS-FADD-CASP8 complex	0.21822
Lymphopenia	5749	MRIT complex	0.21822
Lymphopenia	5799	Death induced signaling complex DISC (FAS FADD CASP8 CFLAR) membrane-associated CD95L	0.18898
Lymphopenia	5800	Death-inducing signaling complex DISC (type I cells associated) stimulated	0.21822
Lymphopenia	5808	DISC complex	0.21822
Lymphopenia	5812	p53-BCL2 complex	0.26726
Lymphopenia	5819	BIM-BCL2xL complex	0.26726
Lymphopenia	5820	tBID-BCL2xL complex	0.26726
Lymphopenia	5859	FAS-FADD-CASP8-CASP10 complex	0.18898
Lymphopenia	5861	FAS-FADD-CASP10 complex	0.21822
Lymphoproliferative disorder	1	BCL6-HDAC4 complex	0.18257
Lymphoproliferative disorder	2	BCL6-HDAC5 complex	0.18257
Lymphoproliferative disorder	3	BCL6-HDAC7 complex	0.18257
Lymphoproliferative disorder	41	Mi-2/NuRD-MTA2 complex	0.11547
Lymphoproliferative disorder	585	Mi2/NuRD-BCL6-MTA3 complex	0.11547
Lymphoproliferative disorder	1508	BCL6-ZBTB17 complex	0.18257
Lymphoproliferative disorder	1615	G protein complex (BTK GNG1 GNG2)	0.14907
Lymphoproliferative disorder	2551	PDGFRA-PLC-gamma-1-PI3K-SHP-2 complex PDGF stimulated	0.1291
Lymphoproliferative disorder	3183	PDGFRA-SHP-2 complex PDGF stimulated	0.18257
Lymphoproliferative disorder	3186	GRB2-SHP-2 complex PDGF stimulated	0.18257
Lymphoproliferative disorder	5564	LMO4-gp130 complex	0.11547
Lysosomal storage disease	5830	DJ-1-SNCA complex high molecular weight complex	0.35355
Macular degeneration	1088	PRNP-ApolopoproteinE3 complex	0.14744
Macular degeneration	1714	TICAM1-TICAM2-TLR4 complex	0.12039
Macular degeneration	2972	ITGA9-ITGB1-VEGFA complex	0.12039

Macular degeneration	4062	NRP1-VEGFR2-VEGF(165) complex	0.12039
Macular degeneration	5696	VEGFA(165)-KDR-NRP1 complex	0.12039
Macular degeneration	5698	VEGFA(165)-VEGFR2-NRP1 complex	0.12039
Macular degeneration	5701	NRP1-VEGF(165/121) complex	0.14744
Malaria	552	IFNB1-IFNAR1-IFNAR2- complex	0.10721
Malaria	668	BKCA-beta2AR-AKAP79 signaling complex	0.10721
Malaria	672	BKCA-beta2AR complex	0.13131
Malaria	687	CFTR-NHERF-beta(2)AR signaling complex	0.10721
Malaria	1088	PRNP-ApolopoproteinE3 complex	0.13131
Malaria	2254	CTGF/Hcs24-actin complex	0.10721
Malaria	2456	MET-CIN85-SH3GL3-CBL complex HGF stimulated	0.09285
Malaria	2489	NCR3-CD247 complex	0.13131
Malaria	2541	HGF-Met complex	0.13131
Malaria	2972	ITGA9-ITGB1-VEGFA complex	0.10721
Malaria	3830	ADRB2 homodimer complex	0.1857
Malaria	4062	NRP1-VEGFR2-VEGF(165) complex	0.10721
Malaria	5696	VEGFA(165)-KDR-NRP1 complex	0.10721
Malaria	5698	VEGFA(165)-VEGFR2-NRP1 complex	0.10721
Malaria	5701	NRP1-VEGF(165/121) complex	0.13131
Malignant glioma	1056	ZNF198-SUMO1 complex	0.13131
Malignant glioma	1223	H2AX complex isolated from cells without IR exposure	0.0515
Malignant glioma	1226	H2AX complex I	0.07019
Malignant glioma	1227	H2AX complex II	0.05872
Malignant glioma	1514	IL4-IL4R complex	0.26261
Malignant glioma	1515	IL4-IL4R-IL2RG complex	0.21442
Malignant glioma	1519	IL6ST-PRKCD-STAT3 complex	0.10721
Malignant glioma	2383	ITGA5-ITGB1-FN1-TGM2 complex	0.09285
Malignant glioma	2384	ITGA5-ITGB1-ADAM15 complex	0.10721
Malignant glioma	2385	ITGA5-ITGB4 complex	0.13131
Malignant glioma	2388	Itga5-Itgb1-Fn1-Sfrp2 complex	0.09285
Malignant glioma	2850	ITGA5-ITGB1-FN-1-NOV complex	0.09285
Malignant glioma	2853	ITGA5-ITGB1-CAL4A3 complex	0.10721
Malignant glioma	2882	ITGA5-ITGB3-COL6A3 complex	0.10721
Malignant glioma	3112	ITGA5-ITGB1-SPP1 complex	0.10721
Malignant glioma	3229	Heterodimer complex (CDK9 IL6ST)	0.13131
Malignant glioma	5165	AP1G1-PACS1-FURIN complex	0.10721
Malignant glioma	5217	Calreticulin oligomer complex	0.1857
Malignant glioma	5260	TCF4-CTNNB1-SUMO1-EP300-HADAC6 complex	0.08305
Malignant glioma	5564	LMO4-gp130 complex	0.08305
Malignant glioma	5579	CNTF-CNTFR-gp130-LIFR complex	0.09285
Malignant glioma	5582	LIFR-LIF-gp130 complex	0.10721
Malignant glioma	5755	SUMO1-SUA1-UBA2 complex	0.10721
Malignant glioma	5830	DJ-1-SNCA complex high molecular weight complex	0.13131
Malignant glioma	5837	PPD complex	0.10721
Melanoma	120	Lymphotoxin beta receptor complex	0.09623
Melanoma	159	Condensin I-PARP-1-XRCC1 complex	0.0315
Melanoma	227	Ubiquitin E3 ligase (SKP1A BTRC CUL1)	0.04811

Melanoma	244	BRAFT complex	0.02311
Melanoma	245	FA core complex (Fanconi anemia core complex)	0.02778
Melanoma	246	BLM complex III	0.04167
Melanoma	475	STAGA complex (SPT3-TAF9-GCN5 acetyltransferase complex)	0.02406
Melanoma	476	STAGA complex (SPT3-TAF9-GCN5 acetyltransferase complex)	0.02311
Melanoma	552	IFNB1-IFNAR1-IFNAR2- complex	0.04811
Melanoma	725	P2X7 receptor signalling complex	0.02406
Melanoma	947	CDC7-DBF7 complex	0.05893
Melanoma	959	LLGL1-PAR-6B-PRKCI complex	0.04811
Melanoma	999	p23 protein complex	0.04811
Melanoma	1004	RC complex during S-phase of cell cycle	0.02311
Melanoma	1005	RC complex during G2/M-phase of cell cycle	0.02311
Melanoma	1041	Alpha-dystrobrevin-ZO-1-actin complex	0.04167
Melanoma	1067	CD8A-LCK complex	0.05893
Melanoma	1141	CF IIAm complex (Cleavage factor IIAm complex)	0.02083
Melanoma	1193	Rap1 complex	0.0315
Melanoma	1223	H2AX complex isolated from cells without IR exposure	0.02311
Melanoma	1226	H2AX complex I	0.06299
Melanoma	1227	H2AX complex II	0.02635
Melanoma	1306	PIN1-AUF1 complex	0.05893
Melanoma	1307	Multiprotein complex (mRNA turnover)	0.03727
Melanoma	1308	PABPC1-HSPA8-HNRPD-EIF4G1 complex	0.03727
Melanoma	1519	IL6ST-PRKCD-STAT3 complex	0.04811
Melanoma	1539	G protein complex (GNG2 GNB2L1 RAF1)	0.04811
Melanoma	1707	IL2-IL2RA-IL2RB complex	0.04811
Melanoma	1714	TICAM1-TICAM2-TLR4 complex	0.04811
Melanoma	1728	CTCF-nucleophosmin-PARP-HIS-KPNA-LMNA-TOP complex	0.02778
Melanoma	1729	TLE1 corepressor complex (MASH1 promoter-corepressor complex)	0.02635
Melanoma	1774	MICA-KLRK1-HCST complex	0.04811
Melanoma	1810	ITGA4-PXN-GIT1 complex	0.04811
Melanoma	1826	SMAD3-HEF1-APC10-CDH1 complex	0.04167
Melanoma	2016	IL12A homodimer complex	0.08333
Melanoma	2018	IL12A-IL12B complex	0.05893
Melanoma	2019	IL12A-IL12B-IL12RB1 complex	0.04811
Melanoma	2021	IL12A-IL12B-IL12RB2 complex	0.04811
Melanoma	2153	ITGAM-ITGB2-CD11 complex	0.09623
Melanoma	2187	Ubiquitin E3 ligase (NFKBIA FBXW11 BTRC CUL1 SKP1A)	0.03727
Melanoma	2188	Ubiquitin E3 ligase (CDC34 NEDD8 BTRC CUL1 SKP1A RBX1)	0.03727
Melanoma	2189	Ubiquitin E3 ligase (SMAD3 BTRC CUL1 SKP1A RBX1)	0.03727
Melanoma	2222	BLM complex II	0.03402
Melanoma	2417	ITGA4-ITGB1-EMILIN1 complex	0.04811
Melanoma	2418	ITGA4-ITGB1 complex	0.05893

Melanoma	2419	ITGA4-ITGB1-CD81 complex	0.04811
Melanoma	2420	ITGA4-ITGB1-CD53 complex	0.04811
Melanoma	2421	ITGA4-ITGB1-VCAM1 complex	0.04811
Melanoma	2422	ITGA4-ITGB1-JAM2 complex	0.04811
Melanoma	2423	ITGA4-ITGB1-CD47 complex	0.04811
Melanoma	2424	ITGA4-ITGB1-CD63 complex	0.04811
Melanoma	2425	ITGA4-ITGB1-PXN complex	0.04811
Melanoma	2426	ITGA4-ITGB1-THBS1 complex	0.04811
Melanoma	2428	ITGA4-ITGB1-THBS2 complex	0.04811
Melanoma	2429	ITGA2-ITGB1-CD47 complex	0.04811
Melanoma	2430	ITGA2-ITGB1-CHAD complex	0.04811
Melanoma	2431	ITGA2-ITGB1-COL6A3 complex	0.04811
Melanoma	2432	ITGA2-ITGB1 complex	0.05893
Melanoma	2537	PKC-alpha-PLD1-PLC-gamma-2 signaling complex lacritin stimulated	0.04811
Melanoma	2625	CDK8-MED6-PARP1 complex	0.04811
Melanoma	2709	MMP-9-TIMP-1-LRP complex	0.04811
Melanoma	2724	Ubiquitin E3 ligase (NFKBIA BTRC CUL1 SKP1A)	0.04167
Melanoma	2739	FA complex (Fanconi anemia complex)	0.02513
Melanoma	2851	ING2 complex	0.02406
Melanoma	2880	SCF subcomplex (WEE1 SKP2 BTRC)	0.04811
Melanoma	3044	SKI-NCOR1-SIN3A-HDAC1 complex	0.04167
Melanoma	3137	MASH1 promoter-coactivator complex	0.02513
Melanoma	3142	CAMK2-delta-MASH1 promoter-coactivator complex	0.02946
Melanoma	3162	TF-FVIIa-FXa-TFPI complex	0.08333
Melanoma	3197	SMAD4-SNO-SKI complex	0.09623
Melanoma	3198	SMAD2-SKI complex	0.05893
Melanoma	3199	SMAD3-SKI complex	0.05893
Melanoma	3200	SMAD4-SKI complex	0.05893
Melanoma	3204	SMAD2-SKI-NCOR complex	0.04811
Melanoma	3205	SMAD3-SKI-NCOR complex	0.04811
Melanoma	3206	SMAD4-SKI-NCOR complex	0.04811
Melanoma	3229	Heterodimer complex (CDK9 IL6ST)	0.05893
Melanoma	3634	NR3C2-UBC9-SRC-1 complex	0.04811
Melanoma	3729	SKI-SMAD2 hexameric complex	0.05893
Melanoma	3733	SKI-SMAD3 hexameric complex	0.05893
Melanoma	3739	SKI-SMAD2-SMAD4 pentameric complex	0.04811
Melanoma	3740	SKI-SMAD3-SMAD4 pentameric complex	0.04811
Melanoma	3959	SMAD3-SMAD4-cSKI TGF(beta)-dependent	0.04811
Melanoma	3961	SMAD3-cSKI-SIN3A-HDAC1 complex	0.04167
Melanoma	3972	SMURF2-SMAD3-SnoN complex TGF(beta)-	0.04811
Melanoma	4977	gp78-p97/VCP-DERL1 complex	0.04811
Melanoma	5772	ZO1-(beta)cadherin-(VE)cadherin-VEGFR2 complex	0.04167
Melanoma	5179	NCOA6-DNA-PK-Ku-PARP1 complex	0.03727
Melanoma	5196	TNF-alpha/NF-kappa B signaling complex (CHUK BTRC NFKB2 PPP6C REL CUL1 IKBKE SAPS2 SAPS1 ANKRD28 RELA SKP1)	0.02406
Melanoma	5199	Kinase maturation complex 1	0.02083

Melanoma	5217	Calreticulin oligomer complex	0.08333
Melanoma	5235	WRN-Ku70-Ku80-PARP1 complex	0.04167
Melanoma	5276	HIF1A-OS9-EGLN1 complex	0.04811
Melanoma	5277	HIF1A-OS9-EGLN3 complex	0.04811
Melanoma	5383	TRIB3-DDIT3 complex	0.05893
Melanoma	5530	Homodimeric complex LTBR	0.08333
Melanoma	5548	IL-12 heterodimer complex	0.05893
Melanoma	5564	LMO4-gp130 complex	0.1118
Melanoma	5579	CNTF-CNTFR-gp130-LIFR complex	0.04167
Melanoma	5582	LIFR-LIF-gp130 complex	0.04811
Melanoma	5660	PlexinC1-SEMA7A complex	0.05893
Melanoma	5822	MCL1-NOXA complex	0.05893
Melanoma	5823	MCL1-BAK1 complex	0.05893
Meningioma	277	RFC complex	0.11547
Meningioma	279	RFC complex (activator A 1 complex)	0.11547
Meningioma	433	BASC complex (BRCA1-associated genome surveillance complex)	0.07454
Meningioma	434	BASC (Ab 80) complex (BRCA1-associated genome surveillance complex)	0.09129
Meningioma	435	BASC (Ab 81) complex (BRCA1-associated genome surveillance complex)	0.10541
Meningioma	1003	RC complex (Replication competent complex)	0.08607
Meningioma	1004	RC complex during S-phase of cell cycle	0.07161
Meningioma	1005	RC complex during G2/M-phase of cell cycle	0.07161
Meningioma	1098	DNA synthesome complex (13 subunits)	0.06901
Meningioma	1099	DNA synthesome complex (17 subunits)	0.06086
Meningioma	1107	DNA synthesome core complex	0.08165
Meningioma	1108	DNA synthesome complex (15 subunits)	0.06667
Meningioma	2203	BRD4-RFC complex	0.10541
Meningioma	2318	ITGA6-ITGB4-Laminin10/12 complex	0.11547
Meningioma	2709	MMP-9-TIMP-1-LRP complex	0.14907
Meningioma	3162	TF-FVIIa-FXa-TFPI complex	0.1291
Mental retardation	63	Mitotic 14S cohesin 1 complex	0.06455
Mental retardation	64	Mitotic 14S cohesin 2 complex	0.06455
Mental retardation	66	TRAP complex	0.04564
Mental retardation	100	hNURF complex	0.06455
Mental retardation	163	Cohesin-SA2 complex	0.06455
Mental retardation	164	Cohesin-SA1 complex	0.06455
Mental retardation	220	ARF-Mule complex	0.07454
Mental retardation	230	Mediator complex	0.02282
Mental retardation	232	ARC complex	0.03333
Mental retardation	282	SNF2h-cohesin-NuRD complex	0.03227
Mental retardation	287	ARC-L complex	0.0345
Mental retardation	288	ARC complex	0.03333
Mental retardation	301	SMCC complex	0.03131
Mental retardation	302	INO80 chromatin remodeling complex	0.03581
Mental retardation	441	TFTC-type histone acetyl transferase complex	0.03892
Mental retardation	535	TRAP complex	0.03227

Mental retardation	547	SMCC complex	0.0345
Mental retardation	548	DRIP complex	0.0345
Mental retardation	909	ARC92-Mediator complex	0.03581
Mental retardation	917	CERF complex (CECR2-containing remodeling factor complex)	0.09129
Mental retardation	923	SNF2L-RSF1 complex	0.09129
Mental retardation	1057	DIPA-MCRS1 complex	0.09129
Mental retardation	1232	REST-CoREST-mSIN3A complex	0.07454
Mental retardation	1335	SNW1 complex	0.03043
Mental retardation	1617	G protein complex (CACNA1A GNB1 GNG2)	0.07454
Mental retardation	1856	CDCA5-PDS5A-RAD21-SMC1A-PDS5B-SMC3	0.0527
Mental retardation	2319	ITGA6-ITGB4-Laminin10/12 complex	0.05774
Mental retardation	2600	BRD4 complex	0.04564
Mental retardation	2839	ATRX-DAXX complex	0.09129
Mental retardation	3011	APC-IQGAP1-Rac1 complex	0.07454
Mental retardation	3055	Nop56p-associated pre-rRNA complex	0.02532
Mental retardation	5241	SMC1-SMC3 complex	0.09129
Mental retardation	5342	ELMO1-DOCK1-RAC1 complex	0.07454
Mental retardation	5386	MLL1-WDR5 complex	0.02485
Mental retardation	5432	Sororin-cohesin complex	0.0527
Mesothelioma	819	20S methylosome-SmD complex	0.20412
Mesothelioma	825	JBP1-pICln complex	0.2357
Mesothelioma	832	Anti-Sm protein complex	0.1543
Mesothelioma	833	6S methyltransferase complex	0.2357
Mesothelioma	834	20S methylosome and RG-containing Sm protein complex	0.16667
Mesothelioma	835	6S methyltransferase and RG-containing Sm proteins complex	0.14434
Mesothelioma	836	20S methyltransferase core complex	0.28868
Mesothelioma	837	20S methyltransferase complex	0.2357
Mesothelioma	2247	Dynactin complex (DCTN1 DCTN2 DCTN3 DCTN4 DCTN6 CAPZA1 CAPZB ACTR1A)	0.14434
Mesothelioma	2429	ITGA2-ITGB1-CD47 complex	0.2357
Mesothelioma	2430	ITGA2-ITGB1-CHAD complex	0.2357
Mesothelioma	2431	ITGA2-ITGB1-COL6A3 complex	0.2357
Mesothelioma	2432	ITGA2-ITGB1 complex	0.28868
Mesothelioma	2838	AR coactivator complex	0.2357
Metabolic syndrome X	541	IGF1-IGFBP3-ALS complex	0.20412
Metabolism disease	298	VEGF transcriptional complex	0.06537
Metabolism disease	531	XPA-ERCC1-ERCC4 complex	0.09245
Metabolism disease	668	BKCA-beta2AR-AKAP79 signaling complex	0.09245
Metabolism disease	672	BKCA-beta2AR complex	0.11323
Metabolism disease	687	CFTR-NHERF-beta(2)AR signaling complex	0.09245
Metabolism disease	1028	HNF4A-SUB1 complex	0.11323
Metabolism disease	1728	CTCF-nucleophosmin-PARP-HIS-KPNA-LMNA-TOP complex	0.05338
Metabolism disease	2589	PGC-1-SRp40-SRp55-SRp75 complex	0.08006
Metabolism disease	2599	POLR2A-CCNT1-CDK9-NCL-LEM6-CPSF2 complex	0.06537

Metabolism disease	2709	MMP-9-TIMP-1-LRP complex	0.09245
Metabolism disease	3138	POSH-AKT2 complex	0.11323
Metabolism disease	3830	ADRB2 homodimer complex	0.16013
Metabolism disease	3848	TCL1(trimer)-AKT2 complex	0.11323
Metabolism disease	5273	VHL-TBP1-HIF1A complex	0.09245
Metabolism disease	5276	HIF1A-OS9-EGLN1 complex	0.09245
Metabolism disease	5277	HIF1A-OS9-EGLN3 complex	0.09245
Metabolism disease	5382	ARNT-HIF1A complex	0.11323
Metabolism disease	5608	Emerin architectural complex	0.06537
Metabolism disease	5611	Emerin complex 24	0.04134
Metaplastic polyp	244	BRAFT complex	0.09245
Metaplastic polyp	246	BLM complex III	0.16667
Metaplastic polyp	285	PCNA-MLH1-PMS1 complex	0.19245
Metaplastic polyp	290	MSH2-MLH1-PMS2-PCNA DNA-repair initiation complex	0.16667
Metaplastic polyp	291	MSH2-MLH1-PMS2 DNA-repair initiation complex	0.19245
Metaplastic polyp	292	MutL-alpha complex	0.2357
Metaplastic polyp	310	Cell cycle kinase complex CDC2	0.13608
Metaplastic polyp	311	Cell cycle kinase complex CDK2	0.16667
Metaplastic polyp	312	Cell cycle kinase complex CDK4	0.16667
Metaplastic polyp	313	Cell cycle kinase complex CDK5	0.14907
Metaplastic polyp	314	PCNA-p21 complex	0.2357
Metaplastic polyp	369	MSH2-MSH6-PMS2-MLH1 complex	0.16667
Metaplastic polyp	370	MSH2-MSH6-PMS1-MLH1 complex	0.16667
Metaplastic polyp	376	PCNA-MutS-alpha-MutL-alpha-DNA complex	0.14907
Metaplastic polyp	380	MutL-beta complex	0.2357
Metaplastic polyp	415	EXO1-MLH1-PMS2 complex	0.19245
Metaplastic polyp	424	EXO1-MLH1-PCNA complex	0.19245
Metaplastic polyp	433	BASC complex (BRCA1-associated genome surveillance complex)	0.09623
Metaplastic polyp	434	BASC (Ab 80) complex (BRCA1-associated genome surveillance complex)	0.11785
Metaplastic polyp	435	BASC (Ab 81) complex (BRCA1-associated genome surveillance complex)	0.13608
Metaplastic polyp	1439	PTGS2 homodimer complex	0.33333
Metaplastic polyp	1634	CyclinD1-CDK4-p21 complex	0.19245
Metaplastic polyp	2230	PCNA complex	0.12599
Metaplastic polyp	2817	BRCA1-BARD1-BACH1-DNA damage complex I	0.13608
Metaplastic polyp	5101	CyclinD3-CDK4-CDK6-p21 complex	0.16667
Metaplastic polyp	5622	HSP90-CIP1-FKBPL complex	0.19245
Metaplastic polyp	5877	MAP2K1-BRAF-RAF1-YWHAE-KSR1 complex	0.14907
Metaplastic polyp	5872	BRAF-MAP2K1-MAP2K2-YWHAE complex	0.16667
Metaplastic polyp	5919	BRAF-RAF1-14-3-3 complex	0.11111
Metaplastic polyp	5921	KSR1-BRAF-MEK complex	0.16667
Metaplastic polyp	5923	RAF1-BRAF complex RAS stimulated	0.2357
Metaplastic polyp	5925	BRAF-CNK1 complex not RAS stimulated	0.2357
Metastasis to lymph nodes	959	LLGL1-PAR-6B-PRKCI complex	0.11323
Metastasis to lymph nodes	1094	Frataxin complex	0.07412

Metastasis to lymph nodes	2254	CTGF/Hcs24-actin complex	0.11323
Metastasis to lymph nodes	2709	MMP-9-TIMP-1-LRP complex	0.11323
Metastasis to lymph nodes	2755	17S U2 snRNP	0.03414
Metastasis to lymph nodes	5389	SERPINA3-CTSG complex	0.13868
Metastasis to lymph nodes	5564	LMO4-gp130 complex	0.08771
Metastasis to lymph nodes	5613	Emerin complex 25	0.04903
Migraine	120	Lymphotoxin beta receptor complex	0.10721
Migraine	426	Meprin A	0.13131
Migraine	441	TFTC-type histone acetyl transferase complex	0.05599
Migraine	725	P2X7 receptor signalling complex	0.05361
Migraine	1054	ESR1-RELA-BCL3-NCOA3 complex	0.09285
Migraine	1093	SNX complex (SNX1a SNX2 SNX4 INSR)	0.09285
Migraine	1300	CRLR-RAMP1 complex	0.13131
Migraine	1617	G protein complex (CACNA1A GNB1 GNG2)	0.10721
Migraine	2124	IKK-alpha--ER-alpha-AIB1 complex	0.10721
Migraine	2153	ITGAM-ITGB2-CD11 complex	0.10721
Migraine	2159	AR-AKT-APPL complex	0.10721
Migraine	2160	AOF2-AR complex	0.13131
Migraine	2470	p130Cas-ER-alpha-cSrc-kinase- PI3-kinase p85-subunit complex	0.09285
Migraine	2577	Sam68-p85 P13K-IRS-1-IR signaling complex	0.09285
Migraine	2657	ESR1-CDK7-CCNH-MNAT1-MTA1-HDAC2 complex	0.07581
Migraine	2670	Er-alpha-p53-hdm2 complex	0.10721
Migraine	2699	ER-alpha-GRIP1-c-Jun complex	0.10721
Migraine	2700	ER-alpha-c-Jun complex	0.13131
Migraine	3139	CRLR-RAMP1-ARRB2 complex	0.10721
Migraine	5414	HTR1A-HTR1D complex	0.13131
Migraine	5416	HTR1A-HTR1B complex	0.13131
Migraine	5418	GABBR2-HTR1A complex	0.13131
Migraine	5419	HTR1A-GPR26 complex	0.13131
Migraine	5420	HTR1A-EDG3 complex	0.13131
Migraine	5421	HTR1A homodimer complex	0.1857
Migraine	5422	HTR1A-EDG1 complex	0.13131
Migraine	5862	CAV1-VDAC1-ESR1 complex	0.10721
Mitochondrial encephalomyopathies	2936	Ecsit complex (ECSIT MT-CO2 GAPDH TRAF6 NDUFAF1)	0.22361
Mitochondrial encephalomyopathies	2939	Ecsit complex (ECSIT MT-CO2 NDUFA1 MT-ND1 TRAF6 NDUFAF1)	0.20412
Mitral valve disease	1707	IL2-IL2RA-IL2RB complex	0.2582
Movement disorder	668	BKCA-beta2AR-AKAP79 signaling complex	0.12039
Movement disorder	672	BKCA-beta2AR complex	0.14744
Movement disorder	749	MeCP2-SIN3A-HDAC complex	0.10426
Movement disorder	753	UTM-SGCE-DAG1-CAV1-NOS3 complex	0.09325
Movement disorder	1000	TorsinA-TorsinB complex	0.14744
Movement disorder	5158	SMARCA2/BRM-BAF57-MECP2 complex	0.12039
Movement disorder	5184	SWI/SNF chromatin-remodeling complex	0.09325
Movement disorder	5209	Ubiquilin-proteasome complex	0.08513
Movement disorder	5423	HSP70-BAG5-PARK2 complex	0.10426

Movement disorder	5830	DJ-1-SNCA complex high molecular weight complex	0.14744
Movement disorder	5837	PPD complex	0.12039
Moyamoya disease	298	VEGF transcriptional complex	0.16667
Moyamoya disease	1986	Endoglin homodimer complex	0.40825
Moyamoya disease	5273	VHL-TBP1-HIF1A complex	0.2357
Moyamoya disease	5276	HIF1A-OS9-EGLN1 complex	0.2357
Moyamoya disease	5277	HIF1A-OS9-EGLN3 complex	0.2357
Moyamoya disease	5382	ARNT-HIF1A complex	0.28868
Mucocutaneous lymph node syndrome	1514	IL4-IL4R complex	0.1543
Mucocutaneous lymph node syndrome	1515	IL4-IL4R-IL2RG complex	0.12599
Mucocutaneous lymph node syndrome	2709	MMP-9-TIMP-1-LRP complex	0.25198
Mucocutaneous lymph node syndrome	2972	ITGA9-ITGB1-VEGFA complex	0.12599
Mucocutaneous lymph node syndrome	4062	NRP1-VEGFR2-VEGF(165) complex	0.25198
Mucocutaneous lymph node syndrome	5772	ZO1-(beta)cadherin-(VE)cadherin-VEGFR2 complex	0.10911
Mucocutaneous lymph node syndrome	5696	VEGFA(165)-KDR-NRP1 complex	0.25198
Mucocutaneous lymph node syndrome	5698	VEGFA(165)-VEGFR2-NRP1 complex	0.25198
Mucocutaneous lymph node syndrome	5701	NRP1-VEGF(165/121) complex	0.1543
Mucopolysaccharidosis	4	Multisubunit ACTR coactivator complex	0.28868
Mucopolysaccharidosis	305	40S ribosomal subunit cytoplasmic	0.09901
Mucopolysaccharidosis	306	Ribosome cytoplasmic	0.06415
Mucopolysaccharidosis	570	p300-CBP-p270-SWI/SNF complex	0.21822
Mucopolysaccharidosis	571	p300-CBP-p270 complex	0.33333
Mucopolysaccharidosis	1379	GALNS-lysosomal hydrolase 1.27 MDa complex	0.28868
Mucopolysaccharidosis	2638	HES1 promoter corepressor complex	0.2357
Mucopolysaccharidosis	2641	p300/CBP-PCAF-MyoD complex	0.28868
Mucopolysaccharidosis	2727	SRC-3 complex	0.21822
Mucopolysaccharidosis	2728	SRC-1 complex	0.28868
Mucopolysaccharidosis	2829	RSmad complex	0.18257
Mucopolysaccharidosis	2958	SMAD1-CBP complex	0.40825
Mucopolysaccharidosis	3061	RNA polymerase II complex (CBP PCAF RPB1 BAF47 CYCC CDK8) chromatin structure modifying	0.2357
Mucopolysaccharidosis	3062	RNA polymerase II complex incomplete (CBP RPBI PCAF BAF47) chromatin structure modifying	0.28868
Mucopolysaccharidosis	3066	RNA polymerase II complex chromatin structure modifying	0.16013
Mucopolysaccharidosis	3137	MASH1 promoter-coactivator complex	0.17408
Mucopolysaccharidosis	3142	CAMK2-delta-MASH1 promoter-coactivator complex	0.20412
Mucopolysaccharidosis	3749	CREBBP-SMAD2 hexameric complex	0.40825
Mucopolysaccharidosis	3750	CREBBP-SMAD3 hexameric complex	0.40825
Mucopolysaccharidosis	3753	CREBBP-SMAD2-SMAD4 pentameric complex	0.33333
Mucopolysaccharidosis	3754	CREBBP-SMAD3-SMAD4 pentameric complex	0.33333

Mucopolysaccharidosis	5198	CBP-RARA-RXRA-DNA complex ligand stimulated	0.33333
Mucopolysaccharidosis	5264	TCF4-CTNNB1-CREBBP complex	0.33333
Mucopolysaccharidosis	5573	Stat1-alpha-dimer-CBP DNA-protein complex	0.40825
Multiple endocrine neoplasia	244	BRAFT complex	0.09245
Multiple endocrine neoplasia	903	RET-Rai complex	0.2357
Multiple endocrine neoplasia	1004	RC complex during S-phase of cell cycle	0.09245
Multiple endocrine neoplasia	1005	RC complex during G2/M-phase of cell cycle	0.09245
Multiple endocrine neoplasia	1254	Menin-associated histone methyltransferase complex	0.12599
Multiple endocrine neoplasia	1256	MLL-HCF complex	0.12599
Multiple endocrine neoplasia	1656	p27-cyclinE-CDK2 complex	0.19245
Multiple endocrine neoplasia	2222	BLM complex II	0.13608
Multiple endocrine neoplasia	3015	p27-cyclinE-Cdk2 - Ubiquitin E3 ligase (SKP1A SKP2 CUL1 CKS1B RBX1) complex	0.11785
Multiple endocrine neoplasia	5229	RPA complex	0.19245
Multiple endocrine neoplasia	5231	53BP1-containing complex	0.14907
Multiple myeloma	92	CD28-transactivation complex	0.09285
Multiple myeloma	120	Lymphotoxin beta receptor complex	0.07581
Multiple myeloma	206	DNA ligase IV-XRCC4 complex	0.1857
Multiple myeloma	213	DNA ligase IV-XRCC1 complex	0.09285
Multiple myeloma	336	DNA ligase IV-XRCC4-AHNK complex	0.15162
Multiple myeloma	344	DNA ligase IV-XRCC4 complex (LX complex)	0.1857
Multiple myeloma	350	DNA ligase IV-XRCC4-PNK complex	0.15162
Multiple myeloma	353	DNA ligase IV-condensin complex	0.07581
Multiple myeloma	359	DNA ligase IV-XRCC4-XLF complex	0.15162
Multiple myeloma	552	IFNB1-IFNAR1-IFNAR2- complex	0.15162
Multiple myeloma	1189	DNA double-strand break end-joining complex	0.09926
Multiple myeloma	1519	IL6ST-PRKCD-STAT3 complex	0.07581
Multiple myeloma	1703	IGHM-VPREB1-IGLL1 complex	0.07581
Multiple myeloma	1774	MICA-KLRK1-HCST complex	0.07581
Multiple myeloma	2020	IL12B-IL12RB1-IL12RB2 complex	0.07581
Multiple myeloma	2021	IL12A-IL12B-IL12RB2 complex	0.07581
Multiple myeloma	2026	IL12RB1-IL12RB2 complex	0.09285
Multiple myeloma	2028	JAK2-IL12RB2 complex	0.09285
Multiple myeloma	2489	NCR3-CD247 complex	0.09285
Multiple myeloma	2536	PLC-gamma-2-SLP-76-Lyn-Grb2 complex	0.06565
Multiple myeloma	2563	FGFR2-c-Cbl-Lyn-Fyn complex	0.06565
Multiple myeloma	2564	p21(ras)GAP-Fyn-Lyn-Yes complex thrombin	0.06565
Multiple myeloma	2565	CD20-LCK-LYN-FYN-p75/80 complex (Raji human B cell line)	0.06565
Multiple myeloma	2709	MMP-9-TIMP-1-LRP complex	0.07581
Multiple myeloma	2910	PLC-gamma-2-Lyn-FcR-gamma complex	0.07581
Multiple myeloma	3229	Heterodimer complex (CDK9 IL6ST)	0.09285
Multiple myeloma	5210	TANK-TRAF2-TRAF3 complex	0.07581
Multiple myeloma	5564	LMO4-gp130 complex	0.05872
Multiple myeloma	5579	CNTF-CNTFR-gp130-LIFR complex	0.06565
Multiple myeloma	5582	LIFR-LIF-gp130 complex	0.07581
Multiple myeloma	5822	MCL1-NOXA complex	0.09285
Multiple sclerosis	92	CD28-transactivation complex	0.07293

Multiple sclerosis	178	Respiratory chain complex I (holoenzyme)	0.01555
Multiple sclerosis	563	F1F0-ATP synthase (EC 3.6.3.14) mitochondrial	0.05157
Multiple sclerosis	1054	ESR1-RELA-BCL3-NCOA3 complex	0.05157
Multiple sclerosis	1067	CD8A-LCK complex	0.07293
Multiple sclerosis	1091	SNX complex (SNX1a SNX2 SNX4 LEPR)	0.05157
Multiple sclerosis	1707	IL2-IL2RA-IL2RB complex	0.05955
Multiple sclerosis	1714	TICAM1-TICAM2-TLR4 complex	0.05955
Multiple sclerosis	1772	MICB-KLRK1-HCST complex	0.05955
Multiple sclerosis	1787	Nogo-potassium channel complex	0.05157
Multiple sclerosis	1992	LEPR homodimer complex	0.10314
Multiple sclerosis	1993	SLC1A2 homotrimer complex	0.10314
Multiple sclerosis	2018	IL12A-IL12B complex	0.07293
Multiple sclerosis	2019	IL12A-IL12B-IL12RB1 complex	0.05955
Multiple sclerosis	2020	IL12B-IL12RB1-IL12RB2 complex	0.05955
Multiple sclerosis	2021	IL12A-IL12B-IL12RB2 complex	0.05955
Multiple sclerosis	2084	NFKB1-NFKB2-REL-RELA-RELB complex	0.04613
Multiple sclerosis	2086	NFKB1-NFKB2-RELA-RELB complex	0.05157
Multiple sclerosis	2105	IkappaB kinase complex (IKBKB CHUK IKBKAP NFKBIA RELA MAP3K14)	0.04211
Multiple sclerosis	2347	ITGAV-ITGB5-SPP1 complex	0.05955
Multiple sclerosis	2352	ITGAV-ITGB6-SPP1 complex	0.05955
Multiple sclerosis	2358	ITGAV-ITGB3-SPP1 complex	0.05955
Multiple sclerosis	2434	ITGA1-ITGB1-COL6A3 complex	0.05955
Multiple sclerosis	2435	ITGA1-ITGB1-PTPN2 complex	0.05955
Multiple sclerosis	2709	MMP-9-TIMP-1-LRP complex	0.05955
Multiple sclerosis	2710	LRP-1-Alpha-2-M-annexin VI complex	0.05955
Multiple sclerosis	2885	ITGAV-ITGB1-SPP1 complex	0.05955
Multiple sclerosis	2914	Respiratory chain complex I (beta subunit)	0.02579
Multiple sclerosis	3045	hs4 enhancer complex (faster migrating complex)	0.04613
Multiple sclerosis	3111	ITGA9-ITGB1-SPP1 complex	0.05955
Multiple sclerosis	3112	ITGA5-ITGB1-SPP1 complex	0.05955
Multiple sclerosis	5193	TNF-alpha/NF-kappa B signaling complex (CHUK KPNA3 NFKB2 NFKBIB REL IKBKG NFKB1 NFKBIE RELB NFKBIA RELA TNIP2)	0.02977
Multiple sclerosis	5194	TNF-alpha/NF-kappa B signaling complex (SEC16A CHUK IKBKB NFKB2 REL IKBKG MAP3K14 RELA FBXW7 USP2)	0.03262
Multiple sclerosis	5196	TNF-alpha/NF-kappa B signaling complex (CHUK BTRC NFKB2 PPP6C REL CUL1 IKBKE SAPS2 SAPS1 ANKRD28 RELA SKP1)	0.02977
Multiple sclerosis	5220	CHUK-IQGAP2-AKAP8L-RELA-TNIP2 complex	0.04613
Multiple sclerosis	5228	REL-MAP3K8-RELA-TNIP2-PAPOLA complex	0.04613
Multiple sclerosis	5230	CHUK-NFKB2-REL-IKBKG-SPAG9-NFKB1-NFKBIE-COPB2-TNIP1-NFKBIA-RELA-TNIP2	0.02977
Multiple sclerosis	5232	TNF-alpha/Nf-kappa B signaling complex (RPL6 RPL30 RPS13 CHUK DDX3X NFKB2 NFKBIB REL IKBKG NFKB1 MAP3K8 RELB GLG1 NFKBIA RELA TNIP2 GTF2I)	0.02502
Multiple sclerosis	5233	TNF-alpha/NF-kappa B signaling complex 5	0.02063

Multiple sclerosis	5317	LATS1-HTRA2-BIRC4 complex	0.05955
Multiple sclerosis	5460	p50-p65 NF(kappa)B complex	0.07293
Multiple sclerosis	5461	p50-p65 NF(kappa)B-SRC1 complex	0.05955
Multiple sclerosis	5464	I(kappa)B(alpha)-NF(kappa)Bp50-NF(kappa)Bp65 complex	0.05955
Multiple sclerosis	5465	IKB(epsilon)-RELA-cREL complex	0.05955
Multiple sclerosis	5466	IKB(beta)-RELA-cREL complex	0.05955
Multiple sclerosis	5467	IKB(alpha)-RELA-cREL complex	0.05955
Multiple sclerosis	5475	MURR1-NF(kappa)Bp65-IKBA complex	0.05955
Multiple sclerosis	5492	IKBA-NF(kappa)Bp65-NF(kappa)Bp50 complex	0.05955
Multiple sclerosis	5529	TRAF2-cIAP1/BIRC2 complex	0.07293
Multiple sclerosis	5531	Tumor necrosis factor receptor 1 signaling complex	0.05157
Multiple sclerosis	5548	IL-12 heterodimer complex	0.07293
Multiple sclerosis	5549	IL-12 subunit p40 homodimer complex	0.10314
Multiple sclerosis	5749	MRIT complex	0.05955
Multiple sclerosis	5812	p53-BCL2 complex	0.07293
Multiple sclerosis	5819	BIM-BCL2xL complex	0.07293
Multiple sclerosis	5820	tBID-BCL2xL complex	0.07293
Muscular atrophy	1728	CTCF-nucleophosmin-PARP-HIS-KPNA-LMNA-TOP complex	0.09245
Muscular atrophy	2159	AR-AKT-APPL complex	0.16013
Muscular atrophy	2160	AOF2-AR complex	0.19612
Muscular atrophy	2453	Multiprotein complex (monoubiquitination)	0.13868
Muscular atrophy	2590	FOXO1-FHL2-SIRT1 complex	0.16013
Muscular atrophy	2761	SMAD3-SMAD4-FOXO1 complex	0.16013
Muscular atrophy	2945	RBP-Jkappa-RING1-KyoT2 complex	0.16013
Muscular atrophy	5171	SH3KBP1-CBLB-EGFR complex	0.16013
Muscular atrophy	5604	Emerin complex 1	0.09806
Muscular atrophy	5606	Emerin-actin-NMI-(alphaII)spectrin complex	0.13868
Muscular atrophy	5607	Emerin-actin-NMI complex	0.16013
Muscular atrophy	5608	Emerin architectural complex	0.22646
Muscular atrophy	5609	Emerin regulatory complex	0.09245
Muscular atrophy	5611	Emerin complex 24	0.14322
Muscular atrophy	5613	Emerin complex 25	0.06934
Muscular atrophy	5614	Emerin complex 32	0.05913
Muscular atrophy	5615	Emerin complex 52	0.05783
Muscular dystrophies	63	Mitotic 14S cohesin 1 complex	0.09806
Muscular dystrophies	64	Mitotic 14S cohesin 2 complex	0.09806
Muscular dystrophies	163	Cohesin-SA2 complex	0.09806
Muscular dystrophies	164	Cohesin-SA1 complex	0.09806
Muscular dystrophies	282	SNF2h-cohesin-NuRD complex	0.04903
Muscular dystrophies	422	Beta-dystroglycan-caveolin-3 complex	0.13868
Muscular dystrophies	824	Anti-SMN protein complex	0.08771
Muscular dystrophies	832	Anti-Sm protein complex	0.07412
Muscular dystrophies	999	p23 protein complex	0.11323
Muscular dystrophies	1093	SNX complex (SNX1a SNX2 SNX4 INSR)	0.09806
Muscular dystrophies	1142	SMN complex	0.06202
Muscular dystrophies	1143	SMN complex	0.04903

Muscular dystrophies	1181	C complex spliceosome	0.04385
Muscular dystrophies	1728	CTCF-nucleophosmin-PARP-HIS-KPNA-LMNA-TOP complex	0.06537
Muscular dystrophies	1746	SMN containing complex	0.06934
Muscular dystrophies	1751	SMN complex	0.06202
Muscular dystrophies	1752	SMN complex	0.07412
Muscular dystrophies	1856	CDCA5-PDS5A-RAD21-SMC1A-PDS5B-SMC3	0.08006
Muscular dystrophies	2254	CTGF/Hcs24-actin complex	0.11323
Muscular dystrophies	2395	ITGA7-ITGB1-CD151 complex	0.11323
Muscular dystrophies	2396	ITGA7-ITGB1-CD9 complex	0.11323
Muscular dystrophies	2397	ITGA7-ITGB1-ITGB1BP3 complex	0.11323
Muscular dystrophies	2577	Sam68-p85 P13K-IRS-1-IR signaling complex	0.09806
Muscular dystrophies	3055	Nop56p-associated pre-rRNA complex	0.01923
Muscular dystrophies	3098	TIM50a-SMN1 complex	0.13868
Muscular dystrophies	3118	SMN1-SIP1-SNRP complex	0.07412
Muscular dystrophies	3284	SMN complex (GEMIN5 2 3 4 SMN)	0.08771
Muscular dystrophies	3298	SMN complex (GEMIN2 5 SMN)	0.11323
Muscular dystrophies	5241	SMC1-SMC3 complex	0.13868
Muscular dystrophies	5432	Sororin-cohesin complex	0.08006
Muscular dystrophies	5604	Emerin complex 1	0.06934
Muscular dystrophies	5606	Emerin-actin-NMI-(alphaII)spectrin complex	0.09806
Muscular dystrophies	5607	Emerin-actin-NMI complex	0.11323
Muscular dystrophies	5608	Emerin architectural complex	0.16013
Muscular dystrophies	5609	Emerin regulatory complex	0.06537
Muscular dystrophies	5611	Emerin complex 24	0.10127
Muscular dystrophies	5613	Emerin complex 25	0.04903
Muscular dystrophies	5614	Emerin complex 32	0.04181
Muscular dystrophies	5615	Emerin complex 52	0.04089
Muscular dystrophy	343	Sarcoglycan-sarcospan complex SG-SPN	0.24807
Muscular dystrophy	2709	MMP-9-TIMP-1-LRP complex	0.16013
Muscular dystrophy	2798	MMP-2-claudin-1 complex	0.19612
Muscular dystrophy	5749	MRIT complex	0.16013
Muscular dystrophy	5798	Death induced signaling complex II (FADD CASP8 CFLAR) cytosolic CD95L induced	0.16013
Muscular dystrophy	5799	Death induced signaling complex DISC (FAS FADD CASP8 CFLAR) membrane-associated CD95L	0.13868
Myasthenia Gravis	441	TFTC-type histone acetyl transferase complex	0.1066
Myasthenia Gravis	1054	ESR1-RELA-BCL3-NCOA3 complex	0.17678
Myasthenia Gravis	2018	IL12A-IL12B complex	0.25
Myasthenia Gravis	2019	IL12A-IL12B-IL12RB1 complex	0.20412
Myasthenia Gravis	2020	IL12B-IL12RB1-IL12RB2 complex	0.20412
Myasthenia Gravis	2021	IL12A-IL12B-IL12RB2 complex	0.20412
Myasthenia Gravis	2124	IKK-alpha--ER-alpha-AIB1 complex	0.20412
Myasthenia Gravis	2470	p130Cas-ER-alpha-cSrc-kinase- PI3-kinase p85-subunit complex	0.17678
Myasthenia Gravis	2657	ESR1-CDK7-CCNH-MNAT1-MTA1-HDAC2 complex	0.14434
Myasthenia Gravis	2670	Er-alpha-p53-hdm2 complex	0.20412
Myasthenia Gravis	2699	ER-alpha-GRIP1-c-Jun complex	0.20412

Myasthenia Gravis	2700	ER-alpha-c-Jun complex	0.25
Myasthenia Gravis	5548	IL-12 heterodimer complex	0.25
Myasthenia Gravis	5549	IL-12 subunit p40 homodimer complex	0.35355
Myasthenia Gravis	5862	CAV1-VDAC1-ESR1 complex	0.20412
Mycoses	1088	PRNP-ApolopoproteinE3 complex	0.31623
Mycoses	1714	TICAM1-TICAM2-TLR4 complex	0.2582
Mycosis fungoides	1714	TICAM1-TICAM2-TLR4 complex	0.1291
Myelofibrosis	1	BCL6-HDAC4 complex	0.2132
Myelofibrosis	55	HDAC4-ERK1 complex	0.2132
Myelofibrosis	57	HDAC4-ERK2 complex	0.2132
Myelofibrosis	1620	G protein complex (HDAC4 GNB1 GNG2)	0.17408
Myelofibrosis	4977	gp78-p97/VCP-DERL1 complex	0.17408
Myelofibrosis	5268	TNF-alpha/NF-kappa B signaling complex 7	0.1066
Myelofibrosis	5269	TNF-alpha/NF-kappa B signaling complex 8	0.12309
Myelofibrosis	5285	TNF-alpha/NF-kappa B signaling complex 9	0.13484
Myeloproliferative disease	4	Multisubunit ACTR coactivator complex	0.13363
Myeloproliferative disease	98	p300-MDM2-p53 protein complex	0.1543
Myeloproliferative disease	570	p300-CBP-p270-SWI/SNF complex	0.10102
Myeloproliferative disease	571	p300-CBP-p270 complex	0.1543
Myeloproliferative disease	1158	p33ING1b-p300 complex	0.18898
Myeloproliferative disease	1160	ING1-p300-PCNA complex	0.1543
Myeloproliferative disease	1471	pRb2/p130-multimolecular complex (RB2 E2F5 HDAC1 SUV39H1 P300)	0.11952
Myeloproliferative disease	1521	p300-SMAD1-STAT3 complex	0.1543
Myeloproliferative disease	1831	PIAS3-SMAD3-P300 complex	0.1543
Myeloproliferative disease	2028	JAK2-IL12RB2 complex	0.18898
Myeloproliferative disease	2551	PDGFRA-PLC-gamma-1-PI3K-SHP-2 complex PDGF stimulated	0.13363
Myeloproliferative disease	2638	HES1 promoter corepressor complex	0.10911
Myeloproliferative disease	2639	HES1 promoter-Notch enhancer complex	0.07412
Myeloproliferative disease	2641	p300/CBP-PCAF-MyoD complex	0.13363
Myeloproliferative disease	2642	SMAD1-P300 complex	0.18898
Myeloproliferative disease	2954	Smad1-Notch1-p300-Pcaf complex	0.13363
Myeloproliferative disease	3183	PDGFRA-SHP-2 complex PDGF stimulated	0.18898
Myeloproliferative disease	3186	GRB2-SHP-2 complex PDGF stimulated	0.18898
Myeloproliferative disease	5118	pRb2/p130-multimolecular complex (RB2 E2F4 HDAC1 SUV39H1 P300)	0.11952
Myeloproliferative disease	5178	JAK2-PAFR-TYK2 complex	0.1543
Myeloproliferative disease	5260	TCF4-CTNNB1-SUMO1-EP300-HADAC6 complex	0.11952
Myeloproliferative disease	5261	TCF4-CTNNB1-EP300 complex	0.1543
Myeloproliferative disease	5375	EGR-EP300 complex	0.18898
Myeloproliferative disease	5442	EPOR receptor complex	0.26726
Myeloproliferative disease	5446	EPO-EPOR complex	0.18898
Myeloproliferative disease	5564	LMO4-gp130 complex	0.11952
Myopathy	422	Beta-dystroglycan-caveolin-3 complex	0.08575
Myopathy	668	BKCA-beta2AR-AKAP79 signaling complex	0.07001
Myopathy	672	BKCA-beta2AR complex	0.08575
Myopathy	687	CFTR-NHERF-beta(2)AR signaling complex	0.07001

Myopathy	824	Anti-SMN protein complex	0.05423
Myopathy	832	Anti-Sm protein complex	0.04583
Myopathy	903	RET-Rai complex	0.08575
Myopathy	1142	SMN complex	0.03835
Myopathy	1143	SMN complex	0.03032
Myopathy	1379	GALNS-lysosomal hydrolase 1.27 MDa complex	0.06063
Myopathy	1617	G protein complex (CACNA1A GNB1 GNG2)	0.07001
Myopathy	1746	SMN containing complex	0.04287
Myopathy	1751	SMN complex	0.03835
Myopathy	1752	SMN complex	0.04583
Myopathy	2143	MAP2K5-PRKCI-SQSTM1 complex	0.07001
Myopathy	2395	ITGA7-ITGB1-CD151 complex	0.07001
Myopathy	2396	ITGA7-ITGB1-CD9 complex	0.07001
Myopathy	2397	ITGA7-ITGB1-ITGB1BP3 complex	0.07001
Myopathy	2453	Multiprotein complex (monoubiquitination)	0.06063
Myopathy	2863	Serine-palmitoyltransferase (SPT) complex	0.07001
Myopathy	2945	RBP-Jkappa-RING1-KyoT2 complex	0.07001
Myopathy	3098	TIM50a-SMN1 complex	0.08575
Myopathy	3118	SMN1-SIP1-SNRP complex	0.04583
Myopathy	3284	SMN complex (GEMIN5 2 3 4 SMN)	0.05423
Myopathy	3298	SMN complex (GEMIN2 5 SMN)	0.07001
Myopathy	3335	Homotetrameric complex NIAP	0.12127
Myopathy	3830	ADRB2 homodimer complex	0.12127
Myopathy	5276	HIF1A-OS9-EGLN1 complex	0.07001
Myopathy	5277	HIF1A-OS9-EGLN3 complex	0.07001
Myopathy	5604	Emerin complex 1	0.08575
Myopathy	5606	Emerin-actin-NMI-(alphaII)spectrin complex	0.12127
Myopathy	5607	Emerin-actin-NMI complex	0.07001
Myopathy	5608	Emerin architectural complex	0.09901
Myopathy	5609	Emerin regulatory complex	0.04042
Myopathy	5611	Emerin complex 24	0.03131
Myopathy	5613	Emerin complex 25	0.03032
Myopathy	5614	Emerin complex 32	0.05171
Myopathy	5615	Emerin complex 52	0.02529
Myopathy	5830	DJ-1-SNCA complex high molecular weight complex	0.08575
Myopathy	5877	MAP2K1-BRAF-RAF1-YWHAE-KSR1 complex	0.05423
Myopathy	5872	BRAF-MAP2K1-MAP2K2-YWHAE complex	0.06063
Myopathy	5919	BRAF-RAF1-14-3-3 complex	0.04042
Myopathy	5921	KSR1-BRAF-MEK complex	0.06063
Myopathy	5923	RAF1-BRAF complex RAS stimulated	0.08575
Myopathy	5925	BRAF-CNK1 complex not RAS stimulated	0.08575
Myotonic disorder	86	NUMAC complex (nucleosomal methylation activator complex)	0.09535
Myotonic disorder	149	PBAF complex (Polybromo- and BAF containing complex)	0.08704
Myotonic disorder	178	Respiratory chain complex I (holoenzyme)	0.09091
Myotonic disorder	189	BAF complex	0.08362
Myotonic disorder	566	BAF complex	0.09091

Myotonic disorder	725	P2X7 receptor signalling complex	0.08704
Myotonic disorder	778	LARC complex (LCR-associated remodeling complex)	0.06917
Myotonic disorder	1041	Alpha-dystrobrevin-ZO-1-actin complex	0.15076
Myotonic disorder	1166	p400-associated complex	0.11396
Myotonic disorder	1617	G protein complex (CACNA1A GNB1 GNG2)	0.17408
Myotonic disorder	1729	TLE1 corepressor complex (MASH1 promoter-corepressor complex)	0.09535
Myotonic disorder	2254	CTGF/Hcs24-actin complex	0.17408
Myotonic disorder	2255	Cofilin-actin-CAP1 complex	0.17408
Myotonic disorder	2300	Profilin 2 complex	0.1005
Myotonic disorder	2884	Respiratory chain complex I (early intermediate NDUFAF1 assembly) mitochondrial	0.11396
Myotonic disorder	2886	Respiratory chain complex I (incomplete intermediate ND1 ND2 ND3 CIA30 assembly) mitochondrial	0.30151
Myotonic disorder	2901	Respiratory chain complex I (intermediate IV/310kD) mitochondrial	0.15076
Myotonic disorder	2903	Respiratory chain complex I (intermediate V/380kD and VI/480kD) mitochondrial	0.13484
Myotonic disorder	2904	Respiratory chain complex I (intermediate VII/650kD) mitochondrial	0.09535
Myotonic disorder	2919	Respiratory chain complex I (gamma subunit) mitochondrial	0.16725
Myotonic disorder	2939	Ecsit complex (ECSIT MT-CO2 NDUFA1 MT-ND1 TRAF6 NDUFAF1)	0.12309
Myotonic disorder	2943	Respiratory chain complex I (incomplete NDUFAF1 assembly) mitochondrial	0.2132
Myotonic disorder	5604	Emerin complex 1	0.1066
Myotonic disorder	5606	Emerin-actin-NMI-(alphaII)spectrin complex	0.15076
Myotonic disorder	5607	Emerin-actin-NMI complex	0.17408
Myotonic disorder	5608	Emerin architectural complex	0.12309
Myotonic disorder	5609	Emerin regulatory complex	0.1005
Myotonic disorder	5613	Emerin complex 25	0.07538
Myotonic disorder	5614	Emerin complex 32	0.06428
Myotonic disorder	5615	Emerin complex 52	0.06287
Myotonic disorder	5736	Pre-initiation complex (PIC)	0.17408
Nasopharyngeal cancer	927	CENP-A nucleosome associated complex	0.07857
Nasopharyngeal cancer	929	CEN complex	0.03164
Nasopharyngeal cancer	1179	CENP-A NAC-CAD complex	0.05338
Nasopharyngeal cancer	1714	TICAM1-TICAM2-TLR4 complex	0.11111
Nasopharyngeal cancer	1774	MICA-KLRK1-HCST complex	0.11111
Nasopharyngeal cancer	4039	PAR4-BACE1 complex	0.13608
Nasopharyngeal cancer	5210	TANK-TRAF2-TRAF3 complex	0.11111
Neck cancer	252	RAD51C-XRCC3 complex	0.22361
Neck cancer	261	RAD51B-RAD51C-RAD51D-XRCC2-XRCC3	0.14142
Neck cancer	310	Cell cycle kinase complex CDC2	0.2582
Neck cancer	311	Cell cycle kinase complex CDK2	0.31623
Neck cancer	312	Cell cycle kinase complex CDK4	0.31623
Neck cancer	313	Cell cycle kinase complex CDK5	0.28284
Neck cancer	314	PCNA-p21 complex	0.22361

Neck cancer	903	RET-Rai complex	0.22361
Neck cancer	1633	CyclinD1-CDK4-CDK6 complex	0.18257
Neck cancer	1634	CyclinD1-CDK4-p21 complex	0.36515
Neck cancer	2230	PCNA complex	0.11952
Neck cancer	2635	BETA2-Cyclin D1 complex	0.22361
Neck cancer	3084	CCND1-CDK4 complex	0.22361
Neck cancer	3087	CCND1-CDK6 complex	0.22361
Neck cancer	5101	CyclinD3-CDK4-CDK6-p21 complex	0.15811
Neck cancer	5287	CDK4-CCND1 complex	0.22361
Neck cancer	5622	HSP90-CIP1-FKBPL complex	0.18257
Neck cancer	5749	MRIT complex	0.18257
Neck cancer	5812	p53-BCL2 complex	0.22361
Neck cancer	5819	BIM-BCL2xL complex	0.22361
Neck cancer	5820	tBID-BCL2xL complex	0.22361
Necrotizing enterocolitis	1514	IL4-IL4R complex	0.28868
Necrotizing enterocolitis	1515	IL4-IL4R-IL2RG complex	0.2357
Necrotizing enterocolitis	1714	TICAM1-TICAM2-TLR4 complex	0.2357
Neoplasm metastasis	41	Mi-2/NuRD-MTA2 complex	0.03639
Neoplasm metastasis	63	Mitotic 14S cohesin 1 complex	0.04069
Neoplasm metastasis	64	Mitotic 14S cohesin 2 complex	0.04069
Neoplasm metastasis	75	TSC1-TSC2 complex	0.05754
Neoplasm metastasis	86	NUMAC complex (nucleosomal methylation activator complex)	0.02573
Neoplasm metastasis	149	PBAF complex (Polybromo- and BAF containing complex)	0.02349
Neoplasm metastasis	163	Cohesin-SA2 complex	0.04069
Neoplasm metastasis	164	Cohesin-SA1 complex	0.04069
Neoplasm metastasis	189	BAF complex	0.02257
Neoplasm metastasis	217	CRSP complex	0.02877
Neoplasm metastasis	229	NAT complex	0.03076
Neoplasm metastasis	230	Mediator complex	0.01439
Neoplasm metastasis	232	ARC complex	0.02101
Neoplasm metastasis	238	SWI-SNF chromatin remodeling-related-BRCA1	0.02454
Neoplasm metastasis	282	SNF2h-cohesin-NuRD complex	0.02034
Neoplasm metastasis	284	CRSP complex	0.02454
Neoplasm metastasis	287	ARC-L complex	0.02175
Neoplasm metastasis	288	ARC complex	0.02101
Neoplasm metastasis	5856	AK2-FADD-caspase-10 (AFAC10) complex	0.04698
Neoplasm metastasis	375	MSH2-MSH3 complex	0.05754
Neoplasm metastasis	378	MutS-beta complex	0.05754
Neoplasm metastasis	404	Isocitrate dehydrogenase cytoplasmic	0.08138
Neoplasm metastasis	441	TFTC-type histone acetyl transferase complex	0.02454
Neoplasm metastasis	521	Polycystin-1-E-cadherin-beta-catenin complex	0.04698
Neoplasm metastasis	522	Polycystin-1-E-cadherin-beta-catenin-Flotillin-2	0.04069
Neoplasm metastasis	548	DRIP complex	0.02175
Neoplasm metastasis	554	PBAF complex (Polybromo- and BAF containing complex)	0.02573
Neoplasm metastasis	555	BAF complex	0.02713

Neoplasm metastasis	564	BAF complex	0.02454
Neoplasm metastasis	565	PBAF complex (Polybromo- and BAF containing complex)	0.02454
Neoplasm metastasis	566	BAF complex	0.02454
Neoplasm metastasis	570	p300-CBP-p270-SWI/SNF complex	0.03076
Neoplasm metastasis	585	Mi2/NuRD-BCL6-MTA3 complex	0.03639
Neoplasm metastasis	587	NuRD.1 complex	0.02877
Neoplasm metastasis	710	Brg1-associated complex I	0.02454
Neoplasm metastasis	711	Brm-associated complex	0.02454
Neoplasm metastasis	713	BRG1-SIN3A complex	0.02175
Neoplasm metastasis	714	BRM-SIN3A complex	0.02101
Neoplasm metastasis	739	SIN3-ING1b complex II	0.02034
Neoplasm metastasis	778	LARC complex (LCR-associated remodeling complex)	0.01867
Neoplasm metastasis	803	BRG1-SIN3A-HDAC containing SWI/SNF remodeling complex I	0.02454
Neoplasm metastasis	806	BRM-SIN3A-HDAC complex	0.02349
Neoplasm metastasis	807	BRG1-associated complex	0.02713
Neoplasm metastasis	808	BRM-associated complex	0.02573
Neoplasm metastasis	845	PCI-PSA-SCG2 complex	0.04698
Neoplasm metastasis	895	hMediator complex (MED23 CDK8 CCNC MED7)	0.04069
Neoplasm metastasis	896	hMediator complex (MED23 CDK8 CCNC)	0.04698
Neoplasm metastasis	898	Mediator complex 1	0.03639
Neoplasm metastasis	900	Mediator complex 2	0.04698
Neoplasm metastasis	909	ARC92-Mediator complex	0.02257
Neoplasm metastasis	910	CRSP-Mediator 2 complex	0.02573
Neoplasm metastasis	926	SCRIB-LGL2 complex	0.05754
Neoplasm metastasis	955	LLGL2-PAR-6B-PRKCI complex	0.04698
Neoplasm metastasis	1045	Snurportin-CRM1-RanGTP complex	0.04698
Neoplasm metastasis	1091	SNX complex (SNX1a SNX2 SNX4 LEPR)	0.04069
Neoplasm metastasis	1176	CRM1-RAN-PHAX-CBC complex (cap binding	0.03639
Neoplasm metastasis	1230	WINAC complex	0.02175
Neoplasm metastasis	1239	EBAFb complex	0.02573
Neoplasm metastasis	1252	EBAFa complex	0.02573
Neoplasm metastasis	1256	MLL-HCF complex	0.03076
Neoplasm metastasis	1257	ALL-1 supercomplex	0.04614
Neoplasm metastasis	1401	MOF complex	0.02573
Neoplasm metastasis	1413	NCOR1 complex	0.02573
Neoplasm metastasis	1551	IPO13-RAN-EIF1AX complex	0.04698
Neoplasm metastasis	1552	TNPO2-RAN-NXF1 complex	0.04698
Neoplasm metastasis	1554	RANBP1-RAN-KPNB1 complex	0.04698
Neoplasm metastasis	1729	TLE1 corepressor complex (MASH1 promoter-corepressor complex)	0.02573
Neoplasm metastasis	1856	CDCA5-PDS5A-RAD21-SMC1A-PDS5B-SMC3	0.03322
Neoplasm metastasis	1982	CACY homodimer complex	0.08138
Neoplasm metastasis	1992	LEPR homodimer complex	0.08138
Neoplasm metastasis	2016	IL12A homodimer complex	0.08138
Neoplasm metastasis	2018	IL12A-IL12B complex	0.05754
Neoplasm metastasis	2019	IL12A-IL12B-IL12RB1 complex	0.04698

Neoplasm metastasis	2021	IL12A-IL12B-IL12RB2 complex	0.04698
Neoplasm metastasis	2055	CASP8-CHUK-IKBKB-MALT1-BCL10 complex	0.03639
Neoplasm metastasis	2056	BCL10-CHUK-BCL10-IKBKB complex	0.04069
Neoplasm metastasis	2100	CHUK-IKBKB-MAP3K14 complex	0.04698
Neoplasm metastasis	2101	IKKA-IKKB complex	0.05754
Neoplasm metastasis	2105	IkappaB kinase complex (IKBKB CHUK IKBKAP NFKBIA RELA MAP3K14)	0.03322
Neoplasm metastasis	2118	CHUK-ERC1-IKBKB-IKBKG	0.04069
Neoplasm metastasis	2121	CHUK-IKBKB-IKBKG complex	0.04698
Neoplasm metastasis	2124	IKK-alpha--ER-alpha-AIB1 complex	0.04698
Neoplasm metastasis	2258	VILIP-1-AChR-alpha-4-AChR-beta-2 complex	0.04698
Neoplasm metastasis	2318	ITGA6-ITGB4-Laminin10/12 complex	0.03639
Neoplasm metastasis	2342	ITGAV-ITGB8-MMP14-TGFB1 complex	0.04069
Neoplasm metastasis	2351	ITGB6-FYN-FN1 complex	0.04698
Neoplasm metastasis	2352	ITGAV-ITGB6-SPP1 complex	0.04698
Neoplasm metastasis	2353	ITGAV-ITGB6-TGFB3 complex	0.04698
Neoplasm metastasis	2354	ITGAV-ITGB6 complex	0.05754
Neoplasm metastasis	2390	CD98-LAT2-ITGB1 complex	0.04069
Neoplasm metastasis	2416	ITGB1-RAP1A-PKD1 complex	0.04698
Neoplasm metastasis	2471	SRC-PRKCD-CDCP1 complex	0.04698
Neoplasm metastasis	2589	PGC-1-SRp40-SRp55-SRp75 complex	0.04069
Neoplasm metastasis	2599	POLR2A-CCNT1-CDK9-NCL-LEM6-CPSF2 complex	0.03322
Neoplasm metastasis	2721	HCF-1 complex	0.01867
Neoplasm metastasis	2727	SRC-3 complex	0.03076
Neoplasm metastasis	2829	RSmad complex	0.02573
Neoplasm metastasis	2851	ING2 complex	0.02349
Neoplasm metastasis	2852	Brg1-based SWI/SNF chromatin remodeling complex	0.04069
Neoplasm metastasis	2966	NuMA-LGN-G-alpha-i-1 complex	0.04698
Neoplasm metastasis	3063	Brg1-associated complex II	0.03076
Neoplasm metastasis	3064	RNA polymerase II complex chromatin structure modifying	0.01867
Neoplasm metastasis	3065	RNA polymerase II complex chromatin structure modifying	0.02454
Neoplasm metastasis	3067	RNA polymerase II complex incomplete (CDK8 complex) chromatin structure modifying	0.02877
Neoplasm metastasis	3103	ITGAV-ITGB3-SLC3A2 complex	0.04698
Neoplasm metastasis	3110	ITGAV-P2RY2-GNA12 complex	0.04698
Neoplasm metastasis	3162	TF-FVIIa-FXa-TFPI complex	0.04069
Neoplasm metastasis	4977	gp78-p97/VCP-DERL1 complex	0.04698
Neoplasm metastasis	5165	AP1G1-PACS1-FURIN complex	0.04698
Neoplasm metastasis	5177	Polycystin-1 multiprotein complex (ACTN1 CDH1 SRC JUP VCL CTNNB1 PXN BCAR1 PKD1	0.02454
Neoplasm metastasis	5193	TNF-alpha/NF-kappa B signaling complex (CHUK KPNA3 NFKB2 NFKBIB REL IKBKG NFKB1 NFKBIE RELB NFKBIA RELA TNIP2)	0.02349
Neoplasm metastasis	5194	TNF-alpha/NF-kappa B signaling complex (SEC16A CHUK IKBKB NFKB2 REL IKBKG MAP3K14 RELA FBXW7 USP2)	0.02573

Neoplasm metastasis	5196	TNF-alpha/NF-kappa B signaling complex (CHUK BTRC NFKB2 PPP6C REL CUL1 IKBKE SAPS2 SAPS1 ANKRD28 RELA SKP1)	0.02349
Neoplasm metastasis	5199	Kinase maturation complex 1	0.04069
Neoplasm metastasis	5220	CHUK-IQGAP2-AKAP8L-RELA-TNIP2 complex	0.03639
Neoplasm metastasis	5230	CHUK-NFKB2-REL-IKBKG-SPAG9-NFKB1-NFKBIE-COPB2-TNIP1-NFKBIA-RELA-TNIP2	0.02349
Neoplasm metastasis	5232	TNF-alpha/Nf-kappa B signaling complex (RPL6 RPL30 RPS13 CHUK DDX3X NFKB2 NFKBIB REL IKBKG NFKB1 MAP3K8 RELB GLG1 NFKBIA RELA TNIP2 GTF2I)	0.01974
Neoplasm metastasis	5233	TNF-alpha/NF-kappa B signaling complex 5	0.01628
Neoplasm metastasis	5266	TNF-alpha/NF-kappa B signaling complex 6	0.02175
Neoplasm metastasis	5285	TNF-alpha/NF-kappa B signaling complex 9	0.03639
Neoplasm metastasis	5286	TNF-alpha/NF-kappa B signaling complex 10	0.02573
Neoplasm metastasis	5378	TRBP containing complex (DICER TRBP AGO2 RPL7A EIF6 MOV10)	0.03322
Neoplasm metastasis	5380	TRBP containing complex (DICER RPL7A EIF6 MOV10 and subunits of the 60S ribosomal particle)	0.01628
Neoplasm metastasis	5386	MLL1-WDR5 complex	0.04698
Neoplasm metastasis	5423	HSP70-BAG5-PARK2 complex	0.04069
Neoplasm metastasis	5432	Sororin-cohesin complex	0.03322
Neoplasm metastasis	5450	Mediator complex	0.01566
Neoplasm metastasis	5548	IL-12 heterodimer complex	0.05754
Neoplasm metastasis	5614	Emerin complex 32	0.01735
Neoplasm metastasis	5762	CRMP-MICAL-PlexinA1 complex induced by	0.04698
Neoplasm metastasis	5829	IKBKG-CHUK complex	0.05754
Neoplasm metastasis	5844	I-kappa-B kinase (IKK) complex	0.04698
Neoplasm metastasis	5859	FAS-FADD-CASP8-CASP10 complex	0.04069
Neoplasm metastasis	5861	FAS-FADD-CASP10 complex	0.04698
Nephroblastoma	351	Spliceosome	0.03161
Nephroblastoma	929	CEN complex	0.06214
Nephroblastoma	1728	CTCF-nucleophosmin-PARP-HIS-KPNA-LMNA-TOP complex	0.12599
Nephroblastoma	2705	SMAD3-SMAD4-CTCF protein-DNA complex	0.21822
Nephroblastoma	5153	CTFC-TAF1 complex	0.26726
Nephroblastoma	5154	CTCF-nucleophosmin complex	0.26726
Nephrosis	1054	ESR1-RELA-BCL3-NCOA3 complex	0.1118
Nephrosis	1088	PRNP-ApolopoproteinE3 complex	0.15811
Nephrosis	1091	SNX complex (SNX1a SNX2 SNX4 LEPR)	0.1118
Nephrosis	1992	LEPR homodimer complex	0.22361
Nephrosis	2084	NFKB1-NFKB2-REL-RELA-RELB complex	0.1
Nephrosis	2086	NFKB1-NFKB2-RELA-RELB complex	0.1118
Nephrosis	2105	IkappaB kinase complex (IKBKB CHUK IKBKAP NFKBIA RELA MAP3K14)	0.09129
Nephrosis	2319	ITGA6-ITGB4-Laminin10/12 complex	0.1
Nephrosis	3045	hs4 enhancer complex (faster migrating complex)	0.1
Nephrosis	3162	TF-FVIIa-FXa-TFPI complex	0.1118

Nephrosis	5193	TNF-alpha/NF-kappa B signaling complex (CHUK KPNA3 NFKB2 NFKBIB REL IKBKG NFKB1 NFKBIE RELB NFKBIA RELA TNIP2)	0.06455
Nephrosis	5194	TNF-alpha/NF-kappa B signaling complex (SEC16A CHUK IKBKB NFKB2 REL IKBKG MAP3K14 RELA FBXW7 USP2)	0.07071
Nephrosis	5196	TNF-alpha/NF-kappa B signaling complex (CHUK BTRC NFKB2 PPP6C REL CUL1 IKBKE SAPS2 SAPS1 ANKRD28 RELA SKP1)	0.06455
Nephrosis	5220	CHUK-IQGAP2-AKAP8L-RELA-TNIP2 complex	0.1
Nephrosis	5228	REL-MAP3K8-RELA-TNIP2-PAPOLA complex	0.1
Nephrosis	5230	CHUK-NFKB2-REL-IKBKG-SPAG9-NFKB1-NFKBIE-COPB2-TNIP1-NFKBIA-RELA-TNIP2	0.06455
Nephrosis	5232	TNF-alpha/Nf-kappa B signaling complex (RPL6 RPL30 RPS13 CHUK DDX3X NFKB2 NFKBIB REL IKBKG NFKB1 MAP3K8 RELB GLG1 NFKBIA RELA TNIP2 GTF2I)	0.05423
Nephrosis	5233	TNF-alpha/NF-kappa B signaling complex 5	0.04472
Nephrosis	5460	p50-p65 NF(kappa)B complex	0.15811
Nephrosis	5461	p50-p65 NF(kappa)B-SRC1 complex	0.1291
Nephrosis	5464	I(kappa)B(alpha)-NF(kappa)Bp50-NF(kappa)Bp65 complex	0.1291
Nephrosis	5465	IKB(epsilon)-RELA-cREL complex	0.1291
Nephrosis	5466	IKB(beta)-RELA-cREL complex	0.1291
Nephrosis	5467	IKB(alpha)-RELA-cREL complex	0.1291
Nephrosis	5475	MURR1-NF(kappa)Bp65-IKBA complex	0.1291
Nephrosis	5492	IKBA-NF(kappa)Bp65-NF(kappa)Bp50 complex	0.1291
Nephrosis	5877	MAP2K1-BRAF-RAF1-YWHAE-KSR1 complex	0.1
Nephrosis	5872	BRAF-MAP2K1-MAP2K2-YWHAE complex	0.1118
Nephrosis	5919	BRAF-RAF1-14-3-3 complex	0.07454
Nephrosis	5921	KSR1-BRAF-MEK complex	0.1118
Nephrosis	5923	RAF1-BRAF complex RAS stimulated	0.15811
Nephrosis	5925	BRAF-CNK1 complex not RAS stimulated	0.15811
Nervous system disease	143	APP-FE65-LRP complex	0.14907
Nervous system disease	749	MeCP2-SIN3A-HDAC complex	0.1291
Nervous system disease	2711	Amyloid beta protein oligomer	0.2582
Nervous system disease	3092	APP-TOMM40 complex	0.18257
Nervous system disease	3093	APP-TIMM23 complex	0.18257
Nervous system disease	5158	SMARCA2/BRM-BAF57-MECP2 complex	0.14907
Nervous system disease	5184	SWI/SNF chromatin-remodeling complex	0.11547
Nervous system disease	5473	FAS-FADD-CASP8 complex	0.14907
Nervous system disease	5747	2AR-mGluR2 complex	0.18257
Nervous system disease	5799	Death induced signaling complex DISC (FAS FADD CASP8 CFLAR) membrane-associated CD95L	0.1291
Nervous system disease	5800	Death-inducing signaling complex DISC (type I cells associated) stimulated	0.14907
Nervous system disease	5808	DISC complex	0.14907
Nervous system disease	5830	DJ-1-SNCA complex high molecular weight complex	0.18257
Nervous system disease	5859	FAS-FADD-CASP8-CASP10 complex	0.1291

Nervous system disease	5861	FAS-FADD-CASP10 complex	0.14907
Nervous system tumor	443	BP-SMAD complex	0.10206
Nervous system tumor	2363	ITGAV-ITGB3-PXN-PTK2b complex	0.14434
Nervous system tumor	2443	ITGA9-ITGB1-TNC complex	0.16667
Nervous system tumor	5709	ArgBP2a-CBL-PTK2B complex	0.16667
Neurilemmoma	706	SNARE complex (HGS SNAP25 STX13)	0.2582
Neurilemmoma	3677	RIN1-STAM2-HRS complex	0.2582
Neuritis	1345	Septin complex	0.15811
Neuritis	5473	FAS-FADD-CASP8 complex	0.20412
Neuritis	5799	Death induced signaling complex DISC (FAS FADD CASP8 CFLAR) membrane-associated CD95L	0.17678
Neuritis	5800	Death-inducing signaling complex DISC (type I cells associated) stimulated	0.20412
Neuritis	5808	DISC complex	0.20412
Neuritis	5859	FAS-FADD-CASP8-CASP10 complex	0.17678
Neuritis	5861	FAS-FADD-CASP10 complex	0.20412
Neuroblastoma	4	Multisubunit ACTR coactivator complex	0.06868
Neuroblastoma	74	TRPC1-Homer3-IP3R complex	0.07931
Neuroblastoma	351	Spliceosome	0.01149
Neuroblastoma	536	TRPC1-TRPC3-TRPC7 complex	0.07931
Neuroblastoma	553	RHOA-IP3R-TRPC1 complex	0.07931
Neuroblastoma	557	TRP1-G alpha-11-IP3R3-CAV1 signaling complex	0.06868
Neuroblastoma	570	p300-CBP-p270-SWI/SNF complex	0.05192
Neuroblastoma	571	p300-CBP-p270 complex	0.07931
Neuroblastoma	828	TRPC1-STIM1-ORAI1 complex	0.07931
Neuroblastoma	839	LIN9-BMYB complex	0.09713
Neuroblastoma	924	Toposome	0.05192
Neuroblastoma	1055	ZNF198-PML complex	0.09713
Neuroblastoma	1079	P-TEFb.1 complex	0.09713
Neuroblastoma	1080	P-TEFb.2 complex	0.09713
Neuroblastoma	1083	P-TEFb.4 complex	0.09713
Neuroblastoma	1179	CENP-A NAC-CAD complex	0.0381
Neuroblastoma	1191	RNA pol II containing coactivator complex Tat-SF	0.06143
Neuroblastoma	1255	Ubiquitin E3 ligase (SIAH1 SIP SKP1A TBL1X)	0.06868
Neuroblastoma	1306	PIN1-AUF1 complex	0.09713
Neuroblastoma	1332	Large Drosha complex	0.03071
Neuroblastoma	1714	TICAM1-TICAM2-TLR4 complex	0.07931
Neuroblastoma	1749	SMN-PolII-RHA complex	0.04344
Neuroblastoma	1827	PML-SMAD2/3-SARA complex	0.06868
Neuroblastoma	2029	BNIP2-ARHGAP8 complex	0.09713
Neuroblastoma	2230	PCNA complex	0.05192
Neuroblastoma	2255	Cofilin-actin-CAP1 complex	0.07931
Neuroblastoma	2599	POLR2A-CCNT1-CDK9-NCL-LEM6-CPSF2 complex	0.05608
Neuroblastoma	2600	BRD4 complex	0.04856
Neuroblastoma	2601	P-TEFb-BRD4-TRAP220 complex	0.06868
Neuroblastoma	2602	P-TEFb-7SKRNA-HEXIM1 complex	0.07931
Neuroblastoma	2603	Transcription elongation factor complex (SUPT5H CDK9 CCNT1)	0.07931

Neuroblastoma	2604	P-TEFb-SKP2 complex	0.07931
Neuroblastoma	2605	Heterotrimeric complex (CCNT1 CDK9 GRN)	0.07931
Neuroblastoma	2638	HES1 promoter corepressor complex	0.05608
Neuroblastoma	2639	HES1 promoter-Notch enhancer complex	0.0381
Neuroblastoma	2641	p300/CBP-PCAF-MyoD complex	0.06868
Neuroblastoma	2727	SRC-3 complex	0.05192
Neuroblastoma	2728	SRC-1 complex	0.06868
Neuroblastoma	2822	BRCA1-BARD1-UbcH5c complex	0.07931
Neuroblastoma	2829	RSmad complex	0.04344
Neuroblastoma	2875	BRD4-P-TEFb complex	0.07931
Neuroblastoma	2958	SMAD1-CBP complex	0.09713
Neuroblastoma	3055	Nop56p-associated pre-rRNA complex	0.01347
Neuroblastoma	3061	RNA polymerase II complex (CBP PCAF RPB1 BAF47 CYCC CDK8) chromatin structure modifying	0.05608
Neuroblastoma	3062	RNA polymerase II complex incomplete (CBP RPBI PCAF BAF47) chromatin structure modifying	0.06868
Neuroblastoma	3066	RNA polymerase II complex chromatin structure modifying	0.0381
Neuroblastoma	3082	DGCR8 multiprotein complex	0.04142
Neuroblastoma	3102	DHX9-ADAR-vigilin-DNA-PK-Ku antigen complex	0.05608
Neuroblastoma	3129	STAT6-p100-RHA complex	0.07931
Neuroblastoma	3137	MASH1 promoter-coactivator complex	0.04142
Neuroblastoma	3142	CAMK2-delta-MASH1 promoter-coactivator complex	0.04856
Neuroblastoma	3229	Heterodimer complex (CDK9 IL6ST)	0.09713
Neuroblastoma	3618	GammaH2AFX-NDHII-Ku70-DNA complex	0.07931
Neuroblastoma	3749	CREBBP-SMAD2 hexameric complex	0.09713
Neuroblastoma	3750	CREBBP-SMAD3 hexameric complex	0.09713
Neuroblastoma	3753	CREBBP-SMAD2-SMAD4 pentameric complex	0.07931
Neuroblastoma	3754	CREBBP-SMAD3-SMAD4 pentameric complex	0.07931
Neuroblastoma	4039	PAR4-BACE1 complex	0.09713
Neuroblastoma	5198	CBP-RARA-RXRA-DNA complex ligand stimulated	0.07931
Neuroblastoma	5211	RAF1-PPP2-PIN1 complex	0.06143
Neuroblastoma	5264	TCF4-CTNNB1-CREBBP complex	0.07931
Neuroblastoma	5573	Stat1-alpha-dimer-CBP DNA-protein complex	0.09713
Neuroblastoma	5589	LINC complex S-phase	0.05192
Neuroblastoma	5816	Apoptosome-procaspase 9 complex	0.07931
Neuroblastoma	5822	MCL1-NOXA complex	0.09713
Neurodegenerative disorder	143	APP-FE65-LRP complex	0.09492
Neurodegenerative disorder	280	HMGB1-HMGB2-HSC70-ERP60-GAPDH complex	0.07352
Neurodegenerative disorder	351	Spliceosome	0.01375
Neurodegenerative disorder	432	N-NOS-CHIP-HSP70-1 complex	0.09492
Neurodegenerative disorder	668	BKCA-beta2AR-AKAP79 signaling complex	0.09492
Neurodegenerative disorder	672	BKCA-beta2AR complex	0.11625
Neurodegenerative disorder	1056	ZNF198-SUMO1 complex	0.11625
Neurodegenerative disorder	1062	BAR-BCL2-CASP8 complex	0.09492
Neurodegenerative disorder	1068	12S U11 snRNP	0.04245
Neurodegenerative disorder	1088	PRNP-ApolopoproteinE3 complex	0.11625
Neurodegenerative disorder	1186	ESCRT-III complex	0.05199

Neurodegenerative disorder	1306	PIN1-AUF1 complex	0.11625
Neurodegenerative disorder	2054	CASP8-FADD-MALT1-BCL10 complex	0.0822
Neurodegenerative disorder	2055	CASP8-CHUK-IKBKB-MALT1-BCL10 complex	0.07352
Neurodegenerative disorder	2056	BCL10-CHUK-BCL10-IKBKB complex	0.0822
Neurodegenerative disorder	2143	MAP2K5-PRKCI-SQSTM1 complex	0.09492
Neurodegenerative disorder	2711	Amyloid beta protein oligomer	0.1644
Neurodegenerative disorder	2936	Ecsit complex (ECSIT MT-CO2 GAPDH TRAF6 NDUFAF1)	0.07352
Neurodegenerative disorder	3092	APP-TOMM40 complex	0.11625
Neurodegenerative disorder	3093	APP-TIMM23 complex	0.11625
Neurodegenerative disorder	5211	RAF1-PPP2-PIN1 complex	0.07352
Neurodegenerative disorder	5260	TCF4-CTNNB1-SUMO1-EP300-HADAC6 complex	0.07352
Neurodegenerative disorder	5385	GAIT complex	0.0822
Neurodegenerative disorder	5473	FAS-FADD-CASP8 complex	0.09492
Neurodegenerative disorder	5749	MRIT complex	0.09492
Neurodegenerative disorder	5755	SUMO1-SUA1-UBA2 complex	0.09492
Neurodegenerative disorder	5798	Death induced signaling complex II (FADD CASP8 CFLAR) cytosolic CD95L induced	0.09492
Neurodegenerative disorder	5799	Death induced signaling complex DISC (FAS FADD CASP8 CFLAR) membrane-associated CD95L	0.0822
Neurodegenerative disorder	5800	Death-inducing signaling complex DISC (type I cells associated) stimulated	0.09492
Neurodegenerative disorder	5808	DISC complex	0.09492
Neurodegenerative disorder	5830	DJ-1-SNCA complex high molecular weight complex	0.2325
Neurodegenerative disorder	5832	PINK1-MIRO2-Milton complex	0.09492
Neurodegenerative disorder	5837	PPD complex	0.18983
Neurodegenerative disorder	5859	FAS-FADD-CASP8-CASP10 complex	0.0822
Neurofibromatosis	5401	PLEKHM2-KIF5B complex	0.22361
Neuropathy	178	Respiratory chain complex I (holoenzyme)	0.04828
Neuropathy	824	Anti-SMN protein complex	0.07161
Neuropathy	832	Anti-Sm protein complex	0.06052
Neuropathy	873	SNARE complex (STX1A SNAP29)	0.11323
Neuropathy	1142	SMN complex	0.05064
Neuropathy	1143	SMN complex	0.04003
Neuropathy	1728	CTCF-nucleophosmin-PARP-HIS-KPNA-LMNA-TOP complex	0.05338
Neuropathy	1746	SMN containing complex	0.05661
Neuropathy	1751	SMN complex	0.05064
Neuropathy	1752	SMN complex	0.06052
Neuropathy	2863	Serine-palmitoyltransferase (SPT) complex	0.1849
Neuropathy	2884	Respiratory chain complex I (early intermediate NDUFAF1 assembly) mitochondrial	0.06052
Neuropathy	2886	Respiratory chain complex I (incomplete intermediate ND1 ND2 ND3 CIA30 assembly) mitochondrial	0.08006
Neuropathy	2901	Respiratory chain complex I (intermediate IV/310kD) mitochondrial	0.08006
Neuropathy	2903	Respiratory chain complex I (intermediate V/380kD and VI/480kD) mitochondrial	0.07161

Neuropathy	2904	Respiratory chain complex I (intermediate VII/650kD) mitochondrial	0.05064
Neuropathy	2919	Respiratory chain complex I (gamma subunit) mitochondrial	0.08882
Neuropathy	2939	Ecsit complex (ECSIT MT-CO2 NDUFA1 MT-ND1 TRAF6 NDUFAF1)	0.06537
Neuropathy	2943	Respiratory chain complex I (incomplete NDUFAF1 assembly) mitochondrial	0.11323
Neuropathy	3098	TIM50a-SMN1 complex	0.11323
Neuropathy	3118	SMN1-SIP1-SNRP complex	0.06052
Neuropathy	3284	SMN complex (GEMIN5 2 3 4 SMN)	0.07161
Neuropathy	3298	SMN complex (GEMIN2 5 SMN)	0.09245
Neuropathy	5608	Emerin architectural complex	0.06537
Neuropathy	5611	Emerin complex 24	0.04134
Neuropathy	5615	Emerin complex 52	0.03339
Neuropathy	5830	DJ-1-SNCA complex high molecular weight complex	0.11323
Neurotic disorder	2489	NCR3-CD247 complex	0.22361
Neutropenia	5388	SERPINA1-ELA2 complex	0.28868
Nevus	1379	GALNS-lysosomal hydrolase 1.27 MDa complex	0.17678
Nevus	3162	TF-FVIIa-FXa-TFPI complex	0.17678
Obesity	143	APP-FE65-LRP complex	0.04441
Obesity	298	VEGF transcriptional complex	0.0314
Obesity	310	Cell cycle kinase complex CDC2	0.0314
Obesity	311	Cell cycle kinase complex CDK2	0.03846
Obesity	312	Cell cycle kinase complex CDK4	0.03846
Obesity	313	Cell cycle kinase complex CDK5	0.0344
Obesity	351	Spliceosome	0.00643
Obesity	441	TFTC-type histone acetyl transferase complex	0.02319
Obesity	541	IGF1-IGFBP3-ALS complex	0.08882
Obesity	668	BKCA-beta2AR-AKAP79 signaling complex	0.04441
Obesity	672	BKCA-beta2AR complex	0.05439
Obesity	687	CFTR-NHERF-beta(2)AR signaling complex	0.04441
Obesity	725	P2X7 receptor signalling complex	0.02221
Obesity	746	C/EBPalpha-HNF6 complex	0.05439
Obesity	879	PRKAC-AKAP5-ADRB1 complex	0.0344
Obesity	1054	ESR1-RELA-BCL3-NCOA3 complex	0.03846
Obesity	1091	SNX complex (SNX1a SNX2 SNX4 LEPR)	0.03846
Obesity	1093	SNX complex (SNX1a SNX2 SNX4 INSR)	0.03846
Obesity	1519	IL6ST-PRKCD-STAT3 complex	0.04441
Obesity	1633	CyclinD1-CDK4-CDK6 complex	0.04441
Obesity	1634	CyclinD1-CDK4-p21 complex	0.04441
Obesity	5718	eNOS-HSP90-AKT complex VEGF induced	0.04441
Obesity	1992	LEPR homodimer complex	0.07692
Obesity	2055	CASP8-CHUK-IKBKB-MALT1-BCL10 complex	0.0344
Obesity	2056	BCL10-CHUK-BCL10-IKBKB complex	0.03846
Obesity	2100	CHUK-IKBKB-MAP3K14 complex	0.04441
Obesity	2101	IKKA-IKKB complex	0.05439

Obesity	2105	IkappaB kinase complex (IKBKB CHUK IKBKAP NFKBIA RELA MAP3K14)	0.0314
Obesity	2118	CHUK-ERC1-IKBKB-IKBKG	0.03846
Obesity	2121	CHUK-IKBKB-IKBKG complex	0.04441
Obesity	2124	IKK-alpha--ER-alpha-AIB1 complex	0.08882
Obesity	2153	ITGAM-ITGB2-CD11 complex	0.04441
Obesity	2156	YBX1-AKT1 complex	0.05439
Obesity	2159	AR-AKT-APPL complex	0.04441
Obesity	2347	ITGAV-ITGB5-SPP1 complex	0.04441
Obesity	2352	ITGAV-ITGB6-SPP1 complex	0.04441
Obesity	2358	ITGAV-ITGB3-SPP1 complex	0.04441
Obesity	2470	p130Cas-ER-alpha-cSrc-kinase- PI3-kinase p85-subunit complex	0.03846
Obesity	2577	Sam68-p85 P13K-IRS-1-IR signaling complex	0.07692
Obesity	2635	BETA2-Cyclin D1 complex	0.05439
Obesity	2657	ESR1-CDK7-CCNH-MNAT1-MTA1-HDAC2 complex	0.0314
Obesity	2670	Er-alpha-p53-hdm2 complex	0.04441
Obesity	2699	ER-alpha-GRIP1-c-Jun complex	0.04441
Obesity	2700	ER-alpha-c-Jun complex	0.05439
Obesity	2709	MMP-9-TIMP-1-LRP complex	0.04441
Obesity	2710	LRP-1-Alpha-2-M-annexin VI complex	0.04441
Obesity	2711	Amyloid beta protein oligomer	0.07692
Obesity	2727	SRC-3 complex	0.02907
Obesity	2798	MMP-2-claudin-1 complex	0.05439
Obesity	2885	ITGAV-ITGB1-SPP1 complex	0.04441
Obesity	2972	ITGA9-ITGB1-VEGFA complex	0.04441
Obesity	3084	CCND1-CDK4 complex	0.05439
Obesity	3087	CCND1-CDK6 complex	0.05439
Obesity	3092	APP-TOMM40 complex	0.05439
Obesity	3093	APP-TIMM23 complex	0.05439
Obesity	3111	ITGA9-ITGB1-SPP1 complex	0.04441
Obesity	3112	ITGA5-ITGB1-SPP1 complex	0.04441
Obesity	3138	POSH-AKT2 complex	0.05439
Obesity	3229	Heterodimer complex (CDK9 IL6ST)	0.05439
Obesity	3335	Homotetrameric complex NIAP	0.07692
Obesity	3830	ADRB2 homodimer complex	0.07692
Obesity	3847	TCL1(trimer)-AKT1 complex	0.05439
Obesity	3848	TCL1(trimer)-AKT2 complex	0.05439
Obesity	4062	NRP1-VEGFR2-VEGF(165) complex	0.04441
Obesity	4869	beta(1)-AR receptosome (ADRB1-SAP97-AKAP79-PRKAR2A)	0.03846
Obesity	5193	TNF-alpha/NF-kappa B signaling complex (CHUK KPNA3 NFKB2 NFKBIB REL IKBKG NFKB1 NFKBIE RELB NFKBIA RELA TNIP2)	0.02221
Obesity	5194	TNF-alpha/NF-kappa B signaling complex (SEC16A CHUK IKBKB NFKB2 REL IKBKG MAP3K14 RELA FBXW7 USP2)	0.02433

Obesity	5196	TNF-alpha/NF-kappa B signaling complex (CHUK BTRC NFKB2 PPP6C REL CUL1 IKBKE SAPS2 SAPS1 ANKRD28 RELA SKP1)	0.02221
Obesity	5198	CBP-RARA-RXRA-DNA complex ligand stimulated	0.04441
Obesity	5220	CHUK-IQGAP2-AKAP8L-RELA-TNIP2 complex	0.0344
Obesity	5230	CHUK-NFKB2-REL-IKBKG-SPAG9-NFKB1-NFKBIE-COPB2-TNIP1-NFKBIA-RELA-TNIP2	0.02221
Obesity	5232	TNF-alpha/Nf-kappa B signaling complex (RPL6 RPL30 RPS13 CHUK DDX3X NFKB2 NFKBIB REL IKBKG NFKB1 MAP3K8 RELB GLG1 NFKBIA RELA TNIP2 GTF2I)	0.01866
Obesity	5233	TNF-alpha/NF-kappa B signaling complex 5	0.01538
Obesity	5266	TNF-alpha/NF-kappa B signaling complex 6	0.02056
Obesity	5273	VHL-TBP1-HIF1A complex	0.04441
Obesity	5276	HIF1A-OS9-EGLN1 complex	0.04441
Obesity	5277	HIF1A-OS9-EGLN3 complex	0.04441
Obesity	5285	TNF-alpha/NF-kappa B signaling complex 9	0.0344
Obesity	5286	TNF-alpha/NF-kappa B signaling complex 10	0.02433
Obesity	5287	CDK4-CCND1 complex	0.05439
Obesity	5382	ARNT-HIF1A complex	0.05439
Obesity	5473	FAS-FADD-CASP8 complex	0.04441
Obesity	5564	LMO4-gp130 complex	0.0688
Obesity	5579	CNTF-CNTFR-gp130-LIFR complex	0.03846
Obesity	5582	LIFR-LIF-gp130 complex	0.04441
Obesity	5696	VEGFA(165)-KDR-NRP1 complex	0.04441
Obesity	5698	VEGFA(165)-VEGFR2-NRP1 complex	0.04441
Obesity	5701	NRP1-VEGF(165/121) complex	0.05439
Obesity	5747	2AR-mGluR2 complex	0.05439
Obesity	5799	Death induced signaling complex DISC (FAS FADD CASP8 CFLAR) membrane-associated CD95L	0.03846
Obesity	5800	Death-inducing signaling complex DISC (type I cells associated) stimulated	0.04441
Obesity	5808	DISC complex	0.04441
Obesity	5829	IKBKG-CHUK complex	0.05439
Obesity	5832	PINK1-MIRO2-Milton complex	0.04441
Obesity	5837	PPD complex	0.04441
Obesity	5844	I-kappa-B kinase (IKK) complex	0.04441
Obesity	5859	FAS-FADD-CASP8-CASP10 complex	0.03846
Obesity	5861	FAS-FADD-CASP10 complex	0.04441
Obesity	5862	CAV1-VDAC1-ESR1 complex	0.04441
Obsessive-compulsive disorder	753	UTM-SGCE-DAG1-CAV1-NOS3 complex	0.13484
Obsessive-compulsive disorder	1000	TorsinA-TorsinB complex	0.2132
Oligospermia	681	(C-CFTR)2-NHERF-ezrin complex	0.14907
Oligospermia	682	C-CFTR-NHERF(PDZ1 domain)-ezrin complex	0.14907
Oligospermia	683	C-CFTR-NHERF(PDZ2 domain)-ezrin complex	0.14907
Oligospermia	687	CFTR-NHERF-beta(2)AR signaling complex	0.14907
Oligospermia	871	BRAF53-BRCA2 complex	0.09759
Oligospermia	1154	DSS1 complex	0.07161
Oligospermia	2159	AR-AKT-APPL complex	0.14907

Oligospermia	2160	AOF2-AR complex	0.18257
Oligospermia	2818	BRCA1-BARD1-BRCA2-DNA damage complex III	0.14907
Oligospermia	5400	BRCC complex	0.11547
Optic atrophy	178	Respiratory chain complex I (holoenzyme)	0.06742
Optic atrophy	1095	SNX complex (SNX1a SNX2 SNX4 EGFR)	0.22361
Optic atrophy	1185	EGFR-containing signaling complex	0.22361
Optic atrophy	2369	ITGAV-ITGB3-EGFR complex	0.2582
Optic atrophy	2453	Multiprotein complex (monoubiquitination)	0.22361
Optic atrophy	2454	CIN85-CBL-SH3GL2-EGFR complex EGF stimulated	0.22361
Optic atrophy	2542	EGFR-CBL-GRB2 complex	0.2582
Optic atrophy	2919	Respiratory chain complex I (gamma subunit) mitochondrial	0.12403
Optic atrophy	3678	RIN1-STAM2-EGFR complex EGF stimulated	0.2582
Optic atrophy	5171	SH3KBP1-CBLB-EGFR complex	0.2582
Oral cancer	68	BCDX2 complex	0.06804
Oral cancer	115	Polycomb repressive complex 1 (PRC1 hPRC-H)	0.03928
Oral cancer	116	Polycomb repressive complex 1 (PRC1 hPRC-H)	0.03774
Oral cancer	142	CD147-gamma-secretase complex (APH-1a PS-1 PEN-2 NCT variant)	0.06086
Oral cancer	159	Condensin I-PARP-1-XRCC1 complex	0.05143
Oral cancer	205	Ubiquitin E3 ligase (VHL TCEB1 TCEB2 CUL2	0.06086
Oral cancer	206	DNA ligase IV-XRCC4 complex	0.09623
Oral cancer	212	DNA ligase III-XRCC1 complex	0.09623
Oral cancer	213	DNA ligase IV-XRCC1 complex	0.09623
Oral cancer	220	ARF-Mule complex	0.07857
Oral cancer	252	RAD51C-XRCC3 complex	0.09623
Oral cancer	261	RAD51B-RAD51C-RAD51D-XRCC2-XRCC3	0.12172
Oral cancer	262	RAD51L3-XRCC2 complex	0.09623
Oral cancer	310	Cell cycle kinase complex CDC2	0.11111
Oral cancer	311	Cell cycle kinase complex CDK2	0.13608
Oral cancer	312	Cell cycle kinase complex CDK4	0.13608
Oral cancer	313	Cell cycle kinase complex CDK5	0.12172
Oral cancer	314	PCNA-p21 complex	0.09623
Oral cancer	336	DNA ligase IV-XRCC4-AHNK complex	0.07857
Oral cancer	344	DNA ligase IV-XRCC4 complex (LX complex)	0.09623
Oral cancer	350	DNA ligase IV-XRCC4-PNK complex	0.07857
Oral cancer	359	DNA ligase IV-XRCC4-XLF complex	0.07857
Oral cancer	362	DNA ligase III-XRCC1-PNK-DNA-pol III multiprotein complex	0.06804
Oral cancer	521	Polycystin-1-E-cadherin-beta-catenin complex	0.07857
Oral cancer	522	Polycystin-1-E-cadherin-beta-catenin-Flotillin-2	0.06804
Oral cancer	550	NOS3-CAV1-NOSTRIN complex	0.07857
Oral cancer	557	TRP1-G alpha-11-IP3R3-CAV1 signaling complex	0.06804
Oral cancer	622	Ubiquitin E3 ligase (VHL TCEB1 TCEB2 CUL2)	0.06804
Oral cancer	753	UTM-SGCE-DAG1-CAV1-NOS3 complex	0.06086
Oral cancer	826	PAR-3-VE-cadherin-beta-catenin complex	0.07857
Oral cancer	929	CEN complex	0.02237

Oral cancer	1085	DNA repair complex NEIL2-PNK-Pol(beta)-LigIII(alpha)-XRCC1	0.06086
Oral cancer	1086	DNA repair complex NEIL1-PNK-Pol(beta)-LigIII(alpha)-XRCC1	0.06086
Oral cancer	1095	SNX complex (SNX1a SNX2 SNX4 EGFR)	0.06804
Oral cancer	1170	cMYC-ATPase-helicase complex	0.06086
Oral cancer	1171	c-MYC-ATPase-helicase complex	0.06086
Oral cancer	1185	EGFR-containing signaling complex	0.13608
Oral cancer	1189	DNA double-strand break end-joining complex	0.05143
Oral cancer	1224	Ubiquitin E3 ligase (BMI1 SPOP CUL3)	0.07857
Oral cancer	1439	PTGS2 homodimer complex	0.13608
Oral cancer	1462	hPRC1L complex	0.06804
Oral cancer	1514	IL4-IL4R complex	0.09623
Oral cancer	1515	IL4-IL4R-IL2RG complex	0.07857
Oral cancer	1618	G protein complex (PTHR1 GNB1 GNG2)	0.07857
Oral cancer	1633	CyclinD1-CDK4-CDK6 complex	0.07857
Oral cancer	1634	CyclinD1-CDK4-p21 complex	0.15713
Oral cancer	1642	p16-cyclin D2-CDK4 complex	0.07857
Oral cancer	1656	p27-cyclinE-CDK2 complex	0.07857
Oral cancer	1714	TICAM1-TICAM2-TLR4 complex	0.07857
Oral cancer	1816	JUN-TCF4-CTNNB1 complex	0.07857
Oral cancer	1839	SDCBP-CTNNB1-CTNNA1-CDH1 complex	0.06804
Oral cancer	2228	BLM-RAD51L3-XRCC2 complex	0.07857
Oral cancer	2230	PCNA complex	0.05143
Oral cancer	2369	ITGAV-ITGB3-EGFR complex	0.07857
Oral cancer	2398	ITGA3-ITGB1-BSG complex	0.07857
Oral cancer	2429	ITGA2-ITGB1-CD47 complex	0.07857
Oral cancer	2430	ITGA2-ITGB1-CHAD complex	0.07857
Oral cancer	2431	ITGA2-ITGB1-COL6A3 complex	0.07857
Oral cancer	2432	ITGA2-ITGB1 complex	0.09623
Oral cancer	2443	ITGA9-ITGB1-TNC complex	0.07857
Oral cancer	2453	Multiprotein complex (monoubiquitination)	0.06804
Oral cancer	2454	CIN85-CBL-SH3GL2-EGFR complex EGF stimulated	0.06804
Oral cancer	5714	eNOS-CAV1 complex	0.09623
Oral cancer	2462	Caveolin-1 homodimer complex	0.13608
Oral cancer	2528	ERBB2-MEMO-SHC complex	0.07857
Oral cancer	2542	EGFR-CBL-GRB2 complex	0.07857
Oral cancer	2635	BETA2-Cyclin D1 complex	0.09623
Oral cancer	2649	MYC-DNMT3A-ZBTB17 complex	0.07857
Oral cancer	2653	MYC-MAX-BLOC1S1 complex	0.07857
Oral cancer	2655	MYC-MAX complex	0.09623
Oral cancer	2709	MMP-9-TIMP-1-LRP complex	0.07857
Oral cancer	2998	Axin-PP2A A-PP2A C-GSK3-beta-beta-catenin	0.06804
Oral cancer	3004	APC-Axin-1-beta-catenin complex	0.07857
Oral cancer	3015	p27-cyclinE-Cdk2 - Ubiquitin E3 ligase (SKP1A SKP2 CUL1 CKS1B RBX1) complex	0.04811
Oral cancer	3084	CCND1-CDK4 complex	0.09623
Oral cancer	3087	CCND1-CDK6 complex	0.09623

Oral cancer	3155	Bipartite complex (TFC4 CTNNB1)	0.09623
Oral cancer	3166	AXIN-APC-betaCatenin-GSK3B complex	0.06804
Oral cancer	3678	RIN1-STAM2-EGFR complex EGF stimulated	0.07857
Oral cancer	4095	Catulin (alpha) - catenin (beta) complex	0.09623
Oral cancer	4096	Catenin (alpha) - catenin (beta) complex	0.09623
Oral cancer	5772	ZO1-(beta)cadherin-(VE)cadherin-VEGFR2 complex	0.06804
Oral cancer	5101	CyclinD3-CDK4-CDK6-p21 complex	0.06804
Oral cancer	5171	SH3KBP1-CBLB-EGFR complex	0.07857
Oral cancer	5177	Polycystin-1 multiprotein complex (ACTN1 CDH1 SRC JUP VCL CTNNB1 PXN BCAR1 PKD1	0.04103
Oral cancer	5243	XRCC1-LIG3-PNK-TDP1 complex	0.06804
Oral cancer	5260	TCF4-CTNNB1-SUMO1-EP300-HADAC6 complex	0.06086
Oral cancer	5261	TCF4-CTNNB1-EP300 complex	0.07857
Oral cancer	5262	TCF4-CTNNB1 complex	0.09623
Oral cancer	5264	TCF4-CTNNB1-CREBBP complex	0.07857
Oral cancer	5267	VHL-VDU1-TCEB1-TCEB2 complex	0.06804
Oral cancer	5270	VHL-TCEB1-TCEB2 complex	0.07857
Oral cancer	5273	VHL-TBP1-HIF1A complex	0.07857
Oral cancer	5281	Cell-cell junction complex (CDH1-CTNNB1)	0.09623
Oral cancer	5287	CDK4-CCND1 complex	0.09623
Oral cancer	5513	Polycomb repressive complex	0.06804
Oral cancer	5518	BMI1-HPH1-HPH2 complex	0.07857
Oral cancer	5622	HSP90-CIP1-FKBPL complex	0.07857
Oral cancer	5683	hRAD51C-hXRCC3 complex	0.09623
Oral cancer	5862	CAV1-VDAC1-ESR1 complex	0.07857
Osteitis deformans	310	Cell cycle kinase complex CDC2	0.16667
Osteitis deformans	311	Cell cycle kinase complex CDK2	0.20412
Osteitis deformans	312	Cell cycle kinase complex CDK4	0.20412
Osteitis deformans	313	Cell cycle kinase complex CDK5	0.18257
Osteitis deformans	1185	EGFR-containing signaling complex	0.20412
Osteitis deformans	1633	CyclinD1-CDK4-CDK6 complex	0.2357
Osteitis deformans	1634	CyclinD1-CDK4-p21 complex	0.2357
Osteitis deformans	2143	MAP2K5-PRKCI-SQSTM1 complex	0.2357
Osteitis deformans	2528	ERBB2-MEMO-SHC complex	0.2357
Osteitis deformans	2635	BETA2-Cyclin D1 complex	0.28868
Osteitis deformans	3084	CCND1-CDK4 complex	0.28868
Osteitis deformans	3087	CCND1-CDK6 complex	0.28868
Osteitis deformans	5287	CDK4-CCND1 complex	0.28868
Osteomyelitis	1256	MLL-HCF complex	0.12599
Osteomyelitis	1257	ALL-1 supercomplex	0.06299
Osteomyelitis	1401	MOF complex	0.10541
Osteomyelitis	1539	G protein complex (GNG2 GNB2L1 RAF1)	0.19245
Osteomyelitis	2000	BAX homo-oligomer complex	0.33333
Osteomyelitis	3162	TF-FVIIa-FXa-TFPI complex	0.16667
Osteomyelitis	3492	Bax homooligomeric complex after apoptotic	0.33333
Osteomyelitis	5211	RAF1-PPP2-PIN1 complex	0.14907
Osteomyelitis	5386	MLL1-WDR5 complex	0.06415

Osteomyelitis	5770	RUNX1-CBF-beta-DNA complex	0.2357
Osteomyelitis	5877	MAP2K1-BRAF-RAF1-YWHAE-KSR1 complex	0.14907
Osteomyelitis	5873	RAF1-MAP2K1-YWHAE complex	0.19245
Osteomyelitis	5919	BRAF-RAF1-14-3-3 complex	0.11111
Osteomyelitis	5920	KSR1-RAF1-MEK complex	0.16667
Osteomyelitis	5922	RAF1-RAS complex EGF induced	0.16667
Osteomyelitis	5923	RAF1-BRAF complex RAS stimulated	0.2357
Osteomyelitis	5924	RAF1-CNK1 complex RAS stimulated	0.2357
Osteomyelitis	5928	CNK1-SRC-RAF1 complex	0.19245
Osteoporosis	441	TFTC-type histone acetyl transferase complex	0.04767
Osteoporosis	541	IGF1-IGFBP3-ALS complex	0.09129
Osteoporosis	748	TRAF6-TAK1 complex	0.1118
Osteoporosis	1054	ESR1-RELA-BCL3-NCOA3 complex	0.07906
Osteoporosis	2073	TNFRSF11A-TRAF6-SRC complex	0.09129
Osteoporosis	2074	TRAF6 oligomer complex	0.15811
Osteoporosis	2124	IKK-alpha--ER-alpha-AIB1 complex	0.09129
Osteoporosis	2159	AR-AKT-APPL complex	0.09129
Osteoporosis	2160	AOF2-AR complex	0.1118
Osteoporosis	2318	ITGA6-ITGB4-Laminin10/12 complex	0.07071
Osteoporosis	2319	ITGA6-ITGB4-Laminin10/12 complex	0.07071
Osteoporosis	2320	ITGA6-ITGB4-CD151 complex	0.09129
Osteoporosis	2321	ITGA6-ITGB4-FYN complex	0.09129
Osteoporosis	2322	ITGA6-ITGB4-LAMA5 complex	0.09129
Osteoporosis	2323	ITGA6-ITGB4 complex	0.1118
Osteoporosis	2347	ITGAV-ITGB5-SPP1 complex	0.09129
Osteoporosis	2352	ITGAV-ITGB6-SPP1 complex	0.09129
Osteoporosis	2358	ITGAV-ITGB3-SPP1 complex	0.09129
Osteoporosis	2363	ITGAV-ITGB3-PXN-PTK2b complex	0.07906
Osteoporosis	2411	ITGA6-ITGB1-CD151 complex	0.09129
Osteoporosis	2413	ITGA6-ITGB1 complex	0.1118
Osteoporosis	2434	ITGA1-ITGB1-COL6A3 complex	0.09129
Osteoporosis	2435	ITGA1-ITGB1-PTPN2 complex	0.09129
Osteoporosis	2437	ITGA6-ITGB1-CYR61 complex	0.09129
Osteoporosis	2470	p130Cas-ER-alpha-cSrc-kinase- PI3-kinase p85-subunit complex	0.07906
Osteoporosis	5709	ArgBP2a-CBL-PTK2B complex	0.09129
Osteoporosis	2657	ESR1-CDK7-CCNH-MNAT1-MTA1-HDAC2 complex	0.06455
Osteoporosis	2670	Er-alpha-p53-hdm2 complex	0.09129
Osteoporosis	2699	ER-alpha-GRIP1-c-Jun complex	0.09129
Osteoporosis	2700	ER-alpha-c-Jun complex	0.1118
Osteoporosis	2743	TRAF6-MALT1 complex	0.1118
Osteoporosis	2745	Ubiquitin ligase complex (TRAF6 TAB2 MALT1 UEV1A BCL10)	0.07071
Osteoporosis	2770	ITGA6-ITGB4-CD9 complex	0.09129
Osteoporosis	2885	ITGAV-ITGB1-SPP1 complex	0.09129
Osteoporosis	2936	Ecsit complex (ECSIT MT-CO2 GAPDH TRAF6 NDUFAF1)	0.07071

Osteoporosis	2939	Ecsit complex (ECSIT MT-CO2 NDUFA1 MT-ND1 TRAF6 NDUFAF1)	0.06455
Osteoporosis	3043	BMP2-BRIA complex	0.09129
Osteoporosis	3096	ITGA6-ITGB4-SHC1-GRB2 complex	0.07906
Osteoporosis	3111	ITGA9-ITGB1-SPP1 complex	0.09129
Osteoporosis	3112	ITGA5-ITGB1-SPP1 complex	0.09129
Osteoporosis	3710	CHL2-BMP2 complex	0.1118
Osteoporosis	3711	CHL2-BMP2-TSG complex	0.09129
Osteoporosis	5862	CAV1-VDAC1-ESR1 complex	0.09129
Osteosarcoma	190	Mitotic checkpoint complex (MCC)	0.1066
Osteosarcoma	305	40S ribosomal subunit cytoplasmic	0.03656
Osteosarcoma	306	Ribosome cytoplasmic	0.02369
Osteosarcoma	486	WIP-WASp-actin-myosin-IIa complex	0.08704
Osteosarcoma	541	IGF1-IGFBP3-ALS complex	0.12309
Osteosarcoma	1056	ZNF198-SUMO1 complex	0.15076
Osteosarcoma	1774	MICA-KLRK1-HCST complex	0.12309
Osteosarcoma	1810	ITGA4-PXN-GIT1 complex	0.12309
Osteosarcoma	1851	BUB1-BUB3 complex	0.15076
Osteosarcoma	1976	MTNR1A homodimer complex	0.2132
Osteosarcoma	1978	MTNR1A-MTNR1B complex	0.15076
Osteosarcoma	2254	CTGF/Hcs24-actin complex	0.12309
Osteosarcoma	2417	ITGA4-ITGB1-EMILIN1 complex	0.12309
Osteosarcoma	2418	ITGA4-ITGB1 complex	0.15076
Osteosarcoma	2419	ITGA4-ITGB1-CD81 complex	0.12309
Osteosarcoma	2420	ITGA4-ITGB1-CD53 complex	0.12309
Osteosarcoma	2421	ITGA4-ITGB1-VCAM1 complex	0.12309
Osteosarcoma	2422	ITGA4-ITGB1-JAM2 complex	0.12309
Osteosarcoma	2423	ITGA4-ITGB1-CD47 complex	0.12309
Osteosarcoma	2424	ITGA4-ITGB1-CD63 complex	0.12309
Osteosarcoma	2425	ITGA4-ITGB1-PXN complex	0.12309
Osteosarcoma	2426	ITGA4-ITGB1-THBS1 complex	0.12309
Osteosarcoma	2428	ITGA4-ITGB1-THBS2 complex	0.12309
Osteosarcoma	3055	Nop56p-associated pre-rRNA complex	0.02091
Osteosarcoma	5190	TIAM1-EFNB1-EPHA2 complex	0.12309
Osteosarcoma	5260	TCF4-CTNNB1-SUMO1-EP300-HADAC6 complex	0.09535
Osteosarcoma	5655	Ternary complex (LRRC7 CAMK2a ACTN4)	0.12309
Osteosarcoma	5755	SUMO1-SUA1-UBA2 complex	0.12309
Otitis media	1	BCL6-HDAC4 complex	0.2357
Otitis media	2	BCL6-HDAC5 complex	0.2357
Otitis media	3	BCL6-HDAC7 complex	0.2357
Otitis media	41	Mi-2/NuRD-MTA2 complex	0.14907
Otitis media	585	Mi2/NuRD-BCL6-MTA3 complex	0.14907
Otitis media	1260	Neddylin ligase (FBXO11 SKP1 CUL1 RBX1)	0.16667
Otitis media	1508	BCL6-ZBTB17 complex	0.2357
Ovarian cancer	68	BCDX2 complex	0.05025
Ovarian cancer	91	FA complex (Fanconi anemia complex) cytoplasmic	0.1005
Ovarian cancer	103	RNA polymerase II holoenzyme complex	0.02052

Ovarian cancer	107	TFIIH transcription factor complex	0.0335
Ovarian cancer	240	BRCA1-CTIP-ZBRK1 repressor complex	0.05803
Ovarian cancer	244	BRAFT complex	0.05575
Ovarian cancer	245	FA core complex (Fanconi anemia core complex)	0.067
Ovarian cancer	261	RAD51B-RAD51C-RAD51D-XRCC2-XRCC3	0.04495
Ovarian cancer	262	RAD51L3-XRCC2 complex	0.07107
Ovarian cancer	375	MSH2-MSH3 complex	0.07107
Ovarian cancer	378	MutS-beta complex	0.07107
Ovarian cancer	387	MCM complex	0.04103
Ovarian cancer	443	BP-SMAD complex	0.03553
Ovarian cancer	1009	TFIIH transcription factor complex	0.03178
Ovarian cancer	1029	TFIIH transcription factor complex	0.03178
Ovarian cancer	1037	TFIIH transcription factor core complex	0.04495
Ovarian cancer	1088	PRNP-ApolopoproteinE3 complex	0.07107
Ovarian cancer	1093	SNX complex (SNX1a SNX2 SNX4 INSR)	0.05025
Ovarian cancer	1141	CF IIAm complex (Cleavage factor IIAm complex)	0.02513
Ovarian cancer	1152	FA complex (Fanconi anemia complex)	0.07107
Ovarian cancer	1192	ESCRT-I complex	0.05803
Ovarian cancer	1514	IL4-IL4R complex	0.07107
Ovarian cancer	1515	IL4-IL4R-IL2RG complex	0.05803
Ovarian cancer	1623	FA core complex 1 (Fanconi anemia core complex 1)	0.08989
Ovarian cancer	1624	FA core complex (Fanconi anemia core complex)	0.067
Ovarian cancer	1625	FA core complex (Fanconi anemia core complex)	0.07597
Ovarian cancer	1839	SDCBP-CTNNB1-CTNNA1-CDH1 complex	0.05025
Ovarian cancer	1844	APC-IQGAP1 complex	0.07107
Ovarian cancer	1845	APC-IQGAP1-CLIP-170 complex	0.11605
Ovarian cancer	1863	TSG101-VPS37B-VPS28 complex	0.05803
Ovarian cancer	2220	RAD52-ERCC4-ERCC1 complex	0.05803
Ovarian cancer	2228	BLM-RAD51L3-XRCC2 complex	0.05803
Ovarian cancer	2254	CTGF/Hcs24-actin complex	0.05803
Ovarian cancer	2383	ITGA5-ITGB1-FN1-TGM2 complex	0.05025
Ovarian cancer	2384	ITGA5-ITGB1-ADAM15 complex	0.05803
Ovarian cancer	2385	ITGA5-ITGB4 complex	0.07107
Ovarian cancer	2388	Itga5-Itgb1-Fn1-Sfrp2 complex	0.05025
Ovarian cancer	2577	Sam68-p85 P13K-IRS-1-IR signaling complex	0.05025
Ovarian cancer	2589	PGC-1-SRp40-SRp55-SRp75 complex	0.05025
Ovarian cancer	2599	POLR2A-CCNT1-CDK9-NCL-LEM6-CPSF2 complex	0.04103
Ovarian cancer	2739	FA complex (Fanconi anemia complex)	0.06061
Ovarian cancer	2825	BRCA1-RNA polymerase II complex	0.01971
Ovarian cancer	2826	ITGB3-ITGAV-VTN complex	0.05803
Ovarian cancer	2850	ITGA5-ITGB1-FN-1-NOV complex	0.05025
Ovarian cancer	2853	ITGA5-ITGB1-CAL4A3 complex	0.05803
Ovarian cancer	2882	ITGA5-ITGB3-COL6A3 complex	0.05803
Ovarian cancer	2936	Ecsit complex (ECSIT MT-CO2 GAPDH TRAF6 NDUFAF1)	0.04495
Ovarian cancer	2939	Ecsit complex (ECSIT MT-CO2 NDUFA1 MT-ND1 TRAF6 NDUFAF1)	0.04103
Ovarian cancer	3008	60S APC containing complex	0.03799

Ovarian cancer	3011	APC-IQGAP1-Rac1 complex	0.05803
Ovarian cancer	3012	APC-IQGAP1-Cdc42 complex	0.05803
Ovarian cancer	3066	RNA polymerase II complex chromatin structure modifying	0.02787
Ovarian cancer	3083	Nucleic and chromatin Fanconi complex	0.08989
Ovarian cancer	3112	ITGA5-ITGB1-SPP1 complex	0.05803
Ovarian cancer	3117	ITGB5-ITGAV-VTN complex	0.05803
Ovarian cancer	4096	Catenin (alpha) - catenin (beta) complex	0.07107
Ovarian cancer	5274	Cell-cell junction complex (ARHGAP10-CTNNA1)	0.07107
Ovarian cancer	5423	HSP70-BAG5-PARK2 complex	0.05025
Ovarian cancer	5495	TFIIH transcription factor complex (ERCC2 ERCC3 GTF2H1 CDK7 CCNH GTF2H2)	0.04103
Ovarian cancer	5582	LIFR-LIF-gp130 complex	0.05803
Ovarian cancer	5615	Emerin complex 52	0.02096
Ovarian cancer	5683	hRAD51C-hXRCC3 complex	0.07107
Ovarian cancer	5770	RUNX1-CBF-beta-DNA complex	0.07107
Ovarian cancer	5837	PPD complex	0.05803
Ovarian disease	368	ERCC1-ERCC4-MSH2 complex	0.13608
Ovarian disease	371	Structure-specific endonulease complex	0.2357
Ovarian disease	441	TFTC-type histone acetyl transferase complex	0.07107
Ovarian disease	531	XPA-ERCC1-ERCC4 complex	0.27217
Ovarian disease	725	P2X7 receptor signalling complex	0.06804
Ovarian disease	1054	ESR1-RELA-BCL3-NCOA3 complex	0.11785
Ovarian disease	2124	IKK-alpha--ER-alpha-AIB1 complex	0.13608
Ovarian disease	2220	RAD52-ERCC4-ERCC1 complex	0.13608
Ovarian disease	2470	p130Cas-ER-alpha-cSrc-kinase- PI3-kinase p85-subunit complex	0.11785
Ovarian disease	2657	ESR1-CDK7-CCNH-MNAT1-MTA1-HDAC2 complex	0.09623
Ovarian disease	2670	Er-alpha-p53-hdm2 complex	0.13608
Ovarian disease	2699	ER-alpha-GRIP1-c-Jun complex	0.13608
Ovarian disease	2700	ER-alpha-c-Jun complex	0.16667
Ovarian disease	2709	MMP-9-TIMP-1-LRP complex	0.13608
Ovarian disease	3917	Ternary complex (GATA4 SRF MYOCD)	0.13608
Ovarian disease	5655	Ternary complex (LRRC7 CAMK2a ACTN4)	0.13608
Ovarian disease	5862	CAV1-VDAC1-ESR1 complex	0.13608
Ovarian failure	425	MSH4-MSH5-GPS2 complex	0.26491
Ovarian failure	441	TFTC-type histone acetyl transferase complex	0.06917
Ovarian failure	1054	ESR1-RELA-BCL3-NCOA3 complex	0.11471
Ovarian failure	2124	IKK-alpha--ER-alpha-AIB1 complex	0.13245
Ovarian failure	2470	p130Cas-ER-alpha-cSrc-kinase- PI3-kinase p85-subunit complex	0.11471
Ovarian failure	2657	ESR1-CDK7-CCNH-MNAT1-MTA1-HDAC2 complex	0.09366
Ovarian failure	2670	Er-alpha-p53-hdm2 complex	0.13245
Ovarian failure	2699	ER-alpha-GRIP1-c-Jun complex	0.13245
Ovarian failure	2700	ER-alpha-c-Jun complex	0.16222
Ovarian failure	5862	CAV1-VDAC1-ESR1 complex	0.13245
Ovary cancer	267	Checkpoint 9-1-1 complex	0.2582
Ovary cancer	268	Checkpoint Rad complex	0.15811

Ovary cancer	274	RAD17-RFC-9-1-1 checkpoint supercomplex	0.15811
Ovary cancer	1990	PGRMC1-SCAP complex	0.15811
Ovary cancer	2196	LIG1-9-1-1 complex	0.22361
Ovary cancer	2197	FEN1-9-1-1 complex	0.22361
Ovary cancer	2198	RAD9-RAD1-HUS1-POLB complex	0.22361
Ovary cancer	2709	MMP-9-TIMP-1-LRP complex	0.1291
Ovary cancer	2808	RAD9-RAD1-HUS1-APE1 complex	0.22361
Ovary cancer	2809	9-1-1 complex	0.2582
Ovary cancer	5153	CTFC-TAF1 complex	0.15811
Ovary cancer	5165	AP1G1-PACS1-FURIN complex	0.1291
Overnutrition	1810	ITGA4-PXN-GIT1 complex	0.14907
Overnutrition	2417	ITGA4-ITGB1-EMILIN1 complex	0.14907
Overnutrition	2418	ITGA4-ITGB1 complex	0.18257
Overnutrition	2419	ITGA4-ITGB1-CD81 complex	0.14907
Overnutrition	2420	ITGA4-ITGB1-CD53 complex	0.14907
Overnutrition	2421	ITGA4-ITGB1-VCAM1 complex	0.14907
Overnutrition	2422	ITGA4-ITGB1-JAM2 complex	0.14907
Overnutrition	2423	ITGA4-ITGB1-CD47 complex	0.14907
Overnutrition	2424	ITGA4-ITGB1-CD63 complex	0.14907
Overnutrition	2425	ITGA4-ITGB1-PXN complex	0.14907
Overnutrition	2426	ITGA4-ITGB1-THBS1 complex	0.14907
Overnutrition	2428	ITGA4-ITGB1-THBS2 complex	0.14907
Overnutrition	2470	p130Cas-ER-alpha-cSrc-kinase- PI3-kinase p85-subunit complex	0.1291
Overnutrition	2480	CIN85 complex (CIN85 CRK BCAR1 CBL PIK3R1 GRB2 SOS1)	0.09759
Overnutrition	2529	LAT-PLC-gamma-1-p85-GRB2-CBL-VAV-SLP-76 signaling complex C305 activated	0.09759
Overnutrition	2551	PDGFRA-PLC-gamma-1-PI3K-SHP-2 complex PDGF stimulated	0.1291
Overnutrition	2574	CD19-Vav-PI 3-kinase (p85 subunit) complex	0.14907
Overnutrition	2577	Sam68-p85 P13K-IRS-1-IR signaling complex	0.1291
Overnutrition	2922	LAT-PLC-gamma-1-p85-GRB2-SOS signaling complex C305 activated	0.11547
Overnutrition	2944	Notch1-p56lck-PI3K complex	0.14907
Overnutrition	3133	Phosphatidylinositol 3-kinase (PIK3CA PIK3R1)	0.18257
PEComa	75	TSC1-TSC2 complex	0.5
Palmoplantar keratosis	873	SNARE complex (STX1A SNAP29)	0.31623
Pancreas cancer	68	BCDX2 complex	0.05185
Pancreas cancer	75	TSC1-TSC2 complex	0.07332
Pancreas cancer	91	FA complex (Fanconi anemia complex) cytoplasmic	0.05185
Pancreas cancer	244	BRAFT complex	0.02876
Pancreas cancer	245	FA core complex (Fanconi anemia core complex)	0.03457
Pancreas cancer	261	RAD51B-RAD51C-RAD51D-XRCC2-XRCC3	0.04637
Pancreas cancer	262	RAD51L3-XRCC2 complex	0.07332
Pancreas cancer	285	PCNA-MLH1-PMS1 complex	0.05987
Pancreas cancer	306	Ribosome cytoplasmic	0.01152
Pancreas cancer	308	60S ribosomal subunit cytoplasmic	0.01513

Pancreas cancer	351	Spliceosome	0.01734
Pancreas cancer	370	MSH2-MSH6-PMS1-MLH1 complex	0.05185
Pancreas cancer	380	MutL-beta complex	0.07332
Pancreas cancer	443	BP-SMAD complex	0.03666
Pancreas cancer	552	IFNB1-IFNAR1-IFNAR2- complex	0.11974
Pancreas cancer	740	Exon junction complex EIF4A3-MLN51-MAGOH-Y14 (RNA-protein complex)	0.05185
Pancreas cancer	744	Exon junction complex (EIF4A3-MLN51-MAGOH-	0.05185
Pancreas cancer	754	Exon junction complex (EIF4A3-MLN51-UPF3B-MAGOH-Y14)	0.04637
Pancreas cancer	755	Exon junction complex (F4A3-MLN51-UPF3B-MAGOH-Y14-PYM)	0.04233
Pancreas cancer	769	Exon junction complex (EIF4A3-MLN51-MAGOH-Y14) RNA-protein complex	0.05185
Pancreas cancer	772	eIF4AIII-Btz complex	0.07332
Pancreas cancer	785	Exon junction complex	0.03666
Pancreas cancer	999	p23 protein complex	0.05987
Pancreas cancer	1141	CF IIAm complex (Cleavage factor IIAm complex)	0.02592
Pancreas cancer	1152	FA complex (Fanconi anemia complex)	0.03666
Pancreas cancer	1181	C complex spliceosome	0.03478
Pancreas cancer	1255	Ubiquitin E3 ligase (SIAH1 SIP SKP1A TBL1X)	0.05185
Pancreas cancer	1297	MKK4-ARRB2-ASK1 complex	0.05987
Pancreas cancer	1514	IL4-IL4R complex	0.14665
Pancreas cancer	1515	IL4-IL4R-IL2RG complex	0.11974
Pancreas cancer	1521	p300-SMAD1-STAT3 complex	0.05987
Pancreas cancer	1623	FA core complex 1 (Fanconi anemia core complex 1)	0.04637
Pancreas cancer	1624	FA core complex (Fanconi anemia core complex)	0.03457
Pancreas cancer	1625	FA core complex (Fanconi anemia core complex)	0.03919
Pancreas cancer	1707	IL2-IL2RA-IL2RB complex	0.05987
Pancreas cancer	1783	TGF-beta receptor I-SMAD7-SMURF1 complex	0.05987
Pancreas cancer	1972	BMP4-BGN complex	0.07332
Pancreas cancer	1982	CACY homodimer complex	0.1037
Pancreas cancer	2228	BLM-RAD51L3-XRCC2 complex	0.05987
Pancreas cancer	2254	CTGF/Hcs24-actin complex	0.05987
Pancreas cancer	2429	ITGA2-ITGB1-CD47 complex	0.05987
Pancreas cancer	2430	ITGA2-ITGB1-CHAD complex	0.05987
Pancreas cancer	2431	ITGA2-ITGB1-COL6A3 complex	0.05987
Pancreas cancer	2432	ITGA2-ITGB1 complex	0.07332
Pancreas cancer	2486	GIPC1-LHCGR complex	0.07332
Pancreas cancer	2487	GIPC1-NTRK1-RGS19 complex	0.05987
Pancreas cancer	2642	SMAD1-P300 complex	0.07332
Pancreas cancer	2739	FA complex (Fanconi anemia complex)	0.03127
Pancreas cancer	2755	17S U2 snRNP	0.01805
Pancreas cancer	2958	SMAD1-CBP complex	0.07332
Pancreas cancer	2959	SMAD1-OAZ-HsN3 complex	0.05987
Pancreas cancer	2989	ITGA9-ITGB1-ADAM8 complex	0.05987
Pancreas cancer	2996	SMAD7-SMURF1 complex	0.07332
Pancreas cancer	2997	SMAD7-SMURF1-TGF-beta receptor complex	0.05185

Pancreas cancer	3055	Nop56p-associated pre-rRNA complex	0.02034
Pancreas cancer	3083	Nucleic and chromatin Fanconi complex	0.04637
Pancreas cancer	3162	TF-FVIIa-FXa-TFPI complex	0.05185
Pancreas cancer	4039	PAR4-BACE1 complex	0.07332
Pancreas cancer	5215	CS-MAP3K7IP1-MAP3K7IP2 complex	0.05987
Pancreas cancer	5380	TRBP containing complex (DICER RPL7A EIF6 MOV10 and subunits of the 60S ribosomal particle)	0.02074
Pancreas cancer	5579	CNTF-CNTFR-gp130-LIFR complex	0.05185
Pancreas cancer	5582	LIFR-LIF-gp130 complex	0.11974
Pancreas cancer	5683	hRAD51C-hXRCC3 complex	0.07332
Pancreas disease	306	Ribosome cytoplasmic	0.02317
Pancreas disease	308	60S ribosomal subunit cytoplasmic	0.03041
Pancreas disease	725	P2X7 receptor signalling complex	0.06019
Pancreas disease	1028	HNF4A-SUB1 complex	0.14744
Pancreas disease	1700	ABL2-HRAS-RIN1 complex	0.12039
Pancreas disease	3055	Nop56p-associated pre-rRNA complex	0.02045
Pancreas disease	5380	TRBP containing complex (DICER RPL7A EIF6 MOV10 and subunits of the 60S ribosomal particle)	0.0417
Pancreas disease	5383	TRIB3-DDIT3 complex	0.14744
Pancreas disease	5655	Ternary complex (LRRC7 CAMK2a ACTN4)	0.12039
Pancreas disease	5922	RAF1-RAS complex EGF induced	0.10426
Pancreatitis	120	Lymphotoxin beta receptor complex	0.09017
Pancreatitis	220	ARF-Mule complex	0.09017
Pancreatitis	655	HSF1-HSF2 complex	0.11043
Pancreatitis	1054	ESR1-RELA-BCL3-NCOA3 complex	0.07809
Pancreatitis	1642	p16-cyclin D2-CDK4 complex	0.09017
Pancreatitis	1707	IL2-IL2RA-IL2RB complex	0.09017
Pancreatitis	1714	TICAM1-TICAM2-TLR4 complex	0.09017
Pancreatitis	1986	Endoglin homodimer complex	0.15617
Pancreatitis	2084	NFKB1-NFKB2-REL-RELA-RELB complex	0.06984
Pancreatitis	2086	NFKB1-NFKB2-RELA-RELB complex	0.07809
Pancreatitis	2105	IkappaB kinase complex (IKBKB CHUK IKBKAP NFKBIA RELA MAP3K14)	0.06376
Pancreatitis	2145	HSF1-YWHAE complex	0.11043
Pancreatitis	2347	ITGAV-ITGB5-SPP1 complex	0.09017
Pancreatitis	2352	ITGAV-ITGB6-SPP1 complex	0.09017
Pancreatitis	2358	ITGAV-ITGB3-SPP1 complex	0.09017
Pancreatitis	2456	MET-CIN85-SH3GL3-CBL complex HGF stimulated	0.07809
Pancreatitis	2541	HGF-Met complex	0.11043
Pancreatitis	2710	LRP-1-Alpha-2-M-annexin VI complex	0.09017
Pancreatitis	2885	ITGAV-ITGB1-SPP1 complex	0.09017
Pancreatitis	3045	hs4 enhancer complex (faster migrating complex)	0.06984
Pancreatitis	3111	ITGA9-ITGB1-SPP1 complex	0.09017
Pancreatitis	3112	ITGA5-ITGB1-SPP1 complex	0.09017
Pancreatitis	5193	TNF-alpha/NF-kappa B signaling complex (CHUK KPNA3 NFKB2 NFKBIB REL IKBKG NFKB1 NFKBIE RELB NFKBIA RELA TNIP2)	0.04508

Pancreatitis	5194	TNF-alpha/NF-kappa B signaling complex (SEC16A CHUK IKBKB NFKB2 REL IKBKG MAP3K14 RELA FBXW7 USP2)	0.04939
Pancreatitis	5196	TNF-alpha/NF-kappa B signaling complex (CHUK BTRC NFKB2 PPP6C REL CUL1 IKBKE SAPS2 SAPS1 ANKRD28 RELA SKP1)	0.04508
Pancreatitis	5220	CHUK-IQGAP2-AKAP8L-RELA-TNIP2 complex	0.06984
Pancreatitis	5228	REL-MAP3K8-RELA-TNIP2-PAPOLA complex	0.06984
Pancreatitis	5230	CHUK-NFKB2-REL-IKBKG-SPAG9-NFKB1-NFKBIE-COPB2-TNIP1-NFKBIA-RELA-TNIP2	0.04508
Pancreatitis	5232	TNF-alpha/Nf-kappa B signaling complex (RPL6 RPL30 RPS13 CHUK DDX3X NFKB2 NFKBIB REL IKBKG NFKB1 MAP3K8 RELB GLG1 NFKBIA RELA TNIP2 GTF2I)	0.03788
Pancreatitis	5233	TNF-alpha/NF-kappa B signaling complex 5	0.03123
Pancreatitis	5460	p50-p65 NF(kappa)B complex	0.11043
Pancreatitis	5461	p50-p65 NF(kappa)B-SRC1 complex	0.09017
Pancreatitis	5464	I(kappa)B(alpha)-NF(kappa)Bp50-NF(kappa)Bp65 complex	0.09017
Pancreatitis	5465	IKB(epsilon)-RELA-cREL complex	0.09017
Pancreatitis	5466	IKB(beta)-RELA-cREL complex	0.09017
Pancreatitis	5467	IKB(alpha)-RELA-cREL complex	0.09017
Pancreatitis	5475	MURR1-NF(kappa)Bp65-IKBA complex	0.09017
Pancreatitis	5492	IKBA-NF(kappa)Bp65-NF(kappa)Bp50 complex	0.09017
Pancreatitis	5922	RAF1-RAS complex EGF induced	0.07809
Panic disorder	441	TFTC-type histone acetyl transferase complex	0.0658
Panic disorder	1054	ESR1-RELA-BCL3-NCOA3 complex	0.10911
Panic disorder	2124	IKK-alpha--ER-alpha-AIB1 complex	0.12599
Panic disorder	2470	p130Cas-ER-alpha-cSrc-kinase- PI3-kinase p85-subunit complex	0.10911
Panic disorder	2657	ESR1-CDK7-CCNH-MNAT1-MTA1-HDAC2 complex	0.08909
Panic disorder	2670	Er-alpha-p53-hdm2 complex	0.12599
Panic disorder	2699	ER-alpha-GRIP1-c-Jun complex	0.12599
Panic disorder	2700	ER-alpha-c-Jun complex	0.1543
Panic disorder	5414	HTR1A-HTR1D complex	0.1543
Panic disorder	5416	HTR1A-HTR1B complex	0.1543
Panic disorder	5418	GABBR2-HTR1A complex	0.1543
Panic disorder	5419	HTR1A-GPR26 complex	0.1543
Panic disorder	5420	HTR1A-EDG3 complex	0.1543
Panic disorder	5421	HTR1A homodimer complex	0.21822
Panic disorder	5422	HTR1A-EDG1 complex	0.1543
Panic disorder	5862	CAV1-VDAC1-ESR1 complex	0.12599
Papillary adenocarcinoma	1095	SNX complex (SNX1a SNX2 SNX4 EGFR)	0.28868
Papillary adenocarcinoma	1185	EGFR-containing signaling complex	0.28868
Papillary adenocarcinoma	2369	ITGAV-ITGB3-EGFR complex	0.33333
Papillary adenocarcinoma	2453	Multiprotein complex (monoubiquitination)	0.28868
Papillary adenocarcinoma	2454	CIN85-CBL-SH3GL2-EGFR complex EGF stimulated	0.28868
Papillary adenocarcinoma	2542	EGFR-CBL-GRB2 complex	0.33333
Papillary adenocarcinoma	3678	RIN1-STAM2-EGFR complex EGF stimulated	0.33333

Papillary adenocarcinoma	5171	SH3KBP1-CBLB-EGFR complex	0.33333
Papillary cancer	520	KCNQ1 macromolecular complex	0.1118
Papillary cancer	1474	SMAD3/4-E2F4/5-p107-DP1 complex	0.14434
Papillary cancer	1661	E2F4-p107-cyclinA complex	0.20412
Papillary cancer	2975	SMAD3-E2F4/5-p107-DP1 complex	0.15811
Papillary cancer	5144	E2F1-p107-cyclinA complex	0.20412
Papillary cancer	5159	E2F4-p107-cyclinE complex	0.20412
Papillary cancer	5183	DNA-PK-Ku-eIF2-NF90-NF45 complex	0.125
Papillary cancer	5589	LINC complex S-phase	0.13363
Papillary cancer	5609	Emerin regulatory complex	0.11785
Papillary cancer	5614	Emerin complex 32	0.07538
Papillomavirus infection	220	ARF-Mule complex	0.33333
Papillomavirus infection	310	Cell cycle kinase complex CDC2	0.2357
Papillomavirus infection	311	Cell cycle kinase complex CDK2	0.28868
Papillomavirus infection	312	Cell cycle kinase complex CDK4	0.28868
Papillomavirus infection	313	Cell cycle kinase complex CDK5	0.2582
Papillomavirus infection	314	PCNA-p21 complex	0.40825
Papillomavirus infection	1185	EGFR-containing signaling complex	0.28868
Papillomavirus infection	1634	CyclinD1-CDK4-p21 complex	0.33333
Papillomavirus infection	1642	p16-cyclin D2-CDK4 complex	0.33333
Papillomavirus infection	2230	PCNA complex	0.21822
Papillomavirus infection	2528	ERBB2-MEMO-SHC complex	0.33333
Papillomavirus infection	5101	CyclinD3-CDK4-CDK6-p21 complex	0.28868
Papillomavirus infection	5622	HSP90-CIP1-FKBPL complex	0.33333
Parasitic disease	1087	BIRC5-AURKB-INCENP-EVI5 complex	0.25
Parasitic disease	1116	CRM1-Survivin-AuroraB mitotic complex	0.28868
Parasitic disease	1117	CRM1-Survivin mitotic complex	0.35355
Parasitic disease	1118	Chromosomal passenger complex CPC (INCENP CDCA8 BIRC5 AURKB)	0.25
Parasitic disease	1120	Chromosomal passenger complex CPC (INCENP CDCA8 BIRC5)	0.28868
Parasitic disease	2579	Chromosomal passenger complex CPC (INCENP BIRC5 AURKB)	0.28868
Parasitic disease	2580	Survivin homodimer complex	0.5
Parasitic disease	2581	RasGAP-AURKA/AURKB-survivin complex	0.25
Parasitic disease	2582	Chromosomal passenger complex CPC (CDCA8 AURKB BIRC5)	0.28868
Parkinson disease	75	TSC1-TSC2 complex	0.07809
Parkinson disease	159	Condensin I-PARP-1-XRCC1 complex	0.04174
Parkinson disease	178	Respiratory chain complex I (holoenzyme)	0.04994
Parkinson disease	220	ARF-Mule complex	0.06376
Parkinson disease	441	TFTC-type histone acetyl transferase complex	0.0333
Parkinson disease	722	MRG15-PAM14-RB complex	0.06376
Parkinson disease	723	MAF1 complex	0.06376
Parkinson disease	1004	RC complex during S-phase of cell cycle	0.03063
Parkinson disease	1005	RC complex during G2/M-phase of cell cycle	0.03063
Parkinson disease	1054	ESR1-RELA-BCL3-NCOA3 complex	0.05522
Parkinson disease	1094	Frataxin complex	0.04174

Parkinson disease	1193	Rap1 complex	0.04174
Parkinson disease	1226	H2AX complex I	0.04174
Parkinson disease	1250	pRB-E2F-1 complex	0.15617
Parkinson disease	1255	Ubiquitin E3 ligase (SIAH1 SIP SKP1A TBL1X)	0.05522
Parkinson disease	1297	MKK4-ARRB2-ASK1 complex	0.06376
Parkinson disease	1372	Rb-tal-1-E2A-Lmo2-Ldb1 complex	0.04939
Parkinson disease	1439	PTGS2 homodimer complex	0.11043
Parkinson disease	1488	DNMT1-RB1-HDAC1-E2F1 complex	0.11043
Parkinson disease	1642	p16-cyclin D2-CDK4 complex	0.06376
Parkinson disease	1728	CTCF-nucleophosmin-PARP-HIS-KPNA-LMNA-TOP complex	0.03681
Parkinson disease	1729	TLE1 corepressor complex (MASH1 promoter-corepressor complex)	0.03492
Parkinson disease	1784	RNF11-SMURF2-STAMBP complex	0.06376
Parkinson disease	1826	SMAD3-HEF1-APC10-CDH1 complex	0.05522
Parkinson disease	2124	IKK-alpha--ER-alpha-AIB1 complex	0.06376
Parkinson disease	2258	VILIP-1-AChR-alpha-4-AChR-beta-2 complex	0.06376
Parkinson disease	2347	ITGAV-ITGB5-SPP1 complex	0.06376
Parkinson disease	2352	ITGAV-ITGB6-SPP1 complex	0.06376
Parkinson disease	2358	ITGAV-ITGB3-SPP1 complex	0.06376
Parkinson disease	2470	p130Cas-ER-alpha-cSrc-kinase- PI3-kinase p85-subunit complex	0.05522
Parkinson disease	2625	CDK8-MED6-PARP1 complex	0.06376
Parkinson disease	2657	ESR1-CDK7-CCNH-MNAT1-MTA1-HDAC2 complex	0.04508
Parkinson disease	2670	Er-alpha-p53-hdm2 complex	0.06376
Parkinson disease	2699	ER-alpha-GRIP1-c-Jun complex	0.06376
Parkinson disease	2700	ER-alpha-c-Jun complex	0.07809
Parkinson disease	2709	MMP-9-TIMP-1-LRP complex	0.06376
Parkinson disease	2885	ITGAV-ITGB1-SPP1 complex	0.06376
Parkinson disease	2886	Respiratory chain complex I (incomplete intermediate ND1 ND2 ND3 CIA30 assembly) mitochondrial	0.05522
Parkinson disease	2914	Respiratory chain complex I (beta subunit)	0.02761
Parkinson disease	2919	Respiratory chain complex I (gamma subunit) mitochondrial	0.06126
Parkinson disease	2939	Ecsit complex (ECSIT MT-CO2 NDUFA1 MT-ND1 TRAF6 NDUFAF1)	0.04508
Parkinson disease	3111	ITGA9-ITGB1-SPP1 complex	0.06376
Parkinson disease	3112	ITGA5-ITGB1-SPP1 complex	0.06376
Parkinson disease	3137	MASH1 promoter-coactivator complex	0.0333
Parkinson disease	3142	CAMK2-delta-MASH1 promoter-coactivator complex	0.03904
Parkinson disease	3269	RB1-HDAC1-BRG1 complex	0.06376
Parkinson disease	3852	Rb-HDAC1 complex	0.07809
Parkinson disease	3900	GABP(gamma)1-E2F1-DP1 complex	0.06376
Parkinson disease	5099	RB1(hypophosphorylated)-E2F4 complex	0.07809
Parkinson disease	5143	E2F1-Rb complex	0.15617
Parkinson disease	5144	E2F1-p107-cyclinA complex	0.06376
Parkinson disease	5146	RB1-TFAP2A complex	0.07809
Parkinson disease	5179	NCOA6-DNA-PK-Ku-PARP1 complex	0.04939

Parkinson disease	5235	WRN-Ku70-Ku80-PARP1 complex	0.05522
Parkinson disease	5317	LATS1-HTRA2-BIRC4 complex	0.06376
Parkinson disease	5473	FAS-FADD-CASP8 complex	0.06376
Parkinson disease	5611	Emerin complex 24	0.02851
Parkinson disease	5656	CEBPE-E2F1-RB1 complex	0.12752
Parkinson disease	5663	TRIM27-RB1 complex	0.07809
Parkinson disease	5749	MRIT complex	0.06376
Parkinson disease	5799	Death induced signaling complex DISC (FAS FADD CASP8 CFLAR) membrane-associated CD95L	0.05522
Parkinson disease	5800	Death-inducing signaling complex DISC (type I cells associated) stimulated	0.06376
Parkinson disease	5808	DISC complex	0.06376
Parkinson disease	5812	p53-BCL2 complex	0.07809
Parkinson disease	5816	Apoptosome-procaspase 9 complex	0.06376
Parkinson disease	5819	BIM-BCL2xL complex	0.07809
Parkinson disease	5820	tBID-BCL2xL complex	0.07809
Parkinson disease	5830	DJ-1-SNCA complex high molecular weight complex	0.07809
Parkinson disease	5832	PINK1-MIRO2-Milton complex	0.06376
Parkinson disease	5837	PPD complex	0.12752
Parkinson disease	5859	FAS-FADD-CASP8-CASP10 complex	0.05522
Parkinson disease	5861	FAS-FADD-CASP10 complex	0.06376
Parkinson disease	5862	CAV1-VDAC1-ESR1 complex	0.06376
Pelvic inflammatory disease	41	Mi-2/NuRD-MTA2 complex	0.2
Pelvic inflammatory disease	61	Mi2/NuRD complex	0.16903
Pelvic inflammatory disease	62	MeCP1 complex	0.15811
Pelvic inflammatory disease	282	SNF2h-cohesin-NuRD complex	0.1118
Pelvic inflammatory disease	632	Anti-HDAC2 complex	0.10541
Pelvic inflammatory disease	646	HDAC1-associated protein complex	0.14907
Pelvic inflammatory disease	649	HDAC1-associated core complex cII	0.14142
Pelvic inflammatory disease	650	HDAC2-asscociated core complex	0.15811
Pelvic inflammatory disease	659	MeCP1 complex	0.14907
Pelvic inflammatory disease	685	MeCP1 complex	0.14907
Pelvic inflammatory disease	778	LARC complex (LCR-associated remodeling complex)	0.1026
Pelvic inflammatory disease	888	MTA2 complex	0.14907
Pelvic inflammatory disease	1257	ALL-1 supercomplex	0.08452
Pelvic inflammatory disease	1495	PID complex	0.2
Pelvic inflammatory disease	5830	DJ-1-SNCA complex high molecular weight complex	0.31623
Penile disease	1700	ABL2-HRAS-RIN1 complex	0.20412
Penile disease	2355	ITGAV-ITGB3-CD47-FCER2 complex	0.17678
Penile disease	2356	ITGB3-ITGAV-CD47 complex	0.20412
Penile disease	2358	ITGAV-ITGB3-SPP1 complex	0.20412
Penile disease	2359	ITGAV-ITGB3-ADAM15 complex	0.20412
Penile disease	2362	ITAGV-ITGB3-F11R complex	0.20412
Penile disease	2363	ITGAV-ITGB3-PXN-PTK2b complex	0.17678
Penile disease	2364	ITGAV-ITGB3-ADAM23 complex	0.20412
Penile disease	2365	ITGAV-ITGB3-COL4A3 complex	0.20412
Penile disease	2366	ITGAV-ITGB3-PPAP2b complex	0.20412
Penile disease	2369	ITGAV-ITGB3-EGFR complex	0.20412

Penile disease	2370	ITGA2b-ITGB3-CD9 complex	0.20412
Penile disease	2374	ITGAV-ITGB3-LAMA4 complex	0.20412
Penile disease	2376	ITGA2B-ITGB3-FN1-TGM2 complex	0.17678
Penile disease	2377	ITGA2b-ITGB3-CD47-SRC complex	0.17678
Penile disease	2378	ITGA2b-ITGB3-TLN1 complex	0.20412
Penile disease	2379	ITGA2B-ITGB3-CIB1 complex	0.20412
Penile disease	2381	ITGA2B-ITGB3 complex	0.25
Penile disease	2382	ITGA2B-ITGB3-F11R complex	0.20412
Penile disease	2434	ITGA1-ITGB1-COL6A3 complex	0.20412
Penile disease	2435	ITGA1-ITGB1-PTPN2 complex	0.20412
Penile disease	2816	ITGAV-ITGB3 complex	0.25
Penile disease	2826	ITGB3-ITGAV-VTN complex	0.20412
Penile disease	2846	ITGAV-ITGB3-THBS1 complex	0.20412
Penile disease	2849	ITGAV-ITGB3-NOV complex	0.20412
Penile disease	2872	ITGA2b-ITGB3-CD9-GP1b-CD47 complex	0.14434
Penile disease	2882	ITGA5-ITGB3-COL6A3 complex	0.20412
Penile disease	2896	ITGA2b-ITGB3-CD47-FAK complex	0.17678
Penile disease	3103	ITGAV-ITGB3-SLC3A2 complex	0.20412
Penile disease	3115	ITGA2B-ITGB3-ICAM4 complex	0.20412
Penile disease	5922	RAF1-RAS complex EGF induced	0.35355
Peptic esophagitis	103	RNA polymerase II holoenzyme complex	0.06455
Peptic esophagitis	107	TFIIH transcription factor complex	0.10541
Peptic esophagitis	159	Condensin I-PARP-1-XRCC1 complex	0.11952
Peptic esophagitis	212	DNA ligase III-XRCC1 complex	0.22361
Peptic esophagitis	213	DNA ligase IV-XRCC1 complex	0.22361
Peptic esophagitis	362	DNA ligase III-XRCC1-PNK-DNA-pol III multiprotein complex	0.15811
Peptic esophagitis	1009	TFIIH transcription factor complex	0.1
Peptic esophagitis	1029	TFIIH transcription factor complex	0.1
Peptic esophagitis	1030	CAK-ERCC2 complex	0.15811
Peptic esophagitis	1085	DNA repair complex NEIL2-PNK-Pol(beta)-LigIII(alpha)-XRCC1	0.14142
Peptic esophagitis	1086	DNA repair complex NEIL1-PNK-Pol(beta)-LigIII(alpha)-XRCC1	0.14142
Peptic esophagitis	2660	ERCC2/CAK complex	0.15811
Peptic esophagitis	2825	BRCA1-RNA polymerase II complex	0.06202
Peptic esophagitis	5243	XRCC1-LIG3-PNK-TDP1 complex	0.15811
Peptic esophagitis	5495	TFIIH transcription factor complex (ERCC2 ERCC3 GTF2H1 CDK7 CCNH GTF2H2)	0.1291
Peptic ulcer	120	Lymphotoxin beta receptor complex	0.13608
Peptic ulcer	845	PCI-PSA-SCG2 complex	0.13608
Peptic ulcer	1146	Cleavage stimulation factor	0.13608
Peptic ulcer	1147	Polyadenylation complex (CSTF1 CSTF2 CSTF3 SYMPK CPSF1 CPSF2 CPSF3)	0.08909
Peptic ulcer	2001	NOD1 homodimer complex	0.2357
Peptic ulcer	2018	IL12A-IL12B complex	0.16667
Peptic ulcer	2019	IL12A-IL12B-IL12RB1 complex	0.13608
Peptic ulcer	2020	IL12B-IL12RB1-IL12RB2 complex	0.13608

Peptic ulcer	2021	IL12A-IL12B-IL12RB2 complex	0.13608
Peptic ulcer	2211	BARD1-BRCA1-CSTF complex	0.10541
Peptic ulcer	2783	BARD1-BRCA1-CSTF64 complex	0.13608
Peptic ulcer	2972	ITGA9-ITGB1-VEGFA complex	0.13608
Peptic ulcer	4062	NRP1-VEGFR2-VEGF(165) complex	0.13608
Peptic ulcer	5548	IL-12 heterodimer complex	0.16667
Peptic ulcer	5549	IL-12 subunit p40 homodimer complex	0.2357
Peptic ulcer	5696	VEGFA(165)-KDR-NRP1 complex	0.13608
Peptic ulcer	5698	VEGFA(165)-VEGFR2-NRP1 complex	0.13608
Peptic ulcer	5701	NRP1-VEGF(165/121) complex	0.16667
Periodontitis	1439	PTGS2 homodimer complex	0.1525
Periodontitis	1514	IL4-IL4R complex	0.10783
Periodontitis	1515	IL4-IL4R-IL2RG complex	0.08805
Periodontitis	1707	IL2-IL2RA-IL2RB complex	0.08805
Periodontitis	1714	TICAM1-TICAM2-TLR4 complex	0.08805
Periodontitis	2020	IL12B-IL12RB1-IL12RB2 complex	0.08805
Periodontitis	2021	IL12A-IL12B-IL12RB2 complex	0.08805
Periodontitis	2026	IL12RB1-IL12RB2 complex	0.10783
Periodontitis	2028	JAK2-IL12RB2 complex	0.10783
Periodontitis	2709	MMP-9-TIMP-1-LRP complex	0.08805
Periodontitis	2798	MMP-2-claudin-1 complex	0.10783
Periodontitis	5389	SERPINA3-CTSG complex	0.10783
Periodontitis	5391	SERPINA1-CTSG complex	0.10783
Periodontitis	5691	TALL1 homo-oligomer complex	0.1525
Pertussis	725	P2X7 receptor signalling complex	0.11785
Pertussis	2153	ITGAM-ITGB2-CD11 complex	0.4714
Pertussis	2383	ITGA5-ITGB1-FN1-TGM2 complex	0.20412
Pertussis	2384	ITGA5-ITGB1-ADAM15 complex	0.2357
Pertussis	2385	ITGA5-ITGB4 complex	0.28868
Pertussis	2388	Itga5-Itgb1-Fn1-Sfrp2 complex	0.20412
Pertussis	2850	ITGA5-ITGB1-FN-1-NOV complex	0.20412
Pertussis	2853	ITGA5-ITGB1-CAL4A3 complex	0.2357
Pertussis	2882	ITGA5-ITGB3-COL6A3 complex	0.2357
Pertussis	3112	ITGA5-ITGB1-SPP1 complex	0.2357
Pervasive development disorder	933	SCRIB-APC complex	0.18257
Pervasive development disorder	1844	APC-IQGAP1 complex	0.18257
Pervasive development disorder	1845	APC-IQGAP1-CLIP-170 complex	0.14907
Pervasive development disorder	1909	APC-DLG4 complex	0.18257
Pervasive development disorder	2456	MET-CIN85-SH3GL3-CBL complex HGF stimulated	0.1291
Pervasive development disorder	2541	HGF-Met complex	0.18257
Pervasive development disorder	3004	APC-Axin-1-beta-catenin complex	0.14907
Pervasive development disorder	3008	60S APC containing complex	0.09759
Pervasive development disorder	3011	APC-IQGAP1-Rac1 complex	0.14907
Pervasive development disorder	3012	APC-IQGAP1-Cdc42 complex	0.14907
Pervasive development disorder	3166	AXIN-APC-betaCatenin-GSK3B complex	0.1291
Phobic anxiety disorder	2159	AR-AKT-APPL complex	0.28868
Phobic anxiety disorder	2160	AOF2-AR complex	0.35355

Pick disease of the brain	3151	Sulphiredoxin-peroxiredoxin complex	0.31623
Pituitary tumor	159	Condensin I-PARP-1-XRCC1 complex	0.10483
Pituitary tumor	1004	RC complex during S-phase of cell cycle	0.07692
Pituitary tumor	1005	RC complex during G2/M-phase of cell cycle	0.07692
Pituitary tumor	1091	SNX complex (SNX1a SNX2 SNX4 LEPR)	0.13868
Pituitary tumor	1193	Rap1 complex	0.10483
Pituitary tumor	1226	H2AX complex I	0.10483
Pituitary tumor	1714	TICAM1-TICAM2-TLR4 complex	0.16013
Pituitary tumor	1728	CTCF-nucleophosmin-PARP-HIS-KPNA-LMNA-TOP complex	0.09245
Pituitary tumor	1729	TLE1 corepressor complex (MASH1 promoter-corepressor complex)	0.08771
Pituitary tumor	1992	LEPR homodimer complex	0.27735
Pituitary tumor	2625	CDK8-MED6-PARP1 complex	0.16013
Pituitary tumor	2635	BETA2-Cyclin D1 complex	0.19612
Pituitary tumor	3137	MASH1 promoter-coactivator complex	0.08362
Pituitary tumor	3142	CAMK2-delta-MASH1 promoter-coactivator complex	0.09806
Pituitary tumor	5179	NCOA6-DNA-PK-Ku-PARP1 complex	0.12403
Pituitary tumor	5235	WRN-Ku70-Ku80-PARP1 complex	0.13868
Pituitary tumor	5546	CDC2-PCNA-CCNB1-GADD45G complex	0.13868
Pneumoconiosis	1185	EGFR-containing signaling complex	0.35355
Pneumoconiosis	2528	ERBB2-MEMO-SHC complex	0.40825
Polyarthritis	298	VEGF transcriptional complex	0.04652
Polyarthritis	310	Cell cycle kinase complex CDC2	0.04652
Polyarthritis	311	Cell cycle kinase complex CDK2	0.05698
Polyarthritis	312	Cell cycle kinase complex CDK4	0.05698
Polyarthritis	313	Cell cycle kinase complex CDK5	0.05096
Polyarthritis	314	PCNA-p21 complex	0.08058
Polyarthritis	441	TFTC-type histone acetyl transferase complex	0.03436
Polyarthritis	472	Prolyl 4-hydroxylase (alpha(I)-type)	0.08058
Polyarthritis	577	FHL2-p53-HIPK2 complex	0.0658
Polyarthritis	1054	ESR1-RELA-BCL3-NCOA3 complex	0.11396
Polyarthritis	1379	GALNS-lysosomal hydrolase 1.27 MDa complex	0.05698
Polyarthritis	1439	PTGS2 homodimer complex	0.11396
Polyarthritis	1474	SMAD3/4-E2F4/5-p107-DP1 complex	0.04652
Polyarthritis	1634	CyclinD1-CDK4-p21 complex	0.0658
Polyarthritis	1714	TICAM1-TICAM2-TLR4 complex	0.0658
Polyarthritis	1826	SMAD3-HEF1-APC10-CDH1 complex	0.05698
Polyarthritis	1827	PML-SMAD2/3-SARA complex	0.05698
Polyarthritis	1828	TGF-beta receptor I-Axin-SMAD3 complex	0.0658
Polyarthritis	1831	PIAS3-SMAD3-P300 complex	0.0658
Polyarthritis	1970	BMP4-TWSG1 complex	0.08058
Polyarthritis	1972	BMP4-BGN complex	0.08058
Polyarthritis	2055	CASP8-CHUK-IKBKB-MALT1-BCL10 complex	0.10193
Polyarthritis	2056	BCL10-CHUK-BCL10-IKBKB complex	0.11396
Polyarthritis	2084	NFKB1-NFKB2-REL-RELA-RELB complex	0.05096
Polyarthritis	2086	NFKB1-NFKB2-RELA-RELB complex	0.05698
Polyarthritis	2100	CHUK-IKBKB-MAP3K14 complex	0.13159

Polyarthritis	2101	IKKA-IKKB complex	0.16116
Polyarthritis	2104	IKKB-NIK complex	0.08058
Polyarthritis	2105	IkappaB kinase complex (IKBKB CHUK IKBKAP NFKBIA RELA MAP3K14)	0.13957
Polyarthritis	2118	CHUK-ERC1-IKBKB-IKBKG	0.11396
Polyarthritis	2121	CHUK-IKBKB-IKBKG complex	0.13159
Polyarthritis	2124	IKK-alpha--ER-alpha-AIB1 complex	0.13159
Polyarthritis	2189	Ubiquitin E3 ligase (SMAD3 BTRC CUL1 SKP1A RBX1)	0.05096
Polyarthritis	2230	PCNA complex	0.04307
Polyarthritis	2383	ITGA5-ITGB1-FN1-TGM2 complex	0.05698
Polyarthritis	2384	ITGA5-ITGB1-ADAM15 complex	0.0658
Polyarthritis	2385	ITGA5-ITGB4 complex	0.08058
Polyarthritis	2388	Itga5-Itgb1-Fn1-Sfrp2 complex	0.05698
Polyarthritis	2443	ITGA9-ITGB1-TNC complex	0.0658
Polyarthritis	2470	p130Cas-ER-alpha-cSrc-kinase- PI3-kinase p85-subunit complex	0.05698
Polyarthritis	2489	NCR3-CD247 complex	0.08058
Polyarthritis	2590	FOXO1-FHL2-SIRT1 complex	0.0658
Polyarthritis	2657	ESR1-CDK7-CCNH-MNAT1-MTA1-HDAC2 complex	0.04652
Polyarthritis	2670	Er-alpha-p53-hdm2 complex	0.0658
Polyarthritis	2692	SMAD3-SMAD4-cJun-cFos complex	0.05698
Polyarthritis	2699	ER-alpha-GRIP1-c-Jun complex	0.0658
Polyarthritis	2700	ER-alpha-c-Jun complex	0.08058
Polyarthritis	2705	SMAD3-SMAD4-CTCF protein-DNA complex	0.0658
Polyarthritis	2706	SMAD3-SMAD4-SP1 complex	0.0658
Polyarthritis	2707	SMAD3-SMAD4-FOXO3-FOXG1 complex	0.05698
Polyarthritis	2708	SMAD3-SMAD4-cJUN complex	0.0658
Polyarthritis	2727	SRC-3 complex	0.08615
Polyarthritis	2754	JUND-FOSB-SMAD3-SMAD4 complex	0.05698
Polyarthritis	2760	SMAD3-SMAD4-FOXO3 complex	0.0658
Polyarthritis	2761	SMAD3-SMAD4-FOXO1 complex	0.0658
Polyarthritis	2762	SMAD3-SMAD4-FOXO4 complex	0.0658
Polyarthritis	2813	BRCA1-SMAD3 complex	0.08058
Polyarthritis	2829	RSmad complex	0.03604
Polyarthritis	2830	TIF1gamma-SMAD2-SMAD3 complex	0.0658
Polyarthritis	2834	SMAD4-SMAD2-SMAD3 complex	0.0658
Polyarthritis	2850	ITGA5-ITGB1-FN-1-NOV complex	0.05698
Polyarthritis	2853	ITGA5-ITGB1-CAL4A3 complex	0.0658
Polyarthritis	2882	ITGA5-ITGB3-COL6A3 complex	0.0658
Polyarthritis	2968	Axin-SMAD3 complex	0.08058
Polyarthritis	2975	SMAD3-E2F4/5-p107-DP1 complex	0.05096
Polyarthritis	3043	BMP2-BRIA complex	0.0658
Polyarthritis	3045	hs4 enhancer complex (faster migrating complex)	0.05096
Polyarthritis	3112	ITGA5-ITGB1-SPP1 complex	0.0658
Polyarthritis	3182	FHL2 homodimer complex	0.11396
Polyarthritis	3187	FHL2-FHL3 complex	0.08058
Polyarthritis	3188	FHL2-ACT complex	0.08058

Polyarthritis	3189	FHL2-CREB complex	0.08058
Polyarthritis	3199	SMAD3-SKI complex	0.08058
Polyarthritis	3205	SMAD3-SKI-NCOR complex	0.0658
Polyarthritis	3733	SKI-SMAD3 hexameric complex	0.08058
Polyarthritis	3740	SKI-SMAD3-SMAD4 pentameric complex	0.0658
Polyarthritis	3750	CREBBP-SMAD3 hexameric complex	0.08058
Polyarthritis	3754	CREBBP-SMAD3-SMAD4 pentameric complex	0.0658
Polyarthritis	3959	SMAD3-SMAD4-cSKI TGF(beta)-dependent	0.0658
Polyarthritis	3961	SMAD3-cSKI-SIN3A-HDAC1 complex	0.05698
Polyarthritis	3971	SMURF2-SMAD3 complex TGF(beta)-dependent	0.08058
Polyarthritis	3972	SMURF2-SMAD3-SnoN complex TGF(beta)-	0.0658
Polyarthritis	4043	NEMO-HIF2(alpha)-ARNT complex	0.0658
Polyarthritis	5101	CyclinD3-CDK4-CDK6-p21 complex	0.05698
Polyarthritis	5193	TNF-alpha/NF-kappa B signaling complex (CHUK KPNA3 NFKB2 NFKBIB REL IKBKG NFKB1 NFKBIE RELB NFKBIA RELA TNIP2)	0.0658
Polyarthritis	5194	TNF-alpha/NF-kappa B signaling complex (SEC16A CHUK IKBKB NFKB2 REL IKBKG MAP3K14 RELA FBXW7 USP2)	0.10811
Polyarthritis	5196	TNF-alpha/NF-kappa B signaling complex (CHUK BTRC NFKB2 PPP6C REL CUL1 IKBKE SAPS2 SAPS1 ANKRD28 RELA SKP1)	0.09869
Polyarthritis	5220	CHUK-IQGAP2-AKAP8L-RELA-TNIP2 complex	0.10193
Polyarthritis	5228	REL-MAP3K8-RELA-TNIP2-PAPOLA complex	0.05096
Polyarthritis	5230	CHUK-NFKB2-REL-IKBKG-SPAG9-NFKB1-NFKBIE-COPB2-TNIP1-NFKBIA-RELA-TNIP2	0.0658
Polyarthritis	5232	TNF-alpha/Nf-kappa B signaling complex (RPL6 RPL30 RPS13 CHUK DDX3X NFKB2 NFKBIB REL IKBKG NFKB1 MAP3K8 RELB GLG1 NFKBIA RELA TNIP2 GTF2I)	0.05528
Polyarthritis	5233	TNF-alpha/NF-kappa B signaling complex 5	0.06838
Polyarthritis	5234	IKBKB-CDC37-KIAA1967-HSP90AB1-HSP90AA1 complex	0.05096
Polyarthritis	5266	TNF-alpha/NF-kappa B signaling complex 6	0.06091
Polyarthritis	5269	TNF-alpha/NF-kappa B signaling complex 8	0.04652
Polyarthritis	5273	VHL-TBP1-HIF1A complex	0.0658
Polyarthritis	5276	HIF1A-OS9-EGLN1 complex	0.0658
Polyarthritis	5277	HIF1A-OS9-EGLN3 complex	0.0658
Polyarthritis	5285	TNF-alpha/NF-kappa B signaling complex 9	0.05096
Polyarthritis	5286	TNF-alpha/NF-kappa B signaling complex 10	0.03604
Polyarthritis	5382	ARNT-HIF1A complex	0.08058
Polyarthritis	5460	p50-p65 NF(kappa)B complex	0.08058
Polyarthritis	5461	p50-p65 NF(kappa)B-SRC1 complex	0.0658
Polyarthritis	5464	I(kappa)B(alpha)-NF(kappa)Bp50-NF(kappa)Bp65 complex	0.0658
Polyarthritis	5465	IKB(epsilon)-RELA-cREL complex	0.0658
Polyarthritis	5466	IKB(beta)-RELA-cREL complex	0.0658
Polyarthritis	5467	IKB(alpha)-RELA-cREL complex	0.0658
Polyarthritis	5475	MURR1-NF(kappa)Bp65-IKBA complex	0.0658

Polyarthritis	5492	IKBA-NF(kappa)Bp65-NF(kappa)Bp50 complex	0.0658
Polyarthritis	5545	CDC2-PCNA-CCNB1-GADD45B complex	0.05698
Polyarthritis	5564	LMO4-gp130 complex	0.05096
Polyarthritis	5622	HSP90-CIP1-FKBPL complex	0.0658
Polyarthritis	5735	TGF-beta receptor-SMAD3 complex	0.0658
Polyarthritis	5749	MRIT complex	0.0658
Polyarthritis	5812	p53-BCL2 complex	0.08058
Polyarthritis	5819	BIM-BCL2xL complex	0.08058
Polyarthritis	5820	tBID-BCL2xL complex	0.08058
Polyarthritis	5828	IKBKG-IKBKB complex	0.08058
Polyarthritis	5829	IKBKG-CHUK complex	0.08058
Polyarthritis	5844	I-kappa-B kinase (IKK) complex	0.13159
Polyarthritis	5862	CAV1-VDAC1-ESR1 complex	0.0658
Polycystic kidney	75	TSC1-TSC2 complex	0.18898
Polycystic kidney	521	Polycystin-1-E-cadherin-beta-catenin complex	0.1543
Polycystic kidney	522	Polycystin-1-E-cadherin-beta-catenin-Flotillin-2	0.13363
Polycystic kidney	541	IGF1-IGFBP3-ALS complex	0.1543
Polycystic kidney	681	(C-CFTR)2-NHERF-ezrin complex	0.1543
Polycystic kidney	682	C-CFTR-NHERF(PDZ1 domain)-ezrin complex	0.1543
Polycystic kidney	683	C-CFTR-NHERF(PDZ2 domain)-ezrin complex	0.1543
Polycystic kidney	687	CFTR-NHERF-beta(2)AR signaling complex	0.1543
Polycystic kidney	1071	PKD2-FPC complex	0.18898
Polycystic kidney	1088	PRNP-ApolopoproteinE3 complex	0.18898
Polycystic kidney	2416	ITGB1-RAP1A-PKD1 complex	0.1543
Polycystic kidney	5177	Polycystin-1 multiprotein complex (ACTN1 CDH1 SRC JUP VCL CTNNB1 PXN BCAR1 PKD1	0.08058
Polycystic ovary syndrome	201	HUIC complex	0.09366
Polycystic ovary syndrome	202	BRCA1-RAD50-MRE11-NBS1 complex	0.06623
Polycystic ovary syndrome	238	SWI-SNF chromatin remodeling-related-BRCA1	0.03994
Polycystic ovary syndrome	240	BRCA1-CTIP-ZBRK1 repressor complex	0.07647
Polycystic ovary syndrome	242	BRCA1-BACH1 complex	0.09366
Polycystic ovary syndrome	433	BASC complex (BRCA1-associated genome surveillance complex)	0.03824
Polycystic ovary syndrome	434	BASC (Ab 80) complex (BRCA1-associated genome surveillance complex)	0.04683
Polycystic ovary syndrome	435	BASC (Ab 81) complex (BRCA1-associated genome surveillance complex)	0.05407
Polycystic ovary syndrome	436	BASC (Ab C-20) complex (BRCA1-associated genome surveillance complex)	0.07647
Polycystic ovary syndrome	438	GCN5-TRRAP histone acetyltransferase complex	0.04189
Polycystic ovary syndrome	668	BKCA-beta2AR-AKAP79 signaling complex	0.07647
Polycystic ovary syndrome	672	BKCA-beta2AR complex	0.09366
Polycystic ovary syndrome	687	CFTR-NHERF-beta(2)AR signaling complex	0.07647
Polycystic ovary syndrome	845	PCI-PSA-SCG2 complex	0.07647
Polycystic ovary syndrome	1093	SNX complex (SNX1a SNX2 SNX4 INSR)	0.06623
Polycystic ovary syndrome	5718	eNOS-HSP90-AKT complex VEGF induced	0.07647
Polycystic ovary syndrome	2156	YBX1-AKT1 complex	0.09366
Polycystic ovary syndrome	2159	AR-AKT-APPL complex	0.07647

Polycystic ovary syndrome	2210	BRCA1-IRIS-pre-replication complex	0.06623
Polycystic ovary syndrome	2211	BARD1-BRCA1-CSTF complex	0.05923
Polycystic ovary syndrome	2213	BRCA1-BARD1-POLR2A complex	0.07647
Polycystic ovary syndrome	2214	LMO4-BRCA1-CTIP-LDB1 complex	0.06623
Polycystic ovary syndrome	2215	BRCA1-LMO4-CTIP complex	0.07647
Polycystic ovary syndrome	2343	ITGAV-ITGB5-PLAUR complex	0.07647
Polycystic ovary syndrome	2348	ITGAV-ITGB5-CYR61 complex	0.07647
Polycystic ovary syndrome	2437	ITGA6-ITGB1-CYR61 complex	0.07647
Polycystic ovary syndrome	2480	CIN85 complex (CIN85 CRK BCAR1 CBL PIK3R1 GRB2 SOS1)	0.05006
Polycystic ovary syndrome	2547	PLC-gamma-1-SLP-76-SOS1-LAT complex	0.06623
Polycystic ovary syndrome	2564	p21(ras)GAP-Fyn-Lyn-Yes complex thrombin	0.06623
Polycystic ovary syndrome	2577	Sam68-p85 P13K-IRS-1-IR signaling complex	0.13245
Polycystic ovary syndrome	2578	Sam68-p120GAP complex	0.09366
Polycystic ovary syndrome	2581	RasGAP-AURKA/AURKB-survivin complex	0.06623
Polycystic ovary syndrome	2589	PGC-1-SRp40-SRp55-SRp75 complex	0.06623
Polycystic ovary syndrome	2599	POLR2A-CCNT1-CDK9-NCL-LEM6-CPSF2 complex	0.05407
Polycystic ovary syndrome	2686	BRCA1-core RNA polymerase II complex	0.03674
Polycystic ovary syndrome	2709	MMP-9-TIMP-1-LRP complex	0.07647
Polycystic ovary syndrome	2776	RAD50-BRCA1 complex	0.09366
Polycystic ovary syndrome	2783	BARD1-BRCA1-CSTF64 complex	0.07647
Polycystic ovary syndrome	2786	BRCA1 A complex	0.06623
Polycystic ovary syndrome	2787	BRCA1 C complex	0.06623
Polycystic ovary syndrome	2788	BRCA1 B complex	0.07647
Polycystic ovary syndrome	2798	MMP-2-claudin-1 complex	0.09366
Polycystic ovary syndrome	2811	BRCA1-cABL complex	0.09366
Polycystic ovary syndrome	2813	BRCA1-SMAD3 complex	0.09366
Polycystic ovary syndrome	2814	BRCA1-HDAC1-HDAC2 complex	0.07647
Polycystic ovary syndrome	2815	BRCA1-BARD1-BACH1-DNA damage complex II	0.04683
Polycystic ovary syndrome	2817	BRCA1-BARD1-BACH1-DNA damage complex I	0.05407
Polycystic ovary syndrome	2818	BRCA1-BARD1-BRCA2-DNA damage complex III	0.07647
Polycystic ovary syndrome	2819	BRCA1-CtIP-CtBP complex	0.07647
Polycystic ovary syndrome	2820	BRCA1-VCP complex	0.09366
Polycystic ovary syndrome	2822	BRCA1-BARD1-UbcH5c complex	0.07647
Polycystic ovary syndrome	2823	BRCA1-BARD1-UbcH7c complex	0.07647
Polycystic ovary syndrome	2824	BRCA1-RAD51 complex	0.09366
Polycystic ovary syndrome	2825	BRCA1-RNA polymerase II complex	0.02598
Polycystic ovary syndrome	2893	BCR-ABL (p210 fusion protein)-GRB2-SOS1 complex	0.09366
Polycystic ovary syndrome	2917	Grb2-Sos complex Fc receptor gamma-R1 stimulated	0.09366
Polycystic ovary syndrome	2922	LAT-PLC-gamma-1-p85-GRB2-SOS signaling complex C305 activated	0.05923
Polycystic ovary syndrome	3144	Sos1-Grb2 complex	0.09366
Polycystic ovary syndrome	3830	ADRB2 homodimer complex	0.13245
Polycystic ovary syndrome	3847	TCL1(trimer)-AKT1 complex	0.09366
Polycystic ovary syndrome	5400	BRCC complex	0.05923
Polycystic ovary syndrome	5564	LMO4-gp130 complex	0.05923
Polycystic ovary syndrome	5922	RAF1-RAS complex EGF induced	0.06623
Polycythemia	205	Ubiquitin E3 ligase (VHL TCEB1 TCEB2 CUL2	0.14907

Polycythemia	298	VEGF transcriptional complex	0.13608
Polycythemia	541	IGF1-IGFBP3-ALS complex	0.19245
Polycythemia	622	Ubiquitin E3 ligase (VHL TCEB1 TCEB2 CUL2)	0.16667
Polycythemia	2028	JAK2-IL12RB2 complex	0.2357
Polycythemia	5178	JAK2-PAFR-TYK2 complex	0.19245
Polycythemia	5267	VHL-VDU1-TCEB1-TCEB2 complex	0.16667
Polycythemia	5270	VHL-TCEB1-TCEB2 complex	0.19245
Polycythemia	5273	VHL-TBP1-HIF1A complex	0.3849
Polycythemia	5276	HIF1A-OS9-EGLN1 complex	0.19245
Polycythemia	5277	HIF1A-OS9-EGLN3 complex	0.19245
Polycythemia	5382	ARNT-HIF1A complex	0.2357
Polycythemia	5442	EPOR receptor complex	0.33333
Polycythemia	5446	EPO-EPOR complex	0.4714
Polymyositis	143	APP-FE65-LRP complex	0.14907
Polymyositis	2004	C1D homodimer protein	0.2582
Polymyositis	5870	FE65-TSHZ3-HDAC1 complex	0.14907
Polyneuropathy	2972	ITGA9-ITGB1-VEGFA complex	0.1543
Polyneuropathy	4062	NRP1-VEGFR2-VEGF(165) complex	0.1543
Polyneuropathy	5696	VEGFA(165)-KDR-NRP1 complex	0.1543
Polyneuropathy	5698	VEGFA(165)-VEGFR2-NRP1 complex	0.1543
Polyneuropathy	5701	NRP1-VEGF(165/121) complex	0.18898
Polyneuropathy	5830	DJ-1-SNCA complex high molecular weight complex	0.18898
Polyneuropathy	5837	PPD complex	0.1543
Porcine reproductive and respiratory syndrome	1714	TICAM1-TICAM2-TLR4 complex	0.2357
Porcine reproductive and respiratory syndrome	2001	NOD1 homodimer complex	0.40825
Pre-Eclampsia	298	VEGF transcriptional complex	0.04811
Pre-Eclampsia	1069	FIF-FGR2 complex	0.08333
Pre-Eclampsia	1223	H2AX complex isolated from cells without IR exposure	0.03269
Pre-Eclampsia	1226	H2AX complex I	0.04454
Pre-Eclampsia	1227	H2AX complex II	0.03727
Pre-Eclampsia	1514	IL4-IL4R complex	0.08333
Pre-Eclampsia	1515	IL4-IL4R-IL2RG complex	0.06804
Pre-Eclampsia	1714	TICAM1-TICAM2-TLR4 complex	0.06804
Pre-Eclampsia	1972	BMP4-BGN complex	0.08333
Pre-Eclampsia	1986	Endoglin homodimer complex	0.11785
Pre-Eclampsia	2348	ITGAV-ITGB5-CYR61 complex	0.06804
Pre-Eclampsia	2437	ITGA6-ITGB1-CYR61 complex	0.06804
Pre-Eclampsia	2709	MMP-9-TIMP-1-LRP complex	0.06804
Pre-Eclampsia	2972	ITGA9-ITGB1-VEGFA complex	0.06804
Pre-Eclampsia	3055	Nop56p-associated pre-rRNA complex	0.01156
Pre-Eclampsia	4062	NRP1-VEGFR2-VEGF(165) complex	0.06804
Pre-Eclampsia	5217	Calreticulin oligomer complex	0.11785
Pre-Eclampsia	5273	VHL-TBP1-HIF1A complex	0.06804
Pre-Eclampsia	5276	HIF1A-OS9-EGLN1 complex	0.06804
Pre-Eclampsia	5277	HIF1A-OS9-EGLN3 complex	0.06804
Pre-Eclampsia	5382	ARNT-HIF1A complex	0.08333

Pre-Eclampsia	5473	FAS-FADD-CASP8 complex	0.06804
Pre-Eclampsia	5582	LIFR-LIF-gp130 complex	0.06804
Pre-Eclampsia	5696	VEGFA(165)-KDR-NRP1 complex	0.06804
Pre-Eclampsia	5698	VEGFA(165)-VEGFR2-NRP1 complex	0.06804
Pre-Eclampsia	5701	NRP1-VEGF(165/121) complex	0.08333
Pre-Eclampsia	5799	Death induced signaling complex DISC (FAS FADD CASP8 CFLAR) membrane-associated CD95L	0.05893
Pre-Eclampsia	5800	Death-inducing signaling complex DISC (type I cells associated) stimulated	0.06804
Pre-Eclampsia	5808	DISC complex	0.06804
Pre-Eclampsia	5859	FAS-FADD-CASP8-CASP10 complex	0.05893
Pre-Eclampsia	5861	FAS-FADD-CASP10 complex	0.06804
Premature birth	120	Lymphotoxin beta receptor complex	0.14003
Premature birth	668	BKCA-beta2AR-AKAP79 signaling complex	0.14003
Premature birth	672	BKCA-beta2AR complex	0.1715
Premature birth	687	CFTR-NHERF-beta(2)AR signaling complex	0.14003
Premature birth	1714	TICAM1-TICAM2-TLR4 complex	0.14003
Premature birth	3830	ADRB2 homodimer complex	0.24254
Premature birth	5446	EPO-EPOR complex	0.1715
Primary biliary cirrhosis	115	Polycomb repressive complex 1 (PRC1 hPRC-H)	0.05185
Primary biliary cirrhosis	116	Polycomb repressive complex 1 (PRC1 hPRC-H)	0.04981
Primary biliary cirrhosis	441	TFTC-type histone acetyl transferase complex	0.05415
Primary biliary cirrhosis	550	NOS3-CAV1-NOSTRIN complex	0.1037
Primary biliary cirrhosis	557	TRP1-G alpha-11-IP3R3-CAV1 signaling complex	0.0898
Primary biliary cirrhosis	753	UTM-SGCE-DAG1-CAV1-NOS3 complex	0.08032
Primary biliary cirrhosis	929	CEN complex	0.02953
Primary biliary cirrhosis	1054	ESR1-RELA-BCL3-NCOA3 complex	0.0898
Primary biliary cirrhosis	1224	Ubiquitin E3 ligase (BMI1 SPOP CUL3)	0.1037
Primary biliary cirrhosis	1462	hPRC1L complex	0.0898
Primary biliary cirrhosis	1714	TICAM1-TICAM2-TLR4 complex	0.1037
Primary biliary cirrhosis	2028	JAK2-IL12RB2 complex	0.127
Primary biliary cirrhosis	2124	IKK-alpha--ER-alpha-AIB1 complex	0.1037
Primary biliary cirrhosis	5714	eNOS-CAV1 complex	0.127
Primary biliary cirrhosis	2462	Caveolin-1 homodimer complex	0.17961
Primary biliary cirrhosis	2470	p130Cas-ER-alpha-cSrc-kinase- PI3-kinase p85-subunit complex	0.0898
Primary biliary cirrhosis	2657	ESR1-CDK7-CCNH-MNAT1-MTA1-HDAC2 complex	0.07332
Primary biliary cirrhosis	2670	Er-alpha-p53-hdm2 complex	0.1037
Primary biliary cirrhosis	2699	ER-alpha-GRIP1-c-Jun complex	0.1037
Primary biliary cirrhosis	2700	ER-alpha-c-Jun complex	0.127
Primary biliary cirrhosis	5178	JAK2-PAFR-TYK2 complex	0.1037
Primary biliary cirrhosis	5473	FAS-FADD-CASP8 complex	0.1037
Primary biliary cirrhosis	5513	Polycomb repressive complex	0.0898
Primary biliary cirrhosis	5518	BMI1-HPH1-HPH2 complex	0.1037
Primary biliary cirrhosis	5799	Death induced signaling complex DISC (FAS FADD CASP8 CFLAR) membrane-associated CD95L	0.0898
Primary biliary cirrhosis	5800	Death-inducing signaling complex DISC (type I cells associated) stimulated	0.1037

Primary biliary cirrhosis	5808	DISC complex	0.1037
Primary biliary cirrhosis	5830	DJ-1-SNCA complex high molecular weight complex	0.127
Primary biliary cirrhosis	5859	FAS-FADD-CASP8-CASP10 complex	0.0898
Primary biliary cirrhosis	5861	FAS-FADD-CASP10 complex	0.1037
Primary biliary cirrhosis	5862	CAV1-VDAC1-ESR1 complex	0.20739
Primary hyperparathyroidism	520	KCNQ1 macromolecular complex	0.11952
Primary hyperparathyroidism	903	RET-Rai complex	0.26726
Primary hyperparathyroidism	1095	SNX complex (SNX1a SNX2 SNX4 EGFR)	0.18898
Primary hyperparathyroidism	1185	EGFR-containing signaling complex	0.18898
Primary hyperparathyroidism	5712	FAK-beta5 integrin complex VEGF induced	0.26726
Primary hyperparathyroidism	2369	ITGAV-ITGB3-EGFR complex	0.21822
Primary hyperparathyroidism	2383	ITGA5-ITGB1-FN1-TGM2 complex	0.18898
Primary hyperparathyroidism	2384	ITGA5-ITGB1-ADAM15 complex	0.21822
Primary hyperparathyroidism	2385	ITGA5-ITGB4 complex	0.26726
Primary hyperparathyroidism	2388	Itga5-Itgb1-Fn1-Sfrp2 complex	0.18898
Primary hyperparathyroidism	2390	CD98-LAT2-ITGB1 complex	0.18898
Primary hyperparathyroidism	2395	ITGA7-ITGB1-CD151 complex	0.21822
Primary hyperparathyroidism	2396	ITGA7-ITGB1-CD9 complex	0.21822
Primary hyperparathyroidism	2397	ITGA7-ITGB1-ITGB1BP3 complex	0.21822
Primary hyperparathyroidism	2398	ITGA3-ITGB1-BSG complex	0.21822
Primary hyperparathyroidism	2399	ITGA3-ITGB1-CD63 complex	0.21822
Primary hyperparathyroidism	2400	ITGA3-ITGB1-CD151 complex	0.21822
Primary hyperparathyroidism	2401	ITGA3-ITGB1-THBS1 complex	0.21822
Primary hyperparathyroidism	2406	ITGA3-ITGB1 complex	0.26726
Primary hyperparathyroidism	2411	ITGA6-ITGB1-CD151 complex	0.21822
Primary hyperparathyroidism	2413	ITGA6-ITGB1 complex	0.26726
Primary hyperparathyroidism	2416	ITGB1-RAP1A-PKD1 complex	0.21822
Primary hyperparathyroidism	2417	ITGA4-ITGB1-EMILIN1 complex	0.21822
Primary hyperparathyroidism	2418	ITGA4-ITGB1 complex	0.26726
Primary hyperparathyroidism	2419	ITGA4-ITGB1-CD81 complex	0.21822
Primary hyperparathyroidism	2420	ITGA4-ITGB1-CD53 complex	0.21822
Primary hyperparathyroidism	2421	ITGA4-ITGB1-VCAM1 complex	0.21822
Primary hyperparathyroidism	2422	ITGA4-ITGB1-JAM2 complex	0.21822
Primary hyperparathyroidism	2423	ITGA4-ITGB1-CD47 complex	0.21822
Primary hyperparathyroidism	2424	ITGA4-ITGB1-CD63 complex	0.21822
Primary hyperparathyroidism	2425	ITGA4-ITGB1-PXN complex	0.21822
Primary hyperparathyroidism	2426	ITGA4-ITGB1-THBS1 complex	0.21822
Primary hyperparathyroidism	2428	ITGA4-ITGB1-THBS2 complex	0.21822
Primary hyperparathyroidism	2429	ITGA2-ITGB1-CD47 complex	0.21822
Primary hyperparathyroidism	2430	ITGA2-ITGB1-CHAD complex	0.21822
Primary hyperparathyroidism	2431	ITGA2-ITGB1-COL6A3 complex	0.21822
Primary hyperparathyroidism	2432	ITGA2-ITGB1 complex	0.26726
Primary hyperparathyroidism	2434	ITGA1-ITGB1-COL6A3 complex	0.21822
Primary hyperparathyroidism	2435	ITGA1-ITGB1-PTPN2 complex	0.21822
Primary hyperparathyroidism	2436	ITGAV-ITGB1 complex	0.26726
Primary hyperparathyroidism	2437	ITGA6-ITGB1-CYR61 complex	0.21822
Primary hyperparathyroidism	2439	ITGA8-ITGB1 complex	0.26726

Primary hyperparathyroidism	2440	ITGA9-ITGB1-ADAM9 complex	0.21822
Primary hyperparathyroidism	2441	Itga9-Itgb1-Adam2 complex	0.21822
Primary hyperparathyroidism	2442	ITGA9-ITGB1-VCAM1 complex	0.21822
Primary hyperparathyroidism	2443	ITGA9-ITGB1-TNC complex	0.21822
Primary hyperparathyroidism	2444	ITGB1-ITGA9 complex	0.26726
Primary hyperparathyroidism	2445	ITGA9-ITGB1-ADAM15 complex	0.21822
Primary hyperparathyroidism	2446	ITGA9-ITGB1-FIGF complex	0.21822
Primary hyperparathyroidism	2447	ITGA9-ITGB1-ADAM12 complex	0.21822
Primary hyperparathyroidism	2453	Multiprotein complex (monoubiquitination)	0.18898
Primary hyperparathyroidism	2454	CIN85-CBL-SH3GL2-EGFR complex EGF stimulated	0.18898
Primary hyperparathyroidism	2542	EGFR-CBL-GRB2 complex	0.21822
Primary hyperparathyroidism	2726	PXN-ITGB5-PTK2 complex	0.21822
Primary hyperparathyroidism	2850	ITGA5-ITGB1-FN-1-NOV complex	0.18898
Primary hyperparathyroidism	2853	ITGA5-ITGB1-CAL4A3 complex	0.21822
Primary hyperparathyroidism	2885	ITGAV-ITGB1-SPP1 complex	0.21822
Primary hyperparathyroidism	2896	ITGA2b-ITGB3-CD47-FAK complex	0.18898
Primary hyperparathyroidism	2964	ITGA9-ITGB1-ADAM1 complex	0.21822
Primary hyperparathyroidism	2965	ITGA9-ITGB1-ADAM3 complex	0.21822
Primary hyperparathyroidism	2971	ITGA9-ITGB1-VEGFC complex	0.21822
Primary hyperparathyroidism	2972	ITGA9-ITGB1-VEGFA complex	0.21822
Primary hyperparathyroidism	2989	ITGA9-ITGB1-ADAM8 complex	0.21822
Primary hyperparathyroidism	3035	LAT2-ITGB1 complex	0.26726
Primary hyperparathyroidism	3057	ITGA10-ITGB1 complex	0.26726
Primary hyperparathyroidism	3058	ITGA11-ITGB1 complex	0.26726
Primary hyperparathyroidism	3059	ITGA11-ITGB1-COL1A1 complex	0.21822
Primary hyperparathyroidism	3104	ITGB1-NRP1 complex	0.26726
Primary hyperparathyroidism	3111	ITGA9-ITGB1-SPP1 complex	0.21822
Primary hyperparathyroidism	3112	ITGA5-ITGB1-SPP1 complex	0.21822
Primary hyperparathyroidism	3678	RIN1-STAM2-EGFR complex EGF stimulated	0.21822
Primary hyperparathyroidism	5171	SH3KBP1-CBLB-EGFR complex	0.21822
Primary hyperparathyroidism	5177	Polycystin-1 multiprotein complex (ACTN1 CDH1 SRC JUP VCL CTNNB1 PXN BCAR1 PKD1	0.11396
Primary hyperparathyroidism	5282	CAS-SRC-FAK complex	0.21822
Primary tumor	387	MCM complex	0.07217
Primary tumor	541	IGF1-IGFBP3-ALS complex	0.10206
Primary tumor	1067	CD8A-LCK complex	0.125
Primary tumor	1079	P-TEFb.1 complex	0.125
Primary tumor	1080	P-TEFb.2 complex	0.125
Primary tumor	1083	P-TEFb.4 complex	0.125
Primary tumor	1191	RNA pol II containing coactivator complex Tat-SF	0.07906
Primary tumor	1452	MCM2-MCM6-MCM7 complex	0.10206
Primary tumor	2258	VILIP-1-AChR-alpha-4-AChR-beta-2 complex	0.10206
Primary tumor	2486	GIPC1-LHCGR complex	0.125
Primary tumor	2487	GIPC1-NTRK1-RGS19 complex	0.10206
Primary tumor	2559	p56(LCK)-CAML complex	0.125
Primary tumor	2565	CD20-LCK-LYN-FYN-p75/80 complex (Raji human B cell line)	0.08839
Primary tumor	2599	POLR2A-CCNT1-CDK9-NCL-LEM6-CPSF2 complex	0.07217

Primary tumor	2600	BRD4 complex	0.0625
Primary tumor	2601	P-TEFb-BRD4-TRAP220 complex	0.08839
Primary tumor	2602	P-TEFb-7SKRNA-HEXIM1 complex	0.10206
Primary tumor	2603	Transcription elongation factor complex (SUPT5H CDK9 CCNT1)	0.10206
Primary tumor	2604	P-TEFb-SKP2 complex	0.10206
Primary tumor	2605	Heterotrimeric complex (CCNT1 CDK9 GRN)	0.10206
Primary tumor	2639	HES1 promoter-Notch enhancer complex	0.04903
Primary tumor	2709	MMP-9-TIMP-1-LRP complex	0.10206
Primary tumor	2791	MCM4-MCM6-MCM7 complex	0.10206
Primary tumor	2792	MCM2-MCM4-MCM6-MCM7 complex	0.08839
Primary tumor	2875	BRD4-P-TEFb complex	0.10206
Primary tumor	2879	CD20-LCK-FYN-p75/80 complex	0.10206
Primary tumor	2944	Notch1-p56lck-PI3K complex	0.10206
Primary tumor	2955	LCK-SLP76-PLC-gamma-1-LAT complex pervanadate-activated	0.08839
Primary tumor	3162	TF-FVIIa-FXa-TFPI complex	0.08839
Primary tumor	3229	Heterodimer complex (CDK9 IL6ST)	0.125
Primary tumor	4977	gp78-p97/VCP-DERL1 complex	0.10206
Primary tumor	5423	HSP70-BAG5-PARK2 complex	0.08839
Primary tumor	5611	Emerin complex 24	0.04564
Primary tumor	5837	PPD complex	0.10206
Prion disease	143	APP-FE65-LRP complex	0.2357
Prion disease	1094	Frataxin complex	0.1543
Prion disease	1439	PTGS2 homodimer complex	0.40825
Prion disease	2710	LRP-1-Alpha-2-M-annexin VI complex	0.2357
Prion disease	2711	Amyloid beta protein oligomer	0.40825
Prion disease	2755	17S U2 snRNP	0.07107
Prion disease	3092	APP-TOMM40 complex	0.28868
Prion disease	3093	APP-TIMM23 complex	0.28868
Prion disease	5189	YWHAQ-CALM1-CABIN1 complex	0.2357
Prion disease	5199	Kinase maturation complex 1	0.10206
Prion disease	5615	Emerin complex 52	0.08513
Prion disease	5919	BRAF-RAF1-14-3-3 complex	0.13608
Prostate cancer	4	Multisubunit ACTR coactivator complex	0.0522
Prostate cancer	58	SMRT complex	0.02334
Prostate cancer	63	Mitotic 14S cohesin 1 complex	0.0261
Prostate cancer	64	Mitotic 14S cohesin 2 complex	0.0261
Prostate cancer	74	TRPC1-Homer3-IP3R complex	0.03014
Prostate cancer	86	NUMAC complex (nucleosomal methylation activator complex)	0.03301
Prostate cancer	98	p300-MDM2-p53 protein complex	0.03014
Prostate cancer	115	Polycomb repressive complex 1 (PRC1 hPRC-H)	0.03014
Prostate cancer	116	Polycomb repressive complex 1 (PRC1 hPRC-H)	0.02896
Prostate cancer	149	PBAF complex (Polybromo- and BAF containing complex)	0.01507
Prostate cancer	163	Cohesin-SA2 complex	0.0261
Prostate cancer	164	Cohesin-SA1 complex	0.0261

Prostate cancer	189	BAF complex	0.02896
Prostate cancer	227	Ubiquitin E3 ligase (SKP1A BTRC CUL1)	0.03014
Prostate cancer	238	SWI-SNF chromatin remodeling-related-BRCA1	0.03148
Prostate cancer	267	Checkpoint 9-1-1 complex	0.03014
Prostate cancer	268	Checkpoint Rad complex	0.01846
Prostate cancer	274	RAD17-RFC-9-1-1 checkpoint supercomplex	0.01846
Prostate cancer	280	HMGB1-HMGB2-HSC70-ERP60-GAPDH complex	0.02334
Prostate cancer	282	SNF2h-cohesin-NuRD complex	0.01305
Prostate cancer	304	SRCAP-associated chromatin remodeling complex	0.01651
Prostate cancer	305	40S ribosomal subunit cytoplasmic	0.00895
Prostate cancer	306	Ribosome cytoplasmic	0.0174
Prostate cancer	308	60S ribosomal subunit cytoplasmic	0.01523
Prostate cancer	351	Spliceosome	0.00437
Prostate cancer	368	ERCC1-ERCC4-MSH2 complex	0.03014
Prostate cancer	371	Structure-specific endonulease complex	0.0522
Prostate cancer	375	MSH2-MSH3 complex	0.03691
Prostate cancer	378	MutS-beta complex	0.03691
Prostate cancer	430	18S U11/U12 snRNP	0.01066
Prostate cancer	432	N-NOS-CHIP-HSP70-1 complex	0.03014
Prostate cancer	531	XPA-ERCC1-ERCC4 complex	0.03014
Prostate cancer	541	IGF1-IGFBP3-ALS complex	0.03014
Prostate cancer	548	DRIP complex	0.01395
Prostate cancer	552	IFNB1-IFNAR1-IFNAR2- complex	0.03014
Prostate cancer	553	RHOA-IP3R-TRPC1 complex	0.03014
Prostate cancer	554	PBAF complex (Polybromo- and BAF containing complex)	0.01651
Prostate cancer	555	BAF complex	0.0174
Prostate cancer	561	LSm1-7 complex	0.01973
Prostate cancer	563	F1F0-ATP synthase (EC 3.6.3.14) mitochondrial	0.01305
Prostate cancer	564	BAF complex	0.03148
Prostate cancer	565	PBAF complex (Polybromo- and BAF containing complex)	0.01574
Prostate cancer	566	BAF complex	0.01574
Prostate cancer	570	p300-CBP-p270-SWI/SNF complex	0.05919
Prostate cancer	571	p300-CBP-p270 complex	0.06027
Prostate cancer	577	FHL2-p53-HIPK2 complex	0.03014
Prostate cancer	626	LSD1 complex	0.01448
Prostate cancer	668	BKCA-beta2AR-AKAP79 signaling complex	0.06027
Prostate cancer	672	BKCA-beta2AR complex	0.07382
Prostate cancer	687	CFTR-NHERF-beta(2)AR signaling complex	0.03014
Prostate cancer	710	Brg1-associated complex I	0.01574
Prostate cancer	711	Brm-associated complex	0.03148
Prostate cancer	713	BRG1-SIN3A complex	0.01395
Prostate cancer	714	BRM-SIN3A complex	0.02696
Prostate cancer	725	P2X7 receptor signalling complex	0.01507
Prostate cancer	739	SIN3-ING1b complex II	0.01305
Prostate cancer	745	NCOR-SIN3-RPD3 complex	0.0261
Prostate cancer	747	NCOR-SIN3-HDAC1 complex	0.03014

Prostate cancer	749	MeCP2-SIN3A-HDAC complex	0.0261
Prostate cancer	752	SMRT core complex	0.03014
Prostate cancer	778	LARC complex (LCR-associated remodeling complex)	0.01198
Prostate cancer	803	BRG1-SIN3A-HDAC containing SWI/SNF remodeling complex I	0.01574
Prostate cancer	806	BRM-SIN3A-HDAC complex	0.03014
Prostate cancer	807	BRG1-associated complex	0.0174
Prostate cancer	808	BRM-associated complex	0.03301
Prostate cancer	810	FCP1-associated protein complex	0.01973
Prostate cancer	828	TRPC1-STIM1-ORAI1 complex	0.03014
Prostate cancer	837	20S methyltransferase complex	0.03014
Prostate cancer	845	PCI-PSA-SCG2 complex	0.06027
Prostate cancer	871	BRAF53-BRCA2 complex	0.01973
Prostate cancer	924	Toposome	0.01973
Prostate cancer	929	CEN complex	0.00858
Prostate cancer	1055	ZNF198-PML complex	0.03691
Prostate cancer	1056	ZNF198-SUMO1 complex	0.03691
Prostate cancer	1088	PRNP-ApolopoproteinE3 complex	0.03691
Prostate cancer	1094	Frataxin complex	0.01973
Prostate cancer	1149	Histone H3.1 complex	0.01651
Prostate cancer	1154	DSS1 complex	0.01448
Prostate cancer	1158	p33ING1b-p300 complex	0.03691
Prostate cancer	1160	ING1-p300-PCNA complex	0.03014
Prostate cancer	1178	BCOR complex	0.01846
Prostate cancer	1181	C complex spliceosome	0.01167
Prostate cancer	1192	ESCRT-I complex	0.03014
Prostate cancer	1194	E2F-6 complex	0.01507
Prostate cancer	1195	Exon junction complex (mRNA splicing-dependent)	0.03691
Prostate cancer	1210	Sec61-Sec62-Sec63 complex	0.02131
Prostate cancer	1230	WINAC complex	0.04185
Prostate cancer	1239	EBAFb complex	0.01651
Prostate cancer	1252	EBAFa complex	0.01651
Prostate cancer	1257	ALL-1 supercomplex	0.01973
Prostate cancer	1259	Chromatin assembly complex (CAF-1 complex)	0.03014
Prostate cancer	1306	PIN1-AUF1 complex	0.03691
Prostate cancer	1308	PABPC1-HSPA8-HNRPD-EIF4G1 complex	0.02334
Prostate cancer	1335	SNW1 complex	0.0123
Prostate cancer	1413	NCOR1 complex	0.01651
Prostate cancer	1462	hPRC1L complex	0.0261
Prostate cancer	1471	pRb2/p130-multimolecular complex (RB2 E2F5 HDAC1 SUV39H1 P300)	0.02334
Prostate cancer	1474	SMAD3/4-E2F4/5-p107-DP1 complex	0.04262
Prostate cancer	1505	NCOR2 complex	0.01973
Prostate cancer	1514	IL4-IL4R complex	0.03691
Prostate cancer	1515	IL4-IL4R-IL2RG complex	0.03014
Prostate cancer	1521	p300-SMAD1-STAT3 complex	0.03014
Prostate cancer	1661	E2F4-p107-cyclinA complex	0.03014
Prostate cancer	1707	IL2-IL2RA-IL2RB complex	0.03014

Prostate cancer	1714	TICAM1-TICAM2-TLR4 complex	0.03014
Prostate cancer	1826	SMAD3-HEF1-APC10-CDH1 complex	0.0261
Prostate cancer	1827	PML-SMAD2/3-SARA complex	0.0522
Prostate cancer	1828	TGF-beta receptor I-Axin-SMAD3 complex	0.03014
Prostate cancer	1831	PIAS3-SMAD3-P300 complex	0.06027
Prostate cancer	1856	CDCA5-PDS5A-RAD21-SMC1A-PDS5B-SMC3	0.02131
Prostate cancer	1863	TSG101-VPS37B-VPS28 complex	0.03014
Prostate cancer	1890	ELK1-SRF-ELK4 complex	0.06027
Prostate cancer	2055	CASP8-CHUK-IKBKB-MALT1-BCL10 complex	0.04669
Prostate cancer	2056	BCL10-CHUK-BCL10-IKBKB complex	0.0522
Prostate cancer	2084	NFKB1-NFKB2-REL-RELA-RELB complex	0.02334
Prostate cancer	2086	NFKB1-NFKB2-RELA-RELB complex	0.0261
Prostate cancer	2100	CHUK-IKBKB-MAP3K14 complex	0.06027
Prostate cancer	2101	IKKA-IKKB complex	0.07382
Prostate cancer	2104	IKKB-NIK complex	0.03691
Prostate cancer	2105	IkappaB kinase complex (IKBKB CHUK IKBKAP NFKBIA RELA MAP3K14)	0.04262
Prostate cancer	2112	CDC37-HSP90AA1-HSP90AB1-MAP3K11 complex	0.0261
Prostate cancer	2118	CHUK-ERC1-IKBKB-IKBKG	0.0522
Prostate cancer	2121	CHUK-IKBKB-IKBKG complex	0.06027
Prostate cancer	2124	IKK-alpha--ER-alpha-AIB1 complex	0.03014
Prostate cancer	2143	MAP2K5-PRKCI-SQSTM1 complex	0.03014
Prostate cancer	2159	AR-AKT-APPL complex	0.03014
Prostate cancer	2160	AOF2-AR complex	0.03691
Prostate cancer	2187	Ubiquitin E3 ligase (NFKBIA FBXW11 BTRC CUL1 SKP1A)	0.02334
Prostate cancer	2188	Ubiquitin E3 ligase (CDC34 NEDD8 BTRC CUL1 SKP1A RBX1)	0.02334
Prostate cancer	2189	Ubiquitin E3 ligase (SMAD3 BTRC CUL1 SKP1A RBX1)	0.04669
Prostate cancer	2196	LIG1-9-1-1 complex	0.0261
Prostate cancer	2197	FEN1-9-1-1 complex	0.0261
Prostate cancer	2198	RAD9-RAD1-HUS1-POLB complex	0.0261
Prostate cancer	2220	RAD52-ERCC4-ERCC1 complex	0.03014
Prostate cancer	2233	Replication-coupled CAF-1-MBD1-ETDB1 complex	0.03014
Prostate cancer	2235	ASF1-interacting protein complex	0.02131
Prostate cancer	2318	ITGA6-ITGB4-Laminin10/12 complex	0.07003
Prostate cancer	2319	ITGA6-ITGB4-Laminin10/12 complex	0.04669
Prostate cancer	2322	ITGA6-ITGB4-LAMA5 complex	0.03014
Prostate cancer	2363	ITGAV-ITGB3-PXN-PTK2b complex	0.0261
Prostate cancer	2383	ITGA5-ITGB1-FN1-TGM2 complex	0.0261
Prostate cancer	2384	ITGA5-ITGB1-ADAM15 complex	0.03014
Prostate cancer	2385	ITGA5-ITGB4 complex	0.03691
Prostate cancer	2388	Itga5-Itgb1-Fn1-Sfrp2 complex	0.0261
Prostate cancer	2395	ITGA7-ITGB1-CD151 complex	0.03014
Prostate cancer	2396	ITGA7-ITGB1-CD9 complex	0.03014
Prostate cancer	2397	ITGA7-ITGB1-ITGB1BP3 complex	0.03014
Prostate cancer	2429	ITGA2-ITGB1-CD47 complex	0.03014

Prostate cancer	2430	ITGA2-ITGB1-CHAD complex	0.03014
Prostate cancer	2431	ITGA2-ITGB1-COL6A3 complex	0.03014
Prostate cancer	2432	ITGA2-ITGB1 complex	0.03691
Prostate cancer	2471	SRC-PRKCD-CDCP1 complex	0.03014
Prostate cancer	5709	ArgBP2a-CBL-PTK2B complex	0.03014
Prostate cancer	2528	ERBB2-MEMO-SHC complex	0.03014
Prostate cancer	2529	LAT-PLC-gamma-1-p85-GRB2-CBL-VAV-SLP-76 signaling complex C305 activated	0.01973
Prostate cancer	2535	SLP-76-Cbl-Grb2-Shc complex Fc receptor gamma-R1 stimulated	0.0261
Prostate cancer	2547	PLC-gamma-1-SLP-76-SOS1-LAT complex	0.0261
Prostate cancer	2551	PDGFRA-PLC-gamma-1-PI3K-SHP-2 complex PDGF stimulated	0.0261
Prostate cancer	2590	FOXO1-FHL2-SIRT1 complex	0.06027
Prostate cancer	2638	HES1 promoter corepressor complex	0.04262
Prostate cancer	2639	HES1 promoter-Notch enhancer complex	0.01448
Prostate cancer	2641	p300/CBP-PCAF-MyoD complex	0.0522
Prostate cancer	2642	SMAD1-P300 complex	0.03691
Prostate cancer	2692	SMAD3-SMAD4-cJun-cFos complex	0.0261
Prostate cancer	2694	ERG-JUN-FOS DNA-protein complex	0.03014
Prostate cancer	2705	SMAD3-SMAD4-CTCF protein-DNA complex	0.03014
Prostate cancer	2706	SMAD3-SMAD4-SP1 complex	0.03014
Prostate cancer	2707	SMAD3-SMAD4-FOXO3-FOXG1 complex	0.0261
Prostate cancer	2708	SMAD3-SMAD4-cJUN complex	0.03014
Prostate cancer	2709	MMP-9-TIMP-1-LRP complex	0.03014
Prostate cancer	2710	LRP-1-Alpha-2-M-annexin VI complex	0.03014
Prostate cancer	2724	Ubiquitin E3 ligase (NFKBIA BTRC CUL1 SKP1A)	0.0261
Prostate cancer	2727	SRC-3 complex	0.07892
Prostate cancer	2728	SRC-1 complex	0.0522
Prostate cancer	2754	JUND-FOSB-SMAD3-SMAD4 complex	0.0522
Prostate cancer	2755	17S U2 snRNP	0.00909
Prostate cancer	2760	SMAD3-SMAD4-FOXO3 complex	0.03014
Prostate cancer	2761	SMAD3-SMAD4-FOXO1 complex	0.06027
Prostate cancer	2762	SMAD3-SMAD4-FOXO4 complex	0.03014
Prostate cancer	2789	ETS2-ERG complex	0.03691
Prostate cancer	2808	RAD9-RAD1-HUS1-APE1 complex	0.0261
Prostate cancer	2809	9-1-1 complex	0.03014
Prostate cancer	2813	BRCA1-SMAD3 complex	0.03691
Prostate cancer	2818	BRCA1-BARD1-BRCA2-DNA damage complex III	0.03014
Prostate cancer	2829	RSmad complex	0.04952
Prostate cancer	2830	TIF1gamma-SMAD2-SMAD3 complex	0.03014
Prostate cancer	2834	SMAD4-SMAD2-SMAD3 complex	0.03014
Prostate cancer	2838	AR coactivator complex	0.03014
Prostate cancer	2850	ITGA5-ITGB1-FN-1-NOV complex	0.0261
Prostate cancer	2852	Brg1-based SWI/SNF chromatin remodeling complex	0.0261
Prostate cancer	2853	ITGA5-ITGB1-CAL4A3 complex	0.03014
Prostate cancer	2880	SCF subcomplex (WEE1 SKP2 BTRC)	0.03014
Prostate cancer	2882	ITGA5-ITGB3-COL6A3 complex	0.03014

Prostate cancer	2895	SHC-GRB2 complex	0.03691
Prostate cancer	2911	SMRT-SKIP-CBF1 complex	0.03014
Prostate cancer	2922	LAT-PLC-gamma-1-p85-GRB2-SOS signaling complex C305 activated	0.02334
Prostate cancer	2936	Ecsit complex (ECSIT MT-CO2 GAPDH TRAF6 NDUFAF1)	0.04669
Prostate cancer	2939	Ecsit complex (ECSIT MT-CO2 NDUFA1 MT-ND1 TRAF6 NDUFAF1)	0.02131
Prostate cancer	2945	RBP-Jkappa-RING1-KyoT2 complex	0.03014
Prostate cancer	2954	Smad1-Notch1-p300-Pcaf complex	0.0261
Prostate cancer	2955	LCK-SLP76-PLC-gamma-1-LAT complex pervanadate-activated	0.0261
Prostate cancer	2956	PLC-gamma-1-LAT-c-CBL complex OKT3 stimulated	0.03014
Prostate cancer	2958	SMAD1-CBP complex	0.03691
Prostate cancer	2960	SLP-76-PLC-gamma-1-ITK complex alpha-TCR stimulated	0.03014
Prostate cancer	2961	SLP-76-PLC-gamma-1-VAV complex alpha-TCR stimulated	0.03014
Prostate cancer	2968	Axin-SMAD3 complex	0.03691
Prostate cancer	2975	SMAD3-E2F4/5-p107-DP1 complex	0.04669
Prostate cancer	3046	hs4 enhancer complex (slow migrating complex)	0.03691
Prostate cancer	3047	Parvulin-associated pre-rRNP complex	0.00663
Prostate cancer	3055	Nop56p-associated pre-rRNA complex	0.01536
Prostate cancer	3060	RNA polymerase II complex (RPB1 RAP74 CDK8 CYCC SRB7 BAF190 BAF47) chromatin structure modifying	0.01846
Prostate cancer	3061	RNA polymerase II complex (CBP PCAF RPB1 BAF47 CYCC CDK8) chromatin structure modifying	0.02131
Prostate cancer	3062	RNA polymerase II complex incomplete (CBP RPBI PCAF BAF47) chromatin structure modifying	0.0261
Prostate cancer	3063	Brg1-associated complex II	0.01973
Prostate cancer	3064	RNA polymerase II complex chromatin structure modifying	0.02395
Prostate cancer	3065	RNA polymerase II complex chromatin structure modifying	0.01574
Prostate cancer	3066	RNA polymerase II complex chromatin structure modifying	0.02896
Prostate cancer	3067	RNA polymerase II complex incomplete (CDK8 complex) chromatin structure modifying	0.01846
Prostate cancer	3096	ITGA6-ITGB4-SHC1-GRB2 complex	0.0261
Prostate cancer	3110	ITGAV-P2RY2-GNA12 complex	0.03014
Prostate cancer	3112	ITGA5-ITGB1-SPP1 complex	0.03014
Prostate cancer	3127	TLE-Histone H3 complex	0.01973
Prostate cancer	3137	MASH1 promoter-coactivator complex	0.01574
Prostate cancer	3142	CAMK2-delta-MASH1 promoter-coactivator complex	0.01846
Prostate cancer	3149	NK-3-Groucho-HIPK2-SIN3A-RbpA48-HDAC1	0.01574
Prostate cancer	3150	NK-3-Groucho complex	0.01973
Prostate cancer	3156	CBF1-HDAC1-SMRT complex	0.03014
Prostate cancer	3162	TF-FVIIa-FXa-TFPI complex	0.0261
Prostate cancer	3167	NCOR-SIN3-HDAC-HESX1 complex	0.02131

Prostate cancer	3182	FHL2 homodimer complex	0.0522
Prostate cancer	3187	FHL2-FHL3 complex	0.03691
Prostate cancer	3188	FHL2-ACT complex	0.03691
Prostate cancer	3189	FHL2-CREB complex	0.03691
Prostate cancer	3199	SMAD3-SKI complex	0.03691
Prostate cancer	3205	SMAD3-SKI-NCOR complex	0.03014
Prostate cancer	3263	HERP1/HEY2-NCOR-SIN3A complex	0.0261
Prostate cancer	3714	Pericentrin-GCP complex	0.03014
Prostate cancer	3733	SKI-SMAD3 hexameric complex	0.03691
Prostate cancer	3740	SKI-SMAD3-SMAD4 pentameric complex	0.03014
Prostate cancer	3749	CREBBP-SMAD2 hexameric complex	0.03691
Prostate cancer	3750	CREBBP-SMAD3 hexameric complex	0.07382
Prostate cancer	3753	CREBBP-SMAD2-SMAD4 pentameric complex	0.03014
Prostate cancer	3754	CREBBP-SMAD3-SMAD4 pentameric complex	0.06027
Prostate cancer	3830	ADRB2 homodimer complex	0.0522
Prostate cancer	3959	SMAD3-SMAD4-cSKI TGF(beta)-dependent	0.03014
Prostate cancer	3961	SMAD3-cSKI-SIN3A-HDAC1 complex	0.0261
Prostate cancer	3971	SMURF2-SMAD3 complex TGF(beta)-dependent	0.03691
Prostate cancer	3972	SMURF2-SMAD3-SnoN complex TGF(beta)-	0.03014
Prostate cancer	4089	SMAD6-HOXC8 complex	0.03691
Prostate cancer	5118	pRb2/p130-multimolecular complex (RB2 E2F4 HDAC1 SUV39H1 P300)	0.02334
Prostate cancer	5144	E2F1-p107-cyclinA complex	0.03014
Prostate cancer	5153	CTFC-TAF1 complex	0.03691
Prostate cancer	5158	SMARCA2/BRM-BAF57-MECP2 complex	0.06027
Prostate cancer	5159	E2F4-p107-cyclinE complex	0.03014
Prostate cancer	5162	ELK1-SRF-ELK3 complex	0.03014
Prostate cancer	5178	JAK2-PAFR-TYK2 complex	0.03014
Prostate cancer	5184	SWI/SNF chromatin-remodeling complex	0.04669
Prostate cancer	5193	TNF-alpha/NF-kappa B signaling complex (CHUK KPNA3 NFKB2 NFKBIB REL IKBKG NFKB1 NFKBIE RELB NFKBIA RELA TNIP2)	0.03014
Prostate cancer	5194	TNF-alpha/NF-kappa B signaling complex (SEC16A CHUK IKBKB NFKB2 REL IKBKG MAP3K14 RELA FBXW7 USP2)	0.04952
Prostate cancer	5196	TNF-alpha/NF-kappa B signaling complex (CHUK BTRC NFKB2 PPP6C REL CUL1 IKBKE SAPS2 SAPS1 ANKRD28 RELA SKP1)	0.03014
Prostate cancer	5198	CBP-RARA-RXRA-DNA complex ligand stimulated	0.06027
Prostate cancer	5199	Kinase maturation complex 1	0.01305
Prostate cancer	5211	RAF1-PPP2-PIN1 complex	0.02334
Prostate cancer	5212	Kinase maturation complex 2	0.01846
Prostate cancer	5220	CHUK-IQGAP2-AKAP8L-RELA-TNIP2 complex	0.02334
Prostate cancer	5230	CHUK-NFKB2-REL-IKBKG-SPAG9-NFKB1-NFKBIE-COPB2-TNIP1-NFKBIA-RELA-TNIP2	0.01507
Prostate cancer	5232	TNF-alpha/Nf-kappa B signaling complex (RPL6 RPL30 RPS13 CHUK DDX3X NFKB2 NFKBIB REL IKBKG NFKB1 MAP3K8 RELB GLG1 NFKBIA RELA TNIP2 GTF2I)	0.02532

Prostate cancer	5233	TNF-alpha/NF-kappa B signaling complex 5	0.02088
Prostate cancer	5234	IKBKB-CDC37-KIAA1967-HSP90AB1-HSP90AA1 complex	0.04669
Prostate cancer	5260	TCF4-CTNNB1-SUMO1-EP300-HADAC6 complex	0.04669
Prostate cancer	5261	TCF4-CTNNB1-EP300 complex	0.03014
Prostate cancer	5264	TCF4-CTNNB1-CREBBP complex	0.03014
Prostate cancer	5266	TNF-alpha/NF-kappa B signaling complex 6	0.04185
Prostate cancer	5268	TNF-alpha/NF-kappa B signaling complex 7	0.01846
Prostate cancer	5269	TNF-alpha/NF-kappa B signaling complex 8	0.02131
Prostate cancer	5285	TNF-alpha/NF-kappa B signaling complex 9	0.04669
Prostate cancer	5286	TNF-alpha/NF-kappa B signaling complex 10	0.03301
Prostate cancer	5367	THRB-RXRB complex	0.03691
Prostate cancer	5375	EGR-EP300 complex	0.07382
Prostate cancer	5380	TRBP containing complex (DICER RPL7A EIF6 MOV10 and subunits of the 60S ribosomal particle)	0.01044
Prostate cancer	5385	GAIT complex	0.0261
Prostate cancer	5386	MLL1-WDR5 complex	0.01005
Prostate cancer	5389	SERPINA3-CTSG complex	0.03691
Prostate cancer	5400	BRCC complex	0.02334
Prostate cancer	5423	HSP70-BAG5-PARK2 complex	0.0261
Prostate cancer	5432	Sororin-cohesin complex	0.02131
Prostate cancer	5513	Polycomb repressive complex	0.0261
Prostate cancer	5564	LMO4-gp130 complex	0.04669
Prostate cancer	5573	Stat1-alpha-dimer-CBP DNA-protein complex	0.03691
Prostate cancer	5589	LINC complex S-phase	0.01973
Prostate cancer	5609	Emerin regulatory complex	0.0174
Prostate cancer	5613	Emerin complex 25	0.01305
Prostate cancer	5614	Emerin complex 32	0.02226
Prostate cancer	5735	TGF-beta receptor-SMAD3 complex	0.03014
Prostate cancer	5755	SUMO1-SUA1-UBA2 complex	0.03014
Prostate cancer	5828	IKBKG-IKBKB complex	0.03691
Prostate cancer	5829	IKBKG-CHUK complex	0.03691
Prostate cancer	5843	AIF-CYPA-DNA complex	0.03691
Prostate cancer	5844	I-kappa-B kinase (IKK) complex	0.06027
Prostate cancer	5849	HSP90-CDC37-LRRK2 complex	0.03014
Protein-energy malnutrition	220	ARF-Mule complex	0.19245
Protein-energy malnutrition	387	MCM complex	0.13608
Protein-energy malnutrition	541	IGF1-IGFBP3-ALS complex	0.19245
Protein-energy malnutrition	1091	SNX complex (SNX1a SNX2 SNX4 LEPR)	0.16667
Protein-energy malnutrition	1452	MCM2-MCM6-MCM7 complex	0.19245
Protein-energy malnutrition	1642	p16-cyclin D2-CDK4 complex	0.19245
Protein-energy malnutrition	1992	LEPR homodimer complex	0.33333
Protein-energy malnutrition	2370	ITGA2b-ITGB3-CD9 complex	0.19245
Protein-energy malnutrition	2396	ITGA7-ITGB1-CD9 complex	0.19245
Protein-energy malnutrition	2770	ITGA6-ITGB4-CD9 complex	0.19245
Protein-energy malnutrition	2792	MCM2-MCM4-MCM6-MCM7 complex	0.16667
Protein-energy malnutrition	2872	ITGA2b-ITGB3-CD9-GP1b-CD47 complex	0.13608
Protein-energy malnutrition	5611	Emerin complex 24	0.08607

Proteinuria	725	P2X7 receptor signalling complex	0.06455
Proteinuria	786	MR-UBC9-SRC1 complex	0.1291
Proteinuria	1656	p27-cyclinE-CDK2 complex	0.1291
Proteinuria	2254	CTGF/Hcs24-actin complex	0.1291
Proteinuria	3015	p27-cyclinE-Cdk2 - Ubiquitin E3 ligase (SKP1A SKP2 CUL1 CKS1B RBX1) complex	0.07906
Proteinuria	3162	TF-FVIIa-FXa-TFPI complex	0.1118
Proteinuria	3634	NR3C2-UBC9-SRC-1 complex	0.1291
Proteinuria	5655	Ternary complex (LRRC7 CAMK2a ACTN4)	0.1291
Psoriasis	120	Lymphotoxin beta receptor complex	0.13608
Psoriasis	1514	IL4-IL4R complex	0.16667
Psoriasis	1515	IL4-IL4R-IL2RG complex	0.13608
Psoriasis	1774	MICA-KLRK1-HCST complex	0.13608
Psoriasis	1986	Endoglin homodimer complex	0.2357
Psychotic disorder	143	APP-FE65-LRP complex	0.09245
Psychotic disorder	1000	TorsinA-TorsinB complex	0.11323
Psychotic disorder	1088	PRNP-ApolopoproteinE3 complex	0.11323
Psychotic disorder	1186	ESCRT-III complex	0.05064
Psychotic disorder	5718	eNOS-HSP90-AKT complex VEGF induced	0.09245
Psychotic disorder	2143	MAP2K5-PRKCI-SQSTM1 complex	0.09245
Psychotic disorder	2156	YBX1-AKT1 complex	0.11323
Psychotic disorder	2159	AR-AKT-APPL complex	0.09245
Psychotic disorder	2258	VILIP-1-AChR-alpha-4-AChR-beta-2 complex	0.1849
Psychotic disorder	2272	PICK1-GRIP1-GLUR2 complex	0.09245
Psychotic disorder	2711	Amyloid beta protein oligomer	0.16013
Psychotic disorder	3092	APP-TOMM40 complex	0.11323
Psychotic disorder	3093	APP-TIMM23 complex	0.11323
Psychotic disorder	3847	TCL1(trimer)-AKT1 complex	0.11323
Psychotic disorder	4039	PAR4-BACE1 complex	0.11323
Psychotic disorder	5389	SERPINA3-CTSG complex	0.11323
Ptosis	2318	ITGA6-ITGB4-Laminin10/12 complex	0.14907
Ptosis	2319	ITGA6-ITGB4-Laminin10/12 complex	0.14907
Ptosis	2798	MMP-2-claudin-1 complex	0.2357
Pulmonary fibrosis	541	IGF1-IGFBP3-ALS complex	0.10911
Pulmonary fibrosis	550	NOS3-CAV1-NOSTRIN complex	0.10911
Pulmonary fibrosis	557	TRP1-G alpha-11-IP3R3-CAV1 signaling complex	0.09449
Pulmonary fibrosis	753	UTM-SGCE-DAG1-CAV1-NOS3 complex	0.08452
Pulmonary fibrosis	801	SNARE complex (STX2 SNAP23)	0.13363
Pulmonary fibrosis	1185	EGFR-containing signaling complex	0.09449
Pulmonary fibrosis	5714	eNOS-CAV1 complex	0.13363
Pulmonary fibrosis	2462	Caveolin-1 homodimer complex	0.18898
Pulmonary fibrosis	2528	ERBB2-MEMO-SHC complex	0.10911
Pulmonary fibrosis	2798	MMP-2-claudin-1 complex	0.13363
Pulmonary fibrosis	5473	FAS-FADD-CASP8 complex	0.10911
Pulmonary fibrosis	5799	Death induced signaling complex DISC (FAS FADD CASP8 CFLAR) membrane-associated CD95L	0.09449
Pulmonary fibrosis	5800	Death-inducing signaling complex DISC (type I cells associated) stimulated	0.10911

Pulmonary fibrosis	5808	DISC complex	0.10911
Pulmonary fibrosis	5859	FAS-FADD-CASP8-CASP10 complex	0.09449
Pulmonary fibrosis	5861	FAS-FADD-CASP10 complex	0.10911
Pulmonary fibrosis	5862	CAV1-VDAC1-ESR1 complex	0.10911
Rabies	74	TRPC1-Homer3-IP3R complex	0.06415
Rabies	143	APP-FE65-LRP complex	0.06415
Rabies	201	HUIC complex	0.07857
Rabies	202	BRCA1-RAD50-MRE11-NBS1 complex	0.05556
Rabies	205	Ubiquitin E3 ligase (VHL TCEB1 TCEB2 CUL2	0.04969
Rabies	238	SWI-SNF chromatin remodeling-related-BRCA1	0.0335
Rabies	240	BRCA1-CTIP-ZBRK1 repressor complex	0.06415
Rabies	242	BRCA1-BACH1 complex	0.07857
Rabies	433	BASC complex (BRCA1-associated genome surveillance complex)	0.03208
Rabies	434	BASC (Ab 80) complex (BRCA1-associated genome surveillance complex)	0.03928
Rabies	435	BASC (Ab 81) complex (BRCA1-associated genome surveillance complex)	0.04536
Rabies	436	BASC (Ab C-20) complex (BRCA1-associated genome surveillance complex)	0.06415
Rabies	438	GCN5-TRRAP histone acetyltransferase complex	0.03514
Rabies	441	TFTC-type histone acetyl transferase complex	0.0335
Rabies	520	KCNQ1 macromolecular complex	0.03514
Rabies	528	NuA4/Tip60 HAT complex	0.02869
Rabies	529	NuA4/Tip60 HAT complex	0.02778
Rabies	536	TRPC1-TRPC3-TRPC7 complex	0.06415
Rabies	550	NOS3-CAV1-NOSTRIN complex	0.06415
Rabies	553	RHOA-IP3R-TRPC1 complex	0.06415
Rabies	557	TRP1-G alpha-11-IP3R3-CAV1 signaling complex	0.11111
Rabies	563	F1F0-ATP synthase (EC 3.6.3.14) mitochondrial	0.02778
Rabies	622	Ubiquitin E3 ligase (VHL TCEB1 TCEB2 CUL2)	0.05556
Rabies	722	MRG15-PAM14-RB complex	0.06415
Rabies	723	MAF1 complex	0.06415
Rabies	724	MAF2 complex	0.07857
Rabies	753	UTM-SGCE-DAG1-CAV1-NOS3 complex	0.04969
Rabies	787	NuA4/Tip60-HAT complex B	0.03928
Rabies	798	NuA4/Tip60-HAT complex A	0.02869
Rabies	828	TRPC1-STIM1-ORAI1 complex	0.06415
Rabies	999	p23 protein complex	0.06415
Rabies	1054	ESR1-RELA-BCL3-NCOA3 complex	0.05556
Rabies	1223	H2AX complex isolated from cells without IR exposure	0.03082
Rabies	1226	H2AX complex I	0.042
Rabies	1227	H2AX complex II	0.03514
Rabies	1288	DCS complex (PTBP1 PTBP2 HNRPH1 HNRPF)	0.05556
Rabies	1474	SMAD3/4-E2F4/5-p107-DP1 complex	0.04536
Rabies	1826	SMAD3-HEF1-APC10-CDH1 complex	0.05556
Rabies	1827	PML-SMAD2/3-SARA complex	0.05556
Rabies	1828	TGF-beta receptor I-Axin-SMAD3 complex	0.06415

Rabies	1831	PIAS3-SMAD3-P300 complex	0.06415
Rabies	2124	IKK-alpha--ER-alpha-AIB1 complex	0.06415
Rabies	2159	AR-AKT-APPL complex	0.06415
Rabies	2160	AOF2-AR complex	0.07857
Rabies	2189	Ubiquitin E3 ligase (SMAD3 BTRC CUL1 SKP1A RBX1)	0.04969
Rabies	2210	BRCA1-IRIS-pre-replication complex	0.05556
Rabies	2211	BARD1-BRCA1-CSTF complex	0.04969
Rabies	2213	BRCA1-BARD1-POLR2A complex	0.06415
Rabies	2214	LMO4-BRCA1-CTIP-LDB1 complex	0.05556
Rabies	2215	BRCA1-LMO4-CTIP complex	0.06415
Rabies	2395	ITGA7-ITGB1-CD151 complex	0.06415
Rabies	2396	ITGA7-ITGB1-CD9 complex	0.06415
Rabies	2397	ITGA7-ITGB1-ITGB1BP3 complex	0.06415
Rabies	5714	eNOS-CAV1 complex	0.07857
Rabies	2462	Caveolin-1 homodimer complex	0.11111
Rabies	2470	p130Cas-ER-alpha-cSrc-kinase- PI3-kinase p85-subunit complex	0.05556
Rabies	2589	PGC-1-SRp40-SRp55-SRp75 complex	0.05556
Rabies	2599	POLR2A-CCNT1-CDK9-NCL-LEM6-CPSF2 complex	0.04536
Rabies	2657	ESR1-CDK7-CCNH-MNAT1-MTA1-HDAC2 complex	0.04536
Rabies	2670	Er-alpha-p53-hdm2 complex	0.06415
Rabies	2686	BRCA1-core RNA polymerase II complex	0.03082
Rabies	2692	SMAD3-SMAD4-cJun-cFos complex	0.05556
Rabies	2699	ER-alpha-GRIP1-c-Jun complex	0.06415
Rabies	2700	ER-alpha-c-Jun complex	0.07857
Rabies	2705	SMAD3-SMAD4-CTCF protein-DNA complex	0.06415
Rabies	2706	SMAD3-SMAD4-SP1 complex	0.06415
Rabies	2707	SMAD3-SMAD4-FOXO3-FOXG1 complex	0.05556
Rabies	2708	SMAD3-SMAD4-cJUN complex	0.06415
Rabies	2711	Amyloid beta protein oligomer	0.11111
Rabies	2754	JUND-FOSB-SMAD3-SMAD4 complex	0.05556
Rabies	2760	SMAD3-SMAD4-FOXO3 complex	0.06415
Rabies	2761	SMAD3-SMAD4-FOXO1 complex	0.06415
Rabies	2762	SMAD3-SMAD4-FOXO4 complex	0.06415
Rabies	2776	RAD50-BRCA1 complex	0.07857
Rabies	2783	BARD1-BRCA1-CSTF64 complex	0.06415
Rabies	2786	BRCA1 A complex	0.05556
Rabies	2787	BRCA1 C complex	0.05556
Rabies	2788	BRCA1 B complex	0.06415
Rabies	2811	BRCA1-cABL complex	0.07857
Rabies	2813	BRCA1-SMAD3 complex	0.15713
Rabies	2814	BRCA1-HDAC1-HDAC2 complex	0.06415
Rabies	2815	BRCA1-BARD1-BACH1-DNA damage complex II	0.03928
Rabies	2817	BRCA1-BARD1-BACH1-DNA damage complex I	0.04536
Rabies	2818	BRCA1-BARD1-BRCA2-DNA damage complex III	0.06415
Rabies	2819	BRCA1-CtIP-CtBP complex	0.06415
Rabies	2820	BRCA1-VCP complex	0.07857

Rabies	2822	BRCA1-BARD1-UbcH5c complex	0.06415
Rabies	2823	BRCA1-BARD1-UbcH7c complex	0.06415
Rabies	2824	BRCA1-RAD51 complex	0.07857
Rabies	2825	BRCA1-RNA polymerase II complex	0.02179
Rabies	2829	RSmad complex	0.03514
Rabies	2830	TIF1gamma-SMAD2-SMAD3 complex	0.06415
Rabies	2834	SMAD4-SMAD2-SMAD3 complex	0.06415
Rabies	2857	NuA4/Tip60 HAT complex	0.0297
Rabies	2968	Axin-SMAD3 complex	0.07857
Rabies	2975	SMAD3-E2F4/5-p107-DP1 complex	0.04969
Rabies	3092	APP-TOMM40 complex	0.07857
Rabies	3093	APP-TIMM23 complex	0.07857
Rabies	3162	TF-FVIIa-FXa-TFPI complex	0.05556
Rabies	3199	SMAD3-SKI complex	0.07857
Rabies	3205	SMAD3-SKI-NCOR complex	0.06415
Rabies	3733	SKI-SMAD3 hexameric complex	0.07857
Rabies	3740	SKI-SMAD3-SMAD4 pentameric complex	0.06415
Rabies	3750	CREBBP-SMAD3 hexameric complex	0.07857
Rabies	3754	CREBBP-SMAD3-SMAD4 pentameric complex	0.06415
Rabies	3959	SMAD3-SMAD4-cSKI TGF(beta)-dependent	0.06415
Rabies	3961	SMAD3-cSKI-SIN3A-HDAC1 complex	0.05556
Rabies	3971	SMURF2-SMAD3 complex TGF(beta)-dependent	0.07857
Rabies	3972	SMURF2-SMAD3-SnoN complex TGF(beta)-	0.06415
Rabies	5165	AP1G1-PACS1-FURIN complex	0.06415
Rabies	5217	Calreticulin oligomer complex	0.11111
Rabies	5267	VHL-VDU1-TCEB1-TCEB2 complex	0.05556
Rabies	5270	VHL-TCEB1-TCEB2 complex	0.06415
Rabies	5273	VHL-TBP1-HIF1A complex	0.06415
Rabies	5280	RAB9-TIP47-MPRI complex	0.06415
Rabies	5400	BRCC complex	0.04969
Rabies	5735	TGF-beta receptor-SMAD3 complex	0.06415
Rabies	5749	MRIT complex	0.06415
Rabies	5812	p53-BCL2 complex	0.07857
Rabies	5819	BIM-BCL2xL complex	0.07857
Rabies	5820	tBID-BCL2xL complex	0.07857
Rabies	5830	DJ-1-SNCA complex high molecular weight complex	0.07857
Rabies	5862	CAV1-VDAC1-ESR1 complex	0.1283
Rectum cancer	1088	PRNP-ApolopoproteinE3 complex	0.5
Rectum cancer	3162	TF-FVIIa-FXa-TFPI complex	0.35355
Renal Cell cancer	36	AP1 adaptor complex	0.0411
Renal Cell cancer	144	Gamma-BAR-AP1 complex	0.03875
Renal Cell cancer	186	Wave-2 complex	0.05199
Renal Cell cancer	527	TRPC3-TRPC4 channel complex redox-sensitive	0.0822
Renal Cell cancer	541	IGF1-IGFBP3-ALS complex	0.06712
Renal Cell cancer	552	IFNB1-IFNAR1-IFNAR2- complex	0.06712
Renal Cell cancer	786	MR-UBC9-SRC1 complex	0.06712
Renal Cell cancer	845	PCI-PSA-SCG2 complex	0.06712

Renal Cell cancer	1045	Snurportin-CRM1-RanGTP complex	0.06712
Renal Cell cancer	1067	CD8A-LCK complex	0.0822
Renal Cell cancer	1176	CRM1-RAN-PHAX-CBC complex (cap binding	0.05199
Renal Cell cancer	1255	Ubiquitin E3 ligase (SIAH1 SIP SKP1A TBL1X)	0.05812
Renal Cell cancer	1257	ALL-1 supercomplex	0.02197
Renal Cell cancer	1352	ING4 complex (ING4 MYST2 C1orf149 PHF17)	0.05812
Renal Cell cancer	1551	IPO13-RAN-EIF1AX complex	0.06712
Renal Cell cancer	1552	TNPO2-RAN-NXF1 complex	0.06712
Renal Cell cancer	1554	RANBP1-RAN-KPNB1 complex	0.06712
Renal Cell cancer	1707	IL2-IL2RA-IL2RB complex	0.06712
Renal Cell cancer	1729	TLE1 corepressor complex (MASH1 promoter-corepressor complex)	0.03676
Renal Cell cancer	2374	ITGAV-ITGB3-LAMA4 complex	0.06712
Renal Cell cancer	2489	NCR3-CD247 complex	0.0822
Renal Cell cancer	2559	p56(LCK)-CAML complex	0.0822
Renal Cell cancer	2565	CD20-LCK-LYN-FYN-p75/80 complex (Raji human B cell line)	0.05812
Renal Cell cancer	2721	HCF-1 complex	0.02667
Renal Cell cancer	2858	HBO1 complex	0.04394
Renal Cell cancer	2859	ING5 complex	0.03505
Renal Cell cancer	2879	CD20-LCK-FYN-p75/80 complex	0.06712
Renal Cell cancer	2944	Notch1-p56lck-PI3K complex	0.06712
Renal Cell cancer	2955	LCK-SLP76-PLC-gamma-1-LAT complex pervanadate-activated	0.05812
Renal Cell cancer	3634	NR3C2-UBC9-SRC-1 complex	0.06712
Renal Cell cancer	3979	mTORC2 complex (mTOR/FRAP1 LST8 mAVO3/RICTOR SIN1)	0.05812
Renal Cell cancer	5199	Kinase maturation complex 1	0.02906
Renal Cell cancer	5317	LATS1-HTRA2-BIRC4 complex	0.06712
Renal Cell cancer	5386	MLL1-WDR5 complex	0.02237
Renal Cell cancer	5423	HSP70-BAG5-PARK2 complex	0.05812
Renal Cell cancer	5661	PlexinC1-SEMA7A complex	0.0822
Renal Cell cancer	5670	PlexinB1-Nrp1 complex	0.0822
Renal tubular acidosis	66	TRAP complex	0.11785
Renal tubular acidosis	86	NUMAC complex (nucleosomal methylation activator complex)	0.10541
Renal tubular acidosis	149	PBAF complex (Polybromo- and BAF containing complex)	0.09623
Renal tubular acidosis	159	Condensin I-PARP-1-XRCC1 complex	0.12599
Renal tubular acidosis	189	BAF complex	0.09245
Renal tubular acidosis	230	Mediator complex	0.05893
Renal tubular acidosis	232	ARC complex	0.08607
Renal tubular acidosis	238	SWI-SNF chromatin remodeling-related-BRCA1	0.1005
Renal tubular acidosis	287	ARC-L complex	0.08909
Renal tubular acidosis	288	ARC complex	0.08607
Renal tubular acidosis	301	SMCC complex	0.08085
Renal tubular acidosis	441	TFTC-type histone acetyl transferase complex	0.20101
Renal tubular acidosis	535	TRAP complex	0.08333

Renal tubular acidosis	547	SMCC complex	0.08909
Renal tubular acidosis	548	DRIP complex	0.08909
Renal tubular acidosis	554	PBAF complex (Polybromo- and BAF containing complex)	0.10541
Renal tubular acidosis	555	BAF complex	0.11111
Renal tubular acidosis	564	BAF complex	0.1005
Renal tubular acidosis	565	PBAF complex (Polybromo- and BAF containing complex)	0.1005
Renal tubular acidosis	566	BAF complex	0.1005
Renal tubular acidosis	570	p300-CBP-p270-SWI/SNF complex	0.12599
Renal tubular acidosis	710	Brg1-associated complex I	0.1005
Renal tubular acidosis	713	BRG1-SIN3A complex	0.08909
Renal tubular acidosis	739	SIN3-ING1b complex II	0.08333
Renal tubular acidosis	778	LARC complex (LCR-associated remodeling complex)	0.07647
Renal tubular acidosis	803	BRG1-SIN3A-HDAC containing SWI/SNF remodeling complex I	0.1005
Renal tubular acidosis	807	BRG1-associated complex	0.11111
Renal tubular acidosis	909	ARC92-Mediator complex	0.09245
Renal tubular acidosis	1004	RC complex during S-phase of cell cycle	0.09245
Renal tubular acidosis	1005	RC complex during G2/M-phase of cell cycle	0.09245
Renal tubular acidosis	1054	ESR1-RELA-BCL3-NCOA3 complex	0.16667
Renal tubular acidosis	1056	ZNF198-SUMO1 complex	0.2357
Renal tubular acidosis	1193	Rap1 complex	0.12599
Renal tubular acidosis	1226	H2AX complex I	0.12599
Renal tubular acidosis	1230	WINAC complex	0.08909
Renal tubular acidosis	1239	EBAFb complex	0.10541
Renal tubular acidosis	1252	EBAFa complex	0.10541
Renal tubular acidosis	1413	NCOR1 complex	0.10541
Renal tubular acidosis	1728	CTCF-nucleophosmin-PARP-HIS-KPNA-LMNA-TOP complex	0.11111
Renal tubular acidosis	1729	TLE1 corepressor complex (MASH1 promoter-corepressor complex)	0.10541
Renal tubular acidosis	2124	IKK-alpha--ER-alpha-AIB1 complex	0.19245
Renal tubular acidosis	2470	p130Cas-ER-alpha-cSrc-kinase- PI3-kinase p85-subunit complex	0.16667
Renal tubular acidosis	2600	BRD4 complex	0.11785
Renal tubular acidosis	2625	CDK8-MED6-PARP1 complex	0.19245
Renal tubular acidosis	2638	HES1 promoter corepressor complex	0.13608
Renal tubular acidosis	2639	HES1 promoter-Notch enhancer complex	0.09245
Renal tubular acidosis	2657	ESR1-CDK7-CCNH-MNAT1-MTA1-HDAC2 complex	0.13608
Renal tubular acidosis	2670	Er-alpha-p53-hdm2 complex	0.19245
Renal tubular acidosis	2699	ER-alpha-GRIP1-c-Jun complex	0.19245
Renal tubular acidosis	2700	ER-alpha-c-Jun complex	0.2357
Renal tubular acidosis	2829	RSmad complex	0.10541
Renal tubular acidosis	2890	Notch1-fraction 30 complex	0.19245
Renal tubular acidosis	2897	RBPJ-NotchIC-Mastermind complex	0.19245
Renal tubular acidosis	2907	RBP-Jkappa-Notch1 complex	0.2357
Renal tubular acidosis	2908	RBP-Jkappa-SHARP complex	0.2357

Renal tubular acidosis	2911	SMRT-SKIP-CBF1 complex	0.19245
Renal tubular acidosis	2930	SHARP-CtIP-RBP-Jkappa complex	0.19245
Renal tubular acidosis	2931	SHARP-CtBP1-CtIP-RBP-Jkappa corepressor complex	0.16667
Renal tubular acidosis	2945	RBP-Jkappa-RING1-KyoT2 complex	0.19245
Renal tubular acidosis	2946	YY1-Notch1-RBP-Jkappa complex	0.19245
Renal tubular acidosis	3060	RNA polymerase II complex (RPB1 RAP74 CDK8 CYCC SRB7 BAF190 BAF47) chromatin structure modifying	0.11785
Renal tubular acidosis	3063	Brg1-associated complex II	0.12599
Renal tubular acidosis	3064	RNA polymerase II complex chromatin structure modifying	0.07647
Renal tubular acidosis	3066	RNA polymerase II complex chromatin structure modifying	0.09245
Renal tubular acidosis	3113	MAML1-RBP-Jkappa-Notch1 complex	0.19245
Renal tubular acidosis	3137	MASH1 promoter-coactivator complex	0.1005
Renal tubular acidosis	3142	CAMK2-delta-MASH1 promoter-coactivator complex	0.11785
Renal tubular acidosis	3156	CBF1-HDAC1-SMRT complex	0.19245
Renal tubular acidosis	3269	RB1-HDAC1-BRG1 complex	0.19245
Renal tubular acidosis	5179	NCOA6-DNA-PK-Ku-PARP1 complex	0.14907
Renal tubular acidosis	5235	WRN-Ku70-Ku80-PARP1 complex	0.16667
Renal tubular acidosis	5260	TCF4-CTNNB1-SUMO1-EP300-HADAC6 complex	0.14907
Renal tubular acidosis	5293	ETS2-SMARCA4-INI1 complex	0.19245
Renal tubular acidosis	5755	SUMO1-SUA1-UBA2 complex	0.19245
Renal tubular acidosis	5862	CAV1-VDAC1-ESR1 complex	0.19245
Respiratory distress syndrome	3162	TF-FVIIa-FXa-TFPI complex	0.13868
Respiratory distress syndrome	5473	FAS-FADD-CASP8 complex	0.16013
Respiratory distress syndrome	5799	Death induced signaling complex DISC (FAS FADD CASP8 CFLAR) membrane-associated CD95L	0.13868
Respiratory distress syndrome	5800	Death-inducing signaling complex DISC (type I cells associated) stimulated	0.16013
Respiratory distress syndrome	5808	DISC complex	0.16013
Respiratory distress syndrome	5859	FAS-FADD-CASP8-CASP10 complex	0.13868
Respiratory distress syndrome	5861	FAS-FADD-CASP10 complex	0.16013
Respiratory failure	115	Polycomb repressive complex 1 (PRC1 hPRC-H)	0.10206
Respiratory failure	116	Polycomb repressive complex 1 (PRC1 hPRC-H)	0.09806
Respiratory failure	343	Sarcoglycan-sarcospan complex SG-SPN	0.15811
Respiratory failure	432	N-NOS-CHIP-HSP70-1 complex	0.20412
Respiratory failure	626	LSD1 complex	0.09806
Respiratory failure	725	P2X7 receptor signalling complex	0.10206
Respiratory failure	1308	PABPC1-HSPA8-HNRPD-EIF4G1 complex	0.15811
Respiratory failure	1729	TLE1 corepressor complex (MASH1 promoter-corepressor complex)	0.1118
Respiratory failure	2443	ITGA9-ITGB1-TNC complex	0.20412
Respiratory failure	5423	HSP70-BAG5-PARK2 complex	0.17678
Respiratory tract disease	10	13S condensin complex	0.19069
Respiratory tract disease	49	DNMT3B complex	0.16116
Respiratory tract disease	157	Condensin I complex	0.19069
Respiratory tract disease	159	Condensin I-PARP-1-XRCC1 complex	0.16116
Respiratory tract disease	160	Condensin II	0.19069

Respiratory tract disease	299	IRF3-CBP complex	0.1066
Respiratory tract disease	353	DNA ligase IV-condensin complex	0.24618
Respiratory tract disease	548	DRIP complex	0.05698
Respiratory tract disease	552	IFNB1-IFNAR1-IFNAR2- complex	0.12309
Respiratory tract disease	681	(C-CFTR)2-NHERF-ezrin complex	0.12309
Respiratory tract disease	682	C-CFTR-NHERF(PDZ1 domain)-ezrin complex	0.12309
Respiratory tract disease	683	C-CFTR-NHERF(PDZ2 domain)-ezrin complex	0.12309
Respiratory tract disease	687	CFTR-NHERF-beta(2)AR signaling complex	0.12309
Respiratory tract disease	2709	MMP-9-TIMP-1-LRP complex	0.12309
Respiratory tract disease	5239	CAP(C)-CAP(E) complex	0.30151
Respiratory tract disease	5367	THRB-RXRB complex	0.15076
Reticulosarcoma	5816	Apoptosome-procaspase 9 complex	0.20412
Retinal disease	95	Ku antigen-NARG1 complex	0.08085
Retinal disease	298	VEGF transcriptional complex	0.05717
Retinal disease	351	Spliceosome	0.01171
Retinal disease	445	TFTC complex (TATA-binding protein-free TAF-II-containing complex)	0.03501
Retinal disease	470	TFTC complex (TATA-binding protein-free TAF-II-containing complex)	0.03396
Retinal disease	476	STAGA complex (SPT3-TAF9-GCN5 acetyltransferase complex)	0.03884
Retinal disease	513	TFTC complex (TATA-binding protein-free TAF-II-containing complex)	0.03396
Retinal disease	702	ARD1-NATH complex	0.09901
Retinal disease	725	P2X7 receptor signalling complex	0.04042
Retinal disease	4062	NRP1-VEGFR2-VEGF(165) complex	0.08085
Retinal disease	5772	ZO1-(beta)cadherin-(VE)cadherin-VEGFR2 complex	0.07001
Retinal disease	5273	VHL-TBP1-HIF1A complex	0.08085
Retinal disease	5276	HIF1A-OS9-EGLN1 complex	0.08085
Retinal disease	5277	HIF1A-OS9-EGLN3 complex	0.08085
Retinal disease	5382	ARNT-HIF1A complex	0.09901
Retinal disease	5654	SEMA4A-PlexinD1 complex	0.09901
Retinal disease	5696	VEGFA(165)-KDR-NRP1 complex	0.08085
Retinal disease	5698	VEGFA(165)-VEGFR2-NRP1 complex	0.08085
Retinitis pigmentosa	63	Mitotic 14S cohesin 1 complex	0.1
Retinitis pigmentosa	64	Mitotic 14S cohesin 2 complex	0.1
Retinitis pigmentosa	163	Cohesin-SA2 complex	0.1
Retinitis pigmentosa	164	Cohesin-SA1 complex	0.1
Retinitis pigmentosa	282	SNF2h-cohesin-NuRD complex	0.05
Retinitis pigmentosa	351	Spliceosome	0.05017
Retinitis pigmentosa	430	18S U11/U12 snRNP	0.04082
Retinitis pigmentosa	461	MPP4-MPP5-CRB1 complex	0.11547
Retinitis pigmentosa	924	Toposome	0.07559
Retinitis pigmentosa	1181	C complex spliceosome	0.02236
Retinitis pigmentosa	1195	Exon junction complex (mRNA splicing-dependent)	0.14142
Retinitis pigmentosa	1335	SNW1 complex	0.04714
Retinitis pigmentosa	1856	CDCA5-PDS5A-RAD21-SMC1A-PDS5B-SMC3	0.08165
Retinitis pigmentosa	5241	SMC1-SMC3 complex	0.14142

Retinitis pigmentosa	5386	MLL1-WDR5 complex	0.03849
Retinitis pigmentosa	5432	Sororin-cohesin complex	0.08165
Rett syndrome	749	MeCP2-SIN3A-HDAC complex	0.15076
Rett syndrome	2233	Replication-coupled CAF-1-MBD1-ETDB1 complex	0.17408
Rett syndrome	2238	MBD1-MCAF1-SETDB1 complex	0.17408
Rett syndrome	2707	SMAD3-SMAD4-FOXO3-FOXG1 complex	0.15076
Rett syndrome	2759	MBD1-MCAF complex	0.2132
Rett syndrome	2763	MBD1-Suv39h1-HP1 complex	0.17408
Rett syndrome	5158	SMARCA2/BRM-BAF57-MECP2 complex	0.17408
Rett syndrome	5184	SWI/SNF chromatin-remodeling complex	0.13484
Rhabdomyosarcoma	4	Multisubunit ACTR coactivator complex	0.15076
Rhabdomyosarcoma	471	PCAF complex	0.09535
Rhabdomyosarcoma	1338	FOXO3-PCAF complex oxidative stress stimulated	0.2132
Rhabdomyosarcoma	2641	p300/CBP-PCAF-MyoD complex	0.30151
Rhabdomyosarcoma	2954	Smad1-Notch1-p300-Pcaf complex	0.15076
Rhabdomyosarcoma	3061	RNA polymerase II complex (CBP PCAF RPB1 BAF47 CYCC CDK8) chromatin structure modifying	0.12309
Rhabdomyosarcoma	3062	RNA polymerase II complex incomplete (CBP RPBI PCAF BAF47) chromatin structure modifying	0.15076
Rhabdomyosarcoma	3066	RNA polymerase II complex chromatin structure modifying	0.08362
Rhabdomyosarcoma	3137	MASH1 promoter-coactivator complex	0.09091
Rhabdomyosarcoma	3142	CAMK2-delta-MASH1 promoter-coactivator complex	0.1066
Rhabdomyosarcoma	5373	Chromatin remodeling complex (TACC2 TACC3	0.17408
Rhabdomyosarcoma	5691	TALL1 homo-oligomer complex	0.30151
Rheumatic fever	2343	ITGAV-ITGB5-PLAUR complex	0.14907
Rheumatism	4	Multisubunit ACTR coactivator complex	0.11785
Rheumatism	570	p300-CBP-p270-SWI/SNF complex	0.08909
Rheumatism	571	p300-CBP-p270 complex	0.13608
Rheumatism	824	Anti-SMN protein complex	0.10541
Rheumatism	832	Anti-Sm protein complex	0.08909
Rheumatism	1142	SMN complex	0.07454
Rheumatism	1143	SMN complex	0.05893
Rheumatism	1306	PIN1-AUF1 complex	0.16667
Rheumatism	1307	Multiprotein complex (mRNA turnover)	0.10541
Rheumatism	1308	PABPC1-HSPA8-HNRPD-EIF4G1 complex	0.10541
Rheumatism	1618	G protein complex (PTHR1 GNB1 GNG2)	0.13608
Rheumatism	1746	SMN containing complex	0.08333
Rheumatism	1751	SMN complex	0.07454
Rheumatism	1752	SMN complex	0.08909
Rheumatism	2638	HES1 promoter corepressor complex	0.09623
Rheumatism	2641	p300/CBP-PCAF-MyoD complex	0.11785
Rheumatism	2727	SRC-3 complex	0.08909
Rheumatism	2728	SRC-1 complex	0.11785
Rheumatism	2829	RSmad complex	0.07454
Rheumatism	2958	SMAD1-CBP complex	0.16667
Rheumatism	3061	RNA polymerase II complex (CBP PCAF RPB1 BAF47 CYCC CDK8) chromatin structure modifying	0.09623

Rheumatism	3062	RNA polymerase II complex incomplete (CBP RPBI PCAF BAF47) chromatin structure modifying	0.11785
Rheumatism	3066	RNA polymerase II complex chromatin structure modifying	0.06537
Rheumatism	3098	TIM50a-SMN1 complex	0.16667
Rheumatism	3118	SMN1-SIP1-SNRP complex	0.08909
Rheumatism	3137	MASH1 promoter-coactivator complex	0.07107
Rheumatism	3142	CAMK2-delta-MASH1 promoter-coactivator complex	0.08333
Rheumatism	3162	TF-FVIIa-FXa-TFPI complex	0.11785
Rheumatism	3284	SMN complex (GEMIN5 2 3 4 SMN)	0.10541
Rheumatism	3298	SMN complex (GEMIN2 5 SMN)	0.13608
Rheumatism	3749	CREBBP-SMAD2 hexameric complex	0.16667
Rheumatism	3750	CREBBP-SMAD3 hexameric complex	0.16667
Rheumatism	3753	CREBBP-SMAD2-SMAD4 pentameric complex	0.13608
Rheumatism	3754	CREBBP-SMAD3-SMAD4 pentameric complex	0.13608
Rheumatism	5198	CBP-RARA-RXRA-DNA complex ligand stimulated	0.13608
Rheumatism	5264	TCF4-CTNNB1-CREBBP complex	0.13608
Rheumatism	5573	Stat1-alpha-dimer-CBP DNA-protein complex	0.16667
Rheumatoid arthritis	74	TRPC1-Homer3-IP3R complex	0.03533
Rheumatoid arthritis	92	CD28-transactivation complex	0.04327
Rheumatoid arthritis	98	p300-MDM2-p53 protein complex	0.03533
Rheumatoid arthritis	107	TFIIH transcription factor complex	0.0408
Rheumatoid arthritis	120	Lymphotoxin beta receptor complex	0.03533
Rheumatoid arthritis	142	CD147-gamma-secretase complex (APH-1a PS-1 PEN-2 NCT variant)	0.02737
Rheumatoid arthritis	159	Condensin I-PARP-1-XRCC1 complex	0.04626
Rheumatoid arthritis	212	DNA ligase III-XRCC1 complex	0.04327
Rheumatoid arthritis	213	DNA ligase IV-XRCC1 complex	0.04327
Rheumatoid arthritis	286	PCNA-MSH2-MSH6 complex	0.03533
Rheumatoid arthritis	299	IRF3-CBP complex	0.0306
Rheumatoid arthritis	310	Cell cycle kinase complex CDC2	0.02498
Rheumatoid arthritis	311	Cell cycle kinase complex CDK2	0.0306
Rheumatoid arthritis	312	Cell cycle kinase complex CDK4	0.0306
Rheumatoid arthritis	313	Cell cycle kinase complex CDK5	0.05474
Rheumatoid arthritis	314	PCNA-p21 complex	0.04327
Rheumatoid arthritis	320	55S ribosome mitochondrial	0.00693
Rheumatoid arthritis	324	39S ribosomal subunit mitochondrial	0.00883
Rheumatoid arthritis	5856	AK2-FADD-caspase-10 (AFAC10) complex	0.03533
Rheumatoid arthritis	362	DNA ligase III-XRCC1-PNK-DNA-pol III multiprotein complex	0.0306
Rheumatoid arthritis	369	MSH2-MSH6-PMS2-MLH1 complex	0.0306
Rheumatoid arthritis	370	MSH2-MSH6-PMS1-MLH1 complex	0.0306
Rheumatoid arthritis	374	MSH2-MSH6 complex	0.04327
Rheumatoid arthritis	376	PCNA-MutS-alpha-MutL-alpha-DNA complex	0.02737
Rheumatoid arthritis	377	PCNA-MutS-alpha-DNA initial complex	0.03533
Rheumatoid arthritis	433	BASC complex (BRCA1-associated genome surveillance complex)	0.03533

Rheumatoid arthritis	434	BASC (Ab 80) complex (BRCA1-associated genome surveillance complex)	0.02164
Rheumatoid arthritis	435	BASC (Ab 81) complex (BRCA1-associated genome surveillance complex)	0.02498
Rheumatoid arthritis	436	BASC (Ab C-20) complex (BRCA1-associated genome surveillance complex)	0.03533
Rheumatoid arthritis	438	GCN5-TRRAP histone acetyltransferase complex	0.01935
Rheumatoid arthritis	536	TRPC1-TRPC3-TRPC7 complex	0.03533
Rheumatoid arthritis	553	RHOA-IP3R-TRPC1 complex	0.07067
Rheumatoid arthritis	557	TRP1-G alpha-11-IP3R3-CAV1 signaling complex	0.0306
Rheumatoid arthritis	668	BKCA-beta2AR-AKAP79 signaling complex	0.03533
Rheumatoid arthritis	672	BKCA-beta2AR complex	0.04327
Rheumatoid arthritis	687	CFTR-NHERF-beta(2)AR signaling complex	0.03533
Rheumatoid arthritis	722	MRG15-PAM14-RB complex	0.03533
Rheumatoid arthritis	723	MAF1 complex	0.03533
Rheumatoid arthritis	725	P2X7 receptor signalling complex	0.01767
Rheumatoid arthritis	815	MRIP-MBS-RHOA complex	0.03533
Rheumatoid arthritis	816	MRIP-RHOA complex	0.04327
Rheumatoid arthritis	828	TRPC1-STIM1-ORAI1 complex	0.03533
Rheumatoid arthritis	903	RET-Rai complex	0.04327
Rheumatoid arthritis	999	p23 protein complex	0.03533
Rheumatoid arthritis	1004	RC complex during S-phase of cell cycle	0.01697
Rheumatoid arthritis	1005	RC complex during G2/M-phase of cell cycle	0.01697
Rheumatoid arthritis	1007	CAK core complex (Cdk-activating kinase core	0.04327
Rheumatoid arthritis	1008	CAK complex (Cdk-activating kinase complex)	0.07067
Rheumatoid arthritis	1009	TFIIH transcription factor complex	0.03871
Rheumatoid arthritis	1029	TFIIH transcription factor complex	0.03871
Rheumatoid arthritis	1030	CAK-ERCC2 complex	0.0612
Rheumatoid arthritis	1055	ZNF198-PML complex	0.04327
Rheumatoid arthritis	1056	ZNF198-SUMO1 complex	0.04327
Rheumatoid arthritis	1062	BAR-BCL2-CASP8 complex	0.03533
Rheumatoid arthritis	1085	DNA repair complex NEIL2-PNK-Pol(beta)-LigIII(alpha)-XRCC1	0.02737
Rheumatoid arthritis	1086	DNA repair complex NEIL1-PNK-Pol(beta)-LigIII(alpha)-XRCC1	0.02737
Rheumatoid arthritis	1087	BIRC5-AURKB-INCENP-EVI5 complex	0.0306
Rheumatoid arthritis	1088	PRNP-ApolopoproteinE3 complex	0.04327
Rheumatoid arthritis	1091	SNX complex (SNX1a SNX2 SNX4 LEPR)	0.0306
Rheumatoid arthritis	1094	Frataxin complex	0.02313
Rheumatoid arthritis	1116	CRM1-Survivin-AuroraB mitotic complex	0.03533
Rheumatoid arthritis	1117	CRM1-Survivin mitotic complex	0.04327
Rheumatoid arthritis	1118	Chromosomal passenger complex CPC (INCENP CDCA8 BIRC5 AURKB)	0.0306
Rheumatoid arthritis	1120	Chromosomal passenger complex CPC (INCENP CDCA8 BIRC5)	0.03533
Rheumatoid arthritis	1170	cMYC-ATPase-helicase complex	0.02737
Rheumatoid arthritis	1171	c-MYC-ATPase-helicase complex	0.02737
Rheumatoid arthritis	1181	C complex spliceosome	0.00684
Rheumatoid arthritis	1185	EGFR-containing signaling complex	0.0306

Rheumatoid arthritis	1193	Rap1 complex	0.02313
Rheumatoid arthritis	1226	H2AX complex I	0.02313
Rheumatoid arthritis	1250	pRB-E2F-1 complex	0.04327
Rheumatoid arthritis	1297	MKK4-ARRB2-ASK1 complex	0.03533
Rheumatoid arthritis	1298	MKK4-ARRB2-JNK3 complex	0.03533
Rheumatoid arthritis	1300	CRLR-RAMP1 complex	0.04327
Rheumatoid arthritis	1308	PABPC1-HSPA8-HNRPD-EIF4G1 complex	0.02737
Rheumatoid arthritis	1372	Rb-tal-1-E2A-Lmo2-Ldb1 complex	0.02737
Rheumatoid arthritis	1439	PTGS2 homodimer complex	0.0612
Rheumatoid arthritis	1488	DNMT1-RB1-HDAC1-E2F1 complex	0.0306
Rheumatoid arthritis	1514	IL4-IL4R complex	0.04327
Rheumatoid arthritis	1515	IL4-IL4R-IL2RG complex	0.03533
Rheumatoid arthritis	1634	CyclinD1-CDK4-p21 complex	0.03533
Rheumatoid arthritis	1707	IL2-IL2RA-IL2RB complex	0.03533
Rheumatoid arthritis	1728	CTCF-nucleophosmin-PARP-HIS-KPNA-LMNA-TOP complex	0.0204
Rheumatoid arthritis	1729	TLE1 corepressor complex (MASH1 promoter-corepressor complex)	0.01935
Rheumatoid arthritis	1810	ITGA4-PXN-GIT1 complex	0.03533
Rheumatoid arthritis	1816	JUN-TCF4-CTNNB1 complex	0.03533
Rheumatoid arthritis	1826	SMAD3-HEF1-APC10-CDH1 complex	0.0306
Rheumatoid arthritis	1827	PML-SMAD2/3-SARA complex	0.0306
Rheumatoid arthritis	1970	BMP4-TWSG1 complex	0.04327
Rheumatoid arthritis	1972	BMP4-BGN complex	0.04327
Rheumatoid arthritis	1977	MTNR1B homodimer complex	0.0612
Rheumatoid arthritis	1978	MTNR1A-MTNR1B complex	0.04327
Rheumatoid arthritis	1992	LEPR homodimer complex	0.0612
Rheumatoid arthritis	2018	IL12A-IL12B complex	0.04327
Rheumatoid arthritis	2019	IL12A-IL12B-IL12RB1 complex	0.03533
Rheumatoid arthritis	2020	IL12B-IL12RB1-IL12RB2 complex	0.03533
Rheumatoid arthritis	2021	IL12A-IL12B-IL12RB2 complex	0.03533
Rheumatoid arthritis	2028	JAK2-IL12RB2 complex	0.04327
Rheumatoid arthritis	2153	ITGAM-ITGB2-CD11 complex	0.03533
Rheumatoid arthritis	2159	AR-AKT-APPL complex	0.03533
Rheumatoid arthritis	2160	AOF2-AR complex	0.04327
Rheumatoid arthritis	2217	MDC1-MRN-ATM-FANCD2 complex	0.02498
Rheumatoid arthritis	2224	MSH2/6-BLM-p53-RAD51 complex	0.02737
Rheumatoid arthritis	2226	MutS-alpha-PK-zeta complex	0.03533
Rheumatoid arthritis	2230	PCNA complex	0.02313
Rheumatoid arthritis	2342	ITGAV-ITGB8-MMP14-TGFB1 complex	0.0306
Rheumatoid arthritis	2343	ITGAV-ITGB5-PLAUR complex	0.03533
Rheumatoid arthritis	2345	ITGAV-ITGB5-ICAM4 complex	0.03533
Rheumatoid arthritis	2346	ITGAV-ITGB5-ADAM9 complex	0.03533
Rheumatoid arthritis	2347	ITGAV-ITGB5-SPP1 complex	0.07067
Rheumatoid arthritis	2348	ITGAV-ITGB5-CYR61 complex	0.07067
Rheumatoid arthritis	2350	ITGAV-ITGB5 complex	0.04327
Rheumatoid arthritis	2352	ITGAV-ITGB6-SPP1 complex	0.07067
Rheumatoid arthritis	2353	ITGAV-ITGB6-TGFB3 complex	0.03533

Rheumatoid arthritis	2354	ITGAV-ITGB6 complex	0.04327
Rheumatoid arthritis	2355	ITGAV-ITGB3-CD47-FCER2 complex	0.0306
Rheumatoid arthritis	2356	ITGB3-ITGAV-CD47 complex	0.03533
Rheumatoid arthritis	2358	ITGAV-ITGB3-SPP1 complex	0.07067
Rheumatoid arthritis	2359	ITGAV-ITGB3-ADAM15 complex	0.03533
Rheumatoid arthritis	2362	ITAGV-ITGB3-F11R complex	0.03533
Rheumatoid arthritis	2363	ITGAV-ITGB3-PXN-PTK2b complex	0.0306
Rheumatoid arthritis	2364	ITGAV-ITGB3-ADAM23 complex	0.03533
Rheumatoid arthritis	2365	ITGAV-ITGB3-COL4A3 complex	0.03533
Rheumatoid arthritis	2366	ITGAV-ITGB3-PPAP2b complex	0.03533
Rheumatoid arthritis	2369	ITGAV-ITGB3-EGFR complex	0.03533
Rheumatoid arthritis	2370	ITGA2b-ITGB3-CD9 complex	0.03533
Rheumatoid arthritis	2374	ITGAV-ITGB3-LAMA4 complex	0.03533
Rheumatoid arthritis	2383	ITGA5-ITGB1-FN1-TGM2 complex	0.0306
Rheumatoid arthritis	2384	ITGA5-ITGB1-ADAM15 complex	0.03533
Rheumatoid arthritis	2385	ITGA5-ITGB4 complex	0.04327
Rheumatoid arthritis	2388	Itga5-Itgb1-Fn1-Sfrp2 complex	0.0306
Rheumatoid arthritis	2390	CD98-LAT2-ITGB1 complex	0.0306
Rheumatoid arthritis	2395	ITGA7-ITGB1-CD151 complex	0.03533
Rheumatoid arthritis	2396	ITGA7-ITGB1-CD9 complex	0.07067
Rheumatoid arthritis	2397	ITGA7-ITGB1-ITGB1BP3 complex	0.03533
Rheumatoid arthritis	2398	ITGA3-ITGB1-BSG complex	0.07067
Rheumatoid arthritis	2399	ITGA3-ITGB1-CD63 complex	0.03533
Rheumatoid arthritis	2400	ITGA3-ITGB1-CD151 complex	0.03533
Rheumatoid arthritis	2401	ITGA3-ITGB1-THBS1 complex	0.03533
Rheumatoid arthritis	2406	ITGA3-ITGB1 complex	0.04327
Rheumatoid arthritis	2411	ITGA6-ITGB1-CD151 complex	0.03533
Rheumatoid arthritis	2413	ITGA6-ITGB1 complex	0.04327
Rheumatoid arthritis	2416	ITGB1-RAP1A-PKD1 complex	0.03533
Rheumatoid arthritis	2417	ITGA4-ITGB1-EMILIN1 complex	0.07067
Rheumatoid arthritis	2418	ITGA4-ITGB1 complex	0.08655
Rheumatoid arthritis	2419	ITGA4-ITGB1-CD81 complex	0.07067
Rheumatoid arthritis	2420	ITGA4-ITGB1-CD53 complex	0.07067
Rheumatoid arthritis	2421	ITGA4-ITGB1-VCAM1 complex	0.07067
Rheumatoid arthritis	2422	ITGA4-ITGB1-JAM2 complex	0.07067
Rheumatoid arthritis	2423	ITGA4-ITGB1-CD47 complex	0.07067
Rheumatoid arthritis	2424	ITGA4-ITGB1-CD63 complex	0.07067
Rheumatoid arthritis	2425	ITGA4-ITGB1-PXN complex	0.07067
Rheumatoid arthritis	2426	ITGA4-ITGB1-THBS1 complex	0.07067
Rheumatoid arthritis	2428	ITGA4-ITGB1-THBS2 complex	0.07067
Rheumatoid arthritis	2429	ITGA2-ITGB1-CD47 complex	0.03533
Rheumatoid arthritis	2430	ITGA2-ITGB1-CHAD complex	0.03533
Rheumatoid arthritis	2431	ITGA2-ITGB1-COL6A3 complex	0.03533
Rheumatoid arthritis	2432	ITGA2-ITGB1 complex	0.04327
Rheumatoid arthritis	2434	ITGA1-ITGB1-COL6A3 complex	0.07067
Rheumatoid arthritis	2435	ITGA1-ITGB1-PTPN2 complex	0.07067
Rheumatoid arthritis	2436	ITGAV-ITGB1 complex	0.08655

Rheumatoid arthritis	2437	ITGA6-ITGB1-CYR61 complex	0.07067
Rheumatoid arthritis	2439	ITGA8-ITGB1 complex	0.04327
Rheumatoid arthritis	2440	ITGA9-ITGB1-ADAM9 complex	0.03533
Rheumatoid arthritis	2441	Itga9-Itgb1-Adam2 complex	0.03533
Rheumatoid arthritis	2442	ITGA9-ITGB1-VCAM1 complex	0.03533
Rheumatoid arthritis	2443	ITGA9-ITGB1-TNC complex	0.03533
Rheumatoid arthritis	2444	ITGB1-ITGA9 complex	0.04327
Rheumatoid arthritis	2445	ITGA9-ITGB1-ADAM15 complex	0.03533
Rheumatoid arthritis	2446	ITGA9-ITGB1-FIGF complex	0.03533
Rheumatoid arthritis	2447	ITGA9-ITGB1-ADAM12 complex	0.03533
Rheumatoid arthritis	2489	NCR3-CD247 complex	0.04327
Rheumatoid arthritis	2528	ERBB2-MEMO-SHC complex	0.03533
Rheumatoid arthritis	2529	LAT-PLC-gamma-1-p85-GRB2-CBL-VAV-SLP-76 signaling complex C305 activated	0.02313
Rheumatoid arthritis	2534	Cbl-SLP-76-Grb2 complex Fc receptor gamma-R1 stimulated	0.03533
Rheumatoid arthritis	2535	SLP-76-Cbl-Grb2-Shc complex Fc receptor gamma-R1 stimulated	0.0306
Rheumatoid arthritis	2536	PLC-gamma-2-SLP-76-Lyn-Grb2 complex	0.0306
Rheumatoid arthritis	2547	PLC-gamma-1-SLP-76-SOS1-LAT complex	0.0306
Rheumatoid arthritis	2579	Chromosomal passenger complex CPC (INCENP BIRC5 AURKB)	0.03533
Rheumatoid arthritis	2580	Survivin homodimer complex	0.0612
Rheumatoid arthritis	2581	RasGAP-AURKA/AURKB-survivin complex	0.0306
Rheumatoid arthritis	2582	Chromosomal passenger complex CPC (CDCA8 AURKB BIRC5)	0.03533
Rheumatoid arthritis	2625	CDK8-MED6-PARP1 complex	0.03533
Rheumatoid arthritis	2649	MYC-DNMT3A-ZBTB17 complex	0.03533
Rheumatoid arthritis	2653	MYC-MAX-BLOC1S1 complex	0.03533
Rheumatoid arthritis	2655	MYC-MAX complex	0.04327
Rheumatoid arthritis	2657	ESR1-CDK7-CCNH-MNAT1-MTA1-HDAC2 complex	0.04997
Rheumatoid arthritis	2660	ERCC2/CAK complex	0.0612
Rheumatoid arthritis	2670	Er-alpha-p53-hdm2 complex	0.03533
Rheumatoid arthritis	2692	SMAD3-SMAD4-cJun-cFos complex	0.0306
Rheumatoid arthritis	2693	NFAT-JUN-FOS DNA-protein complex	0.03533
Rheumatoid arthritis	2694	ERG-JUN-FOS DNA-protein complex	0.03533
Rheumatoid arthritis	2695	ETS2-FOS-JUN complex	0.03533
Rheumatoid arthritis	2699	ER-alpha-GRIP1-c-Jun complex	0.03533
Rheumatoid arthritis	2700	ER-alpha-c-Jun complex	0.04327
Rheumatoid arthritis	2708	SMAD3-SMAD4-cJUN complex	0.03533
Rheumatoid arthritis	2723	ATM-NBS1 complex	0.04327
Rheumatoid arthritis	2740	MutS-alpha complex	0.04327
Rheumatoid arthritis	2755	17S U2 snRNP	0.01065
Rheumatoid arthritis	2770	ITGA6-ITGB4-CD9 complex	0.03533
Rheumatoid arthritis	2798	MMP-2-claudin-1 complex	0.04327
Rheumatoid arthritis	2816	ITGAV-ITGB3 complex	0.04327
Rheumatoid arthritis	2817	BRCA1-BARD1-BACH1-DNA damage complex I	0.02498
Rheumatoid arthritis	2826	ITGB3-ITGAV-VTN complex	0.03533

Rheumatoid arthritis	2846	ITGAV-ITGB3-THBS1 complex	0.03533
Rheumatoid arthritis	2849	ITGAV-ITGB3-NOV complex	0.03533
Rheumatoid arthritis	2850	ITGA5-ITGB1-FN-1-NOV complex	0.0306
Rheumatoid arthritis	2853	ITGA5-ITGB1-CAL4A3 complex	0.03533
Rheumatoid arthritis	2872	ITGA2b-ITGB3-CD9-GP1b-CD47 complex	0.02498
Rheumatoid arthritis	2885	ITGAV-ITGB1-SPP1 complex	0.106
Rheumatoid arthritis	2912	PLC-gamma-2-SLP-76 complex	0.04327
Rheumatoid arthritis	2955	LCK-SLP76-PLC-gamma-1-LAT complex pervanadate-activated	0.0306
Rheumatoid arthritis	2960	SLP-76-PLC-gamma-1-ITK complex alpha-TCR stimulated	0.03533
Rheumatoid arthritis	2961	SLP-76-PLC-gamma-1-VAV complex alpha-TCR stimulated	0.03533
Rheumatoid arthritis	2963	ITK-SLP-76 complex anti-TCR stimulated	0.04327
Rheumatoid arthritis	2964	ITGA9-ITGB1-ADAM1 complex	0.03533
Rheumatoid arthritis	2965	ITGA9-ITGB1-ADAM3 complex	0.03533
Rheumatoid arthritis	2971	ITGA9-ITGB1-VEGFC complex	0.07067
Rheumatoid arthritis	2972	ITGA9-ITGB1-VEGFA complex	0.03533
Rheumatoid arthritis	2989	ITGA9-ITGB1-ADAM8 complex	0.03533
Rheumatoid arthritis	3011	APC-IQGAP1-Rac1 complex	0.03533
Rheumatoid arthritis	3035	LAT2-ITGB1 complex	0.04327
Rheumatoid arthritis	3043	BMP2-BRIA complex	0.03533
Rheumatoid arthritis	3057	ITGA10-ITGB1 complex	0.04327
Rheumatoid arthritis	3058	ITGA11-ITGB1 complex	0.04327
Rheumatoid arthritis	3059	ITGA11-ITGB1-COL1A1 complex	0.03533
Rheumatoid arthritis	3067	RNA polymerase II complex incomplete (CDK8 complex) chromatin structure modifying	0.02164
Rheumatoid arthritis	3086	CCND3-CDK4 complex	0.04327
Rheumatoid arthritis	3089	CCND3-CDK6 complex	0.04327
Rheumatoid arthritis	3103	ITGAV-ITGB3-SLC3A2 complex	0.03533
Rheumatoid arthritis	3104	ITGB1-NRP1 complex	0.04327
Rheumatoid arthritis	3110	ITGAV-P2RY2-GNA12 complex	0.03533
Rheumatoid arthritis	3111	ITGA9-ITGB1-SPP1 complex	0.07067
Rheumatoid arthritis	3112	ITGA5-ITGB1-SPP1 complex	0.07067
Rheumatoid arthritis	3117	ITGB5-ITGAV-VTN complex	0.03533
Rheumatoid arthritis	3137	MASH1 promoter-coactivator complex	0.01845
Rheumatoid arthritis	3139	CRLR-RAMP1-ARRB2 complex	0.03533
Rheumatoid arthritis	3140	CRLR-RAMP2 complex	0.04327
Rheumatoid arthritis	3141	CRLR-RAMP3 complex	0.04327
Rheumatoid arthritis	3142	CAMK2-delta-MASH1 promoter-coactivator complex	0.02164
Rheumatoid arthritis	3153	GNAQ-GEFT-RHOA complex	0.03533
Rheumatoid arthritis	3154	Notch2(N-TM)-Notch2(N-EC)-Delta complex	0.0306
Rheumatoid arthritis	3172	NUMB-TP53-MDM2 complex	0.03533
Rheumatoid arthritis	3269	RB1-HDAC1-BRG1 complex	0.03533
Rheumatoid arthritis	3270	Delta1 homodimer complex	0.0612
Rheumatoid arthritis	3271	Gamma-secretase-Delta1 complex	0.02737
Rheumatoid arthritis	3830	ADRB2 homodimer complex	0.0612
Rheumatoid arthritis	3852	Rb-HDAC1 complex	0.04327

Rheumatoid arthritis	4043	NEMO-HIF2(alpha)-ARNT complex	0.03533
Rheumatoid arthritis	4997	p97/VCP-VIMP-DERL1 complex	0.03533
Rheumatoid arthritis	4998	p97/VCP-VIMP-DERL2 complex	0.03533
Rheumatoid arthritis	4999	p97/VCP-VIMP-DERL1-DERL2-HRD1-SEL1L	0.02498
Rheumatoid arthritis	5099	RB1(hypophosphorylated)-E2F4 complex	0.04327
Rheumatoid arthritis	5100	CyclinD3-CDK4-CDK6 complex	0.03533
Rheumatoid arthritis	5101	CyclinD3-CDK4-CDK6-p21 complex	0.0612
Rheumatoid arthritis	5143	E2F1-Rb complex	0.04327
Rheumatoid arthritis	5146	RB1-TFAP2A complex	0.04327
Rheumatoid arthritis	5178	JAK2-PAFR-TYK2 complex	0.03533
Rheumatoid arthritis	5179	NCOA6-DNA-PK-Ku-PARP1 complex	0.02737
Rheumatoid arthritis	5190	TIAM1-EFNB1-EPHA2 complex	0.03533
Rheumatoid arthritis	5196	TNF-alpha/NF-kappa B signaling complex (CHUK BTRC NFKB2 PPP6C REL CUL1 IKBKE SAPS2 SAPS1 ANKRD28 RELA SKP1)	0.01767
Rheumatoid arthritis	5198	CBP-RARA-RXRA-DNA complex ligand stimulated	0.03533
Rheumatoid arthritis	5235	WRN-Ku70-Ku80-PARP1 complex	0.0306
Rheumatoid arthritis	5243	XRCC1-LIG3-PNK-TDP1 complex	0.0306
Rheumatoid arthritis	5253	MNK1-eIF4F complex	0.02737
Rheumatoid arthritis	5260	TCF4-CTNNB1-SUMO1-EP300-HADAC6 complex	0.02737
Rheumatoid arthritis	5269	TNF-alpha/NF-kappa B signaling complex 8	0.02498
Rheumatoid arthritis	5331	YY1-MDM2-p53 complex	0.03533
Rheumatoid arthritis	5342	ELMO1-DOCK1-RAC1 complex	0.03533
Rheumatoid arthritis	5369	ATM homodimer complex	0.0612
Rheumatoid arthritis	5375	EGR-EP300 complex	0.04327
Rheumatoid arthritis	5389	SERPINA3-CTSG complex	0.04327
Rheumatoid arthritis	5391	SERPINA1-CTSG complex	0.04327
Rheumatoid arthritis	5495	TFIIH transcription factor complex (ERCC2 ERCC3 GTF2H1 CDK7 CCNH GTF2H2)	0.02498
Rheumatoid arthritis	5526	CALM1-FKBP38-BCL2 complex	0.03533
Rheumatoid arthritis	5548	IL-12 heterodimer complex	0.04327
Rheumatoid arthritis	5549	IL-12 subunit p40 homodimer complex	0.0612
Rheumatoid arthritis	5611	Emerin complex 24	0.0158
Rheumatoid arthritis	5622	HSP90-CIP1-FKBPL complex	0.03533
Rheumatoid arthritis	5656	CEBPE-E2F1-RB1 complex	0.03533
Rheumatoid arthritis	5659	SEMA3C-PlexinD1-Nrp1 complex	0.03533
Rheumatoid arthritis	5663	TRIM27-RB1 complex	0.04327
Rheumatoid arthritis	5684	Membrane protein complex (DERL1 SELS VCP)	0.03533
Rheumatoid arthritis	5731	NRP1-VEGFC complex heparin dependent	0.04327
Rheumatoid arthritis	5732	NRP2-VEGFC complex	0.04327
Rheumatoid arthritis	5749	MRIT complex	0.07067
Rheumatoid arthritis	5755	SUMO1-SUA1-UBA2 complex	0.03533
Rheumatoid arthritis	5770	RUNX1-CBF-beta-DNA complex	0.04327
Rheumatoid arthritis	5798	Death induced signaling complex II (FADD CASP8 CFLAR) cytosolic CD95L induced	0.03533
Rheumatoid arthritis	5799	Death induced signaling complex DISC (FAS FADD CASP8 CFLAR) membrane-associated CD95L	0.0306
Rheumatoid arthritis	5811	p53-BCL2 complex	0.04327

Rheumatoid arthritis	5812	p53-BCL2 complex	0.04327
Rheumatoid arthritis	5817	tBID-BCL2 complex	0.04327
Rheumatoid arthritis	5818	BIM-BCL2 complex	0.04327
Rheumatoid arthritis	5819	BIM-BCL2xL complex	0.04327
Rheumatoid arthritis	5820	tBID-BCL2xL complex	0.04327
Rheumatoid arthritis	5822	MCL1-NOXA complex	0.04327
Rheumatoid arthritis	5823	MCL1-BAK1 complex	0.04327
Rheumatoid arthritis	5859	FAS-FADD-CASP8-CASP10 complex	0.0306
Rheumatoid arthritis	5861	FAS-FADD-CASP10 complex	0.03533
Salmonella infection	27	Arp2/3 protein complex	0.26726
Salmonella infection	149	PBAF complex (Polybromo- and BAF containing complex)	0.10206
Salmonella infection	189	BAF complex	0.09806
Salmonella infection	1041	Alpha-dystrobrevin-ZO-1-actin complex	0.17678
Salmonella infection	1844	APC-IQGAP1 complex	0.25
Salmonella infection	1845	APC-IQGAP1-CLIP-170 complex	0.20412
Salmonella infection	2018	IL12A-IL12B complex	0.25
Salmonella infection	2019	IL12A-IL12B-IL12RB1 complex	0.20412
Salmonella infection	2020	IL12B-IL12RB1-IL12RB2 complex	0.20412
Salmonella infection	2021	IL12A-IL12B-IL12RB2 complex	0.20412
Salmonella infection	2254	CTGF/Hcs24-actin complex	0.20412
Salmonella infection	2837	Profilin 1 complex	0.14434
Salmonella infection	3008	60S APC containing complex	0.13363
Salmonella infection	3011	APC-IQGAP1-Rac1 complex	0.40825
Salmonella infection	3012	APC-IQGAP1-Cdc42 complex	0.20412
Salmonella infection	5342	ELMO1-DOCK1-RAC1 complex	0.20412
Salmonella infection	5548	IL-12 heterodimer complex	0.25
Salmonella infection	5549	IL-12 subunit p40 homodimer complex	0.35355
Salmonella infection	5615	Emerin complex 52	0.07372
Sarcoidosis	120	Lymphotoxin beta receptor complex	0.11323
Sarcoidosis	310	Cell cycle kinase complex CDC2	0.08006
Sarcoidosis	311	Cell cycle kinase complex CDK2	0.09806
Sarcoidosis	312	Cell cycle kinase complex CDK4	0.09806
Sarcoidosis	313	Cell cycle kinase complex CDK5	0.08771
Sarcoidosis	314	PCNA-p21 complex	0.13868
Sarcoidosis	1634	CyclinD1-CDK4-p21 complex	0.11323
Sarcoidosis	2001	NOD1 homodimer complex	0.19612
Sarcoidosis	2230	PCNA complex	0.07412
Sarcoidosis	2443	ITGA9-ITGB1-TNC complex	0.11323
Sarcoidosis	5101	CyclinD3-CDK4-CDK6-p21 complex	0.09806
Sarcoidosis	5178	JAK2-PAFR-TYK2 complex	0.11323
Sarcoidosis	5622	HSP90-CIP1-FKBPL complex	0.11323
Sarcoma	1217	WRN-TRF2 complex	0.1715
Sarcoma	1982	CACY homodimer complex	0.24254
Sarcoma	4082	Ku70/Ku86/Werner complex	0.14003
Sarcoma	5235	WRN-Ku70-Ku80-PARP1 complex	0.12127
Sarcoma	5383	TRIB3-DDIT3 complex	0.1715

Schistosomiasis	98	p300-MDM2-p53 protein complex	0.28868
Schistosomiasis	1062	BAR-BCL2-CASP8 complex	0.28868
Schistosomiasis	2670	Er-alpha-p53-hdm2 complex	0.28868
Schistosomiasis	3172	NUMB-TP53-MDM2 complex	0.28868
Schistosomiasis	5331	YY1-MDM2-p53 complex	0.28868
Schistosomiasis	5526	CALM1-FKBP38-BCL2 complex	0.28868
Schistosomiasis	5811	p53-BCL2 complex	0.35355
Schistosomiasis	5817	tBID-BCL2 complex	0.35355
Schistosomiasis	5818	BIM-BCL2 complex	0.35355
Schizophrenia	66	TRAP complex	0.02688
Schizophrenia	120	Lymphotoxin beta receptor complex	0.0439
Schizophrenia	178	Respiratory chain complex I (holoenzyme)	0.03439
Schizophrenia	189	BAF complex	0.02109
Schizophrenia	230	Mediator complex	0.01344
Schizophrenia	232	ARC complex	0.01963
Schizophrenia	238	SWI-SNF chromatin remodeling-related-BRCA1	0.02292
Schizophrenia	287	ARC-L complex	0.02032
Schizophrenia	288	ARC complex	0.01963
Schizophrenia	301	SMCC complex	0.01844
Schizophrenia	441	TFTC-type histone acetyl transferase complex	0.04585
Schizophrenia	535	TRAP complex	0.01901
Schizophrenia	541	IGF1-IGFBP3-ALS complex	0.0439
Schizophrenia	547	SMCC complex	0.02032
Schizophrenia	548	DRIP complex	0.04064
Schizophrenia	564	BAF complex	0.02292
Schizophrenia	706	SNARE complex (HGS SNAP25 STX13)	0.0439
Schizophrenia	707	SNARE complex (VAMP2 SNAP25 STX13)	0.0439
Schizophrenia	711	Brm-associated complex	0.02292
Schizophrenia	714	BRM-SIN3A complex	0.01963
Schizophrenia	793	SNARE complex (VAMP2 SNAP25 STX1a CPLX1)	0.03801
Schizophrenia	794	SNARE complex (VAMP2 SNAP25 STX1a CPLX2)	0.07603
Schizophrenia	806	BRM-SIN3A-HDAC complex	0.02195
Schizophrenia	808	BRM-associated complex	0.02404
Schizophrenia	827	NgR-TROY-LINGO1 complex	0.0439
Schizophrenia	909	ARC92-Mediator complex	0.02109
Schizophrenia	933	SCRIB-APC complex	0.05376
Schizophrenia	1054	ESR1-RELA-BCL3-NCOA3 complex	0.03801
Schizophrenia	1091	SNX complex (SNX1a SNX2 SNX4 LEPR)	0.03801
Schizophrenia	1095	SNX complex (SNX1a SNX2 SNX4 EGFR)	0.03801
Schizophrenia	1137	SNARE complex (VAMP2 SNAP25 STX1a CPLX1 CPLX3)	0.034
Schizophrenia	1138	SNARE complex (VAMP2 SNAP25 STX1a CPLX3 CPLX4)	0.034
Schizophrenia	1139	SNARE complex (VAMP2 SNAP25 STX1a STX3 CPLX1 CPLX3 CPLX4)	0.02874
Schizophrenia	1185	EGFR-containing signaling complex	0.03801
Schizophrenia	1230	WINAC complex	0.02032
Schizophrenia	1256	MLL-HCF complex	0.02874

Schizophrenia	1257	ALL-1 supercomplex	0.02874
Schizophrenia	1401	MOF complex	0.02404
Schizophrenia	1514	IL4-IL4R complex	0.05376
Schizophrenia	1515	IL4-IL4R-IL2RG complex	0.0439
Schizophrenia	1707	IL2-IL2RA-IL2RB complex	0.0439
Schizophrenia	1728	CTCF-nucleophosmin-PARP-HIS-KPNA-LMNA-TOP complex	0.02534
Schizophrenia	1772	MICB-KLRK1-HCST complex	0.0439
Schizophrenia	1787	Nogo-potassium channel complex	0.03801
Schizophrenia	1793	LINGO-1-Nogo-66-p75 signaling complex	0.0439
Schizophrenia	5718	eNOS-HSP90-AKT complex VEGF induced	0.0439
Schizophrenia	1816	JUN-TCF4-CTNNB1 complex	0.0439
Schizophrenia	1844	APC-IQGAP1 complex	0.05376
Schizophrenia	1845	APC-IQGAP1-CLIP-170 complex	0.0439
Schizophrenia	1874	SNARE complex (SNAP25 VAMP3 VAMP2 NAPB STX13)	0.034
Schizophrenia	1909	APC-DLG4 complex	0.05376
Schizophrenia	1992	LEPR homodimer complex	0.07603
Schizophrenia	2018	IL12A-IL12B complex	0.05376
Schizophrenia	2019	IL12A-IL12B-IL12RB1 complex	0.0439
Schizophrenia	2020	IL12B-IL12RB1-IL12RB2 complex	0.0439
Schizophrenia	2021	IL12A-IL12B-IL12RB2 complex	0.0439
Schizophrenia	2124	IKK-alpha--ER-alpha-AIB1 complex	0.0439
Schizophrenia	2156	YBX1-AKT1 complex	0.05376
Schizophrenia	2159	AR-AKT-APPL complex	0.08779
Schizophrenia	2160	AOF2-AR complex	0.05376
Schizophrenia	2237	SP1-MCAF2 complex	0.05376
Schizophrenia	2272	PICK1-GRIP1-GLUR2 complex	0.0439
Schizophrenia	2369	ITGAV-ITGB3-EGFR complex	0.0439
Schizophrenia	2370	ITGA2b-ITGB3-CD9 complex	0.0439
Schizophrenia	2376	ITGA2B-ITGB3-FN1-TGM2 complex	0.03801
Schizophrenia	2377	ITGA2b-ITGB3-CD47-SRC complex	0.03801
Schizophrenia	2378	ITGA2b-ITGB3-TLN1 complex	0.0439
Schizophrenia	2379	ITGA2B-ITGB3-CIB1 complex	0.0439
Schizophrenia	2381	ITGA2B-ITGB3 complex	0.05376
Schizophrenia	2382	ITGA2B-ITGB3-F11R complex	0.0439
Schizophrenia	2453	Multiprotein complex (monoubiquitination)	0.03801
Schizophrenia	2454	CIN85-CBL-SH3GL2-EGFR complex EGF stimulated	0.03801
Schizophrenia	2470	p130Cas-ER-alpha-cSrc-kinase- PI3-kinase p85-subunit complex	0.03801
Schizophrenia	2542	EGFR-CBL-GRB2 complex	0.0439
Schizophrenia	2600	BRD4 complex	0.02688
Schizophrenia	2657	ESR1-CDK7-CCNH-MNAT1-MTA1-HDAC2 complex	0.03104
Schizophrenia	2670	Er-alpha-p53-hdm2 complex	0.0439
Schizophrenia	2679	p53-SP1 complex	0.05376
Schizophrenia	2692	SMAD3-SMAD4-cJun-cFos complex	0.03801
Schizophrenia	2693	NFAT-JUN-FOS DNA-protein complex	0.0439
Schizophrenia	2694	ERG-JUN-FOS DNA-protein complex	0.0439

Schizophrenia	2695	ETS2-FOS-JUN complex	0.0439
Schizophrenia	2699	ER-alpha-GRIP1-c-Jun complex	0.08779
Schizophrenia	2700	ER-alpha-c-Jun complex	0.10752
Schizophrenia	2706	SMAD3-SMAD4-SP1 complex	0.0439
Schizophrenia	2708	SMAD3-SMAD4-cJUN complex	0.0439
Schizophrenia	2721	HCF-1 complex	0.01744
Schizophrenia	2736	TAJ-NgR1-LINGO-1 signaling complex	0.0439
Schizophrenia	2772	Ubiquitin E3 ligase (CRY1 SKP1A CUL1 FBXL3)	0.03801
Schizophrenia	2872	ITGA2b-ITGB3-CD9-GP1b-CD47 complex	0.03104
Schizophrenia	2884	Respiratory chain complex I (early intermediate NDUFAF1 assembly) mitochondrial	0.02874
Schizophrenia	2886	Respiratory chain complex I (incomplete intermediate ND1 ND2 ND3 CIA30 assembly) mitochondrial	0.03801
Schizophrenia	2896	ITGA2b-ITGB3-CD47-FAK complex	0.03801
Schizophrenia	2901	Respiratory chain complex I (intermediate IV/310kD) mitochondrial	0.03801
Schizophrenia	2903	Respiratory chain complex I (intermediate V/380kD and VI/480kD) mitochondrial	0.034
Schizophrenia	2904	Respiratory chain complex I (intermediate VII/650kD) mitochondrial	0.04808
Schizophrenia	2906	Respiratory chain complex I (intermediate II/230kD) mitochondrial	0.0439
Schizophrenia	2914	Respiratory chain complex I (beta subunit)	0.01901
Schizophrenia	2919	Respiratory chain complex I (gamma subunit) mitochondrial	0.02109
Schizophrenia	2920	Respiratory chain complex I (lambda subunit) mitochondrial	0.01901
Schizophrenia	2939	Ecsit complex (ECSIT MT-CO2 NDUFA1 MT-ND1 TRAF6 NDUFAF1)	0.03104
Schizophrenia	2943	Respiratory chain complex I (incomplete NDUFAF1 assembly) mitochondrial	0.05376
Schizophrenia	2948	Respiratory chain complex I (incomplete intermediate) mitochondrial	0.02292
Schizophrenia	3004	APC-Axin-1-beta-catenin complex	0.0439
Schizophrenia	3008	60S APC containing complex	0.02874
Schizophrenia	3011	APC-IQGAP1-Rac1 complex	0.0439
Schizophrenia	3012	APC-IQGAP1-Cdc42 complex	0.0439
Schizophrenia	3060	RNA polymerase II complex (RPB1 RAP74 CDK8 CYCC SRB7 BAF190 BAF47) chromatin structure modifying	0.02688
Schizophrenia	3064	RNA polymerase II complex chromatin structure modifying	0.01744
Schizophrenia	3066	RNA polymerase II complex chromatin structure modifying	0.02109
Schizophrenia	3115	ITGA2B-ITGB3-ICAM4 complex	0.0439
Schizophrenia	3166	AXIN-APC-betaCatenin-GSK3B complex	0.03801
Schizophrenia	3678	RIN1-STAM2-EGFR complex EGF stimulated	0.0439
Schizophrenia	3838	SP1-E2F2 complex	0.05376
Schizophrenia	3839	SP1-E2F3 complex	0.05376
Schizophrenia	3847	TCL1(trimer)-AKT1 complex	0.05376

Schizophrenia	5158	SMARCA2/BRM-BAF57-MECP2 complex	0.0439
Schizophrenia	5171	SH3KBP1-CBLB-EGFR complex	0.0439
Schizophrenia	5184	SWI/SNF chromatin-remodeling complex	0.034
Schizophrenia	5193	TNF-alpha/NF-kappa B signaling complex (CHUK KPNA3 NFKB2 NFKBIB REL IKBKG NFKB1 NFKBIE RELB NFKBIA RELA TNIP2)	0.02195
Schizophrenia	5367	THRB-RXRB complex	0.05376
Schizophrenia	5386	MLL1-WDR5 complex	0.01463
Schizophrenia	5529	TRAF2-cIAP1/BIRC2 complex	0.05376
Schizophrenia	5531	Tumor necrosis factor receptor 1 signaling complex	0.03801
Schizophrenia	5548	IL-12 heterodimer complex	0.05376
Schizophrenia	5549	IL-12 subunit p40 homodimer complex	0.07603
Schizophrenia	5647	FARP2-NRP1-PlexinA2 complex	0.0439
Schizophrenia	5655	Ternary complex (LRRC7 CAMK2a ACTN4)	0.0439
Schizophrenia	5746	PlexinA1-NRP1-SEMA3A complex	0.0439
Schizophrenia	5747	2AR-mGluR2 complex	0.05376
Schizophrenia	5758	PLXNA2-RANBPM complex	0.05376
Schizophrenia	5862	CAV1-VDAC1-ESR1 complex	0.0439
Scleroderma	4	Multisubunit ACTR coactivator complex	0.1066
Scleroderma	98	p300-MDM2-p53 protein complex	0.12309
Scleroderma	550	NOS3-CAV1-NOSTRIN complex	0.12309
Scleroderma	557	TRP1-G alpha-11-IP3R3-CAV1 signaling complex	0.1066
Scleroderma	570	p300-CBP-p270-SWI/SNF complex	0.08058
Scleroderma	571	p300-CBP-p270 complex	0.12309
Scleroderma	753	UTM-SGCE-DAG1-CAV1-NOS3 complex	0.09535
Scleroderma	788	Exosome	0.06742
Scleroderma	789	Exosome	0.12856
Scleroderma	822	mRNA decay complex (UPF1 UPF2 UPF3B DCP2 XRN1 XRN2 EXOSC2 EXOSC4 EXOSC10 PARN)	0.06742
Scleroderma	1158	p33ING1b-p300 complex	0.15076
Scleroderma	1160	ING1-p300-PCNA complex	0.12309
Scleroderma	1471	pRb2/p130-multimolecular complex (RB2 E2F5 HDAC1 SUV39H1 P300)	0.09535
Scleroderma	1474	SMAD3/4-E2F4/5-p107-DP1 complex	0.08704
Scleroderma	1521	p300-SMAD1-STAT3 complex	0.12309
Scleroderma	1777	TGF-beta-receptor-SMAD7-SMURF2 complex	0.1066
Scleroderma	1783	TGF-beta receptor I-SMAD7-SMURF1 complex	0.24618
Scleroderma	1826	SMAD3-HEF1-APC10-CDH1 complex	0.1066
Scleroderma	1827	PML-SMAD2/3-SARA complex	0.1066
Scleroderma	1828	TGF-beta receptor I-Axin-SMAD3 complex	0.12309
Scleroderma	1831	PIAS3-SMAD3-P300 complex	0.24618
Scleroderma	1986	Endoglin homodimer complex	0.2132
Scleroderma	2004	C1D homodimer protein	0.2132
Scleroderma	2189	Ubiquitin E3 ligase (SMAD3 BTRC CUL1 SKP1A RBX1)	0.09535
Scleroderma	2254	CTGF/Hcs24-actin complex	0.12309
Scleroderma	2300	Profilin 2 complex	0.07107
Scleroderma	5714	eNOS-CAV1 complex	0.15076

Scleroderma	2462	Caveolin-1 homodimer complex	0.2132
Scleroderma	2638	HES1 promoter corepressor complex	0.08704
Scleroderma	2639	HES1 promoter-Notch enhancer complex	0.05913
Scleroderma	2641	p300/CBP-PCAF-MyoD complex	0.1066
Scleroderma	2642	SMAD1-P300 complex	0.15076
Scleroderma	2692	SMAD3-SMAD4-cJun-cFos complex	0.1066
Scleroderma	2705	SMAD3-SMAD4-CTCF protein-DNA complex	0.12309
Scleroderma	2706	SMAD3-SMAD4-SP1 complex	0.12309
Scleroderma	2707	SMAD3-SMAD4-FOXO3-FOXG1 complex	0.1066
Scleroderma	2708	SMAD3-SMAD4-cJUN complex	0.12309
Scleroderma	2754	JUND-FOSB-SMAD3-SMAD4 complex	0.1066
Scleroderma	2760	SMAD3-SMAD4-FOXO3 complex	0.12309
Scleroderma	2761	SMAD3-SMAD4-FOXO1 complex	0.12309
Scleroderma	2762	SMAD3-SMAD4-FOXO4 complex	0.12309
Scleroderma	2813	BRCA1-SMAD3 complex	0.15076
Scleroderma	2829	RSmad complex	0.06742
Scleroderma	2830	TIF1gamma-SMAD2-SMAD3 complex	0.12309
Scleroderma	2834	SMAD4-SMAD2-SMAD3 complex	0.12309
Scleroderma	2954	Smad1-Notch1-p300-Pcaf complex	0.1066
Scleroderma	2968	Axin-SMAD3 complex	0.15076
Scleroderma	2975	SMAD3-E2F4/5-p107-DP1 complex	0.09535
Scleroderma	2992	SMAD7-SMURF2 complex	0.15076
Scleroderma	2996	SMAD7-SMURF1 complex	0.30151
Scleroderma	2997	SMAD7-SMURF1-TGF-beta receptor complex	0.2132
Scleroderma	3044	SKI-NCOR1-SIN3A-HDAC1 complex	0.1066
Scleroderma	3197	SMAD4-SNO-SKI complex	0.24618
Scleroderma	3198	SMAD2-SKI complex	0.15076
Scleroderma	3199	SMAD3-SKI complex	0.30151
Scleroderma	3200	SMAD4-SKI complex	0.15076
Scleroderma	3204	SMAD2-SKI-NCOR complex	0.12309
Scleroderma	3205	SMAD3-SKI-NCOR complex	0.24618
Scleroderma	3206	SMAD4-SKI-NCOR complex	0.12309
Scleroderma	3729	SKI-SMAD2 hexameric complex	0.15076
Scleroderma	3733	SKI-SMAD3 hexameric complex	0.30151
Scleroderma	3739	SKI-SMAD2-SMAD4 pentameric complex	0.12309
Scleroderma	3740	SKI-SMAD3-SMAD4 pentameric complex	0.24618
Scleroderma	3750	CREBBP-SMAD3 hexameric complex	0.15076
Scleroderma	3754	CREBBP-SMAD3-SMAD4 pentameric complex	0.12309
Scleroderma	3959	SMAD3-SMAD4-cSKI TGF(beta)-dependent	0.24618
Scleroderma	3961	SMAD3-cSKI-SIN3A-HDAC1 complex	0.2132
Scleroderma	3971	SMURF2-SMAD3 complex TGF(beta)-dependent	0.15076
Scleroderma	3972	SMURF2-SMAD3-SnoN complex TGF(beta)-	0.24618
Scleroderma	5118	pRb2/p130-multimolecular complex (RB2 E2F4 HDAC1 SUV39H1 P300)	0.09535
Scleroderma	5260	TCF4-CTNNB1-SUMO1-EP300-HADAC6 complex	0.09535
Scleroderma	5261	TCF4-CTNNB1-EP300 complex	0.12309
Scleroderma	5375	EGR-EP300 complex	0.15076

Scleroderma	5735	TGF-beta receptor-SMAD3 complex	0.12309
Scleroderma	5862	CAV1-VDAC1-ESR1 complex	0.12309
Sella turcica tumor	706	SNARE complex (HGS SNAP25 STX13)	0.21822
Sella turcica tumor	3677	RIN1-STAM2-HRS complex	0.21822
Sella turcica tumor	5582	LIFR-LIF-gp130 complex	0.21822
Serous cancer	1042	SRA-SRC-1 ribonucleoprotein complex	0.35355
Severe acute respiratory syndrome	313	Cell cycle kinase complex CDK5	0.14142
Severe acute respiratory syndrome	722	MRG15-PAM14-RB complex	0.18257
Severe acute respiratory syndrome	723	MAF1 complex	0.18257
Severe acute respiratory syndrome	1250	pRB-E2F-1 complex	0.22361
Severe acute respiratory syndrome	1372	Rb-tal-1-E2A-Lmo2-Ldb1 complex	0.14142
Severe acute respiratory syndrome	1488	DNMT1-RB1-HDAC1-E2F1 complex	0.15811
Severe acute respiratory syndrome	3086	CCND3-CDK4 complex	0.22361
Severe acute respiratory syndrome	3089	CCND3-CDK6 complex	0.22361
Severe acute respiratory syndrome	3269	RB1-HDAC1-BRG1 complex	0.18257
Severe acute respiratory syndrome	3852	Rb-HDAC1 complex	0.22361
Severe acute respiratory syndrome	5099	RB1(hypophosphorylated)-E2F4 complex	0.22361
Severe acute respiratory syndrome	5100	CyclinD3-CDK4-CDK6 complex	0.18257
Severe acute respiratory syndrome	5101	CyclinD3-CDK4-CDK6-p21 complex	0.15811
Severe acute respiratory syndrome	5143	E2F1-Rb complex	0.22361
Severe acute respiratory syndrome	5146	RB1-TFAP2A complex	0.22361
Severe acute respiratory syndrome	5611	Emerin complex 24	0.08165
Severe acute respiratory syndrome	5656	CEBPE-E2F1-RB1 complex	0.18257
Severe acute respiratory syndrome	5663	TRIM27-RB1 complex	0.22361
Severe acute respiratory syndrome	5749	MRIT complex	0.18257
Severe acute respiratory syndrome	5812	p53-BCL2 complex	0.22361
Severe acute respiratory syndrome	5819	BIM-BCL2xL complex	0.22361
Severe acute respiratory syndrome	5820	tBID-BCL2xL complex	0.22361
Shigella infection	2383	ITGA5-ITGB1-FN1-TGM2 complex	0.20412
Shigella infection	2384	ITGA5-ITGB1-ADAM15 complex	0.2357

Shigella infection	2385	ITGA5-ITGB4 complex	0.28868
Shigella infection	2388	Itga5-Itgb1-Fn1-Sfrp2 complex	0.20412
Shigella infection	2390	CD98-LAT2-ITGB1 complex	0.20412
Shigella infection	2395	ITGA7-ITGB1-CD151 complex	0.2357
Shigella infection	2396	ITGA7-ITGB1-CD9 complex	0.2357
Shigella infection	2397	ITGA7-ITGB1-ITGB1BP3 complex	0.2357
Shigella infection	2398	ITGA3-ITGB1-BSG complex	0.2357
Shigella infection	2399	ITGA3-ITGB1-CD63 complex	0.2357
Shigella infection	2400	ITGA3-ITGB1-CD151 complex	0.2357
Shigella infection	2401	ITGA3-ITGB1-THBS1 complex	0.2357
Shigella infection	2406	ITGA3-ITGB1 complex	0.28868
Shigella infection	2411	ITGA6-ITGB1-CD151 complex	0.2357
Shigella infection	2413	ITGA6-ITGB1 complex	0.28868
Shigella infection	2416	ITGB1-RAP1A-PKD1 complex	0.2357
Shigella infection	2417	ITGA4-ITGB1-EMILIN1 complex	0.2357
Shigella infection	2418	ITGA4-ITGB1 complex	0.28868
Shigella infection	2419	ITGA4-ITGB1-CD81 complex	0.2357
Shigella infection	2420	ITGA4-ITGB1-CD53 complex	0.2357
Shigella infection	2421	ITGA4-ITGB1-VCAM1 complex	0.2357
Shigella infection	2422	ITGA4-ITGB1-JAM2 complex	0.2357
Shigella infection	2423	ITGA4-ITGB1-CD47 complex	0.2357
Shigella infection	2424	ITGA4-ITGB1-CD63 complex	0.2357
Shigella infection	2425	ITGA4-ITGB1-PXN complex	0.2357
Shigella infection	2426	ITGA4-ITGB1-THBS1 complex	0.2357
Shigella infection	2428	ITGA4-ITGB1-THBS2 complex	0.2357
Shigella infection	2429	ITGA2-ITGB1-CD47 complex	0.2357
Shigella infection	2430	ITGA2-ITGB1-CHAD complex	0.2357
Shigella infection	2431	ITGA2-ITGB1-COL6A3 complex	0.2357
Shigella infection	2432	ITGA2-ITGB1 complex	0.28868
Shigella infection	2434	ITGA1-ITGB1-COL6A3 complex	0.2357
Shigella infection	2435	ITGA1-ITGB1-PTPN2 complex	0.2357
Shigella infection	2436	ITGAV-ITGB1 complex	0.28868
Shigella infection	2437	ITGA6-ITGB1-CYR61 complex	0.2357
Shigella infection	2439	ITGA8-ITGB1 complex	0.28868
Shigella infection	2440	ITGA9-ITGB1-ADAM9 complex	0.2357
Shigella infection	2441	Itga9-Itgb1-Adam2 complex	0.2357
Shigella infection	2442	ITGA9-ITGB1-VCAM1 complex	0.2357
Shigella infection	2443	ITGA9-ITGB1-TNC complex	0.2357
Shigella infection	2444	ITGB1-ITGA9 complex	0.28868
Shigella infection	2445	ITGA9-ITGB1-ADAM15 complex	0.2357
Shigella infection	2446	ITGA9-ITGB1-FIGF complex	0.2357
Shigella infection	2447	ITGA9-ITGB1-ADAM12 complex	0.2357
Shigella infection	2850	ITGA5-ITGB1-FN-1-NOV complex	0.20412
Shigella infection	2853	ITGA5-ITGB1-CAL4A3 complex	0.2357
Shigella infection	2885	ITGAV-ITGB1-SPP1 complex	0.2357
Shigella infection	2964	ITGA9-ITGB1-ADAM1 complex	0.2357
Shigella infection	2965	ITGA9-ITGB1-ADAM3 complex	0.2357

Shigella infection	2971	ITGA9-ITGB1-VEGFC complex	0.2357
Shigella infection	2972	ITGA9-ITGB1-VEGFA complex	0.2357
Shigella infection	2989	ITGA9-ITGB1-ADAM8 complex	0.2357
Shigella infection	3035	LAT2-ITGB1 complex	0.28868
Shigella infection	3057	ITGA10-ITGB1 complex	0.28868
Shigella infection	3058	ITGA11-ITGB1 complex	0.28868
Shigella infection	3059	ITGA11-ITGB1-COL1A1 complex	0.2357
Shigella infection	3104	ITGB1-NRP1 complex	0.28868
Shigella infection	3111	ITGA9-ITGB1-SPP1 complex	0.2357
Shigella infection	3112	ITGA5-ITGB1-SPP1 complex	0.2357
Shigella infection	5337	ELMO1-DOCK1 complex	0.28868
Shigella infection	5341	ELMO1-DOCK2 complex	0.28868
Shigella infection	5342	ELMO1-DOCK1-RAC1 complex	0.2357
Shigella infection	5343	ELMO1-DOCK1-CRKII complex	0.2357
Shigella infection	5388	SERPINA1-ELA2 complex	0.28868
Sicca syndrome	74	TRPC1-Homer3-IP3R complex	0.11323
Sicca syndrome	552	IFNB1-IFNAR1-IFNAR2- complex	0.11323
Sicca syndrome	553	RHOA-IP3R-TRPC1 complex	0.11323
Sicca syndrome	1297	MKK4-ARRB2-ASK1 complex	0.11323
Sicca syndrome	1298	MKK4-ARRB2-JNK3 complex	0.11323
Sicca syndrome	1816	JUN-TCF4-CTNNB1 complex	0.11323
Sicca syndrome	2051	MALT1 oligomer complex	0.19612
Sicca syndrome	2053	BCL10-MALT1 complex	0.13868
Sicca syndrome	2054	CASP8-FADD-MALT1-BCL10 complex	0.09806
Sicca syndrome	2055	CASP8-CHUK-IKBKB-MALT1-BCL10 complex	0.08771
Sicca syndrome	2692	SMAD3-SMAD4-cJun-cFos complex	0.09806
Sicca syndrome	2693	NFAT-JUN-FOS DNA-protein complex	0.11323
Sicca syndrome	2694	ERG-JUN-FOS DNA-protein complex	0.11323
Sicca syndrome	2695	ETS2-FOS-JUN complex	0.11323
Sicca syndrome	2699	ER-alpha-GRIP1-c-Jun complex	0.11323
Sicca syndrome	2700	ER-alpha-c-Jun complex	0.13868
Sicca syndrome	2708	SMAD3-SMAD4-cJUN complex	0.11323
Sicca syndrome	2743	TRAF6-MALT1 complex	0.13868
Sicca syndrome	2744	TRAF2-MALT1 complex	0.13868
Sicca syndrome	2745	Ubiquitin ligase complex (TRAF6 TAB2 MALT1 UEV1A BCL10)	0.08771
Sicca syndrome	2752	CARMA1-BCL10-MALT1 complex	0.11323
Sicca syndrome	2753	FYB-CARMA1-BCL-10-MALT1 complex	0.09806
Sicca syndrome	5473	FAS-FADD-CASP8 complex	0.11323
Sicca syndrome	5604	Emerin complex 1	0.06934
Sicca syndrome	5606	Emerin-actin-NMI-(alphaII)spectrin complex	0.09806
Sicca syndrome	5608	Emerin architectural complex	0.08006
Sicca syndrome	5614	Emerin complex 32	0.04181
Sicca syndrome	5691	TALL1 homo-oligomer complex	0.19612
Sicca syndrome	5799	Death induced signaling complex DISC (FAS FADD CASP8 CFLAR) membrane-associated CD95L	0.09806
Sicca syndrome	5800	Death-inducing signaling complex DISC (type I cells associated) stimulated	0.11323

Sicca syndrome	5808	DISC complex	0.11323
Sicca syndrome	5859	FAS-FADD-CASP8-CASP10 complex	0.09806
Sicca syndrome	5861	FAS-FADD-CASP10 complex	0.11323
Sickle cell disease	541	IGF1-IGFBP3-ALS complex	0.22222
Sickle cell disease	668	BKCA-beta2AR-AKAP79 signaling complex	0.11111
Sickle cell disease	672	BKCA-beta2AR complex	0.13608
Sickle cell disease	687	CFTR-NHERF-beta(2)AR signaling complex	0.11111
Sickle cell disease	1439	PTGS2 homodimer complex	0.19245
Sickle cell disease	1810	ITGA4-PXN-GIT1 complex	0.11111
Sickle cell disease	2345	ITGAV-ITGB5-ICAM4 complex	0.11111
Sickle cell disease	2383	ITGA5-ITGB1-FN1-TGM2 complex	0.09623
Sickle cell disease	2384	ITGA5-ITGB1-ADAM15 complex	0.11111
Sickle cell disease	2385	ITGA5-ITGB4 complex	0.13608
Sickle cell disease	2388	Itga5-Itgb1-Fn1-Sfrp2 complex	0.09623
Sickle cell disease	2390	CD98-LAT2-ITGB1 complex	0.09623
Sickle cell disease	2395	ITGA7-ITGB1-CD151 complex	0.11111
Sickle cell disease	2396	ITGA7-ITGB1-CD9 complex	0.11111
Sickle cell disease	2397	ITGA7-ITGB1-ITGB1BP3 complex	0.11111
Sickle cell disease	2398	ITGA3-ITGB1-BSG complex	0.11111
Sickle cell disease	2399	ITGA3-ITGB1-CD63 complex	0.11111
Sickle cell disease	2400	ITGA3-ITGB1-CD151 complex	0.11111
Sickle cell disease	2401	ITGA3-ITGB1-THBS1 complex	0.11111
Sickle cell disease	2406	ITGA3-ITGB1 complex	0.13608
Sickle cell disease	2411	ITGA6-ITGB1-CD151 complex	0.11111
Sickle cell disease	2413	ITGA6-ITGB1 complex	0.13608
Sickle cell disease	2416	ITGB1-RAP1A-PKD1 complex	0.11111
Sickle cell disease	2417	ITGA4-ITGB1-EMILIN1 complex	0.22222
Sickle cell disease	2418	ITGA4-ITGB1 complex	0.27217
Sickle cell disease	2419	ITGA4-ITGB1-CD81 complex	0.22222
Sickle cell disease	2420	ITGA4-ITGB1-CD53 complex	0.22222
Sickle cell disease	2421	ITGA4-ITGB1-VCAM1 complex	0.22222
Sickle cell disease	2422	ITGA4-ITGB1-JAM2 complex	0.22222
Sickle cell disease	2423	ITGA4-ITGB1-CD47 complex	0.22222
Sickle cell disease	2424	ITGA4-ITGB1-CD63 complex	0.22222
Sickle cell disease	2425	ITGA4-ITGB1-PXN complex	0.22222
Sickle cell disease	2426	ITGA4-ITGB1-THBS1 complex	0.22222
Sickle cell disease	2428	ITGA4-ITGB1-THBS2 complex	0.22222
Sickle cell disease	2429	ITGA2-ITGB1-CD47 complex	0.11111
Sickle cell disease	2430	ITGA2-ITGB1-CHAD complex	0.11111
Sickle cell disease	2431	ITGA2-ITGB1-COL6A3 complex	0.11111
Sickle cell disease	2432	ITGA2-ITGB1 complex	0.13608
Sickle cell disease	2434	ITGA1-ITGB1-COL6A3 complex	0.11111
Sickle cell disease	2435	ITGA1-ITGB1-PTPN2 complex	0.11111
Sickle cell disease	2436	ITGAV-ITGB1 complex	0.13608
Sickle cell disease	2437	ITGA6-ITGB1-CYR61 complex	0.11111
Sickle cell disease	2439	ITGA8-ITGB1 complex	0.13608
Sickle cell disease	2440	ITGA9-ITGB1-ADAM9 complex	0.11111

Sickle cell disease	2441	Itga9-Itgb1-Adam2 complex	0.11111
Sickle cell disease	2442	ITGA9-ITGB1-VCAM1 complex	0.11111
Sickle cell disease	2443	ITGA9-ITGB1-TNC complex	0.11111
Sickle cell disease	2444	ITGB1-ITGA9 complex	0.13608
Sickle cell disease	2445	ITGA9-ITGB1-ADAM15 complex	0.11111
Sickle cell disease	2446	ITGA9-ITGB1-FIGF complex	0.11111
Sickle cell disease	2447	ITGA9-ITGB1-ADAM12 complex	0.11111
Sickle cell disease	2850	ITGA5-ITGB1-FN-1-NOV complex	0.09623
Sickle cell disease	2853	ITGA5-ITGB1-CAL4A3 complex	0.11111
Sickle cell disease	2885	ITGAV-ITGB1-SPP1 complex	0.11111
Sickle cell disease	2964	ITGA9-ITGB1-ADAM1 complex	0.11111
Sickle cell disease	2965	ITGA9-ITGB1-ADAM3 complex	0.11111
Sickle cell disease	2971	ITGA9-ITGB1-VEGFC complex	0.11111
Sickle cell disease	2972	ITGA9-ITGB1-VEGFA complex	0.11111
Sickle cell disease	2989	ITGA9-ITGB1-ADAM8 complex	0.11111
Sickle cell disease	3035	LAT2-ITGB1 complex	0.13608
Sickle cell disease	3057	ITGA10-ITGB1 complex	0.13608
Sickle cell disease	3058	ITGA11-ITGB1 complex	0.13608
Sickle cell disease	3059	ITGA11-ITGB1-COL1A1 complex	0.11111
Sickle cell disease	3104	ITGB1-NRP1 complex	0.13608
Sickle cell disease	3111	ITGA9-ITGB1-SPP1 complex	0.11111
Sickle cell disease	3112	ITGA5-ITGB1-SPP1 complex	0.11111
Sickle cell disease	3115	ITGA2B-ITGB3-ICAM4 complex	0.11111
Sickle cell disease	3830	ADRB2 homodimer complex	0.19245
Silicosis	2018	IL12A-IL12B complex	0.26726
Silicosis	2019	IL12A-IL12B-IL12RB1 complex	0.21822
Silicosis	2020	IL12B-IL12RB1-IL12RB2 complex	0.21822
Silicosis	2021	IL12A-IL12B-IL12RB2 complex	0.21822
Silicosis	5473	FAS-FADD-CASP8 complex	0.21822
Silicosis	5548	IL-12 heterodimer complex	0.26726
Silicosis	5549	IL-12 subunit p40 homodimer complex	0.37796
Silicosis	5799	Death induced signaling complex DISC (FAS FADD CASP8 CFLAR) membrane-associated CD95L	0.18898
Silicosis	5800	Death-inducing signaling complex DISC (type I cells associated) stimulated	0.21822
Silicosis	5808	DISC complex	0.21822
Silicosis	5859	FAS-FADD-CASP8-CASP10 complex	0.18898
Silicosis	5861	FAS-FADD-CASP10 complex	0.21822
Sinusitis	681	(C-CFTR)2-NHERF-ezrin complex	0.1291
Sinusitis	682	C-CFTR-NHERF(PDZ1 domain)-ezrin complex	0.1291
Sinusitis	683	C-CFTR-NHERF(PDZ2 domain)-ezrin complex	0.1291
Sinusitis	687	CFTR-NHERF-beta(2)AR signaling complex	0.1291
Sinusitis	1514	IL4-IL4R complex	0.15811
Sinusitis	1515	IL4-IL4R-IL2RG complex	0.1291
Sinusitis	2016	IL12A homodimer complex	0.22361
Sinusitis	2018	IL12A-IL12B complex	0.31623
Sinusitis	2019	IL12A-IL12B-IL12RB1 complex	0.2582
Sinusitis	2020	IL12B-IL12RB1-IL12RB2 complex	0.1291

Sinusitis	2021	IL12A-IL12B-IL12RB2 complex	0.2582
Sinusitis	2709	MMP-9-TIMP-1-LRP complex	0.1291
Sinusitis	2798	MMP-2-claudin-1 complex	0.15811
Sinusitis	5548	IL-12 heterodimer complex	0.31623
Sinusitis	5549	IL-12 subunit p40 homodimer complex	0.22361
Sinusitis	5691	TALL1 homo-oligomer complex	0.22361
Skin cancer	159	Condensin I-PARP-1-XRCC1 complex	0.08671
Skin cancer	212	DNA ligase III-XRCC1 complex	0.16222
Skin cancer	213	DNA ligase IV-XRCC1 complex	0.16222
Skin cancer	362	DNA ligase III-XRCC1-PNK-DNA-pol III multiprotein complex	0.11471
Skin cancer	1085	DNA repair complex NEIL2-PNK-Pol(beta)-LigIII(alpha)-XRCC1	0.1026
Skin cancer	1086	DNA repair complex NEIL1-PNK-Pol(beta)-LigIII(alpha)-XRCC1	0.1026
Skin cancer	1095	SNX complex (SNX1a SNX2 SNX4 EGFR)	0.11471
Skin cancer	1185	EGFR-containing signaling complex	0.11471
Skin cancer	1746	SMN containing complex	0.08111
Skin cancer	1827	PML-SMAD2/3-SARA complex	0.11471
Skin cancer	2369	ITGAV-ITGB3-EGFR complex	0.13245
Skin cancer	2453	Multiprotein complex (monoubiquitination)	0.11471
Skin cancer	2454	CIN85-CBL-SH3GL2-EGFR complex EGF stimulated	0.11471
Skin cancer	2542	EGFR-CBL-GRB2 complex	0.13245
Skin cancer	2551	PDGFRA-PLC-gamma-1-PI3K-SHP-2 complex PDGF stimulated	0.11471
Skin cancer	2709	MMP-9-TIMP-1-LRP complex	0.26491
Skin cancer	2798	MMP-2-claudin-1 complex	0.16222
Skin cancer	2829	RSmad complex	0.07255
Skin cancer	2830	TIF1gamma-SMAD2-SMAD3 complex	0.13245
Skin cancer	2834	SMAD4-SMAD2-SMAD3 complex	0.13245
Skin cancer	3038	SMAD2-SMAD4-FAST1 complex	0.13245
Skin cancer	3039	SMAD2-FAST1 complex	0.16222
Skin cancer	3183	PDGFRA-SHP-2 complex PDGF stimulated	0.16222
Skin cancer	3186	GRB2-SHP-2 complex PDGF stimulated	0.16222
Skin cancer	3198	SMAD2-SKI complex	0.16222
Skin cancer	3204	SMAD2-SKI-NCOR complex	0.13245
Skin cancer	3233	SMAD2-SMAD4-FAST1-TGIF complex TGF(beta) induced	0.11471
Skin cancer	3234	SMAD2-SMAD4-FAST1-TGIF-HDAC1 complex TGF(beta) induced	0.1026
Skin cancer	3678	RIN1-STAM2-EGFR complex EGF stimulated	0.13245
Skin cancer	3729	SKI-SMAD2 hexameric complex	0.16222
Skin cancer	3739	SKI-SMAD2-SMAD4 pentameric complex	0.13245
Skin cancer	3749	CREBBP-SMAD2 hexameric complex	0.16222
Skin cancer	3753	CREBBP-SMAD2-SMAD4 pentameric complex	0.13245
Skin cancer	3967	SMURF2-SMAD2 complex TGF(beta)-dependent	0.16222
Skin cancer	5171	SH3KBP1-CBLB-EGFR complex	0.13245
Skin cancer	5243	XRCC1-LIG3-PNK-TDP1 complex	0.11471

Skin cancer	5564	LMO4-gp130 complex	0.1026
Skin disease	75	TSC1-TSC2 complex	0.12127
Skin disease	80	Ubiquitin E3 ligase (Skp1A Skp2 Cul1 Rbx1)	0.08575
Skin disease	201	HUIC complex	0.12127
Skin disease	202	BRCA1-RAD50-MRE11-NBS1 complex	0.08575
Skin disease	226	Ubiquitin E3 ligase (SKP1A SKP2 CUL1)	0.09901
Skin disease	238	SWI-SNF chromatin remodeling-related-BRCA1	0.05171
Skin disease	240	BRCA1-CTIP-ZBRK1 repressor complex	0.09901
Skin disease	242	BRCA1-BACH1 complex	0.12127
Skin disease	387	MCM complex	0.07001
Skin disease	433	BASC complex (BRCA1-associated genome surveillance complex)	0.04951
Skin disease	434	BASC (Ab 80) complex (BRCA1-associated genome surveillance complex)	0.06063
Skin disease	435	BASC (Ab 81) complex (BRCA1-associated genome surveillance complex)	0.07001
Skin disease	436	BASC (Ab C-20) complex (BRCA1-associated genome surveillance complex)	0.09901
Skin disease	438	GCN5-TRRAP histone acetyltransferase complex	0.05423
Skin disease	443	BP-SMAD complex	0.06063
Skin disease	781	URI complex (Unconventional prefoldin RPB5 Interactor)	0.05717
Skin disease	1051	Ubiquitin E3 ligase (SKP1A SKP2 CUL1 RBX1)	0.08575
Skin disease	1194	E2F-6 complex	0.04951
Skin disease	1707	IL2-IL2RA-IL2RB complex	0.09901
Skin disease	2210	BRCA1-IRIS-pre-replication complex	0.08575
Skin disease	2211	BARD1-BRCA1-CSTF complex	0.0767
Skin disease	2213	BRCA1-BARD1-POLR2A complex	0.09901
Skin disease	2214	LMO4-BRCA1-CTIP-LDB1 complex	0.08575
Skin disease	2215	BRCA1-LMO4-CTIP complex	0.09901
Skin disease	2604	P-TEFb-SKP2 complex	0.09901
Skin disease	2686	BRCA1-core RNA polymerase II complex	0.04757
Skin disease	2709	MMP-9-TIMP-1-LRP complex	0.09901
Skin disease	2715	Ubiquitin E3 ligase (CSN1 CSN8 HRT1 SKP1 SKP2 CUL1 CUL2 CUL3)	0.06063
Skin disease	2776	RAD50-BRCA1 complex	0.12127
Skin disease	2783	BARD1-BRCA1-CSTF64 complex	0.09901
Skin disease	2786	BRCA1 A complex	0.08575
Skin disease	2787	BRCA1 C complex	0.08575
Skin disease	2788	BRCA1 B complex	0.09901
Skin disease	2811	BRCA1-cABL complex	0.12127
Skin disease	2813	BRCA1-SMAD3 complex	0.12127
Skin disease	2814	BRCA1-HDAC1-HDAC2 complex	0.09901
Skin disease	2815	BRCA1-BARD1-BACH1-DNA damage complex II	0.06063
Skin disease	2817	BRCA1-BARD1-BACH1-DNA damage complex I	0.07001
Skin disease	2818	BRCA1-BARD1-BRCA2-DNA damage complex III	0.09901
Skin disease	2819	BRCA1-CtIP-CtBP complex	0.09901
Skin disease	2820	BRCA1-VCP complex	0.12127

Skin disease	2822	BRCA1-BARD1-UbcH5c complex	0.09901
Skin disease	2823	BRCA1-BARD1-UbcH7c complex	0.09901
Skin disease	2824	BRCA1-RAD51 complex	0.12127
Skin disease	2825	BRCA1-RNA polymerase II complex	0.03363
Skin disease	2880	SCF subcomplex (WEE1 SKP2 BTRC)	0.09901
Skin disease	3015	p27-cyclinE-Cdk2 - Ubiquitin E3 ligase (SKP1A SKP2 CUL1 CKS1B RBX1) complex	0.06063
Skin disease	3036	Ubiquitin E3 ligase (SKP1A SKP2 CUL1 CKS1B RBX1)	0.0767
Skin disease	5320	CDH1-SKP2 complex	0.12127
Skin disease	5400	BRCC complex	0.0767
Skin tumor	2529	LAT-PLC-gamma-1-p85-GRB2-CBL-VAV-SLP-76 signaling complex C305 activated	0.14286
Skin tumor	2547	PLC-gamma-1-SLP-76-SOS1-LAT complex	0.18898
Skin tumor	2909	PLC-gamma-2-Syk-LAT-FcR-gamma complex	0.18898
Skin tumor	2913	PLC-gamma-2-LAT complex	0.26726
Skin tumor	2922	LAT-PLC-gamma-1-p85-GRB2-SOS signaling complex C305 activated	0.16903
Skin tumor	2955	LCK-SLP76-PLC-gamma-1-LAT complex pervanadate-activated	0.18898
Skin tumor	2956	PLC-gamma-1-LAT-c-CBL complex OKT3 stimulated	0.21822
Skin tumor	2957	LAT-GRB2 complex Fyn-mLck(KA) or Syk kinase activated	0.26726
Skin tumor	3070	CTF18-cohesion-RFC-POLH complex	0.13363
Solid tumor	91	FA complex (Fanconi anemia complex) cytoplasmic	0.14434
Solid tumor	244	BRAFT complex	0.08006
Solid tumor	245	FA core complex (Fanconi anemia core complex)	0.09623
Solid tumor	541	IGF1-IGFBP3-ALS complex	0.16667
Solid tumor	1152	FA complex (Fanconi anemia complex)	0.10206
Solid tumor	1514	IL4-IL4R complex	0.20412
Solid tumor	1515	IL4-IL4R-IL2RG complex	0.16667
Solid tumor	1623	FA core complex 1 (Fanconi anemia core complex 1)	0.1291
Solid tumor	1624	FA core complex (Fanconi anemia core complex)	0.09623
Solid tumor	1625	FA core complex (Fanconi anemia core complex)	0.10911
Solid tumor	2551	PDGFRA-PLC-gamma-1-PI3K-SHP-2 complex PDGF stimulated	0.14434
Solid tumor	2739	FA complex (Fanconi anemia complex)	0.08704
Solid tumor	3040	Multisynthetase complex	0.08704
Solid tumor	3083	Nucleic and chromatin Fanconi complex	0.1291
Solid tumor	3162	TF-FVIIa-FXa-TFPI complex	0.14434
Solid tumor	3183	PDGFRA-SHP-2 complex PDGF stimulated	0.20412
Solid tumor	3186	GRB2-SHP-2 complex PDGF stimulated	0.20412
Solid tumor	5564	LMO4-gp130 complex	0.1291
Spinal cord disease	351	Spliceosome	0.0187
Spinal cord disease	541	IGF1-IGFBP3-ALS complex	0.1291
Spinal cord disease	824	Anti-SMN protein complex	0.1
Spinal cord disease	832	Anti-Sm protein complex	0.08452
Spinal cord disease	1142	SMN complex	0.07071
Spinal cord disease	1143	SMN complex	0.0559

Spinal cord disease	1261	SRm160/300 complex	0.1
Spinal cord disease	1745	SMN complex	0.05774
Spinal cord disease	1749	SMN-PolII-RHA complex	0.07071
Spinal cord disease	1750	PPP4C-PPP4R2-Gemin3-Gemin4 complex	0.1118
Spinal cord disease	1751	SMN complex	0.07071
Spinal cord disease	1752	SMN complex	0.08452
Spinal cord disease	2247	Dynactin complex (DCTN1 DCTN2 DCTN3 DCTN4 DCTN6 CAPZA1 CAPZB ACTR1A)	0.07906
Spinal cord disease	3284	SMN complex (GEMIN5 2 3 4 SMN)	0.1
Spinal cord disease	3296	SMN complex (GEMIN5 4 3) SMN-independent intermediate	0.1291
Spinal dysraphism	201	HUIC complex	0.16222
Spinal dysraphism	202	BRCA1-RAD50-MRE11-NBS1 complex	0.11471
Spinal dysraphism	238	SWI-SNF chromatin remodeling-related-BRCA1	0.06917
Spinal dysraphism	240	BRCA1-CTIP-ZBRK1 repressor complex	0.13245
Spinal dysraphism	242	BRCA1-BACH1 complex	0.16222
Spinal dysraphism	433	BASC complex (BRCA1-associated genome surveillance complex)	0.06623
Spinal dysraphism	434	BASC (Ab 80) complex (BRCA1-associated genome surveillance complex)	0.08111
Spinal dysraphism	435	BASC (Ab 81) complex (BRCA1-associated genome surveillance complex)	0.09366
Spinal dysraphism	436	BASC (Ab C-20) complex (BRCA1-associated genome surveillance complex)	0.13245
Spinal dysraphism	438	GCN5-TRRAP histone acetyltransferase complex	0.07255
Spinal dysraphism	1091	SNX complex (SNX1a SNX2 SNX4 LEPR)	0.11471
Spinal dysraphism	1970	BMP4-TWSG1 complex	0.16222
Spinal dysraphism	1972	BMP4-BGN complex	0.16222
Spinal dysraphism	1992	LEPR homodimer complex	0.22942
Spinal dysraphism	2210	BRCA1-IRIS-pre-replication complex	0.11471
Spinal dysraphism	2211	BARD1-BRCA1-CSTF complex	0.1026
Spinal dysraphism	2213	BRCA1-BARD1-POLR2A complex	0.13245
Spinal dysraphism	2214	LMO4-BRCA1-CTIP-LDB1 complex	0.11471
Spinal dysraphism	2215	BRCA1-LMO4-CTIP complex	0.13245
Spinal dysraphism	2255	Cofilin-actin-CAP1 complex	0.13245
Spinal dysraphism	2686	BRCA1-core RNA polymerase II complex	0.06363
Spinal dysraphism	2776	RAD50-BRCA1 complex	0.16222
Spinal dysraphism	2783	BARD1-BRCA1-CSTF64 complex	0.13245
Spinal dysraphism	2786	BRCA1 A complex	0.11471
Spinal dysraphism	2787	BRCA1 C complex	0.11471
Spinal dysraphism	2788	BRCA1 B complex	0.13245
Spinal dysraphism	2811	BRCA1-cABL complex	0.16222
Spinal dysraphism	2813	BRCA1-SMAD3 complex	0.16222
Spinal dysraphism	2814	BRCA1-HDAC1-HDAC2 complex	0.13245
Spinal dysraphism	2815	BRCA1-BARD1-BACH1-DNA damage complex II	0.08111
Spinal dysraphism	2817	BRCA1-BARD1-BACH1-DNA damage complex I	0.09366
Spinal dysraphism	2818	BRCA1-BARD1-BRCA2-DNA damage complex III	0.13245
Spinal dysraphism	2819	BRCA1-CtIP-CtBP complex	0.13245

Spinal dysraphism	2820	BRCA1-VCP complex	0.16222
Spinal dysraphism	2822	BRCA1-BARD1-UbcH5c complex	0.13245
Spinal dysraphism	2823	BRCA1-BARD1-UbcH7c complex	0.13245
Spinal dysraphism	2824	BRCA1-RAD51 complex	0.16222
Spinal dysraphism	2825	BRCA1-RNA polymerase II complex	0.04499
Spinal dysraphism	3043	BMP2-BRIA complex	0.13245
Spinal dysraphism	5400	BRCC complex	0.1026
Spinocerebellar ataxias	74	TRPC1-Homer3-IP3R complex	0.16667
Spinocerebellar ataxias	432	N-NOS-CHIP-HSP70-1 complex	0.16667
Spinocerebellar ataxias	445	TFTC complex (TATA-binding protein-free TAF-II-containing complex)	0.07217
Spinocerebellar ataxias	470	TFTC complex (TATA-binding protein-free TAF-II-containing complex)	0.07001
Spinocerebellar ataxias	476	STAGA complex (SPT3-TAF9-GCN5 acetyltransferase complex)	0.08006
Spinocerebellar ataxias	513	TFTC complex (TATA-binding protein-free TAF-II-containing complex)	0.07001
Spinocerebellar ataxias	553	RHOA-IP3R-TRPC1 complex	0.16667
Spinocerebellar ataxias	1617	G protein complex (CACNA1A GNB1 GNG2)	0.16667
Squamous cell cancer	75	TSC1-TSC2 complex	0.07217
Squamous cell cancer	126	CCT micro-complex	0.03608
Squamous cell cancer	531	XPA-ERCC1-ERCC4 complex	0.05893
Squamous cell cancer	1056	ZNF198-SUMO1 complex	0.07217
Squamous cell cancer	1085	DNA repair complex NEIL2-PNK-Pol(beta)-LigIII(alpha)-XRCC1	0.04564
Squamous cell cancer	1306	PIN1-AUF1 complex	0.07217
Squamous cell cancer	1308	PABPC1-HSPA8-HNRPD-EIF4G1 complex	0.04564
Squamous cell cancer	1514	IL4-IL4R complex	0.07217
Squamous cell cancer	1515	IL4-IL4R-IL2RG complex	0.05893
Squamous cell cancer	1707	IL2-IL2RA-IL2RB complex	0.05893
Squamous cell cancer	1774	MICA-KLRK1-HCST complex	0.05893
Squamous cell cancer	2084	NFKB1-NFKB2-REL-RELA-RELB complex	0.04564
Squamous cell cancer	2188	Ubiquitin E3 ligase (CDC34 NEDD8 BTRC CUL1 SKP1A RBX1)	0.04564
Squamous cell cancer	2254	CTGF/Hcs24-actin complex	0.05893
Squamous cell cancer	2256	RIAM-Rap1-GTP complex	0.07217
Squamous cell cancer	2351	ITGB6-FYN-FN1 complex	0.05893
Squamous cell cancer	2352	ITGAV-ITGB6-SPP1 complex	0.05893
Squamous cell cancer	2353	ITGAV-ITGB6-TGFB3 complex	0.05893
Squamous cell cancer	2354	ITGAV-ITGB6 complex	0.07217
Squamous cell cancer	2383	ITGA5-ITGB1-FN1-TGM2 complex	0.05103
Squamous cell cancer	2384	ITGA5-ITGB1-ADAM15 complex	0.05893
Squamous cell cancer	2385	ITGA5-ITGB4 complex	0.07217
Squamous cell cancer	2388	Itga5-Itgb1-Fn1-Sfrp2 complex	0.05103
Squamous cell cancer	2416	ITGB1-RAP1A-PKD1 complex	0.05893
Squamous cell cancer	2850	ITGA5-ITGB1-FN-1-NOV complex	0.05103
Squamous cell cancer	2853	ITGA5-ITGB1-CAL4A3 complex	0.05893
Squamous cell cancer	2882	ITGA5-ITGB3-COL6A3 complex	0.05893

Squamous cell cancer	3112	ITGA5-ITGB1-SPP1 complex	0.05893
Squamous cell cancer	3137	MASH1 promoter-coactivator complex	0.03077
Squamous cell cancer	3142	CAMK2-delta-MASH1 promoter-coactivator complex	0.03608
Squamous cell cancer	3158	RIAM-Rap1-GTP-profilin complex	0.05893
Squamous cell cancer	5107	p34(SEI-1)-CDK4-CyclinD2 complex	0.05893
Squamous cell cancer	5193	TNF-alpha/NF-kappa B signaling complex (CHUK KPNA3 NFKB2 NFKBIB REL IKBKG NFKB1 NFKBIE RELB NFKBIA RELA TNIP2)	0.02946
Squamous cell cancer	5194	TNF-alpha/NF-kappa B signaling complex (SEC16A CHUK IKBKB NFKB2 REL IKBKG MAP3K14 RELA FBXW7 USP2)	0.03227
Squamous cell cancer	5196	TNF-alpha/NF-kappa B signaling complex (CHUK BTRC NFKB2 PPP6C REL CUL1 IKBKE SAPS2 SAPS1 ANKRD28 RELA SKP1)	0.05893
Squamous cell cancer	5211	RAF1-PPP2-PIN1 complex	0.04564
Squamous cell cancer	5228	REL-MAP3K8-RELA-TNIP2-PAPOLA complex	0.04564
Squamous cell cancer	5230	CHUK-NFKB2-REL-IKBKG-SPAG9-NFKB1-NFKBIE-COPB2-TNIP1-NFKBIA-RELA-TNIP2	0.02946
Squamous cell cancer	5232	TNF-alpha/Nf-kappa B signaling complex (RPL6 RPL30 RPS13 CHUK DDX3X NFKB2 NFKBIB REL IKBKG NFKB1 MAP3K8 RELB GLG1 NFKBIA RELA TNIP2 GTF2I)	0.02475
Squamous cell cancer	5233	TNF-alpha/NF-kappa B signaling complex 5	0.02041
Squamous cell cancer	5253	MNK1-eIF4F complex	0.04564
Squamous cell cancer	5260	TCF4-CTNNB1-SUMO1-EP300-HADAC6 complex	0.04564
Squamous cell cancer	5269	TNF-alpha/NF-kappa B signaling complex 8	0.04167
Squamous cell cancer	5465	IKB(epsilon)-RELA-cREL complex	0.05893
Squamous cell cancer	5466	IKB(beta)-RELA-cREL complex	0.05893
Squamous cell cancer	5467	IKB(alpha)-RELA-cREL complex	0.05893
Squamous cell cancer	5613	Emerin complex 25	0.02552
Squamous cell cancer	5755	SUMO1-SUA1-UBA2 complex	0.05893
Squamous cell cancer	5823	MCL1-BAK1 complex	0.07217
Stomach cancer	27	Arp2/3 protein complex	0.03183
Stomach cancer	71	MRN complex (MRE11-RAD50-NBS1 complex)	0.09724
Stomach cancer	72	R/M complex (RAD50-MRE11 complex)	0.1191
Stomach cancer	73	MRN complex (MRE11-RAD50-NBN complex)	0.09724
Stomach cancer	120	Lymphotoxin beta receptor complex	0.04862
Stomach cancer	129	PIDDsome complex	0.04862
Stomach cancer	138	Telosome complex	0.03438
Stomach cancer	202	BRCA1-RAD50-MRE11-NBS1 complex	0.08422
Stomach cancer	227	Ubiquitin E3 ligase (SKP1A BTRC CUL1)	0.04862
Stomach cancer	244	BRAFT complex	0.02336
Stomach cancer	246	BLM complex III	0.04211
Stomach cancer	281	NELF complex (Negative elongation factor complex)	0.04211
Stomach cancer	305	40S ribosomal subunit cytoplasmic	0.02889
Stomach cancer	306	Ribosome cytoplasmic	0.04679
Stomach cancer	308	60S ribosomal subunit cytoplasmic	0.03685
Stomach cancer	351	Spliceosome	0.00704

Stomach cancer	362	DNA ligase III-XRCC1-PNK-DNA-pol III multiprotein complex	0.04211
Stomach cancer	433	BASC complex (BRCA1-associated genome surveillance complex)	0.07293
Stomach cancer	434	BASC (Ab 80) complex (BRCA1-associated genome surveillance complex)	0.02977
Stomach cancer	436	BASC (Ab C-20) complex (BRCA1-associated genome surveillance complex)	0.04862
Stomach cancer	521	Polycystin-1-E-cadherin-beta-catenin complex	0.04862
Stomach cancer	522	Polycystin-1-E-cadherin-beta-catenin-Flotillin-2	0.04211
Stomach cancer	557	TRP1-G alpha-11-IP3R3-CAV1 signaling complex	0.04211
Stomach cancer	619	MRE11A-RAD50-NBN-TRF2 complex	0.08422
Stomach cancer	627	MRN-TRRAP complex (MRE11A-RAD50-NBN-TRRAP complex)	0.08422
Stomach cancer	629	BLM-TRF2 complex	0.05955
Stomach cancer	710	Brg1-associated complex I	0.02539
Stomach cancer	711	Brm-associated complex	0.02539
Stomach cancer	713	BRG1-SIN3A complex	0.02251
Stomach cancer	714	BRM-SIN3A complex	0.02174
Stomach cancer	740	Exon junction complex EIF4A3-MLN51-MAGOH-Y14 (RNA-protein complex)	0.04211
Stomach cancer	744	Exon junction complex (EIF4A3-MLN51-MAGOH-	0.04211
Stomach cancer	754	Exon junction complex (EIF4A3-MLN51-UPF3B-MAGOH-Y14)	0.03766
Stomach cancer	755	Exon junction complex (F4A3-MLN51-UPF3B-MAGOH-Y14-PYM)	0.03438
Stomach cancer	769	Exon junction complex (EIF4A3-MLN51-MAGOH-Y14) RNA-protein complex	0.04211
Stomach cancer	772	eIF4AIII-Btz complex	0.05955
Stomach cancer	784	SMG-1-Upf1-eRF1-eRF3 complex (SURF)	0.03766
Stomach cancer	785	Exon junction complex	0.02977
Stomach cancer	803	BRG1-SIN3A-HDAC containing SWI/SNF remodeling complex I	0.02539
Stomach cancer	806	BRM-SIN3A-HDAC complex	0.02431
Stomach cancer	807	BRG1-associated complex	0.02807
Stomach cancer	808	BRM-associated complex	0.02663
Stomach cancer	810	FCP1-associated protein complex	0.03183
Stomach cancer	812	Upf complex (UPF1 UPF2 UPF3a)	0.04862
Stomach cancer	813	Upf complex (UPF1 UPF2 UPF3b)	0.04862
Stomach cancer	814	Postsplicing complex	0.03183
Stomach cancer	819	20S methylosome-SmD complex	0.04211
Stomach cancer	822	mRNA decay complex (UPF1 UPF2 UPF3B DCP2 XRN1 XRN2 EXOSC2 EXOSC4 EXOSC10 PARN)	0.02663
Stomach cancer	825	JBP1-pICln complex	0.04862
Stomach cancer	832	Anti-Sm protein complex	0.03183
Stomach cancer	834	20S methylosome and RG-containing Sm protein complex	0.03438
Stomach cancer	836	20S methyltransferase core complex	0.05955
Stomach cancer	837	20S methyltransferase complex	0.04862

Stomach cancer	1085	DNA repair complex NEIL2-PNK-Pol(beta)-LigIII(alpha)-XRCC1	0.03766
Stomach cancer	1086	DNA repair complex NEIL1-PNK-Pol(beta)-LigIII(alpha)-XRCC1	0.03766
Stomach cancer	1091	SNX complex (SNX1a SNX2 SNX4 LEPR)	0.04211
Stomach cancer	1141	CF IIAm complex (Cleavage factor IIAm complex)	0.02105
Stomach cancer	1181	C complex spliceosome	0.00942
Stomach cancer	1189	DNA double-strand break end-joining complex	0.06366
Stomach cancer	1193	Rap1 complex	0.06366
Stomach cancer	1197	TRF1-TIN2 complex	0.03438
Stomach cancer	1198	TIN2 complex	0.04211
Stomach cancer	1202	TRF1 telomere length regulation complex	0.04862
Stomach cancer	1204	Rap1 complex	0.06366
Stomach cancer	1206	TRF-Rap1 complex I 2MD	0.03438
Stomach cancer	1207	TRF2-Rap1 complex II	0.04211
Stomach cancer	1211	Ubiquitin E3 ligase (AHR ARNT DDB1 TBL3 CUL4B RBX1)	0.03438
Stomach cancer	1218	BLM-TRF2 complex	0.05955
Stomach cancer	1231	FIB-associated protein complex	0.03438
Stomach cancer	1255	Ubiquitin E3 ligase (SIAH1 SIP SKP1A TBL1X)	0.08422
Stomach cancer	1306	PIN1-AUF1 complex	0.05955
Stomach cancer	1474	SMAD3/4-E2F4/5-p107-DP1 complex	0.03438
Stomach cancer	5757	PLXNA1-RANBPM complex	0.05955
Stomach cancer	1714	TICAM1-TICAM2-TLR4 complex	0.04862
Stomach cancer	1729	TLE1 corepressor complex (MASH1 promoter-corepressor complex)	0.02663
Stomach cancer	1748	PRMT5 complex	0.08422
Stomach cancer	1826	SMAD3-HEF1-APC10-CDH1 complex	0.04211
Stomach cancer	1827	PML-SMAD2/3-SARA complex	0.04211
Stomach cancer	1828	TGF-beta receptor I-Axin-SMAD3 complex	0.04862
Stomach cancer	1831	PIAS3-SMAD3-P300 complex	0.04862
Stomach cancer	1944	IRAK1-IRAK2 complex	0.05955
Stomach cancer	1945	IRAK1-IRAK3 complex	0.05955
Stomach cancer	1982	CACY homodimer complex	0.08422
Stomach cancer	1992	LEPR homodimer complex	0.08422
Stomach cancer	2016	IL12A homodimer complex	0.08422
Stomach cancer	2018	IL12A-IL12B complex	0.1191
Stomach cancer	2019	IL12A-IL12B-IL12RB1 complex	0.09724
Stomach cancer	2020	IL12B-IL12RB1-IL12RB2 complex	0.04862
Stomach cancer	2021	IL12A-IL12B-IL12RB2 complex	0.09724
Stomach cancer	2051	MALT1 oligomer complex	0.08422
Stomach cancer	2053	BCL10-MALT1 complex	0.05955
Stomach cancer	2054	CASP8-FADD-MALT1-BCL10 complex	0.04211
Stomach cancer	2055	CASP8-CHUK-IKBKB-MALT1-BCL10 complex	0.03766
Stomach cancer	2187	Ubiquitin E3 ligase (NFKBIA FBXW11 BTRC CUL1 SKP1A)	0.03766
Stomach cancer	2188	Ubiquitin E3 ligase (CDC34 NEDD8 BTRC CUL1 SKP1A RBX1)	0.03766

Stomach cancer	2189	Ubiquitin E3 ligase (SMAD3 BTRC CUL1 SKP1A RBX1)	0.07532
Stomach cancer	2198	RAD9-RAD1-HUS1-POLB complex	0.04211
Stomach cancer	2217	MDC1-MRN-ATM-FANCD2 complex	0.06876
Stomach cancer	2218	MDC1-MRE11-RAD50-NBS1 complex	0.08422
Stomach cancer	2222	BLM complex II	0.03438
Stomach cancer	2223	BLM-TOP3A complex	0.05955
Stomach cancer	2224	MSH2/6-BLM-p53-RAD51 complex	0.03766
Stomach cancer	2228	BLM-RAD51L3-XRCC2 complex	0.04862
Stomach cancer	2254	CTGF/Hcs24-actin complex	0.04862
Stomach cancer	2416	ITGB1-RAP1A-PKD1 complex	0.04862
Stomach cancer	2429	ITGA2-ITGB1-CD47 complex	0.04862
Stomach cancer	2430	ITGA2-ITGB1-CHAD complex	0.04862
Stomach cancer	2431	ITGA2-ITGB1-COL6A3 complex	0.04862
Stomach cancer	2432	ITGA2-ITGB1 complex	0.05955
Stomach cancer	2489	NCR3-CD247 complex	0.05955
Stomach cancer	2536	PLC-gamma-2-SLP-76-Lyn-Grb2 complex	0.04211
Stomach cancer	2537	PKC-alpha-PLD1-PLC-gamma-2 signaling complex lacritin stimulated	0.04862
Stomach cancer	2692	SMAD3-SMAD4-cJun-cFos complex	0.04211
Stomach cancer	2705	SMAD3-SMAD4-CTCF protein-DNA complex	0.04862
Stomach cancer	2706	SMAD3-SMAD4-SP1 complex	0.04862
Stomach cancer	2707	SMAD3-SMAD4-FOXO3-FOXG1 complex	0.04211
Stomach cancer	2708	SMAD3-SMAD4-cJUN complex	0.04862
Stomach cancer	2709	MMP-9-TIMP-1-LRP complex	0.04862
Stomach cancer	2724	Ubiquitin E3 ligase (NFKBIA BTRC CUL1 SKP1A)	0.04211
Stomach cancer	2739	FA complex (Fanconi anemia complex)	0.02539
Stomach cancer	2743	TRAF6-MALT1 complex	0.05955
Stomach cancer	2744	TRAF2-MALT1 complex	0.05955
Stomach cancer	2745	Ubiquitin ligase complex (TRAF6 TAB2 MALT1 UEV1A BCL10)	0.03766
Stomach cancer	2752	CARMA1-BCL10-MALT1 complex	0.04862
Stomach cancer	2753	FYB-CARMA1-BCL-10-MALT1 complex	0.04211
Stomach cancer	2754	JUND-FOSB-SMAD3-SMAD4 complex	0.04211
Stomach cancer	2760	SMAD3-SMAD4-FOXO3 complex	0.04862
Stomach cancer	2761	SMAD3-SMAD4-FOXO1 complex	0.04862
Stomach cancer	2762	SMAD3-SMAD4-FOXO4 complex	0.04862
Stomach cancer	2766	TERF2-RAP1 complex	0.09549
Stomach cancer	2767	RAD50-MRE11-NBN-p200-p350 complex	0.09724
Stomach cancer	2776	RAD50-BRCA1 complex	0.05955
Stomach cancer	2813	BRCA1-SMAD3 complex	0.05955
Stomach cancer	2815	BRCA1-BARD1-BACH1-DNA damage complex II	0.05955
Stomach cancer	2829	RSmad complex	0.02663
Stomach cancer	2830	TIF1gamma-SMAD2-SMAD3 complex	0.04862
Stomach cancer	2834	SMAD4-SMAD2-SMAD3 complex	0.04862
Stomach cancer	2838	AR coactivator complex	0.04862
Stomach cancer	2880	SCF subcomplex (WEE1 SKP2 BTRC)	0.04862
Stomach cancer	2909	PLC-gamma-2-Syk-LAT-FcR-gamma complex	0.04211

Stomach cancer	2910	PLC-gamma-2-Lyn-FcR-gamma complex	0.04862
Stomach cancer	2912	PLC-gamma-2-SLP-76 complex	0.05955
Stomach cancer	2913	PLC-gamma-2-LAT complex	0.05955
Stomach cancer	2968	Axin-SMAD3 complex	0.05955
Stomach cancer	2975	SMAD3-E2F4/5-p107-DP1 complex	0.03766
Stomach cancer	3055	Nop56p-associated pre-rRNA complex	0.04129
Stomach cancer	3063	Brg1-associated complex II	0.03183
Stomach cancer	3162	TF-FVIIa-FXa-TFPI complex	0.04211
Stomach cancer	3199	SMAD3-SKI complex	0.05955
Stomach cancer	3205	SMAD3-SKI-NCOR complex	0.04862
Stomach cancer	3733	SKI-SMAD3 hexameric complex	0.05955
Stomach cancer	3740	SKI-SMAD3-SMAD4 pentameric complex	0.04862
Stomach cancer	3750	CREBBP-SMAD3 hexameric complex	0.05955
Stomach cancer	3754	CREBBP-SMAD3-SMAD4 pentameric complex	0.04862
Stomach cancer	3959	SMAD3-SMAD4-cSKI TGF(beta)-dependent	0.04862
Stomach cancer	3961	SMAD3-cSKI-SIN3A-HDAC1 complex	0.04211
Stomach cancer	3971	SMURF2-SMAD3 complex TGF(beta)-dependent	0.05955
Stomach cancer	3972	SMURF2-SMAD3-SnoN complex TGF(beta)-	0.04862
Stomach cancer	5177	Polycystin-1 multiprotein complex (ACTN1 CDH1 SRC JUP VCL CTNNB1 PXN BCAR1 PKD1	0.02539
Stomach cancer	5190	TIAM1-EFNB1-EPHA2 complex	0.04862
Stomach cancer	5196	TNF-alpha/NF-kappa B signaling complex (CHUK BTRC NFKB2 PPP6C REL CUL1 IKBKE SAPS2 SAPS1 ANKRD28 RELA SKP1)	0.02431
Stomach cancer	5197	PTIP-DNA damage response complex	0.10314
Stomach cancer	5211	RAF1-PPP2-PIN1 complex	0.03766
Stomach cancer	5220	CHUK-IQGAP2-AKAP8L-RELA-TNIP2 complex	0.03766
Stomach cancer	5232	TNF-alpha/Nf-kappa B signaling complex (RPL6 RPL30 RPS13 CHUK DDX3X NFKB2 NFKBIB REL IKBKG NFKB1 MAP3K8 RELB GLG1 NFKBIA RELA TNIP2 GTF2I)	0.04085
Stomach cancer	5233	TNF-alpha/NF-kappa B signaling complex 5	0.01684
Stomach cancer	5266	TNF-alpha/NF-kappa B signaling complex 6	0.04501
Stomach cancer	5380	TRBP containing complex (DICER RPL7A EIF6 MOV10 and subunits of the 60S ribosomal particle)	0.01684
Stomach cancer	5383	TRIB3-DDIT3 complex	0.05955
Stomach cancer	5548	IL-12 heterodimer complex	0.1191
Stomach cancer	5549	IL-12 subunit p40 homodimer complex	0.08422
Stomach cancer	5646	FARP2-NRP1-PlexinA1 complex	0.04862
Stomach cancer	5668	PlexinA1-Nrp1 complex	0.05955
Stomach cancer	5673	PlexinA1-Nrp2 complex	0.05955
Stomach cancer	5689	SEMA6D-PlexinA1-NRP1 complex	0.04862
Stomach cancer	5735	TGF-beta receptor-SMAD3 complex	0.04862
Stomach cancer	5745	PlexinA1-NRP1 complex	0.05955
Stomach cancer	5746	PlexinA1-NRP1-SEMA3A complex	0.04862
Stomach cancer	5762	CRMP-MICAL-PlexinA1 complex induced by	0.04862
Stomach cancer	5770	RUNX1-CBF-beta-DNA complex	0.1191
Stomach cancer	5823	MCL1-BAK1 complex	0.05955

Stomach disease	1	BCL6-HDAC4 complex	0.25
Stomach disease	2	BCL6-HDAC5 complex	0.25
Stomach disease	3	BCL6-HDAC7 complex	0.25
Stomach disease	41	Mi-2/NuRD-MTA2 complex	0.15811
Stomach disease	585	Mi2/NuRD-BCL6-MTA3 complex	0.15811
Stomach disease	1508	BCL6-ZBTB17 complex	0.25
Stomach disease	1772	MICB-KLRK1-HCST complex	0.20412
Stroke	103	RNA polymerase II holoenzyme complex	0.02311
Stroke	107	TFIIH transcription factor complex	0.03774
Stroke	120	Lymphotoxin beta receptor complex	0.06537
Stroke	159	Condensin I-PARP-1-XRCC1 complex	0.0428
Stroke	212	DNA ligase III-XRCC1 complex	0.08006
Stroke	213	DNA ligase IV-XRCC1 complex	0.08006
Stroke	298	VEGF transcriptional complex	0.09245
Stroke	362	DNA ligase III-XRCC1-PNK-DNA-pol III multiprotein complex	0.05661
Stroke	441	TFTC-type histone acetyl transferase complex	0.03414
Stroke	541	IGF1-IGFBP3-ALS complex	0.06537
Stroke	725	P2X7 receptor signalling complex	0.03269
Stroke	1009	TFIIH transcription factor complex	0.03581
Stroke	1029	TFIIH transcription factor complex	0.03581
Stroke	1030	CAK-ERCC2 complex	0.05661
Stroke	1054	ESR1-RELA-BCL3-NCOA3 complex	0.11323
Stroke	1062	BAR-BCL2-CASP8 complex	0.06537
Stroke	1069	FIF-FGR2 complex	0.08006
Stroke	1085	DNA repair complex NEIL2-PNK-Pol(beta)-LigIII(alpha)-XRCC1	0.05064
Stroke	1086	DNA repair complex NEIL1-PNK-Pol(beta)-LigIII(alpha)-XRCC1	0.05064
Stroke	1095	SNX complex (SNX1a SNX2 SNX4 EGFR)	0.05661
Stroke	1185	EGFR-containing signaling complex	0.05661
Stroke	1439	PTGS2 homodimer complex	0.11323
Stroke	1514	IL4-IL4R complex	0.08006
Stroke	1515	IL4-IL4R-IL2RG complex	0.06537
Stroke	1714	TICAM1-TICAM2-TLR4 complex	0.06537
Stroke	5721	CIN85-CBL-SH3GL2 complex	0.06537
Stroke	5713	SH3P2/OSTF1-CBL-SRC complex	0.06537
Stroke	2028	JAK2-IL12RB2 complex	0.08006
Stroke	2084	NFKB1-NFKB2-REL-RELA-RELB complex	0.05064
Stroke	2086	NFKB1-NFKB2-RELA-RELB complex	0.05661
Stroke	2105	IkappaB kinase complex (IKBKB CHUK IKBKAP NFKBIA RELA MAP3K14)	0.04623
Stroke	2124	IKK-alpha--ER-alpha-AIB1 complex	0.06537
Stroke	2153	ITGAM-ITGB2-CD11 complex	0.06537
Stroke	2355	ITGAV-ITGB3-CD47-FCER2 complex	0.05661
Stroke	2356	ITGB3-ITGAV-CD47 complex	0.06537
Stroke	2358	ITGAV-ITGB3-SPP1 complex	0.06537
Stroke	2359	ITGAV-ITGB3-ADAM15 complex	0.06537

Stroke	2362	ITAGV-ITGB3-F11R complex	0.06537
Stroke	2363	ITGAV-ITGB3-PXN-PTK2b complex	0.05661
Stroke	2364	ITGAV-ITGB3-ADAM23 complex	0.06537
Stroke	2365	ITGAV-ITGB3-COL4A3 complex	0.06537
Stroke	2366	ITGAV-ITGB3-PPAP2b complex	0.06537
Stroke	2369	ITGAV-ITGB3-EGFR complex	0.13074
Stroke	2370	ITGA2b-ITGB3-CD9 complex	0.13074
Stroke	2374	ITGAV-ITGB3-LAMA4 complex	0.06537
Stroke	2376	ITGA2B-ITGB3-FN1-TGM2 complex	0.11323
Stroke	2377	ITGA2b-ITGB3-CD47-SRC complex	0.11323
Stroke	2378	ITGA2b-ITGB3-TLN1 complex	0.13074
Stroke	2379	ITGA2B-ITGB3-CIB1 complex	0.13074
Stroke	2381	ITGA2B-ITGB3 complex	0.16013
Stroke	2382	ITGA2B-ITGB3-F11R complex	0.13074
Stroke	2429	ITGA2-ITGB1-CD47 complex	0.06537
Stroke	2430	ITGA2-ITGB1-CHAD complex	0.06537
Stroke	2431	ITGA2-ITGB1-COL6A3 complex	0.06537
Stroke	2432	ITGA2-ITGB1 complex	0.08006
Stroke	2453	Multiprotein complex (monoubiquitination)	0.05661
Stroke	2454	CIN85-CBL-SH3GL2-EGFR complex EGF stimulated	0.11323
Stroke	2456	MET-CIN85-SH3GL3-CBL complex HGF stimulated	0.05661
Stroke	2470	p130Cas-ER-alpha-cSrc-kinase- PI3-kinase p85-subunit complex	0.05661
Stroke	2480	CIN85 complex (CIN85 CRK BCAR1 CBL PIK3R1 GRB2 SOS1)	0.0428
Stroke	5709	ArgBP2a-CBL-PTK2B complex	0.06537
Stroke	5720	CIN85-CBL complex	0.08006
Stroke	2529	LAT-PLC-gamma-1-p85-GRB2-CBL-VAV-SLP-76 signaling complex C305 activated	0.0428
Stroke	2534	Cbl-SLP-76-Grb2 complex Fc receptor gamma-R1 stimulated	0.06537
Stroke	2535	SLP-76-Cbl-Grb2-Shc complex Fc receptor gamma-R1 stimulated	0.05661
Stroke	2542	EGFR-CBL-GRB2 complex	0.13074
Stroke	2563	FGFR2-c-Cbl-Lyn-Fyn complex	0.05661
Stroke	2657	ESR1-CDK7-CCNH-MNAT1-MTA1-HDAC2 complex	0.04623
Stroke	2660	ERCC2/CAK complex	0.05661
Stroke	2670	Er-alpha-p53-hdm2 complex	0.06537
Stroke	2699	ER-alpha-GRIP1-c-Jun complex	0.06537
Stroke	2700	ER-alpha-c-Jun complex	0.08006
Stroke	2709	MMP-9-TIMP-1-LRP complex	0.06537
Stroke	2808	RAD9-RAD1-HUS1-APE1 complex	0.05661
Stroke	2816	ITGAV-ITGB3 complex	0.08006
Stroke	2825	BRCA1-RNA polymerase II complex	0.02221
Stroke	2826	ITGB3-ITGAV-VTN complex	0.06537
Stroke	2846	ITGAV-ITGB3-THBS1 complex	0.06537
Stroke	2849	ITGAV-ITGB3-NOV complex	0.06537
Stroke	2872	ITGA2b-ITGB3-CD9-GP1b-CD47 complex	0.09245

Stroke	2882	ITGA5-ITGB3-COL6A3 complex	0.06537
Stroke	2896	ITGA2b-ITGB3-CD47-FAK complex	0.11323
Stroke	2956	PLC-gamma-1-LAT-c-CBL complex OKT3 stimulated	0.06537
Stroke	3045	hs4 enhancer complex (faster migrating complex)	0.05064
Stroke	3103	ITGAV-ITGB3-SLC3A2 complex	0.06537
Stroke	3115	ITGA2B-ITGB3-ICAM4 complex	0.13074
Stroke	3175	CIN85-c-CBL complex	0.08006
Stroke	3678	RIN1-STAM2-EGFR complex EGF stimulated	0.06537
Stroke	4997	p97/VCP-VIMP-DERL1 complex	0.06537
Stroke	4998	p97/VCP-VIMP-DERL2 complex	0.06537
Stroke	4999	p97/VCP-VIMP-DERL1-DERL2-HRD1-SEL1L	0.04623
Stroke	5171	SH3KBP1-CBLB-EGFR complex	0.06537
Stroke	5178	JAK2-PAFR-TYK2 complex	0.06537
Stroke	5193	TNF-alpha/NF-kappa B signaling complex (CHUK KPNA3 NFKB2 NFKBIB REL IKBKG NFKB1 NFKBIE RELB NFKBIA RELA TNIP2)	0.03269
Stroke	5194	TNF-alpha/NF-kappa B signaling complex (SEC16A CHUK IKBKB NFKB2 REL IKBKG MAP3K14 RELA FBXW7 USP2)	0.03581
Stroke	5196	TNF-alpha/NF-kappa B signaling complex (CHUK BTRC NFKB2 PPP6C REL CUL1 IKBKE SAPS2 SAPS1 ANKRD28 RELA SKP1)	0.03269
Stroke	5220	CHUK-IQGAP2-AKAP8L-RELA-TNIP2 complex	0.05064
Stroke	5228	REL-MAP3K8-RELA-TNIP2-PAPOLA complex	0.05064
Stroke	5230	CHUK-NFKB2-REL-IKBKG-SPAG9-NFKB1-NFKBIE-COPB2-TNIP1-NFKBIA-RELA-TNIP2	0.03269
Stroke	5232	TNF-alpha/Nf-kappa B signaling complex (RPL6 RPL30 RPS13 CHUK DDX3X NFKB2 NFKBIB REL IKBKG NFKB1 MAP3K8 RELB GLG1 NFKBIA RELA TNIP2 GTF2I)	0.02746
Stroke	5233	TNF-alpha/NF-kappa B signaling complex 5	0.02265
Stroke	5243	XRCC1-LIG3-PNK-TDP1 complex	0.05661
Stroke	5273	VHL-TBP1-HIF1A complex	0.06537
Stroke	5276	HIF1A-OS9-EGLN1 complex	0.06537
Stroke	5277	HIF1A-OS9-EGLN3 complex	0.06537
Stroke	5382	ARNT-HIF1A complex	0.08006
Stroke	5389	SERPINA3-CTSG complex	0.08006
Stroke	5414	HTR1A-HTR1D complex	0.08006
Stroke	5416	HTR1A-HTR1B complex	0.08006
Stroke	5418	GABBR2-HTR1A complex	0.08006
Stroke	5419	HTR1A-GPR26 complex	0.08006
Stroke	5420	HTR1A-EDG3 complex	0.08006
Stroke	5421	HTR1A homodimer complex	0.11323
Stroke	5422	HTR1A-EDG1 complex	0.08006
Stroke	5460	p50-p65 NF(kappa)B complex	0.08006
Stroke	5461	p50-p65 NF(kappa)B-SRC1 complex	0.06537
Stroke	5464	I(kappa)B(alpha)-NF(kappa)Bp50-NF(kappa)Bp65 complex	0.06537
Stroke	5465	IKB(epsilon)-RELA-cREL complex	0.06537

Stroke	5466	IKB(beta)-RELA-cREL complex	0.06537
Stroke	5467	IKB(alpha)-RELA-cREL complex	0.06537
Stroke	5473	FAS-FADD-CASP8 complex	0.06537
Stroke	5475	MURR1-NF(kappa)Bp65-IKBA complex	0.06537
Stroke	5492	IKBA-NF(kappa)Bp65-NF(kappa)Bp50 complex	0.06537
Stroke	5495	TFIIH transcription factor complex (ERCC2 ERCC3 GTF2H1 CDK7 CCNH GTF2H2)	0.04623
Stroke	5526	CALM1-FKBP38-BCL2 complex	0.06537
Stroke	5684	Membrane protein complex (DERL1 SELS VCP)	0.06537
Stroke	5724	CIN85-SH3GL3-CBL complex	0.06537
Stroke	5799	Death induced signaling complex DISC (FAS FADD CASP8 CFLAR) membrane-associated CD95L	0.05661
Stroke	5800	Death-inducing signaling complex DISC (type I cells associated) stimulated	0.06537
Stroke	5808	DISC complex	0.06537
Stroke	5811	p53-BCL2 complex	0.08006
Stroke	5817	tBID-BCL2 complex	0.08006
Stroke	5818	BIM-BCL2 complex	0.08006
Stroke	5859	FAS-FADD-CASP8-CASP10 complex	0.05661
Stroke	5861	FAS-FADD-CASP10 complex	0.06537
Stroke	5862	CAV1-VDAC1-ESR1 complex	0.06537
Subacute sclerosing panencephalitis	2709	MMP-9-TIMP-1-LRP complex	0.57735
Subarachnoid hemorrhage	668	BKCA-beta2AR-AKAP79 signaling complex	0.17408
Subarachnoid hemorrhage	672	BKCA-beta2AR complex	0.2132
Subarachnoid hemorrhage	687	CFTR-NHERF-beta(2)AR signaling complex	0.17408
Subarachnoid hemorrhage	879	PRKAC-AKAP5-ADRB1 complex	0.13484
Subarachnoid hemorrhage	1088	PRNP-ApolopoproteinE3 complex	0.2132
Subarachnoid hemorrhage	2355	ITGAV-ITGB3-CD47-FCER2 complex	0.15076
Subarachnoid hemorrhage	2356	ITGB3-ITGAV-CD47 complex	0.17408
Subarachnoid hemorrhage	2358	ITGAV-ITGB3-SPP1 complex	0.17408
Subarachnoid hemorrhage	2359	ITGAV-ITGB3-ADAM15 complex	0.17408
Subarachnoid hemorrhage	2362	ITAGV-ITGB3-F11R complex	0.17408
Subarachnoid hemorrhage	2363	ITGAV-ITGB3-PXN-PTK2b complex	0.15076
Subarachnoid hemorrhage	2364	ITGAV-ITGB3-ADAM23 complex	0.17408
Subarachnoid hemorrhage	2365	ITGAV-ITGB3-COL4A3 complex	0.17408
Subarachnoid hemorrhage	2366	ITGAV-ITGB3-PPAP2b complex	0.17408
Subarachnoid hemorrhage	2369	ITGAV-ITGB3-EGFR complex	0.17408
Subarachnoid hemorrhage	2370	ITGA2b-ITGB3-CD9 complex	0.17408
Subarachnoid hemorrhage	2374	ITGAV-ITGB3-LAMA4 complex	0.17408
Subarachnoid hemorrhage	2376	ITGA2B-ITGB3-FN1-TGM2 complex	0.15076
Subarachnoid hemorrhage	2377	ITGA2b-ITGB3-CD47-SRC complex	0.15076
Subarachnoid hemorrhage	2378	ITGA2b-ITGB3-TLN1 complex	0.17408
Subarachnoid hemorrhage	2379	ITGA2B-ITGB3-CIB1 complex	0.17408
Subarachnoid hemorrhage	2381	ITGA2B-ITGB3 complex	0.2132
Subarachnoid hemorrhage	2382	ITGA2B-ITGB3-F11R complex	0.17408
Subarachnoid hemorrhage	2816	ITGAV-ITGB3 complex	0.2132
Subarachnoid hemorrhage	2826	ITGB3-ITGAV-VTN complex	0.17408

Subarachnoid hemorrhage	2846	ITGAV-ITGB3-THBS1 complex	0.17408
Subarachnoid hemorrhage	2849	ITGAV-ITGB3-NOV complex	0.17408
Subarachnoid hemorrhage	2872	ITGA2b-ITGB3-CD9-GP1b-CD47 complex	0.12309
Subarachnoid hemorrhage	2882	ITGA5-ITGB3-COL6A3 complex	0.17408
Subarachnoid hemorrhage	2896	ITGA2b-ITGB3-CD47-FAK complex	0.15076
Subarachnoid hemorrhage	3103	ITGAV-ITGB3-SLC3A2 complex	0.17408
Subarachnoid hemorrhage	3115	ITGA2B-ITGB3-ICAM4 complex	0.17408
Subarachnoid hemorrhage	3830	ADRB2 homodimer complex	0.30151
Subarachnoid hemorrhage	4869	beta(1)-AR receptosome (ADRB1-SAP97-AKAP79-PRKAR2A)	0.15076
Sudden infant death syndrome	422	Beta-dystroglycan-caveolin-3 complex	0.1543
Sudden infant death syndrome	520	KCNQ1 macromolecular complex	0.06901
Sudden infant death syndrome	1042	SRA-SRC-1 ribonucleoprotein complex	0.1543
Sudden infant death syndrome	5414	HTR1A-HTR1D complex	0.1543
Sudden infant death syndrome	5416	HTR1A-HTR1B complex	0.1543
Sudden infant death syndrome	5418	GABBR2-HTR1A complex	0.1543
Sudden infant death syndrome	5419	HTR1A-GPR26 complex	0.1543
Sudden infant death syndrome	5420	HTR1A-EDG3 complex	0.1543
Sudden infant death syndrome	5421	HTR1A homodimer complex	0.21822
Sudden infant death syndrome	5422	HTR1A-EDG1 complex	0.1543
Synovial sarcoma	1729	TLE1 corepressor complex (MASH1 promoter-corepressor complex)	0.15811
Synovial sarcoma	3120	OCT1-OBF1-DNA-TLE1 complex	0.28868
Synovial sarcoma	3124	TLE1-TLE2 complex	0.35355
Synovial sarcoma	3125	TLE1 homodimer complex	0.5
Synovial sarcoma	3127	TLE-Histone H3 complex	0.18898
Synovial sarcoma	3131	Hes1-TLE1 complex	0.35355
Synovial sarcoma	3149	NK-3-Groucho-HIPK2-SIN3A-RbpA48-HDAC1	0.15076
Synovial sarcoma	3150	NK-3-Groucho complex	0.18898
Synovial sarcoma	3164	HESX1-TLE1 complex	0.35355
Synovitis	441	TFTC-type histone acetyl transferase complex	0.1066
Synovitis	1054	ESR1-RELA-BCL3-NCOA3 complex	0.17678
Synovitis	1170	cMYC-ATPase-helicase complex	0.15811
Synovitis	1171	c-MYC-ATPase-helicase complex	0.15811
Synovitis	2124	IKK-alpha--ER-alpha-AIB1 complex	0.20412
Synovitis	2470	p130Cas-ER-alpha-cSrc-kinase- PI3-kinase p85-subunit complex	0.17678
Synovitis	2649	MYC-DNMT3A-ZBTB17 complex	0.20412
Synovitis	2653	MYC-MAX-BLOC1S1 complex	0.20412
Synovitis	2655	MYC-MAX complex	0.25
Synovitis	2657	ESR1-CDK7-CCNH-MNAT1-MTA1-HDAC2 complex	0.14434
Synovitis	2670	Er-alpha-p53-hdm2 complex	0.20412
Synovitis	2699	ER-alpha-GRIP1-c-Jun complex	0.20412
Synovitis	2700	ER-alpha-c-Jun complex	0.25
Synovitis	5862	CAV1-VDAC1-ESR1 complex	0.20412
Systemic infection	1094	Frataxin complex	0.04336
Systemic infection	1707	IL2-IL2RA-IL2RB complex	0.06623
Systemic infection	1714	TICAM1-TICAM2-TLR4 complex	0.06623

Systemic infection	5718	eNOS-HSP90-AKT complex VEGF induced	0.06623
Systemic infection	1810	ITGA4-PXN-GIT1 complex	0.06623
Systemic infection	2084	NFKB1-NFKB2-REL-RELA-RELB complex	0.0513
Systemic infection	2086	NFKB1-NFKB2-RELA-RELB complex	0.05735
Systemic infection	2153	ITGAM-ITGB2-CD11 complex	0.06623
Systemic infection	2156	YBX1-AKT1 complex	0.08111
Systemic infection	2159	AR-AKT-APPL complex	0.06623
Systemic infection	2383	ITGA5-ITGB1-FN1-TGM2 complex	0.05735
Systemic infection	2384	ITGA5-ITGB1-ADAM15 complex	0.06623
Systemic infection	2385	ITGA5-ITGB4 complex	0.08111
Systemic infection	2388	Itga5-Itgb1-Fn1-Sfrp2 complex	0.05735
Systemic infection	2390	CD98-LAT2-ITGB1 complex	0.05735
Systemic infection	2395	ITGA7-ITGB1-CD151 complex	0.06623
Systemic infection	2396	ITGA7-ITGB1-CD9 complex	0.06623
Systemic infection	2397	ITGA7-ITGB1-ITGB1BP3 complex	0.06623
Systemic infection	2398	ITGA3-ITGB1-BSG complex	0.06623
Systemic infection	2399	ITGA3-ITGB1-CD63 complex	0.06623
Systemic infection	2400	ITGA3-ITGB1-CD151 complex	0.06623
Systemic infection	2401	ITGA3-ITGB1-THBS1 complex	0.06623
Systemic infection	2406	ITGA3-ITGB1 complex	0.08111
Systemic infection	2411	ITGA6-ITGB1-CD151 complex	0.06623
Systemic infection	2413	ITGA6-ITGB1 complex	0.08111
Systemic infection	2416	ITGB1-RAP1A-PKD1 complex	0.06623
Systemic infection	2417	ITGA4-ITGB1-EMILIN1 complex	0.13245
Systemic infection	2418	ITGA4-ITGB1 complex	0.16222
Systemic infection	2419	ITGA4-ITGB1-CD81 complex	0.13245
Systemic infection	2420	ITGA4-ITGB1-CD53 complex	0.13245
Systemic infection	2421	ITGA4-ITGB1-VCAM1 complex	0.13245
Systemic infection	2422	ITGA4-ITGB1-JAM2 complex	0.13245
Systemic infection	2423	ITGA4-ITGB1-CD47 complex	0.13245
Systemic infection	2424	ITGA4-ITGB1-CD63 complex	0.13245
Systemic infection	2425	ITGA4-ITGB1-PXN complex	0.13245
Systemic infection	2426	ITGA4-ITGB1-THBS1 complex	0.13245
Systemic infection	2428	ITGA4-ITGB1-THBS2 complex	0.13245
Systemic infection	2429	ITGA2-ITGB1-CD47 complex	0.06623
Systemic infection	2430	ITGA2-ITGB1-CHAD complex	0.06623
Systemic infection	2431	ITGA2-ITGB1-COL6A3 complex	0.06623
Systemic infection	2432	ITGA2-ITGB1 complex	0.08111
Systemic infection	2434	ITGA1-ITGB1-COL6A3 complex	0.06623
Systemic infection	2435	ITGA1-ITGB1-PTPN2 complex	0.06623
Systemic infection	2436	ITGAV-ITGB1 complex	0.08111
Systemic infection	2437	ITGA6-ITGB1-CYR61 complex	0.06623
Systemic infection	2439	ITGA8-ITGB1 complex	0.08111
Systemic infection	2440	ITGA9-ITGB1-ADAM9 complex	0.06623
Systemic infection	2441	Itga9-Itgb1-Adam2 complex	0.06623
Systemic infection	2442	ITGA9-ITGB1-VCAM1 complex	0.06623
Systemic infection	2443	ITGA9-ITGB1-TNC complex	0.06623

Systemic infection	2444	ITGB1-ITGA9 complex	0.08111
Systemic infection	2445	ITGA9-ITGB1-ADAM15 complex	0.06623
Systemic infection	2446	ITGA9-ITGB1-FIGF complex	0.06623
Systemic infection	2447	ITGA9-ITGB1-ADAM12 complex	0.06623
Systemic infection	2709	MMP-9-TIMP-1-LRP complex	0.13245
Systemic infection	2755	17S U2 snRNP	0.01997
Systemic infection	2798	MMP-2-claudin-1 complex	0.08111
Systemic infection	2850	ITGA5-ITGB1-FN-1-NOV complex	0.05735
Systemic infection	2853	ITGA5-ITGB1-CAL4A3 complex	0.06623
Systemic infection	2885	ITGAV-ITGB1-SPP1 complex	0.06623
Systemic infection	2909	PLC-gamma-2-Syk-LAT-FcR-gamma complex	0.05735
Systemic infection	2910	PLC-gamma-2-Lyn-FcR-gamma complex	0.06623
Systemic infection	2964	ITGA9-ITGB1-ADAM1 complex	0.06623
Systemic infection	2965	ITGA9-ITGB1-ADAM3 complex	0.06623
Systemic infection	2971	ITGA9-ITGB1-VEGFC complex	0.06623
Systemic infection	2972	ITGA9-ITGB1-VEGFA complex	0.13245
Systemic infection	2989	ITGA9-ITGB1-ADAM8 complex	0.06623
Systemic infection	3035	LAT2-ITGB1 complex	0.08111
Systemic infection	3046	hs4 enhancer complex (slow migrating complex)	0.08111
Systemic infection	3057	ITGA10-ITGB1 complex	0.08111
Systemic infection	3058	ITGA11-ITGB1 complex	0.08111
Systemic infection	3059	ITGA11-ITGB1-COL1A1 complex	0.06623
Systemic infection	3104	ITGB1-NRP1 complex	0.08111
Systemic infection	3111	ITGA9-ITGB1-SPP1 complex	0.06623
Systemic infection	3112	ITGA5-ITGB1-SPP1 complex	0.06623
Systemic infection	3162	TF-FVIIa-FXa-TFPI complex	0.11471
Systemic infection	3847	TCL1(trimer)-AKT1 complex	0.08111
Systemic infection	4062	NRP1-VEGFR2-VEGF(165) complex	0.06623
Systemic infection	5193	TNF-alpha/NF-kappa B signaling complex (CHUK KPNA3 NFKB2 NFKBIB REL IKBKG NFKB1 NFKBIE RELB NFKBIA RELA TNIP2)	0.03311
Systemic infection	5232	TNF-alpha/Nf-kappa B signaling complex (RPL6 RPL30 RPS13 CHUK DDX3X NFKB2 NFKBIB REL IKBKG NFKB1 MAP3K8 RELB GLG1 NFKBIA RELA TNIP2 GTF2I)	0.02782
Systemic infection	5388	SERPINA1-ELA2 complex	0.08111
Systemic infection	5696	VEGFA(165)-KDR-NRP1 complex	0.06623
Systemic infection	5698	VEGFA(165)-VEGFR2-NRP1 complex	0.06623
Systemic infection	5701	NRP1-VEGF(165/121) complex	0.08111
Systemic scleroderma	4	Multisubunit ACTR coactivator complex	0.05241
Systemic scleroderma	98	p300-MDM2-p53 protein complex	0.06052
Systemic scleroderma	521	Polycystin-1-E-cadherin-beta-catenin complex	0.06052
Systemic scleroderma	522	Polycystin-1-E-cadherin-beta-catenin-Flotillin-2	0.05241
Systemic scleroderma	550	NOS3-CAV1-NOSTRIN complex	0.06052
Systemic scleroderma	557	TRP1-G alpha-11-IP3R3-CAV1 signaling complex	0.05241
Systemic scleroderma	570	p300-CBP-p270-SWI/SNF complex	0.03962
Systemic scleroderma	571	p300-CBP-p270 complex	0.06052
Systemic scleroderma	753	UTM-SGCE-DAG1-CAV1-NOS3 complex	0.04688

Systemic scleroderma	788	Exosome	0.03315
Systemic scleroderma	789	Exosome	0.06321
Systemic scleroderma	822	mRNA decay complex (UPF1 UPF2 UPF3B DCP2 XRN1 XRN2 EXOSC2 EXOSC4 EXOSC10 PARN)	0.03315
Systemic scleroderma	826	PAR-3-VE-cadherin-beta-catenin complex	0.06052
Systemic scleroderma	845	PCI-PSA-SCG2 complex	0.06052
Systemic scleroderma	1062	BAR-BCL2-CASP8 complex	0.06052
Systemic scleroderma	1069	FIF-FGR2 complex	0.07412
Systemic scleroderma	1093	SNX complex (SNX1a SNX2 SNX4 INSR)	0.05241
Systemic scleroderma	1158	p33ING1b-p300 complex	0.07412
Systemic scleroderma	1160	ING1-p300-PCNA complex	0.06052
Systemic scleroderma	1217	WRN-TRF2 complex	0.07412
Systemic scleroderma	1471	pRb2/p130-multimolecular complex (RB2 E2F5 HDAC1 SUV39H1 P300)	0.04688
Systemic scleroderma	1474	SMAD3/4-E2F4/5-p107-DP1 complex	0.0428
Systemic scleroderma	1514	IL4-IL4R complex	0.07412
Systemic scleroderma	1515	IL4-IL4R-IL2RG complex	0.06052
Systemic scleroderma	1521	p300-SMAD1-STAT3 complex	0.06052
Systemic scleroderma	1700	ABL2-HRAS-RIN1 complex	0.06052
Systemic scleroderma	1707	IL2-IL2RA-IL2RB complex	0.06052
Systemic scleroderma	1777	TGF-beta-receptor-SMAD7-SMURF2 complex	0.05241
Systemic scleroderma	1783	TGF-beta receptor I-SMAD7-SMURF1 complex	0.12105
Systemic scleroderma	5718	eNOS-HSP90-AKT complex VEGF induced	0.06052
Systemic scleroderma	1816	JUN-TCF4-CTNNB1 complex	0.06052
Systemic scleroderma	1826	SMAD3-HEF1-APC10-CDH1 complex	0.05241
Systemic scleroderma	1827	PML-SMAD2/3-SARA complex	0.05241
Systemic scleroderma	1828	TGF-beta receptor I-Axin-SMAD3 complex	0.06052
Systemic scleroderma	1831	PIAS3-SMAD3-P300 complex	0.12105
Systemic scleroderma	1839	SDCBP-CTNNB1-CTNNA1-CDH1 complex	0.05241
Systemic scleroderma	1986	Endoglin homodimer complex	0.10483
Systemic scleroderma	2004	C1D homodimer protein	0.10483
Systemic scleroderma	2054	CASP8-FADD-MALT1-BCL10 complex	0.05241
Systemic scleroderma	2055	CASP8-CHUK-IKBKB-MALT1-BCL10 complex	0.04688
Systemic scleroderma	2056	BCL10-CHUK-BCL10-IKBKB complex	0.05241
Systemic scleroderma	2156	YBX1-AKT1 complex	0.07412
Systemic scleroderma	2159	AR-AKT-APPL complex	0.06052
Systemic scleroderma	2189	Ubiquitin E3 ligase (SMAD3 BTRC CUL1 SKP1A RBX1)	0.04688
Systemic scleroderma	2254	CTGF/Hcs24-actin complex	0.06052
Systemic scleroderma	2300	Profilin 2 complex	0.03494
Systemic scleroderma	2456	MET-CIN85-SH3GL3-CBL complex HGF stimulated	0.05241
Systemic scleroderma	5714	eNOS-CAV1 complex	0.07412
Systemic scleroderma	2462	Caveolin-1 homodimer complex	0.10483
Systemic scleroderma	2541	HGF-Met complex	0.07412
Systemic scleroderma	2574	CD19-Vav-PI 3-kinase (p85 subunit) complex	0.06052
Systemic scleroderma	2577	Sam68-p85 P13K-IRS-1-IR signaling complex	0.05241
Systemic scleroderma	2638	HES1 promoter corepressor complex	0.0428
Systemic scleroderma	2639	HES1 promoter-Notch enhancer complex	0.02907

Systemic scleroderma	2641	p300/CBP-PCAF-MyoD complex	0.05241
Systemic scleroderma	2642	SMAD1-P300 complex	0.07412
Systemic scleroderma	2692	SMAD3-SMAD4-cJun-cFos complex	0.05241
Systemic scleroderma	2705	SMAD3-SMAD4-CTCF protein-DNA complex	0.06052
Systemic scleroderma	2706	SMAD3-SMAD4-SP1 complex	0.06052
Systemic scleroderma	2707	SMAD3-SMAD4-FOXO3-FOXG1 complex	0.05241
Systemic scleroderma	2708	SMAD3-SMAD4-cJUN complex	0.06052
Systemic scleroderma	2709	MMP-9-TIMP-1-LRP complex	0.12105
Systemic scleroderma	2754	JUND-FOSB-SMAD3-SMAD4 complex	0.05241
Systemic scleroderma	2760	SMAD3-SMAD4-FOXO3 complex	0.06052
Systemic scleroderma	2761	SMAD3-SMAD4-FOXO1 complex	0.06052
Systemic scleroderma	2762	SMAD3-SMAD4-FOXO4 complex	0.06052
Systemic scleroderma	2798	MMP-2-claudin-1 complex	0.07412
Systemic scleroderma	2813	BRCA1-SMAD3 complex	0.07412
Systemic scleroderma	2829	RSmad complex	0.03315
Systemic scleroderma	2830	TIF1gamma-SMAD2-SMAD3 complex	0.06052
Systemic scleroderma	2834	SMAD4-SMAD2-SMAD3 complex	0.06052
Systemic scleroderma	2954	Smad1-Notch1-p300-Pcaf complex	0.05241
Systemic scleroderma	2968	Axin-SMAD3 complex	0.07412
Systemic scleroderma	2975	SMAD3-E2F4/5-p107-DP1 complex	0.04688
Systemic scleroderma	2992	SMAD7-SMURF2 complex	0.07412
Systemic scleroderma	2996	SMAD7-SMURF1 complex	0.14825
Systemic scleroderma	2997	SMAD7-SMURF1-TGF-beta receptor complex	0.10483
Systemic scleroderma	2998	Axin-PP2A A-PP2A C-GSK3-beta-beta-catenin	0.05241
Systemic scleroderma	3004	APC-Axin-1-beta-catenin complex	0.06052
Systemic scleroderma	3044	SKI-NCOR1-SIN3A-HDAC1 complex	0.05241
Systemic scleroderma	3154	Notch2(N-TM)-Notch2(N-EC)-Delta complex	0.05241
Systemic scleroderma	3155	Bipartite complex (TFC4 CTNNB1)	0.07412
Systemic scleroderma	3162	TF-FVIIa-FXa-TFPI complex	0.05241
Systemic scleroderma	3166	AXIN-APC-betaCatenin-GSK3B complex	0.05241
Systemic scleroderma	3197	SMAD4-SNO-SKI complex	0.12105
Systemic scleroderma	3198	SMAD2-SKI complex	0.07412
Systemic scleroderma	3199	SMAD3-SKI complex	0.14825
Systemic scleroderma	3200	SMAD4-SKI complex	0.07412
Systemic scleroderma	3204	SMAD2-SKI-NCOR complex	0.06052
Systemic scleroderma	3205	SMAD3-SKI-NCOR complex	0.12105
Systemic scleroderma	3206	SMAD4-SKI-NCOR complex	0.06052
Systemic scleroderma	3270	Delta1 homodimer complex	0.10483
Systemic scleroderma	3271	Gamma-secretase-Delta1 complex	0.04688
Systemic scleroderma	3729	SKI-SMAD2 hexameric complex	0.07412
Systemic scleroderma	3733	SKI-SMAD3 hexameric complex	0.14825
Systemic scleroderma	3739	SKI-SMAD2-SMAD4 pentameric complex	0.06052
Systemic scleroderma	3740	SKI-SMAD3-SMAD4 pentameric complex	0.12105
Systemic scleroderma	3750	CREBBP-SMAD3 hexameric complex	0.07412
Systemic scleroderma	3754	CREBBP-SMAD3-SMAD4 pentameric complex	0.06052
Systemic scleroderma	3847	TCL1(trimer)-AKT1 complex	0.07412
Systemic scleroderma	3959	SMAD3-SMAD4-cSKI TGF(beta)-dependent	0.12105

Systemic scleroderma	3961	SMAD3-cSKI-SIN3A-HDAC1 complex	0.10483
Systemic scleroderma	3971	SMURF2-SMAD3 complex TGF(beta)-dependent	0.07412
Systemic scleroderma	3972	SMURF2-SMAD3-SnoN complex TGF(beta)-	0.12105
Systemic scleroderma	4082	Ku70/Ku86/Werner complex	0.06052
Systemic scleroderma	4095	Catulin (alpha) - catenin (beta) complex	0.07412
Systemic scleroderma	4096	Catenin (alpha) - catenin (beta) complex	0.07412
Systemic scleroderma	5772	ZO1-(beta)cadherin-(VE)cadherin-VEGFR2 complex	0.05241
Systemic scleroderma	5118	pRb2/p130-multimolecular complex (RB2 E2F4 HDAC1 SUV39H1 P300)	0.04688
Systemic scleroderma	5177	Polycystin-1 multiprotein complex (ACTN1 CDH1 SRC JUP VCL CTNNB1 PXN BCAR1 PKD1	0.03161
Systemic scleroderma	5230	CHUK-NFKB2-REL-IKBKG-SPAG9-NFKB1-NFKBIE-COPB2-TNIP1-NFKBIA-RELA-TNIP2	0.03026
Systemic scleroderma	5235	WRN-Ku70-Ku80-PARP1 complex	0.05241
Systemic scleroderma	5260	TCF4-CTNNB1-SUMO1-EP300-HADAC6 complex	0.09376
Systemic scleroderma	5261	TCF4-CTNNB1-EP300 complex	0.12105
Systemic scleroderma	5262	TCF4-CTNNB1 complex	0.07412
Systemic scleroderma	5264	TCF4-CTNNB1-CREBBP complex	0.06052
Systemic scleroderma	5274	Cell-cell junction complex (ARHGAP10-CTNNA1)	0.07412
Systemic scleroderma	5281	Cell-cell junction complex (CDH1-CTNNB1)	0.07412
Systemic scleroderma	5375	EGR-EP300 complex	0.07412
Systemic scleroderma	5389	SERPINA3-CTSG complex	0.07412
Systemic scleroderma	5391	SERPINA1-CTSG complex	0.07412
Systemic scleroderma	5473	FAS-FADD-CASP8 complex	0.12105
Systemic scleroderma	5691	TALL1 homo-oligomer complex	0.10483
Systemic scleroderma	5735	TGF-beta receptor-SMAD3 complex	0.06052
Systemic scleroderma	5749	MRIT complex	0.06052
Systemic scleroderma	5798	Death induced signaling complex II (FADD CASP8 CFLAR) cytosolic CD95L induced	0.06052
Systemic scleroderma	5799	Death induced signaling complex DISC (FAS FADD CASP8 CFLAR) membrane-associated CD95L	0.10483
Systemic scleroderma	5800	Death-inducing signaling complex DISC (type I cells associated) stimulated	0.12105
Systemic scleroderma	5808	DISC complex	0.12105
Systemic scleroderma	5822	MCL1-NOXA complex	0.07412
Systemic scleroderma	5830	DJ-1-SNCA complex high molecular weight complex	0.07412
Systemic scleroderma	5859	FAS-FADD-CASP8-CASP10 complex	0.10483
Systemic scleroderma	5861	FAS-FADD-CASP10 complex	0.06052
Systemic scleroderma	5862	CAV1-VDAC1-ESR1 complex	0.06052
Systemic scleroderma	5922	RAF1-RAS complex EGF induced	0.05241
Takayasu's arteritis	1774	MICA-KLRK1-HCST complex	0.18257
Takayasu's arteritis	2355	ITGAV-ITGB3-CD47-FCER2 complex	0.15811
Takayasu's arteritis	2356	ITGB3-ITGAV-CD47 complex	0.18257
Takayasu's arteritis	2358	ITGAV-ITGB3-SPP1 complex	0.18257
Takayasu's arteritis	2359	ITGAV-ITGB3-ADAM15 complex	0.18257
Takayasu's arteritis	2362	ITAGV-ITGB3-F11R complex	0.18257
Takayasu's arteritis	2363	ITGAV-ITGB3-PXN-PTK2b complex	0.15811
Takayasu's arteritis	2364	ITGAV-ITGB3-ADAM23 complex	0.18257

Takayasu's arteritis	2365	ITGAV-ITGB3-COL4A3 complex	0.18257
Takayasu's arteritis	2366	ITGAV-ITGB3-PPAP2b complex	0.18257
Takayasu's arteritis	2369	ITGAV-ITGB3-EGFR complex	0.18257
Takayasu's arteritis	2370	ITGA2b-ITGB3-CD9 complex	0.36515
Takayasu's arteritis	2374	ITGAV-ITGB3-LAMA4 complex	0.18257
Takayasu's arteritis	2376	ITGA2B-ITGB3-FN1-TGM2 complex	0.31623
Takayasu's arteritis	2377	ITGA2b-ITGB3-CD47-SRC complex	0.31623
Takayasu's arteritis	2378	ITGA2b-ITGB3-TLN1 complex	0.36515
Takayasu's arteritis	2379	ITGA2B-ITGB3-CIB1 complex	0.36515
Takayasu's arteritis	2381	ITGA2B-ITGB3 complex	0.44721
Takayasu's arteritis	2382	ITGA2B-ITGB3-F11R complex	0.36515
Takayasu's arteritis	2709	MMP-9-TIMP-1-LRP complex	0.18257
Takayasu's arteritis	2798	MMP-2-claudin-1 complex	0.22361
Takayasu's arteritis	2816	ITGAV-ITGB3 complex	0.22361
Takayasu's arteritis	2826	ITGB3-ITGAV-VTN complex	0.18257
Takayasu's arteritis	2846	ITGAV-ITGB3-THBS1 complex	0.18257
Takayasu's arteritis	2849	ITGAV-ITGB3-NOV complex	0.18257
Takayasu's arteritis	2872	ITGA2b-ITGB3-CD9-GP1b-CD47 complex	0.2582
Takayasu's arteritis	2882	ITGA5-ITGB3-COL6A3 complex	0.18257
Takayasu's arteritis	2896	ITGA2b-ITGB3-CD47-FAK complex	0.31623
Takayasu's arteritis	3103	ITGAV-ITGB3-SLC3A2 complex	0.18257
Takayasu's arteritis	3115	ITGA2B-ITGB3-ICAM4 complex	0.36515
Temporal arteritis	1514	IL4-IL4R complex	0.31623
Temporal arteritis	1515	IL4-IL4R-IL2RG complex	0.2582
Temporal arteritis	2355	ITGAV-ITGB3-CD47-FCER2 complex	0.22361
Temporal arteritis	2356	ITGB3-ITGAV-CD47 complex	0.2582
Temporal arteritis	2358	ITGAV-ITGB3-SPP1 complex	0.2582
Temporal arteritis	2359	ITGAV-ITGB3-ADAM15 complex	0.2582
Temporal arteritis	2362	ITAGV-ITGB3-F11R complex	0.2582
Temporal arteritis	2363	ITGAV-ITGB3-PXN-PTK2b complex	0.22361
Temporal arteritis	2364	ITGAV-ITGB3-ADAM23 complex	0.2582
Temporal arteritis	2365	ITGAV-ITGB3-COL4A3 complex	0.2582
Temporal arteritis	2366	ITGAV-ITGB3-PPAP2b complex	0.2582
Temporal arteritis	2369	ITGAV-ITGB3-EGFR complex	0.2582
Temporal arteritis	2370	ITGA2b-ITGB3-CD9 complex	0.2582
Temporal arteritis	2374	ITGAV-ITGB3-LAMA4 complex	0.2582
Temporal arteritis	2376	ITGA2B-ITGB3-FN1-TGM2 complex	0.22361
Temporal arteritis	2377	ITGA2b-ITGB3-CD47-SRC complex	0.22361
Temporal arteritis	2378	ITGA2b-ITGB3-TLN1 complex	0.2582
Temporal arteritis	2379	ITGA2B-ITGB3-CIB1 complex	0.2582
Temporal arteritis	2381	ITGA2B-ITGB3 complex	0.31623
Temporal arteritis	2382	ITGA2B-ITGB3-F11R complex	0.2582
Temporal arteritis	2816	ITGAV-ITGB3 complex	0.31623
Temporal arteritis	2826	ITGB3-ITGAV-VTN complex	0.2582
Temporal arteritis	2846	ITGAV-ITGB3-THBS1 complex	0.2582
Temporal arteritis	2849	ITGAV-ITGB3-NOV complex	0.2582
Temporal arteritis	2872	ITGA2b-ITGB3-CD9-GP1b-CD47 complex	0.18257

Temporal arteritis	2882	ITGA5-ITGB3-COL6A3 complex	0.2582
Temporal arteritis	2896	ITGA2b-ITGB3-CD47-FAK complex	0.22361
Temporal arteritis	3103	ITGAV-ITGB3-SLC3A2 complex	0.2582
Temporal arteritis	3115	ITGA2B-ITGB3-ICAM4 complex	0.2582
Testicular dysfunction	159	Condensin I-PARP-1-XRCC1 complex	0.05399
Testicular dysfunction	212	DNA ligase III-XRCC1 complex	0.10102
Testicular dysfunction	213	DNA ligase IV-XRCC1 complex	0.10102
Testicular dysfunction	244	BRAFT complex	0.03962
Testicular dysfunction	246	BLM complex III	0.07143
Testicular dysfunction	280	HMGB1-HMGB2-HSC70-ERP60-GAPDH complex	0.06389
Testicular dysfunction	285	PCNA-MLH1-PMS1 complex	0.08248
Testicular dysfunction	286	PCNA-MSH2-MSH6 complex	0.08248
Testicular dysfunction	290	MSH2-MLH1-PMS2-PCNA DNA-repair initiation complex	0.14286
Testicular dysfunction	291	MSH2-MLH1-PMS2 DNA-repair initiation complex	0.16496
Testicular dysfunction	292	MutL-alpha complex	0.10102
Testicular dysfunction	362	DNA ligase III-XRCC1-PNK-DNA-pol III multiprotein complex	0.07143
Testicular dysfunction	368	ERCC1-ERCC4-MSH2 complex	0.08248
Testicular dysfunction	369	MSH2-MSH6-PMS2-MLH1 complex	0.14286
Testicular dysfunction	370	MSH2-MSH6-PMS1-MLH1 complex	0.14286
Testicular dysfunction	374	MSH2-MSH6 complex	0.10102
Testicular dysfunction	375	MSH2-MSH3 complex	0.10102
Testicular dysfunction	376	PCNA-MutS-alpha-MutL-alpha-DNA complex	0.12778
Testicular dysfunction	377	PCNA-MutS-alpha-DNA initial complex	0.08248
Testicular dysfunction	378	MutS-beta complex	0.10102
Testicular dysfunction	380	MutL-beta complex	0.10102
Testicular dysfunction	415	EXO1-MLH1-PMS2 complex	0.08248
Testicular dysfunction	424	EXO1-MLH1-PCNA complex	0.08248
Testicular dysfunction	425	MSH4-MSH5-GPS2 complex	0.08248
Testicular dysfunction	433	BASC complex (BRCA1-associated genome surveillance complex)	0.08248
Testicular dysfunction	434	BASC (Ab 80) complex (BRCA1-associated genome surveillance complex)	0.10102
Testicular dysfunction	435	BASC (Ab 81) complex (BRCA1-associated genome surveillance complex)	0.05832
Testicular dysfunction	438	GCN5-TRRAP histone acetyltransferase complex	0.04518
Testicular dysfunction	541	IGF1-IGFBP3-ALS complex	0.08248
Testicular dysfunction	681	(C-CFTR)2-NHERF-ezrin complex	0.08248
Testicular dysfunction	682	C-CFTR-NHERF(PDZ1 domain)-ezrin complex	0.08248
Testicular dysfunction	683	C-CFTR-NHERF(PDZ2 domain)-ezrin complex	0.08248
Testicular dysfunction	687	CFTR-NHERF-beta(2)AR signaling complex	0.08248
Testicular dysfunction	1056	ZNF198-SUMO1 complex	0.10102
Testicular dysfunction	1085	DNA repair complex NEIL2-PNK-Pol(beta)-LigIII(alpha)-XRCC1	0.06389
Testicular dysfunction	1086	DNA repair complex NEIL1-PNK-Pol(beta)-LigIII(alpha)-XRCC1	0.06389
Testicular dysfunction	1087	BIRC5-AURKB-INCENP-EVI5 complex	0.07143
Testicular dysfunction	1095	SNX complex (SNX1a SNX2 SNX4 EGFR)	0.07143

Testicular dysfunction	1116	CRM1-Survivin-AuroraB mitotic complex	0.08248
Testicular dysfunction	1117	CRM1-Survivin mitotic complex	0.10102
Testicular dysfunction	1118	Chromosomal passenger complex CPC (INCENP CDCA8 BIRC5 AURKB)	0.07143
Testicular dysfunction	1120	Chromosomal passenger complex CPC (INCENP CDCA8 BIRC5)	0.08248
Testicular dysfunction	1185	EGFR-containing signaling complex	0.14286
Testicular dysfunction	1335	SNW1 complex	0.03367
Testicular dysfunction	1642	p16-cyclin D2-CDK4 complex	0.08248
Testicular dysfunction	1656	p27-cyclinE-CDK2 complex	0.16496
Testicular dysfunction	2217	MDC1-MRN-ATM-FANCD2 complex	0.05832
Testicular dysfunction	2218	MDC1-MRE11-RAD50-NBS1 complex	0.07143
Testicular dysfunction	2224	MSH2/6-BLM-p53-RAD51 complex	0.06389
Testicular dysfunction	2226	MutS-alpha-PK-zeta complex	0.08248
Testicular dysfunction	2369	ITGAV-ITGB3-EGFR complex	0.08248
Testicular dysfunction	2453	Multiprotein complex (monoubiquitination)	0.07143
Testicular dysfunction	2454	CIN85-CBL-SH3GL2-EGFR complex EGF stimulated	0.07143
Testicular dysfunction	2528	ERBB2-MEMO-SHC complex	0.08248
Testicular dysfunction	2542	EGFR-CBL-GRB2 complex	0.08248
Testicular dysfunction	2579	Chromosomal passenger complex CPC (INCENP BIRC5 AURKB)	0.08248
Testicular dysfunction	2580	Survivin homodimer complex	0.14286
Testicular dysfunction	2581	RasGAP-AURKA/AURKB-survivin complex	0.07143
Testicular dysfunction	2582	Chromosomal passenger complex CPC (CDCA8 AURKB BIRC5)	0.08248
Testicular dysfunction	2740	MutS-alpha complex	0.10102
Testicular dysfunction	2774	MDC1-H2AFX-TP53BP1 complex	0.08248
Testicular dysfunction	2775	MDC1-p53BP1-SMC1 complex	0.08248
Testicular dysfunction	2817	BRCA1-BARD1-BACH1-DNA damage complex I	0.05832
Testicular dysfunction	2936	Ecsit complex (ECSIT MT-CO2 GAPDH TRAF6 NDUFAF1)	0.06389
Testicular dysfunction	3015	p27-cyclinE-Cdk2 - Ubiquitin E3 ligase (SKP1A SKP2 CUL1 CKS1B RBX1) complex	0.10102
Testicular dysfunction	3085	CCND2-CDK4 complex	0.10102
Testicular dysfunction	3088	CCND2-CDK6 complex	0.10102
Testicular dysfunction	3678	RIN1-STAM2-EGFR complex EGF stimulated	0.08248
Testicular dysfunction	5107	p34(SEI-1)-CDK4-CyclinD2 complex	0.08248
Testicular dysfunction	5159	E2F4-p107-cyclinE complex	0.08248
Testicular dysfunction	5171	SH3KBP1-CBLB-EGFR complex	0.08248
Testicular dysfunction	5176	MGC1-DNA-PKcs-Ku complex	0.07143
Testicular dysfunction	5230	CHUK-NFKB2-REL-IKBKG-SPAG9-NFKB1-NFKBIE-COPB2-TNIP1-NFKBIA-RELA-TNIP2	0.04124
Testicular dysfunction	5243	XRCC1-LIG3-PNK-TDP1 complex	0.07143
Testicular dysfunction	5260	TCF4-CTNNB1-SUMO1-EP300-HADAC6 complex	0.06389
Testicular dysfunction	5385	GAIT complex	0.07143
Testicular dysfunction	5473	FAS-FADD-CASP8 complex	0.08248
Testicular dysfunction	5560	CDK2-CCNE1 complex	0.10102
Testicular dysfunction	5655	Ternary complex (LRRC7 CAMK2a ACTN4)	0.08248
Testicular dysfunction	5755	SUMO1-SUA1-UBA2 complex	0.08248

Testicular dysfunction	5799	Death induced signaling complex DISC (FAS FADD CASP8 CFLAR) membrane-associated CD95L	0.07143
Testicular dysfunction	5800	Death-inducing signaling complex DISC (type I cells associated) stimulated	0.08248
Testicular dysfunction	5808	DISC complex	0.08248
Testicular dysfunction	5822	MCL1-NOXA complex	0.10102
Testicular dysfunction	5823	MCL1-BAK1 complex	0.10102
Testicular dysfunction	5877	MAP2K1-BRAF-RAF1-YWHAE-KSR1 complex	0.06389
Testicular dysfunction	5859	FAS-FADD-CASP8-CASP10 complex	0.07143
Testicular dysfunction	5861	FAS-FADD-CASP10 complex	0.08248
Testicular dysfunction	5872	BRAF-MAP2K1-MAP2K2-YWHAE complex	0.07143
Testicular dysfunction	5919	BRAF-RAF1-14-3-3 complex	0.04762
Testicular dysfunction	5921	KSR1-BRAF-MEK complex	0.07143
Testicular dysfunction	5922	RAF1-RAS complex EGF induced	0.07143
Testicular dysfunction	5923	RAF1-BRAF complex RAS stimulated	0.10102
Testicular dysfunction	5925	BRAF-CNK1 complex not RAS stimulated	0.10102
Testicular tumor	368	ERCC1-ERCC4-MSH2 complex	0.40825
Testicular tumor	371	Structure-specific endonulease complex	0.35355
Testicular tumor	531	XPA-ERCC1-ERCC4 complex	0.61237
Testicular tumor	710	Brg1-associated complex I	0.1066
Testicular tumor	711	Brm-associated complex	0.1066
Testicular tumor	713	BRG1-SIN3A complex	0.09449
Testicular tumor	714	BRM-SIN3A complex	0.09129
Testicular tumor	803	BRG1-SIN3A-HDAC containing SWI/SNF remodeling complex I	0.1066
Testicular tumor	806	BRM-SIN3A-HDAC complex	0.10206
Testicular tumor	807	BRG1-associated complex	0.11785
Testicular tumor	808	BRM-associated complex	0.1118
Testicular tumor	810	FCP1-associated protein complex	0.26726
Testicular tumor	819	20S methylosome-SmD complex	0.17678
Testicular tumor	825	JBP1-pICln complex	0.20412
Testicular tumor	832	Anti-Sm protein complex	0.13363
Testicular tumor	834	20S methylosome and RG-containing Sm protein complex	0.14434
Testicular tumor	836	20S methyltransferase core complex	0.25
Testicular tumor	837	20S methyltransferase complex	0.40825
Testicular tumor	1231	FIB-associated protein complex	0.14434
Testicular tumor	1748	PRMT5 complex	0.35355
Testicular tumor	2220	RAD52-ERCC4-ERCC1 complex	0.40825
Testicular tumor	2838	AR coactivator complex	0.40825
Testicular tumor	3063	Brg1-associated complex II	0.13363
Thalassemia	189	BAF complex	0.06537
Thalassemia	238	SWI-SNF chromatin remodeling-related-BRCA1	0.07107
Thalassemia	541	IGF1-IGFBP3-ALS complex	0.13608
Thalassemia	1239	EBAFb complex	0.07454
Thalassemia	1810	ITGA4-PXN-GIT1 complex	0.13608
Thalassemia	2383	ITGA5-ITGB1-FN1-TGM2 complex	0.11785
Thalassemia	2384	ITGA5-ITGB1-ADAM15 complex	0.13608

Thalassemia	2385	ITGA5-ITGB4 complex	0.16667
Thalassemia	2388	Itga5-Itgb1-Fn1-Sfrp2 complex	0.11785
Thalassemia	2390	CD98-LAT2-ITGB1 complex	0.11785
Thalassemia	2395	ITGA7-ITGB1-CD151 complex	0.13608
Thalassemia	2396	ITGA7-ITGB1-CD9 complex	0.13608
Thalassemia	2397	ITGA7-ITGB1-ITGB1BP3 complex	0.13608
Thalassemia	2398	ITGA3-ITGB1-BSG complex	0.13608
Thalassemia	2399	ITGA3-ITGB1-CD63 complex	0.13608
Thalassemia	2400	ITGA3-ITGB1-CD151 complex	0.13608
Thalassemia	2401	ITGA3-ITGB1-THBS1 complex	0.13608
Thalassemia	2406	ITGA3-ITGB1 complex	0.16667
Thalassemia	2411	ITGA6-ITGB1-CD151 complex	0.13608
Thalassemia	2413	ITGA6-ITGB1 complex	0.16667
Thalassemia	2416	ITGB1-RAP1A-PKD1 complex	0.13608
Thalassemia	2417	ITGA4-ITGB1-EMILIN1 complex	0.27217
Thalassemia	2418	ITGA4-ITGB1 complex	0.33333
Thalassemia	2419	ITGA4-ITGB1-CD81 complex	0.27217
Thalassemia	2420	ITGA4-ITGB1-CD53 complex	0.27217
Thalassemia	2421	ITGA4-ITGB1-VCAM1 complex	0.27217
Thalassemia	2422	ITGA4-ITGB1-JAM2 complex	0.27217
Thalassemia	2423	ITGA4-ITGB1-CD47 complex	0.27217
Thalassemia	2424	ITGA4-ITGB1-CD63 complex	0.27217
Thalassemia	2425	ITGA4-ITGB1-PXN complex	0.27217
Thalassemia	2426	ITGA4-ITGB1-THBS1 complex	0.27217
Thalassemia	2428	ITGA4-ITGB1-THBS2 complex	0.27217
Thalassemia	2429	ITGA2-ITGB1-CD47 complex	0.13608
Thalassemia	2430	ITGA2-ITGB1-CHAD complex	0.13608
Thalassemia	2431	ITGA2-ITGB1-COL6A3 complex	0.13608
Thalassemia	2432	ITGA2-ITGB1 complex	0.16667
Thalassemia	2434	ITGA1-ITGB1-COL6A3 complex	0.13608
Thalassemia	2435	ITGA1-ITGB1-PTPN2 complex	0.13608
Thalassemia	2436	ITGAV-ITGB1 complex	0.16667
Thalassemia	2437	ITGA6-ITGB1-CYR61 complex	0.13608
Thalassemia	2439	ITGA8-ITGB1 complex	0.16667
Thalassemia	2440	ITGA9-ITGB1-ADAM9 complex	0.13608
Thalassemia	2441	Itga9-Itgb1-Adam2 complex	0.13608
Thalassemia	2442	ITGA9-ITGB1-VCAM1 complex	0.13608
Thalassemia	2443	ITGA9-ITGB1-TNC complex	0.13608
Thalassemia	2444	ITGB1-ITGA9 complex	0.16667
Thalassemia	2445	ITGA9-ITGB1-ADAM15 complex	0.13608
Thalassemia	2446	ITGA9-ITGB1-FIGF complex	0.13608
Thalassemia	2447	ITGA9-ITGB1-ADAM12 complex	0.13608
Thalassemia	2829	RSmad complex	0.07454
Thalassemia	2850	ITGA5-ITGB1-FN-1-NOV complex	0.11785
Thalassemia	2853	ITGA5-ITGB1-CAL4A3 complex	0.13608
Thalassemia	2885	ITGAV-ITGB1-SPP1 complex	0.13608
Thalassemia	2964	ITGA9-ITGB1-ADAM1 complex	0.13608

Thalassemia	2965	ITGA9-ITGB1-ADAM3 complex	0.13608
Thalassemia	2971	ITGA9-ITGB1-VEGFC complex	0.13608
Thalassemia	2972	ITGA9-ITGB1-VEGFA complex	0.13608
Thalassemia	2989	ITGA9-ITGB1-ADAM8 complex	0.13608
Thalassemia	3035	LAT2-ITGB1 complex	0.16667
Thalassemia	3057	ITGA10-ITGB1 complex	0.16667
Thalassemia	3058	ITGA11-ITGB1 complex	0.16667
Thalassemia	3059	ITGA11-ITGB1-COL1A1 complex	0.13608
Thalassemia	3104	ITGB1-NRP1 complex	0.16667
Thalassemia	3111	ITGA9-ITGB1-SPP1 complex	0.13608
Thalassemia	3112	ITGA5-ITGB1-SPP1 complex	0.13608
Thromboangiitis obliterans	2429	ITGA2-ITGB1-CD47 complex	0.2357
Thromboangiitis obliterans	2430	ITGA2-ITGB1-CHAD complex	0.2357
Thromboangiitis obliterans	2431	ITGA2-ITGB1-COL6A3 complex	0.2357
Thromboangiitis obliterans	2432	ITGA2-ITGB1 complex	0.28868
Thrombocytopenia	486	WIP-WASp-actin-myosin-IIa complex	0.30619
Thrombocytopenia	2355	ITGAV-ITGB3-CD47-FCER2 complex	0.125
Thrombocytopenia	2356	ITGB3-ITGAV-CD47 complex	0.14434
Thrombocytopenia	2358	ITGAV-ITGB3-SPP1 complex	0.14434
Thrombocytopenia	2359	ITGAV-ITGB3-ADAM15 complex	0.14434
Thrombocytopenia	2362	ITAGV-ITGB3-F11R complex	0.14434
Thrombocytopenia	2363	ITGAV-ITGB3-PXN-PTK2b complex	0.125
Thrombocytopenia	2364	ITGAV-ITGB3-ADAM23 complex	0.14434
Thrombocytopenia	2365	ITGAV-ITGB3-COL4A3 complex	0.14434
Thrombocytopenia	2366	ITGAV-ITGB3-PPAP2b complex	0.14434
Thrombocytopenia	2369	ITGAV-ITGB3-EGFR complex	0.14434
Thrombocytopenia	2370	ITGA2b-ITGB3-CD9 complex	0.28868
Thrombocytopenia	2374	ITGAV-ITGB3-LAMA4 complex	0.14434
Thrombocytopenia	2376	ITGA2B-ITGB3-FN1-TGM2 complex	0.25
Thrombocytopenia	2377	ITGA2b-ITGB3-CD47-SRC complex	0.25
Thrombocytopenia	2378	ITGA2b-ITGB3-TLN1 complex	0.28868
Thrombocytopenia	2379	ITGA2B-ITGB3-CIB1 complex	0.28868
Thrombocytopenia	2381	ITGA2B-ITGB3 complex	0.35355
Thrombocytopenia	2382	ITGA2B-ITGB3-F11R complex	0.28868
Thrombocytopenia	2510	ZAP70-CRKL-WIPF1-WAS complex	0.25
Thrombocytopenia	2511	CRKL-WIPF1-WAS complex	0.28868
Thrombocytopenia	2816	ITGAV-ITGB3 complex	0.17678
Thrombocytopenia	2826	ITGB3-ITGAV-VTN complex	0.14434
Thrombocytopenia	2846	ITGAV-ITGB3-THBS1 complex	0.14434
Thrombocytopenia	2849	ITGAV-ITGB3-NOV complex	0.14434
Thrombocytopenia	2872	ITGA2b-ITGB3-CD9-GP1b-CD47 complex	0.20412
Thrombocytopenia	2882	ITGA5-ITGB3-COL6A3 complex	0.14434
Thrombocytopenia	2896	ITGA2b-ITGB3-CD47-FAK complex	0.25
Thrombocytopenia	3103	ITGAV-ITGB3-SLC3A2 complex	0.14434
Thrombocytopenia	3115	ITGA2B-ITGB3-ICAM4 complex	0.28868
Thrombocytopenia	5473	FAS-FADD-CASP8 complex	0.14434
Thrombocytopenia	5604	Emerin complex 1	0.08839

Thrombocytopenia	5770	RUNX1-CBF-beta-DNA complex	0.17678
Thrombocytopenia	5799	Death induced signaling complex DISC (FAS FADD CASP8 CFLAR) membrane-associated CD95L	0.125
Thrombocytopenia	5800	Death-inducing signaling complex DISC (type I cells associated) stimulated	0.14434
Thrombocytopenia	5808	DISC complex	0.14434
Thrombocytopenia	5859	FAS-FADD-CASP8-CASP10 complex	0.125
Thrombocytopenia	5861	FAS-FADD-CASP10 complex	0.14434
Thrombophilia	725	P2X7 receptor signalling complex	0.06804
Thrombophilia	2028	JAK2-IL12RB2 complex	0.16667
Thrombophilia	2153	ITGAM-ITGB2-CD11 complex	0.27217
Thrombophilia	3162	TF-FVIIa-FXa-TFPI complex	0.11785
Thrombophilia	5178	JAK2-PAFR-TYK2 complex	0.13608
Thrombophlebitis	1095	SNX complex (SNX1a SNX2 SNX4 EGFR)	0.15076
Thrombophlebitis	1185	EGFR-containing signaling complex	0.15076
Thrombophlebitis	2237	SP1-MCAF2 complex	0.2132
Thrombophlebitis	2369	ITGAV-ITGB3-EGFR complex	0.17408
Thrombophlebitis	2453	Multiprotein complex (monoubiquitination)	0.15076
Thrombophlebitis	2454	CIN85-CBL-SH3GL2-EGFR complex EGF stimulated	0.15076
Thrombophlebitis	2542	EGFR-CBL-GRB2 complex	0.17408
Thrombophlebitis	2679	p53-SP1 complex	0.2132
Thrombophlebitis	2706	SMAD3-SMAD4-SP1 complex	0.17408
Thrombophlebitis	2721	HCF-1 complex	0.06917
Thrombophlebitis	3678	RIN1-STAM2-EGFR complex EGF stimulated	0.17408
Thrombophlebitis	3838	SP1-E2F2 complex	0.2132
Thrombophlebitis	3839	SP1-E2F3 complex	0.2132
Thrombophlebitis	5171	SH3KBP1-CBLB-EGFR complex	0.17408
Thymoma	280	HMGB1-HMGB2-HSC70-ERP60-GAPDH complex	0.14142
Thymoma	441	TFTC-type histone acetyl transferase complex	0.09535
Thymoma	1054	ESR1-RELA-BCL3-NCOA3 complex	0.15811
Thymoma	1700	ABL2-HRAS-RIN1 complex	0.18257
Thymoma	1774	MICA-KLRK1-HCST complex	0.18257
Thymoma	1985	AIRE homodimer complex	0.31623
Thymoma	2018	IL12A-IL12B complex	0.22361
Thymoma	2019	IL12A-IL12B-IL12RB1 complex	0.18257
Thymoma	2020	IL12B-IL12RB1-IL12RB2 complex	0.18257
Thymoma	2021	IL12A-IL12B-IL12RB2 complex	0.18257
Thymoma	2124	IKK-alpha--ER-alpha-AIB1 complex	0.18257
Thymoma	2470	p130Cas-ER-alpha-cSrc-kinase- PI3-kinase p85-subunit complex	0.15811
Thymoma	2657	ESR1-CDK7-CCNH-MNAT1-MTA1-HDAC2 complex	0.1291
Thymoma	2670	Er-alpha-p53-hdm2 complex	0.18257
Thymoma	2699	ER-alpha-GRIP1-c-Jun complex	0.18257
Thymoma	2700	ER-alpha-c-Jun complex	0.22361
Thymoma	2936	Ecsit complex (ECSIT MT-CO2 GAPDH TRAF6 NDUFAF1)	0.14142
Thymoma	5385	GAIT complex	0.15811
Thymoma	5548	IL-12 heterodimer complex	0.22361

Thymoma	5549	IL-12 subunit p40 homodimer complex	0.31623
Thymoma	5862	CAV1-VDAC1-ESR1 complex	0.18257
Thyroid cancer	95	Ku antigen-NARG1 complex	0.1005
Thyroid cancer	280	HMGB1-HMGB2-HSC70-ERP60-GAPDH complex	0.07785
Thyroid cancer	702	ARD1-NATH complex	0.12309
Thyroid cancer	1039	PCNA-PAF complex	0.12309
Thyroid cancer	1040	p33ING1b-PCNA complex	0.12309
Thyroid cancer	1041	Alpha-dystrobrevin-ZO-1-actin complex	0.08704
Thyroid cancer	1141	CF IIAm complex (Cleavage factor IIAm complex)	0.04352
Thyroid cancer	1474	SMAD3/4-E2F4/5-p107-DP1 complex	0.07107
Thyroid cancer	1514	IL4-IL4R complex	0.12309
Thyroid cancer	1515	IL4-IL4R-IL2RG complex	0.1005
Thyroid cancer	1661	E2F4-p107-cyclinA complex	0.1005
Thyroid cancer	2709	MMP-9-TIMP-1-LRP complex	0.1005
Thyroid cancer	2936	Ecsit complex (ECSIT MT-CO2 GAPDH TRAF6 NDUFAF1)	0.07785
Thyroid cancer	2975	SMAD3-E2F4/5-p107-DP1 complex	0.07785
Thyroid cancer	3110	ITGAV-P2RY2-GNA12 complex	0.1005
Thyroid cancer	5772	ZO1-(beta)cadherin-(VE)cadherin-VEGFR2 complex	0.08704
Thyroid cancer	5144	E2F1-p107-cyclinA complex	0.1005
Thyroid cancer	5159	E2F4-p107-cyclinE complex	0.1005
Thyroid cancer	5183	DNA-PK-Ku-eIF2-NF90-NF45 complex	0.06155
Thyroid cancer	5385	GAIT complex	0.08704
Thyroid cancer	5589	LINC complex S-phase	0.0658
Thyroid cancer	5609	Emerin regulatory complex	0.05803
Thyroid cancer	5614	Emerin complex 32	0.03711
Thyroid cancer	5830	DJ-1-SNCA complex high molecular weight complex	0.12309
Thyroid cancer	5837	PPD complex	0.1005
Thyroid gland disease	903	RET-Rai complex	0.16222
Thyroid gland disease	1062	BAR-BCL2-CASP8 complex	0.13245
Thyroid gland disease	1439	PTGS2 homodimer complex	0.22942
Thyroid gland disease	1746	SMN containing complex	0.16222
Thyroid gland disease	2000	BAX homo-oligomer complex	0.22942
Thyroid gland disease	3492	Bax homooligomeric complex after apoptotic	0.22942
Thyroid gland disease	5526	CALM1-FKBP38-BCL2 complex	0.13245
Thyroid gland disease	5749	MRIT complex	0.13245
Thyroid gland disease	5811	p53-BCL2 complex	0.16222
Thyroid gland disease	5812	p53-BCL2 complex	0.16222
Thyroid gland disease	5817	tBID-BCL2 complex	0.16222
Thyroid gland disease	5818	BIM-BCL2 complex	0.16222
Thyroid gland disease	5819	BIM-BCL2xL complex	0.16222
Thyroid gland disease	5820	tBID-BCL2xL complex	0.16222
Thyroid gland disease	5823	MCL1-BAK1 complex	0.16222
Thyroid gland disease	5877	MAP2K1-BRAF-RAF1-YWHAE-KSR1 complex	0.1026
Thyroid gland disease	5872	BRAF-MAP2K1-MAP2K2-YWHAE complex	0.11471
Thyroid gland disease	5919	BRAF-RAF1-14-3-3 complex	0.07647
Thyroid gland disease	5921	KSR1-BRAF-MEK complex	0.11471

Thyroid gland disease	5923	RAF1-BRAF complex RAS stimulated	0.16222
Thyroid gland disease	5925	BRAF-CNK1 complex not RAS stimulated	0.16222
Tic disorder	201	HUIC complex	0.5
Tic disorder	202	BRCA1-RAD50-MRE11-NBS1 complex	0.35355
Tic disorder	238	SWI-SNF chromatin remodeling-related-BRCA1	0.2132
Tic disorder	240	BRCA1-CTIP-ZBRK1 repressor complex	0.40825
Tic disorder	242	BRCA1-BACH1 complex	0.5
Tic disorder	433	BASC complex (BRCA1-associated genome surveillance complex)	0.20412
Tic disorder	434	BASC (Ab 80) complex (BRCA1-associated genome surveillance complex)	0.25
Tic disorder	435	BASC (Ab 81) complex (BRCA1-associated genome surveillance complex)	0.28868
Tic disorder	436	BASC (Ab C-20) complex (BRCA1-associated genome surveillance complex)	0.40825
Tic disorder	438	GCN5-TRRAP histone acetyltransferase complex	0.22361
Tic disorder	1094	Frataxin complex	0.26726
Tic disorder	2210	BRCA1-IRIS-pre-replication complex	0.35355
Tic disorder	2211	BARD1-BRCA1-CSTF complex	0.31623
Tic disorder	2213	BRCA1-BARD1-POLR2A complex	0.40825
Tic disorder	2214	LMO4-BRCA1-CTIP-LDB1 complex	0.35355
Tic disorder	2215	BRCA1-LMO4-CTIP complex	0.40825
Tic disorder	2686	BRCA1-core RNA polymerase II complex	0.19612
Tic disorder	2755	17S U2 snRNP	0.12309
Tic disorder	2776	RAD50-BRCA1 complex	0.5
Tic disorder	2783	BARD1-BRCA1-CSTF64 complex	0.40825
Tic disorder	2786	BRCA1 A complex	0.35355
Tic disorder	2787	BRCA1 C complex	0.35355
Tic disorder	2788	BRCA1 B complex	0.40825
Tic disorder	2811	BRCA1-cABL complex	0.5
Tic disorder	2813	BRCA1-SMAD3 complex	0.5
Tic disorder	2814	BRCA1-HDAC1-HDAC2 complex	0.40825
Tic disorder	2815	BRCA1-BARD1-BACH1-DNA damage complex II	0.25
Tic disorder	2817	BRCA1-BARD1-BACH1-DNA damage complex I	0.28868
Tic disorder	2818	BRCA1-BARD1-BRCA2-DNA damage complex III	0.40825
Tic disorder	2819	BRCA1-CtIP-CtBP complex	0.40825
Tic disorder	2820	BRCA1-VCP complex	0.5
Tic disorder	2822	BRCA1-BARD1-UbcH5c complex	0.40825
Tic disorder	2823	BRCA1-BARD1-UbcH7c complex	0.40825
Tic disorder	2824	BRCA1-RAD51 complex	0.5
Tic disorder	2825	BRCA1-RNA polymerase II complex	0.13868
Tic disorder	5400	BRCC complex	0.31623
Transient hypertension of pregnancy	2709	MMP-9-TIMP-1-LRP complex	0.36515
Transient hypertension of pregnancy	2798	MMP-2-claudin-1 complex	0.22361
Tropical spastic paraparesis	1772	MICB-KLRK1-HCST complex	0.21822
Tropical spastic paraparesis	1774	MICA-KLRK1-HCST complex	0.21822

Tropical spastic paraparesis	5718	eNOS-HSP90-AKT complex VEGF induced	0.21822
Tropical spastic paraparesis	2156	YBX1-AKT1 complex	0.26726
Tropical spastic paraparesis	2159	AR-AKT-APPL complex	0.21822
Tropical spastic paraparesis	2233	Replication-coupled CAF-1-MBD1-ETDB1 complex	0.21822
Tropical spastic paraparesis	2238	MBD1-MCAF1-SETDB1 complex	0.21822
Tropical spastic paraparesis	2709	MMP-9-TIMP-1-LRP complex	0.21822
Tropical spastic paraparesis	2749	SETDB1-containing HMTase complex	0.26726
Tropical spastic paraparesis	3847	TCL1(trimer)-AKT1 complex	0.26726
Tuberculosis	1054	ESR1-RELA-BCL3-NCOA3 complex	0.06742
Tuberculosis	1062	BAR-BCL2-CASP8 complex	0.07785
Tuberculosis	1707	IL2-IL2RA-IL2RB complex	0.07785
Tuberculosis	2084	NFKB1-NFKB2-REL-RELA-RELB complex	0.0603
Tuberculosis	2086	NFKB1-NFKB2-RELA-RELB complex	0.06742
Tuberculosis	2105	IkappaB kinase complex (IKBKB CHUK IKBKAP NFKBIA RELA MAP3K14)	0.05505
Tuberculosis	2443	ITGA9-ITGB1-TNC complex	0.07785
Tuberculosis	2489	NCR3-CD247 complex	0.09535
Tuberculosis	3045	hs4 enhancer complex (faster migrating complex)	0.0603
Tuberculosis	5193	TNF-alpha/NF-kappa B signaling complex (CHUK KPNA3 NFKB2 NFKBIB REL IKBKG NFKB1 NFKBIE RELB NFKBIA RELA TNIP2)	0.03892
Tuberculosis	5194	TNF-alpha/NF-kappa B signaling complex (SEC16A CHUK IKBKB NFKB2 REL IKBKG MAP3K14 RELA FBXW7 USP2)	0.04264
Tuberculosis	5196	TNF-alpha/NF-kappa B signaling complex (CHUK BTRC NFKB2 PPP6C REL CUL1 IKBKE SAPS2 SAPS1 ANKRD28 RELA SKP1)	0.03892
Tuberculosis	5209	Ubiquilin-proteasome complex	0.05505
Tuberculosis	5220	CHUK-IQGAP2-AKAP8L-RELA-TNIP2 complex	0.0603
Tuberculosis	5228	REL-MAP3K8-RELA-TNIP2-PAPOLA complex	0.0603
Tuberculosis	5230	CHUK-NFKB2-REL-IKBKG-SPAG9-NFKB1-NFKBIE-COPB2-TNIP1-NFKBIA-RELA-TNIP2	0.03892
Tuberculosis	5232	TNF-alpha/Nf-kappa B signaling complex (RPL6 RPL30 RPS13 CHUK DDX3X NFKB2 NFKBIB REL IKBKG NFKB1 MAP3K8 RELB GLG1 NFKBIA RELA TNIP2 GTF2I)	0.0327
Tuberculosis	5233	TNF-alpha/NF-kappa B signaling complex 5	0.02697
Tuberculosis	5389	SERPINA3-CTSG complex	0.09535
Tuberculosis	5391	SERPINA1-CTSG complex	0.09535
Tuberculosis	5423	HSP70-BAG5-PARK2 complex	0.06742
Tuberculosis	5460	p50-p65 NF(kappa)B complex	0.09535
Tuberculosis	5461	p50-p65 NF(kappa)B-SRC1 complex	0.07785
Tuberculosis	5464	I(kappa)B(alpha)-NF(kappa)Bp50-NF(kappa)Bp65 complex	0.07785
Tuberculosis	5465	IKB(epsilon)-RELA-cREL complex	0.07785
Tuberculosis	5466	IKB(beta)-RELA-cREL complex	0.07785
Tuberculosis	5467	IKB(alpha)-RELA-cREL complex	0.07785
Tuberculosis	5475	MURR1-NF(kappa)Bp65-IKBA complex	0.07785
Tuberculosis	5492	IKBA-NF(kappa)Bp65-NF(kappa)Bp50 complex	0.07785

Tuberculosis	5526	CALM1-FKBP38-BCL2 complex	0.07785
Tuberculosis	5564	LMO4-gp130 complex	0.0603
Tuberculosis	5811	p53-BCL2 complex	0.09535
Tuberculosis	5817	tBID-BCL2 complex	0.09535
Tuberculosis	5818	BIM-BCL2 complex	0.09535
Tuberculosis	5822	MCL1-NOXA complex	0.09535
Tuberculosis	5823	MCL1-BAK1 complex	0.09535
Tuberculosis	5837	PPD complex	0.07785
Tuberous sclerosis	54	SIN3 complex	0.09759
Tuberous sclerosis	61	Mi2/NuRD complex	0.09759
Tuberous sclerosis	62	MeCP1 complex	0.09129
Tuberous sclerosis	75	TSC1-TSC2 complex	0.36515
Tuberous sclerosis	100	hNURF complex	0.1291
Tuberous sclerosis	105	Polycomb repressive complex 2 (PRC 2)	0.11547
Tuberous sclerosis	282	SNF2h-cohesin-NuRD complex	0.06455
Tuberous sclerosis	283	Sin3 complex	0.09759
Tuberous sclerosis	285	PCNA-MLH1-PMS1 complex	0.14907
Tuberous sclerosis	286	PCNA-MSH2-MSH6 complex	0.14907
Tuberous sclerosis	290	MSH2-MLH1-PMS2-PCNA DNA-repair initiation complex	0.1291
Tuberous sclerosis	297	PCNA-DNA polymerase delta complex	0.11547
Tuberous sclerosis	305	40S ribosomal subunit cytoplasmic	0.04428
Tuberous sclerosis	306	Ribosome cytoplasmic	0.02869
Tuberous sclerosis	310	Cell cycle kinase complex CDC2	0.10541
Tuberous sclerosis	311	Cell cycle kinase complex CDK2	0.1291
Tuberous sclerosis	312	Cell cycle kinase complex CDK4	0.1291
Tuberous sclerosis	313	Cell cycle kinase complex CDK5	0.11547
Tuberous sclerosis	314	PCNA-p21 complex	0.18257
Tuberous sclerosis	376	PCNA-MutS-alpha-MutL-alpha-DNA complex	0.11547
Tuberous sclerosis	377	PCNA-MutS-alpha-DNA initial complex	0.14907
Tuberous sclerosis	424	EXO1-MLH1-PCNA complex	0.14907
Tuberous sclerosis	521	Polycystin-1-E-cadherin-beta-catenin complex	0.14907
Tuberous sclerosis	522	Polycystin-1-E-cadherin-beta-catenin-Flotillin-2	0.1291
Tuberous sclerosis	587	NuRD.1 complex	0.09129
Tuberous sclerosis	591	SAP complex (Sin3-associated protein complex)	0.09129
Tuberous sclerosis	592	SAP complex (Sin3-associated protein complex)	0.08607
Tuberous sclerosis	596	SIN3-HDAC-SAP30-ARID4 complex	0.09759
Tuberous sclerosis	614	NRD complex (Nucleosome remodeling and deacetylation complex)	0.09759
Tuberous sclerosis	632	Anti-HDAC2 complex	0.06086
Tuberous sclerosis	646	HDAC1-associated protein complex	0.08607
Tuberous sclerosis	649	HDAC1-associated core complex cII	0.08165
Tuberous sclerosis	650	HDAC2-asscociated core complex	0.09129
Tuberous sclerosis	659	MeCP1 complex	0.08607
Tuberous sclerosis	685	MeCP1 complex	0.08607
Tuberous sclerosis	691	SIN3-SAP25 complex	0.07785
Tuberous sclerosis	696	BRMS1-SIN3-HDAC complex	0.09129
Tuberous sclerosis	713	BRG1-SIN3A complex	0.06901

Tuberous sclerosis	714	BRM-SIN3A complex	0.06667
Tuberous sclerosis	738	SIN3-ING1b complex I	0.08607
Tuberous sclerosis	739	SIN3-ING1b complex II	0.06455
Tuberous sclerosis	778	LARC complex (LCR-associated remodeling complex)	0.05923
Tuberous sclerosis	860	DNMT1-G9a-PCNA complex	0.14907
Tuberous sclerosis	886	MTA1 complex	0.10541
Tuberous sclerosis	888	MTA2 complex	0.08607
Tuberous sclerosis	889	MTA1-HDAC core complex	0.11547
Tuberous sclerosis	974	EED-EZH2 complex	0.11547
Tuberous sclerosis	995	Polycomb repressive complex 3 (PRC3)	0.11547
Tuberous sclerosis	996	Polycomb repressive complex 2 (PRC2)	0.11547
Tuberous sclerosis	997	KIN17-PCNA-RPA70 complex	0.14907
Tuberous sclerosis	1039	PCNA-PAF complex	0.18257
Tuberous sclerosis	1092	PCNA-KU antigen complex	0.14907
Tuberous sclerosis	1098	DNA synthesome complex (13 subunits)	0.06901
Tuberous sclerosis	1099	DNA synthesome complex (17 subunits)	0.06086
Tuberous sclerosis	1107	DNA synthesome core complex	0.08165
Tuberous sclerosis	1108	DNA synthesome complex (15 subunits)	0.06667
Tuberous sclerosis	1149	Histone H3.1 complex	0.08165
Tuberous sclerosis	1150	Histone H3.3 complex	0.08607
Tuberous sclerosis	1160	ING1-p300-PCNA complex	0.14907
Tuberous sclerosis	1163	ING1-PCNA complex	0.18257
Tuberous sclerosis	1257	ALL-1 supercomplex	0.0488
Tuberous sclerosis	1259	Chromatin assembly complex (CAF-1 complex)	0.14907
Tuberous sclerosis	1495	PID complex	0.11547
Tuberous sclerosis	1897	RAPTOR-mTOR complex	0.14907
Tuberous sclerosis	2055	CASP8-CHUK-IKBKB-MALT1-BCL10 complex	0.11547
Tuberous sclerosis	2056	BCL10-CHUK-BCL10-IKBKB complex	0.1291
Tuberous sclerosis	2100	CHUK-IKBKB-MAP3K14 complex	0.14907
Tuberous sclerosis	2101	IKKA-IKKB complex	0.18257
Tuberous sclerosis	2104	IKKB-NIK complex	0.18257
Tuberous sclerosis	2105	IkappaB kinase complex (IKBKB CHUK IKBKAP NFKBIA RELA MAP3K14)	0.10541
Tuberous sclerosis	2118	CHUK-ERC1-IKBKB-IKBKG	0.1291
Tuberous sclerosis	2121	CHUK-IKBKB-IKBKG complex	0.14907
Tuberous sclerosis	2201	PCNA-RFC2-5 complex	0.11547
Tuberous sclerosis	2230	PCNA complex	0.09759
Tuberous sclerosis	2231	PCNA homotrimer complex	0.2582
Tuberous sclerosis	2416	ITGB1-RAP1A-PKD1 complex	0.14907
Tuberous sclerosis	2577	Sam68-p85 P13K-IRS-1-IR signaling complex	0.1291
Tuberous sclerosis	2721	HCF-1 complex	0.05923
Tuberous sclerosis	2727	SRC-3 complex	0.09759
Tuberous sclerosis	2797	PCNA-CHL12-RFC2-5 complex	0.10541
Tuberous sclerosis	2851	ING2 complex	0.07454
Tuberous sclerosis	2970	mTORC1 complex (mTOR/FRAP1 LST8 RAPTOR)	0.14907
Tuberous sclerosis	2985	mTOR-signaling complex	0.18257
Tuberous sclerosis	2990	mTOR-signaling complex (FRAP1/mTOR GBL RAPTOR)	0.14907

Tuberous sclerosis	2991	mTOR-signaling complex (mTOR/FRAP1 RAPTOR)	0.18257
Tuberous sclerosis	3048	mSin3A complex	0.11547
Tuberous sclerosis	3055	Nop56p-associated pre-rRNA complex	0.02532
Tuberous sclerosis	3149	NK-3-Groucho-HIPK2-SIN3A-RbpA48-HDAC1	0.07785
Tuberous sclerosis	3980	mTOR-RAPTOR complex	0.14907
Tuberous sclerosis	5177	Polycystin-1 multiprotein complex (ACTN1 CDH1 SRC JUP VCL CTNNB1 PXN BCAR1 PKD1	0.07785
Tuberous sclerosis	5194	TNF-alpha/NF-kappa B signaling complex (SEC16A CHUK IKBKB NFKB2 REL IKBKG MAP3K14 RELA FBXW7 USP2)	0.08165
Tuberous sclerosis	5233	TNF-alpha/NF-kappa B signaling complex 5	0.05164
Tuberous sclerosis	5234	IKBKB-CDC37-KIAA1967-HSP90AB1-HSP90AA1 complex	0.11547
Tuberous sclerosis	5266	TNF-alpha/NF-kappa B signaling complex 6	0.06901
Tuberous sclerosis	5544	CDC2-PCNA-CCNB1-GADD45A complex	0.1291
Tuberous sclerosis	5545	CDC2-PCNA-CCNB1-GADD45B complex	0.1291
Tuberous sclerosis	5546	CDC2-PCNA-CCNB1-GADD45G complex	0.1291
Tuberous sclerosis	5589	LINC complex S-phase	0.09759
Tuberous sclerosis	5593	LINC core complex	0.11547
Tuberous sclerosis	5596	LINC complex quiescent cells	0.09759
Tuberous sclerosis	5828	IKBKG-IKBKB complex	0.18257
Tuberous sclerosis	5844	I-kappa-B kinase (IKK) complex	0.14907
Tumor virus infection	5473	FAS-FADD-CASP8 complex	0.57735
Tumor virus infection	5799	Death induced signaling complex DISC (FAS FADD CASP8 CFLAR) membrane-associated CD95L	0.5
Tumor virus infection	5800	Death-inducing signaling complex DISC (type I cells associated) stimulated	0.57735
Tumor virus infection	5808	DISC complex	0.57735
Tumor virus infection	5859	FAS-FADD-CASP8-CASP10 complex	0.5
Tumor virus infection	5861	FAS-FADD-CASP10 complex	0.57735
Turner's syndrome	2529	LAT-PLC-gamma-1-p85-GRB2-CBL-VAV-SLP-76 signaling complex C305 activated	0.21822
Turner's syndrome	2547	PLC-gamma-1-SLP-76-SOS1-LAT complex	0.28868
Turner's syndrome	2551	PDGFRA-PLC-gamma-1-PI3K-SHP-2 complex PDGF stimulated	0.28868
Turner's syndrome	2922	LAT-PLC-gamma-1-p85-GRB2-SOS signaling complex C305 activated	0.2582
Turner's syndrome	2955	LCK-SLP76-PLC-gamma-1-LAT complex pervanadate-activated	0.28868
Turner's syndrome	2956	PLC-gamma-1-LAT-c-CBL complex OKT3 stimulated	0.33333
Turner's syndrome	2960	SLP-76-PLC-gamma-1-ITK complex alpha-TCR stimulated	0.33333
Turner's syndrome	2961	SLP-76-PLC-gamma-1-VAV complex alpha-TCR stimulated	0.33333
Ulcerative colitis	220	ARF-Mule complex	0.05955
Ulcerative colitis	244	BRAFT complex	0.02861
Ulcerative colitis	246	BLM complex III	0.05157
Ulcerative colitis	285	PCNA-MLH1-PMS1 complex	0.05955
Ulcerative colitis	290	MSH2-MLH1-PMS2-PCNA DNA-repair initiation complex	0.05157

Ulcerative colitis	291	MSH2-MLH1-PMS2 DNA-repair initiation complex	0.05955
Ulcerative colitis	292	MutL-alpha complex	0.07293
Ulcerative colitis	369	MSH2-MSH6-PMS2-MLH1 complex	0.05157
Ulcerative colitis	370	MSH2-MSH6-PMS1-MLH1 complex	0.05157
Ulcerative colitis	376	PCNA-MutS-alpha-MutL-alpha-DNA complex	0.04613
Ulcerative colitis	380	MutL-beta complex	0.07293
Ulcerative colitis	415	EXO1-MLH1-PMS2 complex	0.05955
Ulcerative colitis	424	EXO1-MLH1-PCNA complex	0.05955
Ulcerative colitis	433	BASC complex (BRCA1-associated genome surveillance complex)	0.02977
Ulcerative colitis	434	BASC (Ab 80) complex (BRCA1-associated genome surveillance complex)	0.03647
Ulcerative colitis	435	BASC (Ab 81) complex (BRCA1-associated genome surveillance complex)	0.04211
Ulcerative colitis	521	Polycystin-1-E-cadherin-beta-catenin complex	0.05955
Ulcerative colitis	522	Polycystin-1-E-cadherin-beta-catenin-Flotillin-2	0.05157
Ulcerative colitis	826	PAR-3-VE-cadherin-beta-catenin complex	0.05955
Ulcerative colitis	933	SCRIB-APC complex	0.07293
Ulcerative colitis	1003	RC complex (Replication competent complex)	0.03438
Ulcerative colitis	1004	RC complex during S-phase of cell cycle	0.02861
Ulcerative colitis	1005	RC complex during G2/M-phase of cell cycle	0.02861
Ulcerative colitis	1054	ESR1-RELA-BCL3-NCOA3 complex	0.05157
Ulcerative colitis	1091	SNX complex (SNX1a SNX2 SNX4 LEPR)	0.05157
Ulcerative colitis	1514	IL4-IL4R complex	0.07293
Ulcerative colitis	1515	IL4-IL4R-IL2RG complex	0.05955
Ulcerative colitis	1642	p16-cyclin D2-CDK4 complex	0.05955
Ulcerative colitis	1656	p27-cyclinE-CDK2 complex	0.05955
Ulcerative colitis	1661	E2F4-p107-cyclinA complex	0.05955
Ulcerative colitis	1714	TICAM1-TICAM2-TLR4 complex	0.05955
Ulcerative colitis	1772	MICB-KLRK1-HCST complex	0.05955
Ulcerative colitis	1774	MICA-KLRK1-HCST complex	0.05955
Ulcerative colitis	1816	JUN-TCF4-CTNNB1 complex	0.05955
Ulcerative colitis	1839	SDCBP-CTNNB1-CTNNA1-CDH1 complex	0.05157
Ulcerative colitis	1844	APC-IQGAP1 complex	0.07293
Ulcerative colitis	1845	APC-IQGAP1-CLIP-170 complex	0.05955
Ulcerative colitis	1909	APC-DLG4 complex	0.07293
Ulcerative colitis	1945	IRAK1-IRAK3 complex	0.07293
Ulcerative colitis	1992	LEPR homodimer complex	0.10314
Ulcerative colitis	2001	NOD1 homodimer complex	0.10314
Ulcerative colitis	2018	IL12A-IL12B complex	0.07293
Ulcerative colitis	2019	IL12A-IL12B-IL12RB1 complex	0.05955
Ulcerative colitis	2020	IL12B-IL12RB1-IL12RB2 complex	0.05955
Ulcerative colitis	2021	IL12A-IL12B-IL12RB2 complex	0.05955
Ulcerative colitis	2084	NFKB1-NFKB2-REL-RELA-RELB complex	0.04613
Ulcerative colitis	2086	NFKB1-NFKB2-RELA-RELB complex	0.05157
Ulcerative colitis	2105	IkappaB kinase complex (IKBKB CHUK IKBKAP NFKBIA RELA MAP3K14)	0.04211
Ulcerative colitis	2347	ITGAV-ITGB5-SPP1 complex	0.05955

Ulcerative colitis	2352	ITGAV-ITGB6-SPP1 complex	0.05955
Ulcerative colitis	2358	ITGAV-ITGB3-SPP1 complex	0.05955
Ulcerative colitis	2435	ITGA1-ITGB1-PTPN2 complex	0.05955
Ulcerative colitis	2709	MMP-9-TIMP-1-LRP complex	0.05955
Ulcerative colitis	2730	Set1B complex	0.04211
Ulcerative colitis	2731	Set1A complex	0.04211
Ulcerative colitis	2817	BRCA1-BARD1-BACH1-DNA damage complex I	0.04211
Ulcerative colitis	2885	ITGAV-ITGB1-SPP1 complex	0.05955
Ulcerative colitis	2998	Axin-PP2A A-PP2A C-GSK3-beta-beta-catenin	0.05157
Ulcerative colitis	3004	APC-Axin-1-beta-catenin complex	0.1191
Ulcerative colitis	3008	60S APC containing complex	0.03898
Ulcerative colitis	3011	APC-IQGAP1-Rac1 complex	0.05955
Ulcerative colitis	3012	APC-IQGAP1-Cdc42 complex	0.05955
Ulcerative colitis	3015	p27-cyclinE-Cdk2 - Ubiquitin E3 ligase (SKP1A SKP2 CUL1 CKS1B RBX1) complex	0.03647
Ulcerative colitis	3045	hs4 enhancer complex (faster migrating complex)	0.04613
Ulcerative colitis	3075	UTX-MLL2/3 complex	0.02977
Ulcerative colitis	3111	ITGA9-ITGB1-SPP1 complex	0.05955
Ulcerative colitis	3112	ITGA5-ITGB1-SPP1 complex	0.05955
Ulcerative colitis	3155	Bipartite complex (TFC4 CTNNB1)	0.07293
Ulcerative colitis	3166	AXIN-APC-betaCatenin-GSK3B complex	0.10314
Ulcerative colitis	4095	Catulin (alpha) - catenin (beta) complex	0.07293
Ulcerative colitis	4096	Catenin (alpha) - catenin (beta) complex	0.07293
Ulcerative colitis	5772	ZO1-(beta)cadherin-(VE)cadherin-VEGFR2 complex	0.05157
Ulcerative colitis	5144	E2F1-p107-cyclinA complex	0.05955
Ulcerative colitis	5177	Polycystin-1 multiprotein complex (ACTN1 CDH1 SRC JUP VCL CTNNB1 PXN BCAR1 PKD1	0.0311
Ulcerative colitis	5193	TNF-alpha/NF-kappa B signaling complex (CHUK KPNA3 NFKB2 NFKBIB REL IKBKG NFKB1 NFKBIE RELB NFKBIA RELA TNIP2)	0.02977
Ulcerative colitis	5194	TNF-alpha/NF-kappa B signaling complex (SEC16A CHUK IKBKB NFKB2 REL IKBKG MAP3K14 RELA FBXW7 USP2)	0.03262
Ulcerative colitis	5196	TNF-alpha/NF-kappa B signaling complex (CHUK BTRC NFKB2 PPP6C REL CUL1 IKBKE SAPS2 SAPS1 ANKRD28 RELA SKP1)	0.02977
Ulcerative colitis	5220	CHUK-IQGAP2-AKAP8L-RELA-TNIP2 complex	0.04613
Ulcerative colitis	5228	REL-MAP3K8-RELA-TNIP2-PAPOLA complex	0.04613
Ulcerative colitis	5230	CHUK-NFKB2-REL-IKBKG-SPAG9-NFKB1-NFKBIE-COPB2-TNIP1-NFKBIA-RELA-TNIP2	0.02977
Ulcerative colitis	5232	TNF-alpha/Nf-kappa B signaling complex (RPL6 RPL30 RPS13 CHUK DDX3X NFKB2 NFKBIB REL IKBKG NFKB1 MAP3K8 RELB GLG1 NFKBIA RELA TNIP2 GTF2I)	0.02502
Ulcerative colitis	5233	TNF-alpha/NF-kappa B signaling complex 5	0.02063
Ulcerative colitis	5260	TCF4-CTNNB1-SUMO1-EP300-HADAC6 complex	0.04613
Ulcerative colitis	5261	TCF4-CTNNB1-EP300 complex	0.05955
Ulcerative colitis	5262	TCF4-CTNNB1 complex	0.07293
Ulcerative colitis	5264	TCF4-CTNNB1-CREBBP complex	0.05955

Ulcerative colitis	5281	Cell-cell junction complex (CDH1-CTNNB1)	0.07293
Ulcerative colitis	5460	p50-p65 NF(kappa)B complex	0.07293
Ulcerative colitis	5461	p50-p65 NF(kappa)B-SRC1 complex	0.05955
Ulcerative colitis	5464	I(kappa)B(alpha)-NF(kappa)Bp50-NF(kappa)Bp65 complex	0.05955
Ulcerative colitis	5465	IKB(epsilon)-RELA-cREL complex	0.05955
Ulcerative colitis	5466	IKB(beta)-RELA-cREL complex	0.05955
Ulcerative colitis	5467	IKB(alpha)-RELA-cREL complex	0.05955
Ulcerative colitis	5475	MURR1-NF(kappa)Bp65-IKBA complex	0.05955
Ulcerative colitis	5492	IKBA-NF(kappa)Bp65-NF(kappa)Bp50 complex	0.05955
Ulcerative colitis	5548	IL-12 heterodimer complex	0.07293
Ulcerative colitis	5549	IL-12 subunit p40 homodimer complex	0.10314
Ulcerative colitis	5556	CDK2-CCNA2 complex	0.07293
Ulcerative colitis	5557	CDC2-CCNA2 complex	0.07293
Ulcerative colitis	5559	CDC2-CCNA2-CDK2 complex	0.05955
Ulcerative colitis	5564	LMO4-gp130 complex	0.04613
Ulcerative colitis	5877	MAP2K1-BRAF-RAF1-YWHAE-KSR1 complex	0.04613
Ulcerative colitis	5872	BRAF-MAP2K1-MAP2K2-YWHAE complex	0.05157
Ulcerative colitis	5919	BRAF-RAF1-14-3-3 complex	0.03438
Ulcerative colitis	5921	KSR1-BRAF-MEK complex	0.05157
Ulcerative colitis	5923	RAF1-BRAF complex RAS stimulated	0.07293
Ulcerative colitis	5925	BRAF-CNK1 complex not RAS stimulated	0.07293
Urogenital abnormalities	759	Fgfr1-Kal1 complex	0.18898
Urogenital abnormalities	912	Kal1-Fgfr1 complex	0.18898
Urogenital abnormalities	1071	PKD2-FPC complex	0.18898
Urogenital abnormalities	2159	AR-AKT-APPL complex	0.1543
Urogenital abnormalities	2160	AOF2-AR complex	0.18898
Urogenital abnormalities	2486	GIPC1-LHCGR complex	0.18898
Uterine disease	441	TFTC-type histone acetyl transferase complex	0.11396
Uterine disease	521	Polycystin-1-E-cadherin-beta-catenin complex	0.21822
Uterine disease	522	Polycystin-1-E-cadherin-beta-catenin-Flotillin-2	0.18898
Uterine disease	826	PAR-3-VE-cadherin-beta-catenin complex	0.21822
Uterine disease	1054	ESR1-RELA-BCL3-NCOA3 complex	0.18898
Uterine disease	1816	JUN-TCF4-CTNNB1 complex	0.21822
Uterine disease	1839	SDCBP-CTNNB1-CTNNA1-CDH1 complex	0.18898
Uterine disease	2124	IKK-alpha--ER-alpha-AIB1 complex	0.21822
Uterine disease	2470	p130Cas-ER-alpha-cSrc-kinase- PI3-kinase p85-subunit complex	0.18898
Uterine disease	2657	ESR1-CDK7-CCNH-MNAT1-MTA1-HDAC2 complex	0.1543
Uterine disease	2670	Er-alpha-p53-hdm2 complex	0.21822
Uterine disease	2699	ER-alpha-GRIP1-c-Jun complex	0.21822
Uterine disease	2700	ER-alpha-c-Jun complex	0.26726
Uterine disease	2798	MMP-2-claudin-1 complex	0.26726
Uterine disease	2998	Axin-PP2A A-PP2A C-GSK3-beta-beta-catenin	0.18898
Uterine disease	3004	APC-Axin-1-beta-catenin complex	0.21822
Uterine disease	3155	Bipartite complex (TFC4 CTNNB1)	0.26726
Uterine disease	3166	AXIN-APC-betaCatenin-GSK3B complex	0.18898
Uterine disease	4095	Catulin (alpha) - catenin (beta) complex	0.26726

Uterine disease	4096	Catenin (alpha) - catenin (beta) complex	0.26726
Uterine disease	5772	ZO1-(beta)cadherin-(VE)cadherin-VEGFR2 complex	0.18898
Uterine disease	5177	Polycystin-1 multiprotein complex (ACTN1 CDH1 SRC JUP VCL CTNNB1 PXN BCAR1 PKD1	0.11396
Uterine disease	5260	TCF4-CTNNB1-SUMO1-EP300-HADAC6 complex	0.16903
Uterine disease	5261	TCF4-CTNNB1-EP300 complex	0.21822
Uterine disease	5262	TCF4-CTNNB1 complex	0.26726
Uterine disease	5264	TCF4-CTNNB1-CREBBP complex	0.21822
Uterine disease	5281	Cell-cell junction complex (CDH1-CTNNB1)	0.26726
Uterine disease	5862	CAV1-VDAC1-ESR1 complex	0.21822
Uterine fibroids	159	Condensin I-PARP-1-XRCC1 complex	0.13363
Uterine fibroids	1004	RC complex during S-phase of cell cycle	0.09806
Uterine fibroids	1005	RC complex during G2/M-phase of cell cycle	0.09806
Uterine fibroids	1193	Rap1 complex	0.13363
Uterine fibroids	1211	Ubiquitin E3 ligase (AHR ARNT DDB1 TBL3 CUL4B RBX1)	0.28868
Uterine fibroids	1226	H2AX complex I	0.13363
Uterine fibroids	1728	CTCF-nucleophosmin-PARP-HIS-KPNA-LMNA-TOP complex	0.11785
Uterine fibroids	1729	TLE1 corepressor complex (MASH1 promoter-corepressor complex)	0.1118
Uterine fibroids	2152	ARNT-HLF complex	0.25
Uterine fibroids	2318	ITGA6-ITGB4-Laminin10/12 complex	0.15811
Uterine fibroids	2625	CDK8-MED6-PARP1 complex	0.20412
Uterine fibroids	3137	MASH1 promoter-coactivator complex	0.1066
Uterine fibroids	3142	CAMK2-delta-MASH1 promoter-coactivator complex	0.125
Uterine fibroids	4043	NEMO-HIF2(alpha)-ARNT complex	0.20412
Uterine fibroids	5179	NCOA6-DNA-PK-Ku-PARP1 complex	0.15811
Uterine fibroids	5235	WRN-Ku70-Ku80-PARP1 complex	0.17678
Uterine fibroids	5382	ARNT-HIF1A complex	0.25
Uveitis	5473	FAS-FADD-CASP8 complex	0.14434
Uveitis	5564	LMO4-gp130 complex	0.1118
Uveitis	5615	Emerin complex 52	0.05213
Uveitis	5799	Death induced signaling complex DISC (FAS FADD CASP8 CFLAR) membrane-associated CD95L	0.125
Uveitis	5800	Death-inducing signaling complex DISC (type I cells associated) stimulated	0.14434
Uveitis	5808	DISC complex	0.14434
Uveitis	5859	FAS-FADD-CASP8-CASP10 complex	0.125
Uveitis	5861	FAS-FADD-CASP10 complex	0.14434
Vaccinia	280	HMGB1-HMGB2-HSC70-ERP60-GAPDH complex	0.13484
Vaccinia	553	RHOA-IP3R-TRPC1 complex	0.17408
Vaccinia	815	MRIP-MBS-RHOA complex	0.17408
Vaccinia	816	MRIP-RHOA complex	0.2132
Vaccinia	1816	JUN-TCF4-CTNNB1 complex	0.17408
Vaccinia	2000	BAX homo-oligomer complex	0.30151
Vaccinia	2692	SMAD3-SMAD4-cJun-cFos complex	0.15076
Vaccinia	2693	NFAT-JUN-FOS DNA-protein complex	0.17408

Vaccinia	2694	ERG-JUN-FOS DNA-protein complex	0.17408
Vaccinia	2695	ETS2-FOS-JUN complex	0.17408
Vaccinia	2699	ER-alpha-GRIP1-c-Jun complex	0.17408
Vaccinia	2700	ER-alpha-c-Jun complex	0.2132
Vaccinia	2708	SMAD3-SMAD4-cJUN complex	0.17408
Vaccinia	2936	Ecsit complex (ECSIT MT-CO2 GAPDH TRAF6 NDUFAF1)	0.13484
Vaccinia	3153	GNAQ-GEFT-RHOA complex	0.17408
Vaccinia	3492	Bax homooligomeric complex after apoptotic	0.30151
Vaccinia	5385	GAIT complex	0.15076
Vaccinia	5818	BIM-BCL2 complex	0.2132
Vaccinia	5819	BIM-BCL2xL complex	0.2132
Vaccinia	5823	MCL1-BAK1 complex	0.2132
Varicosity	2709	MMP-9-TIMP-1-LRP complex	0.21822
Vascular dementia	1088	PRNP-ApolopoproteinE3 complex	0.2357
Vascular disease	205	Ubiquitin E3 ligase (VHL TCEB1 TCEB2 CUL2	0.08165
Vascular disease	622	Ubiquitin E3 ligase (VHL TCEB1 TCEB2 CUL2)	0.09129
Vascular disease	1474	SMAD3/4-E2F4/5-p107-DP1 complex	0.07454
Vascular disease	2692	SMAD3-SMAD4-cJun-cFos complex	0.09129
Vascular disease	2704	Ectodermin-SMAD4 complex	0.1291
Vascular disease	2705	SMAD3-SMAD4-CTCF protein-DNA complex	0.10541
Vascular disease	2706	SMAD3-SMAD4-SP1 complex	0.10541
Vascular disease	2707	SMAD3-SMAD4-FOXO3-FOXG1 complex	0.09129
Vascular disease	2708	SMAD3-SMAD4-cJUN complex	0.10541
Vascular disease	2754	JUND-FOSB-SMAD3-SMAD4 complex	0.09129
Vascular disease	2760	SMAD3-SMAD4-FOXO3 complex	0.10541
Vascular disease	2761	SMAD3-SMAD4-FOXO1 complex	0.10541
Vascular disease	2762	SMAD3-SMAD4-FOXO4 complex	0.10541
Vascular disease	2829	RSmad complex	0.05774
Vascular disease	2834	SMAD4-SMAD2-SMAD3 complex	0.10541
Vascular disease	3038	SMAD2-SMAD4-FAST1 complex	0.10541
Vascular disease	3197	SMAD4-SNO-SKI complex	0.10541
Vascular disease	3200	SMAD4-SKI complex	0.1291
Vascular disease	3206	SMAD4-SKI-NCOR complex	0.10541
Vascular disease	3233	SMAD2-SMAD4-FAST1-TGIF complex TGF(beta) induced	0.09129
Vascular disease	3234	SMAD2-SMAD4-FAST1-TGIF-HDAC1 complex TGF(beta) induced	0.08165
Vascular disease	3335	Homotetrameric complex NIAP	0.18257
Vascular disease	3739	SKI-SMAD2-SMAD4 pentameric complex	0.10541
Vascular disease	3740	SKI-SMAD3-SMAD4 pentameric complex	0.10541
Vascular disease	3753	CREBBP-SMAD2-SMAD4 pentameric complex	0.10541
Vascular disease	3754	CREBBP-SMAD3-SMAD4 pentameric complex	0.10541
Vascular disease	3959	SMAD3-SMAD4-cSKI TGF(beta)-dependent	0.10541
Vascular disease	5267	VHL-VDU1-TCEB1-TCEB2 complex	0.09129
Vascular disease	5270	VHL-TCEB1-TCEB2 complex	0.10541
Vascular disease	5273	VHL-TBP1-HIF1A complex	0.10541
Vasculitis	298	VEGF transcriptional complex	0.12309

Vasculitis	2798	MMP-2-claudin-1 complex	0.2132
Vasculitis	5273	VHL-TBP1-HIF1A complex	0.17408
Vasculitis	5276	HIF1A-OS9-EGLN1 complex	0.17408
Vasculitis	5277	HIF1A-OS9-EGLN3 complex	0.17408
Vasculitis	5382	ARNT-HIF1A complex	0.2132
Virus disease	240	BRCA1-CTIP-ZBRK1 repressor complex	0.09623
Virus disease	906	ADAR1-CDK2 complex	0.11785
Virus disease	1243	Ubiquitin E3 ligase (SPOP DAXX CUL3)	0.09623
Virus disease	1332	Large Drosha complex	0.03727
Virus disease	1490	DAXX-DNMT1-DMAP1 complex	0.09623
Virus disease	1514	IL4-IL4R complex	0.11785
Virus disease	1515	IL4-IL4R-IL2RG complex	0.09623
Virus disease	1554	RANBP1-RAN-KPNB1 complex	0.09623
Virus disease	1728	CTCF-nucleophosmin-PARP-HIS-KPNA-LMNA-TOP complex	0.05556
Virus disease	1810	ITGA4-PXN-GIT1 complex	0.09623
Virus disease	2019	IL12A-IL12B-IL12RB1 complex	0.09623
Virus disease	2020	IL12B-IL12RB1-IL12RB2 complex	0.09623
Virus disease	2026	IL12RB1-IL12RB2 complex	0.11785
Virus disease	2055	CASP8-CHUK-IKBKB-MALT1-BCL10 complex	0.07454
Virus disease	2056	BCL10-CHUK-BCL10-IKBKB complex	0.08333
Virus disease	2100	CHUK-IKBKB-MAP3K14 complex	0.09623
Virus disease	2101	IKKA-IKKB complex	0.11785
Virus disease	2105	IkappaB kinase complex (IKBKB CHUK IKBKAP NFKBIA RELA MAP3K14)	0.06804
Virus disease	2118	CHUK-ERC1-IKBKB-IKBKG	0.08333
Virus disease	2121	CHUK-IKBKB-IKBKG complex	0.09623
Virus disease	2124	IKK-alpha--ER-alpha-AIB1 complex	0.09623
Virus disease	2214	LMO4-BRCA1-CTIP-LDB1 complex	0.08333
Virus disease	2215	BRCA1-LMO4-CTIP complex	0.09623
Virus disease	2348	ITGAV-ITGB5-CYR61 complex	0.09623
Virus disease	2417	ITGA4-ITGB1-EMILIN1 complex	0.09623
Virus disease	2418	ITGA4-ITGB1 complex	0.11785
Virus disease	2419	ITGA4-ITGB1-CD81 complex	0.09623
Virus disease	2420	ITGA4-ITGB1-CD53 complex	0.09623
Virus disease	2421	ITGA4-ITGB1-VCAM1 complex	0.09623
Virus disease	2422	ITGA4-ITGB1-JAM2 complex	0.09623
Virus disease	2423	ITGA4-ITGB1-CD47 complex	0.09623
Virus disease	2424	ITGA4-ITGB1-CD63 complex	0.09623
Virus disease	2425	ITGA4-ITGB1-PXN complex	0.09623
Virus disease	2426	ITGA4-ITGB1-THBS1 complex	0.09623
Virus disease	2428	ITGA4-ITGB1-THBS2 complex	0.09623
Virus disease	2437	ITGA6-ITGB1-CYR61 complex	0.09623
Virus disease	2572	RAB5-EEA1 complex	0.11785
Virus disease	2573	Class C VPS/HOPS complex	0.06804
Virus disease	2727	SRC-3 complex	0.06299
Virus disease	2787	BRCA1 C complex	0.08333
Virus disease	2815	BRCA1-BARD1-BACH1-DNA damage complex II	0.05893

Virus disease	2819	BRCA1-CtIP-CtBP complex	0.09623
Virus disease	2839	ATRX-DAXX complex	0.11785
Virus disease	2842	DAXX-Axin-p53-HIPK2 complex	0.08333
Virus disease	2923	SHARP-CtBP1-CtIP complex	0.09623
Virus disease	2930	SHARP-CtIP-RBP-Jkappa complex	0.09623
Virus disease	2931	SHARP-CtBP1-CtIP-RBP-Jkappa corepressor complex	0.08333
Virus disease	3055	Nop56p-associated pre-rRNA complex	0.01634
Virus disease	3079	DGCR8-ILF3 complex	0.11785
Virus disease	3080	ILF3-XPO5 complex	0.11785
Virus disease	3082	DGCR8 multiprotein complex	0.05025
Virus disease	3102	DHX9-ADAR-vigilin-DNA-PK-Ku antigen complex	0.06804
Virus disease	3168	DAXX-AXIN complex	0.11785
Virus disease	3170	Daxx-Axin-p53 complex	0.09623
Virus disease	5183	DNA-PK-Ku-eIF2-NF90-NF45 complex	0.05893
Virus disease	5193	TNF-alpha/NF-kappa B signaling complex (CHUK KPNA3 NFKB2 NFKBIB REL IKBKG NFKB1 NFKBIE RELB NFKBIA RELA TNIP2)	0.04811
Virus disease	5194	TNF-alpha/NF-kappa B signaling complex (SEC16A CHUK IKBKB NFKB2 REL IKBKG MAP3K14 RELA FBXW7 USP2)	0.0527
Virus disease	5196	TNF-alpha/NF-kappa B signaling complex (CHUK BTRC NFKB2 PPP6C REL CUL1 IKBKE SAPS2 SAPS1 ANKRD28 RELA SKP1)	0.04811
Virus disease	5210	TANK-TRAF2-TRAF3 complex	0.09623
Virus disease	5212	Kinase maturation complex 2	0.11785
Virus disease	5220	CHUK-IQGAP2-AKAP8L-RELA-TNIP2 complex	0.07454
Virus disease	5230	CHUK-NFKB2-REL-IKBKG-SPAG9-NFKB1-NFKBIE-COPB2-TNIP1-NFKBIA-RELA-TNIP2	0.04811
Virus disease	5232	TNF-alpha/Nf-kappa B signaling complex (RPL6 RPL30 RPS13 CHUK DDX3X NFKB2 NFKBIB REL IKBKG NFKB1 MAP3K8 RELB GLG1 NFKBIA RELA TNIP2 GTF2I)	0.04042
Virus disease	5233	TNF-alpha/NF-kappa B signaling complex 5	0.06667
Virus disease	5266	TNF-alpha/NF-kappa B signaling complex 6	0.04454
Virus disease	5285	TNF-alpha/NF-kappa B signaling complex 9	0.07454
Virus disease	5286	TNF-alpha/NF-kappa B signaling complex 10	0.10541
Virus disease	5608	Emerin architectural complex	0.13608
Virus disease	5611	Emerin complex 24	0.08607
Virus disease	5613	Emerin complex 25	0.04167
Virus disease	5614	Emerin complex 32	0.03553
Virus disease	5615	Emerin complex 52	0.0695
Virus disease	5822	MCL1-NOXA complex	0.11785
Virus disease	5829	IKBKG-CHUK complex	0.11785
Virus disease	5844	I-kappa-B kinase (IKK) complex	0.09623
Vitamin D deficiency	1256	MLL-HCF complex	0.1543
Vitamin D deficiency	1257	ALL-1 supercomplex	0.07715
Vitamin D deficiency	1401	MOF complex	0.1291
Vitamin D deficiency	1539	G protein complex (GNG2 GNB2L1 RAF1)	0.2357
Vitamin D deficiency	3162	TF-FVIIa-FXa-TFPI complex	0.20412

Vitamin D deficiency	5211	RAF1-PPP2-PIN1 complex	0.18257
Vitamin D deficiency	5386	MLL1-WDR5 complex	0.07857
Vitamin D deficiency	5770	RUNX1-CBF-beta-DNA complex	0.28868
Vitamin D deficiency	5877	MAP2K1-BRAF-RAF1-YWHAE-KSR1 complex	0.18257
Vitamin D deficiency	5873	RAF1-MAP2K1-YWHAE complex	0.2357
Vitamin D deficiency	5919	BRAF-RAF1-14-3-3 complex	0.13608
Vitamin D deficiency	5920	KSR1-RAF1-MEK complex	0.20412
Vitamin D deficiency	5922	RAF1-RAS complex EGF induced	0.20412
Vitamin D deficiency	5923	RAF1-BRAF complex RAS stimulated	0.28868
Vitamin D deficiency	5924	RAF1-CNK1 complex RAS stimulated	0.28868
Vitamin D deficiency	5928	CNK1-SRC-RAF1 complex	0.2357
Vitiligo	441	TFTC-type histone acetyl transferase complex	0.07107
Vitiligo	1054	ESR1-RELA-BCL3-NCOA3 complex	0.11785
Vitiligo	5718	eNOS-HSP90-AKT complex VEGF induced	0.13608
Vitiligo	1985	AIRE homodimer complex	0.2357
Vitiligo	2124	IKK-alpha--ER-alpha-AIB1 complex	0.13608
Vitiligo	2156	YBX1-AKT1 complex	0.16667
Vitiligo	2159	AR-AKT-APPL complex	0.13608
Vitiligo	2470	p130Cas-ER-alpha-cSrc-kinase- PI3-kinase p85-subunit complex	0.11785
Vitiligo	2657	ESR1-CDK7-CCNH-MNAT1-MTA1-HDAC2 complex	0.09623
Vitiligo	2670	Er-alpha-p53-hdm2 complex	0.13608
Vitiligo	2699	ER-alpha-GRIP1-c-Jun complex	0.13608
Vitiligo	2700	ER-alpha-c-Jun complex	0.16667
Vitiligo	3847	TCL1(trimer)-AKT1 complex	0.16667
Vitiligo	5165	AP1G1-PACS1-FURIN complex	0.13608
Vitiligo	5862	CAV1-VDAC1-ESR1 complex	0.13608
Von Hippel-Lindau syndrome	1352	ING4 complex (ING4 MYST2 C1orf149 PHF17)	0.35355
Von Hippel-Lindau syndrome	2858	HBO1 complex	0.26726
Von Hippel-Lindau syndrome	2859	ING5 complex	0.2132
Vulvar disease	1439	PTGS2 homodimer complex	0.44721
Vulvar disease	2443	ITGA9-ITGB1-TNC complex	0.2582
Werner syndrome	71	MRN complex (MRE11-RAD50-NBS1 complex)	0.21822
Werner syndrome	73	MRN complex (MRE11-RAD50-NBN complex)	0.21822
Werner syndrome	159	Condensin I-PARP-1-XRCC1 complex	0.14286
Werner syndrome	202	BRCA1-RAD50-MRE11-NBS1 complex	0.18898
Werner syndrome	351	Spliceosome	0.03161
Werner syndrome	362	DNA ligase III-XRCC1-PNK-DNA-pol III multiprotein complex	0.18898
Werner syndrome	433	BASC complex (BRCA1-associated genome surveillance complex)	0.10911
Werner syndrome	619	MRE11A-RAD50-NBN-TRF2 complex	0.18898
Werner syndrome	627	MRN-TRRAP complex (MRE11A-RAD50-NBN-TRRAP complex)	0.18898
Werner syndrome	1004	RC complex during S-phase of cell cycle	0.10483
Werner syndrome	1005	RC complex during G2/M-phase of cell cycle	0.10483
Werner syndrome	1085	DNA repair complex NEIL2-PNK-Pol(beta)-LigIII(alpha)-XRCC1	0.16903

Werner syndrome	1086	DNA repair complex NEIL1-PNK-Pol(beta)-LigIII(alpha)-XRCC1	0.16903
Werner syndrome	1170	cMYC-ATPase-helicase complex	0.16903
Werner syndrome	1171	c-MYC-ATPase-helicase complex	0.16903
Werner syndrome	1181	C complex spliceosome	0.04226
Werner syndrome	1182	CDC5L core complex	0.1543
Werner syndrome	1183	CDC5L complex	0.06901
Werner syndrome	1189	DNA double-strand break end-joining complex	0.14286
Werner syndrome	1193	Rap1 complex	0.14286
Werner syndrome	1217	WRN-TRF2 complex	0.26726
Werner syndrome	1226	H2AX complex I	0.14286
Werner syndrome	1728	CTCF-nucleophosmin-PARP-HIS-KPNA-LMNA-TOP complex	0.12599
Werner syndrome	1729	TLE1 corepressor complex (MASH1 promoter-corepressor complex)	0.11952
Werner syndrome	2198	RAD9-RAD1-HUS1-POLB complex	0.18898
Werner syndrome	2217	MDC1-MRN-ATM-FANCD2 complex	0.1543
Werner syndrome	2218	MDC1-MRE11-RAD50-NBS1 complex	0.18898
Werner syndrome	2625	CDK8-MED6-PARP1 complex	0.21822
Werner syndrome	2649	MYC-DNMT3A-ZBTB17 complex	0.21822
Werner syndrome	2653	MYC-MAX-BLOC1S1 complex	0.21822
Werner syndrome	2655	MYC-MAX complex	0.26726
Werner syndrome	2723	ATM-NBS1 complex	0.26726
Werner syndrome	2767	RAD50-MRE11-NBN-p200-p350 complex	0.21822
Werner syndrome	2815	BRCA1-BARD1-BACH1-DNA damage complex II	0.13363
Werner syndrome	3137	MASH1 promoter-coactivator complex	0.11396
Werner syndrome	3142	CAMK2-delta-MASH1 promoter-coactivator complex	0.13363
Werner syndrome	4082	Ku70/Ku86/Werner complex	0.21822
Werner syndrome	5179	NCOA6-DNA-PK-Ku-PARP1 complex	0.16903
Werner syndrome	5197	PTIP-DNA damage response complex	0.1543
Werner syndrome	5235	WRN-Ku70-Ku80-PARP1 complex	0.37796
Wiskott-Aldrich syndrome	27	Arp2/3 protein complex	0.23905
Wiskott-Aldrich syndrome	486	WIP-WASp-actin-myosin-IIa complex	0.2582
Wiskott-Aldrich syndrome	5712	FAK-beta5 integrin complex VEGF induced	0.22361
Wiskott-Aldrich syndrome	1707	IL2-IL2RA-IL2RB complex	0.18257
Wiskott-Aldrich syndrome	2470	p130Cas-ER-alpha-cSrc-kinase- PI3-kinase p85-subunit complex	0.15811
Wiskott-Aldrich syndrome	2480	CIN85 complex (CIN85 CRK BCAR1 CBL PIK3R1 GRB2 SOS1)	0.11952
Wiskott-Aldrich syndrome	2510	ZAP70-CRKL-WIPF1-WAS complex	0.31623
Wiskott-Aldrich syndrome	2511	CRKL-WIPF1-WAS complex	0.36515
Wiskott-Aldrich syndrome	2726	PXN-ITGB5-PTK2 complex	0.18257
Wiskott-Aldrich syndrome	2896	ITGA2b-ITGB3-CD47-FAK complex	0.15811
Wiskott-Aldrich syndrome	2962	CRK-BCAR1-DOCK1 complex	0.18257
Wiskott-Aldrich syndrome	5177	Polycystin-1 multiprotein complex (ACTN1 CDH1 SRC JUP VCL CTNNB1 PXN BCAR1 PKD1	0.19069
Wiskott-Aldrich syndrome	5282	CAS-SRC-FAK complex	0.36515
Yersinia infection	4	Multisubunit ACTR coactivator complex	0.05934

Yersinia infection	98	p300-MDM2-p53 protein complex	0.06852
Yersinia infection	286	PCNA-MSH2-MSH6 complex	0.13704
Yersinia infection	290	MSH2-MLH1-PMS2-PCNA DNA-repair initiation complex	0.05934
Yersinia infection	291	MSH2-MLH1-PMS2 DNA-repair initiation complex	0.06852
Yersinia infection	310	Cell cycle kinase complex CDC2	0.04845
Yersinia infection	368	ERCC1-ERCC4-MSH2 complex	0.06852
Yersinia infection	369	MSH2-MSH6-PMS2-MLH1 complex	0.11868
Yersinia infection	370	MSH2-MSH6-PMS1-MLH1 complex	0.11868
Yersinia infection	374	MSH2-MSH6 complex	0.16784
Yersinia infection	375	MSH2-MSH3 complex	0.08392
Yersinia infection	376	PCNA-MutS-alpha-MutL-alpha-DNA complex	0.10615
Yersinia infection	377	PCNA-MutS-alpha-DNA initial complex	0.13704
Yersinia infection	378	MutS-beta complex	0.08392
Yersinia infection	433	BASC complex (BRCA1-associated genome surveillance complex)	0.06852
Yersinia infection	434	BASC (Ab 80) complex (BRCA1-associated genome surveillance complex)	0.08392
Yersinia infection	436	BASC (Ab C-20) complex (BRCA1-associated genome surveillance complex)	0.06852
Yersinia infection	438	GCN5-TRRAP histone acetyltransferase complex	0.07506
Yersinia infection	520	KCNQ1 macromolecular complex	0.03753
Yersinia infection	570	p300-CBP-p270-SWI/SNF complex	0.04486
Yersinia infection	571	p300-CBP-p270 complex	0.06852
Yersinia infection	577	FHL2-p53-HIPK2 complex	0.06852
Yersinia infection	746	C/EBPalpha-HNF6 complex	0.08392
Yersinia infection	1088	PRNP-ApolopoproteinE3 complex	0.08392
Yersinia infection	1094	Frataxin complex	0.04486
Yersinia infection	1095	SNX complex (SNX1a SNX2 SNX4 EGFR)	0.05934
Yersinia infection	1158	p33ING1b-p300 complex	0.08392
Yersinia infection	1160	ING1-p300-PCNA complex	0.06852
Yersinia infection	1185	EGFR-containing signaling complex	0.11868
Yersinia infection	1211	Ubiquitin E3 ligase (AHR ARNT DDB1 TBL3 CUL4B RBX1)	0.04845
Yersinia infection	1335	SNW1 complex	0.02797
Yersinia infection	1471	pRb2/p130-multimolecular complex (RB2 E2F5 HDAC1 SUV39H1 P300)	0.05307
Yersinia infection	1521	p300-SMAD1-STAT3 complex	0.06852
Yersinia infection	1774	MICA-KLRK1-HCST complex	0.06852
Yersinia infection	1831	PIAS3-SMAD3-P300 complex	0.06852
Yersinia infection	2055	CASP8-CHUK-IKBKB-MALT1-BCL10 complex	0.05307
Yersinia infection	2056	BCL10-CHUK-BCL10-IKBKB complex	0.05934
Yersinia infection	2100	CHUK-IKBKB-MAP3K14 complex	0.06852
Yersinia infection	2101	IKKA-IKKB complex	0.08392
Yersinia infection	2104	IKKB-NIK complex	0.08392
Yersinia infection	2105	IkappaB kinase complex (IKBKB CHUK IKBKAP NFKBIA RELA MAP3K14)	0.04845
Yersinia infection	2118	CHUK-ERC1-IKBKB-IKBKG	0.05934
Yersinia infection	2121	CHUK-IKBKB-IKBKG complex	0.06852

Yersinia infection	2153	ITGAM-ITGB2-CD11 complex	0.06852
Yersinia infection	2159	AR-AKT-APPL complex	0.06852
Yersinia infection	2160	AOF2-AR complex	0.08392
Yersinia infection	2224	MSH2/6-BLM-p53-RAD51 complex	0.10615
Yersinia infection	2226	MutS-alpha-PK-zeta complex	0.13704
Yersinia infection	2236	ASF1-histone containing complex	0.04486
Yersinia infection	2237	SP1-MCAF2 complex	0.08392
Yersinia infection	2369	ITGAV-ITGB3-EGFR complex	0.06852
Yersinia infection	2453	Multiprotein complex (monoubiquitination)	0.05934
Yersinia infection	2454	CIN85-CBL-SH3GL2-EGFR complex EGF stimulated	0.05934
Yersinia infection	2489	NCR3-CD247 complex	0.08392
Yersinia infection	2528	ERBB2-MEMO-SHC complex	0.06852
Yersinia infection	2529	LAT-PLC-gamma-1-p85-GRB2-CBL-VAV-SLP-76 signaling complex C305 activated	0.08971
Yersinia infection	2534	Cbl-SLP-76-Grb2 complex Fc receptor gamma-R1 stimulated	0.06852
Yersinia infection	2535	SLP-76-Cbl-Grb2-Shc complex Fc receptor gamma-R1 stimulated	0.05934
Yersinia infection	2536	PLC-gamma-2-SLP-76-Lyn-Grb2 complex	0.05934
Yersinia infection	2542	EGFR-CBL-GRB2 complex	0.06852
Yersinia infection	2547	PLC-gamma-1-SLP-76-SOS1-LAT complex	0.11868
Yersinia infection	2638	HES1 promoter corepressor complex	0.04845
Yersinia infection	2639	HES1 promoter-Notch enhancer complex	0.03292
Yersinia infection	2641	p300/CBP-PCAF-MyoD complex	0.05934
Yersinia infection	2642	SMAD1-P300 complex	0.08392
Yersinia infection	2679	p53-SP1 complex	0.08392
Yersinia infection	2706	SMAD3-SMAD4-SP1 complex	0.06852
Yersinia infection	2721	HCF-1 complex	0.02723
Yersinia infection	2727	SRC-3 complex	0.04486
Yersinia infection	2740	MutS-alpha complex	0.16784
Yersinia infection	2755	17S U2 snRNP	0.02066
Yersinia infection	2817	BRCA1-BARD1-BACH1-DNA damage complex I	0.04845
Yersinia infection	2842	DAXX-Axin-p53-HIPK2 complex	0.05934
Yersinia infection	2844	Axin-p53-HIPK2 complex	0.06852
Yersinia infection	2909	PLC-gamma-2-Syk-LAT-FcR-gamma complex	0.05934
Yersinia infection	2912	PLC-gamma-2-SLP-76 complex	0.08392
Yersinia infection	2913	PLC-gamma-2-LAT complex	0.08392
Yersinia infection	2922	LAT-PLC-gamma-1-p85-GRB2-SOS signaling complex C305 activated	0.05307
Yersinia infection	2954	Smad1-Notch1-p300-Pcaf complex	0.05934
Yersinia infection	2955	LCK-SLP76-PLC-gamma-1-LAT complex pervanadate-activated	0.11868
Yersinia infection	2956	PLC-gamma-1-LAT-c-CBL complex OKT3 stimulated	0.06852
Yersinia infection	2957	LAT-GRB2 complex Fyn-mLck(KA) or Syk kinase activated	0.08392
Yersinia infection	2960	SLP-76-PLC-gamma-1-ITK complex alpha-TCR stimulated	0.06852
Yersinia infection	2961	SLP-76-PLC-gamma-1-VAV complex alpha-TCR stimulated	0.06852

Yersinia infection	2963	ITK-SLP-76 complex anti-TCR stimulated	0.08392
Yersinia infection	3070	CTF18-cohesion-RFC-POLH complex	0.04196
Yersinia infection	3149	NK-3-Groucho-HIPK2-SIN3A-RbpA48-HDAC1	0.03578
Yersinia infection	3678	RIN1-STAM2-EGFR complex EGF stimulated	0.06852
Yersinia infection	3838	SP1-E2F2 complex	0.08392
Yersinia infection	3839	SP1-E2F3 complex	0.08392
Yersinia infection	5118	pRb2/p130-multimolecular complex (RB2 E2F4 HDAC1 SUV39H1 P300)	0.05307
Yersinia infection	5171	SH3KBP1-CBLB-EGFR complex	0.06852
Yersinia infection	5194	TNF-alpha/NF-kappa B signaling complex (SEC16A CHUK IKBKB NFKB2 REL IKBKG MAP3K14 RELA FBXW7 USP2)	0.03753
Yersinia infection	5233	TNF-alpha/NF-kappa B signaling complex 5	0.02374
Yersinia infection	5234	IKBKB-CDC37-KIAA1967-HSP90AB1-HSP90AA1 complex	0.05307
Yersinia infection	5260	TCF4-CTNNB1-SUMO1-EP300-HADAC6 complex	0.05307
Yersinia infection	5261	TCF4-CTNNB1-EP300 complex	0.06852
Yersinia infection	5266	TNF-alpha/NF-kappa B signaling complex 6	0.03172
Yersinia infection	5375	EGR-EP300 complex	0.08392
Yersinia infection	5542	CCNB2-CDC2 complex	0.08392
Yersinia infection	5544	CDC2-PCNA-CCNB1-GADD45A complex	0.05934
Yersinia infection	5655	Ternary complex (LRRC7 CAMK2a ACTN4)	0.06852
Yersinia infection	5828	IKBKG-IKBKB complex	0.08392
Yersinia infection	5844	I-kappa-B kinase (IKK) complex	0.06852
