# Supplementary material for: Inferring drug-disease associations based on known protein complexes
Source: BMC Med Genomics. 2015 May 29;8(Suppl 2):S2. doi: 10.1186/1755-8794-8-S2-S2 (PMC4460611; doi:10.1186/1755-8794-8-S2-S2)
Supplement: Additional file 6 — Table illustrating the information of drug-disease network before being filtered by PPI network and weight. [file 1755-8794-8-S2-S2-S6.PDF]

Drug ID	Disease Name	Weight
DB00117	Brain tumor	0.04016
DB00117	Neoplasm metastasis	0.04069
DB00117	Renal Cell cancer	0.05812
DB00125	Hypertension, Pulmonary	0.07647
DB00125	Adenovirus infection	0.03148
DB00125	Alzheimer's disease	0.05755
DB00125	Atherosclerosis	0.03458
DB00125	Bladder cancer	0.25868
DB00125	Cancer	0.07983
DB00125	Depression	0.03104
DB00125	Diabetes mellitus	0.02373
DB00125	Drug abuse	0.02289
DB00125	Eating disorder	0.11327
DB00125	Embryoma	0.02055
DB00125	Encephalopathies	0.04668
DB00125	Fanconi's anemia	0.10147
DB00125	Gilles de la Tourette syndrome	0.38506
DB00125	Herpes	0.08147
DB00125	Huntington disease	0.11048
DB00125	Hyperglycemia	0.09606
DB00125	Hypertension	0.21199
DB00125	Lung cancer	0.02328
DB00125	Movement disorder	0.10379
DB00125	Obesity	0.04387
DB00125	Obsessive-compulsive disorder	0.23968
DB00125	Oral cancer	0.12919
DB00125	Polycystic ovary syndrome	0.05609
DB00125	Primary biliary cirrhosis	0.23974
DB00125	Prostate cancer	0.0174
DB00125	Psychotic disorder	0.10017
DB00125	Pulmonary fibrosis	0.3124
DB00125	Rabies	0.12049
DB00125	Renal Cell cancer	0.03875
DB00125	Schizophrenia	0.03994
DB00125	Scleroderma	0.14094
DB00125	Sickle cell disease	0.06415
DB00125	Systemic infection	0.04401
DB00125	Systemic scleroderma	0.14013
DB00125	Tropical spastic paraparesis	0.13391
DB00125	Vitiligo	0.11161
DB00155	Adenovirus infection	0.02884
DB00155	Alzheimer's disease	0.07643
DB00155	Arthritis	0.0344
DB00155	Asthma	0.03652
DB00155	Atherosclerosis	0.03168
DB00155	Bladder cancer	0.23699

DB00155	Cancer	0.07314
DB00155	Colon cancer	0.02993
DB00155	Common cold	0.09458
DB00155	Depression	0.02844
DB00155	Diabetes mellitus	0.04349
DB00155	Drug abuse	0.02097
DB00155	Eating disorder	0.10377
DB00155	Embryoma	0.0171
DB00155	Encephalopathies	0.07767
DB00155	Fanconi's anemia	0.09296
DB00155	Gilles de la Tourette syndrome	0.35278
DB00155	Herpes	0.07464
DB00155	Huntington disease	0.10121
DB00155	Hyperglycemia	0.08801
DB00155	Hypertension	0.17008
DB00155	Kidney failure	0.05401
DB00155	Liver cancer	0.0624
DB00155	Movement disorder	0.09508
DB00155	Multiple sclerosis	0.02861
DB00155	Neurodegenerative disorder	0.06788
DB00155	Obesity	0.04019
DB00155	Obsessive-compulsive disorder	0.21958
DB00155	Oral cancer	0.11836
DB00155	Polycystic ovary syndrome	0.05138
DB00155	Primary biliary cirrhosis	0.21963
DB00155	Prostate cancer	0.02294
DB00155	Psychotic disorder	0.09177
DB00155	Pulmonary fibrosis	0.28621
DB00155	Rabies	0.11038
DB00155	Renal Cell cancer	0.03224
DB00155	Respiratory failure	0.15233
DB00155	Rheumatoid arthritis	0.01697
DB00155	Schizophrenia	0.03659
DB00155	Scleroderma	0.12912
DB00155	Spinocerebellar ataxias	0.16711
DB00155	Spondylarthropathies	0.09245
DB00155	Systemic infection	0.04032
DB00155	Systemic scleroderma	0.12838
DB00155	Tropical spastic paraparesis	0.12269
DB00155	Vitiligo	0.10225
DB01110	Adenovirus infection	0.02636
DB01110	Alzheimer's disease	0.0482
DB01110	Arthritis	0.17197
DB01110	Atherosclerosis	0.09339
DB01110	Autistic disorder	0.10268
DB01110	Bladder cancer	0.21663
DB01110	Brain tumor	0.02074

DB01110	Bronchial disease	0.40659
DB01110	Cancer	0.07637
DB01110	Chronic obstructive airway disease	0.13796
DB01110	Cystic fibrosis	0.15029
DB01110	Depression	0.026
DB01110	Dermatitis	0.071
DB01110	Diabetes mellitus	0.0641
DB01110	Drug abuse	0.0984
DB01110	Eating disorder	0.09486
DB01110	Endometrium cancer	0.06262
DB01110	Enteritis	0.02834
DB01110	Epilepsy	0.16303
DB01110	Fanconi's anemia	0.08498
DB01110	Gilles de la Tourette syndrome	0.32247
DB01110	Glaucoma	0.12968
DB01110	Graves' disease	0.1861
DB01110	Heart failure	0.13263
DB01110	Herpes	0.06823
DB01110	Huntington disease	0.09252
DB01110	Hyperglycemia	0.08045
DB01110	Hypertension	0.24195
DB01110	Ischemia	0.14104
DB01110	Long QT syndrome	0.07454
DB01110	Lung cancer	0.0622
DB01110	Malaria	0.19934
DB01110	Metabolism disease	0.18766
DB01110	Migraine	0.04795
DB01110	Movement disorder	0.39039
DB01110	Myopathy	0.12513
DB01110	Neurodegenerative disorder	0.18048
DB01110	Obesity	0.11848
DB01110	Obsessive-compulsive disorder	0.20072
DB01110	Oral cancer	0.10819
DB01110	Polycystic ovary syndrome	0.15147
DB01110	Premature birth	0.28498
DB01110	Primary biliary cirrhosis	0.20077
DB01110	Prostate cancer	0.07946
DB01110	Psychotic disorder	0.12523
DB01110	Pulmonary fibrosis	0.26162
DB01110	Rabies	0.1009
DB01110	Rheumatoid arthritis	0.04455
DB01110	Schizophrenia	0.03344
DB01110	Scleroderma	0.11803
DB01110	Sickle cell disease	0.13859
DB01110	Subarachnoid hemorrhage	0.13917
DB01110	Sudden infant death syndrome	0.05634
DB01110	Systemic infection	0.03685

DB01110	Systemic scleroderma	0.11736
DB01110	Tropical spastic paraparesis	0.11215
DB01110	Ulcerative colitis	0.02663
DB01110	Vitiligo	0.09347
DB01234	Adrenal gland hypofunction	0.28868
DB01234	Adrenoleukodystrophy	0.13363
DB01234	Azoospermia	0.10206
DB01234	Cancer	0.01843
DB01234	Endometrial cancer	0.08704
DB01234	Endometriosis	0.04152
DB01234	Ewings sarcoma	0.16667
DB01234	Hamman-Rich syndrome	0.09623
DB01234	Hepatitis	0.1291
DB01234	Hypertension	0.03953
DB01234	Muscular dystrophies	0.09806
DB01234	Rheumatoid arthritis	0.0306
DB01234	Urogenital abnormalities	0.13363
DB08814	Adenoma	0.01326
DB08814	Brain tumor	0.0198
DB08814	Breast cancer	0.04388
DB08814	Cancer	0.07974
DB08814	Colon cancer	0.00775
DB08814	Congenital abnormality	0.007
DB08814	Dermatitis	0.06632
DB08814	Diabetes mellitus	0.13664
DB08814	Embryoma	0.01723
DB08814	Endometriosis	0.18031
DB08814	Hodgkin's disease	0.11996
DB08814	Infectious lung disease	0.09529
DB08814	Infiltrating cancer	0.26047
DB08814	Leukemia	0.03506
DB08814	Liver cancer	0.08883
DB08814	Lymphoma	0.01424
DB08814	Multiple sclerosis	0.34363
DB08814	Neoplasm metastasis	0.05196
DB08814	Nephrosis	0.47968
DB08814	Obesity	0.05308
DB08814	Pancreatitis	0.42148
DB08814	Polyarthritis	0.2456
DB08814	Prostate cancer	0.06844
DB08814	Schizophrenia	0.01367
DB08814	Squamous cell cancer	0.11505
DB08814	Stomach cancer	0.02085
DB08814	Stroke	0.21085
DB08814	Systemic infection	0.07717
DB08814	Systemic scleroderma	0.012
DB08814	Testicular dysfunction	0.01522

DB08814	Tuberculosis	0.43375
DB08814	Tuberous sclerosis	0.01171
DB08814	Ulcerative colitis	0.2494
DB08814	Virus disease	0.07038
DB08814	Yersinia infection	0.00996
DB00131	Adenoma of thyroid	0.13757
DB00131	Atherosclerosis	0.04139
DB00131	Behavior disease	0.03984
DB00131	Breast cancer	0.02382
DB00131	Cancer	0.0089
DB00131	Cardiovascular disease	0.12018
DB00131	Chronic obstructive airway disease	0.07878
DB00131	Depression	0.0339
DB00131	Diabetes mellitus	0.02718
DB00131	Down syndrome	0.02942
DB00131	Embryoma	0.03498
DB00131	Gastrointestinal cancer	0.17135
DB00131	Leukemia	0.04696
DB00131	Polyarthritis	0.04065
DB00131	Prostate cancer	0.02669
DB00116	Infertility, Male	0.06155
DB00116	Abortion	0.03769
DB00116	Abruption placentae	0.2132
DB00116	Alzheimer's disease	0.04341
DB00116	Antiphospholipid syndrome	0.1005
DB00116	Arteriopathy	0.1005
DB00116	Aseptic necrosis of bone	0.09535
DB00116	Asthma	0.02462
DB00116	Atherosclerosis	0.02111
DB00116	Attention deficit hyperactivity disorder	0.08704
DB00116	Bipolar disorder	0.03414
DB00116	Bladder cancer	0.03892
DB00116	Brain tumor	0.02422
DB00116	Cancer	0.03334
DB00116	Cardiovascular disease	0.04598
DB00116	Cerebrovascular disorder	0.0658
DB00116	Chronic rejection of renal transplant	0.07538
DB00116	Chronic simple glaucoma	0.06428
DB00116	Cirrhosis	0.05096
DB00116	Cleft palate	0.24175
DB00116	Depression	0.03959
DB00116	Diabetes mellitus	0.01587
DB00116	Down syndrome	0.06872
DB00116	Embryoma	0.01859
DB00116	Epilepsy	0.04264
DB00116	Folic acid deficiency	0.12309
DB00116	Glaucoma	0.05025

DB00116	Hepatitis C	0.04446
DB00116	Huntington disease	0.0658
DB00116	Hyperglycemia	0.04828
DB00116	Hyperhomocysteinemia	0.17408
DB00116	Hyperuricemia	0.17408
DB00116	Infertility	0.04142
DB00116	Kidney failure	0.03414
DB00116	Liver disease	0.04652
DB00116	Lupus erythematosus	0.02576
DB00116	Meningioma	0.07785
DB00116	Migraine	0.05599
DB00116	Moyamoya disease	0.12309
DB00116	Obesity	0.04639
DB00116	Osteoporosis	0.04767
DB00116	Parkinson disease	0.0333
DB00116	Polycystic ovary syndrome	0.03994
DB00116	Rheumatoid arthritis	0.0369
DB00116	Schizophrenia	0.04585
DB00116	Sickle cell disease	0.05803
DB00116	Spinal dysraphism	0.20751
DB00116	Takayasu's arteritis	0.09535
DB00116	Thalassemia	0.07107
DB00116	Thrombophilia	0.07107
DB00116	Ulcerative colitis	0.0622
DB00116	Vitamin B deficiency	0.11396
DB00568	Amyotrophic lateral sclerosis	0.04495
DB00568	Atherosclerosis	0.02334
DB00568	Atopic rhinitis	0.08607
DB00568	Breast cancer	0.01604
DB00568	Colon cancer	0.01978
DB00568	Congenital heart disease	0.11111
DB00568	Drug abuse	0.03122
DB00568	Endocrine system disease	0.12599
DB00568	Epilepsy	0.09428
DB00568	Infertility	0.04579
DB00568	Optic atrophy	0.14907
DB00568	Parkinson disease	0.03681
DB00568	Prostate cancer	0.0174
DB00568	Retinal disease	0.04668
DB00568	Schizophrenia	0.02534
DB00661	Hypertension, Pulmonary	0.05735
DB00661	Amyotrophic lateral sclerosis	0.03371
DB00661	Anorexia nervosa	0.05103
DB00661	Atherosclerosis	0.05251
DB00661	Autistic disorder	0.03032
DB00661	Behavior disease	0.03858
DB00661	Bipolar disorder	0.02831

DB00661	Breast cancer	0.01203
DB00661	Cancer	0.00922
DB00661	Cerebellar disease	0.60623
DB00661	Chronic fatigue syndrome	0.08839
DB00661	Chronic obstructive airway disease	0.02795
DB00661	Colon cancer	0.01483
DB00661	Congenital abnormality	0.10517
DB00661	Congenital heart disease	0.08333
DB00661	Depression	0.03283
DB00661	Dermatitis	0.02273
DB00661	Diabetes mellitus	0.01316
DB00661	Drug abuse	0.09366
DB00661	Endocrine system disease	0.09449
DB00661	Epilepsy	0.07071
DB00661	Fibromyalgia	0.08839
DB00661	Generalized anxiety disorder	0.07217
DB00661	Heart disease	0.07538
DB00661	Heart failure	0.0533
DB00661	Herpes	0.03571
DB00661	Hyperglycemia	0.04003
DB00661	Hyperinsulinism	0.04811
DB00661	Infantile spasms	0.09449
DB00661	Intestinal disease	0.0625
DB00661	Ischemia	0.03201
DB00661	Late pregnancy	0.07217
DB00661	Long QT syndrome	0.14434
DB00661	Mental retardation	0.23106
DB00661	Metabolism disease	0.04003
DB00661	Migraine	0.28125
DB00661	Myopathy	0.18383
DB00661	Myotonic disorder	0.28615
DB00661	Neurotic disorder	0.07906
DB00661	Obesity	0.01923
DB00661	Obsessive-compulsive disorder	0.07538
DB00661	Optic atrophy	0.1118
DB00661	Pancreas disease	0.05213
DB00661	Panic disorder	0.05455
DB00661	Pervasive development disorder	0.06455
DB00661	Polycystic ovary syndrome	0.03311
DB00661	Psychotic disorder	0.04003
DB00661	Pulmonary hypertension	0.125
DB00661	Retinal disease	0.03501
DB00661	Spinocerebellar ataxias	0.48907
DB00661	Stroke	0.02831
DB00661	Sudden infant death syndrome	0.16366
DB00661	Ulcerative colitis	0.02579
DB00909	Myoclonic epilepsy, Juvenile	0.07332

DB00909	Stress disorder, post-traumatic	0.0635
DB00909	Anemia	0.07658
DB00909	Anorexia nervosa	0.03666
DB00909	Arteriopathy	0.05987
DB00909	Autistic disorder	0.08712
DB00909	Behavior disease	0.05543
DB00909	Bipolar disorder	0.02034
DB00909	Brain disease	0.03226
DB00909	Breast cancer	0.02592
DB00909	Cancer	0.01986
DB00909	Central nervous system disease	0.0449
DB00909	Cervical cancer	0.02088
DB00909	Colon cancer	0.01066
DB00909	Common cold	0.048
DB00909	Congenital abnormality	0.01354
DB00909	Congenital heart disease	0.05987
DB00909	Dental plaque	0.02281
DB00909	Depression	0.02358
DB00909	Diabetes mellitus	0.00945
DB00909	Down syndrome	0.04094
DB00909	Drug abuse	0.06729
DB00909	Encephalopathies	0.02515
DB00909	Epilepsy	0.0762
DB00909	Fibromyalgia	0.127
DB00909	Generalized anxiety disorder	0.05185
DB00909	Glaucoma	0.02993
DB00909	Heart disease	0.05415
DB00909	Huntington disease	0.03919
DB00909	Infantile spasms	0.06788
DB00909	Intestinal disease	0.0449
DB00909	Intractable epilepsy	0.127
DB00909	Ischemia	0.023
DB00909	Long QT syndrome	0.1037
DB00909	Migraine	0.03335
DB00909	Myopathy	0.06534
DB00909	Nervous system disease	0.04637
DB00909	Neuropathy	0.05752
DB00909	Neurotic disorder	0.0568
DB00909	Obesity	0.01382
DB00909	Pancreas cancer	0.01862
DB00909	Pancreatitis	0.02805
DB00909	Panic disorder	0.03919
DB00909	Prostate cancer	0.01875
DB00909	Psychotic disorder	0.02876
DB00909	Rabies	0.01996
DB00909	Retinitis pigmentosa	0.03592
DB00909	Rheumatism	0.08467

DB00909	Stomach cancer	0.01513
DB00909	Sudden infant death syndrome	0.07839
DB00909	Systemic scleroderma	0.01883
DB00909	Ulcerative colitis	0.01852
DB00909	Vulvar disease	0.08032
DB04841	Abortion	0.02905
DB04841	Achalasia and cardiospasm	0.12469
DB04841	Alzheimer's disease	0.05292
DB04841	Amyloidosis	0.06639
DB04841	Asthma	0.04371
DB04841	Atopic rhinitis	0.11547
DB04841	Autistic disorder	0.06042
DB04841	Autoimmune disease	0.05572
DB04841	Bipolar disorder	0.04432
DB04841	Bladder cancer	0.04542
DB04841	Brain ischemia	0.18262
DB04841	Breast cancer	0.02152
DB04841	Cancer	0.03396
DB04841	Celiac disease	0.08498
DB04841	Colon cancer	0.04374
DB04841	Congenital abnormality	0.03237
DB04841	Congenital heart disease	0.14907
DB04841	Dental plaque	0.06543
DB04841	Diabetes mellitus	0.0125
DB04841	Drug abuse	0.03239
DB04841	Eating disorder	0.10416
DB04841	Epilepsy	0.12649
DB04841	Epstein-Barr virus infection	0.09263
DB04841	Esophageal tumor	0.07695
DB04841	Esophagus cancer	0.03074
DB04841	Esotropia	0.08852
DB04841	Eye cancer	0.10972
DB04841	Fanconi's anemia	0.0433
DB04841	Glaucoma	0.07631
DB04841	Gram-Negative bacterial infection	0.15306
DB04841	Graves' disease	0.10951
DB04841	HIV infection	0.05185
DB04841	Herpes	0.07492
DB04841	Infertility	0.06143
DB04841	Ischemia	0.06354
DB04841	Keratosis	0.08781
DB04841	Leukemia	0.03912
DB04841	Lichen planus	0.17067
DB04841	Lung cancer	0.0366
DB04841	Lupus erythematosus	0.03596
DB04841	Malignant glioma	0.04453
DB04841	Melanoma	0.02281

DB04841	Parkinson disease	0.04939
DB04841	Pre-Eclampsia	0.04147
DB04841	Prion disease	0.14306
DB04841	Prostate cancer	0.02334
DB04841	Rabies	0.02116
DB04841	Rheumatoid arthritis	0.02622
DB04841	Schistosomiasis	0.15421
DB04841	Schizophrenia	0.034
DB04841	Stroke	0.04015
DB04841	Thyroid gland disease	0.09641
DB04841	Tuberculosis	0.0826
DB00201	Myopathies, Nemaline	0.20412
DB00201	Asthma	0.04082
DB00201	Behavior disease	0.07715
DB00201	Cancer	0.01843
DB00201	Chronic obstructive airway disease	0.0559
DB00201	Congenital abnormality	0.03769
DB00201	Cystic fibrosis	0.07143
DB00201	Depression	0.13131
DB00201	Eating disorder	0.08704
DB00201	Endocrine system disease	0.18898
DB00201	Heart failure	0.0533
DB00201	Herpes	0.07143
DB00201	Huntington disease	0.10911
DB00201	Hypertension	0.07906
DB00201	Ischemia	0.06402
DB00201	Kidney failure	0.05661
DB00201	Leukemia	0.02817
DB00201	Myopathy	0.06063
DB00201	Obesity	0.03846
DB00201	Panic disorder	0.10911
DB00201	Phobic anxiety disorder	0.25
DB00201	Pick disease of the brain	0.22361
DB00201	Respiratory tract disease	0.1066
DB00201	Schizophrenia	0.03801
DB00201	Thrombocytosis	0.22361
DB00277	Infertility, Male	0.05682
DB00277	Abortion	0.03622
DB00277	Adenocarcinoma	0.05157
DB00277	Adenovirus infection	0.19128
DB00277	Adrenal gland hyperfunction	0.07408
DB00277	Adrenal gland tumor	0.07452
DB00277	Alzheimer's disease	0.02356
DB00277	Asthma	0.02887
DB00277	Atherosclerosis	0.00971
DB00277	Autistic disorder	0.01894
DB00277	Azoospermia	0.02066

DB00277	Behavior disease	0.08572
DB00277	Bipolar disorder	0.03689
DB00277	Brain disease	0.09829
DB00277	Brain tumor	0.07519
DB00277	Breast cancer	0.19613
DB00277	Bronchial hyperreactivity	0.04292
DB00277	Cancer	0.16365
DB00277	Cerebrovascular disorder	0.0295
DB00277	Cholelithiasis	0.02577
DB00277	Chronic obstructive airway disease	0.07906
DB00277	Colon cancer	0.0237
DB00277	Congenital abnormality	0.01341
DB00277	Corneal disease	0.01733
DB00277	Cystic fibrosis	0.06323
DB00277	Dental plaque	0.01675
DB00277	Depression	0.1041
DB00277	Diabetes mellitus	0.02901
DB00277	Down syndrome	0.01675
DB00277	Drug abuse	0.01639
DB00277	Eating disorder	0.06958
DB00277	Embryoma	0.13885
DB00277	Encephalopathies	0.03636
DB00277	Endometriosis	0.02936
DB00277	Epilepsy	0.03405
DB00277	Fanconi's anemia	0.05932
DB00277	Glaucoma	0.01953
DB00277	Granulomatous disease	0.04164
DB00277	HIV infection	0.05687
DB00277	Heart failure	0.03769
DB00277	Hereditary disease	0.04196
DB00277	Herpes	0.07156
DB00277	Huntington disease	0.07715
DB00277	Hyperlipidemia	0.0327
DB00277	Hypertension	0.09688
DB00277	Hypopituitarism	0.08834
DB00277	Immunologic deficiency syndrome	0.00837
DB00277	Infection	0.03252
DB00277	Infertility	0.01885
DB00277	Ischemia	0.05441
DB00277	Kaposi sarcoma	0.08043
DB00277	Keratoconus	0.0275
DB00277	Kidney failure	0.05658
DB00277	Late pregnancy	0.04051
DB00277	Leukemia	0.04933
DB00277	Leukoencephalopathy	0.0177
DB00277	Liver cancer	0.06189
DB00277	Lung cancer	0.2201

DB00277	Lupus erythematosus	0.02048
DB00277	Lupus vulgaris	0.02503
DB00277	Lymphoma	0.0078
DB00277	Melanoma	0.0086
DB00277	Mental retardation	0.01495
DB00277	Migraine	0.02313
DB00277	Movement disorder	0.04605
DB00277	Muscular dystrophies	0.01372
DB00277	Myasthenia Gravis	0.03132
DB00277	Myotonic disorder	0.01454
DB00277	Neoplasm metastasis	0.0912
DB00277	Nervous system disease	0.03759
DB00277	Obesity	0.03951
DB00277	Oligospermia	0.03761
DB00277	Osteomyelitis	0.0139
DB00277	Osteoporosis	0.01877
DB00277	Ovarian disease	0.02942
DB00277	Ovarian failure	0.03847
DB00277	Panic disorder	0.1067
DB00277	Parkinson disease	0.01432
DB00277	Pelvic inflammatory disease	0.29842
DB00277	Phobic anxiety disorder	0.17678
DB00277	Pick disease of the brain	0.15811
DB00277	Polyarthritis	0.0107
DB00277	Polycystic ovary syndrome	0.02226
DB00277	Primary biliary cirrhosis	0.02243
DB00277	Prostate cancer	0.07835
DB00277	Rabies	0.02722
DB00277	Renal Cell cancer	0.02207
DB00277	Renal tubular acidosis	0.06058
DB00277	Respiratory tract disease	0.07538
DB00277	Retinitis pigmentosa	0.02253
DB00277	Rett syndrome	0.05409
DB00277	Rheumatoid arthritis	0.00949
DB00277	Schizophrenia	0.0671
DB00277	Skin disease	0.02616
DB00277	Spinal dysraphism	0.02835
DB00277	Stomach cancer	0.02448
DB00277	Stroke	0.01028
DB00277	Synovitis	0.03411
DB00277	Testicular tumor	0.0662
DB00277	Thrombocytosis	0.15811
DB00277	Thrombophlebitis	0.0186
DB00277	Thymoma	0.02647
DB00277	Tic disorder	0.03097
DB00277	Tuberous sclerosis	0.30975
DB00277	Uterine disease	0.02356

DB00277	Vitamin D deficiency	0.01561
DB00277	Vitiligo	0.03132
DB00277	Yersinia infection	0.00662
DB00640	Adenocarcinoma	0.07293
DB00640	Asthma	0.04082
DB00640	Brain tumor	0.04016
DB00640	Breast cancer	0.04811
DB00640	Chronic obstructive airway disease	0.0559
DB00640	Colon cancer	0.02967
DB00640	Cystic fibrosis	0.07143
DB00640	Depression	0.06565
DB00640	Diabetes mellitus	0.02632
DB00640	Eating disorder	0.08704
DB00640	Heart failure	0.0533
DB00640	Herpes	0.07143
DB00640	Huntington disease	0.10911
DB00640	Hypertension	0.11859
DB00640	Ischemia	0.12804
DB00640	Kidney failure	0.05661
DB00640	Liver cancer	0.03881
DB00640	Obesity	0.03846
DB00640	Panic disorder	0.10911
DB00640	Phobic anxiety disorder	0.25
DB00640	Pick disease of the brain	0.22361
DB00640	Respiratory tract disease	0.1066
DB00640	Rheumatoid arthritis	0.0306
DB00640	Schizophrenia	0.03801
DB00640	Thrombocytosis	0.22361
DB00651	Asthma	0.02887
DB00651	Atherosclerosis	0.09432
DB00651	Behavior disease	0.05455
DB00651	Cancer	0.02656
DB00651	Cardiovascular disease	0.29506
DB00651	Chronic obstructive airway disease	0.26754
DB00651	Cystic fibrosis	0.05051
DB00651	Depression	0.09285
DB00651	Eating disorder	0.06155
DB00651	Heart failure	0.03769
DB00651	Herpes	0.05051
DB00651	Huntington disease	0.07715
DB00651	Hypertension	0.0559
DB00651	Ischemia	0.04527
DB00651	Kidney failure	0.04003
DB00651	Leukemia	0.11307
DB00651	Obesity	0.0272
DB00651	Panic disorder	0.07715
DB00651	Phobic anxiety disorder	0.17678

DB00651	Pick disease of the brain	0.15811
DB00651	Respiratory tract disease	0.07538
DB00651	Schizophrenia	0.02688
DB00651	Thrombocytosis	0.15811
DB00806	Asthma	0.03333
DB00806	Behavior disease	0.06299
DB00806	Chronic obstructive airway disease	0.09129
DB00806	Cystic fibrosis	0.05832
DB00806	Depression	0.10721
DB00806	Eating disorder	0.07107
DB00806	Heart failure	0.04352
DB00806	Herpes	0.05832
DB00806	Huntington disease	0.08909
DB00806	Hypertension	0.06455
DB00806	Ischemia	0.05227
DB00806	Kidney failure	0.04623
DB00806	Leukemia	0.046
DB00806	Obesity	0.0314
DB00806	Panic disorder	0.08909
DB00806	Phobic anxiety disorder	0.20412
DB00806	Pick disease of the brain	0.18257
DB00806	Respiratory tract disease	0.08704
DB00806	Schizophrenia	0.03104
DB00806	Thrombocytosis	0.18257
DB00996	Eating disorder	0.08704
DB00996	Herpes	0.07143
DB00996	Hypertension	0.03953
DB00996	Ischemia	0.06402
DB00996	Lung cancer	0.03492
DB00996	Obesity	0.03846
DB00996	Peptic ulcer	0.11785
DB00996	Pick disease of the brain	0.22361
DB00996	Respiratory tract disease	0.1066
DB01223	Infertility, Male	0.05682
DB01223	Abortion	0.03622
DB01223	Adenovirus infection	0.19128
DB01223	Adrenal gland hyperfunction	0.07408
DB01223	Adrenal gland tumor	0.07452
DB01223	Alzheimer's disease	0.02356
DB01223	Atherosclerosis	0.00971
DB01223	Autistic disorder	0.01894
DB01223	Azoospermia	0.02066
DB01223	Behavior disease	0.03116
DB01223	Bipolar disorder	0.03689
DB01223	Brain disease	0.09829
DB01223	Brain tumor	0.11535
DB01223	Breast cancer	0.20318

DB01223	Bronchial hyperreactivity	0.04292
DB01223	Cancer	0.16365
DB01223	Cerebrovascular disorder	0.0295
DB01223	Cholelithiasis	0.02577
DB01223	Colon cancer	0.05337
DB01223	Congenital abnormality	0.01341
DB01223	Corneal disease	0.01733
DB01223	Cystic fibrosis	0.01272
DB01223	Dental plaque	0.01675
DB01223	Depression	0.01125
DB01223	Diabetes mellitus	0.0104
DB01223	Down syndrome	0.01675
DB01223	Drug abuse	0.01639
DB01223	Eating disorder	0.09507
DB01223	Embryoma	0.13885
DB01223	Encephalopathies	0.03636
DB01223	Endometriosis	0.02936
DB01223	Epilepsy	0.03405
DB01223	Fanconi's anemia	0.05932
DB01223	Glaucoma	0.01953
DB01223	Granulomatous disease	0.04164
DB01223	HIV infection	0.05687
DB01223	Hereditary disease	0.04196
DB01223	Herpes	0.09248
DB01223	Hyperlipidemia	0.0327
DB01223	Hypertension	0.05256
DB01223	Hypopituitarism	0.08834
DB01223	Immunologic deficiency syndrome	0.00837
DB01223	Infection	0.03252
DB01223	Infertility	0.01885
DB01223	Ischemia	0.13718
DB01223	Kaposi sarcoma	0.08043
DB01223	Keratoconus	0.0275
DB01223	Kidney failure	0.01654
DB01223	Late pregnancy	0.04051
DB01223	Leukemia	0.00948
DB01223	Leukoencephalopathy	0.0177
DB01223	Liver cancer	0.10069
DB01223	Lung cancer	0.2201
DB01223	Lupus erythematosus	0.02048
DB01223	Lupus vulgaris	0.02503
DB01223	Lymphoma	0.0078
DB01223	Melanoma	0.0086
DB01223	Mental retardation	0.01495
DB01223	Migraine	0.02313
DB01223	Movement disorder	0.04605
DB01223	Muscular dystrophies	0.01372

DB01223	Myasthenia Gravis	0.03132
DB01223	Myotonic disorder	0.01454
DB01223	Neoplasm metastasis	0.0912
DB01223	Nervous system disease	0.03759
DB01223	Obesity	0.05077
DB01223	Oligospermia	0.03761
DB01223	Osteomyelitis	0.0139
DB01223	Osteoporosis	0.01877
DB01223	Ovarian disease	0.02942
DB01223	Ovarian failure	0.03847
DB01223	Panic disorder	0.02955
DB01223	Parkinson disease	0.01432
DB01223	Pelvic inflammatory disease	0.29842
DB01223	Pick disease of the brain	0.22361
DB01223	Polyarthritis	0.0107
DB01223	Polycystic ovary syndrome	0.02226
DB01223	Primary biliary cirrhosis	0.02243
DB01223	Prostate cancer	0.07835
DB01223	Rabies	0.02722
DB01223	Renal Cell cancer	0.02207
DB01223	Renal tubular acidosis	0.06058
DB01223	Respiratory tract disease	0.1066
DB01223	Retinitis pigmentosa	0.02253
DB01223	Rett syndrome	0.05409
DB01223	Rheumatoid arthritis	0.04009
DB01223	Schizophrenia	0.04022
DB01223	Skin disease	0.02616
DB01223	Spinal dysraphism	0.02835
DB01223	Stomach cancer	0.02448
DB01223	Stroke	0.01028
DB01223	Synovitis	0.03411
DB01223	Testicular tumor	0.0662
DB01223	Thrombophlebitis	0.0186
DB01223	Thymoma	0.02647
DB01223	Tic disorder	0.03097
DB01223	Tuberous sclerosis	0.30975
DB01223	Uterine disease	0.02356
DB01223	Vitamin D deficiency	0.01561
DB01223	Vitiligo	0.03132
DB01223	Yersinia infection	0.00662
DB01303	Infertility, Male	0.05682
DB01303	Abortion	0.03622
DB01303	Adenovirus infection	0.19128
DB01303	Adrenal gland hyperfunction	0.07408
DB01303	Adrenal gland tumor	0.07452
DB01303	Alzheimer's disease	0.02356
DB01303	Asthma	0.03651

DB01303	Atherosclerosis	0.00971
DB01303	Autistic disorder	0.01894
DB01303	Azoospermia	0.02066
DB01303	Behavior disease	0.03116
DB01303	Bipolar disorder	0.03689
DB01303	Brain disease	0.09829
DB01303	Brain tumor	0.07519
DB01303	Breast cancer	0.17912
DB01303	Bronchial hyperreactivity	0.04292
DB01303	Cancer	0.16365
DB01303	Cerebrovascular disorder	0.0295
DB01303	Cholelithiasis	0.02577
DB01303	Chronic obstructive airway disease	0.1
DB01303	Colon cancer	0.0237
DB01303	Congenital abnormality	0.01341
DB01303	Corneal disease	0.01733
DB01303	Cystic fibrosis	0.07661
DB01303	Dental plaque	0.01675
DB01303	Depression	0.06997
DB01303	Diabetes mellitus	0.0104
DB01303	Down syndrome	0.01675
DB01303	Drug abuse	0.01639
DB01303	Eating disorder	0.08588
DB01303	Embryoma	0.13885
DB01303	Encephalopathies	0.03636
DB01303	Endometriosis	0.02936
DB01303	Epilepsy	0.03405
DB01303	Fanconi's anemia	0.05932
DB01303	Glaucoma	0.01953
DB01303	Granulomatous disease	0.04164
DB01303	HIV infection	0.05687
DB01303	Heart failure	0.04767
DB01303	Hereditary disease	0.04196
DB01303	Herpes	0.08494
DB01303	Huntington disease	0.09759
DB01303	Hyperlipidemia	0.0327
DB01303	Hypertension	0.08374
DB01303	Hypopituitarism	0.08834
DB01303	Immunologic deficiency syndrome	0.00837
DB01303	Infection	0.03252
DB01303	Infertility	0.01885
DB01303	Ischemia	0.0664
DB01303	Kaposi sarcoma	0.08043
DB01303	Keratoconus	0.0275
DB01303	Kidney failure	0.06718
DB01303	Late pregnancy	0.04051
DB01303	Leukemia	0.03468

DB01303	Leukoencephalopathy	0.0177
DB01303	Liver cancer	0.06189
DB01303	Lung cancer	0.2201
DB01303	Lupus erythematosus	0.02048
DB01303	Lupus vulgaris	0.02503
DB01303	Lymphoma	0.0078
DB01303	Melanoma	0.0086
DB01303	Mental retardation	0.01495
DB01303	Migraine	0.02313
DB01303	Movement disorder	0.04605
DB01303	Muscular dystrophies	0.01372
DB01303	Myasthenia Gravis	0.03132
DB01303	Myotonic disorder	0.01454
DB01303	Neoplasm metastasis	0.0912
DB01303	Nervous system disease	0.03759
DB01303	Obesity	0.04671
DB01303	Oligospermia	0.03761
DB01303	Osteomyelitis	0.0139
DB01303	Osteoporosis	0.01877
DB01303	Ovarian disease	0.02942
DB01303	Ovarian failure	0.03847
DB01303	Panic disorder	0.12714
DB01303	Parkinson disease	0.01432
DB01303	Pelvic inflammatory disease	0.29842
DB01303	Phobic anxiety disorder	0.22361
DB01303	Pick disease of the brain	0.2
DB01303	Polyarthritis	0.0107
DB01303	Polycystic ovary syndrome	0.02226
DB01303	Primary biliary cirrhosis	0.02243
DB01303	Prostate cancer	0.07835
DB01303	Rabies	0.02722
DB01303	Renal Cell cancer	0.02207
DB01303	Renal tubular acidosis	0.06058
DB01303	Respiratory tract disease	0.09535
DB01303	Retinitis pigmentosa	0.02253
DB01303	Rett syndrome	0.05409
DB01303	Rheumatoid arthritis	0.00949
DB01303	Schizophrenia	0.07422
DB01303	Skin disease	0.02616
DB01303	Spinal dysraphism	0.02835
DB01303	Stomach cancer	0.02448
DB01303	Stroke	0.01028
DB01303	Synovitis	0.03411
DB01303	Testicular tumor	0.0662
DB01303	Thrombocytosis	0.2
DB01303	Thrombophlebitis	0.0186
DB01303	Thymoma	0.02647

DB01303	Tic disorder	0.03097
DB01303	Tuberous sclerosis	0.30975
DB01303	Uterine disease	0.02356
DB01303	Vitamin D deficiency	0.01561
DB01303	Vitiligo	0.03132
DB01303	Yersinia infection	0.00662
DB01412	Asthma	0.04714
DB01412	Behavior disease	0.08909
DB01412	Chronic obstructive airway disease	0.06455
DB01412	Cystic fibrosis	0.08248
DB01412	Depression	0.15162
DB01412	Eating disorder	0.1005
DB01412	Heart failure	0.06155
DB01412	Herpes	0.08248
DB01412	Huntington disease	0.12599
DB01412	Hypertension	0.09129
DB01412	Ischemia	0.07392
DB01412	Kidney failure	0.06537
DB01412	Leukemia	0.03253
DB01412	Obesity	0.04441
DB01412	Panic disorder	0.12599
DB01412	Phobic anxiety disorder	0.28868
DB01412	Pick disease of the brain	0.2582
DB01412	Respiratory tract disease	0.12309
DB01412	Schizophrenia	0.0439
DB01412	Thrombocytosis	0.2582
DB04932	Adenocarcinoma	0.08422
DB04932	Asthma	0.04714
DB04932	Breast cancer	0.02778
DB04932	Chronic obstructive airway disease	0.06455
DB04932	Cystic fibrosis	0.08248
DB04932	Depression	0.07581
DB04932	Diabetes mellitus	0.03039
DB04932	Eating disorder	0.1005
DB04932	Heart failure	0.06155
DB04932	Herpes	0.08248
DB04932	Huntington disease	0.12599
DB04932	Hypertension	0.13693
DB04932	Ischemia	0.07392
DB04932	Kidney failure	0.06537
DB04932	Obesity	0.04441
DB04932	Panic disorder	0.12599
DB04932	Phobic anxiety disorder	0.28868
DB04932	Pick disease of the brain	0.2582
DB04932	Respiratory tract disease	0.12309
DB04932	Schizophrenia	0.0439
DB04932	Thrombocytosis	0.2582

DB00171	Infertility, Male	0.06935
DB00171	Spastic paraplegia, Hereditary	0.19324
DB00171	Abortion	0.04457
DB00171	Actinic keratosis	0.09768
DB00171	Adenovirus infection	0.13584
DB00171	Alzheimer's disease	0.0974
DB00171	Amyotrophic lateral sclerosis	0.02803
DB00171	Asthma	0.01421
DB00171	Atherosclerosis	0.15172
DB00171	Azoospermia	0.05245
DB00171	Bacterial infection	0.17526
DB00171	Bipolar disorder	0.03942
DB00171	Bone marrow disease	0.27082
DB00171	Brain disease	0.03127
DB00171	Brain tumor	0.03211
DB00171	Breast cancer	0.0244
DB00171	Cancer	0.13843
DB00171	Cardiovascular disease	0.05309
DB00171	Cervical cancer	0.03198
DB00171	Cholelithiasis	0.06058
DB00171	Chronic progressive external ophthalmoplegia	0.07107
DB00171	Colon cancer	0.01411
DB00171	Congenital abnormality	0.03698
DB00171	Connective tissue disease	0.04352
DB00171	Dental plaque	0.02578
DB00171	Depression	0.10812
DB00171	Dermatitis	0.01646
DB00171	Diabetes mellitus	0.10459
DB00171	Down syndrome	0.04562
DB00171	Drug abuse	0.04337
DB00171	Eating disorder	0.06718
DB00171	Embryoma	0.02491
DB00171	Encephalopathies	0.02438
DB00171	Endometriosis	0.04597
DB00171	Eosinophilia	0.13024
DB00171	Fanconi's anemia	0.03009
DB00171	Female reproductive cancer	0.06155
DB00171	Heart failure	0.02436
DB00171	Herpes	0.04832
DB00171	Huntington disease	0.36228
DB00171	Hypercholesterolemia	0.0335
DB00171	Hyperglycemia	0.30985
DB00171	Hypertension	0.15002
DB00171	Hypogonadism	0.19351
DB00171	Infection	0.07491
DB00171	Infertility	0.04431
DB00171	Intestinal disease	0.28451

DB00171	Kidney failure	0.03942
DB00171	Late pregnancy	0.05025
DB00171	Leukemia	0.00981
DB00171	Leukodystrophy NOS	0.19324
DB00171	Liver cancer	0.0487
DB00171	Liver tumor	0.05008
DB00171	Lung cancer	0.02203
DB00171	Lyme disease	0.19324
DB00171	Metastasis to lymph nodes	0.07476
DB00171	Migraine	0.05438
DB00171	Multiple endocrine neoplasia	0.05803
DB00171	Muscular atrophy	0.07661
DB00171	Neuroblastoma	0.03815
DB00171	Neurodegenerative disorder	0.02862
DB00171	Obesity	0.15557
DB00171	Oligospermia	0.0955
DB00171	Osteoporosis	0.04412
DB00171	Ovarian disease	0.04103
DB00171	Pancreas disease	0.11088
DB00171	Parkinson disease	0.05571
DB00171	Penile disease	0.05166
DB00171	Phobic anxiety disorder	0.19351
DB00171	Polycystic ovary syndrome	0.23301
DB00171	Polyneuropathy	0.04652
DB00171	Prion disease	0.05636
DB00171	Prostate cancer	0.02734
DB00171	Psychotic disorder	0.32191
DB00171	Pulmonary alveolar proteinosis	0.06155
DB00171	Rabies	0.05896
DB00171	Renal Cell cancer	0.02024
DB00171	Reticulosarcoma	0.35018
DB00171	Rheumatoid arthritis	0.0261
DB00171	Schizophrenia	0.14297
DB00171	Skin disease	0.05326
DB00171	Spinal dysraphism	0.05771
DB00171	Systemic infection	0.16815
DB00171	Systemic scleroderma	0.14422
DB00171	Thymoma	0.11728
DB00171	Tic disorder	0.09675
DB00171	Tropical spastic paraparesis	0.45889
DB00171	Urogenital abnormalities	0.10814
DB00171	Vitiligo	0.36864
DB00171	Yersinia infection	0.0665
DB00619	Skin disease, Genetic	0.14051
DB00619	Abortion	0.09774
DB00619	Alzheimer's disease	0.06518
DB00619	Atherosclerosis	0.02646

DB00619	Bacterial infection	0.26641
DB00619	Bone marrow disease	0.38777
DB00619	Brain tumor	0.08626
DB00619	Breast cancer	0.01818
DB00619	Cancer	0.08114
DB00619	Colon cancer	0.04796
DB00619	Congenital abnormality	0.05914
DB00619	Congenital heart disease	0.34364
DB00619	Deafness	0.22184
DB00619	Drug abuse	0.03067
DB00619	Embryoma	0.02782
DB00619	Endometriosis	0.03251
DB00619	Enteritis	0.04149
DB00619	Eosinophilia	0.21385
DB00619	Gastritis	0.03902
DB00619	Gastrointestinal tumor	0.10483
DB00619	Hemorrhagic disorder	0.11603
DB00619	Herpes	0.0649
DB00619	Infection	0.14759
DB00619	Influenza	0.14931
DB00619	Leukemia	0.0877
DB00619	Leukoencephalopathy	0.06299
DB00619	Lung disease	0.06682
DB00619	Lymphoproliferative disorder	0.22824
DB00619	Myeloproliferative disease	0.1529
DB00619	Neoplasm metastasis	0.03076
DB00619	Nephritis	0.16903
DB00619	Neurofibromatosis	0.11952
DB00619	Osteoporosis	0.05976
DB00619	Overnutrition	0.07157
DB00619	Pancreas cancer	0.04798
DB00619	Polycystic ovary syndrome	0.05822
DB00619	Primary tumor	0.06505
DB00619	Prostate cancer	0.03811
DB00619	Rabies	0.0417
DB00619	Schizophrenia	0.02874
DB00619	Skin cancer	0.12754
DB00619	Skin disease	0.06842
DB00619	Solid tumor	0.2033
DB00619	Spinal dysraphism	0.07414
DB00619	Testicular dysfunction	0.05399
DB00619	Thyroid gland disease	0.08671
DB00619	Tic disorder	0.081
DB00619	Turner's syndrome	0.12114
DB00619	Vasculitis	0.11396
DB01254	Hemorrhagic fevers, Viral	0.03187
DB01254	Infertility, Male	0.04005

DB01254	Kidney tubular necrosis, acute	0.07064
DB01254	Pemphigoid, Bullous	0.0432
DB01254	Purpura, Thrombocytopenic, Idiopathic	0.07262
DB01254	Skin disease, Genetic	0.08798
DB01254	Abortion	0.02972
DB01254	Actinic keratosis	0.06514
DB01254	Adenovirus infection	0.03679
DB01254	Alzheimer's disease	0.07034
DB01254	Amyotrophic lateral sclerosis	0.02121
DB01254	Aplastic anemia	0.03737
DB01254	Aseptic necrosis of bone	0.04985
DB01254	Asthma	0.01519
DB01254	Atherosclerosis	0.05441
DB01254	Autistic disorder	0.021
DB01254	Autoimmune disease	0.01936
DB01254	Bacterial infection	0.17037
DB01254	Bipolar disorder	0.03207
DB01254	Bone disease	0.02742
DB01254	Bone marrow disease	0.23227
DB01254	Breast cancer	0.10183
DB01254	Bronchial hyperreactivity	0.05826
DB01254	Cancer	0.13545
DB01254	Celiac disease	0.03869
DB01254	Cerebrovascular disorder	0.04005
DB01254	Cervical cancer	0.03697
DB01254	Cholelithiasis	0.03499
DB01254	Chronic obstructive airway disease	0.07686
DB01254	Colon cancer	0.04119
DB01254	Common cold	0.05153
DB01254	Congenital abnormality	0.04653
DB01254	Connective tissue disease	0.07766
DB01254	Craniosynostosis	0.0557
DB01254	Dental plaque	0.02274
DB01254	Depression	0.02448
DB01254	Dermatitis	0.00876
DB01254	Diabetes mellitus	0.12526
DB01254	Down syndrome	0.02275
DB01254	Drug abuse	0.08629
DB01254	Eating disorder	0.0224
DB01254	Ectodermal dysplasia	0.06626
DB01254	Emphysema	0.05194
DB01254	Endometrial cancer	0.02262
DB01254	Endometriosis	0.05141
DB01254	Eosinophilia	0.1472
DB01254	Esophagus cancer	0.02913
DB01254	Esotropia	0.06629
DB01254	Ewings sarcoma	0.06889

DB01254	Fanconi's anemia	0.0322
DB01254	Gastrointestinal tumor	0.12648
DB01254	Generalized anxiety disorder	0.05515
DB01254	Glaucoma	0.02652
DB01254	Heart failure	0.03494
DB01254	Hemorrhagic disorder	0.03528
DB01254	Herpes	0.06229
DB01254	Hyperlipidemia	0.04439
DB01254	Hypertension	0.01769
DB01254	Infection	0.0862
DB01254	Infertility	0.02559
DB01254	Intermediate coronary syndrome	0.03179
DB01254	Ischemia	0.02893
DB01254	Kaposi sarcoma	0.15502
DB01254	Kidney disease	0.0801
DB01254	Kidney failure	0.02246
DB01254	Late pregnancy	0.055
DB01254	Leukemia	0.1118
DB01254	Leukoencephalopathy	0.02227
DB01254	Liver cancer	0.0419
DB01254	Lung cancer	0.05003
DB01254	Lung disease	0.26122
DB01254	Lupus erythematosus	0.12649
DB01254	Lupus vulgaris	0.03398
DB01254	Lymphoma	0.22225
DB01254	Melanoma	0.04496
DB01254	Migraine	0.03141
DB01254	Mucopolysaccharidosis	0.04932
DB01254	Multiple myeloma	0.10353
DB01254	Multiple sclerosis	0.03216
DB01254	Myasthenia Gravis	0.04252
DB01254	Neoplasm metastasis	0.04766
DB01254	Nephritis	0.14142
DB01254	Neuroblastoma	0.02887
DB01254	Neurofibromatosis	0.1
DB01254	Obesity	0.01672
DB01254	Oral cancer	0.0099
DB01254	Osteomyelitis	0.04706
DB01254	Osteoporosis	0.13432
DB01254	Osteosarcoma	0.03729
DB01254	Ovarian disease	0.03994
DB01254	Ovarian failure	0.05223
DB01254	Overnutrition	0.08005
DB01254	Pancreas disease	0.07394
DB01254	Panic disorder	0.04012
DB01254	Parkinson disease	0.04492
DB01254	Penile disease	0.06428

DB01254	Pituitary tumor	0.05941
DB01254	Polyarthritis	0.01453
DB01254	Polycystic kidney	0.02894
DB01254	Polycystic ovary syndrome	0.06135
DB01254	Primary biliary cirrhosis	0.03045
DB01254	Primary hyperparathyroidism	0.03383
DB01254	Primary tumor	0.29405
DB01254	Prostate cancer	0.03306
DB01254	Rabies	0.03695
DB01254	Renal Cell cancer	0.2749
DB01254	Renal tubular acidosis	0.05325
DB01254	Rhabdomyosarcoma	0.06538
DB01254	Rheumatism	0.03937
DB01254	Rheumatoid arthritis	0.03157
DB01254	Schizophrenia	0.03043
DB01254	Skin disease	0.03552
DB01254	Skin tumor	0.05861
DB01254	Spinal dysraphism	0.03848
DB01254	Squamous cell cancer	0.06209
DB01254	Stomach cancer	0.02138
DB01254	Stroke	0.06457
DB01254	Subarachnoid hemorrhage	0.02846
DB01254	Synovitis	0.0463
DB01254	Systemic scleroderma	0.02347
DB01254	Takayasu's arteritis	0.03763
DB01254	Temporal arteritis	0.03111
DB01254	Testicular dysfunction	0.04518
DB01254	Thrombocytopenia	0.03353
DB01254	Thymoma	0.17743
DB01254	Thyroid gland disease	0.07255
DB01254	Tic disorder	0.04204
DB01254	Tuberous sclerosis	0.01134
DB01254	Turner's syndrome	0.06288
DB01254	Ulcerative colitis	0.00995
DB01254	Uterine disease	0.05127
DB01254	Uterine fibroids	0.05952
DB01254	Vitamin D deficiency	0.05285
DB01254	Vitiligo	0.04252
DB01254	Werner syndrome	0.0391
DB01254	Wiskott-Aldrich syndrome	0.14655
DB01254	Yersinia infection	0.02263
DB04868	Abortion	0.13952
DB04868	Alzheimer's disease	0.14344
DB04868	Atherosclerosis	0.04951
DB04868	Bacterial infection	0.59201
DB04868	Bone marrow disease	0.88968
DB04868	Cancer	0.09936

DB04868	Congenital abnormality	0.12798
DB04868	Drug abuse	0.07474
DB04868	Endometriosis	0.07922
DB04868	Eosinophilia	0.45662
DB04868	Gastrointestinal tumor	0.19612
DB04868	Infection	0.16706
DB04868	Nephritis	0.31623
DB04868	Neurofibromatosis	0.22361
DB04868	Osteoporosis	0.1118
DB04868	Polycystic ovary syndrome	0.14187
DB04868	Rabies	0.10161
DB04868	Skin disease	0.16673
DB04868	Spinal dysraphism	0.18065
DB04868	Testicular dysfunction	0.10102
DB04868	Thyroid gland disease	0.16222
DB04868	Tic disorder	0.19738
DB06616	Hemorrhagic fevers, Viral	0.0301
DB06616	Infertility, Male	0.03783
DB06616	Kidney tubular necrosis, acute	0.09497
DB06616	Pemphigoid, Bullous	0.01528
DB06616	Purpura, Thrombocytopenic, Idiopathic	0.03577
DB06616	Skin disease, Genetic	0.15074
DB06616	Abortion	0.02807
DB06616	Adenovirus infection	0.06717
DB06616	Alopecia	0.0208
DB06616	Alzheimer's disease	0.11853
DB06616	Amyotrophic lateral sclerosis	0.02851
DB06616	Angiomyolipoma	0.15713
DB06616	Aortic valve disease	0.03109
DB06616	Aplastic anemia	0.0353
DB06616	Aseptic necrosis of bone	0.04708
DB06616	Asthma	0.01435
DB06616	Atherosclerosis	0.03048
DB06616	Autistic disorder	0.01983
DB06616	Autoimmune disease	0.01829
DB06616	Bacterial infection	0.1656
DB06616	Barrett's esophagus	0.075
DB06616	Bipolar disorder	0.03028
DB06616	Bone disease	0.0259
DB06616	Bone marrow disease	0.30556
DB06616	Brain tumor	0.02477
DB06616	Breast cancer	0.02782
DB06616	Bronchial hyperreactivity	0.05503
DB06616	Cancer	0.16146
DB06616	Celiac disease	0.05201
DB06616	Cerebrovascular disorder	0.10683
DB06616	Cervical cancer	0.03491

DB06616	Cholelithiasis	0.03305
DB06616	Chronic obstructive airway disease	0.04969
DB06616	Chronic rejection of renal transplant	0.04832
DB06616	Chronic simple glaucoma	0.0368
DB06616	Colon cancer	0.02009
DB06616	Common cold	0.2107
DB06616	Congenital abnormality	0.08724
DB06616	Craniosynostosis	0.05261
DB06616	Dental plaque	0.02148
DB06616	Depression	0.02312
DB06616	Dermatitis	0.02198
DB06616	Diabetes mellitus	0.06226
DB06616	Down syndrome	0.04772
DB06616	Drug abuse	0.0396
DB06616	Ectodermal dysplasia	0.06258
DB06616	Emphysema	0.04553
DB06616	Endemic goiter	0.04951
DB06616	Endometrial cancer	0.02137
DB06616	Endometriosis	0.06495
DB06616	Enteritis	0.02905
DB06616	Eosinophilia	0.14432
DB06616	Esophagus cancer	0.02752
DB06616	Esotropia	0.0626
DB06616	Ewings sarcoma	0.09261
DB06616	Fanconi's anemia	0.03994
DB06616	Generalized anxiety disorder	0.03683
DB06616	Glaucoma	0.02505
DB06616	HIV infection	0.0115
DB06616	Hamman-Rich syndrome	0.03453
DB06616	Heart failure	0.04446
DB06616	Helicobacter infection	0.02698
DB06616	Hemolytic-Uremic syndrome	0.03929
DB06616	Hemorrhagic disorder	0.03332
DB06616	Herpes	0.07488
DB06616	Hyperlipidemia	0.04193
DB06616	Hyperparathyroidism	0.11218
DB06616	Hypertension	0.0167
DB06616	Immune complex disease	0.09584
DB06616	Infection	0.08083
DB06616	Infertility	0.02417
DB06616	Intermediate coronary syndrome	0.03003
DB06616	Ischemia	0.03889
DB06616	Kaposi sarcoma	0.28478
DB06616	Keratoconjunctivitis Sicca	0.12265
DB06616	Keratosis	0.04076
DB06616	Kidney disease	0.07565
DB06616	Kidney failure	0.04243

DB06616	Late pregnancy	0.05194
DB06616	Leukemia	0.12926
DB06616	Leukoencephalopathy	0.02103
DB06616	Liver cancer	0.02994
DB06616	Lung cancer	0.04829
DB06616	Lung disease	0.02381
DB06616	Lupus erythematosus	0.15552
DB06616	Lupus vulgaris	0.03209
DB06616	Lymphoma	0.04797
DB06616	Melanoma	0.02907
DB06616	Meningioma	0.06044
DB06616	Metaplastic polyp	0.14026
DB06616	Migraine	0.02966
DB06616	Mucopolysaccharidosis	0.04658
DB06616	Multiple endocrine neoplasia	0.13393
DB06616	Multiple myeloma	0.17166
DB06616	Myasthenia Gravis	0.04016
DB06616	Myeloproliferative disease	0.08452
DB06616	Myopathy	0.09096
DB06616	Neck cancer	0.04369
DB06616	Neoplasm metastasis	0.02717
DB06616	Nephrosis	0.11285
DB06616	Neuroblastoma	0.02726
DB06616	Obesity	0.03158
DB06616	Oral cancer	0.06096
DB06616	Osteitis deformans	0.0437
DB06616	Osteomyelitis	0.18005
DB06616	Osteoporosis	0.05185
DB06616	Ovarian disease	0.03772
DB06616	Ovarian failure	0.04933
DB06616	Overnutrition	0.03509
DB06616	Panic disorder	0.03789
DB06616	Papillomavirus infection	0.04578
DB06616	Parkinson disease	0.05261
DB06616	Penile disease	0.02818
DB06616	Pituitary tumor	0.07986
DB06616	Polyarthritis	0.02745
DB06616	Polycystic kidney	0.02734
DB06616	Polycystic ovary syndrome	0.04872
DB06616	Primary biliary cirrhosis	0.02876
DB06616	Primary hyperparathyroidism	0.03195
DB06616	Primary tumor	0.02762
DB06616	Prostate cancer	0.02221
DB06616	Proteinuria	0.11096
DB06616	Rabies	0.0349
DB06616	Renal Cell cancer	0.02762
DB06616	Renal tubular acidosis	0.06237

DB06616	Rhabdomyosarcoma	0.06175
DB06616	Rheumatism	0.03719
DB06616	Rheumatoid arthritis	0.05261
DB06616	Sarcoidosis	0.04419
DB06616	Schizophrenia	0.02874
DB06616	Skin disease	0.05031
DB06616	Spinal dysraphism	0.03634
DB06616	Squamous cell cancer	0.03117
DB06616	Stomach cancer	0.03168
DB06616	Stroke	0.06099
DB06616	Subarachnoid hemorrhage	0.02688
DB06616	Synovitis	0.04373
DB06616	Systemic infection	0.01828
DB06616	Systemic scleroderma	0.0076
DB06616	Takayasu's arteritis	0.03554
DB06616	Temporal arteritis	0.02938
DB06616	Testicular dysfunction	0.12955
DB06616	Thrombocytopenia	0.03167
DB06616	Thymoma	0.03394
DB06616	Thyroid gland disease	0.1191
DB06616	Tic disorder	0.03971
DB06616	Tuberous sclerosis	0.02848
DB06616	Ulcerative colitis	0.15745
DB06616	Uterine disease	0.04842
DB06616	Uterine fibroids	0.08001
DB06616	Virus disease	0.02707
DB06616	Vitamin D deficiency	0.2022
DB06616	Vitiligo	0.04016
DB06616	Werner syndrome	0.05256
DB06616	Wiskott-Aldrich syndrome	0.13841
DB06616	Yersinia infection	0.01511
DB08896	Skin disease, Genetic	0.45791
DB08896	Abortion	0.04789
DB08896	Actinic keratosis	0.05325
DB08896	Adenoid cystic cancer	0.09953
DB08896	Adrenoleukodystrophy	0.06967
DB08896	Alzheimer's disease	0.18631
DB08896	Amyotrophic lateral sclerosis	0.09999
DB08896	Angiomyolipoma	0.41182
DB08896	Aseptic necrosis of bone	0.02459
DB08896	Asthma	0.09005
DB08896	Atherosclerosis	0.05958
DB08896	Autistic disorder	0.02803
DB08896	Bacterial infection	0.14403
DB08896	Bone marrow disease	0.20303
DB08896	Brain disease	0.02578
DB08896	Brain tumor	0.04226

DB08896	Breast cancer	0.00719
DB08896	Cancer	0.24772
DB08896	Capillaries disease	0.0977
DB08896	Cardiovascular disease	0.22839
DB08896	Chronic obstructive airway disease	0.21112
DB08896	Cleft palate	0.06299
DB08896	Colon cancer	0.03524
DB08896	Common cold	0.35019
DB08896	Congenital abnormality	0.0434
DB08896	Congenital heart disease	0.16837
DB08896	Connective tissue disease	0.0733
DB08896	Craniosynostosis	0.24502
DB08896	Cystic fibrosis	0.0335
DB08896	Deafness	0.10869
DB08896	Dental plaque	0.04066
DB08896	Depression	0.01441
DB08896	Dermatitis	0.08912
DB08896	Diabetes mellitus	0.0381
DB08896	Down syndrome	0.02479
DB08896	Drug abuse	0.06
DB08896	Eating disorder	0.01831
DB08896	Embryoma	0.03781
DB08896	Emphysema	0.07087
DB08896	Endocrine system disease	0.14992
DB08896	Endometriosis	0.13335
DB08896	Eosinophilia	0.12001
DB08896	Esotropia	0.02904
DB08896	Familial Mediterranean fever	0.15474
DB08896	Gastritis	0.01912
DB08896	Gastrointestinal tumor	0.06537
DB08896	Gram-Negative bacterial infection	0.04734
DB08896	Graves' disease	0.04167
DB08896	Heart failure	0.02029
DB08896	Hemorrhagic disorder	0.05685
DB08896	Herpes	0.23277
DB08896	Infection	0.07702
DB08896	Influenza	0.07315
DB08896	Kaposi sarcoma	0.02806
DB08896	Kidney failure	0.07344
DB08896	Leukemia	0.04659
DB08896	Leukoencephalopathy	0.05557
DB08896	Liver cancer	0.03288
DB08896	Liver metastases	0.05676
DB08896	Lung cancer	0.01132
DB08896	Lupus erythematosus	0.08458
DB08896	Lymphoproliferative disorder	0.11183
DB08896	Macular degeneration	0.1997

DB08896	Malaria	0.13329
DB08896	Melanoma	0.03357
DB08896	Metaplastic polyp	0.30489
DB08896	Mucocutaneous lymph node syndrome	0.30795
DB08896	Multiple endocrine neoplasia	0.1569
DB08896	Multiple myeloma	0.02523
DB08896	Myeloproliferative disease	0.13791
DB08896	Myopathy	0.27869
DB08896	Neck cancer	0.1182
DB08896	Nephritis	0.10541
DB08896	Nephrosis	0.28449
DB08896	Neuroblastoma	0.0186
DB08896	Neurodegenerative disorder	0.02383
DB08896	Neurofibromatosis	0.07454
DB08896	Obesity	0.05466
DB08896	Oral cancer	0.01549
DB08896	Osteomyelitis	0.54581
DB08896	Osteoporosis	0.03727
DB08896	Osteosarcoma	0.0352
DB08896	Overnutrition	0.03507
DB08896	Pancreas cancer	0.02351
DB08896	Pancreas disease	0.06044
DB08896	Pancreatitis	0.02633
DB08896	Penile disease	0.03982
DB08896	Peptic ulcer	0.12322
DB08896	Polycystic ovary syndrome	0.0487
DB08896	Polyneuropathy	0.1997
DB08896	Pre-Eclampsia	0.09808
DB08896	Primary hyperparathyroidism	0.11478
DB08896	Primary tumor	0.03187
DB08896	Prion disease	0.04424
DB08896	Prostate cancer	0.01706
DB08896	Rabies	0.02043
DB08896	Retinal disease	0.21654
DB08896	Rheumatoid arthritis	0.03652
DB08896	Skin cancer	0.06249
DB08896	Skin disease	0.03352
DB08896	Solid tumor	0.09961
DB08896	Spinal dysraphism	0.03632
DB08896	Squamous cell cancer	0.02127
DB08896	Stomach cancer	0.02353
DB08896	Stroke	0.01317
DB08896	Systemic infection	0.05482
DB08896	Systemic scleroderma	0.0252
DB08896	Testicular dysfunction	0.20722
DB08896	Thyroid cancer	0.03324
DB08896	Thyroid gland disease	0.45227

DB08896	Tic disorder	0.03969
DB08896	Turner's syndrome	0.05935
DB08896	Ulcerative colitis	0.16065
DB08896	Urogenital abnormalities	0.06299
DB08896	Uterine disease	0.03019
DB08896	Vitamin D deficiency	0.62095
DB08901	Hemorrhagic fevers, Viral	0.03021
DB08901	Infertility, Male	0.03797
DB08901	Kidney tubular necrosis, acute	0.06696
DB08901	Pemphigoid, Bullous	0.01534
DB08901	Purpura, Thrombocytopenic, Idiopathic	0.06089
DB08901	Skin disease, Genetic	0.11947
DB08901	Abortion	0.04809
DB08901	Adenovirus infection	0.03488
DB08901	Adrenoleukodystrophy	0.06997
DB08901	Alopecia	0.02088
DB08901	Alzheimer's disease	0.07311
DB08901	Amyotrophic lateral sclerosis	0.12341
DB08901	Aplastic anemia	0.03543
DB08901	Aseptic necrosis of bone	0.07195
DB08901	Asthma	0.10665
DB08901	Atherosclerosis	0.0923
DB08901	Autistic disorder	0.0199
DB08901	Autoimmune disease	0.01836
DB08901	Bacterial infection	0.15039
DB08901	Bipolar disorder	0.0304
DB08901	Bone disease	0.07028
DB08901	Bone marrow disease	0.27611
DB08901	Brain tumor	0.04244
DB08901	Breast cancer	0.08993
DB08901	Bronchial hyperreactivity	0.05523
DB08901	Cancer	0.21051
DB08901	Capillaries disease	0.09811
DB08901	Cardiovascular disease	0.23264
DB08901	Celiac disease	0.03667
DB08901	Cerebrovascular disorder	0.09431
DB08901	Cervical cancer	0.03504
DB08901	Cholelithiasis	0.03317
DB08901	Chronic obstructive airway disease	0.29175
DB08901	Cleft palate	0.06901
DB08901	Colon cancer	0.06081
DB08901	Common cold	0.04885
DB08901	Congenital abnormality	0.0711
DB08901	Congenital heart disease	0.16908
DB08901	Craniosynostosis	0.39089
DB08901	Deafness	0.10915
DB08901	Dental plaque	0.04311

DB08901	Depression	0.03768
DB08901	Dermatitis	0.09979
DB08901	Diabetes mellitus	0.14998
DB08901	Down syndrome	0.02156
DB08901	Drug abuse	0.02907
DB08901	Ectodermal dysplasia	0.06281
DB08901	Emphysema	0.08502
DB08901	Endocrine system disease	0.15869
DB08901	Endometrial cancer	0.02144
DB08901	Endometriosis	0.15349
DB08901	Enteritis	0.02916
DB08901	Eosinophilia	0.127
DB08901	Esophagus cancer	0.02762
DB08901	Esotropia	0.07603
DB08901	Ewings sarcoma	0.0653
DB08901	Familial Mediterranean fever	0.1554
DB08901	Fanconi's anemia	0.03052
DB08901	Gastritis	0.0192
DB08901	Gastrointestinal tumor	0.07161
DB08901	Generalized anxiety disorder	0.09055
DB08901	Glaucoma	0.02514
DB08901	Graves' disease	0.04564
DB08901	Heart failure	0.05379
DB08901	Hemorrhagic disorder	0.09181
DB08901	Herpes	0.06043
DB08901	Hyperlipidemia	0.04208
DB08901	Hyperopia	0.08607
DB08901	Hypertension	0.01676
DB08901	Immune complex disease	0.09619
DB08901	Infection	0.09602
DB08901	Infertility	0.02426
DB08901	Influenza	0.07347
DB08901	Intermediate coronary syndrome	0.03014
DB08901	Intracranial hypertension	0.18257
DB08901	Ischemia	0.02742
DB08901	Kaposi sarcoma	0.28006
DB08901	Keratosis	0.08607
DB08901	Kidney disease	0.07592
DB08901	Kidney failure	0.09504
DB08901	Late pregnancy	0.05213
DB08901	Leukemia	0.17392
DB08901	Leukoencephalopathy	0.07692
DB08901	Liver cancer	0.02111
DB08901	Liver metastases	0.057
DB08901	Lung cancer	0.04178
DB08901	Lung disease	0.22778
DB08901	Lupus erythematosus	0.24977

DB08901	Lupus vulgaris	0.03221
DB08901	Lymphoma	0.1946
DB08901	Lymphoproliferative disorder	0.1123
DB08901	Macular degeneration	0.20055
DB08901	Malaria	0.13386
DB08901	Melanoma	0.05826
DB08901	Migraine	0.02977
DB08901	Mucocutaneous lymph node syndrome	0.31395
DB08901	Mucopolysaccharidosis	0.04675
DB08901	Multiple endocrine neoplasia	0.16473
DB08901	Multiple myeloma	0.18779
DB08901	Multiple sclerosis	0.03049
DB08901	Myasthenia Gravis	0.04031
DB08901	Myeloproliferative disease	0.21325
DB08901	Myopathy	0.06561
DB08901	Neck cancer	0.1255
DB08901	Neoplasm metastasis	0.02727
DB08901	Nephritis	0.11547
DB08901	Neuroblastoma	0.02736
DB08901	Neurofibromatosis	0.08165
DB08901	Obesity	0.07073
DB08901	Oral cancer	0.02494
DB08901	Osteomyelitis	0.04461
DB08901	Osteoporosis	0.09286
DB08901	Ovarian disease	0.03786
DB08901	Ovarian failure	0.04951
DB08901	Overnutrition	0.1111
DB08901	Panic disorder	0.03803
DB08901	Parkinson disease	0.04258
DB08901	Penile disease	0.02828
DB08901	Peptic ulcer	0.12374
DB08901	Pituitary tumor	0.05631
DB08901	Polyarthritis	0.01377
DB08901	Polycystic kidney	0.02743
DB08901	Polycystic ovary syndrome	0.0489
DB08901	Polyneuropathy	0.20055
DB08901	Pre-Eclampsia	0.0985
DB08901	Primary biliary cirrhosis	0.02887
DB08901	Primary hyperparathyroidism	0.15546
DB08901	Primary tumor	0.25689
DB08901	Prostate cancer	0.04038
DB08901	Ptosis	0.08607
DB08901	Rabies	0.03503
DB08901	Renal Cell cancer	0.24126
DB08901	Renal tubular acidosis	0.05048
DB08901	Retinal disease	0.22047
DB08901	Rhabdomyosarcoma	0.06198

DB08901	Rheumatism	0.03732
DB08901	Rheumatoid arthritis	0.0566
DB08901	Schizophrenia	0.02885
DB08901	Skin cancer	0.06275
DB08901	Skin disease	0.03367
DB08901	Skin tumor	0.05556
DB08901	Solid tumor	0.10003
DB08901	Spinal dysraphism	0.03648
DB08901	Squamous cell cancer	0.03128
DB08901	Stomach cancer	0.0318
DB08901	Stroke	0.06818
DB08901	Subarachnoid hemorrhage	0.02698
DB08901	Synovitis	0.04389
DB08901	Systemic infection	0.07341
DB08901	Systemic scleroderma	0.02029
DB08901	Takayasu's arteritis	0.03567
DB08901	Temporal arteritis	0.02949
DB08901	Testicular dysfunction	0.03689
DB08901	Thrombocytopenia	0.03178
DB08901	Thymoma	0.03407
DB08901	Thyroid cancer	0.03339
DB08901	Thyroid gland disease	0.16339
DB08901	Tic disorder	0.03985
DB08901	Tuberous sclerosis	0.01075
DB08901	Turner's syndrome	0.26828
DB08901	Ulcerative colitis	0.02508
DB08901	Urogenital abnormalities	0.06901
DB08901	Uterine disease	0.07892
DB08901	Uterine fibroids	0.05642
DB08901	Vitamin D deficiency	0.0501
DB08901	Vitiligo	0.04031
DB08901	Werner syndrome	0.03706
DB08901	Wiskott-Aldrich syndrome	0.13891
DB08901	Yersinia infection	0.03715
DB00043	Asthma	0.05774
DB00043	Dermatitis	0.06428
DB00895	Asthma	0.05774
DB00895	Dermatitis	0.06428
DB00055	Abortion	0.10825
DB00055	Abruption placentae	0.10206
DB00055	Adrenal gland hyperfunction	0.23498
DB00055	Aortic aneurysm	0.17881
DB00055	Aseptic necrosis of bone	0.27386
DB00055	Asthma	0.02357
DB00055	Atherosclerosis	0.02021
DB00055	Behcet syndrome	0.08248
DB00055	Breast cancer	0.09275

DB00055	Cancer	0.03018
DB00055	Capillaries disease	0.09129
DB00055	Cardiovascular disease	0.13207
DB00055	Cerebral palsy	0.10206
DB00055	Cerebrovascular disorder	0.06299
DB00055	Chronic simple glaucoma	0.06155
DB00055	Cirrhosis	0.0488
DB00055	Colon cancer	0.01713
DB00055	Deafness	0.04167
DB00055	Dental plaque	0.10999
DB00055	Diabetes mellitus	0.04558
DB00055	Embryoma	0.0534
DB00055	Endometriosis	0.02397
DB00055	Enteritis	0.06337
DB00055	Esophagus cancer	0.04303
DB00055	Glomerulonephritis	0.07217
DB00055	Hemophilia	0.16667
DB00055	Hemorrhagic disorder	0.15309
DB00055	Hyperglycemia	0.04623
DB00055	Hyperhomocysteinemia	0.16667
DB00055	Hyperinsulinism	0.05556
DB00055	Hyperthyroidism	0.09623
DB00055	Infectious lung disease	0.06455
DB00055	Infertility	0.03965
DB00055	Infiltrating cancer	0.07715
DB00055	Influenza	0.05774
DB00055	Leukemia	0.03253
DB00055	Liver cancer	0.04481
DB00055	Liver disease	0.13363
DB00055	Lung cancer	0.02016
DB00055	Lupus erythematosus	0.04933
DB00055	Malignant childhood tumor	0.20412
DB00055	Malignant glioma	0.10721
DB00055	Melanoma	0.02406
DB00055	Metabolism disease	0.04623
DB00055	Multiple sclerosis	0.02977
DB00055	Neoplasm metastasis	0.16864
DB00055	Nephrosis	0.06455
DB00055	Nevus	0.10206
DB00055	Obesity	0.02221
DB00055	Ovarian cancer	0.02901
DB00055	Pancreas cancer	0.02993
DB00055	Peptic ulcer	0.22606
DB00055	Periodontal disease	0.06804
DB00055	Polyarthritis	0.0329
DB00055	Polycystic ovary syndrome	0.20467
DB00055	Prostate cancer	0.11108

DB00055	Pulmonary embolism	0.11785
DB00055	Pulmonary fibrosis	0.05455
DB00055	Rabies	0.03208
DB00055	Renal Cell cancer	0.20897
DB00055	Respiratory distress syndrome	0.08006
DB00055	Retinal disease	0.04042
DB00055	Rheumatoid arthritis	0.01767
DB00055	Sarcoma	0.07001
DB00055	Sickle cell disease	0.05556
DB00055	Stomach cancer	0.02431
DB00055	Stroke	0.06537
DB00055	Systemic infection	0.13245
DB00055	Systemic scleroderma	0.17087
DB00055	Thrombophilia	0.34021
DB00055	Vascular disease	0.0527
DB00100	Hypertension, Pulmonary	0.11145
DB00100	Pleural effusion, Malignant	0.04566
DB00100	Prostatic hypertrophy, Benign	0.25599
DB00100	Abortion	0.04725
DB00100	Abruption placentae	0.605
DB00100	Adenovirus infection	0.12389
DB00100	Alzheimer's disease	0.14252
DB00100	Amnionitis	0.20111
DB00100	Amyloidosis	0.13245
DB00100	Amyotrophic lateral sclerosis	0.10934
DB00100	Aortic aneurysm	0.0539
DB00100	Arteriopathy	0.4597
DB00100	Arthritis	0.09698
DB00100	Aseptic necrosis of bone	0.11952
DB00100	Asthma	0.04189
DB00100	Atherosclerosis	0.27082
DB00100	Autoimmune disease	0.0534
DB00100	Azoospermia	0.08354
DB00100	Bacterial infection	0.20503
DB00100	Behcet syndrome	0.15986
DB00100	Biliary Atresia	0.16253
DB00100	Brain disease	0.08421
DB00100	Brain tumor	0.05115
DB00100	Breast cancer	0.12819
DB00100	Bronchiolitis obliterans	0.4597
DB00100	Bronchopulmonary dysplasia	0.31629
DB00100	Cancer	0.06199
DB00100	Capillaries disease	0.11952
DB00100	Cardiovascular disease	0.26954
DB00100	Central nervous system disease	0.18162
DB00100	Cerebrovascular disorder	0.11044
DB00100	Cervical cancer	0.07781

DB00100	Chronic obstructive airway disease	0.11003
DB00100	Cirrhosis	0.23126
DB00100	Colon cancer	0.03432
DB00100	Cystic fibrosis	0.08476
DB00100	Dental plaque	0.2862
DB00100	Dermatitis	0.04004
DB00100	Diabetes mellitus	0.0762
DB00100	Down syndrome	0.06273
DB00100	Drug abuse	0.03802
DB00100	Eating disorder	0.05349
DB00100	Embryoma	0.10117
DB00100	Emphysema	0.06648
DB00100	Encephalitis	0.16892
DB00100	Endometriosis	0.07793
DB00100	Enteritis	0.11495
DB00100	Esotropia	0.08484
DB00100	Gastritis	0.05586
DB00100	Glaucoma	0.07313
DB00100	Glomerulonephritis	0.25421
DB00100	Growth retardation	0.12012
DB00100	HIV infection	0.1369
DB00100	HTLV-I infection	0.14838
DB00100	Heart failure	0.16586
DB00100	Helicobacter infection	0.07879
DB00100	Hemolytic-Uremic syndrome	0.1405
DB00100	Hemophilia	0.21822
DB00100	Hemorrhagic disorder	0.45324
DB00100	Henoch-Schoenlein purpura	0.4597
DB00100	Hepatitis	0.11491
DB00100	Hepatitis B	0.36203
DB00100	Hereditary disease	0.06118
DB00100	Herpes	0.03848
DB00100	Hodgkin's disease	0.07243
DB00100	Hyperhomocysteinemia	0.10911
DB00100	Hyperlipidemia	0.28617
DB00100	Hypertension	0.06897
DB00100	IGA glomerulonephritis	0.14407
DB00100	Infiltrating cancer	0.14753
DB00100	Intracranial aneurysm	0.27812
DB00100	Ischemia	0.0609
DB00100	Keratoconjunctivitis Sicca	0.25324
DB00100	Kidney disease	0.12567
DB00100	Kidney failure	0.07586
DB00100	Leukemia	0.11236
DB00100	Leukoencephalopathy	0.0574
DB00100	Lichen planus	0.16356
DB00100	Liver cancer	0.07621

DB00100	Liver disease	0.05832
DB00100	Liver metastases	0.37442
DB00100	Lung cancer	0.03508
DB00100	Lupus erythematosus	0.06675
DB00100	Lupus vulgaris	0.13253
DB00100	Lymphatic metastasis	0.605
DB00100	Malignant childhood tumor	0.26726
DB00100	Malignant glioma	0.07019
DB00100	Melanoma	0.15584
DB00100	Meningioma	0.32627
DB00100	Metabolism disease	0.10583
DB00100	Metastasis to lymph nodes	0.18188
DB00100	Mucocutaneous lymph node syndrome	0.17816
DB00100	Multiple myeloma	0.07372
DB00100	Multiple sclerosis	0.12543
DB00100	Muscular dystrophy	0.1784
DB00100	Neoplasm metastasis	0.11679
DB00100	Nephrosis	0.10722
DB00100	Nervous system disease	0.11491
DB00100	Neurodegenerative disorder	0.07785
DB00100	Nevus	0.69506
DB00100	Obesity	0.13829
DB00100	Oral cancer	0.04526
DB00100	Osteomyelitis	0.26365
DB00100	Ovarian disease	0.11015
DB00100	Ovary cancer	0.10888
DB00100	Pancreas cancer	0.07285
DB00100	Pancreatitis	0.07692
DB00100	Parkinson disease	0.05363
DB00100	Periodontitis	0.11211
DB00100	Polycystic ovary syndrome	0.05893
DB00100	Polymyositis	0.23796
DB00100	Pre-Eclampsia	0.08273
DB00100	Primary tumor	0.18207
DB00100	Prion disease	0.27422
DB00100	Prostate cancer	0.08605
DB00100	Proteinuria	0.21313
DB00100	Psychotic disorder	0.10525
DB00100	Pulmonary fibrosis	0.07143
DB00100	Rabies	0.17929
DB00100	Rectum cancer	0.38937
DB00100	Respiratory distress syndrome	0.21919
DB00100	Respiratory tract disease	0.10211
DB00100	Rheumatism	0.1015
DB00100	Sarcoma	0.09167
DB00100	Sinusitis	0.09474
DB00100	Skin cancer	0.10694

DB00100	Skin disease	0.06926
DB00100	Solid tumor	0.25675
DB00100	Stomach cancer	0.07574
DB00100	Stroke	0.08128
DB00100	Subacute sclerosing panencephalitis	0.4597
DB00100	Systemic infection	0.23435
DB00100	Systemic scleroderma	0.18226
DB00100	Takayasu's arteritis	0.07338
DB00100	Thrombophilia	0.50538
DB00100	Thyroid cancer	0.09713
DB00100	Transient hypertension of pregnancy	0.36203
DB00100	Tropical spastic paraparesis	0.1407
DB00100	Ulcerative colitis	0.04552
DB00100	Varicosity	0.4597
DB00100	Vascular disease	0.06901
DB00100	Vitamin D deficiency	0.30891
DB00154	Alzheimer's disease	0.10592
DB00154	Asthma	0.14253
DB00154	Atherosclerosis	0.12306
DB00154	Cancer	0.05415
DB00154	Chronic fatigue syndrome	0.58081
DB00154	Dental plaque	0.21675
DB00154	Endometriosis	0.12534
DB00154	Gouts	0.52146
DB00154	Growth retardation	0.37925
DB00154	Ischemia	0.21381
DB00154	Metaplastic polyp	0.40875
DB00154	Oral cancer	0.18784
DB00154	Parkinson disease	0.18664
DB00154	Periodontitis	0.33479
DB00154	Polyarthritis	0.1617
DB00154	Prion disease	0.56624
DB00154	Rheumatoid arthritis	0.09414
DB00154	Sickle cell disease	0.23758
DB00154	Stroke	0.15796
DB00154	Thyroid gland disease	0.34927
DB00154	Vulvar disease	0.87924
DB00159	Alzheimer's disease	0.07779
DB00159	Asthma	0.11062
DB00159	Atherosclerosis	0.0957
DB00159	Brain tumor	0.0254
DB00159	Cancer	0.03974
DB00159	Chronic fatigue syndrome	0.44261
DB00159	Colon cancer	0.01876
DB00159	Dental plaque	0.16711
DB00159	Embryoma	0.0195
DB00159	Endometriosis	0.09288

DB00159	Gouts	0.41305
DB00159	Growth retardation	0.30403
DB00159	Hyperlipidemia	0.06202
DB00159	Hypertension	0.025
DB00159	Intestinal disease	0.07906
DB00159	Ischemia	0.20425
DB00159	Liver cancer	0.04909
DB00159	Liver tumor	0.0559
DB00159	Mental retardation	0.04082
DB00159	Metaplastic polyp	0.27846
DB00159	Myopathy	0.03835
DB00159	Neuropathy	0.05064
DB00159	Obesity	0.02433
DB00159	Oral cancer	0.13465
DB00159	Parkinson disease	0.14348
DB00159	Periodontitis	0.27518
DB00159	Polyarthritis	0.15319
DB00159	Prion disease	0.40666
DB00159	Rheumatoid arthritis	0.08957
DB00159	Sickle cell disease	0.16235
DB00159	Stroke	0.11371
DB00159	Thyroid gland disease	0.2596
DB00159	Vulvar disease	0.70444
DB00244	Infertility, Male	0.04259
DB00244	Stress disorder, post-traumatic	0.06607
DB00244	AIDS	0.12102
DB00244	Adenocarcinoma	0.06785
DB00244	Adenoma	0.01109
DB00244	Alzheimer's disease	0.10061
DB00244	Aseptic necrosis of bone	0.02565
DB00244	Asthma	0.08571
DB00244	Atherosclerosis	0.11254
DB00244	Autoimmune disease	0.04777
DB00244	Bipolar disorder	0.0341
DB00244	Brain tumor	0.04139
DB00244	Breast cancer	0.22116
DB00244	Bronchial hyperreactivity	0.06195
DB00244	Cancer	0.10744
DB00244	Cerebrovascular disorder	0.04258
DB00244	Cervical cancer	0.0411
DB00244	Cholelithiasis	0.0372
DB00244	Chronic fatigue syndrome	0.23413
DB00244	Chronic simple glaucoma	0.02269
DB00244	Colon cancer	0.00648
DB00244	Congenital abnormality	0.01516
DB00244	Cytomegalovirus infection	0.09129
DB00244	Dental plaque	0.15586

DB00244	Depression	0.02881
DB00244	Dermatitis	0.04988
DB00244	Diabetes mellitus	0.11801
DB00244	Down syndrome	0.02419
DB00244	Eating disorder	0.04132
DB00244	Embryoma	0.02693
DB00244	Endometriosis	0.12817
DB00244	Esophagus cancer	0.02189
DB00244	Fanconi's anemia	0.03702
DB00244	Glaucoma	0.0282
DB00244	Glomerulonephritis	0.08839
DB00244	Gouts	0.20539
DB00244	Growth retardation	0.14826
DB00244	Helicobacter infection	0.07906
DB00244	Hepatoblastoma	0.20412
DB00244	Herpes	0.04462
DB00244	Hodgkin's disease	0.09021
DB00244	Hyperlipidemia	0.11654
DB00244	Hypertension	0.0188
DB00244	Immunologic deficiency syndrome	0.06058
DB00244	Infection	0.01697
DB00244	Infectious lung disease	0.98574
DB00244	Infertility	0.02721
DB00244	Infiltrating cancer	0.19474
DB00244	Intracranial hypertension	0.12852
DB00244	Ischemia	0.1312
DB00244	Kidney failure	0.06391
DB00244	Late pregnancy	0.05848
DB00244	Leukemia	0.38439
DB00244	Leukoencephalopathy	0.01673
DB00244	Liver cancer	0.34736
DB00244	Lung cancer	0.04424
DB00244	Lupus erythematosus	0.01329
DB00244	Lupus vulgaris	0.03613
DB00244	Lymphoma	0.01191
DB00244	Melanoma	0.00877
DB00244	Metaplastic polyp	0.17494
DB00244	Migraine	0.03339
DB00244	Mucopolysaccharidosis	0.03706
DB00244	Multiple sclerosis	0.15818
DB00244	Myasthenia Gravis	0.04521
DB00244	Myelofibrosis	0.04926
DB00244	Neoplasm metastasis	0.37438
DB00244	Nephrosis	0.1699
DB00244	Neuroblastoma	0.02169
DB00244	Neurodegenerative disorder	0.06964
DB00244	Obesity	0.39132

DB00244	Oral cancer	0.07834
DB00244	Osteoporosis	0.02709
DB00244	Ovarian disease	0.04247
DB00244	Ovarian failure	0.05554
DB00244	Pancreas cancer	0.03666
DB00244	Pancreatitis	0.14928
DB00244	Panic disorder	0.04266
DB00244	Parkinson disease	0.09553
DB00244	Periodontal disease	0.08333
DB00244	Periodontitis	0.18271
DB00244	Polyarthritis	0.62869
DB00244	Primary biliary cirrhosis	0.03238
DB00244	Prion disease	0.2359
DB00244	Prostate cancer	0.37137
DB00244	Rabies	0.01627
DB00244	Renal tubular acidosis	0.02451
DB00244	Rheumatism	0.02958
DB00244	Rheumatoid arthritis	0.04326
DB00244	Schizophrenia	0.02427
DB00244	Sicca syndrome	0.0339
DB00244	Sickle cell disease	0.16956
DB00244	Squamous cell cancer	0.0939
DB00244	Stomach cancer	0.05818
DB00244	Stomach disease	0.125
DB00244	Stroke	0.19561
DB00244	Synovitis	0.04924
DB00244	Systemic infection	0.05696
DB00244	Systemic scleroderma	0.04039
DB00244	Testicular dysfunction	0.009
DB00244	Thymoma	0.03821
DB00244	Thyroid gland disease	0.14282
DB00244	Tuberculosis	0.2013
DB00244	Tuberous sclerosis	0.42623
DB00244	Ulcerative colitis	0.08833
DB00244	Uterine disease	0.03401
DB00244	Vasculitis	0.1066
DB00244	Virus disease	0.49899
DB00244	Vitiligo	0.04521
DB00244	Vulvar disease	0.34385
DB00244	Yersinia infection	0.32683
DB00316	Alzheimer's disease	0.10592
DB00316	Asthma	0.14253
DB00316	Atherosclerosis	0.12306
DB00316	Cancer	0.05415
DB00316	Chronic fatigue syndrome	0.58081
DB00316	Dental plaque	0.21675
DB00316	Endometriosis	0.12534

DB00316	Gouts	0.52146
DB00316	Growth retardation	0.37925
DB00316	Ischemia	0.21381
DB00316	Metaplastic polyp	0.40875
DB00316	Oral cancer	0.18784
DB00316	Parkinson disease	0.18664
DB00316	Periodontitis	0.33479
DB00316	Polyarthritis	0.1617
DB00316	Prion disease	0.56624
DB00316	Rheumatoid arthritis	0.09414
DB00316	Sickle cell disease	0.23758
DB00316	Stroke	0.15796
DB00316	Thyroid gland disease	0.34927
DB00316	Vulvar disease	0.87924
DB00328	Alzheimer's disease	0.10944
DB00328	Asthma	0.14652
DB00328	Atherosclerosis	0.12648
DB00328	Autistic disorder	0.04583
DB00328	Cancer	0.04201
DB00328	Cholelithiasis	0.07559
DB00328	Chronic fatigue syndrome	0.46444
DB00328	Cystic fibrosis	0.05399
DB00328	Dental plaque	0.22295
DB00328	Dermatitis	0.03436
DB00328	Diabetes mellitus	0.01989
DB00328	Drug abuse	0.0354
DB00328	Embryoma	0.02331
DB00328	Endometriosis	0.098
DB00328	Gouts	0.43018
DB00328	Growth retardation	0.31591
DB00328	Hyperglycemia	0.06052
DB00328	Ischemia	0.17167
DB00328	Metaplastic polyp	0.29904
DB00328	Multiple sclerosis	0.03898
DB00328	Neoplasm metastasis	0.03076
DB00328	Oral cancer	0.14305
DB00328	Panic disorder	0.08248
DB00328	Parkinson disease	0.1503
DB00328	Periodontitis	0.2846
DB00328	Polyarthritis	0.12419
DB00328	Prion disease	0.43186
DB00328	Prostate cancer	0.01973
DB00328	Renal Cell cancer	0.04394
DB00328	Rheumatoid arthritis	0.074
DB00328	Schizophrenia	0.02874
DB00328	Sickle cell disease	0.17423
DB00328	Stomach cancer	0.03183

DB00328	Stroke	0.16349
DB00328	Thyroid gland disease	0.27376
DB00328	Tuberculosis	0.05096
DB00328	Vascular dementia	0.12599
DB00328	Vulvar disease	0.73205
DB00350	Hyperaldosteronism	0.22361
DB00350	Kidney disease	0.08452
DB00461	Alzheimer's disease	0.10592
DB00461	Asthma	0.14253
DB00461	Atherosclerosis	0.12306
DB00461	Cancer	0.05415
DB00461	Chronic fatigue syndrome	0.58081
DB00461	Dental plaque	0.21675
DB00461	Endometriosis	0.12534
DB00461	Gouts	0.52146
DB00461	Growth retardation	0.37925
DB00461	Ischemia	0.21381
DB00461	Metaplastic polyp	0.40875
DB00461	Oral cancer	0.18784
DB00461	Parkinson disease	0.18664
DB00461	Periodontitis	0.33479
DB00461	Polyarthritis	0.1617
DB00461	Prion disease	0.56624
DB00461	Rheumatoid arthritis	0.09414
DB00461	Sickle cell disease	0.23758
DB00461	Stroke	0.15796
DB00461	Thyroid gland disease	0.34927
DB00461	Vulvar disease	0.87924
DB00465	Alzheimer's disease	0.10592
DB00465	Asthma	0.14253
DB00465	Atherosclerosis	0.12306
DB00465	Cancer	0.05415
DB00465	Chronic fatigue syndrome	0.58081
DB00465	Dental plaque	0.21675
DB00465	Endometriosis	0.12534
DB00465	Gouts	0.52146
DB00465	Growth retardation	0.37925
DB00465	Ischemia	0.21381
DB00465	Metaplastic polyp	0.40875
DB00465	Oral cancer	0.18784
DB00465	Parkinson disease	0.18664
DB00465	Periodontitis	0.33479
DB00465	Polyarthritis	0.1617
DB00465	Prion disease	0.56624
DB00465	Rheumatoid arthritis	0.09414
DB00465	Sickle cell disease	0.23758
DB00465	Stroke	0.15796

DB00465	Thyroid gland disease	0.34927
DB00465	Vulvar disease	0.87924
DB00469	Alzheimer's disease	0.10592
DB00469	Asthma	0.14253
DB00469	Atherosclerosis	0.12306
DB00469	Cancer	0.05415
DB00469	Chronic fatigue syndrome	0.58081
DB00469	Dental plaque	0.21675
DB00469	Endometriosis	0.12534
DB00469	Gouts	0.52146
DB00469	Growth retardation	0.37925
DB00469	Ischemia	0.21381
DB00469	Metaplastic polyp	0.40875
DB00469	Oral cancer	0.18784
DB00469	Parkinson disease	0.18664
DB00469	Periodontitis	0.33479
DB00469	Polyarthritis	0.1617
DB00469	Prion disease	0.56624
DB00469	Rheumatoid arthritis	0.09414
DB00469	Sickle cell disease	0.23758
DB00469	Stroke	0.15796
DB00469	Thyroid gland disease	0.34927
DB00469	Vulvar disease	0.87924
DB00500	Alzheimer's disease	0.10592
DB00500	Asthma	0.14253
DB00500	Atherosclerosis	0.12306
DB00500	Cancer	0.05415
DB00500	Chronic fatigue syndrome	0.58081
DB00500	Dental plaque	0.21675
DB00500	Endometriosis	0.12534
DB00500	Gouts	0.52146
DB00500	Growth retardation	0.37925
DB00500	Ischemia	0.21381
DB00500	Metaplastic polyp	0.40875
DB00500	Oral cancer	0.18784
DB00500	Parkinson disease	0.18664
DB00500	Periodontitis	0.33479
DB00500	Polyarthritis	0.1617
DB00500	Prion disease	0.56624
DB00500	Rheumatoid arthritis	0.09414
DB00500	Sickle cell disease	0.23758
DB00500	Stroke	0.15796
DB00500	Thyroid gland disease	0.34927
DB00500	Vulvar disease	0.87924
DB00554	Alzheimer's disease	0.10592
DB00554	Asthma	0.14253
DB00554	Atherosclerosis	0.12306

DB00554	Cancer	0.05415
DB00554	Chronic fatigue syndrome	0.58081
DB00554	Dental plaque	0.21675
DB00554	Endometriosis	0.12534
DB00554	Gouts	0.52146
DB00554	Growth retardation	0.37925
DB00554	Ischemia	0.21381
DB00554	Metaplastic polyp	0.40875
DB00554	Oral cancer	0.18784
DB00554	Parkinson disease	0.18664
DB00554	Periodontitis	0.33479
DB00554	Polyarthritis	0.1617
DB00554	Prion disease	0.56624
DB00554	Rheumatoid arthritis	0.09414
DB00554	Sickle cell disease	0.23758
DB00554	Stroke	0.15796
DB00554	Thyroid gland disease	0.34927
DB00554	Vulvar disease	0.87924
DB00573	Alzheimer's disease	0.10592
DB00573	Asthma	0.14253
DB00573	Atherosclerosis	0.12306
DB00573	Cancer	0.05415
DB00573	Chronic fatigue syndrome	0.58081
DB00573	Dental plaque	0.21675
DB00573	Endometriosis	0.12534
DB00573	Gouts	0.52146
DB00573	Growth retardation	0.37925
DB00573	Ischemia	0.21381
DB00573	Metaplastic polyp	0.40875
DB00573	Oral cancer	0.18784
DB00573	Parkinson disease	0.18664
DB00573	Periodontitis	0.33479
DB00573	Polyarthritis	0.1617
DB00573	Prion disease	0.56624
DB00573	Rheumatoid arthritis	0.09414
DB00573	Sickle cell disease	0.23758
DB00573	Stroke	0.15796
DB00573	Thyroid gland disease	0.34927
DB00573	Vulvar disease	0.87924
DB00586	Myoclonic epilepsy, Juvenile	0.14434
DB00586	Adenoid cystic cancer	0.17678
DB00586	Alzheimer's disease	0.10592
DB00586	Anxiety disorder	0.20412
DB00586	Asthma	0.14253
DB00586	Atherosclerosis	0.14782
DB00586	Cancer	0.05415
DB00586	Cholelithiasis	0.07071

DB00586	Chronic fatigue syndrome	0.45581
DB00586	Common cold	0.09449
DB00586	Cystic fibrosis	0.05051
DB00586	Cytomegalovirus infection	0.09129
DB00586	Dental plaque	0.26165
DB00586	Diabetes mellitus	0.03722
DB00586	Drug abuse	0.06623
DB00586	Embryoma	0.0218
DB00586	Endometriosis	0.09598
DB00586	Gouts	0.42341
DB00586	Growth retardation	0.31121
DB00586	Ischemia	0.21381
DB00586	Metaplastic polyp	0.2909
DB00586	Myopathy	0.08575
DB00586	Neoplasm metastasis	0.02877
DB00586	Oral cancer	0.13973
DB00586	Parkinson disease	0.1476
DB00586	Periodontitis	0.28088
DB00586	Polyarthritis	0.12141
DB00586	Prion disease	0.4219
DB00586	Rheumatoid arthritis	0.0725
DB00586	Schizophrenia	0.02688
DB00586	Sickle cell disease	0.16953
DB00586	Stomach cancer	0.02977
DB00586	Stroke	0.198
DB00586	Sudden infant death syndrome	0.1543
DB00586	Thyroid gland disease	0.26816
DB00586	Tuberculosis	0.04767
DB00586	Vascular dementia	0.11785
DB00586	Vulvar disease	0.72113
DB00586	Yersinia infection	0.04196
DB00605	Adrenal gland tumor	0.12309
DB00605	Alzheimer's disease	0.07668
DB00605	Asthma	0.13955
DB00605	Atherosclerosis	0.12039
DB00605	Breast cancer	0.02889
DB00605	Cancer	0.03919
DB00605	Chronic fatigue syndrome	0.42868
DB00605	Colon cancer	0.02423
DB00605	Common cold	0.10911
DB00605	Dental plaque	0.16096
DB00605	Dermatitis	0.03711
DB00605	Diabetes mellitus	0.02149
DB00605	Endometriosis	0.09116
DB00605	Gouts	0.39287
DB00605	Growth retardation	0.28758
DB00605	Heart failure	0.07293

DB00605	Ischemia	0.2105
DB00605	Leukemia	0.03426
DB00605	Metabolism disease	0.06537
DB00605	Metaplastic polyp	0.28482
DB00605	Myelofibrosis	0.20293
DB00605	Oral cancer	0.1343
DB00605	Pancreas disease	0.08513
DB00605	Parkinson disease	0.13839
DB00605	Periodontitis	0.25734
DB00605	Polyarthritis	0.11625
DB00605	Prion disease	0.40524
DB00605	Rheumatoid arthritis	0.0687
DB00605	Sickle cell disease	0.1658
DB00605	Stroke	0.11318
DB00605	Thyroid gland disease	0.25443
DB00605	Vulvar disease	0.6665
DB00711	Asthma	0.05774
DB00711	Atherosclerosis	0.04951
DB00711	Cancer	0.02606
DB00711	Cytomegalovirus infection	0.18257
DB00711	Dental plaque	0.0898
DB00711	Diabetes mellitus	0.03722
DB00711	Tuberculosis	0.09535
DB00712	Alzheimer's disease	0.10592
DB00712	Asthma	0.14253
DB00712	Atherosclerosis	0.12306
DB00712	Cancer	0.05415
DB00712	Chronic fatigue syndrome	0.58081
DB00712	Dental plaque	0.21675
DB00712	Endometriosis	0.12534
DB00712	Gouts	0.52146
DB00712	Growth retardation	0.37925
DB00712	Ischemia	0.21381
DB00712	Metaplastic polyp	0.40875
DB00712	Oral cancer	0.18784
DB00712	Parkinson disease	0.18664
DB00712	Periodontitis	0.33479
DB00712	Polyarthritis	0.1617
DB00712	Prion disease	0.56624
DB00712	Rheumatoid arthritis	0.09414
DB00712	Sickle cell disease	0.23758
DB00712	Stroke	0.15796
DB00712	Thyroid gland disease	0.34927
DB00712	Vulvar disease	0.87924
DB00749	Alzheimer's disease	0.09003
DB00749	Aseptic necrosis of bone	0.07398
DB00749	Asthma	0.12184

DB00749	Atherosclerosis	0.10522
DB00749	Breast cancer	0.02418
DB00749	Cancer	0.08158
DB00749	Chronic fatigue syndrome	0.49554
DB00749	Dental plaque	0.18515
DB00749	Dermatitis	0.09371
DB00749	Endometriosis	0.10663
DB00749	Esophagus cancer	0.08929
DB00749	Gouts	0.44673
DB00749	Growth retardation	0.32532
DB00749	Ischemia	0.18252
DB00749	Leukemia	0.04054
DB00749	Leukoencephalopathy	0.06824
DB00749	Lung cancer	0.03612
DB00749	Metaplastic polyp	0.34489
DB00749	Mucopolysaccharidosis	0.10689
DB00749	Neuroblastoma	0.06256
DB00749	Obesity	0.04746
DB00749	Oral cancer	0.15927
DB00749	Parkinson disease	0.15939
DB00749	Periodontitis	0.28797
DB00749	Polyarthritis	0.13725
DB00749	Prion disease	0.48021
DB00749	Prostate cancer	0.03831
DB00749	Rheumatism	0.08533
DB00749	Rheumatoid arthritis	0.10601
DB00749	Sickle cell disease	0.20052
DB00749	Stroke	0.13399
DB00749	Thyroid gland disease	0.29723
DB00749	Vulvar disease	0.75416
DB00784	Alzheimer's disease	0.10592
DB00784	Asthma	0.14253
DB00784	Atherosclerosis	0.12306
DB00784	Cancer	0.05415
DB00784	Chronic fatigue syndrome	0.58081
DB00784	Dental plaque	0.21675
DB00784	Endometriosis	0.12534
DB00784	Gouts	0.52146
DB00784	Growth retardation	0.37925
DB00784	Ischemia	0.21381
DB00784	Metaplastic polyp	0.40875
DB00784	Oral cancer	0.18784
DB00784	Parkinson disease	0.18664
DB00784	Periodontitis	0.33479
DB00784	Polyarthritis	0.1617
DB00784	Prion disease	0.56624
DB00784	Rheumatoid arthritis	0.09414

DB00784	Sickle cell disease	0.23758
DB00784	Stroke	0.15796
DB00784	Thyroid gland disease	0.34927
DB00784	Vulvar disease	0.87924
DB00788	Alzheimer's disease	0.10592
DB00788	Asthma	0.14253
DB00788	Atherosclerosis	0.12306
DB00788	Cancer	0.05415
DB00788	Chronic fatigue syndrome	0.58081
DB00788	Dental plaque	0.21675
DB00788	Endometriosis	0.12534
DB00788	Gouts	0.52146
DB00788	Growth retardation	0.37925
DB00788	Ischemia	0.21381
DB00788	Metaplastic polyp	0.40875
DB00788	Oral cancer	0.18784
DB00788	Parkinson disease	0.18664
DB00788	Periodontitis	0.33479
DB00788	Polyarthritis	0.1617
DB00788	Prion disease	0.56624
DB00788	Rheumatoid arthritis	0.09414
DB00788	Sickle cell disease	0.23758
DB00788	Stroke	0.15796
DB00788	Thyroid gland disease	0.34927
DB00788	Vulvar disease	0.87924
DB00795	Infertility, Male	0.04259
DB00795	Stress disorder, post-traumatic	0.06607
DB00795	AIDS	0.12102
DB00795	Adenocarcinoma	0.06785
DB00795	Adenoma	0.01109
DB00795	Alzheimer's disease	0.07516
DB00795	Aseptic necrosis of bone	0.02565
DB00795	Asthma	0.08571
DB00795	Atherosclerosis	0.08778
DB00795	Autoimmune disease	0.04777
DB00795	Bipolar disorder	0.0341
DB00795	Brain tumor	0.04139
DB00795	Breast cancer	0.20415
DB00795	Bronchial hyperreactivity	0.06195
DB00795	Cancer	0.10744
DB00795	Cerebrovascular disorder	0.04258
DB00795	Cholelithiasis	0.0372
DB00795	Chronic fatigue syndrome	0.23413
DB00795	Chronic simple glaucoma	0.02269
DB00795	Colon cancer	0.00648
DB00795	Congenital abnormality	0.01516
DB00795	Cytomegalovirus infection	0.09129

DB00795	Dental plaque	0.15586
DB00795	Depression	0.02881
DB00795	Dermatitis	0.04988
DB00795	Diabetes mellitus	0.0994
DB00795	Down syndrome	0.02419
DB00795	Eating disorder	0.04132
DB00795	Embryoma	0.02693
DB00795	Endometriosis	0.12817
DB00795	Esophagus cancer	0.02189
DB00795	Fanconi's anemia	0.03702
DB00795	Glaucoma	0.0282
DB00795	Gouts	0.20539
DB00795	Growth retardation	0.14826
DB00795	Herpes	0.04462
DB00795	Hodgkin's disease	0.09021
DB00795	Hyperlipidemia	0.0472
DB00795	Hypertension	0.0188
DB00795	Immunologic deficiency syndrome	0.06058
DB00795	Infection	0.01697
DB00795	Infectious lung disease	0.98574
DB00795	Infertility	0.02721
DB00795	Infiltrating cancer	0.19474
DB00795	Intracranial hypertension	0.12852
DB00795	Ischemia	0.08594
DB00795	Kidney failure	0.02388
DB00795	Late pregnancy	0.05848
DB00795	Leukemia	0.36447
DB00795	Leukoencephalopathy	0.01673
DB00795	Liver cancer	0.34736
DB00795	Lung cancer	0.01955
DB00795	Lupus erythematosus	0.01329
DB00795	Lupus vulgaris	0.03613
DB00795	Lymphoma	0.01191
DB00795	Melanoma	0.00877
DB00795	Metaplastic polyp	0.17494
DB00795	Migraine	0.03339
DB00795	Mucopolysaccharidosis	0.03706
DB00795	Multiple sclerosis	0.12171
DB00795	Myasthenia Gravis	0.04521
DB00795	Myelofibrosis	0.04926
DB00795	Neoplasm metastasis	0.37438
DB00795	Nephrosis	0.1699
DB00795	Neuroblastoma	0.02169
DB00795	Neurodegenerative disorder	0.06964
DB00795	Obesity	0.39132
DB00795	Oral cancer	0.07834
DB00795	Osteoporosis	0.02709

DB00795	Ovarian disease	0.04247
DB00795	Ovarian failure	0.05554
DB00795	Pancreatitis	0.14928
DB00795	Panic disorder	0.04266
DB00795	Parkinson disease	0.09553
DB00795	Periodontitis	0.12879
DB00795	Polyarthritis	0.62869
DB00795	Primary biliary cirrhosis	0.03238
DB00795	Prion disease	0.2359
DB00795	Prostate cancer	0.35291
DB00795	Rabies	0.01627
DB00795	Renal tubular acidosis	0.02451
DB00795	Rheumatism	0.02958
DB00795	Rheumatoid arthritis	0.04326
DB00795	Schizophrenia	0.02427
DB00795	Sicca syndrome	0.0339
DB00795	Sickle cell disease	0.10152
DB00795	Squamous cell cancer	0.0939
DB00795	Stomach cancer	0.05818
DB00795	Stroke	0.15558
DB00795	Synovitis	0.04924
DB00795	Systemic infection	0.0164
DB00795	Systemic scleroderma	0.04039
DB00795	Testicular dysfunction	0.009
DB00795	Thymoma	0.03821
DB00795	Thyroid gland disease	0.14282
DB00795	Tuberculosis	0.2013
DB00795	Tuberous sclerosis	0.42623
DB00795	Ulcerative colitis	0.08833
DB00795	Uterine disease	0.03401
DB00795	Virus disease	0.49899
DB00795	Vitiligo	0.04521
DB00795	Vulvar disease	0.34385
DB00795	Yersinia infection	0.36879
DB00812	Alzheimer's disease	0.09658
DB00812	Asthma	0.13194
DB00812	Atherosclerosis	0.11398
DB00812	Cancer	0.04936
DB00812	Chronic fatigue syndrome	0.53493
DB00812	Dental plaque	0.20027
DB00812	Endometriosis	0.11456
DB00812	Gouts	0.48548
DB00812	Growth retardation	0.35428
DB00812	Ischemia	0.1972
DB00812	Metaplastic polyp	0.3655
DB00812	Oral cancer	0.17018
DB00812	Parkinson disease	0.17231

DB00812	Periodontitis	0.315
DB00812	Polyarthritis	0.14691
DB00812	Prion disease	0.51326
DB00812	Rheumatoid arthritis	0.0862
DB00812	Sickle cell disease	0.2126
DB00812	Stroke	0.14327
DB00812	Thyroid gland disease	0.3195
DB00812	Vulvar disease	0.82122
DB00814	Alzheimer's disease	0.10592
DB00814	Asthma	0.14253
DB00814	Atherosclerosis	0.12306
DB00814	Cancer	0.05415
DB00814	Chronic fatigue syndrome	0.58081
DB00814	Dental plaque	0.21675
DB00814	Endometriosis	0.12534
DB00814	Gouts	0.52146
DB00814	Growth retardation	0.37925
DB00814	Ischemia	0.21381
DB00814	Metaplastic polyp	0.40875
DB00814	Oral cancer	0.18784
DB00814	Parkinson disease	0.18664
DB00814	Periodontitis	0.33479
DB00814	Polyarthritis	0.1617
DB00814	Prion disease	0.56624
DB00814	Rheumatoid arthritis	0.09414
DB00814	Sickle cell disease	0.23758
DB00814	Stroke	0.15796
DB00814	Thyroid gland disease	0.34927
DB00814	Vulvar disease	0.87924
DB00821	Alzheimer's disease	0.10592
DB00821	Asthma	0.14253
DB00821	Atherosclerosis	0.12306
DB00821	Cancer	0.05415
DB00821	Chronic fatigue syndrome	0.58081
DB00821	Dental plaque	0.21675
DB00821	Endometriosis	0.12534
DB00821	Gouts	0.52146
DB00821	Growth retardation	0.37925
DB00821	Ischemia	0.21381
DB00821	Metaplastic polyp	0.40875
DB00821	Oral cancer	0.18784
DB00821	Parkinson disease	0.18664
DB00821	Periodontitis	0.33479
DB00821	Polyarthritis	0.1617
DB00821	Prion disease	0.56624
DB00821	Rheumatoid arthritis	0.09414
DB00821	Sickle cell disease	0.23758

DB00821	Stroke	0.15796
DB00821	Thyroid gland disease	0.34927
DB00821	Vulvar disease	0.87924
DB00861	Alzheimer's disease	0.10592
DB00861	Asthma	0.14253
DB00861	Atherosclerosis	0.12306
DB00861	Cancer	0.05415
DB00861	Chronic fatigue syndrome	0.58081
DB00861	Dental plaque	0.21675
DB00861	Endometriosis	0.12534
DB00861	Gouts	0.52146
DB00861	Growth retardation	0.37925
DB00861	Ischemia	0.21381
DB00861	Metaplastic polyp	0.40875
DB00861	Oral cancer	0.18784
DB00861	Parkinson disease	0.18664
DB00861	Periodontitis	0.33479
DB00861	Polyarthritis	0.1617
DB00861	Prion disease	0.56624
DB00861	Rheumatoid arthritis	0.09414
DB00861	Sickle cell disease	0.23758
DB00861	Stroke	0.15796
DB00861	Thyroid gland disease	0.34927
DB00861	Vulvar disease	0.87924
DB00870	Alzheimer's disease	0.10592
DB00870	Asthma	0.14253
DB00870	Atherosclerosis	0.12306
DB00870	Cancer	0.05415
DB00870	Chronic fatigue syndrome	0.58081
DB00870	Dental plaque	0.21675
DB00870	Endometriosis	0.12534
DB00870	Gouts	0.52146
DB00870	Growth retardation	0.37925
DB00870	Ischemia	0.21381
DB00870	Metaplastic polyp	0.40875
DB00870	Oral cancer	0.18784
DB00870	Parkinson disease	0.18664
DB00870	Periodontitis	0.33479
DB00870	Polyarthritis	0.1617
DB00870	Prion disease	0.56624
DB00870	Rheumatoid arthritis	0.09414
DB00870	Sickle cell disease	0.23758
DB00870	Stroke	0.15796
DB00870	Thyroid gland disease	0.34927
DB00870	Vulvar disease	0.87924
DB00936	Alzheimer's disease	0.09658
DB00936	Asthma	0.13194

DB00936	Atherosclerosis	0.11398
DB00936	Cancer	0.07065
DB00936	Chronic fatigue syndrome	0.53493
DB00936	Dental plaque	0.20027
DB00936	Endometriosis	0.11456
DB00936	Glaucoma	0.09623
DB00936	Gouts	0.48548
DB00936	Growth retardation	0.35428
DB00936	Ischemia	0.1972
DB00936	Metaplastic polyp	0.3655
DB00936	Obesity	0.04441
DB00936	Oral cancer	0.17018
DB00936	Parkinson disease	0.17231
DB00936	Periodontitis	0.315
DB00936	Polyarthritis	0.14691
DB00936	Prion disease	0.51326
DB00936	Rheumatoid arthritis	0.0862
DB00936	Sickle cell disease	0.2126
DB00936	Stroke	0.14327
DB00936	Thyroid gland disease	0.3195
DB00936	Vulvar disease	0.82122
DB00939	Myoclonic epilepsy, Juvenile	0.18257
DB00939	Alzheimer's disease	0.08721
DB00939	Asthma	0.15783
DB00939	Atherosclerosis	0.13618
DB00939	Cancer	0.06105
DB00939	Chronic fatigue syndrome	0.48892
DB00939	Cytomegalovirus infection	0.11547
DB00939	Dental plaque	0.24054
DB00939	Diabetes mellitus	0.02354
DB00939	Endometriosis	0.10376
DB00939	Gouts	0.44938
DB00939	Growth retardation	0.32924
DB00939	Ischemia	0.18053
DB00939	Metaplastic polyp	0.32212
DB00939	Myopathy	0.05423
DB00939	Oral cancer	0.15248
DB00939	Parkinson disease	0.15794
DB00939	Periodontitis	0.29516
DB00939	Polyarthritis	0.13208
DB00939	Prion disease	0.46013
DB00939	Rheumatoid arthritis	0.07823
DB00939	Sickle cell disease	0.18756
DB00939	Stroke	0.12854
DB00939	Sudden infant death syndrome	0.19518
DB00939	Thyroid gland disease	0.28965
DB00939	Tuberculosis	0.0603

DB00939	Vulvar disease	0.76302
DB00939	Yersinia infection	0.05307
DB00945	Alzheimer's disease	0.09658
DB00945	Asthma	0.13194
DB00945	Atherosclerosis	0.11398
DB00945	Cancer	0.07065
DB00945	Chronic fatigue syndrome	0.53493
DB00945	Dental plaque	0.20027
DB00945	Endometriosis	0.11456
DB00945	Glaucoma	0.09623
DB00945	Gouts	0.48548
DB00945	Growth retardation	0.35428
DB00945	Ischemia	0.1972
DB00945	Metaplastic polyp	0.3655
DB00945	Obesity	0.04441
DB00945	Oral cancer	0.17018
DB00945	Parkinson disease	0.17231
DB00945	Periodontitis	0.315
DB00945	Polyarthritis	0.14691
DB00945	Prion disease	0.51326
DB00945	Rheumatoid arthritis	0.0862
DB00945	Sickle cell disease	0.2126
DB00945	Stroke	0.14327
DB00945	Thyroid gland disease	0.3195
DB00945	Vulvar disease	0.82122
DB00963	Alzheimer's disease	0.10592
DB00963	Asthma	0.14253
DB00963	Atherosclerosis	0.12306
DB00963	Cancer	0.05415
DB00963	Chronic fatigue syndrome	0.58081
DB00963	Dental plaque	0.21675
DB00963	Endometriosis	0.12534
DB00963	Gouts	0.52146
DB00963	Growth retardation	0.37925
DB00963	Ischemia	0.21381
DB00963	Metaplastic polyp	0.40875
DB00963	Oral cancer	0.18784
DB00963	Parkinson disease	0.18664
DB00963	Periodontitis	0.33479
DB00963	Polyarthritis	0.1617
DB00963	Prion disease	0.56624
DB00963	Rheumatoid arthritis	0.09414
DB00963	Sickle cell disease	0.23758
DB00963	Stroke	0.15796
DB00963	Thyroid gland disease	0.34927
DB00963	Vulvar disease	0.87924
DB00991	Alzheimer's disease	0.10592

DB00991	Asthma	0.14253
DB00991	Atherosclerosis	0.12306
DB00991	Cancer	0.05415
DB00991	Chronic fatigue syndrome	0.58081
DB00991	Dental plaque	0.21675
DB00991	Endometriosis	0.12534
DB00991	Gouts	0.52146
DB00991	Growth retardation	0.37925
DB00991	Ischemia	0.21381
DB00991	Metaplastic polyp	0.40875
DB00991	Oral cancer	0.18784
DB00991	Parkinson disease	0.18664
DB00991	Periodontitis	0.33479
DB00991	Polyarthritis	0.1617
DB00991	Prion disease	0.56624
DB00991	Rheumatoid arthritis	0.09414
DB00991	Sickle cell disease	0.23758
DB00991	Stroke	0.15796
DB00991	Thyroid gland disease	0.34927
DB00991	Vulvar disease	0.87924
DB01009	Alzheimer's disease	0.09658
DB01009	Arthritis	0.07161
DB01009	Asthma	0.13194
DB01009	Atherosclerosis	0.11398
DB01009	Bronchiectasis	0.2357
DB01009	Cancer	0.04936
DB01009	Chronic fatigue syndrome	0.53493
DB01009	Colon cancer	0.03426
DB01009	Congenital abnormality	0.04352
DB01009	Dental plaque	0.20027
DB01009	Endometriosis	0.16251
DB01009	Gouts	0.48548
DB01009	Growth retardation	0.35428
DB01009	Hepatitis	0.14907
DB01009	Infection	0.05384
DB01009	Ischemia	0.1972
DB01009	Leukemia	0.03253
DB01009	Melanoma	0.04811
DB01009	Metaplastic polyp	0.3655
DB01009	Myelofibrosis	0.17408
DB01009	Myeloid metaplasia	0.33333
DB01009	Oral cancer	0.17018
DB01009	Parkinson disease	0.17231
DB01009	Peptic esophagitis	0.18257
DB01009	Periodontitis	0.315
DB01009	Polyarthritis	0.21271
DB01009	Prion disease	0.51326

DB01009	Rheumatoid arthritis	0.0862
DB01009	Sickle cell disease	0.2126
DB01009	Stroke	0.14327
DB01009	Thyroid gland disease	0.3195
DB01009	Vulvar disease	0.82122
DB01014	Alzheimer's disease	0.09101
DB01014	Asthma	0.16645
DB01014	Atherosclerosis	0.14357
DB01014	Cancer	0.06494
DB01014	Chronic fatigue syndrome	0.50759
DB01014	Cytomegalovirus infection	0.1291
DB01014	Dental plaque	0.25395
DB01014	Diabetes mellitus	0.02632
DB01014	Endometriosis	0.10814
DB01014	Gouts	0.46402
DB01014	Growth retardation	0.3394
DB01014	Ischemia	0.18729
DB01014	Metaplastic polyp	0.33971
DB01014	Oral cancer	0.15966
DB01014	Parkinson disease	0.16377
DB01014	Periodontitis	0.30321
DB01014	Polyarthritis	0.1381
DB01014	Prion disease	0.48168
DB01014	Rheumatoid arthritis	0.08146
DB01014	Sickle cell disease	0.19772
DB01014	Stroke	0.13451
DB01014	Thyroid gland disease	0.30176
DB01014	Tuberculosis	0.06742
DB01014	Vulvar disease	0.78662
DB01050	Infertility, Male	0.33733
DB01050	AIDS	0.08119
DB01050	Alimentary system disease	0.57067
DB01050	Alzheimer's disease	0.26241
DB01050	Antiphospholipid syndrome	0.11785
DB01050	Arthritis	0.0582
DB01050	Asthma	0.24392
DB01050	Atherosclerosis	0.10908
DB01050	Autistic disorder	0.27482
DB01050	Autoimmune disease	0.23557
DB01050	Azoospermia	0.27271
DB01050	Bone disease	0.2258
DB01050	Brain ischemia	0.75411
DB01050	Breast cancer	0.05638
DB01050	Bronchial disease	0.11642
DB01050	Cancer	0.12603
DB01050	Celiac disease	0.33545
DB01050	Chronic fatigue syndrome	0.29486

DB01050	Chronic obstructive airway disease	0.04669
DB01050	Conduct disorder	0.63169
DB01050	Congenital abnormality	0.18813
DB01050	Cystic fibrosis	0.3557
DB01050	Dental plaque	0.36853
DB01050	Dermatitis	0.02403
DB01050	Diabetes mellitus	0.11205
DB01050	Drug abuse	0.15747
DB01050	Eating disorder	0.24371
DB01050	Endometriosis	0.12281
DB01050	Enteritis	0.21516
DB01050	Epstein-Barr virus infection	0.37948
DB01050	Esotropia	0.39096
DB01050	Eye cancer	0.6081
DB01050	Glaucoma	0.35187
DB01050	Gouts	0.26512
DB01050	Gram-Negative bacterial infection	0.56202
DB01050	Graves' disease	0.48289
DB01050	Growth retardation	0.19291
DB01050	HIV infection	0.14817
DB01050	Heart failure	0.19677
DB01050	Hemorrhagic disorder	0.0625
DB01050	Herpes	0.18154
DB01050	Hyperlipidemia	0.06934
DB01050	Hypertension	0.1868
DB01050	Immunologic deficiency syndrome	0.04064
DB01050	Infection	0.07563
DB01050	Infertility	0.21797
DB01050	Intracranial hypertension	0.08622
DB01050	Ischemia	0.40647
DB01050	Keratosis	0.40444
DB01050	Kidney disease	0.04226
DB01050	Late pregnancy	0.10206
DB01050	Leukemia	0.01992
DB01050	Lichen planus	0.682
DB01050	Liver cancer	0.11254
DB01050	Liver disease	0.05455
DB01050	Lung cancer	0.04575
DB01050	Lupus erythematosus	0.14755
DB01050	Lymphoma	0.04635
DB01050	Malaria	0.06747
DB01050	Metabolism disease	0.06351
DB01050	Metaplastic polyp	0.20671
DB01050	Multiple sclerosis	0.03647
DB01050	Myopathy	0.04235
DB01050	Neurodegenerative disorder	0.04672
DB01050	Obesity	0.05486

DB01050	Oligospermia	0.45642
DB01050	Oral cancer	0.09516
DB01050	Ovarian cancer	0.03553
DB01050	Pancreas cancer	0.03666
DB01050	Parkinson disease	0.09479
DB01050	Periodontitis	0.22437
DB01050	Polyarthritis	0.08194
DB01050	Polycystic kidney	0.41223
DB01050	Polycystic ovary syndrome	0.0822
DB01050	Premature birth	0.09645
DB01050	Prion disease	0.28686
DB01050	Prostate cancer	0.01579
DB01050	Respiratory tract disease	0.32049
DB01050	Rhabdomyosarcoma	0.1066
DB01050	Rheumatoid arthritis	0.19167
DB01050	Schistosomiasis	0.68008
DB01050	Sickle cell disease	0.15024
DB01050	Sinusitis	0.30648
DB01050	Stroke	0.33117
DB01050	Subarachnoid hemorrhage	0.0471
DB01050	Systemic infection	0.04056
DB01050	Systemic scleroderma	0.02209
DB01050	Testicular dysfunction	0.16261
DB01050	Thyroid gland disease	0.57292
DB01050	Tuberculosis	0.31725
DB01050	Ulcerative colitis	0.03647
DB01050	Vaccinia	0.07225
DB01050	Vulvar disease	0.44721
DB01283	Alzheimer's disease	0.10592
DB01283	Asthma	0.14253
DB01283	Atherosclerosis	0.12306
DB01283	Cancer	0.05415
DB01283	Chronic fatigue syndrome	0.58081
DB01283	Dental plaque	0.21675
DB01283	Endometriosis	0.12534
DB01283	Gouts	0.52146
DB01283	Growth retardation	0.37925
DB01283	Ischemia	0.21381
DB01283	Metaplastic polyp	0.40875
DB01283	Oral cancer	0.18784
DB01283	Parkinson disease	0.18664
DB01283	Periodontitis	0.33479
DB01283	Polyarthritis	0.1617
DB01283	Prion disease	0.56624
DB01283	Rheumatoid arthritis	0.09414
DB01283	Sickle cell disease	0.23758
DB01283	Stroke	0.15796

DB01283	Thyroid gland disease	0.34927
DB01283	Vulvar disease	0.87924
DB01397	Alzheimer's disease	0.10592
DB01397	Asthma	0.14253
DB01397	Atherosclerosis	0.12306
DB01397	Cancer	0.05415
DB01397	Chronic fatigue syndrome	0.58081
DB01397	Dental plaque	0.21675
DB01397	Endometriosis	0.12534
DB01397	Gouts	0.52146
DB01397	Growth retardation	0.37925
DB01397	Ischemia	0.21381
DB01397	Metaplastic polyp	0.40875
DB01397	Oral cancer	0.18784
DB01397	Parkinson disease	0.18664
DB01397	Periodontitis	0.33479
DB01397	Polyarthritis	0.1617
DB01397	Prion disease	0.56624
DB01397	Rheumatoid arthritis	0.09414
DB01397	Sickle cell disease	0.23758
DB01397	Stroke	0.15796
DB01397	Thyroid gland disease	0.34927
DB01397	Vulvar disease	0.87924
DB01398	Alzheimer's disease	0.10592
DB01398	Asthma	0.14253
DB01398	Atherosclerosis	0.12306
DB01398	Cancer	0.05415
DB01398	Chronic fatigue syndrome	0.58081
DB01398	Dental plaque	0.21675
DB01398	Endometriosis	0.12534
DB01398	Gouts	0.52146
DB01398	Growth retardation	0.37925
DB01398	Ischemia	0.21381
DB01398	Metaplastic polyp	0.40875
DB01398	Oral cancer	0.18784
DB01398	Parkinson disease	0.18664
DB01398	Periodontitis	0.33479
DB01398	Polyarthritis	0.1617
DB01398	Prion disease	0.56624
DB01398	Rheumatoid arthritis	0.09414
DB01398	Sickle cell disease	0.23758
DB01398	Stroke	0.15796
DB01398	Thyroid gland disease	0.34927
DB01398	Vulvar disease	0.87924
DB01399	Alzheimer's disease	0.10592
DB01399	Asthma	0.14253
DB01399	Atherosclerosis	0.12306

DB01399	Cancer	0.05415
DB01399	Chronic fatigue syndrome	0.58081
DB01399	Dental plaque	0.21675
DB01399	Endometriosis	0.12534
DB01399	Gouts	0.52146
DB01399	Growth retardation	0.37925
DB01399	Ischemia	0.21381
DB01399	Metaplastic polyp	0.40875
DB01399	Oral cancer	0.18784
DB01399	Parkinson disease	0.18664
DB01399	Periodontitis	0.33479
DB01399	Polyarthritis	0.1617
DB01399	Prion disease	0.56624
DB01399	Rheumatoid arthritis	0.09414
DB01399	Sickle cell disease	0.23758
DB01399	Stroke	0.15796
DB01399	Thyroid gland disease	0.34927
DB01399	Vulvar disease	0.87924
DB01401	Alzheimer's disease	0.10592
DB01401	Asthma	0.14253
DB01401	Atherosclerosis	0.12306
DB01401	Cancer	0.05415
DB01401	Chronic fatigue syndrome	0.58081
DB01401	Dental plaque	0.21675
DB01401	Endometriosis	0.12534
DB01401	Gouts	0.52146
DB01401	Growth retardation	0.37925
DB01401	Ischemia	0.21381
DB01401	Metaplastic polyp	0.40875
DB01401	Oral cancer	0.18784
DB01401	Parkinson disease	0.18664
DB01401	Periodontitis	0.33479
DB01401	Polyarthritis	0.1617
DB01401	Prion disease	0.56624
DB01401	Rheumatoid arthritis	0.09414
DB01401	Sickle cell disease	0.23758
DB01401	Stroke	0.15796
DB01401	Thyroid gland disease	0.34927
DB01401	Vulvar disease	0.87924
DB01419	Alzheimer's disease	0.10592
DB01419	Asthma	0.14253
DB01419	Atherosclerosis	0.12306
DB01419	Cancer	0.05415
DB01419	Chronic fatigue syndrome	0.58081
DB01419	Dental plaque	0.21675
DB01419	Endometriosis	0.12534
DB01419	Gouts	0.52146

DB01419	Growth retardation	0.37925
DB01419	Ischemia	0.21381
DB01419	Metaplastic polyp	0.40875
DB01419	Oral cancer	0.18784
DB01419	Parkinson disease	0.18664
DB01419	Periodontitis	0.33479
DB01419	Polyarthritis	0.1617
DB01419	Prion disease	0.56624
DB01419	Rheumatoid arthritis	0.09414
DB01419	Sickle cell disease	0.23758
DB01419	Stroke	0.15796
DB01419	Thyroid gland disease	0.34927
DB01419	Vulvar disease	0.87924
DB01435	Alzheimer's disease	0.10592
DB01435	Asthma	0.14253
DB01435	Atherosclerosis	0.12306
DB01435	Cancer	0.05415
DB01435	Chronic fatigue syndrome	0.58081
DB01435	Dental plaque	0.21675
DB01435	Endometriosis	0.12534
DB01435	Gouts	0.52146
DB01435	Growth retardation	0.37925
DB01435	Ischemia	0.21381
DB01435	Metaplastic polyp	0.40875
DB01435	Oral cancer	0.18784
DB01435	Parkinson disease	0.18664
DB01435	Periodontitis	0.33479
DB01435	Polyarthritis	0.1617
DB01435	Prion disease	0.56624
DB01435	Rheumatoid arthritis	0.09414
DB01435	Sickle cell disease	0.23758
DB01435	Stroke	0.15796
DB01435	Thyroid gland disease	0.34927
DB01435	Vulvar disease	0.87924
DB01600	Alzheimer's disease	0.10592
DB01600	Asthma	0.14253
DB01600	Atherosclerosis	0.12306
DB01600	Cancer	0.05415
DB01600	Chronic fatigue syndrome	0.58081
DB01600	Dental plaque	0.21675
DB01600	Endometriosis	0.12534
DB01600	Gouts	0.52146
DB01600	Growth retardation	0.37925
DB01600	Ischemia	0.21381
DB01600	Metaplastic polyp	0.40875
DB01600	Oral cancer	0.18784
DB01600	Parkinson disease	0.18664

DB01600	Periodontitis	0.33479
DB01600	Polyarthritis	0.1617
DB01600	Prion disease	0.56624
DB01600	Rheumatoid arthritis	0.09414
DB01600	Sickle cell disease	0.23758
DB01600	Stroke	0.15796
DB01600	Thyroid gland disease	0.34927
DB01600	Vulvar disease	0.87924
DB04552	Alzheimer's disease	0.08441
DB04552	Asthma	0.11813
DB04552	Atherosclerosis	0.10214
DB04552	Bacterial infection	0.10911
DB04552	Cancer	0.04313
DB04552	Chronic fatigue syndrome	0.47515
DB04552	Colon cancer	0.02423
DB04552	Conduct disorder	0.13608
DB04552	Deafness	0.05893
DB04552	Dental plaque	0.17879
DB04552	Diabetes mellitus	0.02149
DB04552	Endometriosis	0.10052
DB04552	Gouts	0.5518
DB04552	Growth retardation	0.32174
DB04552	Hyperaldosteronism	0.1291
DB04552	Hypertension	0.03227
DB04552	Ischemia	0.17555
DB04552	Leukemia	0.023
DB04552	Liver cancer	0.03169
DB04552	Metaplastic polyp	0.30913
DB04552	Oral cancer	0.14717
DB04552	Parkinson disease	0.15364
DB04552	Periodontitis	0.28922
DB04552	Polyarthritis	0.17417
DB04552	Prion disease	0.44423
DB04552	Rheumatoid arthritis	0.10083
DB04552	Schizophrenia	0.03104
DB04552	Sickle cell disease	0.18006
DB04552	Stroke	0.12412
DB04552	Thyroid gland disease	0.28071
DB04552	Vulvar disease	0.74559
DB06725	Alzheimer's disease	0.10592
DB06725	Asthma	0.14253
DB06725	Atherosclerosis	0.12306
DB06725	Cancer	0.05415
DB06725	Chronic fatigue syndrome	0.58081
DB06725	Dental plaque	0.21675
DB06725	Endometriosis	0.12534
DB06725	Gouts	0.52146

DB06725	Growth retardation	0.37925
DB06725	Ischemia	0.21381
DB06725	Metaplastic polyp	0.40875
DB06725	Oral cancer	0.18784
DB06725	Parkinson disease	0.18664
DB06725	Periodontitis	0.33479
DB06725	Polyarthritis	0.1617
DB06725	Prion disease	0.56624
DB06725	Rheumatoid arthritis	0.09414
DB06725	Sickle cell disease	0.23758
DB06725	Stroke	0.15796
DB06725	Thyroid gland disease	0.34927
DB06725	Vulvar disease	0.87924
DB06802	Alzheimer's disease	0.10592
DB06802	Asthma	0.14253
DB06802	Atherosclerosis	0.12306
DB06802	Cancer	0.05415
DB06802	Chronic fatigue syndrome	0.58081
DB06802	Dental plaque	0.21675
DB06802	Endometriosis	0.12534
DB06802	Gouts	0.52146
DB06802	Growth retardation	0.37925
DB06802	Ischemia	0.21381
DB06802	Metaplastic polyp	0.40875
DB06802	Oral cancer	0.18784
DB06802	Parkinson disease	0.18664
DB06802	Periodontitis	0.33479
DB06802	Polyarthritis	0.1617
DB06802	Prion disease	0.56624
DB06802	Rheumatoid arthritis	0.09414
DB06802	Sickle cell disease	0.23758
DB06802	Stroke	0.15796
DB06802	Thyroid gland disease	0.34927
DB06802	Vulvar disease	0.87924
DB01017	Labor, Premature	0.08607
DB01017	Pemphigoid, Bullous	0.03339
DB01017	Pleural effusion, Malignant	0.16885
DB01017	Purpura, Thrombocytopenic, Idiopathic	0.03191
DB01017	Adenovirus infection	0.02475
DB01017	Alimentary system disease	0.07274
DB01017	Alopecia	0.03937
DB01017	Alveolar bone loss	0.12599
DB01017	Alzheimer's disease	0.07382
DB01017	Amnionitis	0.33541
DB01017	Amyotrophic lateral sclerosis	0.23929
DB01017	Aortic aneurysm	0.08068
DB01017	Arteriopathy	0.45513

DB01017	Arthritis	0.15527
DB01017	Asthma	0.17982
DB01017	Atherosclerosis	0.28791
DB01017	Autoimmune disease	0.07612
DB01017	Azoospermia	0.06252
DB01017	Bacterial vaginosis	0.13608
DB01017	Behcet syndrome	0.17446
DB01017	Biliary Atresia	0.12163
DB01017	Bipolar disorder	0.03774
DB01017	Brain tumor	0.03828
DB01017	Breast cancer	0.03515
DB01017	Bronchiolitis obliterans	0.45513
DB01017	Bronchopulmonary dysplasia	0.43771
DB01017	Cancer	0.17601
DB01017	Capillaries disease	0.42759
DB01017	Cardiovascular disease	0.28277
DB01017	Celiac disease	0.0548
DB01017	Central nervous system disease	0.08333
DB01017	Cervical cancer	0.09698
DB01017	Cholestasis	0.09245
DB01017	Chronic obstructive airway disease	0.5763
DB01017	Colon cancer	0.02569
DB01017	Cystic fibrosis	0.15867
DB01017	Cytomegalovirus infection	0.08607
DB01017	Dental plaque	0.19337
DB01017	Depression	0.10162
DB01017	Dermatitis	0.18047
DB01017	Diabetes mellitus	0.16024
DB01017	Down syndrome	0.03799
DB01017	Drug abuse	0.05445
DB01017	Embryoma	0.02581
DB01017	Emphysema	0.11779
DB01017	Endometriosis	0.1655
DB01017	Enteritis	0.05498
DB01017	Epilepsy	0.04714
DB01017	Epstein-Barr virus infection	0.07274
DB01017	Esophagitis	0.14907
DB01017	Esotropia	0.15971
DB01017	Familial Mediterranean fever	0.59637
DB01017	Gastritis	0.1048
DB01017	Glaucoma	0.16584
DB01017	Glomerulonephritis	0.27357
DB01017	Gouts	0.09245
DB01017	Graves' disease	0.05893
DB01017	Growth retardation	0.15404
DB01017	HIV infection	0.02512
DB01017	HTLV-I infection	0.22215

DB01017	Heart failure	0.1338
DB01017	Helicobacter infection	0.28257
DB01017	Henoch-Schoenlein purpura	0.55484
DB01017	Hepatitis	0.08607
DB01017	Hepatitis B	0.35177
DB01017	Hepatitis C	0.04915
DB01017	Hereditary nonpolyposis colorectal tumor	0.33333
DB01017	Hodgkin's disease	0.11313
DB01017	Hydrocephalus	0.11111
DB01017	Hypertension	0.10432
DB01017	IGA glomerulonephritis	0.16498
DB01017	Infection	0.03143
DB01017	Infectious lung disease	0.07454
DB01017	Infertility	0.04579
DB01017	Infiltrating cancer	0.1104
DB01017	Intestinal disease	0.28723
DB01017	Intracranial aneurysm	0.30864
DB01017	Ischemia	0.08825
DB01017	Keratoconjunctivitis Sicca	0.30062
DB01017	Kidney disease	0.09404
DB01017	Kidney failure	0.31767
DB01017	Leukemia	0.03962
DB01017	Leukoencephalopathy	0.23882
DB01017	Lichen planus	0.24026
DB01017	Liver cancer	0.07016
DB01017	Liver metastases	0.1241
DB01017	Lung cancer	0.07579
DB01017	Lupus erythematosus	0.1859
DB01017	Lupus vulgaris	0.16218
DB01017	Lyme disease	0.11111
DB01017	Macular degeneration	0.72805
DB01017	Malaria	0.50145
DB01017	Melanoma	0.08962
DB01017	Meningioma	0.10814
DB01017	Metabolism disease	0.13258
DB01017	Metastasis to lymph nodes	0.13611
DB01017	Moyamoya disease	0.13608
DB01017	Mucocutaneous lymph node syndrome	0.89761
DB01017	Multiple myeloma	0.09894
DB01017	Multiple sclerosis	0.11569
DB01017	Muscular dystrophy	0.13351
DB01017	Neoplasm metastasis	0.02713
DB01017	Nephrosis	0.07454
DB01017	Neuroblastoma	0.04547
DB01017	Obesity	0.26602
DB01017	Oral cancer	0.12459
DB01017	Otitis media	0.11111

DB01017	Ovarian cancer	0.0335
DB01017	Ovarian disease	0.161
DB01017	Ovary cancer	0.15601
DB01017	Pancreas disease	0.0695
DB01017	Parkinson disease	0.11707
DB01017	Peptic ulcer	0.4849
DB01017	Periodontitis	0.2364
DB01017	Polycystic ovary syndrome	0.0441
DB01017	Polymyositis	0.08607
DB01017	Polyneuropathy	0.74763
DB01017	Pre-Eclampsia	0.46393
DB01017	Premature birth	0.08085
DB01017	Primary hyperparathyroidism	0.03972
DB01017	Primary tumor	0.06035
DB01017	Prostate cancer	0.01969
DB01017	Psoriasis	0.07857
DB01017	Rabies	0.03704
DB01017	Respiratory distress syndrome	0.09245
DB01017	Respiratory tract disease	0.14748
DB01017	Reticulosarcoma	0.46187
DB01017	Retinal disease	0.26968
DB01017	Retinoblastoma	0.19245
DB01017	Rheumatoid arthritis	0.0392
DB01017	Sarcoidosis	0.06537
DB01017	Schizophrenia	0.02534
DB01017	Shigella infection	0.17816
DB01017	Sicca syndrome	0.06537
DB01017	Sickle cell disease	0.03752
DB01017	Sinusitis	0.14543
DB01017	Skin cancer	0.1565
DB01017	Skin disease	0.10899
DB01017	Squamous cell cancer	0.03402
DB01017	Stomach cancer	0.05317
DB01017	Stroke	0.10428
DB01017	Subacute sclerosing panencephalitis	0.51069
DB01017	Sudden infant death syndrome	0.07274
DB01017	Synovial sarcoma	0.16667
DB01017	Systemic infection	0.32405
DB01017	Systemic scleroderma	0.14379
DB01017	Takayasu's arteritis	0.16033
DB01017	Thalassemia	0.03934
DB01017	Thyroid cancer	0.07269
DB01017	Transient hypertension of pregnancy	0.37634
DB01017	Tropical spastic paraparesis	0.23129
DB01017	Tuberculosis	0.08989
DB01017	Ulcerative colitis	0.06844
DB01017	Varicosity	0.47001

DB01017	Vulvar disease	0.14907
DB00246	Supranuclear palsy, progressive	0.23094
DB00246	Abortion	0.06924
DB00246	Adenocarcinoma	0.02917
DB00246	Amyotrophic lateral sclerosis	0.24974
DB00246	Anorexia nervosa	0.63893
DB00246	Asthma	0.03266
DB00246	Atopic rhinitis	0.05164
DB00246	Attention deficit hyperactivity disorder	0.59178
DB00246	Autistic disorder	0.02425
DB00246	Behavior disease	0.82495
DB00246	Bipolar disorder	0.31769
DB00246	Bladder cancer	0.02582
DB00246	Central nervous system disease	0.05
DB00246	Choriocarcinoma	0.05774
DB00246	Chronic fatigue syndrome	0.07071
DB00246	Colon cancer	0.0356
DB00246	Common cold	0.05345
DB00246	Depression	0.40151
DB00246	Dermatitis	0.23619
DB00246	Diabetes mellitus	0.15603
DB00246	Drug abuse	0.09846
DB00246	Drug-Induced dyskinesia	0.07559
DB00246	Eating disorder	0.03482
DB00246	Embryoma	0.01233
DB00246	Epilepsy	0.40759
DB00246	Gilles de la Tourette syndrome	0.07559
DB00246	Hepatitis C	0.43796
DB00246	Herpes	0.02857
DB00246	Hypertension	0.53071
DB00246	Infertility	0.02747
DB00246	Kidney failure	0.04529
DB00246	Migraine	0.5065
DB00246	Nervous system disease	0.12028
DB00246	Neuroblastoma	0.05494
DB00246	Obesity	0.08907
DB00246	Obsessive-compulsive disorder	0.1206
DB00246	Panic disorder	0.68303
DB00246	Parkinson disease	0.02209
DB00246	Polycystic ovary syndrome	0.02649
DB00246	Prostate cancer	0.01044
DB00246	Psychotic disorder	0.06405
DB00246	Rheumatoid arthritis	0.01224
DB00246	Schizophrenia	0.1315
DB00246	Stroke	0.23733
DB00246	Sudden infant death syndrome	0.70377
DB00248	Abortion	0.06924

DB00248	Adenocarcinoma	0.03766
DB00248	Amyotrophic lateral sclerosis	0.25759
DB00248	Anorexia nervosa	0.67457
DB00248	Attention deficit hyperactivity disorder	0.62538
DB00248	Autistic disorder	0.03131
DB00248	Behavior disease	0.83001
DB00248	Bipolar disorder	0.31481
DB00248	Choriocarcinoma	0.07454
DB00248	Chronic fatigue syndrome	0.09129
DB00248	Colon cancer	0.03064
DB00248	Common cold	0.06901
DB00248	Depression	0.39817
DB00248	Dermatitis	0.24677
DB00248	Diabetes mellitus	0.15163
DB00248	Drug abuse	0.1281
DB00248	Drug-Induced dyskinesia	0.09759
DB00248	Epilepsy	0.41583
DB00248	Hepatitis C	0.44654
DB00248	Hypertension	0.53589
DB00248	Migraine	0.52811
DB00248	Nervous system disease	0.13531
DB00248	Obesity	0.10698
DB00248	Obsessive-compulsive disorder	0.07785
DB00248	Panic disorder	0.66478
DB00248	Polycystic ovary syndrome	0.0342
DB00248	Psychotic disorder	0.04134
DB00248	Rheumatoid arthritis	0.0158
DB00248	Schizophrenia	0.06433
DB00248	Stroke	0.25051
DB00248	Sudden infant death syndrome	0.71647
DB00268	Abortion	0.06924
DB00268	Amyotrophic lateral sclerosis	0.25881
DB00268	Anorexia nervosa	0.68011
DB00268	Attention deficit hyperactivity disorder	0.63062
DB00268	Autistic disorder	0.03241
DB00268	Behavior disease	0.8356
DB00268	Bipolar disorder	0.31789
DB00268	Choriocarcinoma	0.07715
DB00268	Chronic fatigue syndrome	0.09449
DB00268	Colon cancer	0.03172
DB00268	Common cold	0.07143
DB00268	Depression	0.40174
DB00268	Dermatitis	0.24842
DB00268	Diabetes mellitus	0.15258
DB00268	Drug abuse	0.12979
DB00268	Drug-Induced dyskinesia	0.10102
DB00268	Epilepsy	0.41711

DB00268	Hepatitis C	0.44788
DB00268	Hypertension	0.5205
DB00268	Migraine	0.53148
DB00268	Nervous system disease	0.13765
DB00268	Obesity	0.10977
DB00268	Obsessive-compulsive disorder	0.08058
DB00268	Panic disorder	0.66874
DB00268	Polycystic ovary syndrome	0.0354
DB00268	Psychotic disorder	0.0428
DB00268	Rheumatoid arthritis	0.01636
DB00268	Schizophrenia	0.04538
DB00268	Stroke	0.25256
DB00268	Sudden infant death syndrome	0.71845
DB00334	Supranuclear palsy, progressive	0.23094
DB00334	Abortion	0.06924
DB00334	Adenocarcinoma	0.02917
DB00334	Amyotrophic lateral sclerosis	0.24974
DB00334	Anorexia nervosa	0.63893
DB00334	Asthma	0.03266
DB00334	Atopic rhinitis	0.05164
DB00334	Attention deficit hyperactivity disorder	0.59178
DB00334	Autistic disorder	0.02425
DB00334	Behavior disease	0.82495
DB00334	Bipolar disorder	0.31769
DB00334	Bladder cancer	0.02582
DB00334	Central nervous system disease	0.05
DB00334	Choriocarcinoma	0.05774
DB00334	Chronic fatigue syndrome	0.07071
DB00334	Colon cancer	0.0356
DB00334	Common cold	0.05345
DB00334	Depression	0.40151
DB00334	Dermatitis	0.23619
DB00334	Diabetes mellitus	0.15603
DB00334	Drug abuse	0.09846
DB00334	Drug-Induced dyskinesia	0.07559
DB00334	Eating disorder	0.03482
DB00334	Embryoma	0.01233
DB00334	Epilepsy	0.40759
DB00334	Gilles de la Tourette syndrome	0.07559
DB00334	Hepatitis C	0.43796
DB00334	Herpes	0.02857
DB00334	Hypertension	0.53071
DB00334	Infertility	0.02747
DB00334	Kidney failure	0.04529
DB00334	Migraine	0.5065
DB00334	Nervous system disease	0.12028
DB00334	Neuroblastoma	0.05494

DB00334	Obesity	0.08907
DB00334	Obsessive-compulsive disorder	0.1206
DB00334	Panic disorder	0.68303
DB00334	Parkinson disease	0.02209
DB00334	Polycystic ovary syndrome	0.02649
DB00334	Prostate cancer	0.01044
DB00334	Psychotic disorder	0.06405
DB00334	Rheumatoid arthritis	0.01224
DB00334	Schizophrenia	0.1315
DB00334	Stroke	0.23733
DB00334	Sudden infant death syndrome	0.70377
DB00363	Supranuclear palsy, progressive	0.22646
DB00363	Abortion	0.06924
DB00363	Adenocarcinoma	0.02861
DB00363	Amyotrophic lateral sclerosis	0.24922
DB00363	Anorexia nervosa	0.63655
DB00363	Arthritis	0.02433
DB00363	Asthma	0.03203
DB00363	Atopic rhinitis	0.05064
DB00363	Attention deficit hyperactivity disorder	0.58954
DB00363	Autistic disorder	0.02378
DB00363	Behavior disease	0.82195
DB00363	Bipolar disorder	0.31593
DB00363	Bladder cancer	0.02532
DB00363	Central nervous system disease	0.04903
DB00363	Choriocarcinoma	0.05661
DB00363	Chronic fatigue syndrome	0.06934
DB00363	Colon cancer	0.04655
DB00363	Common cold	0.05241
DB00363	Depression	0.39947
DB00363	Dermatitis	0.23549
DB00363	Diabetes mellitus	0.16574
DB00363	Drug abuse	0.0981
DB00363	Drug-Induced dyskinesia	0.07412
DB00363	Eating disorder	0.03414
DB00363	Embryoma	0.01209
DB00363	Epilepsy	0.40705
DB00363	Gilles de la Tourette syndrome	0.07412
DB00363	Hepatitis C	0.43739
DB00363	Herpes	0.02802
DB00363	Hypertension	0.52764
DB00363	Infertility	0.02694
DB00363	Kidney failure	0.04441
DB00363	Migraine	0.50505
DB00363	Nervous system disease	0.11928
DB00363	Neuroblastoma	0.05388
DB00363	Obesity	0.08788

DB00363	Obsessive-compulsive disorder	0.11826
DB00363	Pancreas cancer	0.02034
DB00363	Panic disorder	0.68048
DB00363	Parkinson disease	0.02166
DB00363	Polycystic ovary syndrome	0.02598
DB00363	Prostate cancer	0.01024
DB00363	Psychotic disorder	0.06281
DB00363	Rheumatoid arthritis	0.024
DB00363	Schizophrenia	0.12944
DB00363	Stroke	0.23645
DB00363	Sudden infant death syndrome	0.70292
DB00397	Alzheimer's disease	0.0933
DB00397	Attention deficit hyperactivity disorder	0.34242
DB00397	Glaucoma	0.23753
DB00397	Heart failure	0.17827
DB00397	Hypertension	0.29818
DB00397	Kidney failure	0.05661
DB00397	Nervous system disease	0.30996
DB00397	Obesity	0.24667
DB00397	Prostate cancer	0.0261
DB00397	Schizophrenia	0.10406
DB00397	Subarachnoid hemorrhage	0.31622
DB00408	Anorexia nervosa	0.20412
DB00408	Autistic disorder	0.06063
DB00408	Behavior disease	0.1543
DB00408	Bipolar disorder	0.11323
DB00408	Choriocarcinoma	0.14434
DB00408	Chronic fatigue syndrome	0.17678
DB00408	Colon cancer	0.02967
DB00408	Depression	0.06565
DB00408	Dermatitis	0.04545
DB00408	Drug-Induced dyskinesia	0.18898
DB00408	Hypertension	0.07906
DB00408	Migraine	0.09285
DB00408	Obesity	0.07692
DB00408	Obsessive-compulsive disorder	0.15076
DB00408	Panic disorder	0.10911
DB00408	Psychotic disorder	0.08006
DB00408	Rheumatoid arthritis	0.0306
DB00408	Stroke	0.05661
DB00413	Abortion	0.06924
DB00413	Amyotrophic lateral sclerosis	0.25881
DB00413	Anorexia nervosa	0.68011
DB00413	Attention deficit hyperactivity disorder	0.63062
DB00413	Autistic disorder	0.03241
DB00413	Behavior disease	0.8356
DB00413	Bipolar disorder	0.31789

DB00413	Choriocarcinoma	0.07715
DB00413	Chronic fatigue syndrome	0.09449
DB00413	Colon cancer	0.03172
DB00413	Common cold	0.07143
DB00413	Depression	0.40174
DB00413	Dermatitis	0.24842
DB00413	Diabetes mellitus	0.15258
DB00413	Drug abuse	0.12979
DB00413	Drug-Induced dyskinesia	0.10102
DB00413	Epilepsy	0.41711
DB00413	Hepatitis C	0.44788
DB00413	Hypertension	0.5205
DB00413	Migraine	0.53148
DB00413	Nervous system disease	0.13765
DB00413	Obesity	0.10977
DB00413	Obsessive-compulsive disorder	0.08058
DB00413	Panic disorder	0.66874
DB00413	Polycystic ovary syndrome	0.0354
DB00413	Psychotic disorder	0.0428
DB00413	Rheumatoid arthritis	0.01636
DB00413	Schizophrenia	0.04538
DB00413	Stroke	0.25256
DB00413	Sudden infant death syndrome	0.71845
DB00420	Supranuclear palsy, progressive	0.30861
DB00420	Anorexia nervosa	0.10911
DB00420	Asthma	0.04364
DB00420	Atopic rhinitis	0.06901
DB00420	Autistic disorder	0.03241
DB00420	Behavior disease	0.12372
DB00420	Bipolar disorder	0.09078
DB00420	Bladder cancer	0.0345
DB00420	Central nervous system disease	0.06682
DB00420	Choriocarcinoma	0.07715
DB00420	Chronic fatigue syndrome	0.09449
DB00420	Colon cancer	0.03172
DB00420	Depression	0.07019
DB00420	Dermatitis	0.0243
DB00420	Diabetes mellitus	0.01407
DB00420	Drug-Induced dyskinesia	0.10102
DB00420	Hypertension	0.06339
DB00420	Infertility	0.03671
DB00420	Kidney failure	0.09078
DB00420	Migraine	0.04963
DB00420	Neuroblastoma	0.07342
DB00420	Obesity	0.04112
DB00420	Obsessive-compulsive disorder	0.08058
DB00420	Panic disorder	0.05832

DB00420	Parkinson disease	0.02951
DB00420	Prostate cancer	0.01395
DB00420	Psychotic disorder	0.0428
DB00420	Rheumatoid arthritis	0.01636
DB00420	Schizophrenia	0.08128
DB00420	Stroke	0.03026
DB00477	Abortion	0.05124
DB00477	Amyotrophic lateral sclerosis	0.34358
DB00477	Anorexia nervosa	0.22081
DB00477	Atopic rhinitis	0.09759
DB00477	Attention deficit hyperactivity disorder	0.11127
DB00477	Autistic disorder	0.04583
DB00477	Behavior disease	0.78185
DB00477	Bipolar disorder	0.12098
DB00477	Choriocarcinoma	0.10911
DB00477	Chronic fatigue syndrome	0.13363
DB00477	Colon cancer	0.04486
DB00477	Depression	0.39332
DB00477	Dermatitis	0.3312
DB00477	Diabetes mellitus	0.18336
DB00477	Drug abuse	0.02745
DB00477	Drug-Induced dyskinesia	0.14286
DB00477	Epilepsy	0.55168
DB00477	Hepatitis C	0.59226
DB00477	Hypertension	0.43022
DB00477	Infertility	0.05192
DB00477	Kidney failure	0.08559
DB00477	Migraine	0.63791
DB00477	Obesity	0.02907
DB00477	Obsessive-compulsive disorder	0.11396
DB00477	Panic disorder	0.89015
DB00477	Parkinson disease	0.04174
DB00477	Prostate cancer	0.01973
DB00477	Psychotic disorder	0.06052
DB00477	Rheumatoid arthritis	0.02313
DB00477	Schizophrenia	0.02874
DB00477	Stroke	0.33784
DB00477	Sudden infant death syndrome	0.94957
DB00502	Anorexia nervosa	0.09129
DB00502	Autistic disorder	0.05423
DB00502	Behavior disease	0.06901
DB00502	Bipolar disorder	0.05064
DB00502	Choriocarcinoma	0.1291
DB00502	Chronic fatigue syndrome	0.15811
DB00502	Colon cancer	0.02654
DB00502	Depression	0.05872
DB00502	Dermatitis	0.04066

DB00502	Drug-Induced dyskinesia	0.16903
DB00502	Hypertension	0.03536
DB00502	Obesity	0.0344
DB00502	Obsessive-compulsive disorder	0.13484
DB00502	Panic disorder	0.09759
DB00502	Psychotic disorder	0.07161
DB00502	Rheumatoid arthritis	0.02737
DB00502	Stroke	0.05064
DB00508	Supranuclear palsy, progressive	0.2582
DB00508	Asthma	0.03651
DB00508	Behavior disease	0.06901
DB00508	Bipolar disorder	0.05064
DB00508	Bladder cancer	0.05774
DB00508	Central nervous system disease	0.1118
DB00508	Colon cancer	0.02654
DB00508	Depression	0.05872
DB00508	Drug abuse	0.04189
DB00508	Hypertension	0.03536
DB00508	Neuroblastoma	0.06143
DB00508	Schizophrenia	0.034
DB00543	Hypertension, Pulmonary	0.08111
DB00543	Supranuclear palsy, progressive	0.20412
DB00543	Anorexia nervosa	0.14434
DB00543	Asthma	0.02887
DB00543	Atherosclerosis	0.02475
DB00543	Attention deficit hyperactivity disorder	0.32687
DB00543	Autistic disorder	0.04287
DB00543	Behavior disease	0.10911
DB00543	Bipolar disorder	0.08006
DB00543	Chronic fatigue syndrome	0.125
DB00543	Chronic obstructive airway disease	0.03953
DB00543	Colon cancer	0.02098
DB00543	Congenital heart disease	0.11785
DB00543	Depression	0.04642
DB00543	Dermatitis	0.03214
DB00543	Diabetes mellitus	0.01861
DB00543	Drug abuse	0.06623
DB00543	Epilepsy	0.05
DB00543	Fibromyalgia	0.125
DB00543	Generalized anxiety disorder	0.10206
DB00543	Heart failure	0.03769
DB00543	Herpes	0.05051
DB00543	Hypertension	0.17097
DB00543	Kidney failure	0.04003
DB00543	Migraine	0.06565
DB00543	Multiple endocrine neoplasia	0.11785
DB00543	Nervous system disease	0.29654

DB00543	Neuroblastoma	0.04856
DB00543	Neuroendocrine tumor	0.125
DB00543	Neurotic disorder	0.1118
DB00543	Obesity	0.13673
DB00543	Obsessive-compulsive disorder	0.1066
DB00543	Panic disorder	0.1543
DB00543	Pervasive development disorder	0.09129
DB00543	Prostate cancer	0.01846
DB00543	Psychotic disorder	0.05661
DB00543	Pulmonary hypertension	0.17678
DB00543	Schizophrenia	0.12871
DB00543	Stroke	0.04003
DB00543	Sudden infant death syndrome	0.07715
DB00543	Ulcerative colitis	0.03647
DB00589	Abortion	0.06924
DB00589	Amyotrophic lateral sclerosis	0.25881
DB00589	Anorexia nervosa	0.68011
DB00589	Attention deficit hyperactivity disorder	0.63062
DB00589	Autistic disorder	0.03241
DB00589	Behavior disease	0.8356
DB00589	Bipolar disorder	0.31789
DB00589	Choriocarcinoma	0.07715
DB00589	Chronic fatigue syndrome	0.09449
DB00589	Colon cancer	0.03172
DB00589	Common cold	0.07143
DB00589	Depression	0.40174
DB00589	Dermatitis	0.24842
DB00589	Diabetes mellitus	0.15258
DB00589	Drug abuse	0.12979
DB00589	Drug-Induced dyskinesia	0.10102
DB00589	Epilepsy	0.41711
DB00589	Hepatitis C	0.44788
DB00589	Hypertension	0.5205
DB00589	Migraine	0.53148
DB00589	Nervous system disease	0.13765
DB00589	Obesity	0.10977
DB00589	Obsessive-compulsive disorder	0.08058
DB00589	Panic disorder	0.66874
DB00589	Polycystic ovary syndrome	0.0354
DB00589	Psychotic disorder	0.0428
DB00589	Rheumatoid arthritis	0.01636
DB00589	Schizophrenia	0.04538
DB00589	Stroke	0.25256
DB00589	Sudden infant death syndrome	0.71845
DB00623	Abortion	0.02905
DB00623	Achalasia and cardiospasm	0.12469
DB00623	Alzheimer's disease	0.05292

DB00623	Amyloidosis	0.06639
DB00623	Asthma	0.04371
DB00623	Autistic disorder	0.06042
DB00623	Autoimmune disease	0.05572
DB00623	Bipolar disorder	0.04432
DB00623	Bladder cancer	0.04542
DB00623	Brain ischemia	0.18262
DB00623	Cancer	0.03396
DB00623	Celiac disease	0.08498
DB00623	Colon cancer	0.0172
DB00623	Congenital abnormality	0.03237
DB00623	Dental plaque	0.06543
DB00623	Diabetes mellitus	0.0125
DB00623	Drug abuse	0.03239
DB00623	Eating disorder	0.10416
DB00623	Epstein-Barr virus infection	0.09263
DB00623	Esophageal tumor	0.07695
DB00623	Esophagus cancer	0.03074
DB00623	Esotropia	0.08852
DB00623	Eye cancer	0.10972
DB00623	Fanconi's anemia	0.0433
DB00623	Glaucoma	0.07631
DB00623	Gram-Negative bacterial infection	0.15306
DB00623	Graves' disease	0.10951
DB00623	HIV infection	0.05185
DB00623	Herpes	0.07492
DB00623	Ischemia	0.06354
DB00623	Keratosis	0.08781
DB00623	Leukemia	0.03912
DB00623	Lichen planus	0.17067
DB00623	Lung cancer	0.0366
DB00623	Lupus erythematosus	0.03596
DB00623	Malignant glioma	0.04453
DB00623	Melanoma	0.02281
DB00623	Pre-Eclampsia	0.04147
DB00623	Prion disease	0.14306
DB00623	Rabies	0.02116
DB00623	Rheumatoid arthritis	0.02622
DB00623	Schistosomiasis	0.15421
DB00623	Stroke	0.04015
DB00623	Thyroid gland disease	0.09641
DB00623	Tuberculosis	0.0826
DB00679	Anorexia nervosa	0.08333
DB00679	Autistic disorder	0.04951
DB00679	Behavior disease	0.06299
DB00679	Bipolar disorder	0.04623
DB00679	Cancer	0.01505

DB00679	Choriocarcinoma	0.11785
DB00679	Chronic fatigue syndrome	0.14434
DB00679	Colon cancer	0.02423
DB00679	Depression	0.05361
DB00679	Dermatitis	0.03711
DB00679	Drug abuse	0.03824
DB00679	Drug-Induced dyskinesia	0.1543
DB00679	Heart failure	0.04352
DB00679	Hypertension	0.06455
DB00679	Kidney failure	0.09245
DB00679	Long QT syndrome	0.11785
DB00679	Obesity	0.0314
DB00679	Obsessive-compulsive disorder	0.12309
DB00679	Panic disorder	0.08909
DB00679	Prostate cancer	0.02131
DB00679	Psychotic disorder	0.06537
DB00679	Rheumatoid arthritis	0.02498
DB00679	Stroke	0.04623
DB00679	Sudden infant death syndrome	0.08909
DB00714	Abortion	0.06924
DB00714	Amyotrophic lateral sclerosis	0.25759
DB00714	Anorexia nervosa	0.67457
DB00714	Attention deficit hyperactivity disorder	0.62538
DB00714	Autistic disorder	0.03131
DB00714	Behavior disease	0.83001
DB00714	Bipolar disorder	0.31481
DB00714	Choriocarcinoma	0.07454
DB00714	Chronic fatigue syndrome	0.09129
DB00714	Colon cancer	0.03064
DB00714	Common cold	0.06901
DB00714	Depression	0.39817
DB00714	Dermatitis	0.24677
DB00714	Diabetes mellitus	0.15163
DB00714	Drug abuse	0.1281
DB00714	Drug-Induced dyskinesia	0.09759
DB00714	Epilepsy	0.41583
DB00714	Hepatitis C	0.44654
DB00714	Hypertension	0.51548
DB00714	Migraine	0.52811
DB00714	Nervous system disease	0.13531
DB00714	Obesity	0.10698
DB00714	Obsessive-compulsive disorder	0.07785
DB00714	Panic disorder	0.66478
DB00714	Polycystic ovary syndrome	0.0342
DB00714	Psychotic disorder	0.04134
DB00714	Rheumatoid arthritis	0.0158
DB00714	Schizophrenia	0.04469

DB00714	Stroke	0.25051
DB00714	Sudden infant death syndrome	0.71647
DB00726	Hypertension, Pulmonary	0.06917
DB00726	Stress disorder, post-traumatic	0.1066
DB00726	Abortion	0.05124
DB00726	Amyotrophic lateral sclerosis	0.33327
DB00726	Anorexia nervosa	0.32829
DB00726	Atherosclerosis	0.02111
DB00726	Atopic rhinitis	0.07785
DB00726	Attention deficit hyperactivity disorder	0.11127
DB00726	Autistic disorder	0.07313
DB00726	Behavior disease	0.89783
DB00726	Bipolar disorder	0.21474
DB00726	Brain disease	0.05415
DB00726	Breast cancer	0.01451
DB00726	Choriocarcinoma	0.08704
DB00726	Chronic fatigue syndrome	0.2132
DB00726	Chronic obstructive airway disease	0.03371
DB00726	Colon cancer	0.05367
DB00726	Congenital heart disease	0.1005
DB00726	Depression	0.45242
DB00726	Dermatitis	0.34471
DB00726	Diabetes mellitus	0.21107
DB00726	Drug abuse	0.11217
DB00726	Drug-Induced dyskinesia	0.11396
DB00726	Epilepsy	0.58351
DB00726	Fibromyalgia	0.1066
DB00726	Generalized anxiety disorder	0.08704
DB00726	Gilles de la Tourette syndrome	0.11396
DB00726	Heart failure	0.03214
DB00726	Hepatitis C	0.58099
DB00726	Herpes	0.08615
DB00726	Hypertension	0.45976
DB00726	Infertility	0.04142
DB00726	Kidney failure	0.06828
DB00726	Migraine	0.73569
DB00726	Multiple endocrine neoplasia	0.1005
DB00726	Neuroendocrine tumor	0.1066
DB00726	Neurotic disorder	0.09535
DB00726	Obesity	0.09277
DB00726	Obsessive-compulsive disorder	0.18182
DB00726	Panic disorder	0.98837
DB00726	Parkinson disease	0.0333
DB00726	Pervasive development disorder	0.07785
DB00726	Polycystic ovary syndrome	0.03994
DB00726	Prostate cancer	0.01574
DB00726	Psychotic disorder	0.14484

DB00726	Pulmonary hypertension	0.15076
DB00726	Rheumatoid arthritis	0.01845
DB00726	Schizophrenia	0.02292
DB00726	Stroke	0.35467
DB00726	Sudden infant death syndrome	0.99868
DB00726	Ulcerative colitis	0.0311
DB00734	Abortion	0.07791
DB00734	Amyotrophic lateral sclerosis	0.27302
DB00734	Anorexia nervosa	0.69757
DB00734	Atopic rhinitis	0.06901
DB00734	Attention deficit hyperactivity disorder	0.65281
DB00734	Autistic disorder	0.03241
DB00734	Behavior disease	0.72648
DB00734	Bipolar disorder	0.1794
DB00734	Choriocarcinoma	0.07715
DB00734	Chronic fatigue syndrome	0.09449
DB00734	Colon cancer	0.03172
DB00734	Common cold	0.07143
DB00734	Depression	0.33664
DB00734	Dermatitis	0.26117
DB00734	Diabetes mellitus	0.16052
DB00734	Drug abuse	0.04173
DB00734	Drug-Induced dyskinesia	0.10102
DB00734	Epilepsy	0.4413
DB00734	Hepatitis C	0.47393
DB00734	Hypertension	0.46817
DB00734	Infertility	0.03671
DB00734	Kidney failure	0.06052
DB00734	Migraine	0.55905
DB00734	Nervous system disease	0.14624
DB00734	Obesity	0.11322
DB00734	Obsessive-compulsive disorder	0.08058
DB00734	Panic disorder	0.70396
DB00734	Parkinson disease	0.02951
DB00734	Polycystic ovary syndrome	0.0354
DB00734	Prostate cancer	0.01395
DB00734	Psychotic disorder	0.0428
DB00734	Rheumatoid arthritis	0.01636
DB00734	Schizophrenia	0.06884
DB00734	Stroke	0.26481
DB00734	Sudden infant death syndrome	0.76056
DB00777	Supranuclear palsy, progressive	0.30861
DB00777	Anorexia nervosa	0.10911
DB00777	Asthma	0.04364
DB00777	Atopic rhinitis	0.06901
DB00777	Autistic disorder	0.03241
DB00777	Behavior disease	0.12372

DB00777	Bipolar disorder	0.09078
DB00777	Bladder cancer	0.0345
DB00777	Central nervous system disease	0.06682
DB00777	Choriocarcinoma	0.07715
DB00777	Chronic fatigue syndrome	0.09449
DB00777	Colon cancer	0.03172
DB00777	Depression	0.07019
DB00777	Dermatitis	0.0243
DB00777	Diabetes mellitus	0.01407
DB00777	Drug-Induced dyskinesia	0.10102
DB00777	Hypertension	0.06339
DB00777	Infertility	0.03671
DB00777	Kidney failure	0.09078
DB00777	Migraine	0.04963
DB00777	Neuroblastoma	0.07342
DB00777	Obesity	0.04112
DB00777	Obsessive-compulsive disorder	0.08058
DB00777	Panic disorder	0.05832
DB00777	Parkinson disease	0.02951
DB00777	Prostate cancer	0.01395
DB00777	Psychotic disorder	0.0428
DB00777	Rheumatoid arthritis	0.01636
DB00777	Schizophrenia	0.08128
DB00777	Stroke	0.03026
DB00800	Attention deficit hyperactivity disorder	0.43208
DB00800	Common cold	0.11952
DB00800	Diabetes mellitus	0.02354
DB00800	Hypertension	0.18812
DB00800	Nervous system disease	0.39211
DB00800	Obesity	0.17978
DB00800	Polycystic ovary syndrome	0.05923
DB00800	Schizophrenia	0.13502
DB00805	Hypertension, Pulmonary	0.07647
DB00805	Supranuclear palsy, progressive	0.19245
DB00805	Anorexia nervosa	0.27217
DB00805	Asthma	0.02722
DB00805	Atherosclerosis	0.02334
DB00805	Autistic disorder	0.12127
DB00805	Behavior disease	0.20574
DB00805	Bipolar disorder	0.15097
DB00805	Brain tumor	0.02677
DB00805	Breast cancer	0.01604
DB00805	Choriocarcinoma	0.09623
DB00805	Chronic fatigue syndrome	0.2357
DB00805	Chronic obstructive airway disease	0.03727
DB00805	Colon cancer	0.07912
DB00805	Congenital heart disease	0.11111

DB00805	Depression	0.13131
DB00805	Dermatitis	0.06061
DB00805	Down syndrome	0.03799
DB00805	Drug abuse	0.09366
DB00805	Drug-Induced dyskinesia	0.12599
DB00805	Epilepsy	0.04714
DB00805	Fibromyalgia	0.2357
DB00805	Generalized anxiety disorder	0.19245
DB00805	Heart failure	0.03553
DB00805	Herpes	0.04762
DB00805	Hypertension	0.07906
DB00805	Lung cancer	0.02328
DB00805	Migraine	0.1238
DB00805	Neuroblastoma	0.04579
DB00805	Neurodegenerative disorder	0.0548
DB00805	Neurotic disorder	0.21082
DB00805	Obesity	0.10256
DB00805	Obsessive-compulsive disorder	0.20101
DB00805	Ovarian cancer	0.0335
DB00805	Panic disorder	0.21822
DB00805	Pervasive development disorder	0.08607
DB00805	Psychotic disorder	0.10675
DB00805	Pulmonary hypertension	0.16667
DB00805	Rheumatoid arthritis	0.0204
DB00805	Schizophrenia	0.02534
DB00805	Stroke	0.07549
DB00805	Sudden infant death syndrome	0.14548
DB00805	Ulcerative colitis	0.03438
DB00805	Vitiligo	0.07857
DB00850	Abortion	0.02905
DB00850	Achalasia and cardiospasm	0.12469
DB00850	Alzheimer's disease	0.05292
DB00850	Amyloidosis	0.06639
DB00850	Asthma	0.04371
DB00850	Autistic disorder	0.06042
DB00850	Autoimmune disease	0.05572
DB00850	Bipolar disorder	0.04432
DB00850	Bladder cancer	0.04542
DB00850	Brain ischemia	0.18262
DB00850	Cancer	0.03396
DB00850	Celiac disease	0.08498
DB00850	Colon cancer	0.0172
DB00850	Congenital abnormality	0.03237
DB00850	Dental plaque	0.06543
DB00850	Diabetes mellitus	0.0125
DB00850	Drug abuse	0.03239
DB00850	Eating disorder	0.10416

DB00850	Epstein-Barr virus infection	0.09263
DB00850	Esophageal tumor	0.07695
DB00850	Esophagus cancer	0.03074
DB00850	Esotropia	0.08852
DB00850	Eye cancer	0.10972
DB00850	Fanconi's anemia	0.0433
DB00850	Glaucoma	0.07631
DB00850	Gram-Negative bacterial infection	0.15306
DB00850	Graves' disease	0.10951
DB00850	HIV infection	0.05185
DB00850	Herpes	0.07492
DB00850	Ischemia	0.06354
DB00850	Keratosis	0.08781
DB00850	Leukemia	0.03912
DB00850	Lichen planus	0.17067
DB00850	Lung cancer	0.0366
DB00850	Lupus erythematosus	0.03596
DB00850	Malignant glioma	0.04453
DB00850	Melanoma	0.02281
DB00850	Pre-Eclampsia	0.04147
DB00850	Prion disease	0.14306
DB00850	Rabies	0.02116
DB00850	Rheumatoid arthritis	0.02622
DB00850	Schistosomiasis	0.15421
DB00850	Stroke	0.04015
DB00850	Thyroid gland disease	0.09641
DB00850	Tuberculosis	0.0826
DB00875	Supranuclear palsy, progressive	0.2582
DB00875	Anorexia nervosa	0.09129
DB00875	Asthma	0.03651
DB00875	Autistic disorder	0.05423
DB00875	Behavior disease	0.06901
DB00875	Bipolar disorder	0.05064
DB00875	Choriocarcinoma	0.1291
DB00875	Chronic fatigue syndrome	0.15811
DB00875	Colon cancer	0.02654
DB00875	Depression	0.05872
DB00875	Dermatitis	0.04066
DB00875	Drug-Induced dyskinesia	0.16903
DB00875	Hypertension	0.07071
DB00875	Kidney failure	0.05064
DB00875	Neuroblastoma	0.06143
DB00875	Obesity	0.0344
DB00875	Obsessive-compulsive disorder	0.13484
DB00875	Panic disorder	0.09759
DB00875	Prostate cancer	0.02334
DB00875	Psychotic disorder	0.07161

DB00875	Rheumatoid arthritis	0.02737
DB00875	Schizophrenia	0.034
DB00875	Stroke	0.05064
DB00988	Stress disorder, post-traumatic	0.26726
DB00988	Autoimmune disease	0.041
DB00988	Behavior disease	0.11664
DB00988	Bipolar disorder	0.0428
DB00988	Brain disease	0.06788
DB00988	Breast cancer	0.01818
DB00988	Depression	0.04963
DB00988	Drug abuse	0.0354
DB00988	Epilepsy	0.05345
DB00988	Gilles de la Tourette syndrome	0.14286
DB00988	Herpes	0.05399
DB00988	Hypertension	0.02988
DB00988	Migraine	0.07019
DB00988	Multiple system atrophy	0.21822
DB00988	Obesity	0.02907
DB00988	Psychotic disorder	0.12105
DB01049	Abortion	0.066
DB01049	Adenocarcinoma	0.02278
DB01049	Adenovirus infection	0.02033
DB01049	Amyotrophic lateral sclerosis	0.23341
DB01049	Anorexia nervosa	0.58793
DB01049	Attention deficit hyperactivity disorder	0.58929
DB01049	Autistic disorder	0.01894
DB01049	Behavior disease	0.73567
DB01049	Bipolar disorder	0.26953
DB01049	Breast cancer	0.01503
DB01049	Choriocarcinoma	0.04508
DB01049	Chronic fatigue syndrome	0.05522
DB01049	Colon cancer	0.0278
DB01049	Common cold	0.04174
DB01049	Depression	0.34412
DB01049	Dermatitis	0.21888
DB01049	Diabetes mellitus	0.13507
DB01049	Drug abuse	0.11988
DB01049	Drug-Induced dyskinesia	0.05903
DB01049	Eating disorder	0.05437
DB01049	Embryoma	0.00963
DB01049	Epilepsy	0.38366
DB01049	Gilles de la Tourette syndrome	0.11806
DB01049	Hepatitis C	0.41239
DB01049	Herpes	0.04462
DB01049	Hypertension	0.51567
DB01049	Hypogonadism	0.06984
DB01049	Kidney failure	0.05305

DB01049	Migraine	0.47
DB01049	Nervous system disease	0.10575
DB01049	Obesity	0.0743
DB01049	Obsessive-compulsive disorder	0.09418
DB01049	Panic disorder	0.62851
DB01049	Polycystic ovary syndrome	0.02069
DB01049	Prostate cancer	0.00815
DB01049	Psychotic disorder	0.05002
DB01049	Rheumatoid arthritis	0.00956
DB01049	Schizophrenia	0.07139
DB01049	Stroke	0.21842
DB01049	Sudden infant death syndrome	0.66333
DB01049	Yersinia infection	0.01853
DB01186	Abortion	0.06924
DB01186	Amyotrophic lateral sclerosis	0.25548
DB01186	Anorexia nervosa	0.66497
DB01186	Attention deficit hyperactivity disorder	0.61634
DB01186	Autistic disorder	0.02941
DB01186	Behavior disease	0.82034
DB01186	Bipolar disorder	0.30949
DB01186	Choriocarcinoma	0.07001
DB01186	Chronic fatigue syndrome	0.08575
DB01186	Colon cancer	0.02878
DB01186	Common cold	0.06482
DB01186	Depression	0.392
DB01186	Dermatitis	0.24393
DB01186	Diabetes mellitus	0.14998
DB01186	Drug abuse	0.12516
DB01186	Drug-Induced dyskinesia	0.09167
DB01186	Epilepsy	0.41361
DB01186	Hepatitis C	0.44423
DB01186	Hypertension	0.52599
DB01186	Kidney failure	0.08239
DB01186	Migraine	0.52229
DB01186	Nervous system disease	0.13126
DB01186	Obesity	0.10216
DB01186	Obsessive-compulsive disorder	0.07313
DB01186	Panic disorder	0.65795
DB01186	Polycystic ovary syndrome	0.03212
DB01186	Prostate cancer	0.01266
DB01186	Psychotic disorder	0.03884
DB01186	Rheumatoid arthritis	0.01484
DB01186	Schizophrenia	0.0435
DB01186	Stroke	0.24696
DB01186	Sudden infant death syndrome	0.71305
DB01200	Abortion	0.06924
DB01200	Adenocarcinoma	0.03438

DB01200	Amyotrophic lateral sclerosis	0.25456
DB01200	Anorexia nervosa	0.66079
DB01200	Attention deficit hyperactivity disorder	0.6124
DB01200	Autistic disorder	0.02858
DB01200	Behavior disease	0.81612
DB01200	Bipolar disorder	0.30717
DB01200	Choriocarcinoma	0.06804
DB01200	Chronic fatigue syndrome	0.08333
DB01200	Colon cancer	0.02797
DB01200	Common cold	0.06299
DB01200	Depression	0.38931
DB01200	Dermatitis	0.24268
DB01200	Diabetes mellitus	0.14926
DB01200	Drug abuse	0.12388
DB01200	Drug-Induced dyskinesia	0.08909
DB01200	Epilepsy	0.41264
DB01200	Hepatitis C	0.44322
DB01200	Hypertension	0.5403
DB01200	Kidney failure	0.08006
DB01200	Migraine	0.51976
DB01200	Nervous system disease	0.1295
DB01200	Obesity	0.10006
DB01200	Obsessive-compulsive disorder	0.07107
DB01200	Panic disorder	0.65497
DB01200	Polycystic ovary syndrome	0.03122
DB01200	Prostate cancer	0.0123
DB01200	Psychotic disorder	0.03774
DB01200	Rheumatoid arthritis	0.01442
DB01200	Schizophrenia	0.0609
DB01200	Stroke	0.24542
DB01200	Sudden infant death syndrome	0.71156
DB01224	Supranuclear palsy, progressive	0.22646
DB01224	Abortion	0.06924
DB01224	Adenocarcinoma	0.02861
DB01224	Amyotrophic lateral sclerosis	0.24922
DB01224	Anorexia nervosa	0.63655
DB01224	Asthma	0.03203
DB01224	Atopic rhinitis	0.05064
DB01224	Attention deficit hyperactivity disorder	0.58954
DB01224	Autistic disorder	0.02378
DB01224	Behavior disease	0.82195
DB01224	Bipolar disorder	0.31593
DB01224	Bladder cancer	0.02532
DB01224	Central nervous system disease	0.04903
DB01224	Choriocarcinoma	0.05661
DB01224	Chronic fatigue syndrome	0.06934
DB01224	Colon cancer	0.03491

DB01224	Common cold	0.05241
DB01224	Depression	0.39947
DB01224	Dermatitis	0.23549
DB01224	Diabetes mellitus	0.15542
DB01224	Drug abuse	0.0981
DB01224	Drug-Induced dyskinesia	0.07412
DB01224	Eating disorder	0.03414
DB01224	Embryoma	0.01209
DB01224	Epilepsy	0.40705
DB01224	Gilles de la Tourette syndrome	0.07412
DB01224	Hepatitis C	0.43739
DB01224	Herpes	0.02802
DB01224	Hypertension	0.52764
DB01224	Infertility	0.02694
DB01224	Kidney failure	0.06662
DB01224	Migraine	0.50505
DB01224	Nervous system disease	0.11928
DB01224	Neuroblastoma	0.05388
DB01224	Obesity	0.08788
DB01224	Obsessive-compulsive disorder	0.11826
DB01224	Panic disorder	0.68048
DB01224	Parkinson disease	0.02166
DB01224	Polycystic ovary syndrome	0.02598
DB01224	Prostate cancer	0.01024
DB01224	Psychotic disorder	0.06281
DB01224	Rheumatoid arthritis	0.012
DB01224	Schizophrenia	0.12944
DB01224	Stroke	0.23645
DB01224	Sudden infant death syndrome	0.70292
DB01238	Supranuclear palsy, progressive	0.23094
DB01238	Abortion	0.06924
DB01238	Adenocarcinoma	0.02917
DB01238	Amyotrophic lateral sclerosis	0.24974
DB01238	Anorexia nervosa	0.63893
DB01238	Asthma	0.03266
DB01238	Atopic rhinitis	0.05164
DB01238	Attention deficit hyperactivity disorder	0.59178
DB01238	Autistic disorder	0.02425
DB01238	Behavior disease	0.82495
DB01238	Bipolar disorder	0.31769
DB01238	Bladder cancer	0.02582
DB01238	Central nervous system disease	0.05
DB01238	Choriocarcinoma	0.05774
DB01238	Chronic fatigue syndrome	0.07071
DB01238	Colon cancer	0.0356
DB01238	Common cold	0.05345
DB01238	Depression	0.40151

DB01238	Dermatitis	0.23619
DB01238	Diabetes mellitus	0.15603
DB01238	Drug abuse	0.09846
DB01238	Drug-Induced dyskinesia	0.07559
DB01238	Eating disorder	0.03482
DB01238	Embryoma	0.01233
DB01238	Epilepsy	0.40759
DB01238	Gilles de la Tourette syndrome	0.07559
DB01238	Hepatitis C	0.43796
DB01238	Herpes	0.02857
DB01238	Hypertension	0.53071
DB01238	Infertility	0.02747
DB01238	Kidney failure	0.04529
DB01238	Migraine	0.5065
DB01238	Nervous system disease	0.12028
DB01238	Neuroblastoma	0.05494
DB01238	Obesity	0.08907
DB01238	Obsessive-compulsive disorder	0.1206
DB01238	Panic disorder	0.68303
DB01238	Parkinson disease	0.02209
DB01238	Polycystic ovary syndrome	0.02649
DB01238	Prostate cancer	0.01044
DB01238	Psychotic disorder	0.06405
DB01238	Rheumatoid arthritis	0.01224
DB01238	Schizophrenia	0.1315
DB01238	Stroke	0.23733
DB01238	Sudden infant death syndrome	0.70377
DB01239	Supranuclear palsy, progressive	0.33333
DB01239	Anorexia nervosa	0.11785
DB01239	Asthma	0.04714
DB01239	Atopic rhinitis	0.07454
DB01239	Autistic disorder	0.03501
DB01239	Behavior disease	0.13363
DB01239	Bipolar disorder	0.09806
DB01239	Bladder cancer	0.03727
DB01239	Central nervous system disease	0.07217
DB01239	Choriocarcinoma	0.08333
DB01239	Chronic fatigue syndrome	0.10206
DB01239	Colon cancer	0.05139
DB01239	Depression	0.07581
DB01239	Dermatitis	0.02624
DB01239	Diabetes mellitus	0.01519
DB01239	Drug abuse	0.02704
DB01239	Drug-Induced dyskinesia	0.10911
DB01239	Hypertension	0.06847
DB01239	Infertility	0.03965
DB01239	Migraine	0.05361

DB01239	Neuroblastoma	0.07931
DB01239	Obesity	0.04441
DB01239	Obsessive-compulsive disorder	0.08704
DB01239	Panic disorder	0.06299
DB01239	Parkinson disease	0.03188
DB01239	Psychotic disorder	0.04623
DB01239	Rheumatoid arthritis	0.01767
DB01239	Schizophrenia	0.08779
DB01239	Stroke	0.03269
DB01267	Abortion	0.07791
DB01267	Amyotrophic lateral sclerosis	0.26969
DB01267	Anorexia nervosa	0.68243
DB01267	Atopic rhinitis	0.06262
DB01267	Attention deficit hyperactivity disorder	0.63854
DB01267	Autistic disorder	0.02941
DB01267	Behavior disease	0.71504
DB01267	Bipolar disorder	0.1738
DB01267	Choriocarcinoma	0.07001
DB01267	Chronic fatigue syndrome	0.08575
DB01267	Colon cancer	0.02878
DB01267	Common cold	0.06482
DB01267	Depression	0.33015
DB01267	Dermatitis	0.25667
DB01267	Diabetes mellitus	0.15792
DB01267	Drug abuse	0.04173
DB01267	Drug-Induced dyskinesia	0.09167
DB01267	Epilepsy	0.43781
DB01267	Hepatitis C	0.47029
DB01267	Hypertension	0.45644
DB01267	Infertility	0.03331
DB01267	Kidney failure	0.05492
DB01267	Migraine	0.54987
DB01267	Nervous system disease	0.13986
DB01267	Obesity	0.10561
DB01267	Obsessive-compulsive disorder	0.07313
DB01267	Panic disorder	0.69317
DB01267	Parkinson disease	0.02678
DB01267	Polycystic ovary syndrome	0.03212
DB01267	Prostate cancer	0.01266
DB01267	Psychotic disorder	0.03884
DB01267	Rheumatoid arthritis	0.01484
DB01267	Schizophrenia	0.06508
DB01267	Stroke	0.25921
DB01267	Sudden infant death syndrome	0.75516
DB01403	Supranuclear palsy, progressive	0.26491
DB01403	Anorexia nervosa	0.09366
DB01403	Asthma	0.03746

DB01403	Atopic rhinitis	0.05923
DB01403	Attention deficit hyperactivity disorder	0.36921
DB01403	Autistic disorder	0.02782
DB01403	Behavior disease	0.1062
DB01403	Bipolar disorder	0.07793
DB01403	Bladder cancer	0.02962
DB01403	Central nervous system disease	0.05735
DB01403	Choriocarcinoma	0.06623
DB01403	Chronic fatigue syndrome	0.08111
DB01403	Colon cancer	0.02723
DB01403	Common cold	0.06131
DB01403	Depression	0.06025
DB01403	Dermatitis	0.02086
DB01403	Diabetes mellitus	0.02415
DB01403	Drug-Induced dyskinesia	0.08671
DB01403	Hypertension	0.2081
DB01403	Infertility	0.03151
DB01403	Kidney failure	0.07793
DB01403	Migraine	0.0426
DB01403	Nervous system disease	0.33587
DB01403	Neuroblastoma	0.06303
DB01403	Obesity	0.18157
DB01403	Obsessive-compulsive disorder	0.06917
DB01403	Panic disorder	0.05006
DB01403	Parkinson disease	0.02533
DB01403	Polycystic ovary syndrome	0.03039
DB01403	Prostate cancer	0.01198
DB01403	Psychotic disorder	0.03674
DB01403	Rheumatoid arthritis	0.01404
DB01403	Schizophrenia	0.18823
DB01403	Stroke	0.02598
DB01608	Attention deficit hyperactivity disorder	0.46965
DB01608	Hypertension	0.16306
DB01608	Kidney failure	0.06537
DB01608	Nervous system disease	0.42571
DB01608	Obesity	0.15539
DB01608	Schizophrenia	0.14491
DB01614	Abortion	0.05124
DB01614	Amyotrophic lateral sclerosis	0.34767
DB01614	Anorexia nervosa	0.22699
DB01614	Attention deficit hyperactivity disorder	0.11127
DB01614	Autistic disorder	0.04951
DB01614	Behavior disease	0.7912
DB01614	Bipolar disorder	0.12441
DB01614	Choriocarcinoma	0.11785
DB01614	Chronic fatigue syndrome	0.14434
DB01614	Colon cancer	0.02423

DB01614	Depression	0.40127
DB01614	Dermatitis	0.33671
DB01614	Diabetes mellitus	0.18495
DB01614	Drug abuse	0.02745
DB01614	Drug-Induced dyskinesia	0.1543
DB01614	Epilepsy	0.55597
DB01614	Hepatitis C	0.59673
DB01614	Hypertension	0.43741
DB01614	Kidney failure	0.09245
DB01614	Migraine	0.64354
DB01614	Obesity	0.0314
DB01614	Obsessive-compulsive disorder	0.12309
DB01614	Panic disorder	0.90336
DB01614	Prostate cancer	0.02131
DB01614	Psychotic disorder	0.06537
DB01614	Rheumatoid arthritis	0.02498
DB01614	Stroke	0.3447
DB01614	Sudden infant death syndrome	0.95617
DB01621	Abortion	0.05124
DB01621	Amyotrophic lateral sclerosis	0.36004
DB01621	Anorexia nervosa	0.24572
DB01621	Attention deficit hyperactivity disorder	0.11127
DB01621	Autistic disorder	0.06063
DB01621	Behavior disease	0.81951
DB01621	Bipolar disorder	0.1348
DB01621	Choriocarcinoma	0.14434
DB01621	Chronic fatigue syndrome	0.17678
DB01621	Colon cancer	0.02967
DB01621	Depression	0.42537
DB01621	Dermatitis	0.35339
DB01621	Diabetes mellitus	0.18978
DB01621	Drug abuse	0.02745
DB01621	Drug-Induced dyskinesia	0.18898
DB01621	Epilepsy	0.56894
DB01621	Hepatitis C	0.61025
DB01621	Hypertension	0.41964
DB01621	Migraine	0.66057
DB01621	Obesity	0.03846
DB01621	Obsessive-compulsive disorder	0.15076
DB01621	Panic disorder	0.94341
DB01621	Psychotic disorder	0.08006
DB01621	Rheumatoid arthritis	0.0306
DB01621	Stroke	0.36547
DB01621	Sudden infant death syndrome	0.9762
DB01622	Abortion	0.05124
DB01622	Amyotrophic lateral sclerosis	0.34767
DB01622	Anorexia nervosa	0.22699

DB01622	Attention deficit hyperactivity disorder	0.11127
DB01622	Autistic disorder	0.04951
DB01622	Behavior disease	0.7912
DB01622	Bipolar disorder	0.12441
DB01622	Choriocarcinoma	0.11785
DB01622	Chronic fatigue syndrome	0.14434
DB01622	Colon cancer	0.02423
DB01622	Depression	0.40127
DB01622	Dermatitis	0.33671
DB01622	Diabetes mellitus	0.18495
DB01622	Drug abuse	0.02745
DB01622	Drug-Induced dyskinesia	0.1543
DB01622	Epilepsy	0.55597
DB01622	Hepatitis C	0.59673
DB01622	Hypertension	0.43741
DB01622	Kidney failure	0.09245
DB01622	Migraine	0.64354
DB01622	Obesity	0.0314
DB01622	Obsessive-compulsive disorder	0.12309
DB01622	Panic disorder	0.90336
DB01622	Prostate cancer	0.02131
DB01622	Psychotic disorder	0.06537
DB01622	Rheumatoid arthritis	0.02498
DB01622	Stroke	0.3447
DB01622	Sudden infant death syndrome	0.95617
DB01623	Anorexia nervosa	0.11785
DB01623	Autistic disorder	0.07001
DB01623	Behavior disease	0.08909
DB01623	Bipolar disorder	0.06537
DB01623	Choriocarcinoma	0.16667
DB01623	Chronic fatigue syndrome	0.20412
DB01623	Colon cancer	0.03426
DB01623	Depression	0.07581
DB01623	Dermatitis	0.05249
DB01623	Drug-Induced dyskinesia	0.21822
DB01623	Hypertension	0.04564
DB01623	Obesity	0.04441
DB01623	Obsessive-compulsive disorder	0.17408
DB01623	Panic disorder	0.12599
DB01623	Psychotic disorder	0.09245
DB01623	Rheumatoid arthritis	0.03533
DB01623	Stroke	0.06537
DB04946	Abortion	0.05124
DB04946	Adenocarcinoma	0.04398
DB04946	Amyotrophic lateral sclerosis	0.33327
DB04946	Anorexia nervosa	0.2052
DB04946	Atopic rhinitis	0.07785

DB04946	Attention deficit hyperactivity disorder	0.11127
DB04946	Autistic disorder	0.03656
DB04946	Behavior disease	0.75826
DB04946	Bipolar disorder	0.11232
DB04946	Choriocarcinoma	0.08704
DB04946	Chronic fatigue syndrome	0.1066
DB04946	Colon cancer	0.03578
DB04946	Common cold	0.08058
DB04946	Depression	0.37324
DB04946	Dermatitis	0.3173
DB04946	Diabetes mellitus	0.17934
DB04946	Drug abuse	0.02745
DB04946	Drug-Induced dyskinesia	0.11396
DB04946	Epilepsy	0.54087
DB04946	Hepatitis C	0.58099
DB04946	Hypertension	0.45976
DB04946	Infertility	0.04142
DB04946	Kidney failure	0.03414
DB04946	Migraine	0.62372
DB04946	Obesity	0.02319
DB04946	Obsessive-compulsive disorder	0.09091
DB04946	Panic disorder	0.85678
DB04946	Parkinson disease	0.0333
DB04946	Prostate cancer	0.01574
DB04946	Psychotic disorder	0.09656
DB04946	Rheumatoid arthritis	0.01845
DB04946	Schizophrenia	0.04585
DB04946	Stroke	0.32053
DB04946	Sudden infant death syndrome	0.93288
DB05271	Abortion	0.05124
DB05271	Amyotrophic lateral sclerosis	0.34358
DB05271	Anorexia nervosa	0.14366
DB05271	Attention deficit hyperactivity disorder	0.11127
DB05271	Behavior disease	0.72353
DB05271	Bipolar disorder	0.07818
DB05271	Depression	0.34369
DB05271	Dermatitis	0.29684
DB05271	Diabetes mellitus	0.20325
DB05271	Drug abuse	0.02745
DB05271	Epilepsy	0.55168
DB05271	Hepatitis C	0.59226
DB05271	Hypertension	0.40034
DB05271	Migraine	0.63791
DB05271	Obesity	0.02907
DB05271	Panic disorder	0.80767
DB05271	Polycystic ovary syndrome	0.05006
DB05271	Stroke	0.29504

DB05271	Sudden infant death syndrome	0.94957
DB06216	Infertility, Male	0.05233
DB06216	Abortion	0.03363
DB06216	Adenocarcinoma	0.03262
DB06216	Alimentary system disease	0.09739
DB06216	Alzheimer's disease	0.0501
DB06216	Amyotrophic lateral sclerosis	0.23473
DB06216	Anorexia nervosa	0.27984
DB06216	Arthritis	0.25548
DB06216	Atherosclerosis	0.10098
DB06216	Atopic rhinitis	0.11547
DB06216	Attention deficit hyperactivity disorder	0.28362
DB06216	Autistic disorder	0.19021
DB06216	Azoospermia	0.03958
DB06216	Behavior disease	0.73651
DB06216	Bipolar disorder	0.27514
DB06216	Breast cancer	0.01113
DB06216	Bronchial disease	0.50975
DB06216	Choriocarcinoma	0.06455
DB06216	Chronic fatigue syndrome	0.07906
DB06216	Chronic obstructive airway disease	0.2077
DB06216	Colon cancer	0.05307
DB06216	Common cold	0.05976
DB06216	Conduct disorder	0.1014
DB06216	Cystic fibrosis	0.26773
DB06216	Depression	0.35265
DB06216	Dermatitis	0.33864
DB06216	Diabetes mellitus	0.20823
DB06216	Drug abuse	0.20596
DB06216	Drug-Induced dyskinesia	0.08452
DB06216	Enteritis	0.0348
DB06216	Epilepsy	0.45514
DB06216	Glaucoma	0.30312
DB06216	Gram-Negative bacterial infection	0.06949
DB06216	Graves' disease	0.28598
DB06216	Heart failure	0.24647
DB06216	Hepatitis C	0.40807
DB06216	Hypertension	0.71071
DB06216	Infertility	0.09486
DB06216	Ischemia	0.06418
DB06216	Kidney failure	0.02532
DB06216	Liver cancer	0.02221
DB06216	Lung cancer	0.09799
DB06216	Malaria	0.30551
DB06216	Melanoma	0.01863
DB06216	Metabolism disease	0.28433
DB06216	Migraine	0.47995

DB06216	Movement disorder	0.14838
DB06216	Myopathy	0.19283
DB06216	Nervous system disease	0.12441
DB06216	Neurodegenerative disorder	0.08205
DB06216	Obesity	0.27403
DB06216	Obsessive-compulsive disorder	0.06742
DB06216	Oligospermia	0.07206
DB06216	Panic disorder	0.60458
DB06216	Parkinson disease	0.02469
DB06216	Polycystic kidney	0.06271
DB06216	Polycystic ovary syndrome	0.19762
DB06216	Premature birth	0.43164
DB06216	Prostate cancer	0.09772
DB06216	Psychotic disorder	0.07161
DB06216	Respiratory tract disease	0.04837
DB06216	Rheumatoid arthritis	0.08637
DB06216	Schizophrenia	0.07535
DB06216	Sickle cell disease	0.16076
DB06216	Sinusitis	0.04488
DB06216	Stroke	0.22699
DB06216	Subarachnoid hemorrhage	0.38014
DB06216	Sudden infant death syndrome	0.65499
DB06216	Testicular dysfunction	0.02212
DB01099	Adenovirus infection	0.02274
DB01099	Alzheimer's disease	0.01869
DB01099	Aortic valve disease	0.07209
DB01099	Breast cancer	0.01444
DB01099	Cancer	0.06052
DB01099	Colon cancer	0.02111
DB01099	Congenital abnormality	0.02133
DB01099	Emphysema	0.05278
DB01099	Endometriosis	0.02263
DB01099	Eye cancer	0.07231
DB01099	Gastritis	0.03841
DB01099	HIV infection	0.04973
DB01099	Helicobacter infection	0.06256
DB01099	Parkinson disease	0.05215
DB01099	Rheumatoid arthritis	0.01728
DB01099	Severe acute respiratory syndrome	0.06563
DB01099	Tuberous sclerosis	0.04119
DB01099	Virus disease	0.04438
DB00139	Spastic paraplegia, Hereditary	0.25715
DB00139	Abortion	0.02451
DB00139	Actinic keratosis	0.07412
DB00139	Amyotrophic lateral sclerosis	0.04568
DB00139	Atherosclerosis	0.01987
DB00139	Breast cancer	0.01285

DB00139	Cancer	0.04373
DB00139	Cervical cancer	0.04256
DB00139	Colon cancer	0.01877
DB00139	Dental plaque	0.0343
DB00139	Dermatitis	0.0219
DB00139	Down syndrome	0.03431
DB00139	Embryoma	0.01886
DB00139	Gastrointestinal tumor	0.05439
DB00139	Heart failure	0.03242
DB00139	Hypertension	0.0155
DB00139	Leukodystrophy NOS	0.25715
DB00139	Liver cancer	0.02564
DB00139	Liver tumor	0.06665
DB00139	Lyme disease	0.25715
DB00139	Metastasis to lymph nodes	0.09948
DB00139	Parkinson disease	0.02933
DB00139	Polyarthritis	0.0857
DB00139	Prion disease	0.075
DB00139	Prostate cancer	0.01439
DB00139	Rheumatoid arthritis	0.01374
DB00139	Systemic infection	0.02529
DB00139	Tic disorder	0.04485
DB00139	Yersinia infection	0.02413
DB00398	Skin disease, Genetic	0.4712
DB00398	Actinic keratosis	0.06372
DB00398	Adenoid cystic cancer	0.1191
DB00398	Adrenoleukodystrophy	0.08337
DB00398	Alzheimer's disease	0.18143
DB00398	Amyotrophic lateral sclerosis	0.12426
DB00398	Angiomyolipoma	0.50571
DB00398	Aseptic necrosis of bone	0.02943
DB00398	Asthma	0.11055
DB00398	Atherosclerosis	0.07369
DB00398	Autistic disorder	0.03354
DB00398	Brain disease	0.03085
DB00398	Breast cancer	0.0086
DB00398	Cancer	0.23455
DB00398	Capillaries disease	0.11691
DB00398	Cardiovascular disease	0.27851
DB00398	Chronic obstructive airway disease	0.26028
DB00398	Cleft palate	0.08452
DB00398	Colon cancer	0.01406
DB00398	Common cold	0.42818
DB00398	Congenital abnormality	0.0127
DB00398	Craniosynostosis	0.1291
DB00398	Cystic fibrosis	0.04008
DB00398	Dental plaque	0.04866

DB00398	Depression	0.01725
DB00398	Dermatitis	0.10975
DB00398	Diabetes mellitus	0.04559
DB00398	Down syndrome	0.02967
DB00398	Drug abuse	0.01272
DB00398	Eating disorder	0.02191
DB00398	Embryoma	0.02894
DB00398	Endocrine system disease	0.19232
DB00398	Endometriosis	0.10636
DB00398	Familial Mediterranean fever	0.18517
DB00398	Gastrointestinal tumor	0.08771
DB00398	Gram-Negative bacterial infection	0.05664
DB00398	Graves' disease	0.0559
DB00398	Heart failure	0.02428
DB00398	Herpes	0.24528
DB00398	Kidney failure	0.08788
DB00398	Leukoencephalopathy	0.0665
DB00398	Liver cancer	0.01717
DB00398	Liver metastases	0.06792
DB00398	Lung cancer	0.01355
DB00398	Lupus erythematosus	0.09002
DB00398	Macular degeneration	0.23896
DB00398	Malaria	0.1595
DB00398	Melanoma	0.04017
DB00398	Metaplastic polyp	0.37623
DB00398	Mucocutaneous lymph node syndrome	0.37596
DB00398	Multiple endocrine neoplasia	0.19914
DB00398	Myeloproliferative disease	0.08452
DB00398	Myopathy	0.34178
DB00398	Neck cancer	0.15225
DB00398	Nephritis	0.14142
DB00398	Nephrosis	0.34807
DB00398	Neuroblastoma	0.02226
DB00398	Neurodegenerative disorder	0.02852
DB00398	Neurofibromatosis	0.1
DB00398	Obesity	0.0654
DB00398	Oral cancer	0.01854
DB00398	Osteomyelitis	0.66453
DB00398	Osteoporosis	0.05
DB00398	Pancreas disease	0.07232
DB00398	Pancreatitis	0.0315
DB00398	Penile disease	0.04766
DB00398	Peptic ulcer	0.14744
DB00398	Polycystic ovary syndrome	0.02414
DB00398	Polyneuropathy	0.23896
DB00398	Pre-Eclampsia	0.11737
DB00398	Primary hyperparathyroidism	0.15027

DB00398	Prion disease	0.05294
DB00398	Prostate cancer	0.00964
DB00398	Retinal disease	0.26391
DB00398	Rheumatoid arthritis	0.03391
DB00398	Squamous cell cancer	0.02545
DB00398	Stomach cancer	0.01229
DB00398	Systemic infection	0.0656
DB00398	Systemic scleroderma	0.03016
DB00398	Testicular dysfunction	0.25773
DB00398	Thyroid cancer	0.03978
DB00398	Thyroid gland disease	0.56473
DB00398	Ulcerative colitis	0.19577
DB00398	Urogenital abnormalities	0.08452
DB00398	Uterine disease	0.03613
DB00398	Vitamin D deficiency	0.75701
DB01268	Skin disease, Genetic	0.12691
DB01268	Abortion	0.03657
DB01268	Adrenoleukodystrophy	0.12845
DB01268	Alzheimer's disease	0.01715
DB01268	Amyotrophic lateral sclerosis	0.17342
DB01268	Aseptic necrosis of bone	0.04534
DB01268	Asthma	0.15941
DB01268	Atherosclerosis	0.10418
DB01268	Autistic disorder	0.05167
DB01268	Brain tumor	0.07791
DB01268	Breast cancer	0.01701
DB01268	Cancer	0.12375
DB01268	Capillaries disease	0.18011
DB01268	Cardiovascular disease	0.40871
DB01268	Chronic obstructive airway disease	0.33159
DB01268	Colon cancer	0.06497
DB01268	Congenital abnormality	0.01957
DB01268	Congenital heart disease	0.3104
DB01268	Deafness	0.20038
DB01268	Dental plaque	0.03957
DB01268	Depression	0.02657
DB01268	Dermatitis	0.15693
DB01268	Diabetes mellitus	0.07024
DB01268	Endometriosis	0.13191
DB01268	Enteritis	0.03881
DB01268	Familial Mediterranean fever	0.28528
DB01268	Gastritis	0.03525
DB01268	Gastrointestinal tumor	0.09806
DB01268	Heart failure	0.0374
DB01268	Hemorrhagic disorder	0.10481
DB01268	Herpes	0.05862
DB01268	Infection	0.06399

DB01268	Influenza	0.13487
DB01268	Kidney failure	0.13539
DB01268	Leukemia	0.0799
DB01268	Leukoencephalopathy	0.16138
DB01268	Liver metastases	0.10464
DB01268	Lupus erythematosus	0.12727
DB01268	Lymphoproliferative disorder	0.20616
DB01268	Macular degeneration	0.36816
DB01268	Malaria	0.24573
DB01268	Melanoma	0.02872
DB01268	Mucocutaneous lymph node syndrome	0.55006
DB01268	Myeloproliferative disease	0.13811
DB01268	Neoplasm metastasis	0.02877
DB01268	Nephritis	0.15811
DB01268	Neurofibromatosis	0.1118
DB01268	Obesity	0.10076
DB01268	Oral cancer	0.02856
DB01268	Osteoporosis	0.0559
DB01268	Overnutrition	0.06465
DB01268	Peptic ulcer	0.22716
DB01268	Polyneuropathy	0.36816
DB01268	Pre-Eclampsia	0.18083
DB01268	Prostate cancer	0.03506
DB01268	Retinal disease	0.38787
DB01268	Skin cancer	0.1152
DB01268	Solid tumor	0.18364
DB01268	Systemic infection	0.10107
DB01268	Systemic scleroderma	0.02323
DB01268	Testicular dysfunction	0.05051
DB01268	Thyroid cancer	0.06129
DB01268	Thyroid gland disease	0.08111
DB01268	Turner's syndrome	0.10942
DB01268	Ulcerative colitis	0.02872
DB01268	Uterine disease	0.05566
DB06589	Skin disease, Genetic	0.11353
DB06589	Abortion	0.03271
DB06589	Adenocarcinoma	0.04613
DB06589	Adrenoleukodystrophy	0.1149
DB06589	Advanced cancer	0.1
DB06589	Alzheimer's disease	0.03811
DB06589	Amyotrophic lateral sclerosis	0.15513
DB06589	Aseptic necrosis of bone	0.04056
DB06589	Asthma	0.22937
DB06589	Atherosclerosis	0.09319
DB06589	Autistic disorder	0.04623
DB06589	Bone disease	0.05423
DB06589	Brain tumor	0.0697

DB06589	Cancer	0.12235
DB06589	Capillaries disease	0.16112
DB06589	Cardiovascular disease	0.36561
DB06589	Chronic obstructive airway disease	0.29662
DB06589	Cleft palate	0.08452
DB06589	Colon cancer	0.0805
DB06589	Congenital abnormality	0.01751
DB06589	Congenital heart disease	0.27767
DB06589	Craniosynostosis	0.1291
DB06589	Deafness	0.17925
DB06589	Dental plaque	0.0354
DB06589	Depression	0.02377
DB06589	Dermatitis	0.14038
DB06589	Diabetes mellitus	0.06284
DB06589	Endometriosis	0.14426
DB06589	Familial Mediterranean fever	0.25521
DB06589	Gastritis	0.03153
DB06589	Gastrointestinal tumor	0.08771
DB06589	Generalized anxiety disorder	0.15596
DB06589	HIV infection	0.07896
DB06589	Heart failure	0.03346
DB06589	Hemorrhagic disorder	0.09376
DB06589	Herpes	0.05244
DB06589	Hyperopia	0.10541
DB06589	Infection	0.05724
DB06589	Influenza	0.12065
DB06589	Intracranial aneurysm	0.09535
DB06589	Intracranial hypertension	0.22361
DB06589	Keratosis	0.10541
DB06589	Kidney failure	0.12112
DB06589	Leukemia	0.05366
DB06589	Leukoencephalopathy	0.09165
DB06589	Liver metastases	0.0936
DB06589	Lupus erythematosus	0.11385
DB06589	Lymphoproliferative disorder	0.18443
DB06589	Macular degeneration	0.32934
DB06589	Malaria	0.21982
DB06589	Melanoma	0.02569
DB06589	Mucocutaneous lymph node syndrome	0.49206
DB06589	Myeloproliferative disease	0.12355
DB06589	Nephritis	0.14142
DB06589	Neurofibromatosis	0.1
DB06589	Obesity	0.09014
DB06589	Oral cancer	0.02555
DB06589	Osteoporosis	0.05
DB06589	Ovarian cancer	0.03178
DB06589	Overnutrition	0.05783

DB06589	Peptic ulcer	0.20321
DB06589	Polyneuropathy	0.32934
DB06589	Pre-Eclampsia	0.16176
DB06589	Prostate cancer	0.04851
DB06589	Ptosis	0.10541
DB06589	Retinal disease	0.34697
DB06589	Rheumatoid arthritis	0.03644
DB06589	Skin cancer	0.10305
DB06589	Solid tumor	0.16428
DB06589	Systemic infection	0.09041
DB06589	Systemic scleroderma	0.02078
DB06589	Testicular dysfunction	0.04518
DB06589	Thyroid cancer	0.05483
DB06589	Thyroid gland disease	0.07255
DB06589	Turner's syndrome	0.39349
DB06589	Ulcerative colitis	0.02569
DB06589	Uterine disease	0.04979
DB06589	Yersinia infection	0.06398
DB06626	Adrenoleukodystrophy	0.16996
DB06626	Alzheimer's disease	0.02269
DB06626	Amyotrophic lateral sclerosis	0.24424
DB06626	Aseptic necrosis of bone	0.05999
DB06626	Asthma	0.21988
DB06626	Atherosclerosis	0.10509
DB06626	Autistic disorder	0.06838
DB06626	Cancer	0.12563
DB06626	Capillaries disease	0.23833
DB06626	Cardiovascular disease	0.55751
DB06626	Chronic obstructive airway disease	0.45101
DB06626	Colon cancer	0.02866
DB06626	Congenital abnormality	0.0259
DB06626	Dental plaque	0.05236
DB06626	Depression	0.03516
DB06626	Dermatitis	0.21761
DB06626	Diabetes mellitus	0.09295
DB06626	Endometriosis	0.18365
DB06626	Familial Mediterranean fever	0.37749
DB06626	Heart failure	0.04949
DB06626	Kidney failure	0.17915
DB06626	Leukoencephalopathy	0.13557
DB06626	Liver metastases	0.13846
DB06626	Lupus erythematosus	0.17777
DB06626	Macular degeneration	0.48715
DB06626	Malaria	0.32515
DB06626	Melanoma	0.038
DB06626	Mucocutaneous lymph node syndrome	0.75176
DB06626	Obesity	0.13333

DB06626	Oral cancer	0.03779
DB06626	Peptic ulcer	0.30058
DB06626	Polyneuropathy	0.48715
DB06626	Pre-Eclampsia	0.23927
DB06626	Retinal disease	0.52858
DB06626	Systemic infection	0.13373
DB06626	Systemic scleroderma	0.03074
DB06626	Thyroid cancer	0.0811
DB06626	Ulcerative colitis	0.038
DB06626	Uterine disease	0.07365
DB00384	Bronchiectasis	0.40825
DB00384	Cystic fibrosis	0.14286
DB00384	Hypertension	0.07906
DB00384	Kidney disease	0.11952
DB00384	Melanoma	0.04167
DB00384	Respiratory failure	0.17678
DB00384	Ulcerative colitis	0.10314
DB00384	Yersinia infection	0.05934
DB00594	Adenoid cystic cancer	0.16667
DB00594	Alzheimer's disease	0.02399
DB00594	Anxiety disorder	0.19245
DB00594	Asthma	0.02722
DB00594	Atherosclerosis	0.02334
DB00594	Brain tumor	0.02677
DB00594	Breast cancer	0.01604
DB00594	Bronchiectasis	0.27217
DB00594	Cancer	0.01229
DB00594	Celiac disease	0.0548
DB00594	Cervical cancer	0.03875
DB00594	Chronic obstructive airway disease	0.03727
DB00594	Cystic fibrosis	0.14286
DB00594	Dermatitis	0.0303
DB00594	Endometriosis	0.02768
DB00594	Hamman-Rich syndrome	0.06415
DB00594	Hypertension	0.0527
DB00594	Ischemia	0.08536
DB00594	Kidney disease	0.07968
DB00594	Late pregnancy	0.09623
DB00594	Melanoma	0.05556
DB00594	Mitral valve disease	0.14907
DB00594	Oral cancer	0.04536
DB00594	Parkinson disease	0.03681
DB00594	Peptic ulcer	0.07857
DB00594	Periodontal disease	0.07857
DB00594	Polyarthritis	0.03799
DB00594	Prostate cancer	0.0174
DB00594	Rabies	0.03704

DB00594	Respiratory failure	0.11785
DB00594	Rheumatoid arthritis	0.0204
DB00594	Stomach cancer	0.02807
DB00594	Stroke	0.03774
DB00594	Systemic scleroderma	0.03494
DB00594	Ulcerative colitis	0.06876
DB00594	Yersinia infection	0.03956
DB00048	Pemphigoid, Bullous	0.09706
DB00048	Pleural effusion, Malignant	0.09933
DB00048	Purpura, Thrombocytopenic, Idiopathic	0.09276
DB00048	Alopecia	0.11444
DB00048	Alzheimer's disease	0.05913
DB00048	Aortic aneurysm	0.11726
DB00048	Cancer	0.04268
DB00048	Depression	0.0916
DB00048	Diabetes mellitus	0.05425
DB00048	Drug abuse	0.06753
DB00048	Embryoma	0.07501
DB00048	Lupus erythematosus	0.07496
DB00048	Primary hyperparathyroidism	0.11546
DB00048	Rheumatoid arthritis	0.05466
DB00048	Shigella infection	0.12232
DB00048	Sickle cell disease	0.10906
DB00048	Systemic infection	0.10058
DB00048	Thalassemia	0.11434
DB00309	Abortion	0.02286
DB00309	Adenovirus infection	0.06738
DB00309	Alzheimer's disease	0.01072
DB00309	Aortic aneurysm	0.02127
DB00309	Aplastic anemia	0.0498
DB00309	Brain tumor	0.02018
DB00309	Breast cancer	0.06373
DB00309	Bronchial disease	0.07653
DB00309	Cancer	0.03965
DB00309	Carcinoma	0.03517
DB00309	Cervical cancer	0.0307
DB00309	Cholestasis	0.0723
DB00309	Colon cancer	0.01354
DB00309	Diabetes mellitus	0.00984
DB00309	Embryoma	0.08833
DB00309	HIV infection	0.01324
DB00309	Infection	0.06899
DB00309	Keratoconjunctivitis Sicca	0.09991
DB00309	Leukemia	0.01098
DB00309	Leukoencephalopathy	0.01849
DB00309	Liver cancer	0.03203
DB00309	Lung cancer	0.01384

DB00309	Lung disease	0.02743
DB00309	Mental retardation	0.05099
DB00309	Muscular dystrophies	0.03309
DB00309	Neuroblastoma	0.02397
DB00309	Osteosarcoma	0.03514
DB00309	Pancreas cancer	0.03319
DB00309	Pancreas disease	0.06967
DB00309	Pre-Eclampsia	0.03264
DB00309	Prostate cancer	0.01798
DB00309	Stomach cancer	0.09103
DB00309	Testicular tumor	0.14898
DB00309	Tuberous sclerosis	0.02047
DB00309	Virus disease	0.02205
DB01229	AIDS	0.11497
DB01229	Abortion	0.00837
DB01229	Adenovirus infection	0.0778
DB01229	Alzheimer's disease	0.16439
DB01229	Amyotrophic lateral sclerosis	0.05505
DB01229	Aortic aneurysm	0.00778
DB01229	Aplastic anemia	0.01822
DB01229	Asthma	0.23534
DB01229	Autistic disorder	0.32876
DB01229	Autoimmune disease	0.32358
DB01229	Behavior disease	0.06299
DB01229	Brain disease	0.07332
DB01229	Brain ischemia	1.0266
DB01229	Brain tumor	0.00738
DB01229	Breast cancer	0.02332
DB01229	Bronchial disease	0.028
DB01229	Cancer	0.1295
DB01229	Carcinoma	0.01287
DB01229	Celiac disease	0.45986
DB01229	Central nervous system disease	0.10206
DB01229	Cervical cancer	0.01123
DB01229	Cholestasis	0.02645
DB01229	Colon cancer	0.00495
DB01229	Congenital abnormality	0.18036
DB01229	Dental plaque	0.40611
DB01229	Depression	0.05361
DB01229	Diabetes mellitus	0.02479
DB01229	Down syndrome	0.04652
DB01229	Drug abuse	0.18795
DB01229	Eating disorder	0.32904
DB01229	Embryoma	0.03232
DB01229	Endometrium cancer	0.09901
DB01229	Enteritis	0.04481
DB01229	Epilepsy	0.05774

DB01229	Epstein-Barr virus infection	0.51723
DB01229	Esotropia	0.52698
DB01229	Eye cancer	0.7958
DB01229	Glaucoma	0.42073
DB01229	Gram-Negative bacterial infection	0.15269
DB01229	Graves' disease	0.5783
DB01229	HIV infection	0.20582
DB01229	Heart failure	0.04352
DB01229	Herpes	0.24389
DB01229	Hydrocephalus	0.13608
DB01229	Immunologic deficiency syndrome	0.05755
DB01229	Infection	0.02524
DB01229	Intracranial hypertension	0.1221
DB01229	Ischemia	0.39821
DB01229	Keratoconjunctivitis Sicca	0.03655
DB01229	Keratosis	0.54194
DB01229	Leukemia	0.00402
DB01229	Leukoencephalopathy	0.00677
DB01229	Lichen planus	0.93313
DB01229	Liver cancer	0.01172
DB01229	Lung cancer	0.00506
DB01229	Lung disease	0.01004
DB01229	Lupus erythematosus	0.20106
DB01229	Lymphoma	0.06563
DB01229	Mental retardation	0.01865
DB01229	Muscular dystrophies	0.01211
DB01229	Myopathy	0.04951
DB01229	Nervous system disease	0.10541
DB01229	Neuroblastoma	0.00877
DB01229	Neurodegenerative disorder	0.13328
DB01229	Neuropathy	0.06537
DB01229	Oral cancer	0.05556
DB01229	Osteosarcoma	0.0999
DB01229	Pancreas cancer	0.01214
DB01229	Pancreas disease	0.02549
DB01229	Pre-Eclampsia	0.01194
DB01229	Prion disease	0.16667
DB01229	Prostate cancer	0.00658
DB01229	Rheumatism	0.09623
DB01229	Rheumatoid arthritis	0.14616
DB01229	Schistosomiasis	0.91688
DB01229	Schizophrenia	0.03104
DB01229	Stomach cancer	0.0333
DB01229	Stroke	0.2318
DB01229	Systemic scleroderma	0.03129
DB01229	Testicular tumor	0.05451
DB01229	Thyroid gland disease	0.53925

DB01229	Tuberculosis	0.43681
DB01229	Tuberous sclerosis	0.00749
DB01229	Ulcerative colitis	0.04211
DB01229	Vaccinia	0.10232
DB01229	Virus disease	0.00807
DB01248	AIDS	0.11497
DB01248	Abortion	0.00837
DB01248	Adenovirus infection	0.02465
DB01248	Alzheimer's disease	0.16719
DB01248	Amyotrophic lateral sclerosis	0.0603
DB01248	Aortic aneurysm	0.00778
DB01248	Aplastic anemia	0.01822
DB01248	Asthma	0.23852
DB01248	Autistic disorder	0.33348
DB01248	Autoimmune disease	0.32781
DB01248	Behavior disease	0.06901
DB01248	Brain disease	0.08032
DB01248	Brain ischemia	1.04402
DB01248	Brain tumor	0.00738
DB01248	Breast cancer	0.02332
DB01248	Bronchial disease	0.028
DB01248	Cancer	0.13237
DB01248	Carcinoma	0.01287
DB01248	Celiac disease	0.46626
DB01248	Central nervous system disease	0.1118
DB01248	Cervical cancer	0.01123
DB01248	Cholestasis	0.02645
DB01248	Colon cancer	0.00495
DB01248	Congenital abnormality	0.1833
DB01248	Dental plaque	0.416
DB01248	Depression	0.05872
DB01248	Diabetes mellitus	0.02479
DB01248	Down syndrome	0.05096
DB01248	Drug abuse	0.1916
DB01248	Eating disorder	0.33583
DB01248	Embryoma	0.03232
DB01248	Epstein-Barr virus infection	0.52573
DB01248	Esotropia	0.53823
DB01248	Eye cancer	0.82335
DB01248	Glaucoma	0.42723
DB01248	Gram-Negative bacterial infection	0.15269
DB01248	Graves' disease	0.58519
DB01248	HIV infection	0.20956
DB01248	Heart failure	0.04767
DB01248	Herpes	0.24945
DB01248	Hydrocephalus	0.14907
DB01248	Immunologic deficiency syndrome	0.05755

DB01248	Infection	0.02524
DB01248	Intracranial hypertension	0.1221
DB01248	Ischemia	0.40819
DB01248	Keratoconjunctivitis Sicca	0.03655
DB01248	Keratosis	0.55493
DB01248	Leukemia	0.00402
DB01248	Leukoencephalopathy	0.00677
DB01248	Lichen planus	0.9469
DB01248	Liver cancer	0.01172
DB01248	Lung cancer	0.00506
DB01248	Lung disease	0.01004
DB01248	Lupus erythematosus	0.20439
DB01248	Lymphoma	0.06563
DB01248	Mental retardation	0.01865
DB01248	Muscular dystrophies	0.01211
DB01248	Myopathy	0.05423
DB01248	Nervous system disease	0.11547
DB01248	Neuroblastoma	0.00877
DB01248	Neurodegenerative disorder	0.13968
DB01248	Neuropathy	0.07161
DB01248	Oral cancer	0.06086
DB01248	Osteosarcoma	0.01286
DB01248	Pancreas cancer	0.01214
DB01248	Pancreas disease	0.02549
DB01248	Pre-Eclampsia	0.01194
DB01248	Prion disease	0.18257
DB01248	Prostate cancer	0.00658
DB01248	Rheumatism	0.10541
DB01248	Rheumatoid arthritis	0.14854
DB01248	Schistosomiasis	0.93636
DB01248	Schizophrenia	0.034
DB01248	Stomach cancer	0.0333
DB01248	Stroke	0.23621
DB01248	Systemic scleroderma	0.03129
DB01248	Testicular tumor	0.05451
DB01248	Thyroid gland disease	0.54819
DB01248	Tuberculosis	0.44206
DB01248	Tuberous sclerosis	0.00749
DB01248	Vaccinia	0.10232
DB01248	Virus disease	0.00807
DB01394	Abortion	0.01537
DB01394	Adenovirus infection	0.0453
DB01394	Alzheimer's disease	0.00721
DB01394	Aortic aneurysm	0.0143
DB01394	Aplastic anemia	0.03349
DB01394	Bipolar disorder	0.05638
DB01394	Brain tumor	0.01357

DB01394	Breast cancer	0.12646
DB01394	Bronchial disease	0.05146
DB01394	Cancer	0.05923
DB01394	Carcinoma	0.02365
DB01394	Cervical cancer	0.02064
DB01394	Cholestasis	0.04861
DB01394	Clear cell carcinoma	0.29014
DB01394	Colon cancer	0.0091
DB01394	Diabetes mellitus	0.02252
DB01394	Down syndrome	0.04
DB01394	Embryoma	0.05939
DB01394	Female reproductive cancer	0.05523
DB01394	Gastritis	0.03562
DB01394	HIV infection	0.03959
DB01394	Infection	0.04639
DB01394	Keratoconjunctivitis Sicca	0.06718
DB01394	Kidney cancer	0.12795
DB01394	Leukemia	0.00739
DB01394	Leukoencephalopathy	0.01243
DB01394	Liver cancer	0.16929
DB01394	Lung cancer	0.00931
DB01394	Lung disease	0.01844
DB01394	Mental retardation	0.09385
DB01394	Muscular dystrophies	0.02225
DB01394	Neuroblastoma	0.01612
DB01394	Osteosarcoma	0.02363
DB01394	Pancreas cancer	0.02231
DB01394	Pancreas disease	0.04684
DB01394	Pre-Eclampsia	0.02194
DB01394	Prostate cancer	0.02993
DB01394	Retinitis pigmentosa	0.08783
DB01394	Stomach cancer	0.0612
DB01394	Testicular dysfunction	0.02977
DB01394	Testicular tumor	0.10017
DB01394	Tuberous sclerosis	0.01376
DB01394	Virus disease	0.01483
DB01394	Yersinia infection	0.02813
DB06772	Abortion	0.0173
DB06772	Adenovirus infection	0.05098
DB06772	Alzheimer's disease	0.00811
DB06772	Aortic aneurysm	0.01609
DB06772	Aplastic anemia	0.03768
DB06772	Brain tumor	0.01527
DB06772	Breast cancer	0.1205
DB06772	Bronchial disease	0.05791
DB06772	Cancer	0.07864
DB06772	Carcinoma	0.02662

DB06772	Cervical cancer	0.02323
DB06772	Cholestasis	0.0547
DB06772	Colon cancer	0.01025
DB06772	Diabetes mellitus	0.00744
DB06772	Embryoma	0.06684
DB06772	HIV infection	0.01002
DB06772	Infection	0.05221
DB06772	Keratoconjunctivitis Sicca	0.0756
DB06772	Leukemia	0.00831
DB06772	Leukoencephalopathy	0.01399
DB06772	Liver cancer	0.08073
DB06772	Lung cancer	0.01047
DB06772	Lung disease	0.02076
DB06772	Mental retardation	0.03858
DB06772	Muscular dystrophies	0.02504
DB06772	Neuroblastoma	0.01814
DB06772	Osteosarcoma	0.02659
DB06772	Pancreas cancer	0.02511
DB06772	Pancreas disease	0.05272
DB06772	Pre-Eclampsia	0.0247
DB06772	Prostate cancer	0.01361
DB06772	Stomach cancer	0.06888
DB06772	Testicular tumor	0.11273
DB06772	Tuberous sclerosis	0.01549
DB06772	Virus disease	0.01669
DB00120	Pemphigoid, Bullous	0.14034
DB00120	Pleural effusion, Malignant	0.14363
DB00120	Purpura, Thrombocytopenic, Idiopathic	0.13412
DB00120	Alopecia	0.16547
DB00120	Alzheimer's disease	0.08549
DB00120	Aortic aneurysm	0.16955
DB00120	Cancer	0.05189
DB00120	Depression	0.13245
DB00120	Diabetes mellitus	0.07844
DB00120	Drug abuse	0.09765
DB00120	Encephalopathies	0.05293
DB00120	Lupus erythematosus	0.10839
DB00120	Neoplasm metastasis	0.0588
DB00120	Primary hyperparathyroidism	0.16695
DB00120	Rheumatoid arthritis	0.07903
DB00120	Shigella infection	0.17687
DB00120	Sickle cell disease	0.1577
DB00120	Systemic infection	0.14543
DB00120	Thalassemia	0.16534
DB00138	Kidney disease	0.17928
DB00740	Breast cancer	0.03402
DB00740	Congenital abnormality	0.0533

DB00740	Drug abuse	0.06623
DB00740	Heart disease	0.2132
DB00740	Intestinal disease	0.17678
DB00740	Ischemia	0.09054
DB00740	Long QT syndrome	0.20412
DB00740	Sudden infant death syndrome	0.1543
DB00162	Amaurosis congenita of leber I	0.08839
DB00162	Cancer	0.00922
DB00162	Cervical cancer	0.02906
DB00162	Cirrhosis	0.04226
DB00162	Common cold	0.06682
DB00162	Dermatitis	0.02273
DB00162	Drug abuse	0.02341
DB00162	Eye disease	0.04642
DB00162	Liver cancer	0.0194
DB00162	Renal Cell cancer	0.02906
DB00162	Retinal disease	0.07001
DB00162	Retinitis pigmentosa	0.05
DB00162	Rheumatoid arthritis	0.0306
DB00162	Squamous cell cancer	0.02552
DB00162	Thyroid cancer	0.04352
DB00162	Uterine fibroids	0.08839
DB00162	Uveitis	0.0625
DB00030	Hemorrhagic fevers, Viral	0.04888
DB00030	Pemphigoid, Bullous	0.0463
DB00030	Pleural effusion, Malignant	0.04738
DB00030	Purpura, Thrombocytopenic, Idiopathic	0.04425
DB00030	Adenovirus infection	0.2888
DB00030	Adrenal gland hyperfunction	0.06007
DB00030	Alopecia	0.02534
DB00030	Alzheimer's disease	0.11421
DB00030	Aortic aneurysm	0.03671
DB00030	Aplastic anemia	0.04053
DB00030	Asthma	0.02329
DB00030	Atherosclerosis	0.04879
DB00030	Autistic disorder	0.0322
DB00030	Barrett's esophagus	0.0527
DB00030	Bone metastases	0.06701
DB00030	Brain disease	0.05185
DB00030	Breast cancer	0.03572
DB00030	Cancer	0.17652
DB00030	Carcinoma	0.02217
DB00030	Cervical cancer	0.03747
DB00030	Charcot-Marie-Tooth disease	0.06027
DB00030	Cockayne syndrome	0.038
DB00030	Colon cancer	0.04844
DB00030	Congenital abnormality	0.25353

DB00030	Creutzfeldt-Jakob syndrome	0.14434
DB00030	Depression	0.05818
DB00030	Diabetes mellitus	0.07146
DB00030	Drug abuse	0.03222
DB00030	Eating disorder	0.05025
DB00030	Embryoma	0.0356
DB00030	Endometriosis	0.26985
DB00030	Eye cancer	0.98984
DB00030	Gastritis	0.47192
DB00030	Graves' disease	0.05103
DB00030	HIV infection	0.0889
DB00030	Heart disease	0.08688
DB00030	Hemolytic-Uremic syndrome	0.02853
DB00030	Hemorrhagic disorder	0.03826
DB00030	Hereditary disease	0.04923
DB00030	Herpes	0.0214
DB00030	Infection	0.03737
DB00030	Infertility	0.0572
DB00030	Intermediate coronary syndrome	0.04876
DB00030	Keratosis	0.09623
DB00030	Kidney disease	0.0345
DB00030	Leukemia	0.02748
DB00030	Leukoencephalopathy	0.02606
DB00030	Lipodystrophy	0.04185
DB00030	Liver metastases	0.16013
DB00030	Lung cancer	0.03706
DB00030	Lupus erythematosus	0.03576
DB00030	Lymphoma	0.0658
DB00030	Malignant glioma	0.04278
DB00030	Metabolism disease	0.02632
DB00030	Muscular atrophy	0.04291
DB00030	Muscular dystrophies	0.0295
DB00030	Myopathy	0.01755
DB00030	Neuropathy	0.07085
DB00030	Obesity	0.02221
DB00030	Oral cancer	0.03928
DB00030	Osteoporosis	0.04564
DB00030	Ovarian cancer	0.02811
DB00030	Parkinson disease	0.49243
DB00030	Penile disease	0.04575
DB00030	Pertussis	0.06891
DB00030	Polyarthritis	0.0193
DB00030	Primary hyperparathyroidism	0.02556
DB00030	Primary tumor	0.02005
DB00030	Prostate cancer	0.04281
DB00030	Protein-energy malnutrition	0.03256
DB00030	Rabies	0.04694

DB00030	Renal tubular acidosis	0.03535
DB00030	Retinal disease	0.04042
DB00030	Rheumatoid arthritis	0.24915
DB00030	Sella turcica tumor	0.10911
DB00030	Septicemia	0.1291
DB00030	Severe acute respiratory syndrome	0.80439
DB00030	Shigella infection	0.02708
DB00030	Sickle cell disease	0.0797
DB00030	Squamous cell cancer	0.03345
DB00030	Stroke	0.0214
DB00030	Subarachnoid hemorrhage	0.04364
DB00030	Systemic infection	0.02227
DB00030	Takayasu's arteritis	0.04081
DB00030	Temporal arteritis	0.04771
DB00030	Thalassemia	0.02531
DB00030	Thrombocytopenia	0.03636
DB00030	Thyroid gland disease	0.06623
DB00030	Virus disease	0.01966
DB00046	Atherosclerosis	0.04951
DB00046	Cancer	0.02606
DB00046	Depression	0.09285
DB00046	Graves' disease	0.125
DB00046	Infertility	0.09713
DB00046	Oral cancer	0.09623
DB00046	Osteoporosis	0.1118
DB00046	Retinal disease	0.09901
DB00046	Septicemia	0.31623
DB00046	Sickle cell disease	0.13608
DB00047	Atherosclerosis	0.04951
DB00047	Cancer	0.02606
DB00047	Depression	0.09285
DB00047	Graves' disease	0.125
DB00047	Infertility	0.09713
DB00047	Oral cancer	0.09623
DB00047	Osteoporosis	0.1118
DB00047	Retinal disease	0.09901
DB00047	Septicemia	0.31623
DB00047	Sickle cell disease	0.13608
DB00071	Hemorrhagic fevers, Viral	0.04888
DB00071	Pemphigoid, Bullous	0.0463
DB00071	Pleural effusion, Malignant	0.04738
DB00071	Purpura, Thrombocytopenic, Idiopathic	0.04425
DB00071	Adenovirus infection	0.28601
DB00071	Adrenal gland hyperfunction	0.06007
DB00071	Alopecia	0.02534
DB00071	Alzheimer's disease	0.11267
DB00071	Aortic aneurysm	0.03671

DB00071	Aplastic anemia	0.09626
DB00071	Asthma	0.04512
DB00071	Atherosclerosis	0.04729
DB00071	Autistic disorder	0.0322
DB00071	Autoimmune disease	0.02899
DB00071	Azoospermia	0.05455
DB00071	Barrett's esophagus	0.0488
DB00071	Bone metastases	0.06701
DB00071	Brain disease	0.048
DB00071	Breast cancer	0.03469
DB00071	Cancer	0.17415
DB00071	Carcinoma	0.02217
DB00071	Celiac disease	0.04394
DB00071	Cervical cancer	0.03747
DB00071	Charcot-Marie-Tooth disease	0.06027
DB00071	Cockayne syndrome	0.038
DB00071	Colon cancer	0.04844
DB00071	Common wart	0.11952
DB00071	Congenital abnormality	0.25192
DB00071	Creutzfeldt-Jakob syndrome	0.13363
DB00071	Depression	0.05537
DB00071	Dermatitis	0.0243
DB00071	Diabetes mellitus	0.08214
DB00071	Drug abuse	0.03222
DB00071	Eating disorder	0.04652
DB00071	Embryoma	0.03296
DB00071	Endometriosis	0.26807
DB00071	Enteritis	0.02934
DB00071	Eye cancer	0.9747
DB00071	Gastritis	0.46787
DB00071	Graves' disease	0.04725
DB00071	HIV infection	0.0889
DB00071	Heart disease	0.08688
DB00071	Hemolytic-Uremic syndrome	0.02853
DB00071	Hemorrhagic disorder	0.03826
DB00071	Hereditary disease	0.04923
DB00071	Herpes	0.0214
DB00071	Hodgkin's disease	0.04725
DB00071	Infection	0.03537
DB00071	Infertility	0.05426
DB00071	Intermediate coronary syndrome	0.04876
DB00071	Keratosis	0.08909
DB00071	Kidney disease	0.03194
DB00071	Leukemia	0.02748
DB00071	Leukoencephalopathy	0.02606
DB00071	Lipodystrophy	0.04185
DB00071	Liver cancer	0.02074

DB00071	Liver metastases	0.14825
DB00071	Lung cancer	0.03556
DB00071	Lupus erythematosus	0.05859
DB00071	Lymphoma	0.06091
DB00071	Malignant glioma	0.04278
DB00071	Metabolism disease	0.02632
DB00071	Muscular atrophy	0.04291
DB00071	Muscular dystrophies	0.0295
DB00071	Myopathy	0.01755
DB00071	Narcolepsy	0.10102
DB00071	Neuropathy	0.06743
DB00071	Obesity	0.02056
DB00071	Oral cancer	0.03637
DB00071	Osteoporosis	0.04226
DB00071	Ovarian cancer	0.02811
DB00071	Parkinson disease	0.49006
DB00071	Penile disease	0.04575
DB00071	Pertussis	0.06891
DB00071	Polyarthritis	0.0193
DB00071	Primary hyperparathyroidism	0.02556
DB00071	Primary tumor	0.02005
DB00071	Prostate cancer	0.04057
DB00071	Protein-energy malnutrition	0.03256
DB00071	Rabies	0.04694
DB00071	Renal tubular acidosis	0.03535
DB00071	Retinal disease	0.03742
DB00071	Rheumatic fever	0.06901
DB00071	Rheumatoid arthritis	0.24653
DB00071	Schizophrenia	0.02032
DB00071	Sella turcica tumor	0.10102
DB00071	Septicemia	0.11952
DB00071	Severe acute respiratory syndrome	0.79762
DB00071	Shigella infection	0.02708
DB00071	Sickle cell disease	0.07558
DB00071	Silicosis	0.10102
DB00071	Squamous cell cancer	0.03345
DB00071	Stomach cancer	0.02251
DB00071	Stroke	0.0214
DB00071	Subarachnoid hemorrhage	0.04364
DB00071	Systemic infection	0.02227
DB00071	Takayasu's arteritis	0.04081
DB00071	Temporal arteritis	0.04771
DB00071	Thalassemia	0.08831
DB00071	Thrombocytopenia	0.03636
DB00071	Thyroid gland disease	0.06131
DB00071	Virus disease	0.01966
DB01277	Skin disease, Genetic	0.21795

DB01277	Acromegaly	0.87377
DB01277	Advanced cancer	0.21687
DB01277	Alopecia	0.08092
DB01277	Alzheimer's disease	0.11961
DB01277	Amyotrophic lateral sclerosis	0.06866
DB01277	Antiphospholipid syndrome	0.70711
DB01277	Atherosclerosis	0.1259
DB01277	Bone disease	0.19291
DB01277	Breast cancer	0.05109
DB01277	Cancer	0.09797
DB01277	Cardiovascular disease	0.19374
DB01277	Cerebral palsy	0.88388
DB01277	Cervical cancer	0.11969
DB01277	Cirrhosis	0.38179
DB01277	Colon cancer	0.0528
DB01277	Depression	0.06565
DB01277	Diabetes mellitus	0.12935
DB01277	Embryoma	0.05304
DB01277	Endocrine system disease	0.19332
DB01277	Endometriosis	0.11311
DB01277	Esophagus cancer	0.09433
DB01277	Esotropia	0.1305
DB01277	Fibroid tumor	0.39377
DB01277	Fibromyalgia	0.88388
DB01277	Folic acid deficiency	0.70711
DB01277	Gastrointestinal stromal tumor	0.70711
DB01277	Gestational diabetes	0.65085
DB01277	Graves' disease	0.08839
DB01277	Growth retardation	0.35753
DB01277	Hamman-Rich syndrome	0.25132
DB01277	Heart failure	0.09117
DB01277	Hepatitis C	0.1259
DB01277	Hyperglycemia	0.15525
DB01277	Hyperthyroidism	0.70711
DB01277	Hypothyroidism	0.60437
DB01277	Infertility	0.06868
DB01277	Late pregnancy	0.2333
DB01277	Leukemia	0.04283
DB01277	Liver cancer	0.07211
DB01277	Liver disease	0.37153
DB01277	Lung cancer	0.05396
DB01277	Metabolic syndrome X	0.88388
DB01277	Obesity	0.13874
DB01277	Oral cancer	0.06804
DB01277	Osteoporosis	0.18714
DB01277	Osteosarcoma	0.137
DB01277	Polycystic kidney	0.20359

DB01277	Polycythemia	0.17229
DB01277	Primary tumor	0.12404
DB01277	Prostate cancer	0.04047
DB01277	Protein-energy malnutrition	0.20141
DB01277	Pulmonary fibrosis	0.16833
DB01277	Rabies	0.06492
DB01277	Renal Cell cancer	0.12404
DB01277	Retinal disease	0.07001
DB01277	Schizophrenia	0.06454
DB01277	Septicemia	0.22361
DB01277	Sickle cell disease	0.30151
DB01277	Solid tumor	0.18542
DB01277	Spinal cord disease	0.22611
DB01277	Stroke	0.1158
DB01277	Testicular dysfunction	0.07183
DB01277	Thalassemia	0.08085
DB08912	Skin disease, Genetic	0.55586
DB08912	Actinic keratosis	0.07312
DB08912	Adenoid cystic cancer	0.13667
DB08912	Alzheimer's disease	0.20149
DB08912	Angiomyolipoma	0.6122
DB08912	Brain disease	0.0354
DB08912	Breast cancer	0.03139
DB08912	Cancer	0.16028
DB08912	Common cold	0.51389
DB08912	Cystic fibrosis	0.046
DB08912	Dental plaque	0.02636
DB08912	Down syndrome	0.03404
DB08912	Drug abuse	0.01459
DB08912	Eating disorder	0.02514
DB08912	Embryoma	0.03321
DB08912	Endometriosis	0.01547
DB08912	Gram-Negative bacterial infection	0.065
DB08912	Herpes	0.29352
DB08912	Liver cancer	0.01971
DB08912	Lung cancer	0.01554
DB08912	Melanoma	0.0247
DB08912	Metaplastic polyp	0.45986
DB08912	Myopathy	0.31079
DB08912	Nephrosis	0.41828
DB08912	Neuroblastoma	0.02554
DB08912	Neurodegenerative disorder	0.03272
DB08912	Osteomyelitis	0.79068
DB08912	Pancreas disease	0.083
DB08912	Pancreatitis	0.03615
DB08912	Penile disease	0.05469
DB08912	Polycystic ovary syndrome	0.0277

DB08912	Prion disease	0.06076
DB08912	Prostate cancer	0.03441
DB08912	Squamous cell cancer	0.0292
DB08912	Stomach cancer	0.0141
DB08912	Systemic scleroderma	0.0173
DB08912	Testicular dysfunction	0.25596
DB08912	Thyroid gland disease	0.43853
DB08912	Ulcerative colitis	0.21161
DB08912	Vitamin D deficiency	0.90313
DB05777	Abortion	0.17678
DB05777	Abruption placentae	0.25
DB05777	Aseptic necrosis of bone	0.44721
DB05777	Behcet syndrome	0.20203
DB05777	Cardiovascular disease	0.21567
DB05777	Cerebral palsy	0.25
DB05777	Cerebrovascular disorder	0.1543
DB05777	Deafness	0.10206
DB05777	Dental plaque	0.0898
DB05777	Diabetes mellitus	0.07443
DB05777	Embryoma	0.0872
DB05777	Endometriosis	0.05872
DB05777	Enteritis	0.15523
DB05777	Hemorrhagic disorder	0.25
DB05777	Hyperhomocysteinemia	0.40825
DB05777	Infertility	0.09713
DB05777	Liver cancer	0.05488
DB05777	Liver disease	0.21822
DB05777	Lupus erythematosus	0.06041
DB05777	Malignant glioma	0.13131
DB05777	Neoplasm metastasis	0.05754
DB05777	Nephrosis	0.15811
DB05777	Nevus	0.25
DB05777	Polycystic ovary syndrome	0.09366
DB05777	Pulmonary embolism	0.28868
DB05777	Pulmonary fibrosis	0.13363
DB05777	Retinal disease	0.09901
DB05777	Sickle cell disease	0.13608
DB05777	Systemic infection	0.16222
DB05777	Systemic scleroderma	0.14825
DB05777	Thrombophilia	0.33333
DB00147	Alzheimer's disease	0.07359
DB00147	Azoospermia	0.22618
DB00147	Barrett's esophagus	0.15643
DB00147	Breast cancer	0.06359
DB00147	Cancer	0.046
DB00147	Colon cancer	0.04647
DB00147	Endometriosis	0.0891

DB00147	Esophageal disease	0.45119
DB00147	Heart failure	0.16048
DB00147	Ischemia	0.16488
DB00147	Oral cancer	0.12254
DB00147	Peptic esophagitis	0.37207
DB00147	Rheumatoid arthritis	0.06803
DB00147	Skin cancer	0.20475
DB00147	Squamous cell cancer	0.09404
DB00147	Stomach cancer	0.09082
DB00147	Stroke	0.10419
DB00147	Testicular dysfunction	0.12643
DB00147	Werner syndrome	0.22282
DB00165	Alzheimer's disease	0.07359
DB00165	Azoospermia	0.22618
DB00165	Barrett's esophagus	0.15643
DB00165	Breast cancer	0.06359
DB00165	Cancer	0.046
DB00165	Colon cancer	0.04647
DB00165	Endometriosis	0.0891
DB00165	Esophageal disease	0.45119
DB00165	Heart failure	0.16048
DB00165	Ischemia	0.16488
DB00165	Oral cancer	0.12254
DB00165	Peptic esophagitis	0.37207
DB00165	Rheumatoid arthritis	0.06803
DB00165	Skin cancer	0.20475
DB00165	Squamous cell cancer	0.09404
DB00165	Stomach cancer	0.09082
DB00165	Stroke	0.10419
DB00165	Testicular dysfunction	0.12643
DB00165	Werner syndrome	0.22282
DB00559	Atherosclerosis	0.09901
DB00559	Brain tumor	0.0568
DB00559	Breast cancer	0.06804
DB00559	Bronchial disease	0.1715
DB00559	Capillaries disease	0.22361
DB00559	Diabetes mellitus	0.03722
DB00559	Down syndrome	0.08058
DB00559	Glaucoma	0.11785
DB00559	Heart failure	0.15076
DB00559	Hypertension	0.0559
DB00559	Intraocular melanoma	0.22361
DB00559	Kaposi sarcoma	0.39223
DB00559	Leukemia	0.03984
DB00559	Lung cancer	0.04939
DB00559	Nasopharyngeal cancer	0.13608
DB00559	Neoplasm metastasis	0.05754

DB00559	Ovarian cancer	0.07107
DB00559	Pancreatitis	0.11043
DB00559	Polycystic kidney	0.18898
DB00559	Pre-Eclampsia	0.08333
DB00559	Primary tumor	0.125
DB00559	Prostate cancer	0.03691
DB00559	Renal Cell cancer	0.1644
DB00559	Retinal disease	0.09901
DB00559	Squamous cell cancer	0.07217
DB06268	Atherosclerosis	0.09901
DB06268	Brain tumor	0.0568
DB06268	Breast cancer	0.06804
DB06268	Bronchial disease	0.1715
DB06268	Capillaries disease	0.22361
DB06268	Diabetes mellitus	0.03722
DB06268	Down syndrome	0.08058
DB06268	Glaucoma	0.11785
DB06268	Heart failure	0.15076
DB06268	Hypertension	0.0559
DB06268	Intraocular melanoma	0.22361
DB06268	Kaposi sarcoma	0.39223
DB06268	Leukemia	0.03984
DB06268	Lung cancer	0.04939
DB06268	Nasopharyngeal cancer	0.13608
DB06268	Neoplasm metastasis	0.05754
DB06268	Ovarian cancer	0.07107
DB06268	Pancreatitis	0.11043
DB06268	Polycystic kidney	0.18898
DB06268	Pre-Eclampsia	0.08333
DB06268	Primary tumor	0.125
DB06268	Prostate cancer	0.03691
DB06268	Renal Cell cancer	0.1644
DB06268	Retinal disease	0.09901
DB06268	Squamous cell cancer	0.07217
DB08932	Atherosclerosis	0.09901
DB08932	Brain tumor	0.0568
DB08932	Breast cancer	0.06804
DB08932	Bronchial disease	0.1715
DB08932	Capillaries disease	0.22361
DB08932	Diabetes mellitus	0.03722
DB08932	Down syndrome	0.08058
DB08932	Glaucoma	0.11785
DB08932	Heart failure	0.15076
DB08932	Hypertension	0.0559
DB08932	Intraocular melanoma	0.22361
DB08932	Kaposi sarcoma	0.39223
DB08932	Leukemia	0.03984

DB08932	Lung cancer	0.04939
DB08932	Nasopharyngeal cancer	0.13608
DB08932	Neoplasm metastasis	0.05754
DB08932	Ovarian cancer	0.07107
DB08932	Pancreatitis	0.11043
DB08932	Polycystic kidney	0.18898
DB08932	Pre-Eclampsia	0.08333
DB08932	Primary tumor	0.125
DB08932	Prostate cancer	0.03691
DB08932	Renal Cell cancer	0.1644
DB08932	Retinal disease	0.09901
DB08932	Squamous cell cancer	0.07217
DB00185	Supranuclear palsy, progressive	0.40825
DB00185	Asthma	0.11547
DB00185	Diabetes mellitus	0.03722
DB00185	Neuroblastoma	0.19426
DB00185	Schizophrenia	0.05376
DB00193	Hypertension, Pulmonary	0.08111
DB00193	Anorexia nervosa	0.21651
DB00193	Asthma	0.02887
DB00193	Atherosclerosis	0.02475
DB00193	Autistic disorder	0.04287
DB00193	Behavior disease	0.16366
DB00193	Bipolar disorder	0.1201
DB00193	Chronic fatigue syndrome	0.125
DB00193	Chronic obstructive airway disease	0.03953
DB00193	Colon cancer	0.02098
DB00193	Congenital heart disease	0.11785
DB00193	Depression	0.04642
DB00193	Dermatitis	0.03214
DB00193	Diabetes mellitus	0.03722
DB00193	Drug abuse	0.06623
DB00193	Epilepsy	0.05
DB00193	Fibromyalgia	0.125
DB00193	Generalized anxiety disorder	0.10206
DB00193	Heart failure	0.03769
DB00193	Herpes	0.05051
DB00193	Hypertension	0.0559
DB00193	Migraine	0.13131
DB00193	Multiple endocrine neoplasia	0.11785
DB00193	Neuroblastoma	0.04856
DB00193	Neuroendocrine tumor	0.125
DB00193	Neurotic disorder	0.1118
DB00193	Obesity	0.05439
DB00193	Obsessive-compulsive disorder	0.1066
DB00193	Panic disorder	0.1543
DB00193	Pervasive development disorder	0.09129

DB00193	Psychotic disorder	0.05661
DB00193	Pulmonary hypertension	0.17678
DB00193	Stroke	0.04003
DB00193	Sudden infant death syndrome	0.07715
DB00193	Ulcerative colitis	0.03647
DB00202	Supranuclear palsy, progressive	0.28868
DB00202	Asthma	0.08165
DB00202	Behavior disease	0.07715
DB00202	Bipolar disorder	0.05661
DB00202	Bladder cancer	0.06455
DB00202	Central nervous system disease	0.125
DB00202	Depression	0.06565
DB00202	Diabetes mellitus	0.02632
DB00202	Neuroblastoma	0.13736
DB00202	Schizophrenia	0.03801
DB00280	Supranuclear palsy, progressive	0.2357
DB00280	Asthma	0.16751
DB00280	Behavior disease	0.06299
DB00280	Bipolar disorder	0.04623
DB00280	Bladder cancer	0.0527
DB00280	Breast cancer	0.01964
DB00280	Central nervous system disease	0.10206
DB00280	Congenital abnormality	0.03077
DB00280	Depression	0.05361
DB00280	Diabetes mellitus	0.02149
DB00280	Drug abuse	0.07647
DB00280	Heart disease	0.12309
DB00280	Heart failure	0.04352
DB00280	Intestinal disease	0.10206
DB00280	Ischemia	0.05227
DB00280	Long QT syndrome	0.94134
DB00280	Neuroblastoma	0.11215
DB00280	Schizophrenia	0.03104
DB00280	Sudden infant death syndrome	0.08909
DB00321	Hypertension, Pulmonary	0.05006
DB00321	Supranuclear palsy, progressive	0.25198
DB00321	Abortion	0.04038
DB00321	Alzheimer's disease	0.01339
DB00321	Amyotrophic lateral sclerosis	0.28202
DB00321	Anorexia nervosa	0.24684
DB00321	Aortic aneurysm	0.02656
DB00321	Asthma	0.06482
DB00321	Atherosclerosis	0.01528
DB00321	Atopic rhinitis	0.05634
DB00321	Attention deficit hyperactivity disorder	0.23838
DB00321	Autistic disorder	0.05293
DB00321	Behavior disease	0.69259

DB00321	Bipolar disorder	0.20402
DB00321	Bladder cancer	0.02817
DB00321	Cancer	0.01771
DB00321	Central nervous system disease	0.05455
DB00321	Choriocarcinoma	0.06299
DB00321	Chronic fatigue syndrome	0.1543
DB00321	Chronic obstructive airway disease	0.0244
DB00321	Colon cancer	0.03885
DB00321	Congenital heart disease	0.07274
DB00321	Depression	0.36714
DB00321	Dermatitis	0.26637
DB00321	Diabetes mellitus	0.16328
DB00321	Drug abuse	0.08295
DB00321	Drug-Induced dyskinesia	0.08248
DB00321	Embryoma	0.03661
DB00321	Epilepsy	0.48958
DB00321	Fibromyalgia	0.07715
DB00321	Generalized anxiety disorder	0.06299
DB00321	Heart failure	0.11109
DB00321	Hepatitis C	0.45501
DB00321	Herpes	0.03117
DB00321	Hypertension	0.38871
DB00321	Infertility	0.02997
DB00321	Kidney failure	0.04942
DB00321	Leukemia	0.01372
DB00321	Long QT syndrome	0.29611
DB00321	Lymphoma	0.03107
DB00321	Migraine	0.52846
DB00321	Multiple endocrine neoplasia	0.07274
DB00321	Multiple sclerosis	0.0309
DB00321	Myopathy	0.02646
DB00321	Nervous system disease	0.13641
DB00321	Neuroblastoma	0.05995
DB00321	Neuroendocrine tumor	0.07715
DB00321	Neurotic disorder	0.06901
DB00321	Obesity	0.08248
DB00321	Obsessive-compulsive disorder	0.13159
DB00321	Pancreas cancer	0.03384
DB00321	Panic disorder	0.76198
DB00321	Parkinson disease	0.0241
DB00321	Pervasive development disorder	0.05634
DB00321	Primary tumor	0.04588
DB00321	Prostate cancer	0.01139
DB00321	Psychotic disorder	0.06989
DB00321	Pulmonary hypertension	0.10911
DB00321	Rheumatoid arthritis	0.01335
DB00321	Schizophrenia	0.13286

DB00321	Stroke	0.27292
DB00321	Sudden infant death syndrome	0.82619
DB00321	Ulcerative colitis	0.02251
DB00332	Supranuclear palsy, progressive	0.33333
DB00332	Asthma	0.09428
DB00332	Behavior disease	0.08909
DB00332	Bipolar disorder	0.06537
DB00332	Bladder cancer	0.07454
DB00332	Central nervous system disease	0.14434
DB00332	Depression	0.07581
DB00332	Diabetes mellitus	0.03039
DB00332	Neuroblastoma	0.15861
DB00332	Schizophrenia	0.0439
DB00340	Supranuclear palsy, progressive	0.5164
DB00340	Asthma	0.07303
DB00340	Behavior disease	0.06901
DB00340	Bipolar disorder	0.05064
DB00340	Bladder cancer	0.05774
DB00340	Central nervous system disease	0.1118
DB00340	Depression	0.05872
DB00340	Diabetes mellitus	0.02354
DB00340	Neuroblastoma	0.12286
DB00340	Schizophrenia	0.102
DB00342	Asthma	0.04714
DB00342	Atopic rhinitis	0.14907
DB00342	Cancer	0.02128
DB00342	Colon cancer	0.03426
DB00342	Diabetes mellitus	0.03039
DB00342	Drug abuse	0.05407
DB00342	Heart failure	0.06155
DB00342	Infertility	0.07931
DB00342	Long QT syndrome	0.16667
DB00342	Neuroblastoma	0.07931
DB00342	Parkinson disease	0.06376
DB00342	Schizophrenia	0.0439
DB00342	Sudden infant death syndrome	0.12599
DB00376	Supranuclear palsy, progressive	0.5164
DB00376	Asthma	0.07303
DB00376	Behavior disease	0.06901
DB00376	Bipolar disorder	0.05064
DB00376	Bladder cancer	0.05774
DB00376	Central nervous system disease	0.1118
DB00376	Depression	0.05872
DB00376	Diabetes mellitus	0.02354
DB00376	Neuroblastoma	0.12286
DB00376	Schizophrenia	0.102
DB00383	Supranuclear palsy, progressive	0.33333

DB00383	Asthma	0.09428
DB00383	Behavior disease	0.08909
DB00383	Bipolar disorder	0.06537
DB00383	Bladder cancer	0.07454
DB00383	Central nervous system disease	0.14434
DB00383	Depression	0.07581
DB00383	Diabetes mellitus	0.03039
DB00383	Neuroblastoma	0.15861
DB00383	Schizophrenia	0.0439
DB00387	Supranuclear palsy, progressive	0.57735
DB00387	Asthma	0.08165
DB00387	Behavior disease	0.07715
DB00387	Bipolar disorder	0.05661
DB00387	Bladder cancer	0.06455
DB00387	Central nervous system disease	0.125
DB00387	Depression	0.06565
DB00387	Diabetes mellitus	0.02632
DB00387	Neuroblastoma	0.13736
DB00387	Schizophrenia	0.07603
DB00424	Supranuclear palsy, progressive	0.57735
DB00424	Asthma	0.08165
DB00424	Behavior disease	0.07715
DB00424	Bipolar disorder	0.05661
DB00424	Bladder cancer	0.06455
DB00424	Central nervous system disease	0.125
DB00424	Depression	0.06565
DB00424	Diabetes mellitus	0.02632
DB00424	Neuroblastoma	0.13736
DB00424	Schizophrenia	0.07603
DB00434	Supranuclear palsy, progressive	0.2357
DB00434	Anorexia nervosa	0.16667
DB00434	Asthma	0.06667
DB00434	Atopic rhinitis	0.10541
DB00434	Autistic disorder	0.04951
DB00434	Behavior disease	0.18898
DB00434	Bipolar disorder	0.13868
DB00434	Bladder cancer	0.0527
DB00434	Central nervous system disease	0.10206
DB00434	Choriocarcinoma	0.11785
DB00434	Chronic fatigue syndrome	0.14434
DB00434	Colon cancer	0.04845
DB00434	Depression	0.10721
DB00434	Dermatitis	0.03711
DB00434	Diabetes mellitus	0.02149
DB00434	Drug-Induced dyskinesia	0.1543
DB00434	Hypertension	0.06455
DB00434	Infertility	0.05608

DB00434	Migraine	0.07581
DB00434	Neuroblastoma	0.11215
DB00434	Obesity	0.06281
DB00434	Obsessive-compulsive disorder	0.12309
DB00434	Panic disorder	0.08909
DB00434	Parkinson disease	0.04508
DB00434	Psychotic disorder	0.06537
DB00434	Rheumatoid arthritis	0.02498
DB00434	Schizophrenia	0.06208
DB00434	Stroke	0.04623
DB00458	Hypertension, Pulmonary	0.06131
DB00458	Supranuclear palsy, progressive	0.30861
DB00458	Anorexia nervosa	0.16366
DB00458	Asthma	0.14449
DB00458	Atherosclerosis	0.01871
DB00458	Atopic rhinitis	0.06901
DB00458	Autistic disorder	0.06482
DB00458	Behavior disease	0.16496
DB00458	Bipolar disorder	0.12105
DB00458	Bladder cancer	0.0345
DB00458	Central nervous system disease	0.06682
DB00458	Choriocarcinoma	0.07715
DB00458	Chronic fatigue syndrome	0.18898
DB00458	Chronic obstructive airway disease	0.02988
DB00458	Colon cancer	0.04758
DB00458	Congenital heart disease	0.08909
DB00458	Depression	0.10528
DB00458	Dermatitis	0.04859
DB00458	Diabetes mellitus	0.02813
DB00458	Drug abuse	0.07509
DB00458	Drug-Induced dyskinesia	0.10102
DB00458	Epilepsy	0.0378
DB00458	Fibromyalgia	0.09449
DB00458	Generalized anxiety disorder	0.07715
DB00458	Heart failure	0.05698
DB00458	Herpes	0.03818
DB00458	Hypertension	0.06339
DB00458	Infertility	0.03671
DB00458	Kidney failure	0.06052
DB00458	Long QT syndrome	0.74209
DB00458	Migraine	0.04963
DB00458	Multiple endocrine neoplasia	0.08909
DB00458	Neuroblastoma	0.07342
DB00458	Neuroendocrine tumor	0.09449
DB00458	Neurotic disorder	0.08452
DB00458	Obesity	0.04112
DB00458	Obsessive-compulsive disorder	0.16116

DB00458	Panic disorder	0.17496
DB00458	Parkinson disease	0.02951
DB00458	Pervasive development disorder	0.06901
DB00458	Prostate cancer	0.01395
DB00458	Psychotic disorder	0.08559
DB00458	Pulmonary hypertension	0.13363
DB00458	Rheumatoid arthritis	0.01636
DB00458	Schizophrenia	0.08128
DB00458	Stroke	0.06052
DB00458	Sudden infant death syndrome	0.05832
DB00458	Ulcerative colitis	0.02757
DB00462	Supranuclear palsy, progressive	0.33333
DB00462	Asthma	0.09428
DB00462	Behavior disease	0.08909
DB00462	Bipolar disorder	0.06537
DB00462	Bladder cancer	0.07454
DB00462	Central nervous system disease	0.14434
DB00462	Depression	0.07581
DB00462	Diabetes mellitus	0.03039
DB00462	Neuroblastoma	0.15861
DB00462	Schizophrenia	0.0439
DB00496	Supranuclear palsy, progressive	0.5164
DB00496	Asthma	0.07303
DB00496	Behavior disease	0.06901
DB00496	Bipolar disorder	0.05064
DB00496	Bladder cancer	0.05774
DB00496	Central nervous system disease	0.1118
DB00496	Depression	0.05872
DB00496	Diabetes mellitus	0.02354
DB00496	Neuroblastoma	0.12286
DB00496	Schizophrenia	0.102
DB00517	Supranuclear palsy, progressive	0.33333
DB00517	Asthma	0.09428
DB00517	Behavior disease	0.08909
DB00517	Bipolar disorder	0.06537
DB00517	Bladder cancer	0.07454
DB00517	Central nervous system disease	0.14434
DB00517	Depression	0.07581
DB00517	Diabetes mellitus	0.03039
DB00517	Neuroblastoma	0.15861
DB00517	Schizophrenia	0.0439
DB00540	Hypertension, Pulmonary	0.06623
DB00540	Supranuclear palsy, progressive	0.33333
DB00540	Abortion	0.05124
DB00540	Amyotrophic lateral sclerosis	0.33154
DB00540	Anorexia nervosa	0.32043
DB00540	Asthma	0.04714

DB00540	Atherosclerosis	0.02021
DB00540	Atopic rhinitis	0.07454
DB00540	Attention deficit hyperactivity disorder	0.11127
DB00540	Autistic disorder	0.07001
DB00540	Behavior disease	0.88792
DB00540	Bipolar disorder	0.20893
DB00540	Bladder cancer	0.03727
DB00540	Central nervous system disease	0.07217
DB00540	Choriocarcinoma	0.08333
DB00540	Chronic fatigue syndrome	0.20412
DB00540	Chronic obstructive airway disease	0.03227
DB00540	Colon cancer	0.05139
DB00540	Congenital heart disease	0.09623
DB00540	Depression	0.44568
DB00540	Dermatitis	0.34121
DB00540	Diabetes mellitus	0.20905
DB00540	Drug abuse	0.08152
DB00540	Drug-Induced dyskinesia	0.10911
DB00540	Epilepsy	0.57988
DB00540	Fibromyalgia	0.10206
DB00540	Generalized anxiety disorder	0.08333
DB00540	Heart failure	0.03077
DB00540	Hepatitis C	0.5791
DB00540	Herpes	0.04124
DB00540	Hypertension	0.43187
DB00540	Infertility	0.03965
DB00540	Kidney failure	0.06537
DB00540	Migraine	0.67494
DB00540	Multiple endocrine neoplasia	0.09623
DB00540	Neuroblastoma	0.07931
DB00540	Neuroendocrine tumor	0.10206
DB00540	Neurotic disorder	0.09129
DB00540	Obesity	0.04441
DB00540	Obsessive-compulsive disorder	0.17408
DB00540	Panic disorder	0.97717
DB00540	Parkinson disease	0.03188
DB00540	Pervasive development disorder	0.07454
DB00540	Prostate cancer	0.01507
DB00540	Psychotic disorder	0.09245
DB00540	Pulmonary hypertension	0.14434
DB00540	Rheumatoid arthritis	0.01767
DB00540	Schizophrenia	0.08779
DB00540	Stroke	0.3503
DB00540	Sudden infant death syndrome	0.99308
DB00540	Ulcerative colitis	0.02977
DB00572	Supranuclear palsy, progressive	0.5164
DB00572	Asthma	0.07303

DB00572	Behavior disease	0.06901
DB00572	Bipolar disorder	0.05064
DB00572	Bladder cancer	0.05774
DB00572	Central nervous system disease	0.1118
DB00572	Depression	0.05872
DB00572	Diabetes mellitus	0.02354
DB00572	Neuroblastoma	0.12286
DB00572	Schizophrenia	0.102
DB00622	Supranuclear palsy, progressive	0.29814
DB00622	Abortion	0.02905
DB00622	Achalasia and cardiospasm	0.12469
DB00622	Alzheimer's disease	0.05292
DB00622	Amyloidosis	0.06639
DB00622	Asthma	0.08587
DB00622	Atherosclerosis	0.03616
DB00622	Autistic disorder	0.06042
DB00622	Autoimmune disease	0.05572
DB00622	Behavior disease	0.03984
DB00622	Bipolar disorder	0.07356
DB00622	Bladder cancer	0.07876
DB00622	Brain ischemia	0.18262
DB00622	Cancer	0.03396
DB00622	Celiac disease	0.08498
DB00622	Central nervous system disease	0.06455
DB00622	Colon cancer	0.0172
DB00622	Congenital abnormality	0.03237
DB00622	Dental plaque	0.06543
DB00622	Depression	0.06781
DB00622	Diabetes mellitus	0.02609
DB00622	Drug abuse	0.03239
DB00622	Eating disorder	0.10416
DB00622	Epstein-Barr virus infection	0.09263
DB00622	Esophageal tumor	0.07695
DB00622	Esophagus cancer	0.03074
DB00622	Esotropia	0.08852
DB00622	Eye cancer	0.10972
DB00622	Fanconi's anemia	0.0433
DB00622	Glaucoma	0.07631
DB00622	Gram-Negative bacterial infection	0.15306
DB00622	Graves' disease	0.10951
DB00622	HIV infection	0.05185
DB00622	Herpes	0.07492
DB00622	Hypertension	0.02041
DB00622	Ischemia	0.06354
DB00622	Keratosis	0.08781
DB00622	Kidney failure	0.08771
DB00622	Leukemia	0.03912

DB00622	Lichen planus	0.17067
DB00622	Lung cancer	0.0366
DB00622	Lupus erythematosus	0.03596
DB00622	Malignant glioma	0.04453
DB00622	Melanoma	0.02281
DB00622	Neuroblastoma	0.07093
DB00622	Pre-Eclampsia	0.04147
DB00622	Prion disease	0.14306
DB00622	Prostate cancer	0.01348
DB00622	Rabies	0.02116
DB00622	Rheumatoid arthritis	0.02622
DB00622	Schistosomiasis	0.15421
DB00622	Schizophrenia	0.05889
DB00622	Stroke	0.04015
DB00622	Thyroid gland disease	0.09641
DB00622	Tuberculosis	0.0826
DB00715	Hypertension, Pulmonary	0.08111
DB00715	Supranuclear palsy, progressive	0.40825
DB00715	Anorexia nervosa	0.21651
DB00715	Asthma	0.05774
DB00715	Atherosclerosis	0.02475
DB00715	Autistic disorder	0.08575
DB00715	Behavior disease	0.21822
DB00715	Bipolar disorder	0.16013
DB00715	Bladder cancer	0.04564
DB00715	Central nervous system disease	0.08839
DB00715	Choriocarcinoma	0.10206
DB00715	Chronic fatigue syndrome	0.25
DB00715	Chronic obstructive airway disease	0.03953
DB00715	Colon cancer	0.04196
DB00715	Congenital heart disease	0.11785
DB00715	Depression	0.13927
DB00715	Dermatitis	0.06428
DB00715	Diabetes mellitus	0.03722
DB00715	Drug abuse	0.06623
DB00715	Drug-Induced dyskinesia	0.13363
DB00715	Epilepsy	0.05
DB00715	Fibromyalgia	0.125
DB00715	Generalized anxiety disorder	0.10206
DB00715	Heart failure	0.03769
DB00715	Herpes	0.05051
DB00715	Hypertension	0.0559
DB00715	Migraine	0.06565
DB00715	Multiple endocrine neoplasia	0.11785
DB00715	Neuroblastoma	0.09713
DB00715	Neuroendocrine tumor	0.125
DB00715	Neurotic disorder	0.1118

DB00715	Obesity	0.05439
DB00715	Obsessive-compulsive disorder	0.2132
DB00715	Panic disorder	0.23146
DB00715	Pervasive development disorder	0.09129
DB00715	Psychotic disorder	0.11323
DB00715	Pulmonary hypertension	0.17678
DB00715	Rheumatoid arthritis	0.02164
DB00715	Schizophrenia	0.08064
DB00715	Stroke	0.08006
DB00715	Sudden infant death syndrome	0.07715
DB00715	Ulcerative colitis	0.03647
DB00725	Supranuclear palsy, progressive	0.5164
DB00725	Asthma	0.07303
DB00725	Behavior disease	0.06901
DB00725	Bipolar disorder	0.05064
DB00725	Bladder cancer	0.05774
DB00725	Central nervous system disease	0.1118
DB00725	Depression	0.05872
DB00725	Diabetes mellitus	0.02354
DB00725	Neuroblastoma	0.12286
DB00725	Schizophrenia	0.102
DB00729	Asthma	0.08165
DB00729	Diabetes mellitus	0.05263
DB00729	Neuroblastoma	0.13736
DB00747	Supranuclear palsy, progressive	0.4714
DB00747	Asthma	0.06667
DB00747	Behavior disease	0.06299
DB00747	Bipolar disorder	0.04623
DB00747	Bladder cancer	0.0527
DB00747	Central nervous system disease	0.10206
DB00747	Depression	0.05361
DB00747	Diabetes mellitus	0.04297
DB00747	Neuroblastoma	0.11215
DB00747	Schizophrenia	0.09312
DB00785	Supranuclear palsy, progressive	0.5164
DB00785	Asthma	0.07303
DB00785	Behavior disease	0.06901
DB00785	Bipolar disorder	0.05064
DB00785	Bladder cancer	0.05774
DB00785	Central nervous system disease	0.1118
DB00785	Depression	0.05872
DB00785	Diabetes mellitus	0.02354
DB00785	Neuroblastoma	0.12286
DB00785	Schizophrenia	0.102
DB00809	Supranuclear palsy, progressive	0.57735
DB00809	Asthma	0.08165
DB00809	Behavior disease	0.07715

DB00809	Bipolar disorder	0.05661
DB00809	Bladder cancer	0.06455
DB00809	Central nervous system disease	0.125
DB00809	Depression	0.06565
DB00809	Diabetes mellitus	0.02632
DB00809	Neuroblastoma	0.13736
DB00809	Schizophrenia	0.07603
DB00835	Supranuclear palsy, progressive	0.4714
DB00835	Asthma	0.06667
DB00835	Atopic rhinitis	0.10541
DB00835	Behavior disease	0.06299
DB00835	Bipolar disorder	0.04623
DB00835	Bladder cancer	0.0527
DB00835	Central nervous system disease	0.10206
DB00835	Colon cancer	0.02423
DB00835	Depression	0.05361
DB00835	Diabetes mellitus	0.02149
DB00835	Infertility	0.05608
DB00835	Neuroblastoma	0.11215
DB00835	Parkinson disease	0.04508
DB00835	Schizophrenia	0.12415
DB00934	Supranuclear palsy, progressive	0.40825
DB00934	Anorexia nervosa	0.07217
DB00934	Asthma	0.05774
DB00934	Atopic rhinitis	0.09129
DB00934	Behavior disease	0.10911
DB00934	Bipolar disorder	0.08006
DB00934	Bladder cancer	0.04564
DB00934	Central nervous system disease	0.08839
DB00934	Colon cancer	0.02098
DB00934	Depression	0.04642
DB00934	Diabetes mellitus	0.03722
DB00934	Drug abuse	0.03311
DB00934	Hypertension	0.0559
DB00934	Infertility	0.04856
DB00934	Kidney failure	0.04003
DB00934	Multiple endocrine neoplasia	0.11785
DB00934	Neuroblastoma	0.09713
DB00934	Neuroendocrine tumor	0.125
DB00934	Panic disorder	0.07715
DB00934	Parkinson disease	0.03904
DB00934	Prostate cancer	0.01846
DB00934	Schizophrenia	0.10752
DB01036	Supranuclear palsy, progressive	0.5164
DB01036	Asthma	0.07303
DB01036	Behavior disease	0.06901
DB01036	Bipolar disorder	0.05064

DB01036	Bladder cancer	0.05774
DB01036	Central nervous system disease	0.1118
DB01036	Depression	0.05872
DB01036	Diabetes mellitus	0.02354
DB01036	Neuroblastoma	0.12286
DB01036	Schizophrenia	0.102
DB01062	Supranuclear palsy, progressive	0.33333
DB01062	Asthma	0.09428
DB01062	Behavior disease	0.08909
DB01062	Bipolar disorder	0.06537
DB01062	Bladder cancer	0.07454
DB01062	Central nervous system disease	0.14434
DB01062	Depression	0.07581
DB01062	Diabetes mellitus	0.03039
DB01062	Neuroblastoma	0.15861
DB01062	Schizophrenia	0.0439
DB01069	Supranuclear palsy, progressive	0.36515
DB01069	Abortion	0.02905
DB01069	Achalasia and cardiospasm	0.12469
DB01069	Alzheimer's disease	0.05292
DB01069	Amyloidosis	0.06639
DB01069	Anorexia nervosa	0.06455
DB01069	Asthma	0.09535
DB01069	Atopic rhinitis	0.08165
DB01069	Autistic disorder	0.09877
DB01069	Autoimmune disease	0.05572
DB01069	Behavior disease	0.09759
DB01069	Bipolar disorder	0.11594
DB01069	Bladder cancer	0.08625
DB01069	Brain ischemia	0.18262
DB01069	Cancer	0.03396
DB01069	Celiac disease	0.08498
DB01069	Central nervous system disease	0.07906
DB01069	Choriocarcinoma	0.09129
DB01069	Chronic fatigue syndrome	0.1118
DB01069	Colon cancer	0.05473
DB01069	Congenital abnormality	0.03237
DB01069	Dental plaque	0.06543
DB01069	Depression	0.08305
DB01069	Dermatitis	0.02875
DB01069	Diabetes mellitus	0.02914
DB01069	Drug abuse	0.03239
DB01069	Drug-Induced dyskinesia	0.11952
DB01069	Eating disorder	0.10416
DB01069	Epstein-Barr virus infection	0.09263
DB01069	Esophageal tumor	0.07695
DB01069	Esophagus cancer	0.03074

DB01069	Esotropia	0.08852
DB01069	Eye cancer	0.10972
DB01069	Fanconi's anemia	0.0433
DB01069	Glaucoma	0.07631
DB01069	Gram-Negative bacterial infection	0.15306
DB01069	Graves' disease	0.10951
DB01069	HIV infection	0.05185
DB01069	Herpes	0.07492
DB01069	Hypertension	0.05
DB01069	Infertility	0.04344
DB01069	Ischemia	0.06354
DB01069	Keratosis	0.08781
DB01069	Kidney failure	0.03581
DB01069	Leukemia	0.03912
DB01069	Lichen planus	0.17067
DB01069	Lung cancer	0.0366
DB01069	Lupus erythematosus	0.03596
DB01069	Malignant glioma	0.04453
DB01069	Melanoma	0.02281
DB01069	Neuroblastoma	0.08687
DB01069	Obesity	0.02433
DB01069	Obsessive-compulsive disorder	0.09535
DB01069	Panic disorder	0.06901
DB01069	Parkinson disease	0.03492
DB01069	Pre-Eclampsia	0.04147
DB01069	Prion disease	0.14306
DB01069	Prostate cancer	0.01651
DB01069	Psychotic disorder	0.05064
DB01069	Rabies	0.02116
DB01069	Rheumatoid arthritis	0.04557
DB01069	Schistosomiasis	0.15421
DB01069	Schizophrenia	0.09617
DB01069	Stroke	0.07596
DB01069	Thyroid gland disease	0.09641
DB01069	Tuberculosis	0.0826
DB01085	Supranuclear palsy, progressive	0.33333
DB01085	Asthma	0.09428
DB01085	Behavior disease	0.08909
DB01085	Bipolar disorder	0.06537
DB01085	Bladder cancer	0.07454
DB01085	Central nervous system disease	0.14434
DB01085	Depression	0.07581
DB01085	Diabetes mellitus	0.03039
DB01085	Neuroblastoma	0.15861
DB01085	Schizophrenia	0.0439
DB01142	Hypertension, Pulmonary	0.0513
DB01142	Supranuclear palsy, progressive	0.2582

DB01142	Abortion	0.0481
DB01142	Amyotrophic lateral sclerosis	0.30483
DB01142	Anorexia nervosa	0.31742
DB01142	Asthma	0.03651
DB01142	Atherosclerosis	0.01566
DB01142	Atopic rhinitis	0.11547
DB01142	Attention deficit hyperactivity disorder	0.27345
DB01142	Autistic disorder	0.05423
DB01142	Behavior disease	0.83145
DB01142	Bipolar disorder	0.19998
DB01142	Bladder cancer	0.02887
DB01142	Central nervous system disease	0.0559
DB01142	Choriocarcinoma	0.06455
DB01142	Chronic fatigue syndrome	0.15811
DB01142	Chronic obstructive airway disease	0.025
DB01142	Colon cancer	0.06634
DB01142	Common cold	0.05976
DB01142	Congenital heart disease	0.07454
DB01142	Depression	0.39348
DB01142	Dermatitis	0.30737
DB01142	Diabetes mellitus	0.20052
DB01142	Drug abuse	0.08859
DB01142	Drug-Induced dyskinesia	0.08452
DB01142	Epilepsy	0.53093
DB01142	Fibromyalgia	0.07906
DB01142	Generalized anxiety disorder	0.06455
DB01142	Heart failure	0.02384
DB01142	Hepatitis C	0.53661
DB01142	Herpes	0.03194
DB01142	Hypertension	0.50167
DB01142	Infertility	0.06143
DB01142	Kidney failure	0.07596
DB01142	Melanoma	0.01863
DB01142	Migraine	0.65749
DB01142	Multiple endocrine neoplasia	0.07454
DB01142	Nervous system disease	0.1531
DB01142	Neuroblastoma	0.06143
DB01142	Neuroendocrine tumor	0.07906
DB01142	Neurotic disorder	0.07071
DB01142	Obesity	0.12426
DB01142	Obsessive-compulsive disorder	0.13484
DB01142	Panic disorder	0.87591
DB01142	Parkinson disease	0.02469
DB01142	Pervasive development disorder	0.05774
DB01142	Polycystic ovary syndrome	0.02962
DB01142	Prostate cancer	0.01167
DB01142	Psychotic disorder	0.07161

DB01142	Pulmonary hypertension	0.1118
DB01142	Rheumatoid arthritis	0.01368
DB01142	Schizophrenia	0.11983
DB01142	Stroke	0.31274
DB01142	Sudden infant death syndrome	0.91152
DB01142	Ulcerative colitis	0.02306
DB01151	Hypertension, Pulmonary	0.06131
DB01151	Infertility, Male	0.09355
DB01151	Supranuclear palsy, progressive	0.30861
DB01151	Alimentary system disease	0.17411
DB01151	Alzheimer's disease	0.08002
DB01151	Anorexia nervosa	0.16366
DB01151	Arthritis	0.44028
DB01151	Asthma	0.04364
DB01151	Atherosclerosis	0.18996
DB01151	Atopic rhinitis	0.06901
DB01151	Autistic disorder	0.34032
DB01151	Azoospermia	0.07075
DB01151	Behavior disease	0.16496
DB01151	Bipolar disorder	0.12105
DB01151	Bladder cancer	0.0345
DB01151	Breast cancer	0.03275
DB01151	Bronchial disease	0.87914
DB01151	Central nervous system disease	0.06682
DB01151	Choriocarcinoma	0.07715
DB01151	Chronic fatigue syndrome	0.18898
DB01151	Chronic obstructive airway disease	0.38637
DB01151	Colon cancer	0.04758
DB01151	Conduct disorder	0.18128
DB01151	Congenital heart disease	0.08909
DB01151	Cystic fibrosis	0.4597
DB01151	Depression	0.10528
DB01151	Dermatitis	0.26528
DB01151	Diabetes mellitus	0.14689
DB01151	Drug abuse	0.20542
DB01151	Drug-Induced dyskinesia	0.10102
DB01151	Encephalopathies	0.03742
DB01151	Enteritis	0.06222
DB01151	Epilepsy	0.17223
DB01151	Fibromyalgia	0.09449
DB01151	Generalized anxiety disorder	0.07715
DB01151	Glaucoma	0.49772
DB01151	Gram-Negative bacterial infection	0.12423
DB01151	Graves' disease	0.48783
DB01151	Heart failure	0.44087
DB01151	Herpes	0.03818
DB01151	Histiocytosis	0.08909

DB01151	Hypertension	0.39808
DB01151	Infertility	0.09648
DB01151	Ischemia	0.11474
DB01151	Kidney failure	0.03026
DB01151	Liver cancer	0.0397
DB01151	Lung cancer	0.16593
DB01151	Malaria	0.52155
DB01151	Metabolism disease	0.48707
DB01151	Migraine	0.04963
DB01151	Movement disorder	0.26525
DB01151	Multiple endocrine neoplasia	0.08909
DB01151	Myopathy	0.32865
DB01151	Neuroblastoma	0.07342
DB01151	Neurodegenerative disorder	0.14667
DB01151	Neuroendocrine tumor	0.09449
DB01151	Neurotic disorder	0.08452
DB01151	Obesity	0.3398
DB01151	Obsessive-compulsive disorder	0.16116
DB01151	Oligospermia	0.12882
DB01151	Panic disorder	0.17496
DB01151	Parkinson disease	0.02951
DB01151	Pervasive development disorder	0.06901
DB01151	Polycystic kidney	0.1121
DB01151	Polycystic ovary syndrome	0.28279
DB01151	Premature birth	0.7395
DB01151	Prostate cancer	0.16086
DB01151	Psychotic disorder	0.08559
DB01151	Pulmonary hypertension	0.13363
DB01151	Respiratory tract disease	0.08648
DB01151	Rheumatoid arthritis	0.13819
DB01151	Schizophrenia	0.08128
DB01151	Sickle cell disease	0.2619
DB01151	Sinusitis	0.08024
DB01151	Stroke	0.06052
DB01151	Subarachnoid hemorrhage	0.59968
DB01151	Sudden infant death syndrome	0.05832
DB01151	Systemic infection	0.03066
DB01151	Testicular dysfunction	0.03955
DB01151	Ulcerative colitis	0.02757
DB01226	Alzheimer's disease	0.03599
DB01226	Asthma	0.04082
DB01226	Atherosclerosis	0.03501
DB01226	Behavior disease	0.07715
DB01226	Bipolar disorder	0.05661
DB01226	Bladder cancer	0.06455
DB01226	Central nervous system disease	0.125
DB01226	Colon cancer	0.02967

DB01226	Depression	0.06565
DB01226	Diabetes mellitus	0.05263
DB01226	Epilepsy	0.07071
DB01226	Neuroblastoma	0.06868
DB01226	Obesity	0.03846
DB01226	Vitiligo	0.11785
DB01231	Supranuclear palsy, progressive	0.33333
DB01231	Asthma	0.09428
DB01231	Behavior disease	0.08909
DB01231	Bipolar disorder	0.06537
DB01231	Bladder cancer	0.07454
DB01231	Central nervous system disease	0.14434
DB01231	Depression	0.07581
DB01231	Diabetes mellitus	0.03039
DB01231	Neuroblastoma	0.15861
DB01231	Schizophrenia	0.0439
DB01337	Asthma	0.04714
DB01337	Behavior disease	0.08909
DB01337	Bipolar disorder	0.06537
DB01337	Bladder cancer	0.07454
DB01337	Central nervous system disease	0.14434
DB01337	Depression	0.07581
DB01337	Diabetes mellitus	0.03039
DB01337	Epilepsy	0.08165
DB01337	Neuroblastoma	0.07931
DB01338	Asthma	0.04714
DB01338	Behavior disease	0.08909
DB01338	Bipolar disorder	0.06537
DB01338	Bladder cancer	0.07454
DB01338	Central nervous system disease	0.14434
DB01338	Depression	0.07581
DB01338	Diabetes mellitus	0.03039
DB01338	Epilepsy	0.08165
DB01338	Neuroblastoma	0.07931
DB01409	Supranuclear palsy, progressive	0.33333
DB01409	Asthma	0.09428
DB01409	Behavior disease	0.08909
DB01409	Bipolar disorder	0.06537
DB01409	Bladder cancer	0.07454
DB01409	Central nervous system disease	0.14434
DB01409	Depression	0.07581
DB01409	Diabetes mellitus	0.03039
DB01409	Neuroblastoma	0.15861
DB01409	Schizophrenia	0.0439
DB01591	Supranuclear palsy, progressive	0.5164
DB01591	Asthma	0.07303
DB01591	Behavior disease	0.06901

DB01591	Bipolar disorder	0.05064
DB01591	Bladder cancer	0.05774
DB01591	Central nervous system disease	0.1118
DB01591	Depression	0.05872
DB01591	Diabetes mellitus	0.02354
DB01591	Neuroblastoma	0.12286
DB01591	Schizophrenia	0.102
DB01625	Supranuclear palsy, progressive	0.40825
DB01625	Asthma	0.05774
DB01625	Diabetes mellitus	0.03722
DB01625	Neuroblastoma	0.09713
DB01625	Schizophrenia	0.05376
DB04843	Supranuclear palsy, progressive	0.40825
DB04843	Asthma	0.11547
DB04843	Diabetes mellitus	0.03722
DB04843	Neuroblastoma	0.19426
DB04843	Schizophrenia	0.05376
DB06702	Supranuclear palsy, progressive	0.5164
DB06702	Asthma	0.07303
DB06702	Behavior disease	0.06901
DB06702	Bipolar disorder	0.05064
DB06702	Bladder cancer	0.05774
DB06702	Central nervous system disease	0.1118
DB06702	Depression	0.05872
DB06702	Diabetes mellitus	0.02354
DB06702	Neuroblastoma	0.12286
DB06702	Schizophrenia	0.102
DB06709	Asthma	0.08165
DB06709	Diabetes mellitus	0.05263
DB06709	Neuroblastoma	0.13736
DB08897	Supranuclear palsy, progressive	0.5164
DB08897	Asthma	0.07303
DB08897	Behavior disease	0.06901
DB08897	Bipolar disorder	0.05064
DB08897	Bladder cancer	0.05774
DB08897	Central nervous system disease	0.1118
DB08897	Depression	0.05872
DB08897	Diabetes mellitus	0.02354
DB08897	Neuroblastoma	0.12286
DB08897	Schizophrenia	0.102
DB00119	Autistic disorder	0.03241
DB00119	Breast cancer	0.01286
DB00119	Cancer	0.00985
DB00119	Chronic obstructive airway disease	0.02988
DB00119	Colon cancer	0.03172
DB00119	Diabetes mellitus	0.0422
DB00119	Embryoma	0.01648

DB00119	Gilles de la Tourette syndrome	0.10102
DB00119	Hemolytic anemia	0.13363
DB00119	Hyperinsulinism	0.05143
DB00119	Hypoglycemia	0.10911
DB00119	Intestinal disease	0.06682
DB00119	Lymphoma	0.03046
DB00119	Malaria	0.04963
DB00119	Obesity	0.04112
DB00119	Obsessive-compulsive disorder	0.08058
DB00119	Skin disease	0.04583
DB00761	Breast cancer	0.03928
DB00761	Deafness	0.05893
DB00761	Female reproductive cancer	0.43301
DB00761	Hyperaldosteronism	0.1291
DB00761	Liver cancer	0.03169
DB00761	Liver tumor	0.07217
DB00761	Neuropathy	0.06537
DB00761	Polycystic kidney	0.10911
DB00887	Infertility, Male	0.57185
DB00887	Alimentary system disease	0.99202
DB00887	Alzheimer's disease	0.15044
DB00887	Arthritis	0.10548
DB00887	Atherosclerosis	0.03952
DB00887	Autistic disorder	0.06298
DB00887	Azoospermia	0.45473
DB00887	Bone disease	0.40922
DB00887	Breast cancer	0.10217
DB00887	Bronchial disease	0.21098
DB00887	Cancer	0.04527
DB00887	Chronic obstructive airway disease	0.08462
DB00887	Conduct disorder	1.08032
DB00887	Congenital abnormality	0.10122
DB00887	Cystic fibrosis	0.61701
DB00887	Dermatitis	0.04355
DB00887	Diabetes mellitus	0.10849
DB00887	Drug abuse	0.03377
DB00887	Endometriosis	0.10738
DB00887	Enteritis	0.3687
DB00887	Female reproductive cancer	0.15811
DB00887	Glaucoma	0.07955
DB00887	Gram-Negative bacterial infection	0.76729
DB00887	Graves' disease	0.11415
DB00887	Heart failure	0.33599
DB00887	Hyperaldosteronism	0.14142
DB00887	Hypertension	0.27259
DB00887	Infection	0.13706
DB00887	Infertility	0.36846

DB00887	Liver cancer	0.23867
DB00887	Liver tumor	0.07906
DB00887	Lung cancer	0.03816
DB00887	Malaria	0.12227
DB00887	Metabolism disease	0.11511
DB00887	Myopathy	0.07675
DB00887	Obesity	0.05014
DB00887	Oligospermia	0.77721
DB00887	Polycystic kidney	0.8149
DB00887	Polycystic ovary syndrome	0.0641
DB00887	Premature birth	0.1748
DB00887	Prostate cancer	0.02862
DB00887	Respiratory tract disease	0.53958
DB00887	Rheumatoid arthritis	0.02733
DB00887	Sickle cell disease	0.05453
DB00887	Sinusitis	0.51218
DB00887	Subarachnoid hemorrhage	0.08536
DB00887	Testicular dysfunction	0.26705
DB00001	Abortion	0.125
DB00001	Aseptic necrosis of bone	0.31623
DB00001	Behcet syndrome	0.14286
DB00001	Cardiovascular disease	0.1525
DB00001	Dental plaque	0.127
DB00001	Diabetes mellitus	0.05263
DB00001	Embryoma	0.06166
DB00001	Endometriosis	0.08305
DB00001	Enteritis	0.10976
DB00001	Hemorrhagic disorder	0.17678
DB00001	Hyperhomocysteinemia	0.28868
DB00001	Liver cancer	0.07762
DB00001	Liver disease	0.1543
DB00001	Lupus erythematosus	0.08544
DB00001	Malignant glioma	0.1857
DB00001	Neoplasm metastasis	0.08138
DB00001	Nevus	0.35355
DB00001	Pulmonary fibrosis	0.18898
DB00001	Systemic infection	0.11471
DB00001	Systemic scleroderma	0.10483
DB00001	Thrombophilia	0.2357
DB00006	Abortion	0.125
DB00006	Aseptic necrosis of bone	0.31623
DB00006	Behcet syndrome	0.14286
DB00006	Cardiovascular disease	0.1525
DB00006	Dental plaque	0.127
DB00006	Diabetes mellitus	0.05263
DB00006	Embryoma	0.06166
DB00006	Endometriosis	0.08305

DB00006	Enteritis	0.10976
DB00006	Hemorrhagic disorder	0.17678
DB00006	Hyperhomocysteinemia	0.28868
DB00006	Liver cancer	0.07762
DB00006	Liver disease	0.1543
DB00006	Lupus erythematosus	0.08544
DB00006	Malignant glioma	0.1857
DB00006	Neoplasm metastasis	0.08138
DB00006	Nevus	0.35355
DB00006	Pulmonary fibrosis	0.18898
DB00006	Systemic infection	0.11471
DB00006	Systemic scleroderma	0.10483
DB00006	Thrombophilia	0.2357
DB00170	Hypertension, Pulmonary	0.18422
DB00170	Abortion	0.03467
DB00170	Abruption placentae	1
DB00170	Adenocarcinoma	0.04046
DB00170	Adenovirus infection	0.10307
DB00170	Antiphospholipid syndrome	0.09245
DB00170	Aseptic necrosis of bone	0.08771
DB00170	Atherosclerosis	0.09297
DB00170	Bacterial infection	0.3389
DB00170	Behcet syndrome	0.07924
DB00170	Bone disease	0.04757
DB00170	Breast cancer	0.12238
DB00170	Cardiovascular disease	0.33957
DB00170	Cirrhosis	0.27665
DB00170	Dental plaque	0.16217
DB00170	Diabetes mellitus	0.07968
DB00170	Drug abuse	0.06285
DB00170	Embryoma	0.08691
DB00170	Endometriosis	0.02303
DB00170	Enteritis	0.03044
DB00170	Heart failure	0.11999
DB00170	Hemolytic-Uremic syndrome	0.23223
DB00170	Hemorrhagic disorder	0.39308
DB00170	Hyperhomocysteinemia	0.08006
DB00170	Hyperlipidemia	0.40488
DB00170	Kidney failure	0.12539
DB00170	Leukemia	0.05636
DB00170	Leukoencephalopathy	0.09488
DB00170	Liver cancer	0.02153
DB00170	Liver disease	0.0428
DB00170	Liver metastases	0.33569
DB00170	Lupus erythematosus	0.0237
DB00170	Lymphatic metastasis	1
DB00170	Malignant glioma	0.0515

DB00170	Melanoma	0.15341
DB00170	Meningioma	0.29252
DB00170	Multiple myeloma	0.03642
DB00170	Neoplasm metastasis	0.1365
DB00170	Nephrosis	0.17722
DB00170	Nevus	0.90322
DB00170	Osteomyelitis	0.32
DB00170	Osteoporosis	0.04385
DB00170	Pancreas cancer	0.14917
DB00170	Pre-Eclampsia	0.03269
DB00170	Primary tumor	0.16324
DB00170	Prostate cancer	0.05327
DB00170	Protein deficiency	0.16013
DB00170	Proteinuria	0.35228
DB00170	Pulmonary fibrosis	0.05241
DB00170	Rabies	0.1779
DB00170	Rectum cancer	0.64359
DB00170	Respiratory distress syndrome	0.36229
DB00170	Rheumatism	0.16777
DB00170	Solid tumor	0.32409
DB00170	Stomach cancer	0.0679
DB00170	Stroke	0.0314
DB00170	Systemic infection	0.196
DB00170	Systemic scleroderma	0.13268
DB00170	Thrombophilia	0.52432
DB00170	Vascular disease	0.05064
DB00170	Vitamin D deficiency	0.36877
DB00278	Abortion	0.125
DB00278	Aseptic necrosis of bone	0.31623
DB00278	Behcet syndrome	0.14286
DB00278	Cardiovascular disease	0.1525
DB00278	Dental plaque	0.127
DB00278	Diabetes mellitus	0.05263
DB00278	Embryoma	0.06166
DB00278	Endometriosis	0.08305
DB00278	Enteritis	0.10976
DB00278	Hemorrhagic disorder	0.17678
DB00278	Hyperhomocysteinemia	0.28868
DB00278	Liver cancer	0.07762
DB00278	Liver disease	0.1543
DB00278	Lupus erythematosus	0.08544
DB00278	Malignant glioma	0.1857
DB00278	Neoplasm metastasis	0.08138
DB00278	Nevus	0.35355
DB00278	Pulmonary fibrosis	0.18898
DB00278	Systemic infection	0.11471
DB00278	Systemic scleroderma	0.10483

DB00278	Thrombophilia	0.2357
DB01123	Abortion	0.07217
DB01123	Aseptic necrosis of bone	0.18257
DB01123	Behcet syndrome	0.08248
DB01123	Cardiovascular disease	0.08805
DB01123	Dental plaque	0.07332
DB01123	Diabetes mellitus	0.03039
DB01123	Embryoma	0.0356
DB01123	Endometriosis	0.04795
DB01123	Enteritis	0.06337
DB01123	Hemorrhagic disorder	0.10206
DB01123	Hyperhomocysteinemia	0.16667
DB01123	Liver cancer	0.04481
DB01123	Liver disease	0.08909
DB01123	Lupus erythematosus	0.04933
DB01123	Malignant glioma	0.10721
DB01123	Neoplasm metastasis	0.04698
DB01123	Nevus	0.20412
DB01123	Pulmonary fibrosis	0.10911
DB01123	Systemic infection	0.06623
DB01123	Systemic scleroderma	0.06052
DB01123	Thrombophilia	0.13608
DB04786	Myopathies, Nemaline	0.1543
DB04786	Pleural effusion, Malignant	0.09933
DB04786	Abortion	0.04725
DB04786	Alzheimer's disease	0.08633
DB04786	Aseptic necrosis of bone	0.11952
DB04786	Atherosclerosis	0.16471
DB04786	Azoospermia	0.18172
DB04786	Behcet syndrome	0.05399
DB04786	Brain tumor	0.14162
DB04786	Breast cancer	0.05109
DB04786	Cancer	0.04411
DB04786	Cardiovascular disease	0.05764
DB04786	Cholelithiasis	0.07559
DB04786	Congenital abnormality	0.09597
DB04786	Cystic fibrosis	0.05399
DB04786	Dental plaque	0.096
DB04786	Diabetes mellitus	0.03979
DB04786	Drug abuse	0.10293
DB04786	Embryoma	0.04661
DB04786	Endocrine system disease	0.14286
DB04786	Endometriosis	0.03139
DB04786	Enteritis	0.04149
DB04786	Hemorrhagic disorder	0.06682
DB04786	Herpes	0.0837
DB04786	Hyperaldosteronism	0.35266

DB04786	Hyperhomocysteinemia	0.10911
DB04786	Leukemia	0.06057
DB04786	Liver cancer	0.02934
DB04786	Liver disease	0.05832
DB04786	Lung cancer	0.10271
DB04786	Lupus erythematosus	0.03229
DB04786	Malignant glioma	0.07019
DB04786	Myopathy	0.04583
DB04786	Neoplasm metastasis	0.15968
DB04786	Nevus	0.13363
DB04786	Prostate cancer	0.05724
DB04786	Pulmonary fibrosis	0.07143
DB04786	Rheumatoid arthritis	0.05466
DB04786	Schizophrenia	0.02874
DB04786	Stomach cancer	0.03183
DB04786	Stroke	0.0428
DB04786	Systemic infection	0.04336
DB04786	Systemic scleroderma	0.03962
DB04786	Thrombophilia	0.08909
DB04786	Thyroid cancer	0.27709
DB04786	Vascular dementia	0.12599
DB04898	Abortion	0.125
DB04898	Aseptic necrosis of bone	0.31623
DB04898	Behcet syndrome	0.14286
DB04898	Cardiovascular disease	0.1525
DB04898	Dental plaque	0.127
DB04898	Diabetes mellitus	0.05263
DB04898	Embryoma	0.06166
DB04898	Endometriosis	0.08305
DB04898	Enteritis	0.10976
DB04898	Hemorrhagic disorder	0.17678
DB04898	Hyperhomocysteinemia	0.28868
DB04898	Liver cancer	0.07762
DB04898	Liver disease	0.1543
DB04898	Lupus erythematosus	0.08544
DB04898	Malignant glioma	0.1857
DB04898	Neoplasm metastasis	0.08138
DB04898	Nevus	0.35355
DB04898	Pulmonary fibrosis	0.18898
DB04898	Systemic infection	0.11471
DB04898	Systemic scleroderma	0.10483
DB04898	Thrombophilia	0.2357
DB06695	Abortion	0.125
DB06695	Aseptic necrosis of bone	0.31623
DB06695	Behcet syndrome	0.14286
DB06695	Cardiovascular disease	0.1525
DB06695	Dental plaque	0.127

DB06695	Diabetes mellitus	0.05263
DB06695	Embryoma	0.06166
DB06695	Endometriosis	0.08305
DB06695	Enteritis	0.10976
DB06695	Hemorrhagic disorder	0.17678
DB06695	Hyperhomocysteinemia	0.28868
DB06695	Liver cancer	0.07762
DB06695	Liver disease	0.1543
DB06695	Lupus erythematosus	0.08544
DB06695	Malignant glioma	0.1857
DB06695	Neoplasm metastasis	0.08138
DB06695	Nevus	0.35355
DB06695	Pulmonary fibrosis	0.18898
DB06695	Systemic infection	0.11471
DB06695	Systemic scleroderma	0.10483
DB06695	Thrombophilia	0.2357
DB00855	Diabetes mellitus	0.05263
DB00855	Hypothyroidism	0.2357
DB00855	Liver cancer	0.07762
DB00855	Liver tumor	0.17678
DB00855	Parasitic disease	0.5
DB01108	Hypertension, Pulmonary	0.15392
DB01108	Infertility, Male	0.78954
DB01108	Adenovirus infection	0.03828
DB01108	Alzheimer's disease	0.24115
DB01108	Atherosclerosis	0.29616
DB01108	Autistic disorder	0.02299
DB01108	Bipolar disorder	0.60699
DB01108	Bladder cancer	0.1378
DB01108	Breast cancer	0.00932
DB01108	Bronchial hyperreactivity	1.17678
DB01108	Cancer	0.15649
DB01108	Cerebrovascular disorder	0.79651
DB01108	Cholelithiasis	0.80055
DB01108	Cirrhosis	0.1039
DB01108	Congenital abnormality	0.03769
DB01108	Craniosynostosis	0.25982
DB01108	Dental plaque	0.51729
DB01108	Depression	0.32774
DB01108	Diabetes mellitus	0.20848
DB01108	Down syndrome	0.44739
DB01108	Eating disorder	0.04067
DB01108	Endometriosis	0.31
DB01108	Epilepsy	0.05425
DB01108	Gestational trophoblastic tumor	0.35355
DB01108	Glaucoma	0.62185
DB01108	Hepatitis	0.19437

DB01108	Hepatitis C	0.06223
DB01108	Herpes	0.32512
DB01108	Hodgkin's disease	0.04769
DB01108	Hyperlipidemia	0.86002
DB01108	Hypertension	0.44014
DB01108	Infectious lung disease	0.05753
DB01108	Infertility	0.5079
DB01108	Kidney failure	0.44212
DB01108	Late pregnancy	1.23268
DB01108	Leukemia	0.02117
DB01108	Leukoencephalopathy	0.03564
DB01108	Lung cancer	0.02667
DB01108	Lupus erythematosus	0.28458
DB01108	Lupus vulgaris	0.67776
DB01108	Lymphoma	0.04152
DB01108	Mental retardation	0.03628
DB01108	Migraine	0.63189
DB01108	Multiple sclerosis	0.04129
DB01108	Myasthenia Gravis	1.08341
DB01108	Neoplasm metastasis	0.05223
DB01108	Nephrosis	0.05764
DB01108	Obesity	0.34239
DB01108	Oral cancer	0.03441
DB01108	Osteoporosis	0.59543
DB01108	Ovarian disease	0.92125
DB01108	Ovarian failure	1.01118
DB01108	Overnutrition	0.06746
DB01108	Pancreatitis	0.05065
DB01108	Panic disorder	0.79766
DB01108	Parkinson disease	0.44419
DB01108	Polyarthritis	0.33334
DB01108	Polycystic ovary syndrome	0.06623
DB01108	Primary biliary cirrhosis	0.64351
DB01108	Prostate cancer	0.0722
DB01108	Ptosis	0.16667
DB01108	Pulmonary fibrosis	0.08321
DB01108	Rabies	0.33384
DB01108	Renal tubular acidosis	0.57474
DB01108	Rheumatoid arthritis	0.08159
DB01108	Schistosomiasis	0.11237
DB01108	Schizophrenia	0.37784
DB01108	Scleroderma	0.03754
DB01108	Sicca syndrome	0.13578
DB01108	Stroke	0.30847
DB01108	Synovitis	0.97154
DB01108	Systemic scleroderma	0.02799
DB01108	Temporal arteritis	0.22361

DB01108	Testicular dysfunction	0.07143
DB01108	Thymoma	0.77495
DB01108	Tuberculosis	0.05212
DB01108	Ulcerative colitis	0.02997
DB01108	Uterine disease	0.73797
DB01108	Vaccinia	0.16627
DB01108	Virus disease	0.04249
DB01108	Vitiligo	0.8477
DB01108	Wiskott-Aldrich syndrome	0.07541
DB00123	Cancer	0.02181
DB00123	Hypertension	0.03536
DB00123	Solid tumor	0.1895
DB00129	Hepatitis, Autoimmune	0.1543
DB00129	Hypertension, Pulmonary	0.06131
DB00129	Asthma	0.02182
DB00129	Encephalopathies	0.03742
DB00129	Heart failure	0.02849
DB00129	Hypertension	0.04226
DB00129	Liver cancer	0.02074
DB00129	Liver metastases	0.07412
DB00129	Lung cancer	0.09498
DB00129	Pancreas cancer	0.12939
DB00129	Prostate cancer	0.01395
DB00129	Sickle cell disease	0.10287
DB00129	Stroke	0.03026
DB00233	Infertility, Male	0.05756
DB00233	Stress disorder, post-traumatic	0.08929
DB00233	AIDS	0.11566
DB00233	Adenocarcinoma	0.09171
DB00233	Adenoma	0.0106
DB00233	Alzheimer's disease	0.08521
DB00233	Aseptic necrosis of bone	0.02451
DB00233	Asthma	0.11084
DB00233	Atherosclerosis	0.11436
DB00233	Autoimmune disease	0.04566
DB00233	Bipolar disorder	0.04608
DB00233	Brain tumor	0.04946
DB00233	Breast cancer	0.15311
DB00233	Bronchial hyperreactivity	0.08373
DB00233	Cancer	0.12028
DB00233	Cerebrovascular disorder	0.05756
DB00233	Cholelithiasis	0.05028
DB00233	Chronic fatigue syndrome	0.30562
DB00233	Chronic simple glaucoma	0.03067
DB00233	Colon cancer	0.0062
DB00233	Congenital abnormality	0.01726
DB00233	Cytomegalovirus infection	0.11547

DB00233	Dental plaque	0.20287
DB00233	Depression	0.03894
DB00233	Dermatitis	0.05873
DB00233	Diabetes mellitus	0.11368
DB00233	Down syndrome	0.03269
DB00233	Eating disorder	0.02817
DB00233	Embryoma	0.02769
DB00233	Endometriosis	0.15766
DB00233	Esophagus cancer	0.02092
DB00233	Fanconi's anemia	0.02524
DB00233	Glaucoma	0.03811
DB00233	Gouts	0.2691
DB00233	Growth retardation	0.19449
DB00233	Herpes	0.04032
DB00233	Hodgkin's disease	0.10622
DB00233	Hyperlipidemia	0.0638
DB00233	Hypertension	0.02542
DB00233	Immunologic deficiency syndrome	0.0579
DB00233	Infection	0.02293
DB00233	Infectious lung disease	0.88328
DB00233	Infertility	0.03678
DB00233	Infiltrating cancer	0.22929
DB00233	Intracranial hypertension	0.12283
DB00233	Ischemia	0.11223
DB00233	Kidney failure	0.03228
DB00233	Late pregnancy	0.07904
DB00233	Leukemia	0.33063
DB00233	Leukoencephalopathy	0.01599
DB00233	Liver cancer	0.25335
DB00233	Lung cancer	0.02111
DB00233	Lupus erythematosus	0.01796
DB00233	Lupus vulgaris	0.04884
DB00233	Lymphoma	0.01138
DB00233	Melanoma	0.01186
DB00233	Metaplastic polyp	0.22623
DB00233	Migraine	0.04513
DB00233	Mucopolysaccharidosis	0.03542
DB00233	Multiple sclerosis	0.1404
DB00233	Myasthenia Gravis	0.06111
DB00233	Myelofibrosis	0.06658
DB00233	Neoplasm metastasis	0.40543
DB00233	Nephrosis	0.19599
DB00233	Neuroblastoma	0.02073
DB00233	Neurodegenerative disorder	0.06656
DB00233	Obesity	0.4232
DB00233	Oral cancer	0.10171
DB00233	Osteoporosis	0.03662

DB00233	Ovarian disease	0.0574
DB00233	Ovarian failure	0.07506
DB00233	Pancreatitis	0.17221
DB00233	Panic disorder	0.05765
DB00233	Parkinson disease	0.12574
DB00233	Periodontitis	0.1694
DB00233	Polyarthritis	0.60015
DB00233	Primary biliary cirrhosis	0.04377
DB00233	Prion disease	0.30633
DB00233	Prostate cancer	0.32187
DB00233	Rabies	0.022
DB00233	Renal tubular acidosis	0.03313
DB00233	Rheumatism	0.02827
DB00233	Rheumatoid arthritis	0.0566
DB00233	Schizophrenia	0.0328
DB00233	Sicca syndrome	0.0324
DB00233	Sickle cell disease	0.13132
DB00233	Squamous cell cancer	0.11056
DB00233	Stomach cancer	0.06457
DB00233	Stroke	0.19157
DB00233	Synovitis	0.06655
DB00233	Systemic infection	0.02217
DB00233	Systemic scleroderma	0.04107
DB00233	Testicular dysfunction	0.01217
DB00233	Thymoma	0.05165
DB00233	Thyroid gland disease	0.186
DB00233	Tuberculosis	0.23753
DB00233	Tuberous sclerosis	0.25548
DB00233	Ulcerative colitis	0.1019
DB00233	Uterine disease	0.04597
DB00233	Virus disease	0.54443
DB00233	Vitiligo	0.06111
DB00233	Vulvar disease	0.45104
DB00233	Yersinia infection	0.21729
DB00145	Bipolar disorder	0.02067
DB00145	Cancer	0.01346
DB00145	Charcot-Marie-Tooth disease	0.04189
DB00145	Drug abuse	0.0171
DB00145	Encephalopathies	0.02557
DB00145	Heart failure	0.01946
DB00145	Hyperglycemia	0.02924
DB00145	Hyperopia	0.06086
DB00145	Kidney disease	0.02182
DB00145	Liver cancer	0.01417
DB00145	Liver disease	0.02817
DB00145	Liver failure	0.06901
DB00145	Mental retardation	0.02357

DB00145	Neurotic disorder	0.05774
DB00145	Psychotic disorder	0.02924
DB00145	Schizophrenia	0.01388
DB00431	Hyperopia	0.14907
DB00431	Neurotic disorder	0.14142
DB00786	Pleural effusion, Malignant	0.1521
DB00786	Acne	0.42799
DB00786	Actinic keratosis	0.07881
DB00786	Adenoma	0.03576
DB00786	Alveolar bone loss	0.07881
DB00786	Alzheimer's disease	0.08083
DB00786	Amnionitis	0.26843
DB00786	Amyloidosis	0.03941
DB00786	Amyotrophic lateral sclerosis	0.09422
DB00786	Aortic aneurysm	0.14536
DB00786	Arteriopathy	0.55088
DB00786	Arthritis	0.30352
DB00786	Asthma	0.11461
DB00786	Atherosclerosis	0.25085
DB00786	Autoimmune disease	0.07854
DB00786	Azoospermia	0.08748
DB00786	Bacterial vaginosis	0.08513
DB00786	Behcet syndrome	0.3062
DB00786	Biliary Atresia	0.24391
DB00786	Bone disease	0.1966
DB00786	Brain tumor	0.01675
DB00786	Breast cancer	0.05469
DB00786	Bronchiectasis	0.08513
DB00786	Bronchiolitis obliterans	0.62038
DB00786	Bronchopulmonary dysplasia	0.75303
DB00786	CNS metastases	0.40907
DB00786	Cancer	0.15014
DB00786	Capillaries disease	0.17161
DB00786	Cardiovascular disease	0.19333
DB00786	Cervical cancer	0.10572
DB00786	Cholangiocarcinoma	0.07881
DB00786	Cholestasis	0.47001
DB00786	Chondrosarcoma	0.07881
DB00786	Chronic obstructive airway disease	0.38294
DB00786	Chronic rejection of renal transplant	0.23307
DB00786	Cirrhosis	0.03525
DB00786	Colon cancer	0.08543
DB00786	Congenital abnormality	0.01572
DB00786	Connective tissue disease	0.05213
DB00786	Cystic fibrosis	0.11854
DB00786	Degenerative disc disease	0.61785
DB00786	Dental enamel hypoplasia	0.08513

DB00786	Dental plaque	0.17231
DB00786	Dermatitis	0.04193
DB00786	Diabetes mellitus	0.15659
DB00786	Disseminated cancer	0.06594
DB00786	Drug abuse	0.02815
DB00786	Embryoma	0.03611
DB00786	Emphysema	0.51322
DB00786	Endometrial cancer	0.0363
DB00786	Endometriosis	0.24374
DB00786	Enteritis	0.1227
DB00786	Esophagus cancer	0.03108
DB00786	Esotropia	0.20922
DB00786	Ewings sarcoma	0.0695
DB00786	Extramammary Paget's disease	0.20851
DB00786	Gastritis	0.0979
DB00786	Gingival overgrowth	0.07881
DB00786	Glaucoma	0.11134
DB00786	Glomerulonephritis	0.60842
DB00786	Gouts	0.05783
DB00786	Growth retardation	0.3601
DB00786	HIV infection	0.03515
DB00786	HTLV-I infection	0.22488
DB00786	Hamman-Rich syndrome	0.20957
DB00786	Heart failure	0.15446
DB00786	Helicobacter infection	0.2768
DB00786	Hematopoietic system disease	0.48548
DB00786	Henoch-Schoenlein purpura	0.54731
DB00786	Hepatitis B	0.81914
DB00786	Herpes	0.03489
DB00786	Hodgkin's disease	0.07584
DB00786	Huntington disease	0.19437
DB00786	Hyperlipidemia	0.30892
DB00786	Hypertension	0.08871
DB00786	IGA glomerulonephritis	0.18662
DB00786	Infection	0.10774
DB00786	Infiltrating cancer	0.15448
DB00786	Intracranial aneurysm	0.29123
DB00786	Ischemia	0.25048
DB00786	Keratoconjunctivitis Sicca	0.33468
DB00786	Kidney disease	0.13159
DB00786	Leukemia	0.02916
DB00786	Lichen planus	0.60221
DB00786	Liver cancer	0.08146
DB00786	Liver metastases	0.17364
DB00786	Lung cancer	0.08042
DB00786	Lupus erythematosus	0.03608
DB00786	Lupus vulgaris	0.17819

DB00786	Lyme disease	0.13901
DB00786	Lymphatic metastasis	0.14744
DB00786	Lymphoma	0.02376
DB00786	Macular degeneration	0.04348
DB00786	Malaria	0.03872
DB00786	Malignant glioma	0.03872
DB00786	Melanoma	0.08241
DB00786	Meningioma	0.15131
DB00786	Metabolism disease	0.1776
DB00786	Metastasis to lymph nodes	0.27224
DB00786	Mucocutaneous lymph node syndrome	0.32307
DB00786	Multiple myeloma	0.07719
DB00786	Multiple sclerosis	0.10869
DB00786	Muscular dystrophy	0.47344
DB00786	Myelofibrosis	0.12574
DB00786	Necrotizing enterocolitis	0.08513
DB00786	Neoplasm metastasis	0.05789
DB00786	Obesity	0.15551
DB00786	Oral cancer	0.16089
DB00786	Osteoporosis	0.03297
DB00786	Ovarian cancer	0.02096
DB00786	Ovarian disease	0.16449
DB00786	Ovary cancer	0.11401
DB00786	Parkinson disease	0.05615
DB00786	Periodontal disease	0.04915
DB00786	Periodontitis	0.42017
DB00786	Peripheral nerve sheath cancer	0.10426
DB00786	Pneumoconiosis	0.14744
DB00786	Polyarthritis	0.07129
DB00786	Polycystic ovary syndrome	0.19252
DB00786	Pre-Eclampsia	0.1112
DB00786	Primary tumor	0.08444
DB00786	Prostate cancer	0.08197
DB00786	Ptosis	0.49299
DB00786	Pulmonary fibrosis	0.21916
DB00786	Rabies	0.02317
DB00786	Renal Cell cancer	0.02424
DB00786	Respiratory tract disease	0.15138
DB00786	Rheumatoid arthritis	0.10605
DB00786	Sinusitis	0.36058
DB00786	Skin cancer	0.30777
DB00786	Skin disease	0.10828
DB00786	Skin tumor	0.07881
DB00786	Squamous cell cancer	0.04256
DB00786	Stomach cancer	0.05269
DB00786	Stroke	0.11112
DB00786	Subacute sclerosing panencephalitis	0.58563

DB00786	Synovitis	0.07372
DB00786	Systemic infection	0.17752
DB00786	Systemic scleroderma	0.16883
DB00786	Takayasu's arteritis	0.36877
DB00786	Thromboangiitis obliterans	0.08513
DB00786	Thyroid cancer	0.10171
DB00786	Transient hypertension of pregnancy	0.84988
DB00786	Tropical spastic paraparesis	0.22615
DB00786	Tuberculosis	0.02812
DB00786	Ulcerative colitis	0.11218
DB00786	Uterine disease	0.19193
DB00786	Varicosity	0.639
DB00786	Vasculitis	0.30048
DB01197	Pleural effusion, Malignant	0.24067
DB01197	Acne	0.71323
DB01197	Alzheimer's disease	0.09762
DB01197	Amnionitis	0.45344
DB01197	Amyotrophic lateral sclerosis	0.16992
DB01197	Anemia	0.12309
DB01197	Ankylosing spondylitis	0.21822
DB01197	Aortic aneurysm	0.07862
DB01197	Arteriopathy	0.86289
DB01197	Arthritis	0.52951
DB01197	Asthma	0.23019
DB01197	Atherosclerosis	0.13221
DB01197	Autoimmune disease	0.1405
DB01197	Azoospermia	0.12183
DB01197	Behcet syndrome	0.59093
DB01197	Biliary Atresia	0.23704
DB01197	Bone disease	0.32303
DB01197	Breast cancer	0.06203
DB01197	Bronchiolitis obliterans	0.86289
DB01197	Bronchopulmonary dysplasia	1.3959
DB01197	CNS metastases	0.67118
DB01197	Cancer	0.10622
DB01197	Capillaries disease	0.32976
DB01197	Cardiovascular disease	0.40107
DB01197	Cervical cancer	0.11348
DB01197	Cholestasis	0.32731
DB01197	Chronic fatigue syndrome	0.20412
DB01197	Chronic obstructive airway disease	0.28957
DB01197	Chronic rejection of renal transplant	0.54068
DB01197	Chronic simple glaucoma	0.12309
DB01197	Colon cancer	0.08432
DB01197	Connective tissue disease	0.14434
DB01197	Cystic fibrosis	0.20609
DB01197	Degenerative disc disease	0.97767

DB01197	Dental plaque	0.20266
DB01197	Depression	0.07581
DB01197	Dermatitis	0.11088
DB01197	Diabetes mellitus	0.1113
DB01197	Embryoma	0.08589
DB01197	Emphysema	0.45139
DB01197	Endometrial cancer	0.1005
DB01197	Endometriosis	0.16376
DB01197	Enteritis	0.10714
DB01197	Esophagus cancer	0.08607
DB01197	Esotropia	0.2904
DB01197	Familial Mediterranean fever	0.14907
DB01197	Fibroid tumor	0.17408
DB01197	Gastritis	0.19057
DB01197	Glaucoma	0.20289
DB01197	Glomerulonephritis	0.99086
DB01197	Growth retardation	0.61197
DB01197	HIV infection	0.04895
DB01197	HTLV-I infection	0.40886
DB01197	Hamman-Rich syndrome	0.29121
DB01197	Heart disease	0.17408
DB01197	Heart failure	0.1838
DB01197	Helicobacter infection	0.51384
DB01197	Hematopoietic system disease	0.33808
DB01197	Henoch-Schoenlein purpura	0.85301
DB01197	Hepatitis B	1.28006
DB01197	Hodgkin's disease	0.10563
DB01197	Huntington disease	0.33333
DB01197	Hyperhomocysteinemia	0.16667
DB01197	Hypertension	0.19188
DB01197	Hypoglycemia	0.2357
DB01197	IGA glomerulonephritis	0.40814
DB01197	Infection	0.07503
DB01197	Infectious lung disease	0.1291
DB01197	Infiltrating cancer	0.21516
DB01197	Intracranial aneurysm	0.40562
DB01197	Ischemia	0.16273
DB01197	Keratoconjunctivitis Sicca	0.56178
DB01197	Kidney disease	0.25228
DB01197	Leukemia	0.07314
DB01197	Lichen planus	0.93896
DB01197	Liver cancer	0.11318
DB01197	Liver metastases	0.24184
DB01197	Lung cancer	0.05116
DB01197	Lupus erythematosus	0.09958
DB01197	Lupus vulgaris	0.41151
DB01197	Macular degeneration	0.12039

DB01197	Melanoma	0.06638
DB01197	Meningioma	0.21074
DB01197	Metabolic syndrome X	0.20412
DB01197	Metabolism disease	0.33925
DB01197	Metastasis to lymph nodes	0.26525
DB01197	Migraine	0.10721
DB01197	Mucocutaneous lymph node syndrome	0.51181
DB01197	Multiple myeloma	0.10751
DB01197	Multiple sclerosis	0.09147
DB01197	Muscular dystrophy	0.73897
DB01197	Neoplasm metastasis	0.04698
DB01197	Obesity	0.28281
DB01197	Oral cancer	0.14457
DB01197	Ovarian disease	0.29672
DB01197	Ovary cancer	0.15879
DB01197	Parkinson disease	0.14197
DB01197	Periodontitis	0.53986
DB01197	Polycystic ovary syndrome	0.26768
DB01197	Polycythemia	0.19245
DB01197	Pre-Eclampsia	0.18869
DB01197	Premature birth	0.14003
DB01197	Primary tumor	0.11761
DB01197	Prostate cancer	0.06851
DB01197	Proteinuria	0.1291
DB01197	Psoriasis	0.13608
DB01197	Ptosis	0.68546
DB01197	Pulmonary fibrosis	0.30459
DB01197	Respiratory distress syndrome	0.16013
DB01197	Respiratory tract disease	0.27201
DB01197	Rheumatoid arthritis	0.08021
DB01197	Sarcoidosis	0.11323
DB01197	Schizophrenia	0.0439
DB01197	Severe acute respiratory syndrome	0.18257
DB01197	Sinusitis	0.56559
DB01197	Skin cancer	0.56031
DB01197	Skin disease	0.20002
DB01197	Stomach cancer	0.09754
DB01197	Stroke	0.12149
DB01197	Subacute sclerosing panencephalitis	1.24779
DB01197	Systemic infection	0.3793
DB01197	Systemic scleroderma	0.32538
DB01197	Takayasu's arteritis	0.60325
DB01197	Testicular dysfunction	0.08248
DB01197	Thrombophilia	0.13608
DB01197	Thyroid cancer	0.14166
DB01197	Transient hypertension of pregnancy	1.54772
DB01197	Tropical spastic paraparesis	0.42342

DB01197	Ulcerative colitis	0.12593
DB01197	Uterine disease	0.37577
DB01197	Varicosity	0.88866
DB01197	Vascular dementia	0.19245
DB01197	Vasculitis	0.50501
DB01296	Mycobacterium infection, Atypical	0.16667
DB01296	Pleural effusion, Malignant	0.19329
DB01296	Purpura, Thrombocytopenic, Idiopathic	0.13363
DB01296	Abortion	0.0625
DB01296	Adenoma	0.01894
DB01296	Alcoholic liver disease	0.20412
DB01296	Alopecia	0.09129
DB01296	Alzheimer's disease	0.10778
DB01296	Amnionitis	0.32599
DB01296	Amyloidosis	0.09449
DB01296	Amyotrophic lateral sclerosis	0.12622
DB01296	Anemia	0.2132
DB01296	Angiomyolipoma	0.18898
DB01296	Anorexia nervosa	0.20412
DB01296	Aortic aneurysm	0.05021
DB01296	Aplastic anemia	0.10426
DB01296	Arteriopathy	0.59483
DB01296	Arthritis	0.27638
DB01296	Asthma	0.12066
DB01296	Atherosclerosis	0.16364
DB01296	Autoimmune disease	0.1582
DB01296	Azoospermia	0.07781
DB01296	Basal cell carcinoma	0.13868
DB01296	Behcet syndrome	0.31289
DB01296	Biliary Atresia	0.15138
DB01296	Bladder cancer	0.06455
DB01296	Brain tumor	0.0283
DB01296	Breast cancer	0.16599
DB01296	Bronchiectasis	0.20412
DB01296	Bronchiolitis obliterans	0.7615
DB01296	Bronchopulmonary dysplasia	0.44535
DB01296	Brucellosis	0.15076
DB01296	Cancer	0.09997
DB01296	Celiac disease	0.1644
DB01296	Cervical cancer	0.18872
DB01296	Choriocarcinoma	0.14434
DB01296	Chronic obstructive airway disease	0.15838
DB01296	Colon cancer	0.10238
DB01296	Communicable disease	0.10911
DB01296	Congenital abnormality	0.03084
DB01296	Cystic fibrosis	0.15037
DB01296	Dental plaque	0.1461

DB01296	Dermatitis	0.26205
DB01296	Diabetes mellitus	0.17933
DB01296	Down syndrome	0.05698
DB01296	Embryoma	0.1184
DB01296	Emphysema	0.26604
DB01296	Endometriosis	0.30644
DB01296	Enteritis	0.17819
DB01296	Esotropia	0.22335
DB01296	Familial Mediterranean fever	0.2582
DB01296	Fanconi's anemia	0.2
DB01296	Gastritis	0.14652
DB01296	Glaucoma	0.15145
DB01296	Glomerulonephritis	0.36177
DB01296	Granulomatous disease	0.14434
DB01296	Growth retardation	0.20811
DB01296	HIV infection	0.07916
DB01296	HTLV-I infection	0.30487
DB01296	Heart failure	0.18467
DB01296	Helicobacter infection	0.29699
DB01296	Hemolytic anemia	0.125
DB01296	Henoch-Schoenlein purpura	0.58628
DB01296	Hepatitis B	0.57973
DB01296	Hepatitis C	0.07372
DB01296	Histiocytosis	0.16667
DB01296	Hodgkin's disease	0.30955
DB01296	Hypertension	0.10377
DB01296	Hyperthyroidism	0.16667
DB01296	Hypothyroidism	0.11785
DB01296	IGA glomerulonephritis	0.30568
DB01296	Infection	0.06619
DB01296	Infectious lung disease	0.22768
DB01296	Infertility	0.06868
DB01296	Infiltrating cancer	0.65389
DB01296	Influenza	0.1
DB01296	Intermediate coronary syndrome	0.16667
DB01296	Intracranial aneurysm	0.25904
DB01296	Intraocular melanoma	0.15811
DB01296	Ischemia	0.12074
DB01296	Keratoconjunctivitis Sicca	0.40253
DB01296	Kidney disease	0.17681
DB01296	Leprosy	0.13363
DB01296	Leukemia	0.16624
DB01296	Lichen planus	0.68267
DB01296	Lipodystrophy	0.13868
DB01296	Liver cancer	0.27213
DB01296	Liver disease	0.07715
DB01296	Liver metastases	0.15445

DB01296	Lung cancer	0.08393
DB01296	Lupus erythematosus	0.11753
DB01296	Lupus vulgaris	0.21793
DB01296	Lymphoma	0.02034
DB01296	Malaria	0.09285
DB01296	Malignant glioma	0.09285
DB01296	Melanoma	0.10525
DB01296	Meningioma	0.13459
DB01296	Metabolism disease	0.17864
DB01296	Metastasis to lymph nodes	0.1694
DB01296	Migraine	0.09285
DB01296	Mucocutaneous lymph node syndrome	0.38416
DB01296	Multiple myeloma	0.19997
DB01296	Multiple sclerosis	0.42177
DB01296	Muscular dystrophies	0.09806
DB01296	Muscular dystrophy	0.16616
DB01296	Mycoses	0.22361
DB01296	Narcolepsy	0.18898
DB01296	Nasopharyngeal cancer	0.19245
DB01296	Necrotizing enterocolitis	0.20412
DB01296	Neoplasm metastasis	0.15898
DB01296	Nephrosis	0.47505
DB01296	Neutropenia	0.20412
DB01296	Obesity	0.24069
DB01296	Oral cancer	0.11019
DB01296	Otitis media	0.16667
DB01296	Ovarian cancer	0.05025
DB01296	Ovarian disease	0.22044
DB01296	Ovary cancer	0.21321
DB01296	Pancreas cancer	0.1037
DB01296	Pancreatitis	0.47534
DB01296	Parkinson disease	0.10516
DB01296	Pelvic inflammatory disease	0.22361
DB01296	Periodontitis	0.33317
DB01296	Polyarthritis	0.23185
DB01296	Polycystic ovary syndrome	0.12111
DB01296	Pre-Eclampsia	0.1949
DB01296	Primary tumor	0.07511
DB01296	Prostate cancer	0.21726
DB01296	Psoriasis	0.11785
DB01296	Pulmonary fibrosis	0.09449
DB01296	Renal Cell cancer	0.05812
DB01296	Respiratory tract disease	0.2017
DB01296	Rheumatoid arthritis	0.0423
DB01296	Sarcoidosis	0.09806
DB01296	Schizophrenia	0.05756
DB01296	Serous cancer	0.25

DB01296	Silicosis	0.18898
DB01296	Sinusitis	0.20004
DB01296	Skin cancer	0.21431
DB01296	Skin disease	0.15025
DB01296	Solid tumor	0.14434
DB01296	Squamous cell cancer	0.29887
DB01296	Stomach cancer	0.1192
DB01296	Stroke	0.30873
DB01296	Subacute sclerosing panencephalitis	0.67816
DB01296	Systemic infection	0.3456
DB01296	Systemic scleroderma	0.11882
DB01296	Takayasu's arteritis	0.22646
DB01296	Testicular dysfunction	0.02175
DB01296	Thrombophilia	0.11785
DB01296	Thyroid cancer	0.17751
DB01296	Transient hypertension of pregnancy	0.49531
DB01296	Tropical spastic paraparesis	0.32003
DB01296	Tuberculosis	0.39588
DB01296	Tuberous sclerosis	0.1723
DB01296	Ulcerative colitis	0.3344
DB01296	Uveitis	0.125
DB01296	Vaccinia	0.15076
DB01296	Varicosity	0.61715
DB01296	Virus disease	0.23859
DB01296	Yersinia infection	0.03674
DB00126	Hypertension, Pulmonary	0.22047
DB00126	Skin disease, Genetic	0.03666
DB00126	Stress disorder, post-traumatic	0.07217
DB00126	Amyotrophic lateral sclerosis	0.06623
DB00126	Aseptic necrosis of bone	0.17408
DB00126	Atherosclerosis	0.08803
DB00126	Autoimmune disease	0.02214
DB00126	Barrett's esophagus	0.13997
DB00126	Behavior disease	0.0315
DB00126	Cancer	0.03361
DB00126	Colon cancer	0.01211
DB00126	Congenital abnormality	0.07515
DB00126	Dermatitis	0.09701
DB00126	Diabetes mellitus	0.06042
DB00126	Drug abuse	0.07522
DB00126	Encephalopathies	0.26931
DB00126	Epilepsy	0.02887
DB00126	Hyperglycemia	0.24453
DB00126	Hypertension	0.01614
DB00126	Infection	0.10177
DB00126	Ischemia	0.14753
DB00126	Lymphoma	0.02326

DB00126	Melanoma	0.11027
DB00126	Metabolism disease	0.2564
DB00126	Moyamoya disease	0.43144
DB00126	Multiple system atrophy	0.11785
DB00126	Myopathy	0.17097
DB00126	Obesity	0.11168
DB00126	Polyarthritis	0.17979
DB00126	Polycythemia	0.27136
DB00126	Pre-Eclampsia	0.20042
DB00126	Prostate cancer	0.02131
DB00126	Psychotic disorder	0.03269
DB00126	Retinal disease	0.29102
DB00126	Stroke	0.09323
DB00126	Vascular disease	0.03727
DB00126	Vasculitis	0.44886
DB00172	Adenovirus infection	0.00522
DB00172	Amyotrophic lateral sclerosis	0.05959
DB00172	Barrett's esophagus	0.00912
DB00172	Brain tumor	0.04828
DB00172	Breast cancer	0.00371
DB00172	Cancer	0.01208
DB00172	Carcinoma	0.01408
DB00172	Colon cancer	0.06683
DB00172	Connective tissue disease	0.02928
DB00172	Embryoma	0.00544
DB00172	Esophageal tumor	0.02424
DB00172	Esophagus cancer	0.00968
DB00172	Eye disease	0.03458
DB00172	Gigantism	0.27923
DB00172	Huntington disease	0.1279
DB00172	Infection	0.00663
DB00172	Ischemia	0.0813
DB00172	Leigh disease	0.021
DB00172	Leukoencephalopathy	0.0074
DB00172	Lung cancer	0.04683
DB00172	Lupus erythematosus	0.00544
DB00172	Lupus vulgaris	0.0148
DB00172	Nephroblastoma	0.03438
DB00172	Neuroblastoma	0.00959
DB00172	Neurodegenerative disorder	0.08578
DB00172	Obesity	0.02406
DB00172	Pancreas cancer	0.01328
DB00172	Polyarthritis	0.10323
DB00172	Prostate cancer	0.06412
DB00172	Retinal disease	0.01897
DB00172	Retinitis pigmentosa	0.03767
DB00172	Solid tumor	0.06862

DB00172	Spinal cord disease	0.02321
DB00172	Stomach cancer	0.0053
DB00172	Testicular dysfunction	0.04408
DB00172	Thymoma	0.09356
DB00172	Thyroid cancer	0.09169
DB00172	Vaccinia	0.0928
DB00172	Werner syndrome	0.01299
DB01275	Amyotrophic lateral sclerosis	0.10747
DB01275	Polyarthritis	0.17705
DB00177	Abortion	0.125
DB00177	Adenocarcinoma	0.14586
DB00177	Adenoma	0.1715
DB00177	Adrenal gland tumor	0.30151
DB00177	Atherosclerosis	0.07001
DB00177	Brain ischemia	0.44721
DB00177	Breast cancer	0.04811
DB00177	Cardiovascular disease	0.1525
DB00177	Depression	0.13131
DB00177	Diabetes mellitus	0.05263
DB00177	Embryoma	0.06166
DB00177	Esotropia	0.28868
DB00177	Hamman-Rich syndrome	0.19245
DB00177	Heart disease	0.30151
DB00177	Hyperaldosteronism	0.31623
DB00177	Hypercholesterolemia	0.19245
DB00177	Hypertension	0.07906
DB00177	IGA glomerulonephritis	0.1715
DB00177	Ischemia	0.12804
DB00177	Kidney disease	0.11952
DB00177	Lupus erythematosus	0.08544
DB00177	Lupus vulgaris	0.18898
DB00177	Mucocutaneous lymph node syndrome	0.21822
DB00177	Obesity	0.07692
DB00177	Ovarian cancer	0.1005
DB00177	Premature birth	0.24254
DB00177	Rheumatoid arthritis	0.0612
DB00177	Stomach cancer	0.08422
DB00177	Systemic scleroderma	0.10483
DB00275	Abortion	0.125
DB00275	Adenocarcinoma	0.14586
DB00275	Adenoma	0.1715
DB00275	Adrenal gland tumor	0.30151
DB00275	Atherosclerosis	0.07001
DB00275	Brain ischemia	0.44721
DB00275	Breast cancer	0.04811
DB00275	Cardiovascular disease	0.1525
DB00275	Depression	0.13131

DB00275	Diabetes mellitus	0.05263
DB00275	Embryoma	0.06166
DB00275	Esotropia	0.28868
DB00275	Hamman-Rich syndrome	0.19245
DB00275	Heart disease	0.30151
DB00275	Hyperaldosteronism	0.31623
DB00275	Hypercholesterolemia	0.19245
DB00275	Hypertension	0.07906
DB00275	IGA glomerulonephritis	0.1715
DB00275	Ischemia	0.12804
DB00275	Kidney disease	0.11952
DB00275	Lupus erythematosus	0.08544
DB00275	Lupus vulgaris	0.18898
DB00275	Mucocutaneous lymph node syndrome	0.21822
DB00275	Obesity	0.07692
DB00275	Ovarian cancer	0.1005
DB00275	Premature birth	0.24254
DB00275	Rheumatoid arthritis	0.0612
DB00275	Stomach cancer	0.08422
DB00275	Systemic scleroderma	0.10483
DB00678	Abortion	0.125
DB00678	Adenocarcinoma	0.14586
DB00678	Adenoma	0.1715
DB00678	Adrenal gland tumor	0.30151
DB00678	Atherosclerosis	0.07001
DB00678	Brain ischemia	0.44721
DB00678	Breast cancer	0.04811
DB00678	Cardiovascular disease	0.1525
DB00678	Depression	0.13131
DB00678	Diabetes mellitus	0.05263
DB00678	Embryoma	0.06166
DB00678	Esotropia	0.28868
DB00678	Hamman-Rich syndrome	0.19245
DB00678	Heart disease	0.30151
DB00678	Hyperaldosteronism	0.31623
DB00678	Hypercholesterolemia	0.19245
DB00678	Hypertension	0.07906
DB00678	IGA glomerulonephritis	0.1715
DB00678	Ischemia	0.12804
DB00678	Kidney disease	0.11952
DB00678	Lupus erythematosus	0.08544
DB00678	Lupus vulgaris	0.18898
DB00678	Mucocutaneous lymph node syndrome	0.21822
DB00678	Obesity	0.07692
DB00678	Ovarian cancer	0.1005
DB00678	Premature birth	0.24254
DB00678	Rheumatoid arthritis	0.0612

DB00678	Stomach cancer	0.08422
DB00678	Systemic scleroderma	0.10483
DB00796	Abortion	0.125
DB00796	Adenocarcinoma	0.14586
DB00796	Adenoma	0.1715
DB00796	Adrenal gland tumor	0.30151
DB00796	Atherosclerosis	0.07001
DB00796	Brain ischemia	0.44721
DB00796	Breast cancer	0.04811
DB00796	Cardiovascular disease	0.1525
DB00796	Depression	0.13131
DB00796	Diabetes mellitus	0.05263
DB00796	Embryoma	0.06166
DB00796	Esotropia	0.28868
DB00796	Hamman-Rich syndrome	0.19245
DB00796	Heart disease	0.30151
DB00796	Hyperaldosteronism	0.31623
DB00796	Hypercholesterolemia	0.19245
DB00796	Hypertension	0.07906
DB00796	IGA glomerulonephritis	0.1715
DB00796	Ischemia	0.12804
DB00796	Kidney disease	0.11952
DB00796	Lupus erythematosus	0.08544
DB00796	Lupus vulgaris	0.18898
DB00796	Mucocutaneous lymph node syndrome	0.21822
DB00796	Obesity	0.07692
DB00796	Ovarian cancer	0.1005
DB00796	Premature birth	0.24254
DB00796	Rheumatoid arthritis	0.0612
DB00796	Stomach cancer	0.08422
DB00796	Systemic scleroderma	0.10483
DB00876	Abortion	0.125
DB00876	Adenocarcinoma	0.14586
DB00876	Adenoma	0.1715
DB00876	Adrenal gland tumor	0.30151
DB00876	Atherosclerosis	0.07001
DB00876	Brain ischemia	0.44721
DB00876	Breast cancer	0.04811
DB00876	Cardiovascular disease	0.1525
DB00876	Depression	0.13131
DB00876	Diabetes mellitus	0.05263
DB00876	Embryoma	0.06166
DB00876	Esotropia	0.28868
DB00876	Hamman-Rich syndrome	0.19245
DB00876	Heart disease	0.30151
DB00876	Hyperaldosteronism	0.31623
DB00876	Hypercholesterolemia	0.19245

DB00876	Hypertension	0.07906
DB00876	IGA glomerulonephritis	0.1715
DB00876	Ischemia	0.12804
DB00876	Kidney disease	0.11952
DB00876	Lupus erythematosus	0.08544
DB00876	Lupus vulgaris	0.18898
DB00876	Mucocutaneous lymph node syndrome	0.21822
DB00876	Obesity	0.07692
DB00876	Ovarian cancer	0.1005
DB00876	Premature birth	0.24254
DB00876	Rheumatoid arthritis	0.0612
DB00876	Stomach cancer	0.08422
DB00876	Systemic scleroderma	0.10483
DB00966	Abortion	0.08839
DB00966	Adenocarcinoma	0.10314
DB00966	Adenoma	0.12127
DB00966	Adrenal gland tumor	0.2132
DB00966	Atherosclerosis	0.04951
DB00966	Brain ischemia	0.31623
DB00966	Breast cancer	0.03402
DB00966	Cardiovascular disease	0.10783
DB00966	Depression	0.09285
DB00966	Diabetes mellitus	0.03722
DB00966	Embryoma	0.0436
DB00966	Esotropia	0.20412
DB00966	Hamman-Rich syndrome	0.13608
DB00966	Heart disease	0.2132
DB00966	Hyperaldosteronism	0.22361
DB00966	Hypercholesterolemia	0.13608
DB00966	Hypertension	0.0559
DB00966	IGA glomerulonephritis	0.12127
DB00966	Ischemia	0.09054
DB00966	Kidney disease	0.08452
DB00966	Lupus erythematosus	0.06041
DB00966	Lupus vulgaris	0.13363
DB00966	Mucocutaneous lymph node syndrome	0.1543
DB00966	Obesity	0.05439
DB00966	Ovarian cancer	0.07107
DB00966	Premature birth	0.1715
DB00966	Rheumatoid arthritis	0.04327
DB00966	Stomach cancer	0.05955
DB00966	Systemic scleroderma	0.07412
DB01029	Hypertension, Pulmonary	0.72528
DB01029	Infertility, Male	0.18793
DB01029	Abortion	0.08839
DB01029	Adenocarcinoma	0.10314
DB01029	Adenoma	0.20505

DB01029	Adrenal gland tumor	0.2132
DB01029	Alzheimer's disease	0.08782
DB01029	Amyotrophic lateral sclerosis	0.0637
DB01029	Aplastic anemia	0.05574
DB01029	Aseptic necrosis of bone	0.05495
DB01029	Atherosclerosis	0.11133
DB01029	Bipolar disorder	0.15045
DB01029	Bladder cancer	0.0693
DB01029	Brain ischemia	0.31623
DB01029	Breast cancer	0.06994
DB01029	Bronchial hyperreactivity	0.27337
DB01029	Cancer	0.13562
DB01029	Capillaries disease	0.11759
DB01029	Cardiovascular disease	0.10783
DB01029	Cerebrovascular disorder	0.18791
DB01029	Cholelithiasis	0.16417
DB01029	Choriocarcinoma	0.12126
DB01029	Colon cancer	0.02625
DB01029	Congenital abnormality	0.02372
DB01029	Craniosynostosis	1.2391
DB01029	Dental plaque	0.10669
DB01029	Depression	0.1967
DB01029	Dermatitis	0.08777
DB01029	Diabetes mellitus	0.24866
DB01029	Down syndrome	0.1547
DB01029	Eating disorder	0.07634
DB01029	Embryoma	0.09281
DB01029	Endometriosis	0.08115
DB01029	Esophagus cancer	0.0469
DB01029	Esotropia	0.20412
DB01029	Ewings sarcoma	0.1109
DB01029	Glaucoma	0.12443
DB01029	Hamman-Rich syndrome	0.13608
DB01029	Heart disease	0.2132
DB01029	Hepatitis	0.89358
DB01029	Herpes	0.12038
DB01029	Hyperaldosteronism	0.22361
DB01029	Hypercholesterolemia	0.13608
DB01029	Hyperlipidemia	0.2083
DB01029	Hypertension	0.13888
DB01029	IGA glomerulonephritis	0.12127
DB01029	Infertility	0.12007
DB01029	Ischemia	0.09054
DB01029	Kidney disease	0.08452
DB01029	Kidney failure	0.10538
DB01029	Late pregnancy	0.25806
DB01029	Leukemia	0.02129

DB01029	Leukoencephalopathy	0.03584
DB01029	Lupus erythematosus	0.36158
DB01029	Lupus vulgaris	0.29308
DB01029	Lymphoma	0.04822
DB01029	Migraine	0.14736
DB01029	Mucocutaneous lymph node syndrome	0.1543
DB01029	Myasthenia Gravis	0.19952
DB01029	Obesity	0.13283
DB01029	Oral cancer	0.03461
DB01029	Osteoporosis	0.11955
DB01029	Ovarian cancer	0.07107
DB01029	Ovarian disease	0.18741
DB01029	Ovarian failure	0.24506
DB01029	Panic disorder	0.18823
DB01029	Parkinson disease	0.09124
DB01029	Polyarthritis	0.12536
DB01029	Premature birth	0.1715
DB01029	Primary biliary cirrhosis	0.14289
DB01029	Prostate cancer	0.05767
DB01029	Rabies	0.13205
DB01029	Renal tubular acidosis	0.10816
DB01029	Rheumatoid arthritis	0.24202
DB01029	Schizophrenia	0.34692
DB01029	Scleroderma	0.07046
DB01029	Sicca syndrome	0.63536
DB01029	Stomach cancer	0.10742
DB01029	Stroke	0.06547
DB01029	Synovitis	0.21726
DB01029	Systemic scleroderma	0.15482
DB01029	Thymoma	0.16862
DB01029	Ulcerative colitis	0.03481
DB01029	Uterine disease	0.21753
DB01029	Vaccinia	0.82145
DB01029	Vascular disease	0.12144
DB01029	Vitiligo	0.19952
DB01342	Abortion	0.125
DB01342	Adenocarcinoma	0.14586
DB01342	Adenoma	0.1715
DB01342	Adrenal gland tumor	0.30151
DB01342	Atherosclerosis	0.07001
DB01342	Brain ischemia	0.44721
DB01342	Breast cancer	0.04811
DB01342	Cardiovascular disease	0.1525
DB01342	Depression	0.13131
DB01342	Diabetes mellitus	0.05263
DB01342	Embryoma	0.06166
DB01342	Esotropia	0.28868

DB01342	Hamman-Rich syndrome	0.19245
DB01342	Heart disease	0.30151
DB01342	Hyperaldosteronism	0.31623
DB01342	Hypercholesterolemia	0.19245
DB01342	Hypertension	0.07906
DB01342	IGA glomerulonephritis	0.1715
DB01342	Ischemia	0.12804
DB01342	Kidney disease	0.11952
DB01342	Lupus erythematosus	0.08544
DB01342	Lupus vulgaris	0.18898
DB01342	Mucocutaneous lymph node syndrome	0.21822
DB01342	Obesity	0.07692
DB01342	Ovarian cancer	0.1005
DB01342	Premature birth	0.24254
DB01342	Rheumatoid arthritis	0.0612
DB01342	Stomach cancer	0.08422
DB01342	Systemic scleroderma	0.10483
DB01347	Abortion	0.125
DB01347	Adenocarcinoma	0.14586
DB01347	Adenoma	0.1715
DB01347	Adrenal gland tumor	0.30151
DB01347	Atherosclerosis	0.07001
DB01347	Brain ischemia	0.44721
DB01347	Breast cancer	0.04811
DB01347	Cardiovascular disease	0.1525
DB01347	Depression	0.13131
DB01347	Diabetes mellitus	0.05263
DB01347	Embryoma	0.06166
DB01347	Esotropia	0.28868
DB01347	Hamman-Rich syndrome	0.19245
DB01347	Heart disease	0.30151
DB01347	Hyperaldosteronism	0.31623
DB01347	Hypercholesterolemia	0.19245
DB01347	Hypertension	0.07906
DB01347	IGA glomerulonephritis	0.1715
DB01347	Ischemia	0.12804
DB01347	Kidney disease	0.11952
DB01347	Lupus erythematosus	0.08544
DB01347	Lupus vulgaris	0.18898
DB01347	Mucocutaneous lymph node syndrome	0.21822
DB01347	Obesity	0.07692
DB01347	Ovarian cancer	0.1005
DB01347	Premature birth	0.24254
DB01347	Rheumatoid arthritis	0.0612
DB01347	Stomach cancer	0.08422
DB01347	Systemic scleroderma	0.10483
DB01349	Abortion	0.08839

DB01349	Adenocarcinoma	0.10314
DB01349	Adenoma	0.12127
DB01349	Adrenal gland tumor	0.4264
DB01349	Atherosclerosis	0.04951
DB01349	Brain ischemia	0.31623
DB01349	Breast cancer	0.06804
DB01349	Cardiovascular disease	0.21567
DB01349	Depression	0.09285
DB01349	Diabetes mellitus	0.07443
DB01349	Embryoma	0.0872
DB01349	Esotropia	0.20412
DB01349	Hamman-Rich syndrome	0.13608
DB01349	Heart disease	0.2132
DB01349	Hyperaldosteronism	0.22361
DB01349	Hypercholesterolemia	0.13608
DB01349	Hypertension	0.0559
DB01349	IGA glomerulonephritis	0.24254
DB01349	Ischemia	0.18107
DB01349	Kidney disease	0.08452
DB01349	Lupus erythematosus	0.06041
DB01349	Lupus vulgaris	0.13363
DB01349	Mental retardation	0.09129
DB01349	Mucocutaneous lymph node syndrome	0.1543
DB01349	Obesity	0.05439
DB01349	Ovarian cancer	0.07107
DB01349	Premature birth	0.1715
DB01349	Rheumatoid arthritis	0.04327
DB01349	Stomach cancer	0.05955
DB01349	Systemic scleroderma	0.07412
DB01349	Tuberculosis	0.09535
DB08822	Abortion	0.125
DB08822	Adenocarcinoma	0.14586
DB08822	Adenoma	0.1715
DB08822	Adrenal gland tumor	0.30151
DB08822	Atherosclerosis	0.07001
DB08822	Brain ischemia	0.44721
DB08822	Breast cancer	0.04811
DB08822	Cardiovascular disease	0.1525
DB08822	Depression	0.13131
DB08822	Diabetes mellitus	0.05263
DB08822	Embryoma	0.06166
DB08822	Esotropia	0.28868
DB08822	Hamman-Rich syndrome	0.19245
DB08822	Heart disease	0.30151
DB08822	Hyperaldosteronism	0.31623
DB08822	Hypercholesterolemia	0.19245
DB08822	Hypertension	0.07906

DB08822	IGA glomerulonephritis	0.1715
DB08822	Ischemia	0.12804
DB08822	Kidney disease	0.11952
DB08822	Lupus erythematosus	0.08544
DB08822	Lupus vulgaris	0.18898
DB08822	Mucocutaneous lymph node syndrome	0.21822
DB08822	Obesity	0.07692
DB08822	Ovarian cancer	0.1005
DB08822	Premature birth	0.24254
DB08822	Rheumatoid arthritis	0.0612
DB08822	Stomach cancer	0.08422
DB08822	Systemic scleroderma	0.10483
DB00133	Alzheimer's disease	0.02721
DB00133	Atherosclerosis	0.02646
DB00133	Bipolar disorder	0.0428
DB00133	Down syndrome	0.04307
DB00133	Embryoma	0.02331
DB00133	Encephalopathies	0.05293
DB00133	Hyperhomocysteinemia	0.10911
DB00133	Kidney disease	0.04518
DB00133	Lung cancer	0.0264
DB00133	Meningioma	0.09759
DB00133	Myopathy	0.19934
DB00133	Neuropathy	0.42572
DB00133	Schizophrenia	0.02874
DB00133	Stroke	0.0428
DB01088	Antiphospholipid syndrome	0.12599
DB01088	Atherosclerosis	0.12249
DB01088	Behavior disease	0.05832
DB01088	Cancer	0.02656
DB01088	Cardiovascular disease	0.29878
DB01088	Chronic obstructive airway disease	0.23347
DB01088	Depression	0.04963
DB01088	Diabetes mellitus	0.01989
DB01088	Hemorrhagic disorder	0.06682
DB01088	Hypertension	0.02988
DB01088	Ischemia	0.04839
DB01088	Late pregnancy	0.10911
DB01088	Leukemia	0.13849
DB01088	Liver disease	0.05832
DB01088	Lung cancer	0.0264
DB01088	Multiple sclerosis	0.03898
DB01088	Ovarian cancer	0.03799
DB01088	Pancreas cancer	0.03919
DB01088	Periodontitis	0.05764
DB01088	Polycystic ovary syndrome	0.05006
DB01088	Rheumatoid arthritis	0.02313

DB01088	Stroke	0.0428
DB01088	Systemic infection	0.04336
DB00073	Pemphigoid, Bullous	0.09535
DB00073	Purpura, Thrombocytopenic, Idiopathic	0.17018
DB00073	Abortion	0.03769
DB00073	Alopecia	0.16559
DB00073	Alzheimer's disease	0.0217
DB00073	Antiphospholipid syndrome	0.1005
DB00073	Asthma	0.02462
DB00073	Atherosclerosis	0.04222
DB00073	Autoimmune disease	0.09811
DB00073	Breast cancer	0.09287
DB00073	Brucellosis	0.09091
DB00073	Cancer	0.04026
DB00073	Celiac disease	0.04957
DB00073	Communicable disease	0.0658
DB00073	Cystic fibrosis	0.04307
DB00073	Diabetes mellitus	0.0524
DB00073	Embryoma	0.01859
DB00073	Enteritis	0.22055
DB00073	Generalized anxiety disorder	0.10488
DB00073	Glomerulonephritis	0.07538
DB00073	IGA glomerulonephritis	0.05171
DB00073	Immune complex disease	0.72242
DB00073	Infection by cryptococcus neoformans	0.34816
DB00073	Kaposi sarcoma	0.17225
DB00073	Kidney disease	0.03604
DB00073	Kidney failure	0.03414
DB00073	Leukemia	0.0585
DB00073	Lung disease	0.14609
DB00073	Lupus erythematosus	0.26633
DB00073	Lupus vulgaris	0.05698
DB00073	Lymphoma	0.13249
DB00073	Melanoma	0.02513
DB00073	Multiple myeloma	0.1549
DB00073	Multiple sclerosis	0.0311
DB00073	Neoplasm metastasis	0.02454
DB00073	Periodontal disease	0.07107
DB00073	Periodontitis	0.09196
DB00073	Primary tumor	0.16944
DB00073	Renal Cell cancer	0.20449
DB00073	Rheumatic fever	0.07785
DB00073	Rheumatoid arthritis	0.01845
DB00073	Skin tumor	0.15762
DB00073	Stomach cancer	0.07048
DB00073	Systemic infection	0.13174
DB00073	Thrombocytopenia	0.07538

DB00073	Vascular disease	0.05505
DB00073	Yersinia infection	0.04303
DB00078	Pemphigoid, Bullous	0.09535
DB00078	Purpura, Thrombocytopenic, Idiopathic	0.17018
DB00078	Abortion	0.03769
DB00078	Alopecia	0.16559
DB00078	Alzheimer's disease	0.0217
DB00078	Antiphospholipid syndrome	0.1005
DB00078	Asthma	0.02462
DB00078	Atherosclerosis	0.04222
DB00078	Autoimmune disease	0.09811
DB00078	Breast cancer	0.09287
DB00078	Brucellosis	0.09091
DB00078	Cancer	0.04026
DB00078	Celiac disease	0.04957
DB00078	Communicable disease	0.0658
DB00078	Cystic fibrosis	0.04307
DB00078	Diabetes mellitus	0.0524
DB00078	Embryoma	0.01859
DB00078	Enteritis	0.22055
DB00078	Generalized anxiety disorder	0.10488
DB00078	Glomerulonephritis	0.07538
DB00078	IGA glomerulonephritis	0.05171
DB00078	Immune complex disease	0.72242
DB00078	Infection by cryptococcus neoformans	0.34816
DB00078	Kaposi sarcoma	0.17225
DB00078	Kidney disease	0.03604
DB00078	Kidney failure	0.03414
DB00078	Leukemia	0.0585
DB00078	Lung disease	0.14609
DB00078	Lupus erythematosus	0.26633
DB00078	Lupus vulgaris	0.05698
DB00078	Lymphoma	0.13249
DB00078	Melanoma	0.02513
DB00078	Multiple myeloma	0.1549
DB00078	Multiple sclerosis	0.0311
DB00078	Neoplasm metastasis	0.02454
DB00078	Periodontal disease	0.07107
DB00078	Periodontitis	0.09196
DB00078	Primary tumor	0.16944
DB00078	Renal Cell cancer	0.20449
DB00078	Rheumatic fever	0.07785
DB00078	Rheumatoid arthritis	0.01845
DB00078	Skin tumor	0.15762
DB00078	Stomach cancer	0.07048
DB00078	Systemic infection	0.13174
DB00078	Thrombocytopenia	0.07538

DB00078	Vascular disease	0.05505
DB00078	Yersinia infection	0.04303
DB00081	Pemphigoid, Bullous	0.1
DB00081	Purpura, Thrombocytopenic, Idiopathic	0.17411
DB00081	Abortion	0.03953
DB00081	Alopecia	0.16828
DB00081	Alzheimer's disease	0.02276
DB00081	Antiphospholipid syndrome	0.10541
DB00081	Asthma	0.02582
DB00081	Atherosclerosis	0.04428
DB00081	Autoimmune disease	0.1029
DB00081	Breast cancer	0.09499
DB00081	Brucellosis	0.09535
DB00081	Cancer	0.0408
DB00081	Celiac disease	0.05199
DB00081	Communicable disease	0.06901
DB00081	Cystic fibrosis	0.04518
DB00081	Diabetes mellitus	0.0524
DB00081	Embryoma	0.0195
DB00081	Enteritis	0.22378
DB00081	Generalized anxiety disorder	0.10488
DB00081	Glomerulonephritis	0.07906
DB00081	IGA glomerulonephritis	0.05423
DB00081	Immune complex disease	0.73283
DB00081	Infection by cryptococcus neoformans	0.36515
DB00081	Kaposi sarcoma	0.17225
DB00081	Kidney disease	0.0378
DB00081	Kidney failure	0.03581
DB00081	Leukemia	0.0585
DB00081	Lung disease	0.14609
DB00081	Lupus erythematosus	0.27261
DB00081	Lupus vulgaris	0.05976
DB00081	Lymphoma	0.13249
DB00081	Melanoma	0.02635
DB00081	Multiple myeloma	0.1549
DB00081	Multiple sclerosis	0.03262
DB00081	Neoplasm metastasis	0.02573
DB00081	Periodontal disease	0.07454
DB00081	Periodontitis	0.09645
DB00081	Primary tumor	0.16944
DB00081	Renal Cell cancer	0.2062
DB00081	Rheumatic fever	0.08165
DB00081	Rheumatoid arthritis	0.01935
DB00081	Skin tumor	0.15762
DB00081	Stomach cancer	0.07048
DB00081	Systemic infection	0.13343
DB00081	Thrombocytopenia	0.07906

DB00081	Vascular disease	0.05774
DB00081	Yersinia infection	0.04303
DB08935	Purpura, Thrombocytopenic, Idiopathic	0.39397
DB08935	Breast cancer	0.06979
DB08935	Cancer	0.07808
DB08935	Diabetes mellitus	0.0741
DB08935	Kaposi sarcoma	0.11305
DB08935	Leukemia	0.0384
DB08935	Lung disease	0.2066
DB08935	Lupus erythematosus	0.04752
DB08935	Lymphoma	0.18737
DB08935	Multiple myeloma	0.10167
DB08935	Primary tumor	0.23962
DB08935	Renal Cell cancer	0.23962
DB03147	Stress disorder, post-traumatic	0.04527
DB03147	Adenoma	0.02196
DB03147	Adenovirus infection	0.01711
DB03147	Alzheimer's disease	0.03143
DB03147	Anorexia nervosa	0.02614
DB03147	Asthma	0.0589
DB03147	Atherosclerosis	0.00896
DB03147	Autistic disorder	0.04548
DB03147	Behavior disease	0.03951
DB03147	Bipolar disorder	0.02899
DB03147	Breast cancer	0.02447
DB03147	Cirrhosis	0.02164
DB03147	Colon cancer	0.09591
DB03147	Common cold	0.12545
DB03147	Depression	0.01681
DB03147	Diabetes mellitus	0.02884
DB03147	Down syndrome	0.01459
DB03147	Drug abuse	0.01199
DB03147	Embryoma	0.0079
DB03147	Encephalopathies	0.01793
DB03147	Fibromyalgia	0.09054
DB03147	Generalized anxiety disorder	0.03696
DB03147	Huntington disease	0.02794
DB03147	Hypertension	0.01012
DB03147	Ischemia	0.10265
DB03147	Kidney failure	0.08613
DB03147	Leukoencephalopathy	0.02134
DB03147	Liver cancer	0.05422
DB03147	Liver failure	0.04839
DB03147	Lung cancer	0.05863
DB03147	Mental retardation	0.04726
DB03147	Neoplasm metastasis	0.02334
DB03147	Neurodegenerative disorder	0.09003

DB03147	Neurotic disorder	0.04049
DB03147	Obesity	0.00985
DB03147	Panic disorder	0.02794
DB03147	Pervasive development disorder	0.03306
DB03147	Prostate cancer	0.07438
DB03147	Psychotic disorder	0.041
DB03147	Renal tubular acidosis	0.03288
DB03147	Respiratory failure	0.20205
DB03147	Rheumatoid arthritis	0.00784
DB03147	Schizophrenia	0.0217
DB03147	Sicca syndrome	0.02511
DB03147	Spinocerebellar ataxias	0.22164
DB03147	Stomach disease	0.04527
DB03147	Sudden infant death syndrome	0.02794
DB00121	Breast cancer	0.01604
DB00121	Lung cancer	0.04656
DB00755	Prostatic hypertrophy, Benign	0.08333
DB00755	Adenovirus infection	0.02564
DB00755	Adrenal gland hypofunction	0.20412
DB00755	Autistic disorder	0.04489
DB00755	Azoospermia	0.07217
DB00755	Breast cancer	0.0182
DB00755	Cancer	0.02606
DB00755	Dermatitis	0.06428
DB00755	Drug abuse	0.15396
DB00755	Embryoma	0.0218
DB00755	Endometrial cancer	0.06155
DB00755	Endometriosis	0.02936
DB00755	Esophagus cancer	0.0527
DB00755	Ewings sarcoma	0.11785
DB00755	Hyperlipidemia	0.06934
DB00755	Hypertension	0.02795
DB00755	Liver cancer	0.02744
DB00755	Mental retardation	0.07083
DB00755	Neoplasm metastasis	0.03498
DB00755	Pancreas cancer	0.03666
DB00755	Prostate cancer	0.11127
DB00755	Renal tubular acidosis	0.04928
DB00755	Respiratory tract disease	0.36393
DB00755	Rheumatoid arthritis	0.04327
DB00755	Schizophrenia	0.16074
DB00755	Thyroid cancer	0.06155
DB00755	Urogenital abnormalities	0.09449
DB00755	Uterine fibroids	0.125
DB00052	Acromegaly	0.2357
DB00052	Adenoma	0.12127
DB00052	Breast cancer	0.03402

DB00052	Congenital abnormality	0.0533
DB00052	Diabetes mellitus	0.03722
DB00052	Embryoma	0.0436
DB00052	Hypertension	0.0559
DB00052	Lung cancer	0.04939
DB00052	Pituitary tumor	0.19612
DB00052	Prostate cancer	0.03691
DB00052	Stroke	0.08006
DB00082	Acromegaly	0.33333
DB00082	Adenoma	0.1715
DB00082	Breast cancer	0.04811
DB00082	Congenital abnormality	0.07538
DB00082	Diabetes mellitus	0.05263
DB00082	Embryoma	0.06166
DB00082	Hypertension	0.07906
DB00082	Lung cancer	0.06984
DB00082	Pituitary tumor	0.27735
DB00082	Prostate cancer	0.0522
DB00082	Stroke	0.11323
DB00163	Skin disease, Genetic	0.06425
DB00163	Adenoid cystic cancer	0.25072
DB00163	Adenovirus infection	0.04117
DB00163	Alzheimer's disease	0.02964
DB00163	Asthma	0.02582
DB00163	Atherosclerosis	0.02214
DB00163	Brain disease	0.06494
DB00163	Brain tumor	0.05092
DB00163	Breast cancer	0.04149
DB00163	Cancer	0.02235
DB00163	Cytomegalovirus infection	0.08165
DB00163	Dental plaque	0.08852
DB00163	Diabetes mellitus	0.01664
DB00163	Embryoma	0.02659
DB00163	Endometrium cancer	0.0767
DB00163	Enteritis	0.03471
DB00163	Epilepsy	0.04472
DB00163	Herpes	0.02967
DB00163	Liver cancer	0.03615
DB00163	Melanoma	0.07167
DB00163	Neuroblastoma	0.04686
DB00163	Neurodegenerative disorder	0.06003
DB00163	Osteomyelitis	0.08668
DB00163	Osteosarcoma	0.06742
DB00163	Prostate cancer	0.02029
DB00163	Squamous cell cancer	0.05357
DB00163	Stomach cancer	0.05926
DB00163	Tuberculosis	0.04264

DB00163	Ulcerative colitis	0.03262
DB00163	Vitamin D deficiency	0.09735
DB00993	Arthritis	0.12403
DB00993	Ataxia telangiectasia	0.44721
DB00993	Colon cancer	0.05934
DB00993	Embryoma	0.06166
DB00993	Encephalopathies	0.14003
DB00993	Gouts	0.27735
DB00993	Kidney failure	0.11323
DB00993	Leukemia	0.05634
DB00993	Malaria	0.1857
DB01033	Arthritis	0.12403
DB01033	Ataxia telangiectasia	0.44721
DB01033	Colon cancer	0.05934
DB01033	Embryoma	0.06166
DB01033	Encephalopathies	0.14003
DB01033	Gouts	0.27735
DB01033	Kidney failure	0.11323
DB01033	Leukemia	0.05634
DB01033	Malaria	0.1857
DB00210	Prostatic hypertrophy, Benign	0.09623
DB00210	Adenovirus infection	0.01734
DB00210	Aseptic necrosis of bone	0.11511
DB00210	Autistic disorder	0.03036
DB00210	Breast cancer	0.07014
DB00210	Cancer	0.05232
DB00210	Congenital abnormality	0.03077
DB00210	Dermatitis	0.13837
DB00210	Drug abuse	0.09759
DB00210	Esophagus cancer	0.19979
DB00210	Hyperlipidemia	0.08006
DB00210	Leukemia	0.08608
DB00210	Leukoencephalopathy	0.17423
DB00210	Lung cancer	0.0562
DB00210	Mental retardation	0.04792
DB00210	Mucopolysaccharidosis	0.16633
DB00210	Neoplasm metastasis	0.02366
DB00210	Neuroblastoma	0.09735
DB00210	Obesity	0.10525
DB00210	Pancreas cancer	0.04233
DB00210	Prostate cancer	0.17385
DB00210	Renal tubular acidosis	0.03333
DB00210	Respiratory tract disease	0.28224
DB00210	Rheumatism	0.13277
DB00210	Rheumatoid arthritis	0.11521
DB00210	Schizophrenia	0.12159
DB00210	Thyroid cancer	0.07107

DB00307	Adenovirus infection	0.02032
DB00307	Aseptic necrosis of bone	0.09534
DB00307	Autistic disorder	0.03557
DB00307	Breast cancer	0.04559
DB00307	Cancer	0.03969
DB00307	Dermatitis	0.1581
DB00307	Drug abuse	0.1236
DB00307	Esophagus cancer	0.11508
DB00307	Hyperlipidemia	0.11323
DB00307	Leukemia	0.05225
DB00307	Leukoencephalopathy	0.08795
DB00307	Lung cancer	0.04655
DB00307	Mental retardation	0.05613
DB00307	Mucopolysaccharidosis	0.13776
DB00307	Neoplasm metastasis	0.02772
DB00307	Neuroblastoma	0.08063
DB00307	Obesity	0.06117
DB00307	Prostate cancer	0.17019
DB00307	Renal tubular acidosis	0.03905
DB00307	Respiratory tract disease	0.35174
DB00307	Rheumatism	0.10997
DB00307	Rheumatoid arthritis	0.03334
DB00307	Schizophrenia	0.14997
DB00307	Thyroid cancer	0.1005
DB00459	Prostatic hypertrophy, Benign	0.08909
DB00459	Adenovirus infection	0.01734
DB00459	Aseptic necrosis of bone	0.11511
DB00459	Autistic disorder	0.03036
DB00459	Breast cancer	0.06868
DB00459	Cancer	0.06402
DB00459	Cirrhosis	0.06389
DB00459	Congenital abnormality	0.02849
DB00459	Dermatitis	0.13287
DB00459	Drug abuse	0.09475
DB00459	Esophagus cancer	0.19528
DB00459	Hyperlipidemia	0.07412
DB00459	Leukemia	0.08438
DB00459	Leukoencephalopathy	0.16918
DB00459	Lung cancer	0.0562
DB00459	Mental retardation	0.04792
DB00459	Mucopolysaccharidosis	0.16633
DB00459	Neoplasm metastasis	0.02366
DB00459	Neuroblastoma	0.09735
DB00459	Obesity	0.10292
DB00459	Pancreas cancer	0.03919
DB00459	Prostate cancer	0.1691
DB00459	Renal tubular acidosis	0.03333

DB00459	Respiratory tract disease	0.27579
DB00459	Rheumatism	0.13277
DB00459	Rheumatoid arthritis	0.13278
DB00459	Schizophrenia	0.11929
DB00459	Thyroid cancer	0.0658
DB00523	Prostatic hypertrophy, Benign	0.09623
DB00523	Adenovirus infection	0.01734
DB00523	Aseptic necrosis of bone	0.11511
DB00523	Autistic disorder	0.03036
DB00523	Breast cancer	0.07014
DB00523	Cancer	0.05232
DB00523	Congenital abnormality	0.03077
DB00523	Dermatitis	0.13837
DB00523	Drug abuse	0.09759
DB00523	Esophagus cancer	0.19979
DB00523	Hyperlipidemia	0.08006
DB00523	Leukemia	0.08608
DB00523	Leukoencephalopathy	0.17423
DB00523	Lung cancer	0.0562
DB00523	Mental retardation	0.04792
DB00523	Mucopolysaccharidosis	0.16633
DB00523	Neoplasm metastasis	0.02366
DB00523	Neuroblastoma	0.09735
DB00523	Obesity	0.10525
DB00523	Pancreas cancer	0.04233
DB00523	Prostate cancer	0.17385
DB00523	Renal tubular acidosis	0.03333
DB00523	Respiratory tract disease	0.28224
DB00523	Rheumatism	0.13277
DB00523	Rheumatoid arthritis	0.11521
DB00523	Schizophrenia	0.12159
DB00523	Thyroid cancer	0.07107
DB00799	Prostatic hypertrophy, Benign	0.11785
DB00799	Adenovirus infection	0.02032
DB00799	Aseptic necrosis of bone	0.09534
DB00799	Autistic disorder	0.03557
DB00799	Breast cancer	0.06964
DB00799	Cancer	0.03684
DB00799	Congenital abnormality	0.03769
DB00799	Dermatitis	0.05313
DB00799	Drug abuse	0.11635
DB00799	Esophagus cancer	0.18961
DB00799	Leukemia	0.08042
DB00799	Leukoencephalopathy	0.17129
DB00799	Lung cancer	0.04655
DB00799	Mental retardation	0.05613
DB00799	Mucopolysaccharidosis	0.13776

DB00799	Neoplasm metastasis	0.02772
DB00799	Neuroblastoma	0.08063
DB00799	Obesity	0.09963
DB00799	Pancreas cancer	0.05185
DB00799	Prostate cancer	0.16211
DB00799	Renal tubular acidosis	0.03905
DB00799	Respiratory tract disease	0.33525
DB00799	Rheumatism	0.10997
DB00799	Rheumatoid arthritis	0.12514
DB00799	Schizophrenia	0.14408
DB00151	Alzheimer's disease	0.01996
DB00151	Atherosclerosis	0.03884
DB00151	Breast cancer	0.01334
DB00151	Bronchial disease	0.06727
DB00151	Cancer	0.01022
DB00151	Chronic obstructive airway disease	0.03101
DB00151	Colon cancer	0.01646
DB00151	Congenital abnormality	0.02091
DB00151	Diabetes mellitus	0.0146
DB00151	Down syndrome	0.03161
DB00151	Embryoma	0.0171
DB00151	Encephalopathies	0.03884
DB00151	Esophageal disease	0.11323
DB00151	Hyperhomocysteinemia	0.08006
DB00151	Hypertension	0.04385
DB00151	Lung cancer	0.03874
DB00151	Lung disease	0.04903
DB00151	Meningioma	0.07161
DB00151	Mycosis fungoides	0.06202
DB00151	Oral cancer	0.03774
DB00151	Reticulosarcoma	0.09806
DB00151	Schizophrenia	0.02109
DB00151	Stroke	0.0314
DB00148	Adenovirus infection	0.04324
DB00148	Alzheimer's disease	0.06492
DB00148	Behavior disease	0.06299
DB00148	Breast cancer	0.04342
DB00148	Cancer	0.04055
DB00148	Carcinoma	0.11655
DB00148	Chronic obstructive airway disease	0.04564
DB00148	Colon cancer	0.0691
DB00148	Congenital abnormality	0.04055
DB00148	Disseminated cancer	0.18647
DB00148	Embryoma	0.04508
DB00148	Gram-Negative bacterial infection	0.19177
DB00148	HIV infection	0.04388
DB00148	Hemolytic-Uremic syndrome	0.14997

DB00148	Infection	0.05491
DB00148	Leukemia	0.023
DB00148	Lung cancer	0.04586
DB00148	Muscular atrophy	0.1595
DB00148	Muscular dystrophies	0.10966
DB00148	Myopathy	0.09225
DB00148	Myotonic disorder	0.12667
DB00148	Neuropathy	0.19484
DB00148	Ovarian cancer	0.07632
DB00148	Polymyositis	0.10541
DB00148	Prion disease	0.17924
DB00148	Salmonella infection	0.13685
DB00148	Uveitis	0.33138
DB00148	Virus disease	0.10333
DB00087	Pemphigoid, Bullous	0.1
DB00087	Abortion	0.03953
DB00087	Alopecia	0.21406
DB00087	Alzheimer's disease	0.02276
DB00087	Antiphospholipid syndrome	0.10541
DB00087	Asthma	0.02582
DB00087	Atherosclerosis	0.04428
DB00087	Autoimmune disease	0.1029
DB00087	Breast cancer	0.04564
DB00087	Brucellosis	0.09535
DB00087	Celiac disease	0.05199
DB00087	Communicable disease	0.06901
DB00087	Cystic fibrosis	0.04518
DB00087	Embryoma	0.0195
DB00087	Enteritis	0.28772
DB00087	Generalized anxiety disorder	0.14832
DB00087	Glomerulonephritis	0.07906
DB00087	IGA glomerulonephritis	0.05423
DB00087	Immune complex disease	0.94376
DB00087	Infection by cryptococcus neoformans	0.36515
DB00087	Kaposi sarcoma	0.13054
DB00087	Kidney disease	0.0378
DB00087	Kidney failure	0.03581
DB00087	Leukemia	0.06216
DB00087	Lupus erythematosus	0.2799
DB00087	Lupus vulgaris	0.05976
DB00087	Melanoma	0.02635
DB00087	Multiple myeloma	0.11739
DB00087	Multiple sclerosis	0.03262
DB00087	Neoplasm metastasis	0.02573
DB00087	Overnutrition	0.08165
DB00087	Periodontal disease	0.07454
DB00087	Periodontitis	0.09645

DB00087	Renal Cell cancer	0.03676
DB00087	Rheumatic fever	0.08165
DB00087	Rheumatoid arthritis	0.01935
DB00087	Skin tumor	0.2229
DB00087	Stomach cancer	0.09968
DB00087	Systemic infection	0.17367
DB00087	Thrombocytopenia	0.07906
DB00087	Vascular disease	0.05774
DB00087	Yersinia infection	0.06085
DB00604	Anorexia nervosa	0.10206
DB00604	Autistic disorder	0.06063
DB00604	Behavior disease	0.07715
DB00604	Bipolar disorder	0.05661
DB00604	Cancer	0.01843
DB00604	Choriocarcinoma	0.14434
DB00604	Chronic fatigue syndrome	0.17678
DB00604	Colon cancer	0.05934
DB00604	Depression	0.06565
DB00604	Dermatitis	0.04545
DB00604	Drug abuse	0.04683
DB00604	Drug-Induced dyskinesia	0.18898
DB00604	Eating disorder	0.08704
DB00604	Embryoma	0.03083
DB00604	Gilles de la Tourette syndrome	0.18898
DB00604	Heart failure	0.0533
DB00604	Herpes	0.07143
DB00604	Hypertension	0.07906
DB00604	Leukemia	0.02817
DB00604	Long QT syndrome	0.14434
DB00604	Obesity	0.03846
DB00604	Obsessive-compulsive disorder	0.30151
DB00604	Overnutrition	0.1291
DB00604	Panic disorder	0.21822
DB00604	Psychotic disorder	0.08006
DB00604	Rheumatoid arthritis	0.0306
DB00604	Schizophrenia	0.03801
DB00604	Stroke	0.05661
DB00604	Sudden infant death syndrome	0.10911
DB00904	Abortion	0.0424
DB00904	Amyotrophic lateral sclerosis	0.31825
DB00904	Anorexia nervosa	0.23774
DB00904	Attention deficit hyperactivity disorder	0.18415
DB00904	Behavior disease	0.89264
DB00904	Bipolar disorder	0.30179
DB00904	Colon cancer	0.02654
DB00904	Depression	0.45103
DB00904	Dermatitis	0.27203

DB00904	Diabetes mellitus	0.16763
DB00904	Drug abuse	0.13006
DB00904	Eating disorder	0.07785
DB00904	Embryoma	0.02758
DB00904	Epilepsy	0.50244
DB00904	Gilles de la Tourette syndrome	0.16903
DB00904	Hepatitis C	0.53889
DB00904	Herpes	0.06389
DB00904	Hypertension	0.49243
DB00904	Leukemia	0.0252
DB00904	Migraine	0.5835
DB00904	Obsessive-compulsive disorder	0.13484
DB00904	Overnutrition	0.11547
DB00904	Panic disorder	0.83444
DB00904	Schizophrenia	0.034
DB00904	Stroke	0.27299
DB00904	Sudden infant death syndrome	0.86193
DB01233	Supranuclear palsy, progressive	0.33333
DB01233	Asthma	0.04714
DB01233	Leukemia	0.03253
DB01233	Neuroblastoma	0.07931
DB01233	Overnutrition	0.14907
DB01233	Schizophrenia	0.0439
DB08810	Abortion	0.05124
DB08810	Amyotrophic lateral sclerosis	0.37047
DB08810	Anorexia nervosa	0.26151
DB08810	Attention deficit hyperactivity disorder	0.11127
DB08810	Autistic disorder	0.07001
DB08810	Behavior disease	0.84338
DB08810	Bipolar disorder	0.14355
DB08810	Choriocarcinoma	0.16667
DB08810	Chronic fatigue syndrome	0.20412
DB08810	Colon cancer	0.03426
DB08810	Depression	0.44568
DB08810	Dermatitis	0.36745
DB08810	Diabetes mellitus	0.19385
DB08810	Drug abuse	0.02745
DB08810	Drug-Induced dyskinesia	0.21822
DB08810	Epilepsy	0.57988
DB08810	Hepatitis C	0.62166
DB08810	Hypertension	0.43187
DB08810	Leukemia	0.03253
DB08810	Migraine	0.67494
DB08810	Obesity	0.04441
DB08810	Obsessive-compulsive disorder	0.17408
DB08810	Overnutrition	0.14907
DB08810	Panic disorder	0.97717

DB08810	Psychotic disorder	0.09245
DB08810	Rheumatoid arthritis	0.03533
DB08810	Stroke	0.38299
DB08810	Sudden infant death syndrome	0.99308
DB00204	Brain ischemia	0.2582
DB00204	Cancer	0.02128
DB00204	Depression	0.07581
DB00204	Drug abuse	0.05407
DB00204	Glycogen storage disease	0.2357
DB00204	Heart failure	0.06155
DB00204	Ischemia	0.07392
DB00204	Long QT syndrome	0.16667
DB00204	Overnutrition	0.14907
DB00204	Sudden infant death syndrome	0.12599
DB00276	Kidney tubular necrosis, acute	0.12007
DB00276	Amyotrophic lateral sclerosis	0.03604
DB00276	Aortic valve disease	0.11062
DB00276	Brain tumor	0.02262
DB00276	Breast cancer	0.04151
DB00276	Cancer	0.05199
DB00276	Celiac disease	0.06576
DB00276	Charcot-Marie-Tooth disease	0.19569
DB00276	Chronic obstructive airway disease	0.06282
DB00276	Cockayne syndrome	0.12336
DB00276	Colon cancer	0.08471
DB00276	Common cold	0.08759
DB00276	Diabetes mellitus	0.02014
DB00276	Down syndrome	0.07642
DB00276	Drug abuse	0.06623
DB00276	Embryoma	0.01525
DB00276	Emphysema	0.08099
DB00276	Epilepsy	0.03155
DB00276	Ewings sarcoma	0.11709
DB00276	Eye disease	0.09687
DB00276	Fanconi's anemia	0.03869
DB00276	HIV infection	0.0409
DB00276	Heart disease	0.28207
DB00276	Heart failure	0.12324
DB00276	Helicobacter infection	0.09599
DB00276	Hereditary disease	0.0494
DB00276	Herpes	0.01702
DB00276	Infection	0.01858
DB00276	Infertility	0.05698
DB00276	Ischemia	0.04917
DB00276	Kaposi sarcoma	0.06619
DB00276	Kidney cancer	0.08874
DB00276	Leigh disease	0.05883

DB00276	Leukemia	0.02248
DB00276	Leukoencephalopathy	0.04291
DB00276	Lipodystrophy	0.13587
DB00276	Liver cancer	0.03785
DB00276	Long QT syndrome	0.20412
DB00276	Lung cancer	0.04835
DB00276	Lymphoma	0.07682
DB00276	Melanoma	0.03675
DB00276	Meningioma	0.17605
DB00276	Metabolism disease	0.08545
DB00276	Muscular atrophy	0.09852
DB00276	Muscular dystrophies	0.06773
DB00276	Nephroblastoma	0.17583
DB00276	Neuroblastoma	0.05563
DB00276	Neuropathy	0.07997
DB00276	Parkinson disease	0.0433
DB00276	Pituitary tumor	0.10097
DB00276	Prostate cancer	0.02409
DB00276	Renal tubular acidosis	0.05133
DB00276	Retinitis pigmentosa	0.12611
DB00276	Rheumatoid arthritis	0.02029
DB00276	Schizophrenia	0.03388
DB00276	Sudden infant death syndrome	0.1543
DB00276	Tuberous sclerosis	0.0632
DB00276	Uterine fibroids	0.10116
DB00276	Virus disease	0.04513
DB00276	Werner syndrome	0.10284
DB00308	Atherosclerosis	0.04428
DB00308	Cancer	0.01166
DB00308	Diabetes mellitus	0.01664
DB00308	Drug abuse	0.02962
DB00308	Heart failure	0.03371
DB00308	Hyperglycemia	0.05064
DB00308	Hyperinsulinism	0.06086
DB00308	Infantile spasms	0.11952
DB00308	Late pregnancy	0.09129
DB00308	Long QT syndrome	0.09129
DB00308	Metabolism disease	0.05064
DB00308	Pancreas disease	0.06594
DB00308	Polycystic ovary syndrome	0.04189
DB00308	Sudden infant death syndrome	0.06901
DB00457	Cancer	0.01505
DB00457	Drug abuse	0.03824
DB00457	Heart failure	0.04352
DB00457	Hypertension	0.03227
DB00457	Kidney failure	0.13868
DB00457	Long QT syndrome	0.11785

DB00457	Prostate cancer	0.02131
DB00457	Sudden infant death syndrome	0.08909
DB00489	Infertility, Male	0.09355
DB00489	Alimentary system disease	0.17411
DB00489	Alzheimer's disease	0.10234
DB00489	Arthritis	0.47874
DB00489	Atherosclerosis	0.19296
DB00489	Autistic disorder	0.3131
DB00489	Azoospermia	0.07075
DB00489	Breast cancer	0.01989
DB00489	Bronchial disease	0.95435
DB00489	Cancer	0.02128
DB00489	Chronic obstructive airway disease	0.39116
DB00489	Conduct disorder	0.18128
DB00489	Cystic fibrosis	0.504
DB00489	Dermatitis	0.22058
DB00489	Diabetes mellitus	0.13507
DB00489	Drug abuse	0.23848
DB00489	Enteritis	0.06222
DB00489	Epilepsy	0.13443
DB00489	Glaucoma	0.60108
DB00489	Gram-Negative bacterial infection	0.12423
DB00489	Graves' disease	0.54265
DB00489	Heart failure	0.54003
DB00489	Hypertension	0.38372
DB00489	Infertility	0.05977
DB00489	Ischemia	0.11474
DB00489	Liver cancer	0.0397
DB00489	Long QT syndrome	0.16667
DB00489	Lung cancer	0.18759
DB00489	Malaria	0.57914
DB00489	Metabolism disease	0.53673
DB00489	Movement disorder	0.26525
DB00489	Myopathy	0.36626
DB00489	Neurodegenerative disorder	0.14667
DB00489	Obesity	0.34639
DB00489	Oligospermia	0.12882
DB00489	Polycystic kidney	0.1121
DB00489	Polycystic ovary syndrome	0.32386
DB00489	Premature birth	0.81471
DB00489	Prostate cancer	0.16309
DB00489	Respiratory tract disease	0.08648
DB00489	Rheumatoid arthritis	0.14081
DB00489	Sickle cell disease	0.32158
DB00489	Sinusitis	0.08024
DB00489	Subarachnoid hemorrhage	0.78667
DB00489	Sudden infant death syndrome	0.12599

DB00489	Testicular dysfunction	0.03955
DB00590	Cancer	0.01505
DB00590	Drug abuse	0.03824
DB00590	Heart failure	0.04352
DB00590	Hypertension	0.03227
DB00590	Kidney failure	0.13868
DB00590	Long QT syndrome	0.11785
DB00590	Prostate cancer	0.02131
DB00590	Sudden infant death syndrome	0.08909
DB00637	Atopic rhinitis	0.18257
DB00637	Cancer	0.02606
DB00637	Colon cancer	0.04196
DB00637	Drug abuse	0.06623
DB00637	Heart failure	0.07538
DB00637	Infertility	0.09713
DB00637	Long QT syndrome	0.20412
DB00637	Parkinson disease	0.07809
DB00637	Schizophrenia	0.05376
DB00637	Sudden infant death syndrome	0.1543
DB00908	Breast cancer	0.02406
DB00908	Cancer	0.01843
DB00908	Congenital abnormality	0.03769
DB00908	Drug abuse	0.09366
DB00908	Heart disease	0.15076
DB00908	Heart failure	0.0533
DB00908	Intestinal disease	0.125
DB00908	Ischemia	0.06402
DB00908	Long QT syndrome	0.28868
DB00908	Sudden infant death syndrome	0.21822
DB01100	Abortion	0.02905
DB01100	Achalasia and cardiospasm	0.12469
DB01100	Alzheimer's disease	0.05292
DB01100	Amyloidosis	0.06639
DB01100	Asthma	0.04371
DB01100	Autistic disorder	0.06042
DB01100	Autoimmune disease	0.05572
DB01100	Bipolar disorder	0.04432
DB01100	Bladder cancer	0.04542
DB01100	Brain ischemia	0.18262
DB01100	Cancer	0.05239
DB01100	Celiac disease	0.08498
DB01100	Colon cancer	0.0172
DB01100	Congenital abnormality	0.03237
DB01100	Dental plaque	0.06543
DB01100	Diabetes mellitus	0.0125
DB01100	Drug abuse	0.07922
DB01100	Eating disorder	0.10416

DB01100	Epstein-Barr virus infection	0.09263
DB01100	Esophageal tumor	0.07695
DB01100	Esophagus cancer	0.03074
DB01100	Esotropia	0.08852
DB01100	Eye cancer	0.10972
DB01100	Fanconi's anemia	0.0433
DB01100	Glaucoma	0.07631
DB01100	Gram-Negative bacterial infection	0.15306
DB01100	Graves' disease	0.10951
DB01100	HIV infection	0.05185
DB01100	Heart failure	0.0533
DB01100	Herpes	0.07492
DB01100	Ischemia	0.06354
DB01100	Keratosis	0.08781
DB01100	Leukemia	0.03912
DB01100	Lichen planus	0.17067
DB01100	Long QT syndrome	0.14434
DB01100	Lung cancer	0.0366
DB01100	Lupus erythematosus	0.03596
DB01100	Malignant glioma	0.04453
DB01100	Melanoma	0.02281
DB01100	Pre-Eclampsia	0.04147
DB01100	Prion disease	0.14306
DB01100	Rabies	0.02116
DB01100	Rheumatoid arthritis	0.02622
DB01100	Schistosomiasis	0.15421
DB01100	Stroke	0.04015
DB01100	Sudden infant death syndrome	0.10911
DB01100	Thyroid gland disease	0.09641
DB01100	Tuberculosis	0.0826
DB01118	Alzheimer's disease	0.11172
DB01118	Breast cancer	0.02406
DB01118	Cancer	0.01843
DB01118	Congenital heart disease	0.16667
DB01118	Drug abuse	0.04683
DB01118	Epilepsy	0.07071
DB01118	Glaucoma	0.28711
DB01118	Heart failure	0.27175
DB01118	Hypertension	0.17542
DB01118	Long QT syndrome	0.14434
DB01118	Lung cancer	0.03492
DB01118	Obesity	0.16691
DB01118	Peptic ulcer	0.11785
DB01118	Prostate cancer	0.0261
DB01118	Subarachnoid hemorrhage	0.36943
DB01118	Sudden infant death syndrome	0.10911
DB01136	Infertility, Male	0.06307

DB01136	Pemphigoid, Bullous	0.07642
DB01136	Pleural effusion, Malignant	0.07821
DB01136	Purpura, Thrombocytopenic, Idiopathic	0.08434
DB01136	Alimentary system disease	0.11738
DB01136	Alopecia	0.15097
DB01136	Alzheimer's disease	0.11153
DB01136	Amyotrophic lateral sclerosis	0.07646
DB01136	Aortic aneurysm	0.09233
DB01136	Arthritis	0.31581
DB01136	Asthma	0.09566
DB01136	Atherosclerosis	0.2579
DB01136	Autistic disorder	0.24472
DB01136	Azoospermia	0.0477
DB01136	Basal cell carcinoma	0.03097
DB01136	Bipolar disorder	0.01318
DB01136	Breast cancer	0.01691
DB01136	Bronchial disease	0.62982
DB01136	Cancer	0.12054
DB01136	Capillaries disease	0.35121
DB01136	Cardiovascular disease	0.21574
DB01136	Cervical cancer	0.03084
DB01136	Chronic obstructive airway disease	0.58914
DB01136	Conduct disorder	0.12221
DB01136	Cystic fibrosis	0.35302
DB01136	Deafness	0.03252
DB01136	Demyelinating disease	0.07507
DB01136	Dental plaque	0.04233
DB01136	Depression	0.07213
DB01136	Dermatitis	0.21221
DB01136	Diabetes mellitus	0.23677
DB01136	Down syndrome	0.00935
DB01136	Drug abuse	0.20348
DB01136	Encephalopathies	0.02344
DB01136	Endometriosis	0.05636
DB01136	Enteritis	0.04194
DB01136	Epilepsy	0.10126
DB01136	Esophagus cancer	0.03501
DB01136	Eye disease	0.03265
DB01136	Familial Mediterranean fever	0.47539
DB01136	Glaucoma	0.44215
DB01136	Gram-Negative bacterial infection	0.08375
DB01136	Graves' disease	0.35595
DB01136	Heart failure	0.38172
DB01136	Hypertension	0.28686
DB01136	Infertility	0.05774
DB01136	Intestinal disease	0.06837
DB01136	Ischemia	0.07735

DB01136	Kidney failure	0.26025
DB01136	Leigh disease	0.04435
DB01136	Leukemia	0.0159
DB01136	Leukoencephalopathy	0.19537
DB01136	Liver cancer	0.02677
DB01136	Long QT syndrome	0.09623
DB01136	Lung cancer	0.12256
DB01136	Lung disease	0.03969
DB01136	Lupus erythematosus	0.11805
DB01136	Macular degeneration	0.57193
DB01136	Malaria	0.77729
DB01136	Melanoma	0.02599
DB01136	Metabolism disease	0.35289
DB01136	Movement disorder	0.17882
DB01136	Mucocutaneous lymph node syndrome	0.54425
DB01136	Multiple sclerosis	0.02485
DB01136	Myopathy	0.24014
DB01136	Myotonic disorder	0.02043
DB01136	Neurodegenerative disorder	0.09888
DB01136	Neuropathy	0.02088
DB01136	Obesity	0.38808
DB01136	Oligospermia	0.08684
DB01136	Optic atrophy	0.02392
DB01136	Osteosarcoma	0.05085
DB01136	Overnutrition	0.0585
DB01136	Palmoplantar keratosis	0.14907
DB01136	Parkinson disease	0.02813
DB01136	Peptic ulcer	0.38857
DB01136	Polycystic kidney	0.07557
DB01136	Polycystic ovary syndrome	0.21093
DB01136	Polyneuropathy	0.59152
DB01136	Pre-Eclampsia	0.28606
DB01136	Premature birth	0.53568
DB01136	Primary hyperparathyroidism	0.09091
DB01136	Prostate cancer	0.12443
DB01136	Respiratory tract disease	0.0583
DB01136	Retinal disease	0.20575
DB01136	Rheumatoid arthritis	0.14151
DB01136	Schizophrenia	0.02201
DB01136	Shigella infection	0.09631
DB01136	Sickle cell disease	0.3058
DB01136	Sinusitis	0.05409
DB01136	Subarachnoid hemorrhage	0.49663
DB01136	Sudden infant death syndrome	0.07274
DB01136	Systemic infection	0.25375
DB01136	Testicular dysfunction	0.02666
DB01136	Thalassemia	0.10396

DB01136	Vascular disease	0.06086
DB01136	Virus disease	0.03191
DB01162	Cancer	0.01505
DB01162	Drug abuse	0.03824
DB01162	Heart failure	0.04352
DB01162	Hypertension	0.03227
DB01162	Kidney failure	0.13868
DB01162	Long QT syndrome	0.11785
DB01162	Prostate cancer	0.02131
DB01162	Sudden infant death syndrome	0.08909
DB01182	Breast cancer	0.03402
DB01182	Cancer	0.02606
DB01182	Congenital abnormality	0.0533
DB01182	Drug abuse	0.13245
DB01182	Heart disease	0.2132
DB01182	Heart failure	0.07538
DB01182	Intestinal disease	0.17678
DB01182	Ischemia	0.09054
DB01182	Long QT syndrome	0.40825
DB01182	Sudden infant death syndrome	0.30861
DB01218	Cancer	0.03686
DB01218	Drug abuse	0.09366
DB01218	Heart failure	0.1066
DB01218	Long QT syndrome	0.28868
DB01218	Sudden infant death syndrome	0.21822
DB04855	Myoclonic epilepsy, Juvenile	0.09623
DB04855	Alzheimer's disease	0.07427
DB04855	Amyotrophic lateral sclerosis	0.03178
DB04855	Atherosclerosis	0.0165
DB04855	Attention deficit hyperactivity disorder	0.26613
DB04855	Autistic disorder	0.02858
DB04855	Brain disease	0.04233
DB04855	Brain ischemia	0.10541
DB04855	Cancer	0.00869
DB04855	Central nervous system disease	0.05893
DB04855	Common cold	0.06299
DB04855	Depression	0.03095
DB04855	Diabetes mellitus	0.01241
DB04855	Drug abuse	0.06623
DB04855	Endocrine system disease	0.08909
DB04855	Glaucoma	0.19348
DB04855	Heart failure	0.17522
DB04855	Hypertension	0.25413
DB04855	Infantile spasms	0.08909
DB04855	Intractable epilepsy	0.08333
DB04855	Ischemia	0.03018
DB04855	Kidney failure	0.08006

DB04855	Long QT syndrome	0.06804
DB04855	Migraine	0.04377
DB04855	Myopathy	0.02858
DB04855	Nervous system disease	0.30258
DB04855	Neuropathy	0.03774
DB04855	Obesity	0.22414
DB04855	Optic atrophy	0.10541
DB04855	Polycystic ovary syndrome	0.03122
DB04855	Prostate cancer	0.0123
DB04855	Retinal disease	0.033
DB04855	Rheumatism	0.05556
DB04855	Schizophrenia	0.08396
DB04855	Subarachnoid hemorrhage	0.23654
DB04855	Sudden infant death syndrome	0.05143
DB06144	Anorexia nervosa	0.14434
DB06144	Autistic disorder	0.04287
DB06144	Behavior disease	0.10911
DB06144	Bipolar disorder	0.08006
DB06144	Cancer	0.01303
DB06144	Choriocarcinoma	0.10206
DB06144	Chronic fatigue syndrome	0.125
DB06144	Colon cancer	0.02098
DB06144	Depression	0.04642
DB06144	Dermatitis	0.03214
DB06144	Drug abuse	0.03311
DB06144	Drug-Induced dyskinesia	0.13363
DB06144	Heart failure	0.03769
DB06144	Hypertension	0.1118
DB06144	Kidney failure	0.1201
DB06144	Long QT syndrome	0.10206
DB06144	Migraine	0.06565
DB06144	Obesity	0.05439
DB06144	Obsessive-compulsive disorder	0.1066
DB06144	Panic disorder	0.07715
DB06144	Prostate cancer	0.01846
DB06144	Psychotic disorder	0.11323
DB06144	Rheumatoid arthritis	0.02164
DB06144	Stroke	0.04003
DB06144	Sudden infant death syndrome	0.07715
DB00762	Kidney tubular necrosis, acute	0.14968
DB00762	Abortion	0.01031
DB00762	Adenovirus infection	0.00589
DB00762	Alzheimer's disease	0.00484
DB00762	Amyotrophic lateral sclerosis	0.04493
DB00762	Aortic aneurysm	0.00959
DB00762	Aortic valve disease	0.14514
DB00762	Aplastic anemia	0.02246

DB00762	Barrett's esophagus	0.05816
DB00762	Brain tumor	0.0091
DB00762	Breast cancer	0.0352
DB00762	Bronchial disease	0.03452
DB00762	Cancer	0.05103
DB00762	Carcinoma	0.01586
DB00762	Celiac disease	0.08198
DB00762	Cervical cancer	0.01385
DB00762	Cholestasis	0.03261
DB00762	Chronic obstructive airway disease	0.07831
DB00762	Chronic rejection of renal transplant	0.09677
DB00762	Chronic simple glaucoma	0.0737
DB00762	Colon cancer	0.07198
DB00762	Common cold	0.10919
DB00762	Dermatitis	0.02746
DB00762	Diabetes mellitus	0.02954
DB00762	Down syndrome	0.12039
DB00762	Embryoma	0.01227
DB00762	Emphysema	0.10627
DB00762	Endometriosis	0.02257
DB00762	Ewings sarcoma	0.14596
DB00762	Fanconi's anemia	0.04824
DB00762	HIV infection	0.05963
DB00762	Heart failure	0.05966
DB00762	Helicobacter infection	0.12595
DB00762	Hemolytic-Uremic syndrome	0.07868
DB00762	Hyperparathyroidism	0.06758
DB00762	Infection	0.02881
DB00762	Ischemia	0.0613
DB00762	Kaposi sarcoma	0.08252
DB00762	Keratoconjunctivitis Sicca	0.04506
DB00762	Keratosis	0.05772
DB00762	Kidney cancer	0.21023
DB00762	Leukemia	0.03298
DB00762	Leukoencephalopathy	0.04049
DB00762	Liver cancer	0.06332
DB00762	Lung cancer	0.05656
DB00762	Lung disease	0.01237
DB00762	Lymphoma	0.12102
DB00762	Melanoma	0.04581
DB00762	Meningioma	0.27734
DB00762	Mental retardation	0.023
DB00762	Metaplastic polyp	0.05863
DB00762	Multiple endocrine neoplasia	0.1629
DB00762	Muscular dystrophies	0.01493
DB00762	Neck cancer	0.06187
DB00762	Neuroblastoma	0.05249

DB00762	Oral cancer	0.03104
DB00762	Osteosarcoma	0.01585
DB00762	Pancreas cancer	0.01497
DB00762	Pancreas disease	0.03142
DB00762	Papillomavirus infection	0.09169
DB00762	Parkinson disease	0.05398
DB00762	Pituitary tumor	0.12587
DB00762	Polyarthritis	0.02748
DB00762	Pre-Eclampsia	0.01472
DB00762	Prostate cancer	0.00811
DB00762	Renal tubular acidosis	0.06399
DB00762	Rheumatoid arthritis	0.04253
DB00762	Sarcoidosis	0.0885
DB00762	Stomach cancer	0.01335
DB00762	Tuberous sclerosis	0.09216
DB00762	Ulcerative colitis	0.04582
DB00762	Uterine fibroids	0.12611
DB00762	Virus disease	0.00995
DB00762	Werner syndrome	0.08284
DB01030	Kidney tubular necrosis, acute	0.14968
DB01030	Abortion	0.01031
DB01030	Adenovirus infection	0.00589
DB01030	Alzheimer's disease	0.00484
DB01030	Amyotrophic lateral sclerosis	0.04493
DB01030	Aortic aneurysm	0.00959
DB01030	Aortic valve disease	0.14514
DB01030	Aplastic anemia	0.02246
DB01030	Barrett's esophagus	0.05816
DB01030	Brain tumor	0.0091
DB01030	Breast cancer	0.0352
DB01030	Bronchial disease	0.03452
DB01030	Cancer	0.05103
DB01030	Carcinoma	0.01586
DB01030	Celiac disease	0.08198
DB01030	Cervical cancer	0.01385
DB01030	Cholestasis	0.03261
DB01030	Chronic obstructive airway disease	0.07831
DB01030	Chronic rejection of renal transplant	0.09677
DB01030	Chronic simple glaucoma	0.0737
DB01030	Colon cancer	0.07198
DB01030	Common cold	0.10919
DB01030	Dermatitis	0.02746
DB01030	Diabetes mellitus	0.02954
DB01030	Down syndrome	0.12039
DB01030	Embryoma	0.01227
DB01030	Emphysema	0.10627
DB01030	Endometriosis	0.02257

DB01030	Ewings sarcoma	0.14596
DB01030	Fanconi's anemia	0.04824
DB01030	HIV infection	0.05963
DB01030	Heart failure	0.05966
DB01030	Helicobacter infection	0.12595
DB01030	Hemolytic-Uremic syndrome	0.07868
DB01030	Hyperparathyroidism	0.06758
DB01030	Infection	0.02881
DB01030	Ischemia	0.0613
DB01030	Kaposi sarcoma	0.08252
DB01030	Keratoconjunctivitis Sicca	0.04506
DB01030	Keratosis	0.05772
DB01030	Kidney cancer	0.21023
DB01030	Leukemia	0.03298
DB01030	Leukoencephalopathy	0.04049
DB01030	Liver cancer	0.06332
DB01030	Lung cancer	0.05656
DB01030	Lung disease	0.01237
DB01030	Lymphoma	0.12102
DB01030	Melanoma	0.04581
DB01030	Meningioma	0.27734
DB01030	Mental retardation	0.023
DB01030	Metaplastic polyp	0.05863
DB01030	Multiple endocrine neoplasia	0.1629
DB01030	Muscular dystrophies	0.01493
DB01030	Neck cancer	0.06187
DB01030	Neuroblastoma	0.05249
DB01030	Oral cancer	0.03104
DB01030	Osteosarcoma	0.01585
DB01030	Pancreas cancer	0.01497
DB01030	Pancreas disease	0.03142
DB01030	Papillomavirus infection	0.09169
DB01030	Parkinson disease	0.05398
DB01030	Pituitary tumor	0.12587
DB01030	Polyarthritis	0.02748
DB01030	Pre-Eclampsia	0.01472
DB01030	Prostate cancer	0.00811
DB01030	Renal tubular acidosis	0.06399
DB01030	Rheumatoid arthritis	0.04253
DB01030	Sarcoidosis	0.0885
DB01030	Stomach cancer	0.01335
DB01030	Tuberous sclerosis	0.09216
DB01030	Ulcerative colitis	0.04582
DB01030	Uterine fibroids	0.12611
DB01030	Virus disease	0.00995
DB01030	Werner syndrome	0.08284
DB00209	Supranuclear palsy, progressive	0.57735

DB00209	Asthma	0.08165
DB00209	Neuroblastoma	0.13736
DB00209	Schizophrenia	0.07603
DB00215	Hypertension, Pulmonary	0.11471
DB00215	Supranuclear palsy, progressive	0.28868
DB00215	Anorexia nervosa	0.10206
DB00215	Asthma	0.04082
DB00215	Atherosclerosis	0.03501
DB00215	Atopic rhinitis	0.1291
DB00215	Autistic disorder	0.06063
DB00215	Behavior disease	0.07715
DB00215	Bipolar disorder	0.05661
DB00215	Chronic fatigue syndrome	0.17678
DB00215	Chronic obstructive airway disease	0.0559
DB00215	Colon cancer	0.05934
DB00215	Congenital heart disease	0.16667
DB00215	Depression	0.06565
DB00215	Dermatitis	0.04545
DB00215	Drug abuse	0.04683
DB00215	Epilepsy	0.07071
DB00215	Fibromyalgia	0.17678
DB00215	Generalized anxiety disorder	0.14434
DB00215	Heart failure	0.0533
DB00215	Herpes	0.07143
DB00215	Hypertension	0.03953
DB00215	Infertility	0.06868
DB00215	Kidney failure	0.05661
DB00215	Migraine	0.09285
DB00215	Neuroblastoma	0.06868
DB00215	Neurotic disorder	0.15811
DB00215	Obesity	0.03846
DB00215	Obsessive-compulsive disorder	0.15076
DB00215	Panic disorder	0.10911
DB00215	Parkinson disease	0.05522
DB00215	Pervasive development disorder	0.1291
DB00215	Prostate cancer	0.0261
DB00215	Psychotic disorder	0.08006
DB00215	Pulmonary hypertension	0.25
DB00215	Schizophrenia	0.07603
DB00215	Stroke	0.05661
DB00215	Sudden infant death syndrome	0.10911
DB00215	Ulcerative colitis	0.05157
DB00219	Supranuclear palsy, progressive	0.57735
DB00219	Asthma	0.08165
DB00219	Neuroblastoma	0.13736
DB00219	Schizophrenia	0.07603
DB00245	Stress disorder, post-traumatic	0.20412

DB00245	Supranuclear palsy, progressive	0.33333
DB00245	Asthma	0.04714
DB00245	Atopic rhinitis	0.14907
DB00245	Behavior disease	0.08909
DB00245	Bipolar disorder	0.06537
DB00245	Brain disease	0.1037
DB00245	Breast cancer	0.02778
DB00245	Colon cancer	0.03426
DB00245	Depression	0.07581
DB00245	Drug abuse	0.05407
DB00245	Gilles de la Tourette syndrome	0.21822
DB00245	Herpes	0.08248
DB00245	Infertility	0.07931
DB00245	Migraine	0.10721
DB00245	Neuroblastoma	0.07931
DB00245	Obesity	0.04441
DB00245	Parkinson disease	0.06376
DB00245	Psychotic disorder	0.09245
DB00245	Schizophrenia	0.08779
DB00354	Supranuclear palsy, progressive	0.40825
DB00354	Asthma	0.05774
DB00354	Atopic rhinitis	0.18257
DB00354	Colon cancer	0.04196
DB00354	Infertility	0.09713
DB00354	Neuroblastoma	0.09713
DB00354	Parkinson disease	0.07809
DB00354	Schizophrenia	0.10752
DB00366	Supranuclear palsy, progressive	0.40825
DB00366	Asthma	0.05774
DB00366	Atopic rhinitis	0.18257
DB00366	Colon cancer	0.04196
DB00366	Infertility	0.09713
DB00366	Neuroblastoma	0.09713
DB00366	Parkinson disease	0.07809
DB00366	Schizophrenia	0.10752
DB00392	Supranuclear palsy, progressive	0.33333
DB00392	Asthma	0.04714
DB00392	Behavior disease	0.08909
DB00392	Bipolar disorder	0.06537
DB00392	Bladder cancer	0.07454
DB00392	Central nervous system disease	0.14434
DB00392	Depression	0.07581
DB00392	Neuroblastoma	0.07931
DB00392	Schizophrenia	0.0439
DB00411	Supranuclear palsy, progressive	0.33333
DB00411	Asthma	0.04714
DB00411	Behavior disease	0.08909

DB00411	Bipolar disorder	0.06537
DB00411	Bladder cancer	0.07454
DB00411	Central nervous system disease	0.14434
DB00411	Depression	0.07581
DB00411	Epilepsy	0.08165
DB00411	Neuroblastoma	0.07931
DB00411	Schizophrenia	0.0439
DB00670	Supranuclear palsy, progressive	0.57735
DB00670	Asthma	0.08165
DB00670	Neuroblastoma	0.13736
DB00670	Schizophrenia	0.07603
DB00771	Supranuclear palsy, progressive	0.57735
DB00771	Asthma	0.08165
DB00771	Neuroblastoma	0.13736
DB00771	Schizophrenia	0.07603
DB00782	Supranuclear palsy, progressive	0.57735
DB00782	Asthma	0.08165
DB00782	Neuroblastoma	0.13736
DB00782	Schizophrenia	0.07603
DB00804	Supranuclear palsy, progressive	0.40825
DB00804	Asthma	0.05774
DB00804	Behavior disease	0.10911
DB00804	Bipolar disorder	0.08006
DB00804	Bladder cancer	0.09129
DB00804	Central nervous system disease	0.17678
DB00804	Depression	0.09285
DB00804	Neuroblastoma	0.09713
DB00804	Schizophrenia	0.05376
DB00810	Supranuclear palsy, progressive	0.40825
DB00810	Asthma	0.05774
DB00810	Epilepsy	0.1
DB00810	Neuroblastoma	0.09713
DB00810	Schizophrenia	0.05376
DB00907	Hypertension, Pulmonary	0.08111
DB00907	Stress disorder, post-traumatic	0.125
DB00907	Supranuclear palsy, progressive	0.20412
DB00907	Anorexia nervosa	0.14434
DB00907	Asthma	0.02887
DB00907	Atherosclerosis	0.02475
DB00907	Autistic disorder	0.04287
DB00907	Behavior disease	0.21822
DB00907	Bipolar disorder	0.16013
DB00907	Bladder cancer	0.04564
DB00907	Brain disease	0.0635
DB00907	Breast cancer	0.03402
DB00907	Central nervous system disease	0.08839
DB00907	Chronic fatigue syndrome	0.125

DB00907	Chronic obstructive airway disease	0.03953
DB00907	Colon cancer	0.02098
DB00907	Congenital abnormality	0.02665
DB00907	Congenital heart disease	0.11785
DB00907	Depression	0.13927
DB00907	Dermatitis	0.03214
DB00907	Diabetes mellitus	0.01861
DB00907	Drug abuse	0.13245
DB00907	Epilepsy	0.05
DB00907	Fibromyalgia	0.125
DB00907	Generalized anxiety disorder	0.10206
DB00907	Gilles de la Tourette syndrome	0.13363
DB00907	Heart disease	0.1066
DB00907	Heart failure	0.03769
DB00907	Herpes	0.10102
DB00907	Hypertension	0.02795
DB00907	Intestinal disease	0.08839
DB00907	Ischemia	0.04527
DB00907	Long QT syndrome	0.10206
DB00907	Migraine	0.13131
DB00907	Multiple endocrine neoplasia	0.11785
DB00907	Neuroblastoma	0.04856
DB00907	Neuroendocrine tumor	0.125
DB00907	Neurotic disorder	0.1118
DB00907	Obesity	0.05439
DB00907	Obsessive-compulsive disorder	0.1066
DB00907	Panic disorder	0.1543
DB00907	Pervasive development disorder	0.09129
DB00907	Psychotic disorder	0.11323
DB00907	Pulmonary hypertension	0.17678
DB00907	Schizophrenia	0.02688
DB00907	Stroke	0.04003
DB00907	Sudden infant death syndrome	0.1543
DB00907	Ulcerative colitis	0.03647
DB00940	Supranuclear palsy, progressive	0.40825
DB00940	Asthma	0.05774
DB00940	Atopic rhinitis	0.18257
DB00940	Colon cancer	0.04196
DB00940	Infertility	0.09713
DB00940	Melanoma	0.05893
DB00940	Neuroblastoma	0.09713
DB00940	Schizophrenia	0.05376
DB00942	Supranuclear palsy, progressive	0.57735
DB00942	Asthma	0.08165
DB00942	Neuroblastoma	0.13736
DB00942	Schizophrenia	0.07603
DB00979	Supranuclear palsy, progressive	0.57735

DB00979	Asthma	0.08165
DB00979	Neuroblastoma	0.13736
DB00979	Schizophrenia	0.07603
DB00986	Supranuclear palsy, progressive	0.57735
DB00986	Asthma	0.08165
DB00986	Neuroblastoma	0.13736
DB00986	Schizophrenia	0.07603
DB01148	Supranuclear palsy, progressive	0.40825
DB01148	Asthma	0.05774
DB01148	Behavior disease	0.10911
DB01148	Bipolar disorder	0.08006
DB01148	Bladder cancer	0.09129
DB01148	Central nervous system disease	0.17678
DB01148	Depression	0.09285
DB01148	Neuroblastoma	0.09713
DB01148	Schizophrenia	0.05376
DB01175	Hypertension, Pulmonary	0.09366
DB01175	Stress disorder, post-traumatic	0.14434
DB01175	Supranuclear palsy, progressive	0.2357
DB01175	Anorexia nervosa	0.16667
DB01175	Asthma	0.03333
DB01175	Atherosclerosis	0.02858
DB01175	Atopic rhinitis	0.10541
DB01175	Autistic disorder	0.04951
DB01175	Behavior disease	0.18898
DB01175	Bipolar disorder	0.13868
DB01175	Brain disease	0.07332
DB01175	Breast cancer	0.01964
DB01175	Chronic fatigue syndrome	0.14434
DB01175	Chronic obstructive airway disease	0.04564
DB01175	Colon cancer	0.04845
DB01175	Congenital heart disease	0.13608
DB01175	Depression	0.10721
DB01175	Dermatitis	0.03711
DB01175	Diabetes mellitus	0.02149
DB01175	Drug abuse	0.11471
DB01175	Epilepsy	0.05774
DB01175	Fibromyalgia	0.14434
DB01175	Generalized anxiety disorder	0.11785
DB01175	Gilles de la Tourette syndrome	0.1543
DB01175	Heart failure	0.04352
DB01175	Herpes	0.11664
DB01175	Hypertension	0.06455
DB01175	Infertility	0.05608
DB01175	Kidney failure	0.04623
DB01175	Migraine	0.15162
DB01175	Multiple endocrine neoplasia	0.13608

DB01175	Neuroblastoma	0.05608
DB01175	Neuroendocrine tumor	0.14434
DB01175	Neurotic disorder	0.1291
DB01175	Obesity	0.06281
DB01175	Obsessive-compulsive disorder	0.12309
DB01175	Panic disorder	0.17817
DB01175	Parkinson disease	0.04508
DB01175	Pervasive development disorder	0.10541
DB01175	Prostate cancer	0.02131
DB01175	Psychotic disorder	0.13074
DB01175	Pulmonary hypertension	0.20412
DB01175	Schizophrenia	0.06208
DB01175	Stroke	0.04623
DB01175	Sudden infant death syndrome	0.08909
DB01175	Ulcerative colitis	0.04211
DB01618	Supranuclear palsy, progressive	0.28868
DB01618	Abortion	0.05124
DB01618	Amyotrophic lateral sclerosis	0.36004
DB01618	Anorexia nervosa	0.24572
DB01618	Asthma	0.04082
DB01618	Attention deficit hyperactivity disorder	0.11127
DB01618	Autistic disorder	0.06063
DB01618	Behavior disease	0.81951
DB01618	Bipolar disorder	0.1348
DB01618	Choriocarcinoma	0.14434
DB01618	Chronic fatigue syndrome	0.17678
DB01618	Colon cancer	0.02967
DB01618	Depression	0.42537
DB01618	Dermatitis	0.35339
DB01618	Diabetes mellitus	0.18978
DB01618	Drug abuse	0.02745
DB01618	Drug-Induced dyskinesia	0.18898
DB01618	Epilepsy	0.56894
DB01618	Hepatitis C	0.61025
DB01618	Hypertension	0.41964
DB01618	Migraine	0.66057
DB01618	Neuroblastoma	0.06868
DB01618	Obesity	0.03846
DB01618	Obsessive-compulsive disorder	0.15076
DB01618	Panic disorder	0.94341
DB01618	Psychotic disorder	0.08006
DB01618	Rheumatoid arthritis	0.0306
DB01618	Schizophrenia	0.03801
DB01618	Stroke	0.36547
DB01618	Sudden infant death syndrome	0.9762
DB00583	Mycobacterium infection, Atypical	0.1005
DB00583	Alzheimer's disease	0.0217

DB00583	Asthma	0.02462
DB00583	Atherosclerosis	0.04222
DB00583	Breast cancer	0.02901
DB00583	Cancer	0.01111
DB00583	Cervical cancer	0.03505
DB00583	Diabetes mellitus	0.04761
DB00583	Embryoma	0.01859
DB00583	Enteritis	0.06619
DB00583	Glomerulonephritis	0.07538
DB00583	Helicobacter infection	0.06742
DB00583	Hepatoblastoma	0.17408
DB00583	Hyperlipidemia	0.05913
DB00583	Hypertension	0.02384
DB00583	Ischemia	0.0386
DB00583	Kidney failure	0.03414
DB00583	Leukemia	0.01699
DB00583	Lung cancer	0.02106
DB00583	Multiple sclerosis	0.0311
DB00583	Pancreas cancer	0.03127
DB00583	Periodontal disease	0.07107
DB00583	Periodontitis	0.04598
DB00583	Prostate cancer	0.01574
DB00583	Rheumatoid arthritis	0.0369
DB00583	Sicca syndrome	0.05913
DB00583	Sickle cell disease	0.05803
DB00583	Stomach disease	0.2132
DB00583	Stroke	0.03414
DB00583	Systemic infection	0.03459
DB00583	Ulcerative colitis	0.0622
DB00583	Vasculitis	0.09091
DB00716	Stress disorder, post-traumatic	0.20595
DB00716	Adenocarcinoma	0.21153
DB00716	Adenovirus infection	0.03261
DB00716	Alzheimer's disease	0.22227
DB00716	Asthma	0.07303
DB00716	Atherosclerosis	0.02564
DB00716	Bipolar disorder	0.02479
DB00716	Brain tumor	0.07866
DB00716	Breast cancer	0.055
DB00716	Cancer	0.14706
DB00716	Chronic rejection of renal transplant	0.14266
DB00716	Chronic simple glaucoma	0.16379
DB00716	Colon cancer	0.03616
DB00716	Corneal disease	0.03237
DB00716	Cystic fibrosis	0.02376
DB00716	Depression	0.03921
DB00716	Dermatitis	0.06891

DB00716	Diabetes mellitus	0.02459
DB00716	Drug abuse	0.01819
DB00716	Eating disorder	0.4366
DB00716	Endometriosis	0.02322
DB00716	Epilepsy	0.02
DB00716	Fanconi's anemia	0.41082
DB00716	Gram-Negative bacterial infection	0.04482
DB00716	HIV infection	0.00941
DB00716	Hemolytic-Uremic syndrome	0.08095
DB00716	Herpes	0.33251
DB00716	Huntington disease	0.08191
DB00716	Hyperglycemia	0.07122
DB00716	Hyperparathyroidism	0.06952
DB00716	Hypertension	0.03441
DB00716	Infection	0.09676
DB00716	Infectious lung disease	0.10697
DB00716	Ischemia	0.03568
DB00716	Keratoconus	0.05137
DB00716	Keratosis	0.05938
DB00716	Leukemia	0.1506
DB00716	Liver cancer	0.0684
DB00716	Lung cancer	0.02055
DB00716	Melanoma	0.01391
DB00716	Metaplastic polyp	0.06032
DB00716	Myelofibrosis	0.15356
DB00716	Neck cancer	0.06365
DB00716	Neoplasm metastasis	0.06515
DB00716	Obesity	0.0654
DB00716	Oral cancer	0.03193
DB00716	Papillomavirus infection	0.09433
DB00716	Polyarthritis	0.10508
DB00716	Polycystic ovary syndrome	0.04158
DB00716	Prion disease	0.04189
DB00716	Prostate cancer	0.13542
DB00716	Psychotic disorder	0.07426
DB00716	Renal Cell cancer	0.04725
DB00716	Rheumatoid arthritis	0.03027
DB00716	Sarcoidosis	0.09105
DB00716	Schizophrenia	0.04137
DB00716	Squamous cell cancer	0.03466
DB00716	Stomach cancer	0.01549
DB00716	Systemic infection	0.03263
DB00716	Systemic scleroderma	0.02598
DB00716	Thrombophlebitis	0.03475
DB00716	Tropical spastic paraparesis	0.09928
DB00716	Tuberous sclerosis	0.05984
DB00716	Virus disease	0.08382

DB00716	Vitiligo	0.08274
DB00716	Yersinia infection	0.0509
DB00118	Alzheimer's disease	0.02939
DB00118	Atherosclerosis	0.02858
DB00118	Bipolar disorder	0.04623
DB00118	Cancer	0.01505
DB00118	Down syndrome	0.04652
DB00118	Embryoma	0.02517
DB00118	Encephalopathies	0.05717
DB00118	Hyperhomocysteinemia	0.11785
DB00118	Liver cancer	0.03169
DB00118	Liver disease	0.06299
DB00118	Lung cancer	0.02851
DB00118	Meningioma	0.10541
DB00118	Prostate cancer	0.02131
DB00118	Schizophrenia	0.03104
DB00118	Stroke	0.04623
DB00160	Pemphigoid, Bullous	0.14034
DB00160	Pleural effusion, Malignant	0.14363
DB00160	Purpura, Thrombocytopenic, Idiopathic	0.13412
DB00160	Alopecia	0.16547
DB00160	Alzheimer's disease	0.08549
DB00160	Aortic aneurysm	0.16955
DB00160	Autistic disorder	0.03363
DB00160	Cancer	0.05189
DB00160	Depression	0.13245
DB00160	Diabetes mellitus	0.07844
DB00160	Drug abuse	0.09765
DB00160	Kidney disease	0.03315
DB00160	Lupus erythematosus	0.10839
DB00160	Neoplasm metastasis	0.0588
DB00160	Primary hyperparathyroidism	0.16695
DB00160	Rheumatoid arthritis	0.07903
DB00160	Shigella infection	0.17687
DB00160	Sickle cell disease	0.1577
DB00160	Systemic infection	0.14543
DB00160	Thalassemia	0.16534
DB00973	Acne	0.2357
DB00973	Alzheimer's disease	0.04156
DB00973	Cancer	0.02128
DB00973	Coronavirus infection	0.28868
DB00973	Encephalopathies	0.08085
DB00973	Histiocytosis	0.19245
DB00973	Hypercholesterolemia	0.11111
DB00973	Hypertension	0.04564
DB00973	Infection	0.05384
DB00973	Rabies	0.06415

DB00973	Renal Cell cancer	0.06712
DB01094	Alzheimer's disease	0.04156
DB01094	Atherosclerosis	0.04042
DB01094	Diabetes mellitus	0.03039
DB01094	Encephalopathies	0.08085
DB01094	Hypercholesterolemia	0.11111
DB01094	Liver cancer	0.04481
DB01094	Liver tumor	0.10206
DB01094	Obesity	0.04441
DB01094	Rabies	0.06415
DB01094	Renal Cell cancer	0.06712
DB00113	Autoimmune disease	0.10847
DB00113	Cancer	0.03686
DB00113	Gram-Negative bacterial infection	0.28868
DB00113	Metaplastic polyp	0.33333
DB00141	Connective tissue disease	0.08839
DB00141	Disseminated cancer	0.1118
DB00141	Hydrocephalus	0.11785
DB00141	Nervous system disease	0.09129
DB00208	Atherosclerosis	0.07001
DB00208	Lupus erythematosus	0.08544
DB00208	Pulmonary embolism	0.40825
DB00208	Vascular disease	0.18257
DB00374	Atherosclerosis	0.04042
DB00374	Lupus erythematosus	0.04933
DB00374	Pulmonary embolism	0.2357
DB00374	Vascular disease	0.10541
DB00758	Atherosclerosis	0.07001
DB00758	Lupus erythematosus	0.08544
DB00758	Pulmonary embolism	0.40825
DB00758	Vascular disease	0.18257
DB01240	Atherosclerosis	0.04042
DB01240	Lupus erythematosus	0.04933
DB01240	Pulmonary embolism	0.2357
DB01240	Vascular disease	0.10541
DB06209	Atherosclerosis	0.07001
DB06209	Lupus erythematosus	0.08544
DB06209	Pulmonary embolism	0.40825
DB06209	Vascular disease	0.18257
DB08816	Atherosclerosis	0.07001
DB08816	Lupus erythematosus	0.08544
DB08816	Pulmonary embolism	0.40825
DB08816	Vascular disease	0.18257
DB00272	Atopic rhinitis	0.2582
DB00272	Colon cancer	0.05934
DB00272	Infertility	0.13736
DB00272	Melanoma	0.08333

DB00501	Atopic rhinitis	0.2582
DB00501	Colon cancer	0.05934
DB00501	Infertility	0.13736
DB00501	Melanoma	0.08333
DB00585	Atopic rhinitis	0.2582
DB00585	Colon cancer	0.05934
DB00585	Infertility	0.13736
DB00585	Melanoma	0.08333
DB00667	Arthritis	0.06202
DB00667	Atopic rhinitis	0.2582
DB00667	Colon cancer	0.08901
DB00667	Diabetes mellitus	0.02632
DB00667	Infertility	0.13736
DB00667	Melanoma	0.04167
DB00667	Obesity	0.03846
DB00667	Pancreas cancer	0.1037
DB00667	Parkinson disease	0.05522
DB00667	Rheumatoid arthritis	0.0306
DB00667	Schizophrenia	0.03801
DB00751	Adenocarcinoma	0.05955
DB00751	Anorexia nervosa	0.08333
DB00751	Atopic rhinitis	0.21082
DB00751	Attention deficit hyperactivity disorder	0.42084
DB00751	Autistic disorder	0.04951
DB00751	Behavior disease	0.06299
DB00751	Bipolar disorder	0.04623
DB00751	Choriocarcinoma	0.11785
DB00751	Chronic fatigue syndrome	0.14434
DB00751	Colon cancer	0.07268
DB00751	Depression	0.05361
DB00751	Dermatitis	0.03711
DB00751	Drug-Induced dyskinesia	0.1543
DB00751	Hypertension	0.24651
DB00751	Infertility	0.11215
DB00751	Kidney failure	0.04623
DB00751	Melanoma	0.03402
DB00751	Nervous system disease	0.38205
DB00751	Obesity	0.17378
DB00751	Obsessive-compulsive disorder	0.12309
DB00751	Panic disorder	0.08909
DB00751	Parkinson disease	0.04508
DB00751	Prostate cancer	0.02131
DB00751	Psychotic disorder	0.06537
DB00751	Rheumatoid arthritis	0.02498
DB00751	Schizophrenia	0.19413
DB00751	Stroke	0.04623
DB00863	Atopic rhinitis	0.2582

DB00863	Colon cancer	0.05934
DB00863	Infertility	0.13736
DB00863	Melanoma	0.08333
DB00927	Atopic rhinitis	0.2582
DB00927	Colon cancer	0.05934
DB00927	Infertility	0.13736
DB00927	Melanoma	0.08333
DB08806	Atopic rhinitis	0.2582
DB08806	Colon cancer	0.05934
DB08806	Infertility	0.13736
DB08806	Melanoma	0.08333
DB00987	Adenoma	0.06106
DB00987	Alzheimer's disease	0.17908
DB00987	Azoospermia	0.2423
DB00987	Barrett's esophagus	0.16757
DB00987	Breast cancer	0.14065
DB00987	Cancer	0.04624
DB00987	Carcinoma	0.09272
DB00987	Colon cancer	0.03193
DB00987	Endometriosis	0.09544
DB00987	Esophageal disease	0.48333
DB00987	Heart failure	0.34016
DB00987	Ischemia	0.17662
DB00987	Lung cancer	0.03648
DB00987	Oral cancer	0.13127
DB00987	Ovary cancer	0.16013
DB00987	Peptic esophagitis	0.39858
DB00987	Prostate cancer	0.02736
DB00987	Rheumatoid arthritis	0.07288
DB00987	Skin cancer	0.21933
DB00987	Squamous cell cancer	0.06462
DB00987	Stomach cancer	0.21639
DB00987	Stroke	0.11161
DB00987	Testicular dysfunction	0.13544
DB00987	Werner syndrome	0.70225
DB00756	Actinic keratosis	0.21822
DB00756	Cancer	0.02128
DB00756	Encephalopathies	0.08085
DB00756	Hyperinsulinism	0.11111
DB00756	Pancreas disease	0.12039
DB00173	Abortion	0.03769
DB00173	Arthritis	0.0374
DB00173	Cancer	0.01111
DB00173	Colon cancer	0.01789
DB00173	Diabetes mellitus	0.01587
DB00173	Embryoma	0.01859
DB00173	Encephalopathies	0.04222

DB00173	Lung cancer	0.02106
DB00173	Neuroblastoma	0.04142
DB00173	Tuberculosis	0.04066
DB00182	Stress disorder, post-traumatic	0.17678
DB00182	Behavior disease	0.1543
DB00182	Bipolar disorder	0.11323
DB00182	Brain disease	0.17961
DB00182	Breast cancer	0.02406
DB00182	Depression	0.06565
DB00182	Drug abuse	0.09366
DB00182	Gilles de la Tourette syndrome	0.18898
DB00182	Herpes	0.07143
DB00182	Migraine	0.09285
DB00182	Multiple endocrine neoplasia	0.16667
DB00182	Obesity	0.07692
DB00182	Psychotic disorder	0.16013
DB00206	Behavior disease	0.1543
DB00206	Bipolar disorder	0.11323
DB00206	Brain disease	0.17961
DB00206	Multiple endocrine neoplasia	0.33333
DB00206	Psychotic disorder	0.16013
DB00386	Behavior disease	0.1543
DB00386	Bipolar disorder	0.11323
DB00386	Brain disease	0.17961
DB00386	Multiple endocrine neoplasia	0.33333
DB00386	Psychotic disorder	0.16013
DB00865	Stress disorder, post-traumatic	0.17678
DB00865	Attention deficit hyperactivity disorder	0.44732
DB00865	Behavior disease	0.1543
DB00865	Bipolar disorder	0.11323
DB00865	Brain disease	0.17961
DB00865	Breast cancer	0.02406
DB00865	Depression	0.06565
DB00865	Drug abuse	0.04683
DB00865	Gilles de la Tourette syndrome	0.18898
DB00865	Herpes	0.07143
DB00865	Hypertension	0.19647
DB00865	Kidney failure	0.05661
DB00865	Migraine	0.09285
DB00865	Multiple endocrine neoplasia	0.16667
DB00865	Nervous system disease	0.40574
DB00865	Obesity	0.1879
DB00865	Prostate cancer	0.0261
DB00865	Psychotic disorder	0.16013
DB00865	Schizophrenia	0.13903
DB01089	Behavior disease	0.1543
DB01089	Bipolar disorder	0.11323

DB01089	Brain disease	0.17961
DB01089	Multiple endocrine neoplasia	0.33333
DB01089	Psychotic disorder	0.16013
DB01363	Hypertension, Pulmonary	0.06363
DB01363	Infertility, Male	0.08591
DB01363	Stress disorder, post-traumatic	0.19612
DB01363	Alimentary system disease	0.1599
DB01363	Alzheimer's disease	0.07579
DB01363	Amnionitis	0.07692
DB01363	Anorexia nervosa	0.16984
DB01363	Arthritis	0.4083
DB01363	Atherosclerosis	0.19834
DB01363	Attention deficit hyperactivity disorder	0.19995
DB01363	Autistic disorder	0.32415
DB01363	Azoospermia	0.06497
DB01363	Behavior disease	0.25678
DB01363	Bipolar disorder	0.18842
DB01363	Brain disease	0.09963
DB01363	Breast cancer	0.04495
DB01363	Bronchial disease	0.81512
DB01363	Cholelithiasis	0.05547
DB01363	Chronic fatigue syndrome	0.09806
DB01363	Chronic obstructive airway disease	0.36197
DB01363	Colon cancer	0.01646
DB01363	Common cold	0.07412
DB01363	Conduct disorder	0.16648
DB01363	Congenital heart disease	0.09245
DB01363	Cystic fibrosis	0.42674
DB01363	Depression	0.10925
DB01363	Dermatitis	0.2048
DB01363	Diabetes mellitus	0.13993
DB01363	Down syndrome	0.03161
DB01363	Drug abuse	0.24957
DB01363	Encephalopathies	0.03884
DB01363	Endometrium cancer	0.06727
DB01363	Enteritis	0.05714
DB01363	Epilepsy	0.16268
DB01363	Fibromyalgia	0.29417
DB01363	Generalized anxiety disorder	0.16013
DB01363	Gilles de la Tourette syndrome	0.10483
DB01363	Glaucoma	0.46773
DB01363	Gram-Negative bacterial infection	0.11409
DB01363	Graves' disease	0.45366
DB01363	Heart failure	0.41509
DB01363	Herpes	0.07924
DB01363	Huntington disease	0.06052
DB01363	Hypertension	0.44668

DB01363	Infertility	0.05489
DB01363	Ischemia	0.10537
DB01363	Liver cancer	0.03646
DB01363	Lung cancer	0.15462
DB01363	Malaria	0.48491
DB01363	Metabolism disease	0.49684
DB01363	Migraine	0.10301
DB01363	Movement disorder	0.2436
DB01363	Multiple endocrine neoplasia	0.27735
DB01363	Myopathy	0.3057
DB01363	Nervous system disease	0.18108
DB01363	Neurodegenerative disorder	0.1347
DB01363	Neuroendocrine tumor	0.09806
DB01363	Neurotic disorder	0.17541
DB01363	Obesity	0.4298
DB01363	Obsessive-compulsive disorder	0.08362
DB01363	Oligospermia	0.1183
DB01363	Panic disorder	0.18157
DB01363	Pervasive development disorder	0.07161
DB01363	Polycystic kidney	0.10295
DB01363	Polycystic ovary syndrome	0.30067
DB01363	Pre-Eclampsia	0.03269
DB01363	Premature birth	0.68688
DB01363	Prostate cancer	0.13658
DB01363	Psychotic disorder	0.17765
DB01363	Pulmonary hypertension	0.13868
DB01363	Respiratory tract disease	0.07942
DB01363	Rheumatoid arthritis	0.13082
DB01363	Schizophrenia	0.08214
DB01363	Sickle cell disease	0.24666
DB01363	Sinusitis	0.07369
DB01363	Stroke	0.0314
DB01363	Subarachnoid hemorrhage	0.56997
DB01363	Sudden infant death syndrome	0.12105
DB01363	Systemic infection	0.03181
DB01363	Testicular dysfunction	0.03632
DB01363	Ulcerative colitis	0.02861
DB01364	Anorexia nervosa	0.10206
DB01364	Behavior disease	0.1543
DB01364	Bipolar disorder	0.11323
DB01364	Brain disease	0.0898
DB01364	Brain tumor	0.04016
DB01364	Colon cancer	0.02967
DB01364	Diabetes mellitus	0.02632
DB01364	Drug abuse	0.04683
DB01364	Hypertension	0.07906
DB01364	Kidney failure	0.05661

DB01364	Lung cancer	0.03492
DB01364	Multiple endocrine neoplasia	0.33333
DB01364	Neurodegenerative disorder	0.0822
DB01364	Neuroendocrine tumor	0.17678
DB01364	Ovarian cancer	0.05025
DB01364	Panic disorder	0.10911
DB01364	Prostate cancer	0.0261
DB01364	Psychotic disorder	0.08006
DB01364	Vitiligo	0.11785
DB01576	Stress disorder, post-traumatic	0.14434
DB01576	Anorexia nervosa	0.08333
DB01576	Behavior disease	0.18898
DB01576	Bipolar disorder	0.13868
DB01576	Brain disease	0.14665
DB01576	Breast cancer	0.01964
DB01576	Depression	0.05361
DB01576	Diabetes mellitus	0.02149
DB01576	Drug abuse	0.07647
DB01576	Gilles de la Tourette syndrome	0.1543
DB01576	Herpes	0.05832
DB01576	Hypertension	0.06455
DB01576	Kidney failure	0.09245
DB01576	Migraine	0.07581
DB01576	Multiple endocrine neoplasia	0.27217
DB01576	Neuroendocrine tumor	0.14434
DB01576	Obesity	0.0314
DB01576	Panic disorder	0.08909
DB01576	Prostate cancer	0.02131
DB01576	Psychotic disorder	0.13074
DB01577	Hypertension, Pulmonary	0.06917
DB01577	Stress disorder, post-traumatic	0.2132
DB01577	Anorexia nervosa	0.18464
DB01577	Atherosclerosis	0.02111
DB01577	Attention deficit hyperactivity disorder	0.39002
DB01577	Autistic disorder	0.07313
DB01577	Behavior disease	0.27915
DB01577	Bipolar disorder	0.20484
DB01577	Brain disease	0.10831
DB01577	Breast cancer	0.02901
DB01577	Chronic fatigue syndrome	0.1066
DB01577	Chronic obstructive airway disease	0.03371
DB01577	Colon cancer	0.01789
DB01577	Common cold	0.08058
DB01577	Congenital heart disease	0.1005
DB01577	Depression	0.11877
DB01577	Dermatitis	0.02741
DB01577	Diabetes mellitus	0.03174

DB01577	Down syndrome	0.03436
DB01577	Drug abuse	0.11296
DB01577	Encephalopathies	0.04222
DB01577	Epilepsy	0.04264
DB01577	Fibromyalgia	0.3198
DB01577	Generalized anxiety disorder	0.17408
DB01577	Gilles de la Tourette syndrome	0.11396
DB01577	Heart failure	0.03214
DB01577	Herpes	0.08615
DB01577	Huntington disease	0.0658
DB01577	Hypertension	0.18892
DB01577	Migraine	0.11198
DB01577	Multiple endocrine neoplasia	0.30151
DB01577	Nervous system disease	0.35449
DB01577	Neuroendocrine tumor	0.1066
DB01577	Neurotic disorder	0.19069
DB01577	Obesity	0.22694
DB01577	Obsessive-compulsive disorder	0.09091
DB01577	Panic disorder	0.19739
DB01577	Pervasive development disorder	0.07785
DB01577	Polycystic ovary syndrome	0.03994
DB01577	Psychotic disorder	0.19312
DB01577	Pulmonary hypertension	0.15076
DB01577	Schizophrenia	0.14686
DB01577	Stroke	0.03414
DB01577	Sudden infant death syndrome	0.13159
DB01577	Ulcerative colitis	0.0311
DB04844	Behavior disease	0.10911
DB04844	Bipolar disorder	0.08006
DB04844	Brain disease	0.127
DB04844	Multiple endocrine neoplasia	0.2357
DB04844	Psychotic disorder	0.11323
DB06706	Behavior disease	0.10911
DB06706	Bipolar disorder	0.08006
DB06706	Brain disease	0.127
DB06706	Hypertension	0.0559
DB06706	Kidney failure	0.08006
DB06706	Multiple endocrine neoplasia	0.2357
DB06706	Prostate cancer	0.03691
DB06706	Psychotic disorder	0.11323
DB06714	Behavior disease	0.10911
DB06714	Bipolar disorder	0.08006
DB06714	Brain disease	0.127
DB06714	Multiple endocrine neoplasia	0.2357
DB06714	Psychotic disorder	0.11323
DB01016	Infertility, Male	0.55273
DB01016	Alimentary system disease	0.97158

DB01016	Alzheimer's disease	0.16915
DB01016	Arthritis	0.10548
DB01016	Atherosclerosis	0.08903
DB01016	Autistic disorder	0.06298
DB01016	Azoospermia	0.43561
DB01016	Bone disease	0.40922
DB01016	Breast cancer	0.10217
DB01016	Bronchial disease	0.21098
DB01016	Cancer	0.0583
DB01016	Cardiovascular disease	0.05392
DB01016	Chronic obstructive airway disease	0.08462
DB01016	Conduct disorder	1.0491
DB01016	Congenital abnormality	0.10122
DB01016	Cystic fibrosis	0.60363
DB01016	Dermatitis	0.04355
DB01016	Diabetes mellitus	0.14571
DB01016	Drug abuse	0.03377
DB01016	Endometriosis	0.10738
DB01016	Enteritis	0.35842
DB01016	Glaucoma	0.07955
DB01016	Gram-Negative bacterial infection	0.74025
DB01016	Graves' disease	0.11415
DB01016	Heart failure	0.326
DB01016	Hyperaldosteronism	0.1118
DB01016	Hypercholesterolemia	0.06804
DB01016	Hyperglycemia	0.05661
DB01016	Hyperinsulinism	0.06804
DB01016	Hypertension	0.26518
DB01016	Infantile spasms	0.13363
DB01016	Infection	0.13706
DB01016	Infertility	0.35559
DB01016	Kidney disease	0.04226
DB01016	Kidney failure	0.04003
DB01016	Late pregnancy	0.20412
DB01016	Liver cancer	0.20395
DB01016	Lung cancer	0.06285
DB01016	Malaria	0.12227
DB01016	Metabolism disease	0.17172
DB01016	Myopathy	0.07675
DB01016	Obesity	0.07734
DB01016	Oligospermia	0.75303
DB01016	Pancreas disease	0.07372
DB01016	Polycystic kidney	0.67034
DB01016	Polycystic ovary syndrome	0.11093
DB01016	Polyneuropathy	0.09449
DB01016	Premature birth	0.1748
DB01016	Prostate cancer	0.02862

DB01016	Respiratory tract disease	0.51961
DB01016	Rheumatoid arthritis	0.02733
DB01016	Schizophrenia	0.02688
DB01016	Sickle cell disease	0.05453
DB01016	Sinusitis	0.49123
DB01016	Subarachnoid hemorrhage	0.08536
DB01016	Testicular dysfunction	0.25367
DB01016	Yersinia infection	0.04196
DB01599	Alzheimer's disease	0.0509
DB01599	Atherosclerosis	0.04951
DB01599	Cancer	0.05213
DB01599	Cardiovascular disease	0.10783
DB01599	Diabetes mellitus	0.03722
DB01599	Embryoma	0.0436
DB01599	Hypercholesterolemia	0.13608
DB01599	Kidney failure	0.08006
DB01599	Obesity	0.05439
DB01599	Polyneuropathy	0.18898
DB01599	Yersinia infection	0.08392
DB00360	Adenovirus infection	0.03148
DB00360	Alzheimer's disease	0.05755
DB00360	Atherosclerosis	0.03458
DB00360	Autistic disorder	0.06063
DB00360	Behavior disease	0.07715
DB00360	Bipolar disorder	0.05661
DB00360	Bladder cancer	0.25868
DB00360	Cancer	0.07983
DB00360	Depression	0.0967
DB00360	Diabetes mellitus	0.02373
DB00360	Drug abuse	0.02289
DB00360	Eating disorder	0.11327
DB00360	Encephalopathies	0.07001
DB00360	Fanconi's anemia	0.10147
DB00360	Gilles de la Tourette syndrome	0.38506
DB00360	Herpes	0.08147
DB00360	Huntington disease	0.11048
DB00360	Hyperglycemia	0.09606
DB00360	Hypertension	0.22517
DB00360	Migraine	0.09285
DB00360	Movement disorder	0.10379
DB00360	Obesity	0.04387
DB00360	Obsessive-compulsive disorder	0.23968
DB00360	Oral cancer	0.12919
DB00360	Polycystic ovary syndrome	0.05609
DB00360	Primary biliary cirrhosis	0.23974
DB00360	Psychotic disorder	0.10017
DB00360	Pulmonary fibrosis	0.3124

DB00360	Rabies	0.12049
DB00360	Schizophrenia	0.03994
DB00360	Scleroderma	0.14094
DB00360	Systemic infection	0.04401
DB00360	Systemic scleroderma	0.14013
DB00360	Thalassemia	0.11785
DB00360	Tropical spastic paraparesis	0.13391
DB00360	Vitiligo	0.11161
DB00255	Hypertension, Pulmonary	0.15392
DB00255	Infertility, Male	0.80533
DB00255	Adenovirus infection	0.03828
DB00255	Alzheimer's disease	0.25229
DB00255	Atherosclerosis	0.30699
DB00255	Autistic disorder	0.02299
DB00255	Bipolar disorder	0.61575
DB00255	Bladder cancer	0.1378
DB00255	Breast cancer	0.00932
DB00255	Bronchial hyperreactivity	1.20412
DB00255	Cancer	0.16219
DB00255	Cerebrovascular disorder	0.81339
DB00255	Cholelithiasis	0.83149
DB00255	Cirrhosis	0.1039
DB00255	Craniosynostosis	0.25982
DB00255	Dental plaque	0.53694
DB00255	Depression	0.3379
DB00255	Dermatitis	0.05249
DB00255	Diabetes mellitus	0.21255
DB00255	Down syndrome	0.4562
DB00255	Eating disorder	0.04067
DB00255	Endometrial cancer	0.1005
DB00255	Endometriosis	0.32285
DB00255	Epilepsy	0.05425
DB00255	Glaucoma	0.64763
DB00255	Hepatitis	0.19437
DB00255	Hepatitis C	0.06223
DB00255	Herpes	0.33617
DB00255	Hodgkin's disease	0.04769
DB00255	Hyperlipidemia	0.87519
DB00255	Hypertension	0.41284
DB00255	Infectious lung disease	0.05753
DB00255	Infertility	0.51853
DB00255	Kidney failure	0.45088
DB00255	Late pregnancy	1.27734
DB00255	Leukemia	0.02117
DB00255	Leukoencephalopathy	0.03564
DB00255	Lung cancer	0.02667
DB00255	Lupus erythematosus	0.29119

DB00255	Lupus vulgaris	0.69238
DB00255	Lymphoma	0.04152
DB00255	Mental retardation	0.03628
DB00255	Migraine	0.64625
DB00255	Multiple sclerosis	0.04129
DB00255	Myasthenia Gravis	1.1381
DB00255	Neoplasm metastasis	0.05223
DB00255	Nephrosis	0.05764
DB00255	Obesity	0.34834
DB00255	Oral cancer	0.03441
DB00255	Osteoporosis	0.61989
DB00255	Ovarian disease	0.95771
DB00255	Ovarian failure	1.02892
DB00255	Overnutrition	0.06746
DB00255	Pancreatitis	0.05065
DB00255	Panic disorder	0.81454
DB00255	Parkinson disease	0.46127
DB00255	Polyarthritis	0.34215
DB00255	Primary biliary cirrhosis	0.6574
DB00255	Prostate cancer	0.02
DB00255	Ptosis	0.19245
DB00255	Pulmonary fibrosis	0.08321
DB00255	Rabies	0.34243
DB00255	Renal tubular acidosis	0.60053
DB00255	Rheumatoid arthritis	0.08159
DB00255	Schistosomiasis	0.11237
DB00255	Schizophrenia	0.3896
DB00255	Scleroderma	0.03754
DB00255	Sicca syndrome	0.13578
DB00255	Stroke	0.31722
DB00255	Synovitis	0.99888
DB00255	Systemic scleroderma	0.02799
DB00255	Temporal arteritis	0.2582
DB00255	Testicular dysfunction	0.08248
DB00255	Thymoma	0.79941
DB00255	Tuberculosis	0.05212
DB00255	Ulcerative colitis	0.02997
DB00255	Uterine disease	0.7672
DB00255	Vaccinia	0.16627
DB00255	Virus disease	0.04249
DB00255	Vitiligo	0.86594
DB00255	Wiskott-Aldrich syndrome	0.07541
DB00269	Hypertension, Pulmonary	0.15392
DB00269	Infertility, Male	0.8916
DB00269	Adenovirus infection	0.03828
DB00269	Alzheimer's disease	0.24115
DB00269	Atherosclerosis	0.29616

DB00269	Autistic disorder	0.02299
DB00269	Bipolar disorder	0.6636
DB00269	Bladder cancer	0.1378
DB00269	Breast cancer	0.00932
DB00269	Bronchial hyperreactivity	1.35355
DB00269	Cancer	0.15649
DB00269	Cerebrovascular disorder	0.90562
DB00269	Cholelithiasis	0.80055
DB00269	Cirrhosis	0.1039
DB00269	Craniosynostosis	0.25982
DB00269	Dental plaque	0.51729
DB00269	Depression	0.39339
DB00269	Diabetes mellitus	0.2348
DB00269	Down syndrome	0.50437
DB00269	Eating disorder	0.04067
DB00269	Endometriosis	0.31
DB00269	Epilepsy	0.05425
DB00269	Glaucoma	0.62185
DB00269	Hepatitis	0.19437
DB00269	Hepatitis C	0.06223
DB00269	Herpes	0.39655
DB00269	Hodgkin's disease	0.04769
DB00269	Hyperlipidemia	0.95808
DB00269	Hypertension	0.40061
DB00269	Infectious lung disease	0.05753
DB00269	Infertility	0.57658
DB00269	Kidney failure	0.49873
DB00269	Late pregnancy	1.23268
DB00269	Leukemia	0.02117
DB00269	Leukoencephalopathy	0.03564
DB00269	Lung cancer	0.02667
DB00269	Lupus erythematosus	0.3273
DB00269	Lupus vulgaris	0.77225
DB00269	Lymphoma	0.04152
DB00269	Mental retardation	0.03628
DB00269	Migraine	0.72474
DB00269	Multiple sclerosis	0.04129
DB00269	Myasthenia Gravis	1.08341
DB00269	Neoplasm metastasis	0.05223
DB00269	Nephrosis	0.05764
DB00269	Obesity	0.38085
DB00269	Oral cancer	0.03441
DB00269	Osteoporosis	0.59543
DB00269	Ovarian disease	0.92125
DB00269	Ovarian failure	1.12588
DB00269	Overnutrition	0.06746
DB00269	Pancreatitis	0.05065

DB00269	Panic disorder	0.90677
DB00269	Parkinson disease	0.44419
DB00269	Polyarthritis	0.39032
DB00269	Primary biliary cirrhosis	0.73331
DB00269	Prostate cancer	0.02
DB00269	Pulmonary fibrosis	0.08321
DB00269	Rabies	0.38939
DB00269	Renal tubular acidosis	0.74141
DB00269	Rheumatoid arthritis	0.08159
DB00269	Schistosomiasis	0.11237
DB00269	Schizophrenia	0.37784
DB00269	Scleroderma	0.03754
DB00269	Sicca syndrome	0.13578
DB00269	Stroke	0.36508
DB00269	Synovitis	1.14831
DB00269	Systemic scleroderma	0.02799
DB00269	Thymoma	0.93306
DB00269	Tuberculosis	0.05212
DB00269	Ulcerative colitis	0.02997
DB00269	Uterine disease	0.92695
DB00269	Vaccinia	0.16627
DB00269	Virus disease	0.04249
DB00269	Vitiligo	0.96556
DB00269	Wiskott-Aldrich syndrome	0.07541
DB00286	Hypertension, Pulmonary	0.15392
DB00286	Infertility, Male	0.8916
DB00286	Adenovirus infection	0.03828
DB00286	Alzheimer's disease	0.24115
DB00286	Atherosclerosis	0.29616
DB00286	Autistic disorder	0.02299
DB00286	Bipolar disorder	0.6636
DB00286	Bladder cancer	0.1378
DB00286	Breast cancer	0.00932
DB00286	Bronchial hyperreactivity	1.35355
DB00286	Cancer	0.15649
DB00286	Cerebrovascular disorder	0.90562
DB00286	Cholelithiasis	0.80055
DB00286	Cirrhosis	0.1039
DB00286	Craniosynostosis	0.25982
DB00286	Dental plaque	0.51729
DB00286	Depression	0.39339
DB00286	Diabetes mellitus	0.2348
DB00286	Down syndrome	0.50437
DB00286	Eating disorder	0.04067
DB00286	Endometriosis	0.31
DB00286	Epilepsy	0.05425
DB00286	Glaucoma	0.62185

DB00286	Hepatitis	0.19437
DB00286	Hepatitis C	0.06223
DB00286	Herpes	0.39655
DB00286	Hodgkin's disease	0.04769
DB00286	Hyperlipidemia	0.95808
DB00286	Hypertension	0.40061
DB00286	Infectious lung disease	0.05753
DB00286	Infertility	0.57658
DB00286	Kidney failure	0.49873
DB00286	Late pregnancy	1.23268
DB00286	Leukemia	0.02117
DB00286	Leukoencephalopathy	0.03564
DB00286	Lung cancer	0.02667
DB00286	Lupus erythematosus	0.3273
DB00286	Lupus vulgaris	0.77225
DB00286	Lymphoma	0.04152
DB00286	Mental retardation	0.03628
DB00286	Migraine	0.72474
DB00286	Multiple sclerosis	0.04129
DB00286	Myasthenia Gravis	1.08341
DB00286	Neoplasm metastasis	0.05223
DB00286	Nephrosis	0.05764
DB00286	Obesity	0.38085
DB00286	Oral cancer	0.03441
DB00286	Osteoporosis	0.59543
DB00286	Ovarian disease	0.92125
DB00286	Ovarian failure	1.12588
DB00286	Overnutrition	0.06746
DB00286	Pancreatitis	0.05065
DB00286	Panic disorder	0.90677
DB00286	Parkinson disease	0.44419
DB00286	Polyarthritis	0.39032
DB00286	Primary biliary cirrhosis	0.73331
DB00286	Prostate cancer	0.02
DB00286	Pulmonary fibrosis	0.08321
DB00286	Rabies	0.38939
DB00286	Renal tubular acidosis	0.74141
DB00286	Rheumatoid arthritis	0.08159
DB00286	Schistosomiasis	0.11237
DB00286	Schizophrenia	0.37784
DB00286	Scleroderma	0.03754
DB00286	Sicca syndrome	0.13578
DB00286	Stroke	0.36508
DB00286	Synovitis	1.14831
DB00286	Systemic scleroderma	0.02799
DB00286	Thymoma	0.93306
DB00286	Tuberculosis	0.05212

DB00286	Ulcerative colitis	0.02997
DB00286	Uterine disease	0.92695
DB00286	Vaccinia	0.16627
DB00286	Virus disease	0.04249
DB00286	Vitiligo	0.96556
DB00286	Wiskott-Aldrich syndrome	0.07541
DB00294	Hypertension, Pulmonary	0.15392
DB00294	Infertility, Male	0.83182
DB00294	Adenovirus infection	0.03828
DB00294	Alzheimer's disease	0.22007
DB00294	Atherosclerosis	0.27565
DB00294	Autistic disorder	0.02299
DB00294	Bipolar disorder	0.63044
DB00294	Bladder cancer	0.1378
DB00294	Breast cancer	0.00932
DB00294	Bronchial hyperreactivity	1.25
DB00294	Cancer	0.1457
DB00294	Cerebrovascular disorder	0.8417
DB00294	Cholelithiasis	0.74197
DB00294	Cirrhosis	0.1039
DB00294	Craniosynostosis	0.25982
DB00294	Dental plaque	0.4801
DB00294	Depression	0.35494
DB00294	Diabetes mellitus	0.21938
DB00294	Down syndrome	0.47099
DB00294	Eating disorder	0.04067
DB00294	Endometriosis	0.28568
DB00294	Epilepsy	0.05425
DB00294	Glaucoma	0.57304
DB00294	Hepatitis	0.19437
DB00294	Hepatitis C	0.06223
DB00294	Herpes	0.35471
DB00294	Hodgkin's disease	0.04769
DB00294	Hyperlipidemia	0.90064
DB00294	Hypertension	0.37746
DB00294	Infectious lung disease	0.05753
DB00294	Infertility	0.53635
DB00294	Kidney failure	0.46557
DB00294	Late pregnancy	1.14813
DB00294	Leukemia	0.02117
DB00294	Leukoencephalopathy	0.03564
DB00294	Lung cancer	0.02667
DB00294	Lupus erythematosus	0.30228
DB00294	Lupus vulgaris	0.7169
DB00294	Lymphoma	0.04152
DB00294	Mental retardation	0.03628
DB00294	Migraine	0.67035

DB00294	Multiple sclerosis	0.04129
DB00294	Myasthenia Gravis	0.97985
DB00294	Neoplasm metastasis	0.05223
DB00294	Nephrosis	0.05764
DB00294	Obesity	0.35832
DB00294	Oral cancer	0.03441
DB00294	Osteoporosis	0.54912
DB00294	Ovarian disease	0.85221
DB00294	Ovarian failure	1.05869
DB00294	Overnutrition	0.06746
DB00294	Pancreatitis	0.05065
DB00294	Panic disorder	0.84285
DB00294	Parkinson disease	0.41185
DB00294	Polyarthritis	0.35694
DB00294	Primary biliary cirrhosis	0.68071
DB00294	Prostate cancer	0.02
DB00294	Pulmonary fibrosis	0.08321
DB00294	Rabies	0.35685
DB00294	Renal tubular acidosis	0.64378
DB00294	Rheumatoid arthritis	0.08159
DB00294	Schistosomiasis	0.11237
DB00294	Schizophrenia	0.35557
DB00294	Scleroderma	0.03754
DB00294	Sicca syndrome	0.13578
DB00294	Stroke	0.33192
DB00294	Synovitis	1.04476
DB00294	Systemic scleroderma	0.02799
DB00294	Thymoma	0.84044
DB00294	Tuberculosis	0.05212
DB00294	Ulcerative colitis	0.02997
DB00294	Uterine disease	0.81625
DB00294	Vaccinia	0.16627
DB00294	Virus disease	0.04249
DB00294	Vitiligo	0.89652
DB00294	Wiskott-Aldrich syndrome	0.07541
DB00304	Hypertension, Pulmonary	0.15392
DB00304	Infertility, Male	0.83182
DB00304	Adenovirus infection	0.03828
DB00304	Alzheimer's disease	0.22007
DB00304	Atherosclerosis	0.27565
DB00304	Autistic disorder	0.02299
DB00304	Bipolar disorder	0.63044
DB00304	Bladder cancer	0.1378
DB00304	Breast cancer	0.00932
DB00304	Bronchial hyperreactivity	1.25
DB00304	Cancer	0.1457
DB00304	Cerebrovascular disorder	0.8417

DB00304	Cholelithiasis	0.74197
DB00304	Cirrhosis	0.1039
DB00304	Craniosynostosis	0.25982
DB00304	Dental plaque	0.4801
DB00304	Depression	0.35494
DB00304	Diabetes mellitus	0.21938
DB00304	Down syndrome	0.47099
DB00304	Eating disorder	0.04067
DB00304	Endometriosis	0.28568
DB00304	Epilepsy	0.05425
DB00304	Glaucoma	0.57304
DB00304	Hepatitis	0.19437
DB00304	Hepatitis C	0.06223
DB00304	Herpes	0.35471
DB00304	Hodgkin's disease	0.04769
DB00304	Hyperlipidemia	0.90064
DB00304	Hypertension	0.37746
DB00304	Infectious lung disease	0.05753
DB00304	Infertility	0.53635
DB00304	Kidney failure	0.46557
DB00304	Late pregnancy	1.14813
DB00304	Leukemia	0.02117
DB00304	Leukoencephalopathy	0.03564
DB00304	Lung cancer	0.02667
DB00304	Lupus erythematosus	0.30228
DB00304	Lupus vulgaris	0.7169
DB00304	Lymphoma	0.04152
DB00304	Mental retardation	0.03628
DB00304	Migraine	0.67035
DB00304	Multiple sclerosis	0.04129
DB00304	Myasthenia Gravis	0.97985
DB00304	Neoplasm metastasis	0.05223
DB00304	Nephrosis	0.05764
DB00304	Obesity	0.35832
DB00304	Oral cancer	0.03441
DB00304	Osteoporosis	0.54912
DB00304	Ovarian disease	0.85221
DB00304	Ovarian failure	1.05869
DB00304	Overnutrition	0.06746
DB00304	Pancreatitis	0.05065
DB00304	Panic disorder	0.84285
DB00304	Parkinson disease	0.41185
DB00304	Polyarthritis	0.35694
DB00304	Primary biliary cirrhosis	0.68071
DB00304	Prostate cancer	0.02
DB00304	Pulmonary fibrosis	0.08321
DB00304	Rabies	0.35685

DB00304	Renal tubular acidosis	0.64378
DB00304	Rheumatoid arthritis	0.08159
DB00304	Schistosomiasis	0.11237
DB00304	Schizophrenia	0.35557
DB00304	Scleroderma	0.03754
DB00304	Sicca syndrome	0.13578
DB00304	Stroke	0.33192
DB00304	Synovitis	1.04476
DB00304	Systemic scleroderma	0.02799
DB00304	Thymoma	0.84044
DB00304	Tuberculosis	0.05212
DB00304	Ulcerative colitis	0.02997
DB00304	Uterine disease	0.81625
DB00304	Vaccinia	0.16627
DB00304	Virus disease	0.04249
DB00304	Vitiligo	0.89652
DB00304	Wiskott-Aldrich syndrome	0.07541
DB00367	Hypertension, Pulmonary	0.13649
DB00367	Infertility, Male	0.97941
DB00367	Adenovirus infection	0.05624
DB00367	Alzheimer's disease	0.186
DB00367	Amyotrophic lateral sclerosis	0.13437
DB00367	Atherosclerosis	0.33701
DB00367	Autistic disorder	0.02039
DB00367	Azoospermia	0.22736
DB00367	Bipolar disorder	0.54465
DB00367	Bladder cancer	0.12219
DB00367	Breast cancer	0.00827
DB00367	Bronchial hyperreactivity	1.06352
DB00367	Cancer	0.16819
DB00367	Cerebrovascular disorder	0.71865
DB00367	Cholelithiasis	0.87725
DB00367	Cirrhosis	0.09213
DB00367	Craniosynostosis	0.23039
DB00367	Dental plaque	0.40959
DB00367	Depression	0.42687
DB00367	Diabetes mellitus	0.25921
DB00367	Down syndrome	0.40317
DB00367	Eating disorder	0.03607
DB00367	Endometriosis	0.33386
DB00367	Epilepsy	0.0481
DB00367	Glaucoma	0.48696
DB00367	Hepatitis	0.17235
DB00367	Hepatitis C	0.05518
DB00367	Herpes	0.29639
DB00367	Hodgkin's disease	0.04229
DB00367	Huntington disease	0.07826

DB00367	Hyperglycemia	0.06805
DB00367	Hyperlipidemia	0.77372
DB00367	Hypertension	0.3576
DB00367	Hypogonadism	0.68588
DB00367	Infectious lung disease	0.05102
DB00367	Infertility	0.63269
DB00367	Kidney failure	0.39846
DB00367	Late pregnancy	0.98142
DB00367	Leukemia	0.01877
DB00367	Leukoencephalopathy	0.0316
DB00367	Lung cancer	0.02365
DB00367	Lupus erythematosus	0.25719
DB00367	Lupus vulgaris	0.6117
DB00367	Lymphoma	0.03681
DB00367	Mental retardation	0.03217
DB00367	Migraine	0.79359
DB00367	Multiple sclerosis	0.03662
DB00367	Muscular atrophy	0.32168
DB00367	Myasthenia Gravis	0.82397
DB00367	Neoplasm metastasis	0.04631
DB00367	Nephrosis	0.05111
DB00367	Obesity	0.33909
DB00367	Oligospermia	0.35724
DB00367	Oral cancer	0.03051
DB00367	Osteoporosis	0.65129
DB00367	Ovarian disease	0.72575
DB00367	Ovarian failure	0.90964
DB00367	Overnutrition	0.05982
DB00367	Pancreatitis	0.04491
DB00367	Panic disorder	0.71967
DB00367	Parkinson disease	0.35117
DB00367	Phobic anxiety disorder	0.71227
DB00367	Polyarthritis	0.30204
DB00367	Polycystic ovary syndrome	0.03973
DB00367	Primary biliary cirrhosis	0.58079
DB00367	Prostate cancer	0.0833
DB00367	Psychotic disorder	0.07096
DB00367	Pulmonary fibrosis	0.07378
DB00367	Rabies	0.42127
DB00367	Renal tubular acidosis	0.52853
DB00367	Rheumatoid arthritis	0.14072
DB00367	Schistosomiasis	0.09964
DB00367	Schizophrenia	0.41948
DB00367	Scleroderma	0.03329
DB00367	Sicca syndrome	0.1204
DB00367	Stroke	0.27994
DB00367	Synovitis	0.88152

DB00367	Systemic infection	0.03117
DB00367	Systemic scleroderma	0.04964
DB00367	Thymoma	0.70509
DB00367	Tropical spastic paraparesis	0.09486
DB00367	Tuberculosis	0.04622
DB00367	Ulcerative colitis	0.02658
DB00367	Urogenital abnormalities	0.39197
DB00367	Uterine disease	0.67579
DB00367	Vaccinia	0.14744
DB00367	Virus disease	0.03768
DB00367	Vitiligo	0.8441
DB00367	Wiskott-Aldrich syndrome	0.06687
DB00367	Yersinia infection	0.12552
DB00396	Hypertension, Pulmonary	0.13799
DB00396	Infertility, Male	0.71836
DB00396	Adenovirus infection	0.03432
DB00396	Alopecia	0.163
DB00396	Alzheimer's disease	0.18765
DB00396	Atherosclerosis	0.23774
DB00396	Autistic disorder	0.02061
DB00396	Bipolar disorder	0.55001
DB00396	Bladder cancer	0.12353
DB00396	Breast cancer	0.06443
DB00396	Bronchial hyperreactivity	1.07324
DB00396	Cancer	0.12568
DB00396	Cerebrovascular disorder	0.72534
DB00396	Cholelithiasis	0.63837
DB00396	Cirrhosis	0.09314
DB00396	Craniosynostosis	0.23292
DB00396	Dental plaque	0.41338
DB00396	Depression	0.30061
DB00396	Diabetes mellitus	0.18962
DB00396	Down syndrome	0.40697
DB00396	Eating disorder	0.03646
DB00396	Endometriosis	0.24498
DB00396	Epilepsy	0.04863
DB00396	Glaucoma	0.49139
DB00396	Heart failure	0.1341
DB00396	Hepatitis	0.17424
DB00396	Hepatitis C	0.05579
DB00396	Herpes	0.29886
DB00396	Hodgkin's disease	0.04275
DB00396	Hyperlipidemia	0.78113
DB00396	Hypertension	0.4339
DB00396	Hypoglycemia	0.55309
DB00396	Infectious lung disease	0.05158
DB00396	Infertility	0.46243

DB00396	Kidney failure	0.40221
DB00396	Late pregnancy	0.99061
DB00396	Leukemia	0.01898
DB00396	Leukoencephalopathy	0.03195
DB00396	Lung cancer	0.02391
DB00396	Lupus erythematosus	0.25954
DB00396	Lupus vulgaris	0.61737
DB00396	Lymphoma	0.18015
DB00396	Melanoma	0.03102
DB00396	Mental retardation	0.03253
DB00396	Migraine	0.57608
DB00396	Multiple sclerosis	0.03702
DB00396	Myasthenia Gravis	0.83107
DB00396	Neoplasm metastasis	0.04682
DB00396	Nephrosis	0.05168
DB00396	Obesity	0.31093
DB00396	Oral cancer	0.03085
DB00396	Osteoporosis	0.4711
DB00396	Ovarian disease	0.73242
DB00396	Ovarian failure	0.91836
DB00396	Overnutrition	0.06048
DB00396	Pancreatitis	0.0454
DB00396	Panic disorder	0.72637
DB00396	Parkinson disease	0.35442
DB00396	Polyarthritis	0.30473
DB00396	Primary biliary cirrhosis	0.58618
DB00396	Prostate cancer	0.01793
DB00396	Proteinuria	0.34902
DB00396	Pulmonary fibrosis	0.07459
DB00396	Rabies	0.30503
DB00396	Renal Cell cancer	0.16805
DB00396	Renal tubular acidosis	0.5325
DB00396	Rheumatoid arthritis	0.07314
DB00396	Schistosomiasis	0.10074
DB00396	Schizophrenia	0.30858
DB00396	Scleroderma	0.03365
DB00396	Sicca syndrome	0.12172
DB00396	Stroke	0.28239
DB00396	Synovitis	0.88925
DB00396	Systemic scleroderma	0.02509
DB00396	Thymoma	0.71109
DB00396	Tuberculosis	0.04673
DB00396	Ulcerative colitis	0.02687
DB00396	Uterine disease	0.68113
DB00396	Vaccinia	0.14906
DB00396	Virus disease	0.03809
DB00396	Vitiligo	0.77214

DB00396	Wiskott-Aldrich syndrome	0.0676
DB00481	Hypertension, Pulmonary	0.15392
DB00481	Infertility, Male	0.83182
DB00481	Adenovirus infection	0.03828
DB00481	Alzheimer's disease	0.27097
DB00481	Atherosclerosis	0.32516
DB00481	Autistic disorder	0.02299
DB00481	Bipolar disorder	0.63044
DB00481	Bladder cancer	0.1378
DB00481	Breast cancer	0.00932
DB00481	Bronchial hyperreactivity	1.25
DB00481	Cancer	0.17176
DB00481	Cerebrovascular disorder	0.8417
DB00481	Cholelithiasis	0.88339
DB00481	Cirrhosis	0.1039
DB00481	Craniosynostosis	0.25982
DB00481	Dental plaque	0.5699
DB00481	Depression	0.35494
DB00481	Diabetes mellitus	0.21938
DB00481	Down syndrome	0.47099
DB00481	Eating disorder	0.04067
DB00481	Endometriosis	0.3444
DB00481	Epilepsy	0.05425
DB00481	Glaucoma	0.69089
DB00481	Hepatitis	0.19437
DB00481	Hepatitis C	0.06223
DB00481	Herpes	0.35471
DB00481	Hodgkin's disease	0.04769
DB00481	Hyperlipidemia	0.90064
DB00481	Hypertension	0.43336
DB00481	Infectious lung disease	0.05753
DB00481	Infertility	0.53635
DB00481	Kidney failure	0.46557
DB00481	Late pregnancy	1.35225
DB00481	Leukemia	0.02117
DB00481	Leukoencephalopathy	0.03564
DB00481	Lung cancer	0.02667
DB00481	Lupus erythematosus	0.30228
DB00481	Lupus vulgaris	0.7169
DB00481	Lymphoma	0.04152
DB00481	Mental retardation	0.03628
DB00481	Migraine	0.67035
DB00481	Multiple sclerosis	0.04129
DB00481	Myasthenia Gravis	1.22985
DB00481	Neoplasm metastasis	0.05223
DB00481	Nephrosis	0.05764
DB00481	Obesity	0.35832

DB00481	Oral cancer	0.03441
DB00481	Osteoporosis	0.66092
DB00481	Ovarian disease	1.01888
DB00481	Ovarian failure	1.05869
DB00481	Overnutrition	0.06746
DB00481	Pancreatitis	0.05065
DB00481	Panic disorder	0.84285
DB00481	Parkinson disease	0.48993
DB00481	Polyarthritis	0.35694
DB00481	Primary biliary cirrhosis	0.68071
DB00481	Prostate cancer	0.02
DB00481	Ptosis	0.2357
DB00481	Pulmonary fibrosis	0.08321
DB00481	Rabies	0.35685
DB00481	Renal tubular acidosis	0.64378
DB00481	Rheumatoid arthritis	0.08159
DB00481	Schistosomiasis	0.11237
DB00481	Schizophrenia	0.40933
DB00481	Scleroderma	0.03754
DB00481	Sicca syndrome	0.13578
DB00481	Stroke	0.33192
DB00481	Synovitis	1.04476
DB00481	Systemic scleroderma	0.02799
DB00481	Temporal arteritis	0.31623
DB00481	Testicular dysfunction	0.10102
DB00481	Thymoma	0.84044
DB00481	Tuberculosis	0.05212
DB00481	Ulcerative colitis	0.02997
DB00481	Uterine disease	0.81625
DB00481	Vaccinia	0.16627
DB00481	Virus disease	0.04249
DB00481	Vitiligo	0.89652
DB00481	Wiskott-Aldrich syndrome	0.07541
DB00539	Hypertension, Pulmonary	0.15392
DB00539	Infertility, Male	0.8916
DB00539	Adenovirus infection	0.03828
DB00539	Alzheimer's disease	0.24115
DB00539	Atherosclerosis	0.29616
DB00539	Autistic disorder	0.02299
DB00539	Bipolar disorder	0.6636
DB00539	Bladder cancer	0.1378
DB00539	Breast cancer	0.00932
DB00539	Bronchial hyperreactivity	1.35355
DB00539	Cancer	0.15649
DB00539	Cerebrovascular disorder	0.90562
DB00539	Cholelithiasis	0.80055
DB00539	Cirrhosis	0.1039

DB00539	Craniosynostosis	0.25982
DB00539	Dental plaque	0.51729
DB00539	Depression	0.39339
DB00539	Diabetes mellitus	0.2348
DB00539	Down syndrome	0.50437
DB00539	Eating disorder	0.04067
DB00539	Endometriosis	0.31
DB00539	Epilepsy	0.05425
DB00539	Glaucoma	0.62185
DB00539	Hepatitis	0.19437
DB00539	Hepatitis C	0.06223
DB00539	Herpes	0.39655
DB00539	Hodgkin's disease	0.04769
DB00539	Hyperlipidemia	0.95808
DB00539	Hypertension	0.40061
DB00539	Infectious lung disease	0.05753
DB00539	Infertility	0.57658
DB00539	Kidney failure	0.49873
DB00539	Late pregnancy	1.23268
DB00539	Leukemia	0.02117
DB00539	Leukoencephalopathy	0.03564
DB00539	Lung cancer	0.02667
DB00539	Lupus erythematosus	0.3273
DB00539	Lupus vulgaris	0.77225
DB00539	Lymphoma	0.04152
DB00539	Mental retardation	0.03628
DB00539	Migraine	0.72474
DB00539	Multiple sclerosis	0.04129
DB00539	Myasthenia Gravis	1.08341
DB00539	Neoplasm metastasis	0.05223
DB00539	Nephrosis	0.05764
DB00539	Obesity	0.38085
DB00539	Oral cancer	0.03441
DB00539	Osteoporosis	0.59543
DB00539	Ovarian disease	0.92125
DB00539	Ovarian failure	1.12588
DB00539	Overnutrition	0.06746
DB00539	Pancreatitis	0.05065
DB00539	Panic disorder	0.90677
DB00539	Parkinson disease	0.44419
DB00539	Polyarthritis	0.39032
DB00539	Primary biliary cirrhosis	0.73331
DB00539	Prostate cancer	0.02
DB00539	Pulmonary fibrosis	0.08321
DB00539	Rabies	0.38939
DB00539	Renal tubular acidosis	0.74141
DB00539	Rheumatoid arthritis	0.08159

DB00539	Schistosomiasis	0.11237
DB00539	Schizophrenia	0.37784
DB00539	Scleroderma	0.03754
DB00539	Sicca syndrome	0.13578
DB00539	Stroke	0.36508
DB00539	Synovitis	1.14831
DB00539	Systemic scleroderma	0.02799
DB00539	Thymoma	0.93306
DB00539	Tuberculosis	0.05212
DB00539	Ulcerative colitis	0.02997
DB00539	Uterine disease	0.92695
DB00539	Vaccinia	0.16627
DB00539	Virus disease	0.04249
DB00539	Vitiligo	0.96556
DB00539	Wiskott-Aldrich syndrome	0.07541
DB00603	Hypertension, Pulmonary	0.15392
DB00603	Infertility, Male	0.83182
DB00603	Adenovirus infection	0.03828
DB00603	Alzheimer's disease	0.22007
DB00603	Atherosclerosis	0.27565
DB00603	Autistic disorder	0.02299
DB00603	Bipolar disorder	0.63044
DB00603	Bladder cancer	0.1378
DB00603	Breast cancer	0.00932
DB00603	Bronchial hyperreactivity	1.25
DB00603	Cancer	0.1457
DB00603	Cerebrovascular disorder	0.8417
DB00603	Cholelithiasis	0.74197
DB00603	Cirrhosis	0.1039
DB00603	Craniosynostosis	0.25982
DB00603	Dental plaque	0.4801
DB00603	Depression	0.35494
DB00603	Diabetes mellitus	0.21938
DB00603	Down syndrome	0.47099
DB00603	Eating disorder	0.04067
DB00603	Endometriosis	0.28568
DB00603	Epilepsy	0.05425
DB00603	Glaucoma	0.57304
DB00603	Hepatitis	0.19437
DB00603	Hepatitis C	0.06223
DB00603	Herpes	0.35471
DB00603	Hodgkin's disease	0.04769
DB00603	Hyperlipidemia	0.90064
DB00603	Hypertension	0.37746
DB00603	Infectious lung disease	0.05753
DB00603	Infertility	0.53635
DB00603	Kidney failure	0.46557

DB00603	Late pregnancy	1.14813
DB00603	Leukemia	0.02117
DB00603	Leukoencephalopathy	0.03564
DB00603	Lung cancer	0.02667
DB00603	Lupus erythematosus	0.30228
DB00603	Lupus vulgaris	0.7169
DB00603	Lymphoma	0.04152
DB00603	Mental retardation	0.03628
DB00603	Migraine	0.67035
DB00603	Multiple sclerosis	0.04129
DB00603	Myasthenia Gravis	0.97985
DB00603	Neoplasm metastasis	0.05223
DB00603	Nephrosis	0.05764
DB00603	Obesity	0.35832
DB00603	Oral cancer	0.03441
DB00603	Osteoporosis	0.54912
DB00603	Ovarian disease	0.85221
DB00603	Ovarian failure	1.05869
DB00603	Overnutrition	0.06746
DB00603	Pancreatitis	0.05065
DB00603	Panic disorder	0.84285
DB00603	Parkinson disease	0.41185
DB00603	Polyarthritis	0.35694
DB00603	Primary biliary cirrhosis	0.68071
DB00603	Prostate cancer	0.02
DB00603	Pulmonary fibrosis	0.08321
DB00603	Rabies	0.35685
DB00603	Renal tubular acidosis	0.64378
DB00603	Rheumatoid arthritis	0.08159
DB00603	Schistosomiasis	0.11237
DB00603	Schizophrenia	0.35557
DB00603	Scleroderma	0.03754
DB00603	Sicca syndrome	0.13578
DB00603	Stroke	0.33192
DB00603	Synovitis	1.04476
DB00603	Systemic scleroderma	0.02799
DB00603	Thymoma	0.84044
DB00603	Tuberculosis	0.05212
DB00603	Ulcerative colitis	0.02997
DB00603	Uterine disease	0.81625
DB00603	Vaccinia	0.16627
DB00603	Virus disease	0.04249
DB00603	Vitiligo	0.89652
DB00603	Wiskott-Aldrich syndrome	0.07541
DB00655	Hypertension, Pulmonary	0.15392
DB00655	Infertility, Male	0.8916
DB00655	Adenovirus infection	0.03828

DB00655	Alzheimer's disease	0.24115
DB00655	Atherosclerosis	0.29616
DB00655	Autistic disorder	0.02299
DB00655	Bipolar disorder	0.6636
DB00655	Bladder cancer	0.1378
DB00655	Breast cancer	0.00932
DB00655	Bronchial hyperreactivity	1.35355
DB00655	Cancer	0.15649
DB00655	Cerebrovascular disorder	0.90562
DB00655	Cholelithiasis	0.80055
DB00655	Cirrhosis	0.1039
DB00655	Craniosynostosis	0.25982
DB00655	Dental plaque	0.51729
DB00655	Depression	0.39339
DB00655	Diabetes mellitus	0.2348
DB00655	Down syndrome	0.50437
DB00655	Eating disorder	0.04067
DB00655	Endometriosis	0.31
DB00655	Epilepsy	0.05425
DB00655	Glaucoma	0.62185
DB00655	Hepatitis	0.19437
DB00655	Hepatitis C	0.06223
DB00655	Herpes	0.39655
DB00655	Hodgkin's disease	0.04769
DB00655	Hyperlipidemia	0.95808
DB00655	Hypertension	0.40061
DB00655	Infectious lung disease	0.05753
DB00655	Infertility	0.57658
DB00655	Kidney failure	0.49873
DB00655	Late pregnancy	1.23268
DB00655	Leukemia	0.02117
DB00655	Leukoencephalopathy	0.03564
DB00655	Lung cancer	0.02667
DB00655	Lupus erythematosus	0.3273
DB00655	Lupus vulgaris	0.77225
DB00655	Lymphoma	0.04152
DB00655	Mental retardation	0.03628
DB00655	Migraine	0.72474
DB00655	Multiple sclerosis	0.04129
DB00655	Myasthenia Gravis	1.08341
DB00655	Neoplasm metastasis	0.05223
DB00655	Nephrosis	0.05764
DB00655	Obesity	0.38085
DB00655	Oral cancer	0.03441
DB00655	Osteoporosis	0.59543
DB00655	Ovarian disease	0.92125
DB00655	Ovarian failure	1.12588

DB00655	Overnutrition	0.06746
DB00655	Pancreatitis	0.05065
DB00655	Panic disorder	0.90677
DB00655	Parkinson disease	0.44419
DB00655	Polyarthritis	0.39032
DB00655	Primary biliary cirrhosis	0.73331
DB00655	Prostate cancer	0.02
DB00655	Pulmonary fibrosis	0.08321
DB00655	Rabies	0.38939
DB00655	Renal tubular acidosis	0.74141
DB00655	Rheumatoid arthritis	0.08159
DB00655	Schistosomiasis	0.11237
DB00655	Schizophrenia	0.37784
DB00655	Scleroderma	0.03754
DB00655	Sicca syndrome	0.13578
DB00655	Stroke	0.36508
DB00655	Synovitis	1.14831
DB00655	Systemic scleroderma	0.02799
DB00655	Thymoma	0.93306
DB00655	Tuberculosis	0.05212
DB00655	Ulcerative colitis	0.02997
DB00655	Uterine disease	0.92695
DB00655	Vaccinia	0.16627
DB00655	Virus disease	0.04249
DB00655	Vitiligo	0.96556
DB00655	Wiskott-Aldrich syndrome	0.07541
DB00675	Hypertension, Pulmonary	0.15392
DB00675	Infertility, Male	0.83182
DB00675	Adenovirus infection	0.03828
DB00675	Alzheimer's disease	0.27097
DB00675	Atherosclerosis	0.32516
DB00675	Autistic disorder	0.02299
DB00675	Bipolar disorder	0.63044
DB00675	Bladder cancer	0.1378
DB00675	Breast cancer	0.00932
DB00675	Bronchial hyperreactivity	1.25
DB00675	Cancer	0.17176
DB00675	Cerebrovascular disorder	0.8417
DB00675	Cholelithiasis	0.88339
DB00675	Cirrhosis	0.1039
DB00675	Craniosynostosis	0.25982
DB00675	Dental plaque	0.5699
DB00675	Depression	0.35494
DB00675	Diabetes mellitus	0.21938
DB00675	Down syndrome	0.47099
DB00675	Eating disorder	0.04067
DB00675	Endometriosis	0.3444

DB00675	Epilepsy	0.05425
DB00675	Glaucoma	0.69089
DB00675	Hepatitis	0.19437
DB00675	Hepatitis C	0.06223
DB00675	Herpes	0.35471
DB00675	Hodgkin's disease	0.04769
DB00675	Hyperlipidemia	0.90064
DB00675	Hypertension	0.43336
DB00675	Infectious lung disease	0.05753
DB00675	Infertility	0.53635
DB00675	Kidney failure	0.46557
DB00675	Late pregnancy	1.35225
DB00675	Leukemia	0.02117
DB00675	Leukoencephalopathy	0.03564
DB00675	Lung cancer	0.02667
DB00675	Lupus erythematosus	0.30228
DB00675	Lupus vulgaris	0.7169
DB00675	Lymphoma	0.04152
DB00675	Mental retardation	0.03628
DB00675	Migraine	0.67035
DB00675	Multiple sclerosis	0.04129
DB00675	Myasthenia Gravis	1.22985
DB00675	Neoplasm metastasis	0.05223
DB00675	Nephrosis	0.05764
DB00675	Obesity	0.35832
DB00675	Oral cancer	0.03441
DB00675	Osteoporosis	0.66092
DB00675	Ovarian disease	1.01888
DB00675	Ovarian failure	1.05869
DB00675	Overnutrition	0.06746
DB00675	Pancreatitis	0.05065
DB00675	Panic disorder	0.84285
DB00675	Parkinson disease	0.48993
DB00675	Polyarthritis	0.35694
DB00675	Primary biliary cirrhosis	0.68071
DB00675	Prostate cancer	0.02
DB00675	Ptosis	0.2357
DB00675	Pulmonary fibrosis	0.08321
DB00675	Rabies	0.35685
DB00675	Renal tubular acidosis	0.64378
DB00675	Rheumatoid arthritis	0.08159
DB00675	Schistosomiasis	0.11237
DB00675	Schizophrenia	0.40933
DB00675	Scleroderma	0.03754
DB00675	Sicca syndrome	0.13578
DB00675	Stroke	0.33192
DB00675	Synovitis	1.04476

DB00675	Systemic scleroderma	0.02799
DB00675	Temporal arteritis	0.31623
DB00675	Testicular dysfunction	0.10102
DB00675	Thymoma	0.84044
DB00675	Tuberculosis	0.05212
DB00675	Ulcerative colitis	0.02997
DB00675	Uterine disease	0.81625
DB00675	Vaccinia	0.16627
DB00675	Virus disease	0.04249
DB00675	Vitiligo	0.89652
DB00675	Wiskott-Aldrich syndrome	0.07541
DB00783	Hypertension, Pulmonary	0.15392
DB00783	Infertility, Male	0.80533
DB00783	Adenovirus infection	0.11344
DB00783	Alzheimer's disease	0.25229
DB00783	Atherosclerosis	0.30699
DB00783	Autistic disorder	0.02299
DB00783	Bipolar disorder	0.61575
DB00783	Bladder cancer	0.1378
DB00783	Breast cancer	0.00932
DB00783	Bronchial hyperreactivity	1.20412
DB00783	Cancer	0.16219
DB00783	Cerebrovascular disorder	0.81339
DB00783	Cholelithiasis	0.83149
DB00783	Cirrhosis	0.1039
DB00783	Craniosynostosis	0.25982
DB00783	Dental plaque	0.53694
DB00783	Depression	0.3379
DB00783	Diabetes mellitus	0.21255
DB00783	Down syndrome	0.4562
DB00783	Eating disorder	0.04067
DB00783	Endometriosis	0.32285
DB00783	Endometrium cancer	0.14003
DB00783	Enteritis	0.06337
DB00783	Epilepsy	0.13589
DB00783	Glaucoma	0.64763
DB00783	Hepatitis	0.19437
DB00783	Hepatitis C	0.06223
DB00783	Herpes	0.33617
DB00783	Hodgkin's disease	0.04769
DB00783	Hyperlipidemia	0.87519
DB00783	Hypertension	0.41284
DB00783	Infectious lung disease	0.05753
DB00783	Infertility	0.51853
DB00783	Kidney failure	0.45088
DB00783	Late pregnancy	1.27734
DB00783	Leukemia	0.02117

DB00783	Leukoencephalopathy	0.03564
DB00783	Lung cancer	0.02667
DB00783	Lupus erythematosus	0.29119
DB00783	Lupus vulgaris	0.69238
DB00783	Lymphoma	0.04152
DB00783	Mental retardation	0.03628
DB00783	Migraine	0.64625
DB00783	Multiple sclerosis	0.04129
DB00783	Myasthenia Gravis	1.1381
DB00783	Neoplasm metastasis	0.05223
DB00783	Nephrosis	0.05764
DB00783	Obesity	0.34834
DB00783	Oral cancer	0.03441
DB00783	Osteoporosis	0.61989
DB00783	Osteosarcoma	0.12309
DB00783	Ovarian disease	0.95771
DB00783	Ovarian failure	1.02892
DB00783	Overnutrition	0.06746
DB00783	Pancreatitis	0.05065
DB00783	Panic disorder	0.81454
DB00783	Parkinson disease	0.46127
DB00783	Polyarthritis	0.34215
DB00783	Primary biliary cirrhosis	0.6574
DB00783	Prostate cancer	0.02
DB00783	Ptosis	0.19245
DB00783	Pulmonary fibrosis	0.08321
DB00783	Rabies	0.34243
DB00783	Renal tubular acidosis	0.60053
DB00783	Rheumatoid arthritis	0.08159
DB00783	Schistosomiasis	0.11237
DB00783	Schizophrenia	0.3896
DB00783	Scleroderma	0.03754
DB00783	Sicca syndrome	0.13578
DB00783	Stroke	0.31722
DB00783	Synovitis	0.99888
DB00783	Systemic scleroderma	0.02799
DB00783	Temporal arteritis	0.2582
DB00783	Testicular dysfunction	0.08248
DB00783	Thymoma	0.79941
DB00783	Tuberculosis	0.05212
DB00783	Ulcerative colitis	0.08952
DB00783	Uterine disease	0.7672
DB00783	Vaccinia	0.16627
DB00783	Virus disease	0.04249
DB00783	Vitiligo	0.86594
DB00783	Wiskott-Aldrich syndrome	0.07541
DB00823	Hypertension, Pulmonary	0.15392

DB00823	Infertility, Male	0.83182
DB00823	Adenovirus infection	0.03828
DB00823	Alzheimer's disease	0.22007
DB00823	Atherosclerosis	0.27565
DB00823	Autistic disorder	0.02299
DB00823	Bipolar disorder	0.63044
DB00823	Bladder cancer	0.1378
DB00823	Breast cancer	0.00932
DB00823	Bronchial hyperreactivity	1.25
DB00823	Cancer	0.1457
DB00823	Cerebrovascular disorder	0.8417
DB00823	Cholelithiasis	0.74197
DB00823	Cirrhosis	0.1039
DB00823	Craniosynostosis	0.25982
DB00823	Dental plaque	0.4801
DB00823	Depression	0.35494
DB00823	Diabetes mellitus	0.21938
DB00823	Down syndrome	0.47099
DB00823	Eating disorder	0.04067
DB00823	Endometriosis	0.28568
DB00823	Epilepsy	0.05425
DB00823	Glaucoma	0.57304
DB00823	Hepatitis	0.19437
DB00823	Hepatitis C	0.06223
DB00823	Herpes	0.35471
DB00823	Hodgkin's disease	0.04769
DB00823	Hyperlipidemia	0.90064
DB00823	Hypertension	0.37746
DB00823	Infectious lung disease	0.05753
DB00823	Infertility	0.53635
DB00823	Kidney failure	0.46557
DB00823	Late pregnancy	1.14813
DB00823	Leukemia	0.02117
DB00823	Leukoencephalopathy	0.03564
DB00823	Lung cancer	0.02667
DB00823	Lupus erythematosus	0.30228
DB00823	Lupus vulgaris	0.7169
DB00823	Lymphoma	0.04152
DB00823	Mental retardation	0.03628
DB00823	Migraine	0.67035
DB00823	Multiple sclerosis	0.04129
DB00823	Myasthenia Gravis	0.97985
DB00823	Neoplasm metastasis	0.05223
DB00823	Nephrosis	0.05764
DB00823	Obesity	0.35832
DB00823	Oral cancer	0.03441
DB00823	Osteoporosis	0.54912

DB00823	Ovarian disease	0.85221
DB00823	Ovarian failure	1.05869
DB00823	Overnutrition	0.06746
DB00823	Pancreatitis	0.05065
DB00823	Panic disorder	0.84285
DB00823	Parkinson disease	0.41185
DB00823	Polyarthritis	0.35694
DB00823	Primary biliary cirrhosis	0.68071
DB00823	Prostate cancer	0.02
DB00823	Pulmonary fibrosis	0.08321
DB00823	Rabies	0.35685
DB00823	Renal tubular acidosis	0.64378
DB00823	Rheumatoid arthritis	0.08159
DB00823	Schistosomiasis	0.11237
DB00823	Schizophrenia	0.35557
DB00823	Scleroderma	0.03754
DB00823	Sicca syndrome	0.13578
DB00823	Stroke	0.33192
DB00823	Synovitis	1.04476
DB00823	Systemic scleroderma	0.02799
DB00823	Thymoma	0.84044
DB00823	Tuberculosis	0.05212
DB00823	Ulcerative colitis	0.02997
DB00823	Uterine disease	0.81625
DB00823	Vaccinia	0.16627
DB00823	Virus disease	0.04249
DB00823	Vitiligo	0.89652
DB00823	Wiskott-Aldrich syndrome	0.07541
DB00882	Hypertension, Pulmonary	0.15392
DB00882	Infertility, Male	0.8916
DB00882	Adenovirus infection	0.03828
DB00882	Alzheimer's disease	0.24115
DB00882	Atherosclerosis	0.29616
DB00882	Autistic disorder	0.02299
DB00882	Bipolar disorder	0.6636
DB00882	Bladder cancer	0.1378
DB00882	Breast cancer	0.00932
DB00882	Bronchial hyperreactivity	1.35355
DB00882	Cancer	0.15649
DB00882	Cerebrovascular disorder	0.90562
DB00882	Cholelithiasis	0.80055
DB00882	Cirrhosis	0.1039
DB00882	Craniosynostosis	0.25982
DB00882	Dental plaque	0.51729
DB00882	Depression	0.39339
DB00882	Diabetes mellitus	0.2348
DB00882	Down syndrome	0.50437

DB00882	Eating disorder	0.04067
DB00882	Endometriosis	0.31
DB00882	Epilepsy	0.05425
DB00882	Glaucoma	0.62185
DB00882	Hepatitis	0.19437
DB00882	Hepatitis C	0.06223
DB00882	Herpes	0.39655
DB00882	Hodgkin's disease	0.04769
DB00882	Hyperlipidemia	0.95808
DB00882	Hypertension	0.40061
DB00882	Infectious lung disease	0.05753
DB00882	Infertility	0.57658
DB00882	Kidney failure	0.49873
DB00882	Late pregnancy	1.23268
DB00882	Leukemia	0.02117
DB00882	Leukoencephalopathy	0.03564
DB00882	Lung cancer	0.02667
DB00882	Lupus erythematosus	0.3273
DB00882	Lupus vulgaris	0.77225
DB00882	Lymphoma	0.04152
DB00882	Mental retardation	0.03628
DB00882	Migraine	0.72474
DB00882	Multiple sclerosis	0.04129
DB00882	Myasthenia Gravis	1.08341
DB00882	Neoplasm metastasis	0.05223
DB00882	Nephrosis	0.05764
DB00882	Obesity	0.38085
DB00882	Oral cancer	0.03441
DB00882	Osteoporosis	0.59543
DB00882	Ovarian disease	0.92125
DB00882	Ovarian failure	1.12588
DB00882	Overnutrition	0.06746
DB00882	Pancreatitis	0.05065
DB00882	Panic disorder	0.90677
DB00882	Parkinson disease	0.44419
DB00882	Polyarthritis	0.39032
DB00882	Primary biliary cirrhosis	0.73331
DB00882	Prostate cancer	0.02
DB00882	Pulmonary fibrosis	0.08321
DB00882	Rabies	0.38939
DB00882	Renal tubular acidosis	0.74141
DB00882	Rheumatoid arthritis	0.08159
DB00882	Schistosomiasis	0.11237
DB00882	Schizophrenia	0.37784
DB00882	Scleroderma	0.03754
DB00882	Sicca syndrome	0.13578
DB00882	Stroke	0.36508

DB00882	Synovitis	1.14831
DB00882	Systemic scleroderma	0.02799
DB00882	Thymoma	0.93306
DB00882	Tuberculosis	0.05212
DB00882	Ulcerative colitis	0.02997
DB00882	Uterine disease	0.92695
DB00882	Vaccinia	0.16627
DB00882	Virus disease	0.04249
DB00882	Vitiligo	0.96556
DB00882	Wiskott-Aldrich syndrome	0.07541
DB00890	Hypertension, Pulmonary	0.15392
DB00890	Infertility, Male	0.8916
DB00890	Adenovirus infection	0.03828
DB00890	Alzheimer's disease	0.24115
DB00890	Atherosclerosis	0.29616
DB00890	Autistic disorder	0.02299
DB00890	Bipolar disorder	0.6636
DB00890	Bladder cancer	0.1378
DB00890	Breast cancer	0.00932
DB00890	Bronchial hyperreactivity	1.35355
DB00890	Cancer	0.15649
DB00890	Cerebrovascular disorder	0.90562
DB00890	Cholelithiasis	0.80055
DB00890	Cirrhosis	0.1039
DB00890	Craniosynostosis	0.25982
DB00890	Dental plaque	0.51729
DB00890	Depression	0.39339
DB00890	Diabetes mellitus	0.2348
DB00890	Down syndrome	0.50437
DB00890	Eating disorder	0.04067
DB00890	Endometriosis	0.31
DB00890	Epilepsy	0.05425
DB00890	Glaucoma	0.62185
DB00890	Hepatitis	0.19437
DB00890	Hepatitis C	0.06223
DB00890	Herpes	0.39655
DB00890	Hodgkin's disease	0.04769
DB00890	Hyperlipidemia	0.95808
DB00890	Hypertension	0.40061
DB00890	Infectious lung disease	0.05753
DB00890	Infertility	0.57658
DB00890	Kidney failure	0.49873
DB00890	Late pregnancy	1.23268
DB00890	Leukemia	0.02117
DB00890	Leukoencephalopathy	0.03564
DB00890	Lung cancer	0.02667
DB00890	Lupus erythematosus	0.3273

DB00890	Lupus vulgaris	0.77225
DB00890	Lymphoma	0.04152
DB00890	Mental retardation	0.03628
DB00890	Migraine	0.72474
DB00890	Multiple sclerosis	0.04129
DB00890	Myasthenia Gravis	1.08341
DB00890	Neoplasm metastasis	0.05223
DB00890	Nephrosis	0.05764
DB00890	Obesity	0.38085
DB00890	Oral cancer	0.03441
DB00890	Osteoporosis	0.59543
DB00890	Ovarian disease	0.92125
DB00890	Ovarian failure	1.12588
DB00890	Overnutrition	0.06746
DB00890	Pancreatitis	0.05065
DB00890	Panic disorder	0.90677
DB00890	Parkinson disease	0.44419
DB00890	Polyarthritis	0.39032
DB00890	Primary biliary cirrhosis	0.73331
DB00890	Prostate cancer	0.02
DB00890	Pulmonary fibrosis	0.08321
DB00890	Rabies	0.38939
DB00890	Renal tubular acidosis	0.74141
DB00890	Rheumatoid arthritis	0.08159
DB00890	Schistosomiasis	0.11237
DB00890	Schizophrenia	0.37784
DB00890	Scleroderma	0.03754
DB00890	Sicca syndrome	0.13578
DB00890	Stroke	0.36508
DB00890	Synovitis	1.14831
DB00890	Systemic scleroderma	0.02799
DB00890	Thymoma	0.93306
DB00890	Tuberculosis	0.05212
DB00890	Ulcerative colitis	0.02997
DB00890	Uterine disease	0.92695
DB00890	Vaccinia	0.16627
DB00890	Virus disease	0.04249
DB00890	Vitiligo	0.96556
DB00890	Wiskott-Aldrich syndrome	0.07541
DB00947	Hypertension, Pulmonary	0.15392
DB00947	Infertility, Male	0.8916
DB00947	Adenovirus infection	0.03828
DB00947	Alzheimer's disease	0.24115
DB00947	Atherosclerosis	0.29616
DB00947	Autistic disorder	0.02299
DB00947	Bipolar disorder	0.6636
DB00947	Bladder cancer	0.1378

DB00947	Breast cancer	0.00932
DB00947	Bronchial hyperreactivity	1.35355
DB00947	Cancer	0.15649
DB00947	Cerebrovascular disorder	0.90562
DB00947	Cholelithiasis	0.80055
DB00947	Cirrhosis	0.1039
DB00947	Craniosynostosis	0.25982
DB00947	Dental plaque	0.51729
DB00947	Depression	0.39339
DB00947	Diabetes mellitus	0.2348
DB00947	Down syndrome	0.50437
DB00947	Eating disorder	0.04067
DB00947	Endometriosis	0.31
DB00947	Epilepsy	0.05425
DB00947	Glaucoma	0.62185
DB00947	Hepatitis	0.19437
DB00947	Hepatitis C	0.06223
DB00947	Herpes	0.39655
DB00947	Hodgkin's disease	0.04769
DB00947	Hyperlipidemia	0.95808
DB00947	Hypertension	0.40061
DB00947	Infectious lung disease	0.05753
DB00947	Infertility	0.57658
DB00947	Kidney failure	0.49873
DB00947	Late pregnancy	1.23268
DB00947	Leukemia	0.02117
DB00947	Leukoencephalopathy	0.03564
DB00947	Lung cancer	0.02667
DB00947	Lupus erythematosus	0.3273
DB00947	Lupus vulgaris	0.77225
DB00947	Lymphoma	0.04152
DB00947	Mental retardation	0.03628
DB00947	Migraine	0.72474
DB00947	Multiple sclerosis	0.04129
DB00947	Myasthenia Gravis	1.08341
DB00947	Neoplasm metastasis	0.05223
DB00947	Nephrosis	0.05764
DB00947	Obesity	0.38085
DB00947	Oral cancer	0.03441
DB00947	Osteoporosis	0.59543
DB00947	Ovarian disease	0.92125
DB00947	Ovarian failure	1.12588
DB00947	Overnutrition	0.06746
DB00947	Pancreatitis	0.05065
DB00947	Panic disorder	0.90677
DB00947	Parkinson disease	0.44419
DB00947	Polyarthritis	0.39032

DB00947	Primary biliary cirrhosis	0.73331
DB00947	Prostate cancer	0.02
DB00947	Pulmonary fibrosis	0.08321
DB00947	Rabies	0.38939
DB00947	Renal tubular acidosis	0.74141
DB00947	Rheumatoid arthritis	0.08159
DB00947	Schistosomiasis	0.11237
DB00947	Schizophrenia	0.37784
DB00947	Scleroderma	0.03754
DB00947	Sicca syndrome	0.13578
DB00947	Stroke	0.36508
DB00947	Synovitis	1.14831
DB00947	Systemic scleroderma	0.02799
DB00947	Thymoma	0.93306
DB00947	Tuberculosis	0.05212
DB00947	Ulcerative colitis	0.02997
DB00947	Uterine disease	0.92695
DB00947	Vaccinia	0.16627
DB00947	Virus disease	0.04249
DB00947	Vitiligo	0.96556
DB00947	Wiskott-Aldrich syndrome	0.07541
DB00957	Hypertension, Pulmonary	0.15392
DB00957	Infertility, Male	0.83182
DB00957	Adenovirus infection	0.03828
DB00957	Alzheimer's disease	0.22007
DB00957	Atherosclerosis	0.27565
DB00957	Autistic disorder	0.02299
DB00957	Bipolar disorder	0.63044
DB00957	Bladder cancer	0.1378
DB00957	Breast cancer	0.00932
DB00957	Bronchial hyperreactivity	1.25
DB00957	Cancer	0.1457
DB00957	Cerebrovascular disorder	0.8417
DB00957	Cholelithiasis	0.74197
DB00957	Cirrhosis	0.1039
DB00957	Craniosynostosis	0.25982
DB00957	Dental plaque	0.4801
DB00957	Depression	0.35494
DB00957	Diabetes mellitus	0.21938
DB00957	Down syndrome	0.47099
DB00957	Eating disorder	0.04067
DB00957	Endometriosis	0.28568
DB00957	Epilepsy	0.05425
DB00957	Glaucoma	0.57304
DB00957	Hepatitis	0.19437
DB00957	Hepatitis C	0.06223
DB00957	Herpes	0.35471

DB00957	Hodgkin's disease	0.04769
DB00957	Hyperlipidemia	0.90064
DB00957	Hypertension	0.37746
DB00957	Infectious lung disease	0.05753
DB00957	Infertility	0.53635
DB00957	Kidney failure	0.46557
DB00957	Late pregnancy	1.14813
DB00957	Leukemia	0.02117
DB00957	Leukoencephalopathy	0.03564
DB00957	Lung cancer	0.02667
DB00957	Lupus erythematosus	0.30228
DB00957	Lupus vulgaris	0.7169
DB00957	Lymphoma	0.04152
DB00957	Mental retardation	0.03628
DB00957	Migraine	0.67035
DB00957	Multiple sclerosis	0.04129
DB00957	Myasthenia Gravis	0.97985
DB00957	Neoplasm metastasis	0.05223
DB00957	Nephrosis	0.05764
DB00957	Obesity	0.35832
DB00957	Oral cancer	0.03441
DB00957	Osteoporosis	0.54912
DB00957	Ovarian disease	0.85221
DB00957	Ovarian failure	1.05869
DB00957	Overnutrition	0.06746
DB00957	Pancreatitis	0.05065
DB00957	Panic disorder	0.84285
DB00957	Parkinson disease	0.41185
DB00957	Polyarthritis	0.35694
DB00957	Primary biliary cirrhosis	0.68071
DB00957	Prostate cancer	0.02
DB00957	Pulmonary fibrosis	0.08321
DB00957	Rabies	0.35685
DB00957	Renal tubular acidosis	0.64378
DB00957	Rheumatoid arthritis	0.08159
DB00957	Schistosomiasis	0.11237
DB00957	Schizophrenia	0.35557
DB00957	Scleroderma	0.03754
DB00957	Sicca syndrome	0.13578
DB00957	Stroke	0.33192
DB00957	Synovitis	1.04476
DB00957	Systemic scleroderma	0.02799
DB00957	Thymoma	0.84044
DB00957	Tuberculosis	0.05212
DB00957	Ulcerative colitis	0.02997
DB00957	Uterine disease	0.81625
DB00957	Vaccinia	0.16627

DB00957	Virus disease	0.04249
DB00957	Vitiligo	0.89652
DB00957	Wiskott-Aldrich syndrome	0.07541
DB00977	Hypertension, Pulmonary	0.15392
DB00977	Infertility, Male	0.83182
DB00977	Adenovirus infection	0.13034
DB00977	Alzheimer's disease	0.22007
DB00977	Atherosclerosis	0.27565
DB00977	Autistic disorder	0.02299
DB00977	Bipolar disorder	0.63044
DB00977	Bladder cancer	0.1378
DB00977	Breast cancer	0.00932
DB00977	Bronchial hyperreactivity	1.25
DB00977	Cancer	0.1457
DB00977	Cerebrovascular disorder	0.8417
DB00977	Cholelithiasis	0.74197
DB00977	Cirrhosis	0.1039
DB00977	Craniosynostosis	0.25982
DB00977	Dental plaque	0.4801
DB00977	Depression	0.35494
DB00977	Diabetes mellitus	0.21938
DB00977	Down syndrome	0.47099
DB00977	Eating disorder	0.04067
DB00977	Endometriosis	0.28568
DB00977	Endometrium cancer	0.1715
DB00977	Enteritis	0.07762
DB00977	Epilepsy	0.15425
DB00977	Glaucoma	0.57304
DB00977	Hepatitis	0.19437
DB00977	Hepatitis C	0.06223
DB00977	Herpes	0.35471
DB00977	Hodgkin's disease	0.04769
DB00977	Hyperlipidemia	0.90064
DB00977	Hypertension	0.37746
DB00977	Infectious lung disease	0.05753
DB00977	Infertility	0.53635
DB00977	Kidney failure	0.46557
DB00977	Late pregnancy	1.14813
DB00977	Leukemia	0.02117
DB00977	Leukoencephalopathy	0.03564
DB00977	Lung cancer	0.02667
DB00977	Lupus erythematosus	0.30228
DB00977	Lupus vulgaris	0.7169
DB00977	Lymphoma	0.04152
DB00977	Mental retardation	0.03628
DB00977	Migraine	0.67035
DB00977	Multiple sclerosis	0.04129

DB00977	Myasthenia Gravis	0.97985
DB00977	Neoplasm metastasis	0.05223
DB00977	Nephrosis	0.05764
DB00977	Obesity	0.35832
DB00977	Oral cancer	0.03441
DB00977	Osteoporosis	0.54912
DB00977	Osteosarcoma	0.15076
DB00977	Ovarian disease	0.85221
DB00977	Ovarian failure	1.05869
DB00977	Overnutrition	0.06746
DB00977	Pancreatitis	0.05065
DB00977	Panic disorder	0.84285
DB00977	Parkinson disease	0.41185
DB00977	Polyarthritis	0.35694
DB00977	Primary biliary cirrhosis	0.68071
DB00977	Prostate cancer	0.02
DB00977	Pulmonary fibrosis	0.08321
DB00977	Rabies	0.35685
DB00977	Renal tubular acidosis	0.64378
DB00977	Rheumatoid arthritis	0.08159
DB00977	Schistosomiasis	0.11237
DB00977	Schizophrenia	0.35557
DB00977	Scleroderma	0.03754
DB00977	Sicca syndrome	0.13578
DB00977	Stroke	0.33192
DB00977	Synovitis	1.04476
DB00977	Systemic scleroderma	0.02799
DB00977	Thymoma	0.84044
DB00977	Tuberculosis	0.05212
DB00977	Ulcerative colitis	0.1029
DB00977	Uterine disease	0.81625
DB00977	Vaccinia	0.16627
DB00977	Virus disease	0.04249
DB00977	Vitiligo	0.89652
DB00977	Wiskott-Aldrich syndrome	0.07541
DB01065	Hypertension, Pulmonary	0.09419
DB01065	Infertility, Male	0.48873
DB01065	Kidney tubular necrosis, acute	0.04529
DB01065	Abortion	0.14
DB01065	Achalasia and cardiospasm	0.57119
DB01065	Adenovirus infection	0.02342
DB01065	Alzheimer's disease	0.24406
DB01065	Amyloidosis	0.28774
DB01065	Amyotrophic lateral sclerosis	0.0136
DB01065	Asthma	0.01949
DB01065	Atherosclerosis	0.18506
DB01065	Autistic disorder	0.08143

DB01065	Autoimmune disease	0.02485
DB01065	Bipolar disorder	0.45353
DB01065	Bladder cancer	0.28124
DB01065	Brain ischemia	0.08143
DB01065	Breast cancer	0.0289
DB01065	Bronchial hyperreactivity	0.72979
DB01065	Cancer	0.1113
DB01065	Celiac disease	0.0627
DB01065	Cerebrovascular disorder	0.49338
DB01065	Cervical cancer	0.03875
DB01065	Cholelithiasis	0.43416
DB01065	Chronic obstructive airway disease	0.0237
DB01065	Cirrhosis	0.06358
DB01065	Colon cancer	0.07802
DB01065	Common cold	0.03304
DB01065	Congenital abnormality	0.01443
DB01065	Congenital heart disease	0.11111
DB01065	Craniosynostosis	0.15899
DB01065	Dental plaque	0.31034
DB01065	Depression	0.31753
DB01065	Diabetes mellitus	0.17281
DB01065	Down syndrome	0.27689
DB01065	Drug abuse	0.01444
DB01065	Eating disorder	0.07133
DB01065	Endometriosis	0.16656
DB01065	Epilepsy	0.03319
DB01065	Epstein-Barr virus infection	0.04131
DB01065	Esophageal tumor	0.04852
DB01065	Esophagus cancer	0.01938
DB01065	Esotropia	0.03947
DB01065	Ewings sarcoma	0.04417
DB01065	Eye cancer	0.04892
DB01065	Fanconi's anemia	0.03391
DB01065	Glaucoma	0.36812
DB01065	Glomerulonephritis	0.08333
DB01065	Gram-Negative bacterial infection	0.06825
DB01065	Graves' disease	0.04883
DB01065	HIV infection	0.04549
DB01065	Heart failure	0.01805
DB01065	Helicobacter infection	0.07454
DB01065	Hepatitis	0.11894
DB01065	Hepatitis C	0.03808
DB01065	Hepatoblastoma	0.19245
DB01065	Herpes	0.23632
DB01065	Hodgkin's disease	0.02918
DB01065	Hyperlipidemia	0.59702
DB01065	Hypertension	0.22312

DB01065	Infectious lung disease	0.03521
DB01065	Infertility	0.31456
DB01065	Ischemia	0.08956
DB01065	Kaposi sarcoma	0.02497
DB01065	Keratosis	0.03916
DB01065	Kidney failure	0.31139
DB01065	Late pregnancy	0.67389
DB01065	Leukemia	0.11331
DB01065	Leukoencephalopathy	0.02181
DB01065	Lichen planus	0.0761
DB01065	Liver cancer	0.01428
DB01065	Lung cancer	0.05641
DB01065	Lupus erythematosus	0.19262
DB01065	Lupus vulgaris	0.41992
DB01065	Lymphoma	0.02541
DB01065	Malignant glioma	0.21264
DB01065	Melanoma	0.11209
DB01065	Mental retardation	0.0222
DB01065	Migraine	0.39176
DB01065	Multiple sclerosis	0.05965
DB01065	Myasthenia Gravis	0.56447
DB01065	Neoplasm metastasis	0.03196
DB01065	Nephrosis	0.03527
DB01065	Obesity	0.21163
DB01065	Oral cancer	0.02106
DB01065	Osteoporosis	0.32031
DB01065	Osteosarcoma	0.21678
DB01065	Ovarian disease	0.49808
DB01065	Ovarian failure	0.62505
DB01065	Overnutrition	0.04128
DB01065	Pancreas cancer	0.03457
DB01065	Pancreatitis	0.03099
DB01065	Panic disorder	0.49409
DB01065	Parkinson disease	0.25738
DB01065	Periodontal disease	0.07857
DB01065	Periodontitis	0.05083
DB01065	Pervasive development disorder	0.08607
DB01065	Pituitary tumor	0.03809
DB01065	Polyarthritis	0.2071
DB01065	Pre-Eclampsia	0.17965
DB01065	Primary biliary cirrhosis	0.3987
DB01065	Prion disease	0.06379
DB01065	Prostate cancer	0.02964
DB01065	Pulmonary fibrosis	0.05092
DB01065	Rabies	0.31637
DB01065	Renal tubular acidosis	0.3802
DB01065	Rheumatoid arthritis	0.13152

DB01065	Schistosomiasis	0.13753
DB01065	Schizophrenia	0.21003
DB01065	Scleroderma	0.02297
DB01065	Sicca syndrome	0.08309
DB01065	Sickle cell disease	0.06415
DB01065	Stomach disease	0.11785
DB01065	Stroke	0.24753
DB01065	Synovitis	0.60419
DB01065	Systemic infection	0.03824
DB01065	Systemic scleroderma	0.01713
DB01065	Thymoma	0.48287
DB01065	Thyroid gland disease	0.04299
DB01065	Tuberculosis	0.06873
DB01065	Ulcerative colitis	0.01834
DB01065	Uterine disease	0.46193
DB01065	Uterine fibroids	0.03816
DB01065	Vaccinia	0.10175
DB01065	Vasculitis	0.1005
DB01065	Virus disease	0.026
DB01065	Vitiligo	0.52519
DB01065	Werner syndrome	0.02507
DB01065	Wiskott-Aldrich syndrome	0.04615
DB01183	Hypertension, Pulmonary	0.11734
DB01183	Infertility, Male	0.60744
DB01183	Adenocarcinoma	0.19124
DB01183	Adenoma of thyroid	0.09628
DB01183	Adenovirus infection	0.02918
DB01183	Alzheimer's disease	0.2035
DB01183	Anemia	0.16876
DB01183	Asthma	0.05761
DB01183	Atherosclerosis	0.25063
DB01183	Autistic disorder	0.01753
DB01183	Autoimmune disease	0.07523
DB01183	Behcet syndrome	0.11968
DB01183	Bipolar disorder	0.46581
DB01183	Bladder cancer	0.10505
DB01183	Breast cancer	0.02378
DB01183	Bronchial hyperreactivity	0.9067
DB01183	Brucellosis	0.27147
DB01183	Cancer	0.10625
DB01183	Celiac disease	0.11432
DB01183	Cerebrovascular disorder	0.61313
DB01183	Cholelithiasis	0.53948
DB01183	Chronic obstructive airway disease	0.09074
DB01183	Cirrhosis	0.07921
DB01183	Colon cancer	0.04412
DB01183	Communicable disease	0.16067

DB01183	Congenital abnormality	0.04875
DB01183	Craniosynostosis	0.19807
DB01183	Cystic fibrosis	0.10745
DB01183	Cytomegalovirus infection	0.17902
DB01183	Dental plaque	0.34939
DB01183	Depression	0.25341
DB01183	Diabetes mellitus	0.19647
DB01183	Down syndrome	0.34415
DB01183	Eating disorder	0.03101
DB01183	Embryoma	0.06964
DB01183	Endometriosis	0.20693
DB01183	Enteritis	0.08739
DB01183	Epilepsy	0.04135
DB01183	Gastritis	0.10953
DB01183	Gastrointestinal cancer	0.11991
DB01183	Glaucoma	0.41506
DB01183	Heart failure	0.07788
DB01183	Helicobacter infection	0.13696
DB01183	Hepatitis	0.14818
DB01183	Hepatitis C	0.15508
DB01183	Herpes	0.25173
DB01183	Hodgkin's disease	0.03636
DB01183	Hypercholesterolemia	0.15868
DB01183	Hyperlipidemia	0.66095
DB01183	Hypertension	0.27742
DB01183	Infectious lung disease	0.04386
DB01183	Infertility	0.39092
DB01183	Influenza	0.16925
DB01183	Intermediate coronary syndrome	0.18691
DB01183	Kidney failure	0.42225
DB01183	Late pregnancy	0.83752
DB01183	Leukemia	0.01614
DB01183	Leukoencephalopathy	0.02717
DB01183	Lung cancer	0.06979
DB01183	Lupus erythematosus	0.21927
DB01183	Lupus vulgaris	0.52181
DB01183	Lymphoma	0.03165
DB01183	Macular degeneration	0.18275
DB01183	Melanoma	0.0604
DB01183	Mental retardation	0.02766
DB01183	Migraine	0.48675
DB01183	Multiple sclerosis	0.10994
DB01183	Myasthenia Gravis	0.70075
DB01183	Mycoses	0.36121
DB01183	Mycosis fungoides	0.35774
DB01183	Nasopharyngeal cancer	0.1902
DB01183	Necrotizing enterocolitis	0.31505

DB01183	Neoplasm metastasis	0.03982
DB01183	Nephrosis	0.04394
DB01183	Neuroblastoma	0.0913
DB01183	Obesity	0.26311
DB01183	Oral cancer	0.10802
DB01183	Osteoporosis	0.39794
DB01183	Ovarian disease	0.61886
DB01183	Ovarian failure	0.77709
DB01183	Overnutrition	0.05143
DB01183	Pancreatitis	0.14696
DB01183	Panic disorder	0.61401
DB01183	Parkinson disease	0.29953
DB01183	Periodontitis	0.12724
DB01183	Pituitary tumor	0.18571
DB01183	Polyarthritis	0.35562
DB01183	Porcine reproductive and respiratory syndrome	0.32787
DB01183	Pre-Eclampsia	0.09606
DB01183	Premature birth	0.19217
DB01183	Primary biliary cirrhosis	0.61752
DB01183	Prostate cancer	0.07049
DB01183	Pulmonary fibrosis	0.06343
DB01183	Rabies	0.25751
DB01183	Renal tubular acidosis	0.44718
DB01183	Rheumatoid arthritis	0.0622
DB01183	Schistosomiasis	0.08567
DB01183	Schizophrenia	0.26113
DB01183	Scleroderma	0.02862
DB01183	Sicca syndrome	0.10351
DB01183	Stomach cancer	0.05382
DB01183	Stroke	0.30678
DB01183	Synovitis	0.75023
DB01183	Systemic infection	0.07363
DB01183	Systemic scleroderma	0.02134
DB01183	Thymoma	0.59935
DB01183	Tuberculosis	0.03974
DB01183	Ulcerative colitis	0.09134
DB01183	Uterine disease	0.57283
DB01183	Vaccinia	0.12676
DB01183	Virus disease	0.0324
DB01183	Vitiligo	0.65263
DB01183	Wiskott-Aldrich syndrome	0.05749
DB01185	Hypertension, Pulmonary	0.11529
DB01185	Infertility, Male	0.85898
DB01185	Adenovirus infection	0.04751
DB01185	Alzheimer's disease	0.1627
DB01185	Amyotrophic lateral sclerosis	0.12397
DB01185	Atherosclerosis	0.29554

DB01185	Autistic disorder	0.01722
DB01185	Azoospermia	0.2079
DB01185	Bipolar disorder	0.46884
DB01185	Bladder cancer	0.10321
DB01185	Breast cancer	0.00698
DB01185	Bronchial hyperreactivity	0.92577
DB01185	Cancer	0.14779
DB01185	Cerebrovascular disorder	0.62396
DB01185	Cholelithiasis	0.77205
DB01185	Cirrhosis	0.07782
DB01185	Craniosynostosis	0.1946
DB01185	Dental plaque	0.35583
DB01185	Depression	0.38096
DB01185	Diabetes mellitus	0.22712
DB01185	Down syndrome	0.34939
DB01185	Eating disorder	0.03046
DB01185	Endometriosis	0.2949
DB01185	Epilepsy	0.04063
DB01185	Glaucoma	0.42426
DB01185	Hepatitis	0.14558
DB01185	Hepatitis C	0.04661
DB01185	Herpes	0.26144
DB01185	Hodgkin's disease	0.03572
DB01185	Huntington disease	0.0661
DB01185	Hyperglycemia	0.05748
DB01185	Hyperlipidemia	0.66876
DB01185	Hypertension	0.30819
DB01185	Hypogonadism	0.61407
DB01185	Infectious lung disease	0.04309
DB01185	Infertility	0.55574
DB01185	Kidney failure	0.34535
DB01185	Late pregnancy	0.85139
DB01185	Leukemia	0.01586
DB01185	Leukoencephalopathy	0.02669
DB01185	Lung cancer	0.01998
DB01185	Lupus erythematosus	0.22387
DB01185	Lupus vulgaris	0.53135
DB01185	Lymphoma	0.0311
DB01185	Mental retardation	0.02718
DB01185	Migraine	0.69916
DB01185	Multiple sclerosis	0.03093
DB01185	Muscular atrophy	0.29325
DB01185	Myasthenia Gravis	0.72343
DB01185	Neoplasm metastasis	0.03912
DB01185	Nephrosis	0.04317
DB01185	Obesity	0.29239
DB01185	Oligospermia	0.3218

DB01185	Oral cancer	0.02577
DB01185	Osteoporosis	0.57468
DB01185	Ovarian disease	0.63132
DB01185	Ovarian failure	0.78615
DB01185	Overnutrition	0.05053
DB01185	Pancreatitis	0.03794
DB01185	Panic disorder	0.62483
DB01185	Parkinson disease	0.3052
DB01185	Phobic anxiety disorder	0.64046
DB01185	Polyarthritis	0.26397
DB01185	Polycystic ovary syndrome	0.03356
DB01185	Primary biliary cirrhosis	0.50452
DB01185	Prostate cancer	0.07442
DB01185	Psychotic disorder	0.05993
DB01185	Pulmonary fibrosis	0.06232
DB01185	Rabies	0.37309
DB01185	Renal tubular acidosis	0.47231
DB01185	Rheumatoid arthritis	0.12361
DB01185	Schistosomiasis	0.08416
DB01185	Schizophrenia	0.36613
DB01185	Scleroderma	0.02812
DB01185	Sicca syndrome	0.10169
DB01185	Stroke	0.24525
DB01185	Synovitis	0.77204
DB01185	Systemic infection	0.02633
DB01185	Systemic scleroderma	0.04193
DB01185	Thymoma	0.62012
DB01185	Tropical spastic paraparesis	0.08012
DB01185	Tuberculosis	0.03904
DB01185	Ulcerative colitis	0.02245
DB01185	Urogenital abnormalities	0.35183
DB01185	Uterine disease	0.60017
DB01185	Vaccinia	0.12454
DB01185	Virus disease	0.03183
DB01185	Vitiligo	0.73128
DB01185	Wiskott-Aldrich syndrome	0.05648
DB01185	Yersinia infection	0.11524
DB01196	Hypertension, Pulmonary	0.15392
DB01196	Infertility, Male	0.78954
DB01196	Adenovirus infection	0.03828
DB01196	Alzheimer's disease	0.24115
DB01196	Atherosclerosis	0.29616
DB01196	Autistic disorder	0.02299
DB01196	Bipolar disorder	0.60699
DB01196	Bladder cancer	0.1378
DB01196	Breast cancer	0.00932
DB01196	Bronchial hyperreactivity	1.17678

DB01196	Cancer	0.17492
DB01196	Cerebrovascular disorder	0.79651
DB01196	Cholelithiasis	0.80055
DB01196	Cirrhosis	0.1039
DB01196	Craniosynostosis	0.25982
DB01196	Dental plaque	0.51729
DB01196	Depression	0.32774
DB01196	Diabetes mellitus	0.20848
DB01196	Down syndrome	0.44739
DB01196	Eating disorder	0.04067
DB01196	Endometriosis	0.31
DB01196	Epilepsy	0.05425
DB01196	Glaucoma	0.62185
DB01196	Hepatitis	0.19437
DB01196	Hepatitis C	0.06223
DB01196	Herpes	0.32512
DB01196	Hodgkin's disease	0.04769
DB01196	Hyperlipidemia	0.86002
DB01196	Hypertension	0.40061
DB01196	Infectious lung disease	0.05753
DB01196	Infertility	0.5079
DB01196	Kidney failure	0.44212
DB01196	Late pregnancy	1.23268
DB01196	Leukemia	0.02117
DB01196	Leukoencephalopathy	0.03564
DB01196	Lung cancer	0.02667
DB01196	Lupus erythematosus	0.28458
DB01196	Lupus vulgaris	0.67776
DB01196	Lymphoma	0.04152
DB01196	Mental retardation	0.03628
DB01196	Migraine	0.63189
DB01196	Multiple sclerosis	0.04129
DB01196	Myasthenia Gravis	1.08341
DB01196	Neoplasm metastasis	0.05223
DB01196	Nephrosis	0.05764
DB01196	Obesity	0.34239
DB01196	Oral cancer	0.10245
DB01196	Osteoporosis	0.59543
DB01196	Ovarian disease	0.92125
DB01196	Ovarian failure	1.01118
DB01196	Overnutrition	0.06746
DB01196	Pancreatitis	0.05065
DB01196	Panic disorder	0.79766
DB01196	Parkinson disease	0.44419
DB01196	Polyarthritis	0.33334
DB01196	Primary biliary cirrhosis	0.64351
DB01196	Prostate cancer	0.02

DB01196	Ptosis	0.16667
DB01196	Pulmonary fibrosis	0.08321
DB01196	Rabies	0.33384
DB01196	Renal tubular acidosis	0.57474
DB01196	Rheumatoid arthritis	0.08159
DB01196	Schistosomiasis	0.11237
DB01196	Schizophrenia	0.41585
DB01196	Scleroderma	0.03754
DB01196	Sicca syndrome	0.13578
DB01196	Stroke	0.30847
DB01196	Synovitis	0.97154
DB01196	Systemic scleroderma	0.02799
DB01196	Temporal arteritis	0.22361
DB01196	Testicular dysfunction	0.07143
DB01196	Thymoma	0.77495
DB01196	Tuberculosis	0.05212
DB01196	Ulcerative colitis	0.02997
DB01196	Uterine disease	0.73797
DB01196	Vaccinia	0.16627
DB01196	Virus disease	0.04249
DB01196	Vitiligo	0.8477
DB01196	Wiskott-Aldrich syndrome	0.07541
DB01357	Hypertension, Pulmonary	0.15392
DB01357	Infertility, Male	0.8916
DB01357	Adenovirus infection	0.03828
DB01357	Alzheimer's disease	0.24115
DB01357	Atherosclerosis	0.29616
DB01357	Autistic disorder	0.02299
DB01357	Bipolar disorder	0.6636
DB01357	Bladder cancer	0.1378
DB01357	Breast cancer	0.00932
DB01357	Bronchial hyperreactivity	1.35355
DB01357	Cancer	0.15649
DB01357	Cerebrovascular disorder	0.90562
DB01357	Cholelithiasis	0.80055
DB01357	Cirrhosis	0.1039
DB01357	Craniosynostosis	0.25982
DB01357	Dental plaque	0.51729
DB01357	Depression	0.39339
DB01357	Diabetes mellitus	0.2348
DB01357	Down syndrome	0.50437
DB01357	Eating disorder	0.04067
DB01357	Endometriosis	0.31
DB01357	Epilepsy	0.05425
DB01357	Glaucoma	0.62185
DB01357	Hepatitis	0.19437
DB01357	Hepatitis C	0.06223

DB01357	Herpes	0.39655
DB01357	Hodgkin's disease	0.04769
DB01357	Hyperlipidemia	0.95808
DB01357	Hypertension	0.40061
DB01357	Infectious lung disease	0.05753
DB01357	Infertility	0.57658
DB01357	Kidney failure	0.49873
DB01357	Late pregnancy	1.23268
DB01357	Leukemia	0.02117
DB01357	Leukoencephalopathy	0.03564
DB01357	Lung cancer	0.02667
DB01357	Lupus erythematosus	0.3273
DB01357	Lupus vulgaris	0.77225
DB01357	Lymphoma	0.04152
DB01357	Mental retardation	0.03628
DB01357	Migraine	0.72474
DB01357	Multiple sclerosis	0.04129
DB01357	Myasthenia Gravis	1.08341
DB01357	Neoplasm metastasis	0.05223
DB01357	Nephrosis	0.05764
DB01357	Obesity	0.38085
DB01357	Oral cancer	0.03441
DB01357	Osteoporosis	0.59543
DB01357	Ovarian disease	0.92125
DB01357	Ovarian failure	1.12588
DB01357	Overnutrition	0.06746
DB01357	Pancreatitis	0.05065
DB01357	Panic disorder	0.90677
DB01357	Parkinson disease	0.44419
DB01357	Polyarthritis	0.39032
DB01357	Primary biliary cirrhosis	0.73331
DB01357	Prostate cancer	0.02
DB01357	Pulmonary fibrosis	0.08321
DB01357	Rabies	0.38939
DB01357	Renal tubular acidosis	0.74141
DB01357	Rheumatoid arthritis	0.08159
DB01357	Schistosomiasis	0.11237
DB01357	Schizophrenia	0.37784
DB01357	Scleroderma	0.03754
DB01357	Sicca syndrome	0.13578
DB01357	Stroke	0.36508
DB01357	Synovitis	1.14831
DB01357	Systemic scleroderma	0.02799
DB01357	Thymoma	0.93306
DB01357	Tuberculosis	0.05212
DB01357	Ulcerative colitis	0.02997
DB01357	Uterine disease	0.92695

DB01357	Vaccinia	0.16627
DB01357	Virus disease	0.04249
DB01357	Vitiligo	0.96556
DB01357	Wiskott-Aldrich syndrome	0.07541
DB01406	Hemorrhagic fevers, Viral	0.18257
DB01406	Hypertension, Pulmonary	0.13649
DB01406	Infertility, Male	0.95786
DB01406	Labor, Premature	0.11547
DB01406	Mycobacterium infection, Atypical	0.14907
DB01406	Pleural effusion, Malignant	0.13484
DB01406	Adenovirus infection	0.05624
DB01406	Alopecia	0.08165
DB01406	Alzheimer's disease	0.21439
DB01406	Amyotrophic lateral sclerosis	0.18756
DB01406	Arthritis	0.05547
DB01406	Atherosclerosis	0.36093
DB01406	Autistic disorder	0.02039
DB01406	Autoimmune disease	0.04851
DB01406	Azoospermia	0.21659
DB01406	Bipolar disorder	0.58931
DB01406	Bladder cancer	0.12219
DB01406	Brain tumor	0.03592
DB01406	Breast cancer	0.00827
DB01406	Bronchial hyperreactivity	1.04485
DB01406	Bronchiolitis	0.15811
DB01406	CNS metastases	0.15811
DB01406	Cancer	0.1643
DB01406	Cerebrovascular disorder	0.70713
DB01406	Cholelithiasis	0.85614
DB01406	Cirrhosis	0.09213
DB01406	Craniosynostosis	0.23039
DB01406	Dental plaque	0.40288
DB01406	Depression	0.47173
DB01406	Diabetes mellitus	0.27719
DB01406	Down syndrome	0.44812
DB01406	Eating disorder	0.03607
DB01406	Endometriosis	0.36224
DB01406	Epilepsy	0.0481
DB01406	Epstein-Barr virus infection	0.09759
DB01406	Esophagus cancer	0.06667
DB01406	Glaucoma	0.47817
DB01406	Growth retardation	0.08607
DB01406	HIV infection	0.04284
DB01406	Heart failure	0.04767
DB01406	Hepatitis	0.17235
DB01406	Hepatitis B	0.10847
DB01406	Hepatitis C	0.12112

DB01406	Herpes	0.28885
DB01406	Hodgkin's disease	0.04229
DB01406	Huntington disease	0.07826
DB01406	Hypercholesterolemia	0.08607
DB01406	Hyperglycemia	0.06805
DB01406	Hyperhomocysteinemia	0.1291
DB01406	Hyperinsulinism	0.08607
DB01406	Hyperlipidemia	0.76337
DB01406	Hypertension	0.35342
DB01406	Hypogonadism	0.66227
DB01406	Hypothyroidism	0.10541
DB01406	IGA glomerulonephritis	0.0767
DB01406	Infectious lung disease	0.05102
DB01406	Infertility	0.67961
DB01406	Influenza	0.08944
DB01406	Intractable epilepsy	0.15811
DB01406	Kidney failure	0.44312
DB01406	Late pregnancy	0.96618
DB01406	Leukemia	0.04397
DB01406	Leukoencephalopathy	0.0316
DB01406	Lipodystrophy	0.12403
DB01406	Liver cancer	0.03471
DB01406	Liver disease	0.06901
DB01406	Lung cancer	0.02365
DB01406	Lupus erythematosus	0.25268
DB01406	Lupus vulgaris	0.68624
DB01406	Lyme disease	0.14907
DB01406	Lymphoma	0.03681
DB01406	Macular degeneration	0.09325
DB01406	Melanoma	0.03727
DB01406	Mental retardation	0.03217
DB01406	Migraine	0.77398
DB01406	Multiple sclerosis	0.03662
DB01406	Muscular atrophy	0.30704
DB01406	Myasthenia Gravis	0.8053
DB01406	Nasopharyngeal cancer	0.08607
DB01406	Neoplasm metastasis	0.04631
DB01406	Nephritis	0.2
DB01406	Nephrosis	0.05111
DB01406	Neuroblastoma	0.06143
DB01406	Obesity	0.36944
DB01406	Oligospermia	0.34361
DB01406	Oral cancer	0.03051
DB01406	Osteoporosis	0.6346
DB01406	Ovarian disease	0.71331
DB01406	Ovarian failure	0.89753
DB01406	Overnutrition	0.05982

DB01406	Pancreatitis	0.11476
DB01406	Panic disorder	0.70815
DB01406	Parkinson disease	0.34534
DB01406	Penile disease	0.15811
DB01406	Peptic esophagitis	0.14142
DB01406	Phobic anxiety disorder	0.68588
DB01406	Polyarthritis	0.29602
DB01406	Polycystic ovary syndrome	0.03973
DB01406	Primary biliary cirrhosis	0.57131
DB01406	Prostate cancer	0.10389
DB01406	Proteinuria	0.1
DB01406	Psychotic disorder	0.07096
DB01406	Pulmonary fibrosis	0.1583
DB01406	Rabies	0.40954
DB01406	Renal tubular acidosis	0.51093
DB01406	Rheumatoid arthritis	0.13749
DB01406	Schistosomiasis	0.09964
DB01406	Schizophrenia	0.44545
DB01406	Scleroderma	0.03329
DB01406	Sicca syndrome	0.1204
DB01406	Squamous cell cancer	0.04564
DB01406	Stomach cancer	0.03766
DB01406	Stroke	0.27396
DB01406	Synovitis	0.86286
DB01406	Systemic infection	0.03117
DB01406	Systemic scleroderma	0.09652
DB01406	Thymoma	0.68839
DB01406	Tropical spastic paraparesis	0.09486
DB01406	Tuberculosis	0.10652
DB01406	Tuberous sclerosis	0.11547
DB01406	Ulcerative colitis	0.02658
DB01406	Urogenital abnormalities	0.37786
DB01406	Uterine disease	0.65584
DB01406	Vaccinia	0.14744
DB01406	Virus disease	0.03768
DB01406	Vitiligo	0.83166
DB01406	Wiskott-Aldrich syndrome	0.06687
DB01406	Yersinia infection	0.11925
DB01431	Hypertension, Pulmonary	0.15392
DB01431	Infertility, Male	0.83182
DB01431	Adenovirus infection	0.03828
DB01431	Alzheimer's disease	0.22007
DB01431	Atherosclerosis	0.27565
DB01431	Autistic disorder	0.02299
DB01431	Bipolar disorder	0.63044
DB01431	Bladder cancer	0.1378
DB01431	Breast cancer	0.00932

DB01431	Bronchial hyperreactivity	1.25
DB01431	Cancer	0.1457
DB01431	Cerebrovascular disorder	0.8417
DB01431	Cholelithiasis	0.74197
DB01431	Cirrhosis	0.1039
DB01431	Craniosynostosis	0.25982
DB01431	Dental plaque	0.4801
DB01431	Depression	0.35494
DB01431	Diabetes mellitus	0.21938
DB01431	Down syndrome	0.47099
DB01431	Eating disorder	0.04067
DB01431	Endometriosis	0.28568
DB01431	Epilepsy	0.05425
DB01431	Glaucoma	0.57304
DB01431	Hepatitis	0.19437
DB01431	Hepatitis C	0.06223
DB01431	Herpes	0.35471
DB01431	Hodgkin's disease	0.04769
DB01431	Hyperlipidemia	0.90064
DB01431	Hypertension	0.37746
DB01431	Infectious lung disease	0.05753
DB01431	Infertility	0.53635
DB01431	Kidney failure	0.46557
DB01431	Late pregnancy	1.14813
DB01431	Leukemia	0.02117
DB01431	Leukoencephalopathy	0.03564
DB01431	Lung cancer	0.02667
DB01431	Lupus erythematosus	0.30228
DB01431	Lupus vulgaris	0.7169
DB01431	Lymphoma	0.04152
DB01431	Mental retardation	0.03628
DB01431	Migraine	0.67035
DB01431	Multiple sclerosis	0.04129
DB01431	Myasthenia Gravis	0.97985
DB01431	Neoplasm metastasis	0.05223
DB01431	Nephrosis	0.05764
DB01431	Obesity	0.35832
DB01431	Oral cancer	0.03441
DB01431	Osteoporosis	0.54912
DB01431	Ovarian disease	0.85221
DB01431	Ovarian failure	1.05869
DB01431	Overnutrition	0.06746
DB01431	Pancreatitis	0.05065
DB01431	Panic disorder	0.84285
DB01431	Parkinson disease	0.41185
DB01431	Polyarthritis	0.35694
DB01431	Primary biliary cirrhosis	0.68071

DB01431	Prostate cancer	0.02
DB01431	Pulmonary fibrosis	0.08321
DB01431	Rabies	0.35685
DB01431	Renal tubular acidosis	0.64378
DB01431	Rheumatoid arthritis	0.08159
DB01431	Schistosomiasis	0.11237
DB01431	Schizophrenia	0.35557
DB01431	Scleroderma	0.03754
DB01431	Sicca syndrome	0.13578
DB01431	Stroke	0.33192
DB01431	Synovitis	1.04476
DB01431	Systemic scleroderma	0.02799
DB01431	Thymoma	0.84044
DB01431	Tuberculosis	0.05212
DB01431	Ulcerative colitis	0.02997
DB01431	Uterine disease	0.81625
DB01431	Vaccinia	0.16627
DB01431	Virus disease	0.04249
DB01431	Vitiligo	0.89652
DB01431	Wiskott-Aldrich syndrome	0.07541
DB04573	Hypertension, Pulmonary	0.15392
DB04573	Infertility, Male	0.83182
DB04573	Adenovirus infection	0.03828
DB04573	Alzheimer's disease	0.27097
DB04573	Atherosclerosis	0.32516
DB04573	Autistic disorder	0.02299
DB04573	Bipolar disorder	0.63044
DB04573	Bladder cancer	0.1378
DB04573	Breast cancer	0.00932
DB04573	Bronchial hyperreactivity	1.25
DB04573	Cancer	0.17176
DB04573	Cerebrovascular disorder	0.8417
DB04573	Cholelithiasis	0.88339
DB04573	Cirrhosis	0.1039
DB04573	Craniosynostosis	0.25982
DB04573	Dental plaque	0.5699
DB04573	Depression	0.35494
DB04573	Diabetes mellitus	0.21938
DB04573	Down syndrome	0.47099
DB04573	Eating disorder	0.04067
DB04573	Endometriosis	0.3444
DB04573	Epilepsy	0.05425
DB04573	Glaucoma	0.69089
DB04573	Hepatitis	0.19437
DB04573	Hepatitis C	0.06223
DB04573	Herpes	0.35471
DB04573	Hodgkin's disease	0.04769

DB04573	Hyperlipidemia	0.90064
DB04573	Hypertension	0.43336
DB04573	Infectious lung disease	0.05753
DB04573	Infertility	0.53635
DB04573	Kidney failure	0.46557
DB04573	Late pregnancy	1.35225
DB04573	Leukemia	0.02117
DB04573	Leukoencephalopathy	0.03564
DB04573	Lung cancer	0.02667
DB04573	Lupus erythematosus	0.30228
DB04573	Lupus vulgaris	0.7169
DB04573	Lymphoma	0.04152
DB04573	Mental retardation	0.03628
DB04573	Migraine	0.67035
DB04573	Multiple sclerosis	0.04129
DB04573	Myasthenia Gravis	1.22985
DB04573	Neoplasm metastasis	0.05223
DB04573	Nephrosis	0.05764
DB04573	Obesity	0.35832
DB04573	Oral cancer	0.03441
DB04573	Osteoporosis	0.66092
DB04573	Ovarian disease	1.01888
DB04573	Ovarian failure	1.05869
DB04573	Overnutrition	0.06746
DB04573	Pancreatitis	0.05065
DB04573	Panic disorder	0.84285
DB04573	Parkinson disease	0.48993
DB04573	Polyarthritis	0.35694
DB04573	Primary biliary cirrhosis	0.68071
DB04573	Prostate cancer	0.02
DB04573	Ptosis	0.2357
DB04573	Pulmonary fibrosis	0.08321
DB04573	Rabies	0.35685
DB04573	Renal tubular acidosis	0.64378
DB04573	Rheumatoid arthritis	0.08159
DB04573	Schistosomiasis	0.11237
DB04573	Schizophrenia	0.40933
DB04573	Scleroderma	0.03754
DB04573	Sicca syndrome	0.13578
DB04573	Stroke	0.33192
DB04573	Synovitis	1.04476
DB04573	Systemic scleroderma	0.02799
DB04573	Temporal arteritis	0.31623
DB04573	Testicular dysfunction	0.10102
DB04573	Thymoma	0.84044
DB04573	Tuberculosis	0.05212
DB04573	Ulcerative colitis	0.02997

DB04573	Uterine disease	0.81625
DB04573	Vaccinia	0.16627
DB04573	Virus disease	0.04249
DB04573	Vitiligo	0.89652
DB04573	Wiskott-Aldrich syndrome	0.07541
DB04574	Hypertension, Pulmonary	0.15392
DB04574	Infertility, Male	0.83182
DB04574	Adenovirus infection	0.03828
DB04574	Alzheimer's disease	0.27097
DB04574	Atherosclerosis	0.32516
DB04574	Autistic disorder	0.02299
DB04574	Bipolar disorder	0.63044
DB04574	Bladder cancer	0.1378
DB04574	Breast cancer	0.00932
DB04574	Bronchial hyperreactivity	1.25
DB04574	Cancer	0.17176
DB04574	Cerebrovascular disorder	0.8417
DB04574	Cholelithiasis	0.88339
DB04574	Cirrhosis	0.1039
DB04574	Craniosynostosis	0.25982
DB04574	Dental plaque	0.5699
DB04574	Depression	0.35494
DB04574	Diabetes mellitus	0.21938
DB04574	Down syndrome	0.47099
DB04574	Eating disorder	0.04067
DB04574	Endometriosis	0.3444
DB04574	Epilepsy	0.05425
DB04574	Glaucoma	0.69089
DB04574	Hepatitis	0.19437
DB04574	Hepatitis C	0.06223
DB04574	Herpes	0.35471
DB04574	Hodgkin's disease	0.04769
DB04574	Hyperlipidemia	0.90064
DB04574	Hypertension	0.43336
DB04574	Infectious lung disease	0.05753
DB04574	Infertility	0.53635
DB04574	Kidney failure	0.46557
DB04574	Late pregnancy	1.35225
DB04574	Leukemia	0.02117
DB04574	Leukoencephalopathy	0.03564
DB04574	Lung cancer	0.02667
DB04574	Lupus erythematosus	0.30228
DB04574	Lupus vulgaris	0.7169
DB04574	Lymphoma	0.04152
DB04574	Mental retardation	0.03628
DB04574	Migraine	0.67035
DB04574	Multiple sclerosis	0.04129

DB04574	Myasthenia Gravis	1.22985
DB04574	Neoplasm metastasis	0.05223
DB04574	Nephrosis	0.05764
DB04574	Obesity	0.35832
DB04574	Oral cancer	0.03441
DB04574	Osteoporosis	0.66092
DB04574	Ovarian disease	1.01888
DB04574	Ovarian failure	1.05869
DB04574	Overnutrition	0.06746
DB04574	Pancreatitis	0.05065
DB04574	Panic disorder	0.84285
DB04574	Parkinson disease	0.48993
DB04574	Polyarthritis	0.35694
DB04574	Primary biliary cirrhosis	0.68071
DB04574	Prostate cancer	0.02
DB04574	Ptosis	0.2357
DB04574	Pulmonary fibrosis	0.08321
DB04574	Rabies	0.35685
DB04574	Renal tubular acidosis	0.64378
DB04574	Rheumatoid arthritis	0.08159
DB04574	Schistosomiasis	0.11237
DB04574	Schizophrenia	0.40933
DB04574	Scleroderma	0.03754
DB04574	Sicca syndrome	0.13578
DB04574	Stroke	0.33192
DB04574	Synovitis	1.04476
DB04574	Systemic scleroderma	0.02799
DB04574	Temporal arteritis	0.31623
DB04574	Testicular dysfunction	0.10102
DB04574	Thymoma	0.84044
DB04574	Tuberculosis	0.05212
DB04574	Ulcerative colitis	0.02997
DB04574	Uterine disease	0.81625
DB04574	Vaccinia	0.16627
DB04574	Virus disease	0.04249
DB04574	Vitiligo	0.89652
DB04574	Wiskott-Aldrich syndrome	0.07541
DB04575	Hypertension, Pulmonary	0.15392
DB04575	Infertility, Male	0.8916
DB04575	Adenovirus infection	0.03828
DB04575	Alzheimer's disease	0.24115
DB04575	Atherosclerosis	0.29616
DB04575	Autistic disorder	0.02299
DB04575	Bipolar disorder	0.6636
DB04575	Bladder cancer	0.1378
DB04575	Breast cancer	0.00932
DB04575	Bronchial hyperreactivity	1.35355

DB04575	Cancer	0.15649
DB04575	Cerebrovascular disorder	0.90562
DB04575	Cholelithiasis	0.80055
DB04575	Cirrhosis	0.1039
DB04575	Craniosynostosis	0.25982
DB04575	Dental plaque	0.51729
DB04575	Depression	0.39339
DB04575	Diabetes mellitus	0.2348
DB04575	Down syndrome	0.50437
DB04575	Eating disorder	0.04067
DB04575	Endometriosis	0.31
DB04575	Epilepsy	0.05425
DB04575	Glaucoma	0.62185
DB04575	Hepatitis	0.19437
DB04575	Hepatitis C	0.06223
DB04575	Herpes	0.39655
DB04575	Hodgkin's disease	0.04769
DB04575	Hyperlipidemia	0.95808
DB04575	Hypertension	0.40061
DB04575	Infectious lung disease	0.05753
DB04575	Infertility	0.57658
DB04575	Kidney failure	0.49873
DB04575	Late pregnancy	1.23268
DB04575	Leukemia	0.02117
DB04575	Leukoencephalopathy	0.03564
DB04575	Lung cancer	0.02667
DB04575	Lupus erythematosus	0.3273
DB04575	Lupus vulgaris	0.77225
DB04575	Lymphoma	0.04152
DB04575	Mental retardation	0.03628
DB04575	Migraine	0.72474
DB04575	Multiple sclerosis	0.04129
DB04575	Myasthenia Gravis	1.08341
DB04575	Neoplasm metastasis	0.05223
DB04575	Nephrosis	0.05764
DB04575	Obesity	0.38085
DB04575	Oral cancer	0.03441
DB04575	Osteoporosis	0.59543
DB04575	Ovarian disease	0.92125
DB04575	Ovarian failure	1.12588
DB04575	Overnutrition	0.06746
DB04575	Pancreatitis	0.05065
DB04575	Panic disorder	0.90677
DB04575	Parkinson disease	0.44419
DB04575	Polyarthritis	0.39032
DB04575	Primary biliary cirrhosis	0.73331
DB04575	Prostate cancer	0.02

DB04575	Pulmonary fibrosis	0.08321
DB04575	Rabies	0.38939
DB04575	Renal tubular acidosis	0.74141
DB04575	Rheumatoid arthritis	0.08159
DB04575	Schistosomiasis	0.11237
DB04575	Schizophrenia	0.37784
DB04575	Scleroderma	0.03754
DB04575	Sicca syndrome	0.13578
DB04575	Stroke	0.36508
DB04575	Synovitis	1.14831
DB04575	Systemic scleroderma	0.02799
DB04575	Thymoma	0.93306
DB04575	Tuberculosis	0.05212
DB04575	Ulcerative colitis	0.02997
DB04575	Uterine disease	0.92695
DB04575	Vaccinia	0.16627
DB04575	Virus disease	0.04249
DB04575	Vitiligo	0.96556
DB04575	Wiskott-Aldrich syndrome	0.07541
DB04938	Hypertension, Pulmonary	0.15392
DB04938	Infertility, Male	0.8916
DB04938	Adenovirus infection	0.03828
DB04938	Alzheimer's disease	0.24115
DB04938	Atherosclerosis	0.29616
DB04938	Autistic disorder	0.02299
DB04938	Bipolar disorder	0.6636
DB04938	Bladder cancer	0.1378
DB04938	Breast cancer	0.00932
DB04938	Bronchial hyperreactivity	1.35355
DB04938	Cancer	0.15649
DB04938	Cerebrovascular disorder	0.90562
DB04938	Cholelithiasis	0.80055
DB04938	Cirrhosis	0.1039
DB04938	Craniosynostosis	0.25982
DB04938	Dental plaque	0.51729
DB04938	Depression	0.39339
DB04938	Diabetes mellitus	0.2348
DB04938	Down syndrome	0.50437
DB04938	Eating disorder	0.04067
DB04938	Endometriosis	0.31
DB04938	Epilepsy	0.05425
DB04938	Glaucoma	0.62185
DB04938	Hepatitis	0.19437
DB04938	Hepatitis C	0.06223
DB04938	Herpes	0.39655
DB04938	Hodgkin's disease	0.04769
DB04938	Hyperlipidemia	0.95808

DB04938	Hypertension	0.40061
DB04938	Infectious lung disease	0.05753
DB04938	Infertility	0.57658
DB04938	Kidney failure	0.49873
DB04938	Late pregnancy	1.23268
DB04938	Leukemia	0.02117
DB04938	Leukoencephalopathy	0.03564
DB04938	Lung cancer	0.02667
DB04938	Lupus erythematosus	0.3273
DB04938	Lupus vulgaris	0.77225
DB04938	Lymphoma	0.04152
DB04938	Mental retardation	0.03628
DB04938	Migraine	0.72474
DB04938	Multiple sclerosis	0.04129
DB04938	Myasthenia Gravis	1.08341
DB04938	Neoplasm metastasis	0.05223
DB04938	Nephrosis	0.05764
DB04938	Obesity	0.38085
DB04938	Oral cancer	0.03441
DB04938	Osteoporosis	0.59543
DB04938	Ovarian disease	0.92125
DB04938	Ovarian failure	1.12588
DB04938	Overnutrition	0.06746
DB04938	Pancreatitis	0.05065
DB04938	Panic disorder	0.90677
DB04938	Parkinson disease	0.44419
DB04938	Polyarthritis	0.39032
DB04938	Primary biliary cirrhosis	0.73331
DB04938	Prostate cancer	0.02
DB04938	Pulmonary fibrosis	0.08321
DB04938	Rabies	0.38939
DB04938	Renal tubular acidosis	0.74141
DB04938	Rheumatoid arthritis	0.08159
DB04938	Schistosomiasis	0.11237
DB04938	Schizophrenia	0.37784
DB04938	Scleroderma	0.03754
DB04938	Sicca syndrome	0.13578
DB04938	Stroke	0.36508
DB04938	Synovitis	1.14831
DB04938	Systemic scleroderma	0.02799
DB04938	Thymoma	0.93306
DB04938	Tuberculosis	0.05212
DB04938	Ulcerative colitis	0.02997
DB04938	Uterine disease	0.92695
DB04938	Vaccinia	0.16627
DB04938	Virus disease	0.04249
DB04938	Vitiligo	0.96556

DB04938	Wiskott-Aldrich syndrome	0.07541
DB01025	Adenocarcinoma	0.07293
DB01025	Advanced cancer	0.15811
DB01025	Alzheimer's disease	0.07198
DB01025	Arthritis	0.06202
DB01025	Brain tumor	0.04016
DB01025	Cancer	0.01843
DB01025	Cleft palate	0.13363
DB01025	Endometriosis	0.08305
DB01025	Enteritis	0.05488
DB01025	Intracranial aneurysm	0.15076
DB01025	Mucocutaneous lymph node syndrome	0.10911
DB01025	Ovarian cancer	0.05025
DB01025	Polyneuropathy	0.13363
DB01025	Prostate cancer	0.0261
DB01025	Psoriasis	0.11785
DB00231	Breast cancer	0.00802
DB00231	Cancer	0.00614
DB00231	Drug abuse	0.01561
DB00231	Hypertension	0.01318
DB00231	Hypogonadism	0.07454
DB00231	Panic disorder	0.03637
DB00231	Yersinia infection	0.01978
DB00683	Breast cancer	0.01104
DB00683	Drug abuse	0.02149
DB00683	Hypogonadism	0.1026
DB00683	Yersinia infection	0.02723
DB00690	Breast cancer	0.01104
DB00690	Drug abuse	0.02149
DB00690	Hypogonadism	0.1026
DB00690	Yersinia infection	0.02723
DB00801	Breast cancer	0.00851
DB00801	Drug abuse	0.01656
DB00801	Hypogonadism	0.07906
DB00801	Yersinia infection	0.02098
DB00829	Breast cancer	0.00825
DB00829	Cancer	0.00632
DB00829	Drug abuse	0.01606
DB00829	Hypertension	0.01356
DB00829	Hypogonadism	0.0767
DB00829	Panic disorder	0.03742
DB00829	Yersinia infection	0.02035
DB00842	Breast cancer	0.00813
DB00842	Drug abuse	0.01583
DB00842	Hypogonadism	0.07559
DB00842	Yersinia infection	0.02006
DB00897	Breast cancer	0.00802

DB00897	Cancer	0.00614
DB00897	Drug abuse	0.01561
DB00897	Hypertension	0.01318
DB00897	Hypogonadism	0.07454
DB00897	Panic disorder	0.03637
DB00897	Yersinia infection	0.01978
DB01215	Breast cancer	0.00851
DB01215	Drug abuse	0.01656
DB01215	Hypogonadism	0.07906
DB01215	Yersinia infection	0.02098
DB01558	Breast cancer	0.01104
DB01558	Drug abuse	0.02149
DB01558	Hypogonadism	0.1026
DB01558	Yersinia infection	0.02723
DB01559	Breast cancer	0.01203
DB01559	Drug abuse	0.02341
DB01559	Hypogonadism	0.1118
DB01559	Yersinia infection	0.02967
DB01567	Breast cancer	0.01203
DB01567	Drug abuse	0.02341
DB01567	Hypogonadism	0.1118
DB01567	Yersinia infection	0.02967
DB01588	Breast cancer	0.00851
DB01588	Drug abuse	0.01656
DB01588	Hypogonadism	0.07906
DB01588	Yersinia infection	0.02098
DB01589	Breast cancer	0.00864
DB01589	Drug abuse	0.01682
DB01589	Hypogonadism	0.08032
DB01589	Yersinia infection	0.02132
DB01594	Breast cancer	0.01203
DB01594	Drug abuse	0.02341
DB01594	Hypogonadism	0.1118
DB01594	Yersinia infection	0.02967
DB01595	Myoclonic epilepsy, Juvenile	0.09129
DB01595	Autistic disorder	0.02712
DB01595	Brain disease	0.04016
DB01595	Breast cancer	0.01076
DB01595	Central nervous system disease	0.0559
DB01595	Drug abuse	0.04189
DB01595	Hypogonadism	0.1
DB01595	Infantile spasms	0.08452
DB01595	Intractable epilepsy	0.07906
DB01595	Migraine	0.04152
DB01595	Myopathy	0.02712
DB01595	Nervous system disease	0.05774
DB01595	Neuropathy	0.03581

DB01595	Rheumatism	0.0527
DB01595	Yersinia infection	0.02654
DB00358	Asthma	0.05774
DB00358	Chronic obstructive airway disease	0.07906
DB00358	Cystic fibrosis	0.10102
DB00358	Depression	0.09285
DB00358	Heart failure	0.07538
DB00358	Huntington disease	0.1543
DB00358	Hypertension	0.0559
DB00358	Kidney failure	0.08006
DB00358	Panic disorder	0.1543
DB00358	Phobic anxiety disorder	0.35355
DB00358	Schizophrenia	0.05376
DB00358	Thrombocytosis	0.31623
DB00421	Infertility, Male	0.40441
DB00421	Adenovirus infection	0.03501
DB00421	Alopecia	0.24045
DB00421	Amyotrophic lateral sclerosis	0.20045
DB00421	Atherosclerosis	0.15251
DB00421	Azoospermia	0.34103
DB00421	Breast cancer	0.08373
DB00421	Cancer	0.06539
DB00421	Cholelithiasis	0.36861
DB00421	Depression	0.192
DB00421	Diabetes mellitus	0.10791
DB00421	Endometriosis	0.1362
DB00421	Heart failure	0.20084
DB00421	Huntington disease	0.12285
DB00421	Hyperglycemia	0.10682
DB00421	Hypertension	0.21075
DB00421	Hypoglycemia	0.83053
DB00421	Hypogonadism	1.0419
DB00421	Infertility	0.26329
DB00421	Lymphoma	0.21405
DB00421	Melanoma	0.04817
DB00421	Migraine	0.33523
DB00421	Muscular atrophy	0.48339
DB00421	Obesity	0.04879
DB00421	Oligospermia	0.5407
DB00421	Osteoporosis	0.27724
DB00421	Phobic anxiety disorder	1.07922
DB00421	Polycystic ovary syndrome	0.06237
DB00421	Prostate cancer	0.09886
DB00421	Proteinuria	0.52646
DB00421	Psychotic disorder	0.11139
DB00421	Rabies	0.17795
DB00421	Renal Cell cancer	0.25288

DB00421	Rheumatoid arthritis	0.10244
DB00421	Schizophrenia	0.17271
DB00421	Systemic infection	0.04893
DB00421	Systemic scleroderma	0.03896
DB00421	Tropical spastic paraparesis	0.14891
DB00421	Urogenital abnormalities	0.59452
DB00421	Vitiligo	0.12411
DB00421	Yersinia infection	0.1878
DB00499	Infertility, Male	0.45653
DB00499	Adenovirus infection	0.04202
DB00499	Amyotrophic lateral sclerosis	0.22152
DB00499	Atherosclerosis	0.17315
DB00499	Azoospermia	0.38045
DB00499	Breast cancer	0.06386
DB00499	Cancer	0.07327
DB00499	Cholelithiasis	0.41414
DB00499	Depression	0.21187
DB00499	Diabetes mellitus	0.14478
DB00499	Endometriosis	0.254
DB00499	HIV infection	0.03015
DB00499	Huntington disease	0.14747
DB00499	Hyperglycemia	0.12823
DB00499	Hypertension	0.06196
DB00499	Hypogonadism	1.18733
DB00499	Infertility	0.4871
DB00499	Leukemia	0.07521
DB00499	Lung cancer	0.0809
DB00499	Lymphoma	0.13723
DB00499	Migraine	0.3761
DB00499	Muscular atrophy	0.54096
DB00499	Obesity	0.05857
DB00499	Oligospermia	0.61248
DB00499	Osteoporosis	0.3104
DB00499	Phobic anxiety disorder	1.22466
DB00499	Polycystic ovary syndrome	0.07487
DB00499	Prostate cancer	0.11128
DB00499	Psychotic disorder	0.13371
DB00499	Rabies	0.19786
DB00499	Rheumatoid arthritis	0.11429
DB00499	Schizophrenia	0.19654
DB00499	Stomach cancer	0.08968
DB00499	Systemic infection	0.05874
DB00499	Systemic scleroderma	0.04677
DB00499	Tropical spastic paraparesis	0.17876
DB00499	Urogenital abnormalities	0.67579
DB00499	Uterine fibroids	0.40917
DB00499	Vitiligo	0.14898

DB00499	Yersinia infection	0.33217
DB00621	Infertility, Male	0.56252
DB00621	Adenovirus infection	0.04824
DB00621	Amyotrophic lateral sclerosis	0.27968
DB00621	Atherosclerosis	0.21195
DB00621	Azoospermia	0.47517
DB00621	Cancer	0.09105
DB00621	Cholelithiasis	0.51307
DB00621	Depression	0.26794
DB00621	Diabetes mellitus	0.15005
DB00621	Endometriosis	0.18982
DB00621	Huntington disease	0.16929
DB00621	Hyperglycemia	0.1472
DB00621	Hypertension	0.07113
DB00621	Hypogonadism	1.44721
DB00621	Infertility	0.36633
DB00621	Migraine	0.46671
DB00621	Muscular atrophy	0.67322
DB00621	Obesity	0.06723
DB00621	Oligospermia	0.75171
DB00621	Osteoporosis	0.38609
DB00621	Phobic anxiety disorder	1.5
DB00621	Polycystic ovary syndrome	0.08595
DB00621	Prostate cancer	0.13757
DB00621	Psychotic disorder	0.1535
DB00621	Rabies	0.24806
DB00621	Rheumatoid arthritis	0.14273
DB00621	Schizophrenia	0.23994
DB00621	Systemic infection	0.06743
DB00621	Systemic scleroderma	0.05369
DB00621	Tropical spastic paraparesis	0.20521
DB00621	Urogenital abnormalities	0.8261
DB00621	Vitiligo	0.17102
DB00621	Yersinia infection	0.26183
DB00624	Infertility, Male	0.56252
DB00624	Adenovirus infection	0.04824
DB00624	Amyotrophic lateral sclerosis	0.27968
DB00624	Atherosclerosis	0.21195
DB00624	Azoospermia	0.47517
DB00624	Cancer	0.09105
DB00624	Cholelithiasis	0.51307
DB00624	Depression	0.26794
DB00624	Diabetes mellitus	0.15005
DB00624	Endometriosis	0.18982
DB00624	Huntington disease	0.16929
DB00624	Hyperglycemia	0.1472
DB00624	Hypertension	0.07113

DB00624	Hypogonadism	1.44721
DB00624	Infertility	0.36633
DB00624	Migraine	0.46671
DB00624	Muscular atrophy	0.67322
DB00624	Obesity	0.06723
DB00624	Oligospermia	0.75171
DB00624	Osteoporosis	0.38609
DB00624	Phobic anxiety disorder	1.5
DB00624	Polycystic ovary syndrome	0.08595
DB00624	Prostate cancer	0.13757
DB00624	Psychotic disorder	0.1535
DB00624	Rabies	0.24806
DB00624	Rheumatoid arthritis	0.14273
DB00624	Schizophrenia	0.23994
DB00624	Systemic infection	0.06743
DB00624	Systemic scleroderma	0.05369
DB00624	Tropical spastic paraparesis	0.20521
DB00624	Urogenital abnormalities	0.8261
DB00624	Vitiligo	0.17102
DB00624	Yersinia infection	0.26183
DB00665	Infertility, Male	0.56252
DB00665	Adenovirus infection	0.04824
DB00665	Amyotrophic lateral sclerosis	0.27968
DB00665	Atherosclerosis	0.21195
DB00665	Azoospermia	0.47517
DB00665	Cancer	0.09105
DB00665	Cholelithiasis	0.51307
DB00665	Depression	0.26794
DB00665	Diabetes mellitus	0.15005
DB00665	Endometriosis	0.18982
DB00665	Huntington disease	0.16929
DB00665	Hyperglycemia	0.1472
DB00665	Hypertension	0.07113
DB00665	Hypogonadism	1.44721
DB00665	Infertility	0.36633
DB00665	Migraine	0.46671
DB00665	Muscular atrophy	0.67322
DB00665	Obesity	0.06723
DB00665	Oligospermia	0.75171
DB00665	Osteoporosis	0.38609
DB00665	Phobic anxiety disorder	1.5
DB00665	Polycystic ovary syndrome	0.08595
DB00665	Prostate cancer	0.13757
DB00665	Psychotic disorder	0.1535
DB00665	Rabies	0.24806
DB00665	Rheumatoid arthritis	0.14273
DB00665	Schizophrenia	0.23994

DB00665	Systemic infection	0.06743
DB00665	Systemic scleroderma	0.05369
DB00665	Tropical spastic paraparesis	0.20521
DB00665	Urogenital abnormalities	0.8261
DB00665	Vitiligo	0.17102
DB00665	Yersinia infection	0.26183
DB00687	Infertility, Male	0.34684
DB00687	Adenovirus infection	0.03082
DB00687	Alopecia	0.20346
DB00687	Amyotrophic lateral sclerosis	0.17039
DB00687	Atherosclerosis	0.13111
DB00687	Azoospermia	0.29104
DB00687	Breast cancer	0.07155
DB00687	Cancer	0.05591
DB00687	Cholelithiasis	0.31551
DB00687	Depression	0.16311
DB00687	Diabetes mellitus	0.09263
DB00687	Endometriosis	0.11617
DB00687	Heart failure	0.17201
DB00687	Huntington disease	0.10817
DB00687	Hyperglycemia	0.09405
DB00687	Hypertension	0.18199
DB00687	Hypoglycemia	0.7128
DB00687	Hypogonadism	0.89714
DB00687	Infertility	0.22561
DB00687	Lymphoma	0.18331
DB00687	Melanoma	0.04241
DB00687	Migraine	0.28676
DB00687	Muscular atrophy	0.41307
DB00687	Obesity	0.04296
DB00687	Oligospermia	0.4644
DB00687	Osteoporosis	0.23695
DB00687	Phobic anxiety disorder	0.92762
DB00687	Polycystic ovary syndrome	0.05491
DB00687	Prostate cancer	0.08469
DB00687	Proteinuria	0.45342
DB00687	Psychotic disorder	0.09808
DB00687	Rabies	0.15165
DB00687	Renal Cell cancer	0.2174
DB00687	Rheumatoid arthritis	0.08742
DB00687	Schizophrenia	0.14863
DB00687	Systemic infection	0.04309
DB00687	Systemic scleroderma	0.03431
DB00687	Tropical spastic paraparesis	0.13112
DB00687	Urogenital abnormalities	0.51137
DB00687	Vitiligo	0.10927
DB00687	Yersinia infection	0.15999

DB00858	Infertility, Male	0.56252
DB00858	Adenovirus infection	0.04824
DB00858	Amyotrophic lateral sclerosis	0.27968
DB00858	Atherosclerosis	0.21195
DB00858	Azoospermia	0.47517
DB00858	Cancer	0.09105
DB00858	Cholelithiasis	0.51307
DB00858	Depression	0.26794
DB00858	Diabetes mellitus	0.15005
DB00858	Endometriosis	0.18982
DB00858	Huntington disease	0.16929
DB00858	Hyperglycemia	0.1472
DB00858	Hypertension	0.07113
DB00858	Hypogonadism	1.44721
DB00858	Infertility	0.36633
DB00858	Migraine	0.46671
DB00858	Muscular atrophy	0.67322
DB00858	Obesity	0.06723
DB00858	Oligospermia	0.75171
DB00858	Osteoporosis	0.38609
DB00858	Phobic anxiety disorder	1.5
DB00858	Polycystic ovary syndrome	0.08595
DB00858	Prostate cancer	0.13757
DB00858	Psychotic disorder	0.1535
DB00858	Rabies	0.24806
DB00858	Rheumatoid arthritis	0.14273
DB00858	Schizophrenia	0.23994
DB00858	Systemic infection	0.06743
DB00858	Systemic scleroderma	0.05369
DB00858	Tropical spastic paraparesis	0.20521
DB00858	Urogenital abnormalities	0.8261
DB00858	Vitiligo	0.17102
DB00858	Yersinia infection	0.26183
DB00984	Infertility, Male	0.56252
DB00984	Adenovirus infection	0.04824
DB00984	Amyotrophic lateral sclerosis	0.27968
DB00984	Atherosclerosis	0.21195
DB00984	Azoospermia	0.47517
DB00984	Cancer	0.09105
DB00984	Cholelithiasis	0.51307
DB00984	Depression	0.26794
DB00984	Diabetes mellitus	0.15005
DB00984	Endometriosis	0.18982
DB00984	Huntington disease	0.16929
DB00984	Hyperglycemia	0.1472
DB00984	Hypertension	0.07113
DB00984	Hypogonadism	1.44721

DB00984	Infertility	0.36633
DB00984	Migraine	0.46671
DB00984	Muscular atrophy	0.67322
DB00984	Obesity	0.06723
DB00984	Oligospermia	0.75171
DB00984	Osteoporosis	0.38609
DB00984	Phobic anxiety disorder	1.5
DB00984	Polycystic ovary syndrome	0.08595
DB00984	Prostate cancer	0.13757
DB00984	Psychotic disorder	0.1535
DB00984	Rabies	0.24806
DB00984	Rheumatoid arthritis	0.14273
DB00984	Schizophrenia	0.23994
DB00984	Systemic infection	0.06743
DB00984	Systemic scleroderma	0.05369
DB00984	Tropical spastic paraparesis	0.20521
DB00984	Urogenital abnormalities	0.8261
DB00984	Vitiligo	0.17102
DB00984	Yersinia infection	0.26183
DB01128	Infertility, Male	0.56252
DB01128	Adenovirus infection	0.04824
DB01128	Amyotrophic lateral sclerosis	0.27968
DB01128	Atherosclerosis	0.21195
DB01128	Azoospermia	0.47517
DB01128	Cancer	0.09105
DB01128	Cholelithiasis	0.51307
DB01128	Depression	0.26794
DB01128	Diabetes mellitus	0.15005
DB01128	Endometriosis	0.18982
DB01128	Huntington disease	0.16929
DB01128	Hyperglycemia	0.1472
DB01128	Hypertension	0.07113
DB01128	Hypogonadism	1.44721
DB01128	Infertility	0.36633
DB01128	Migraine	0.46671
DB01128	Muscular atrophy	0.67322
DB01128	Obesity	0.06723
DB01128	Oligospermia	0.75171
DB01128	Osteoporosis	0.38609
DB01128	Phobic anxiety disorder	1.5
DB01128	Polycystic ovary syndrome	0.08595
DB01128	Prostate cancer	0.13757
DB01128	Psychotic disorder	0.1535
DB01128	Rabies	0.24806
DB01128	Rheumatoid arthritis	0.14273
DB01128	Schizophrenia	0.23994
DB01128	Systemic infection	0.06743

DB01128	Systemic scleroderma	0.05369
DB01128	Tropical spastic paraparesis	0.20521
DB01128	Urogenital abnormalities	0.8261
DB01128	Vitiligo	0.17102
DB01128	Yersinia infection	0.26183
DB01395	Infertility, Male	0.37793
DB01395	Adenovirus infection	0.03501
DB01395	Alopecia	0.21676
DB01395	Amyotrophic lateral sclerosis	0.18296
DB01395	Atherosclerosis	0.14342
DB01395	Azoospermia	0.31454
DB01395	Breast cancer	0.07749
DB01395	Cancer	0.06061
DB01395	Cholelithiasis	0.34266
DB01395	Depression	0.17496
DB01395	Diabetes mellitus	0.10108
DB01395	Endometriosis	0.12543
DB01395	Heart failure	0.18701
DB01395	Huntington disease	0.12285
DB01395	Hyperglycemia	0.10682
DB01395	Hypertension	0.20049
DB01395	Hypoglycemia	0.77756
DB01395	Hypogonadism	0.98387
DB01395	Infertility	0.24547
DB01395	Lymphoma	0.19926
DB01395	Melanoma	0.04817
DB01395	Migraine	0.31113
DB01395	Muscular atrophy	0.4474
DB01395	Obesity	0.04879
DB01395	Oligospermia	0.5072
DB01395	Osteoporosis	0.25673
DB01395	Phobic anxiety disorder	1.01435
DB01395	Polycystic ovary syndrome	0.06237
DB01395	Prostate cancer	0.09209
DB01395	Proteinuria	0.49744
DB01395	Psychotic disorder	0.11139
DB01395	Rabies	0.16353
DB01395	Renal Cell cancer	0.2378
DB01395	Rheumatoid arthritis	0.09449
DB01395	Schizophrenia	0.16284
DB01395	Systemic infection	0.04893
DB01395	Systemic scleroderma	0.03896
DB01395	Tropical spastic paraparesis	0.14891
DB01395	Urogenital abnormalities	0.55984
DB01395	Vitiligo	0.12411
DB01395	Yersinia infection	0.1724
DB01420	Infertility, Male	0.56252

DB01420	Adenovirus infection	0.04824
DB01420	Amyotrophic lateral sclerosis	0.27968
DB01420	Atherosclerosis	0.21195
DB01420	Azoospermia	0.47517
DB01420	Cancer	0.09105
DB01420	Cholelithiasis	0.51307
DB01420	Depression	0.26794
DB01420	Diabetes mellitus	0.15005
DB01420	Endometriosis	0.18982
DB01420	Huntington disease	0.16929
DB01420	Hyperglycemia	0.1472
DB01420	Hypertension	0.07113
DB01420	Hypogonadism	1.44721
DB01420	Infertility	0.36633
DB01420	Migraine	0.46671
DB01420	Muscular atrophy	0.67322
DB01420	Obesity	0.06723
DB01420	Oligospermia	0.75171
DB01420	Osteoporosis	0.38609
DB01420	Phobic anxiety disorder	1.5
DB01420	Polycystic ovary syndrome	0.08595
DB01420	Prostate cancer	0.13757
DB01420	Psychotic disorder	0.1535
DB01420	Rabies	0.24806
DB01420	Rheumatoid arthritis	0.14273
DB01420	Schizophrenia	0.23994
DB01420	Systemic infection	0.06743
DB01420	Systemic scleroderma	0.05369
DB01420	Tropical spastic paraparesis	0.20521
DB01420	Urogenital abnormalities	0.8261
DB01420	Vitiligo	0.17102
DB01420	Yersinia infection	0.26183
DB04839	Infertility, Male	0.56252
DB04839	Adenovirus infection	0.04824
DB04839	Amyotrophic lateral sclerosis	0.27968
DB04839	Atherosclerosis	0.21195
DB04839	Azoospermia	0.47517
DB04839	Cancer	0.09105
DB04839	Cholelithiasis	0.51307
DB04839	Depression	0.26794
DB04839	Diabetes mellitus	0.15005
DB04839	Endometriosis	0.18982
DB04839	Huntington disease	0.16929
DB04839	Hyperglycemia	0.1472
DB04839	Hypertension	0.07113
DB04839	Hypogonadism	1.44721
DB04839	Infertility	0.36633

DB04839	Migraine	0.46671
DB04839	Muscular atrophy	0.67322
DB04839	Obesity	0.06723
DB04839	Oligospermia	0.75171
DB04839	Osteoporosis	0.38609
DB04839	Phobic anxiety disorder	1.5
DB04839	Polycystic ovary syndrome	0.08595
DB04839	Prostate cancer	0.13757
DB04839	Psychotic disorder	0.1535
DB04839	Rabies	0.24806
DB04839	Rheumatoid arthritis	0.14273
DB04839	Schizophrenia	0.23994
DB04839	Systemic infection	0.06743
DB04839	Systemic scleroderma	0.05369
DB04839	Tropical spastic paraparesis	0.20521
DB04839	Urogenital abnormalities	0.8261
DB04839	Vitiligo	0.17102
DB04839	Yersinia infection	0.26183
DB06710	Infertility, Male	0.56252
DB06710	Adenovirus infection	0.04824
DB06710	Amyotrophic lateral sclerosis	0.27968
DB06710	Atherosclerosis	0.21195
DB06710	Azoospermia	0.47517
DB06710	Cancer	0.09105
DB06710	Cholelithiasis	0.51307
DB06710	Depression	0.26794
DB06710	Diabetes mellitus	0.15005
DB06710	Endometriosis	0.18982
DB06710	Huntington disease	0.16929
DB06710	Hyperglycemia	0.1472
DB06710	Hypertension	0.07113
DB06710	Hypogonadism	1.44721
DB06710	Infertility	0.36633
DB06710	Migraine	0.46671
DB06710	Muscular atrophy	0.67322
DB06710	Obesity	0.06723
DB06710	Oligospermia	0.75171
DB06710	Osteoporosis	0.38609
DB06710	Phobic anxiety disorder	1.5
DB06710	Polycystic ovary syndrome	0.08595
DB06710	Prostate cancer	0.13757
DB06710	Psychotic disorder	0.1535
DB06710	Rabies	0.24806
DB06710	Rheumatoid arthritis	0.14273
DB06710	Schizophrenia	0.23994
DB06710	Systemic infection	0.06743
DB06710	Systemic scleroderma	0.05369

DB06710	Tropical spastic paraparesis	0.20521
DB06710	Urogenital abnormalities	0.8261
DB06710	Vitiligo	0.17102
DB06710	Yersinia infection	0.26183
DB08804	Infertility, Male	0.56252
DB08804	Adenovirus infection	0.04824
DB08804	Amyotrophic lateral sclerosis	0.27968
DB08804	Atherosclerosis	0.21195
DB08804	Azoospermia	0.47517
DB08804	Cancer	0.09105
DB08804	Cholelithiasis	0.51307
DB08804	Depression	0.26794
DB08804	Diabetes mellitus	0.15005
DB08804	Endometriosis	0.18982
DB08804	Huntington disease	0.16929
DB08804	Hyperglycemia	0.1472
DB08804	Hypertension	0.07113
DB08804	Hypogonadism	1.44721
DB08804	Infertility	0.36633
DB08804	Migraine	0.46671
DB08804	Muscular atrophy	0.67322
DB08804	Obesity	0.06723
DB08804	Oligospermia	0.75171
DB08804	Osteoporosis	0.38609
DB08804	Phobic anxiety disorder	1.5
DB08804	Polycystic ovary syndrome	0.08595
DB08804	Prostate cancer	0.13757
DB08804	Psychotic disorder	0.1535
DB08804	Rabies	0.24806
DB08804	Rheumatoid arthritis	0.14273
DB08804	Schizophrenia	0.23994
DB08804	Systemic infection	0.06743
DB08804	Systemic scleroderma	0.05369
DB08804	Tropical spastic paraparesis	0.20521
DB08804	Urogenital abnormalities	0.8261
DB08804	Vitiligo	0.17102
DB08804	Yersinia infection	0.26183
DB08899	Infertility, Male	0.56252
DB08899	Adenovirus infection	0.04824
DB08899	Amyotrophic lateral sclerosis	0.27968
DB08899	Atherosclerosis	0.21195
DB08899	Azoospermia	0.47517
DB08899	Cancer	0.09105
DB08899	Cholelithiasis	0.51307
DB08899	Depression	0.26794
DB08899	Diabetes mellitus	0.15005
DB08899	Endometriosis	0.18982

DB08899	Huntington disease	0.16929
DB08899	Hyperglycemia	0.1472
DB08899	Hypertension	0.07113
DB08899	Hypogonadism	1.44721
DB08899	Infertility	0.36633
DB08899	Migraine	0.46671
DB08899	Muscular atrophy	0.67322
DB08899	Obesity	0.06723
DB08899	Oligospermia	0.75171
DB08899	Osteoporosis	0.38609
DB08899	Phobic anxiety disorder	1.5
DB08899	Polycystic ovary syndrome	0.08595
DB08899	Prostate cancer	0.13757
DB08899	Psychotic disorder	0.1535
DB08899	Rabies	0.24806
DB08899	Rheumatoid arthritis	0.14273
DB08899	Schizophrenia	0.23994
DB08899	Systemic infection	0.06743
DB08899	Systemic scleroderma	0.05369
DB08899	Tropical spastic paraparesis	0.20521
DB08899	Urogenital abnormalities	0.8261
DB08899	Vitiligo	0.17102
DB08899	Yersinia infection	0.26183
DB00536	Adenoma	0.07001
DB00536	Alcoholic liver disease	0.16667
DB00536	Atherosclerosis	0.02858
DB00536	Barrett's esophagus	0.1032
DB00536	Cancer	0.02478
DB00536	Colon cancer	0.02423
DB00536	Conduct disorder	0.13608
DB00536	Congenital abnormality	0.05541
DB00536	Drug abuse	0.03824
DB00536	Esophageal tumor	0.08909
DB00536	Heart disease	0.12309
DB00536	Hypertension	0.03227
DB00536	Kidney failure	0.04623
DB00536	Liver cancer	0.03169
DB00536	Lung cancer	0.02851
DB00536	Neoplasm metastasis	0.03322
DB00536	Pancreas disease	0.08513
DB00536	Pancreatitis	0.06376
DB00536	Pervasive development disorder	0.24442
DB00536	Rabies	0.04536
DB00536	Schizophrenia	0.07495
DB00536	Squamous cell cancer	0.04167
DB00536	Stomach cancer	0.03438
DB00536	Ulcerative colitis	0.0813

DB00822	Stress disorder, post-traumatic	0.25
DB00822	Adenoma	0.12127
DB00822	Alcoholic liver disease	0.28868
DB00822	Atherosclerosis	0.04951
DB00822	Autoimmune disease	0.0767
DB00822	Behavior disease	0.10911
DB00822	Colon cancer	0.04196
DB00822	Conduct disorder	0.2357
DB00822	Drug abuse	0.06623
DB00822	Epilepsy	0.1
DB00822	Esophageal tumor	0.1543
DB00822	Heart disease	0.2132
DB00822	Hypertension	0.1118
DB00822	Kidney failure	0.08006
DB00822	Liver cancer	0.05488
DB00822	Lung cancer	0.04939
DB00822	Multiple system atrophy	0.40825
DB00822	Pancreas disease	0.14744
DB00822	Pancreatitis	0.11043
DB00822	Psychotic disorder	0.11323
DB00822	Squamous cell cancer	0.07217
DB00014	Adrenal gland tumor	0.5858
DB00014	Alzheimer's disease	0.11633
DB00014	Breast cancer	0.09056
DB00014	Cushing syndrome	1.2132
DB00014	Embryoma	0.08302
DB00014	Endometrium cancer	0.67543
DB00014	Pancreas cancer	0.1432
DB00014	Primary tumor	0.19413
DB00014	Urogenital abnormalities	0.60362
DB00032	Adrenal gland tumor	0.5858
DB00032	Alzheimer's disease	0.11633
DB00032	Breast cancer	0.09056
DB00032	Cushing syndrome	1.2132
DB00032	Embryoma	0.08302
DB00032	Endometrium cancer	0.67543
DB00032	Pancreas cancer	0.1432
DB00032	Primary tumor	0.19413
DB00032	Urogenital abnormalities	0.60362
DB00044	Adrenal gland tumor	0.67411
DB00044	Alzheimer's disease	0.13742
DB00044	Breast cancer	0.10465
DB00044	Cushing syndrome	1.30151
DB00044	Embryoma	0.08302
DB00044	Endometrium cancer	0.74647
DB00044	Pancreas cancer	0.1432
DB00044	Primary tumor	0.19413

DB00044	Urogenital abnormalities	0.6819
DB00050	Adrenal gland tumor	0.5858
DB00050	Alzheimer's disease	0.11633
DB00050	Breast cancer	0.09056
DB00050	Cushing syndrome	1.2132
DB00050	Embryoma	0.08302
DB00050	Endometrium cancer	0.67543
DB00050	Pancreas cancer	0.1432
DB00050	Primary tumor	0.19413
DB00050	Urogenital abnormalities	0.60362
DB00097	Adrenal gland tumor	0.5858
DB00097	Alzheimer's disease	0.11633
DB00097	Breast cancer	0.09056
DB00097	Cushing syndrome	1.2132
DB00097	Embryoma	0.08302
DB00097	Endometrium cancer	0.67543
DB00097	Pancreas cancer	0.1432
DB00097	Primary tumor	0.19413
DB00097	Urogenital abnormalities	0.60362
DB06719	Adrenal gland tumor	0.5858
DB06719	Alzheimer's disease	0.11633
DB06719	Breast cancer	0.09056
DB06719	Cushing syndrome	1.2132
DB06719	Embryoma	0.08302
DB06719	Endometrium cancer	0.67543
DB06719	Pancreas cancer	0.1432
DB06719	Primary tumor	0.19413
DB06719	Urogenital abnormalities	0.60362
DB00252	Myoclonic epilepsy, Juvenile	0.28868
DB00252	Autistic disorder	0.08575
DB00252	Brain disease	0.127
DB00252	Breast cancer	0.03402
DB00252	Central nervous system disease	0.17678
DB00252	Congenital abnormality	0.0533
DB00252	Drug abuse	0.13245
DB00252	Heart disease	0.2132
DB00252	Infantile spasms	0.26726
DB00252	Intestinal disease	0.17678
DB00252	Intractable epilepsy	0.25
DB00252	Ischemia	0.09054
DB00252	Long QT syndrome	0.20412
DB00252	Migraine	0.13131
DB00252	Myopathy	0.08575
DB00252	Nervous system disease	0.18257
DB00252	Neuropathy	0.11323
DB00252	Rheumatism	0.16667
DB00252	Sudden infant death syndrome	0.1543

DB00273	Myoclonic epilepsy, Juvenile	0.08909
DB00273	Anemia	0.04652
DB00273	Autistic disorder	0.02646
DB00273	Brain disease	0.03919
DB00273	Breast cancer	0.0105
DB00273	Central nervous system disease	0.05455
DB00273	Down syndrome	0.02487
DB00273	Drug abuse	0.04088
DB00273	Hypogonadism	0.09759
DB00273	Infantile spasms	0.08248
DB00273	Intractable epilepsy	0.07715
DB00273	Migraine	0.04052
DB00273	Myopathy	0.02646
DB00273	Nervous system disease	0.05634
DB00273	Neuropathy	0.03494
DB00273	Pancreas cancer	0.02263
DB00273	Pancreatitis	0.03408
DB00273	Retinitis pigmentosa	0.04364
DB00273	Rheumatism	0.05143
DB00273	Ulcerative colitis	0.02251
DB00273	Yersinia infection	0.0259
DB01121	Myoclonic epilepsy, Juvenile	0.40825
DB01121	Autistic disorder	0.12127
DB01121	Brain disease	0.17961
DB01121	Central nervous system disease	0.25
DB01121	Drug abuse	0.09366
DB01121	Infantile spasms	0.37796
DB01121	Intractable epilepsy	0.35355
DB01121	Migraine	0.1857
DB01121	Myopathy	0.12127
DB01121	Nervous system disease	0.2582
DB01121	Neuropathy	0.16013
DB01121	Rheumatism	0.2357
DB01438	Myoclonic epilepsy, Juvenile	0.40825
DB01438	Autistic disorder	0.12127
DB01438	Brain disease	0.17961
DB01438	Central nervous system disease	0.25
DB01438	Drug abuse	0.09366
DB01438	Infantile spasms	0.37796
DB01438	Intractable epilepsy	0.35355
DB01438	Migraine	0.1857
DB01438	Myopathy	0.12127
DB01438	Nervous system disease	0.2582
DB01438	Neuropathy	0.16013
DB01438	Rheumatism	0.2357
DB04930	Myoclonic epilepsy, Juvenile	0.40825
DB04930	Autistic disorder	0.12127

DB04930	Brain disease	0.17961
DB04930	Central nervous system disease	0.25
DB04930	Drug abuse	0.09366
DB04930	Infantile spasms	0.37796
DB04930	Intractable epilepsy	0.35355
DB04930	Migraine	0.1857
DB04930	Myopathy	0.12127
DB04930	Nervous system disease	0.2582
DB04930	Neuropathy	0.16013
DB04930	Rheumatism	0.2357
DB00361	Bipolar disorder	0.07617
DB00361	Breast cancer	0.11506
DB00361	Cancer	0.044
DB00361	Clear cell carcinoma	0.39196
DB00361	Diabetes mellitus	0.02148
DB00361	Down syndrome	0.05403
DB00361	Female reproductive cancer	0.07461
DB00361	Gastritis	0.04812
DB00361	HIV infection	0.04089
DB00361	Kidney cancer	0.17285
DB00361	Liver cancer	0.2013
DB00361	Mental retardation	0.07871
DB00361	Prostate cancer	0.02266
DB00361	Retinitis pigmentosa	0.11865
DB00361	Testicular dysfunction	0.04022
DB00361	Yersinia infection	0.03801
DB00541	Bipolar disorder	0.05992
DB00541	Breast cancer	0.13499
DB00541	Cancer	0.07006
DB00541	Clear cell carcinoma	0.30834
DB00541	Diabetes mellitus	0.0169
DB00541	Down syndrome	0.04251
DB00541	Female reproductive cancer	0.0587
DB00541	Gastritis	0.03785
DB00541	HIV infection	0.03217
DB00541	Kidney cancer	0.13598
DB00541	Liver cancer	0.17857
DB00541	Mental retardation	0.06192
DB00541	Prostate cancer	0.01783
DB00541	Retinitis pigmentosa	0.09334
DB00541	Testicular dysfunction	0.03164
DB00541	Yersinia infection	0.0299
DB00570	Hypertension, Pulmonary	0.56926
DB00570	Infertility, Male	0.15874
DB00570	Abortion	0.04134
DB00570	Adenoma	0.07077
DB00570	Adenovirus infection	0.01874

DB00570	Alzheimer's disease	0.07716
DB00570	Amyotrophic lateral sclerosis	0.05381
DB00570	Aortic aneurysm	0.00591
DB00570	Aplastic anemia	0.06256
DB00570	Aseptic necrosis of bone	0.04642
DB00570	Atherosclerosis	0.05222
DB00570	Barrett's esophagus	0.03455
DB00570	Bipolar disorder	0.15041
DB00570	Bladder cancer	0.05854
DB00570	Brain tumor	0.00561
DB00570	Breast cancer	0.10427
DB00570	Bronchial disease	0.02128
DB00570	Bronchial hyperreactivity	0.23091
DB00570	Cancer	0.15934
DB00570	Capillaries disease	0.09933
DB00570	Carcinoma	0.00978
DB00570	Cerebrovascular disorder	0.15872
DB00570	Cervical cancer	0.00854
DB00570	Cholelithiasis	0.13867
DB00570	Cholestasis	0.0201
DB00570	Choriocarcinoma	0.10242
DB00570	Clear cell carcinoma	0.12
DB00570	Colon cancer	0.02594
DB00570	Congenital abnormality	0.03917
DB00570	Craniosynostosis	0.96947
DB00570	Dental plaque	0.09012
DB00570	Depression	0.08772
DB00570	Dermatitis	0.07413
DB00570	Diabetes mellitus	0.17803
DB00570	Disseminated cancer	0.08531
DB00570	Down syndrome	0.14721
DB00570	Eating disorder	0.06448
DB00570	Embryoma	0.06654
DB00570	Endometriosis	0.06855
DB00570	Esophagus cancer	0.03961
DB00570	Ewings sarcoma	0.09367
DB00570	Female reproductive cancer	0.02284
DB00570	Gastritis	0.01473
DB00570	Glaucoma	0.1051
DB00570	HIV infection	0.01637
DB00570	Hepatitis	0.70598
DB00570	Herpes	0.10168
DB00570	Hyperlipidemia	0.17594
DB00570	Hypertension	0.07009
DB00570	Infection	0.01919
DB00570	Infertility	0.10142
DB00570	Keratoconjunctivitis Sicca	0.02778

DB00570	Kidney cancer	0.05292
DB00570	Kidney failure	0.08902
DB00570	Late pregnancy	0.21798
DB00570	Leukemia	0.02104
DB00570	Leukoencephalopathy	0.03542
DB00570	Lewy body disease	0.29398
DB00570	Liver cancer	0.07901
DB00570	Lung cancer	0.00385
DB00570	Lung disease	0.00763
DB00570	Lupus erythematosus	0.23824
DB00570	Lupus vulgaris	0.13468
DB00570	Lymphoma	0.04073
DB00570	Mental retardation	0.03882
DB00570	Migraine	0.12447
DB00570	Muscular dystrophies	0.0092
DB00570	Myasthenia Gravis	0.16853
DB00570	Neuroblastoma	0.00667
DB00570	Obesity	0.06625
DB00570	Oral cancer	0.02924
DB00570	Osteoporosis	0.10098
DB00570	Osteosarcoma	0.00977
DB00570	Ovarian cancer	0.03491
DB00570	Ovarian disease	0.1583
DB00570	Ovarian failure	0.207
DB00570	Pancreas cancer	0.00923
DB00570	Pancreas disease	0.01937
DB00570	Panic disorder	0.15899
DB00570	Parkinson disease	0.07707
DB00570	Pervasive development disorder	0.08184
DB00570	Polyarthritis	0.10589
DB00570	Pre-Eclampsia	0.00908
DB00570	Primary biliary cirrhosis	0.12069
DB00570	Prostate cancer	0.06136
DB00570	Rabies	0.11154
DB00570	Renal tubular acidosis	0.09136
DB00570	Retinitis pigmentosa	0.03632
DB00570	Rheumatoid arthritis	0.15631
DB00570	Salmonella infection	0.06261
DB00570	Schizophrenia	0.30629
DB00570	Scleroderma	0.05951
DB00570	Sicca syndrome	0.4996
DB00570	Stomach cancer	0.06639
DB00570	Stroke	0.0553
DB00570	Synovitis	0.18351
DB00570	Systemic scleroderma	0.06816
DB00570	Testicular dysfunction	0.01231
DB00570	Testicular tumor	0.04143

DB00570	Thymoma	0.14243
DB00570	Tuberous sclerosis	0.00569
DB00570	Ulcerative colitis	0.05748
DB00570	Uterine disease	0.18374
DB00570	Vaccinia	0.63686
DB00570	Vascular disease	0.10258
DB00570	Virus disease	0.00613
DB00570	Vitiligo	0.16853
DB00570	Yersinia infection	0.01164
DB01179	Kidney tubular necrosis, acute	0.09182
DB01179	Amyotrophic lateral sclerosis	0.02756
DB01179	Aortic valve disease	0.08459
DB01179	Bipolar disorder	0.03861
DB01179	Brain tumor	0.0173
DB01179	Breast cancer	0.13154
DB01179	Cancer	0.06946
DB01179	Celiac disease	0.05029
DB01179	Charcot-Marie-Tooth disease	0.14965
DB01179	Chronic obstructive airway disease	0.04804
DB01179	Clear cell carcinoma	0.19868
DB01179	Cockayne syndrome	0.09434
DB01179	Colon cancer	0.06478
DB01179	Common cold	0.06698
DB01179	Diabetes mellitus	0.02629
DB01179	Down syndrome	0.08583
DB01179	Embryoma	0.01166
DB01179	Emphysema	0.06194
DB01179	Epilepsy	0.02413
DB01179	Ewings sarcoma	0.08954
DB01179	Eye disease	0.07408
DB01179	Fanconi's anemia	0.02959
DB01179	Female reproductive cancer	0.03782
DB01179	Gastritis	0.02439
DB01179	HIV infection	0.05275
DB01179	Heart disease	0.21571
DB01179	Heart failure	0.0366
DB01179	Helicobacter infection	0.07341
DB01179	Hereditary disease	0.03778
DB01179	Herpes	0.01301
DB01179	Infection	0.01421
DB01179	Infertility	0.04358
DB01179	Ischemia	0.0376
DB01179	Kaposi sarcoma	0.05062
DB01179	Kidney cancer	0.15548
DB01179	Leigh disease	0.04499
DB01179	Leukemia	0.01719
DB01179	Leukoencephalopathy	0.03282

DB01179	Lipodystrophy	0.1039
DB01179	Liver cancer	0.15352
DB01179	Lung cancer	0.03698
DB01179	Lymphoma	0.05875
DB01179	Melanoma	0.0281
DB01179	Meningioma	0.13463
DB01179	Mental retardation	0.0399
DB01179	Metabolism disease	0.06535
DB01179	Muscular atrophy	0.07534
DB01179	Muscular dystrophies	0.0518
DB01179	Nephroblastoma	0.13446
DB01179	Neuroblastoma	0.04254
DB01179	Neuropathy	0.06115
DB01179	Parkinson disease	0.03311
DB01179	Pituitary tumor	0.07722
DB01179	Prostate cancer	0.02991
DB01179	Renal tubular acidosis	0.03925
DB01179	Retinitis pigmentosa	0.15658
DB01179	Rheumatoid arthritis	0.01552
DB01179	Schizophrenia	0.02591
DB01179	Testicular dysfunction	0.02039
DB01179	Tuberous sclerosis	0.04833
DB01179	Uterine fibroids	0.07736
DB01179	Virus disease	0.03451
DB01179	Werner syndrome	0.07865
DB01179	Yersinia infection	0.01926
DB00313	Autistic disorder	0.05423
DB00313	Rabies	0.04969
DB00534	Hyperaldosteronism	0.22361
DB01346	Attention deficit hyperactivity disorder	0.18672
DB01346	Breast cancer	0.02152
DB01346	Congenital abnormality	0.03371
DB01346	Drug abuse	0.04189
DB01346	Heart disease	0.13484
DB01346	Hypertension	0.07236
DB01346	Intestinal disease	0.1118
DB01346	Ischemia	0.05726
DB01346	Long QT syndrome	0.1291
DB01346	Nervous system disease	0.17049
DB01346	Obesity	0.06839
DB01346	Psychotic disorder	0.12749
DB01346	Schizophrenia	0.11308
DB01346	Sudden infant death syndrome	0.09759
DB00409	Alzheimer's disease	0.03219
DB00409	Anorexia nervosa	0.09129
DB00409	Autistic disorder	0.05423
DB00409	Behavior disease	0.06901

DB00409	Bipolar disorder	0.05064
DB00409	Breast cancer	0.02152
DB00409	Choriocarcinoma	0.1291
DB00409	Chronic fatigue syndrome	0.15811
DB00409	Colon cancer	0.02654
DB00409	Depression	0.05872
DB00409	Dermatitis	0.04066
DB00409	Drug-Induced dyskinesia	0.16903
DB00409	Hypertension	0.03536
DB00409	Obesity	0.0344
DB00409	Obsessive-compulsive disorder	0.13484
DB00409	Panic disorder	0.09759
DB00409	Psychotic disorder	0.07161
DB00409	Rheumatoid arthritis	0.02737
DB00409	Stroke	0.05064
DB00514	Hypertension, Pulmonary	0.11471
DB00514	Alzheimer's disease	0.03599
DB00514	Anorexia nervosa	0.10206
DB00514	Atherosclerosis	0.03501
DB00514	Autistic disorder	0.06063
DB00514	Behavior disease	0.07715
DB00514	Bipolar disorder	0.05661
DB00514	Breast cancer	0.02406
DB00514	Chronic fatigue syndrome	0.17678
DB00514	Chronic obstructive airway disease	0.0559
DB00514	Colon cancer	0.02967
DB00514	Congenital heart disease	0.16667
DB00514	Depression	0.06565
DB00514	Dermatitis	0.04545
DB00514	Drug abuse	0.04683
DB00514	Epilepsy	0.14142
DB00514	Fibromyalgia	0.17678
DB00514	Generalized anxiety disorder	0.14434
DB00514	Heart failure	0.0533
DB00514	Herpes	0.07143
DB00514	Migraine	0.09285
DB00514	Neurotic disorder	0.15811
DB00514	Obesity	0.03846
DB00514	Obsessive-compulsive disorder	0.15076
DB00514	Panic disorder	0.10911
DB00514	Pervasive development disorder	0.1291
DB00514	Psychotic disorder	0.08006
DB00514	Pulmonary hypertension	0.25
DB00514	Stroke	0.05661
DB00514	Sudden infant death syndrome	0.10911
DB00514	Ulcerative colitis	0.05157
DB00652	Alzheimer's disease	0.04156

DB00652	Breast cancer	0.02778
DB00152	Brain disease	0.127
DB00152	Vitamin B deficiency	0.26726
DB00112	Pemphigoid, Bullous	0.13645
DB00112	Pleural effusion, Malignant	0.0373
DB00112	Purpura, Thrombocytopenic, Idiopathic	0.03483
DB00112	Abortion	0.03953
DB00112	Alopecia	0.18091
DB00112	Alzheimer's disease	0.04497
DB00112	Amyotrophic lateral sclerosis	0.1094
DB00112	Antiphospholipid syndrome	0.10541
DB00112	Aortic aneurysm	0.04404
DB00112	Asthma	0.12848
DB00112	Atherosclerosis	0.22151
DB00112	Autoimmune disease	0.1029
DB00112	Breast cancer	0.04564
DB00112	Brucellosis	0.09535
DB00112	Cancer	0.10665
DB00112	Capillaries disease	0.4517
DB00112	Cardiovascular disease	0.30868
DB00112	Celiac disease	0.05199
DB00112	Chronic obstructive airway disease	0.45199
DB00112	Communicable disease	0.06901
DB00112	Cystic fibrosis	0.04518
DB00112	Depression	0.0344
DB00112	Dermatitis	0.09813
DB00112	Diabetes mellitus	0.13282
DB00112	Drug abuse	0.02536
DB00112	Embryoma	0.0195
DB00112	Endometriosis	0.08065
DB00112	Enteritis	0.18141
DB00112	Familial Mediterranean fever	0.6387
DB00112	Generalized anxiety disorder	0.07609
DB00112	Glomerulonephritis	0.07906
DB00112	IGA glomerulonephritis	0.05423
DB00112	Immune complex disease	0.59304
DB00112	Infection by cryptococcus neoformans	0.36515
DB00112	Kaposi sarcoma	0.06697
DB00112	Kidney disease	0.0378
DB00112	Kidney failure	0.33598
DB00112	Leukemia	0.02275
DB00112	Leukoencephalopathy	0.25275
DB00112	Lupus erythematosus	0.32467
DB00112	Lupus vulgaris	0.05976
DB00112	Macular degeneration	0.78482
DB00112	Malaria	0.53854
DB00112	Melanoma	0.02635

DB00112	Mucocutaneous lymph node syndrome	0.74364
DB00112	Multiple myeloma	0.06022
DB00112	Multiple sclerosis	0.03262
DB00112	Neoplasm metastasis	0.02573
DB00112	Obesity	0.22108
DB00112	Peptic ulcer	0.51809
DB00112	Periodontal disease	0.07454
DB00112	Periodontitis	0.09645
DB00112	Polyneuropathy	0.80339
DB00112	Pre-Eclampsia	0.39036
DB00112	Primary hyperparathyroidism	0.04336
DB00112	Renal Cell cancer	0.03676
DB00112	Retinal disease	0.29439
DB00112	Rheumatic fever	0.08165
DB00112	Rheumatoid arthritis	0.03988
DB00112	Shigella infection	0.04594
DB00112	Sickle cell disease	0.04096
DB00112	Skin tumor	0.11435
DB00112	Stomach cancer	0.05113
DB00112	Systemic infection	0.35864
DB00112	Thalassemia	0.04294
DB00112	Thrombocytopenia	0.07906
DB00112	Vascular disease	0.05774
DB00112	Yersinia infection	0.03121
DB01120	Pemphigoid, Bullous	0.04246
DB01120	Pleural effusion, Malignant	0.04346
DB01120	Purpura, Thrombocytopenic, Idiopathic	0.04058
DB01120	Alopecia	0.05007
DB01120	Alzheimer's disease	0.02587
DB01120	Amyotrophic lateral sclerosis	0.12745
DB01120	Aortic aneurysm	0.0513
DB01120	Asthma	0.11959
DB01120	Atherosclerosis	0.23018
DB01120	Cancer	0.13673
DB01120	Capillaries disease	0.63332
DB01120	Cardiovascular disease	0.3596
DB01120	Chronic obstructive airway disease	0.56443
DB01120	Depression	0.04008
DB01120	Dermatitis	0.11432
DB01120	Diabetes mellitus	0.17256
DB01120	Drug abuse	0.02955
DB01120	Endometriosis	0.09395
DB01120	Familial Mediterranean fever	0.83152
DB01120	Kidney failure	0.38805
DB01120	Leukoencephalopathy	0.3509
DB01120	Lupus erythematosus	0.13118
DB01120	Macular degeneration	0.98491

DB01120	Malaria	0.69028
DB01120	Mucocutaneous lymph node syndrome	0.94023
DB01120	Obesity	0.28361
DB01120	Peptic ulcer	0.6834
DB01120	Polyneuropathy	1.02645
DB01120	Pre-Eclampsia	0.49467
DB01120	Primary hyperparathyroidism	0.05051
DB01120	Retinal disease	0.34295
DB01120	Rheumatoid arthritis	0.02391
DB01120	Shigella infection	0.05351
DB01120	Sickle cell disease	0.04771
DB01120	Systemic infection	0.33205
DB01120	Thalassemia	0.05002
DB01270	Pemphigoid, Bullous	0.04246
DB01270	Pleural effusion, Malignant	0.04346
DB01270	Purpura, Thrombocytopenic, Idiopathic	0.04058
DB01270	Alopecia	0.05007
DB01270	Alzheimer's disease	0.02587
DB01270	Amyotrophic lateral sclerosis	0.12745
DB01270	Aortic aneurysm	0.0513
DB01270	Asthma	0.11959
DB01270	Atherosclerosis	0.25068
DB01270	Cancer	0.14753
DB01270	Capillaries disease	0.72594
DB01270	Cardiovascular disease	0.3596
DB01270	Chronic obstructive airway disease	0.59717
DB01270	Depression	0.04008
DB01270	Dermatitis	0.11432
DB01270	Diabetes mellitus	0.18797
DB01270	Drug abuse	0.02955
DB01270	Endometriosis	0.09395
DB01270	Familial Mediterranean fever	0.90715
DB01270	Kidney failure	0.42121
DB01270	Leukoencephalopathy	0.39972
DB01270	Lupus erythematosus	0.13118
DB01270	Macular degeneration	1.04598
DB01270	Malaria	0.74467
DB01270	Mucocutaneous lymph node syndrome	1.00414
DB01270	Obesity	0.30614
DB01270	Peptic ulcer	0.75243
DB01270	Polyneuropathy	1.10473
DB01270	Pre-Eclampsia	0.52919
DB01270	Primary hyperparathyroidism	0.05051
DB01270	Retinal disease	0.34295
DB01270	Rheumatoid arthritis	0.02391
DB01270	Shigella infection	0.05351
DB01270	Sickle cell disease	0.04771

DB01270	Systemic infection	0.36565
DB01270	Thalassemia	0.05002
DB05294	Hemorrhagic fevers, Viral	0.05368
DB05294	Pemphigoid, Bullous	0.05449
DB05294	Pleural effusion, Malignant	0.40034
DB05294	Purpura, Thrombocytopenic, Idiopathic	0.05208
DB05294	Abortion	0.03065
DB05294	Adenovirus infection	0.21586
DB05294	Alopecia	0.03213
DB05294	Alzheimer's disease	0.0332
DB05294	Amyotrophic lateral sclerosis	0.08178
DB05294	Angiomyolipoma	0.74461
DB05294	Aortic aneurysm	0.03292
DB05294	Aplastic anemia	0.04451
DB05294	Asthma	0.321
DB05294	Atherosclerosis	0.18381
DB05294	Atopic rhinitis	0.89607
DB05294	Autistic disorder	0.03536
DB05294	Autoimmune disease	0.06085
DB05294	Barrett's esophagus	0.35464
DB05294	Breast cancer	0.03648
DB05294	Cancer	0.18708
DB05294	Capillaries disease	0.42102
DB05294	Cardiovascular disease	0.23075
DB05294	Charcot-Marie-Tooth disease	0.12818
DB05294	Cholelithiasis	0.53982
DB05294	Chronic obstructive airway disease	0.36736
DB05294	Colon cancer	0.01815
DB05294	Common wart	0.90473
DB05294	Cytomegalovirus infection	0.70795
DB05294	Depression	0.02572
DB05294	Dermatitis	0.07336
DB05294	Diabetes mellitus	0.27335
DB05294	Drug abuse	0.22411
DB05294	Endometriosis	0.25181
DB05294	Esotropia	0.57669
DB05294	Familial Mediterranean fever	0.54552
DB05294	Gastrointestinal tumor	0.56077
DB05294	Glaucoma	0.41669
DB05294	Hemorrhagic disorder	0.04201
DB05294	Herpes	0.0235
DB05294	Hypercholesterolemia	0.72623
DB05294	Intermediate coronary syndrome	0.05355
DB05294	Kidney failure	0.25424
DB05294	Leukemia	0.06346
DB05294	Leukoencephalopathy	0.23288
DB05294	Lupus erythematosus	0.29426

DB05294	Lymphoma	0.05698
DB05294	Macular degeneration	0.64165
DB05294	Malaria	0.45153
DB05294	Mucocutaneous lymph node syndrome	0.61343
DB05294	Muscular atrophy	0.13904
DB05294	Myopathy	0.03732
DB05294	Obesity	0.18554
DB05294	Optic atrophy	0.9551
DB05294	Oral cancer	0.28597
DB05294	Osteitis deformans	0.06748
DB05294	Papillary adenocarcinoma	1.05564
DB05294	Papillomavirus infection	0.0707
DB05294	Penile disease	0.05024
DB05294	Peptic ulcer	0.44943
DB05294	Pneumoconiosis	0.17798
DB05294	Polyneuropathy	0.67102
DB05294	Pre-Eclampsia	0.32287
DB05294	Primary hyperparathyroidism	0.46333
DB05294	Pulmonary fibrosis	0.05788
DB05294	Retinal disease	0.22007
DB05294	Rheumatoid arthritis	0.04398
DB05294	Schizophrenia	0.22928
DB05294	Shigella infection	0.03434
DB05294	Sickle cell disease	0.03062
DB05294	Skin cancer	0.4594
DB05294	Squamous cell cancer	0.05103
DB05294	Stroke	0.26296
DB05294	Subarachnoid hemorrhage	0.04793
DB05294	Systemic infection	0.21838
DB05294	Takayasu's arteritis	0.04481
DB05294	Temporal arteritis	0.05239
DB05294	Testicular dysfunction	0.29629
DB05294	Thalassemia	0.0321
DB05294	Thrombocytopenia	0.03993
DB05294	Thrombophlebitis	0.71563
DB05294	Yersinia infection	0.2718
DB06779	Hypertension, Pulmonary	0.06946
DB06779	Pemphigoid, Bullous	0.03933
DB06779	Pleural effusion, Malignant	0.04025
DB06779	Purpura, Thrombocytopenic, Idiopathic	0.03758
DB06779	Abruption placentae	0.37708
DB06779	Adenovirus infection	0.02525
DB06779	Adrenal gland hyperfunction	0.18898
DB06779	Alopecia	0.04637
DB06779	Alzheimer's disease	0.02396
DB06779	Amyotrophic lateral sclerosis	0.11804
DB06779	Aortic aneurysm	0.04751

DB06779	Asthma	0.11077
DB06779	Atherosclerosis	0.23008
DB06779	Bacterial infection	0.12779
DB06779	Breast cancer	0.03105
DB06779	CNS lymphoma	0.5
DB06779	Cancer	0.12093
DB06779	Capillaries disease	0.53758
DB06779	Cardiovascular disease	0.50545
DB06779	Chronic obstructive airway disease	0.50544
DB06779	Cirrhosis	0.18883
DB06779	Colon cancer	0.02967
DB06779	Common cold	0.13363
DB06779	Dental plaque	0.11137
DB06779	Depression	0.10277
DB06779	Dermatitis	0.15134
DB06779	Diabetes mellitus	0.19708
DB06779	Drug abuse	0.05106
DB06779	Embryoma	0.02632
DB06779	Endometriosis	0.08702
DB06779	Familial Mediterranean fever	0.73015
DB06779	Heart failure	0.04525
DB06779	Hemolytic-Uremic syndrome	0.08757
DB06779	Hemorrhagic disorder	0.07427
DB06779	Hypercholesterolemia	0.09623
DB06779	Hyperlipidemia	0.13216
DB06779	Hypertension	0.03953
DB06779	Ischemia	0.06402
DB06779	Kidney disease	0.05976
DB06779	Kidney failure	0.38914
DB06779	Leukemia	0.02125
DB06779	Leukoencephalopathy	0.33496
DB06779	Liver metastases	0.12658
DB06779	Lung cancer	0.03492
DB06779	Lupus erythematosus	0.16421
DB06779	Lymphatic metastasis	0.37708
DB06779	Macular degeneration	0.87991
DB06779	Malaria	0.61056
DB06779	Melanoma	0.04913
DB06779	Meningioma	0.1103
DB06779	Mucocutaneous lymph node syndrome	0.83702
DB06779	Neoplasm metastasis	0.07514
DB06779	Nephrosis	0.06683
DB06779	Nevus	0.26663
DB06779	Obesity	0.25076
DB06779	Osteomyelitis	0.0858
DB06779	Pancreas cancer	0.0454
DB06779	Pancreatitis	0.07809

DB06779	Peptic ulcer	0.59644
DB06779	Polyneuropathy	0.90928
DB06779	Pre-Eclampsia	0.4399
DB06779	Primary hyperparathyroidism	0.04678
DB06779	Primary tumor	0.06156
DB06779	Prostate cancer	0.04618
DB06779	Proteinuria	0.13284
DB06779	Rabies	0.08777
DB06779	Rectum cancer	0.24268
DB06779	Respiratory distress syndrome	0.13661
DB06779	Retinal disease	0.31764
DB06779	Rheumatism	0.06326
DB06779	Rheumatoid arthritis	0.05275
DB06779	Shigella infection	0.04956
DB06779	Sickle cell disease	0.14042
DB06779	Solid tumor	0.09202
DB06779	Stomach cancer	0.02561
DB06779	Systemic infection	0.34107
DB06779	Systemic scleroderma	0.08052
DB06779	Takayasu's arteritis	0.15811
DB06779	Thalassemia	0.04633
DB06779	Thrombophilia	0.26626
DB06779	Vitamin D deficiency	0.09636
DB08885	Pemphigoid, Bullous	0.04246
DB08885	Pleural effusion, Malignant	0.04346
DB08885	Purpura, Thrombocytopenic, Idiopathic	0.04058
DB08885	Alopecia	0.05007
DB08885	Alzheimer's disease	0.02587
DB08885	Amyotrophic lateral sclerosis	0.12745
DB08885	Aortic aneurysm	0.0513
DB08885	Asthma	0.11959
DB08885	Atherosclerosis	0.22109
DB08885	Cancer	0.15323
DB08885	Capillaries disease	0.59229
DB08885	Cardiovascular disease	0.3596
DB08885	Chronic obstructive airway disease	0.54992
DB08885	Depression	0.04008
DB08885	Dermatitis	0.11432
DB08885	Diabetes mellitus	0.16573
DB08885	Drug abuse	0.02955
DB08885	Endometriosis	0.09395
DB08885	Familial Mediterranean fever	0.79802
DB08885	Kidney failure	0.37335
DB08885	Leukoencephalopathy	0.32928
DB08885	Liver cancer	0.04481
DB08885	Lupus erythematosus	0.13118
DB08885	Macular degeneration	0.95786

DB08885	Malaria	0.66618
DB08885	Mucocutaneous lymph node syndrome	0.91192
DB08885	Obesity	0.27363
DB08885	Peptic ulcer	0.65281
DB08885	Polyneuropathy	0.99177
DB08885	Pre-Eclampsia	0.47938
DB08885	Primary hyperparathyroidism	0.05051
DB08885	Retinal disease	0.34295
DB08885	Rheumatoid arthritis	0.02391
DB08885	Shigella infection	0.05351
DB08885	Sickle cell disease	0.04771
DB08885	Systemic infection	0.31717
DB08885	Thalassemia	0.05002
DB00035	Autistic disorder	0.07001
DB00035	Conduct disorder	0.19245
DB00035	Hypertension	0.04564
DB00035	Kidney failure	0.06537
DB00035	Pervasive development disorder	0.14907
DB00035	Psychotic disorder	0.09245
DB00035	Rabies	0.06415
DB00067	Autistic disorder	0.07001
DB00067	Conduct disorder	0.19245
DB00067	Hypertension	0.04564
DB00067	Kidney failure	0.06537
DB00067	Pervasive development disorder	0.14907
DB00067	Psychotic disorder	0.09245
DB00067	Rabies	0.06415
DB00093	Autistic disorder	0.12127
DB00093	Conduct disorder	0.33333
DB00093	Hypertension	0.07906
DB00093	Pervasive development disorder	0.2582
DB00872	Autistic disorder	0.08575
DB00872	Conduct disorder	0.2357
DB00872	Hypertension	0.0559
DB00872	Kidney failure	0.08006
DB00872	Pervasive development disorder	0.18257
DB00872	Rabies	0.07857
DB02638	Autistic disorder	0.07001
DB02638	Conduct disorder	0.19245
DB02638	Hypertension	0.04564
DB02638	Kidney failure	0.06537
DB02638	Pervasive development disorder	0.14907
DB02638	Psychotic disorder	0.09245
DB02638	Rabies	0.06415
DB06212	Autistic disorder	0.08575
DB06212	Conduct disorder	0.2357
DB06212	Hypertension	0.0559

DB06212	Kidney failure	0.08006
DB06212	Pervasive development disorder	0.18257
DB06212	Rabies	0.07857
DB00290	Kidney tubular necrosis, acute	0.06191
DB00290	Adenoma	0.08813
DB00290	Alzheimer's disease	0.0569
DB00290	Amyotrophic lateral sclerosis	0.01859
DB00290	Aortic valve disease	0.03519
DB00290	Azoospermia	0.32624
DB00290	Barrett's esophagus	0.24968
DB00290	Breast cancer	0.05894
DB00290	Cancer	0.06983
DB00290	Carcinoma	0.06692
DB00290	Celiac disease	0.03391
DB00290	Chronic obstructive airway disease	0.03239
DB00290	Colon cancer	0.05064
DB00290	Common cold	0.04516
DB00290	Diabetes mellitus	0.01038
DB00290	Down syndrome	0.05043
DB00290	Emphysema	0.02577
DB00290	Endometriosis	0.12851
DB00290	Esophageal disease	0.65078
DB00290	Ewings sarcoma	0.06037
DB00290	Fanconi's anemia	0.01995
DB00290	HIV infection	0.01301
DB00290	Heart failure	0.14875
DB00290	Helicobacter infection	0.03054
DB00290	Ischemia	0.26317
DB00290	Kaposi sarcoma	0.03413
DB00290	Leukemia	0.01159
DB00290	Liver cancer	0.01952
DB00290	Lung cancer	0.05453
DB00290	Lymphoma	0.05069
DB00290	Melanoma	0.01895
DB00290	Meningioma	0.11617
DB00290	Multiple endocrine neoplasia	0.06738
DB00290	Oral cancer	0.17674
DB00290	Ovary cancer	0.11557
DB00290	Parkinson disease	0.02233
DB00290	Peptic esophagitis	0.53667
DB00290	Pituitary tumor	0.05206
DB00290	Prostate cancer	0.01975
DB00290	Renal tubular acidosis	0.02647
DB00290	Rheumatoid arthritis	0.10859
DB00290	Skin cancer	0.29532
DB00290	Squamous cell cancer	0.04663
DB00290	Stomach cancer	0.07022

DB00290	Stroke	0.15028
DB00290	Testicular dysfunction	0.18236
DB00290	Tuberous sclerosis	0.02011
DB00290	Ulcerative colitis	0.01895
DB00290	Uterine fibroids	0.05216
DB00290	Werner syndrome	0.20653
DB00187	Alzheimer's disease	0.14772
DB00187	Glaucoma	0.37044
DB00187	Heart failure	0.27175
DB00187	Hypertension	0.21495
DB00187	Obesity	0.20537
DB00187	Subarachnoid hemorrhage	0.52019
DB00195	Infertility, Male	0.09355
DB00195	Alimentary system disease	0.17411
DB00195	Alzheimer's disease	0.11168
DB00195	Arthritis	0.49484
DB00195	Atherosclerosis	0.20204
DB00195	Autistic disorder	0.32884
DB00195	Azoospermia	0.07075
DB00195	Breast cancer	0.01989
DB00195	Bronchial disease	0.98582
DB00195	Chronic obstructive airway disease	0.40566
DB00195	Conduct disorder	0.18128
DB00195	Cystic fibrosis	0.52253
DB00195	Dermatitis	0.23238
DB00195	Diabetes mellitus	0.1419
DB00195	Drug abuse	0.19656
DB00195	Enteritis	0.06222
DB00195	Epilepsy	0.13443
DB00195	Glaucoma	0.64434
DB00195	Gram-Negative bacterial infection	0.12423
DB00195	Graves' disease	0.56559
DB00195	Heart failure	0.50615
DB00195	Hypertension	0.40424
DB00195	Infertility	0.05977
DB00195	Ischemia	0.11474
DB00195	Liver cancer	0.0397
DB00195	Lung cancer	0.19665
DB00195	Malaria	0.60323
DB00195	Metabolism disease	0.55751
DB00195	Movement disorder	0.26525
DB00195	Myopathy	0.38199
DB00195	Neurodegenerative disorder	0.14667
DB00195	Obesity	0.36636
DB00195	Oligospermia	0.12882
DB00195	Polycystic kidney	0.1121
DB00195	Polycystic ovary syndrome	0.34105

DB00195	Premature birth	0.84618
DB00195	Prostate cancer	0.16986
DB00195	Respiratory tract disease	0.08648
DB00195	Rheumatoid arthritis	0.14875
DB00195	Sickle cell disease	0.34655
DB00195	Sinusitis	0.08024
DB00195	Subarachnoid hemorrhage	0.86492
DB00195	Testicular dysfunction	0.03955
DB00221	Alzheimer's disease	0.14772
DB00221	Glaucoma	0.37044
DB00221	Heart failure	0.27175
DB00221	Hypertension	0.21495
DB00221	Obesity	0.20537
DB00221	Subarachnoid hemorrhage	0.52019
DB00264	Infertility, Male	0.09355
DB00264	Alimentary system disease	0.17411
DB00264	Alzheimer's disease	0.11168
DB00264	Arthritis	0.49484
DB00264	Atherosclerosis	0.20204
DB00264	Autistic disorder	0.32884
DB00264	Azoospermia	0.07075
DB00264	Breast cancer	0.01989
DB00264	Bronchial disease	0.98582
DB00264	Chronic obstructive airway disease	0.40566
DB00264	Conduct disorder	0.18128
DB00264	Cystic fibrosis	0.52253
DB00264	Dermatitis	0.23238
DB00264	Diabetes mellitus	0.1419
DB00264	Drug abuse	0.19656
DB00264	Enteritis	0.06222
DB00264	Epilepsy	0.13443
DB00264	Glaucoma	0.64434
DB00264	Gram-Negative bacterial infection	0.12423
DB00264	Graves' disease	0.56559
DB00264	Heart failure	0.50615
DB00264	Hypertension	0.40424
DB00264	Infertility	0.05977
DB00264	Ischemia	0.11474
DB00264	Liver cancer	0.0397
DB00264	Lung cancer	0.19665
DB00264	Malaria	0.60323
DB00264	Metabolism disease	0.55751
DB00264	Movement disorder	0.26525
DB00264	Myopathy	0.38199
DB00264	Neurodegenerative disorder	0.14667
DB00264	Obesity	0.36636
DB00264	Oligospermia	0.12882

DB00264	Polycystic kidney	0.1121
DB00264	Polycystic ovary syndrome	0.34105
DB00264	Premature birth	0.84618
DB00264	Prostate cancer	0.16986
DB00264	Respiratory tract disease	0.08648
DB00264	Rheumatoid arthritis	0.14875
DB00264	Sickle cell disease	0.34655
DB00264	Sinusitis	0.08024
DB00264	Subarachnoid hemorrhage	0.86492
DB00264	Testicular dysfunction	0.03955
DB00335	Alzheimer's disease	0.14772
DB00335	Glaucoma	0.37044
DB00335	Heart failure	0.27175
DB00335	Hypertension	0.21495
DB00335	Obesity	0.20537
DB00335	Subarachnoid hemorrhage	0.52019
DB00368	Infertility, Male	0.08591
DB00368	Alimentary system disease	0.1599
DB00368	Alzheimer's disease	0.07859
DB00368	Amnionitis	0.08771
DB00368	Arthritis	0.41312
DB00368	Atherosclerosis	0.18437
DB00368	Attention deficit hyperactivity disorder	0.21118
DB00368	Autistic disorder	0.26159
DB00368	Azoospermia	0.06497
DB00368	Breast cancer	0.01827
DB00368	Bronchial disease	0.82455
DB00368	Cholelithiasis	0.06325
DB00368	Chronic obstructive airway disease	0.33531
DB00368	Common cold	0.08452
DB00368	Conduct disorder	0.16648
DB00368	Cystic fibrosis	0.43229
DB00368	Dermatitis	0.18312
DB00368	Diabetes mellitus	0.12943
DB00368	Drug abuse	0.14931
DB00368	Encephalopathies	0.04428
DB00368	Endometrium cancer	0.0767
DB00368	Enteritis	0.05714
DB00368	Epilepsy	0.12346
DB00368	Glaucoma	0.48069
DB00368	Gram-Negative bacterial infection	0.11409
DB00368	Graves' disease	0.46053
DB00368	Heart failure	0.39381
DB00368	Hypertension	0.46512
DB00368	Infertility	0.05489
DB00368	Ischemia	0.10537
DB00368	Kidney failure	0.10742

DB00368	Liver cancer	0.03646
DB00368	Lung cancer	0.15733
DB00368	Malaria	0.49213
DB00368	Metabolism disease	0.50929
DB00368	Movement disorder	0.2436
DB00368	Myopathy	0.31041
DB00368	Nervous system disease	0.19111
DB00368	Neurodegenerative disorder	0.1347
DB00368	Obesity	0.37776
DB00368	Oligospermia	0.1183
DB00368	Polycystic kidney	0.10295
DB00368	Polycystic ovary syndrome	0.31097
DB00368	Pre-Eclampsia	0.03727
DB00368	Premature birth	0.69631
DB00368	Prostate cancer	0.15512
DB00368	Respiratory tract disease	0.07942
DB00368	Rheumatoid arthritis	0.13558
DB00368	Schizophrenia	0.06401
DB00368	Sickle cell disease	0.25415
DB00368	Sinusitis	0.07369
DB00368	Subarachnoid hemorrhage	0.59342
DB00368	Systemic infection	0.03627
DB00368	Testicular dysfunction	0.03632
DB00373	Infertility, Male	0.09355
DB00373	Alimentary system disease	0.17411
DB00373	Alzheimer's disease	0.10234
DB00373	Arthritis	0.47874
DB00373	Atherosclerosis	0.19296
DB00373	Autistic disorder	0.3131
DB00373	Azoospermia	0.07075
DB00373	Breast cancer	0.01989
DB00373	Bronchial disease	0.95435
DB00373	Chronic obstructive airway disease	0.39116
DB00373	Conduct disorder	0.18128
DB00373	Cystic fibrosis	0.504
DB00373	Dermatitis	0.22058
DB00373	Diabetes mellitus	0.13507
DB00373	Drug abuse	0.1844
DB00373	Enteritis	0.06222
DB00373	Epilepsy	0.13443
DB00373	Glaucoma	0.60108
DB00373	Gram-Negative bacterial infection	0.12423
DB00373	Graves' disease	0.54265
DB00373	Heart failure	0.47849
DB00373	Hypertension	0.38372
DB00373	Infertility	0.05977
DB00373	Ischemia	0.11474

DB00373	Liver cancer	0.0397
DB00373	Lung cancer	0.18759
DB00373	Malaria	0.57914
DB00373	Metabolism disease	0.53673
DB00373	Movement disorder	0.26525
DB00373	Myopathy	0.36626
DB00373	Neurodegenerative disorder	0.14667
DB00373	Obesity	0.34639
DB00373	Oligospermia	0.12882
DB00373	Polycystic kidney	0.1121
DB00373	Polycystic ovary syndrome	0.32386
DB00373	Premature birth	0.81471
DB00373	Prostate cancer	0.16309
DB00373	Respiratory tract disease	0.08648
DB00373	Rheumatoid arthritis	0.14081
DB00373	Sickle cell disease	0.32158
DB00373	Sinusitis	0.08024
DB00373	Subarachnoid hemorrhage	0.78667
DB00373	Testicular dysfunction	0.03955
DB00521	Infertility, Male	0.09355
DB00521	Alimentary system disease	0.17411
DB00521	Alzheimer's disease	0.11168
DB00521	Arthritis	0.49484
DB00521	Atherosclerosis	0.20204
DB00521	Autistic disorder	0.32884
DB00521	Azoospermia	0.07075
DB00521	Breast cancer	0.01989
DB00521	Bronchial disease	0.98582
DB00521	Chronic obstructive airway disease	0.40566
DB00521	Conduct disorder	0.18128
DB00521	Cystic fibrosis	0.52253
DB00521	Dermatitis	0.23238
DB00521	Diabetes mellitus	0.1419
DB00521	Drug abuse	0.19656
DB00521	Enteritis	0.06222
DB00521	Epilepsy	0.13443
DB00521	Glaucoma	0.64434
DB00521	Gram-Negative bacterial infection	0.12423
DB00521	Graves' disease	0.56559
DB00521	Heart failure	0.50615
DB00521	Hypertension	0.40424
DB00521	Infertility	0.05977
DB00521	Ischemia	0.11474
DB00521	Liver cancer	0.0397
DB00521	Lung cancer	0.19665
DB00521	Malaria	0.60323
DB00521	Metabolism disease	0.55751

DB00521	Movement disorder	0.26525
DB00521	Myopathy	0.38199
DB00521	Neurodegenerative disorder	0.14667
DB00521	Obesity	0.36636
DB00521	Oligospermia	0.12882
DB00521	Polycystic kidney	0.1121
DB00521	Polycystic ovary syndrome	0.34105
DB00521	Premature birth	0.84618
DB00521	Prostate cancer	0.16986
DB00521	Respiratory tract disease	0.08648
DB00521	Rheumatoid arthritis	0.14875
DB00521	Sickle cell disease	0.34655
DB00521	Sinusitis	0.08024
DB00521	Subarachnoid hemorrhage	0.86492
DB00521	Testicular dysfunction	0.03955
DB00571	Infertility, Male	0.05392
DB00571	Abortion	0.03465
DB00571	Alimentary system disease	0.10035
DB00571	Alzheimer's disease	0.06723
DB00571	Amnionitis	0.12403
DB00571	Amyotrophic lateral sclerosis	0.27109
DB00571	Anorexia nervosa	0.19428
DB00571	Arthritis	0.29013
DB00571	Atherosclerosis	0.15054
DB00571	Attention deficit hyperactivity disorder	0.15048
DB00571	Autistic disorder	0.19434
DB00571	Azoospermia	0.04078
DB00571	Behavior disease	0.75469
DB00571	Bipolar disorder	0.25587
DB00571	Breast cancer	0.01146
DB00571	Bronchial disease	0.57782
DB00571	Cholelithiasis	0.08944
DB00571	Chronic obstructive airway disease	0.23825
DB00571	Conduct disorder	0.10448
DB00571	Cystic fibrosis	0.30684
DB00571	Depression	0.39005
DB00571	Dermatitis	0.3674
DB00571	Diabetes mellitus	0.22525
DB00571	Drug abuse	0.23125
DB00571	Endometrium cancer	0.10847
DB00571	Enteritis	0.03586
DB00571	Epilepsy	0.49963
DB00571	Glaucoma	0.3846
DB00571	Gram-Negative bacterial infection	0.0716
DB00571	Graves' disease	0.333
DB00571	Heart failure	0.30019
DB00571	Hepatitis C	0.45243

DB00571	Hypertension	0.66133
DB00571	Infertility	0.03445
DB00571	Ischemia	0.06613
DB00571	Liver cancer	0.02288
DB00571	Lung cancer	0.11611
DB00571	Malaria	0.35505
DB00571	Metabolism disease	0.39929
DB00571	Migraine	0.49201
DB00571	Movement disorder	0.15289
DB00571	Myopathy	0.22498
DB00571	Neurodegenerative disorder	0.08454
DB00571	Obesity	0.21726
DB00571	Oligospermia	0.07425
DB00571	Panic disorder	0.61998
DB00571	Polycystic kidney	0.06461
DB00571	Polycystic ovary syndrome	0.20182
DB00571	Pre-Eclampsia	0.0527
DB00571	Premature birth	0.49733
DB00571	Prostate cancer	0.09998
DB00571	Respiratory tract disease	0.04984
DB00571	Rheumatoid arthritis	0.11553
DB00571	Sickle cell disease	0.20737
DB00571	Sinusitis	0.04625
DB00571	Stroke	0.23234
DB00571	Subarachnoid hemorrhage	0.52243
DB00571	Sudden infant death syndrome	0.7222
DB00571	Systemic infection	0.0513
DB00571	Testicular dysfunction	0.02279
DB00598	Infertility, Male	0.09355
DB00598	Alimentary system disease	0.17411
DB00598	Alzheimer's disease	0.09678
DB00598	Arthritis	0.46915
DB00598	Atherosclerosis	0.18754
DB00598	Autistic disorder	0.30372
DB00598	Azoospermia	0.07075
DB00598	Breast cancer	0.01989
DB00598	Bronchial disease	0.93559
DB00598	Chronic obstructive airway disease	0.38251
DB00598	Conduct disorder	0.18128
DB00598	Cystic fibrosis	0.49295
DB00598	Dermatitis	0.21355
DB00598	Diabetes mellitus	0.131
DB00598	Drug abuse	0.17716
DB00598	Enteritis	0.06222
DB00598	Epilepsy	0.13443
DB00598	Glaucoma	0.5753
DB00598	Gram-Negative bacterial infection	0.12423

DB00598	Graves' disease	0.52897
DB00598	Heart failure	0.462
DB00598	Hypertension	0.41102
DB00598	Infertility	0.05977
DB00598	Ischemia	0.11474
DB00598	Kidney failure	0.11323
DB00598	Liver cancer	0.0397
DB00598	Lung cancer	0.18219
DB00598	Malaria	0.56477
DB00598	Metabolism disease	0.52434
DB00598	Movement disorder	0.26525
DB00598	Myopathy	0.35688
DB00598	Neurodegenerative disorder	0.14667
DB00598	Obesity	0.33449
DB00598	Oligospermia	0.12882
DB00598	Polycystic kidney	0.1121
DB00598	Polycystic ovary syndrome	0.31362
DB00598	Premature birth	0.79595
DB00598	Prostate cancer	0.18515
DB00598	Respiratory tract disease	0.08648
DB00598	Rheumatoid arthritis	0.13608
DB00598	Sickle cell disease	0.30669
DB00598	Sinusitis	0.08024
DB00598	Subarachnoid hemorrhage	0.74003
DB00598	Testicular dysfunction	0.03955
DB00612	Infertility, Male	0.09355
DB00612	Alimentary system disease	0.17411
DB00612	Alzheimer's disease	0.11168
DB00612	Arthritis	0.49484
DB00612	Atherosclerosis	0.20204
DB00612	Autistic disorder	0.32884
DB00612	Azoospermia	0.07075
DB00612	Breast cancer	0.01989
DB00612	Bronchial disease	0.98582
DB00612	Chronic obstructive airway disease	0.40566
DB00612	Conduct disorder	0.18128
DB00612	Cystic fibrosis	0.52253
DB00612	Dermatitis	0.23238
DB00612	Diabetes mellitus	0.1419
DB00612	Drug abuse	0.19656
DB00612	Enteritis	0.06222
DB00612	Epilepsy	0.13443
DB00612	Glaucoma	0.64434
DB00612	Gram-Negative bacterial infection	0.12423
DB00612	Graves' disease	0.56559
DB00612	Heart failure	0.50615
DB00612	Hypertension	0.40424

DB00612	Infertility	0.05977
DB00612	Ischemia	0.11474
DB00612	Liver cancer	0.0397
DB00612	Lung cancer	0.19665
DB00612	Malaria	0.60323
DB00612	Metabolism disease	0.55751
DB00612	Movement disorder	0.26525
DB00612	Myopathy	0.38199
DB00612	Neurodegenerative disorder	0.14667
DB00612	Obesity	0.36636
DB00612	Oligospermia	0.12882
DB00612	Polycystic kidney	0.1121
DB00612	Polycystic ovary syndrome	0.34105
DB00612	Premature birth	0.84618
DB00612	Prostate cancer	0.16986
DB00612	Respiratory tract disease	0.08648
DB00612	Rheumatoid arthritis	0.14875
DB00612	Sickle cell disease	0.34655
DB00612	Sinusitis	0.08024
DB00612	Subarachnoid hemorrhage	0.86492
DB00612	Testicular dysfunction	0.03955
DB00668	Infertility, Male	0.08591
DB00668	Alimentary system disease	0.1599
DB00668	Alzheimer's disease	0.08127
DB00668	Arthritis	0.41775
DB00668	Atherosclerosis	0.16484
DB00668	Attention deficit hyperactivity disorder	0.22195
DB00668	Autistic disorder	0.26612
DB00668	Azoospermia	0.06497
DB00668	Breast cancer	0.01827
DB00668	Bronchial disease	0.83361
DB00668	Chronic obstructive airway disease	0.33948
DB00668	Conduct disorder	0.16648
DB00668	Cystic fibrosis	0.43762
DB00668	Dermatitis	0.18652
DB00668	Diabetes mellitus	0.13336
DB00668	Drug abuse	0.1528
DB00668	Encephalopathies	0.04951
DB00668	Enteritis	0.05714
DB00668	Epilepsy	0.12346
DB00668	Glaucoma	0.49313
DB00668	Gram-Negative bacterial infection	0.11409
DB00668	Graves' disease	0.46713
DB00668	Heart failure	0.40177
DB00668	Hypertension	0.45488
DB00668	Infertility	0.05489
DB00668	Ischemia	0.10537

DB00668	Kidney failure	0.1201
DB00668	Liver cancer	0.03646
DB00668	Lung cancer	0.15994
DB00668	Malaria	0.49906
DB00668	Metabolism disease	0.46463
DB00668	Movement disorder	0.2436
DB00668	Myopathy	0.31494
DB00668	Nervous system disease	0.20075
DB00668	Neurodegenerative disorder	0.1347
DB00668	Obesity	0.38925
DB00668	Oligospermia	0.1183
DB00668	Polycystic kidney	0.10295
DB00668	Polycystic ovary syndrome	0.32086
DB00668	Premature birth	0.70536
DB00668	Prostate cancer	0.15901
DB00668	Respiratory tract disease	0.07942
DB00668	Rheumatoid arthritis	0.11851
DB00668	Schizophrenia	0.06685
DB00668	Sickle cell disease	0.26133
DB00668	Sinusitis	0.07369
DB00668	Subarachnoid hemorrhage	0.61592
DB00668	Testicular dysfunction	0.03632
DB00841	Infertility, Male	0.09355
DB00841	Alimentary system disease	0.17411
DB00841	Alzheimer's disease	0.11168
DB00841	Arthritis	0.49484
DB00841	Atherosclerosis	0.20204
DB00841	Autistic disorder	0.32884
DB00841	Azoospermia	0.07075
DB00841	Breast cancer	0.01989
DB00841	Bronchial disease	0.98582
DB00841	Chronic obstructive airway disease	0.40566
DB00841	Conduct disorder	0.18128
DB00841	Cystic fibrosis	0.52253
DB00841	Dermatitis	0.23238
DB00841	Diabetes mellitus	0.1419
DB00841	Drug abuse	0.19656
DB00841	Enteritis	0.06222
DB00841	Epilepsy	0.13443
DB00841	Glaucoma	0.64434
DB00841	Gram-Negative bacterial infection	0.12423
DB00841	Graves' disease	0.56559
DB00841	Heart failure	0.50615
DB00841	Hypertension	0.40424
DB00841	Infertility	0.05977
DB00841	Ischemia	0.11474
DB00841	Liver cancer	0.0397

DB00841	Lung cancer	0.19665
DB00841	Malaria	0.60323
DB00841	Metabolism disease	0.55751
DB00841	Movement disorder	0.26525
DB00841	Myopathy	0.38199
DB00841	Neurodegenerative disorder	0.14667
DB00841	Obesity	0.36636
DB00841	Oligospermia	0.12882
DB00841	Polycystic kidney	0.1121
DB00841	Polycystic ovary syndrome	0.34105
DB00841	Premature birth	0.84618
DB00841	Prostate cancer	0.16986
DB00841	Respiratory tract disease	0.08648
DB00841	Rheumatoid arthritis	0.14875
DB00841	Sickle cell disease	0.34655
DB00841	Sinusitis	0.08024
DB00841	Subarachnoid hemorrhage	0.86492
DB00841	Testicular dysfunction	0.03955
DB00852	Hypertension, Pulmonary	0.08671
DB00852	Infertility, Male	0.08591
DB00852	Stress disorder, post-traumatic	0.13363
DB00852	Alimentary system disease	0.1599
DB00852	Alzheimer's disease	0.08303
DB00852	Anorexia nervosa	0.1543
DB00852	Arthritis	0.42078
DB00852	Atherosclerosis	0.19301
DB00852	Attention deficit hyperactivity disorder	0.229
DB00852	Autistic disorder	0.31492
DB00852	Azoospermia	0.06497
DB00852	Behavior disease	0.17496
DB00852	Bipolar disorder	0.12839
DB00852	Brain disease	0.06788
DB00852	Breast cancer	0.03645
DB00852	Bronchial disease	0.83953
DB00852	Chronic fatigue syndrome	0.13363
DB00852	Chronic obstructive airway disease	0.38447
DB00852	Colon cancer	0.02243
DB00852	Conduct disorder	0.16648
DB00852	Congenital heart disease	0.12599
DB00852	Cystic fibrosis	0.44111
DB00852	Depression	0.09926
DB00852	Dermatitis	0.2231
DB00852	Diabetes mellitus	0.13593
DB00852	Drug abuse	0.26129
DB00852	Enteritis	0.05714
DB00852	Epilepsy	0.17691
DB00852	Fibromyalgia	0.13363

DB00852	Generalized anxiety disorder	0.10911
DB00852	Gilles de la Tourette syndrome	0.14286
DB00852	Glaucoma	0.50127
DB00852	Gram-Negative bacterial infection	0.11409
DB00852	Graves' disease	0.47144
DB00852	Heart failure	0.44726
DB00852	Herpes	0.10799
DB00852	Hypertension	0.46453
DB00852	Infertility	0.05489
DB00852	Ischemia	0.10537
DB00852	Kidney failure	0.0428
DB00852	Liver cancer	0.03646
DB00852	Lung cancer	0.16164
DB00852	Malaria	0.50359
DB00852	Metabolism disease	0.46854
DB00852	Migraine	0.14037
DB00852	Movement disorder	0.2436
DB00852	Multiple endocrine neoplasia	0.12599
DB00852	Myopathy	0.3179
DB00852	Nervous system disease	0.20705
DB00852	Neurodegenerative disorder	0.1347
DB00852	Neuroendocrine tumor	0.13363
DB00852	Neurotic disorder	0.11952
DB00852	Obesity	0.42583
DB00852	Obsessive-compulsive disorder	0.11396
DB00852	Oligospermia	0.1183
DB00852	Panic disorder	0.16496
DB00852	Pervasive development disorder	0.09759
DB00852	Polycystic kidney	0.10295
DB00852	Polycystic ovary syndrome	0.27726
DB00852	Premature birth	0.71128
DB00852	Prostate cancer	0.16156
DB00852	Psychotic disorder	0.12105
DB00852	Pulmonary hypertension	0.18898
DB00852	Respiratory tract disease	0.07942
DB00852	Rheumatoid arthritis	0.12
DB00852	Schizophrenia	0.06871
DB00852	Sickle cell disease	0.26603
DB00852	Sinusitis	0.07369
DB00852	Stroke	0.0428
DB00852	Subarachnoid hemorrhage	0.63064
DB00852	Sudden infant death syndrome	0.08248
DB00852	Testicular dysfunction	0.03632
DB00852	Ulcerative colitis	0.03898
DB00866	Infertility, Male	0.06068
DB00866	Abortion	0.03899
DB00866	Alimentary system disease	0.11295

DB00866	Alzheimer's disease	0.08099
DB00866	Amyotrophic lateral sclerosis	0.30055
DB00866	Anorexia nervosa	0.10933
DB00866	Arthritis	0.33572
DB00866	Atherosclerosis	0.13937
DB00866	Attention deficit hyperactivity disorder	0.08468
DB00866	Autistic disorder	0.2277
DB00866	Azoospermia	0.04589
DB00866	Behavior disease	0.59534
DB00866	Bipolar disorder	0.0595
DB00866	Breast cancer	0.0129
DB00866	Bronchial disease	0.66828
DB00866	Chronic obstructive airway disease	0.27642
DB00866	Conduct disorder	0.1176
DB00866	Cystic fibrosis	0.35592
DB00866	Depression	0.2996
DB00866	Dermatitis	0.41377
DB00866	Diabetes mellitus	0.25309
DB00866	Drug abuse	0.15951
DB00866	Enteritis	0.04036
DB00866	Epilepsy	0.54817
DB00866	Glaucoma	0.45753
DB00866	Gram-Negative bacterial infection	0.08059
DB00866	Graves' disease	0.38787
DB00866	Heart failure	0.35364
DB00866	Hepatitis C	0.49345
DB00866	Hypertension	0.58583
DB00866	Infertility	0.03877
DB00866	Ischemia	0.07443
DB00866	Liver cancer	0.02575
DB00866	Lung cancer	0.13585
DB00866	Malaria	0.41335
DB00866	Metabolism disease	0.38065
DB00866	Migraine	0.53928
DB00866	Movement disorder	0.17207
DB00866	Myopathy	0.26219
DB00866	Neurodegenerative disorder	0.09515
DB00866	Obesity	0.25591
DB00866	Oligospermia	0.08356
DB00866	Panic disorder	0.67789
DB00866	Polycystic kidney	0.07272
DB00866	Polycystic ovary syndrome	0.23695
DB00866	Premature birth	0.57769
DB00866	Prostate cancer	0.11638
DB00866	Respiratory tract disease	0.0561
DB00866	Rheumatoid arthritis	0.10376
DB00866	Sickle cell disease	0.24764

DB00866	Sinusitis	0.05205
DB00866	Stroke	0.25734
DB00866	Subarachnoid hemorrhage	0.63262
DB00866	Sudden infant death syndrome	0.78588
DB00866	Testicular dysfunction	0.02565
DB00960	Infertility, Male	0.05392
DB00960	Abortion	0.03465
DB00960	Alimentary system disease	0.10035
DB00960	Alzheimer's disease	0.07103
DB00960	Amyotrophic lateral sclerosis	0.27821
DB00960	Anorexia nervosa	0.19428
DB00960	Arthritis	0.29668
DB00960	Atherosclerosis	0.12292
DB00960	Attention deficit hyperactivity disorder	0.15048
DB00960	Autistic disorder	0.20074
DB00960	Azoospermia	0.04078
DB00960	Behavior disease	0.77098
DB00960	Bipolar disorder	0.26185
DB00960	Breast cancer	0.01146
DB00960	Bronchial disease	0.59062
DB00960	Chronic obstructive airway disease	0.24415
DB00960	Conduct disorder	0.10448
DB00960	Cystic fibrosis	0.31438
DB00960	Depression	0.40391
DB00960	Dermatitis	0.377
DB00960	Diabetes mellitus	0.2308
DB00960	Drug abuse	0.24113
DB00960	Enteritis	0.03586
DB00960	Epilepsy	0.50709
DB00960	Glaucoma	0.40219
DB00960	Gram-Negative bacterial infection	0.0716
DB00960	Graves' disease	0.34233
DB00960	Heart failure	0.31144
DB00960	Hepatitis C	0.46022
DB00960	Hypertension	0.64267
DB00960	Infertility	0.03445
DB00960	Ischemia	0.06613
DB00960	Liver cancer	0.02288
DB00960	Lung cancer	0.1198
DB00960	Malaria	0.36485
DB00960	Metabolism disease	0.33613
DB00960	Migraine	0.50181
DB00960	Movement disorder	0.15289
DB00960	Myopathy	0.23138
DB00960	Neurodegenerative disorder	0.08454
DB00960	Obesity	0.22538
DB00960	Oligospermia	0.07425

DB00960	Panic disorder	0.6315
DB00960	Polycystic kidney	0.06461
DB00960	Polycystic ovary syndrome	0.20882
DB00960	Premature birth	0.51013
DB00960	Prostate cancer	0.10273
DB00960	Respiratory tract disease	0.04984
DB00960	Rheumatoid arthritis	0.0914
DB00960	Sickle cell disease	0.21753
DB00960	Sinusitis	0.04625
DB00960	Stroke	0.23832
DB00960	Subarachnoid hemorrhage	0.55426
DB00960	Sudden infant death syndrome	0.73372
DB00960	Testicular dysfunction	0.02279
DB01001	Infertility, Male	0.09355
DB01001	Alimentary system disease	0.17411
DB01001	Alzheimer's disease	0.11168
DB01001	Arthritis	0.49484
DB01001	Atherosclerosis	0.20204
DB01001	Autistic disorder	0.32884
DB01001	Azoospermia	0.07075
DB01001	Breast cancer	0.01989
DB01001	Bronchial disease	0.98582
DB01001	Chronic obstructive airway disease	0.40566
DB01001	Conduct disorder	0.18128
DB01001	Cystic fibrosis	0.52253
DB01001	Dermatitis	0.23238
DB01001	Diabetes mellitus	0.1419
DB01001	Drug abuse	0.19656
DB01001	Enteritis	0.06222
DB01001	Epilepsy	0.13443
DB01001	Glaucoma	0.64434
DB01001	Gram-Negative bacterial infection	0.12423
DB01001	Graves' disease	0.56559
DB01001	Heart failure	0.50615
DB01001	Hypertension	0.40424
DB01001	Infertility	0.05977
DB01001	Ischemia	0.11474
DB01001	Liver cancer	0.0397
DB01001	Lung cancer	0.19665
DB01001	Malaria	0.60323
DB01001	Metabolism disease	0.55751
DB01001	Movement disorder	0.26525
DB01001	Myopathy	0.38199
DB01001	Neurodegenerative disorder	0.14667
DB01001	Obesity	0.36636
DB01001	Oligospermia	0.12882
DB01001	Polycystic kidney	0.1121

DB01001	Polycystic ovary syndrome	0.34105
DB01001	Premature birth	0.84618
DB01001	Prostate cancer	0.16986
DB01001	Respiratory tract disease	0.08648
DB01001	Rheumatoid arthritis	0.14875
DB01001	Sickle cell disease	0.34655
DB01001	Sinusitis	0.08024
DB01001	Subarachnoid hemorrhage	0.86492
DB01001	Testicular dysfunction	0.03955
DB01064	Infertility, Male	0.10962
DB01064	Skin disease, Genetic	0.03837
DB01064	Abortion	0.02669
DB01064	Alimentary system disease	0.10934
DB01064	Alzheimer's disease	0.0779
DB01064	Amnionitis	0.10483
DB01064	Arthritis	0.30256
DB01064	Atherosclerosis	0.16545
DB01064	Autistic disorder	0.19849
DB01064	Azoospermia	0.04443
DB01064	Bipolar disorder	0.04073
DB01064	Brain tumor	0.02356
DB01064	Breast cancer	0.0511
DB01064	Bronchial disease	0.60306
DB01064	Bronchial hyperreactivity	0.074
DB01064	Cancer	0.08781
DB01064	Cerebrovascular disorder	0.05087
DB01064	Cholelithiasis	0.12004
DB01064	Choriocarcinoma	0.07303
DB01064	Chronic obstructive airway disease	0.24736
DB01064	Colon cancer	0.05771
DB01064	Common variable immunodeficiency	0.12656
DB01064	Conduct disorder	0.11384
DB01064	Congenital heart disease	0.09385
DB01064	Cystic fibrosis	0.3187
DB01064	Deafness	0.06059
DB01064	Dental plaque	0.02888
DB01064	Depression	0.0194
DB01064	Dermatitis	0.13992
DB01064	Diabetes mellitus	0.12187
DB01064	Down syndrome	0.02889
DB01064	Drug abuse	0.11724
DB01064	Embryoma	0.02621
DB01064	Endometriosis	0.01516
DB01064	Endometrium cancer	0.09167
DB01064	Enteritis	0.03907
DB01064	Epilepsy	0.08442
DB01064	Esotropia	0.05908

DB01064	Gastritis	0.01945
DB01064	Generalized anxiety disorder	0.09872
DB01064	Glaucoma	0.41629
DB01064	Gram-Negative bacterial infection	0.07802
DB01064	Graves' disease	0.3435
DB01064	HIV infection	0.01546
DB01064	Heart failure	0.34246
DB01064	Hemorrhagic disorder	0.03169
DB01064	Herpes	0.03545
DB01064	Hyperglycemia	0.10701
DB01064	Hyperinsulinism	0.14643
DB01064	Hyperlipidemia	0.11278
DB01064	Hypertension	0.3183
DB01064	Hypogammaglobulinemia	0.24449
DB01064	Infection	0.01935
DB01064	Infertility	0.07004
DB01064	Influenza	0.05262
DB01064	Ischemia	0.07205
DB01064	Kidney failure	0.02853
DB01064	Late pregnancy	0.06986
DB01064	Leukemia	0.06516
DB01064	Liver cancer	0.02493
DB01064	Lung cancer	0.11888
DB01064	Lung disease	0.03698
DB01064	Lupus erythematosus	0.04207
DB01064	Lupus vulgaris	0.04316
DB01064	Lymphoma	0.03354
DB01064	Lymphoproliferative disorder	0.06233
DB01064	Malaria	0.36655
DB01064	Metabolism disease	0.40005
DB01064	Migraine	0.07978
DB01064	Movement disorder	0.16658
DB01064	Muscular dystrophies	0.03864
DB01064	Myasthenia Gravis	0.05401
DB01064	Myelofibrosis	0.10744
DB01064	Myeloproliferative disease	0.04176
DB01064	Myopathy	0.23187
DB01064	Neurodegenerative disorder	0.09211
DB01064	Obesity	0.27272
DB01064	Oligospermia	0.0809
DB01064	Osteoporosis	0.03236
DB01064	Ovarian cancer	0.02689
DB01064	Ovarian disease	0.05073
DB01064	Ovarian failure	0.06634
DB01064	Overnutrition	0.52844
DB01064	Panic disorder	0.05096
DB01064	Parkinson disease	0.0247

DB01064	Polyarthritis	0.01846
DB01064	Polycystic kidney	0.0704
DB01064	Polycystic ovary syndrome	0.29055
DB01064	Pre-Eclampsia	0.04454
DB01064	Premature birth	0.51536
DB01064	Primary biliary cirrhosis	0.03868
DB01064	Primary tumor	0.04289
DB01064	Prostate cancer	0.13574
DB01064	Rabies	0.01944
DB01064	Renal Cell cancer	0.04289
DB01064	Renal tubular acidosis	0.02928
DB01064	Respiratory tract disease	0.05431
DB01064	Rheumatoid arthritis	0.12125
DB01064	Schizophrenia	0.01933
DB01064	Sickle cell disease	0.20491
DB01064	Sinusitis	0.05039
DB01064	Skin cancer	0.03483
DB01064	Skin tumor	0.12286
DB01064	Solid tumor	0.05552
DB01064	Stroke	0.04452
DB01064	Subarachnoid hemorrhage	0.5033
DB01064	Synovitis	0.05882
DB01064	Systemic infection	0.04336
DB01064	Systemic scleroderma	0.03654
DB01064	Testicular dysfunction	0.02484
DB01064	Thymoma	0.04565
DB01064	Tuberous sclerosis	0.02389
DB01064	Turner's syndrome	0.21169
DB01064	Uterine disease	0.04063
DB01064	Vitiligo	0.05401
DB01064	Wiskott-Aldrich syndrome	0.09263
DB01064	Yersinia infection	0.0405
DB01102	Infertility, Male	0.09355
DB01102	Alimentary system disease	0.17411
DB01102	Alzheimer's disease	0.10234
DB01102	Amnionitis	0.16013
DB01102	Arthritis	0.47874
DB01102	Atherosclerosis	0.23338
DB01102	Autistic disorder	0.3131
DB01102	Azoospermia	0.07075
DB01102	Breast cancer	0.01989
DB01102	Bronchial disease	0.95435
DB01102	Cholelithiasis	0.11547
DB01102	Chronic obstructive airway disease	0.39116
DB01102	Conduct disorder	0.18128
DB01102	Cystic fibrosis	0.504
DB01102	Dermatitis	0.22058

DB01102	Diabetes mellitus	0.13507
DB01102	Drug abuse	0.1844
DB01102	Endometrium cancer	0.14003
DB01102	Enteritis	0.06222
DB01102	Epilepsy	0.13443
DB01102	Glaucoma	0.60108
DB01102	Gram-Negative bacterial infection	0.12423
DB01102	Graves' disease	0.54265
DB01102	Heart failure	0.47849
DB01102	Hypertension	0.42936
DB01102	Infertility	0.05977
DB01102	Ischemia	0.11474
DB01102	Liver cancer	0.0397
DB01102	Lung cancer	0.18759
DB01102	Malaria	0.57914
DB01102	Metabolism disease	0.62918
DB01102	Movement disorder	0.26525
DB01102	Myopathy	0.36626
DB01102	Neurodegenerative disorder	0.14667
DB01102	Obesity	0.34639
DB01102	Oligospermia	0.12882
DB01102	Polycystic kidney	0.1121
DB01102	Polycystic ovary syndrome	0.32386
DB01102	Pre-Eclampsia	0.06804
DB01102	Premature birth	0.81471
DB01102	Prostate cancer	0.16309
DB01102	Respiratory tract disease	0.08648
DB01102	Rheumatoid arthritis	0.17615
DB01102	Sickle cell disease	0.32158
DB01102	Sinusitis	0.08024
DB01102	Subarachnoid hemorrhage	0.78667
DB01102	Systemic infection	0.06623
DB01102	Testicular dysfunction	0.03955
DB01193	Infertility, Male	0.09355
DB01193	Alimentary system disease	0.17411
DB01193	Alzheimer's disease	0.11168
DB01193	Arthritis	0.49484
DB01193	Atherosclerosis	0.20204
DB01193	Autistic disorder	0.32884
DB01193	Azoospermia	0.07075
DB01193	Breast cancer	0.01989
DB01193	Bronchial disease	0.98582
DB01193	Chronic obstructive airway disease	0.40566
DB01193	Conduct disorder	0.18128
DB01193	Cystic fibrosis	0.52253
DB01193	Dermatitis	0.23238
DB01193	Diabetes mellitus	0.1419

DB01193	Drug abuse	0.19656
DB01193	Enteritis	0.06222
DB01193	Epilepsy	0.13443
DB01193	Glaucoma	0.64434
DB01193	Gram-Negative bacterial infection	0.12423
DB01193	Graves' disease	0.56559
DB01193	Heart failure	0.50615
DB01193	Hypertension	0.40424
DB01193	Infertility	0.05977
DB01193	Ischemia	0.11474
DB01193	Liver cancer	0.0397
DB01193	Lung cancer	0.19665
DB01193	Malaria	0.60323
DB01193	Metabolism disease	0.55751
DB01193	Movement disorder	0.26525
DB01193	Myopathy	0.38199
DB01193	Neurodegenerative disorder	0.14667
DB01193	Obesity	0.36636
DB01193	Oligospermia	0.12882
DB01193	Polycystic kidney	0.1121
DB01193	Polycystic ovary syndrome	0.34105
DB01193	Premature birth	0.84618
DB01193	Prostate cancer	0.16986
DB01193	Respiratory tract disease	0.08648
DB01193	Rheumatoid arthritis	0.14875
DB01193	Sickle cell disease	0.34655
DB01193	Sinusitis	0.08024
DB01193	Subarachnoid hemorrhage	0.86492
DB01193	Testicular dysfunction	0.03955
DB01203	Infertility, Male	0.09355
DB01203	Alimentary system disease	0.17411
DB01203	Alzheimer's disease	0.11168
DB01203	Arthritis	0.49484
DB01203	Atherosclerosis	0.20204
DB01203	Autistic disorder	0.32884
DB01203	Azoospermia	0.07075
DB01203	Breast cancer	0.01989
DB01203	Bronchial disease	0.98582
DB01203	Chronic obstructive airway disease	0.40566
DB01203	Conduct disorder	0.18128
DB01203	Cystic fibrosis	0.52253
DB01203	Dermatitis	0.23238
DB01203	Diabetes mellitus	0.1419
DB01203	Drug abuse	0.19656
DB01203	Enteritis	0.06222
DB01203	Epilepsy	0.13443
DB01203	Glaucoma	0.64434

DB01203	Gram-Negative bacterial infection	0.12423
DB01203	Graves' disease	0.56559
DB01203	Heart failure	0.50615
DB01203	Hypertension	0.40424
DB01203	Infertility	0.05977
DB01203	Ischemia	0.11474
DB01203	Liver cancer	0.0397
DB01203	Lung cancer	0.19665
DB01203	Malaria	0.60323
DB01203	Metabolism disease	0.55751
DB01203	Movement disorder	0.26525
DB01203	Myopathy	0.38199
DB01203	Neurodegenerative disorder	0.14667
DB01203	Obesity	0.36636
DB01203	Oligospermia	0.12882
DB01203	Polycystic kidney	0.1121
DB01203	Polycystic ovary syndrome	0.34105
DB01203	Premature birth	0.84618
DB01203	Prostate cancer	0.16986
DB01203	Respiratory tract disease	0.08648
DB01203	Rheumatoid arthritis	0.14875
DB01203	Sickle cell disease	0.34655
DB01203	Sinusitis	0.08024
DB01203	Subarachnoid hemorrhage	0.86492
DB01203	Testicular dysfunction	0.03955
DB01210	Infertility, Male	0.09355
DB01210	Alimentary system disease	0.17411
DB01210	Alzheimer's disease	0.11168
DB01210	Arthritis	0.49484
DB01210	Atherosclerosis	0.20204
DB01210	Autistic disorder	0.32884
DB01210	Azoospermia	0.07075
DB01210	Breast cancer	0.01989
DB01210	Bronchial disease	0.98582
DB01210	Chronic obstructive airway disease	0.40566
DB01210	Conduct disorder	0.18128
DB01210	Cystic fibrosis	0.52253
DB01210	Dermatitis	0.23238
DB01210	Diabetes mellitus	0.1419
DB01210	Drug abuse	0.19656
DB01210	Enteritis	0.06222
DB01210	Epilepsy	0.13443
DB01210	Glaucoma	0.64434
DB01210	Gram-Negative bacterial infection	0.12423
DB01210	Graves' disease	0.56559
DB01210	Heart failure	0.50615
DB01210	Hypertension	0.40424

DB01210	Infertility	0.05977
DB01210	Ischemia	0.11474
DB01210	Liver cancer	0.0397
DB01210	Lung cancer	0.19665
DB01210	Malaria	0.60323
DB01210	Metabolism disease	0.55751
DB01210	Movement disorder	0.26525
DB01210	Myopathy	0.38199
DB01210	Neurodegenerative disorder	0.14667
DB01210	Obesity	0.36636
DB01210	Oligospermia	0.12882
DB01210	Polycystic kidney	0.1121
DB01210	Polycystic ovary syndrome	0.34105
DB01210	Premature birth	0.84618
DB01210	Prostate cancer	0.16986
DB01210	Respiratory tract disease	0.08648
DB01210	Rheumatoid arthritis	0.14875
DB01210	Sickle cell disease	0.34655
DB01210	Sinusitis	0.08024
DB01210	Subarachnoid hemorrhage	0.86492
DB01210	Testicular dysfunction	0.03955
DB01214	Infertility, Male	0.09355
DB01214	Alimentary system disease	0.17411
DB01214	Alzheimer's disease	0.11168
DB01214	Arthritis	0.49484
DB01214	Atherosclerosis	0.20204
DB01214	Autistic disorder	0.32884
DB01214	Azoospermia	0.07075
DB01214	Breast cancer	0.01989
DB01214	Bronchial disease	0.98582
DB01214	Chronic obstructive airway disease	0.40566
DB01214	Conduct disorder	0.18128
DB01214	Cystic fibrosis	0.52253
DB01214	Dermatitis	0.23238
DB01214	Diabetes mellitus	0.1419
DB01214	Drug abuse	0.19656
DB01214	Enteritis	0.06222
DB01214	Epilepsy	0.13443
DB01214	Glaucoma	0.64434
DB01214	Gram-Negative bacterial infection	0.12423
DB01214	Graves' disease	0.56559
DB01214	Heart failure	0.50615
DB01214	Hypertension	0.40424
DB01214	Infertility	0.05977
DB01214	Ischemia	0.11474
DB01214	Liver cancer	0.0397
DB01214	Lung cancer	0.19665

DB01214	Malaria	0.60323
DB01214	Metabolism disease	0.55751
DB01214	Movement disorder	0.26525
DB01214	Myopathy	0.38199
DB01214	Neurodegenerative disorder	0.14667
DB01214	Obesity	0.36636
DB01214	Oligospermia	0.12882
DB01214	Polycystic kidney	0.1121
DB01214	Polycystic ovary syndrome	0.34105
DB01214	Premature birth	0.84618
DB01214	Prostate cancer	0.16986
DB01214	Respiratory tract disease	0.08648
DB01214	Rheumatoid arthritis	0.14875
DB01214	Sickle cell disease	0.34655
DB01214	Sinusitis	0.08024
DB01214	Subarachnoid hemorrhage	0.86492
DB01214	Testicular dysfunction	0.03955
DB01288	Infertility, Male	0.09355
DB01288	Alimentary system disease	0.17411
DB01288	Alzheimer's disease	0.10234
DB01288	Amnionitis	0.16013
DB01288	Arthritis	0.47874
DB01288	Atherosclerosis	0.23338
DB01288	Autistic disorder	0.3131
DB01288	Azoospermia	0.07075
DB01288	Breast cancer	0.01989
DB01288	Bronchial disease	0.95435
DB01288	Cholelithiasis	0.11547
DB01288	Chronic obstructive airway disease	0.39116
DB01288	Conduct disorder	0.18128
DB01288	Cystic fibrosis	0.504
DB01288	Dermatitis	0.22058
DB01288	Diabetes mellitus	0.13507
DB01288	Drug abuse	0.1844
DB01288	Endometrium cancer	0.14003
DB01288	Enteritis	0.06222
DB01288	Epilepsy	0.13443
DB01288	Glaucoma	0.60108
DB01288	Gram-Negative bacterial infection	0.12423
DB01288	Graves' disease	0.54265
DB01288	Heart failure	0.47849
DB01288	Hypertension	0.42936
DB01288	Infertility	0.05977
DB01288	Ischemia	0.11474
DB01288	Liver cancer	0.0397
DB01288	Lung cancer	0.18759
DB01288	Malaria	0.57914

DB01288	Metabolism disease	0.62918
DB01288	Movement disorder	0.26525
DB01288	Myopathy	0.36626
DB01288	Neurodegenerative disorder	0.14667
DB01288	Obesity	0.34639
DB01288	Oligospermia	0.12882
DB01288	Polycystic kidney	0.1121
DB01288	Polycystic ovary syndrome	0.32386
DB01288	Pre-Eclampsia	0.06804
DB01288	Premature birth	0.81471
DB01288	Prostate cancer	0.16309
DB01288	Respiratory tract disease	0.08648
DB01288	Rheumatoid arthritis	0.17615
DB01288	Sickle cell disease	0.32158
DB01288	Sinusitis	0.08024
DB01288	Subarachnoid hemorrhage	0.78667
DB01288	Systemic infection	0.06623
DB01288	Testicular dysfunction	0.03955
DB01291	Infertility, Male	0.09355
DB01291	Alimentary system disease	0.17411
DB01291	Alzheimer's disease	0.11168
DB01291	Arthritis	0.49484
DB01291	Atherosclerosis	0.20204
DB01291	Autistic disorder	0.32884
DB01291	Azoospermia	0.07075
DB01291	Breast cancer	0.01989
DB01291	Bronchial disease	0.98582
DB01291	Chronic obstructive airway disease	0.40566
DB01291	Conduct disorder	0.18128
DB01291	Cystic fibrosis	0.52253
DB01291	Dermatitis	0.23238
DB01291	Diabetes mellitus	0.1419
DB01291	Drug abuse	0.19656
DB01291	Enteritis	0.06222
DB01291	Epilepsy	0.13443
DB01291	Glaucoma	0.64434
DB01291	Gram-Negative bacterial infection	0.12423
DB01291	Graves' disease	0.56559
DB01291	Heart failure	0.50615
DB01291	Hypertension	0.40424
DB01291	Infertility	0.05977
DB01291	Ischemia	0.11474
DB01291	Liver cancer	0.0397
DB01291	Lung cancer	0.19665
DB01291	Malaria	0.60323
DB01291	Metabolism disease	0.55751
DB01291	Movement disorder	0.26525

DB01291	Myopathy	0.38199
DB01291	Neurodegenerative disorder	0.14667
DB01291	Obesity	0.36636
DB01291	Oligospermia	0.12882
DB01291	Polycystic kidney	0.1121
DB01291	Polycystic ovary syndrome	0.34105
DB01291	Premature birth	0.84618
DB01291	Prostate cancer	0.16986
DB01291	Respiratory tract disease	0.08648
DB01291	Rheumatoid arthritis	0.14875
DB01291	Sickle cell disease	0.34655
DB01291	Sinusitis	0.08024
DB01291	Subarachnoid hemorrhage	0.86492
DB01291	Testicular dysfunction	0.03955
DB01295	Infertility, Male	0.09355
DB01295	Alimentary system disease	0.17411
DB01295	Alzheimer's disease	0.10234
DB01295	Arthritis	0.47874
DB01295	Atherosclerosis	0.19296
DB01295	Autistic disorder	0.3131
DB01295	Azoospermia	0.07075
DB01295	Breast cancer	0.01989
DB01295	Bronchial disease	0.95435
DB01295	Chronic obstructive airway disease	0.39116
DB01295	Conduct disorder	0.18128
DB01295	Cystic fibrosis	0.504
DB01295	Dermatitis	0.22058
DB01295	Diabetes mellitus	0.13507
DB01295	Drug abuse	0.1844
DB01295	Enteritis	0.06222
DB01295	Epilepsy	0.13443
DB01295	Glaucoma	0.60108
DB01295	Gram-Negative bacterial infection	0.12423
DB01295	Graves' disease	0.54265
DB01295	Heart failure	0.47849
DB01295	Hypertension	0.42936
DB01295	Infertility	0.05977
DB01295	Ischemia	0.11474
DB01295	Kidney failure	0.06537
DB01295	Liver cancer	0.0397
DB01295	Lung cancer	0.18759
DB01295	Malaria	0.57914
DB01295	Metabolism disease	0.53673
DB01295	Movement disorder	0.26525
DB01295	Myopathy	0.36626
DB01295	Neurodegenerative disorder	0.14667
DB01295	Obesity	0.34639

DB01295	Oligospermia	0.12882
DB01295	Polycystic kidney	0.1121
DB01295	Polycystic ovary syndrome	0.32386
DB01295	Premature birth	0.81471
DB01295	Prostate cancer	0.19323
DB01295	Respiratory tract disease	0.08648
DB01295	Rheumatoid arthritis	0.14081
DB01295	Sickle cell disease	0.32158
DB01295	Sinusitis	0.08024
DB01295	Subarachnoid hemorrhage	0.78667
DB01295	Testicular dysfunction	0.03955
DB01297	Alzheimer's disease	0.14772
DB01297	Glaucoma	0.37044
DB01297	Heart failure	0.27175
DB01297	Hypertension	0.21495
DB01297	Obesity	0.20537
DB01297	Subarachnoid hemorrhage	0.52019
DB01359	Infertility, Male	0.06068
DB01359	Abortion	0.03899
DB01359	Alimentary system disease	0.11295
DB01359	Alzheimer's disease	0.08099
DB01359	Amyotrophic lateral sclerosis	0.30055
DB01359	Anorexia nervosa	0.10933
DB01359	Arthritis	0.33572
DB01359	Atherosclerosis	0.13937
DB01359	Attention deficit hyperactivity disorder	0.08468
DB01359	Autistic disorder	0.2277
DB01359	Azoospermia	0.04589
DB01359	Behavior disease	0.59534
DB01359	Bipolar disorder	0.0595
DB01359	Breast cancer	0.0129
DB01359	Bronchial disease	0.66828
DB01359	Chronic obstructive airway disease	0.27642
DB01359	Conduct disorder	0.1176
DB01359	Cystic fibrosis	0.35592
DB01359	Depression	0.2996
DB01359	Dermatitis	0.41377
DB01359	Diabetes mellitus	0.25309
DB01359	Drug abuse	0.15951
DB01359	Enteritis	0.04036
DB01359	Epilepsy	0.54817
DB01359	Glaucoma	0.45753
DB01359	Gram-Negative bacterial infection	0.08059
DB01359	Graves' disease	0.38787
DB01359	Heart failure	0.35364
DB01359	Hepatitis C	0.49345
DB01359	Hypertension	0.58583

DB01359	Infertility	0.03877
DB01359	Ischemia	0.07443
DB01359	Liver cancer	0.02575
DB01359	Lung cancer	0.13585
DB01359	Malaria	0.41335
DB01359	Metabolism disease	0.38065
DB01359	Migraine	0.53928
DB01359	Movement disorder	0.17207
DB01359	Myopathy	0.26219
DB01359	Neurodegenerative disorder	0.09515
DB01359	Obesity	0.25591
DB01359	Oligospermia	0.08356
DB01359	Panic disorder	0.67789
DB01359	Polycystic kidney	0.07272
DB01359	Polycystic ovary syndrome	0.23695
DB01359	Premature birth	0.57769
DB01359	Prostate cancer	0.11638
DB01359	Respiratory tract disease	0.0561
DB01359	Rheumatoid arthritis	0.10376
DB01359	Sickle cell disease	0.24764
DB01359	Sinusitis	0.05205
DB01359	Stroke	0.25734
DB01359	Subarachnoid hemorrhage	0.63262
DB01359	Sudden infant death syndrome	0.78588
DB01359	Testicular dysfunction	0.02565
DB01407	Infertility, Male	0.07581
DB01407	Alcoholic liver disease	0.18257
DB01407	Alimentary system disease	0.14109
DB01407	Alzheimer's disease	0.11364
DB01407	Amnionitis	0.12403
DB01407	Amyloidosis	0.08452
DB01407	Anemia	0.09535
DB01407	Anorexia nervosa	0.09129
DB01407	Aplastic anemia	0.09325
DB01407	Arthritis	0.44086
DB01407	Asthma	0.03651
DB01407	Atherosclerosis	0.21754
DB01407	Autistic disorder	0.25122
DB01407	Autoimmune disease	0.04851
DB01407	Azoospermia	0.05733
DB01407	Basal cell carcinoma	0.12403
DB01407	Behcet syndrome	0.06389
DB01407	Bladder cancer	0.05774
DB01407	Breast cancer	0.03763
DB01407	Bronchial disease	0.76835
DB01407	Celiac disease	0.07352
DB01407	Cervical cancer	0.05199

DB01407	Cholelithiasis	0.08944
DB01407	Chronic obstructive airway disease	0.31466
DB01407	Colon cancer	0.02654
DB01407	Conduct disorder	0.1469
DB01407	Cystic fibrosis	0.40546
DB01407	Dermatitis	0.21753
DB01407	Diabetes mellitus	0.13191
DB01407	Down syndrome	0.05096
DB01407	Drug abuse	0.1475
DB01407	Embryoma	0.02758
DB01407	Endometriosis	0.03714
DB01407	Endometrium cancer	0.10847
DB01407	Enteritis	0.09951
DB01407	Epilepsy	0.10893
DB01407	Familial Mediterranean fever	0.11547
DB01407	Fanconi's anemia	0.08944
DB01407	Glaucoma	0.48021
DB01407	Gram-Negative bacterial infection	0.10067
DB01407	Granulomatous disease	0.1291
DB01407	Graves' disease	0.43608
DB01407	HIV infection	0.04284
DB01407	Heart failure	0.43101
DB01407	Hepatitis B	0.10847
DB01407	Histiocytosis	0.14907
DB01407	Hypertension	0.34304
DB01407	Hypothyroidism	0.10541
DB01407	Infertility	0.10986
DB01407	Intermediate coronary syndrome	0.14907
DB01407	Ischemia	0.09298
DB01407	Kidney disease	0.05345
DB01407	Leprosy	0.11952
DB01407	Leukemia	0.0252
DB01407	Lichen planus	0.15811
DB01407	Lipodystrophy	0.12403
DB01407	Liver cancer	0.06688
DB01407	Lung cancer	0.15057
DB01407	Lupus erythematosus	0.03821
DB01407	Malaria	0.54851
DB01407	Malignant glioma	0.08305
DB01407	Melanoma	0.03727
DB01407	Metabolism disease	0.50324
DB01407	Migraine	0.08305
DB01407	Movement disorder	0.21495
DB01407	Mucocutaneous lymph node syndrome	0.09759
DB01407	Multiple myeloma	0.05872
DB01407	Multiple sclerosis	0.04613
DB01407	Muscular dystrophies	0.08771

DB01407	Mycoses	0.2
DB01407	Myopathy	0.29429
DB01407	Narcolepsy	0.16903
DB01407	Nasopharyngeal cancer	0.08607
DB01407	Necrotizing enterocolitis	0.18257
DB01407	Nephrosis	0.1
DB01407	Neurodegenerative disorder	0.11886
DB01407	Obesity	0.31192
DB01407	Oligospermia	0.10439
DB01407	Otitis media	0.14907
DB01407	Ovary cancer	0.1
DB01407	Pancreas cancer	0.04637
DB01407	Pancreatitis	0.06984
DB01407	Periodontitis	0.0682
DB01407	Polycystic kidney	0.09084
DB01407	Polycystic ovary syndrome	0.31894
DB01407	Pre-Eclampsia	0.0527
DB01407	Premature birth	0.65519
DB01407	Prostate cancer	0.15443
DB01407	Psoriasis	0.10541
DB01407	Pulmonary fibrosis	0.08452
DB01407	Renal Cell cancer	0.05199
DB01407	Respiratory tract disease	0.07008
DB01407	Rheumatoid arthritis	0.16758
DB01407	Sarcoidosis	0.08771
DB01407	Schizophrenia	0.034
DB01407	Sickle cell disease	0.25662
DB01407	Silicosis	0.16903
DB01407	Sinusitis	0.06502
DB01407	Stomach cancer	0.03766
DB01407	Stroke	0.05064
DB01407	Subarachnoid hemorrhage	0.62503
DB01407	Systemic infection	0.1026
DB01407	Testicular dysfunction	0.03205
DB01407	Thrombophilia	0.10541
DB01407	Thyroid cancer	0.07785
DB01407	Tuberculosis	0.0603
DB01407	Tuberous sclerosis	0.11547
DB01407	Ulcerative colitis	0.04613
DB01580	Infertility, Male	0.09355
DB01580	Alimentary system disease	0.17411
DB01580	Alzheimer's disease	0.11168
DB01580	Arthritis	0.49484
DB01580	Atherosclerosis	0.20204
DB01580	Autistic disorder	0.32884
DB01580	Azoospermia	0.07075
DB01580	Breast cancer	0.01989

DB01580	Bronchial disease	0.98582
DB01580	Chronic obstructive airway disease	0.40566
DB01580	Conduct disorder	0.18128
DB01580	Cystic fibrosis	0.52253
DB01580	Dermatitis	0.23238
DB01580	Diabetes mellitus	0.1419
DB01580	Drug abuse	0.19656
DB01580	Enteritis	0.06222
DB01580	Epilepsy	0.13443
DB01580	Glaucoma	0.64434
DB01580	Gram-Negative bacterial infection	0.12423
DB01580	Graves' disease	0.56559
DB01580	Heart failure	0.50615
DB01580	Hypertension	0.40424
DB01580	Infertility	0.05977
DB01580	Ischemia	0.11474
DB01580	Liver cancer	0.0397
DB01580	Lung cancer	0.19665
DB01580	Malaria	0.60323
DB01580	Metabolism disease	0.55751
DB01580	Movement disorder	0.26525
DB01580	Myopathy	0.38199
DB01580	Neurodegenerative disorder	0.14667
DB01580	Obesity	0.36636
DB01580	Oligospermia	0.12882
DB01580	Polycystic kidney	0.1121
DB01580	Polycystic ovary syndrome	0.34105
DB01580	Premature birth	0.84618
DB01580	Prostate cancer	0.16986
DB01580	Respiratory tract disease	0.08648
DB01580	Rheumatoid arthritis	0.14875
DB01580	Sickle cell disease	0.34655
DB01580	Sinusitis	0.08024
DB01580	Subarachnoid hemorrhage	0.86492
DB01580	Testicular dysfunction	0.03955
DB04861	Infertility, Male	0.09355
DB04861	Alimentary system disease	0.17411
DB04861	Alzheimer's disease	0.11168
DB04861	Arthritis	0.49484
DB04861	Atherosclerosis	0.20204
DB04861	Autistic disorder	0.32884
DB04861	Azoospermia	0.07075
DB04861	Breast cancer	0.01989
DB04861	Bronchial disease	0.98582
DB04861	Chronic obstructive airway disease	0.40566
DB04861	Conduct disorder	0.18128
DB04861	Cystic fibrosis	0.52253

DB04861	Dermatitis	0.23238
DB04861	Diabetes mellitus	0.1419
DB04861	Drug abuse	0.19656
DB04861	Enteritis	0.06222
DB04861	Epilepsy	0.13443
DB04861	Glaucoma	0.64434
DB04861	Gram-Negative bacterial infection	0.12423
DB04861	Graves' disease	0.56559
DB04861	Heart failure	0.50615
DB04861	Hypertension	0.40424
DB04861	Infertility	0.05977
DB04861	Ischemia	0.11474
DB04861	Liver cancer	0.0397
DB04861	Lung cancer	0.19665
DB04861	Malaria	0.60323
DB04861	Metabolism disease	0.55751
DB04861	Movement disorder	0.26525
DB04861	Myopathy	0.38199
DB04861	Neurodegenerative disorder	0.14667
DB04861	Obesity	0.36636
DB04861	Oligospermia	0.12882
DB04861	Polycystic kidney	0.1121
DB04861	Polycystic ovary syndrome	0.34105
DB04861	Premature birth	0.84618
DB04861	Prostate cancer	0.16986
DB04861	Respiratory tract disease	0.08648
DB04861	Rheumatoid arthritis	0.14875
DB04861	Sickle cell disease	0.34655
DB04861	Sinusitis	0.08024
DB04861	Subarachnoid hemorrhage	0.86492
DB04861	Testicular dysfunction	0.03955
DB06262	Infertility, Male	0.08591
DB06262	Alimentary system disease	0.1599
DB06262	Alzheimer's disease	0.07859
DB06262	Amnionitis	0.08771
DB06262	Arthritis	0.41312
DB06262	Atherosclerosis	0.18437
DB06262	Attention deficit hyperactivity disorder	0.21118
DB06262	Autistic disorder	0.26159
DB06262	Azoospermia	0.06497
DB06262	Breast cancer	0.01827
DB06262	Bronchial disease	0.82455
DB06262	Cholelithiasis	0.06325
DB06262	Chronic obstructive airway disease	0.33531
DB06262	Common cold	0.08452
DB06262	Conduct disorder	0.16648
DB06262	Cystic fibrosis	0.43229

DB06262	Dermatitis	0.18312
DB06262	Diabetes mellitus	0.12943
DB06262	Drug abuse	0.14931
DB06262	Encephalopathies	0.04428
DB06262	Endometrium cancer	0.0767
DB06262	Enteritis	0.05714
DB06262	Epilepsy	0.12346
DB06262	Glaucoma	0.48069
DB06262	Gram-Negative bacterial infection	0.11409
DB06262	Graves' disease	0.46053
DB06262	Heart failure	0.39381
DB06262	Hypertension	0.46512
DB06262	Infertility	0.05489
DB06262	Ischemia	0.10537
DB06262	Kidney failure	0.10742
DB06262	Liver cancer	0.03646
DB06262	Lung cancer	0.15733
DB06262	Malaria	0.49213
DB06262	Metabolism disease	0.50929
DB06262	Movement disorder	0.2436
DB06262	Myopathy	0.31041
DB06262	Nervous system disease	0.19111
DB06262	Neurodegenerative disorder	0.1347
DB06262	Obesity	0.37776
DB06262	Oligospermia	0.1183
DB06262	Polycystic kidney	0.10295
DB06262	Polycystic ovary syndrome	0.31097
DB06262	Pre-Eclampsia	0.03727
DB06262	Premature birth	0.69631
DB06262	Prostate cancer	0.15512
DB06262	Respiratory tract disease	0.07942
DB06262	Rheumatoid arthritis	0.13558
DB06262	Schizophrenia	0.06401
DB06262	Sickle cell disease	0.25415
DB06262	Sinusitis	0.07369
DB06262	Subarachnoid hemorrhage	0.59342
DB06262	Systemic infection	0.03627
DB06262	Testicular dysfunction	0.03632
DB08807	Infertility, Male	0.05392
DB08807	Abortion	0.03465
DB08807	Alimentary system disease	0.10035
DB08807	Alzheimer's disease	0.06723
DB08807	Amnionitis	0.12403
DB08807	Amyotrophic lateral sclerosis	0.27109
DB08807	Anorexia nervosa	0.19428
DB08807	Arthritis	0.29013
DB08807	Atherosclerosis	0.15054

DB08807	Attention deficit hyperactivity disorder	0.15048
DB08807	Autistic disorder	0.19434
DB08807	Azoospermia	0.04078
DB08807	Behavior disease	0.75469
DB08807	Bipolar disorder	0.25587
DB08807	Breast cancer	0.01146
DB08807	Bronchial disease	0.57782
DB08807	Cholelithiasis	0.08944
DB08807	Chronic obstructive airway disease	0.23825
DB08807	Conduct disorder	0.10448
DB08807	Cystic fibrosis	0.30684
DB08807	Depression	0.39005
DB08807	Dermatitis	0.3674
DB08807	Diabetes mellitus	0.22525
DB08807	Drug abuse	0.23125
DB08807	Endometrium cancer	0.10847
DB08807	Enteritis	0.03586
DB08807	Epilepsy	0.49963
DB08807	Glaucoma	0.3846
DB08807	Gram-Negative bacterial infection	0.0716
DB08807	Graves' disease	0.333
DB08807	Heart failure	0.30019
DB08807	Hepatitis C	0.45243
DB08807	Hypertension	0.66133
DB08807	Infertility	0.03445
DB08807	Ischemia	0.06613
DB08807	Liver cancer	0.02288
DB08807	Lung cancer	0.11611
DB08807	Malaria	0.35505
DB08807	Metabolism disease	0.39929
DB08807	Migraine	0.49201
DB08807	Movement disorder	0.15289
DB08807	Myopathy	0.22498
DB08807	Neurodegenerative disorder	0.08454
DB08807	Obesity	0.21726
DB08807	Oligospermia	0.07425
DB08807	Panic disorder	0.61998
DB08807	Polycystic kidney	0.06461
DB08807	Polycystic ovary syndrome	0.20182
DB08807	Pre-Eclampsia	0.0527
DB08807	Premature birth	0.49733
DB08807	Prostate cancer	0.09998
DB08807	Respiratory tract disease	0.04984
DB08807	Rheumatoid arthritis	0.11553
DB08807	Sickle cell disease	0.20737
DB08807	Sinusitis	0.04625
DB08807	Stroke	0.23234

DB08807	Subarachnoid hemorrhage	0.52243
DB08807	Sudden infant death syndrome	0.7222
DB08807	Systemic infection	0.0513
DB08807	Testicular dysfunction	0.02279
DB08808	Infertility, Male	0.09355
DB08808	Alimentary system disease	0.17411
DB08808	Alzheimer's disease	0.10234
DB08808	Amnionitis	0.16013
DB08808	Arthritis	0.47874
DB08808	Atherosclerosis	0.23338
DB08808	Autistic disorder	0.3131
DB08808	Azoospermia	0.07075
DB08808	Breast cancer	0.01989
DB08808	Bronchial disease	0.95435
DB08808	Cholelithiasis	0.11547
DB08808	Chronic obstructive airway disease	0.39116
DB08808	Conduct disorder	0.18128
DB08808	Cystic fibrosis	0.504
DB08808	Dermatitis	0.22058
DB08808	Diabetes mellitus	0.13507
DB08808	Drug abuse	0.1844
DB08808	Endometrium cancer	0.14003
DB08808	Enteritis	0.06222
DB08808	Epilepsy	0.13443
DB08808	Glaucoma	0.60108
DB08808	Gram-Negative bacterial infection	0.12423
DB08808	Graves' disease	0.54265
DB08808	Heart failure	0.47849
DB08808	Hypertension	0.42936
DB08808	Infertility	0.05977
DB08808	Ischemia	0.11474
DB08808	Liver cancer	0.0397
DB08808	Lung cancer	0.18759
DB08808	Malaria	0.57914
DB08808	Metabolism disease	0.62918
DB08808	Movement disorder	0.26525
DB08808	Myopathy	0.36626
DB08808	Neurodegenerative disorder	0.14667
DB08808	Obesity	0.34639
DB08808	Oligospermia	0.12882
DB08808	Polycystic kidney	0.1121
DB08808	Polycystic ovary syndrome	0.32386
DB08808	Pre-Eclampsia	0.06804
DB08808	Premature birth	0.81471
DB08808	Prostate cancer	0.16309
DB08808	Respiratory tract disease	0.08648
DB08808	Rheumatoid arthritis	0.17615

DB08808	Sickle cell disease	0.32158
DB08808	Sinusitis	0.08024
DB08808	Subarachnoid hemorrhage	0.78667
DB08808	Systemic infection	0.06623
DB08808	Testicular dysfunction	0.03955
DB00281	Hemorrhagic fevers, Viral	0.06999
DB00281	Pemphigoid, Bullous	0.03553
DB00281	Pleural effusion, Malignant	0.43975
DB00281	Purpura, Thrombocytopenic, Idiopathic	0.03395
DB00281	Abortion	0.03996
DB00281	Adenovirus infection	0.26167
DB00281	Alzheimer's disease	0.02164
DB00281	Angiomyolipoma	0.91342
DB00281	Aplastic anemia	0.05803
DB00281	Arteriopathy	0.16667
DB00281	Asthma	0.30593
DB00281	Atherosclerosis	0.04091
DB00281	Atopic rhinitis	1.1291
DB00281	Autistic disorder	0.04611
DB00281	Autoimmune disease	0.07934
DB00281	Barrett's esophagus	0.43465
DB00281	Breast cancer	0.04025
DB00281	Cancer	0.12001
DB00281	Charcot-Marie-Tooth disease	0.16712
DB00281	Cholelithiasis	0.67345
DB00281	Colon cancer	0.02367
DB00281	Common wart	1.11168
DB00281	Congenital abnormality	0.03769
DB00281	Cytomegalovirus infection	0.88382
DB00281	Diabetes mellitus	0.20067
DB00281	Drug abuse	0.29994
DB00281	Endometriosis	0.23709
DB00281	Esotropia	0.70805
DB00281	Gastrointestinal tumor	0.68901
DB00281	Glaucoma	0.51798
DB00281	Heart disease	0.15076
DB00281	Hemorrhagic disorder	0.05478
DB00281	Herpes	0.03064
DB00281	Hypercholesterolemia	0.91765
DB00281	Intermediate coronary syndrome	0.06982
DB00281	Intestinal disease	0.125
DB00281	Ischemia	0.06402
DB00281	Leukemia	0.08274
DB00281	Long QT syndrome	0.14434
DB00281	Lupus erythematosus	0.2608
DB00281	Muscular atrophy	0.18128
DB00281	Myopathy	0.04866

DB00281	Optic atrophy	1.17735
DB00281	Oral cancer	0.35218
DB00281	Osteitis deformans	0.08799
DB00281	Papillary adenocarcinoma	1.28868
DB00281	Papillomavirus infection	0.09218
DB00281	Penile disease	0.06551
DB00281	Pneumoconiosis	0.23205
DB00281	Primary hyperparathyroidism	0.50443
DB00281	Prostate cancer	0.0261
DB00281	Pulmonary fibrosis	0.07546
DB00281	Rheumatoid arthritis	0.03733
DB00281	Schizophrenia	0.28739
DB00281	Skin cancer	0.56413
DB00281	Stroke	0.32566
DB00281	Subarachnoid hemorrhage	0.06249
DB00281	Sudden infant death syndrome	0.10911
DB00281	Takayasu's arteritis	0.05843
DB00281	Temporal arteritis	0.06831
DB00281	Testicular dysfunction	0.36461
DB00281	Thrombocytopenia	0.05206
DB00281	Thrombophlebitis	0.88726
DB00281	Yersinia infection	0.33635
DB00473	Breast cancer	0.03402
DB00473	Congenital abnormality	0.0533
DB00473	Drug abuse	0.06623
DB00473	Heart disease	0.2132
DB00473	Intestinal disease	0.17678
DB00473	Ischemia	0.09054
DB00473	Long QT syndrome	0.20412
DB00473	Sudden infant death syndrome	0.1543
DB00527	Abortion	0.02905
DB00527	Achalasia and cardiospasm	0.12469
DB00527	Alzheimer's disease	0.05292
DB00527	Amyloidosis	0.06639
DB00527	Asthma	0.04371
DB00527	Autistic disorder	0.06042
DB00527	Autoimmune disease	0.05572
DB00527	Bipolar disorder	0.04432
DB00527	Bladder cancer	0.04542
DB00527	Brain ischemia	0.18262
DB00527	Breast cancer	0.02778
DB00527	Cancer	0.03396
DB00527	Celiac disease	0.08498
DB00527	Colon cancer	0.0172
DB00527	Congenital abnormality	0.07589
DB00527	Dental plaque	0.06543
DB00527	Diabetes mellitus	0.0125

DB00527	Drug abuse	0.08647
DB00527	Eating disorder	0.10416
DB00527	Epstein-Barr virus infection	0.09263
DB00527	Esophageal tumor	0.07695
DB00527	Esophagus cancer	0.03074
DB00527	Esotropia	0.08852
DB00527	Eye cancer	0.10972
DB00527	Fanconi's anemia	0.0433
DB00527	Glaucoma	0.07631
DB00527	Gram-Negative bacterial infection	0.15306
DB00527	Graves' disease	0.10951
DB00527	HIV infection	0.05185
DB00527	Heart disease	0.17408
DB00527	Herpes	0.07492
DB00527	Intestinal disease	0.14434
DB00527	Ischemia	0.13746
DB00527	Keratosis	0.08781
DB00527	Leukemia	0.03912
DB00527	Lichen planus	0.17067
DB00527	Long QT syndrome	0.16667
DB00527	Lung cancer	0.0366
DB00527	Lupus erythematosus	0.03596
DB00527	Malignant glioma	0.04453
DB00527	Melanoma	0.02281
DB00527	Pre-Eclampsia	0.04147
DB00527	Prion disease	0.14306
DB00527	Rabies	0.02116
DB00527	Rheumatoid arthritis	0.02622
DB00527	Schistosomiasis	0.15421
DB00527	Stroke	0.04015
DB00527	Sudden infant death syndrome	0.12599
DB00527	Thyroid gland disease	0.09641
DB00527	Tuberculosis	0.0826
DB00721	Stress disorder, post-traumatic	0.15811
DB00721	Behavior disease	0.06901
DB00721	Bipolar disorder	0.05064
DB00721	Brain disease	0.08032
DB00721	Breast cancer	0.02152
DB00721	Colon cancer	0.02654
DB00721	Depression	0.05872
DB00721	Drug abuse	0.04189
DB00721	Eating disorder	0.07785
DB00721	Embryoma	0.02758
DB00721	Epilepsy	0.06325
DB00721	Gilles de la Tourette syndrome	0.33806
DB00721	Herpes	0.12778
DB00721	Hypertension	0.03536

DB00721	Migraine	0.08305
DB00721	Obesity	0.0344
DB00721	Obsessive-compulsive disorder	0.13484
DB00721	Panic disorder	0.09759
DB00721	Psychotic disorder	0.07161
DB00721	Schizophrenia	0.034
DB01161	Stress disorder, post-traumatic	0.15811
DB01161	Behavior disease	0.06901
DB01161	Bipolar disorder	0.05064
DB01161	Brain disease	0.08032
DB01161	Breast cancer	0.02152
DB01161	Colon cancer	0.02654
DB01161	Depression	0.05872
DB01161	Drug abuse	0.04189
DB01161	Eating disorder	0.07785
DB01161	Embryoma	0.02758
DB01161	Gilles de la Tourette syndrome	0.33806
DB01161	Herpes	0.12778
DB01161	Hypertension	0.03536
DB01161	Migraine	0.08305
DB01161	Obesity	0.0344
DB01161	Obsessive-compulsive disorder	0.13484
DB01161	Panic disorder	0.09759
DB01161	Psychotic disorder	0.07161
DB01161	Schizophrenia	0.034
DB01173	Anorexia nervosa	0.07715
DB01173	Atopic rhinitis	0.09759
DB01173	Behavior disease	0.05832
DB01173	Bipolar disorder	0.0428
DB01173	Cancer	0.0334
DB01173	Colon cancer	0.02243
DB01173	Connective tissue disease	0.44649
DB01173	Diabetes mellitus	0.01989
DB01173	Drug abuse	0.0354
DB01173	Hypertension	0.02988
DB01173	Infertility	0.05192
DB01173	Multiple endocrine neoplasia	0.12599
DB01173	Neuroendocrine tumor	0.13363
DB01173	Panic disorder	0.08248
DB01173	Parkinson disease	0.04174
DB01173	Schizophrenia	0.02874
DB06218	Arteriopathy	0.19245
DB06218	Autistic disorder	0.07001
DB06218	Prostate cancer	0.03014
DB00975	Asthma	0.04082
DB00975	Atherosclerosis	0.03501
DB00975	Autistic disorder	0.06063

DB00975	Chronic obstructive airway disease	0.0559
DB00975	Combined immunodeficiency	0.20412
DB00975	Diabetes mellitus	0.02632
DB00975	Hydatidiform mole	0.35355
DB00975	Hypertension	0.03953
DB00975	Kidney failure	0.05661
DB00975	Leukemia	0.02817
DB00975	Lupus erythematosus	0.04272
DB00975	Mental retardation	0.06455
DB00975	Nephrosis	0.1118
DB00975	Peptic ulcer	0.11785
DB00975	Pre-Eclampsia	0.05893
DB00143	Hepatitis, Autoimmune	0.09366
DB00143	Infertility, Male	0.03311
DB00143	Actinic keratosis	0.06131
DB00143	Amnionitis	0.04499
DB00143	Amyotrophic lateral sclerosis	0.02187
DB00143	Aplastic anemia	0.10148
DB00143	Asthma	0.05298
DB00143	Atherosclerosis	0.03407
DB00143	Autistic disorder	0.03934
DB00143	Autoimmune disease	0.0176
DB00143	Azoospermia	0.06623
DB00143	Barrett's esophagus	0.08885
DB00143	Behcet syndrome	0.06952
DB00143	Breast cancer	0.0078
DB00143	Cancer	0.02392
DB00143	Cardiovascular disease	0.02474
DB00143	Cerebrovascular disorder	0.1062
DB00143	Chronic obstructive airway disease	0.07255
DB00143	Chronic simple glaucoma	0.06917
DB00143	Colon cancer	0.01925
DB00143	Cystic fibrosis	0.02317
DB00143	Deafness	0.07024
DB00143	Dermatitis	0.04424
DB00143	Diabetes mellitus	0.03415
DB00143	Drug abuse	0.03039
DB00143	Drug-Induced dyskinesia	0.12263
DB00143	Eating disorder	0.02824
DB00143	Embryoma	0.03001
DB00143	Emphysema	0.03311
DB00143	Endometriosis	0.04042
DB00143	Fatty liver	0.04683
DB00143	Gallbaldder cancer	0.07255
DB00143	Glucose intolerance	0.03722
DB00143	Hepatitis	0.12566
DB00143	Herpes	0.02317

DB00143	Hyperglycemia	0.02598
DB00143	Hypertension	0.01282
DB00143	Infertility	0.02228
DB00143	Intractable epilepsy	0.05735
DB00143	Kidney failure	0.0551
DB00143	Kuhnt-Junius degeneration	0.19868
DB00143	Larynx cancer	0.0513
DB00143	Leukemia	0.01828
DB00143	Liver cancer	0.02518
DB00143	Liver disease	0.05006
DB00143	Lung cancer	0.01133
DB00143	Multiple sclerosis	0.03346
DB00143	Obesity	0.01248
DB00143	Oligospermia	0.08377
DB00143	Oral cancer	0.0883
DB00143	Ovarian cancer	0.0163
DB00143	Pancreatitis	0.076
DB00143	Panic disorder	0.0354
DB00143	Parkinson disease	0.01791
DB00143	Peptic esophagitis	0.1539
DB00143	Periodontitis	0.04948
DB00143	Polycystic ovary syndrome	0.04297
DB00143	Pre-Eclampsia	0.03824
DB00143	Premature birth	0.07869
DB00143	Primary biliary cirrhosis	0.02914
DB00143	Prostate cancer	0.01694
DB00143	Psychotic disorder	0.02598
DB00143	Renal Cell cancer	0.01886
DB00143	Rheumatoid arthritis	0.01986
DB00143	Schizophrenia	0.02467
DB00143	Silicosis	0.06131
DB00143	Sinusitis	0.03627
DB00143	Squamous cell cancer	0.01656
DB00143	Stroke	0.03674
DB00143	Testicular dysfunction	0.04635
DB00143	Tuberculosis	0.06562
DB00143	Uveitis	0.04056
DB00143	Varicosity	0.06131
DB00143	Vascular dementia	0.05407
DB00143	Vascular disease	0.05923
DB00143	Vitiligo	0.07647
DB00128	Abortion	0.02728
DB00128	Amyloidosis	0.04124
DB00128	Cancer	0.02985
DB00128	Embryoma	0.01346
DB00128	Encephalopathies	0.06111
DB00128	Heart failure	0.02326

DB00128	Hypertension	0.01725
DB00128	Leukemia	0.02459
DB00128	Leukoencephalopathy	0.03637
DB00128	Metabolism disease	0.03494
DB00128	Multiple sclerosis	0.02251
DB00128	Neoplasm metastasis	0.01776
DB00128	Obsessive-compulsive disorder	0.0658
DB00128	Rabies	0.04849
DB00128	Renal Cell cancer	0.02537
DB00128	Schizophrenia	0.01659
DB00128	Solid tumor	0.1895
DB00128	Stomach cancer	0.01838
DB00144	Brain tumor	0.11126
DB00144	Breast cancer	0.05109
DB00144	CNS metastases	0.1118
DB00144	Melanoma	0.09901
DB00144	Stomach cancer	0.07297
DB02187	Breast cancer	0.04811
DB02187	Endometriosis	0.08305
DB02187	Endometrium cancer	0.24254
DB02187	Esotropia	0.28868
DB02187	Polycystic ovary syndrome	0.13245
DB02187	Prostate cancer	0.0522
DB02187	Stroke	0.11323
DB00192	Breast cancer	0.04811
DB00192	Congenital abnormality	0.07538
DB00192	Drug abuse	0.09366
DB00192	Heart disease	0.30151
DB00192	Intestinal disease	0.25
DB00192	Ischemia	0.12804
DB00192	Long QT syndrome	0.28868
DB00192	Sudden infant death syndrome	0.21822
DB00243	Arteriopathy	0.2357
DB00243	Breast cancer	0.03402
DB00243	Congenital abnormality	0.0533
DB00243	Drug abuse	0.06623
DB00243	Heart disease	0.2132
DB00243	Intestinal disease	0.17678
DB00243	Ischemia	0.09054
DB00243	Long QT syndrome	0.20412
DB00243	Prostate cancer	0.03691
DB00243	Sudden infant death syndrome	0.1543
DB00379	Breast cancer	0.12879
DB00379	Congenital abnormality	0.0533
DB00379	Diabetes mellitus	0.04562
DB00379	Drug abuse	0.06623
DB00379	Endometriosis	0.14385

DB00379	HIV infection	0.0614
DB00379	Heart disease	0.2132
DB00379	Infertility	0.27969
DB00379	Intestinal disease	0.17678
DB00379	Ischemia	0.09054
DB00379	Leukemia	0.11187
DB00379	Long QT syndrome	0.20412
DB00379	Lung cancer	0.11356
DB00379	Lymphoma	0.19592
DB00379	Stomach cancer	0.12091
DB00379	Sudden infant death syndrome	0.1543
DB00379	Uterine fibroids	0.5741
DB00379	Yersinia infection	0.16462
DB00564	Breast cancer	0.04811
DB00564	Congenital abnormality	0.07538
DB00564	Drug abuse	0.09366
DB00564	Heart disease	0.30151
DB00564	Intestinal disease	0.25
DB00564	Ischemia	0.12804
DB00564	Long QT syndrome	0.28868
DB00564	Sudden infant death syndrome	0.21822
DB00680	Breast cancer	0.04811
DB00680	Congenital abnormality	0.07538
DB00680	Drug abuse	0.09366
DB00680	Heart disease	0.30151
DB00680	Intestinal disease	0.25
DB00680	Ischemia	0.12804
DB00680	Long QT syndrome	0.28868
DB00680	Sudden infant death syndrome	0.21822
DB00750	Breast cancer	0.04811
DB00750	Congenital abnormality	0.07538
DB00750	Drug abuse	0.09366
DB00750	Heart disease	0.30151
DB00750	Intestinal disease	0.25
DB00750	Ischemia	0.12804
DB00750	Long QT syndrome	0.28868
DB00750	Sudden infant death syndrome	0.21822
DB00754	Breast cancer	0.04811
DB00754	Congenital abnormality	0.07538
DB00754	Drug abuse	0.09366
DB00754	Heart disease	0.30151
DB00754	Intestinal disease	0.25
DB00754	Ischemia	0.12804
DB00754	Long QT syndrome	0.28868
DB00754	Sudden infant death syndrome	0.21822
DB00776	Breast cancer	0.04811
DB00776	Congenital abnormality	0.07538

DB00776	Drug abuse	0.09366
DB00776	Heart disease	0.30151
DB00776	Intestinal disease	0.25
DB00776	Ischemia	0.12804
DB00776	Long QT syndrome	0.28868
DB00776	Sudden infant death syndrome	0.21822
DB00868	Breast cancer	0.04811
DB00868	Congenital abnormality	0.07538
DB00868	Drug abuse	0.09366
DB00868	Heart disease	0.30151
DB00868	Intestinal disease	0.25
DB00868	Ischemia	0.12804
DB00868	Long QT syndrome	0.28868
DB00868	Sudden infant death syndrome	0.21822
DB01035	Adenovirus infection	0.02274
DB01035	Alzheimer's disease	0.01869
DB01035	Aortic valve disease	0.07209
DB01035	Breast cancer	0.04846
DB01035	Cancer	0.06052
DB01035	Colon cancer	0.02111
DB01035	Congenital abnormality	0.07463
DB01035	Drug abuse	0.06623
DB01035	Emphysema	0.05278
DB01035	Endometriosis	0.02263
DB01035	Eye cancer	0.07231
DB01035	Gastritis	0.03841
DB01035	HIV infection	0.04973
DB01035	Heart disease	0.2132
DB01035	Helicobacter infection	0.06256
DB01035	Intestinal disease	0.17678
DB01035	Ischemia	0.09054
DB01035	Long QT syndrome	0.20412
DB01035	Parkinson disease	0.05215
DB01035	Rheumatoid arthritis	0.01728
DB01035	Severe acute respiratory syndrome	0.06563
DB01035	Sudden infant death syndrome	0.1543
DB01035	Tuberous sclerosis	0.04119
DB01035	Virus disease	0.04438
DB01056	Breast cancer	0.04811
DB01056	Congenital abnormality	0.07538
DB01056	Drug abuse	0.09366
DB01056	Heart disease	0.30151
DB01056	Intestinal disease	0.25
DB01056	Ischemia	0.12804
DB01056	Long QT syndrome	0.28868
DB01056	Sudden infant death syndrome	0.21822
DB01195	Breast cancer	0.03402

DB01195	Common cold	0.18898
DB01195	Congenital abnormality	0.0533
DB01195	Drug abuse	0.13245
DB01195	Heart disease	0.2132
DB01195	Intestinal disease	0.17678
DB01195	Ischemia	0.09054
DB01195	Long QT syndrome	0.20412
DB01195	Myopathy	0.08575
DB01195	Sudden infant death syndrome	0.1543
DB01228	Breast cancer	0.04811
DB01228	Congenital abnormality	0.07538
DB01228	Drug abuse	0.09366
DB01228	Heart disease	0.30151
DB01228	Intestinal disease	0.25
DB01228	Ischemia	0.12804
DB01228	Long QT syndrome	0.28868
DB01228	Sudden infant death syndrome	0.21822
DB01320	Breast cancer	0.04811
DB01320	Congenital abnormality	0.07538
DB01320	Drug abuse	0.09366
DB01320	Heart disease	0.30151
DB01320	Intestinal disease	0.25
DB01320	Ischemia	0.12804
DB01320	Long QT syndrome	0.28868
DB01320	Sudden infant death syndrome	0.21822
DB01426	Breast cancer	0.04811
DB01426	Congenital abnormality	0.07538
DB01426	Drug abuse	0.09366
DB01426	Heart disease	0.30151
DB01426	Intestinal disease	0.25
DB01426	Ischemia	0.12804
DB01426	Long QT syndrome	0.28868
DB01426	Sudden infant death syndrome	0.21822
DB01429	Abortion	0.02905
DB01429	Achalasia and cardiospasm	0.12469
DB01429	Alzheimer's disease	0.05292
DB01429	Amyloidosis	0.06639
DB01429	Asthma	0.04371
DB01429	Autistic disorder	0.06042
DB01429	Autoimmune disease	0.05572
DB01429	Bipolar disorder	0.04432
DB01429	Bladder cancer	0.04542
DB01429	Brain ischemia	0.18262
DB01429	Breast cancer	0.03402
DB01429	Cancer	0.03396
DB01429	Celiac disease	0.08498
DB01429	Colon cancer	0.0172

DB01429	Congenital abnormality	0.08567
DB01429	Dental plaque	0.06543
DB01429	Diabetes mellitus	0.0125
DB01429	Drug abuse	0.09862
DB01429	Eating disorder	0.10416
DB01429	Epstein-Barr virus infection	0.09263
DB01429	Esophageal tumor	0.07695
DB01429	Esophagus cancer	0.03074
DB01429	Esotropia	0.08852
DB01429	Eye cancer	0.10972
DB01429	Fanconi's anemia	0.0433
DB01429	Glaucoma	0.07631
DB01429	Gram-Negative bacterial infection	0.15306
DB01429	Graves' disease	0.10951
DB01429	HIV infection	0.05185
DB01429	Heart disease	0.2132
DB01429	Herpes	0.07492
DB01429	Intestinal disease	0.17678
DB01429	Ischemia	0.15408
DB01429	Keratosis	0.08781
DB01429	Leukemia	0.03912
DB01429	Lichen planus	0.17067
DB01429	Long QT syndrome	0.20412
DB01429	Lung cancer	0.0366
DB01429	Lupus erythematosus	0.03596
DB01429	Malignant glioma	0.04453
DB01429	Melanoma	0.02281
DB01429	Pre-Eclampsia	0.04147
DB01429	Prion disease	0.14306
DB01429	Rabies	0.02116
DB01429	Rheumatoid arthritis	0.02622
DB01429	Schistosomiasis	0.15421
DB01429	Stroke	0.04015
DB01429	Sudden infant death syndrome	0.1543
DB01429	Thyroid gland disease	0.09641
DB01429	Tuberculosis	0.0826
DB00149	Advanced cancer	0.1291
DB00149	Cancer	0.03686
DB00149	Colon cancer	0.02423
DB00149	Gastrointestinal stromal tumor	0.16667
DB00149	Nasopharyngeal cancer	0.07857
DB00149	Rabies	0.04536
DB00149	Solid tumor	0.1895
DB00102	Prostatic hypertrophy, Benign	0.38966
DB00102	Skin disease, Genetic	0.17254
DB00102	Abortion	0.04971
DB00102	Alzheimer's disease	0.07963

DB00102	Atherosclerosis	0.03599
DB00102	Brain tumor	0.10592
DB00102	Cancer	0.03754
DB00102	Colon cancer	0.05889
DB00102	Congenital heart disease	0.42198
DB00102	Deafness	0.27241
DB00102	Gastritis	0.04792
DB00102	HIV infection	0.03325
DB00102	Hemorrhagic disorder	0.14248
DB00102	Herpes	0.07969
DB00102	Infection	0.08699
DB00102	Influenza	0.18335
DB00102	Leukemia	0.08155
DB00102	Lymphoproliferative disorder	0.28028
DB00102	Multiple sclerosis	0.12167
DB00102	Myeloproliferative disease	0.18776
DB00102	Obesity	0.09007
DB00102	Overnutrition	0.08789
DB00102	Pancreatitis	0.16636
DB00102	Prion disease	0.37152
DB00102	Prostate cancer	0.07877
DB00102	Skin cancer	0.15661
DB00102	Solid tumor	0.24965
DB00102	Turner's syndrome	0.14876
DB04942	Aseptic necrosis of bone	0.1563
DB04942	Breast cancer	0.08511
DB04942	Cancer	0.05624
DB04942	Congenital abnormality	0.0533
DB04942	Dermatitis	0.0871
DB04942	Esophagus cancer	0.29407
DB04942	Leukemia	0.1255
DB04942	Leukoencephalopathy	0.26204
DB04942	Lung cancer	0.07631
DB04942	Mucopolysaccharidosis	0.22585
DB04942	Neuroblastoma	0.13218
DB04942	Obesity	0.15467
DB04942	Prostate cancer	0.11786
DB04942	Rheumatism	0.18028
DB04942	Rheumatoid arthritis	0.14121
DB00222	Atherosclerosis	0.04042
DB00222	Diabetes mellitus	0.03039
DB00222	Hyperaldosteronism	0.18257
DB00222	Hyperglycemia	0.09245
DB00222	Hyperinsulinism	0.11111
DB00222	Infantile spasms	0.21822
DB00222	Kidney disease	0.06901
DB00222	Late pregnancy	0.16667

DB00222	Metabolism disease	0.09245
DB00222	Pancreas disease	0.12039
DB00222	Polycystic ovary syndrome	0.07647
DB01124	Hyperaldosteronism	0.22361
DB01124	Kidney disease	0.08452
DB01251	Congenital abnormality	0.0533
DB01251	Late pregnancy	0.20412
DB01382	Hyperaldosteronism	0.22361
DB01382	Kidney disease	0.08452
DB00009	Pleural effusion, Malignant	0.09933
DB00009	Abortion	0.0625
DB00009	Adrenoleukodystrophy	0.13363
DB00009	Alzheimer's disease	0.05913
DB00009	Amyloidosis	0.09449
DB00009	Aseptic necrosis of bone	0.15811
DB00009	Asthma	0.04082
DB00009	Atherosclerosis	0.17191
DB00009	Breast cancer	0.02406
DB00009	Cancer	0.06111
DB00009	Cardiovascular disease	0.07625
DB00009	Cerebrovascular disorder	0.10911
DB00009	Chronic simple glaucoma	0.1066
DB00009	Colon cancer	0.02967
DB00009	Congenital abnormality	0.03769
DB00009	Dental plaque	0.0635
DB00009	Dermatitis	0.13256
DB00009	Diabetes mellitus	0.02632
DB00009	Drug abuse	0.06753
DB00009	Embryoma	0.06166
DB00009	Esophagus cancer	0.07454
DB00009	Glomerulonephritis	0.125
DB00009	HIV infection	0.12091
DB00009	Hemorrhagic disorder	0.17678
DB00009	Herpes	0.0837
DB00009	Hyperglycemia	0.08006
DB00009	Hyperinsulinism	0.09623
DB00009	Hyperthyroidism	0.16667
DB00009	Infectious lung disease	0.1118
DB00009	Infiltrating cancer	0.13363
DB00009	Influenza	0.1
DB00009	Leukemia	0.02817
DB00009	Liver cancer	0.03881
DB00009	Liver disease	0.07715
DB00009	Lung cancer	0.06984
DB00009	Malignant glioma	0.09285
DB00009	Melanoma	0.04167
DB00009	Metabolism disease	0.08006

DB00009	Multiple sclerosis	0.05157
DB00009	Neoplasm metastasis	0.04069
DB00009	Obesity	0.07692
DB00009	Ovarian cancer	0.05025
DB00009	Pancreas cancer	0.1037
DB00009	Periodontal disease	0.11785
DB00009	Polyarthritis	0.05698
DB00009	Polycystic ovary syndrome	0.26065
DB00009	Prostate cancer	0.0522
DB00009	Respiratory distress syndrome	0.13868
DB00009	Rheumatic fever	1.1291
DB00009	Rheumatoid arthritis	0.08526
DB00009	Stomach cancer	0.04211
DB00009	Systemic infection	0.05735
DB00009	Thrombophilia	0.11785
DB00013	Pleural effusion, Malignant	0.07024
DB00013	Abortion	0.03953
DB00013	Adrenal gland hyperfunction	0.16616
DB00013	Adrenoleukodystrophy	0.08452
DB00013	Alzheimer's disease	0.06457
DB00013	Antiphospholipid syndrome	0.21082
DB00013	Aortic aneurysm	0.15034
DB00013	Aseptic necrosis of bone	0.1
DB00013	Asthma	0.05164
DB00013	Atherosclerosis	0.16323
DB00013	Breast cancer	0.06655
DB00013	Cancer	0.07558
DB00013	Cardiovascular disease	0.04822
DB00013	Chronic obstructive airway disease	0.03536
DB00013	Chronic simple glaucoma	0.06742
DB00013	Colon cancer	0.01876
DB00013	Congenital abnormality	0.04767
DB00013	Cystic fibrosis	0.04518
DB00013	Dental plaque	0.04016
DB00013	Dermatitis	0.11909
DB00013	Diabetes mellitus	0.06657
DB00013	Drug abuse	0.04775
DB00013	Embryoma	0.039
DB00013	Endometrial cancer	0.05505
DB00013	Endometriosis	0.02626
DB00013	Esophagus cancer	0.04714
DB00013	Glomerulonephritis	0.07906
DB00013	HIV infection	0.08192
DB00013	Hamman-Rich syndrome	0.06086
DB00013	Hemorrhagic disorder	0.1118
DB00013	Herpes	0.05919
DB00013	Hyperglycemia	0.05064

DB00013	Hyperinsulinism	0.06086
DB00013	Hypertension	0.025
DB00013	Hyperthyroidism	0.10541
DB00013	Infectious lung disease	0.07071
DB00013	Infiltrating cancer	0.08452
DB00013	Influenza	0.06325
DB00013	Ischemia	0.04049
DB00013	Kidney disease	0.0378
DB00013	Late pregnancy	0.18257
DB00013	Leukemia	0.03563
DB00013	Liver cancer	0.02454
DB00013	Liver disease	0.09759
DB00013	Lung cancer	0.06626
DB00013	Lupus erythematosus	0.02702
DB00013	Malignant glioma	0.05872
DB00013	Melanoma	0.02635
DB00013	Metabolism disease	0.05064
DB00013	Mitral valve disease	0.14142
DB00013	Multiple sclerosis	0.06523
DB00013	Neoplasm metastasis	0.12088
DB00013	Neuropathy	0.05064
DB00013	Obesity	0.02433
DB00013	Oral cancer	0.04303
DB00013	Ovarian cancer	0.06356
DB00013	Pancreas cancer	0.09837
DB00013	Peptic ulcer	0.23439
DB00013	Periodontal disease	0.14907
DB00013	Periodontitis	0.04822
DB00013	Polyarthritis	0.07207
DB00013	Polycystic ovary syndrome	0.30695
DB00013	Prostate cancer	0.13977
DB00013	Renal Cell cancer	0.1608
DB00013	Respiratory distress syndrome	0.08771
DB00013	Rheumatic fever	0.78876
DB00013	Rheumatoid arthritis	0.11606
DB00013	Scleroderma	0.06742
DB00013	Stomach cancer	0.02663
DB00013	Stroke	0.03581
DB00013	Systemic infection	0.07255
DB00013	Systemic scleroderma	0.15608
DB00013	Thrombophilia	0.07454
DB00013	Thyroid gland disease	0.07255
DB00015	Pleural effusion, Malignant	0.09933
DB00015	Abortion	0.0625
DB00015	Adrenoleukodystrophy	0.13363
DB00015	Alzheimer's disease	0.05913
DB00015	Amyloidosis	0.09449

DB00015	Aseptic necrosis of bone	0.15811
DB00015	Asthma	0.04082
DB00015	Atherosclerosis	0.17191
DB00015	Breast cancer	0.02406
DB00015	Cancer	0.06111
DB00015	Cardiovascular disease	0.07625
DB00015	Cerebrovascular disorder	0.10911
DB00015	Chronic simple glaucoma	0.1066
DB00015	Colon cancer	0.02967
DB00015	Congenital abnormality	0.03769
DB00015	Dental plaque	0.0635
DB00015	Dermatitis	0.13256
DB00015	Diabetes mellitus	0.02632
DB00015	Drug abuse	0.06753
DB00015	Embryoma	0.06166
DB00015	Esophagus cancer	0.07454
DB00015	Glomerulonephritis	0.125
DB00015	HIV infection	0.12091
DB00015	Hemorrhagic disorder	0.17678
DB00015	Herpes	0.0837
DB00015	Hyperglycemia	0.08006
DB00015	Hyperinsulinism	0.09623
DB00015	Hyperthyroidism	0.16667
DB00015	Infectious lung disease	0.1118
DB00015	Infiltrating cancer	0.13363
DB00015	Influenza	0.1
DB00015	Leukemia	0.02817
DB00015	Liver cancer	0.03881
DB00015	Liver disease	0.07715
DB00015	Lung cancer	0.06984
DB00015	Malignant glioma	0.09285
DB00015	Melanoma	0.04167
DB00015	Metabolism disease	0.08006
DB00015	Multiple sclerosis	0.05157
DB00015	Neoplasm metastasis	0.04069
DB00015	Obesity	0.07692
DB00015	Ovarian cancer	0.05025
DB00015	Pancreas cancer	0.1037
DB00015	Periodontal disease	0.11785
DB00015	Polyarthritis	0.05698
DB00015	Polycystic ovary syndrome	0.26065
DB00015	Prostate cancer	0.0522
DB00015	Respiratory distress syndrome	0.13868
DB00015	Rheumatic fever	1.1291
DB00015	Rheumatoid arthritis	0.08526
DB00015	Stomach cancer	0.04211
DB00015	Systemic infection	0.05735

DB00015	Thrombophilia	0.11785
DB00029	Pleural effusion, Malignant	0.09933
DB00029	Abortion	0.0625
DB00029	Adrenoleukodystrophy	0.13363
DB00029	Alzheimer's disease	0.05913
DB00029	Amyloidosis	0.09449
DB00029	Aseptic necrosis of bone	0.15811
DB00029	Asthma	0.04082
DB00029	Atherosclerosis	0.17191
DB00029	Breast cancer	0.02406
DB00029	Cancer	0.06111
DB00029	Cardiovascular disease	0.07625
DB00029	Cerebrovascular disorder	0.10911
DB00029	Chronic simple glaucoma	0.1066
DB00029	Colon cancer	0.02967
DB00029	Congenital abnormality	0.03769
DB00029	Dental plaque	0.0635
DB00029	Dermatitis	0.13256
DB00029	Diabetes mellitus	0.02632
DB00029	Drug abuse	0.06753
DB00029	Embryoma	0.06166
DB00029	Esophagus cancer	0.07454
DB00029	Glomerulonephritis	0.125
DB00029	HIV infection	0.12091
DB00029	Hemorrhagic disorder	0.17678
DB00029	Herpes	0.0837
DB00029	Hyperglycemia	0.08006
DB00029	Hyperinsulinism	0.09623
DB00029	Hyperthyroidism	0.16667
DB00029	Infectious lung disease	0.1118
DB00029	Infiltrating cancer	0.13363
DB00029	Influenza	0.1
DB00029	Leukemia	0.02817
DB00029	Liver cancer	0.03881
DB00029	Liver disease	0.07715
DB00029	Lung cancer	0.06984
DB00029	Malignant glioma	0.09285
DB00029	Melanoma	0.04167
DB00029	Metabolism disease	0.08006
DB00029	Multiple sclerosis	0.05157
DB00029	Neoplasm metastasis	0.04069
DB00029	Obesity	0.07692
DB00029	Ovarian cancer	0.05025
DB00029	Pancreas cancer	0.1037
DB00029	Periodontal disease	0.11785
DB00029	Polyarthritis	0.05698
DB00029	Polycystic ovary syndrome	0.26065

DB00029	Prostate cancer	0.0522
DB00029	Respiratory distress syndrome	0.13868
DB00029	Rheumatic fever	1.1291
DB00029	Rheumatoid arthritis	0.08526
DB00029	Stomach cancer	0.04211
DB00029	Systemic infection	0.05735
DB00029	Thrombophilia	0.11785
DB00031	Kidney tubular necrosis, acute	0.07777
DB00031	Pleural effusion, Malignant	0.07296
DB00031	Prostatic hypertrophy, Benign	0.2045
DB00031	Abortion	0.23347
DB00031	Achalasia and cardiospasm	0.81347
DB00031	Adenocarcinoma	0.04398
DB00031	Adenovirus infection	0.02642
DB00031	Adrenoleukodystrophy	0.16116
DB00031	Alzheimer's disease	0.1366
DB00031	Amnionitis	0.24429
DB00031	Amyloidosis	0.5811
DB00031	Amyotrophic lateral sclerosis	0.11108
DB00031	Antiphospholipid syndrome	0.20101
DB00031	Aortic aneurysm	0.04306
DB00031	Arteriopathy	0.36724
DB00031	Arthritis	0.07748
DB00031	Aseptic necrosis of bone	0.19069
DB00031	Asthma	0.0827
DB00031	Atherosclerosis	0.20642
DB00031	Autoimmune disease	0.04266
DB00031	Azoospermia	0.06674
DB00031	Behcet syndrome	0.08457
DB00031	Biliary Atresia	0.12984
DB00031	Bipolar disorder	0.11888
DB00031	Bladder cancer	0.28613
DB00031	Brain disease	0.06727
DB00031	Brain tumor	0.04086
DB00031	Breast cancer	0.06006
DB00031	Bronchiolitis obliterans	0.36724
DB00031	Bronchopulmonary dysplasia	0.25268
DB00031	Cancer	0.10657
DB00031	Cardiovascular disease	0.09196
DB00031	Celiac disease	0.04259
DB00031	Central nervous system disease	0.14509
DB00031	Cerebrovascular disorder	0.15403
DB00031	Cervical cancer	0.06216
DB00031	Chronic obstructive airway disease	0.13026
DB00031	Chronic simple glaucoma	0.06428
DB00031	Cirrhosis	0.05096
DB00031	Colon cancer	0.15684

DB00031	Common cold	0.05673
DB00031	Congenital abnormality	0.04545
DB00031	Cystic fibrosis	0.06771
DB00031	Dental plaque	0.161
DB00031	Dermatitis	0.09139
DB00031	Diabetes mellitus	0.09135
DB00031	Down syndrome	0.08447
DB00031	Drug abuse	0.0248
DB00031	Eating disorder	0.04273
DB00031	Embryoma	0.08332
DB00031	Emphysema	0.05311
DB00031	Encephalitis	0.13494
DB00031	Endometrial cancer	0.05249
DB00031	Endometriosis	0.03718
DB00031	Enteritis	0.09178
DB00031	Esophageal tumor	0.05892
DB00031	Esophagus cancer	0.06848
DB00031	Esotropia	0.06777
DB00031	Ewings sarcoma	0.07584
DB00031	Fanconi's anemia	0.02506
DB00031	Gastritis	0.04462
DB00031	Glaucoma	0.05842
DB00031	Glomerulonephritis	0.27846
DB00031	Growth retardation	0.09596
DB00031	HIV infection	0.21015
DB00031	HTLV-I infection	0.11854
DB00031	Heart failure	0.09924
DB00031	Helicobacter infection	0.06294
DB00031	Hemorrhagic disorder	0.1599
DB00031	Henoch-Schoenlein purpura	0.36724
DB00031	Hepatitis	0.0918
DB00031	Hepatitis B	0.28921
DB00031	Hepatitis C	0.04446
DB00031	Hereditary disease	0.04887
DB00031	Herpes	0.06148
DB00031	Hodgkin's disease	0.05786
DB00031	Hyperglycemia	0.04828
DB00031	Hyperinsulinism	0.05803
DB00031	Hypertension	0.0551
DB00031	Hyperthyroidism	0.1005
DB00031	IGA glomerulonephritis	0.11509
DB00031	Infectious lung disease	0.06742
DB00031	Infiltrating cancer	0.19844
DB00031	Influenza	0.0603
DB00031	Intracranial aneurysm	0.22218
DB00031	Ischemia	0.08049
DB00031	Kaposi sarcoma	0.04287

DB00031	Keratoconjunctivitis Sicca	0.2023
DB00031	Kidney disease	0.10039
DB00031	Leukemia	0.16149
DB00031	Lichen planus	0.13067
DB00031	Liver cancer	0.08537
DB00031	Liver disease	0.09305
DB00031	Liver metastases	0.13247
DB00031	Lung cancer	0.0912
DB00031	Lupus erythematosus	0.05329
DB00031	Lupus vulgaris	0.10588
DB00031	Malignant glioma	0.35433
DB00031	Melanoma	0.24739
DB00031	Meningioma	0.11544
DB00031	Metabolism disease	0.13283
DB00031	Metastasis to lymph nodes	0.14529
DB00031	Mucocutaneous lymph node syndrome	0.14233
DB00031	Multiple myeloma	0.05889
DB00031	Multiple sclerosis	0.1313
DB00031	Muscular dystrophies	0.05913
DB00031	Muscular dystrophy	0.14252
DB00031	Neoplasm metastasis	0.02454
DB00031	Nervous system disease	0.0918
DB00031	Neurodegenerative disorder	0.06219
DB00031	Obesity	0.15687
DB00031	Oral cancer	0.03615
DB00031	Ovarian cancer	0.0303
DB00031	Ovarian disease	0.08799
DB00031	Ovary cancer	0.08698
DB00031	Pancreas cancer	0.0938
DB00031	Pancreatitis	0.10854
DB00031	Parkinson disease	0.07088
DB00031	Periodontal disease	0.07107
DB00031	Periodontitis	0.08956
DB00031	Pituitary tumor	0.0654
DB00031	Polyarthritis	0.03436
DB00031	Polycystic ovary syndrome	0.17403
DB00031	Polymyositis	0.1901
DB00031	Pre-Eclampsia	0.32729
DB00031	Primary tumor	0.11772
DB00031	Prion disease	0.21906
DB00031	Prostate cancer	0.07352
DB00031	Psychotic disorder	0.08408
DB00031	Rabies	0.18236
DB00031	Renal tubular acidosis	0.03325
DB00031	Respiratory distress syndrome	0.08362
DB00031	Respiratory tract disease	0.08157
DB00031	Retinoblastoma	0.17408

DB00031	Rheumatic fever	0.44509
DB00031	Rheumatoid arthritis	0.05167
DB00031	Scleroderma	0.06428
DB00031	Sinusitis	0.07568
DB00031	Skin cancer	0.08543
DB00031	Skin disease	0.05533
DB00031	Stomach cancer	0.05219
DB00031	Stroke	0.03074
DB00031	Subacute sclerosing panencephalitis	0.36724
DB00031	Systemic infection	0.08682
DB00031	Systemic scleroderma	0.0732
DB00031	Takayasu's arteritis	0.05862
DB00031	Thrombophilia	0.07107
DB00031	Thyroid cancer	0.07759
DB00031	Transient hypertension of pregnancy	0.28921
DB00031	Tropical spastic paraparesis	0.1124
DB00031	Ulcerative colitis	0.06746
DB00031	Uterine fibroids	0.06552
DB00031	Varicosity	0.36724
DB00031	Werner syndrome	0.04304
DB00086	Adrenoleukodystrophy	0.18898
DB00086	Cancer	0.02606
DB00086	Congenital abnormality	0.0533
DB00086	Embryoma	0.0436
DB00086	Encephalitis	0.19612
DB00086	Endometriosis	0.05872
DB00086	HIV infection	0.06773
DB00086	Hemorrhagic disorder	0.125
DB00086	Lung cancer	0.04939
DB00086	Pancreas cancer	0.07332
DB00086	Premature birth	0.1715
DB00086	Prostate cancer	0.03691
DB00086	Pulmonary fibrosis	0.13363
DB00086	Stroke	0.08006
DB00086	Systemic infection	0.08111
DB00302	Adrenoleukodystrophy	0.26726
DB00302	Congenital abnormality	0.07538
DB00302	Embryoma	0.06166
DB00302	Hemorrhagic disorder	0.17678
DB00302	Lung cancer	0.06984
DB00302	Pancreas cancer	0.1037
DB00302	Prostate cancer	0.0522
DB00513	Adrenoleukodystrophy	0.18898
DB00513	Antiphospholipid syndrome	0.2357
DB00513	Atherosclerosis	0.04951
DB00513	Congenital abnormality	0.0533
DB00513	Diabetes mellitus	0.03722

DB00513	Embryoma	0.0436
DB00513	Hemorrhagic disorder	0.25
DB00513	Hypertension	0.0559
DB00513	Ischemia	0.09054
DB00513	Late pregnancy	0.20412
DB00513	Leukemia	0.03984
DB00513	Liver disease	0.10911
DB00513	Lung cancer	0.09877
DB00513	Multiple sclerosis	0.07293
DB00513	Ovarian cancer	0.07107
DB00513	Pancreas cancer	0.14665
DB00513	Periodontitis	0.10783
DB00513	Polycystic ovary syndrome	0.09366
DB00513	Prostate cancer	0.03691
DB00513	Rheumatoid arthritis	0.04327
DB00513	Stroke	0.08006
DB00513	Systemic infection	0.08111
DB06692	Adrenoleukodystrophy	0.1543
DB06692	Colon cancer	0.03426
DB06692	Congenital abnormality	0.08704
DB06692	Embryoma	0.0356
DB06692	Glomerulonephritis	0.14434
DB06692	Hemorrhagic disorder	0.10206
DB06692	Hypertension	0.04564
DB06692	Kidney failure	0.06537
DB06692	Lung cancer	0.04032
DB06692	Pancreas cancer	0.05987
DB06692	Prostate cancer	0.03014
DB00412	Colon cancer	0.04196
DB00412	Embryoma	0.0436
DB00412	Liver cancer	0.05488
DB00412	Mental retardation	0.09129
DB00412	Myopathy	0.08575
DB00025	Hypertension, Pulmonary	0.05271
DB00025	Kidney tubular necrosis, acute	0.09209
DB00025	Pleural effusion, Malignant	0.03054
DB00025	Prostatic hypertrophy, Benign	0.17123
DB00025	Abortion	0.19942
DB00025	Abruption placentae	0.28615
DB00025	Achalasia and cardiospasm	0.82909
DB00025	Adenovirus infection	0.08933
DB00025	Alzheimer's disease	0.10906
DB00025	Amnionitis	0.13452
DB00025	Amyloidosis	0.51666
DB00025	Amyotrophic lateral sclerosis	0.10384
DB00025	Aortic aneurysm	0.03606
DB00025	Arteriopathy	0.30749

DB00025	Arthritis	0.06487
DB00025	Asthma	0.02802
DB00025	Atherosclerosis	0.15796
DB00025	Autoimmune disease	0.03572
DB00025	Azoospermia	0.05588
DB00025	Bacterial infection	0.17756
DB00025	Behcet syndrome	0.11389
DB00025	Biliary Atresia	0.10872
DB00025	Bipolar disorder	0.27371
DB00025	Bladder cancer	0.29182
DB00025	Brain disease	0.05632
DB00025	Brain tumor	0.05755
DB00025	Breast cancer	0.11226
DB00025	Bronchiolitis obliterans	0.30749
DB00025	Bronchopulmonary dysplasia	0.21157
DB00025	Cancer	0.10784
DB00025	Cardiovascular disease	0.07296
DB00025	Celiac disease	0.05043
DB00025	Central nervous system disease	0.12149
DB00025	Cerebrovascular disorder	0.07388
DB00025	Cervical cancer	0.05205
DB00025	Chronic obstructive airway disease	0.12885
DB00025	Cirrhosis	0.07916
DB00025	Clear cell carcinoma	0.12426
DB00025	Colon cancer	0.17861
DB00025	Common cold	0.06718
DB00025	Congenital abnormality	0.02273
DB00025	Corneal disease	0.03069
DB00025	Cystic fibrosis	0.07922
DB00025	Dental plaque	0.13962
DB00025	Dermatitis	0.02678
DB00025	Diabetes mellitus	0.13698
DB00025	Down syndrome	0.05909
DB00025	Drug abuse	0.02624
DB00025	Eating disorder	0.05
DB00025	Embryoma	0.06166
DB00025	Emphysema	0.04447
DB00025	Encephalitis	0.11299
DB00025	Endometriosis	0.03113
DB00025	Enteritis	0.04914
DB00025	Epilepsy	0.03255
DB00025	Esophageal tumor	0.06976
DB00025	Esophagus cancer	0.02787
DB00025	Esotropia	0.05675
DB00025	Ewings sarcoma	0.0898
DB00025	Fanconi's anemia	0.04295
DB00025	Female reproductive cancer	0.02365

DB00025	Gastritis	0.05262
DB00025	Glaucoma	0.04892
DB00025	Glomerulonephritis	0.17004
DB00025	Glycogen storage disease	0.12309
DB00025	Growth retardation	0.08035
DB00025	HIV infection	0.23156
DB00025	HTLV-I infection	0.09925
DB00025	Heart failure	0.13256
DB00025	Helicobacter infection	0.0527
DB00025	Hemolytic-Uremic syndrome	0.06645
DB00025	Hemorrhagic disorder	0.21626
DB00025	Henoch-Schoenlein purpura	0.30749
DB00025	Hepatitis	0.15471
DB00025	Hepatitis B	0.24216
DB00025	Hereditary disease	0.04092
DB00025	Herpes	0.05254
DB00025	Hodgkin's disease	0.04845
DB00025	Hypercholesterolemia	0.05803
DB00025	Hyperlipidemia	0.10029
DB00025	Hypertension	0.06997
DB00025	IGA glomerulonephritis	0.09637
DB00025	Infection	0.04728
DB00025	Infiltrating cancer	0.09868
DB00025	Intracranial aneurysm	0.18603
DB00025	Ischemia	0.096
DB00025	Kaposi sarcoma	0.05077
DB00025	Keratoconjunctivitis Sicca	0.16939
DB00025	Keratoconus	0.04869
DB00025	Kidney cancer	0.0548
DB00025	Kidney disease	0.08406
DB00025	Kidney failure	0.03588
DB00025	Leukemia	0.17645
DB00025	Leukoencephalopathy	0.04352
DB00025	Lichen planus	0.10941
DB00025	Liver cancer	0.09373
DB00025	Liver metastases	0.20698
DB00025	Lung cancer	0.03279
DB00025	Lupus erythematosus	0.02305
DB00025	Lupus vulgaris	0.08865
DB00025	Lymphatic metastasis	0.28615
DB00025	Malignant glioma	0.30392
DB00025	Melanoma	0.2849
DB00025	Meningioma	0.18036
DB00025	Mental retardation	0.02495
DB00025	Metabolism disease	0.07079
DB00025	Metastasis to lymph nodes	0.12166
DB00025	Mucocutaneous lymph node syndrome	0.11917

DB00025	Multiple myeloma	0.04931
DB00025	Multiple sclerosis	0.0839
DB00025	Muscular dystrophy	0.11933
DB00025	Nasopharyngeal cancer	0.03668
DB00025	Neoplasm metastasis	0.06267
DB00025	Nephroblastoma	0.04148
DB00025	Nephrosis	0.05071
DB00025	Nervous system disease	0.07686
DB00025	Neuroblastoma	0.02123
DB00025	Neurodegenerative disorder	0.05207
DB00025	Nevus	0.30894
DB00025	Obesity	0.09251
DB00025	Oral cancer	0.03889
DB00025	Osteomyelitis	0.16562
DB00025	Ovarian disease	0.07368
DB00025	Ovary cancer	0.07283
DB00025	Pancreas cancer	0.03446
DB00025	Pancreatitis	0.05145
DB00025	Parkinson disease	0.07012
DB00025	Periodontitis	0.07499
DB00025	Pituitary tumor	0.07744
DB00025	Polycystic ovary syndrome	0.03942
DB00025	Polymyositis	0.15917
DB00025	Pre-Eclampsia	0.32263
DB00025	Primary biliary cirrhosis	0.016
DB00025	Primary tumor	0.10065
DB00025	Prion disease	0.18342
DB00025	Prostate cancer	0.06264
DB00025	Proteinuria	0.10081
DB00025	Psychotic disorder	0.0704
DB00025	Rabies	0.23821
DB00025	Rectum cancer	0.18417
DB00025	Renal Cell cancer	0.02143
DB00025	Renal tubular acidosis	0.03937
DB00025	Respiratory distress syndrome	0.10367
DB00025	Respiratory tract disease	0.0683
DB00025	Retinitis pigmentosa	0.03761
DB00025	Rheumatism	0.04801
DB00025	Rheumatoid arthritis	0.01556
DB00025	Schizophrenia	0.01115
DB00025	Sinusitis	0.06337
DB00025	Skin cancer	0.07153
DB00025	Skin disease	0.04633
DB00025	Solid tumor	0.15687
DB00025	Stomach cancer	0.04187
DB00025	Stroke	0.02574
DB00025	Subacute sclerosing panencephalitis	0.30749

DB00025	Systemic infection	0.1162
DB00025	Systemic scleroderma	0.08856
DB00025	Takayasu's arteritis	0.04909
DB00025	Testicular dysfunction	0.01275
DB00025	Thrombophilia	0.18369
DB00025	Thrombophlebitis	0.03294
DB00025	Thyroid cancer	0.06497
DB00025	Transient hypertension of pregnancy	0.24216
DB00025	Tropical spastic paraparesis	0.09412
DB00025	Tuberous sclerosis	0.01379
DB00025	Ulcerative colitis	0.03045
DB00025	Uterine fibroids	0.18419
DB00025	Varicosity	0.30749
DB00025	Virus disease	0.01952
DB00025	Vitamin D deficiency	0.19622
DB00025	Werner syndrome	0.05096
DB00025	Yersinia infection	0.05956
DB00036	Hypertension, Pulmonary	0.27787
DB00036	Abruption placentae	1.14434
DB00036	Adenovirus infection	0.12011
DB00036	Atherosclerosis	0.10214
DB00036	Bacterial infection	0.44801
DB00036	Breast cancer	0.14127
DB00036	Cardiovascular disease	0.31723
DB00036	Cirrhosis	0.34565
DB00036	Dental plaque	0.17879
DB00036	Diabetes mellitus	0.07197
DB00036	Drug abuse	0.10108
DB00036	Embryoma	0.09498
DB00036	Heart failure	0.16351
DB00036	Hemolytic-Uremic syndrome	0.33124
DB00036	Hemorrhagic disorder	0.3413
DB00036	Hyperlipidemia	0.51062
DB00036	Kidney failure	0.17161
DB00036	Leukemia	0.07937
DB00036	Leukoencephalopathy	0.16292
DB00036	Liver metastases	0.44892
DB00036	Lymphatic metastasis	1.28868
DB00036	Melanoma	0.19834
DB00036	Meningioma	0.39793
DB00036	Neoplasm metastasis	0.12458
DB00036	Nephrosis	0.26851
DB00036	Nevus	0.85144
DB00036	Osteomyelitis	0.36363
DB00036	Pancreas cancer	0.16275
DB00036	Primary tumor	0.23541
DB00036	Prostate cancer	0.09589

DB00036	Proteinuria	0.44357
DB00036	Rabies	0.13081
DB00036	Rectum cancer	0.93227
DB00036	Renal Cell cancer	0.04746
DB00036	Respiratory distress syndrome	0.47552
DB00036	Rheumatism	0.264
DB00036	Solid tumor	0.36188
DB00036	Stomach cancer	0.10229
DB00036	Systemic infection	0.22603
DB00036	Systemic scleroderma	0.11732
DB00036	Thrombophilia	0.4898
DB00036	Vitamin D deficiency	0.42221
DB00569	Hypertension, Pulmonary	0.18422
DB00569	Abruption placentae	1
DB00569	Adenovirus infection	0.15901
DB00569	Atherosclerosis	0.07356
DB00569	Bacterial infection	0.3389
DB00569	Breast cancer	0.11636
DB00569	CNS lymphoma	0.70711
DB00569	Cardiovascular disease	0.25497
DB00569	Cirrhosis	0.27665
DB00569	Dental plaque	0.12695
DB00569	Diabetes mellitus	0.05048
DB00569	Drug abuse	0.06285
DB00569	Embryoma	0.06981
DB00569	Heart failure	0.11999
DB00569	Hemolytic-Uremic syndrome	0.23223
DB00569	Hemorrhagic disorder	0.32197
DB00569	Hyperlipidemia	0.35049
DB00569	Kidney disease	0.08452
DB00569	Kidney failure	0.12539
DB00569	Leukemia	0.05636
DB00569	Leukoencephalopathy	0.09488
DB00569	Liver metastases	0.33569
DB00569	Lymphatic metastasis	1
DB00569	Melanoma	0.18923
DB00569	Meningioma	0.29252
DB00569	Neoplasm metastasis	0.09136
DB00569	Nephrosis	0.17722
DB00569	Nevus	0.95711
DB00569	Osteomyelitis	0.46325
DB00569	Pancreas cancer	0.12041
DB00569	Primary tumor	0.16324
DB00569	Prostate cancer	0.09018
DB00569	Proteinuria	0.35228
DB00569	Rabies	0.16401
DB00569	Rectum cancer	0.64359

DB00569	Respiratory distress syndrome	0.36229
DB00569	Rheumatism	0.16777
DB00569	Solid tumor	0.44815
DB00569	Stomach cancer	0.0679
DB00569	Systemic infection	0.13237
DB00569	Systemic scleroderma	0.14865
DB00569	Thrombophilia	0.56024
DB00569	Vitamin D deficiency	0.54422
DB01109	Hypertension, Pulmonary	0.18422
DB01109	Abruption placentae	1
DB01109	Adenovirus infection	0.14212
DB01109	Adrenal gland hyperfunction	0.21822
DB01109	Atherosclerosis	0.07356
DB01109	Bacterial infection	0.3389
DB01109	Breast cancer	0.11012
DB01109	CNS lymphoma	0.57735
DB01109	Cardiovascular disease	0.34302
DB01109	Cirrhosis	0.37424
DB01109	Colon cancer	0.03426
DB01109	Common cold	0.1543
DB01109	Dental plaque	0.20027
DB01109	Depression	0.07581
DB01109	Dermatitis	0.05249
DB01109	Diabetes mellitus	0.08087
DB01109	Drug abuse	0.06285
DB01109	Embryoma	0.06981
DB01109	Heart failure	0.11999
DB01109	Hemolytic-Uremic syndrome	0.23223
DB01109	Hemorrhagic disorder	0.29903
DB01109	Hypercholesterolemia	0.11111
DB01109	Hyperlipidemia	0.35049
DB01109	Hypertension	0.04564
DB01109	Ischemia	0.07392
DB01109	Kidney disease	0.06901
DB01109	Kidney failure	0.12539
DB01109	Leukemia	0.05636
DB01109	Leukoencephalopathy	0.09488
DB01109	Liver metastases	0.33569
DB01109	Lung cancer	0.04032
DB01109	Lupus erythematosus	0.04933
DB01109	Lymphatic metastasis	1
DB01109	Melanoma	0.17841
DB01109	Meningioma	0.29252
DB01109	Neoplasm metastasis	0.13834
DB01109	Nephrosis	0.17722
DB01109	Nevus	0.91123
DB01109	Osteomyelitis	0.42

DB01109	Pancreas cancer	0.12041
DB01109	Pancreatitis	0.09017
DB01109	Primary tumor	0.16324
DB01109	Prostate cancer	0.0834
DB01109	Proteinuria	0.35228
DB01109	Rabies	0.1496
DB01109	Rectum cancer	0.64359
DB01109	Respiratory distress syndrome	0.36229
DB01109	Rheumatism	0.16777
DB01109	Rheumatoid arthritis	0.03533
DB01109	Sickle cell disease	0.11111
DB01109	Solid tumor	0.4107
DB01109	Stomach cancer	0.0679
DB01109	Systemic infection	0.13237
DB01109	Systemic scleroderma	0.19557
DB01109	Takayasu's arteritis	0.18257
DB01109	Thrombophilia	0.52966
DB01109	Vitamin D deficiency	0.49124
DB01225	Hypertension, Pulmonary	0.18422
DB01225	Abruption placentae	1
DB01225	Adenovirus infection	0.15901
DB01225	Atherosclerosis	0.07356
DB01225	Bacterial infection	0.3389
DB01225	Breast cancer	0.11636
DB01225	CNS lymphoma	0.70711
DB01225	Cardiovascular disease	0.25497
DB01225	Cirrhosis	0.27665
DB01225	Dental plaque	0.12695
DB01225	Diabetes mellitus	0.05048
DB01225	Drug abuse	0.06285
DB01225	Embryoma	0.06981
DB01225	Heart failure	0.11999
DB01225	Hemolytic-Uremic syndrome	0.23223
DB01225	Hemorrhagic disorder	0.32197
DB01225	Hyperlipidemia	0.35049
DB01225	Kidney disease	0.08452
DB01225	Kidney failure	0.12539
DB01225	Leukemia	0.05636
DB01225	Leukoencephalopathy	0.09488
DB01225	Liver metastases	0.33569
DB01225	Lymphatic metastasis	1
DB01225	Melanoma	0.18923
DB01225	Meningioma	0.29252
DB01225	Neoplasm metastasis	0.09136
DB01225	Nephrosis	0.17722
DB01225	Nevus	0.95711
DB01225	Osteomyelitis	0.46325

DB01225	Pancreas cancer	0.12041
DB01225	Primary tumor	0.16324
DB01225	Prostate cancer	0.09018
DB01225	Proteinuria	0.35228
DB01225	Rabies	0.16401
DB01225	Rectum cancer	0.64359
DB01225	Respiratory distress syndrome	0.36229
DB01225	Rheumatism	0.16777
DB01225	Solid tumor	0.44815
DB01225	Stomach cancer	0.0679
DB01225	Systemic infection	0.13237
DB01225	Systemic scleroderma	0.14865
DB01225	Thrombophilia	0.56024
DB01225	Vitamin D deficiency	0.54422
DB06228	Hypertension, Pulmonary	0.18422
DB06228	Abruption placentae	1
DB06228	Adenovirus infection	0.19715
DB06228	Atherosclerosis	0.07356
DB06228	Bacterial infection	0.3389
DB06228	Breast cancer	0.13046
DB06228	Cardiovascular disease	0.25497
DB06228	Cirrhosis	0.27665
DB06228	Dental plaque	0.12695
DB06228	Diabetes mellitus	0.05048
DB06228	Drug abuse	0.06285
DB06228	Embryoma	0.06981
DB06228	Heart failure	0.11999
DB06228	Hemolytic-Uremic syndrome	0.23223
DB06228	Hemorrhagic disorder	0.37374
DB06228	Hyperlipidemia	0.35049
DB06228	Kidney failure	0.12539
DB06228	Leukemia	0.05636
DB06228	Leukoencephalopathy	0.09488
DB06228	Liver metastases	0.33569
DB06228	Lymphatic metastasis	1
DB06228	Melanoma	0.21363
DB06228	Meningioma	0.29252
DB06228	Neoplasm metastasis	0.09136
DB06228	Nephrosis	0.17722
DB06228	Nevus	1.06066
DB06228	Osteomyelitis	0.56088
DB06228	Pancreas cancer	0.12041
DB06228	Primary tumor	0.16324
DB06228	Prostate cancer	0.05327
DB06228	Proteinuria	0.35228
DB06228	Rabies	0.08545
DB06228	Rectum cancer	0.64359

DB06228	Respiratory distress syndrome	0.36229
DB06228	Rheumatism	0.16777
DB06228	Solid tumor	0.53271
DB06228	Stomach cancer	0.0679
DB06228	Systemic infection	0.13237
DB06228	Systemic scleroderma	0.17936
DB06228	Thrombophilia	0.39358
DB06228	Vitamin D deficiency	0.66379
DB06605	Hypertension, Pulmonary	0.18422
DB06605	Abruption placentae	1
DB06605	Adenovirus infection	0.19715
DB06605	Atherosclerosis	0.07356
DB06605	Bacterial infection	0.3389
DB06605	Breast cancer	0.13046
DB06605	Cardiovascular disease	0.25497
DB06605	Cirrhosis	0.27665
DB06605	Dental plaque	0.12695
DB06605	Diabetes mellitus	0.05048
DB06605	Drug abuse	0.06285
DB06605	Embryoma	0.06981
DB06605	Heart failure	0.11999
DB06605	Hemolytic-Uremic syndrome	0.23223
DB06605	Hemorrhagic disorder	0.37374
DB06605	Hyperlipidemia	0.35049
DB06605	Kidney failure	0.12539
DB06605	Leukemia	0.05636
DB06605	Leukoencephalopathy	0.09488
DB06605	Liver metastases	0.33569
DB06605	Lymphatic metastasis	1
DB06605	Melanoma	0.21363
DB06605	Meningioma	0.29252
DB06605	Neoplasm metastasis	0.09136
DB06605	Nephrosis	0.17722
DB06605	Nevus	1.06066
DB06605	Osteomyelitis	0.56088
DB06605	Pancreas cancer	0.12041
DB06605	Primary tumor	0.16324
DB06605	Prostate cancer	0.05327
DB06605	Proteinuria	0.35228
DB06605	Rabies	0.08545
DB06605	Rectum cancer	0.64359
DB06605	Respiratory distress syndrome	0.36229
DB06605	Rheumatism	0.16777
DB06605	Solid tumor	0.53271
DB06605	Stomach cancer	0.0679
DB06605	Systemic infection	0.13237
DB06605	Systemic scleroderma	0.17936

DB06605	Thrombophilia	0.39358
DB06605	Vitamin D deficiency	0.66379
DB00181	Amyotrophic lateral sclerosis	0.10747
DB00181	Behavior disease	0.22932
DB00181	Depression	0.10137
DB00181	Dermatitis	0.0964
DB00181	Diabetes mellitus	0.06003
DB00181	Epilepsy	0.34291
DB00181	Hepatitis C	0.19704
DB00181	Hypertension	0.11741
DB00181	Migraine	0.2085
DB00181	Panic disorder	0.26633
DB00181	Stroke	0.09264
DB00181	Sudden infant death syndrome	0.31844
DB01080	Autistic disorder	0.08575
DB00228	Alzheimer's disease	0.04229
DB00228	Amyotrophic lateral sclerosis	0.0463
DB00228	Aortic aneurysm	0.05591
DB00228	Atherosclerosis	0.01884
DB00228	Bipolar disorder	0.09172
DB00228	Breast cancer	0.01003
DB00228	Cancer	0.02803
DB00228	Cardiovascular disease	0.06532
DB00228	Deafness	0.06821
DB00228	Depression	0.04368
DB00228	Drug abuse	0.01953
DB00228	Eating disorder	0.0363
DB00228	Embryoma	0.03577
DB00228	Endometrium cancer	0.05057
DB00228	Epilepsy	0.074
DB00228	Heart failure	0.10917
DB00228	Herpes	0.02979
DB00228	Hyperopia	0.0695
DB00228	Hypertension	0.02529
DB00228	Hypogonadism	0.09325
DB00228	Ischemia	0.03158
DB00228	Kidney failure	0.03212
DB00228	Leigh disease	0.09757
DB00228	Leukemia	0.02888
DB00228	Lymphoma	0.0654
DB00228	Multiple sclerosis	0.11267
DB00228	Neurotic disorder	0.06594
DB00228	Prostate cancer	0.01365
DB00228	Rabies	0.02189
DB00228	Schizophrenia	0.04352
DB00228	Sickle cell disease	0.04013
DB00228	Yersinia infection	0.02475

DB00753	Abortion	0.0206
DB00753	Achalasia and cardiospasm	0.08841
DB00753	Alzheimer's disease	0.06365
DB00753	Amyloidosis	0.04707
DB00753	Amyotrophic lateral sclerosis	0.0286
DB00753	Aortic aneurysm	0.03454
DB00753	Asthma	0.03099
DB00753	Atherosclerosis	0.01164
DB00753	Autistic disorder	0.04284
DB00753	Autoimmune disease	0.03951
DB00753	Bipolar disorder	0.08809
DB00753	Bladder cancer	0.03221
DB00753	Brain ischemia	0.12948
DB00753	Breast cancer	0.01003
DB00753	Cancer	0.04483
DB00753	Cardiovascular disease	0.04035
DB00753	Celiac disease	0.06025
DB00753	Colon cancer	0.0122
DB00753	Congenital abnormality	0.02295
DB00753	Deafness	0.04214
DB00753	Dental plaque	0.04639
DB00753	Depression	0.02698
DB00753	Diabetes mellitus	0.00886
DB00753	Drug abuse	0.0425
DB00753	Eating disorder	0.11015
DB00753	Embryoma	0.02209
DB00753	Epilepsy	0.04571
DB00753	Epstein-Barr virus infection	0.06568
DB00753	Esophageal tumor	0.05456
DB00753	Esophagus cancer	0.02179
DB00753	Esotropia	0.06277
DB00753	Eye cancer	0.0778
DB00753	Fanconi's anemia	0.0307
DB00753	Glaucoma	0.05411
DB00753	Gram-Negative bacterial infection	0.10852
DB00753	Graves' disease	0.07765
DB00753	HIV infection	0.03676
DB00753	Heart failure	0.07593
DB00753	Herpes	0.08291
DB00753	Hyperopia	0.0695
DB00753	Hypertension	0.01562
DB00753	Hypogonadism	0.09325
DB00753	Ischemia	0.06456
DB00753	Keratosis	0.06226
DB00753	Kidney failure	0.01984
DB00753	Leigh disease	0.06027
DB00753	Leukemia	0.04557

DB00753	Lichen planus	0.12101
DB00753	Lung cancer	0.02595
DB00753	Lupus erythematosus	0.02549
DB00753	Lymphoma	0.0404
DB00753	Malignant glioma	0.03157
DB00753	Melanoma	0.01618
DB00753	Multiple sclerosis	0.0696
DB00753	Neurotic disorder	0.06594
DB00753	Pre-Eclampsia	0.0294
DB00753	Prion disease	0.10144
DB00753	Prostate cancer	0.00843
DB00753	Rabies	0.02852
DB00753	Rheumatoid arthritis	0.01859
DB00753	Schistosomiasis	0.10934
DB00753	Schizophrenia	0.02688
DB00753	Stroke	0.02847
DB00753	Thyroid gland disease	0.06836
DB00753	Tuberculosis	0.05857
DB00753	Yersinia infection	0.02475
DB01028	Alzheimer's disease	0.0235
DB01028	Amyotrophic lateral sclerosis	0.02573
DB01028	Aortic aneurysm	0.03107
DB01028	Atherosclerosis	0.01047
DB01028	Basal cell carcinoma	0.20027
DB01028	Bipolar disorder	0.09858
DB01028	Breast cancer	0.03516
DB01028	Cancer	0.01899
DB01028	Cardiovascular disease	0.0363
DB01028	Cervical cancer	0.05015
DB01028	Deafness	0.03791
DB01028	Depression	0.02427
DB01028	Diabetes mellitus	0.11691
DB01028	Down syndrome	0.1296
DB01028	Drug abuse	0.01953
DB01028	Eating disorder	0.0363
DB01028	Ectodermal dysplasia	0.07448
DB01028	Embryoma	0.03611
DB01028	Encephalopathies	0.53044
DB01028	Epilepsy	0.10992
DB01028	Eye disease	0.21119
DB01028	Heart failure	0.07055
DB01028	Herpes	0.02979
DB01028	Hyperopia	0.0695
DB01028	Hypertension	0.26244
DB01028	Hypogonadism	0.09325
DB01028	Infection	0.01977
DB01028	Intestinal disease	0.02998

DB01028	Ischemia	0.01755
DB01028	Kidney failure	0.01785
DB01028	Leigh disease	0.41009
DB01028	Leukemia	0.01605
DB01028	Lymphoma	0.03635
DB01028	Mitochondrial encephalomyopathies	0.17774
DB01028	Multiple sclerosis	0.07361
DB01028	Myotonic disorder	0.5234
DB01028	Neuropathy	0.47788
DB01028	Neurotic disorder	0.06594
DB01028	Optic atrophy	0.0792
DB01028	Osteoporosis	0.03307
DB01028	Ovarian cancer	0.02748
DB01028	Parkinson disease	0.0989
DB01028	Prostate cancer	0.01997
DB01028	Rabies	0.01216
DB01028	Schizophrenia	0.23272
DB01028	Yersinia infection	0.02475
DB01159	Skin disease, Genetic	0.03951
DB01159	Alzheimer's disease	0.02407
DB01159	Arthritis	0.10232
DB01159	Asthma	0.01421
DB01159	Atherosclerosis	0.0458
DB01159	Autistic disorder	0.06109
DB01159	Autoimmune disease	0.01888
DB01159	Basal cell carcinoma	0.1427
DB01159	Bipolar disorder	0.03392
DB01159	Bone disease	0.05948
DB01159	Breast cancer	0.04579
DB01159	Bronchial disease	0.20466
DB01159	Bronchial hyperreactivity	0.06155
DB01159	Cancer	0.01974
DB01159	Cardiovascular disease	0.02586
DB01159	Cerebellar disease	0.10779
DB01159	Cervical cancer	0.03573
DB01159	Chronic obstructive airway disease	0.08209
DB01159	Common cold	0.04652
DB01159	Common variable immunodeficiency	0.11285
DB01159	Congenital abnormality	0.01471
DB01159	Cystic fibrosis	0.12962
DB01159	Deafness	0.02701
DB01159	Dermatitis	0.05807
DB01159	Diabetes mellitus	0.11095
DB01159	Down syndrome	0.14193
DB01159	Drug abuse	0.04906
DB01159	Eating disorder	0.0303
DB01159	Ectodermal dysplasia	0.05307

DB01159	Embryoma	0.02792
DB01159	Encephalopathies	0.38151
DB01159	Endemic goiter	0.1005
DB01159	Endometrium cancer	0.04222
DB01159	Epilepsy	0.14891
DB01159	Eye disease	0.15047
DB01159	Glaucoma	0.07716
DB01159	Graves' disease	0.11073
DB01159	Heart failure	0.11876
DB01159	Herpes	0.04312
DB01159	Hyperopia	0.05803
DB01159	Hypertension	0.24069
DB01159	Hypogonadism	0.07785
DB01159	Immunologic deficiency syndrome	0.03211
DB01159	Infection	0.01409
DB01159	Intestinal disease	0.02136
DB01159	Ischemia	0.09904
DB01159	Kidney failure	0.04209
DB01159	Leigh disease	0.29219
DB01159	Leukemia	0.0132
DB01159	Lung cancer	0.06133
DB01159	Lymphoproliferative disorder	0.06418
DB01159	Malaria	0.11861
DB01159	Melanoma	0.02158
DB01159	Mental retardation	0.04334
DB01159	Metabolism disease	0.11166
DB01159	Migraine	0.04107
DB01159	Mitochondrial encephalomyopathies	0.12664
DB01159	Movement disorder	0.18483
DB01159	Multiple sclerosis	0.02631
DB01159	Myelofibrosis	0.07822
DB01159	Myopathy	0.10792
DB01159	Myotonic disorder	0.42683
DB01159	Neurodegenerative disorder	0.11075
DB01159	Neuropathy	0.34458
DB01159	Neurotic disorder	0.05505
DB01159	Obesity	0.04864
DB01159	Optic atrophy	0.05643
DB01159	Oral cancer	0.02146
DB01159	Osteomyelitis	0.05331
DB01159	Osteoporosis	0.02356
DB01159	Ovarian cancer	0.01958
DB01159	Overnutrition	0.08989
DB01159	Panic disorder	0.03799
DB01159	Parkinson disease	0.07047
DB01159	Polycystic ovary syndrome	0.06218
DB01159	Premature birth	0.16957

DB01159	Prostate cancer	0.0632
DB01159	Rabies	0.00867
DB01159	Respiratory failure	0.06155
DB01159	Retinal disease	0.02438
DB01159	Retinitis pigmentosa	0.03482
DB01159	Rheumatism	0.0393
DB01159	Rheumatoid arthritis	0.02651
DB01159	Schizophrenia	0.15047
DB01159	Sickle cell disease	0.0864
DB01159	Spinocerebellar ataxias	0.09089
DB01159	Subarachnoid hemorrhage	0.0828
DB01159	Vitamin D deficiency	0.05987
DB01159	Yersinia infection	0.02066
DB01189	Alzheimer's disease	0.02469
DB01189	Amyotrophic lateral sclerosis	0.02703
DB01189	Aortic aneurysm	0.03264
DB01189	Atherosclerosis	0.011
DB01189	Basal cell carcinoma	0.21036
DB01189	Bipolar disorder	0.10355
DB01189	Breast cancer	0.03665
DB01189	Cancer	0.01974
DB01189	Cardiovascular disease	0.03813
DB01189	Cervical cancer	0.05267
DB01189	Deafness	0.03982
DB01189	Depression	0.0255
DB01189	Diabetes mellitus	0.12249
DB01189	Down syndrome	0.13613
DB01189	Drug abuse	0.01997
DB01189	Eating disorder	0.03711
DB01189	Ectodermal dysplasia	0.07823
DB01189	Embryoma	0.03793
DB01189	Encephalopathies	0.55635
DB01189	Epilepsy	0.11546
DB01189	Eye disease	0.22183
DB01189	Heart failure	0.07348
DB01189	Herpes	0.03046
DB01189	Hyperopia	0.07107
DB01189	Hypertension	0.2752
DB01189	Hypogonadism	0.09535
DB01189	Infection	0.02077
DB01189	Intestinal disease	0.03149
DB01189	Ischemia	0.01843
DB01189	Kidney failure	0.01875
DB01189	Leigh disease	0.43075
DB01189	Leukemia	0.01686
DB01189	Lymphoma	0.03818
DB01189	Mitochondrial encephalomyopathies	0.1867

DB01189	Multiple sclerosis	0.07732
DB01189	Myotonic disorder	0.54802
DB01189	Neuropathy	0.50102
DB01189	Neurotic disorder	0.06742
DB01189	Optic atrophy	0.08319
DB01189	Osteoporosis	0.03474
DB01189	Ovarian cancer	0.02886
DB01189	Parkinson disease	0.10389
DB01189	Prostate cancer	0.02097
DB01189	Rabies	0.01278
DB01189	Schizophrenia	0.244
DB01189	Yersinia infection	0.0253
DB01236	Alzheimer's disease	0.0235
DB01236	Amyotrophic lateral sclerosis	0.02573
DB01236	Aortic aneurysm	0.03107
DB01236	Atherosclerosis	0.01047
DB01236	Basal cell carcinoma	0.20027
DB01236	Bipolar disorder	0.09858
DB01236	Breast cancer	0.03516
DB01236	Cancer	0.01899
DB01236	Cardiovascular disease	0.0363
DB01236	Cervical cancer	0.05015
DB01236	Deafness	0.03791
DB01236	Depression	0.02427
DB01236	Diabetes mellitus	0.11691
DB01236	Down syndrome	0.1296
DB01236	Drug abuse	0.01953
DB01236	Eating disorder	0.0363
DB01236	Ectodermal dysplasia	0.07448
DB01236	Embryoma	0.03611
DB01236	Encephalopathies	0.53044
DB01236	Epilepsy	0.10992
DB01236	Eye disease	0.21119
DB01236	Heart failure	0.07055
DB01236	Herpes	0.02979
DB01236	Hyperopia	0.0695
DB01236	Hypertension	0.26244
DB01236	Hypogonadism	0.09325
DB01236	Infection	0.01977
DB01236	Intestinal disease	0.02998
DB01236	Ischemia	0.01755
DB01236	Kidney failure	0.01785
DB01236	Leigh disease	0.41009
DB01236	Leukemia	0.01605
DB01236	Lymphoma	0.03635
DB01236	Mitochondrial encephalomyopathies	0.17774
DB01236	Multiple sclerosis	0.07361

DB01236	Myotonic disorder	0.5234
DB01236	Neuropathy	0.47788
DB01236	Neurotic disorder	0.06594
DB01236	Optic atrophy	0.0792
DB01236	Osteoporosis	0.03307
DB01236	Ovarian cancer	0.02748
DB01236	Parkinson disease	0.0989
DB01236	Prostate cancer	0.01997
DB01236	Rabies	0.01216
DB01236	Schizophrenia	0.23272
DB01236	Yersinia infection	0.02475
DB00174	Brain tumor	0.03279
DB00174	Kidney disease	0.0488
DB00174	Leukemia	0.023
DB00174	Neoplasm metastasis	0.03322
DB00174	Pre-Eclampsia	0.04811
DB00174	Renal Cell cancer	0.04746
DB01087	Cancer	0.02606
DB01087	Ovarian disease	0.16667
DB01087	Ulcerative colitis	0.07293
DB00178	Alzheimer's disease	0.07198
DB00178	Anemia	0.2132
DB00178	Ankylosing spondylitis	0.37796
DB00178	Arthritis	0.12403
DB00178	Asthma	0.08165
DB00178	Behcet syndrome	0.14286
DB00178	Breast cancer	0.04811
DB00178	Bronchopulmonary dysplasia	0.30151
DB00178	Cardiovascular disease	0.1525
DB00178	Chronic fatigue syndrome	0.35355
DB00178	Chronic obstructive airway disease	0.1118
DB00178	Chronic rejection of renal transplant	0.25
DB00178	Chronic simple glaucoma	0.2132
DB00178	Colon cancer	0.05934
DB00178	Connective tissue disease	0.25
DB00178	Depression	0.13131
DB00178	Dermatitis	0.09091
DB00178	Embryoma	0.06166
DB00178	Endometrial cancer	0.17408
DB00178	Endometriosis	0.08305
DB00178	Esophagus cancer	0.14907
DB00178	Familial Mediterranean fever	0.2582
DB00178	Fibroid tumor	0.30151
DB00178	Heart disease	0.30151
DB00178	Hyperhomocysteinemia	0.28868
DB00178	Hypertension	0.07906
DB00178	Hypoglycemia	0.40825

DB00178	IGA glomerulonephritis	0.1715
DB00178	Infectious lung disease	0.22361
DB00178	Kidney disease	0.11952
DB00178	Leukemia	0.05634
DB00178	Liver cancer	0.07762
DB00178	Lupus erythematosus	0.08544
DB00178	Lupus vulgaris	0.18898
DB00178	Macular degeneration	0.20851
DB00178	Metabolic syndrome X	0.35355
DB00178	Metabolism disease	0.16013
DB00178	Migraine	0.1857
DB00178	Mucocutaneous lymph node syndrome	0.21822
DB00178	Neoplasm metastasis	0.08138
DB00178	Obesity	0.07692
DB00178	Parkinson disease	0.11043
DB00178	Polycythemia	0.33333
DB00178	Premature birth	0.24254
DB00178	Prostate cancer	0.0522
DB00178	Proteinuria	0.22361
DB00178	Psoriasis	0.2357
DB00178	Respiratory distress syndrome	0.27735
DB00178	Sarcoidosis	0.19612
DB00178	Schizophrenia	0.07603
DB00178	Severe acute respiratory syndrome	0.31623
DB00178	Stomach cancer	0.08422
DB00178	Subacute sclerosing panencephalitis	0.5
DB00178	Systemic infection	0.11471
DB00178	Systemic scleroderma	0.10483
DB00178	Testicular dysfunction	0.14286
DB00178	Thrombophilia	0.2357
DB00178	Transient hypertension of pregnancy	0.31623
DB00178	Ulcerative colitis	0.10314
DB00178	Vascular dementia	0.33333
DB00492	Alzheimer's disease	0.07198
DB00492	Anemia	0.2132
DB00492	Ankylosing spondylitis	0.37796
DB00492	Arthritis	0.12403
DB00492	Asthma	0.08165
DB00492	Behcet syndrome	0.14286
DB00492	Breast cancer	0.04811
DB00492	Bronchopulmonary dysplasia	0.30151
DB00492	Cardiovascular disease	0.1525
DB00492	Chronic fatigue syndrome	0.35355
DB00492	Chronic obstructive airway disease	0.1118
DB00492	Chronic rejection of renal transplant	0.25
DB00492	Chronic simple glaucoma	0.2132
DB00492	Colon cancer	0.05934

DB00492	Connective tissue disease	0.25
DB00492	Depression	0.13131
DB00492	Dermatitis	0.09091
DB00492	Embryoma	0.06166
DB00492	Endometrial cancer	0.17408
DB00492	Endometriosis	0.08305
DB00492	Esophagus cancer	0.14907
DB00492	Familial Mediterranean fever	0.2582
DB00492	Fibroid tumor	0.30151
DB00492	Heart disease	0.30151
DB00492	Hyperhomocysteinemia	0.28868
DB00492	Hypertension	0.07906
DB00492	Hypoglycemia	0.40825
DB00492	IGA glomerulonephritis	0.1715
DB00492	Infectious lung disease	0.22361
DB00492	Kidney disease	0.11952
DB00492	Leukemia	0.05634
DB00492	Liver cancer	0.07762
DB00492	Lupus erythematosus	0.08544
DB00492	Lupus vulgaris	0.18898
DB00492	Macular degeneration	0.20851
DB00492	Metabolic syndrome X	0.35355
DB00492	Metabolism disease	0.16013
DB00492	Migraine	0.1857
DB00492	Mucocutaneous lymph node syndrome	0.21822
DB00492	Neoplasm metastasis	0.08138
DB00492	Obesity	0.07692
DB00492	Parkinson disease	0.11043
DB00492	Polycythemia	0.33333
DB00492	Premature birth	0.24254
DB00492	Prostate cancer	0.0522
DB00492	Proteinuria	0.22361
DB00492	Psoriasis	0.2357
DB00492	Respiratory distress syndrome	0.27735
DB00492	Sarcoidosis	0.19612
DB00492	Schizophrenia	0.07603
DB00492	Severe acute respiratory syndrome	0.31623
DB00492	Stomach cancer	0.08422
DB00492	Subacute sclerosing panencephalitis	0.5
DB00492	Systemic infection	0.11471
DB00492	Systemic scleroderma	0.10483
DB00492	Testicular dysfunction	0.14286
DB00492	Thrombophilia	0.2357
DB00492	Transient hypertension of pregnancy	0.31623
DB00492	Ulcerative colitis	0.10314
DB00492	Vascular dementia	0.33333
DB00519	Alzheimer's disease	0.07198

DB00519	Anemia	0.2132
DB00519	Ankylosing spondylitis	0.37796
DB00519	Arthritis	0.12403
DB00519	Asthma	0.08165
DB00519	Behcet syndrome	0.14286
DB00519	Breast cancer	0.04811
DB00519	Bronchopulmonary dysplasia	0.30151
DB00519	Cardiovascular disease	0.1525
DB00519	Chronic fatigue syndrome	0.35355
DB00519	Chronic obstructive airway disease	0.1118
DB00519	Chronic rejection of renal transplant	0.25
DB00519	Chronic simple glaucoma	0.2132
DB00519	Colon cancer	0.05934
DB00519	Connective tissue disease	0.25
DB00519	Depression	0.13131
DB00519	Dermatitis	0.09091
DB00519	Embryoma	0.06166
DB00519	Endometrial cancer	0.17408
DB00519	Endometriosis	0.08305
DB00519	Esophagus cancer	0.14907
DB00519	Familial Mediterranean fever	0.2582
DB00519	Fibroid tumor	0.30151
DB00519	Heart disease	0.30151
DB00519	Hyperhomocysteinemia	0.28868
DB00519	Hypertension	0.07906
DB00519	Hypoglycemia	0.40825
DB00519	IGA glomerulonephritis	0.1715
DB00519	Infectious lung disease	0.22361
DB00519	Kidney disease	0.11952
DB00519	Leukemia	0.05634
DB00519	Liver cancer	0.07762
DB00519	Lupus erythematosus	0.08544
DB00519	Lupus vulgaris	0.18898
DB00519	Macular degeneration	0.20851
DB00519	Metabolic syndrome X	0.35355
DB00519	Metabolism disease	0.16013
DB00519	Migraine	0.1857
DB00519	Mucocutaneous lymph node syndrome	0.21822
DB00519	Neoplasm metastasis	0.08138
DB00519	Obesity	0.07692
DB00519	Parkinson disease	0.11043
DB00519	Polycythemia	0.33333
DB00519	Premature birth	0.24254
DB00519	Prostate cancer	0.0522
DB00519	Proteinuria	0.22361
DB00519	Psoriasis	0.2357
DB00519	Respiratory distress syndrome	0.27735

DB00519	Sarcoidosis	0.19612
DB00519	Schizophrenia	0.07603
DB00519	Severe acute respiratory syndrome	0.31623
DB00519	Stomach cancer	0.08422
DB00519	Subacute sclerosing panencephalitis	0.5
DB00519	Systemic infection	0.11471
DB00519	Systemic scleroderma	0.10483
DB00519	Testicular dysfunction	0.14286
DB00519	Thrombophilia	0.2357
DB00519	Transient hypertension of pregnancy	0.31623
DB00519	Ulcerative colitis	0.10314
DB00519	Vascular dementia	0.33333
DB00542	Alzheimer's disease	0.07198
DB00542	Anemia	0.2132
DB00542	Ankylosing spondylitis	0.37796
DB00542	Arthritis	0.12403
DB00542	Asthma	0.08165
DB00542	Behcet syndrome	0.14286
DB00542	Breast cancer	0.04811
DB00542	Bronchopulmonary dysplasia	0.30151
DB00542	Cardiovascular disease	0.1525
DB00542	Chronic fatigue syndrome	0.35355
DB00542	Chronic obstructive airway disease	0.1118
DB00542	Chronic rejection of renal transplant	0.25
DB00542	Chronic simple glaucoma	0.2132
DB00542	Colon cancer	0.05934
DB00542	Connective tissue disease	0.25
DB00542	Depression	0.13131
DB00542	Dermatitis	0.09091
DB00542	Embryoma	0.06166
DB00542	Endometrial cancer	0.17408
DB00542	Endometriosis	0.08305
DB00542	Esophagus cancer	0.14907
DB00542	Familial Mediterranean fever	0.2582
DB00542	Fibroid tumor	0.30151
DB00542	Heart disease	0.30151
DB00542	Hyperhomocysteinemia	0.28868
DB00542	Hypertension	0.07906
DB00542	Hypoglycemia	0.40825
DB00542	IGA glomerulonephritis	0.1715
DB00542	Infectious lung disease	0.22361
DB00542	Kidney disease	0.11952
DB00542	Leukemia	0.05634
DB00542	Liver cancer	0.07762
DB00542	Lupus erythematosus	0.08544
DB00542	Lupus vulgaris	0.18898
DB00542	Macular degeneration	0.20851

DB00542	Metabolic syndrome X	0.35355
DB00542	Metabolism disease	0.16013
DB00542	Migraine	0.1857
DB00542	Mucocutaneous lymph node syndrome	0.21822
DB00542	Neoplasm metastasis	0.08138
DB00542	Obesity	0.07692
DB00542	Parkinson disease	0.11043
DB00542	Polycythemia	0.33333
DB00542	Premature birth	0.24254
DB00542	Prostate cancer	0.0522
DB00542	Proteinuria	0.22361
DB00542	Psoriasis	0.2357
DB00542	Respiratory distress syndrome	0.27735
DB00542	Sarcoidosis	0.19612
DB00542	Schizophrenia	0.07603
DB00542	Severe acute respiratory syndrome	0.31623
DB00542	Stomach cancer	0.08422
DB00542	Subacute sclerosing panencephalitis	0.5
DB00542	Systemic infection	0.11471
DB00542	Systemic scleroderma	0.10483
DB00542	Testicular dysfunction	0.14286
DB00542	Thrombophilia	0.2357
DB00542	Transient hypertension of pregnancy	0.31623
DB00542	Ulcerative colitis	0.10314
DB00542	Vascular dementia	0.33333
DB00584	Alzheimer's disease	0.07198
DB00584	Anemia	0.2132
DB00584	Ankylosing spondylitis	0.37796
DB00584	Arthritis	0.12403
DB00584	Asthma	0.08165
DB00584	Behcet syndrome	0.14286
DB00584	Breast cancer	0.04811
DB00584	Bronchopulmonary dysplasia	0.30151
DB00584	Cardiovascular disease	0.1525
DB00584	Chronic fatigue syndrome	0.35355
DB00584	Chronic obstructive airway disease	0.1118
DB00584	Chronic rejection of renal transplant	0.25
DB00584	Chronic simple glaucoma	0.2132
DB00584	Colon cancer	0.05934
DB00584	Connective tissue disease	0.25
DB00584	Depression	0.13131
DB00584	Dermatitis	0.09091
DB00584	Embryoma	0.06166
DB00584	Endometrial cancer	0.17408
DB00584	Endometriosis	0.08305
DB00584	Esophagus cancer	0.14907
DB00584	Familial Mediterranean fever	0.2582

DB00584	Fibroid tumor	0.30151
DB00584	Heart disease	0.30151
DB00584	Hyperhomocysteinemia	0.28868
DB00584	Hypertension	0.07906
DB00584	Hypoglycemia	0.40825
DB00584	IGA glomerulonephritis	0.1715
DB00584	Infectious lung disease	0.22361
DB00584	Kidney disease	0.11952
DB00584	Leukemia	0.05634
DB00584	Liver cancer	0.07762
DB00584	Lupus erythematosus	0.08544
DB00584	Lupus vulgaris	0.18898
DB00584	Macular degeneration	0.20851
DB00584	Metabolic syndrome X	0.35355
DB00584	Metabolism disease	0.16013
DB00584	Migraine	0.1857
DB00584	Mucocutaneous lymph node syndrome	0.21822
DB00584	Neoplasm metastasis	0.08138
DB00584	Obesity	0.07692
DB00584	Parkinson disease	0.11043
DB00584	Polycythemia	0.33333
DB00584	Premature birth	0.24254
DB00584	Prostate cancer	0.0522
DB00584	Proteinuria	0.22361
DB00584	Psoriasis	0.2357
DB00584	Respiratory distress syndrome	0.27735
DB00584	Sarcoidosis	0.19612
DB00584	Schizophrenia	0.07603
DB00584	Severe acute respiratory syndrome	0.31623
DB00584	Stomach cancer	0.08422
DB00584	Subacute sclerosing panencephalitis	0.5
DB00584	Systemic infection	0.11471
DB00584	Systemic scleroderma	0.10483
DB00584	Testicular dysfunction	0.14286
DB00584	Thrombophilia	0.2357
DB00584	Transient hypertension of pregnancy	0.31623
DB00584	Ulcerative colitis	0.10314
DB00584	Vascular dementia	0.33333
DB00616	Abortion	0.08839
DB00616	Acne	0.28868
DB00616	Alzheimer's disease	0.1018
DB00616	Amyloidosis	0.13363
DB00616	Anemia	0.15076
DB00616	Ankylosing spondylitis	0.26726
DB00616	Arthritis	0.08771
DB00616	Asthma	0.05774
DB00616	Behcet syndrome	0.10102

DB00616	Breast cancer	0.03402
DB00616	Bronchopulmonary dysplasia	0.2132
DB00616	Cancer	0.02606
DB00616	Cardiovascular disease	0.10783
DB00616	Cholestasis	0.19612
DB00616	Chronic fatigue syndrome	0.25
DB00616	Chronic obstructive airway disease	0.07906
DB00616	Chronic rejection of renal transplant	0.17678
DB00616	Chronic simple glaucoma	0.15076
DB00616	Colon cancer	0.04196
DB00616	Connective tissue disease	0.17678
DB00616	Depression	0.09285
DB00616	Dermatitis	0.06428
DB00616	Diabetes mellitus	0.03722
DB00616	Embryoma	0.0436
DB00616	Endometrial cancer	0.12309
DB00616	Endometriosis	0.05872
DB00616	Esophagus cancer	0.10541
DB00616	Familial Mediterranean fever	0.18257
DB00616	Fibroid tumor	0.2132
DB00616	HIV infection	0.06773
DB00616	Heart disease	0.2132
DB00616	Hyperhomocysteinemia	0.20412
DB00616	Hypertension	0.0559
DB00616	Hypoglycemia	0.28868
DB00616	IGA glomerulonephritis	0.12127
DB00616	Infectious lung disease	0.15811
DB00616	Kidney disease	0.16903
DB00616	Leukemia	0.03984
DB00616	Liver cancer	0.05488
DB00616	Lupus erythematosus	0.06041
DB00616	Lupus vulgaris	0.13363
DB00616	Macular degeneration	0.14744
DB00616	Metabolic syndrome X	0.25
DB00616	Metabolism disease	0.11323
DB00616	Migraine	0.13131
DB00616	Mucocutaneous lymph node syndrome	0.1543
DB00616	Neoplasm metastasis	0.05754
DB00616	Obesity	0.05439
DB00616	Parkinson disease	0.07809
DB00616	Polycythemia	0.2357
DB00616	Premature birth	0.1715
DB00616	Prostate cancer	0.03691
DB00616	Proteinuria	0.15811
DB00616	Psoriasis	0.16667
DB00616	Respiratory distress syndrome	0.19612
DB00616	Sarcoidosis	0.13868

DB00616	Schizophrenia	0.05376
DB00616	Severe acute respiratory syndrome	0.22361
DB00616	Stomach cancer	0.05955
DB00616	Subacute sclerosing panencephalitis	0.35355
DB00616	Systemic infection	0.08111
DB00616	Systemic scleroderma	0.07412
DB00616	Testicular dysfunction	0.10102
DB00616	Thrombophilia	0.16667
DB00616	Transient hypertension of pregnancy	0.22361
DB00616	Ulcerative colitis	0.07293
DB00616	Vascular dementia	0.2357
DB00691	Alzheimer's disease	0.0509
DB00691	Anemia	0.15076
DB00691	Ankylosing spondylitis	0.26726
DB00691	Arthritis	0.08771
DB00691	Asthma	0.05774
DB00691	Behcet syndrome	0.10102
DB00691	Breast cancer	0.03402
DB00691	Bronchopulmonary dysplasia	0.2132
DB00691	Cardiovascular disease	0.10783
DB00691	Chronic fatigue syndrome	0.25
DB00691	Chronic obstructive airway disease	0.07906
DB00691	Chronic rejection of renal transplant	0.17678
DB00691	Chronic simple glaucoma	0.15076
DB00691	Colon cancer	0.04196
DB00691	Connective tissue disease	0.17678
DB00691	Coronavirus infection	0.35355
DB00691	Depression	0.09285
DB00691	Dermatitis	0.06428
DB00691	Embryoma	0.0436
DB00691	Endometrial cancer	0.12309
DB00691	Endometriosis	0.05872
DB00691	Esophagus cancer	0.10541
DB00691	Familial Mediterranean fever	0.18257
DB00691	Fibroid tumor	0.2132
DB00691	Heart disease	0.2132
DB00691	Hyperhomocysteinemia	0.20412
DB00691	Hypertension	0.1118
DB00691	Hypoglycemia	0.28868
DB00691	IGA glomerulonephritis	0.12127
DB00691	Infectious lung disease	0.15811
DB00691	Kidney disease	0.16903
DB00691	Leukemia	0.03984
DB00691	Liver cancer	0.05488
DB00691	Lupus erythematosus	0.06041
DB00691	Lupus vulgaris	0.13363
DB00691	Macular degeneration	0.14744

DB00691	Metabolic syndrome X	0.25
DB00691	Metabolism disease	0.11323
DB00691	Migraine	0.13131
DB00691	Mucocutaneous lymph node syndrome	0.1543
DB00691	Neoplasm metastasis	0.05754
DB00691	Obesity	0.05439
DB00691	Parkinson disease	0.07809
DB00691	Polycythemia	0.2357
DB00691	Premature birth	0.1715
DB00691	Prostate cancer	0.03691
DB00691	Proteinuria	0.31623
DB00691	Psoriasis	0.16667
DB00691	Respiratory distress syndrome	0.19612
DB00691	Sarcoidosis	0.27735
DB00691	Schizophrenia	0.05376
DB00691	Severe acute respiratory syndrome	0.22361
DB00691	Stomach cancer	0.05955
DB00691	Subacute sclerosing panencephalitis	0.35355
DB00691	Systemic infection	0.08111
DB00691	Systemic scleroderma	0.07412
DB00691	Testicular dysfunction	0.10102
DB00691	Thrombophilia	0.16667
DB00691	Transient hypertension of pregnancy	0.22361
DB00691	Ulcerative colitis	0.07293
DB00691	Vascular dementia	0.2357
DB00722	Alzheimer's disease	0.0509
DB00722	Anemia	0.15076
DB00722	Ankylosing spondylitis	0.26726
DB00722	Arthritis	0.08771
DB00722	Asthma	0.05774
DB00722	Behcet syndrome	0.10102
DB00722	Breast cancer	0.03402
DB00722	Bronchopulmonary dysplasia	0.2132
DB00722	Cardiovascular disease	0.10783
DB00722	Chronic fatigue syndrome	0.25
DB00722	Chronic obstructive airway disease	0.07906
DB00722	Chronic rejection of renal transplant	0.17678
DB00722	Chronic simple glaucoma	0.15076
DB00722	Colon cancer	0.04196
DB00722	Connective tissue disease	0.17678
DB00722	Coronavirus infection	0.35355
DB00722	Depression	0.09285
DB00722	Dermatitis	0.06428
DB00722	Embryoma	0.0436
DB00722	Endometrial cancer	0.12309
DB00722	Endometriosis	0.05872
DB00722	Esophagus cancer	0.10541

DB00722	Familial Mediterranean fever	0.18257
DB00722	Fibroid tumor	0.2132
DB00722	Heart disease	0.2132
DB00722	Hyperhomocysteinemia	0.20412
DB00722	Hypertension	0.1118
DB00722	Hypoglycemia	0.28868
DB00722	IGA glomerulonephritis	0.12127
DB00722	Infectious lung disease	0.15811
DB00722	Kidney disease	0.16903
DB00722	Leukemia	0.03984
DB00722	Liver cancer	0.05488
DB00722	Lupus erythematosus	0.06041
DB00722	Lupus vulgaris	0.13363
DB00722	Macular degeneration	0.14744
DB00722	Metabolic syndrome X	0.25
DB00722	Metabolism disease	0.11323
DB00722	Migraine	0.13131
DB00722	Mucocutaneous lymph node syndrome	0.1543
DB00722	Neoplasm metastasis	0.05754
DB00722	Obesity	0.05439
DB00722	Parkinson disease	0.07809
DB00722	Polycythemia	0.2357
DB00722	Premature birth	0.1715
DB00722	Prostate cancer	0.03691
DB00722	Proteinuria	0.31623
DB00722	Psoriasis	0.16667
DB00722	Respiratory distress syndrome	0.19612
DB00722	Sarcoidosis	0.27735
DB00722	Schizophrenia	0.05376
DB00722	Severe acute respiratory syndrome	0.22361
DB00722	Stomach cancer	0.05955
DB00722	Subacute sclerosing panencephalitis	0.35355
DB00722	Systemic infection	0.08111
DB00722	Systemic scleroderma	0.07412
DB00722	Testicular dysfunction	0.10102
DB00722	Thrombophilia	0.16667
DB00722	Transient hypertension of pregnancy	0.22361
DB00722	Ulcerative colitis	0.07293
DB00722	Vascular dementia	0.2357
DB00790	Alzheimer's disease	0.07198
DB00790	Anemia	0.2132
DB00790	Ankylosing spondylitis	0.37796
DB00790	Arthritis	0.12403
DB00790	Asthma	0.08165
DB00790	Behcet syndrome	0.14286
DB00790	Breast cancer	0.04811
DB00790	Bronchopulmonary dysplasia	0.30151

DB00790	Cardiovascular disease	0.1525
DB00790	Chronic fatigue syndrome	0.35355
DB00790	Chronic obstructive airway disease	0.1118
DB00790	Chronic rejection of renal transplant	0.25
DB00790	Chronic simple glaucoma	0.2132
DB00790	Colon cancer	0.05934
DB00790	Connective tissue disease	0.25
DB00790	Depression	0.13131
DB00790	Dermatitis	0.09091
DB00790	Embryoma	0.06166
DB00790	Endometrial cancer	0.17408
DB00790	Endometriosis	0.08305
DB00790	Esophagus cancer	0.14907
DB00790	Familial Mediterranean fever	0.2582
DB00790	Fibroid tumor	0.30151
DB00790	Heart disease	0.30151
DB00790	Hyperhomocysteinemia	0.28868
DB00790	Hypertension	0.07906
DB00790	Hypoglycemia	0.40825
DB00790	IGA glomerulonephritis	0.1715
DB00790	Infectious lung disease	0.22361
DB00790	Kidney disease	0.11952
DB00790	Leukemia	0.05634
DB00790	Liver cancer	0.07762
DB00790	Lupus erythematosus	0.08544
DB00790	Lupus vulgaris	0.18898
DB00790	Macular degeneration	0.20851
DB00790	Metabolic syndrome X	0.35355
DB00790	Metabolism disease	0.16013
DB00790	Migraine	0.1857
DB00790	Mucocutaneous lymph node syndrome	0.21822
DB00790	Neoplasm metastasis	0.08138
DB00790	Obesity	0.07692
DB00790	Parkinson disease	0.11043
DB00790	Polycythemia	0.33333
DB00790	Premature birth	0.24254
DB00790	Prostate cancer	0.0522
DB00790	Proteinuria	0.22361
DB00790	Psoriasis	0.2357
DB00790	Respiratory distress syndrome	0.27735
DB00790	Sarcoidosis	0.19612
DB00790	Schizophrenia	0.07603
DB00790	Severe acute respiratory syndrome	0.31623
DB00790	Stomach cancer	0.08422
DB00790	Subacute sclerosing panencephalitis	0.5
DB00790	Systemic infection	0.11471
DB00790	Systemic scleroderma	0.10483

DB00790	Testicular dysfunction	0.14286
DB00790	Thrombophilia	0.2357
DB00790	Transient hypertension of pregnancy	0.31623
DB00790	Ulcerative colitis	0.10314
DB00790	Vascular dementia	0.33333
DB00881	Alzheimer's disease	0.07198
DB00881	Anemia	0.2132
DB00881	Ankylosing spondylitis	0.37796
DB00881	Arthritis	0.12403
DB00881	Asthma	0.08165
DB00881	Behcet syndrome	0.14286
DB00881	Breast cancer	0.04811
DB00881	Bronchopulmonary dysplasia	0.30151
DB00881	Cardiovascular disease	0.1525
DB00881	Chronic fatigue syndrome	0.35355
DB00881	Chronic obstructive airway disease	0.1118
DB00881	Chronic rejection of renal transplant	0.25
DB00881	Chronic simple glaucoma	0.2132
DB00881	Colon cancer	0.05934
DB00881	Connective tissue disease	0.25
DB00881	Depression	0.13131
DB00881	Dermatitis	0.09091
DB00881	Embryoma	0.06166
DB00881	Endometrial cancer	0.17408
DB00881	Endometriosis	0.08305
DB00881	Esophagus cancer	0.14907
DB00881	Familial Mediterranean fever	0.2582
DB00881	Fibroid tumor	0.30151
DB00881	Heart disease	0.30151
DB00881	Hyperhomocysteinemia	0.28868
DB00881	Hypertension	0.07906
DB00881	Hypoglycemia	0.40825
DB00881	IGA glomerulonephritis	0.1715
DB00881	Infectious lung disease	0.22361
DB00881	Kidney disease	0.11952
DB00881	Leukemia	0.05634
DB00881	Liver cancer	0.07762
DB00881	Lupus erythematosus	0.08544
DB00881	Lupus vulgaris	0.18898
DB00881	Macular degeneration	0.20851
DB00881	Metabolic syndrome X	0.35355
DB00881	Metabolism disease	0.16013
DB00881	Migraine	0.1857
DB00881	Mucocutaneous lymph node syndrome	0.21822
DB00881	Neoplasm metastasis	0.08138
DB00881	Obesity	0.07692
DB00881	Parkinson disease	0.11043

DB00881	Polycythemia	0.33333
DB00881	Premature birth	0.24254
DB00881	Prostate cancer	0.0522
DB00881	Proteinuria	0.22361
DB00881	Psoriasis	0.2357
DB00881	Respiratory distress syndrome	0.27735
DB00881	Sarcoidosis	0.19612
DB00881	Schizophrenia	0.07603
DB00881	Severe acute respiratory syndrome	0.31623
DB00881	Stomach cancer	0.08422
DB00881	Subacute sclerosing panencephalitis	0.5
DB00881	Systemic infection	0.11471
DB00881	Systemic scleroderma	0.10483
DB00881	Testicular dysfunction	0.14286
DB00881	Thrombophilia	0.2357
DB00881	Transient hypertension of pregnancy	0.31623
DB00881	Ulcerative colitis	0.10314
DB00881	Vascular dementia	0.33333
DB01180	Alzheimer's disease	0.07198
DB01180	Anemia	0.2132
DB01180	Ankylosing spondylitis	0.37796
DB01180	Arthritis	0.12403
DB01180	Asthma	0.08165
DB01180	Behcet syndrome	0.14286
DB01180	Breast cancer	0.04811
DB01180	Bronchopulmonary dysplasia	0.30151
DB01180	Cardiovascular disease	0.1525
DB01180	Chronic fatigue syndrome	0.35355
DB01180	Chronic obstructive airway disease	0.1118
DB01180	Chronic rejection of renal transplant	0.25
DB01180	Chronic simple glaucoma	0.2132
DB01180	Colon cancer	0.05934
DB01180	Connective tissue disease	0.25
DB01180	Depression	0.13131
DB01180	Dermatitis	0.09091
DB01180	Embryoma	0.06166
DB01180	Endometrial cancer	0.17408
DB01180	Endometriosis	0.08305
DB01180	Esophagus cancer	0.14907
DB01180	Familial Mediterranean fever	0.2582
DB01180	Fibroid tumor	0.30151
DB01180	Heart disease	0.30151
DB01180	Hyperhomocysteinemia	0.28868
DB01180	Hypertension	0.07906
DB01180	Hypoglycemia	0.40825
DB01180	IGA glomerulonephritis	0.1715
DB01180	Infectious lung disease	0.22361

DB01180	Kidney disease	0.11952
DB01180	Leukemia	0.05634
DB01180	Liver cancer	0.07762
DB01180	Lupus erythematosus	0.08544
DB01180	Lupus vulgaris	0.18898
DB01180	Macular degeneration	0.20851
DB01180	Metabolic syndrome X	0.35355
DB01180	Metabolism disease	0.16013
DB01180	Migraine	0.1857
DB01180	Mucocutaneous lymph node syndrome	0.21822
DB01180	Neoplasm metastasis	0.08138
DB01180	Obesity	0.07692
DB01180	Parkinson disease	0.11043
DB01180	Polycythemia	0.33333
DB01180	Premature birth	0.24254
DB01180	Prostate cancer	0.0522
DB01180	Proteinuria	0.22361
DB01180	Psoriasis	0.2357
DB01180	Respiratory distress syndrome	0.27735
DB01180	Sarcoidosis	0.19612
DB01180	Schizophrenia	0.07603
DB01180	Severe acute respiratory syndrome	0.31623
DB01180	Stomach cancer	0.08422
DB01180	Subacute sclerosing panencephalitis	0.5
DB01180	Systemic infection	0.11471
DB01180	Systemic scleroderma	0.10483
DB01180	Testicular dysfunction	0.14286
DB01180	Thrombophilia	0.2357
DB01180	Transient hypertension of pregnancy	0.31623
DB01180	Ulcerative colitis	0.10314
DB01180	Vascular dementia	0.33333
DB01340	Alzheimer's disease	0.07198
DB01340	Anemia	0.2132
DB01340	Ankylosing spondylitis	0.37796
DB01340	Arthritis	0.12403
DB01340	Asthma	0.08165
DB01340	Behcet syndrome	0.14286
DB01340	Breast cancer	0.04811
DB01340	Bronchopulmonary dysplasia	0.30151
DB01340	Cardiovascular disease	0.1525
DB01340	Chronic fatigue syndrome	0.35355
DB01340	Chronic obstructive airway disease	0.1118
DB01340	Chronic rejection of renal transplant	0.25
DB01340	Chronic simple glaucoma	0.2132
DB01340	Colon cancer	0.05934
DB01340	Connective tissue disease	0.25
DB01340	Depression	0.13131

DB01340	Dermatitis	0.09091
DB01340	Embryoma	0.06166
DB01340	Endometrial cancer	0.17408
DB01340	Endometriosis	0.08305
DB01340	Esophagus cancer	0.14907
DB01340	Familial Mediterranean fever	0.2582
DB01340	Fibroid tumor	0.30151
DB01340	Heart disease	0.30151
DB01340	Hyperhomocysteinemia	0.28868
DB01340	Hypertension	0.07906
DB01340	Hypoglycemia	0.40825
DB01340	IGA glomerulonephritis	0.1715
DB01340	Infectious lung disease	0.22361
DB01340	Kidney disease	0.11952
DB01340	Leukemia	0.05634
DB01340	Liver cancer	0.07762
DB01340	Lupus erythematosus	0.08544
DB01340	Lupus vulgaris	0.18898
DB01340	Macular degeneration	0.20851
DB01340	Metabolic syndrome X	0.35355
DB01340	Metabolism disease	0.16013
DB01340	Migraine	0.1857
DB01340	Mucocutaneous lymph node syndrome	0.21822
DB01340	Neoplasm metastasis	0.08138
DB01340	Obesity	0.07692
DB01340	Parkinson disease	0.11043
DB01340	Polycythemia	0.33333
DB01340	Premature birth	0.24254
DB01340	Prostate cancer	0.0522
DB01340	Proteinuria	0.22361
DB01340	Psoriasis	0.2357
DB01340	Respiratory distress syndrome	0.27735
DB01340	Sarcoidosis	0.19612
DB01340	Schizophrenia	0.07603
DB01340	Severe acute respiratory syndrome	0.31623
DB01340	Stomach cancer	0.08422
DB01340	Subacute sclerosing panencephalitis	0.5
DB01340	Systemic infection	0.11471
DB01340	Systemic scleroderma	0.10483
DB01340	Testicular dysfunction	0.14286
DB01340	Thrombophilia	0.2357
DB01340	Transient hypertension of pregnancy	0.31623
DB01340	Ulcerative colitis	0.10314
DB01340	Vascular dementia	0.33333
DB01348	Alzheimer's disease	0.07198
DB01348	Anemia	0.2132
DB01348	Ankylosing spondylitis	0.37796

DB01348	Arthritis	0.12403
DB01348	Asthma	0.08165
DB01348	Behcet syndrome	0.14286
DB01348	Breast cancer	0.04811
DB01348	Bronchopulmonary dysplasia	0.30151
DB01348	Cardiovascular disease	0.1525
DB01348	Chronic fatigue syndrome	0.35355
DB01348	Chronic obstructive airway disease	0.1118
DB01348	Chronic rejection of renal transplant	0.25
DB01348	Chronic simple glaucoma	0.2132
DB01348	Colon cancer	0.05934
DB01348	Connective tissue disease	0.25
DB01348	Depression	0.13131
DB01348	Dermatitis	0.09091
DB01348	Embryoma	0.06166
DB01348	Endometrial cancer	0.17408
DB01348	Endometriosis	0.08305
DB01348	Esophagus cancer	0.14907
DB01348	Familial Mediterranean fever	0.2582
DB01348	Fibroid tumor	0.30151
DB01348	Heart disease	0.30151
DB01348	Hyperhomocysteinemia	0.28868
DB01348	Hypertension	0.07906
DB01348	Hypoglycemia	0.40825
DB01348	IGA glomerulonephritis	0.1715
DB01348	Infectious lung disease	0.22361
DB01348	Kidney disease	0.11952
DB01348	Leukemia	0.05634
DB01348	Liver cancer	0.07762
DB01348	Lupus erythematosus	0.08544
DB01348	Lupus vulgaris	0.18898
DB01348	Macular degeneration	0.20851
DB01348	Metabolic syndrome X	0.35355
DB01348	Metabolism disease	0.16013
DB01348	Migraine	0.1857
DB01348	Mucocutaneous lymph node syndrome	0.21822
DB01348	Neoplasm metastasis	0.08138
DB01348	Obesity	0.07692
DB01348	Parkinson disease	0.11043
DB01348	Polycythemia	0.33333
DB01348	Premature birth	0.24254
DB01348	Prostate cancer	0.0522
DB01348	Proteinuria	0.22361
DB01348	Psoriasis	0.2357
DB01348	Respiratory distress syndrome	0.27735
DB01348	Sarcoidosis	0.19612
DB01348	Schizophrenia	0.07603

DB01348	Severe acute respiratory syndrome	0.31623
DB01348	Stomach cancer	0.08422
DB01348	Subacute sclerosing panencephalitis	0.5
DB01348	Systemic infection	0.11471
DB01348	Systemic scleroderma	0.10483
DB01348	Testicular dysfunction	0.14286
DB01348	Thrombophilia	0.2357
DB01348	Transient hypertension of pregnancy	0.31623
DB01348	Ulcerative colitis	0.10314
DB01348	Vascular dementia	0.33333
DB00161	Advanced cancer	0.18257
DB00161	Colon cancer	0.03426
DB00161	Nasopharyngeal cancer	0.11111
DB00898	Atherosclerosis	0.02646
DB00898	Cancer	0.02786
DB00898	Drug abuse	0.0708
DB00898	Hyperopia	0.12599
DB00898	Movement disorder	0.07881
DB00898	Neurotic disorder	0.11952
DB00898	Pancreatitis	0.05903
DB00898	Parkinson disease	0.04174
DB00898	Stroke	0.0428
DB01213	AIDS	0.18898
DB01213	Adenocarcinoma	0.07293
DB01213	Adenovirus infection	0.06509
DB01213	Asthma	0.04082
DB01213	Atherosclerosis	0.03501
DB01213	Brain tumor	0.04016
DB01213	Breast cancer	0.02406
DB01213	Cancer	0.03686
DB01213	Chronic obstructive airway disease	0.0559
DB01213	Deafness	0.07217
DB01213	Diabetes mellitus	0.02632
DB01213	Drug abuse	0.09366
DB01213	Embryoma	0.03083
DB01213	Endometriosis	0.04152
DB01213	Henoch-Schoenlein purpura	0.15811
DB01213	Hypertension	0.03953
DB01213	Lupus erythematosus	0.04272
DB01213	Malnutrition	0.17678
DB01213	Movement disorder	0.10426
DB01213	Pancreatitis	0.07809
DB01213	Parkinson disease	0.05522
DB01213	Rheumatoid arthritis	0.0306
DB01213	Squamous cell cancer	0.05103
DB01213	Stroke	0.05661
DB01213	Vitiligo	0.11785

DB00606	Anemia	0.2132
DB00606	Cancer	0.01843
DB00606	Diabetes mellitus	0.02632
DB00606	Down syndrome	0.05698
DB00606	Pancreas cancer	0.05185
DB00606	Pancreatitis	0.07809
DB00606	Retinitis pigmentosa	0.1
DB00606	Ulcerative colitis	0.05157
DB08827	Atherosclerosis	0.07001
DB08827	Diabetes mellitus	0.05263
DB08827	Encephalopathies	0.14003
DB08827	Hypercholesterolemia	0.19245
DB08827	Liver cancer	0.07762
DB08827	Liver tumor	0.17678
DB08827	Obesity	0.07692
DB00257	Endometrium cancer	0.1715
DB00257	Sickle cell disease	0.13608
DB04794	Breast cancer	0.03402
DB04794	HIV infection	0.06773
DB04794	Polyarthritis	0.08058
DB04794	Prostate cancer	0.03691
DB00200	Infertility, Male	0.06804
DB00200	Abruption placentae	0.11785
DB00200	Alzheimer's disease	0.04799
DB00200	Atherosclerosis	0.02334
DB00200	Bipolar disorder	0.03774
DB00200	Bladder cancer	0.04303
DB00200	Brain tumor	0.02677
DB00200	Breast cancer	0.01604
DB00200	Cleft palate	0.08909
DB00200	Congenital abnormality	0.05025
DB00200	Down syndrome	0.07597
DB00200	Embryoma	0.02055
DB00200	Encephalopathies	0.04668
DB00200	Enteritis	0.03659
DB00200	Hyperhomocysteinemia	0.19245
DB00200	Leukemia	0.01878
DB00200	Lupus erythematosus	0.02848
DB00200	Malignant glioma	0.0619
DB00200	Meningioma	0.17213
DB00200	Multiple myeloma	0.04377
DB00200	Obesity	0.05128
DB00200	Pancreas cancer	0.03457
DB00200	Prostate cancer	0.0174
DB00200	Rheumatoid arthritis	0.0204
DB00200	Schizophrenia	0.02534
DB00200	Spinal dysraphism	0.15294

DB00200	Ulcerative colitis	0.03438
DB05269	Alzheimer's disease	0.07198
DB05269	Atherosclerosis	0.07001
DB05269	Common cold	0.26726
DB05269	Diabetes mellitus	0.05263
DB05269	Hyperglycemia	0.16013
DB05269	Hyperinsulinism	0.19245
DB05269	Hypertension	0.07906
DB05269	Leukemia	0.05634
DB05269	Lung cancer	0.06984
DB05269	Metabolism disease	0.16013
DB05269	Pancreatitis	0.15617
DB05269	Skin disease	0.1715
DB05269	Ulcerative colitis	0.10314
DB06439	Alzheimer's disease	0.07198
DB06439	Atherosclerosis	0.07001
DB06439	Common cold	0.26726
DB06439	Diabetes mellitus	0.05263
DB06439	Hyperglycemia	0.16013
DB06439	Hyperinsulinism	0.19245
DB06439	Hypertension	0.07906
DB06439	Leukemia	0.05634
DB06439	Lung cancer	0.06984
DB06439	Metabolism disease	0.16013
DB06439	Pancreatitis	0.15617
DB06439	Skin disease	0.1715
DB06439	Ulcerative colitis	0.10314
DB00824	Adenocarcinoma	0.08422
DB00824	Behavior disease	0.08909
DB00824	Breast cancer	0.02778
DB00824	Chronic obstructive airway disease	0.06455
DB00824	Depression	0.07581
DB00824	Diabetes mellitus	0.03039
DB00824	Hypertension	0.04564
DB00824	Leukemia	0.06506
DB01367	Stress disorder, post-traumatic	0.25
DB01367	AIDS	0.12354
DB01367	Alzheimer's disease	0.19174
DB01367	Asthma	0.27479
DB01367	Autistic disorder	0.3858
DB01367	Autoimmune disease	0.37681
DB01367	Behavior disease	0.10911
DB01367	Brain ischemia	1.22313
DB01367	Cancer	0.11721
DB01367	Celiac disease	0.53825
DB01367	Congenital abnormality	0.21404
DB01367	Dental plaque	0.41474

DB01367	Diabetes mellitus	0.02277
DB01367	Drug abuse	0.2271
DB01367	Eating disorder	0.40029
DB01367	Encephalopathies	0.09901
DB01367	Epstein-Barr virus infection	0.61434
DB01367	Esotropia	0.64373
DB01367	Eye cancer	1.0449
DB01367	Fibromyalgia	0.25
DB01367	Glaucoma	0.49682
DB01367	Gram-Negative bacterial infection	0.16406
DB01367	Graves' disease	0.66884
DB01367	HIV infection	0.24166
DB01367	Herpes	0.3004
DB01367	Huntington disease	0.1543
DB01367	Immunologic deficiency syndrome	0.06184
DB01367	Intracranial hypertension	0.1312
DB01367	Ischemia	0.40608
DB01367	Keratosis	0.67179
DB01367	Lichen planus	1.09756
DB01367	Lupus erythematosus	0.23897
DB01367	Lymphoma	0.07052
DB01367	Neurodegenerative disorder	0.07109
DB01367	Psychotic disorder	0.11323
DB01367	Rheumatoid arthritis	0.17347
DB01367	Schistosomiasis	1.11941
DB01367	Stroke	0.27946
DB01367	Systemic scleroderma	0.03362
DB01367	Thyroid gland disease	0.64101
DB01367	Tuberculosis	0.50555
DB01367	Vaccinia	0.10995
DB00179	Asthma	0.08165
DB00179	Atherosclerosis	0.07001
DB00179	Cancer	0.03686
DB00179	Cytomegalovirus infection	0.2582
DB00179	Dental plaque	0.127
DB00179	Diabetes mellitus	0.05263
DB00179	Tuberculosis	0.13484
DB00471	Asthma	0.11547
DB00471	Atherosclerosis	0.04951
DB00471	Cancer	0.02606
DB00471	Colon cancer	0.04196
DB00471	Cytomegalovirus infection	0.18257
DB00471	Dental plaque	0.0898
DB00471	Dermatitis	0.06428
DB00471	Diabetes mellitus	0.03722
DB00471	Prostate cancer	0.03691
DB00471	Tuberculosis	0.09535

DB00744	Asthma	0.08165
DB00744	Atherosclerosis	0.07001
DB00744	Cancer	0.03686
DB00744	Cytomegalovirus infection	0.2582
DB00744	Dental plaque	0.127
DB00744	Diabetes mellitus	0.05263
DB00744	Tuberculosis	0.13484
DB00906	Drug abuse	0.09366
DB00242	Kidney tubular necrosis, acute	0.14246
DB00242	Amyotrophic lateral sclerosis	0.04276
DB00242	Aortic valve disease	0.20109
DB00242	Barrett's esophagus	0.07986
DB00242	Breast cancer	0.0225
DB00242	Cancer	0.07837
DB00242	Celiac disease	0.07802
DB00242	Chronic obstructive airway disease	0.07453
DB00242	Colon cancer	0.10925
DB00242	Common cold	0.10392
DB00242	Diabetes mellitus	0.02389
DB00242	Down syndrome	0.2277
DB00242	Emphysema	0.14723
DB00242	Ewings sarcoma	0.13892
DB00242	Fanconi's anemia	0.04591
DB00242	HIV infection	0.07435
DB00242	Heart failure	0.05678
DB00242	Helicobacter infection	0.17451
DB00242	Ischemia	0.05834
DB00242	Kaposi sarcoma	0.07853
DB00242	Leukemia	0.02667
DB00242	Liver cancer	0.04491
DB00242	Lung cancer	0.10627
DB00242	Lymphoma	0.22889
DB00242	Melanoma	0.0436
DB00242	Meningioma	0.52456
DB00242	Multiple endocrine neoplasia	0.15503
DB00242	Parkinson disease	0.05137
DB00242	Pituitary tumor	0.1198
DB00242	Renal tubular acidosis	0.0609
DB00242	Rheumatoid arthritis	0.02407
DB00242	Tuberous sclerosis	0.1149
DB00242	Ulcerative colitis	0.06292
DB00242	Uterine fibroids	0.12002
DB00242	Werner syndrome	0.07884
DB00548	Congenital abnormality	0.03371
DB00548	Glaucoma	0.07454
DB00548	Neoplasm metastasis	0.03639
DB00548	Parkinson disease	0.04939

DB00548	Skin disease	0.0767
DB00548	Uveomeningoencephalitic syndrome	0.2582
DB00600	Congenital abnormality	0.07538
DB00600	Glaucoma	0.16667
DB00600	Neoplasm metastasis	0.08138
DB00600	Parkinson disease	0.11043
DB00600	Skin disease	0.1715
DB00600	Uveomeningoencephalitic syndrome	0.57735
DB01055	Hemorrhagic fevers, Viral	0.2357
DB01055	Labor, Premature	0.14907
DB01055	Mycobacterium infection, Atypical	0.19245
DB01055	Pleural effusion, Malignant	0.17408
DB01055	Alopecia	0.10541
DB01055	Alzheimer's disease	0.04156
DB01055	Amyotrophic lateral sclerosis	0.07785
DB01055	Arthritis	0.07161
DB01055	Atherosclerosis	0.04042
DB01055	Autoimmune disease	0.06262
DB01055	Bipolar disorder	0.06537
DB01055	Brain tumor	0.04637
DB01055	Bronchiolitis	0.20412
DB01055	CNS metastases	0.20412
DB01055	Cancer	0.02128
DB01055	Congenital abnormality	0.04352
DB01055	Depression	0.07581
DB01055	Diabetes mellitus	0.03039
DB01055	Down syndrome	0.0658
DB01055	Endometriosis	0.04795
DB01055	Epstein-Barr virus infection	0.12599
DB01055	Esophagus cancer	0.08607
DB01055	Glaucoma	0.09623
DB01055	Growth retardation	0.11111
DB01055	HIV infection	0.0553
DB01055	Heart failure	0.06155
DB01055	Hepatitis B	0.14003
DB01055	Hepatitis C	0.08513
DB01055	Hypercholesterolemia	0.11111
DB01055	Hyperhomocysteinemia	0.16667
DB01055	Hyperinsulinism	0.11111
DB01055	Hypothyroidism	0.13608
DB01055	IGA glomerulonephritis	0.09901
DB01055	Infertility	0.07931
DB01055	Influenza	0.11547
DB01055	Intractable epilepsy	0.20412
DB01055	Kidney failure	0.06537
DB01055	Leukemia	0.03253
DB01055	Lipodystrophy	0.16013

DB01055	Liver cancer	0.04481
DB01055	Liver disease	0.08909
DB01055	Lupus vulgaris	0.10911
DB01055	Lyme disease	0.19245
DB01055	Macular degeneration	0.12039
DB01055	Melanoma	0.04811
DB01055	Nasopharyngeal cancer	0.11111
DB01055	Neoplasm metastasis	0.04698
DB01055	Nephritis	0.2582
DB01055	Neuroblastoma	0.07931
DB01055	Obesity	0.04441
DB01055	Pancreatitis	0.09017
DB01055	Parkinson disease	0.06376
DB01055	Penile disease	0.20412
DB01055	Peptic esophagitis	0.18257
DB01055	Prostate cancer	0.03014
DB01055	Proteinuria	0.1291
DB01055	Pulmonary fibrosis	0.10911
DB01055	Schizophrenia	0.0439
DB01055	Skin disease	0.09901
DB01055	Squamous cell cancer	0.05893
DB01055	Stomach cancer	0.04862
DB01055	Systemic scleroderma	0.06052
DB01055	Tuberculosis	0.07785
DB01055	Tuberous sclerosis	0.14907
DB01055	Uveomeningoencephalitic syndrome	0.33333
DB00780	Stress disorder, post-traumatic	0.14434
DB00780	Anorexia nervosa	0.08333
DB00780	Autistic disorder	0.09901
DB00780	Behavior disease	0.12599
DB00780	Bipolar disorder	0.04623
DB00780	Breast cancer	0.01964
DB00780	Depression	0.05361
DB00780	Down syndrome	0.04652
DB00780	Drug abuse	0.03824
DB00780	Encephalopathies	0.05717
DB00780	Fibromyalgia	0.28868
DB00780	Generalized anxiety disorder	0.11785
DB00780	Huntington disease	0.08909
DB00780	Neurotic disorder	0.1291
DB00780	Obesity	0.0314
DB00780	Panic disorder	0.08909
DB00780	Psychotic disorder	0.06537
DB00780	Sudden infant death syndrome	0.08909
DB00615	Stress disorder, post-traumatic	0.20595
DB00615	Adenocarcinoma	0.21153
DB00615	Adenovirus infection	0.03261

DB00615	Alzheimer's disease	0.22227
DB00615	Atherosclerosis	0.02564
DB00615	Bacterial infection	0.11952
DB00615	Bipolar disorder	0.07543
DB00615	Brain tumor	0.07866
DB00615	Breast cancer	0.055
DB00615	Cancer	0.16354
DB00615	Carcinoma	0.07559
DB00615	Chronic rejection of renal transplant	0.14266
DB00615	Chronic simple glaucoma	0.16379
DB00615	Colon cancer	0.00962
DB00615	Corneal disease	0.03237
DB00615	Cystic fibrosis	0.02376
DB00615	Depression	0.03921
DB00615	Dermatitis	0.02825
DB00615	Diabetes mellitus	0.02459
DB00615	Drug abuse	0.01819
DB00615	Eating disorder	0.4366
DB00615	Endometriosis	0.02322
DB00615	Epilepsy	0.02
DB00615	Fanconi's anemia	0.41082
DB00615	Gastrointestinal tumor	0.12403
DB00615	Gram-Negative bacterial infection	0.04482
DB00615	HIV infection	0.00941
DB00615	Hemolytic-Uremic syndrome	0.08095
DB00615	Herpes	0.33251
DB00615	Huntington disease	0.08191
DB00615	Hyperglycemia	0.07122
DB00615	Hyperparathyroidism	0.06952
DB00615	Hypertension	0.03441
DB00615	Infection	0.13846
DB00615	Infectious lung disease	0.10697
DB00615	Ischemia	0.03568
DB00615	Keratoconus	0.05137
DB00615	Keratosis	0.05938
DB00615	Leukemia	0.1506
DB00615	Liver cancer	0.0684
DB00615	Lung cancer	0.02055
DB00615	Melanoma	0.01391
DB00615	Metaplastic polyp	0.06032
DB00615	Myelofibrosis	0.15356
DB00615	Neck cancer	0.06365
DB00615	Neoplasm metastasis	0.06515
DB00615	Obesity	0.0654
DB00615	Oral cancer	0.03193
DB00615	Papillomavirus infection	0.09433
DB00615	Polyarthritis	0.10508

DB00615	Polycystic ovary syndrome	0.04158
DB00615	Prion disease	0.04189
DB00615	Prostate cancer	0.11207
DB00615	Psychotic disorder	0.07426
DB00615	Renal Cell cancer	0.04725
DB00615	Rheumatoid arthritis	0.03027
DB00615	Sarcoidosis	0.09105
DB00615	Schizophrenia	0.04137
DB00615	Squamous cell cancer	0.03466
DB00615	Stomach cancer	0.01549
DB00615	Systemic infection	0.03263
DB00615	Systemic scleroderma	0.02598
DB00615	Thrombophlebitis	0.03475
DB00615	Tropical spastic paraparesis	0.09928
DB00615	Tuberous sclerosis	0.05984
DB00615	Virus disease	0.08382
DB00615	Vitiligo	0.08274
DB00615	Yersinia infection	0.0509
DB01045	Adenovirus infection	0.07516
DB01045	Endometrium cancer	0.14003
DB01045	Enteritis	0.06337
DB01045	Epilepsy	0.08165
DB01045	Osteosarcoma	0.12309
DB01045	Ulcerative colitis	0.05955
DB01220	Adenovirus infection	0.09206
DB01220	Endometrium cancer	0.1715
DB01220	Enteritis	0.07762
DB01220	Epilepsy	0.1
DB01220	Osteosarcoma	0.15076
DB01220	Ulcerative colitis	0.07293
DB00811	Amaurosis congenita of leber I	0.15811
DB00811	Retinal disease	0.06262
DB00811	Retinitis pigmentosa	0.08944
DB00480	Adrenoleukodystrophy	0.16405
DB00480	Alzheimer's disease	0.127
DB00480	Amyotrophic lateral sclerosis	0.03598
DB00480	Aseptic necrosis of bone	0.12477
DB00480	Asthma	0.15777
DB00480	Atherosclerosis	0.09357
DB00480	Cancer	0.07017
DB00480	Cardiovascular disease	0.10151
DB00480	Chronic fatigue syndrome	0.44018
DB00480	Chronic obstructive airway disease	0.0627
DB00480	Colon cancer	0.02766
DB00480	Congenital abnormality	0.05387
DB00480	Dental plaque	0.21512
DB00480	Depression	0.07312

DB00480	Dermatitis	0.08414
DB00480	Diabetes mellitus	0.0201
DB00480	Endometriosis	0.16425
DB00480	Gouts	0.39773
DB00480	Growth retardation	0.28984
DB00480	Heart failure	0.04777
DB00480	Ischemia	0.16217
DB00480	Liver metastases	0.13364
DB00480	Lupus erythematosus	0.02777
DB00480	Melanoma	0.03668
DB00480	Metaplastic polyp	0.30445
DB00480	Mucocutaneous lymph node syndrome	0.10153
DB00480	Oral cancer	0.21958
DB00480	Parkinson disease	0.14165
DB00480	Periodontitis	0.25696
DB00480	Polyarthritis	0.12157
DB00480	Prion disease	0.42512
DB00480	Retinal disease	0.09681
DB00480	Rheumatoid arthritis	0.0711
DB00480	Sickle cell disease	0.17704
DB00480	Stroke	0.11864
DB00480	Systemic scleroderma	0.06393
DB00480	Thyroid cancer	0.07828
DB00480	Thyroid gland disease	0.26364
DB00480	Ulcerative colitis	0.07904
DB00480	Uterine disease	0.15317
DB00480	Vulvar disease	0.67189
DB00482	Alzheimer's disease	0.12871
DB00482	Asthma	0.17766
DB00482	Atherosclerosis	0.15353
DB00482	Cancer	0.06578
DB00482	Chronic fatigue syndrome	0.71784
DB00482	Dental plaque	0.26933
DB00482	Endometriosis	0.15293
DB00482	Gouts	0.65623
DB00482	Growth retardation	0.47998
DB00482	Ischemia	0.26487
DB00482	Metaplastic polyp	0.48043
DB00482	Oral cancer	0.22579
DB00482	Parkinson disease	0.23161
DB00482	Periodontitis	0.4288
DB00482	Polyarthritis	0.1953
DB00482	Prion disease	0.6812
DB00482	Rheumatoid arthritis	0.11521
DB00482	Sickle cell disease	0.27961
DB00482	Stroke	0.19023
DB00482	Thyroid gland disease	0.42675

DB00482	Vulvar disease	1.11245
DB01041	Adenoma	0.01277
DB01041	Alcoholic liver disease	0.18257
DB01041	Alzheimer's disease	0.09394
DB01041	Amyloidosis	0.08452
DB01041	Anemia	0.09535
DB01041	Anorexia nervosa	0.09129
DB01041	Aplastic anemia	0.09325
DB01041	Arthritis	0.05547
DB01041	Asthma	0.11858
DB01041	Atherosclerosis	0.10214
DB01041	Autoimmune disease	0.04851
DB01041	Basal cell carcinoma	0.12403
DB01041	Behcet syndrome	0.06389
DB01041	Bladder cancer	0.05774
DB01041	Brain tumor	0.01908
DB01041	Breast cancer	0.06378
DB01041	Cancer	0.13243
DB01041	Celiac disease	0.07352
DB01041	Cervical cancer	0.05199
DB01041	Chronic fatigue syndrome	0.33582
DB01041	Colon cancer	0.034
DB01041	Congenital abnormality	0.00675
DB01041	Craniosynostosis	0.26609
DB01041	Dental plaque	0.12499
DB01041	Dermatitis	0.10454
DB01041	Diabetes mellitus	0.15516
DB01041	Down syndrome	0.05096
DB01041	Embryoma	0.04417
DB01041	Emphysema	0.12743
DB01041	Endometriosis	0.33878
DB01041	Enteritis	0.04909
DB01041	Esotropia	0.04613
DB01041	Familial Mediterranean fever	0.11547
DB01041	Fanconi's anemia	0.08944
DB01041	Gouts	0.29881
DB01041	Granulomatous disease	0.1291
DB01041	Growth retardation	0.21669
DB01041	HIV infection	0.04284
DB01041	Heart failure	0.04767
DB01041	Hepatitis B	0.10847
DB01041	Histiocytosis	0.14907
DB01041	Hodgkin's disease	0.11555
DB01041	Hypothyroidism	0.10541
DB01041	Infectious lung disease	0.09179
DB01041	Infertility	0.06143
DB01041	Infiltrating cancer	0.2509

DB01041	Intermediate coronary syndrome	0.14907
DB01041	Ischemia	0.12348
DB01041	Kaposi sarcoma	0.04457
DB01041	Kidney disease	0.05345
DB01041	Leprosy	0.11952
DB01041	Leukemia	0.08081
DB01041	Lichen planus	0.15811
DB01041	Lipodystrophy	0.12403
DB01041	Liver cancer	0.12027
DB01041	Lupus erythematosus	0.05694
DB01041	Lymphoma	0.01371
DB01041	Malaria	0.08305
DB01041	Malignant glioma	0.08305
DB01041	Melanoma	0.03727
DB01041	Metaplastic polyp	0.24203
DB01041	Migraine	0.08305
DB01041	Mucocutaneous lymph node syndrome	0.09759
DB01041	Multiple myeloma	0.09881
DB01041	Multiple sclerosis	0.37713
DB01041	Muscular dystrophies	0.08771
DB01041	Mycoses	0.2
DB01041	Narcolepsy	0.16903
DB01041	Nasopharyngeal cancer	0.08607
DB01041	Necrotizing enterocolitis	0.18257
DB01041	Neoplasm metastasis	0.05005
DB01041	Nephrosis	0.56206
DB01041	Obesity	0.08553
DB01041	Oral cancer	0.11007
DB01041	Otitis media	0.14907
DB01041	Ovary cancer	0.1
DB01041	Pancreas cancer	0.04637
DB01041	Pancreatitis	0.47583
DB01041	Parkinson disease	0.1077
DB01041	Periodontitis	0.25832
DB01041	Polyarthritis	0.33135
DB01041	Polycystic ovary syndrome	0.05923
DB01041	Prion disease	0.33168
DB01041	Prostate cancer	0.08927
DB01041	Psoriasis	0.10541
DB01041	Pulmonary fibrosis	0.08452
DB01041	Renal Cell cancer	0.05199
DB01041	Rheumatoid arthritis	0.08206
DB01041	Sarcoidosis	0.08771
DB01041	Schizophrenia	0.04717
DB01041	Sickle cell disease	0.14059
DB01041	Silicosis	0.16903
DB01041	Squamous cell cancer	0.11083

DB01041	Stomach cancer	0.05775
DB01041	Stroke	0.36714
DB01041	Systemic infection	0.12564
DB01041	Systemic scleroderma	0.01156
DB01041	Testicular dysfunction	0.01466
DB01041	Thrombophilia	0.10541
DB01041	Thyroid cancer	0.07785
DB01041	Thyroid gland disease	0.20308
DB01041	Tuberculosis	0.47812
DB01041	Tuberous sclerosis	0.12675
DB01041	Ulcerative colitis	0.28636
DB01041	Virus disease	0.06779
DB01041	Vulvar disease	0.50244
DB01041	Yersinia infection	0.0096
DB01404	Hemorrhagic fevers, Viral	0.2357
DB01404	Abortion	0.07217
DB01404	Adenoma	0.09901
DB01404	Adrenal gland tumor	0.17408
DB01404	Alimentary system disease	0.12599
DB01404	Alopecia	0.10541
DB01404	Alzheimer's disease	0.14869
DB01404	Amnionitis	0.16013
DB01404	Anemia	0.12309
DB01404	Anorexia nervosa	0.11785
DB01404	Arteriopathy	0.19245
DB01404	Asthma	0.1482
DB01404	Atherosclerosis	0.12808
DB01404	Autoimmune disease	0.06262
DB01404	Barrett's esophagus	0.10541
DB01404	Basal cell carcinoma	0.16013
DB01404	Bone metastases	0.20412
DB01404	Brain tumor	0.04637
DB01404	Breast cancer	0.08827
DB01404	Bronchiolitis obliterans	0.19245
DB01404	Brucellosis	0.17408
DB01404	Cancer	0.05475
DB01404	Cardiovascular disease	0.08805
DB01404	Cerebral palsy	0.20412
DB01404	Cervical cancer	0.06712
DB01404	Chronic fatigue syndrome	0.59836
DB01404	Chronic obstructive airway disease	0.06455
DB01404	Cushing syndrome	0.17408
DB01404	Dental plaque	0.29793
DB01404	Dermatitis	0.05249
DB01404	Diabetes mellitus	0.02456
DB01404	Down syndrome	0.0658
DB01404	Embryoma	0.0356

DB01404	Endometrial cancer	0.1005
DB01404	Endometriosis	0.27081
DB01404	Enteritis	0.06337
DB01404	Esophagitis	0.2582
DB01404	Generalized anxiety disorder	0.16667
DB01404	Glomerulonephritis	0.14434
DB01404	Gouts	0.54785
DB01404	Growth retardation	0.4009
DB01404	HIV infection	0.03306
DB01404	HTLV-I infection	0.19245
DB01404	Hamman-Rich syndrome	0.11111
DB01404	Heart disease	0.17408
DB01404	Henoch-Schoenlein purpura	0.18257
DB01404	Hepatitis C	0.08513
DB01404	Hypercholesterolemia	0.11111
DB01404	Hyperglycemia	0.09245
DB01404	IGA glomerulonephritis	0.09901
DB01404	Infertility	0.2569
DB01404	Intracranial aneurysm	0.17408
DB01404	Ischemia	0.22083
DB01404	Keratoconjunctivitis Sicca	0.19245
DB01404	Kidney failure	0.06537
DB01404	Larynx cancer	0.18257
DB01404	Leukemia	0.10384
DB01404	Leukoencephalopathy	0.09623
DB01404	Liver disease	0.08909
DB01404	Lung cancer	0.1152
DB01404	Lupus vulgaris	0.10911
DB01404	Lyme disease	0.19245
DB01404	Lymphoma	0.1279
DB01404	Malnutrition	0.20412
DB01404	Metabolism disease	0.09245
DB01404	Metaplastic polyp	0.39868
DB01404	Migraine	0.10721
DB01404	Nasopharyngeal cancer	0.11111
DB01404	Neuroendocrine tumor	0.20412
DB01404	Obesity	0.04441
DB01404	Obsessive-compulsive disorder	0.17408
DB01404	Oral cancer	0.18775
DB01404	Ovarian cancer	0.05803
DB01404	Parkinson disease	0.19313
DB01404	Periodontitis	0.35852
DB01404	Pituitary tumor	0.16013
DB01404	Polyarthritis	0.16246
DB01404	Polycystic ovary syndrome	0.07647
DB01404	Prion disease	0.56648
DB01404	Prostate cancer	0.03014

DB01404	Renal Cell cancer	0.06712
DB01404	Renal tubular acidosis	0.19245
DB01404	Respiratory distress syndrome	0.16013
DB01404	Rheumatoid arthritis	0.09595
DB01404	Septicemia	0.2582
DB01404	Severe acute respiratory syndrome	0.18257
DB01404	Sickle cell disease	0.23206
DB01404	Stomach cancer	0.08166
DB01404	Stroke	0.15821
DB01404	Subacute sclerosing panencephalitis	0.28868
DB01404	Sudden infant death syndrome	0.12599
DB01404	Systemic infection	0.06623
DB01404	Thalassemia	0.13608
DB01404	Thrombocytopenia	0.14434
DB01404	Thrombocytosis	0.2582
DB01404	Thyroid gland disease	0.35536
DB01404	Tropical spastic paraparesis	0.21822
DB01404	Ulcerative colitis	0.05955
DB01404	Uterine fibroids	0.58275
DB01404	Vulvar disease	0.92916
DB01404	Yersinia infection	0.18049
DB01628	Alzheimer's disease	0.1498
DB01628	Asthma	0.20157
DB01628	Atherosclerosis	0.17404
DB01628	Cancer	0.07658
DB01628	Chronic fatigue syndrome	0.82139
DB01628	Dental plaque	0.30653
DB01628	Endometriosis	0.17725
DB01628	Gouts	0.73746
DB01628	Growth retardation	0.53635
DB01628	Ischemia	0.30237
DB01628	Metaplastic polyp	0.57806
DB01628	Oral cancer	0.26565
DB01628	Parkinson disease	0.26395
DB01628	Periodontitis	0.47347
DB01628	Polyarthritis	0.22868
DB01628	Prion disease	0.80078
DB01628	Rheumatoid arthritis	0.13313
DB01628	Sickle cell disease	0.33598
DB01628	Stroke	0.22339
DB01628	Thyroid gland disease	0.49394
DB01628	Vulvar disease	1.24344
DB08910	Alcoholic liver disease	0.2357
DB08910	Alzheimer's disease	0.16093
DB08910	Amyloidosis	0.10911
DB08910	Anemia	0.12309
DB08910	Anorexia nervosa	0.11785

DB08910	Aplastic anemia	0.12039
DB08910	Arthritis	0.07161
DB08910	Asthma	0.2142
DB08910	Atherosclerosis	0.18487
DB08910	Autoimmune disease	0.06262
DB08910	Basal cell carcinoma	0.16013
DB08910	Behcet syndrome	0.08248
DB08910	Bladder cancer	0.07454
DB08910	Breast cancer	0.02778
DB08910	Cancer	0.061
DB08910	Celiac disease	0.09492
DB08910	Cervical cancer	0.06712
DB08910	Chronic fatigue syndrome	0.67196
DB08910	Colon cancer	0.03426
DB08910	Dental plaque	0.25285
DB08910	Dermatitis	0.05249
DB08910	Diabetes mellitus	0.03039
DB08910	Down syndrome	0.0658
DB08910	Embryoma	0.0356
DB08910	Endometriosis	0.1901
DB08910	Enteritis	0.06337
DB08910	Familial Mediterranean fever	0.14907
DB08910	Fanconi's anemia	0.11547
DB08910	Gouts	0.62024
DB08910	Granulomatous disease	0.16667
DB08910	Growth retardation	0.45501
DB08910	HIV infection	0.0553
DB08910	Heart failure	0.06155
DB08910	Hepatitis B	0.14003
DB08910	Histiocytosis	0.19245
DB08910	Hypothyroidism	0.13608
DB08910	Infertility	0.07931
DB08910	Intermediate coronary syndrome	0.19245
DB08910	Ischemia	0.24826
DB08910	Kidney disease	0.06901
DB08910	Leprosy	0.1543
DB08910	Leukemia	0.03253
DB08910	Lichen planus	0.20412
DB08910	Lipodystrophy	0.16013
DB08910	Liver cancer	0.04481
DB08910	Lupus erythematosus	0.04933
DB08910	Malaria	0.10721
DB08910	Malignant glioma	0.10721
DB08910	Melanoma	0.04811
DB08910	Metaplastic polyp	0.43718
DB08910	Migraine	0.10721
DB08910	Mucocutaneous lymph node syndrome	0.12599

DB08910	Multiple myeloma	0.07581
DB08910	Multiple sclerosis	0.05955
DB08910	Muscular dystrophies	0.11323
DB08910	Mycoses	0.2582
DB08910	Narcolepsy	0.21822
DB08910	Nasopharyngeal cancer	0.11111
DB08910	Necrotizing enterocolitis	0.2357
DB08910	Nephrosis	0.1291
DB08910	Obesity	0.04441
DB08910	Oral cancer	0.20813
DB08910	Otitis media	0.19245
DB08910	Ovary cancer	0.1291
DB08910	Pancreas cancer	0.05987
DB08910	Pancreatitis	0.09017
DB08910	Parkinson disease	0.21728
DB08910	Periodontitis	0.49706
DB08910	Polyarthritis	0.18051
DB08910	Polycystic ovary syndrome	0.07647
DB08910	Prion disease	0.62823
DB08910	Prostate cancer	0.03014
DB08910	Psoriasis	0.13608
DB08910	Pulmonary fibrosis	0.10911
DB08910	Renal Cell cancer	0.06712
DB08910	Rheumatoid arthritis	0.1426
DB08910	Sarcoidosis	0.11323
DB08910	Schizophrenia	0.0439
DB08910	Sickle cell disease	0.25464
DB08910	Silicosis	0.21822
DB08910	Stomach cancer	0.04862
DB08910	Stroke	0.24091
DB08910	Systemic infection	0.06623
DB08910	Thrombophilia	0.13608
DB08910	Thyroid cancer	0.1005
DB08910	Thyroid gland disease	0.39698
DB08910	Tuberculosis	0.07785
DB08910	Tuberous sclerosis	0.14907
DB08910	Ulcerative colitis	0.05955
DB08910	Vulvar disease	1.05442
DB00232	Anemia	0.2132
DB00232	Cancer	0.01843
DB00232	Diabetes mellitus	0.02632
DB00232	Down syndrome	0.05698
DB00232	Hyperaldosteronism	0.15811
DB00232	Pancreas cancer	0.05185
DB00232	Pancreatitis	0.07809
DB00232	Retinitis pigmentosa	0.1
DB00232	Ulcerative colitis	0.05157

DB00311	Anemia	0.2132
DB00311	Cancer	0.01843
DB00311	Diabetes mellitus	0.02632
DB00311	Down syndrome	0.05698
DB00311	Pancreas cancer	0.05185
DB00311	Pancreatitis	0.07809
DB00311	Retinitis pigmentosa	0.1
DB00311	Ulcerative colitis	0.05157
DB00381	Amyotrophic lateral sclerosis	0.04767
DB00381	Anemia	0.07538
DB00381	Atherosclerosis	0.02475
DB00381	Cancer	0.01303
DB00381	Diabetes mellitus	0.01861
DB00381	Endocrine system disease	0.13363
DB00423	Anemia	0.2132
DB00423	Cancer	0.03686
DB00423	Diabetes mellitus	0.05263
DB00436	Anemia	0.19069
DB00436	Arthritis	0.31467
DB00436	Atherosclerosis	0.11789
DB00436	Autistic disorder	0.18788
DB00436	Bronchial disease	0.62939
DB00436	Cancer	0.01648
DB00436	Chronic obstructive airway disease	0.25244
DB00436	Cystic fibrosis	0.275
DB00436	Dermatitis	0.12992
DB00436	Diabetes mellitus	0.12799
DB00436	Down syndrome	0.05096
DB00436	Drug abuse	0.10073
DB00436	Epilepsy	0.29474
DB00436	Glaucoma	0.23729
DB00436	Graves' disease	0.34053
DB00436	Heart failure	0.19232
DB00436	Hypertension	0.1936
DB00436	Ischemia	0.25484
DB00436	Lung cancer	0.11382
DB00436	Malaria	0.36475
DB00436	Metabolism disease	0.34338
DB00436	Movement disorder	0.55003
DB00436	Myopathy	0.22897
DB00436	Neurodegenerative disorder	0.3261
DB00436	Obesity	0.14957
DB00436	Pancreas cancer	0.04637
DB00436	Pancreatitis	0.06984
DB00436	Polycystic ovary syndrome	0.19121
DB00436	Premature birth	0.52146
DB00436	Prostate cancer	0.14408

DB00436	Retinitis pigmentosa	0.08944
DB00436	Rheumatoid arthritis	0.08153
DB00436	Sickle cell disease	0.16267
DB00436	Subarachnoid hemorrhage	0.25465
DB00436	Ulcerative colitis	0.04613
DB00562	Anemia	0.17408
DB00562	Cancer	0.0301
DB00562	Cervical cancer	0.04746
DB00562	Diabetes mellitus	0.04297
DB00562	Down syndrome	0.04652
DB00562	Glaucoma	0.06804
DB00562	Hypertension	0.03227
DB00562	Pancreas cancer	0.04233
DB00562	Pancreatitis	0.06376
DB00562	Retinitis pigmentosa	0.08165
DB00562	Stomach cancer	0.03438
DB00562	Ulcerative colitis	0.04211
DB00562	Vulvar disease	0.18257
DB00703	Anemia	0.2132
DB00703	Cancer	0.01843
DB00703	Diabetes mellitus	0.02632
DB00703	Down syndrome	0.05698
DB00703	Pancreas cancer	0.05185
DB00703	Pancreatitis	0.07809
DB00703	Retinitis pigmentosa	0.1
DB00703	Ulcerative colitis	0.05157
DB00774	Anemia	0.15076
DB00774	Arthritis	0.31467
DB00774	Atherosclerosis	0.11789
DB00774	Autistic disorder	0.18788
DB00774	Bronchial disease	0.62939
DB00774	Cancer	0.02606
DB00774	Cervical cancer	0.0411
DB00774	Chronic obstructive airway disease	0.25244
DB00774	Cystic fibrosis	0.275
DB00774	Dermatitis	0.12992
DB00774	Diabetes mellitus	0.09952
DB00774	Down syndrome	0.04029
DB00774	Drug abuse	0.10073
DB00774	Epilepsy	0.2815
DB00774	Glaucoma	0.29622
DB00774	Graves' disease	0.34053
DB00774	Growth retardation	0.06804
DB00774	Heart failure	0.19232
DB00774	Hyperaldosteronism	0.1118
DB00774	Hypertension	0.1862
DB00774	Ischemia	0.24285

DB00774	Lung cancer	0.11382
DB00774	Malaria	0.36475
DB00774	Metabolism disease	0.34338
DB00774	Movement disorder	0.5305
DB00774	Myopathy	0.22897
DB00774	Neurodegenerative disorder	0.3107
DB00774	Obesity	0.14957
DB00774	Pancreas cancer	0.03666
DB00774	Pancreatitis	0.05522
DB00774	Polycystic ovary syndrome	0.19121
DB00774	Premature birth	0.52146
DB00774	Prostate cancer	0.13919
DB00774	Retinitis pigmentosa	0.07071
DB00774	Rheumatoid arthritis	0.10316
DB00774	Sickle cell disease	0.16267
DB00774	Stomach cancer	0.02977
DB00774	Subarachnoid hemorrhage	0.25465
DB00774	Ulcerative colitis	0.03647
DB00774	Vulvar disease	0.15811
DB00819	Anemia	0.16116
DB00819	Brain disease	0.06788
DB00819	Cancer	0.02786
DB00819	Diabetes mellitus	0.01989
DB00819	Down syndrome	0.04307
DB00819	Intractable epilepsy	0.13363
DB00819	Migraine	0.07019
DB00819	Pancreas cancer	0.03919
DB00819	Pancreatitis	0.05903
DB00819	Retinitis pigmentosa	0.07559
DB00819	Subarachnoid hemorrhage	0.11396
DB00819	Ulcerative colitis	0.03898
DB00819	Vascular disease	0.06901
DB00869	Anemia	0.24618
DB00869	Cancer	0.02128
DB00869	Diabetes mellitus	0.03039
DB00869	Down syndrome	0.0658
DB00869	Pancreas cancer	0.05987
DB00869	Pancreatitis	0.09017
DB00869	Retinitis pigmentosa	0.11547
DB00869	Ulcerative colitis	0.05955
DB00880	Anemia	0.2132
DB00880	Cancer	0.01843
DB00880	Diabetes mellitus	0.05263
DB00880	Down syndrome	0.05698
DB00880	Hypertension	0.03953
DB00880	Pancreas cancer	0.05185
DB00880	Pancreatitis	0.07809

DB00880	Retinitis pigmentosa	0.1
DB00880	Ulcerative colitis	0.05157
DB00999	Anemia	0.16116
DB00999	Arthritis	0.31467
DB00999	Atherosclerosis	0.11789
DB00999	Autistic disorder	0.18788
DB00999	Bronchial disease	0.62939
DB00999	Cancer	0.02786
DB00999	Cervical cancer	0.04394
DB00999	Chronic obstructive airway disease	0.25244
DB00999	Cystic fibrosis	0.275
DB00999	Dermatitis	0.12992
DB00999	Diabetes mellitus	0.1207
DB00999	Down syndrome	0.04307
DB00999	Drug abuse	0.10073
DB00999	Epilepsy	0.28495
DB00999	Glaucoma	0.30029
DB00999	Graves' disease	0.34053
DB00999	Heart failure	0.19232
DB00999	Hypertension	0.18813
DB00999	Ischemia	0.24598
DB00999	Lung cancer	0.11382
DB00999	Malaria	0.36475
DB00999	Metabolism disease	0.34338
DB00999	Movement disorder	0.53559
DB00999	Myopathy	0.22897
DB00999	Neurodegenerative disorder	0.31472
DB00999	Obesity	0.14957
DB00999	Pancreas cancer	0.03919
DB00999	Pancreatitis	0.05903
DB00999	Polycystic ovary syndrome	0.19121
DB00999	Premature birth	0.52146
DB00999	Prostate cancer	0.14046
DB00999	Retinitis pigmentosa	0.07559
DB00999	Rheumatoid arthritis	0.08153
DB00999	Sickle cell disease	0.16267
DB00999	Stomach cancer	0.03183
DB00999	Subarachnoid hemorrhage	0.25465
DB00999	Ulcerative colitis	0.03898
DB00999	Vulvar disease	0.16903
DB01021	Anemia	0.19069
DB01021	Cancer	0.01648
DB01021	Diabetes mellitus	0.02354
DB01021	Down syndrome	0.05096
DB01021	Growth retardation	0.08607
DB01021	Hyperaldosteronism	0.14142
DB01021	Hypertension	0.03536

DB01021	Pancreas cancer	0.04637
DB01021	Pancreatitis	0.06984
DB01021	Retinitis pigmentosa	0.08944
DB01021	Rheumatoid arthritis	0.02737
DB01021	Ulcerative colitis	0.04613
DB01031	Anemia	0.30151
DB01031	Cancer	0.02606
DB01031	Diabetes mellitus	0.03722
DB01031	Down syndrome	0.08058
DB01031	Pancreas cancer	0.07332
DB01031	Pancreatitis	0.11043
DB01031	Ulcerative colitis	0.07293
DB01119	Anemia	0.17408
DB01119	Arthritis	0.31467
DB01119	Atherosclerosis	0.14648
DB01119	Autistic disorder	0.18788
DB01119	Bronchial disease	0.62939
DB01119	Cancer	0.01505
DB01119	Chronic obstructive airway disease	0.25244
DB01119	Cystic fibrosis	0.275
DB01119	Dermatitis	0.12992
DB01119	Diabetes mellitus	0.14537
DB01119	Down syndrome	0.04652
DB01119	Drug abuse	0.10073
DB01119	Epilepsy	0.28923
DB01119	Glaucoma	0.23729
DB01119	Graves' disease	0.34053
DB01119	Growth retardation	0.07857
DB01119	Heart failure	0.19232
DB01119	Hyperglycemia	0.06537
DB01119	Hyperinsulinism	0.07857
DB01119	Hypertension	0.2228
DB01119	Infantile spasms	0.1543
DB01119	Ischemia	0.24985
DB01119	Late pregnancy	0.11785
DB01119	Lung cancer	0.11382
DB01119	Malaria	0.36475
DB01119	Metabolism disease	0.40876
DB01119	Movement disorder	0.54191
DB01119	Myopathy	0.22897
DB01119	Neurodegenerative disorder	0.31969
DB01119	Obesity	0.14957
DB01119	Pancreas cancer	0.04233
DB01119	Pancreas disease	0.08513
DB01119	Pancreatitis	0.06376
DB01119	Polycystic ovary syndrome	0.24528
DB01119	Premature birth	0.52146

DB01119	Prostate cancer	0.14205
DB01119	Rheumatoid arthritis	0.10651
DB01119	Sickle cell disease	0.16267
DB01119	Subarachnoid hemorrhage	0.25465
DB01119	Ulcerative colitis	0.04211
DB01144	Anemia	0.2132
DB01144	Cancer	0.01843
DB01144	Diabetes mellitus	0.02632
DB01144	Down syndrome	0.05698
DB01144	Pancreas cancer	0.05185
DB01144	Pancreatitis	0.07809
DB01144	Retinitis pigmentosa	0.1
DB01144	Ulcerative colitis	0.05157
DB01194	Anemia	0.2132
DB01194	Cancer	0.01843
DB01194	Diabetes mellitus	0.02632
DB01194	Down syndrome	0.05698
DB01194	Pancreas cancer	0.05185
DB01194	Pancreatitis	0.07809
DB01194	Retinitis pigmentosa	0.1
DB01194	Ulcerative colitis	0.05157
DB01325	Anemia	0.19069
DB01325	Cancer	0.01648
DB01325	Diabetes mellitus	0.04708
DB01325	Down syndrome	0.05096
DB01325	Hyperaldosteronism	0.14142
DB01325	Hypertension	0.03536
DB01325	Liver cancer	0.03471
DB01325	Liver tumor	0.07906
DB01325	Pancreas cancer	0.04637
DB01325	Pancreatitis	0.06984
DB01325	Polycystic kidney	0.11952
DB01325	Ulcerative colitis	0.04613
DB00212	Labor, Premature	0.2582
DB00212	Diabetes mellitus	0.05263
DB00212	Drug abuse	0.09366
DB00212	Hyperglycemia	0.16013
DB00212	Hyperinsulinism	0.19245
DB00212	Hyperlipidemia	0.19612
DB00212	Hypertension	0.07906
DB00212	Kidney failure	0.11323
DB00212	Leukemia	0.05634
DB00212	Obesity	0.07692
DB00212	Premature birth	0.24254
DB00212	Stroke	0.11323
DB01258	Labor, Premature	0.2582
DB01258	Diabetes mellitus	0.05263

DB01258	Drug abuse	0.09366
DB01258	Hyperglycemia	0.16013
DB01258	Hyperinsulinism	0.19245
DB01258	Hyperlipidemia	0.19612
DB01258	Hypertension	0.07906
DB01258	Kidney failure	0.11323
DB01258	Leukemia	0.05634
DB01258	Obesity	0.07692
DB01258	Premature birth	0.24254
DB01258	Stroke	0.11323
DB00075	Pemphigoid, Bullous	0.08452
DB00075	Pleural effusion, Malignant	0.14978
DB00075	Abortion	0.03341
DB00075	Alopecia	0.17026
DB00075	Alzheimer's disease	0.01924
DB00075	Antiphospholipid syndrome	0.08909
DB00075	Asthma	0.02182
DB00075	Atherosclerosis	0.03742
DB00075	Autoimmune disease	0.08697
DB00075	Bladder cancer	0.0345
DB00075	Breast cancer	0.03858
DB00075	Brucellosis	0.08058
DB00075	Celiac disease	0.04394
DB00075	Clear cell carcinoma	0.10102
DB00075	Combined immunodeficiency	0.21822
DB00075	Communicable disease	0.05832
DB00075	Congenital abnormality	0.06715
DB00075	Cystic fibrosis	0.03818
DB00075	Embryoma	0.08522
DB00075	Enteritis	0.22829
DB00075	Generalized anxiety disorder	0.11525
DB00075	Glomerulonephritis	0.06682
DB00075	HTLV-I infection	0.08909
DB00075	Hyperopia	0.3706
DB00075	IGA glomerulonephritis	0.04583
DB00075	Immune complex disease	0.74855
DB00075	Infection by cryptococcus neoformans	0.30861
DB00075	Kaposi sarcoma	0.10143
DB00075	Kidney cancer	0.1543
DB00075	Kidney disease	0.03194
DB00075	Kidney failure	0.03026
DB00075	Leukemia	0.03445
DB00075	Lung cancer	0.01867
DB00075	Lupus erythematosus	0.30389
DB00075	Lupus vulgaris	0.24303
DB00075	Malaria	0.17036
DB00075	Melanoma	0.02227

DB00075	Multiple myeloma	0.20293
DB00075	Multiple sclerosis	0.02757
DB00075	Neoplasm metastasis	0.02175
DB00075	Neurotic disorder	0.71401
DB00075	Periodontal disease	0.06299
DB00075	Periodontitis	0.08151
DB00075	Polyarthritis	0.09118
DB00075	Renal Cell cancer	0.18434
DB00075	Rheumatic fever	0.06901
DB00075	Rheumatoid arthritis	0.05443
DB00075	Skin tumor	0.1732
DB00075	Stomach cancer	0.15079
DB00075	Systemic infection	0.13741
DB00075	Thrombocytopenia	0.06682
DB00075	Tuberculosis	0.156
DB00075	Vascular disease	0.0488
DB00075	Yersinia infection	0.14586
DB00798	Diabetes mellitus	0.03722
DB00798	Kidney disease	0.08452
DB00798	Neuropathy	0.11323
DB00798	Prostate cancer	0.03691
DB00798	Rheumatoid arthritis	0.04327
DB00798	Thyroid gland disease	0.16222
DB00407	CNS lymphoma	0.70711
DB00407	Cancer	0.03453
DB00407	Congenital abnormality	0.0546
DB00407	Endocrine system disease	0.22121
DB00407	Granulomatous disease	0.59278
DB00407	Hyperaldosteronism	0.28534
DB00407	Hyperparathyroidism	0.17342
DB00407	Immunologic deficiency syndrome	0.11919
DB00407	Kidney disease	0.08452
DB00407	Leukemia	0.04901
DB00407	Lymphoma	0.11098
DB00407	Multiple endocrine neoplasia	0.28483
DB00407	Neoplasm metastasis	0.07943
DB00407	Osteomyelitis	0.19784
DB00407	Prostate cancer	0.03691
DB00407	Rabies	0.07857
DB00407	Schizophrenia	0.07385
DB00407	Thrombophilia	0.16667
DB00407	Vitamin D deficiency	0.22218
DB06271	CNS lymphoma	0.70711
DB06271	Cancer	0.03453
DB06271	Congenital abnormality	0.0546
DB06271	Endocrine system disease	0.22121
DB06271	Granulomatous disease	0.59278

DB06271	Hyperaldosteronism	0.28534
DB06271	Hyperparathyroidism	0.17342
DB06271	Immunologic deficiency syndrome	0.11919
DB06271	Kidney disease	0.08452
DB06271	Leukemia	0.04901
DB06271	Lymphoma	0.11098
DB06271	Multiple endocrine neoplasia	0.28483
DB06271	Neoplasm metastasis	0.07943
DB06271	Osteomyelitis	0.19784
DB06271	Prostate cancer	0.03691
DB06271	Rabies	0.07857
DB06271	Schizophrenia	0.07385
DB06271	Thrombophilia	0.16667
DB06271	Vitamin D deficiency	0.22218
DB06822	Pemphigoid, Bullous	0.3116
DB06822	Pleural effusion, Malignant	0.3189
DB06822	Purpura, Thrombocytopenic, Idiopathic	0.60348
DB06822	Alopecia	0.3674
DB06822	Alzheimer's disease	0.18982
DB06822	Aortic aneurysm	0.37645
DB06822	Arthritis	0.07161
DB06822	Asthma	0.36574
DB06822	Atherosclerosis	0.04042
DB06822	Bacterial infection	0.10416
DB06822	Behcet syndrome	0.08248
DB06822	CNS lymphoma	0.57735
DB06822	Cancer	0.13102
DB06822	Demyelinating disease	1.2582
DB06822	Depression	0.29408
DB06822	Diabetes mellitus	0.20454
DB06822	Drug abuse	0.21681
DB06822	Esophagus cancer	0.55249
DB06822	HIV infection	0.0553
DB06822	Huntington disease	0.07222
DB06822	Immunologic deficiency syndrome	0.1037
DB06822	Kidney disease	0.06901
DB06822	Leukemia	0.2443
DB06822	Leukoencephalopathy	0.09623
DB06822	Lung disease	0.63085
DB06822	Lupus erythematosus	0.28998
DB06822	Melanoma	0.39428
DB06822	Multiple sclerosis	0.05955
DB06822	Oral cancer	0.07857
DB06822	Osteosarcoma	0.80053
DB06822	Overnutrition	0.92833
DB06822	Polyarthritis	0.0658
DB06822	Primary hyperparathyroidism	0.37068

DB06822	Prostate cancer	0.03014
DB06822	Pulmonary fibrosis	0.10911
DB06822	Rabies	0.06415
DB06822	Rheumatoid arthritis	0.33535
DB06822	Shigella infection	0.39269
DB06822	Sickle cell disease	0.63923
DB06822	Systemic infection	0.55328
DB06822	Thalassemia	0.68979
DB06822	Thrombophilia	0.13608
DB06822	Virus disease	0.52132
DB06822	Wiskott-Aldrich syndrome	0.18257
DB08813	Hypertension, Pulmonary	0.26036
DB08813	Adenoma	0.03763
DB08813	Adenovirus infection	0.18195
DB08813	Adrenal gland hyperfunction	0.18898
DB08813	Alzheimer's disease	0.02012
DB08813	Amyotrophic lateral sclerosis	0.02861
DB08813	Aplastic anemia	0.05394
DB08813	Bladder cancer	0.06706
DB08813	Breast cancer	0.03476
DB08813	CNS lymphoma	0.5
DB08813	Cancer	0.11147
DB08813	Capillaries disease	0.05281
DB08813	Cardiovascular disease	0.07625
DB08813	Choriocarcinoma	0.11734
DB08813	Cirrhosis	0.08452
DB08813	Colon cancer	0.05507
DB08813	Common cold	0.13363
DB08813	Craniosynostosis	0.43947
DB08813	Dental plaque	0.0635
DB08813	Depression	0.06565
DB08813	Dermatitis	0.07112
DB08813	Diabetes mellitus	0.09766
DB08813	Down syndrome	0.04642
DB08813	Eating disorder	0.03429
DB08813	Embryoma	0.0221
DB08813	Epstein-Barr virus infection	0.42277
DB08813	Esophagus cancer	0.04538
DB08813	Ewings sarcoma	0.10732
DB08813	Hemophilia	1.03201
DB08813	Hepatitis	0.32877
DB08813	Herpes	0.02466
DB08813	Hypercholesterolemia	0.09623
DB08813	Hypertension	0.03953
DB08813	Ischemia	0.06402
DB08813	Kidney disease	0.05976
DB08813	Kidney failure	0.27545

DB08813	Leukemia	0.02061
DB08813	Leukoencephalopathy	0.28361
DB08813	Lung cancer	0.09268
DB08813	Lupus erythematosus	0.14131
DB08813	Lymphoma	0.04666
DB08813	Neoplasm metastasis	0.04069
DB08813	Oral cancer	0.22794
DB08813	Pancreatitis	0.07809
DB08813	Polyarthritis	0.02568
DB08813	Prostate cancer	0.06244
DB08813	Rabies	0.08261
DB08813	Rheumatoid arthritis	0.22186
DB08813	Schizophrenia	0.12005
DB08813	Scleroderma	0.03164
DB08813	Sicca syndrome	0.22966
DB08813	Sickle cell disease	0.09623
DB08813	Stomach cancer	0.0215
DB08813	Synovitis	0.62792
DB08813	Systemic scleroderma	0.07601
DB08813	Takayasu's arteritis	0.15811
DB08813	Thrombophilia	0.11785
DB08813	Vaccinia	0.28125
DB08813	Vascular disease	0.05454
DB08813	Werner syndrome	0.47973
DB00217	Attention deficit hyperactivity disorder	0.44732
DB00217	Common cold	0.13363
DB00217	Diabetes mellitus	0.02632
DB00217	Hyperaldosteronism	0.15811
DB00217	Hypertension	0.19647
DB00217	Kidney disease	0.05976
DB00217	Nervous system disease	0.40574
DB00217	Obesity	0.1879
DB00217	Polycystic ovary syndrome	0.06623
DB00217	Schizophrenia	0.13903
DB00320	Abortion	0.04696
DB00320	Amyotrophic lateral sclerosis	0.07234
DB00320	Anorexia nervosa	0.74566
DB00320	Attention deficit hyperactivity disorder	0.8896
DB00320	Behavior disease	0.49502
DB00320	Bipolar disorder	0.33476
DB00320	Colon cancer	0.02967
DB00320	Depression	0.25038
DB00320	Dermatitis	0.06489
DB00320	Diabetes mellitus	0.04041
DB00320	Drug abuse	0.19131
DB00320	Epilepsy	0.11561
DB00320	Hepatitis C	0.13263

DB00320	Hypertension	0.37205
DB00320	Migraine	0.14034
DB00320	Nervous system disease	0.2222
DB00320	Obesity	0.07581
DB00320	Panic disorder	0.17927
DB00320	Schizophrenia	0.07201
DB00320	Stroke	0.06236
DB00320	Sudden infant death syndrome	0.21435
DB00370	Anorexia nervosa	0.16667
DB00370	Atopic rhinitis	0.10541
DB00370	Attention deficit hyperactivity disorder	0.42084
DB00370	Autistic disorder	0.04951
DB00370	Behavior disease	0.12599
DB00370	Bipolar disorder	0.09245
DB00370	Choriocarcinoma	0.11785
DB00370	Chronic fatigue syndrome	0.14434
DB00370	Colon cancer	0.07268
DB00370	Depression	0.05361
DB00370	Dermatitis	0.03711
DB00370	Drug-Induced dyskinesia	0.1543
DB00370	Eating disorder	0.07107
DB00370	Embryoma	0.02517
DB00370	Gilles de la Tourette syndrome	0.1543
DB00370	Herpes	0.05832
DB00370	Hypertension	0.24651
DB00370	Infertility	0.05608
DB00370	Migraine	0.07581
DB00370	Nervous system disease	0.38205
DB00370	Obesity	0.20519
DB00370	Obsessive-compulsive disorder	0.24618
DB00370	Panic disorder	0.17817
DB00370	Parkinson disease	0.04508
DB00370	Psychotic disorder	0.06537
DB00370	Rheumatoid arthritis	0.02498
DB00370	Schizophrenia	0.19413
DB00370	Stroke	0.04623
DB00449	Infertility, Male	0.09664
DB00449	Alimentary system disease	0.17987
DB00449	Alzheimer's disease	0.05977
DB00449	Arthritis	0.48262
DB00449	Atherosclerosis	0.2276
DB00449	Attention deficit hyperactivity disorder	0.2792
DB00449	Autistic disorder	0.31176
DB00449	Azoospermia	0.07309
DB00449	Breast cancer	0.02055
DB00449	Bronchial disease	0.96254
DB00449	Chronic obstructive airway disease	0.39332

DB00449	Colon cancer	0.02967
DB00449	Conduct disorder	0.18728
DB00449	Cystic fibrosis	0.50689
DB00449	Dermatitis	0.21911
DB00449	Diabetes mellitus	0.16078
DB00449	Drug abuse	0.18147
DB00449	Enteritis	0.06428
DB00449	Epilepsy	0.13888
DB00449	Glaucoma	0.40051
DB00449	Gram-Negative bacterial infection	0.12834
DB00449	Graves' disease	0.54355
DB00449	Heart failure	0.3351
DB00449	Hypertension	0.4029
DB00449	Infertility	0.06174
DB00449	Ischemia	0.11853
DB00449	Kidney failure	0.05661
DB00449	Liver cancer	0.04102
DB00449	Lung cancer	0.18706
DB00449	Malaria	0.58039
DB00449	Metabolism disease	0.53904
DB00449	Movement disorder	0.27403
DB00449	Myopathy	0.36668
DB00449	Nervous system disease	0.25224
DB00449	Neurodegenerative disorder	0.15153
DB00449	Obesity	0.3647
DB00449	Oligospermia	0.13308
DB00449	Polycystic kidney	0.11581
DB00449	Polycystic ovary syndrome	0.3218
DB00449	Premature birth	0.81827
DB00449	Prostate cancer	0.18955
DB00449	Respiratory tract disease	0.08934
DB00449	Rheumatoid arthritis	0.13957
DB00449	Schizophrenia	0.08298
DB00449	Sickle cell disease	0.31366
DB00449	Sinusitis	0.08289
DB00449	Subarachnoid hemorrhage	0.49112
DB00449	Testicular dysfunction	0.04086
DB00449	Vitiligo	0.11785
DB00484	Attention deficit hyperactivity disorder	0.46965
DB00484	Common cold	0.1543
DB00484	Diabetes mellitus	0.03039
DB00484	Hypertension	0.2087
DB00484	Nervous system disease	0.42571
DB00484	Obesity	0.1998
DB00484	Polycystic ovary syndrome	0.07647
DB00484	Schizophrenia	0.14491
DB00575	Attention deficit hyperactivity disorder	0.46965

DB00575	Common cold	0.1543
DB00575	Diabetes mellitus	0.03039
DB00575	Hypertension	0.2087
DB00575	Nervous system disease	0.42571
DB00575	Obesity	0.1998
DB00575	Polycystic ovary syndrome	0.07647
DB00575	Schizophrenia	0.14491
DB00629	Attention deficit hyperactivity disorder	0.59166
DB00629	Hypertension	0.19647
DB00629	Nervous system disease	0.53484
DB00629	Obesity	0.1879
DB00629	Schizophrenia	0.17704
DB00633	Attention deficit hyperactivity disorder	0.59166
DB00633	Hypertension	0.19647
DB00633	Nervous system disease	0.53484
DB00633	Obesity	0.1879
DB00633	Schizophrenia	0.17704
DB00656	Hypertension, Pulmonary	0.08671
DB00656	Abortion	0.0481
DB00656	Amyotrophic lateral sclerosis	0.32564
DB00656	Anorexia nervosa	0.36631
DB00656	Atherosclerosis	0.02646
DB00656	Atopic rhinitis	0.09759
DB00656	Attention deficit hyperactivity disorder	0.31801
DB00656	Autistic disorder	0.09167
DB00656	Behavior disease	0.85771
DB00656	Bipolar disorder	0.20178
DB00656	Choriocarcinoma	0.10911
DB00656	Chronic fatigue syndrome	0.26726
DB00656	Chronic obstructive airway disease	0.04226
DB00656	Colon cancer	0.06728
DB00656	Congenital heart disease	0.12599
DB00656	Depression	0.42492
DB00656	Dermatitis	0.34947
DB00656	Diabetes mellitus	0.17334
DB00656	Drug abuse	0.06117
DB00656	Drug-Induced dyskinesia	0.14286
DB00656	Epilepsy	0.57459
DB00656	Fibromyalgia	0.13363
DB00656	Generalized anxiety disorder	0.10911
DB00656	Heart failure	0.04029
DB00656	Hepatitis C	0.55937
DB00656	Herpes	0.05399
DB00656	Hypertension	0.50966
DB00656	Infertility	0.05192
DB00656	Kidney failure	0.0428
DB00656	Migraine	0.74348

DB00656	Nervous system disease	0.19296
DB00656	Neurotic disorder	0.11952
DB00656	Obesity	0.15456
DB00656	Obsessive-compulsive disorder	0.22792
DB00656	Panic disorder	0.92817
DB00656	Parkinson disease	0.04174
DB00656	Pervasive development disorder	0.09759
DB00656	Prostate cancer	0.01973
DB00656	Psychotic disorder	0.12105
DB00656	Pulmonary hypertension	0.18898
DB00656	Rheumatoid arthritis	0.02313
DB00656	Schizophrenia	0.0923
DB00656	Stroke	0.36517
DB00656	Sudden infant death syndrome	0.97889
DB00656	Ulcerative colitis	0.03898
DB00692	Attention deficit hyperactivity disorder	0.50711
DB00692	Hypertension	0.22922
DB00692	Kidney failure	0.08006
DB00692	Nervous system disease	0.45921
DB00692	Obesity	0.16537
DB00692	Prostate cancer	0.03691
DB00692	Schizophrenia	0.15478
DB00696	Abortion	0.04696
DB00696	Amyotrophic lateral sclerosis	0.07234
DB00696	Anorexia nervosa	0.83724
DB00696	Attention deficit hyperactivity disorder	0.7835
DB00696	Autistic disorder	0.03835
DB00696	Behavior disease	0.56426
DB00696	Bipolar disorder	0.38556
DB00696	Choriocarcinoma	0.09129
DB00696	Chronic fatigue syndrome	0.1118
DB00696	Colon cancer	0.01876
DB00696	Depression	0.26777
DB00696	Dermatitis	0.09363
DB00696	Diabetes mellitus	0.0737
DB00696	Drug abuse	0.15689
DB00696	Drug-Induced dyskinesia	0.11952
DB00696	Epilepsy	0.11561
DB00696	Hepatitis C	0.13263
DB00696	Hypertension	0.40346
DB00696	Kidney failure	0.10742
DB00696	Migraine	0.14034
DB00696	Multiple endocrine neoplasia	0.10541
DB00696	Nervous system disease	0.17475
DB00696	Neuroendocrine tumor	0.1118
DB00696	Obesity	0.11033
DB00696	Obsessive-compulsive disorder	0.09535

DB00696	Panic disorder	0.31728
DB00696	Polycystic ovary syndrome	0.04189
DB00696	Prostate cancer	0.01651
DB00696	Psychotic disorder	0.05064
DB00696	Rheumatoid arthritis	0.01935
DB00696	Schizophrenia	0.05804
DB00696	Stroke	0.09816
DB00696	Sudden infant death syndrome	0.21435
DB00697	Attention deficit hyperactivity disorder	0.44732
DB00697	Common cold	0.13363
DB00697	Diabetes mellitus	0.02632
DB00697	Heart failure	0.0533
DB00697	Hypertension	0.236
DB00697	Nervous system disease	0.40574
DB00697	Obesity	0.1879
DB00697	Polycystic ovary syndrome	0.06623
DB00697	Schizophrenia	0.13903
DB00797	Atopic rhinitis	0.14907
DB00797	Attention deficit hyperactivity disorder	0.46965
DB00797	Colon cancer	0.03426
DB00797	Hypertension	0.2087
DB00797	Infertility	0.07931
DB00797	Kidney failure	0.06537
DB00797	Nervous system disease	0.42571
DB00797	Obesity	0.15539
DB00797	Parkinson disease	0.06376
DB00797	Prostate cancer	0.03014
DB00797	Schizophrenia	0.18881
DB00925	Infertility, Male	0.07405
DB00925	Abortion	0.01866
DB00925	Achalasia and cardiospasm	0.08012
DB00925	Alimentary system disease	0.13783
DB00925	Alzheimer's disease	0.05223
DB00925	Amyloidosis	0.04266
DB00925	Arthritis	0.37293
DB00925	Asthma	0.02808
DB00925	Atherosclerosis	0.14933
DB00925	Attention deficit hyperactivity disorder	0.22119
DB00925	Autistic disorder	0.28076
DB00925	Autoimmune disease	0.0358
DB00925	Azoospermia	0.05601
DB00925	Bipolar disorder	0.02848
DB00925	Bladder cancer	0.02919
DB00925	Brain ischemia	0.11734
DB00925	Breast cancer	0.01574
DB00925	Bronchial disease	0.74364
DB00925	Cancer	0.02182

DB00925	Celiac disease	0.0546
DB00925	Chronic obstructive airway disease	0.30419
DB00925	Colon cancer	0.01105
DB00925	Common cold	0.10911
DB00925	Conduct disorder	0.1435
DB00925	Congenital abnormality	0.0208
DB00925	Cystic fibrosis	0.392
DB00925	Dental plaque	0.04204
DB00925	Dermatitis	0.17018
DB00925	Diabetes mellitus	0.13388
DB00925	Drug abuse	0.16222
DB00925	Eating disorder	0.06692
DB00925	Enteritis	0.04925
DB00925	Epilepsy	0.10642
DB00925	Epstein-Barr virus infection	0.05952
DB00925	Esophageal tumor	0.04944
DB00925	Esophagus cancer	0.01975
DB00925	Esotropia	0.05688
DB00925	Eye cancer	0.0705
DB00925	Fanconi's anemia	0.02782
DB00925	Glaucoma	0.36011
DB00925	Gram-Negative bacterial infection	0.19669
DB00925	Graves' disease	0.49131
DB00925	HIV infection	0.03331
DB00925	Heart failure	0.25945
DB00925	Herpes	0.04814
DB00925	Hypertension	0.34696
DB00925	Infertility	0.04731
DB00925	Ischemia	0.13165
DB00925	Keratosis	0.05642
DB00925	Kidney failure	0.04623
DB00925	Leukemia	0.02513
DB00925	Lichen planus	0.10966
DB00925	Liver cancer	0.03143
DB00925	Lung cancer	0.16861
DB00925	Lupus erythematosus	0.0231
DB00925	Malaria	0.44939
DB00925	Malignant glioma	0.02861
DB00925	Melanoma	0.01466
DB00925	Metabolism disease	0.41707
DB00925	Movement disorder	0.20998
DB00925	Myopathy	0.28402
DB00925	Nervous system disease	0.19976
DB00925	Neurodegenerative disorder	0.11611
DB00925	Obesity	0.28526
DB00925	Oligospermia	0.10197
DB00925	Polycystic kidney	0.08874

DB00925	Polycystic ovary syndrome	0.30399
DB00925	Pre-Eclampsia	0.02664
DB00925	Premature birth	0.6331
DB00925	Prion disease	0.09192
DB00925	Prostate cancer	0.14787
DB00925	Rabies	0.01359
DB00925	Respiratory tract disease	0.06846
DB00925	Rheumatoid arthritis	0.12533
DB00925	Schistosomiasis	0.09909
DB00925	Schizophrenia	0.06549
DB00925	Sickle cell disease	0.24518
DB00925	Sinusitis	0.06352
DB00925	Stroke	0.0258
DB00925	Subarachnoid hemorrhage	0.3839
DB00925	Testicular dysfunction	0.03131
DB00925	Thyroid gland disease	0.06195
DB00925	Tuberculosis	0.05307
DB00935	Attention deficit hyperactivity disorder	0.50711
DB00935	Hypertension	0.22922
DB00935	Kidney failure	0.08006
DB00935	Nervous system disease	0.45921
DB00935	Obesity	0.16537
DB00935	Prostate cancer	0.03691
DB00935	Schizophrenia	0.15478
DB00964	Attention deficit hyperactivity disorder	0.50711
DB00964	Hypertension	0.22922
DB00964	Kidney failure	0.08006
DB00964	Nervous system disease	0.45921
DB00964	Obesity	0.16537
DB00964	Prostate cancer	0.03691
DB00964	Schizophrenia	0.15478
DB00968	Attention deficit hyperactivity disorder	0.59166
DB00968	Hypertension	0.19647
DB00968	Nervous system disease	0.53484
DB00968	Obesity	0.1879
DB00968	Schizophrenia	0.17704
DB01018	Attention deficit hyperactivity disorder	0.59166
DB01018	Hypertension	0.19647
DB01018	Nervous system disease	0.53484
DB01018	Obesity	0.1879
DB01018	Schizophrenia	0.17704
DB01149	Hypertension, Pulmonary	0.07647
DB01149	Stress disorder, post-traumatic	0.11785
DB01149	Abortion	0.0481
DB01149	Amyotrophic lateral sclerosis	0.31963
DB01149	Anorexia nervosa	0.40702
DB01149	Atherosclerosis	0.02334

DB01149	Attention deficit hyperactivity disorder	0.30513
DB01149	Autistic disorder	0.08085
DB01149	Behavior disease	0.93303
DB01149	Bipolar disorder	0.2621
DB01149	Brain disease	0.05987
DB01149	Breast cancer	0.01604
DB01149	Choriocarcinoma	0.09623
DB01149	Chronic fatigue syndrome	0.2357
DB01149	Chronic obstructive airway disease	0.03727
DB01149	Colon cancer	0.03956
DB01149	Congenital heart disease	0.11111
DB01149	Depression	0.45111
DB01149	Dermatitis	0.3373
DB01149	Diabetes mellitus	0.18853
DB01149	Drug abuse	0.11942
DB01149	Drug-Induced dyskinesia	0.12599
DB01149	Epilepsy	0.56197
DB01149	Fibromyalgia	0.11785
DB01149	Generalized anxiety disorder	0.09623
DB01149	Gilles de la Tourette syndrome	0.12599
DB01149	Heart failure	0.03553
DB01149	Hepatitis C	0.55279
DB01149	Herpes	0.09524
DB01149	Hypertension	0.51837
DB01149	Kidney failure	0.07549
DB01149	Migraine	0.78052
DB01149	Multiple endocrine neoplasia	0.11111
DB01149	Nervous system disease	0.18143
DB01149	Neuroendocrine tumor	0.11785
DB01149	Neurotic disorder	0.10541
DB01149	Obesity	0.16646
DB01149	Obsessive-compulsive disorder	0.20101
DB01149	Panic disorder	0.97169
DB01149	Pervasive development disorder	0.08607
DB01149	Prostate cancer	0.0174
DB01149	Psychotic disorder	0.16013
DB01149	Pulmonary hypertension	0.16667
DB01149	Rheumatoid arthritis	0.0204
DB01149	Schizophrenia	0.06017
DB01149	Stroke	0.35001
DB01149	Sudden infant death syndrome	0.95941
DB01149	Ulcerative colitis	0.03438
DB01392	Abortion	0.06924
DB01392	Amyotrophic lateral sclerosis	0.26541
DB01392	Anorexia nervosa	0.7101
DB01392	Attention deficit hyperactivity disorder	0.65889
DB01392	Autistic disorder	0.03835

DB01392	Behavior disease	0.86582
DB01392	Bipolar disorder	0.33452
DB01392	Choriocarcinoma	0.09129
DB01392	Chronic fatigue syndrome	0.1118
DB01392	Colon cancer	0.01876
DB01392	Common cold	0.08452
DB01392	Depression	0.42103
DB01392	Dermatitis	0.25733
DB01392	Diabetes mellitus	0.15774
DB01392	Drug abuse	0.10935
DB01392	Drug-Induced dyskinesia	0.11952
DB01392	Epilepsy	0.42403
DB01392	Hepatitis C	0.4551
DB01392	Hypertension	0.52259
DB01392	Migraine	0.54966
DB01392	Nervous system disease	0.15029
DB01392	Obesity	0.12484
DB01392	Obsessive-compulsive disorder	0.09535
DB01392	Panic disorder	0.69011
DB01392	Polycystic ovary syndrome	0.04189
DB01392	Psychotic disorder	0.05064
DB01392	Rheumatoid arthritis	0.01935
DB01392	Schizophrenia	0.04911
DB01392	Stroke	0.26365
DB01392	Sudden infant death syndrome	0.72913
DB04948	Attention deficit hyperactivity disorder	0.59166
DB04948	Hypertension	0.19647
DB04948	Nervous system disease	0.53484
DB04948	Obesity	0.1879
DB04948	Schizophrenia	0.17704
DB06148	Hypertension, Pulmonary	0.09366
DB06148	Anorexia nervosa	0.33333
DB06148	Atherosclerosis	0.02858
DB06148	Atopic rhinitis	0.10541
DB06148	Attention deficit hyperactivity disorder	0.42084
DB06148	Autistic disorder	0.09901
DB06148	Behavior disease	0.25198
DB06148	Bipolar disorder	0.1849
DB06148	Choriocarcinoma	0.11785
DB06148	Chronic fatigue syndrome	0.28868
DB06148	Chronic obstructive airway disease	0.04564
DB06148	Colon cancer	0.07268
DB06148	Congenital heart disease	0.13608
DB06148	Depression	0.10721
DB06148	Dermatitis	0.07423
DB06148	Diabetes mellitus	0.02149
DB06148	Drug abuse	0.07647

DB06148	Drug-Induced dyskinesia	0.1543
DB06148	Epilepsy	0.05774
DB06148	Fibromyalgia	0.14434
DB06148	Generalized anxiety disorder	0.11785
DB06148	Heart failure	0.04352
DB06148	Herpes	0.05832
DB06148	Hypertension	0.24651
DB06148	Infertility	0.05608
DB06148	Migraine	0.15162
DB06148	Multiple endocrine neoplasia	0.13608
DB06148	Nervous system disease	0.38205
DB06148	Neuroendocrine tumor	0.14434
DB06148	Neurotic disorder	0.1291
DB06148	Obesity	0.23659
DB06148	Obsessive-compulsive disorder	0.24618
DB06148	Panic disorder	0.26726
DB06148	Parkinson disease	0.04508
DB06148	Pervasive development disorder	0.10541
DB06148	Psychotic disorder	0.13074
DB06148	Pulmonary hypertension	0.20412
DB06148	Rheumatoid arthritis	0.02498
DB06148	Schizophrenia	0.16309
DB06148	Stroke	0.09245
DB06148	Sudden infant death syndrome	0.08909
DB06148	Ulcerative colitis	0.04211
DB06623	Attention deficit hyperactivity disorder	0.59166
DB06623	Hypertension	0.19647
DB06623	Nervous system disease	0.53484
DB06623	Obesity	0.1879
DB06623	Schizophrenia	0.17704
DB06694	Attention deficit hyperactivity disorder	0.42084
DB06694	Common cold	0.10911
DB06694	Diabetes mellitus	0.02149
DB06694	Hypertension	0.21424
DB06694	Kidney failure	0.13868
DB06694	Nervous system disease	0.38205
DB06694	Obesity	0.17378
DB06694	Polycystic ovary syndrome	0.05407
DB06694	Prostate cancer	0.02131
DB06694	Schizophrenia	0.13205
DB06711	Attention deficit hyperactivity disorder	0.50711
DB06711	Hypertension	0.22922
DB06711	Kidney failure	0.08006
DB06711	Nervous system disease	0.45921
DB06711	Obesity	0.16537
DB06711	Prostate cancer	0.03691
DB06711	Schizophrenia	0.15478

DB08815	Abortion	0.0481
DB08815	Adenocarcinoma	0.05955
DB08815	Amyotrophic lateral sclerosis	0.32973
DB08815	Anorexia nervosa	0.21818
DB08815	Attention deficit hyperactivity disorder	0.32675
DB08815	Autistic disorder	0.04951
DB08815	Behavior disease	0.75042
DB08815	Bipolar disorder	0.11962
DB08815	Choriocarcinoma	0.11785
DB08815	Chronic fatigue syndrome	0.14434
DB08815	Colon cancer	0.02423
DB08815	Common cold	0.10911
DB08815	Depression	0.38325
DB08815	Dermatitis	0.32062
DB08815	Diabetes mellitus	0.17493
DB08815	Drug abuse	0.02577
DB08815	Drug-Induced dyskinesia	0.1543
DB08815	Epilepsy	0.52542
DB08815	Hepatitis C	0.56384
DB08815	Hypertension	0.48935
DB08815	Migraine	0.60873
DB08815	Nervous system disease	0.20078
DB08815	Obesity	0.10107
DB08815	Obsessive-compulsive disorder	0.12309
DB08815	Panic disorder	0.85891
DB08815	Psychotic disorder	0.06537
DB08815	Rheumatoid arthritis	0.02498
DB08815	Schizophrenia	0.0969
DB08815	Stroke	0.32923
DB08815	Sudden infant death syndrome	0.90302
DB00216	Abortion	0.07147
DB00216	Adenocarcinoma	0.05513
DB00216	Amyotrophic lateral sclerosis	0.28093
DB00216	Anorexia nervosa	0.61028
DB00216	Attention deficit hyperactivity disorder	0.52205
DB00216	Behavior disease	0.80893
DB00216	Bipolar disorder	0.27723
DB00216	Colon cancer	0.02243
DB00216	Depression	0.40529
DB00216	Dermatitis	0.24064
DB00216	Diabetes mellitus	0.14836
DB00216	Drug abuse	0.15311
DB00216	Epilepsy	0.44501
DB00216	Hepatitis C	0.47738
DB00216	Hypertension	0.47397
DB00216	Migraine	0.51636
DB00216	Panic disorder	0.6524

DB00216	Schizophrenia	0.02874
DB00216	Stroke	0.24103
DB00216	Sudden infant death syndrome	0.76391
DB00247	Abortion	0.05124
DB00247	Adenocarcinoma	0.06523
DB00247	Amyotrophic lateral sclerosis	0.35292
DB00247	Anorexia nervosa	0.32623
DB00247	Attention deficit hyperactivity disorder	0.11127
DB00247	Autistic disorder	0.05423
DB00247	Behavior disease	0.87223
DB00247	Bipolar disorder	0.17946
DB00247	Choriocarcinoma	0.1291
DB00247	Chronic fatigue syndrome	0.15811
DB00247	Colon cancer	0.05307
DB00247	Depression	0.4115
DB00247	Dermatitis	0.34379
DB00247	Diabetes mellitus	0.187
DB00247	Drug abuse	0.06933
DB00247	Drug-Induced dyskinesia	0.16903
DB00247	Epilepsy	0.56148
DB00247	Hepatitis C	0.60247
DB00247	Hypertension	0.51736
DB00247	Migraine	0.73382
DB00247	Obesity	0.0688
DB00247	Obsessive-compulsive disorder	0.13484
DB00247	Panic disorder	0.92037
DB00247	Psychotic disorder	0.07161
DB00247	Rheumatoid arthritis	0.02737
DB00247	Schizophrenia	0.034
DB00247	Stroke	0.35352
DB00247	Sudden infant death syndrome	0.96468
DB00315	Abortion	0.07147
DB00315	Amyotrophic lateral sclerosis	0.29739
DB00315	Anorexia nervosa	0.63519
DB00315	Attention deficit hyperactivity disorder	0.55728
DB00315	Behavior disease	0.8466
DB00315	Bipolar disorder	0.29105
DB00315	Depression	0.43734
DB00315	Dermatitis	0.25173
DB00315	Diabetes mellitus	0.15478
DB00315	Drug abuse	0.12914
DB00315	Epilepsy	0.46227
DB00315	Hepatitis C	0.49538
DB00315	Hypertension	0.43351
DB00315	Migraine	0.53902
DB00315	Panic disorder	0.67903
DB00315	Stroke	0.25485

DB00315	Sudden infant death syndrome	0.79054
DB00490	Abortion	0.05124
DB00490	Amyotrophic lateral sclerosis	0.38796
DB00490	Anorexia nervosa	0.14366
DB00490	Attention deficit hyperactivity disorder	0.11127
DB00490	Behavior disease	0.77432
DB00490	Bipolar disorder	0.07818
DB00490	Depression	0.38691
DB00490	Dermatitis	0.32676
DB00490	Diabetes mellitus	0.20068
DB00490	Drug abuse	0.02745
DB00490	Epilepsy	0.59823
DB00490	Hepatitis C	0.64079
DB00490	Hypertension	0.39648
DB00490	Migraine	0.69903
DB00490	Panic disorder	0.87949
DB00490	Stroke	0.33231
DB00490	Sudden infant death syndrome	1.02139
DB00669	Abortion	0.07147
DB00669	Amyotrophic lateral sclerosis	0.29739
DB00669	Anorexia nervosa	0.63519
DB00669	Attention deficit hyperactivity disorder	0.55728
DB00669	Behavior disease	0.8466
DB00669	Bipolar disorder	0.29105
DB00669	Depression	0.43734
DB00669	Dermatitis	0.25173
DB00669	Diabetes mellitus	0.15478
DB00669	Drug abuse	0.12914
DB00669	Epilepsy	0.46227
DB00669	Hepatitis C	0.49538
DB00669	Hypertension	0.43351
DB00669	Migraine	0.53902
DB00669	Panic disorder	0.67903
DB00669	Stroke	0.25485
DB00669	Sudden infant death syndrome	0.79054
DB00952	Abortion	0.07147
DB00952	Amyotrophic lateral sclerosis	0.29739
DB00952	Anorexia nervosa	0.63519
DB00952	Attention deficit hyperactivity disorder	0.55728
DB00952	Behavior disease	0.8466
DB00952	Bipolar disorder	0.29105
DB00952	Depression	0.43734
DB00952	Dermatitis	0.25173
DB00952	Diabetes mellitus	0.15478
DB00952	Drug abuse	0.12914
DB00952	Epilepsy	0.46227
DB00952	Hepatitis C	0.49538

DB00952	Hypertension	0.43351
DB00952	Migraine	0.53902
DB00952	Panic disorder	0.67903
DB00952	Stroke	0.25485
DB00952	Sudden infant death syndrome	0.79054
DB01616	Abortion	0.05124
DB01616	Amyotrophic lateral sclerosis	0.42746
DB01616	Anorexia nervosa	0.14366
DB01616	Attention deficit hyperactivity disorder	0.11127
DB01616	Behavior disease	0.81951
DB01616	Bipolar disorder	0.07818
DB01616	Depression	0.42537
DB01616	Dermatitis	0.35339
DB01616	Diabetes mellitus	0.2161
DB01616	Drug abuse	0.02745
DB01616	Epilepsy	0.63965
DB01616	Hepatitis C	0.68398
DB01616	Hypertension	0.41964
DB01616	Migraine	0.75342
DB01616	Panic disorder	0.94341
DB01616	Stroke	0.36547
DB01616	Sudden infant death syndrome	1.0853
DB06684	Abortion	0.05124
DB06684	Amyotrophic lateral sclerosis	0.42746
DB06684	Anorexia nervosa	0.14366
DB06684	Attention deficit hyperactivity disorder	0.11127
DB06684	Behavior disease	0.81951
DB06684	Bipolar disorder	0.07818
DB06684	Depression	0.42537
DB06684	Dermatitis	0.35339
DB06684	Diabetes mellitus	0.2161
DB06684	Drug abuse	0.02745
DB06684	Epilepsy	0.63965
DB06684	Hepatitis C	0.68398
DB06684	Hypertension	0.41964
DB06684	Migraine	0.75342
DB06684	Panic disorder	0.94341
DB06684	Stroke	0.36547
DB06684	Sudden infant death syndrome	1.0853
DB00393	Alopecia	0.19684
DB00393	Amyotrophic lateral sclerosis	0.04264
DB00393	Atherosclerosis	0.02214
DB00393	Breast cancer	0.12482
DB00393	Diabetes mellitus	0.02331
DB00393	Drug abuse	0.02962
DB00393	Endocrine system disease	0.11952
DB00393	Endometriosis	0.06977

DB00393	HIV infection	0.03138
DB00393	Heart failure	0.19044
DB00393	Hypertension	0.15396
DB00393	Hypoglycemia	0.80601
DB00393	Infertility	0.13674
DB00393	Leukemia	0.05463
DB00393	Lung cancer	0.05488
DB00393	Lymphoma	0.29775
DB00393	Melanoma	0.06017
DB00393	Optic atrophy	0.14142
DB00393	Proteinuria	0.53086
DB00393	Renal Cell cancer	0.24999
DB00393	Retinal disease	0.04428
DB00393	Stomach cancer	0.05799
DB00393	Uterine fibroids	0.27745
DB00393	Yersinia infection	0.07878
DB00653	Amyotrophic lateral sclerosis	0.05505
DB00653	Atherosclerosis	0.02858
DB00653	Endocrine system disease	0.1543
DB00377	Colon cancer	0.05934
DB00377	Eating disorder	0.17408
DB00377	Embryoma	0.06166
DB00377	Gilles de la Tourette syndrome	0.37796
DB00377	Herpes	0.14286
DB00377	Hypertension	0.07906
DB00377	Obsessive-compulsive disorder	0.30151
DB00377	Panic disorder	0.21822
DB00377	Schizophrenia	0.07603
DB00728	Behavior disease	0.08909
DB00728	Bipolar disorder	0.06537
DB00728	Bladder cancer	0.07454
DB00728	Central nervous system disease	0.14434
DB00728	Colon cancer	0.03426
DB00728	Depression	0.07581
DB00728	Eating disorder	0.1005
DB00728	Embryoma	0.0356
DB00728	Epilepsy	0.08165
DB00728	Gilles de la Tourette syndrome	0.21822
DB00728	Herpes	0.08248
DB00728	Hypertension	0.04564
DB00728	Obsessive-compulsive disorder	0.17408
DB00728	Panic disorder	0.12599
DB00728	Schizophrenia	0.0439
DB00757	Colon cancer	0.05934
DB00757	Eating disorder	0.17408
DB00757	Embryoma	0.06166
DB00757	Gilles de la Tourette syndrome	0.37796

DB00757	Herpes	0.14286
DB00757	Hypertension	0.07906
DB00757	Obsessive-compulsive disorder	0.30151
DB00757	Panic disorder	0.21822
DB00757	Schizophrenia	0.07603
DB00889	Colon cancer	0.05934
DB00889	Eating disorder	0.17408
DB00889	Embryoma	0.06166
DB00889	Gilles de la Tourette syndrome	0.37796
DB00889	Herpes	0.14286
DB00889	Hypertension	0.07906
DB00889	Obsessive-compulsive disorder	0.30151
DB00889	Panic disorder	0.21822
DB00889	Schizophrenia	0.07603
DB00969	Colon cancer	0.05934
DB00969	Eating disorder	0.17408
DB00969	Embryoma	0.06166
DB00969	Gilles de la Tourette syndrome	0.37796
DB00969	Herpes	0.14286
DB00969	Hypertension	0.07906
DB00969	Obsessive-compulsive disorder	0.30151
DB00969	Panic disorder	0.21822
DB00969	Schizophrenia	0.07603
DB01043	Colon cancer	0.02967
DB01043	Eating disorder	0.08704
DB01043	Embryoma	0.03083
DB01043	Gilles de la Tourette syndrome	0.18898
DB01043	Herpes	0.07143
DB01043	Hypertension	0.03953
DB01043	Obsessive-compulsive disorder	0.15076
DB01043	Panic disorder	0.10911
DB01043	Schizophrenia	0.03801
DB01199	Brain tumor	0.04637
DB01199	Colon cancer	0.06852
DB01199	Eating disorder	0.1005
DB01199	Embryoma	0.0356
DB01199	Epilepsy	0.08165
DB01199	Gilles de la Tourette syndrome	0.21822
DB01199	Herpes	0.08248
DB01199	Hypertension	0.04564
DB01199	Lung cancer	0.04032
DB01199	Neurodegenerative disorder	0.09492
DB01199	Obsessive-compulsive disorder	0.17408
DB01199	Ovarian cancer	0.05803
DB01199	Panic disorder	0.12599
DB01199	Schizophrenia	0.0439
DB01199	Vitiligo	0.13608

DB06204	Hypertension, Pulmonary	0.09366
DB06204	Anorexia nervosa	0.16667
DB06204	Atherosclerosis	0.02858
DB06204	Autistic disorder	0.04951
DB06204	Behavior disease	0.12599
DB06204	Bipolar disorder	0.09245
DB06204	Chronic fatigue syndrome	0.14434
DB06204	Chronic obstructive airway disease	0.04564
DB06204	Colon cancer	0.04845
DB06204	Congenital heart disease	0.13608
DB06204	Depression	0.05361
DB06204	Dermatitis	0.03711
DB06204	Diabetes mellitus	0.02149
DB06204	Drug abuse	0.07647
DB06204	Eating disorder	0.07107
DB06204	Embryoma	0.02517
DB06204	Epilepsy	0.05774
DB06204	Fibromyalgia	0.14434
DB06204	Generalized anxiety disorder	0.11785
DB06204	Gilles de la Tourette syndrome	0.1543
DB06204	Heart failure	0.04352
DB06204	Herpes	0.11664
DB06204	Hypertension	0.06455
DB06204	Migraine	0.07581
DB06204	Multiple endocrine neoplasia	0.13608
DB06204	Neuroendocrine tumor	0.14434
DB06204	Neurotic disorder	0.1291
DB06204	Obesity	0.0314
DB06204	Obsessive-compulsive disorder	0.24618
DB06204	Panic disorder	0.26726
DB06204	Pervasive development disorder	0.10541
DB06204	Psychotic disorder	0.06537
DB06204	Pulmonary hypertension	0.20412
DB06204	Schizophrenia	0.03104
DB06204	Stroke	0.04623
DB06204	Sudden infant death syndrome	0.08909
DB06204	Ulcerative colitis	0.04211
DB00279	Adenovirus infection	0.01602
DB00279	Alzheimer's disease	0.02895
DB00279	Autistic disorder	0.08118
DB00279	Breast cancer	0.04966
DB00279	Cancer	0.01502
DB00279	Carcinoma	0.04318
DB00279	Colon cancer	0.01662
DB00279	Congenital abnormality	0.01502
DB00279	Disseminated cancer	0.06908
DB00279	Drug abuse	0.05099

DB00279	Embryoma	0.0167
DB00279	Gram-Negative bacterial infection	0.07105
DB00279	HIV infection	0.01626
DB00279	Hemolytic-Uremic syndrome	0.05556
DB00279	Infection	0.02034
DB00279	Lung cancer	0.01699
DB00279	Mental retardation	0.12811
DB00279	Muscular atrophy	0.05909
DB00279	Muscular dystrophies	0.04063
DB00279	Myopathy	0.03418
DB00279	Myotonic disorder	0.04693
DB00279	Neuropathy	0.04797
DB00279	Ovarian cancer	0.02827
DB00279	Prion disease	0.06641
DB00279	Prostate cancer	0.04322
DB00279	Renal tubular acidosis	0.08912
DB00279	Respiratory tract disease	0.1677
DB00279	Salmonella infection	0.0507
DB00279	Schizophrenia	0.12774
DB00279	Uveitis	0.08496
DB00279	Virus disease	0.03828
DB00451	Adenovirus infection	0.01602
DB00451	Alzheimer's disease	0.02895
DB00451	Autistic disorder	0.08118
DB00451	Breast cancer	0.04966
DB00451	Cancer	0.01502
DB00451	Carcinoma	0.04318
DB00451	Colon cancer	0.01662
DB00451	Congenital abnormality	0.01502
DB00451	Disseminated cancer	0.06908
DB00451	Drug abuse	0.05099
DB00451	Embryoma	0.0167
DB00451	Gram-Negative bacterial infection	0.07105
DB00451	HIV infection	0.01626
DB00451	Hemolytic-Uremic syndrome	0.05556
DB00451	Infection	0.02034
DB00451	Lung cancer	0.01699
DB00451	Mental retardation	0.12811
DB00451	Muscular atrophy	0.05909
DB00451	Muscular dystrophies	0.04063
DB00451	Myopathy	0.03418
DB00451	Myotonic disorder	0.04693
DB00451	Neuropathy	0.04797
DB00451	Ovarian cancer	0.02827
DB00451	Prion disease	0.06641
DB00451	Prostate cancer	0.04322
DB00451	Renal tubular acidosis	0.08912

DB00451	Respiratory tract disease	0.1677
DB00451	Salmonella infection	0.0507
DB00451	Schizophrenia	0.12774
DB00451	Uveitis	0.08496
DB00451	Virus disease	0.03828
DB00509	Adenovirus infection	0.01602
DB00509	Alzheimer's disease	0.02895
DB00509	Autistic disorder	0.08118
DB00509	Breast cancer	0.04966
DB00509	Cancer	0.01502
DB00509	Carcinoma	0.04318
DB00509	Colon cancer	0.01662
DB00509	Congenital abnormality	0.01502
DB00509	Disseminated cancer	0.06908
DB00509	Drug abuse	0.05099
DB00509	Embryoma	0.0523
DB00509	Gram-Negative bacterial infection	0.07105
DB00509	HIV infection	0.01626
DB00509	Hemolytic-Uremic syndrome	0.05556
DB00509	Hypothyroidism	0.13608
DB00509	Infection	0.07418
DB00509	Kidney disease	0.06901
DB00509	Leukemia	0.03253
DB00509	Lung cancer	0.01699
DB00509	Mental retardation	0.12811
DB00509	Muscular atrophy	0.05909
DB00509	Muscular dystrophies	0.04063
DB00509	Myopathy	0.03418
DB00509	Myotonic disorder	0.04693
DB00509	Neoplasm metastasis	0.04698
DB00509	Neuropathy	0.04797
DB00509	Ovarian cancer	0.02827
DB00509	Prion disease	0.06641
DB00509	Prostate cancer	0.04322
DB00509	Renal tubular acidosis	0.08912
DB00509	Respiratory tract disease	0.1677
DB00509	Salmonella infection	0.0507
DB00509	Schizophrenia	0.12774
DB00509	Thrombocytosis	0.2582
DB00509	Thyroid gland disease	0.13245
DB00509	Uveitis	0.08496
DB00509	Virus disease	0.03828
DB01583	Adenovirus infection	0.01602
DB01583	Alzheimer's disease	0.02895
DB01583	Autistic disorder	0.08118
DB01583	Breast cancer	0.04966
DB01583	Cancer	0.01502

DB01583	Carcinoma	0.04318
DB01583	Colon cancer	0.01662
DB01583	Congenital abnormality	0.01502
DB01583	Disseminated cancer	0.06908
DB01583	Drug abuse	0.05099
DB01583	Embryoma	0.0167
DB01583	Gram-Negative bacterial infection	0.07105
DB01583	HIV infection	0.01626
DB01583	Hemolytic-Uremic syndrome	0.05556
DB01583	Infection	0.02034
DB01583	Lung cancer	0.01699
DB01583	Mental retardation	0.12811
DB01583	Muscular atrophy	0.05909
DB01583	Muscular dystrophies	0.04063
DB01583	Myopathy	0.03418
DB01583	Myotonic disorder	0.04693
DB01583	Neuropathy	0.04797
DB01583	Ovarian cancer	0.02827
DB01583	Prion disease	0.06641
DB01583	Prostate cancer	0.04322
DB01583	Renal tubular acidosis	0.08912
DB01583	Respiratory tract disease	0.1677
DB01583	Salmonella infection	0.0507
DB01583	Schizophrenia	0.12774
DB01583	Uveitis	0.08496
DB01583	Virus disease	0.03828
DB00021	Cancer	0.03686
DB00115	Infertility, Male	0.16667
DB00115	Abortion	0.05103
DB00115	Abruption placentae	0.28868
DB00115	Alzheimer's disease	0.08816
DB00115	Antiphospholipid syndrome	0.13608
DB00115	Arteriopathy	0.13608
DB00115	Aseptic necrosis of bone	0.1291
DB00115	Asthma	0.03333
DB00115	Atherosclerosis	0.02858
DB00115	Attention deficit hyperactivity disorder	0.11785
DB00115	Bipolar disorder	0.09245
DB00115	Bladder cancer	0.0527
DB00115	Brain tumor	0.03279
DB00115	Breast cancer	0.01964
DB00115	Cancer	0.01505
DB00115	Cardiovascular disease	0.06226
DB00115	Cerebrovascular disorder	0.08909
DB00115	Chronic rejection of renal transplant	0.10206
DB00115	Chronic simple glaucoma	0.08704
DB00115	Cirrhosis	0.06901

DB00115	Cleft palate	0.21822
DB00115	Congenital abnormality	0.03077
DB00115	Depression	0.05361
DB00115	Diabetes mellitus	0.02149
DB00115	Down syndrome	0.13957
DB00115	Embryoma	0.02517
DB00115	Encephalopathies	0.05717
DB00115	Enteritis	0.04481
DB00115	Epilepsy	0.05774
DB00115	Folic acid deficiency	0.16667
DB00115	Glaucoma	0.06804
DB00115	Hepatitis C	0.06019
DB00115	Huntington disease	0.08909
DB00115	Hyperglycemia	0.06537
DB00115	Hyperhomocysteinemia	0.35355
DB00115	Hyperuricemia	0.2357
DB00115	Infertility	0.05608
DB00115	Kidney failure	0.04623
DB00115	Leukemia	0.023
DB00115	Liver disease	0.06299
DB00115	Lupus erythematosus	0.03488
DB00115	Malignant glioma	0.07581
DB00115	Meningioma	0.21082
DB00115	Migraine	0.07581
DB00115	Moyamoya disease	0.16667
DB00115	Multiple myeloma	0.05361
DB00115	Obesity	0.09421
DB00115	Osteoporosis	0.06455
DB00115	Pancreas cancer	0.04233
DB00115	Parkinson disease	0.04508
DB00115	Polycystic ovary syndrome	0.05407
DB00115	Prostate cancer	0.02131
DB00115	Rheumatoid arthritis	0.04997
DB00115	Schizophrenia	0.06208
DB00115	Sickle cell disease	0.07857
DB00115	Spinal dysraphism	0.28098
DB00115	Takayasu's arteritis	0.1291
DB00115	Thalassemia	0.09623
DB00115	Thrombophilia	0.09623
DB00115	Ulcerative colitis	0.08422
DB00695	Anemia	0.15076
DB00695	Down syndrome	0.08058
DB00695	Hyperaldosteronism	0.22361
DB00695	Pancreas cancer	0.07332
DB00695	Pancreatitis	0.11043
DB00695	Ulcerative colitis	0.07293
DB00293	Abortion	0.08839

DB00293	Cancer	0.02606
DB00293	Central nervous system disease	0.17678
DB00293	Cytomegalovirus infection	0.18257
DB00293	Folic acid deficiency	0.28868
DB00293	Hypertension	0.0559
DB00293	Rheumatoid arthritis	0.04327
DB00293	Thrombophlebitis	0.2132
DB00322	Abortion	0.125
DB00322	Cancer	0.03686
DB00322	Central nervous system disease	0.25
DB00322	Cytomegalovirus infection	0.2582
DB00322	Folic acid deficiency	0.40825
DB00322	Hypertension	0.07906
DB00322	Rheumatoid arthritis	0.0612
DB00322	Thrombophlebitis	0.30151
DB00432	Abortion	0.125
DB00432	Cancer	0.03686
DB00432	Central nervous system disease	0.25
DB00432	Cytomegalovirus infection	0.2582
DB00432	Folic acid deficiency	0.40825
DB00432	Hypertension	0.07906
DB00432	Rheumatoid arthritis	0.0612
DB00432	Thrombophlebitis	0.30151
DB00440	Abortion	0.08839
DB00440	Cancer	0.02606
DB00440	Central nervous system disease	0.17678
DB00440	Cytomegalovirus infection	0.18257
DB00440	Folic acid deficiency	0.28868
DB00440	Hypertension	0.0559
DB00440	Rheumatoid arthritis	0.04327
DB00440	Thrombophlebitis	0.2132
DB00441	Abortion	0.07217
DB00441	Cancer	0.04256
DB00441	Central nervous system disease	0.14434
DB00441	Cytomegalovirus infection	0.14907
DB00441	Folic acid deficiency	0.2357
DB00441	Hypertension	0.04564
DB00441	Rheumatoid arthritis	0.03533
DB00441	Thrombophlebitis	0.17408
DB00544	Abortion	0.125
DB00544	Cancer	0.03686
DB00544	Central nervous system disease	0.25
DB00544	Cytomegalovirus infection	0.2582
DB00544	Folic acid deficiency	0.40825
DB00544	Hypertension	0.07906
DB00544	Rheumatoid arthritis	0.0612
DB00544	Thrombophlebitis	0.30151

DB00642	Abortion	0.0625
DB00642	Alzheimer's disease	0.03599
DB00642	Cancer	0.01843
DB00642	Central nervous system disease	0.125
DB00642	Cytomegalovirus infection	0.1291
DB00642	Folic acid deficiency	0.20412
DB00642	Hypertension	0.03953
DB00642	Rheumatoid arthritis	0.0612
DB00642	Thrombophlebitis	0.15076
DB00650	Abortion	0.125
DB00650	Cancer	0.03686
DB00650	Central nervous system disease	0.25
DB00650	Cytomegalovirus infection	0.2582
DB00650	Folic acid deficiency	0.40825
DB00650	Hypertension	0.07906
DB00650	Rheumatoid arthritis	0.0612
DB00650	Thrombophlebitis	0.30151
DB01101	Abortion	0.125
DB01101	Cancer	0.03686
DB01101	Central nervous system disease	0.25
DB01101	Cytomegalovirus infection	0.2582
DB01101	Folic acid deficiency	0.40825
DB01101	Hypertension	0.07906
DB01101	Rheumatoid arthritis	0.0612
DB01101	Thrombophlebitis	0.30151
DB06813	Abortion	0.08839
DB06813	Cancer	0.02606
DB06813	Central nervous system disease	0.17678
DB06813	Cytomegalovirus infection	0.18257
DB06813	Folic acid deficiency	0.28868
DB06813	Hypertension	0.0559
DB06813	Rheumatoid arthritis	0.04327
DB06813	Thrombophlebitis	0.2132
DB00631	Kidney tubular necrosis, acute	0.15039
DB00631	Amyotrophic lateral sclerosis	0.04515
DB00631	Aortic valve disease	0.17835
DB00631	Barrett's esophagus	0.09355
DB00631	Breast cancer	0.02375
DB00631	Cancer	0.07917
DB00631	Celiac disease	0.08237
DB00631	Chronic obstructive airway disease	0.07869
DB00631	Colon cancer	0.10214
DB00631	Common cold	0.10971
DB00631	Diabetes mellitus	0.02522
DB00631	Down syndrome	0.22479
DB00631	Emphysema	0.13059
DB00631	Ewings sarcoma	0.14666

DB00631	Fanconi's anemia	0.04847
DB00631	HIV infection	0.06594
DB00631	Heart failure	0.05995
DB00631	Helicobacter infection	0.15477
DB00631	Ischemia	0.06159
DB00631	Kaposi sarcoma	0.08291
DB00631	Leukemia	0.02816
DB00631	Liver cancer	0.04741
DB00631	Lung cancer	0.06707
DB00631	Lymphoma	0.22596
DB00631	Melanoma	0.04603
DB00631	Meningioma	0.51783
DB00631	Multiple endocrine neoplasia	0.16367
DB00631	Parkinson disease	0.05424
DB00631	Pituitary tumor	0.12647
DB00631	Renal tubular acidosis	0.06429
DB00631	Rheumatoid arthritis	0.02541
DB00631	Tuberous sclerosis	0.10191
DB00631	Ulcerative colitis	0.0737
DB00631	Uterine fibroids	0.12671
DB00631	Werner syndrome	0.08323
DB01005	Cancer	0.03686
DB01073	Kidney tubular necrosis, acute	0.15039
DB01073	Amyotrophic lateral sclerosis	0.04515
DB01073	Aortic valve disease	0.17835
DB01073	Barrett's esophagus	0.09355
DB01073	Breast cancer	0.02375
DB01073	Cancer	0.09567
DB01073	Celiac disease	0.08237
DB01073	Chronic obstructive airway disease	0.07869
DB01073	Colon cancer	0.10214
DB01073	Common cold	0.10971
DB01073	Diabetes mellitus	0.02522
DB01073	Down syndrome	0.22479
DB01073	Emphysema	0.13059
DB01073	Ewings sarcoma	0.14666
DB01073	Fanconi's anemia	0.04847
DB01073	HIV infection	0.06594
DB01073	Heart failure	0.05995
DB01073	Helicobacter infection	0.15477
DB01073	Ischemia	0.06159
DB01073	Kaposi sarcoma	0.08291
DB01073	Leukemia	0.02816
DB01073	Liver cancer	0.04741
DB01073	Lung cancer	0.06707
DB01073	Lymphoma	0.22596
DB01073	Melanoma	0.04603

DB01073	Meningioma	0.51783
DB01073	Multiple endocrine neoplasia	0.16367
DB01073	Parkinson disease	0.05424
DB01073	Pituitary tumor	0.12647
DB01073	Renal tubular acidosis	0.06429
DB01073	Rheumatoid arthritis	0.02541
DB01073	Tuberous sclerosis	0.10191
DB01073	Ulcerative colitis	0.0737
DB01073	Uterine fibroids	0.12671
DB01073	Werner syndrome	0.08323
DB00686	Adenocarcinoma	0.08422
DB00686	Advanced cancer	0.18257
DB00686	Alzheimer's disease	0.04156
DB00686	Anemia	0.46251
DB00686	Arthritis	0.30509
DB00686	Asthma	0.14798
DB00686	Breast cancer	0.02778
DB00686	Cancer	0.05468
DB00686	Cirrhosis	0.42658
DB00686	Cleft palate	0.1543
DB00686	Diabetes mellitus	0.09042
DB00686	Eating disorder	0.22929
DB00686	Endometriosis	0.17511
DB00686	Gestational diabetes	0.94609
DB00686	Heart failure	0.20424
DB00686	Hepatitis C	0.28217
DB00686	Herpes	0.17511
DB00686	Intracranial aneurysm	0.17408
DB00686	Leukemia	0.03253
DB00686	Ovarian cancer	0.05803
DB00686	Pre-Eclampsia	0.26719
DB00686	Prostate cancer	0.03014
DB00686	Stroke	0.15801
DB00686	Systemic scleroderma	0.14915
DB00686	Testicular dysfunction	0.08248
DB00980	Alzheimer's disease	0.13639
DB00980	Depression	0.2253
DB00980	Osteosarcoma	0.43091
DB00980	Rheumatoid arthritis	0.12231
DB06594	Alzheimer's disease	0.12705
DB06594	Anorexia nervosa	0.11785
DB06594	Behavior disease	0.08909
DB06594	Bipolar disorder	0.06537
DB06594	Depression	0.20826
DB06594	Hypertension	0.04564
DB06594	Migraine	0.10721
DB06594	Obesity	0.04441

DB06594	Osteosarcoma	0.40325
DB06594	Rheumatoid arthritis	0.11437
DB00635	Adenoma	0.12127
DB00635	Adrenal gland tumor	0.2132
DB00635	Colon cancer	0.04196
DB00635	Congenital abnormality	0.0533
DB00635	Cushing syndrome	0.2132
DB00635	Diabetes mellitus	0.03722
DB00635	Hypertension	0.0559
DB00635	Obesity	0.05439
DB00635	Osteoporosis	0.1118
DB00213	Gastritis	0.18898
DB00338	Gastritis	0.18898
DB00448	Gastritis	0.18898
DB00736	Gastritis	0.18898
DB01129	Gastritis	0.18898
DB00230	Cerebellar disease	0.94164
DB00230	Congenital abnormality	0.14286
DB00230	Mental retardation	0.32788
DB00230	Migraine	0.3741
DB00230	Myopathy	0.27478
DB00230	Myotonic disorder	0.51228
DB00230	Spinocerebellar ataxias	0.70557
DB00836	Abortion	0.07722
DB00836	Achalasia and cardiospasm	0.11243
DB00836	Adrenal gland hyperfunction	0.1543
DB00836	Alopecia	0.07454
DB00836	Alzheimer's disease	0.04772
DB00836	Amyloidosis	0.05986
DB00836	Asthma	0.03941
DB00836	Autistic disorder	0.05448
DB00836	Autoimmune disease	0.05024
DB00836	Bipolar disorder	0.03996
DB00836	Bladder cancer	0.04095
DB00836	Brain ischemia	0.16466
DB00836	Cancer	0.03062
DB00836	Celiac disease	0.07662
DB00836	Cerebellar disease	0.3964
DB00836	Colon cancer	0.01551
DB00836	Congenital abnormality	0.08914
DB00836	Cushing syndrome	0.12309
DB00836	Dental plaque	0.059
DB00836	Depression	0.05361
DB00836	Diabetes mellitus	0.03276
DB00836	Drug abuse	0.06744
DB00836	Eating disorder	0.09391
DB00836	Epstein-Barr virus infection	0.08352

DB00836	Esophageal tumor	0.06938
DB00836	Esophagus cancer	0.02772
DB00836	Esotropia	0.07981
DB00836	Eye cancer	0.09893
DB00836	Fanconi's anemia	0.03904
DB00836	Glaucoma	0.0688
DB00836	Gram-Negative bacterial infection	0.138
DB00836	Graves' disease	0.09874
DB00836	HIV infection	0.04675
DB00836	Herpes	0.06755
DB00836	Infection	0.03807
DB00836	Ischemia	0.05729
DB00836	Keratosis	0.07918
DB00836	Leukemia	0.03527
DB00836	Lichen planus	0.15388
DB00836	Lung cancer	0.033
DB00836	Lupus erythematosus	0.03242
DB00836	Malignant glioma	0.04015
DB00836	Melanoma	0.05459
DB00836	Mental retardation	0.13867
DB00836	Mesothelioma	0.16667
DB00836	Migraine	0.15729
DB00836	Myopathy	0.1159
DB00836	Myotonic disorder	0.21425
DB00836	Obesity	0.0314
DB00836	Osteoporosis	0.06455
DB00836	Pre-Eclampsia	0.0855
DB00836	Prion disease	0.12899
DB00836	Rabies	0.01908
DB00836	Rheumatoid arthritis	0.02364
DB00836	Schistosomiasis	0.13905
DB00836	Schizophrenia	0.03104
DB00836	Sella turcica tumor	0.1543
DB00836	Spinocerebellar ataxias	0.29815
DB00836	Stroke	0.0362
DB00836	Thyroid gland disease	0.08693
DB00836	Tuberculosis	0.07448
DB00836	Vitiligo	0.09623
DB01244	Abortion	0.02495
DB01244	Achalasia and cardiospasm	0.10708
DB01244	Alzheimer's disease	0.04545
DB01244	Amyloidosis	0.05701
DB01244	Asthma	0.03753
DB01244	Atherosclerosis	0.02334
DB01244	Autistic disorder	0.05188
DB01244	Autoimmune disease	0.04785
DB01244	Bipolar disorder	0.03806

DB01244	Bladder cancer	0.03901
DB01244	Brain ischemia	0.15682
DB01244	Breast cancer	0.02756
DB01244	Cancer	0.02917
DB01244	Celiac disease	0.07297
DB01244	Cerebellar disease	0.35272
DB01244	Colon cancer	0.01477
DB01244	Congenital abnormality	0.08071
DB01244	Congenital heart disease	0.11111
DB01244	Deafness	0.11267
DB01244	Dental plaque	0.05619
DB01244	Depression	0.04377
DB01244	Diabetes mellitus	0.01073
DB01244	Drug abuse	0.02782
DB01244	Eating disorder	0.08944
DB01244	Epilepsy	0.04714
DB01244	Epstein-Barr virus infection	0.07955
DB01244	Esophageal tumor	0.06608
DB01244	Esophagus cancer	0.0264
DB01244	Esotropia	0.07602
DB01244	Eye cancer	0.09422
DB01244	Fanconi's anemia	0.03719
DB01244	Glaucoma	0.06553
DB01244	Gram-Negative bacterial infection	0.13144
DB01244	Graves' disease	0.09404
DB01244	Growth retardation	0.06415
DB01244	HIV infection	0.04452
DB01244	Heart failure	0.06462
DB01244	Herpes	0.06434
DB01244	Hypertension	0.02635
DB01244	Ischemia	0.05456
DB01244	Keratosis	0.07541
DB01244	Leukemia	0.03359
DB01244	Lichen planus	0.14656
DB01244	Long QT syndrome	0.26568
DB01244	Lung cancer	0.05471
DB01244	Lung disease	0.09305
DB01244	Lupus erythematosus	0.03088
DB01244	Malignant glioma	0.03824
DB01244	Melanoma	0.01959
DB01244	Mental retardation	0.12491
DB01244	Migraine	0.1395
DB01244	Myopathy	0.10365
DB01244	Myotonic disorder	0.18732
DB01244	Papillary cancer	0.08383
DB01244	Peptic ulcer	0.07857
DB01244	Pre-Eclampsia	0.03561

DB01244	Primary hyperparathyroidism	0.15204
DB01244	Prion disease	0.12285
DB01244	Prostate cancer	0.0174
DB01244	Rabies	0.07592
DB01244	Rheumatoid arthritis	0.04291
DB01244	Schistosomiasis	0.13243
DB01244	Spinocerebellar ataxias	0.26795
DB01244	Stroke	0.03448
DB01244	Sudden infant death syndrome	0.13766
DB01244	Thyroid gland disease	0.08279
DB01244	Tuberculosis	0.07093
DB01244	Yersinia infection	0.06121
DB00454	Cancer	0.02478
DB00454	Connective tissue disease	0.33128
DB04896	Hypertension, Pulmonary	0.13245
DB04896	Anorexia nervosa	0.2357
DB04896	Atherosclerosis	0.04042
DB04896	Autistic disorder	0.07001
DB04896	Behavior disease	0.17817
DB04896	Bipolar disorder	0.13074
DB04896	Cancer	0.0334
DB04896	Chronic fatigue syndrome	0.20412
DB04896	Chronic obstructive airway disease	0.06455
DB04896	Colon cancer	0.03426
DB04896	Congenital heart disease	0.19245
DB04896	Connective tissue disease	0.44649
DB04896	Depression	0.07581
DB04896	Dermatitis	0.05249
DB04896	Diabetes mellitus	0.03039
DB04896	Drug abuse	0.10815
DB04896	Epilepsy	0.08165
DB04896	Fibromyalgia	0.20412
DB04896	Generalized anxiety disorder	0.16667
DB04896	Heart failure	0.06155
DB04896	Herpes	0.08248
DB04896	Hypertension	0.04564
DB04896	Migraine	0.10721
DB04896	Multiple endocrine neoplasia	0.19245
DB04896	Neuroendocrine tumor	0.20412
DB04896	Neurotic disorder	0.18257
DB04896	Obesity	0.04441
DB04896	Obsessive-compulsive disorder	0.17408
DB04896	Panic disorder	0.25198
DB04896	Pervasive development disorder	0.14907
DB04896	Psychotic disorder	0.09245
DB04896	Pulmonary hypertension	0.28868
DB04896	Stroke	0.06537

DB04896	Sudden infant death syndrome	0.12599
DB04896	Ulcerative colitis	0.05955
DB00218	Kidney tubular necrosis, acute	0.12007
DB00218	Amyotrophic lateral sclerosis	0.03604
DB00218	Aortic valve disease	0.11062
DB00218	Brain tumor	0.02262
DB00218	Breast cancer	0.04151
DB00218	Cancer	0.02592
DB00218	Celiac disease	0.06576
DB00218	Charcot-Marie-Tooth disease	0.19569
DB00218	Chronic obstructive airway disease	0.06282
DB00218	Cockayne syndrome	0.12336
DB00218	Colon cancer	0.08471
DB00218	Common cold	0.08759
DB00218	Diabetes mellitus	0.02014
DB00218	Down syndrome	0.07642
DB00218	Embryoma	0.01525
DB00218	Emphysema	0.08099
DB00218	Epilepsy	0.03155
DB00218	Ewings sarcoma	0.11709
DB00218	Eye disease	0.09687
DB00218	Fanconi's anemia	0.03869
DB00218	HIV infection	0.0409
DB00218	Heart disease	0.28207
DB00218	Heart failure	0.04786
DB00218	Helicobacter infection	0.09599
DB00218	Hereditary disease	0.0494
DB00218	Herpes	0.01702
DB00218	Infection	0.01858
DB00218	Infertility	0.05698
DB00218	Ischemia	0.04917
DB00218	Kaposi sarcoma	0.06619
DB00218	Kidney cancer	0.08874
DB00218	Leigh disease	0.05883
DB00218	Leukemia	0.02248
DB00218	Leukoencephalopathy	0.04291
DB00218	Lipodystrophy	0.13587
DB00218	Liver cancer	0.03785
DB00218	Lung cancer	0.04835
DB00218	Lymphoma	0.07682
DB00218	Melanoma	0.03675
DB00218	Meningioma	0.17605
DB00218	Metabolism disease	0.08545
DB00218	Muscular atrophy	0.09852
DB00218	Muscular dystrophies	0.06773
DB00218	Nephroblastoma	0.17583
DB00218	Neuroblastoma	0.05563

DB00218	Neuropathy	0.07997
DB00218	Parkinson disease	0.0433
DB00218	Pituitary tumor	0.10097
DB00218	Prostate cancer	0.02409
DB00218	Renal tubular acidosis	0.05133
DB00218	Retinitis pigmentosa	0.12611
DB00218	Rheumatoid arthritis	0.02029
DB00218	Schizophrenia	0.03388
DB00218	Tuberous sclerosis	0.0632
DB00218	Uterine fibroids	0.10116
DB00218	Virus disease	0.04513
DB00218	Werner syndrome	0.10284
DB00467	Kidney tubular necrosis, acute	0.12007
DB00467	Amyotrophic lateral sclerosis	0.03604
DB00467	Aortic valve disease	0.11062
DB00467	Brain tumor	0.02262
DB00467	Breast cancer	0.04151
DB00467	Cancer	0.02592
DB00467	Celiac disease	0.06576
DB00467	Charcot-Marie-Tooth disease	0.19569
DB00467	Chronic obstructive airway disease	0.06282
DB00467	Cockayne syndrome	0.12336
DB00467	Colon cancer	0.08471
DB00467	Common cold	0.08759
DB00467	Diabetes mellitus	0.02014
DB00467	Down syndrome	0.07642
DB00467	Embryoma	0.01525
DB00467	Emphysema	0.08099
DB00467	Epilepsy	0.03155
DB00467	Ewings sarcoma	0.11709
DB00467	Eye disease	0.09687
DB00467	Fanconi's anemia	0.03869
DB00467	HIV infection	0.0409
DB00467	Heart disease	0.28207
DB00467	Heart failure	0.04786
DB00467	Helicobacter infection	0.09599
DB00467	Hereditary disease	0.0494
DB00467	Herpes	0.01702
DB00467	Infection	0.01858
DB00467	Infertility	0.05698
DB00467	Ischemia	0.04917
DB00467	Kaposi sarcoma	0.06619
DB00467	Kidney cancer	0.08874
DB00467	Leigh disease	0.05883
DB00467	Leukemia	0.02248
DB00467	Leukoencephalopathy	0.04291
DB00467	Lipodystrophy	0.13587

DB00467	Liver cancer	0.03785
DB00467	Lung cancer	0.04835
DB00467	Lymphoma	0.07682
DB00467	Melanoma	0.03675
DB00467	Meningioma	0.17605
DB00467	Metabolism disease	0.08545
DB00467	Muscular atrophy	0.09852
DB00467	Muscular dystrophies	0.06773
DB00467	Nephroblastoma	0.17583
DB00467	Neuroblastoma	0.05563
DB00467	Neuropathy	0.07997
DB00467	Parkinson disease	0.0433
DB00467	Pituitary tumor	0.10097
DB00467	Prostate cancer	0.02409
DB00467	Renal tubular acidosis	0.05133
DB00467	Retinitis pigmentosa	0.12611
DB00467	Rheumatoid arthritis	0.02029
DB00467	Schizophrenia	0.03388
DB00467	Tuberous sclerosis	0.0632
DB00467	Uterine fibroids	0.10116
DB00467	Virus disease	0.04513
DB00467	Werner syndrome	0.10284
DB00487	Kidney tubular necrosis, acute	0.12007
DB00487	Amyotrophic lateral sclerosis	0.03604
DB00487	Aortic valve disease	0.11062
DB00487	Brain tumor	0.02262
DB00487	Breast cancer	0.04151
DB00487	Cancer	0.02592
DB00487	Celiac disease	0.06576
DB00487	Charcot-Marie-Tooth disease	0.19569
DB00487	Chronic obstructive airway disease	0.06282
DB00487	Cockayne syndrome	0.12336
DB00487	Colon cancer	0.08471
DB00487	Common cold	0.08759
DB00487	Diabetes mellitus	0.02014
DB00487	Down syndrome	0.07642
DB00487	Embryoma	0.01525
DB00487	Emphysema	0.08099
DB00487	Epilepsy	0.03155
DB00487	Ewings sarcoma	0.11709
DB00487	Eye disease	0.09687
DB00487	Fanconi's anemia	0.03869
DB00487	HIV infection	0.0409
DB00487	Heart disease	0.28207
DB00487	Heart failure	0.04786
DB00487	Helicobacter infection	0.09599
DB00487	Hereditary disease	0.0494

DB00487	Herpes	0.01702
DB00487	Infection	0.01858
DB00487	Infertility	0.05698
DB00487	Ischemia	0.04917
DB00487	Kaposi sarcoma	0.06619
DB00487	Kidney cancer	0.08874
DB00487	Leigh disease	0.05883
DB00487	Leukemia	0.02248
DB00487	Leukoencephalopathy	0.04291
DB00487	Lipodystrophy	0.13587
DB00487	Liver cancer	0.03785
DB00487	Lung cancer	0.04835
DB00487	Lymphoma	0.07682
DB00487	Melanoma	0.03675
DB00487	Meningioma	0.17605
DB00487	Metabolism disease	0.08545
DB00487	Muscular atrophy	0.09852
DB00487	Muscular dystrophies	0.06773
DB00487	Nephroblastoma	0.17583
DB00487	Neuroblastoma	0.05563
DB00487	Neuropathy	0.07997
DB00487	Parkinson disease	0.0433
DB00487	Pituitary tumor	0.10097
DB00487	Prostate cancer	0.02409
DB00487	Renal tubular acidosis	0.05133
DB00487	Retinitis pigmentosa	0.12611
DB00487	Rheumatoid arthritis	0.02029
DB00487	Schizophrenia	0.03388
DB00487	Tuberous sclerosis	0.0632
DB00487	Uterine fibroids	0.10116
DB00487	Virus disease	0.04513
DB00487	Werner syndrome	0.10284
DB00537	Kidney tubular necrosis, acute	0.12007
DB00537	Amyotrophic lateral sclerosis	0.03604
DB00537	Aortic valve disease	0.11062
DB00537	Brain tumor	0.02262
DB00537	Breast cancer	0.04151
DB00537	Cancer	0.02592
DB00537	Celiac disease	0.06576
DB00537	Charcot-Marie-Tooth disease	0.19569
DB00537	Chronic obstructive airway disease	0.06282
DB00537	Cockayne syndrome	0.12336
DB00537	Colon cancer	0.08471
DB00537	Common cold	0.08759
DB00537	Diabetes mellitus	0.02014
DB00537	Down syndrome	0.07642
DB00537	Embryoma	0.01525

DB00537	Emphysema	0.08099
DB00537	Epilepsy	0.03155
DB00537	Ewings sarcoma	0.11709
DB00537	Eye disease	0.09687
DB00537	Fanconi's anemia	0.03869
DB00537	HIV infection	0.0409
DB00537	Heart disease	0.28207
DB00537	Heart failure	0.04786
DB00537	Helicobacter infection	0.09599
DB00537	Hereditary disease	0.0494
DB00537	Herpes	0.01702
DB00537	Infection	0.01858
DB00537	Infertility	0.05698
DB00537	Ischemia	0.04917
DB00537	Kaposi sarcoma	0.06619
DB00537	Kidney cancer	0.08874
DB00537	Leigh disease	0.05883
DB00537	Leukemia	0.02248
DB00537	Leukoencephalopathy	0.04291
DB00537	Lipodystrophy	0.13587
DB00537	Liver cancer	0.03785
DB00537	Lung cancer	0.04835
DB00537	Lymphoma	0.07682
DB00537	Melanoma	0.03675
DB00537	Meningioma	0.17605
DB00537	Metabolism disease	0.08545
DB00537	Muscular atrophy	0.09852
DB00537	Muscular dystrophies	0.06773
DB00537	Nephroblastoma	0.17583
DB00537	Neuroblastoma	0.05563
DB00537	Neuropathy	0.07997
DB00537	Parkinson disease	0.0433
DB00537	Pituitary tumor	0.10097
DB00537	Prostate cancer	0.02409
DB00537	Renal tubular acidosis	0.05133
DB00537	Retinitis pigmentosa	0.12611
DB00537	Rheumatoid arthritis	0.02029
DB00537	Schizophrenia	0.03388
DB00537	Tuberous sclerosis	0.0632
DB00537	Uterine fibroids	0.10116
DB00537	Virus disease	0.04513
DB00537	Werner syndrome	0.10284
DB00685	Kidney tubular necrosis, acute	0.12007
DB00685	Amyotrophic lateral sclerosis	0.03604
DB00685	Aortic valve disease	0.11062
DB00685	Brain tumor	0.02262
DB00685	Breast cancer	0.04151

DB00685	Cancer	0.02592
DB00685	Celiac disease	0.06576
DB00685	Charcot-Marie-Tooth disease	0.19569
DB00685	Chronic obstructive airway disease	0.06282
DB00685	Cockayne syndrome	0.12336
DB00685	Colon cancer	0.08471
DB00685	Common cold	0.08759
DB00685	Diabetes mellitus	0.02014
DB00685	Down syndrome	0.07642
DB00685	Embryoma	0.01525
DB00685	Emphysema	0.08099
DB00685	Epilepsy	0.03155
DB00685	Ewings sarcoma	0.11709
DB00685	Eye disease	0.09687
DB00685	Fanconi's anemia	0.03869
DB00685	HIV infection	0.0409
DB00685	Heart disease	0.28207
DB00685	Heart failure	0.04786
DB00685	Helicobacter infection	0.09599
DB00685	Hereditary disease	0.0494
DB00685	Herpes	0.01702
DB00685	Infection	0.01858
DB00685	Infertility	0.05698
DB00685	Ischemia	0.04917
DB00685	Kaposi sarcoma	0.06619
DB00685	Kidney cancer	0.08874
DB00685	Leigh disease	0.05883
DB00685	Leukemia	0.02248
DB00685	Leukoencephalopathy	0.04291
DB00685	Lipodystrophy	0.13587
DB00685	Liver cancer	0.03785
DB00685	Lung cancer	0.04835
DB00685	Lymphoma	0.07682
DB00685	Melanoma	0.03675
DB00685	Meningioma	0.17605
DB00685	Metabolism disease	0.08545
DB00685	Muscular atrophy	0.09852
DB00685	Muscular dystrophies	0.06773
DB00685	Nephroblastoma	0.17583
DB00685	Neuroblastoma	0.05563
DB00685	Neuropathy	0.07997
DB00685	Parkinson disease	0.0433
DB00685	Pituitary tumor	0.10097
DB00685	Prostate cancer	0.02409
DB00685	Renal tubular acidosis	0.05133
DB00685	Retinitis pigmentosa	0.12611
DB00685	Rheumatoid arthritis	0.02029

DB00685	Schizophrenia	0.03388
DB00685	Tuberous sclerosis	0.0632
DB00685	Uterine fibroids	0.10116
DB00685	Virus disease	0.04513
DB00685	Werner syndrome	0.10284
DB00978	Kidney tubular necrosis, acute	0.12007
DB00978	Amyotrophic lateral sclerosis	0.03604
DB00978	Aortic valve disease	0.11062
DB00978	Brain tumor	0.02262
DB00978	Breast cancer	0.04151
DB00978	Cancer	0.02592
DB00978	Celiac disease	0.06576
DB00978	Charcot-Marie-Tooth disease	0.19569
DB00978	Chronic obstructive airway disease	0.06282
DB00978	Cockayne syndrome	0.12336
DB00978	Colon cancer	0.08471
DB00978	Common cold	0.08759
DB00978	Diabetes mellitus	0.02014
DB00978	Down syndrome	0.07642
DB00978	Embryoma	0.01525
DB00978	Emphysema	0.08099
DB00978	Epilepsy	0.03155
DB00978	Ewings sarcoma	0.11709
DB00978	Eye disease	0.09687
DB00978	Fanconi's anemia	0.03869
DB00978	HIV infection	0.0409
DB00978	Heart disease	0.28207
DB00978	Heart failure	0.04786
DB00978	Helicobacter infection	0.09599
DB00978	Hereditary disease	0.0494
DB00978	Herpes	0.01702
DB00978	Infection	0.01858
DB00978	Infertility	0.05698
DB00978	Ischemia	0.04917
DB00978	Kaposi sarcoma	0.06619
DB00978	Kidney cancer	0.08874
DB00978	Leigh disease	0.05883
DB00978	Leukemia	0.02248
DB00978	Leukoencephalopathy	0.04291
DB00978	Lipodystrophy	0.13587
DB00978	Liver cancer	0.03785
DB00978	Lung cancer	0.04835
DB00978	Lymphoma	0.07682
DB00978	Melanoma	0.03675
DB00978	Meningioma	0.17605
DB00978	Metabolism disease	0.08545
DB00978	Muscular atrophy	0.09852

DB00978	Muscular dystrophies	0.06773
DB00978	Nephroblastoma	0.17583
DB00978	Neuroblastoma	0.05563
DB00978	Neuropathy	0.07997
DB00978	Parkinson disease	0.0433
DB00978	Pituitary tumor	0.10097
DB00978	Prostate cancer	0.02409
DB00978	Renal tubular acidosis	0.05133
DB00978	Retinitis pigmentosa	0.12611
DB00978	Rheumatoid arthritis	0.02029
DB00978	Schizophrenia	0.03388
DB00978	Tuberous sclerosis	0.0632
DB00978	Uterine fibroids	0.10116
DB00978	Virus disease	0.04513
DB00978	Werner syndrome	0.10284
DB01059	Kidney tubular necrosis, acute	0.12007
DB01059	Amyotrophic lateral sclerosis	0.03604
DB01059	Aortic valve disease	0.11062
DB01059	Brain tumor	0.02262
DB01059	Breast cancer	0.04151
DB01059	Cancer	0.02592
DB01059	Celiac disease	0.06576
DB01059	Charcot-Marie-Tooth disease	0.19569
DB01059	Chronic obstructive airway disease	0.06282
DB01059	Cockayne syndrome	0.12336
DB01059	Colon cancer	0.08471
DB01059	Common cold	0.08759
DB01059	Diabetes mellitus	0.02014
DB01059	Down syndrome	0.07642
DB01059	Embryoma	0.01525
DB01059	Emphysema	0.08099
DB01059	Epilepsy	0.03155
DB01059	Ewings sarcoma	0.11709
DB01059	Eye disease	0.09687
DB01059	Fanconi's anemia	0.03869
DB01059	HIV infection	0.0409
DB01059	Heart disease	0.28207
DB01059	Heart failure	0.04786
DB01059	Helicobacter infection	0.09599
DB01059	Hereditary disease	0.0494
DB01059	Herpes	0.01702
DB01059	Infection	0.01858
DB01059	Infertility	0.05698
DB01059	Ischemia	0.04917
DB01059	Kaposi sarcoma	0.06619
DB01059	Kidney cancer	0.08874
DB01059	Leigh disease	0.05883

DB01059	Leukemia	0.02248
DB01059	Leukoencephalopathy	0.04291
DB01059	Lipodystrophy	0.13587
DB01059	Liver cancer	0.03785
DB01059	Lung cancer	0.04835
DB01059	Lymphoma	0.07682
DB01059	Melanoma	0.03675
DB01059	Meningioma	0.17605
DB01059	Metabolism disease	0.08545
DB01059	Muscular atrophy	0.09852
DB01059	Muscular dystrophies	0.06773
DB01059	Nephroblastoma	0.17583
DB01059	Neuroblastoma	0.05563
DB01059	Neuropathy	0.07997
DB01059	Parkinson disease	0.0433
DB01059	Pituitary tumor	0.10097
DB01059	Prostate cancer	0.02409
DB01059	Renal tubular acidosis	0.05133
DB01059	Retinitis pigmentosa	0.12611
DB01059	Rheumatoid arthritis	0.02029
DB01059	Schizophrenia	0.03388
DB01059	Tuberous sclerosis	0.0632
DB01059	Uterine fibroids	0.10116
DB01059	Virus disease	0.04513
DB01059	Werner syndrome	0.10284
DB01137	Kidney tubular necrosis, acute	0.12007
DB01137	Amyotrophic lateral sclerosis	0.03604
DB01137	Aortic valve disease	0.11062
DB01137	Brain tumor	0.02262
DB01137	Breast cancer	0.04151
DB01137	Cancer	0.02592
DB01137	Celiac disease	0.06576
DB01137	Charcot-Marie-Tooth disease	0.19569
DB01137	Chronic obstructive airway disease	0.06282
DB01137	Cockayne syndrome	0.12336
DB01137	Colon cancer	0.08471
DB01137	Common cold	0.08759
DB01137	Diabetes mellitus	0.02014
DB01137	Down syndrome	0.07642
DB01137	Embryoma	0.01525
DB01137	Emphysema	0.08099
DB01137	Epilepsy	0.03155
DB01137	Ewings sarcoma	0.11709
DB01137	Eye disease	0.09687
DB01137	Fanconi's anemia	0.03869
DB01137	HIV infection	0.0409
DB01137	Heart disease	0.28207

DB01137	Heart failure	0.04786
DB01137	Helicobacter infection	0.09599
DB01137	Hereditary disease	0.0494
DB01137	Herpes	0.01702
DB01137	Infection	0.01858
DB01137	Infertility	0.05698
DB01137	Ischemia	0.04917
DB01137	Kaposi sarcoma	0.06619
DB01137	Kidney cancer	0.08874
DB01137	Leigh disease	0.05883
DB01137	Leukemia	0.02248
DB01137	Leukoencephalopathy	0.04291
DB01137	Lipodystrophy	0.13587
DB01137	Liver cancer	0.03785
DB01137	Lung cancer	0.04835
DB01137	Lymphoma	0.07682
DB01137	Melanoma	0.03675
DB01137	Meningioma	0.17605
DB01137	Metabolism disease	0.08545
DB01137	Muscular atrophy	0.09852
DB01137	Muscular dystrophies	0.06773
DB01137	Nephroblastoma	0.17583
DB01137	Neuroblastoma	0.05563
DB01137	Neuropathy	0.07997
DB01137	Parkinson disease	0.0433
DB01137	Pituitary tumor	0.10097
DB01137	Prostate cancer	0.02409
DB01137	Renal tubular acidosis	0.05133
DB01137	Retinitis pigmentosa	0.12611
DB01137	Rheumatoid arthritis	0.02029
DB01137	Schizophrenia	0.03388
DB01137	Tuberous sclerosis	0.0632
DB01137	Uterine fibroids	0.10116
DB01137	Virus disease	0.04513
DB01137	Werner syndrome	0.10284
DB01165	Kidney tubular necrosis, acute	0.12007
DB01165	Amyotrophic lateral sclerosis	0.03604
DB01165	Aortic valve disease	0.11062
DB01165	Brain tumor	0.02262
DB01165	Breast cancer	0.04151
DB01165	Cancer	0.02592
DB01165	Celiac disease	0.06576
DB01165	Charcot-Marie-Tooth disease	0.19569
DB01165	Chronic obstructive airway disease	0.06282
DB01165	Cockayne syndrome	0.12336
DB01165	Colon cancer	0.08471
DB01165	Common cold	0.08759

DB01165	Diabetes mellitus	0.02014
DB01165	Down syndrome	0.07642
DB01165	Embryoma	0.01525
DB01165	Emphysema	0.08099
DB01165	Epilepsy	0.03155
DB01165	Ewings sarcoma	0.11709
DB01165	Eye disease	0.09687
DB01165	Fanconi's anemia	0.03869
DB01165	HIV infection	0.0409
DB01165	Heart disease	0.28207
DB01165	Heart failure	0.04786
DB01165	Helicobacter infection	0.09599
DB01165	Hereditary disease	0.0494
DB01165	Herpes	0.01702
DB01165	Infection	0.01858
DB01165	Infertility	0.05698
DB01165	Ischemia	0.04917
DB01165	Kaposi sarcoma	0.06619
DB01165	Kidney cancer	0.08874
DB01165	Leigh disease	0.05883
DB01165	Leukemia	0.02248
DB01165	Leukoencephalopathy	0.04291
DB01165	Lipodystrophy	0.13587
DB01165	Liver cancer	0.03785
DB01165	Lung cancer	0.04835
DB01165	Lymphoma	0.07682
DB01165	Melanoma	0.03675
DB01165	Meningioma	0.17605
DB01165	Metabolism disease	0.08545
DB01165	Muscular atrophy	0.09852
DB01165	Muscular dystrophies	0.06773
DB01165	Nephroblastoma	0.17583
DB01165	Neuroblastoma	0.05563
DB01165	Neuropathy	0.07997
DB01165	Parkinson disease	0.0433
DB01165	Pituitary tumor	0.10097
DB01165	Prostate cancer	0.02409
DB01165	Renal tubular acidosis	0.05133
DB01165	Retinitis pigmentosa	0.12611
DB01165	Rheumatoid arthritis	0.02029
DB01165	Schizophrenia	0.03388
DB01165	Tuberous sclerosis	0.0632
DB01165	Uterine fibroids	0.10116
DB01165	Virus disease	0.04513
DB01165	Werner syndrome	0.10284
DB01208	Kidney tubular necrosis, acute	0.12007
DB01208	Amyotrophic lateral sclerosis	0.03604

DB01208	Aortic valve disease	0.11062
DB01208	Brain tumor	0.02262
DB01208	Breast cancer	0.04151
DB01208	Cancer	0.02592
DB01208	Celiac disease	0.06576
DB01208	Charcot-Marie-Tooth disease	0.19569
DB01208	Chronic obstructive airway disease	0.06282
DB01208	Cockayne syndrome	0.12336
DB01208	Colon cancer	0.08471
DB01208	Common cold	0.08759
DB01208	Diabetes mellitus	0.02014
DB01208	Down syndrome	0.07642
DB01208	Embryoma	0.01525
DB01208	Emphysema	0.08099
DB01208	Epilepsy	0.03155
DB01208	Ewings sarcoma	0.11709
DB01208	Eye disease	0.09687
DB01208	Fanconi's anemia	0.03869
DB01208	HIV infection	0.0409
DB01208	Heart disease	0.28207
DB01208	Heart failure	0.04786
DB01208	Helicobacter infection	0.09599
DB01208	Hereditary disease	0.0494
DB01208	Herpes	0.01702
DB01208	Infection	0.01858
DB01208	Infertility	0.05698
DB01208	Ischemia	0.04917
DB01208	Kaposi sarcoma	0.06619
DB01208	Kidney cancer	0.08874
DB01208	Leigh disease	0.05883
DB01208	Leukemia	0.02248
DB01208	Leukoencephalopathy	0.04291
DB01208	Lipodystrophy	0.13587
DB01208	Liver cancer	0.03785
DB01208	Lung cancer	0.04835
DB01208	Lymphoma	0.07682
DB01208	Melanoma	0.03675
DB01208	Meningioma	0.17605
DB01208	Metabolism disease	0.08545
DB01208	Muscular atrophy	0.09852
DB01208	Muscular dystrophies	0.06773
DB01208	Nephroblastoma	0.17583
DB01208	Neuroblastoma	0.05563
DB01208	Neuropathy	0.07997
DB01208	Parkinson disease	0.0433
DB01208	Pituitary tumor	0.10097
DB01208	Prostate cancer	0.02409

DB01208	Renal tubular acidosis	0.05133
DB01208	Retinitis pigmentosa	0.12611
DB01208	Rheumatoid arthritis	0.02029
DB01208	Schizophrenia	0.03388
DB01208	Tuberous sclerosis	0.0632
DB01208	Uterine fibroids	0.10116
DB01208	Virus disease	0.04513
DB01208	Werner syndrome	0.10284
DB04576	Kidney tubular necrosis, acute	0.12007
DB04576	Amyotrophic lateral sclerosis	0.03604
DB04576	Aortic valve disease	0.11062
DB04576	Brain tumor	0.02262
DB04576	Breast cancer	0.04151
DB04576	Cancer	0.02592
DB04576	Celiac disease	0.06576
DB04576	Charcot-Marie-Tooth disease	0.19569
DB04576	Chronic obstructive airway disease	0.06282
DB04576	Cockayne syndrome	0.12336
DB04576	Colon cancer	0.08471
DB04576	Common cold	0.08759
DB04576	Diabetes mellitus	0.02014
DB04576	Down syndrome	0.07642
DB04576	Embryoma	0.01525
DB04576	Emphysema	0.08099
DB04576	Epilepsy	0.03155
DB04576	Ewings sarcoma	0.11709
DB04576	Eye disease	0.09687
DB04576	Fanconi's anemia	0.03869
DB04576	HIV infection	0.0409
DB04576	Heart disease	0.28207
DB04576	Heart failure	0.04786
DB04576	Helicobacter infection	0.09599
DB04576	Hereditary disease	0.0494
DB04576	Herpes	0.01702
DB04576	Infection	0.01858
DB04576	Infertility	0.05698
DB04576	Ischemia	0.04917
DB04576	Kaposi sarcoma	0.06619
DB04576	Kidney cancer	0.08874
DB04576	Leigh disease	0.05883
DB04576	Leukemia	0.02248
DB04576	Leukoencephalopathy	0.04291
DB04576	Lipodystrophy	0.13587
DB04576	Liver cancer	0.03785
DB04576	Lung cancer	0.04835
DB04576	Lymphoma	0.07682
DB04576	Melanoma	0.03675

DB04576	Meningioma	0.17605
DB04576	Metabolism disease	0.08545
DB04576	Muscular atrophy	0.09852
DB04576	Muscular dystrophies	0.06773
DB04576	Nephroblastoma	0.17583
DB04576	Neuroblastoma	0.05563
DB04576	Neuropathy	0.07997
DB04576	Parkinson disease	0.0433
DB04576	Pituitary tumor	0.10097
DB04576	Prostate cancer	0.02409
DB04576	Renal tubular acidosis	0.05133
DB04576	Retinitis pigmentosa	0.12611
DB04576	Rheumatoid arthritis	0.02029
DB04576	Schizophrenia	0.03388
DB04576	Tuberous sclerosis	0.0632
DB04576	Uterine fibroids	0.10116
DB04576	Virus disease	0.04513
DB04576	Werner syndrome	0.10284
DB08875	Adrenoleukodystrophy	0.1431
DB08875	Alzheimer's disease	0.01911
DB08875	Amyotrophic lateral sclerosis	0.37155
DB08875	Aseptic necrosis of bone	0.05051
DB08875	Asthma	0.19258
DB08875	Atherosclerosis	0.08848
DB08875	Autistic disorder	0.16829
DB08875	Cancer	0.17695
DB08875	Capillaries disease	0.20066
DB08875	Cardiovascular disease	0.48331
DB08875	Cerebellar disease	0.64394
DB08875	Chronic obstructive airway disease	0.38993
DB08875	Colon cancer	0.02413
DB08875	Congenital abnormality	0.02181
DB08875	Dental plaque	0.04408
DB08875	Depression	0.0296
DB08875	Dermatitis	0.19151
DB08875	Diabetes mellitus	0.07826
DB08875	Emphysema	0.23067
DB08875	Endocrine system disease	0.34317
DB08875	Endometriosis	0.1622
DB08875	Esotropia	0.05964
DB08875	Familial Mediterranean fever	0.31783
DB08875	Fatty liver	0.44561
DB08875	Heart failure	0.04167
DB08875	Kidney failure	0.15084
DB08875	Leukemia	0.01957
DB08875	Leukoencephalopathy	0.11414
DB08875	Liver metastases	0.11657

DB08875	Lupus erythematosus	0.15747
DB08875	Macular degeneration	0.41016
DB08875	Malaria	0.57176
DB08875	Melanoma	0.032
DB08875	Mucocutaneous lymph node syndrome	0.65285
DB08875	Multiple endocrine neoplasia	0.35334
DB08875	Myopathy	0.14017
DB08875	Neck cancer	0.27225
DB08875	Obesity	0.11226
DB08875	Oral cancer	0.03182
DB08875	Pancreatitis	0.22072
DB08875	Peptic ulcer	0.25307
DB08875	Pervasive development disorder	0.38131
DB08875	Polyneuropathy	0.41016
DB08875	Pre-Eclampsia	0.20145
DB08875	Primary hyperparathyroidism	0.27099
DB08875	Retinal disease	0.45781
DB08875	Rheumatoid arthritis	0.06031
DB08875	Stroke	0.02705
DB08875	Systemic infection	0.1126
DB08875	Systemic scleroderma	0.14889
DB08875	Thyroid cancer	0.06828
DB08875	Thyroid gland disease	0.22431
DB08875	Ulcerative colitis	0.032
DB08875	Uterine disease	0.06201
DB00130	Alzheimer's disease	0.04156
DB00130	Embryoma	0.0356
DB00130	Epilepsy	0.08165
DB00130	Liver cancer	0.04481
DB00091	Breast cancer	0.03998
DB00091	Colon cancer	0.05843
DB00091	Diabetes mellitus	0.04245
DB00091	Ischemia	0.10366
DB00091	Leukemia	0.07993
DB00091	Lung cancer	0.05972
DB00091	Lung disease	0.11835
DB00091	Lymphoma	0.10733
DB00091	Primary tumor	0.13727
DB00091	Prostate cancer	0.04479
DB00091	Renal Cell cancer	0.13727
DB00241	Myoclonic epilepsy, Juvenile	0.08006
DB00241	Alzheimer's disease	0.0237
DB00241	Attention deficit hyperactivity disorder	0.13439
DB00241	Autistic disorder	0.11897
DB00241	Behavior disease	0.17798
DB00241	Bipolar disorder	0.02221
DB00241	Brain tumor	0.04459

DB00241	Breast cancer	0.00944
DB00241	Colon cancer	0.01164
DB00241	Down syndrome	0.12204
DB00241	Drug abuse	0.03674
DB00241	Epilepsy	0.11571
DB00241	Hypertension	0.05208
DB00241	Hypogonadism	0.08771
DB00241	Neoplasm metastasis	0.03935
DB00241	Nervous system disease	0.12271
DB00241	Neuroblastoma	0.02694
DB00241	Obesity	0.04923
DB00241	Parkinson disease	0.09007
DB00241	Primary tumor	0.07031
DB00241	Psychotic disorder	0.28758
DB00241	Schizophrenia	0.08139
DB00241	Yersinia infection	0.02327
DB00306	Myoclonic epilepsy, Juvenile	0.08006
DB00306	Alzheimer's disease	0.0237
DB00306	Attention deficit hyperactivity disorder	0.13439
DB00306	Autistic disorder	0.11897
DB00306	Behavior disease	0.17798
DB00306	Bipolar disorder	0.02221
DB00306	Brain tumor	0.04459
DB00306	Breast cancer	0.00944
DB00306	Colon cancer	0.01164
DB00306	Down syndrome	0.12204
DB00306	Drug abuse	0.03674
DB00306	Epilepsy	0.11571
DB00306	Hypertension	0.05208
DB00306	Hypogonadism	0.08771
DB00306	Neoplasm metastasis	0.03935
DB00306	Nervous system disease	0.12271
DB00306	Neuroblastoma	0.02694
DB00306	Obesity	0.04923
DB00306	Parkinson disease	0.09007
DB00306	Primary tumor	0.07031
DB00306	Psychotic disorder	0.28758
DB00306	Schizophrenia	0.08139
DB00306	Yersinia infection	0.02327
DB00312	Myoclonic epilepsy, Juvenile	0.08006
DB00312	Alzheimer's disease	0.0237
DB00312	Attention deficit hyperactivity disorder	0.13439
DB00312	Autistic disorder	0.11897
DB00312	Behavior disease	0.17798
DB00312	Bipolar disorder	0.02221
DB00312	Brain tumor	0.04459
DB00312	Breast cancer	0.00944

DB00312	Colon cancer	0.01164
DB00312	Down syndrome	0.12204
DB00312	Drug abuse	0.03674
DB00312	Epilepsy	0.11571
DB00312	Hypertension	0.05208
DB00312	Hypogonadism	0.08771
DB00312	Neoplasm metastasis	0.03935
DB00312	Nervous system disease	0.12271
DB00312	Neuroblastoma	0.02694
DB00312	Obesity	0.04923
DB00312	Parkinson disease	0.09007
DB00312	Primary tumor	0.07031
DB00312	Psychotic disorder	0.28758
DB00312	Schizophrenia	0.08139
DB00312	Yersinia infection	0.02327
DB00371	Breast cancer	0.01026
DB00371	Drug abuse	0.01997
DB00371	Hypogonadism	0.09535
DB00371	Yersinia infection	0.0253
DB00402	Breast cancer	0.0105
DB00402	Cancer	0.00804
DB00402	Drug abuse	0.02044
DB00402	Hypertension	0.01725
DB00402	Hypogonadism	0.09759
DB00402	Panic disorder	0.04762
DB00402	Yersinia infection	0.0259
DB00418	Myoclonic epilepsy, Juvenile	0.1291
DB00418	Alzheimer's disease	0.02877
DB00418	Attention deficit hyperactivity disorder	0.16313
DB00418	Autistic disorder	0.16337
DB00418	Behavior disease	0.24016
DB00418	Bipolar disorder	0.03581
DB00418	Brain tumor	0.05413
DB00418	Colon cancer	0.01876
DB00418	Down syndrome	0.16596
DB00418	Drug abuse	0.02962
DB00418	Epilepsy	0.15151
DB00418	Hypertension	0.06322
DB00418	Neoplasm metastasis	0.04776
DB00418	Nervous system disease	0.14895
DB00418	Neuroblastoma	0.04344
DB00418	Obesity	0.05975
DB00418	Parkinson disease	0.1266
DB00418	Primary tumor	0.08534
DB00418	Psychotic disorder	0.37412
DB00418	Schizophrenia	0.0988
DB00463	Myoclonic epilepsy, Juvenile	0.08006

DB00463	Alzheimer's disease	0.0237
DB00463	Attention deficit hyperactivity disorder	0.13439
DB00463	Autistic disorder	0.11897
DB00463	Behavior disease	0.17798
DB00463	Bipolar disorder	0.02221
DB00463	Brain tumor	0.04459
DB00463	Breast cancer	0.00944
DB00463	Colon cancer	0.01164
DB00463	Down syndrome	0.12204
DB00463	Drug abuse	0.03674
DB00463	Epilepsy	0.11571
DB00463	Hypertension	0.05208
DB00463	Hypogonadism	0.08771
DB00463	Neoplasm metastasis	0.03935
DB00463	Nervous system disease	0.12271
DB00463	Neuroblastoma	0.02694
DB00463	Obesity	0.04923
DB00463	Parkinson disease	0.09007
DB00463	Primary tumor	0.07031
DB00463	Psychotic disorder	0.28758
DB00463	Schizophrenia	0.08139
DB00463	Yersinia infection	0.02327
DB00599	Myoclonic epilepsy, Juvenile	0.12309
DB00599	Alzheimer's disease	0.02877
DB00599	Attention deficit hyperactivity disorder	0.16313
DB00599	Autistic disorder	0.1598
DB00599	Behavior disease	0.28215
DB00599	Bipolar disorder	0.03414
DB00599	Brain tumor	0.05413
DB00599	Colon cancer	0.01789
DB00599	Down syndrome	0.19697
DB00599	Drug abuse	0.05648
DB00599	Epilepsy	0.14943
DB00599	Huntington disease	0.0658
DB00599	Hypertension	0.06322
DB00599	Neoplasm metastasis	0.04776
DB00599	Nervous system disease	0.14895
DB00599	Neuroblastoma	0.04142
DB00599	Obesity	0.08295
DB00599	Parkinson disease	0.12335
DB00599	Primary tumor	0.08534
DB00599	Psychotic disorder	0.3694
DB00599	Schizophrenia	0.0988
DB00794	Myoclonic epilepsy, Juvenile	0.08006
DB00794	Alzheimer's disease	0.0237
DB00794	Attention deficit hyperactivity disorder	0.13439
DB00794	Autistic disorder	0.11897

DB00794	Behavior disease	0.17798
DB00794	Bipolar disorder	0.02221
DB00794	Brain tumor	0.04459
DB00794	Breast cancer	0.00944
DB00794	Colon cancer	0.01164
DB00794	Down syndrome	0.12204
DB00794	Drug abuse	0.03674
DB00794	Epilepsy	0.11571
DB00794	Hypertension	0.05208
DB00794	Hypogonadism	0.08771
DB00794	Neoplasm metastasis	0.03935
DB00794	Nervous system disease	0.12271
DB00794	Neuroblastoma	0.02694
DB00794	Obesity	0.04923
DB00794	Parkinson disease	0.09007
DB00794	Primary tumor	0.07031
DB00794	Psychotic disorder	0.28758
DB00794	Schizophrenia	0.08139
DB00794	Yersinia infection	0.02327
DB00849	Myoclonic epilepsy, Juvenile	0.1291
DB00849	Alzheimer's disease	0.02877
DB00849	Attention deficit hyperactivity disorder	0.16313
DB00849	Autistic disorder	0.16337
DB00849	Behavior disease	0.24016
DB00849	Bipolar disorder	0.03581
DB00849	Brain tumor	0.05413
DB00849	Colon cancer	0.01876
DB00849	Down syndrome	0.16596
DB00849	Drug abuse	0.02962
DB00849	Epilepsy	0.15151
DB00849	Hypertension	0.06322
DB00849	Neoplasm metastasis	0.04776
DB00849	Nervous system disease	0.14895
DB00849	Neuroblastoma	0.04344
DB00849	Obesity	0.05975
DB00849	Parkinson disease	0.1266
DB00849	Primary tumor	0.08534
DB00849	Psychotic disorder	0.37412
DB00849	Schizophrenia	0.0988
DB01174	Myoclonic epilepsy, Juvenile	0.1291
DB01174	Alzheimer's disease	0.02877
DB01174	Attention deficit hyperactivity disorder	0.16313
DB01174	Autistic disorder	0.16337
DB01174	Behavior disease	0.24016
DB01174	Bipolar disorder	0.03581
DB01174	Brain tumor	0.05413
DB01174	Colon cancer	0.01876

DB01174	Down syndrome	0.16596
DB01174	Drug abuse	0.02962
DB01174	Epilepsy	0.15151
DB01174	Hypertension	0.06322
DB01174	Neoplasm metastasis	0.04776
DB01174	Nervous system disease	0.14895
DB01174	Neuroblastoma	0.04344
DB01174	Obesity	0.05975
DB01174	Parkinson disease	0.1266
DB01174	Primary tumor	0.08534
DB01174	Psychotic disorder	0.37412
DB01174	Schizophrenia	0.0988
DB01198	Cancer	0.01648
DB01198	Hypertension	0.03536
DB01198	Panic disorder	0.09759
DB01351	Myoclonic epilepsy, Juvenile	0.1291
DB01351	Alzheimer's disease	0.02877
DB01351	Attention deficit hyperactivity disorder	0.16313
DB01351	Autistic disorder	0.16337
DB01351	Behavior disease	0.24016
DB01351	Bipolar disorder	0.03581
DB01351	Brain tumor	0.05413
DB01351	Colon cancer	0.01876
DB01351	Down syndrome	0.16596
DB01351	Drug abuse	0.02962
DB01351	Epilepsy	0.15151
DB01351	Hypertension	0.06322
DB01351	Neoplasm metastasis	0.04776
DB01351	Nervous system disease	0.14895
DB01351	Neuroblastoma	0.04344
DB01351	Obesity	0.05975
DB01351	Parkinson disease	0.1266
DB01351	Primary tumor	0.08534
DB01351	Psychotic disorder	0.37412
DB01351	Schizophrenia	0.0988
DB01352	Myoclonic epilepsy, Juvenile	0.1291
DB01352	Alzheimer's disease	0.02877
DB01352	Attention deficit hyperactivity disorder	0.16313
DB01352	Autistic disorder	0.16337
DB01352	Behavior disease	0.24016
DB01352	Bipolar disorder	0.03581
DB01352	Brain tumor	0.05413
DB01352	Colon cancer	0.01876
DB01352	Down syndrome	0.16596
DB01352	Drug abuse	0.02962
DB01352	Epilepsy	0.15151
DB01352	Hypertension	0.06322

DB01352	Neoplasm metastasis	0.04776
DB01352	Nervous system disease	0.14895
DB01352	Neuroblastoma	0.04344
DB01352	Obesity	0.05975
DB01352	Parkinson disease	0.1266
DB01352	Primary tumor	0.08534
DB01352	Psychotic disorder	0.37412
DB01352	Schizophrenia	0.0988
DB01353	Myoclonic epilepsy, Juvenile	0.1291
DB01353	Alzheimer's disease	0.02877
DB01353	Attention deficit hyperactivity disorder	0.16313
DB01353	Autistic disorder	0.16337
DB01353	Behavior disease	0.24016
DB01353	Bipolar disorder	0.03581
DB01353	Brain tumor	0.05413
DB01353	Colon cancer	0.01876
DB01353	Down syndrome	0.16596
DB01353	Drug abuse	0.02962
DB01353	Epilepsy	0.15151
DB01353	Hypertension	0.06322
DB01353	Neoplasm metastasis	0.04776
DB01353	Nervous system disease	0.14895
DB01353	Neuroblastoma	0.04344
DB01353	Obesity	0.05975
DB01353	Parkinson disease	0.1266
DB01353	Primary tumor	0.08534
DB01353	Psychotic disorder	0.37412
DB01353	Schizophrenia	0.0988
DB01354	Myoclonic epilepsy, Juvenile	0.1291
DB01354	Alzheimer's disease	0.02877
DB01354	Attention deficit hyperactivity disorder	0.16313
DB01354	Autistic disorder	0.16337
DB01354	Behavior disease	0.24016
DB01354	Bipolar disorder	0.03581
DB01354	Brain tumor	0.05413
DB01354	Colon cancer	0.01876
DB01354	Down syndrome	0.16596
DB01354	Drug abuse	0.02962
DB01354	Epilepsy	0.15151
DB01354	Hypertension	0.06322
DB01354	Neoplasm metastasis	0.04776
DB01354	Nervous system disease	0.14895
DB01354	Neuroblastoma	0.04344
DB01354	Obesity	0.05975
DB01354	Parkinson disease	0.1266
DB01354	Primary tumor	0.08534
DB01354	Psychotic disorder	0.37412

DB01354	Schizophrenia	0.0988
DB01355	Myoclonic epilepsy, Juvenile	0.1291
DB01355	Alzheimer's disease	0.02877
DB01355	Attention deficit hyperactivity disorder	0.16313
DB01355	Autistic disorder	0.16337
DB01355	Behavior disease	0.24016
DB01355	Bipolar disorder	0.03581
DB01355	Brain tumor	0.05413
DB01355	Colon cancer	0.01876
DB01355	Down syndrome	0.16596
DB01355	Drug abuse	0.02962
DB01355	Epilepsy	0.15151
DB01355	Hypertension	0.06322
DB01355	Neoplasm metastasis	0.04776
DB01355	Nervous system disease	0.14895
DB01355	Neuroblastoma	0.04344
DB01355	Obesity	0.05975
DB01355	Parkinson disease	0.1266
DB01355	Primary tumor	0.08534
DB01355	Psychotic disorder	0.37412
DB01355	Schizophrenia	0.0988
DB01544	Cancer	0.01505
DB01544	Hypertension	0.03227
DB01544	Panic disorder	0.08909
DB01097	Hemorrhagic fevers, Viral	0.10323
DB01097	Pemphigoid, Bullous	0.0524
DB01097	Pleural effusion, Malignant	0.05363
DB01097	Purpura, Thrombocytopenic, Idiopathic	0.05008
DB01097	Alzheimer's disease	0.03192
DB01097	Aplastic anemia	0.08559
DB01097	Asthma	0.04919
DB01097	Atherosclerosis	0.06035
DB01097	Autistic disorder	0.06801
DB01097	Breast cancer	0.05963
DB01097	Cancer	0.02822
DB01097	Diabetes mellitus	0.0532
DB01097	Drug abuse	0.03646
DB01097	Endometriosis	0.09257
DB01097	Esotropia	0.11505
DB01097	HIV infection	0.03219
DB01097	Hemorrhagic disorder	0.0808
DB01097	Herpes	0.04519
DB01097	Hypertension	0.16906
DB01097	Infertility	0.17501
DB01097	Intermediate coronary syndrome	0.10298
DB01097	Leukemia	0.10918
DB01097	Lung cancer	0.07396

DB01097	Lupus erythematosus	0.04047
DB01097	Lymphoma	0.12626
DB01097	Nervous system tumor	0.7904
DB01097	Osteoporosis	0.26909
DB01097	Penile disease	0.09663
DB01097	Prostate cancer	0.09672
DB01097	Rheumatoid arthritis	0.02951
DB01097	Stomach cancer	0.08079
DB01097	Stroke	0.09738
DB01097	Subarachnoid hemorrhage	0.09217
DB01097	Takayasu's arteritis	0.08618
DB01097	Temporal arteritis	0.10076
DB01097	Thrombocytopenia	0.07678
DB01097	Uterine fibroids	0.37402
DB01097	Yersinia infection	0.11083
DB00008	Arthritis	0.21097
DB00008	Autoimmune disease	0.11617
DB00008	Brain tumor	0.11126
DB00008	Colon cancer	0.07466
DB00008	Encephalitis	0.36745
DB00008	Herpes	0.0837
DB00008	Infection	0.15731
DB00008	Liver cancer	0.15686
DB00008	Liver metastases	0.36072
DB00008	Lymphoma	0.21774
DB00008	Malaria	0.37585
DB00008	Melanoma	0.09901
DB00008	Multiple myeloma	0.41248
DB00008	Pancreas cancer	0.32963
DB00008	Prostate cancer	0.05724
DB00008	Renal Cell cancer	0.25761
DB00008	Respiratory tract disease	0.22212
DB00008	Sicca syndrome	0.17462
DB00011	Arthritis	0.21097
DB00011	Autoimmune disease	0.11617
DB00011	Brain tumor	0.11126
DB00011	Colon cancer	0.07466
DB00011	Encephalitis	0.36745
DB00011	Herpes	0.0837
DB00011	Infection	0.15731
DB00011	Liver cancer	0.15686
DB00011	Liver metastases	0.36072
DB00011	Lymphoma	0.21774
DB00011	Malaria	0.37585
DB00011	Melanoma	0.09901
DB00011	Multiple myeloma	0.41248
DB00011	Pancreas cancer	0.32963

DB00011	Prostate cancer	0.05724
DB00011	Renal Cell cancer	0.25761
DB00011	Respiratory tract disease	0.22212
DB00011	Sicca syndrome	0.17462
DB00018	Arthritis	0.21097
DB00018	Autoimmune disease	0.11617
DB00018	Brain tumor	0.11126
DB00018	Colon cancer	0.07466
DB00018	Encephalitis	0.36745
DB00018	Herpes	0.0837
DB00018	Infection	0.15731
DB00018	Liver cancer	0.15686
DB00018	Liver metastases	0.36072
DB00018	Lymphoma	0.21774
DB00018	Malaria	0.37585
DB00018	Melanoma	0.09901
DB00018	Multiple myeloma	0.41248
DB00018	Pancreas cancer	0.32963
DB00018	Prostate cancer	0.05724
DB00018	Renal Cell cancer	0.25761
DB00018	Respiratory tract disease	0.22212
DB00018	Sicca syndrome	0.17462
DB00022	Arthritis	0.21097
DB00022	Autoimmune disease	0.11617
DB00022	Brain tumor	0.11126
DB00022	Colon cancer	0.07466
DB00022	Encephalitis	0.36745
DB00022	Herpes	0.0837
DB00022	Infection	0.15731
DB00022	Liver cancer	0.15686
DB00022	Liver metastases	0.36072
DB00022	Lymphoma	0.21774
DB00022	Malaria	0.37585
DB00022	Melanoma	0.09901
DB00022	Multiple myeloma	0.41248
DB00022	Pancreas cancer	0.32963
DB00022	Prostate cancer	0.05724
DB00022	Renal Cell cancer	0.25761
DB00022	Respiratory tract disease	0.22212
DB00022	Sicca syndrome	0.17462
DB00034	Arthritis	0.21097
DB00034	Autoimmune disease	0.11617
DB00034	Brain tumor	0.11126
DB00034	Colon cancer	0.07466
DB00034	Encephalitis	0.36745
DB00034	Herpes	0.0837
DB00034	Infection	0.15731

DB00034	Liver cancer	0.15686
DB00034	Liver metastases	0.36072
DB00034	Lymphoma	0.21774
DB00034	Malaria	0.37585
DB00034	Melanoma	0.09901
DB00034	Multiple myeloma	0.41248
DB00034	Pancreas cancer	0.32963
DB00034	Prostate cancer	0.05724
DB00034	Renal Cell cancer	0.25761
DB00034	Respiratory tract disease	0.22212
DB00034	Sicca syndrome	0.17462
DB00060	Arthritis	0.21097
DB00060	Autoimmune disease	0.11617
DB00060	Brain tumor	0.11126
DB00060	Colon cancer	0.07466
DB00060	Encephalitis	0.36745
DB00060	Herpes	0.0837
DB00060	Infection	0.15731
DB00060	Liver cancer	0.15686
DB00060	Liver metastases	0.36072
DB00060	Lymphoma	0.21774
DB00060	Malaria	0.37585
DB00060	Melanoma	0.09901
DB00060	Multiple myeloma	0.41248
DB00060	Pancreas cancer	0.32963
DB00060	Prostate cancer	0.05724
DB00060	Renal Cell cancer	0.25761
DB00060	Respiratory tract disease	0.22212
DB00060	Sicca syndrome	0.17462
DB00068	Arthritis	0.21097
DB00068	Autoimmune disease	0.11617
DB00068	Brain tumor	0.11126
DB00068	Colon cancer	0.07466
DB00068	Encephalitis	0.36745
DB00068	Herpes	0.0837
DB00068	Infection	0.15731
DB00068	Liver cancer	0.15686
DB00068	Liver metastases	0.36072
DB00068	Lymphoma	0.21774
DB00068	Malaria	0.37585
DB00068	Melanoma	0.09901
DB00068	Multiple myeloma	0.41248
DB00068	Pancreas cancer	0.32963
DB00068	Prostate cancer	0.05724
DB00068	Renal Cell cancer	0.25761
DB00068	Respiratory tract disease	0.22212
DB00068	Sicca syndrome	0.17462

DB00069	Arthritis	0.21097
DB00069	Autoimmune disease	0.11617
DB00069	Brain tumor	0.11126
DB00069	Colon cancer	0.07466
DB00069	Encephalitis	0.36745
DB00069	Herpes	0.0837
DB00069	Infection	0.15731
DB00069	Liver cancer	0.15686
DB00069	Liver metastases	0.36072
DB00069	Lymphoma	0.21774
DB00069	Malaria	0.37585
DB00069	Melanoma	0.09901
DB00069	Multiple myeloma	0.41248
DB00069	Pancreas cancer	0.32963
DB00069	Prostate cancer	0.05724
DB00069	Renal Cell cancer	0.25761
DB00069	Respiratory tract disease	0.22212
DB00069	Sicca syndrome	0.17462
DB00105	Arthritis	0.21097
DB00105	Autoimmune disease	0.11617
DB00105	Brain tumor	0.11126
DB00105	Colon cancer	0.07466
DB00105	Encephalitis	0.36745
DB00105	Herpes	0.0837
DB00105	Infection	0.15731
DB00105	Liver cancer	0.15686
DB00105	Liver metastases	0.36072
DB00105	Lymphoma	0.21774
DB00105	Malaria	0.37585
DB00105	Melanoma	0.09901
DB00105	Multiple myeloma	0.41248
DB00105	Pancreas cancer	0.32963
DB00105	Prostate cancer	0.05724
DB00105	Renal Cell cancer	0.25761
DB00105	Respiratory tract disease	0.22212
DB00105	Sicca syndrome	0.17462
DB00083	Adenoma	0.0493
DB00083	Attention deficit hyperactivity disorder	0.91333
DB00083	Behavior disease	0.25288
DB00083	Bipolar disorder	0.05752
DB00083	Cancer	0.02606
DB00083	Cystic fibrosis	0.05513
DB00083	Down syndrome	0.43405
DB00083	Drug abuse	0.02607
DB00083	Neurilemmoma	0.27297
DB00083	Polyarthritis	0.08058
DB00083	Schizophrenia	0.30501

DB00083	Sella turcica tumor	0.22288
DB01242	Hypertension, Pulmonary	0.08671
DB01242	Amyotrophic lateral sclerosis	0.05096
DB01242	Anorexia nervosa	0.30861
DB01242	Aplastic anemia	0.07881
DB01242	Asthma	0.03086
DB01242	Atherosclerosis	0.02646
DB01242	Autistic disorder	0.09167
DB01242	Barrett's esophagus	0.06901
DB01242	Behavior disease	0.23328
DB01242	Behcet syndrome	0.05399
DB01242	Bipolar disorder	0.17118
DB01242	Cancer	0.01393
DB01242	Choriocarcinoma	0.10911
DB01242	Chronic fatigue syndrome	0.26726
DB01242	Chronic obstructive airway disease	0.08452
DB01242	Colon cancer	0.06728
DB01242	Congenital heart disease	0.12599
DB01242	Cystic fibrosis	0.05399
DB01242	Deafness	0.05455
DB01242	Depression	0.09926
DB01242	Dermatitis	0.10308
DB01242	Diabetes mellitus	0.03979
DB01242	Drug abuse	0.1416
DB01242	Drug-Induced dyskinesia	0.28571
DB01242	Eating disorder	0.0658
DB01242	Emphysema	0.07715
DB01242	Endometriosis	0.03139
DB01242	Epilepsy	0.05345
DB01242	Fibromyalgia	0.13363
DB01242	Generalized anxiety disorder	0.10911
DB01242	Heart failure	0.04029
DB01242	Herpes	0.10799
DB01242	Hypertension	0.11952
DB01242	Kidney failure	0.0428
DB01242	Kuhnt-Junius degeneration	0.1543
DB01242	Liver disease	0.05832
DB01242	Migraine	0.14037
DB01242	Multiple endocrine neoplasia	0.12599
DB01242	Neuroendocrine tumor	0.13363
DB01242	Neurotic disorder	0.11952
DB01242	Obesity	0.08722
DB01242	Obsessive-compulsive disorder	0.22792
DB01242	Oral cancer	0.05143
DB01242	Pancreatitis	0.05903
DB01242	Panic disorder	0.24744
DB01242	Peptic esophagitis	0.11952

DB01242	Pervasive development disorder	0.09759
DB01242	Pre-Eclampsia	0.04454
DB01242	Primary biliary cirrhosis	0.06788
DB01242	Prostate cancer	0.01973
DB01242	Psychotic disorder	0.18157
DB01242	Pulmonary hypertension	0.18898
DB01242	Rheumatoid arthritis	0.02313
DB01242	Schizophrenia	0.02874
DB01242	Silicosis	0.14286
DB01242	Stroke	0.08559
DB01242	Sudden infant death syndrome	0.08248
DB01242	Testicular dysfunction	0.05399
DB01242	Ulcerative colitis	0.03898
DB00019	Asthma	0.15858
DB00019	Atherosclerosis	0.13698
DB00019	Bladder cancer	0.09129
DB00019	Congenital abnormality	0.0533
DB00019	Connective tissue disease	0.62327
DB00019	Dental plaque	0.24077
DB00019	Disseminated intravascular coagulation	1.5
DB00019	Embryoma	0.0436
DB00019	Glomerulonephritis	0.60951
DB00019	Infectious lung disease	0.34028
DB00019	Leukemia	0.14671
DB00019	Leukoencephalopathy	0.23069
DB00019	Lupus erythematosus	0.14337
DB00019	Neutropenia	1.57735
DB00019	Shigella infection	0.42404
DB00019	Systemic infection	0.19242
DB00058	Asthma	0.18249
DB00058	Atherosclerosis	0.15749
DB00058	Connective tissue disease	0.69649
DB00058	Dental plaque	0.27796
DB00058	Disseminated intravascular coagulation	1.70711
DB00058	Glomerulonephritis	0.68274
DB00058	Infectious lung disease	0.40578
DB00058	Leukemia	0.12337
DB00058	Leukoencephalopathy	0.2795
DB00058	Lupus erythematosus	0.16839
DB00058	Neutropenia	1.40825
DB00058	Shigella infection	0.54362
DB00058	Systemic infection	0.22602
DB00099	Asthma	0.15858
DB00099	Atherosclerosis	0.13698
DB00099	Bladder cancer	0.09129
DB00099	Congenital abnormality	0.0533
DB00099	Connective tissue disease	0.62327

DB00099	Dental plaque	0.24077
DB00099	Disseminated intravascular coagulation	1.5
DB00099	Embryoma	0.0436
DB00099	Glomerulonephritis	0.60951
DB00099	Infectious lung disease	0.34028
DB00099	Leukemia	0.14671
DB00099	Leukoencephalopathy	0.23069
DB00099	Lupus erythematosus	0.14337
DB00099	Neutropenia	1.57735
DB00099	Shigella infection	0.42404
DB00099	Systemic infection	0.19242
DB00168	Brain tumor	0.0568
DB00168	Intestinal disease	0.17678
DB00168	Neuropathy	0.11323
DB00168	Obesity	0.05439
DB00168	Polyarthritis	0.08058
DB00168	Rheumatoid arthritis	0.04327
DB00403	Drug abuse	0.09366
DB00403	Obesity	0.07692
DB00403	Pancreas cancer	0.1037
DB00403	Panic disorder	0.21822
DB00403	Parkinson disease	0.11043
DB00403	Schizophrenia	0.07603
DB01022	Bone disease	0.12127
DB01022	Breast cancer	0.03402
DB01022	Diabetes mellitus	0.03722
DB01022	Multiple myeloma	0.09285
DB01022	Osteoporosis	0.1118
DB01022	Pancreas cancer	0.07332
DB00127	Mental retardation	0.07454
DB00184	Myoclonic epilepsy, Juvenile	0.12309
DB00184	Alzheimer's disease	0.08083
DB00184	Autistic disorder	0.28783
DB00184	Behavior disease	0.43262
DB00184	Bipolar disorder	0.03414
DB00184	Brain tumor	0.11126
DB00184	Colon cancer	0.01789
DB00184	Down syndrome	0.33041
DB00184	Drug abuse	0.02824
DB00184	Epilepsy	0.34741
DB00184	Lung cancer	0.04212
DB00184	Neoplasm metastasis	0.09817
DB00184	Neuroblastoma	0.04142
DB00184	Parkinson disease	0.18324
DB00184	Primary tumor	0.17542
DB00184	Psychotic disorder	0.46862
DB00184	Squamous cell cancer	0.03077

DB00674	Myoclonic epilepsy, Juvenile	0.09623
DB00674	Alzheimer's disease	0.09306
DB00674	Atherosclerosis	0.0165
DB00674	Autistic disorder	0.26389
DB00674	Behavior disease	0.40216
DB00674	Bipolar disorder	0.02669
DB00674	Brain tumor	0.13019
DB00674	Colon cancer	0.04196
DB00674	Diabetes mellitus	0.01241
DB00674	Down syndrome	0.30041
DB00674	Drug abuse	0.02208
DB00674	Epilepsy	0.31949
DB00674	Lung cancer	0.06585
DB00674	Myopathy	0.05717
DB00674	Neoplasm metastasis	0.09817
DB00674	Neuroblastoma	0.03238
DB00674	Neurodegenerative disorder	0.03875
DB00674	Obesity	0.01813
DB00674	Ovarian cancer	0.02369
DB00674	Parkinson disease	0.16871
DB00674	Primary tumor	0.17542
DB00674	Psychotic disorder	0.43701
DB00674	Squamous cell cancer	0.02406
DB00674	Vitiligo	0.11111
DB01090	Alzheimer's disease	0.04156
DB01090	Down syndrome	0.0658
DB01090	Lung cancer	0.04032
DB00468	Endometrium cancer	0.1715
DB00468	Sickle cell disease	0.13608
DB00038	Breast cancer	0.04811
DB00038	Disseminated cancer	0.31623
DB00038	Hodgkin's disease	0.17678
DB00038	Multiple myeloma	0.13131
DB00038	Prostate cancer	0.0522
DB00038	Stomach cancer	0.08422
DB00831	Abortion	0.02905
DB00831	Achalasia and cardiospasm	0.12469
DB00831	Alzheimer's disease	0.05292
DB00831	Amyloidosis	0.06639
DB00831	Asthma	0.04371
DB00831	Autistic disorder	0.06042
DB00831	Autoimmune disease	0.05572
DB00831	Bipolar disorder	0.04432
DB00831	Bladder cancer	0.04542
DB00831	Brain ischemia	0.18262
DB00831	Cancer	0.04901
DB00831	Celiac disease	0.08498

DB00831	Colon cancer	0.0172
DB00831	Congenital abnormality	0.03237
DB00831	Dental plaque	0.06543
DB00831	Diabetes mellitus	0.0125
DB00831	Drug abuse	0.03239
DB00831	Eating disorder	0.10416
DB00831	Epstein-Barr virus infection	0.09263
DB00831	Esophageal tumor	0.07695
DB00831	Esophagus cancer	0.03074
DB00831	Esotropia	0.08852
DB00831	Eye cancer	0.10972
DB00831	Fanconi's anemia	0.0433
DB00831	Glaucoma	0.07631
DB00831	Gram-Negative bacterial infection	0.15306
DB00831	Graves' disease	0.10951
DB00831	HIV infection	0.05185
DB00831	Herpes	0.07492
DB00831	Hypertension	0.03227
DB00831	Ischemia	0.06354
DB00831	Keratosis	0.08781
DB00831	Kidney failure	0.04623
DB00831	Leukemia	0.03912
DB00831	Lichen planus	0.17067
DB00831	Lung cancer	0.0366
DB00831	Lupus erythematosus	0.03596
DB00831	Malignant glioma	0.04453
DB00831	Melanoma	0.02281
DB00831	Pre-Eclampsia	0.04147
DB00831	Prion disease	0.14306
DB00831	Prostate cancer	0.02131
DB00831	Rabies	0.02116
DB00831	Rheumatoid arthritis	0.0512
DB00831	Schistosomiasis	0.15421
DB00831	Stroke	0.04015
DB00831	Thyroid gland disease	0.09641
DB00831	Tuberculosis	0.0826
DB01023	Abortion	0.02404
DB01023	Achalasia and cardiospasm	0.10319
DB01023	Alopecia	0.14149
DB01023	Alzheimer's disease	0.0438
DB01023	Amyloidosis	0.05494
DB01023	Amyotrophic lateral sclerosis	0.0374
DB01023	Asthma	0.03617
DB01023	Atherosclerosis	0.03884
DB01023	Autistic disorder	0.05
DB01023	Autoimmune disease	0.04611
DB01023	Bipolar disorder	0.03668

DB01023	Bladder cancer	0.03759
DB01023	Brain ischemia	0.15113
DB01023	Breast cancer	0.06725
DB01023	Cancer	0.02811
DB01023	Celiac disease	0.07032
DB01023	Colon cancer	0.01424
DB01023	Congenital abnormality	0.02679
DB01023	Congenital heart disease	0.09245
DB01023	Dental plaque	0.05415
DB01023	Depression	0.03642
DB01023	Diabetes mellitus	0.01034
DB01023	Drug abuse	0.02681
DB01023	Eating disorder	0.0862
DB01023	Endocrine system disease	0.10483
DB01023	Epilepsy	0.03922
DB01023	Epstein-Barr virus infection	0.07666
DB01023	Esophageal tumor	0.06368
DB01023	Esophagus cancer	0.02544
DB01023	Esotropia	0.07326
DB01023	Eye cancer	0.0908
DB01023	Fanconi's anemia	0.03584
DB01023	Glaucoma	0.06315
DB01023	Gram-Negative bacterial infection	0.12667
DB01023	Graves' disease	0.09063
DB01023	HIV infection	0.04291
DB01023	Heart failure	0.13193
DB01023	Herpes	0.062
DB01023	Hypertension	0.10615
DB01023	Hypoglycemia	0.55533
DB01023	Ischemia	0.05258
DB01023	Keratosis	0.07267
DB01023	Leukemia	0.03237
DB01023	Lichen planus	0.14124
DB01023	Lung cancer	0.04966
DB01023	Lupus erythematosus	0.02976
DB01023	Lymphoma	0.1405
DB01023	Malignant glioma	0.03685
DB01023	Melanoma	0.05818
DB01023	Peptic ulcer	0.06537
DB01023	Pre-Eclampsia	0.03432
DB01023	Prion disease	0.11839
DB01023	Prostate cancer	0.01448
DB01023	Proteinuria	0.36255
DB01023	Rabies	0.01751
DB01023	Renal Cell cancer	0.1715
DB01023	Rheumatoid arthritis	0.0217
DB01023	Schistosomiasis	0.12762

DB01023	Stroke	0.03323
DB01023	Thyroid gland disease	0.07979
DB01023	Tuberculosis	0.06836
DB01115	Abortion	0.02653
DB01115	Achalasia and cardiospasm	0.11386
DB01115	Alzheimer's disease	0.07075
DB01115	Amyloidosis	0.06062
DB01115	Amyotrophic lateral sclerosis	0.08451
DB01115	Aortic aneurysm	0.04448
DB01115	Asthma	0.03991
DB01115	Atherosclerosis	0.02475
DB01115	Autistic disorder	0.05517
DB01115	Autoimmune disease	0.05088
DB01115	Bipolar disorder	0.11344
DB01115	Bladder cancer	0.04148
DB01115	Brain ischemia	0.16676
DB01115	Breast cancer	0.01701
DB01115	Cancer	0.06087
DB01115	Celiac disease	0.0776
DB01115	Colon cancer	0.01571
DB01115	Congenital abnormality	0.02956
DB01115	Congenital heart disease	0.11785
DB01115	Dental plaque	0.05975
DB01115	Depression	0.03475
DB01115	Diabetes mellitus	0.01141
DB01115	Drug abuse	0.02958
DB01115	Eating disorder	0.09511
DB01115	Embryoma	0.02845
DB01115	Endocrine system disease	0.13363
DB01115	Epilepsy	0.10887
DB01115	Epstein-Barr virus infection	0.08459
DB01115	Esophageal tumor	0.07027
DB01115	Esophagus cancer	0.02807
DB01115	Esotropia	0.08083
DB01115	Eye cancer	0.10019
DB01115	Fanconi's anemia	0.03954
DB01115	Glaucoma	0.06968
DB01115	Gram-Negative bacterial infection	0.13977
DB01115	Graves' disease	0.1
DB01115	HIV infection	0.04735
DB01115	Heart failure	0.10686
DB01115	Herpes	0.06841
DB01115	Ischemia	0.05802
DB01115	Keratosis	0.08019
DB01115	Leukemia	0.05869
DB01115	Lichen planus	0.15585
DB01115	Lung cancer	0.03342

DB01115	Lupus erythematosus	0.03283
DB01115	Lymphoma	0.05203
DB01115	Malignant glioma	0.04066
DB01115	Melanoma	0.02083
DB01115	Multiple sclerosis	0.05175
DB01115	Pre-Eclampsia	0.03786
DB01115	Prion disease	0.13064
DB01115	Prostate cancer	0.01846
DB01115	Rabies	0.01932
DB01115	Rheumatoid arthritis	0.02394
DB01115	Schistosomiasis	0.14082
DB01115	Schizophrenia	0.03462
DB01115	Stroke	0.03666
DB01115	Thyroid gland disease	0.08804
DB01115	Tuberculosis	0.07543
DB00524	Diabetes mellitus	0.05263
DB00524	Hypertension	0.07906
DB01324	Diabetes mellitus	0.05263
DB01324	Hypertension	0.07906
DB00288	Adrenoleukodystrophy	0.18898
DB00288	Cancer	0.02606
DB00288	Hamman-Rich syndrome	0.13608
DB00288	Hepatitis	0.18257
DB00288	Muscular dystrophies	0.13868
DB00288	Rheumatoid arthritis	0.04327
DB00741	Adrenoleukodystrophy	0.18898
DB00741	Cancer	0.02606
DB00741	Hamman-Rich syndrome	0.13608
DB00741	Hepatitis	0.18257
DB00741	Muscular dystrophies	0.13868
DB00741	Rheumatoid arthritis	0.04327
DB00122	Alzheimer's disease	0.02721
DB00122	Atherosclerosis	0.02646
DB00122	Brain tumor	0.20233
DB00122	Breast cancer	0.06927
DB00122	Colon cancer	0.06728
DB00122	Diabetes mellitus	0.01989
DB00122	Ewings sarcoma	0.12599
DB00122	Lung cancer	0.0264
DB00122	Melanoma	0.1305
DB00122	Neoplasm metastasis	0.03076
DB00122	Neurodegenerative disorder	0.06214
DB00122	Obesity	0.02907
DB00122	Ovarian cancer	0.03799
DB00122	Spinal dysraphism	0.08671
DB00122	Stomach cancer	0.07297
DB00122	Vitiligo	0.17817

DB00122	Yersinia infection	0.04486
DB00382	Alzheimer's disease	0.0509
DB00382	Atherosclerosis	0.04951
DB00382	Brain tumor	0.0568
DB00382	Colon cancer	0.08392
DB00382	Diabetes mellitus	0.03722
DB00382	Lung cancer	0.04939
DB00382	Neurodegenerative disorder	0.11625
DB00382	Obesity	0.05439
DB00382	Ovarian cancer	0.07107
DB00382	Vitiligo	0.33333
DB00483	Behavior disease	0.08909
DB00483	Bipolar disorder	0.06537
DB00483	Bladder cancer	0.07454
DB00483	Brain tumor	0.04637
DB00483	Central nervous system disease	0.14434
DB00483	Colon cancer	0.03426
DB00483	Depression	0.07581
DB00483	Epilepsy	0.08165
DB00483	Lung cancer	0.04032
DB00483	Neurodegenerative disorder	0.09492
DB00483	Ovarian cancer	0.05803
DB00483	Vitiligo	0.13608
DB00545	Alzheimer's disease	0.0509
DB00545	Atherosclerosis	0.04951
DB00545	Brain tumor	0.0568
DB00545	Colon cancer	0.08392
DB00545	Diabetes mellitus	0.03722
DB00545	Lung cancer	0.04939
DB00545	Neurodegenerative disorder	0.11625
DB00545	Obesity	0.05439
DB00545	Ovarian cancer	0.07107
DB00545	Vitiligo	0.33333
DB00677	Alzheimer's disease	0.0509
DB00677	Atherosclerosis	0.04951
DB00677	Brain tumor	0.0568
DB00677	Colon cancer	0.08392
DB00677	Diabetes mellitus	0.03722
DB00677	Lung cancer	0.04939
DB00677	Neurodegenerative disorder	0.11625
DB00677	Obesity	0.05439
DB00677	Ovarian cancer	0.07107
DB00677	Vitiligo	0.33333
DB00733	Alzheimer's disease	0.0509
DB00733	Atherosclerosis	0.04951
DB00733	Brain tumor	0.0568
DB00733	Colon cancer	0.08392

DB00733	Diabetes mellitus	0.03722
DB00733	Lung cancer	0.04939
DB00733	Neurodegenerative disorder	0.11625
DB00733	Obesity	0.05439
DB00733	Ovarian cancer	0.07107
DB00733	Vitiligo	0.33333
DB00843	Anorexia nervosa	0.14434
DB00843	Autistic disorder	0.08575
DB00843	Behavior disease	0.10911
DB00843	Bipolar disorder	0.08006
DB00843	Brain tumor	0.0568
DB00843	Choriocarcinoma	0.20412
DB00843	Chronic fatigue syndrome	0.25
DB00843	Colon cancer	0.08392
DB00843	Depression	0.09285
DB00843	Dermatitis	0.06428
DB00843	Drug-Induced dyskinesia	0.26726
DB00843	Hypertension	0.0559
DB00843	Lung cancer	0.04939
DB00843	Neurodegenerative disorder	0.11625
DB00843	Obesity	0.05439
DB00843	Obsessive-compulsive disorder	0.2132
DB00843	Ovarian cancer	0.07107
DB00843	Panic disorder	0.1543
DB00843	Psychotic disorder	0.11323
DB00843	Rheumatoid arthritis	0.04327
DB00843	Stroke	0.08006
DB00843	Vitiligo	0.16667
DB00944	Alzheimer's disease	0.0509
DB00944	Atherosclerosis	0.04951
DB00944	Brain tumor	0.0568
DB00944	Colon cancer	0.08392
DB00944	Diabetes mellitus	0.03722
DB00944	Lung cancer	0.04939
DB00944	Neurodegenerative disorder	0.11625
DB00944	Obesity	0.05439
DB00944	Ovarian cancer	0.07107
DB00944	Vitiligo	0.33333
DB00981	Brain tumor	0.08032
DB00981	Colon cancer	0.05934
DB00981	Lung cancer	0.06984
DB00981	Neurodegenerative disorder	0.1644
DB00981	Ovarian cancer	0.1005
DB00981	Vitiligo	0.2357
DB00989	Alzheimer's disease	0.0509
DB00989	Atherosclerosis	0.04951
DB00989	Brain tumor	0.0568

DB00989	Colon cancer	0.08392
DB00989	Diabetes mellitus	0.03722
DB00989	Lung cancer	0.04939
DB00989	Neurodegenerative disorder	0.11625
DB00989	Obesity	0.05439
DB00989	Ovarian cancer	0.07107
DB00989	Vitiligo	0.33333
DB01010	Alzheimer's disease	0.0509
DB01010	Atherosclerosis	0.04951
DB01010	Brain tumor	0.0568
DB01010	Colon cancer	0.08392
DB01010	Diabetes mellitus	0.03722
DB01010	Lung cancer	0.04939
DB01010	Neurodegenerative disorder	0.11625
DB01010	Obesity	0.05439
DB01010	Ovarian cancer	0.07107
DB01010	Vitiligo	0.33333
DB01122	Brain tumor	0.08032
DB01122	Colon cancer	0.05934
DB01122	Lung cancer	0.06984
DB01122	Neurodegenerative disorder	0.1644
DB01122	Ovarian cancer	0.1005
DB01122	Vitiligo	0.2357
DB01245	Brain tumor	0.0568
DB01245	Colon cancer	0.04196
DB01245	Epilepsy	0.1
DB01245	Lung cancer	0.04939
DB01245	Neurodegenerative disorder	0.11625
DB01245	Ovarian cancer	0.07107
DB01245	Vitiligo	0.16667
DB01400	Brain tumor	0.08032
DB01400	Colon cancer	0.05934
DB01400	Lung cancer	0.06984
DB01400	Neurodegenerative disorder	0.1644
DB01400	Ovarian cancer	0.1005
DB01400	Vitiligo	0.2357
DB01169	Hypertension, Pulmonary	0.34391
DB01169	Infertility, Male	0.12443
DB01169	AIDS	0.07753
DB01169	Adenoma	0.1102
DB01169	Adenovirus infection	0.26667
DB01169	Alzheimer's disease	0.06136
DB01169	Amyotrophic lateral sclerosis	0.04469
DB01169	Aortic valve disease	0.10193
DB01169	Aplastic anemia	0.02546
DB01169	Aseptic necrosis of bone	0.04153
DB01169	Atherosclerosis	0.1173

DB01169	Autoimmune disease	0.03061
DB01169	Azoospermia	0.02918
DB01169	Bipolar disorder	0.06873
DB01169	Bladder cancer	0.03166
DB01169	Brain tumor	0.00827
DB01169	Breast cancer	0.17929
DB01169	Bronchial hyperreactivity	0.12487
DB01169	Cancer	0.21366
DB01169	Capillaries disease	0.05371
DB01169	Cerebrovascular disorder	0.08584
DB01169	Cholelithiasis	0.1087
DB01169	Choriocarcinoma	0.05539
DB01169	Chronic rejection of renal transplant	0.20767
DB01169	Chronic simple glaucoma	0.15818
DB01169	Colon cancer	0.01614
DB01169	Congenital abnormality	0.15266
DB01169	Craniosynostosis	0.58844
DB01169	Dental plaque	0.04874
DB01169	Depression	0.06215
DB01169	Dermatitis	0.11154
DB01169	Diabetes mellitus	0.20138
DB01169	Down syndrome	0.07066
DB01169	Drug abuse	0.14507
DB01169	Eating disorder	0.07668
DB01169	Embryoma	0.05074
DB01169	Emphysema	0.07463
DB01169	Endemic goiter	0.7289
DB01169	Endometriosis	0.23741
DB01169	Esophagus cancer	0.03545
DB01169	Ewings sarcoma	0.05066
DB01169	Fanconi's anemia	0.03746
DB01169	Glaucoma	0.05684
DB01169	Glucose intolerance	0.11758
DB01169	Graves' disease	0.0284
DB01169	HIV infection	0.03768
DB01169	Hamman-Rich syndrome	0.42893
DB01169	Heart failure	0.05072
DB01169	Helicobacter infection	0.08845
DB01169	Hemolytic-Uremic syndrome	0.16886
DB01169	Hepatitis	0.42237
DB01169	Herpes	0.08506
DB01169	Hodgkin's disease	0.02262
DB01169	Huntington disease	0.2629
DB01169	Hyperglycemia	0.2174
DB01169	Hyperlipidemia	0.09515
DB01169	Hyperparathyroidism	0.54028
DB01169	Hypertension	0.14359

DB01169	Hypogonadism	0.10766
DB01169	Immunologic deficiency syndrome	0.03881
DB01169	Infection	0.06183
DB01169	Infectious lung disease	0.52717
DB01169	Infertility	0.0795
DB01169	Infiltrating cancer	0.05428
DB01169	Intracranial hypertension	0.08234
DB01169	Keratosis	0.47986
DB01169	Kidney failure	0.30975
DB01169	Late pregnancy	0.11788
DB01169	Leukemia	0.22841
DB01169	Leukoencephalopathy	0.02709
DB01169	Liver cancer	0.23689
DB01169	Lung cancer	0.01669
DB01169	Lupus erythematosus	0.14226
DB01169	Lupus vulgaris	0.07283
DB01169	Lymphoma	0.02966
DB01169	Metaplastic polyp	0.12583
DB01169	Migraine	0.09756
DB01169	Mucopolysaccharidosis	0.02374
DB01169	Multiple sclerosis	0.03508
DB01169	Muscular atrophy	0.04262
DB01169	Myasthenia Gravis	0.09114
DB01169	Myelofibrosis	0.14113
DB01169	Neck cancer	0.49882
DB01169	Neoplasm metastasis	0.14898
DB01169	Nephrosis	0.04897
DB01169	Neuroblastoma	0.0139
DB01169	Neurodegenerative disorder	0.04461
DB01169	Obesity	0.48066
DB01169	Oligospermia	0.05313
DB01169	Oral cancer	0.25776
DB01169	Osteitis deformans	0.60509
DB01169	Osteoporosis	0.07915
DB01169	Ovarian disease	0.0856
DB01169	Ovarian failure	0.11194
DB01169	Pancreatitis	0.04303
DB01169	Panic disorder	0.08598
DB01169	Papillomavirus infection	0.19678
DB01169	Parkinson disease	0.04168
DB01169	Phobic anxiety disorder	0.10766
DB01169	Pituitary tumor	0.0535
DB01169	Polyarthritis	0.40592
DB01169	Polycystic ovary syndrome	0.14166
DB01169	Primary biliary cirrhosis	0.06527
DB01169	Prostate cancer	0.22133
DB01169	Psychotic disorder	0.22411

DB01169	Rabies	0.07506
DB01169	Renal tubular acidosis	0.0494
DB01169	Rheumatism	0.01895
DB01169	Rheumatoid arthritis	0.16639
DB01169	Sarcoidosis	0.18995
DB01169	Schizophrenia	0.26357
DB01169	Scleroderma	0.03218
DB01169	Severe acute respiratory syndrome	0.02582
DB01169	Sicca syndrome	0.32272
DB01169	Squamous cell cancer	0.0217
DB01169	Stomach cancer	0.04305
DB01169	Stroke	0.05143
DB01169	Synovitis	0.09924
DB01169	Systemic infection	0.11522
DB01169	Systemic scleroderma	0.15481
DB01169	Thymoma	0.07702
DB01169	Tropical spastic paraparesis	0.36156
DB01169	Tuberculosis	0.04428
DB01169	Tuberous sclerosis	0.38552
DB01169	Ulcerative colitis	0.04136
DB01169	Urogenital abnormalities	0.06017
DB01169	Uterine disease	0.09936
DB01169	Vaccinia	0.3918
DB01169	Vascular disease	0.05547
DB01169	Virus disease	0.18786
DB01169	Vitiligo	0.3625
DB01169	Yersinia infection	0.26651
DB00270	Amyotrophic lateral sclerosis	0.05096
DB00270	Atherosclerosis	0.02646
DB00270	Breast cancer	0.01818
DB00270	Congenital heart disease	0.12599
DB00270	Endocrine system disease	0.14286
DB00270	Epilepsy	0.05345
DB00270	Lung cancer	0.0264
DB00270	Peptic ulcer	0.08909
DB00270	Prostate cancer	0.01973
DB00401	Amyotrophic lateral sclerosis	0.0603
DB00401	Atherosclerosis	0.03131
DB00401	Endocrine system disease	0.16903
DB01054	Amyotrophic lateral sclerosis	0.04767
DB01054	Atherosclerosis	0.02475
DB01054	Breast cancer	0.01701
DB01054	Congenital heart disease	0.11785
DB01054	Endocrine system disease	0.13363
DB01054	Epilepsy	0.05
DB01054	Lung cancer	0.02469
DB01054	Peptic ulcer	0.08333

DB01054	Prostate cancer	0.01846
DB04920	Amyotrophic lateral sclerosis	0.06742
DB04920	Atherosclerosis	0.03501
DB04920	Drug abuse	0.04683
DB04920	Endocrine system disease	0.18898
DB04920	Optic atrophy	0.22361
DB04920	Retinal disease	0.07001
DB06712	Amyotrophic lateral sclerosis	0.05505
DB06712	Atherosclerosis	0.02858
DB06712	Endocrine system disease	0.1543
DB06751	Atherosclerosis	0.04951
DB06751	Chronic obstructive airway disease	0.07906
DB06751	Leukemia	0.03984
DB00017	Multiple myeloma	0.13131
DB00017	Osteoporosis	0.15811
DB00017	Primary tumor	0.17678
DB00017	Prostate cancer	0.0522
DB01278	Atherosclerosis	0.11634
DB01278	Hypertension	0.22938
DB01278	Lung cancer	0.07815
DB01278	Migraine	0.28672
DB01278	Multiple myeloma	0.06565
DB01278	Osteoporosis	0.07906
DB01278	Primary tumor	0.08839
DB01278	Prostate cancer	0.0261
DB01278	Rheumatoid arthritis	0.11817
DB01381	Alzheimer's disease	0.02939
DB01381	Anorexia nervosa	0.08333
DB01381	Atherosclerosis	0.02858
DB01381	Behavior disease	0.06299
DB01381	Bipolar disorder	0.04623
DB01381	Cholelithiasis	0.08165
DB01381	Cystic fibrosis	0.05832
DB01381	Dental plaque	0.05185
DB01381	Diabetes mellitus	0.04297
DB01381	Drug abuse	0.07647
DB01381	Embryoma	0.02517
DB01381	Hyperopia	0.13608
DB01381	Hypertension	0.03227
DB01381	Multiple endocrine neoplasia	0.13608
DB01381	Neoplasm metastasis	0.03322
DB01381	Neuroendocrine tumor	0.14434
DB01381	Neurotic disorder	0.1291
DB01381	Panic disorder	0.08909
DB01381	Schizophrenia	0.03104
DB01381	Stomach cancer	0.03438
DB01381	Stroke	0.04623

DB01381	Vascular dementia	0.13608
DB00922	Atherosclerosis	0.03501
DB00922	Congenital abnormality	0.03769
DB00922	Diabetes mellitus	0.02632
DB00922	Hyperglycemia	0.08006
DB00922	Hyperinsulinism	0.09623
DB00922	Infantile spasms	0.18898
DB00922	Late pregnancy	0.28868
DB00922	Metabolism disease	0.08006
DB00922	Pancreas disease	0.10426
DB00922	Polycystic ovary syndrome	0.06623
DB01427	Alcoholic liver disease	0.2357
DB01427	Alzheimer's disease	0.04156
DB01427	Amyloidosis	0.10911
DB01427	Anemia	0.12309
DB01427	Anorexia nervosa	0.11785
DB01427	Aplastic anemia	0.12039
DB01427	Arthritis	0.07161
DB01427	Asthma	0.04714
DB01427	Atherosclerosis	0.04042
DB01427	Autoimmune disease	0.06262
DB01427	Basal cell carcinoma	0.16013
DB01427	Behavior disease	0.08909
DB01427	Behcet syndrome	0.08248
DB01427	Bladder cancer	0.07454
DB01427	Breast cancer	0.02778
DB01427	Celiac disease	0.09492
DB01427	Cervical cancer	0.06712
DB01427	Colon cancer	0.03426
DB01427	Depression	0.07581
DB01427	Dermatitis	0.05249
DB01427	Diabetes mellitus	0.03039
DB01427	Down syndrome	0.0658
DB01427	Embryoma	0.0356
DB01427	Endometriosis	0.04795
DB01427	Enteritis	0.06337
DB01427	Familial Mediterranean fever	0.14907
DB01427	Fanconi's anemia	0.11547
DB01427	Granulomatous disease	0.16667
DB01427	HIV infection	0.0553
DB01427	Heart failure	0.06155
DB01427	Hepatitis B	0.14003
DB01427	Histiocytosis	0.19245
DB01427	Hypothyroidism	0.13608
DB01427	Infertility	0.07931
DB01427	Intermediate coronary syndrome	0.19245
DB01427	Kidney disease	0.06901

DB01427	Leprosy	0.1543
DB01427	Leukemia	0.06506
DB01427	Lichen planus	0.20412
DB01427	Lipodystrophy	0.16013
DB01427	Liver cancer	0.04481
DB01427	Lupus erythematosus	0.04933
DB01427	Malaria	0.10721
DB01427	Malignant glioma	0.10721
DB01427	Melanoma	0.04811
DB01427	Migraine	0.10721
DB01427	Mucocutaneous lymph node syndrome	0.12599
DB01427	Multiple myeloma	0.07581
DB01427	Multiple sclerosis	0.05955
DB01427	Muscular dystrophies	0.11323
DB01427	Mycoses	0.2582
DB01427	Narcolepsy	0.21822
DB01427	Nasopharyngeal cancer	0.11111
DB01427	Necrotizing enterocolitis	0.2357
DB01427	Nephrosis	0.1291
DB01427	Obesity	0.04441
DB01427	Otitis media	0.19245
DB01427	Ovary cancer	0.1291
DB01427	Pancreas cancer	0.05987
DB01427	Pancreatitis	0.09017
DB01427	Periodontitis	0.08805
DB01427	Polycystic ovary syndrome	0.07647
DB01427	Prostate cancer	0.03014
DB01427	Psoriasis	0.13608
DB01427	Pulmonary fibrosis	0.10911
DB01427	Renal Cell cancer	0.06712
DB01427	Rheumatoid arthritis	0.03533
DB01427	Sarcoidosis	0.11323
DB01427	Schizophrenia	0.0439
DB01427	Silicosis	0.21822
DB01427	Stomach cancer	0.04862
DB01427	Stroke	0.06537
DB01427	Systemic infection	0.06623
DB01427	Thrombophilia	0.13608
DB01427	Thyroid cancer	0.1005
DB01427	Tuberculosis	0.07785
DB01427	Tuberous sclerosis	0.14907
DB01427	Ulcerative colitis	0.05955
DB05266	Atherosclerosis	0.10088
DB05266	Behavior disease	0.06901
DB05266	Cancer	0.02656
DB05266	Cardiovascular disease	0.30934
DB05266	Chronic obstructive airway disease	0.24895

DB05266	Depression	0.05872
DB05266	Leukemia	0.1289
DB08811	Chronic obstructive airway disease	0.0559
DB08811	Leukemia	0.02817
DB08811	Systemic infection	0.05735
DB00054	Hemorrhagic fevers, Viral	1.06125
DB00054	Pemphigoid, Bullous	0.65664
DB00054	Pleural effusion, Malignant	0.28244
DB00054	Purpura, Thrombocytopenic, Idiopathic	0.68455
DB00054	Abortion	0.03467
DB00054	Adenovirus infection	0.02572
DB00054	Alopecia	0.08445
DB00054	Alzheimer's disease	0.18334
DB00054	Angiomyolipoma	0.04199
DB00054	Antiphospholipid syndrome	0.09245
DB00054	Aortic aneurysm	0.01857
DB00054	Aplastic anemia	1.03229
DB00054	Asthma	0.50554
DB00054	Atherosclerosis	0.67957
DB00054	Atopic rhinitis	0.05796
DB00054	Autistic disorder	0.65818
DB00054	Autoimmune disease	0.36824
DB00054	Barrett's esophagus	0.0199
DB00054	Biliary Atresia	0.05598
DB00054	Bone metastases	0.04793
DB00054	Brain tumor	0.01762
DB00054	Breast cancer	0.07319
DB00054	Brucellosis	0.08362
DB00054	Cancer	0.22011
DB00054	Celiac disease	0.0456
DB00054	Cervical cancer	0.0268
DB00054	Cholelithiasis	0.03324
DB00054	Colon cancer	0.01182
DB00054	Common wart	0.05147
DB00054	Communicable disease	0.06052
DB00054	Cystic fibrosis	0.03962
DB00054	Cytomegalovirus infection	0.04374
DB00054	Dental plaque	0.0216
DB00054	Diabetes mellitus	0.35858
DB00054	Drug abuse	0.19203
DB00054	Eating disorder	0.01843
DB00054	Embryoma	0.0171
DB00054	Endometriosis	0.01134
DB00054	Enteritis	0.13341
DB00054	Esotropia	0.02922
DB00054	Gastrointestinal tumor	0.0319
DB00054	Generalized anxiety disorder	0.03208

DB00054	Glaucoma	0.02519
DB00054	Glomerulonephritis	0.06934
DB00054	HIV infection	0.01156
DB00054	Heart failure	0.04083
DB00054	Hemorrhagic disorder	0.96336
DB00054	Hepatitis C	0.02819
DB00054	Herpes	0.238
DB00054	Hypercholesterolemia	0.04761
DB00054	Hypertension	0.01455
DB00054	IGA glomerulonephritis	0.04757
DB00054	Immune complex disease	0.35189
DB00054	Infection by cryptococcus neoformans	0.32026
DB00054	Infertility	0.05869
DB00054	Inflammation of the central nervous system	0.06464
DB00054	Intermediate coronary syndrome	1.03816
DB00054	Kaposi sarcoma	0.02824
DB00054	Kidney disease	0.08616
DB00054	Kidney failure	0.05274
DB00054	Leukemia	0.00959
DB00054	Lung cancer	0.01208
DB00054	Lupus erythematosus	0.5679
DB00054	Lupus vulgaris	0.08469
DB00054	Malignant glioma	0.0306
DB00054	Melanoma	0.02311
DB00054	Multiple myeloma	0.02539
DB00054	Multiple sclerosis	0.05021
DB00054	Neoplasm metastasis	0.03812
DB00054	Nervous system tumor	0.07353
DB00054	Obesity	0.01588
DB00054	Optic atrophy	0.05528
DB00054	Oral cancer	0.01559
DB00054	Osteoporosis	0.04516
DB00054	Ovarian cancer	0.09753
DB00054	Pancreatitis	0.0265
DB00054	Papillary adenocarcinoma	0.05796
DB00054	Parkinson disease	0.01847
DB00054	Penile disease	0.98544
DB00054	Periodontal disease	0.06537
DB00054	Periodontitis	0.08459
DB00054	Pertussis	0.04928
DB00054	Polyarthritis	0.0138
DB00054	Primary hyperparathyroidism	0.04128
DB00054	Prostate cancer	0.01691
DB00054	Protein-energy malnutrition	0.10889
DB00054	Renal Cell cancer	0.06002
DB00054	Rheumatic fever	0.07161
DB00054	Rheumatoid arthritis	0.1944

DB00054	Schizophrenia	0.23115
DB00054	Sickle cell disease	0.02442
DB00054	Skin cancer	0.02605
DB00054	Skin tumor	0.04822
DB00054	Squamous cell cancer	0.02393
DB00054	Stomach cancer	0.02156
DB00054	Stroke	0.55346
DB00054	Subarachnoid hemorrhage	0.93011
DB00054	Systemic infection	0.06153
DB00054	Takayasu's arteritis	1.09838
DB00054	Temporal arteritis	1.04936
DB00054	Testicular dysfunction	0.01608
DB00054	Thrombocytopenia	1.0303
DB00054	Thrombophlebitis	0.04269
DB00054	Ulcerative colitis	0.01568
DB00054	Vascular disease	0.05064
DB00054	Wiskott-Aldrich syndrome	0.04832
DB00054	Yersinia infection	0.02836
DB00775	Hemorrhagic fevers, Viral	1.27272
DB00775	Pemphigoid, Bullous	0.72312
DB00775	Pleural effusion, Malignant	0.27226
DB00775	Purpura, Thrombocytopenic, Idiopathic	0.93464
DB00775	Adenovirus infection	0.02707
DB00775	Alzheimer's disease	0.15721
DB00775	Angiomyolipoma	0.04418
DB00775	Aortic aneurysm	0.01954
DB00775	Aplastic anemia	1.24633
DB00775	Asthma	0.53546
DB00775	Atherosclerosis	0.6797
DB00775	Atopic rhinitis	0.06099
DB00775	Autistic disorder	0.73403
DB00775	Autoimmune disease	0.33757
DB00775	Barrett's esophagus	0.02094
DB00775	Biliary Atresia	0.05891
DB00775	Bone metastases	0.05044
DB00775	Brain tumor	0.01854
DB00775	Breast cancer	0.00851
DB00775	Cancer	0.23891
DB00775	Cervical cancer	0.0282
DB00775	Cholelithiasis	0.03497
DB00775	Colon cancer	0.01244
DB00775	Common wart	0.05416
DB00775	Cytomegalovirus infection	0.04603
DB00775	Dental plaque	0.02273
DB00775	Diabetes mellitus	0.41633
DB00775	Drug abuse	0.18511
DB00775	Eating disorder	0.01939

DB00775	Endometriosis	0.01193
DB00775	Enteritis	0.02663
DB00775	Esotropia	0.03075
DB00775	Gastrointestinal tumor	0.03357
DB00775	Glaucoma	0.02651
DB00775	HIV infection	0.01217
DB00775	Heart failure	0.04297
DB00775	Hemorrhagic disorder	1.14818
DB00775	Hepatitis C	0.02967
DB00775	Herpes	0.22942
DB00775	Hypercholesterolemia	0.0501
DB00775	Hypertension	0.01531
DB00775	Infertility	0.06175
DB00775	Inflammation of the central nervous system	0.06803
DB00775	Intermediate coronary syndrome	1.21734
DB00775	Kidney disease	0.05579
DB00775	Kidney failure	0.02245
DB00775	Lung cancer	0.01272
DB00775	Lupus erythematosus	0.46653
DB00775	Lupus vulgaris	0.03397
DB00775	Malignant glioma	0.0322
DB00775	Multiple sclerosis	0.02273
DB00775	Neoplasm metastasis	0.01636
DB00775	Nervous system tumor	0.07737
DB00775	Obesity	0.01671
DB00775	Optic atrophy	0.05817
DB00775	Oral cancer	0.0164
DB00775	Osteoporosis	0.04753
DB00775	Ovarian cancer	0.04232
DB00775	Pancreatitis	0.02788
DB00775	Papillary adenocarcinoma	0.06099
DB00775	Parkinson disease	0.01944
DB00775	Penile disease	1.1711
DB00775	Pertussis	0.05186
DB00775	Polyarthritis	0.01452
DB00775	Primary hyperparathyroidism	0.04344
DB00775	Prostate cancer	0.0178
DB00775	Protein-energy malnutrition	0.11458
DB00775	Renal Cell cancer	0.02923
DB00775	Rheumatoid arthritis	0.17317
DB00775	Schizophrenia	0.27481
DB00775	Sickle cell disease	0.0257
DB00775	Skin cancer	0.02741
DB00775	Squamous cell cancer	0.02518
DB00775	Stroke	0.66942
DB00775	Subarachnoid hemorrhage	1.09184
DB00775	Takayasu's arteritis	1.40524

DB00775	Temporal arteritis	1.2767
DB00775	Testicular dysfunction	0.01693
DB00775	Thrombocytopenia	1.20708
DB00775	Thrombophlebitis	0.04492
DB00775	Ulcerative colitis	0.0165
DB00775	Wiskott-Aldrich syndrome	0.05084
DB00775	Yersinia infection	0.01599
DB00283	Atopic rhinitis	0.2582
DB00283	Colon cancer	0.05934
DB00283	Infertility	0.13736
DB00283	Parkinson disease	0.11043
DB00283	Schizophrenia	0.07603
DB00341	Atopic rhinitis	0.2582
DB00341	Colon cancer	0.05934
DB00341	Infertility	0.13736
DB00341	Parkinson disease	0.11043
DB00341	Schizophrenia	0.07603
DB00405	Atopic rhinitis	0.2582
DB00405	Colon cancer	0.05934
DB00405	Infertility	0.13736
DB00405	Parkinson disease	0.11043
DB00405	Schizophrenia	0.07603
DB00427	Atopic rhinitis	0.2582
DB00427	Colon cancer	0.05934
DB00427	Infertility	0.13736
DB00427	Parkinson disease	0.11043
DB00427	Schizophrenia	0.07603
DB00455	Atopic rhinitis	0.2582
DB00455	Colon cancer	0.05934
DB00455	Infertility	0.13736
DB00455	Parkinson disease	0.11043
DB00455	Schizophrenia	0.07603
DB00557	Atopic rhinitis	0.2582
DB00557	Colon cancer	0.05934
DB00557	Infertility	0.13736
DB00557	Parkinson disease	0.11043
DB00557	Schizophrenia	0.07603
DB00719	Atopic rhinitis	0.2582
DB00719	Colon cancer	0.05934
DB00719	Infertility	0.13736
DB00719	Parkinson disease	0.11043
DB00719	Schizophrenia	0.07603
DB00737	Atopic rhinitis	0.2582
DB00737	Colon cancer	0.05934
DB00737	Infertility	0.13736
DB00737	Parkinson disease	0.11043
DB00737	Schizophrenia	0.07603

DB00748	Atopic rhinitis	0.2582
DB00748	Colon cancer	0.05934
DB00748	Infertility	0.13736
DB00748	Parkinson disease	0.11043
DB00748	Schizophrenia	0.07603
DB00768	Alzheimer's disease	0.02939
DB00768	Anorexia nervosa	0.08333
DB00768	Arthritis	0.05064
DB00768	Atopic rhinitis	0.10541
DB00768	Behavior disease	0.06299
DB00768	Bipolar disorder	0.04623
DB00768	Brain disease	0.07332
DB00768	Brain tumor	0.03279
DB00768	Cancer	0.01505
DB00768	Celiac disease	0.06712
DB00768	Colon cancer	0.02423
DB00768	Demyelinating disease	0.18257
DB00768	Down syndrome	0.04652
DB00768	Endometriosis	0.0339
DB00768	Enteritis	0.04481
DB00768	Growth retardation	0.07857
DB00768	Heart failure	0.04352
DB00768	Infection	0.03807
DB00768	Infertility	0.05608
DB00768	Intraocular melanoma	0.1291
DB00768	Ischemia	0.05227
DB00768	Keratoconus	0.1543
DB00768	Lupus erythematosus	0.03488
DB00768	Meningioma	0.10541
DB00768	Mucocutaneous lymph node syndrome	0.08909
DB00768	Neck cancer	0.1291
DB00768	Neoplasm metastasis	0.03322
DB00768	Parkinson disease	0.04508
DB00768	Polyneuropathy	0.10911
DB00768	Psoriasis	0.09623
DB00768	Psychotic disorder	0.06537
DB00768	Renal Cell cancer	0.04746
DB00768	Respiratory tract disease	0.08704
DB00768	Schizophrenia	0.03104
DB00768	Skin cancer	0.09366
DB00768	Spinal cord disease	0.09129
DB00792	Atopic rhinitis	0.2582
DB00792	Colon cancer	0.05934
DB00792	Infertility	0.13736
DB00792	Parkinson disease	0.11043
DB00792	Schizophrenia	0.07603
DB00902	Atopic rhinitis	0.2582

DB00902	Colon cancer	0.05934
DB00902	Infertility	0.13736
DB00902	Parkinson disease	0.11043
DB00902	Schizophrenia	0.07603
DB00920	Atherosclerosis	0.09171
DB00920	Atopic rhinitis	0.08165
DB00920	Behavior disease	0.0488
DB00920	Cancer	0.02656
DB00920	Cardiovascular disease	0.28936
DB00920	Chronic obstructive airway disease	0.21966
DB00920	Colon cancer	0.01876
DB00920	Depression	0.04152
DB00920	Infertility	0.04344
DB00920	Leukemia	0.10676
DB00920	Parkinson disease	0.03492
DB00920	Schizophrenia	0.02404
DB00950	Atopic rhinitis	0.2582
DB00950	Colon cancer	0.05934
DB00950	Infertility	0.13736
DB00950	Parkinson disease	0.11043
DB00950	Schizophrenia	0.07603
DB00967	Atopic rhinitis	0.2582
DB00967	Colon cancer	0.05934
DB00967	Infertility	0.13736
DB00967	Parkinson disease	0.11043
DB00967	Schizophrenia	0.07603
DB00972	Atopic rhinitis	0.2582
DB00972	Colon cancer	0.05934
DB00972	Infertility	0.13736
DB00972	Parkinson disease	0.11043
DB00972	Schizophrenia	0.07603
DB00985	Atopic rhinitis	0.2582
DB00985	Colon cancer	0.05934
DB00985	Infertility	0.13736
DB00985	Parkinson disease	0.11043
DB00985	Schizophrenia	0.07603
DB01071	Atopic rhinitis	0.2582
DB01071	Colon cancer	0.05934
DB01071	Infertility	0.13736
DB01071	Parkinson disease	0.11043
DB01071	Schizophrenia	0.07603
DB01075	Atopic rhinitis	0.2582
DB01075	Colon cancer	0.05934
DB01075	Infertility	0.13736
DB01075	Parkinson disease	0.11043
DB01075	Schizophrenia	0.07603
DB01084	Atopic rhinitis	0.2582

DB01084	Colon cancer	0.05934
DB01084	Infertility	0.13736
DB01084	Parkinson disease	0.11043
DB01084	Schizophrenia	0.07603
DB01106	Atopic rhinitis	0.18257
DB01106	Colon cancer	0.04196
DB01106	Infertility	0.09713
DB01106	Parkinson disease	0.07809
DB01106	Schizophrenia	0.05376
DB01114	Hypertension, Pulmonary	0.11471
DB01114	Stress disorder, post-traumatic	0.17678
DB01114	Anorexia nervosa	0.20412
DB01114	Atherosclerosis	0.03501
DB01114	Atopic rhinitis	0.1291
DB01114	Autistic disorder	0.06063
DB01114	Behavior disease	0.23146
DB01114	Bipolar disorder	0.16984
DB01114	Brain disease	0.0898
DB01114	Breast cancer	0.02406
DB01114	Chronic fatigue syndrome	0.17678
DB01114	Chronic obstructive airway disease	0.0559
DB01114	Colon cancer	0.05934
DB01114	Congenital heart disease	0.16667
DB01114	Depression	0.13131
DB01114	Dermatitis	0.04545
DB01114	Diabetes mellitus	0.02632
DB01114	Drug abuse	0.14049
DB01114	Epilepsy	0.07071
DB01114	Fibromyalgia	0.17678
DB01114	Generalized anxiety disorder	0.14434
DB01114	Gilles de la Tourette syndrome	0.18898
DB01114	Heart failure	0.0533
DB01114	Herpes	0.14286
DB01114	Hypertension	0.03953
DB01114	Infertility	0.06868
DB01114	Migraine	0.1857
DB01114	Multiple endocrine neoplasia	0.16667
DB01114	Neuroendocrine tumor	0.17678
DB01114	Neurotic disorder	0.15811
DB01114	Obesity	0.07692
DB01114	Obsessive-compulsive disorder	0.15076
DB01114	Panic disorder	0.21822
DB01114	Parkinson disease	0.05522
DB01114	Pervasive development disorder	0.1291
DB01114	Psychotic disorder	0.16013
DB01114	Pulmonary hypertension	0.25
DB01114	Schizophrenia	0.03801

DB01114	Stroke	0.05661
DB01114	Sudden infant death syndrome	0.10911
DB01114	Ulcerative colitis	0.05157
DB01146	Stress disorder, post-traumatic	0.25
DB01146	Atopic rhinitis	0.18257
DB01146	Behavior disease	0.10911
DB01146	Bipolar disorder	0.08006
DB01146	Brain disease	0.127
DB01146	Breast cancer	0.03402
DB01146	Colon cancer	0.04196
DB01146	Depression	0.09285
DB01146	Drug abuse	0.06623
DB01146	Gilles de la Tourette syndrome	0.26726
DB01146	Herpes	0.10102
DB01146	Infertility	0.09713
DB01146	Migraine	0.13131
DB01146	Obesity	0.05439
DB01146	Parkinson disease	0.07809
DB01146	Psychotic disorder	0.11323
DB01146	Schizophrenia	0.05376
DB01176	Atopic rhinitis	0.18257
DB01176	Colon cancer	0.04196
DB01176	Infertility	0.09713
DB01176	Parkinson disease	0.07809
DB01176	Schizophrenia	0.05376
DB01237	Atopic rhinitis	0.2582
DB01237	Colon cancer	0.05934
DB01237	Infertility	0.13736
DB01237	Parkinson disease	0.11043
DB01237	Schizophrenia	0.07603
DB01246	Atopic rhinitis	0.2582
DB01246	Colon cancer	0.05934
DB01246	Infertility	0.13736
DB01246	Parkinson disease	0.11043
DB01246	Schizophrenia	0.07603
DB01615	Atopic rhinitis	0.2582
DB01615	Colon cancer	0.05934
DB01615	Infertility	0.13736
DB01615	Parkinson disease	0.11043
DB01615	Schizophrenia	0.07603
DB01619	Atopic rhinitis	0.2582
DB01619	Colon cancer	0.05934
DB01619	Infertility	0.13736
DB01619	Parkinson disease	0.11043
DB01619	Schizophrenia	0.07603
DB01620	Atopic rhinitis	0.2582
DB01620	Colon cancer	0.05934

DB01620	Infertility	0.13736
DB01620	Parkinson disease	0.11043
DB01620	Schizophrenia	0.07603
DB04837	Atopic rhinitis	0.2582
DB04837	Colon cancer	0.05934
DB04837	Infertility	0.13736
DB04837	Parkinson disease	0.11043
DB04837	Schizophrenia	0.07603
DB04890	Atopic rhinitis	0.2582
DB04890	Colon cancer	0.05934
DB04890	Infertility	0.13736
DB04890	Parkinson disease	0.11043
DB04890	Schizophrenia	0.07603
DB06691	Atopic rhinitis	0.2582
DB06691	Colon cancer	0.05934
DB06691	Infertility	0.13736
DB06691	Parkinson disease	0.11043
DB06691	Schizophrenia	0.07603
DB06698	Atopic rhinitis	0.18257
DB06698	Colon cancer	0.04196
DB06698	Infertility	0.09713
DB06698	Obesity	0.05439
DB06698	Pancreas cancer	0.07332
DB06698	Parkinson disease	0.07809
DB06698	Schizophrenia	0.05376
DB06766	Atopic rhinitis	0.2582
DB06766	Colon cancer	0.05934
DB06766	Infertility	0.13736
DB06766	Parkinson disease	0.11043
DB06766	Schizophrenia	0.07603
DB08799	Atopic rhinitis	0.2582
DB08799	Colon cancer	0.05934
DB08799	Infertility	0.13736
DB08799	Parkinson disease	0.11043
DB08799	Schizophrenia	0.07603
DB08800	Atopic rhinitis	0.2582
DB08800	Colon cancer	0.05934
DB08800	Infertility	0.13736
DB08800	Parkinson disease	0.11043
DB08800	Schizophrenia	0.07603
DB08801	Atopic rhinitis	0.18257
DB08801	Behavior disease	0.10911
DB08801	Bipolar disorder	0.08006
DB08801	Bladder cancer	0.09129
DB08801	Central nervous system disease	0.17678
DB08801	Colon cancer	0.04196
DB08801	Depression	0.09285

DB08801	Infertility	0.09713
DB08801	Parkinson disease	0.07809
DB08801	Schizophrenia	0.05376
DB08802	Atopic rhinitis	0.2582
DB08802	Colon cancer	0.05934
DB08802	Infertility	0.13736
DB08802	Parkinson disease	0.11043
DB08802	Schizophrenia	0.07603
DB08936	Atopic rhinitis	0.2582
DB08936	Colon cancer	0.05934
DB08936	Infertility	0.13736
DB08936	Parkinson disease	0.11043
DB08936	Schizophrenia	0.07603
DB01436	Adenovirus infection	0.03462
DB01436	Adrenal gland hyperfunction	0.11305
DB01436	Adrenal gland tumor	0.16198
DB01436	Autistic disorder	0.0606
DB01436	Autoimmune disease	0.0767
DB01436	Brain disease	0.08812
DB01436	Breast cancer	0.08318
DB01436	Cancer	0.02053
DB01436	Diabetes mellitus	0.03722
DB01436	Drug abuse	0.03249
DB01436	Endometrium cancer	0.1715
DB01436	HIV infection	0.03513
DB01436	Hereditary disease	0.06402
DB01436	Lung cancer	0.04939
DB01436	Mental retardation	0.09563
DB01436	Neoplasm metastasis	0.09446
DB01436	Osteoporosis	0.1118
DB01436	Prostate cancer	0.1148
DB01436	Renal tubular acidosis	0.13306
DB01436	Respiratory tract disease	0.10686
DB01436	Schizophrenia	0.10757
DB01436	Seminoma	0.26726
DB00924	Anorexia nervosa	0.20412
DB00924	Autistic disorder	0.12127
DB00924	Behavior disease	0.1543
DB00924	Bipolar disorder	0.11323
DB00924	Choriocarcinoma	0.28868
DB00924	Chronic fatigue syndrome	0.35355
DB00924	Colon cancer	0.05934
DB00924	Depression	0.13131
DB00924	Dermatitis	0.09091
DB00924	Drug-Induced dyskinesia	0.37796
DB00924	Hypertension	0.07906
DB00924	Obesity	0.07692

DB00924	Obsessive-compulsive disorder	0.30151
DB00924	Panic disorder	0.21822
DB00924	Psychotic disorder	0.16013
DB00924	Rheumatoid arthritis	0.0612
DB00924	Stroke	0.11323
DB00933	Anorexia nervosa	0.14434
DB00933	Autistic disorder	0.08575
DB00933	Behavior disease	0.10911
DB00933	Bipolar disorder	0.08006
DB00933	Choriocarcinoma	0.20412
DB00933	Chronic fatigue syndrome	0.25
DB00933	Colon cancer	0.04196
DB00933	Depression	0.09285
DB00933	Dermatitis	0.06428
DB00933	Drug-Induced dyskinesia	0.26726
DB00933	Hypertension	0.0559
DB00933	Obesity	0.05439
DB00933	Obsessive-compulsive disorder	0.2132
DB00933	Panic disorder	0.1543
DB00933	Psychotic disorder	0.11323
DB00933	Rheumatoid arthritis	0.04327
DB00933	Stroke	0.08006
DB04842	Anorexia nervosa	0.11785
DB04842	Autistic disorder	0.07001
DB04842	Behavior disease	0.08909
DB04842	Bipolar disorder	0.06537
DB04842	Choriocarcinoma	0.16667
DB04842	Chronic fatigue syndrome	0.20412
DB04842	Colon cancer	0.03426
DB04842	Depression	0.07581
DB04842	Dermatitis	0.05249
DB04842	Drug-Induced dyskinesia	0.21822
DB04842	Hypertension	0.04564
DB04842	Obesity	0.04441
DB04842	Obsessive-compulsive disorder	0.17408
DB04842	Panic disorder	0.12599
DB04842	Psychotic disorder	0.09245
DB04842	Rheumatoid arthritis	0.03533
DB04842	Stroke	0.06537
DB06288	Adenocarcinoma	0.07293
DB06288	Anorexia nervosa	0.10206
DB06288	Autistic disorder	0.06063
DB06288	Behavior disease	0.07715
DB06288	Bipolar disorder	0.05661
DB06288	Choriocarcinoma	0.14434
DB06288	Chronic fatigue syndrome	0.17678
DB06288	Colon cancer	0.02967

DB06288	Depression	0.06565
DB06288	Dermatitis	0.04545
DB06288	Drug-Induced dyskinesia	0.18898
DB06288	Hypertension	0.07906
DB06288	Obesity	0.03846
DB06288	Obsessive-compulsive disorder	0.15076
DB06288	Panic disorder	0.10911
DB06288	Psychotic disorder	0.08006
DB06288	Rheumatoid arthritis	0.0306
DB06288	Schizophrenia	0.03801
DB06288	Stroke	0.05661
DB00953	Abortion	0.04986
DB00953	Amyotrophic lateral sclerosis	0.07682
DB00953	Anorexia nervosa	0.80132
DB00953	Attention deficit hyperactivity disorder	0.69606
DB00953	Behavior disease	0.53285
DB00953	Bipolar disorder	0.36075
DB00953	Depression	0.27198
DB00953	Dermatitis	0.06891
DB00953	Diabetes mellitus	0.04291
DB00953	Drug abuse	0.15777
DB00953	Epilepsy	0.12278
DB00953	Hepatitis C	0.14085
DB00953	Hypertension	0.27285
DB00953	Migraine	0.14904
DB00953	Panic disorder	0.19038
DB00953	Stroke	0.06622
DB00953	Sudden infant death syndrome	0.22763
DB00033	Celiac disease	0.11625
DB00033	Communicable disease	0.1543
DB00033	Helicobacter infection	0.15811
DB00033	Infection	0.06594
DB00033	Leukemia	0.03984
DB00033	Lupus erythematosus	0.12082
DB00033	Osteomyelitis	0.2357
DB00033	Parasitic disease	0.35355
DB00033	Pre-Eclampsia	0.08333
DB00033	Rheumatoid arthritis	0.04327
DB00033	Stomach cancer	0.05955
DB01205	Breast cancer	0.01104
DB01205	Drug abuse	0.02149
DB01205	Hypogonadism	0.1026
DB01205	Yersinia infection	0.02723
DB00347	Epilepsy	0.14142
DB00593	Epilepsy	0.14142
DB05246	Epilepsy	0.14142
DB00191	Hypertension, Pulmonary	0.1026

DB00191	Stress disorder, post-traumatic	0.31623
DB00191	Anorexia nervosa	0.27386
DB00191	Atherosclerosis	0.03131
DB00191	Autistic disorder	0.10847
DB00191	Behavior disease	0.34503
DB00191	Bipolar disorder	0.20255
DB00191	Brain disease	0.08032
DB00191	Breast cancer	0.04303
DB00191	Chronic fatigue syndrome	0.15811
DB00191	Chronic obstructive airway disease	0.05
DB00191	Colon cancer	0.02654
DB00191	Congenital heart disease	0.14907
DB00191	Depression	0.17617
DB00191	Dermatitis	0.04066
DB00191	Diabetes mellitus	0.02354
DB00191	Down syndrome	0.05096
DB00191	Drug abuse	0.16754
DB00191	Encephalopathies	0.06262
DB00191	Epilepsy	0.06325
DB00191	Fibromyalgia	0.47434
DB00191	Generalized anxiety disorder	0.2582
DB00191	Gilles de la Tourette syndrome	0.16903
DB00191	Heart failure	0.04767
DB00191	Herpes	0.12778
DB00191	Huntington disease	0.09759
DB00191	Hypertension	0.03536
DB00191	Migraine	0.16609
DB00191	Multiple endocrine neoplasia	0.14907
DB00191	Neuroendocrine tumor	0.15811
DB00191	Neurotic disorder	0.28284
DB00191	Obesity	0.1032
DB00191	Obsessive-compulsive disorder	0.13484
DB00191	Panic disorder	0.29277
DB00191	Pervasive development disorder	0.11547
DB00191	Psychotic disorder	0.21483
DB00191	Pulmonary hypertension	0.22361
DB00191	Stroke	0.05064
DB00191	Sudden infant death syndrome	0.19518
DB00191	Ulcerative colitis	0.04613
DB00226	Anorexia nervosa	0.20412
DB00226	Behavior disease	0.1543
DB00226	Bipolar disorder	0.11323
DB00226	Diabetes mellitus	0.05263
DB00226	Drug abuse	0.09366
DB00226	Hypertension	0.07906
DB00226	Multiple endocrine neoplasia	0.33333
DB00226	Neuroendocrine tumor	0.35355

DB00226	Panic disorder	0.21822
DB00234	Anorexia nervosa	0.20412
DB00234	Behavior disease	0.1543
DB00234	Bipolar disorder	0.11323
DB00234	Diabetes mellitus	0.05263
DB00234	Drug abuse	0.09366
DB00234	Hypertension	0.07906
DB00234	Multiple endocrine neoplasia	0.33333
DB00234	Neuroendocrine tumor	0.35355
DB00234	Panic disorder	0.21822
DB00285	Hypertension, Pulmonary	0.13245
DB00285	Stress disorder, post-traumatic	0.20412
DB00285	Anorexia nervosa	0.2357
DB00285	Atherosclerosis	0.04042
DB00285	Autistic disorder	0.07001
DB00285	Behavior disease	0.26726
DB00285	Bipolar disorder	0.19612
DB00285	Brain disease	0.1037
DB00285	Breast cancer	0.02778
DB00285	Chronic fatigue syndrome	0.20412
DB00285	Chronic obstructive airway disease	0.06455
DB00285	Colon cancer	0.03426
DB00285	Congenital heart disease	0.19245
DB00285	Depression	0.15162
DB00285	Dermatitis	0.05249
DB00285	Diabetes mellitus	0.03039
DB00285	Drug abuse	0.16222
DB00285	Epilepsy	0.08165
DB00285	Fibromyalgia	0.20412
DB00285	Generalized anxiety disorder	0.16667
DB00285	Gilles de la Tourette syndrome	0.21822
DB00285	Heart failure	0.06155
DB00285	Herpes	0.16496
DB00285	Hypertension	0.04564
DB00285	Migraine	0.21442
DB00285	Multiple endocrine neoplasia	0.19245
DB00285	Neuroendocrine tumor	0.20412
DB00285	Neurotic disorder	0.18257
DB00285	Obesity	0.08882
DB00285	Obsessive-compulsive disorder	0.17408
DB00285	Panic disorder	0.25198
DB00285	Pervasive development disorder	0.14907
DB00285	Psychotic disorder	0.1849
DB00285	Pulmonary hypertension	0.28868
DB00285	Stroke	0.06537
DB00285	Sudden infant death syndrome	0.12599
DB00285	Ulcerative colitis	0.05955

DB00289	Hypertension, Pulmonary	0.16222
DB00289	Anorexia nervosa	0.28868
DB00289	Atherosclerosis	0.04951
DB00289	Autistic disorder	0.08575
DB00289	Behavior disease	0.21822
DB00289	Bipolar disorder	0.16013
DB00289	Chronic fatigue syndrome	0.25
DB00289	Chronic obstructive airway disease	0.07906
DB00289	Colon cancer	0.04196
DB00289	Congenital heart disease	0.2357
DB00289	Depression	0.09285
DB00289	Dermatitis	0.06428
DB00289	Diabetes mellitus	0.03722
DB00289	Drug abuse	0.13245
DB00289	Epilepsy	0.1
DB00289	Fibromyalgia	0.25
DB00289	Generalized anxiety disorder	0.20412
DB00289	Heart failure	0.07538
DB00289	Herpes	0.10102
DB00289	Hypertension	0.0559
DB00289	Migraine	0.13131
DB00289	Multiple endocrine neoplasia	0.2357
DB00289	Neuroendocrine tumor	0.25
DB00289	Neurotic disorder	0.22361
DB00289	Obesity	0.05439
DB00289	Obsessive-compulsive disorder	0.2132
DB00289	Panic disorder	0.30861
DB00289	Pervasive development disorder	0.18257
DB00289	Psychotic disorder	0.11323
DB00289	Pulmonary hypertension	0.35355
DB00289	Stroke	0.08006
DB00289	Sudden infant death syndrome	0.1543
DB00289	Ulcerative colitis	0.07293
DB00344	Hypertension, Pulmonary	0.16222
DB00344	Anorexia nervosa	0.28868
DB00344	Atherosclerosis	0.04951
DB00344	Autistic disorder	0.08575
DB00344	Behavior disease	0.21822
DB00344	Bipolar disorder	0.16013
DB00344	Chronic fatigue syndrome	0.25
DB00344	Chronic obstructive airway disease	0.07906
DB00344	Colon cancer	0.04196
DB00344	Congenital heart disease	0.2357
DB00344	Depression	0.09285
DB00344	Dermatitis	0.06428
DB00344	Diabetes mellitus	0.03722
DB00344	Drug abuse	0.13245

DB00344	Epilepsy	0.1
DB00344	Fibromyalgia	0.25
DB00344	Generalized anxiety disorder	0.20412
DB00344	Heart failure	0.07538
DB00344	Herpes	0.10102
DB00344	Hypertension	0.0559
DB00344	Migraine	0.13131
DB00344	Multiple endocrine neoplasia	0.2357
DB00344	Neuroendocrine tumor	0.25
DB00344	Neurotic disorder	0.22361
DB00344	Obesity	0.05439
DB00344	Obsessive-compulsive disorder	0.2132
DB00344	Panic disorder	0.30861
DB00344	Pervasive development disorder	0.18257
DB00344	Psychotic disorder	0.11323
DB00344	Pulmonary hypertension	0.35355
DB00344	Stroke	0.08006
DB00344	Sudden infant death syndrome	0.1543
DB00344	Ulcerative colitis	0.07293
DB00422	Hypertension, Pulmonary	0.13245
DB00422	Stress disorder, post-traumatic	0.20412
DB00422	Anorexia nervosa	0.2357
DB00422	Atherosclerosis	0.04042
DB00422	Autistic disorder	0.07001
DB00422	Behavior disease	0.26726
DB00422	Bipolar disorder	0.19612
DB00422	Brain disease	0.1037
DB00422	Breast cancer	0.02778
DB00422	Chronic fatigue syndrome	0.20412
DB00422	Chronic obstructive airway disease	0.06455
DB00422	Colon cancer	0.03426
DB00422	Congenital heart disease	0.19245
DB00422	Depression	0.15162
DB00422	Dermatitis	0.05249
DB00422	Diabetes mellitus	0.03039
DB00422	Drug abuse	0.16222
DB00422	Epilepsy	0.08165
DB00422	Fibromyalgia	0.20412
DB00422	Generalized anxiety disorder	0.16667
DB00422	Gilles de la Tourette syndrome	0.21822
DB00422	Heart failure	0.06155
DB00422	Herpes	0.16496
DB00422	Hypertension	0.04564
DB00422	Migraine	0.21442
DB00422	Multiple endocrine neoplasia	0.19245
DB00422	Neuroendocrine tumor	0.20412
DB00422	Neurotic disorder	0.18257

DB00422	Obesity	0.08882
DB00422	Obsessive-compulsive disorder	0.17408
DB00422	Panic disorder	0.25198
DB00422	Pervasive development disorder	0.14907
DB00422	Psychotic disorder	0.1849
DB00422	Pulmonary hypertension	0.28868
DB00422	Stroke	0.06537
DB00422	Sudden infant death syndrome	0.12599
DB00422	Ulcerative colitis	0.05955
DB00476	Hypertension, Pulmonary	0.13245
DB00476	Stress disorder, post-traumatic	0.20412
DB00476	Anorexia nervosa	0.2357
DB00476	Atherosclerosis	0.04042
DB00476	Autistic disorder	0.07001
DB00476	Behavior disease	0.26726
DB00476	Bipolar disorder	0.19612
DB00476	Brain disease	0.1037
DB00476	Breast cancer	0.02778
DB00476	Chronic fatigue syndrome	0.20412
DB00476	Chronic obstructive airway disease	0.06455
DB00476	Colon cancer	0.03426
DB00476	Congenital heart disease	0.19245
DB00476	Depression	0.15162
DB00476	Dermatitis	0.05249
DB00476	Diabetes mellitus	0.03039
DB00476	Drug abuse	0.16222
DB00476	Epilepsy	0.08165
DB00476	Fibromyalgia	0.20412
DB00476	Generalized anxiety disorder	0.16667
DB00476	Gilles de la Tourette syndrome	0.21822
DB00476	Heart failure	0.06155
DB00476	Herpes	0.16496
DB00476	Hypertension	0.04564
DB00476	Migraine	0.21442
DB00476	Multiple endocrine neoplasia	0.19245
DB00476	Neuroendocrine tumor	0.20412
DB00476	Neurotic disorder	0.18257
DB00476	Obesity	0.08882
DB00476	Obsessive-compulsive disorder	0.17408
DB00476	Panic disorder	0.25198
DB00476	Pervasive development disorder	0.14907
DB00476	Psychotic disorder	0.1849
DB00476	Pulmonary hypertension	0.28868
DB00476	Stroke	0.06537
DB00476	Sudden infant death syndrome	0.12599
DB00476	Ulcerative colitis	0.05955
DB00579	Hypertension, Pulmonary	0.13245

DB00579	Stress disorder, post-traumatic	0.20412
DB00579	Anorexia nervosa	0.2357
DB00579	Atherosclerosis	0.04042
DB00579	Autistic disorder	0.07001
DB00579	Behavior disease	0.26726
DB00579	Bipolar disorder	0.19612
DB00579	Brain disease	0.1037
DB00579	Breast cancer	0.02778
DB00579	Chronic fatigue syndrome	0.20412
DB00579	Chronic obstructive airway disease	0.06455
DB00579	Colon cancer	0.03426
DB00579	Congenital heart disease	0.19245
DB00579	Depression	0.15162
DB00579	Dermatitis	0.05249
DB00579	Diabetes mellitus	0.03039
DB00579	Drug abuse	0.16222
DB00579	Epilepsy	0.08165
DB00579	Fibromyalgia	0.20412
DB00579	Generalized anxiety disorder	0.16667
DB00579	Gilles de la Tourette syndrome	0.21822
DB00579	Heart failure	0.06155
DB00579	Herpes	0.16496
DB00579	Hypertension	0.04564
DB00579	Migraine	0.21442
DB00579	Multiple endocrine neoplasia	0.19245
DB00579	Neuroendocrine tumor	0.20412
DB00579	Neurotic disorder	0.18257
DB00579	Obesity	0.08882
DB00579	Obsessive-compulsive disorder	0.17408
DB00579	Panic disorder	0.25198
DB00579	Pervasive development disorder	0.14907
DB00579	Psychotic disorder	0.1849
DB00579	Pulmonary hypertension	0.28868
DB00579	Stroke	0.06537
DB00579	Sudden infant death syndrome	0.12599
DB00579	Ulcerative colitis	0.05955
DB00830	Stress disorder, post-traumatic	0.25
DB00830	Anorexia nervosa	0.14434
DB00830	Behavior disease	0.21822
DB00830	Bipolar disorder	0.16013
DB00830	Brain disease	0.127
DB00830	Breast cancer	0.03402
DB00830	Depression	0.09285
DB00830	Diabetes mellitus	0.03722
DB00830	Drug abuse	0.13245
DB00830	Gilles de la Tourette syndrome	0.26726
DB00830	Herpes	0.10102

DB00830	Hypertension	0.0559
DB00830	Migraine	0.13131
DB00830	Multiple endocrine neoplasia	0.2357
DB00830	Neuroendocrine tumor	0.25
DB00830	Obesity	0.05439
DB00830	Panic disorder	0.1543
DB00830	Psychotic disorder	0.11323
DB00937	Stress disorder, post-traumatic	0.25
DB00937	Anorexia nervosa	0.14434
DB00937	Behavior disease	0.21822
DB00937	Bipolar disorder	0.16013
DB00937	Brain disease	0.127
DB00937	Breast cancer	0.03402
DB00937	Depression	0.09285
DB00937	Diabetes mellitus	0.03722
DB00937	Drug abuse	0.13245
DB00937	Gilles de la Tourette syndrome	0.26726
DB00937	Herpes	0.10102
DB00937	Hypertension	0.0559
DB00937	Migraine	0.13131
DB00937	Multiple endocrine neoplasia	0.2357
DB00937	Neuroendocrine tumor	0.25
DB00937	Obesity	0.05439
DB00937	Panic disorder	0.1543
DB00937	Psychotic disorder	0.11323
DB01105	Hypertension, Pulmonary	0.13245
DB01105	Stress disorder, post-traumatic	0.20412
DB01105	Anorexia nervosa	0.2357
DB01105	Atherosclerosis	0.04042
DB01105	Autistic disorder	0.07001
DB01105	Behavior disease	0.26726
DB01105	Bipolar disorder	0.19612
DB01105	Brain disease	0.1037
DB01105	Breast cancer	0.02778
DB01105	Chronic fatigue syndrome	0.20412
DB01105	Chronic obstructive airway disease	0.06455
DB01105	Colon cancer	0.03426
DB01105	Congenital heart disease	0.19245
DB01105	Depression	0.15162
DB01105	Dermatitis	0.05249
DB01105	Diabetes mellitus	0.03039
DB01105	Drug abuse	0.16222
DB01105	Epilepsy	0.08165
DB01105	Fibromyalgia	0.20412
DB01105	Generalized anxiety disorder	0.16667
DB01105	Gilles de la Tourette syndrome	0.21822
DB01105	Heart failure	0.06155

DB01105	Herpes	0.16496
DB01105	Hypertension	0.04564
DB01105	Migraine	0.21442
DB01105	Multiple endocrine neoplasia	0.19245
DB01105	Neuroendocrine tumor	0.20412
DB01105	Neurotic disorder	0.18257
DB01105	Obesity	0.08882
DB01105	Obsessive-compulsive disorder	0.17408
DB01105	Panic disorder	0.25198
DB01105	Pervasive development disorder	0.14907
DB01105	Psychotic disorder	0.1849
DB01105	Pulmonary hypertension	0.28868
DB01105	Stroke	0.06537
DB01105	Sudden infant death syndrome	0.12599
DB01105	Ulcerative colitis	0.05955
DB01156	Stress disorder, post-traumatic	0.20412
DB01156	Alzheimer's disease	0.04156
DB01156	Anorexia nervosa	0.11785
DB01156	Behavior disease	0.17817
DB01156	Bipolar disorder	0.13074
DB01156	Brain disease	0.1037
DB01156	Breast cancer	0.02778
DB01156	Depression	0.07581
DB01156	Diabetes mellitus	0.03039
DB01156	Down syndrome	0.0658
DB01156	Drug abuse	0.10815
DB01156	Gilles de la Tourette syndrome	0.21822
DB01156	Herpes	0.08248
DB01156	Hypertension	0.04564
DB01156	Lung cancer	0.04032
DB01156	Migraine	0.10721
DB01156	Multiple endocrine neoplasia	0.19245
DB01156	Neuroendocrine tumor	0.20412
DB01156	Obesity	0.04441
DB01156	Panic disorder	0.12599
DB01156	Psychotic disorder	0.09245
DB01170	Anorexia nervosa	0.20412
DB01170	Behavior disease	0.1543
DB01170	Bipolar disorder	0.11323
DB01170	Diabetes mellitus	0.05263
DB01170	Drug abuse	0.09366
DB01170	Hypertension	0.07906
DB01170	Multiple endocrine neoplasia	0.33333
DB01170	Neuroendocrine tumor	0.35355
DB01170	Panic disorder	0.21822
DB01579	Anorexia nervosa	0.11785
DB01579	Behavior disease	0.08909

DB01579	Bipolar disorder	0.06537
DB01579	Diabetes mellitus	0.03039
DB01579	Drug abuse	0.05407
DB01579	Hypertension	0.09129
DB01579	Kidney failure	0.13074
DB01579	Multiple endocrine neoplasia	0.19245
DB01579	Neuroendocrine tumor	0.20412
DB01579	Panic disorder	0.12599
DB01579	Prostate cancer	0.03014
DB04840	Anorexia nervosa	0.20412
DB04840	Behavior disease	0.1543
DB04840	Bipolar disorder	0.11323
DB04840	Diabetes mellitus	0.05263
DB04840	Drug abuse	0.09366
DB04840	Hypertension	0.07906
DB04840	Multiple endocrine neoplasia	0.33333
DB04840	Neuroendocrine tumor	0.35355
DB04840	Panic disorder	0.21822
DB06700	Hypertension, Pulmonary	0.16222
DB06700	Anorexia nervosa	0.28868
DB06700	Atherosclerosis	0.04951
DB06700	Autistic disorder	0.08575
DB06700	Behavior disease	0.21822
DB06700	Bipolar disorder	0.16013
DB06700	Chronic fatigue syndrome	0.25
DB06700	Chronic obstructive airway disease	0.07906
DB06700	Colon cancer	0.04196
DB06700	Congenital heart disease	0.2357
DB06700	Depression	0.09285
DB06700	Dermatitis	0.06428
DB06700	Diabetes mellitus	0.03722
DB06700	Drug abuse	0.13245
DB06700	Epilepsy	0.1
DB06700	Fibromyalgia	0.25
DB06700	Generalized anxiety disorder	0.20412
DB06700	Heart failure	0.07538
DB06700	Herpes	0.10102
DB06700	Hypertension	0.0559
DB06700	Migraine	0.13131
DB06700	Multiple endocrine neoplasia	0.2357
DB06700	Neuroendocrine tumor	0.25
DB06700	Neurotic disorder	0.22361
DB06700	Obesity	0.05439
DB06700	Obsessive-compulsive disorder	0.2132
DB06700	Panic disorder	0.30861
DB06700	Pervasive development disorder	0.18257
DB06700	Psychotic disorder	0.11323

DB06700	Pulmonary hypertension	0.35355
DB06700	Stroke	0.08006
DB06700	Sudden infant death syndrome	0.1543
DB06700	Ulcerative colitis	0.07293
DB06701	Hypertension, Pulmonary	0.13245
DB06701	Stress disorder, post-traumatic	0.20412
DB06701	Anorexia nervosa	0.2357
DB06701	Atherosclerosis	0.04042
DB06701	Autistic disorder	0.07001
DB06701	Behavior disease	0.26726
DB06701	Bipolar disorder	0.19612
DB06701	Brain disease	0.1037
DB06701	Breast cancer	0.02778
DB06701	Chronic fatigue syndrome	0.20412
DB06701	Chronic obstructive airway disease	0.06455
DB06701	Colon cancer	0.03426
DB06701	Congenital heart disease	0.19245
DB06701	Depression	0.15162
DB06701	Dermatitis	0.05249
DB06701	Diabetes mellitus	0.03039
DB06701	Drug abuse	0.16222
DB06701	Epilepsy	0.08165
DB06701	Fibromyalgia	0.20412
DB06701	Generalized anxiety disorder	0.16667
DB06701	Gilles de la Tourette syndrome	0.21822
DB06701	Heart failure	0.06155
DB06701	Herpes	0.16496
DB06701	Hypertension	0.04564
DB06701	Migraine	0.21442
DB06701	Multiple endocrine neoplasia	0.19245
DB06701	Neuroendocrine tumor	0.20412
DB06701	Neurotic disorder	0.18257
DB06701	Obesity	0.08882
DB06701	Obsessive-compulsive disorder	0.17408
DB06701	Panic disorder	0.25198
DB06701	Pervasive development disorder	0.14907
DB06701	Psychotic disorder	0.1849
DB06701	Pulmonary hypertension	0.28868
DB06701	Stroke	0.06537
DB06701	Sudden infant death syndrome	0.12599
DB06701	Ulcerative colitis	0.05955
DB06707	Anorexia nervosa	0.14434
DB06707	Behavior disease	0.10911
DB06707	Bipolar disorder	0.08006
DB06707	Diabetes mellitus	0.03722
DB06707	Drug abuse	0.06623
DB06707	Hypertension	0.1118

DB06707	Kidney failure	0.08006
DB06707	Multiple endocrine neoplasia	0.2357
DB06707	Neuroendocrine tumor	0.25
DB06707	Panic disorder	0.1543
DB06707	Prostate cancer	0.03691
DB08918	Hypertension, Pulmonary	0.16222
DB08918	Anorexia nervosa	0.28868
DB08918	Atherosclerosis	0.04951
DB08918	Autistic disorder	0.08575
DB08918	Behavior disease	0.21822
DB08918	Bipolar disorder	0.16013
DB08918	Chronic fatigue syndrome	0.25
DB08918	Chronic obstructive airway disease	0.07906
DB08918	Colon cancer	0.04196
DB08918	Congenital heart disease	0.2357
DB08918	Depression	0.09285
DB08918	Dermatitis	0.06428
DB08918	Diabetes mellitus	0.03722
DB08918	Drug abuse	0.13245
DB08918	Epilepsy	0.1
DB08918	Fibromyalgia	0.25
DB08918	Generalized anxiety disorder	0.20412
DB08918	Heart failure	0.07538
DB08918	Herpes	0.10102
DB08918	Hypertension	0.0559
DB08918	Migraine	0.13131
DB08918	Multiple endocrine neoplasia	0.2357
DB08918	Neuroendocrine tumor	0.25
DB08918	Neurotic disorder	0.22361
DB08918	Obesity	0.05439
DB08918	Obsessive-compulsive disorder	0.2132
DB08918	Panic disorder	0.30861
DB08918	Pervasive development disorder	0.18257
DB08918	Psychotic disorder	0.11323
DB08918	Pulmonary hypertension	0.35355
DB08918	Stroke	0.08006
DB08918	Sudden infant death syndrome	0.1543
DB08918	Ulcerative colitis	0.07293
DB01113	Behavior disease	0.10911
DB01113	Depression	0.09285
DB01113	Leukemia	0.03984
DB01656	Atherosclerosis	0.10457
DB01656	Behavior disease	0.07715
DB01656	Cancer	0.02656
DB01656	Cardiovascular disease	0.31739
DB01656	Chronic obstructive airway disease	0.26076
DB01656	Depression	0.06565

DB01656	Leukemia	0.13782
DB01327	Atherosclerosis	0.02858
DB01327	Diabetes mellitus	0.02149
DB01327	Kidney failure	0.04623
DB01327	Retinal disease	0.05717
DB01327	Schizophrenia	0.03104
DB00808	Adenoma	0.12127
DB00808	Breast cancer	0.03781
DB00808	Deafness	0.41589
DB00808	Heart failure	0.24618
DB00808	Long QT syndrome	0.96414
DB00808	Lung disease	0.23693
DB00808	Papillary cancer	0.27499
DB00808	Primary hyperparathyroidism	0.35271
DB00808	Rabies	0.14652
DB00808	Sudden infant death syndrome	0.52156
DB00808	Yersinia infection	0.15495
DB00024	Abortion	0.125
DB00024	Carcinoma	0.16903
DB00024	Graves' disease	0.17678
DB00024	Hyperthyroidism	0.33333
DB00024	Rabies	0.11111
DB00024	Thyroid gland disease	0.22942
DB00167	Advanced cancer	0.14142
DB00167	Cancer	0.02181
DB00167	Colon cancer	0.02654
DB00167	Nasopharyngeal cancer	0.08607
DB00167	Rabies	0.04969
DB00167	Solid tumor	0.1895
DB00707	Abortion	0.08839
DB00707	Alopecia	0.28543
DB00707	Alzheimer's disease	0.0509
DB00707	Atherosclerosis	0.04951
DB00707	Biliary cancer	0.31623
DB00707	Enteritis	0.29592
DB00707	Esotropia	0.20412
DB00707	Generalized anxiety disorder	0.14832
DB00707	Immune complex disease	1.22015
DB00707	Infection	0.06594
DB00707	Kaposi sarcoma	0.13054
DB00707	Leukemia	0.04434
DB00707	Lupus erythematosus	0.20522
DB00707	Migraine	0.13131
DB00707	Multiple myeloma	0.11739
DB00707	Overnutrition	0.18257
DB00707	Skin tumor	0.2229
DB00707	Stomach cancer	0.09968

DB00707	Stroke	0.08006
DB00707	Systemic infection	0.21851
DB00707	Yersinia infection	0.06085
DB00211	Hypertension	0.0559
DB00211	Kidney failure	0.16013
DB00211	Prostate cancer	0.03691
DB00298	Hypertension	0.04564
DB00298	Kidney failure	0.19612
DB00298	Prostate cancer	0.03014
DB00346	Hypertension	0.04564
DB00346	Kidney failure	0.19612
DB00346	Prostate cancer	0.03014
DB00388	Hypertension	0.04564
DB00388	Kidney failure	0.19612
DB00388	Prostate cancer	0.03014
DB00450	Hypertension	0.0559
DB00450	Kidney failure	0.08006
DB00450	Prostate cancer	0.03691
DB00610	Hypertension	0.07906
DB00610	Kidney failure	0.11323
DB00610	Prostate cancer	0.0522
DB00699	Hypertension	0.07906
DB00699	Kidney failure	0.11323
DB00699	Prostate cancer	0.0522
DB00706	Hypertension	0.04564
DB00706	Kidney failure	0.19612
DB00706	Prostate cancer	0.03014
DB00723	Hypertension	0.0559
DB00723	Kidney failure	0.16013
DB00723	Prostate cancer	0.03691
DB01253	Hypertension	0.07906
DB01253	Kidney failure	0.11323
DB01253	Prostate cancer	0.0522
DB01365	Hypertension	0.07906
DB01365	Kidney failure	0.11323
DB01365	Prostate cancer	0.0522
DB06207	Hypertension	0.04564
DB06207	Kidney failure	0.19612
DB06207	Prostate cancer	0.03014
DB00214	Hyperaldosteronism	0.31623
DB00310	Hyperaldosteronism	0.31623
DB00903	Growth retardation	0.13608
DB00903	Hyperaldosteronism	0.22361
DB00903	Hypertension	0.0559
DB00903	Rheumatoid arthritis	0.04327
DB00389	Embryoma	0.06166
DB00389	Hypothyroidism	0.2357

DB00389	Infection	0.09325
DB00389	Kidney disease	0.11952
DB00389	Leukemia	0.05634
DB00389	Neoplasm metastasis	0.08138
DB00389	Thrombocytosis	0.44721
DB00389	Thyroid gland disease	0.22942
DB00550	Embryoma	0.06166
DB00550	Hypothyroidism	0.2357
DB00550	Infection	0.09325
DB00550	Kidney disease	0.11952
DB00550	Leukemia	0.05634
DB00550	Neoplasm metastasis	0.08138
DB00550	Thrombocytosis	0.44721
DB00550	Thyroid gland disease	0.22942
DB00763	Embryoma	0.06166
DB00763	Hypothyroidism	0.2357
DB00763	Infection	0.09325
DB00763	Kidney disease	0.11952
DB00763	Leukemia	0.05634
DB00763	Neoplasm metastasis	0.08138
DB00763	Thrombocytosis	0.44721
DB00763	Thyroid gland disease	0.22942
DB01012	Atherosclerosis	0.07001
DB01012	Autoimmune disease	0.10847
DB01012	Colon cancer	0.05934
DB01012	Epilepsy	0.14142
DB01012	Hyperparathyroidism	0.33333
DB01012	Kidney failure	0.11323
DB01012	Multiple myeloma	0.13131
DB01012	Pancreatitis	0.15617
DB01012	Primary hyperparathyroidism	0.37796
DB01012	Vitamin D deficiency	0.40825
DB01370	Growth retardation	0.13608
DB01370	Hypertension	0.0559
DB01370	Rheumatoid arthritis	0.04327
DB00072	Hemorrhagic fevers, Viral	0.05714
DB00072	Pemphigoid, Bullous	0.12029
DB00072	Pleural effusion, Malignant	0.33631
DB00072	Purpura, Thrombocytopenic, Idiopathic	0.02772
DB00072	Abortion	0.06871
DB00072	Adenovirus infection	0.20714
DB00072	Alopecia	0.11653
DB00072	Alzheimer's disease	0.03845
DB00072	Angiomyolipoma	0.73399
DB00072	Antiphospholipid syndrome	0.09623
DB00072	Aplastic anemia	0.04738
DB00072	Asthma	0.27581

DB00072	Atherosclerosis	0.09828
DB00072	Atopic rhinitis	0.9371
DB00072	Autistic disorder	0.03765
DB00072	Autoimmune disease	0.15872
DB00072	Barrett's esophagus	0.34888
DB00072	Breast cancer	0.07016
DB00072	Brucellosis	0.08704
DB00072	Cancer	0.11801
DB00072	Celiac disease	0.04746
DB00072	Charcot-Marie-Tooth disease	0.42199
DB00072	Cholelithiasis	0.55237
DB00072	Colon cancer	0.04164
DB00072	Common wart	0.89512
DB00072	Communicable disease	0.06299
DB00072	Cystic fibrosis	0.04124
DB00072	Cytomegalovirus infection	0.72553
DB00072	Diabetes mellitus	0.18188
DB00072	Drug abuse	0.25222
DB00072	Embryoma	0.04022
DB00072	Endometriosis	0.19267
DB00072	Enteritis	0.15249
DB00072	Esotropia	0.56957
DB00072	Gastrointestinal tumor	0.55477
DB00072	Generalized anxiety disorder	0.06055
DB00072	Glaucoma	0.42302
DB00072	Glomerulonephritis	0.07217
DB00072	Hemorrhagic disorder	0.04473
DB00072	Herpes	0.02502
DB00072	Hypercholesterolemia	0.76409
DB00072	Hyperglycemia	0.06562
DB00072	IGA glomerulonephritis	0.04951
DB00072	Immune complex disease	0.49812
DB00072	Infection by cryptococcus neoformans	0.33333
DB00072	Intermediate coronary syndrome	0.057
DB00072	Kaposi sarcoma	0.05329
DB00072	Kidney disease	0.0345
DB00072	Kidney failure	0.07295
DB00072	Leukemia	0.08566
DB00072	Liver cancer	0.03048
DB00072	Lupus erythematosus	0.3978
DB00072	Lupus vulgaris	0.05455
DB00072	Melanoma	0.02406
DB00072	Multiple myeloma	0.04793
DB00072	Multiple sclerosis	0.02977
DB00072	Muscular atrophy	0.14801
DB00072	Myopathy	0.03973
DB00072	Neoplasm metastasis	0.02349

DB00072	Optic atrophy	0.95177
DB00072	Oral cancer	0.35308
DB00072	Osteitis deformans	0.30516
DB00072	Papillary adenocarcinoma	1.02923
DB00072	Papillomavirus infection	0.3629
DB00072	Penile disease	0.05349
DB00072	Periodontal disease	0.06804
DB00072	Periodontitis	0.08805
DB00072	Pneumoconiosis	0.6981
DB00072	Primary hyperparathyroidism	0.3812
DB00072	Prostate cancer	0.01711
DB00072	Pulmonary fibrosis	0.2152
DB00072	Renal Cell cancer	0.03356
DB00072	Rheumatic fever	0.07454
DB00072	Rheumatoid arthritis	0.08876
DB00072	Schizophrenia	0.23705
DB00072	Skin cancer	0.45388
DB00072	Skin tumor	0.091
DB00072	Stomach cancer	0.04069
DB00072	Stroke	0.26475
DB00072	Subarachnoid hemorrhage	0.05102
DB00072	Systemic infection	0.0892
DB00072	Takayasu's arteritis	0.04771
DB00072	Temporal arteritis	0.05577
DB00072	Testicular dysfunction	0.36572
DB00072	Thrombocytopenia	0.11467
DB00072	Thrombophlebitis	0.72232
DB00072	Vascular disease	0.0527
DB00072	Yersinia infection	0.36098
DB01259	Hemorrhagic fevers, Viral	0.0626
DB01259	Pemphigoid, Bullous	0.03178
DB01259	Pleural effusion, Malignant	0.48627
DB01259	Purpura, Thrombocytopenic, Idiopathic	0.03037
DB01259	Abortion	0.03574
DB01259	Adenovirus infection	0.2778
DB01259	Alzheimer's disease	0.01936
DB01259	Angiomyolipoma	0.95178
DB01259	Aplastic anemia	0.0519
DB01259	Asthma	0.30823
DB01259	Atherosclerosis	0.06338
DB01259	Atopic rhinitis	1.12747
DB01259	Autistic disorder	0.04124
DB01259	Autoimmune disease	0.07097
DB01259	Barrett's esophagus	0.45354
DB01259	Breast cancer	0.03121
DB01259	Cancer	0.15809
DB01259	Charcot-Marie-Tooth disease	0.55194

DB01259	Cholelithiasis	0.68327
DB01259	Colon cancer	0.04561
DB01259	Common wart	1.15536
DB01259	Cytomegalovirus infection	0.8957
DB01259	Diabetes mellitus	0.21981
DB01259	Drug abuse	0.34951
DB01259	Embryoma	0.02456
DB01259	Endometriosis	0.24351
DB01259	Esotropia	0.73677
DB01259	Gastrointestinal tumor	0.71613
DB01259	Glaucoma	0.52855
DB01259	Hemorrhagic disorder	0.049
DB01259	Herpes	0.0274
DB01259	Hypercholesterolemia	0.91224
DB01259	Hyperglycemia	0.07188
DB01259	Intermediate coronary syndrome	0.06244
DB01259	Kidney failure	0.04411
DB01259	Leukemia	0.074
DB01259	Liver cancer	0.03339
DB01259	Lupus erythematosus	0.26648
DB01259	Muscular atrophy	0.16214
DB01259	Myopathy	0.04352
DB01259	Optic atrophy	1.21742
DB01259	Oral cancer	0.49317
DB01259	Osteitis deformans	0.49386
DB01259	Papillary adenocarcinoma	1.35314
DB01259	Papillomavirus infection	0.62321
DB01259	Penile disease	0.05859
DB01259	Pneumoconiosis	1.04113
DB01259	Primary hyperparathyroidism	0.56533
DB01259	Prostate cancer	0.01874
DB01259	Pulmonary fibrosis	0.3096
DB01259	Rheumatoid arthritis	0.1018
DB01259	Schizophrenia	0.28939
DB01259	Skin cancer	0.58688
DB01259	Stroke	0.33428
DB01259	Subarachnoid hemorrhage	0.05589
DB01259	Takayasu's arteritis	0.05226
DB01259	Temporal arteritis	0.0611
DB01259	Testicular dysfunction	0.51231
DB01259	Thrombocytopenia	0.04656
DB01259	Thrombophlebitis	0.90911
DB01259	Yersinia infection	0.461
DB05773	Pleural effusion, Malignant	0.06297
DB05773	Adenovirus infection	0.04561
DB05773	Angiomyolipoma	0.16811
DB05773	Asthma	0.05777

DB05773	Atherosclerosis	0.05786
DB05773	Atopic rhinitis	0.23205
DB05773	Barrett's esophagus	0.07968
DB05773	Breast cancer	0.0374
DB05773	Cancer	0.08674
DB05773	Charcot-Marie-Tooth disease	0.9496
DB05773	Cholelithiasis	0.13307
DB05773	Colon cancer	0.05466
DB05773	Common wart	0.20608
DB05773	Cytomegalovirus infection	0.17513
DB05773	Diabetes mellitus	0.0741
DB05773	Drug abuse	0.20529
DB05773	Embryoma	0.05491
DB05773	Endometriosis	0.04538
DB05773	Esotropia	0.117
DB05773	Gastrointestinal tumor	0.12771
DB05773	Glaucoma	0.10086
DB05773	Hypercholesterolemia	0.19061
DB05773	Hyperglycemia	0.16073
DB05773	Kidney failure	0.09864
DB05773	Liver cancer	0.07465
DB05773	Lupus erythematosus	0.04752
DB05773	Optic atrophy	0.22132
DB05773	Oral cancer	0.29881
DB05773	Osteitis deformans	0.78743
DB05773	Papillary adenocarcinoma	0.23205
DB05773	Papillomavirus infection	0.97459
DB05773	Pneumoconiosis	1.70711
DB05773	Primary hyperparathyroidism	0.0732
DB05773	Prostate cancer	0.0419
DB05773	Pulmonary fibrosis	0.51418
DB05773	Rheumatoid arthritis	0.13586
DB05773	Schizophrenia	0.05787
DB05773	Skin cancer	0.10429
DB05773	Stroke	0.05307
DB05773	Testicular dysfunction	0.31076
DB05773	Thrombophlebitis	0.17091
DB05773	Yersinia infection	0.27732
DB06366	Pleural effusion, Malignant	0.06297
DB06366	Adenovirus infection	0.04561
DB06366	Angiomyolipoma	0.16811
DB06366	Asthma	0.05777
DB06366	Atherosclerosis	0.05786
DB06366	Atopic rhinitis	0.23205
DB06366	Barrett's esophagus	0.07968
DB06366	Breast cancer	0.0374
DB06366	Cancer	0.08674

DB06366	Charcot-Marie-Tooth disease	0.9496
DB06366	Cholelithiasis	0.13307
DB06366	Colon cancer	0.05466
DB06366	Common wart	0.20608
DB06366	Cytomegalovirus infection	0.17513
DB06366	Diabetes mellitus	0.0741
DB06366	Drug abuse	0.20529
DB06366	Embryoma	0.05491
DB06366	Endometriosis	0.04538
DB06366	Esotropia	0.117
DB06366	Gastrointestinal tumor	0.12771
DB06366	Glaucoma	0.10086
DB06366	Hypercholesterolemia	0.19061
DB06366	Hyperglycemia	0.16073
DB06366	Kidney failure	0.09864
DB06366	Liver cancer	0.07465
DB06366	Lupus erythematosus	0.04752
DB06366	Optic atrophy	0.22132
DB06366	Oral cancer	0.29881
DB06366	Osteitis deformans	0.78743
DB06366	Papillary adenocarcinoma	0.23205
DB06366	Papillomavirus infection	0.97459
DB06366	Pneumoconiosis	1.70711
DB06366	Primary hyperparathyroidism	0.0732
DB06366	Prostate cancer	0.0419
DB06366	Pulmonary fibrosis	0.51418
DB06366	Rheumatoid arthritis	0.13586
DB06366	Schizophrenia	0.05787
DB06366	Skin cancer	0.10429
DB06366	Stroke	0.05307
DB06366	Testicular dysfunction	0.31076
DB06366	Thrombophlebitis	0.17091
DB06366	Yersinia infection	0.27732
DB08916	Hemorrhagic fevers, Viral	0.0626
DB08916	Pemphigoid, Bullous	0.03178
DB08916	Pleural effusion, Malignant	0.44714
DB08916	Purpura, Thrombocytopenic, Idiopathic	0.03037
DB08916	Abortion	0.03574
DB08916	Adenovirus infection	0.2609
DB08916	Alzheimer's disease	0.06092
DB08916	Angiomyolipoma	0.90274
DB08916	Aplastic anemia	0.0519
DB08916	Asthma	0.29763
DB08916	Atherosclerosis	0.06338
DB08916	Atopic rhinitis	1.09396
DB08916	Autistic disorder	0.04124
DB08916	Autoimmune disease	0.07097

DB08916	Barrett's esophagus	0.42985
DB08916	Breast cancer	0.03121
DB08916	Cancer	0.16981
DB08916	Charcot-Marie-Tooth disease	0.52217
DB08916	Cholelithiasis	0.65732
DB08916	Colon cancer	0.04561
DB08916	Common wart	1.09733
DB08916	Cytomegalovirus infection	0.8622
DB08916	Diabetes mellitus	0.21298
DB08916	Drug abuse	0.32521
DB08916	Embryoma	0.02456
DB08916	Endometriosis	0.23274
DB08916	Esotropia	0.69931
DB08916	Gastrointestinal tumor	0.68014
DB08916	Glaucoma	0.50692
DB08916	Hemorrhagic disorder	0.049
DB08916	Herpes	0.0274
DB08916	Hypercholesterolemia	0.88727
DB08916	Hyperglycemia	0.07188
DB08916	Intermediate coronary syndrome	0.06244
DB08916	Kidney failure	0.04411
DB08916	Leukemia	0.074
DB08916	Liver cancer	0.03339
DB08916	Lupus erythematosus	0.25539
DB08916	Muscular atrophy	0.16214
DB08916	Myopathy	0.04352
DB08916	Optic atrophy	1.15939
DB08916	Oral cancer	0.45785
DB08916	Osteitis deformans	0.44089
DB08916	Papillary adenocarcinoma	1.27823
DB08916	Papillomavirus infection	0.54829
DB08916	Penile disease	0.05859
DB08916	Pneumoconiosis	0.94938
DB08916	Primary hyperparathyroidism	0.51628
DB08916	Prostate cancer	0.01874
DB08916	Pulmonary fibrosis	0.28508
DB08916	Rheumatoid arthritis	0.09386
DB08916	Schizophrenia	0.32342
DB08916	Skin cancer	0.55711
DB08916	Stroke	0.31959
DB08916	Subarachnoid hemorrhage	0.05589
DB08916	Takayasu's arteritis	0.05226
DB08916	Temporal arteritis	0.0611
DB08916	Testicular dysfunction	0.47524
DB08916	Thrombocytopenia	0.04656
DB08916	Thrombophlebitis	0.86999
DB08916	Yersinia infection	0.4302

DB00095	Pemphigoid, Bullous	0.1
DB00095	Abortion	0.07906
DB00095	Alopecia	0.21406
DB00095	Alzheimer's disease	0.02276
DB00095	Antiphospholipid syndrome	0.10541
DB00095	Asthma	0.02582
DB00095	Atherosclerosis	0.06642
DB00095	Autoimmune disease	0.1029
DB00095	Bladder cancer	0.04082
DB00095	Breast cancer	0.04564
DB00095	Brucellosis	0.09535
DB00095	Celiac disease	0.05199
DB00095	Chronic obstructive airway disease	0.03536
DB00095	Communicable disease	0.06901
DB00095	Cystic fibrosis	0.04518
DB00095	Eating disorder	0.05505
DB00095	Embryoma	0.0195
DB00095	Enteritis	0.32243
DB00095	Generalized anxiety disorder	0.14832
DB00095	Glomerulonephritis	0.07906
DB00095	Herpes	0.04518
DB00095	IGA glomerulonephritis	0.05423
DB00095	Immune complex disease	0.94376
DB00095	Immunologic deficiency syndrome	0.0568
DB00095	Infection by cryptococcus neoformans	0.36515
DB00095	Kaposi sarcoma	0.13054
DB00095	Kidney disease	0.0378
DB00095	Kidney failure	0.03581
DB00095	Leukemia	0.06216
DB00095	Lupus erythematosus	0.2799
DB00095	Lupus vulgaris	0.05976
DB00095	Melanoma	0.02635
DB00095	Migraine	0.05872
DB00095	Multiple myeloma	0.11739
DB00095	Multiple sclerosis	0.03262
DB00095	Neoplasm metastasis	0.02573
DB00095	Periodontal disease	0.07454
DB00095	Periodontitis	0.09645
DB00095	Primary biliary cirrhosis	0.0568
DB00095	Renal Cell cancer	0.03676
DB00095	Rheumatic fever	0.08165
DB00095	Rheumatoid arthritis	0.01935
DB00095	Skin tumor	0.2229
DB00095	Stomach cancer	0.09968
DB00095	Systemic infection	0.17367
DB00095	Thrombocytopenia	0.07906
DB00095	Tuberculosis	0.04264

DB00095	Ulcerative colitis	0.03262
DB00095	Vascular disease	0.05774
DB00095	Yersinia infection	0.06085
DB00098	Hemorrhagic fevers, Viral	0.72589
DB00098	Hypertension, Pulmonary	0.01821
DB00098	Pemphigoid, Bullous	1.0865
DB00098	Pleural effusion, Malignant	1.11358
DB00098	Purpura, Thrombocytopenic, Idiopathic	1.09221
DB00098	Abortion	0.04167
DB00098	Adenocarcinoma	0.04547
DB00098	Adenovirus infection	0.0587
DB00098	Alopecia	0.7164
DB00098	Alzheimer's disease	0.58824
DB00098	Angiomyolipoma	0.0345
DB00098	Aortic aneurysm	0.79004
DB00098	Aplastic anemia	0.63978
DB00098	Arthritis	0.01941
DB00098	Asthma	0.45353
DB00098	Atherosclerosis	0.66246
DB00098	Atopic rhinitis	0.04762
DB00098	Autistic disorder	0.42898
DB00098	Autoimmune disease	0.13801
DB00098	Azoospermia	0.03344
DB00098	Bacterial infection	0.0335
DB00098	Barrett's esophagus	0.01635
DB00098	Behcet syndrome	0.08951
DB00098	Biliary Atresia	0.22536
DB00098	Bladder cancer	0.04303
DB00098	Bone disease	0.0251
DB00098	Bone metastases	0.21786
DB00098	Brain tumor	0.02507
DB00098	Breast cancer	0.07301
DB00098	Bronchopulmonary dysplasia	0.04476
DB00098	Cancer	0.41022
DB00098	Capillaries disease	0.01649
DB00098	Cervical cancer	0.12182
DB00098	Cholelithiasis	0.02731
DB00098	Chondrosarcoma	0.092
DB00098	Chronic obstructive airway disease	0.06632
DB00098	Colon cancer	0.11178
DB00098	Common variable immunodeficiency	0.11785
DB00098	Common wart	0.04229
DB00098	Congenital abnormality	0.00621
DB00098	Connective tissue disease	0.03712
DB00098	Cytomegalovirus infection	0.03594
DB00098	Demyelinating disease	0.29536
DB00098	Dental plaque	0.08695

DB00098	Depression	0.58632
DB00098	Dermatitis	0.00801
DB00098	Diabetes mellitus	0.62567
DB00098	Drug abuse	0.72808
DB00098	Eating disorder	0.143
DB00098	Embryoma	0.04281
DB00098	Emphysema	0.06773
DB00098	Endometriosis	0.03519
DB00098	Enteritis	0.17504
DB00098	Esophagus cancer	0.13776
DB00098	Esotropia	0.02401
DB00098	Familial Mediterranean fever	0.02612
DB00098	Gastrointestinal tumor	0.12428
DB00098	Glaucoma	0.0207
DB00098	Graves' disease	0.05893
DB00098	HIV infection	0.08525
DB00098	Heart failure	0.09925
DB00098	Hemorrhagic disorder	0.59727
DB00098	Hepatitis C	0.11349
DB00098	Herpes	0.49613
DB00098	Hyperaldosteronism	0.03244
DB00098	Hypercholesterolemia	0.03912
DB00098	Hyperlipidemia	0.02122
DB00098	Hypertension	0.01195
DB00098	IGA glomerulonephritis	0.05717
DB00098	Immunologic deficiency syndrome	0.05987
DB00098	Infection	0.02711
DB00098	Infertility	0.03823
DB00098	Inflammation of the central nervous system	0.26022
DB00098	Intermediate coronary syndrome	0.69948
DB00098	Ischemia	0.01055
DB00098	Kaposi sarcoma	0.09245
DB00098	Kidney disease	0.02178
DB00098	Kidney failure	0.09839
DB00098	Leukemia	0.10568
DB00098	Leukoencephalopathy	0.00938
DB00098	Liver cancer	0.02815
DB00098	Lung cancer	0.10564
DB00098	Lung disease	0.15618
DB00098	Lupus erythematosus	0.81379
DB00098	Lupus vulgaris	0.12994
DB00098	Macular degeneration	0.03371
DB00098	Malaria	0.0225
DB00098	Malignant glioma	0.13908
DB00098	Melanoma	0.19628
DB00098	Mesothelioma	0.10821
DB00098	Metastasis to lymph nodes	0.06537

DB00098	Migraine	0.0619
DB00098	Mucocutaneous lymph node syndrome	0.02521
DB00098	Multiple sclerosis	0.14664
DB00098	Muscular dystrophies	0.05036
DB00098	Mycosis fungoides	0.07454
DB00098	Myopathy	0.04237
DB00098	Nasopharyngeal cancer	0.06415
DB00098	Neoplasm metastasis	0.10337
DB00098	Nervous system tumor	0.11138
DB00098	Neuropathy	0.05338
DB00098	Obesity	0.07323
DB00098	Optic atrophy	0.04542
DB00098	Oral cancer	0.06982
DB00098	Osteoporosis	0.1891
DB00098	Osteosarcoma	0.20009
DB00098	Ovarian cancer	0.12047
DB00098	Overnutrition	0.23016
DB00098	PEComa	0.2357
DB00098	Pancreas cancer	0.0622
DB00098	Pancreatitis	0.15871
DB00098	Papillary adenocarcinoma	0.04762
DB00098	Parkinson disease	0.07435
DB00098	Penile disease	0.70373
DB00098	Peptic ulcer	0.0208
DB00098	Pertussis	0.224
DB00098	Polyarthritis	0.07075
DB00098	Polycystic kidney	0.02649
DB00098	Polycystic ovary syndrome	0.03538
DB00098	Polyneuropathy	0.03371
DB00098	Pre-Eclampsia	0.04967
DB00098	Primary biliary cirrhosis	0.05987
DB00098	Primary hyperparathyroidism	0.81241
DB00098	Primary tumor	0.05893
DB00098	Prostate cancer	0.09132
DB00098	Protein-energy malnutrition	0.07094
DB00098	Rabies	0.02534
DB00098	Renal Cell cancer	0.02282
DB00098	Respiratory failure	0.03496
DB00098	Rheumatic fever	0.092
DB00098	Rheumatoid arthritis	0.63135
DB00098	Sarcoidosis	0.0912
DB00098	Schizophrenia	0.09253
DB00098	Shigella infection	0.83677
DB00098	Sickle cell disease	0.82806
DB00098	Skin cancer	0.0214
DB00098	Squamous cell cancer	0.20151
DB00098	Stomach cancer	0.03507

DB00098	Stroke	0.37561
DB00098	Subarachnoid hemorrhage	0.62714
DB00098	Systemic infection	0.70759
DB00098	Takayasu's arteritis	0.67963
DB00098	Temporal arteritis	0.72476
DB00098	Testicular dysfunction	0.06084
DB00098	Thalassemia	0.79114
DB00098	Thromboangiitis obliterans	0.1891
DB00098	Thrombocytopenia	0.59491
DB00098	Thrombophlebitis	0.03507
DB00098	Thyroid cancer	0.07746
DB00098	Tuberculosis	0.06079
DB00098	Tuberous sclerosis	0.01038
DB00098	Ulcerative colitis	0.14107
DB00098	Virus disease	0.14793
DB00098	Vulvar disease	0.05566
DB00098	Wiskott-Aldrich syndrome	0.01985
DB00098	Yersinia infection	0.01249
DB00227	Infertility, Male	0.05682
DB00227	Abortion	0.10839
DB00227	Adenovirus infection	0.19128
DB00227	Adrenal gland hyperfunction	0.07408
DB00227	Adrenal gland tumor	0.07452
DB00227	Alzheimer's disease	0.02356
DB00227	Atherosclerosis	0.05013
DB00227	Autistic disorder	0.01894
DB00227	Azoospermia	0.02066
DB00227	Behavior disease	0.03116
DB00227	Bipolar disorder	0.03689
DB00227	Bladder cancer	0.07454
DB00227	Brain disease	0.09829
DB00227	Brain tumor	0.07519
DB00227	Breast cancer	0.17912
DB00227	Bronchial hyperreactivity	0.04292
DB00227	Cancer	0.16365
DB00227	Cerebrovascular disorder	0.0295
DB00227	Cholelithiasis	0.02577
DB00227	Chronic obstructive airway disease	0.06455
DB00227	Colon cancer	0.0237
DB00227	Congenital abnormality	0.01341
DB00227	Corneal disease	0.01733
DB00227	Cystic fibrosis	0.01272
DB00227	Dental plaque	0.01675
DB00227	Depression	0.01125
DB00227	Diabetes mellitus	0.0104
DB00227	Down syndrome	0.01675
DB00227	Drug abuse	0.01639

DB00227	Eating disorder	0.10853
DB00227	Embryoma	0.13885
DB00227	Encephalopathies	0.03636
DB00227	Endometriosis	0.02936
DB00227	Enteritis	0.06337
DB00227	Epilepsy	0.03405
DB00227	Fanconi's anemia	0.05932
DB00227	Glaucoma	0.01953
DB00227	Granulomatous disease	0.04164
DB00227	HIV infection	0.05687
DB00227	Hereditary disease	0.04196
DB00227	Herpes	0.10353
DB00227	Hyperlipidemia	0.0327
DB00227	Hypertension	0.01303
DB00227	Hypopituitarism	0.08834
DB00227	Immunologic deficiency syndrome	0.11207
DB00227	Infection	0.03252
DB00227	Infertility	0.01885
DB00227	Ischemia	0.00914
DB00227	Kaposi sarcoma	0.08043
DB00227	Keratoconus	0.0275
DB00227	Kidney failure	0.01654
DB00227	Late pregnancy	0.04051
DB00227	Leukemia	0.04201
DB00227	Leukoencephalopathy	0.0177
DB00227	Liver cancer	0.06189
DB00227	Lung cancer	0.2201
DB00227	Lupus erythematosus	0.02048
DB00227	Lupus vulgaris	0.02503
DB00227	Lymphoma	0.0078
DB00227	Melanoma	0.0086
DB00227	Mental retardation	0.01495
DB00227	Migraine	0.13034
DB00227	Movement disorder	0.04605
DB00227	Muscular dystrophies	0.01372
DB00227	Myasthenia Gravis	0.03132
DB00227	Myotonic disorder	0.01454
DB00227	Neoplasm metastasis	0.0912
DB00227	Nervous system disease	0.03759
DB00227	Obesity	0.01231
DB00227	Oligospermia	0.03761
DB00227	Osteomyelitis	0.0139
DB00227	Osteoporosis	0.01877
DB00227	Ovarian disease	0.02942
DB00227	Ovarian failure	0.03847
DB00227	Panic disorder	0.02955
DB00227	Parkinson disease	0.01432

DB00227	Pelvic inflammatory disease	0.29842
DB00227	Polyarthritis	0.0107
DB00227	Polycystic ovary syndrome	0.02226
DB00227	Primary biliary cirrhosis	0.12613
DB00227	Prostate cancer	0.07835
DB00227	Rabies	0.02722
DB00227	Renal Cell cancer	0.02207
DB00227	Renal tubular acidosis	0.06058
DB00227	Retinitis pigmentosa	0.02253
DB00227	Rett syndrome	0.05409
DB00227	Rheumatoid arthritis	0.00949
DB00227	Schizophrenia	0.04022
DB00227	Skin disease	0.02616
DB00227	Spinal dysraphism	0.02835
DB00227	Stomach cancer	0.02448
DB00227	Stroke	0.01028
DB00227	Synovitis	0.03411
DB00227	Testicular tumor	0.0662
DB00227	Thrombophlebitis	0.0186
DB00227	Thymoma	0.02647
DB00227	Tic disorder	0.03097
DB00227	Tuberculosis	0.07785
DB00227	Tuberous sclerosis	0.30975
DB00227	Ulcerative colitis	0.05955
DB00227	Uterine disease	0.02356
DB00227	Vitamin D deficiency	0.01561
DB00227	Vitiligo	0.03132
DB00227	Yersinia infection	0.00662
DB00627	Schizophrenia	0.03801
DB00040	Colon cancer	0.03426
DB00040	Diabetes mellitus	0.06077
DB00040	Hypertension	0.04564
DB00040	Pancreas cancer	0.05987
DB00445	Kidney tubular necrosis, acute	0.12007
DB00445	Amyotrophic lateral sclerosis	0.03604
DB00445	Aortic valve disease	0.11062
DB00445	Brain tumor	0.02262
DB00445	Breast cancer	0.04151
DB00445	Cancer	0.02592
DB00445	Celiac disease	0.06576
DB00445	Charcot-Marie-Tooth disease	0.19569
DB00445	Chronic obstructive airway disease	0.06282
DB00445	Cockayne syndrome	0.12336
DB00445	Colon cancer	0.08471
DB00445	Common cold	0.08759
DB00445	Diabetes mellitus	0.02014
DB00445	Down syndrome	0.07642

DB00445	Embryoma	0.01525
DB00445	Emphysema	0.08099
DB00445	Epilepsy	0.03155
DB00445	Ewings sarcoma	0.11709
DB00445	Eye disease	0.09687
DB00445	Fanconi's anemia	0.03869
DB00445	HIV infection	0.0409
DB00445	Heart disease	0.28207
DB00445	Heart failure	0.04786
DB00445	Helicobacter infection	0.09599
DB00445	Hereditary disease	0.0494
DB00445	Herpes	0.01702
DB00445	Infection	0.01858
DB00445	Infertility	0.05698
DB00445	Ischemia	0.04917
DB00445	Kaposi sarcoma	0.06619
DB00445	Kidney cancer	0.08874
DB00445	Leigh disease	0.05883
DB00445	Leukemia	0.02248
DB00445	Leukoencephalopathy	0.04291
DB00445	Lipodystrophy	0.13587
DB00445	Liver cancer	0.03785
DB00445	Lung cancer	0.04835
DB00445	Lymphoma	0.07682
DB00445	Melanoma	0.03675
DB00445	Meningioma	0.17605
DB00445	Metabolism disease	0.08545
DB00445	Muscular atrophy	0.09852
DB00445	Muscular dystrophies	0.06773
DB00445	Nephroblastoma	0.17583
DB00445	Neuroblastoma	0.05563
DB00445	Neuropathy	0.07997
DB00445	Parkinson disease	0.0433
DB00445	Pituitary tumor	0.10097
DB00445	Prostate cancer	0.02409
DB00445	Renal tubular acidosis	0.05133
DB00445	Retinitis pigmentosa	0.12611
DB00445	Rheumatoid arthritis	0.02029
DB00445	Schizophrenia	0.03388
DB00445	Tuberous sclerosis	0.0632
DB00445	Uterine fibroids	0.10116
DB00445	Virus disease	0.04513
DB00445	Werner syndrome	0.10284
DB01191	Hypertension, Pulmonary	0.16222
DB01191	Anorexia nervosa	0.28868
DB01191	Atherosclerosis	0.04951
DB01191	Autistic disorder	0.08575

DB01191	Behavior disease	0.21822
DB01191	Bipolar disorder	0.16013
DB01191	Chronic fatigue syndrome	0.25
DB01191	Chronic obstructive airway disease	0.07906
DB01191	Colon cancer	0.04196
DB01191	Congenital heart disease	0.2357
DB01191	Depression	0.09285
DB01191	Dermatitis	0.06428
DB01191	Drug abuse	0.06623
DB01191	Epilepsy	0.1
DB01191	Fibromyalgia	0.25
DB01191	Generalized anxiety disorder	0.20412
DB01191	Heart failure	0.07538
DB01191	Herpes	0.10102
DB01191	Hypertension	0.0559
DB01191	Migraine	0.26261
DB01191	Neurotic disorder	0.22361
DB01191	Obesity	0.10879
DB01191	Obsessive-compulsive disorder	0.2132
DB01191	Panic disorder	0.1543
DB01191	Pervasive development disorder	0.18257
DB01191	Psychotic disorder	0.11323
DB01191	Pulmonary hypertension	0.35355
DB01191	Stroke	0.08006
DB01191	Sudden infant death syndrome	0.1543
DB01191	Ulcerative colitis	0.07293
DB04871	Anorexia nervosa	0.20412
DB04871	Behavior disease	0.1543
DB04871	Bipolar disorder	0.11323
DB04871	Hypertension	0.07906
DB04871	Migraine	0.1857
DB04871	Obesity	0.07692
DB00364	Amyloidosis	0.08452
DB00364	Anemia	0.43476
DB00364	Arthritis	0.28895
DB00364	Asthma	0.13736
DB00364	Autistic disorder	0.05423
DB00364	Cancer	0.06636
DB00364	Cardiovascular disease	0.1364
DB00364	Cerebrovascular disorder	0.09759
DB00364	Cirrhosis	0.48018
DB00364	Cytomegalovirus infection	0.11547
DB00364	Diabetes mellitus	0.10711
DB00364	Drug abuse	0.04189
DB00364	Eating disorder	0.20663
DB00364	Endometriosis	0.1535
DB00364	Gestational diabetes	0.90008

DB00364	Growth retardation	0.08607
DB00364	Heart failure	0.19037
DB00364	Hemorrhagic disorder	0.23717
DB00364	Hepatitis C	0.26298
DB00364	Herpes	0.15652
DB00364	IGA glomerulonephritis	0.0767
DB00364	Infection	0.0417
DB00364	Infectious lung disease	0.1
DB00364	Keratosis	0.14907
DB00364	Kidney failure	0.05064
DB00364	Liver disease	0.06901
DB00364	Metabolism disease	0.07161
DB00364	Necrotizing enterocolitis	0.18257
DB00364	Obesity	0.0344
DB00364	Pre-Eclampsia	0.25186
DB00364	Prostate cancer	0.02334
DB00364	Rabies	0.04969
DB00364	Rheumatoid arthritis	0.02737
DB00364	Schizophrenia	0.034
DB00364	Stroke	0.14328
DB00364	Systemic infection	0.0513
DB00364	Systemic scleroderma	0.13551
DB00364	Thrombophilia	0.10541
DB01280	Kidney tubular necrosis, acute	0.15039
DB01280	Amyotrophic lateral sclerosis	0.04515
DB01280	Aortic valve disease	0.17835
DB01280	Barrett's esophagus	0.09355
DB01280	Breast cancer	0.02375
DB01280	Cancer	0.0531
DB01280	Celiac disease	0.08237
DB01280	Chronic obstructive airway disease	0.07869
DB01280	Colon cancer	0.10214
DB01280	Common cold	0.10971
DB01280	Diabetes mellitus	0.02522
DB01280	Down syndrome	0.22479
DB01280	Emphysema	0.13059
DB01280	Ewings sarcoma	0.14666
DB01280	Fanconi's anemia	0.04847
DB01280	HIV infection	0.06594
DB01280	Heart failure	0.05995
DB01280	Helicobacter infection	0.15477
DB01280	Ischemia	0.06159
DB01280	Kaposi sarcoma	0.08291
DB01280	Leukemia	0.02816
DB01280	Liver cancer	0.04741
DB01280	Lung cancer	0.06707
DB01280	Lymphoma	0.22596

DB01280	Melanoma	0.04603
DB01280	Meningioma	0.51783
DB01280	Multiple endocrine neoplasia	0.16367
DB01280	Parkinson disease	0.05424
DB01280	Pituitary tumor	0.12647
DB01280	Renal tubular acidosis	0.06429
DB01280	Rheumatoid arthritis	0.02541
DB01280	Tuberous sclerosis	0.10191
DB01280	Ulcerative colitis	0.0737
DB01280	Uterine fibroids	0.12671
DB01280	Werner syndrome	0.08323
DB00356	Arthritis	0.31467
DB00356	Atherosclerosis	0.11789
DB00356	Autistic disorder	0.18788
DB00356	Bronchial disease	0.62939
DB00356	Chronic obstructive airway disease	0.25244
DB00356	Cystic fibrosis	0.275
DB00356	Dermatitis	0.12992
DB00356	Diabetes mellitus	0.08091
DB00356	Drug abuse	0.10073
DB00356	Epilepsy	0.37292
DB00356	Glaucoma	0.23729
DB00356	Graves' disease	0.34053
DB00356	Heart failure	0.19232
DB00356	Hypertension	0.15825
DB00356	Ischemia	0.32562
DB00356	Lung cancer	0.11382
DB00356	Malaria	0.36475
DB00356	Metabolism disease	0.34338
DB00356	Movement disorder	0.6653
DB00356	Myopathy	0.22897
DB00356	Neurodegenerative disorder	0.41698
DB00356	Obesity	0.14957
DB00356	Polycystic ovary syndrome	0.19121
DB00356	Premature birth	0.52146
DB00356	Prostate cancer	0.17293
DB00356	Rheumatoid arthritis	0.08153
DB00356	Sickle cell disease	0.16267
DB00356	Subarachnoid hemorrhage	0.25465
DB01003	Arthritis	0.31467
DB01003	Atherosclerosis	0.11789
DB01003	Autistic disorder	0.18788
DB01003	Bronchial disease	0.62939
DB01003	Cancer	0.02606
DB01003	Chronic obstructive airway disease	0.25244
DB01003	Cystic fibrosis	0.275
DB01003	Dermatitis	0.12992

DB01003	Diabetes mellitus	0.08091
DB01003	Drug abuse	0.10073
DB01003	Epilepsy	0.3315
DB01003	Glaucoma	0.23729
DB01003	Graves' disease	0.34053
DB01003	Heart failure	0.19232
DB01003	Hypertension	0.15825
DB01003	Ischemia	0.28812
DB01003	Lung cancer	0.11382
DB01003	Malaria	0.36475
DB01003	Metabolism disease	0.34338
DB01003	Movement disorder	0.60422
DB01003	Myopathy	0.22897
DB01003	Neurodegenerative disorder	0.36883
DB01003	Obesity	0.14957
DB01003	Pancreas cancer	0.07332
DB01003	Polycystic ovary syndrome	0.19121
DB01003	Premature birth	0.52146
DB01003	Prostate cancer	0.15765
DB01003	Rheumatoid arthritis	0.08153
DB01003	Sickle cell disease	0.16267
DB01003	Subarachnoid hemorrhage	0.25465
DB00588	Alopecia	0.21874
DB00588	Bacterial infection	0.13363
DB00588	Breast cancer	0.08095
DB00588	Colon cancer	0.02967
DB00588	Diabetes mellitus	0.02632
DB00588	Gouts	0.13868
DB00588	Heart failure	0.1969
DB00588	Hypertension	0.15769
DB00588	Hypoglycemia	0.82433
DB00588	Leukemia	0.02817
DB00588	Liver cancer	0.03881
DB00588	Lymphoma	0.20974
DB00588	Melanoma	0.05513
DB00588	Polyarthritis	0.05698
DB00588	Proteinuria	0.53341
DB00588	Renal Cell cancer	0.25349
DB00588	Rheumatoid arthritis	0.0306
DB00588	Schizophrenia	0.03801
DB01034	Cancer	0.01843
DB01034	Obesity	0.03846
DB01034	Osteitis deformans	0.20412
DB01019	Behavior disease	0.1543
DB01019	Bipolar disorder	0.11323
DB01019	Bladder cancer	0.1291
DB01019	Central nervous system disease	0.25

DB01019	Depression	0.13131
DB01135	Behavior disease	0.10911
DB01135	Bipolar disorder	0.08006
DB01135	Bladder cancer	0.09129
DB01135	Central nervous system disease	0.17678
DB01135	Depression	0.09285
DB01135	Epilepsy	0.1
DB01336	Behavior disease	0.10911
DB01336	Bipolar disorder	0.08006
DB01336	Bladder cancer	0.09129
DB01336	Central nervous system disease	0.17678
DB01336	Depression	0.09285
DB01336	Epilepsy	0.1
DB00026	Breast cancer	0.04811
DB00026	Degenerative disc disease	0.40825
DB00026	Endometriosis	0.08305
DB00026	Hepatitis	0.2582
DB00026	Lung cancer	0.06984
DB00026	Mitral valve disease	0.44721
DB00026	Nephrosis	0.22361
DB00026	Rheumatoid arthritis	0.0612
DB00026	Schizophrenia	0.07603
DB00292	Breast cancer	0.01134
DB00292	Diabetes mellitus	0.01241
DB00292	Drug abuse	0.02208
DB00292	Hypertension	0.01863
DB00292	Hypogonadism	0.10541
DB00292	Obesity	0.01813
DB00292	Polycystic ovary syndrome	0.03122
DB00292	Yersinia infection	0.02797
DB00641	Skin disease, Genetic	0.07399
DB00641	Alzheimer's disease	0.04828
DB00641	Asthma	0.0372
DB00641	Brain tumor	0.06423
DB00641	Breast cancer	0.03612
DB00641	Cancer	0.01232
DB00641	Chronic obstructive airway disease	0.34665
DB00641	Colon cancer	0.16001
DB00641	Common cold	0.09633
DB00641	Congenital abnormality	0.02755
DB00641	Diabetes mellitus	0.13117
DB00641	Enteritis	0.27334
DB00641	Epilepsy	0.06336
DB00641	HIV infection	0.15716
DB00641	Herpes	0.03417
DB00641	Infection	0.0373
DB00641	Kidney failure	0.31344

DB00641	Leukemia	0.07418
DB00641	Liver cancer	0.04163
DB00641	Lung cancer	0.03115
DB00641	Lupus erythematosus	0.0612
DB00641	Melanoma	0.21547
DB00641	Migraine	0.36205
DB00641	Myotonic disorder	0.08605
DB00641	Obesity	0.17721
DB00641	Ovarian disease	0.09782
DB00641	Pancreas disease	0.15682
DB00641	Pertussis	0.78079
DB00641	Prostate cancer	0.02337
DB00641	Proteinuria	0.15454
DB00641	Respiratory failure	0.15515
DB00641	Retinal disease	0.10668
DB00641	Rheumatoid arthritis	0.11022
DB00641	Stroke	0.18259
DB00641	Systemic infection	0.08212
DB00641	Thrombophilia	0.83537
DB00641	Yersinia infection	0.07836
DB01076	Breast cancer	0.08853
DB01076	Diabetes mellitus	0.04562
DB01076	Endometriosis	0.13307
DB01076	HIV infection	0.0614
DB01076	Infertility	0.26186
DB01076	Leukemia	0.10456
DB01076	Lung cancer	0.10449
DB01076	Lymphoma	0.18113
DB01076	Stomach cancer	0.10998
DB01076	Uterine fibroids	0.52823
DB01076	Yersinia infection	0.14923
DB00745	Stress disorder, post-traumatic	0.25
DB00745	Behavior disease	0.10911
DB00745	Bipolar disorder	0.08006
DB00745	Brain disease	0.127
DB00745	Breast cancer	0.03402
DB00745	Depression	0.09285
DB00745	Drug abuse	0.06623
DB00745	Gilles de la Tourette syndrome	0.26726
DB00745	Herpes	0.10102
DB00745	Kidney failure	0.08006
DB00745	Migraine	0.13131
DB00745	Obesity	0.05439
DB00745	Psychotic disorder	0.11323
DB01255	Stress disorder, post-traumatic	0.25
DB01255	Behavior disease	0.10911
DB01255	Bipolar disorder	0.08006

DB01255	Brain disease	0.127
DB01255	Breast cancer	0.03402
DB01255	Depression	0.09285
DB01255	Drug abuse	0.06623
DB01255	Gilles de la Tourette syndrome	0.26726
DB01255	Herpes	0.10102
DB01255	Kidney failure	0.08006
DB01255	Migraine	0.13131
DB01255	Obesity	0.05439
DB01255	Psychotic disorder	0.11323
DB00108	Hemorrhagic fevers, Viral	0.12309
DB00108	Labor, Premature	0.07785
DB00108	Pemphigoid, Bullous	0.48092
DB00108	Pleural effusion, Malignant	0.29703
DB00108	Purpura, Thrombocytopenic, Idiopathic	0.49896
DB00108	Abortion	0.03769
DB00108	Alopecia	0.50919
DB00108	Alzheimer's disease	0.22021
DB00108	Antiphospholipid syndrome	0.1005
DB00108	Aortic aneurysm	0.35064
DB00108	Asthma	0.37061
DB00108	Atherosclerosis	0.06333
DB00108	Atopic rhinitis	0.07785
DB00108	Autoimmune disease	0.09811
DB00108	Bacterial infection	0.09702
DB00108	Behcet syndrome	0.04307
DB00108	Biliary Atresia	0.1066
DB00108	Bladder cancer	0.03892
DB00108	Brain tumor	0.02422
DB00108	Breast cancer	0.05803
DB00108	Bronchiolitis	0.1066
DB00108	Brucellosis	0.09091
DB00108	Cancer	0.10222
DB00108	Celiac disease	0.09914
DB00108	Chronic obstructive airway disease	0.03371
DB00108	Colon cancer	0.01789
DB00108	Communicable disease	0.0658
DB00108	Cystic fibrosis	0.08615
DB00108	Demyelinating disease	1.06627
DB00108	Dental plaque	0.03829
DB00108	Depression	0.27392
DB00108	Diabetes mellitus	0.17808
DB00108	Drug abuse	0.20195
DB00108	Embryoma	0.01859
DB00108	Endometriosis	0.02504
DB00108	Enteritis	0.17873
DB00108	Esophagus cancer	0.47939

DB00108	Generalized anxiety disorder	0.05398
DB00108	Glomerulonephritis	0.07538
DB00108	Glucose intolerance	0.06917
DB00108	Graves' disease	0.0533
DB00108	HIV infection	0.02888
DB00108	Heart disease	0.09091
DB00108	Herpes	0.04307
DB00108	Hodgkin's disease	0.0533
DB00108	Huntington disease	0.06727
DB00108	Hyperinsulinism	0.05803
DB00108	IGA glomerulonephritis	0.05171
DB00108	Immune complex disease	0.47527
DB00108	Infection by cryptococcus neoformans	0.34816
DB00108	Inflammation of the central nervous system	0.1066
DB00108	Influenza	0.0603
DB00108	Intraocular melanoma	0.09535
DB00108	Kaposi sarcoma	0.13113
DB00108	Kidney disease	0.03604
DB00108	Kidney failure	0.06828
DB00108	Leukemia	0.24736
DB00108	Liver cancer	0.0234
DB00108	Lung cancer	0.02106
DB00108	Lung disease	0.54583
DB00108	Lupus erythematosus	0.43398
DB00108	Lupus vulgaris	0.05698
DB00108	Macular degeneration	0.06287
DB00108	Malaria	0.05599
DB00108	Melanoma	0.39781
DB00108	Migraine	0.05599
DB00108	Multiple myeloma	0.04272
DB00108	Multiple sclerosis	0.0622
DB00108	Multiple system atrophy	0.17408
DB00108	Neoplasm metastasis	0.04907
DB00108	Nervous system disease	0.07785
DB00108	Obesity	0.02319
DB00108	Osteoporosis	0.04767
DB00108	Osteosarcoma	0.69527
DB00108	Overnutrition	0.80368
DB00108	Periodontal disease	0.07107
DB00108	Periodontitis	0.09196
DB00108	Pertussis	0.12309
DB00108	Pre-Eclampsia	0.03553
DB00108	Primary biliary cirrhosis	0.05415
DB00108	Primary hyperparathyroidism	0.34526
DB00108	Prostate cancer	0.01574
DB00108	Pseudoxanthoma elasticum	0.13484
DB00108	Renal Cell cancer	0.03505

DB00108	Rheumatic fever	0.1557
DB00108	Rheumatoid arthritis	0.30189
DB00108	Schistosoma mansonii infection	0.17408
DB00108	Schizophrenia	0.02292
DB00108	Shigella infection	0.36577
DB00108	Sicca syndrome	0.05913
DB00108	Sickle cell disease	0.54994
DB00108	Skin tumor	0.08112
DB00108	Spinal cord disease	0.06742
DB00108	Stomach cancer	0.06166
DB00108	Stroke	0.03414
DB00108	Systemic infection	0.60982
DB00108	Thalassemia	0.58681
DB00108	Thromboangiitis obliterans	0.12309
DB00108	Thrombocytopenia	0.07538
DB00108	Thyroiditis	0.2132
DB00108	Tuberculosis	0.04066
DB00108	Tuberous sclerosis	0.07785
DB00108	Ulcerative colitis	0.0311
DB00108	Urinary tract infection	0.2132
DB00108	Vascular disease	0.05505
DB00108	Vasculitis	0.09091
DB00108	Virus disease	0.4462
DB00108	Yersinia infection	0.05793
DB00720	Abortion	0.05195
DB00720	Adenovirus infection	0.02965
DB00720	Alzheimer's disease	0.02437
DB00720	Aortic aneurysm	0.04833
DB00720	Aplastic anemia	0.11317
DB00720	Bipolar disorder	0.06537
DB00720	Brain tumor	0.04585
DB00720	Breast cancer	0.07486
DB00720	Bronchial disease	0.1739
DB00720	Cancer	0.03046
DB00720	Carcinoma	0.07993
DB00720	Cervical cancer	0.06976
DB00720	Cholestasis	0.16428
DB00720	Chronic progressive external ophthalmoplegia	0.2357
DB00720	Colon cancer	0.03077
DB00720	Diabetes mellitus	0.02236
DB00720	Embryoma	0.06183
DB00720	HIV infection	0.03009
DB00720	Keratoconjunctivitis Sicca	0.22702
DB00720	Leukemia	0.02496
DB00720	Leukoencephalopathy	0.04202
DB00720	Liver cancer	0.07279
DB00720	Lung cancer	0.03145

DB00720	Lung disease	0.06233
DB00720	Mental retardation	0.11586
DB00720	Muscular dystrophies	0.0752
DB00720	Neuroblastoma	0.05448
DB00720	Osteosarcoma	0.07985
DB00720	Pancreas cancer	0.07541
DB00720	Pancreas disease	0.15831
DB00720	Pre-Eclampsia	0.07416
DB00720	Prostate cancer	0.04086
DB00720	Stomach cancer	0.06724
DB00720	Tuberous sclerosis	0.04651
DB00720	Virus disease	0.05011
DB01586	Barrett's esophagus	0.18257
DB01586	Bladder cancer	0.1291
DB01586	Breast cancer	0.04811
DB01586	Esophageal disease	0.40825
DB01586	Glaucoma	0.16667
DB01133	Breast cancer	0.03402
DB01133	Colon cancer	0.04196
DB01133	Hypertension	0.0559
DB01133	Metabolism disease	0.11323
DB01133	Prostate cancer	0.03691
DB00010	Acromegaly	0.33333
DB00010	Cancer	0.03686
DB00010	Congenital abnormality	0.07538
DB08869	Acromegaly	0.33333
DB08869	Cancer	0.03686
DB08869	Congenital abnormality	0.07538
DB00020	Colon cancer	0.02654
DB00020	Diabetes mellitus	0.02354
DB00020	Enteritis	0.04909
DB00020	Leukemia	0.0504
DB00020	Myeloproliferative disease	0.11952
DB00020	Neoplasm metastasis	0.03639
DB00020	Schizophrenia	0.068
DB00825	Common cold	0.13363
DB00825	Neuroendocrine tumor	0.17678
DB00825	Prostate cancer	0.0261
DB08881	Skin disease, Genetic	0.19851
DB08881	Alzheimer's disease	0.06477
DB08881	Angiomyolipoma	1.00172
DB08881	Cancer	0.1228
DB08881	Common cold	0.82233
DB08881	Gram-Negative bacterial infection	0.07832
DB08881	Herpes	0.09169
DB08881	Lung cancer	0.01873
DB08881	Metaplastic polyp	0.77076

DB08881	Myopathy	0.48237
DB08881	Nephrosis	0.67158
DB08881	Osteomyelitis	0.26783
DB08881	Prion disease	0.0732
DB08881	Testicular dysfunction	0.38181
DB08881	Thyroid gland disease	0.70223
DB08881	Ulcerative colitis	0.33606
DB08881	Vitamin D deficiency	0.30079
DB00414	Hyperaldosteronism	0.31623
DB00414	Kidney disease	0.11952
DB00839	Hyperaldosteronism	0.31623
DB00839	Kidney disease	0.11952
DB01104	Hypertension, Pulmonary	0.16222
DB01104	Stress disorder, post-traumatic	0.25
DB01104	Anorexia nervosa	0.14434
DB01104	Atherosclerosis	0.04951
DB01104	Autistic disorder	0.08575
DB01104	Behavior disease	0.21822
DB01104	Bipolar disorder	0.16013
DB01104	Brain disease	0.127
DB01104	Breast cancer	0.03402
DB01104	Chronic fatigue syndrome	0.25
DB01104	Chronic obstructive airway disease	0.07906
DB01104	Colon cancer	0.04196
DB01104	Congenital heart disease	0.2357
DB01104	Depression	0.1857
DB01104	Dermatitis	0.06428
DB01104	Drug abuse	0.13245
DB01104	Epilepsy	0.1
DB01104	Fibromyalgia	0.25
DB01104	Generalized anxiety disorder	0.20412
DB01104	Gilles de la Tourette syndrome	0.26726
DB01104	Heart failure	0.07538
DB01104	Herpes	0.20203
DB01104	Migraine	0.26261
DB01104	Neurotic disorder	0.22361
DB01104	Obesity	0.10879
DB01104	Obsessive-compulsive disorder	0.2132
DB01104	Panic disorder	0.1543
DB01104	Pervasive development disorder	0.18257
DB01104	Psychotic disorder	0.22646
DB01104	Pulmonary hypertension	0.35355
DB01104	Stroke	0.08006
DB01104	Sudden infant death syndrome	0.1543
DB01104	Ulcerative colitis	0.07293
DB01463	Stress disorder, post-traumatic	0.35355
DB01463	Behavior disease	0.1543

DB01463	Bipolar disorder	0.11323
DB01463	Brain disease	0.17961
DB01463	Breast cancer	0.04811
DB01463	Depression	0.13131
DB01463	Drug abuse	0.09366
DB01463	Gilles de la Tourette syndrome	0.37796
DB01463	Herpes	0.14286
DB01463	Migraine	0.1857
DB01463	Obesity	0.07692
DB01463	Psychotic disorder	0.16013
DB00004	Prostatic hypertrophy, Benign	0.32151
DB00004	Purpura, Thrombocytopenic, Idiopathic	0.05355
DB00004	Stress disorder, post-traumatic	0.39289
DB00004	Abortion	0.07279
DB00004	Alimentary system disease	0.36515
DB00004	Asthma	0.0744
DB00004	Atherosclerosis	0.04563
DB00004	Autoimmune disease	0.26383
DB00004	Behcet syndrome	0.32568
DB00004	Bone metastases	0.17477
DB00004	Breast cancer	0.10003
DB00004	Brucellosis	0.32151
DB00004	Celiac disease	0.14465
DB00004	Cerebrovascular disorder	0.19617
DB00004	Colon cancer	0.04311
DB00004	Dermatitis	0.07112
DB00004	Diabetes mellitus	0.04429
DB00004	Down syndrome	0.17721
DB00004	Embryoma	0.27454
DB00004	Endometriosis	0.14918
DB00004	Enteritis	0.09226
DB00004	Eosinophilia	0.35367
DB00004	Epilepsy	0.08961
DB00004	Filariasis	0.8165
DB00004	Gastritis	0.07015
DB00004	Gouts	0.20185
DB00004	HIV infection	0.19492
DB00004	Hamman-Rich syndrome	0.12663
DB00004	Helminthiasis	0.38708
DB00004	Hemolytic-Uremic syndrome	0.49294
DB00004	Hepatitis C	0.14538
DB00004	Hodgkin's disease	0.09096
DB00004	Hyperglycemia	0.12676
DB00004	Hypothyroidism	0.28089
DB00004	IGA glomerulonephritis	0.18094
DB00004	Immunologic deficiency syndrome	0.18874
DB00004	Infection	0.12922

DB00004	Kidney failure	0.07779
DB00004	Leukemia	0.23302
DB00004	Liver cancer	0.18903
DB00004	Lung cancer	0.1725
DB00004	Lupus erythematosus	0.19925
DB00004	Lymphoma	0.25977
DB00004	Lymphopenia	0.22974
DB00004	Malignant glioma	0.15779
DB00004	Melanoma	0.08084
DB00004	Mitral valve disease	1.0747
DB00004	Mucocutaneous lymph node syndrome	0.15822
DB00004	Multiple sclerosis	0.11139
DB00004	Necrotizing enterocolitis	0.32151
DB00004	Oral cancer	0.05684
DB00004	Ovarian cancer	0.07332
DB00004	Pancreas cancer	0.28398
DB00004	Pancreatitis	0.22679
DB00004	Periodontitis	0.43295
DB00004	Pre-Eclampsia	0.1039
DB00004	Prostate cancer	0.08095
DB00004	Psoriasis	0.23656
DB00004	Renal Cell cancer	0.14322
DB00004	Rheumatoid arthritis	0.0773
DB00004	Schizophrenia	0.12909
DB00004	Sinusitis	0.11899
DB00004	Skin disease	0.22202
DB00004	Solid tumor	0.1514
DB00004	Squamous cell cancer	0.27263
DB00004	Stroke	0.04833
DB00004	Systemic infection	0.14835
DB00004	Systemic scleroderma	0.11326
DB00004	Temporal arteritis	0.10775
DB00004	Thyroid cancer	0.12199
DB00004	Tuberculosis	0.21845
DB00004	Ulcerative colitis	0.05717
DB00004	Virus disease	0.07019
DB00004	Wiskott-Aldrich syndrome	0.20342
DB00041	Prostatic hypertrophy, Benign	0.32151
DB00041	Purpura, Thrombocytopenic, Idiopathic	0.05355
DB00041	Stress disorder, post-traumatic	0.39289
DB00041	Abortion	0.07279
DB00041	Alimentary system disease	0.36515
DB00041	Asthma	0.0744
DB00041	Atherosclerosis	0.04563
DB00041	Autoimmune disease	0.26383
DB00041	Behcet syndrome	0.32568
DB00041	Bone metastases	0.17477

DB00041	Breast cancer	0.10003
DB00041	Brucellosis	0.32151
DB00041	Celiac disease	0.14465
DB00041	Cerebrovascular disorder	0.19617
DB00041	Colon cancer	0.04311
DB00041	Dermatitis	0.07112
DB00041	Diabetes mellitus	0.04429
DB00041	Down syndrome	0.17721
DB00041	Embryoma	0.27454
DB00041	Endometriosis	0.14918
DB00041	Enteritis	0.09226
DB00041	Eosinophilia	0.35367
DB00041	Epilepsy	0.08961
DB00041	Filariasis	0.8165
DB00041	Gastritis	0.07015
DB00041	Gouts	0.20185
DB00041	HIV infection	0.19492
DB00041	Hamman-Rich syndrome	0.12663
DB00041	Helminthiasis	0.38708
DB00041	Hemolytic-Uremic syndrome	0.49294
DB00041	Hepatitis C	0.14538
DB00041	Hodgkin's disease	0.09096
DB00041	Hyperglycemia	0.12676
DB00041	Hypothyroidism	0.28089
DB00041	IGA glomerulonephritis	0.18094
DB00041	Immunologic deficiency syndrome	0.18874
DB00041	Infection	0.12922
DB00041	Kidney failure	0.07779
DB00041	Leukemia	0.23302
DB00041	Liver cancer	0.18903
DB00041	Lung cancer	0.1725
DB00041	Lupus erythematosus	0.19925
DB00041	Lymphoma	0.25977
DB00041	Lymphopenia	0.22974
DB00041	Malignant glioma	0.15779
DB00041	Melanoma	0.08084
DB00041	Mitral valve disease	1.0747
DB00041	Mucocutaneous lymph node syndrome	0.15822
DB00041	Multiple sclerosis	0.11139
DB00041	Necrotizing enterocolitis	0.32151
DB00041	Oral cancer	0.05684
DB00041	Ovarian cancer	0.07332
DB00041	Pancreas cancer	0.28398
DB00041	Pancreatitis	0.22679
DB00041	Periodontitis	0.43295
DB00041	Pre-Eclampsia	0.1039
DB00041	Prostate cancer	0.08095

DB00041	Psoriasis	0.23656
DB00041	Renal Cell cancer	0.14322
DB00041	Rheumatoid arthritis	0.0773
DB00041	Schizophrenia	0.12909
DB00041	Sinusitis	0.11899
DB00041	Skin disease	0.22202
DB00041	Solid tumor	0.1514
DB00041	Squamous cell cancer	0.27263
DB00041	Stroke	0.04833
DB00041	Systemic infection	0.14835
DB00041	Systemic scleroderma	0.11326
DB00041	Temporal arteritis	0.10775
DB00041	Thyroid cancer	0.12199
DB00041	Tuberculosis	0.21845
DB00041	Ulcerative colitis	0.05717
DB00041	Virus disease	0.07019
DB00041	Wiskott-Aldrich syndrome	0.20342
DB00074	Pemphigoid, Bullous	0.09129
DB00074	Stress disorder, post-traumatic	0.3461
DB00074	Abortion	0.03608
DB00074	Alimentary system disease	0.32166
DB00074	Alopecia	0.16131
DB00074	Alzheimer's disease	0.02078
DB00074	Antiphospholipid syndrome	0.09623
DB00074	Asthma	0.02357
DB00074	Atherosclerosis	0.04042
DB00074	Autoimmune disease	0.2088
DB00074	Behcet syndrome	0.16564
DB00074	Breast cancer	0.0923
DB00074	Brucellosis	0.08704
DB00074	Celiac disease	0.17488
DB00074	Cerebrovascular disorder	0.1728
DB00074	Communicable disease	0.06299
DB00074	Cystic fibrosis	0.04124
DB00074	Down syndrome	0.13104
DB00074	Embryoma	0.14685
DB00074	Endometriosis	0.07546
DB00074	Enteritis	0.21504
DB00074	Eosinophilia	0.24524
DB00074	Filariasis	0.71925
DB00074	Generalized anxiety disorder	0.10305
DB00074	Glomerulonephritis	0.07217
DB00074	HIV infection	0.12957
DB00074	Hemolytic-Uremic syndrome	0.2495
DB00074	Hepatitis C	0.12806
DB00074	IGA glomerulonephritis	0.04951
DB00074	Immune complex disease	0.70445

DB00074	Infection	0.06572
DB00074	Infection by cryptococcus neoformans	0.33333
DB00074	Kaposi sarcoma	0.09069
DB00074	Kidney disease	0.0345
DB00074	Kidney failure	0.03269
DB00074	Leukemia	0.13924
DB00074	Liver cancer	0.09575
DB00074	Lung cancer	0.09778
DB00074	Lupus erythematosus	0.32745
DB00074	Lupus vulgaris	0.05455
DB00074	Lymphoma	0.13155
DB00074	Lymphopenia	0.20238
DB00074	Melanoma	0.09527
DB00074	Mitral valve disease	0.84835
DB00074	Multiple myeloma	0.08156
DB00074	Multiple sclerosis	0.1279
DB00074	Neoplasm metastasis	0.02349
DB00074	Pancreas cancer	0.123
DB00074	Pancreatitis	0.16544
DB00074	Periodontal disease	0.06804
DB00074	Periodontitis	0.30748
DB00074	Prostate cancer	0.04117
DB00074	Renal Cell cancer	0.15972
DB00074	Rheumatic fever	0.07454
DB00074	Rheumatoid arthritis	0.05698
DB00074	Schizophrenia	0.06565
DB00074	Skin disease	0.15787
DB00074	Skin tumor	0.15486
DB00074	Squamous cell cancer	0.13815
DB00074	Stomach cancer	0.06925
DB00074	Systemic infection	0.2365
DB00074	Systemic scleroderma	0.0576
DB00074	Thrombocytopenia	0.07217
DB00074	Tuberculosis	0.16278
DB00074	Vascular disease	0.0527
DB00074	Wiskott-Aldrich syndrome	0.17919
DB00074	Yersinia infection	0.04227
DB00111	Pemphigoid, Bullous	0.09535
DB00111	Stress disorder, post-traumatic	0.3461
DB00111	Abortion	0.03769
DB00111	Alimentary system disease	0.32166
DB00111	Alopecia	0.16366
DB00111	Alzheimer's disease	0.0217
DB00111	Antiphospholipid syndrome	0.1005
DB00111	Asthma	0.02462
DB00111	Atherosclerosis	0.04222
DB00111	Autoimmune disease	0.21437

DB00111	Behcet syndrome	0.16564
DB00111	Breast cancer	0.09477
DB00111	Brucellosis	0.09091
DB00111	Celiac disease	0.17699
DB00111	Cerebrovascular disorder	0.1728
DB00111	Communicable disease	0.0658
DB00111	Cystic fibrosis	0.04307
DB00111	Down syndrome	0.1325
DB00111	Embryoma	0.14923
DB00111	Endometriosis	0.07653
DB00111	Enteritis	0.21785
DB00111	Eosinophilia	0.24911
DB00111	Filariasis	0.71925
DB00111	Generalized anxiety disorder	0.10305
DB00111	Glomerulonephritis	0.07538
DB00111	HIV infection	0.13203
DB00111	Hemolytic-Uremic syndrome	0.25262
DB00111	Hepatitis C	0.12806
DB00111	IGA glomerulonephritis	0.05171
DB00111	Immune complex disease	0.71352
DB00111	Infection	0.06572
DB00111	Infection by cryptococcus neoformans	0.34816
DB00111	Kaposi sarcoma	0.09069
DB00111	Kidney disease	0.03604
DB00111	Kidney failure	0.03414
DB00111	Leukemia	0.14068
DB00111	Liver cancer	0.09675
DB00111	Lung cancer	0.09868
DB00111	Lupus erythematosus	0.33403
DB00111	Lupus vulgaris	0.05698
DB00111	Lymphoma	0.13301
DB00111	Lymphopenia	0.20238
DB00111	Melanoma	0.09634
DB00111	Mitral valve disease	0.85409
DB00111	Multiple myeloma	0.08156
DB00111	Multiple sclerosis	0.12922
DB00111	Neoplasm metastasis	0.02454
DB00111	Pancreas cancer	0.12433
DB00111	Pancreatitis	0.16744
DB00111	Periodontal disease	0.07107
DB00111	Periodontitis	0.31335
DB00111	Prostate cancer	0.04117
DB00111	Renal Cell cancer	0.16122
DB00111	Rheumatic fever	0.07785
DB00111	Rheumatoid arthritis	0.05777
DB00111	Schizophrenia	0.06565
DB00111	Skin disease	0.16007

DB00111	Skin tumor	0.15486
DB00111	Squamous cell cancer	0.13946
DB00111	Stomach cancer	0.06925
DB00111	Systemic infection	0.23945
DB00111	Systemic scleroderma	0.0576
DB00111	Thrombocytopenia	0.07538
DB00111	Tuberculosis	0.16451
DB00111	Vascular disease	0.05505
DB00111	Wiskott-Aldrich syndrome	0.17919
DB00111	Yersinia infection	0.04227
DB00012	Alzheimer's disease	0.17365
DB00012	Anemia	0.21845
DB00012	Brain ischemia	0.27117
DB00012	Cancer	0.09857
DB00012	Chronic obstructive airway disease	0.12054
DB00012	Chronic simple glaucoma	0.16649
DB00012	Congenital abnormality	0.04806
DB00012	Deafness	0.2038
DB00012	Diabetes mellitus	0.03864
DB00012	Drug abuse	0.23176
DB00012	Endometriosis	0.22943
DB00012	Ischemia	0.09435
DB00012	Kidney failure	0.09597
DB00012	Liver disease	0.20967
DB00012	Myeloproliferative disease	0.60639
DB00012	Polycythemia	0.83157
DB00012	Premature birth	0.24901
DB00016	Alzheimer's disease	0.17365
DB00016	Anemia	0.21845
DB00016	Brain ischemia	0.27117
DB00016	Cancer	0.09857
DB00016	Chronic obstructive airway disease	0.12054
DB00016	Chronic simple glaucoma	0.16649
DB00016	Congenital abnormality	0.04806
DB00016	Deafness	0.2038
DB00016	Diabetes mellitus	0.03864
DB00016	Drug abuse	0.23176
DB00016	Endometriosis	0.22943
DB00016	Ischemia	0.09435
DB00016	Kidney failure	0.09597
DB00016	Liver disease	0.20967
DB00016	Myeloproliferative disease	0.60639
DB00016	Polycythemia	0.83157
DB00016	Premature birth	0.24901
DB08894	Alzheimer's disease	0.17365
DB08894	Anemia	0.21845
DB08894	Brain ischemia	0.27117

DB08894	Cancer	0.09857
DB08894	Chronic obstructive airway disease	0.12054
DB08894	Chronic simple glaucoma	0.16649
DB08894	Congenital abnormality	0.04806
DB08894	Deafness	0.2038
DB08894	Diabetes mellitus	0.03864
DB08894	Drug abuse	0.23176
DB08894	Endometriosis	0.22943
DB08894	Ischemia	0.09435
DB08894	Kidney failure	0.09597
DB08894	Liver disease	0.20967
DB08894	Myeloproliferative disease	0.60639
DB08894	Polycythemia	0.83157
DB08894	Premature birth	0.24901
DB08923	Alzheimer's disease	0.17365
DB08923	Anemia	0.21845
DB08923	Brain ischemia	0.27117
DB08923	Cancer	0.09857
DB08923	Chronic obstructive airway disease	0.12054
DB08923	Chronic simple glaucoma	0.16649
DB08923	Congenital abnormality	0.04806
DB08923	Deafness	0.2038
DB08923	Diabetes mellitus	0.03864
DB08923	Drug abuse	0.23176
DB08923	Endometriosis	0.22943
DB08923	Ischemia	0.09435
DB08923	Kidney failure	0.09597
DB08923	Liver disease	0.20967
DB08923	Myeloproliferative disease	0.60639
DB08923	Polycythemia	0.83157
DB08923	Premature birth	0.24901
DB01103	Bacterial infection	0.1543
DB01103	Colon cancer	0.03426
DB01103	Diabetes mellitus	0.03039
DB01103	Gouts	0.16013
DB01103	Leukemia	0.03253
DB01103	Liver cancer	0.04481
DB01103	Polyarthritis	0.0658
DB01103	Rheumatoid arthritis	0.03533
DB01103	Schizophrenia	0.0439
DB00918	Abortion	0.04986
DB00918	Amyotrophic lateral sclerosis	0.07682
DB00918	Anorexia nervosa	0.8278
DB00918	Attention deficit hyperactivity disorder	0.73352
DB00918	Behavior disease	0.55287
DB00918	Bipolar disorder	0.37544
DB00918	Depression	0.28902

DB00918	Dermatitis	0.06891
DB00918	Diabetes mellitus	0.04291
DB00918	Drug abuse	0.16993
DB00918	Epilepsy	0.12278
DB00918	Hepatitis C	0.14085
DB00918	Hypertension	0.2831
DB00918	Migraine	0.14904
DB00918	Panic disorder	0.19038
DB00918	Stroke	0.06622
DB00918	Sudden infant death syndrome	0.22763
DB00998	Abortion	0.04986
DB00998	Amyotrophic lateral sclerosis	0.07682
DB00998	Anorexia nervosa	0.8278
DB00998	Attention deficit hyperactivity disorder	0.73352
DB00998	Behavior disease	0.55287
DB00998	Bipolar disorder	0.37544
DB00998	Depression	0.28902
DB00998	Dermatitis	0.06891
DB00998	Diabetes mellitus	0.04291
DB00998	Drug abuse	0.16993
DB00998	Epilepsy	0.12278
DB00998	Hepatitis C	0.14085
DB00998	Hypertension	0.2831
DB00998	Migraine	0.14904
DB00998	Panic disorder	0.19038
DB00998	Stroke	0.06622
DB00998	Sudden infant death syndrome	0.22763
DB05258	Arthritis	0.21097
DB05258	Autoimmune disease	0.11617
DB05258	Brain tumor	0.11126
DB05258	Colon cancer	0.07466
DB05258	Encephalitis	0.36745
DB05258	Herpes	0.0837
DB05258	Infection	0.09137
DB05258	Liver cancer	0.10198
DB05258	Liver metastases	0.36072
DB05258	Lymphoma	0.13716
DB05258	Malaria	0.43024
DB05258	Melanoma	0.09901
DB05258	Multiple myeloma	0.35809
DB05258	Pancreas cancer	0.28668
DB05258	Prostate cancer	0.05724
DB05258	Renal Cell cancer	0.17541
DB05258	Respiratory tract disease	0.22212
DB05258	Sicca syndrome	0.17462
DB00982	Aseptic necrosis of bone	0.1563
DB00982	Breast cancer	0.0992

DB00982	Cancer	0.03018
DB00982	Dermatitis	0.0871
DB00982	Esophagus cancer	0.33773
DB00982	Leukemia	0.142
DB00982	Leukoencephalopathy	0.31086
DB00982	Lung cancer	0.07631
DB00982	Mucopolysaccharidosis	0.22585
DB00982	Neuroblastoma	0.13218
DB00982	Obesity	0.1772
DB00982	Prostate cancer	0.13315
DB00982	Rheumatism	0.18028
DB00982	Rheumatoid arthritis	0.11586
DB00700	Alopecia	0.34442
DB00700	Breast cancer	0.12036
DB00700	Heart failure	0.28895
DB00700	Hypertension	0.2291
DB00700	Hypoglycemia	1.19578
DB00700	Lymphoma	0.30794
DB00700	Melanoma	0.07001
DB00700	Proteinuria	0.75896
DB00700	Renal Cell cancer	0.36432
DB00056	Pemphigoid, Bullous	0.09535
DB00056	Abortion	0.03769
DB00056	Alopecia	0.21138
DB00056	Alzheimer's disease	0.0217
DB00056	Antiphospholipid syndrome	0.1005
DB00056	Asthma	0.02462
DB00056	Atherosclerosis	0.04222
DB00056	Autoimmune disease	0.09811
DB00056	Breast cancer	0.04352
DB00056	Brucellosis	0.09091
DB00056	Celiac disease	0.04957
DB00056	Communicable disease	0.0658
DB00056	Cystic fibrosis	0.04307
DB00056	Embryoma	0.01859
DB00056	Enteritis	0.28449
DB00056	Generalized anxiety disorder	0.14832
DB00056	Glomerulonephritis	0.07538
DB00056	IGA glomerulonephritis	0.05171
DB00056	Immune complex disease	0.93335
DB00056	Infection	0.02812
DB00056	Infection by cryptococcus neoformans	0.34816
DB00056	Kaposi sarcoma	0.13054
DB00056	Kidney disease	0.03604
DB00056	Kidney failure	0.03414
DB00056	Leukemia	0.06133
DB00056	Lupus erythematosus	0.27361

DB00056	Lupus vulgaris	0.05698
DB00056	Melanoma	0.02513
DB00056	Multiple myeloma	0.11739
DB00056	Multiple sclerosis	0.0311
DB00056	Neoplasm metastasis	0.02454
DB00056	Periodontal disease	0.07107
DB00056	Periodontitis	0.09196
DB00056	Renal Cell cancer	0.03505
DB00056	Rheumatic fever	0.07785
DB00056	Rheumatoid arthritis	0.01845
DB00056	Skin tumor	0.2229
DB00056	Stomach cancer	0.09968
DB00056	Systemic infection	0.17198
DB00056	Thrombocytopenia	0.07538
DB00056	Vascular disease	0.05505
DB00056	Yersinia infection	0.06085
DB00914	Congenital abnormality	0.0533
DB00914	Late pregnancy	0.20412
DB01083	Cancer	0.02128
DB01083	Obesity	0.04441
DB01083	Osteitis deformans	0.2357
DB04838	Cancer	0.02606
DB04838	Embryoma	0.0436
DB00816	Infertility, Male	0.10792
DB00816	Alimentary system disease	0.20087
DB00816	Alzheimer's disease	0.02656
DB00816	Arthritis	0.59373
DB00816	Atherosclerosis	0.24599
DB00816	Autistic disorder	0.40171
DB00816	Azoospermia	0.08162
DB00816	Breast cancer	0.02295
DB00816	Bronchial disease	1.182
DB00816	Chronic obstructive airway disease	0.4886
DB00816	Conduct disorder	0.20914
DB00816	Cystic fibrosis	0.62915
DB00816	Dermatitis	0.28484
DB00816	Diabetes mellitus	0.17341
DB00816	Drug abuse	0.24402
DB00816	Enteritis	0.07178
DB00816	Epilepsy	0.15509
DB00816	Glaucoma	0.52087
DB00816	Gram-Negative bacterial infection	0.14332
DB00816	Graves' disease	0.68507
DB00816	Heart failure	0.42129
DB00816	Hypertension	0.338
DB00816	Infertility	0.06895
DB00816	Ischemia	0.13237

DB00816	Liver cancer	0.0458
DB00816	Lung cancer	0.23974
DB00816	Malaria	0.73015
DB00816	Metabolism disease	0.67268
DB00816	Movement disorder	0.30602
DB00816	Myopathy	0.46304
DB00816	Neurodegenerative disorder	0.16921
DB00816	Obesity	0.30018
DB00816	Oligospermia	0.14861
DB00816	Polycystic kidney	0.12932
DB00816	Polycystic ovary syndrome	0.41786
DB00816	Premature birth	1.0209
DB00816	Prostate cancer	0.20559
DB00816	Respiratory tract disease	0.09977
DB00816	Rheumatoid arthritis	0.18289
DB00816	Sickle cell disease	0.43526
DB00816	Sinusitis	0.09257
DB00816	Subarachnoid hemorrhage	0.68161
DB00816	Testicular dysfunction	0.04563
DB00867	Infertility, Male	0.10792
DB00867	Alimentary system disease	0.20087
DB00867	Alzheimer's disease	0.02656
DB00867	Arthritis	0.59373
DB00867	Atherosclerosis	0.24599
DB00867	Autistic disorder	0.40171
DB00867	Azoospermia	0.08162
DB00867	Breast cancer	0.02295
DB00867	Bronchial disease	1.182
DB00867	Chronic obstructive airway disease	0.4886
DB00867	Conduct disorder	0.20914
DB00867	Cystic fibrosis	0.62915
DB00867	Dermatitis	0.28484
DB00867	Diabetes mellitus	0.17341
DB00867	Drug abuse	0.24402
DB00867	Enteritis	0.07178
DB00867	Epilepsy	0.15509
DB00867	Glaucoma	0.52087
DB00867	Gram-Negative bacterial infection	0.14332
DB00867	Graves' disease	0.68507
DB00867	Heart failure	0.42129
DB00867	Hypertension	0.338
DB00867	Infertility	0.06895
DB00867	Ischemia	0.13237
DB00867	Liver cancer	0.0458
DB00867	Lung cancer	0.23974
DB00867	Malaria	0.73015
DB00867	Metabolism disease	0.67268

DB00867	Movement disorder	0.30602
DB00867	Myopathy	0.46304
DB00867	Neurodegenerative disorder	0.16921
DB00867	Obesity	0.30018
DB00867	Oligospermia	0.14861
DB00867	Polycystic kidney	0.12932
DB00867	Polycystic ovary syndrome	0.41786
DB00867	Premature birth	1.0209
DB00867	Prostate cancer	0.20559
DB00867	Respiratory tract disease	0.09977
DB00867	Rheumatoid arthritis	0.18289
DB00867	Sickle cell disease	0.43526
DB00867	Sinusitis	0.09257
DB00867	Subarachnoid hemorrhage	0.68161
DB00867	Testicular dysfunction	0.04563
DB00871	Infertility, Male	0.10792
DB00871	Alimentary system disease	0.20087
DB00871	Alzheimer's disease	0.02656
DB00871	Arthritis	0.59373
DB00871	Atherosclerosis	0.24599
DB00871	Autistic disorder	0.40171
DB00871	Azoospermia	0.08162
DB00871	Breast cancer	0.02295
DB00871	Bronchial disease	1.182
DB00871	Chronic obstructive airway disease	0.4886
DB00871	Conduct disorder	0.20914
DB00871	Cystic fibrosis	0.62915
DB00871	Dermatitis	0.28484
DB00871	Diabetes mellitus	0.17341
DB00871	Drug abuse	0.24402
DB00871	Enteritis	0.07178
DB00871	Epilepsy	0.15509
DB00871	Glaucoma	0.52087
DB00871	Gram-Negative bacterial infection	0.14332
DB00871	Graves' disease	0.68507
DB00871	Heart failure	0.42129
DB00871	Hypertension	0.338
DB00871	Infertility	0.06895
DB00871	Ischemia	0.13237
DB00871	Liver cancer	0.0458
DB00871	Lung cancer	0.23974
DB00871	Malaria	0.73015
DB00871	Metabolism disease	0.67268
DB00871	Movement disorder	0.30602
DB00871	Myopathy	0.46304
DB00871	Neurodegenerative disorder	0.16921
DB00871	Obesity	0.30018

DB00871	Oligospermia	0.14861
DB00871	Polycystic kidney	0.12932
DB00871	Polycystic ovary syndrome	0.41786
DB00871	Premature birth	1.0209
DB00871	Prostate cancer	0.20559
DB00871	Respiratory tract disease	0.09977
DB00871	Rheumatoid arthritis	0.18289
DB00871	Sickle cell disease	0.43526
DB00871	Sinusitis	0.09257
DB00871	Subarachnoid hemorrhage	0.68161
DB00871	Testicular dysfunction	0.04563
DB00938	Infertility, Male	0.10792
DB00938	Alimentary system disease	0.20087
DB00938	Alzheimer's disease	0.02656
DB00938	Arthritis	0.59373
DB00938	Atherosclerosis	0.24599
DB00938	Autistic disorder	0.40171
DB00938	Azoospermia	0.08162
DB00938	Breast cancer	0.02295
DB00938	Bronchial disease	1.182
DB00938	Chronic obstructive airway disease	0.4886
DB00938	Conduct disorder	0.20914
DB00938	Cystic fibrosis	0.62915
DB00938	Dermatitis	0.28484
DB00938	Diabetes mellitus	0.17341
DB00938	Drug abuse	0.24402
DB00938	Enteritis	0.07178
DB00938	Epilepsy	0.15509
DB00938	Glaucoma	0.52087
DB00938	Gram-Negative bacterial infection	0.14332
DB00938	Graves' disease	0.68507
DB00938	Heart failure	0.42129
DB00938	Hypertension	0.338
DB00938	Infertility	0.06895
DB00938	Ischemia	0.13237
DB00938	Liver cancer	0.0458
DB00938	Lung cancer	0.23974
DB00938	Malaria	0.73015
DB00938	Metabolism disease	0.67268
DB00938	Movement disorder	0.30602
DB00938	Myopathy	0.46304
DB00938	Neurodegenerative disorder	0.16921
DB00938	Obesity	0.30018
DB00938	Oligospermia	0.14861
DB00938	Polycystic kidney	0.12932
DB00938	Polycystic ovary syndrome	0.41786
DB00938	Premature birth	1.0209

DB00938	Prostate cancer	0.20559
DB00938	Respiratory tract disease	0.09977
DB00938	Rheumatoid arthritis	0.18289
DB00938	Sickle cell disease	0.43526
DB00938	Sinusitis	0.09257
DB00938	Subarachnoid hemorrhage	0.68161
DB00938	Testicular dysfunction	0.04563
DB00983	Infertility, Male	0.10792
DB00983	Alimentary system disease	0.20087
DB00983	Alzheimer's disease	0.02656
DB00983	Arthritis	0.59373
DB00983	Atherosclerosis	0.24599
DB00983	Autistic disorder	0.40171
DB00983	Azoospermia	0.08162
DB00983	Breast cancer	0.02295
DB00983	Bronchial disease	1.182
DB00983	Chronic obstructive airway disease	0.4886
DB00983	Conduct disorder	0.20914
DB00983	Cystic fibrosis	0.62915
DB00983	Dermatitis	0.28484
DB00983	Diabetes mellitus	0.17341
DB00983	Drug abuse	0.24402
DB00983	Enteritis	0.07178
DB00983	Epilepsy	0.15509
DB00983	Glaucoma	0.52087
DB00983	Gram-Negative bacterial infection	0.14332
DB00983	Graves' disease	0.68507
DB00983	Heart failure	0.42129
DB00983	Hypertension	0.338
DB00983	Infertility	0.06895
DB00983	Ischemia	0.13237
DB00983	Liver cancer	0.0458
DB00983	Lung cancer	0.23974
DB00983	Malaria	0.73015
DB00983	Metabolism disease	0.67268
DB00983	Movement disorder	0.30602
DB00983	Myopathy	0.46304
DB00983	Neurodegenerative disorder	0.16921
DB00983	Obesity	0.30018
DB00983	Oligospermia	0.14861
DB00983	Polycystic kidney	0.12932
DB00983	Polycystic ovary syndrome	0.41786
DB00983	Premature birth	1.0209
DB00983	Prostate cancer	0.20559
DB00983	Respiratory tract disease	0.09977
DB00983	Rheumatoid arthritis	0.18289
DB00983	Sickle cell disease	0.43526

DB00983	Sinusitis	0.09257
DB00983	Subarachnoid hemorrhage	0.68161
DB00983	Testicular dysfunction	0.04563
DB01274	Infertility, Male	0.10792
DB01274	Alimentary system disease	0.20087
DB01274	Alzheimer's disease	0.02656
DB01274	Arthritis	0.59373
DB01274	Atherosclerosis	0.24599
DB01274	Autistic disorder	0.40171
DB01274	Azoospermia	0.08162
DB01274	Breast cancer	0.02295
DB01274	Bronchial disease	1.182
DB01274	Chronic obstructive airway disease	0.4886
DB01274	Conduct disorder	0.20914
DB01274	Cystic fibrosis	0.62915
DB01274	Dermatitis	0.28484
DB01274	Diabetes mellitus	0.17341
DB01274	Drug abuse	0.24402
DB01274	Enteritis	0.07178
DB01274	Epilepsy	0.15509
DB01274	Glaucoma	0.52087
DB01274	Gram-Negative bacterial infection	0.14332
DB01274	Graves' disease	0.68507
DB01274	Heart failure	0.42129
DB01274	Hypertension	0.338
DB01274	Infertility	0.06895
DB01274	Ischemia	0.13237
DB01274	Liver cancer	0.0458
DB01274	Lung cancer	0.23974
DB01274	Malaria	0.73015
DB01274	Metabolism disease	0.67268
DB01274	Movement disorder	0.30602
DB01274	Myopathy	0.46304
DB01274	Neurodegenerative disorder	0.16921
DB01274	Obesity	0.30018
DB01274	Oligospermia	0.14861
DB01274	Polycystic kidney	0.12932
DB01274	Polycystic ovary syndrome	0.41786
DB01274	Premature birth	1.0209
DB01274	Prostate cancer	0.20559
DB01274	Respiratory tract disease	0.09977
DB01274	Rheumatoid arthritis	0.18289
DB01274	Sickle cell disease	0.43526
DB01274	Sinusitis	0.09257
DB01274	Subarachnoid hemorrhage	0.68161
DB01274	Testicular dysfunction	0.04563
DB01366	Infertility, Male	0.10792

DB01366	Alimentary system disease	0.20087
DB01366	Alzheimer's disease	0.02656
DB01366	Arthritis	0.59373
DB01366	Atherosclerosis	0.24599
DB01366	Autistic disorder	0.40171
DB01366	Azoospermia	0.08162
DB01366	Breast cancer	0.02295
DB01366	Bronchial disease	1.182
DB01366	Chronic obstructive airway disease	0.4886
DB01366	Conduct disorder	0.20914
DB01366	Cystic fibrosis	0.62915
DB01366	Dermatitis	0.28484
DB01366	Diabetes mellitus	0.17341
DB01366	Drug abuse	0.24402
DB01366	Enteritis	0.07178
DB01366	Epilepsy	0.15509
DB01366	Glaucoma	0.52087
DB01366	Gram-Negative bacterial infection	0.14332
DB01366	Graves' disease	0.68507
DB01366	Heart failure	0.42129
DB01366	Hypertension	0.338
DB01366	Infertility	0.06895
DB01366	Ischemia	0.13237
DB01366	Liver cancer	0.0458
DB01366	Lung cancer	0.23974
DB01366	Malaria	0.73015
DB01366	Metabolism disease	0.67268
DB01366	Movement disorder	0.30602
DB01366	Myopathy	0.46304
DB01366	Neurodegenerative disorder	0.16921
DB01366	Obesity	0.30018
DB01366	Oligospermia	0.14861
DB01366	Polycystic kidney	0.12932
DB01366	Polycystic ovary syndrome	0.41786
DB01366	Premature birth	1.0209
DB01366	Prostate cancer	0.20559
DB01366	Respiratory tract disease	0.09977
DB01366	Rheumatoid arthritis	0.18289
DB01366	Sickle cell disease	0.43526
DB01366	Sinusitis	0.09257
DB01366	Subarachnoid hemorrhage	0.68161
DB01366	Testicular dysfunction	0.04563
DB01408	Infertility, Male	0.10792
DB01408	Alimentary system disease	0.20087
DB01408	Alzheimer's disease	0.02656
DB01408	Arthritis	0.59373
DB01408	Atherosclerosis	0.24599

DB01408	Autistic disorder	0.40171
DB01408	Azoospermia	0.08162
DB01408	Breast cancer	0.02295
DB01408	Bronchial disease	1.182
DB01408	Chronic obstructive airway disease	0.4886
DB01408	Conduct disorder	0.20914
DB01408	Cystic fibrosis	0.62915
DB01408	Dermatitis	0.28484
DB01408	Diabetes mellitus	0.17341
DB01408	Drug abuse	0.24402
DB01408	Enteritis	0.07178
DB01408	Epilepsy	0.15509
DB01408	Glaucoma	0.52087
DB01408	Gram-Negative bacterial infection	0.14332
DB01408	Graves' disease	0.68507
DB01408	Heart failure	0.42129
DB01408	Hypertension	0.338
DB01408	Infertility	0.06895
DB01408	Ischemia	0.13237
DB01408	Liver cancer	0.0458
DB01408	Lung cancer	0.23974
DB01408	Malaria	0.73015
DB01408	Metabolism disease	0.67268
DB01408	Movement disorder	0.30602
DB01408	Myopathy	0.46304
DB01408	Neurodegenerative disorder	0.16921
DB01408	Obesity	0.30018
DB01408	Oligospermia	0.14861
DB01408	Polycystic kidney	0.12932
DB01408	Polycystic ovary syndrome	0.41786
DB01408	Premature birth	1.0209
DB01408	Prostate cancer	0.20559
DB01408	Respiratory tract disease	0.09977
DB01408	Rheumatoid arthritis	0.18289
DB01408	Sickle cell disease	0.43526
DB01408	Sinusitis	0.09257
DB01408	Subarachnoid hemorrhage	0.68161
DB01408	Testicular dysfunction	0.04563
DB05039	Infertility, Male	0.10792
DB05039	Alimentary system disease	0.20087
DB05039	Alzheimer's disease	0.02656
DB05039	Arthritis	0.59373
DB05039	Atherosclerosis	0.24599
DB05039	Autistic disorder	0.40171
DB05039	Azoospermia	0.08162
DB05039	Breast cancer	0.02295
DB05039	Bronchial disease	1.182

DB05039	Chronic obstructive airway disease	0.4886
DB05039	Conduct disorder	0.20914
DB05039	Cystic fibrosis	0.62915
DB05039	Dermatitis	0.28484
DB05039	Diabetes mellitus	0.17341
DB05039	Drug abuse	0.24402
DB05039	Enteritis	0.07178
DB05039	Epilepsy	0.15509
DB05039	Glaucoma	0.52087
DB05039	Gram-Negative bacterial infection	0.14332
DB05039	Graves' disease	0.68507
DB05039	Heart failure	0.42129
DB05039	Hypertension	0.338
DB05039	Infertility	0.06895
DB05039	Ischemia	0.13237
DB05039	Liver cancer	0.0458
DB05039	Lung cancer	0.23974
DB05039	Malaria	0.73015
DB05039	Metabolism disease	0.67268
DB05039	Movement disorder	0.30602
DB05039	Myopathy	0.46304
DB05039	Neurodegenerative disorder	0.16921
DB05039	Obesity	0.30018
DB05039	Oligospermia	0.14861
DB05039	Polycystic kidney	0.12932
DB05039	Polycystic ovary syndrome	0.41786
DB05039	Premature birth	1.0209
DB05039	Prostate cancer	0.20559
DB05039	Respiratory tract disease	0.09977
DB05039	Rheumatoid arthritis	0.18289
DB05039	Sickle cell disease	0.43526
DB05039	Sinusitis	0.09257
DB05039	Subarachnoid hemorrhage	0.68161
DB05039	Testicular dysfunction	0.04563
DB00337	Bone metastases	0.08162
DB00337	Brain tumor	0.13392
DB00337	Infection	0.29347
DB00337	Renal Cell cancer	0.0473
DB00337	Tuberous sclerosis	0.22661
DB00877	Anemia	0.23366
DB00877	Arthritis	0.14767
DB00877	Asthma	0.07999
DB00877	Bone metastases	0.07717
DB00877	Brain tumor	0.12662
DB00877	Cancer	0.03216
DB00877	Cirrhosis	0.20477
DB00877	Diabetes mellitus	0.04994

DB00877	Eating disorder	0.14246
DB00877	Endometriosis	0.07375
DB00877	Gestational diabetes	0.44584
DB00877	Heart failure	0.10803
DB00877	Hepatitis C	0.14932
DB00877	Herpes	0.11266
DB00877	Infection	0.27746
DB00877	Pre-Eclampsia	0.13292
DB00877	Renal Cell cancer	0.04472
DB00877	Stroke	0.09555
DB00877	Systemic scleroderma	0.0894
DB00877	Tuberous sclerosis	0.21425
DB00005	Pemphigoid, Bullous	0.08771
DB00005	Purpura, Thrombocytopenic, Idiopathic	0.12892
DB00005	Abortion	0.03467
DB00005	Alcoholic liver disease	0.11323
DB00005	Alopecia	0.17678
DB00005	Alzheimer's disease	0.03993
DB00005	Amnionitis	0.33534
DB00005	Amyloidosis	0.05241
DB00005	Anemia	0.05913
DB00005	Anorexia nervosa	0.05661
DB00005	Antiphospholipid syndrome	0.09245
DB00005	Aplastic anemia	0.05783
DB00005	Arthritis	0.0344
DB00005	Asthma	0.12176
DB00005	Atherosclerosis	0.12436
DB00005	Autoimmune disease	0.12033
DB00005	Basal cell carcinoma	0.07692
DB00005	Behcet syndrome	0.03962
DB00005	Bladder cancer	0.18805
DB00005	Breast cancer	0.0969
DB00005	Brucellosis	0.08362
DB00005	Celiac disease	0.09119
DB00005	Cervical cancer	0.03224
DB00005	Colon cancer	0.07702
DB00005	Communicable disease	0.06052
DB00005	Cystic fibrosis	0.03962
DB00005	Dermatitis	0.02521
DB00005	Diabetes mellitus	0.07451
DB00005	Down syndrome	0.03161
DB00005	Embryoma	0.0342
DB00005	Endometriosis	0.08835
DB00005	Enteritis	0.39232
DB00005	Familial Mediterranean fever	0.07161
DB00005	Fanconi's anemia	0.05547
DB00005	Generalized anxiety disorder	0.11968

DB00005	Glomerulonephritis	0.06934
DB00005	Granulomatous disease	0.08006
DB00005	HIV infection	0.02657
DB00005	Heart failure	0.02957
DB00005	Hepatitis	0.14765
DB00005	Hepatitis B	0.06727
DB00005	Histiocytosis	0.09245
DB00005	Hypertension	0.0846
DB00005	Hypothyroidism	0.06537
DB00005	IGA glomerulonephritis	0.04757
DB00005	Immune complex disease	0.7772
DB00005	Infection by cryptococcus neoformans	0.32026
DB00005	Infertility	0.0381
DB00005	Intermediate coronary syndrome	0.09245
DB00005	Kaposi sarcoma	0.10534
DB00005	Kidney disease	0.0663
DB00005	Kidney failure	0.0314
DB00005	Leprosy	0.32597
DB00005	Leukemia	0.0514
DB00005	Lichen planus	0.09806
DB00005	Lipodystrophy	0.07692
DB00005	Liver cancer	0.02153
DB00005	Lung cancer	0.06445
DB00005	Lupus erythematosus	0.25902
DB00005	Lupus vulgaris	0.05241
DB00005	Malaria	0.0515
DB00005	Malignant glioma	0.0515
DB00005	Melanoma	0.15204
DB00005	Migraine	0.21429
DB00005	Mucocutaneous lymph node syndrome	0.06052
DB00005	Multiple myeloma	0.26228
DB00005	Multiple sclerosis	0.05721
DB00005	Muscular dystrophies	0.05439
DB00005	Mycoses	0.12403
DB00005	Narcolepsy	0.10483
DB00005	Nasopharyngeal cancer	0.05338
DB00005	Necrotizing enterocolitis	0.11323
DB00005	Neoplasm metastasis	0.02257
DB00005	Nephrosis	0.06202
DB00005	Obesity	0.02133
DB00005	Otitis media	0.09245
DB00005	Ovary cancer	0.06202
DB00005	Pancreas cancer	0.02876
DB00005	Pancreatitis	0.18547
DB00005	Peptic ulcer	0.19891
DB00005	Periodontal disease	0.06537
DB00005	Periodontitis	0.12689

DB00005	Polycystic ovary syndrome	0.03674
DB00005	Premature birth	0.27378
DB00005	Prostate cancer	0.01448
DB00005	Psoriasis	0.37277
DB00005	Pulmonary fibrosis	0.05241
DB00005	Renal Cell cancer	0.06448
DB00005	Rheumatic fever	0.07161
DB00005	Rheumatoid arthritis	0.08321
DB00005	Sarcoidosis	0.27459
DB00005	Schizophrenia	0.09609
DB00005	Silicosis	0.10483
DB00005	Skin tumor	0.17986
DB00005	Stomach cancer	0.17024
DB00005	Stroke	0.11225
DB00005	Systemic infection	0.17449
DB00005	Thrombocytopenia	0.06934
DB00005	Thrombophilia	0.06537
DB00005	Thyroid cancer	0.04828
DB00005	Tuberculosis	0.0374
DB00005	Tuberous sclerosis	0.07161
DB00005	Ulcerative colitis	0.02861
DB00005	Vascular disease	0.05064
DB00005	Yersinia infection	0.0491
DB00051	Pemphigoid, Bullous	0.09535
DB00051	Abortion	0.03769
DB00051	Alcoholic liver disease	0.12309
DB00051	Alopecia	0.21138
DB00051	Alzheimer's disease	0.04341
DB00051	Amyloidosis	0.05698
DB00051	Anemia	0.06428
DB00051	Anorexia nervosa	0.06155
DB00051	Antiphospholipid syndrome	0.1005
DB00051	Aplastic anemia	0.06287
DB00051	Arthritis	0.0374
DB00051	Asthma	0.04924
DB00051	Atherosclerosis	0.06333
DB00051	Autoimmune disease	0.13081
DB00051	Basal cell carcinoma	0.08362
DB00051	Behcet syndrome	0.04307
DB00051	Bladder cancer	0.03892
DB00051	Breast cancer	0.05803
DB00051	Brucellosis	0.09091
DB00051	Celiac disease	0.09914
DB00051	Cervical cancer	0.03505
DB00051	Colon cancer	0.01789
DB00051	Communicable disease	0.0658
DB00051	Cystic fibrosis	0.04307

DB00051	Dermatitis	0.02741
DB00051	Diabetes mellitus	0.01587
DB00051	Down syndrome	0.03436
DB00051	Embryoma	0.03718
DB00051	Endometriosis	0.02504
DB00051	Enteritis	0.31759
DB00051	Familial Mediterranean fever	0.07785
DB00051	Fanconi's anemia	0.0603
DB00051	Generalized anxiety disorder	0.14832
DB00051	Glomerulonephritis	0.07538
DB00051	Granulomatous disease	0.08704
DB00051	HIV infection	0.02888
DB00051	Heart failure	0.03214
DB00051	Hepatitis B	0.07313
DB00051	Histiocytosis	0.1005
DB00051	Hypothyroidism	0.07107
DB00051	IGA glomerulonephritis	0.05171
DB00051	Immune complex disease	0.93335
DB00051	Infection by cryptococcus neoformans	0.34816
DB00051	Infertility	0.04142
DB00051	Intermediate coronary syndrome	0.1005
DB00051	Kaposi sarcoma	0.13054
DB00051	Kidney disease	0.07207
DB00051	Kidney failure	0.03414
DB00051	Leprosy	0.08058
DB00051	Leukemia	0.06133
DB00051	Lichen planus	0.1066
DB00051	Lipodystrophy	0.08362
DB00051	Liver cancer	0.0234
DB00051	Lupus erythematosus	0.29937
DB00051	Lupus vulgaris	0.05698
DB00051	Malaria	0.05599
DB00051	Malignant glioma	0.05599
DB00051	Melanoma	0.05025
DB00051	Migraine	0.05599
DB00051	Mucocutaneous lymph node syndrome	0.0658
DB00051	Multiple myeloma	0.15698
DB00051	Multiple sclerosis	0.0622
DB00051	Muscular dystrophies	0.05913
DB00051	Mycoses	0.13484
DB00051	Narcolepsy	0.11396
DB00051	Nasopharyngeal cancer	0.05803
DB00051	Necrotizing enterocolitis	0.12309
DB00051	Neoplasm metastasis	0.02454
DB00051	Nephrosis	0.06742
DB00051	Obesity	0.02319
DB00051	Otitis media	0.1005

DB00051	Ovary cancer	0.06742
DB00051	Pancreas cancer	0.03127
DB00051	Pancreatitis	0.04709
DB00051	Periodontal disease	0.07107
DB00051	Periodontitis	0.13794
DB00051	Polycystic ovary syndrome	0.03994
DB00051	Prostate cancer	0.01574
DB00051	Psoriasis	0.07107
DB00051	Pulmonary fibrosis	0.05698
DB00051	Renal Cell cancer	0.0701
DB00051	Rheumatic fever	0.07785
DB00051	Rheumatoid arthritis	0.0369
DB00051	Sarcoidosis	0.05913
DB00051	Schizophrenia	0.02292
DB00051	Silicosis	0.11396
DB00051	Skin tumor	0.2229
DB00051	Stomach cancer	0.12507
DB00051	Stroke	0.03414
DB00051	Systemic infection	0.20657
DB00051	Thrombocytopenia	0.07538
DB00051	Thrombophilia	0.07107
DB00051	Thyroid cancer	0.05249
DB00051	Tuberculosis	0.04066
DB00051	Tuberous sclerosis	0.07785
DB00051	Ulcerative colitis	0.0311
DB00051	Vascular disease	0.05505
DB00051	Yersinia infection	0.06085
DB00065	Alcoholic liver disease	0.40825
DB00065	Alzheimer's disease	0.07198
DB00065	Amyloidosis	0.18898
DB00065	Anemia	0.2132
DB00065	Anorexia nervosa	0.20412
DB00065	Aplastic anemia	0.20851
DB00065	Arthritis	0.12403
DB00065	Asthma	0.08165
DB00065	Atherosclerosis	0.07001
DB00065	Autoimmune disease	0.10847
DB00065	Basal cell carcinoma	0.27735
DB00065	Behcet syndrome	0.14286
DB00065	Bladder cancer	0.1291
DB00065	Breast cancer	0.04811
DB00065	Celiac disease	0.1644
DB00065	Cervical cancer	0.11625
DB00065	Colon cancer	0.05934
DB00065	Dermatitis	0.09091
DB00065	Diabetes mellitus	0.05263
DB00065	Down syndrome	0.11396

DB00065	Embryoma	0.06166
DB00065	Endometriosis	0.08305
DB00065	Enteritis	0.10976
DB00065	Familial Mediterranean fever	0.2582
DB00065	Fanconi's anemia	0.2
DB00065	Granulomatous disease	0.28868
DB00065	HIV infection	0.09578
DB00065	Heart failure	0.1066
DB00065	Hepatitis B	0.24254
DB00065	Histiocytosis	0.33333
DB00065	Hypothyroidism	0.2357
DB00065	Infertility	0.13736
DB00065	Intermediate coronary syndrome	0.33333
DB00065	Kidney disease	0.11952
DB00065	Leprosy	0.26726
DB00065	Leukemia	0.05634
DB00065	Lichen planus	0.35355
DB00065	Lipodystrophy	0.27735
DB00065	Liver cancer	0.07762
DB00065	Lupus erythematosus	0.08544
DB00065	Malaria	0.1857
DB00065	Malignant glioma	0.1857
DB00065	Melanoma	0.08333
DB00065	Migraine	0.1857
DB00065	Mucocutaneous lymph node syndrome	0.21822
DB00065	Multiple myeloma	0.13131
DB00065	Multiple sclerosis	0.10314
DB00065	Muscular dystrophies	0.19612
DB00065	Mycoses	0.44721
DB00065	Narcolepsy	0.37796
DB00065	Nasopharyngeal cancer	0.19245
DB00065	Necrotizing enterocolitis	0.40825
DB00065	Nephrosis	0.22361
DB00065	Obesity	0.07692
DB00065	Otitis media	0.33333
DB00065	Ovary cancer	0.22361
DB00065	Pancreas cancer	0.1037
DB00065	Pancreatitis	0.15617
DB00065	Periodontitis	0.1525
DB00065	Polycystic ovary syndrome	0.13245
DB00065	Prostate cancer	0.0522
DB00065	Psoriasis	0.2357
DB00065	Pulmonary fibrosis	0.18898
DB00065	Renal Cell cancer	0.11625
DB00065	Rheumatoid arthritis	0.0612
DB00065	Sarcoidosis	0.19612
DB00065	Schizophrenia	0.07603

DB00065	Silicosis	0.37796
DB00065	Stomach cancer	0.08422
DB00065	Stroke	0.11323
DB00065	Systemic infection	0.11471
DB00065	Thrombophilia	0.2357
DB00065	Thyroid cancer	0.17408
DB00065	Tuberculosis	0.13484
DB00065	Tuberous sclerosis	0.2582
DB00065	Ulcerative colitis	0.10314
DB00608	Alcoholic liver disease	0.2357
DB00608	Alzheimer's disease	0.04156
DB00608	Amyloidosis	0.10911
DB00608	Anemia	0.12309
DB00608	Anorexia nervosa	0.11785
DB00608	Aplastic anemia	0.12039
DB00608	Arthritis	0.07161
DB00608	Asthma	0.04714
DB00608	Atherosclerosis	0.04042
DB00608	Autoimmune disease	0.06262
DB00608	Basal cell carcinoma	0.16013
DB00608	Behcet syndrome	0.08248
DB00608	Bladder cancer	0.07454
DB00608	Breast cancer	0.02778
DB00608	Celiac disease	0.09492
DB00608	Cervical cancer	0.06712
DB00608	Colon cancer	0.03426
DB00608	Dermatitis	0.05249
DB00608	Diabetes mellitus	0.03039
DB00608	Down syndrome	0.0658
DB00608	Embryoma	0.0356
DB00608	Endometriosis	0.04795
DB00608	Enteritis	0.06337
DB00608	Familial Mediterranean fever	0.14907
DB00608	Fanconi's anemia	0.11547
DB00608	Granulomatous disease	0.16667
DB00608	HIV infection	0.0553
DB00608	Heart failure	0.06155
DB00608	Hepatitis B	0.14003
DB00608	Histiocytosis	0.19245
DB00608	Hypothyroidism	0.13608
DB00608	Infertility	0.07931
DB00608	Intermediate coronary syndrome	0.19245
DB00608	Kidney disease	0.06901
DB00608	Leprosy	0.1543
DB00608	Leukemia	0.03253
DB00608	Lichen planus	0.20412
DB00608	Lipodystrophy	0.16013

DB00608	Liver cancer	0.04481
DB00608	Lupus erythematosus	0.04933
DB00608	Malaria	0.10721
DB00608	Malignant glioma	0.10721
DB00608	Melanoma	0.04811
DB00608	Migraine	0.10721
DB00608	Mucocutaneous lymph node syndrome	0.12599
DB00608	Multiple myeloma	0.07581
DB00608	Multiple sclerosis	0.05955
DB00608	Muscular dystrophies	0.11323
DB00608	Mycoses	0.2582
DB00608	Narcolepsy	0.21822
DB00608	Nasopharyngeal cancer	0.11111
DB00608	Necrotizing enterocolitis	0.2357
DB00608	Nephrosis	0.1291
DB00608	Obesity	0.04441
DB00608	Otitis media	0.19245
DB00608	Ovary cancer	0.1291
DB00608	Pancreas cancer	0.05987
DB00608	Pancreatitis	0.09017
DB00608	Periodontitis	0.08805
DB00608	Polycystic ovary syndrome	0.07647
DB00608	Prostate cancer	0.03014
DB00608	Psoriasis	0.13608
DB00608	Pulmonary fibrosis	0.10911
DB00608	Renal Cell cancer	0.06712
DB00608	Rheumatoid arthritis	0.03533
DB00608	Sarcoidosis	0.11323
DB00608	Schizophrenia	0.0439
DB00608	Silicosis	0.21822
DB00608	Stomach cancer	0.04862
DB00608	Stroke	0.06537
DB00608	Systemic infection	0.06623
DB00608	Thrombophilia	0.13608
DB00608	Thyroid cancer	0.1005
DB00608	Tuberculosis	0.07785
DB00608	Tuberous sclerosis	0.14907
DB00608	Ulcerative colitis	0.05955
DB01411	Adenoma	0.01442
DB01411	Alcoholic liver disease	0.16667
DB01411	Alimentary system disease	0.08909
DB01411	Alzheimer's disease	0.02939
DB01411	Amyloidosis	0.07715
DB01411	Anemia	0.08704
DB01411	Anorexia nervosa	0.08333
DB01411	Aplastic anemia	0.08513
DB01411	Arthritis	0.05064

DB01411	Asthma	0.1
DB01411	Atherosclerosis	0.02858
DB01411	Autoimmune disease	0.04428
DB01411	Barrett's esophagus	0.07454
DB01411	Basal cell carcinoma	0.11323
DB01411	Behcet syndrome	0.05832
DB01411	Bladder cancer	0.0527
DB01411	Brain tumor	0.02155
DB01411	Breast cancer	0.06739
DB01411	Bronchial disease	0.09901
DB01411	Bronchial hyperreactivity	0.14434
DB01411	Cancer	0.10182
DB01411	Celiac disease	0.06712
DB01411	Cervical cancer	0.04746
DB01411	Colon cancer	0.05688
DB01411	Congenital abnormality	0.00762
DB01411	Connective tissue disease	0.10206
DB01411	Dermatitis	0.22062
DB01411	Diabetes mellitus	0.17017
DB01411	Down syndrome	0.04652
DB01411	Embryoma	0.04392
DB01411	Endometriosis	0.23011
DB01411	Enteritis	0.04481
DB01411	Eosinophilia	0.12309
DB01411	Familial Mediterranean fever	0.10541
DB01411	Fanconi's anemia	0.08165
DB01411	Granulomatous disease	0.11785
DB01411	HIV infection	0.0391
DB01411	Heart failure	0.04352
DB01411	Helminthiasis	0.20412
DB01411	Hepatitis B	0.09901
DB01411	Histiocytosis	0.13608
DB01411	Hodgkin's disease	0.13054
DB01411	Hypothyroidism	0.09623
DB01411	Infectious lung disease	0.10369
DB01411	Infertility	0.05608
DB01411	Infiltrating cancer	0.28343
DB01411	Intermediate coronary syndrome	0.13608
DB01411	Kidney disease	0.09759
DB01411	Leprosy	0.10911
DB01411	Leukemia	0.06115
DB01411	Lichen planus	0.14434
DB01411	Lipodystrophy	0.11323
DB01411	Liver cancer	0.12835
DB01411	Lupus erythematosus	0.03488
DB01411	Lymphoma	0.01549
DB01411	Malaria	0.07581

DB01411	Malignant glioma	0.07581
DB01411	Melanoma	0.03402
DB01411	Migraine	0.07581
DB01411	Mucocutaneous lymph node syndrome	0.08909
DB01411	Multiple myeloma	0.05361
DB01411	Multiple sclerosis	0.41604
DB01411	Muscular dystrophies	0.08006
DB01411	Mycoses	0.18257
DB01411	Narcolepsy	0.1543
DB01411	Nasopharyngeal cancer	0.07857
DB01411	Necrotizing enterocolitis	0.16667
DB01411	Neoplasm metastasis	0.05654
DB01411	Nephrosis	0.61327
DB01411	Obesity	0.08916
DB01411	Otitis media	0.13608
DB01411	Ovary cancer	0.09129
DB01411	Pancreas cancer	0.04233
DB01411	Pancreatitis	0.5224
DB01411	Periodontitis	0.06226
DB01411	Polyarthritis	0.26726
DB01411	Polycystic ovary syndrome	0.05407
DB01411	Prostate cancer	0.11709
DB01411	Protozoan infection	0.28868
DB01411	Psoriasis	0.09623
DB01411	Pulmonary fibrosis	0.1543
DB01411	Renal Cell cancer	0.04746
DB01411	Rheumatoid arthritis	0.02498
DB01411	Sarcoidosis	0.08006
DB01411	Schizophrenia	0.04592
DB01411	Silicosis	0.1543
DB01411	Sinusitis	0.09129
DB01411	Squamous cell cancer	0.1252
DB01411	Stomach cancer	0.05707
DB01411	Stroke	0.27567
DB01411	Systemic infection	0.13081
DB01411	Systemic scleroderma	0.01306
DB01411	Testicular dysfunction	0.01656
DB01411	Thrombophilia	0.09623
DB01411	Thyroid cancer	0.07107
DB01411	Tuberculosis	0.52705
DB01411	Tuberous sclerosis	0.11816
DB01411	Ulcerative colitis	0.35561
DB01411	Vasculitis	0.12309
DB01411	Virus disease	0.14463
DB01411	Yersinia infection	0.01084
DB06674	Alcoholic liver disease	0.40825
DB06674	Alzheimer's disease	0.07198

DB06674	Amyloidosis	0.18898
DB06674	Anemia	0.2132
DB06674	Anorexia nervosa	0.20412
DB06674	Aplastic anemia	0.20851
DB06674	Arthritis	0.12403
DB06674	Asthma	0.08165
DB06674	Atherosclerosis	0.07001
DB06674	Autoimmune disease	0.10847
DB06674	Basal cell carcinoma	0.27735
DB06674	Behcet syndrome	0.14286
DB06674	Bladder cancer	0.1291
DB06674	Breast cancer	0.04811
DB06674	Celiac disease	0.1644
DB06674	Cervical cancer	0.11625
DB06674	Colon cancer	0.05934
DB06674	Dermatitis	0.09091
DB06674	Diabetes mellitus	0.05263
DB06674	Down syndrome	0.11396
DB06674	Embryoma	0.06166
DB06674	Endometriosis	0.08305
DB06674	Enteritis	0.10976
DB06674	Familial Mediterranean fever	0.2582
DB06674	Fanconi's anemia	0.2
DB06674	Granulomatous disease	0.28868
DB06674	HIV infection	0.09578
DB06674	Heart failure	0.1066
DB06674	Hepatitis B	0.24254
DB06674	Histiocytosis	0.33333
DB06674	Hypothyroidism	0.2357
DB06674	Infertility	0.13736
DB06674	Intermediate coronary syndrome	0.33333
DB06674	Kidney disease	0.11952
DB06674	Leprosy	0.26726
DB06674	Leukemia	0.05634
DB06674	Lichen planus	0.35355
DB06674	Lipodystrophy	0.27735
DB06674	Liver cancer	0.07762
DB06674	Lupus erythematosus	0.08544
DB06674	Malaria	0.1857
DB06674	Malignant glioma	0.1857
DB06674	Melanoma	0.08333
DB06674	Migraine	0.1857
DB06674	Mucocutaneous lymph node syndrome	0.21822
DB06674	Multiple myeloma	0.13131
DB06674	Multiple sclerosis	0.10314
DB06674	Muscular dystrophies	0.19612
DB06674	Mycoses	0.44721

DB06674	Narcolepsy	0.37796
DB06674	Nasopharyngeal cancer	0.19245
DB06674	Necrotizing enterocolitis	0.40825
DB06674	Nephrosis	0.22361
DB06674	Obesity	0.07692
DB06674	Otitis media	0.33333
DB06674	Ovary cancer	0.22361
DB06674	Pancreas cancer	0.1037
DB06674	Pancreatitis	0.15617
DB06674	Periodontitis	0.1525
DB06674	Polycystic ovary syndrome	0.13245
DB06674	Prostate cancer	0.0522
DB06674	Psoriasis	0.2357
DB06674	Pulmonary fibrosis	0.18898
DB06674	Renal Cell cancer	0.11625
DB06674	Rheumatoid arthritis	0.0612
DB06674	Sarcoidosis	0.19612
DB06674	Schizophrenia	0.07603
DB06674	Silicosis	0.37796
DB06674	Stomach cancer	0.08422
DB06674	Stroke	0.11323
DB06674	Systemic infection	0.11471
DB06674	Thrombophilia	0.2357
DB06674	Thyroid cancer	0.17408
DB06674	Tuberculosis	0.13484
DB06674	Tuberous sclerosis	0.2582
DB06674	Ulcerative colitis	0.10314
DB08904	Alcoholic liver disease	0.40825
DB08904	Alzheimer's disease	0.07198
DB08904	Amyloidosis	0.18898
DB08904	Anemia	0.2132
DB08904	Anorexia nervosa	0.20412
DB08904	Aplastic anemia	0.20851
DB08904	Arthritis	0.12403
DB08904	Asthma	0.08165
DB08904	Atherosclerosis	0.07001
DB08904	Autoimmune disease	0.10847
DB08904	Basal cell carcinoma	0.27735
DB08904	Behcet syndrome	0.14286
DB08904	Bladder cancer	0.1291
DB08904	Breast cancer	0.04811
DB08904	Celiac disease	0.1644
DB08904	Cervical cancer	0.11625
DB08904	Colon cancer	0.05934
DB08904	Dermatitis	0.09091
DB08904	Diabetes mellitus	0.05263
DB08904	Down syndrome	0.11396

DB08904	Embryoma	0.06166
DB08904	Endometriosis	0.08305
DB08904	Enteritis	0.10976
DB08904	Familial Mediterranean fever	0.2582
DB08904	Fanconi's anemia	0.2
DB08904	Granulomatous disease	0.28868
DB08904	HIV infection	0.09578
DB08904	Heart failure	0.1066
DB08904	Hepatitis B	0.24254
DB08904	Histiocytosis	0.33333
DB08904	Hypothyroidism	0.2357
DB08904	Infertility	0.13736
DB08904	Intermediate coronary syndrome	0.33333
DB08904	Kidney disease	0.11952
DB08904	Leprosy	0.26726
DB08904	Leukemia	0.05634
DB08904	Lichen planus	0.35355
DB08904	Lipodystrophy	0.27735
DB08904	Liver cancer	0.07762
DB08904	Lupus erythematosus	0.08544
DB08904	Malaria	0.1857
DB08904	Malignant glioma	0.1857
DB08904	Melanoma	0.08333
DB08904	Migraine	0.1857
DB08904	Mucocutaneous lymph node syndrome	0.21822
DB08904	Multiple myeloma	0.13131
DB08904	Multiple sclerosis	0.10314
DB08904	Muscular dystrophies	0.19612
DB08904	Mycoses	0.44721
DB08904	Narcolepsy	0.37796
DB08904	Nasopharyngeal cancer	0.19245
DB08904	Necrotizing enterocolitis	0.40825
DB08904	Nephrosis	0.22361
DB08904	Obesity	0.07692
DB08904	Otitis media	0.33333
DB08904	Ovary cancer	0.22361
DB08904	Pancreas cancer	0.1037
DB08904	Pancreatitis	0.15617
DB08904	Periodontitis	0.1525
DB08904	Polycystic ovary syndrome	0.13245
DB08904	Prostate cancer	0.0522
DB08904	Psoriasis	0.2357
DB08904	Pulmonary fibrosis	0.18898
DB08904	Renal Cell cancer	0.11625
DB08904	Rheumatoid arthritis	0.0612
DB08904	Sarcoidosis	0.19612
DB08904	Schizophrenia	0.07603

DB08904	Silicosis	0.37796
DB08904	Stomach cancer	0.08422
DB08904	Stroke	0.11323
DB08904	Systemic infection	0.11471
DB08904	Thrombophilia	0.2357
DB08904	Thyroid cancer	0.17408
DB08904	Tuberculosis	0.13484
DB08904	Tuberous sclerosis	0.2582
DB08904	Ulcerative colitis	0.10314
DB00549	Asthma	0.08165
DB00549	Colon cancer	0.05934
DB00549	Dermatitis	0.09091
DB00549	Prostate cancer	0.0522
DB00587	Asthma	0.08165
DB00587	Colon cancer	0.05934
DB00587	Dermatitis	0.09091
DB00587	Prostate cancer	0.0522
DB01154	Atherosclerosis	0.04042
DB01154	Congenital abnormality	0.04352
DB01154	Diabetes mellitus	0.03039
DB01154	Hyperglycemia	0.09245
DB01154	Hyperinsulinism	0.11111
DB01154	Infantile spasms	0.21822
DB01154	Late pregnancy	0.33333
DB01154	Metabolism disease	0.09245
DB01154	Pancreas disease	0.12039
DB01154	Polycystic ovary syndrome	0.07647
DB00002	Hemorrhagic fevers, Viral	0.0626
DB00002	Pemphigoid, Bullous	0.12712
DB00002	Pleural effusion, Malignant	0.34939
DB00002	Purpura, Thrombocytopenic, Idiopathic	0.03037
DB00002	Abortion	0.07343
DB00002	Adenovirus infection	0.21507
DB00002	Alopecia	0.12496
DB00002	Alzheimer's disease	0.04106
DB00002	Angiomyolipoma	0.76192
DB00002	Antiphospholipid syndrome	0.1005
DB00002	Aplastic anemia	0.0519
DB00002	Asthma	0.28635
DB00002	Atherosclerosis	0.07882
DB00002	Atopic rhinitis	0.97228
DB00002	Autistic disorder	0.04124
DB00002	Autoimmune disease	0.16908
DB00002	Barrett's esophagus	0.36216
DB00002	Breast cancer	0.058
DB00002	Brucellosis	0.09091
DB00002	Cancer	0.10197

DB00002	Celiac disease	0.04957
DB00002	Charcot-Marie-Tooth disease	0.14948
DB00002	Cholelithiasis	0.57321
DB00002	Colon cancer	0.02117
DB00002	Common wart	0.92916
DB00002	Communicable disease	0.0658
DB00002	Cystic fibrosis	0.04307
DB00002	Cytomegalovirus infection	0.75289
DB00002	Diabetes mellitus	0.17181
DB00002	Drug abuse	0.21275
DB00002	Embryoma	0.01859
DB00002	Endometriosis	0.19996
DB00002	Enteritis	0.16382
DB00002	Esotropia	0.59124
DB00002	Gastrointestinal tumor	0.57586
DB00002	Generalized anxiety disorder	0.06633
DB00002	Glaucoma	0.43901
DB00002	Glomerulonephritis	0.07538
DB00002	Hemorrhagic disorder	0.049
DB00002	Herpes	0.0274
DB00002	Hypercholesterolemia	0.79273
DB00002	IGA glomerulonephritis	0.05171
DB00002	Immune complex disease	0.53526
DB00002	Infection by cryptococcus neoformans	0.34816
DB00002	Intermediate coronary syndrome	0.06244
DB00002	Kaposi sarcoma	0.05838
DB00002	Kidney disease	0.03604
DB00002	Kidney failure	0.03414
DB00002	Leukemia	0.09383
DB00002	Lupus erythematosus	0.41634
DB00002	Lupus vulgaris	0.05698
DB00002	Melanoma	0.02513
DB00002	Multiple myeloma	0.0525
DB00002	Multiple sclerosis	0.0311
DB00002	Muscular atrophy	0.16214
DB00002	Myopathy	0.04352
DB00002	Neoplasm metastasis	0.02454
DB00002	Optic atrophy	0.9879
DB00002	Oral cancer	0.29518
DB00002	Osteitis deformans	0.0787
DB00002	Papillary adenocarcinoma	1.0685
DB00002	Papillomavirus infection	0.08245
DB00002	Penile disease	0.05859
DB00002	Periodontal disease	0.07107
DB00002	Periodontitis	0.09196
DB00002	Pneumoconiosis	0.20755
DB00002	Primary hyperparathyroidism	0.39611

DB00002	Pulmonary fibrosis	0.0675
DB00002	Renal Cell cancer	0.03505
DB00002	Rheumatic fever	0.07785
DB00002	Rheumatoid arthritis	0.05184
DB00002	Schizophrenia	0.24597
DB00002	Skin cancer	0.47115
DB00002	Skin tumor	0.09969
DB00002	Stomach cancer	0.04458
DB00002	Stroke	0.27478
DB00002	Subarachnoid hemorrhage	0.05589
DB00002	Systemic infection	0.09603
DB00002	Takayasu's arteritis	0.05226
DB00002	Temporal arteritis	0.0611
DB00002	Testicular dysfunction	0.3053
DB00002	Thrombocytopenia	0.12194
DB00002	Thrombophlebitis	0.74965
DB00002	Vascular disease	0.05505
DB00002	Yersinia infection	0.31079
DB00028	Pemphigoid, Bullous	0.1
DB00028	Abortion	0.03953
DB00028	Alopecia	0.2718
DB00028	Alzheimer's disease	0.02276
DB00028	Amyotrophic lateral sclerosis	0.04264
DB00028	Antiphospholipid syndrome	0.10541
DB00028	Aortic valve disease	0.21082
DB00028	Asthma	0.05164
DB00028	Atherosclerosis	0.04428
DB00028	Autistic disorder	0.03835
DB00028	Autoimmune disease	0.1715
DB00028	Breast cancer	0.03043
DB00028	Brucellosis	0.09535
DB00028	Celiac disease	0.05199
DB00028	Chronic obstructive airway disease	0.03536
DB00028	Communicable disease	0.06901
DB00028	Cystic fibrosis	0.04518
DB00028	Dental plaque	0.04016
DB00028	Depression	0.08305
DB00028	Drug abuse	0.02962
DB00028	Embryoma	0.0195
DB00028	Endometriosis	0.05252
DB00028	Enteritis	0.28772
DB00028	Generalized anxiety disorder	0.14832
DB00028	Glomerulonephritis	0.07906
DB00028	Henoch-Schoenlein purpura	0.1
DB00028	Hyperlipidemia	0.06202
DB00028	IGA glomerulonephritis	0.05423
DB00028	Immune complex disease	0.94376

DB00028	Infection	0.05898
DB00028	Infection by cryptococcus neoformans	0.36515
DB00028	Infertility	0.08687
DB00028	Kaposi sarcoma	0.13054
DB00028	Kidney disease	0.07559
DB00028	Kidney failure	0.03581
DB00028	Leukemia	0.04434
DB00028	Lupus erythematosus	0.36095
DB00028	Lupus vulgaris	0.05976
DB00028	Macular degeneration	0.06594
DB00028	Melanoma	0.02635
DB00028	Multiple myeloma	0.11739
DB00028	Multiple sclerosis	0.03262
DB00028	Neoplasm metastasis	0.02573
DB00028	Periodontal disease	0.07454
DB00028	Periodontitis	0.09645
DB00028	Polyarthritis	0.03604
DB00028	Renal Cell cancer	0.03676
DB00028	Rheumatic fever	0.08165
DB00028	Rheumatoid arthritis	0.03871
DB00028	Skin tumor	0.2229
DB00028	Stomach cancer	0.09968
DB00028	Stroke	0.07161
DB00028	Systemic infection	0.17367
DB00028	Thrombocytopenia	0.07906
DB00028	Tuberculosis	0.04264
DB00028	Vascular disease	0.05774
DB00028	Yersinia infection	0.06085
DB00092	Pemphigoid, Bullous	0.1
DB00092	Abortion	0.03953
DB00092	Alopecia	0.21406
DB00092	Alzheimer's disease	0.02276
DB00092	Antiphospholipid syndrome	0.10541
DB00092	Asthma	0.02582
DB00092	Atherosclerosis	0.04428
DB00092	Autoimmune disease	0.1029
DB00092	Breast cancer	0.04564
DB00092	Brucellosis	0.09535
DB00092	Celiac disease	0.05199
DB00092	Communicable disease	0.06901
DB00092	Cystic fibrosis	0.04518
DB00092	Embryoma	0.0195
DB00092	Enteritis	0.28772
DB00092	Generalized anxiety disorder	0.14832
DB00092	Glomerulonephritis	0.07906
DB00092	IGA glomerulonephritis	0.05423
DB00092	Immune complex disease	0.94376

DB00092	Infection by cryptococcus neoformans	0.36515
DB00092	Kaposi sarcoma	0.13054
DB00092	Kidney disease	0.0378
DB00092	Kidney failure	0.03581
DB00092	Leukemia	0.06216
DB00092	Lupus erythematosus	0.2799
DB00092	Lupus vulgaris	0.05976
DB00092	Melanoma	0.02635
DB00092	Multiple myeloma	0.11739
DB00092	Multiple sclerosis	0.03262
DB00092	Neoplasm metastasis	0.02573
DB00092	Periodontal disease	0.07454
DB00092	Periodontitis	0.09645
DB00092	Renal Cell cancer	0.03676
DB00092	Rheumatic fever	0.08165
DB00092	Rheumatoid arthritis	0.01935
DB00092	Skin tumor	0.2229
DB00092	Stomach cancer	0.09968
DB00092	Systemic infection	0.17367
DB00092	Thrombocytopenia	0.07906
DB00092	Vascular disease	0.05774
DB00092	Yersinia infection	0.06085
DB00110	Pemphigoid, Bullous	0.10541
DB00110	Abortion	0.04167
DB00110	Alopecia	0.21719
DB00110	Alzheimer's disease	0.02399
DB00110	Antiphospholipid syndrome	0.11111
DB00110	Asthma	0.02722
DB00110	Atherosclerosis	0.04668
DB00110	Autoimmune disease	0.10847
DB00110	Breast cancer	0.04811
DB00110	Brucellosis	0.1005
DB00110	Celiac disease	0.0548
DB00110	Communicable disease	0.07274
DB00110	Cystic fibrosis	0.04762
DB00110	Embryoma	0.02055
DB00110	Enteritis	0.29148
DB00110	Generalized anxiety disorder	0.14832
DB00110	Glomerulonephritis	0.08333
DB00110	IGA glomerulonephritis	0.05717
DB00110	Immune complex disease	0.95585
DB00110	Infection by cryptococcus neoformans	0.3849
DB00110	Kaposi sarcoma	0.13054
DB00110	Kidney disease	0.03984
DB00110	Kidney failure	0.03774
DB00110	Leukemia	0.04434
DB00110	Lupus erythematosus	0.2872

DB00110	Lupus vulgaris	0.06299
DB00110	Melanoma	0.02778
DB00110	Multiple myeloma	0.11739
DB00110	Multiple sclerosis	0.03438
DB00110	Neoplasm metastasis	0.02713
DB00110	Periodontal disease	0.07857
DB00110	Periodontitis	0.10167
DB00110	Renal Cell cancer	0.03875
DB00110	Rheumatic fever	0.08607
DB00110	Rheumatoid arthritis	0.0204
DB00110	Skin tumor	0.2229
DB00110	Stomach cancer	0.09968
DB00110	Systemic infection	0.17563
DB00110	Thrombocytopenia	0.08333
DB00110	Vascular disease	0.06086
DB00110	Yersinia infection	0.06085
DB00992	Alopecia	0.3389
DB00992	Enteritis	0.32806
DB00992	Generalized anxiety disorder	0.14832
DB00992	Immune complex disease	1.42726
DB00992	Kaposi sarcoma	0.13054
DB00992	Leukemia	0.04434
DB00992	Lupus erythematosus	0.23025
DB00992	Multiple myeloma	0.11739
DB00992	Skin tumor	0.2229
DB00992	Stomach cancer	0.09968
DB00992	Systemic infection	0.2521
DB00992	Yersinia infection	0.06085
DB00266	Atherosclerosis	0.04042
DB00266	Rabies	0.06415
DB00266	Vascular disease	0.10541
DB00498	Atherosclerosis	0.07001
DB00498	Rabies	0.11111
DB00498	Vascular disease	0.18257
DB00682	Atherosclerosis	0.07001
DB00682	Rabies	0.11111
DB00682	Vascular disease	0.18257
DB00946	Atherosclerosis	0.07001
DB00946	Rabies	0.11111
DB00946	Vascular disease	0.18257
DB01418	Atherosclerosis	0.07001
DB01418	Rabies	0.11111
DB01418	Vascular disease	0.18257
DB00851	Aortic valve disease	0.2026
DB00851	Barrett's esophagus	0.03989
DB00851	Cancer	0.04053
DB00851	Colon cancer	0.07659

DB00851	Down syndrome	0.18327
DB00851	Emphysema	0.14834
DB00851	HIV infection	0.0749
DB00851	Helicobacter infection	0.17581
DB00851	Lung cancer	0.03588
DB00851	Lymphoma	0.18423
DB00851	Meningioma	0.4222
DB00851	Tuberous sclerosis	0.11576
DB00851	Ulcerative colitis	0.03142
DB00688	Amaurosis congenita of leber I	0.25
DB00688	Diabetes mellitus	0.03722
DB00688	Osteosarcoma	0.15076
DB00688	Retinal disease	0.09901
DB00688	Retinitis pigmentosa	0.14142
DB01024	Amaurosis congenita of leber I	0.25
DB01024	Diabetes mellitus	0.03722
DB01024	Osteosarcoma	0.15076
DB01024	Retinal disease	0.09901
DB01024	Retinitis pigmentosa	0.14142
DB05260	Labor, Premature	0.1291
DB05260	Alimentary system disease	0.10911
DB05260	Alveolar bone loss	0.18898
DB05260	Alzheimer's disease	0.03599
DB05260	Amnionitis	0.13868
DB05260	Arthritis	0.06202
DB05260	Asthma	0.04082
DB05260	Atherosclerosis	0.03501
DB05260	Bacterial vaginosis	0.20412
DB05260	Behcet syndrome	0.07143
DB05260	Bipolar disorder	0.05661
DB05260	Bone disease	0.08575
DB05260	Breast cancer	0.02406
DB05260	Bronchopulmonary dysplasia	0.15076
DB05260	Cancer	0.01843
DB05260	Celiac disease	0.0822
DB05260	Cervical cancer	0.05812
DB05260	Chronic obstructive airway disease	0.0559
DB05260	Cystic fibrosis	0.07143
DB05260	Dental plaque	0.0635
DB05260	Dermatitis	0.04545
DB05260	Diabetes mellitus	0.02632
DB05260	Drug abuse	0.04683
DB05260	Endometriosis	0.04152
DB05260	Epilepsy	0.07071
DB05260	Epstein-Barr virus infection	0.10911
DB05260	Esophagitis	0.22361
DB05260	Glaucoma	0.08333

DB05260	Gouts	0.13868
DB05260	Graves' disease	0.08839
DB05260	Helicobacter infection	0.1118
DB05260	Henoch-Schoenlein purpura	0.15811
DB05260	Hepatitis	0.1291
DB05260	Hepatitis C	0.07372
DB05260	Hodgkin's disease	0.08839
DB05260	Hypertension	0.03953
DB05260	Infectious lung disease	0.1118
DB05260	Infertility	0.06868
DB05260	Kidney failure	0.05661
DB05260	Leukemia	0.02817
DB05260	Lung cancer	0.03492
DB05260	Lupus erythematosus	0.04272
DB05260	Lyme disease	0.16667
DB05260	Melanoma	0.04167
DB05260	Multiple myeloma	0.13131
DB05260	Multiple sclerosis	0.05157
DB05260	Neoplasm metastasis	0.04069
DB05260	Nephrosis	0.1118
DB05260	Oral cancer	0.06804
DB05260	Osteoporosis	0.07906
DB05260	Otitis media	0.16667
DB05260	Ovarian cancer	0.05025
DB05260	Pancreas cancer	0.05185
DB05260	Parkinson disease	0.05522
DB05260	Periodontitis	0.07625
DB05260	Polymyositis	0.1291
DB05260	Premature birth	0.12127
DB05260	Psoriasis	0.11785
DB05260	Respiratory distress syndrome	0.13868
DB05260	Retinoblastoma	0.28868
DB05260	Rheumatoid arthritis	0.0306
DB05260	Sarcoidosis	0.09806
DB05260	Schizophrenia	0.03801
DB05260	Shigella infection	0.20412
DB05260	Sicca syndrome	0.09806
DB05260	Squamous cell cancer	0.05103
DB05260	Stroke	0.05661
DB05260	Synovial sarcoma	0.25
DB05260	Systemic scleroderma	0.05241
DB05260	Tuberculosis	0.06742
DB05260	Ulcerative colitis	0.05157
DB00390	Growth retardation	0.19245
DB00390	Hypertension	0.07906
DB00390	Rheumatoid arthritis	0.0612
DB00511	Growth retardation	0.19245

DB00511	Hypertension	0.07906
DB00511	Rheumatoid arthritis	0.0612
DB01078	Growth retardation	0.19245
DB01078	Hypertension	0.07906
DB01078	Rheumatoid arthritis	0.0612
DB01092	Growth retardation	0.19245
DB01092	Hypertension	0.07906
DB01092	Rheumatoid arthritis	0.0612
DB01158	Growth retardation	0.19245
DB01158	Hypertension	0.07906
DB01158	Rheumatoid arthritis	0.0612
DB01188	Growth retardation	0.19245
DB01188	Hypertension	0.07906
DB01188	Rheumatoid arthritis	0.0612
DB01396	Growth retardation	0.19245
DB01396	Hypertension	0.07906
DB01396	Rheumatoid arthritis	0.0612
DB01430	Growth retardation	0.19245
DB01430	Hypertension	0.07906
DB01430	Rheumatoid arthritis	0.0612
DB00134	Infertility, Male	0.09129
DB00134	Abruption placentae	0.31623
DB00134	Alzheimer's disease	0.06438
DB00134	Atherosclerosis	0.03131
DB00134	Bipolar disorder	0.05064
DB00134	Bladder cancer	0.05774
DB00134	Brain tumor	0.03592
DB00134	Breast cancer	0.02152
DB00134	Cleft palate	0.11952
DB00134	Colon cancer	0.02654
DB00134	Down syndrome	0.10193
DB00134	Embryoma	0.02758
DB00134	Encephalopathies	0.06262
DB00134	Enteritis	0.04909
DB00134	Fatty liver	0.1291
DB00134	Hamman-Rich syndrome	0.08607
DB00134	Hyperhomocysteinemia	0.3873
DB00134	Leukemia	0.0252
DB00134	Lupus erythematosus	0.03821
DB00134	Malignant glioma	0.08305
DB00134	Meningioma	0.23094
DB00134	Multiple myeloma	0.05872
DB00134	Neoplasm metastasis	0.03639
DB00134	Obesity	0.0688
DB00134	Pancreas cancer	0.04637
DB00134	Prostate cancer	0.02334
DB00134	Rheumatoid arthritis	0.02737

DB00134	Schizophrenia	0.034
DB00134	Spinal dysraphism	0.2052
DB00134	Ulcerative colitis	0.04613
DB00186	Breast cancer	0.01167
DB00186	Cancer	0.00894
DB00186	Drug abuse	0.02272
DB00186	Hypertension	0.01917
DB00186	Hypogonadism	0.10847
DB00186	Panic disorder	0.05293
DB00186	Yersinia infection	0.02878
DB00628	Breast cancer	0.01167
DB00628	Cancer	0.00894
DB00628	Drug abuse	0.02272
DB00628	Hypertension	0.01917
DB00628	Hypogonadism	0.10847
DB00628	Panic disorder	0.05293
DB00628	Yersinia infection	0.02878
DB00962	Cancer	0.02606
DB00962	Hypertension	0.0559
DB00962	Panic disorder	0.1543
DB01068	Breast cancer	0.01167
DB01068	Cancer	0.00894
DB01068	Drug abuse	0.02272
DB01068	Hypertension	0.01917
DB01068	Hypogonadism	0.10847
DB01068	Panic disorder	0.05293
DB01068	Yersinia infection	0.02878
DB01178	Cancer	0.03686
DB01178	Hypertension	0.07906
DB01178	Panic disorder	0.21822
DB01587	Cancer	0.01505
DB01587	Hypertension	0.03227
DB01587	Panic disorder	0.08909
DB00416	Epilepsy	0.14142
DB00565	Epilepsy	0.14142
DB00657	Epilepsy	0.14142
DB00732	Epilepsy	0.14142
DB01339	Epilepsy	0.14142
DB01219	Myopathies, Nemaline	0.40825
DB01219	Cancer	0.03686
DB01219	Congenital abnormality	0.07538
DB01219	Endocrine system disease	0.37796
DB01219	Myopathy	0.12127
DB00380	Kidney tubular necrosis, acute	0.17548
DB00380	Adenovirus infection	0.019
DB00380	Adrenal gland hyperfunction	0.05245
DB00380	Adrenal gland tumor	0.07515

DB00380	Amyotrophic lateral sclerosis	0.05268
DB00380	Aortic valve disease	0.11733
DB00380	Brain disease	0.04089
DB00380	Brain tumor	0.01696
DB00380	Breast cancer	0.06707
DB00380	Cancer	0.04786
DB00380	Celiac disease	0.09611
DB00380	Charcot-Marie-Tooth disease	0.14677
DB00380	Chronic obstructive airway disease	0.09181
DB00380	Cockayne syndrome	0.09252
DB00380	Colon cancer	0.07765
DB00380	Common cold	0.12801
DB00380	Congenital abnormality	0.01782
DB00380	Diabetes mellitus	0.02943
DB00380	Down syndrome	0.08106
DB00380	Drug abuse	0.01784
DB00380	Embryoma	0.03125
DB00380	Emphysema	0.0859
DB00380	Epilepsy	0.02367
DB00380	Ewings sarcoma	0.17112
DB00380	Eye disease	0.07265
DB00380	Fanconi's anemia	0.05655
DB00380	HIV infection	0.08014
DB00380	Heart disease	0.21155
DB00380	Heart failure	0.06995
DB00380	Helicobacter infection	0.10181
DB00380	Hereditary disease	0.1019
DB00380	Herpes	0.01276
DB00380	Hodgkin's disease	0.04161
DB00380	Infection	0.01393
DB00380	Infertility	0.04274
DB00380	Ischemia	0.08762
DB00380	Kaposi sarcoma	0.09674
DB00380	Kidney cancer	0.06656
DB00380	Leigh disease	0.04412
DB00380	Leukemia	0.05154
DB00380	Leukoencephalopathy	0.03218
DB00380	Lipodystrophy	0.1019
DB00380	Liver cancer	0.06746
DB00380	Lung cancer	0.04313
DB00380	Lymphoma	0.08148
DB00380	Melanoma	0.05371
DB00380	Meningioma	0.18673
DB00380	Metabolism disease	0.06409
DB00380	Muscular atrophy	0.07389
DB00380	Muscular dystrophies	0.0508
DB00380	Myotonic disorder	0.05567

DB00380	Neoplasm metastasis	0.04784
DB00380	Nephroblastoma	0.13187
DB00380	Neuroblastoma	0.04172
DB00380	Neuropathy	0.05998
DB00380	Parkinson disease	0.06328
DB00380	Pituitary tumor	0.14757
DB00380	Prostate cancer	0.04171
DB00380	Renal Cell cancer	0.04633
DB00380	Renal tubular acidosis	0.10588
DB00380	Respiratory failure	0.10037
DB00380	Retinitis pigmentosa	0.09458
DB00380	Rheumatoid arthritis	0.02965
DB00380	Schizophrenia	0.04578
DB00380	Stomach cancer	0.01927
DB00380	Synovial sarcoma	0.0891
DB00380	Tuberous sclerosis	0.06704
DB00380	Uterine fibroids	0.14785
DB00380	Virus disease	0.03385
DB00380	Werner syndrome	0.12441
DB00385	Kidney tubular necrosis, acute	0.12007
DB00385	Amyotrophic lateral sclerosis	0.03604
DB00385	Aortic valve disease	0.11062
DB00385	Brain tumor	0.02262
DB00385	Breast cancer	0.04151
DB00385	Cancer	0.02592
DB00385	Celiac disease	0.06576
DB00385	Charcot-Marie-Tooth disease	0.19569
DB00385	Chronic obstructive airway disease	0.06282
DB00385	Cockayne syndrome	0.12336
DB00385	Colon cancer	0.08471
DB00385	Common cold	0.08759
DB00385	Diabetes mellitus	0.02014
DB00385	Down syndrome	0.07642
DB00385	Embryoma	0.01525
DB00385	Emphysema	0.08099
DB00385	Epilepsy	0.03155
DB00385	Ewings sarcoma	0.11709
DB00385	Eye disease	0.09687
DB00385	Fanconi's anemia	0.03869
DB00385	HIV infection	0.0409
DB00385	Heart disease	0.28207
DB00385	Heart failure	0.04786
DB00385	Helicobacter infection	0.09599
DB00385	Hereditary disease	0.0494
DB00385	Herpes	0.01702
DB00385	Infection	0.01858
DB00385	Infertility	0.05698

DB00385	Ischemia	0.04917
DB00385	Kaposi sarcoma	0.06619
DB00385	Kidney cancer	0.08874
DB00385	Leigh disease	0.05883
DB00385	Leukemia	0.02248
DB00385	Leukoencephalopathy	0.04291
DB00385	Lipodystrophy	0.13587
DB00385	Liver cancer	0.03785
DB00385	Lung cancer	0.04835
DB00385	Lymphoma	0.07682
DB00385	Melanoma	0.03675
DB00385	Meningioma	0.17605
DB00385	Metabolism disease	0.08545
DB00385	Muscular atrophy	0.09852
DB00385	Muscular dystrophies	0.06773
DB00385	Nephroblastoma	0.17583
DB00385	Neuroblastoma	0.05563
DB00385	Neuropathy	0.07997
DB00385	Parkinson disease	0.0433
DB00385	Pituitary tumor	0.10097
DB00385	Prostate cancer	0.02409
DB00385	Renal tubular acidosis	0.05133
DB00385	Retinitis pigmentosa	0.12611
DB00385	Rheumatoid arthritis	0.02029
DB00385	Schizophrenia	0.03388
DB00385	Tuberous sclerosis	0.0632
DB00385	Uterine fibroids	0.10116
DB00385	Virus disease	0.04513
DB00385	Werner syndrome	0.10284
DB00444	Kidney tubular necrosis, acute	0.12007
DB00444	Amyotrophic lateral sclerosis	0.03604
DB00444	Aortic valve disease	0.11062
DB00444	Brain tumor	0.02262
DB00444	Breast cancer	0.04151
DB00444	Cancer	0.02592
DB00444	Celiac disease	0.06576
DB00444	Charcot-Marie-Tooth disease	0.19569
DB00444	Chronic obstructive airway disease	0.06282
DB00444	Cockayne syndrome	0.12336
DB00444	Colon cancer	0.08471
DB00444	Common cold	0.08759
DB00444	Diabetes mellitus	0.02014
DB00444	Down syndrome	0.07642
DB00444	Embryoma	0.01525
DB00444	Emphysema	0.08099
DB00444	Epilepsy	0.03155
DB00444	Ewings sarcoma	0.11709

DB00444	Eye disease	0.09687
DB00444	Fanconi's anemia	0.03869
DB00444	HIV infection	0.0409
DB00444	Heart disease	0.28207
DB00444	Heart failure	0.04786
DB00444	Helicobacter infection	0.09599
DB00444	Hereditary disease	0.0494
DB00444	Herpes	0.01702
DB00444	Infection	0.01858
DB00444	Infertility	0.05698
DB00444	Ischemia	0.04917
DB00444	Kaposi sarcoma	0.06619
DB00444	Kidney cancer	0.08874
DB00444	Leigh disease	0.05883
DB00444	Leukemia	0.02248
DB00444	Leukoencephalopathy	0.04291
DB00444	Lipodystrophy	0.13587
DB00444	Liver cancer	0.03785
DB00444	Lung cancer	0.04835
DB00444	Lymphoma	0.07682
DB00444	Melanoma	0.03675
DB00444	Meningioma	0.17605
DB00444	Metabolism disease	0.08545
DB00444	Muscular atrophy	0.09852
DB00444	Muscular dystrophies	0.06773
DB00444	Nephroblastoma	0.17583
DB00444	Neuroblastoma	0.05563
DB00444	Neuropathy	0.07997
DB00444	Parkinson disease	0.0433
DB00444	Pituitary tumor	0.10097
DB00444	Prostate cancer	0.02409
DB00444	Renal tubular acidosis	0.05133
DB00444	Retinitis pigmentosa	0.12611
DB00444	Rheumatoid arthritis	0.02029
DB00444	Schizophrenia	0.03388
DB00444	Tuberous sclerosis	0.0632
DB00444	Uterine fibroids	0.10116
DB00444	Virus disease	0.04513
DB00444	Werner syndrome	0.10284
DB00694	Kidney tubular necrosis, acute	0.17548
DB00694	Adenovirus infection	0.019
DB00694	Adrenal gland hyperfunction	0.05245
DB00694	Adrenal gland tumor	0.07515
DB00694	Amyotrophic lateral sclerosis	0.05268
DB00694	Aortic valve disease	0.11733
DB00694	Brain disease	0.04089
DB00694	Brain tumor	0.01696

DB00694	Breast cancer	0.06707
DB00694	Cancer	0.04786
DB00694	Celiac disease	0.09611
DB00694	Charcot-Marie-Tooth disease	0.14677
DB00694	Chronic obstructive airway disease	0.09181
DB00694	Cockayne syndrome	0.09252
DB00694	Colon cancer	0.07765
DB00694	Common cold	0.12801
DB00694	Congenital abnormality	0.01782
DB00694	Diabetes mellitus	0.02943
DB00694	Down syndrome	0.08106
DB00694	Drug abuse	0.01784
DB00694	Embryoma	0.03125
DB00694	Emphysema	0.0859
DB00694	Epilepsy	0.02367
DB00694	Ewings sarcoma	0.17112
DB00694	Eye disease	0.07265
DB00694	Fanconi's anemia	0.05655
DB00694	HIV infection	0.08014
DB00694	Heart disease	0.21155
DB00694	Heart failure	0.06995
DB00694	Helicobacter infection	0.10181
DB00694	Hereditary disease	0.1019
DB00694	Herpes	0.01276
DB00694	Hodgkin's disease	0.04161
DB00694	Infection	0.01393
DB00694	Infertility	0.04274
DB00694	Ischemia	0.08762
DB00694	Kaposi sarcoma	0.09674
DB00694	Kidney cancer	0.06656
DB00694	Leigh disease	0.04412
DB00694	Leukemia	0.05154
DB00694	Leukoencephalopathy	0.03218
DB00694	Lipodystrophy	0.1019
DB00694	Liver cancer	0.06746
DB00694	Lung cancer	0.04313
DB00694	Lymphoma	0.08148
DB00694	Melanoma	0.05371
DB00694	Meningioma	0.18673
DB00694	Metabolism disease	0.06409
DB00694	Muscular atrophy	0.07389
DB00694	Muscular dystrophies	0.0508
DB00694	Myotonic disorder	0.05567
DB00694	Neoplasm metastasis	0.04784
DB00694	Nephroblastoma	0.13187
DB00694	Neuroblastoma	0.04172
DB00694	Neuropathy	0.05998

DB00694	Parkinson disease	0.06328
DB00694	Pituitary tumor	0.14757
DB00694	Prostate cancer	0.04171
DB00694	Renal Cell cancer	0.04633
DB00694	Renal tubular acidosis	0.10588
DB00694	Respiratory failure	0.10037
DB00694	Retinitis pigmentosa	0.09458
DB00694	Rheumatoid arthritis	0.02965
DB00694	Schizophrenia	0.04578
DB00694	Stomach cancer	0.01927
DB00694	Synovial sarcoma	0.0891
DB00694	Tuberous sclerosis	0.06704
DB00694	Uterine fibroids	0.14785
DB00694	Virus disease	0.03385
DB00694	Werner syndrome	0.12441
DB00773	Kidney tubular necrosis, acute	0.17548
DB00773	Adenovirus infection	0.019
DB00773	Adrenal gland hyperfunction	0.05245
DB00773	Adrenal gland tumor	0.07515
DB00773	Amyotrophic lateral sclerosis	0.05268
DB00773	Aortic valve disease	0.11733
DB00773	Brain disease	0.04089
DB00773	Brain tumor	0.01696
DB00773	Breast cancer	0.06707
DB00773	Cancer	0.04786
DB00773	Celiac disease	0.09611
DB00773	Charcot-Marie-Tooth disease	0.14677
DB00773	Chronic obstructive airway disease	0.09181
DB00773	Cockayne syndrome	0.09252
DB00773	Colon cancer	0.07765
DB00773	Common cold	0.12801
DB00773	Congenital abnormality	0.01782
DB00773	Diabetes mellitus	0.02943
DB00773	Down syndrome	0.08106
DB00773	Drug abuse	0.01784
DB00773	Embryoma	0.03125
DB00773	Emphysema	0.0859
DB00773	Epilepsy	0.02367
DB00773	Ewings sarcoma	0.17112
DB00773	Eye disease	0.07265
DB00773	Fanconi's anemia	0.05655
DB00773	HIV infection	0.08014
DB00773	Heart disease	0.21155
DB00773	Heart failure	0.06995
DB00773	Helicobacter infection	0.10181
DB00773	Hereditary disease	0.1019
DB00773	Herpes	0.01276

DB00773	Hodgkin's disease	0.04161
DB00773	Infection	0.01393
DB00773	Infertility	0.04274
DB00773	Ischemia	0.08762
DB00773	Kaposi sarcoma	0.09674
DB00773	Kidney cancer	0.06656
DB00773	Leigh disease	0.04412
DB00773	Leukemia	0.05154
DB00773	Leukoencephalopathy	0.03218
DB00773	Lipodystrophy	0.1019
DB00773	Liver cancer	0.06746
DB00773	Lung cancer	0.04313
DB00773	Lymphoma	0.08148
DB00773	Melanoma	0.05371
DB00773	Meningioma	0.18673
DB00773	Metabolism disease	0.06409
DB00773	Muscular atrophy	0.07389
DB00773	Muscular dystrophies	0.0508
DB00773	Myotonic disorder	0.05567
DB00773	Neoplasm metastasis	0.04784
DB00773	Nephroblastoma	0.13187
DB00773	Neuroblastoma	0.04172
DB00773	Neuropathy	0.05998
DB00773	Parkinson disease	0.06328
DB00773	Pituitary tumor	0.14757
DB00773	Prostate cancer	0.04171
DB00773	Renal Cell cancer	0.04633
DB00773	Renal tubular acidosis	0.10588
DB00773	Respiratory failure	0.10037
DB00773	Retinitis pigmentosa	0.09458
DB00773	Rheumatoid arthritis	0.02965
DB00773	Schizophrenia	0.04578
DB00773	Stomach cancer	0.01927
DB00773	Synovial sarcoma	0.0891
DB00773	Tuberous sclerosis	0.06704
DB00773	Uterine fibroids	0.14785
DB00773	Virus disease	0.03385
DB00773	Werner syndrome	0.12441
DB00997	Kidney tubular necrosis, acute	0.12007
DB00997	Amyotrophic lateral sclerosis	0.03604
DB00997	Aortic valve disease	0.11062
DB00997	Brain tumor	0.02262
DB00997	Breast cancer	0.04151
DB00997	Cancer	0.02592
DB00997	Celiac disease	0.06576
DB00997	Charcot-Marie-Tooth disease	0.19569
DB00997	Chronic obstructive airway disease	0.06282

DB00997	Cockayne syndrome	0.12336
DB00997	Colon cancer	0.08471
DB00997	Common cold	0.08759
DB00997	Diabetes mellitus	0.02014
DB00997	Down syndrome	0.07642
DB00997	Embryoma	0.01525
DB00997	Emphysema	0.08099
DB00997	Epilepsy	0.03155
DB00997	Ewings sarcoma	0.11709
DB00997	Eye disease	0.09687
DB00997	Fanconi's anemia	0.03869
DB00997	HIV infection	0.0409
DB00997	Heart disease	0.28207
DB00997	Heart failure	0.04786
DB00997	Helicobacter infection	0.09599
DB00997	Hereditary disease	0.0494
DB00997	Herpes	0.01702
DB00997	Infection	0.01858
DB00997	Infertility	0.05698
DB00997	Ischemia	0.04917
DB00997	Kaposi sarcoma	0.06619
DB00997	Kidney cancer	0.08874
DB00997	Leigh disease	0.05883
DB00997	Leukemia	0.02248
DB00997	Leukoencephalopathy	0.04291
DB00997	Lipodystrophy	0.13587
DB00997	Liver cancer	0.03785
DB00997	Lung cancer	0.04835
DB00997	Lymphoma	0.07682
DB00997	Melanoma	0.03675
DB00997	Meningioma	0.17605
DB00997	Metabolism disease	0.08545
DB00997	Muscular atrophy	0.09852
DB00997	Muscular dystrophies	0.06773
DB00997	Nephroblastoma	0.17583
DB00997	Neuroblastoma	0.05563
DB00997	Neuropathy	0.07997
DB00997	Parkinson disease	0.0433
DB00997	Pituitary tumor	0.10097
DB00997	Prostate cancer	0.02409
DB00997	Renal tubular acidosis	0.05133
DB00997	Retinitis pigmentosa	0.12611
DB00997	Rheumatoid arthritis	0.02029
DB00997	Schizophrenia	0.03388
DB00997	Tuberous sclerosis	0.0632
DB00997	Uterine fibroids	0.10116
DB00997	Virus disease	0.04513

DB00997	Werner syndrome	0.10284
DB01177	Kidney tubular necrosis, acute	0.12007
DB01177	Amyotrophic lateral sclerosis	0.03604
DB01177	Aortic valve disease	0.11062
DB01177	Brain tumor	0.02262
DB01177	Breast cancer	0.04151
DB01177	Cancer	0.02592
DB01177	Celiac disease	0.06576
DB01177	Charcot-Marie-Tooth disease	0.19569
DB01177	Chronic obstructive airway disease	0.06282
DB01177	Cockayne syndrome	0.12336
DB01177	Colon cancer	0.08471
DB01177	Common cold	0.08759
DB01177	Diabetes mellitus	0.02014
DB01177	Down syndrome	0.07642
DB01177	Embryoma	0.01525
DB01177	Emphysema	0.08099
DB01177	Epilepsy	0.03155
DB01177	Ewings sarcoma	0.11709
DB01177	Eye disease	0.09687
DB01177	Fanconi's anemia	0.03869
DB01177	HIV infection	0.0409
DB01177	Heart disease	0.28207
DB01177	Heart failure	0.04786
DB01177	Helicobacter infection	0.09599
DB01177	Hereditary disease	0.0494
DB01177	Herpes	0.01702
DB01177	Infection	0.01858
DB01177	Infertility	0.05698
DB01177	Ischemia	0.04917
DB01177	Kaposi sarcoma	0.06619
DB01177	Kidney cancer	0.08874
DB01177	Leigh disease	0.05883
DB01177	Leukemia	0.02248
DB01177	Leukoencephalopathy	0.04291
DB01177	Lipodystrophy	0.13587
DB01177	Liver cancer	0.03785
DB01177	Lung cancer	0.04835
DB01177	Lymphoma	0.07682
DB01177	Melanoma	0.03675
DB01177	Meningioma	0.17605
DB01177	Metabolism disease	0.08545
DB01177	Muscular atrophy	0.09852
DB01177	Muscular dystrophies	0.06773
DB01177	Nephroblastoma	0.17583
DB01177	Neuroblastoma	0.05563
DB01177	Neuropathy	0.07997

DB01177	Parkinson disease	0.0433
DB01177	Pituitary tumor	0.10097
DB01177	Prostate cancer	0.02409
DB01177	Renal tubular acidosis	0.05133
DB01177	Retinitis pigmentosa	0.12611
DB01177	Rheumatoid arthritis	0.02029
DB01177	Schizophrenia	0.03388
DB01177	Tuberous sclerosis	0.0632
DB01177	Uterine fibroids	0.10116
DB01177	Virus disease	0.04513
DB01177	Werner syndrome	0.10284
DB01204	Kidney tubular necrosis, acute	0.12007
DB01204	Amyotrophic lateral sclerosis	0.03604
DB01204	Aortic valve disease	0.11062
DB01204	Brain tumor	0.02262
DB01204	Breast cancer	0.04151
DB01204	Cancer	0.02592
DB01204	Celiac disease	0.06576
DB01204	Charcot-Marie-Tooth disease	0.19569
DB01204	Chronic obstructive airway disease	0.06282
DB01204	Cockayne syndrome	0.12336
DB01204	Colon cancer	0.08471
DB01204	Common cold	0.08759
DB01204	Diabetes mellitus	0.02014
DB01204	Down syndrome	0.07642
DB01204	Embryoma	0.01525
DB01204	Emphysema	0.08099
DB01204	Epilepsy	0.03155
DB01204	Ewings sarcoma	0.11709
DB01204	Eye disease	0.09687
DB01204	Fanconi's anemia	0.03869
DB01204	HIV infection	0.0409
DB01204	Heart disease	0.28207
DB01204	Heart failure	0.04786
DB01204	Helicobacter infection	0.09599
DB01204	Hereditary disease	0.0494
DB01204	Herpes	0.01702
DB01204	Infection	0.01858
DB01204	Infertility	0.05698
DB01204	Ischemia	0.04917
DB01204	Kaposi sarcoma	0.06619
DB01204	Kidney cancer	0.08874
DB01204	Leigh disease	0.05883
DB01204	Leukemia	0.02248
DB01204	Leukoencephalopathy	0.04291
DB01204	Lipodystrophy	0.13587
DB01204	Liver cancer	0.03785

DB01204	Lung cancer	0.04835
DB01204	Lymphoma	0.07682
DB01204	Melanoma	0.03675
DB01204	Meningioma	0.17605
DB01204	Metabolism disease	0.08545
DB01204	Muscular atrophy	0.09852
DB01204	Muscular dystrophies	0.06773
DB01204	Nephroblastoma	0.17583
DB01204	Neuroblastoma	0.05563
DB01204	Neuropathy	0.07997
DB01204	Parkinson disease	0.0433
DB01204	Pituitary tumor	0.10097
DB01204	Prostate cancer	0.02409
DB01204	Renal tubular acidosis	0.05133
DB01204	Retinitis pigmentosa	0.12611
DB01204	Rheumatoid arthritis	0.02029
DB01204	Schizophrenia	0.03388
DB01204	Tuberous sclerosis	0.0632
DB01204	Uterine fibroids	0.10116
DB01204	Virus disease	0.04513
DB01204	Werner syndrome	0.10284
DB04967	Hypertension, Pulmonary	0.04858
DB04967	Kidney tubular necrosis, acute	0.17266
DB04967	Abortion	0.00743
DB04967	Adenovirus infection	0.00424
DB04967	Alzheimer's disease	0.00348
DB04967	Amyotrophic lateral sclerosis	0.18269
DB04967	Aortic aneurysm	0.00691
DB04967	Aortic valve disease	0.13108
DB04967	Aplastic anemia	0.01618
DB04967	Aseptic necrosis of bone	0.03835
DB04967	Atherosclerosis	0.0194
DB04967	Barrett's esophagus	0.07274
DB04967	Brain tumor	0.01877
DB04967	Breast cancer	0.04784
DB04967	Bronchial disease	0.02487
DB04967	Cancer	0.08827
DB04967	Carcinoma	0.06972
DB04967	Celiac disease	0.09456
DB04967	Cervical cancer	0.00998
DB04967	Charcot-Marie-Tooth disease	0.10563
DB04967	Cholestasis	0.02349
DB04967	Chronic obstructive airway disease	0.09034
DB04967	Chronic rejection of renal transplant	0.06972
DB04967	Chronic simple glaucoma	0.05311
DB04967	Cockayne syndrome	0.48373
DB04967	Colon cancer	0.10402

DB04967	Common cold	0.12595
DB04967	Congenital abnormality	0.01656
DB04967	Dermatitis	0.04116
DB04967	Diabetes mellitus	0.04546
DB04967	Down syndrome	0.10537
DB04967	Drug abuse	0.01657
DB04967	Embryoma	0.01811
DB04967	Emphysema	0.09597
DB04967	Endometriosis	0.01626
DB04967	Epilepsy	0.01703
DB04967	Ewings sarcoma	0.16838
DB04967	Eye disease	0.05229
DB04967	Fanconi's anemia	0.05564
DB04967	HIV infection	0.05287
DB04967	Heart disease	0.15226
DB04967	Heart failure	0.06882
DB04967	Helicobacter infection	0.11375
DB04967	Hemolytic-Uremic syndrome	0.05669
DB04967	Hereditary disease	0.02666
DB04967	Herpes	0.00919
DB04967	Hyperglycemia	0.05388
DB04967	Hyperparathyroidism	0.04869
DB04967	Infection	0.05321
DB04967	Infertility	0.03076
DB04967	Ischemia	0.10321
DB04967	Kaposi sarcoma	0.09519
DB04967	Keratoconjunctivitis Sicca	0.03247
DB04967	Keratosis	0.04159
DB04967	Kidney cancer	0.19938
DB04967	Leigh disease	0.03176
DB04967	Leukemia	0.0359
DB04967	Leukoencephalopathy	0.05234
DB04967	Lipodystrophy	0.07334
DB04967	Liver cancer	0.06618
DB04967	Lung cancer	0.08421
DB04967	Lung disease	0.00891
DB04967	Lymphoma	0.10592
DB04967	Melanoma	0.05285
DB04967	Meningioma	0.24274
DB04967	Mental retardation	0.01657
DB04967	Metabolism disease	0.10262
DB04967	Metaplastic polyp	0.04225
DB04967	Moyamoya disease	0.09506
DB04967	Multiple endocrine neoplasia	0.11737
DB04967	Muscular atrophy	0.05318
DB04967	Muscular dystrophies	0.04731
DB04967	Neck cancer	0.04458

DB04967	Nephroblastoma	0.09491
DB04967	Neuroblastoma	0.06785
DB04967	Neuropathy	0.04317
DB04967	Obesity	0.02461
DB04967	Oral cancer	0.02237
DB04967	Osteosarcoma	0.01142
DB04967	Ovary cancer	0.10066
DB04967	Pancreas cancer	0.01079
DB04967	Pancreas disease	0.02264
DB04967	Papillomavirus infection	0.06607
DB04967	Parkinson disease	0.06227
DB04967	Pituitary tumor	0.1452
DB04967	Polyarthritis	0.04119
DB04967	Polycythemia	0.05979
DB04967	Pre-Eclampsia	0.05477
DB04967	Prostate cancer	0.0368
DB04967	Renal tubular acidosis	0.07381
DB04967	Retinal disease	0.06412
DB04967	Retinitis pigmentosa	0.06807
DB04967	Rheumatoid arthritis	0.04159
DB04967	Sarcoidosis	0.06377
DB04967	Schizophrenia	0.01829
DB04967	Stomach cancer	0.00962
DB04967	Stroke	0.12039
DB04967	Tuberous sclerosis	0.0817
DB04967	Ulcerative colitis	0.03301
DB04967	Uterine fibroids	0.14547
DB04967	Vasculitis	0.0989
DB04967	Virus disease	0.03153
DB04967	Werner syndrome	0.1152
DB00039	Pemphigoid, Bullous	0.02473
DB00039	Pleural effusion, Malignant	0.02531
DB00039	Purpura, Thrombocytopenic, Idiopathic	0.02363
DB00039	Alopecia	0.02915
DB00039	Alzheimer's disease	0.04445
DB00039	Amyotrophic lateral sclerosis	0.06059
DB00039	Aortic aneurysm	0.02987
DB00039	Asthma	0.05686
DB00039	Atherosclerosis	0.09804
DB00039	Bone disease	0.07001
DB00039	Brain tumor	0.02314
DB00039	Breast cancer	0.06853
DB00039	Cancer	0.2538
DB00039	Capillaries disease	0.15751
DB00039	Cardiovascular disease	0.17097
DB00039	Chronic obstructive airway disease	0.24013
DB00039	Cleft palate	0.10911

DB00039	Craniosynostosis	0.56019
DB00039	Depression	0.02334
DB00039	Dermatitis	0.05435
DB00039	Diabetes mellitus	0.06149
DB00039	Disseminated cancer	0.1291
DB00039	Drug abuse	0.0172
DB00039	Embryoma	0.01911
DB00039	Emphysema	0.10938
DB00039	Endometriosis	0.09147
DB00039	Esotropia	0.03324
DB00039	Familial Mediterranean fever	0.24949
DB00039	Gingival overgrowth	0.1543
DB00039	Graves' disease	0.07217
DB00039	Hyperglycemia	0.06537
DB00039	Hyperopia	0.13608
DB00039	Intracranial hypertension	0.28868
DB00039	Kaposi sarcoma	0.03212
DB00039	Keratosis	0.13608
DB00039	Kidney failure	0.1184
DB00039	Leukemia	0.01543
DB00039	Leukoencephalopathy	0.0896
DB00039	Lupus erythematosus	0.07937
DB00039	Macular degeneration	0.32196
DB00039	Malaria	0.2149
DB00039	Mucocutaneous lymph node syndrome	0.31492
DB00039	Multiple myeloma	0.02889
DB00039	Muscular dystrophy	0.11323
DB00039	Myeloproliferative disease	0.10911
DB00039	Myotonic disorder	0.12309
DB00039	Obesity	0.08812
DB00039	Peptic ulcer	0.19865
DB00039	Polyneuropathy	0.32196
DB00039	Pre-Eclampsia	0.15814
DB00039	Primary hyperparathyroidism	0.02941
DB00039	Prostate cancer	0.02131
DB00039	Ptosis	0.13608
DB00039	Renal Cell cancer	0.04469
DB00039	Retinal disease	0.16305
DB00039	Rheumatoid arthritis	0.03922
DB00039	Schizophrenia	0.03797
DB00039	Shigella infection	0.03116
DB00039	Sickle cell disease	0.02778
DB00039	Stomach cancer	0.08271
DB00039	Stroke	0.01508
DB00039	Systemic infection	0.11401
DB00039	Thalassemia	0.02913
DB00039	Turner's syndrome	0.2357

DB00039	Urogenital abnormalities	0.10911
DB00176	Hypertension, Pulmonary	0.22942
DB00176	Anorexia nervosa	0.20412
DB00176	Atherosclerosis	0.07001
DB00176	Autistic disorder	0.12127
DB00176	Behavior disease	0.1543
DB00176	Bipolar disorder	0.11323
DB00176	Chronic fatigue syndrome	0.35355
DB00176	Chronic obstructive airway disease	0.1118
DB00176	Colon cancer	0.05934
DB00176	Congenital heart disease	0.33333
DB00176	Depression	0.13131
DB00176	Dermatitis	0.09091
DB00176	Drug abuse	0.09366
DB00176	Epilepsy	0.14142
DB00176	Fibromyalgia	0.35355
DB00176	Generalized anxiety disorder	0.28868
DB00176	Heart failure	0.1066
DB00176	Herpes	0.14286
DB00176	Migraine	0.1857
DB00176	Neurotic disorder	0.31623
DB00176	Obesity	0.07692
DB00176	Obsessive-compulsive disorder	0.30151
DB00176	Panic disorder	0.21822
DB00176	Pervasive development disorder	0.2582
DB00176	Psychotic disorder	0.16013
DB00176	Pulmonary hypertension	0.5
DB00176	Stroke	0.11323
DB00176	Sudden infant death syndrome	0.21822
DB00176	Ulcerative colitis	0.10314
DB00472	Hypertension, Pulmonary	0.22942
DB00472	Anorexia nervosa	0.20412
DB00472	Atherosclerosis	0.07001
DB00472	Autistic disorder	0.12127
DB00472	Behavior disease	0.1543
DB00472	Bipolar disorder	0.11323
DB00472	Chronic fatigue syndrome	0.35355
DB00472	Chronic obstructive airway disease	0.1118
DB00472	Colon cancer	0.05934
DB00472	Congenital heart disease	0.33333
DB00472	Depression	0.13131
DB00472	Dermatitis	0.09091
DB00472	Drug abuse	0.09366
DB00472	Epilepsy	0.14142
DB00472	Fibromyalgia	0.35355
DB00472	Generalized anxiety disorder	0.28868
DB00472	Heart failure	0.1066

DB00472	Herpes	0.14286
DB00472	Migraine	0.1857
DB00472	Neurotic disorder	0.31623
DB00472	Obesity	0.07692
DB00472	Obsessive-compulsive disorder	0.30151
DB00472	Panic disorder	0.21822
DB00472	Pervasive development disorder	0.2582
DB00472	Psychotic disorder	0.16013
DB00472	Pulmonary hypertension	0.5
DB00472	Stroke	0.11323
DB00472	Sudden infant death syndrome	0.21822
DB00472	Ulcerative colitis	0.10314
DB00107	Autistic disorder	0.08575
DB00107	Cancer	0.02606
DB00107	Congenital heart disease	0.2357
DB00107	Depression	0.09285
DB00107	Endometriosis	0.05872
DB00107	Fibroid tumor	0.2132
DB00107	Pervasive development disorder	0.18257
DB01046	Brain tumor	0.08032
DB01046	Epilepsy	0.14142
DB01046	Malaria	0.1857
DB01046	Sinusitis	0.22361
DB00317	Hemorrhagic fevers, Viral	0.06999
DB00317	Pemphigoid, Bullous	0.03553
DB00317	Pleural effusion, Malignant	0.5905
DB00317	Purpura, Thrombocytopenic, Idiopathic	0.03395
DB00317	Abortion	0.03996
DB00317	Adenovirus infection	0.32676
DB00317	Alzheimer's disease	0.02164
DB00317	Angiomyolipoma	1.10241
DB00317	Aplastic anemia	0.05803
DB00317	Asthma	0.34675
DB00317	Atherosclerosis	0.04091
DB00317	Atopic rhinitis	1.2582
DB00317	Autistic disorder	0.04611
DB00317	Autoimmune disease	0.07934
DB00317	Barrett's esophagus	0.52594
DB00317	Breast cancer	0.01619
DB00317	Cancer	0.13844
DB00317	Charcot-Marie-Tooth disease	0.16712
DB00317	Cholelithiasis	0.77345
DB00317	Colon cancer	0.02367
DB00317	Common wart	1.33529
DB00317	Cytomegalovirus infection	1.01292
DB00317	Diabetes mellitus	0.22698
DB00317	Drug abuse	0.29994

DB00317	Endometriosis	0.27862
DB00317	Esotropia	0.85239
DB00317	Gastrointestinal tumor	0.82769
DB00317	Glaucoma	0.60131
DB00317	Hemorrhagic disorder	0.05478
DB00317	Herpes	0.03064
DB00317	Hypercholesterolemia	1.01388
DB00317	Intermediate coronary syndrome	0.06982
DB00317	Leukemia	0.08274
DB00317	Lupus erythematosus	0.30352
DB00317	Muscular atrophy	0.18128
DB00317	Myopathy	0.04866
DB00317	Optic atrophy	1.40096
DB00317	Oral cancer	0.42023
DB00317	Osteitis deformans	0.08799
DB00317	Papillary adenocarcinoma	1.57735
DB00317	Papillomavirus infection	0.09218
DB00317	Penile disease	0.06551
DB00317	Pneumoconiosis	0.23205
DB00317	Primary hyperparathyroidism	0.69341
DB00317	Pulmonary fibrosis	0.07546
DB00317	Rheumatoid arthritis	0.03733
DB00317	Schizophrenia	0.3254
DB00317	Skin cancer	0.67884
DB00317	Stroke	0.38227
DB00317	Subarachnoid hemorrhage	0.06249
DB00317	Takayasu's arteritis	0.05843
DB00317	Temporal arteritis	0.06831
DB00317	Testicular dysfunction	0.43604
DB00317	Thrombocytopenia	0.05206
DB00317	Thrombophlebitis	1.03801
DB00317	Yersinia infection	0.39569
DB00530	Hemorrhagic fevers, Viral	0.06999
DB00530	Pemphigoid, Bullous	0.03553
DB00530	Pleural effusion, Malignant	0.50219
DB00530	Purpura, Thrombocytopenic, Idiopathic	0.03395
DB00530	Abortion	0.03996
DB00530	Adenovirus infection	0.38069
DB00530	Alzheimer's disease	0.02164
DB00530	Angiomyolipoma	0.9917
DB00530	Aplastic anemia	0.05803
DB00530	Asthma	0.32284
DB00530	Atherosclerosis	0.04091
DB00530	Atopic rhinitis	1.18257
DB00530	Autistic disorder	0.04611
DB00530	Autoimmune disease	0.07934
DB00530	Barrett's esophagus	0.47246

DB00530	Breast cancer	0.01619
DB00530	Cancer	0.12764
DB00530	Charcot-Marie-Tooth disease	0.16712
DB00530	Cholelithiasis	0.71487
DB00530	Colon cancer	0.02367
DB00530	Common wart	1.2043
DB00530	Cytomegalovirus infection	0.93729
DB00530	Diabetes mellitus	0.21157
DB00530	Drug abuse	0.27251
DB00530	Endometriosis	0.25429
DB00530	Endometrium cancer	0.1715
DB00530	Enteritis	0.07762
DB00530	Epilepsy	0.1
DB00530	Esotropia	0.76784
DB00530	Gastrointestinal tumor	0.74646
DB00530	Glaucoma	0.5525
DB00530	Hemorrhagic disorder	0.05478
DB00530	Herpes	0.03064
DB00530	Hypercholesterolemia	0.95751
DB00530	Intermediate coronary syndrome	0.06982
DB00530	Leukemia	0.08274
DB00530	Lupus erythematosus	0.27849
DB00530	Muscular atrophy	0.18128
DB00530	Myopathy	0.04866
DB00530	Optic atrophy	1.26998
DB00530	Oral cancer	0.38037
DB00530	Osteitis deformans	0.08799
DB00530	Osteosarcoma	0.15076
DB00530	Papillary adenocarcinoma	1.40825
DB00530	Papillomavirus infection	0.09218
DB00530	Penile disease	0.06551
DB00530	Pneumoconiosis	0.23205
DB00530	Primary hyperparathyroidism	0.58271
DB00530	Pulmonary fibrosis	0.07546
DB00530	Rheumatoid arthritis	0.03733
DB00530	Schizophrenia	0.30314
DB00530	Skin cancer	0.61164
DB00530	Stroke	0.34911
DB00530	Subarachnoid hemorrhage	0.06249
DB00530	Takayasu's arteritis	0.05843
DB00530	Temporal arteritis	0.06831
DB00530	Testicular dysfunction	0.39419
DB00530	Thrombocytopenia	0.05206
DB00530	Thrombophlebitis	0.9497
DB00530	Ulcerative colitis	0.07293
DB00530	Yersinia infection	0.36093
DB01269	Hemorrhagic fevers, Viral	0.06999

DB01269	Pemphigoid, Bullous	0.03553
DB01269	Pleural effusion, Malignant	0.5905
DB01269	Purpura, Thrombocytopenic, Idiopathic	0.03395
DB01269	Abortion	0.03996
DB01269	Adenovirus infection	0.32676
DB01269	Alzheimer's disease	0.02164
DB01269	Angiomyolipoma	1.10241
DB01269	Aplastic anemia	0.05803
DB01269	Asthma	0.34675
DB01269	Atherosclerosis	0.04091
DB01269	Atopic rhinitis	1.2582
DB01269	Autistic disorder	0.04611
DB01269	Autoimmune disease	0.07934
DB01269	Barrett's esophagus	0.52594
DB01269	Breast cancer	0.01619
DB01269	Cancer	0.13844
DB01269	Charcot-Marie-Tooth disease	0.16712
DB01269	Cholelithiasis	0.77345
DB01269	Colon cancer	0.02367
DB01269	Common wart	1.33529
DB01269	Cytomegalovirus infection	1.01292
DB01269	Diabetes mellitus	0.22698
DB01269	Drug abuse	0.29994
DB01269	Endometriosis	0.27862
DB01269	Esotropia	0.85239
DB01269	Gastrointestinal tumor	0.82769
DB01269	Glaucoma	0.60131
DB01269	Hemorrhagic disorder	0.05478
DB01269	Herpes	0.03064
DB01269	Hypercholesterolemia	1.01388
DB01269	Intermediate coronary syndrome	0.06982
DB01269	Leukemia	0.08274
DB01269	Lupus erythematosus	0.30352
DB01269	Muscular atrophy	0.18128
DB01269	Myopathy	0.04866
DB01269	Optic atrophy	1.40096
DB01269	Oral cancer	0.42023
DB01269	Osteitis deformans	0.08799
DB01269	Papillary adenocarcinoma	1.57735
DB01269	Papillomavirus infection	0.09218
DB01269	Penile disease	0.06551
DB01269	Pneumoconiosis	0.23205
DB01269	Primary hyperparathyroidism	0.69341
DB01269	Pulmonary fibrosis	0.07546
DB01269	Rheumatoid arthritis	0.03733
DB01269	Schizophrenia	0.3254
DB01269	Skin cancer	0.67884

DB01269	Stroke	0.38227
DB01269	Subarachnoid hemorrhage	0.06249
DB01269	Takayasu's arteritis	0.05843
DB01269	Temporal arteritis	0.06831
DB01269	Testicular dysfunction	0.43604
DB01269	Thrombocytopenia	0.05206
DB01269	Thrombophlebitis	1.03801
DB01269	Yersinia infection	0.39569
DB01227	Alzheimer's disease	0.04156
DB01227	Down syndrome	0.0658
DB01227	Lung cancer	0.04032
DB00282	Colon cancer	0.05934
DB00282	Osteosarcoma	0.2132
DB00399	Colon cancer	0.04196
DB00399	Osteosarcoma	0.15076
DB00630	Colon cancer	0.02654
DB00630	Osteosarcoma	0.09535
DB00630	Pancreas disease	0.09325
DB00630	Ulcerative colitis	0.04613
DB00710	Colon cancer	0.05934
DB00710	Osteosarcoma	0.2132
DB00884	Colon cancer	0.05934
DB00884	Osteosarcoma	0.2132
DB00136	Adenovirus infection	0.03462
DB00136	Adrenal gland hyperfunction	0.11305
DB00136	Adrenal gland tumor	0.16198
DB00136	Autistic disorder	0.0606
DB00136	Brain disease	0.08812
DB00136	Breast cancer	0.04916
DB00136	Cancer	0.02053
DB00136	Drug abuse	0.03249
DB00136	HIV infection	0.03513
DB00136	Hereditary disease	0.06402
DB00136	Mental retardation	0.09563
DB00136	Neoplasm metastasis	0.09446
DB00136	Prostate cancer	0.07789
DB00136	Renal tubular acidosis	0.13306
DB00136	Respiratory tract disease	0.10686
DB00136	Schizophrenia	0.10757
DB00146	Adenovirus infection	0.03462
DB00146	Adrenal gland hyperfunction	0.11305
DB00146	Adrenal gland tumor	0.16198
DB00146	Autistic disorder	0.0606
DB00146	Brain disease	0.08812
DB00146	Breast cancer	0.04916
DB00146	Cancer	0.02053
DB00146	Drug abuse	0.03249

DB00146	HIV infection	0.03513
DB00146	Hereditary disease	0.06402
DB00146	Mental retardation	0.09563
DB00146	Neoplasm metastasis	0.09446
DB00146	Prostate cancer	0.07789
DB00146	Renal tubular acidosis	0.13306
DB00146	Respiratory tract disease	0.10686
DB00146	Schizophrenia	0.10757
DB00153	Adenovirus infection	0.03462
DB00153	Adrenal gland hyperfunction	0.11305
DB00153	Adrenal gland tumor	0.16198
DB00153	Autistic disorder	0.0606
DB00153	Brain disease	0.08812
DB00153	Breast cancer	0.04916
DB00153	Cancer	0.02053
DB00153	Drug abuse	0.03249
DB00153	HIV infection	0.03513
DB00153	Hereditary disease	0.06402
DB00153	Mental retardation	0.09563
DB00153	Neoplasm metastasis	0.09446
DB00153	Prostate cancer	0.07789
DB00153	Renal tubular acidosis	0.13306
DB00153	Respiratory tract disease	0.10686
DB00153	Schizophrenia	0.10757
DB00169	Adenovirus infection	0.03462
DB00169	Adrenal gland hyperfunction	0.11305
DB00169	Adrenal gland tumor	0.16198
DB00169	Autistic disorder	0.0606
DB00169	Brain disease	0.08812
DB00169	Breast cancer	0.04916
DB00169	Cancer	0.02053
DB00169	Drug abuse	0.03249
DB00169	HIV infection	0.03513
DB00169	Hereditary disease	0.06402
DB00169	Mental retardation	0.09563
DB00169	Neoplasm metastasis	0.09446
DB00169	Prostate cancer	0.07789
DB00169	Renal tubular acidosis	0.13306
DB00169	Respiratory tract disease	0.10686
DB00169	Schizophrenia	0.10757
DB00910	Adenovirus infection	0.03462
DB00910	Adrenal gland hyperfunction	0.11305
DB00910	Adrenal gland tumor	0.16198
DB00910	Autistic disorder	0.0606
DB00910	Brain disease	0.08812
DB00910	Breast cancer	0.04916
DB00910	Cancer	0.02053

DB00910	Drug abuse	0.03249
DB00910	HIV infection	0.03513
DB00910	Hereditary disease	0.06402
DB00910	Mental retardation	0.09563
DB00910	Neoplasm metastasis	0.09446
DB00910	Prostate cancer	0.07789
DB00910	Renal tubular acidosis	0.13306
DB00910	Respiratory tract disease	0.10686
DB00910	Schizophrenia	0.10757
DB01070	Adenovirus infection	0.03462
DB01070	Adrenal gland hyperfunction	0.11305
DB01070	Adrenal gland tumor	0.16198
DB01070	Autistic disorder	0.0606
DB01070	Brain disease	0.08812
DB01070	Breast cancer	0.04916
DB01070	Cancer	0.02053
DB01070	Drug abuse	0.03249
DB01070	HIV infection	0.03513
DB01070	Hereditary disease	0.06402
DB01070	Mental retardation	0.09563
DB01070	Neoplasm metastasis	0.09446
DB01070	Prostate cancer	0.07789
DB01070	Renal tubular acidosis	0.13306
DB01070	Respiratory tract disease	0.10686
DB01070	Schizophrenia	0.10757
DB02300	Adenovirus infection	0.03462
DB02300	Adrenal gland hyperfunction	0.11305
DB02300	Adrenal gland tumor	0.16198
DB02300	Autistic disorder	0.0606
DB02300	Brain disease	0.08812
DB02300	Breast cancer	0.04916
DB02300	Cancer	0.02053
DB02300	Drug abuse	0.03249
DB02300	HIV infection	0.03513
DB02300	Hereditary disease	0.06402
DB02300	Mental retardation	0.09563
DB02300	Neoplasm metastasis	0.09446
DB02300	Prostate cancer	0.07789
DB02300	Renal tubular acidosis	0.13306
DB02300	Respiratory tract disease	0.10686
DB02300	Schizophrenia	0.10757
DB06637	Hypertension, Pulmonary	0.05735
DB06637	Alzheimer's disease	0.04211
DB06637	Amyotrophic lateral sclerosis	0.06916
DB06637	Aortic aneurysm	0.08352
DB06637	Asthma	0.0649
DB06637	Autoimmune disease	0.02712

DB06637	Bipolar disorder	0.13701
DB06637	Brain tumor	0.02008
DB06637	Cancer	0.06726
DB06637	Congenital abnormality	0.01884
DB06637	Depression	0.06524
DB06637	Drug abuse	0.02341
DB06637	Embryoma	0.06884
DB06637	Epilepsy	0.11054
DB06637	Heart failure	0.18318
DB06637	Hypertension	0.01976
DB06637	Leukemia	0.04314
DB06637	Long QT syndrome	0.52263
DB06637	Lupus erythematosus	0.02136
DB06637	Lymphoma	0.12618
DB06637	Multiple sclerosis	0.12296
DB06637	Neurodegenerative disorder	0.0411
DB06637	Pulmonary hypertension	0.125
DB06637	Schizophrenia	0.06501
DB01138	Cancer	0.02606
DB01138	Drug abuse	0.06623
DB01138	Epilepsy	0.1
DB01138	Infection	0.06594
DB01138	Primary biliary cirrhosis	0.127
DB01138	Pseudoxanthoma elasticum	0.31623
DB01138	Tuberous sclerosis	0.18257
DB01047	Bone disease	0.12127
DB01047	Chondrosarcoma	0.26726
DB01047	Hyperglycemia	0.11323
DB01047	Pancreas cancer	0.07332
DB01047	Skin cancer	0.16222
DB01107	Breast cancer	0.01167
DB01107	Drug abuse	0.02272
DB01107	Hypogonadism	0.10847
DB01107	Yersinia infection	0.02878
DB01437	Breast cancer	0.01167
DB01437	Drug abuse	0.02272
DB01437	Hypogonadism	0.10847
DB01437	Yersinia infection	0.02878
DB00237	Myoclonic epilepsy, Juvenile	0.09129
DB00237	Alzheimer's disease	0.02587
DB00237	Attention deficit hyperactivity disorder	0.14669
DB00237	Autistic disorder	0.13217
DB00237	Behavior disease	0.19721
DB00237	Bipolar disorder	0.02532
DB00237	Brain tumor	0.04867
DB00237	Breast cancer	0.01076
DB00237	Colon cancer	0.01327

DB00237	Down syndrome	0.13539
DB00237	Drug abuse	0.04189
DB00237	Epilepsy	0.12765
DB00237	Hypertension	0.05685
DB00237	Hypogonadism	0.1
DB00237	Neoplasm metastasis	0.04295
DB00237	Nervous system disease	0.13394
DB00237	Neuroblastoma	0.03071
DB00237	Obesity	0.05373
DB00237	Parkinson disease	0.10042
DB00237	Primary tumor	0.07674
DB00237	Psychotic disorder	0.31696
DB00237	Schizophrenia	0.08884
DB00237	Yersinia infection	0.02654
DB01356	Alzheimer's disease	0.05956
DB01356	Aseptic necrosis of bone	0.15744
DB01356	Barrett's esophagus	0.0633
DB01356	Bipolar disorder	0.11323
DB01356	Cancer	0.059
DB01356	Congenital abnormality	0.08324
DB01356	Depression	0.09227
DB01356	Dermatitis	0.08774
DB01356	Endometriosis	0.0721
DB01356	Oral cancer	0.09917
DB01356	Pervasive development disorder	0.14992
DB01356	Schizophrenia	0.04597
DB01356	Systemic scleroderma	0.08067
DB01356	Ulcerative colitis	0.12215
DB01356	Uterine disease	0.19327
DB00284	Cancer	0.02464
DB00284	Congenital abnormality	0.03896
DB00284	Diabetes mellitus	0.02632
DB00284	Drug abuse	0.03899
DB00284	Embryoma	0.04331
DB00284	Endometrial cancer	0.12994
DB00284	Granulomatous disease	0.42299
DB00284	Immunologic deficiency syndrome	0.08505
DB00284	Ischemia	0.07648
DB00284	Leukemia	0.04945
DB00284	Liver cancer	0.05888
DB00284	Lymphoma	0.07919
DB00284	Mental retardation	0.11477
DB00284	Neoplasm metastasis	0.09817
DB00284	Osteomyelitis	0.14117
DB00284	Prostate cancer	0.03305
DB00284	Renal Cell cancer	0.10128
DB00284	Retinitis pigmentosa	0.17299

DB00284	Schizophrenia	0.0527
DB00284	Vitamin D deficiency	0.15854
DB00491	Cancer	0.0114
DB00491	Congenital abnormality	0.01802
DB00491	Drug abuse	0.01803
DB00491	Embryoma	0.02003
DB00491	Endometrial cancer	0.0601
DB00491	Granulomatous disease	0.19565
DB00491	Hemolytic anemia	0.125
DB00491	Immunologic deficiency syndrome	0.03934
DB00491	Ischemia	0.03537
DB00491	Leukemia	0.02287
DB00491	Liver cancer	0.02723
DB00491	Lymphoma	0.03663
DB00491	Mental retardation	0.05308
DB00491	Neoplasm metastasis	0.04541
DB00491	Osteomyelitis	0.0653
DB00491	Prostate cancer	0.01528
DB00491	Renal Cell cancer	0.04684
DB00491	Retinitis pigmentosa	0.08001
DB00491	Schizophrenia	0.02438
DB00491	Vitamin D deficiency	0.07333
DB04878	Cancer	0.02464
DB04878	Congenital abnormality	0.03896
DB04878	Drug abuse	0.03899
DB04878	Embryoma	0.04331
DB04878	Endometrial cancer	0.12994
DB04878	Granulomatous disease	0.42299
DB04878	Immunologic deficiency syndrome	0.08505
DB04878	Ischemia	0.07648
DB04878	Leukemia	0.04945
DB04878	Liver cancer	0.05888
DB04878	Lymphoma	0.07919
DB04878	Mental retardation	0.11477
DB04878	Neoplasm metastasis	0.09817
DB04878	Osteomyelitis	0.14117
DB04878	Prostate cancer	0.03305
DB04878	Renal Cell cancer	0.10128
DB04878	Retinitis pigmentosa	0.17299
DB04878	Schizophrenia	0.0527
DB04878	Vitamin D deficiency	0.15854
DB01282	Autistic disorder	0.12127
DB01282	Congenital heart disease	0.33333
DB01282	Endometriosis	0.08305
DB01282	Fibroid tumor	0.30151
DB01281	Adenovirus infection	0.18411
DB01281	Asthma	0.05774

DB01281	Atherosclerosis	0.09901
DB01281	Common variable immunodeficiency	0.25
DB01281	Diabetes mellitus	0.03722
DB01281	Enteritis	0.07762
DB01281	Graves' disease	0.125
DB01281	HIV infection	0.06773
DB01281	Herpes	0.20203
DB01281	IGA glomerulonephritis	0.24254
DB01281	Kaposi sarcoma	0.19612
DB01281	Leukemia	0.03984
DB01281	Liver cancer	0.05488
DB01281	Melanoma	0.11785
DB01281	Multiple sclerosis	0.07293
DB01281	Nasopharyngeal cancer	0.27217
DB01281	Rheumatoid arthritis	0.08655
DB01281	Sarcoidosis	0.27735
DB01281	Squamous cell cancer	0.07217
DB01281	Stomach cancer	0.05955
DB01281	Thyroid cancer	0.12309
DB01281	Thyroiditis	0.5
DB01281	Tuberculosis	0.09535
DB01281	Ulcerative colitis	0.14586
DB06681	Adenovirus infection	0.18411
DB06681	Asthma	0.05774
DB06681	Atherosclerosis	0.09901
DB06681	Common variable immunodeficiency	0.25
DB06681	Diabetes mellitus	0.03722
DB06681	Enteritis	0.07762
DB06681	Graves' disease	0.125
DB06681	HIV infection	0.06773
DB06681	Herpes	0.20203
DB06681	IGA glomerulonephritis	0.24254
DB06681	Kaposi sarcoma	0.19612
DB06681	Leukemia	0.03984
DB06681	Liver cancer	0.05488
DB06681	Melanoma	0.11785
DB06681	Multiple sclerosis	0.07293
DB06681	Nasopharyngeal cancer	0.27217
DB06681	Rheumatoid arthritis	0.08655
DB06681	Sarcoidosis	0.27735
DB06681	Squamous cell cancer	0.07217
DB06681	Stomach cancer	0.05955
DB06681	Thyroid cancer	0.12309
DB06681	Thyroiditis	0.5
DB06681	Tuberculosis	0.09535
DB06681	Ulcerative colitis	0.14586
DB01284	Adenoma	0.1715

DB01284	Adrenal gland hyperfunction	0.37796
DB01284	Drug abuse	0.09366
DB01284	Neurotic disorder	0.31623
DB01284	Sella turcica tumor	0.37796
DB01284	Testicular dysfunction	0.14286
DB01285	Labor, Premature	0.18257
DB01285	Stress disorder, post-traumatic	0.25
DB01285	Adenoma	0.12127
DB01285	Adrenal gland hyperfunction	0.26726
DB01285	Drug abuse	0.13245
DB01285	Melanoma	0.05893
DB01285	Neurotic disorder	0.22361
DB01285	Ovarian cancer	0.07107
DB01285	Panic disorder	0.1543
DB01285	Pre-Eclampsia	0.08333
DB01285	Premature birth	0.1715
DB01285	Rheumatoid arthritis	0.04327
DB01285	Sella turcica tumor	0.26726
DB01285	Testicular dysfunction	0.10102
DB01273	Myoclonic epilepsy, Juvenile	0.20412
DB01273	Alzheimer's disease	0.09512
DB01273	Autistic disorder	0.29941
DB01273	Behavior disease	0.44735
DB01273	Bipolar disorder	0.05661
DB01273	Brain tumor	0.11126
DB01273	Colon cancer	0.02967
DB01273	Down syndrome	0.36391
DB01273	Drug abuse	0.04683
DB01273	Epilepsy	0.2902
DB01273	Lung cancer	0.03492
DB01273	Neoplasm metastasis	0.09817
DB01273	Neuroblastoma	0.06868
DB01273	Parkinson disease	0.22708
DB01273	Primary tumor	0.17542
DB01273	Psychotic disorder	0.48391
DB01257	Aortic valve disease	0.33333
DB01257	Depression	0.13131
DB01257	Endometriosis	0.08305
DB01257	Infertility	0.13736
DB01257	Rheumatoid arthritis	0.0612
DB01257	Stroke	0.11323
DB01271	Alzheimer's disease	0.05913
DB01271	Cancer	0.03018
DB01271	Diabetes mellitus	0.05425
DB01271	Rabies	0.20293
DB01279	Alzheimer's disease	0.05913
DB01279	Cancer	0.03018

DB01279	Diabetes mellitus	0.05425
DB01279	Rabies	0.20293
DB01276	Diabetes mellitus	0.05263
DB01276	Pancreas cancer	0.1037
DB06655	Diabetes mellitus	0.05263
DB06655	Pancreas cancer	0.1037
DB08911	Skin disease, Genetic	0.19533
DB08911	Alzheimer's disease	0.06373
DB08911	Angiomyolipoma	0.30615
DB08911	Cancer	0.06403
DB08911	Common cold	0.27243
DB08911	Herpes	0.09022
DB08911	Metaplastic polyp	0.21469
DB08911	Myopathy	0.17723
DB08911	Nephrosis	0.21987
DB08911	Osteomyelitis	0.26354
DB08911	Testicular dysfunction	0.11728
DB08911	Thyroid gland disease	0.23206
DB08911	Ulcerative colitis	0.11432
DB08911	Vitamin D deficiency	0.29597
DB05278	Alzheimer's disease	0.07198
DB05278	Ankylosing spondylitis	0.37796
DB05278	Arthritis	0.12403
DB05278	Asthma	0.08165
DB05278	Bronchial hyperreactivity	0.35355
DB05278	Capillaries disease	0.31623
DB05278	Cardiovascular disease	0.1525
DB05278	Chronic obstructive airway disease	0.1118
DB05278	Cirrhosis	0.16903
DB05278	Colon cancer	0.05934
DB05278	Dental plaque	0.127
DB05278	Diabetes mellitus	0.05263
DB05278	Embryoma	0.06166
DB05278	Endometrial cancer	0.17408
DB05278	Enteritis	0.10976
DB05278	Esophagus cancer	0.14907
DB05278	Hypercholesterolemia	0.19245
DB05278	Hyperinsulinism	0.19245
DB05278	Hypothyroidism	0.2357
DB05278	Intermediate coronary syndrome	0.33333
DB05278	Ischemia	0.12804
DB05278	Kidney failure	0.11323
DB05278	Late pregnancy	0.28868
DB05278	Liver cancer	0.07762
DB05278	Lupus erythematosus	0.08544
DB05278	Lupus vulgaris	0.18898
DB05278	Macular degeneration	0.20851

DB05278	Metabolic syndrome X	0.35355
DB05278	Metabolism disease	0.16013
DB05278	Mucocutaneous lymph node syndrome	0.21822
DB05278	Obesity	0.07692
DB05278	Ovarian cancer	0.1005
DB05278	Penile disease	0.35355
DB05278	Periodontal disease	0.2357
DB05278	Polycystic ovary syndrome	0.13245
DB05278	Premature birth	0.24254
DB05278	Prostate cancer	0.0522
DB05278	Pulmonary embolism	0.40825
DB05278	Renal Cell cancer	0.11625
DB05278	Septicemia	0.44721
DB05278	Systemic infection	0.11471
DB05278	Systemic scleroderma	0.10483
DB05278	Thrombophilia	0.2357
DB05278	Vulvar disease	0.44721
DB01032	Hyperuricemia	0.28868
DB01032	Kidney disease	0.11952
DB01032	Osteoporosis	0.07906
DB06186	Abortion	0.125
DB06186	Alcoholic liver disease	0.40825
DB06186	Asthma	0.08165
DB06186	Autoimmune disease	0.10847
DB06186	Bipolar disorder	0.11323
DB06186	Breast cancer	0.04811
DB06186	Bronchiolitis	0.35355
DB06186	Celiac disease	0.1644
DB06186	Cervical cancer	0.11625
DB06186	Colon cancer	0.05934
DB06186	Dermatitis	0.09091
DB06186	Diabetes mellitus	0.05263
DB06186	Enteritis	0.10976
DB06186	Generalized anxiety disorder	0.28868
DB06186	Gingival overgrowth	0.37796
DB06186	Glomerulonephritis	0.25
DB06186	Graves' disease	0.17678
DB06186	Hemolytic anemia	0.25
DB06186	Hepatitis C	0.14744
DB06186	Hyperthyroidism	0.33333
DB06186	Leukemia	0.05634
DB06186	Lupus erythematosus	0.08544
DB06186	Lymphoma	0.11396
DB06186	Migraine	0.1857
DB06186	Multiple malignancy	0.44721
DB06186	Mycosis fungoides	0.22361
DB06186	Pancreatitis	0.15617

DB06186	Primary biliary cirrhosis	0.17961
DB06186	Prostate cancer	0.0522
DB06186	Renal Cell cancer	0.11625
DB06186	Stomach cancer	0.08422
DB06186	Systemic scleroderma	0.10483
DB06186	Thymoma	0.31623
DB06186	Ulcerative colitis	0.10314
DB00132	Attention deficit hyperactivity disorder	0.1291
DB00132	Brain tumor	0.03592
DB00132	Breast cancer	0.02152
DB00132	Diabetes mellitus	0.02354
DB00132	Hypertension	0.03536
DB00132	Intestinal disease	0.1118
DB00132	Ischemia	0.05726
DB00132	Neuropathy	0.07161
DB00132	Obesity	0.0688
DB00132	Polyarthritis	0.05096
DB00132	Rheumatoid arthritis	0.02737
DB00928	Adenovirus infection	0.02274
DB00928	Alzheimer's disease	0.01869
DB00928	Aortic valve disease	0.07209
DB00928	Breast cancer	0.01444
DB00928	Cancer	0.06052
DB00928	Colon cancer	0.02111
DB00928	Congenital abnormality	0.02133
DB00928	Emphysema	0.05278
DB00928	Endometriosis	0.02263
DB00928	Eye cancer	0.07231
DB00928	Gastritis	0.03841
DB00928	HIV infection	0.04973
DB00928	Helicobacter infection	0.06256
DB00928	Parkinson disease	0.05215
DB00928	Rheumatoid arthritis	0.01728
DB00928	Severe acute respiratory syndrome	0.06563
DB00928	Tuberous sclerosis	0.04119
DB00928	Virus disease	0.04438
DB01262	Adenovirus infection	0.02274
DB01262	Alzheimer's disease	0.01869
DB01262	Aortic valve disease	0.07209
DB01262	Breast cancer	0.01444
DB01262	Cancer	0.06052
DB01262	Colon cancer	0.02111
DB01262	Congenital abnormality	0.02133
DB01262	Emphysema	0.05278
DB01262	Endometriosis	0.02263
DB01262	Eye cancer	0.07231
DB01262	Gastritis	0.03841

DB01262	HIV infection	0.04973
DB01262	Helicobacter infection	0.06256
DB01262	Parkinson disease	0.05215
DB01262	Rheumatoid arthritis	0.01728
DB01262	Severe acute respiratory syndrome	0.06563
DB01262	Tuberous sclerosis	0.04119
DB01262	Virus disease	0.04438
DB04953	Myoclonic epilepsy, Juvenile	0.20412
DB04953	Conduct disorder	0.16667
DB04953	Deafness	0.07217
DB04953	Myopathy	0.06063
DB04953	Sudden infant death syndrome	0.32733
DB04953	Yersinia infection	0.05934
DB00995	AIDS	0.13561
DB00995	Adenoma	0.01243
DB00995	Alzheimer's disease	0.02055
DB00995	Aseptic necrosis of bone	0.02874
DB00995	Autoimmune disease	0.05353
DB00995	Brain tumor	0.01447
DB00995	Breast cancer	0.23413
DB00995	Cancer	0.04979
DB00995	Colon cancer	0.00727
DB00995	Congenital abnormality	0.00657
DB00995	Dermatitis	0.02188
DB00995	Diabetes mellitus	0.0494
DB00995	Eating disorder	0.04045
DB00995	Embryoma	0.02362
DB00995	Endometriosis	0.0322
DB00995	Esophagus cancer	0.02453
DB00995	Fanconi's anemia	0.03624
DB00995	Herpes	0.0291
DB00995	Hodgkin's disease	0.03957
DB00995	Immunologic deficiency syndrome	0.06788
DB00995	Infectious lung disease	0.93238
DB00995	Infiltrating cancer	0.09494
DB00995	Intracranial hypertension	0.14402
DB00995	Leukemia	0.34068
DB00995	Leukoencephalopathy	0.01875
DB00995	Liver cancer	0.41793
DB00995	Lung cancer	0.01403
DB00995	Lymphoma	0.01335
DB00995	Mucopolysaccharidosis	0.04153
DB00995	Multiple sclerosis	0.06136
DB00995	Neoplasm metastasis	0.26059
DB00995	Nephrosis	0.08565
DB00995	Neuroblastoma	0.02431
DB00995	Neurodegenerative disorder	0.07804

DB00995	Obesity	0.26619
DB00995	Pancreatitis	0.07526
DB00995	Polyarthritis	0.50516
DB00995	Prostate cancer	0.32435
DB00995	Rheumatism	0.03315
DB00995	Sicca syndrome	0.03799
DB00995	Squamous cell cancer	0.03795
DB00995	Stomach cancer	0.0369
DB00995	Stroke	0.03765
DB00995	Systemic scleroderma	0.03691
DB00995	Tuberculosis	0.07745
DB00995	Tuberous sclerosis	0.58433
DB00995	Ulcerative colitis	0.04453
DB00995	Virus disease	0.32859
DB00995	Yersinia infection	0.4256
DB00626	Prostatic hypertrophy, Benign	0.72353
DB00626	Alzheimer's disease	0.13452
DB00626	Atherosclerosis	0.07904
DB00626	Brain disease	0.127
DB00626	Cancer	0.03018
DB00626	Diabetes mellitus	0.03722
DB00626	HIV infection	0.07302
DB00626	Infection	0.06594
DB00626	Multiple sclerosis	0.20936
DB00626	Obesity	0.15467
DB00626	Pancreatitis	0.27776
DB00626	Prion disease	0.58693
DB00626	Prostate cancer	0.09415
DB08888	Hemorrhagic fevers, Viral	0.06862
DB08888	Pemphigoid, Bullous	0.1045
DB08888	Pleural effusion, Malignant	0.0713
DB08888	Prostatic hypertrophy, Benign	0.36686
DB08888	Purpura, Thrombocytopenic, Idiopathic	0.11532
DB08888	Alopecia	0.08214
DB08888	Alzheimer's disease	0.1198
DB08888	Aortic aneurysm	0.11903
DB08888	Aplastic anemia	0.08047
DB08888	Asthma	0.0327
DB08888	Atherosclerosis	0.07396
DB08888	Autistic disorder	0.04521
DB08888	Autoimmune disease	0.04169
DB08888	Bone metastases	0.21728
DB08888	Cancer	0.045
DB08888	Cervical cancer	0.1215
DB08888	Colon cancer	0.08453
DB08888	Depression	0.06575
DB08888	Diabetes mellitus	0.06744

DB08888	Drug abuse	0.04848
DB08888	Emphysema	0.05993
DB08888	HIV infection	0.03026
DB08888	Hemorrhagic disorder	0.17802
DB08888	Herpes	0.03469
DB08888	Intermediate coronary syndrome	0.06845
DB08888	Lung cancer	0.05477
DB08888	Lupus erythematosus	0.08071
DB08888	Malignant glioma	0.13871
DB08888	Multiple sclerosis	0.11609
DB08888	Neoplasm metastasis	0.04068
DB08888	Obesity	0.08597
DB08888	Ovarian cancer	0.09115
DB08888	Pancreatitis	0.15951
DB08888	Penile disease	0.06423
DB08888	Pertussis	0.22341
DB08888	Polyarthritis	0.06257
DB08888	Primary hyperparathyroidism	0.08288
DB08888	Prion disease	0.3593
DB08888	Prostate cancer	0.09494
DB08888	Pulmonary embolism	0.2357
DB08888	Rheumatoid arthritis	0.03923
DB08888	Schizophrenia	0.03276
DB08888	Shigella infection	0.0878
DB08888	Sickle cell disease	0.07828
DB08888	Squamous cell cancer	0.23001
DB08888	Stroke	0.04249
DB08888	Subarachnoid hemorrhage	0.06127
DB08888	Systemic infection	0.0722
DB08888	Takayasu's arteritis	0.08102
DB08888	Temporal arteritis	0.06698
DB08888	Thalassemia	0.08207
DB08888	Thrombocytopenia	0.07218
DB04835	Adenovirus infection	0.13019
DB04835	Alzheimer's disease	0.07198
DB04835	Aortic aneurysm	0.2132
DB04835	Arthritis	0.12403
DB04835	Asthma	0.08165
DB04835	Atherosclerosis	0.07001
DB04835	Behcet syndrome	0.14286
DB04835	Breast cancer	0.04811
DB04835	Brucellosis	0.30151
DB04835	Cervical cancer	0.11625
DB04835	Chronic obstructive airway disease	0.1118
DB04835	Chronic rejection of renal transplant	0.25
DB04835	Colon cancer	0.05934
DB04835	Communicable disease	0.21822

DB04835	Diabetes mellitus	0.05263
DB04835	Drug abuse	0.09366
DB04835	Encephalitis	0.27735
DB04835	Fetal disease	0.31623
DB04835	Graves' disease	0.17678
DB04835	Heart failure	0.1066
DB04835	Hemophilia	0.57735
DB04835	Hepatitis C	0.14744
DB04835	Hyperlipidemia	0.19612
DB04835	Hypertension	0.07906
DB04835	IGA glomerulonephritis	0.1715
DB04835	Kidney failure	0.11323
DB04835	Liver cancer	0.07762
DB04835	Liver disease	0.1543
DB04835	Lung cancer	0.06984
DB04835	Lupus erythematosus	0.08544
DB04835	Melanoma	0.08333
DB04835	Mucocutaneous lymph node syndrome	0.21822
DB04835	Multiple myeloma	0.13131
DB04835	Multiple sclerosis	0.10314
DB04835	Neuritis	0.35355
DB04835	Polymyositis	0.2582
DB04835	Prostate cancer	0.0522
DB04835	Schizophrenia	0.07603
DB04835	Testicular dysfunction	0.14286
DB04835	Viremia	0.40825
DB00198	Cancer	0.02128
DB00198	Embryoma	0.0356
DB00198	Encephalopathies	0.22503
DB00198	HIV infection	0.06795
DB00198	Mucopolysaccharidosis	0.21018
DB00198	Myopathy	0.14286
DB00198	Nevus	0.70711
DB00198	Polyarthritis	0.08112
DB00061	Pemphigoid, Bullous	0.02842
DB00061	Pleural effusion, Malignant	0.02375
DB00061	Skin disease, Genetic	0.09059
DB00061	Adenovirus infection	0.0172
DB00061	Angiomyolipoma	0.0634
DB00061	Asthma	0.02179
DB00061	Atherosclerosis	0.05588
DB00061	Atopic rhinitis	0.08751
DB00061	Barrett's esophagus	0.03005
DB00061	Brain tumor	0.03258
DB00061	Breast cancer	0.03611
DB00061	Cancer	0.03549
DB00061	Cholelithiasis	0.05018

DB00061	Colon cancer	0.0783
DB00061	Common wart	0.07772
DB00061	Congenital heart disease	0.12979
DB00061	Cytomegalovirus infection	0.06605
DB00061	Deafness	0.08378
DB00061	Diabetes mellitus	0.05132
DB00061	Drug abuse	0.01615
DB00061	Embryoma	0.14456
DB00061	Endometriosis	0.01711
DB00061	Esotropia	0.2051
DB00061	Gastritis	0.01902
DB00061	Gastrointestinal tumor	0.08987
DB00061	Generalized anxiety disorder	0.31905
DB00061	Glaucoma	0.03804
DB00061	Hemorrhagic disorder	0.04382
DB00061	Herpes	0.02451
DB00061	Hypercholesterolemia	0.07188
DB00061	Hyperglycemia	0.15521
DB00061	Infection	0.04567
DB00061	Influenza	0.05146
DB00061	Kaposi sarcoma	0.03692
DB00061	Kidney failure	0.09525
DB00061	Leukemia	0.09074
DB00061	Liver cancer	0.07209
DB00061	Lupus erythematosus	0.08101
DB00061	Lymphoproliferative disorder	0.0862
DB00061	Multiple myeloma	0.0332
DB00061	Myeloproliferative disease	0.05775
DB00061	Optic atrophy	0.08346
DB00061	Oral cancer	0.02354
DB00061	Osteoporosis	0.03165
DB00061	Overnutrition	0.11104
DB00061	Papillary adenocarcinoma	0.08751
DB00061	Polycystic ovary syndrome	0.15642
DB00061	Primary hyperparathyroidism	0.0276
DB00061	Prostate cancer	0.06002
DB00061	Rheumatoid arthritis	0.04426
DB00061	Schizophrenia	0.02182
DB00061	Skin cancer	0.0875
DB00061	Skin tumor	0.2231
DB00061	Solid tumor	0.07678
DB00061	Stomach cancer	0.01511
DB00061	Stroke	0.09303
DB00061	Testicular dysfunction	0.02429
DB00061	Thrombophlebitis	0.06445
DB00061	Turner's syndrome	0.1289
DB00061	Wiskott-Aldrich syndrome	0.03899

DB00061	Yersinia infection	0.15386
DB06213	Asthma	0.08165
DB06213	Chronic obstructive airway disease	0.1118
DB06213	Cystic fibrosis	0.14286
DB06213	Depression	0.13131
DB06213	Heart failure	0.1066
DB06213	Huntington disease	0.21822
DB06213	Hypertension	0.07906
DB06213	Kidney failure	0.11323
DB06213	Panic disorder	0.21822
DB06213	Phobic anxiety disorder	0.5
DB06213	Schizophrenia	0.07603
DB06213	Thrombocytosis	0.44721
DB00446	Aplastic anemia	0.14744
DB00446	Breast cancer	0.03402
DB00446	Colon cancer	0.04196
DB00446	Diabetes mellitus	0.03722
DB00446	Esophagus cancer	0.10541
DB00446	Herpes	0.10102
DB00446	Prostate cancer	0.03691
DB00446	Rabies	0.07857
DB00446	Ulcerative colitis	0.07293
DB00062	Stress disorder, post-traumatic	0.73665
DB00062	Amyloidosis	0.10911
DB00062	Amyotrophic lateral sclerosis	0.18532
DB00062	Arthritis	0.07161
DB00062	Autistic disorder	0.20942
DB00062	Biliary cancer	1.2582
DB00062	Breast cancer	0.08431
DB00062	Cardiovascular disease	0.39126
DB00062	Cerebrovascular disorder	0.39187
DB00062	Cholelithiasis	0.34776
DB00062	Chronic simple glaucoma	0.38179
DB00062	Cirrhosis	0.42658
DB00062	Colon cancer	0.03426
DB00062	Dental plaque	0.22429
DB00062	Depression	0.17718
DB00062	Dermatitis	0.14888
DB00062	Diabetes mellitus	0.09042
DB00062	Down syndrome	0.2168
DB00062	Drug abuse	0.12881
DB00062	Drug-Induced dyskinesia	1.21822
DB00062	Embryoma	0.11862
DB00062	Encephalitis	0.56678
DB00062	Encephalopathies	0.34845
DB00062	Familial Mediterranean fever	0.14907
DB00062	Glaucoma	0.27229

DB00062	Glomerulonephritis	0.57708
DB00062	Growth retardation	0.40029
DB00062	Herpes	0.17511
DB00062	Histiocytosis	1.19245
DB00062	Hyperhomocysteinemia	1.16667
DB00062	Hyperlipidemia	0.40795
DB00062	Hypoglycemia	0.85198
DB00062	Infectious lung disease	0.31127
DB00062	Ischemia	0.22052
DB00062	Kidney failure	0.27986
DB00062	Learning disorder	1.18949
DB00062	Liver cancer	0.15767
DB00062	Liver disease	0.41487
DB00062	Lung cancer	0.04032
DB00062	Lupus erythematosus	0.13228
DB00062	Macular degeneration	0.52586
DB00062	Malaria	0.37784
DB00062	Multiple myeloma	0.07581
DB00062	Mycoses	1.00016
DB00062	Neoplasm metastasis	0.04698
DB00062	Nephrosis	0.33985
DB00062	Neurodegenerative disorder	0.28232
DB00062	Obesity	0.04441
DB00062	Ovarian cancer	0.19857
DB00062	Polycystic kidney	0.47295
DB00062	Prostate cancer	0.12362
DB00062	Psychotic disorder	0.34582
DB00062	Rectum cancer	1.17362
DB00062	Rheumatoid arthritis	0.13116
DB00062	Subarachnoid hemorrhage	0.36302
DB00062	Vascular dementia	1.19245
DB00062	Yersinia infection	0.17473
DB00064	Stress disorder, post-traumatic	0.73665
DB00064	Amyloidosis	0.10911
DB00064	Amyotrophic lateral sclerosis	0.18532
DB00064	Arthritis	0.07161
DB00064	Autistic disorder	0.20942
DB00064	Biliary cancer	1.2582
DB00064	Breast cancer	0.08431
DB00064	Cardiovascular disease	0.39126
DB00064	Cerebrovascular disorder	0.39187
DB00064	Cholelithiasis	0.34776
DB00064	Chronic simple glaucoma	0.38179
DB00064	Cirrhosis	0.42658
DB00064	Colon cancer	0.03426
DB00064	Dental plaque	0.22429
DB00064	Depression	0.17718

DB00064	Dermatitis	0.14888
DB00064	Diabetes mellitus	0.09042
DB00064	Down syndrome	0.2168
DB00064	Drug abuse	0.12881
DB00064	Drug-Induced dyskinesia	1.21822
DB00064	Embryoma	0.11862
DB00064	Encephalitis	0.56678
DB00064	Encephalopathies	0.34845
DB00064	Familial Mediterranean fever	0.14907
DB00064	Glaucoma	0.27229
DB00064	Glomerulonephritis	0.57708
DB00064	Growth retardation	0.40029
DB00064	Herpes	0.17511
DB00064	Histiocytosis	1.19245
DB00064	Hyperhomocysteinemia	1.16667
DB00064	Hyperlipidemia	0.40795
DB00064	Hypoglycemia	0.85198
DB00064	Infectious lung disease	0.31127
DB00064	Ischemia	0.22052
DB00064	Kidney failure	0.27986
DB00064	Learning disorder	1.18949
DB00064	Liver cancer	0.15767
DB00064	Liver disease	0.41487
DB00064	Lung cancer	0.04032
DB00064	Lupus erythematosus	0.13228
DB00064	Macular degeneration	0.52586
DB00064	Malaria	0.37784
DB00064	Multiple myeloma	0.07581
DB00064	Mycoses	1.00016
DB00064	Neoplasm metastasis	0.04698
DB00064	Nephrosis	0.33985
DB00064	Neurodegenerative disorder	0.28232
DB00064	Obesity	0.04441
DB00064	Ovarian cancer	0.19857
DB00064	Polycystic kidney	0.47295
DB00064	Prostate cancer	0.12362
DB00064	Psychotic disorder	0.34582
DB00064	Rectum cancer	1.17362
DB00064	Rheumatoid arthritis	0.13116
DB00064	Subarachnoid hemorrhage	0.36302
DB00064	Vascular dementia	1.19245
DB00064	Yersinia infection	0.17473
DB01250	Mycobacterium infection, Atypical	0.2357
DB01250	Purpura, Thrombocytopenic, Idiopathic	0.18898
DB01250	Abortion	0.08839
DB01250	Alopecia	0.1291
DB01250	Alzheimer's disease	0.0509

DB01250	Anemia	0.15076
DB01250	Angiomyolipoma	0.26726
DB01250	Anorexia nervosa	0.14434
DB01250	Arthritis	0.08771
DB01250	Asthma	0.05774
DB01250	Atherosclerosis	0.04951
DB01250	Behcet syndrome	0.10102
DB01250	Breast cancer	0.03402
DB01250	Bronchiectasis	0.28868
DB01250	Bronchiolitis obliterans	0.2357
DB01250	Brucellosis	0.2132
DB01250	Celiac disease	0.11625
DB01250	Cervical cancer	0.0822
DB01250	Choriocarcinoma	0.20412
DB01250	Colon cancer	0.04196
DB01250	Communicable disease	0.1543
DB01250	Dermatitis	0.06428
DB01250	Diabetes mellitus	0.03722
DB01250	Embryoma	0.0436
DB01250	Emphysema	0.14434
DB01250	Endometriosis	0.05872
DB01250	Enteritis	0.07762
DB01250	Familial Mediterranean fever	0.18257
DB01250	Fanconi's anemia	0.14142
DB01250	Helicobacter infection	0.15811
DB01250	Hemolytic anemia	0.17678
DB01250	Hepatitis C	0.10426
DB01250	Hyperhomocysteinemia	0.20412
DB01250	Hyperthyroidism	0.2357
DB01250	IGA glomerulonephritis	0.12127
DB01250	Infection	0.06594
DB01250	Influenza	0.14142
DB01250	Intraocular melanoma	0.22361
DB01250	Leukemia	0.07968
DB01250	Lichen planus	0.25
DB01250	Liver cancer	0.05488
DB01250	Liver disease	0.10911
DB01250	Lung cancer	0.04939
DB01250	Lupus erythematosus	0.06041
DB01250	Multiple myeloma	0.09285
DB01250	Multiple sclerosis	0.14586
DB01250	Nasopharyngeal cancer	0.13608
DB01250	Neoplasm metastasis	0.05754
DB01250	Neutropenia	0.28868
DB01250	Ovarian cancer	0.07107
DB01250	Pancreas cancer	0.07332
DB01250	Pancreatitis	0.11043

DB01250	Parkinson disease	0.07809
DB01250	Pelvic inflammatory disease	0.31623
DB01250	Periodontitis	0.10783
DB01250	Pre-Eclampsia	0.08333
DB01250	Prostate cancer	0.03691
DB01250	Serous cancer	0.35355
DB01250	Solid tumor	0.20412
DB01250	Squamous cell cancer	0.07217
DB01250	Systemic infection	0.08111
DB01250	Ulcerative colitis	0.07293
DB01250	Uveitis	0.17678
DB01250	Vaccinia	0.2132
DB01250	Virus disease	0.11785
DB08864	Adenovirus infection	0.13019
DB08864	Endometrium cancer	0.24254
DB08864	Enteritis	0.10976
DB08864	Epilepsy	0.14142
DB08864	Osteosarcoma	0.2132
DB08864	Ulcerative colitis	0.10314
DB05630	Kidney tubular necrosis, acute	0.14968
DB05630	Abortion	0.01031
DB05630	Adenovirus infection	0.00589
DB05630	Alzheimer's disease	0.00484
DB05630	Amyotrophic lateral sclerosis	0.04493
DB05630	Aortic aneurysm	0.00959
DB05630	Aortic valve disease	0.14514
DB05630	Aplastic anemia	0.02246
DB05630	Barrett's esophagus	0.05816
DB05630	Brain tumor	0.0091
DB05630	Breast cancer	0.0352
DB05630	Bronchial disease	0.03452
DB05630	Cancer	0.05103
DB05630	Carcinoma	0.01586
DB05630	Celiac disease	0.08198
DB05630	Cervical cancer	0.01385
DB05630	Cholestasis	0.03261
DB05630	Chronic obstructive airway disease	0.07831
DB05630	Chronic rejection of renal transplant	0.09677
DB05630	Chronic simple glaucoma	0.0737
DB05630	Colon cancer	0.07198
DB05630	Common cold	0.10919
DB05630	Dermatitis	0.02746
DB05630	Diabetes mellitus	0.02954
DB05630	Down syndrome	0.12039
DB05630	Embryoma	0.01227
DB05630	Emphysema	0.10627
DB05630	Endometriosis	0.02257

DB05630	Ewings sarcoma	0.14596
DB05630	Fanconi's anemia	0.04824
DB05630	HIV infection	0.05963
DB05630	Heart failure	0.05966
DB05630	Helicobacter infection	0.12595
DB05630	Hemolytic-Uremic syndrome	0.07868
DB05630	Hyperparathyroidism	0.06758
DB05630	Infection	0.02881
DB05630	Ischemia	0.0613
DB05630	Kaposi sarcoma	0.08252
DB05630	Keratoconjunctivitis Sicca	0.04506
DB05630	Keratosis	0.05772
DB05630	Kidney cancer	0.21023
DB05630	Leukemia	0.03298
DB05630	Leukoencephalopathy	0.04049
DB05630	Liver cancer	0.06332
DB05630	Lung cancer	0.05656
DB05630	Lung disease	0.01237
DB05630	Lymphoma	0.12102
DB05630	Melanoma	0.04581
DB05630	Meningioma	0.27734
DB05630	Mental retardation	0.023
DB05630	Metaplastic polyp	0.05863
DB05630	Multiple endocrine neoplasia	0.1629
DB05630	Muscular dystrophies	0.01493
DB05630	Neck cancer	0.06187
DB05630	Neuroblastoma	0.05249
DB05630	Oral cancer	0.03104
DB05630	Osteosarcoma	0.01585
DB05630	Pancreas cancer	0.01497
DB05630	Pancreas disease	0.03142
DB05630	Papillomavirus infection	0.09169
DB05630	Parkinson disease	0.05398
DB05630	Pituitary tumor	0.12587
DB05630	Polyarthritis	0.02748
DB05630	Pre-Eclampsia	0.01472
DB05630	Prostate cancer	0.00811
DB05630	Renal tubular acidosis	0.06399
DB05630	Rheumatoid arthritis	0.04253
DB05630	Sarcoidosis	0.0885
DB05630	Stomach cancer	0.01335
DB05630	Tuberous sclerosis	0.09216
DB05630	Ulcerative colitis	0.04582
DB05630	Uterine fibroids	0.12611
DB05630	Virus disease	0.00995
DB05630	Werner syndrome	0.08284
DB06720	Brain disease	0.17961

DB06720	Parkinson disease	0.11043
DB00070	Pleural effusion, Malignant	0.04141
DB00070	Alzheimer's disease	0.02465
DB00070	Atherosclerosis	0.0466
DB00070	Cancer	0.01779
DB00070	Chronic obstructive airway disease	0.07055
DB00070	Diabetes mellitus	0.02261
DB00070	Drug abuse	0.02815
DB00070	Emphysema	0.06028
DB00070	Endometriosis	0.02984
DB00070	Herpes	0.03489
DB00070	Hyperlipidemia	0.11102
DB00070	Ischemia	0.05522
DB00070	Neoplasm metastasis	0.04092
DB00070	Rheumatoid arthritis	0.02279
DB08879	Purpura, Thrombocytopenic, Idiopathic	0.38934
DB08879	Alopecia	0.33319
DB08879	Autoimmune disease	0.26135
DB08879	Common variable immunodeficiency	1.0348
DB08879	Congenital abnormality	0.16418
DB08879	Embryoma	0.16039
DB08879	Endometriosis	0.17725
DB08879	Epstein-Barr virus infection	0.47238
DB08879	IGA glomerulonephritis	0.58395
DB08879	Immune complex disease	1.40092
DB08879	Immunologic deficiency syndrome	0.37347
DB08879	Lupus erythematosus	0.18409
DB08879	Lymphoma	0.29448
DB08879	Periodontitis	0.47347
DB08879	Rhabdomyosarcoma	0.69553
DB08879	Sicca syndrome	0.42592
DB08879	Sinusitis	0.49483
DB08879	Systemic scleroderma	0.21023
DB08893	Amnionitis	0.27735
DB08893	Atherosclerosis	0.07001
DB08893	Cholelithiasis	0.2
DB08893	Endometrium cancer	0.24254
DB08893	Hypertension	0.07906
DB08893	Metabolism disease	0.16013
DB08893	Pre-Eclampsia	0.11785
DB08893	Rheumatoid arthritis	0.0612
DB08893	Systemic infection	0.11471
DB01375	Infertility, Male	0.24028
DB01375	Alzheimer's disease	0.05913
DB01375	Atherosclerosis	0.07904
DB01375	Bipolar disorder	0.19236
DB01375	Bladder cancer	0.19713

DB01375	Bronchial hyperreactivity	0.34951
DB01375	Cancer	0.04268
DB01375	Cerebrovascular disorder	0.24025
DB01375	Cholelithiasis	0.2099
DB01375	Dental plaque	0.13641
DB01375	Depression	0.0916
DB01375	Diabetes mellitus	0.05425
DB01375	Down syndrome	0.13645
DB01375	Endometriosis	0.07158
DB01375	Glaucoma	0.15909
DB01375	Herpes	0.0837
DB01375	Hyperlipidemia	0.26631
DB01375	Hypertension	0.15004
DB01375	Infertility	0.15351
DB01375	Kidney failure	0.13474
DB01375	Late pregnancy	0.32994
DB01375	Lupus erythematosus	0.07496
DB01375	Lupus vulgaris	0.20386
DB01375	Migraine	0.1884
DB01375	Myasthenia Gravis	0.25509
DB01375	Obesity	0.10028
DB01375	Oral cancer	0.09845
DB01375	Osteoporosis	0.15285
DB01375	Ovarian disease	0.23961
DB01375	Ovarian failure	0.31333
DB01375	Panic disorder	0.24066
DB01375	Parkinson disease	0.11665
DB01375	Polyarthritis	0.08717
DB01375	Primary biliary cirrhosis	0.25836
DB01375	Pulmonary fibrosis	0.23806
DB01375	Rabies	0.12985
DB01375	Renal tubular acidosis	0.13828
DB01375	Schizophrenia	0.09128
DB01375	Scleroderma	0.1074
DB01375	Stroke	0.08371
DB01375	Synovitis	0.27778
DB01375	Systemic scleroderma	0.08009
DB01375	Thymoma	0.21559
DB01375	Uterine disease	0.19188
DB01375	Vitiligo	0.25509
DB00428	Diabetes mellitus	0.03722
DB00428	Obesity	0.05439
DB06168	Labor, Premature	0.2582
DB06168	Alimentary system disease	0.21822
DB06168	Alveolar bone loss	0.37796
DB06168	Alzheimer's disease	0.07198
DB06168	Amnionitis	0.27735

DB06168	Arthritis	0.12403
DB06168	Asthma	0.08165
DB06168	Atherosclerosis	0.07001
DB06168	Bacterial vaginosis	0.40825
DB06168	Behcet syndrome	0.14286
DB06168	Bipolar disorder	0.11323
DB06168	Bronchopulmonary dysplasia	0.30151
DB06168	Celiac disease	0.1644
DB06168	Cervical cancer	0.11625
DB06168	Chronic obstructive airway disease	0.1118
DB06168	Cystic fibrosis	0.14286
DB06168	Dental plaque	0.127
DB06168	Dermatitis	0.09091
DB06168	Drug abuse	0.09366
DB06168	Endometriosis	0.08305
DB06168	Epilepsy	0.14142
DB06168	Epstein-Barr virus infection	0.21822
DB06168	Esophagitis	0.44721
DB06168	Glaucoma	0.16667
DB06168	Gouts	0.27735
DB06168	Graves' disease	0.17678
DB06168	Helicobacter infection	0.22361
DB06168	Henoch-Schoenlein purpura	0.31623
DB06168	Hepatitis	0.2582
DB06168	Hepatitis C	0.14744
DB06168	Hodgkin's disease	0.17678
DB06168	Hypertension	0.07906
DB06168	Infectious lung disease	0.22361
DB06168	Infertility	0.13736
DB06168	Kidney failure	0.11323
DB06168	Leukemia	0.05634
DB06168	Lung cancer	0.06984
DB06168	Lupus erythematosus	0.08544
DB06168	Lyme disease	0.33333
DB06168	Melanoma	0.08333
DB06168	Multiple myeloma	0.13131
DB06168	Multiple sclerosis	0.10314
DB06168	Neoplasm metastasis	0.08138
DB06168	Nephrosis	0.22361
DB06168	Oral cancer	0.13608
DB06168	Otitis media	0.33333
DB06168	Ovarian cancer	0.1005
DB06168	Parkinson disease	0.11043
DB06168	Periodontitis	0.1525
DB06168	Polymyositis	0.2582
DB06168	Premature birth	0.24254
DB06168	Psoriasis	0.2357

DB06168	Respiratory distress syndrome	0.27735
DB06168	Retinoblastoma	0.57735
DB06168	Rheumatoid arthritis	0.0612
DB06168	Sarcoidosis	0.19612
DB06168	Schizophrenia	0.07603
DB06168	Shigella infection	0.40825
DB06168	Sicca syndrome	0.19612
DB06168	Squamous cell cancer	0.10206
DB06168	Stroke	0.11323
DB06168	Synovial sarcoma	0.5
DB06168	Systemic scleroderma	0.10483
DB06168	Tuberculosis	0.13484
DB06168	Ulcerative colitis	0.10314
DB06372	Labor, Premature	0.29814
DB06372	Prostatic hypertrophy, Benign	0.13608
DB06372	Purpura, Thrombocytopenic, Idiopathic	0.1543
DB06372	Abortion	0.07217
DB06372	Alcoholic liver disease	0.2357
DB06372	Alimentary system disease	0.12599
DB06372	Alopecia	0.10541
DB06372	Alveolar bone loss	0.21822
DB06372	Alzheimer's disease	0.08312
DB06372	Amnionitis	0.16013
DB06372	Arthritis	0.14322
DB06372	Asthma	0.09428
DB06372	Atherosclerosis	0.12127
DB06372	Attention deficit hyperactivity disorder	0.16667
DB06372	Autoimmune disease	0.06262
DB06372	Bacterial vaginosis	0.4714
DB06372	Behcet syndrome	0.16496
DB06372	Bipolar disorder	0.06537
DB06372	Bladder cancer	0.07454
DB06372	Bronchopulmonary dysplasia	0.34816
DB06372	Brucellosis	0.17408
DB06372	Celiac disease	0.09492
DB06372	Cerebrovascular disorder	0.12599
DB06372	Cervical cancer	0.20135
DB06372	Chronic obstructive airway disease	0.1291
DB06372	Chronic simple glaucoma	0.12309
DB06372	Colon cancer	0.03426
DB06372	Cushing syndrome	0.17408
DB06372	Cystic fibrosis	0.24744
DB06372	Dental plaque	0.07332
DB06372	Dermatitis	0.10497
DB06372	Diabetes mellitus	0.03039
DB06372	Drug abuse	0.10815
DB06372	Embryoma	0.0356

DB06372	Encephalitis	0.16013
DB06372	Endometriosis	0.14384
DB06372	Endometrium cancer	0.14003
DB06372	Enteritis	0.06337
DB06372	Epilepsy	0.08165
DB06372	Epstein-Barr virus infection	0.12599
DB06372	Esophagitis	0.2582
DB06372	Gastritis	0.21822
DB06372	Glaucoma	0.19245
DB06372	Gouts	0.16013
DB06372	Graves' disease	0.10206
DB06372	Hamman-Rich syndrome	0.11111
DB06372	Heart disease	0.17408
DB06372	Helicobacter infection	0.1291
DB06372	Henoch-Schoenlein purpura	0.18257
DB06372	Hepatitis	0.14907
DB06372	Hepatitis C	0.17025
DB06372	Herpes	0.08248
DB06372	Histiocytosis	0.19245
DB06372	Hodgkin's disease	0.20412
DB06372	Hyperglycemia	0.09245
DB06372	Hypertension	0.09129
DB06372	Infectious lung disease	0.1291
DB06372	Infertility	0.15861
DB06372	Ischemia	0.07392
DB06372	Kidney failure	0.19612
DB06372	Leukemia	0.06506
DB06372	Liver metastases	0.16013
DB06372	Lung cancer	0.08065
DB06372	Lupus erythematosus	0.09865
DB06372	Lyme disease	0.19245
DB06372	Malaria	0.10721
DB06372	Malnutrition	0.20412
DB06372	Melanoma	0.04811
DB06372	Migraine	0.10721
DB06372	Multiple myeloma	0.15162
DB06372	Multiple sclerosis	0.05955
DB06372	Neoplasm metastasis	0.04698
DB06372	Nephrosis	0.1291
DB06372	Obesity	0.04441
DB06372	Oral cancer	0.07857
DB06372	Osteoporosis	0.09129
DB06372	Otitis media	0.19245
DB06372	Ovarian cancer	0.17408
DB06372	Pancreas cancer	0.05987
DB06372	Parkinson disease	0.06376
DB06372	Peptic ulcer	0.13608

DB06372	Periodontal disease	0.13608
DB06372	Periodontitis	0.17609
DB06372	Polycystic ovary syndrome	0.15294
DB06372	Polymyositis	0.14907
DB06372	Premature birth	0.28006
DB06372	Prostate cancer	0.03014
DB06372	Psoriasis	0.13608
DB06372	Respiratory distress syndrome	0.16013
DB06372	Retinoblastoma	0.33333
DB06372	Rheumatoid arthritis	0.07067
DB06372	Sarcoidosis	0.11323
DB06372	Schizophrenia	0.13169
DB06372	Scleroderma	0.12309
DB06372	Shigella infection	0.2357
DB06372	Sicca syndrome	0.11323
DB06372	Skin disease	0.09901
DB06372	Solid tumor	0.16667
DB06372	Squamous cell cancer	0.05893
DB06372	Stomach cancer	0.09724
DB06372	Stroke	0.13074
DB06372	Synovial sarcoma	0.28868
DB06372	Systemic infection	0.06623
DB06372	Systemic scleroderma	0.18157
DB06372	Tuberculosis	0.1557
DB06372	Ulcerative colitis	0.1191
DB00535	Alzheimer's disease	0.07198
DB00535	Atherosclerosis	0.07001
DB00535	Breast cancer	0.04811
DB00535	Cervical cancer	0.11625
DB00535	Diabetes mellitus	0.05263
DB00535	Glomerulonephritis	0.25
DB00535	Helicobacter infection	0.22361
DB00535	Hepatoblastoma	0.57735
DB00535	Hyperlipidemia	0.19612
DB00535	Ischemia	0.12804
DB00535	Kidney failure	0.11323
DB00535	Leukemia	0.05634
DB00535	Lung cancer	0.06984
DB00535	Multiple sclerosis	0.10314
DB00535	Pancreas cancer	0.1037
DB00535	Periodontal disease	0.2357
DB00535	Periodontitis	0.1525
DB00535	Prostate cancer	0.0522
DB00535	Sickle cell disease	0.19245
DB00535	Stomach disease	0.35355
DB00535	Stroke	0.11323
DB00535	Systemic infection	0.11471

DB00535	Vasculitis	0.30151
DB06196	Acne	0.28868
DB06196	Alzheimer's disease	0.0509
DB06196	Asthma	0.05774
DB06196	Cancer	0.02606
DB06196	Coronavirus infection	0.35355
DB06196	Hypertension	0.1118
DB06196	Infection	0.06594
DB06196	Kidney disease	0.08452
DB06196	Obesity	0.05439
DB06196	Peptic ulcer	0.16667
DB06196	Stomach cancer	0.05955
DB08818	Hemorrhagic fevers, Viral	0.2357
DB08818	Labor, Premature	0.14907
DB08818	Pemphigoid, Bullous	0.18257
DB08818	Alopecia	0.10541
DB08818	Alzheimer's disease	0.04156
DB08818	Asthma	0.04714
DB08818	Atherosclerosis	0.04042
DB08818	Atopic rhinitis	0.14907
DB08818	Behcet syndrome	0.08248
DB08818	Biliary Atresia	0.20412
DB08818	Bladder cancer	0.07454
DB08818	Brain tumor	0.04637
DB08818	Breast cancer	0.02778
DB08818	Bronchiolitis	0.20412
DB08818	Celiac disease	0.09492
DB08818	Chronic obstructive airway disease	0.06455
DB08818	Colon cancer	0.03426
DB08818	Cystic fibrosis	0.08248
DB08818	Dental plaque	0.07332
DB08818	Diabetes mellitus	0.03039
DB08818	Endometriosis	0.04795
DB08818	Enteritis	0.06337
DB08818	Glucose intolerance	0.13245
DB08818	Graves' disease	0.10206
DB08818	HIV infection	0.0553
DB08818	Heart disease	0.17408
DB08818	Herpes	0.08248
DB08818	Hodgkin's disease	0.10206
DB08818	Hyperinsulinism	0.11111
DB08818	Inflammation of the central nervous system	0.20412
DB08818	Influenza	0.11547
DB08818	Intraocular melanoma	0.18257
DB08818	Kaposi sarcoma	0.16013
DB08818	Kidney failure	0.06537
DB08818	Leukemia	0.03253

DB08818	Liver cancer	0.04481
DB08818	Lung cancer	0.04032
DB08818	Lupus erythematosus	0.04933
DB08818	Macular degeneration	0.12039
DB08818	Malaria	0.10721
DB08818	Melanoma	0.04811
DB08818	Migraine	0.10721
DB08818	Multiple sclerosis	0.05955
DB08818	Multiple system atrophy	0.33333
DB08818	Neoplasm metastasis	0.04698
DB08818	Nervous system disease	0.14907
DB08818	Obesity	0.04441
DB08818	Osteoporosis	0.09129
DB08818	Pertussis	0.2357
DB08818	Pre-Eclampsia	0.06804
DB08818	Primary biliary cirrhosis	0.1037
DB08818	Prostate cancer	0.03014
DB08818	Pseudoxanthoma elasticum	0.2582
DB08818	Rheumatic fever	0.14907
DB08818	Rheumatoid arthritis	0.03533
DB08818	Schistosoma mansonii infection	0.33333
DB08818	Schizophrenia	0.0439
DB08818	Sicca syndrome	0.11323
DB08818	Spinal cord disease	0.1291
DB08818	Stomach cancer	0.04862
DB08818	Stroke	0.06537
DB08818	Systemic infection	0.06623
DB08818	Thromboangiitis obliterans	0.2357
DB08818	Thyroiditis	0.40825
DB08818	Tuberculosis	0.07785
DB08818	Tuberous sclerosis	0.14907
DB08818	Ulcerative colitis	0.05955
DB08818	Urinary tract infection	0.40825
DB08818	Vasculitis	0.17408
DB08818	Yersinia infection	0.06852
DB00759	Stress disorder, post-traumatic	0.34273
DB00759	Amyotrophic lateral sclerosis	0.06916
DB00759	Autistic disorder	0.08972
DB00759	Biliary cancer	0.64359
DB00759	Breast cancer	0.03639
DB00759	Cardiovascular disease	0.19515
DB00759	Cerebrovascular disorder	0.17112
DB00759	Cholelithiasis	0.1495
DB00759	Chronic simple glaucoma	0.16649
DB00759	Cirrhosis	0.21174
DB00759	Dental plaque	0.09716
DB00759	Depression	0.06524

DB00759	Dermatitis	0.06204
DB00759	Diabetes mellitus	0.03864
DB00759	Down syndrome	0.09719
DB00759	Drug abuse	0.0481
DB00759	Drug-Induced dyskinesia	0.64359
DB00759	Embryoma	0.05343
DB00759	Encephalitis	0.26172
DB00759	Encephalopathies	0.17223
DB00759	Glaucoma	0.11331
DB00759	Glomerulonephritis	0.27851
DB00759	Growth retardation	0.18611
DB00759	Herpes	0.05962
DB00759	Histiocytosis	0.64359
DB00759	Hyperhomocysteinemia	0.64359
DB00759	Hyperlipidemia	0.18968
DB00759	Hypoglycemia	0.39663
DB00759	Infectious lung disease	0.11724
DB00759	Ischemia	0.09435
DB00759	Kidney failure	0.09597
DB00759	Learning disorder	0.55102
DB00759	Liver cancer	0.07263
DB00759	Liver disease	0.20967
DB00759	Lupus erythematosus	0.05339
DB00759	Macular degeneration	0.26096
DB00759	Malaria	0.17418
DB00759	Mycoses	0.47752
DB00759	Nephrosis	0.13564
DB00759	Neurodegenerative disorder	0.12061
DB00759	Ovarian cancer	0.09045
DB00759	Polycystic kidney	0.20508
DB00759	Prostate cancer	0.04077
DB00759	Psychotic disorder	0.16307
DB00759	Rectum cancer	0.49259
DB00759	Rheumatoid arthritis	0.03893
DB00759	Subarachnoid hemorrhage	0.1216
DB00759	Vascular dementia	0.64359
DB00759	Yersinia infection	0.06836
DB05013	Alopecia	0.06607
DB05013	Atherosclerosis	0.04563
DB05013	Brain tumor	0.06423
DB05013	Breast cancer	0.02949
DB05013	Cervical cancer	0.09773
DB05013	Colon cancer	0.04311
DB05013	Congenital abnormality	0.03896
DB05013	Dermatitis	0.05029
DB05013	Embryoma	0.04331
DB05013	Heart failure	0.07444

DB05013	Infectious lung disease	0.09504
DB05013	Liver disease	0.16996
DB05013	Lung cancer	0.08812
DB05013	Malignant glioma	0.11157
DB05013	Melanoma	0.11432
DB05013	Multiple myeloma	0.09259
DB05013	Neoplasm metastasis	0.05668
DB05013	Obesity	0.0579
DB05013	Prostate cancer	0.03305
DB05013	Stomach cancer	0.04213
DB06201	Arteriopathy	0.2357
DB06201	Prostate cancer	0.03691
DB00063	Hemorrhagic fevers, Viral	1.39229
DB00063	Pemphigoid, Bullous	0.81574
DB00063	Pleural effusion, Malignant	0.31749
DB00063	Purpura, Thrombocytopenic, Idiopathic	0.82394
DB00063	Adenovirus infection	0.02609
DB00063	Alzheimer's disease	0.18332
DB00063	Angiomyolipoma	0.05152
DB00063	Aortic aneurysm	0.02278
DB00063	Aplastic anemia	1.15996
DB00063	Asthma	0.55938
DB00063	Atherosclerosis	0.6507
DB00063	Atopic rhinitis	0.07112
DB00063	Autistic disorder	0.76955
DB00063	Autoimmune disease	0.21511
DB00063	Barrett's esophagus	0.02442
DB00063	Biliary Atresia	0.0687
DB00063	Bone metastases	0.05882
DB00063	Brain tumor	0.02162
DB00063	Breast cancer	0.00993
DB00063	Cancer	0.2497
DB00063	Cervical cancer	0.03289
DB00063	Cholelithiasis	0.04078
DB00063	Colon cancer	0.01451
DB00063	Common wart	0.06316
DB00063	Cytomegalovirus infection	0.05368
DB00063	Dental plaque	0.02651
DB00063	Diabetes mellitus	0.39453
DB00063	Drug abuse	0.21585
DB00063	Eating disorder	0.02261
DB00063	Endometriosis	0.01391
DB00063	Enteritis	0.03105
DB00063	Esotropia	0.03586
DB00063	Gastrointestinal tumor	0.03914
DB00063	Glaucoma	0.03091
DB00063	HIV infection	0.01419

DB00063	Heart failure	0.05011
DB00063	Hemorrhagic disorder	1.07496
DB00063	Hepatitis C	0.0346
DB00063	Herpes	0.26753
DB00063	Hypercholesterolemia	0.05842
DB00063	Hypertension	0.01785
DB00063	Infertility	0.05092
DB00063	Inflammation of the central nervous system	0.07933
DB00063	Intermediate coronary syndrome	1.31497
DB00063	Kidney disease	0.046
DB00063	Kidney failure	0.02618
DB00063	Lung cancer	0.01483
DB00063	Lupus erythematosus	0.49155
DB00063	Lupus vulgaris	0.03961
DB00063	Malignant glioma	0.03755
DB00063	Multiple sclerosis	0.02651
DB00063	Neoplasm metastasis	0.01907
DB00063	Nervous system tumor	0.09022
DB00063	Obesity	0.01948
DB00063	Optic atrophy	0.06783
DB00063	Oral cancer	0.01913
DB00063	Osteoporosis	0.05542
DB00063	Ovarian cancer	0.04935
DB00063	Pancreatitis	0.03251
DB00063	Papillary adenocarcinoma	0.07112
DB00063	Parkinson disease	0.02267
DB00063	Penile disease	1.27465
DB00063	Pertussis	0.06048
DB00063	Polyarthritis	0.01694
DB00063	Primary hyperparathyroidism	0.04186
DB00063	Prostate cancer	0.02075
DB00063	Protein-energy malnutrition	0.09448
DB00063	Renal Cell cancer	0.03408
DB00063	Rheumatoid arthritis	0.19284
DB00063	Schizophrenia	0.18676
DB00063	Sickle cell disease	0.02119
DB00063	Skin cancer	0.03196
DB00063	Squamous cell cancer	0.02936
DB00063	Stroke	0.62252
DB00063	Subarachnoid hemorrhage	1.18015
DB00063	Takayasu's arteritis	1.27426
DB00063	Temporal arteritis	1.40769
DB00063	Testicular dysfunction	0.01974
DB00063	Thrombocytopenia	1.10352
DB00063	Thrombophlebitis	0.05238
DB00063	Ulcerative colitis	0.01924
DB00063	Wiskott-Aldrich syndrome	0.04192

DB00063	Yersinia infection	0.01865
DB00183	Cancer	0.03686
DB00183	Panic disorder	0.21822
DB00183	Parkinson disease	0.11043
DB00045	Abortion	0.125
DB00045	Alimentary system disease	0.21822
DB00045	Arthritis	0.12403
DB00045	Asthma	0.08165
DB00045	Atherosclerosis	0.07001
DB00045	Bacterial vaginosis	0.40825
DB00045	Bronchiectasis	0.40825
DB00045	Chronic obstructive airway disease	0.1118
DB00045	Communicable disease	0.21822
DB00045	Cystic fibrosis	0.14286
DB00045	Cytomegalovirus infection	0.2582
DB00045	Dermatitis	0.09091
DB00045	Drug abuse	0.09366
DB00045	Enteritis	0.10976
DB00045	Epstein-Barr virus infection	0.21822
DB00045	Gouts	0.27735
DB00045	Hepatitis C	0.14744
DB00045	Inflammation of the central nervous system	0.35355
DB00045	Influenza	0.2
DB00045	Kidney failure	0.11323
DB00045	Leprosy	0.26726
DB00045	Liver disease	0.1543
DB00045	Lyme disease	0.33333
DB00045	Mycosis fungoides	0.22361
DB00045	Periodontitis	0.1525
DB00045	Polyarthritis	0.11396
DB00045	Porcine reproductive and respiratory syndrome	0.40825
DB00045	Pre-Eclampsia	0.11785
DB00045	Rheumatoid arthritis	0.0612
DB00045	Systemic infection	0.11471
DB00045	Ulcerative colitis	0.10314
DB00659	Breast cancer	0.00982
DB00659	Cancer	0.01798
DB00659	Connective tissue disease	0.2404
DB00659	Drug abuse	0.01912
DB00659	Hypogonadism	0.09129
DB00659	Yersinia infection	0.02423
DB04941	Infertility, Male	0.6249
DB04941	Alimentary system disease	1.04873
DB04941	Alzheimer's disease	0.16915
DB04941	Arthritis	0.10548
DB04941	Atherosclerosis	0.03952
DB04941	Autistic disorder	0.06298

DB04941	Azoospermia	0.50778
DB04941	Bone disease	0.40922
DB04941	Breast cancer	0.10217
DB04941	Bronchial disease	0.21098
DB04941	Cancer	0.04527
DB04941	Chronic obstructive airway disease	0.08462
DB04941	Conduct disorder	1.16696
DB04941	Congenital abnormality	0.10122
DB04941	Cystic fibrosis	0.65413
DB04941	Dermatitis	0.04355
DB04941	Diabetes mellitus	0.10849
DB04941	Drug abuse	0.03377
DB04941	Endometriosis	0.10738
DB04941	Enteritis	0.39723
DB04941	Glaucoma	0.07955
DB04941	Gram-Negative bacterial infection	0.84232
DB04941	Graves' disease	0.11415
DB04941	Heart failure	0.36369
DB04941	Hypertension	0.29314
DB04941	Infection	0.13706
DB04941	Infertility	0.40416
DB04941	Liver cancer	0.20395
DB04941	Lung cancer	0.03816
DB04941	Malaria	0.12227
DB04941	Metabolism disease	0.11511
DB04941	Myopathy	0.07675
DB04941	Obesity	0.05014
DB04941	Oligospermia	0.84432
DB04941	Polycystic kidney	0.76484
DB04941	Polycystic ovary syndrome	0.0641
DB04941	Premature birth	0.1748
DB04941	Prostate cancer	0.02862
DB04941	Respiratory tract disease	0.59499
DB04941	Rheumatoid arthritis	0.02733
DB04941	Sickle cell disease	0.05453
DB04941	Sinusitis	0.57029
DB04941	Subarachnoid hemorrhage	0.08536
DB04941	Testicular dysfunction	0.30417
DB08820	Infertility, Male	0.68469
DB08820	Alimentary system disease	1.11265
DB08820	Alzheimer's disease	0.19023
DB08820	Arthritis	0.10548
DB08820	Atherosclerosis	0.03952
DB08820	Autistic disorder	0.06298
DB08820	Azoospermia	0.56757
DB08820	Bone disease	0.40922
DB08820	Breast cancer	0.10217

DB08820	Bronchial disease	0.21098
DB08820	Cancer	0.04527
DB08820	Chronic obstructive airway disease	0.08462
DB08820	Conduct disorder	1.26459
DB08820	Congenital abnormality	0.10122
DB08820	Cystic fibrosis	0.69598
DB08820	Dermatitis	0.04355
DB08820	Diabetes mellitus	0.10849
DB08820	Drug abuse	0.03377
DB08820	Endometriosis	0.10738
DB08820	Enteritis	0.42938
DB08820	Glaucoma	0.07955
DB08820	Gram-Negative bacterial infection	0.92687
DB08820	Graves' disease	0.11415
DB08820	Heart failure	0.39491
DB08820	Hypertension	0.31629
DB08820	Infection	0.13706
DB08820	Infertility	0.44439
DB08820	Liver cancer	0.20395
DB08820	Lung cancer	0.03816
DB08820	Malaria	0.12227
DB08820	Metabolism disease	0.11511
DB08820	Myopathy	0.07675
DB08820	Obesity	0.05014
DB08820	Oligospermia	0.91994
DB08820	Polycystic kidney	0.84311
DB08820	Polycystic ovary syndrome	0.0641
DB08820	Premature birth	0.1748
DB08820	Prostate cancer	0.02862
DB08820	Respiratory tract disease	0.65743
DB08820	Rheumatoid arthritis	0.02733
DB08820	Sickle cell disease	0.05453
DB08820	Sinusitis	0.63578
DB08820	Subarachnoid hemorrhage	0.08536
DB08820	Testicular dysfunction	0.34602
DB00551	Actinic keratosis	0.37796
DB00551	Atherosclerosis	0.07001
DB00551	Cervical cancer	0.11625
DB00551	Chronic obstructive airway disease	0.1118
DB00551	Emphysema	0.20412
DB00551	Endometrial cancer	0.17408
DB00551	Endometriosis	0.08305
DB00551	Lung cancer	0.06984
DB00551	Malignant glioma	0.1857
DB00551	Multiple sclerosis	0.10314
DB00551	Periodontitis	0.1525
DB00551	Prostate cancer	0.0522

DB00518	Abortion	0.08399
DB00518	Adenovirus infection	0.05098
DB00518	Alzheimer's disease	0.00811
DB00518	Aortic aneurysm	0.01609
DB00518	Aplastic anemia	0.03768
DB00518	Barrett's esophagus	0.06648
DB00518	Brain tumor	0.01527
DB00518	Breast cancer	0.07596
DB00518	Bronchial disease	0.05791
DB00518	Cancer	0.07386
DB00518	Carcinoma	0.02662
DB00518	Cervical cancer	0.02323
DB00518	Cholestasis	0.0547
DB00518	Colon cancer	0.01025
DB00518	Congenital abnormality	0.03569
DB00518	Diabetes mellitus	0.00744
DB00518	Disseminated cancer	0.16413
DB00518	Embryoma	0.06684
DB00518	HIV infection	0.01002
DB00518	Infection	0.05221
DB00518	Keratoconjunctivitis Sicca	0.0756
DB00518	Leukemia	0.00831
DB00518	Leukoencephalopathy	0.01399
DB00518	Lewy body disease	0.56562
DB00518	Liver cancer	0.02424
DB00518	Lung cancer	0.01047
DB00518	Lung disease	0.02076
DB00518	Mental retardation	0.03858
DB00518	Muscular dystrophies	0.02504
DB00518	Nasopharyngeal cancer	0.11111
DB00518	Neuroblastoma	0.01814
DB00518	Osteosarcoma	0.02659
DB00518	Ovarian cancer	0.06718
DB00518	Pancreas cancer	0.02511
DB00518	Pancreas disease	0.05272
DB00518	Pervasive development disorder	0.15746
DB00518	Pre-Eclampsia	0.0247
DB00518	Prostate cancer	0.01361
DB00518	Salmonella infection	0.12046
DB00518	Schizophrenia	0.04828
DB00518	Stomach cancer	0.06888
DB00518	Testicular tumor	0.11273
DB00518	Tuberous sclerosis	0.01549
DB00518	Ulcerative colitis	0.05238
DB00518	Virus disease	0.01669
DB00643	Abortion	0.08399
DB00643	Adenovirus infection	0.05098

DB00643	Alzheimer's disease	0.00811
DB00643	Aortic aneurysm	0.01609
DB00643	Aplastic anemia	0.03768
DB00643	Barrett's esophagus	0.06648
DB00643	Brain tumor	0.01527
DB00643	Breast cancer	0.07596
DB00643	Bronchial disease	0.05791
DB00643	Cancer	0.07864
DB00643	Carcinoma	0.02662
DB00643	Cervical cancer	0.02323
DB00643	Cholestasis	0.0547
DB00643	Colon cancer	0.01025
DB00643	Congenital abnormality	0.03569
DB00643	Diabetes mellitus	0.00744
DB00643	Disseminated cancer	0.16413
DB00643	Embryoma	0.06684
DB00643	HIV infection	0.01002
DB00643	Infection	0.05221
DB00643	Keratoconjunctivitis Sicca	0.0756
DB00643	Leukemia	0.00831
DB00643	Leukoencephalopathy	0.01399
DB00643	Lewy body disease	0.56562
DB00643	Liver cancer	0.02424
DB00643	Lung cancer	0.01047
DB00643	Lung disease	0.02076
DB00643	Mental retardation	0.03858
DB00643	Muscular dystrophies	0.02504
DB00643	Nasopharyngeal cancer	0.13608
DB00643	Neuroblastoma	0.01814
DB00643	Osteosarcoma	0.02659
DB00643	Ovarian cancer	0.06718
DB00643	Pancreas cancer	0.02511
DB00643	Pancreas disease	0.05272
DB00643	Pervasive development disorder	0.15746
DB00643	Pre-Eclampsia	0.0247
DB00643	Prostate cancer	0.01361
DB00643	Salmonella infection	0.12046
DB00643	Schizophrenia	0.04828
DB00643	Stomach cancer	0.06888
DB00643	Testicular tumor	0.11273
DB00643	Tuberous sclerosis	0.01549
DB00643	Ulcerative colitis	0.05238
DB00643	Virus disease	0.01669
DB04845	Cancer	0.03686
DB02546	Infertility, Male	0.04235
DB02546	Abortion	0.04609
DB02546	Adenoma	0.01176

DB02546	Adenoma of thyroid	0.042
DB02546	Adenovirus infection	0.23426
DB02546	Adrenal gland hyperfunction	0.09385
DB02546	Adrenal gland tumor	0.08613
DB02546	Alzheimer's disease	0.02902
DB02546	Amyotrophic lateral sclerosis	0.01694
DB02546	Aseptic necrosis of bone	0.01288
DB02546	Asthma	0.01216
DB02546	Atherosclerosis	0.00594
DB02546	Autistic disorder	0.02717
DB02546	Azoospermia	0.01789
DB02546	Barrett's esophagus	0.02315
DB02546	Behavior disease	0.04469
DB02546	Bipolar disorder	0.02635
DB02546	Brain disease	0.09537
DB02546	Brain tumor	0.0776
DB02546	Breast cancer	0.25898
DB02546	Bronchial hyperreactivity	0.02628
DB02546	Cancer	0.24124
DB02546	Capillaries disease	0.01477
DB02546	Cerebrovascular disorder	0.01807
DB02546	Cholelithiasis	0.01578
DB02546	Colon cancer	0.05927
DB02546	Common cold	0.01206
DB02546	Congenital abnormality	0.06397
DB02546	Corneal disease	0.01501
DB02546	Cystic fibrosis	0.01102
DB02546	Dental plaque	0.01026
DB02546	Depression	0.01443
DB02546	Dermatitis	0.02237
DB02546	Diabetes mellitus	0.02683
DB02546	Down syndrome	0.01026
DB02546	Drug abuse	0.01419
DB02546	Eating disorder	0.01793
DB02546	Embryoma	0.17707
DB02546	Encephalopathies	0.05215
DB02546	Endometrial cancer	0.05562
DB02546	Endometriosis	0.05334
DB02546	Epilepsy	0.04281
DB02546	Esophagus cancer	0.02457
DB02546	Eye cancer	0.07519
DB02546	Fanconi's anemia	0.04765
DB02546	Gastritis	0.03994
DB02546	Glaucoma	0.01196
DB02546	Granulomatous disease	0.03607
DB02546	HIV infection	0.11754
DB02546	Hepatoblastoma	0.05552

DB02546	Hereditary disease	0.05315
DB02546	Herpes	0.02278
DB02546	Hyperlipidemia	0.02003
DB02546	Hypertension	0.00798
DB02546	Hypopituitarism	0.07652
DB02546	Immunologic deficiency syndrome	0.00725
DB02546	Infection	0.14228
DB02546	Infertility	0.01154
DB02546	Ischemia	0.00792
DB02546	Kaposi sarcoma	0.11151
DB02546	Keratoconus	0.02382
DB02546	Kidney failure	0.01702
DB02546	Late pregnancy	0.02481
DB02546	Leukemia	0.02448
DB02546	Leukoencephalopathy	0.02774
DB02546	Liver cancer	0.08297
DB02546	Liver tumor	0.02819
DB02546	Lung cancer	0.2347
DB02546	Lupus erythematosus	0.0219
DB02546	Lupus vulgaris	0.01533
DB02546	Lymphoma	0.03599
DB02546	Lymphoproliferative disorder	0.0485
DB02546	Malignant glioma	0.01592
DB02546	Melanoma	0.026
DB02546	Mental retardation	0.01295
DB02546	Migraine	0.01417
DB02546	Movement disorder	0.06605
DB02546	Muscular atrophy	0.03779
DB02546	Muscular dystrophies	0.03786
DB02546	Myasthenia Gravis	0.01918
DB02546	Myeloproliferative disease	0.04874
DB02546	Myopathy	0.02577
DB02546	Myotonic disorder	0.04261
DB02546	Neoplasm metastasis	0.10073
DB02546	Nervous system disease	0.05391
DB02546	Neurodegenerative disorder	0.01395
DB02546	Obesity	0.00754
DB02546	Oligospermia	0.03258
DB02546	Oral cancer	0.00811
DB02546	Osteomyelitis	0.01204
DB02546	Osteoporosis	0.01149
DB02546	Osteosarcoma	0.01596
DB02546	Otitis media	0.06092
DB02546	Ovarian disease	0.01802
DB02546	Ovarian failure	0.02356
DB02546	Panic disorder	0.0181
DB02546	Papillary cancer	0.0529

DB02546	Parkinson disease	0.05221
DB02546	Pelvic inflammatory disease	0.34855
DB02546	Polyarthritis	0.01458
DB02546	Polycystic ovary syndrome	0.01928
DB02546	Polymyositis	0.05505
DB02546	Primary biliary cirrhosis	0.01374
DB02546	Prostate cancer	0.14105
DB02546	Rabies	0.02961
DB02546	Renal Cell cancer	0.01912
DB02546	Renal tubular acidosis	0.09186
DB02546	Respiratory failure	0.01942
DB02546	Respiratory tract disease	0.02187
DB02546	Retinitis pigmentosa	0.01951
DB02546	Rett syndrome	0.07756
DB02546	Rheumatoid arthritis	0.02856
DB02546	Schizophrenia	0.03767
DB02546	Scleroderma	0.05103
DB02546	Severe acute respiratory syndrome	0.06824
DB02546	Sicca syndrome	0.0097
DB02546	Skin cancer	0.01355
DB02546	Skin disease	0.02266
DB02546	Spinal dysraphism	0.02455
DB02546	Squamous cell cancer	0.01245
DB02546	Stomach cancer	0.02339
DB02546	Stomach disease	0.06037
DB02546	Stroke	0.0063
DB02546	Synovial sarcoma	0.01874
DB02546	Synovitis	0.02089
DB02546	Systemic scleroderma	0.04094
DB02546	Testicular dysfunction	0.00837
DB02546	Testicular tumor	0.04499
DB02546	Thrombophlebitis	0.01611
DB02546	Thymoma	0.01621
DB02546	Thyroid cancer	0.03008
DB02546	Tic disorder	0.02682
DB02546	Tuberous sclerosis	0.29418
DB02546	Ulcerative colitis	0.00816
DB02546	Uterine disease	0.03024
DB02546	Vascular disease	0.01525
DB02546	Virus disease	0.00675
DB02546	Vitamin D deficiency	0.01352
DB02546	Vitiligo	0.01918
DB02546	Yersinia infection	0.03508
DB06777	Barrett's esophagus	0.18257
DB06777	Cholelithiasis	0.2
DB06777	Cholestasis	0.27735
DB06777	Esophagitis	0.44721

DB06777	Esophagus cancer	0.14907
DB06777	Prostate cancer	0.0522
DB06777	Skin disease	0.1715
DB00555	Autistic disorder	0.12127
DB00555	Dental plaque	0.127
DB00555	Intractable epilepsy	0.35355
DB00555	Myopathy	0.12127
DB00555	Neuropathy	0.16013
DB00555	Rheumatism	0.2357
DB00818	Autistic disorder	0.02712
DB00818	Breast cancer	0.01076
DB00818	Common cold	0.05976
DB00818	Dental plaque	0.0284
DB00818	Drug abuse	0.04189
DB00818	Hypogonadism	0.1
DB00818	Intractable epilepsy	0.07906
DB00818	Myopathy	0.05423
DB00818	Neuropathy	0.03581
DB00818	Rheumatism	0.0527
DB00818	Yersinia infection	0.02654
DB00357	Adrenal gland tumor	0.2132
DB00357	Breast cancer	0.03402
DB00357	Endometriosis	0.05872
DB00357	Hypertension	0.0559
DB00357	Infiltrating cancer	0.18898
DB00357	Obesity	0.05439
DB00357	Osteoporosis	0.1118
DB00357	Ovarian cancer	0.07107
DB00357	Polyarthritis	0.08058
DB00357	Prostate cancer	0.03691
DB00357	Urogenital abnormalities	0.18898
DB00357	Uterine disease	0.26726
DB00990	Adrenal gland tumor	0.30151
DB00990	Breast cancer	0.04811
DB00990	Endometriosis	0.08305
DB00990	Hypertension	0.07906
DB00990	Infiltrating cancer	0.26726
DB00990	Obesity	0.07692
DB00990	Osteoporosis	0.15811
DB00990	Ovarian cancer	0.1005
DB00990	Polyarthritis	0.11396
DB00990	Prostate cancer	0.0522
DB00990	Urogenital abnormalities	0.26726
DB00990	Uterine disease	0.37796
DB01006	Adrenal gland tumor	0.30151
DB01006	Breast cancer	0.04811
DB01006	Endometriosis	0.08305

DB01006	Hypertension	0.07906
DB01006	Infiltrating cancer	0.26726
DB01006	Obesity	0.07692
DB01006	Osteoporosis	0.15811
DB01006	Ovarian cancer	0.1005
DB01006	Polyarthritis	0.11396
DB01006	Prostate cancer	0.0522
DB01006	Urogenital abnormalities	0.26726
DB01006	Uterine disease	0.37796
DB01217	Adrenal gland tumor	0.30151
DB01217	Breast cancer	0.04811
DB01217	Endometriosis	0.08305
DB01217	Hypertension	0.07906
DB01217	Infiltrating cancer	0.26726
DB01217	Obesity	0.07692
DB01217	Osteoporosis	0.15811
DB01217	Ovarian cancer	0.1005
DB01217	Polyarthritis	0.11396
DB01217	Prostate cancer	0.0522
DB01217	Urogenital abnormalities	0.26726
DB01217	Uterine disease	0.37796
DB04895	Pemphigoid, Bullous	0.0252
DB04895	Pleural effusion, Malignant	0.02579
DB04895	Purpura, Thrombocytopenic, Idiopathic	0.02409
DB04895	Alopecia	0.02972
DB04895	Alzheimer's disease	0.01535
DB04895	Amyotrophic lateral sclerosis	0.06176
DB04895	Aortic aneurysm	0.03045
DB04895	Asthma	0.05796
DB04895	Atherosclerosis	0.0708
DB04895	Brain tumor	0.02359
DB04895	Breast cancer	0.06985
DB04895	Cancer	0.2132
DB04895	Capillaries disease	0.16055
DB04895	Cardiovascular disease	0.17427
DB04895	Chronic obstructive airway disease	0.19824
DB04895	Depression	0.02379
DB04895	Dermatitis	0.0554
DB04895	Diabetes mellitus	0.06267
DB04895	Drug abuse	0.01754
DB04895	Embryoma	0.01948
DB04895	Endometriosis	0.04553
DB04895	Familial Mediterranean fever	0.2543
DB04895	Kidney failure	0.12069
DB04895	Leukoencephalopathy	0.09132
DB04895	Lupus erythematosus	0.06714
DB04895	Macular degeneration	0.32817

DB04895	Malaria	0.21904
DB04895	Mucocutaneous lymph node syndrome	0.32099
DB04895	Obesity	0.08982
DB04895	Peptic ulcer	0.20248
DB04895	Polyneuropathy	0.32817
DB04895	Pre-Eclampsia	0.16119
DB04895	Primary hyperparathyroidism	0.02998
DB04895	Renal Cell cancer	0.04555
DB04895	Retinal disease	0.1662
DB04895	Rheumatoid arthritis	0.03997
DB04895	Schizophrenia	0.0387
DB04895	Shigella infection	0.03176
DB04895	Sickle cell disease	0.02832
DB04895	Stomach cancer	0.0843
DB04895	Systemic infection	0.11621
DB04895	Thalassemia	0.02969
DB08900	Colon cancer	0.05934
DB00042	Attention deficit hyperactivity disorder	0.50171
DB00042	Behavior disease	0.21022
DB00042	Bipolar disorder	0.04782
DB00042	Cystic fibrosis	0.04583
DB00042	Down syndrome	0.25005
DB00042	Schizophrenia	0.17951
DB00190	Autistic disorder	0.12127
DB00190	Bipolar disorder	0.11323
DB00190	Neuroblastoma	0.13736
DB00190	Pancreas disease	0.20851
DB00190	Schizophrenia	0.07603
DB00772	Alzheimer's disease	0.07198
DB00772	Atherosclerosis	0.07001
DB00772	Colon cancer	0.05934
DB00772	Diabetes mellitus	0.05263
DB00772	Obesity	0.07692
DB00772	Vitiligo	0.2357
DB00941	Alzheimer's disease	0.07198
DB00941	Atherosclerosis	0.07001
DB00941	Colon cancer	0.05934
DB00941	Diabetes mellitus	0.05263
DB00941	Obesity	0.07692
DB00941	Vitiligo	0.2357
DB01057	Alzheimer's disease	0.07198
DB01057	Atherosclerosis	0.07001
DB01057	Colon cancer	0.05934
DB01057	Diabetes mellitus	0.05263
DB01057	Obesity	0.07692
DB01057	Vitiligo	0.2357
DB00752	Stress disorder, post-traumatic	0.25

DB00752	Anorexia nervosa	0.14434
DB00752	Autistic disorder	0.08575
DB00752	Behavior disease	0.21822
DB00752	Bipolar disorder	0.08006
DB00752	Breast cancer	0.03402
DB00752	Depression	0.09285
DB00752	Down syndrome	0.08058
DB00752	Drug abuse	0.06623
DB00752	Encephalopathies	0.09901
DB00752	Fibromyalgia	0.5
DB00752	Generalized anxiety disorder	0.20412
DB00752	Huntington disease	0.1543
DB00752	Neurotic disorder	0.22361
DB00752	Obesity	0.05439
DB00752	Panic disorder	0.1543
DB00752	Psychotic disorder	0.11323
DB00752	Sudden infant death syndrome	0.1543
DB01037	Stress disorder, post-traumatic	0.25
DB01037	Anorexia nervosa	0.14434
DB01037	Autistic disorder	0.08575
DB01037	Behavior disease	0.21822
DB01037	Bipolar disorder	0.08006
DB01037	Breast cancer	0.03402
DB01037	Depression	0.09285
DB01037	Down syndrome	0.08058
DB01037	Drug abuse	0.06623
DB01037	Encephalopathies	0.09901
DB01037	Fibromyalgia	0.5
DB01037	Generalized anxiety disorder	0.20412
DB01037	Huntington disease	0.1543
DB01037	Neurotic disorder	0.22361
DB01037	Obesity	0.05439
DB01037	Panic disorder	0.1543
DB01037	Psychotic disorder	0.11323
DB01037	Sudden infant death syndrome	0.1543
DB01247	Stress disorder, post-traumatic	0.25
DB01247	Anorexia nervosa	0.14434
DB01247	Autistic disorder	0.08575
DB01247	Behavior disease	0.21822
DB01247	Bipolar disorder	0.08006
DB01247	Breast cancer	0.03402
DB01247	Depression	0.09285
DB01247	Down syndrome	0.08058
DB01247	Drug abuse	0.06623
DB01247	Encephalopathies	0.09901
DB01247	Fibromyalgia	0.5
DB01247	Generalized anxiety disorder	0.20412

DB01247	Huntington disease	0.1543
DB01247	Neurotic disorder	0.22361
DB01247	Obesity	0.05439
DB01247	Panic disorder	0.1543
DB01247	Psychotic disorder	0.11323
DB01247	Sudden infant death syndrome	0.1543
DB01626	Stress disorder, post-traumatic	0.25
DB01626	Anorexia nervosa	0.14434
DB01626	Autistic disorder	0.08575
DB01626	Behavior disease	0.21822
DB01626	Bipolar disorder	0.08006
DB01626	Breast cancer	0.03402
DB01626	Depression	0.09285
DB01626	Down syndrome	0.08058
DB01626	Drug abuse	0.06623
DB01626	Encephalopathies	0.09901
DB01626	Fibromyalgia	0.5
DB01626	Generalized anxiety disorder	0.20412
DB01626	Huntington disease	0.1543
DB01626	Neurotic disorder	0.22361
DB01626	Obesity	0.05439
DB01626	Panic disorder	0.1543
DB01626	Psychotic disorder	0.11323
DB01626	Sudden infant death syndrome	0.1543
DB01171	Anorexia nervosa	0.20412
DB01171	Autistic disorder	0.12127
DB01171	Behavior disease	0.1543
DB01171	Bipolar disorder	0.11323
DB01171	Breast cancer	0.04811
DB01171	Depression	0.13131
DB01171	Down syndrome	0.11396
DB01171	Drug abuse	0.09366
DB01171	Fibromyalgia	0.35355
DB01171	Generalized anxiety disorder	0.28868
DB01171	Neurotic disorder	0.31623
DB01171	Obesity	0.07692
DB01171	Panic disorder	0.21822
DB01171	Sudden infant death syndrome	0.21822
DB00437	Asthma	0.08165
DB00437	Atherosclerosis	0.07001
DB00437	Breast cancer	0.04811
DB00437	Hypertension	0.07906
DB00437	Sicca syndrome	0.19612
DB00437	Stomach disease	0.35355
DB00552	Asthma	0.08165
DB00552	Atherosclerosis	0.07001
DB00552	Autistic disorder	0.12127

DB00552	Combined immunodeficiency	0.40825
DB00552	Diabetes mellitus	0.05263
DB00552	Hydatidiform mole	0.70711
DB00552	Hypertension	0.07906
DB00552	Kidney failure	0.11323
DB00552	Lupus erythematosus	0.08544
DB00552	Mental retardation	0.1291
DB00552	Nephrosis	0.22361
DB00552	Peptic ulcer	0.2357
DB00552	Pre-Eclampsia	0.11785
DB01590	Bone metastases	0.08162
DB01590	Brain tumor	0.13392
DB01590	Infection	0.29347
DB01590	Renal Cell cancer	0.0473
DB01590	Tuberous sclerosis	0.22661
DB06287	Bone metastases	0.08162
DB06287	Brain tumor	0.13392
DB06287	Infection	0.29347
DB06287	Renal Cell cancer	0.0473
DB06287	Tuberous sclerosis	0.22661
DB08877	Skin disease, Genetic	0.08106
DB08877	Thrombocythemia, Hemorrhagic	0.57741
DB08877	Abortion	0.25034
DB08877	Adenovirus infection	0.13757
DB08877	Alopecia	0.05118
DB08877	Atherosclerosis	0.1864
DB08877	Autoimmune disease	0.19573
DB08877	Brain tumor	0.04976
DB08877	Breast cancer	0.02285
DB08877	Cancer	0.06483
DB08877	Cervical cancer	0.10706
DB08877	Cholangiocarcinoma	0.33575
DB08877	Choriocarcinoma	0.64719
DB08877	Colon cancer	0.07535
DB08877	Combined immunodeficiency	0.98033
DB08877	Congenital abnormality	0.03018
DB08877	Congenital heart disease	0.19824
DB08877	Deafness	0.12798
DB08877	Dermatitis	0.03896
DB08877	Down syndrome	0.25587
DB08877	Embryoma	0.09105
DB08877	Heart failure	0.05767
DB08877	Hematopoietic system disease	0.76461
DB08877	Hemorrhagic disorder	0.06694
DB08877	Herpes	0.03744
DB08877	Infection	0.19019
DB08877	Infectious lung disease	0.07362

DB08877	Leukemia	0.08676
DB08877	Liver disease	0.13166
DB08877	Liver tumor	0.11855
DB08877	Lung cancer	0.04827
DB08877	Lupus erythematosus	0.04328
DB08877	Lymphoproliferative disorder	0.13167
DB08877	Malignant glioma	0.08643
DB08877	Melanoma	0.13562
DB08877	Metastasis to lymph nodes	0.17695
DB08877	Multiple myeloma	0.18513
DB08877	Myeloproliferative disease	0.53054
DB08877	Obesity	0.06343
DB08877	Periodontitis	0.17247
DB08877	Polyarthritis	0.03899
DB08877	Polycystic ovary syndrome	0.05734
DB08877	Polycythemia	0.5487
DB08877	Primary biliary cirrhosis	0.36168
DB08877	Prostate cancer	0.1072
DB08877	Rheumatoid arthritis	0.11349
DB08877	Sarcoidosis	0.16208
DB08877	Skin cancer	0.07357
DB08877	Solid tumor	0.11728
DB08877	Stroke	0.1876
DB08877	Thrombophilia	0.70996
DB08877	Tuberculosis	0.07702
DB08877	Ulcerative colitis	0.04429
DB08877	Uveitis	0.17067
DB08895	Skin disease, Genetic	0.08106
DB08895	Thrombocythemia, Hemorrhagic	0.57741
DB08895	Abortion	0.23412
DB08895	Adenovirus infection	0.12067
DB08895	Alopecia	0.05118
DB08895	Atherosclerosis	0.17731
DB08895	Autoimmune disease	0.18166
DB08895	Bone marrow disease	0.14907
DB08895	Brain tumor	0.04976
DB08895	Breast cancer	0.02285
DB08895	Cancer	0.06005
DB08895	Cervical cancer	0.10706
DB08895	Cholangiocarcinoma	0.33575
DB08895	Choriocarcinoma	0.60973
DB08895	Colon cancer	0.10191
DB08895	Combined immunodeficiency	1.16306
DB08895	Congenital abnormality	0.03018
DB08895	Congenital heart disease	0.19824
DB08895	Deafness	0.12798
DB08895	Dermatitis	0.03896

DB08895	Down syndrome	0.30688
DB08895	Embryoma	0.11865
DB08895	Heart failure	0.05767
DB08895	Hematopoietic system disease	0.72136
DB08895	Hemorrhagic disorder	0.06694
DB08895	Herpes	0.03744
DB08895	Infection	0.17809
DB08895	Infectious lung disease	0.07362
DB08895	Leukemia	0.07945
DB08895	Liver disease	0.13166
DB08895	Liver tumor	0.11855
DB08895	Lung cancer	0.04827
DB08895	Lupus erythematosus	0.04328
DB08895	Lymphoproliferative disorder	0.13167
DB08895	Malignant glioma	0.08643
DB08895	Melanoma	0.17292
DB08895	Metastasis to lymph nodes	0.17695
DB08895	Multiple myeloma	0.18513
DB08895	Myeloproliferative disease	0.65016
DB08895	Obesity	0.06343
DB08895	Periodontitis	0.17247
DB08895	Polyarthritis	0.03899
DB08895	Polycystic ovary syndrome	0.05734
DB08895	Polycythemia	0.50544
DB08895	Primary biliary cirrhosis	0.33838
DB08895	Prostate cancer	0.10043
DB08895	Reticulosarcoma	0.20412
DB08895	Rheumatoid arthritis	0.10555
DB08895	Sarcoidosis	0.16208
DB08895	Skin cancer	0.07357
DB08895	Solid tumor	0.11728
DB08895	Stroke	0.1729
DB08895	Thrombophilia	0.67937
DB08895	Tuberculosis	0.07702
DB08895	Ulcerative colitis	0.04429
DB08895	Uveitis	0.17067
DB00613	Asthma	0.08165
DB00613	Celiac disease	0.1644
DB00613	Intestinal disease	0.25
DB00613	Peptic ulcer	0.2357
DB01289	Congenital abnormality	0.07538
DB01289	Late pregnancy	0.28868
DB06273	Kidney tubular necrosis, acute	0.40825
DB06273	Autoimmune disease	0.10847
DB06273	Endometriosis	0.08305
DB06273	HTLV-I infection	0.33333
DB06273	Hyperparathyroidism	0.33333

DB06273	Hypertension	0.07906
DB06273	Infertility	0.13736
DB06273	Liver cancer	0.07762
DB06273	Liver tumor	0.17678
DB06273	Multiple myeloma	0.13131
DB06273	Neoplasm metastasis	0.08138
DB06273	Pancreas cancer	0.1037
DB06273	Stroke	0.11323
DB05389	Dental plaque	0.0898
DB05389	Diabetes mellitus	0.03722
DB05389	Emphysema	0.27161
DB05389	HIV infection	0.06773
DB05389	Kidney failure	0.08006
DB05389	Leiomyosarcoma	0.40825
DB05389	Spondylarthropathies	0.2357
DB05389	Synovitis	0.25
DB05389	Systemic infection	0.08111
DB05332	Bone disease	0.1715
DB05332	Bone marrow disease	0.2582
DB05332	HIV infection	0.09578
DB05332	Hematopoietic system disease	0.33333
DB05332	Hemorrhagic disorder	0.17678
DB05332	Myeloproliferative disease	0.26726
DB05332	Polycythemia	0.33333
DB05332	Thrombocytopenia	0.25
DB05332	Thrombocytosis	0.44721
DB06210	Bone disease	0.1715
DB06210	Bone marrow disease	0.2582
DB06210	HIV infection	0.09578
DB06210	Hematopoietic system disease	0.33333
DB06210	Hemorrhagic disorder	0.17678
DB06210	Myeloproliferative disease	0.26726
DB06210	Polycythemia	0.33333
DB06210	Thrombocytopenia	0.25
DB06210	Thrombocytosis	0.44721
DB08870	Chronic rejection of renal transplant	0.25
DB08870	Cirrhosis	0.16903
DB08870	Common variable immunodeficiency	0.35355
DB08870	Graves' disease	0.17678
DB08870	HIV infection	0.09578
DB08870	Hepatitis C	0.14744
DB08870	Hodgkin's disease	0.17678
DB08870	Hypertension	0.07906
DB08870	Leukemia	0.05634
DB08870	Lupus erythematosus	0.08544
DB08870	Lymphoma	0.11396
DB08870	Pyelonephritis	1

DB08870	Rheumatoid arthritis	0.0612
DB05829	Bone disease	0.39408
DB05829	Cancer	0.05624
DB05829	Kidney failure	0.2148
DB05829	Oral cancer	0.19467
DB05829	Rheumatism	0.34695
DB06285	Bone disease	0.44431
DB06285	Cancer	0.06704
DB06285	Kidney failure	0.24797
DB06285	Oral cancer	0.23453
DB06285	Rheumatism	0.41598
DB01077	Pancreas disease	0.14744
DB01077	Ulcerative colitis	0.07293
DB00561	Autoimmune disease	0.0767
DB04865	Abortion	0.05824
DB04865	Adenovirus infection	0.01558
DB04865	Alzheimer's disease	0.01281
DB04865	Aortic aneurysm	0.0254
DB04865	Aplastic anemia	0.19623
DB04865	Brain tumor	0.09997
DB04865	Breast cancer	0.05812
DB04865	Bronchial disease	0.19495
DB04865	Cancer	0.05749
DB04865	Carcinoma	0.15208
DB04865	Cervical cancer	0.0782
DB04865	Cholestasis	0.08633
DB04865	Colon cancer	0.05855
DB04865	Diabetes mellitus	0.01175
DB04865	Embryoma	0.11144
DB04865	HIV infection	0.01581
DB04865	Keratoconjunctivitis Sicca	0.11931
DB04865	Leukemia	0.01312
DB04865	Leukoencephalopathy	0.02208
DB04865	Liver cancer	0.09964
DB04865	Lung cancer	0.03526
DB04865	Lung disease	0.06987
DB04865	Mental retardation	0.06089
DB04865	Mucopolysaccharidosis	0.05543
DB04865	Muscular dystrophies	0.03952
DB04865	Neuroblastoma	0.02863
DB04865	Osteosarcoma	0.08951
DB04865	Pancreas cancer	0.11462
DB04865	Pancreas disease	0.30123
DB04865	Pre-Eclampsia	0.03898
DB04865	Prostate cancer	0.07222
DB04865	Stomach cancer	0.11696
DB04865	Tuberous sclerosis	0.05214

DB04865	Virus disease	0.02633
DB01272	Infection	0.09325
DB08828	Bone disease	0.1715
DB08828	Chondrosarcoma	0.37796
DB08828	Hyperglycemia	0.16013
DB08828	Pancreas cancer	0.1037
DB08828	Skin cancer	0.22942
DB00400	Corneal disease	0.37796
DB06689	Adenocarcinoma	0.14586
DB06689	Atherosclerosis	0.07001
DB06689	Hemorrhagic disorder	0.17678
DB06292	Kidney disease	0.11952
DB08907	Kidney disease	0.08452
DB08908	Diabetes mellitus	0.10688
DB08908	Embryoma	0.13668
DB08908	Lung cancer	0.14615
DB00189	Breast cancer	0.01203
DB00189	Drug abuse	0.02341
DB00189	Hypogonadism	0.1118
DB00189	Yersinia infection	0.02967
DB00349	Breast cancer	0.01203
DB00349	Drug abuse	0.02341
DB00349	Hypogonadism	0.1118
DB00349	Yersinia infection	0.02967
DB00404	Breast cancer	0.01203
DB00404	Drug abuse	0.02341
DB00404	Hypogonadism	0.1118
DB00404	Yersinia infection	0.02967
DB00475	Breast cancer	0.01203
DB00475	Drug abuse	0.02341
DB00475	Hypogonadism	0.1118
DB00475	Yersinia infection	0.02967
