# Supplementary material for: Inferring drug-disease associations based on known protein complexes
Source: BMC Med Genomics. 2015 May 29;8(Suppl 2):S2. doi: 10.1186/1755-8794-8-S2-S2 (PMC4460611; doi:10.1186/1755-8794-8-S2-S2)
Supplement: Additional file 7 — Table illustrating the information of drug-disease network after being filtered by PPI network and weight. [file 1755-8794-8-S2-S2-S7.PDF]

**Drug ID**	**Disease Name**	**Weight**
DB00775	Takayasu's arteritis	1.41
DB01259	Papillary adenocarcinoma	1.35
DB00036	Lymphatic metastasis	1.29
DB08916	Papillary adenocarcinoma	1.28
DB00775	Temporal arteritis	1.28
DB00063	Takayasu's arteritis	1.27
DB00775	Hemorrhagic fevers, Viral	1.27
DB00775	Aplastic anemia	1.25
DB01259	Optic atrophy	1.22
DB00775	Intermediate coronary syndrome	1.22
DB00775	Thrombocytopenia	1.21
DB00775	Penile disease	1.17
DB00063	Aplastic anemia	1.16
DB08916	Optic atrophy	1.16
DB01259	Common wart	1.16
DB00775	Hemorrhagic disorder	1.15
DB00036	Abruption placentae	1.14
DB01259	Atopic rhinitis	1.13
DB00098	Pleural effusion, Malignant	1.11
DB00063	Thrombocytopenia	1.10
DB00054	Takayasu's arteritis	1.10
DB08916	Common wart	1.10
DB08916	Atopic rhinitis	1.09
DB00098	Purpura, Thrombocytopenic, Idiopathic	1.09
DB00775	Subarachnoid hemorrhage	1.09
DB00098	Pemphigoid, Bullous	1.09
DB00063	Hemorrhagic disorder	1.07
DB00004	Mitral valve disease	1.07
DB00041	Mitral valve disease	1.07
DB00054	Hemorrhagic fevers, Viral	1.06
DB00054	Temporal arteritis	1.05
DB01259	Pneumoconiosis	1.04
DB00054	Intermediate coronary syndrome	1.04
DB00054	Aplastic anemia	1.03
DB00054	Thrombocytopenia	1.03
DB00072	Papillary adenocarcinoma	1.03
DB01270	Mucocutaneous lymph node syndrome	1.00
DB00170	Abruption placentae	1.00
DB00170	Lymphatic metastasis	1.00
DB00569	Abruption placentae	1.00
DB00569	Lymphatic metastasis	1.00
DB01109	Abruption placentae	1.00
DB01109	Lymphatic metastasis	1.00
DB01225	Abruption placentae	1.00
DB01225	Lymphatic metastasis	1.00
DB06228	Abruption placentae	1.00

DB06228	Lymphatic metastasis	1.00
DB06605	Abruption placentae	1.00
DB06605	Lymphatic metastasis	1.00
DB08870	Pyelonephritis	1.00
DB00244	Infectious lung disease	0.99
DB00795	Infectious lung disease	0.99
DB00054	Penile disease	0.99
DB00808	Long QT syndrome	0.96
DB00054	Hemorrhagic disorder	0.96
DB01259	Angiomyolipoma	0.95
DB00072	Optic atrophy	0.95
DB08916	Pneumoconiosis	0.95
DB01120	Mucocutaneous lymph node syndrome	0.94
DB00072	Atopic rhinitis	0.94
DB00775	Purpura, Thrombocytopenic, Idiopathic	0.93
DB01149	Behavior disease	0.93
DB00995	Infectious lung disease	0.93
DB00036	Rectum cancer	0.93
DB00054	Subarachnoid hemorrhage	0.93
DB01259	Hypercholesterolemia	0.91
DB08885	Mucocutaneous lymph node syndrome	0.91
DB01259	Thrombophlebitis	0.91
DB00170	Nevus	0.90
DB08912	Vitamin D deficiency	0.90
DB08916	Angiomyolipoma	0.90
DB00726	Behavior disease	0.90
DB01017	Mucocutaneous lymph node syndrome	0.90
DB01259	Cytomegalovirus infection	0.90
DB00072	Common wart	0.90
DB00904	Behavior disease	0.89
DB00320	Attention deficit hyperactivity disorder	0.89
DB00540	Behavior disease	0.89
DB08916	Hypercholesterolemia	0.89
DB01277	Cerebral palsy	0.88
DB01277	Fibromyalgia	0.88
DB00233	Infectious lung disease	0.88
DB01277	Acromegaly	0.87
DB00247	Behavior disease	0.87
DB08916	Thrombophlebitis	0.87
DB01392	Behavior disease	0.87
DB08916	Cytomegalovirus infection	0.86
DB00904	Sudden infant death syndrome	0.86
DB00656	Behavior disease	0.86
DB00111	Mitral valve disease	0.85
DB00317	Esotropia	0.85
DB01269	Esotropia	0.85
DB00036	Nevus	0.85

DB00074	Mitral valve disease	0.85
DB00315	Behavior disease	0.85
DB00669	Behavior disease	0.85
DB00952	Behavior disease	0.85
DB00367	Vitiligo	0.84
DB08810	Behavior disease	0.84
DB00696	Anorexia nervosa	0.84
DB06779	Mucocutaneous lymph node syndrome	0.84
DB00098	Shigella infection	0.84
DB00268	Behavior disease	0.84
DB00413	Behavior disease	0.84
DB00589	Behavior disease	0.84
DB00641	Thrombophilia	0.84
DB00904	Panic disorder	0.83
DB01406	Vitiligo	0.83
DB00012	Polycythemia	0.83
DB00016	Polycythemia	0.83
DB08894	Polycythemia	0.83
DB08923	Polycythemia	0.83
DB01142	Behavior disease	0.83
DB00248	Behavior disease	0.83
DB00714	Behavior disease	0.83
DB00098	Sickle cell disease	0.83
DB00918	Anorexia nervosa	0.83
DB00998	Anorexia nervosa	0.83
DB00246	Behavior disease	0.82
DB00334	Behavior disease	0.82
DB01238	Behavior disease	0.82
DB00063	Purpura, Thrombocytopenic, Idiopathic	0.82
DB00363	Behavior disease	0.82
DB01224	Behavior disease	0.82
DB01186	Behavior disease	0.82
DB01621	Behavior disease	0.82
DB01618	Behavior disease	0.82
DB01616	Behavior disease	0.82
DB06684	Behavior disease	0.82
DB00004	Filariasis	0.82
DB00041	Filariasis	0.82
DB01200	Behavior disease	0.82
DB00098	Lupus erythematosus	0.81
DB00098	Primary hyperparathyroidism	0.81
DB00216	Behavior disease	0.81
DB00953	Anorexia nervosa	0.80
DB01614	Behavior disease	0.79
DB01622	Behavior disease	0.79
DB00098	Thalassemia	0.79
DB08912	Osteomyelitis	0.79

DB00315	Sudden infant death syndrome	0.79
DB00669	Sudden infant death syndrome	0.79
DB00952	Sudden infant death syndrome	0.79
DB00098	Aortic aneurysm	0.79
DB00696	Attention deficit hyperactivity disorder	0.78
DB00477	Behavior disease	0.78
DB00641	Pertussis	0.78
DB00490	Behavior disease	0.77
DB00960	Behavior disease	0.77
DB00530	Esotropia	0.77
DB00072	Hypercholesterolemia	0.76
DB00216	Sudden infant death syndrome	0.76
DB00734	Sudden infant death syndrome	0.76
DB04946	Behavior disease	0.76
DB00398	Vitamin D deficiency	0.76
DB01267	Sudden infant death syndrome	0.76
DB00571	Behavior disease	0.75
DB08807	Behavior disease	0.75
DB06626	Mucocutaneous lymph node syndrome	0.75
DB08815	Behavior disease	0.75
DB00320	Anorexia nervosa	0.75
DB00112	Mucocutaneous lymph node syndrome	0.74
DB01259	Esotropia	0.74
DB06216	Behavior disease	0.74
DB01049	Behavior disease	0.74
DB00775	Autistic disorder	0.73
DB00072	Angiomyolipoma	0.73
DB00960	Sudden infant death syndrome	0.73
DB00918	Attention deficit hyperactivity disorder	0.73
DB00998	Attention deficit hyperactivity disorder	0.73
DB00269	Primary biliary cirrhosis	0.73
DB00286	Primary biliary cirrhosis	0.73
DB00539	Primary biliary cirrhosis	0.73
DB00655	Primary biliary cirrhosis	0.73
DB00882	Primary biliary cirrhosis	0.73
DB00890	Primary biliary cirrhosis	0.73
DB00947	Primary biliary cirrhosis	0.73
DB01357	Primary biliary cirrhosis	0.73
DB04575	Primary biliary cirrhosis	0.73
DB04938	Primary biliary cirrhosis	0.73
DB01185	Vitiligo	0.73
DB01392	Sudden infant death syndrome	0.73
DB00098	Drug abuse	0.73
DB00734	Behavior disease	0.73
DB00098	Hemorrhagic fevers, Viral	0.73
DB00072	Cytomegalovirus infection	0.73
DB00098	Temporal arteritis	0.72

DB05271	Behavior disease	0.72
DB00775	Pemphigoid, Bullous	0.72
DB00072	Thrombophlebitis	0.72
DB00571	Sudden infant death syndrome	0.72
DB08807	Sudden infant death syndrome	0.72
DB00074	Filariasis	0.72
DB00111	Filariasis	0.72
DB00268	Sudden infant death syndrome	0.72
DB00413	Sudden infant death syndrome	0.72
DB00589	Sudden infant death syndrome	0.72
DB00248	Sudden infant death syndrome	0.72
DB00714	Sudden infant death syndrome	0.72
DB00098	Alopecia	0.72
DB01259	Gastrointestinal tumor	0.72
DB01267	Behavior disease	0.72
DB01186	Sudden infant death syndrome	0.71
DB01200	Sudden infant death syndrome	0.71
DB06216	Hypertension	0.71
DB01392	Anorexia nervosa	0.71
DB00281	Esotropia	0.71
DB00098	Systemic infection	0.71
DB01277	Antiphospholipid syndrome	0.71
DB01277	Folic acid deficiency	0.71
DB01277	Gastrointestinal stromal tumor	0.71
DB01277	Hyperthyroidism	0.71
DB00569	CNS lymphoma	0.71
DB01225	CNS lymphoma	0.71
DB00407	CNS lymphoma	0.71
DB06271	CNS lymphoma	0.71
DB00198	Nevus	0.71
DB00552	Hydatidiform mole	0.71
DB00734	Panic disorder	0.70
DB00246	Sudden infant death syndrome	0.70
DB00334	Sudden infant death syndrome	0.70
DB01238	Sudden infant death syndrome	0.70
DB00098	Penile disease	0.70
DB00363	Sudden infant death syndrome	0.70
DB01224	Sudden infant death syndrome	0.70
DB00098	Intermediate coronary syndrome	0.70
DB08916	Esotropia	0.70
DB00072	Pneumoconiosis	0.70
DB00734	Anorexia nervosa	0.70
DB00953	Attention deficit hyperactivity disorder	0.70
DB08820	Cystic fibrosis	0.70
DB00100	Nevus	0.70
DB01267	Panic disorder	0.69
DB00321	Behavior disease	0.69

DB01392	Panic disorder	0.69
DB06822	Thalassemia	0.69
DB00054	Purpura, Thrombocytopenic, Idiopathic	0.68
DB01259	Cholelithiasis	0.68
DB00246	Panic disorder	0.68
DB00334	Panic disorder	0.68
DB01238	Panic disorder	0.68
DB01267	Anorexia nervosa	0.68
DB00294	Primary biliary cirrhosis	0.68
DB00304	Primary biliary cirrhosis	0.68
DB00481	Primary biliary cirrhosis	0.68
DB00603	Primary biliary cirrhosis	0.68
DB00675	Primary biliary cirrhosis	0.68
DB00823	Primary biliary cirrhosis	0.68
DB00957	Primary biliary cirrhosis	0.68
DB00977	Primary biliary cirrhosis	0.68
DB01431	Primary biliary cirrhosis	0.68
DB04573	Primary biliary cirrhosis	0.68
DB04574	Primary biliary cirrhosis	0.68
DB00363	Panic disorder	0.68
DB01224	Panic disorder	0.68
DB08916	Gastrointestinal tumor	0.68
DB00268	Anorexia nervosa	0.68
DB00413	Anorexia nervosa	0.68
DB00589	Anorexia nervosa	0.68
DB00775	Atherosclerosis	0.68
DB00098	Takayasu's arteritis	0.68
DB00054	Atherosclerosis	0.68
DB00315	Panic disorder	0.68
DB00669	Panic disorder	0.68
DB00952	Panic disorder	0.68
DB00248	Anorexia nervosa	0.67
DB00714	Anorexia nervosa	0.67
DB00775	Stroke	0.67
DB00268	Panic disorder	0.67
DB00413	Panic disorder	0.67
DB00589	Panic disorder	0.67
DB01186	Anorexia nervosa	0.66
DB00248	Panic disorder	0.66
DB00714	Panic disorder	0.66
DB00398	Osteomyelitis	0.66
DB01049	Sudden infant death syndrome	0.66
DB00098	Atherosclerosis	0.66
DB00571	Hypertension	0.66
DB08807	Hypertension	0.66
DB01200	Anorexia nervosa	0.66
DB01392	Attention deficit hyperactivity disorder	0.66

DB00054	Autistic disorder	0.66
DB01186	Panic disorder	0.66
DB00255	Primary biliary cirrhosis	0.66
DB00783	Primary biliary cirrhosis	0.66
DB08916	Cholelithiasis	0.66
DB00054	Pemphigoid, Bullous	0.66
DB06216	Sudden infant death syndrome	0.65
DB01200	Panic disorder	0.65
DB04941	Cystic fibrosis	0.65
DB01296	Infiltrating cancer	0.65
DB08875	Mucocutaneous lymph node syndrome	0.65
DB00734	Attention deficit hyperactivity disorder	0.65
DB00216	Panic disorder	0.65
DB00290	Esophageal disease	0.65
DB00063	Atherosclerosis	0.65
DB08895	Myeloproliferative disease	0.65
DB00170	Rectum cancer	0.64
DB00569	Rectum cancer	0.64
DB01109	Rectum cancer	0.64
DB01225	Rectum cancer	0.64
DB06228	Rectum cancer	0.64
DB06605	Rectum cancer	0.64
DB00759	Biliary cancer	0.64
DB00759	Drug-Induced dyskinesia	0.64
DB00759	Histiocytosis	0.64
DB00759	Hyperhomocysteinemia	0.64
DB00759	Vascular dementia	0.64
DB01108	Primary biliary cirrhosis	0.64
DB01196	Primary biliary cirrhosis	0.64
DB00960	Hypertension	0.64
DB00098	Aplastic anemia	0.64
DB01616	Epilepsy	0.64
DB06684	Epilepsy	0.64
DB06822	Sickle cell disease	0.64
DB00246	Anorexia nervosa	0.64
DB00334	Anorexia nervosa	0.64
DB01238	Anorexia nervosa	0.64
DB01267	Attention deficit hyperactivity disorder	0.64
DB00363	Anorexia nervosa	0.64
DB01224	Anorexia nervosa	0.64
DB00315	Anorexia nervosa	0.64
DB00669	Anorexia nervosa	0.64
DB00952	Anorexia nervosa	0.64
DB00960	Panic disorder	0.63
DB00098	Rheumatoid arthritis	0.63
DB00268	Attention deficit hyperactivity disorder	0.63
DB00413	Attention deficit hyperactivity disorder	0.63

DB00589	Attention deficit hyperactivity disorder	0.63
DB00816	Cystic fibrosis	0.63
DB00867	Cystic fibrosis	0.63
DB00871	Cystic fibrosis	0.63
DB00938	Cystic fibrosis	0.63
DB00983	Cystic fibrosis	0.63
DB01274	Cystic fibrosis	0.63
DB01366	Cystic fibrosis	0.63
DB01408	Cystic fibrosis	0.63
DB05039	Cystic fibrosis	0.63
DB00244	Polyarthritis	0.63
DB00795	Polyarthritis	0.63
DB01049	Panic disorder	0.63
DB00098	Subarachnoid hemorrhage	0.63
DB00098	Diabetes mellitus	0.63
DB00248	Attention deficit hyperactivity disorder	0.63
DB00714	Attention deficit hyperactivity disorder	0.63
DB01259	Papillomavirus infection	0.62
DB00063	Stroke	0.62
DB08896	Vitamin D deficiency	0.62
DB00571	Panic disorder	0.62
DB08807	Panic disorder	0.62
DB01183	Primary biliary cirrhosis	0.62
DB00887	Cystic fibrosis	0.62
DB01186	Attention deficit hyperactivity disorder	0.62
DB05294	Mucocutaneous lymph node syndrome	0.61
DB01411	Nephrosis	0.61
DB01200	Attention deficit hyperactivity disorder	0.61
DB08912	Angiomyolipoma	0.61
DB00216	Anorexia nervosa	0.61
DB00108	Systemic infection	0.61
DB00100	Abruption placentae	0.61
DB00100	Lymphatic metastasis	0.61
DB06216	Panic disorder	0.60
DB01277	Hypothyroidism	0.60
DB01016	Cystic fibrosis	0.60
DB06822	Purpura, Thrombocytopenic, Idiopathic	0.60
DB00233	Polyarthritis	0.60
DB00490	Epilepsy	0.60
DB00098	Hemorrhagic disorder	0.60
DB01270	Chronic obstructive airway disease	0.60
DB00866	Behavior disease	0.60
DB01359	Behavior disease	0.60
DB00098	Thrombocytopenia	0.59
DB00246	Attention deficit hyperactivity disorder	0.59
DB00334	Attention deficit hyperactivity disorder	0.59
DB01238	Attention deficit hyperactivity disorder	0.59

DB00002	Esotropia	0.59
DB00363	Attention deficit hyperactivity disorder	0.59
DB01224	Attention deficit hyperactivity disorder	0.59
DB01049	Attention deficit hyperactivity disorder	0.59
DB01136	Chronic obstructive airway disease	0.59
DB00098	Alzheimer's disease	0.59
DB01049	Anorexia nervosa	0.59
DB01259	Skin cancer	0.59
DB00108	Thalassemia	0.59
DB00098	Depression	0.59
DB00396	Primary biliary cirrhosis	0.59
DB00866	Hypertension	0.59
DB01359	Hypertension	0.59
DB00726	Epilepsy	0.58
DB00904	Migraine	0.58
DB01404	Uterine fibroids	0.58
DB00031	Amyloidosis	0.58
DB00367	Primary biliary cirrhosis	0.58
DB00540	Epilepsy	0.58
DB08810	Epilepsy	0.58
DB00387	Supranuclear palsy, progressive	0.58
DB00424	Supranuclear palsy, progressive	0.58
DB00809	Supranuclear palsy, progressive	0.58
DB00209	Supranuclear palsy, progressive	0.58
DB00219	Supranuclear palsy, progressive	0.58
DB00670	Supranuclear palsy, progressive	0.58
DB00771	Supranuclear palsy, progressive	0.58
DB00782	Supranuclear palsy, progressive	0.58
DB00942	Supranuclear palsy, progressive	0.58
DB00979	Supranuclear palsy, progressive	0.58
DB00986	Supranuclear palsy, progressive	0.58
DB01109	CNS lymphoma	0.58
DB00600	Uveomeningoencephalitic syndrome	0.58
DB06822	CNS lymphoma	0.58
DB04835	Hemophilia	0.58
DB06168	Retinoblastoma	0.58
DB00535	Hepatoblastoma	0.58
DB05294	Esotropia	0.58
DB01017	Chronic obstructive airway disease	0.58
DB00656	Epilepsy	0.57
DB00379	Uterine fibroids	0.57
DB08875	Malaria	0.57
DB01406	Primary biliary cirrhosis	0.57
DB00072	Esotropia	0.57
DB01621	Epilepsy	0.57
DB01618	Epilepsy	0.57
DB00054	Lupus erythematosus	0.57

DB01259	Primary hyperparathyroidism	0.57
DB00398	Thyroid gland disease	0.56
DB01120	Chronic obstructive airway disease	0.56
DB00696	Behavior disease	0.56
DB01041	Nephrosis	0.56
DB01050	Gram-Negative bacterial infection	0.56
DB01149	Epilepsy	0.56
DB00247	Epilepsy	0.56
DB00569	Thrombophilia	0.56
DB01225	Thrombophilia	0.56
DB00734	Migraine	0.56
DB00315	Attention deficit hyperactivity disorder	0.56
DB00669	Attention deficit hyperactivity disorder	0.56
DB00952	Attention deficit hyperactivity disorder	0.56
DB08916	Skin cancer	0.56
DB01614	Epilepsy	0.56
DB01622	Epilepsy	0.56
DB08912	Skin disease, Genetic	0.56
DB00072	Gastrointestinal tumor	0.55
DB00054	Stroke	0.55
DB06822	Systemic infection	0.55
DB00918	Behavior disease	0.55
DB00998	Behavior disease	0.55
DB00072	Cholelithiasis	0.55
DB01259	Charcot-Marie-Tooth disease	0.55
DB00477	Epilepsy	0.55
DB05271	Epilepsy	0.55
DB00759	Learning disorder	0.55
DB01268	Mucocutaneous lymph node syndrome	0.55
DB00108	Sickle cell disease	0.55
DB08885	Chronic obstructive airway disease	0.55
DB01267	Migraine	0.55
DB01392	Migraine	0.55
DB08916	Papillomavirus infection	0.55
DB00866	Epilepsy	0.55
DB01359	Epilepsy	0.55
DB08896	Osteomyelitis	0.55
DB01136	Mucocutaneous lymph node syndrome	0.54
DB04946	Epilepsy	0.54
DB01200	Hypertension	0.54
DB01169	Hyperparathyroidism	0.54
DB00315	Migraine	0.54
DB00669	Migraine	0.54
DB00952	Migraine	0.54
DB00904	Hepatitis C	0.54
DB00290	Peptic esophagitis	0.54
DB00248	Hypertension	0.54

DB00775	Asthma	0.54
DB00953	Behavior disease	0.53
DB00268	Migraine	0.53
DB00413	Migraine	0.53
DB00589	Migraine	0.53
DB01142	Epilepsy	0.53
DB00246	Hypertension	0.53
DB00334	Hypertension	0.53
DB01238	Hypertension	0.53
DB08877	Myeloproliferative disease	0.53
DB01109	Thrombophilia	0.53
DB01259	Glaucoma	0.53
DB01076	Uterine fibroids	0.53
DB00248	Migraine	0.53
DB00714	Migraine	0.53
DB00363	Hypertension	0.53
DB01224	Hypertension	0.53
DB01169	Infectious lung disease	0.53
DB01411	Tuberculosis	0.53
DB01186	Hypertension	0.53
DB08815	Epilepsy	0.53
DB00170	Thrombophilia	0.52
DB01392	Hypertension	0.52
DB00195	Cystic fibrosis	0.52
DB00264	Cystic fibrosis	0.52
DB00521	Cystic fibrosis	0.52
DB00612	Cystic fibrosis	0.52
DB00841	Cystic fibrosis	0.52
DB01001	Cystic fibrosis	0.52
DB01193	Cystic fibrosis	0.52
DB01203	Cystic fibrosis	0.52
DB01210	Cystic fibrosis	0.52
DB01214	Cystic fibrosis	0.52
DB01291	Cystic fibrosis	0.52
DB01580	Cystic fibrosis	0.52
DB04861	Cystic fibrosis	0.52
DB01411	Pancreatitis	0.52
DB01186	Migraine	0.52
DB08916	Charcot-Marie-Tooth disease	0.52
DB00216	Attention deficit hyperactivity disorder	0.52
DB00268	Hypertension	0.52
DB00413	Hypertension	0.52
DB00589	Hypertension	0.52
DB01200	Migraine	0.52
DB01149	Hypertension	0.52
DB00247	Hypertension	0.52
DB00025	Amyloidosis	0.52

DB00340	Supranuclear palsy, progressive	0.52
DB00376	Supranuclear palsy, progressive	0.52
DB00496	Supranuclear palsy, progressive	0.52
DB00572	Supranuclear palsy, progressive	0.52
DB00725	Supranuclear palsy, progressive	0.52
DB00785	Supranuclear palsy, progressive	0.52
DB01036	Supranuclear palsy, progressive	0.52
DB01591	Supranuclear palsy, progressive	0.52
DB06702	Supranuclear palsy, progressive	0.52
DB08897	Supranuclear palsy, progressive	0.52
DB00216	Migraine	0.52
DB08916	Primary hyperparathyroidism	0.52
DB01049	Hypertension	0.52
DB00714	Hypertension	0.52
DB08912	Common cold	0.51
DB01259	Testicular dysfunction	0.51
DB00036	Hyperlipidemia	0.51
DB00656	Hypertension	0.51
DB00108	Alopecia	0.51
DB00960	Epilepsy	0.51
DB08916	Glaucoma	0.51
DB00449	Cystic fibrosis	0.51
DB00246	Migraine	0.51
DB00334	Migraine	0.51
DB01238	Migraine	0.51
DB00398	Angiomyolipoma	0.51
DB00054	Asthma	0.51
DB06779	Cardiovascular disease	0.51
DB06779	Chronic obstructive airway disease	0.51
DB00100	Thrombophilia	0.51
DB00995	Polyarthritis	0.51
DB00363	Migraine	0.51
DB01224	Migraine	0.51
DB01185	Primary biliary cirrhosis	0.50
DB00489	Cystic fibrosis	0.50
DB00373	Cystic fibrosis	0.50
DB01102	Cystic fibrosis	0.50
DB01288	Cystic fibrosis	0.50
DB01295	Cystic fibrosis	0.50
DB08808	Cystic fibrosis	0.50
DB00904	Epilepsy	0.50
DB00960	Migraine	0.50
DB00042	Attention deficit hyperactivity disorder	0.50
DB01142	Hypertension	0.50
DB00855	Parasitic disease	0.50
DB06779	CNS lymphoma	0.50
DB00178	Subacute sclerosing panencephalitis	0.50

DB00492	Subacute sclerosing panencephalitis	0.50
DB00519	Subacute sclerosing panencephalitis	0.50
DB00542	Subacute sclerosing panencephalitis	0.50
DB00584	Subacute sclerosing panencephalitis	0.50
DB00790	Subacute sclerosing panencephalitis	0.50
DB00881	Subacute sclerosing panencephalitis	0.50
DB01180	Subacute sclerosing panencephalitis	0.50
DB01340	Subacute sclerosing panencephalitis	0.50
DB01348	Subacute sclerosing panencephalitis	0.50
DB08813	CNS lymphoma	0.50
DB00176	Pulmonary hypertension	0.50
DB00472	Pulmonary hypertension	0.50
DB01281	Thyroiditis	0.50
DB06681	Thyroiditis	0.50
DB06213	Phobic anxiety disorder	0.50
DB06168	Synovial sarcoma	0.50
DB00752	Fibromyalgia	0.50
DB01037	Fibromyalgia	0.50
DB01247	Fibromyalgia	0.50
DB01626	Fibromyalgia	0.50
DB00571	Epilepsy	0.50
DB08807	Epilepsy	0.50
DB00244	Virus disease	0.50
DB00795	Virus disease	0.50
DB00108	Purpura, Thrombocytopenic, Idiopathic	0.50
DB01169	Neck cancer	0.50
DB00098	Herpes	0.50
DB00315	Hepatitis C	0.50
DB00669	Hepatitis C	0.50
DB00952	Hepatitis C	0.50
DB00320	Behavior disease	0.50
