# Supplementary material for: Inferring drug-disease associations based on known protein complexes
Source: BMC Med Genomics. 2015 May 29;8(Suppl 2):S2. doi: 10.1186/1755-8794-8-S2-S2 (PMC4460611; doi:10.1186/1755-8794-8-S2-S2)
Supplement: Additional file 8 — Table illustrating the drug-mental disorders relations predicted by our method. [file 1755-8794-8-S2-S2-S8.PDF]

Drug ID	Disease Name	Weight	Drug ID	Disease Name	Weight
DB01149	Mental disorder	0.93303	DB04871	Mental disorder	0.1543
DB00726	Mental disorder	0.89783	DB01170	Mental disorder	0.1543
DB00904	Mental disorder	0.89264	DB00226	Mental disorder	0.1543
DB00540	Mental disorder	0.88792	DB01463	Mental disorder	0.1543
DB00247	Mental disorder	0.87223	DB01364	Mental disorder	0.1543
DB01392	Mental disorder	0.86582	DB01089	Mental disorder	0.1543
DB00656	Mental disorder	0.85771	DB04840	Mental disorder	0.1543
DB00669	Mental disorder	0.8466	DB00924	Mental disorder	0.1543
DB00315	Mental disorder	0.8466	DB01019	Mental disorder	0.1543
DB00952	Mental disorder	0.8466	DB00865	Mental disorder	0.1543
DB08810	Mental disorder	0.84338	DB00182	Mental disorder	0.1543
DB00589	Mental disorder	0.8356	DB00386	Mental disorder	0.1543
DB00268	Mental disorder	0.8356	DB01239	Mental disorder	0.13363
DB00413	Mental disorder	0.8356	DB00370	Mental disorder	0.12599
DB01142	Mental disorder	0.83145	DB06204	Mental disorder	0.12599
DB00714	Mental disorder	0.83001	DB00780	Mental disorder	0.12599
DB00248	Mental disorder	0.83001	DB00777	Mental disorder	0.12372
DB01238	Mental disorder	0.82495	DB00420	Mental disorder	0.12372
DB00246	Mental disorder	0.82495	DB00988	Mental disorder	0.11664
DB00334	Mental disorder	0.82495	DB00934	Mental disorder	0.10911
DB00363	Mental disorder	0.82195	DB00822	Mental disorder	0.10911
DB01224	Mental disorder	0.82195	DB00843	Mental disorder	0.10911
DB01186	Mental disorder	0.82034	DB00745	Mental disorder	0.10911
DB01618	Mental disorder	0.81951	DB04844	Mental disorder	0.10911
DB06684	Mental disorder	0.81951	DB06144	Mental disorder	0.10911
DB01621	Mental disorder	0.81951	DB01367	Mental disorder	0.10911
DB01616	Mental disorder	0.81951	DB06714	Mental disorder	0.10911
DB01200	Mental disorder	0.81612	DB01113	Mental disorder	0.10911
DB00216	Mental disorder	0.80893	DB01336	Mental disorder	0.10911
DB01622	Mental disorder	0.7912	DB00933	Mental disorder	0.10911
DB01614	Mental disorder	0.7912	DB01255	Mental disorder	0.10911
DB00477	Mental disorder	0.78185	DB06707	Mental disorder	0.10911
DB00490	Mental disorder	0.77432	DB06706	Mental disorder	0.10911
DB00960	Mental disorder	0.77098	DB01148	Mental disorder	0.10911
DB04946	Mental disorder	0.75826	DB01135	Mental disorder	0.10911
DB00571	Mental disorder	0.75469	DB01146	Mental disorder	0.10911
DB08807	Mental disorder	0.75469	DB08801	Mental disorder	0.10911
DB08815	Mental disorder	0.75042	DB00804	Mental disorder	0.10911
DB06216	Mental disorder	0.73651	DB00543	Mental disorder	0.10911
DB01049	Mental disorder	0.73567	DB01403	Mental disorder	0.1062
DB00734	Mental disorder	0.72648	DB01069	Mental disorder	0.09759
DB05271	Mental disorder	0.72353	DB01623	Mental disorder	0.08909
DB01267	Mental disorder	0.71504	DB01085	Mental disorder	0.08909
DB00321	Mental disorder	0.69259	DB01337	Mental disorder	0.08909
DB01359	Mental disorder	0.59534	DB00411	Mental disorder	0.08909
DB00866	Mental disorder	0.59534	DB01409	Mental disorder	0.08909
DB00696	Mental disorder	0.56426	DB01412	Mental disorder	0.08909
DB00998	Mental disorder	0.55287	DB00728	Mental disorder	0.08909
DB00918	Mental disorder	0.55287	DB01338	Mental disorder	0.08909
DB00953	Mental disorder	0.53285	DB01579	Mental disorder	0.08909

DB00320	Mental disorder	0.49502	DB00383	Mental disorder	0.08909
DB01273	Mental disorder	0.44735	DB01062	Mental disorder	0.08909
DB00184	Mental disorder	0.43262	DB00462	Mental disorder	0.08909
DB00674	Mental disorder	0.40216	DB00332	Mental disorder	0.08909
DB00191	Mental disorder	0.34503	DB00483	Mental disorder	0.08909
DB00599	Mental disorder	0.28215	DB04842	Mental disorder	0.08909
DB01577	Mental disorder	0.27915	DB00392	Mental disorder	0.08909
DB00579	Mental disorder	0.26726	DB00824	Mental disorder	0.08909
DB00285	Mental disorder	0.26726	DB01231	Mental disorder	0.08909
DB01105	Mental disorder	0.26726	DB00245	Mental disorder	0.08909
DB00422	Mental disorder	0.26726	DB00517	Mental disorder	0.08909
DB00476	Mental disorder	0.26726	DB01427	Mental disorder	0.08909
DB06701	Mental disorder	0.26726	DB06594	Mental disorder	0.08909
DB01363	Mental disorder	0.25678	DB00215	Mental disorder	0.07715
DB06148	Mental disorder	0.25198	DB00809	Mental disorder	0.07715
DB01174	Mental disorder	0.24016	DB00514	Mental disorder	0.07715
DB00418	Mental disorder	0.24016	DB00201	Mental disorder	0.07715
DB00849	Mental disorder	0.24016	DB00360	Mental disorder	0.07715
DB01355	Mental disorder	0.24016	DB00202	Mental disorder	0.07715
DB01354	Mental disorder	0.24016	DB01656	Mental disorder	0.07715
DB01353	Mental disorder	0.24016	DB00387	Mental disorder	0.07715
DB01352	Mental disorder	0.24016	DB01226	Mental disorder	0.07715
DB01351	Mental disorder	0.24016	DB00424	Mental disorder	0.07715
DB01242	Mental disorder	0.23328	DB00604	Mental disorder	0.07715
DB01114	Mental disorder	0.23146	DB06288	Mental disorder	0.07715
DB00181	Mental disorder	0.22932	DB00376	Mental disorder	0.06901
DB01104	Mental disorder	0.21822	DB00721	Mental disorder	0.06901
DB00715	Mental disorder	0.21822	DB00502	Mental disorder	0.06901
DB00907	Mental disorder	0.21822	DB00572	Mental disorder	0.06901
DB00830	Mental disorder	0.21822	DB01248	Mental disorder	0.06901
DB01247	Mental disorder	0.21822	DB00508	Mental disorder	0.06901
DB00752	Mental disorder	0.21822	DB01036	Mental disorder	0.06901
DB01037	Mental disorder	0.21822	DB01591	Mental disorder	0.06901
DB00344	Mental disorder	0.21822	DB00409	Mental disorder	0.06901
DB01626	Mental disorder	0.21822	DB00340	Mental disorder	0.06901
DB08918	Mental disorder	0.21822	DB05266	Mental disorder	0.06901
DB00937	Mental disorder	0.21822	DB00725	Mental disorder	0.06901
DB01191	Mental disorder	0.21822	DB00875	Mental disorder	0.06901
DB06700	Mental disorder	0.21822	DB06702	Mental disorder	0.06901
DB00289	Mental disorder	0.21822	DB00496	Mental disorder	0.06901
DB00042	Mental disorder	0.21022	DB00785	Mental disorder	0.06901
DB00805	Mental disorder	0.20574	DB01161	Mental disorder	0.06901
DB00237	Mental disorder	0.19721	DB08897	Mental disorder	0.06901
DB01576	Mental disorder	0.18898	DB01229	Mental disorder	0.06299
DB00434	Mental disorder	0.18898	DB00679	Mental disorder	0.06299
DB01175	Mental disorder	0.18898	DB01381	Mental disorder	0.06299
DB01156	Mental disorder	0.17817	DB00747	Mental disorder	0.06299
DB04896	Mental disorder	0.17817	DB00806	Mental disorder	0.06299
DB00312	Mental disorder	0.17798	DB00768	Mental disorder	0.06299
DB00306	Mental disorder	0.17798	DB00751	Mental disorder	0.06299
DB00794	Mental disorder	0.17798	DB00280	Mental disorder	0.06299

DB00463	Mental disorder	0.17798	DB00148	Mental disorder	0.06299
DB00241	Mental disorder	0.17798	DB00835	Mental disorder	0.06299
DB00852	Mental disorder	0.17496	DB01173	Mental disorder	0.05832
DB00458	Mental disorder	0.16496	DB01088	Mental disorder	0.05832
DB01151	Mental disorder	0.16496	DB00909	Mental disorder	0.05543
DB00193	Mental disorder	0.16366	DB00651	Mental disorder	0.05455
DB01171	Mental disorder	0.1543	DB00920	Mental disorder	0.0488
DB00176	Mental disorder	0.1543	DB00622	Mental disorder	0.03984
DB00206	Mental disorder	0.1543	DB00131	Mental disorder	0.03984
DB00472	Mental disorder	0.1543	DB03147	Mental disorder	0.03951
DB00234	Mental disorder	0.1543	DB00661	Mental disorder	0.03858
DB00408	Mental disorder	0.1543	DB00126	Mental disorder	0.0315
