# Supplementary material for: Inferring drug-disease associations based on known protein complexes
Source: BMC Med Genomics. 2015 May 29;8(Suppl 2):S2. doi: 10.1186/1755-8794-8-S2-S2 (PMC4460611; doi:10.1186/1755-8794-8-S2-S2)
Supplement: Additional file 9 — Table illustrating 23 clusters got from drug-drug network. [file 1755-8794-8-S2-S2-S9.PDF]

Drug ID	Cluster Lab	Drug ID	Cluster Label	Drug ID	Cluster Label	Drug ID	Cluster Label
DB06204	1	DB01259	2	DB00022	4	DB08881	14
DB00907	1	DB00795	2	DB00018	4	DB06372	15
DB01114	1	DB00244	2	DB00011	4	DB01404	15
DB06701	1	DB06616	2	DB00008	4	DB00108	15
DB01105	1	DB08901	2	DB01197	5	DB01411	15
DB00579	1	DB01254	2	DB01404	5	DB00041	15
DB00476	1	DB01110	2	DB01277	5	DB00004	15
DB00422	1	DB00396	2	DB04835	5	DB06168	15
DB00285	1	DB01183	2	DB00722	5	DB05260	15
DB01104	1	DB04938	2	DB00691	5	DB06186	15
DB00191	1	DB04575	2	DB00616	5	DB08818	15
DB04896	1	DB04574	2	DB00759	5	DB01250	15
DB08918	1	DB04573	2	DB01348	5	DB04835	15
DB01577	1	DB01431	2	DB01340	5	DB00616	15
DB01242	1	DB01357	2	DB01180	5	DB01055	15
DB06700	1	DB00957	2	DB00881	5	DB08904	15
DB00344	1	DB00947	2	DB00790	5	DB06674	15
DB00289	1	DB00890	2	DB00584	5	DB00065	15
DB08815	1	DB00882	2	DB00542	5	DB01296	15
DB01149	1	DB00823	2	DB00519	5	DB01017	15
DB00656	1	DB00675	2	DB00492	5	DB00005	15
DB01392	1	DB00655	2	DB00178	5	DB00111	15
DB00696	1	DB00603	2	DB05278	5	DB00074	15
DB00247	1	DB00539	2	DB00966	5	DB00051	15
DB06148	1	DB00481	2	DB08822	5	DB00045	15
DB00924	1	DB00304	2	DB01349	5	DB01041	15
DB00726	1	DB00294	2	DB01347	5	DB08910	15
DB01622	1	DB00286	2	DB01342	5	DB00608	15
DB01614	1	DB00269	2	DB00876	5	DB01427	15
DB01618	1	DB00255	2	DB00796	5	DB01407	15
DB08810	1	DB01108	2	DB00678	5	DB01136	16
DB01621	1	DB01196	2	DB00275	5	DB01017	16
DB00458	1	DB00977	2	DB00177	5	DB00112	16
DB00805	1	DB00783	2	DB08894	6	DB08885	16
DB00434	1	DB01185	2	DB08923	6	DB01270	16
DB00715	1	DB00367	2	DB00016	6	DB01120	16
DB00604	1	DB00360	2	DB00012	6	DB06779	16
DB00852	1	DB05294	2	DB00252	7	DB06626	16
DB01363	1	DB00063	2	DB04930	7	DB00054	17
DB01151	1	DB01041	2	DB01438	7	DB00098	17
DB06216	1	DB00155	2	DB01121	7	DB00048	17
DB00472	1	DB08813	2	DB08932	8	DB08888	17
DB00176	1	DB00125	2	DB06268	8	DB01136	17
DB00514	1	DB00615	2	DB00559	8	DB00108	17
DB01049	1	DB08814	2	DB01303	9	DB06822	17
DB01142	1	DB00995	2	DB00277	9	DB01097	17

DB00321	1	DB06204	3	DB02546	9	DB00775	17
DB00714	1	DB00907	3	DB00227	9	DB00063	17
DB01191	1	DB01114	3	DB01223	9	DB00398	18
DB01267	1	DB00937	3	DB00108	10	DB08896	18
DB00540	1	DB00830	3	DB00075	10	DB04895	18
DB01238	1	DB06701	3	DB00002	10	DB08875	18
DB01200	1	DB01105	3	DB00005	10	DB00039	18
DB01224	1	DB00579	3	DB00056	10	DB06589	18
DB01186	1	DB00476	3	DB00092	10	DB01268	18
DB00215	1	DB00422	3	DB00110	10	DB06626	18
DB00734	1	DB00285	3	DB00111	10	DB08911	18
DB01175	1	DB01104	3	DB00074	10	DB08912	18
DB00904	1	DB00191	3	DB00095	10	DB00398	19
DB04946	1	DB04896	3	DB00028	10	DB08896	19
DB00193	1	DB08918	3	DB00051	10	DB00102	19
DB00477	1	DB01577	3	DB00112	10	DB04895	19
DB00589	1	DB01242	3	DB00087	10	DB08875	19
DB00413	1	DB06700	3	DB00081	10	DB00039	19
DB00543	1	DB00344	3	DB00078	10	DB00619	19
DB00181	1	DB00289	3	DB00073	10	DB06589	19
DB00320	1	DB01156	3	DB00031	11	DB01268	19
DB00998	1	DB01576	3	DB05777	11	DB06626	19
DB00918	1	DB01149	3	DB00036	11	DB00054	20
DB00953	1	DB00656	3	DB01109	11	DB00098	20
DB00952	1	DB00696	3	DB00759	11	DB00048	20
DB00669	1	DB06148	3	DB00170	11	DB08888	20
DB00315	1	DB00924	3	DB00100	11	DB01136	20
DB00216	1	DB00726	3	DB00055	11	DB00108	20
DB05271	1	DB00458	3	DB00025	11	DB06822	20
DB01359	1	DB00805	3	DB06605	11	DB00775	20
DB00866	1	DB00715	3	DB06228	11	DB08818	20
DB08807	1	DB00604	3	DB01225	11	DB00072	20
DB00571	1	DB00852	3	DB00569	11	DB08916	20
DB00960	1	DB01363	3	DB00061	12	DB01259	20
DB00490	1	DB01151	3	DB00072	12	DB00063	20
DB06684	1	DB00472	3	DB08916	12	DB00061	21
DB01616	1	DB00176	3	DB01259	12	DB06366	21
DB00248	1	DB00514	3	DB00002	12	DB05773	21
DB00363	1	DB01142	3	DB05294	12	DB00013	22
DB00334	1	DB00321	3	DB00281	12	DB00031	22
DB00268	1	DB01191	3	DB06366	12	DB00029	22
DB00246	1	DB00540	3	DB05773	12	DB00015	22
DB00054	2	DB00215	3	DB00530	12	DB00055	22
DB01169	2	DB01175	3	DB01269	12	DB00009	22
DB01406	2	DB01171	3	DB00317	12	DB08896	23
DB00098	2	DB01626	3	DB06695	13	DB01169	23
DB01065	2	DB01247	3	DB04898	13	DB00233	23

DB00233	2	DB01037	3	DB01123	13	DB01064	23
DB00108	2	DB00752	3	DB05777	13	DB00570	23
DB06822	2	DB00193	3	DB00055	13	DB01029	23
DB00775	2	DB00661	3	DB00278	13	DB00171	23
DB01064	2	DB00543	3	DB00006	13	DB00795	23
DB00570	2	DB05258	4	DB00001	13	DB00244	23
DB01411	2	DB00105	4	DB00398	14	DB06616	23
DB00072	2	DB00069	4	DB08896	14	DB08901	23
DB01296	2	DB00068	4	DB06616	14	DB01254	23
DB01029	2	DB00060	4	DB08911	14	DB00995	23
DB08916	2	DB00034	4	DB08912	14		
