# Supplementary material for: Inferring drug-disease associations based on known protein complexes
Source: BMC Med Genomics. 2015 May 29;8(Suppl 2):S2. doi: 10.1186/1755-8794-8-S2-S2 (PMC4460611; doi:10.1186/1755-8794-8-S2-S2)
Supplement: Additional file 10 — Table illustrating the drug-hypertension relations predicted by our method. [file 1755-8794-8-S2-S2-S10.PDF]

| Drug ID | Disease Nam | Weight | Drug ID | Disease Name | Weight | Drug ID | Disease Name | Weight |
|---|---|---|---|---|---|---|---|---|
| DB06216 | Hypertension | 0.71071 | DB00234 | Hypertension | 0.07906 | DB00962 | Hypertension | 0.0559 |
| DB00571 | Hypertension | 0.66133 | DB00275 | Hypertension | 0.07906 | DB00966 | Hypertension | 0.0559 |
| DB08807 | Hypertension | 0.66133 | DB00322 | Hypertension | 0.07906 | DB01133 | Hypertension | 0.0559 |
| DB00960 | Hypertension | 0.64267 | DB00377 | Hypertension | 0.07906 | DB01191 | Hypertension | 0.0559 |
| DB00866 | Hypertension | 0.58583 | DB00384 | Hypertension | 0.07906 | DB01349 | Hypertension | 0.0559 |
| DB01359 | Hypertension | 0.58583 | DB00390 | Hypertension | 0.07906 | DB01370 | Hypertension | 0.0559 |
| DB01200 | Hypertension | 0.5403 | DB00408 | Hypertension | 0.07906 | DB06212 | Hypertension | 0.0559 |
| DB00248 | Hypertension | 0.53589 | DB00432 | Hypertension | 0.07906 | DB06268 | Hypertension | 0.0559 |
| DB00246 | Hypertension | 0.53071 | DB00437 | Hypertension | 0.07906 | DB06700 | Hypertension | 0.0559 |
| DB00334 | Hypertension | 0.53071 | DB00492 | Hypertension | 0.07906 | DB06706 | Hypertension | 0.0559 |
| DB01238 | Hypertension | 0.53071 | DB00511 | Hypertension | 0.07906 | DB06813 | Hypertension | 0.0559 |
| DB00363 | Hypertension | 0.52764 | DB00519 | Hypertension | 0.07906 | DB08918 | Hypertension | 0.0559 |
| DB01224 | Hypertension | 0.52764 | DB00524 | Hypertension | 0.07906 | DB08932 | Hypertension | 0.0559 |
| DB01186 | Hypertension | 0.52599 | DB00542 | Hypertension | 0.07906 | DB00594 | Hypertension | 0.0527 |
| DB01392 | Hypertension | 0.52259 | DB00544 | Hypertension | 0.07906 | DB01069 | Hypertension | 0.05 |
| DB00268 | Hypertension | 0.5205 | DB00552 | Hypertension | 0.07906 | DB00035 | Hypertension | 0.04564 |
| DB00413 | Hypertension | 0.5205 | DB00584 | Hypertension | 0.07906 | DB00040 | Hypertension | 0.04564 |
| DB00589 | Hypertension | 0.5205 | DB00604 | Hypertension | 0.07906 | DB00067 | Hypertension | 0.04564 |
| DB01149 | Hypertension | 0.51837 | DB00610 | Hypertension | 0.07906 | DB00285 | Hypertension | 0.04564 |
| DB00247 | Hypertension | 0.51736 | DB00650 | Hypertension | 0.07906 | DB00298 | Hypertension | 0.04564 |
| DB01049 | Hypertension | 0.51567 | DB00678 | Hypertension | 0.07906 | DB00346 | Hypertension | 0.04564 |
| DB00714 | Hypertension | 0.51548 | DB00699 | Hypertension | 0.07906 | DB00388 | Hypertension | 0.04564 |
| DB00656 | Hypertension | 0.50966 | DB00757 | Hypertension | 0.07906 | DB00422 | Hypertension | 0.04564 |
| DB01142 | Hypertension | 0.50167 | DB00790 | Hypertension | 0.07906 | DB00441 | Hypertension | 0.04564 |
| DB00904 | Hypertension | 0.49243 | DB00796 | Hypertension | 0.07906 | DB00476 | Hypertension | 0.04564 |
| DB08815 | Hypertension | 0.48935 | DB00805 | Hypertension | 0.07906 | DB00579 | Hypertension | 0.04564 |
| DB00216 | Hypertension | 0.47397 | DB00876 | Hypertension | 0.07906 | DB00706 | Hypertension | 0.04564 |
| DB00734 | Hypertension | 0.46817 | DB00881 | Hypertension | 0.07906 | DB00728 | Hypertension | 0.04564 |
| DB00726 | Hypertension | 0.45976 | DB00889 | Hypertension | 0.07906 | DB00824 | Hypertension | 0.04564 |
| DB04946 | Hypertension | 0.45976 | DB00924 | Hypertension | 0.07906 | DB00973 | Hypertension | 0.04564 |
| DB01267 | Hypertension | 0.45644 | DB00969 | Hypertension | 0.07906 | DB01105 | Hypertension | 0.04564 |
| DB01108 | Hypertension | 0.44014 | DB00990 | Hypertension | 0.07906 | DB01109 | Hypertension | 0.04564 |
| DB01614 | Hypertension | 0.43741 | DB01006 | Hypertension | 0.07906 | DB01156 | Hypertension | 0.04564 |
| DB01622 | Hypertension | 0.43741 | DB01078 | Hypertension | 0.07906 | DB01199 | Hypertension | 0.04564 |
| DB00396 | Hypertension | 0.4339 | DB01092 | Hypertension | 0.07906 | DB01623 | Hypertension | 0.04564 |
| DB00315 | Hypertension | 0.43351 | DB01101 | Hypertension | 0.07906 | DB02638 | Hypertension | 0.04564 |
| DB00669 | Hypertension | 0.43351 | DB01158 | Hypertension | 0.07906 | DB04842 | Hypertension | 0.04564 |
| DB00952 | Hypertension | 0.43351 | DB01170 | Hypertension | 0.07906 | DB04896 | Hypertension | 0.04564 |
| DB00481 | Hypertension | 0.43336 | DB01178 | Hypertension | 0.07906 | DB06207 | Hypertension | 0.04564 |
| DB00675 | Hypertension | 0.43336 | DB01180 | Hypertension | 0.07906 | DB06594 | Hypertension | 0.04564 |
| DB04573 | Hypertension | 0.43336 | DB01188 | Hypertension | 0.07906 | DB06692 | Hypertension | 0.04564 |
| DB04574 | Hypertension | 0.43336 | DB01217 | Hypertension | 0.07906 | DB06701 | Hypertension | 0.04564 |
| DB00540 | Hypertension | 0.43187 | DB01253 | Hypertension | 0.07906 | DB00151 | Hypertension | 0.04385 |
| DB08810 | Hypertension | 0.43187 | DB01258 | Hypertension | 0.07906 | DB00129 | Hypertension | 0.04226 |
| DB00477 | Hypertension | 0.43022 | DB01324 | Hypertension | 0.07906 | DB00215 | Hypertension | 0.03953 |
| DB01616 | Hypertension | 0.41964 | DB01340 | Hypertension | 0.07906 | DB00642 | Hypertension | 0.03953 |
| DB01618 | Hypertension | 0.41964 | DB01342 | Hypertension | 0.07906 | DB00880 | Hypertension | 0.03953 |
| DB01621 | Hypertension | 0.41964 | DB01347 | Hypertension | 0.07906 | DB00975 | Hypertension | 0.03953 |
| DB06684 | Hypertension | 0.41964 | DB01348 | Hypertension | 0.07906 | DB00996 | Hypertension | 0.03953 |
| DB00255 | Hypertension | 0.41284 | DB01364 | Hypertension | 0.07906 | DB01043 | Hypertension | 0.03953 |
| DB00783 | Hypertension | 0.41284 | DB01365 | Hypertension | 0.07906 | DB01114 | Hypertension | 0.03953 |
| DB00696 | Hypertension | 0.40346 | DB01396 | Hypertension | 0.07906 | DB01213 | Hypertension | 0.03953 |
| DB00269 | Hypertension | 0.40061 | DB01430 | Hypertension | 0.07906 | DB01234 | Hypertension | 0.03953 |

| | | | | | | | | |
|---|---|---|---|---|---|---|---|---|
| DB00286 | Hypertension | 0.40061 | DB04835 | Hypertension | 0.07906 | DB05260 | Hypertension | 0.03953 |
| DB00539 | Hypertension | 0.40061 | DB04840 | Hypertension | 0.07906 | DB06779 | Hypertension | 0.03953 |
| DB00655 | Hypertension | 0.40061 | DB04871 | Hypertension | 0.07906 | DB08813 | Hypertension | 0.03953 |
| DB00882 | Hypertension | 0.40061 | DB05269 | Hypertension | 0.07906 | DB00123 | Hypertension | 0.03536 |
| DB00890 | Hypertension | 0.40061 | DB06168 | Hypertension | 0.07906 | DB00132 | Hypertension | 0.03536 |
| DB00947 | Hypertension | 0.40061 | DB06213 | Hypertension | 0.07906 | DB00191 | Hypertension | 0.03536 |
| DB01196 | Hypertension | 0.40061 | DB06273 | Hypertension | 0.07906 | DB00409 | Hypertension | 0.03536 |
| DB01357 | Hypertension | 0.40061 | DB06288 | Hypertension | 0.07906 | DB00502 | Hypertension | 0.03536 |
| DB04575 | Hypertension | 0.40061 | DB06439 | Hypertension | 0.07906 | DB00508 | Hypertension | 0.03536 |
| DB04938 | Hypertension | 0.40061 | DB08822 | Hypertension | 0.07906 | DB00721 | Hypertension | 0.03536 |
| DB05271 | Hypertension | 0.40034 | DB08870 | Hypertension | 0.07906 | DB01021 | Hypertension | 0.03536 |
| DB00490 | Hypertension | 0.39648 | DB08893 | Hypertension | 0.07906 | DB01161 | Hypertension | 0.03536 |
| DB00321 | Hypertension | 0.38871 | DB00621 | Hypertension | 0.07113 | DB01198 | Hypertension | 0.03536 |
| DB00294 | Hypertension | 0.37746 | DB00624 | Hypertension | 0.07113 | DB01325 | Hypertension | 0.03536 |
| DB00304 | Hypertension | 0.37746 | DB00665 | Hypertension | 0.07113 | DB00615 | Hypertension | 0.03441 |
| DB00603 | Hypertension | 0.37746 | DB00858 | Hypertension | 0.07113 | DB00716 | Hypertension | 0.03441 |
| DB00823 | Hypertension | 0.37746 | DB00984 | Hypertension | 0.07113 | DB00457 | Hypertension | 0.03227 |
| DB00957 | Hypertension | 0.37746 | DB01128 | Hypertension | 0.07113 | DB00536 | Hypertension | 0.03227 |
| DB00977 | Hypertension | 0.37746 | DB01420 | Hypertension | 0.07113 | DB00562 | Hypertension | 0.03227 |
| DB01431 | Hypertension | 0.37746 | DB04839 | Hypertension | 0.07113 | DB00590 | Hypertension | 0.03227 |
| DB00320 | Hypertension | 0.37205 | DB06710 | Hypertension | 0.07113 | DB00831 | Hypertension | 0.03227 |
| DB00367 | Hypertension | 0.3576 | DB08804 | Hypertension | 0.07113 | DB01162 | Hypertension | 0.03227 |
| DB01406 | Hypertension | 0.35342 | DB08899 | Hypertension | 0.07113 | DB01381 | Hypertension | 0.03227 |
| DB01064 | Hypertension | 0.3183 | DB00875 | Hypertension | 0.07071 | DB01544 | Hypertension | 0.03227 |
| DB01185 | Hypertension | 0.30819 | DB00570 | Hypertension | 0.07009 | DB01587 | Hypertension | 0.03227 |
| DB00918 | Hypertension | 0.2831 | DB01239 | Hypertension | 0.06847 | DB04552 | Hypertension | 0.03227 |
| DB00998 | Hypertension | 0.2831 | DB00434 | Hypertension | 0.06455 | DB00988 | Hypertension | 0.02988 |
| DB01183 | Hypertension | 0.27742 | DB00679 | Hypertension | 0.06455 | DB01088 | Hypertension | 0.02988 |
| DB00953 | Hypertension | 0.27285 | DB00806 | Hypertension | 0.06455 | DB01173 | Hypertension | 0.02988 |
| DB01110 | Hypertension | 0.24195 | DB01175 | Hypertension | 0.06455 | DB00755 | Hypertension | 0.02795 |
| DB01278 | Hypertension | 0.22938 | DB01576 | Hypertension | 0.06455 | DB00907 | Hypertension | 0.02795 |
| DB00360 | Hypertension | 0.22517 | DB06204 | Hypertension | 0.06455 | DB01244 | Hypertension | 0.02635 |
| DB01065 | Hypertension | 0.22312 | DB00420 | Hypertension | 0.06339 | DB00233 | Hypertension | 0.02542 |
| DB00125 | Hypertension | 0.21199 | DB00458 | Hypertension | 0.06339 | DB00013 | Hypertension | 0.025 |
| DB00421 | Hypertension | 0.21075 | DB00777 | Hypertension | 0.06339 | DB00159 | Hypertension | 0.025 |
| DB01395 | Hypertension | 0.20049 | DB00499 | Hypertension | 0.06196 | DB00583 | Hypertension | 0.02384 |
| DB00687 | Hypertension | 0.18199 | DB00052 | Hypertension | 0.0559 | DB00622 | Hypertension | 0.02041 |
| DB00155 | Hypertension | 0.17008 | DB00193 | Hypertension | 0.0559 | DB06637 | Hypertension | 0.01976 |
| DB01169 | Hypertension | 0.14359 | DB00211 | Hypertension | 0.0559 | DB00186 | Hypertension | 0.01917 |
| DB01029 | Hypertension | 0.13888 | DB00289 | Hypertension | 0.0559 | DB00628 | Hypertension | 0.01917 |
| DB04932 | Hypertension | 0.13693 | DB00293 | Hypertension | 0.0559 | DB01068 | Hypertension | 0.01917 |
| DB01242 | Hypertension | 0.11952 | DB00344 | Hypertension | 0.0559 | DB00244 | Hypertension | 0.0188 |
| DB00640 | Hypertension | 0.11859 | DB00357 | Hypertension | 0.0559 | DB00795 | Hypertension | 0.0188 |
| DB00181 | Hypertension | 0.11741 | DB00358 | Hypertension | 0.0559 | DB00292 | Hypertension | 0.01863 |
| DB00691 | Hypertension | 0.1118 | DB00440 | Hypertension | 0.0559 | DB00063 | Hypertension | 0.01785 |
| DB00722 | Hypertension | 0.1118 | DB00450 | Hypertension | 0.0559 | DB01254 | Hypertension | 0.01769 |
| DB00822 | Hypertension | 0.1118 | DB00513 | Hypertension | 0.0559 | DB00128 | Hypertension | 0.01725 |
| DB06144 | Hypertension | 0.1118 | DB00559 | Hypertension | 0.0559 | DB00402 | Hypertension | 0.01725 |
| DB06196 | Hypertension | 0.1118 | DB00616 | Hypertension | 0.0559 | DB08901 | Hypertension | 0.01676 |
| DB06707 | Hypertension | 0.1118 | DB00635 | Hypertension | 0.0559 | DB06616 | Hypertension | 0.0167 |
| DB01412 | Hypertension | 0.09129 | DB00651 | Hypertension | 0.0559 | DB00126 | Hypertension | 0.01614 |
| DB01579 | Hypertension | 0.09129 | DB00715 | Hypertension | 0.0559 | DB00139 | Hypertension | 0.0155 |
| DB06372 | Hypertension | 0.09129 | DB00723 | Hypertension | 0.0559 | DB00775 | Hypertension | 0.01531 |
| DB00082 | Hypertension | 0.07906 | DB00830 | Hypertension | 0.0559 | DB00054 | Hypertension | 0.01455 |

| | | | | | | | | |
|---|---|---|---|---|---|---|---|---|
| DB00093 | Hypertension | 0.07906 | DB00843 | Hypertension | 0.0559 | DB00829 | Hypertension | 0.01356 |
| DB00177 | Hypertension | 0.07906 | DB00872 | Hypertension | 0.0559 | DB00231 | Hypertension | 0.01318 |
| DB00178 | Hypertension | 0.07906 | DB00903 | Hypertension | 0.0559 | DB00897 | Hypertension | 0.01318 |
| DB00201 | Hypertension | 0.07906 | DB00933 | Hypertension | 0.0559 | DB00143 | Hypertension | 0.01282 |
| DB00212 | Hypertension | 0.07906 | DB00934 | Hypertension | 0.0559 | DB00098 | Hypertension | 0.01195 |
| DB00226 | Hypertension | 0.07906 | DB00937 | Hypertension | 0.0559 | DB03147 | Hypertension | 0.01012 |
